# Optimization of Experimental Parameters in Data-Independent Mass Spectrometry Significantly Increases Depth and Reproducibility of Results*

**DOI:** 10.1074/mcp.RA117.000314

**Published:** 2017-10-25

**Authors:** Roland Bruderer, Oliver M. Bernhardt, Tejas Gandhi, Yue Xuan, Julia Sondermann, Manuela Schmidt, David Gomez-Varela, Lukas Reiter

**Affiliations:** From the ‡Biognosys, Wagistrasse 21, 8952 Schlieren, Switzerland.; §Thermo Fisher Scientific, 28199 Bremen, Germany.; ¶Somatosensory Signaling and Systems Biology Group, Max Planck Institute of Experimental Medicine, Hermann-Rein-Strasse 3, 37075 Goettingen, Germany

## Abstract

Comprehensive, reproducible and precise analysis of large sample cohorts is one of the key objectives of quantitative proteomics. Here, we present an implementation of data-independent acquisition using its parallel acquisition nature that surpasses the limitation of serial MS2 acquisition of data-dependent acquisition on a quadrupole ultra-high field Orbitrap mass spectrometer. In deep single shot data-independent acquisition, we identified and quantified 6,383 proteins in human cell lines using 2-or-more peptides/protein and over 7100 proteins when including the 717 proteins that were identified on the basis of a single peptide sequence. 7739 proteins were identified in mouse tissues using 2-or-more peptides/protein and 8121 when including the 382 proteins that were identified based on a single peptide sequence. Missing values for proteins were within 0.3 to 2.1% and median coefficients of variation of 4.7 to 6.2% among technical triplicates. In very complex mixtures, we could quantify 10,780 proteins and 12,192 proteins when including the 1412 proteins that were identified based on a single peptide sequence. Using this optimized DIA, we investigated large-protein networks before and after the critical period for whisker experience-induced synaptic strength in the murine somatosensory cortex 1-barrel field. This work shows that parallel mass spectrometry enables proteome profiling for discovery with high coverage, reproducibility, precision and scalability.

Mass spectrometry based proteomics (1) is a powerful technology to profile proteomes (2–5), discover biomarkers (6, 7), investigate biological regulation through post-translational modifications (8–11), study protein degradation (12), protein-protein interaction (13–16) and protein-ligand interaction or target deconvolution (17–20). For all of these approaches proteome coverage, reproducibility and quantitative precision are key to gain a comprehensive and accurate picture of the biology. Technologically, proteome coverage has significantly improved in recent years (2, 21–27).

Most established proteomics workflows today rely on “bottom-up” proteomics where proteins are first proteolytically cleaved into peptides and the resulting peptide mixture is then analyzed by mass spectrometric acquisition (28). In data-dependent acquisition (DDA), the mass spectrometer alternates between performing a survey scan (MS1) spectrum and a sequence of data-dependent fragment ion scans (MS2). During acquisition, the mass spectrometer interrogates each MS1 spectrum for peptide precursor signals. These peptides are usually selected for fragmentation based on relative signal intensity, giving rise to the MS2 spectra. For data analysis, the MS2 scans are compared with theoretically derived spectra using a database search engine, resulting in peptide identifications (29). Inherently, the acquisition process is limited by the reproducibility (30, 31), sensitivity and speed by which the mass spectrometer can sequentially acquire MS2 spectra (32, 33).

To overcome those limitations alternative acquisition methods were developed. These methods typically slice the peptide ion space into segments for MS2 measurement to counterbalance the complexity of biological samples. The mass spectrometer quickly cycles through those segments such that peaks are resolved along chromatographic retention time. Slicing the ion space can be achieved for instance using a quadrupole (34), an ion trap (35) or ion mobility (36). Today many of these methods exist (37–44) and are often termed data-independent acquisition (DIA)^1^. DIA data were originally analyzed like DDA data using database search engines (35, 38, 39), optionally using preprocessing (45–48). More recently, a peptide-centric analysis (49) was introduced using spectral libraries (SWATH) (40), where high performance DDA is a prerequisite for generation of comprehensive spectral libraries. Existing tools for multiple reaction monitoring (MRM) data processing were applied to this type of DIA analysis (50–52).

DIA has been shown to provide improved reproducibility (6, 42, 53, 54), quantitative precision (6, 42, 55) as well as proteome coverage (6, 42, 54) when compared with label-free DDA. Consequently, DIA is increasingly used for label-free quantitative proteomics (3, 5, 6, 56–58).

Here, we were interested in the achievable single shot performance of DIA with state of the art liquid chromatography, mass spectrometry (33), high precision indexed retention time (iRT) (54) and data processing.

We improved the DIA workflow on multiple levels and used Spectronaut for the targeted analysis (42). Significant improvements were achieved by MS1 resolution and dynamic range increase, using high resolution chromatography, increased sample loading, high precision iRT, spectral library generation and improved targeted analysis (see Suppl. Information and Suppl. Table I). His is an improved version of a manuscript submitted before but this time including protein FDR and a refined decoy model. After these improvements, DIA identified and quantified more peptides than MS2 spectra can be acquired on a Q Exactive HF in fast DDA mode. In a HEK-293 sample, we could quantify 7100 proteins (6739 with two or more peptide sequences) and in mouse brain tissue 8121 proteins (7739 with two or more peptide sequences) with single shot DIA. Further, we compared the performance of an internally generated, project specific spectral library to a resource spectral library, the pan human library (59) and to spectral libraries generated from publicly available data. We found that resource spectral libraries provide 90–103% of the performance in protein identification when compared with the project specific spectral library, while sparing the MS time for library generation. Throughout, the DIA measurements showed excellent reproducibility (missing values for technical replicates of 0.3–2.1%) and quantitative precision (median coefficient of variation (CV) of 4.7 to 6.2%) on protein level. Finally, we applied our DIA workflow to four different neuronal developmental stages in mouse somatosensory cortex 1-barrel field (S1BF) with three replicates to profile 5,930 proteins and gain biological insight into a complex system using one and a half days of LC-MS measurement time with DIA.

## EXPERIMENTAL PROCEDURES

## Materials

Frozen HeLa cell pellets were purchased from Dundee cell products. HEK-293 cell pellets were kindly provided by Dr. Thomas Uhlmann (Dualsystems AG, Schlieren, Germany). *E. coli* and *S. cerevisiae* digests were kindly provided by Dr. Audrey van Drogen. *C. elegans* Bristol strain worm digests were kindly provided by Kapil Dev Singh. Ethanol was purchased from AppliedChem, Darmstadt, Germany. Benzonase, iodoacetamide, tris(2-carboxyethyl)phosphine, trifluoroacetic acid, formic acid, ammonium formate, ACN, HPLC water, ammonium bicarbonate, SDS, dithiothreitol, glycerol, tris-(hydroxymethyl)-aminomethane and urea were purchased from SIGMA-Aldrich, Munich, Germany. Trypsin sequencing grade was purchased from Promega, Madison, WI. RapiGest was purchased from Waters, Milford, MA.

### 

#### 

##### Sample Preparation: Tissue Culture

A 15 cm dish of confluent HEK-293 cells was washed three times with PBS and then lysed by resuspension in 599 μl 8 m urea and 0.1 m ammonium bicarbonate. The HeLa pellet was resuspended in 10 ml 8 m urea and 0.1 m ammonium bicarbonate and Benzonase. The HeLa and HEK-293 lysates were reduced with 5 mm TCEP for 1 h at 37 °C. Subsequently, the lysates were alkylated with 25 mm iodoacetamide for 20 min at 21 °C. The lysates were diluted to 2 m urea and digested with trypsin at a ratio 1:100 (enzyme to protein) at 37 °C for 15 h. The samples were spun at 20,000 × *g* at 4 °C for 10 min. The peptides were desalted using C18 MacroSpin columns (The Nest Group, Southborough, MA) according to manufacturer's instructions. After drying, the peptides were resuspended in 1% ACN and 0.1% formic acid. The iRT kit (Biognosys AG, Schlieren-Zürich, Switzerland) was added to all of the samples according to manufacturer's instructions (required for the DIA analysis using Biognosys' Spectronaut). The peptide concentration was determined using a Spectrostar Nano spectrometer (BGM Labtech, Offenburg, Germany).

##### Sample Preparation: C. elegans

The *C. elegans* worms were washed with M9 buffer. Then the worm pellet was resuspended in 8 m urea and 0.1 m ammonium bicarbonate. Next, the samples were lysed in a bead mill (Eppendorf, Hamburg, Germany) at 30/s for three times 1 min. Then, the samples were spun on a table top centrifuge at maximum speed. Finally, filter aided sample preparation was performed with the cleared supernatant (60). The peptides were desalted as described above.

##### Sample Preparation: Mixed Proteome Samples

For the mixed proteome samples, the proteomes were combined the following way by peptide mass: *H. sapiens* 40%, *C. elegans* 42%, *S. cerevisiae* 12%, and *E. coli* 6%(See supplemental Table S2). For the low fold changes experiment using mixed proteomes, it contained *H. sapiens* constant, *C. elegans* at 10%, *S. cerevisiae* at 20%, and *E. coli* at 30% differential abundance (See supplemental Table S2). For the high fold changes experiment using mixed proteomes, it contained *H. sapiens* constant, *C. elegans* at 60%, *S. cerevisiae* at 100%, and *E. coli* at 300% differential abundance (See supplemental Table S2).

##### Mouse Tissue Preparation

All mouse experiments are approved by the IACUC of the Max Planck Institute of Experimental Medicine. Accordingly, the mice were sacrificed (decapitation for mice at P9, CO_2_ inhalation followed by decapitation for the rest of the ages) and the brain was quickly dissected. The cerebellum was dissected from three 12–14-week-old wild-type C57Bl/6J mice and immediately frozen in liquid nitrogen (three mice pooled to yield enough material). The S1BF was isolated following established procedures, five individual samples pooled for each age stage, to yield enough (61). For that, the coronal cut at the branching point of the middle cerebral artery and a second after ∼2 mm from C57Bl/6J mice of different postnatal ages (P9, P15, P30, and P54). If the section showed the beginning of the hippocampus a 1–2 mm-wide piece of the S1BF was isolated according to the topological information of the mouse brain atlas (http://www.mbl.org/atlas170/atlas170_frame.html) and immediately frozen in liquid nitrogen. The frozen tissue was homogenized with help of a glass/Teflon homogenizer in 4% SDS lysis buffer (4% SDS in 100 mm Tris, 10 mm DTT, 5% glycerol, complete protease inhibitor mixture (Roche, Basel, Switzerland)), pH 7.5 and by shearing with a 25G needle. The homogenate was incubated for 10 min at 70 °C, followed by centrifugation at 10,000 × *g* for 5 min for removal of cell debris (note: all centrifugation steps in this study were performed at room temperature, except otherwise mentioned). The supernatant equals the whole cell lysate. Following, acetone precipitation of the proteins was performed by addition of 5× volume precooled acetone and incubation for 2 h at −20 °C. The precipitated proteins were centrifuged at 14,000 × *g* for 30 min, washed with ice-cold 80% ethanol and centrifuged again at 14,000 × *g* for 30 min. The air-dried proteins were resuspended under constant agitation in 2% SDS lysis buffer. Finally, filter aided sample preparation was performed with the cleared supernatant in three sample preparation replicates. The peptides were desalted as described above.

##### High pH Reversed Phase and Strong Anion Exchange Fractionation

The HeLa and HEK-293 digests were further fractionated using high pH reversed phase chromatography. 50 μg of the digest was adjusted to pH 10 using 0.2 m ammonium formate. Next, the sample was applied to a MicroSpin C18 column. The peptides were eluted at 5, 10, 15, 20, 25, and 50% ACN in 0.05 m ammonium formate. Then the samples were dried and resuspended in 1% ACN in 0.1% formic acid. Additionally, the murine cerebellum sample was fractionated using high pH reversed phase separation on a Dionex UHPLC (Thermo Scientific, Waltham, MA) with a 2.1 × 150 mm Acquity CSH C18 1.7 μm column at 60 °C with 0.3 μl/min flow and a 30 min ACN gradient in 20 mm ammonium formate and 1 min fractions were pooled into 15 fractions using fraction pooling.

The HeLa digest was further fractionated using anion exchange chromatography (62). The eluate was captured on MicroSpin C18 columns. Afterward, the samples were dried and resuspended in 1% ACN in 0.1% formic acid.

##### Mass Spectrometric Acquisition

For the 50 cm column setup, 2 μg of each sample was analyzed using a self-packed analytical PicoFrit column (New Objective, Woburn, MA) (75 μm x 50 cm length) packed with ReproSil-Pur 120A C18-AQ 1.9 μm (Dr. Maisch GmbH, Ammerbuch, Germany) at 50 °C on an EASY-nLC 1200 connected to a Q Exactive HF mass spectrometer (Thermo Scientific). The peptides were separated by a 2 h segmented gradient (supplemental Table S3) or as specified. The flow rate was 250 nl/min for 50 cm and 200 nl/min for 100 cm columns. For DDA MS runs, the method from Scheltema *et al.* was used as described in (33), with the following alteration, the quadrupole isolation width was set to 1.6 Thomson. The full scan was performed between 350 and 1650 *m*/*z*. Stepped collision energy was 10% at 27%. For the 4 h gradient acquisitions 30,000 were used for the MS2. The DIA-MS method consisted of a MS1 scan from 350 to 1650 *m*/*z* or two segments (DIA-method-summary.xlsx) (AGC target of 3 × 10^6^ or 60 ms injection time). Then, DIA segments were acquired at variable resolutions (AGC target 3 × 10^6^ and auto for injection time). Stepped collision energy was 10% at 25%. The spectra were recorded in profile mode. The default charge state for the MS2 was set to 3. The S1BF series was acquired using block randomization to avoid bias.

##### Mass Spectrometric Data Analysis

DIA data were analyzed with Spectronaut 11, a mass spectrometer vendor independent software from Biognosys, Schlieren, Switzerland. The default settings were used for targeted analysis of DIA data in Spectronaut except the decoy generation was set to “mutated” (see supplemental Fig. S1 and supplemental Information). In brief, retention time prediction type was set to dynamic iRT (adapted variable iRT extraction width for varying iRT precision during the gradient) and correction factor for window 1. Mass calibration was set to local mass calibration. Interference correction on MS1 and MS2 level was enabled, removing fragments/isotopes from quantification based on presence of interfering signals but keeping at least three for quantification. The false discovery rate (FDR) was estimated with the mProphet approach (50) and set to 1% at peptide precursor level and at 1% at protein level using an adapted version of Rosenberger and colleages (63) (see supplemental Information). For the analysis of the S1BF DIA runs with the phospho-peptide spectral library, the RAW files were converted into the Spectronaut file format HTRMS, then the HTRMS files were calibrated in the retention time dimension using the global S1BF spectral library. Subsequently, the recalibrated files were then used for the targeted data analysis with the S1BF phosphor-peptide spectral library without new recalibration of the retention time dimension. No special scoring for phosphorylation site localization was implemented in Spectronaut and hence phosphorylation site localization can be ambiguous in the analysis.

The DDA spectra were analyzed with the MaxQuant (Version 1.5.1.2 and 1.6.0.1) analysis software using default settings (Trypsin/P, two missed cleavages). Search criteria included carbamidomethylation of cysteine as a fixed modification, oxidation of methionine and acetyl (protein N terminus) as variable modifications. The initial mass tolerance for the precursor was 4.5 ppm and for the fragment ions was 20 ppm. The DDA files were searched against the human UniProt fasta database (state 11.12.2014, 42,004 entries) or the mouse isoform UniProt fasta database (state 11.12.2014, 24,712 entries), and the Biognosys iRT peptides fasta database (uploaded to the public repository).

##### Calculations, Statistics, Term Usage Definition, and Pathway Analysis

When we use the term peptides in this study, we refer to peptide precursors. When we use stripped sequence, we refer to the amino acid sequence of a peptide. When we use proteins, we refer to protein groups as determined by the ID picker algorithm (64) as implemented in Spectronaut. Proteins were counted as single hit identification, if they were identified by precursors derived from a single peptide sequence. Peptide quantities were calculated in two modes: First, using the summed intensities of the respective fragment ions in the spectral library that were not excluded by the interference correction for MS2. Second, using the summed isotope intensities for MS1. The protein CVs were calculated based on the summed intensities of their respective peptides. The theoretical number of MS2 spectra on the Q Exactive HF mass spectrometer was calculated based on the cycle time of a 2 h TOP15 DDA acquisition of Hela (60,000 MS1 and 15,000 MS2 with fragmentation of up to 15 of the most intense peptides per cycle (TOP15)). Only the complete cycles with one MS1 scan and 15 consecutive MS2 scans were used to calculate a median cycle time of 0.97s (Fig. 2;120min-Top15_DDA.raw). Peak capacity was calculated based on Gilar *et al.* (65). Data points per peak are counted at 6σ peak width (width at 1.2% peak height). In Spectronaut, state comparison analysis on protein level was performed using a *t* test (one sample, null hypothesis, no change, mean μ = 0). The *t* test was performed based on the log2 ratios of the peptide intensities of the individual peptides of a protein. The resulting *p* values were corrected for multiple testing using the q-value approach to control the overall FDR (66). For the comparison of the S1BF data of this study (P30/p9 comparison) and of Butko *et al.* (P30 control *versus* postnatal bilateral whisker trimming) (67), data were analyzed through the use of Qiagen's Ingenuity® Pathway Analysis (IPA®, Qiagen Redwood City, Hilden, Germany).

The peptides identified per scan per run were calculated the following way: total identifications multiplied by eight scans (it is identified in) divided by the number of total MS2 scans.

##### Spectral Library Generation

To generate the spectral libraries, the acquired DDA data was searched with MaxQuant and a spectral library was generated using the spectral library generation functionality of Spectronaut with default settings. In brief, segmented regression to determine iRT in each run was used as described (54). iRTs were calculated derived from median iRTs across all DDA runs. Fragment ions <300m/z and >1800 *m*/*z* as well as fragment ions with less than three amino acid residues were not considered. Fragment ions with neutral losses were included. A suitable peptide was added to the spectral library, if minimally six fragment ions could be detected in the MS2 spectrum. At maximum, the six most intensive fragment ions were kept (for the phospho-peptide spectral library 25 fragments were included for Spectronaut to better discriminate among versions of the same peptide differing in the localization of the phosphate). The pan human spectral library was filtered before the targeted data analysis the following way: fragment ions were selected from 300–1800 *m*/*z*, minimal relative intensity was set to >5% and fragment ion number >3 on. Then, the fragment ions were ranked by relative intensity and precursors with at least 6 fragments were retained and the maximal fragment ion number per precursor was set to 6. Supplemental Table S3 contains an overview of the generated spectral libraries and the figures they were used in.

##### Clustering of Regulated Proteins in the S1BF

The raw intensities for the time profiles of regulated proteins of S1BF development were calculated by summation of the peptide intensities per condition. Subsequently, the protein intensities were log2 transformed and then normalized according to the z statistic so that, for each profile, the mean was zero and standard deviation was one. The normalization of data ensures that proteins with similar temporal patterns are close in Euclidean space. The transformed profiles were then clustered using the Mfuzz toolbox (68), which is based on the open-source statistical language R. We used the fuzzy c-means (FCM) clustering algorithm, which is part of the toolbox. FCM assigns to each profile a membership value in the range [0, 1] for each of the c cluster. The final clustering was performed with the parameters c = 6 and m = 2.5. The GO term enrichment analysis was performed using DAVID bioinformatics resources and top significantly enriched terms were selected (69).

## RESULTS

### 

#### 

##### DIA Method Optimization

First, to optimize single shot DIA identification and quantification, we explored a range of variables for optimal DIA methods implemented on the most recent generation of Orbitrap mass spectrometers containing an ultra-high field Orbitrap mass analyzer. We present a simple procedure for DIA method development using the Spectronaut software that can be applied to any liquid chromatography setup and instrument (Q-TOFs and Orbitrap based instruments). In this procedure, the method cycle time (data points per peak), MS1 resolution, MS2 resolution and the number of precursor selection DIA segments for the MS2 scans (MS2 segments) were optimized. The precursor range (350–1650 Th), gradient length (2 h), liquid chromatography peak capacity (average 674 calculated by (65)) and number of MS1 segments (one segment) were kept constant. The DIA methods were benchmarked based on the number of peptide identifications (at 1% peptide and protein FDR) with CV of MS2 quantification below 10 and 20% over triplicate acquisition of the HeLa cell line. The spectral library was generated as described in the methods.

**Fig. 1.**
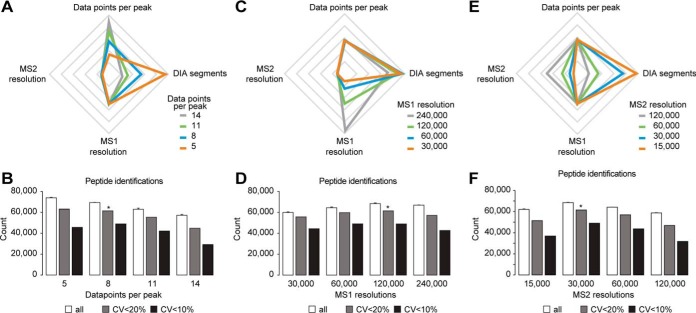
**Systematic optimization of the DIA method on a Q Exactive HF instrument using a HeLa lysate and 2 h gradients.** Triplicate analyses of HeLa digest for DIA method development were performed. *A*, Illustration of the relationship of data points per peak and number of DIA-MS2 segments at constant MS1 and MS2 resolutions. Relative axes were used. *B*, The average identifications of triplicate DIA and the number of peptides with CVs below defined thresholds were calculated. *C*, Illustration of the relationship of the number of DIA-MS2 segments at different MS1 resolutions maintaining a constant cycle time. Relative axes were used. *D*, The average identifications of triplicate DIA and the number of peptides with CVs below defined thresholds were calculated. *E*, Illustration of the relationship of the number DIA-MS2 segments at different MS2 resolutions maintaining a constant cycle time. Relative axes were used. *F*, The average identifications of triplicate DIA and the number of peptides with CVs below defined thresholds were calculated. The error bars display the standard deviations. The asterisk indicates the highest number of peptides with CVs below 20%.

The sampling of chromatographic peaks is pivotal to accurate quantification. We first optimized the cycle time of the method that in turn defines the number of data points per peak. As a basis, we took our previously described DIA method (42). To estimate the number of MS2 segments required for a certain number of data points per peak, we first performed a measurement with a fast DIA scouting method (five DIA segments) resulting in 19 data points per peak (cycle time 600 ms). Based on this, methods for 5, 8, 11, and 14 data points per peak were generated by scaling the MS2 segment number accordingly (Fig. 1*A*). The analysis revealed optimal quantification with high identification rate and reproducibility at eight data points per peak corresponding to the method with 22 MS2 segments and a cycle time of 2.3 s (Fig. 1*B*, supplemental Fig. S2 and S3). Using this method 68,684 peptides were identified and 61,464 peptides with CVs below 20% were observed. Interestingly, the method with the lowest peak sampling performed best as judged only by identifications, but when comparing identifications below 20% CV the method providing eight data points per peak performed best. This set of methods with varying data points per peak represents a tradeoff among high peak sampling but few MS2 segments and low peak sampling with many MS2 segments (for a constant m/z range) (Fig. 1*A*). High peak sampling will provide better quantification because the true peak area can be better approximated. More MS2 segments will provide better quantification because the MS2 spectra resulting from a smaller MS2 segment size will be less complex. Next, we varied the MS1 and MS2 resolutions in the DIA method, whereas the sampling of the chromatographic peaks was kept constant at eight (Fig. 1*C*). To balance the varying time needed for a MS1 scan while changing the resolution, the number of MS2 segments was varied (18, 22, 24, and 25 MS2 segments). The MS1 scan resolution did not have a strong impact on identification, reproducibility and quantitative accuracy (Fig. 1*D*). A resolution of 120,000 was found to perform best, both in terms of peptide identification and peptides with CVs below 20%. It is noteworthy that in Spectronaut, MS1 scores influence the overall performance of the analysis. When looking at MS1 instead of MS2 quantification, we found that using a resolution of 120,000 over 30,000 improved the number of peptides with CVs below 20% by 12% (supplemental Fig. S4).

Subsequently, the MS2 resolution of the DIA method was optimized. Again, the number of MS2 segments was varied to counterbalance the scan time and keep a constant cycle time (6, 11, 22, and 35 MS2 segments, Fig. 1*E*). The method with 30,000 resolution resulted in optimal performance of peptide identification, reproducibility and quantitative precision (Fig. 1*F*). The number of MS2 segments in all the DIA methods tested differed by over 5-fold. Hence, the relative ion current among the different methods also varied by 5-fold. To reach the optimal intra scan dynamic range on a trapping mass spectrometer, it is beneficial that the desired number of ions is trapped before the maximal fill time is reached. In supplemental Fig. S5, the relation between DIA method and the percentage of scans reaching maximal fill time is shown. Large DIA segments result in compressed dynamic range and higher complexity on fragment level but a high resolution, small DIA segments will be undesirable because of lower ion numbers at reduced resolution. A good balance showed to be in the range of 60% of the DIA segments reaching maximal fill time with 22 DIA segments of 30,000 resolution.

##### DIA Performance Compared with Serial MS2 Scan Acquisition Speed of DDA on the Q Exactive HF

It was found that with DDA maximally 20% of the roughly 220,000 detectable peptide features can be identified in a single run even with the newest generation of instruments available (32, 33). This limit is mostly the result of the sequential MS2 acquisition nature of all DDA methods. The theoretical maximum number of MS2 scans can be calculated based on the settings of the DDA method (number of MS2 scans and cycle time) and the acquisition duration (see Methods). In real DDA experiments, fewer MS2 scans are acquired than the theoretical maximum owing to dynamic exclusion of already acquired peaks and the absence of precursor peaks satisfying the criteria of the DDA method (charge state, intensity threshold, etc.). Furthermore, during data analysis, not every MS2 fragmentation spectrum is identified. The best identification rates achieved lie within the range of 70% for short (30 min) and 50% for long gradients (2–4 h) (33).

**Fig. 2.**
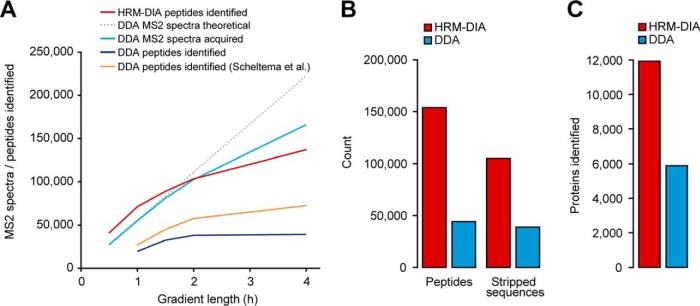
**Parallel acquisition and targeted identification of DIA surpasses serial MS2 acquisition of DDA on the Q Exactive HF.** *A*, The identifications and MS2 spectrum acquisitions were compared at different gradient lengths for a HeLa lysate. The DIA method was optimized for different gradient lengths (red). The theoretical maximal number of MS2 spectra was calculated for a Top15 method (gray, see Methods section). For DDA, empirically derived MS2 spectra (light blue) and number of identifications (dark blue). Additionally, the DDA identifications are shown from Scheltema *et al.* (orange) *B*, A mixed-organism sample was recorded in DIA mode and DDA using a 4h gradient. The peptide and stripped sequence identifications of the DIA single shots were calculated. *C*, The protein identifications were calculated for the mixed-organism acquisitions.

In contrast to DDA, DIA does not have this serial limitation. The parallel fragmentation in DIA of “all” precursors (restricted by the trapping capacity) potentially enables identification and quantification of all trapped and fragmented precursors. To compare peptide identification rates, DIA was compared with an optimized DDA method that is using the fastest MS2 scan speed of the Q Exactive HF and was developed by Scheltema and colleagues (Top15, 60,000 MS1 and 15,000 MS2 resolution) (33). This DDA method displayed the best reported performance in mammalian samples and was used in studies with deepest proteome coverage (2, 23). DDA data from HeLa acquired shuffled with the DIA and data of Scheltema and colleagues were used as external DDA reference data. The chromatographic setup used in this study was analog to the one used by Scheltema *et al.* (75 μm × 50 cm Reprosil Pur chromatography coupled to a Q Exactive HF instrument). DIA methods for gradient lengths other than 2 h were optimized in a similar manner as described above. A comparison of DIA peptide identifications to DDA MS2 spectrum numbers and identifications was performed (Fig. 2*A*). Most strikingly, DIA identifications surpass the theoretical maximal possible number of MS2 spectra that can be acquired on the Q Exactive HF instrument for up to 1.5 h acquisitions when using the optimal DDA method from Scheltema and colleagues. Specifically, the precursor identifications were 40,918 (0.5 h gradient), 71,377 (1 h), 89,068 (1.5 h), 103,639 (2 h) and 137,037 (4 h). On the Q Exactive HF, DIA results exceeded DDA in terms of peptide identifications by a factor of 2.8 at 1 h to 2.0 at 4 h.

To further demonstrate the potential of DIA, an artificially complex sample was generated by combining whole cell lysate peptides from *H. sapiens* (HeLa, liver tissue), *C. elegans*, *S. cerevisiae* and *E. coli* (spectral libraries were generated as described in methods). The targeted analysis of the DIA data resulted in detection of over three times more peptides and over two times more proteins than DDA on the Q Exactive HF (151,599 peptides of 11,931 proteins in DIA (10,347 identified with >2 peptide sequences); 45,812 peptides of 5964 proteins in DDA (4953 identified with >2 peptide sequences) (Fig. 2*B* and 2*C* and supplemental Fig. S6*A* and S6*B*).

**Fig. 3.**
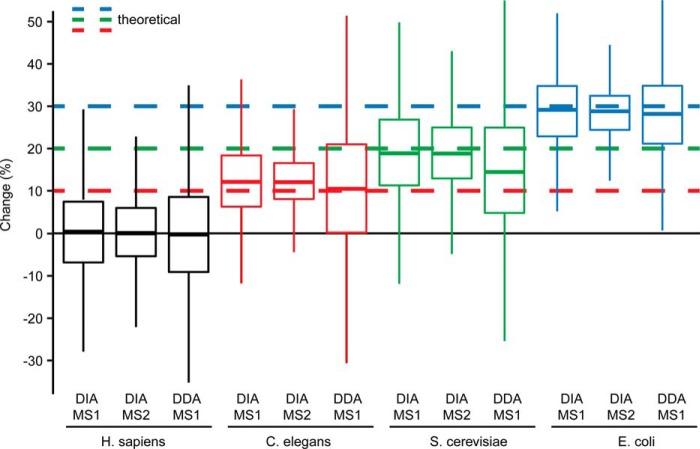
**Controlled quantitative experiment.** Box plot visualization of percent change (based on MS1 and MS2 quantification for DIA and MS1 for DDA) of all identified proteins of the low fold change two sample mixed proteome experiment (comparison S2/S1 for *H. sapiens*, *C. elegans* and *S. cerevisiae* and S1/S2 for *E. coli*). Only overlapping identifications are shown. The theoretical fold changes are indicated for the organisms.

Two controlled, quantitative experiments with triplicate analysis of two mixed proteome samples (as above) were performed in block randomization using DIA and DDA. The quantitative data was analyzed for DDA and DIA (Fig. 3 and supplemental Fig. S6*C* to S6*F*). For DIA, it revealed the ability to significantly identify differential abundance as low as 10%. For the *C. elegans* proteins, with a pipetted fold change of 10%, 1176 out of 3556 proteins statistically tested had a q-value below 5%. Because the ground truth (differentially abundant or not) was known in this specific experiment we could also check the quality of the candidate list in terms of false positives, *i.e.* proteins that are deemed statistically significant (below the cutoff of 5% q-value) but are not changed. In the candidate list filtered with a q-value of 5% we could find 3.6% human proteins that are expected not to be differentially abundant between the two conditions.

##### Optimized Single Shot DIA on a 1 m Column Setup

Label-free proteomics has the advantage of fast sample preparation and scalability to large cohorts of samples. Sample prefractionation in combination with label-free proteomics is problematic because the number of mass spectrometric acquisitions increases by the factor of fractions per sample. This leads to a decreasing quantitative precision because of irreproducibility (70).

**Fig. 4.**
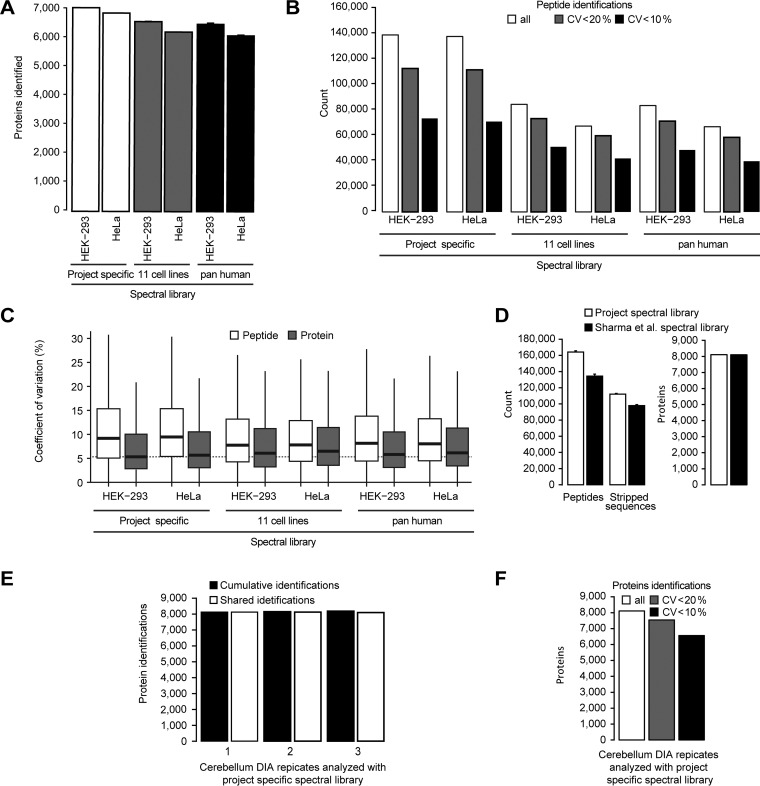
**4 h single shot maximized DIA on 1 m column setup.** *A*, Triplicate DIA of HeLa and HEK-293 lysates were recorded using a 1 m x 75 μm column with Reprosil Pur chromatography with 4 μg loading using a 4 h gradient. The targeted analysis of the DIA was performed using three spectral libraries: a project specific, a library generated from the MS data of the published repository (11 common cell lines, see methods) and the pan human spectral library of Rosenberger *et al.* The average protein identifications were calculated. *B*, The average peptide identifications of a DIA triplicate analysis of HeLa and the number of peptides with CVs below defined thresholds were calculated. *C*, The CVs on peptide and protein level were calculated for the three analyses. Lowest median indicated by the dotted line (HEK-293 project specific). *D*, Triplicate DIAs was performed using a mouse cerebellum lysate and targeted analysis was performed using a project specific and a spectral library generated from the MS data of a published repository (see Methods section). Average identifications were calculated. *E*, The cumulative and shared identifications were calculated for the project specific library analysis. *F*, The average identifications of triplicate DIA and the number of proteins with CVs below defined thresholds were calculated. The error bars display the standard deviations.

For this reason and motivated by the results above, we wanted to explore the current technical limits of single shot DIA. For this purpose, the chromatography was improved for 4 h acquisitions. The column length was increased to 1m and the DIA method was optimized. Again, a spectral library was generated as described before (see Methods). Triplicate DIA of HeLa and HEK-293 was performed. This improved LC-MS setup resulted in the identification and quantification of 152,138 peptides of 7100 proteins in HEK-293 (6383 identified with >2 peptide sequences) and 151,541 peptides of 6978 proteins reproducibly identified in the HeLa triplicate (6141 identified with >2 peptide sequences, improvement over DIA 50 cm setup 11% on peptide and 3% on protein level) (Fig. 4*A*, supplemental Fig. S7*A*). Peptide identifications with CVs below 20% were 112,015 for HEK-293 (median CV was 9.4%) and 111,031 for HeLa (median CV was 9.7%) (Fig. 4*B*, 4*C*).

This maximized LC-MS setup was applied to a mouse cerebellum tissue sample. The targeted analysis of the technical triplicate DIA resulted in the identification of on average 164,101 peptides of 8121 proteins (7739 identified with >2 peptide sequences) (Fig. 4*D*). Remarkably, the number of missing values for proteins was as small as 0.3% (Fig. 4*E*). The high reproducibility of quantification is shown by the fact, that 7525 proteins have a CV of <20% (93% of all) (Fig. 4*F*). Importantly, using the protein inference data from the spectral library, we counted 9818 proteins identified on average (1697 more).

##### Usage of Resource Data for Targeted Analysis of DIA Data

Besides the DIA methods, we also wanted to explore the limits of the project specific spectral libraries that were used for the targeted analysis of DIA data. Different gradients, instruments, sample processing and quality of LC-MS-DIA data have so far hindered the usability of proteome wide spectral libraries derived from DDA resources (6, 42, 71). However, recent approaches improving retention time prediction demonstrate the possibility to use DDA resource proteome wide spectral libraries for the targeted analysis of DIA data (54, 71). To test the limits of these approaches, we generated a spectral library from the data of the “11 common cell lines” publication, that includes HeLa and HEK-293(21) using MaxQuant (72) and Spectronaut (54). In that work, 4 h DDA acquisitions were performed on the identical chromatographic resin as was used for the DIA in this study. The obtained spectral library covered 10,354 proteins and contained 223,700 peptides. Targeted analysis of the 1 m 4 h DIA resulted in the identification of 94,842 peptides for HEK-293 corresponding to 6637 proteins (5978 proteins identified with >2 peptide sequences). For HeLa, 76,385 peptides were identified of 6347 proteins (5458 proteins identified with >2 peptide sequences) (Fig. 4*A*, supplemental Fig. S7*A*). Peptide identifications with CVs below 20% were 72,865 and 59,437, respectively (Fig. 4*B*). Shared peptides with the project specific spectral library showed equal quantitative precision (Suppl. Fig. 7*B*). To further evaluate the robustness with respect to the source of spectral libraries, we used the pan human spectral library published by Rosenberger *et al.* (59). This spectral library was generated on a different instrument class, a time of flight mass spectrometer, and shorter 2 h gradients. Targeted analysis of the HEK-293 and HeLa data using the pan human spectral library resulted in a remarkable 6577 (5895 proteins identified with >2 peptide sequences) and 6256 protein identifications (5372 proteins identified with >2 peptide sequences), respectively (Fig. 4*A*). Peptide identifications with CVs below 20% were 70,861 and 58,267, respectively (Fig. 4*B*). Again, the quantitative precision was like the other experiments (supplemental Fig. S7*B*). Importantly, the CVs of proteins were in all cases lower than the CVs for peptides. The lowest CVs for proteins were recorded for the project specific spectral library analysis (Fig. 4*D*).

The performance of a proteome scale resource spectral library was further evaluated on the mouse cerebellum tissue sample. 269 published mouse sample DDA runs (brain tissues) from Sharma *et al.* (2) were used to generate a spectral library comprising 12,107 mouse proteins. Using the DIA of a murine cerebellum sample from before, targeted data analysis was performed using the resource derived spectral library (Fig. 4*E*). In a single 4 h acquisition on average 8110 proteins (7530 identified with >2 peptide sequences) and cumulatively in triplicate DIA 8156 proteins were identified. The median CV was 4.7% for 8,156 proteins (Fig. 4*F*). For peptides shared between the project specific and the Sharma resource spectral library, the retention times obtained by DIA correlated by 0.9996 for 105,070 shared precursors (Pearson correlation, supplemental Fig. S7*C*).

##### Somatosensory Cortex 1 Barrel Field Profiling

As a demonstration of the application of parallel MS analysis, we studied changes to mouse brain tissue during sensory development. The behavioral adaptation of mammals to environmental alterations underlines neuronal changes in different brain regions at structural, molecular and functional levels. A prime example in the sensory system is the primary somatosensory cortex of mice and the whisker-to-barrel system, which is an established model for characterizing plasticity in cortical development and influence of sensory inputs in this process. However, the longitudinal and system-wide view of the changes in protein networks during neuronal development is uncharacterized.

To investigate changes in the abundance of large-protein networks before and after the critical period for whisker experience-induced synaptic strength (postnatal days 10 to 14, P10 - P14 (73)), samples of the mouse S1BF were dissected at P9, P15, P30, and P54.

**Fig. 5.**
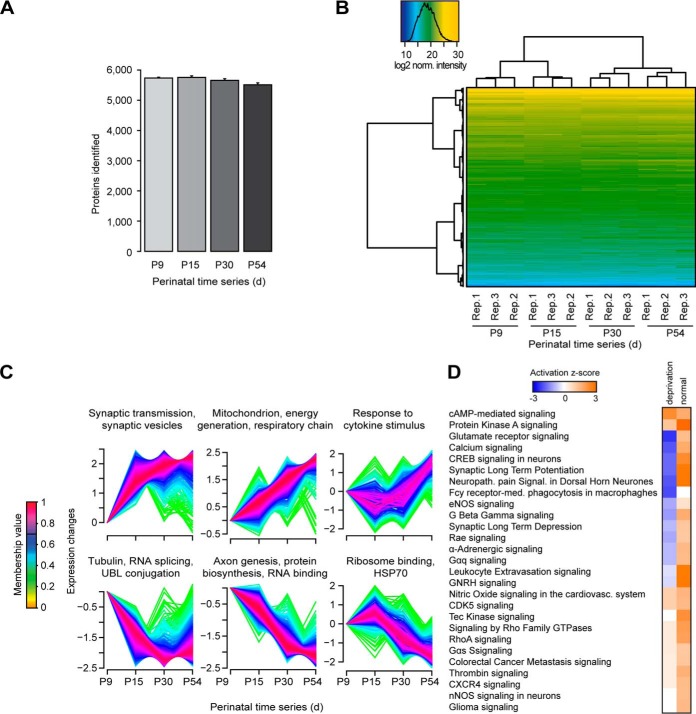
**S1BF sensory development proteome profiling.** *A*, Four important stages of sensory development of the mouse perinatal period were profiled in the S1BF using block randomized DIA and a project specific spectral library. The average protein identifications were plotted for the individual developmental stages with standard deviations. *B*, For exploratory data analysis, a heat map based on normalized intensities was generated using unsupervised clustering. *C*, Fuzzy c-means clustering analysis was performed and visualized with enriched GO terms. The black line in the membership bar shows the threshold above which proteins belong to the cluster. *D*, IPA Pathway activation comparison of the S1BF with normal sensory development and a study with sensory deprivation by postnatal bilateral whisker trimming (Butko *et al.)*. Blue color indicates pathway inactivation and orange depicts its activation.

For the DIA profiling, the samples were prepared in sample preparation replicates and acquired using 2 h single shot measurements in a block randomized manner. Targeted analysis of the DIA data resulted in the quantification of 5930 proteins (5522 identifies with >2 peptide sequences) with 95.5% data set completeness across all four stages (Fig. 5*A* and supplemental Fig. S8*A*, for the spectral library, see Methods section). The median CVs of proteins of the condition replicates were between 7.0 and 10.1% and the CVs of proteins lower than the CVs of peptides (supplemental Fig. S8*B*). Unsupervised clustering clearly separated the critical periods of whisker experience-induced synaptic strength (Fig. 5b). A principal component analysis showed, that the principal component 1 explained 53.4% of the variance and the time points align in the correct order (supplemental Fig. S8*C*). A pairwise statistical testing based on *t*-tests was performed (S1BF-comparison.xlsx). Fuzzy c-means clustering of the significantly differential abundant proteins was performed and resulted in six distinct clusters, three with up-regulation and three with down-regulation of protein expression (Fig. 5*C*).

As expected, synaptic transmission-related proteins displayed an acute increase in expression from P9 to P15 reaching a plateau afterward. A more linear increase of expression over the whole development time was detected for proteins of the mitochondrial respiratory chain and for proteins associated with cytokine stimulus. Conversely, a reduced expression was detected for proteins related to axon genesis, RNA splicing or UBL conjugation processes. These differences serve as molecular portraits of the requirements at the functional, energetic and structural levels during this developmental period (74, 75).

At the level of specific candidates, synaptic proteins significantly regulated during normal development represent attractive candidates. Interestingly, our data partially correlate with previous findings occurring during sensory deprivation in mouse barrel cortex (67) - proteins such as SynGAp1 or GluA1 (reported to play a key role in axonal outgrowth during development (76) and synaptic plasticity (77), respectively) are down-regulated during deprivation and up-regulated during normal development (supplemental Fig. S8*D*). PSD-95 and gephyrin showed increased expression in development, but no significant change during deprivation. A comparative pathway analysis confirmed key neuronal functions to be differentially regulated in these two distinct scenarios (*e.g.* glutamate receptor signaling, synaptic long-term potentiation) (Fig. 5*D*).

In addition, we characterized the longitudinal changes in the phosphorylation status for 141 proteins in this DIA profiling. Fuzzy c-means clustering analysis was performed and clusters detected (supplemental Fig. S8*E*). Our results indicate known neuronal proteins showing both a dual regulation (abundance and phosphorylation; *e.g.* Marcksl1) as well as only changed at the post-translational level (*e.g.* Map2, Tight junction protein ZO-1) (supplemental Fig. S8*F*).

To confirm that high quality profiling studies can be performed with resource spectral libraries, the S1BF DIA data were analyzed using the spectral library from mouse resource data (as described above). The approach profiled over 6132 proteins (5724 identified with >2 peptide sequences) with high reproducibility and quantitative precision (supplemental Fig. S9*A–*S9*D*). The protein identifications were 3% higher than with the project specific spectral library at about 14% lower peptide identifications. Fuzzy c-means clustering resulted in an analogue set of six distinct clusters as with the project specific spectral library (supplemental Fig. S9*E*). Comparison of the differential abundant proteins resulted in an overlap of 1172 proteins from 1666 candidates using the resource spectral library and 1,784 from the project spectral library analysis. Correlation analysis of the candidates resulted good correlation of R^2^ of >0.9 (supplemental Fig. S9*F*).

## DISCUSSION

We introduce a simple framework to optimize DIA methods and show what is achievable with single shot DIA discovery proteomics using current instrumentation. The demonstrated improvements are based on the implementation of improvements in scan resolution, chromatography, spectral library and data analysis. Our data show that roughly 50% of a cell line or tissue proteome can be quantified consistently across samples run at a frequency of approximately ten per day and instrument. The approach features simple sample preparation and an experiment is linearly scalable because no limited sample mixing or (gas phase) fractionation is applied (6). We expect this not only to be relevant to industrial and academic research, but also in clinical research.

Remarkably, we found that single shot DIA can quantify more peptides than MS2 spectra can be theoretically acquired on the Q Exactive HF using state of the art DDA methods. Instruments with faster scanning analyzers (like linear ion trap or time of flight) can acquire higher numbers of MS2 spectra that can lead to an increase or decrease in identifications depending on the spectral quality. Modern DDA software can identify several peptides per MS2 spectrum leading to an increase of identifications. Practically, DIA identified twice as many peptides as the best DDA data of the Q Exactive HF available (33) and in a sample of four mixed proteomes four time as many. This shows that DIA is particularly suited to very complex samples. In contrast to the MS1 alignment in MaxQuant that has no FDR control, the FDR in our DIA is controlled at 1% and no alignment was performed (50). In human cell lines, we could quantify more than 7100 proteins corresponding to 69% of all detectable peptide features in an MS1 map or 152,138 out of 220,000 (33). A calculation of the fraction of the TIC revealed, that over 50% of the ion current can be explained by the targeted analysis, it is clear that a greater fraction would be covered, if peptides < 7 amino acids, additional fragment ions and additional modification would be considered (supplemental Fig. S10). In tissue, more than 8000 proteins could be quantified with a single shot. This corresponds to roughly 53% of the estimated expressed proteome for human cell lines and tissues (2, 78). For technical replicates on the protein level, the number of missing values was 0.3% to 2.1% (supplemental Table S4) and median CVs were in the range of 5%. This confirms the high reproducibility and quantitative precision from our previous study (42). Despite the 4-fold higher resolution of the MS1 scan, the CVs were in median 51% higher than in MS2 quantification (5.9% *versus* 8.8% CV). A possible explanation for this observation could be that, interferences on MS1 are likely to affect multiple members of an isotopic envelope equally.

The DIA data reached a level where proteome scale resource spectral libraries applied to DIA performed with high coverage, reproducibility and quantitative precision. When compared with the project specific library, the coverage was almost identical on protein level, but lower on peptide level. Hence, the performance of proteome scale resource spectral libraries still lags behind extensive project specific spectral libraries. However, when compared with the best DDA data available on the Q Exactive HF, the coverage was 35% higher on peptide level (33). Further, resource spectral libraries have the advantage that no extra project specific spectral library must be generated. It is worth mentioning that the larger “search space” of the resource spectral libraries is controlled for by the protein FDR applied. Importantly, protein inference should be performed based on the identified peptides sequences to prevent inflated protein numbers.

To show the power of single shot DIA in a realistic experiment, we profiled a set of 12 samples representing four stages of mouse S1BF development to a depth of 6132 proteins within one and a half days of LC-MS time. In this profiling, we could directly observe phospho sites of 140 proteins without performing phospho enrichment. The profiling of phosphorylated peptides in the background of the unmodified peptides enabled direct observation of phosphorylation status during the development.

In computing and genomics, parallel processing is the convention nowadays. Similarly, LC-MS is heading into the same direction: in every MS2 on average 12–17 peptides are quantified with single shot DIA (1 - 4 h). Improvements in instrumentation might enable further parallelization of ion processing pushing the boundaries of what is possible today.

## DATA AVAILABILITY

The raw mass spectrometric data, the spectral libraries and the quantitative data tables have been deposited to the ProteomeXchange Consortium via the PRIDE (79) partner repository with the data set identifier PXD005573. The saved projects from Spectronaut can be reviewed with the Spectronaut Viewer (www.biognosys.com/spectronaut-viewer).

## Supplementary Material

Supplemental Data

## Footnotes

^1^ The abbreviations used are:

DIA: Data-independent acquisition
CV: Coefficient of variation
DDA: Data-dependent acquisition
FDR: false discovery rate
MS1: Peptide precursor survey scan
MS2: Fragment ion scan
S1BF: Somatosensory cortex 1 barrel field.
