# Supplementary material for: Optimization of Experimental Parameters in Data-Independent Mass Spectrometry Significantly Increases Depth and Reproducibility of Results
Source: Mol Cell Proteomics. 2017 Oct 25;16(12):2296–309. doi: 10.1074/mcp.RA117.000314 (PMC5724188; doi:10.1074/mcp.RA117.000314)
Supplement: Supplemental Data [file supp_16_12_2296__index.html]

Optimization of Experimental Parameters in Data-Independent Mass Spectrometry Significantly Increases Depth and Reproducibility of Results — Optimizing DIA — Optimization of Experimental Parameters in Data-Independent Mass Spectrometry Significantly Increases Depth and Reproducibility of Results — Optimizing DIA — Supplemental Data 

# Optimization of Experimental Parameters in Data-Independent Mass Spectrometry Significantly Increases Depth and Reproducibility of Results

## Supplemental Data

- Supplementry Information - Supplementary Information, Tables and Figures
- Supplementary File - S1BF comparisons
- Supplementary File - DIA method summary
- Suppl Fig5-phospho spectra XICs - XICs of phospho peptides from Figure 5
