# Supplementary material for: Optimization of Experimental Parameters in Data-Independent Mass Spectrometry Significantly Increases Depth and Reproducibility of Results
Source: Mol Cell Proteomics. 2017 Oct 25;16(12):2296–309. doi: 10.1074/mcp.RA117.000314 (PMC5724188; doi:10.1074/mcp.RA117.000314)

P20357 – _KSEVQAHS[+H+O3+P]PSR_.3

Fig4_MP-DIA-barrel-cortex-p9-1_MHRM.htrms

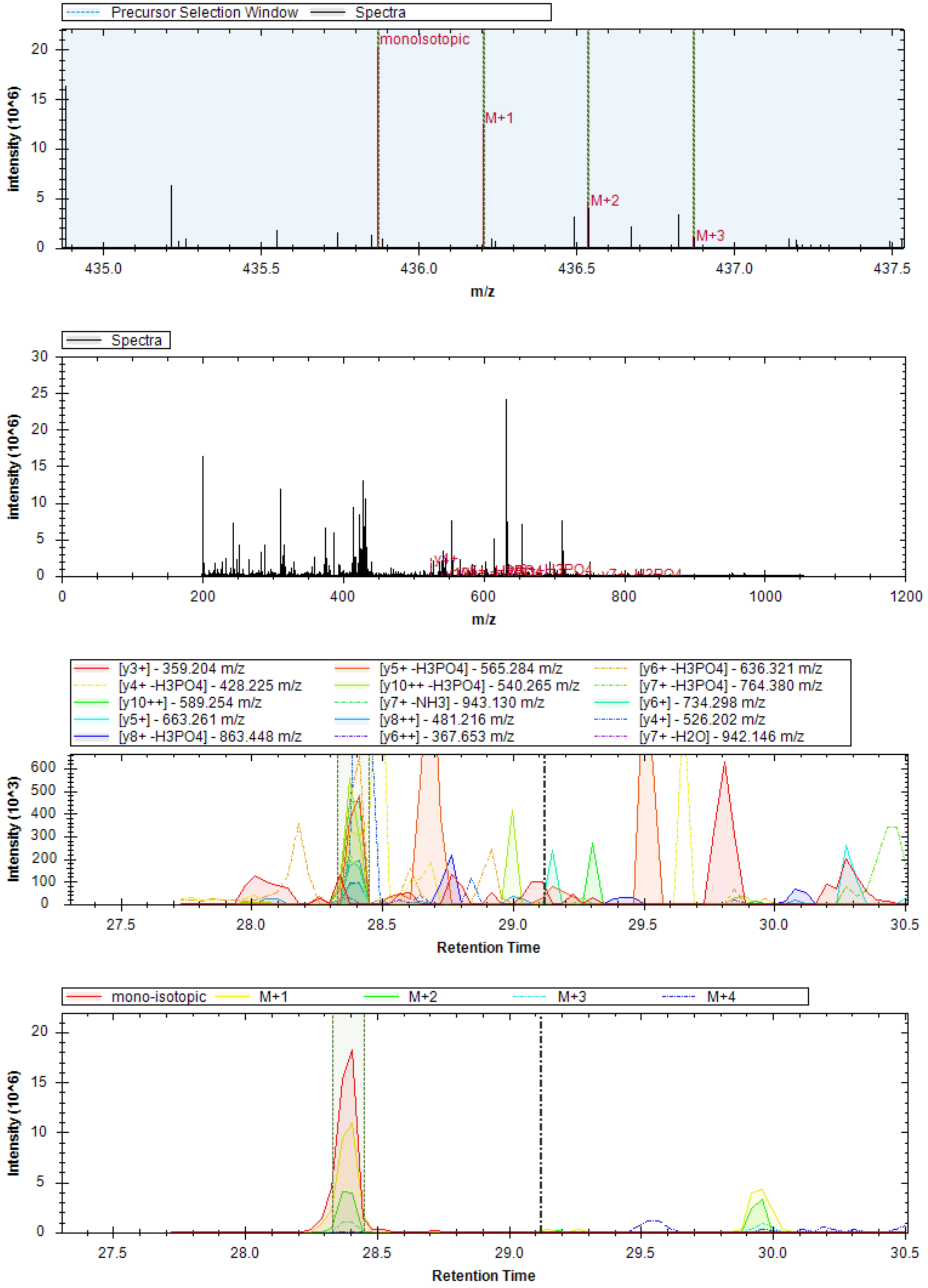

P28667 – _GEVAPKET[+H+O3+P]PKK_.3
Fig4_MP-DIA-barrel-cortex-p9-1_MHRM.htrms

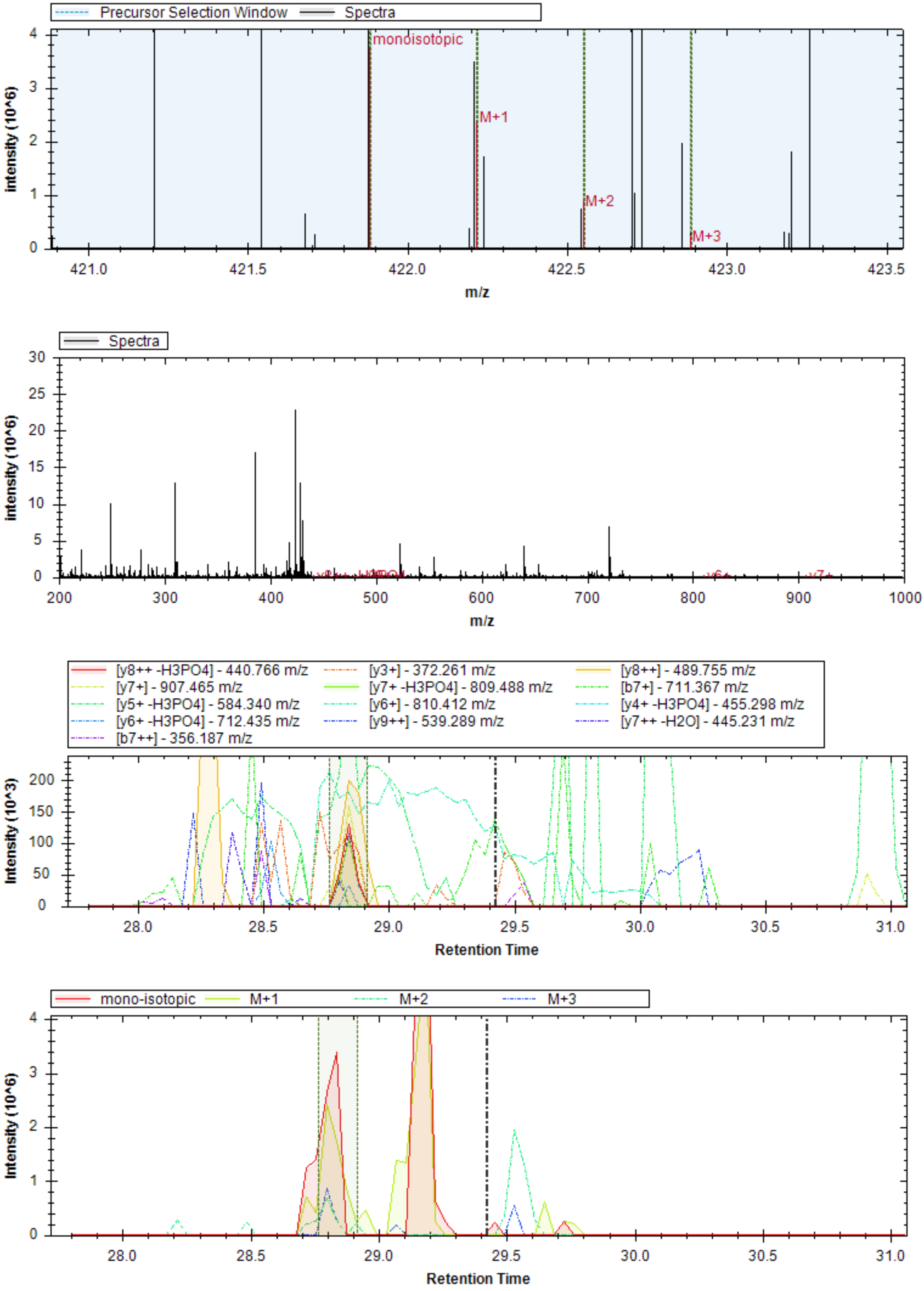

Q52KI8;Q52KI8-2 – _RES[+H+O3+P]PSPAPKPR_.3

Fig4_MP-DIA-barrel-cortex-p9-1_MHRM.htrms

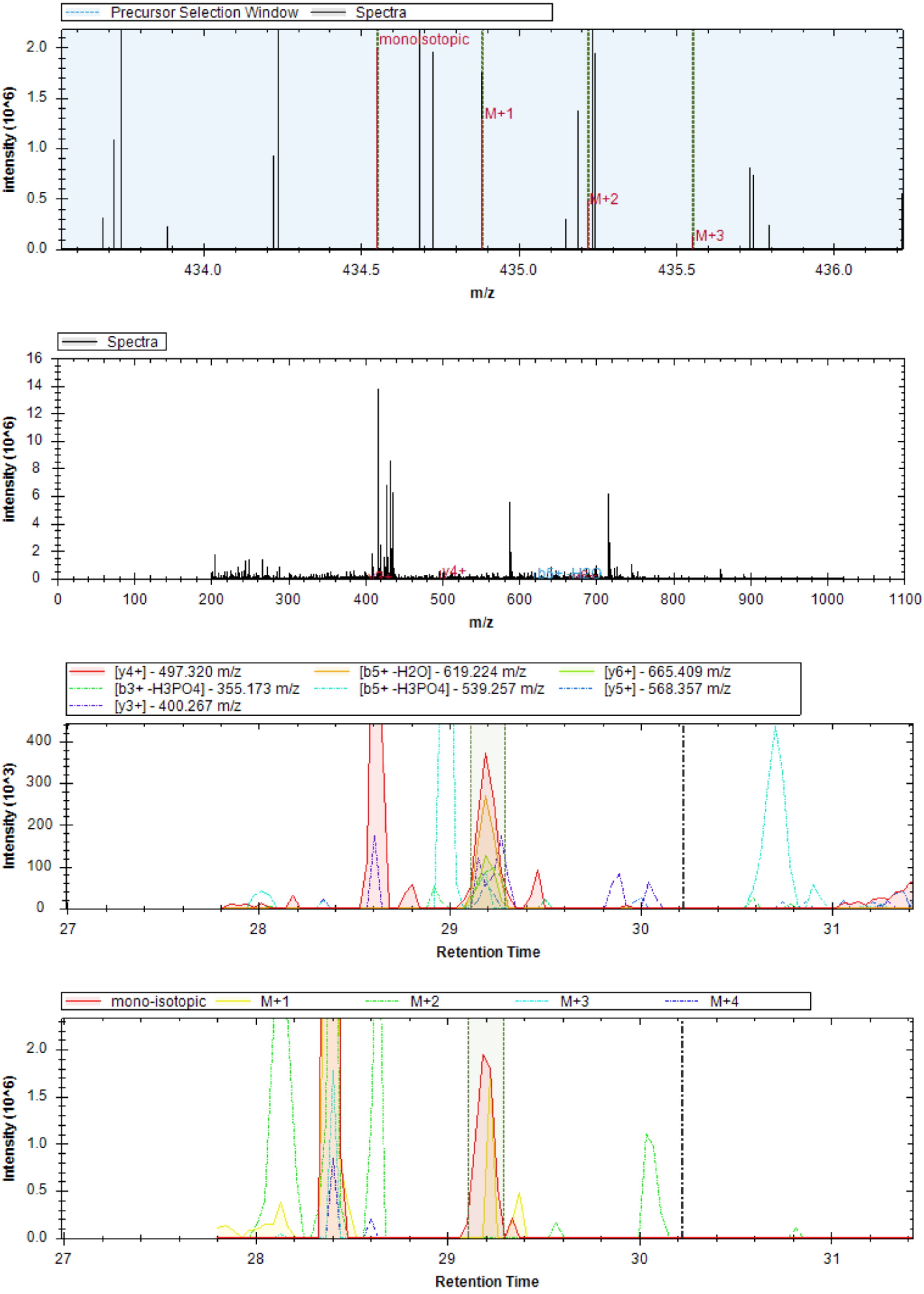

Q62188 – _GS[+H+O3+P]PTRPNPPVR_.3

Fig4_MP-DIA-barrel-cortex-p9-1_MHRM.htrms

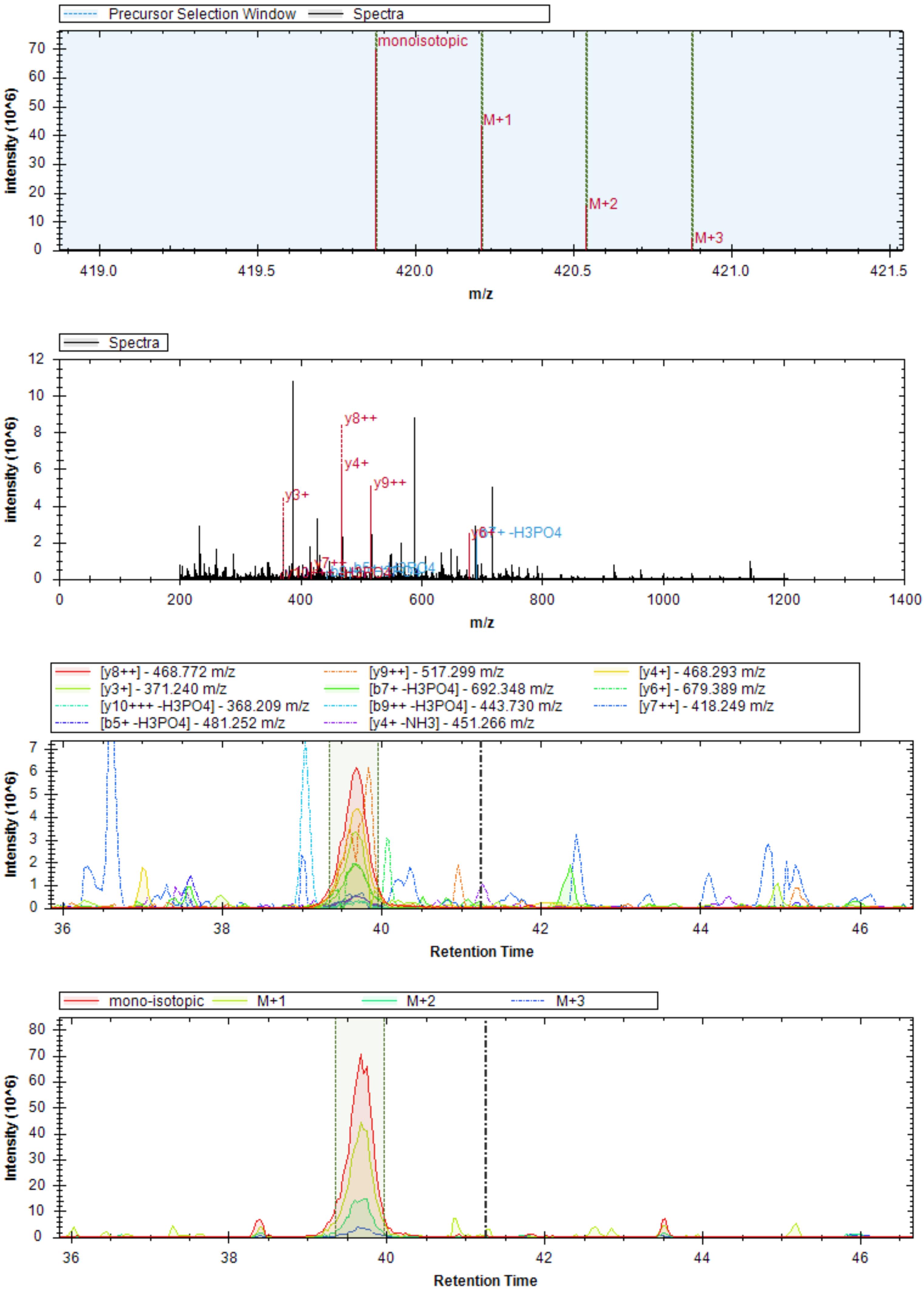

Q93092 – _[+C2+H2+O]SGS[+H+O3+P]PVKR_.2
Fig4_MP-DIA-barrel-cortex-p9-1_MHRM.htrms

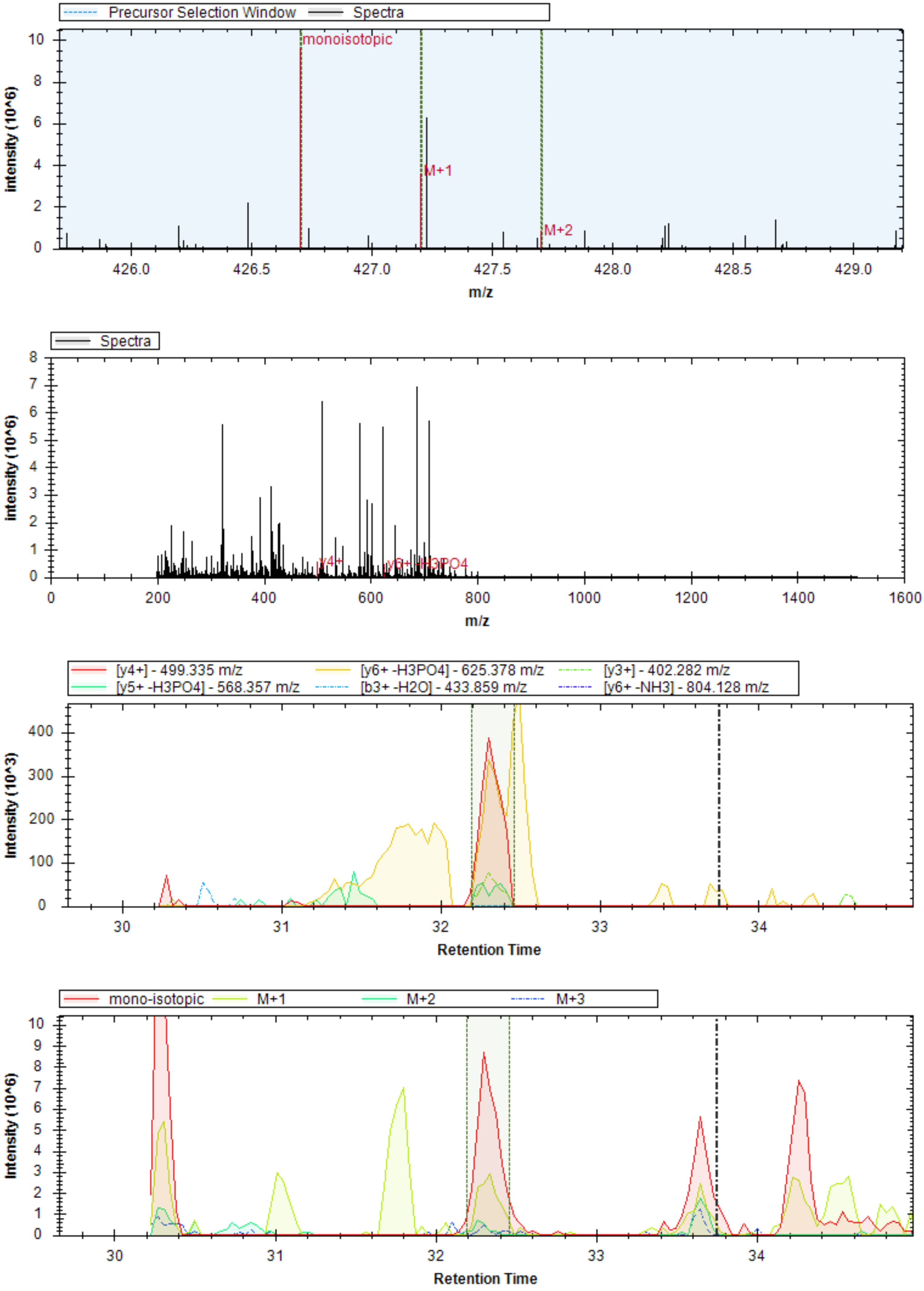

P62484 – _T[+H+O3+P]HKIIAPANLERPVR_.4
Fig4_MP-DIA-barrel-cortex-p9-1_MHRM.htrms

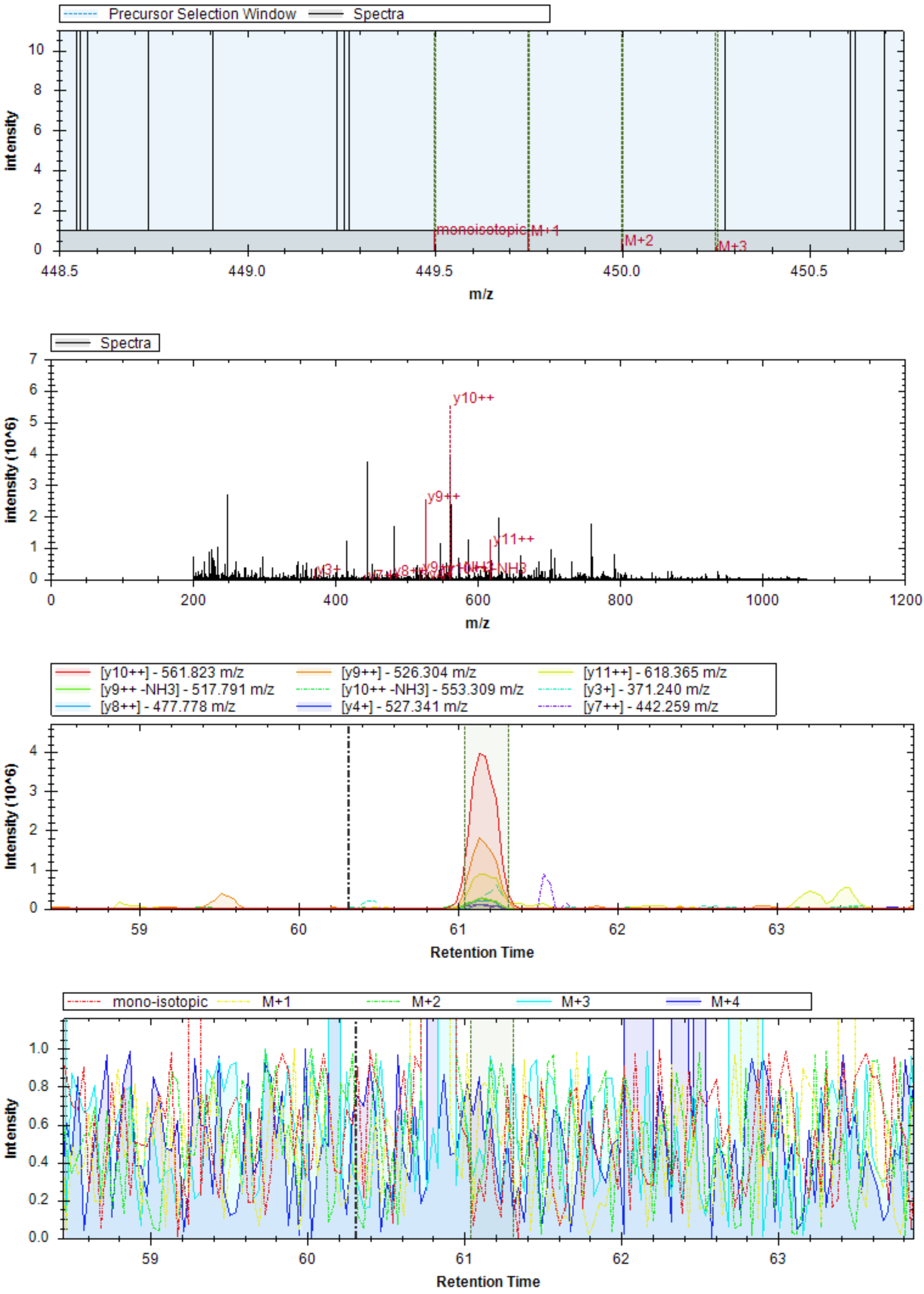

Q9JKK8 – _MLS[+H+O3+P]LWLDFGAK_.3

Fig4_MP-DIA-barrel-cortex-p9-1_MHRM.htrms

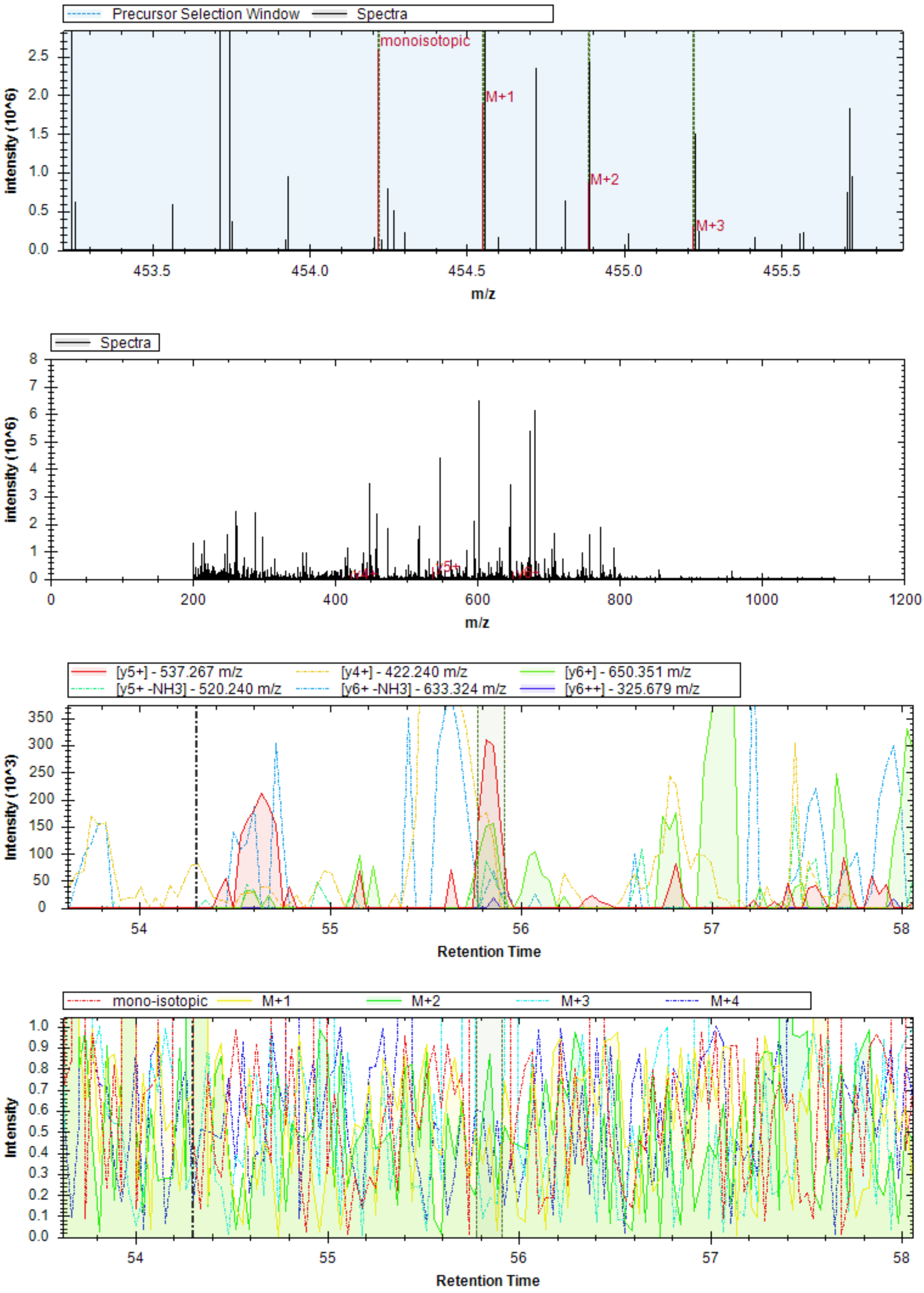

O08553 – _TVT[+H+O3+P]PASSAK_.2

Fig4_MP-DIA-barrel-cortex-p9-1_MHRM.htrms

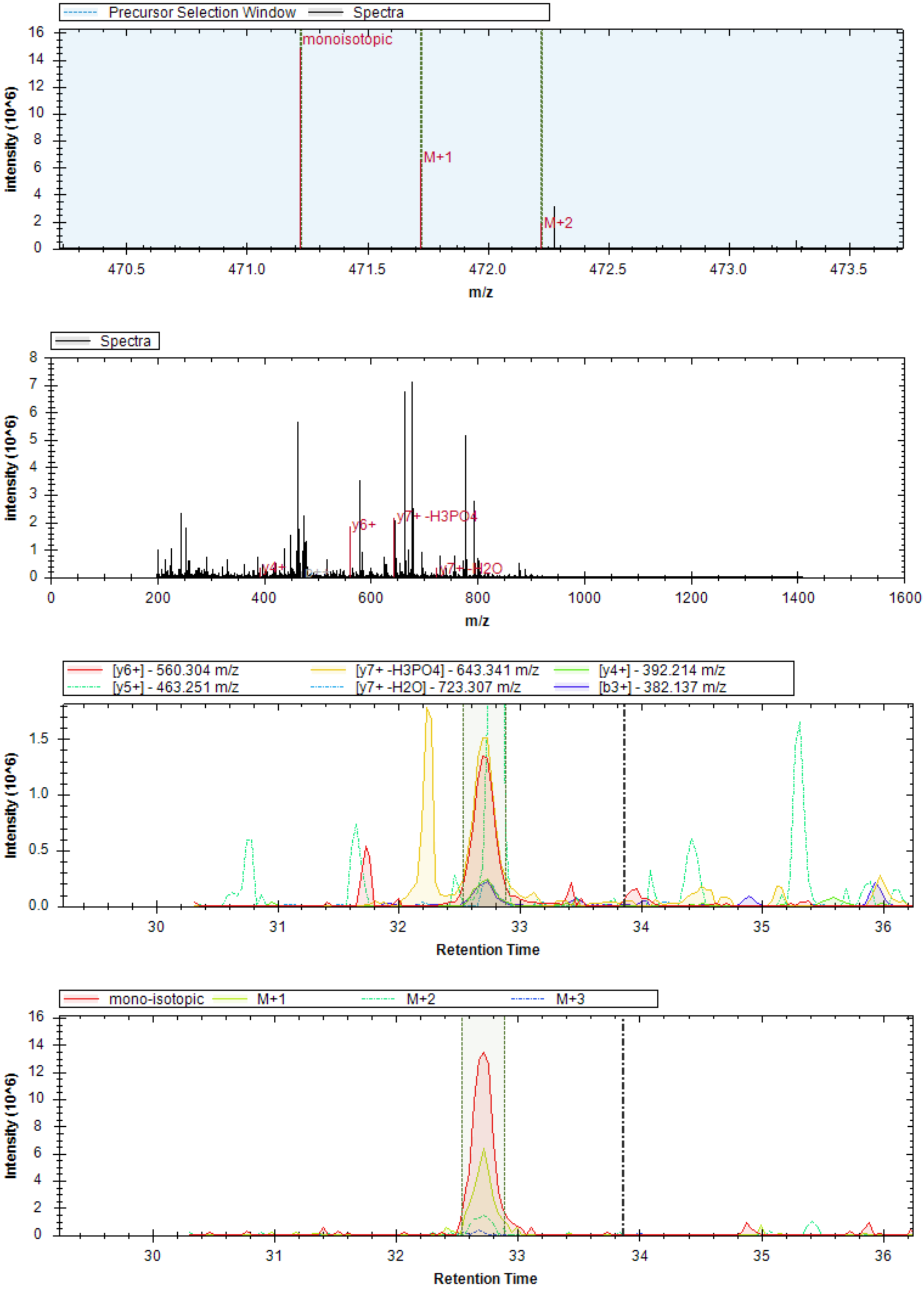

P10637 – _S[+H+O3+P]RTPSLPTPPTR_.3
Fig4_MP-DIA-barrel-cortex-p9-1_MHRM.htrms

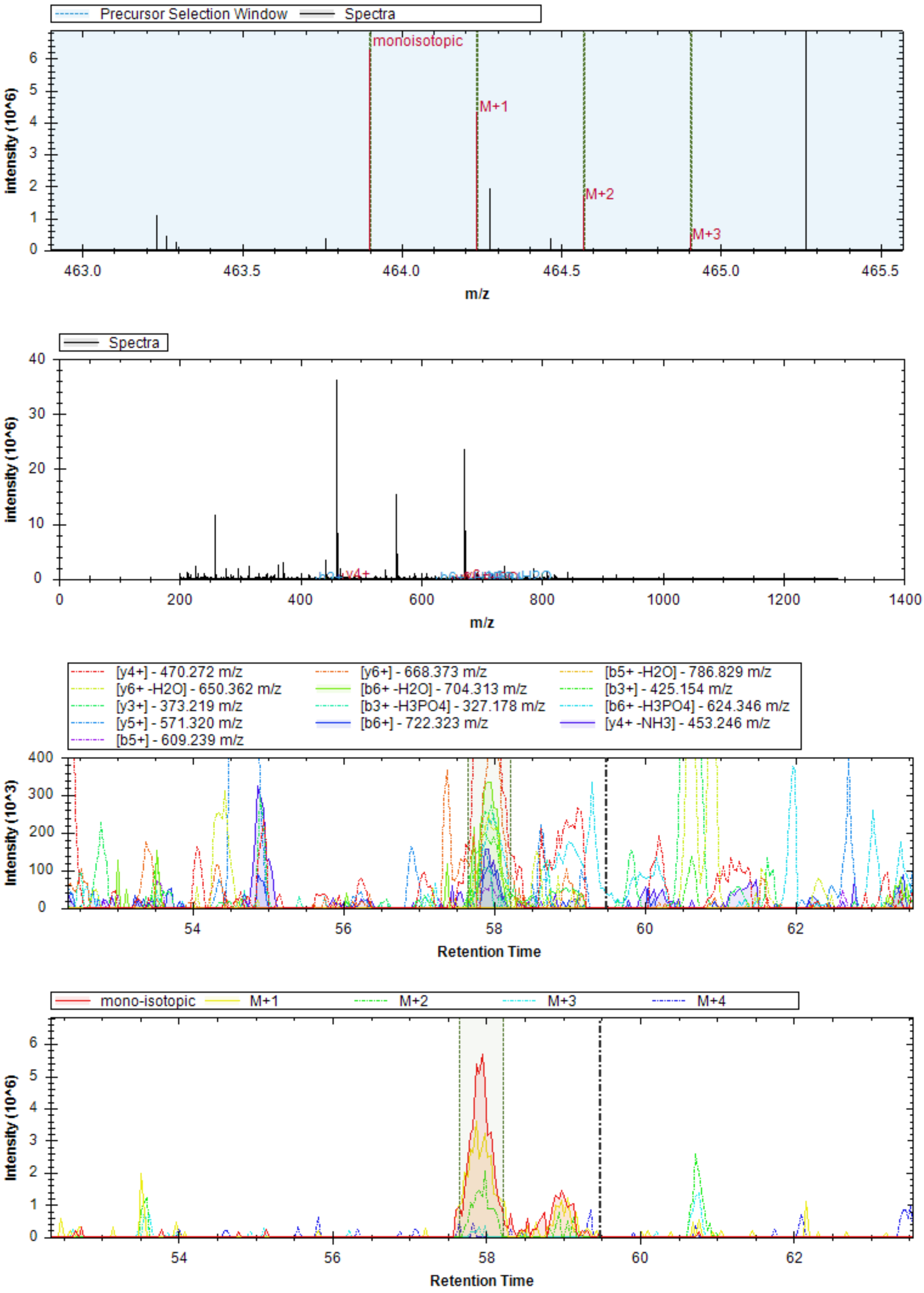

P20357 – _VTDGIS[+H+O3+P]KSPEKR_.3

Fig4_MP-DIA-barrel-cortex-p9-1_MHRM.htrms

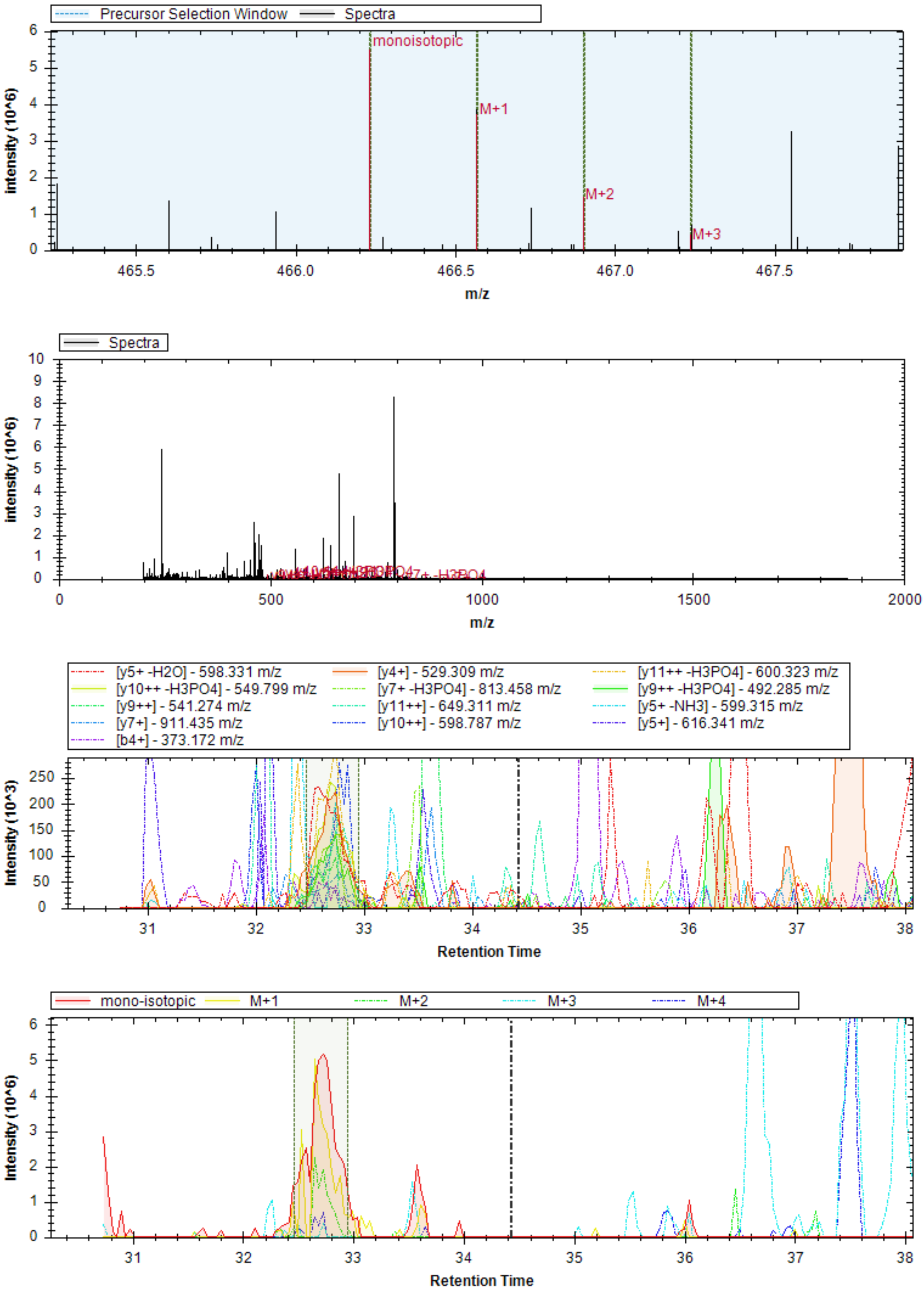

P20357 – _VTDGISKS[+H+O3+P]PEKR_.3

Fig4_MP-DIA-barrel-cortex-p9-1_MHRM.htrms

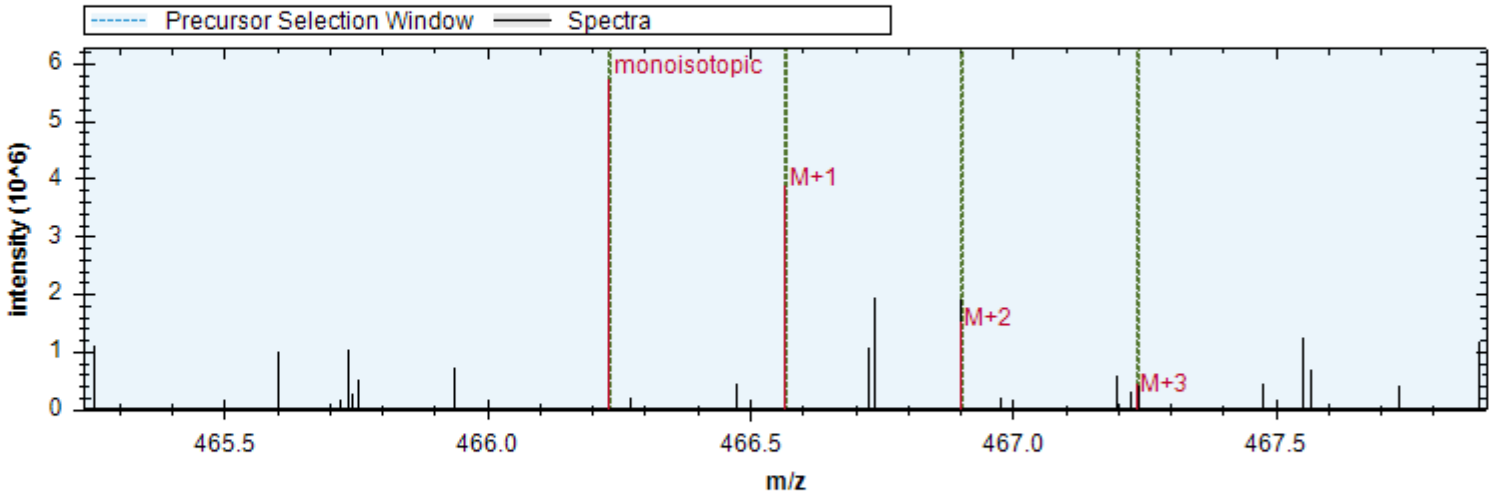

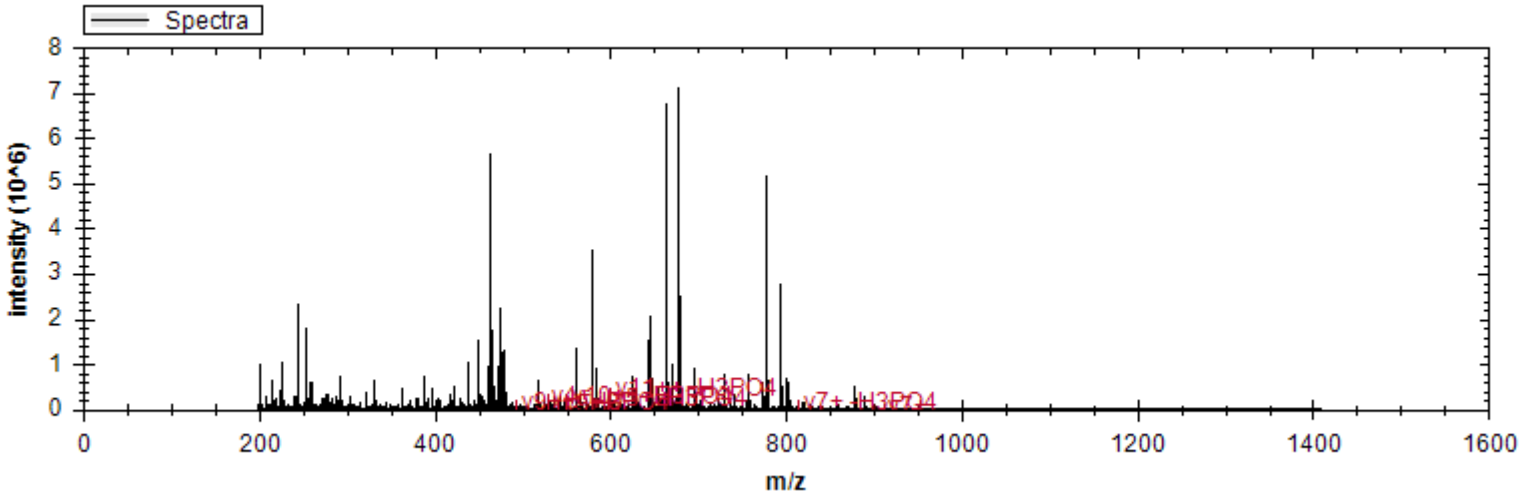

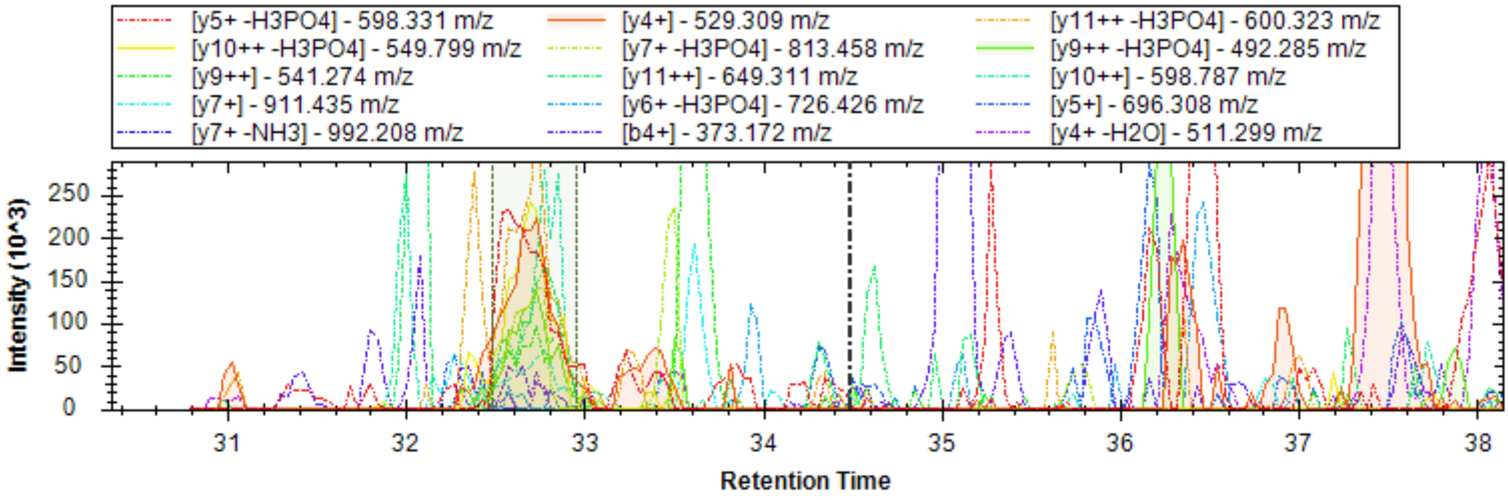

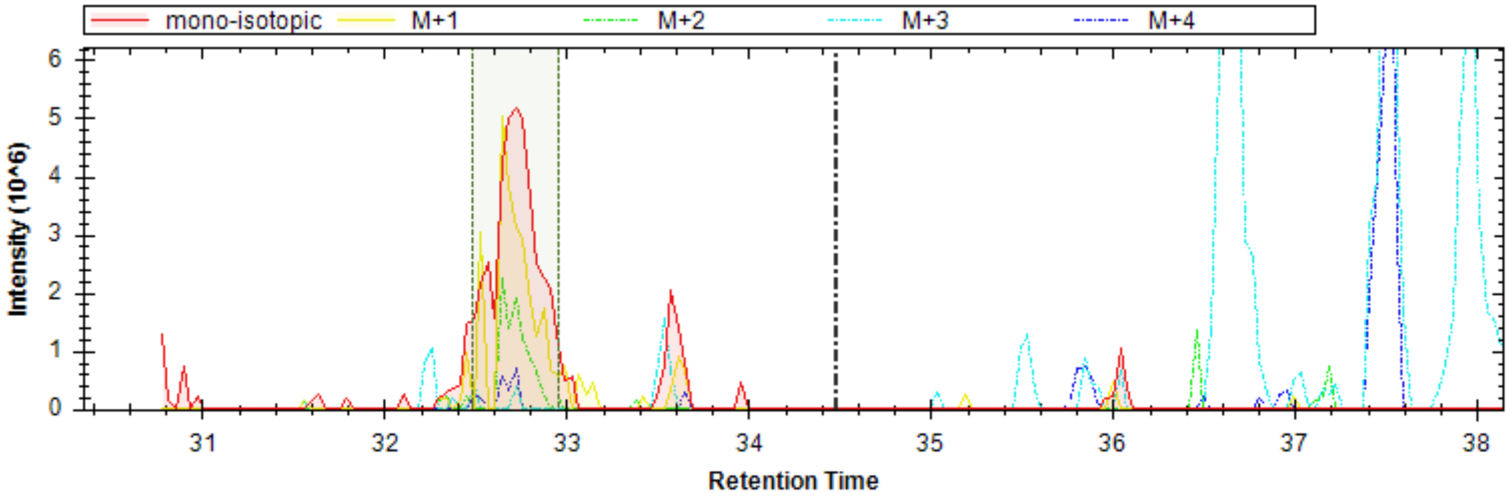

Q9CR81 – _[+C2+H2+O]M[+O]ANHLVKPDS[+H+O3+P]R_.3
Fig4_MP-DIA-barrel-cortex-p9-1_MHRM.htrms

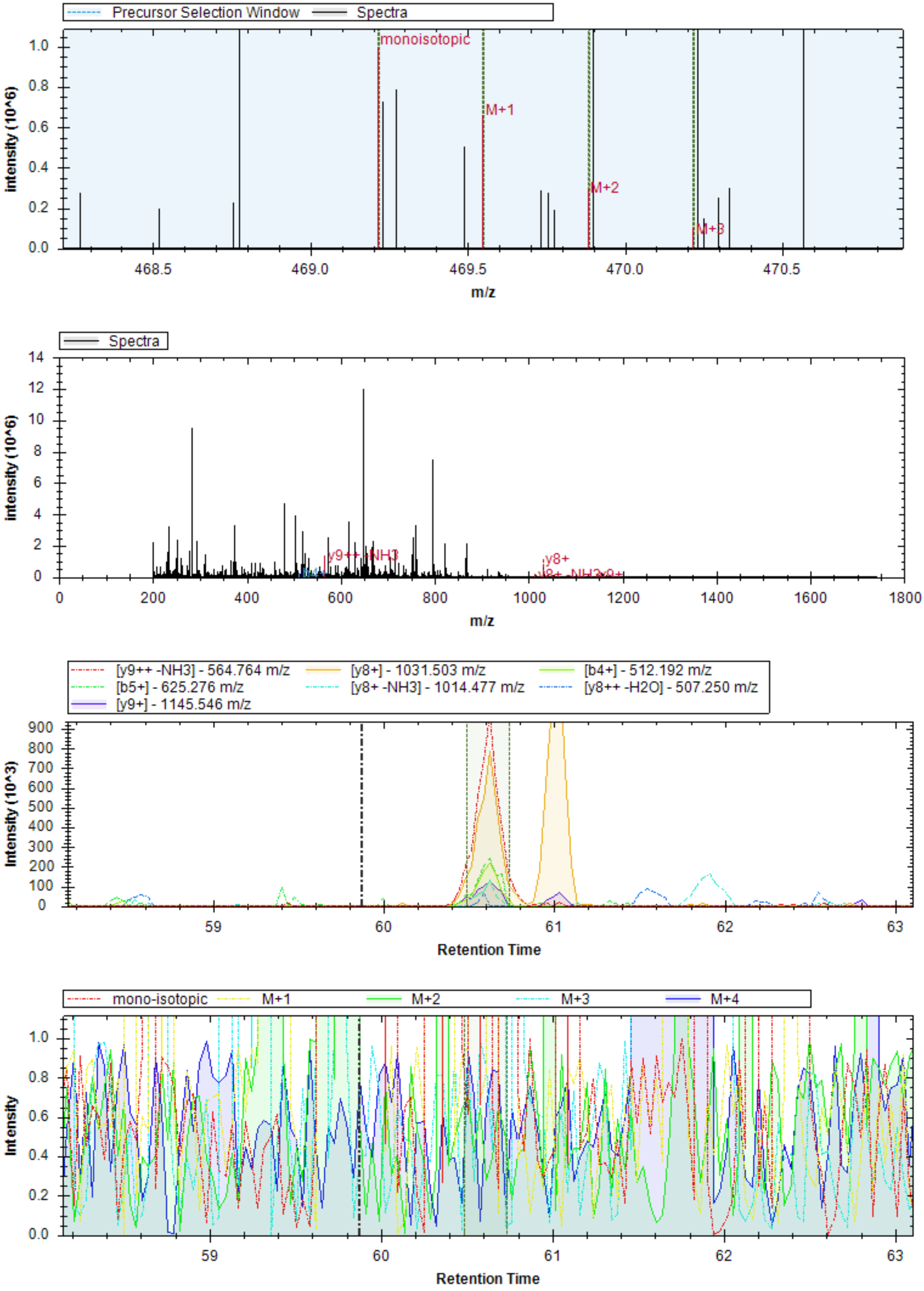

P26645 – _LSGFS[+H+O3+P]FKK_.2
Fig4_MP-DIA-barrel-cortex-p9-1_MHRM.htrms

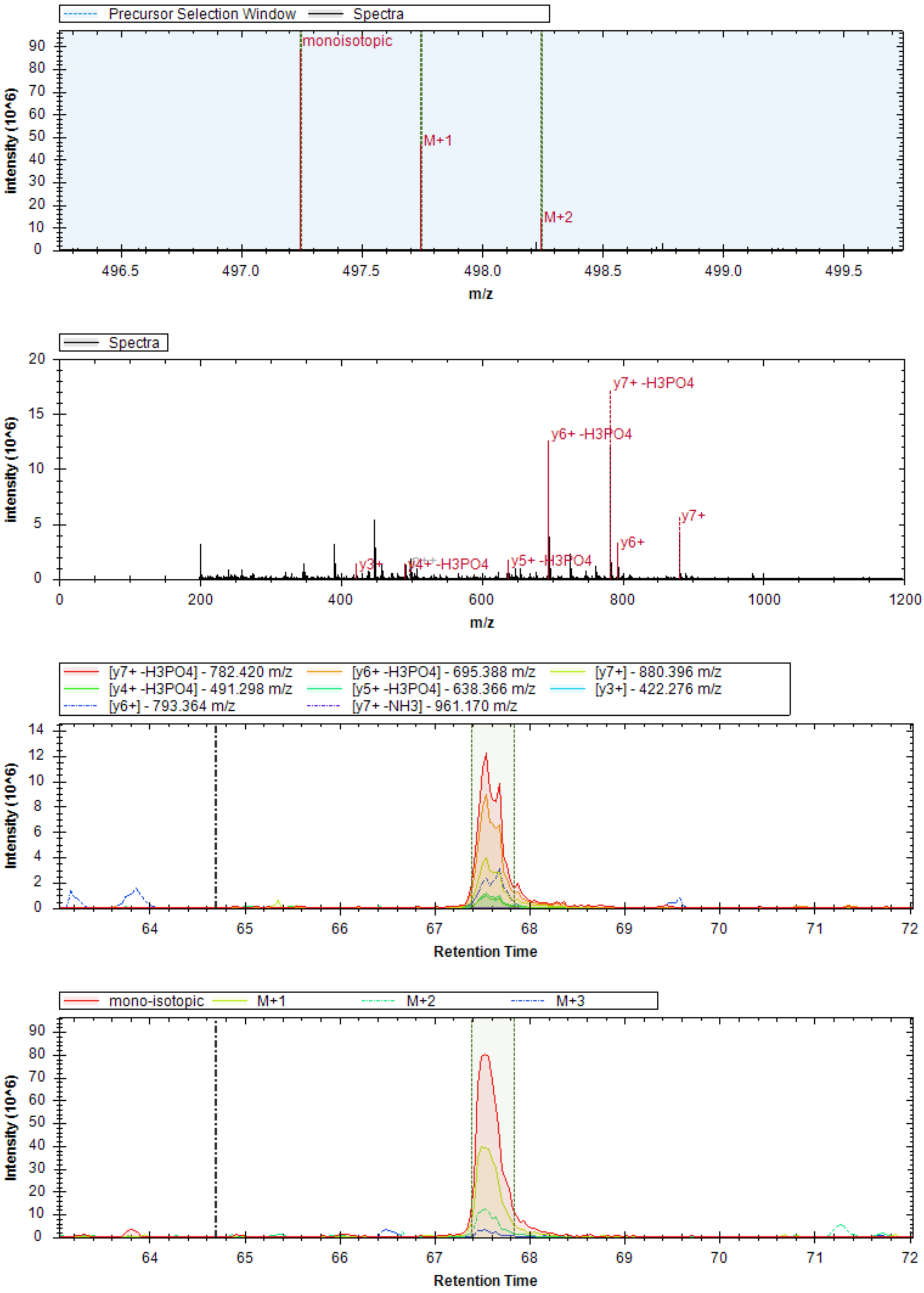

P28667 – _LSGLS[+H+O3+P]FKR_.2
Fig4_MP-DIA-barrel-cortex-p9-1_MHRM.htrms

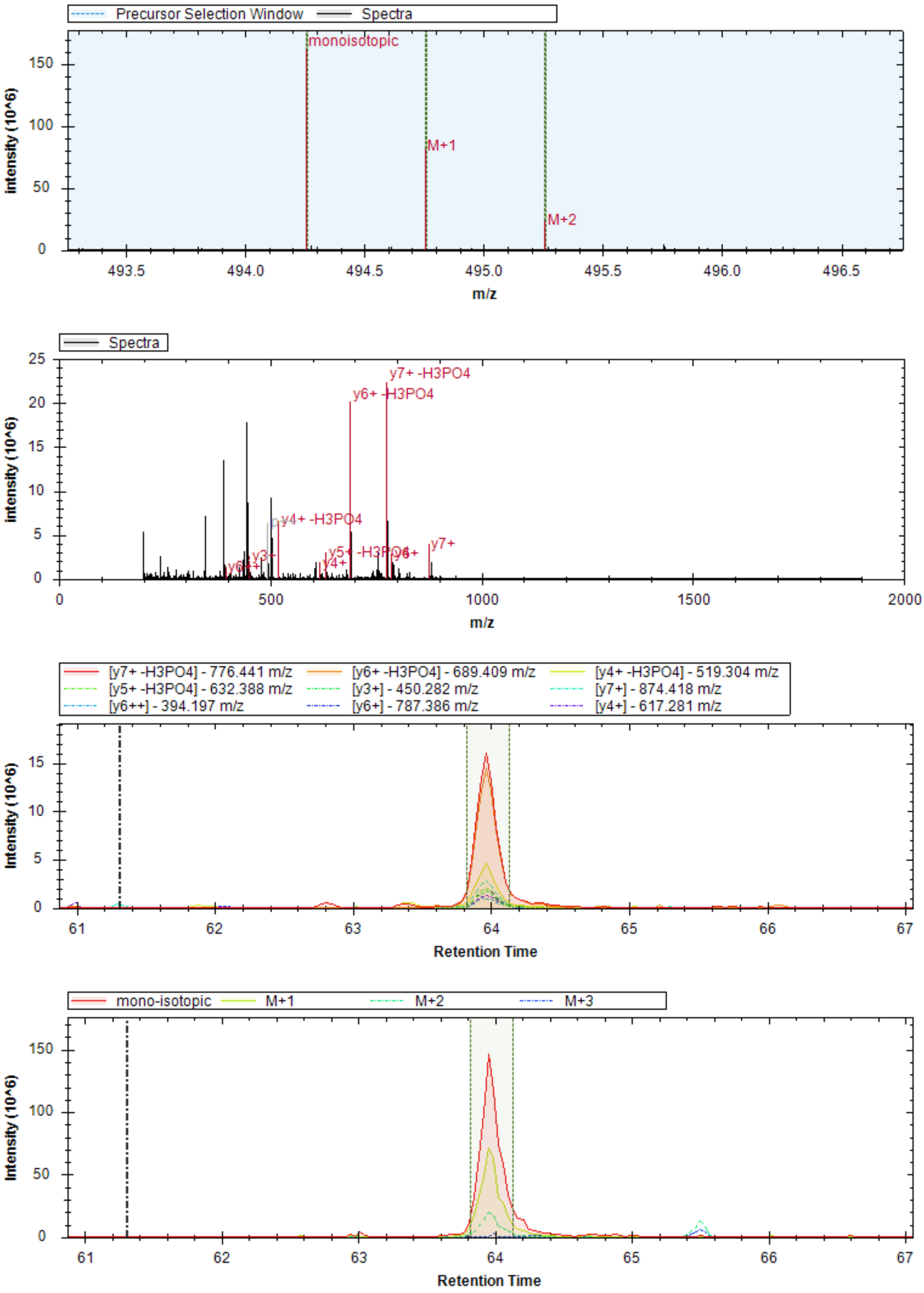

P28738 – _PIRPGHYPAS[+H+O3+P]SPTAVHAVR_.4
Fig4_MP-DIA-barrel-cortex-p9-1_MHRM.htrms

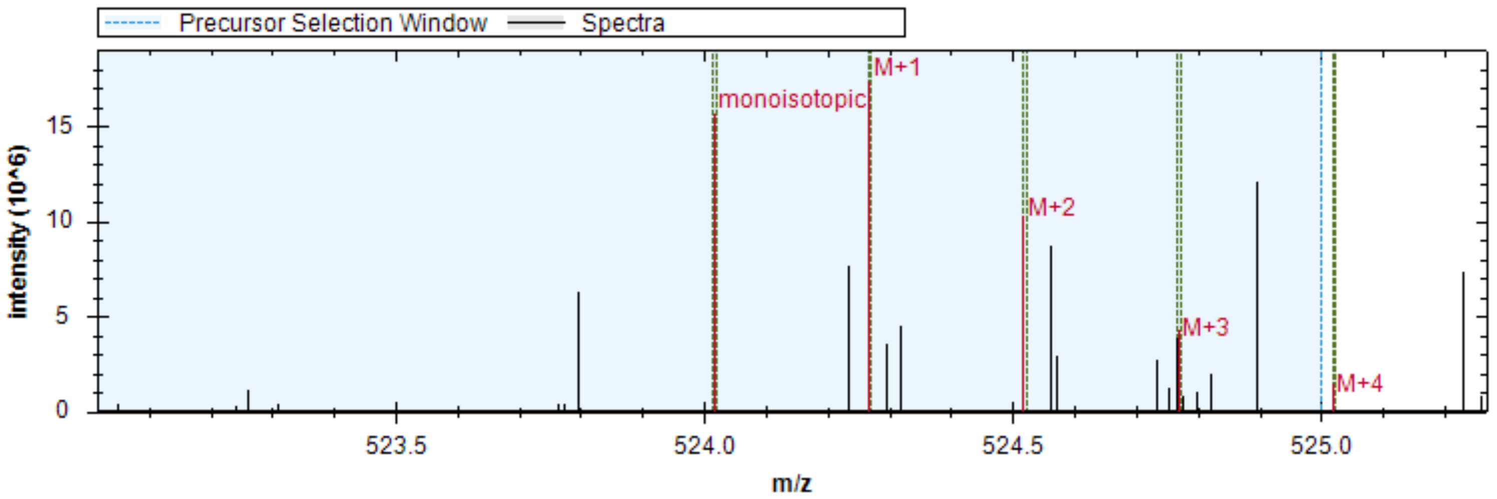

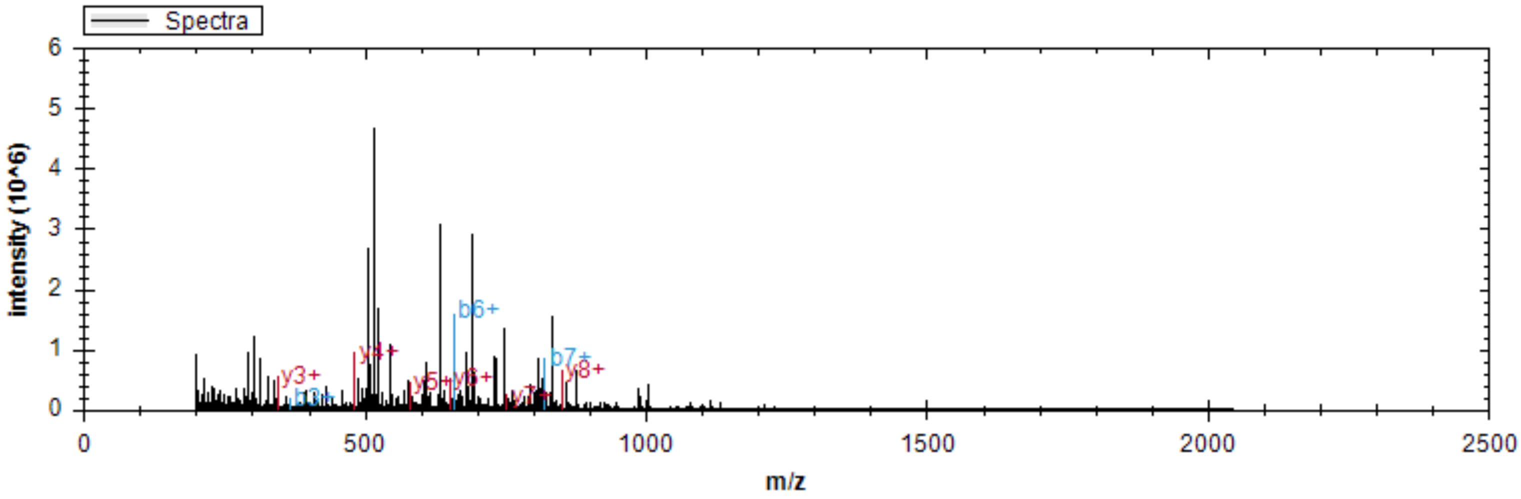

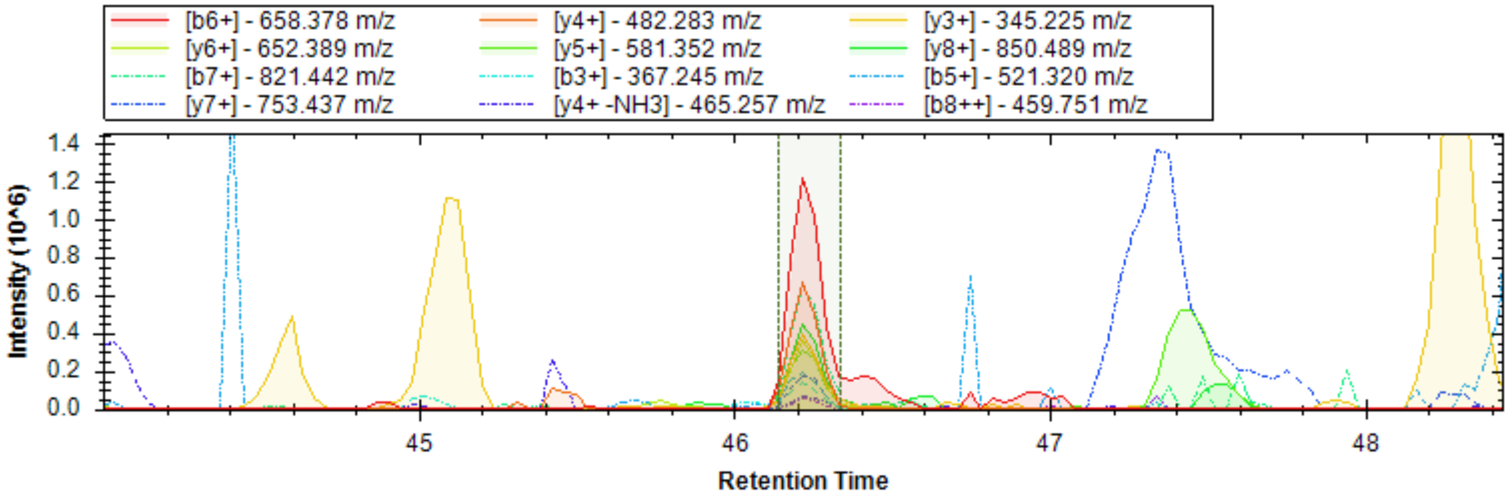

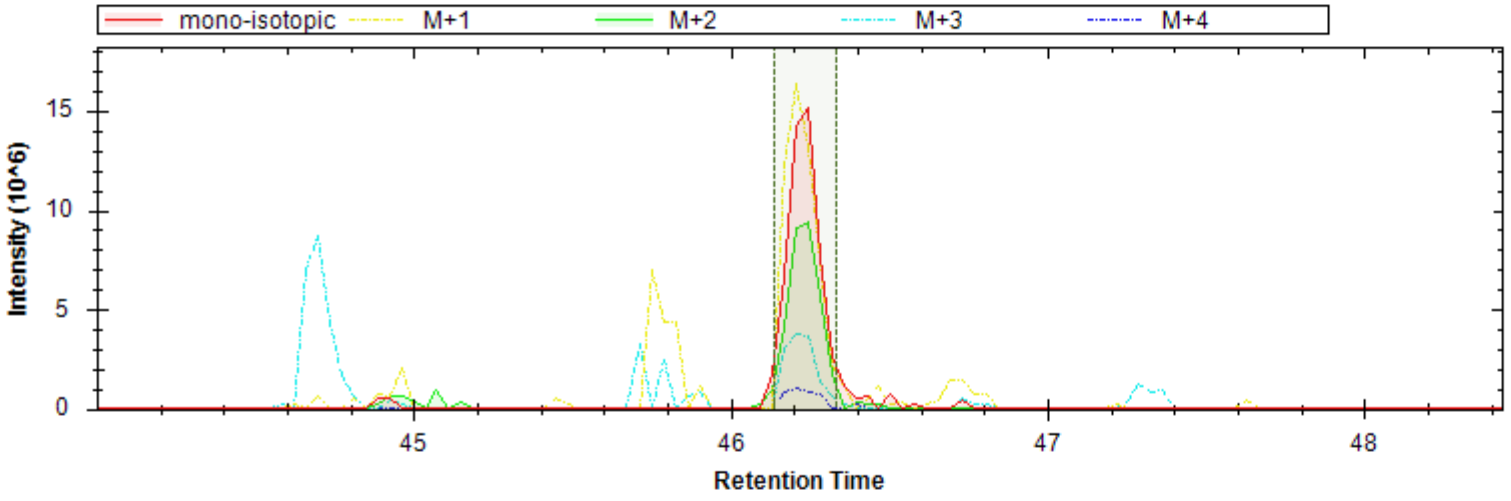

P28738 – _PIRPGHYPASS[+H+O3+P]PTAVHAVR_.4
Fig4_MP-DIA-barrel-cortex-p9-1_MHRM.htrms

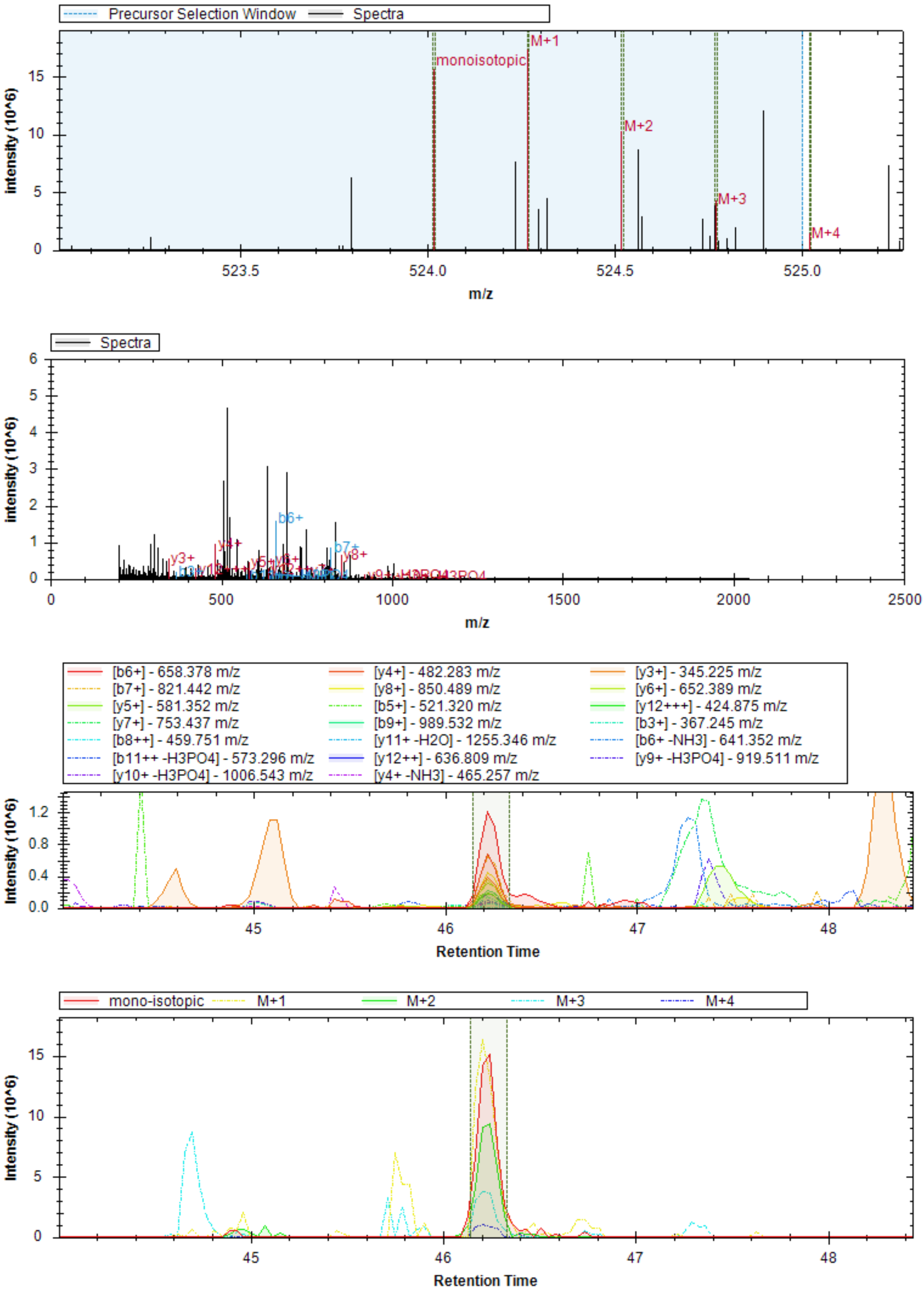

Q8CDY7 – _LVQALC[+C2+H3+N+O]EQIHT[+H+O3+P]R_.3

Fig4_MP-DIA-barrel-cortex-p9-1_MHRM.htrms

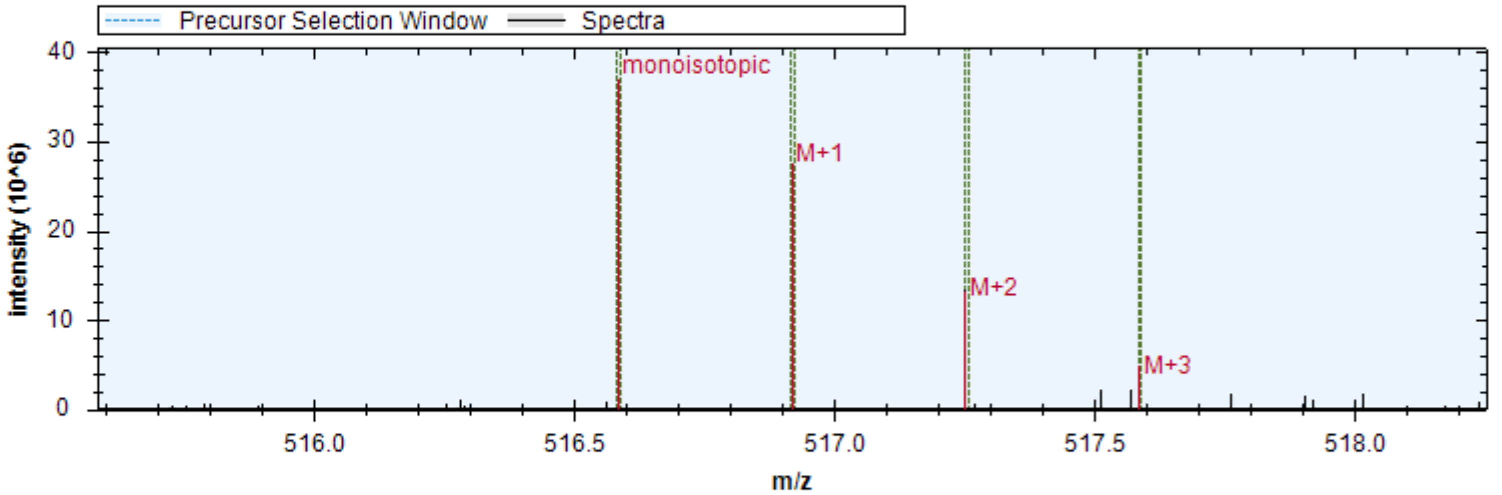

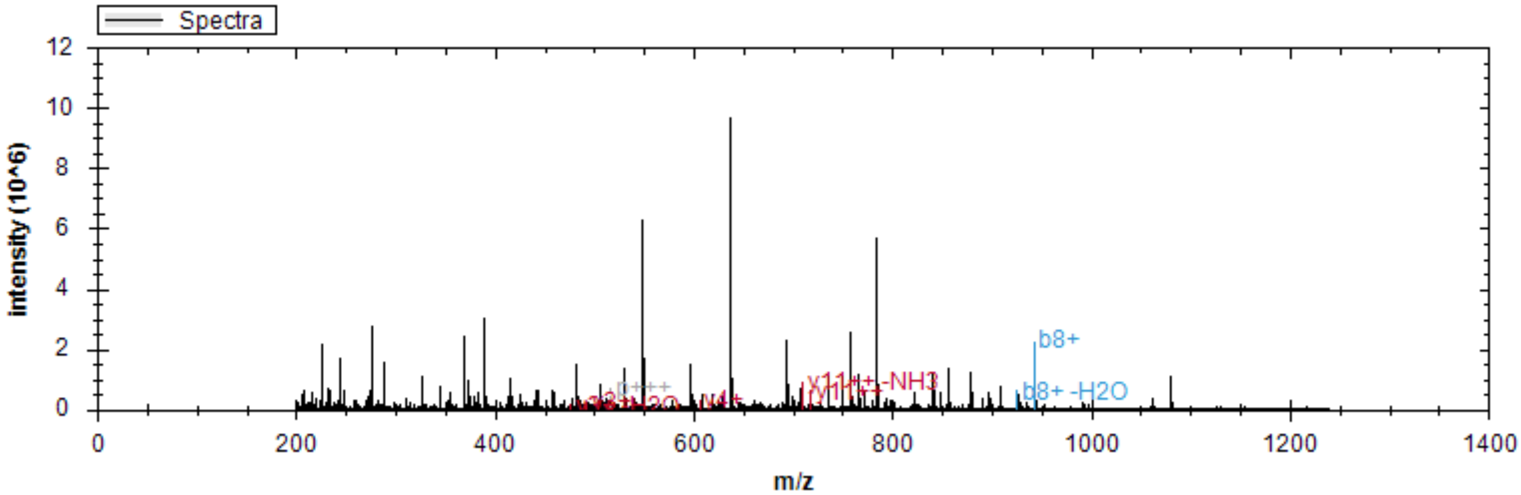

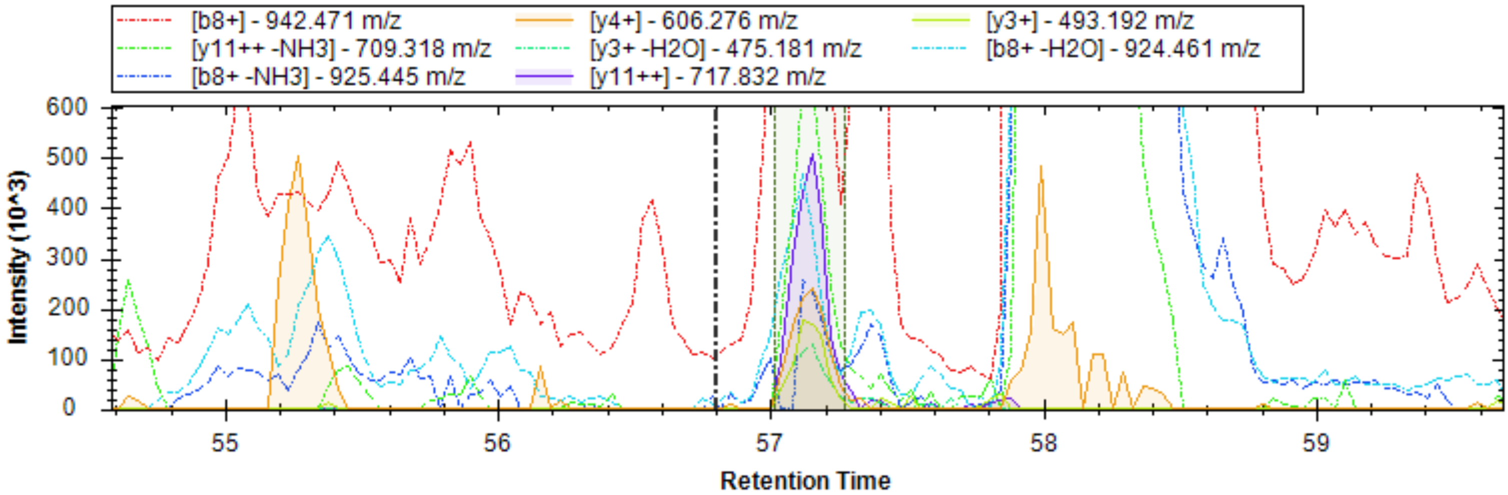

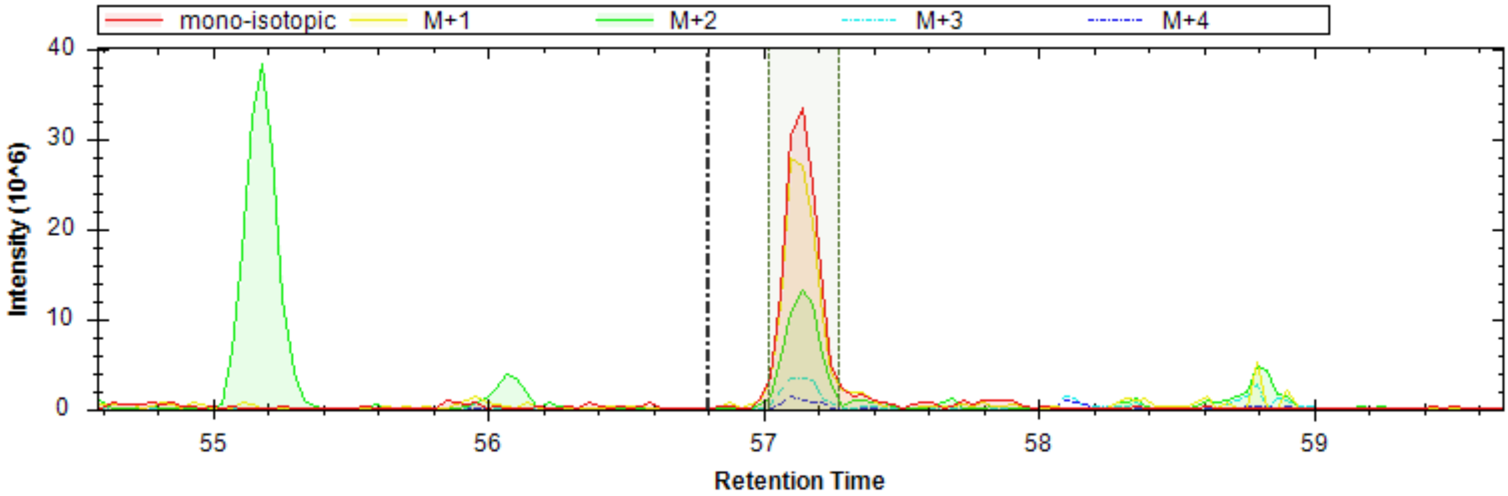

Q9D516;Q9D516-2 – _KLS[+H+O3+P]QGILVIR_.3
Fig4_MP-DIA-barrel-cortex-p9-1_MHRM.htrms

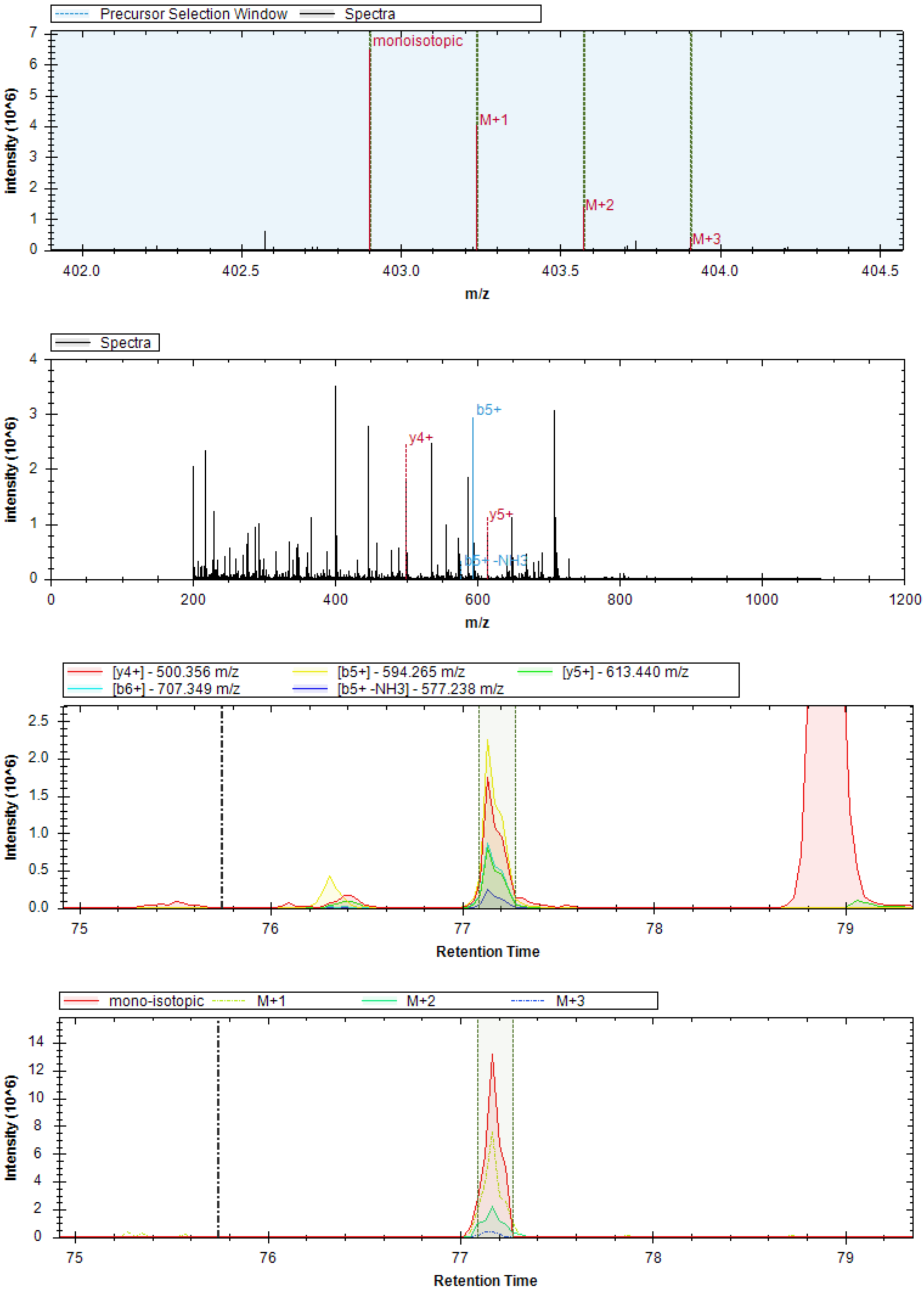

Q9QYC0 – _AAVVT[+H+O3+P]SPPPTTAPHK_.3
Fig4_MP-DIA-barrel-cortex-p9-1_MHRM.htrms

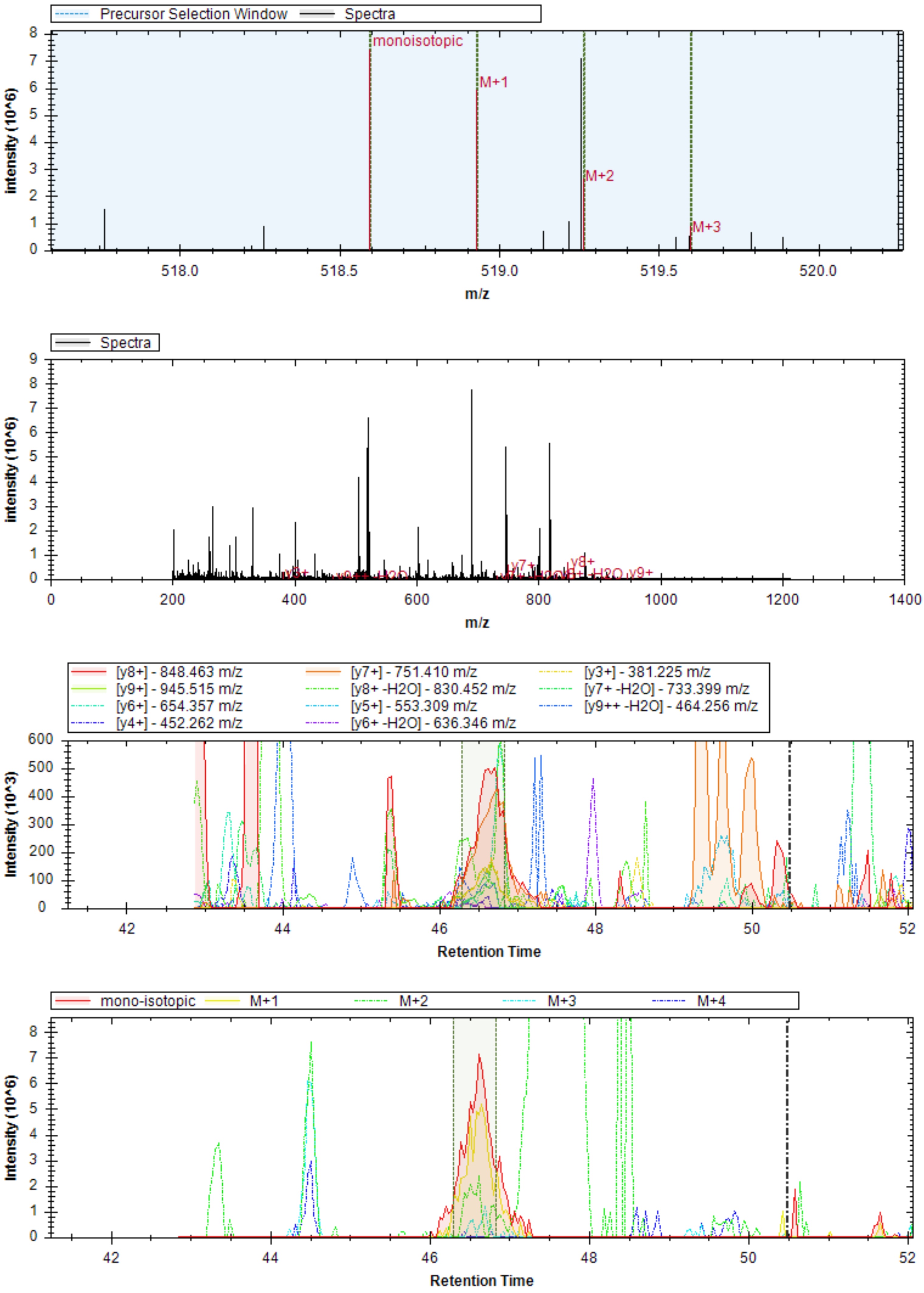

Q9QYC0 – _AAVVTS[+H+O3+P]PPPTTAPHK_.3

Fig4_MP-DIA-barrel-cortex-p9-1_MHRM.htrms

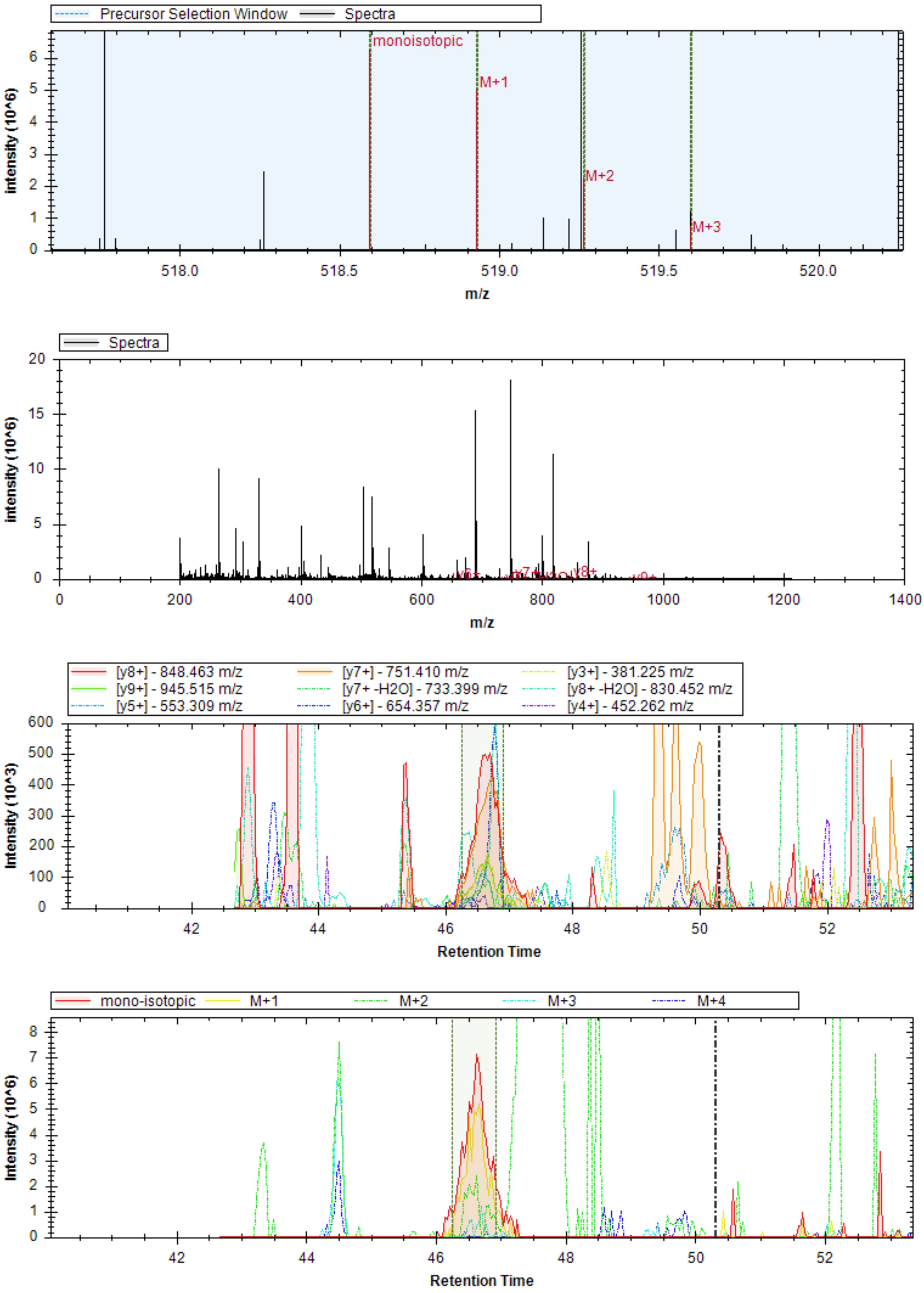

P10637 – _T[+H+O3+P]PPKSPSASK_.2

Fig4_MP-DIA-barrel-cortex-p9-1_MHRM.htrms

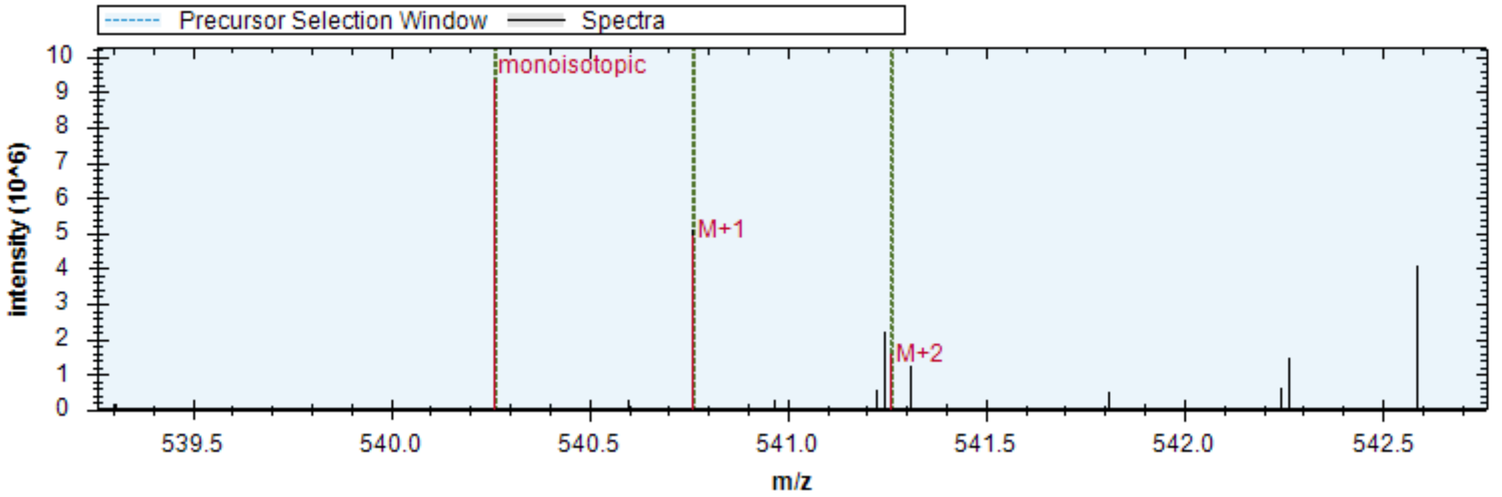

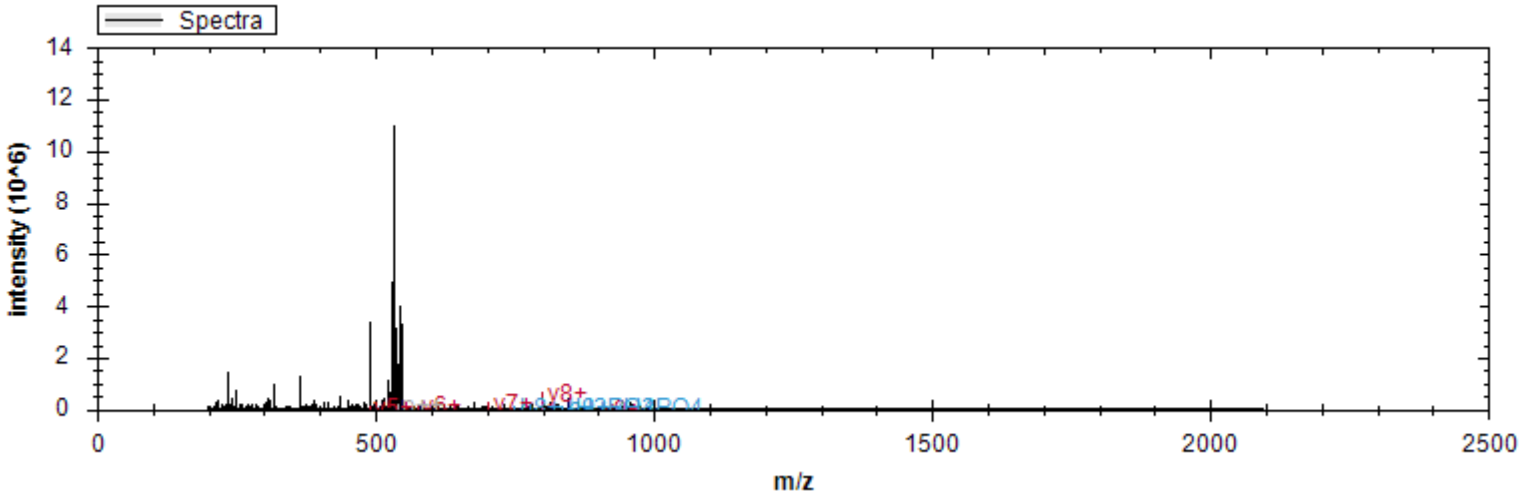

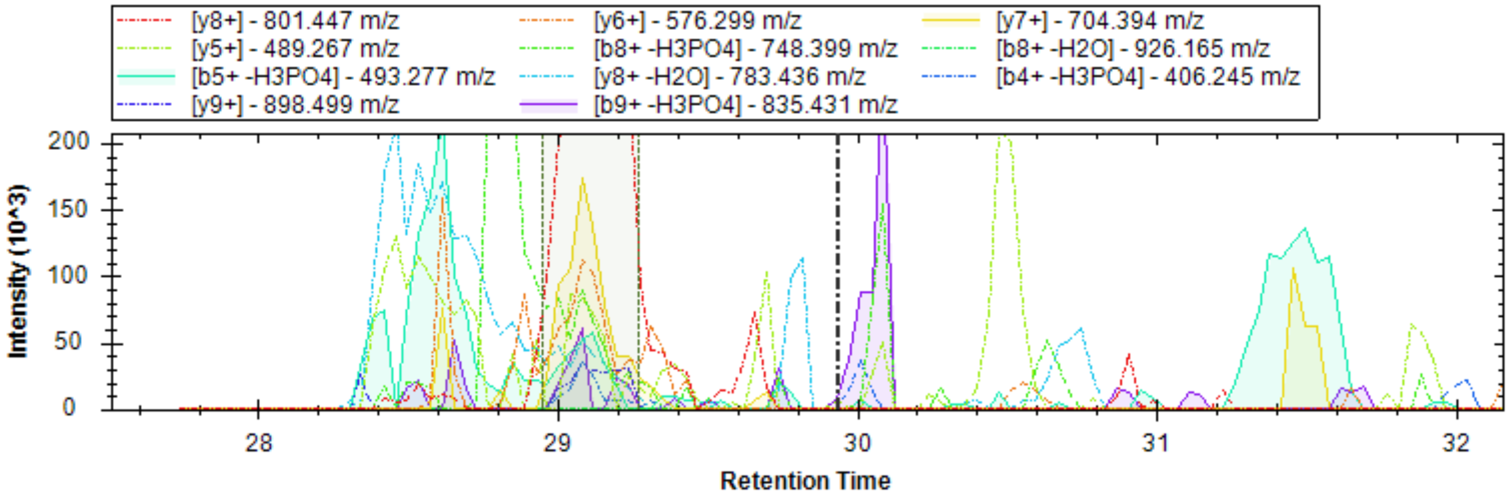

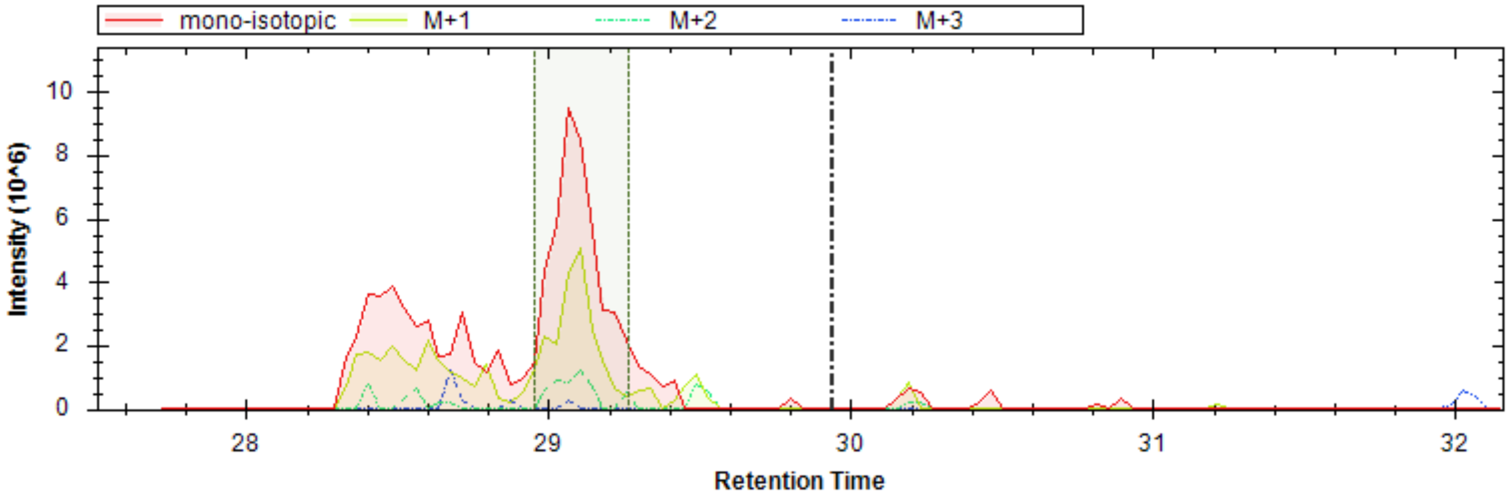

P10637 – _TPPKS[+H+O3+P]PSASK_.2

Fig4_MP-DIA-barrel-cortex-p9-1_MHRM.htrms

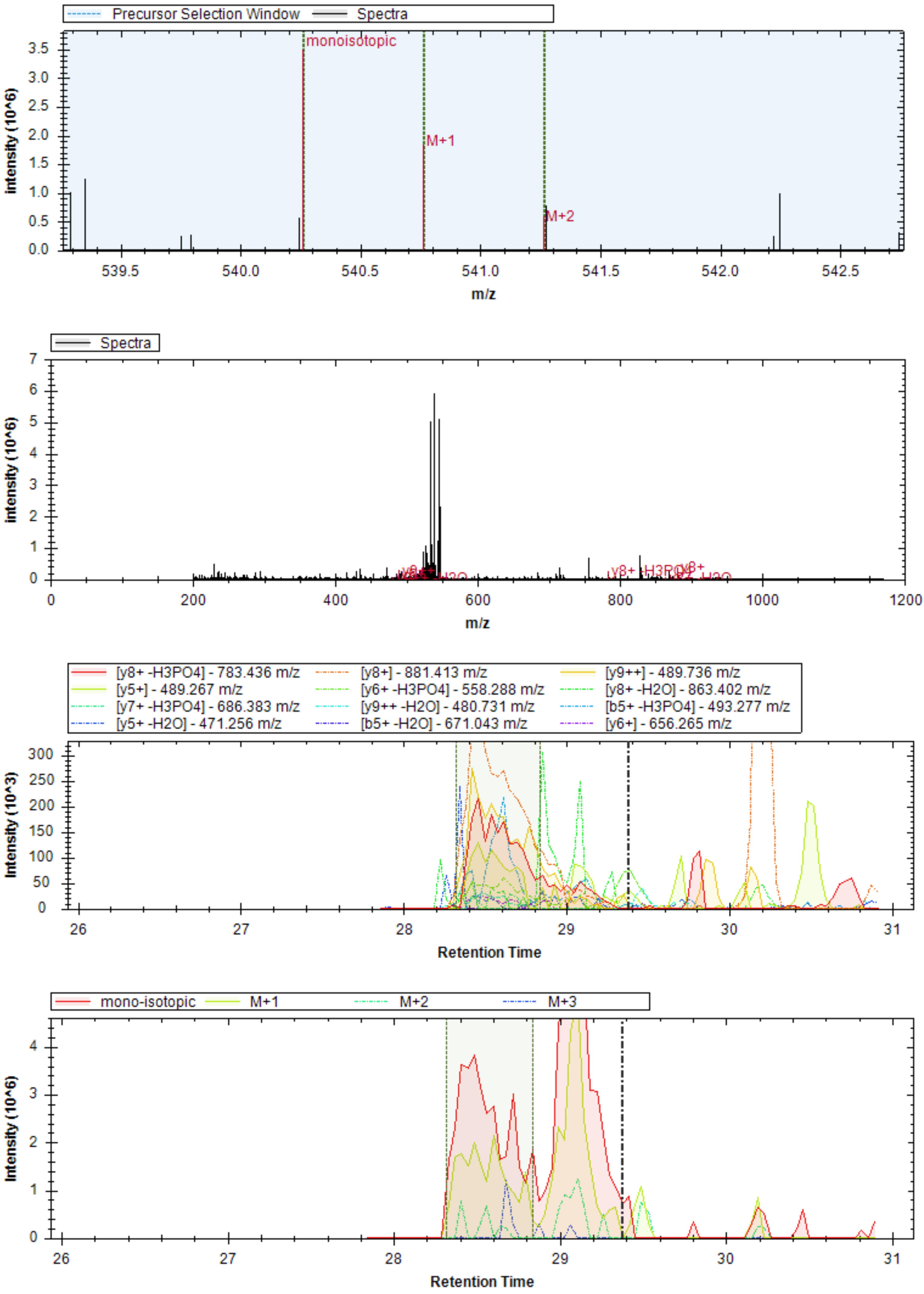

P10637 – _VAVVRT[+H+O3+P]PPKSPSASK_.3
Fig4_MP-DIA-barrel-cortex-p9-1_MHRM.htrms

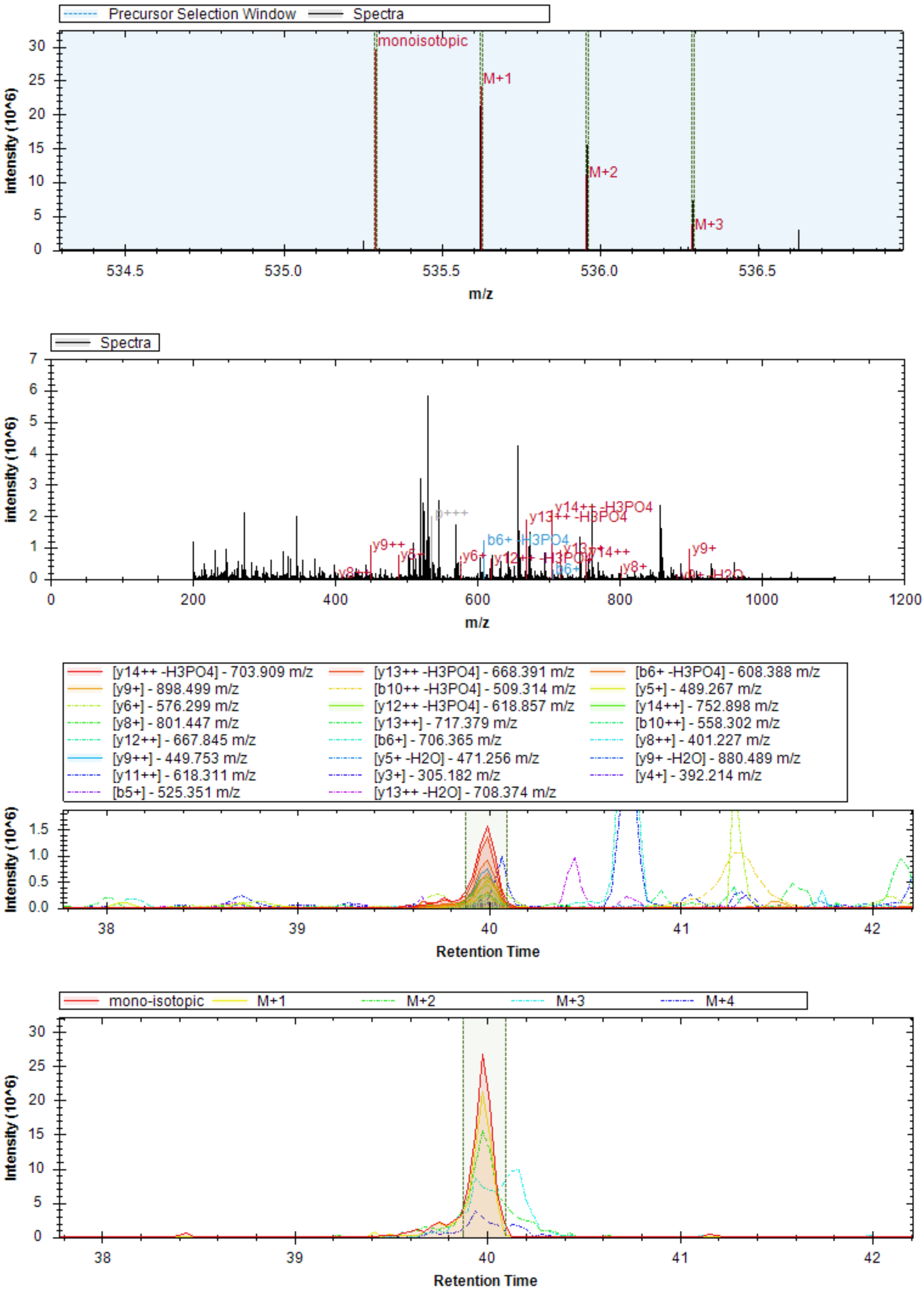

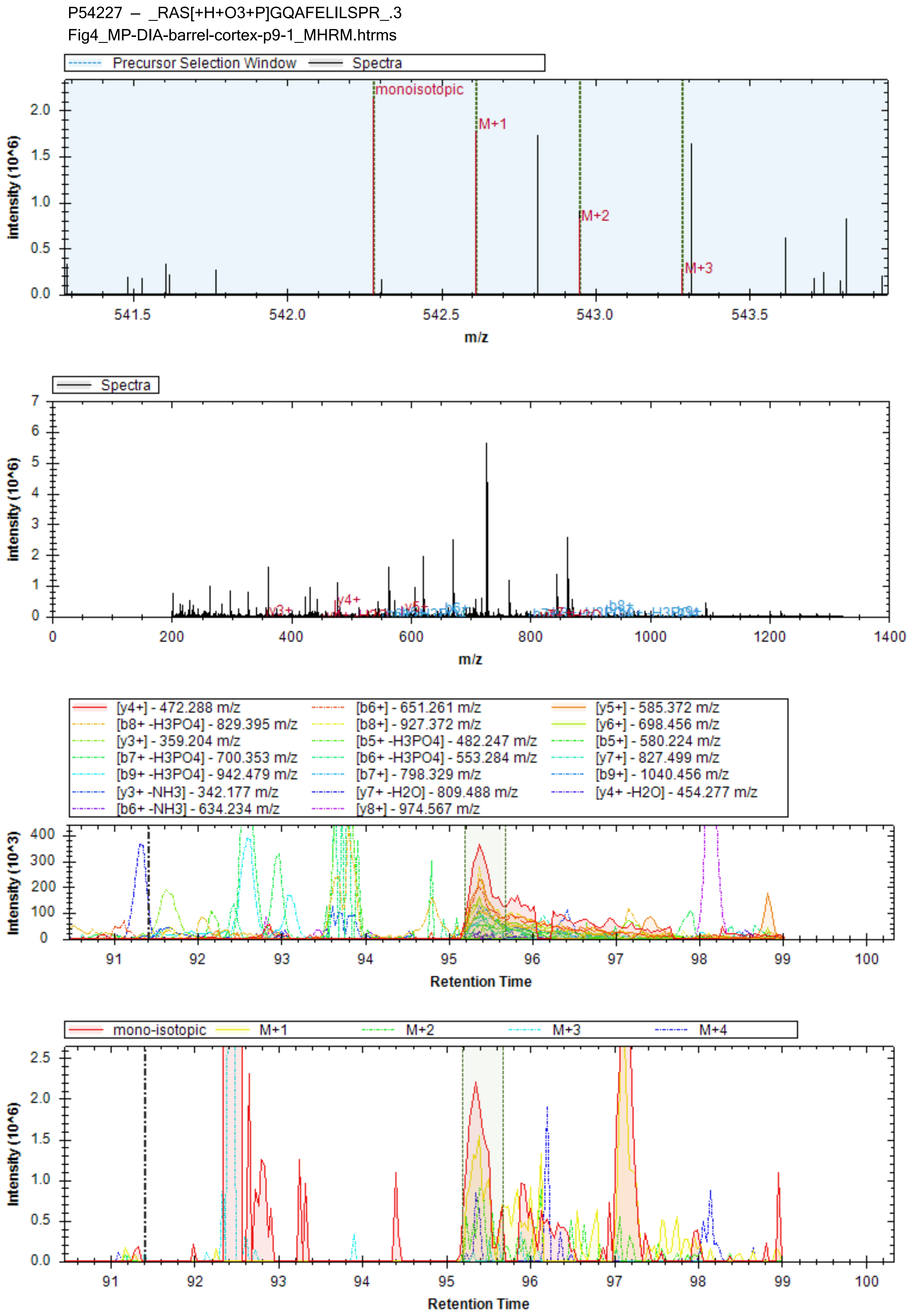
P54227 – _RAS[+H+O3+P]GQAFELILSPR_.3
Fig4_MP-DIA-barrel-cortex-p9-1_MHRM.htrms
Precursor Selection Window
Spectra
monoisotopic
M+1
M+2
M+3
intensity (10^6)
m/z
Spectra
intensity (10^6)
m/z
[y4+] - 472.288 m/z
[b6+] - 651.261 m/z
[y5+] - 585.372 m/z
[b8+ -H3PO4] - 829.395 m/z
[b8+] - 927.372 m/z
[y6+] - 698.456 m/z
[y3+] - 359.204 m/z
[b5+ -H3PO4] - 482.247 m/z
[b5+] - 580.224 m/z
[b7+ -H3PO4] - 700.353 m/z
[b6+ -H3PO4] - 553.284 m/z
[y7+] - 827.499 m/z
[b9+ -H3PO4] - 942.479 m/z
[b7+] - 798.329 m/z
[b9+] - 1040.456 m/z
[y3+ -NH3] - 342.177 m/z
[y7+ -H2O] - 809.488 m/z
[y4+ -H2O] - 454.277 m/z
[b6+ -NH3] - 634.234 m/z
[y8+] - 974.567 m/z
Intensity (10^3)
Retention Time
mono-isotopic
M+1
M+2
M+3
M+4
Intensity (10^6)
Retention Time

P54227 – _SKESVPDFPLS[+H+O3+P]PPK_.3
Fig4_MP-DIA-barrel-cortex-p9-1_MHRM.htrms

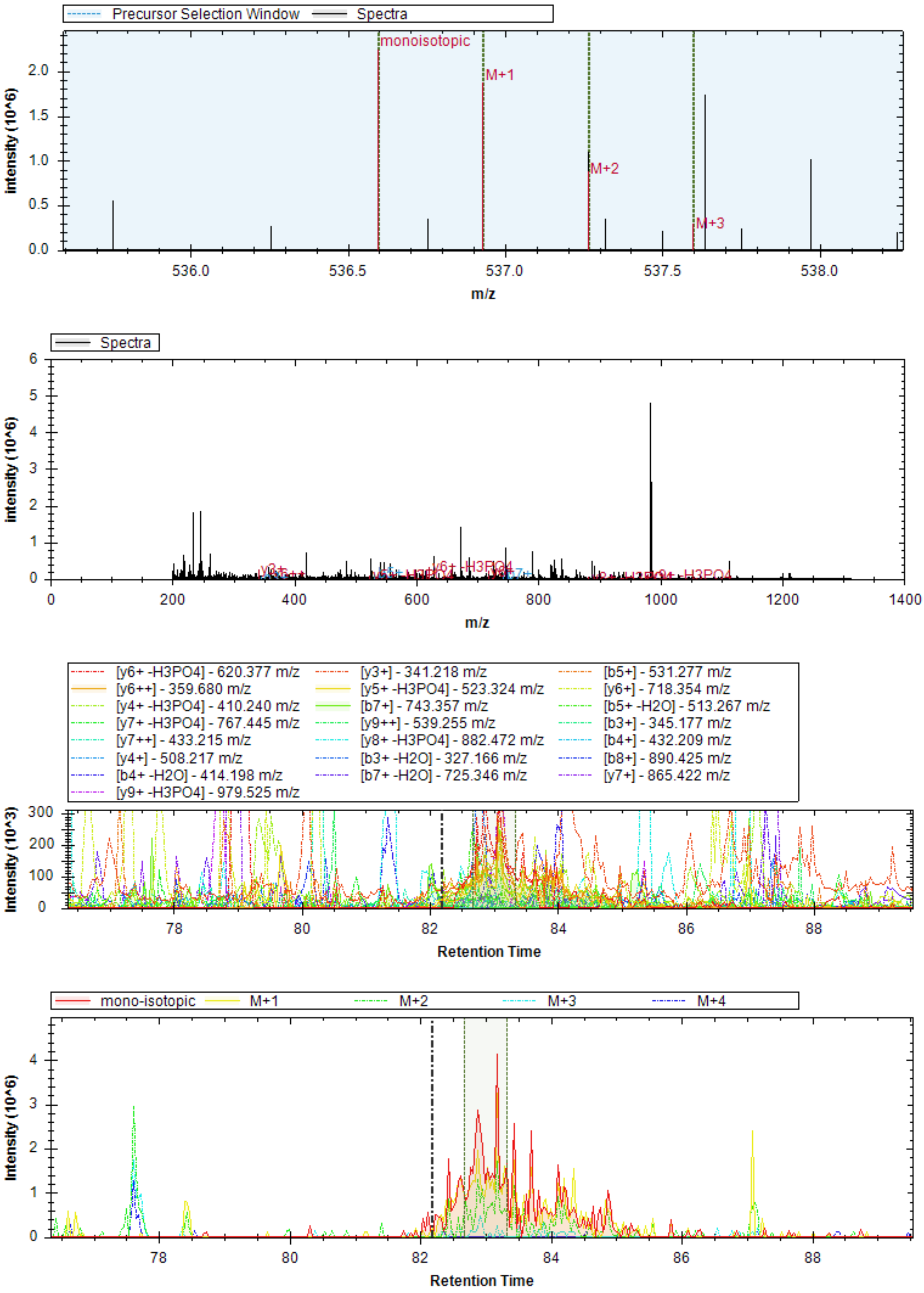

Q7TQD2 – _AANKT[+H+O3+P]PPKSPGDPAR_.3

Fig4_MP-DIA-barrel-cortex-p9-1_MHRM.htrms

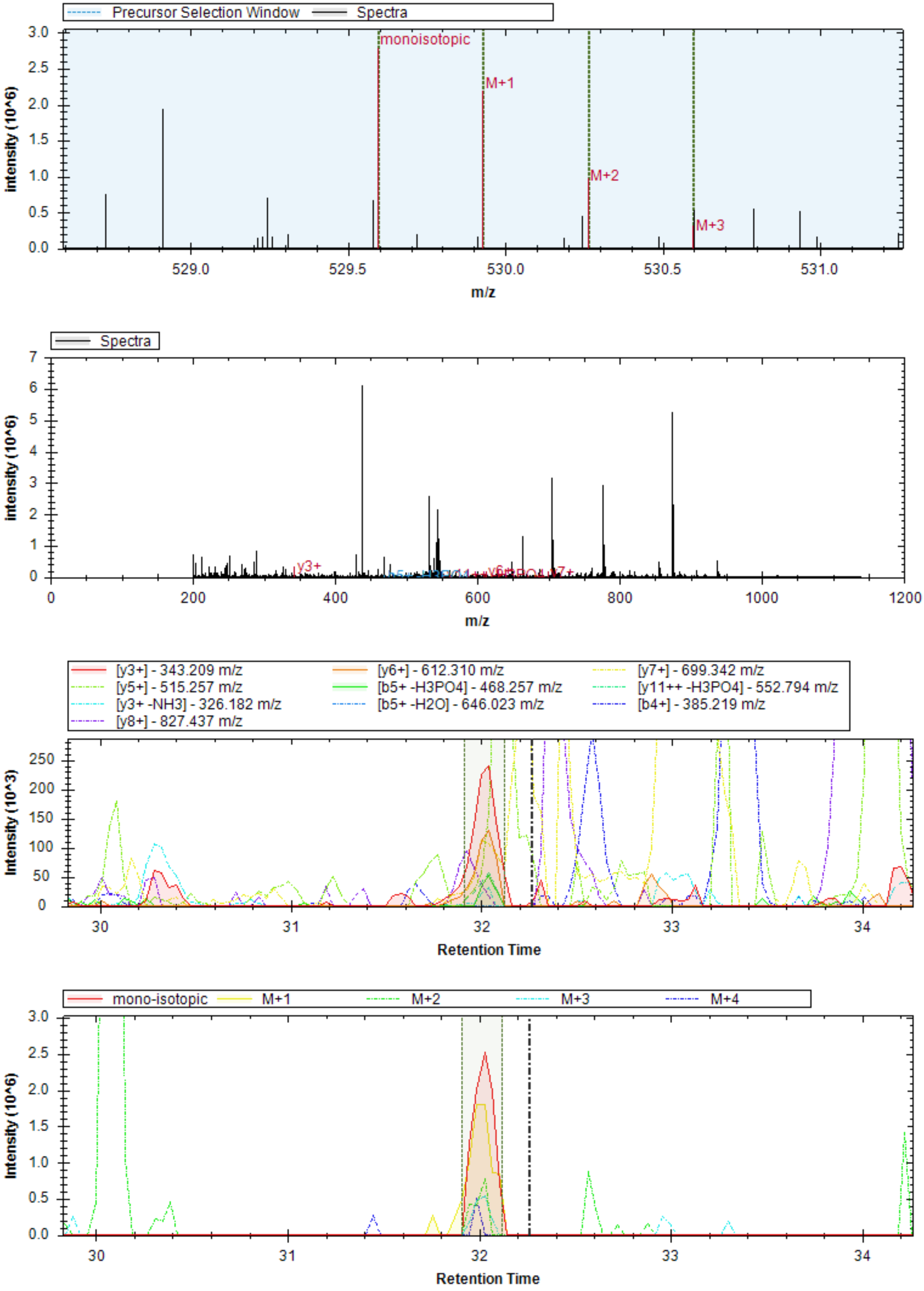

Q921N6;Q921N6-2 – _LIY[+H+O3+P]KPRQAAVTR_.4
Fig4_MP-DIA-barrel-cortex-p9-1_MHRM.htrms

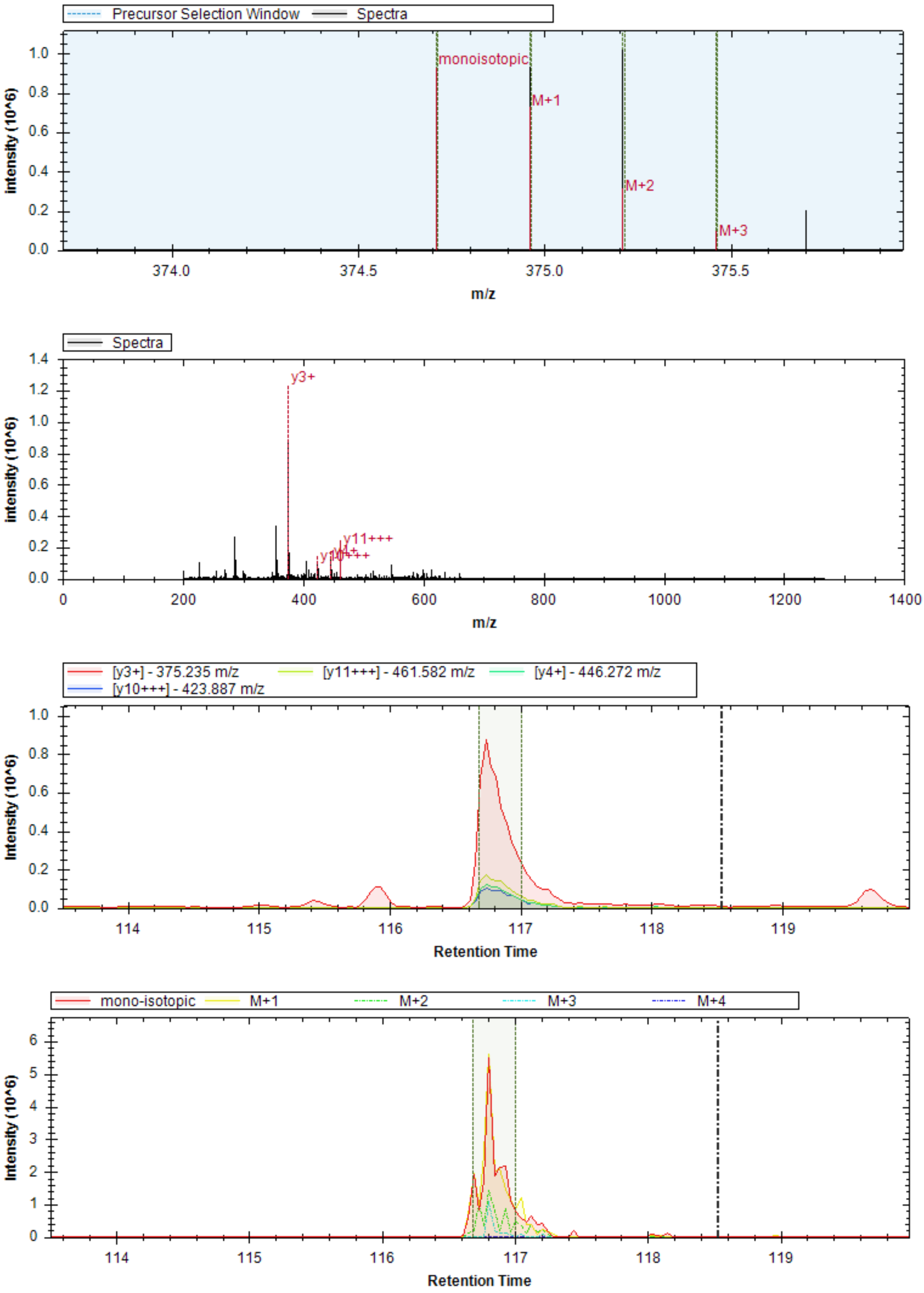

P10637 – _TTPSPKT[+H+O3+P]PPGSGEPPK_.3

Fig4_MP-DIA-barrel-cortex-p9-1_MHRM.htrms

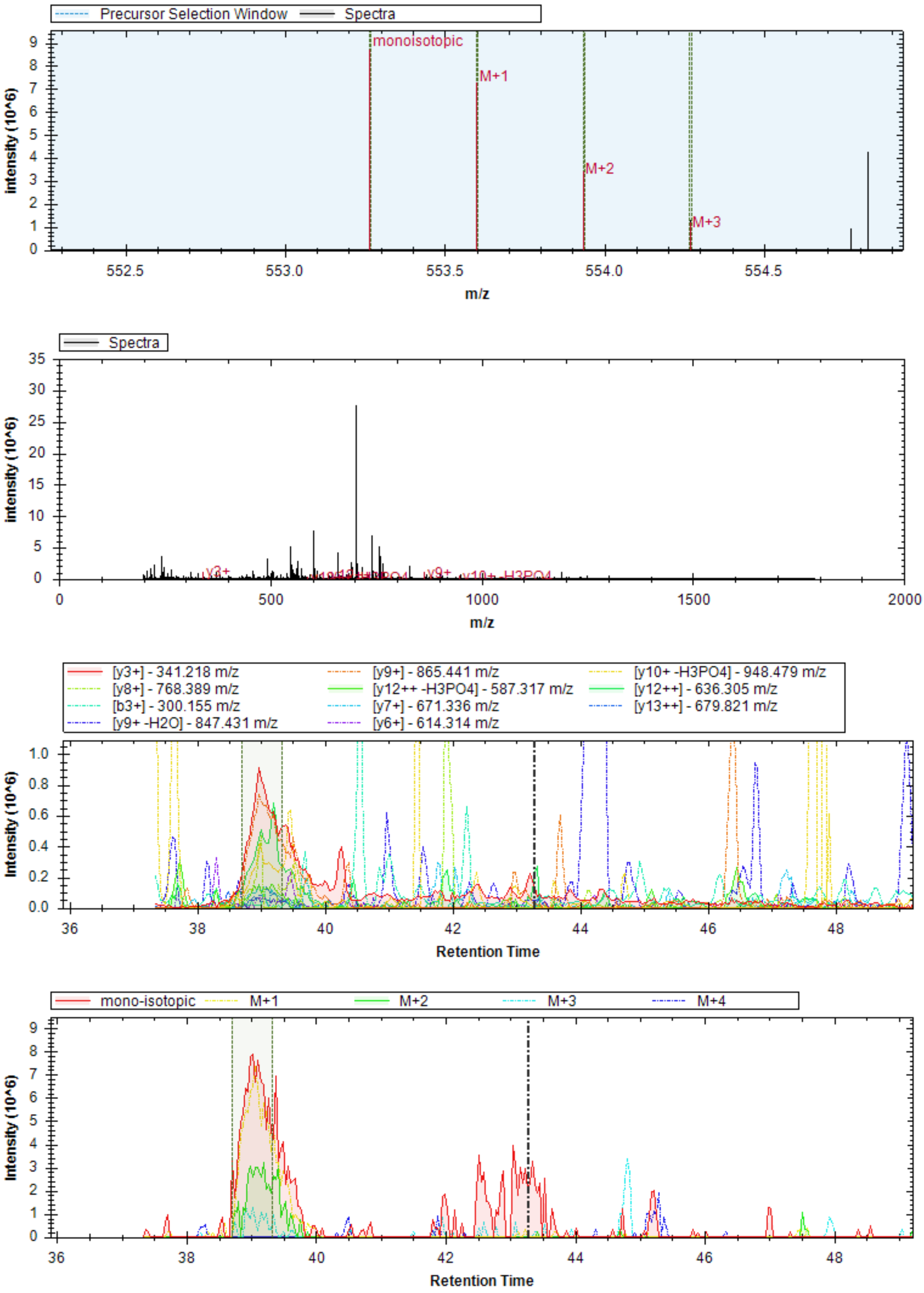

P20357 – _VAIIRT[+H+O3+P]PPKSPATPK_.3

Fig4_MP-DIA-barrel-cortex-p9-1_MHRM.htrms

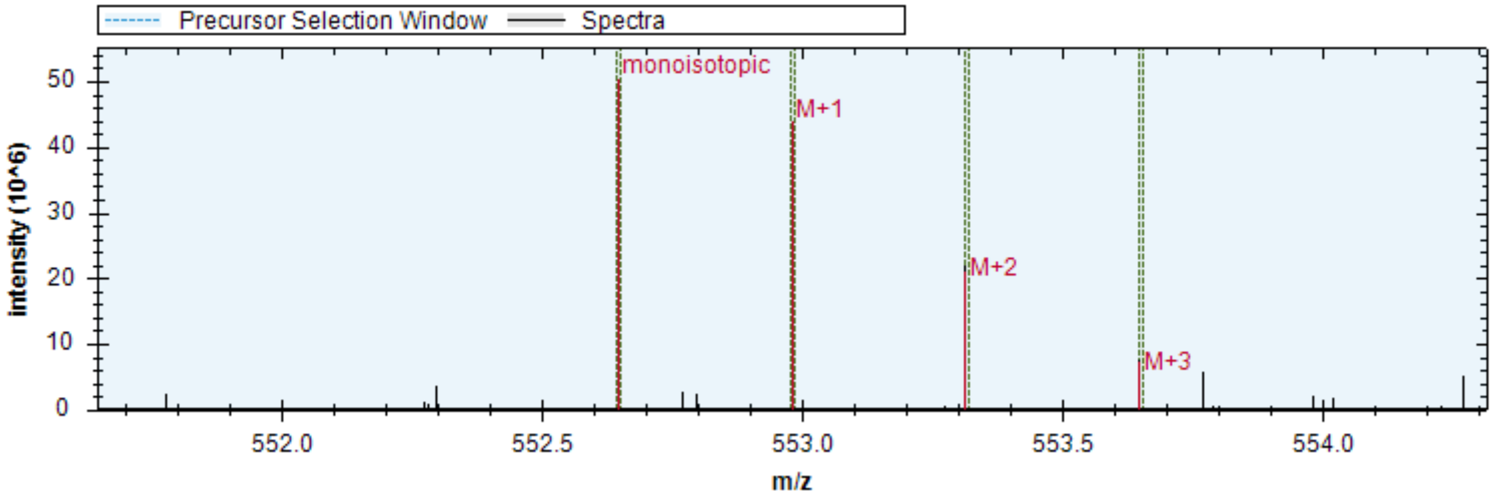

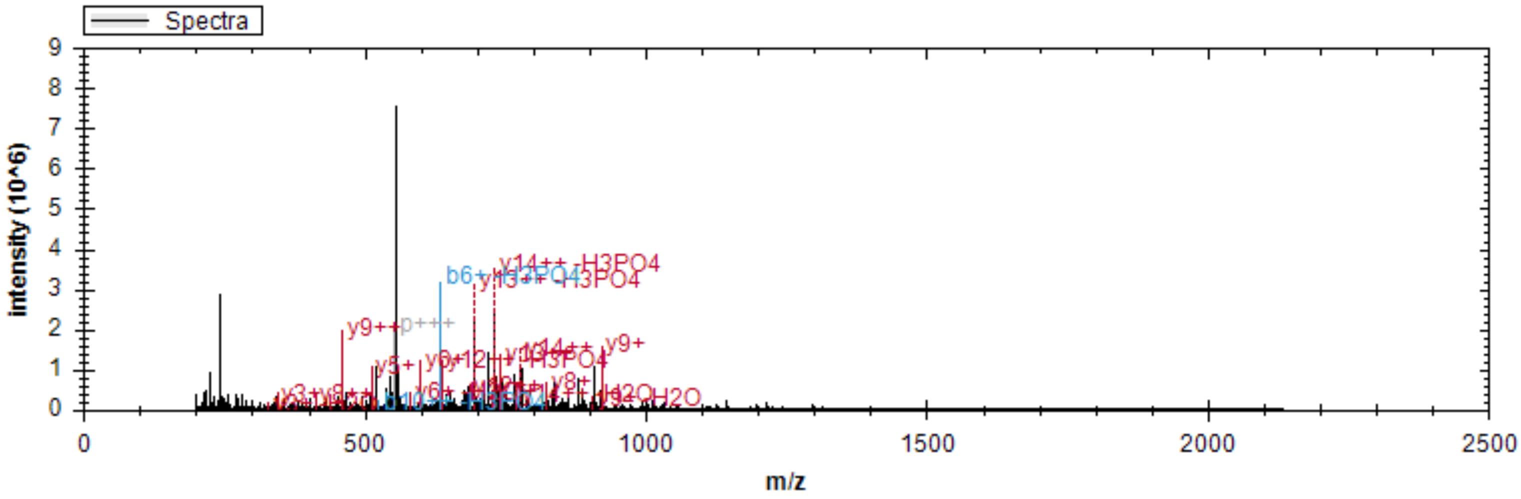

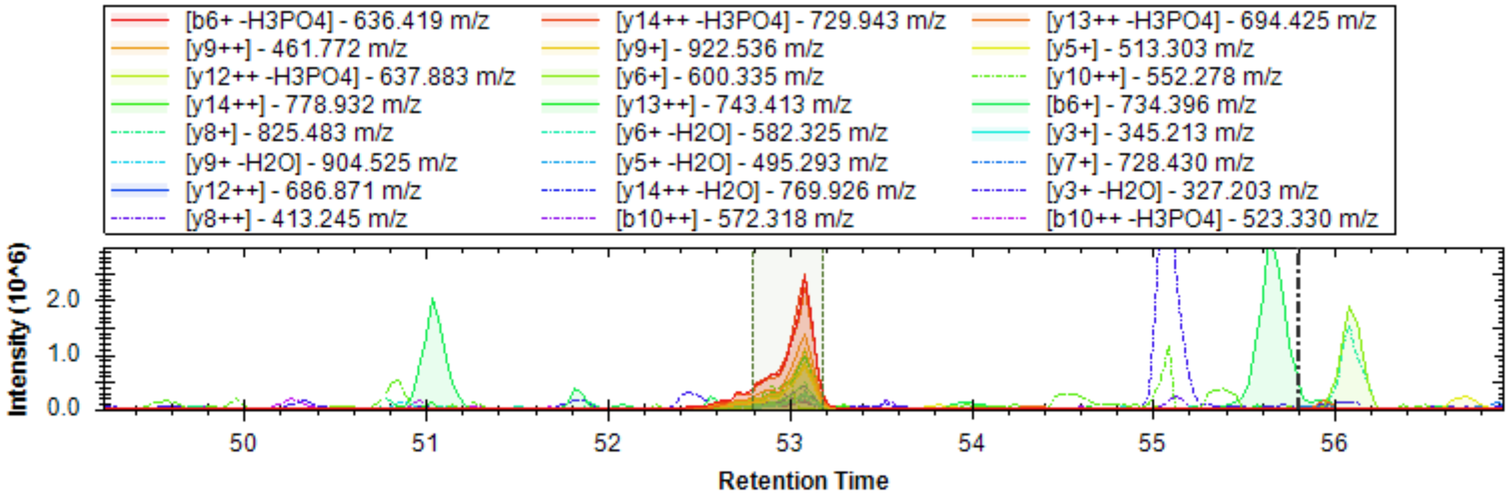

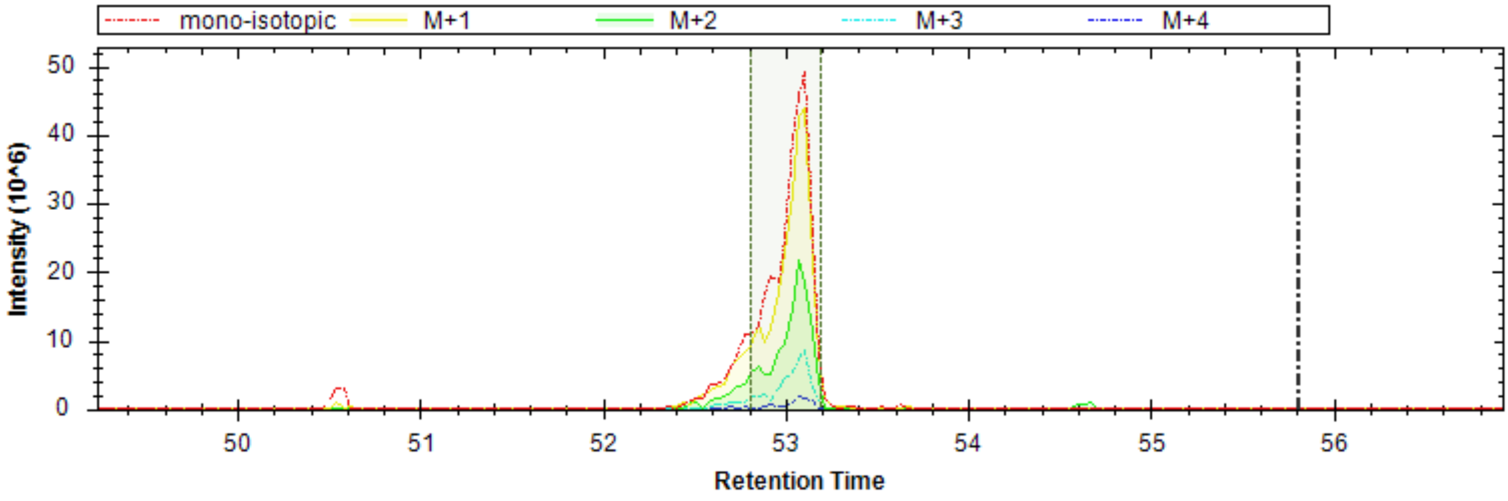

Q9CYT6 – _THTPSPTS[+H+O3+P]PK_.2

Fig4_MP-DIA-barrel-cortex-p9-1_MHRM.htrms

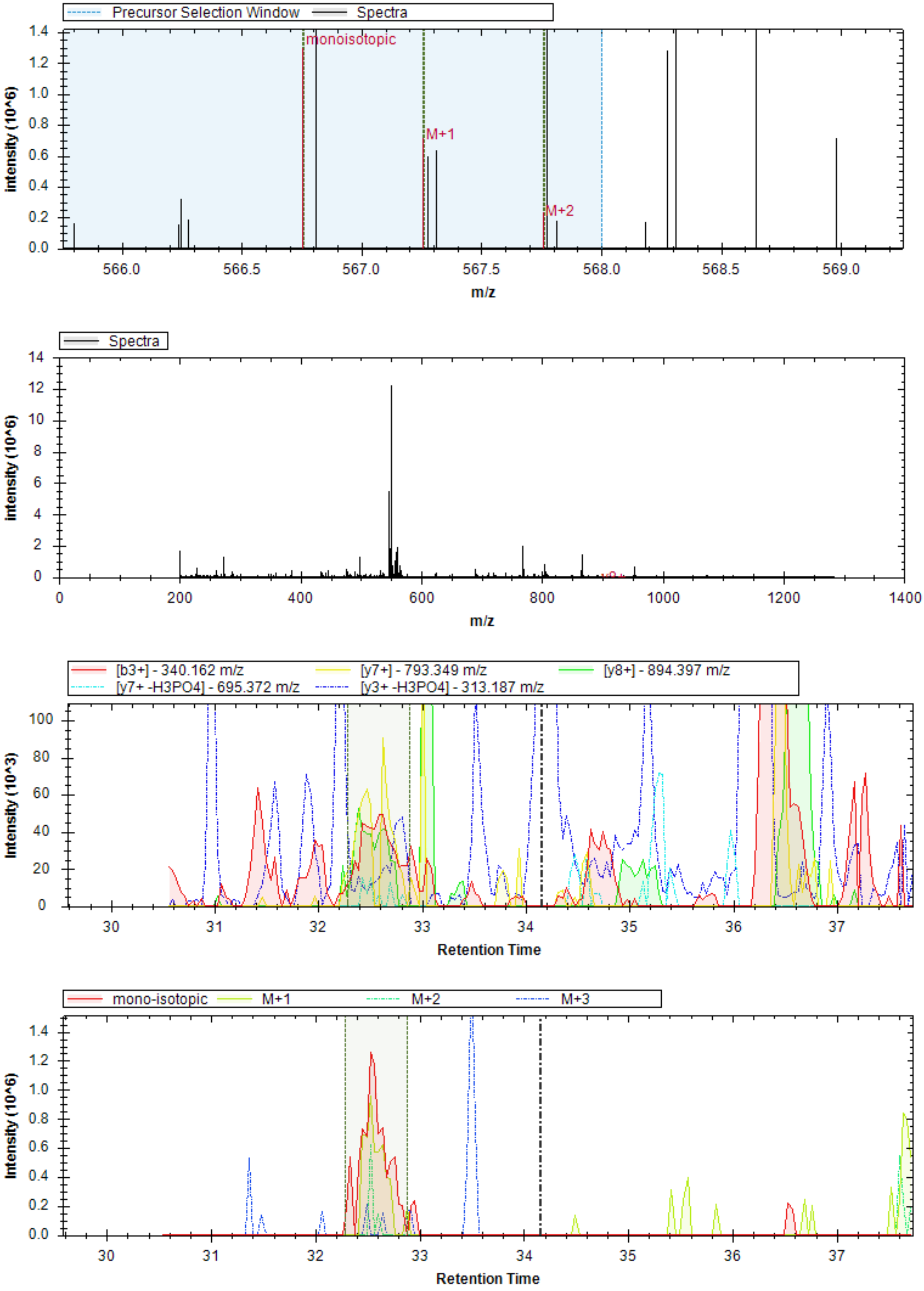

O88935 – _QAS[+H+O3+P]ISGPAPTK_.2

Fig4_MP-DIA-barrel-cortex-p9-1_MHRM.htrms

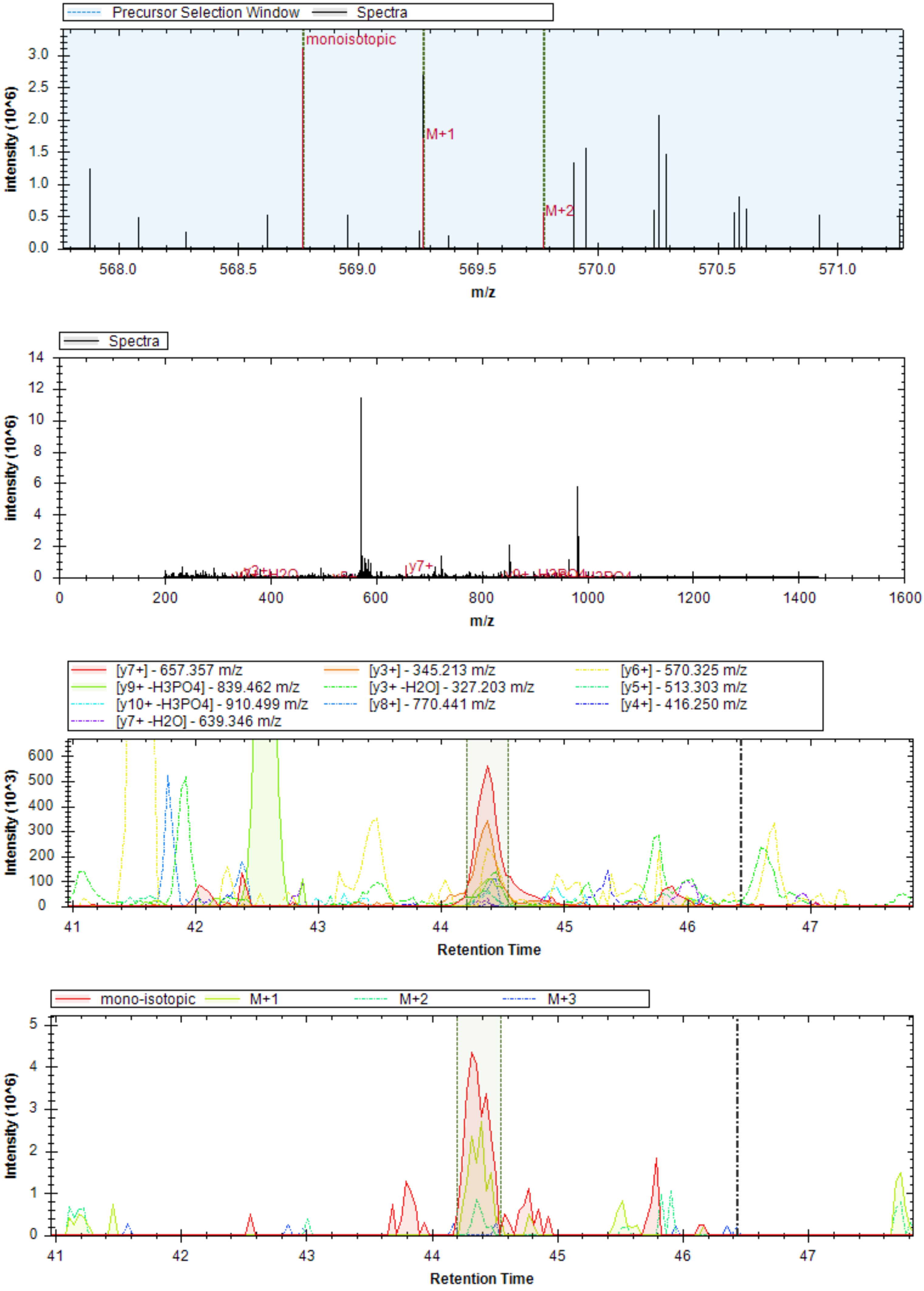

P27546-2 – _T[+H+O3+P]SPSKPSSAPALKPGPK_.3
Fig4_MP-DIA-barrel-cortex-p9-1_MHRM.htrms

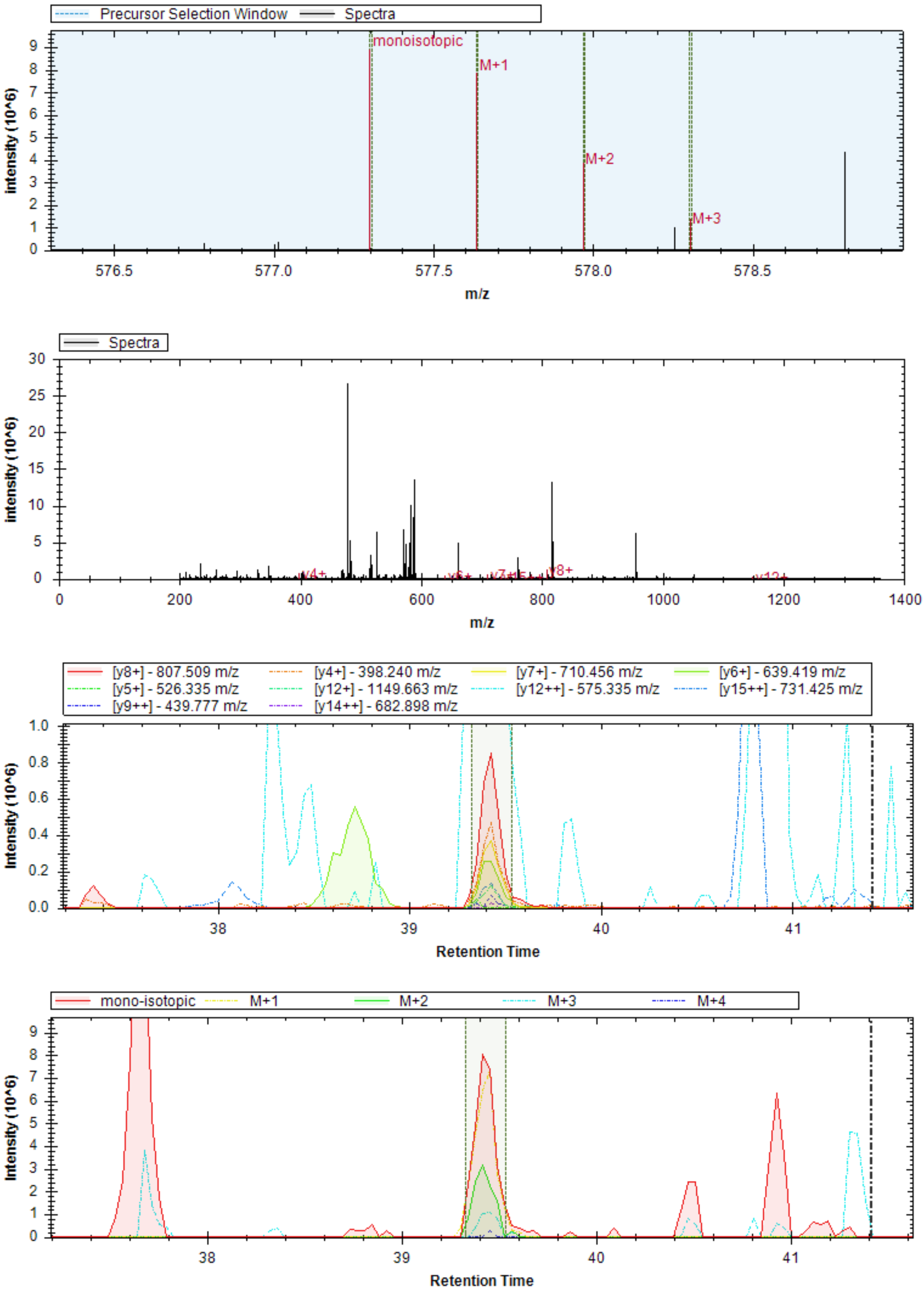

P28667 – _GEVAPKET[+H+O3+P]PK_.2
Fig4_MP-DIA-barrel-cortex-p9-1_MHRM.htrms

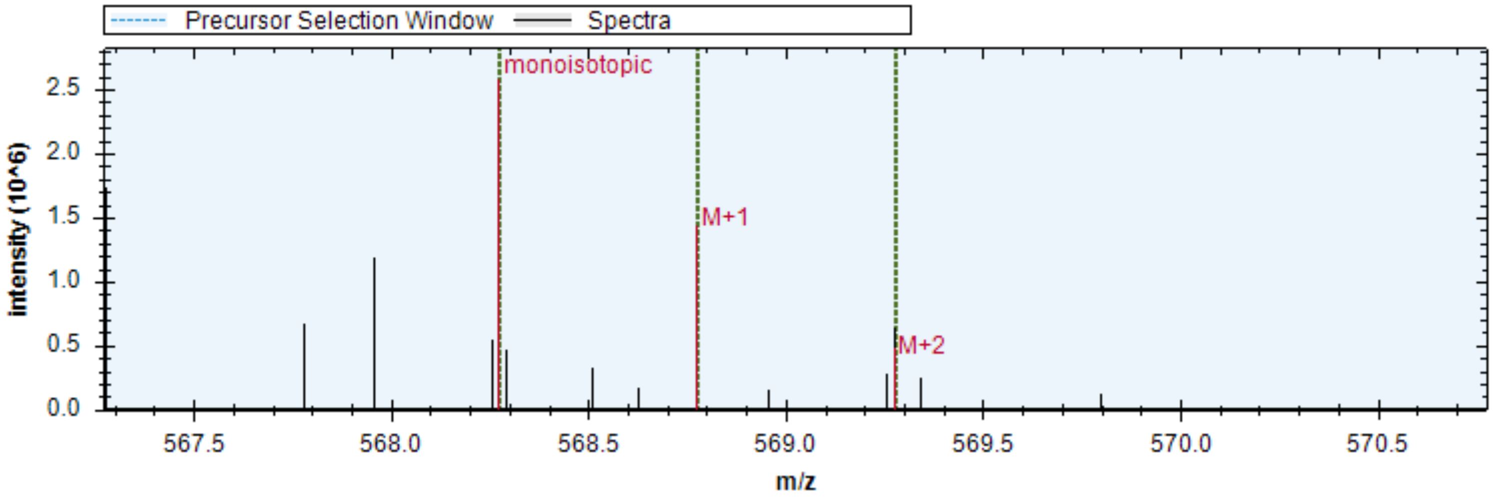

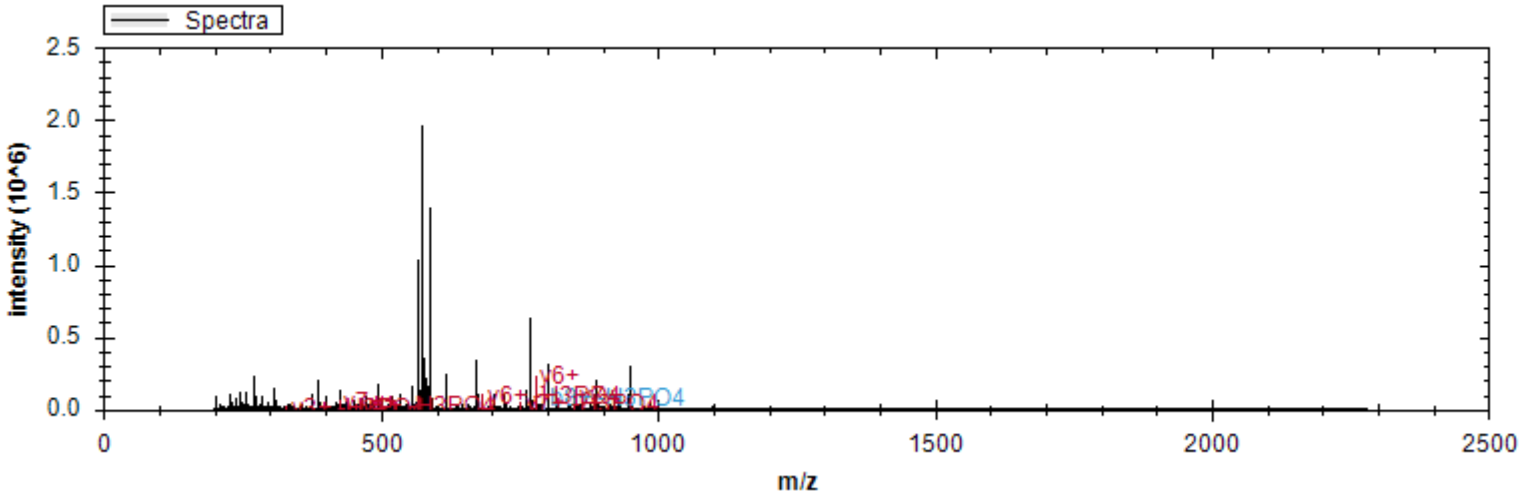

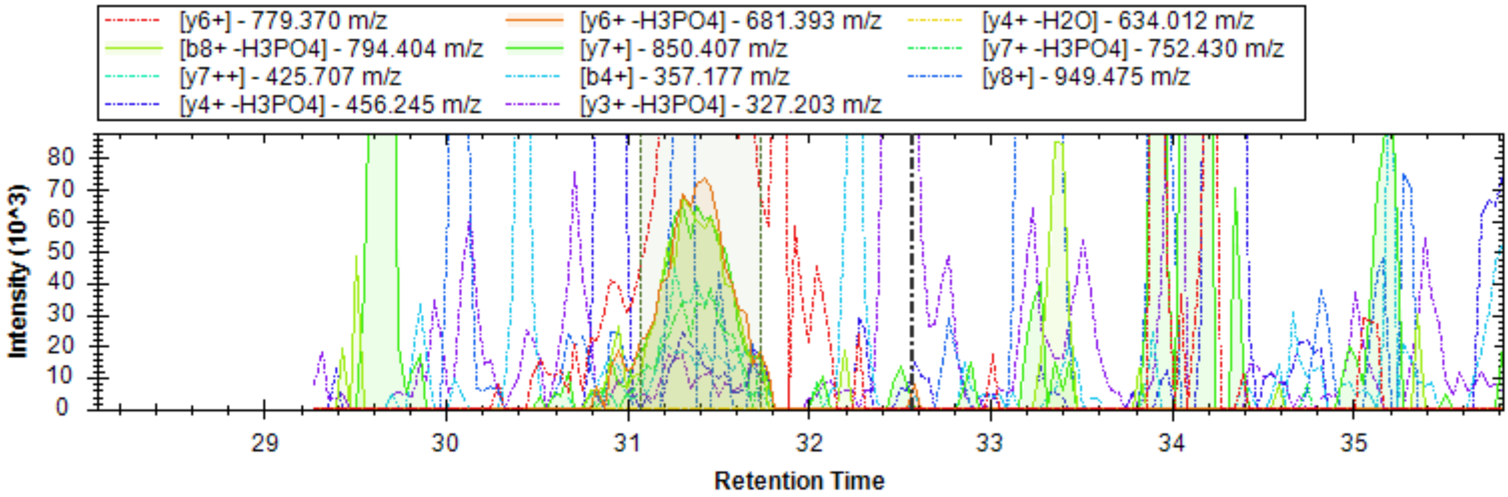

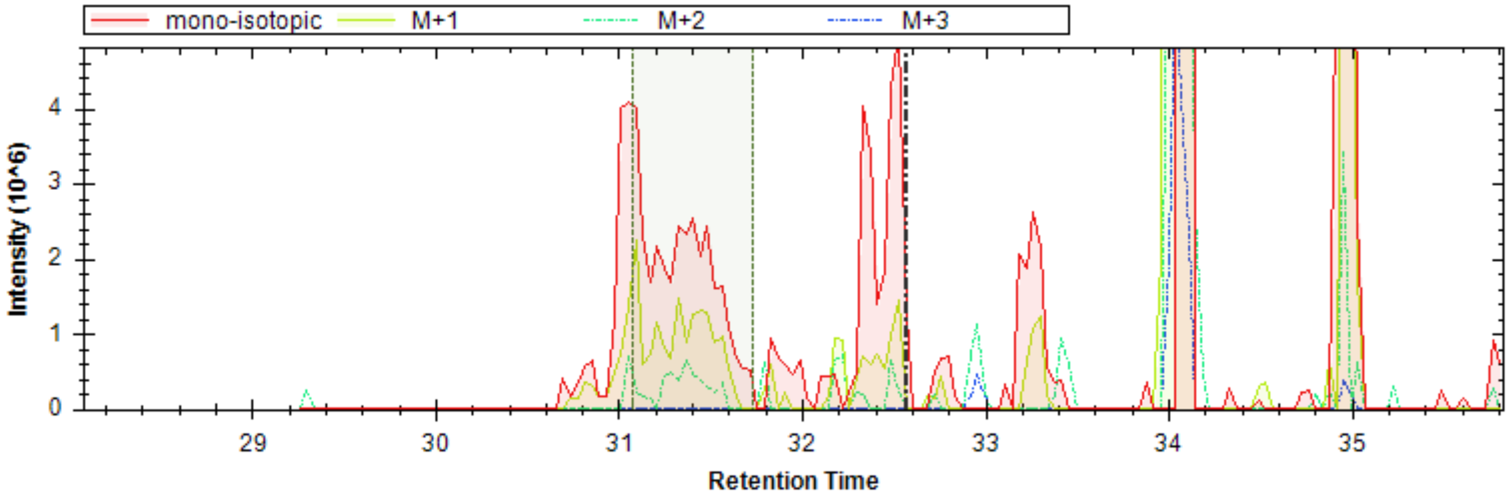

P97792 – _APQS[+H+O3+P]PTLAPAK_.2

Fig4_MP-DIA-barrel-cortex-p9-1_MHRM.htrms

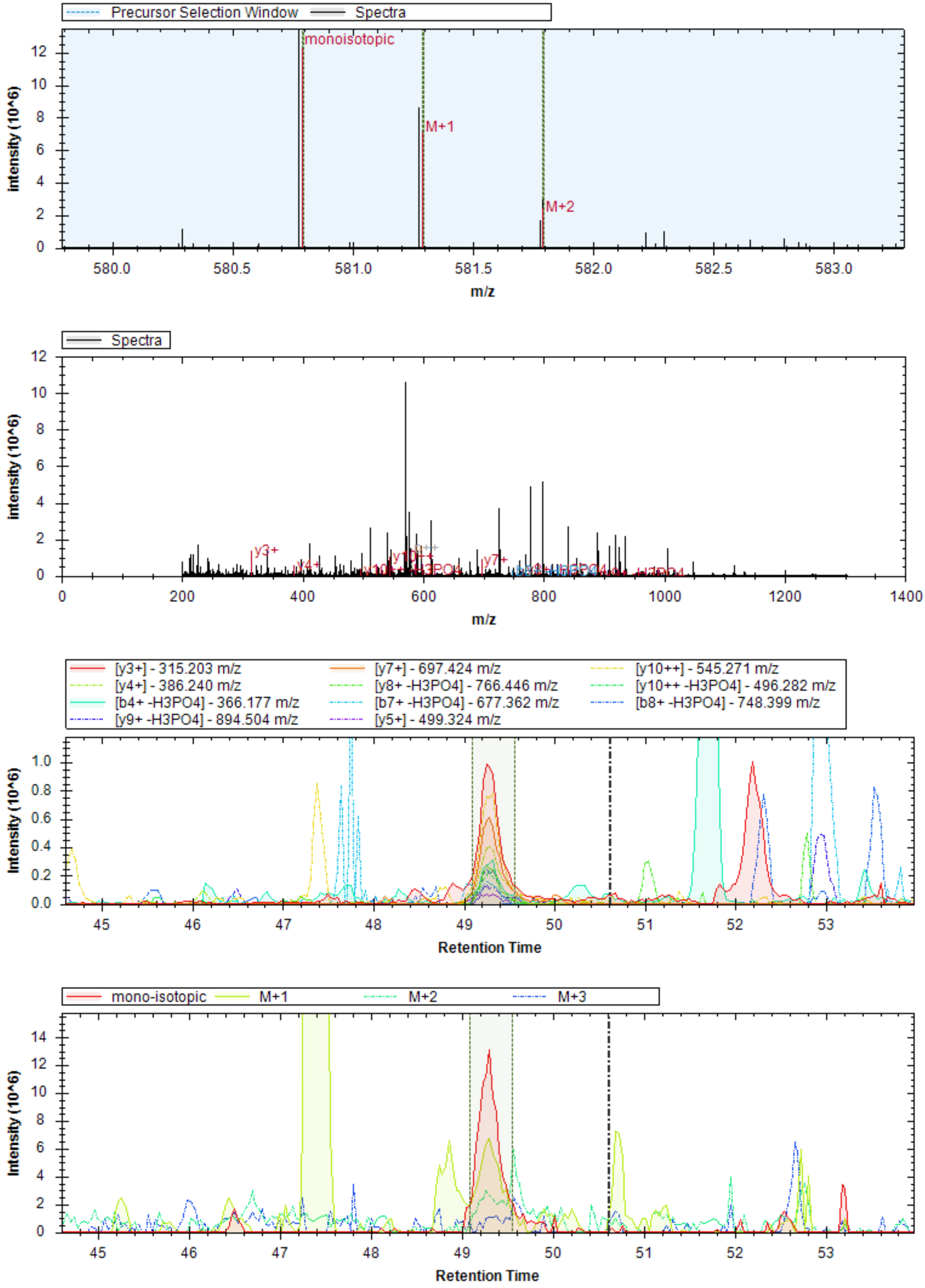

Q03172 – _VALSLLNS[+H+O3+P]KQK_.3
Fig4_MP-DIA-barrel-cortex-p9-1_MHRM.htrms

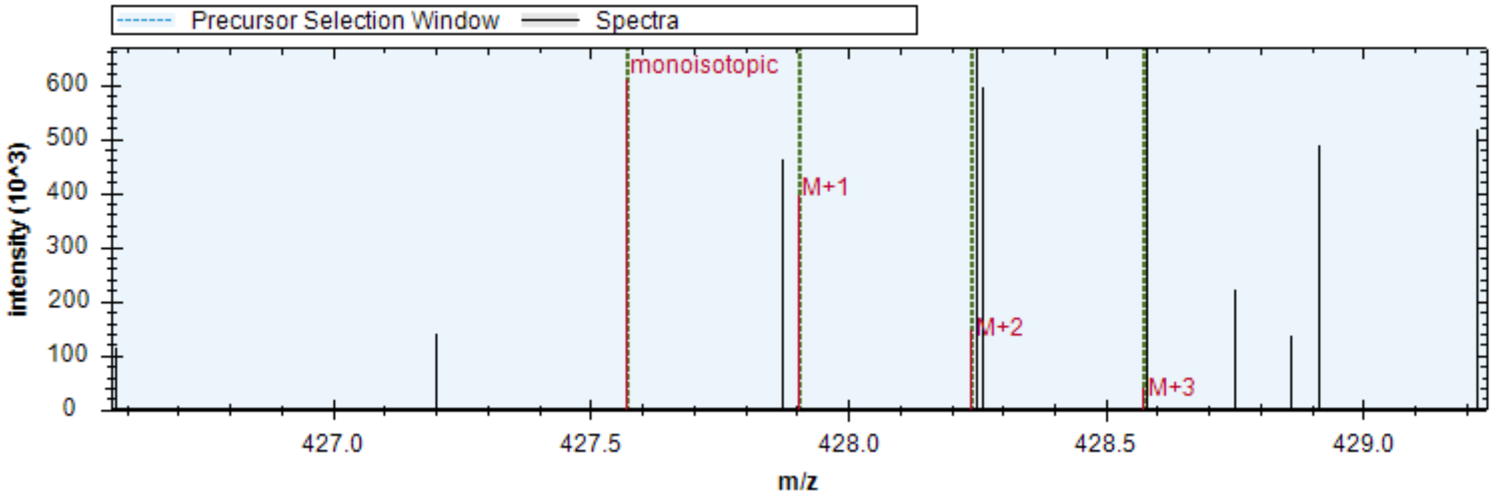

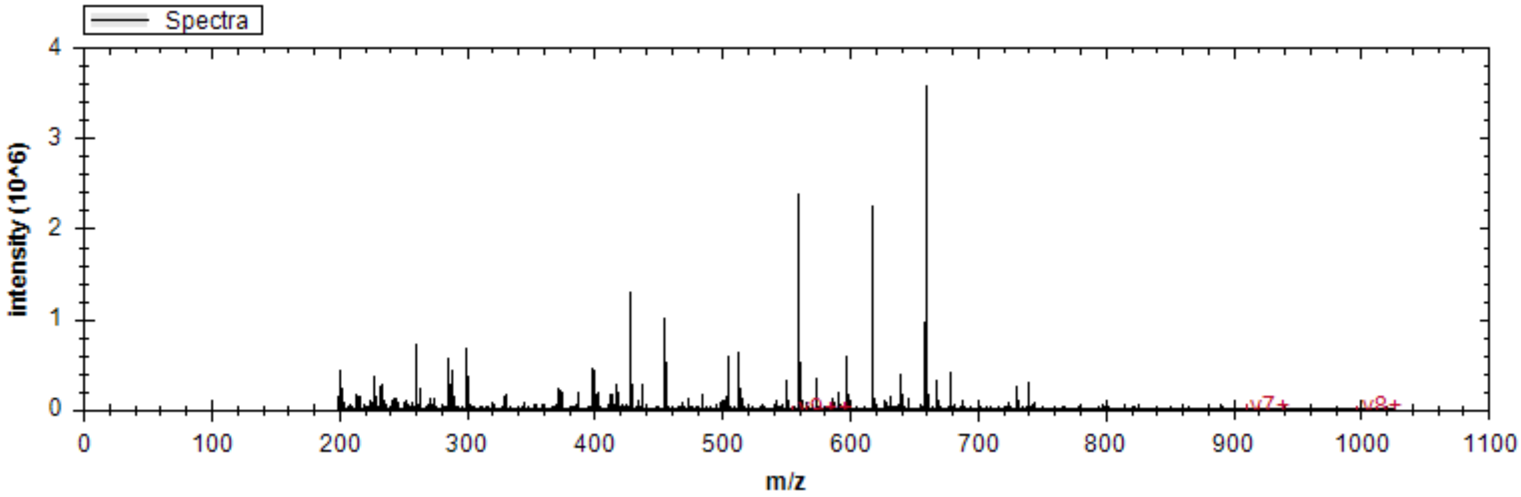

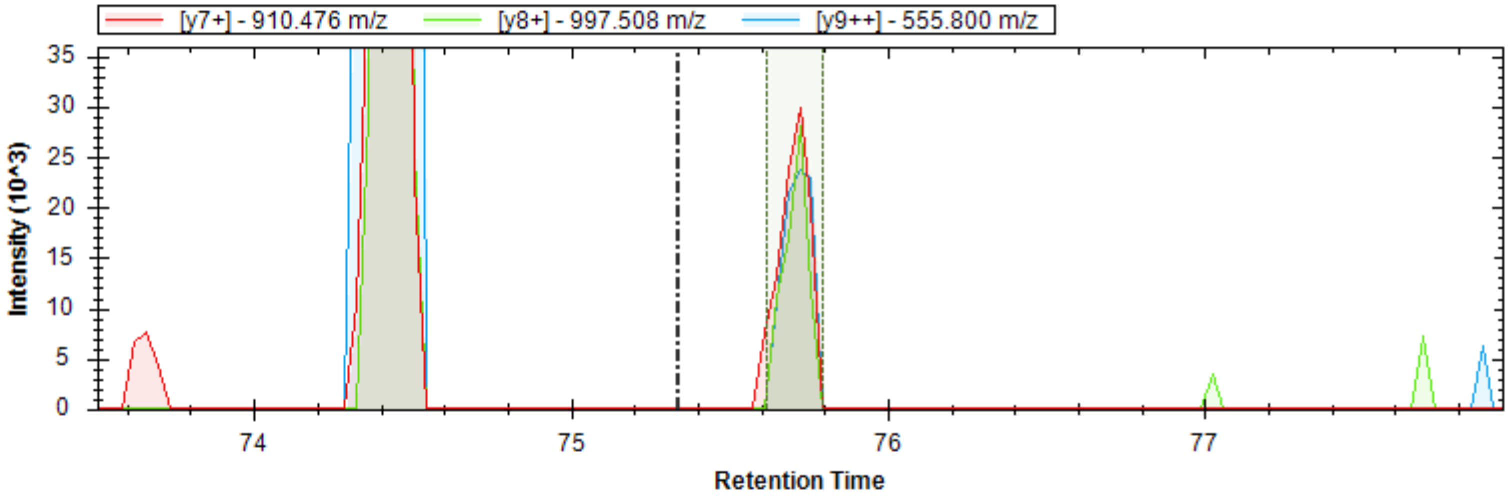

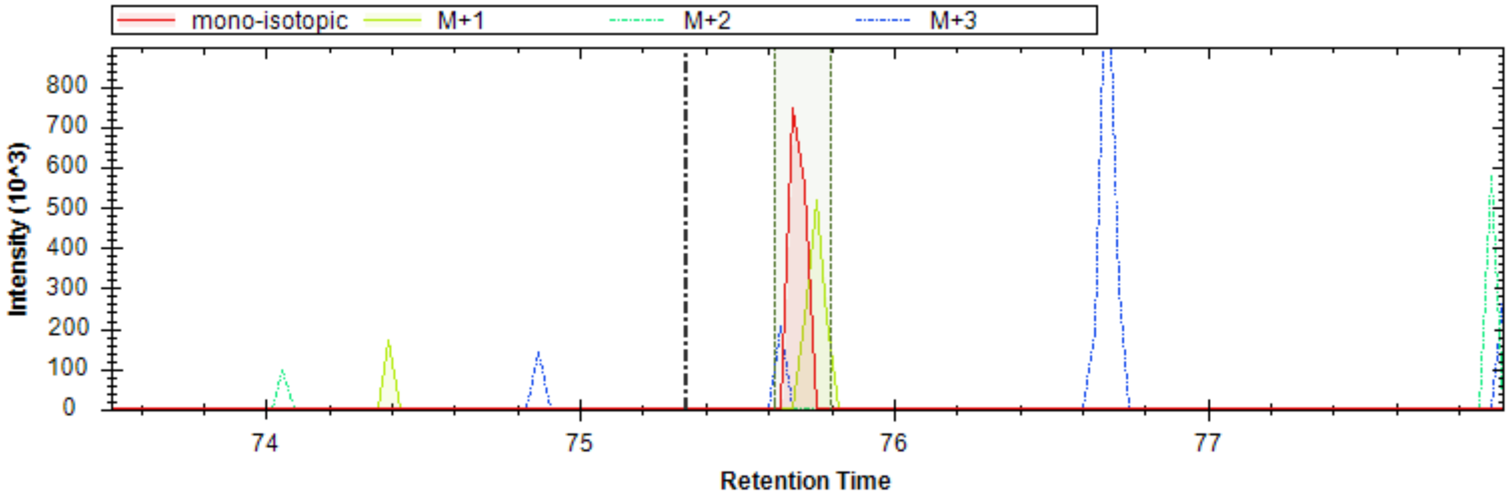

Q64012-2 – _GRLS[+H+O3+P]PVPVPR_.2
Fig4_MP-DIA-barrel-cortex-p9-1_MHRM.htrms

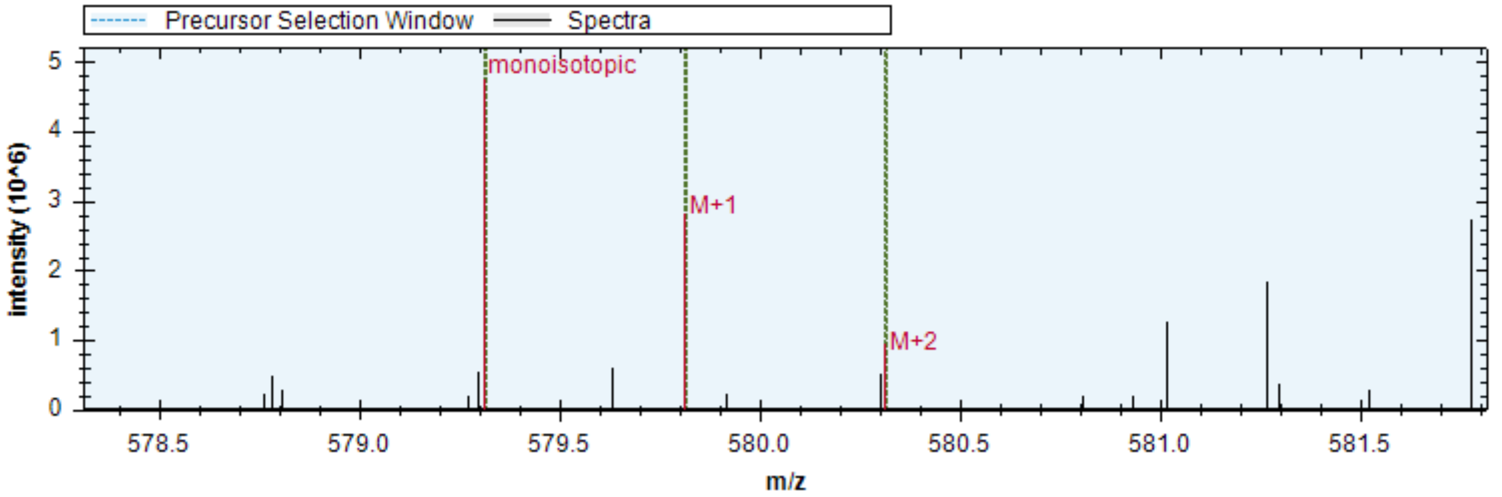

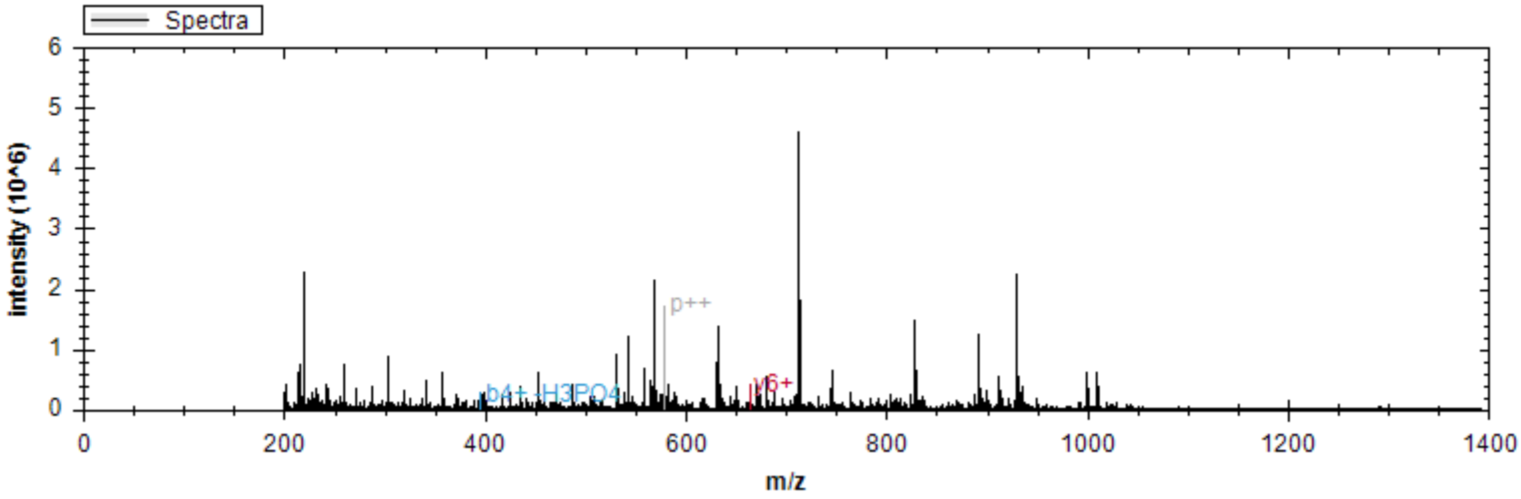

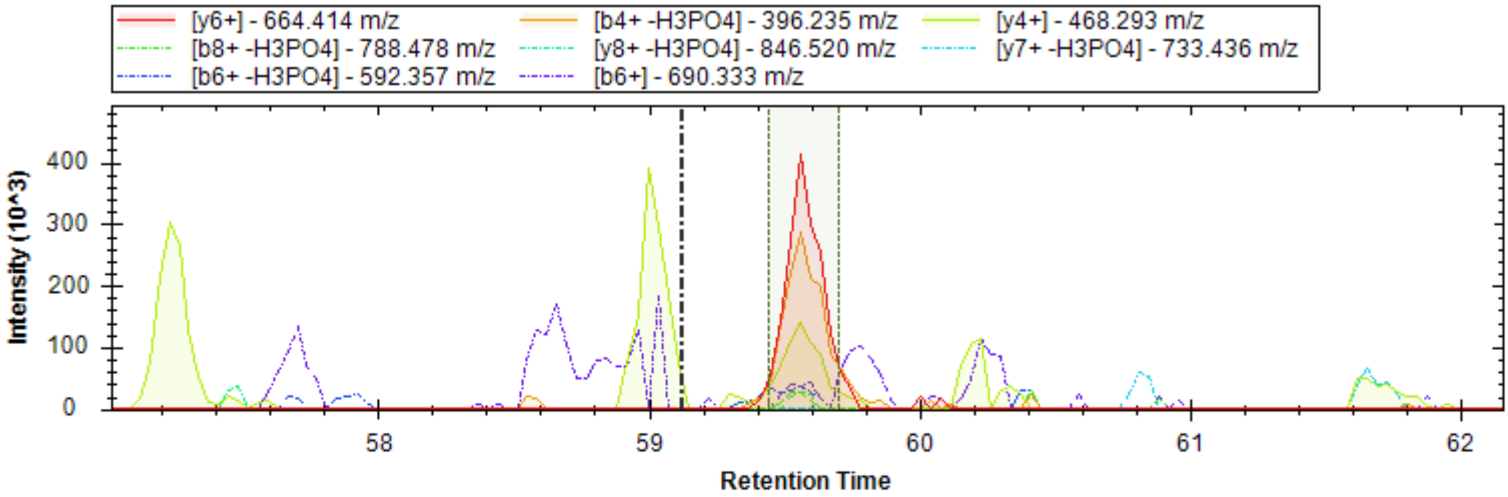

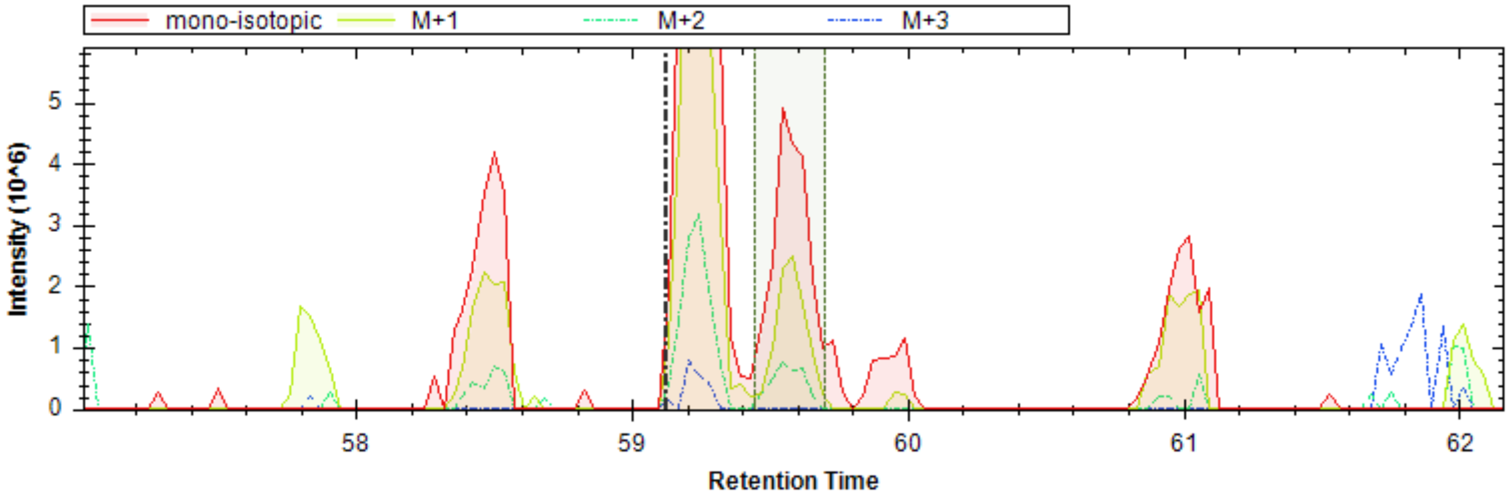

Q8BFU3-4 – _VAS[+H+O3+P]STQVRGASGRELR_.3
Fig4_MP-DIA-barrel-cortex-p9-1_MHRM.htrms

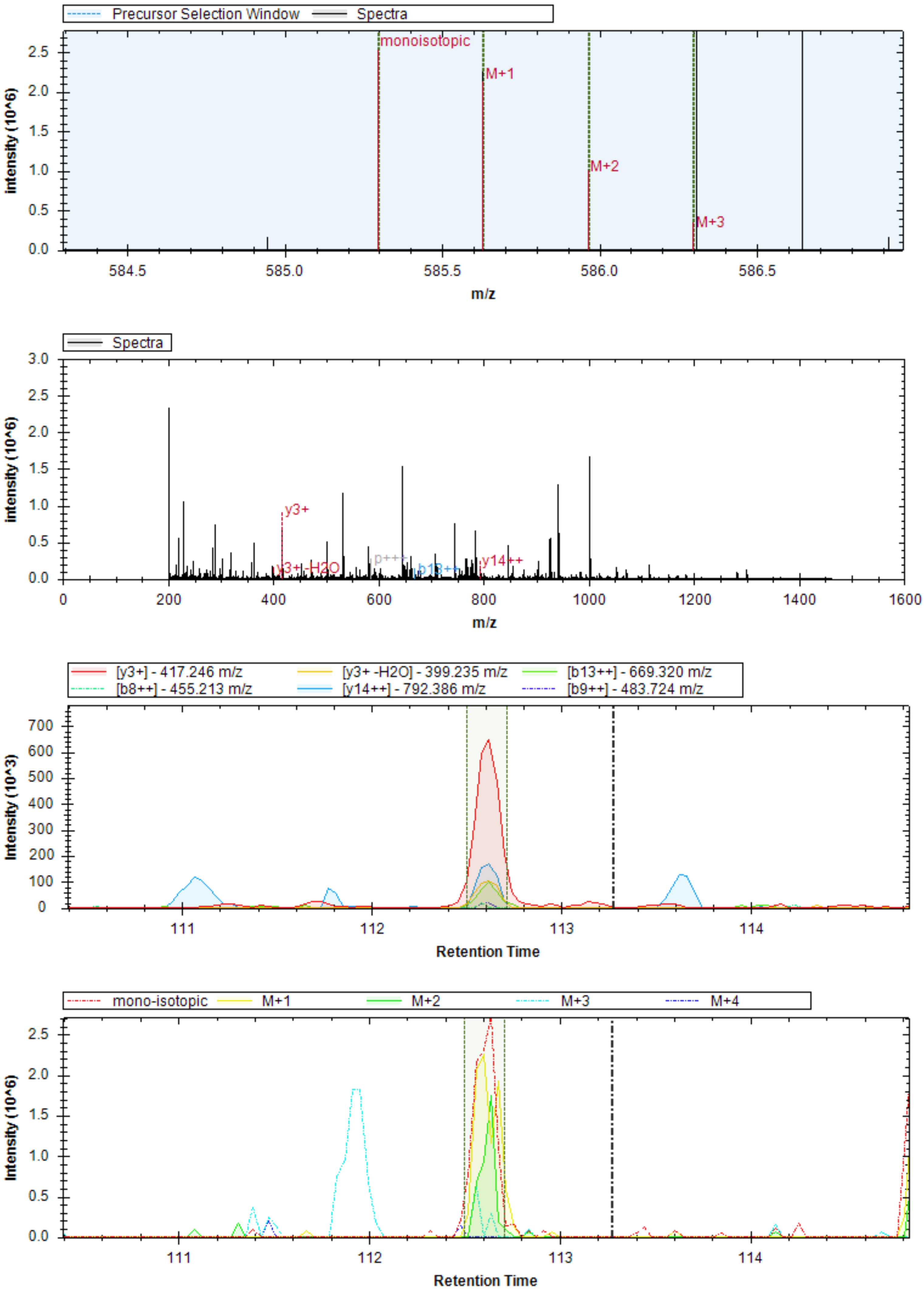

Q8BTI8 – _RVPS[+H+O3+P]PTPVPK_.2
Fig4_MP-DIA-barrel-cortex-p9-1_MHRM.htrms

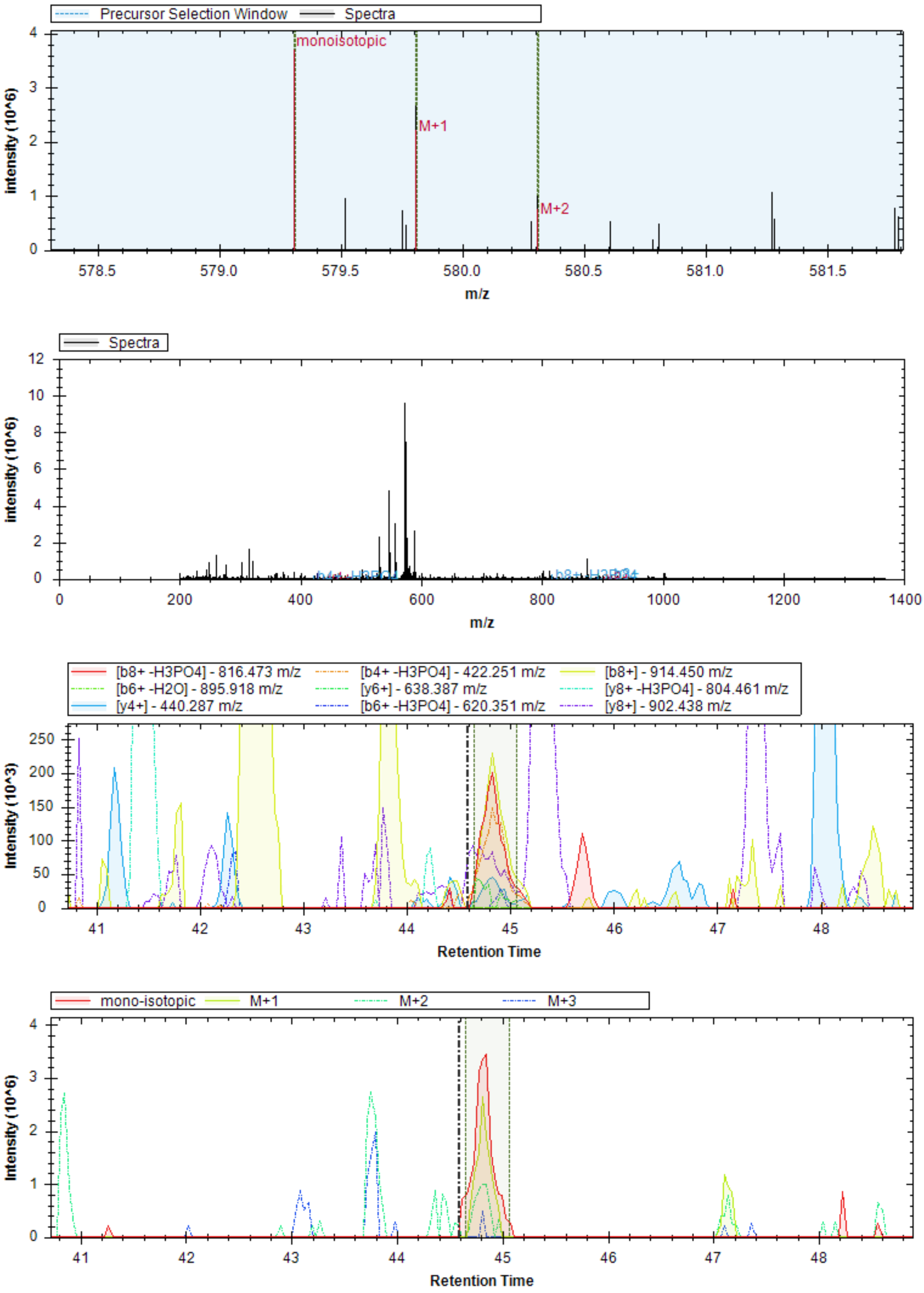

Q9CYT6 – _AQGQIRS[+H+O3+P]PTK_.2
Fig4_MP-DIA-barrel-cortex-p9-1_MHRM.htrms

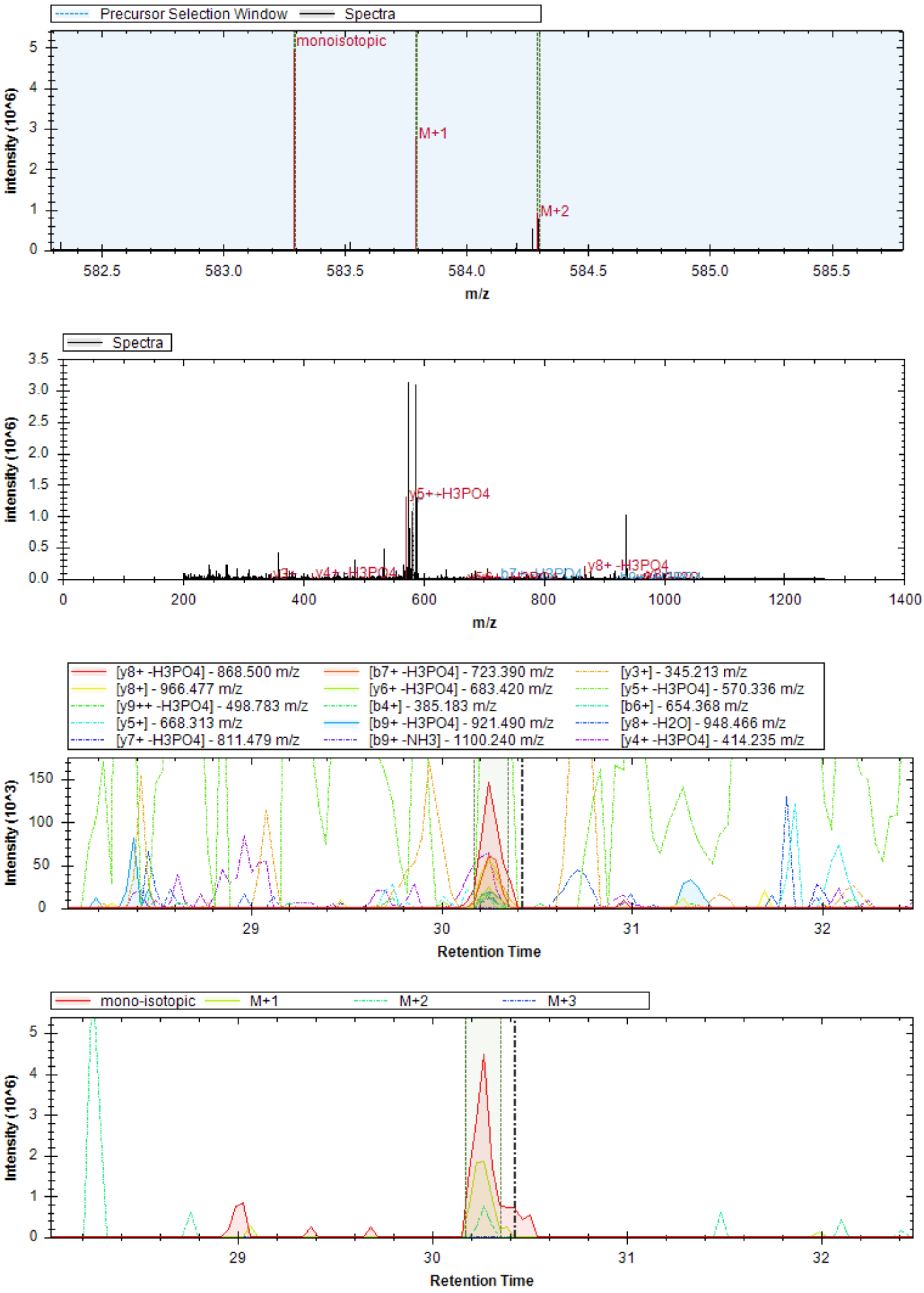

P20357 – _APHWTSASLTEAAAHPHS[+H+O3+P]PEMK_.4
Fig4_MP-DIA-barrel-cortex-p9-1_MHRM.htrms

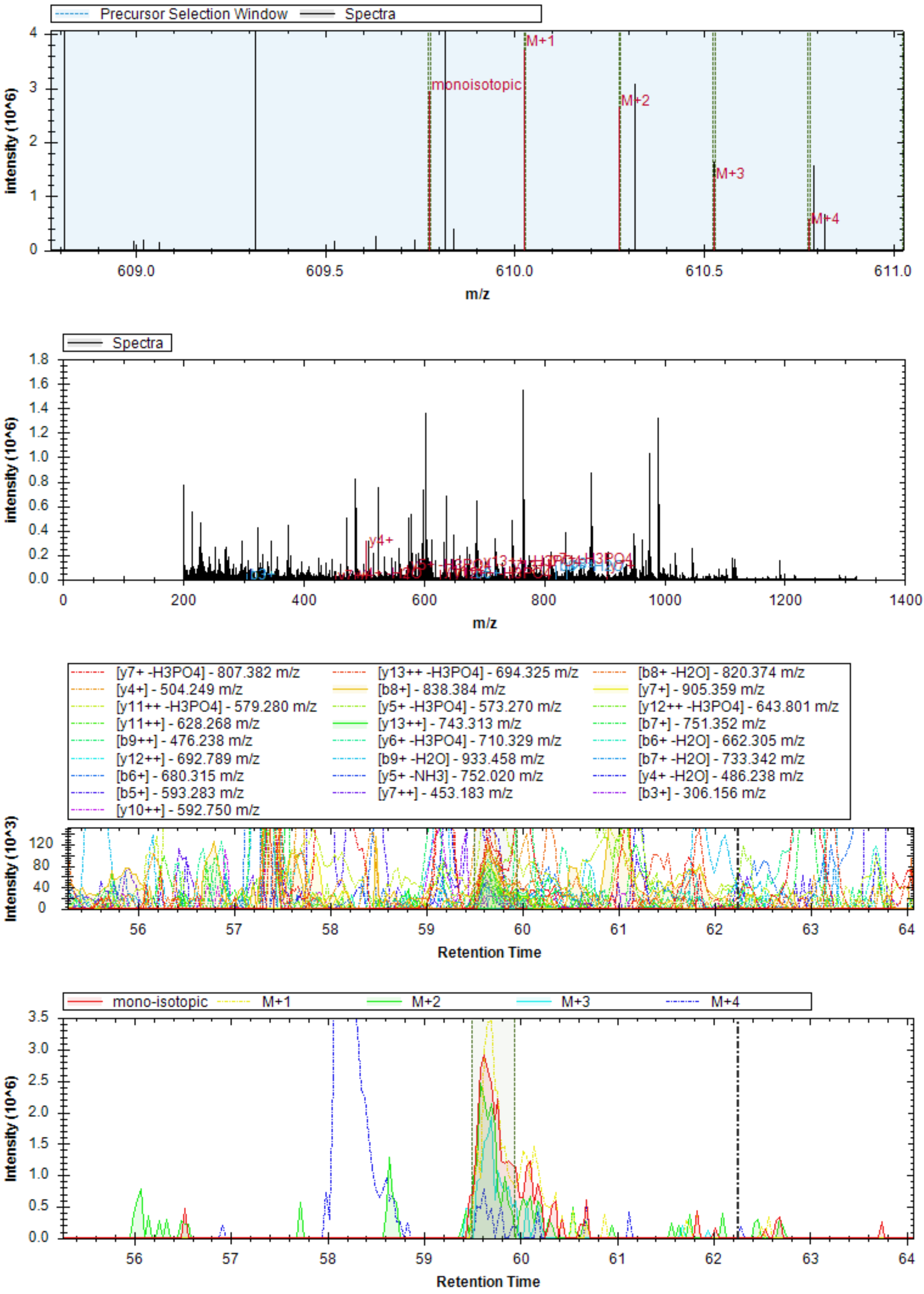

P26645 – _AEDGAAPSPSS[+H+O3+P]ETPKKK_.3

Fig4_MP-DIA-barrel-cortex-p9-1_MHRM.htrms

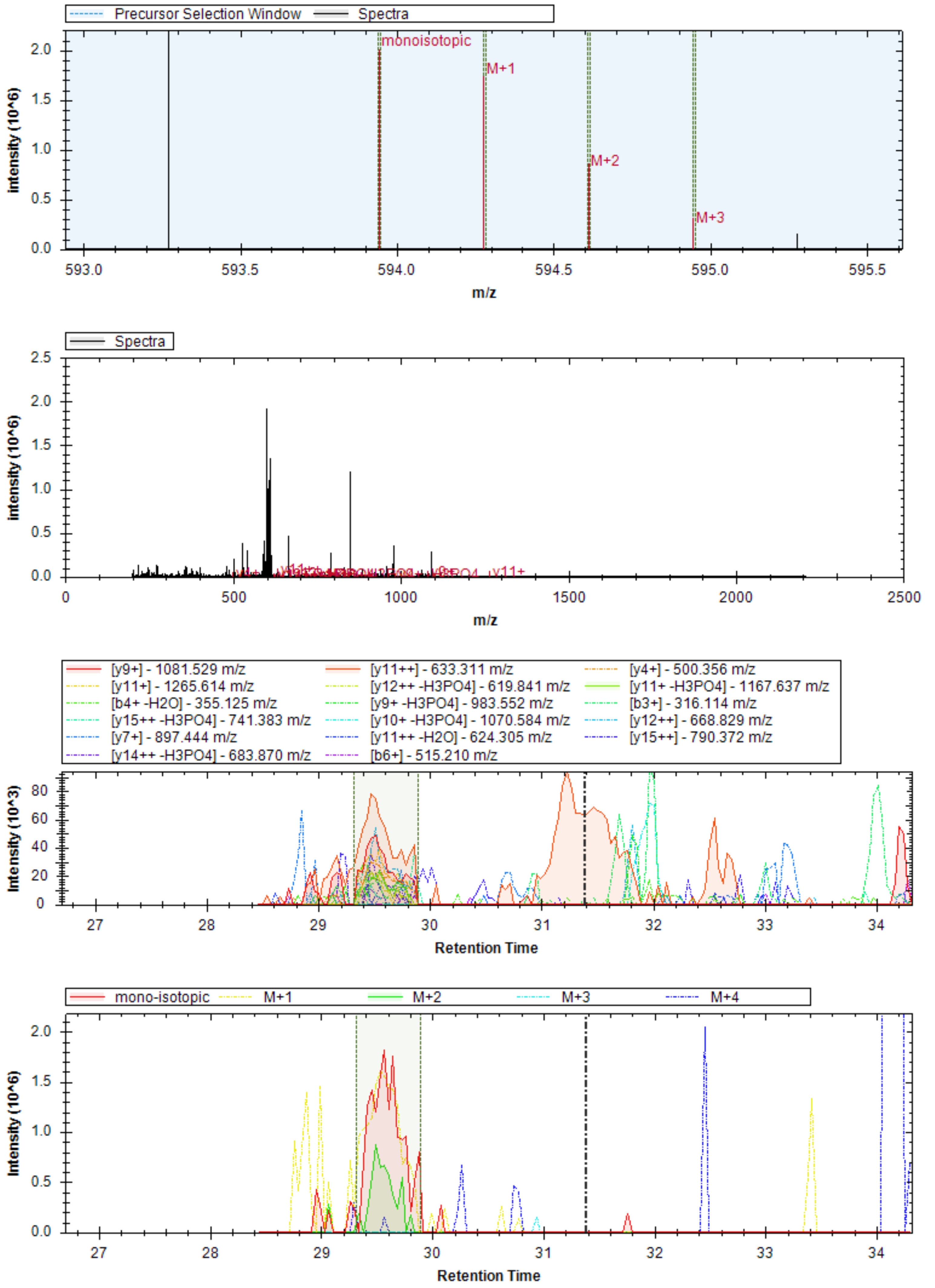

Q62261 – _RPPS[+H+O3+P]PDPNTK_.2
Fig4_MP-DIA-barrel-cortex-p9-1_MHRM.htrms

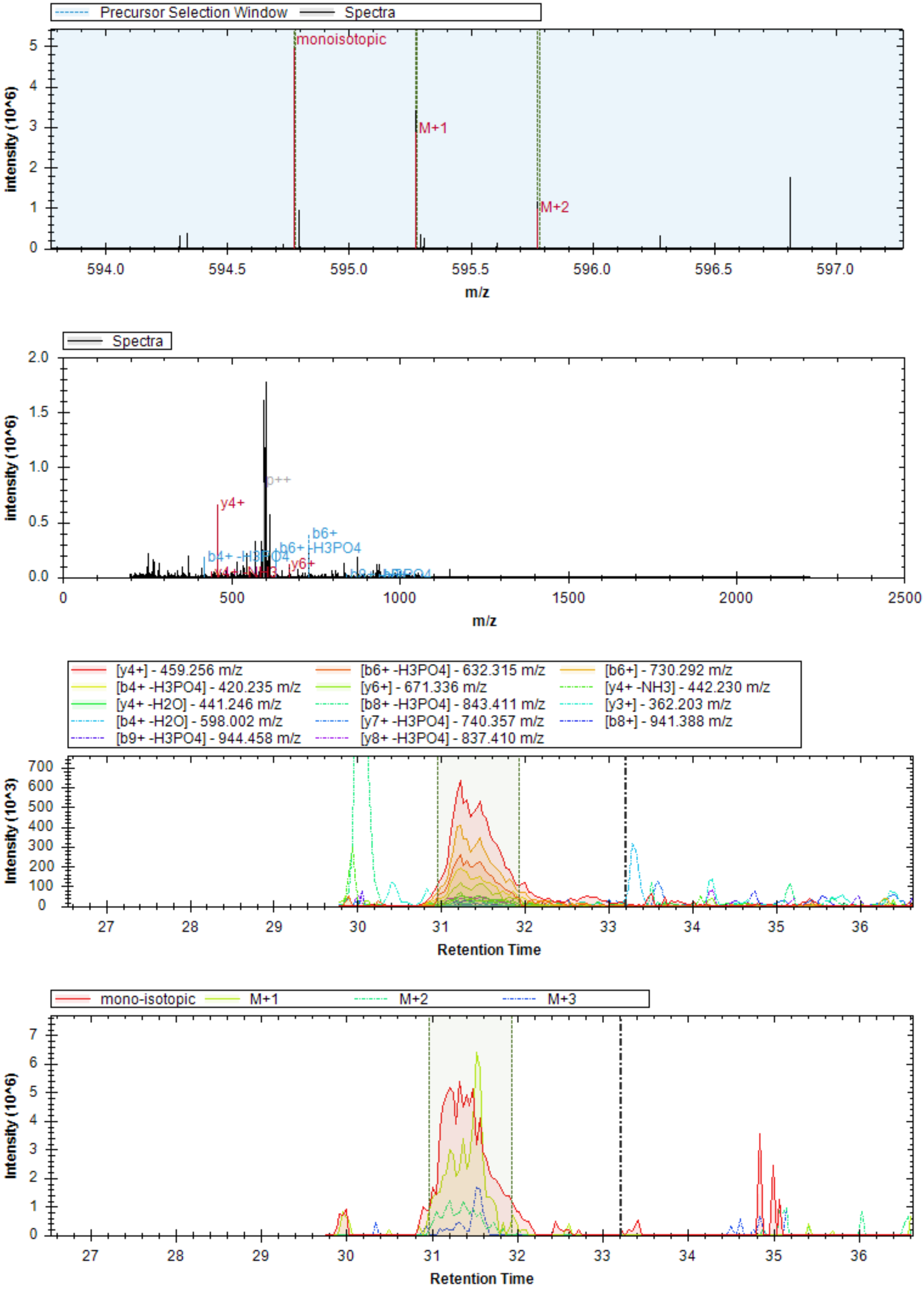

Q80VN0 – _ELTAQIT[+H+O3+P]SIK_.2

Fig4_MP-DIA-barrel-cortex-p9-1_MHRM.htrms

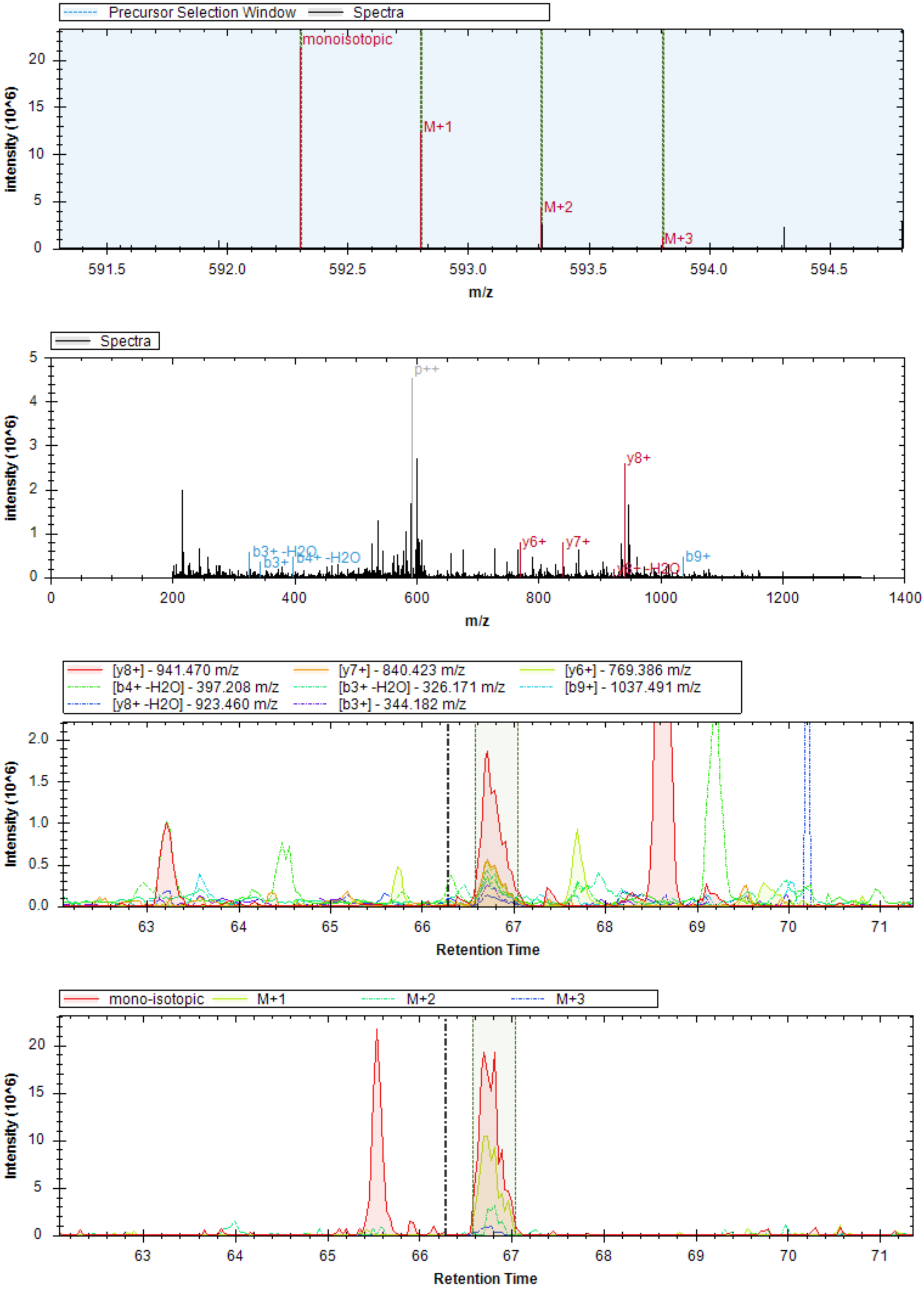

Q99104 – _AIS[+H+O3+P]PTSATSSGR_.2

Fig4_MP-DIA-barrel-cortex-p9-1_MHRM.htrms

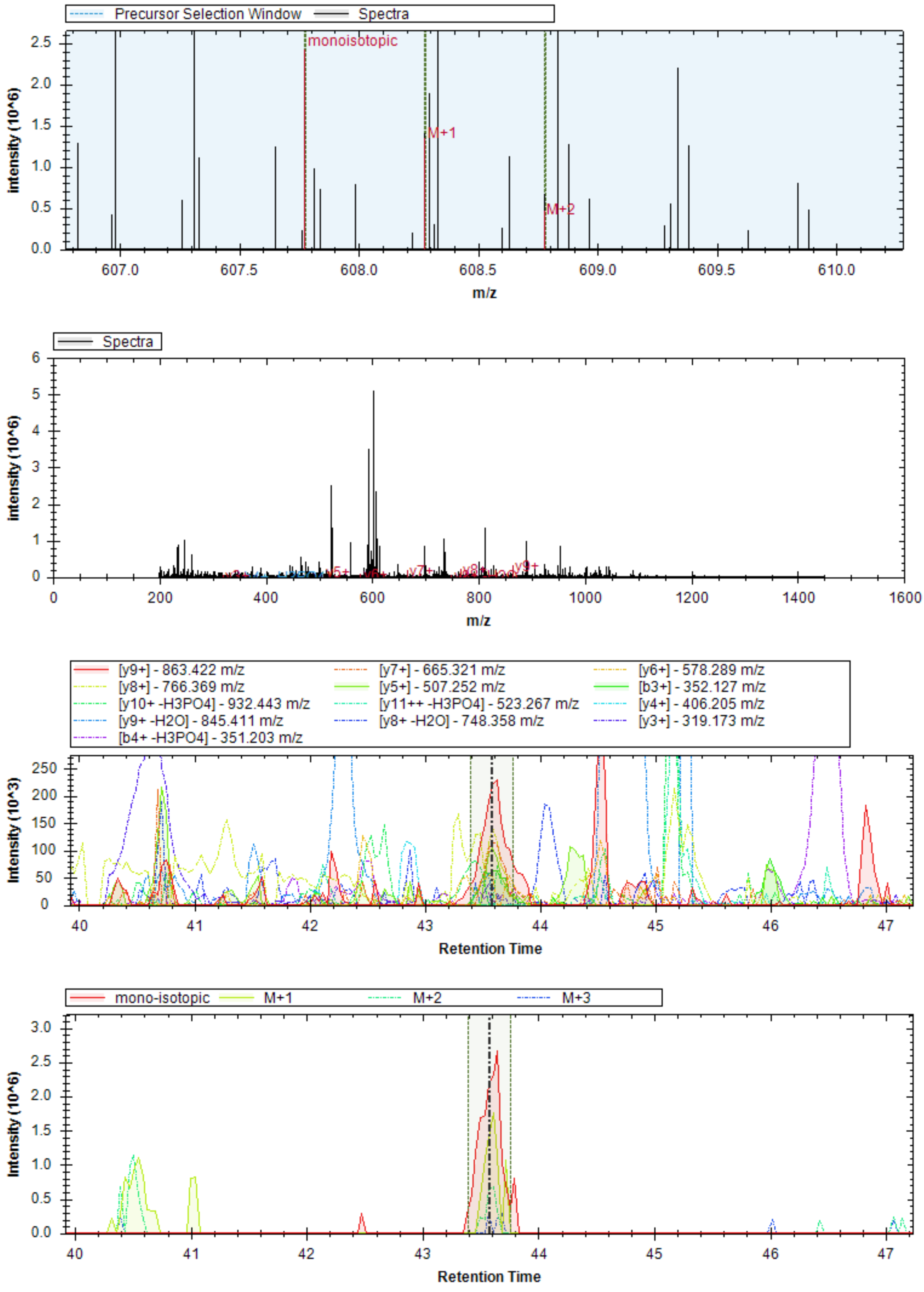

Q9QWI6 – _LSYAGGRPPSYAGS[+H+O3+P]PVHHAAER_.4
Fig4_MP-DIA-barrel-cortex-p9-1_MHRM.htrms

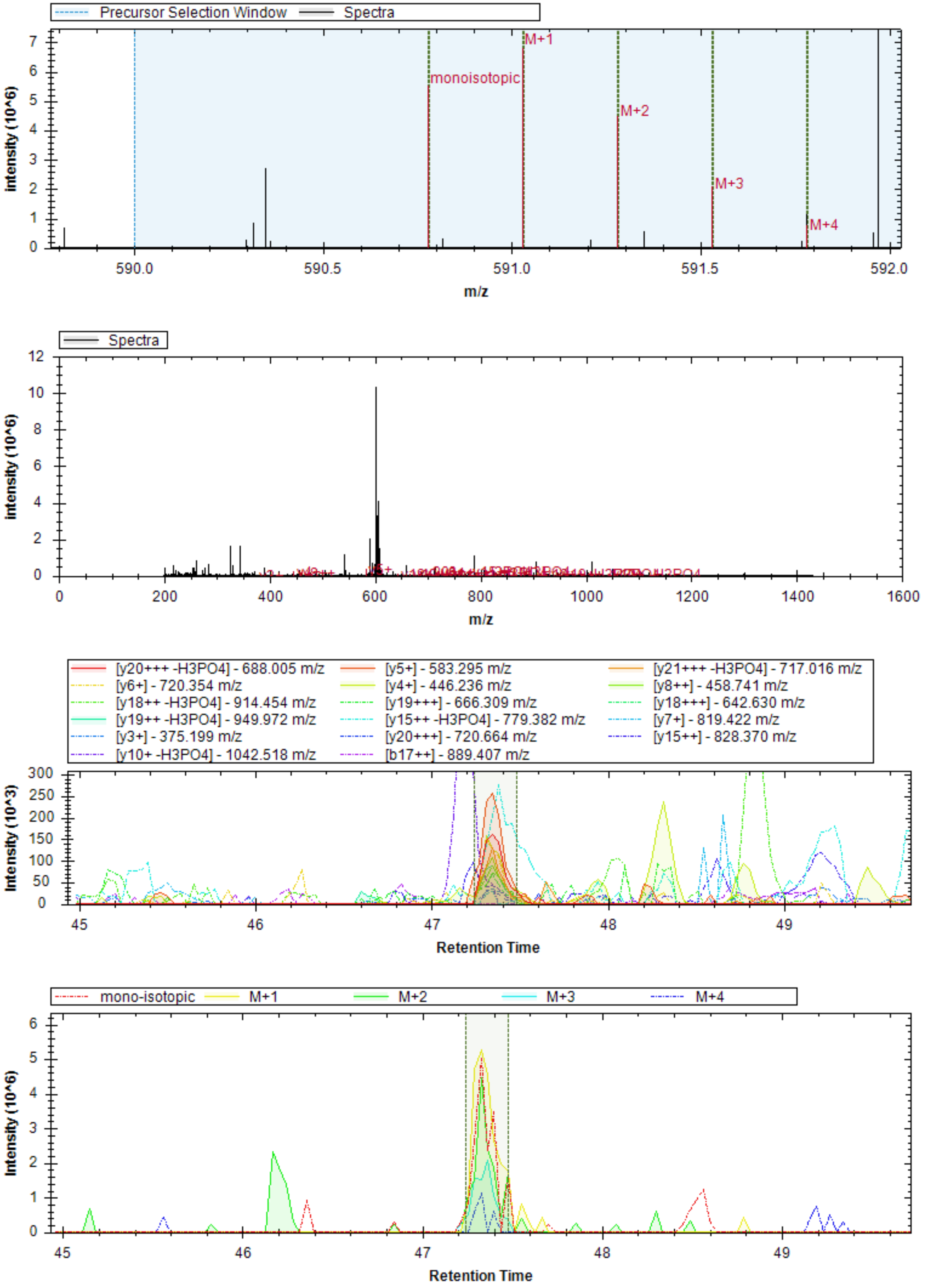

P26645 – _GEATAERPGEAAVASS[+H+O3+P]PSK_.3
Fig4_MP-DIA-barrel-cortex-p9-1_MHRM.htrms

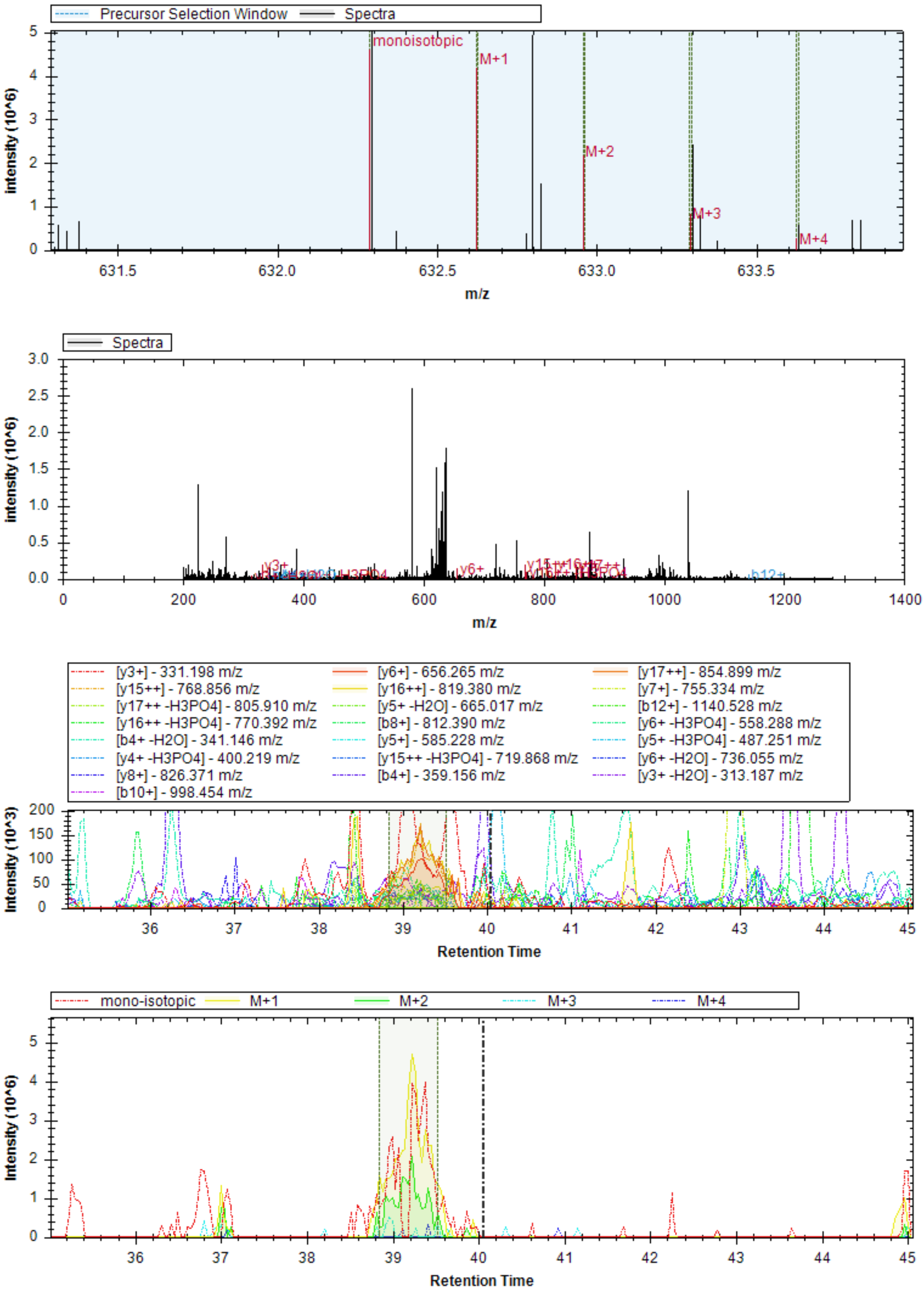

P60904 – _SLS[+H+O3+P]TSGESLYHVLGLDK_.3
Fig4_MP-DIA-barrel-cortex-p9-1_MHRM.htrms

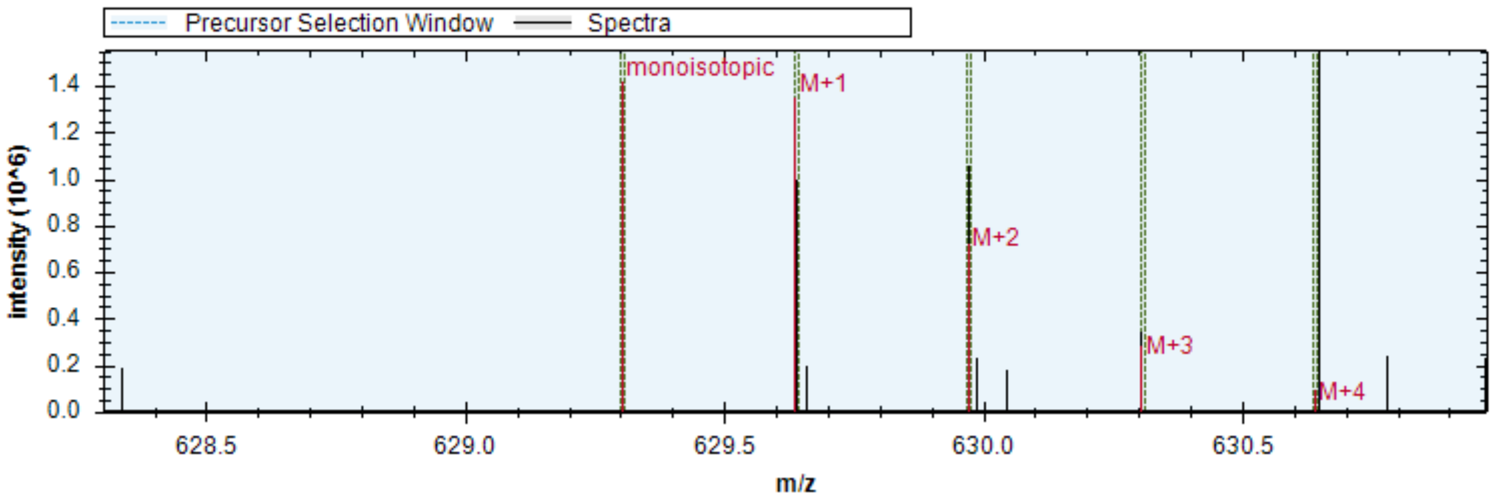

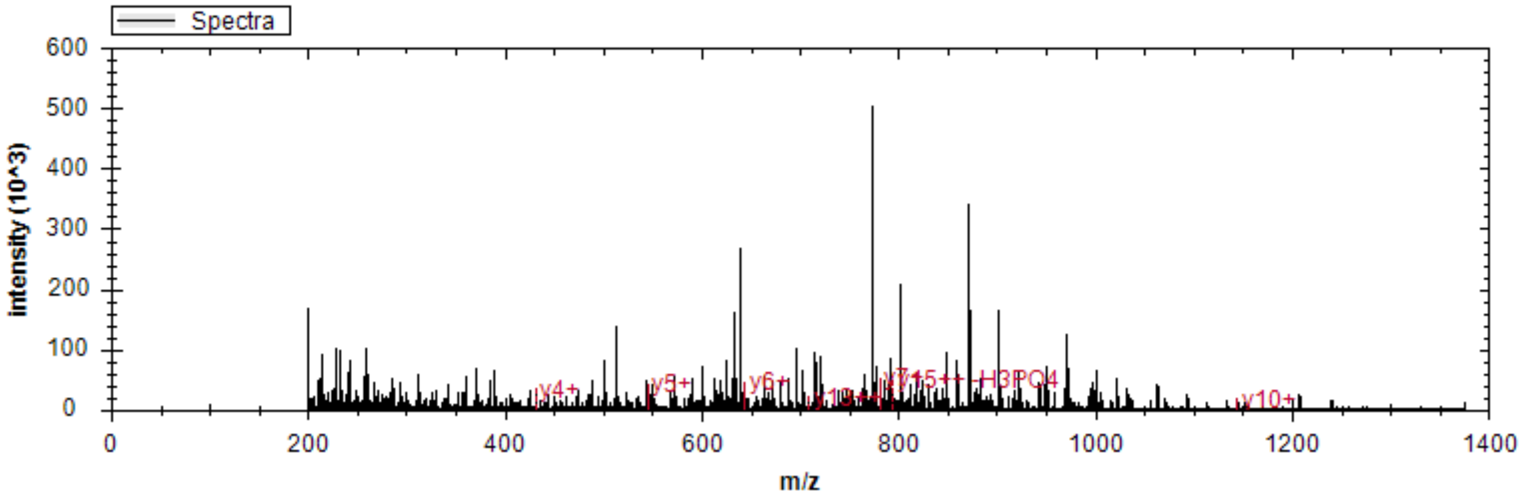

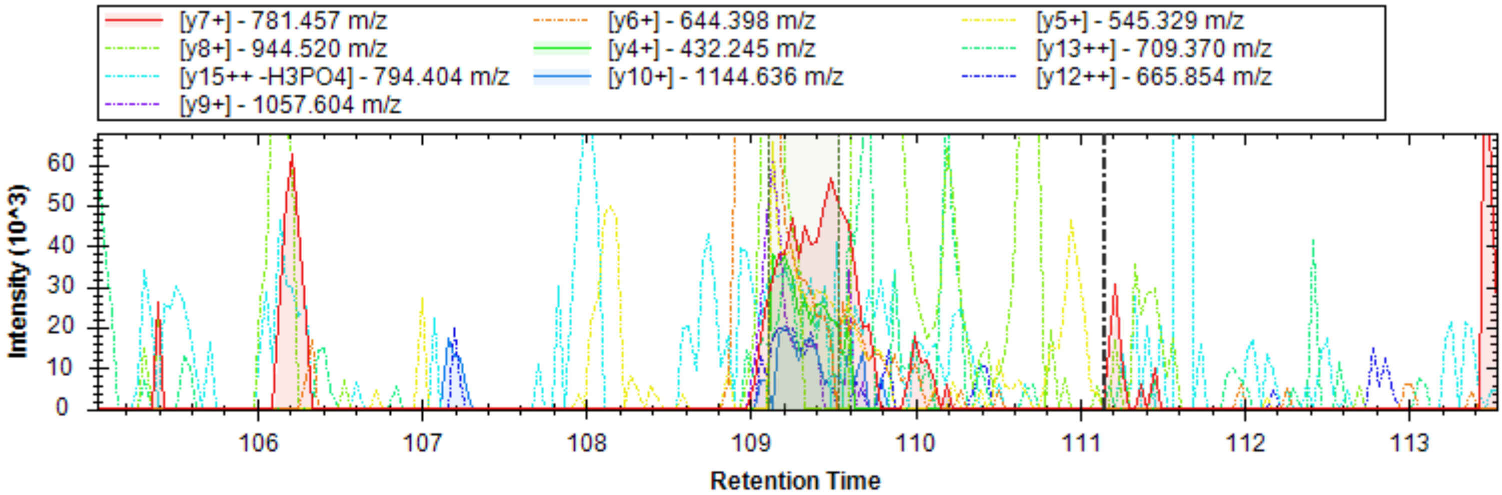

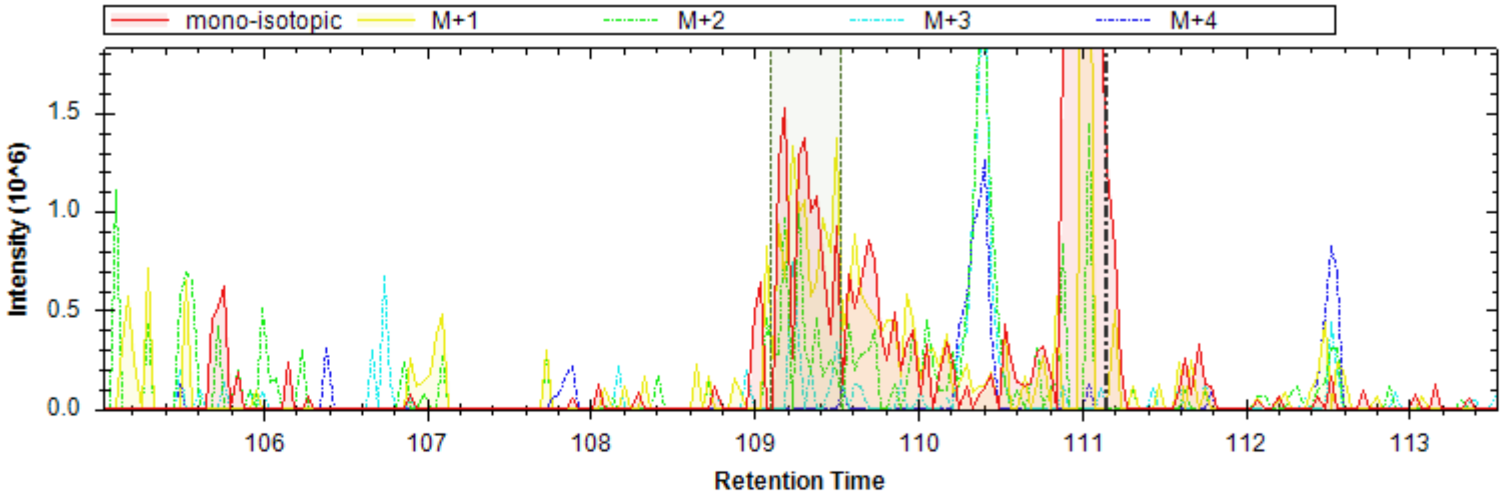

P60904 – _SLST[+H+O3+P]SGESLYHVLGLDK_.3
Fig4_MP-DIA-barrel-cortex-p9-1_MHRM.htrms

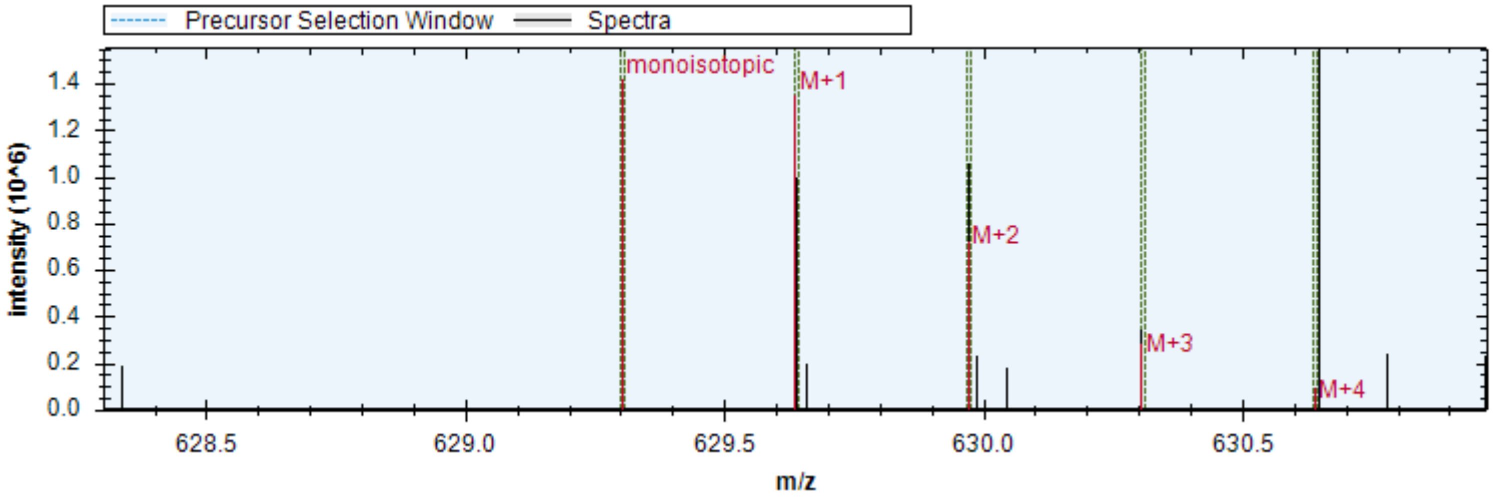

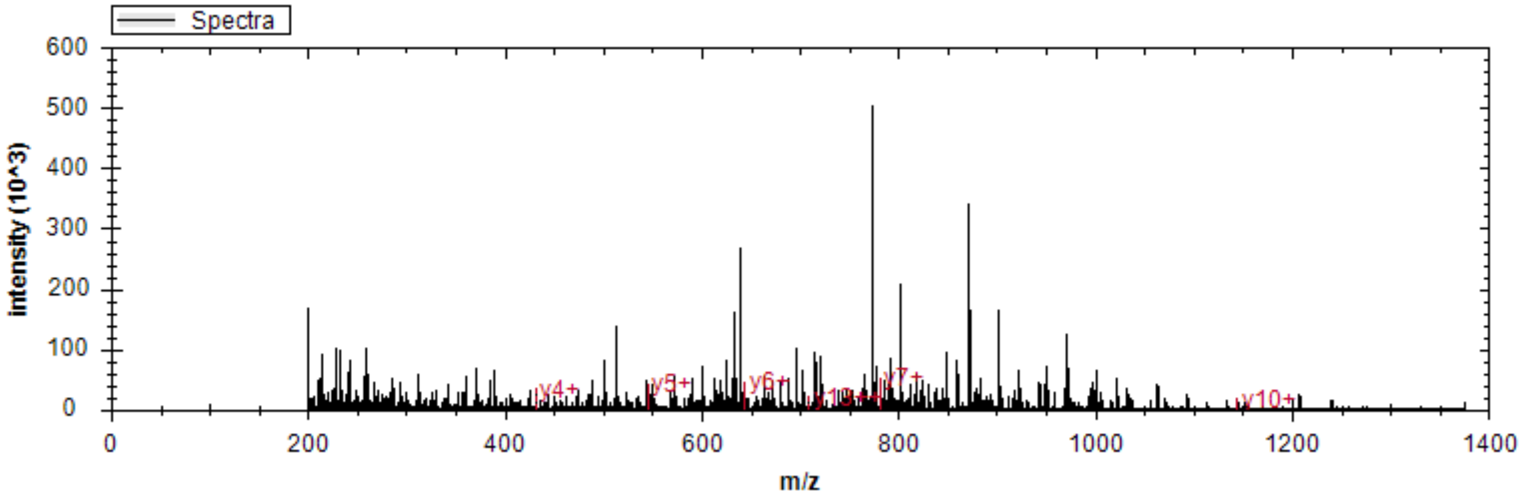

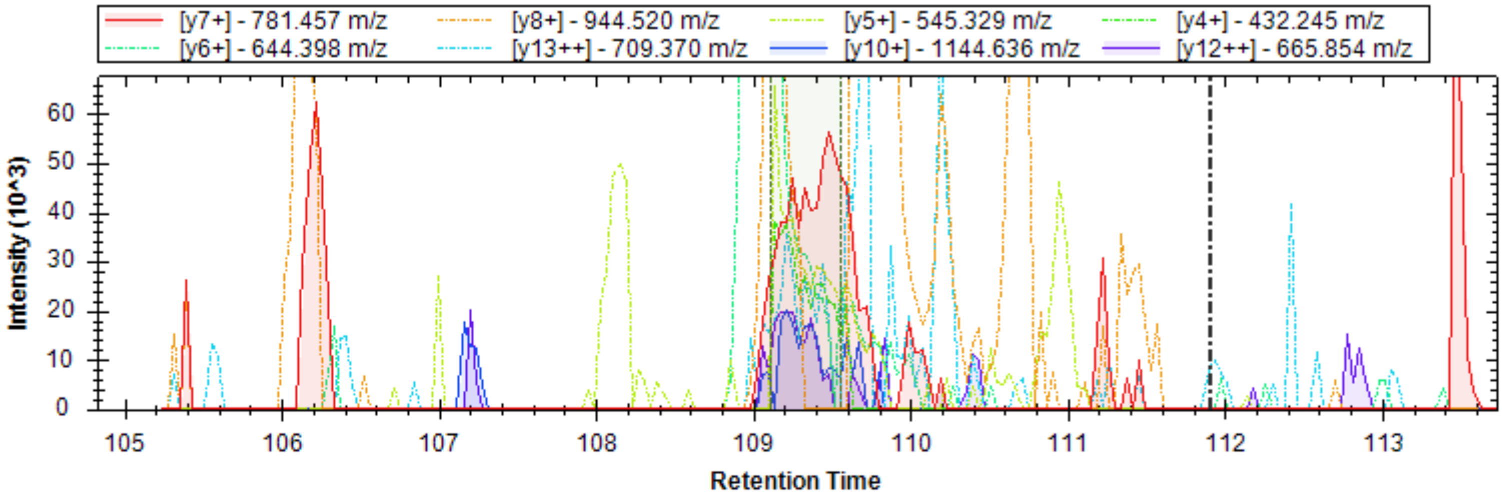

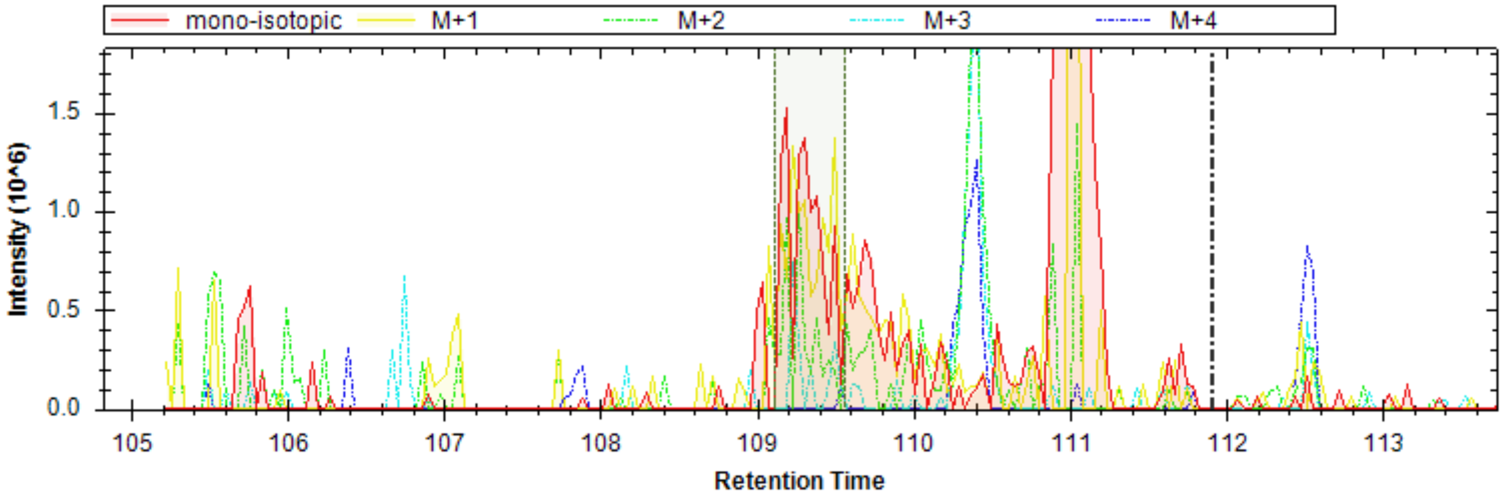

Q9CQS8 – _PGPTPSGTNVGSSGRS[+H+O3+P]PSK_.3

Fig4_MP-DIA-barrel-cortex-p9-1_MHRM.htrms

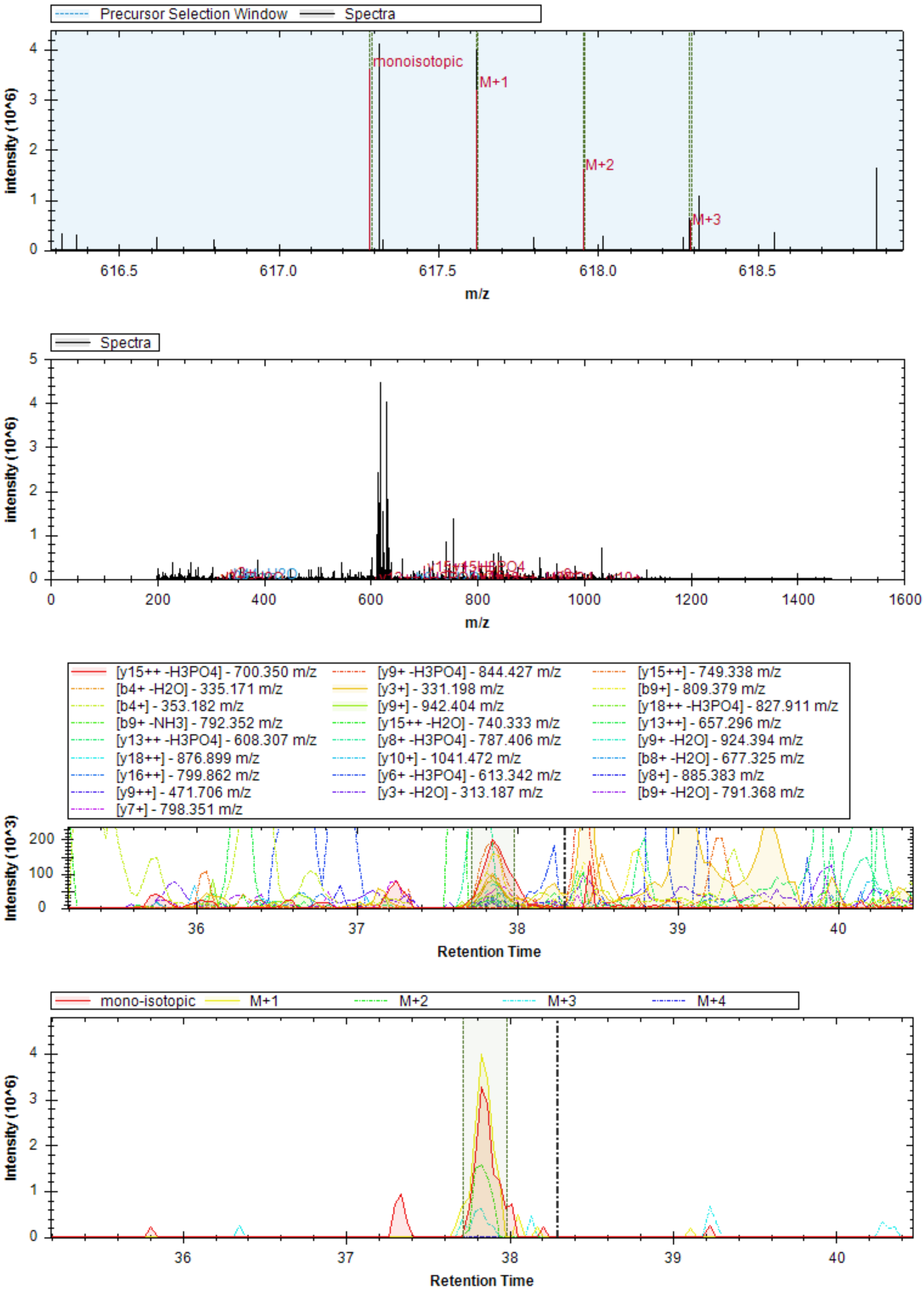

P20357 – _TPGT[+H+O3+P]PGTPSYPR_.2
Fig4_MP-DIA-barrel-cortex-p9-1_MHRM.htrms

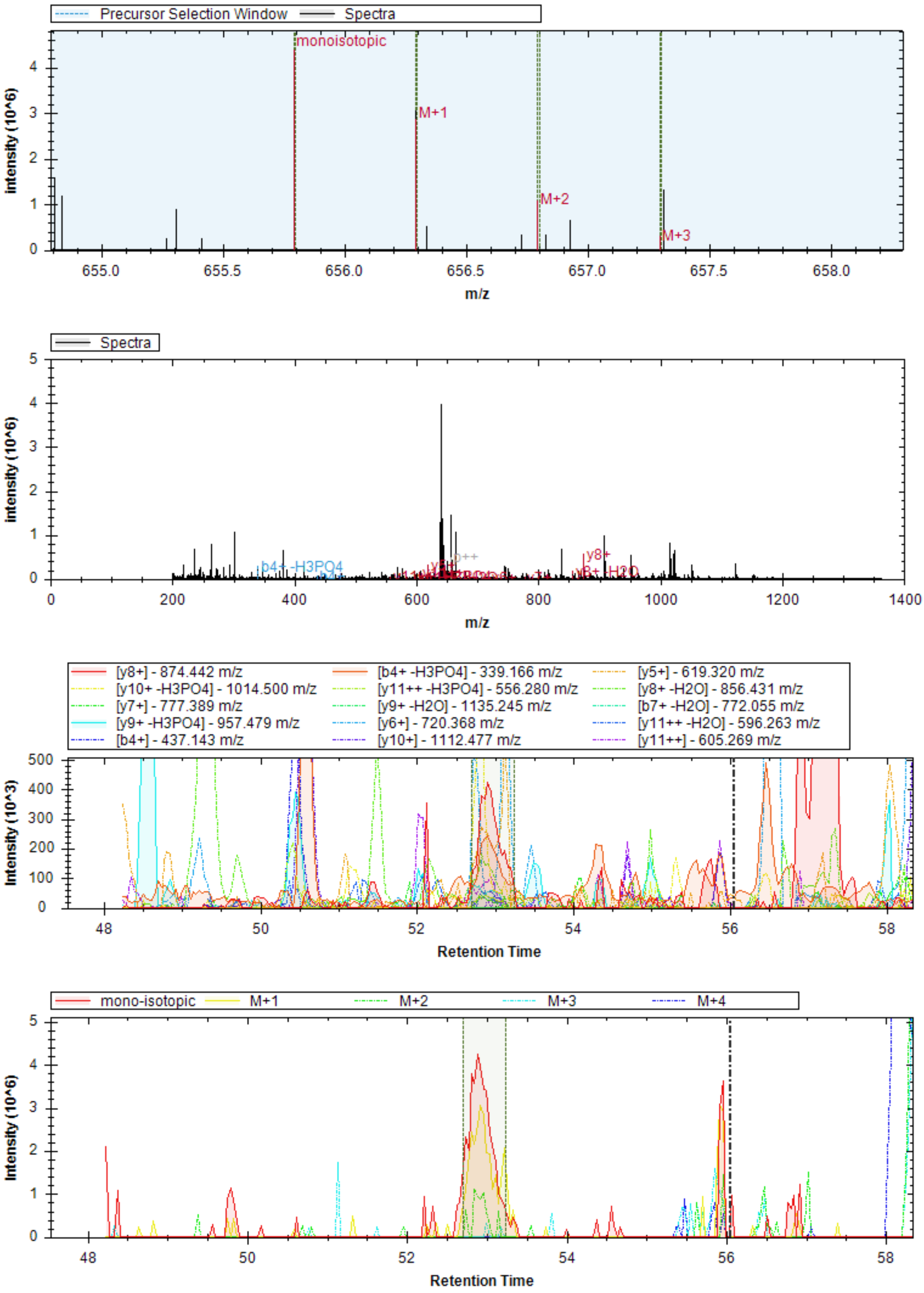

P39447 – _SSGIIRLHT[+H+O3+P]IK_.2
Fig4_MP-DIA-barrel-cortex-p9-1_MHRM.htrms

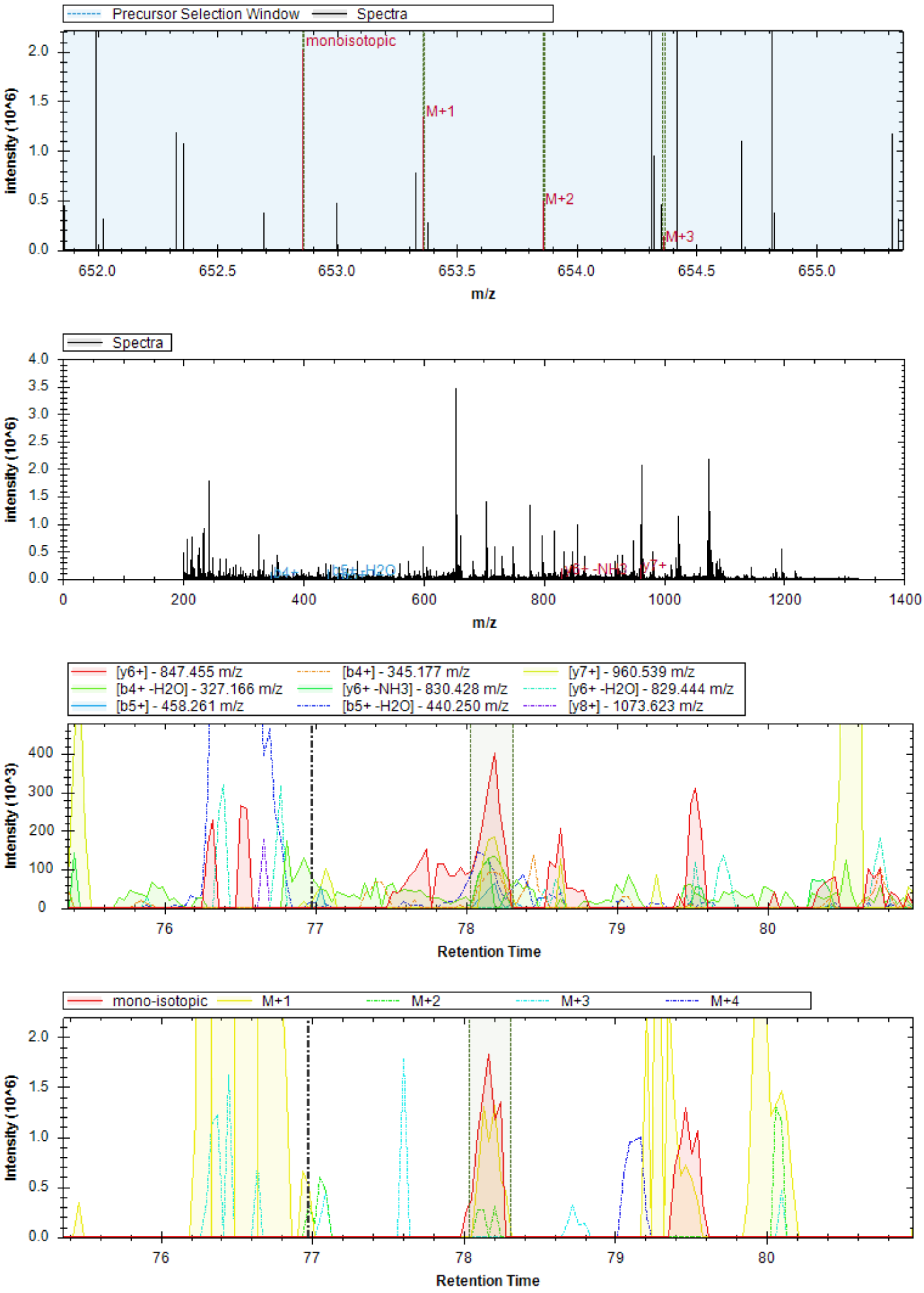

Q8BLJ3 – _M[+O]AS[+H+O3+P]SQGKNELK_.2
Fig4_MP-DIA-barrel-cortex-p9-1_MHRM.htrms

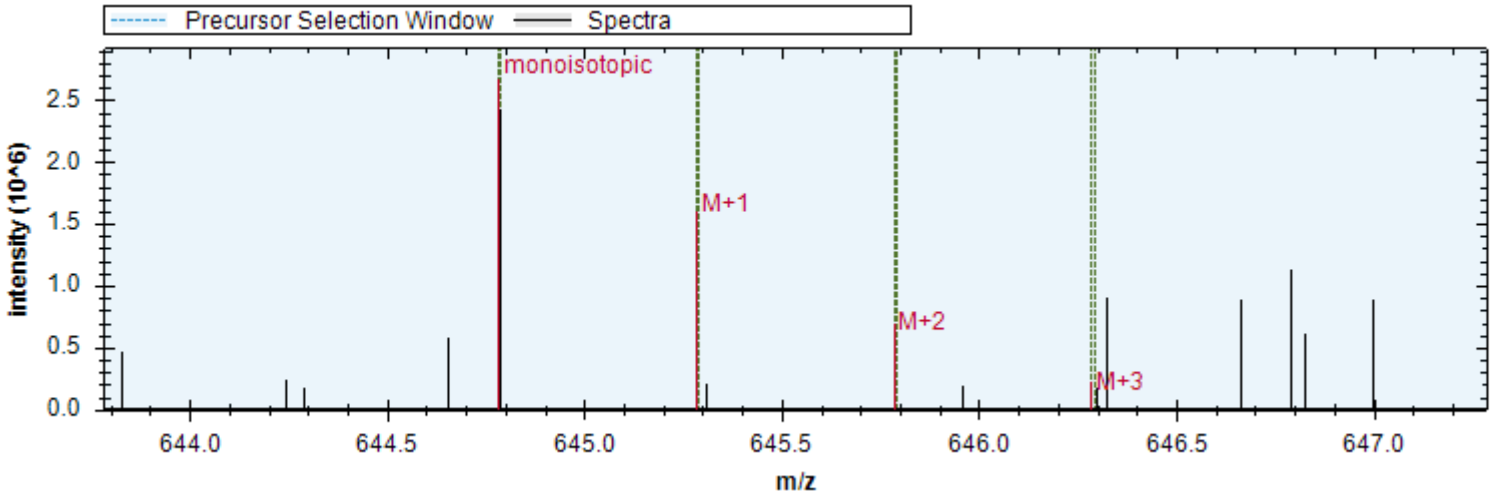

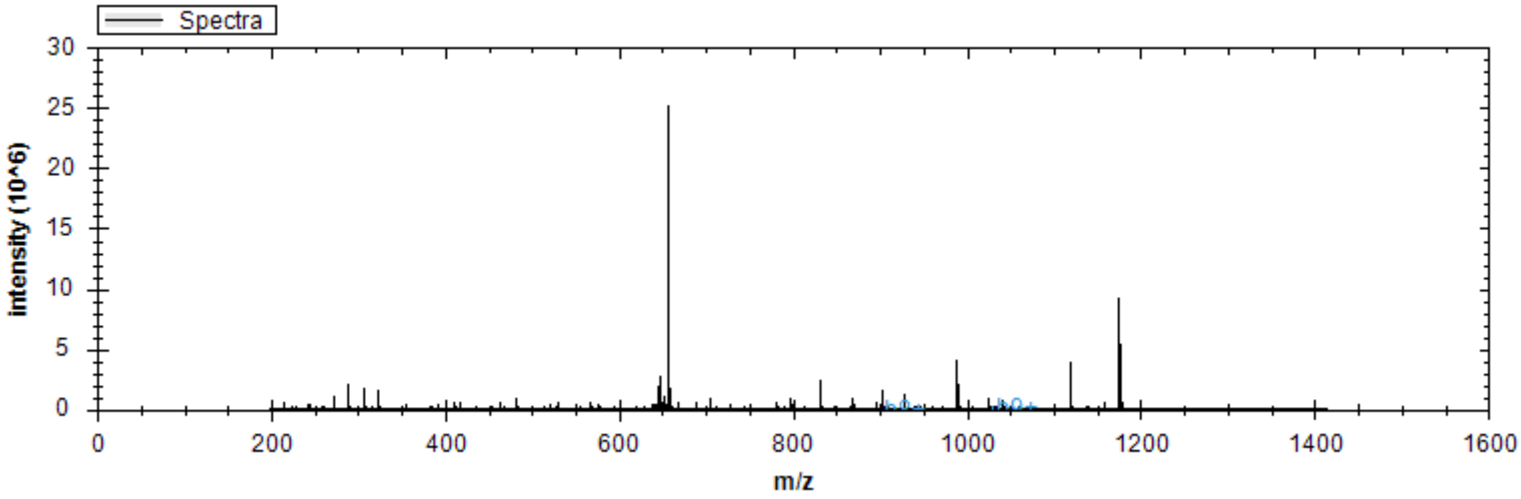

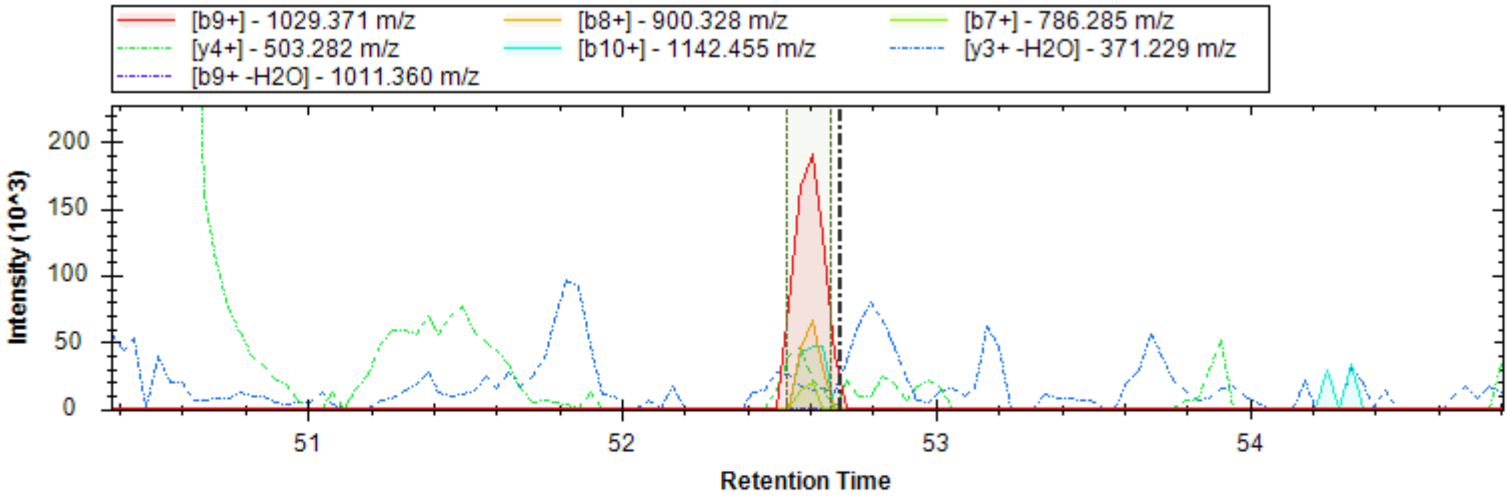

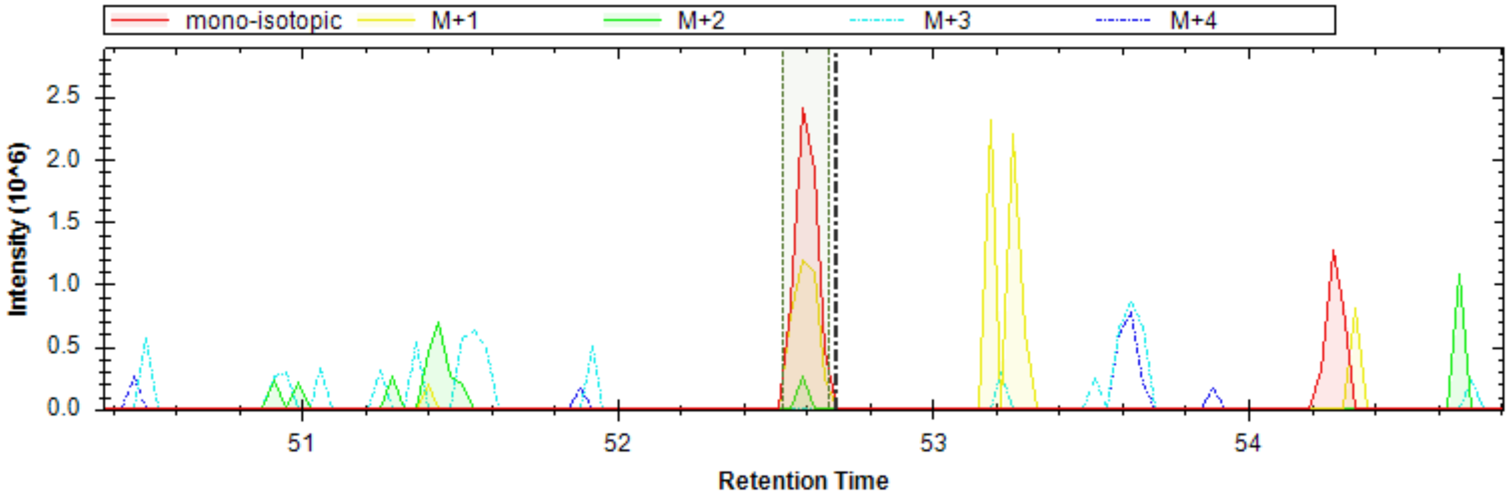

G5E870 – _IIY[+H+O3+P]FADAELLK_.2

Fig4_MP-DIA-barrel-cortex-p9-1_MHRM.htrms

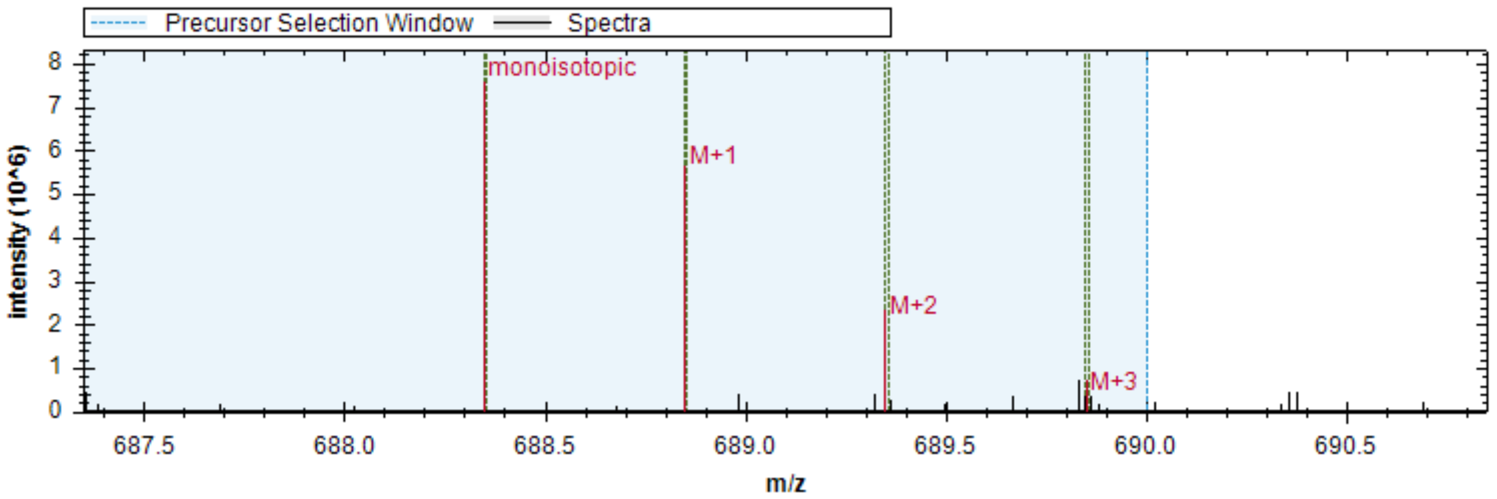

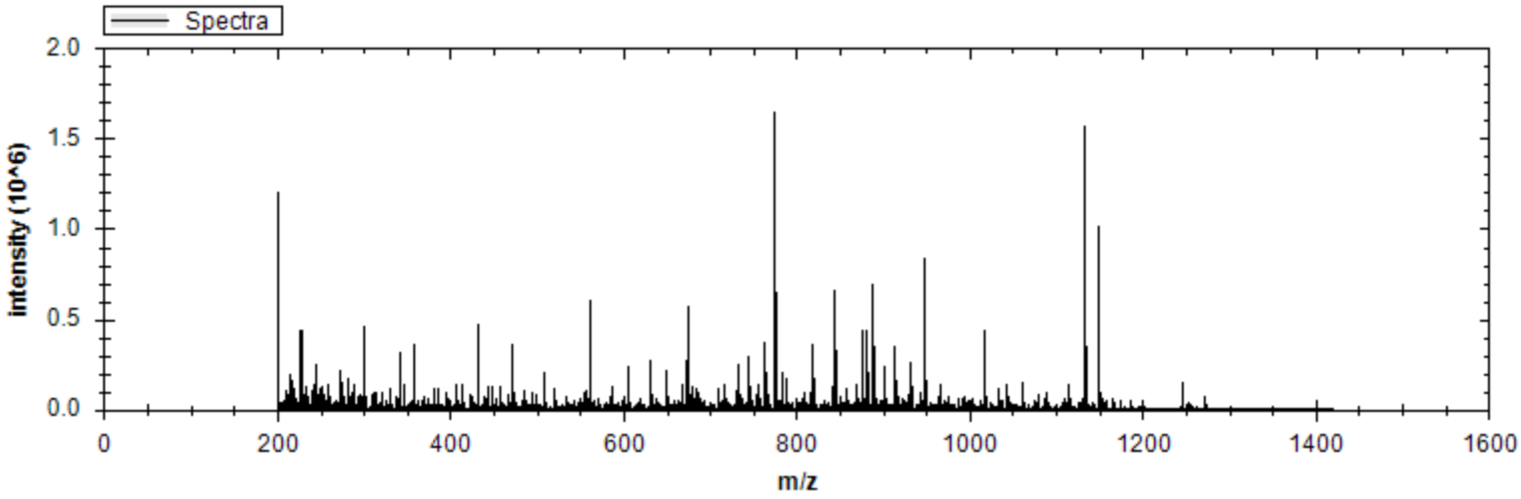

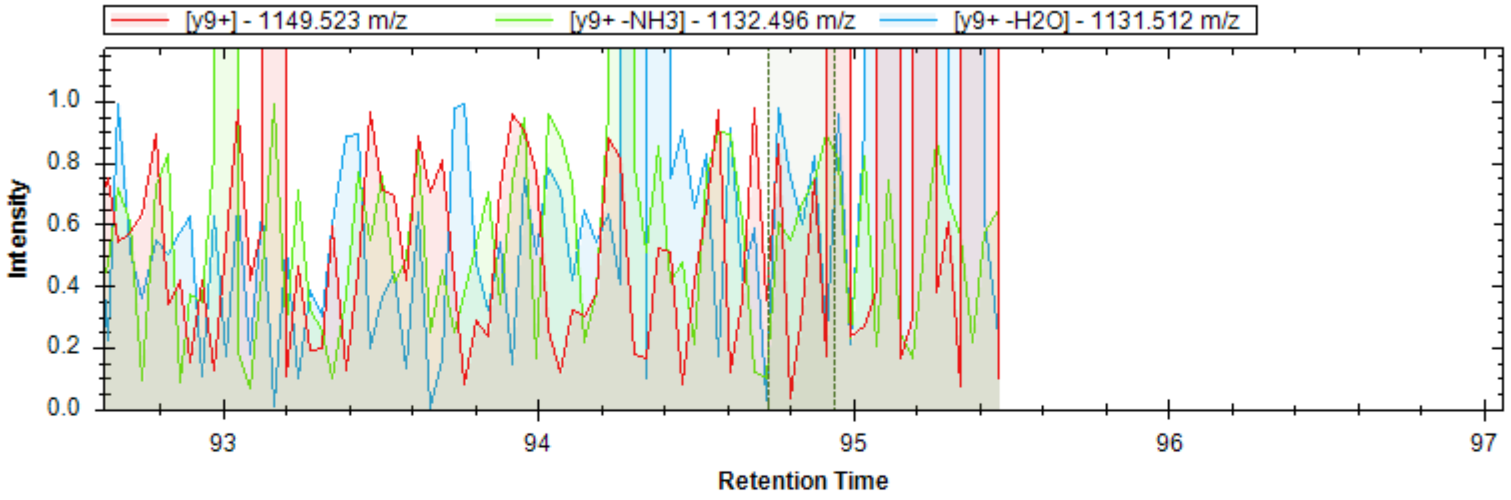

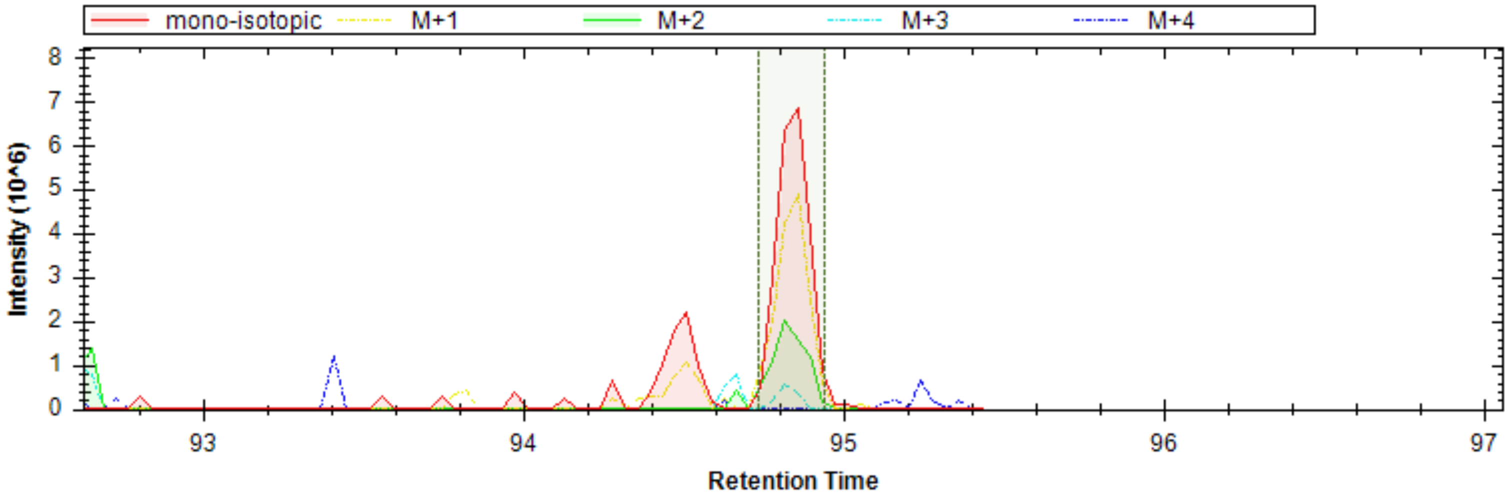

P13595 – _GVTASS[+H+O3+P]SSPASAPK_.2
Fig4_MP-DIA-barrel-cortex-p9-1_MHRM.htrms

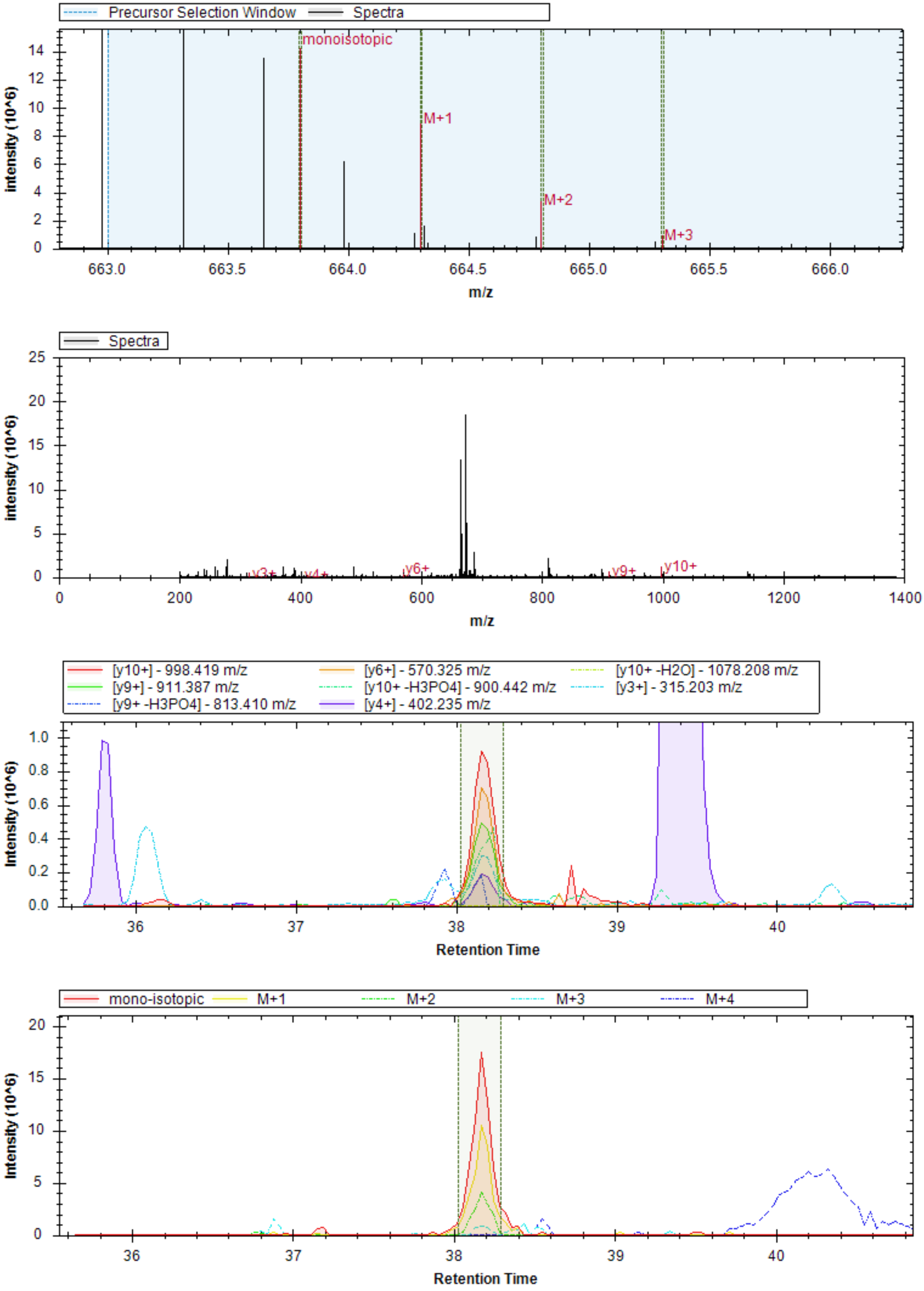

P13595 – _GVTASSS[+H+O3+P]SPASAPK_.2
Fig4_MP-DIA-barrel-cortex-p9-1_MHRM.htrms

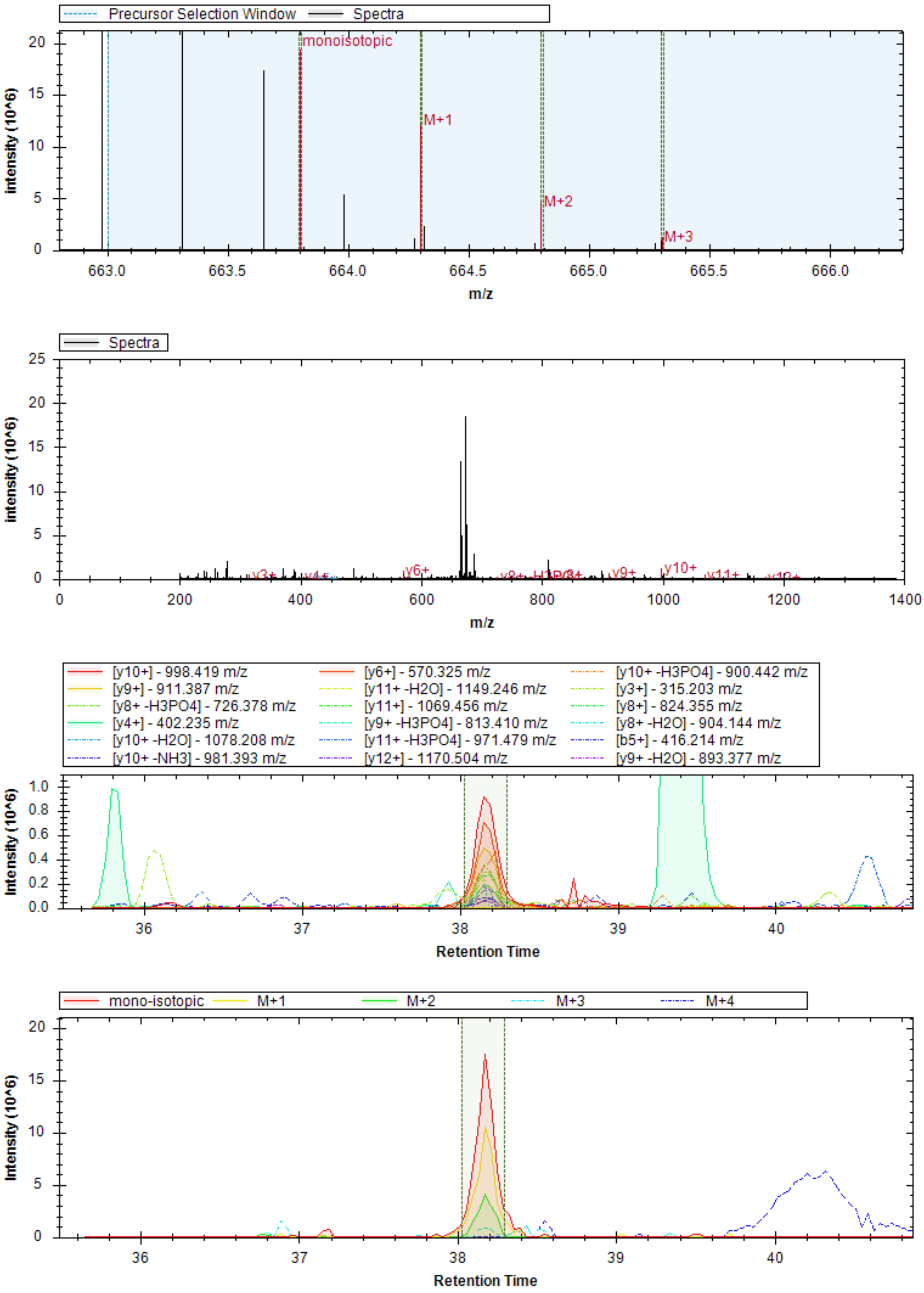

P13595 – _GVTASSSS[+H+O3+P]PASAPK_.2

Fig4_MP-DIA-barrel-cortex-p9-1_MHRM.htrms

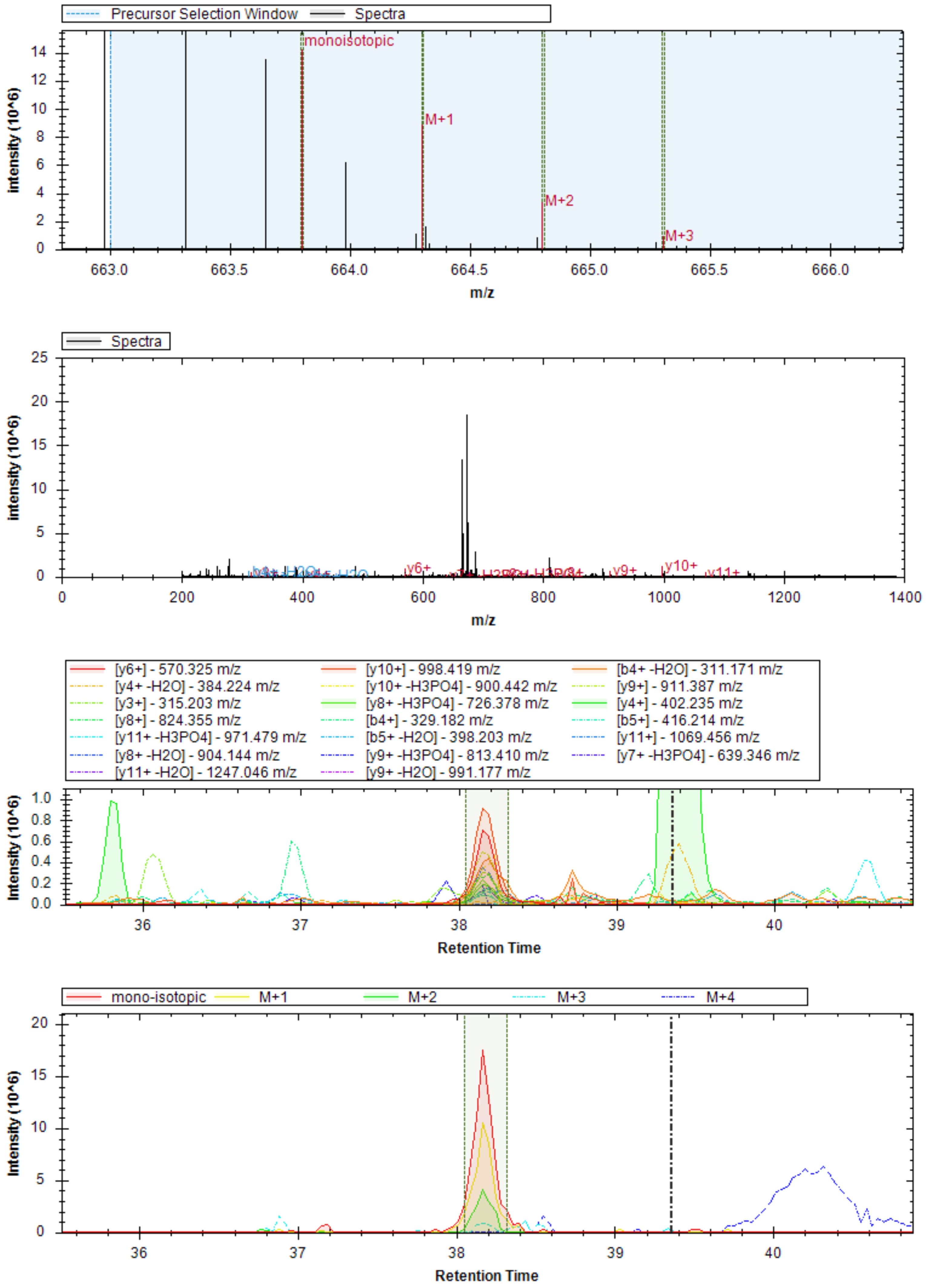

P13595 – _NPPEAATAPAS[+H+O3+P]PK_.2
Fig4_MP-DIA-barrel-cortex-p9-1_MHRM.htrms

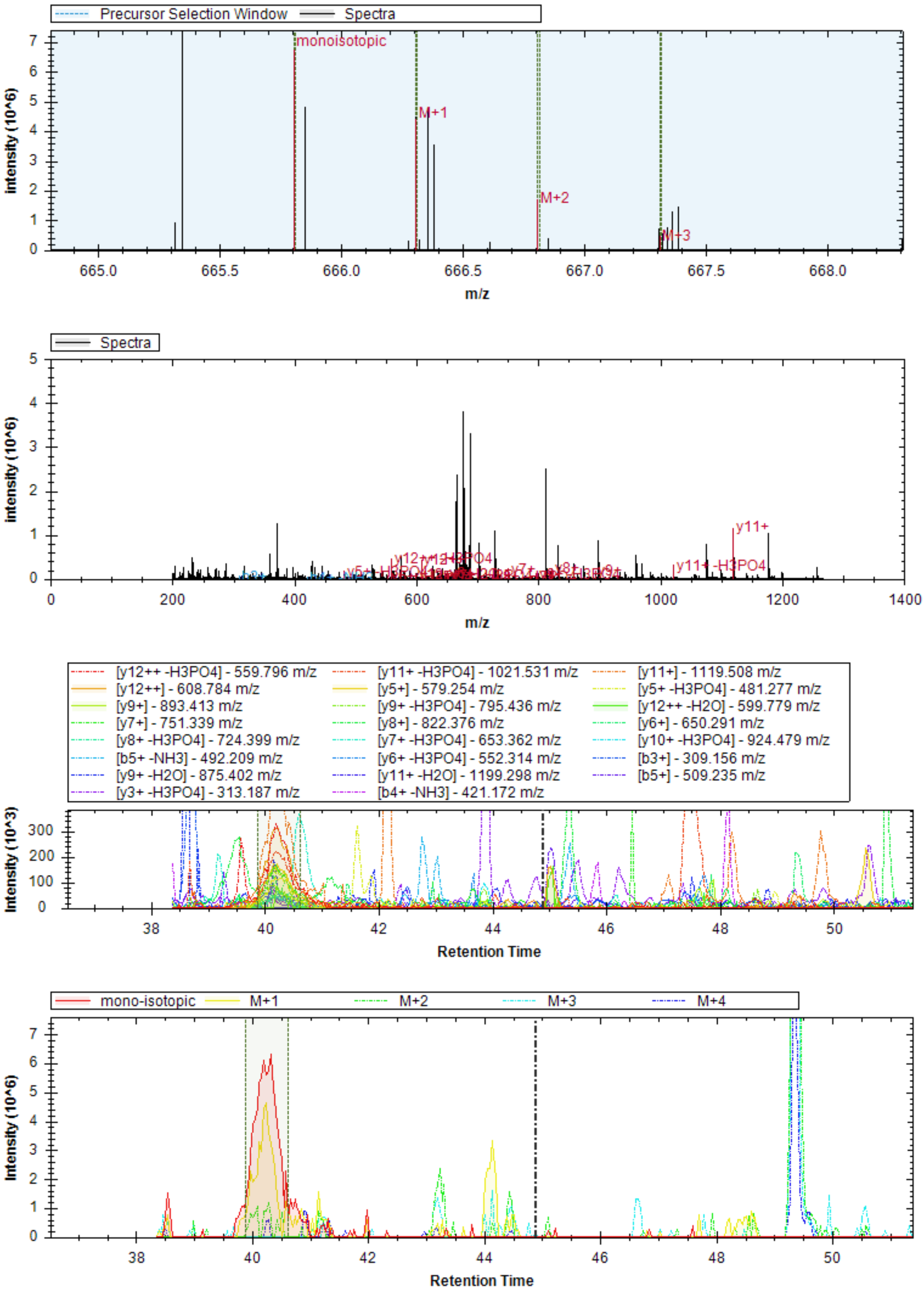

Q3UTZ3 – _SQS[+H+O3+P]FSHQQPSR_.2
Fig4_MP-DIA-barrel-cortex-p9-1_MHRM.htrms

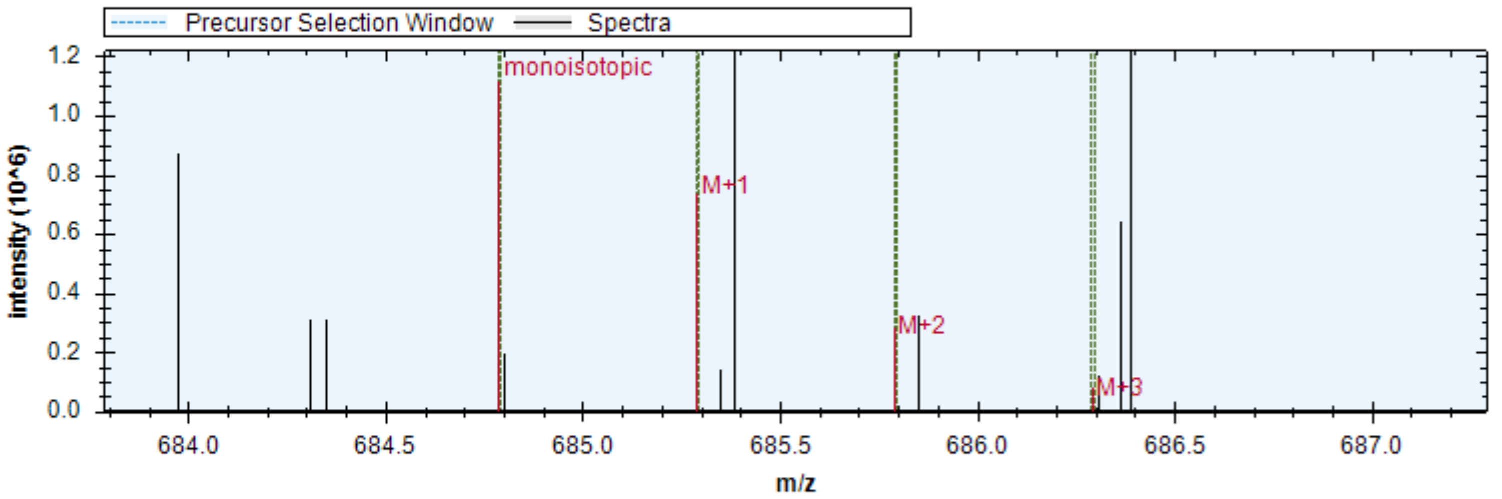

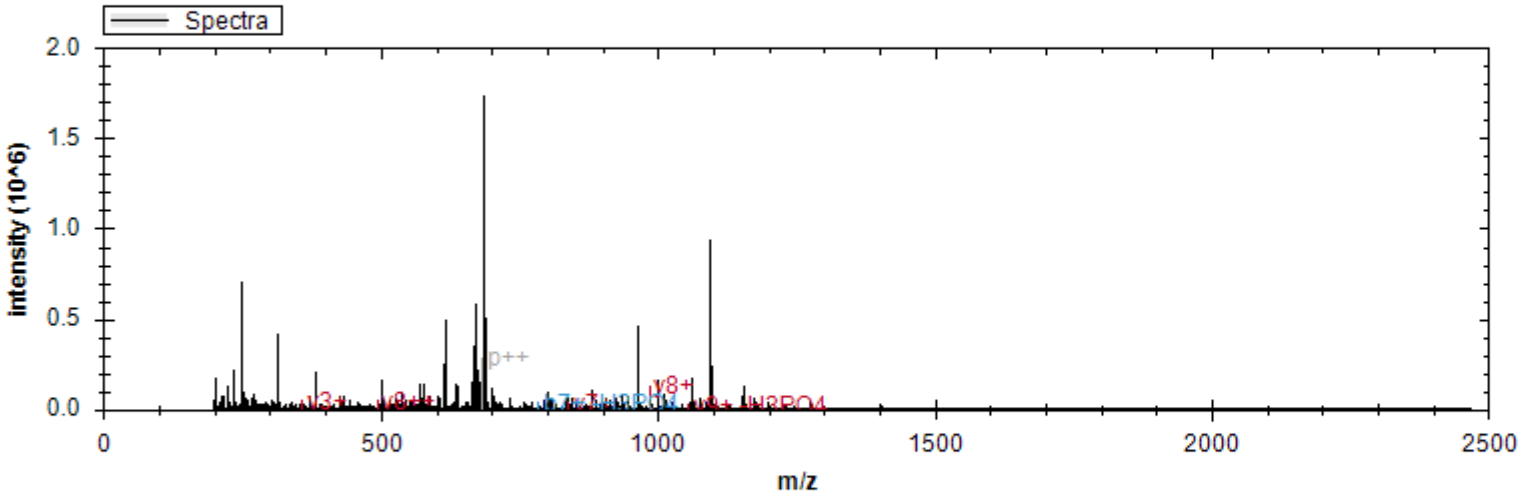

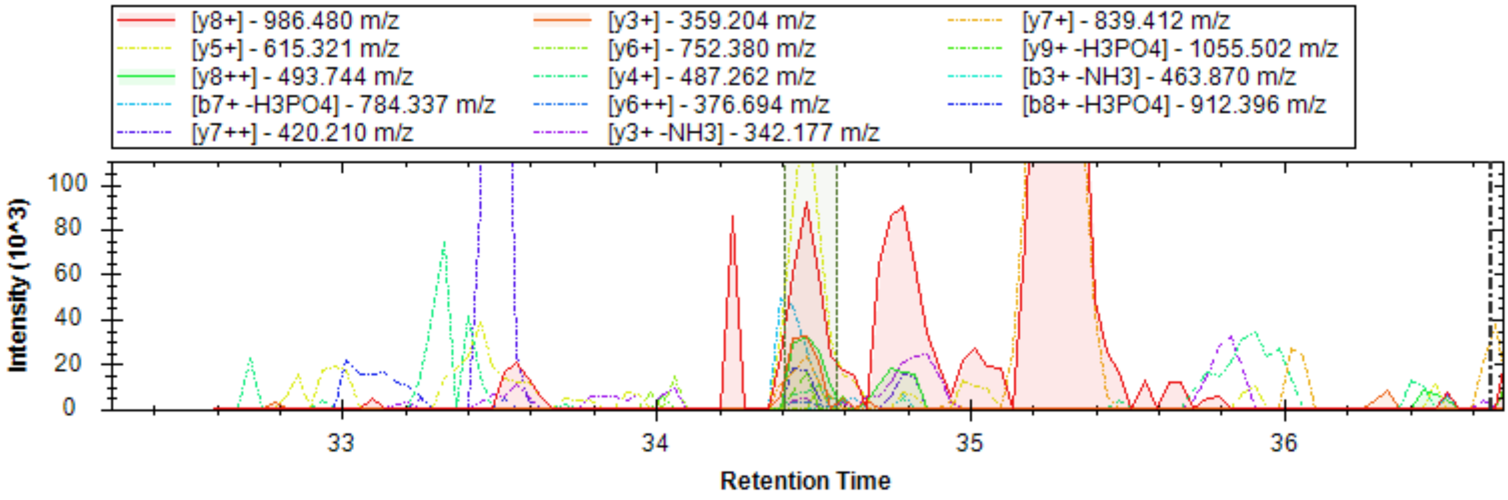

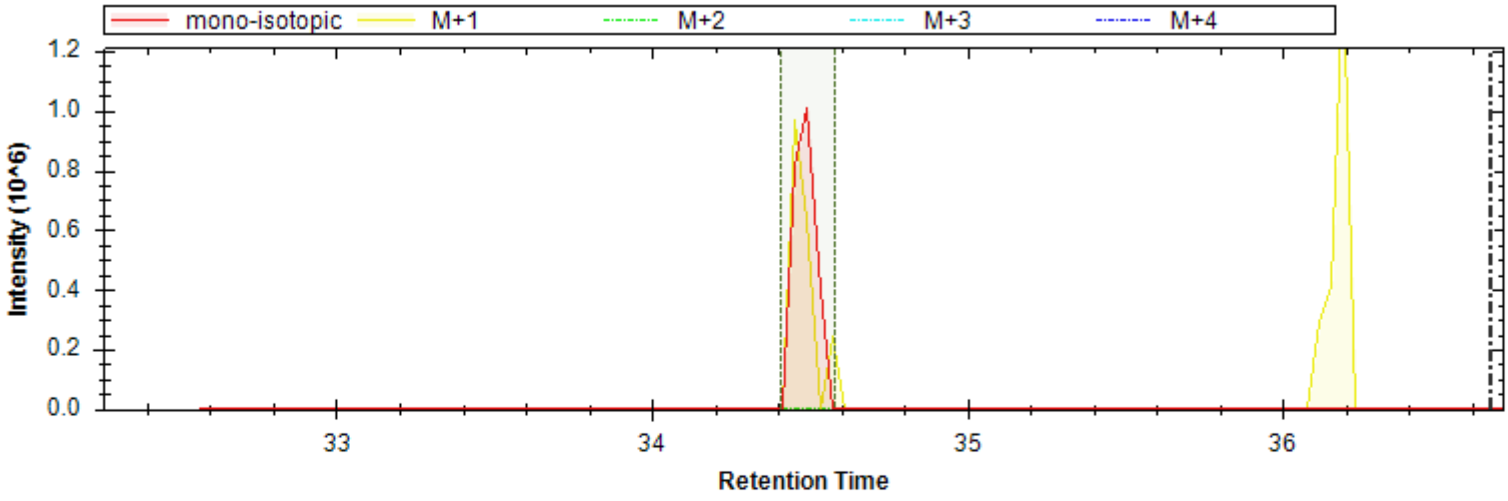

Q6A078 – _T[+H+O3+P]IGLM[+O]KKVVEK_.2

Fig4_MP-DIA-barrel-cortex-p9-1_MHRM.htrms

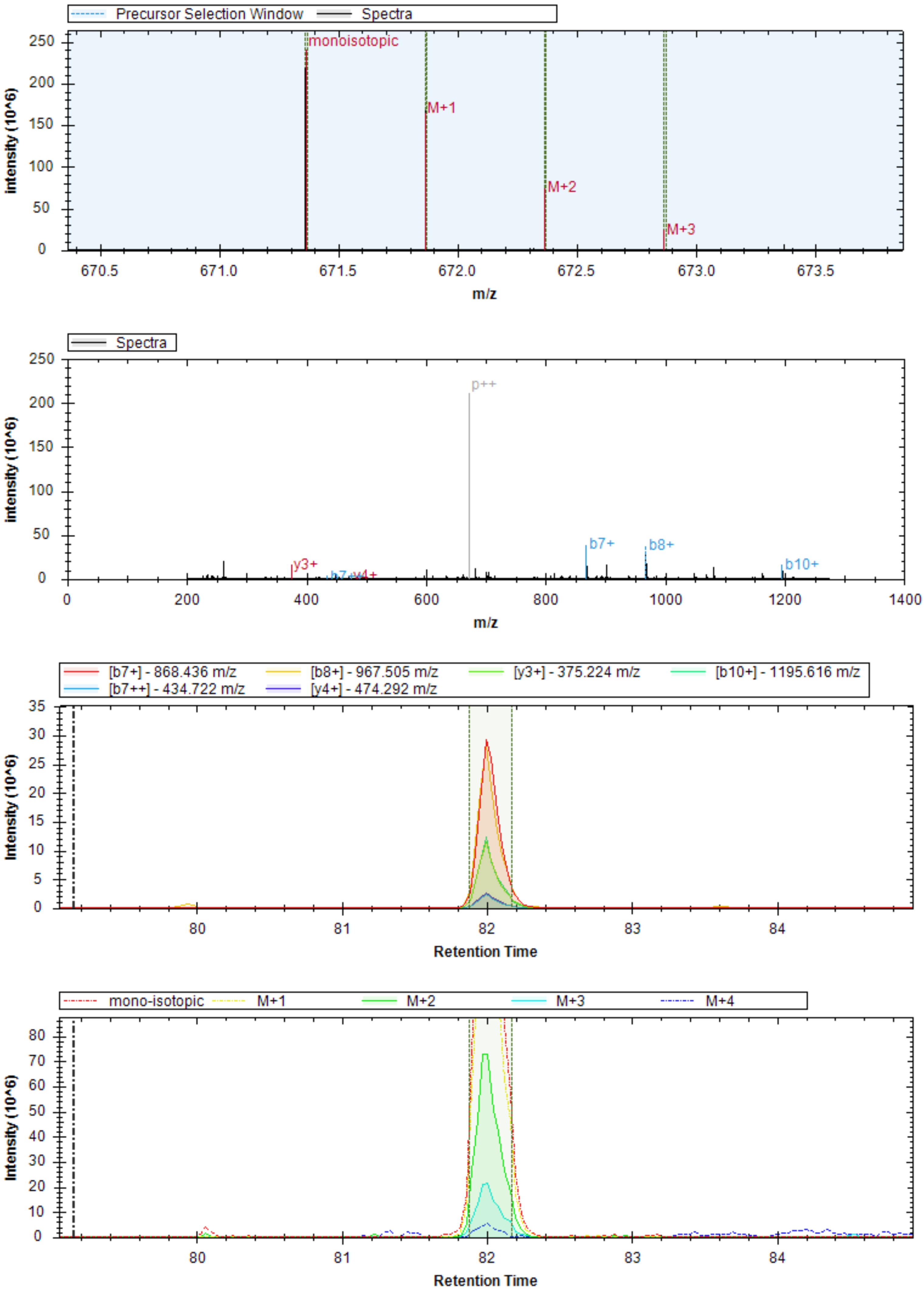

Q8C0E2 – _SMS[+H+O3+P]HQAAIASQR_.2
Fig4_MP-DIA-barrel-cortex-p9-1_MHRM.htrms

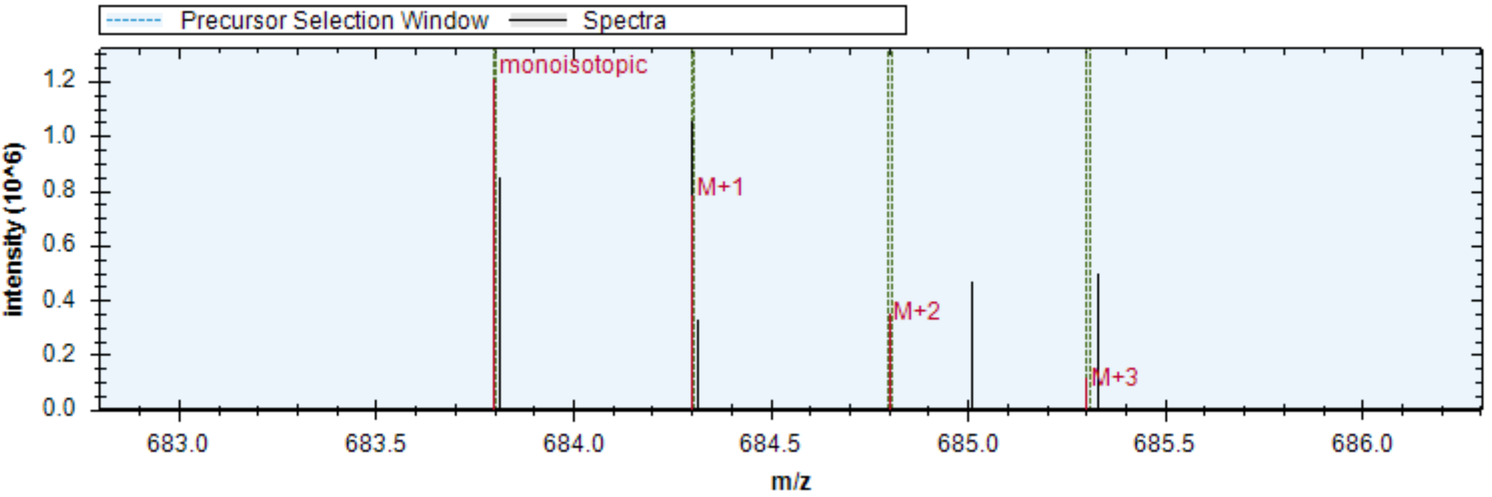

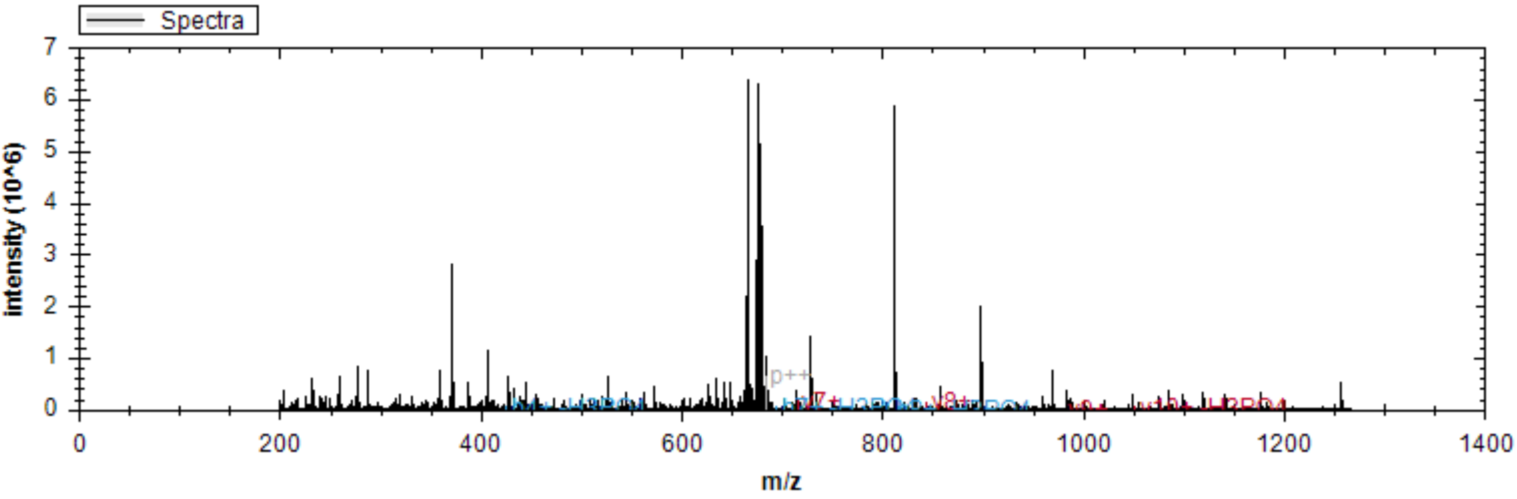

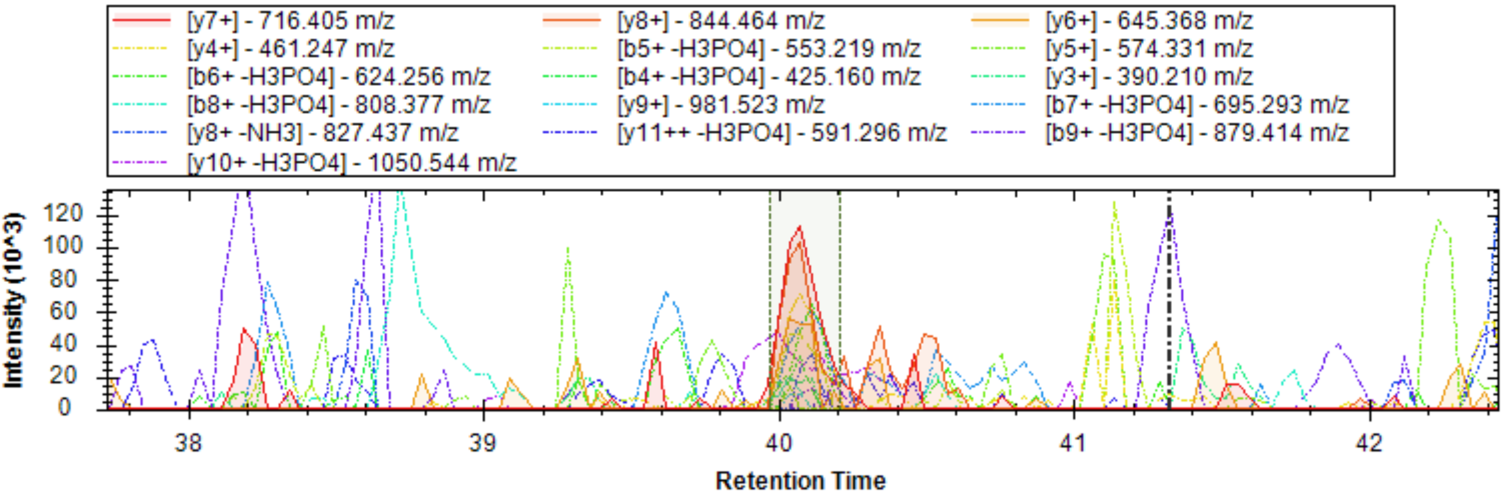

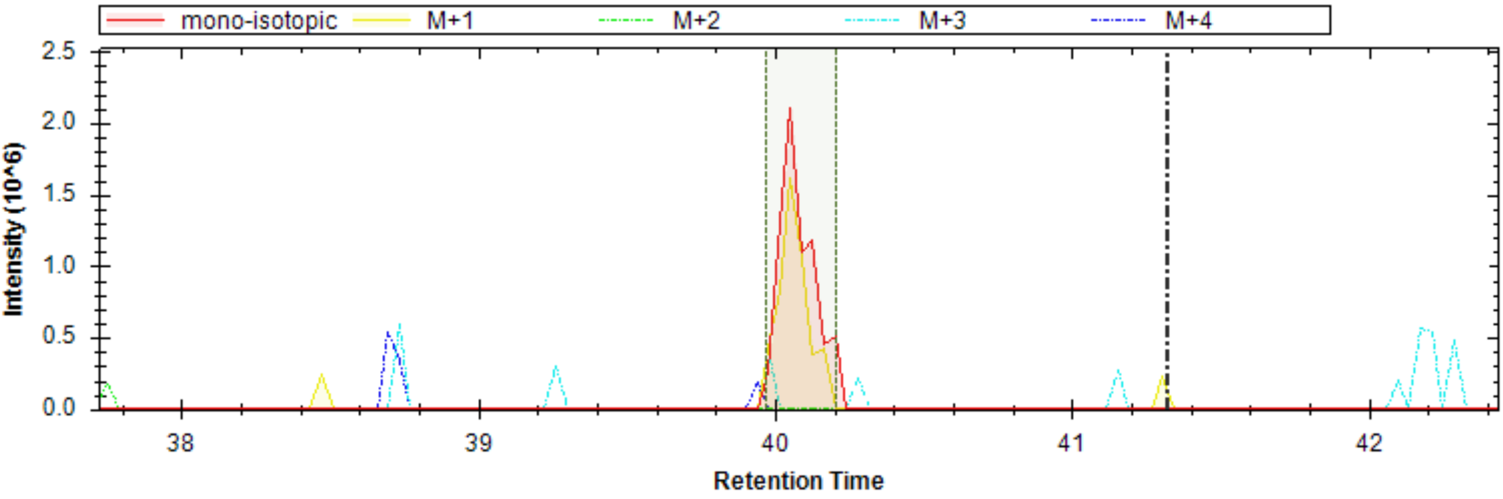

Q8CDU4;Q8CDU4-2 – _[+C2+H2+O]ASLRS[+H+O3+P]ATPRLR_.2
Fig4_MP-DIA-barrel-cortex-p9-1_MHRM.htrms

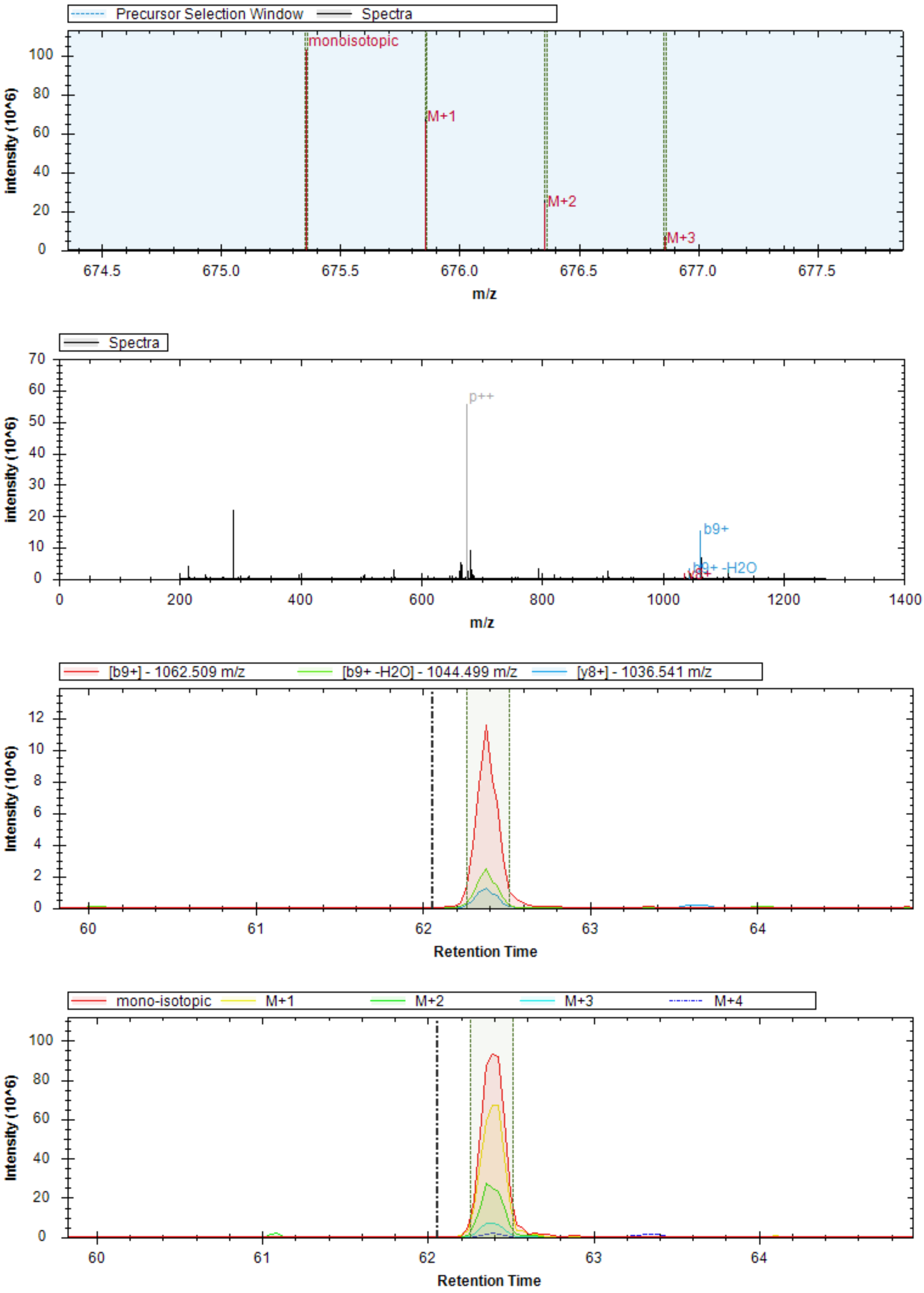

Q8R5L3;Q8R5L3-2 – _LQLY[+H+O3+P]FWKDR_.2

Fig4_MP-DIA-barrel-cortex-p9-1_MHRM.htrms

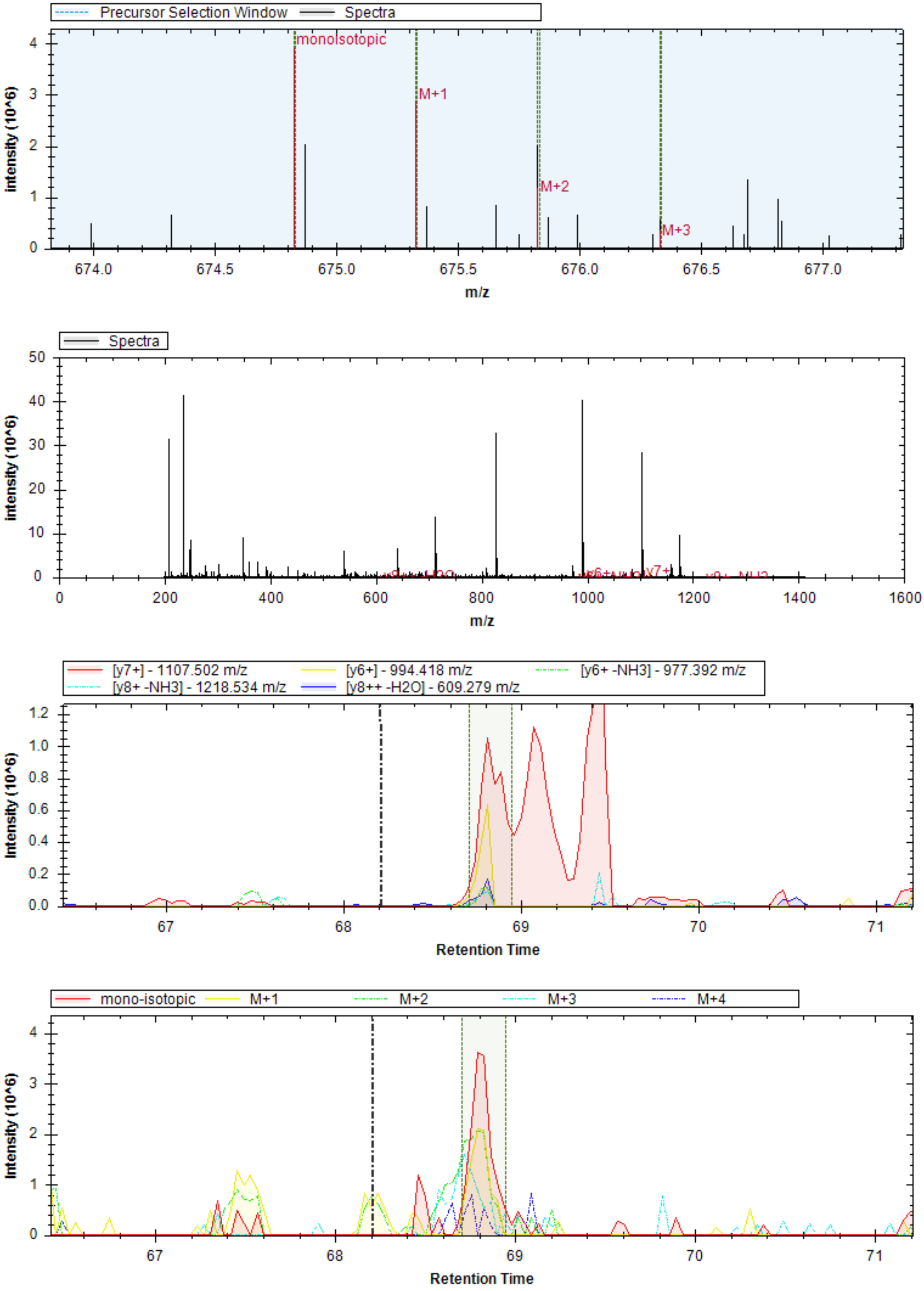

O88935 – _PVAGGPGAPPAARPPAS[+H+O3+P]PSPQR_.3
Fig4_MP-DIA-barrel-cortex-p9-1_MHRM.htrms

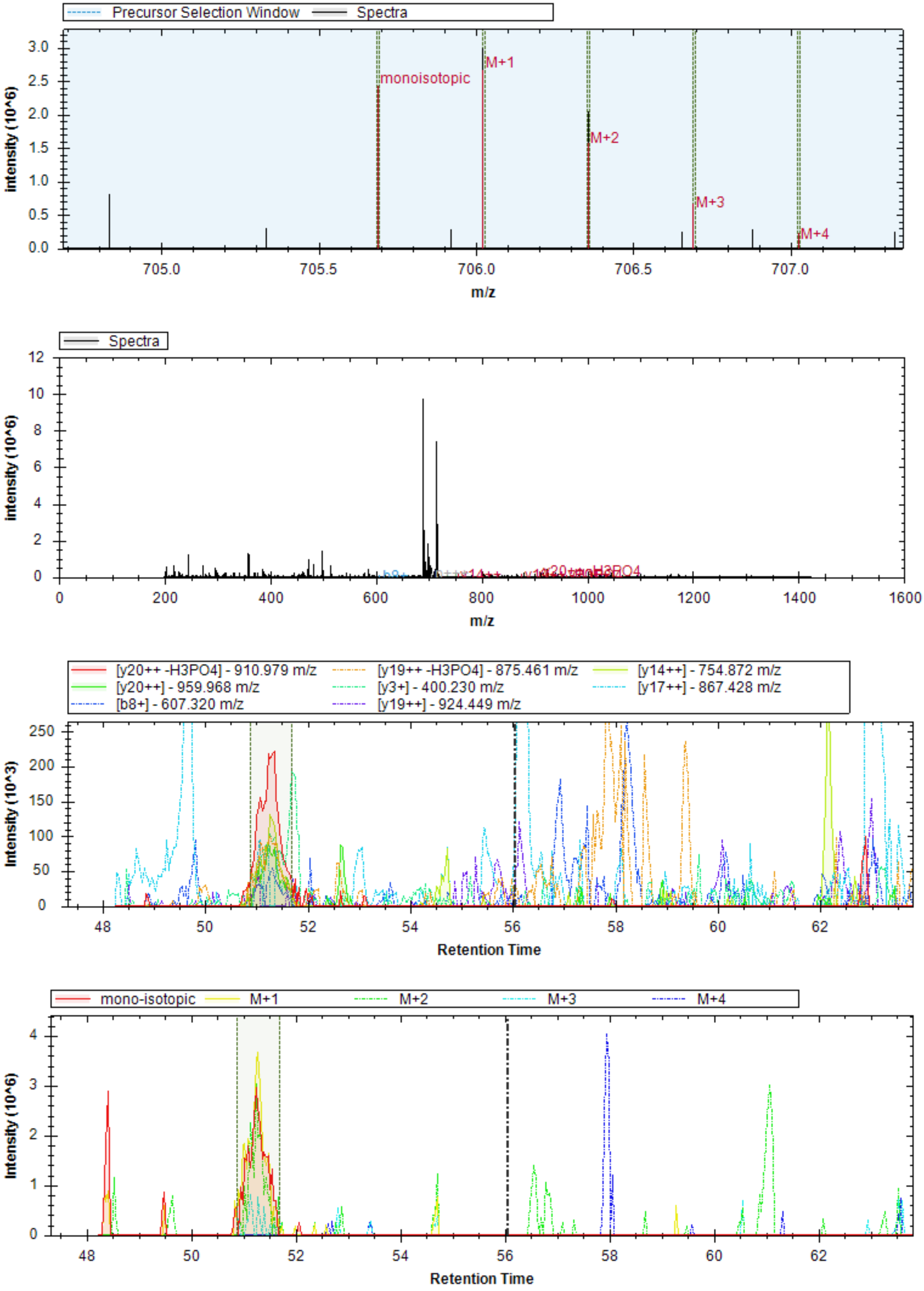

O88935 – _PVAGGPGAPPAARPPASPS[+H+O3+P]PQR_.3
Fig4_MP-DIA-barrel-cortex-p9-1_MHRM.htrms

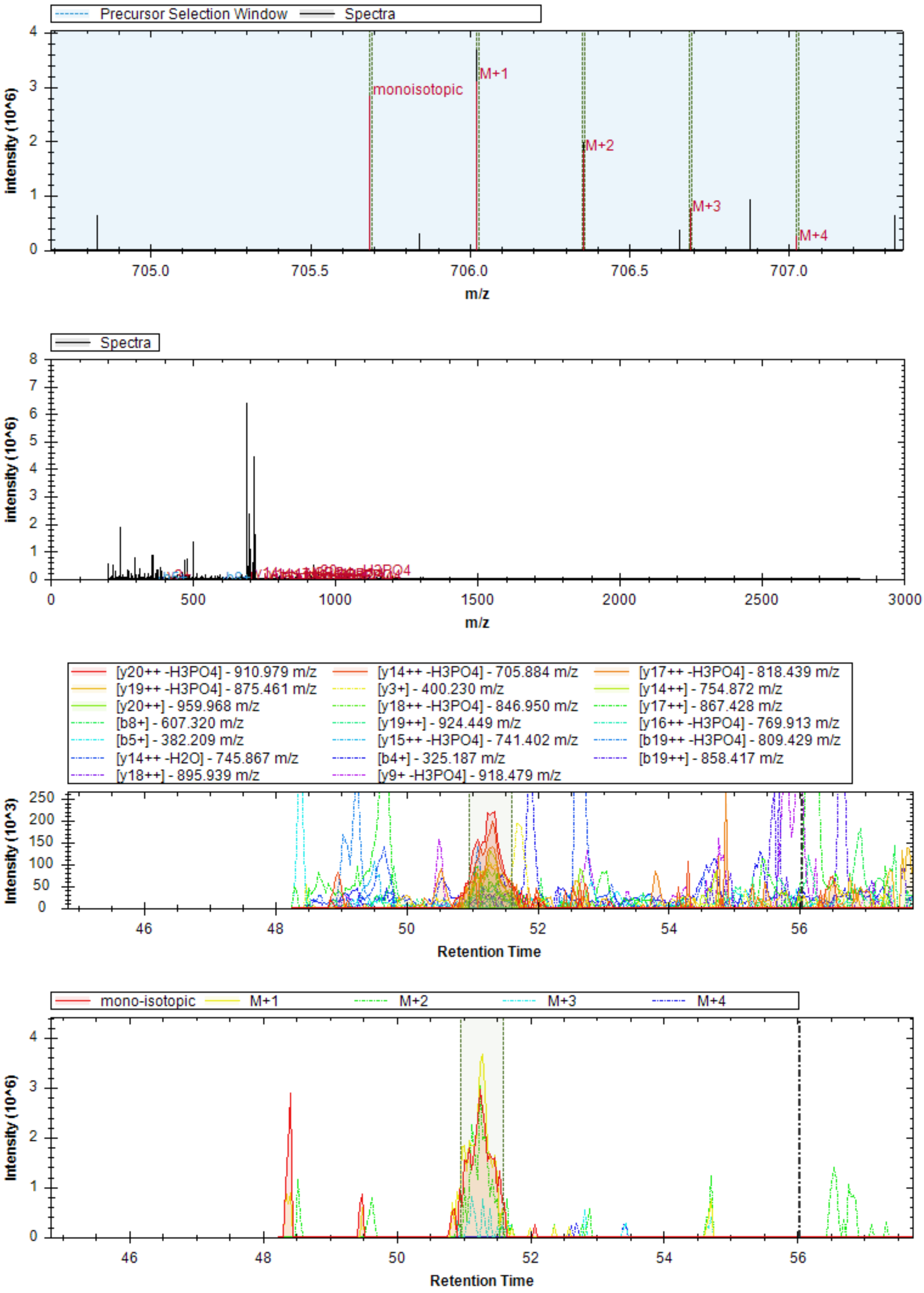

P20357 – _VTDGISKS[+H+O3+P]PEKR_.2
Fig4_MP-DIA-barrel-cortex-p9-1_MHRM.htrms

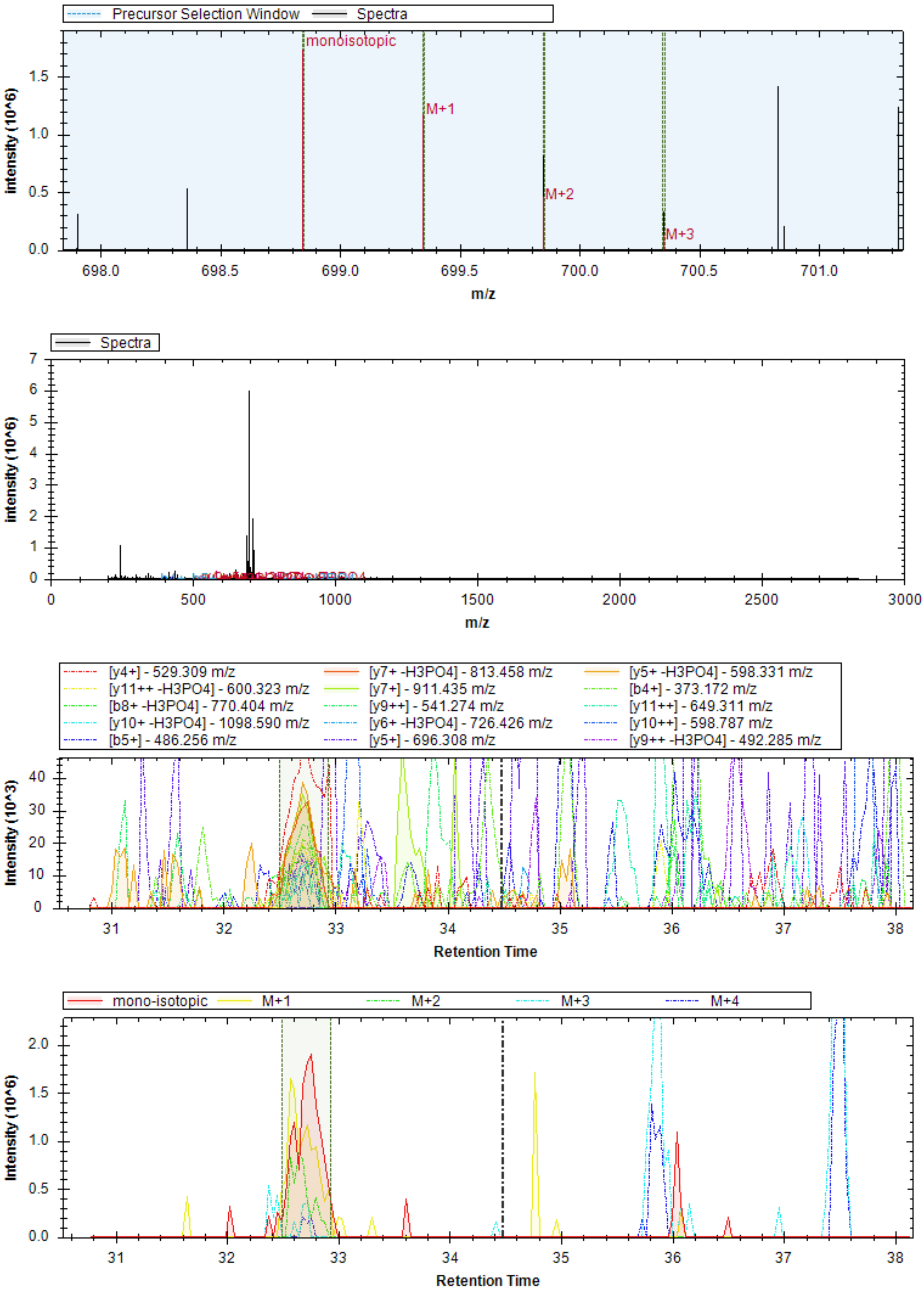

P28738 – _PIRPGHYPAS[+H+O3+P]SPTAVHAVR_.3

Fig4_MP-DIA-barrel-cortex-p9-1_MHRM.htrms

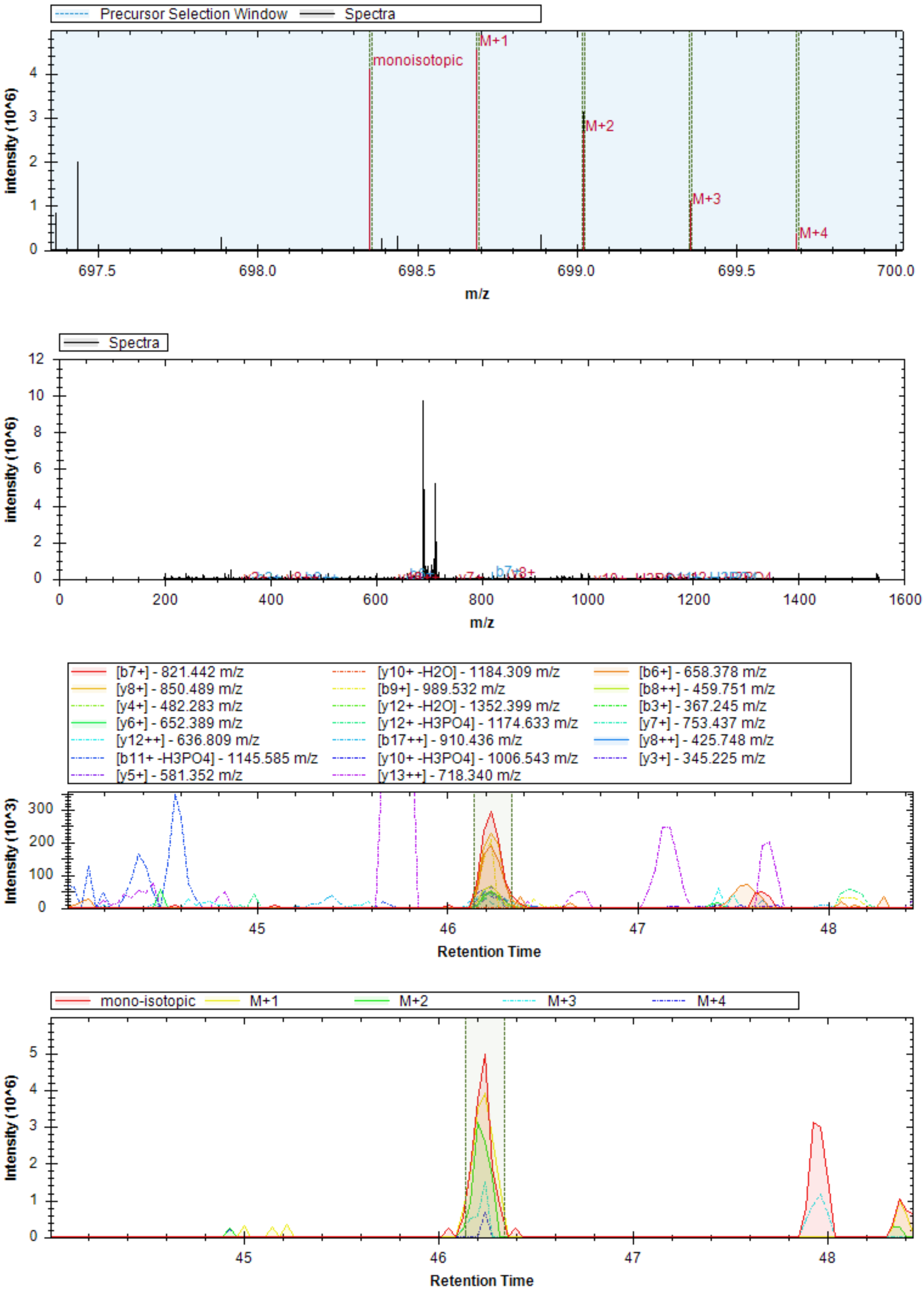

P28738 – _PIRPGHYPASS[+H+O3+P]PTAVHAVR_.3
Fig4_MP-DIA-barrel-cortex-p9-1_MHRM.htrms

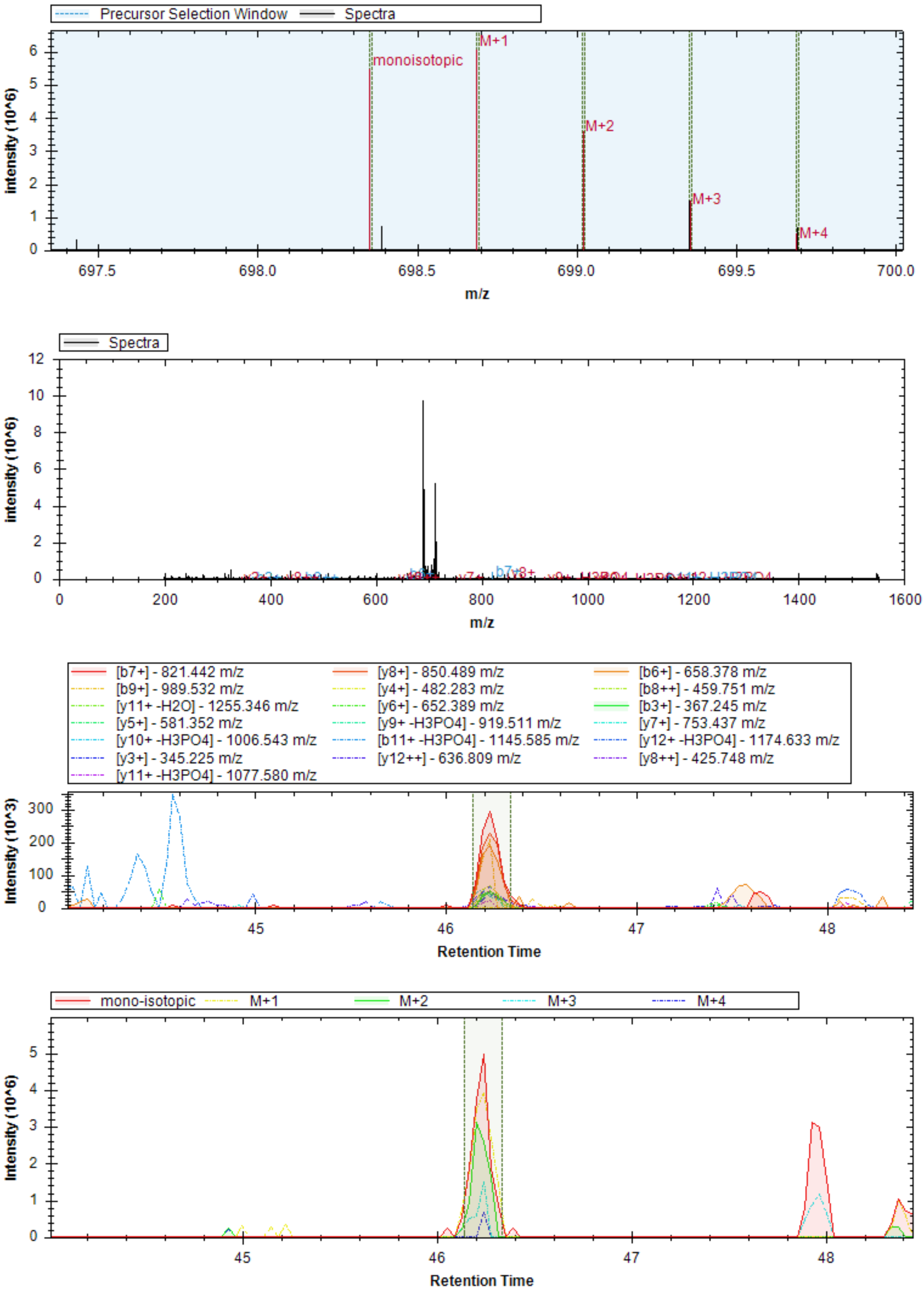

P62751 – _KAY[+H+O3+P]VRLAPDYDALDVANK_.3
Fig4_MP-DIA-barrel-cortex-p9-1_MHRM.htrms

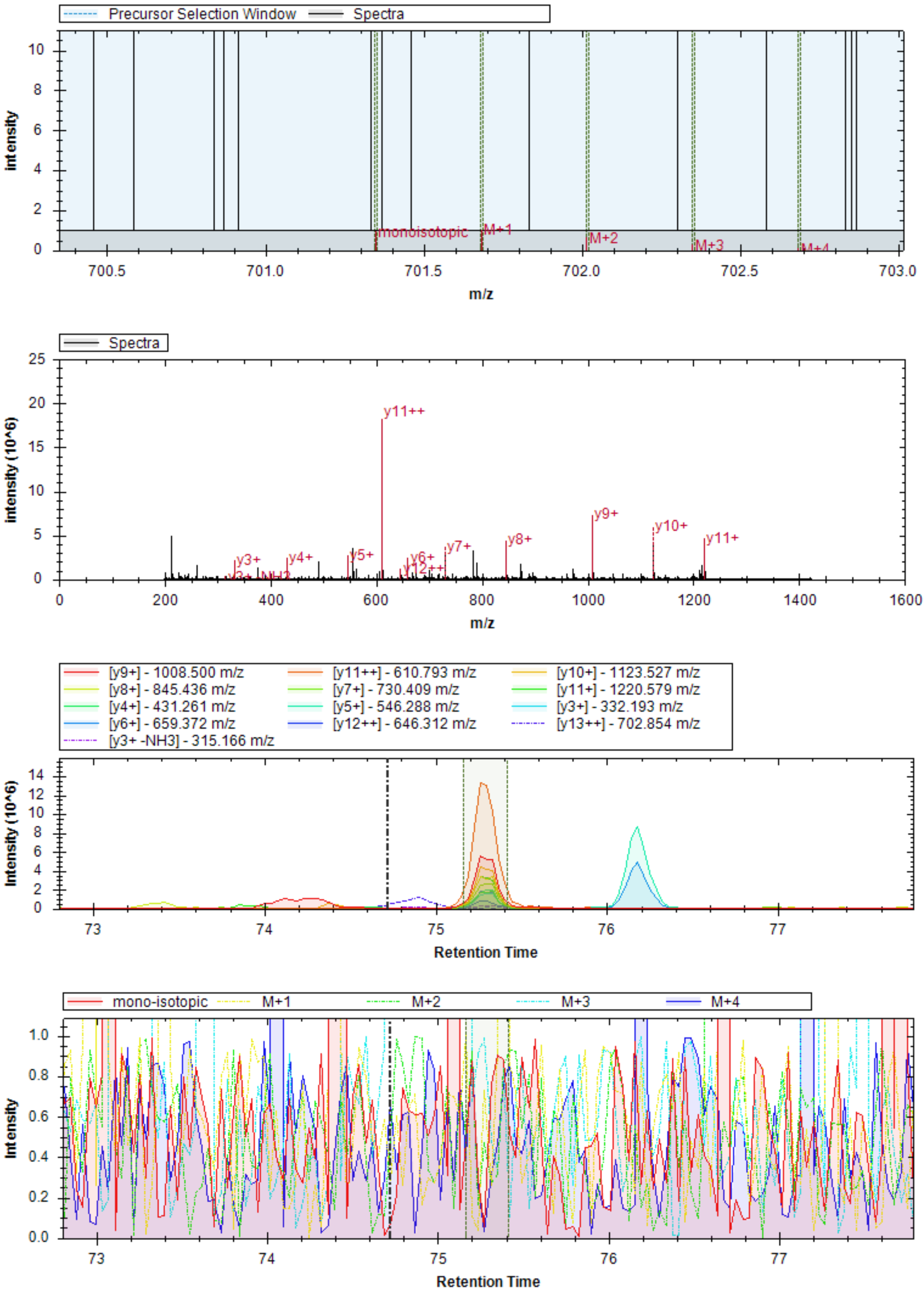

Q3U4B4 – _RHKT[+H+O3+P]LSANEEK_.2

Fig4_MP-DIA-barrel-cortex-p9-1_MHRM.htrms

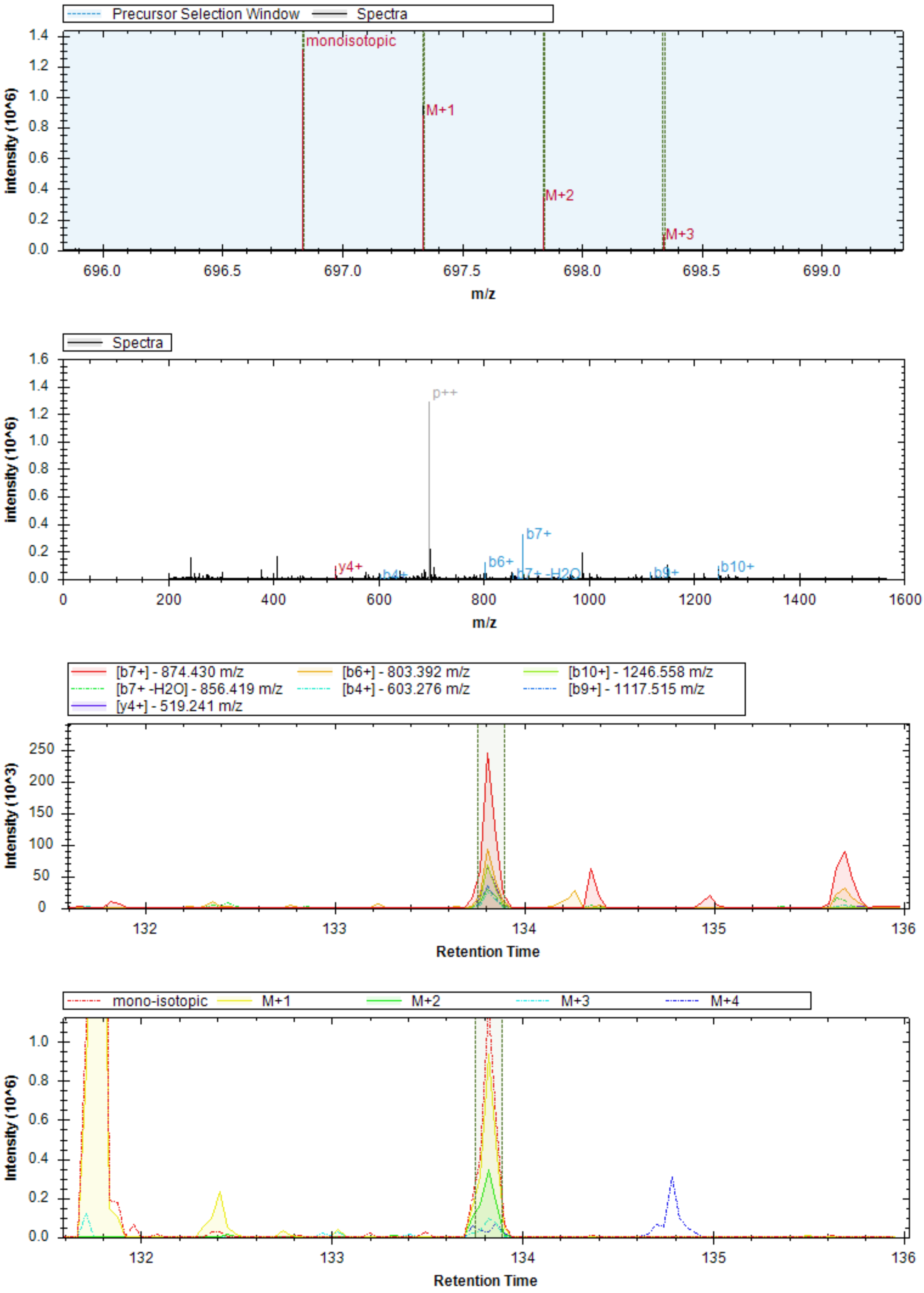

Q9CVB6 – _IIEET[+H+O3+P]LALK_.3
Fig4_MP-DIA-barrel-cortex-p9-1_MHRM.htrms

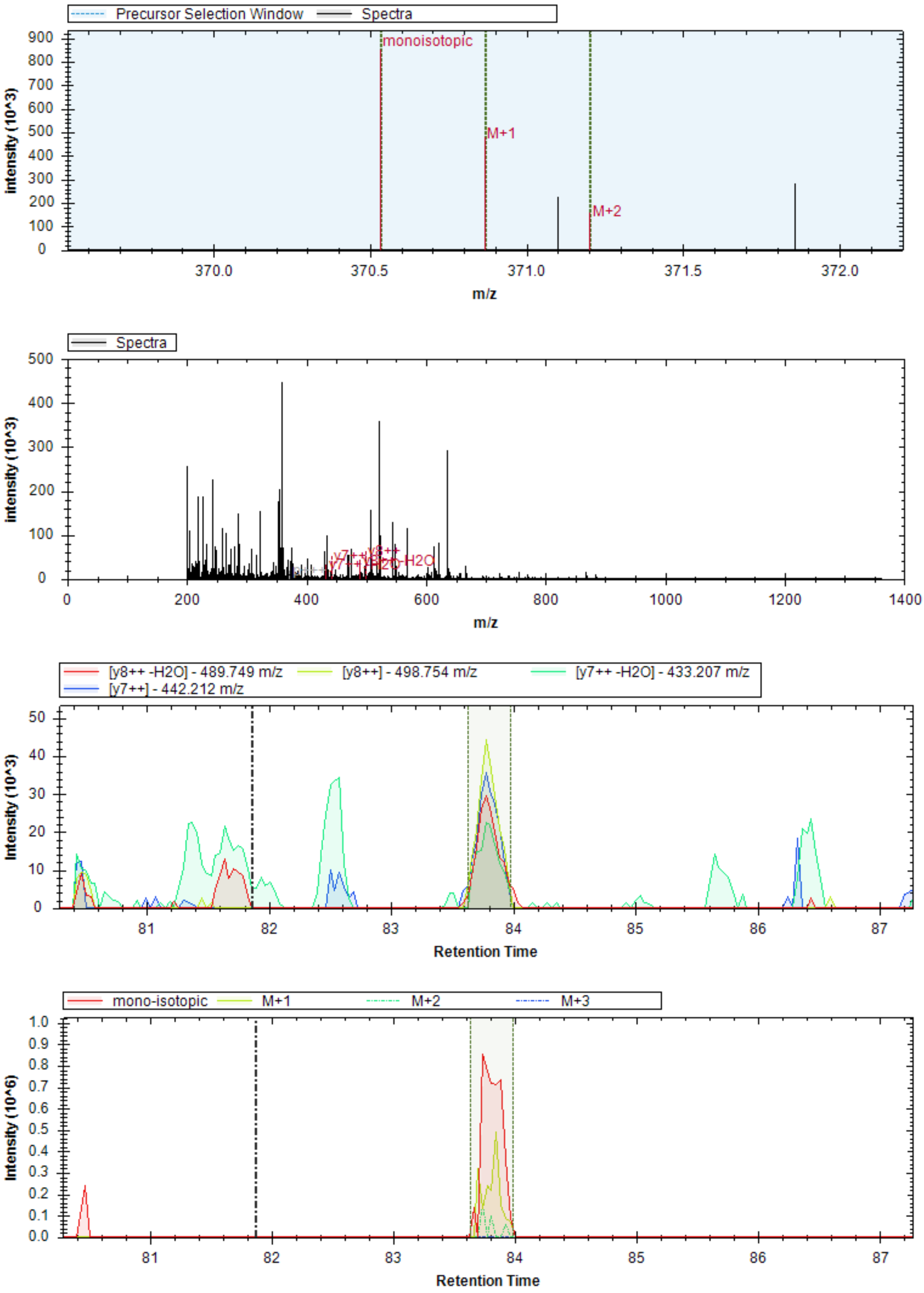

Q9ESE1;Q9ESE1-2 – _VGT[+H+O3+P]VLLIM[+O]HTLK_.2

Fig4_MP-DIA-barrel-cortex-p9-1_MHRM.htrms

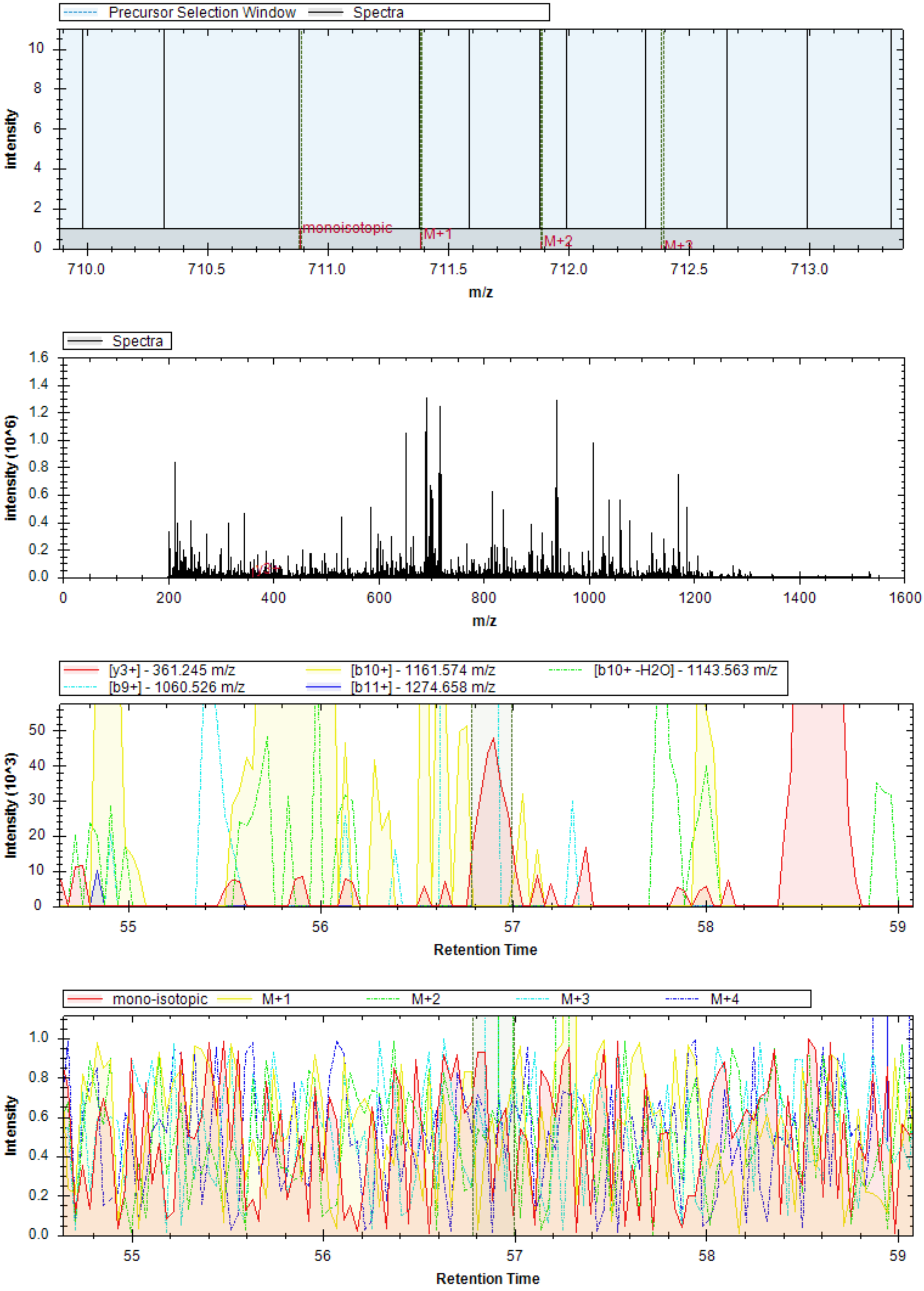

P10637 – _SGYSS[+H+O3+P]PGSPGTPGSR_.2
Fig4_MP-DIA-barrel-cortex-p9-1_MHRM.htrms

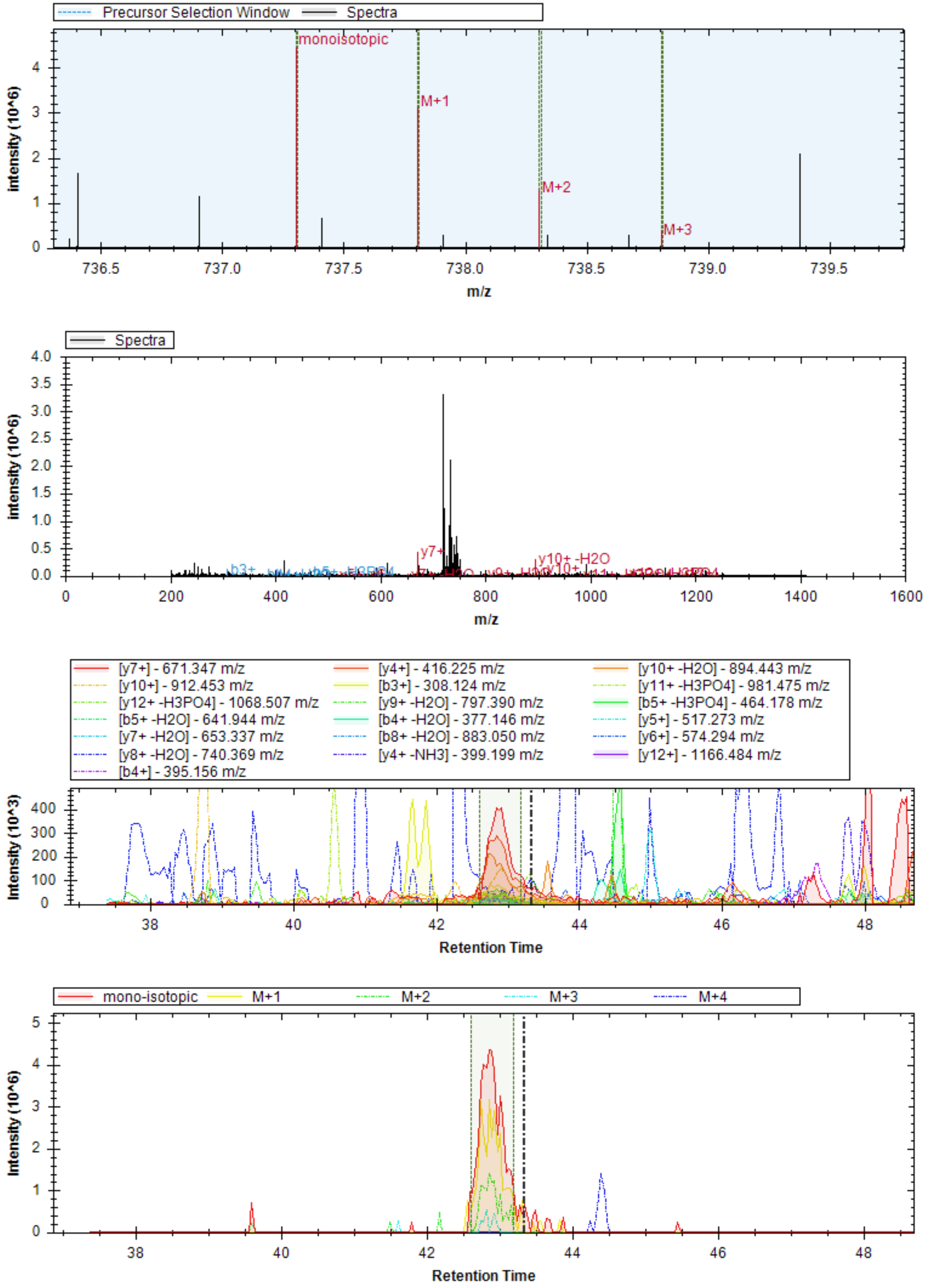

P10637 – _SGYSSPGS[+H+O3+P]PGTPGSR_.2

Fig4_MP-DIA-barrel-cortex-p9-1_MHRM.htrms

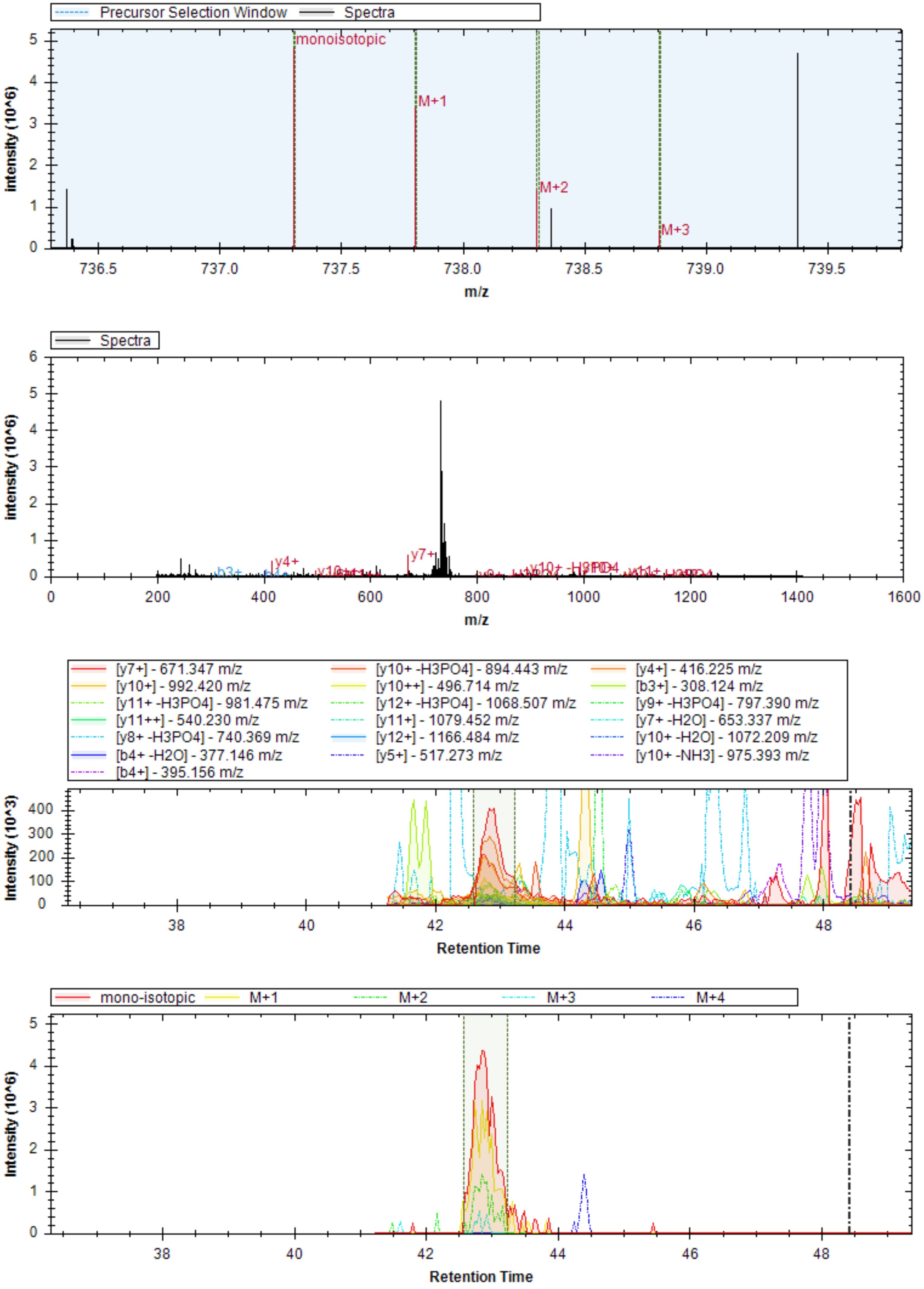

P57776-3 – _ATAPQTQHVS[+H+O3+P]PMR_.2
Fig4_MP-DIA-barrel-cortex-p9-1_MHRM.htrms

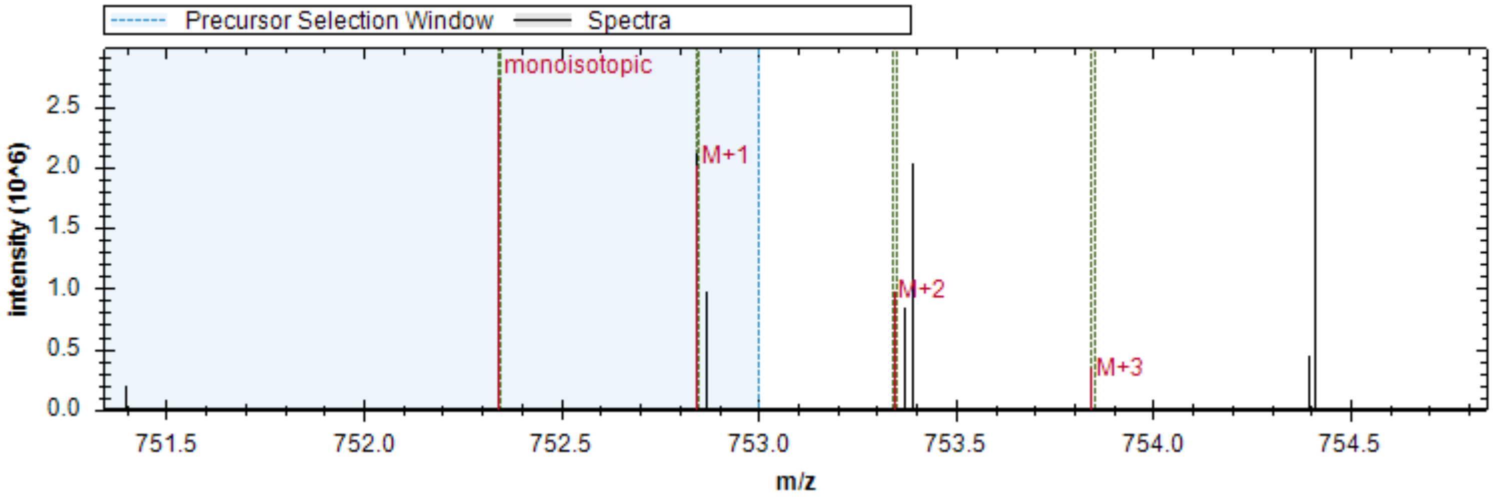

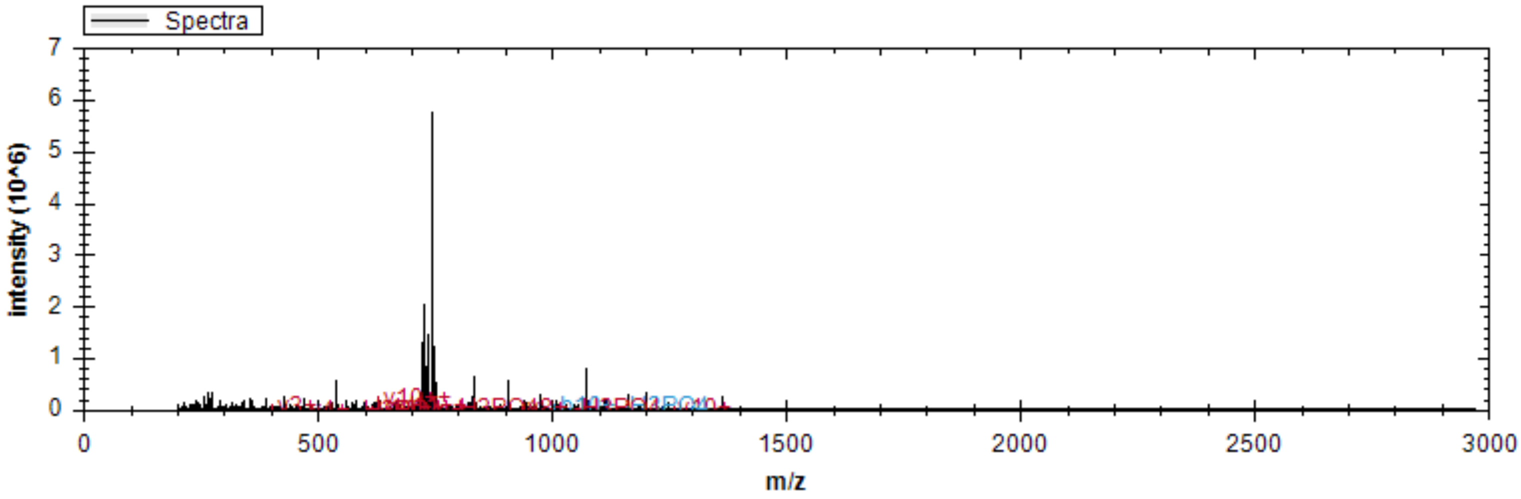

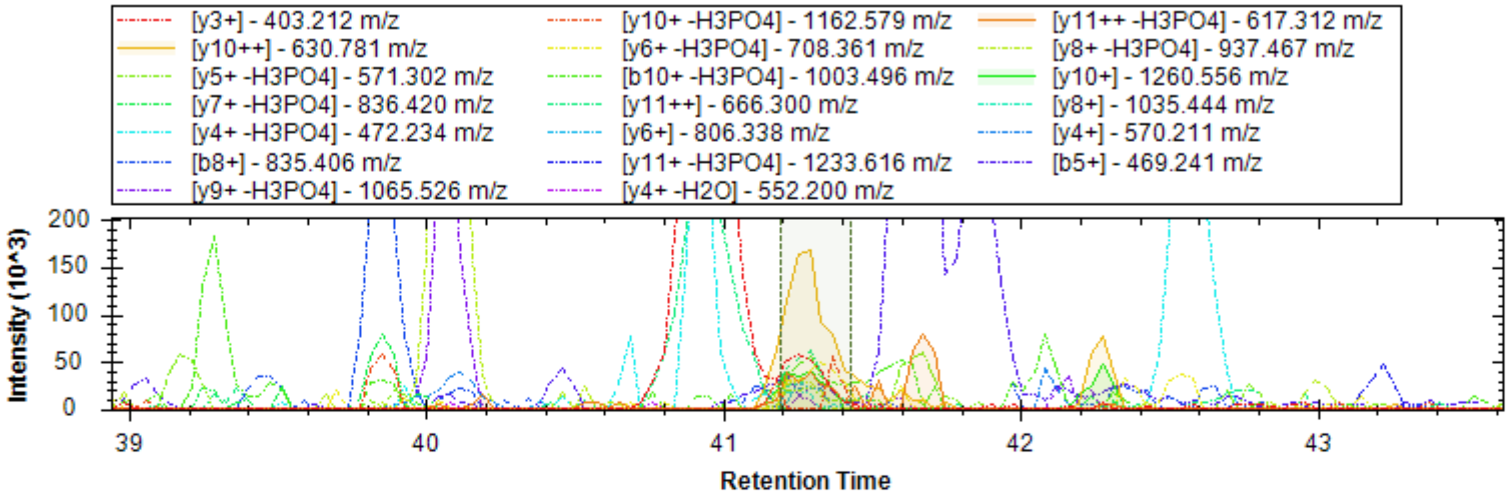

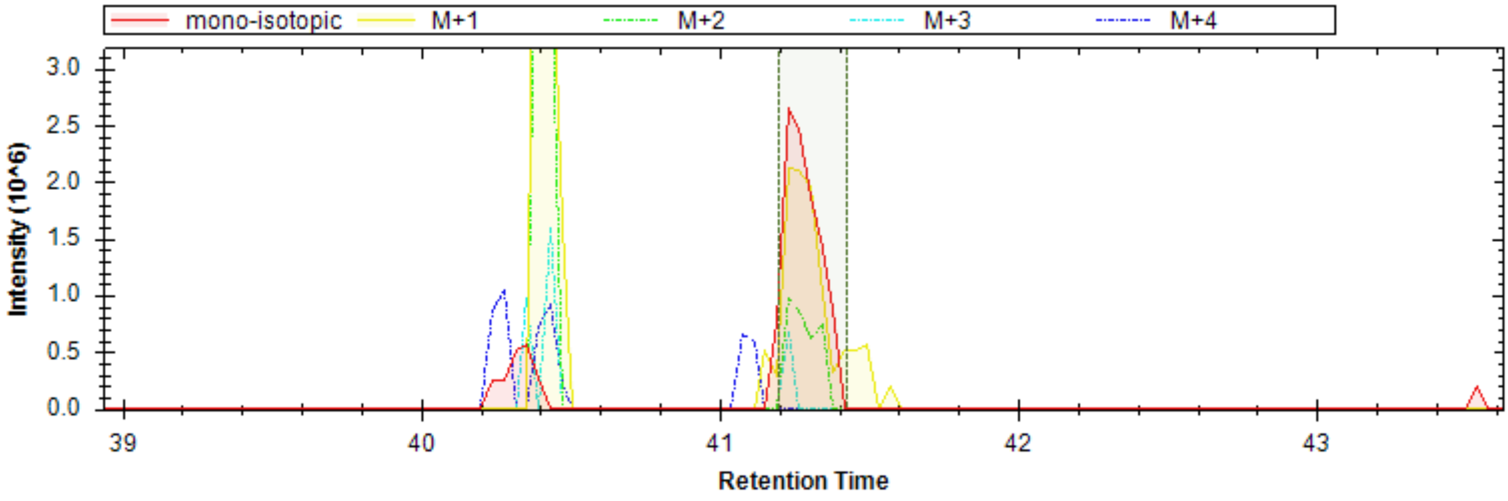

P97798-2 – _NSQDIT[+H+O3+P]PVDNSMDSNIHQR_.3
Fig4_MP-DIA-barrel-cortex-p9-1_MHRM.htrms

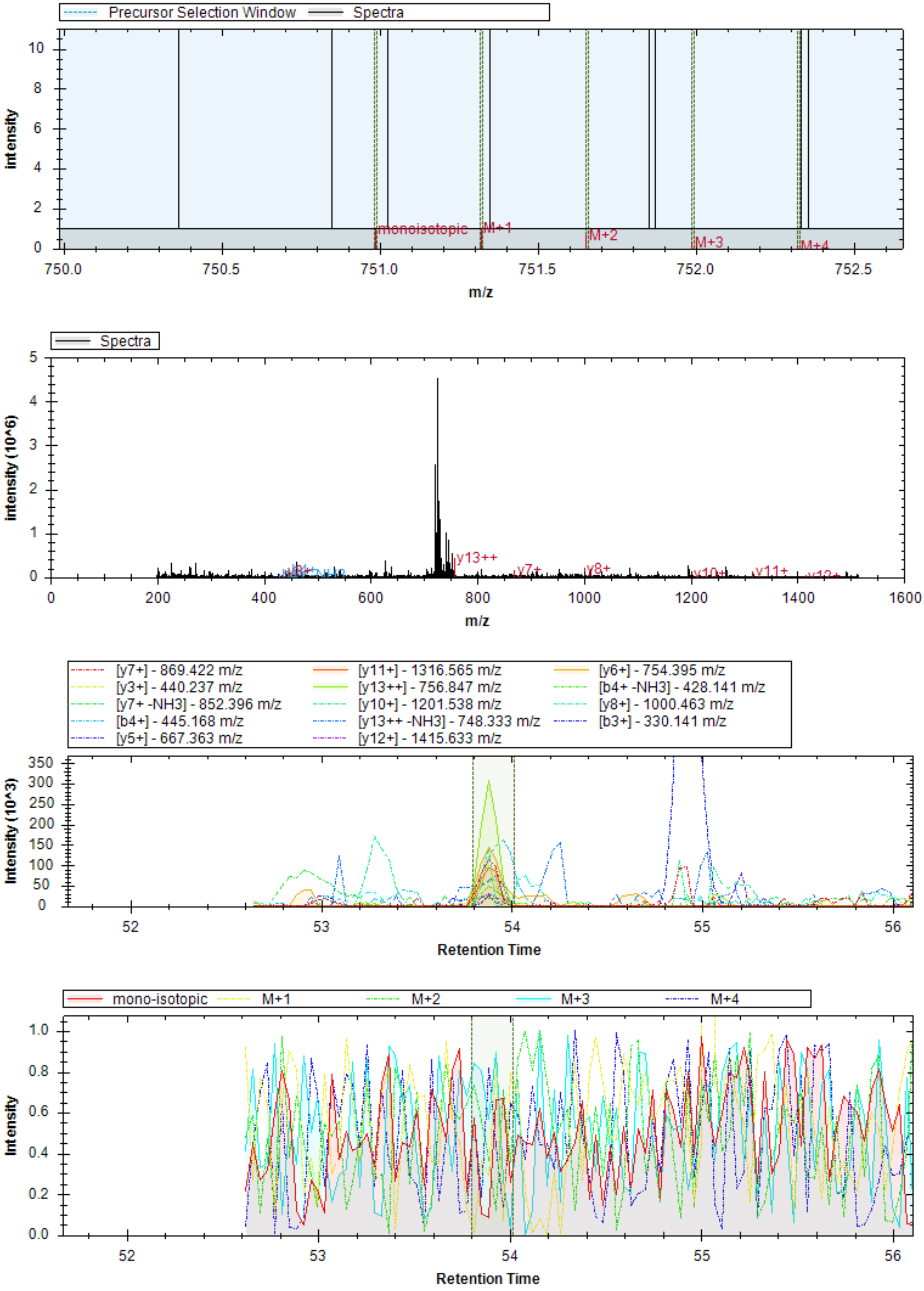

Q8C5R2 – _LAGNEALS[+H+O3+P]PTSPSK_.2
Fig4_MP-DIA-barrel-cortex-p9-1_MHRM.htrms

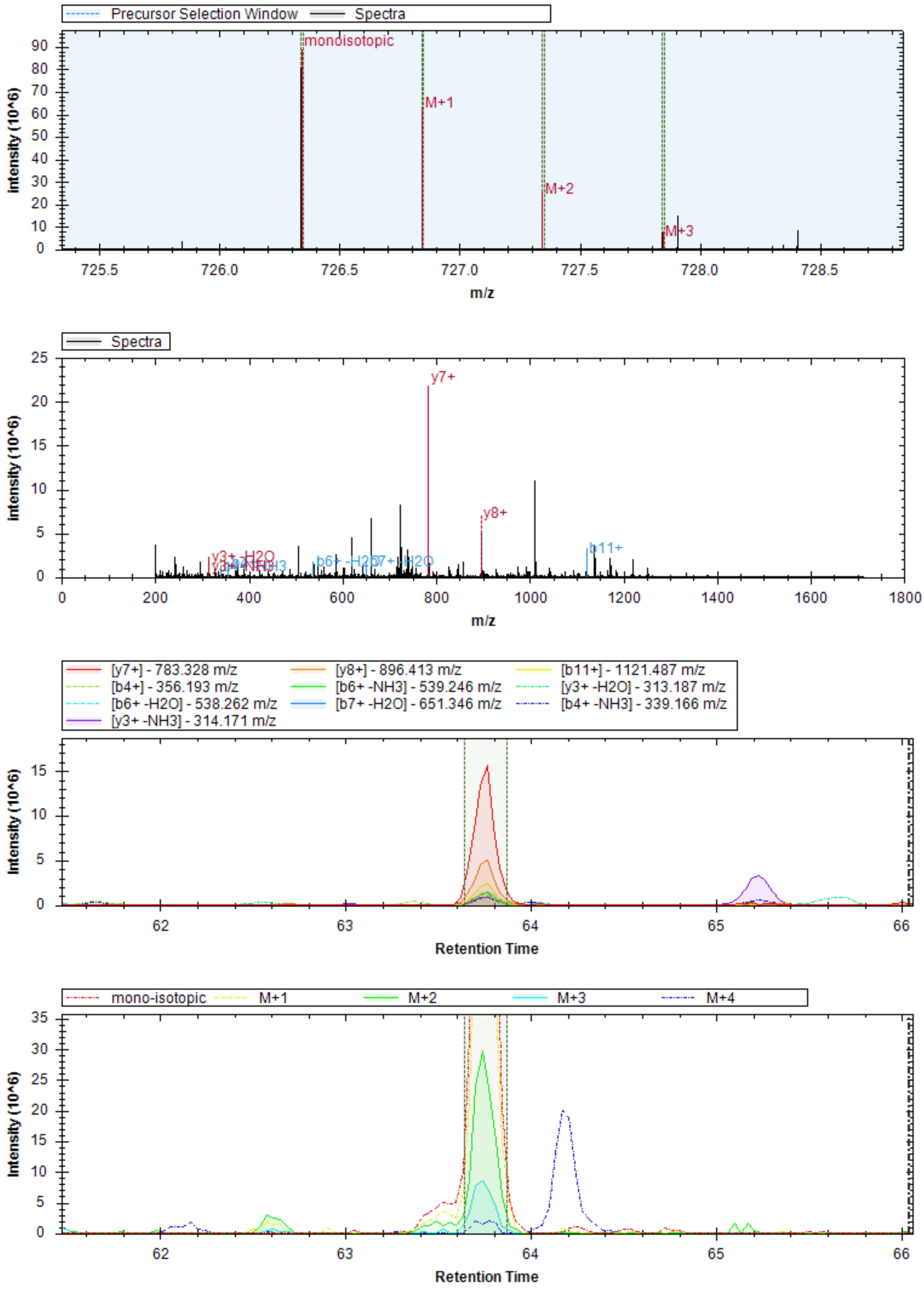

Q8K0U4 – _ETAPTSTYS[+H+O3+P]SPAR_.2
Fig4_MP-DIA-barrel-cortex-p9-1_MHRM.htrms

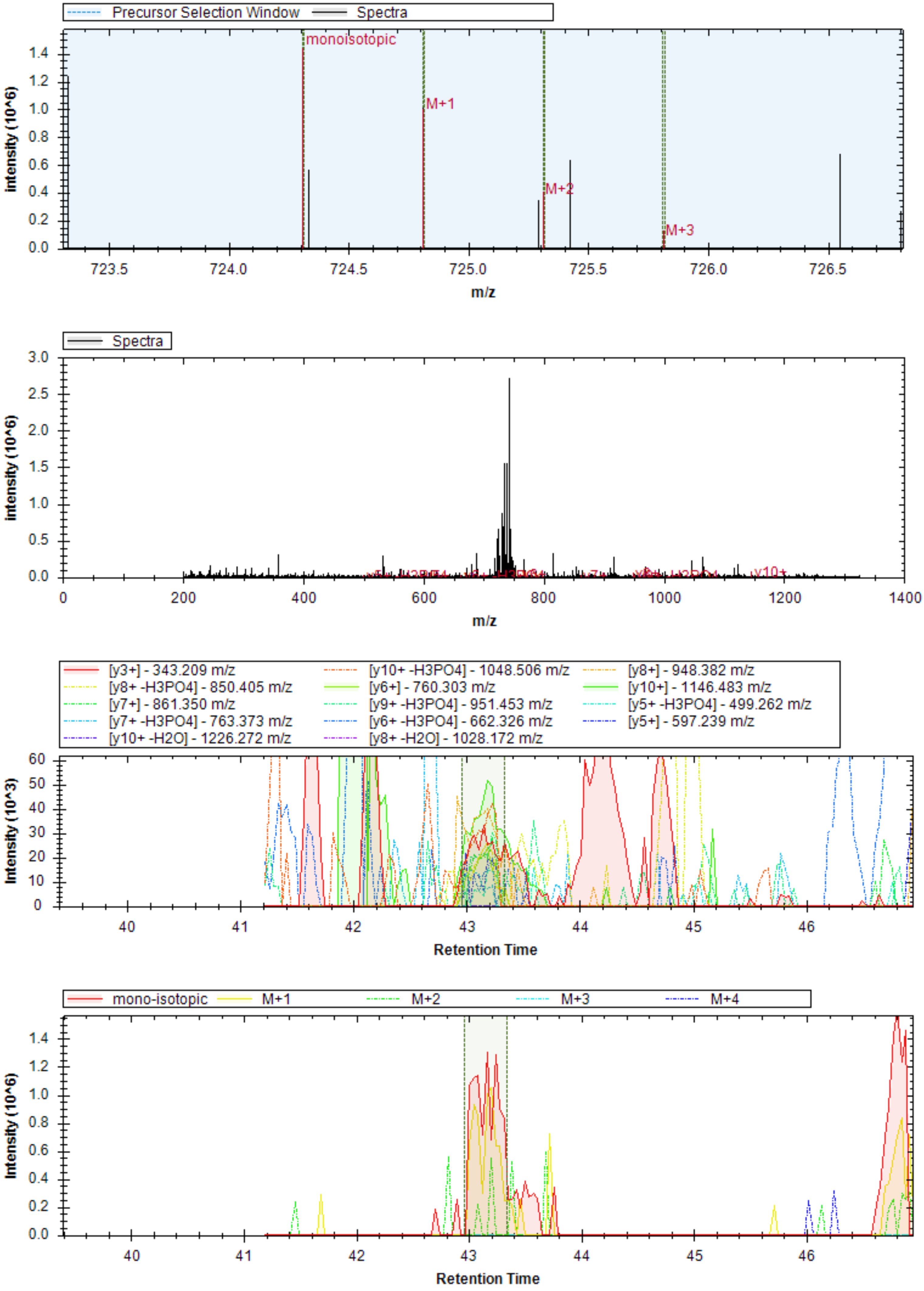

Q8K0U4 – _ETAPTSTYSS[+H+O3+P]PAR_.2

Fig4_MP-DIA-barrel-cortex-p9-1_MHRM.htrms

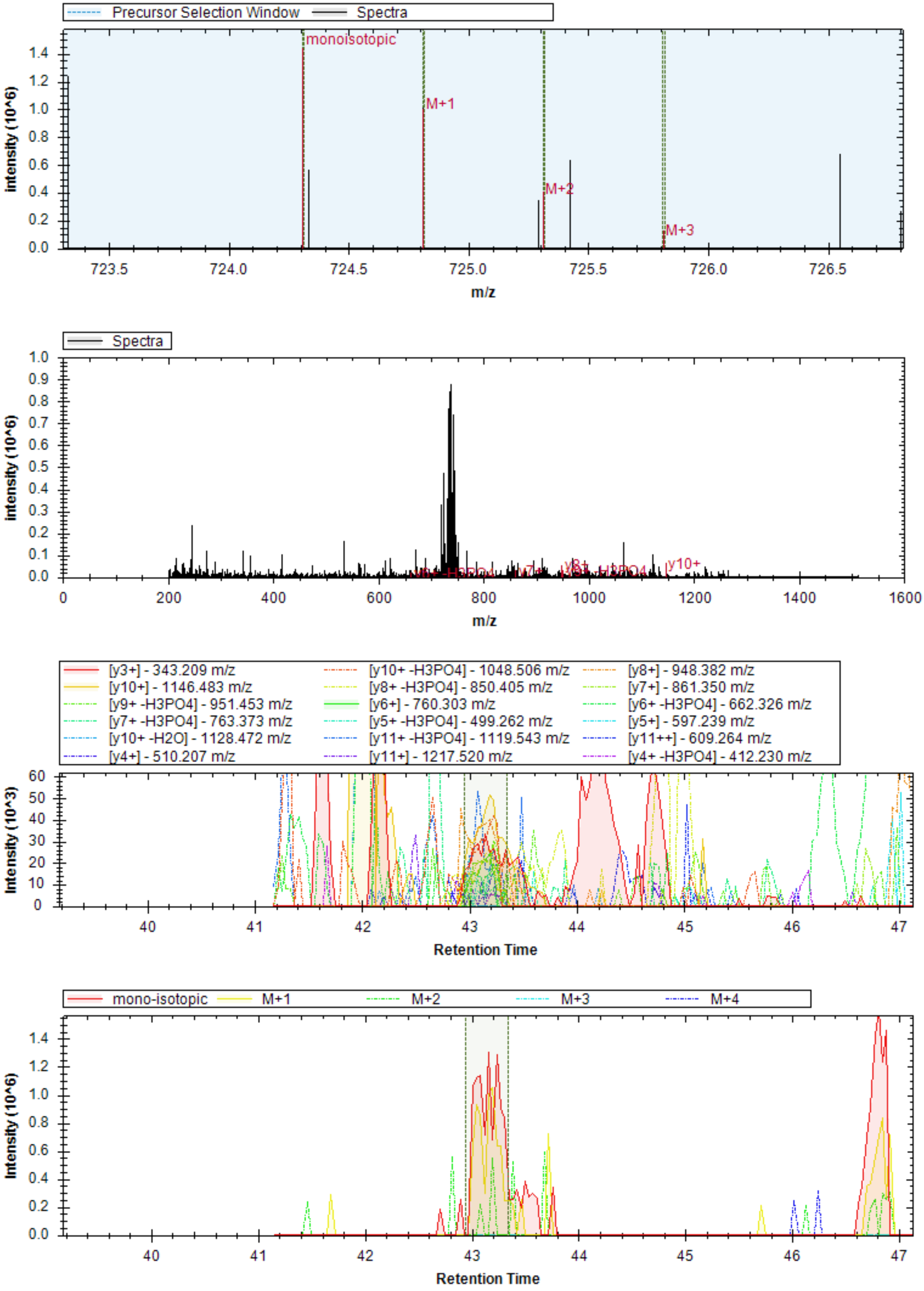

Q8VCI5 – _AKPS[+H+O3+P]PEHAPTISAPDASGPQK_.3
Fig4_MP-DIA-barrel-cortex-p9-1_MHRM.htrms

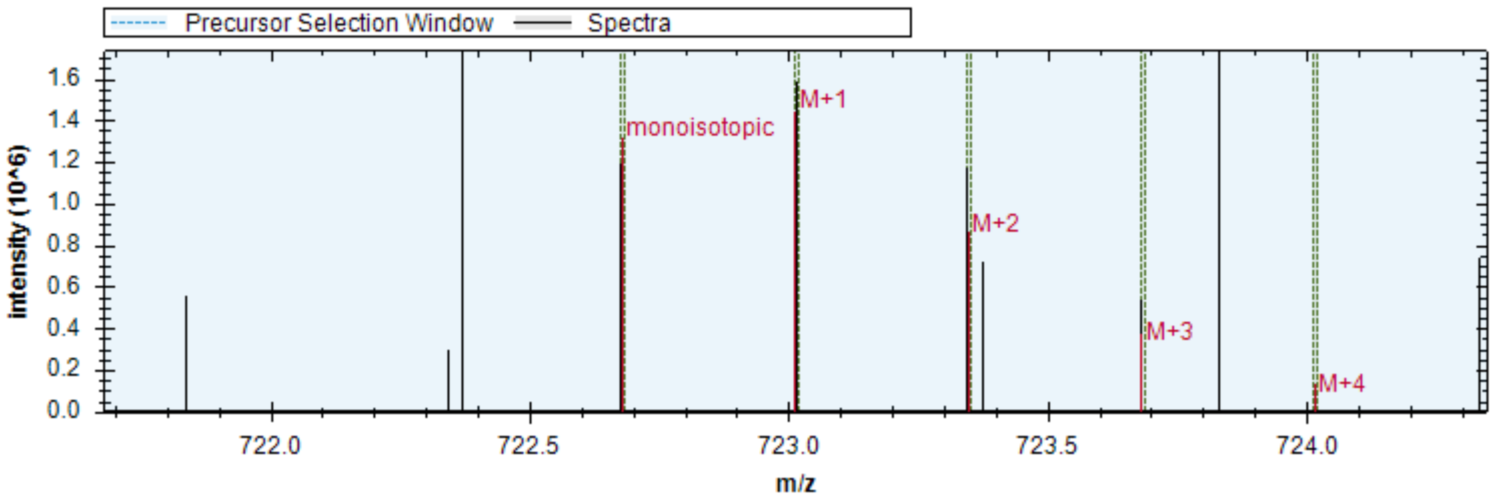

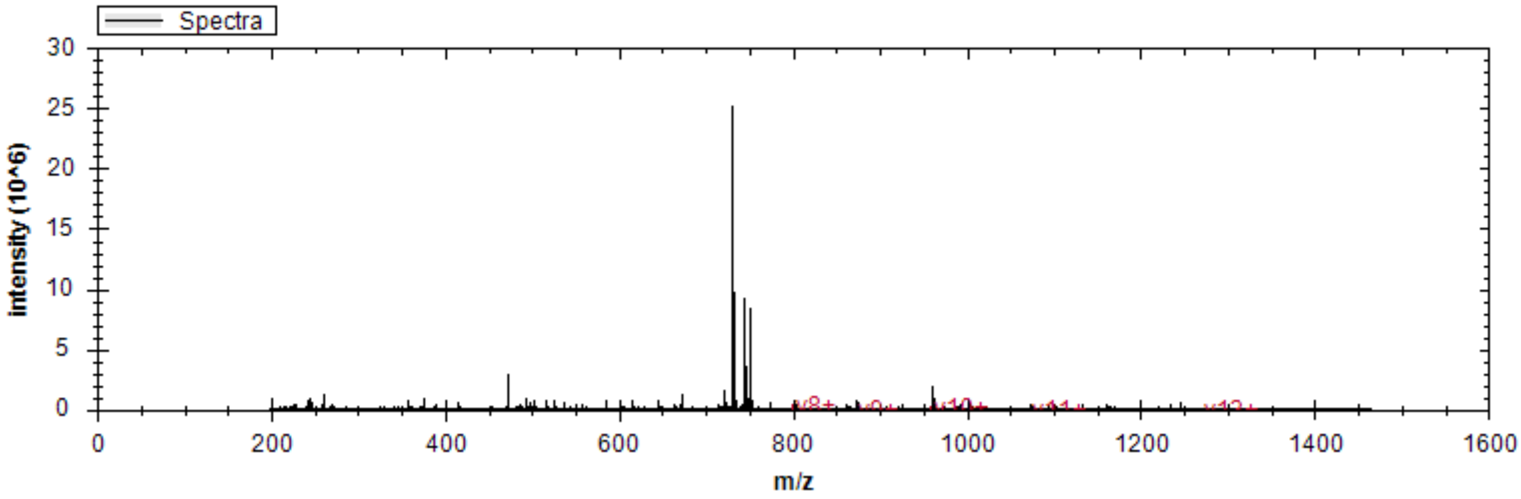

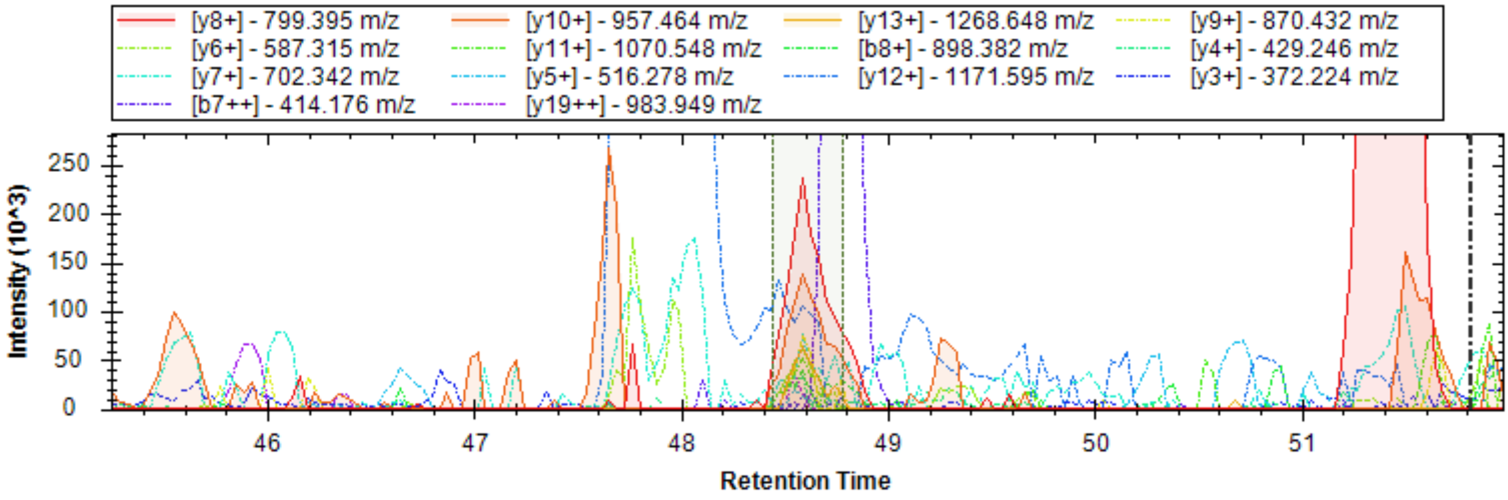

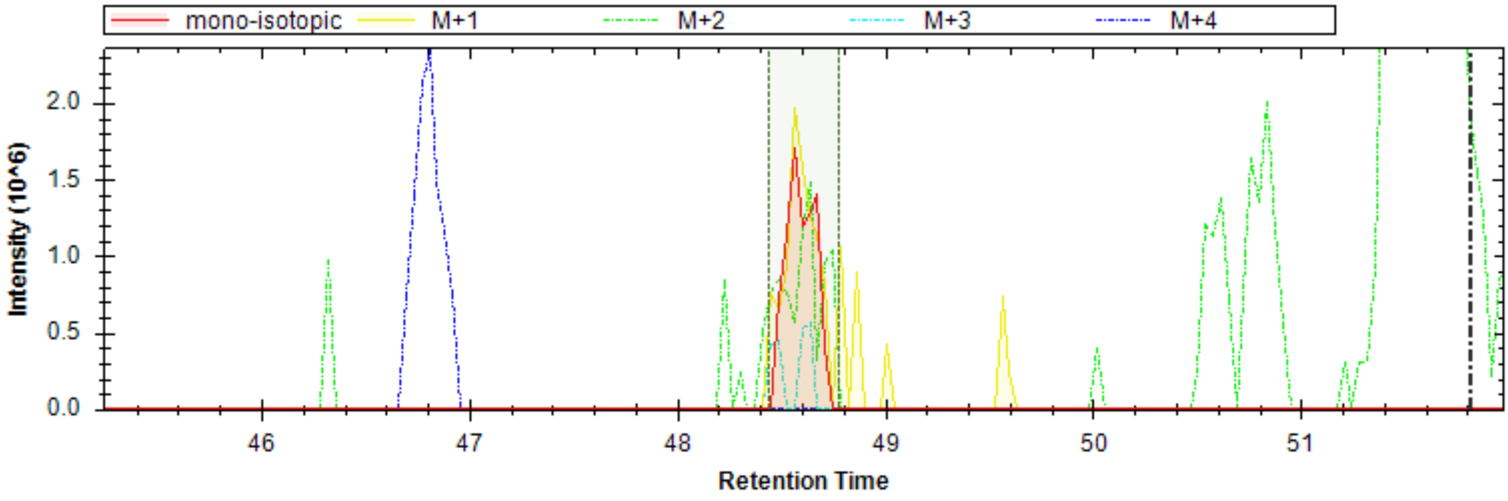

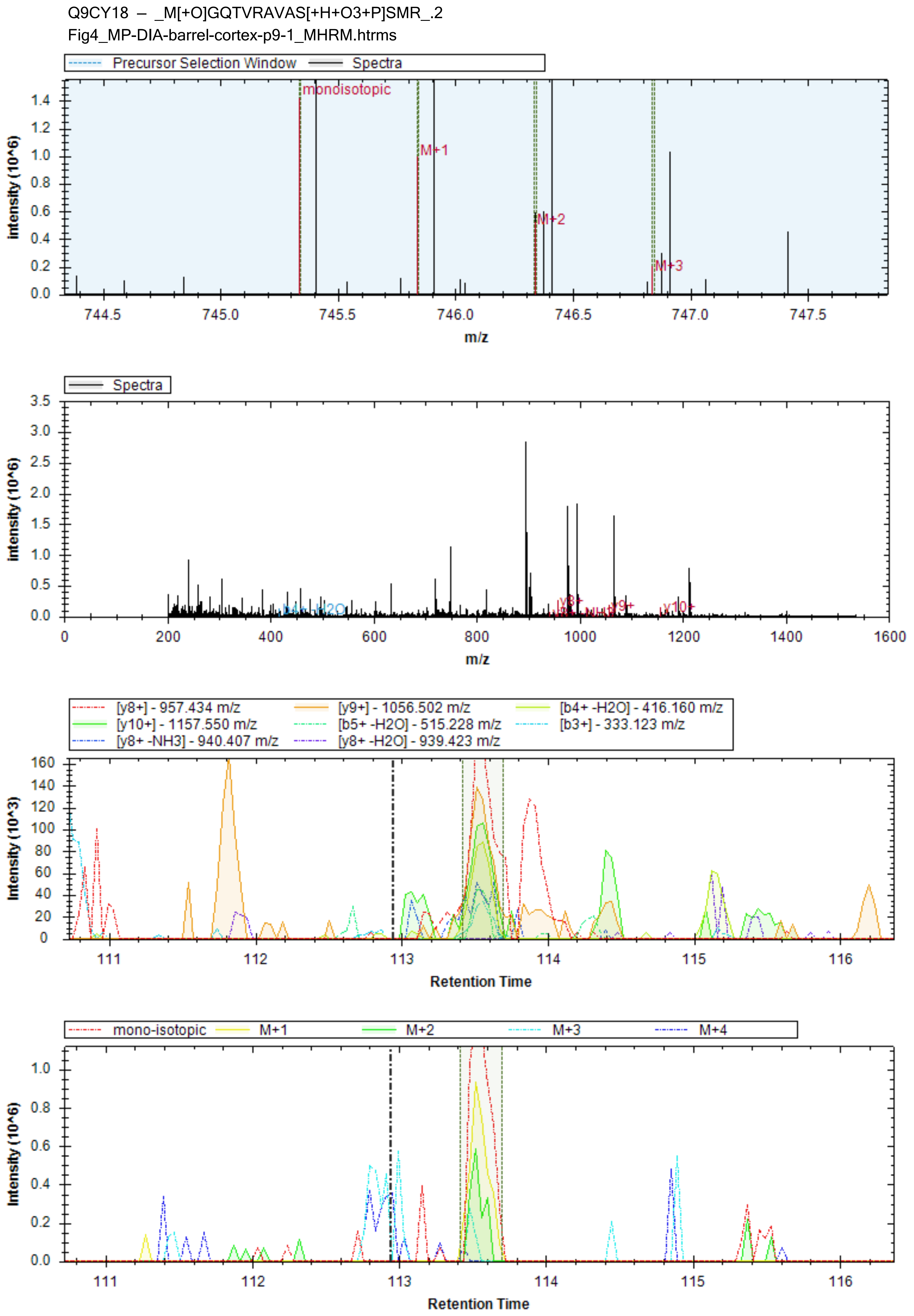
Q9CY18 – _M[+O]GQTVRAVAS[+H+O3+P]SMR_.2
Fig4_MP-DIA-barrel-cortex-p9-1_MHRM.htrms
Precursor Selection Window
Spectra
monoisotopic
M+1
M+2
M+3
intensity (10^6)
m/z
Spectra
intensity (10^6)
m/z
[y8+] - 957.434 m/z
[y9+] - 1056.502 m/z
[b4+ -H2O] - 416.160 m/z
[y10+] - 1157.550 m/z
[b5+ -H2O] - 515.228 m/z
[b3+] - 333.123 m/z
[y8+ -NH3] - 940.407 m/z
[y8+ -H2O] - 939.423 m/z
Intensity (10^3)
Retention Time
mono-isotopic
M+1
M+2
M+3
M+4
Intensity (10^6)
Retention Time

Q9JLM8 – _SKS[+H+O3+P]PASTSSVNGTPGSQLSTPR_.3

Fig4_MP-DIA-barrel-cortex-p9-1_MHRM.htrms

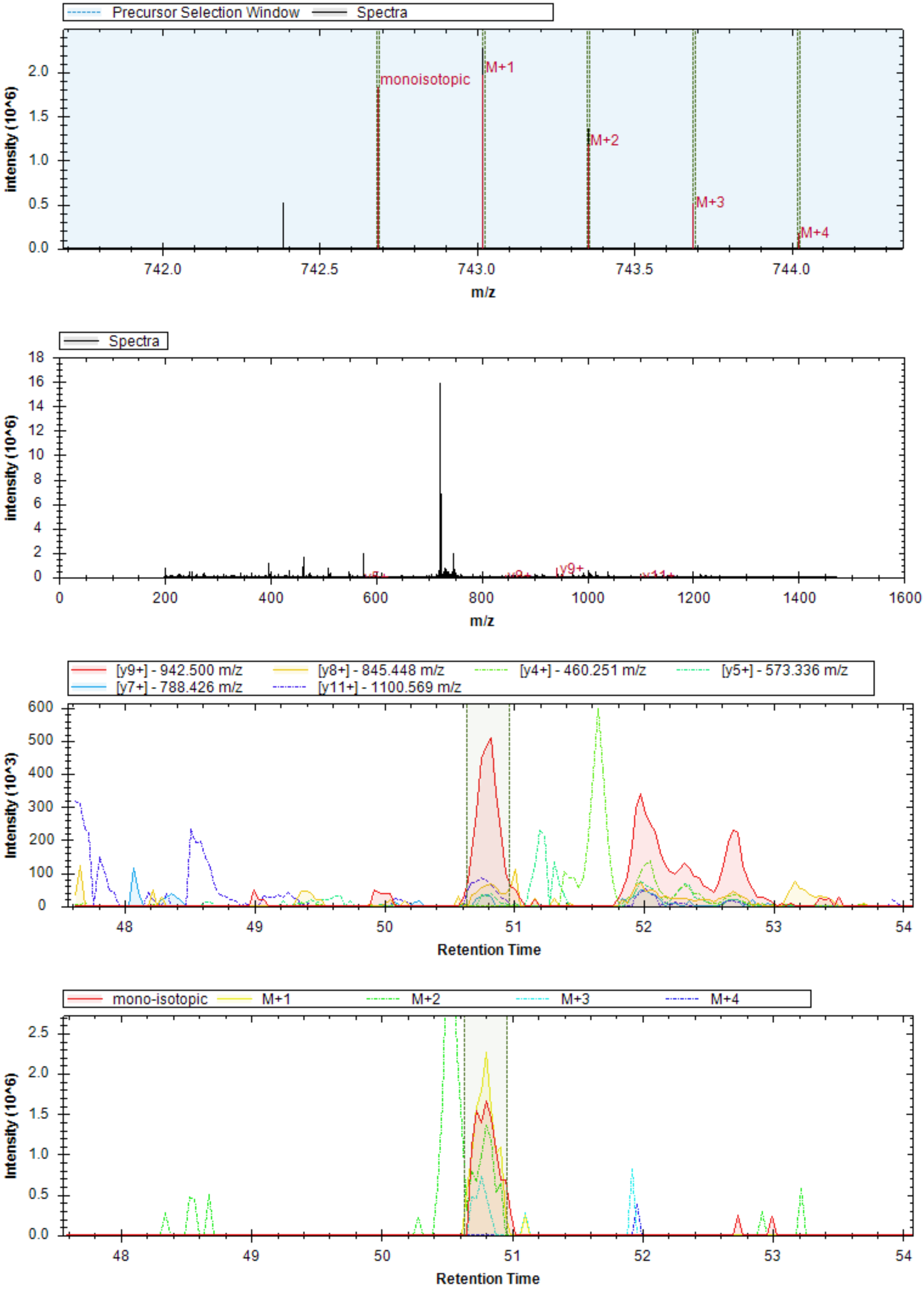

Q9JLM8 – _SPSPSPT[+H+O3+P]SPGSLRK_.2
Fig4_MP-DIA-barrel-cortex-p9-1_MHRM.htrms

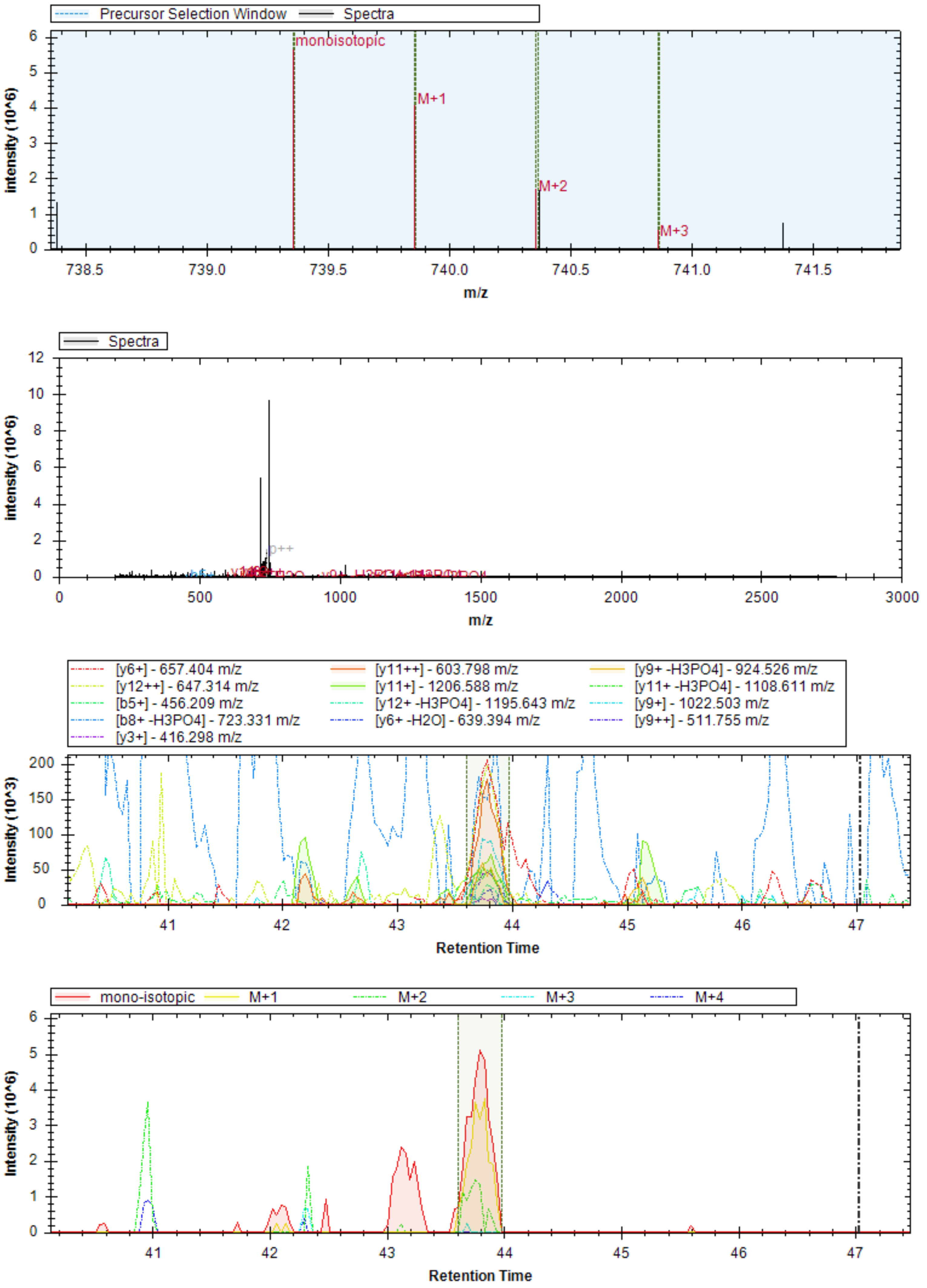

Q9JLM8 – _SPSPSPTS[+H+O3+P]PGSLRK_.2

Fig4_MP-DIA-barrel-cortex-p9-1_MHRM.htrms

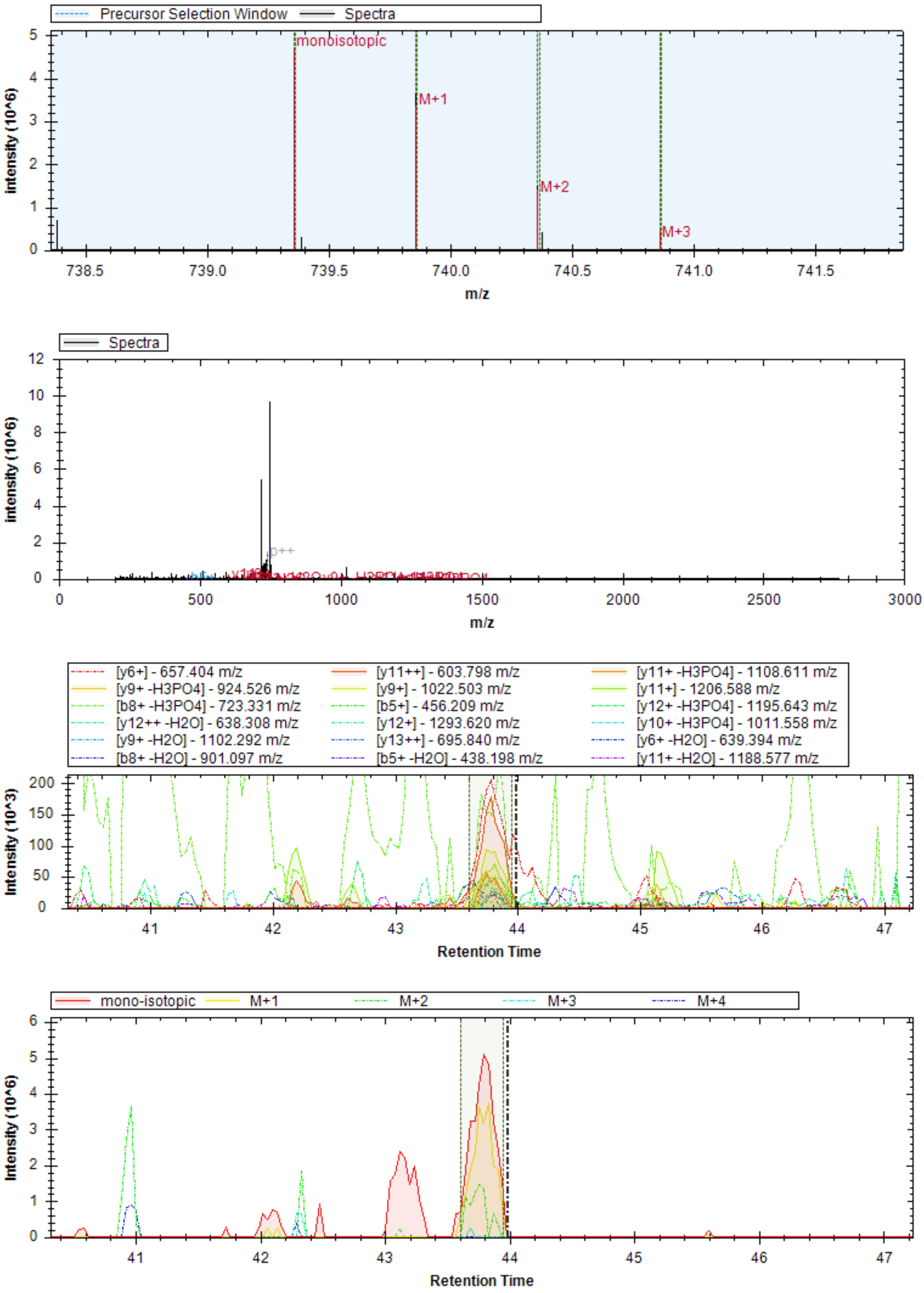

D3Z6S9 – _IPSIEIT[+H+O3+P]ET[+H+O3+P]LPQTQASMLR_.3
Fig4_MP-DIA-barrel-cortex-p9-1_MHRM.htrms

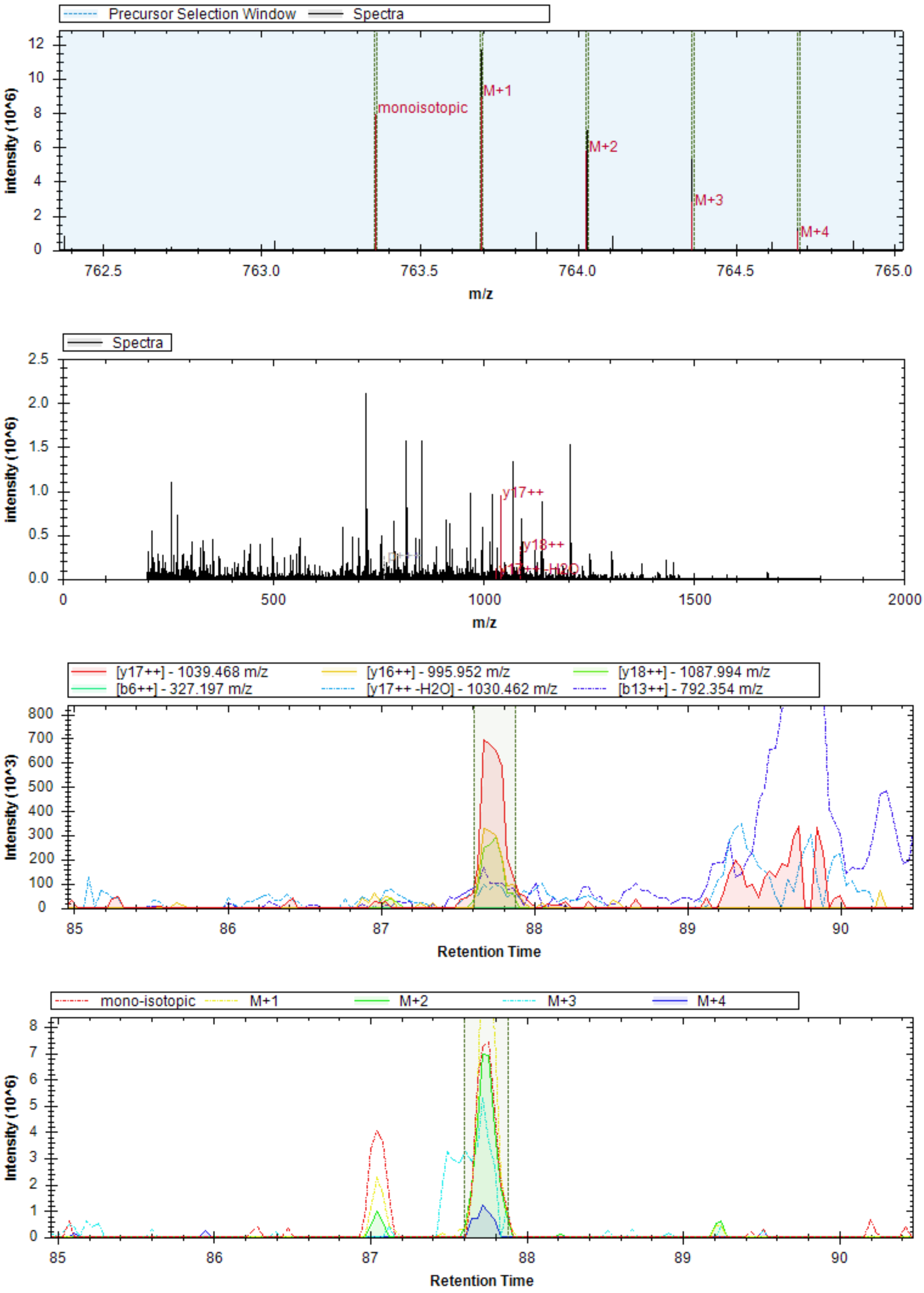

P06837 – _QADVPAAVTDAAAT[+H+O3+P]TPAAEDAATK_.3

Fig4_MP-DIA-barrel-cortex-p9-1_MHRM.htrms

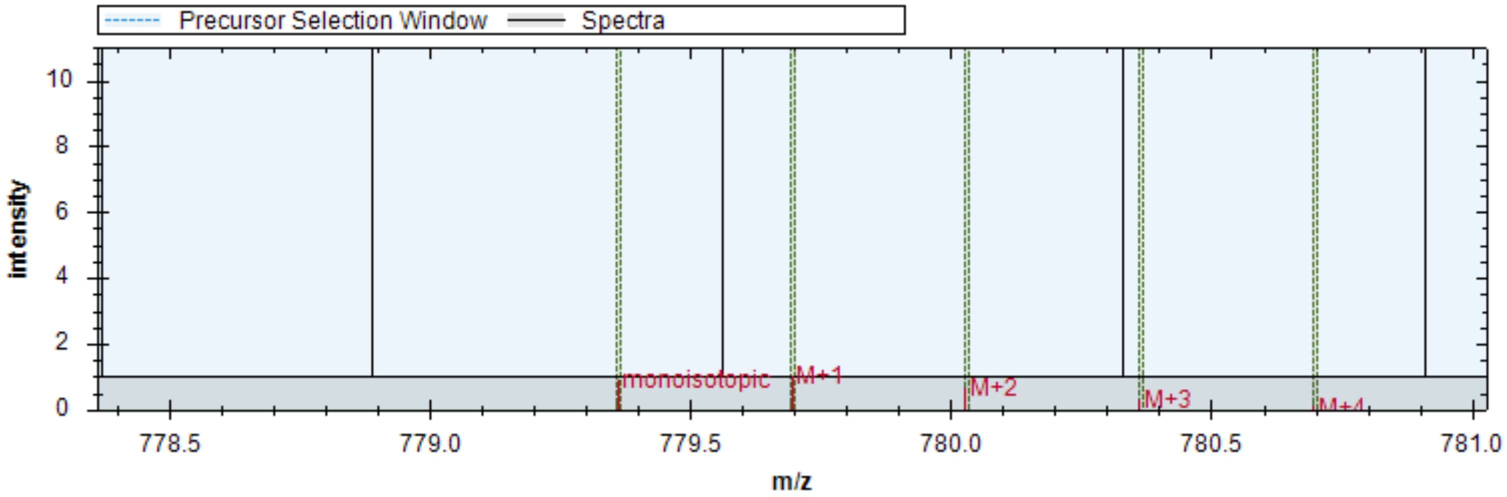

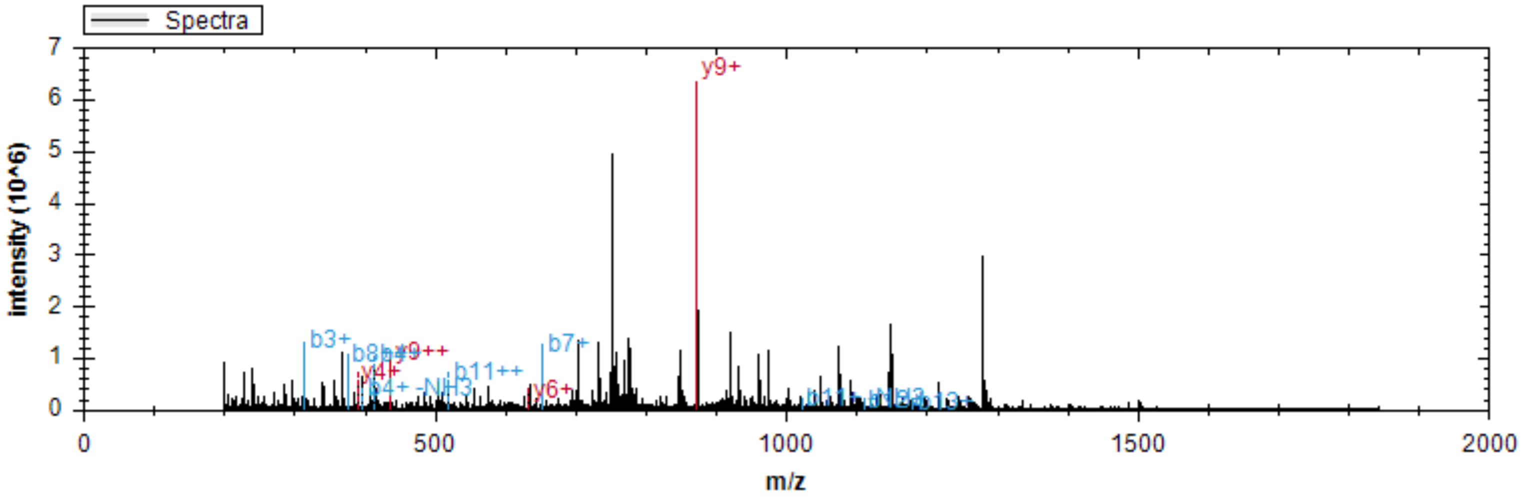

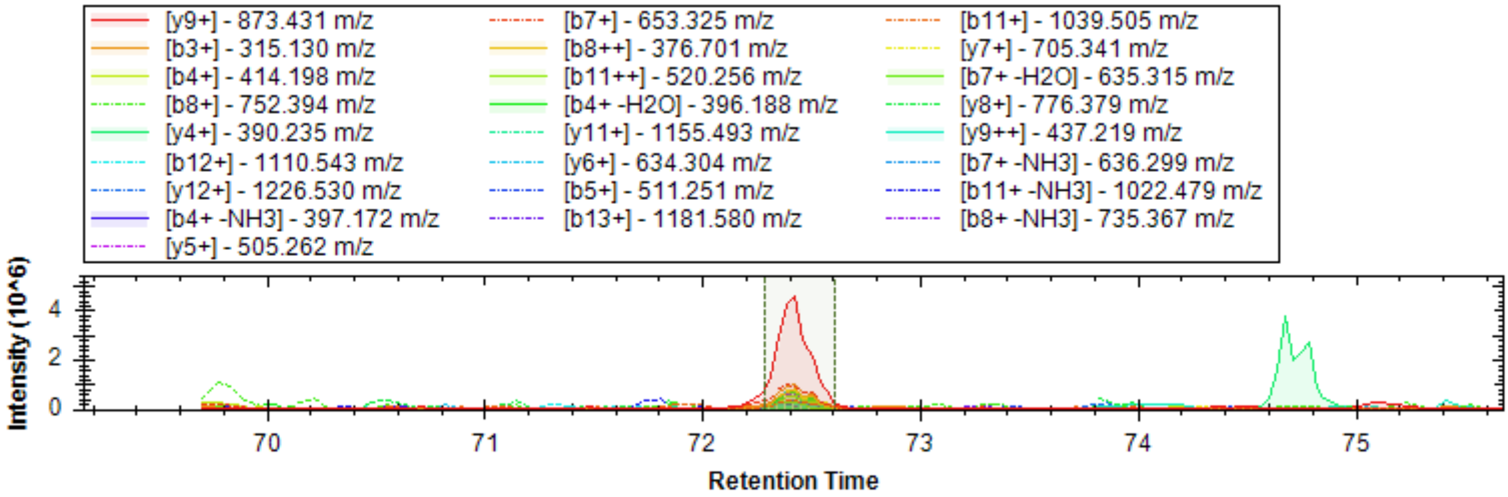

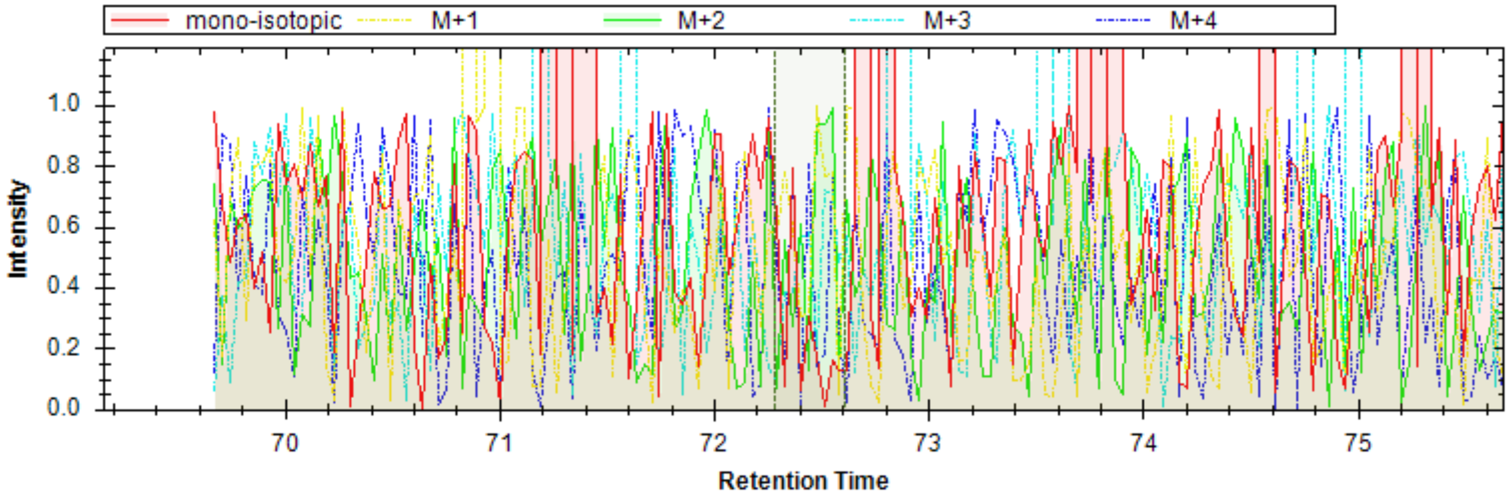

P0C6F1 – _IIDLQM[+O]HDY[+H+O3+P]LR_.2
Fig4_MP-DIA-barrel-cortex-p9-1_MHRM.htrms

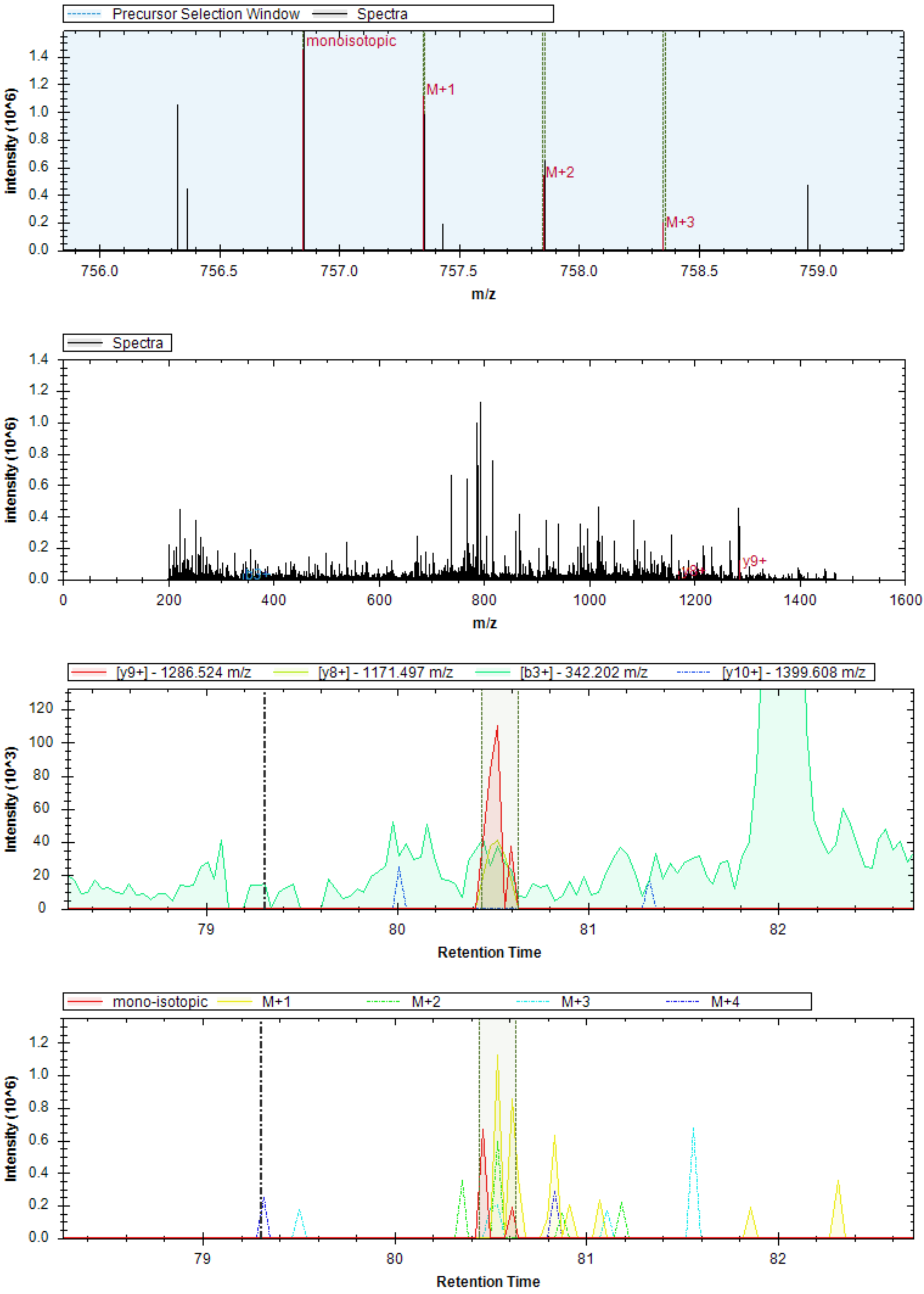

P27546-2 – _AT[+H+O3+P]SPSTLVSTGPSSR_.2
Fig4_MP-DIA-barrel-cortex-p9-1_MHRM.htrms

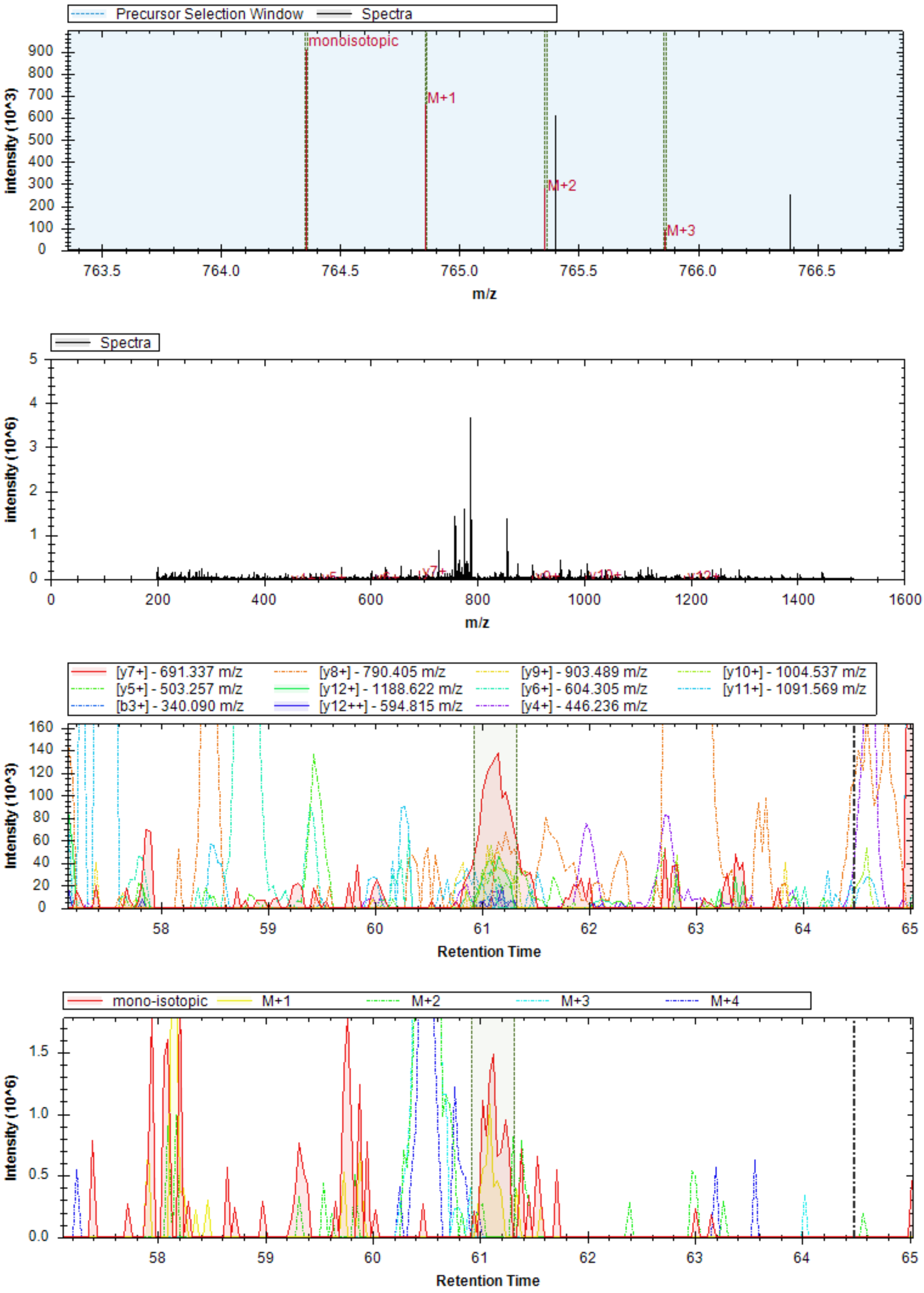

P27546-2 – _ATS[+H+O3+P]PSTLVSTGPSSR_.2
Fig4_MP-DIA-barrel-cortex-p9-1_MHRM.htrms

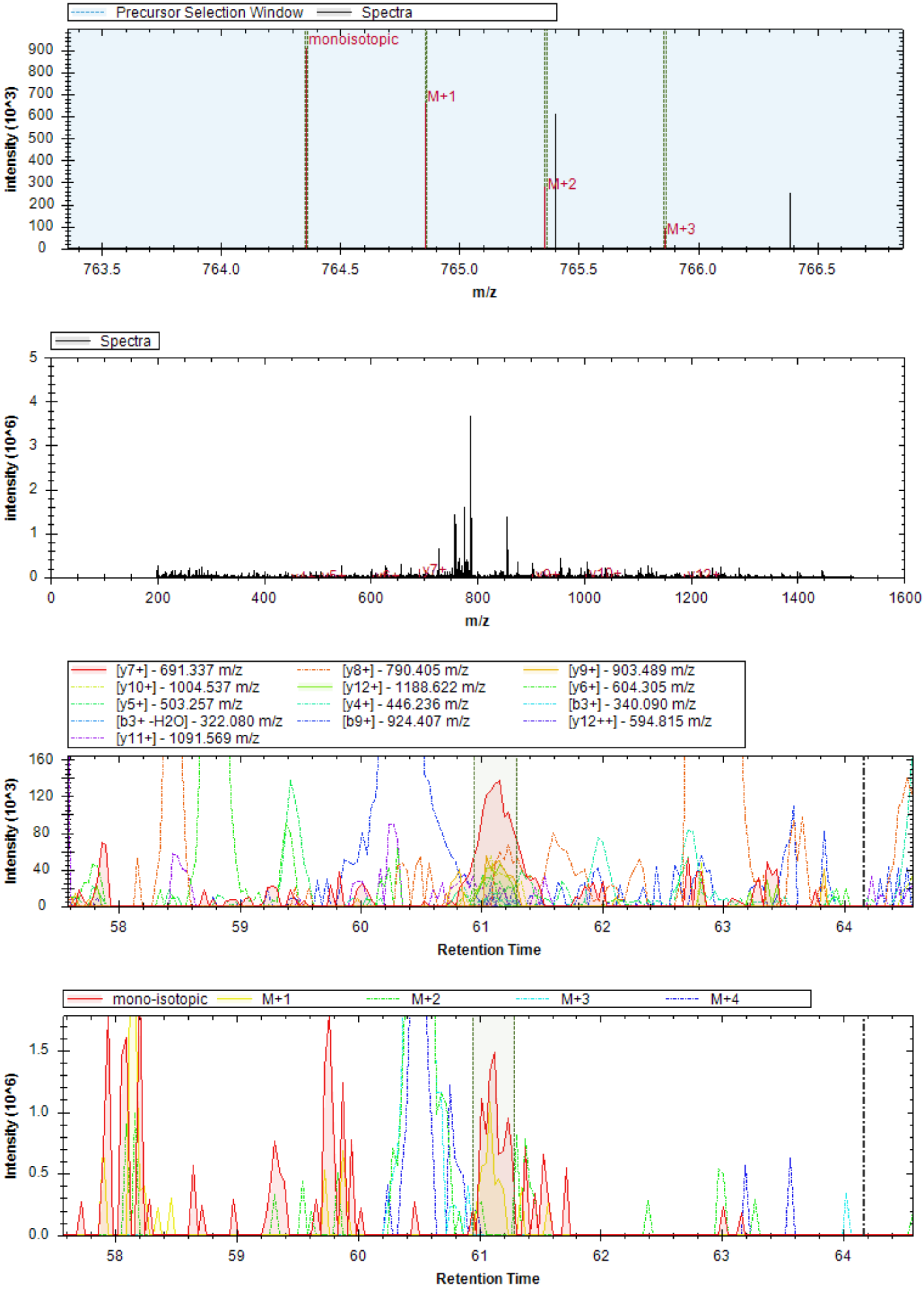

P57776-3 – _ATAPQTQHVS[+H+O3+P]PM[+O]R_.2
Fig4_MP-DIA-barrel-cortex-p9-1_MHRM.htrms

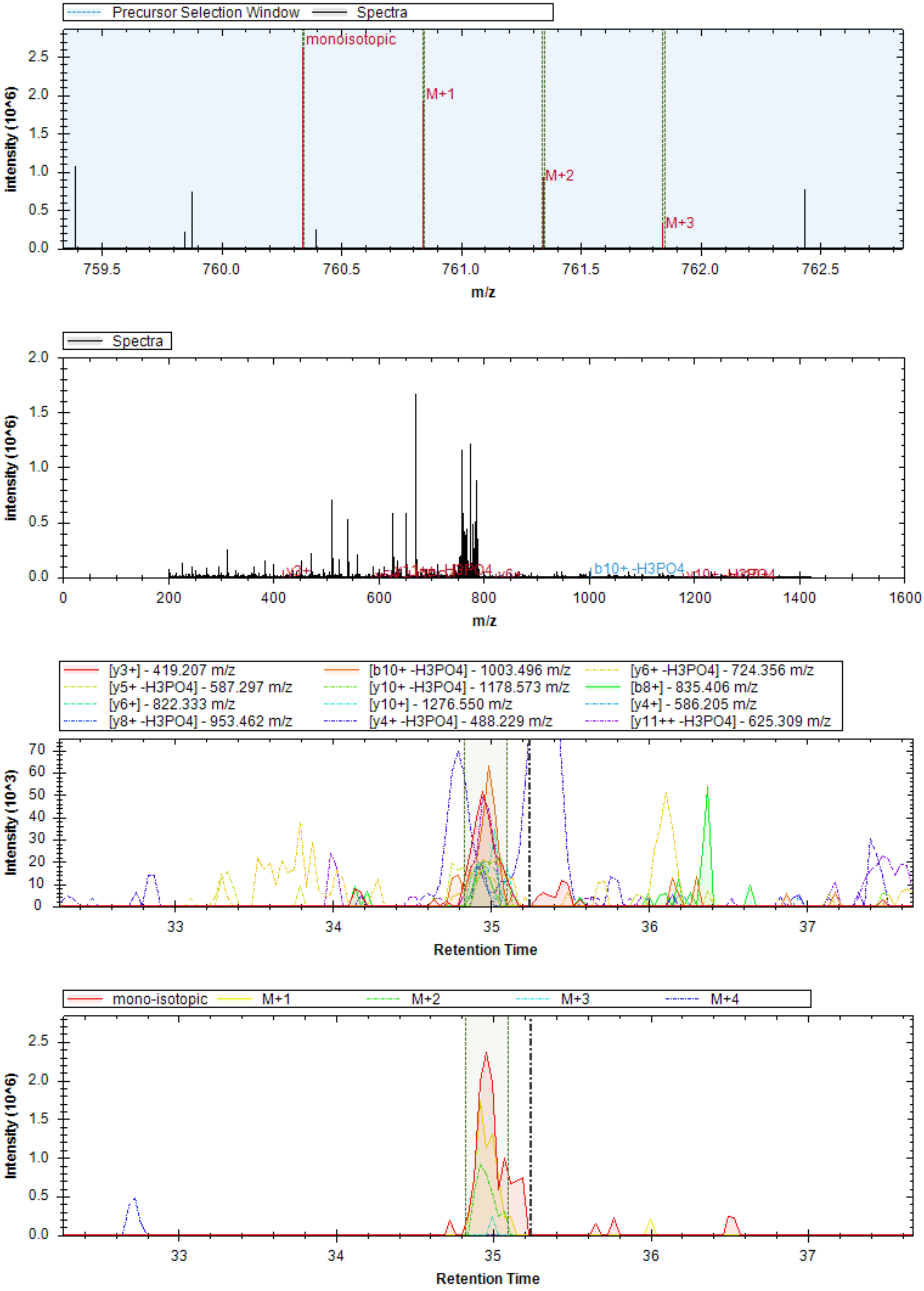

Q3TZM9;Q3TZM9-2;Q3TZM9-3 – _VPDVYIDSM[+O]GYAFT[+H+O3+P]LPLFK_.3
Fig4_MP-DIA-barrel-cortex-p9-1_MHRM.htrms

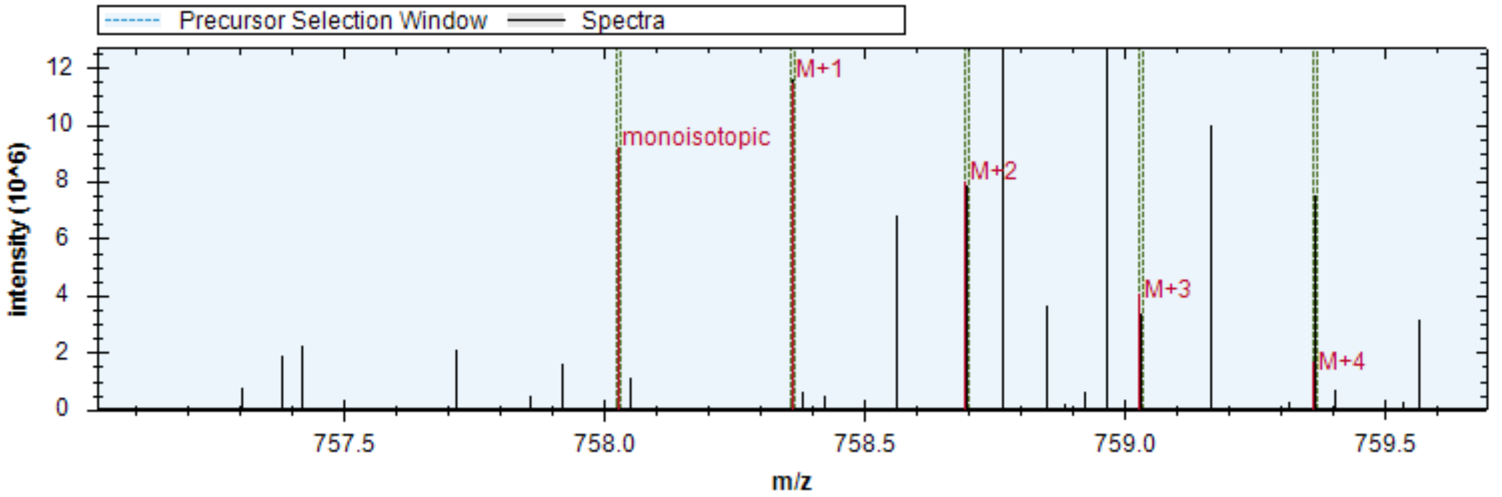

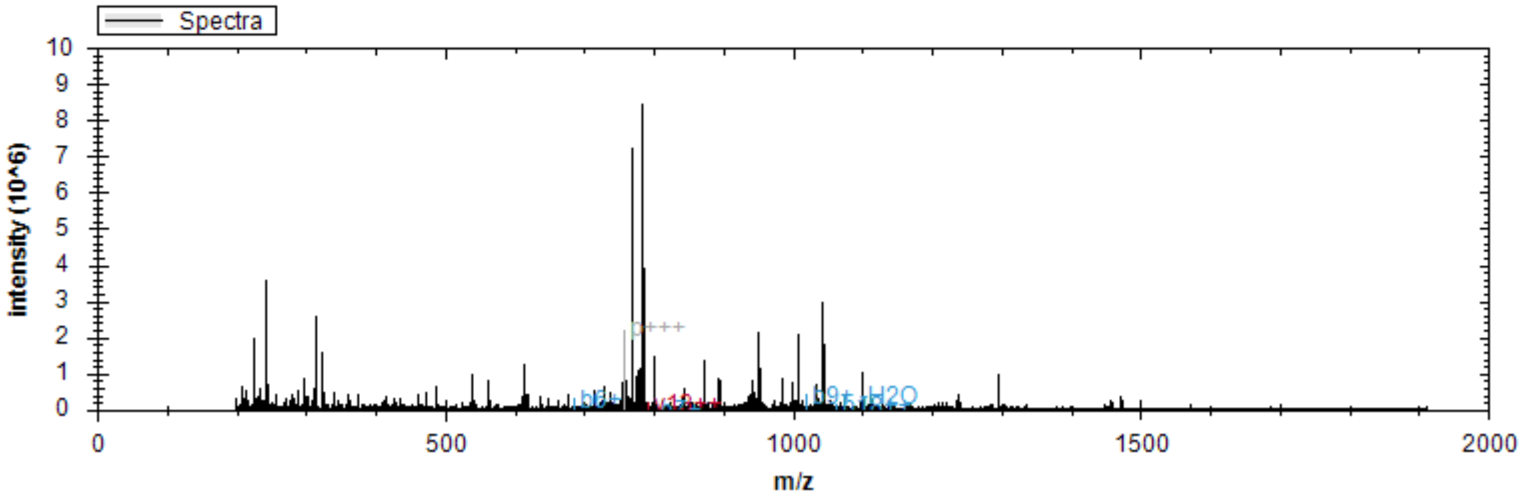

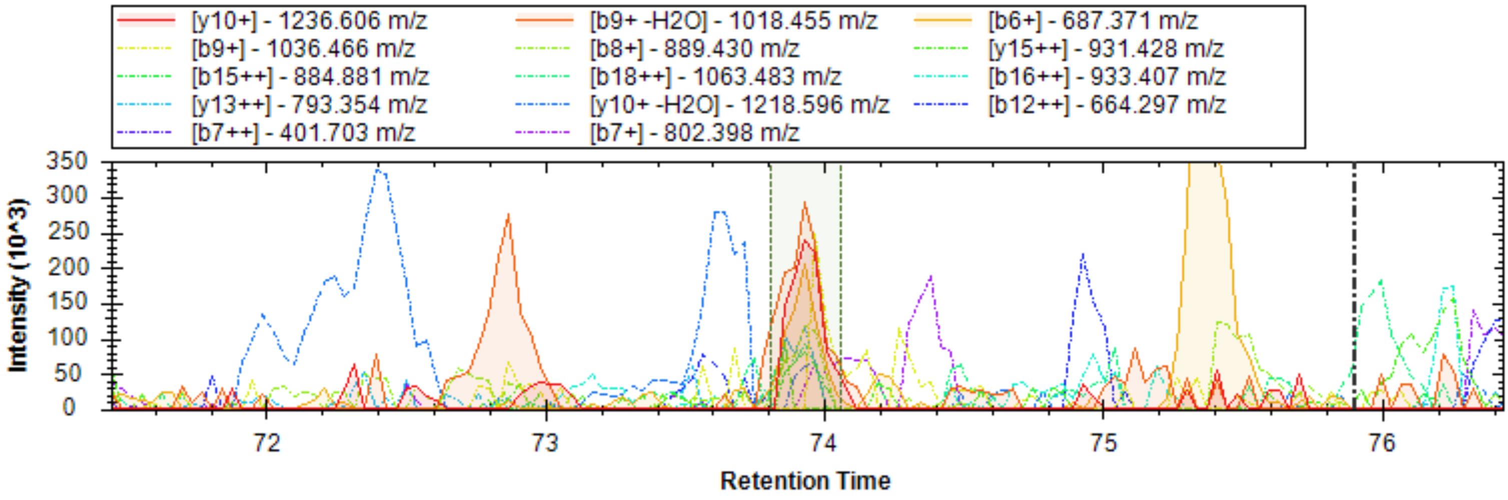

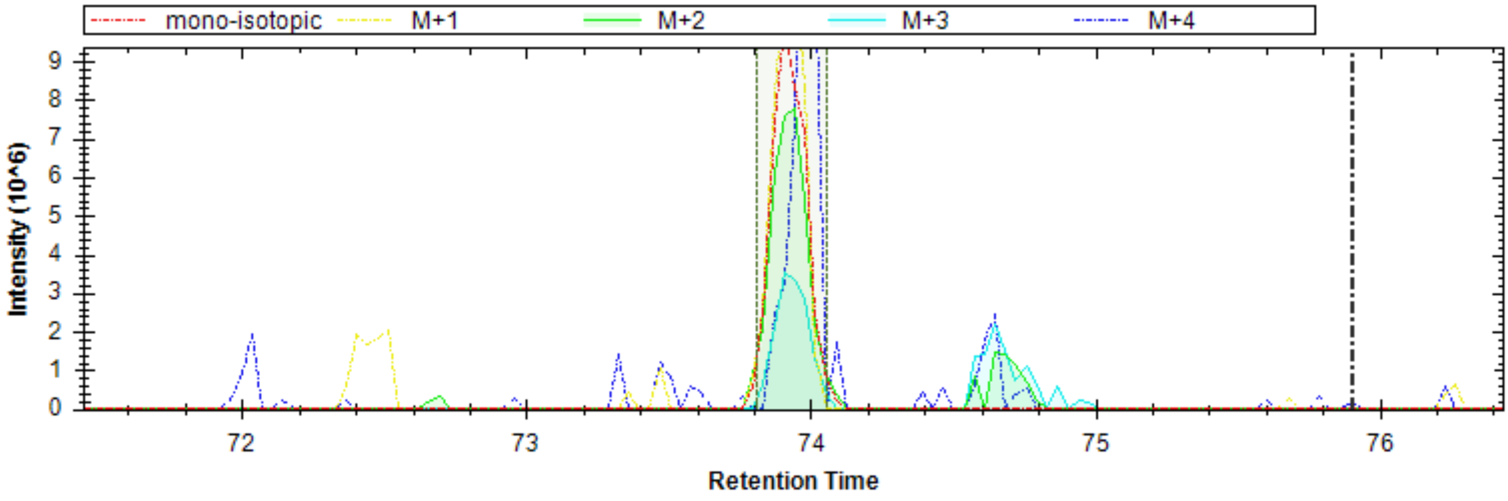

Q68FD9 – _LAQLLS[+H+O3+P]EVPT[+H+O3+P]RR_.2
Fig4_MP-DIA-barrel-cortex-p9-1_MHRM.htrms

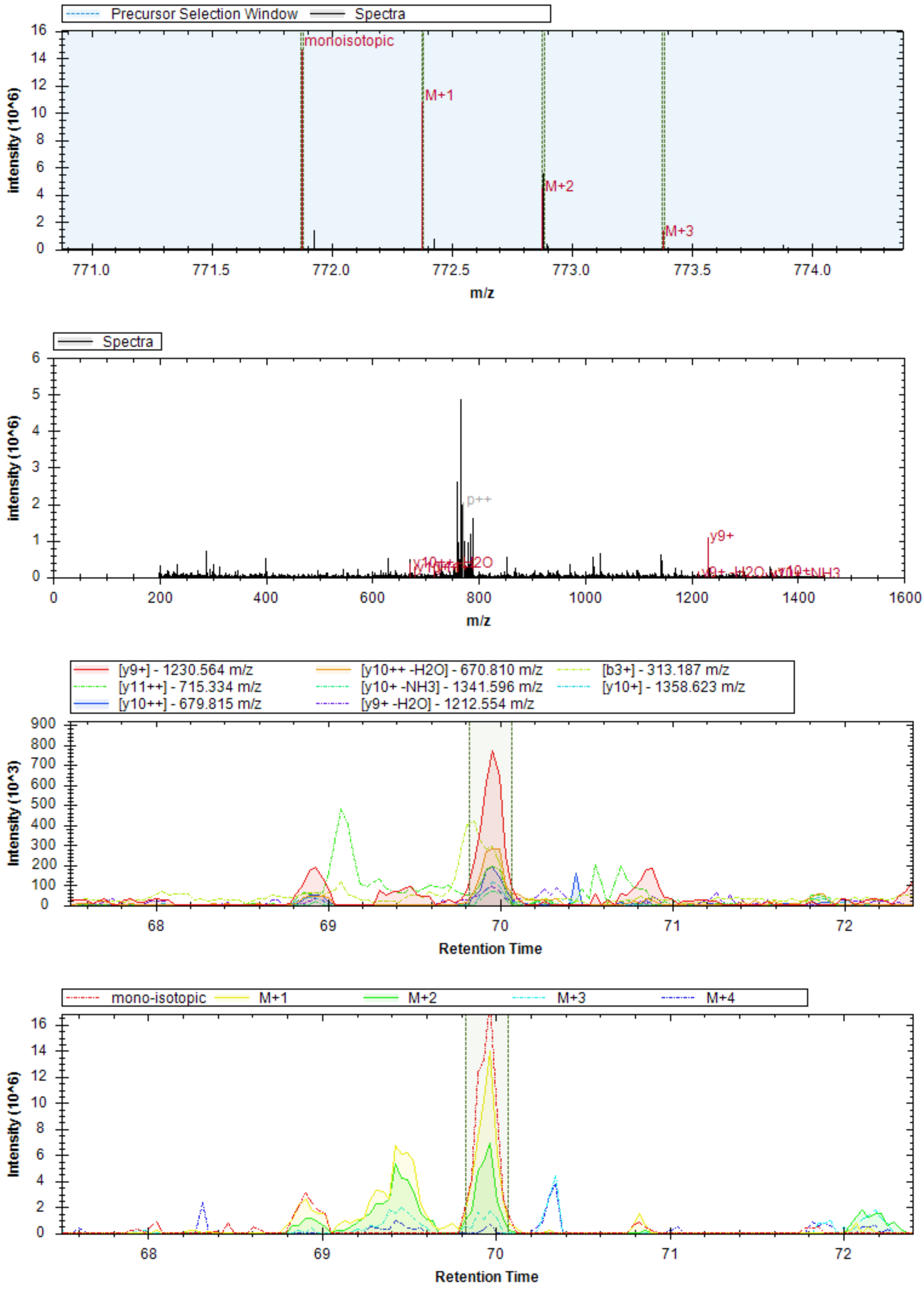

Q8BRT1 – _VLNT[+H+O3+P]GSDVEEAVADALLLGDIR_.3

Fig4_MP-DIA-barrel-cortex-p9-1_MHRM.htrms

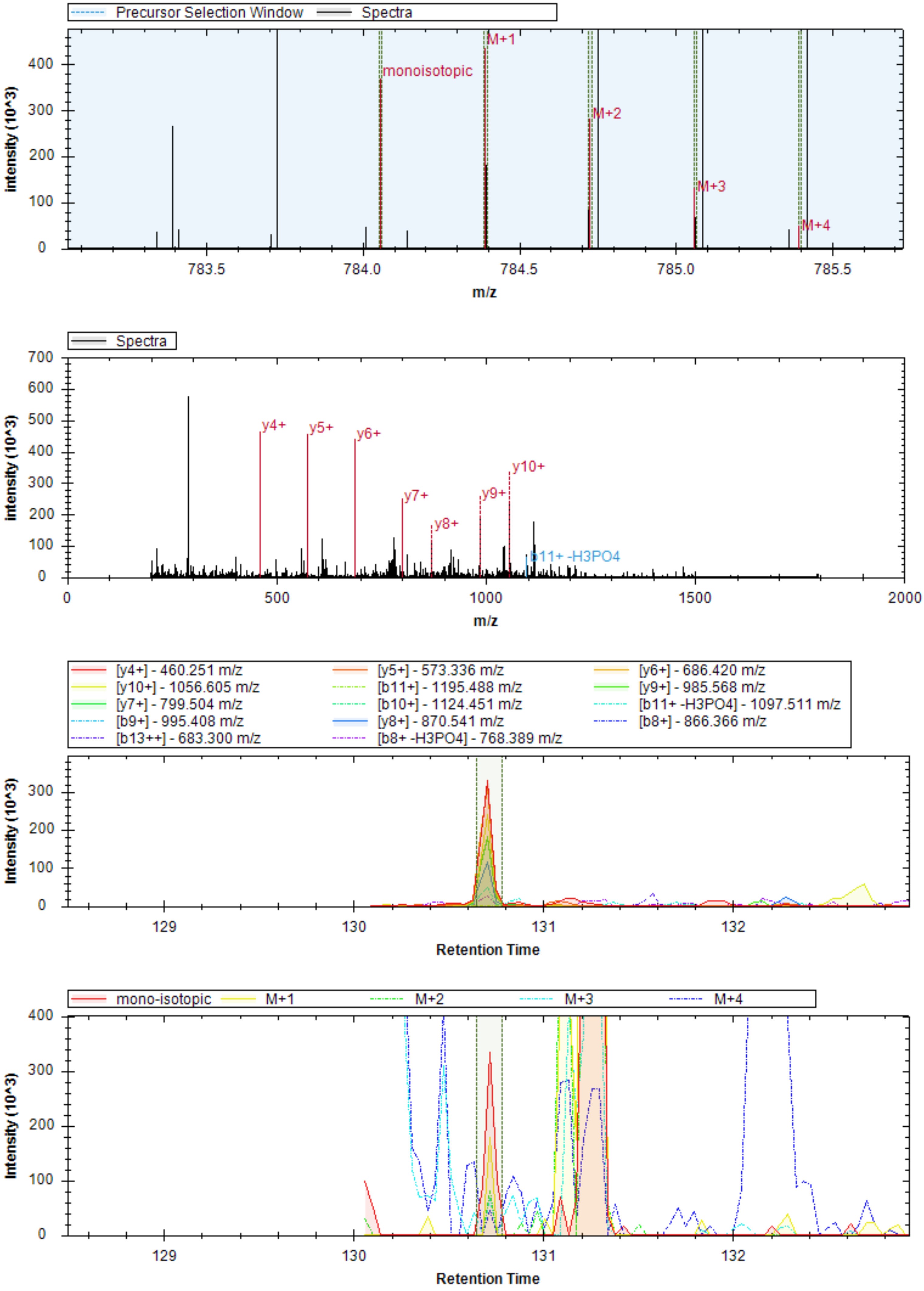

Q8CBW3;Q8CBW3-2;Q8CB[...] – _HNS[+H+O3+P]TTSSTSSGGYR_.2

Fig4_MP-DIA-barrel-cortex-p9-1_MHRM.htrms

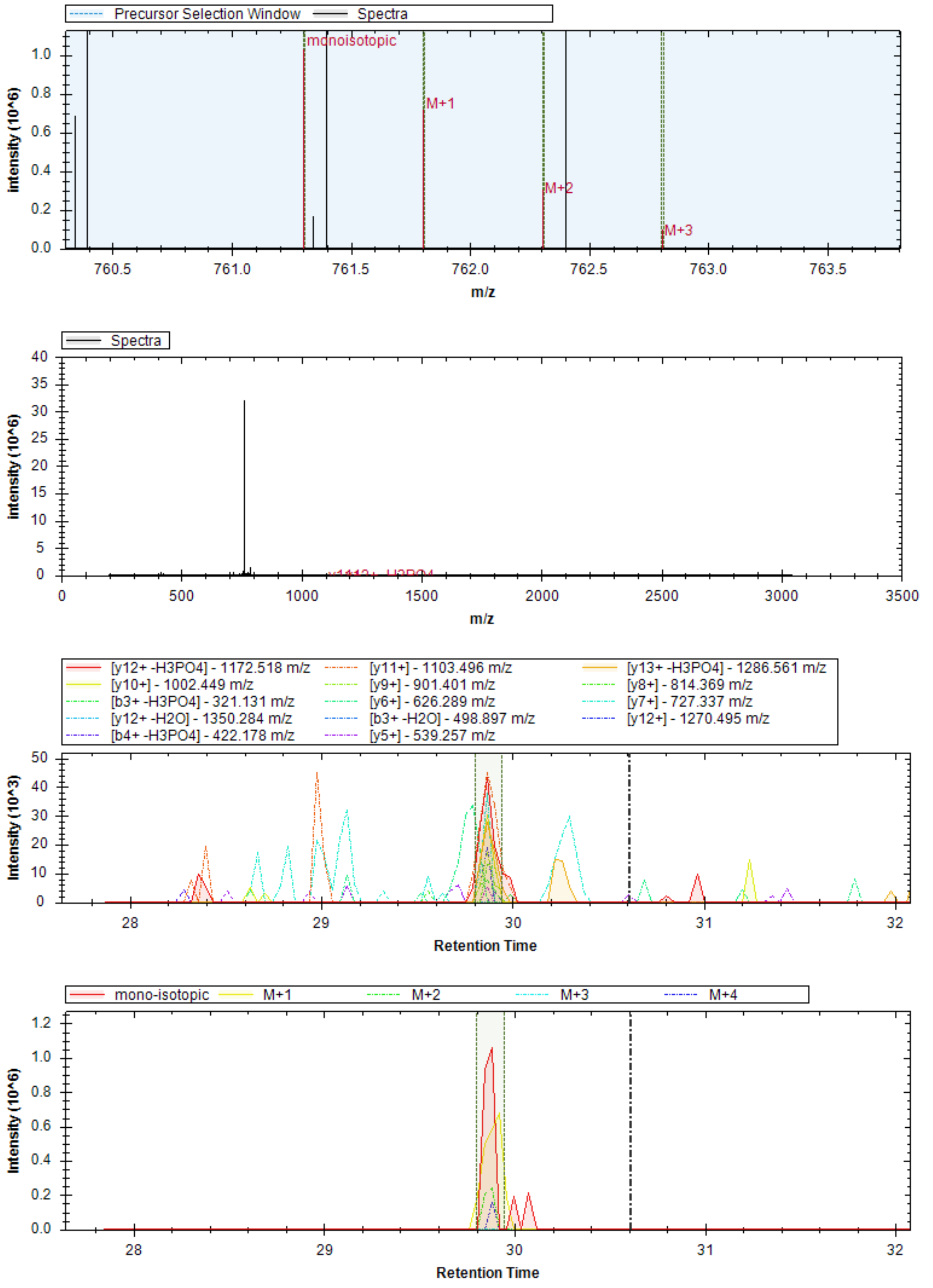

Q9CZX2 – _IHQRY[+H+O3+P]T[+H+O3+P]EIT[+H+O3+P]K_.2

Fig4_MP-DIA-barrel-cortex-p9-1_MHRM.htrms

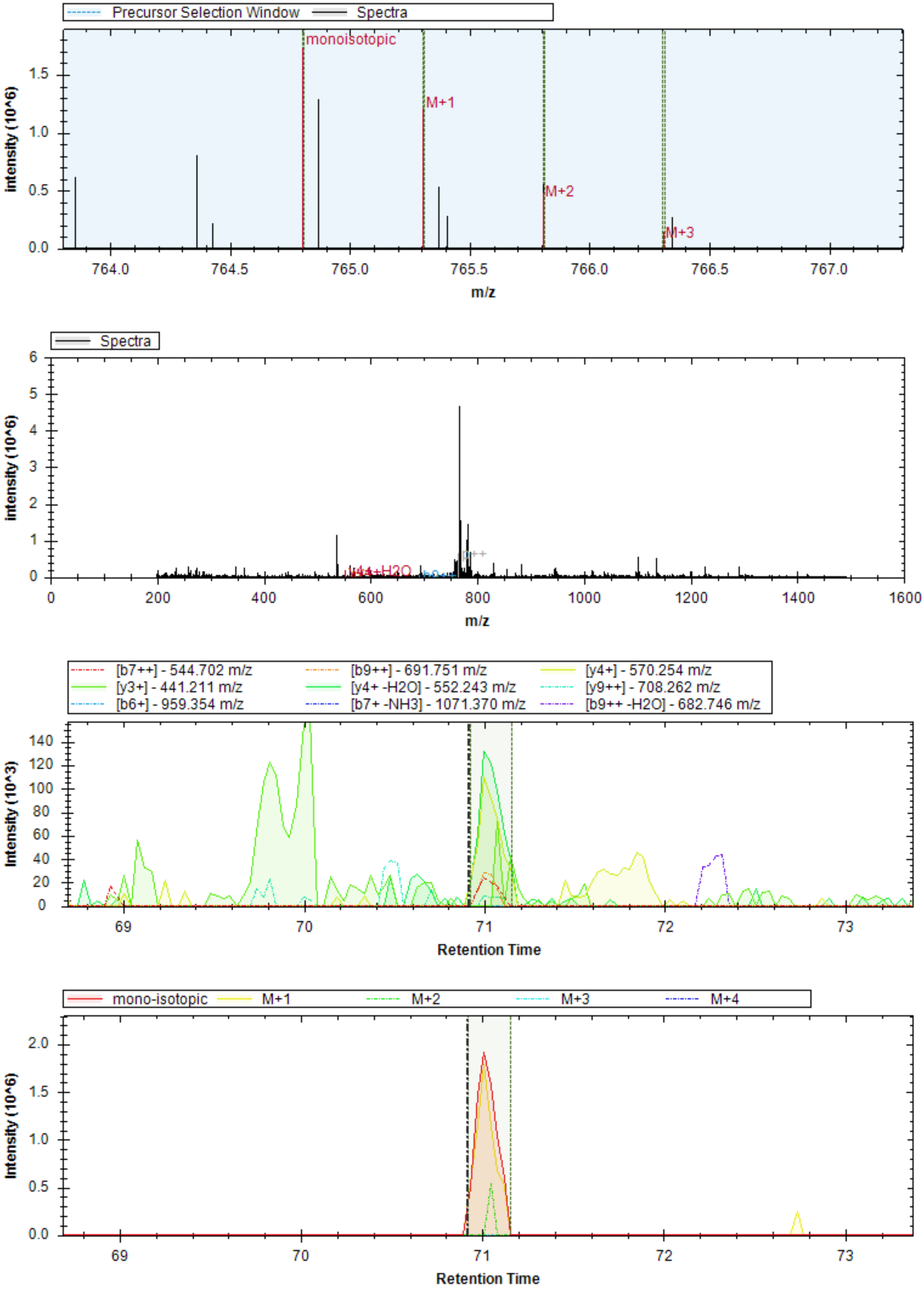

Q9D5R2 – _SNS[+H+O3+P]LPHSAVSNAASK_.2
Fig4_MP-DIA-barrel-cortex-p9-1_MHRM.htrms

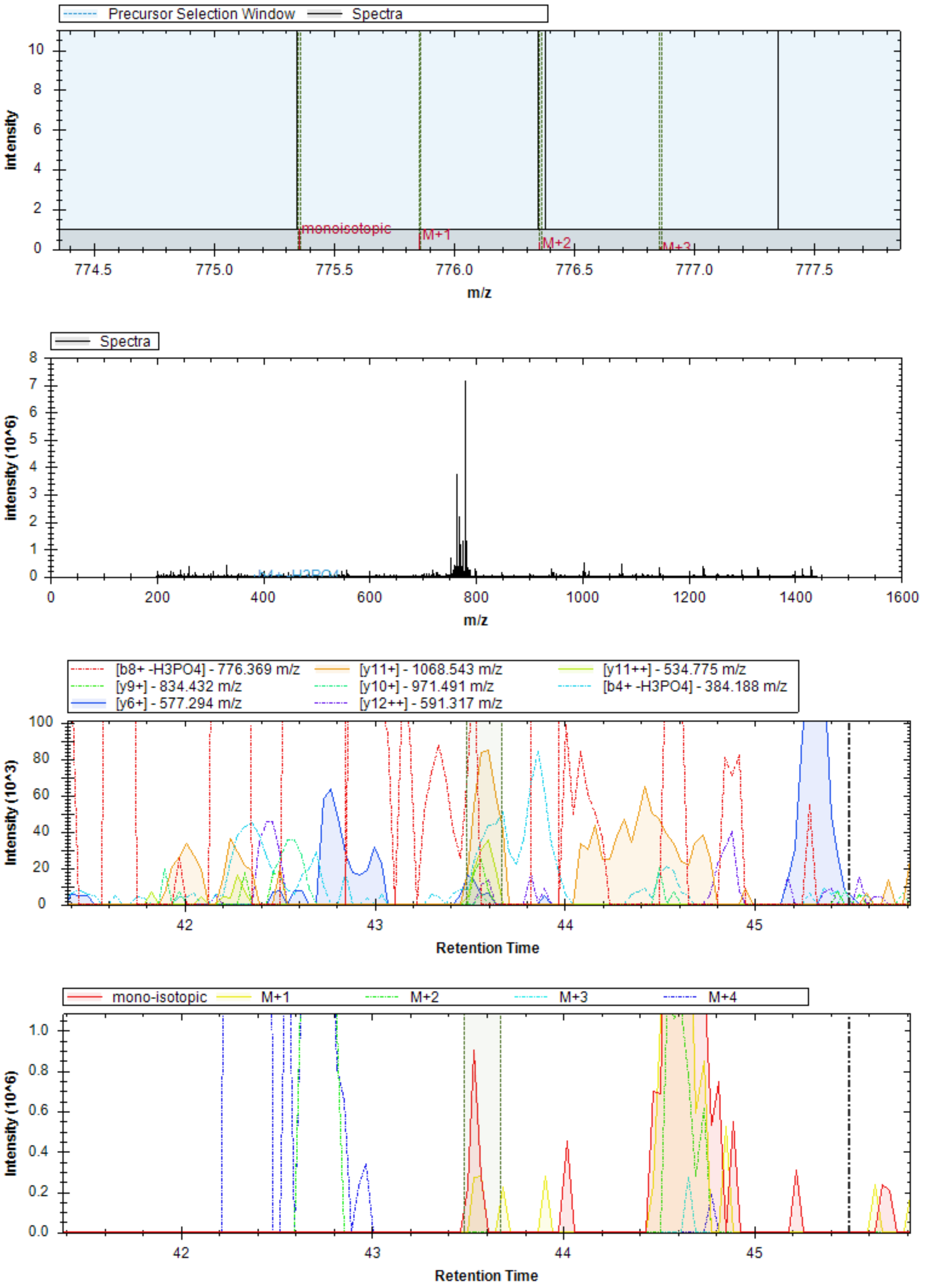

Q9JLM8 – _ISQHGGS[+H+O3+P]STSLSSTK_.2

Fig4_MP-DIA-barrel-cortex-p9-1_MHRM.htrms

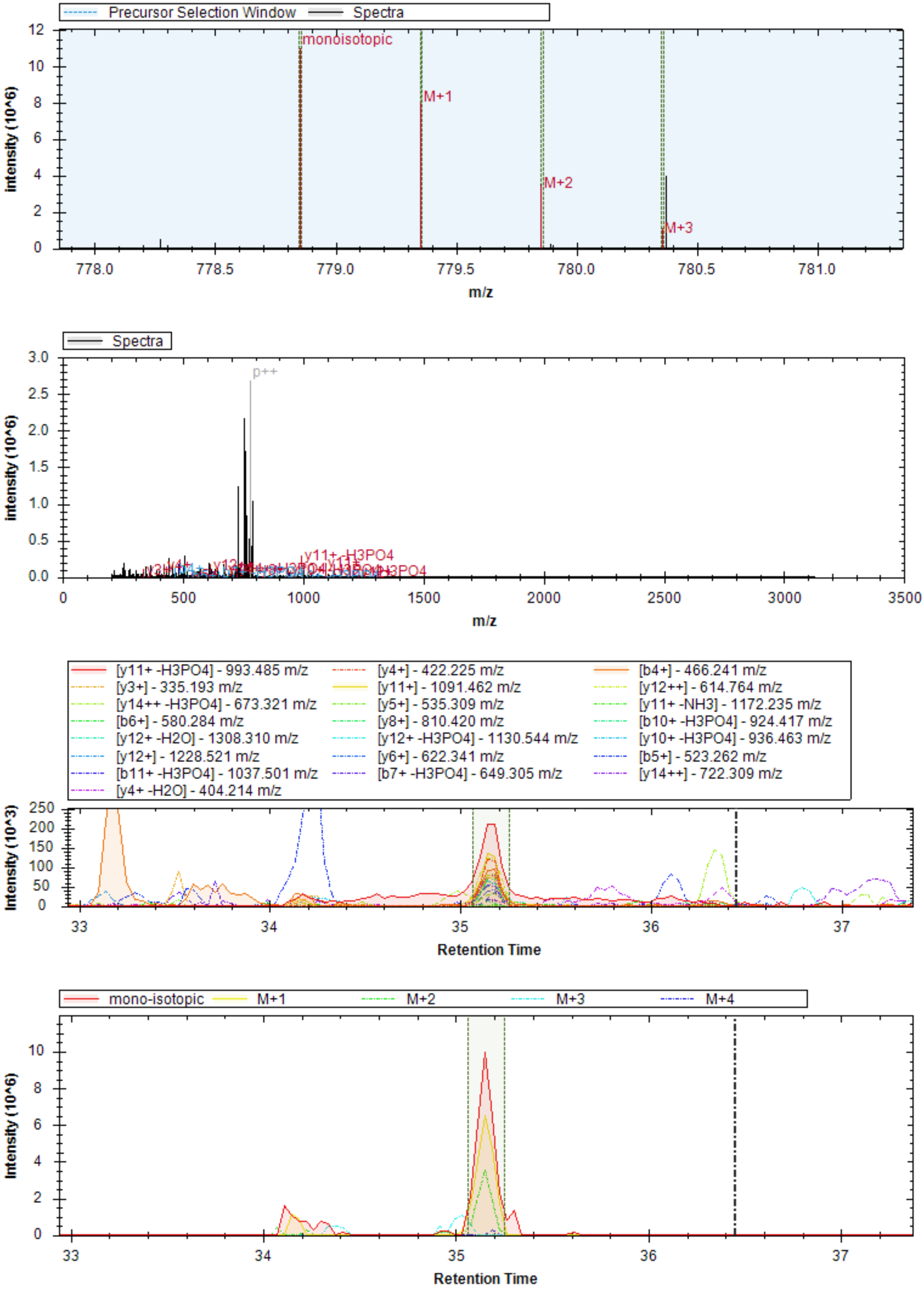

Q9QWI6 – _LSYAGGRPPSYAGS[+H+O3+P]PVHHAAER_.3

Fig4_MP-DIA-barrel-cortex-p9-1_MHRM.htrms

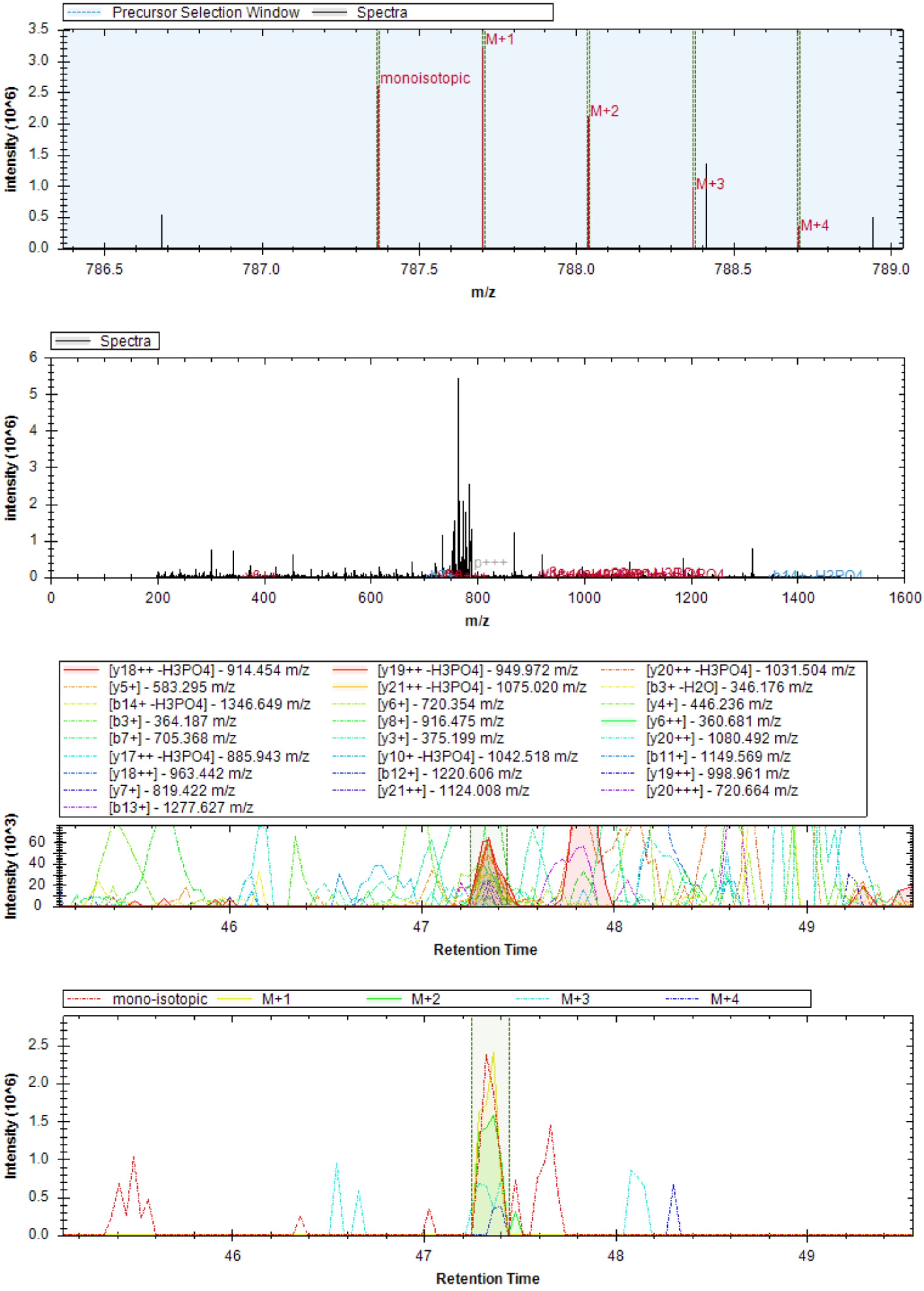

Q9QYC0 – _AAVVTS[+H+O3+P]PPPTTAPHK_.2

Fig4_MP-DIA-barrel-cortex-p9-1_MHRM.htrms

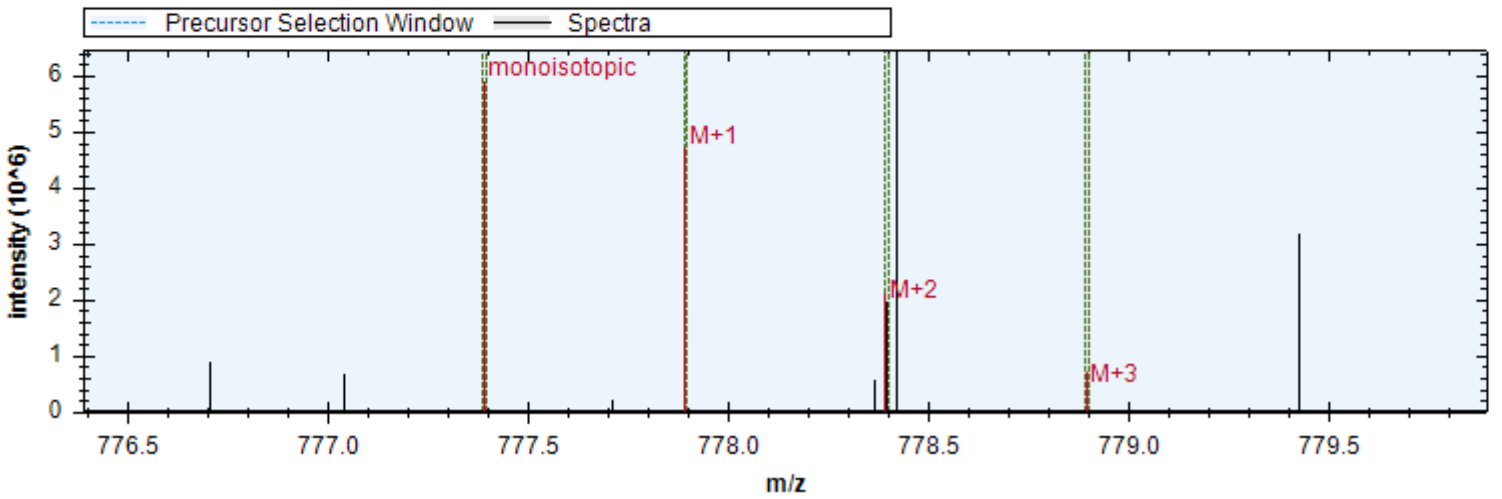

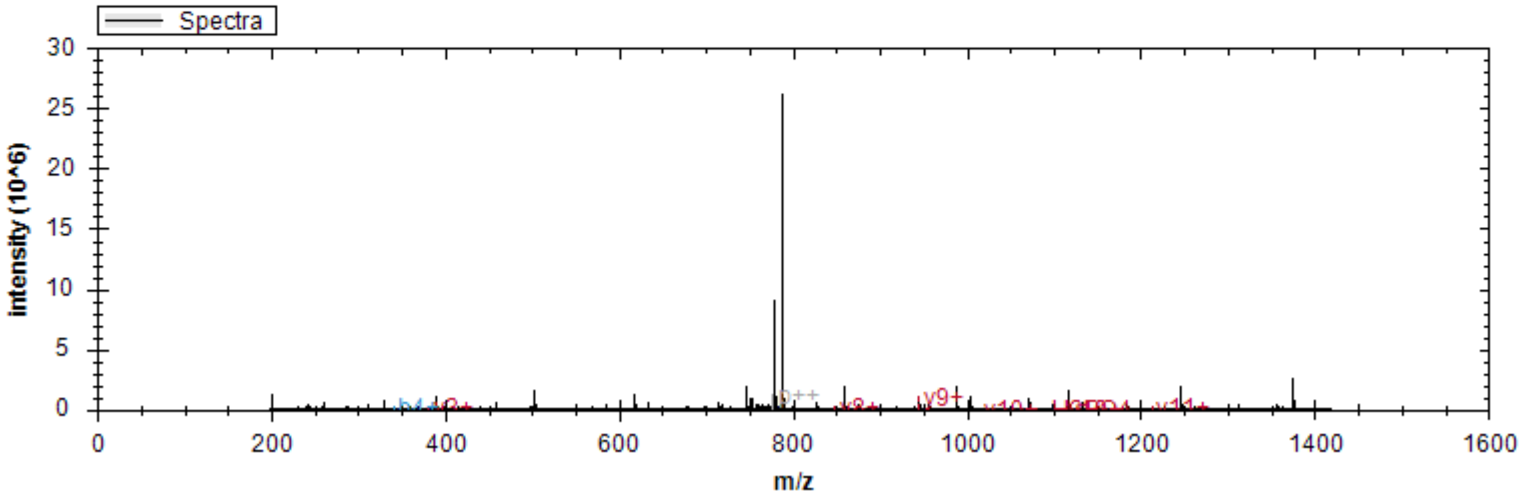

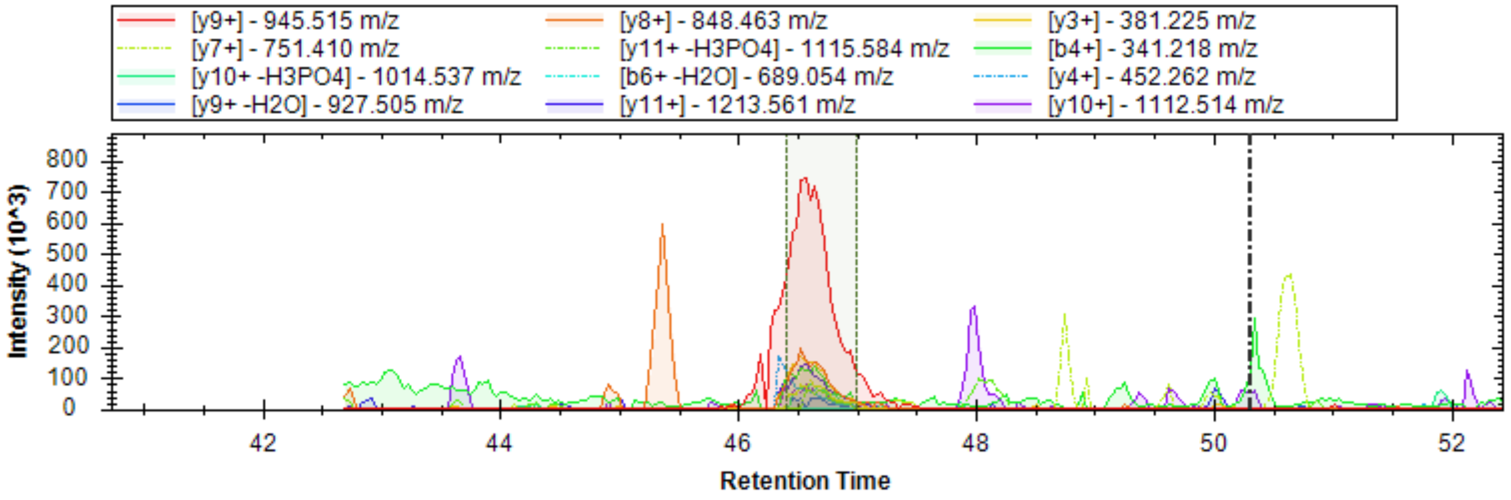

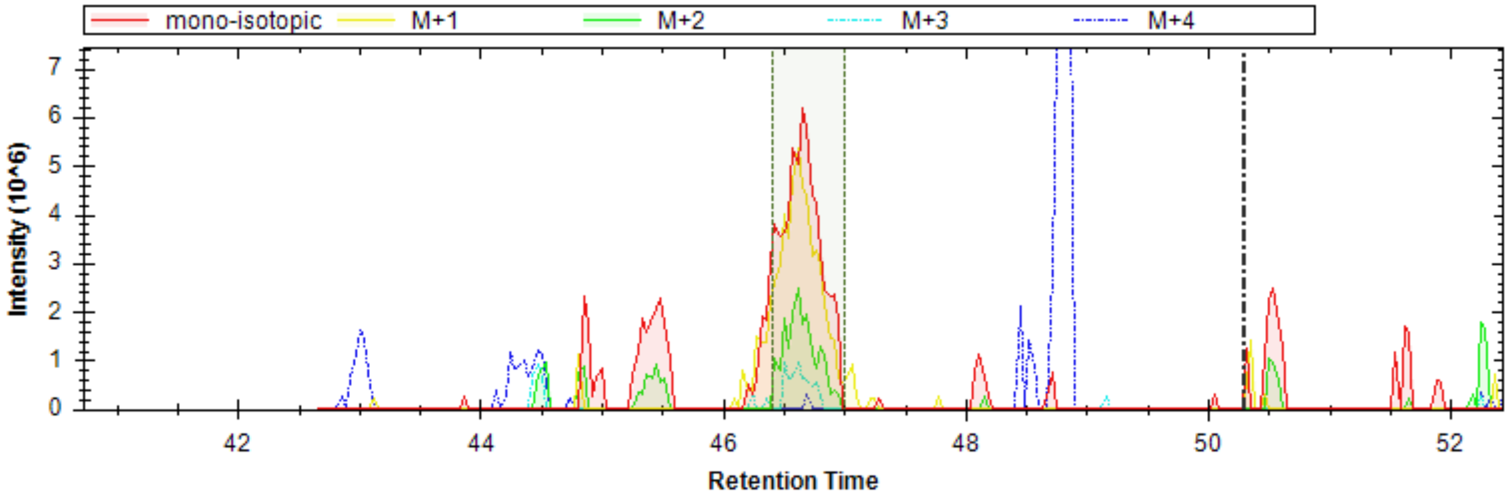

Q9QYR6-2 – _ELSSEPRT[+H+O3+P]PPAQK_.2
Fig4_MP-DIA-barrel-cortex-p9-1_MHRM.htrms

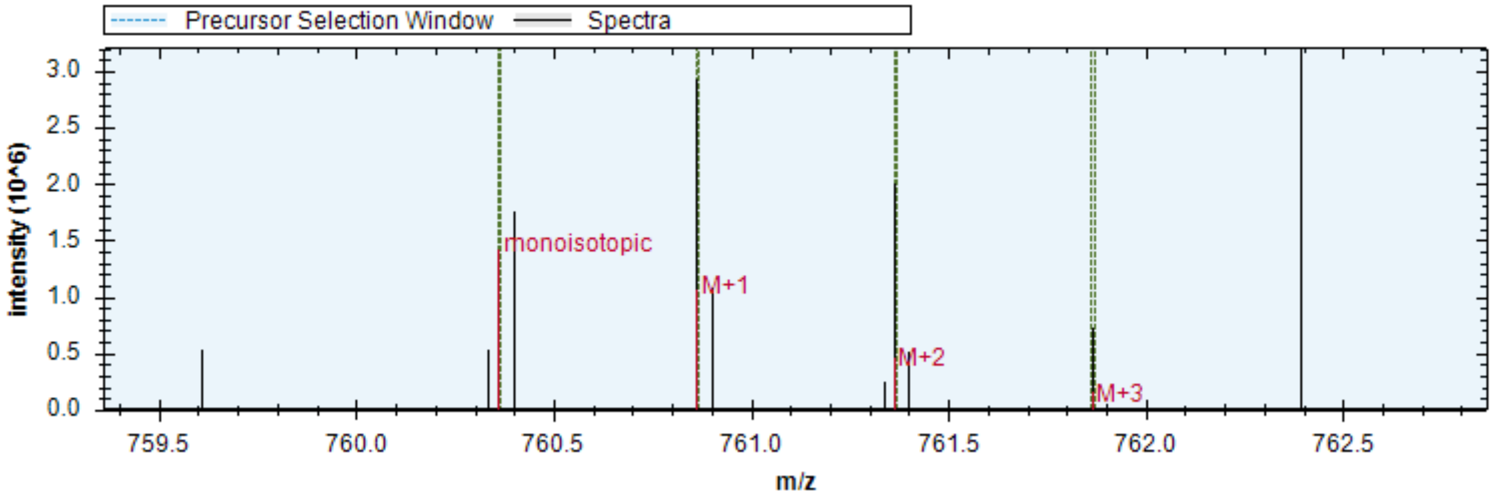

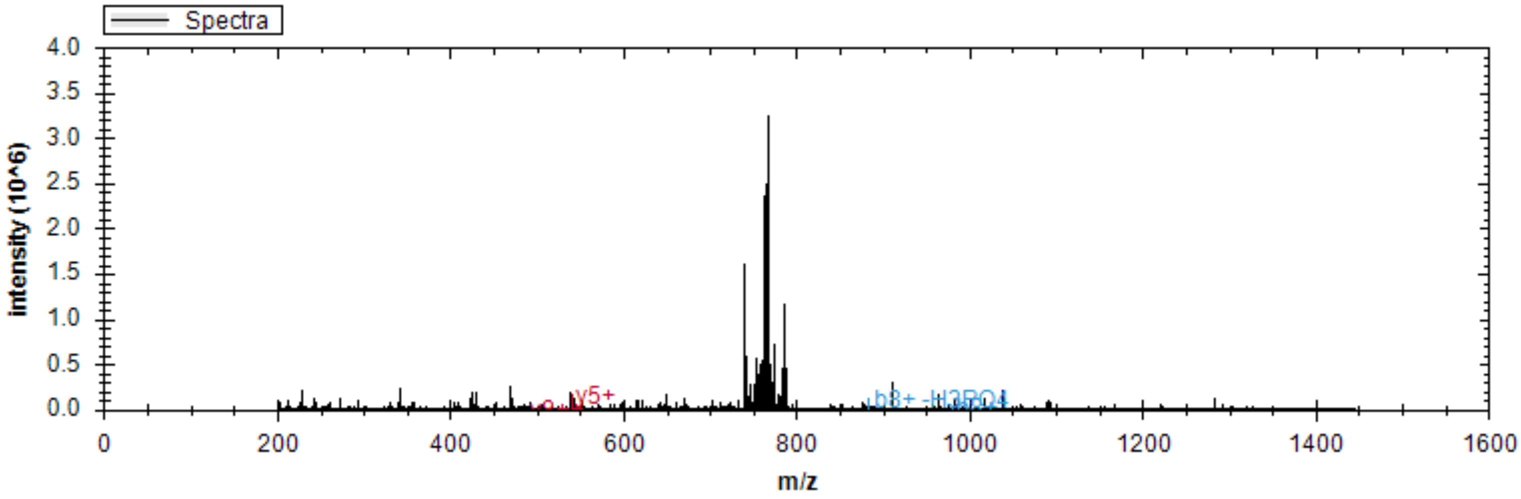

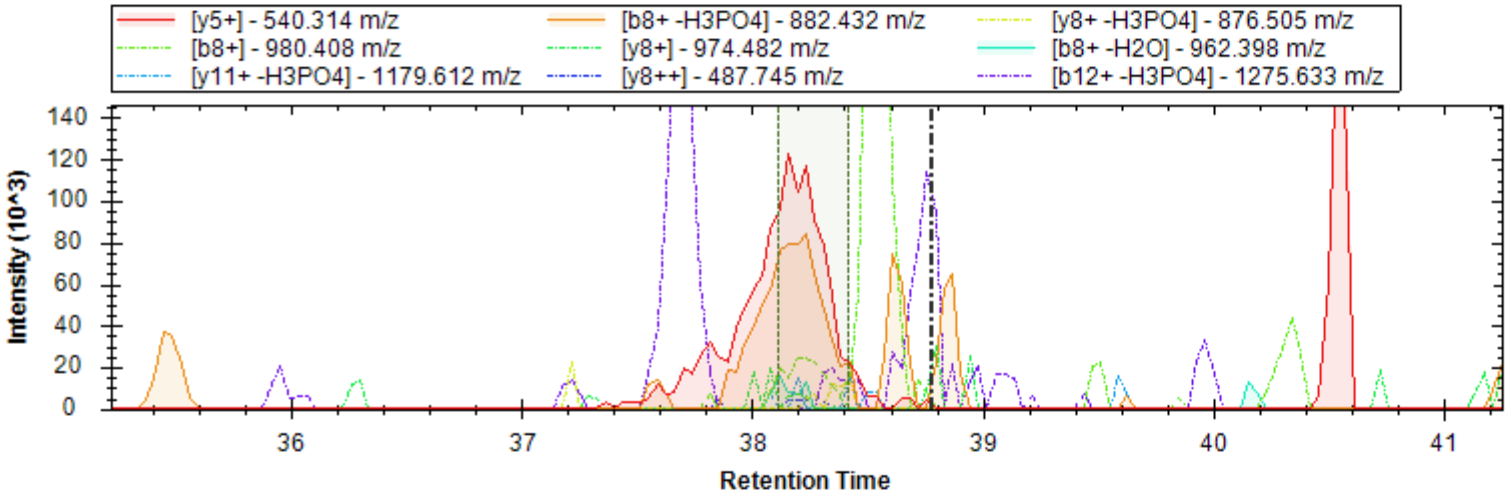

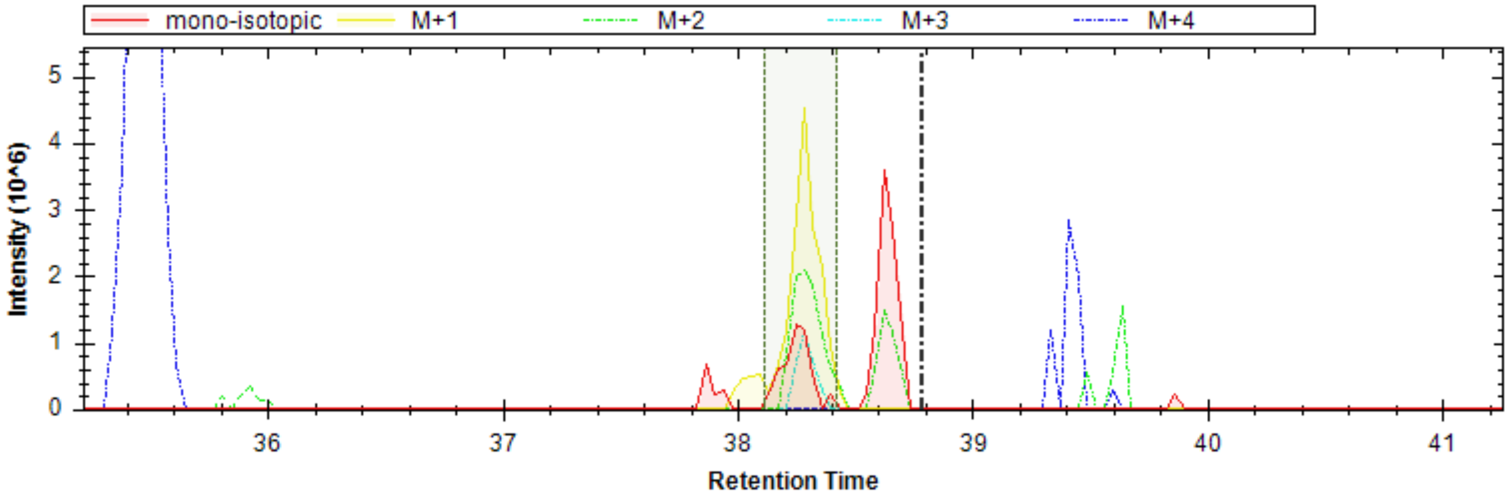

P06837 – _EGDGSATTDAAPAT[+H+O3+P]SPK_.2
Fig4_MP-DIA-barrel-cortex-p9-1_MHRM.htrms

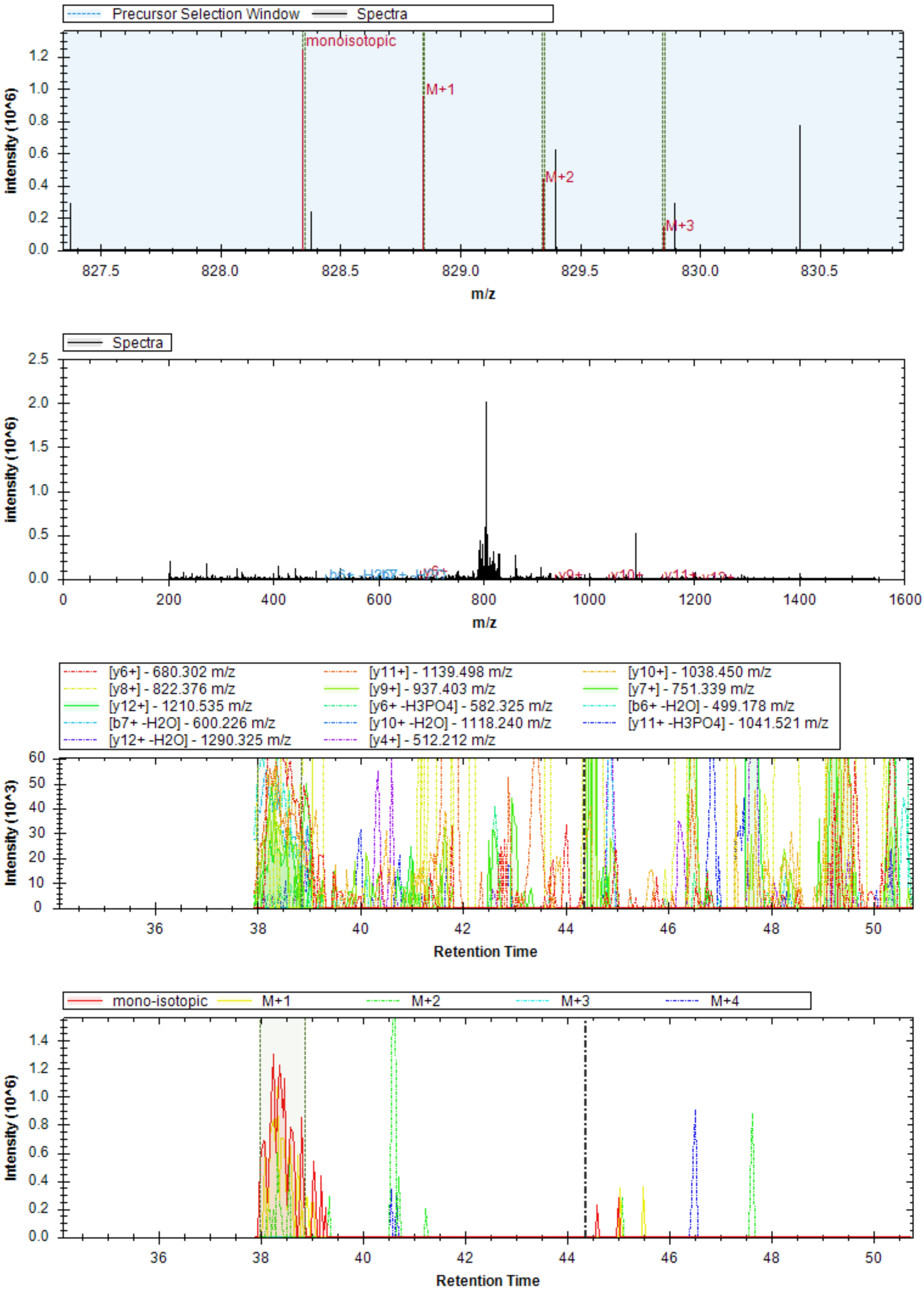

P10637 – _TTPSPKT[+H+O3+P]PPGSGEPPK_.2
Fig4_MP-DIA-barrel-cortex-p9-1_MHRM.htrms

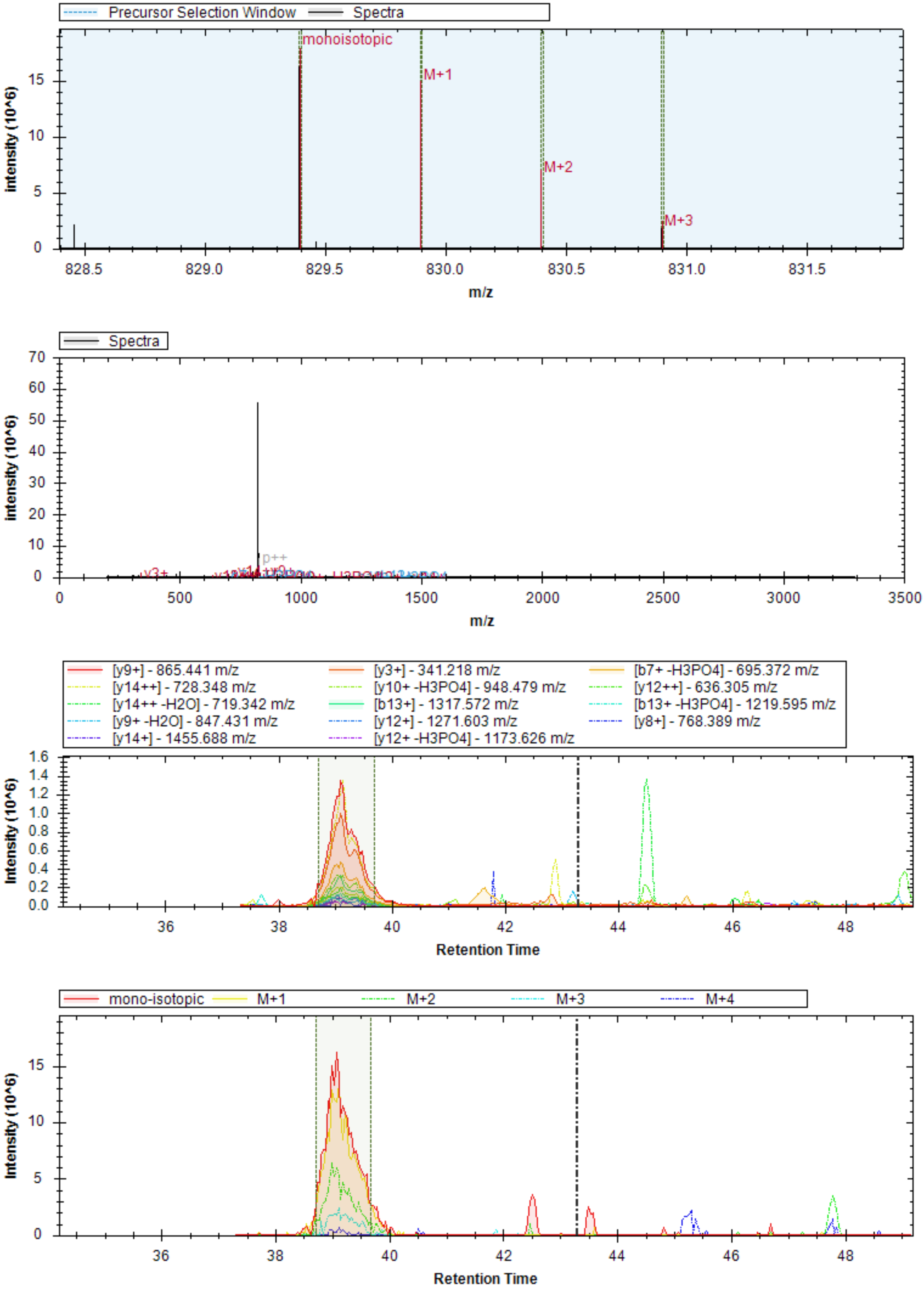

P20357 – _VAIIRT[+H+O3+P]PPKSPATPK_.2
Fig4_MP-DIA-barrel-cortex-p9-1_MHRM.htrms

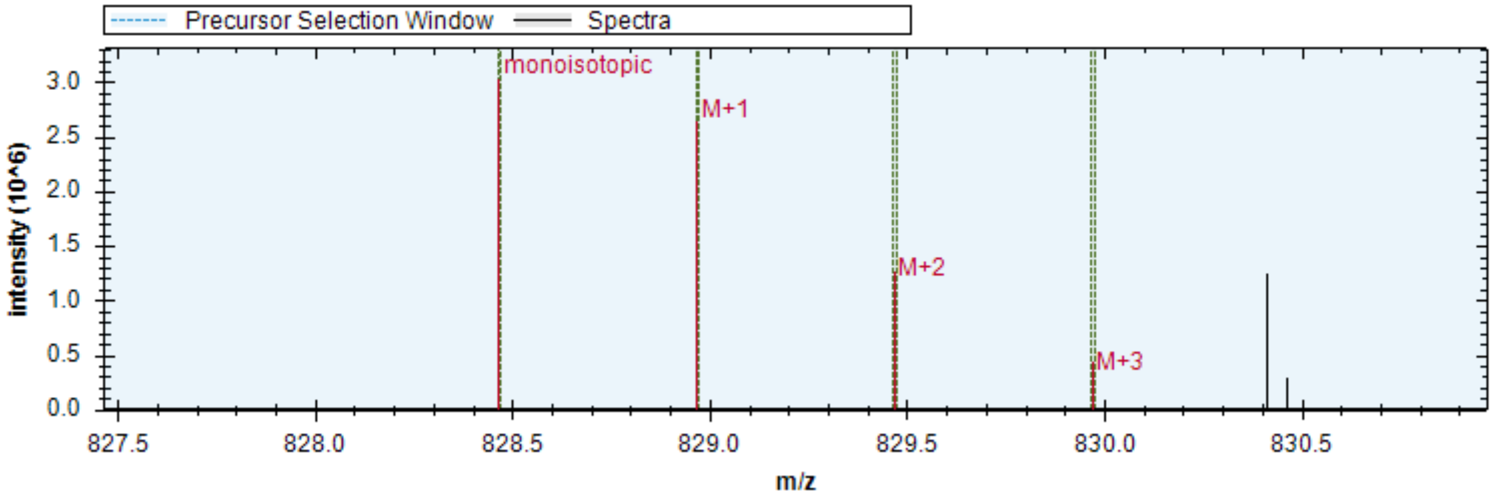

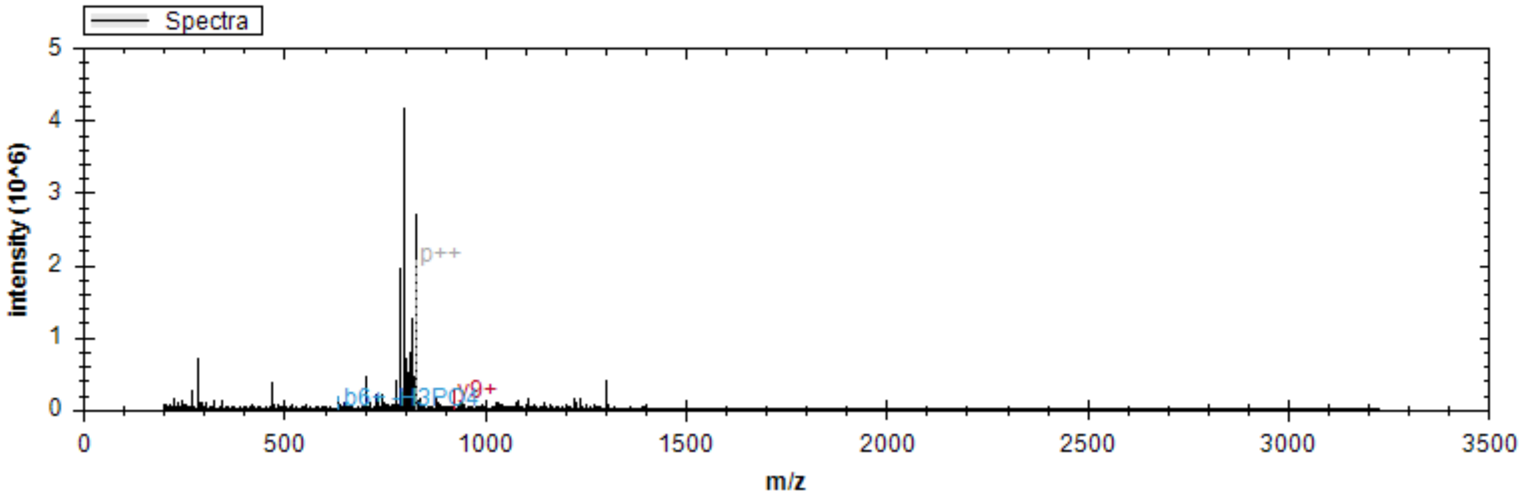

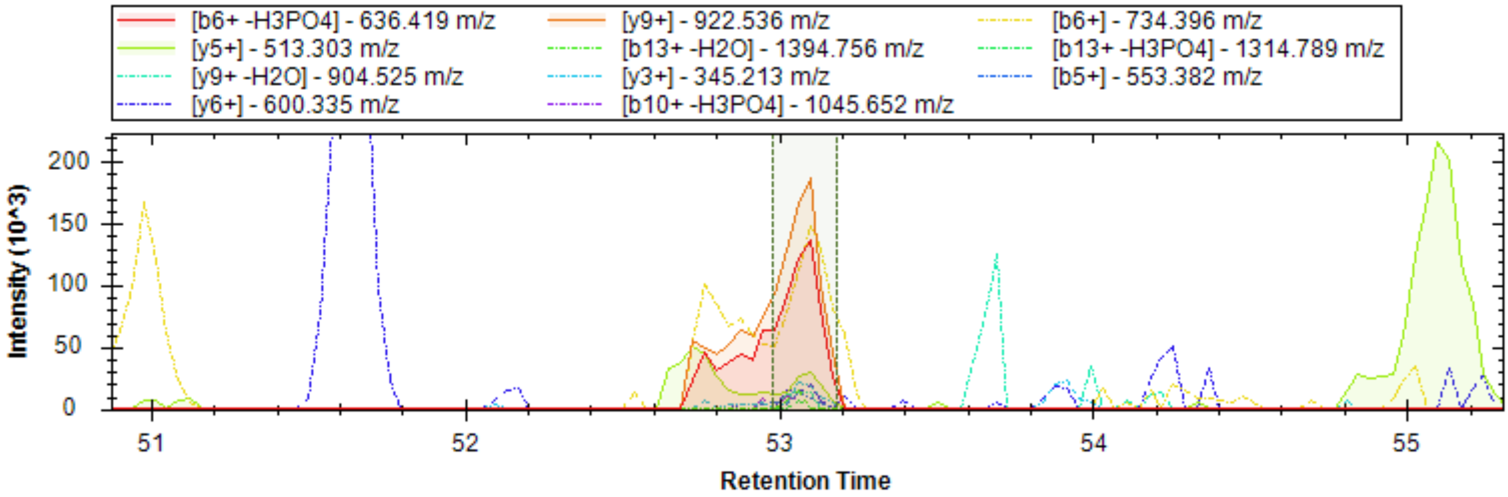

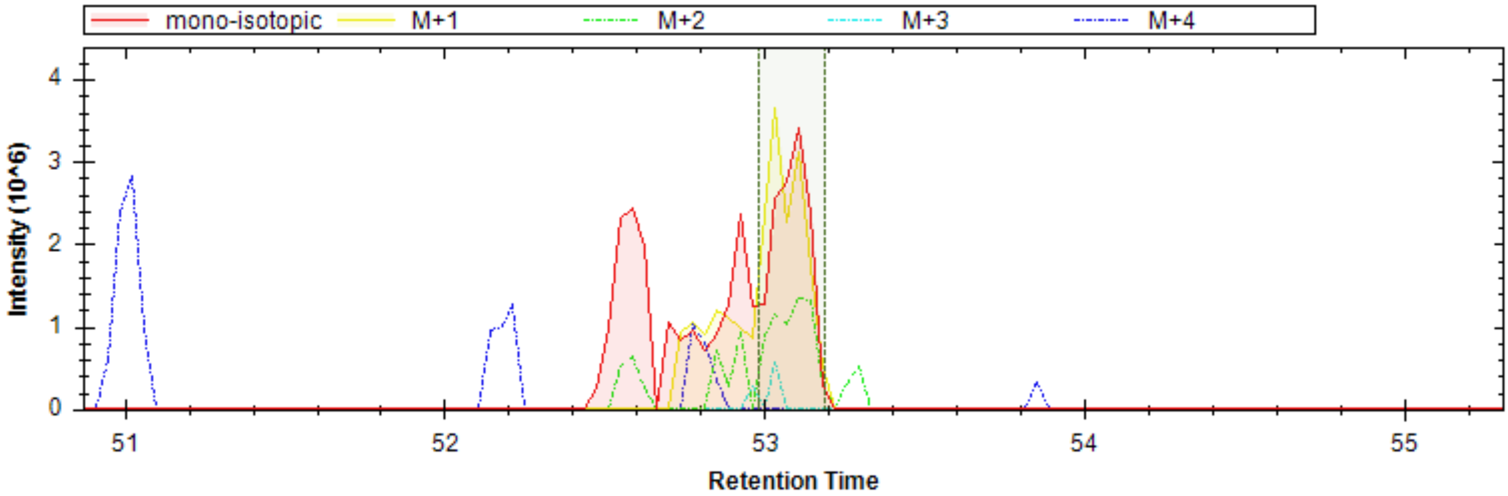

P20357 – _VDHGAEIIT[+H+O3+P]QSPSR_.2

Fig4_MP-DIA-barrel-cortex-p9-1_MHRM.htrms

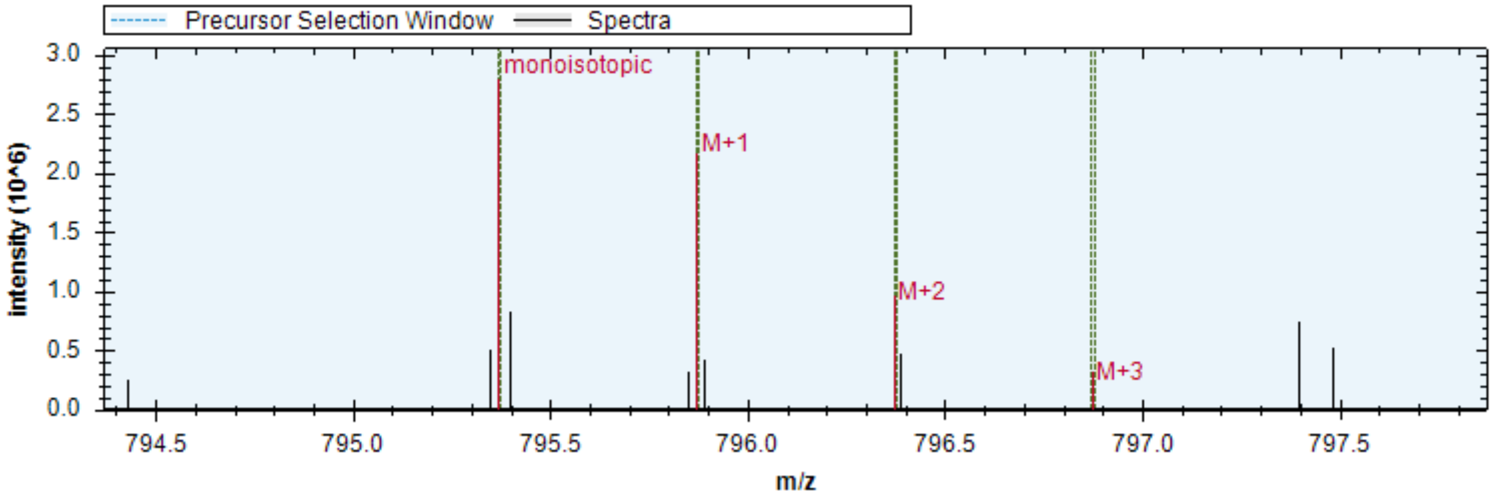

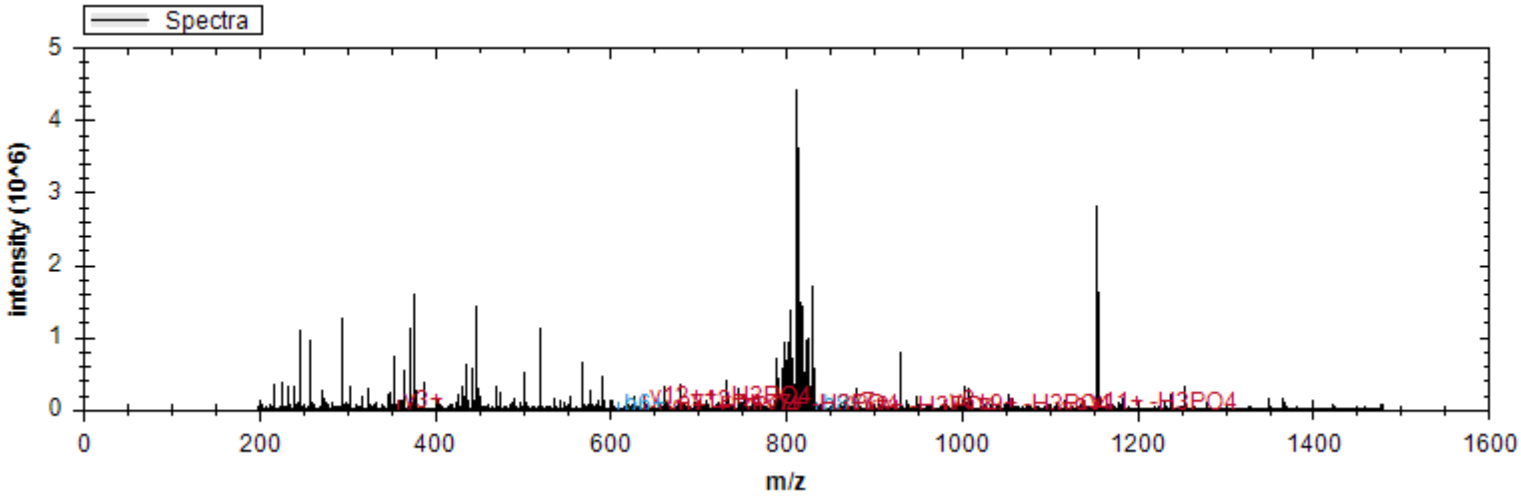

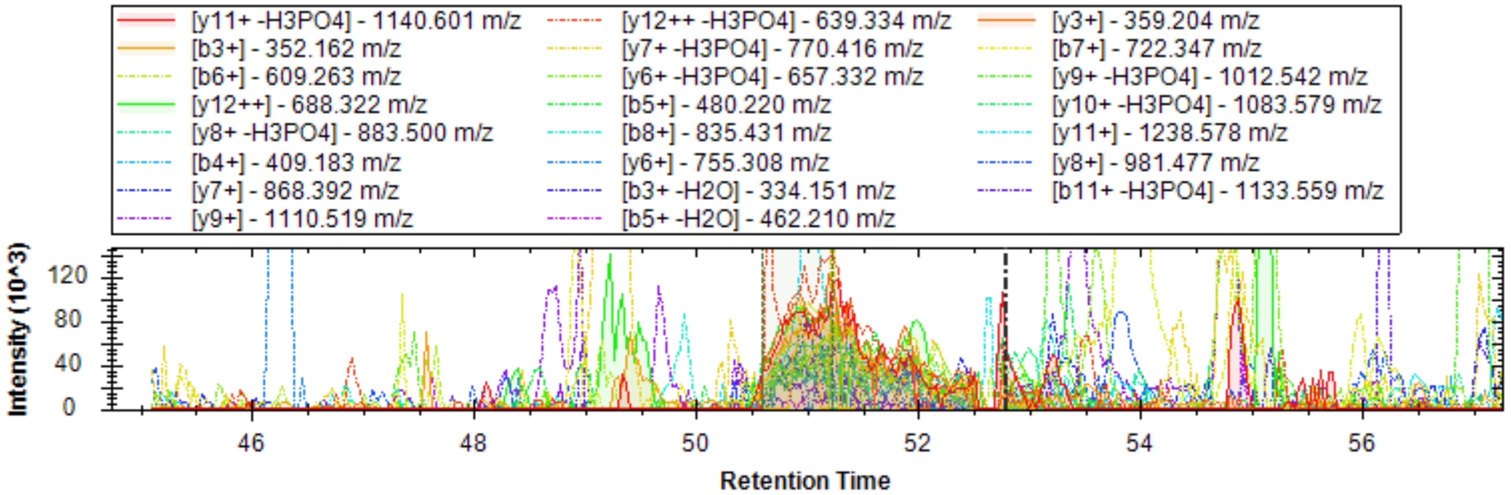

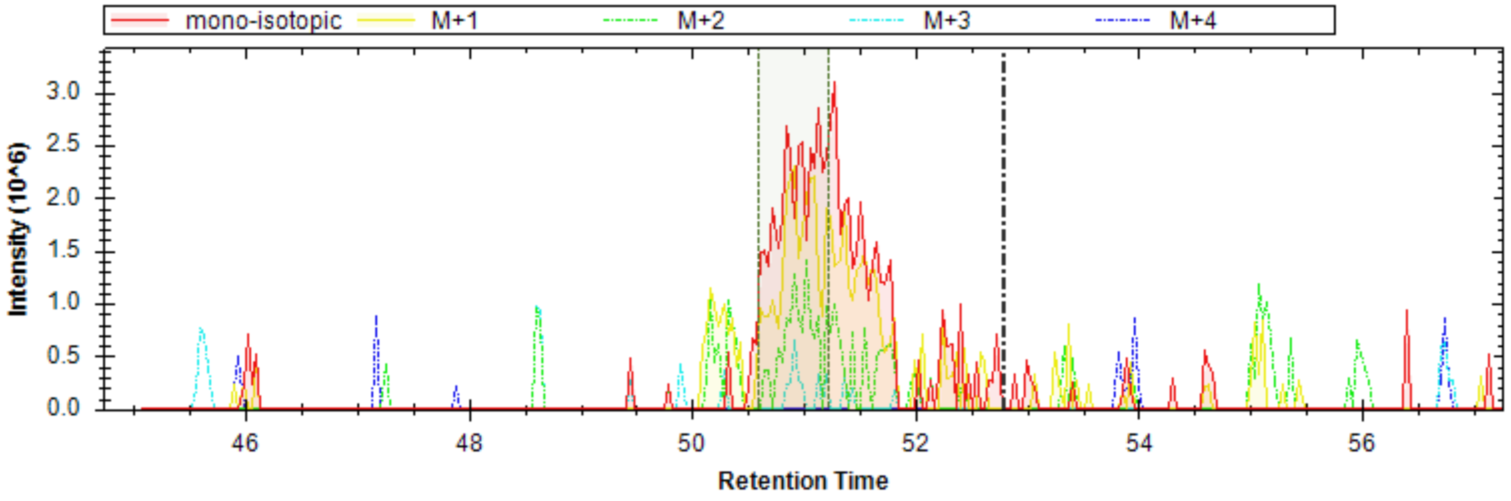

P20357 – _VDHGAEIITQS[+H+O3+P]PSR_.2
Fig4_MP-DIA-barrel-cortex-p9-1_MHRM.htrms

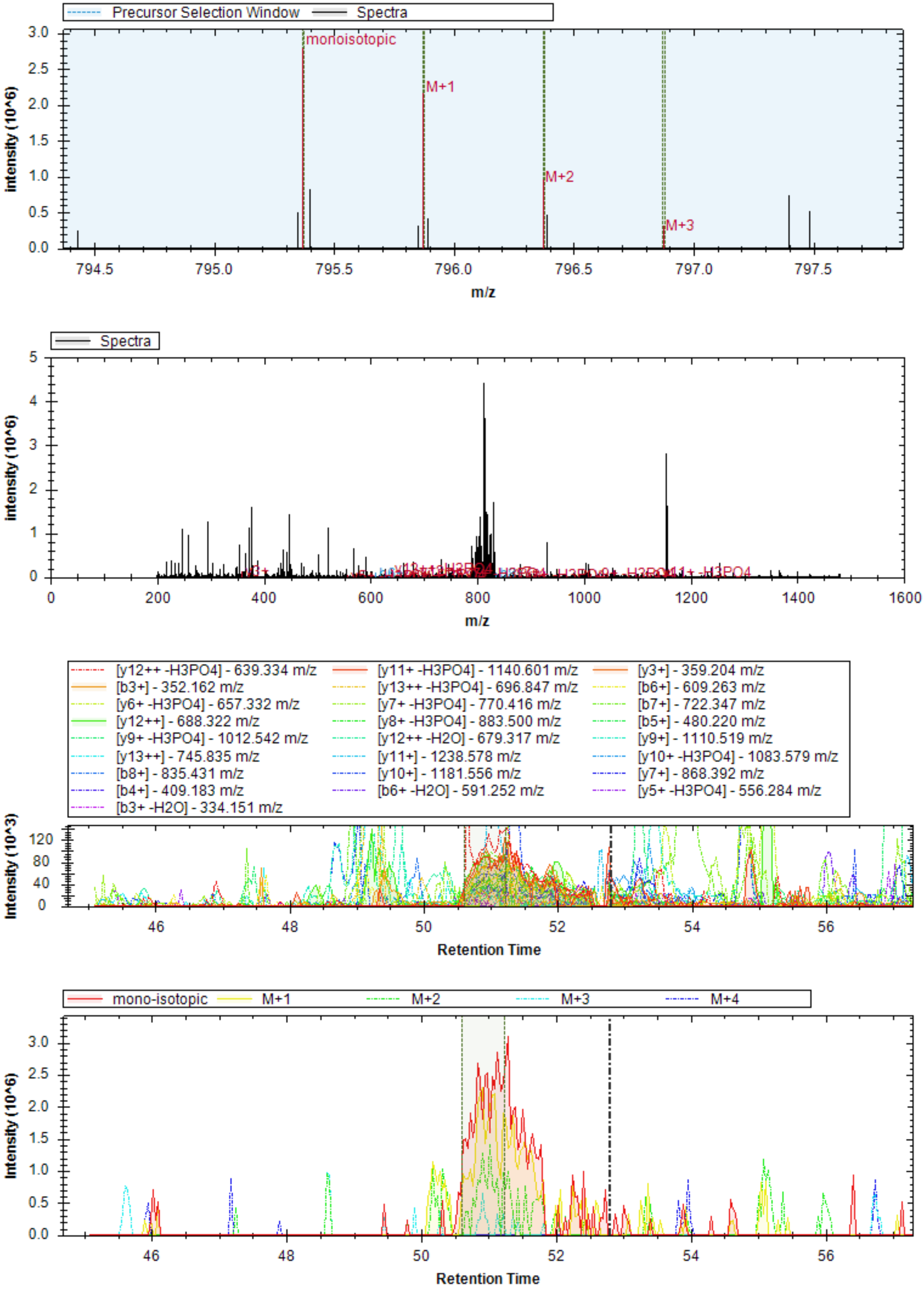

P20357 – _VDHGAEIITQSPS[+H+O3+P]R_.2

Fig4_MP-DIA-barrel-cortex-p9-1_MHRM.htrms

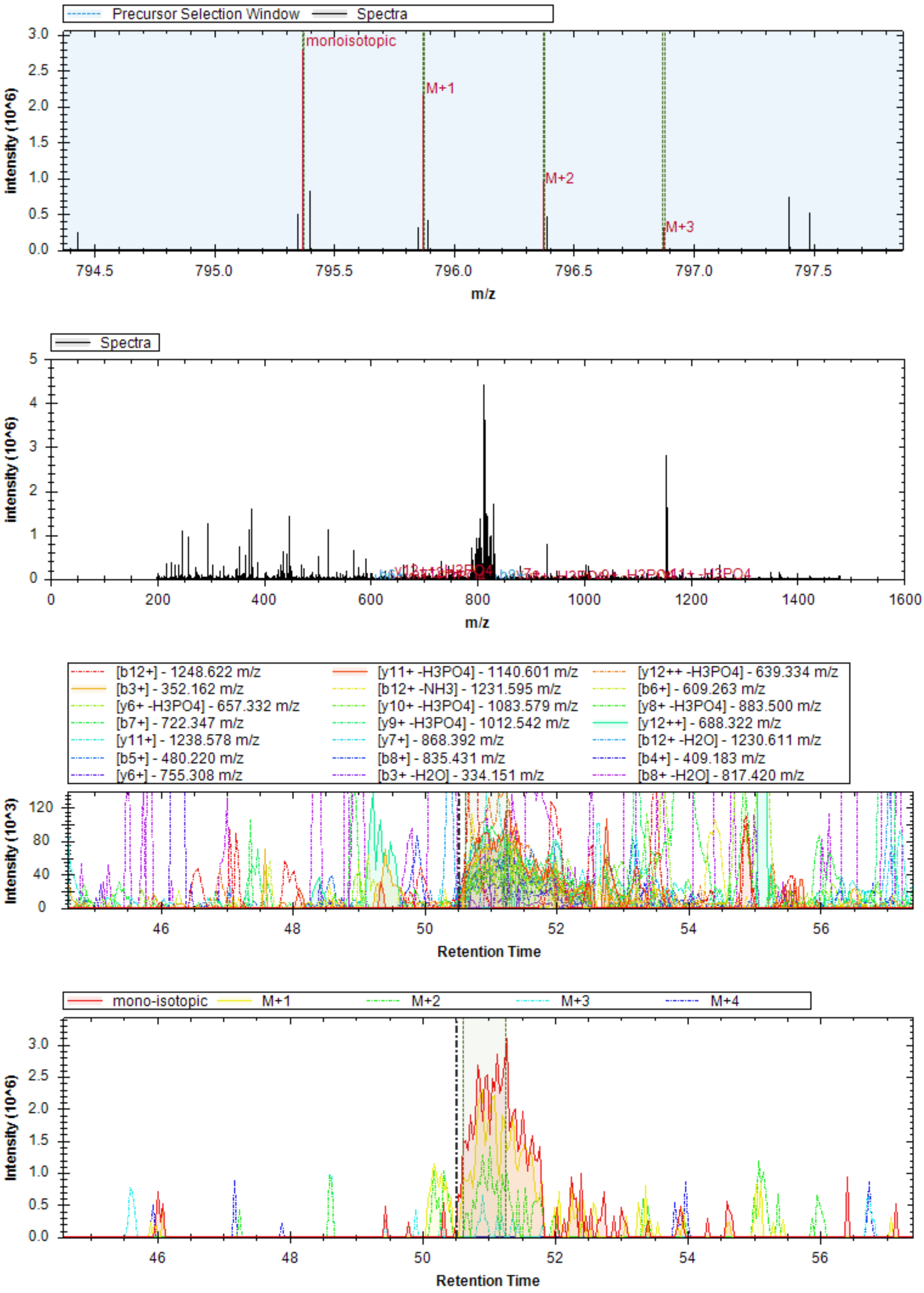

P54227 – _SKESVPDFPLS[+H+O3+P]PPK_.2
Fig4_MP-DIA-barrel-cortex-p9-1_MHRM.htrms

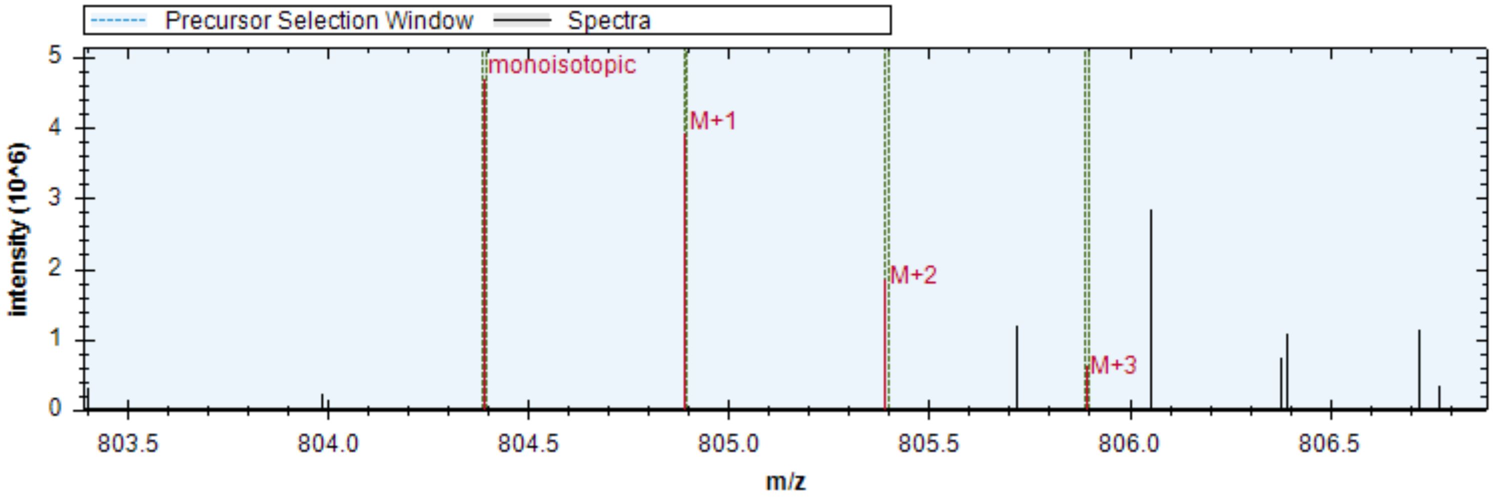

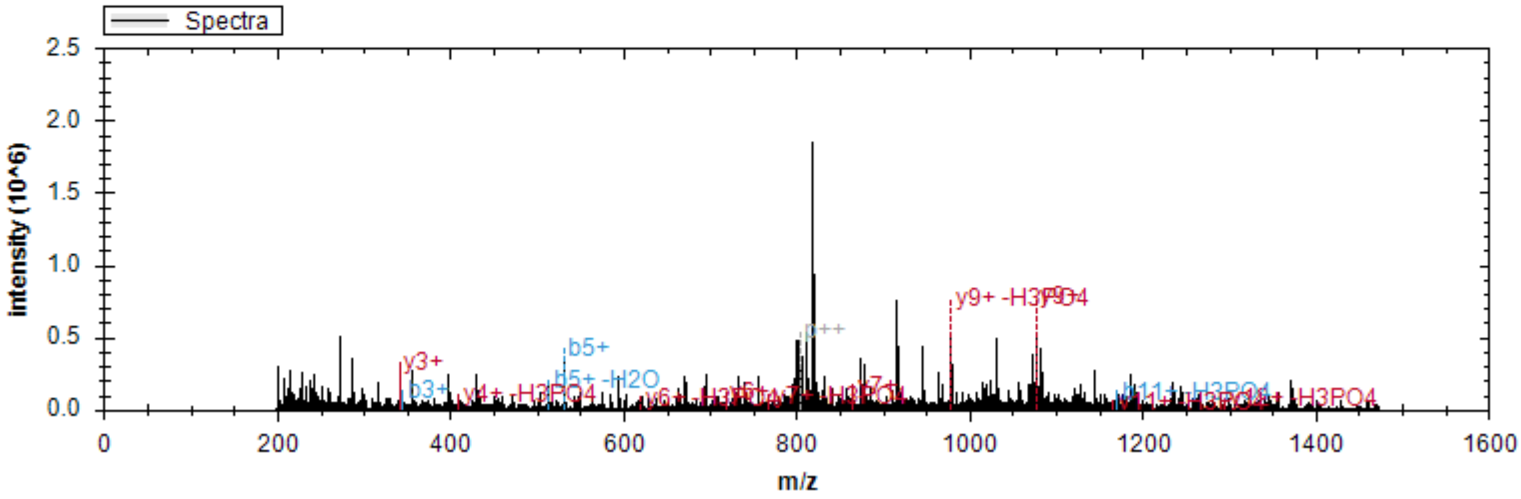

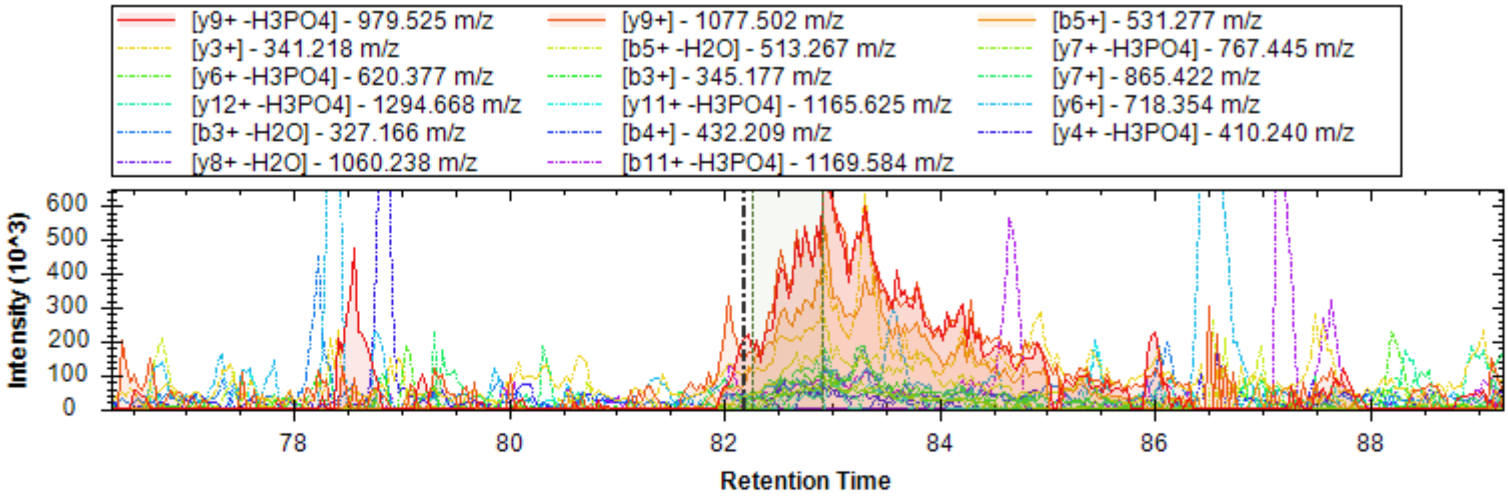

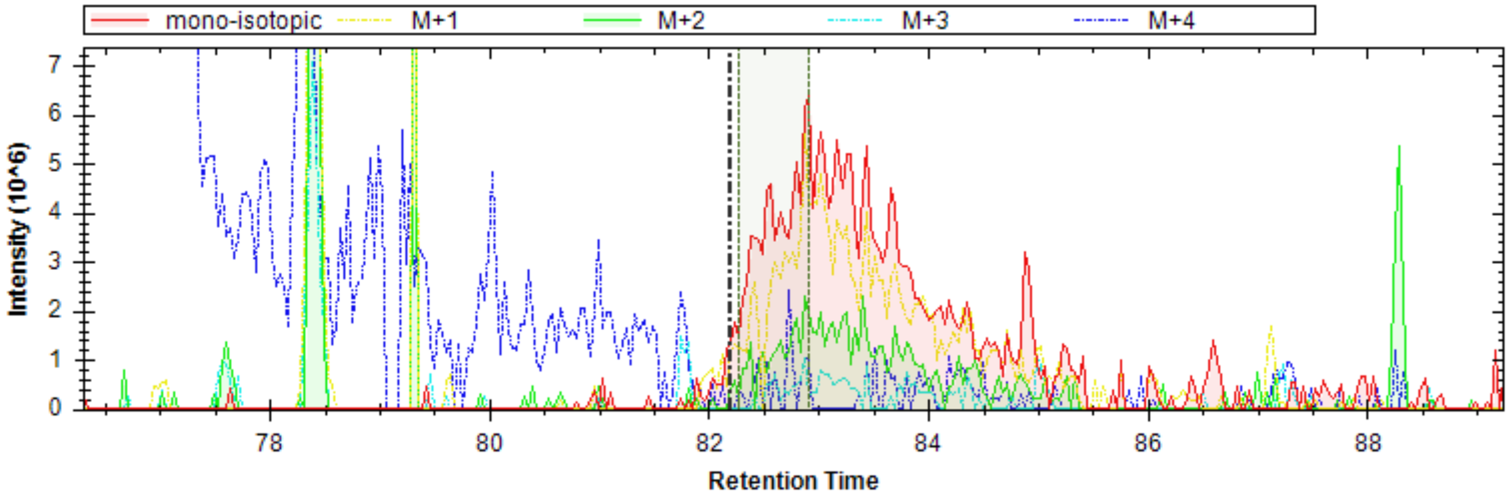

P67871 – _IHPM[+O]AYQLQLQAASNFKS[+H+O3+P]PVK_.3
Fig4_MP-DIA-barrel-cortex-p9-1_MHRM.htrms

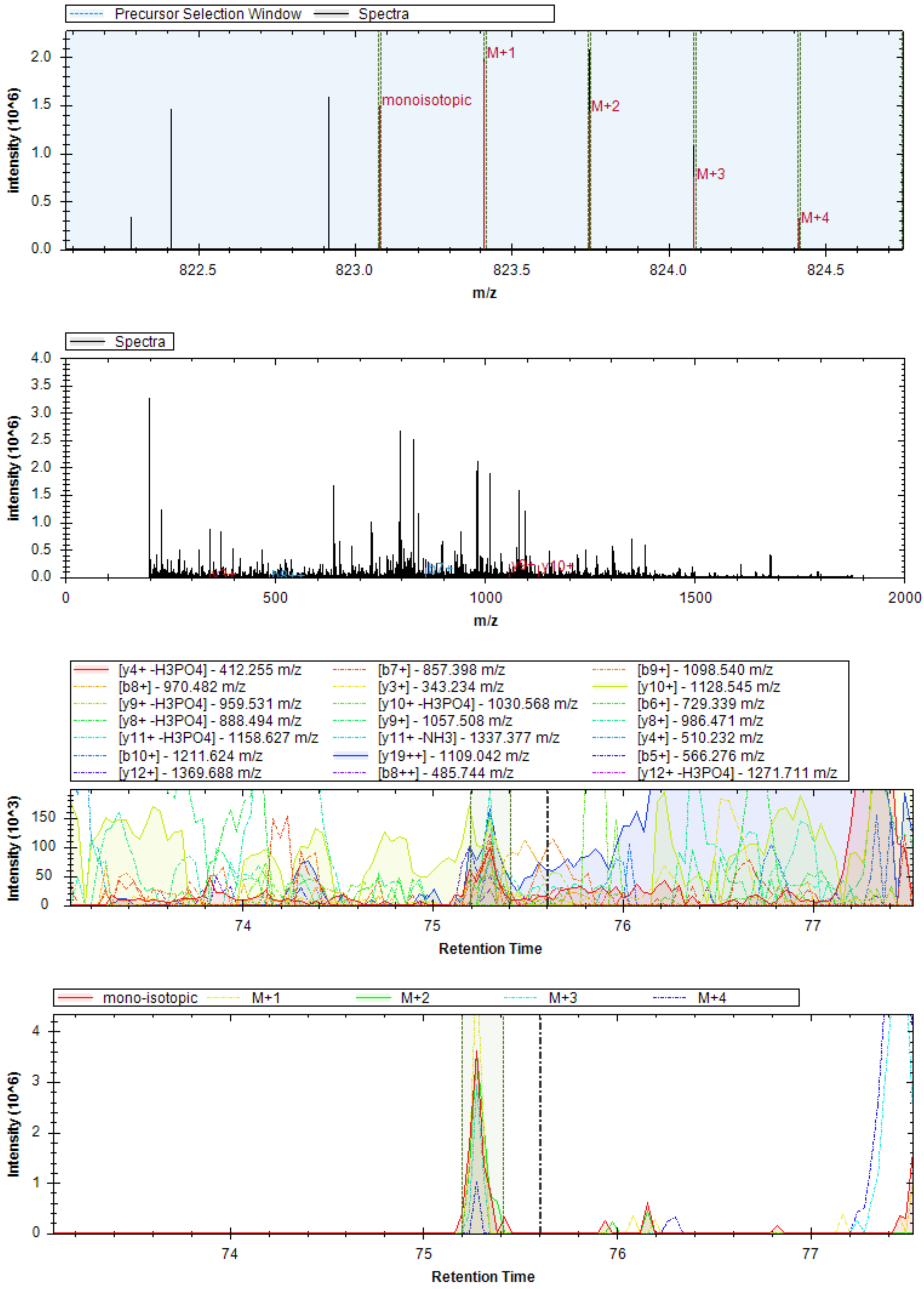

Q61548;Q61548-2;Q61548-3 – _SSPATTVTS[+H+O3+P]PNSTPAK_.2
Fig4_MP-DIA-barrel-cortex-p9-1_MHRM.htrms

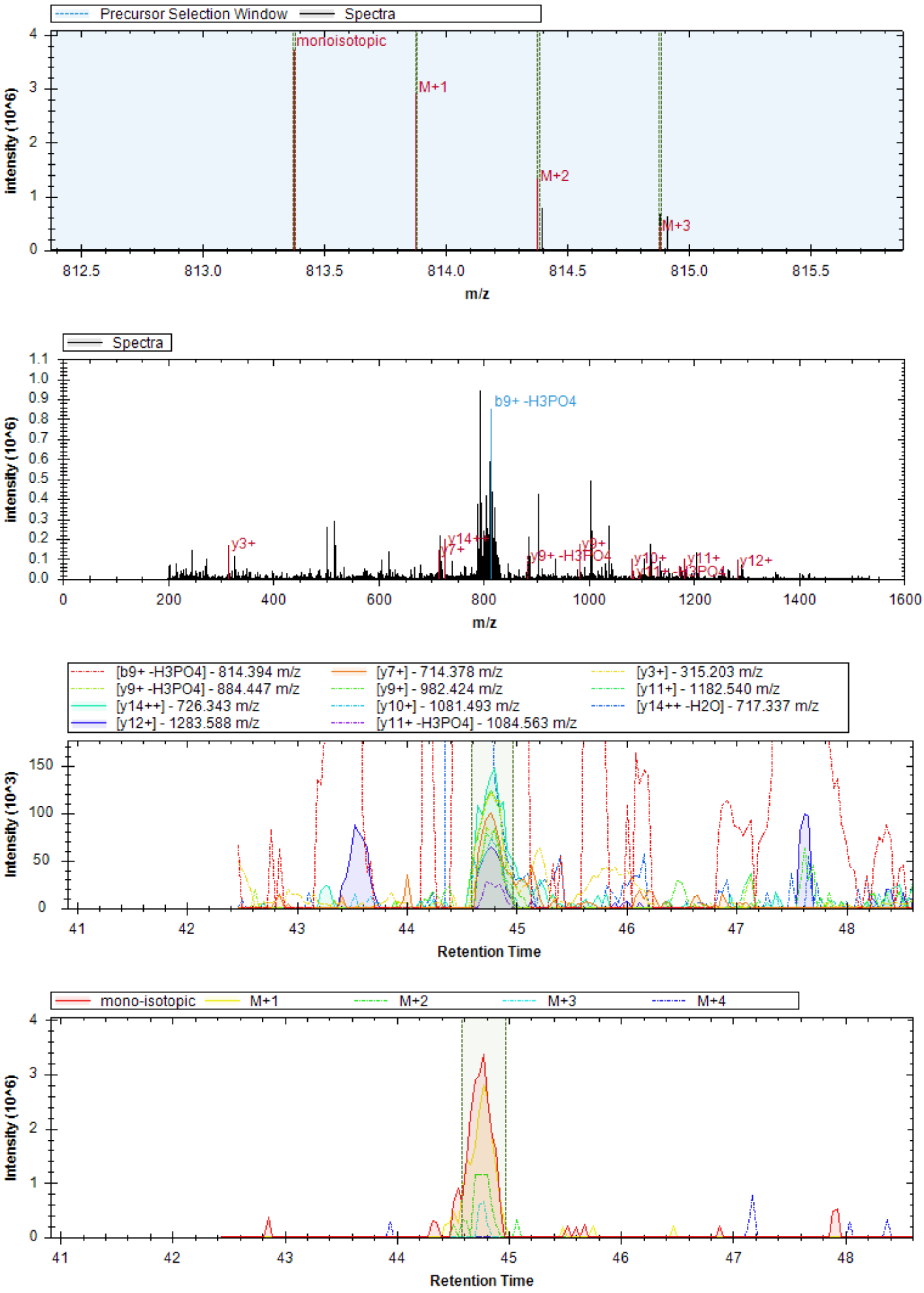

Q64701;Q64701-2 – _LAEILYYKILET[+H+O3+P]IMVQET[+H+O3+P]R_.3
Fig4_MP-DIA-barrel-cortex-p9-1_MHRM.htrms

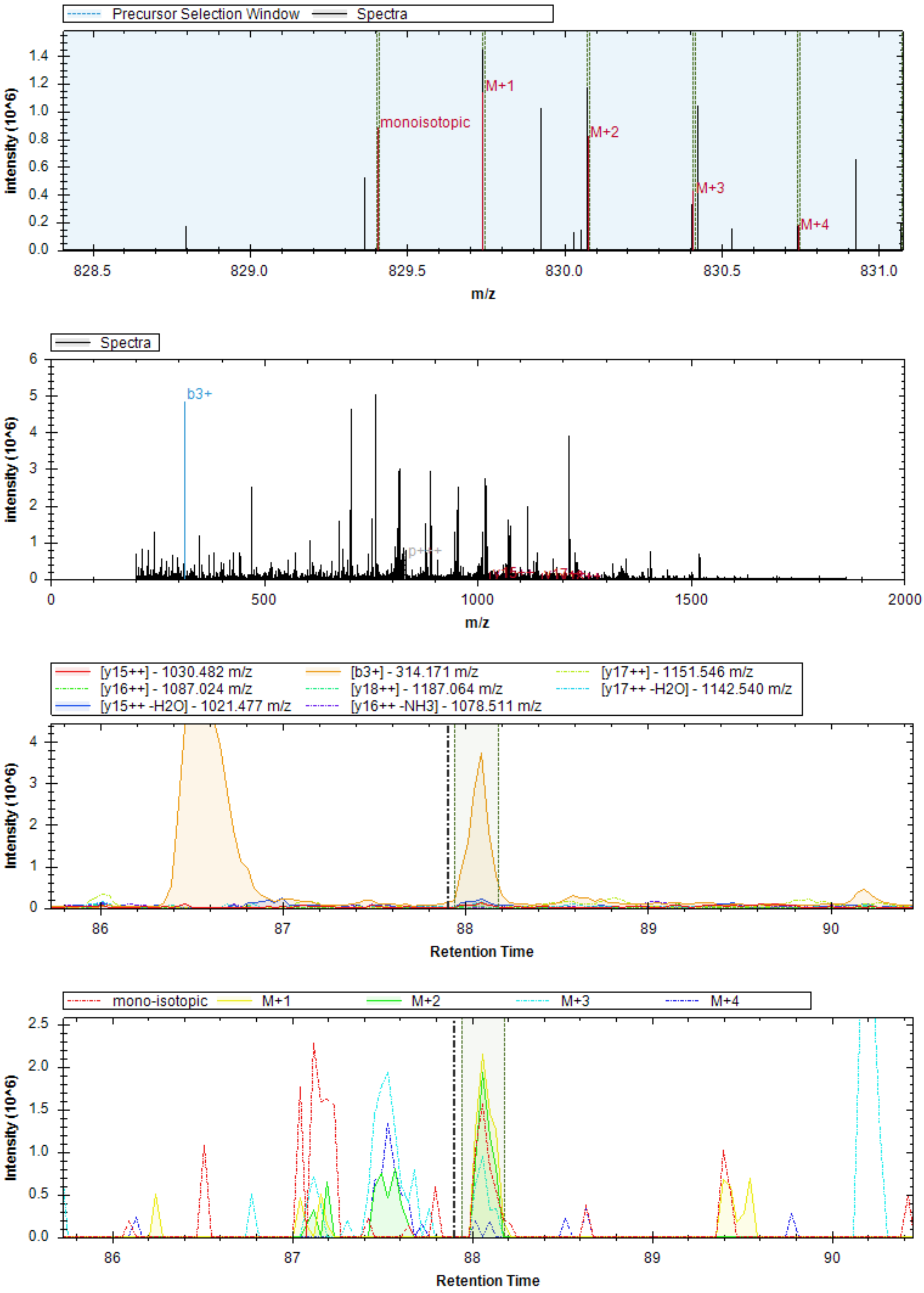

Q91XM9;Q91XM9-2;Q91XM9-7 – _NLSQIENVHGYVLQS[+H+O3+P]HISPLK_.3
Fig4_MP-DIA-barrel-cortex-p9-1_MHRM.htrms

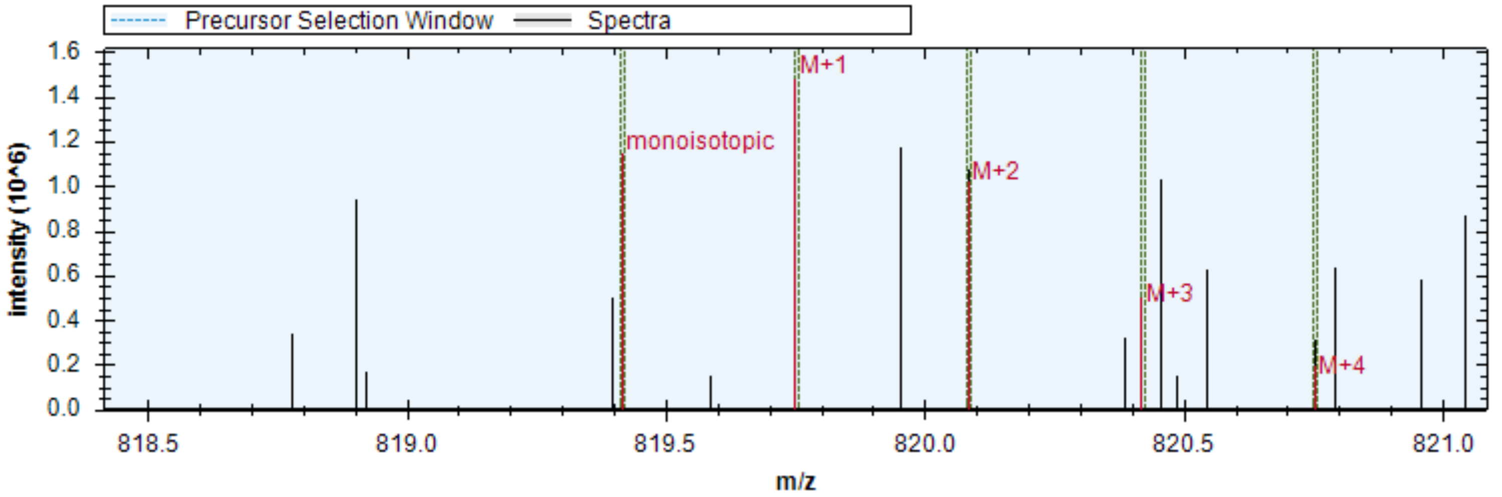

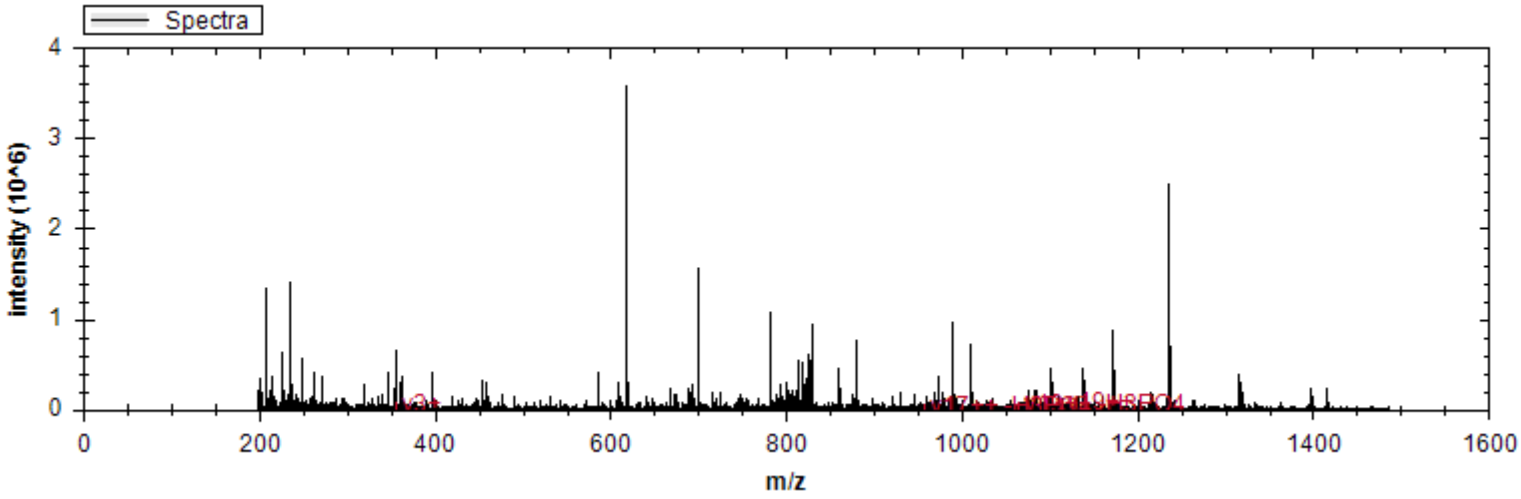

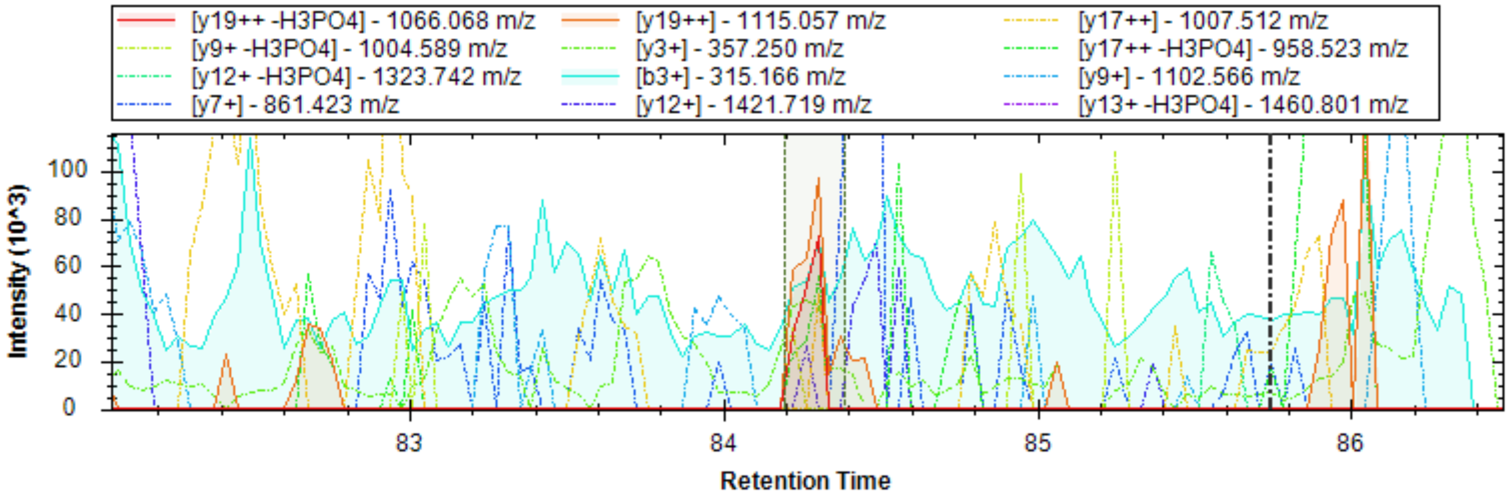

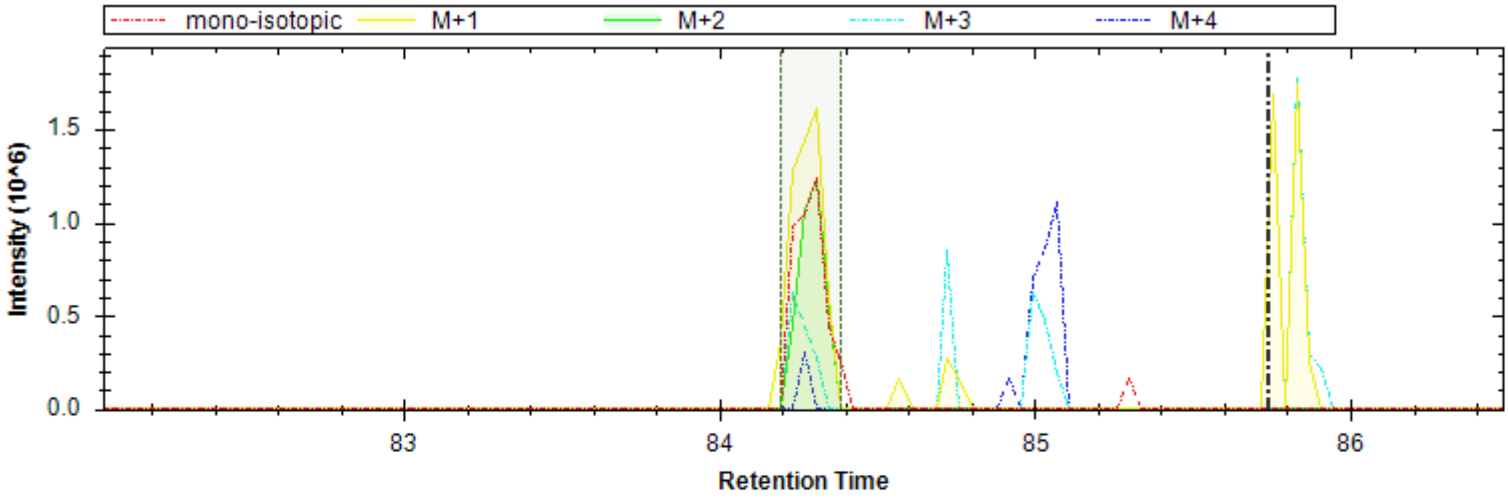

O08784 – _ASAVSPEKAPM[+O]T[+H+O3+P]SKAK_.2
Fig4_MP-DIA-barrel-cortex-p9-1_MHRM.htrms

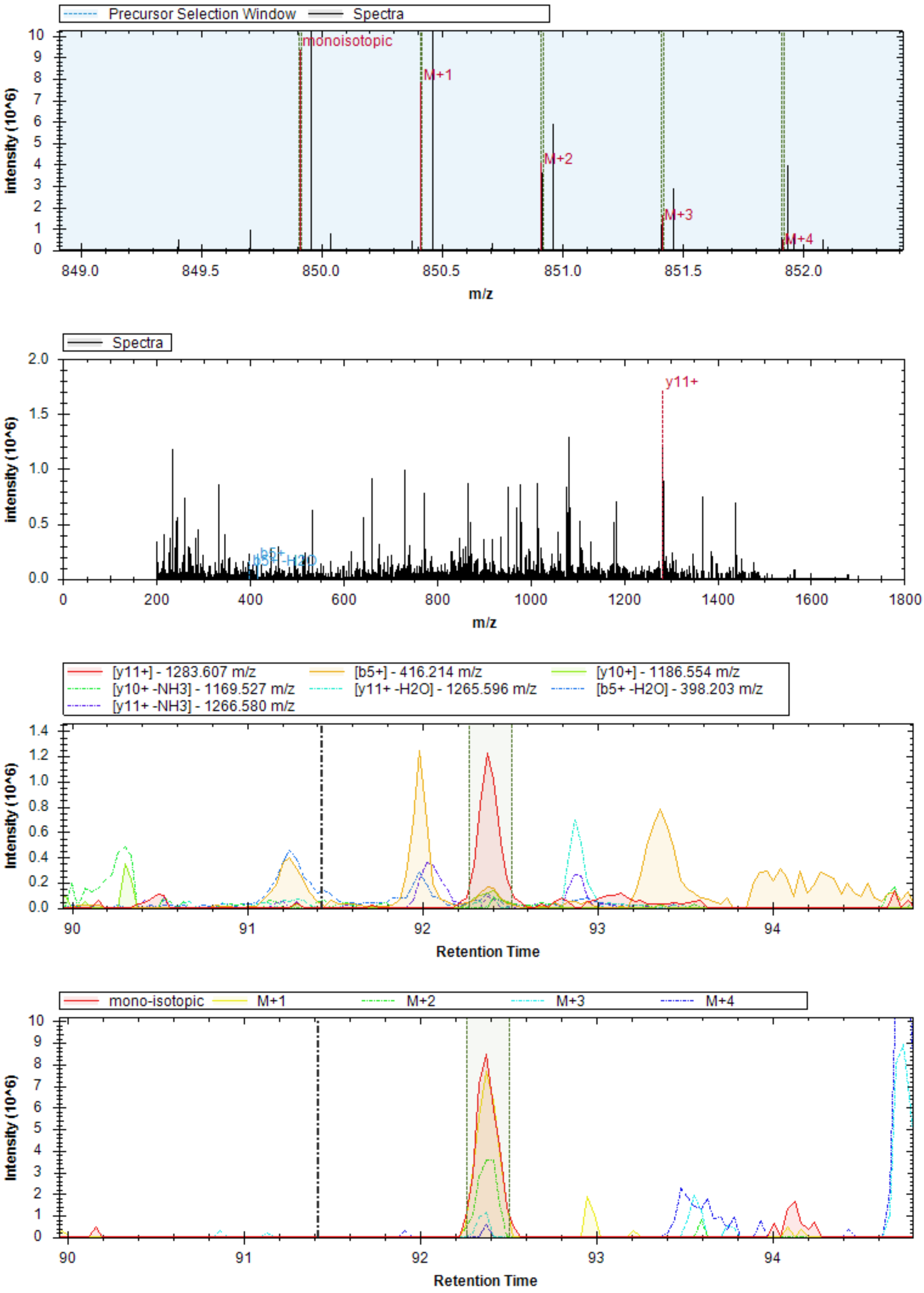

P27546-2 – _AAVGVTGNDITTPPNKEPPPS[+H+O3+P]PEKK_.3
Fig4_MP-DIA-barrel-cortex-p9-1_MHRM.htrms

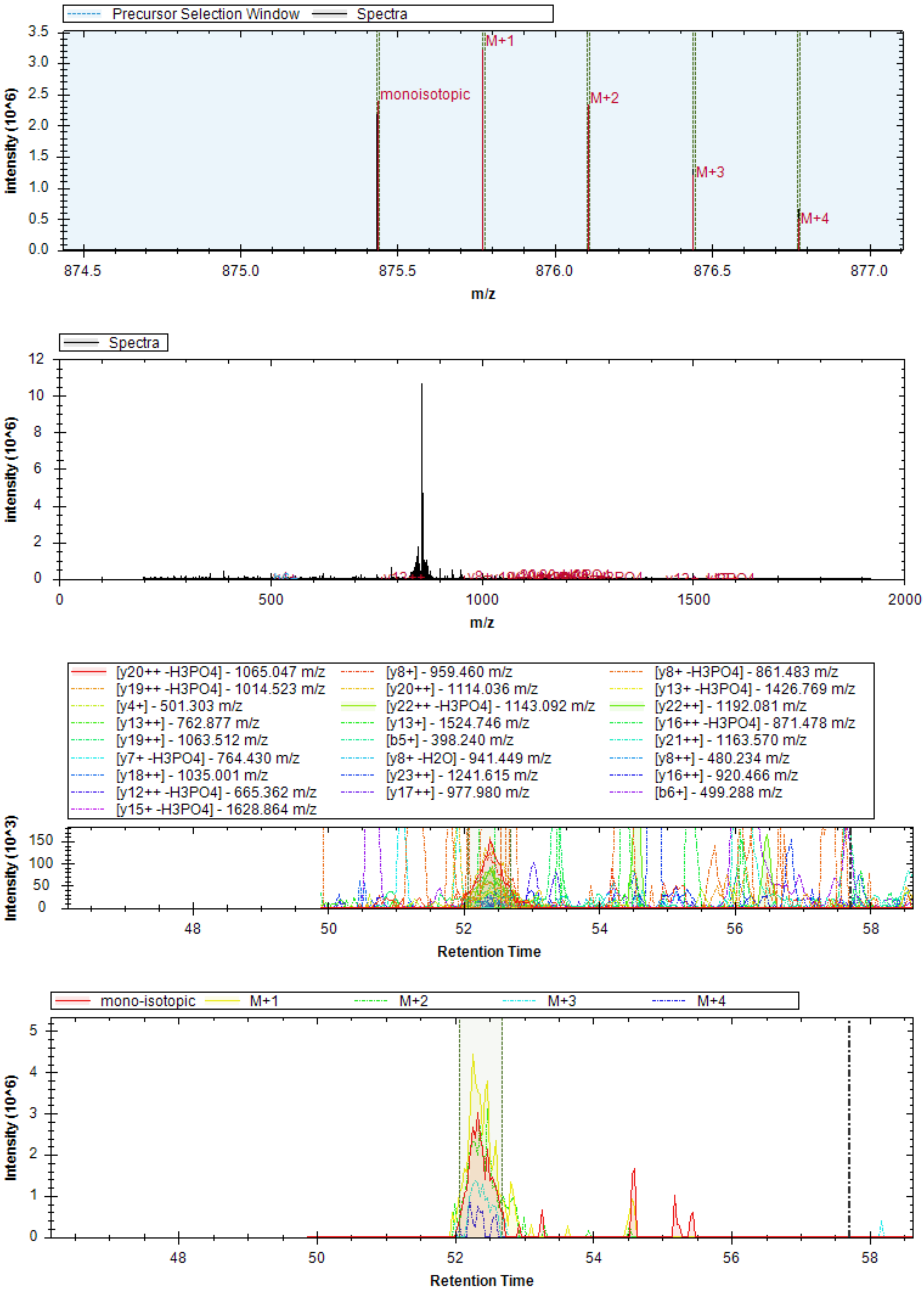

P27546-2 – _NTT[+H+O3+P]PTGAAPPAGMTSTR_.2

Fig4_MP-DIA-barrel-cortex-p9-1_MHRM.htrms

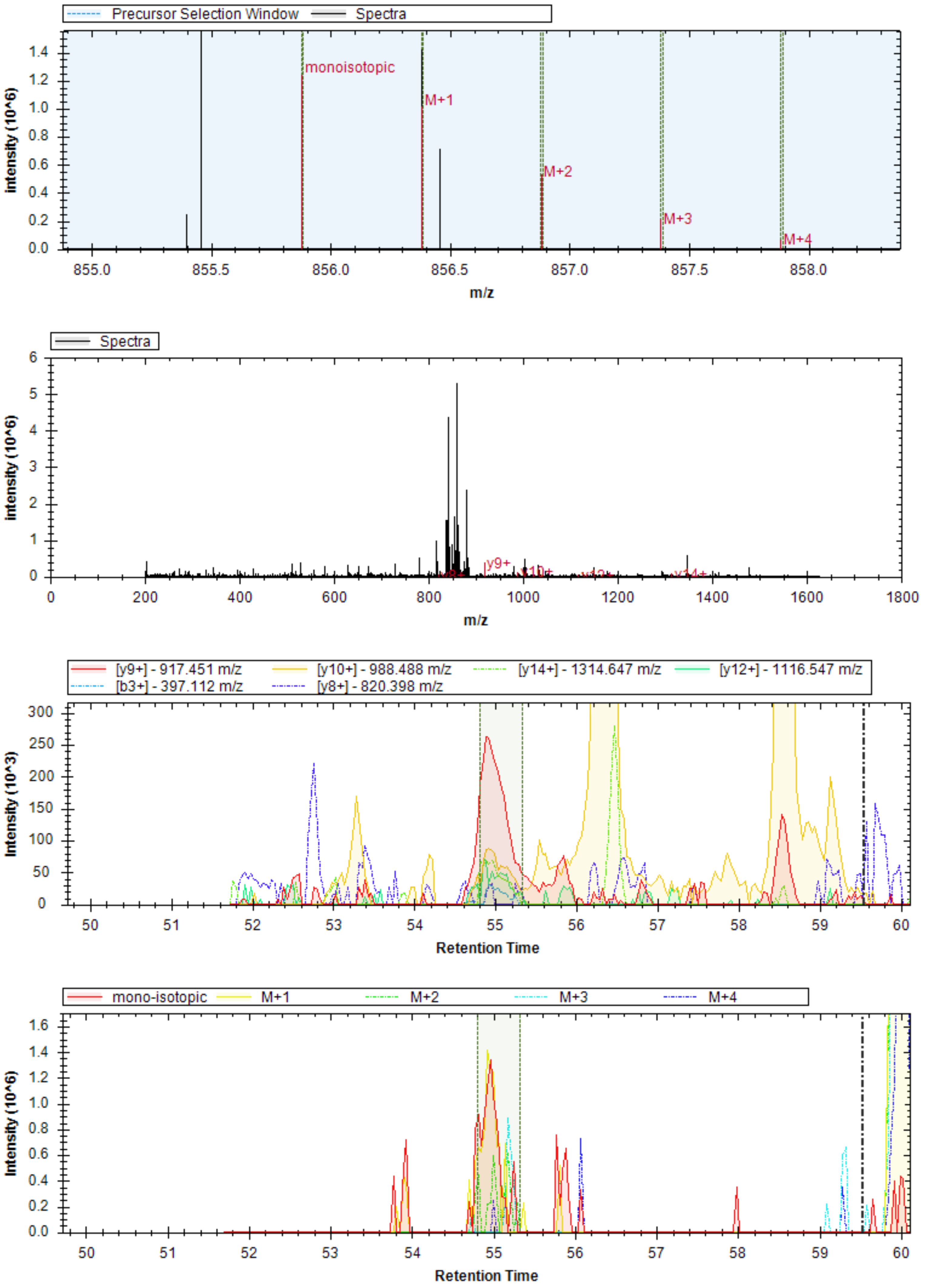

P50538 – _EAEHGYAS[+H+O3+P]M[+O]LPYSK_.2

Fig4_MP-DIA-barrel-cortex-p9-1_MHRM.htrms

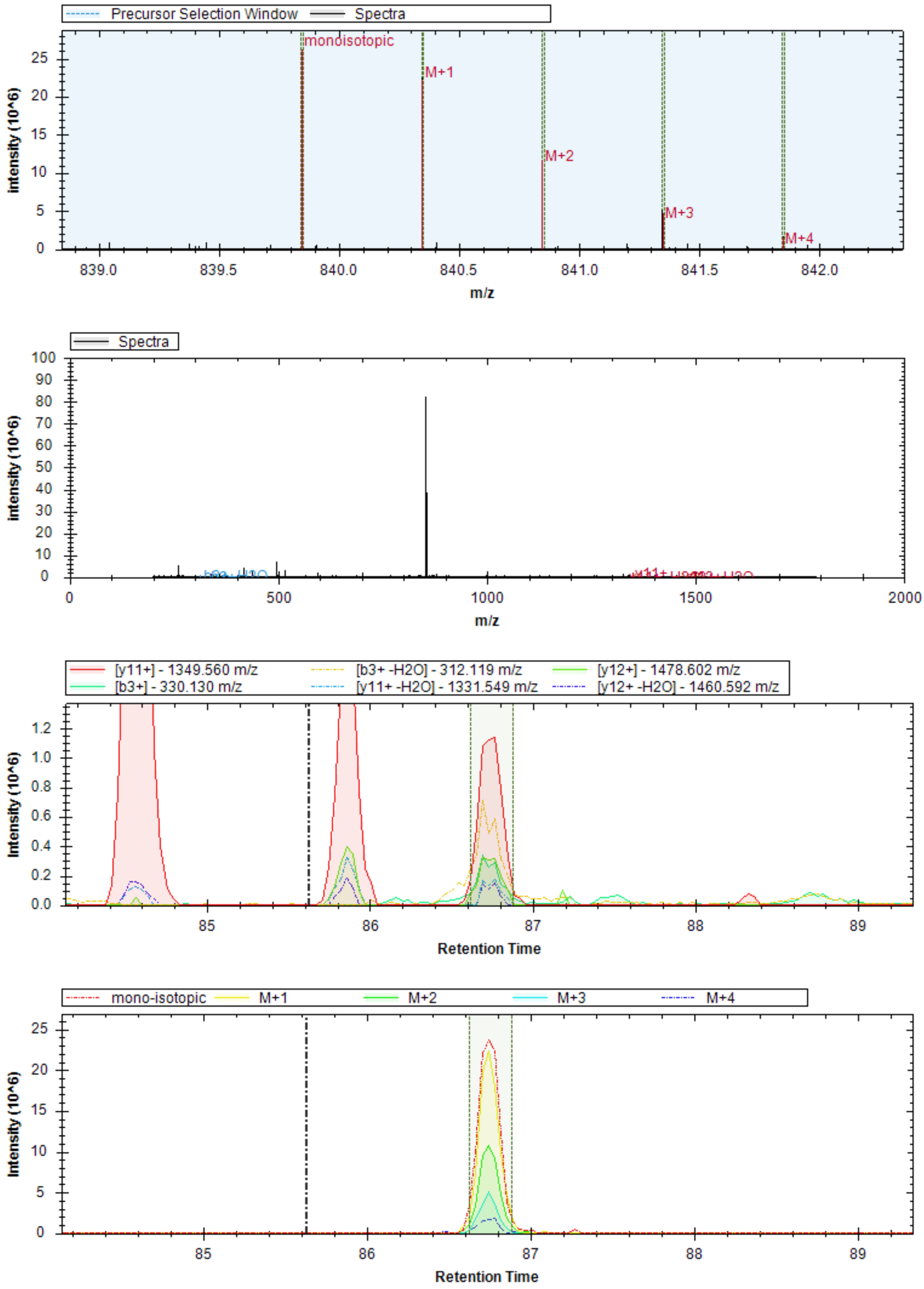

Q61029 – _SST[+H+O3+P]PLPTVSSSAENTR_.2
Fig4_MP-DIA-barrel-cortex-p9-1_MHRM.htrms

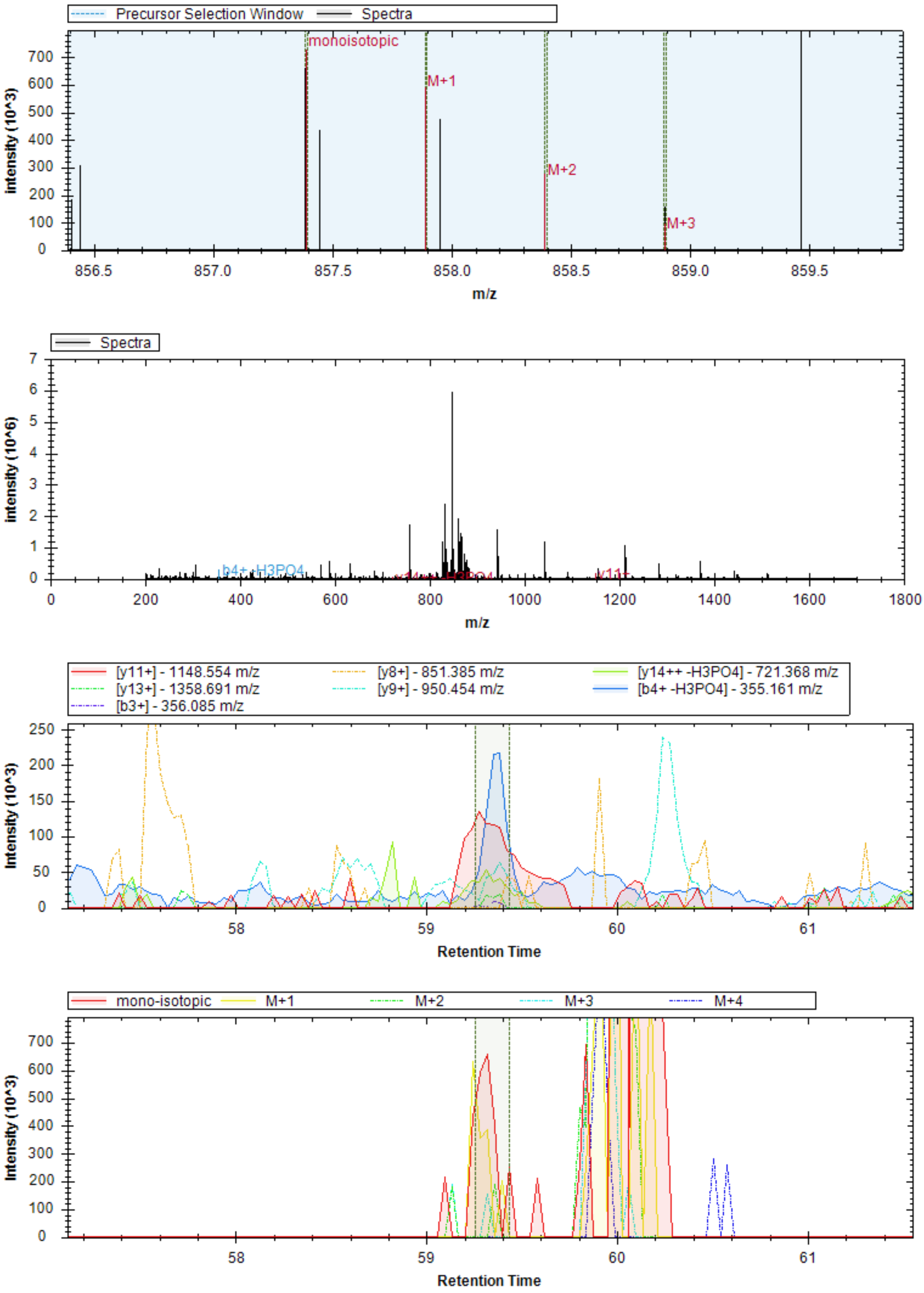

Q61290 – _M[+O]EPS[+H+O3+P]SLPQEIIANAK_.2
Fig4_MP-DIA-barrel-cortex-p9-1_MHRM.htrms

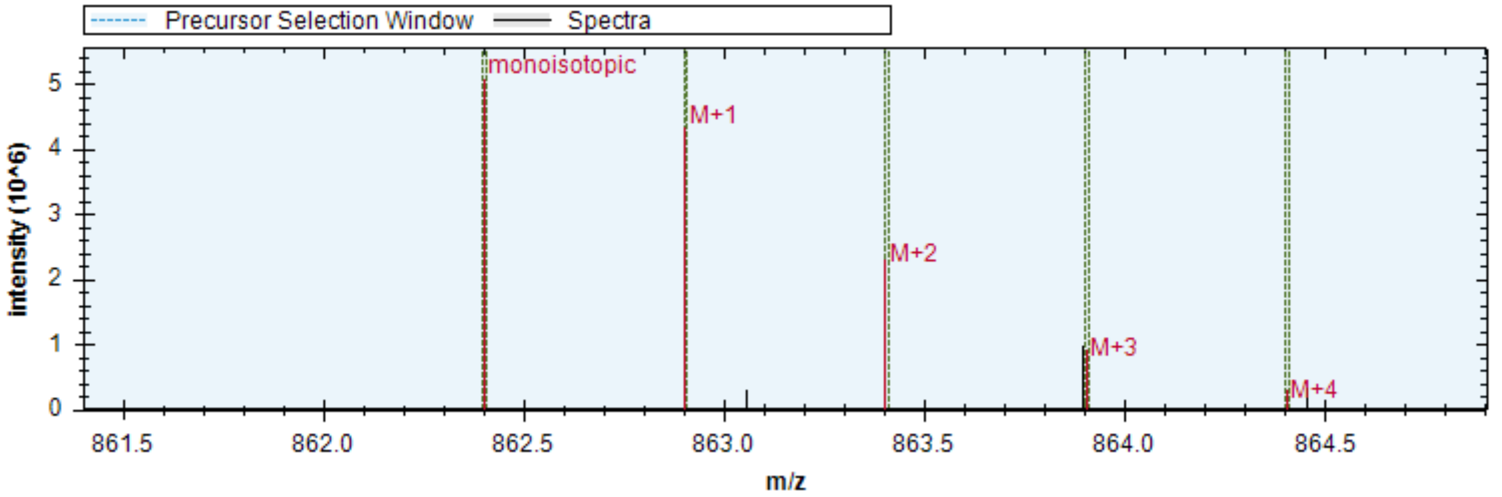

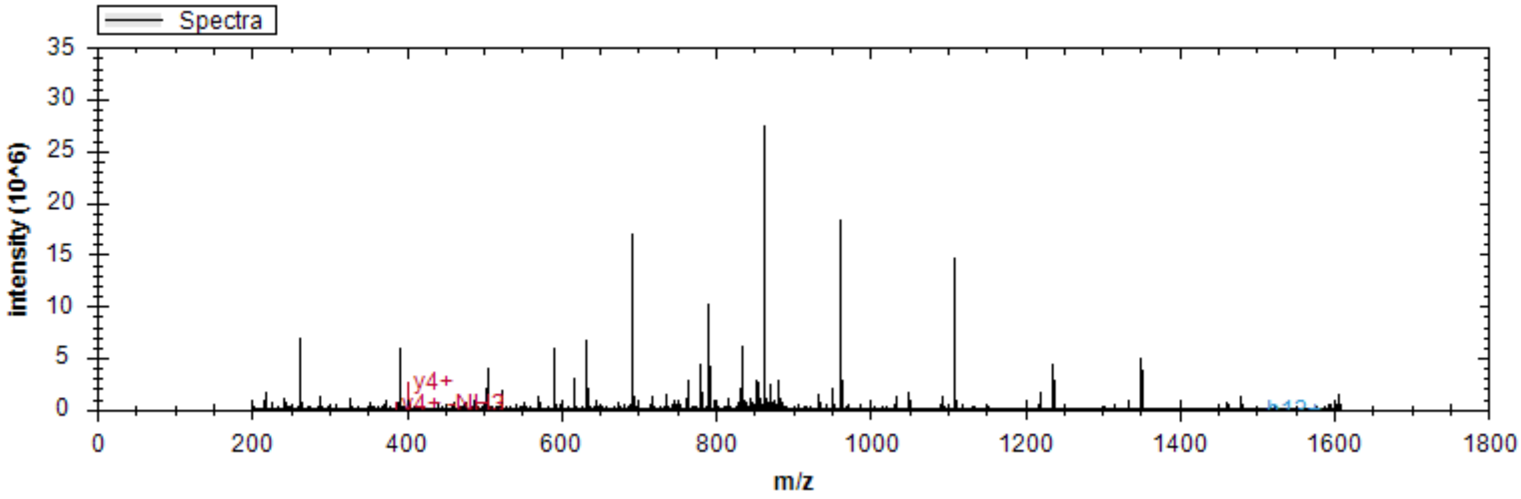

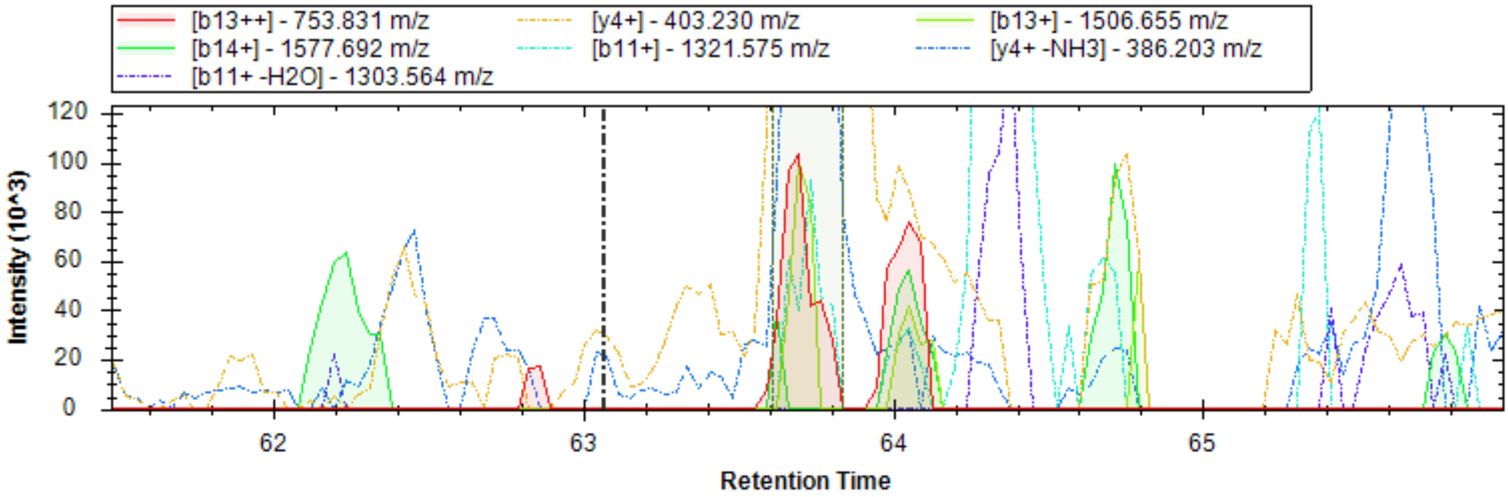

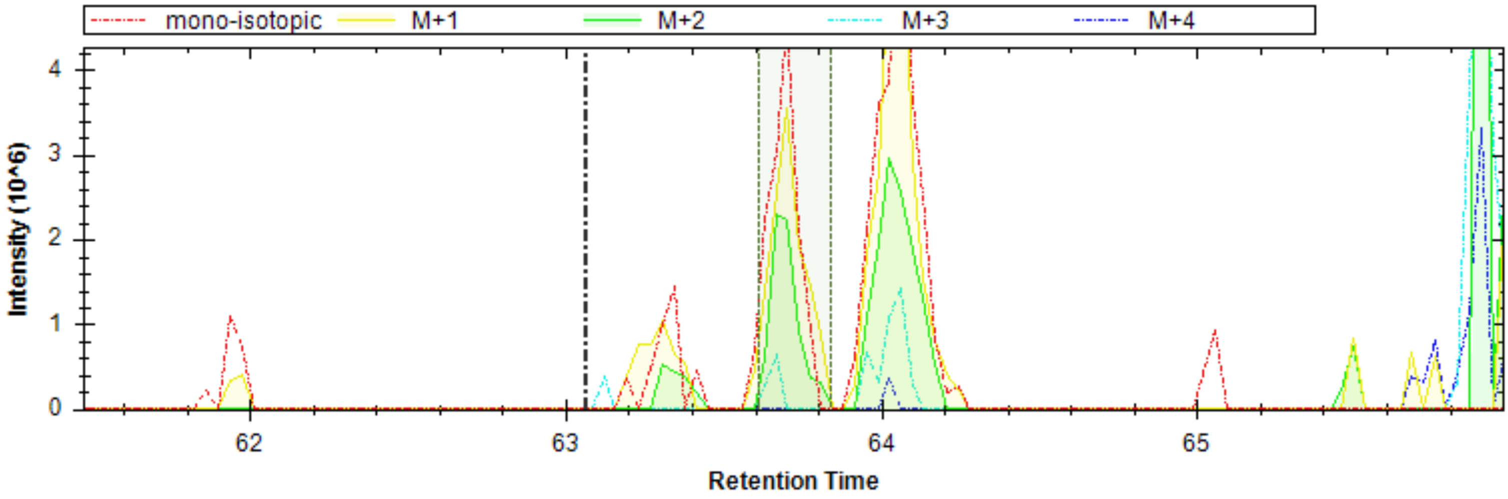

Q62188 – _GM[+O]YDGPVFDLT[+H+O3+P]TTPK_.2
Fig4_MP-DIA-barrel-cortex-p9-1_MHRM.htrms

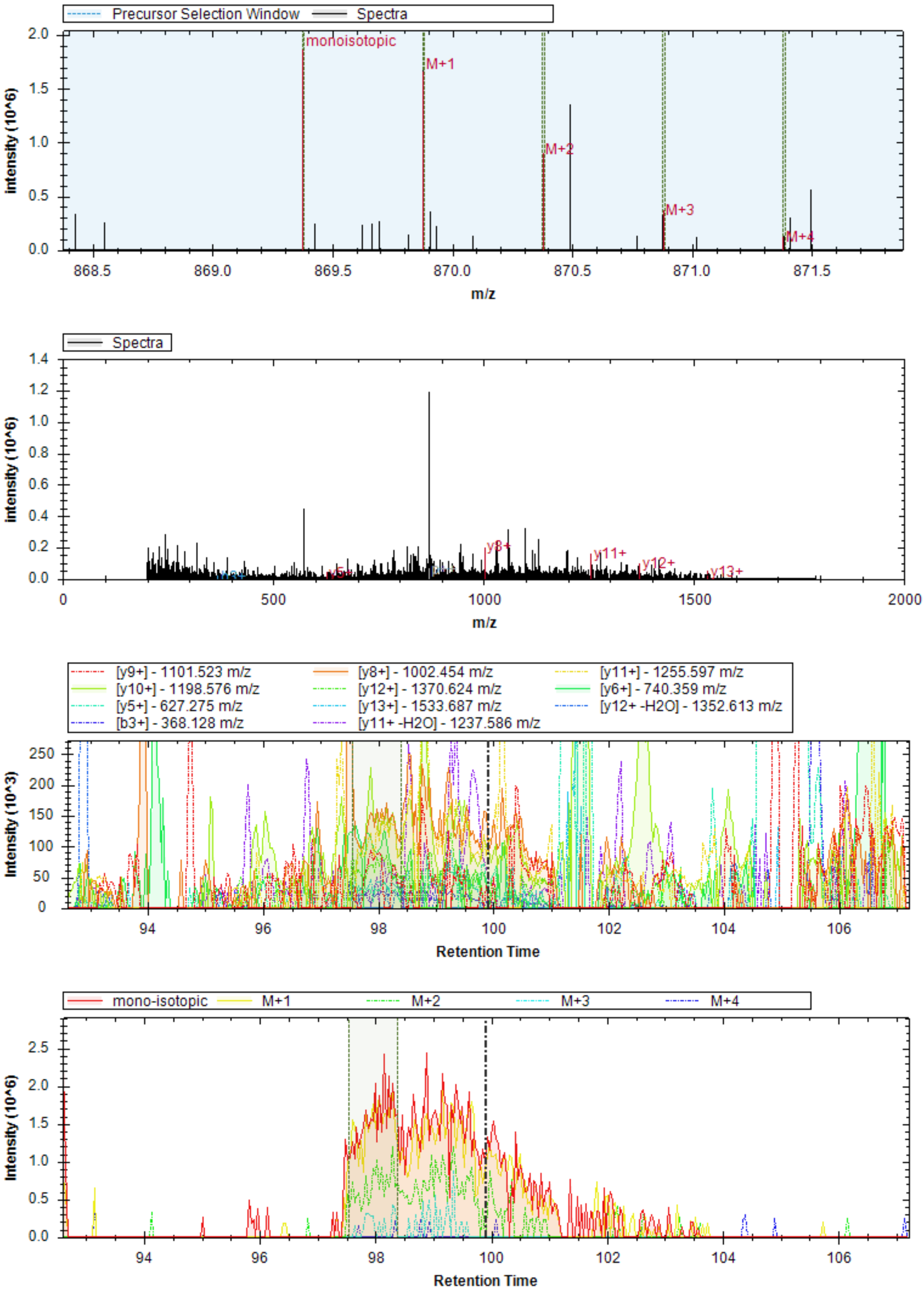

Q62188 – _GM[+O]YDGPVFDLTT[+H+O3+P]TPK_.2
Fig4_MP-DIA-barrel-cortex-p9-1_MHRM.htrms

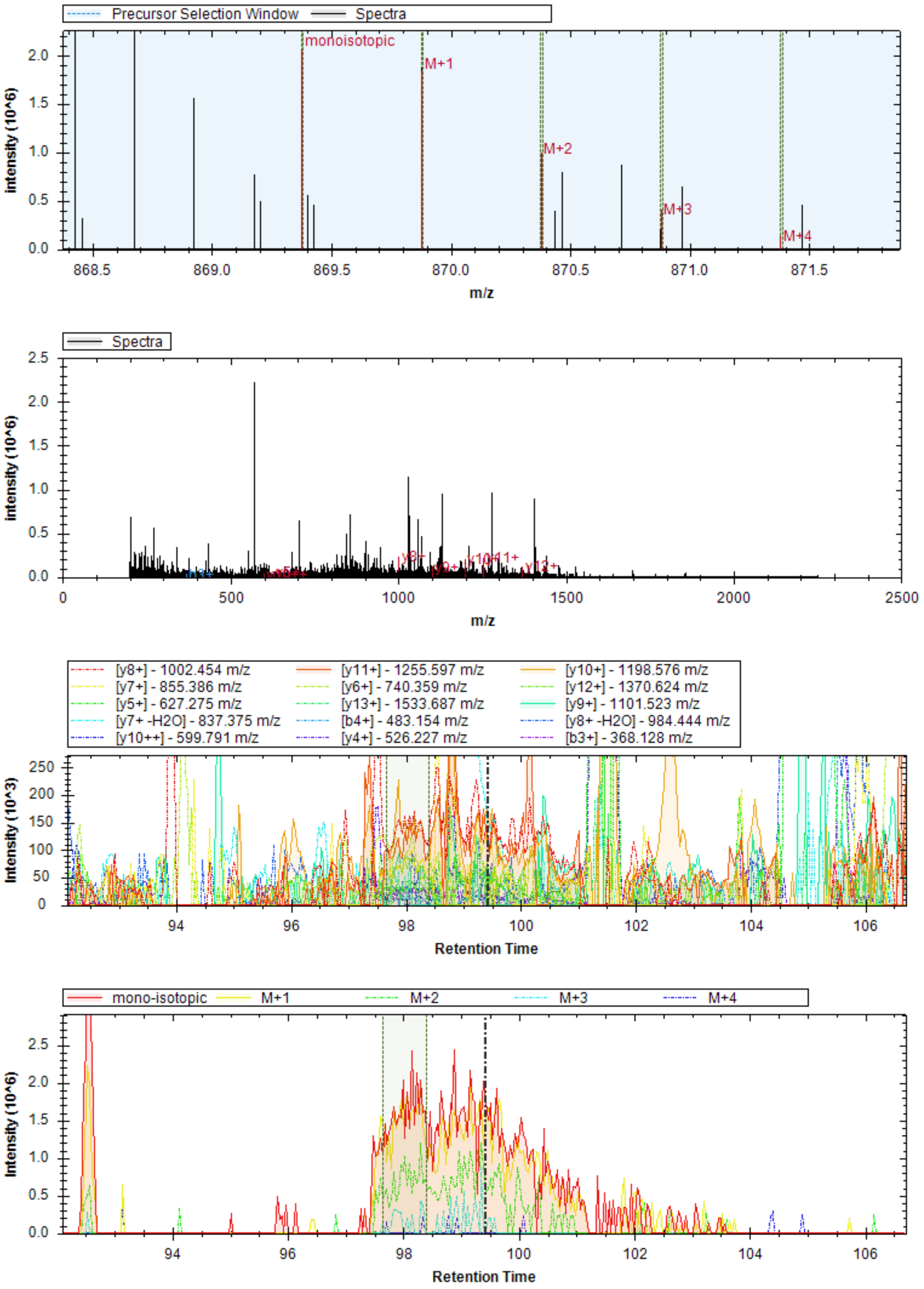

Q9JM96 – _DSSKLPKS[+H+O3+P]LS[+H+O3+P]SSPVK_.2
Fig4_MP-DIA-barrel-cortex-p9-1_MHRM.htrms

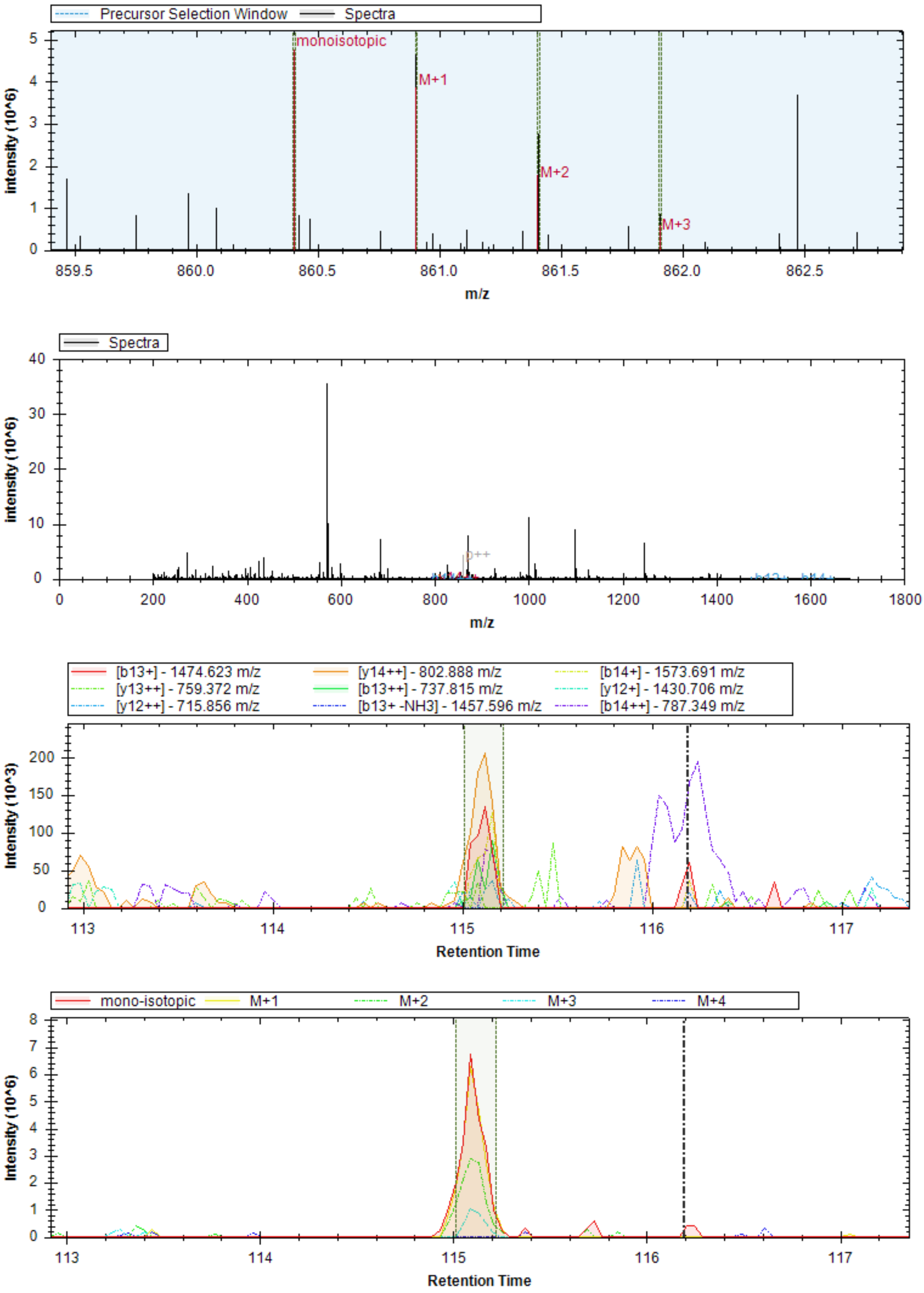

Q9QXS6 – _ASDSGPSSSSSS[+H+O3+P]SSSPPR_.2

Fig4_MP-DIA-barrel-cortex-p9-1_MHRM.htrms

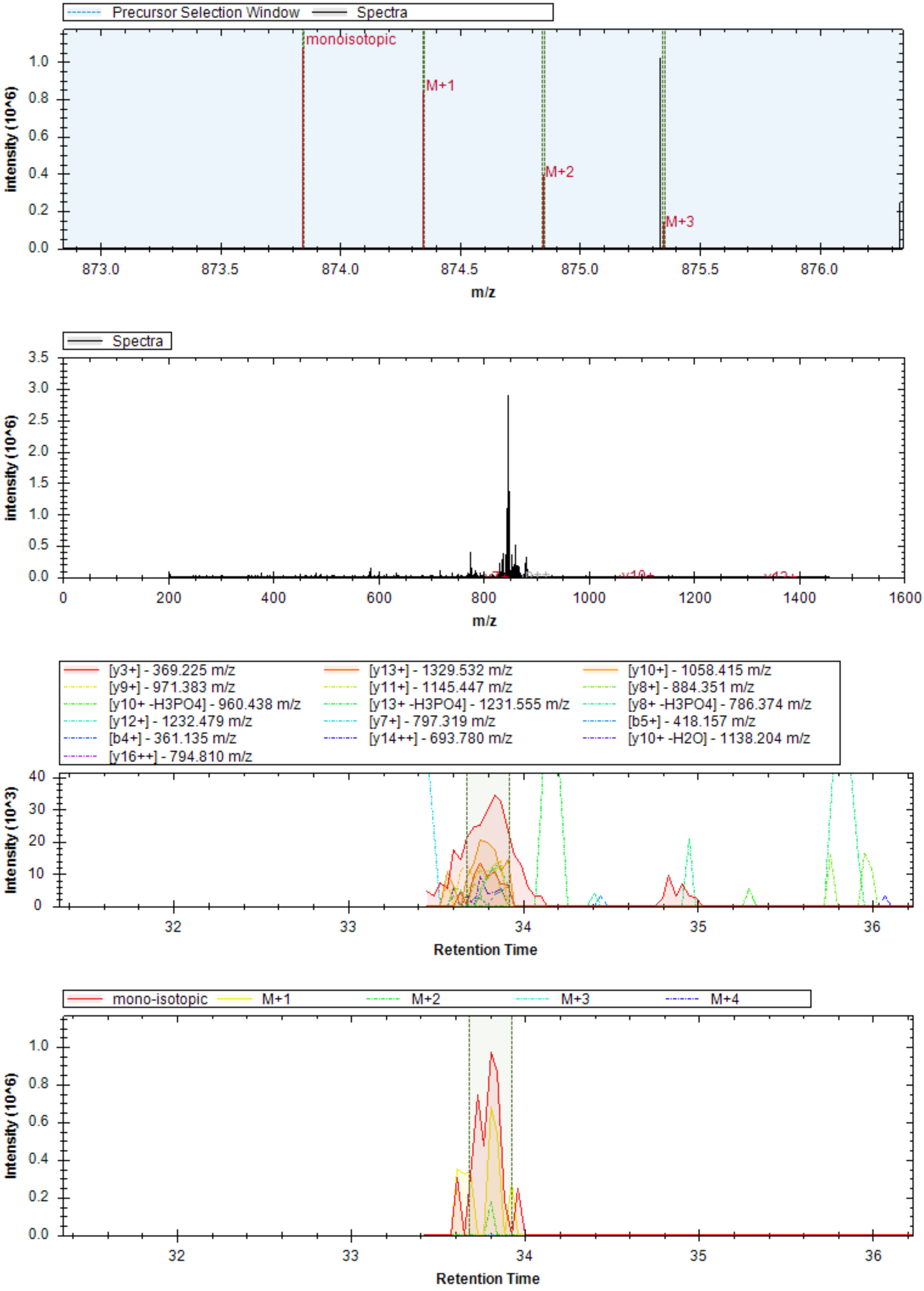

P26645 – _GEATAERPGEAAVASS[+H+O3+P]PSK_.2
Fig4_MP-DIA-barrel-cortex-p9-1_MHRM.htrms

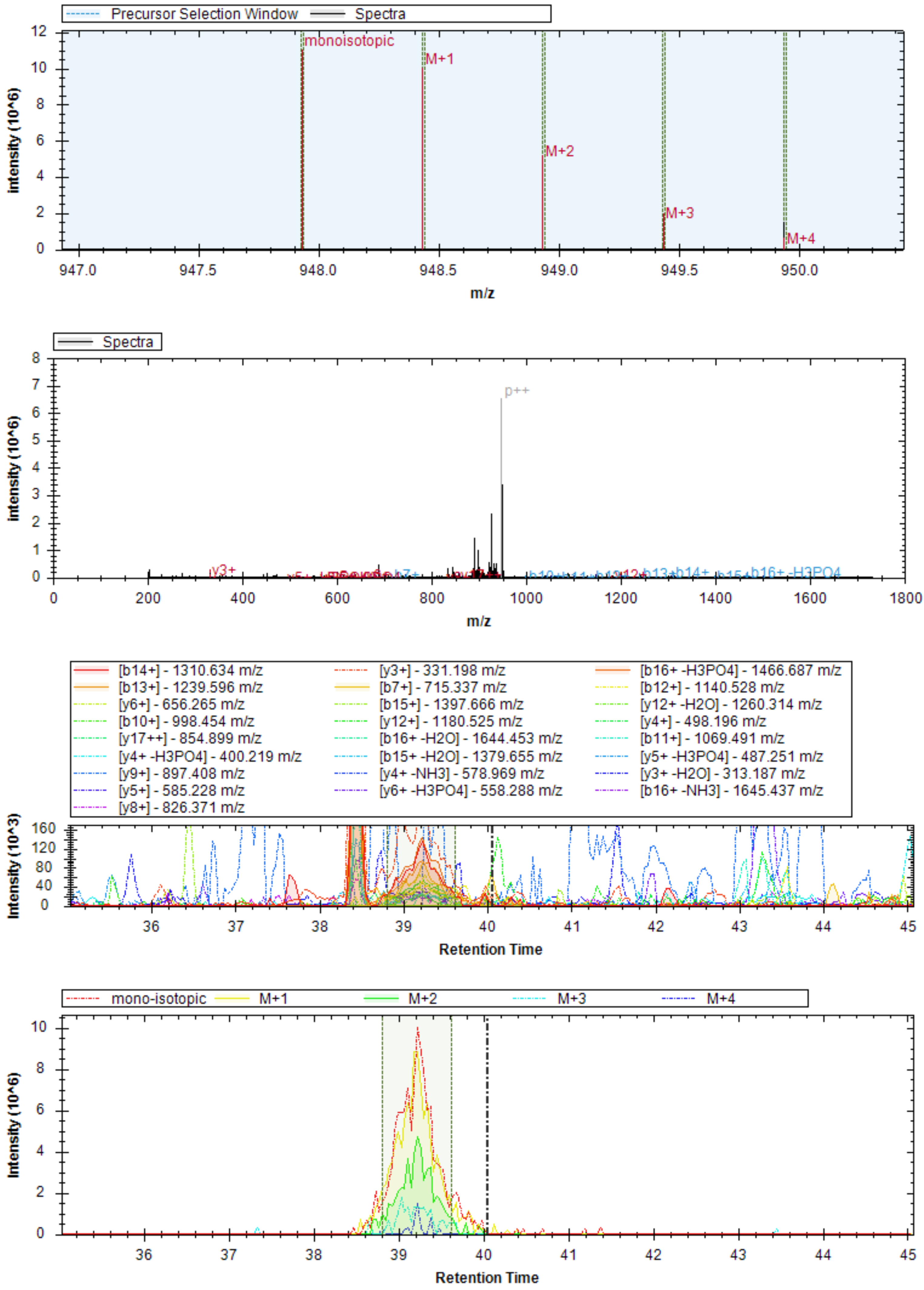

Q8BMA3 – _IIQLC[+C2+H3+N+O]LDLT[+H+O3+P]T[+H+O3+P]AVQK_.2
Fig4_MP-DIA-barrel-cortex-p9-1_MHRM.htrms

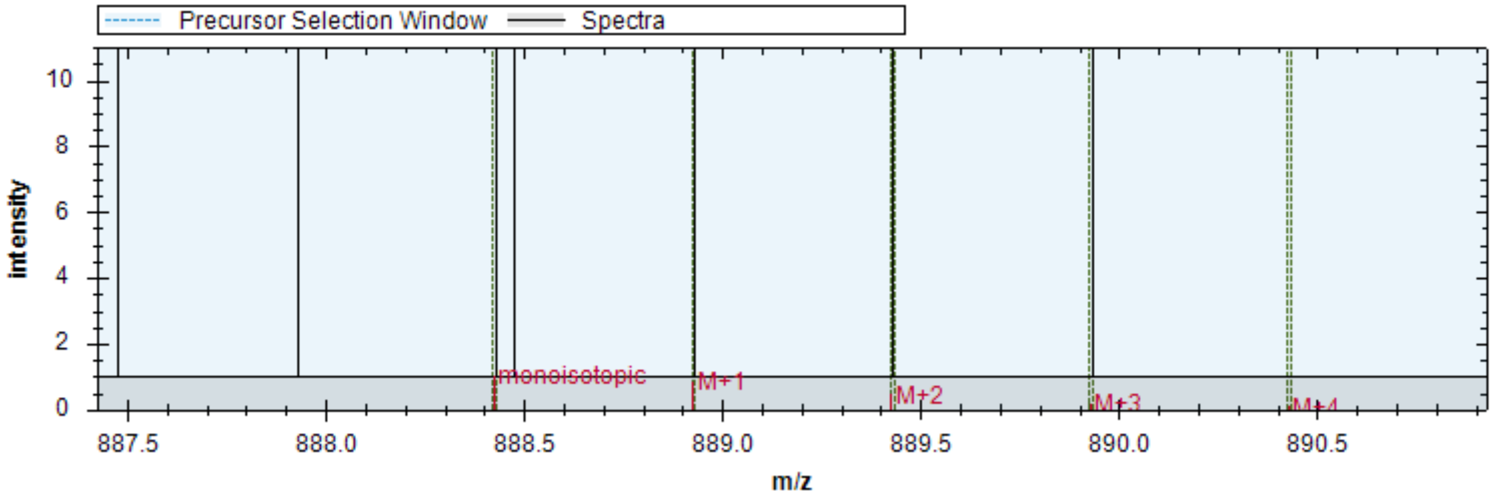

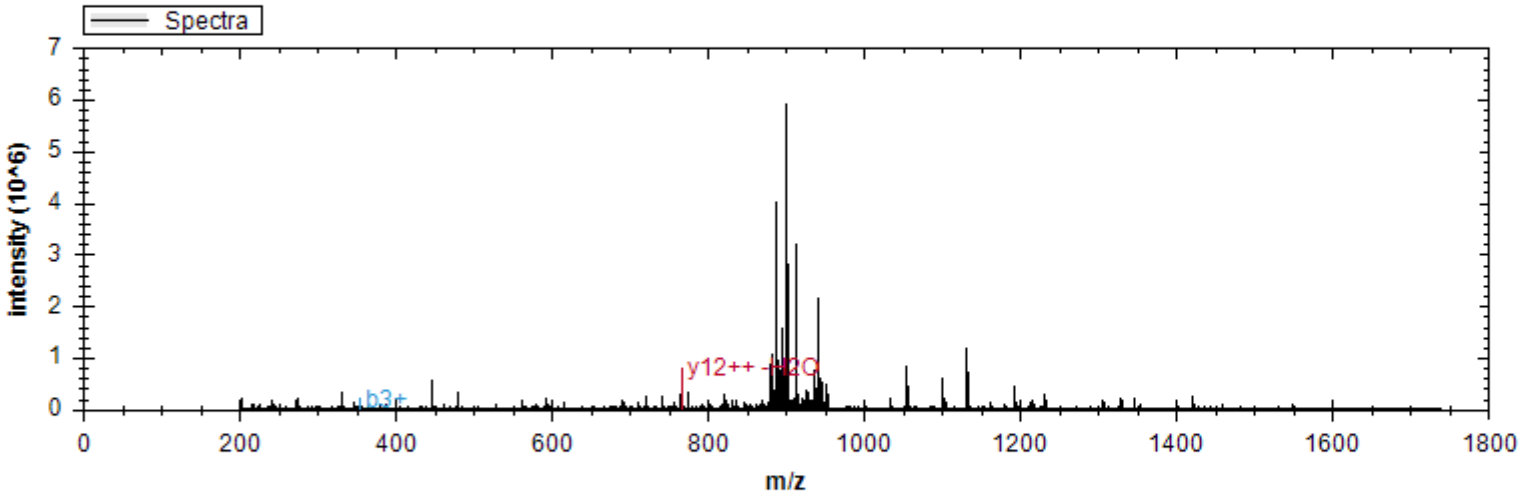

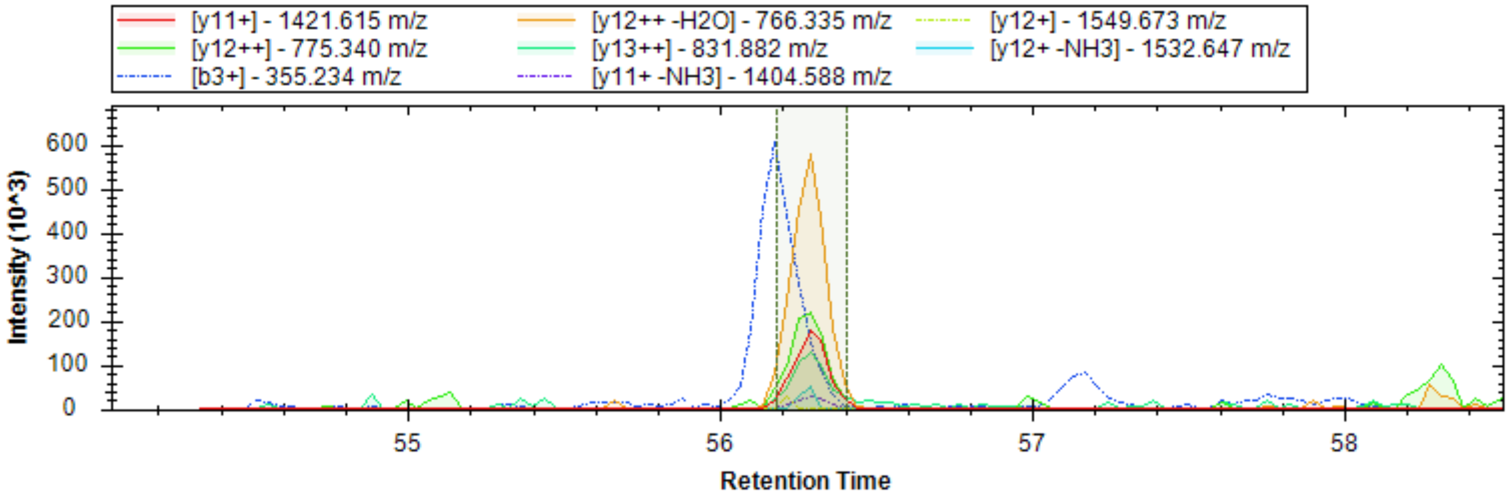

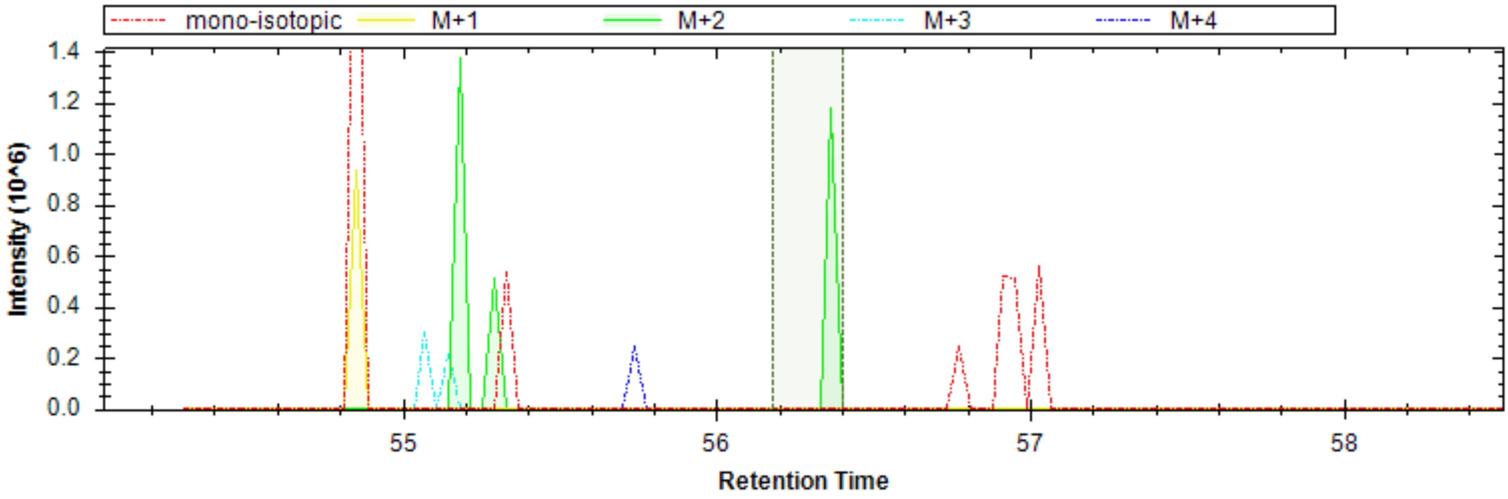

Q8JZP2 – _SQS[+H+O3+P]LTNSLSTSDTSHR_.2
Fig4_MP-DIA-barrel-cortex-p9-1_MHRM.htrms

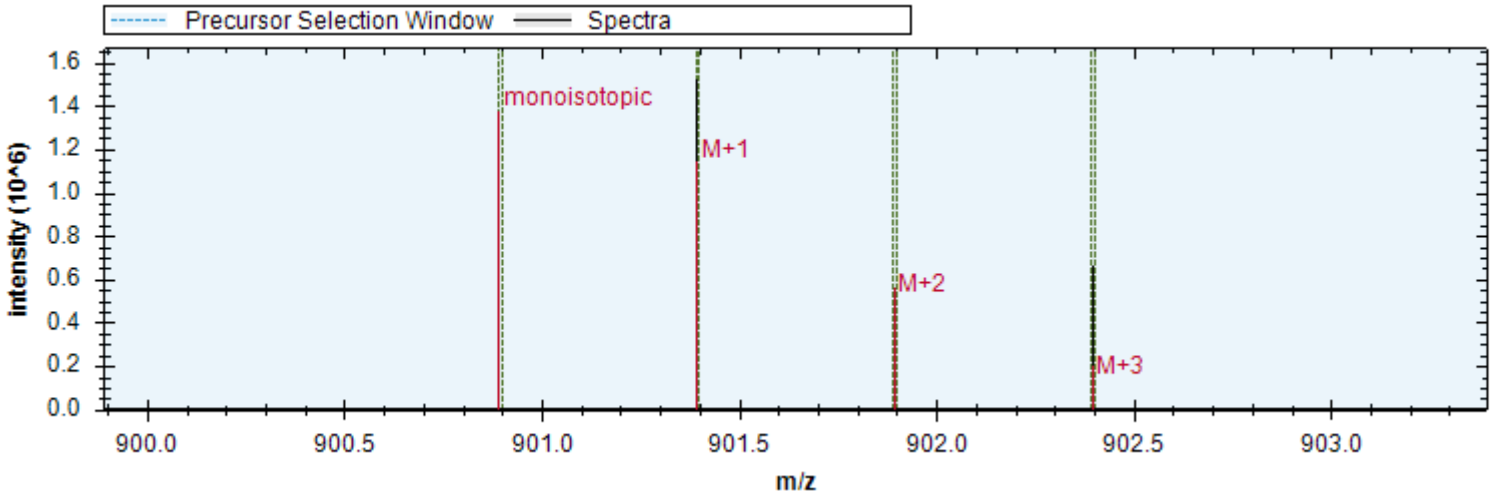

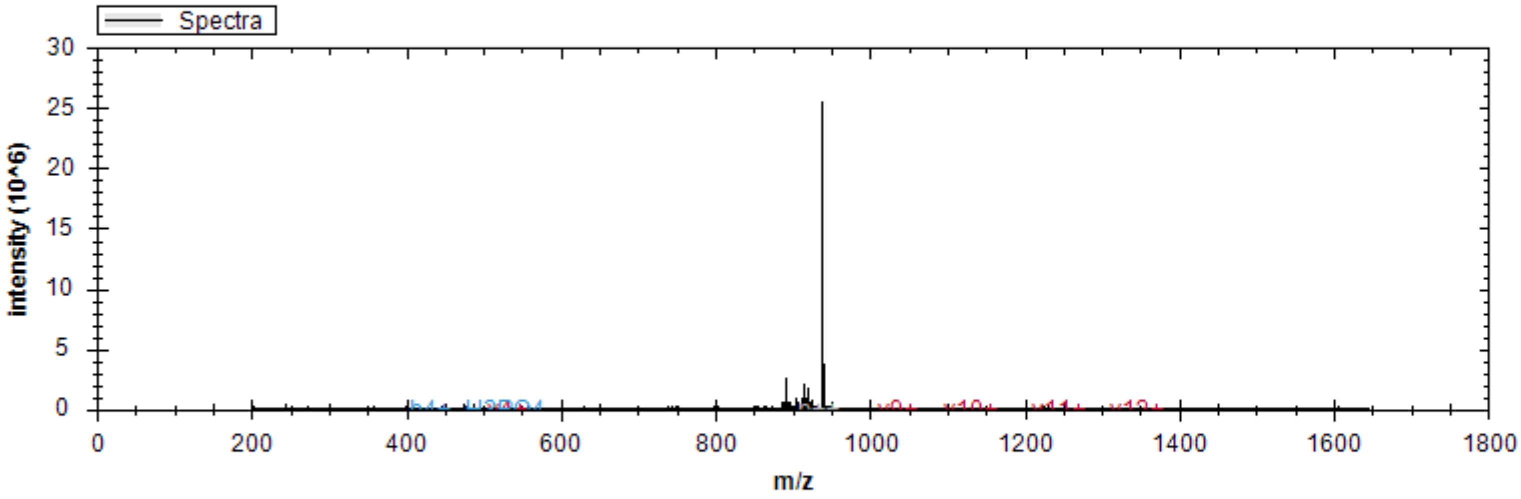

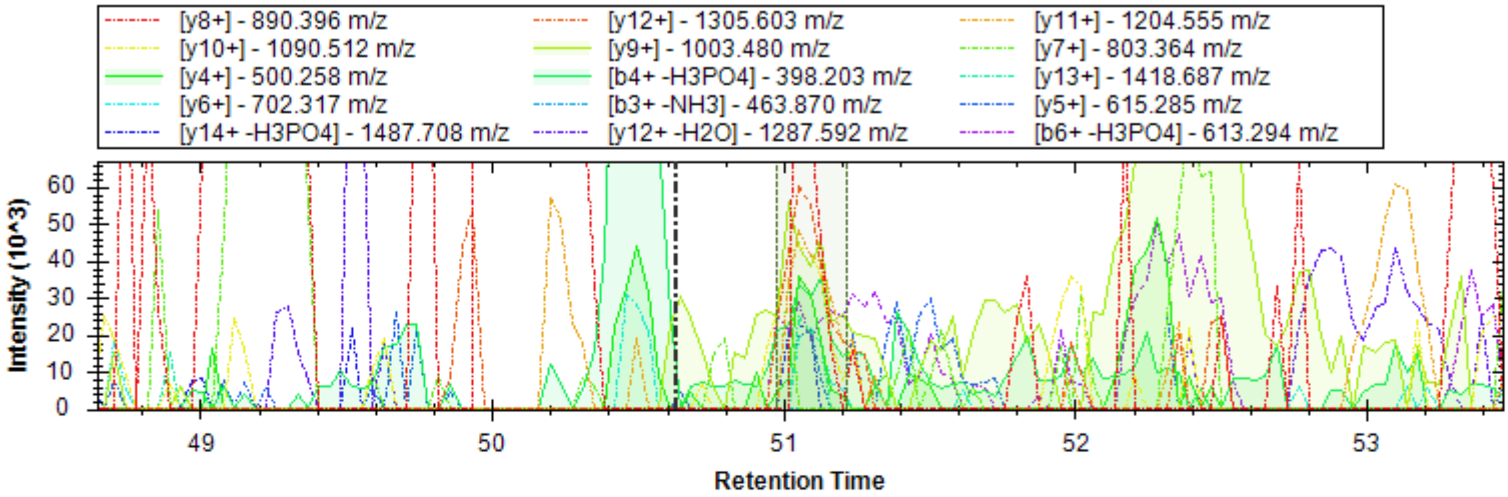

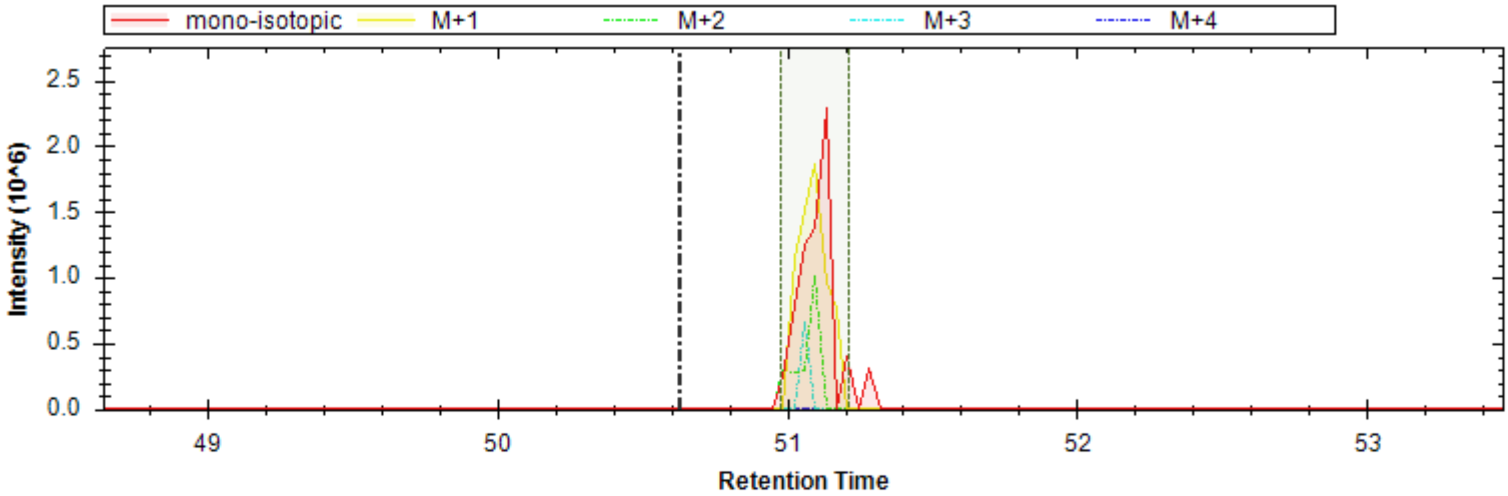

Q91Z31;Q91Z31-2 – _ETSLLAVPGALS[+H+O3+P]PLAIPNAAAAAAAAAAGR_.3
Fig4_MP-DIA-barrel-cortex-p9-1_MHRM.htrms

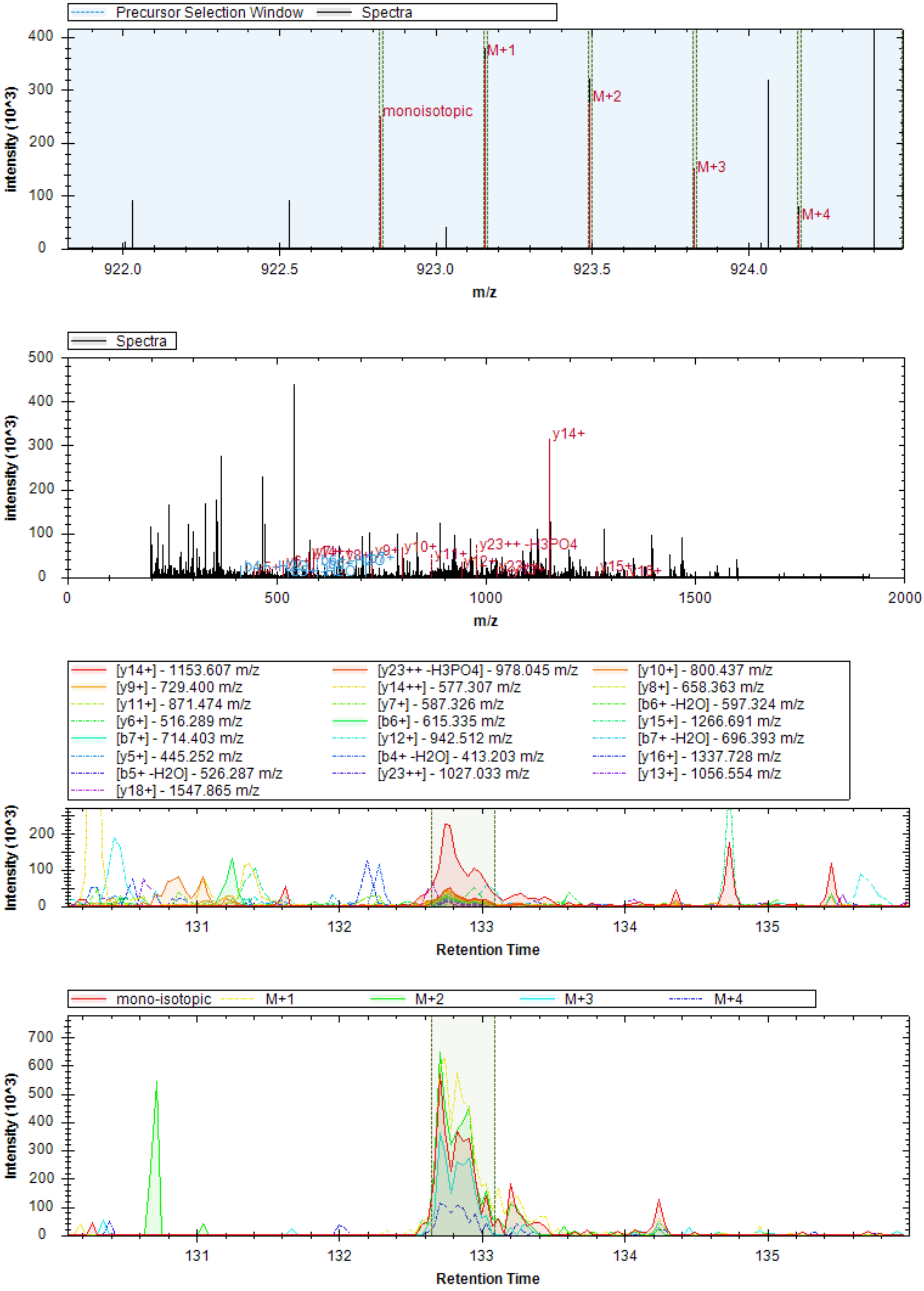

Q9CZT4;Q9CZT4-2 – _KEQM[+O]RAATVLPS[+H+O3+P]VQIK_.2
Fig4_MP-DIA-barrel-cortex-p9-1_MHRM.htrms

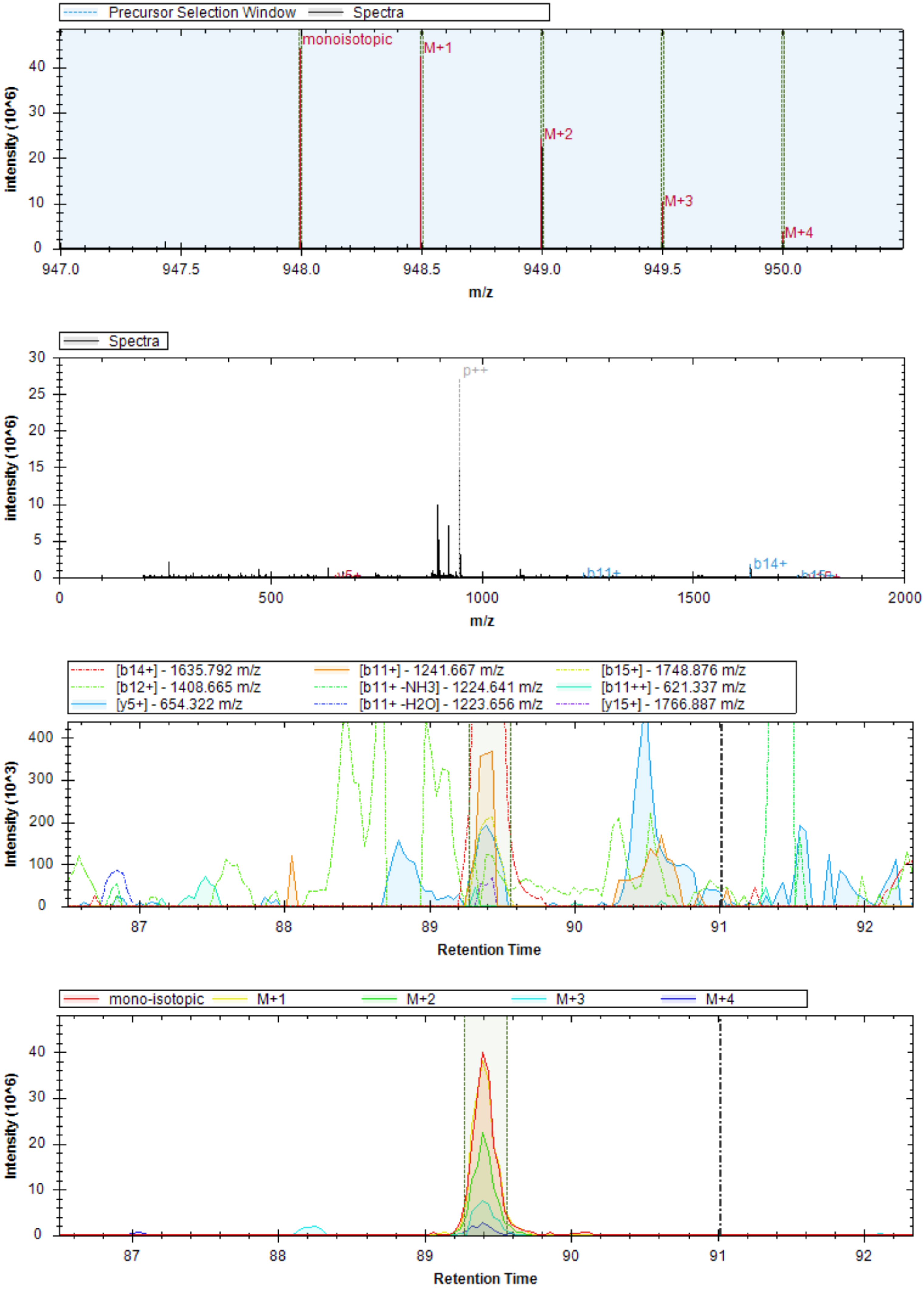

Q9ES97;Q9ES97-2 – _AES[+H+O3+P]SAATQSPSVSSSSSGAEPSALGGGGGSPGAC[+C2+H3+N+O]PALGAK_.4
Fig4_MP-DIA-barrel-cortex-p9-1_MHRM.htrms

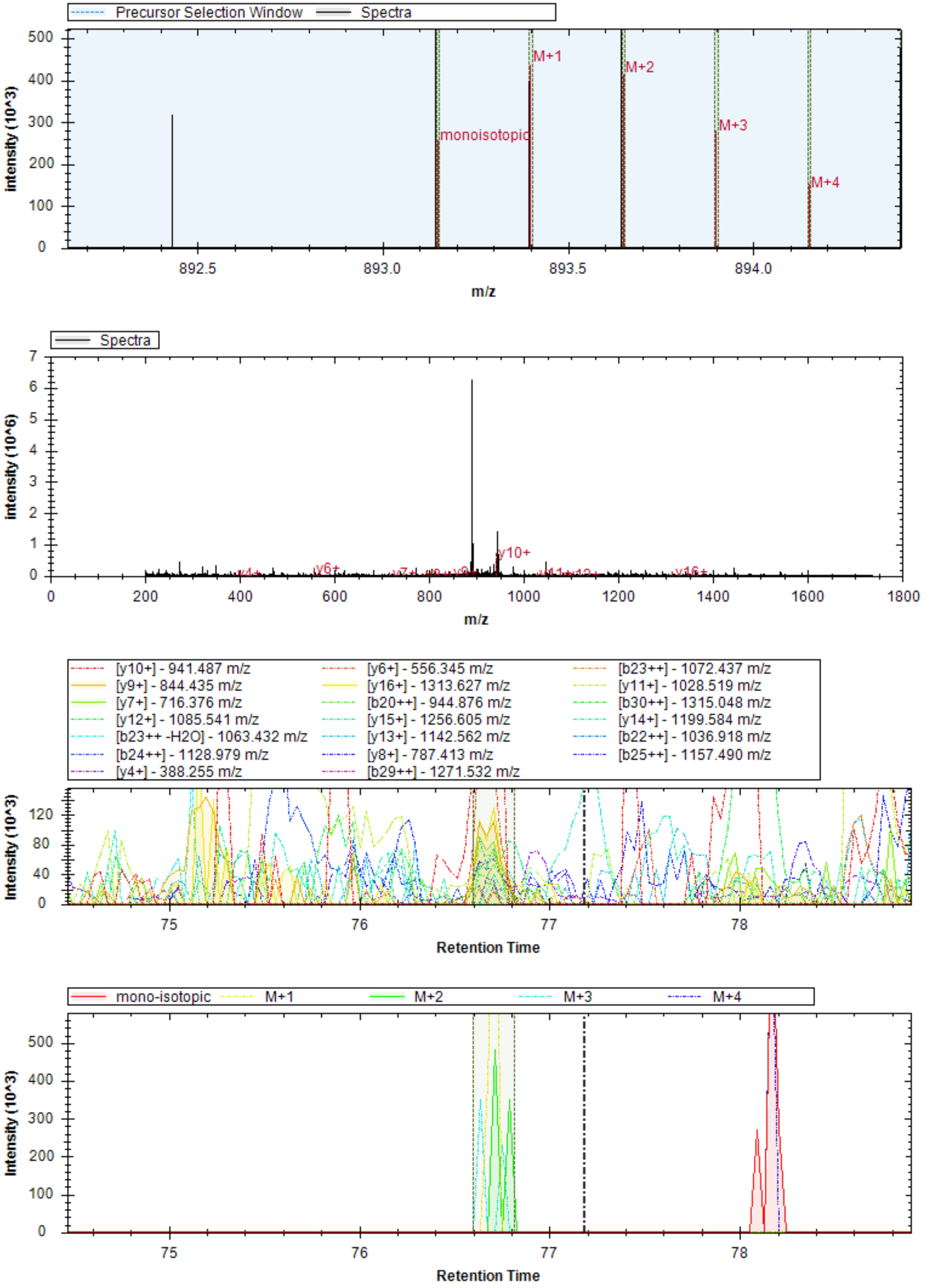

P17742 – _VNPT[+H+O3+P]VFFDITADDEPLGR_.2
Fig4_MP-DIA-barrel-cortex-p9-1_MHRM.htrms

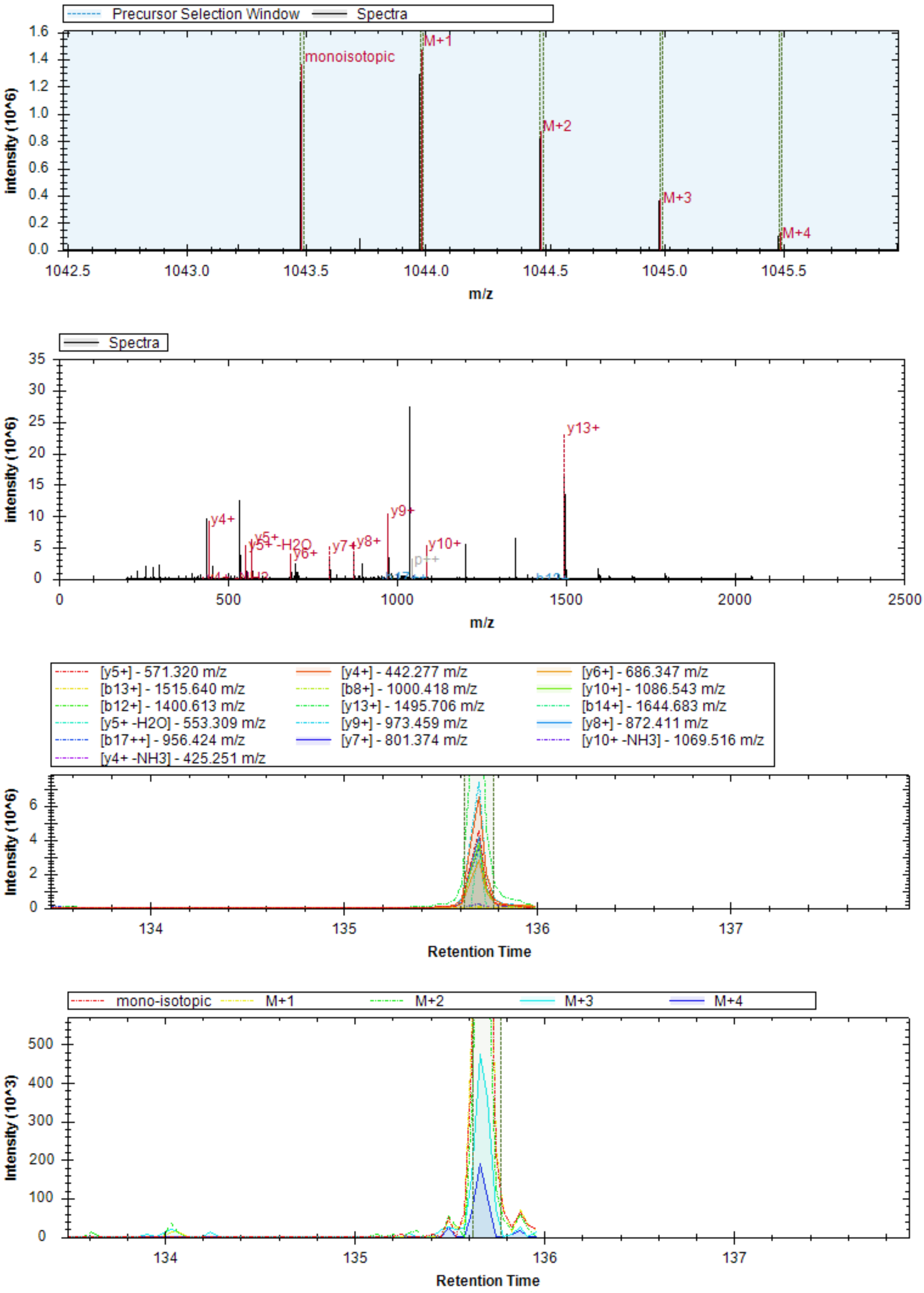

P26645 – _LSGFS[+H+O3+P]FKK_.1

Fig4_MP-DIA-barrel-cortex-p9-1_MHRM.htrms

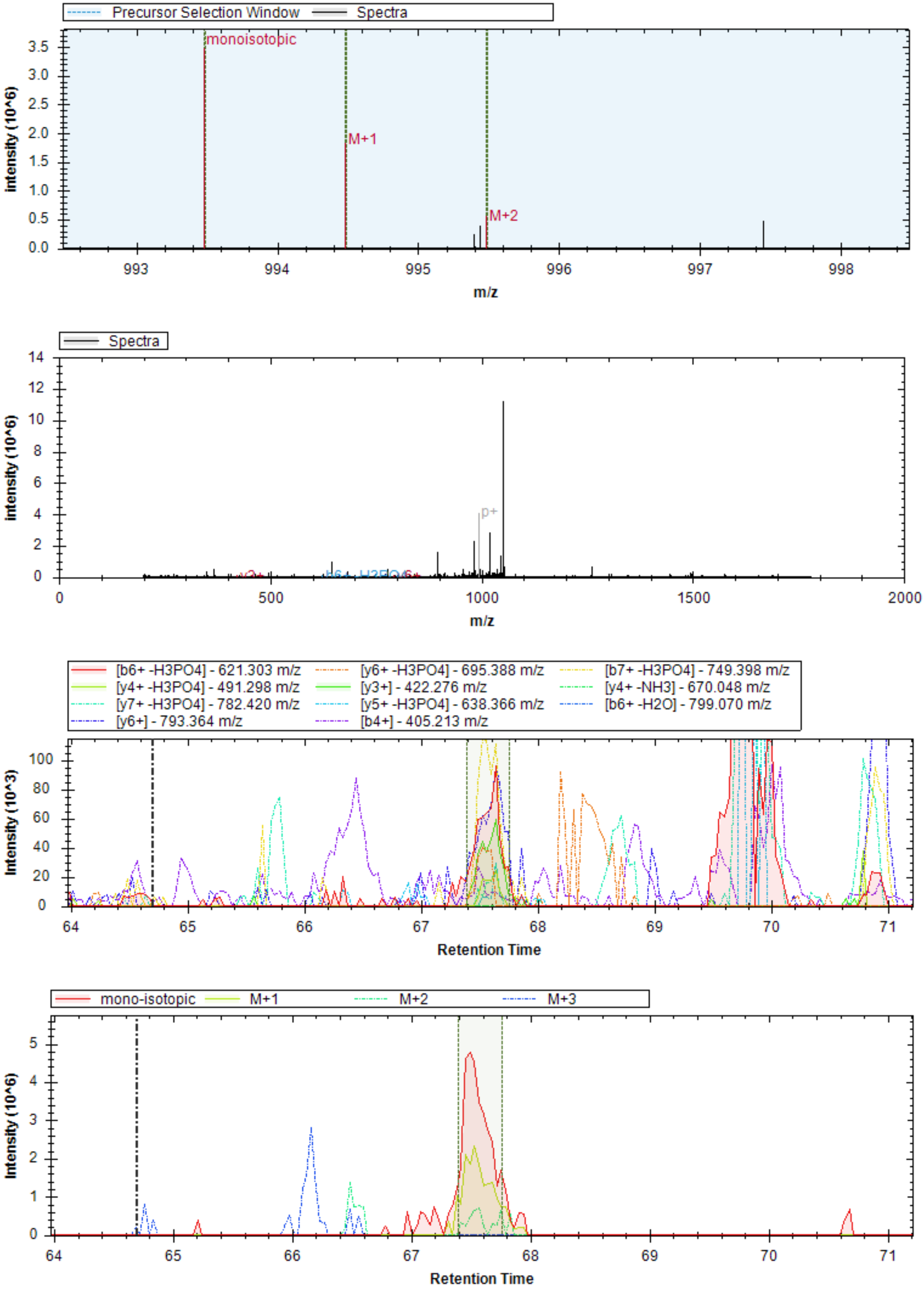

Q8BG05 – _S[+H+O3+P]SGSPYGGGYGSGGGSGGYGSR_.2

Fig4_MP-DIA-barrel-cortex-p9-1_MHRM.htrms

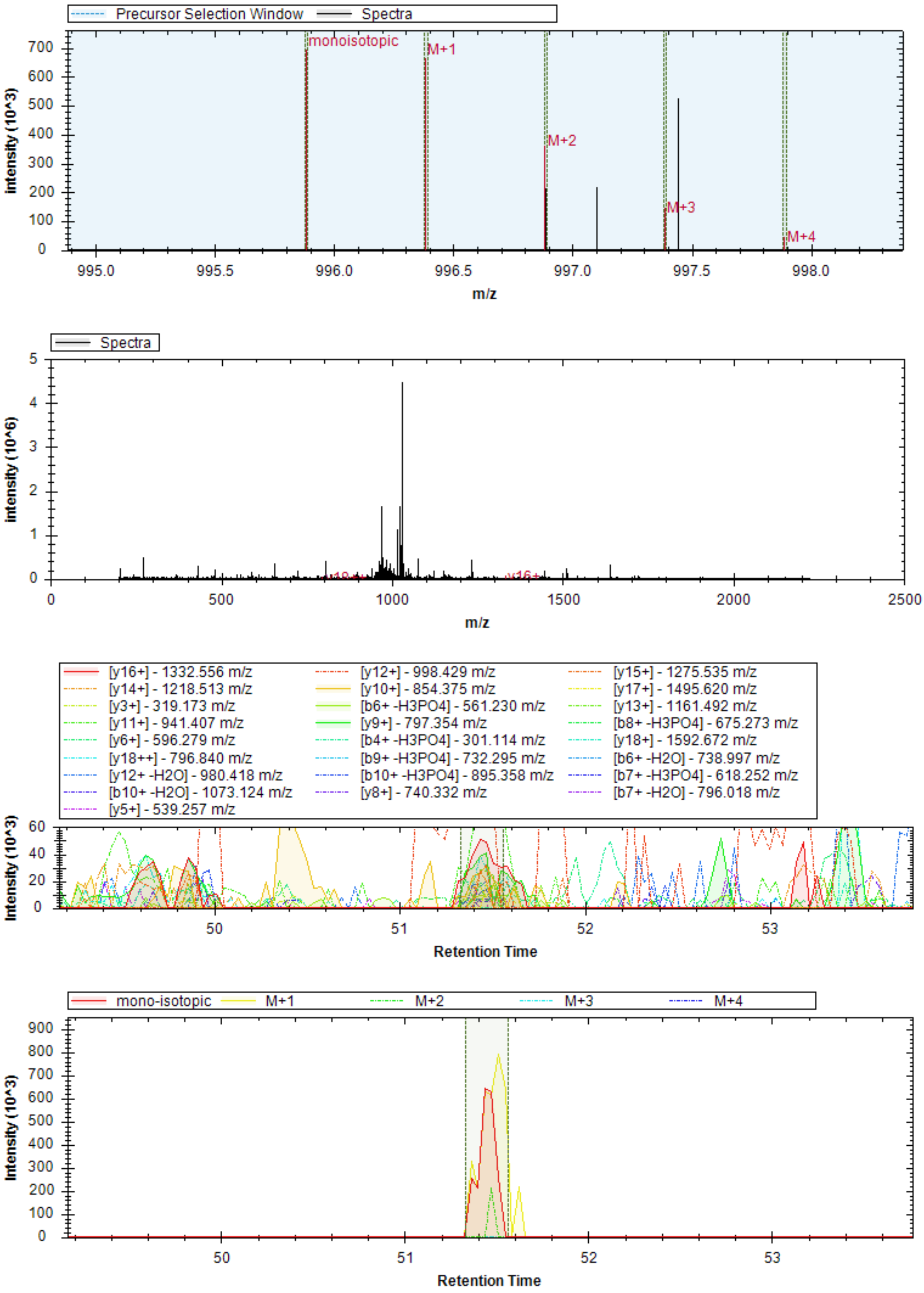

Q8BG05 – _SSGS[+H+O3+P]PYGGGYGSGGGSGGYGSR_.2
Fig4_MP-DIA-barrel-cortex-p9-1_MHRM.htrms

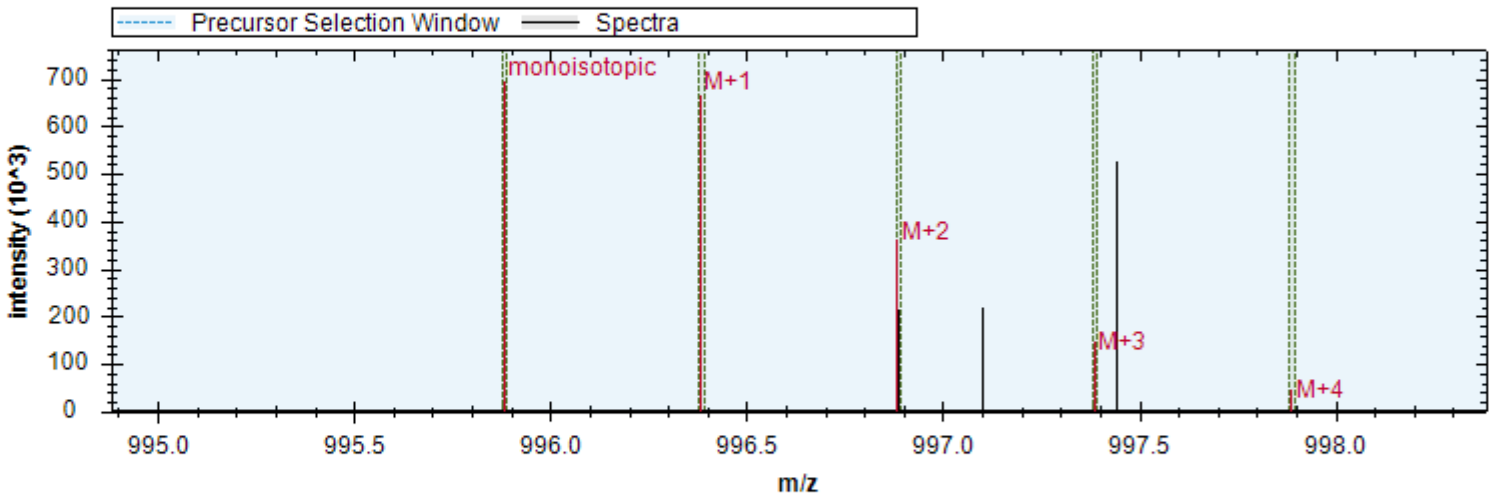

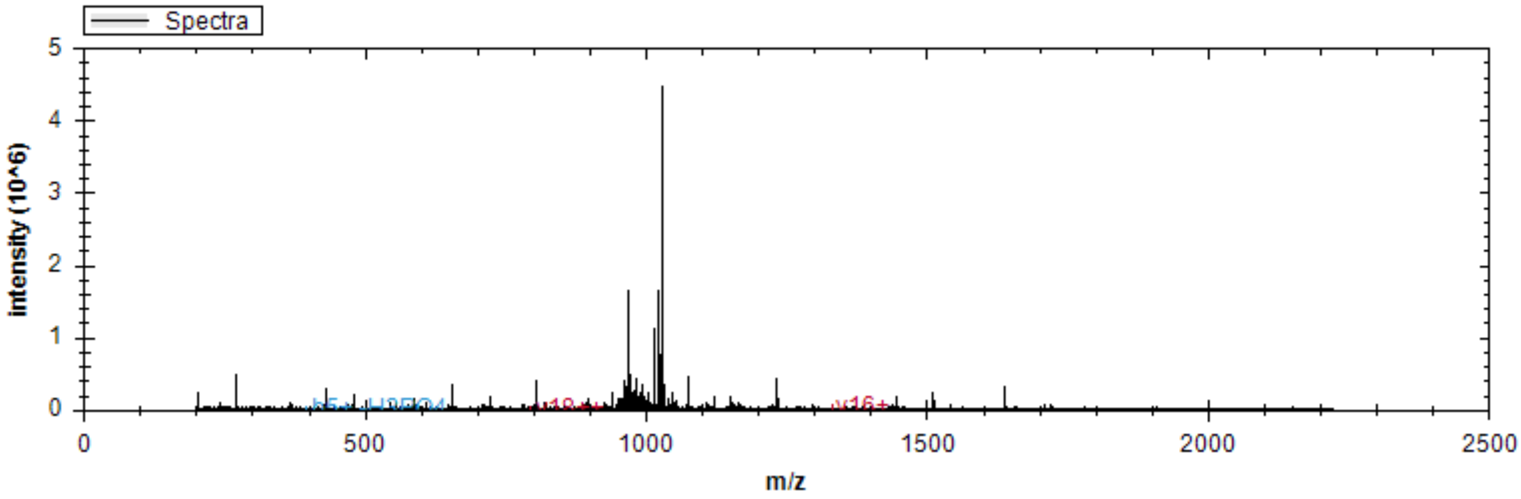

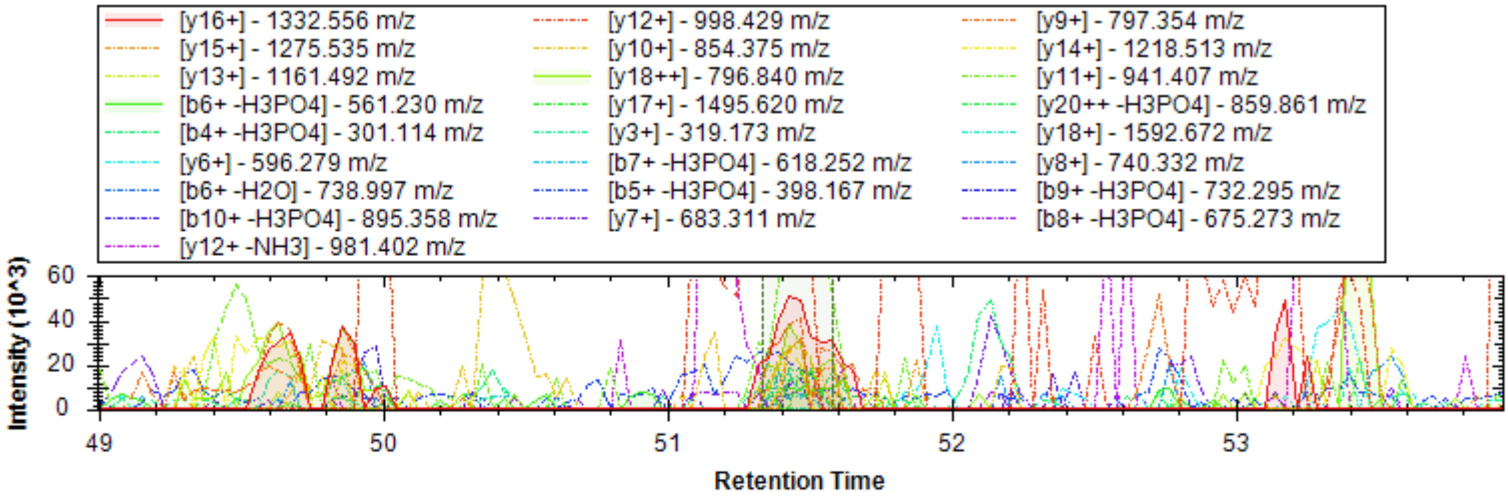

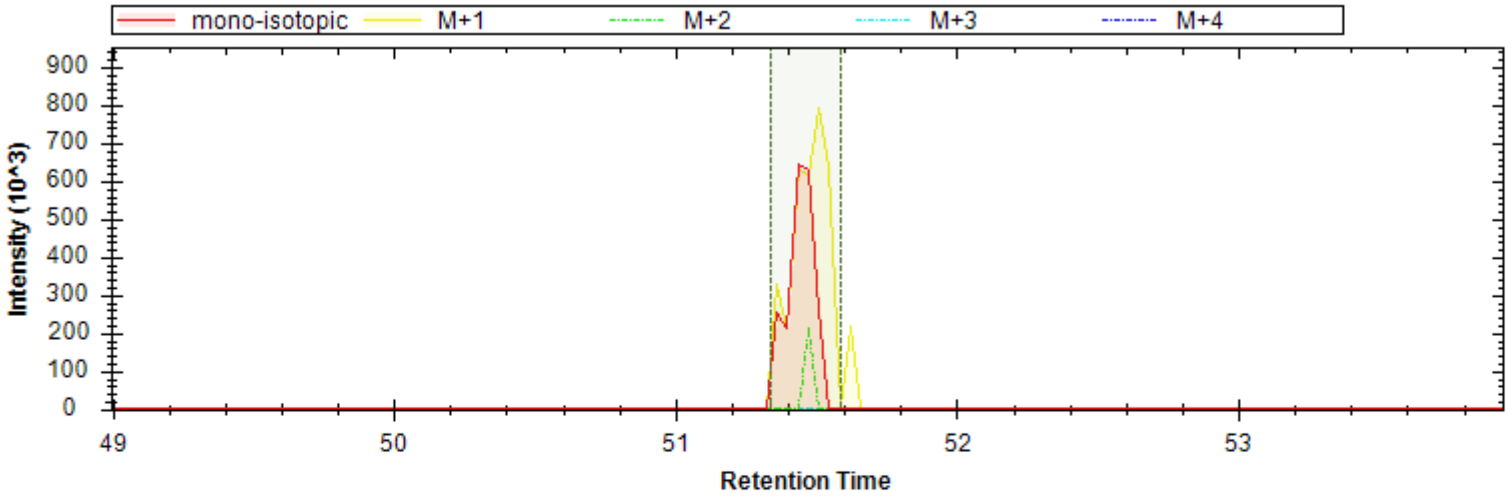

Q8CHK4;Q8CHK4-2;Q8CH[...] – _RGY[+H+O3+P]GKLLIEFS[+H+O3+P]YELSK_.2
Fig4_MP-DIA-barrel-cortex-p9-1_MHRM.htrms

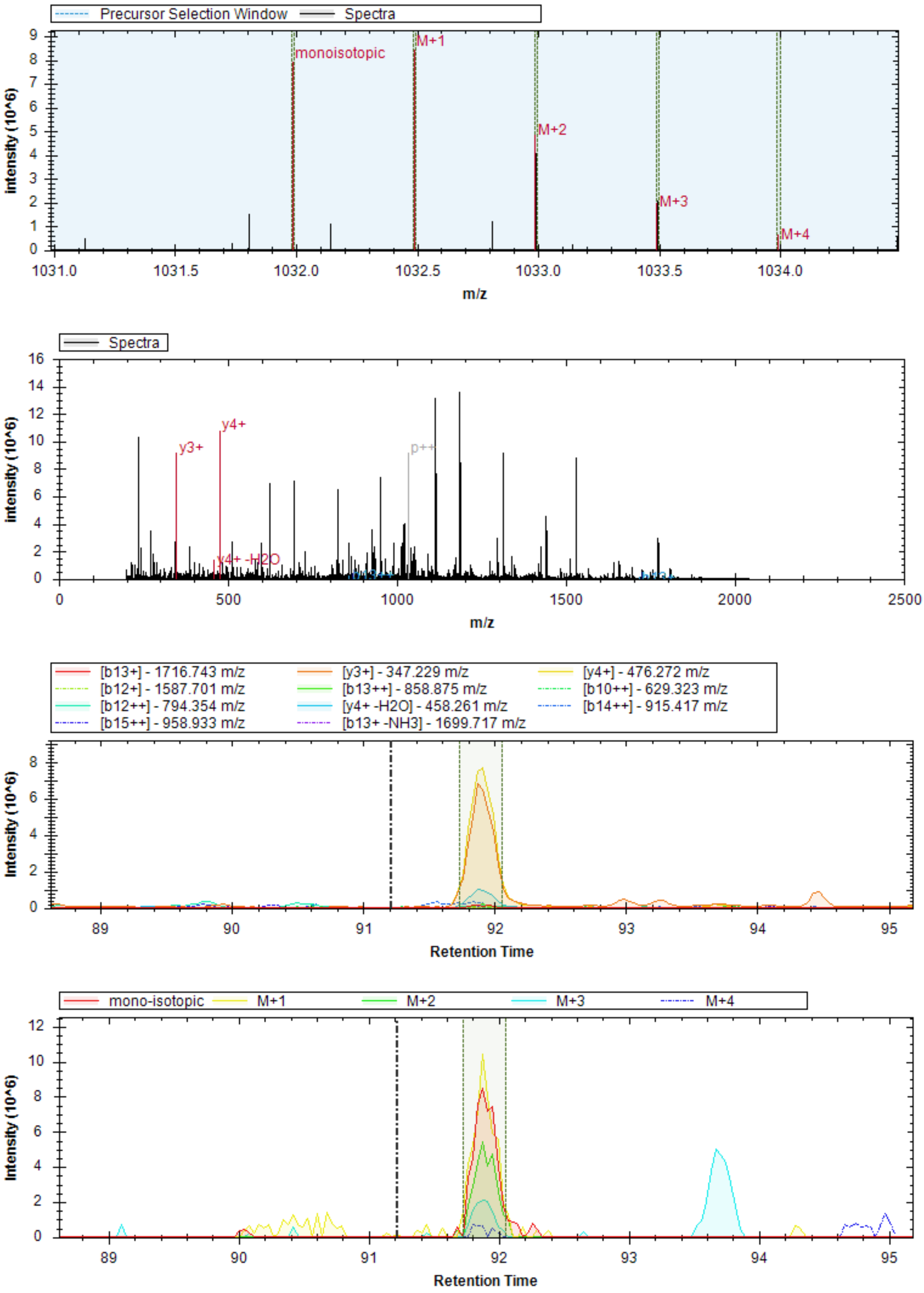

A2ASS6 – _T[+H+O3+P]VS[+H+O3+P]VKVLVLDK_.1
Fig4_MP-DIA-barrel-cortex-p9-1_MHRM.htrms

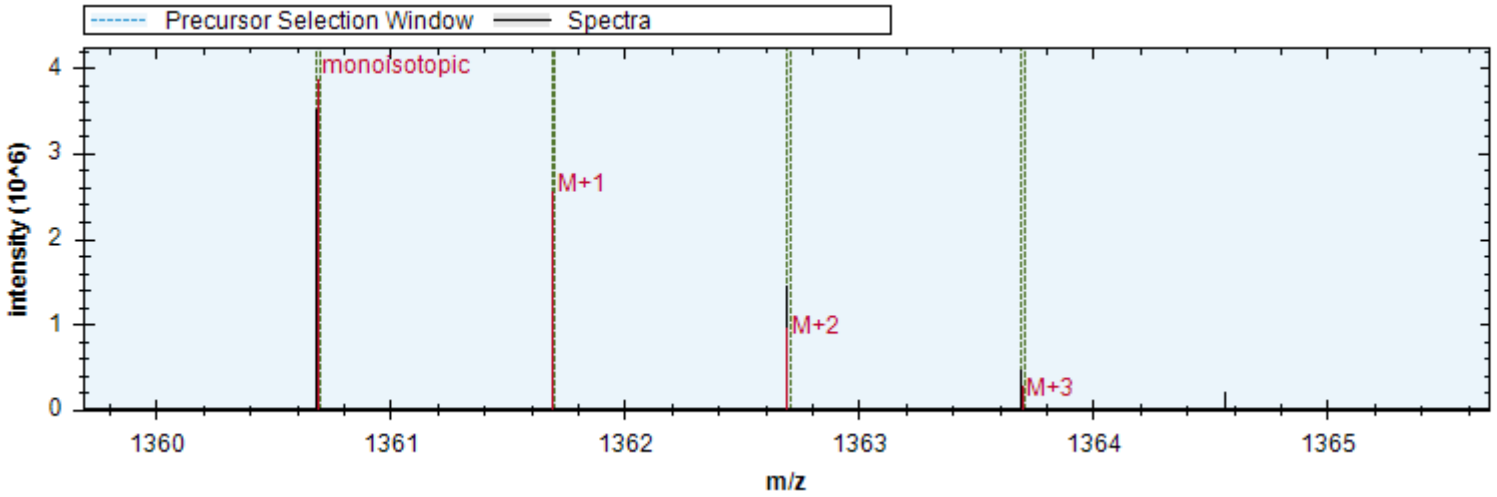

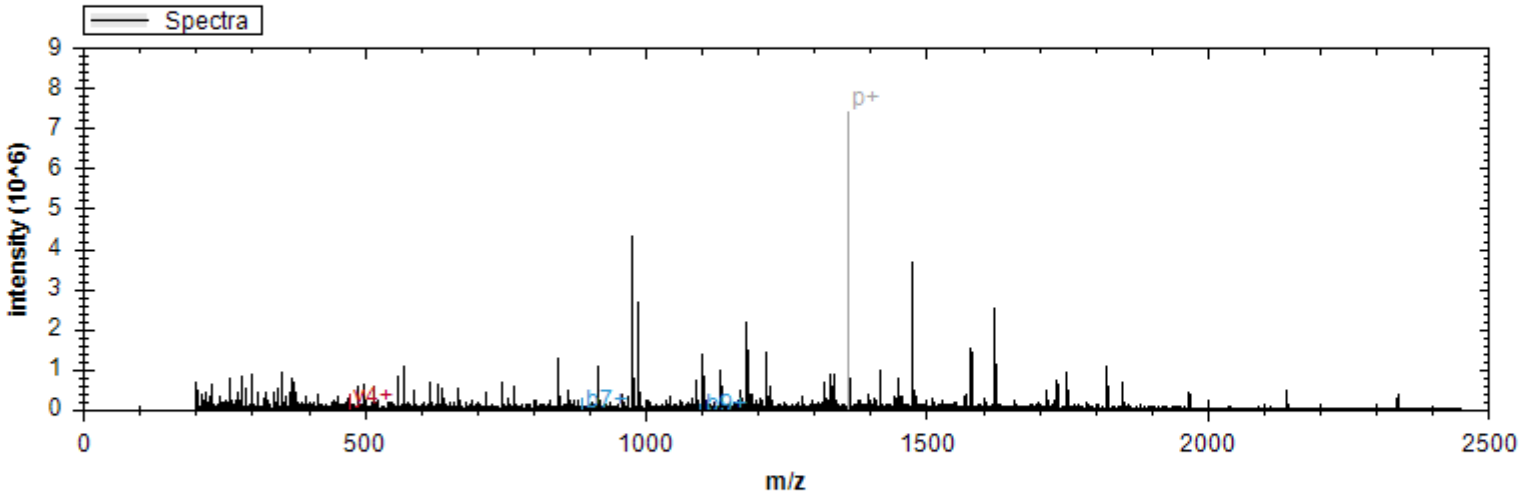

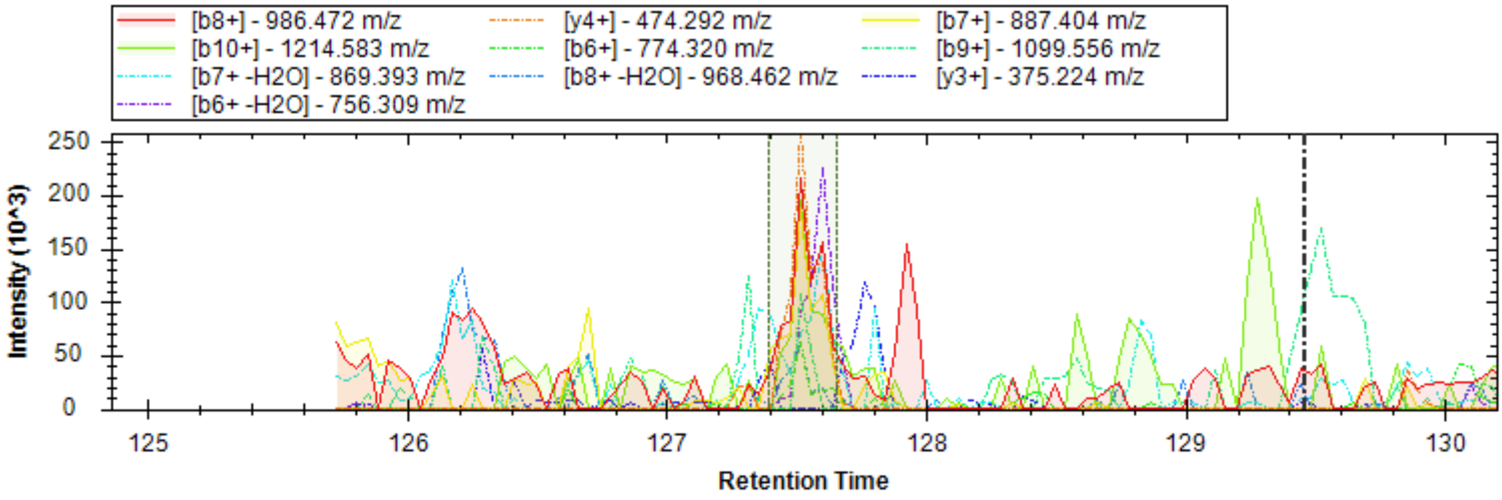

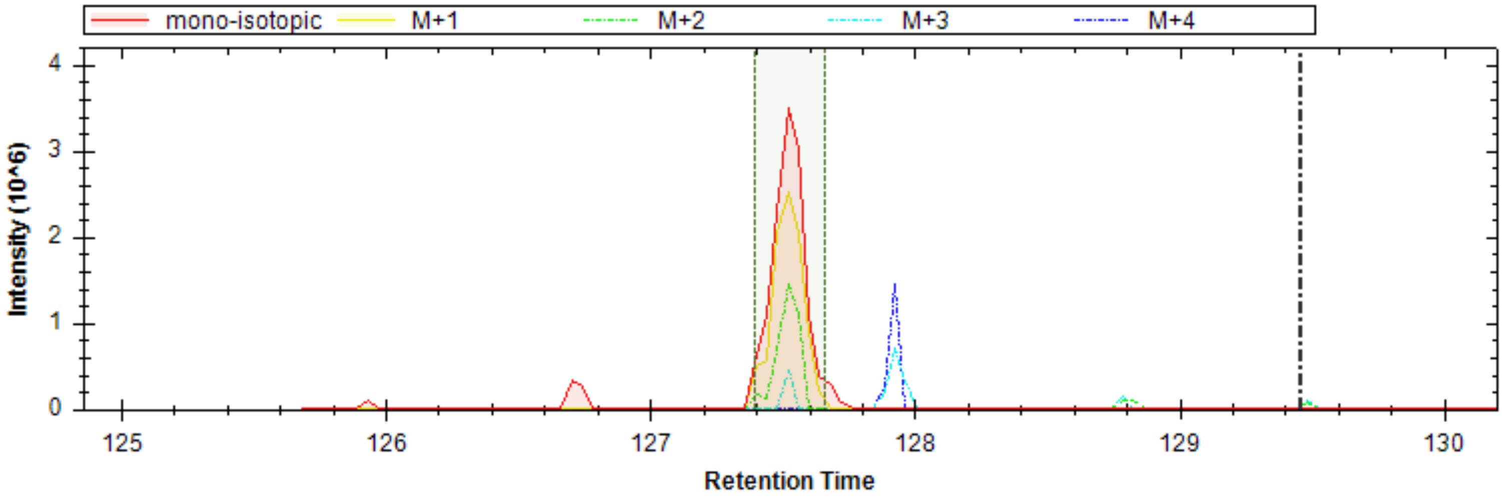

E9Q401 – _MVAEVFIY[+H+O3+P]WSK_.1
Fig4_MP-DIA-barrel-cortex-p9-1_MHRM.htrms

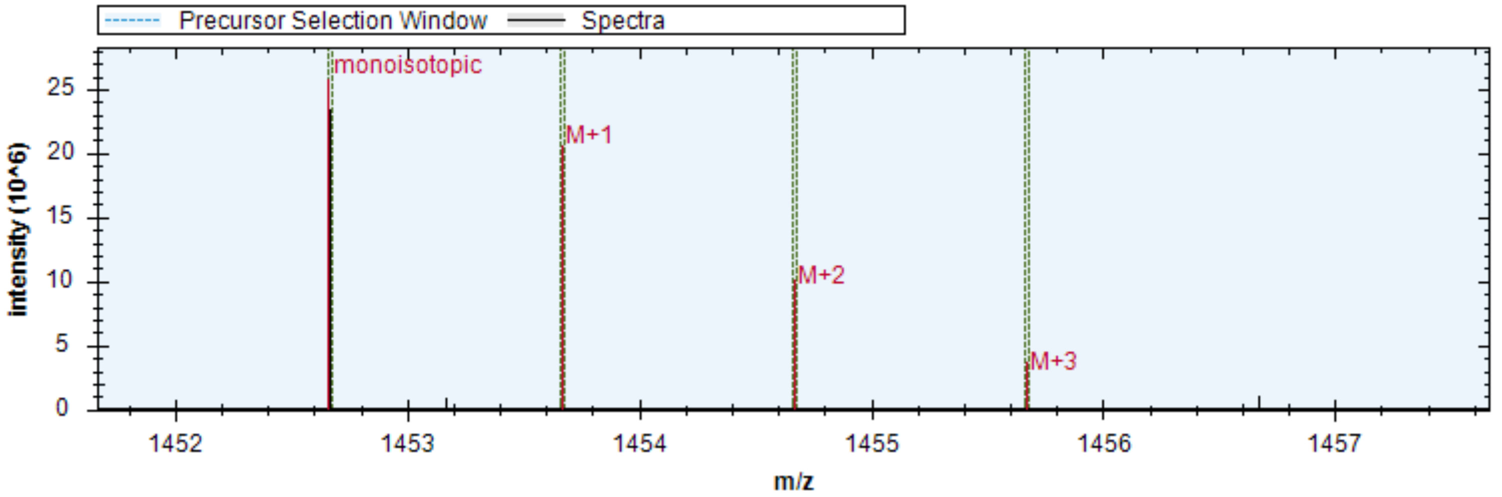

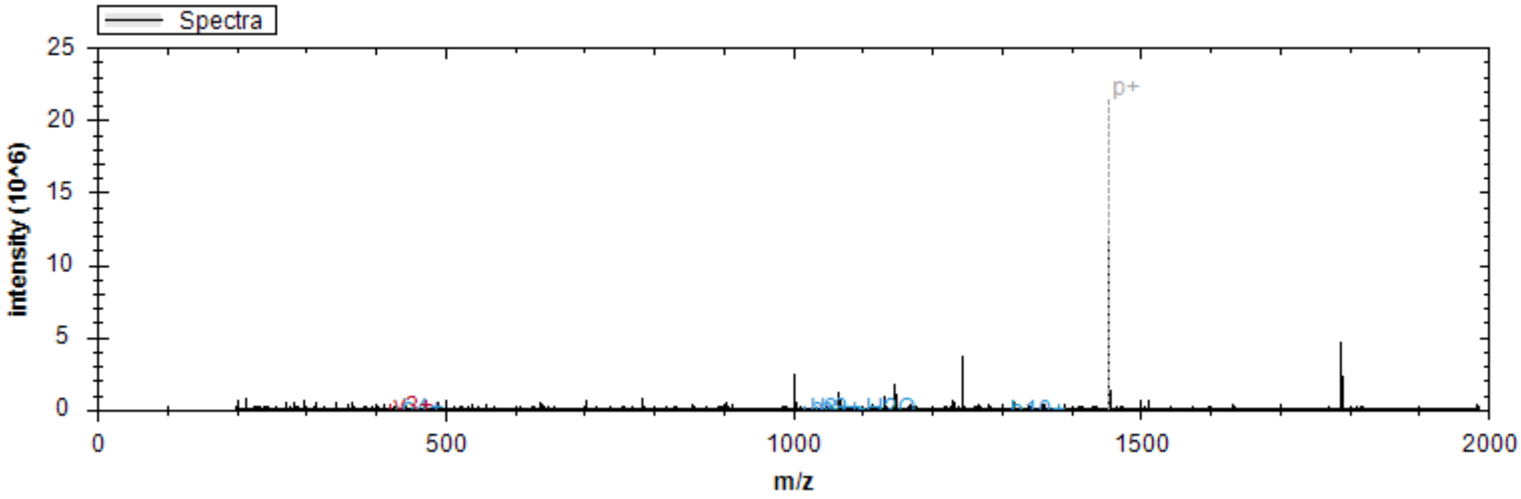

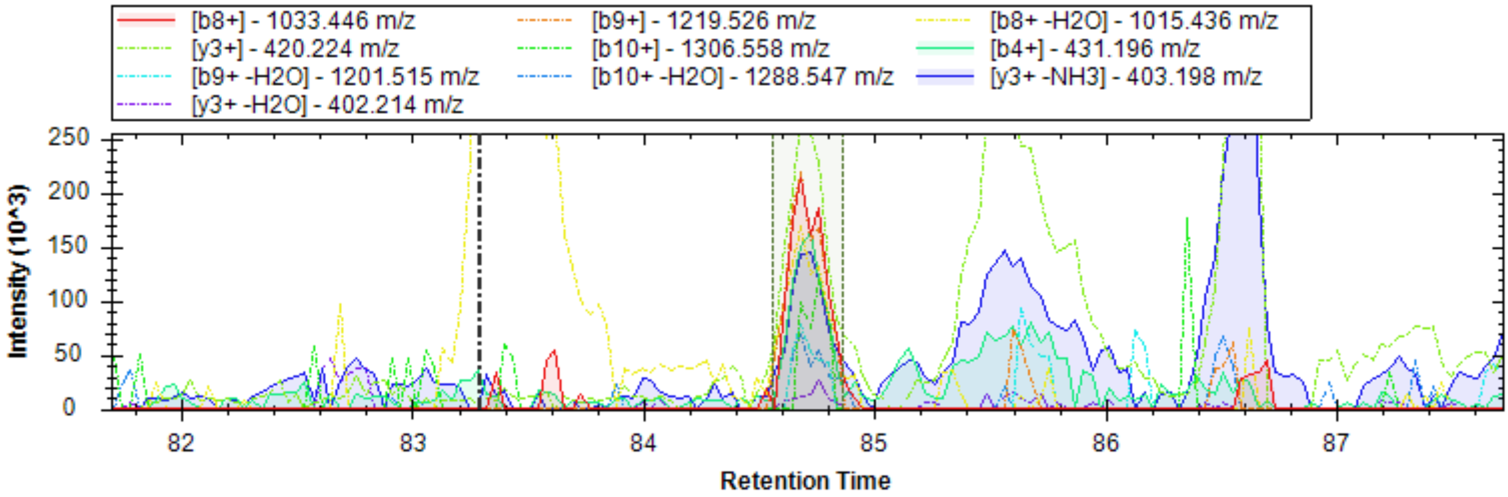

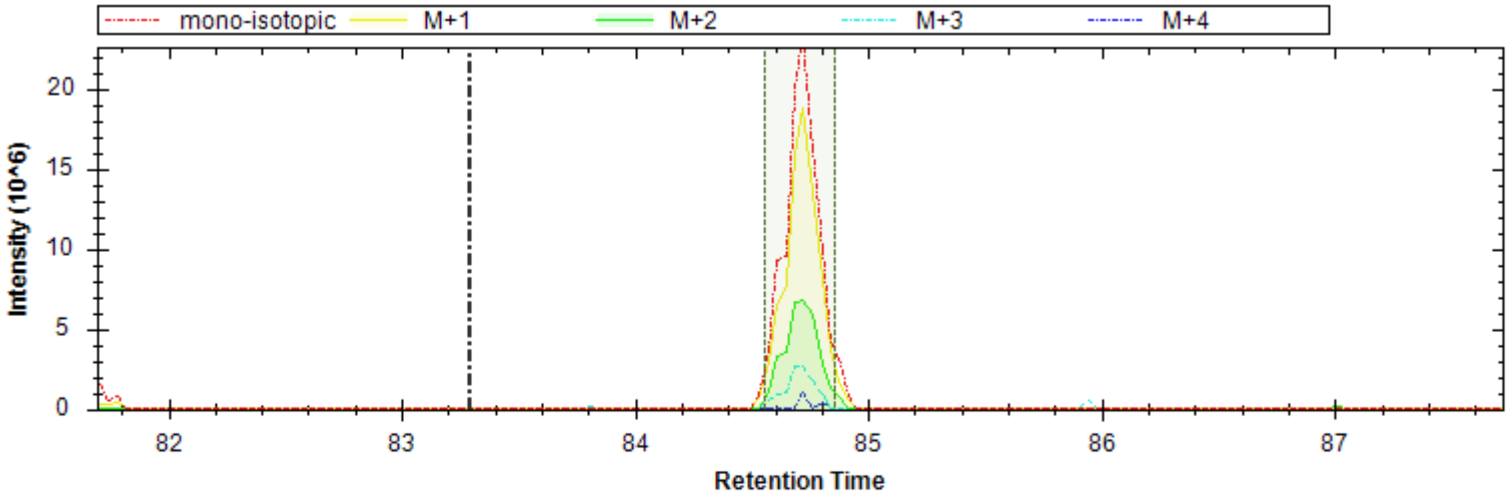

P20357 – _SGTSTPTTPGSTAITPGT[+H+O3+P]PPSYSSR_.2
Fig4_MP-DIA-barrel-cortex-p9-1_MHRM.htrms

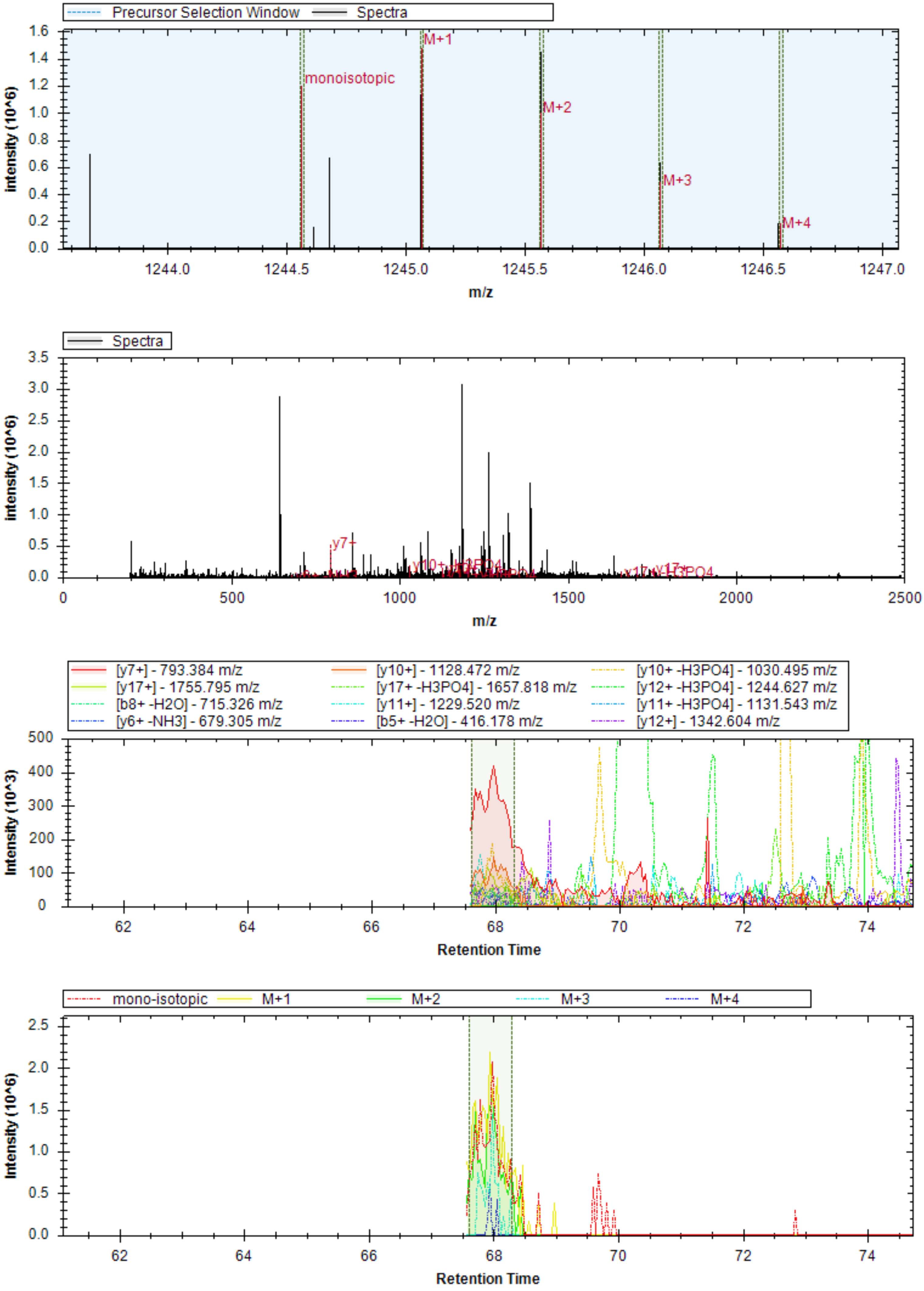

Q8BRT1 – _VLNT[+H+O3+P]GSDVEEAVADALLLGDIR_.2
Fig4_MP-DIA-barrel-cortex-p9-1_MHRM.htrms

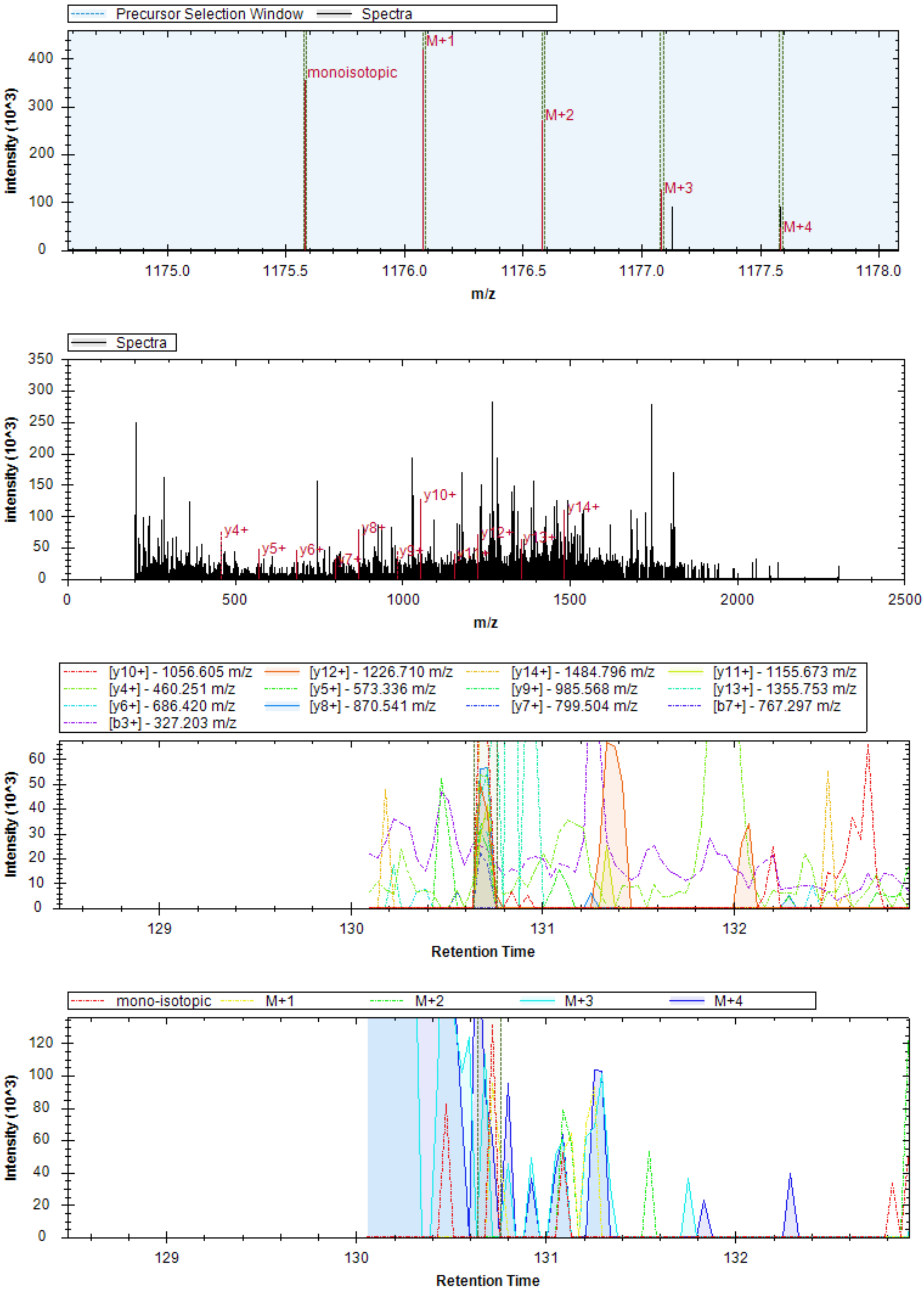

Q8VHY0 – _LLAGPRY[+H+O3+P]GR_.1

Fig4_MP-DIA-barrel-cortex-p9-1_MHRM.htrms

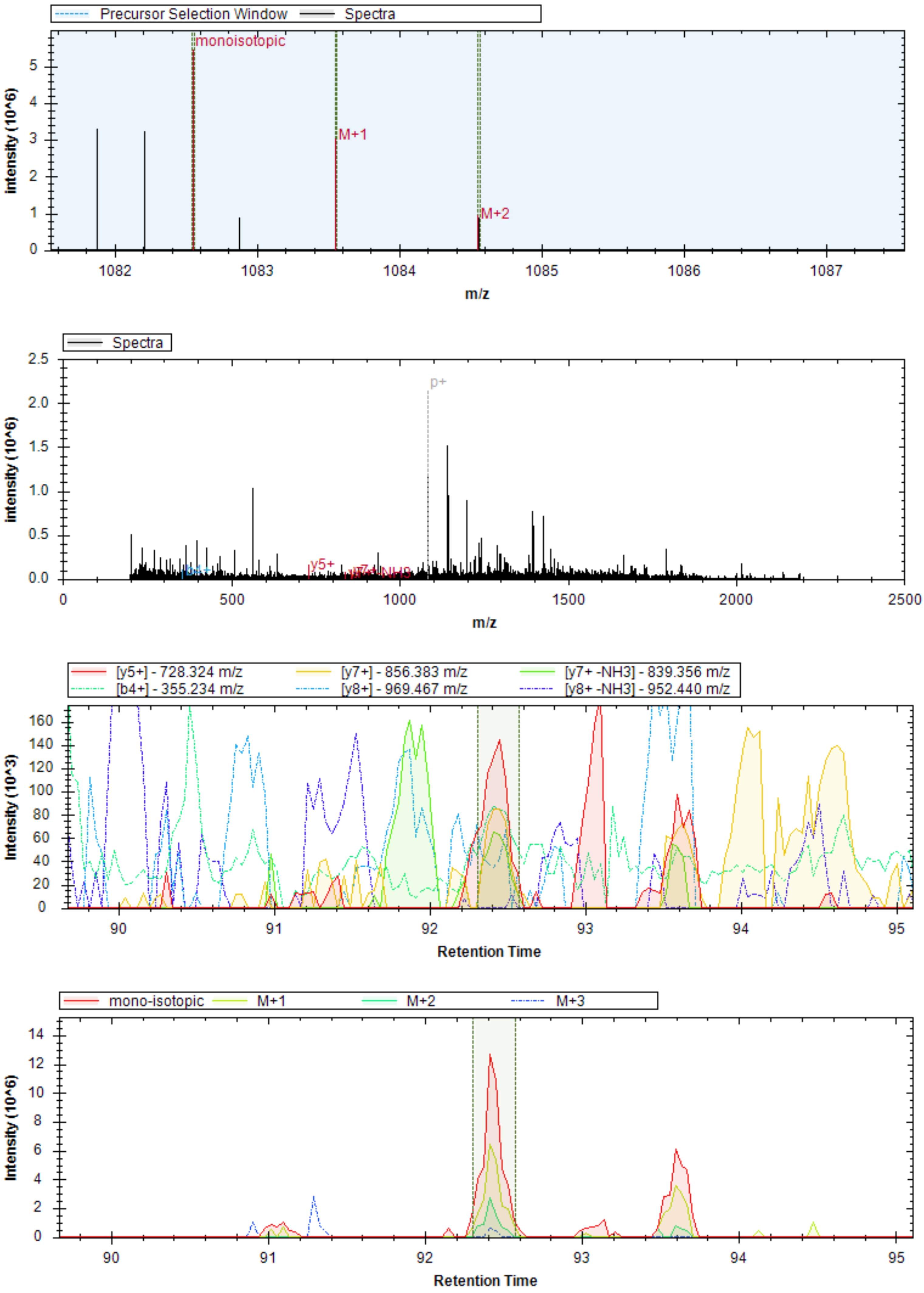

Q921N6;Q921N6-2 – _LIY[+H+O3+P]KPRQAAVTR_.4
Fig4_MP-DIA-barrel-cortex-p9-2_MHRM.htrms

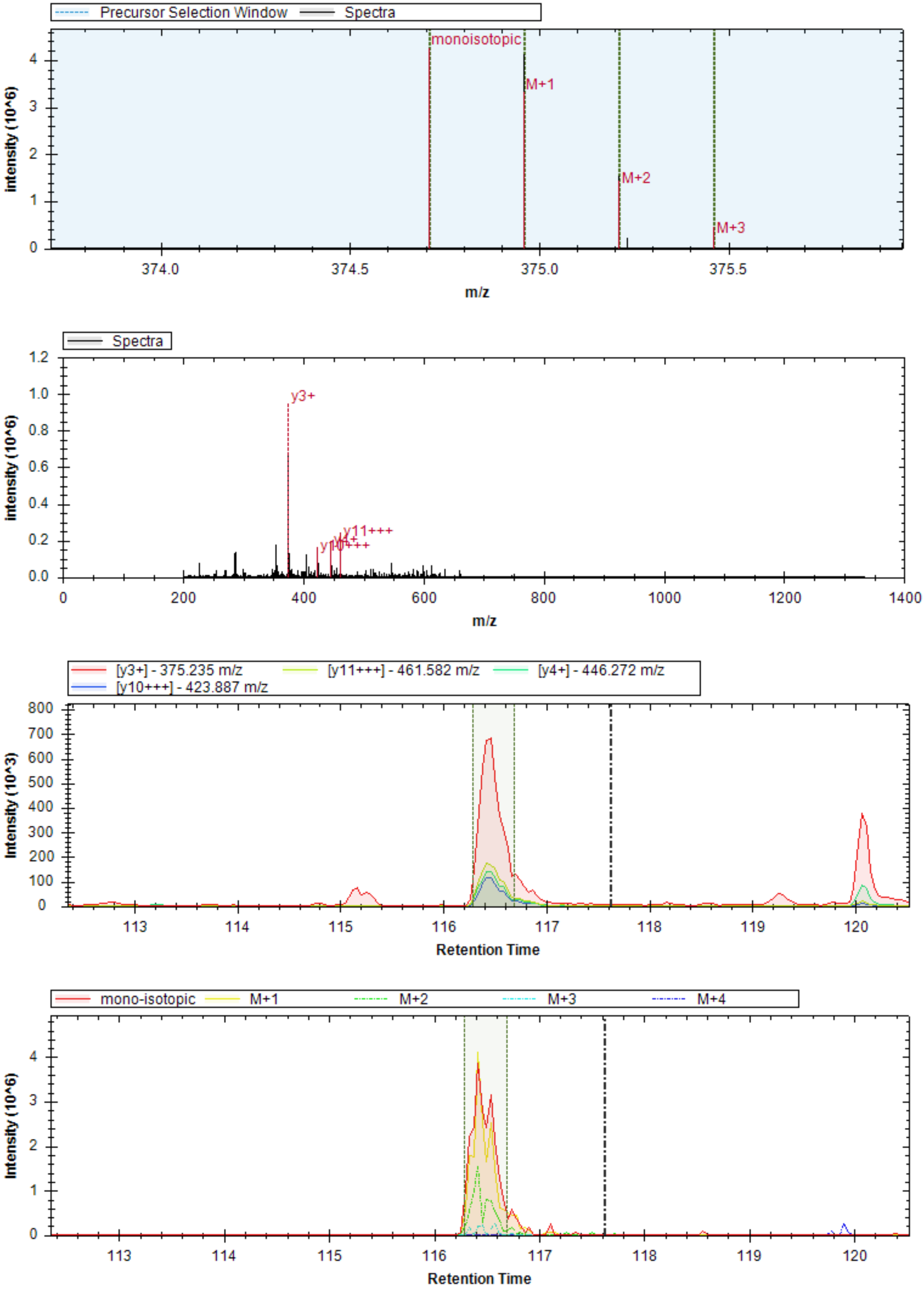

Q9CVB6 – _IIEET[+H+O3+P]LALK_.3
Fig4_MP-DIA-barrel-cortex-p9-2_MHRM.htrms

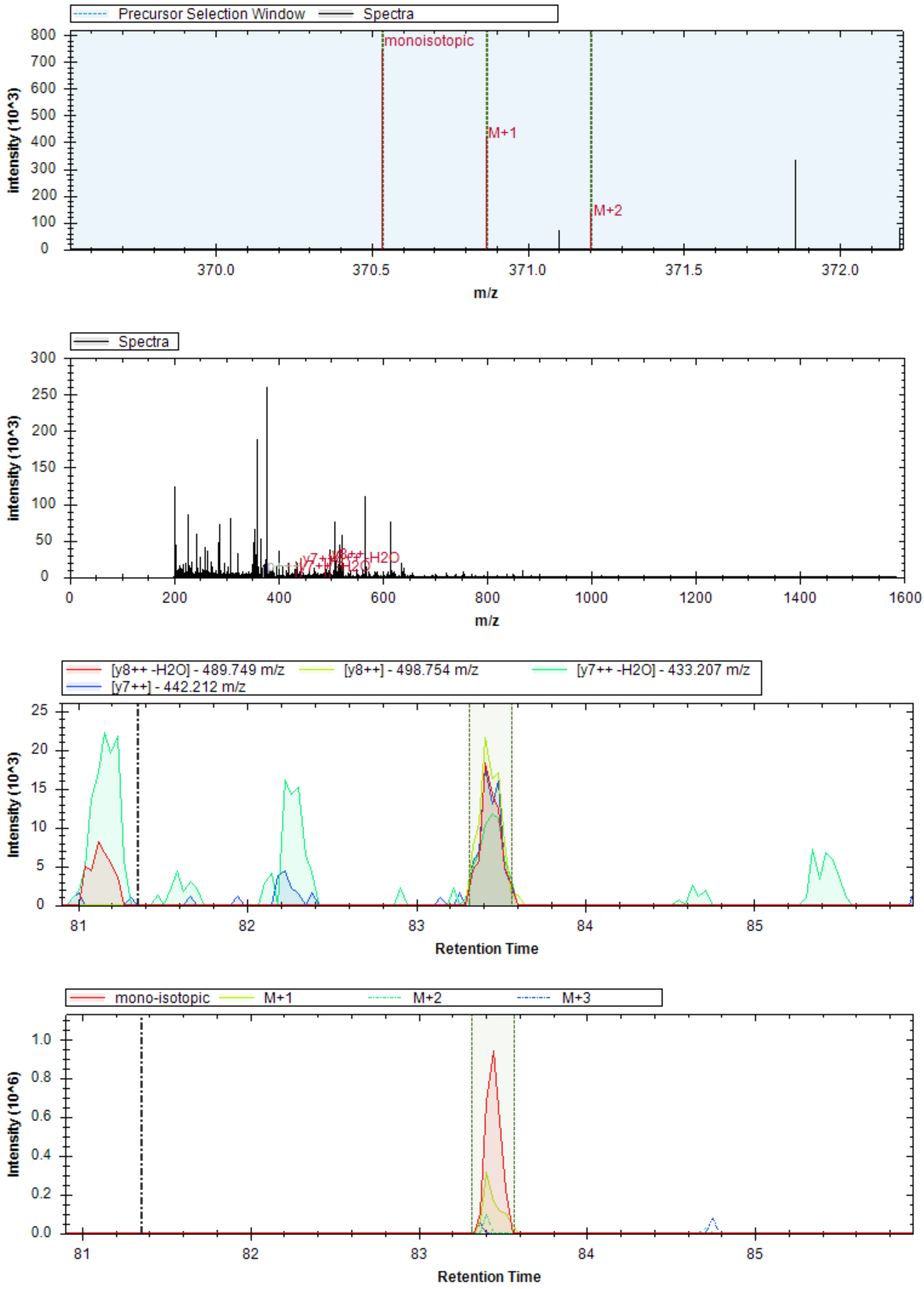

Q9D516;Q9D516-2 – _KLS[+H+O3+P]QGILVIR_.3

Fig4_MP-DIA-barrel-cortex-p9-2_MHRM.htrms

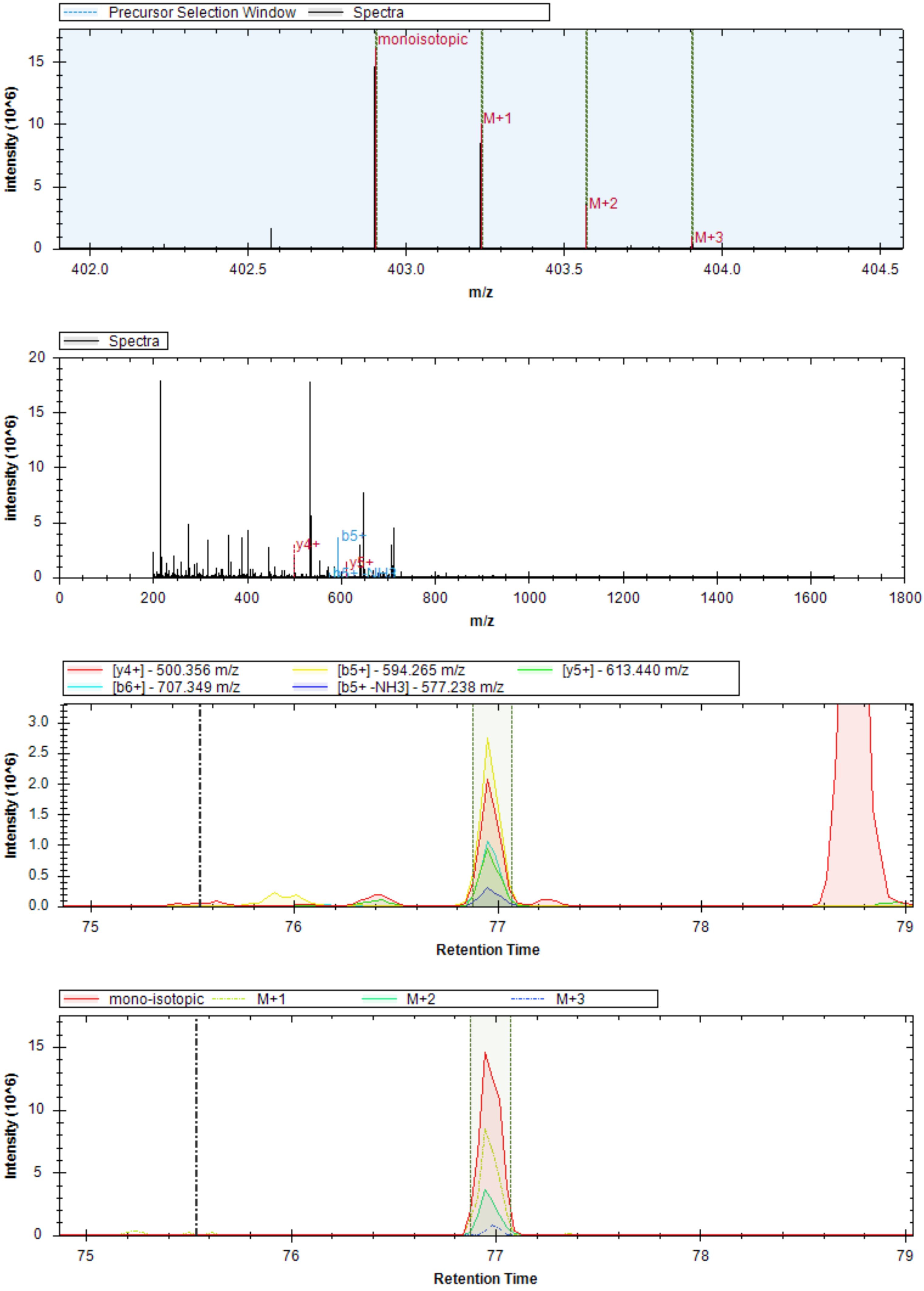

P20357 – _KSEVQAHS[+H+O3+P]PSR_.3

Fig4_MP-DIA-barrel-cortex-p9-2_MHRM.htrms

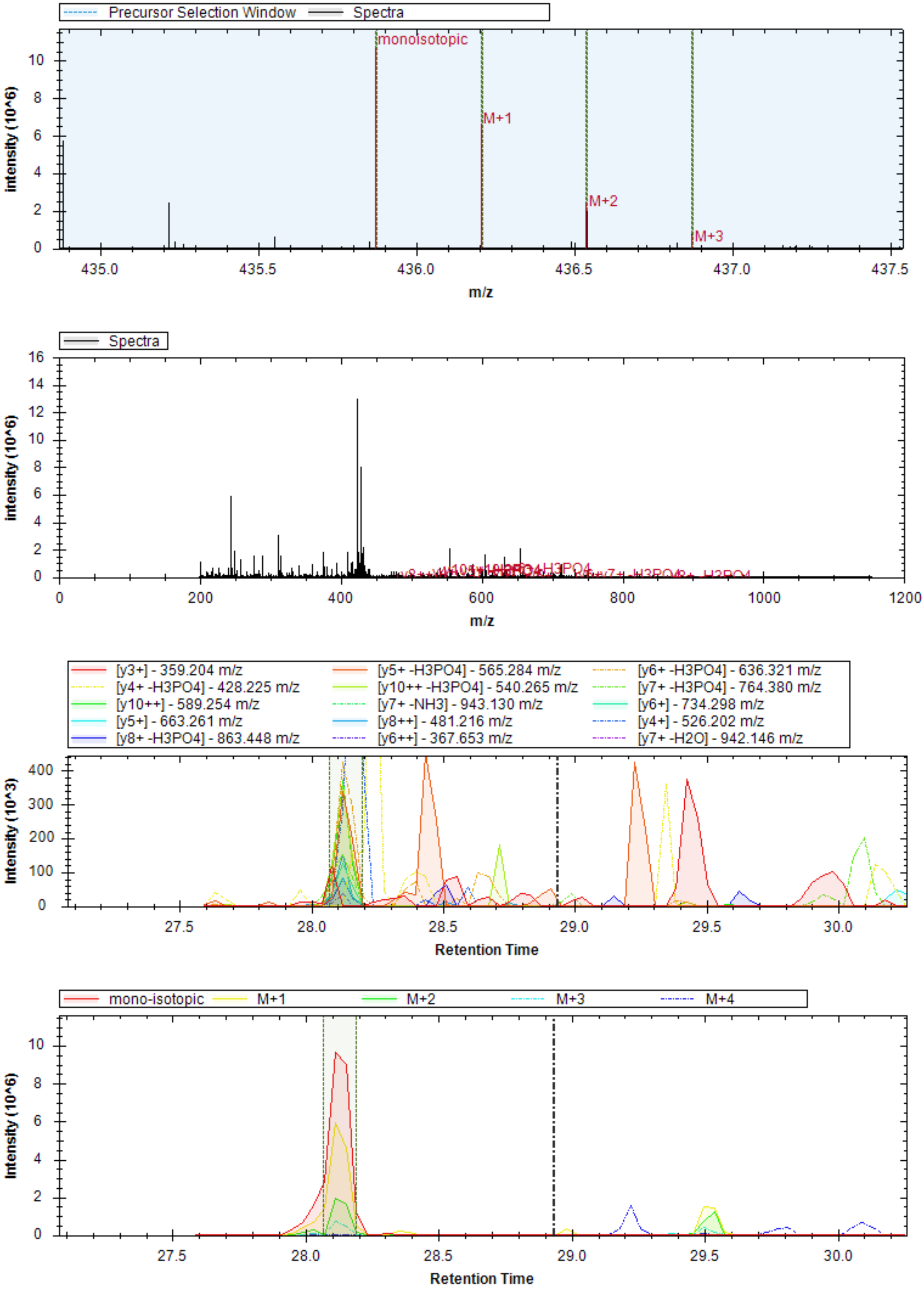

Q03172 – _VALSLLNS[+H+O3+P]KQK_.3
Fig4_MP-DIA-barrel-cortex-p9-2_MHRM.htrms

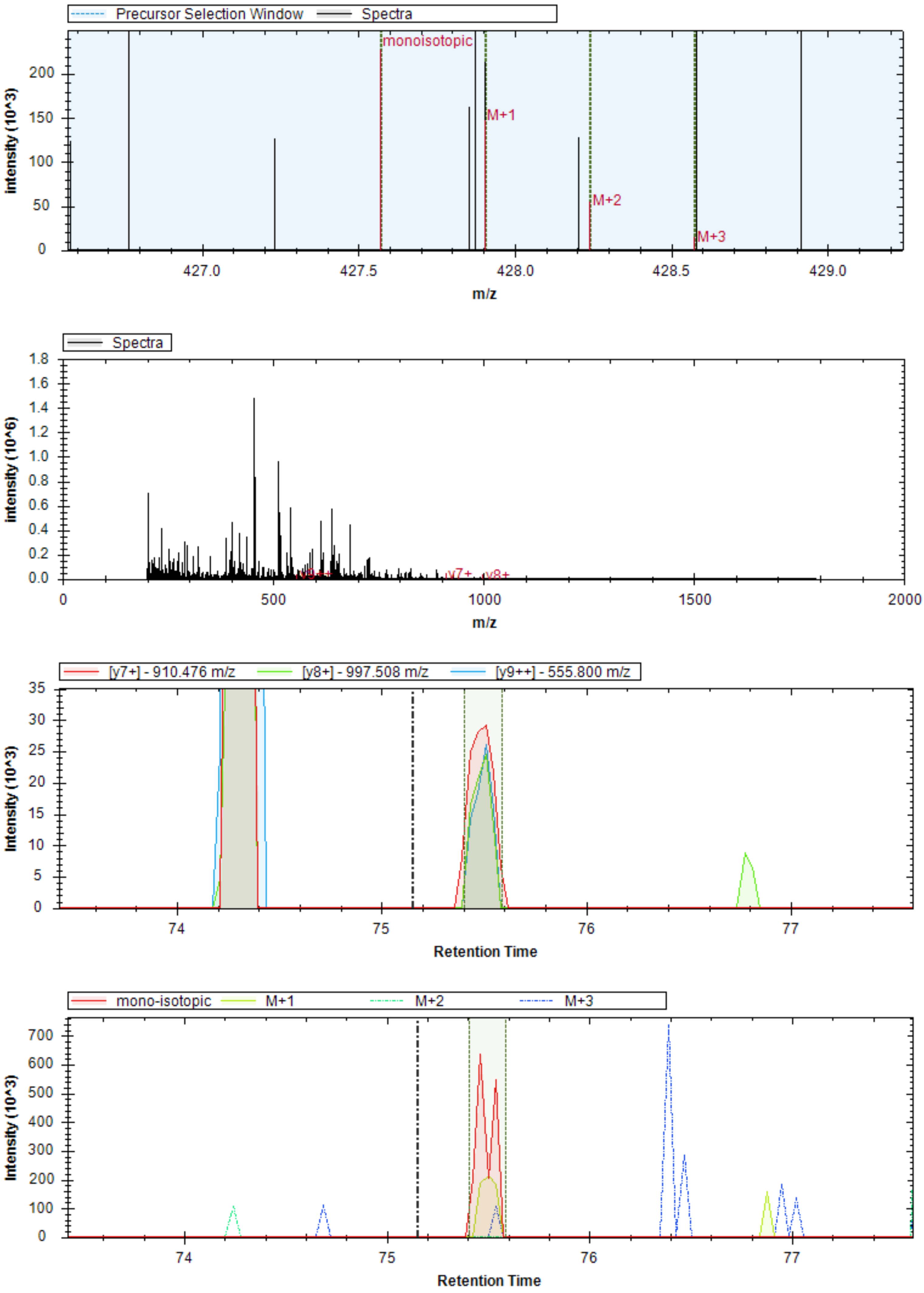

Q52KI8;Q52KI8-2 – _RES[+H+O3+P]PSPAPKPR_.3

Fig4_MP-DIA-barrel-cortex-p9-2_MHRM.htrms

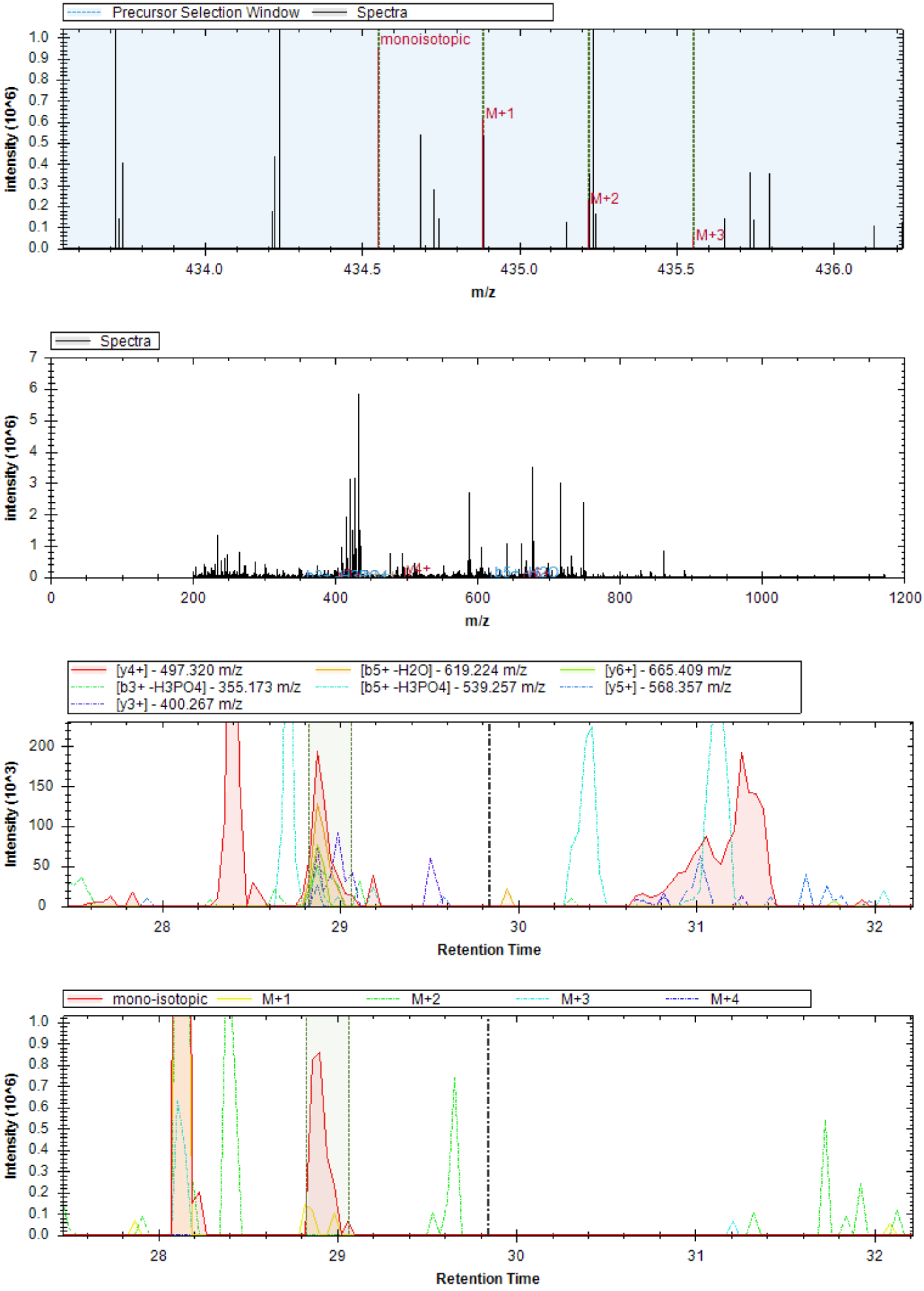

Q62188 – _GS[+H+O3+P]PTRPNPPVR_.3
Fig4_MP-DIA-barrel-cortex-p9-2_MHRM.htrms

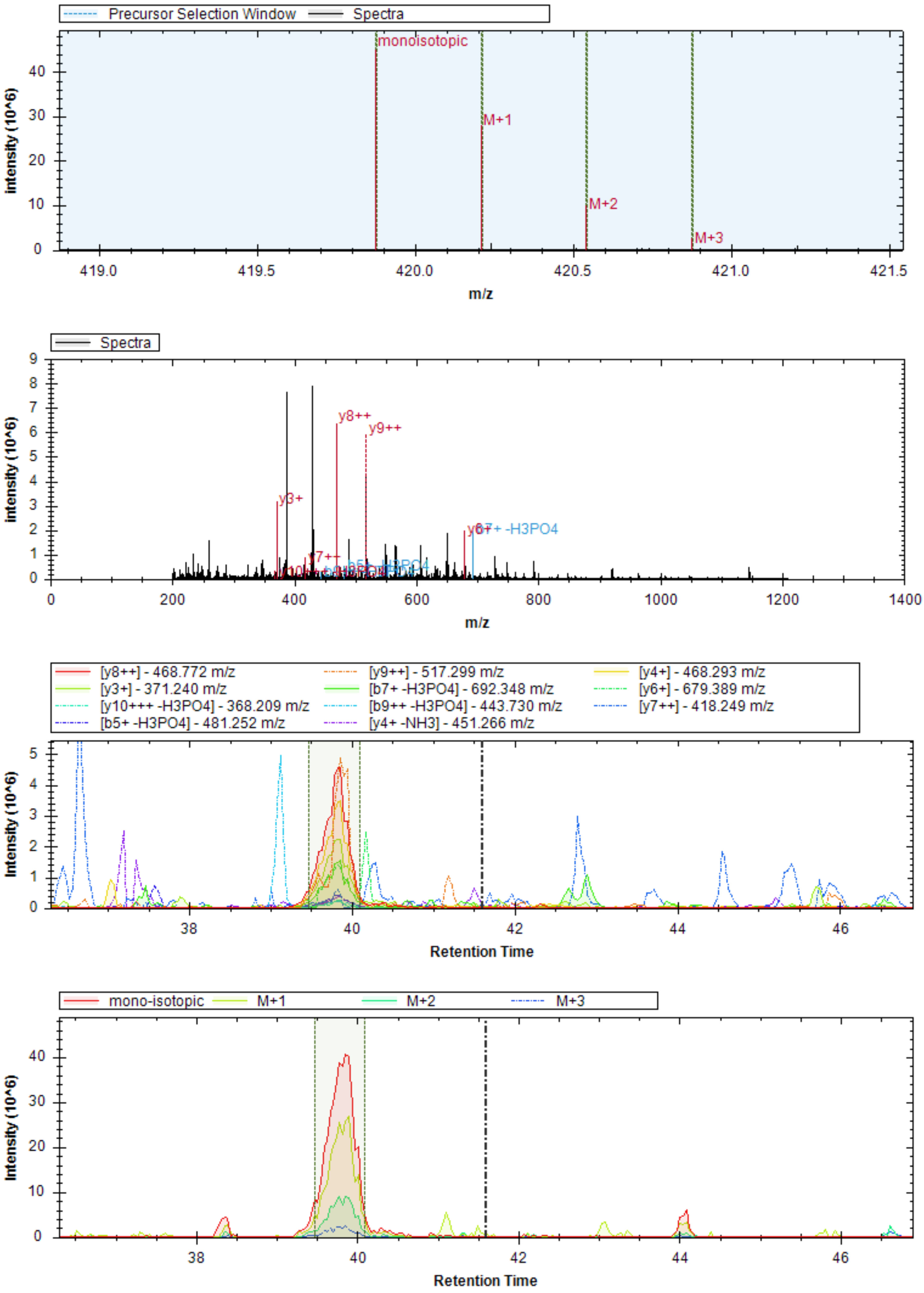

P62484 – _T[+H+O3+P]HKIIAPANLERPVR_.4
Fig4_MP-DIA-barrel-cortex-p9-2_MHRM.htrms

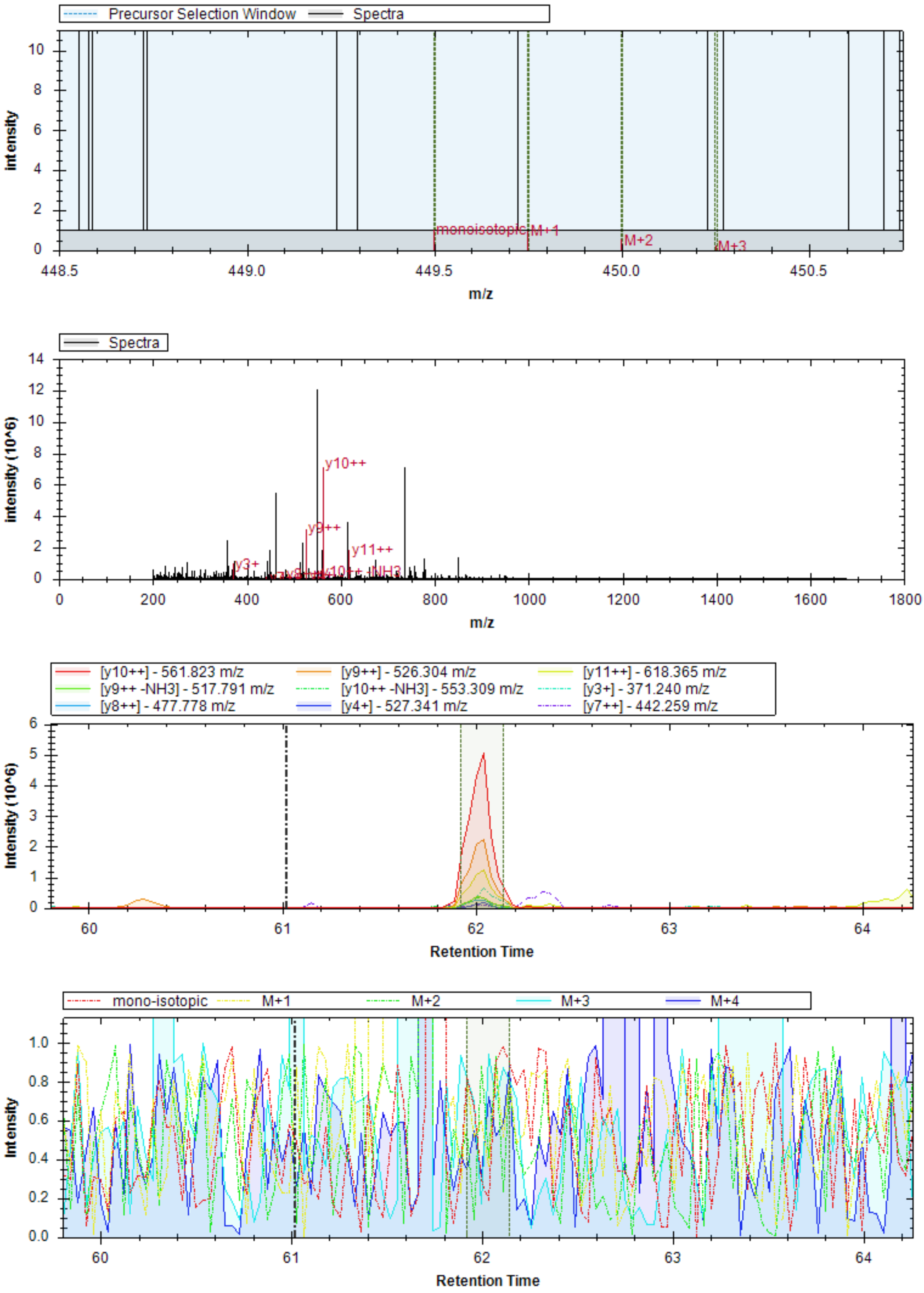

O08553 – _TVT[+H+O3+P]PASSAK_.2
Fig4_MP-DIA-barrel-cortex-p9-2_MHRM.htrms

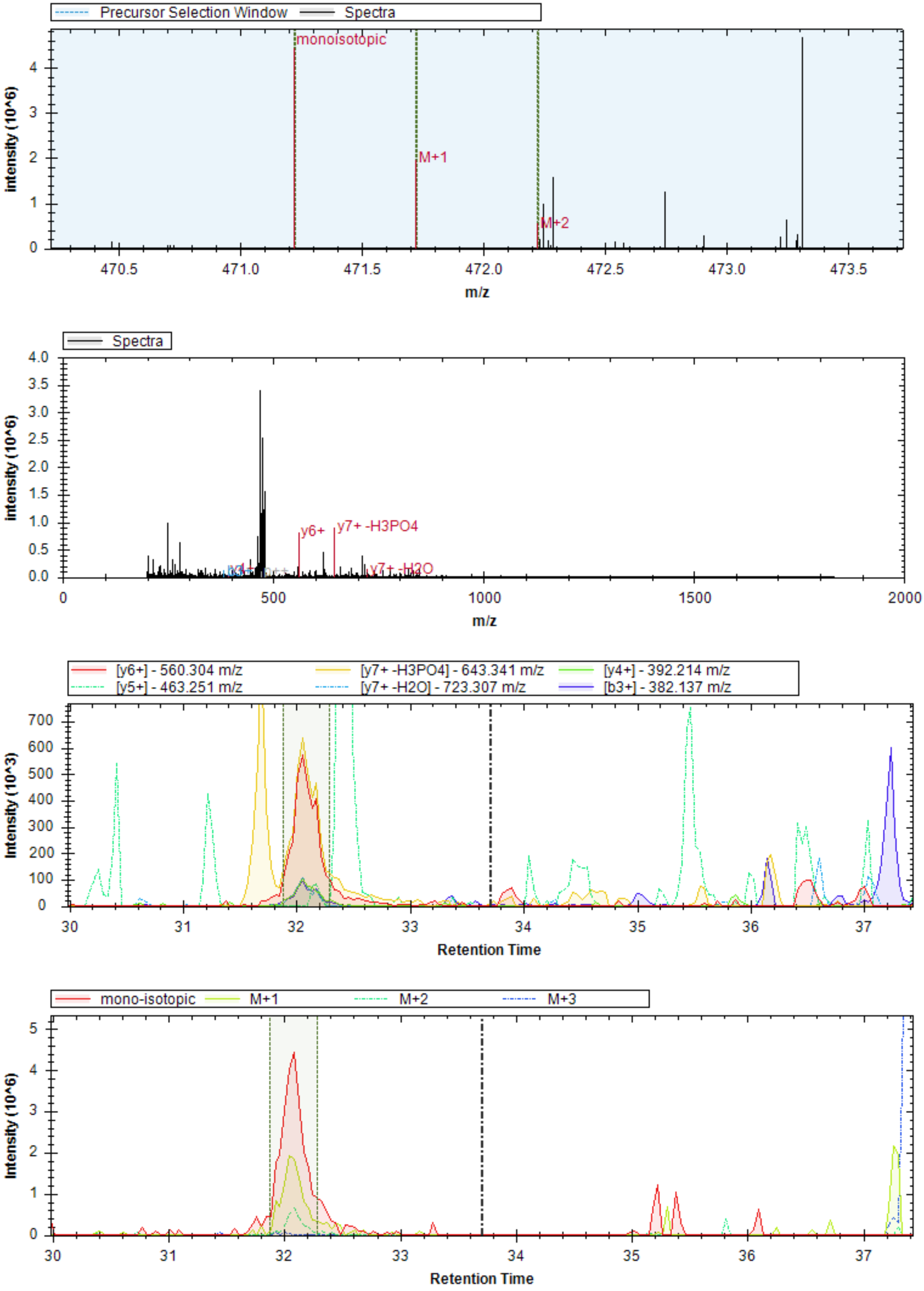

P10637 – _S[+H+O3+P]RTPSLPTPPTR_.3
Fig4_MP-DIA-barrel-cortex-p9-2_MHRM.htrms

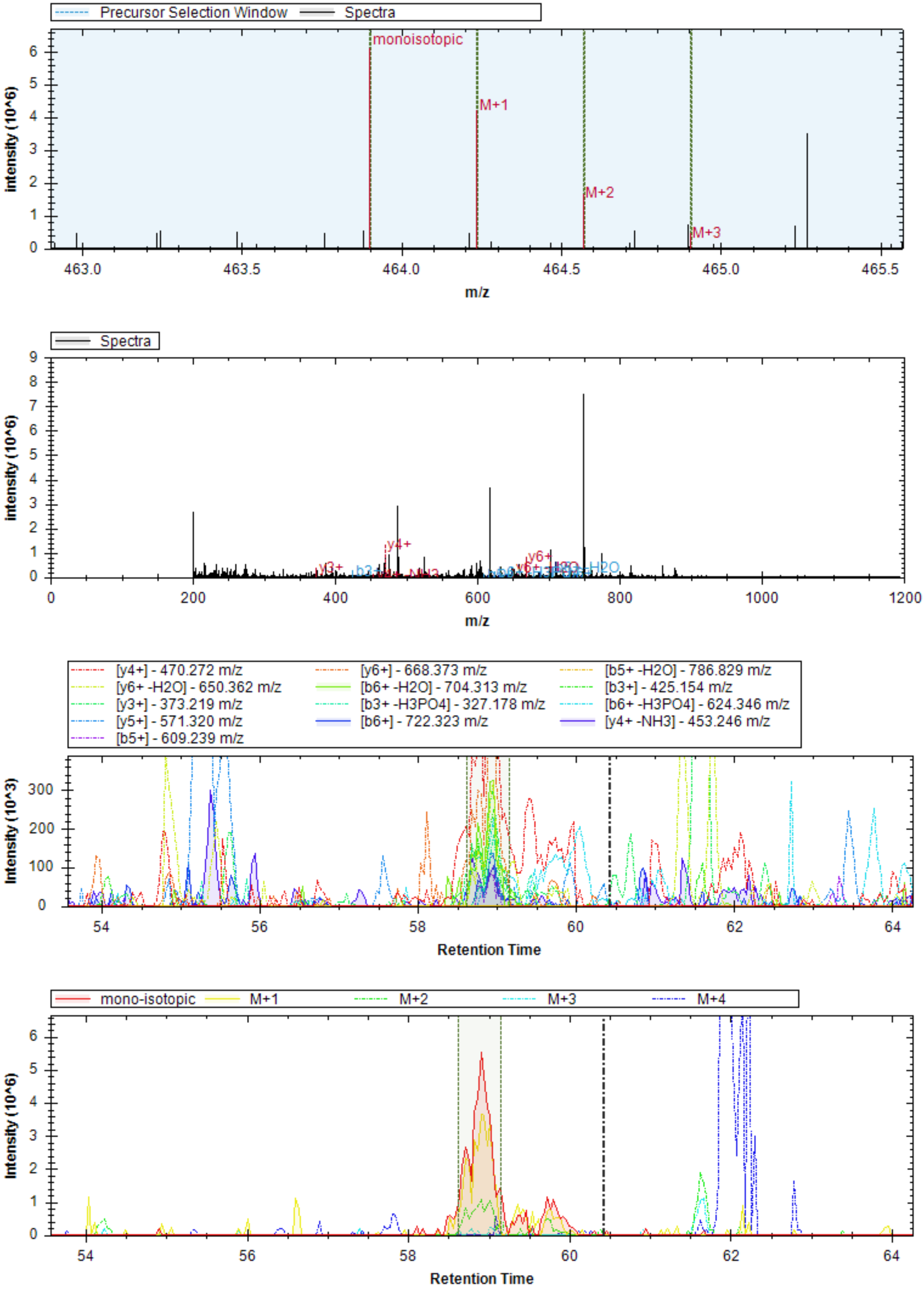

P20357 – _VTDGIS[+H+O3+P]KSPEKR_.3

Fig4_MP-DIA-barrel-cortex-p9-2_MHRM.htrms

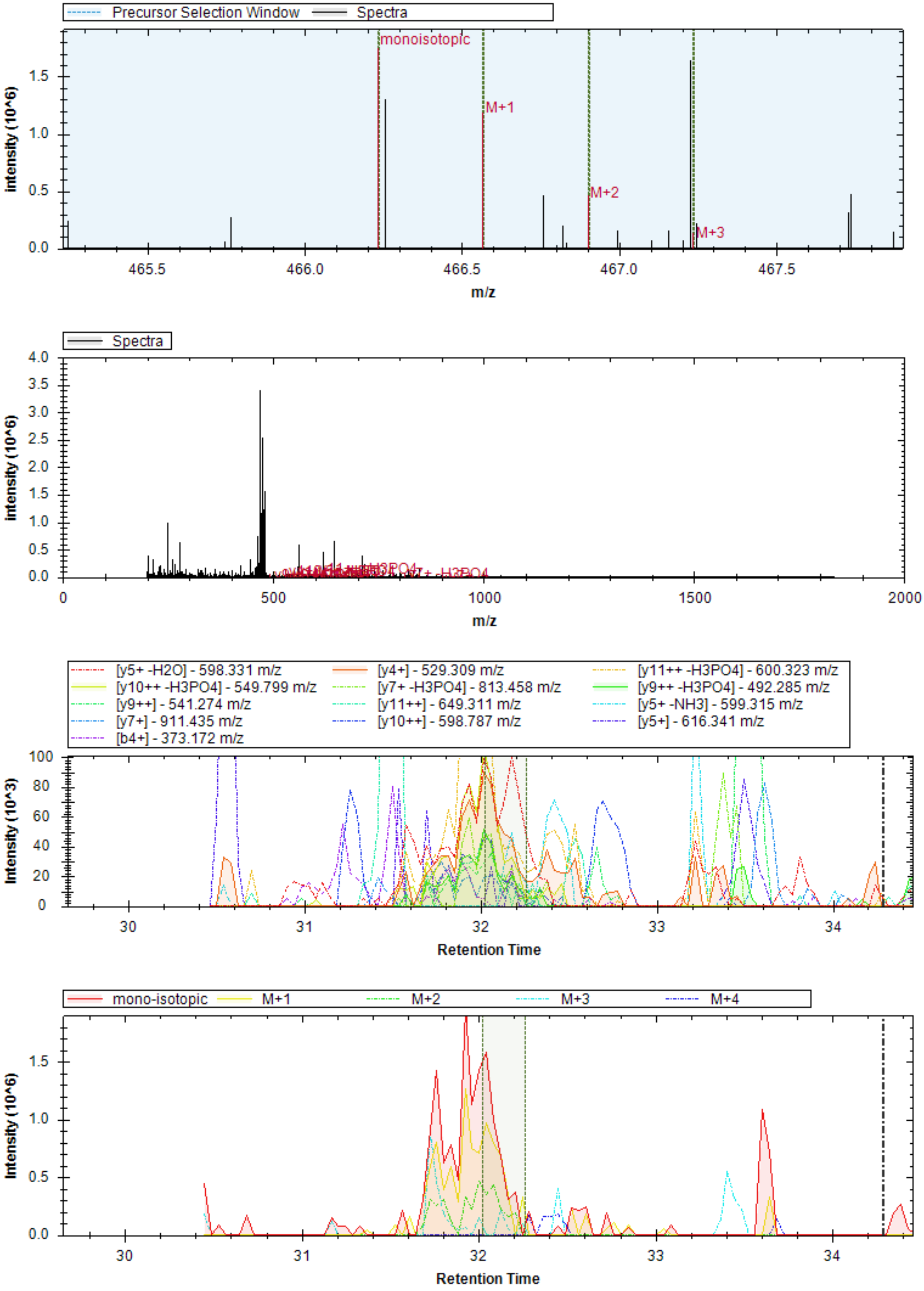

P20357 – _VTDGISKS[+H+O3+P]PEKR_.3
Fig4_MP-DIA-barrel-cortex-p9-2_MHRM.htrms

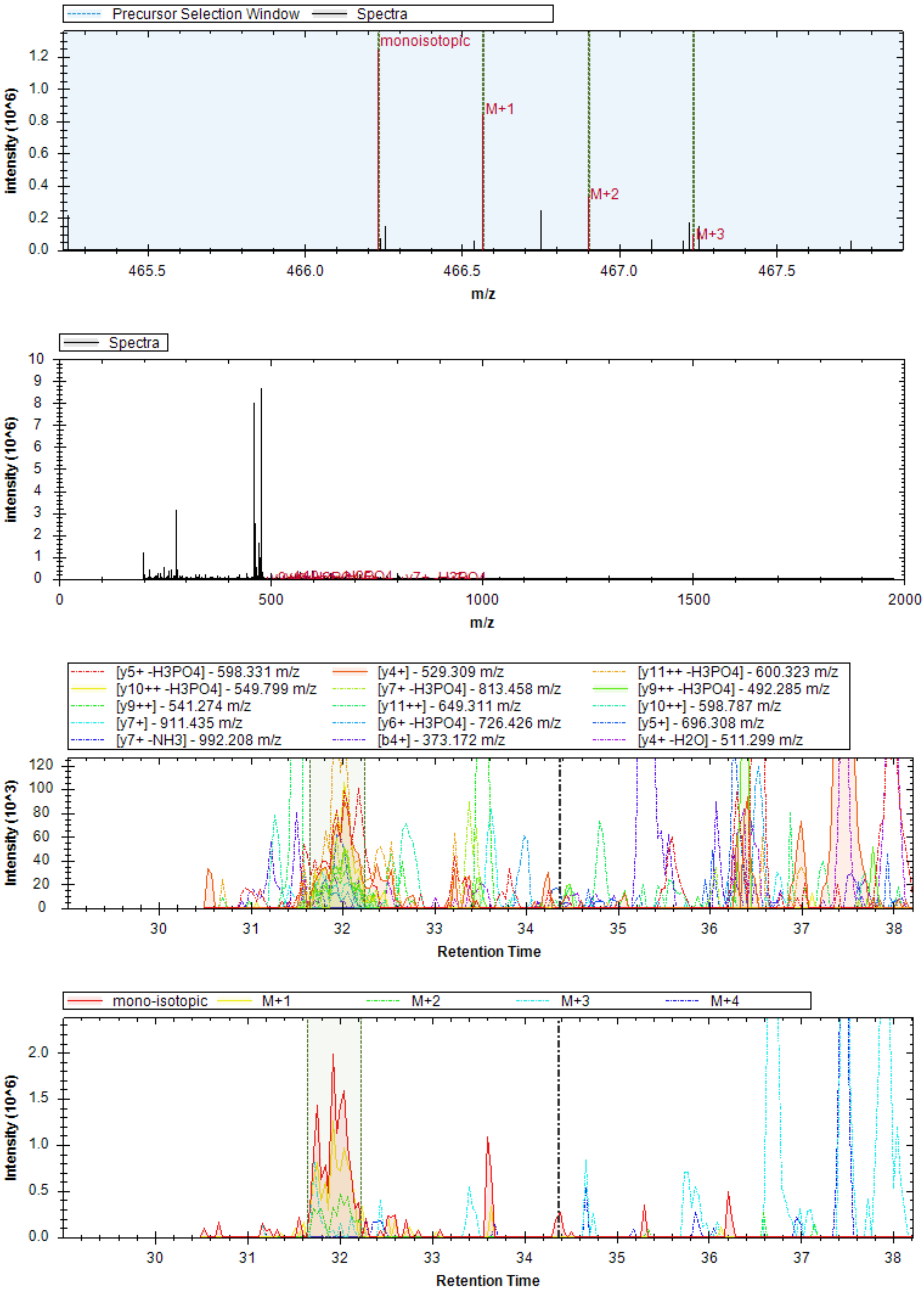

Q9CR81 – _[+C2+H2+O]M[+O]ANHLVKPDS[+H+O3+P]R_.3
Fig4_MP-DIA-barrel-cortex-p9-2_MHRM.htrms

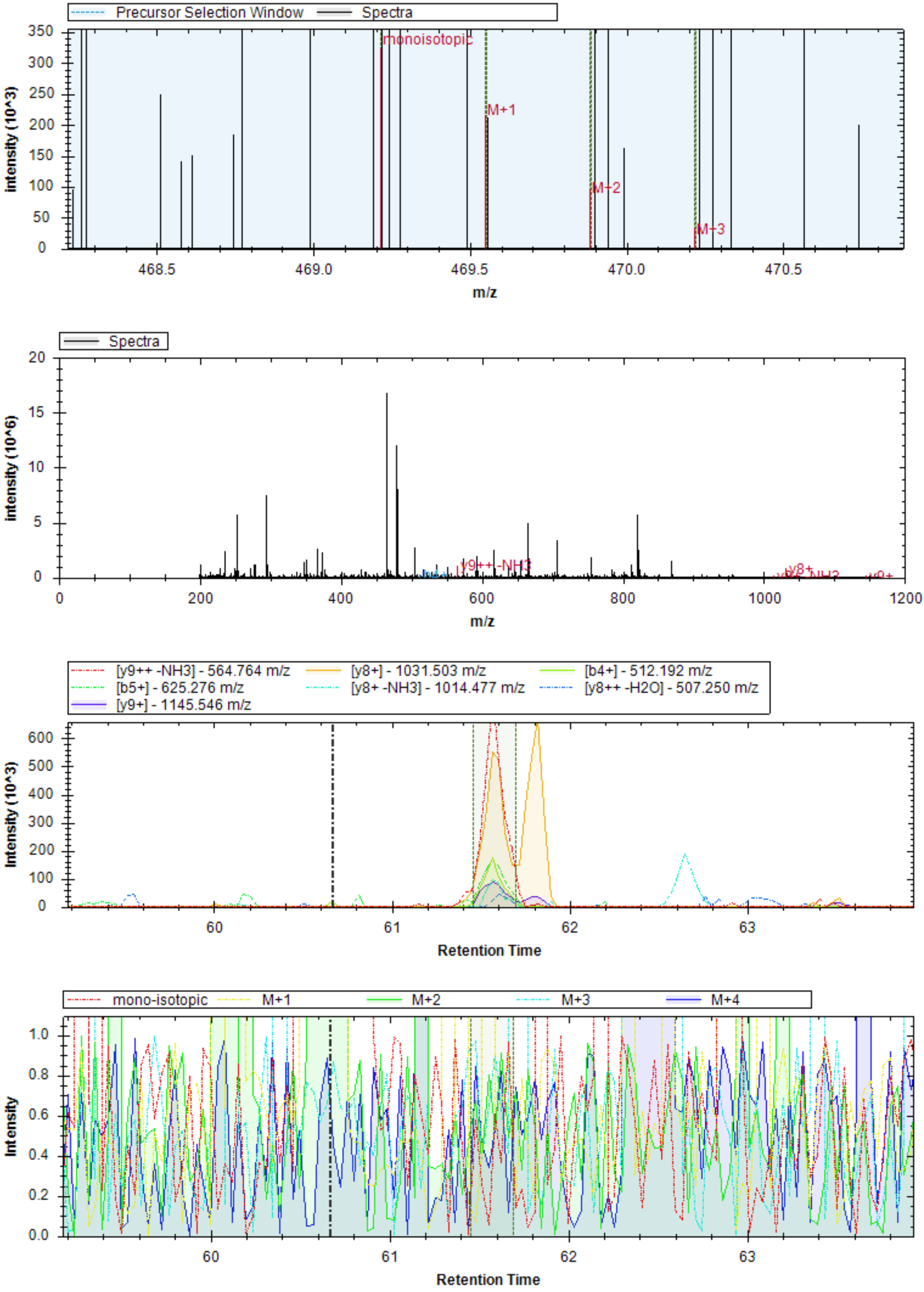

P26645 – _LSGFS[+H+O3+P]FKK_.2

Fig4_MP-DIA-barrel-cortex-p9-2_MHRM.htrms

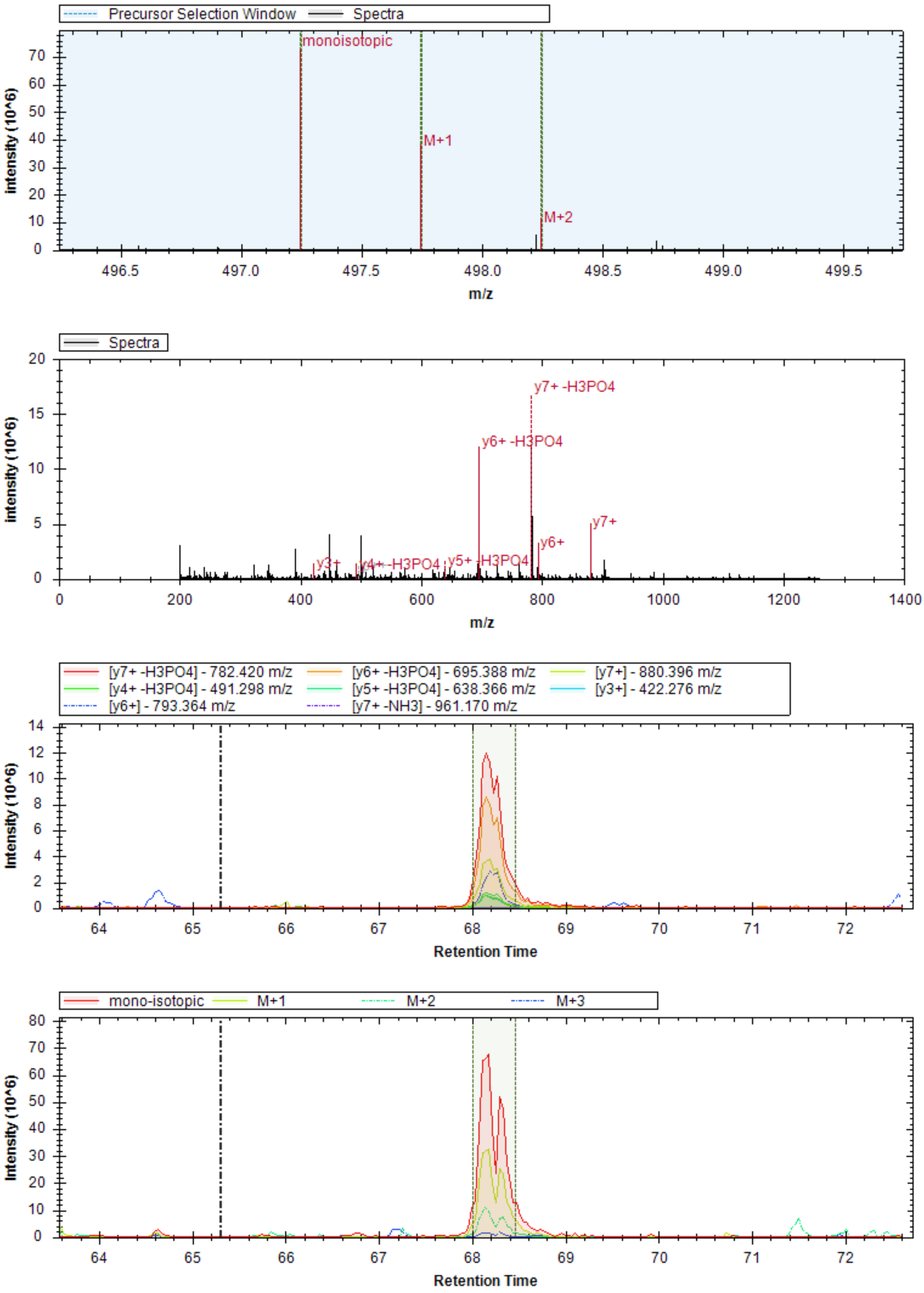

P28667 – _LSGLS[+H+O3+P]FKR_.2

Fig4_MP-DIA-barrel-cortex-p9-2_MHRM.htrms

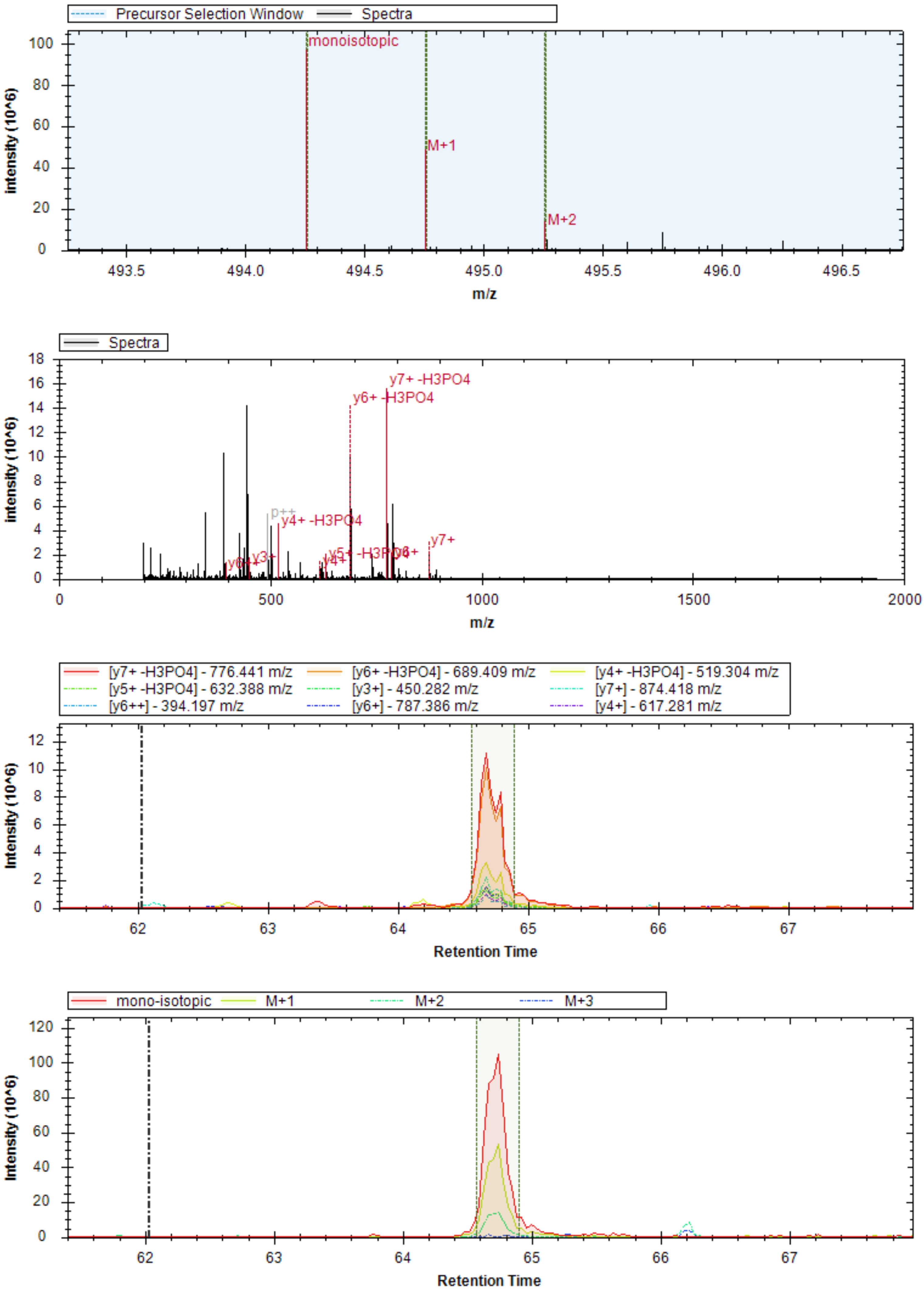

P57776-3 – _ATAPQTQHVS[+H+O3+P]PMR_.3
Fig4_MP-DIA-barrel-cortex-p9-2_MHRM.htrms

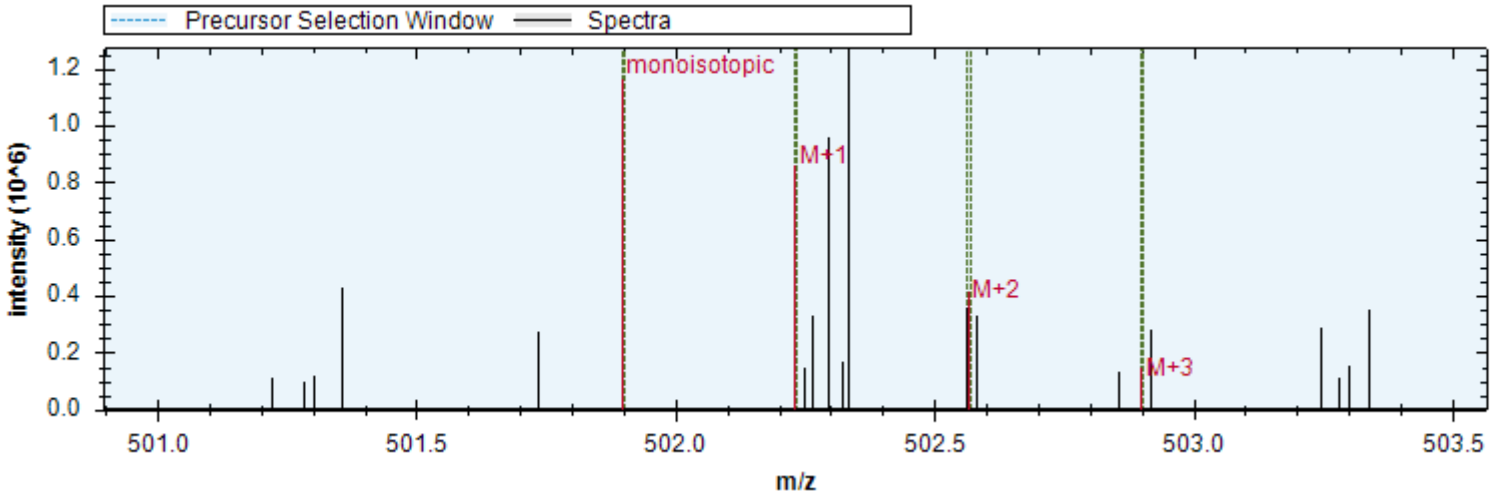

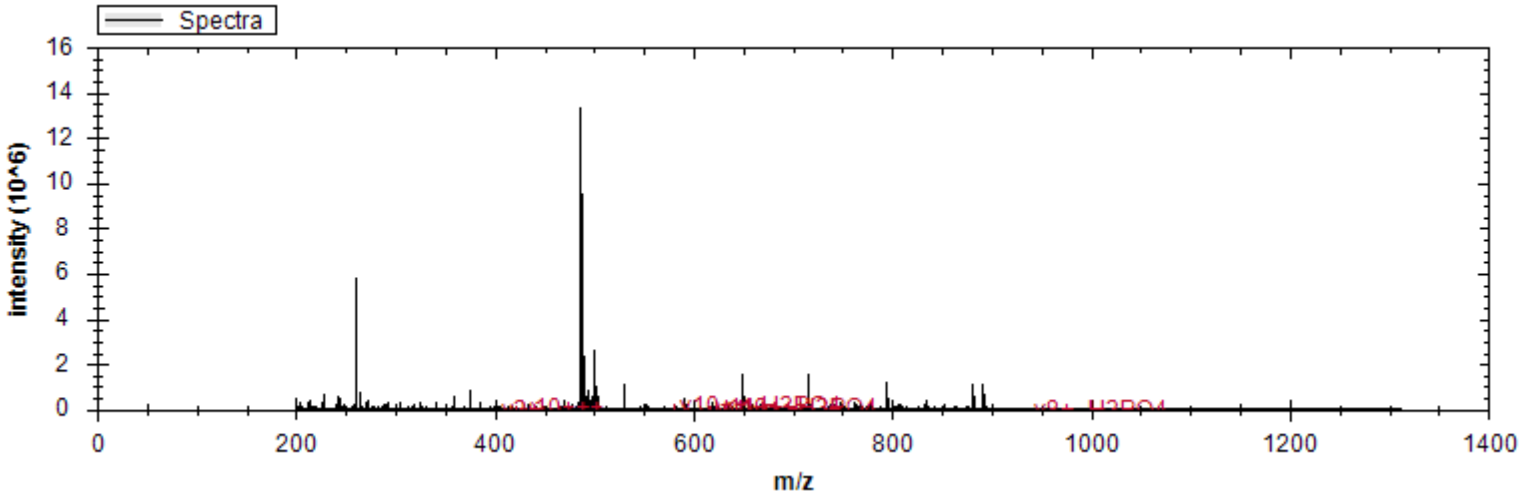

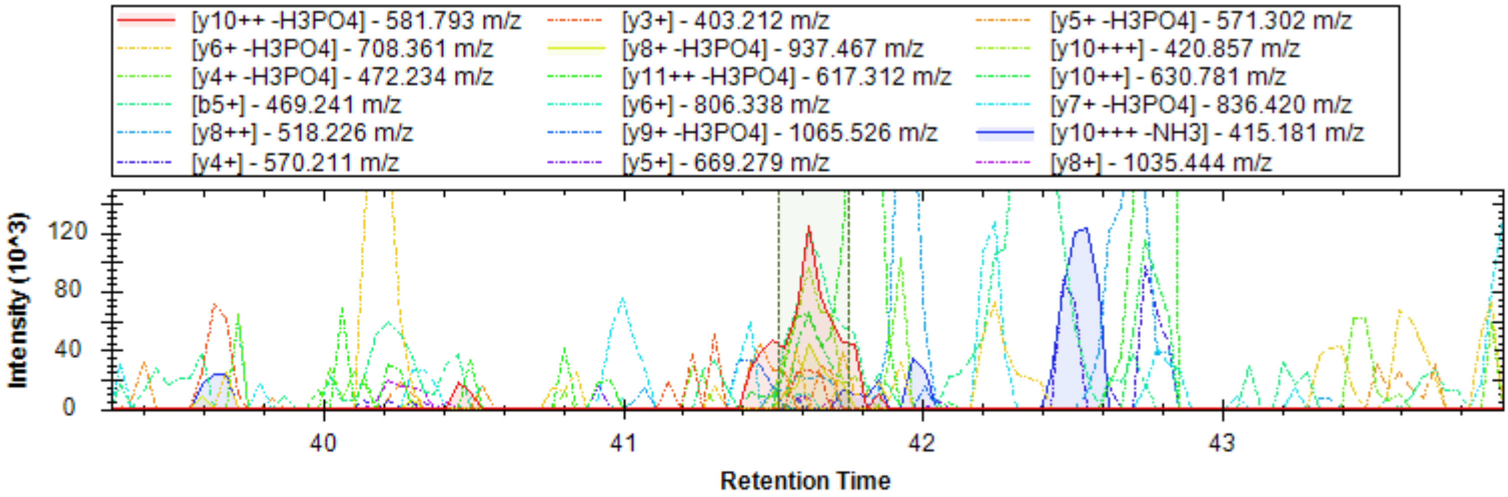

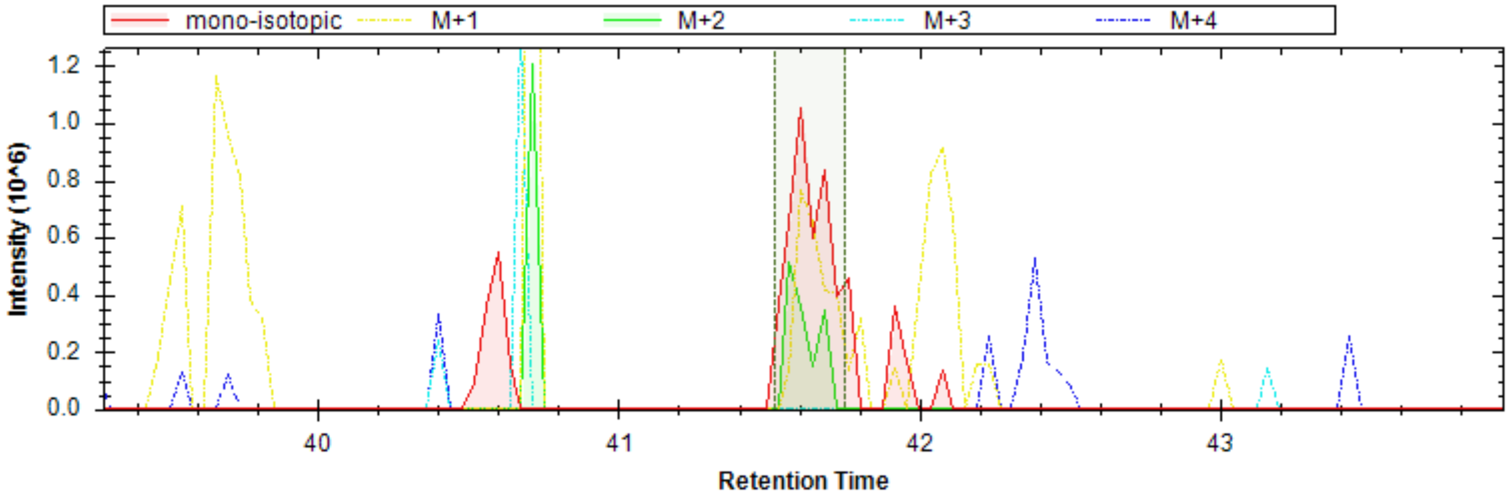

P28738 – _PIRPGHYPAS[+H+O3+P]SPTAVHAVR_.4

Fig4_MP-DIA-barrel-cortex-p9-2_MHRM.htrms

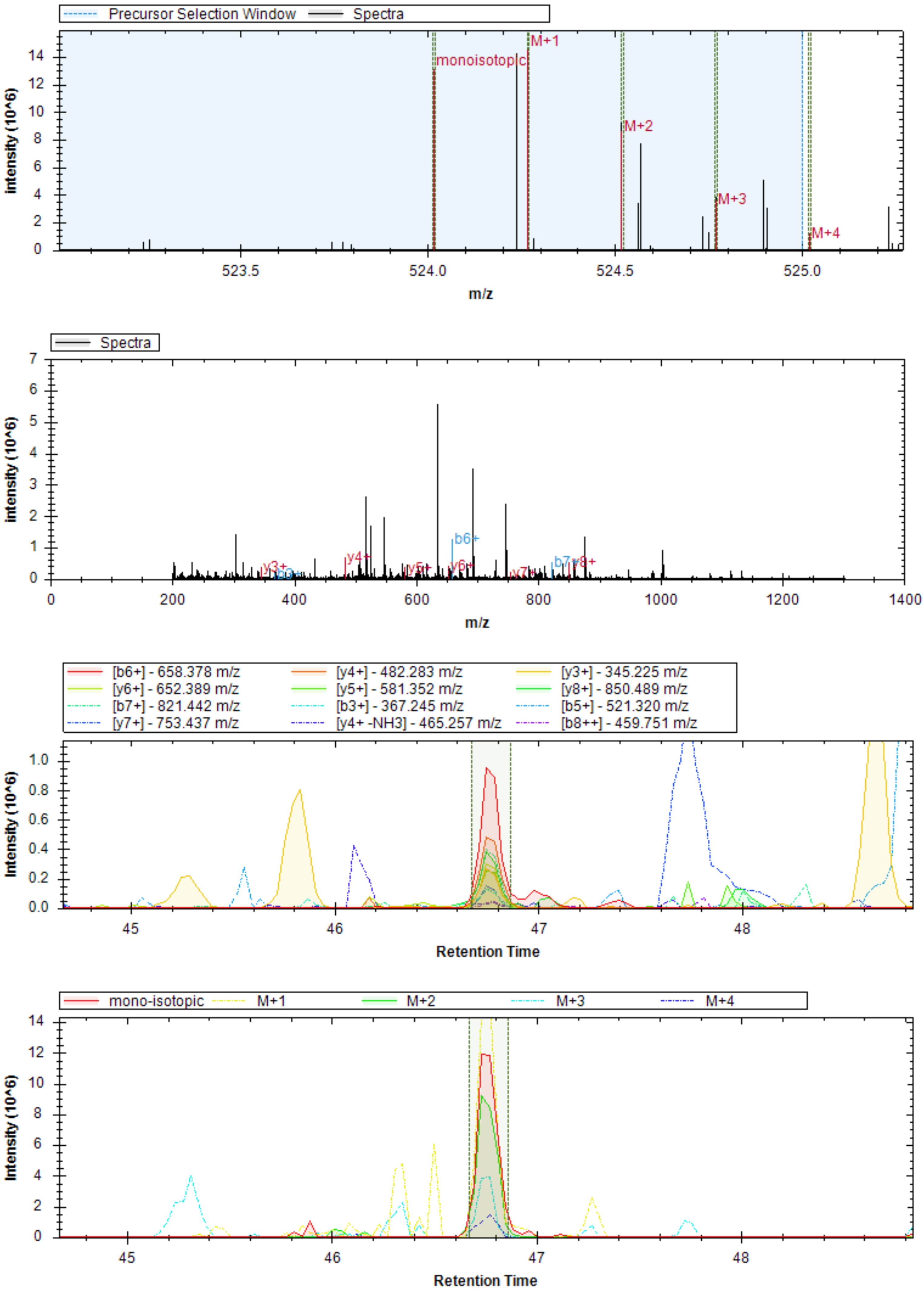

P28738 – _PIRPGHYPASS[+H+O3+P]PTAVHAVR_.4
Fig4_MP-DIA-barrel-cortex-p9-2_MHRM.htrms

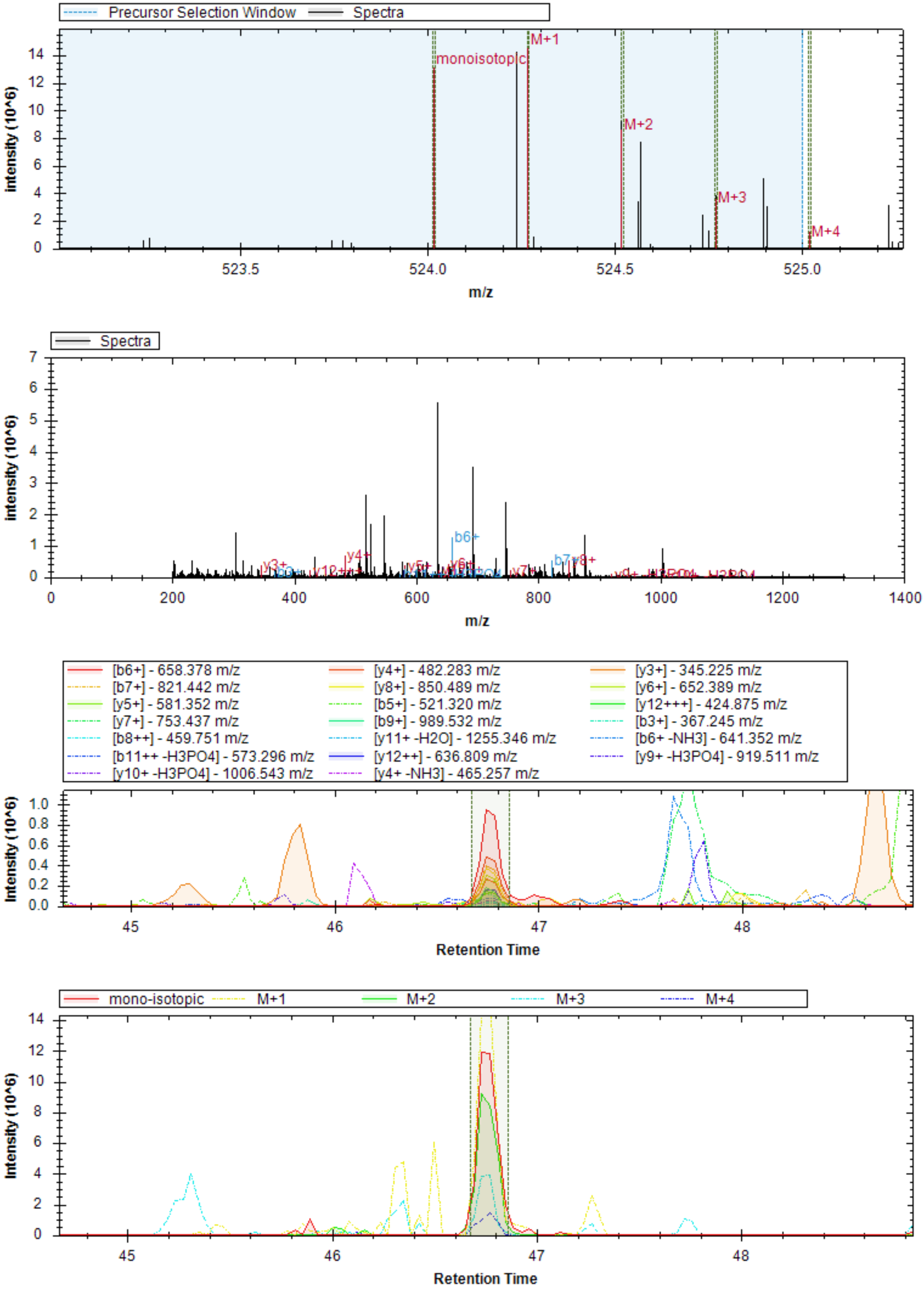

P62996 – _RPHT[+H+O3+P]PTPGIYMGR_.3
Fig4_MP-DIA-barrel-cortex-p9-2_MHRM.htrms

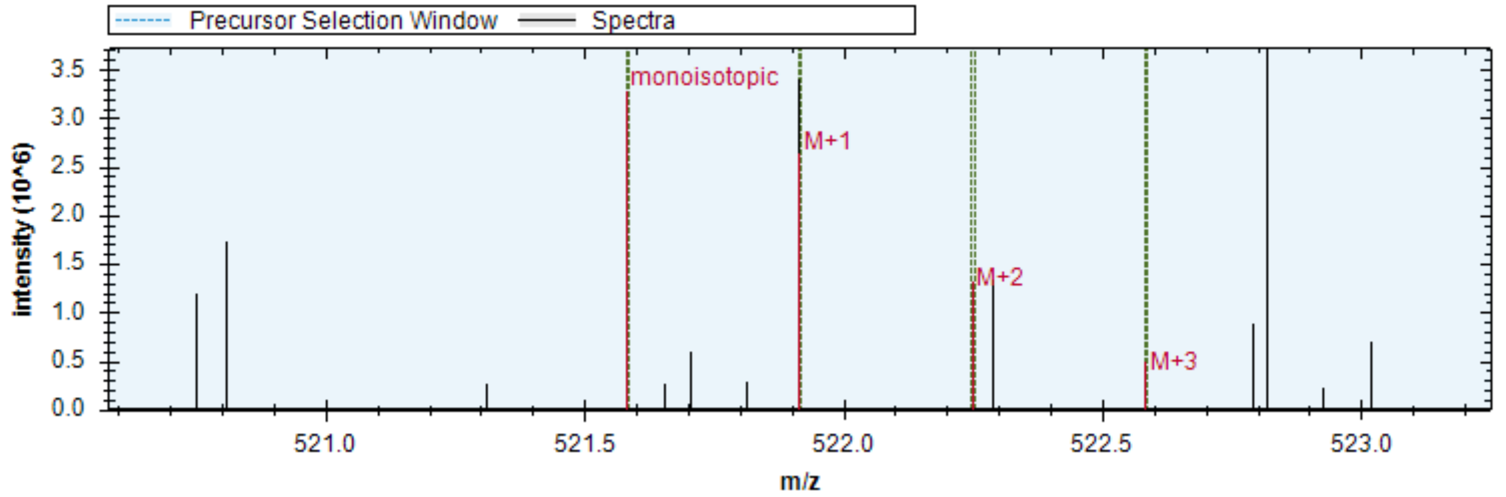

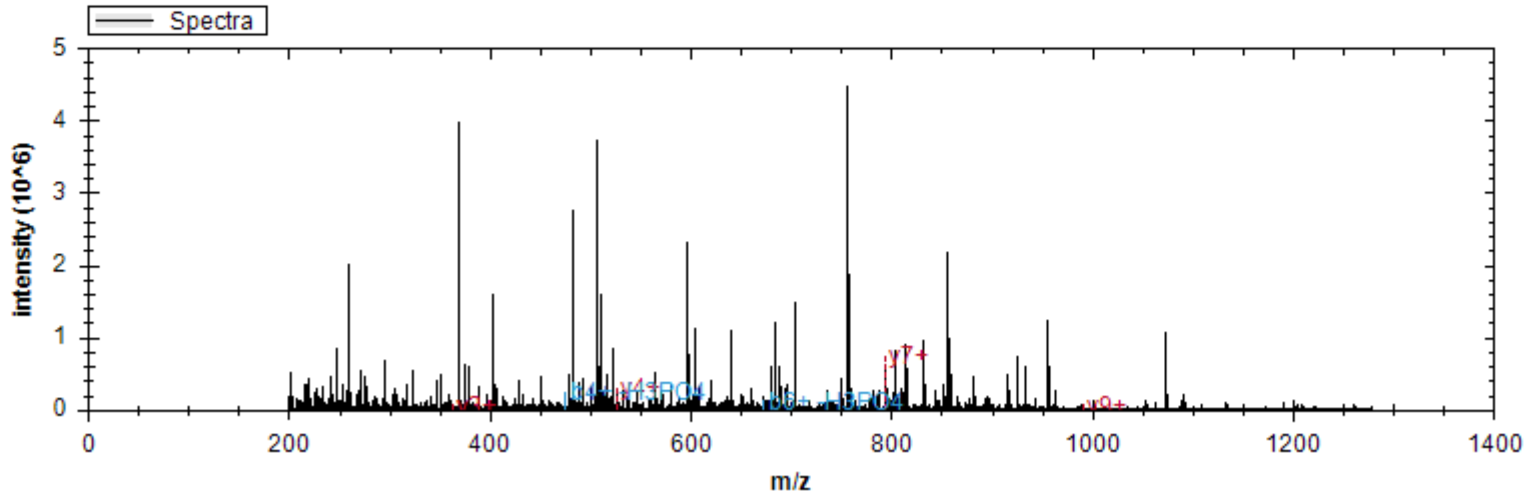

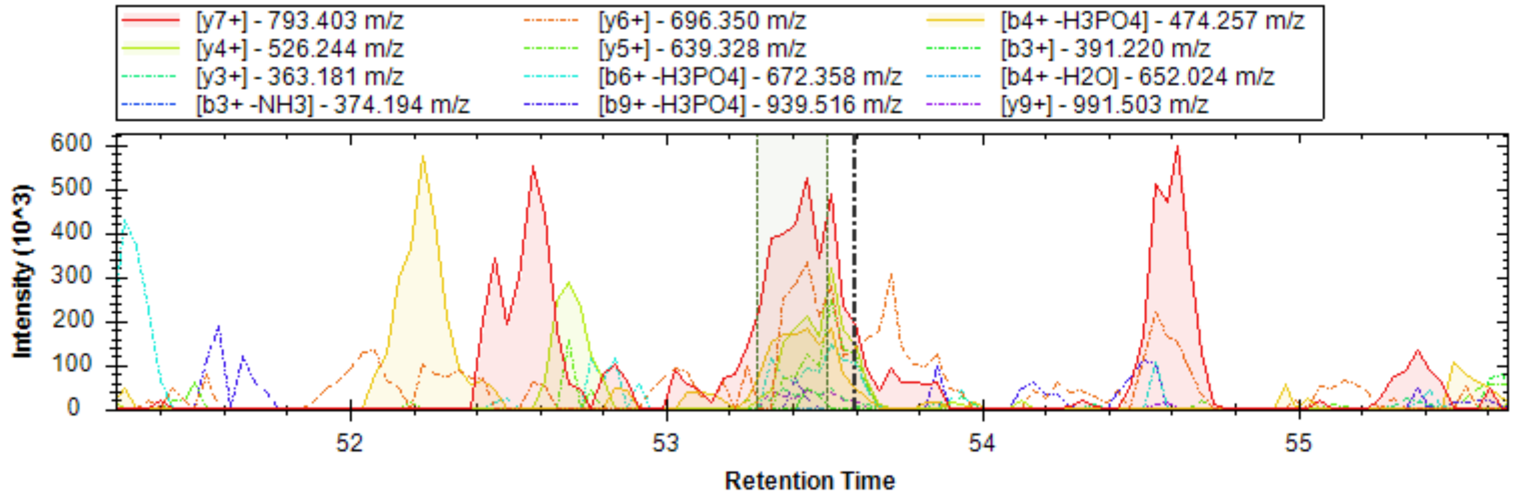

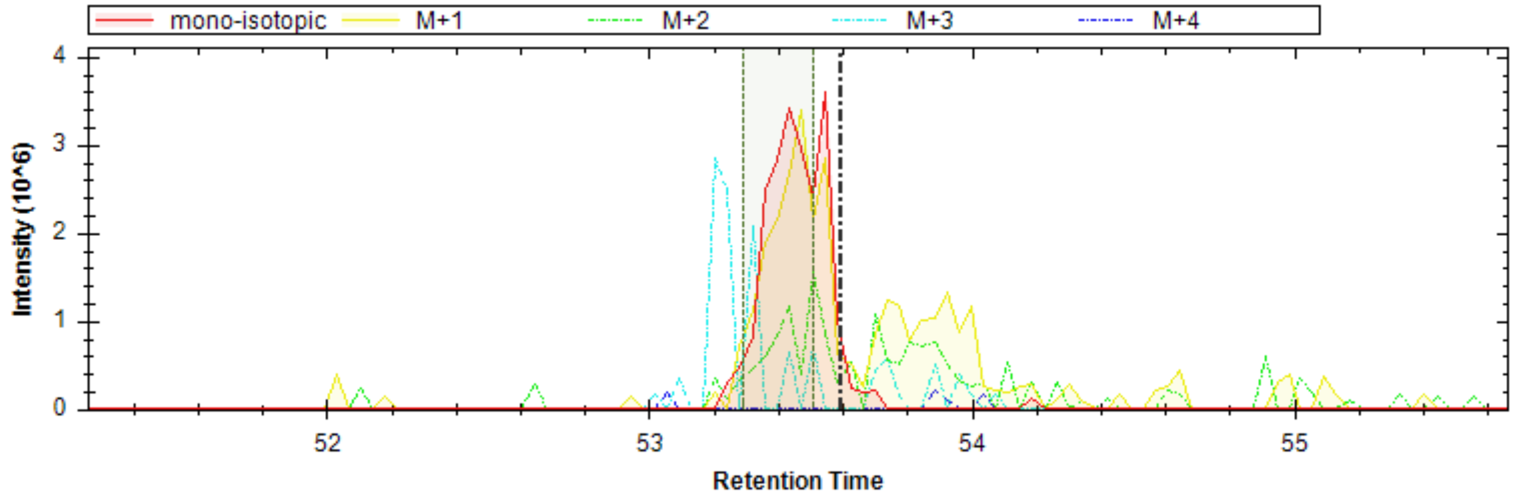

Q8C8R3-7 – _[+C2+H2+O]AHAAAS[+H+O3+P]IKK_.2
Fig4_MP-DIA-barrel-cortex-p9-2_MHRM.htrms

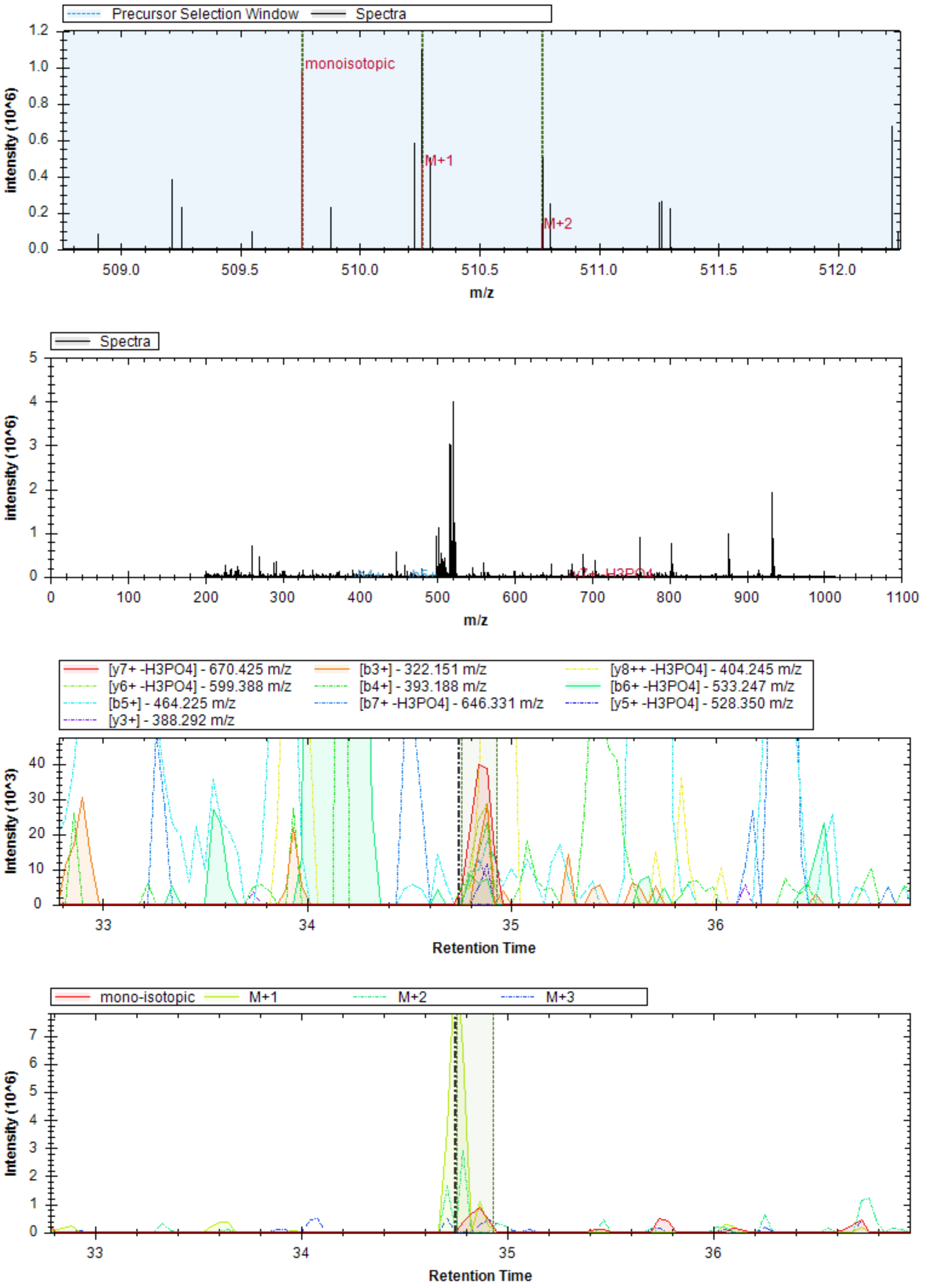

Q8CDY7 – _LVQALC[+C2+H3+N+O]EQIHT[+H+O3+P]R_.3
Fig4_MP-DIA-barrel-cortex-p9-2_MHRM.htrms

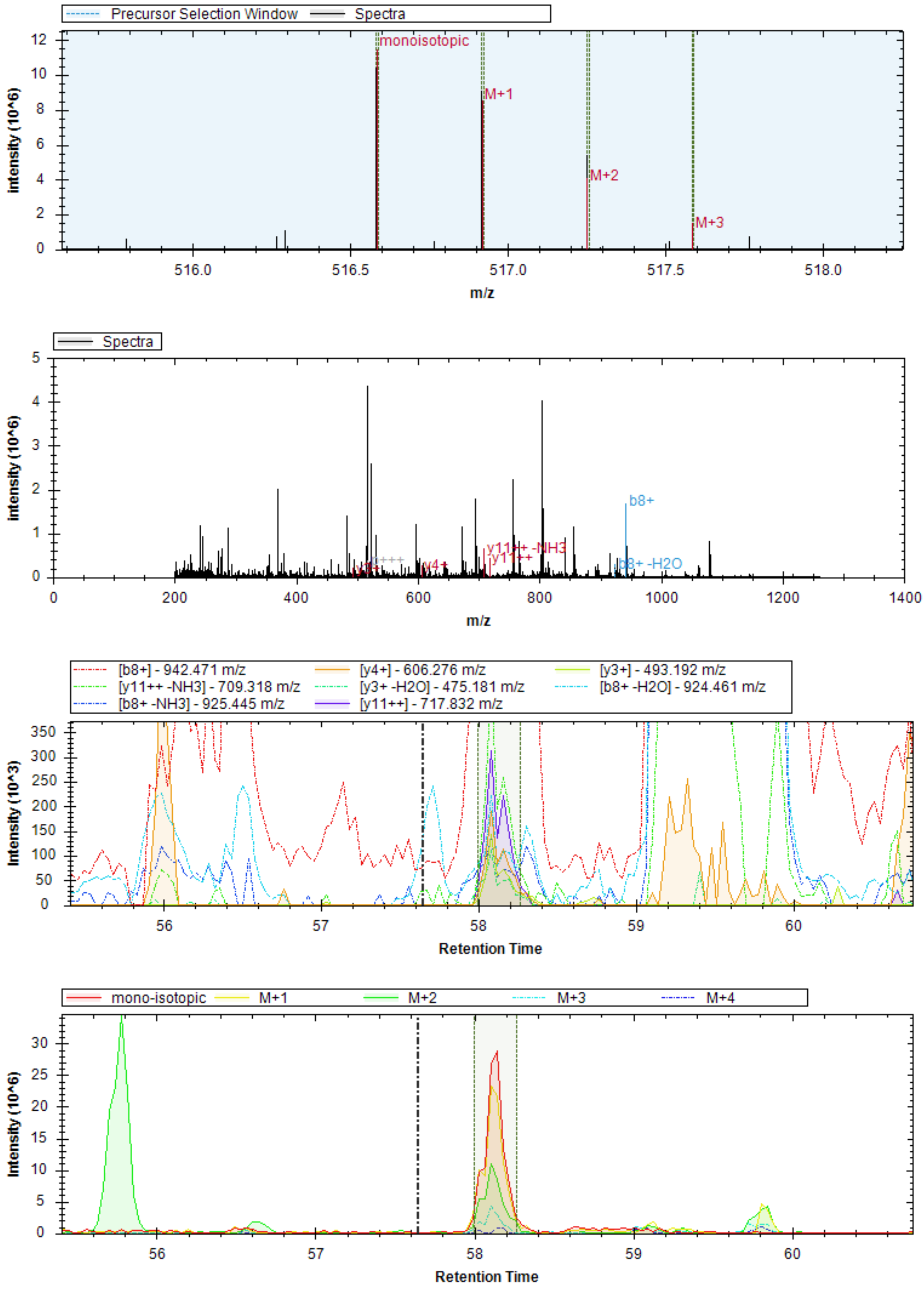

Q9JLM8 – _ISQHGGS[+H+O3+P]STSLSSTK_.3
Fig4_MP-DIA-barrel-cortex-p9-2_MHRM.htrms

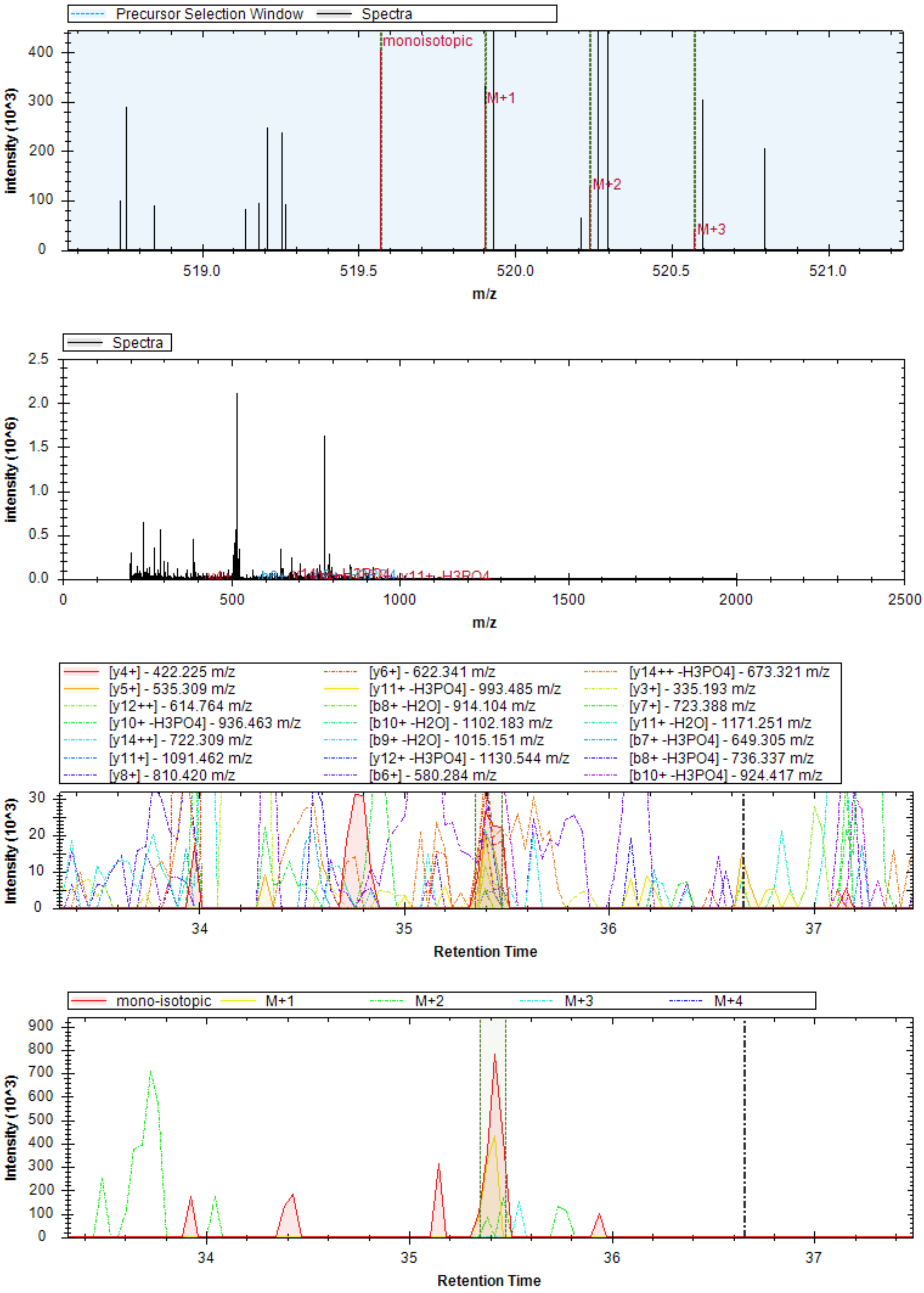

Q9QYC0 – _AAVVT[+H+O3+P]SPPPTTAPHK_.3
Fig4_MP-DIA-barrel-cortex-p9-2_MHRM.htrms

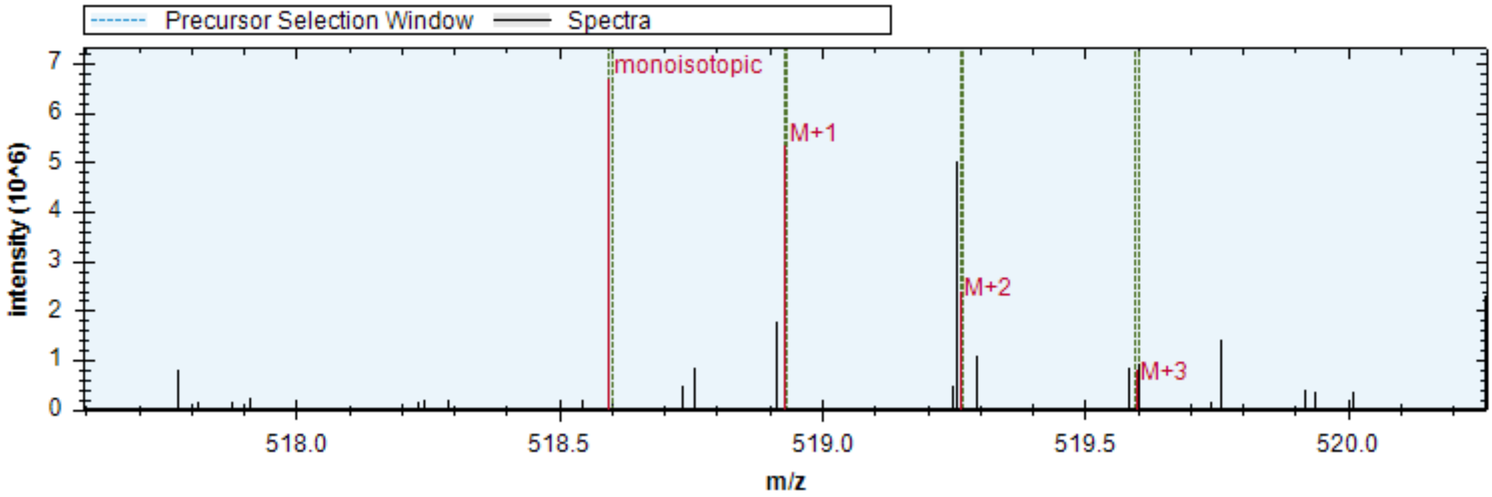

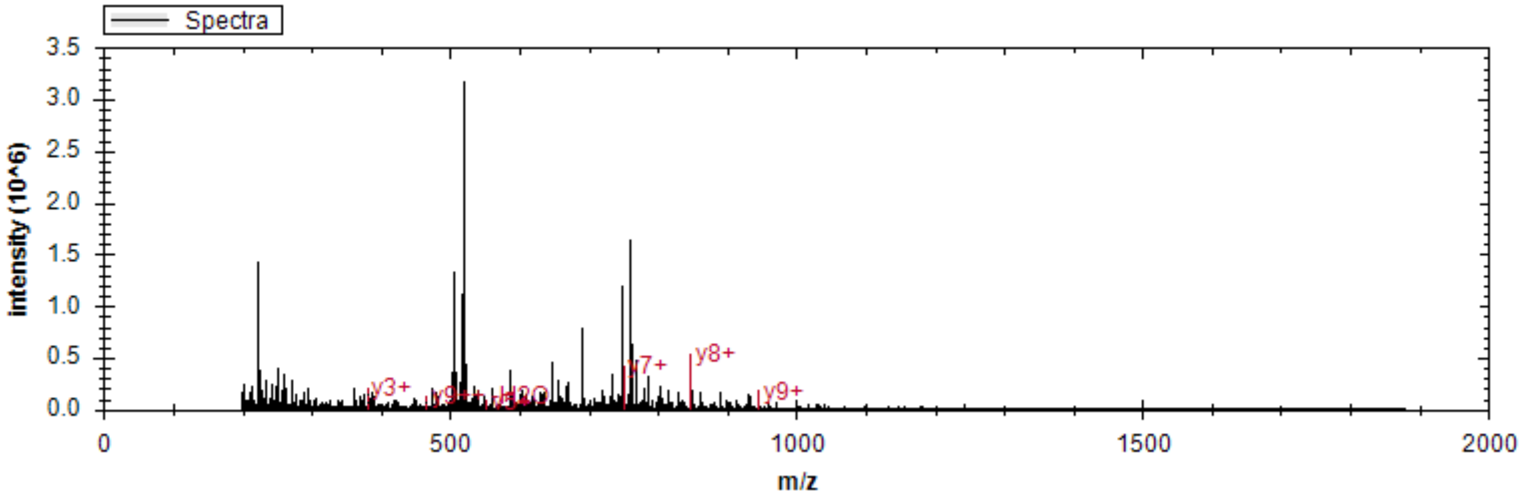

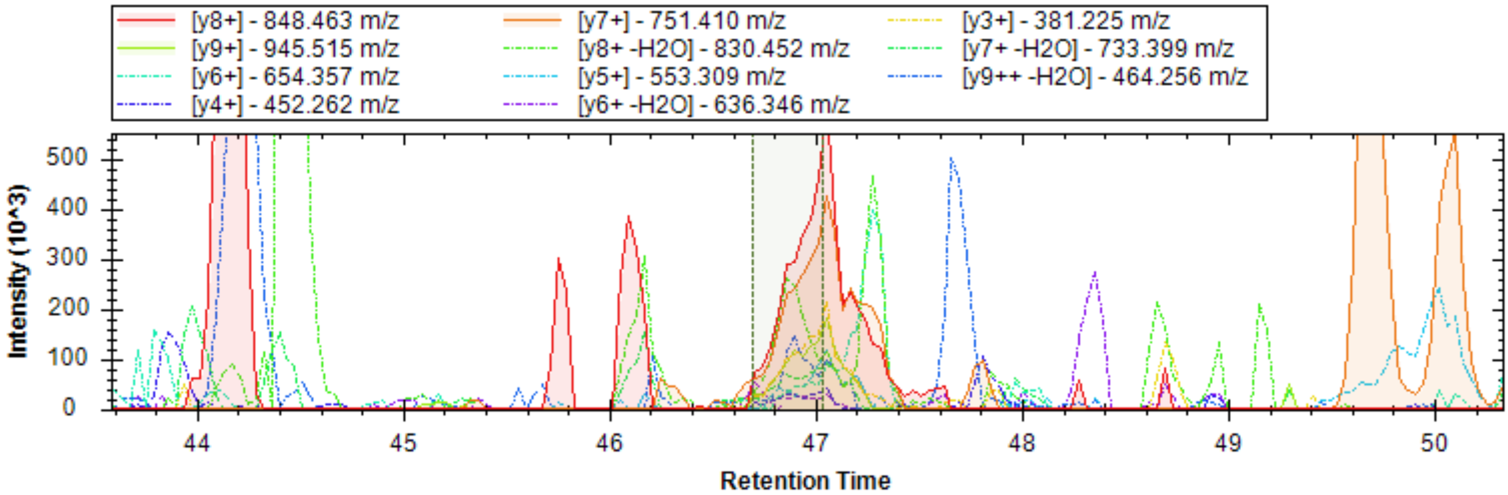

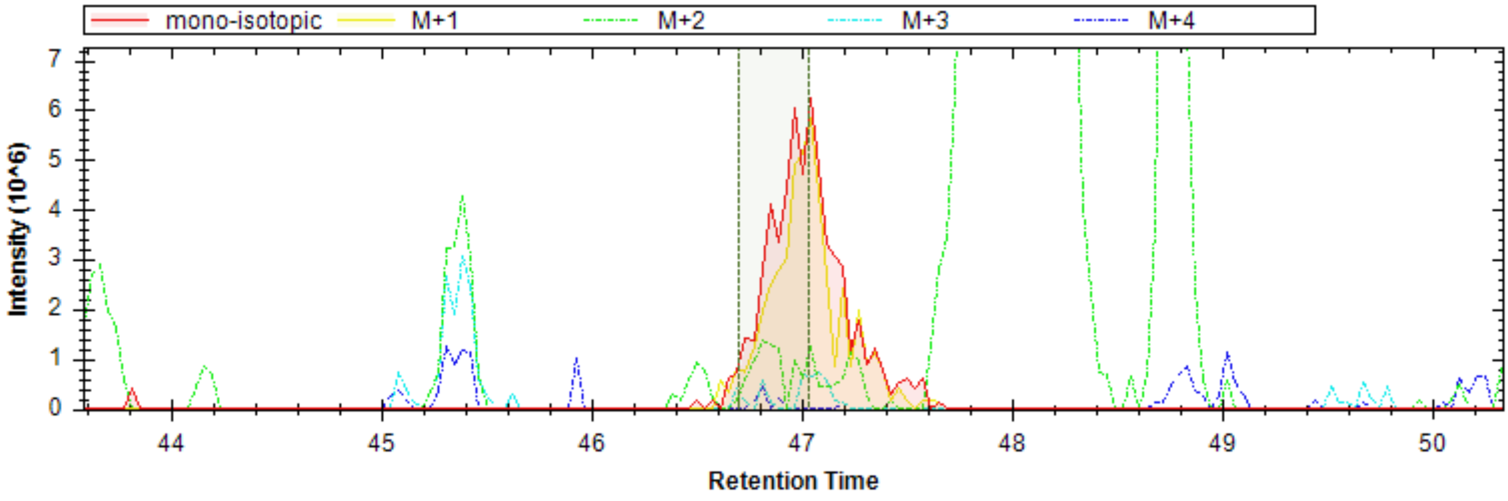

Q9QYC0 – _AAVVTS[+H+O3+P]PPPTTAPHK_.3

Fig4_MP-DIA-barrel-cortex-p9-2_MHRM.htrms

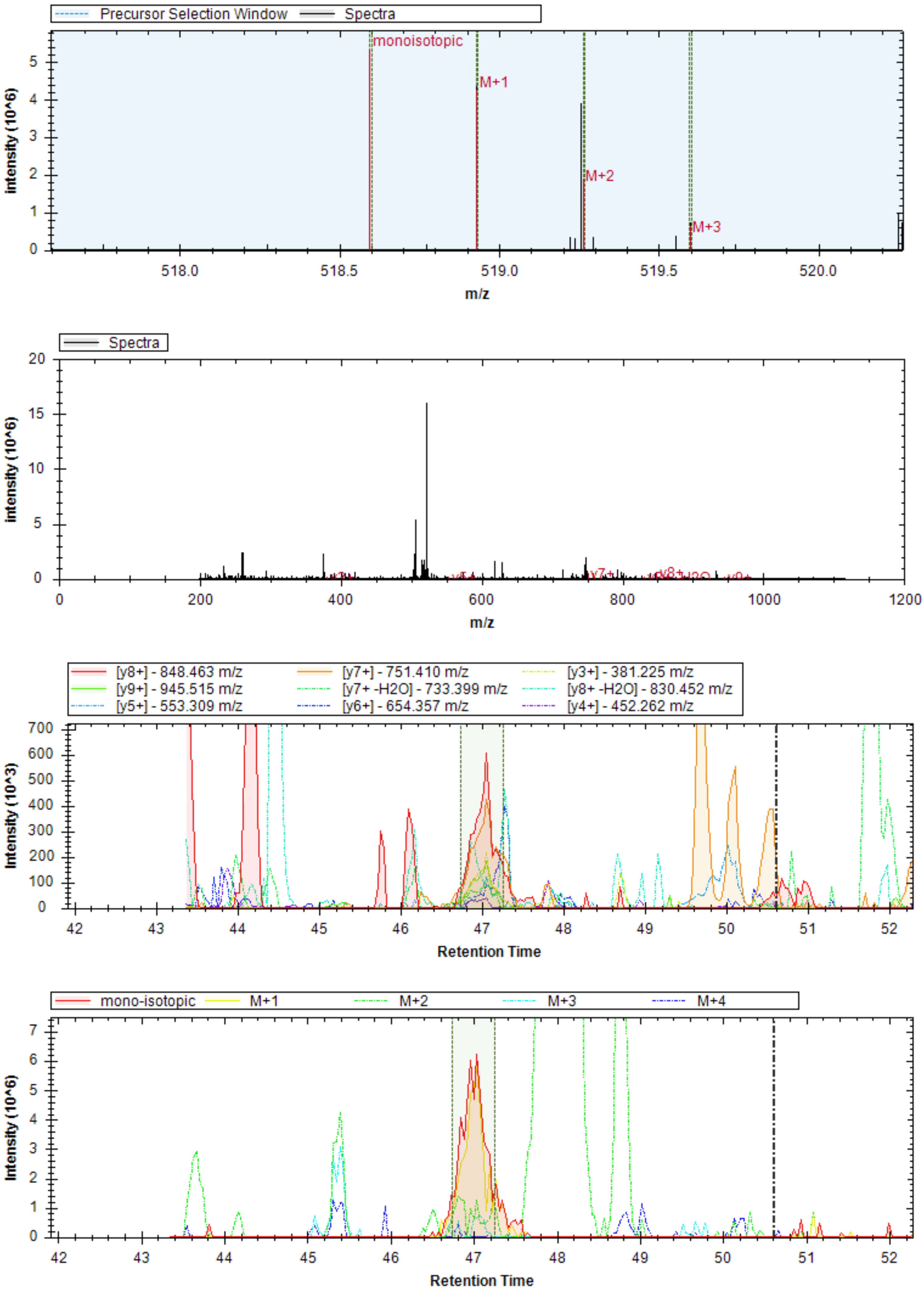

P10637 – _T[+H+O3+P]PPKSPSASK_.2

Fig4_MP-DIA-barrel-cortex-p9-2_MHRM.htrms

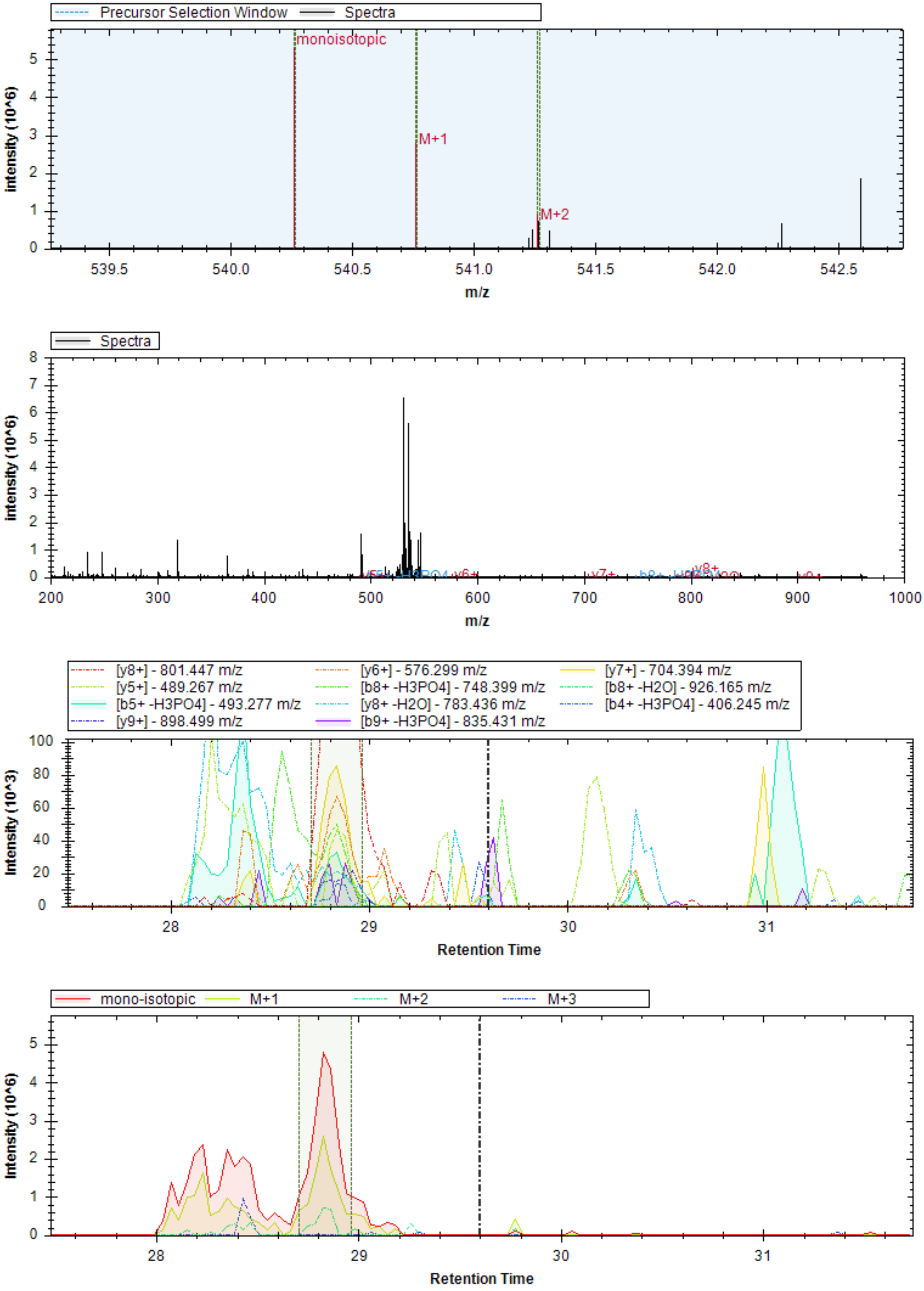

P10637 – _TPPKS[+H+O3+P]PSASK_.2

Fig4_MP-DIA-barrel-cortex-p9-2_MHRM.htrms

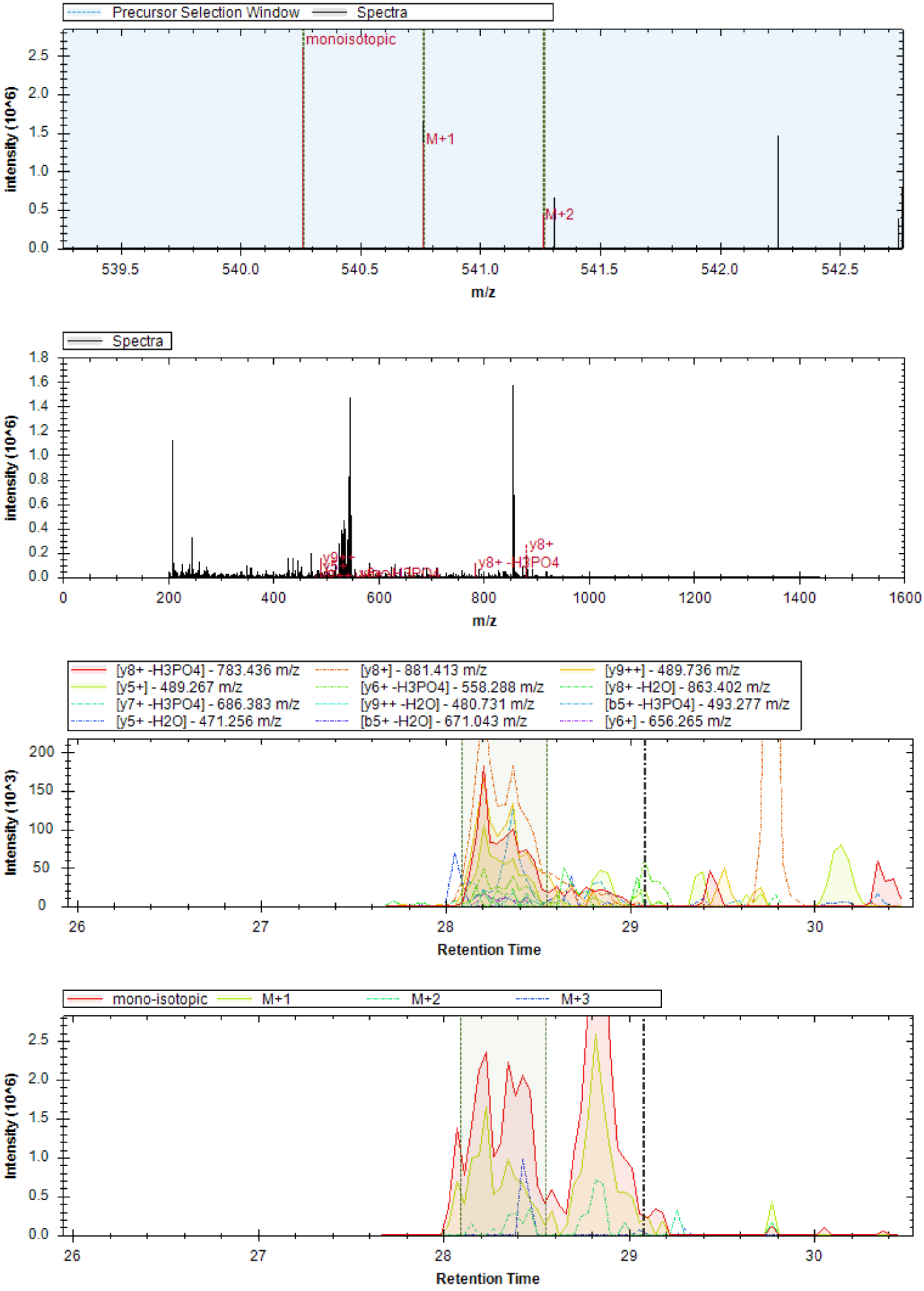

P10637 – _VAVVRT[+H+O3+P]PPKSPSASK_.3

Fig4_MP-DIA-barrel-cortex-p9-2_MHRM.htrms

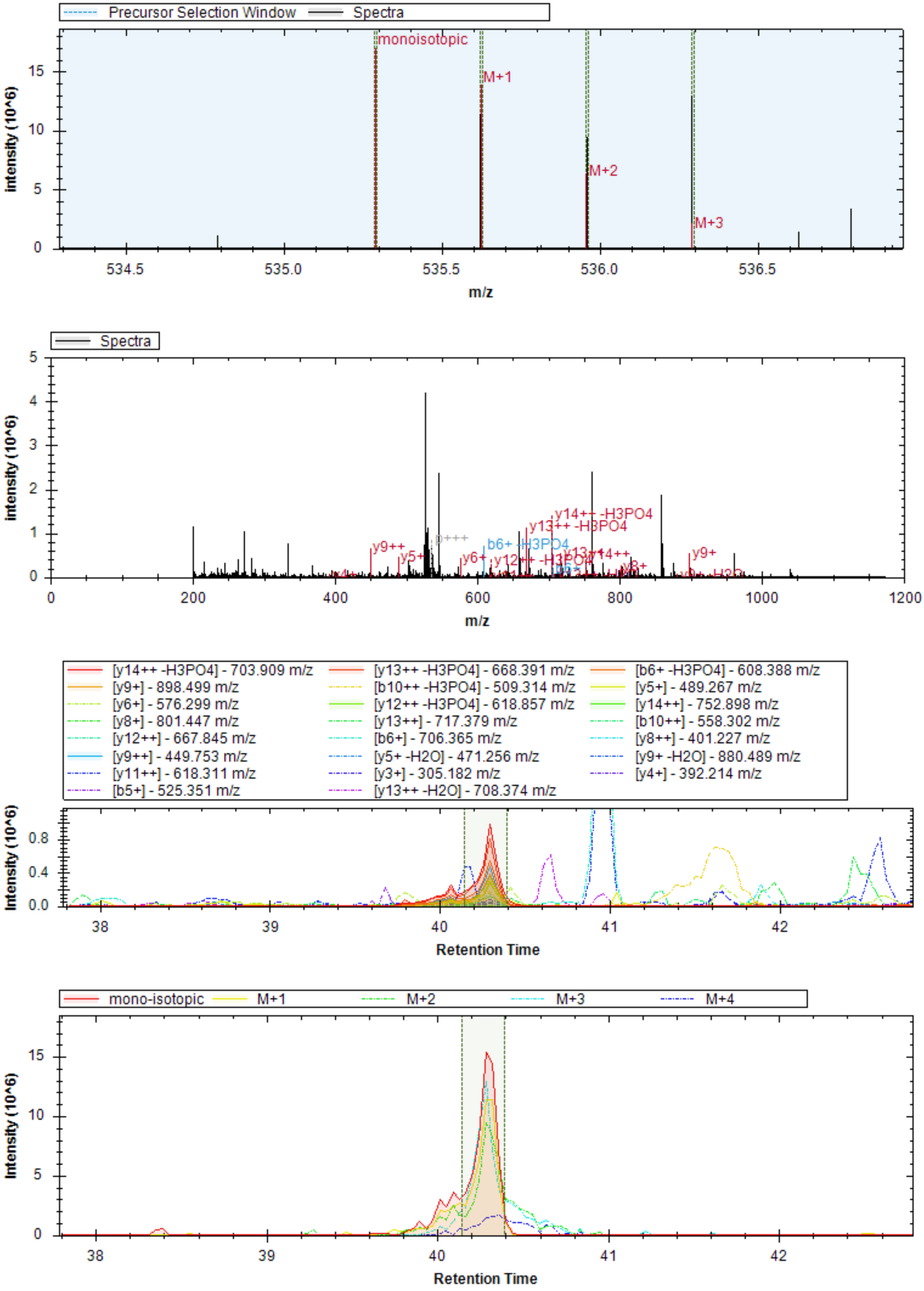

P54227 – _RAS[+H+O3+P]GQAFELILSPR_.3
Fig4_MP-DIA-barrel-cortex-p9-2_MHRM.htrms

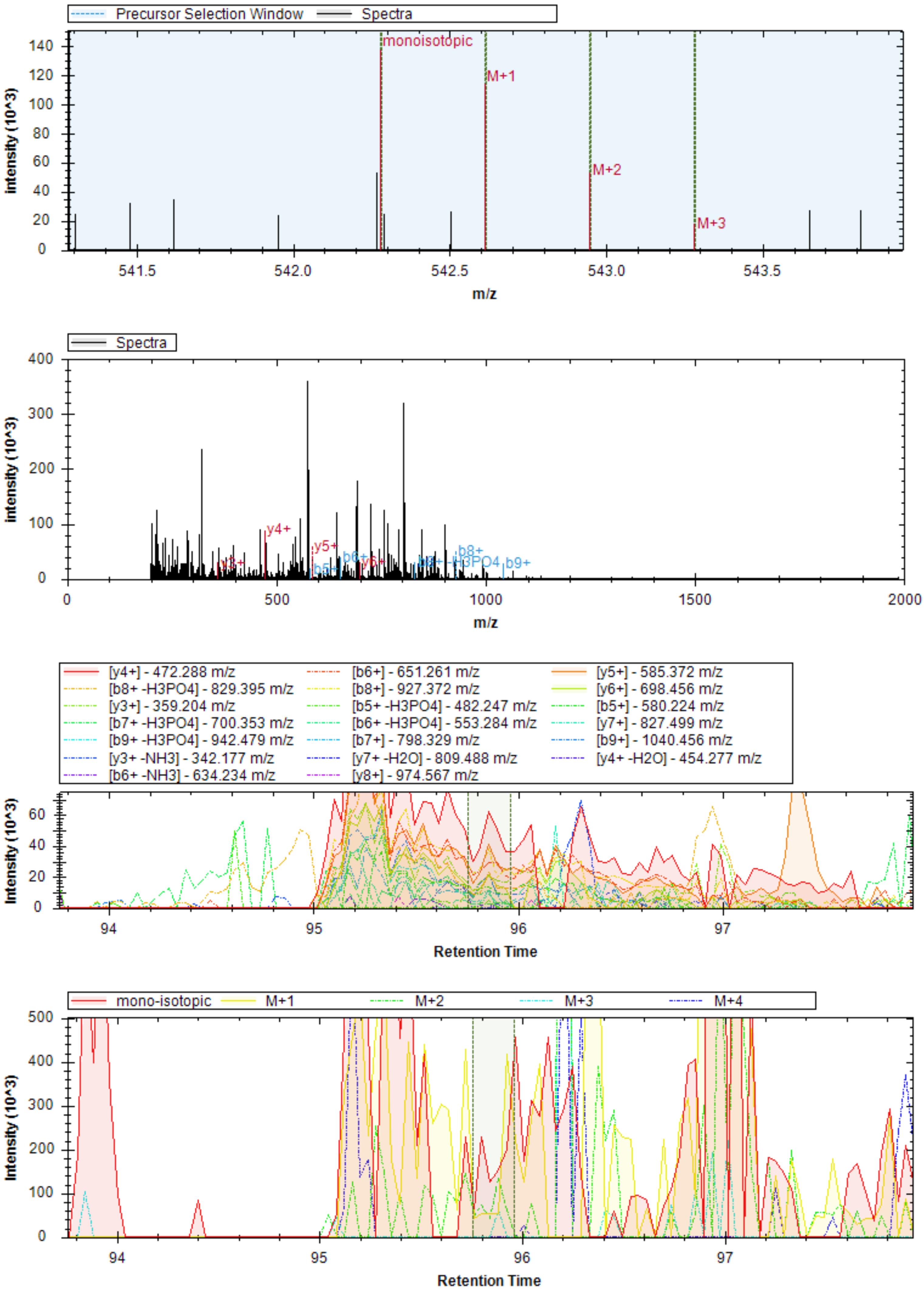

P54227 – _SKESVPDFPLS[+H+O3+P]PPK_.3

Fig4_MP-DIA-barrel-cortex-p9-2_MHRM.htrms

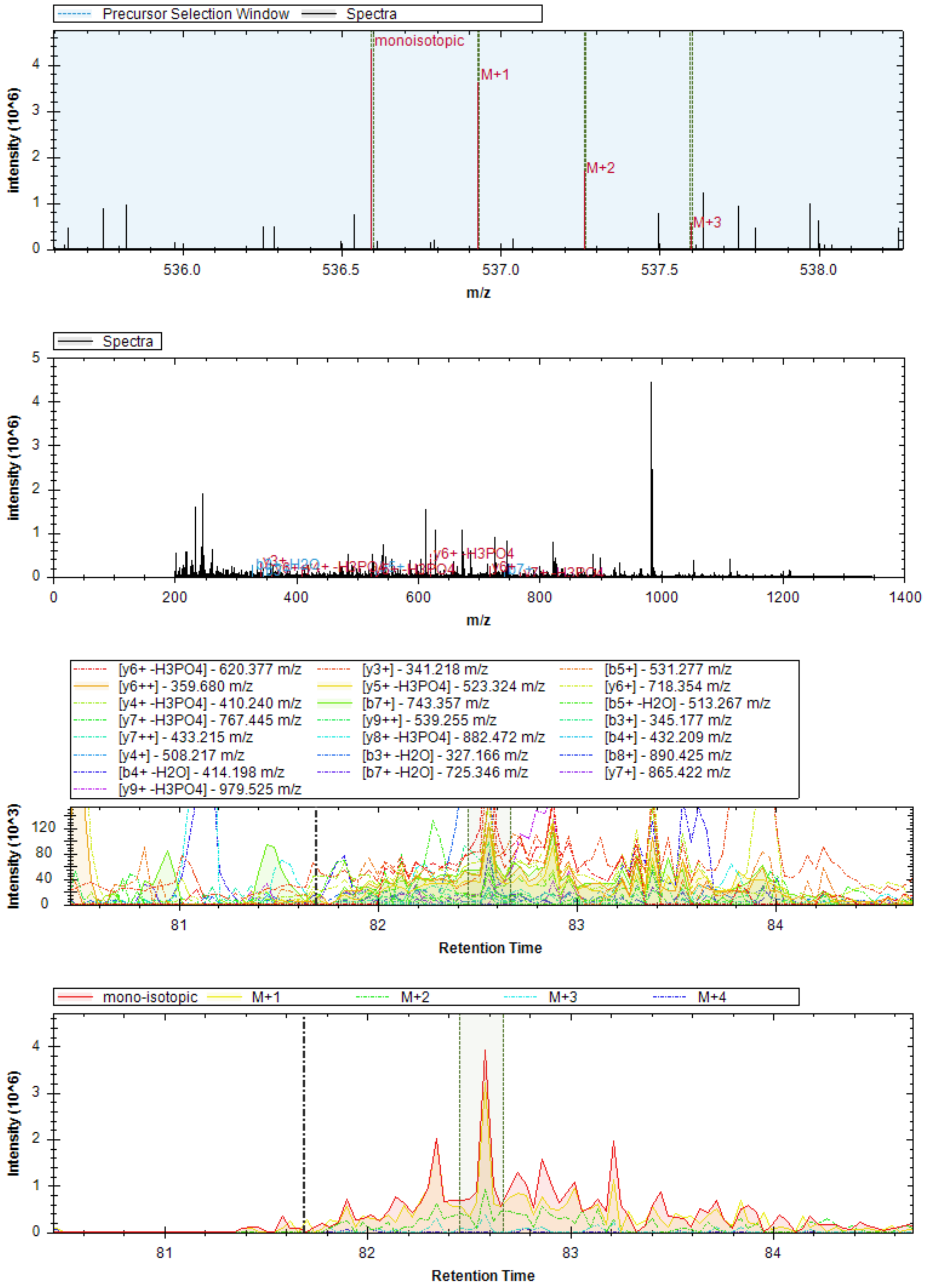

Q7TQD2 – _AANKT[+H+O3+P]PPKSPGDPAR_.3
Fig4_MP-DIA-barrel-cortex-p9-2_MHRM.htrms

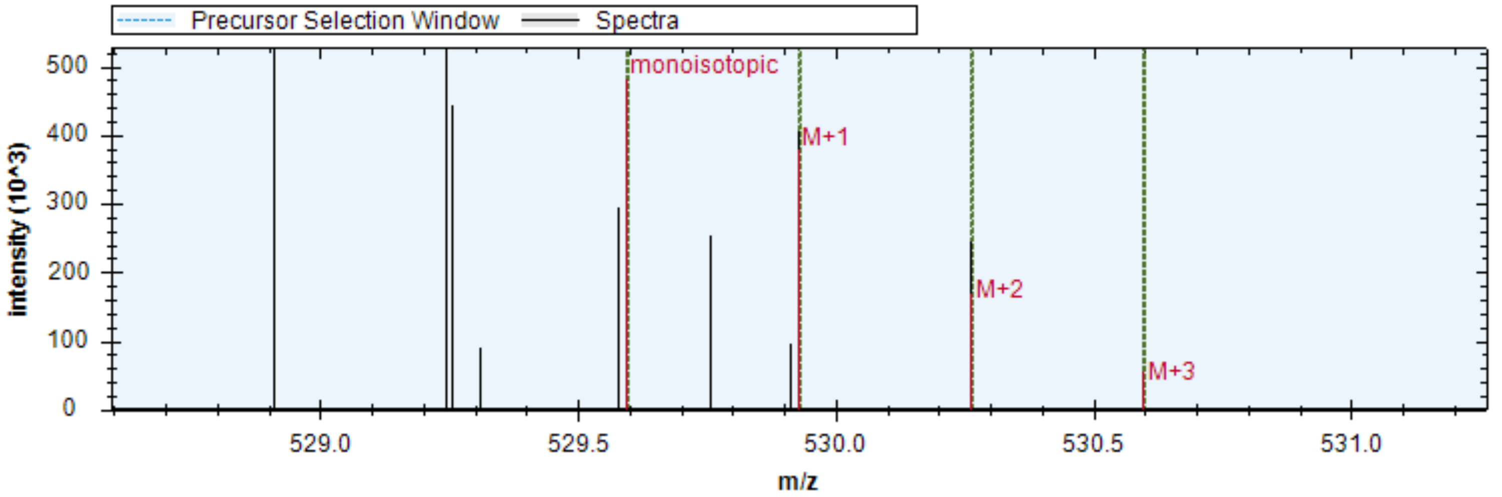

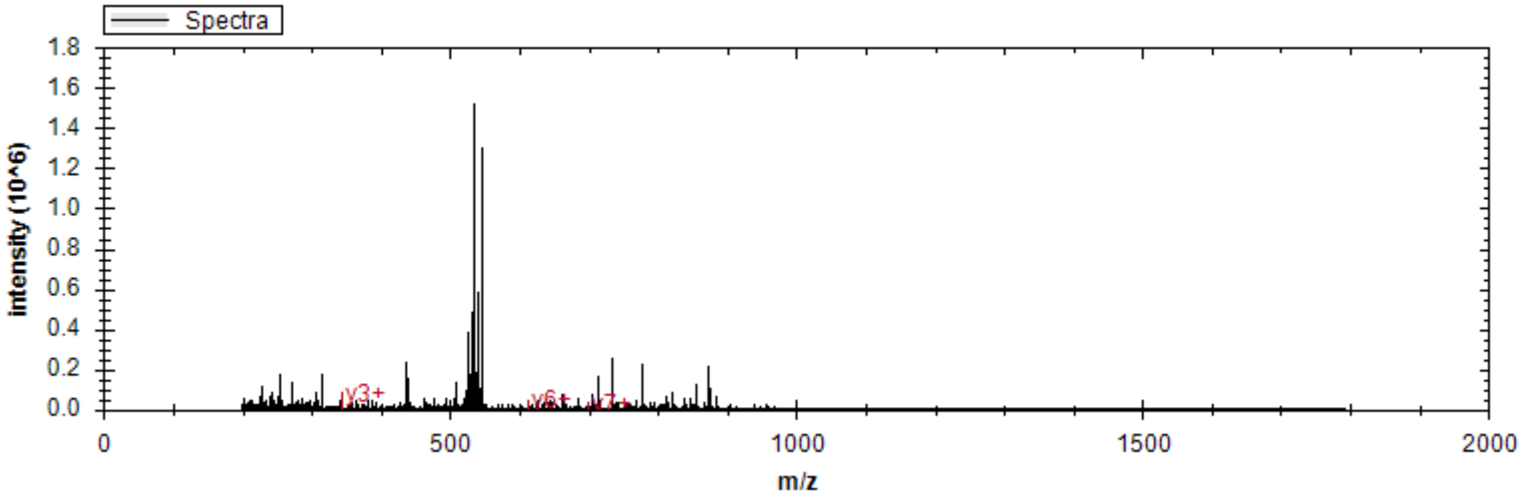

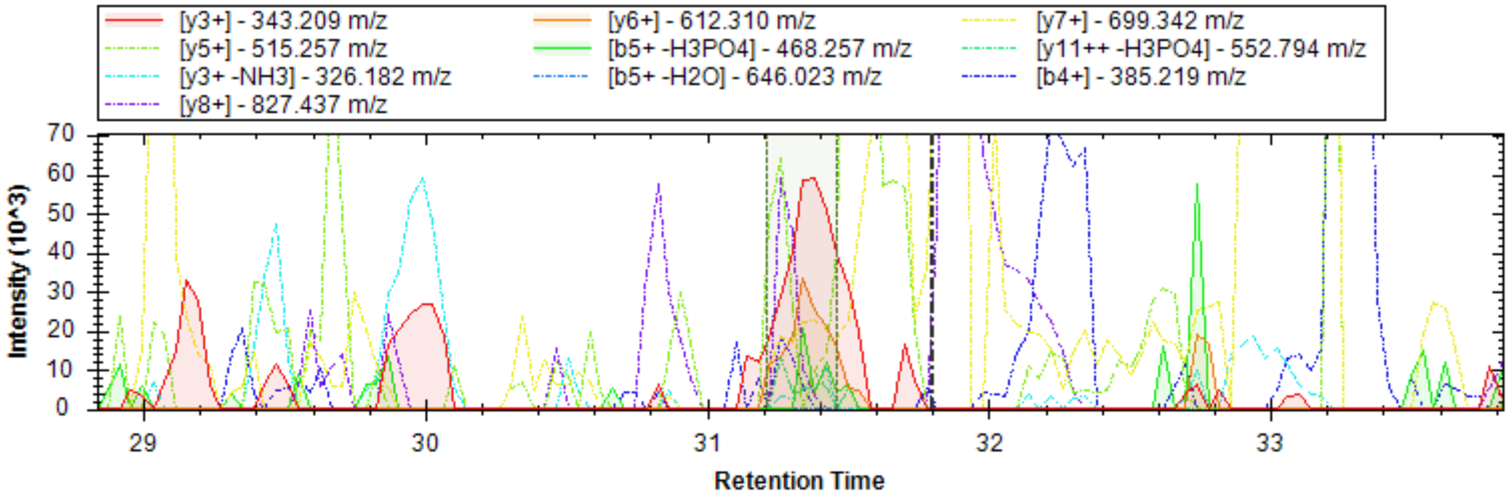

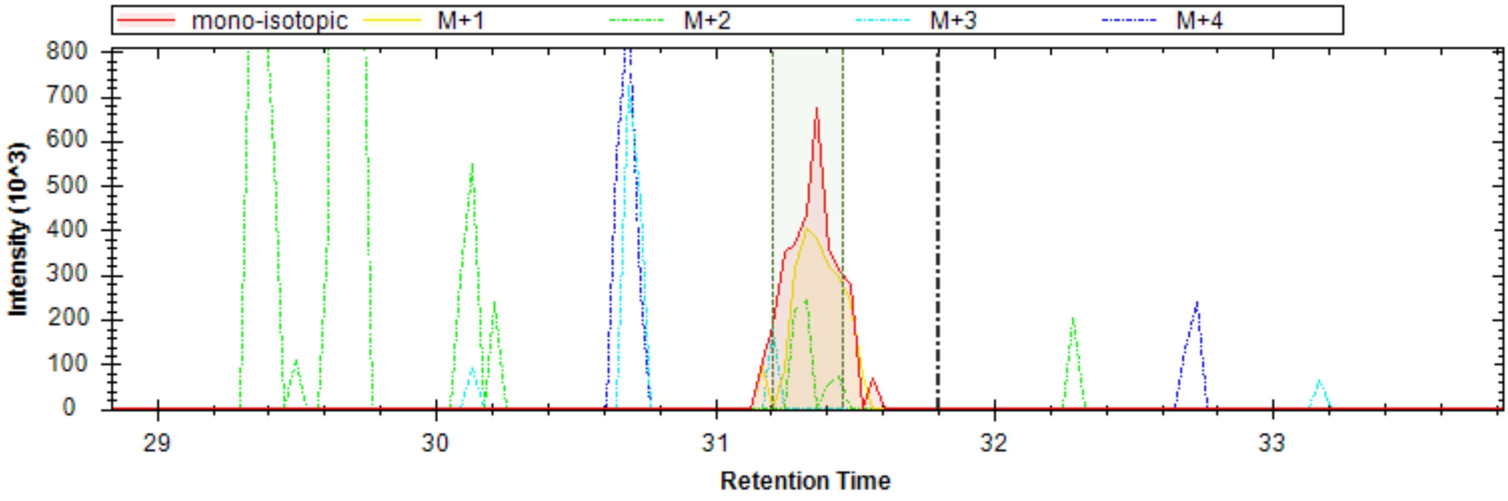

P10637 – _TTPSPKT[+H+O3+P]PPGSGEPPK_.3
Fig4_MP-DIA-barrel-cortex-p9-2_MHRM.htrms

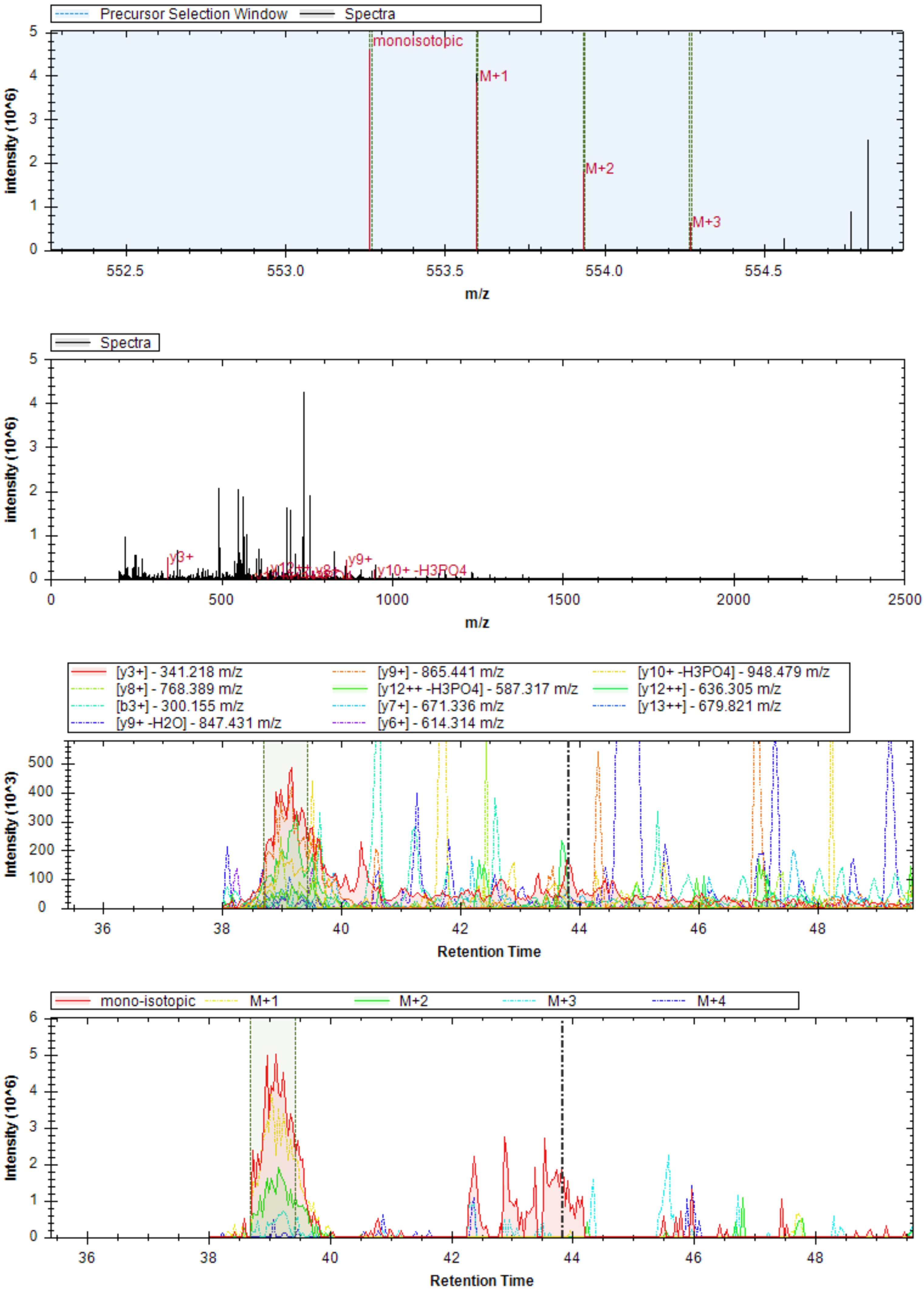

P20357 – _VAIIRT[+H+O3+P]PPKSPATPK_.3

Fig4_MP-DIA-barrel-cortex-p9-2_MHRM.htrms

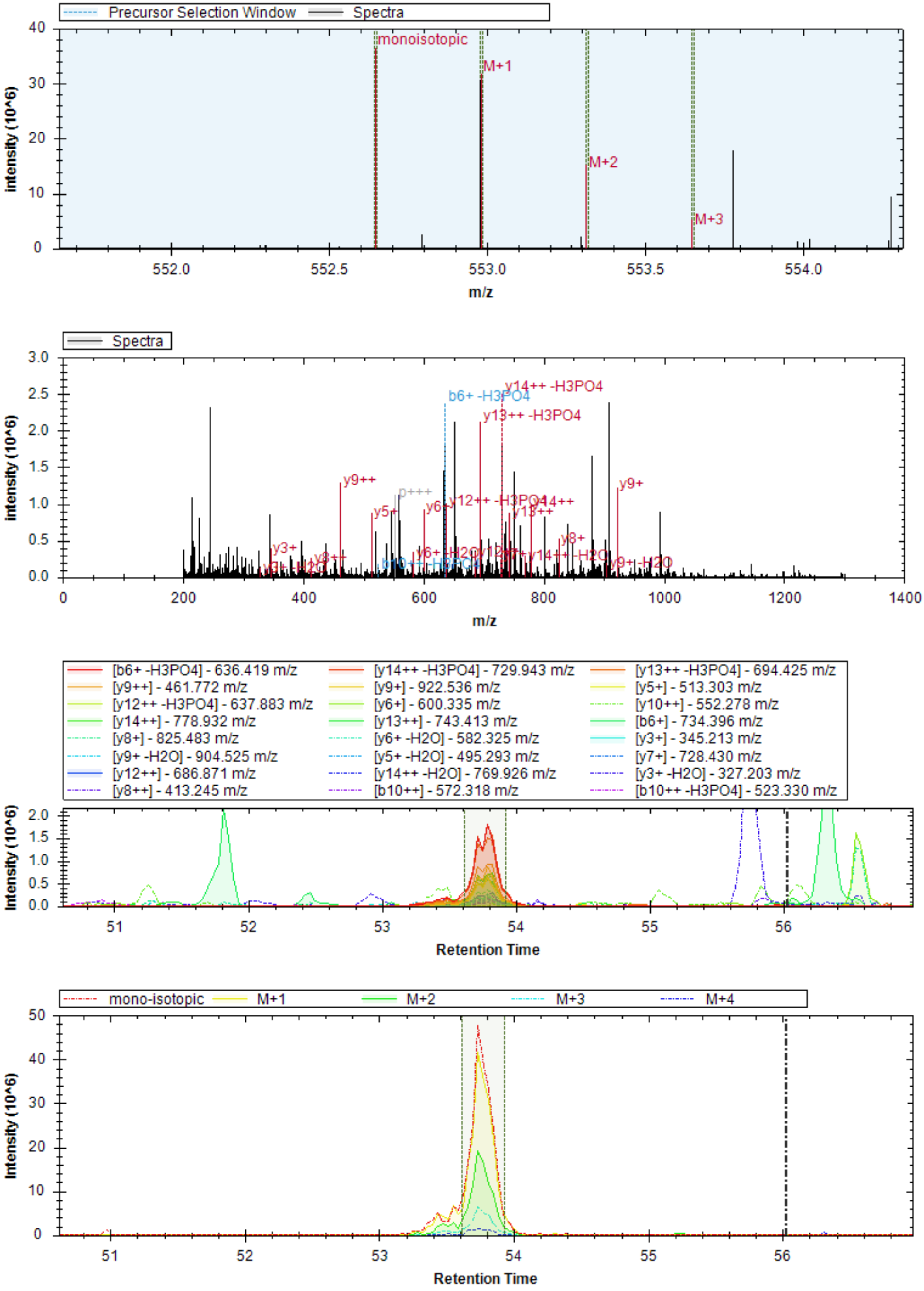

Q9CYT6 – _THTPSPTS[+H+O3+P]PK_.2
Fig4_MP-DIA-barrel-cortex-p9-2_MHRM.htrms

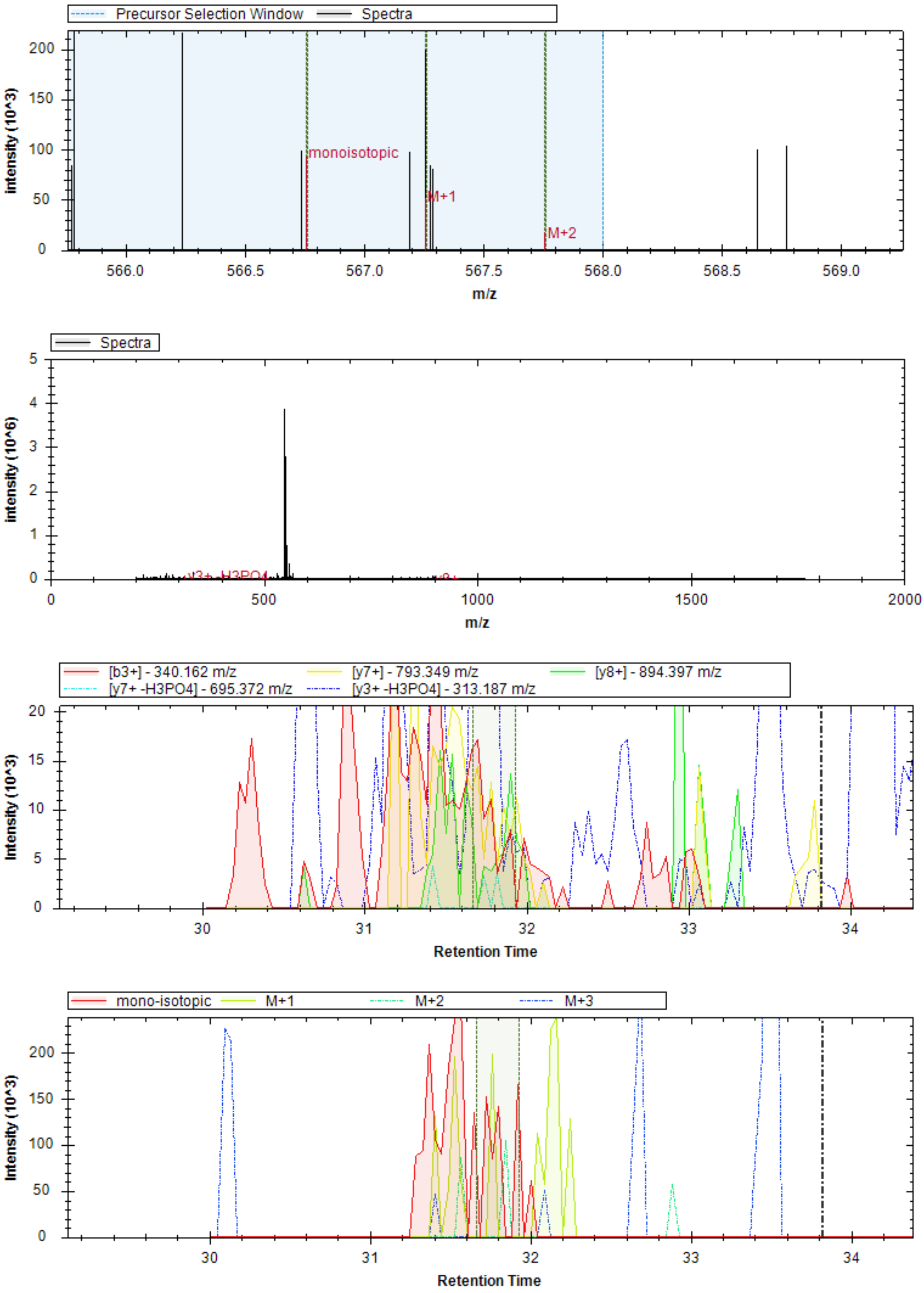

O88935 – _QAS[+H+O3+P]ISGPAPTK_.2

Fig4_MP-DIA-barrel-cortex-p9-2_MHRM.htrms

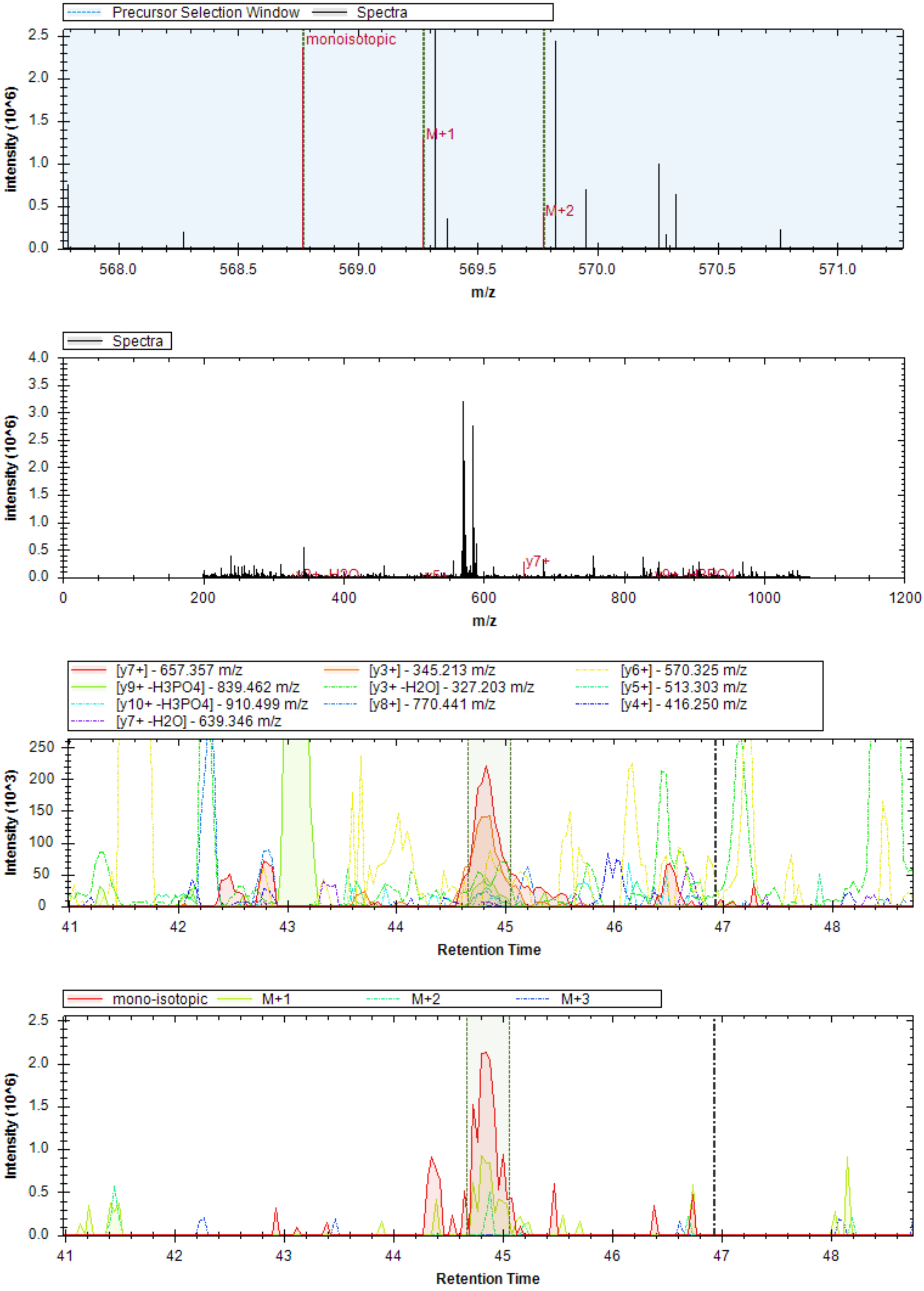

P27546-2 – _T[+H+O3+P]SPSKPSSAPALKPGPK_.3

Fig4_MP-DIA-barrel-cortex-p9-2_MHRM.htrms

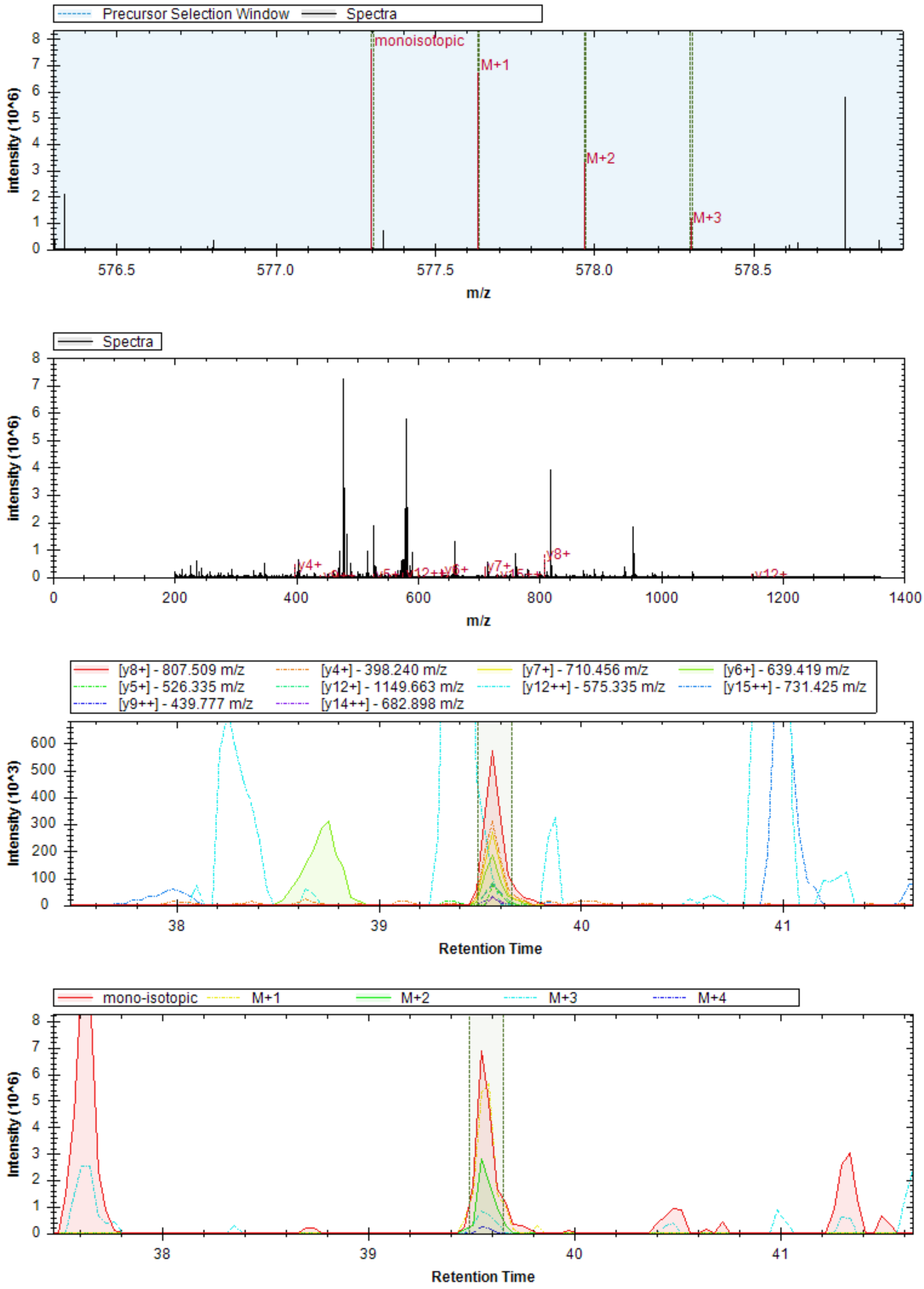

P28667 – _GEVAPKET[+H+O3+P]PK_.2

Fig4_MP-DIA-barrel-cortex-p9-2_MHRM.htrms

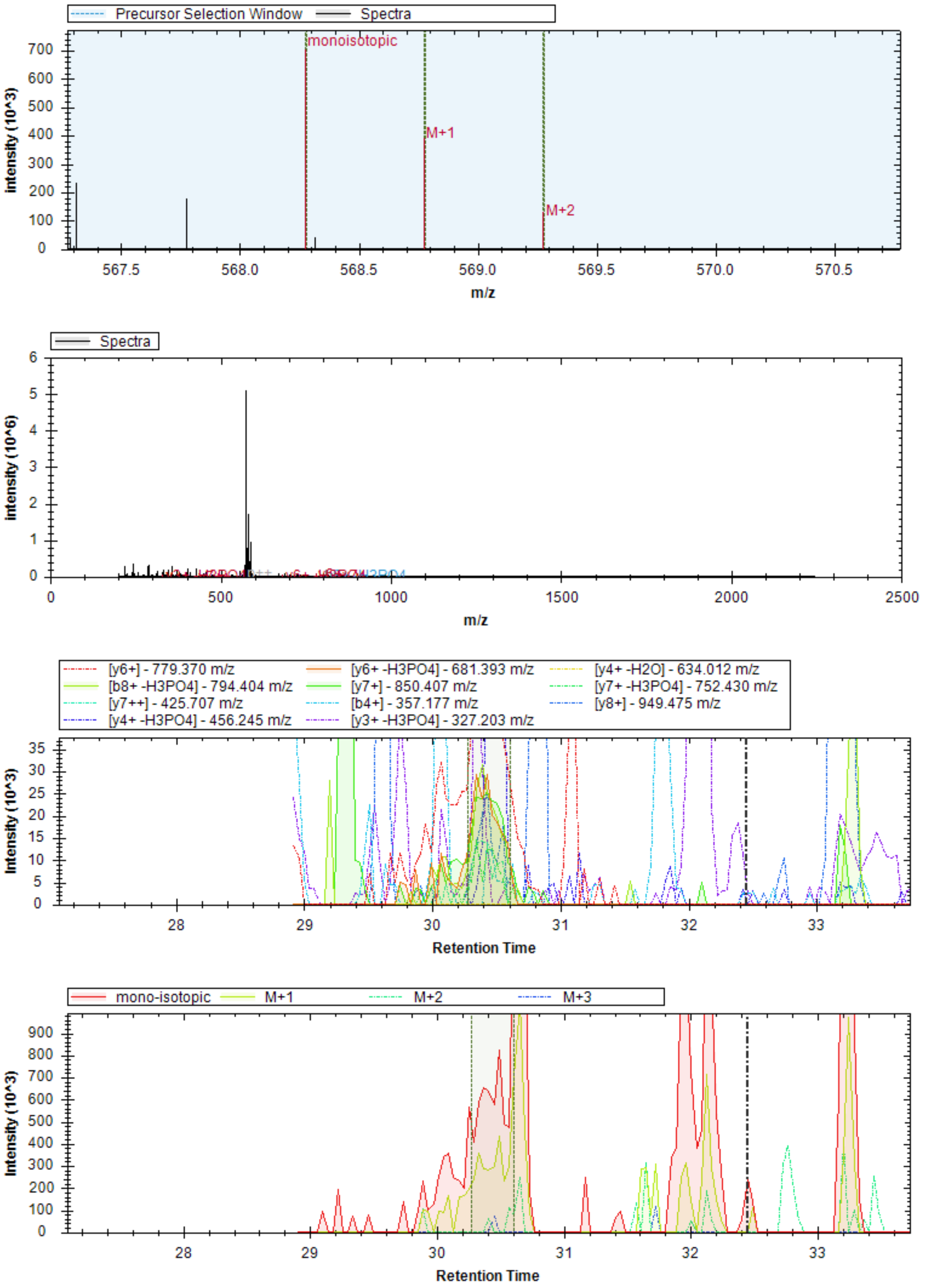

P97792 – _APQS[+H+O3+P]PTLAPAK_.2

Fig4_MP-DIA-barrel-cortex-p9-2_MHRM.htrms

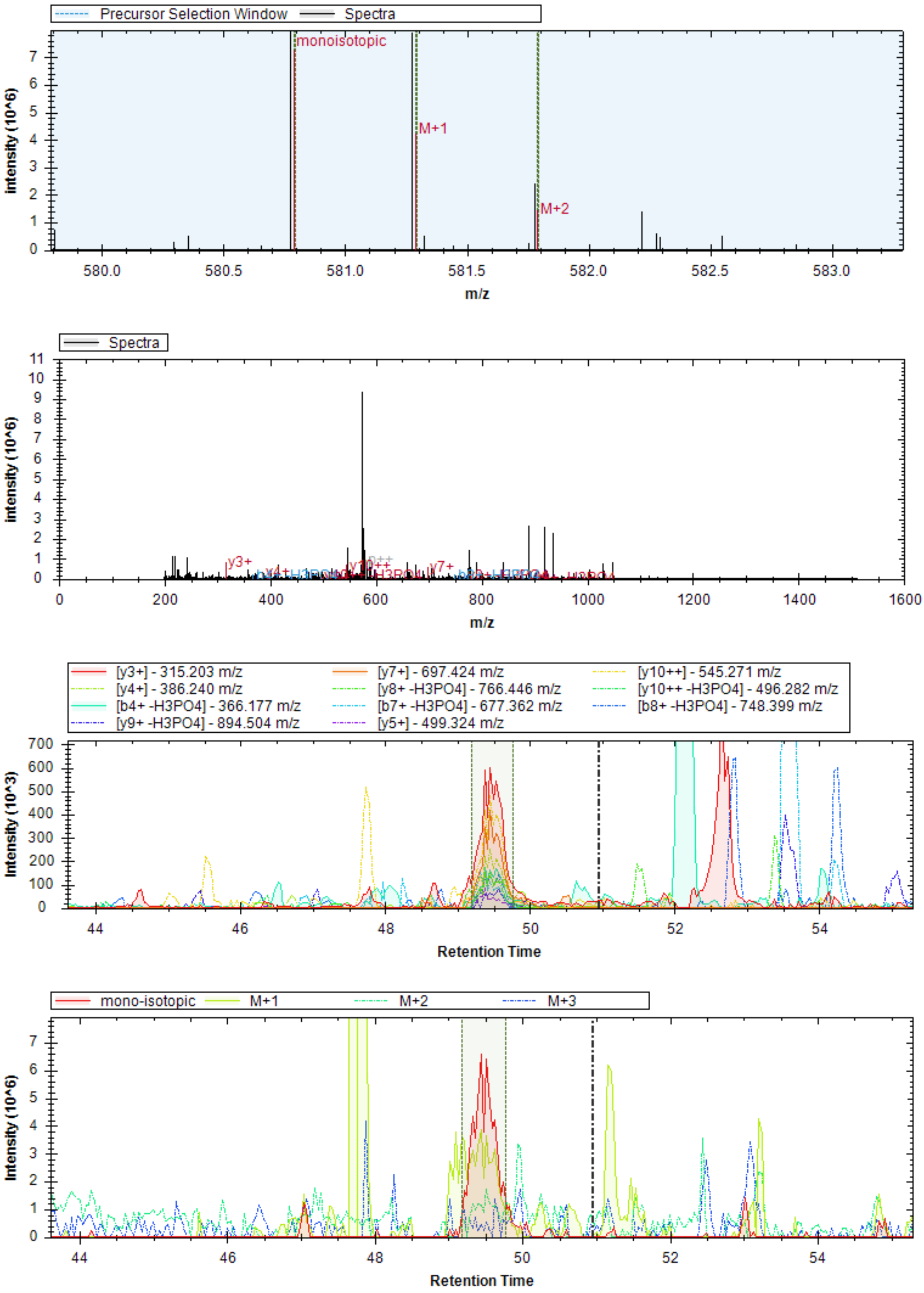

Q64012-2 – _GRLS[+H+O3+P]PVPVPR_.2
Fig4_MP-DIA-barrel-cortex-p9-2_MHRM.htrms

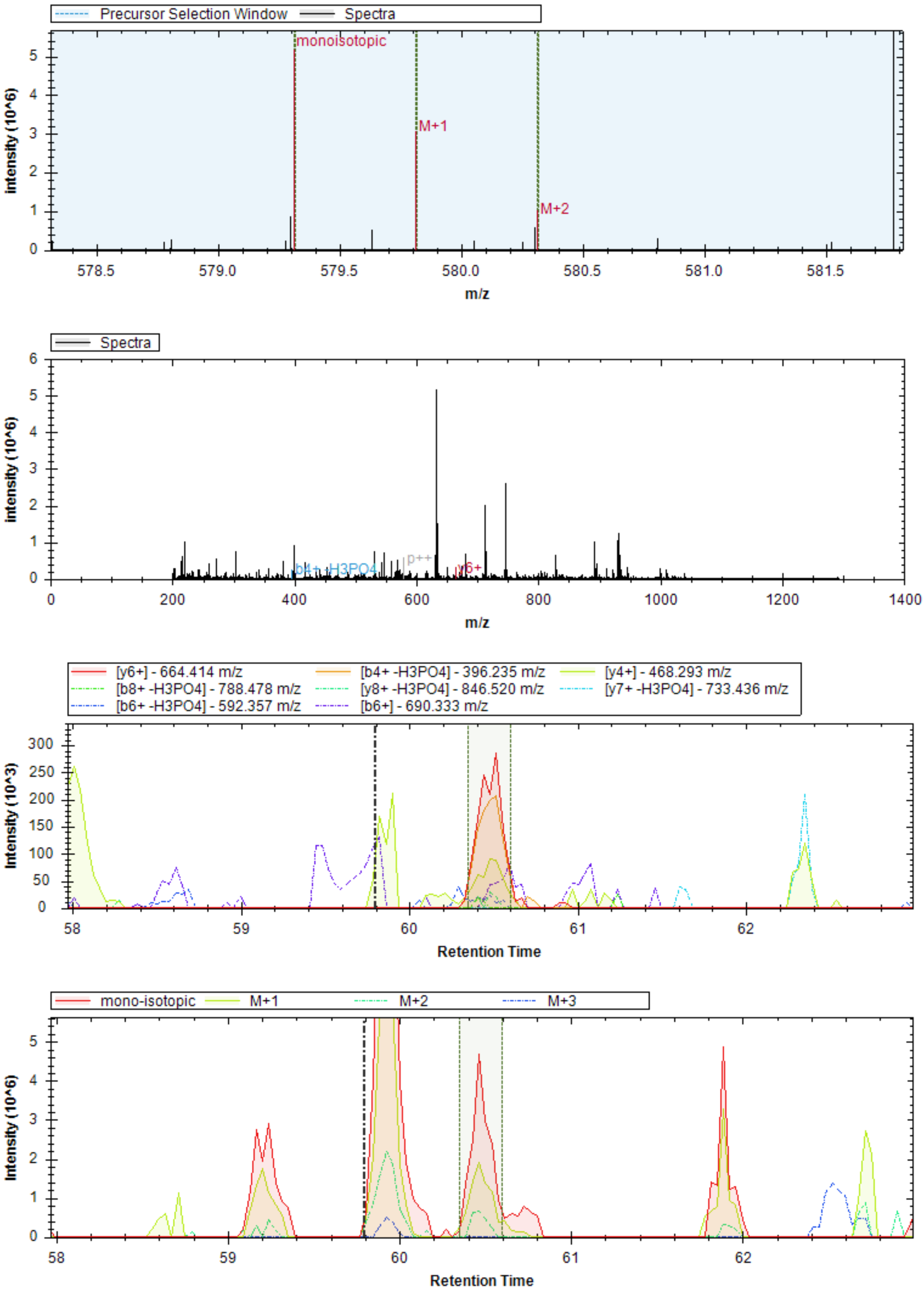

Q8BFU3-4 – _VAS[+H+O3+P]STQVRGASGRELR_.3
Fig4_MP-DIA-barrel-cortex-p9-2_MHRM.htrms

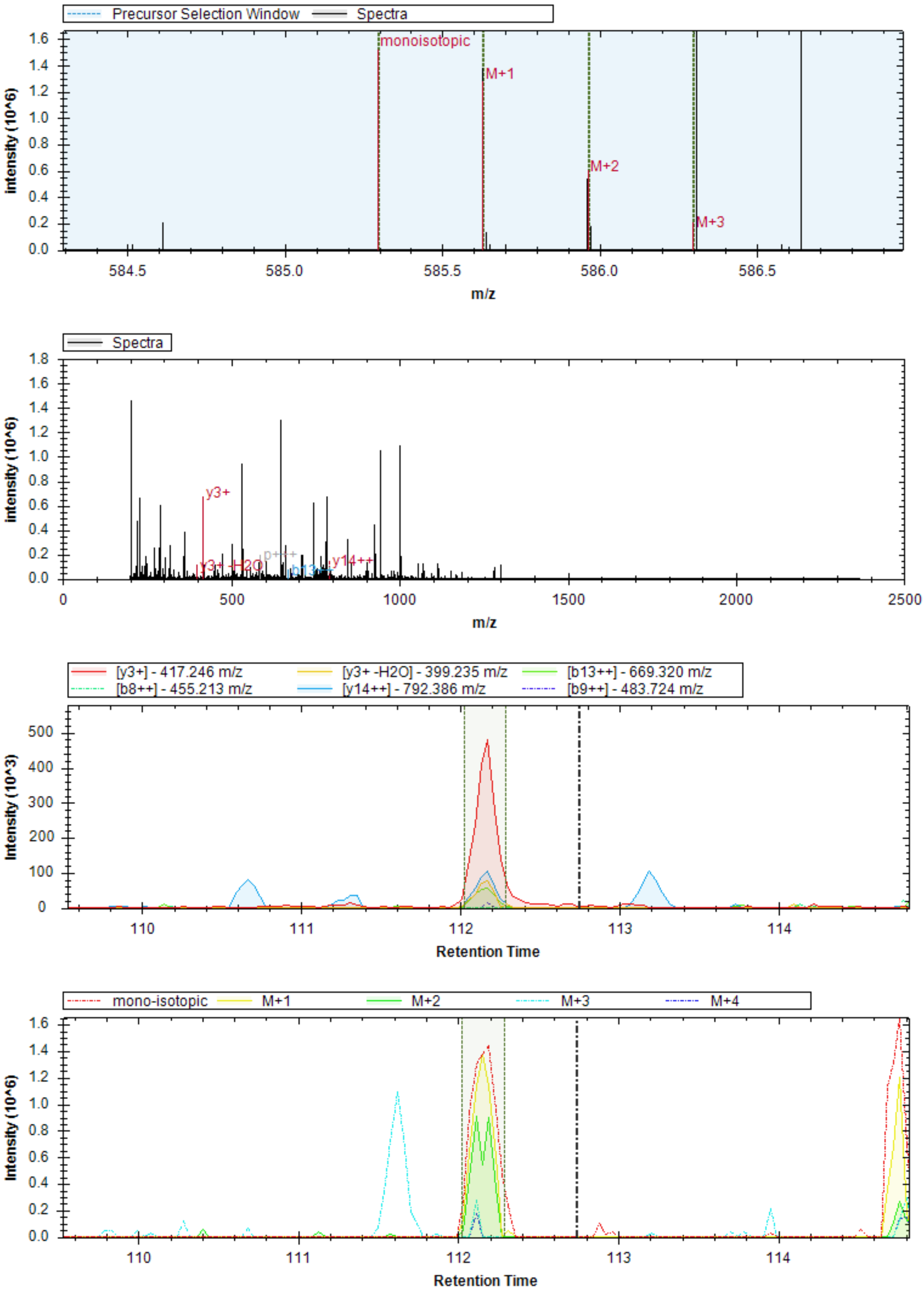

Q8BTI8 – _RVPS[+H+O3+P]PTPVPK_.2

Fig4_MP-DIA-barrel-cortex-p9-2_MHRM.htrms

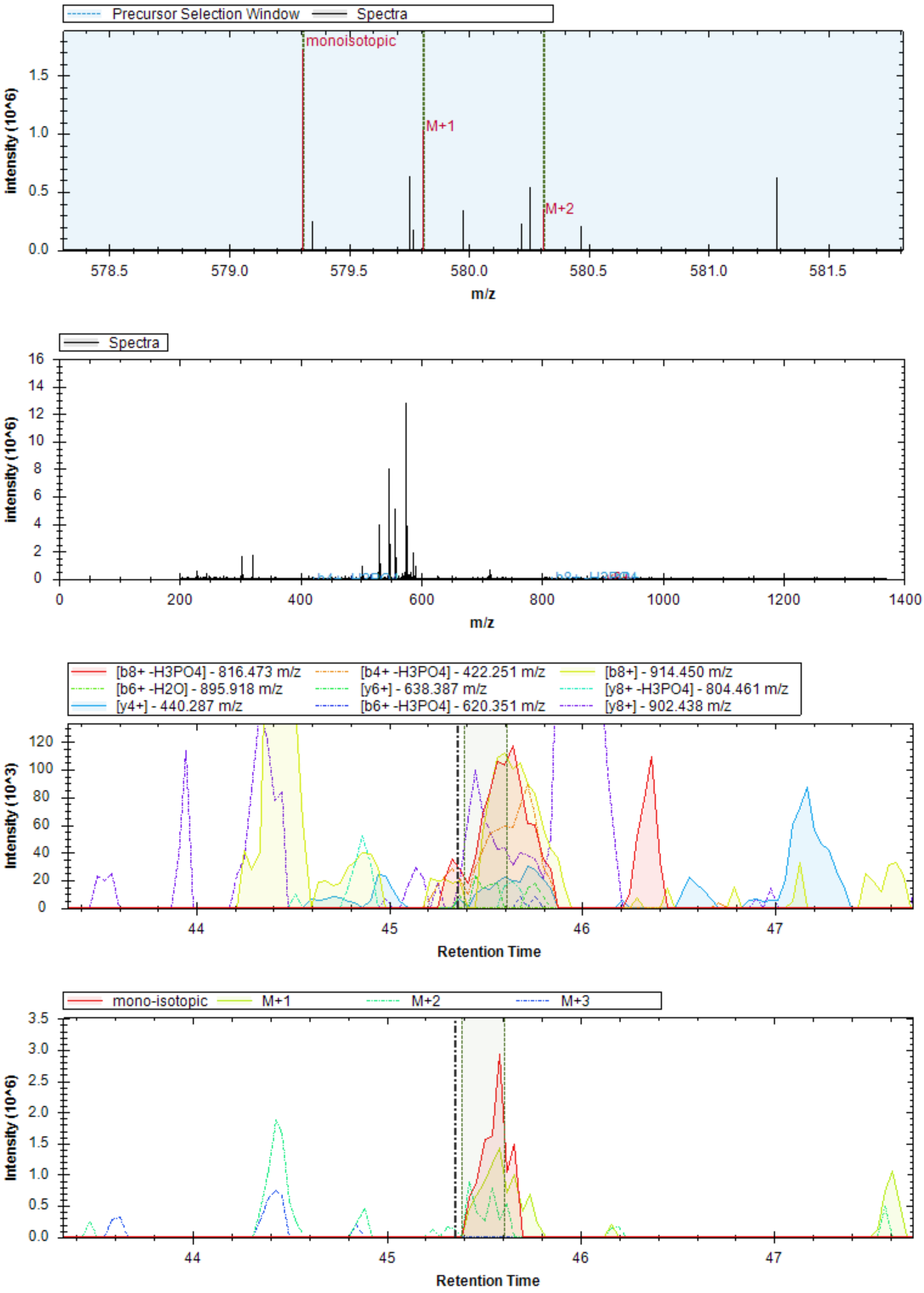

Q9CYT6 – _AQGQIRS[+H+O3+P]PTK_.2
Fig4_MP-DIA-barrel-cortex-p9-2_MHRM.htrms

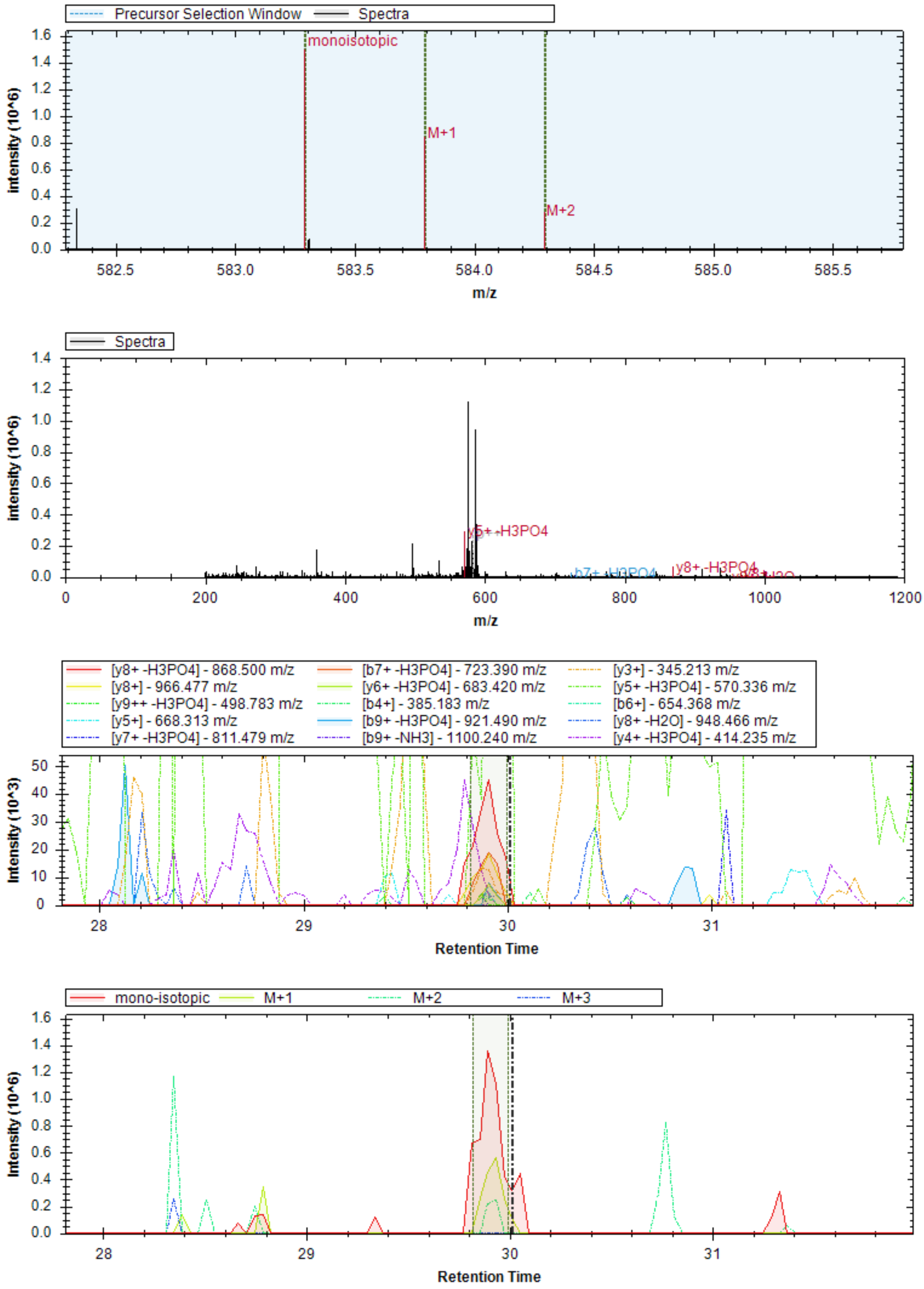

Q9DC63;Q9DC63-2;Q9DC63-3 – _YIDHY[+H+O3+P]AAIK_.2
Fig4_MP-DIA-barrel-cortex-p9-2_MHRM.htrms

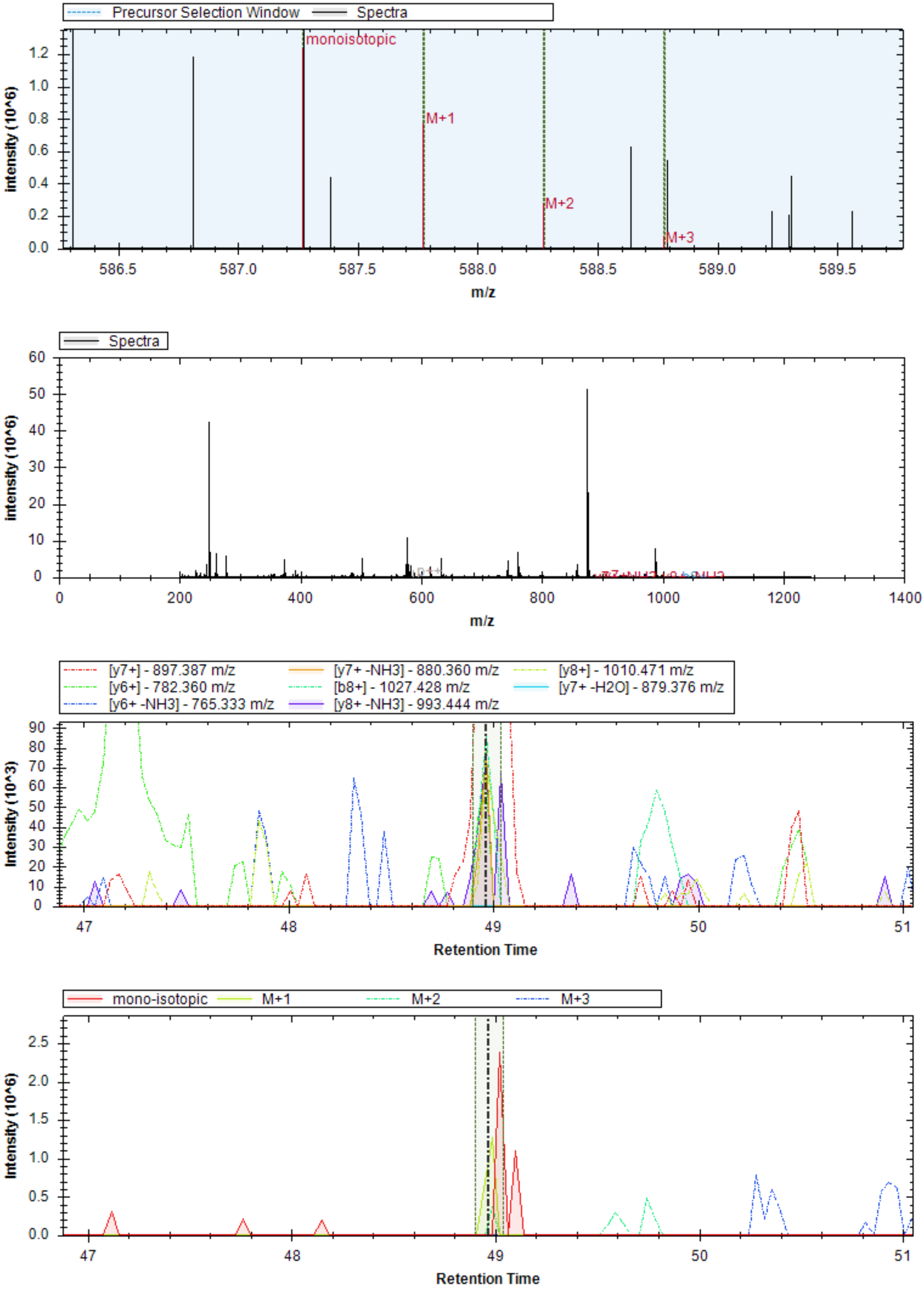

P20357 – _APHWTSASLTEAAAHPHS[+H+O3+P]PEMK_.4

Fig4_MP-DIA-barrel-cortex-p9-2_MHRM.htrms

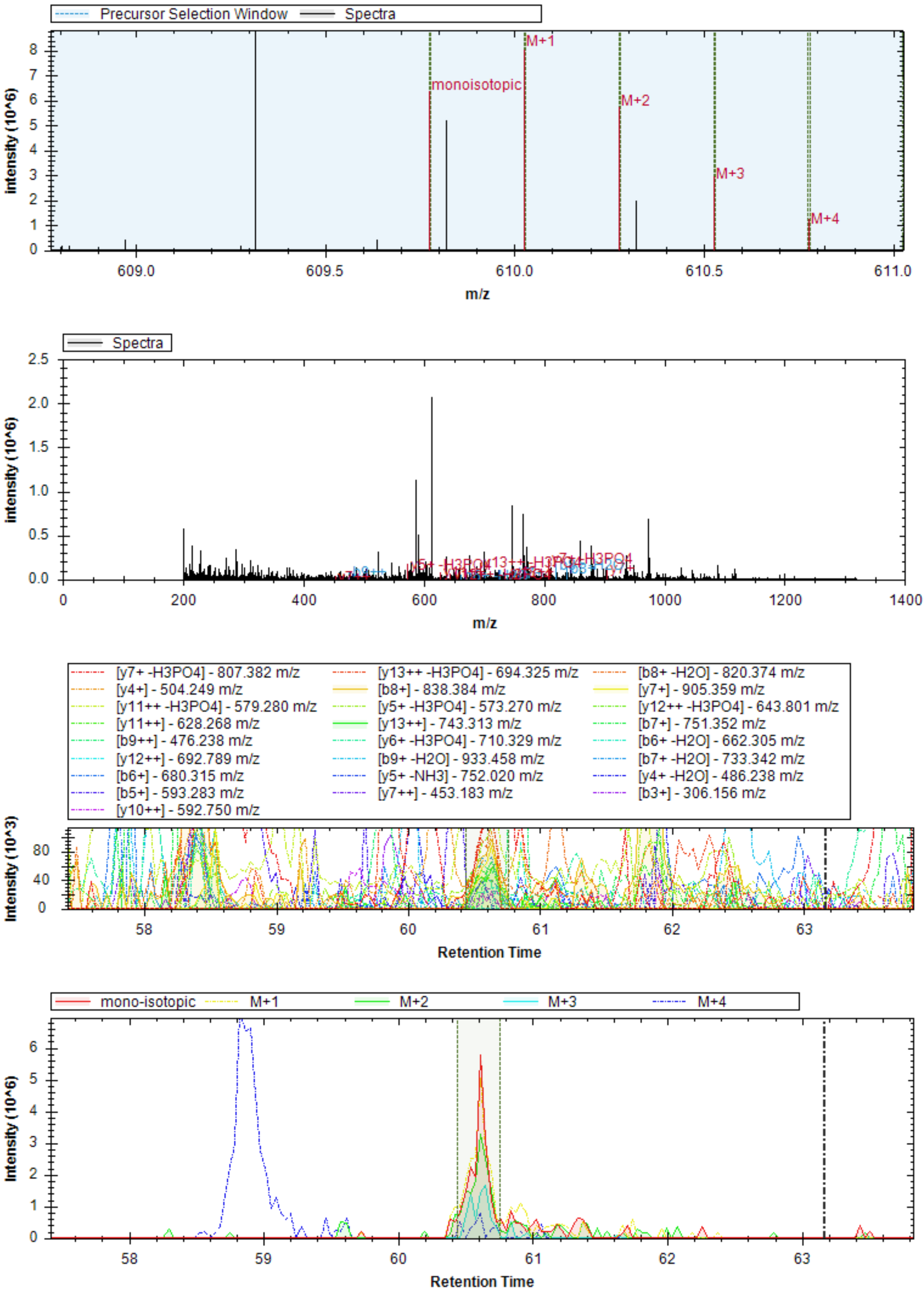

P26645 – _AEDGAAPSPSS[+H+O3+P]ETPKKK_.3
Fig4_MP-DIA-barrel-cortex-p9-2_MHRM.htrms

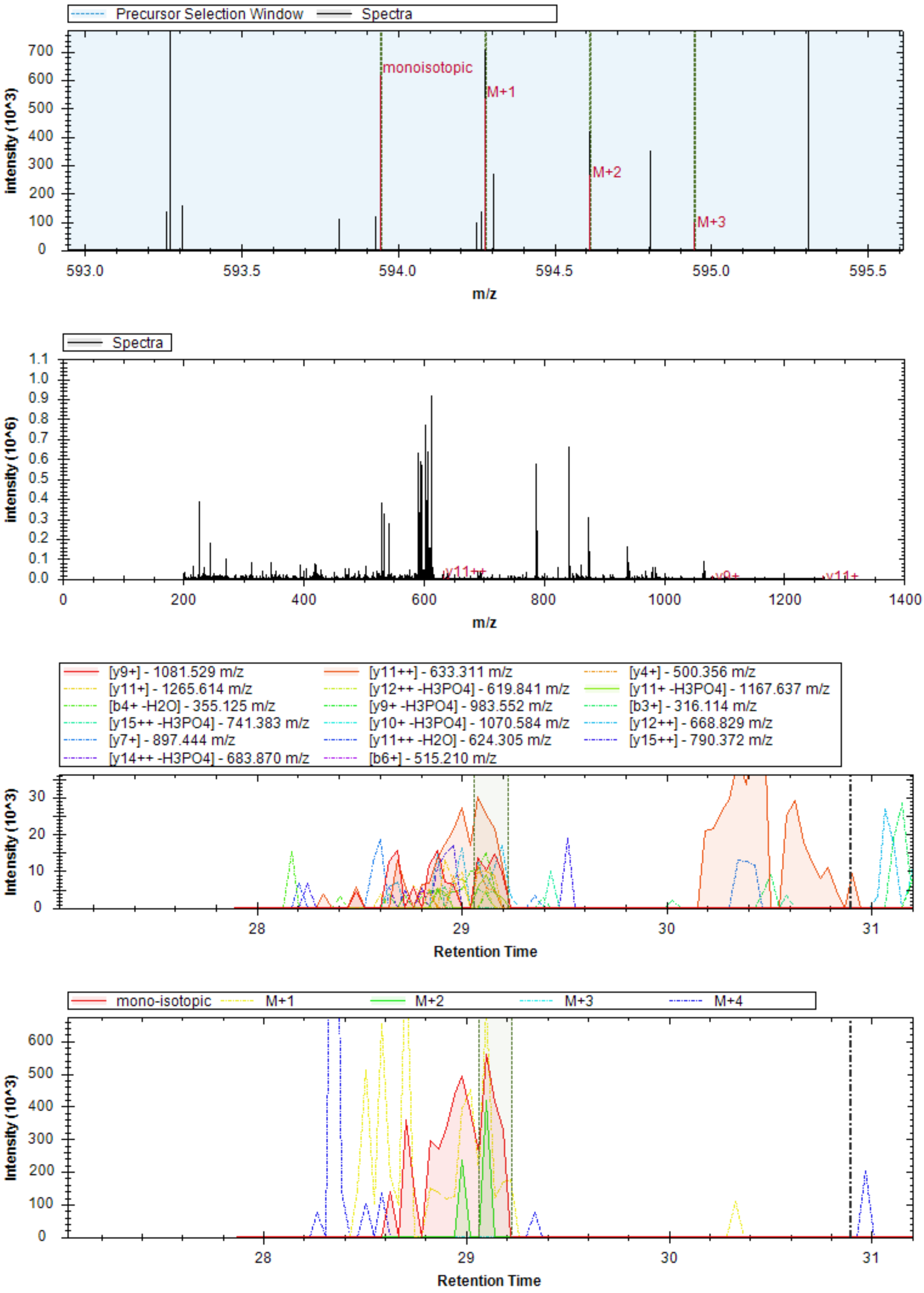

Q62261 – _RPPS[+H+O3+P]PDPNTK_.2
Fig4_MP-DIA-barrel-cortex-p9-2_MHRM.htrms

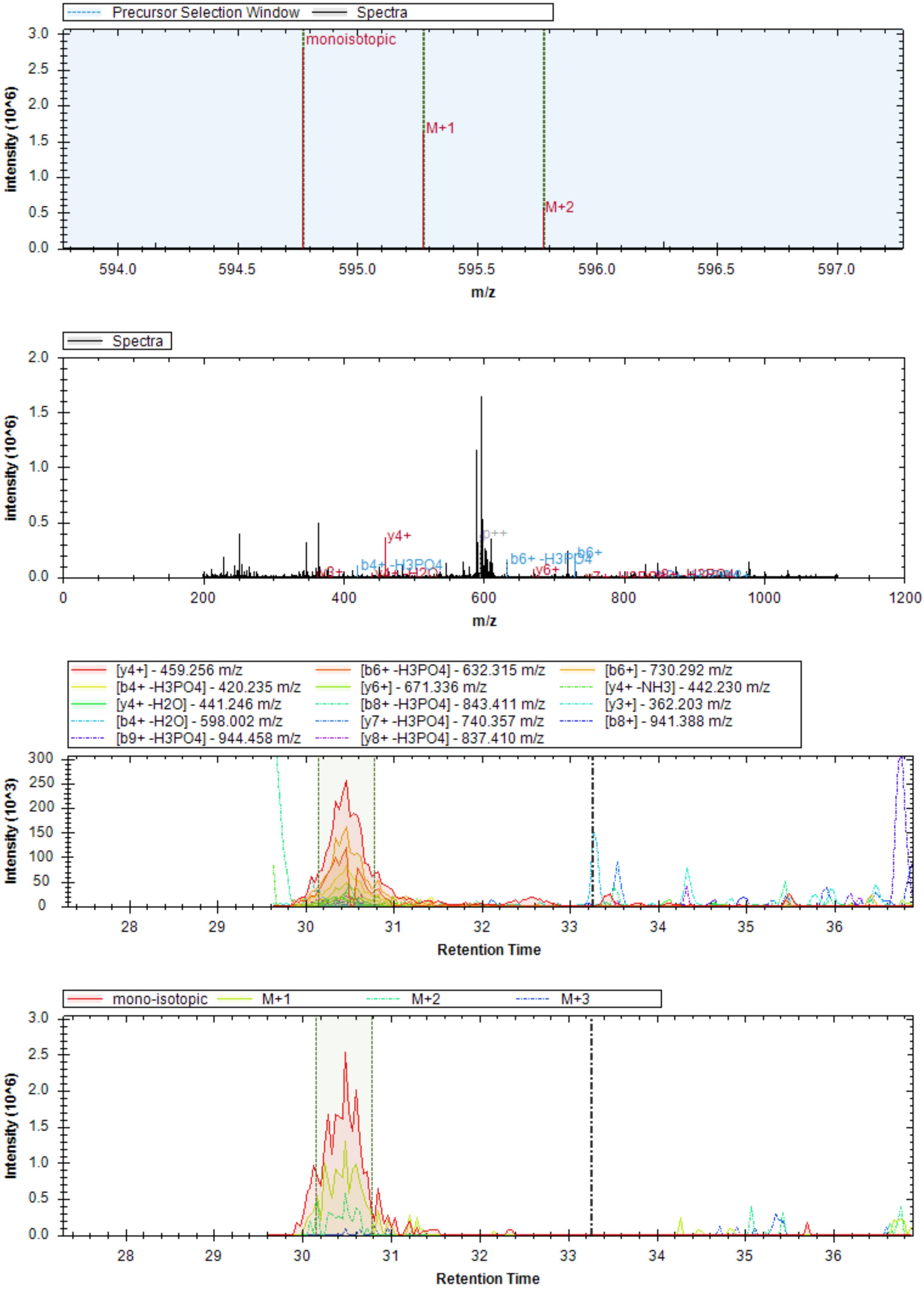

Q80VN0 – _ELTAQIT[+H+O3+P]SIK_.2
Fig4_MP-DIA-barrel-cortex-p9-2_MHRM.htrms

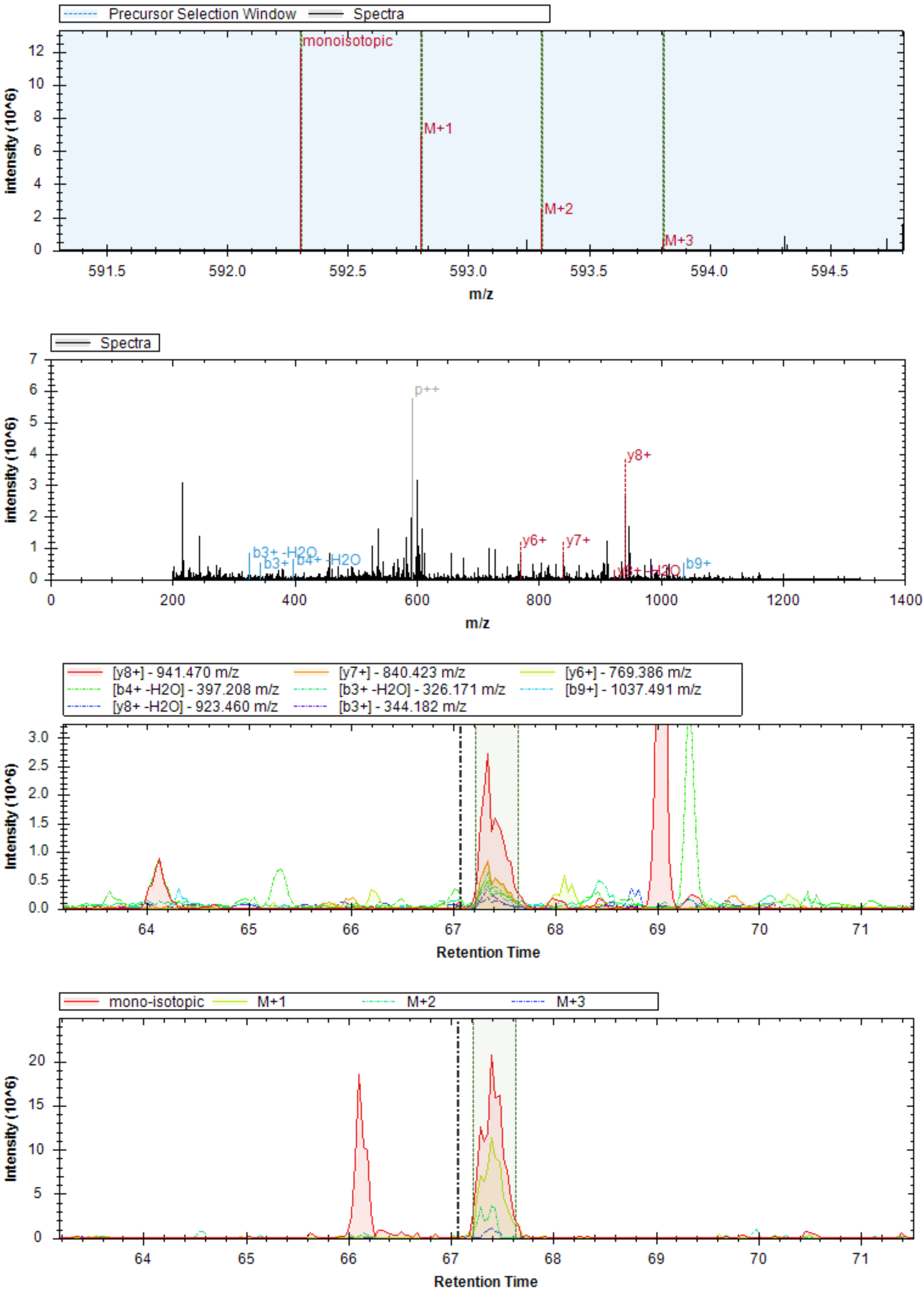

Q99104 – _AIS[+H+O3+P]PTSATSSGR_.2
Fig4_MP-DIA-barrel-cortex-p9-2_MHRM.htrms

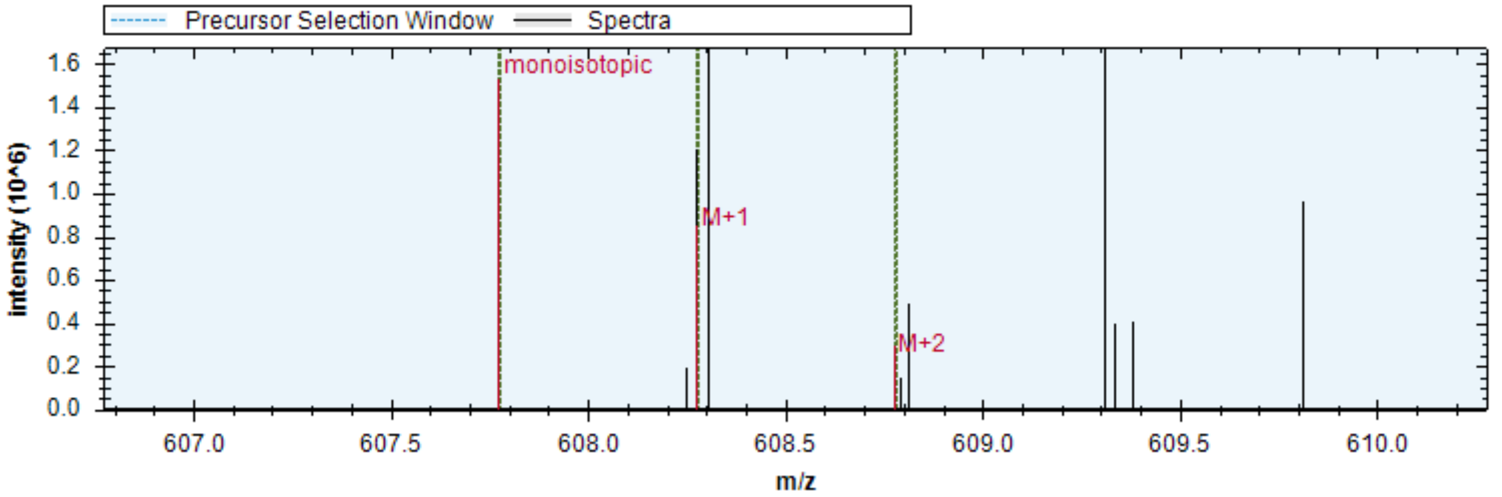

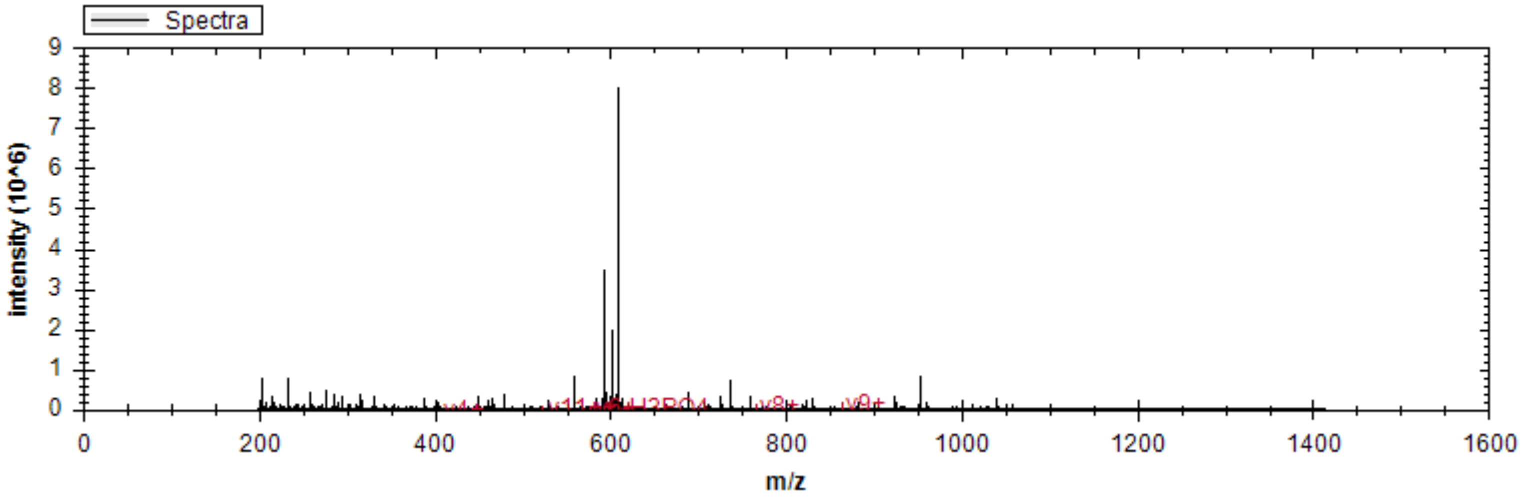

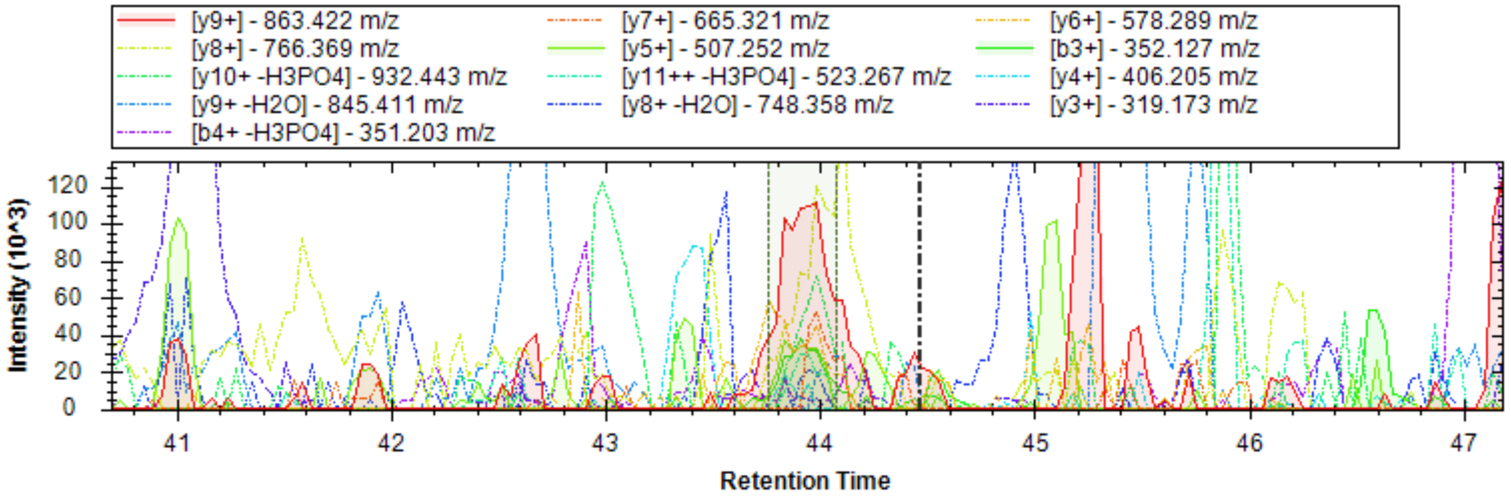

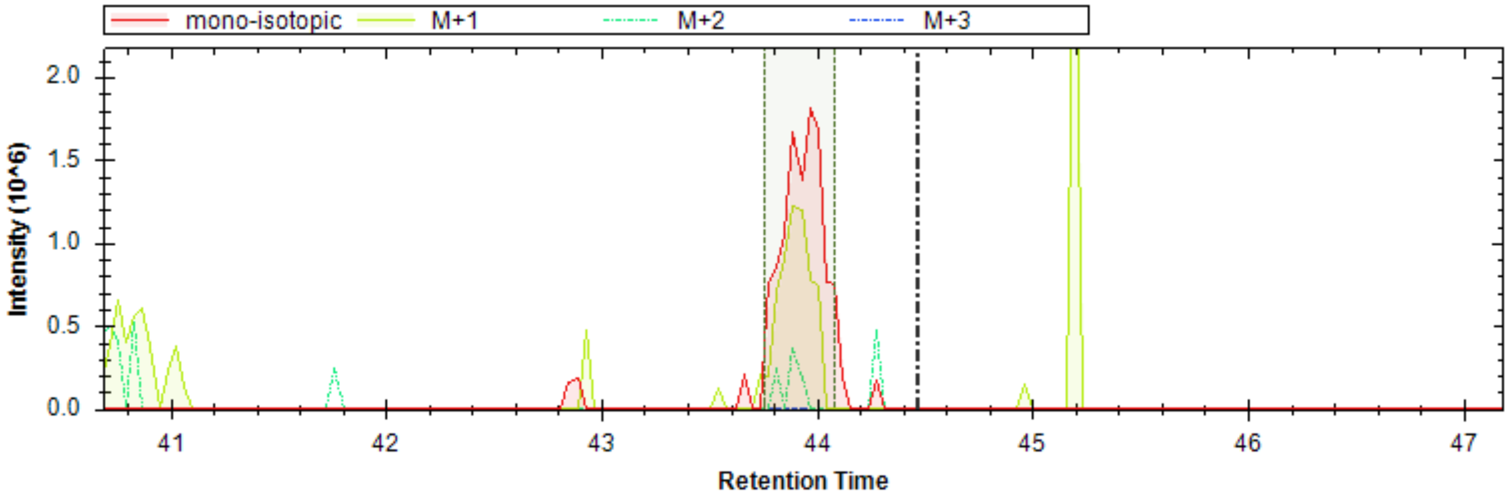

Q9QWI6 – _LSYAGGRPPSYAGS[+H+O3+P]PVHHAAER_.4
Fig4_MP-DIA-barrel-cortex-p9-2_MHRM.htrms

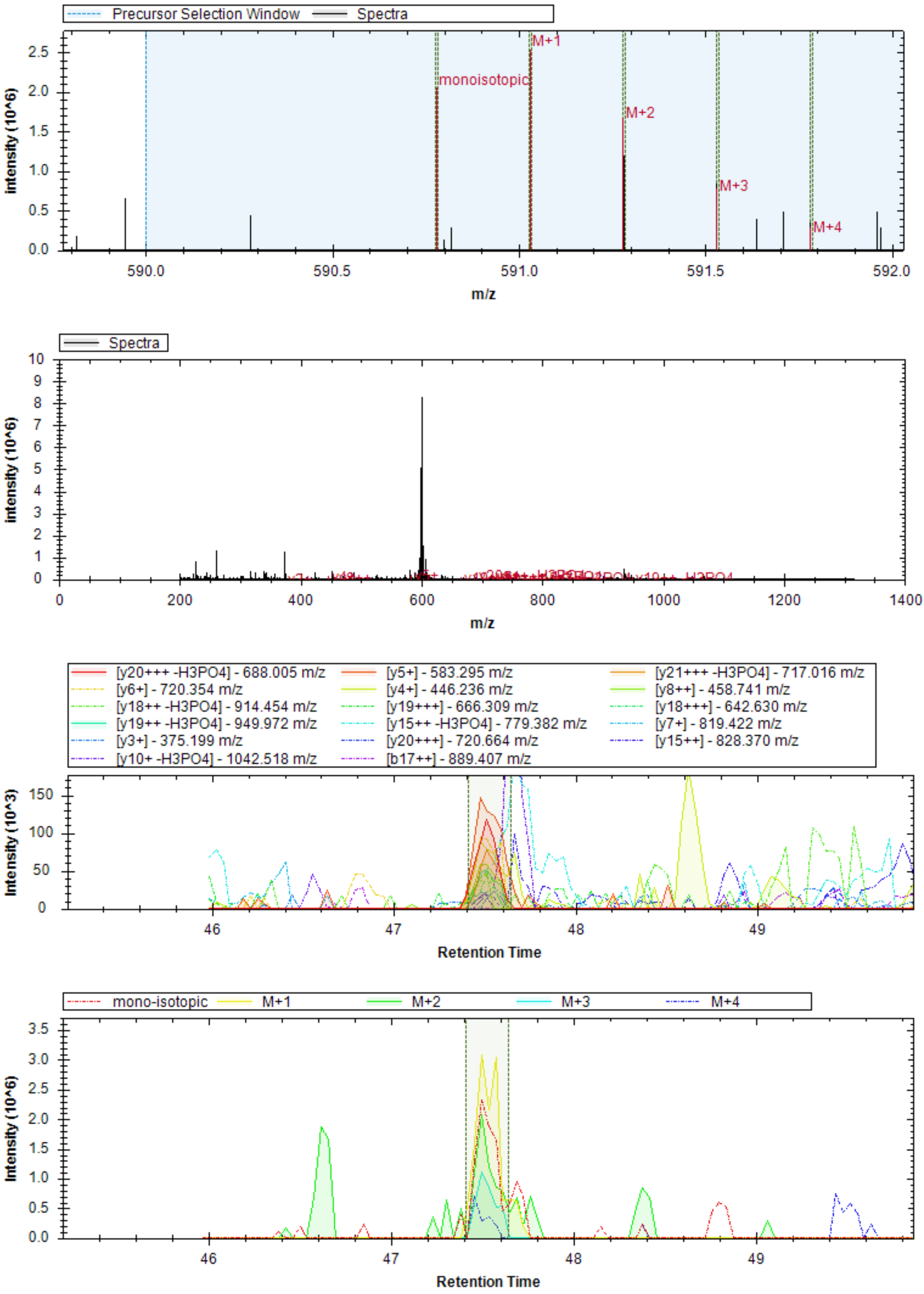

P26645 – _GEATAERPGEAAVASS[+H+O3+P]PSK_.3
Fig4_MP-DIA-barrel-cortex-p9-2_MHRM.htrms

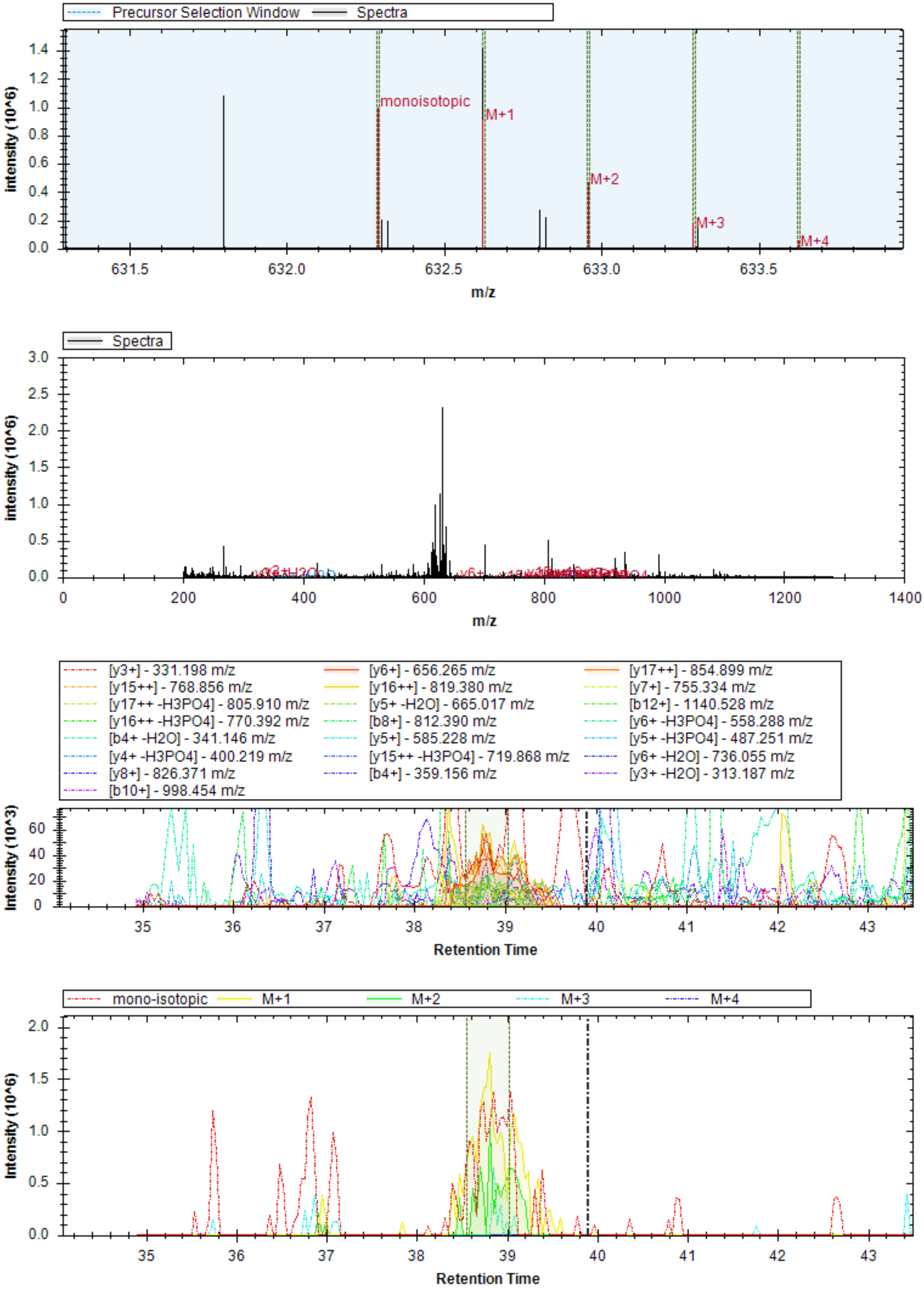

P60904 – _SLS[+H+O3+P]TSGESLYHVLGLDK_.3
Fig4_MP-DIA-barrel-cortex-p9-2_MHRM.htrms

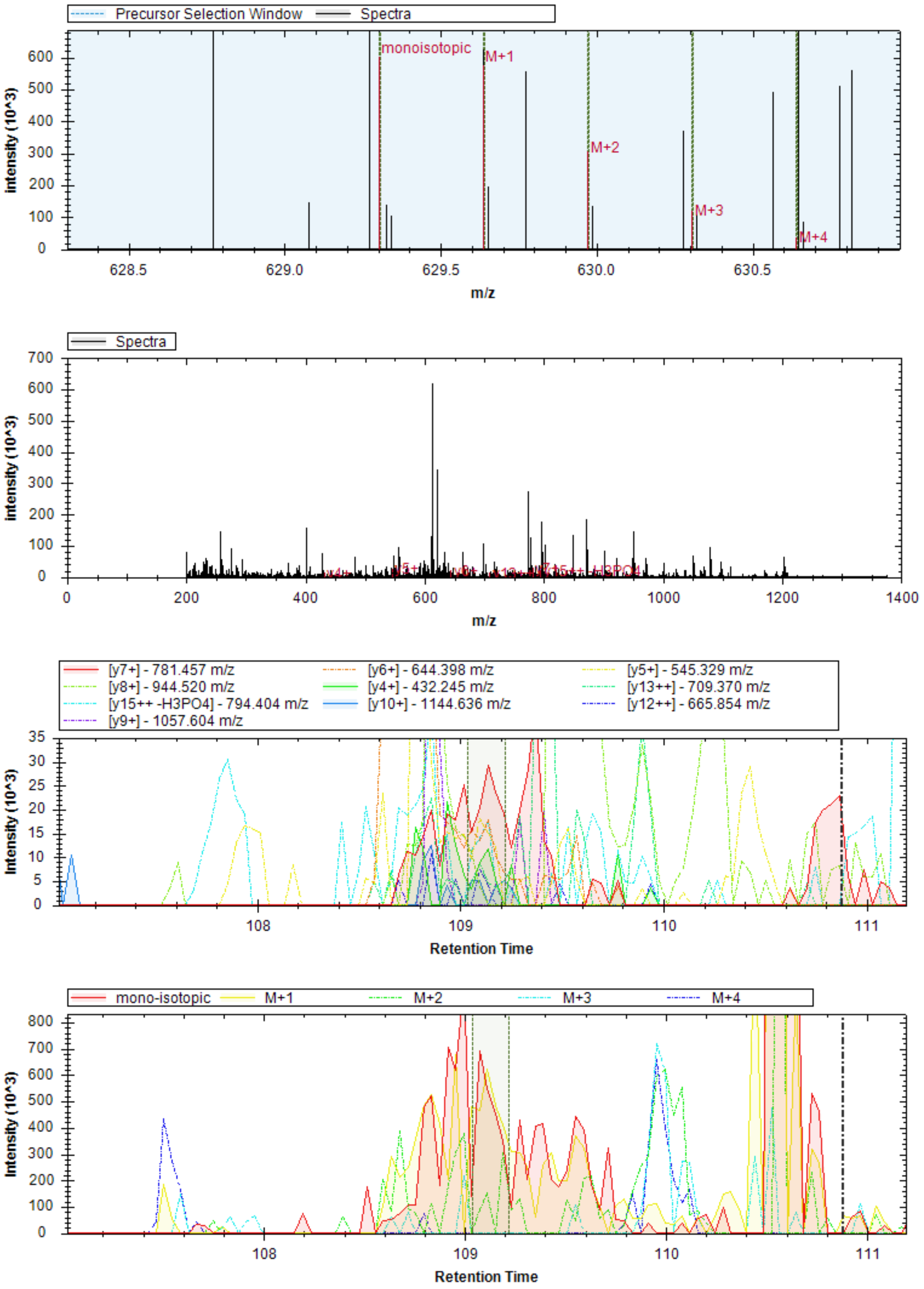

P60904 – _SLST[+H+O3+P]SGESLYHVLGLDK_.3
Fig4_MP-DIA-barrel-cortex-p9-2_MHRM.htrms

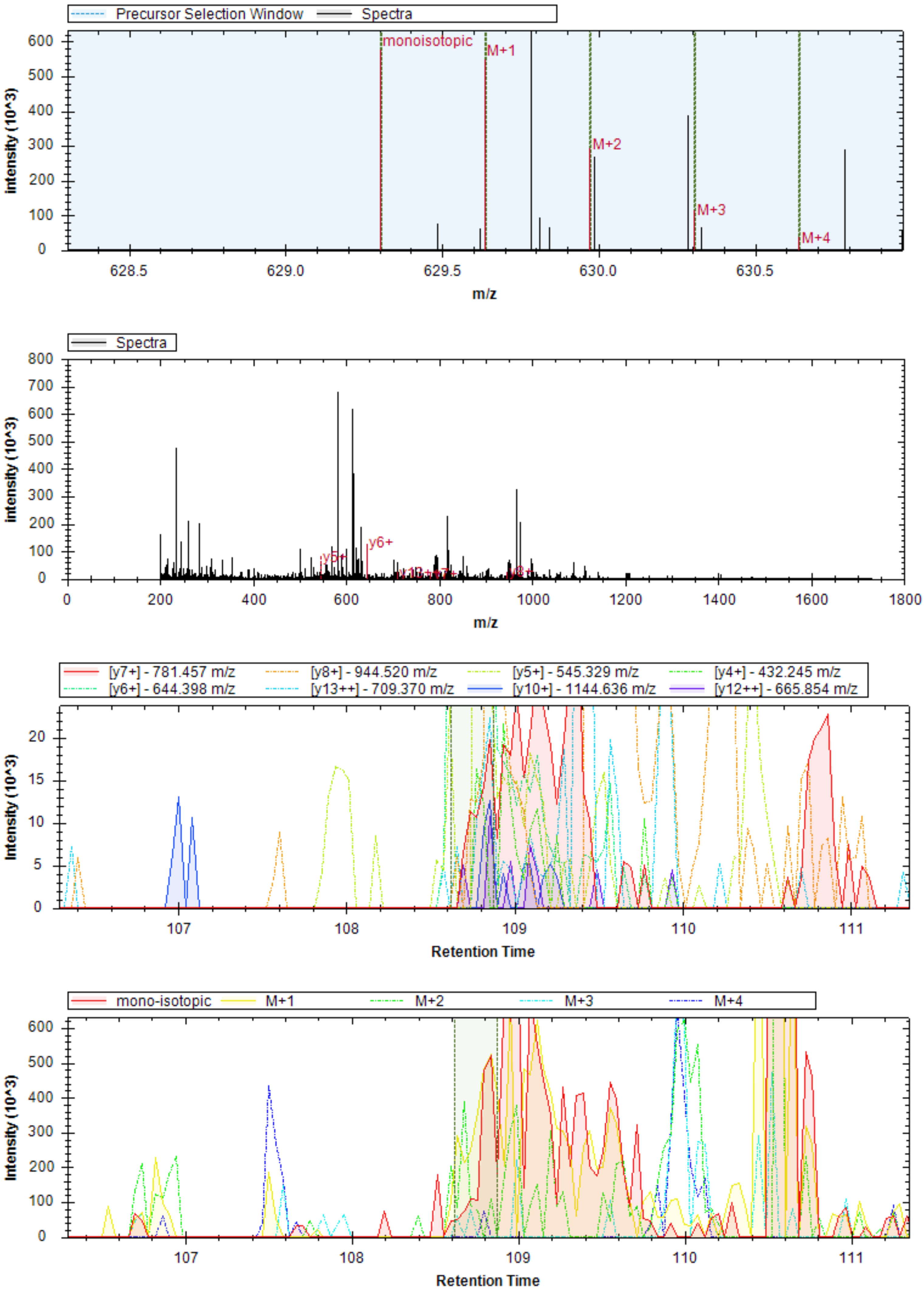

Q9CQS8 – _PGPTPSGTNVGSSGRS[+H+O3+P]PSK_.3

Fig4_MP-DIA-barrel-cortex-p9-2_MHRM.htrms

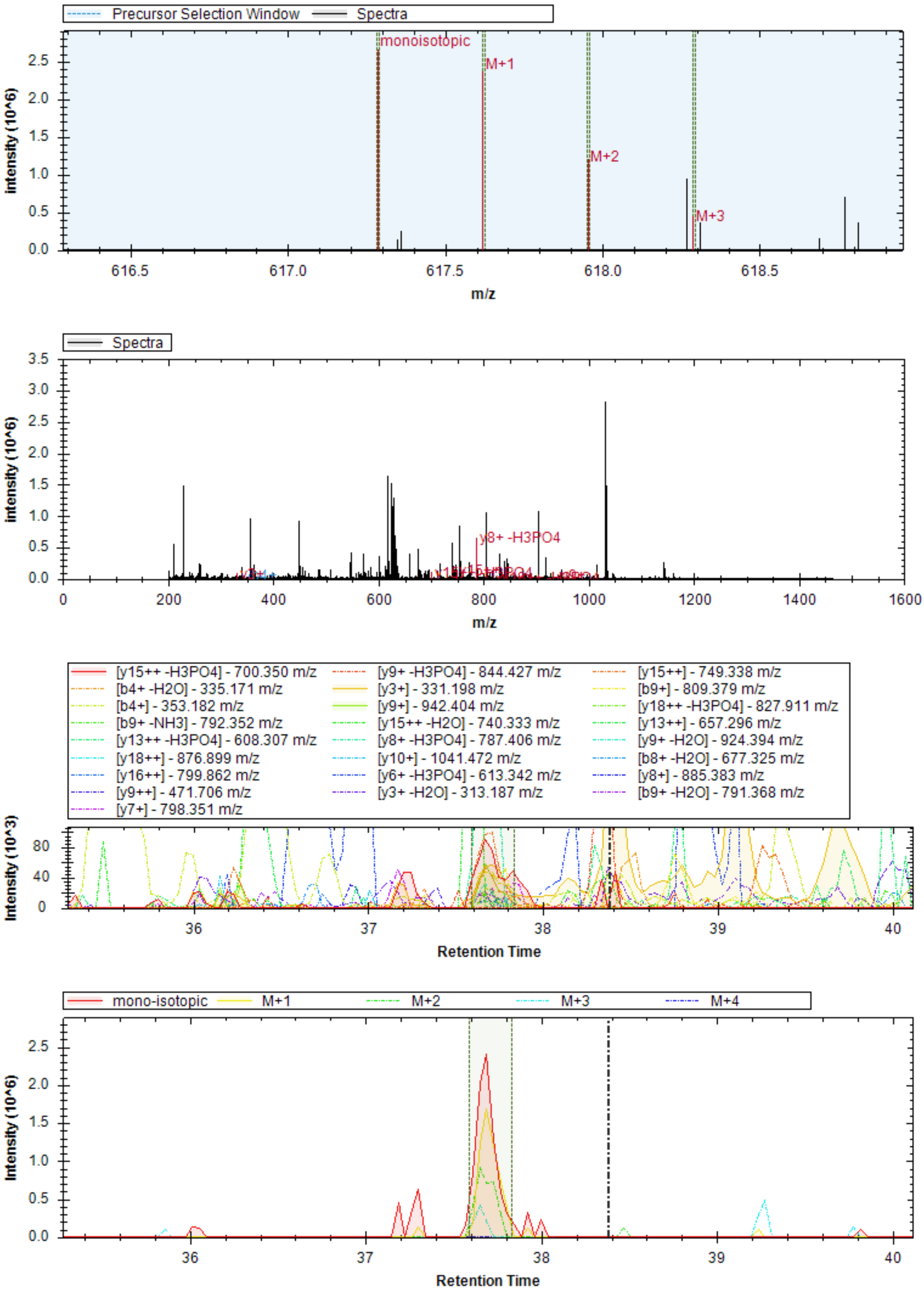

P20357 – _TPGT[+H+O3+P]PGTPSYPR_.2
Fig4_MP-DIA-barrel-cortex-p9-2_MHRM.htrms

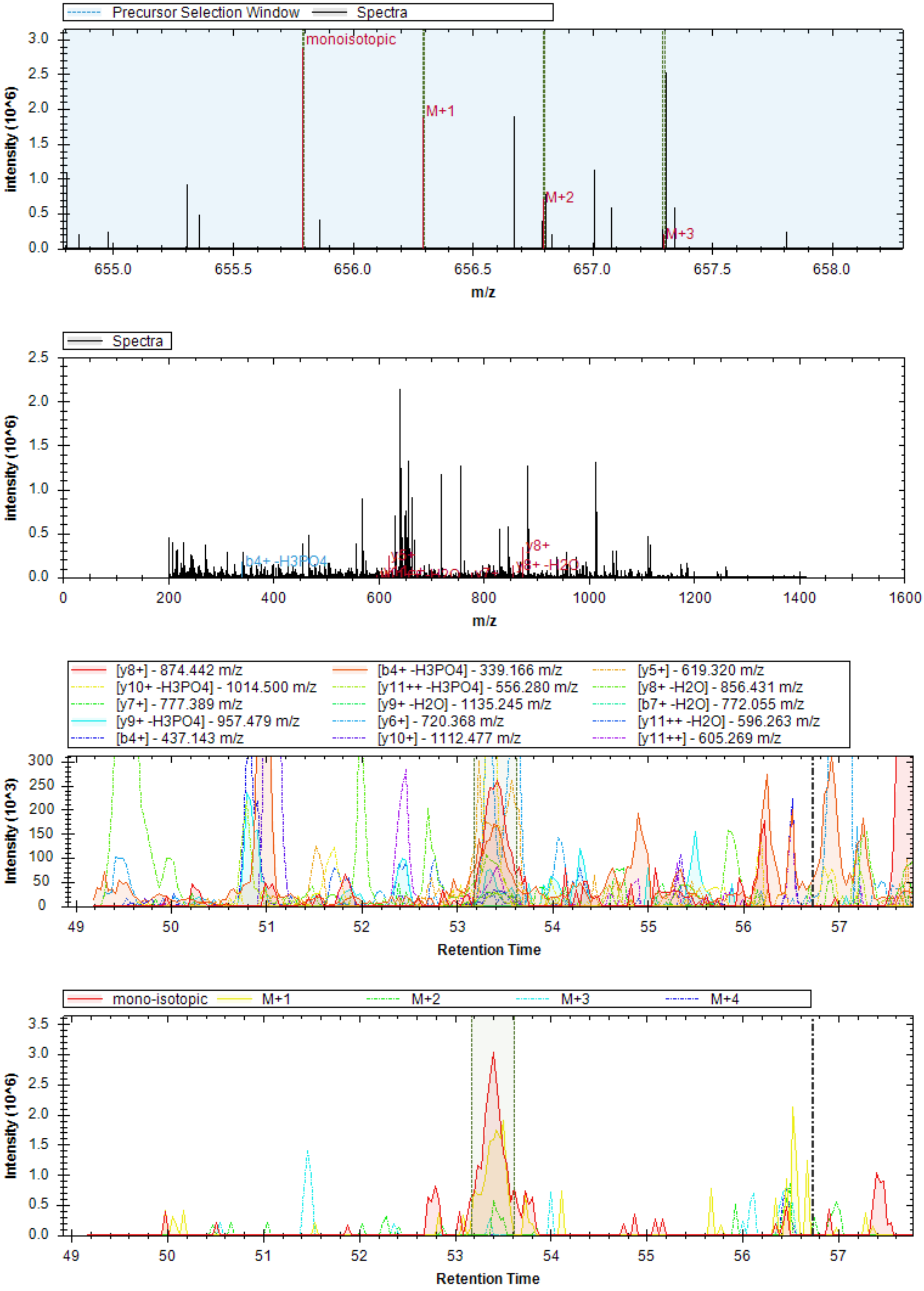

P27546-2 – _AAVGVTGNDITTPPNKEPPPS[+H+O3+P]PEKK_.4

Fig4_MP-DIA-barrel-cortex-p9-2_MHRM.htrms

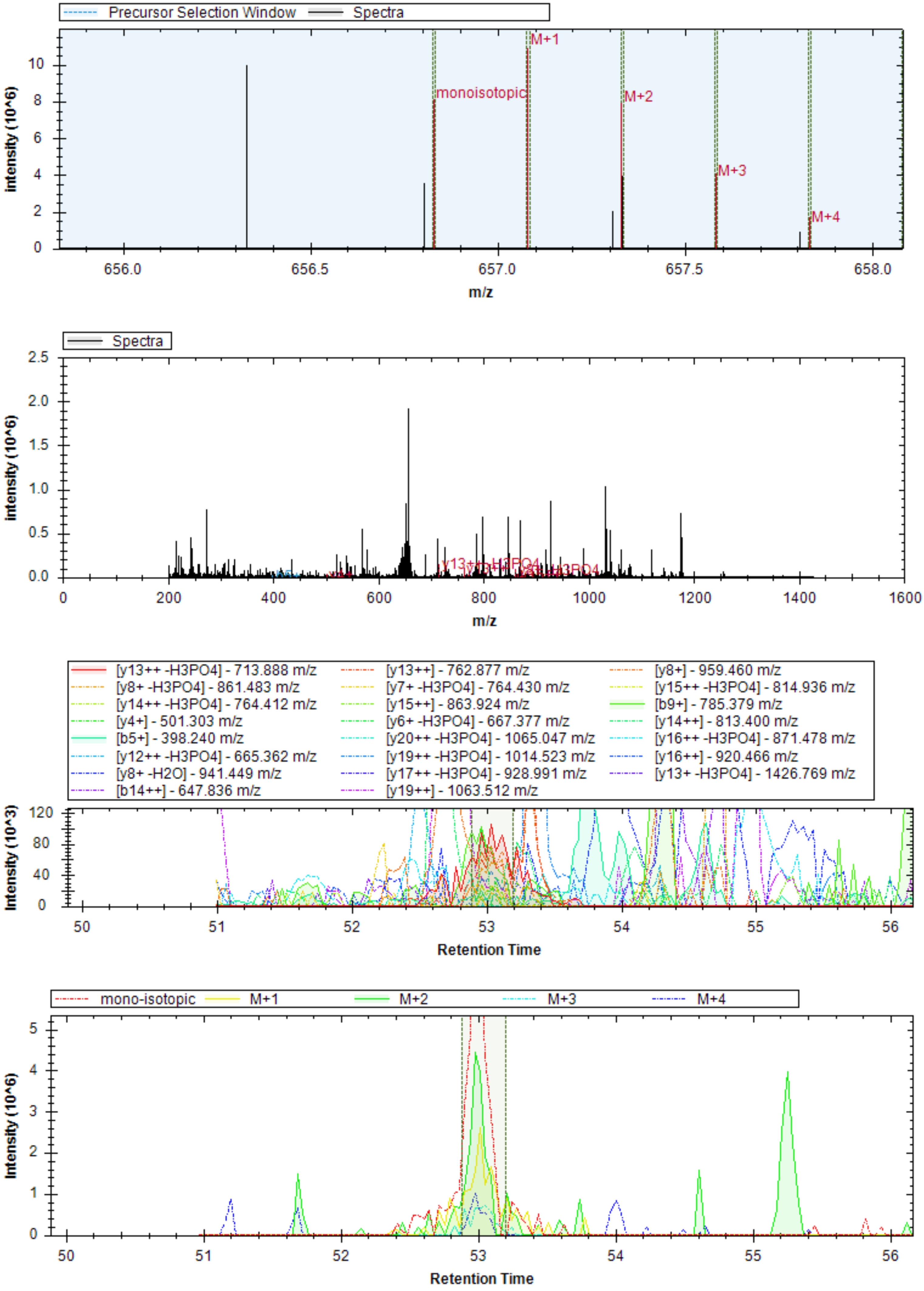

P39447 – _SSGIIRLHT[+H+O3+P]IK_.2
Fig4_MP-DIA-barrel-cortex-p9-2_MHRM.htrms

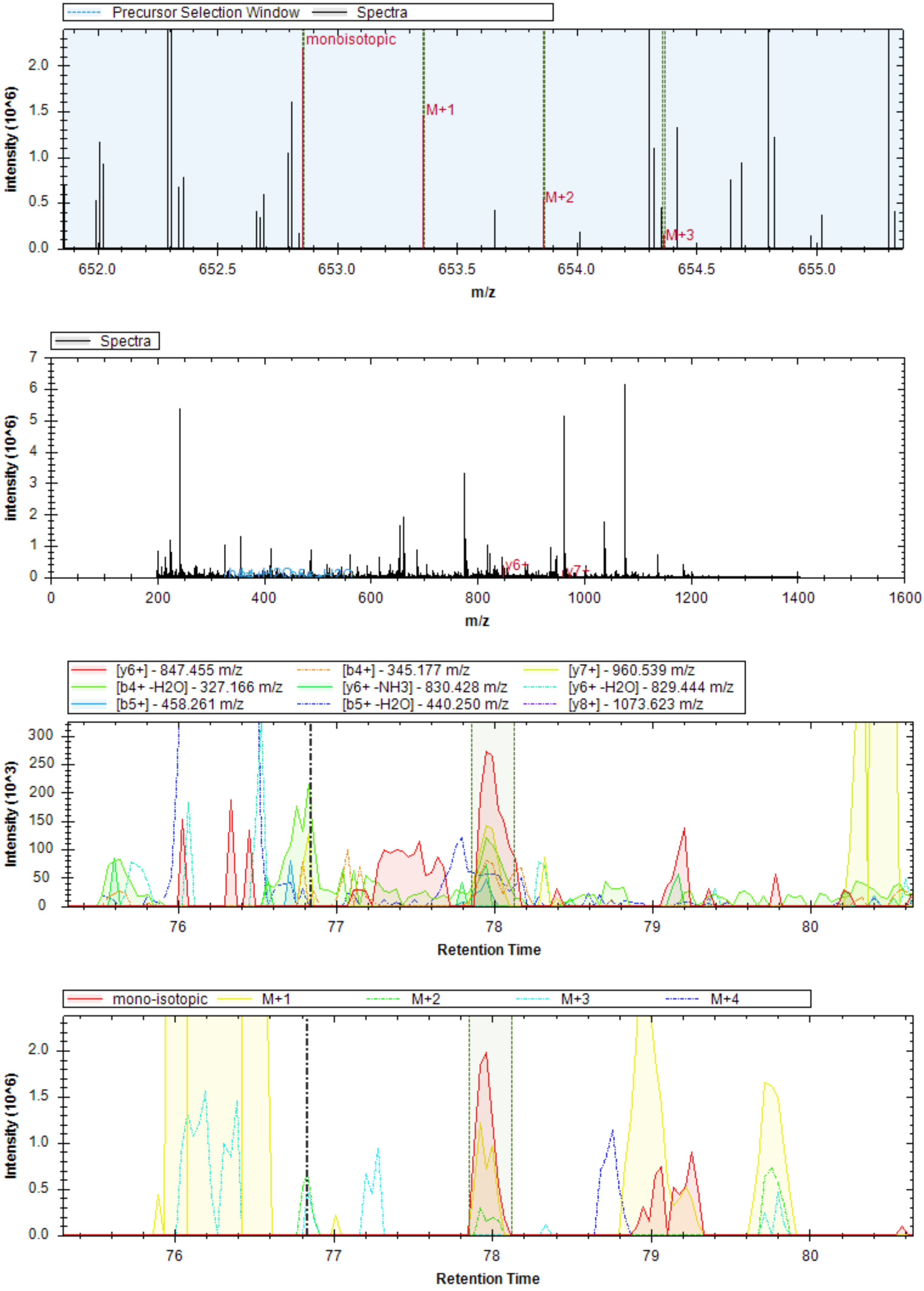

Q8BLJ3 – _M[+O]AS[+H+O3+P]SQGKNELK_.2

Fig4_MP-DIA-barrel-cortex-p9-2_MHRM.htrms

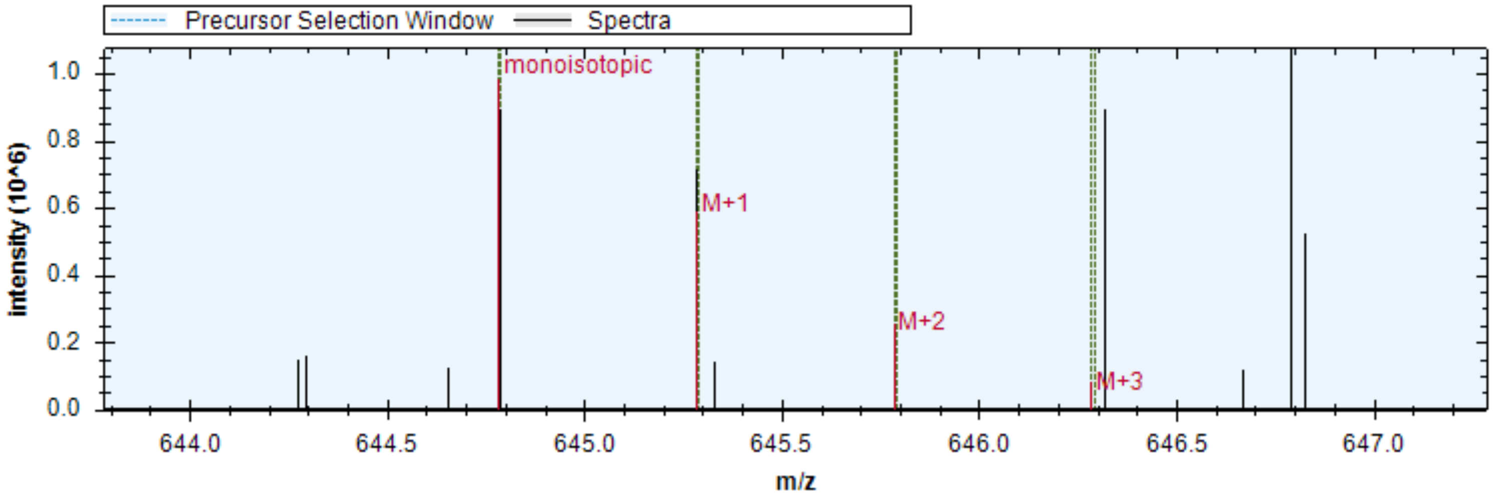

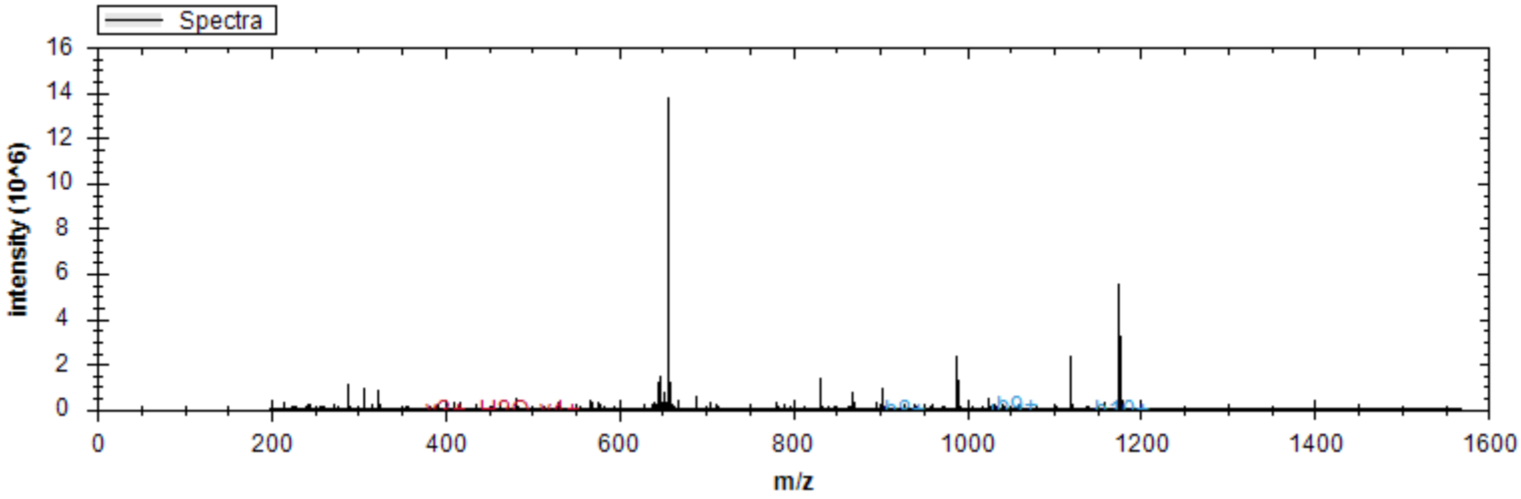

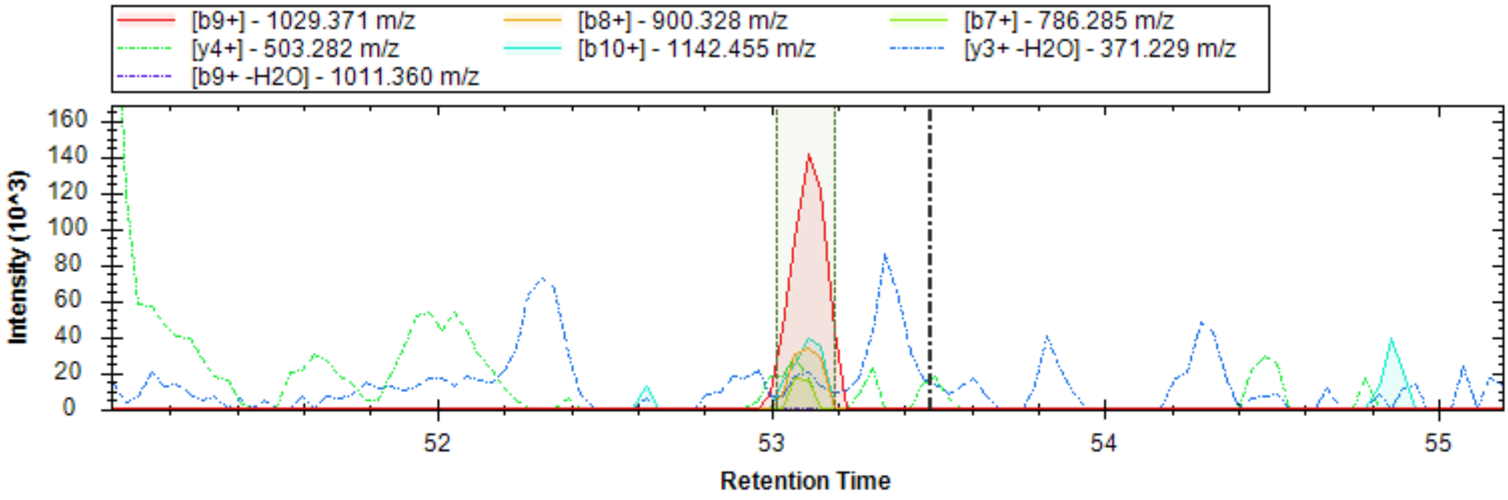

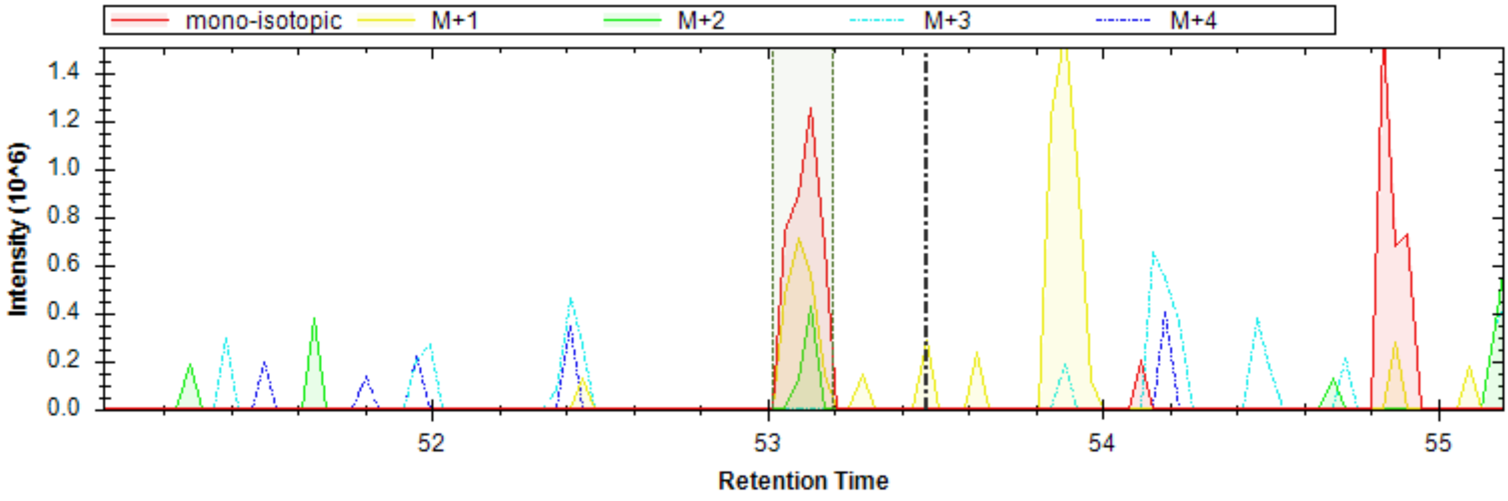

P13595 – _GVTASS[+H+O3+P]SSPASAPK_.2
Fig4_MP-DIA-barrel-cortex-p9-2_MHRM.htrms

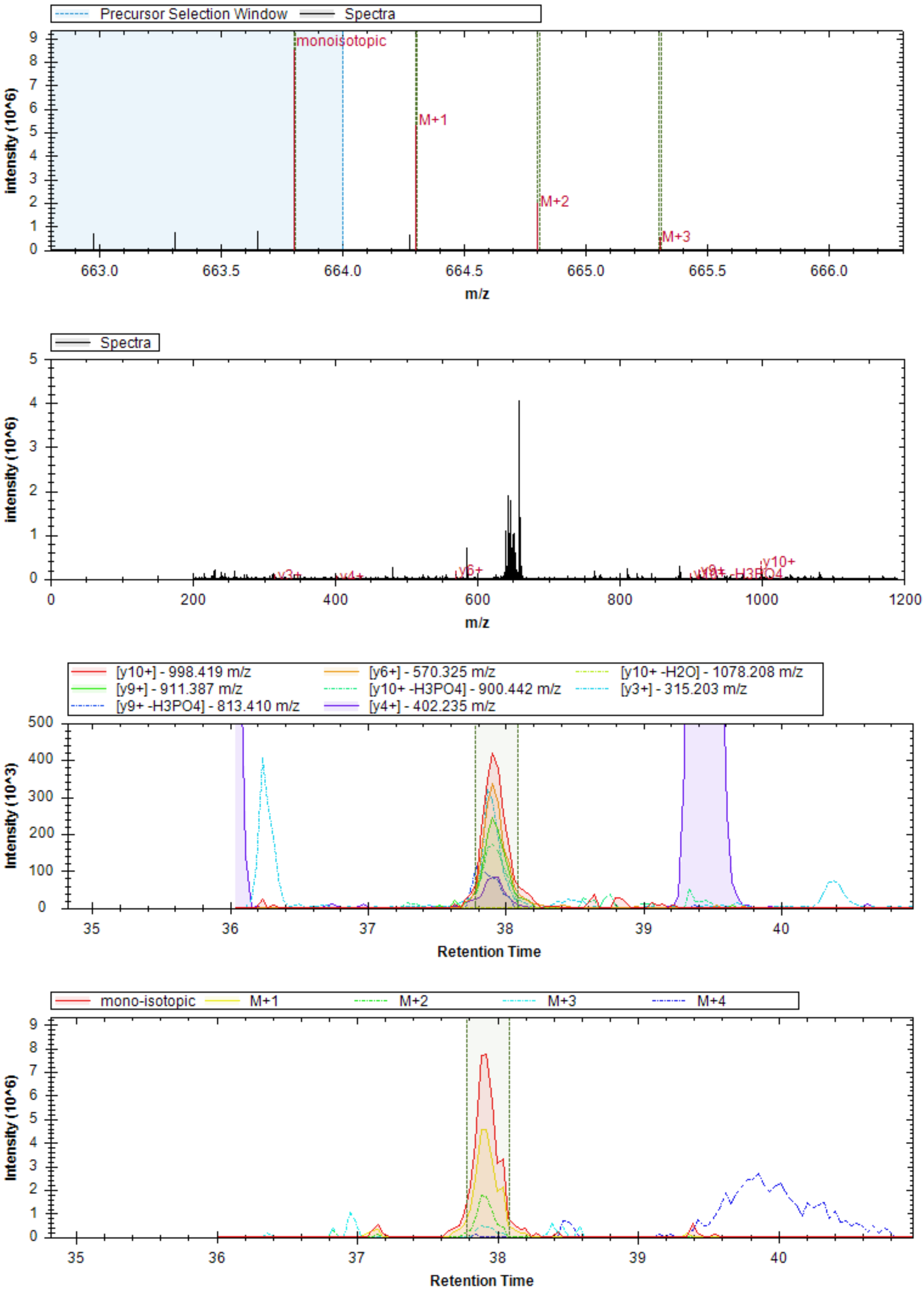

P13595 – _GVTASSS[+H+O3+P]SPASAPK_.2

Fig4_MP-DIA-barrel-cortex-p9-2_MHRM.htrms

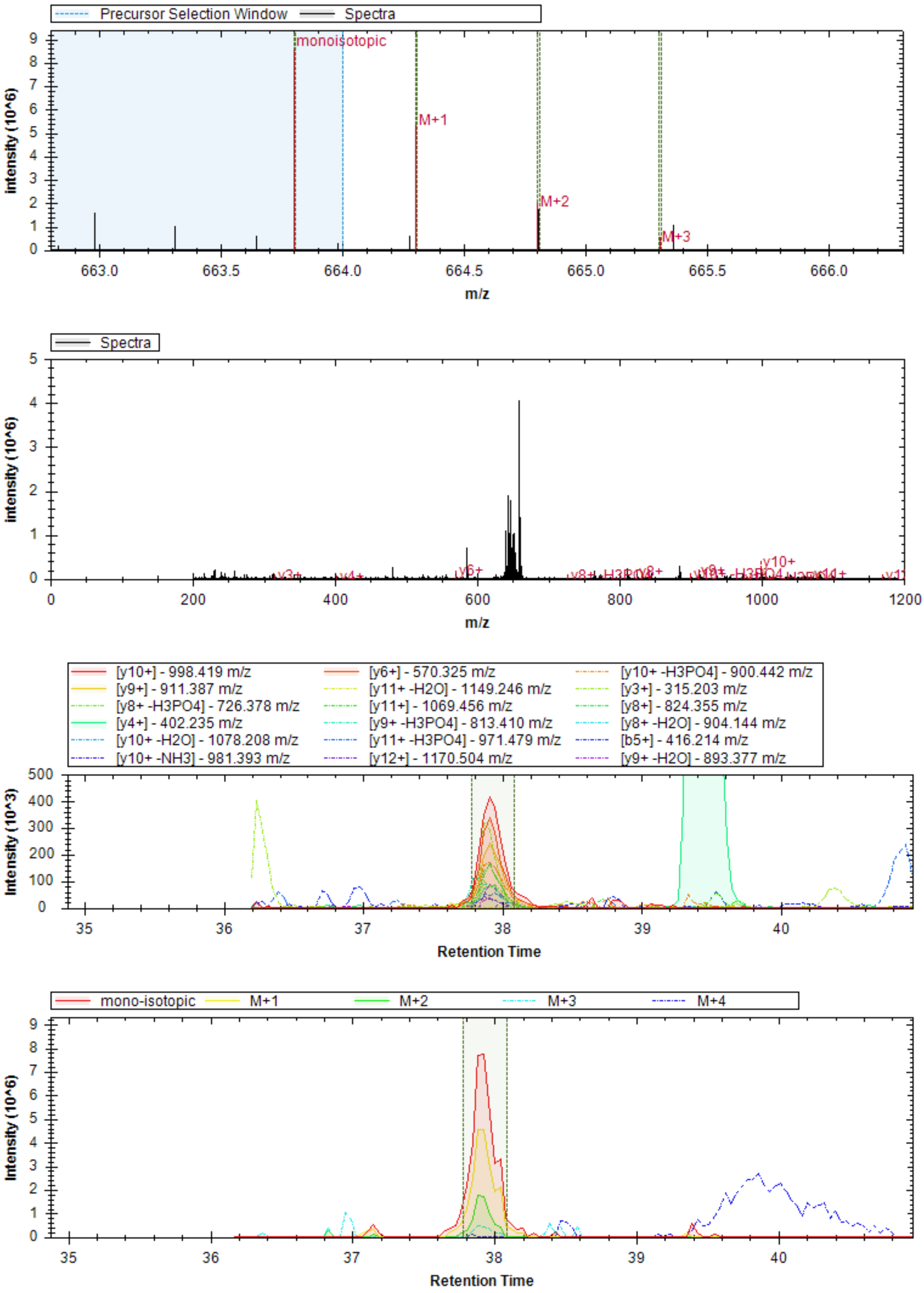

P13595 – _GVTASSSS[+H+O3+P]PASAPK_.2
Fig4_MP-DIA-barrel-cortex-p9-2_MHRM.htrms

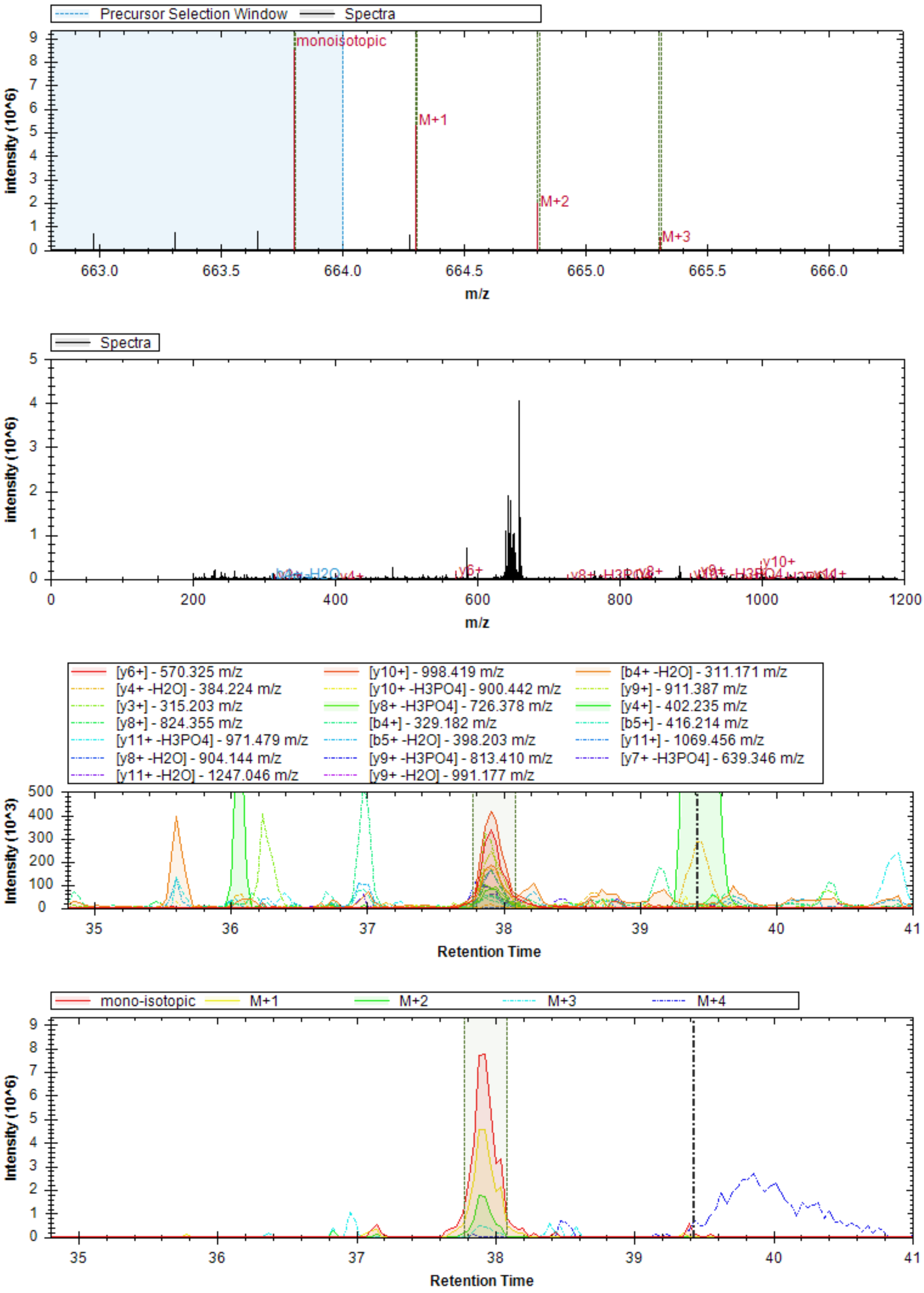

P13595 – _NPPEAATAPAS[+H+O3+P]PK_.2
Fig4_MP-DIA-barrel-cortex-p9-2_MHRM.htrms

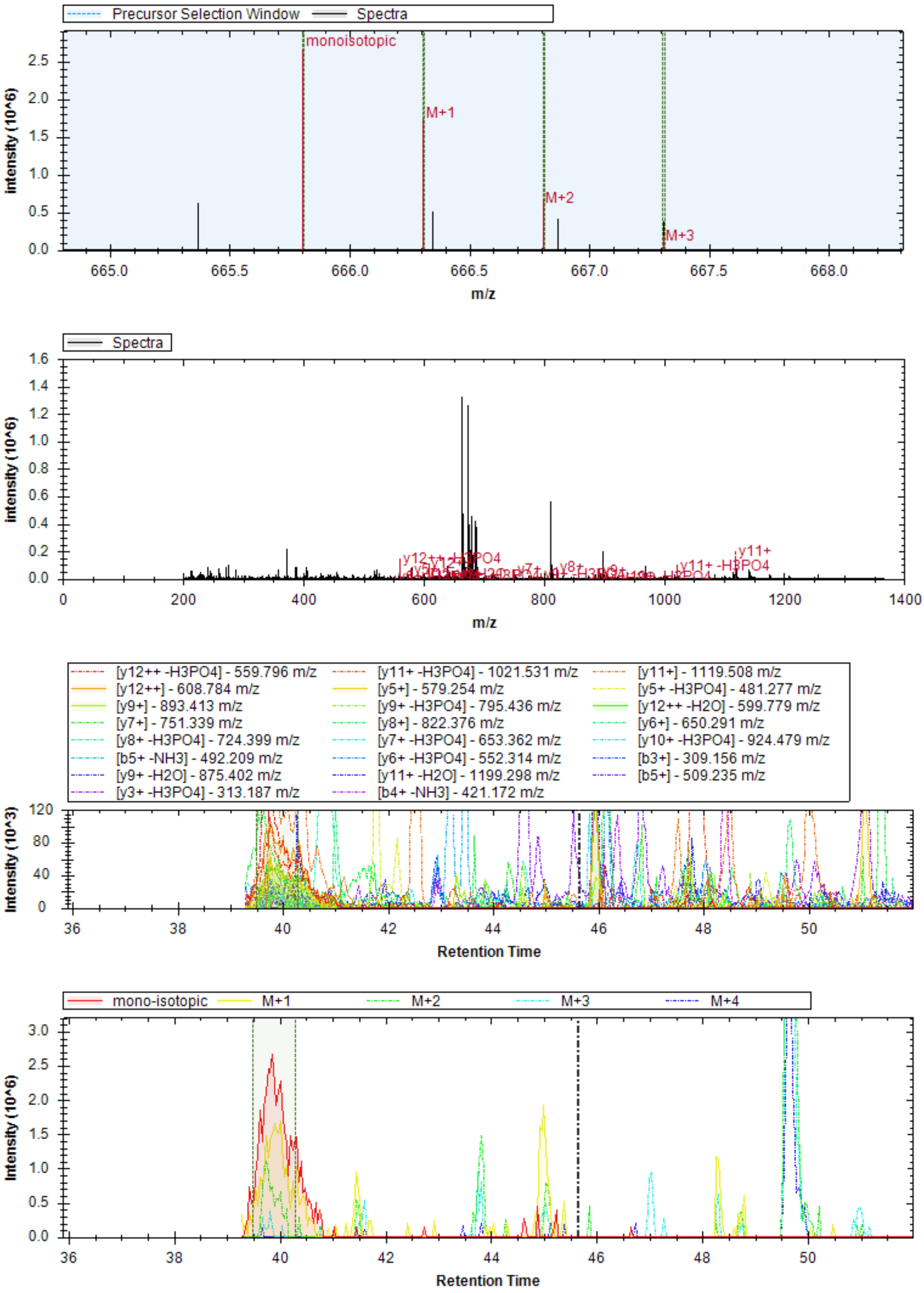

P26361 – _KM[+O]LHS[+H+O3+P]ILHAPMS[+H+O3+P]T[+H+O3+P]ISK_.3

Fig4_MP-DIA-barrel-cortex-p9-2_MHRM.htrms

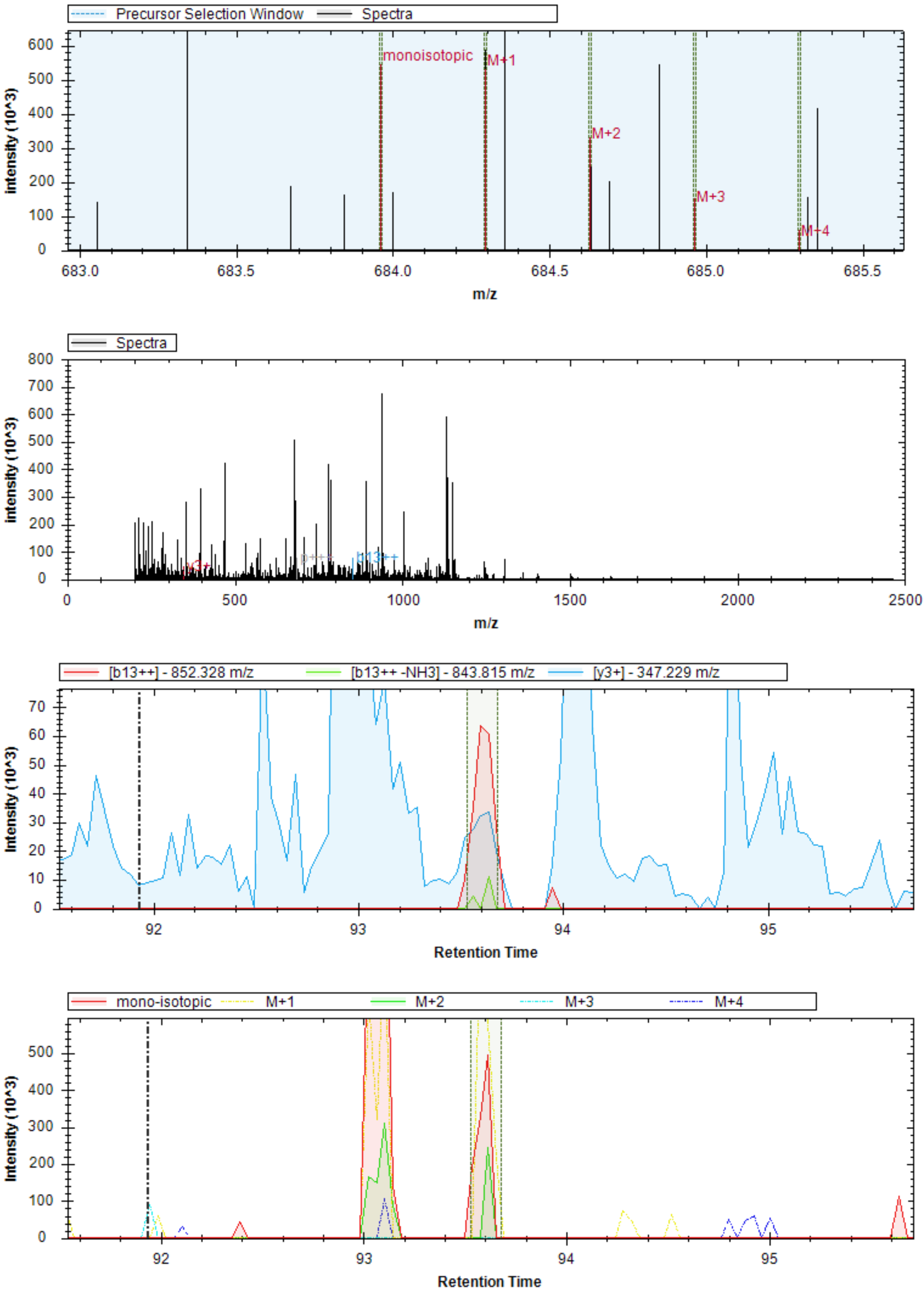

Q3UTZ3 – _SQS[+H+O3+P]FSHQQPSR_.2
Fig4_MP-DIA-barrel-cortex-p9-2_MHRM.htrms

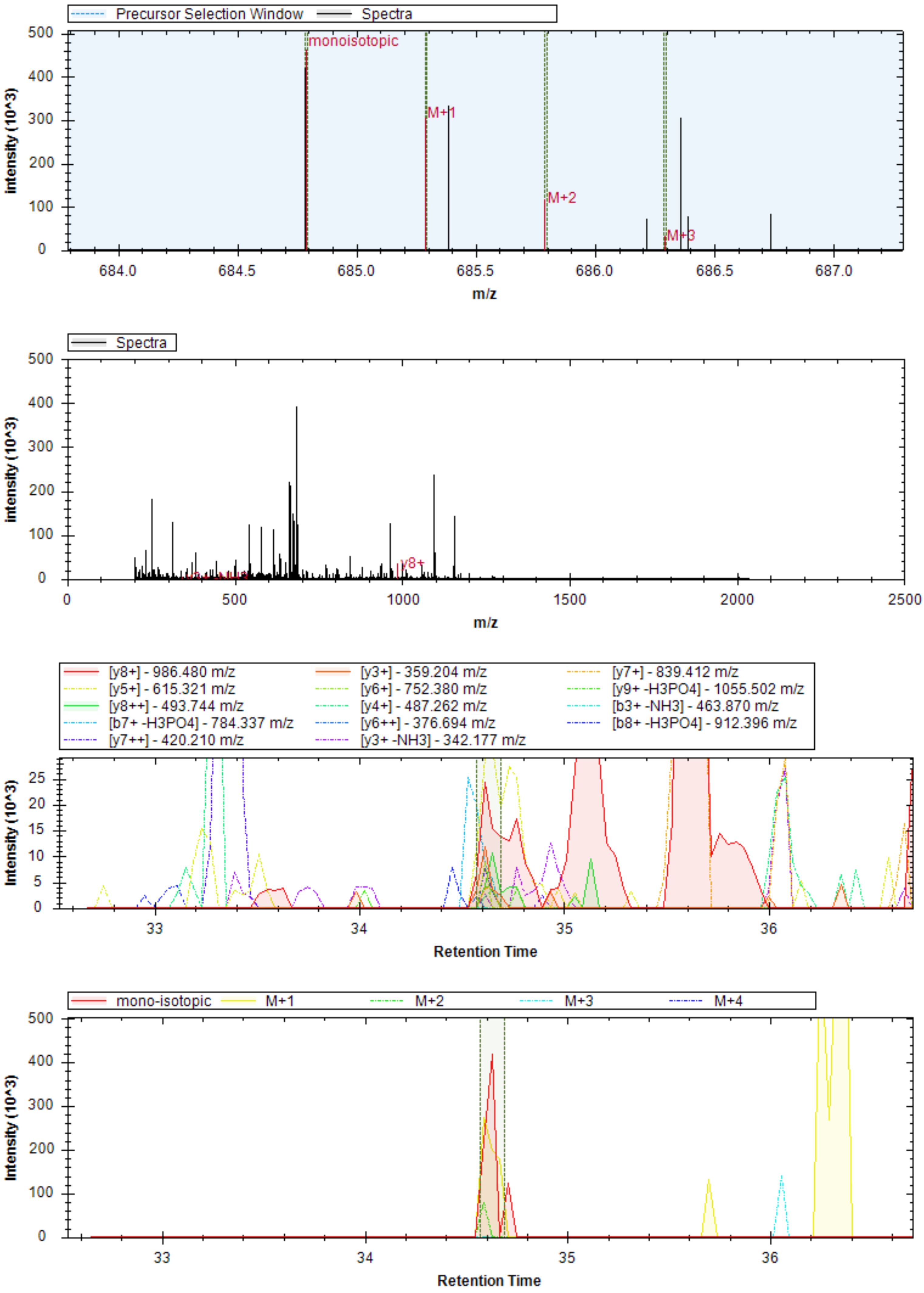

Q6A078 – _T[+H+O3+P]IGLM[+O]KKVVEK_.2
Fig4_MP-DIA-barrel-cortex-p9-2_MHRM.htrms

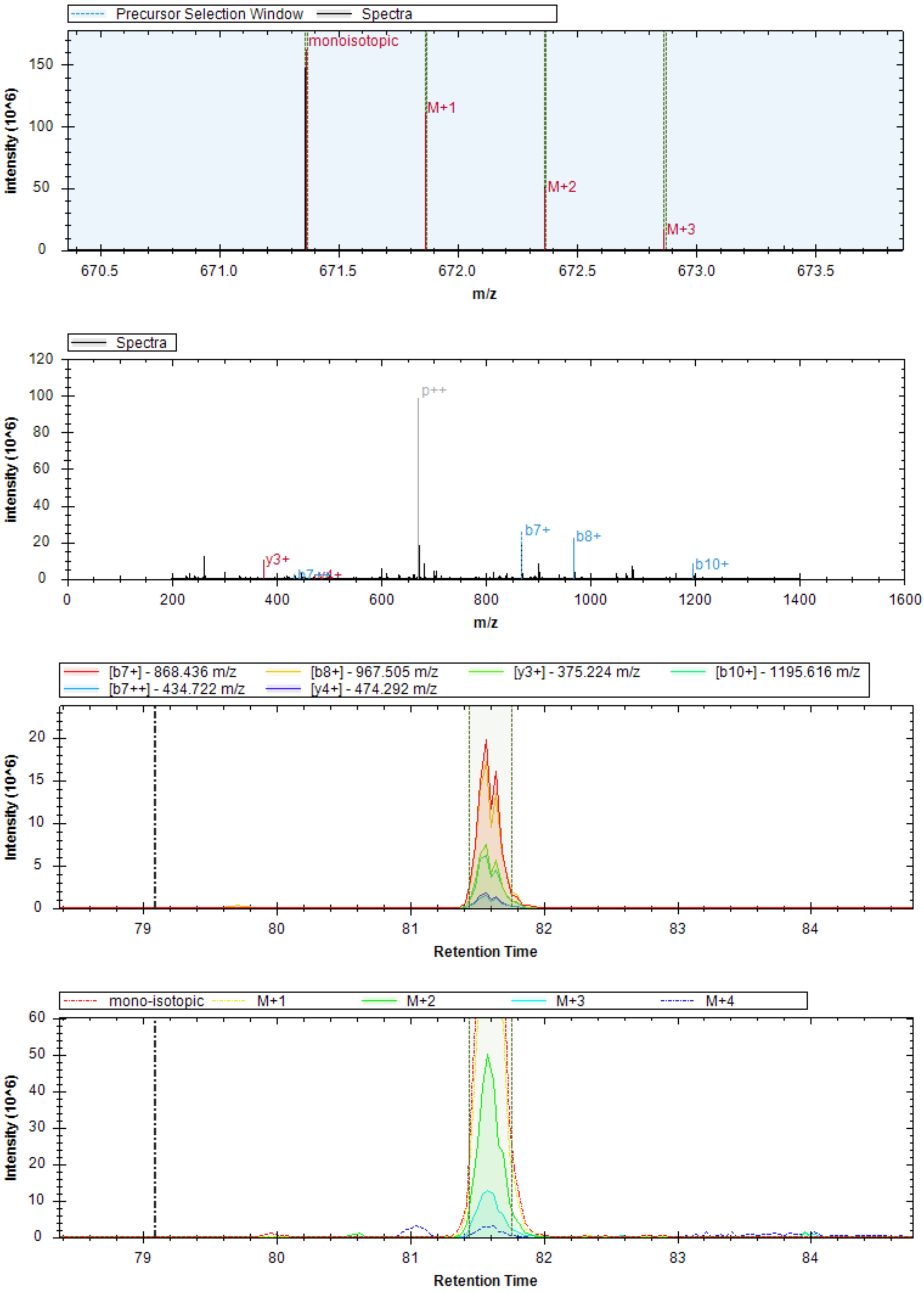

Q8C0E2 – _SMS[+H+O3+P]HQAAIASQR_.2

Fig4_MP-DIA-barrel-cortex-p9-2_MHRM.htrms

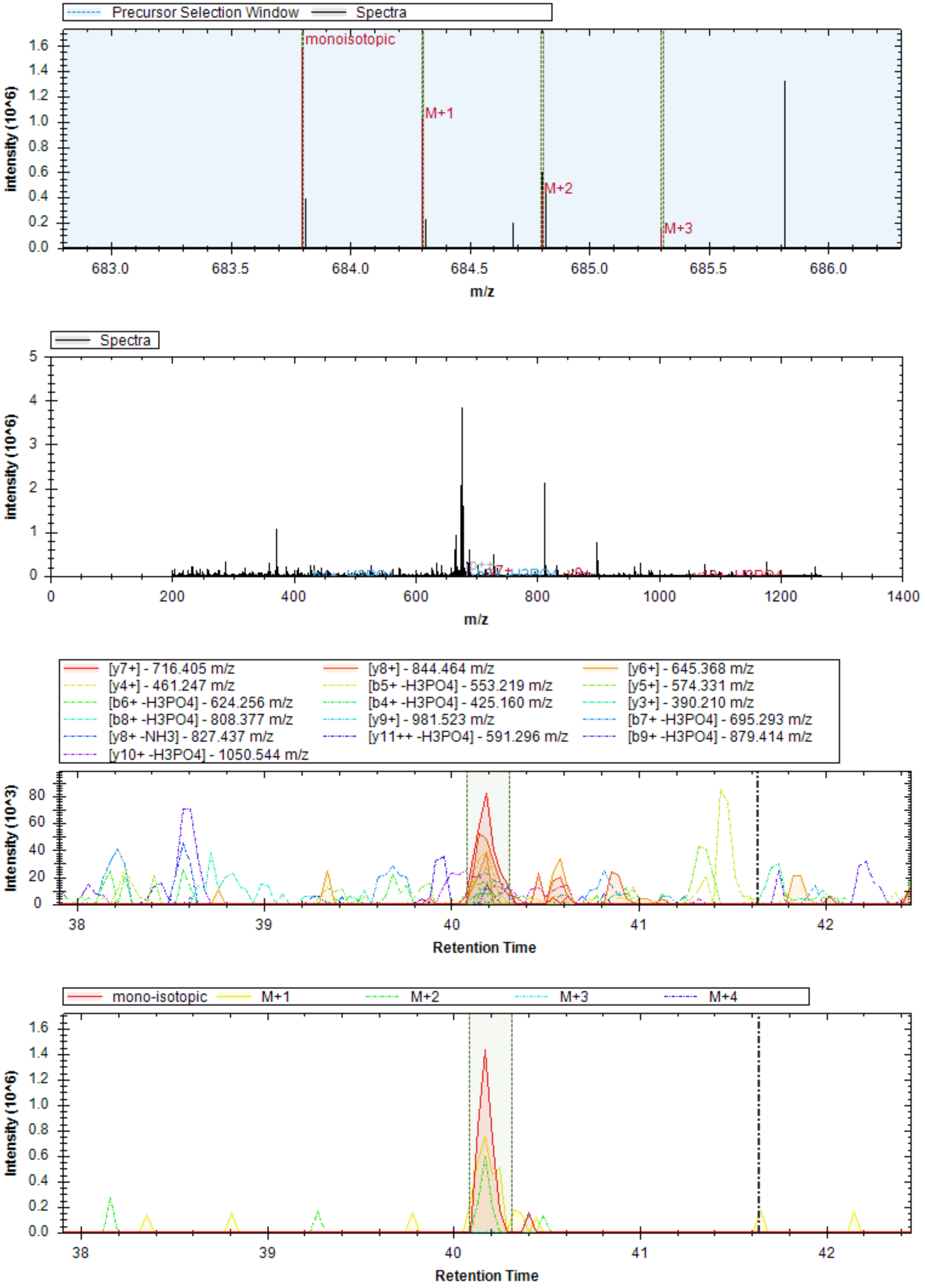

Q8CDU4;Q8CDU4-2 – _[+C2+H2+O]ASLRS[+H+O3+P]ATPRLR_.2
Fig4_MP-DIA-barrel-cortex-p9-2_MHRM.htrms

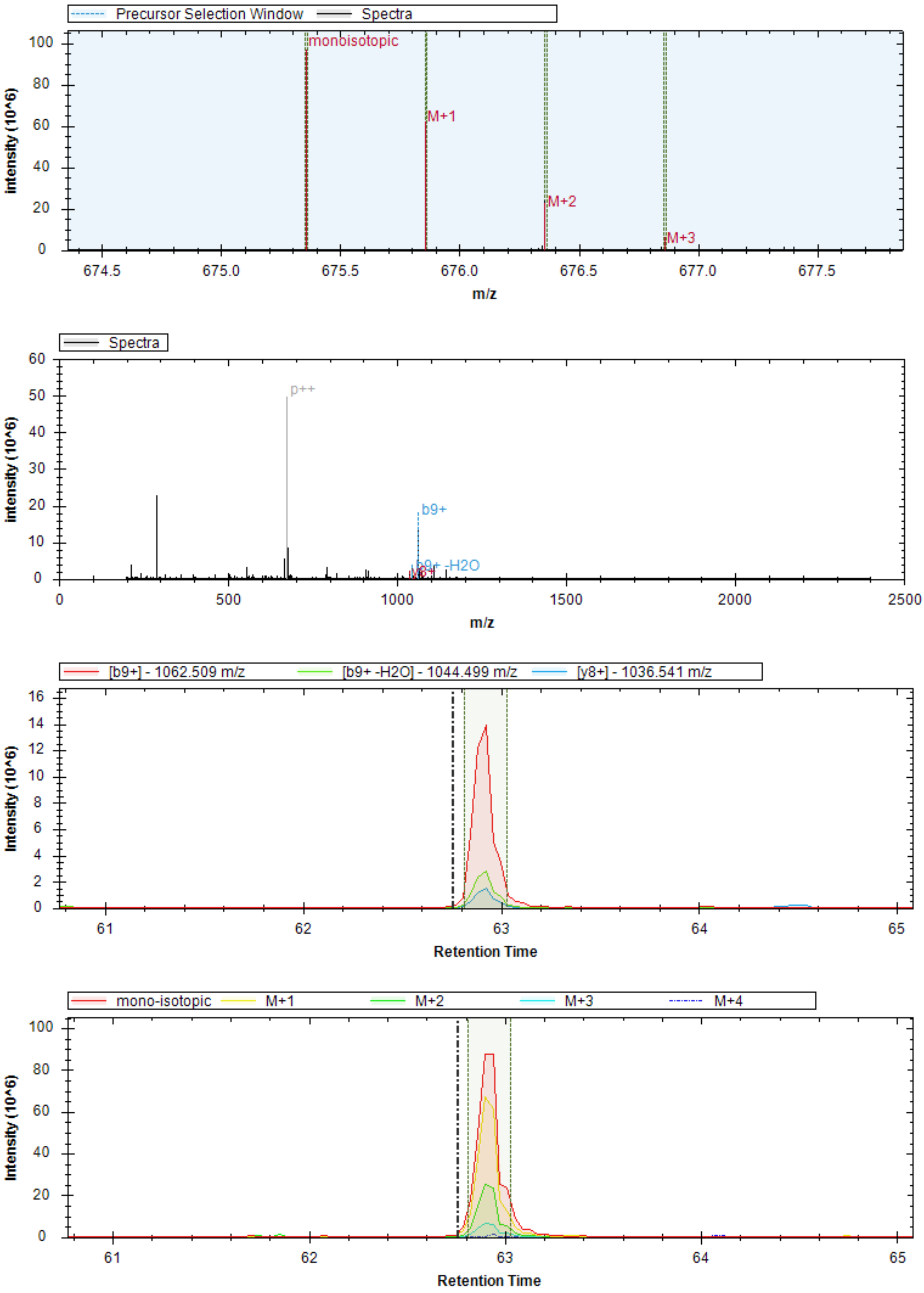

Q9CZU3 – _ESLKNSAT[+H+O3+P]EAAK_.2
Fig4_MP-DIA-barrel-cortex-p9-2_MHRM.htrms

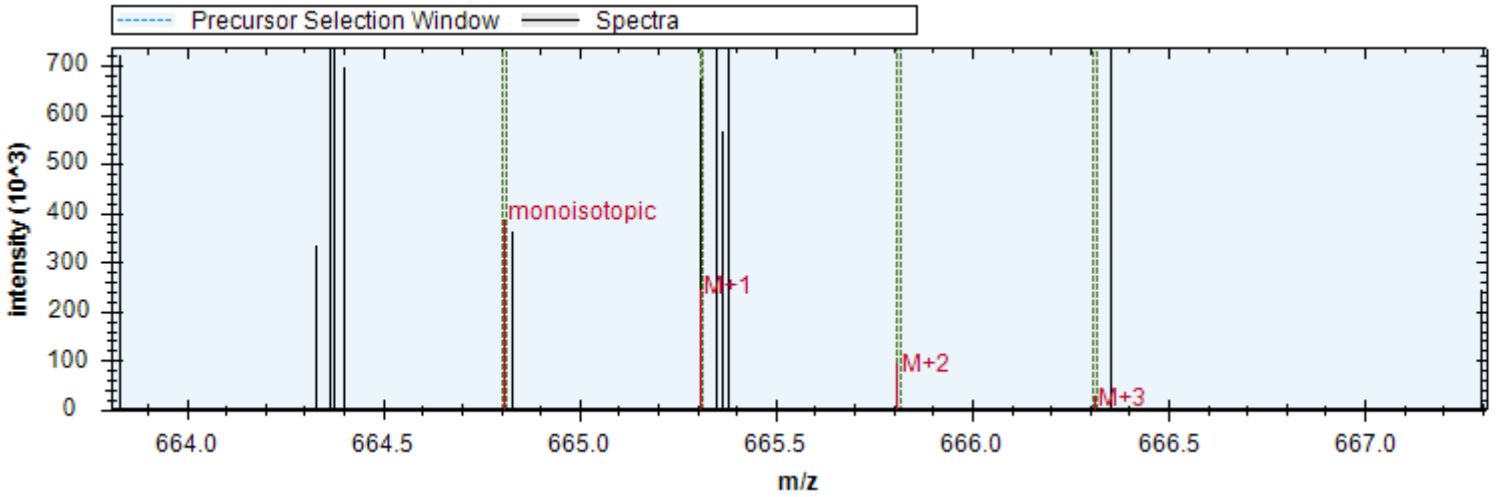

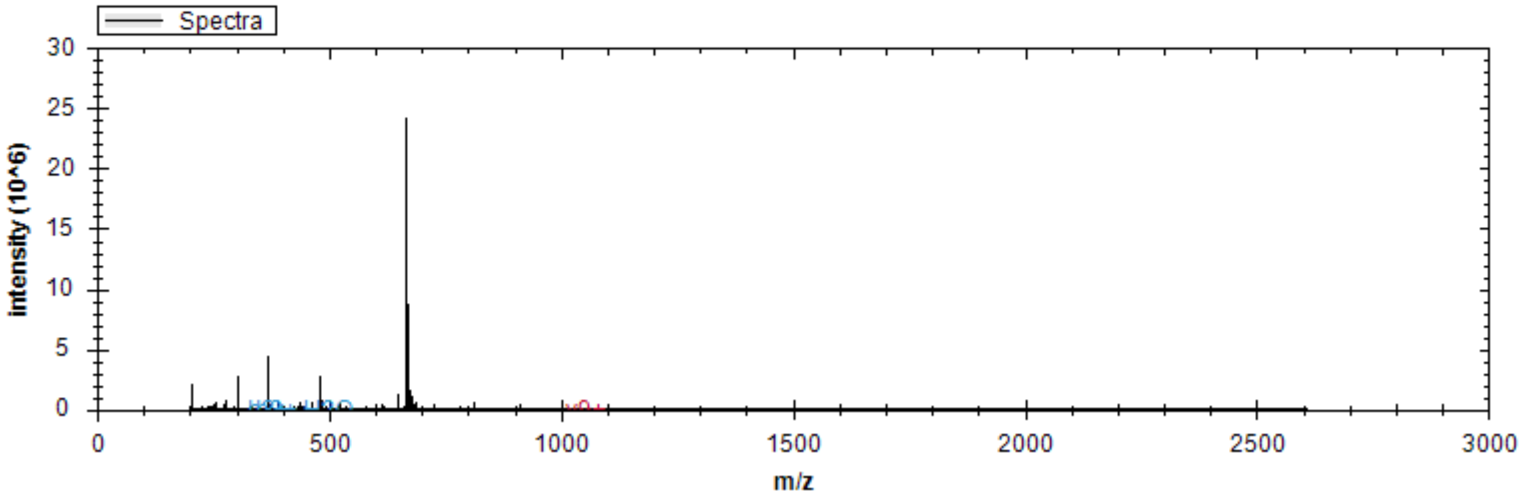

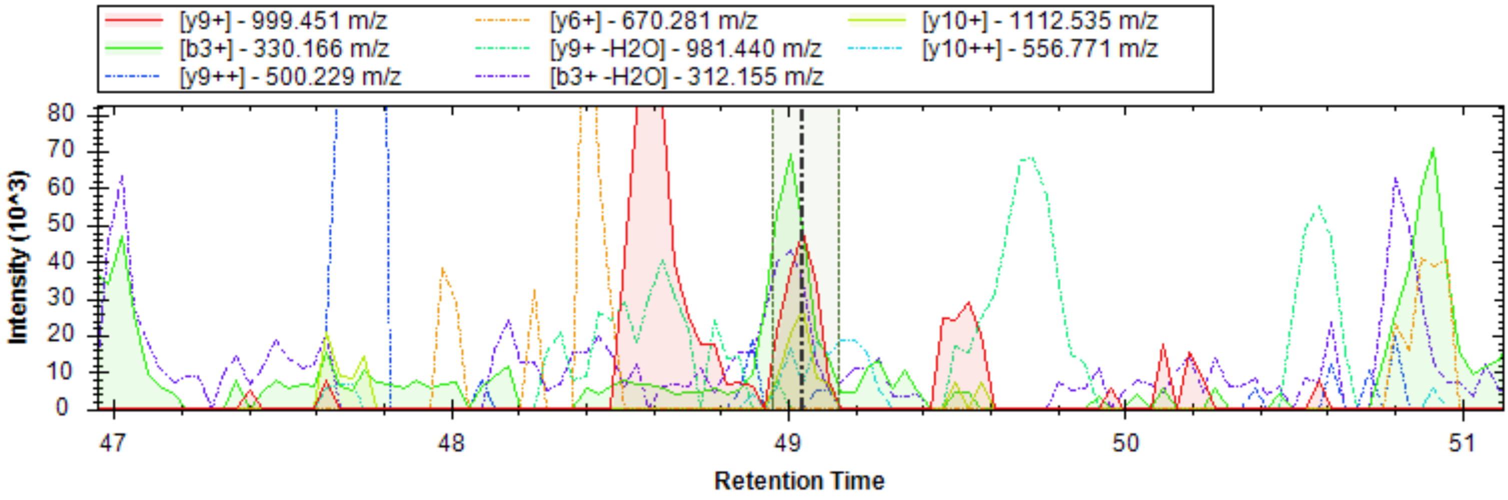

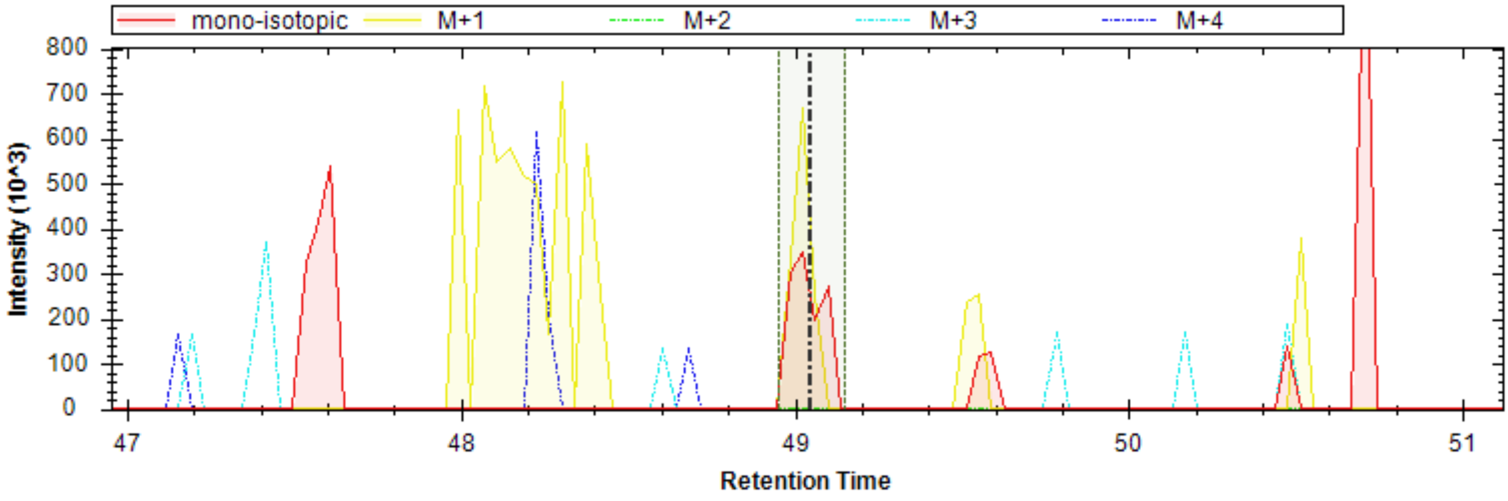

Q9JLM8 – _SPSPSPT[+H+O3+P]SPGSLR_.2
Fig4_MP-DIA-barrel-cortex-p9-2_MHRM.htrms

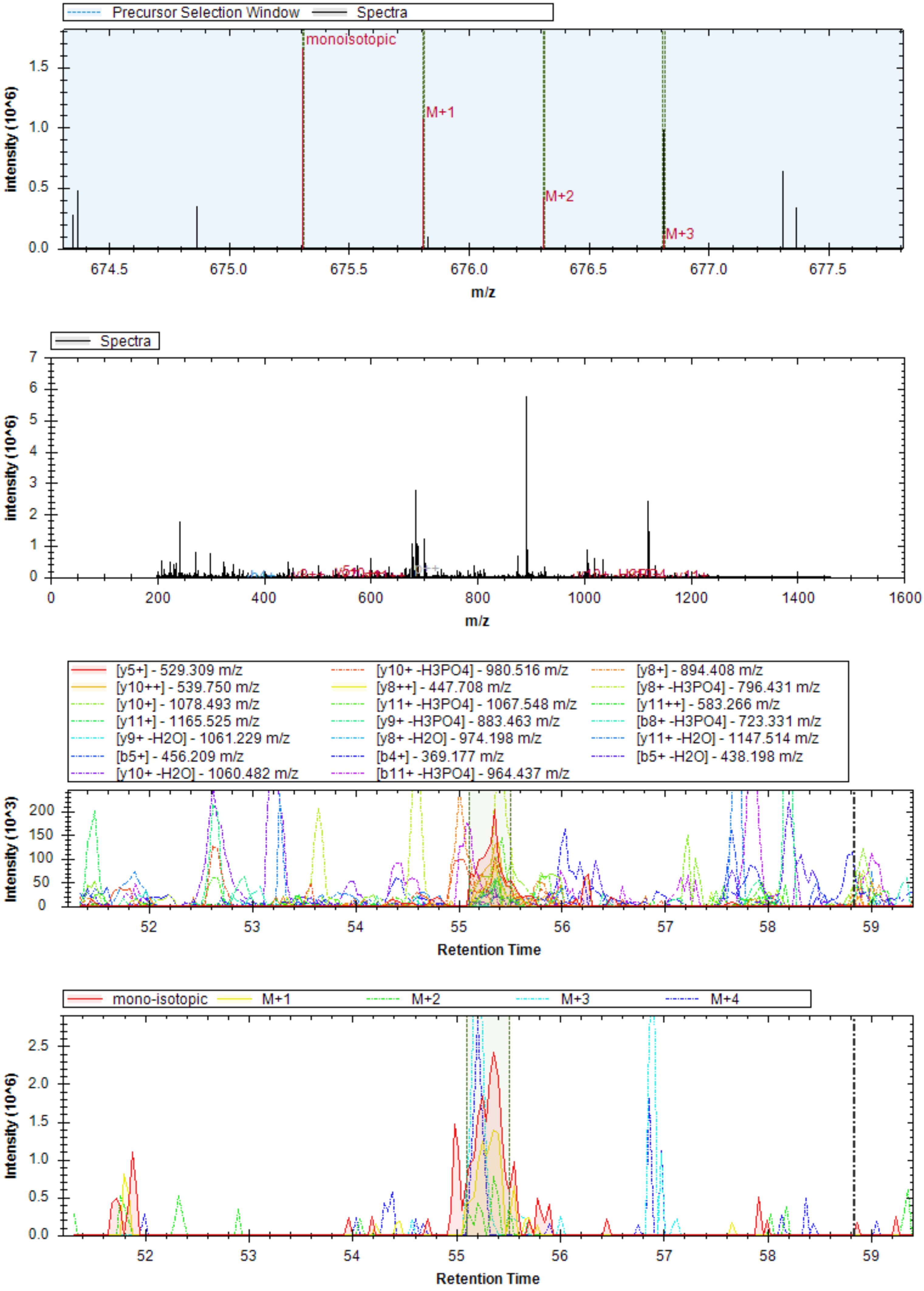

B2RY56 – _LGASNS[+H+O3+P]PGQPNSVK_.2
Fig4_MP-DIA-barrel-cortex-p9-2_MHRM.htrms

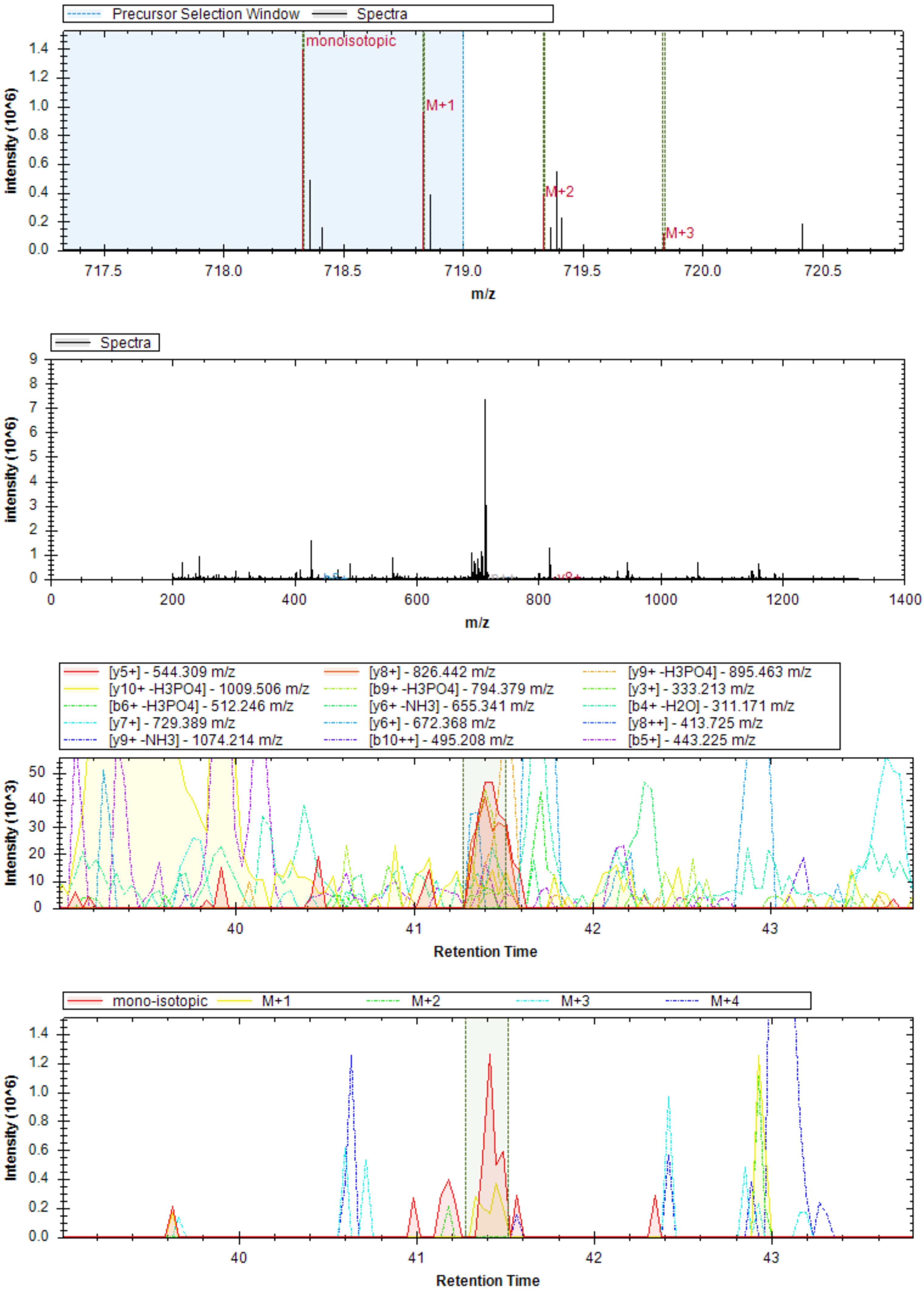

O88935 – _PVAGGPGAPPAARPPAS[+H+O3+P]PSPQR_.3
Fig4_MP-DIA-barrel-cortex-p9-2_MHRM.htrms

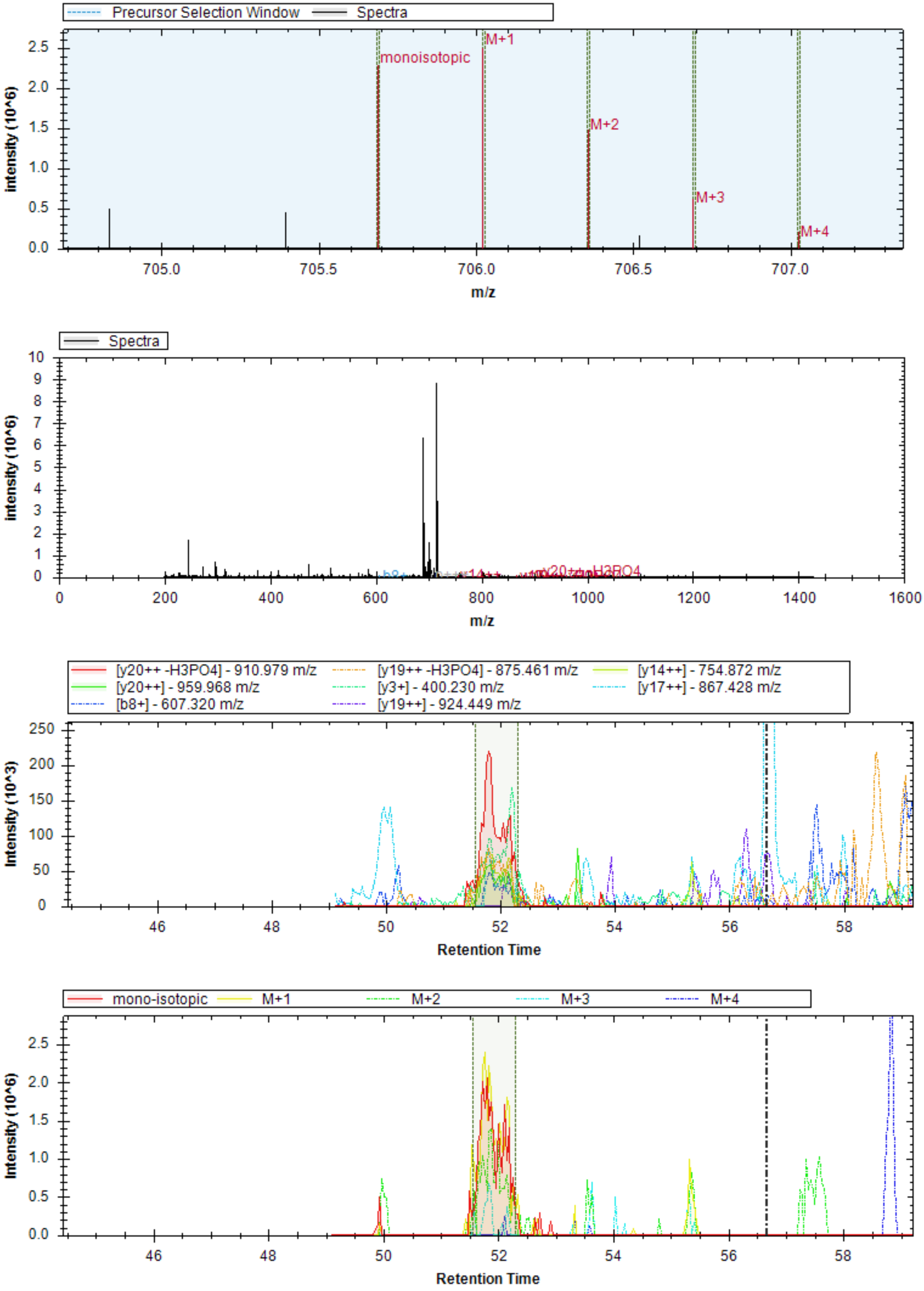

O88935 – _PVAGGPGAPPAARPPASPS[+H+O3+P]PQR_.3

Fig4_MP-DIA-barrel-cortex-p9-2_MHRM.htrms

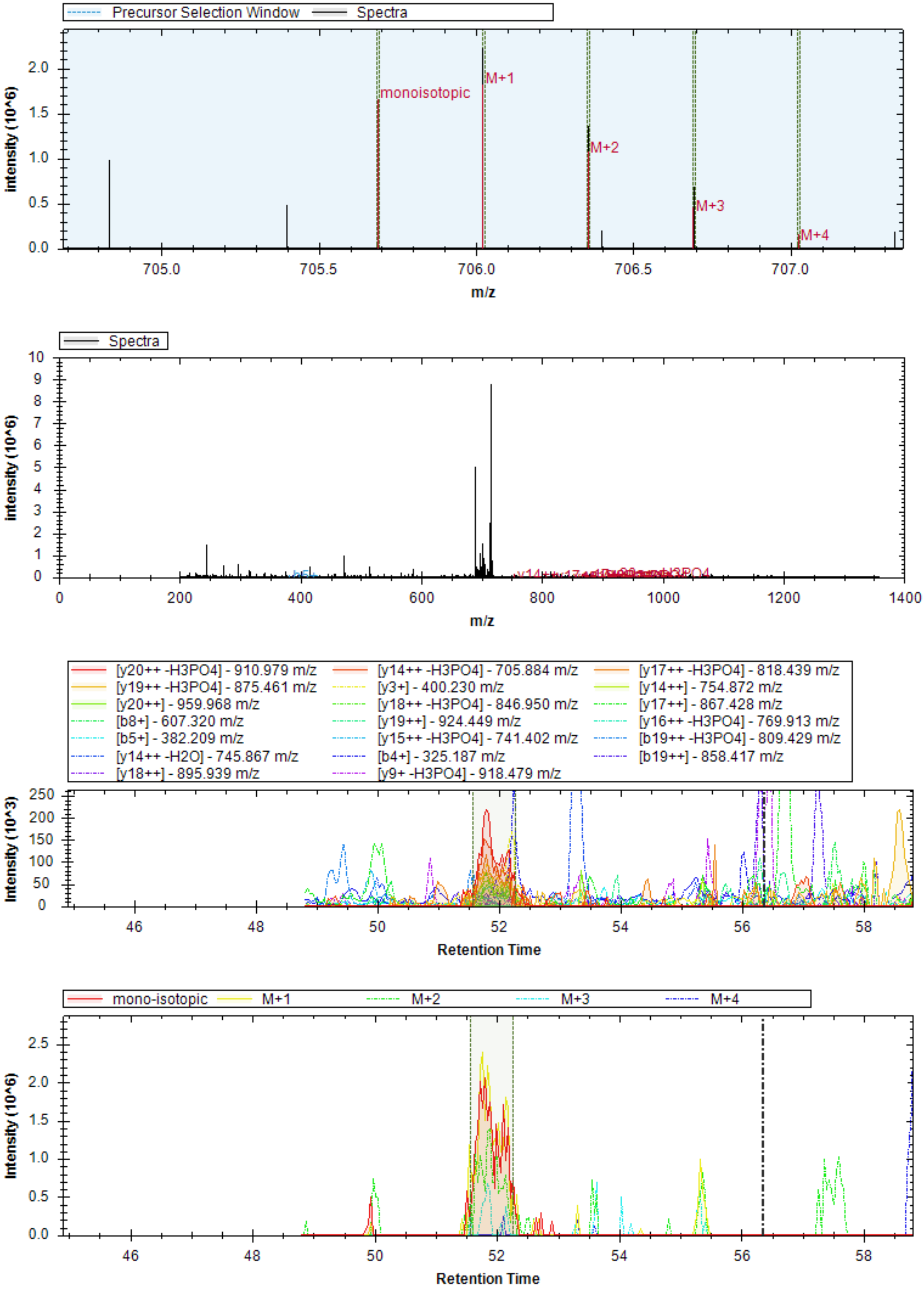

P28738 – _PIRPGHYPAS[+H+O3+P]SPTAVHAVR_.3
Fig4_MP-DIA-barrel-cortex-p9-2_MHRM.htrms

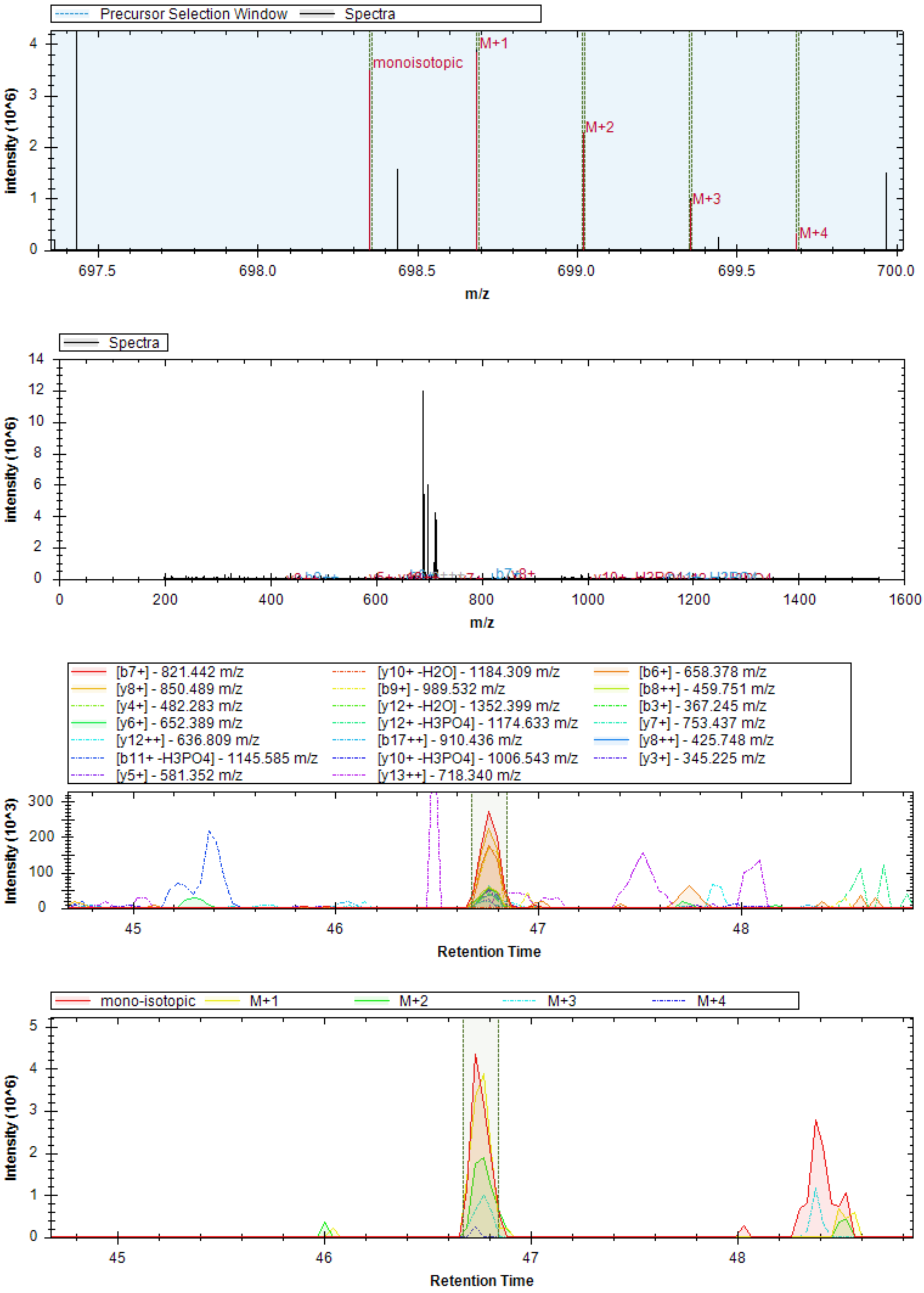

P28738 – _PIRPGHYPASS[+H+O3+P]PTAVHAVR_.3
Fig4_MP-DIA-barrel-cortex-p9-2_MHRM.htrms

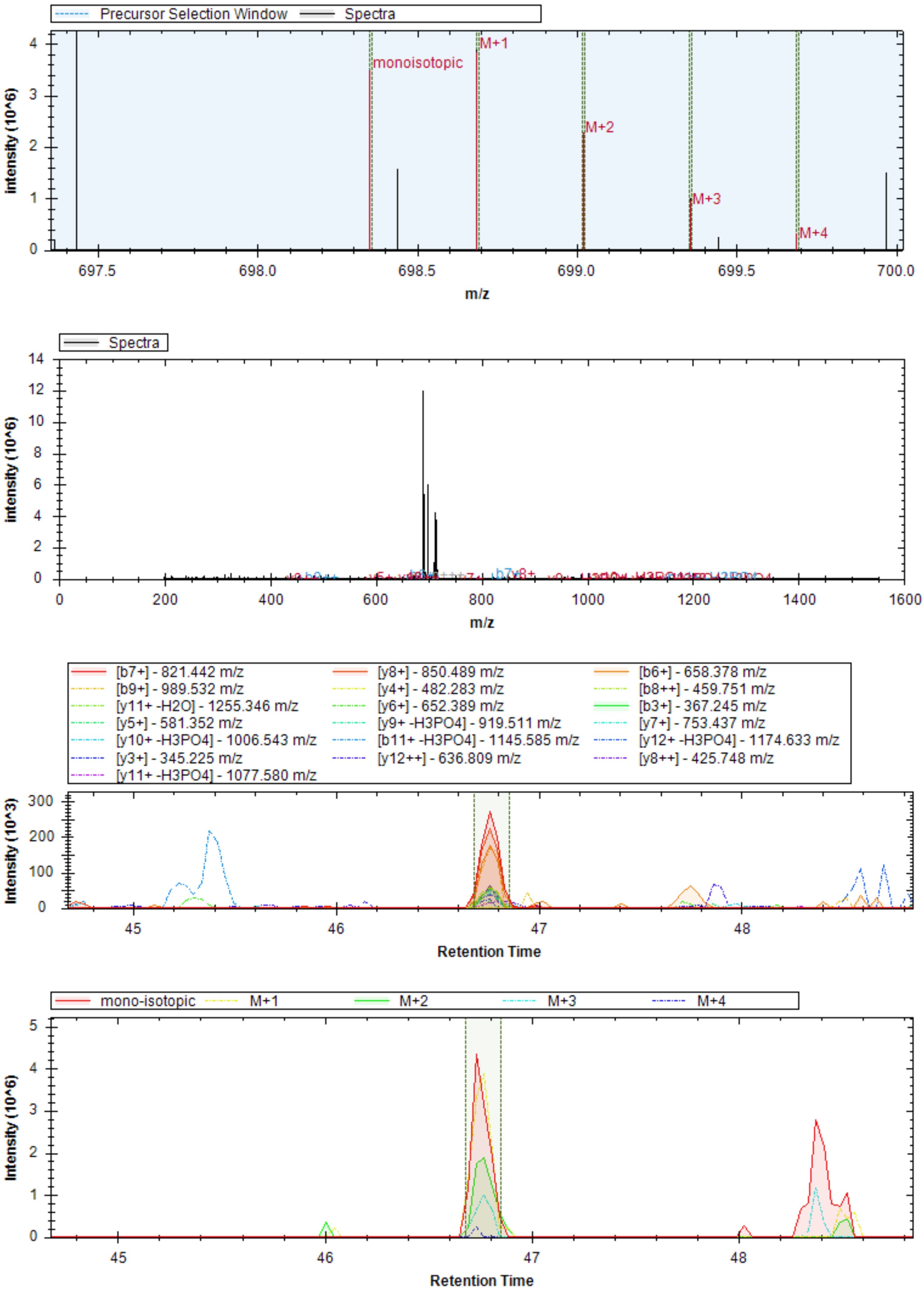

P54227 – _ESVPDFPLS[+H+O3+P]PPK_.2

Fig4_MP-DIA-barrel-cortex-p9-2_MHRM.htrms

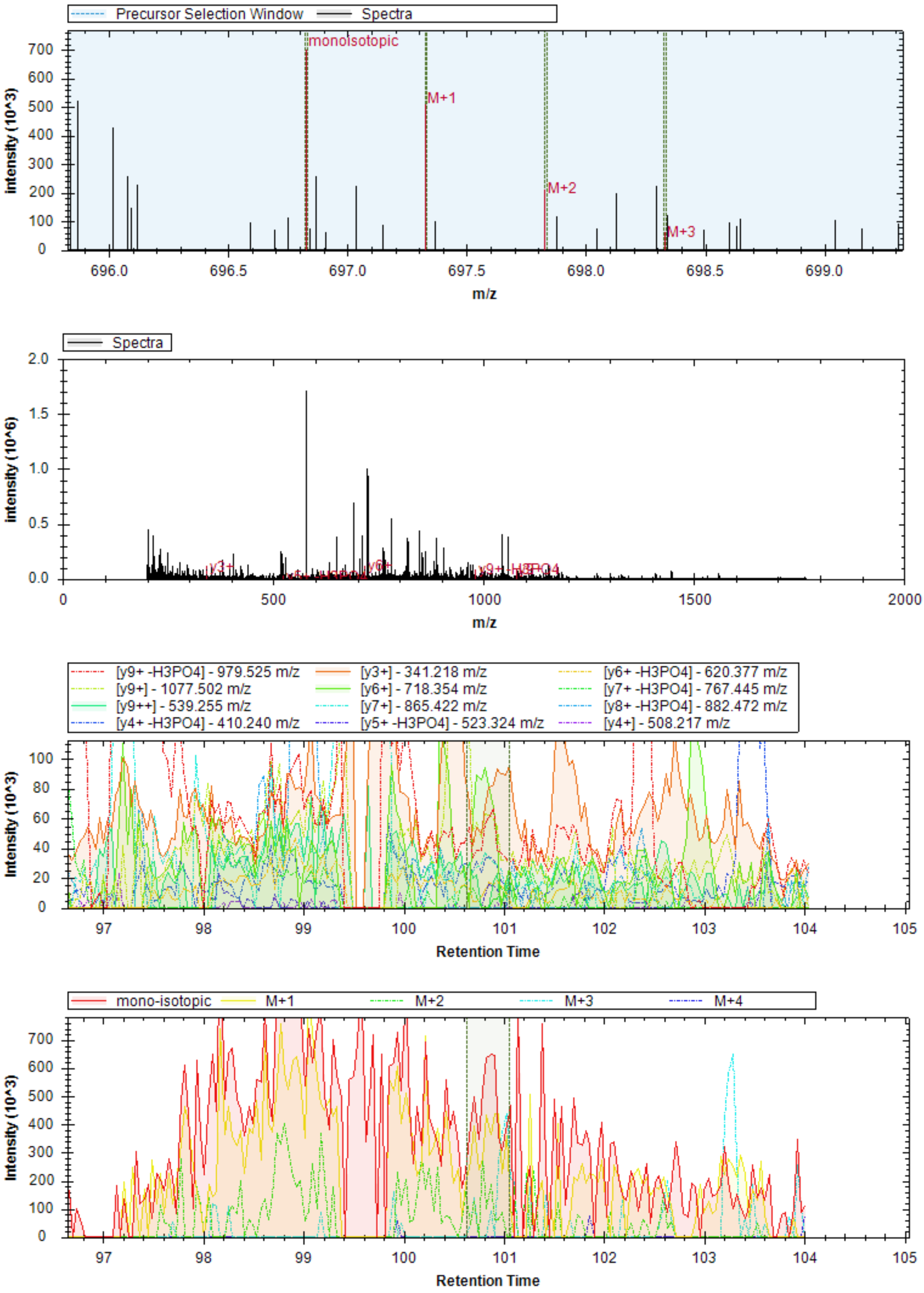

P62751 – _KAY[+H+O3+P]VRLAPDYDALDVANK_.3
Fig4_MP-DIA-barrel-cortex-p9-2_MHRM.htrms

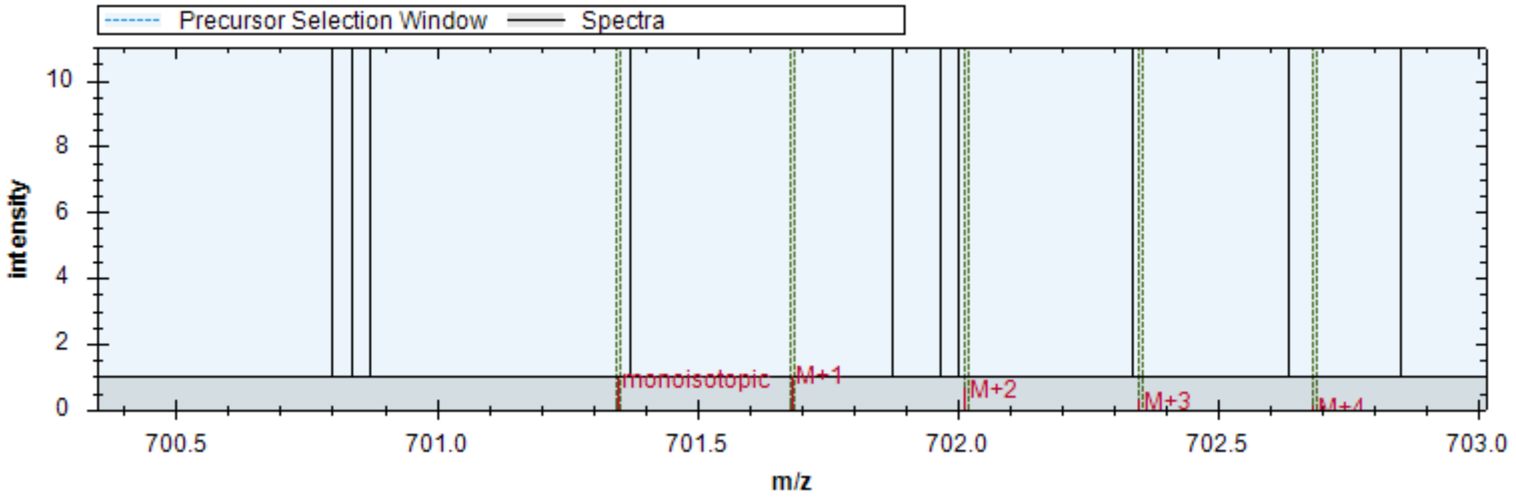

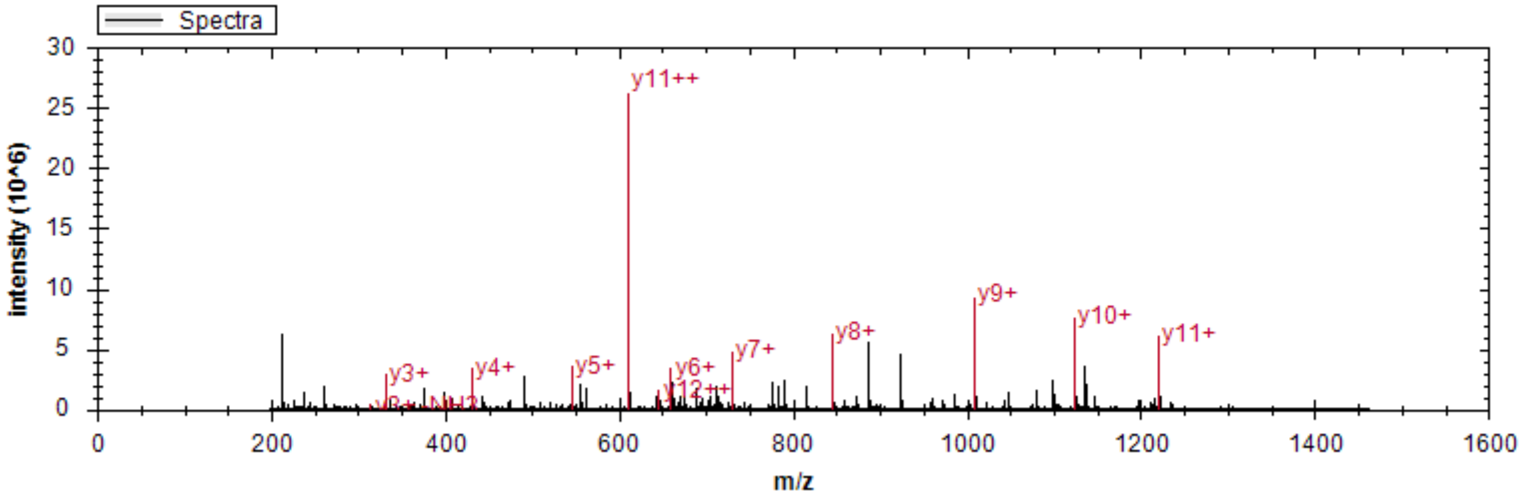

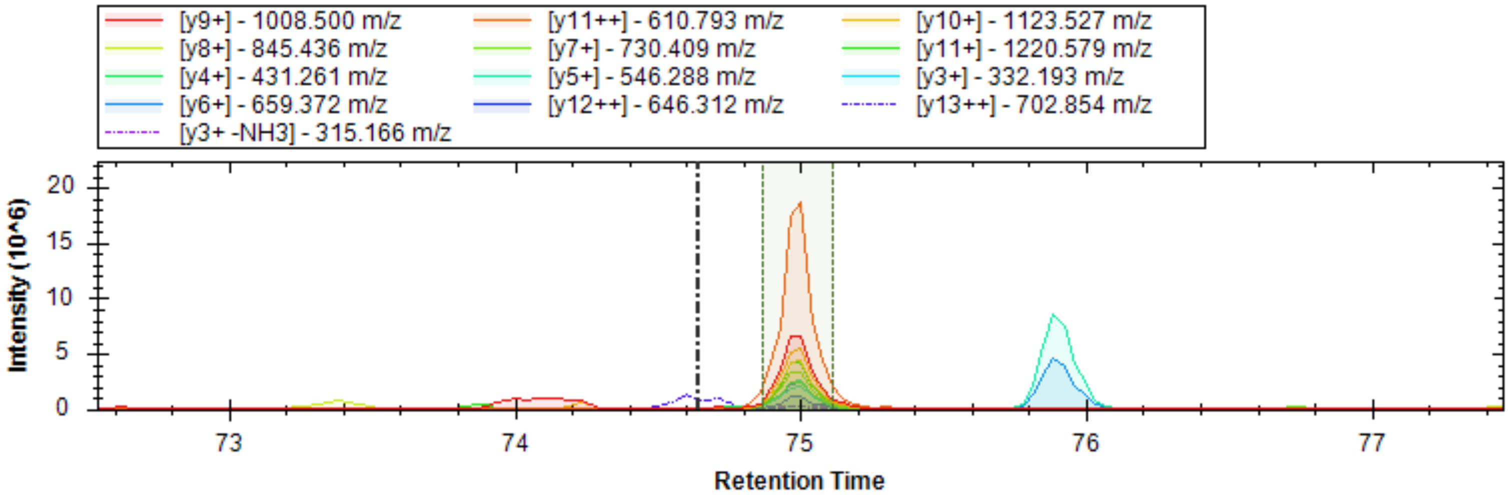

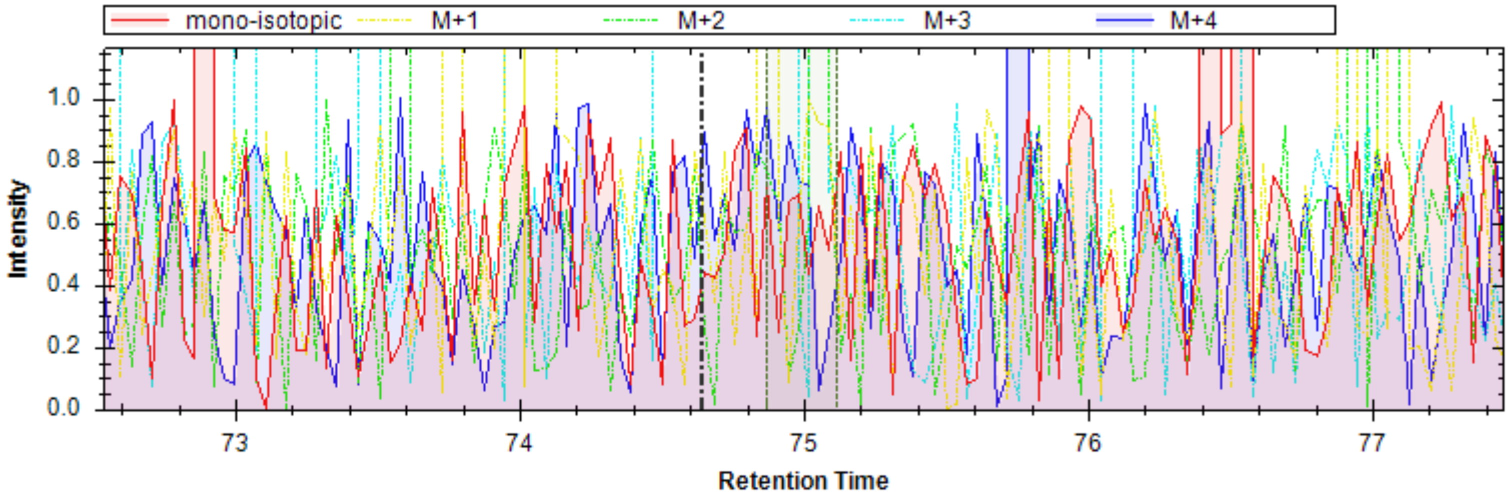

Q3U4B4 – _RHKT[+H+O3+P]LSANEEK_.2
Fig4_MP-DIA-barrel-cortex-p9-2_MHRM.htrms

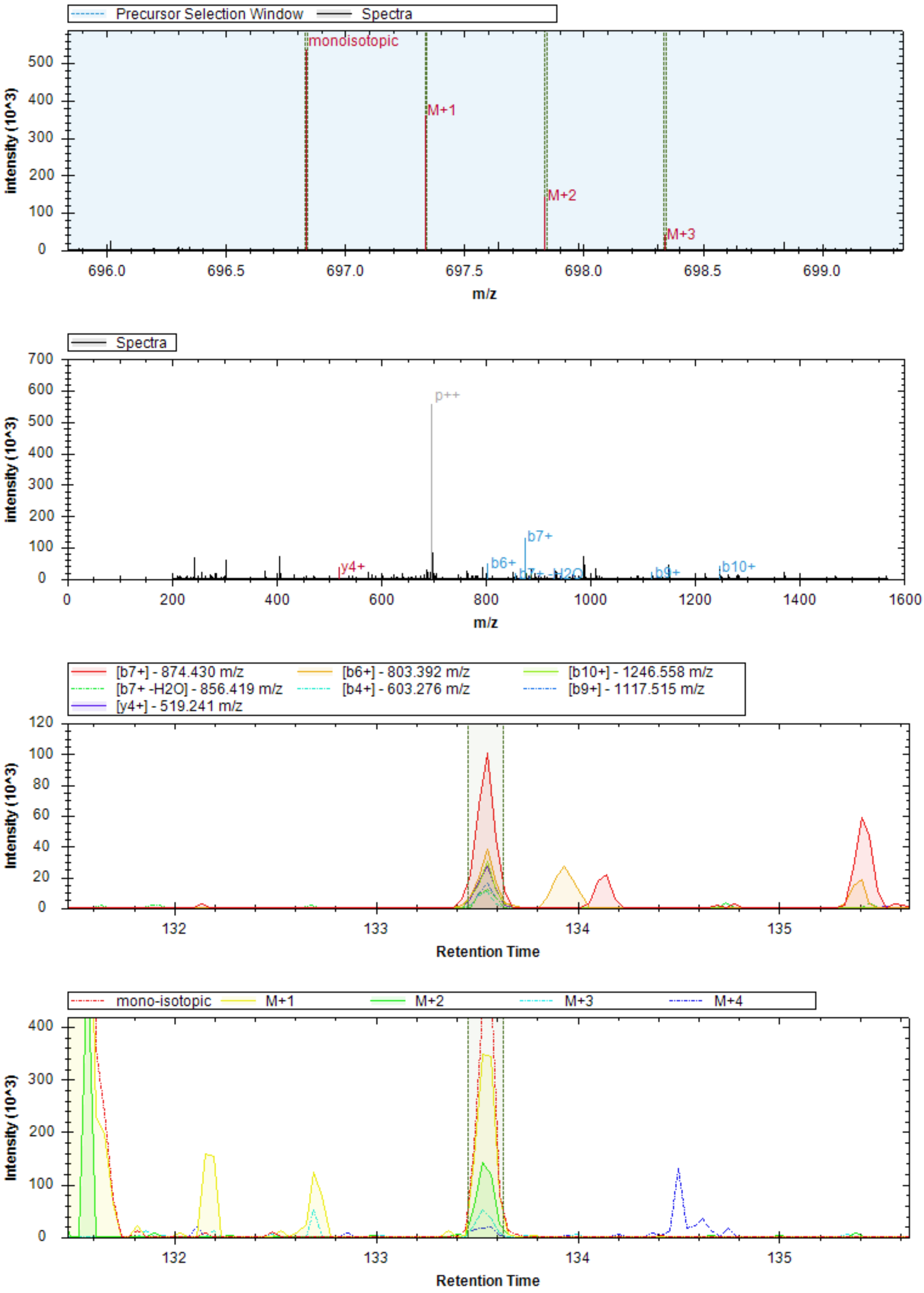

Q9ESE1;Q9ESE1-2 – _VGT[+H+O3+P]VLLIM[+O]HTLK_.2
Fig4_MP-DIA-barrel-cortex-p9-2_MHRM.htrms

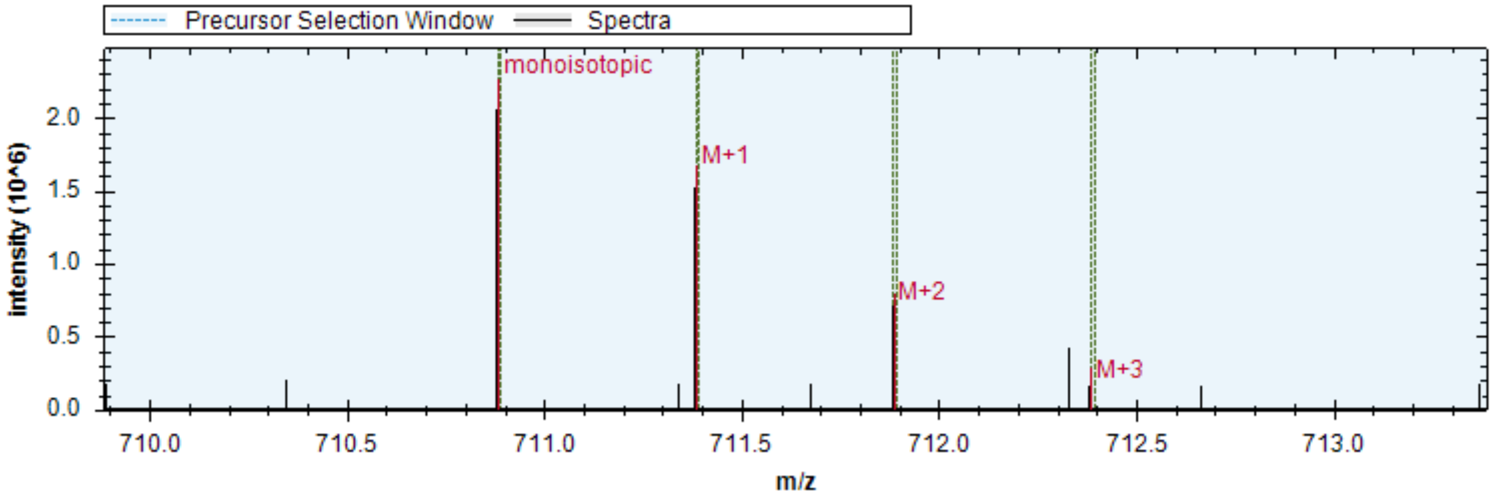

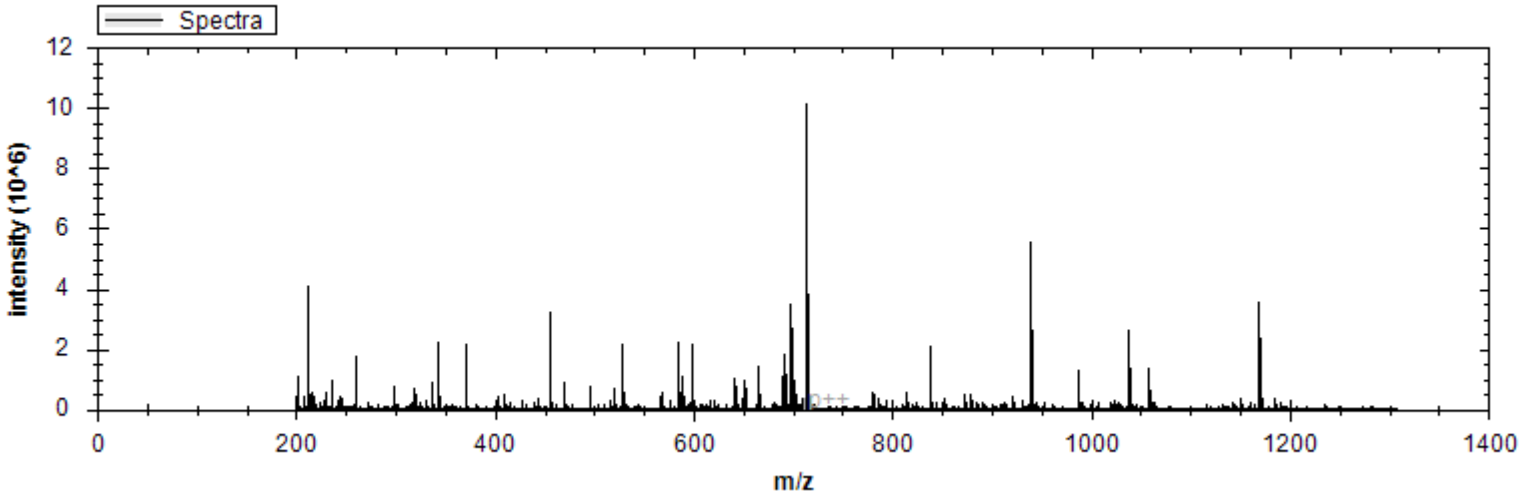

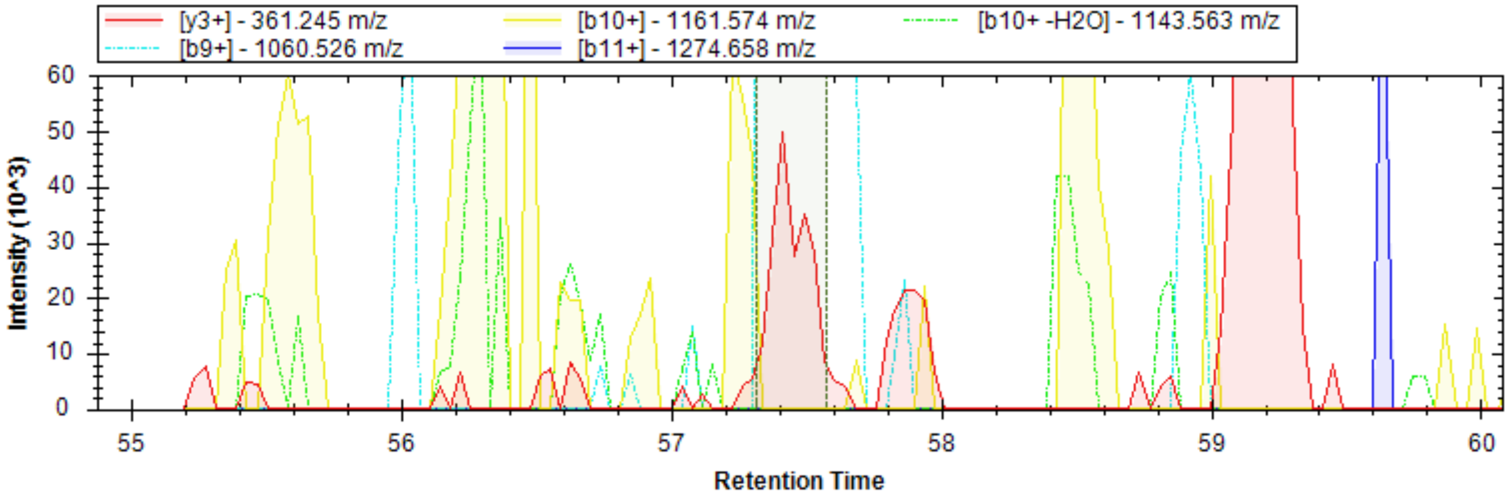

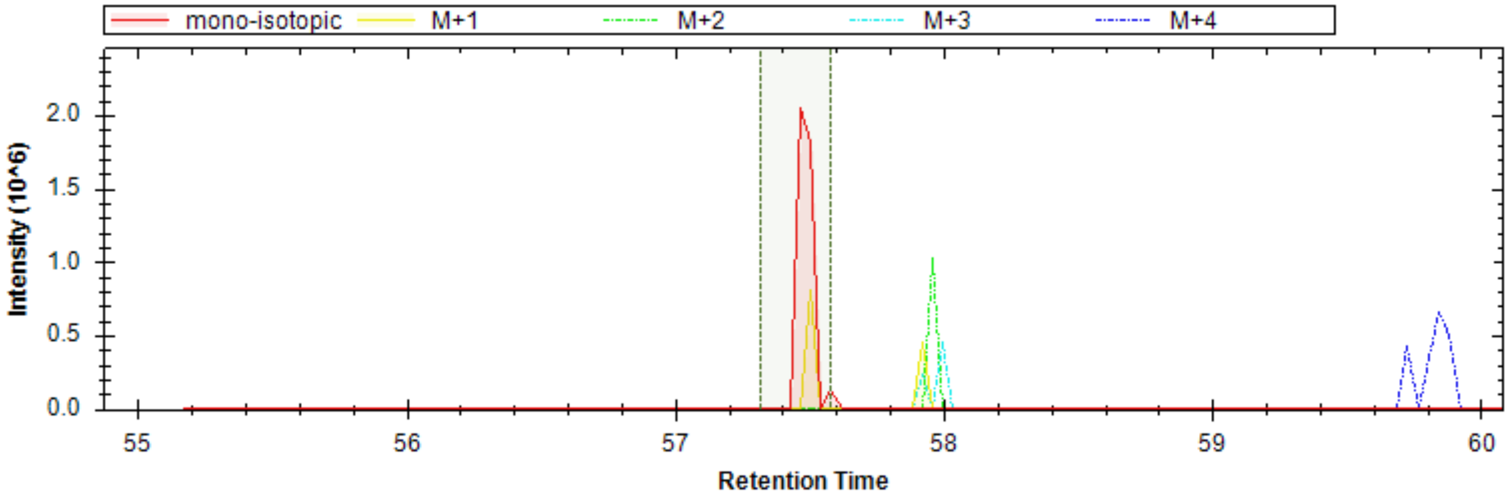

Q9QYC0 – _SPGT[+H+O3+P]PAGEGSGSPPK_.2
Fig4_MP-DIA-barrel-cortex-p9-2_MHRM.htrms

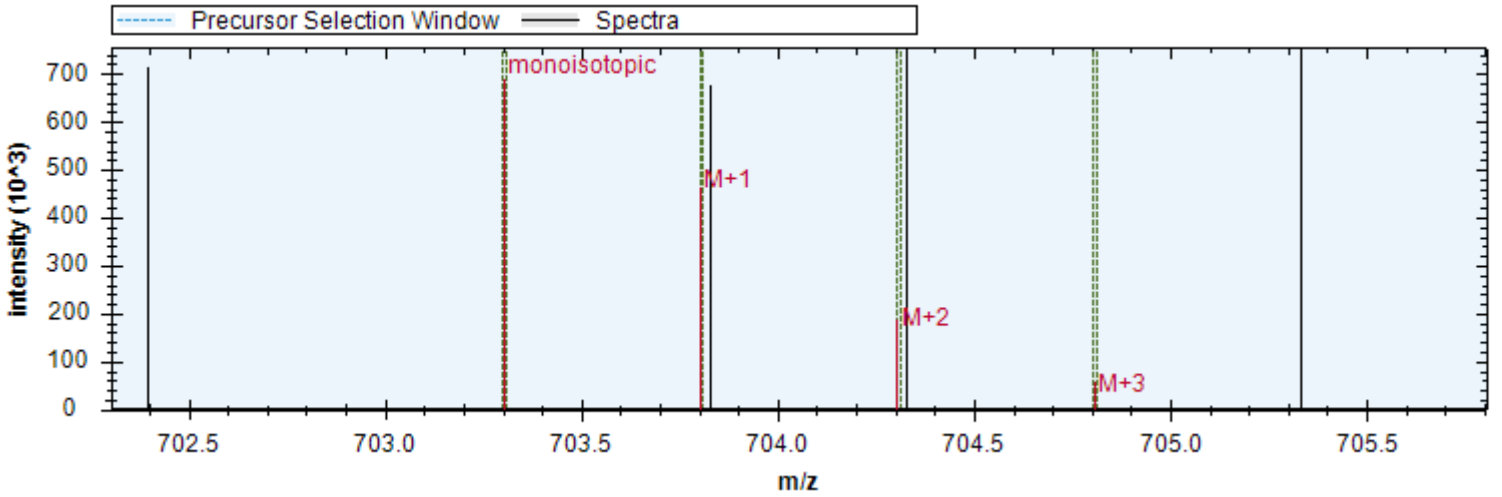

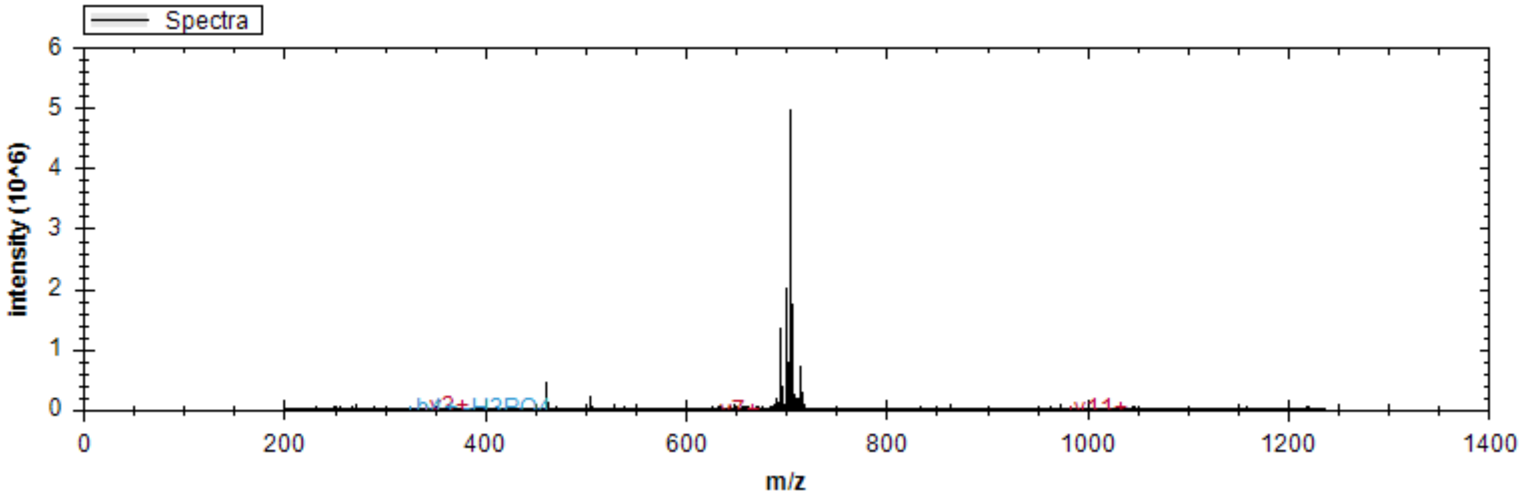

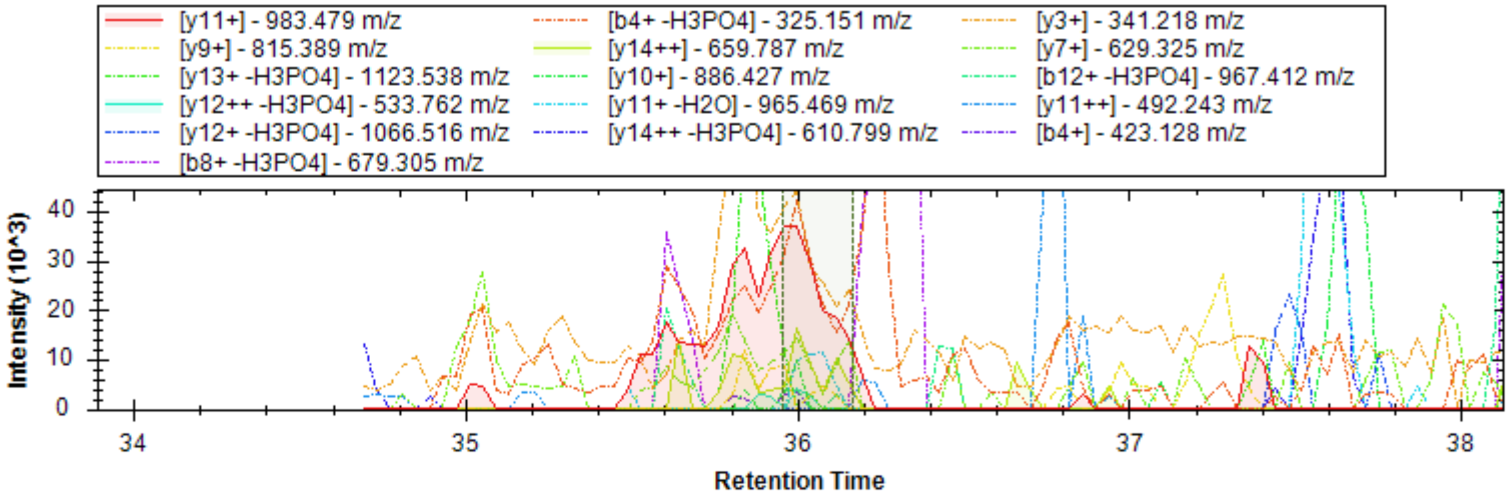

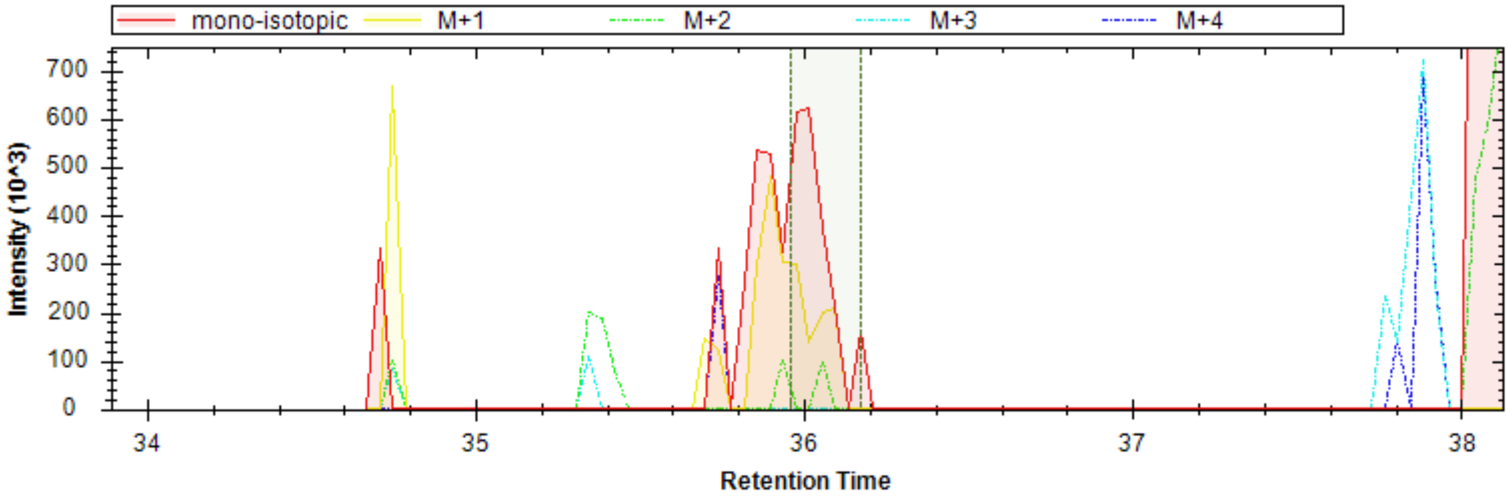

P10637 – _SGYSS[+H+O3+P]PGSPGTPGSR_.2

Fig4_MP-DIA-barrel-cortex-p9-2_MHRM.htrms

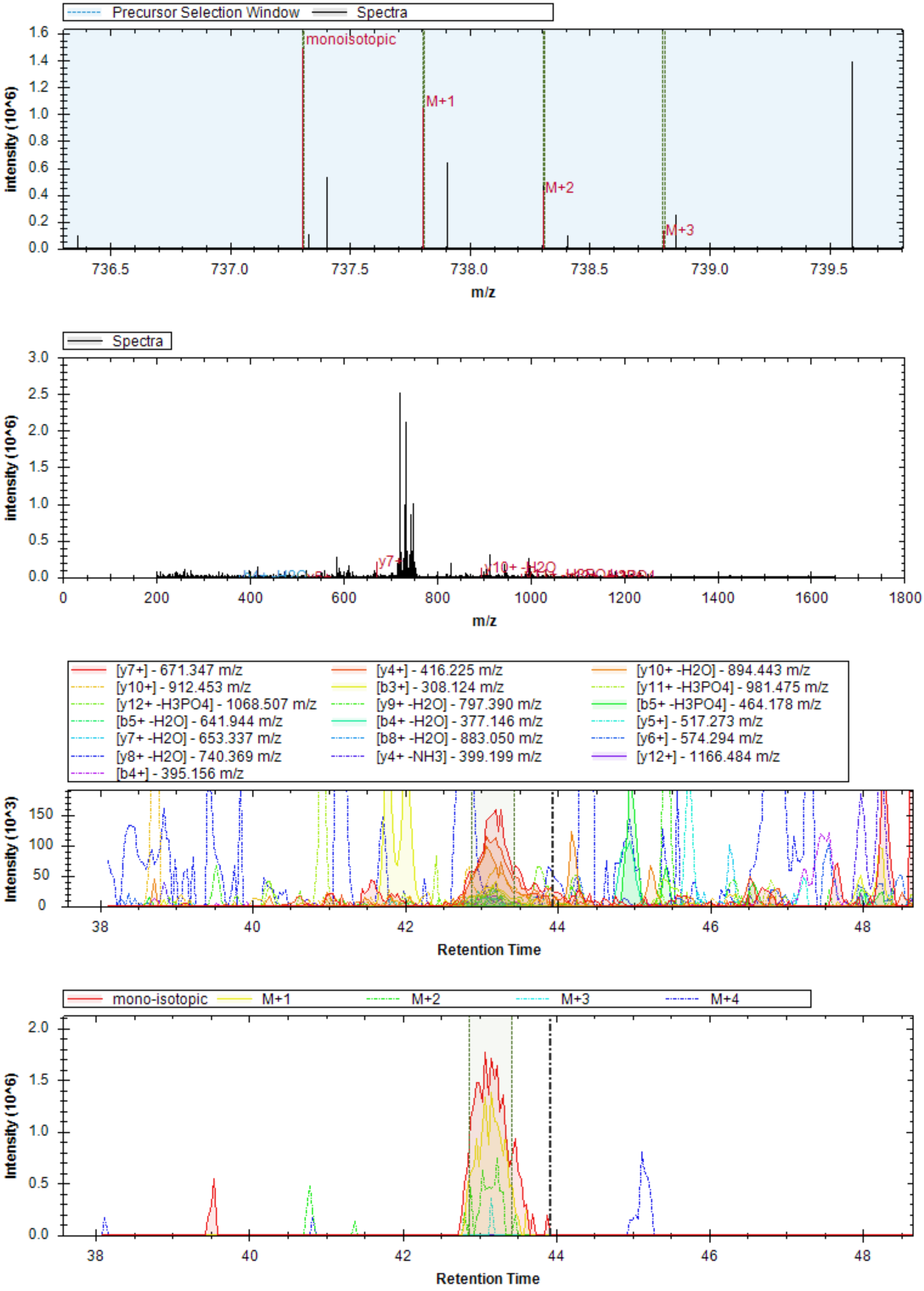

P10637 – _SGYSSPGS[+H+O3+P]PGTPGSR_.2
Fig4_MP-DIA-barrel-cortex-p9-2_MHRM.htrms

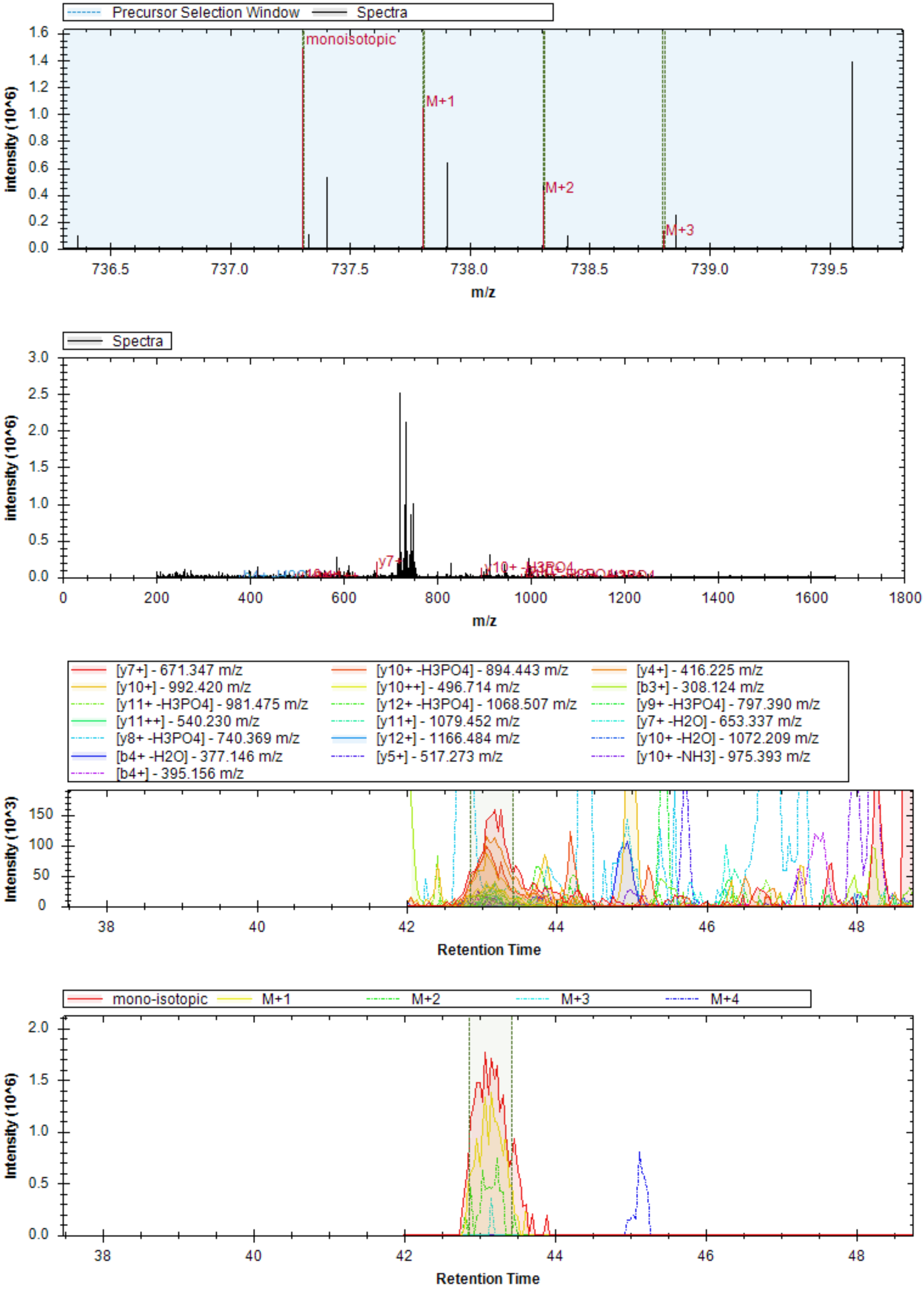

P57776-3 – _ATAPQTQHVS[+H+O3+P]PMR_.2
Fig4_MP-DIA-barrel-cortex-p9-2_MHRM.htrms

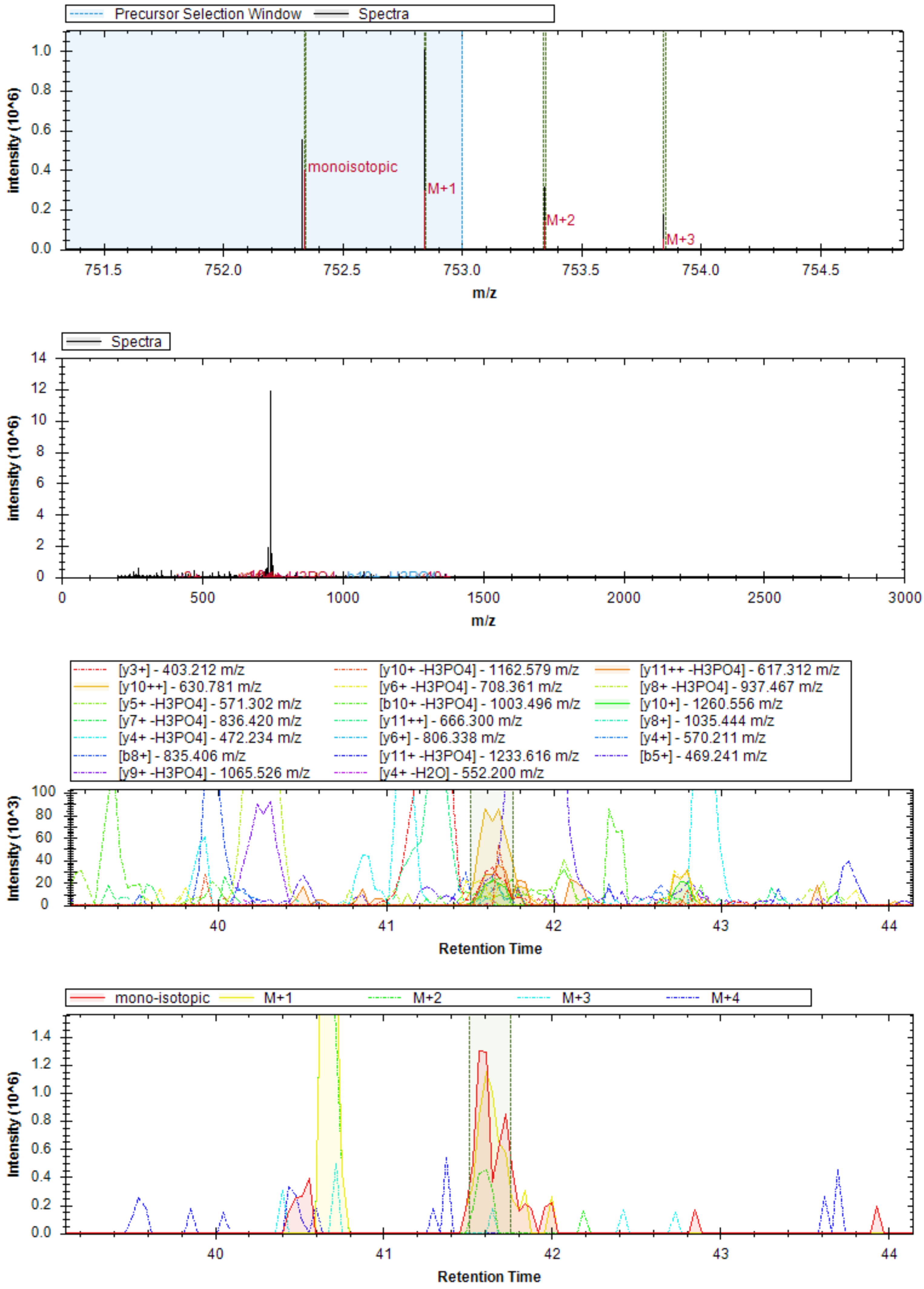

Q3UNH4 – _VITQIPGAT[+H+O3+P]SPGKVETPSLQK_.3

Fig4_MP-DIA-barrel-cortex-p9-2_MHRM.htrms

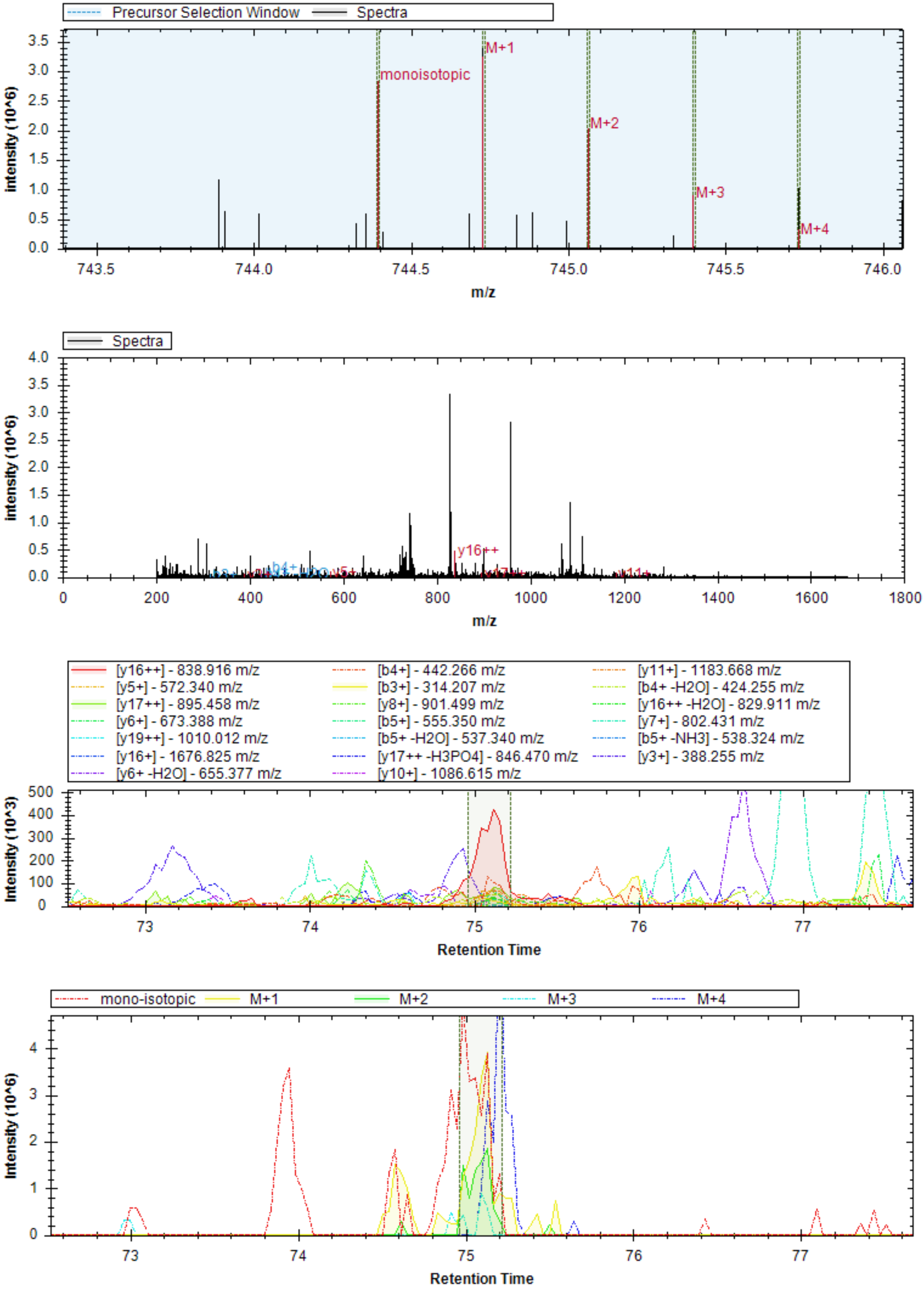

Q3UNH4 – _VITQIPGATS[+H+O3+P]PGKVETPSLQK_.3
Fig4_MP-DIA-barrel-cortex-p9-2_MHRM.htrms

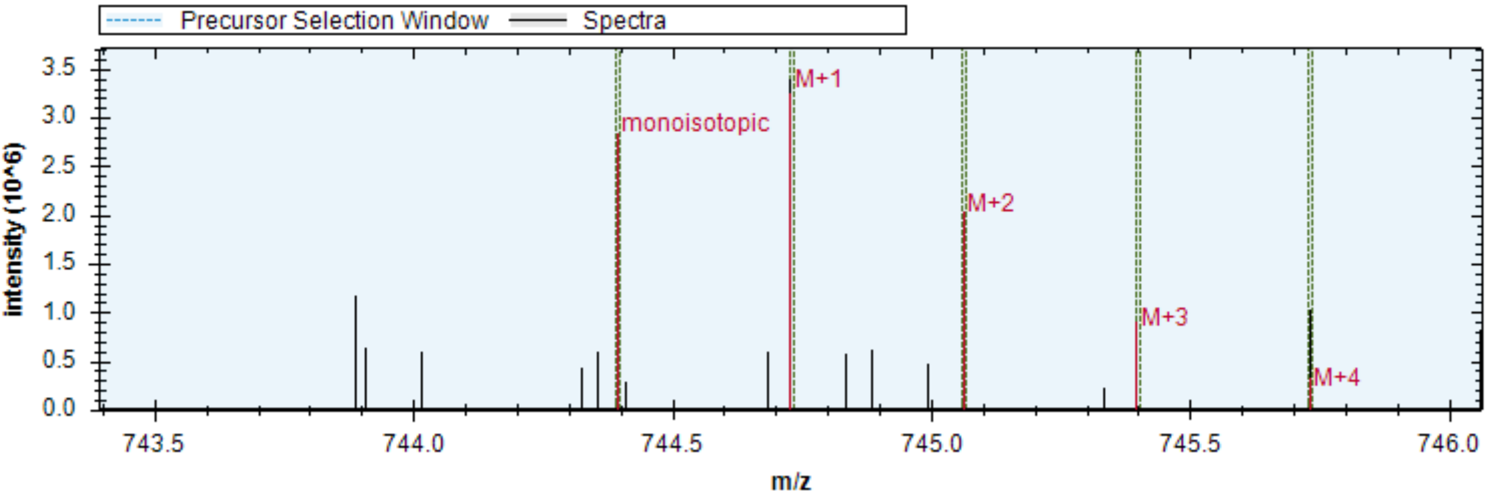

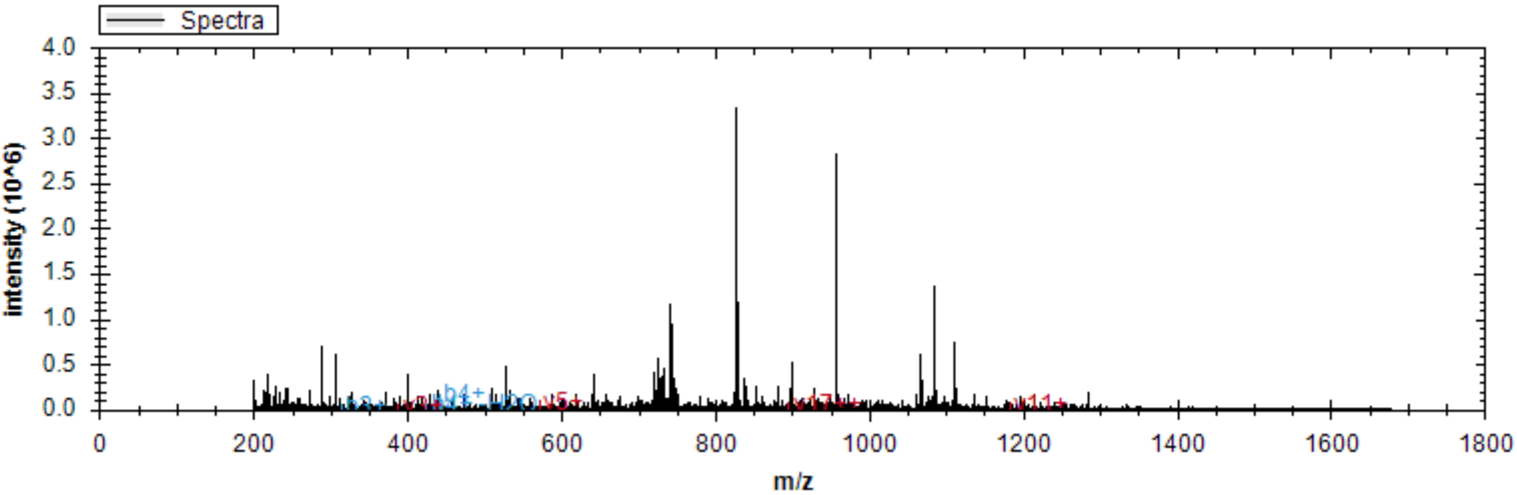

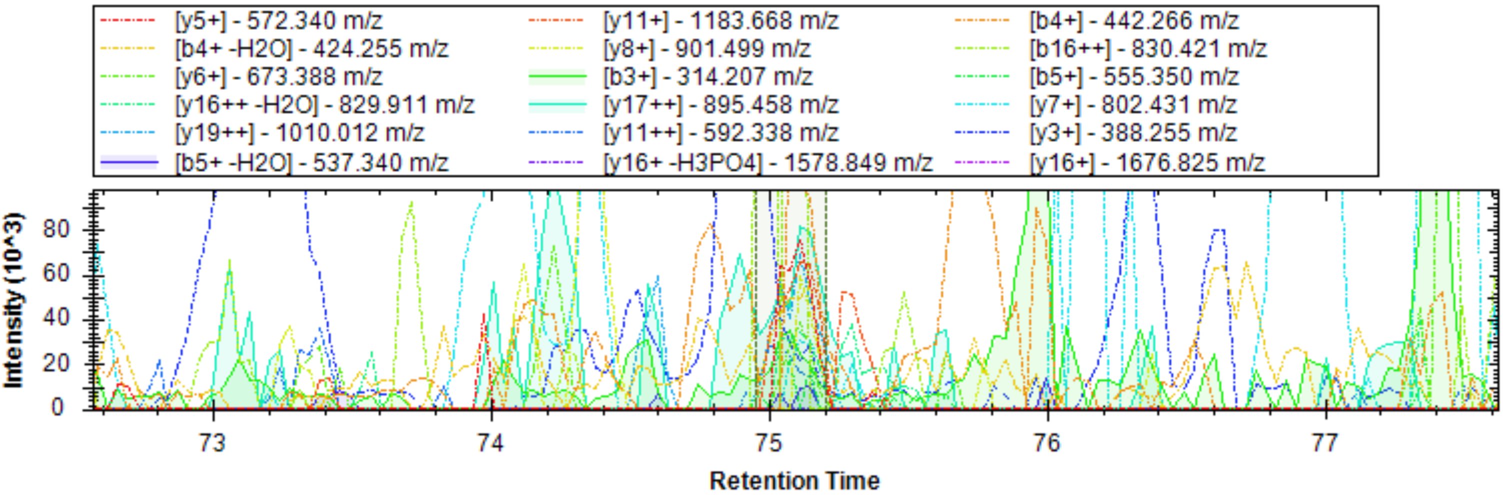

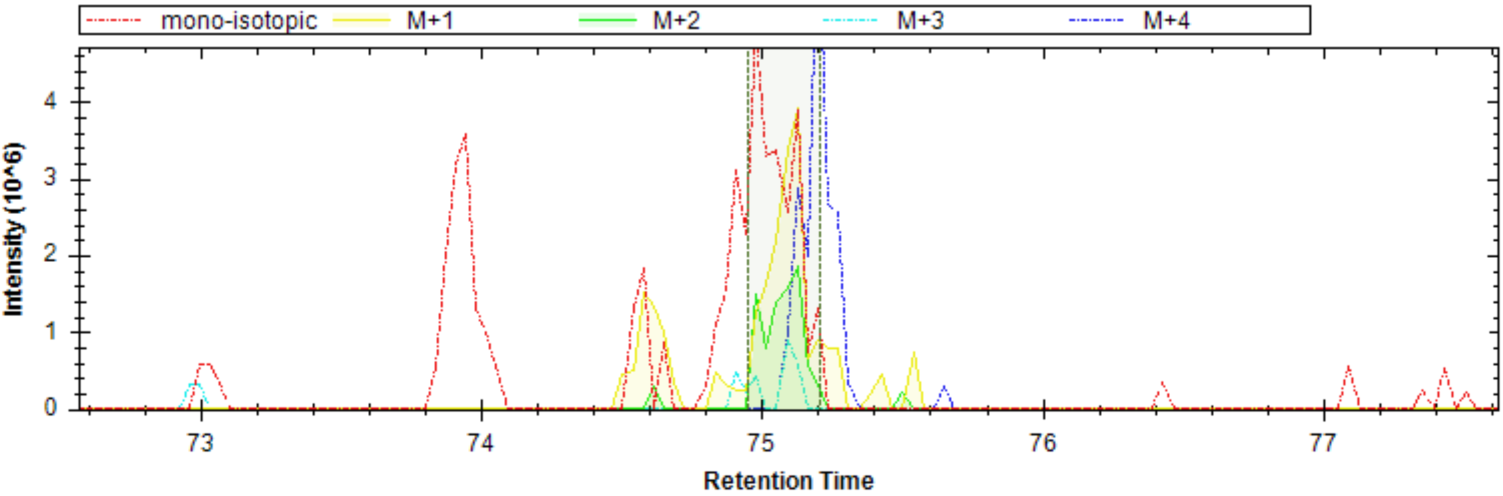

Q8C5R2 – _LAGNEALS[+H+O3+P]PTSPSK_.2
Fig4_MP-DIA-barrel-cortex-p9-2_MHRM.htrms

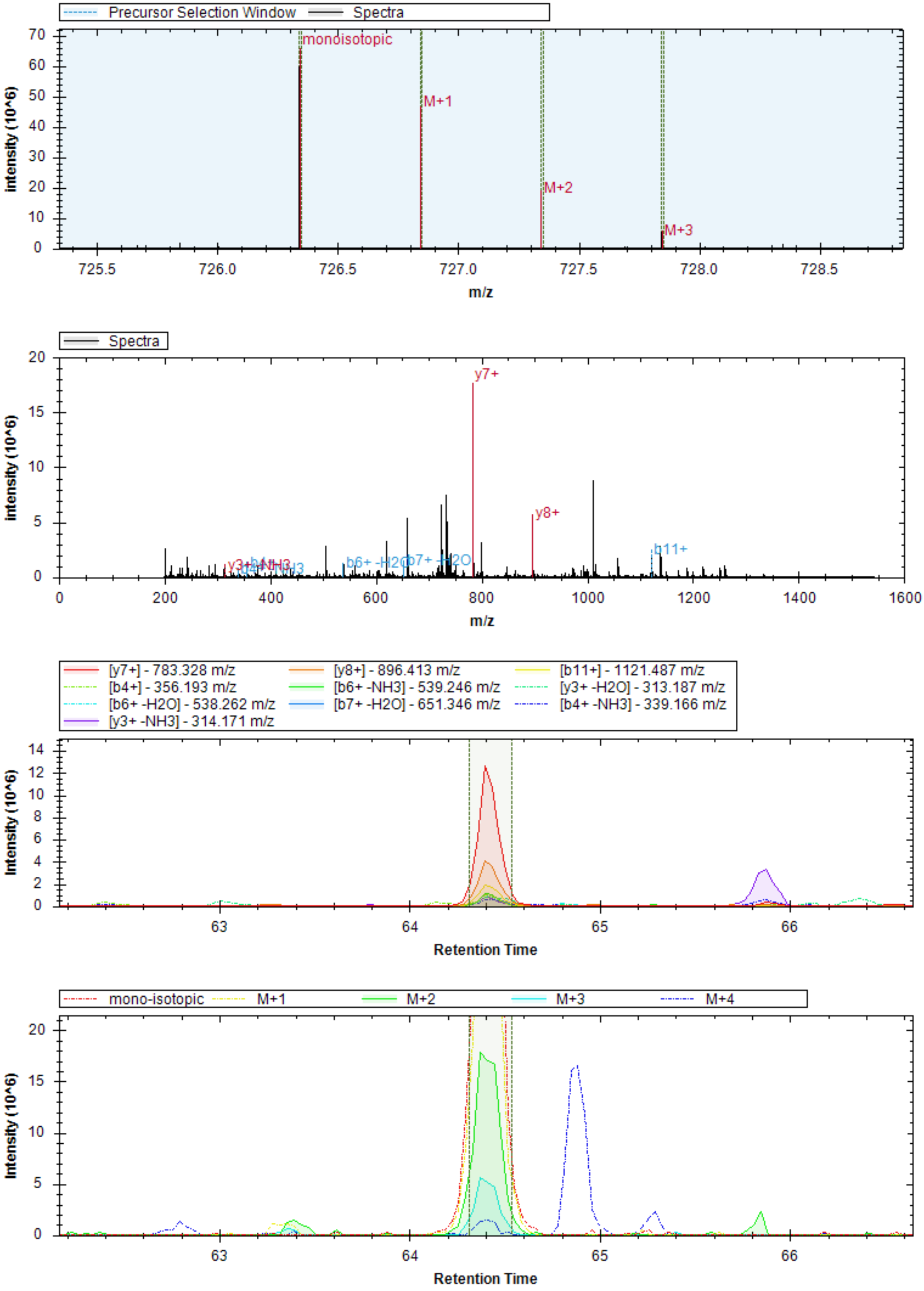

Q8VCI5 – _AKPS[+H+O3+P]PEHAPTISAPDASGPQK_.3

Fig4_MP-DIA-barrel-cortex-p9-2_MHRM.htrms

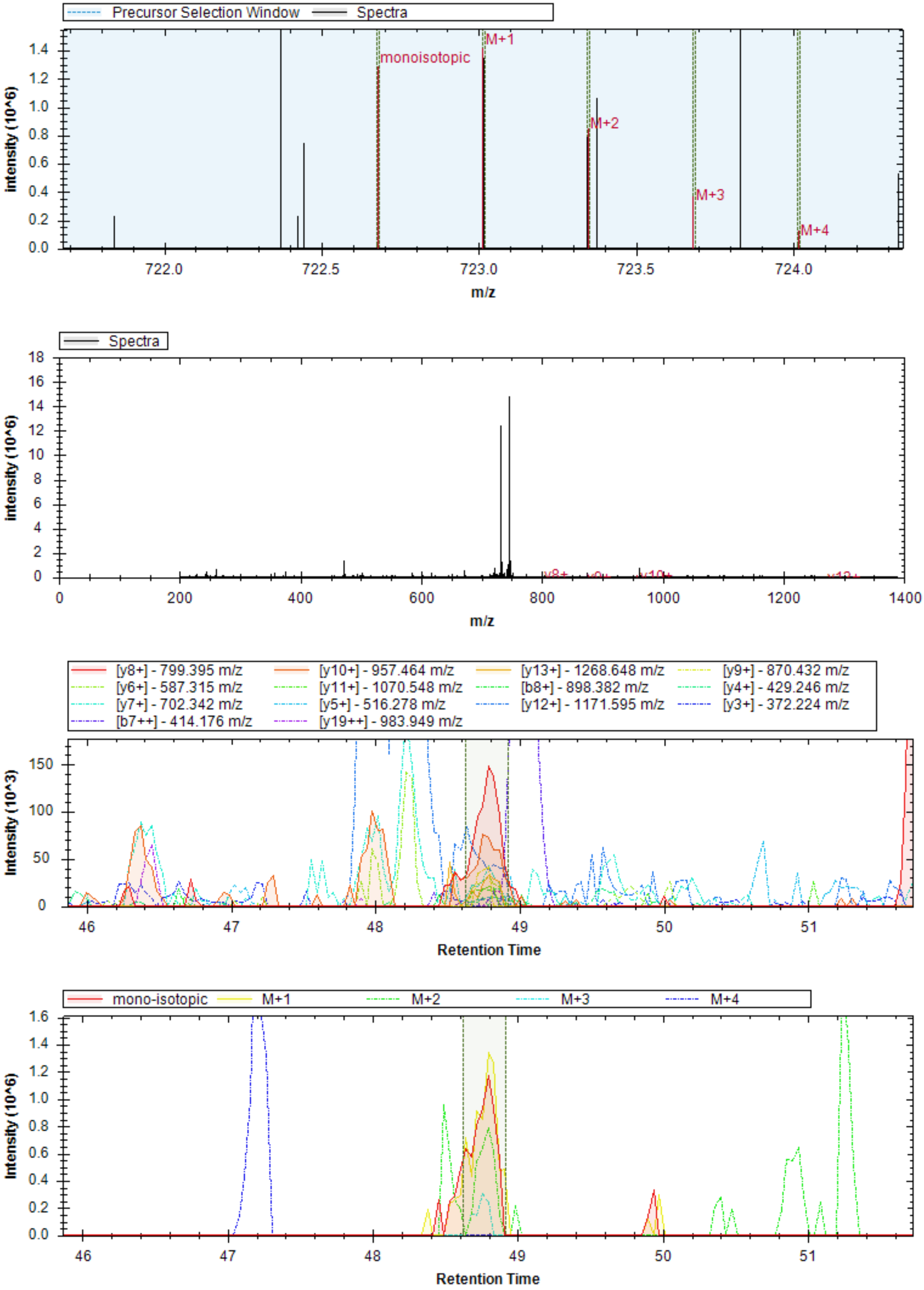

Q9CY18 – _M[+O]GQTVRAVAS[+H+O3+P]SMR_.2
Fig4_MP-DIA-barrel-cortex-p9-2_MHRM.htrms

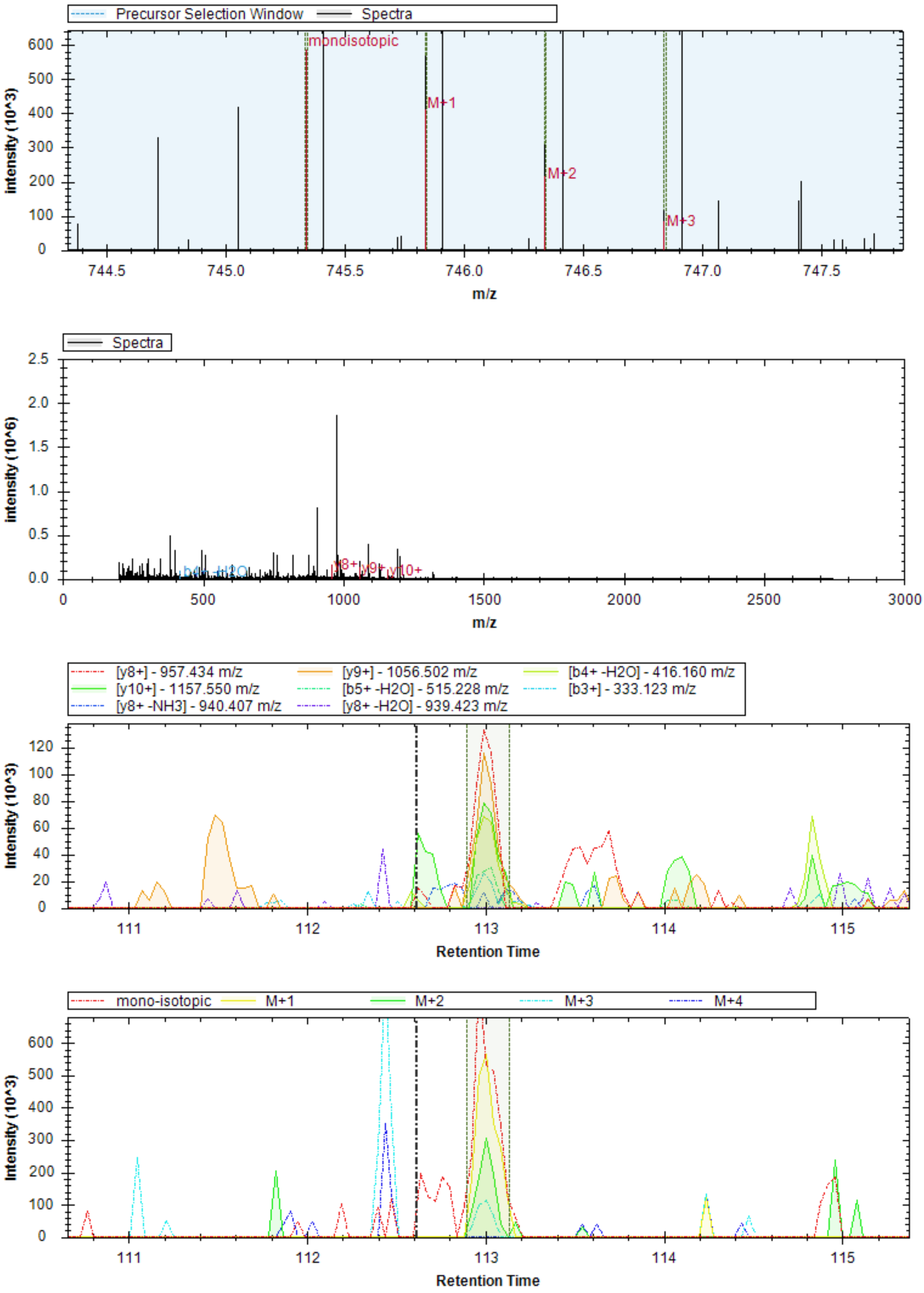

Q9JLM8 – _SKS[+H+O3+P]PASTSSVNGTPGSQLSTPR_.3
Fig4_MP-DIA-barrel-cortex-p9-2_MHRM.htrms

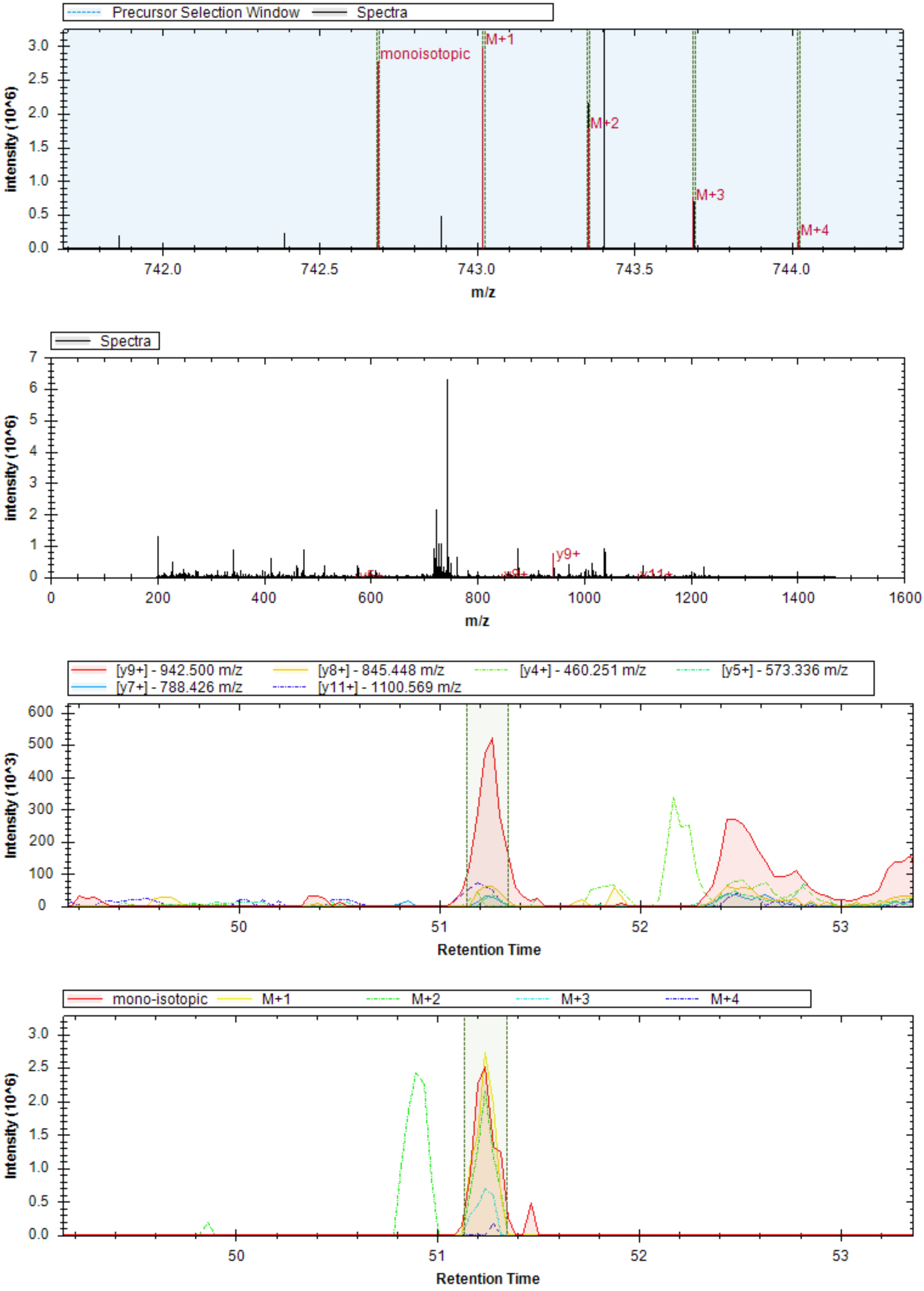

Q9JLM8 – _SPSPSPT[+H+O3+P]SPGSLRK_.2
Fig4_MP-DIA-barrel-cortex-p9-2_MHRM.htrms

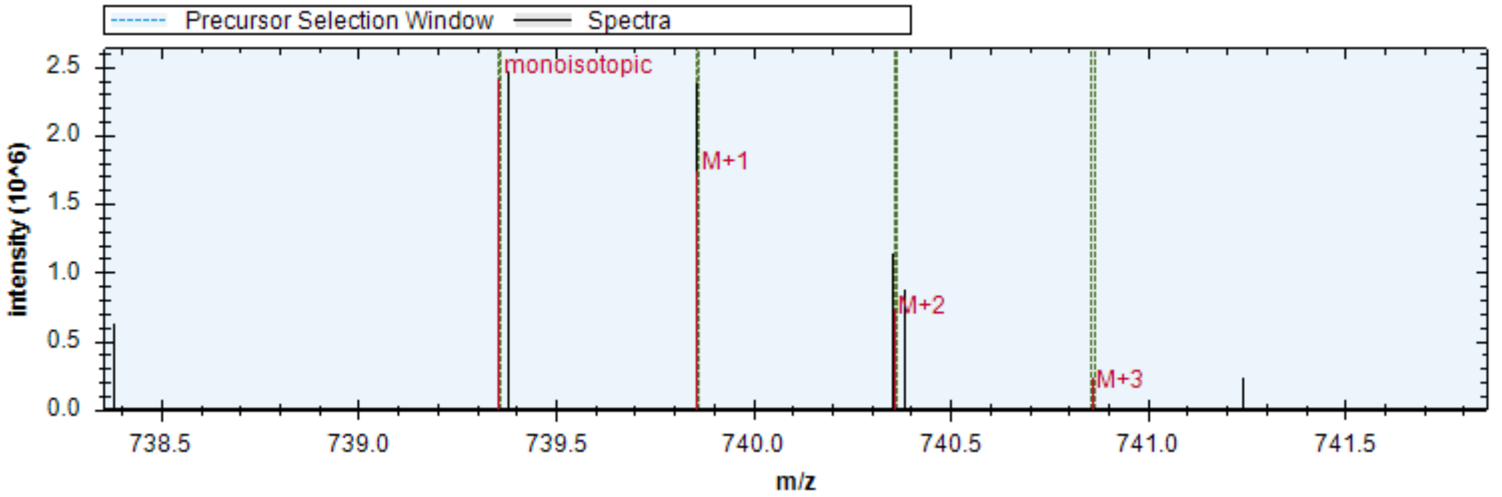

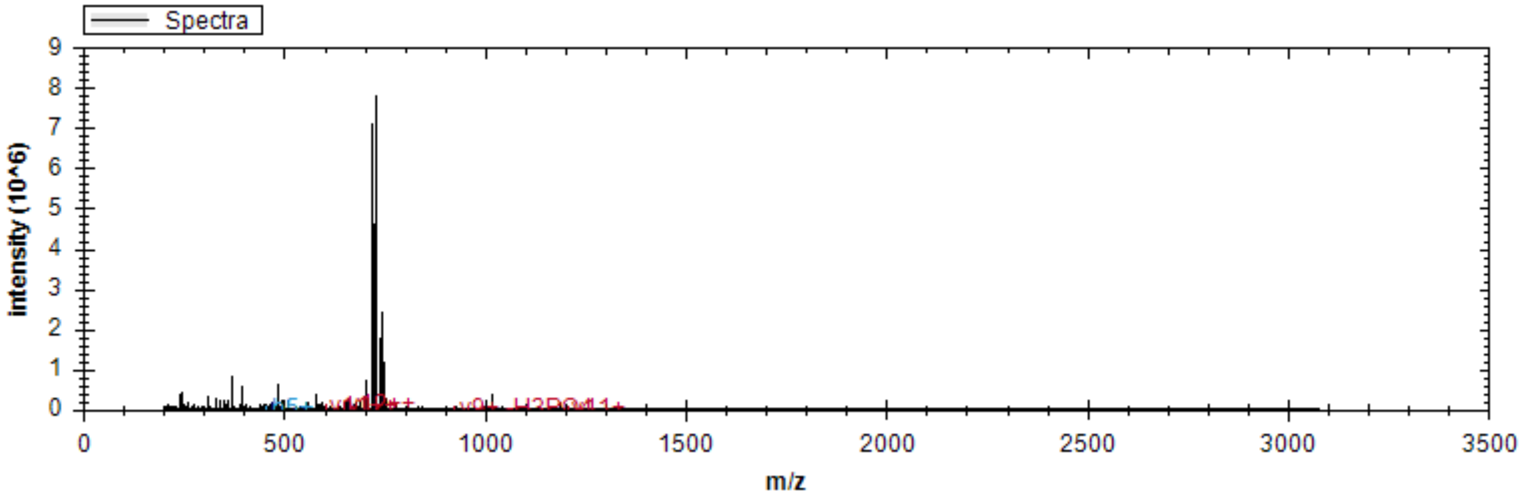

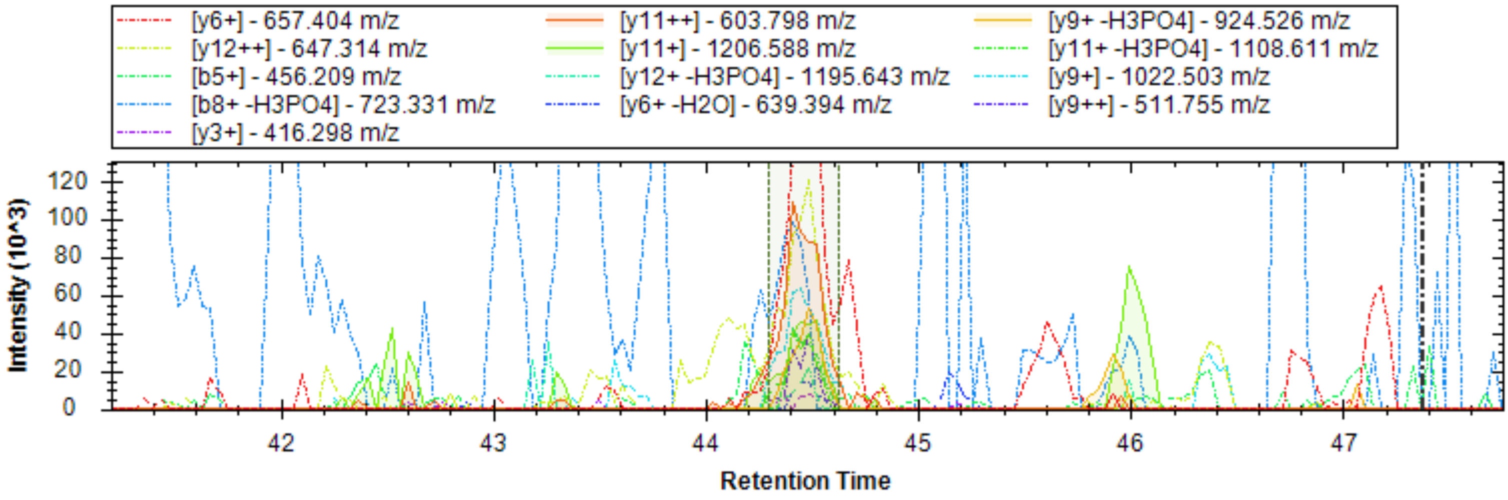

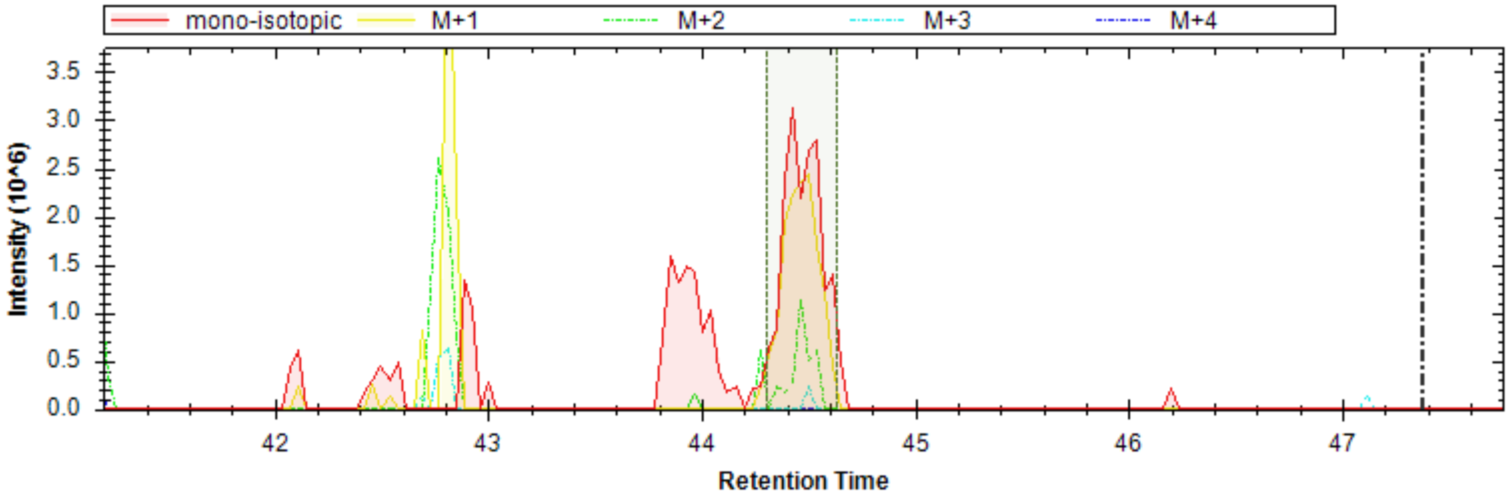

Q9JLM8 – _SPSPSPTS[+H+O3+P]PGSLRK_.2

Fig4_MP-DIA-barrel-cortex-p9-2_MHRM.htrms

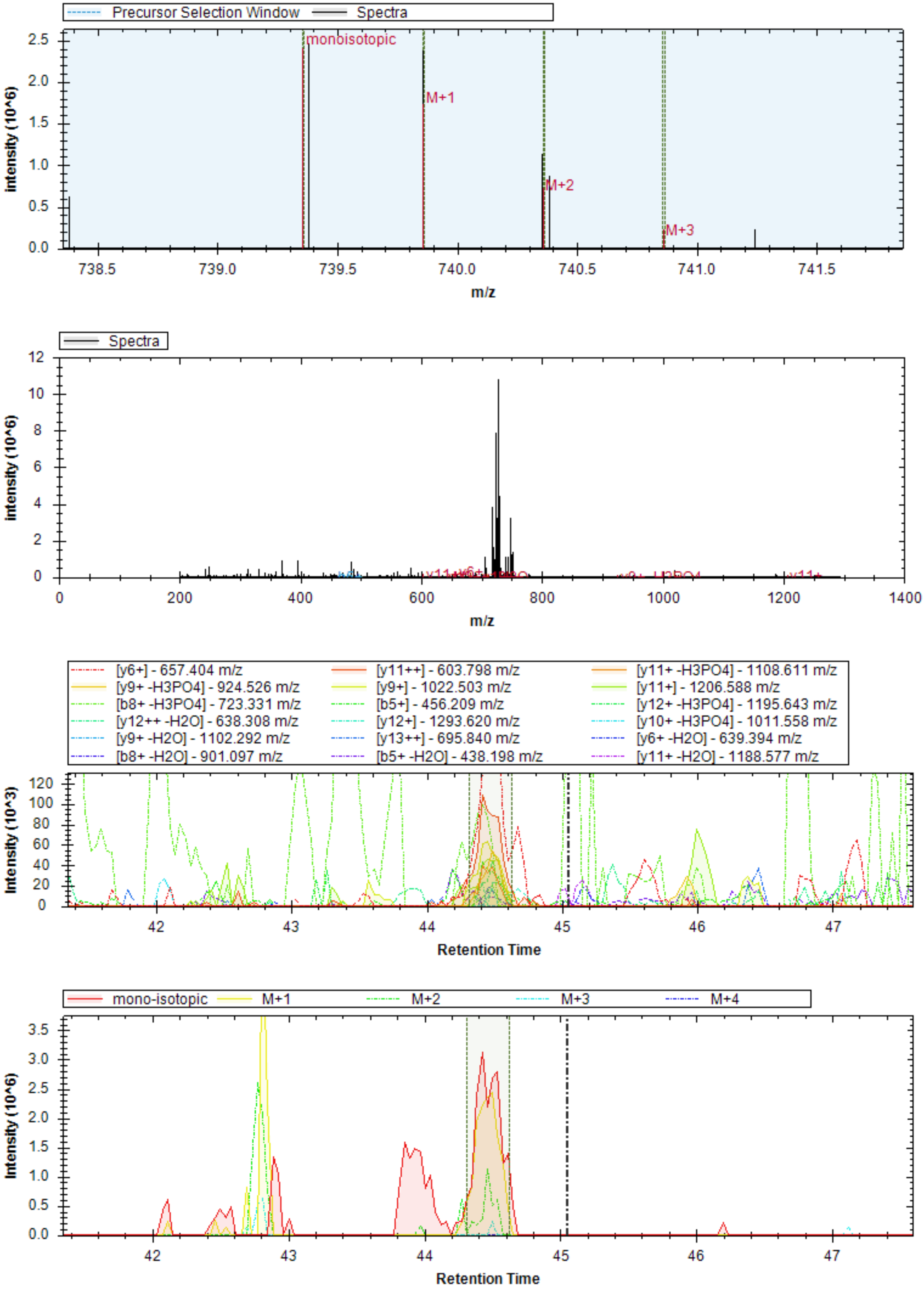

D3Z6S9 – _IPSIEIT[+H+O3+P]ET[+H+O3+P]LPQTQASMLR_.3
Fig4_MP-DIA-barrel-cortex-p9-2_MHRM.htrms

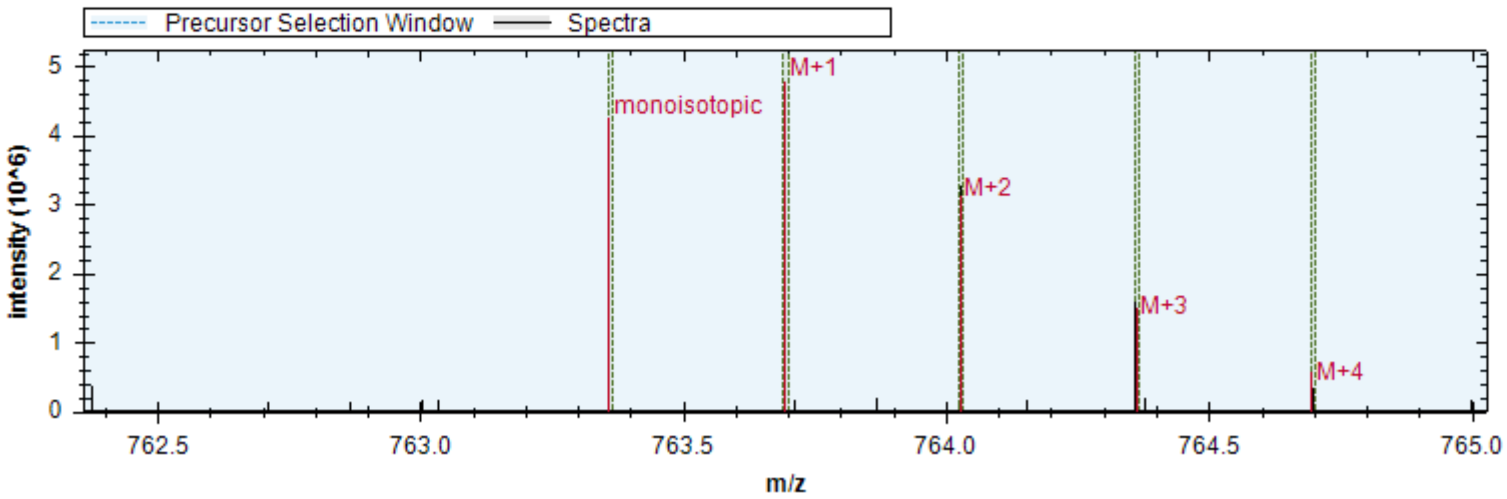

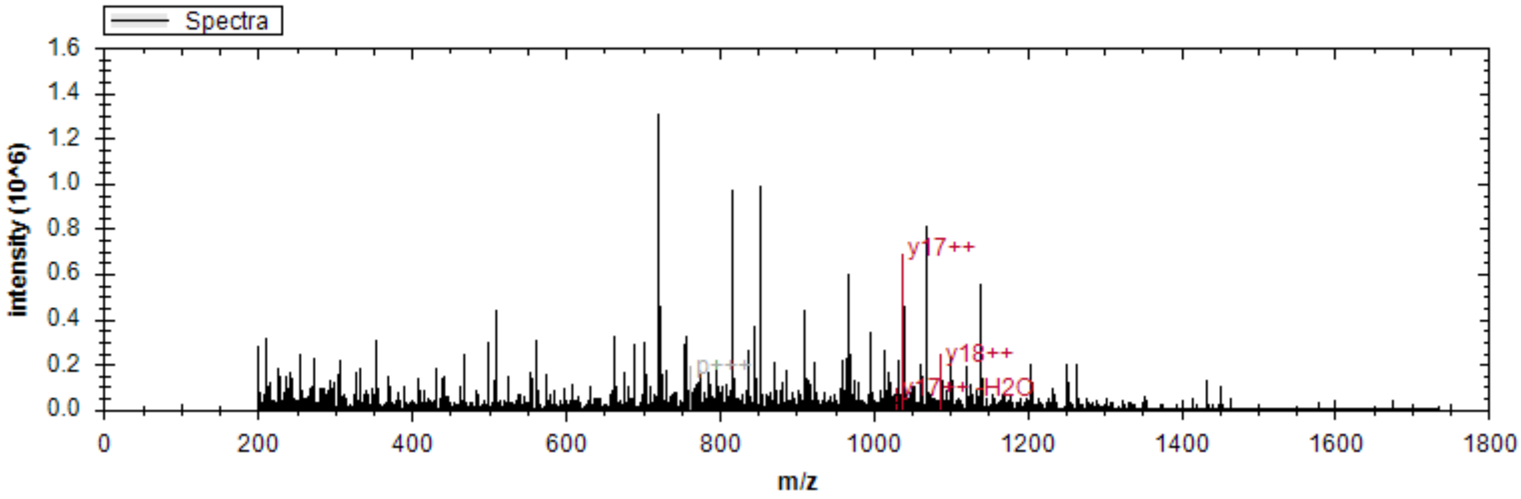

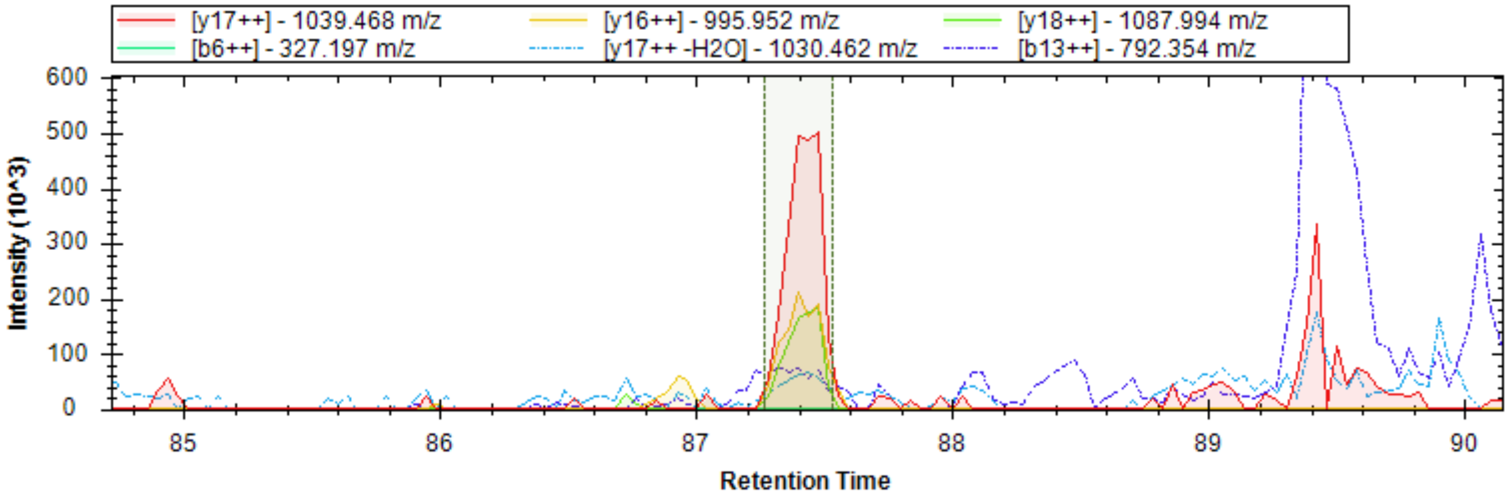

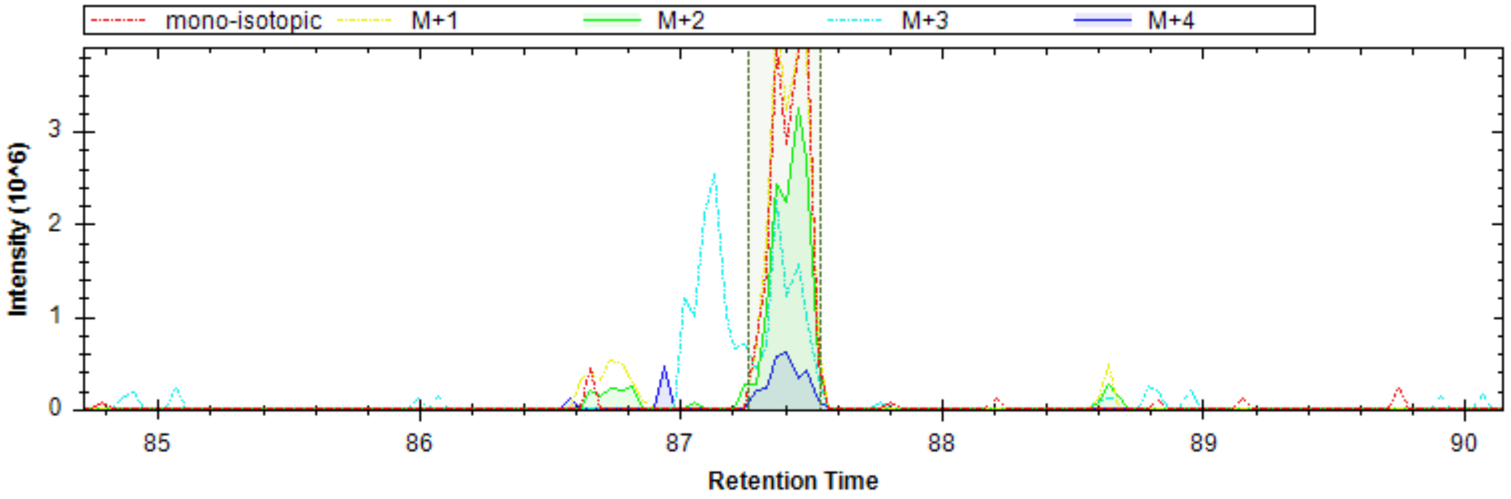

P0C6F1 – _IIDLQM[+O]HDY[+H+O3+P]LR_.2

Fig4_MP-DIA-barrel-cortex-p9-2_MHRM.htrms

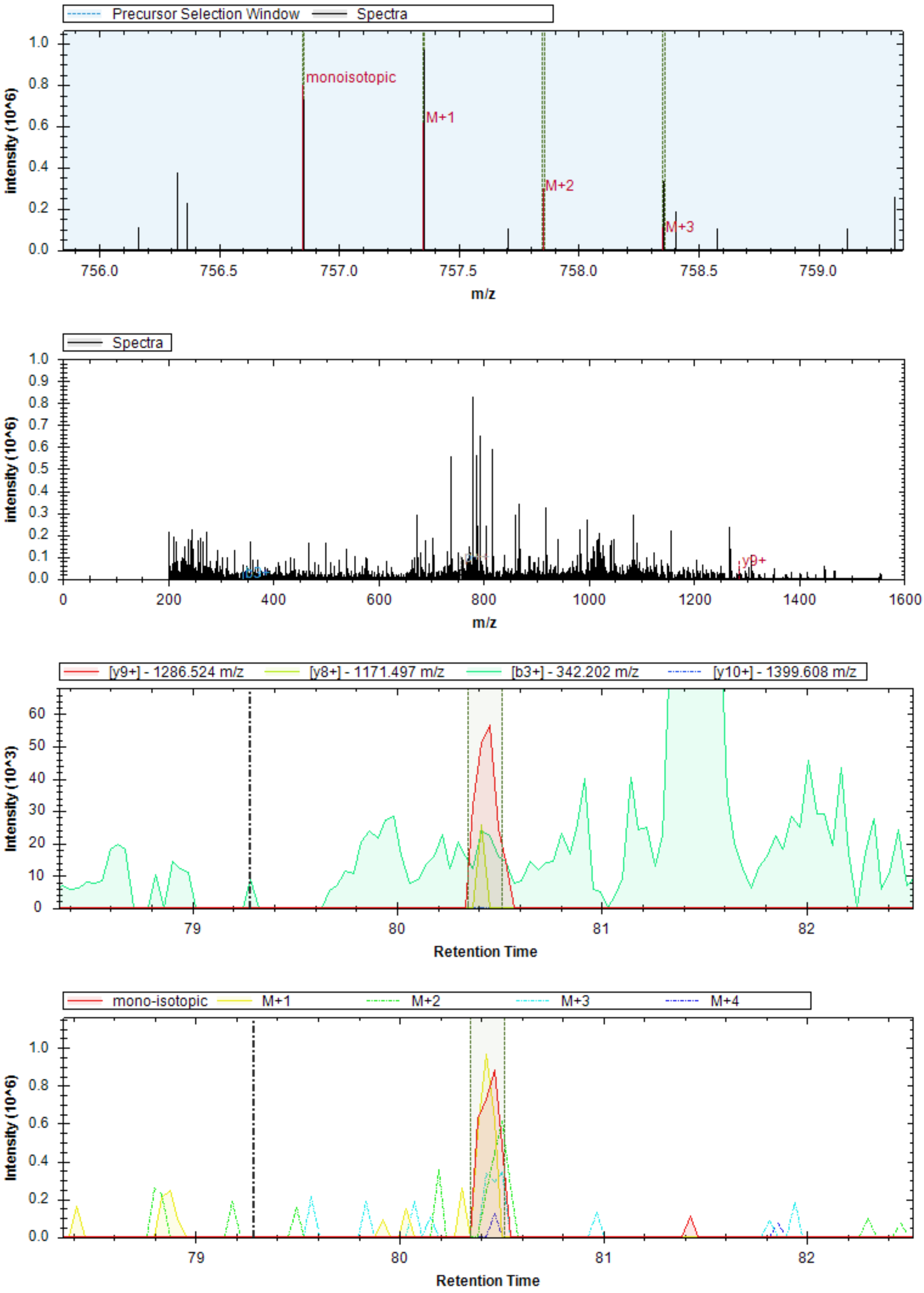

P27546-2 – _AT[+H+O3+P]SPSTLVSTGPSSR_.2
Fig4_MP-DIA-barrel-cortex-p9-2_MHRM.htrms

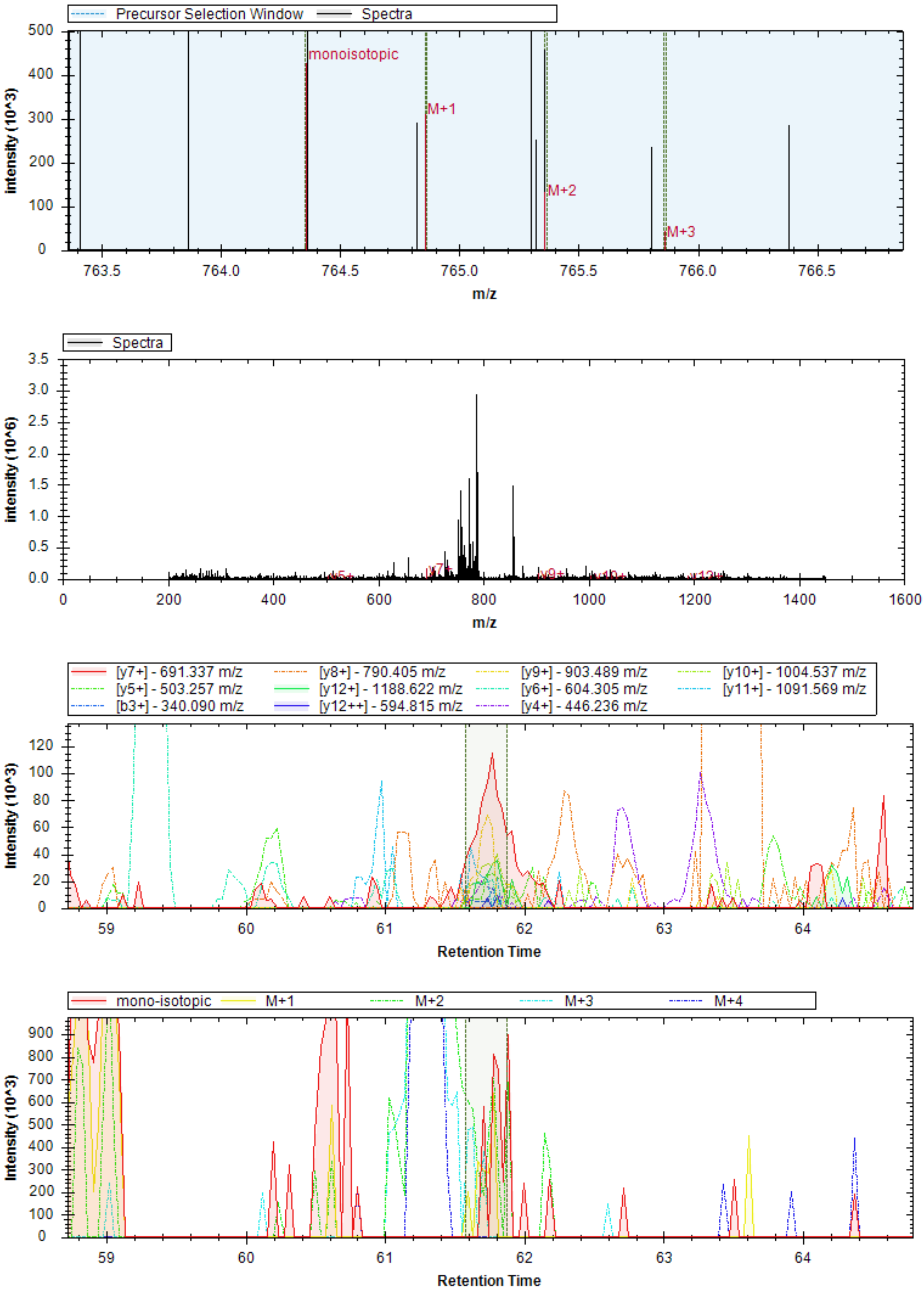

P27546-2 – _ATS[+H+O3+P]PSTLVSTGPSSR_.2
Fig4_MP-DIA-barrel-cortex-p9-2_MHRM.htrms

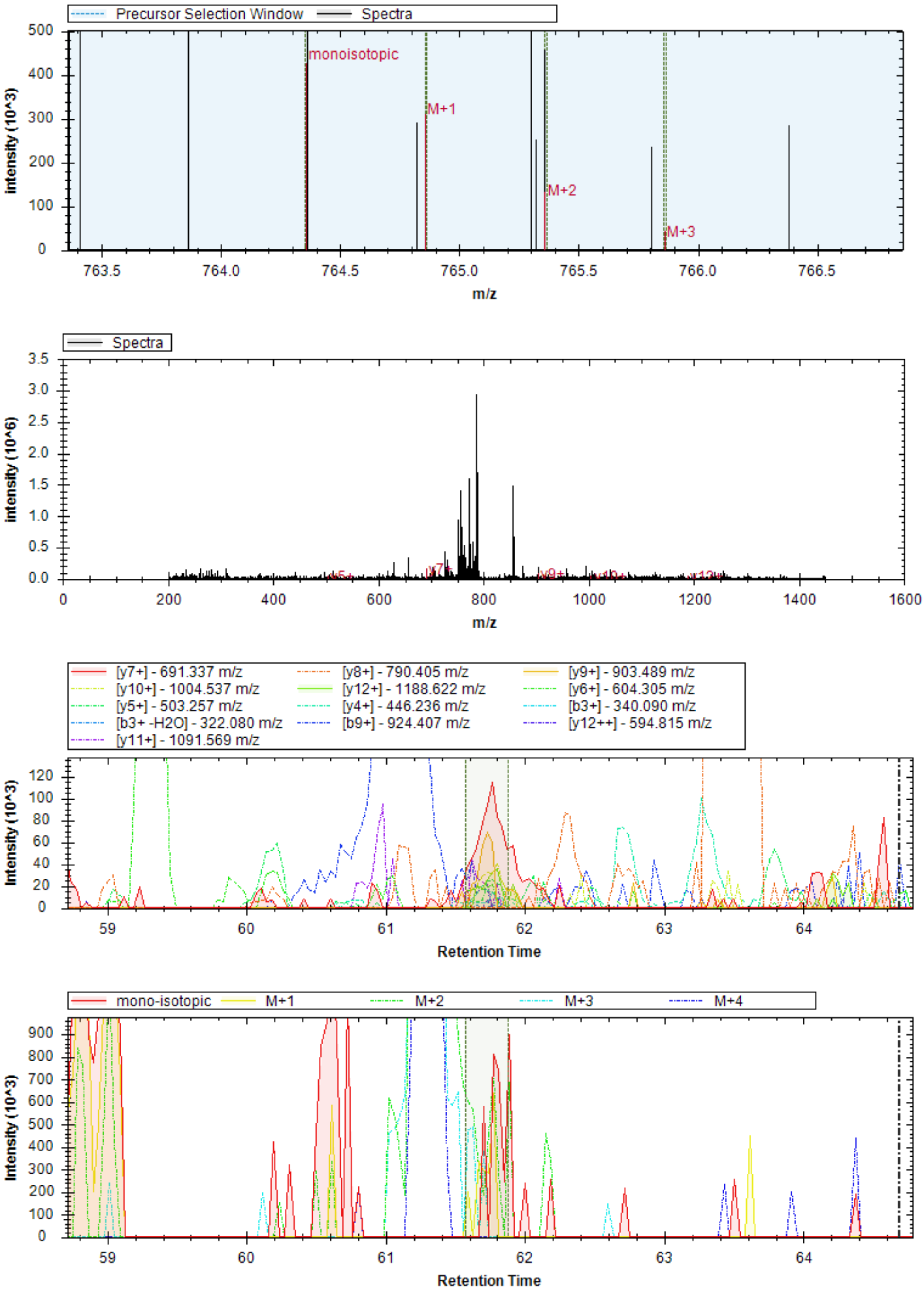

P57776-3 – _ATAPQTQHVS[+H+O3+P]PM[+O]R_.2

Fig4_MP-DIA-barrel-cortex-p9-2_MHRM.htrms

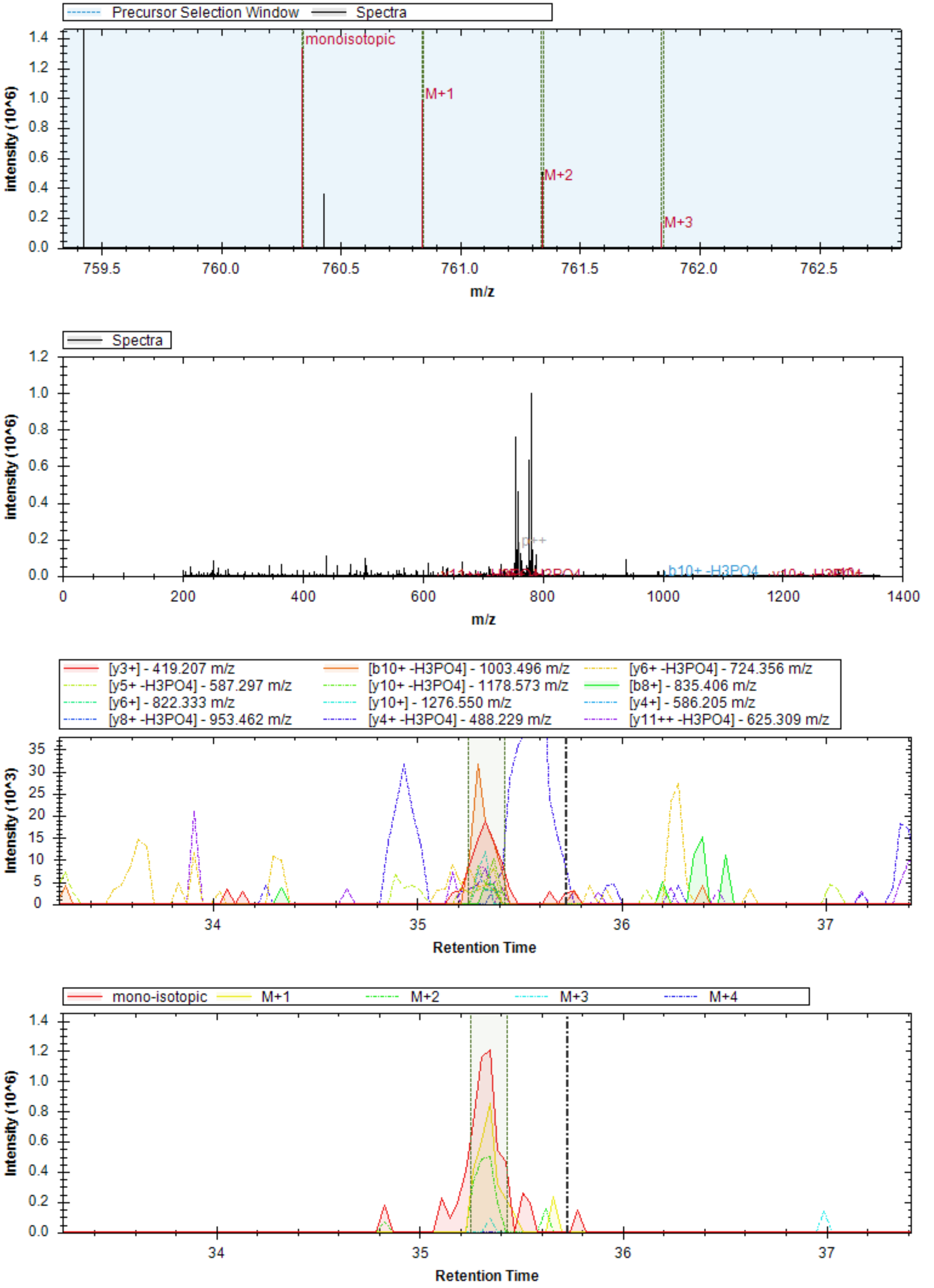

Q68FD9 – _LAQLLS[+H+O3+P]EVPT[+H+O3+P]RR_.2

Fig4_MP-DIA-barrel-cortex-p9-2_MHRM.htrms

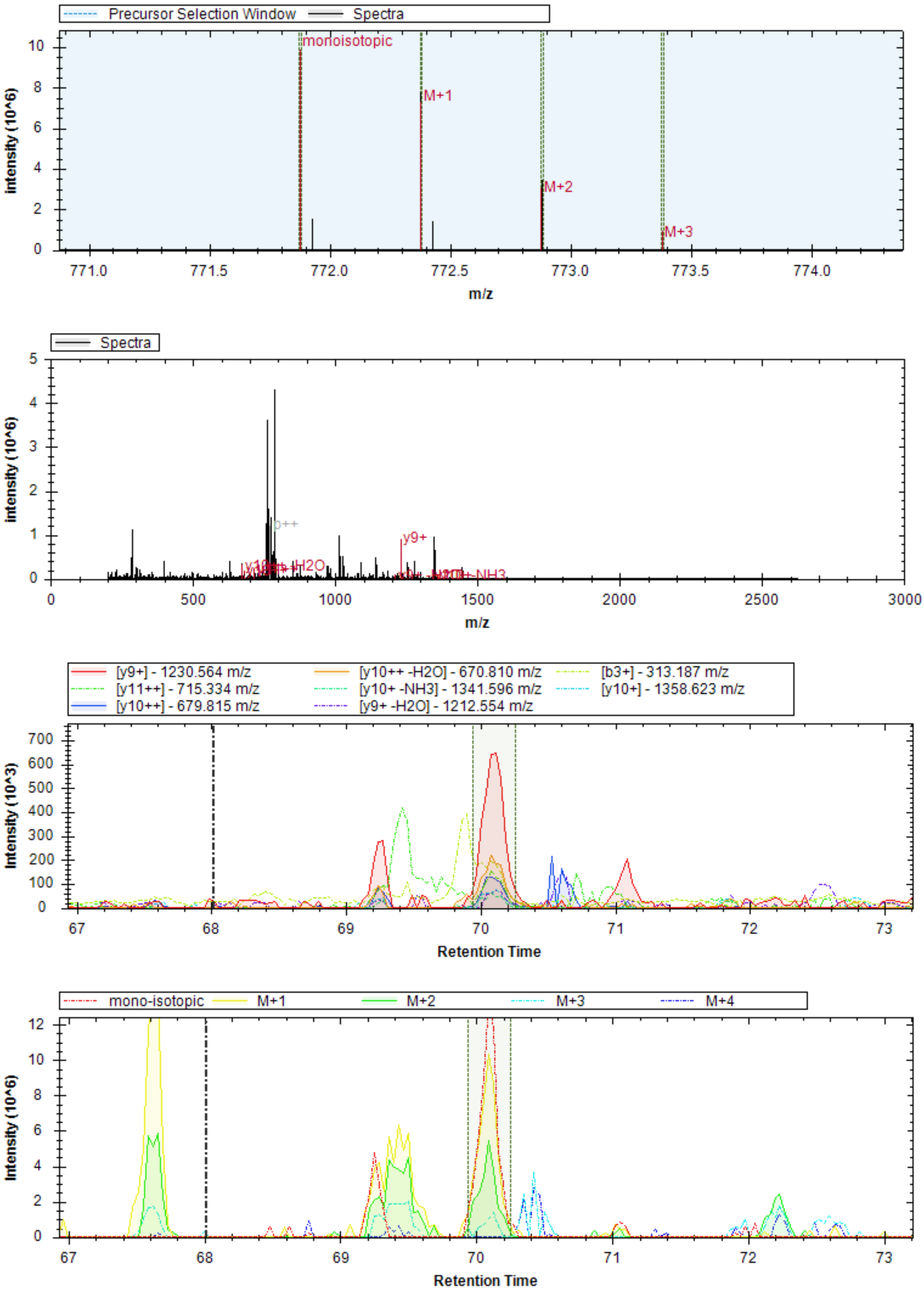

Q6ZQA0;Q6ZQA0-2 – _LHT[+H+O3+P]LLQT[+H+O3+P]AVPTR_.2
Fig4_MP-DIA-barrel-cortex-p9-2_MHRM.htrms

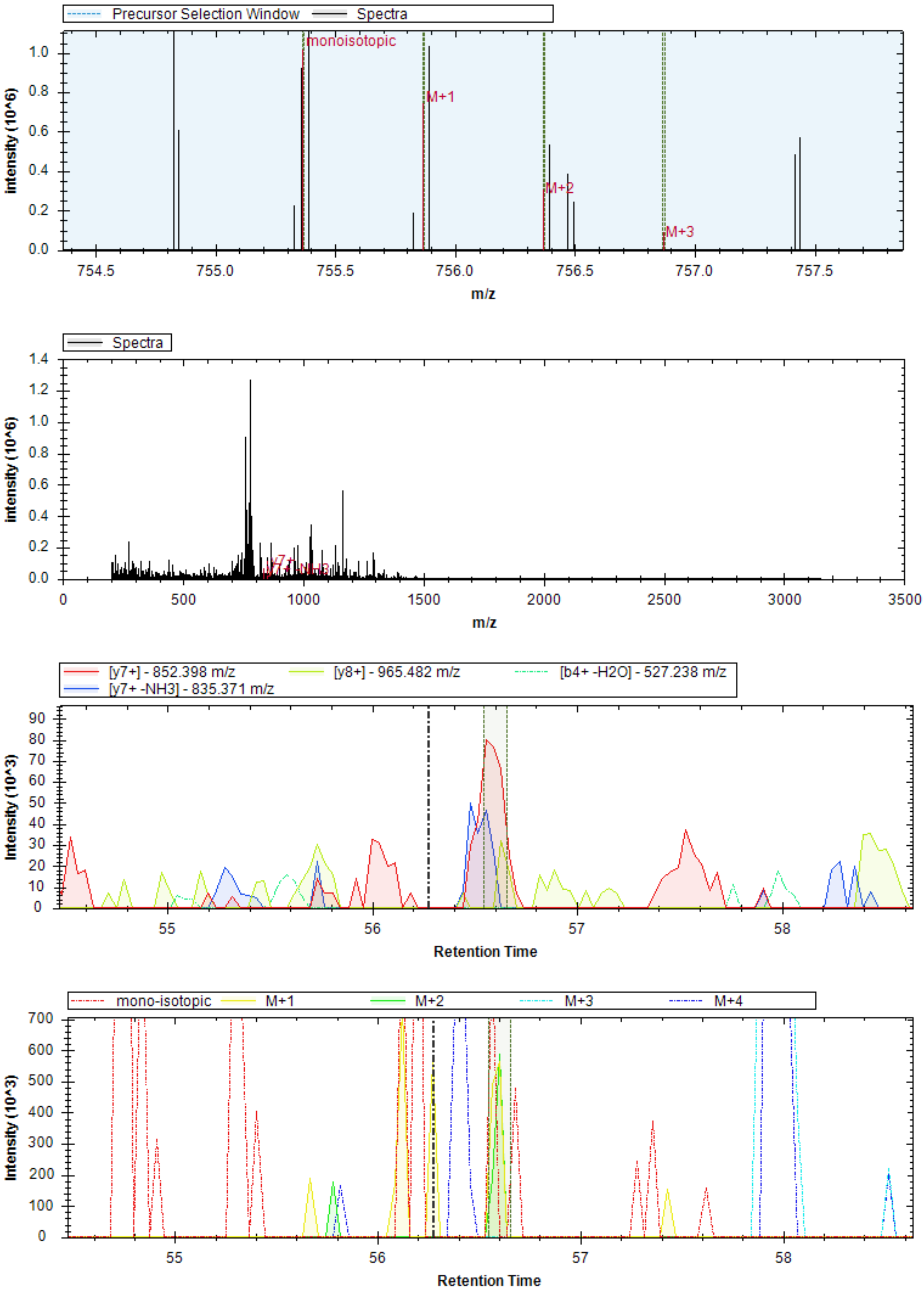

Q8BRT1 – _VLNT[+H+O3+P]GSDVEEAVADALLLGDIR_.3

Fig4_MP-DIA-barrel-cortex-p9-2_MHRM.htrms

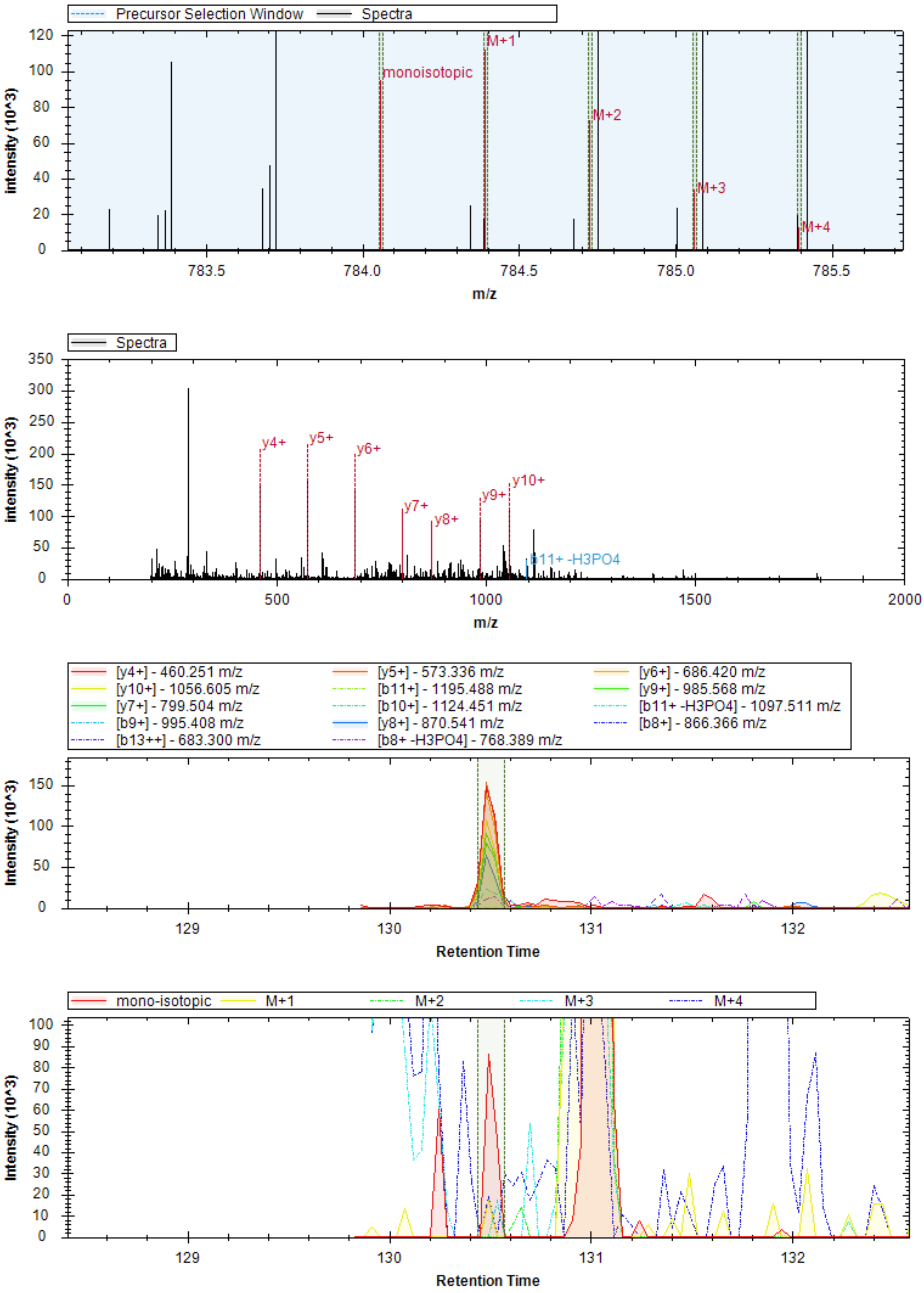

Q9D5R2 – _SNS[+H+O3+P]LPHSAVSNAASK_.2
Fig4_MP-DIA-barrel-cortex-p9-2_MHRM.htrms

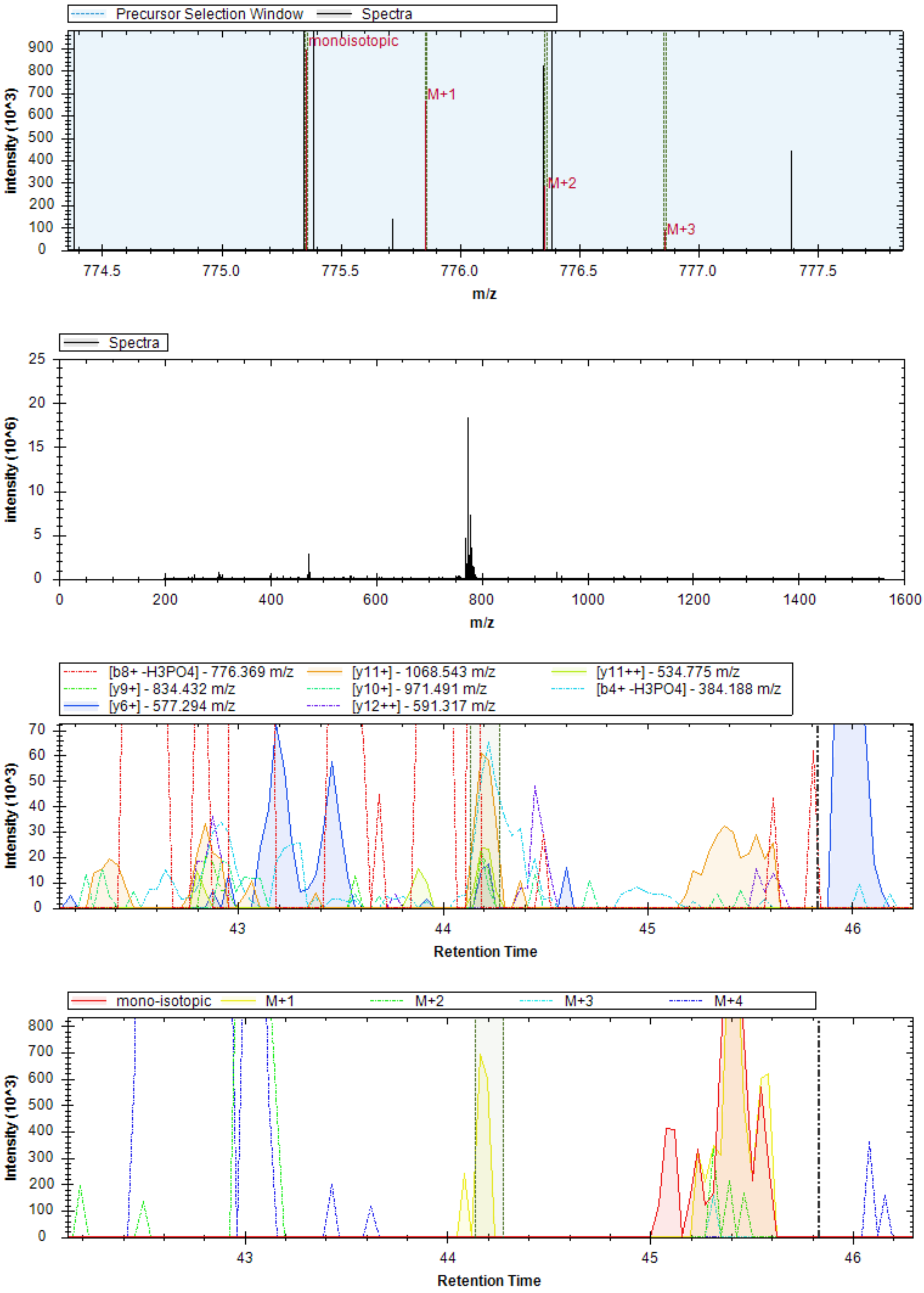

Q9JLM8 – _ISQHGGS[+H+O3+P]STSLSSTK_.2

Fig4_MP-DIA-barrel-cortex-p9-2_MHRM.htrms

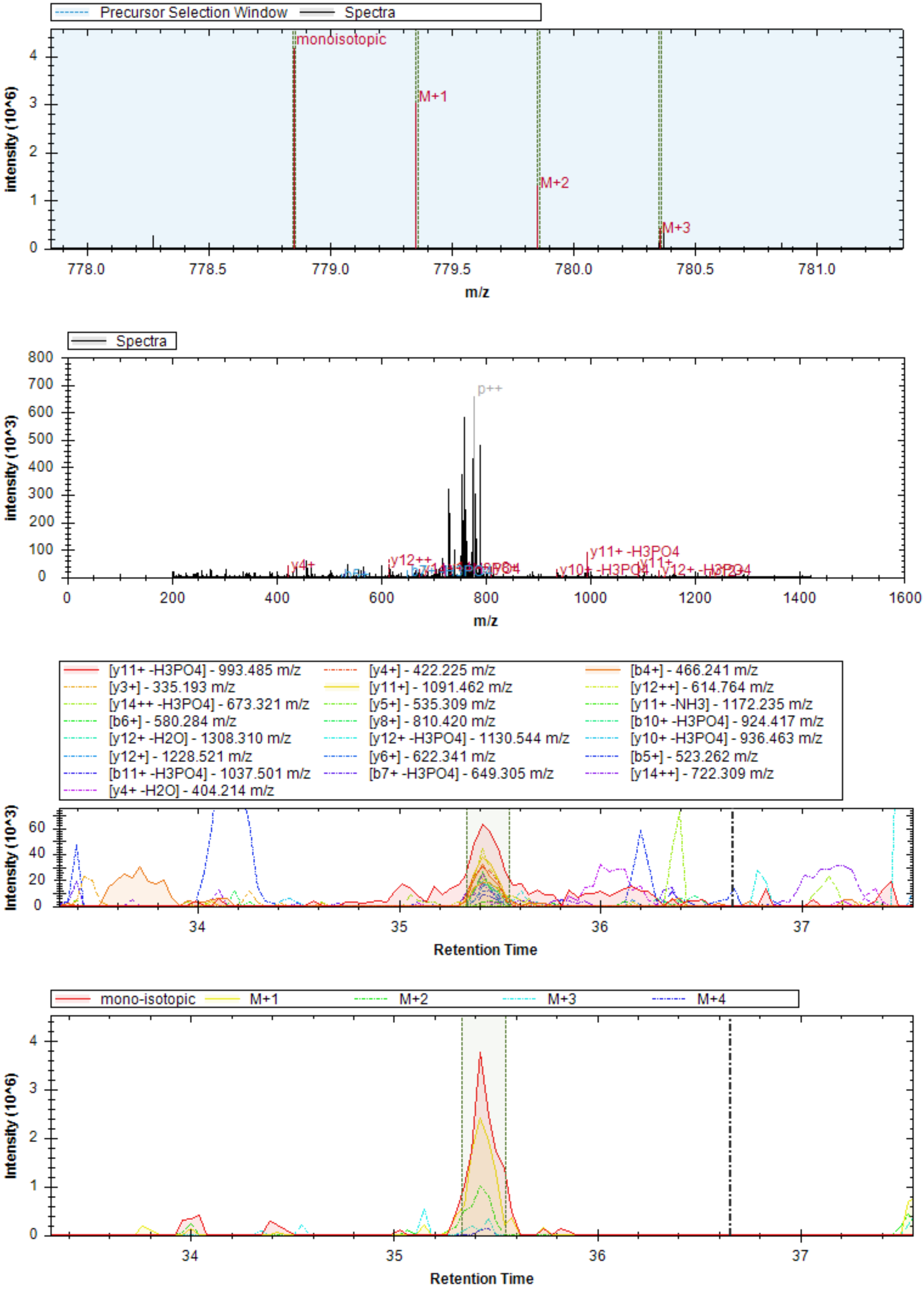

Q9QWI6 – _LSYAGGRPPSYAGS[+H+O3+P]PVHHAAER_.3

Fig4_MP-DIA-barrel-cortex-p9-2_MHRM.htrms

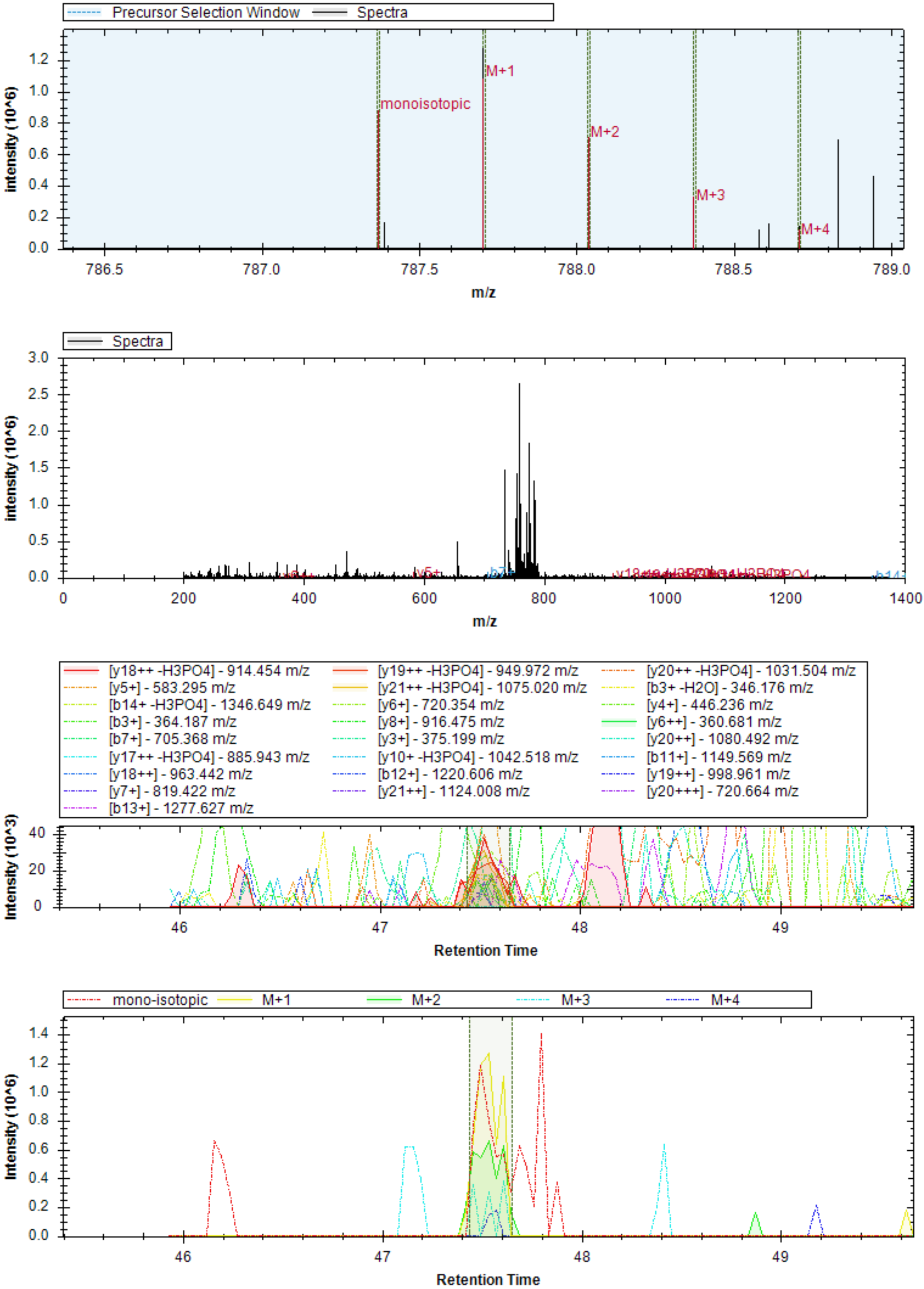

Q9QYC0 – _AAVVTS[+H+O3+P]PPPTTAPHK_.2

Fig4_MP-DIA-barrel-cortex-p9-2_MHRM.htrms

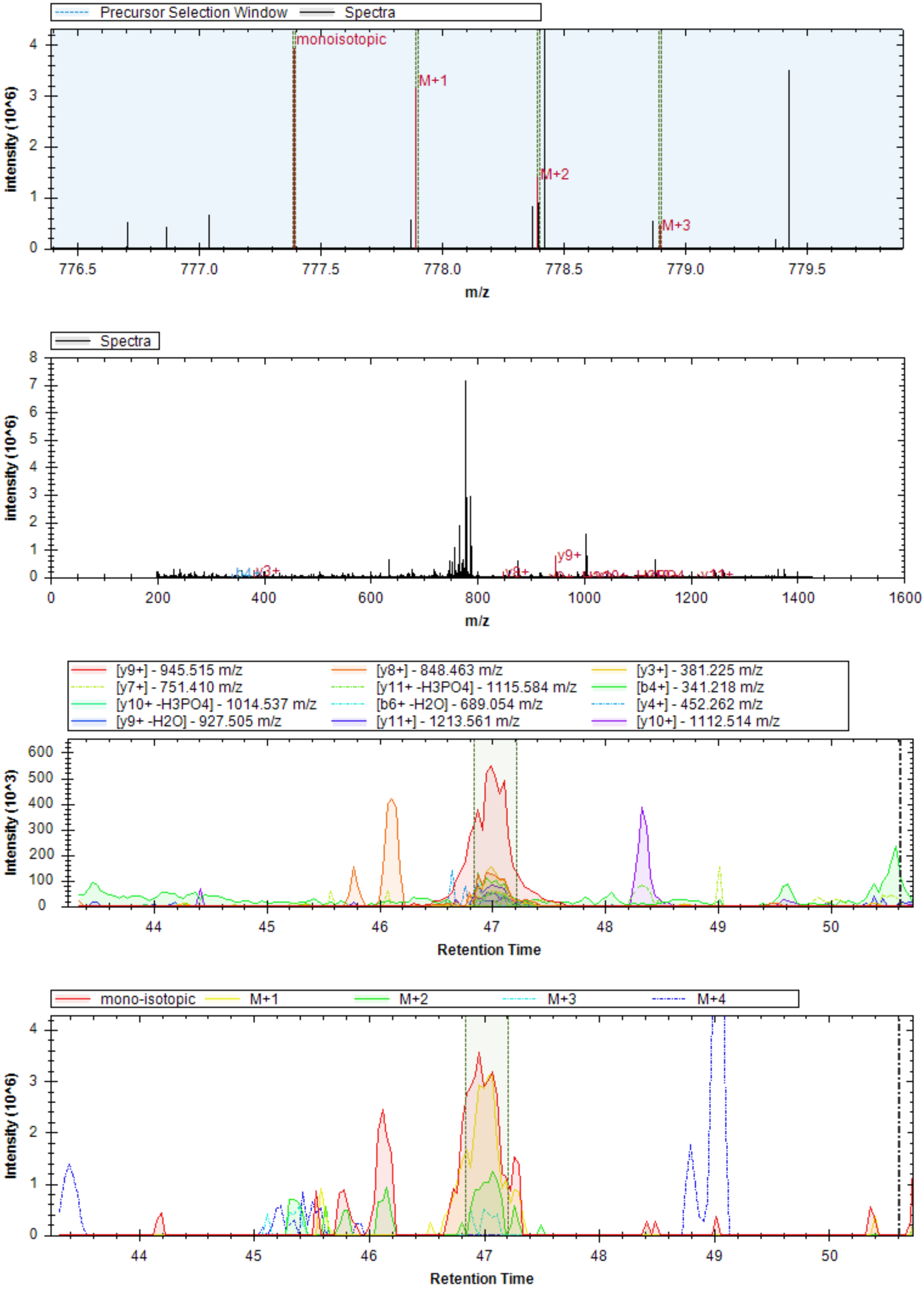

O88935-1 – _PAKPQLAQKPS[+H+O3+P]QDVPPPITAAAGGPPHPQLK_.4

Fig4_MP-DIA-barrel-cortex-p9-2_MHRM.htrms

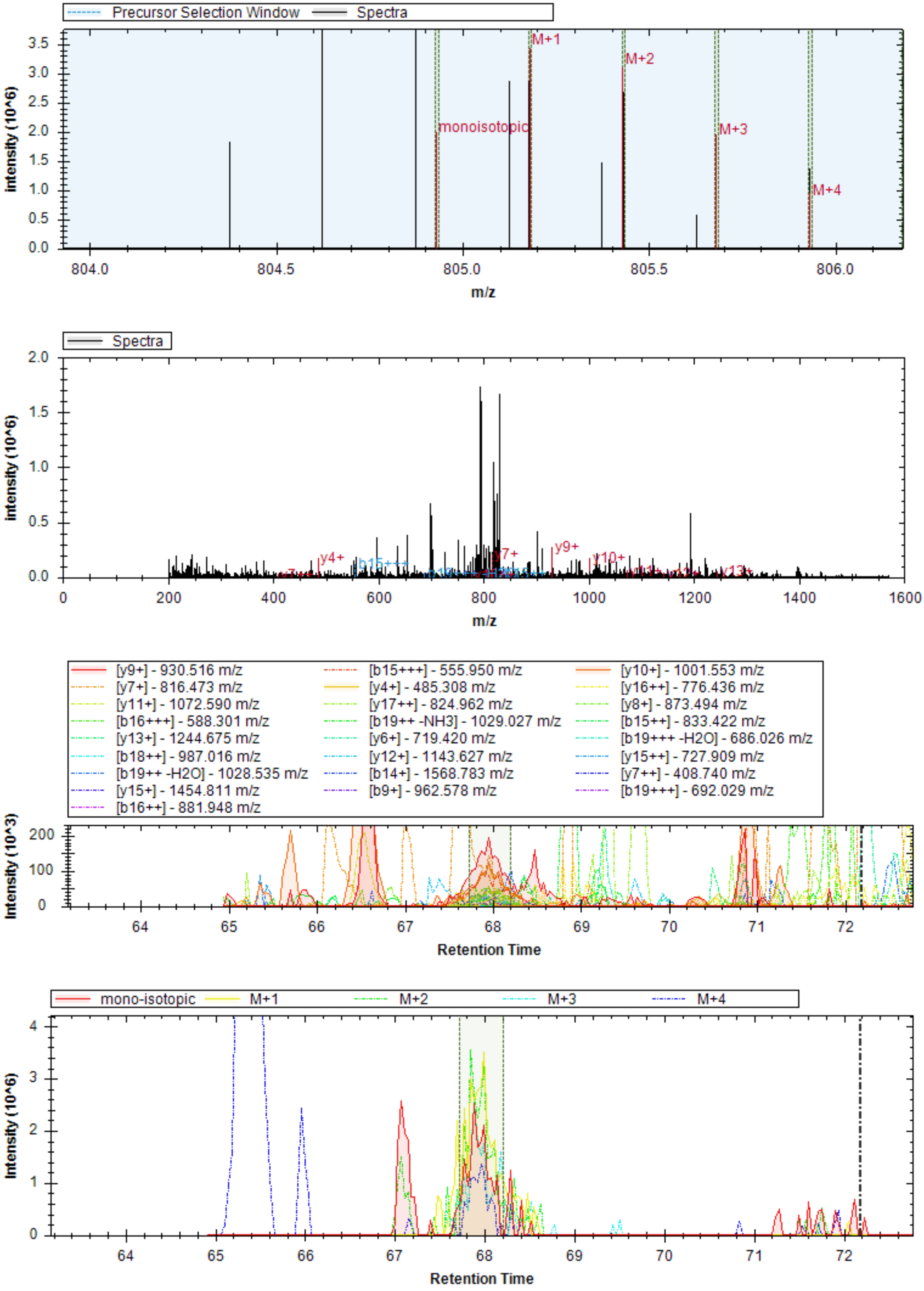

P10637 – _TTPSPKT[+H+O3+P]PPGSGEPPK_.2

Fig4_MP-DIA-barrel-cortex-p9-2_MHRM.htrms

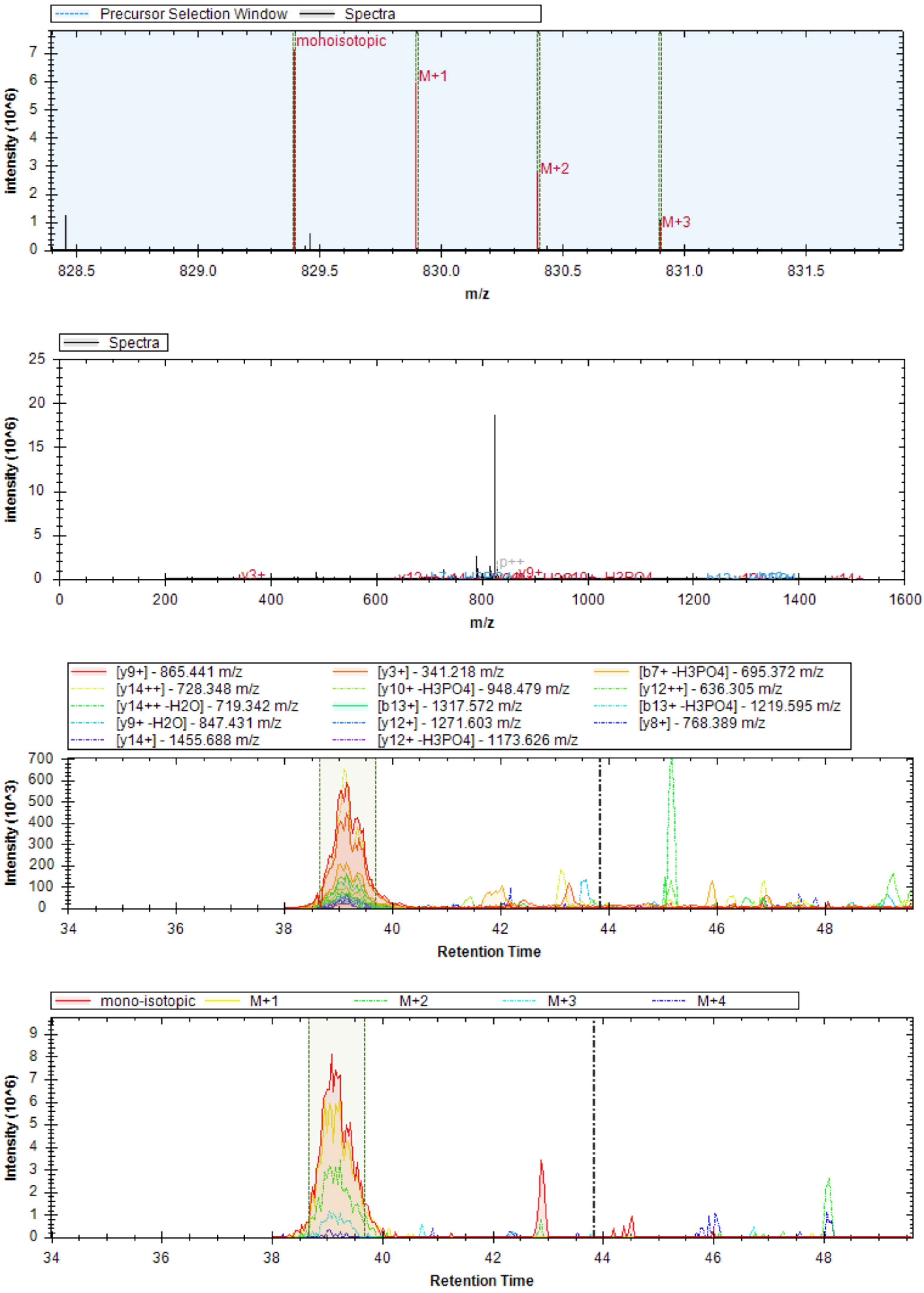

P20357 – _APHWTSASLTEAAAHPHS[+H+O3+P]PEMK_.3
Fig4_MP-DIA-barrel-cortex-p9-2_MHRM.htrms

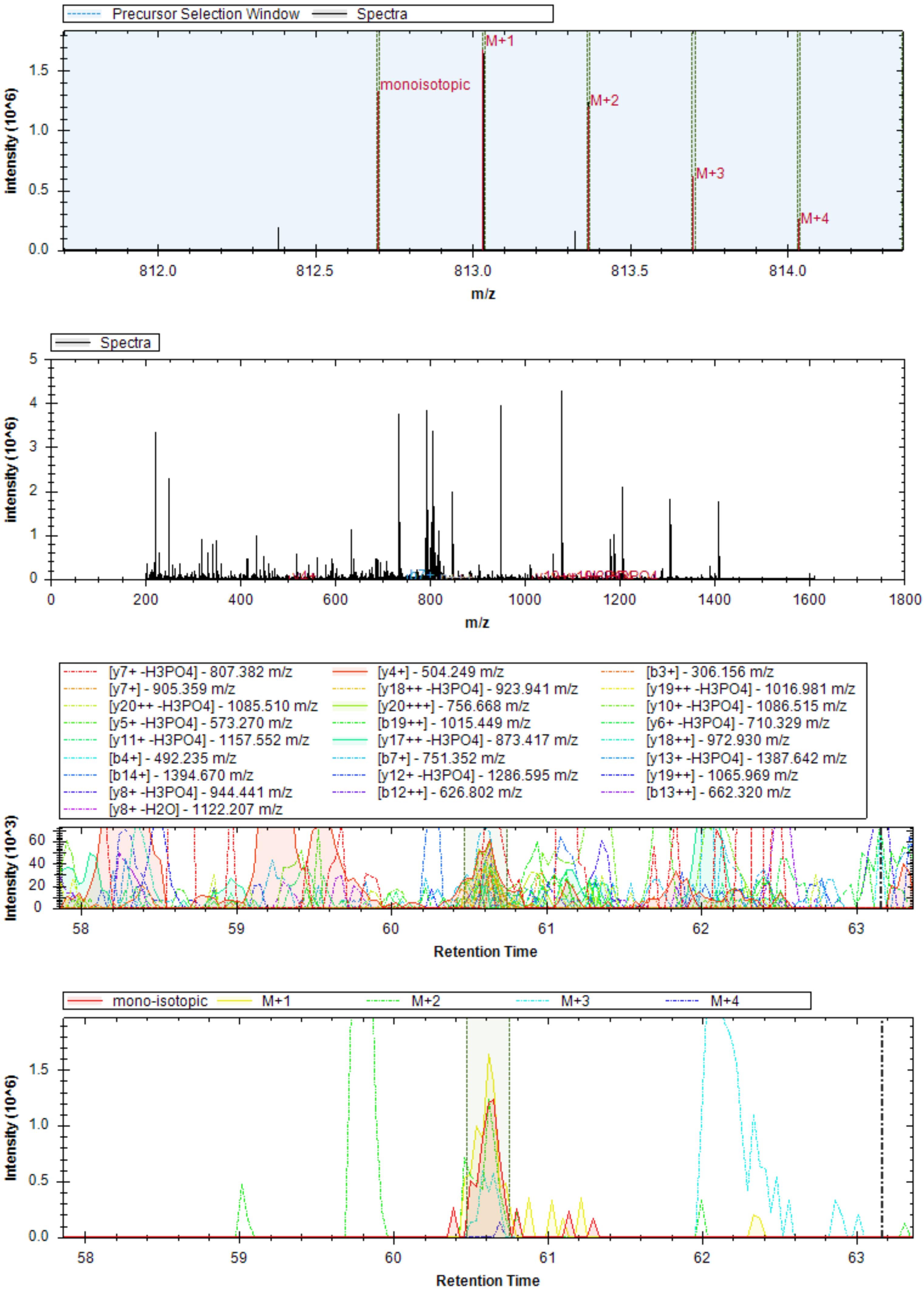

P20357 – _VAIIRT[+H+O3+P]PPKSPATPK_.2
Fig4_MP-DIA-barrel-cortex-p9-2_MHRM.htrms

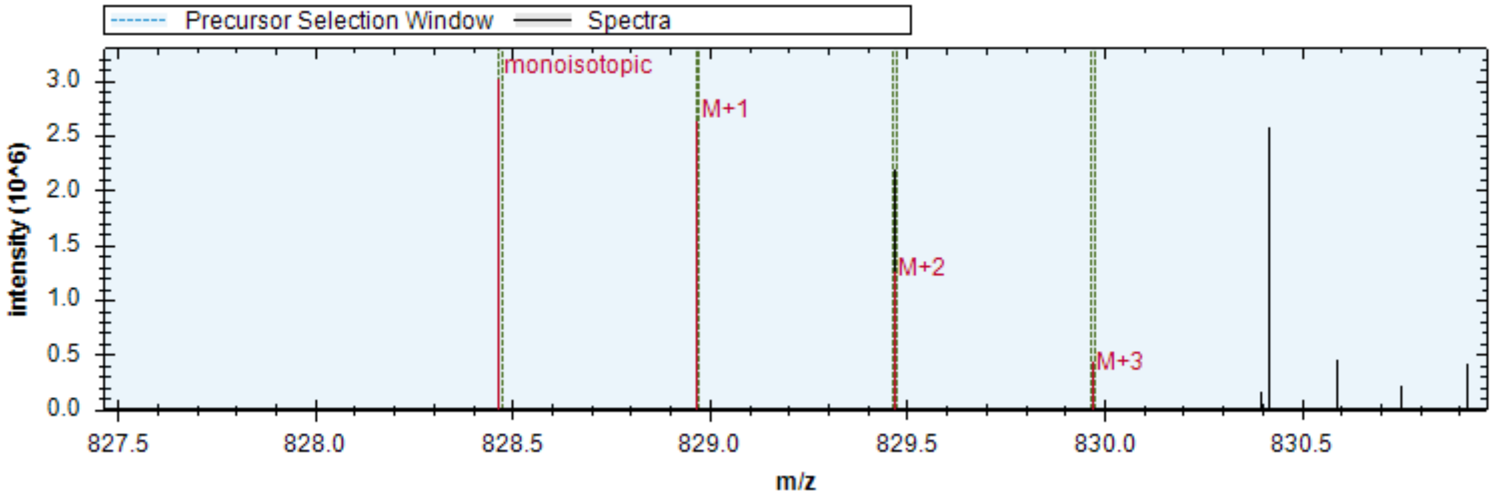

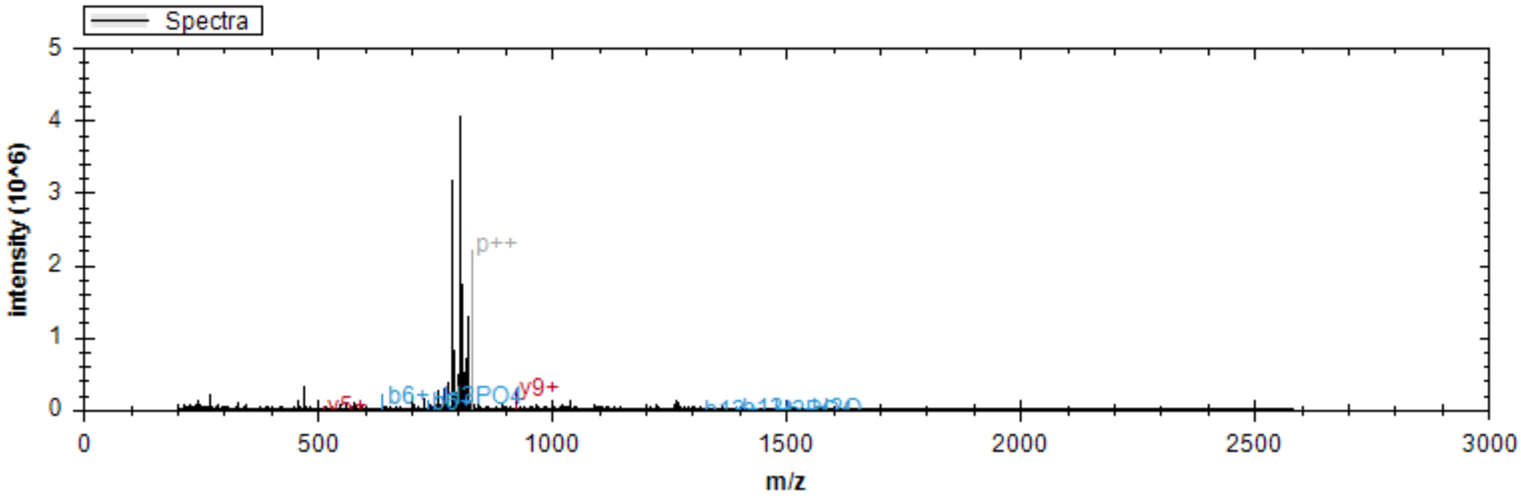

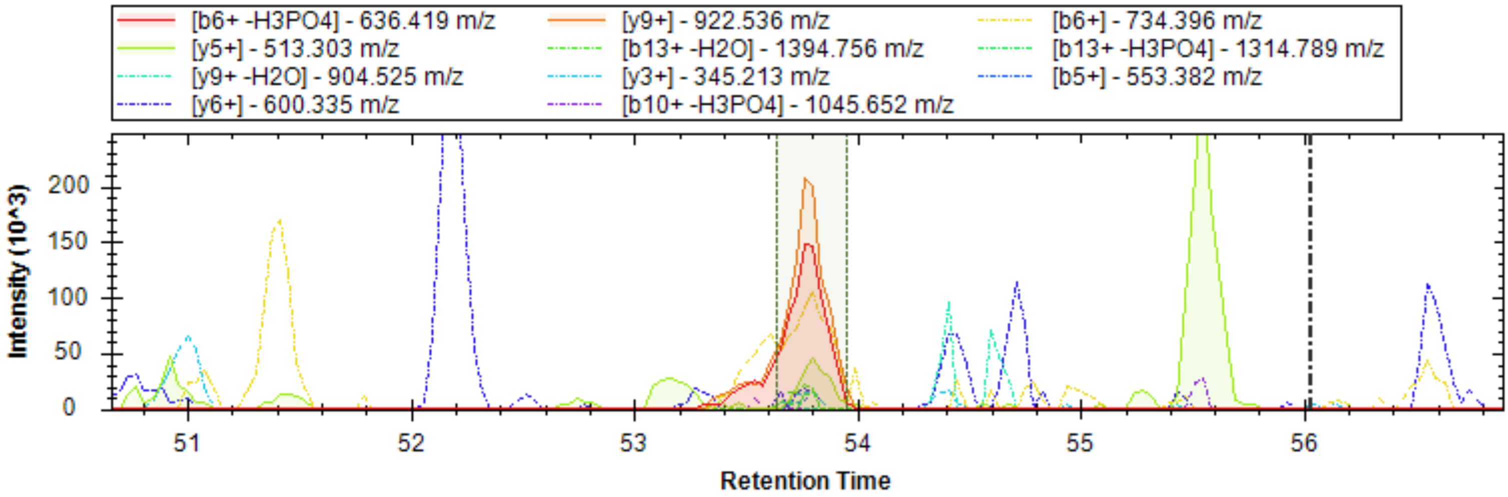

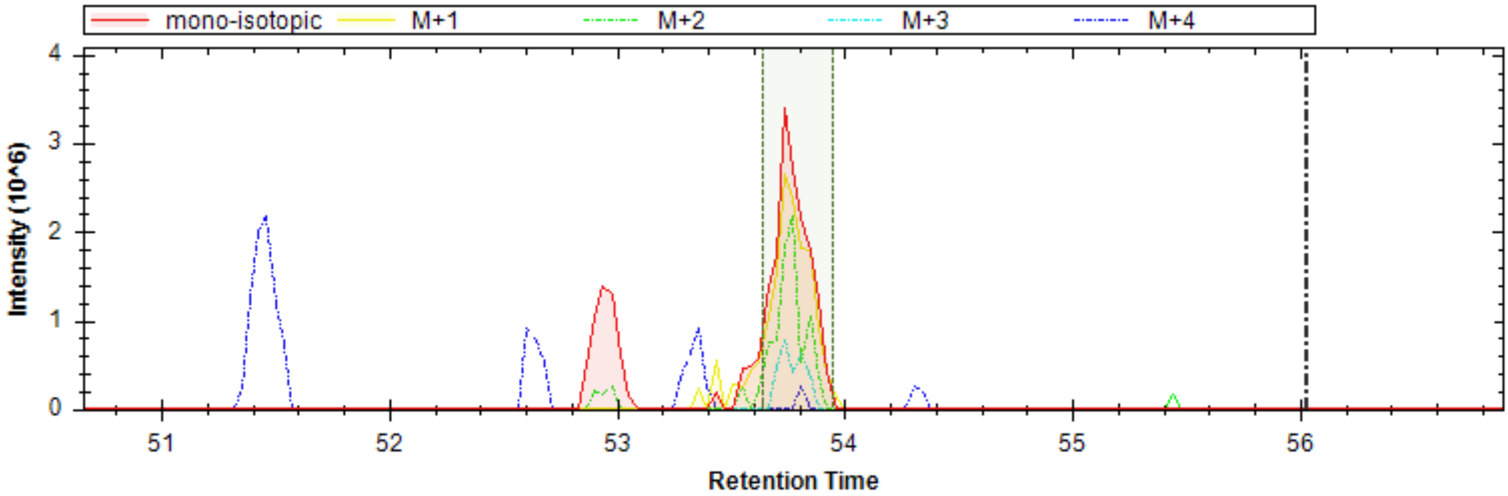

P20357 – _VDHGAEIIT[+H+O3+P]QSPSR_.2

Fig4_MP-DIA-barrel-cortex-p9-2_MHRM.htrms

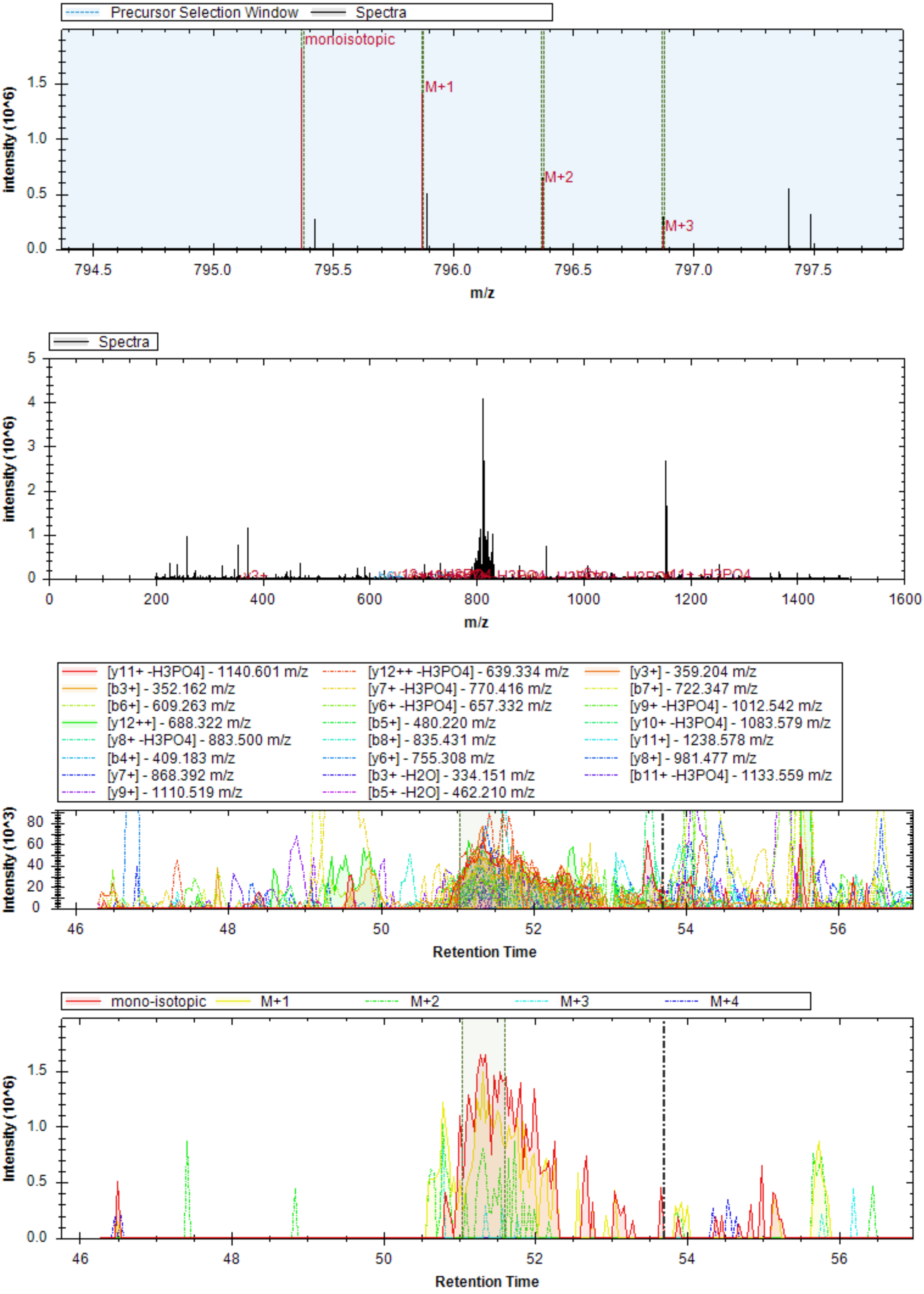

P20357 – _VDHGAEIITQS[+H+O3+P]PSR_.2

Fig4_MP-DIA-barrel-cortex-p9-2_MHRM.htrms

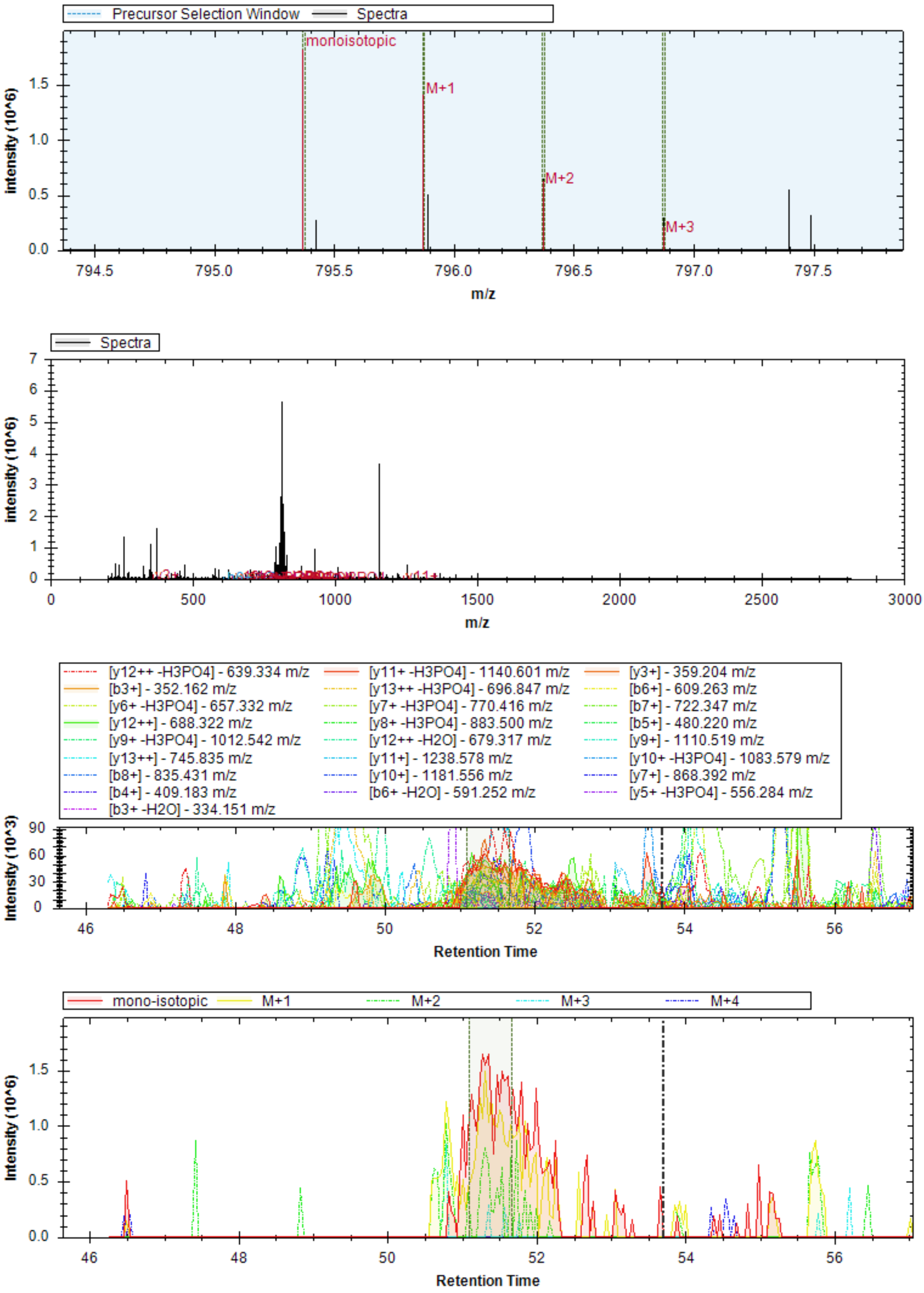

P20357 – _VDHGAEIITQSPS[+H+O3+P]R_.2
Fig4_MP-DIA-barrel-cortex-p9-2_MHRM.htrms

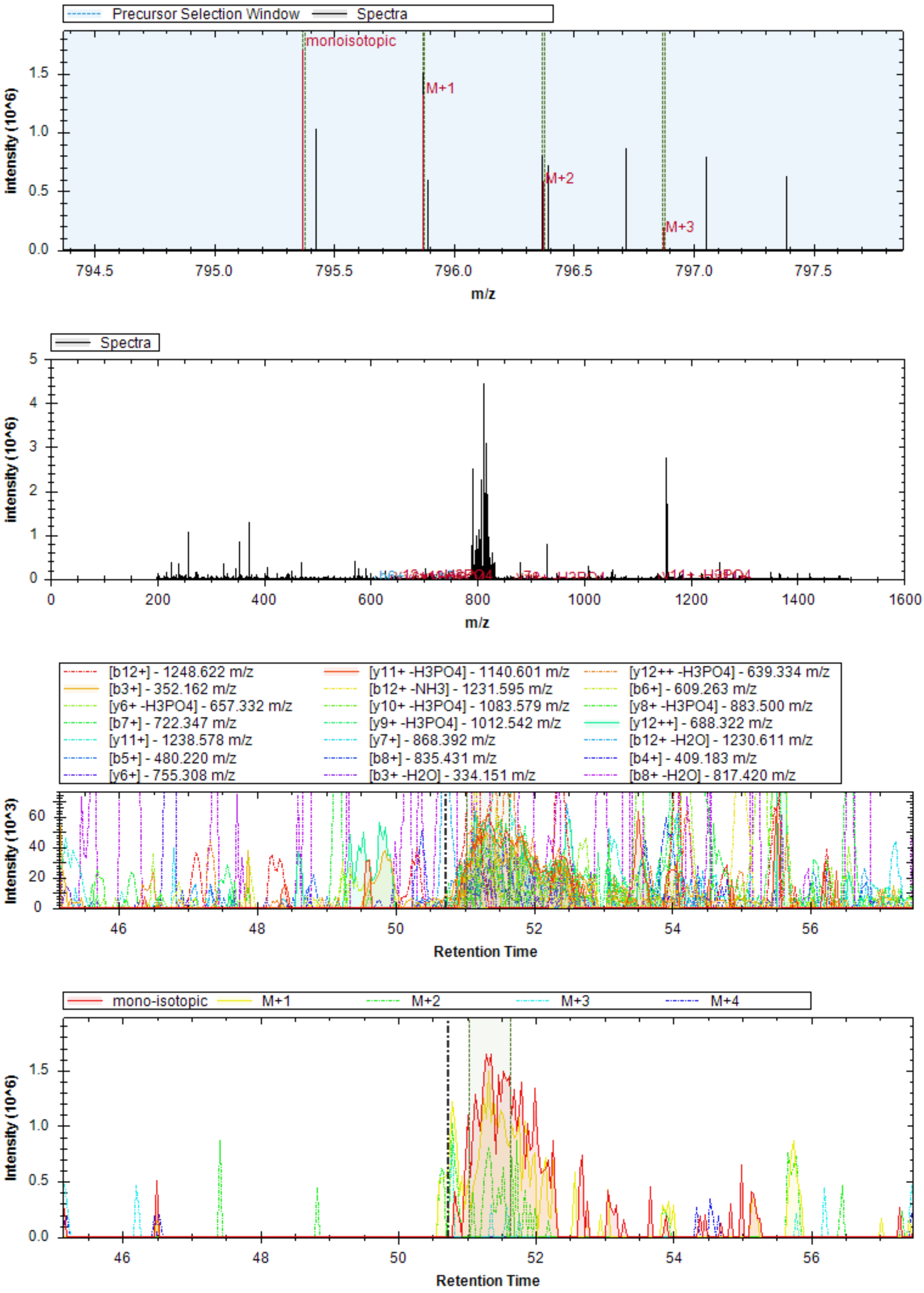

P54227 – _SKESVPDFPLS[+H+O3+P]PPK_.2
Fig4_MP-DIA-barrel-cortex-p9-2_MHRM.htrms

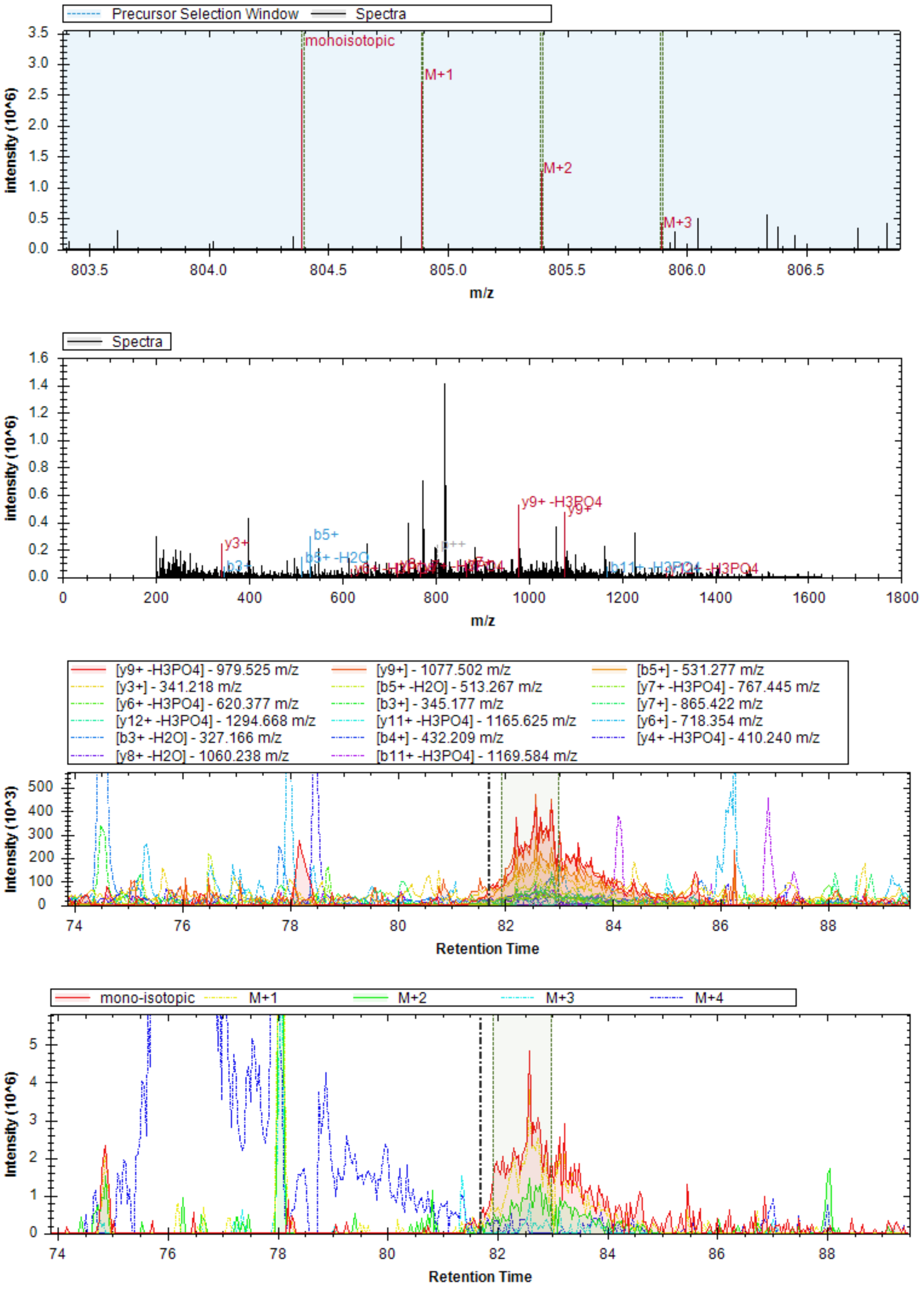

Q45VK7;Q45VK7-2 – _LEEVLAIRT[+H+O3+P]IHEK_.2

Fig4_MP-DIA-barrel-cortex-p9-2_MHRM.htrms

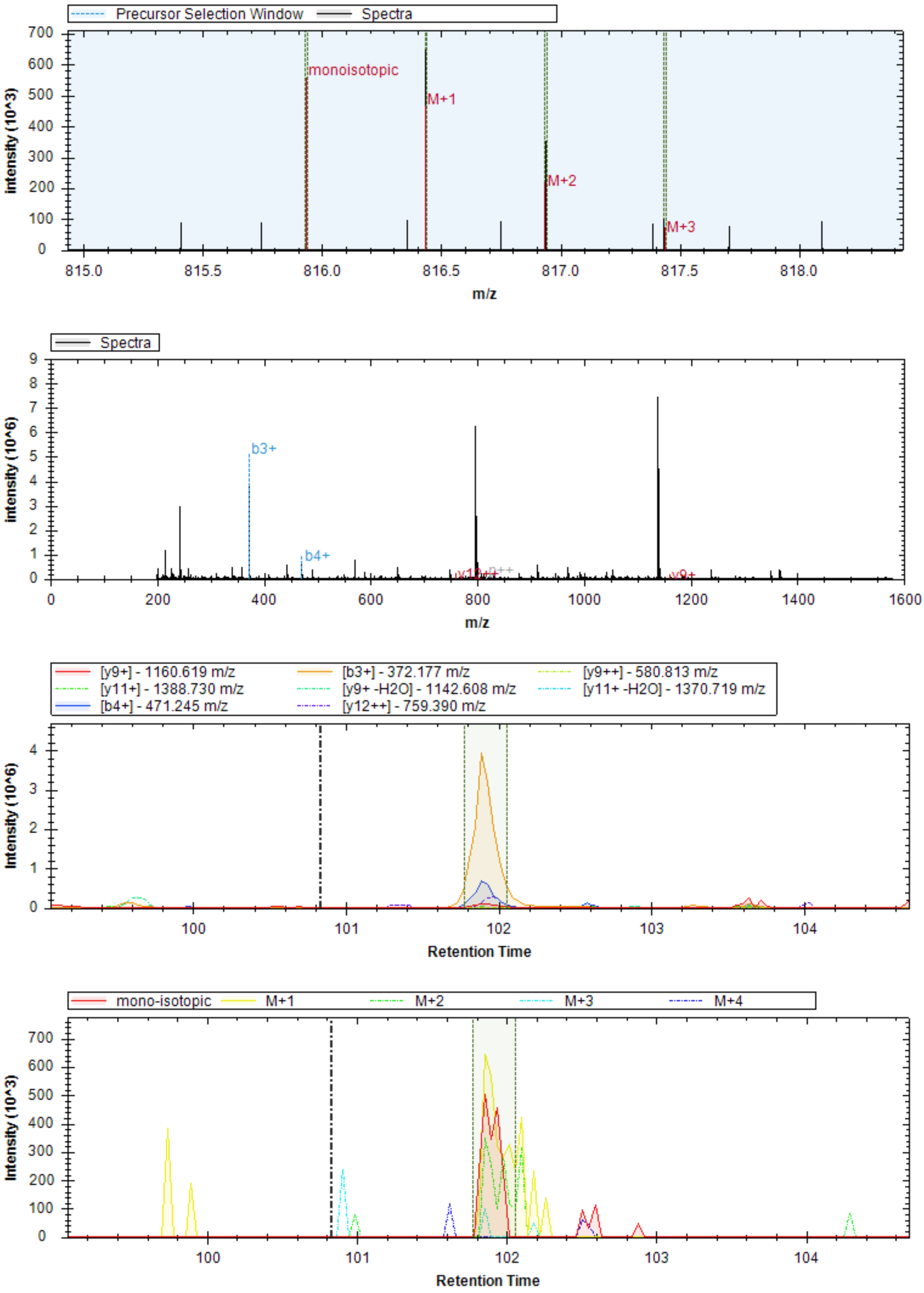

Q61548;Q61548-2;Q61548-3 – _SSPATTVTS[+H+O3+P]PNSTPAK_.2
Fig4_MP-DIA-barrel-cortex-p9-2_MHRM.htrms

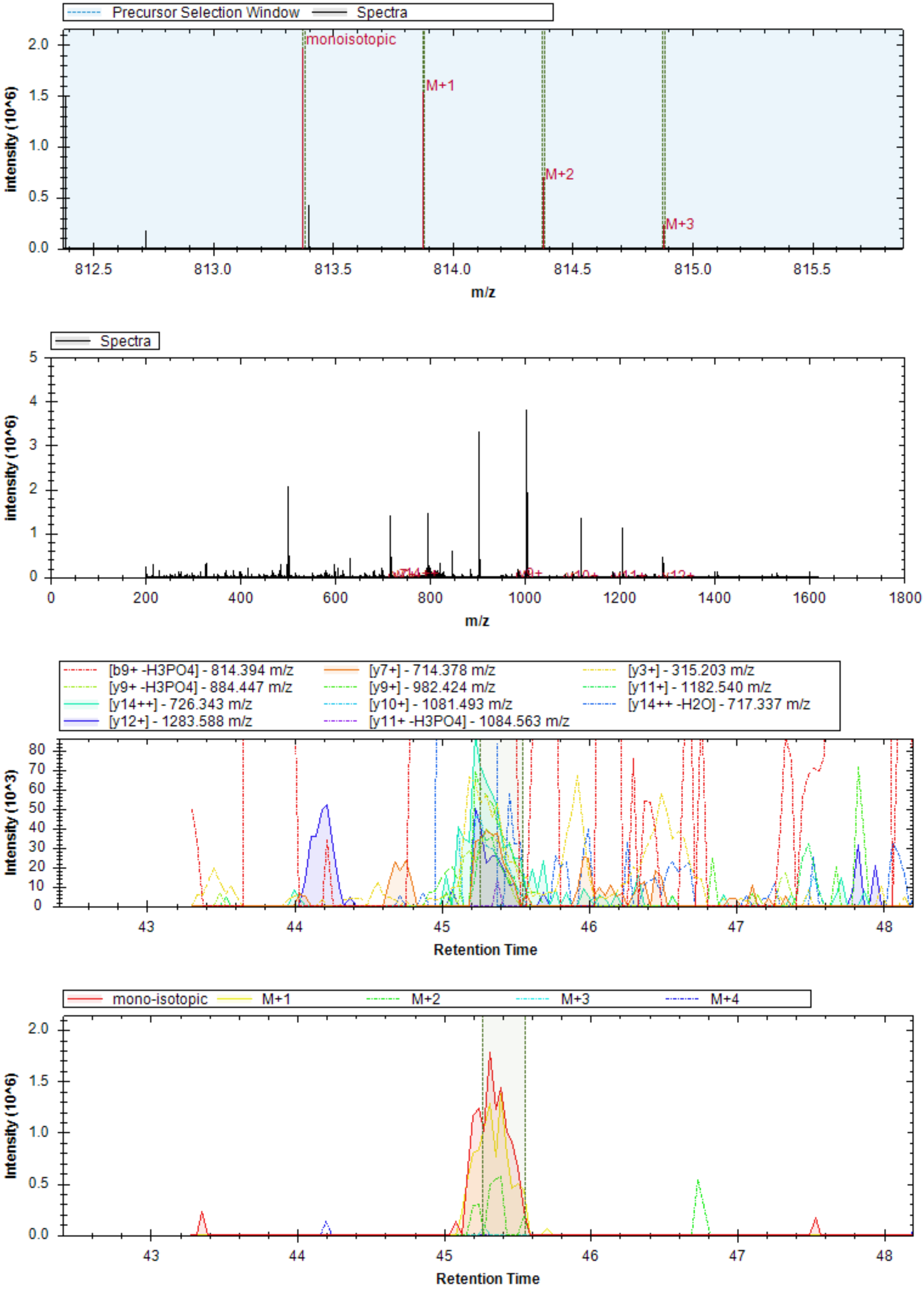

D3YXK2 – _AET[+H+O3+P]LSGLGDASAAGAAAVSSAASETGTR_.3

Fig4_MP-DIA-barrel-cortex-p9-2_MHRM.htrms

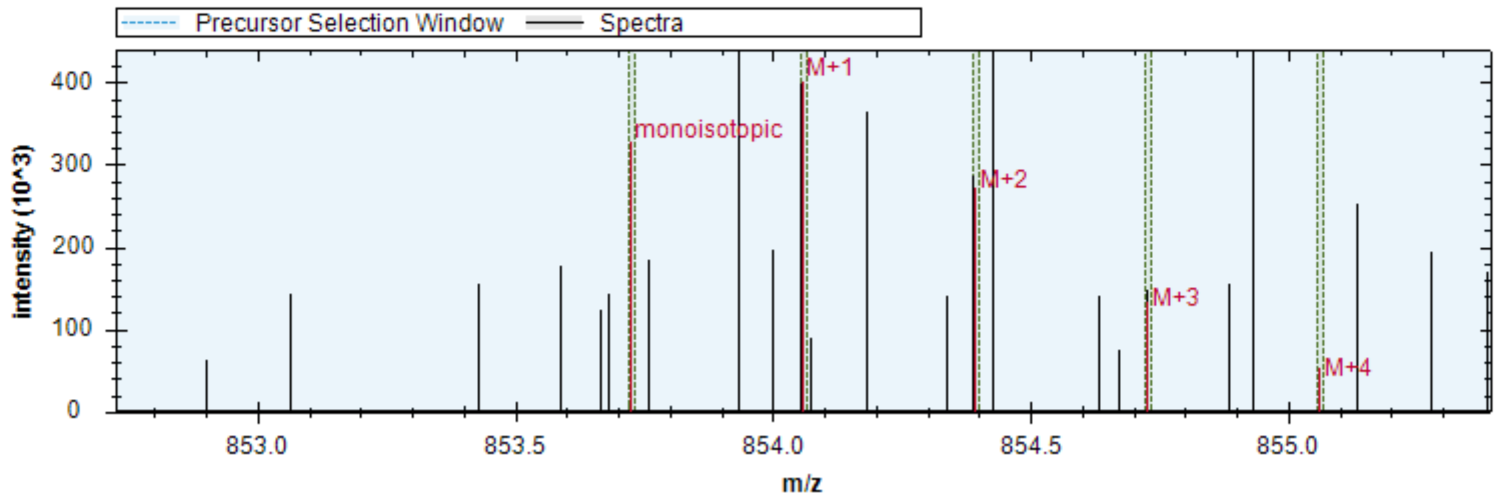

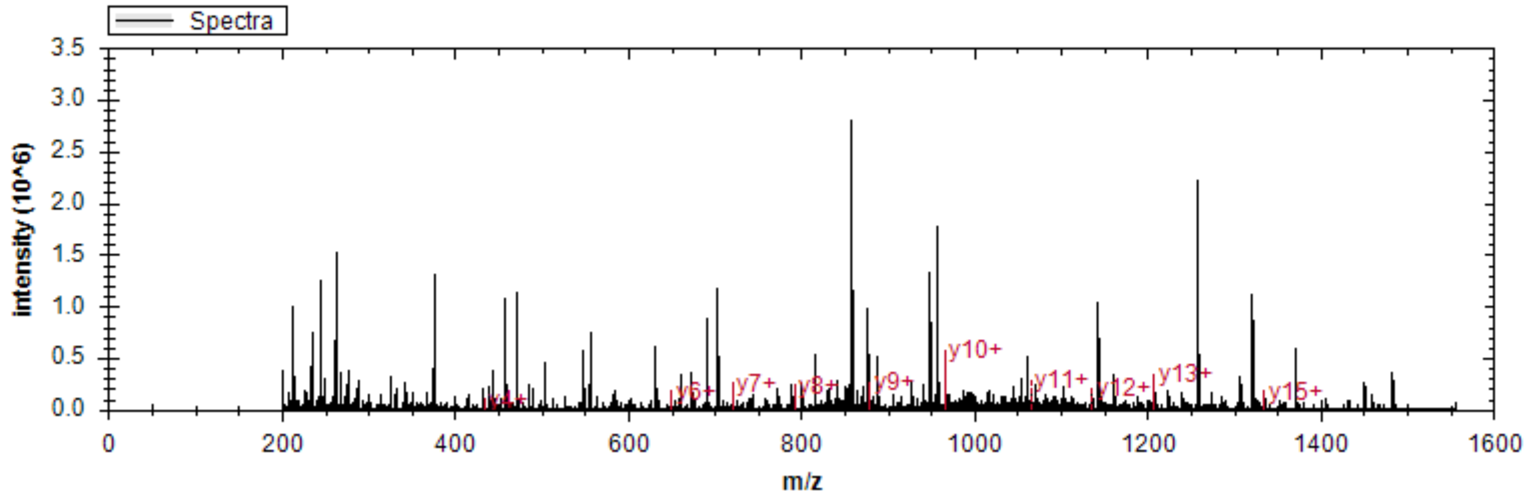

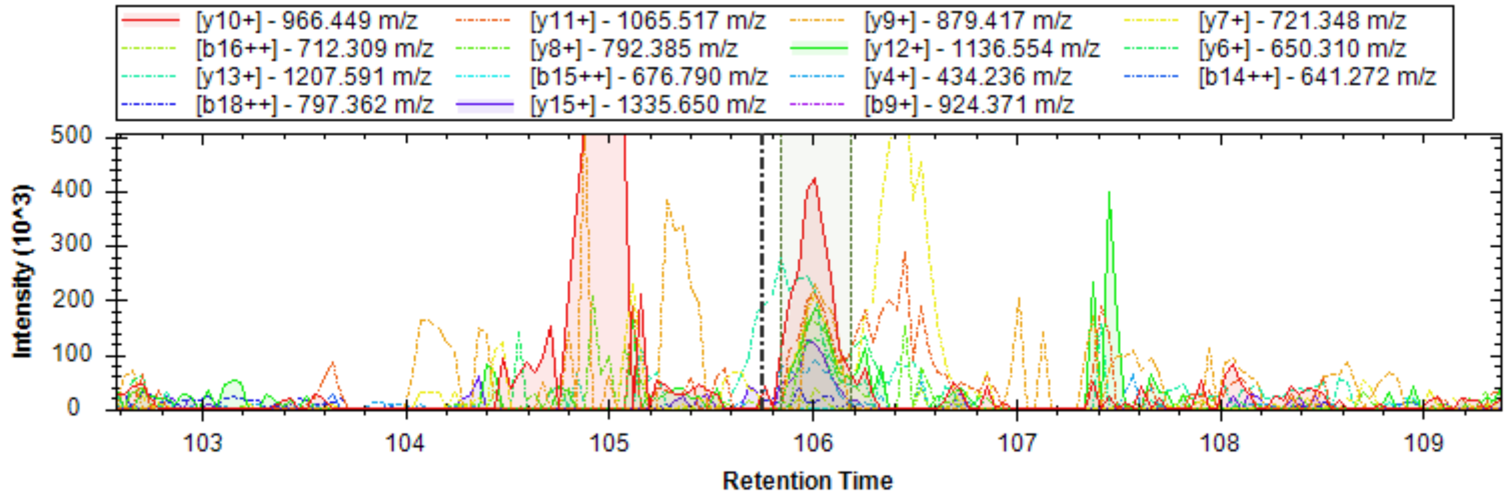

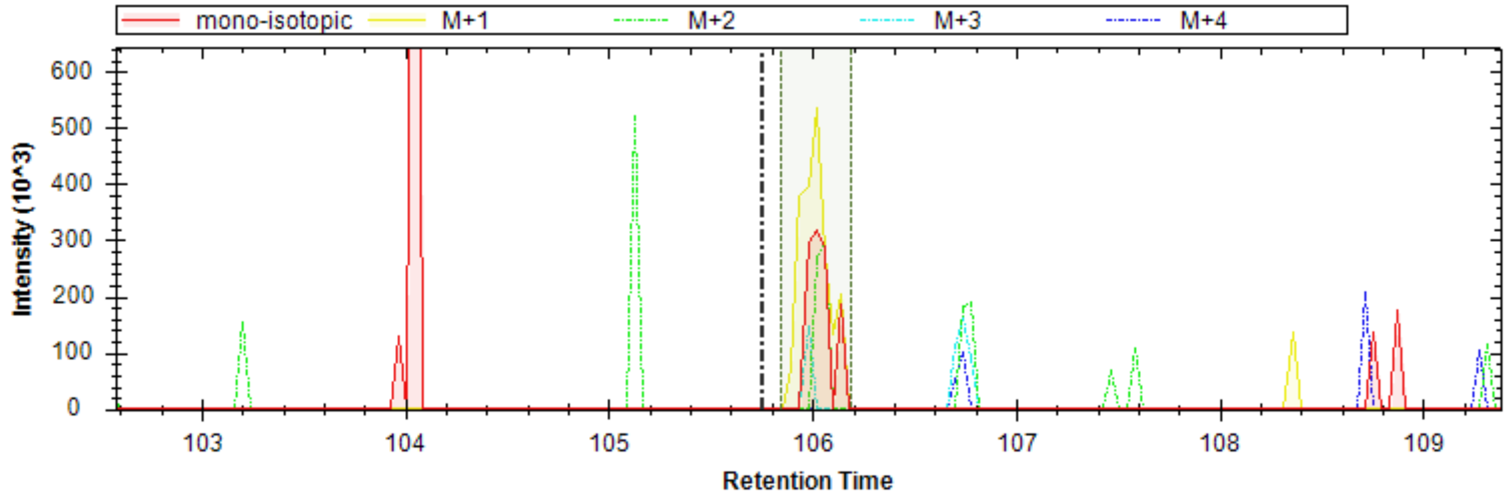

O08784 – _ASAVSPEKAPM[+O]T[+H+O3+P]SKAK_.2
Fig4_MP-DIA-barrel-cortex-p9-2_MHRM.htrms

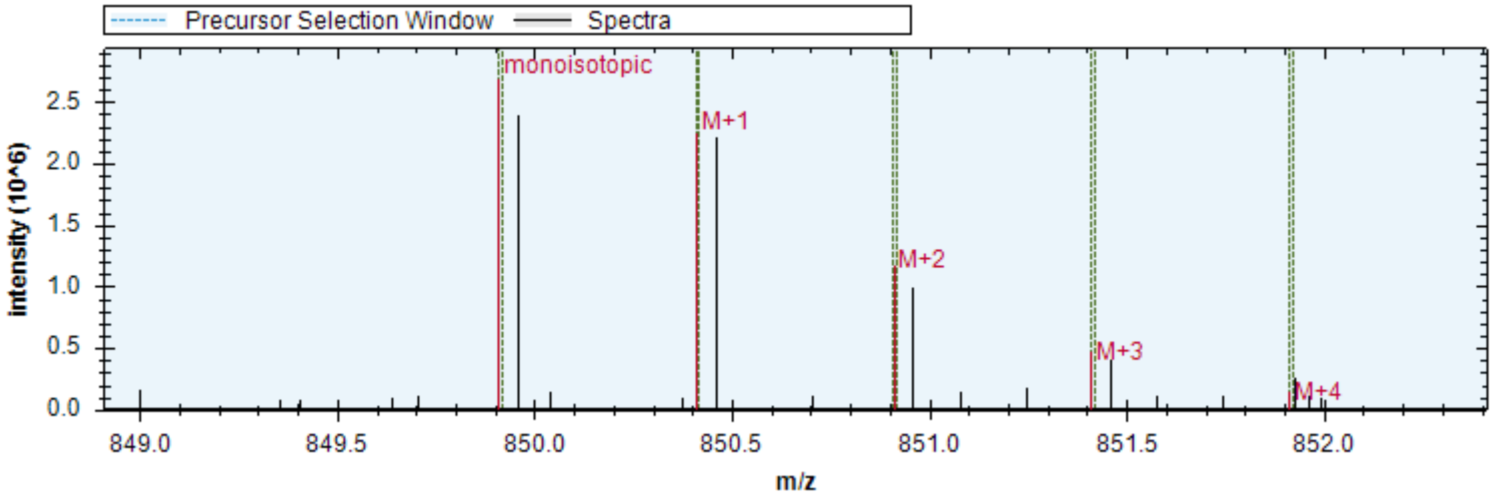

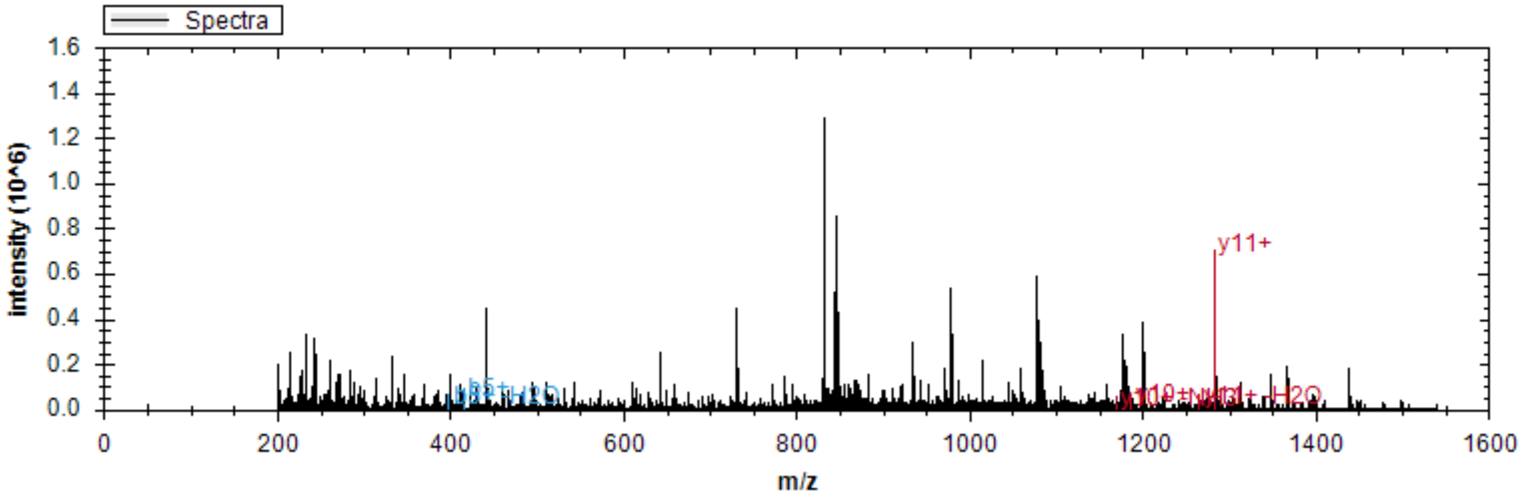

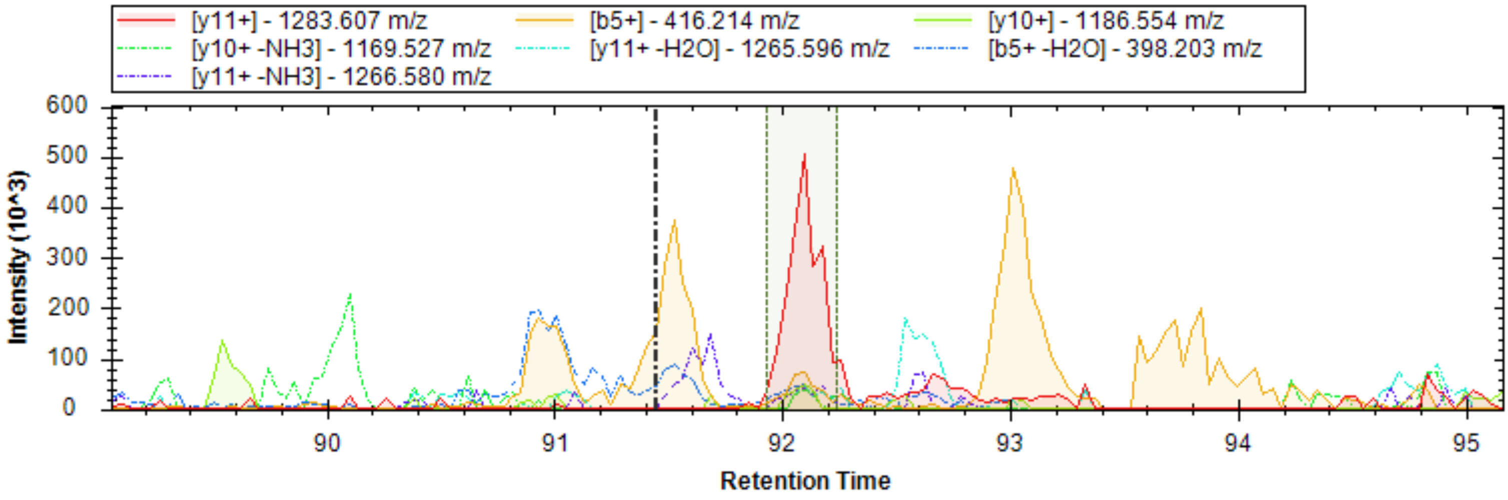

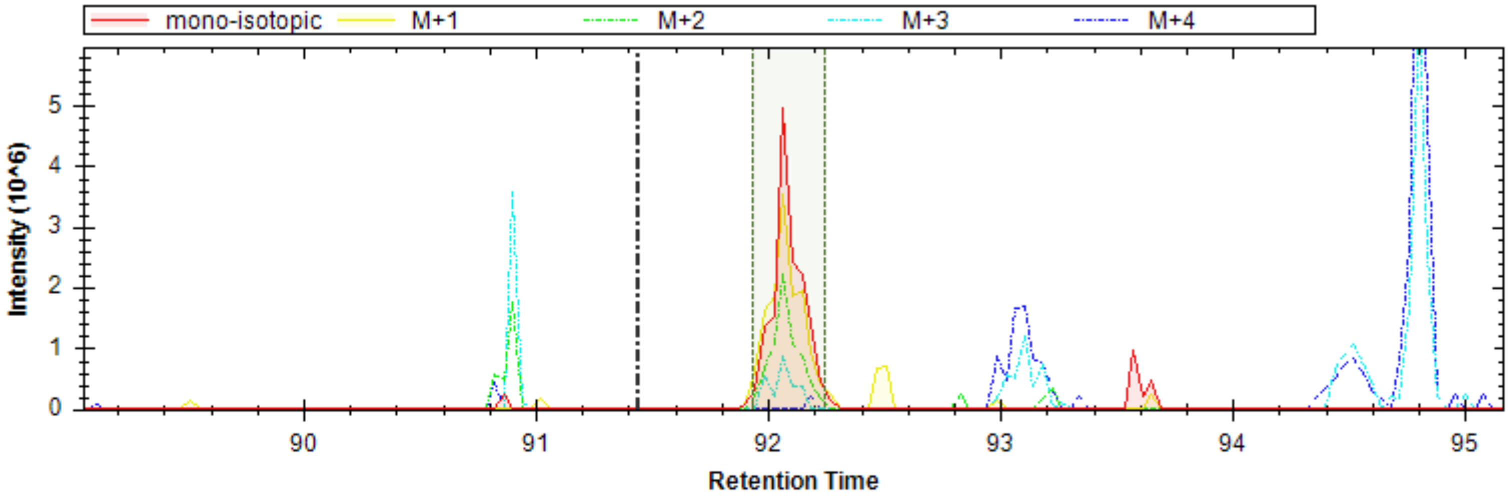

P27546-2 – _AAVGVTGNDITTPPNKEPPPS[+H+O3+P]PEKK_.3

Fig4_MP-DIA-barrel-cortex-p9-2_MHRM.htrms

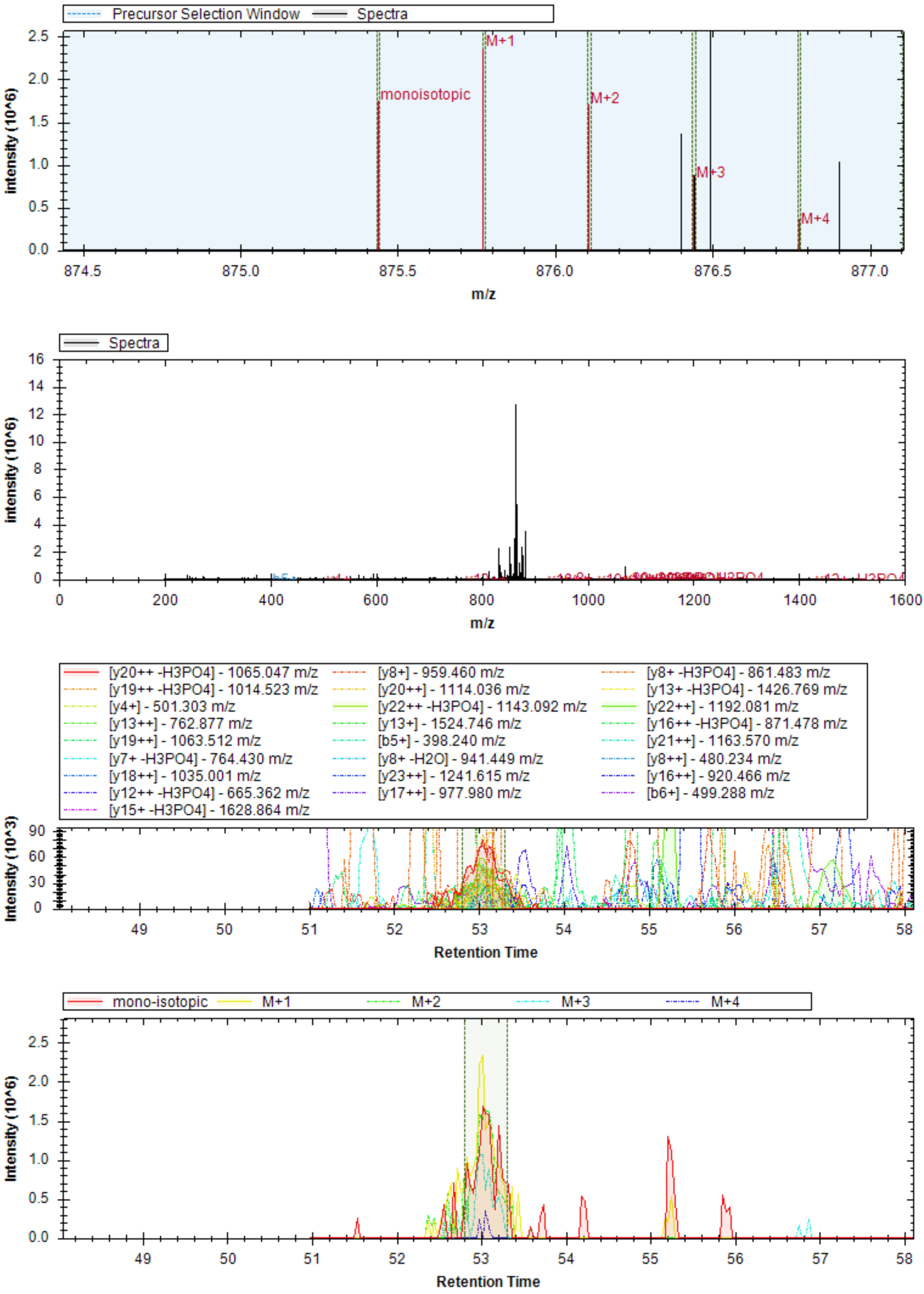

P27546-2 – _NTT[+H+O3+P]PTGAAPPAGMTSTR_.2

Fig4_MP-DIA-barrel-cortex-p9-2_MHRM.htrms

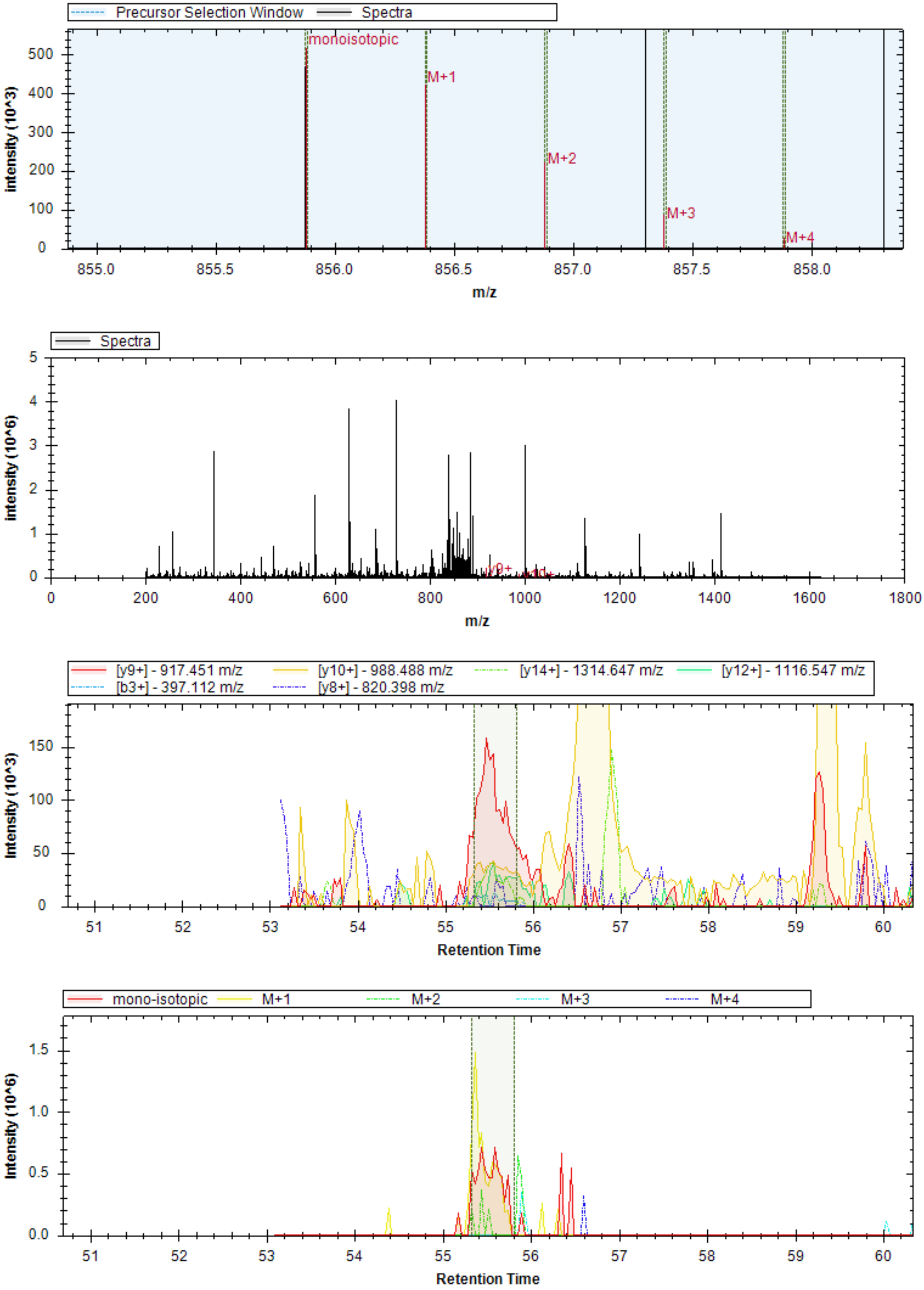

P50538 – _EAEHGYAS[+H+O3+P]M[+O]LPYSK_.2
Fig4_MP-DIA-barrel-cortex-p9-2_MHRM.htrms

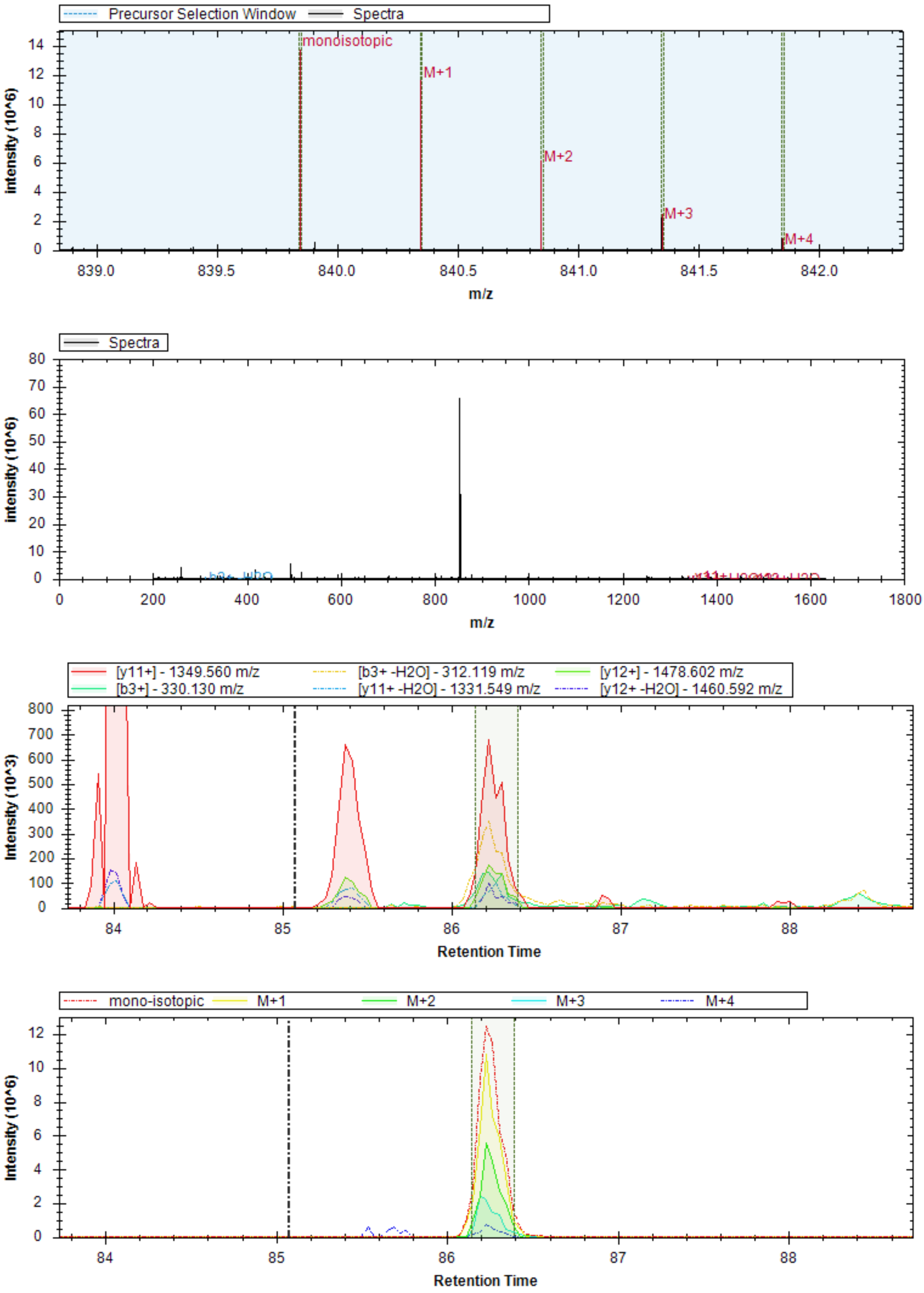

Q61290 – _M[+O]EPS[+H+O3+P]SLPQEIIANAK_.2
Fig4_MP-DIA-barrel-cortex-p9-2_MHRM.htrms

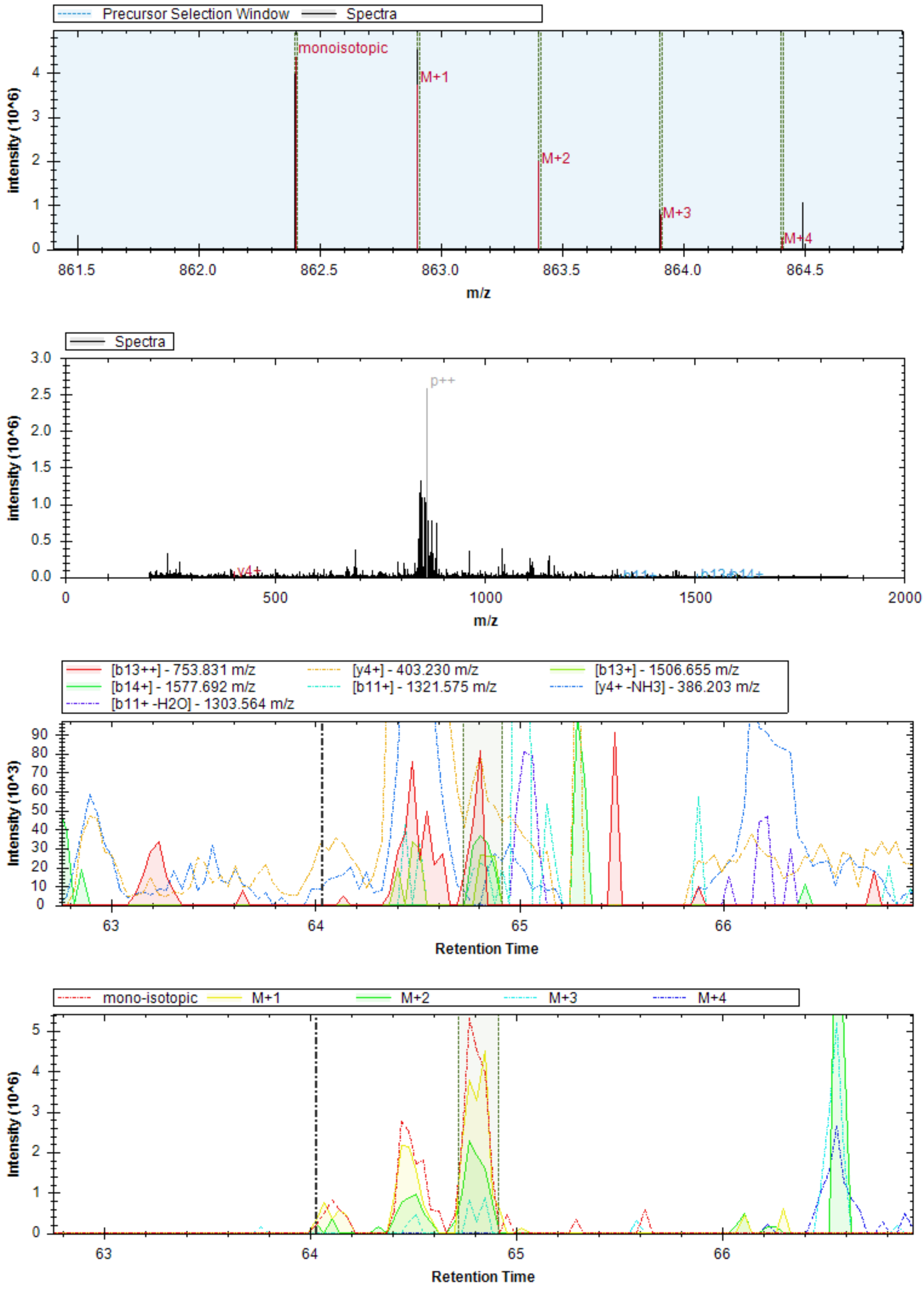

Q62188 – _GM[+O]YDGPVFDLT[+H+O3+P]TTPK_.2
Fig4_MP-DIA-barrel-cortex-p9-2_MHRM.htrms

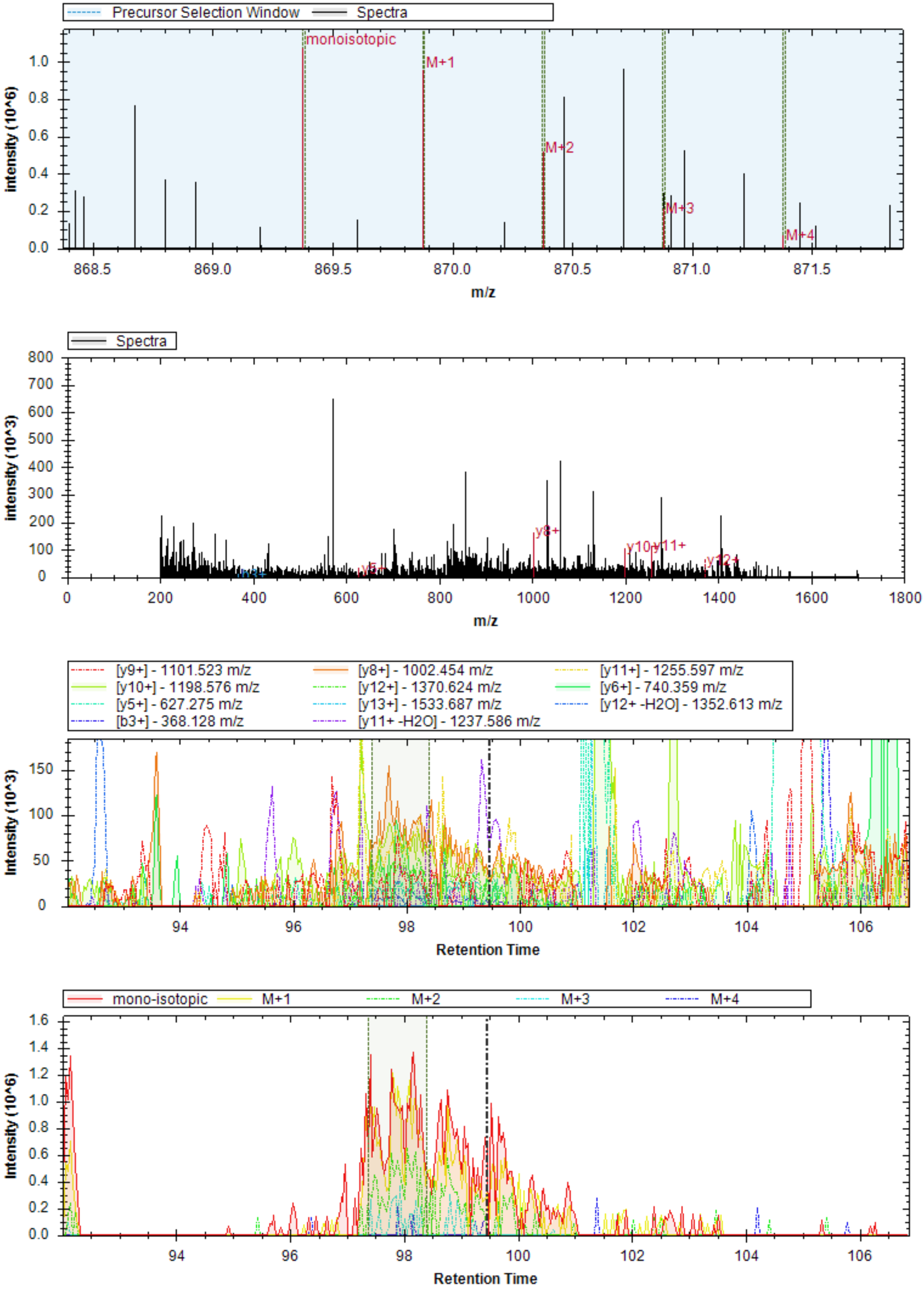

Q62188 – _GM[+O]YDGPVFDLTT[+H+O3+P]TPK_.2
Fig4_MP-DIA-barrel-cortex-p9-2_MHRM.htrms

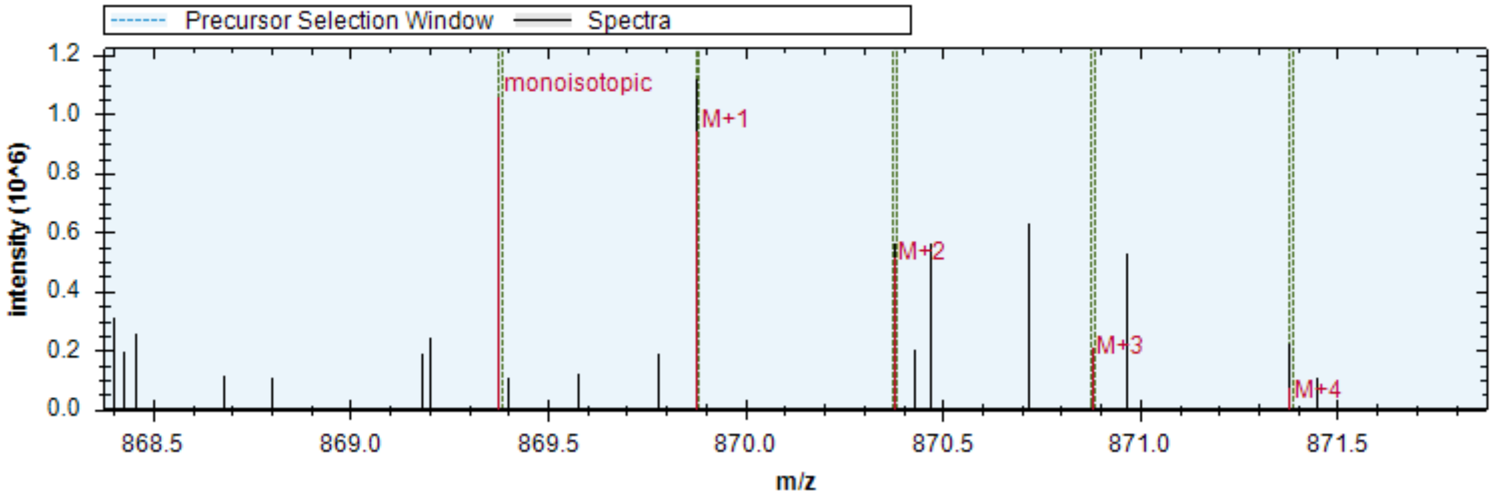

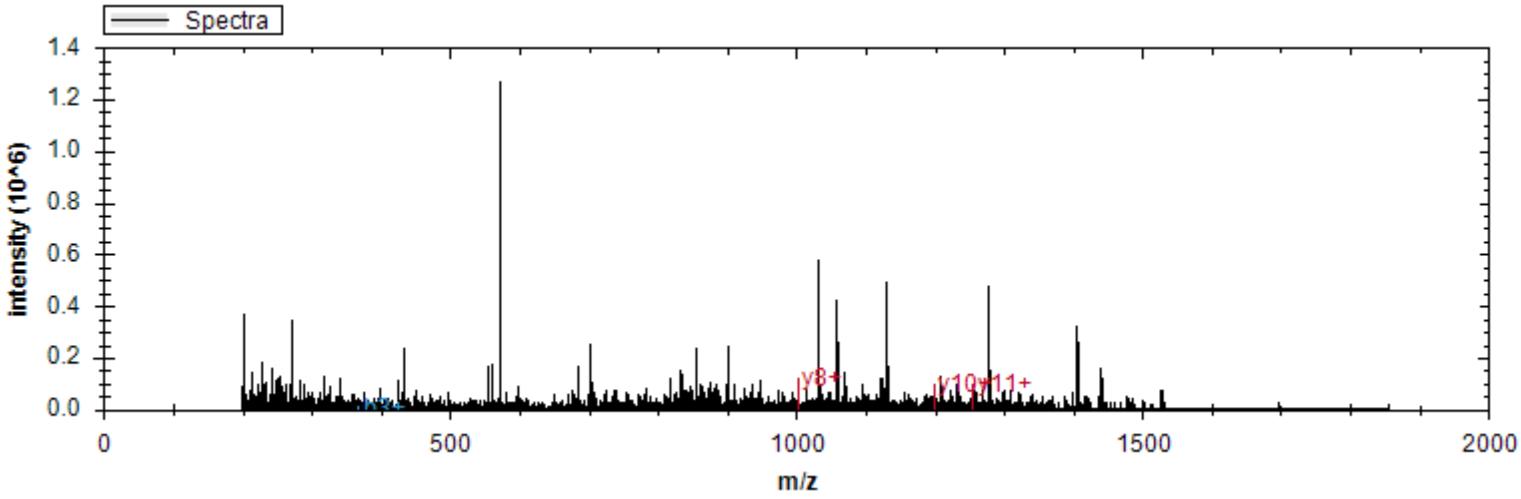

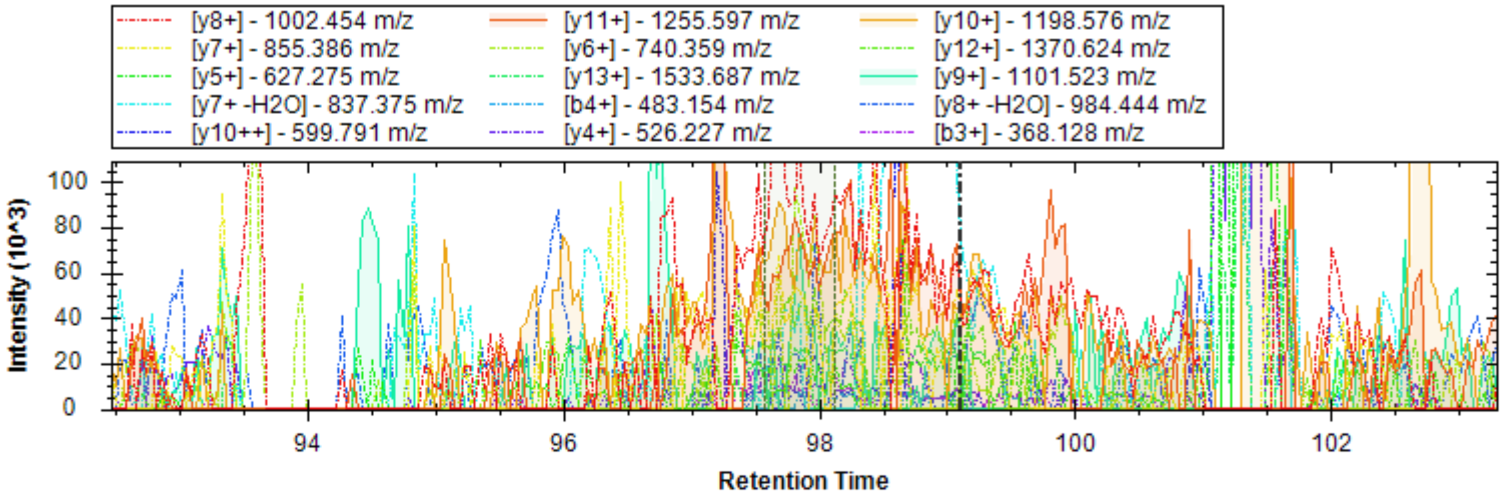

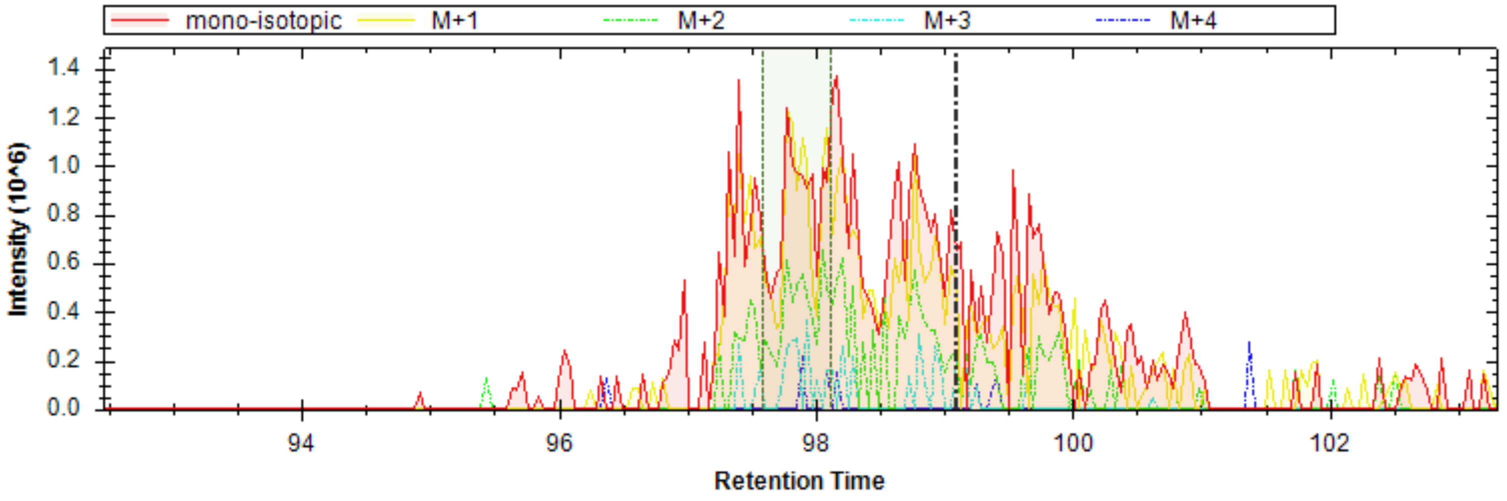

Q62188 – _GM[+O]YDGPVFDLTTT[+H+O3+P]PK_.2

Fig4_MP-DIA-barrel-cortex-p9-2_MHRM.htrms

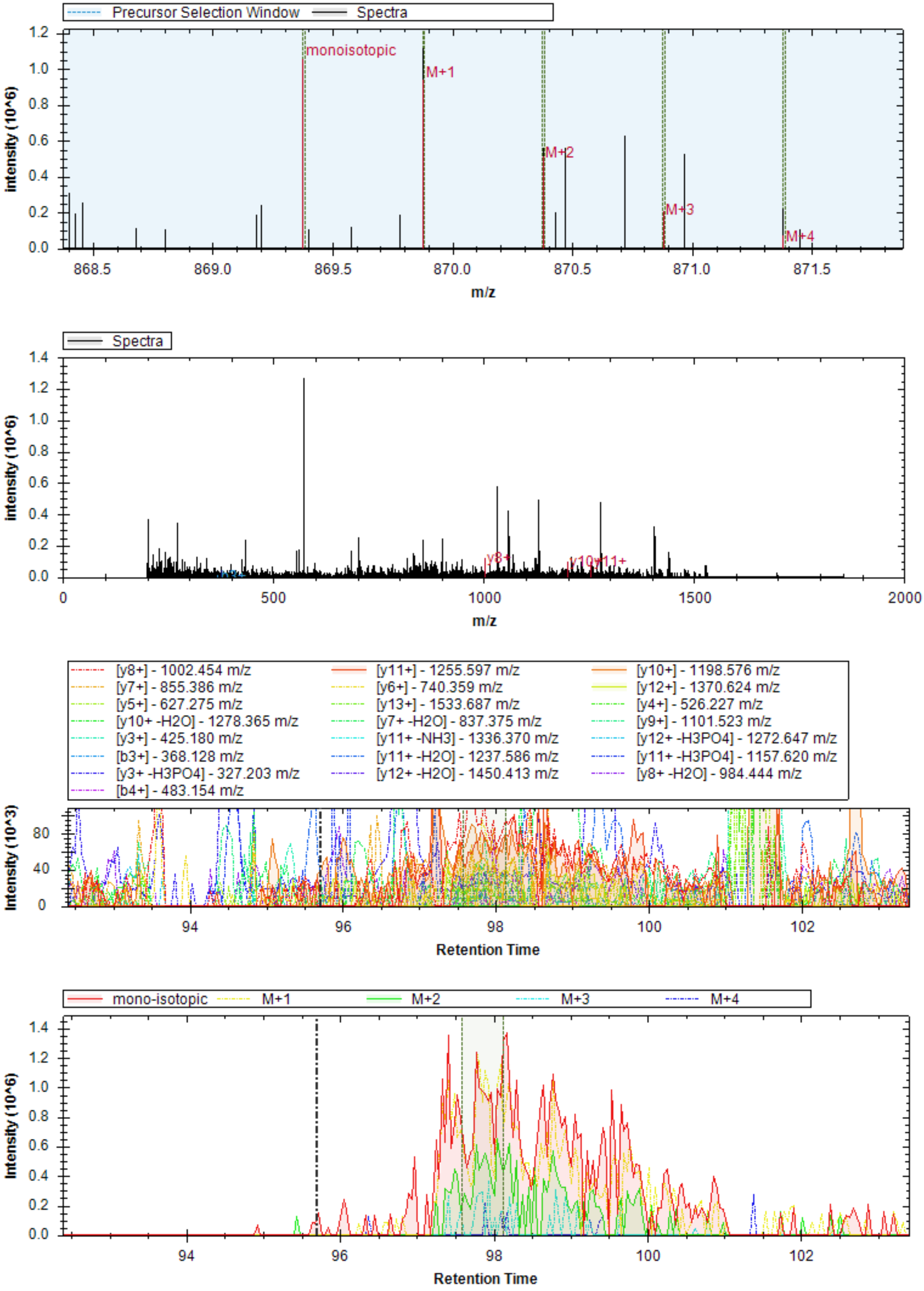

Q9JM96 – _DSSKLPKS[+H+O3+P]LS[+H+O3+P]SSPVK_.2
Fig4_MP-DIA-barrel-cortex-p9-2_MHRM.htrms

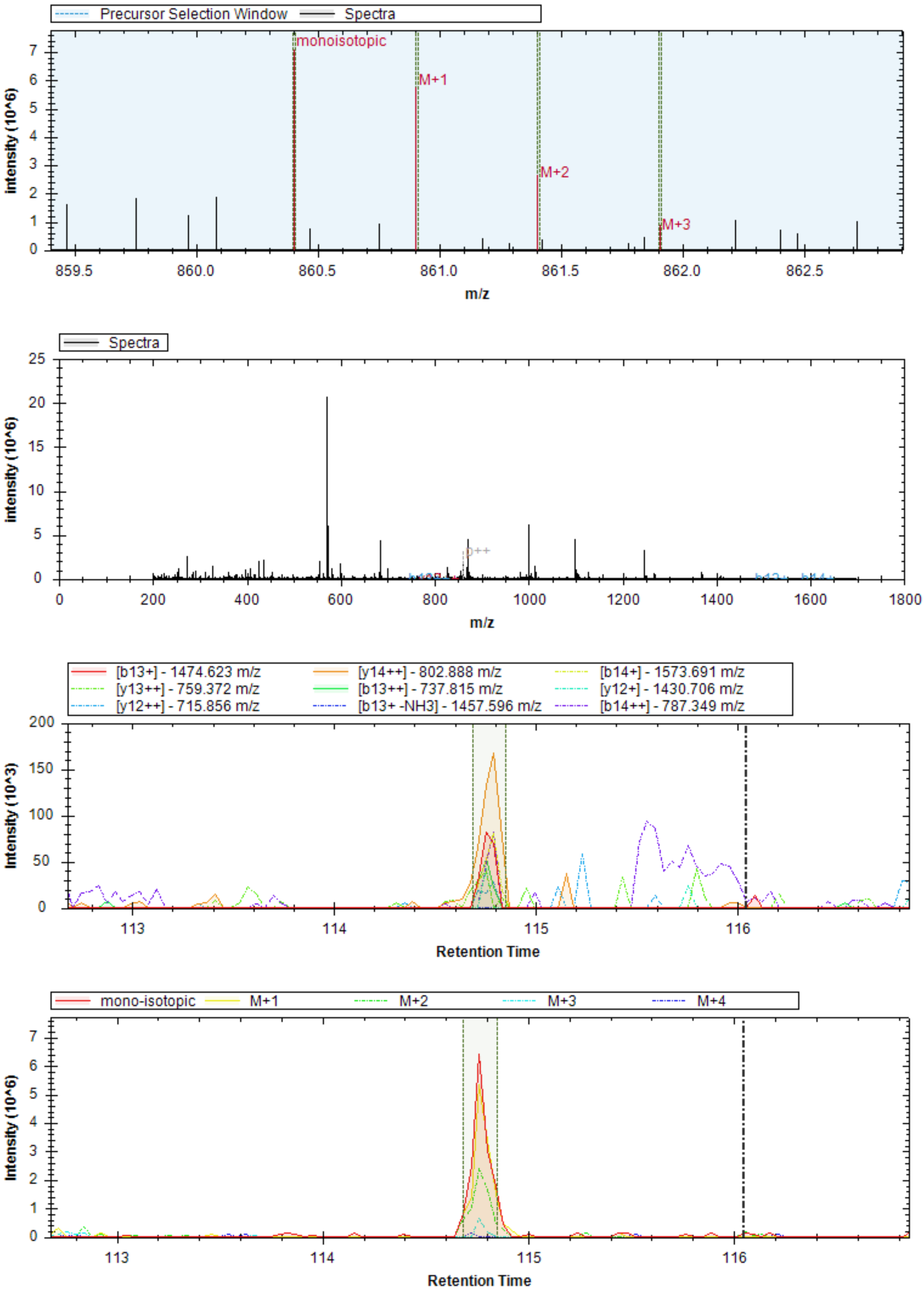

A1A535 – _FLHRGS[+H+O3+P]K_.1

Fig4_MP-DIA-barrel-cortex-p9-2_MHRM.htrms

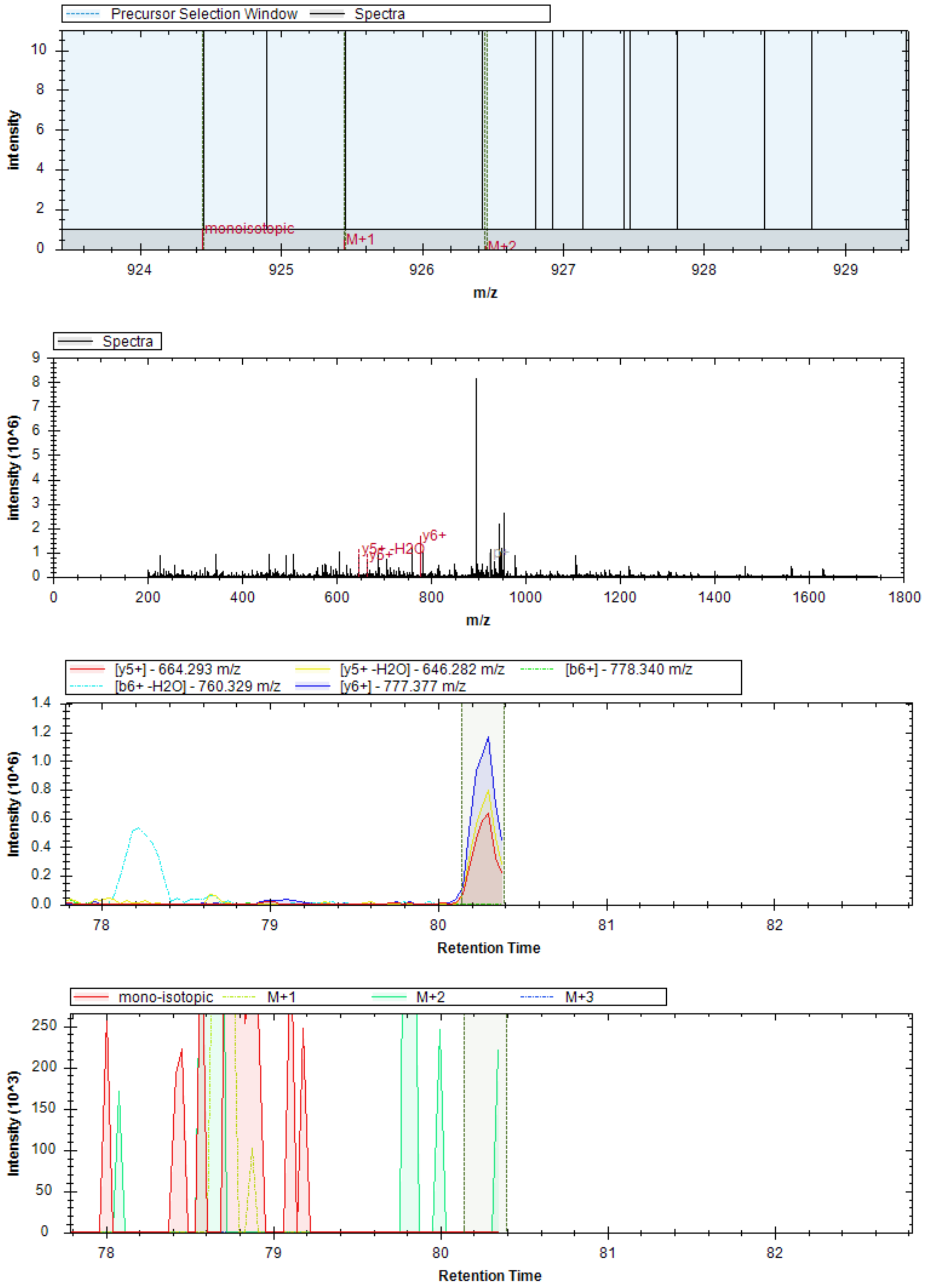

P26645 – _GEATAERPGEAAVASS[+H+O3+P]PSK_.2
Fig4_MP-DIA-barrel-cortex-p9-2_MHRM.htrms

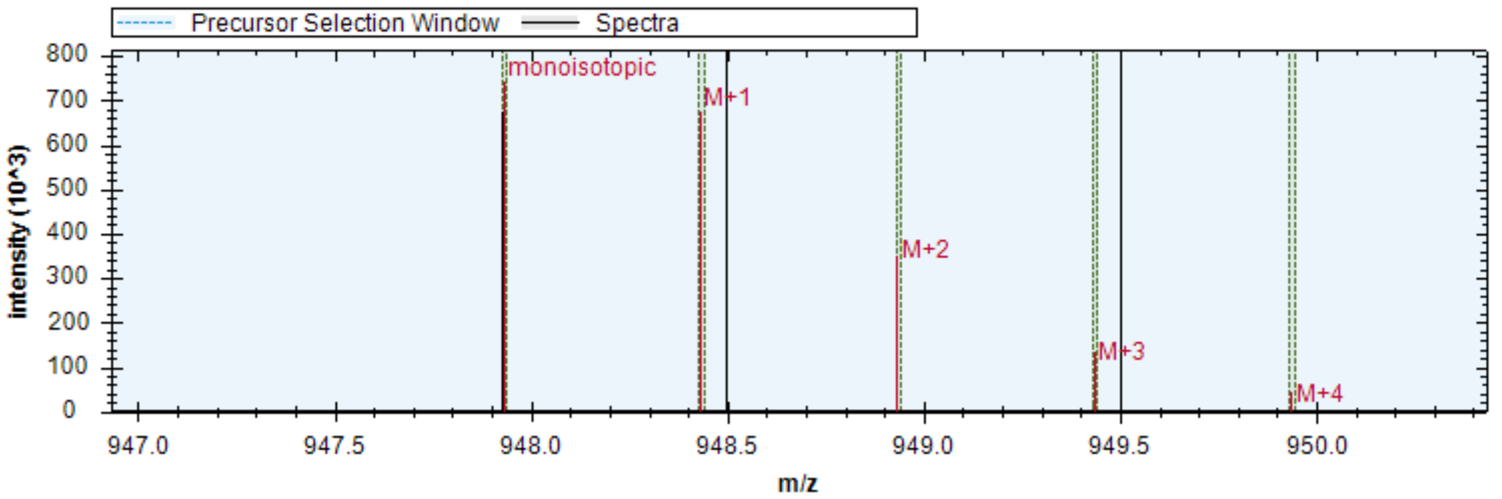

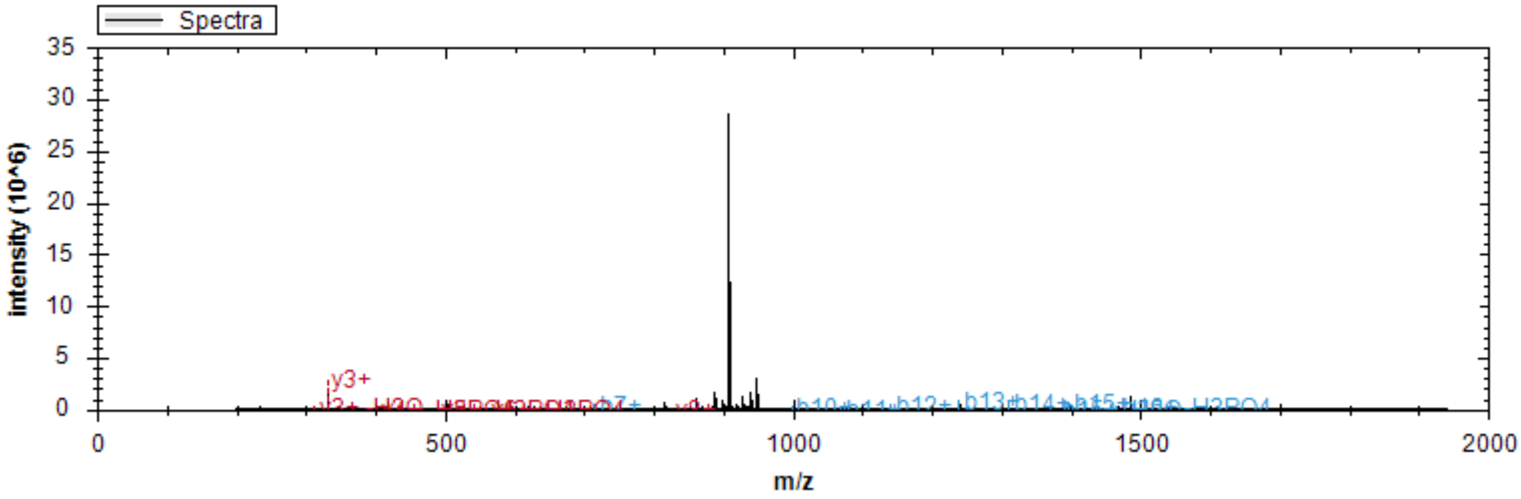

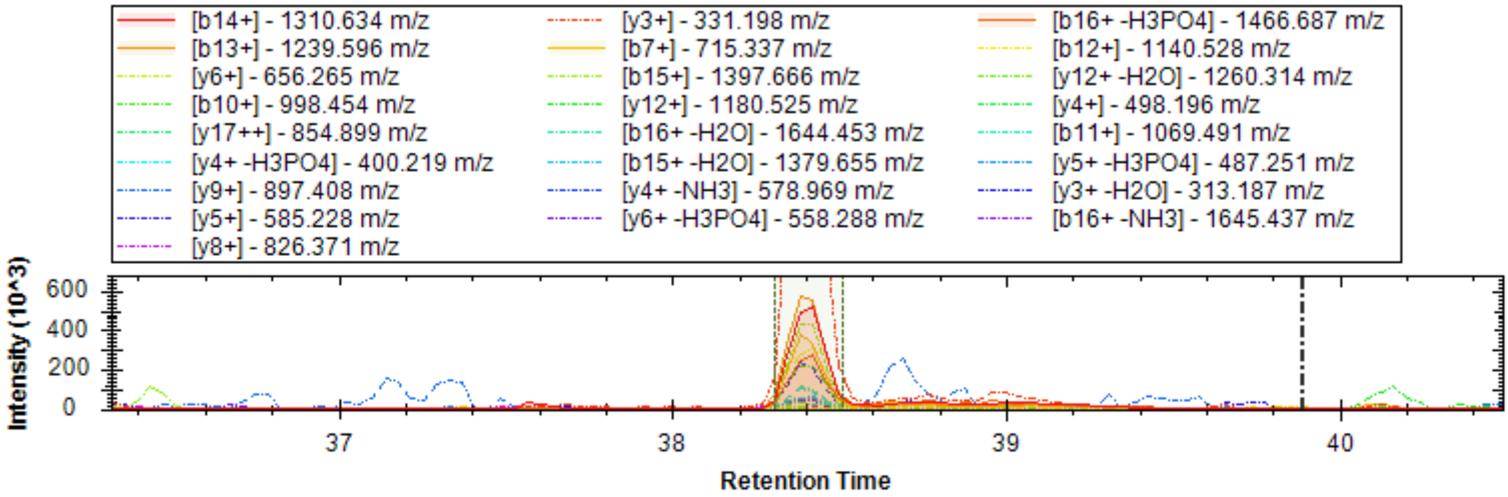

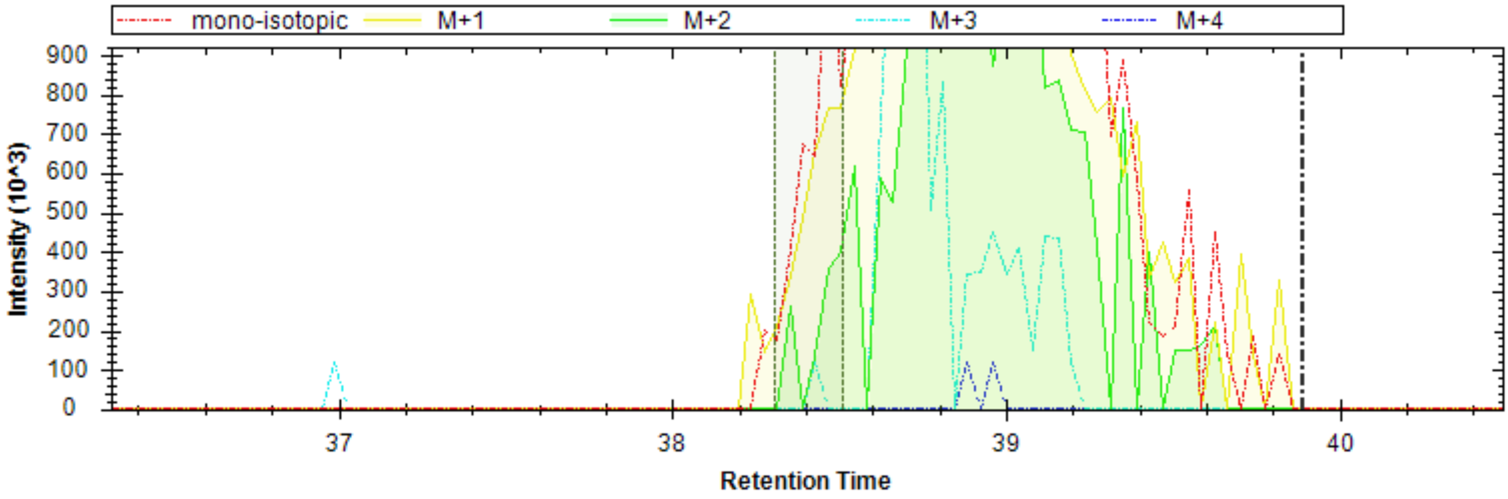

Q8BMA3 – _IIQLC[+C2+H3+N+O]LDLT[+H+O3+P]T[+H+O3+P]AVQK_.2
Fig4_MP-DIA-barrel-cortex-p9-2_MHRM.htrms

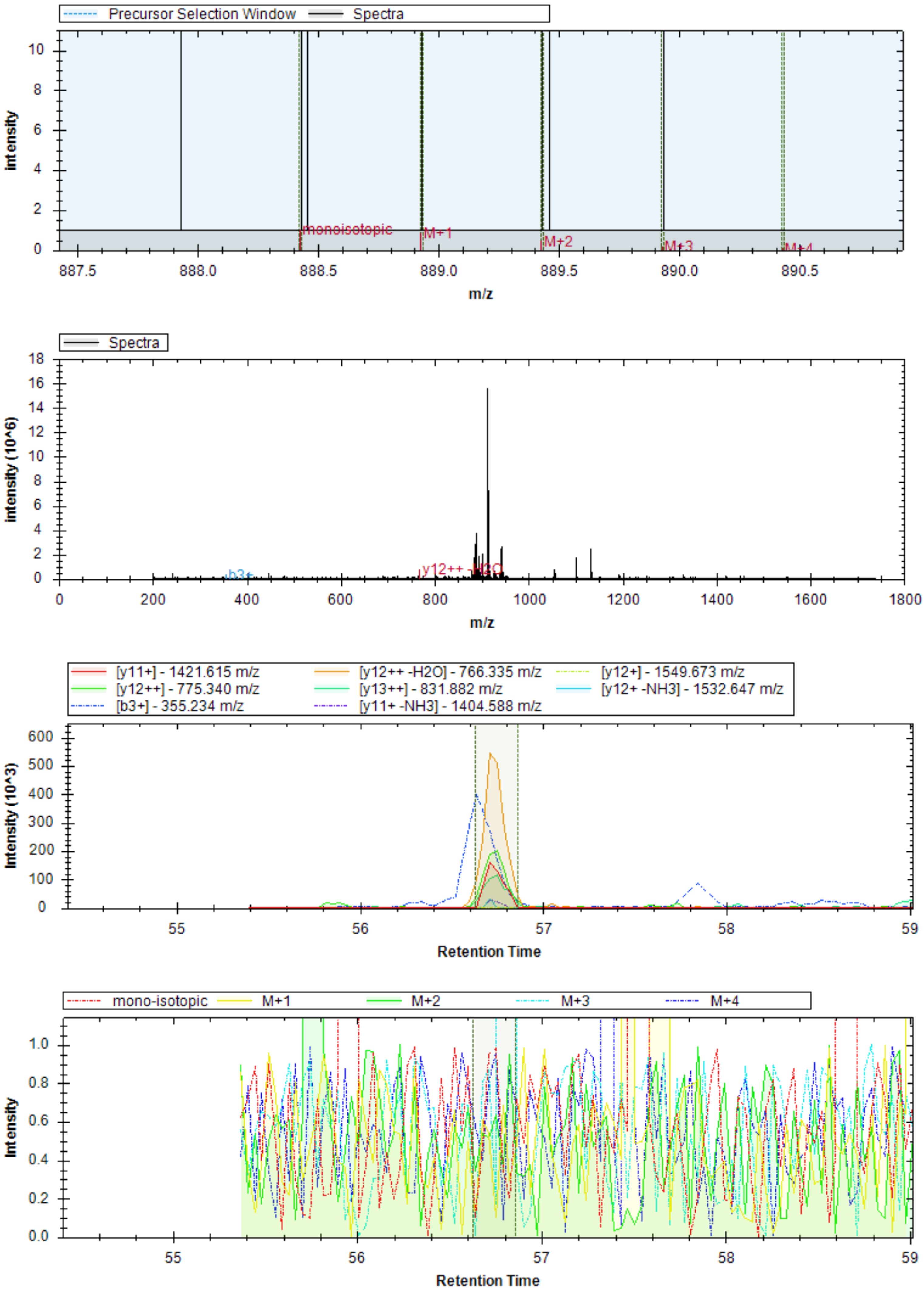

Q91Z31;Q91Z31-2 – _ETSLLAVPGALS[+H+O3+P]PLAIPNAAAAAAAAAAGR_.3

Fig4_MP-DIA-barrel-cortex-p9-2_MHRM.htrms

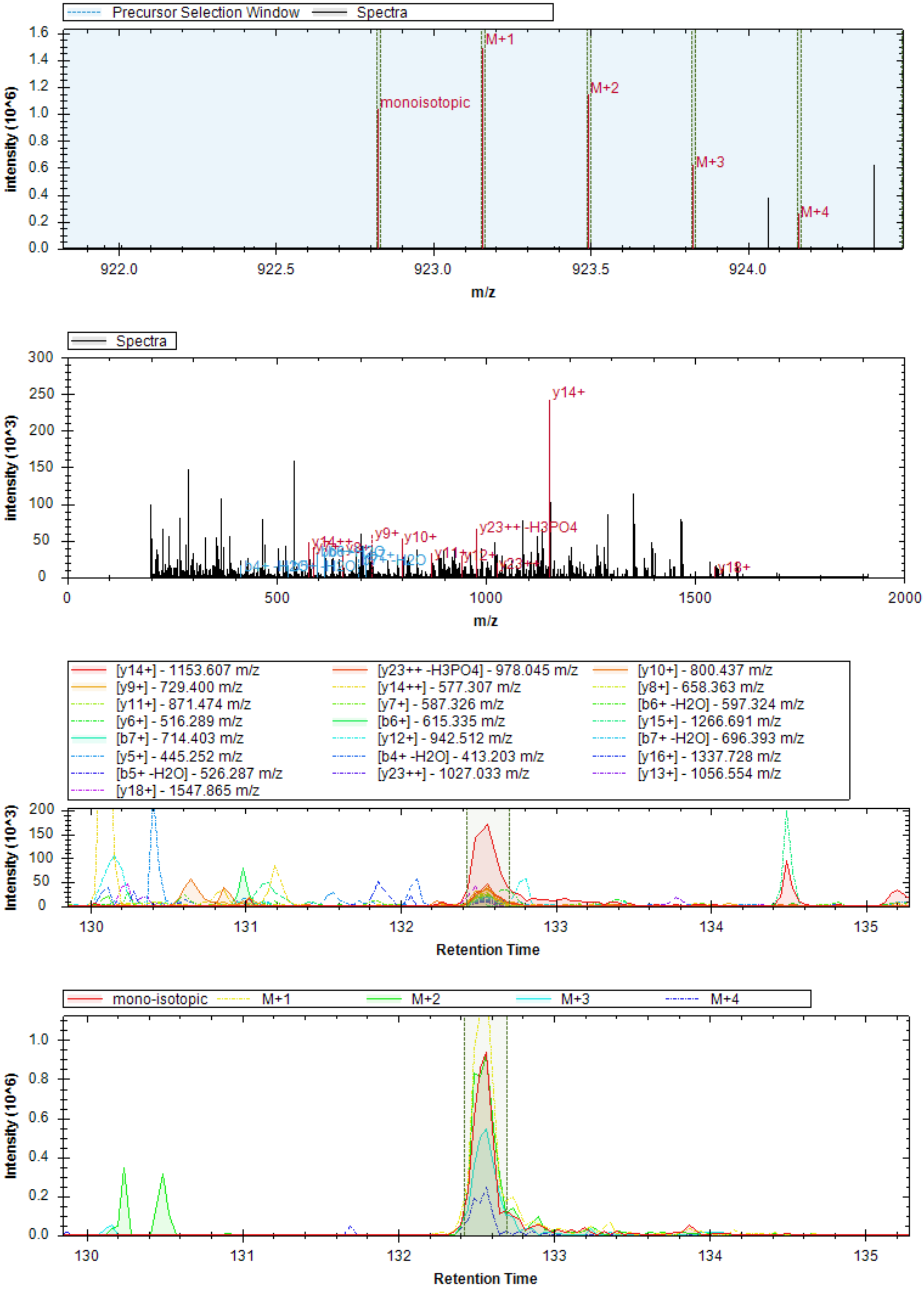

Q99MR1 – _[+C2+H2+O]M[+O]AAET[+H+O3+P]LNFGPEWLR_.2
Fig4_MP-DIA-barrel-cortex-p9-2_MHRM.htrms

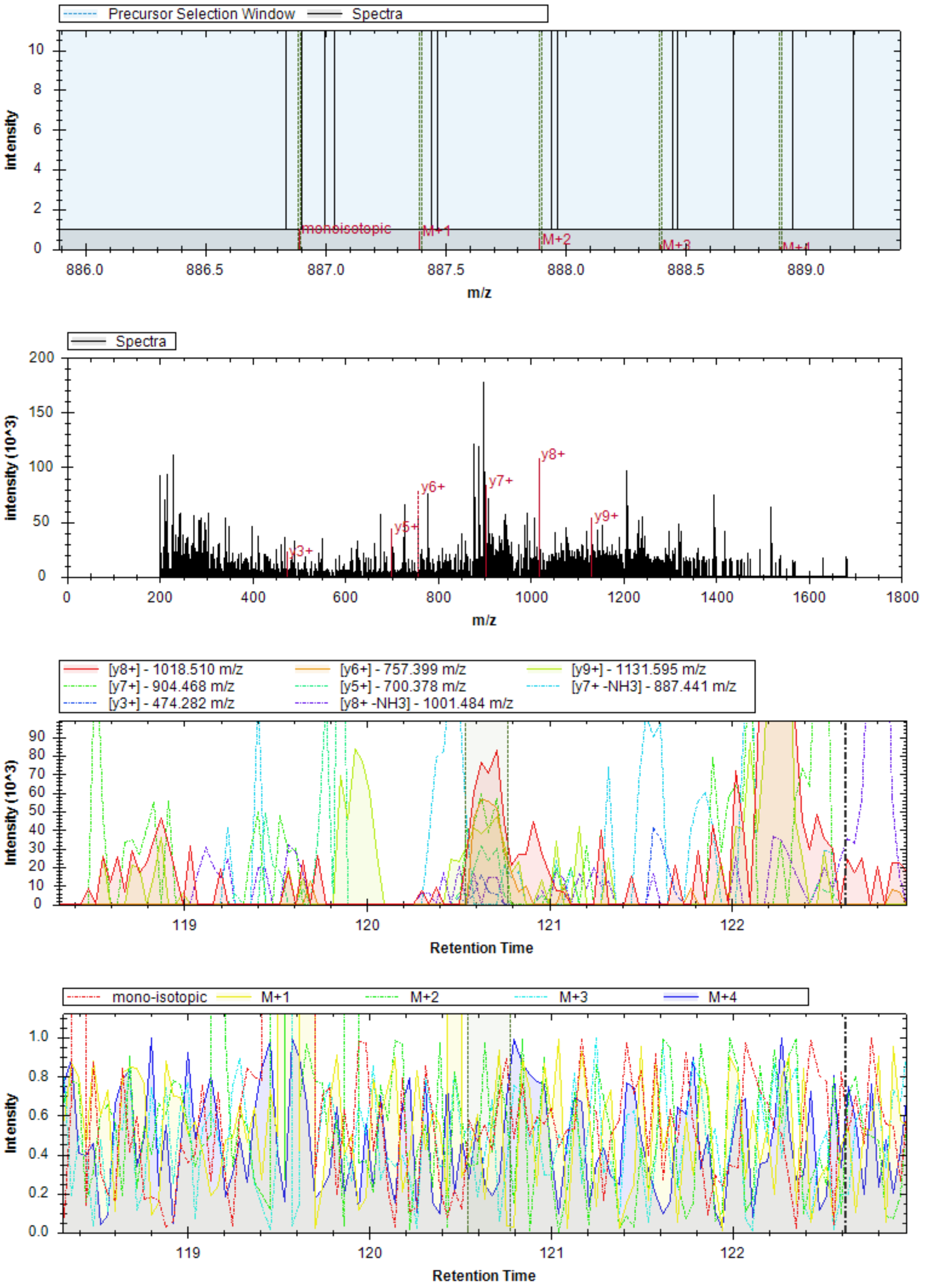

Q9CZT4;Q9CZT4-2 – _KEQM[+O]RAATVLPS[+H+O3+P]VQIK_.2
Fig4_MP-DIA-barrel-cortex-p9-2_MHRM.htrms

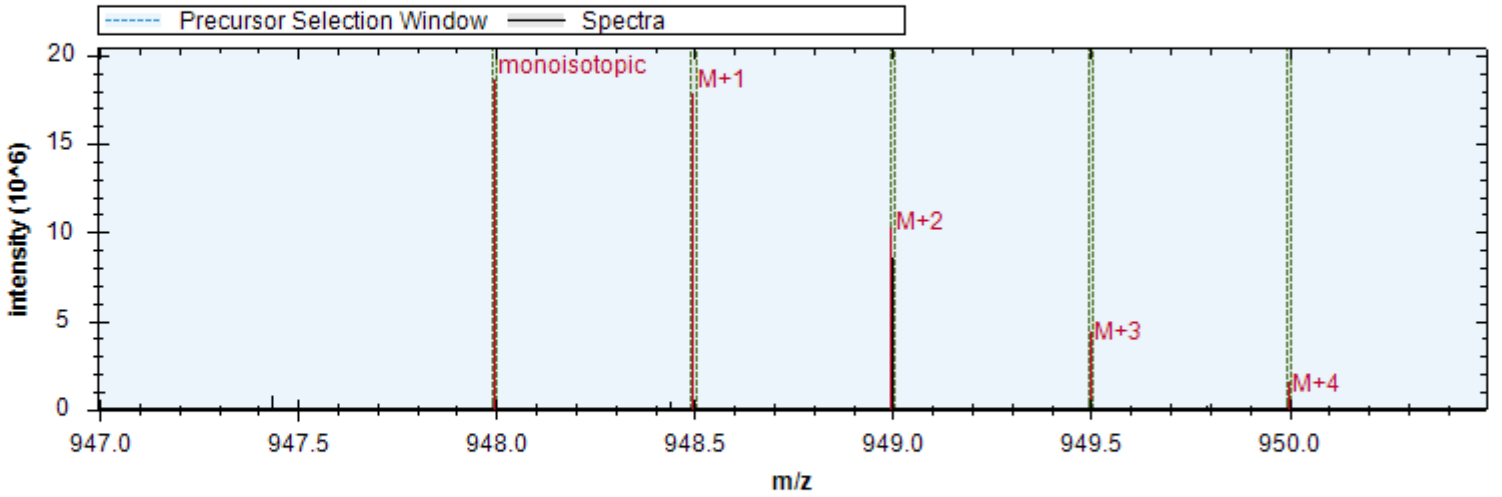

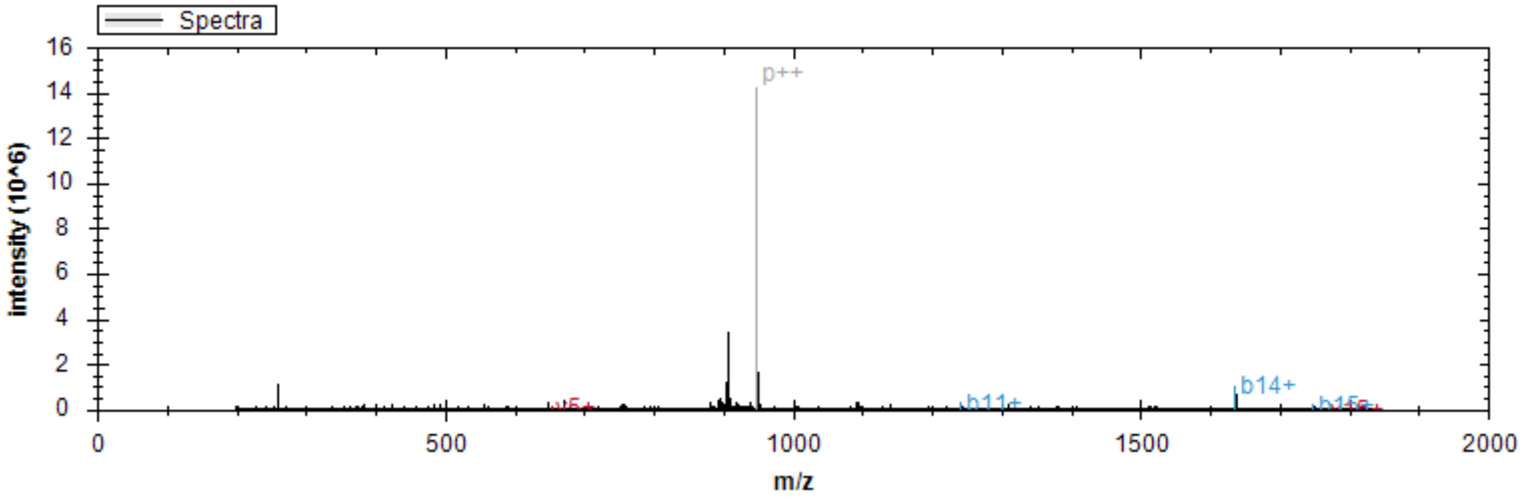

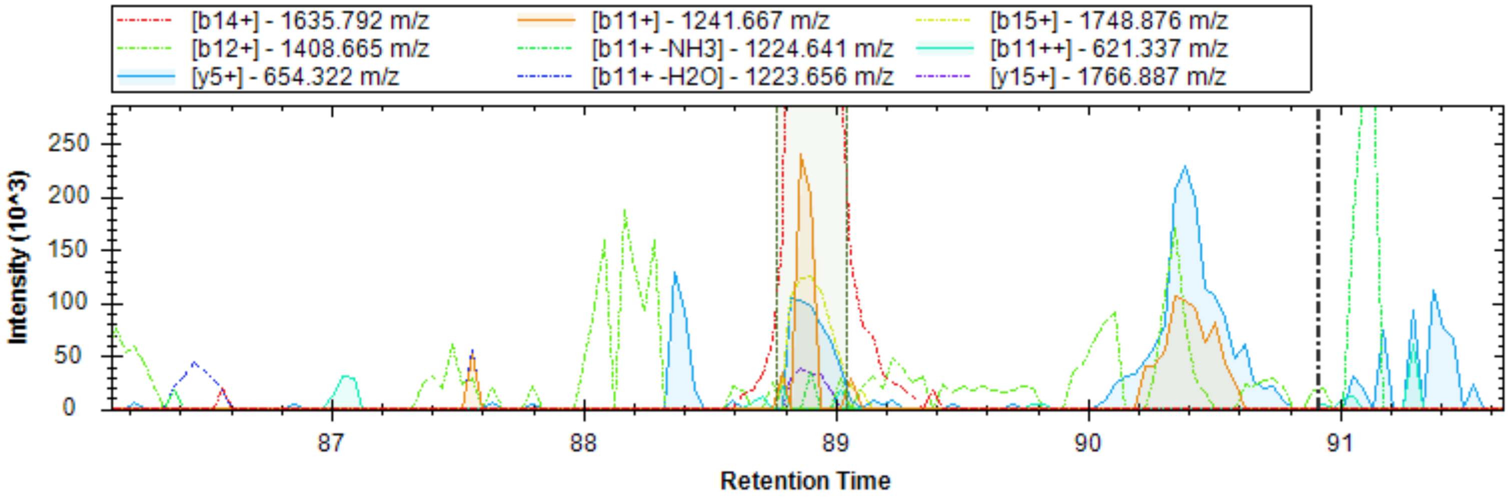

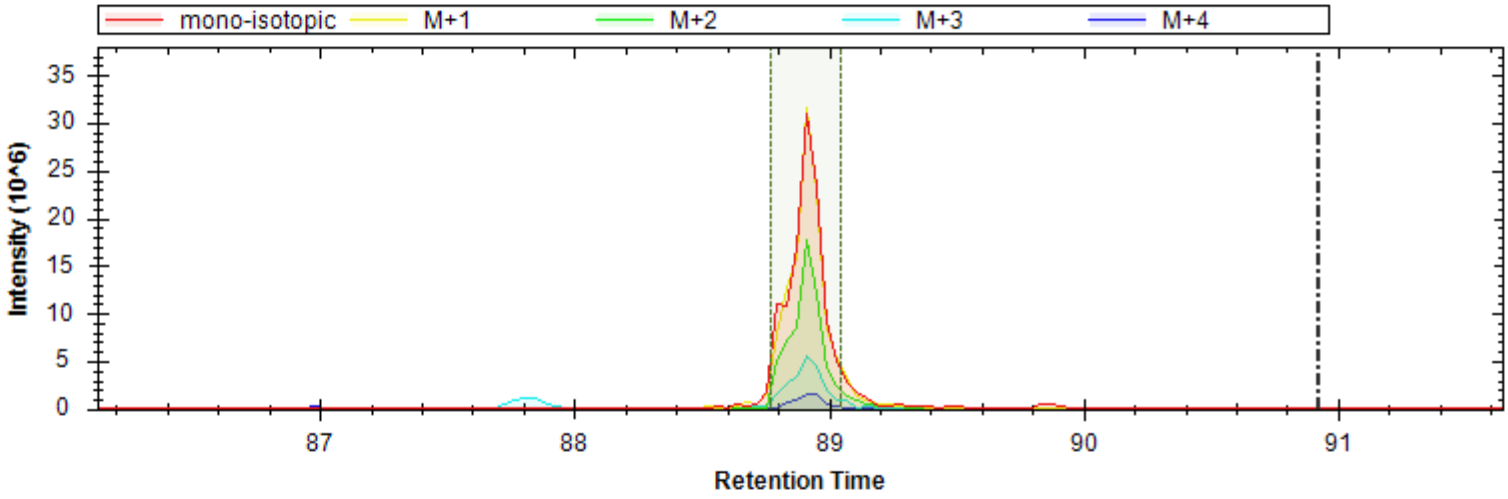

Q9ES97;Q9ES97-2 – _AES[+H+O3+P]SAATQSPSVSSSSGAEPSALGGGGGSPGAC[+C2+H3+N+O]PALGAK_.4
Fig4_MP-DIA-barrel-cortex-p9-2_MHRM.htrms

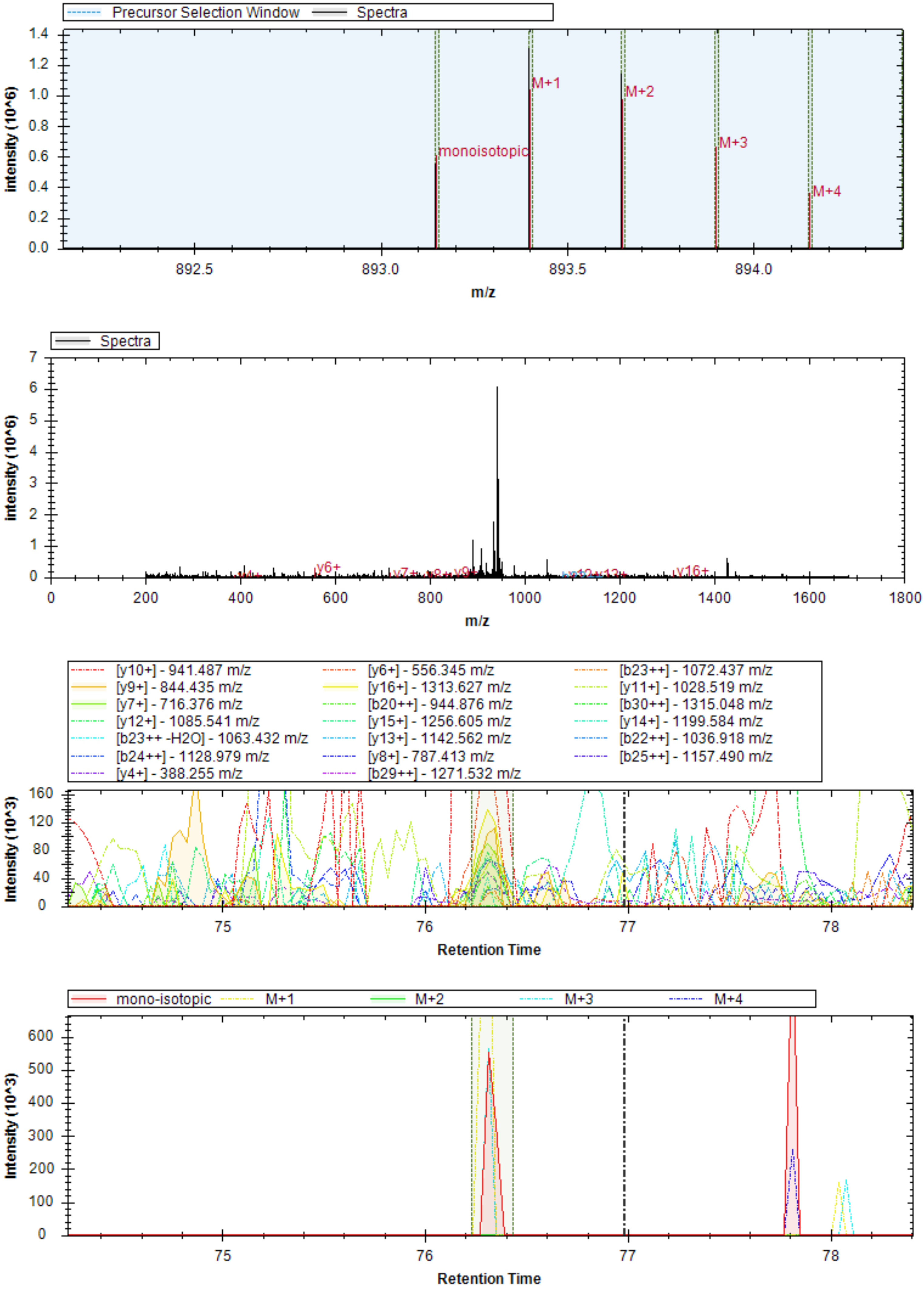

P17742 – _VNPT[+H+O3+P]VFFDITADDEPLGR_.2

Fig4_MP-DIA-barrel-cortex-p9-2_MHRM.htrms

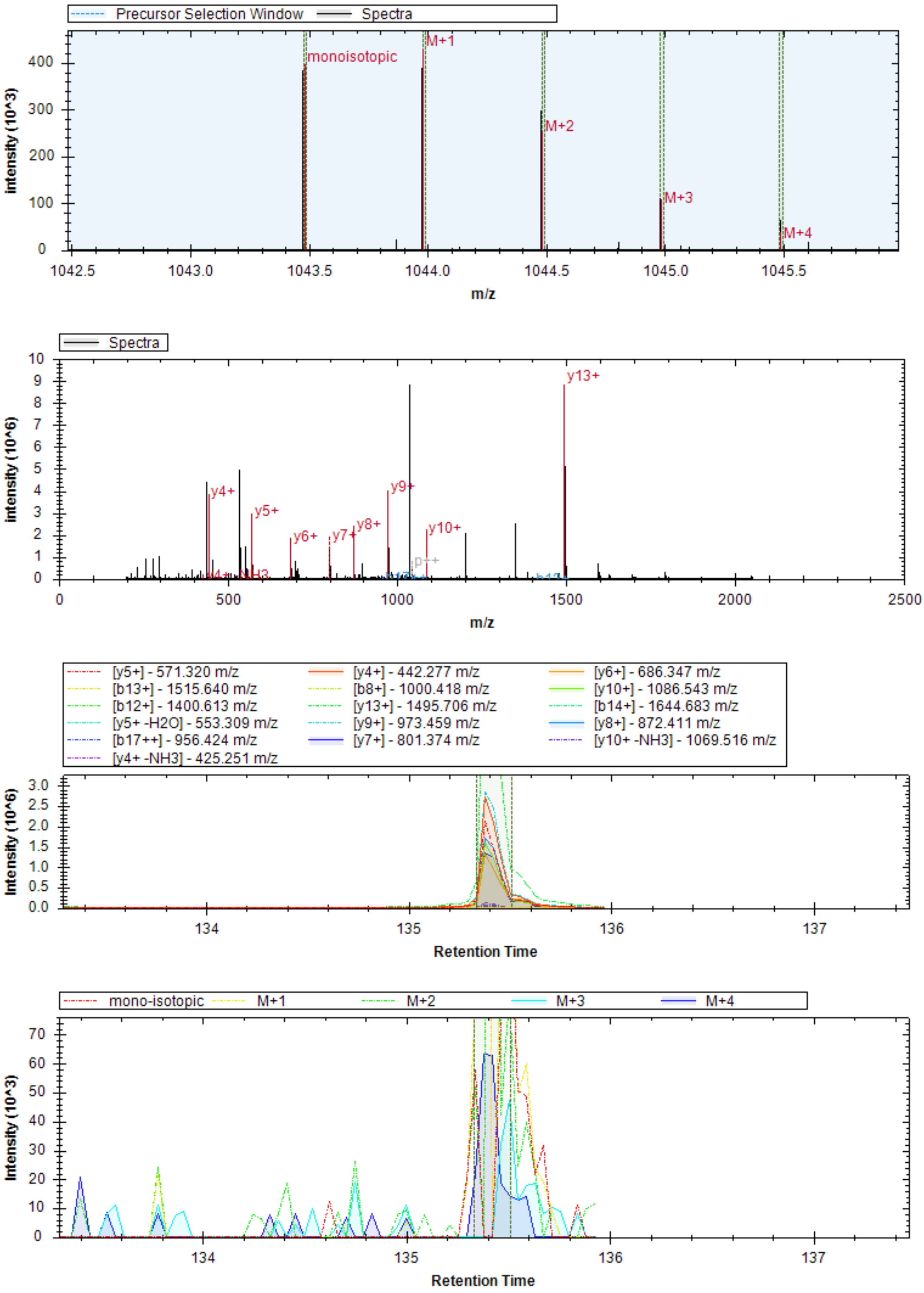

P26645 – _LSGFS[+H+O3+P]FKK_.1

Fig4_MP-DIA-barrel-cortex-p9-2_MHRM.htrms

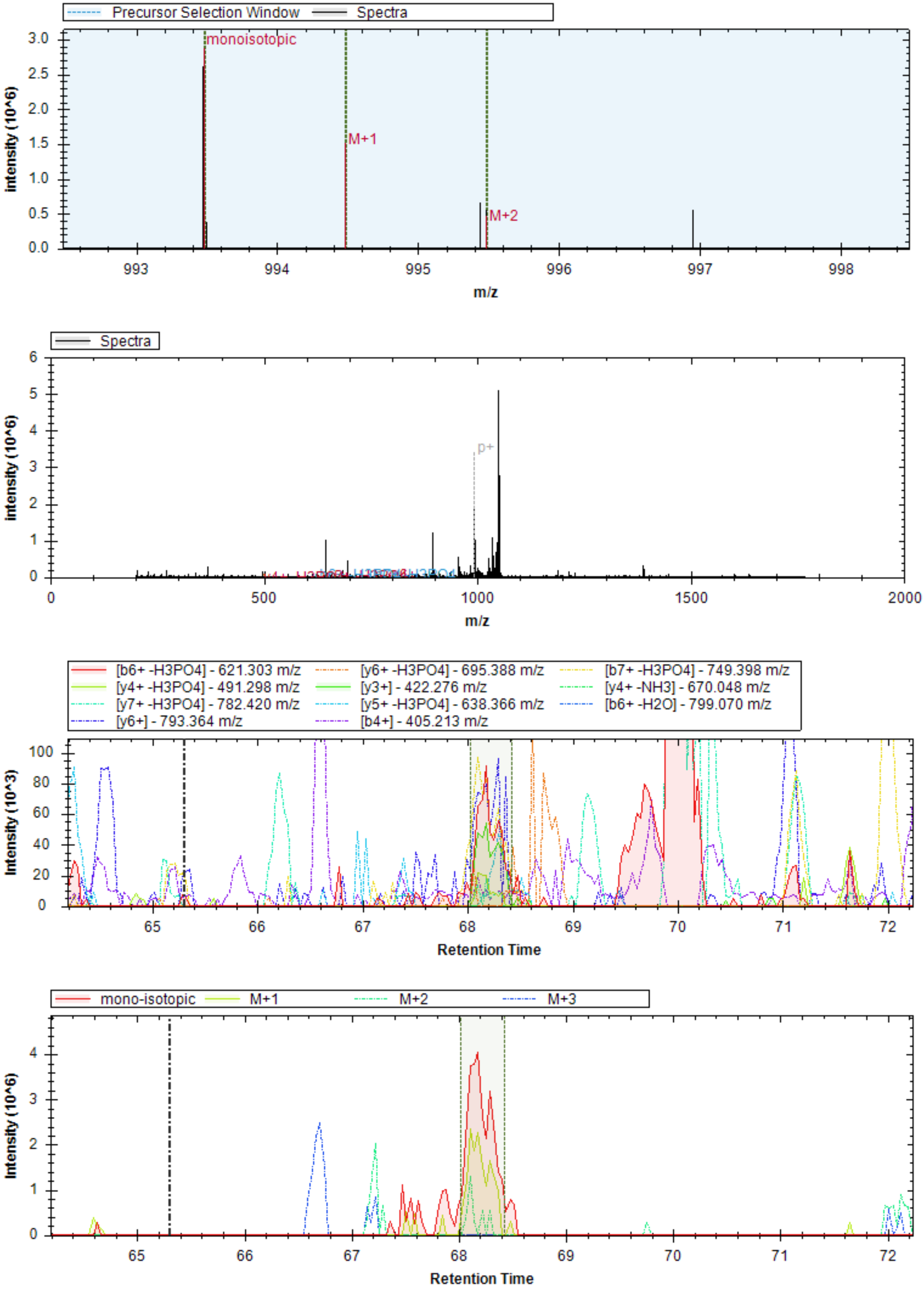

Q8CHK4;Q8CHK4-2;Q8CH[...] – _RGY[+H+O3+P]GKLLIEFS[+H+O3+P]YELSK_.2
Fig4_MP-DIA-barrel-cortex-p9-2_MHRM.htrms

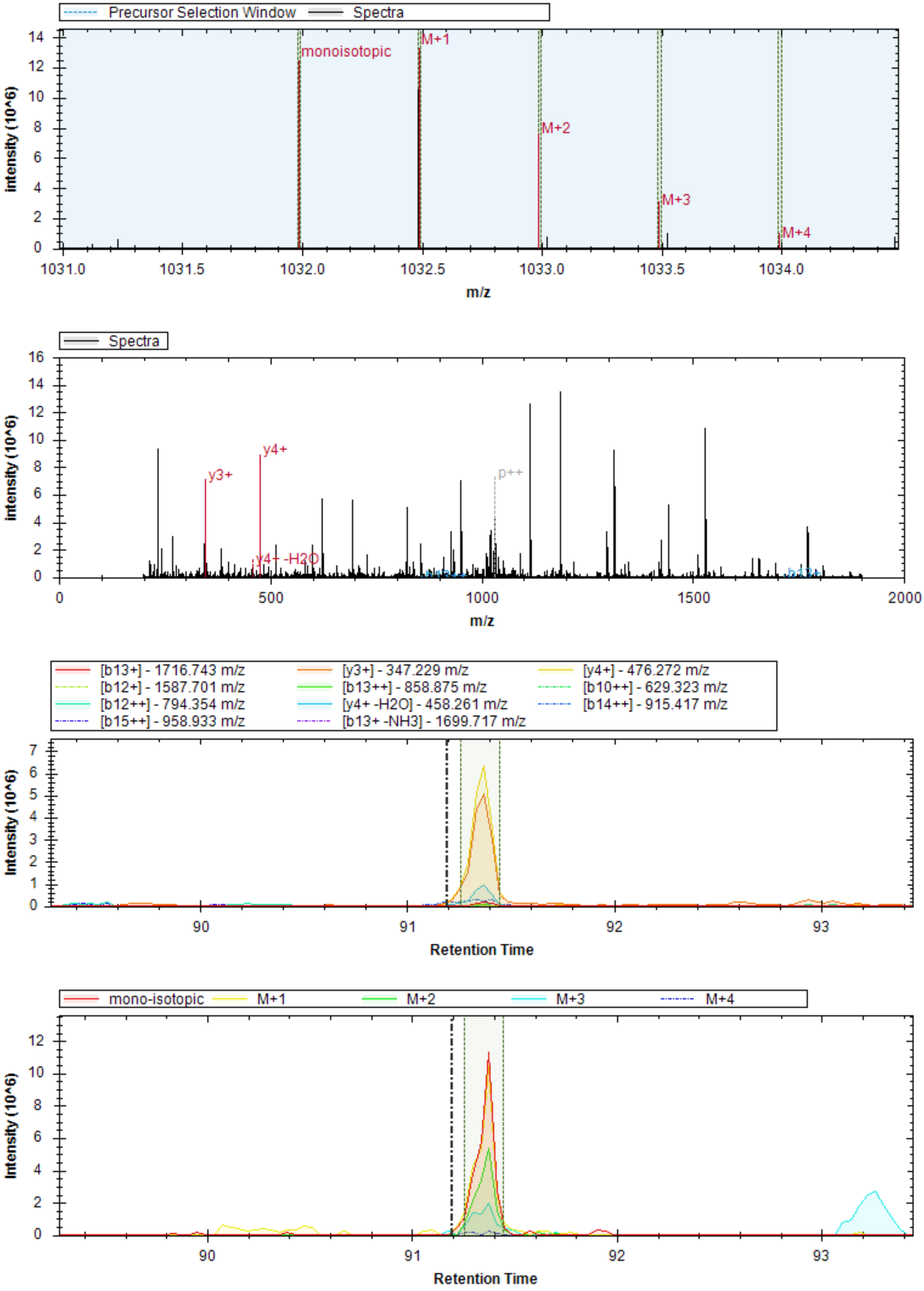

E9Q401 – _MVAEVFIY[+H+O3+P]WSK_.1
Fig4_MP-DIA-barrel-cortex-p9-2_MHRM.htrms

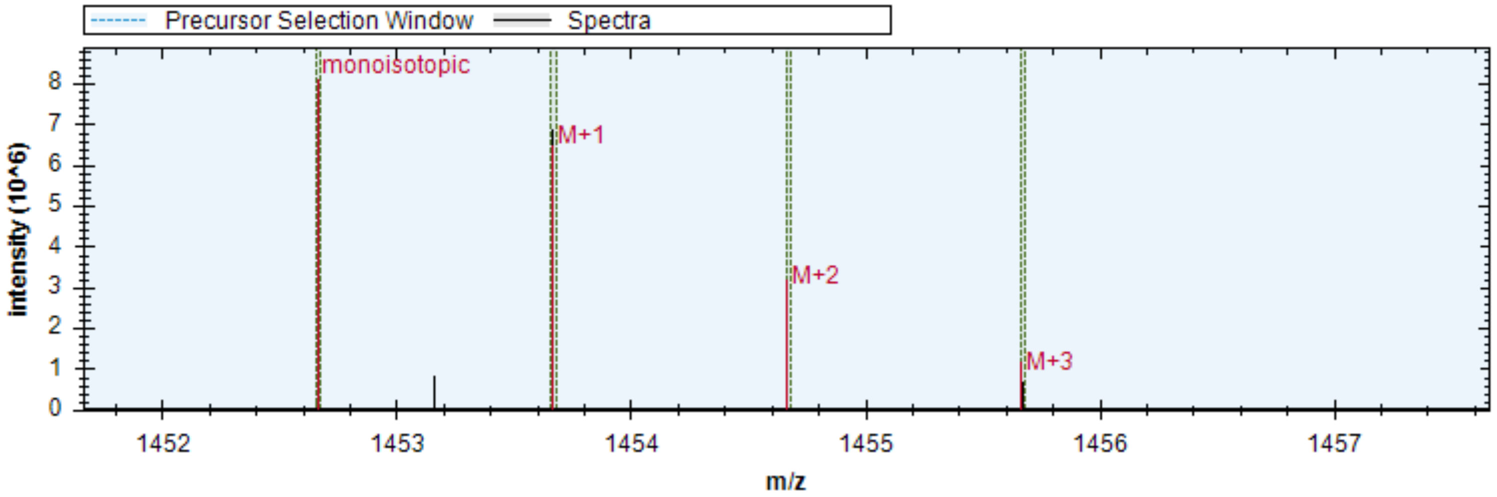

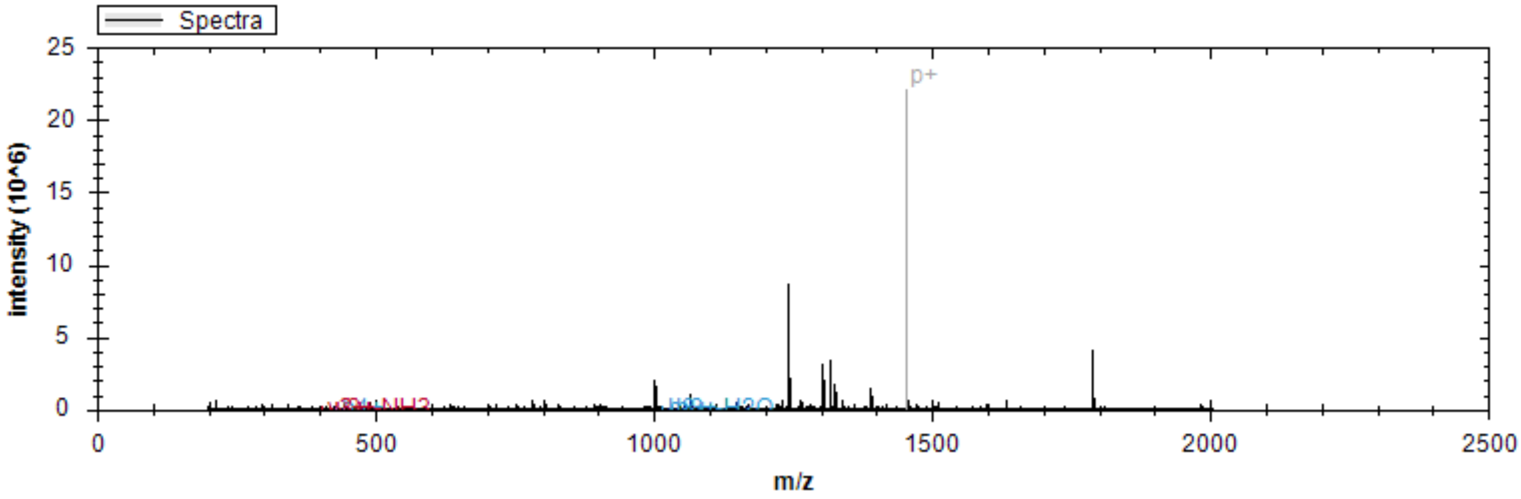

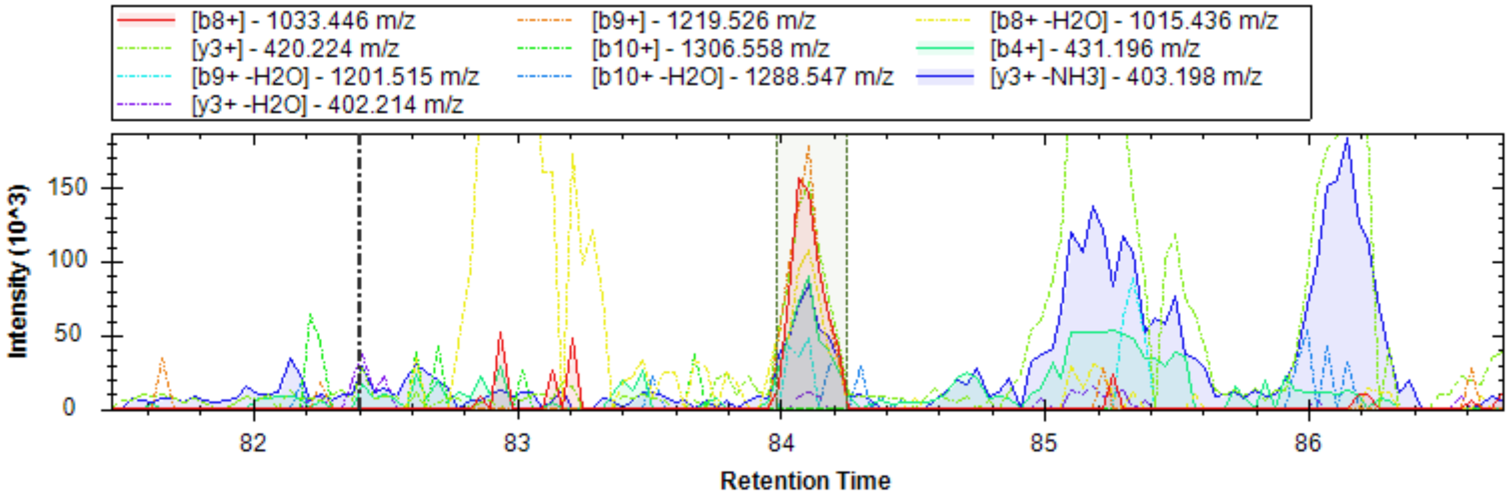

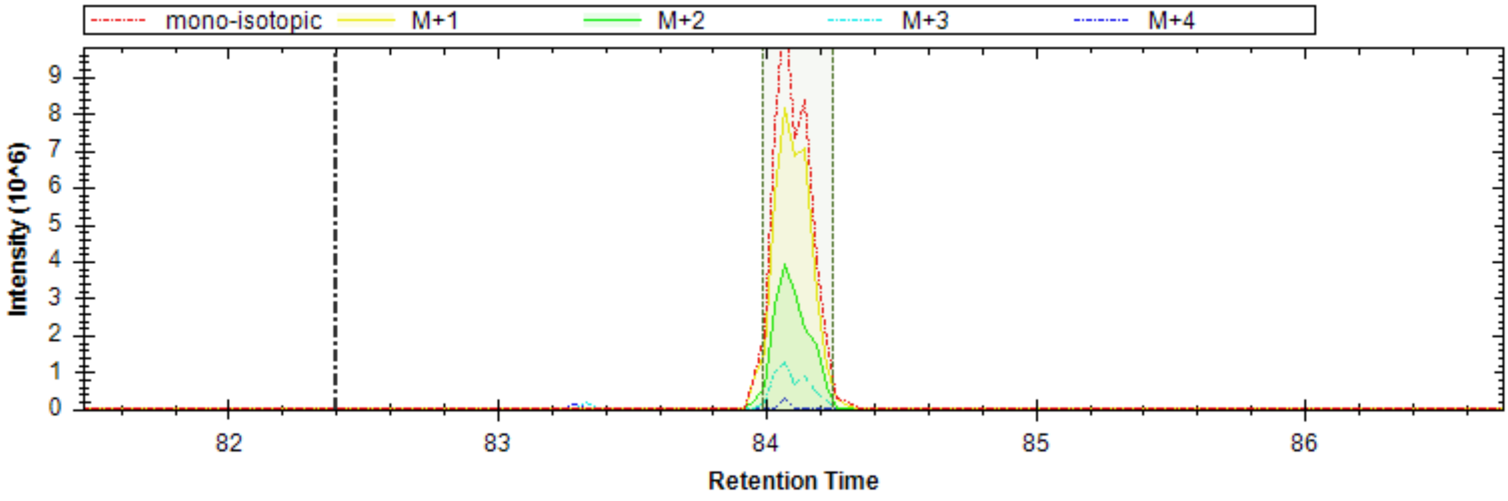

Q8BRT1 – _VLNT[+H+O3+P]GSDVEEAVADALLLGDIR_.2
Fig4_MP-DIA-barrel-cortex-p9-2_MHRM.htrms

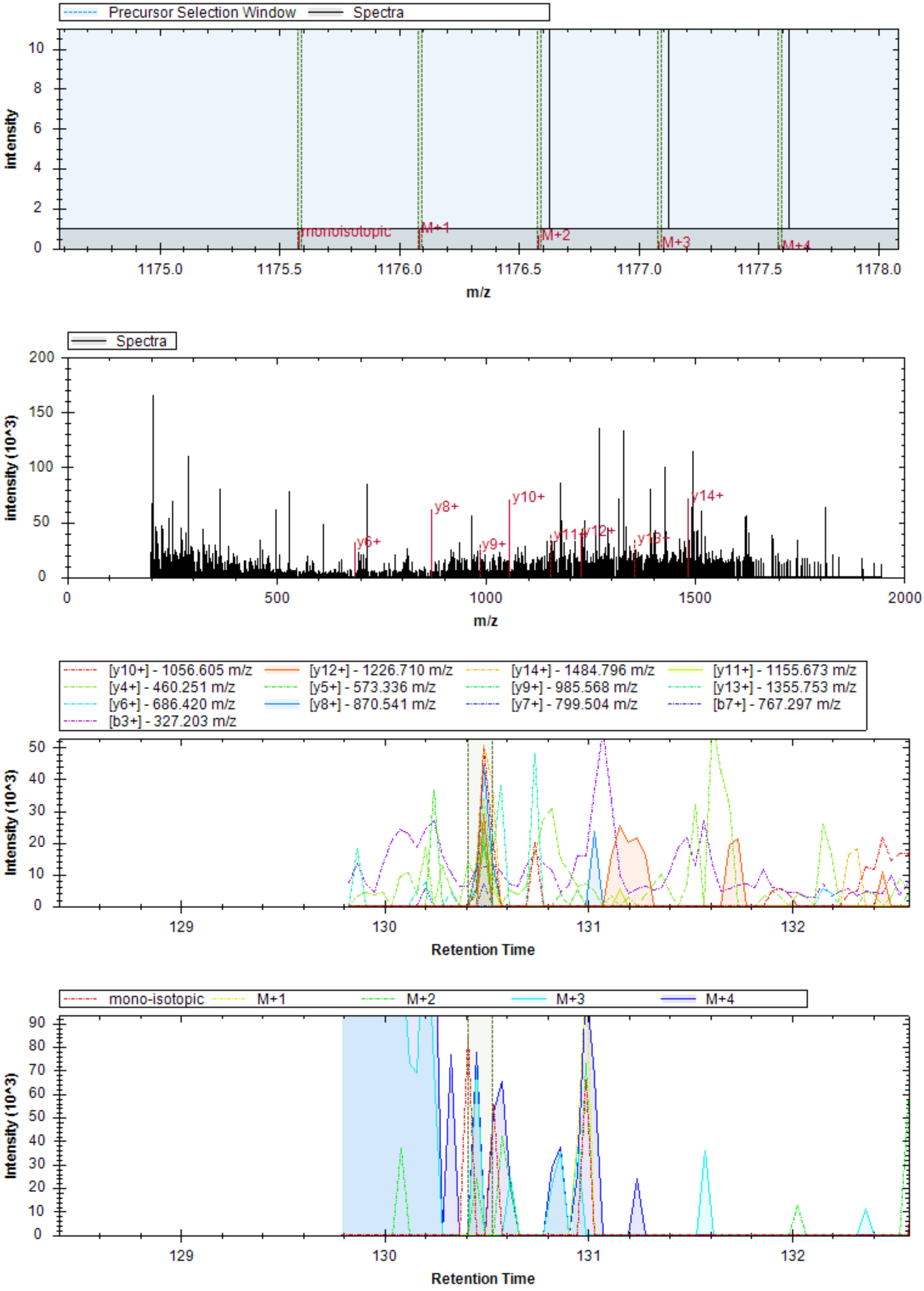

Q8VHY0 – _LLAGPRY[+H+O3+P]GR_.1
Fig4_MP-DIA-barrel-cortex-p9-2_MHRM.htrms

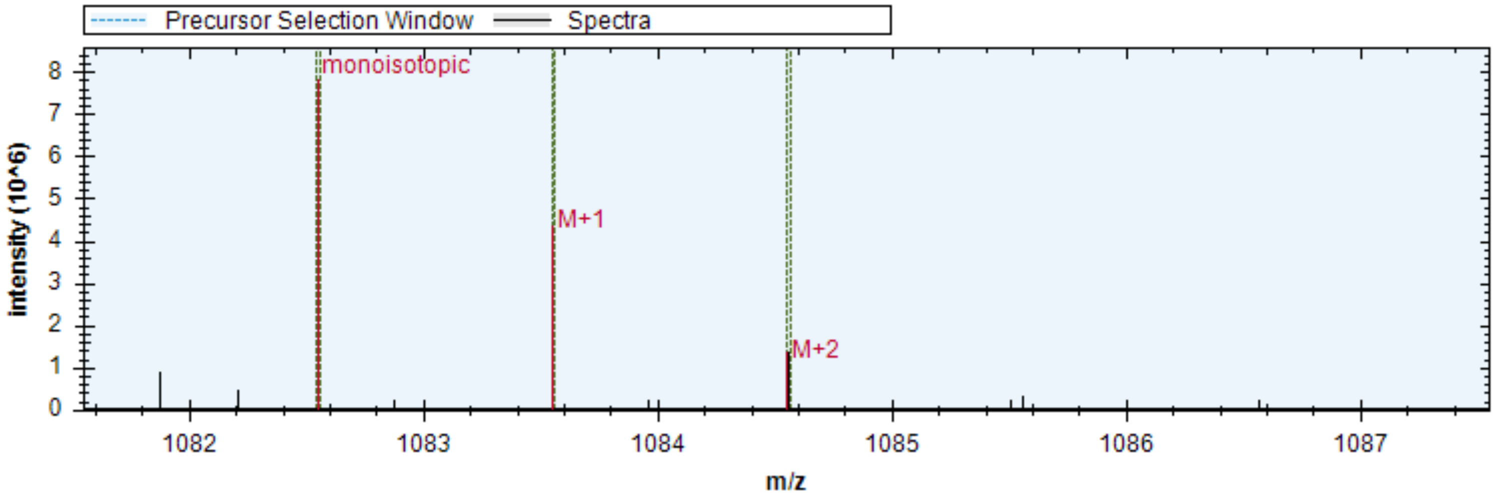

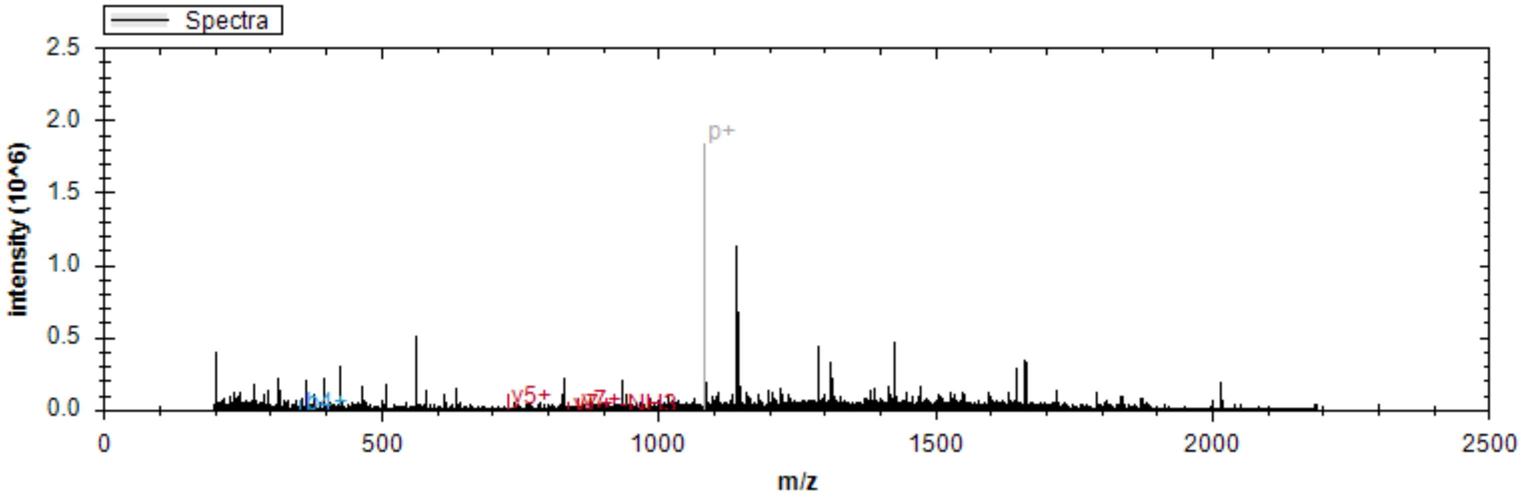

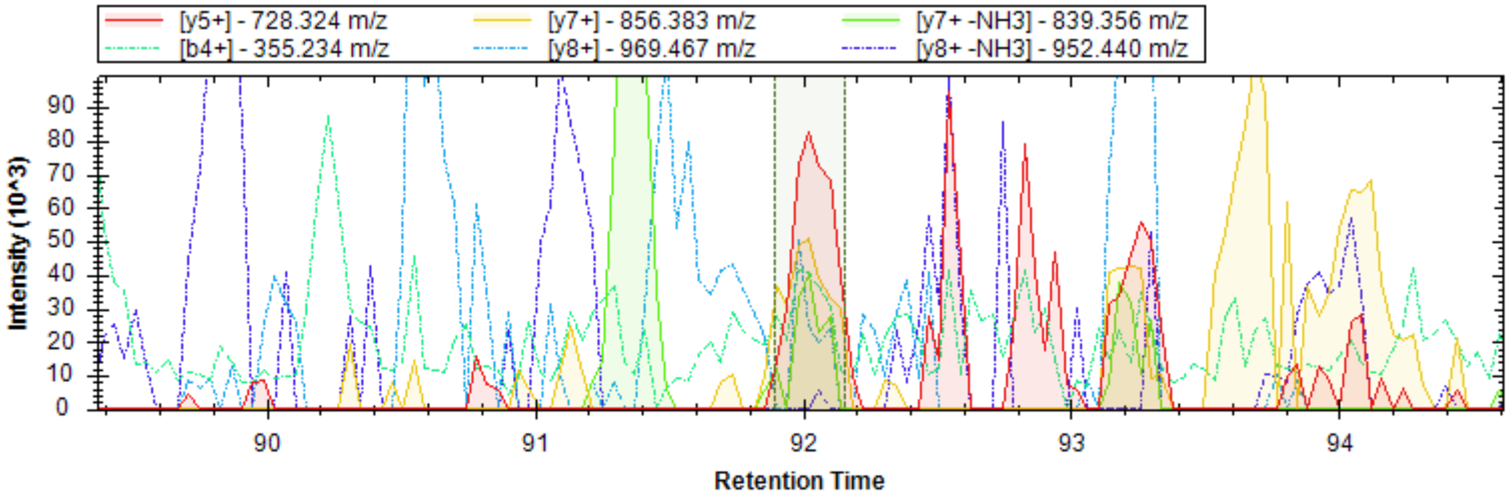

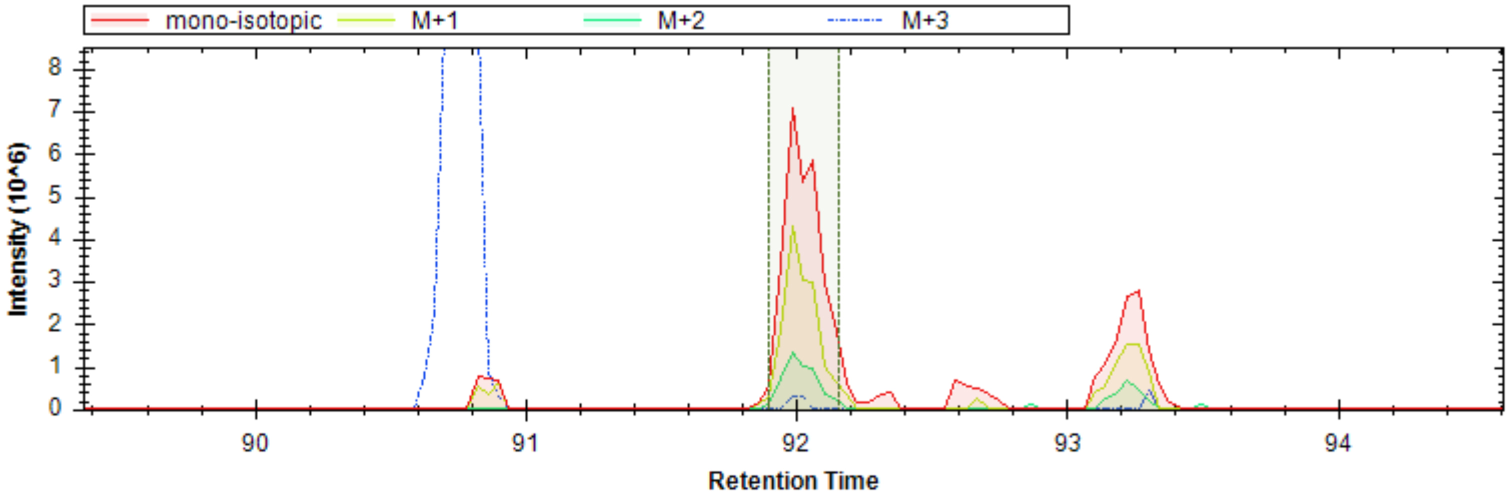

Q921N6;Q921N6-2 – _LIY[+H+O3+P]KPRQAAVTR_.4
Fig4_MP-DIA-barrel-cortex-p9-3_MHRM.htrms

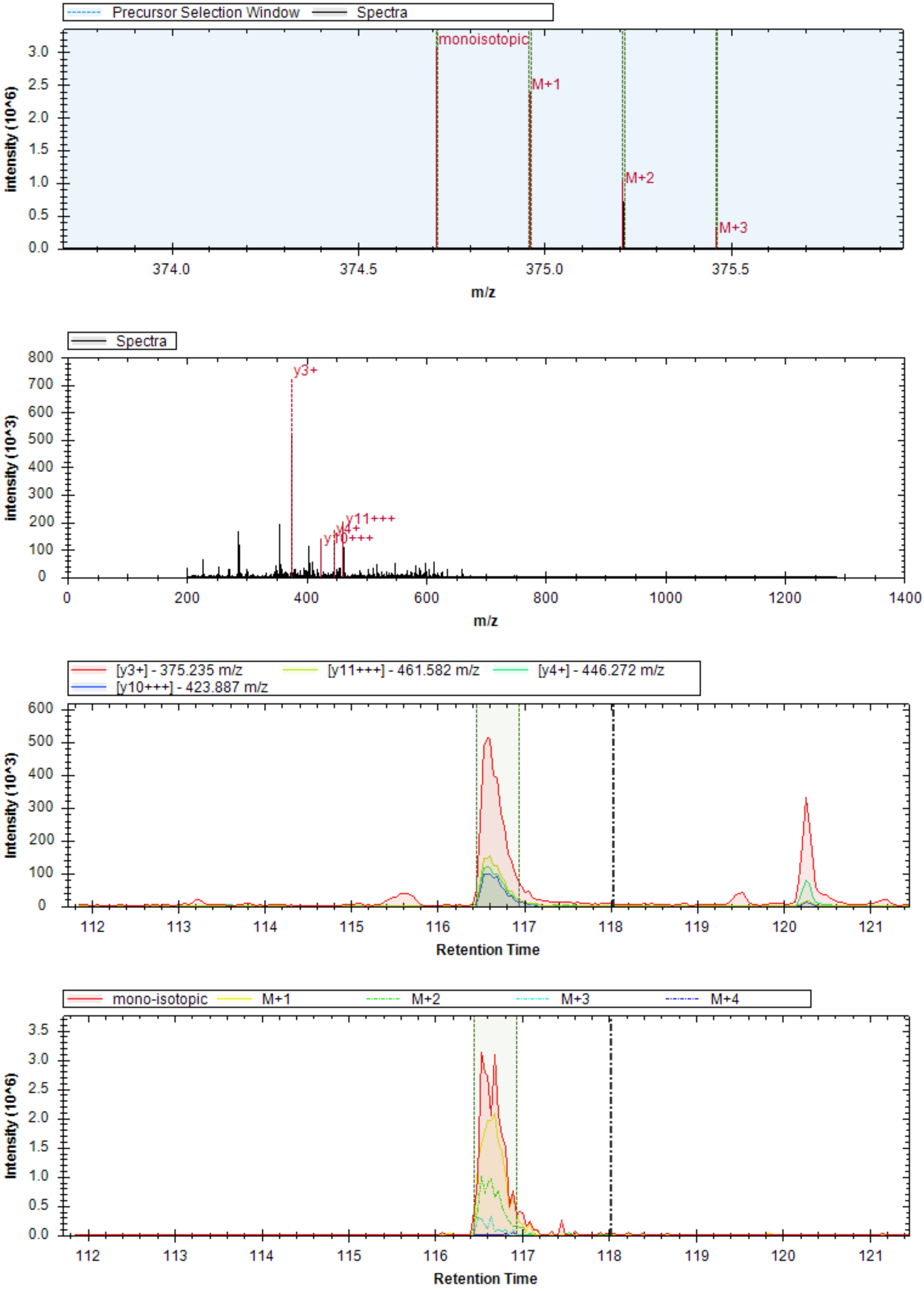

Q9CVB6 – _IIEET[+H+O3+P]LALK_.3

Fig4_MP-DIA-barrel-cortex-p9-3_MHRM.htrms

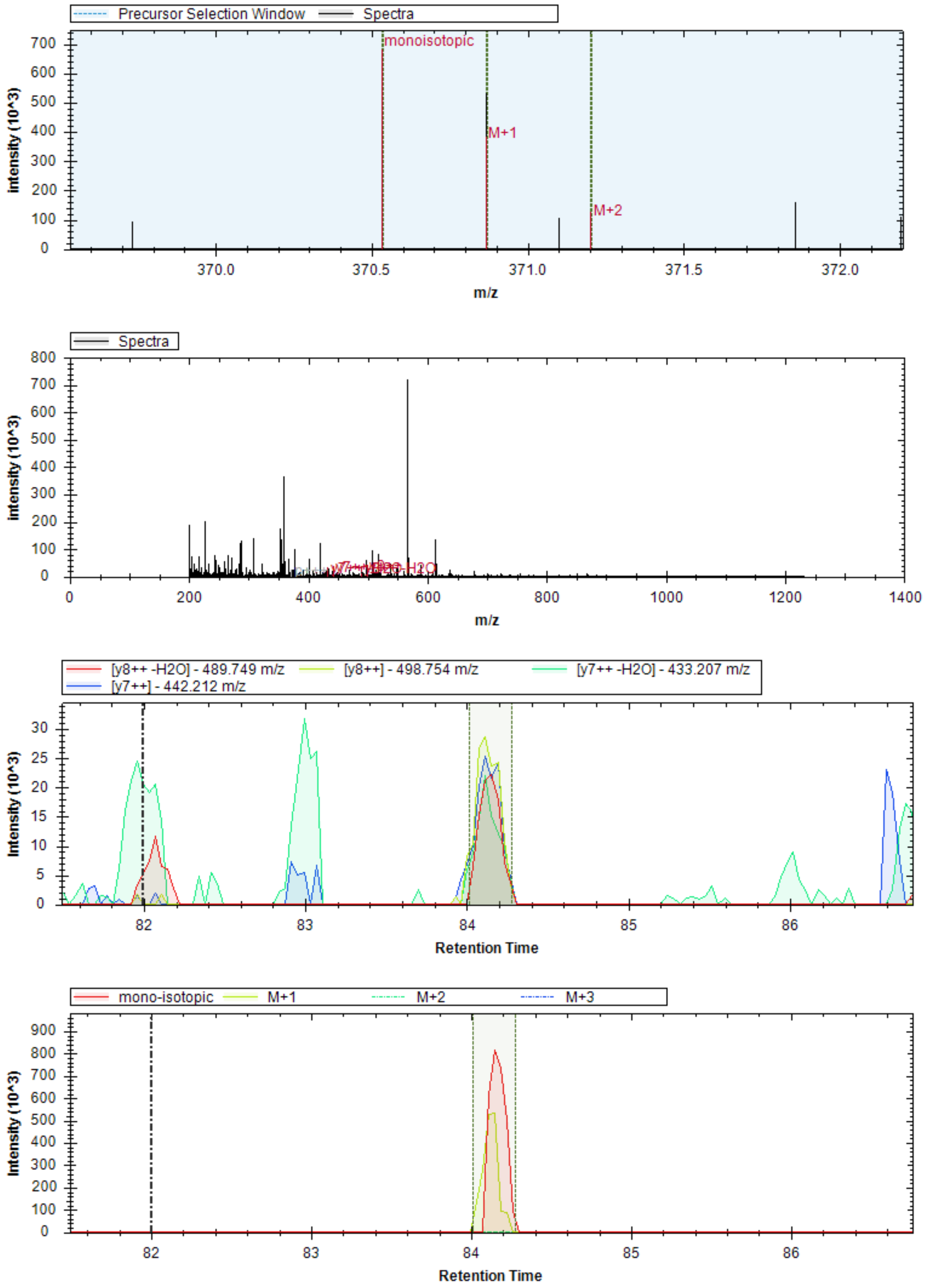

Q9D516;Q9D516-2 – _KLS[+H+O3+P]QGILVIR_.3
Fig4_MP-DIA-barrel-cortex-p9-3_MHRM.htrms

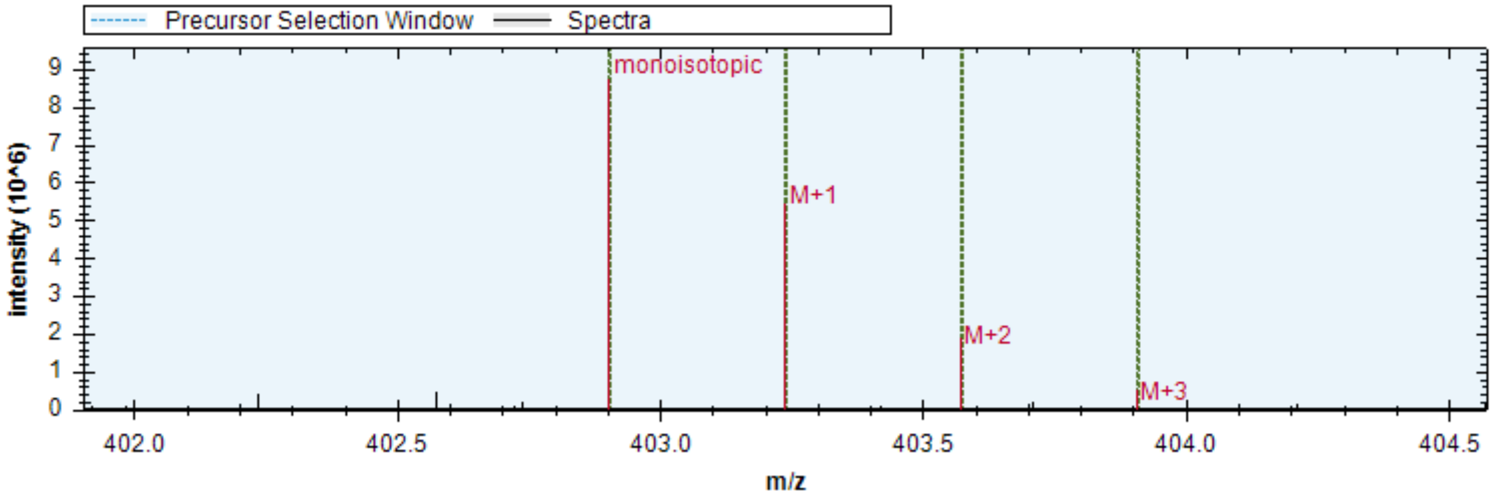

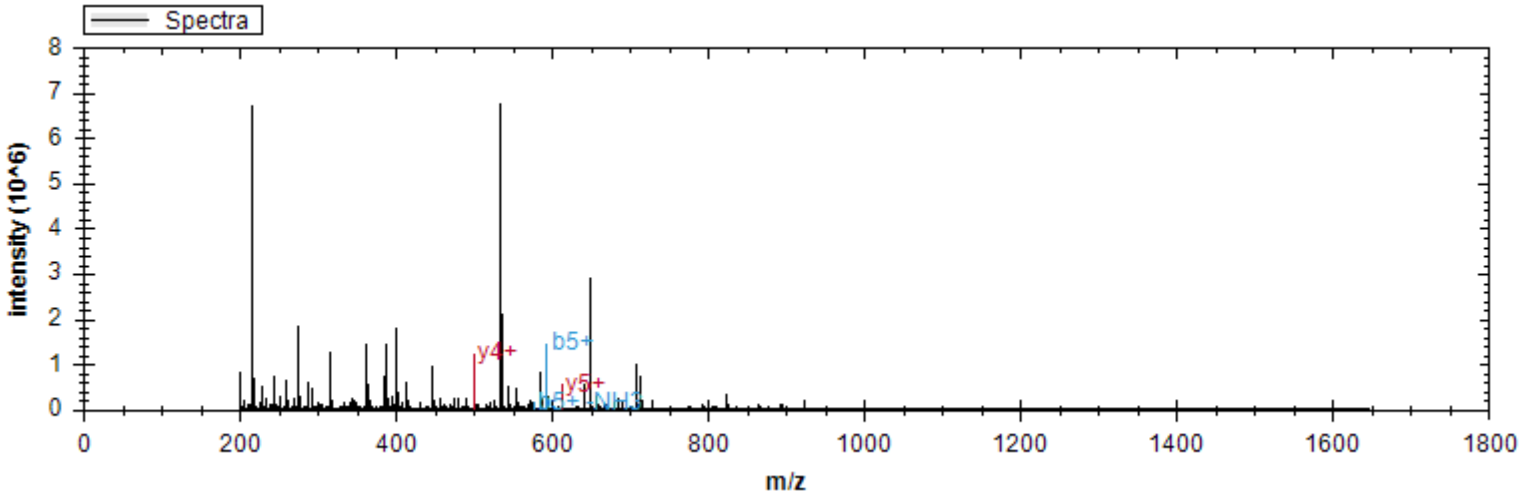

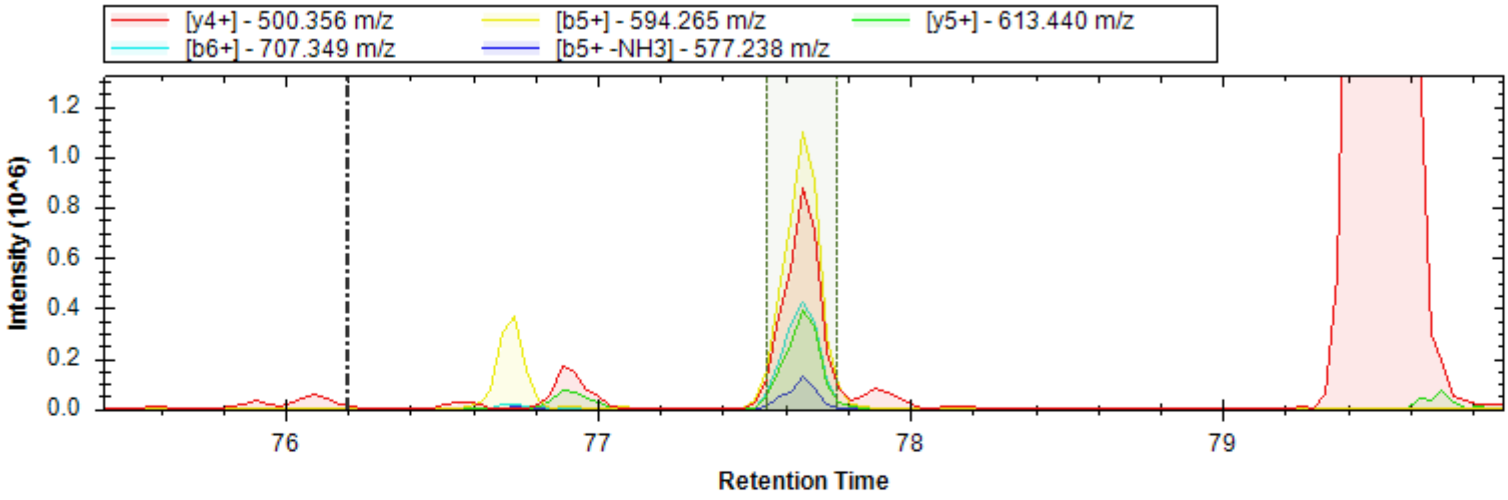

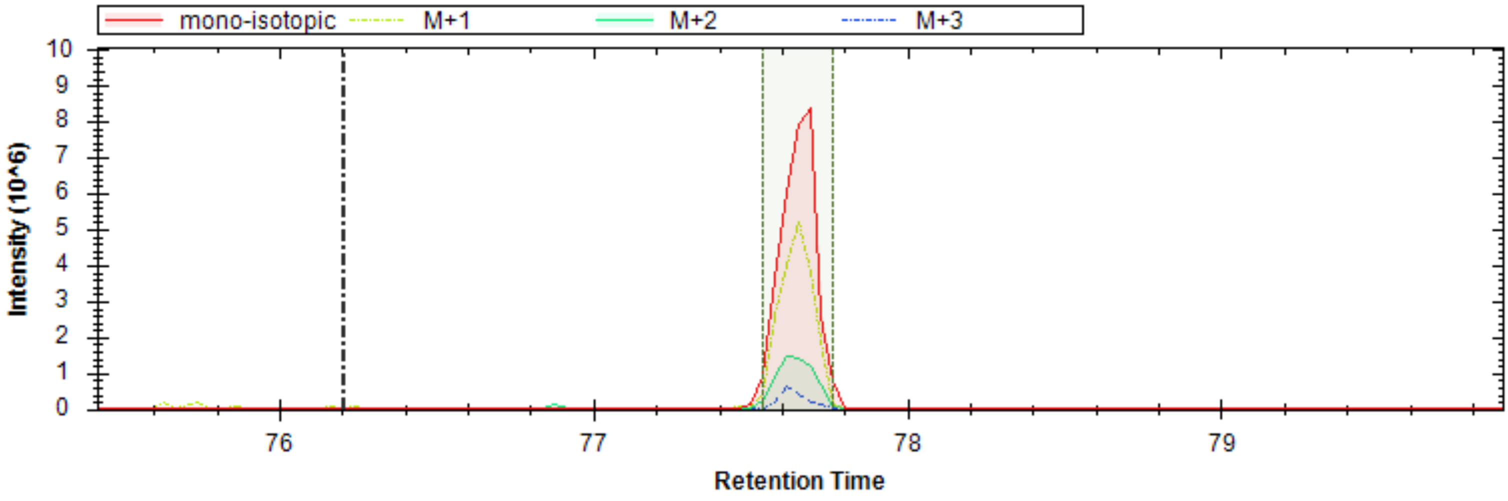

P20357 – _KSEVQAHS[+H+O3+P]PSR_.3
Fig4_MP-DIA-barrel-cortex-p9-3_MHRM.htrms

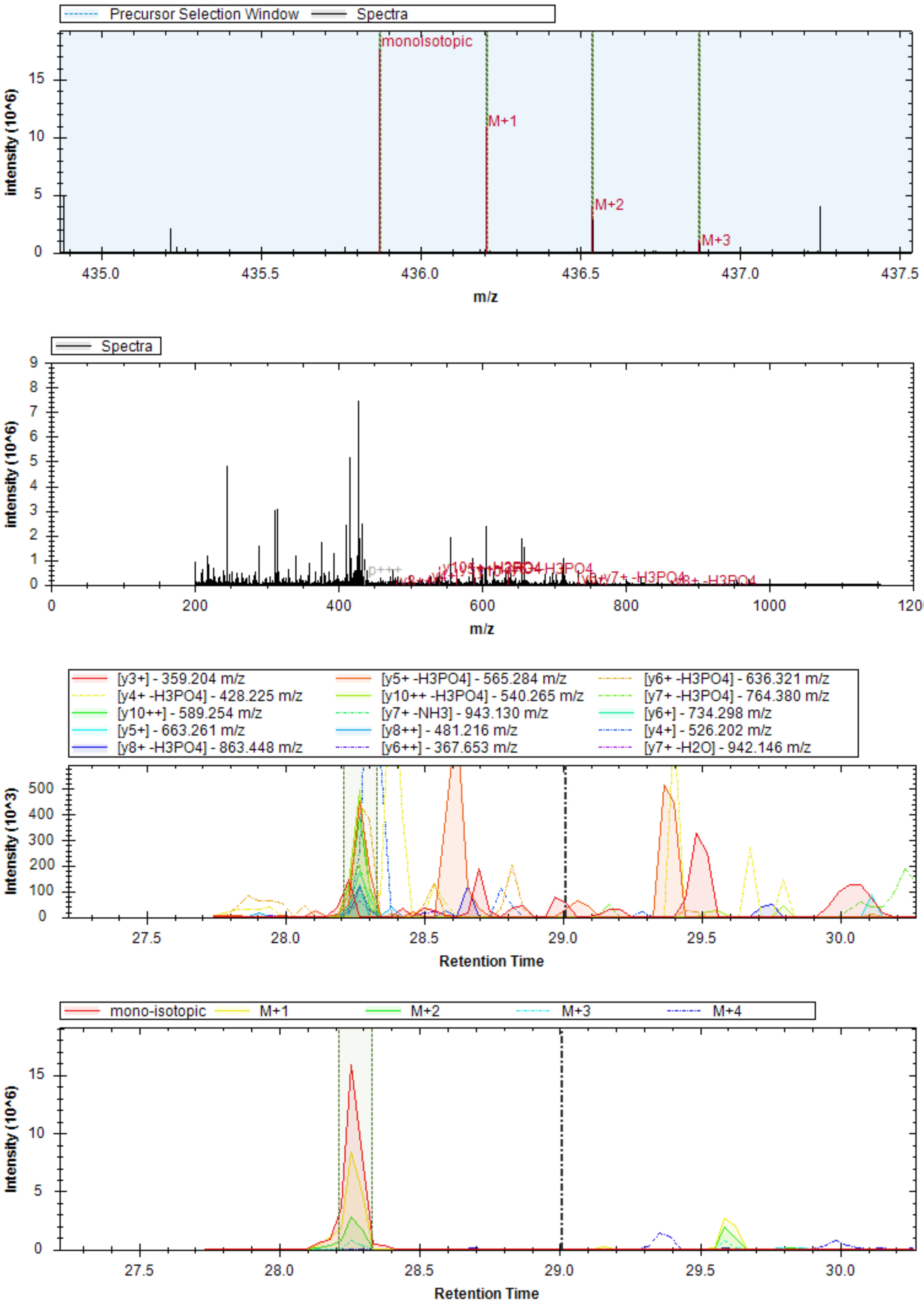

P28667 – _GEVAPKET[+H+O3+P]PKK_.3
Fig4_MP-DIA-barrel-cortex-p9-3_MHRM.htrms

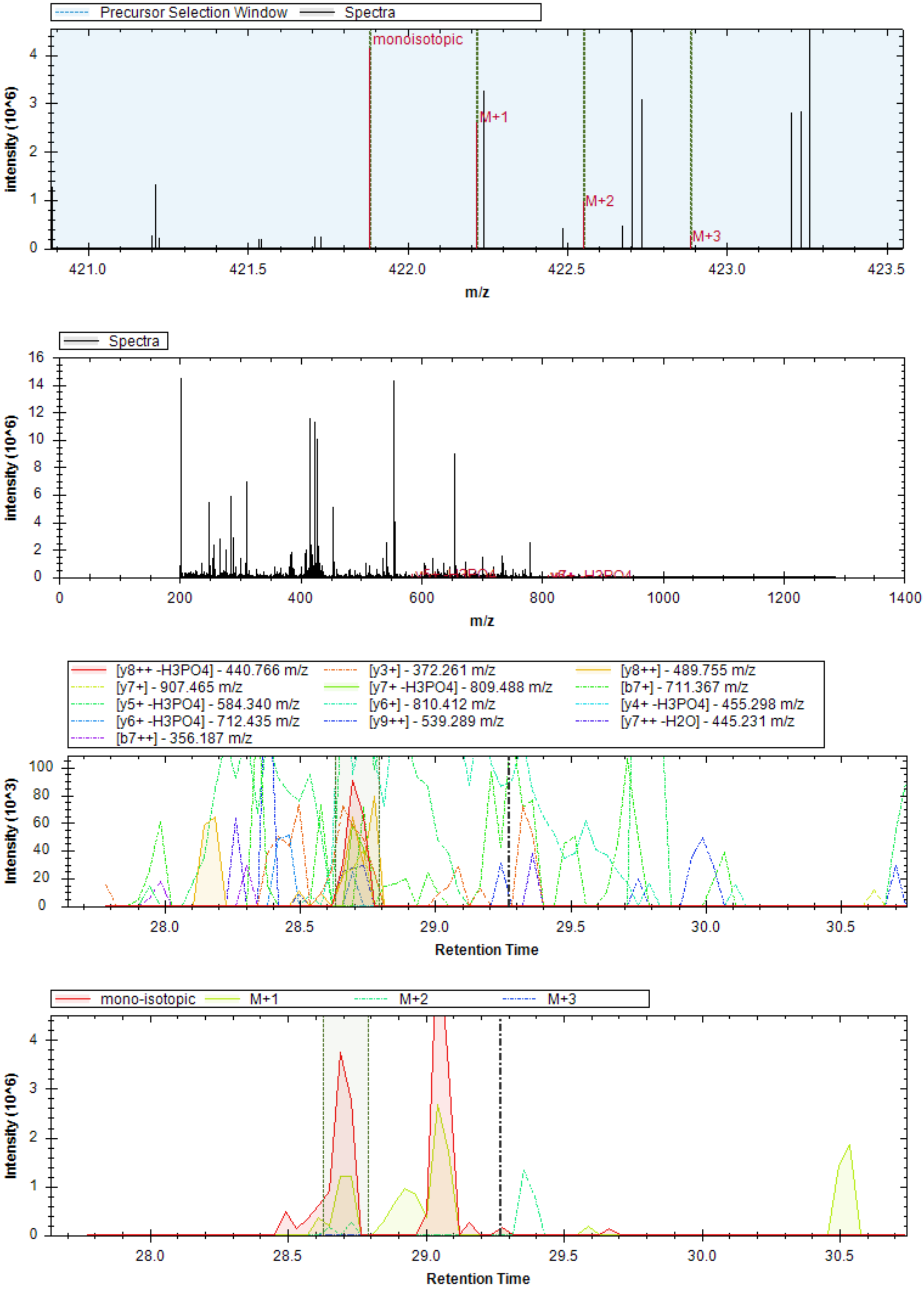

Q03172 – _VALSLLNS[+H+O3+P]KQK_.3

Fig4_MP-DIA-barrel-cortex-p9-3_MHRM.htrms

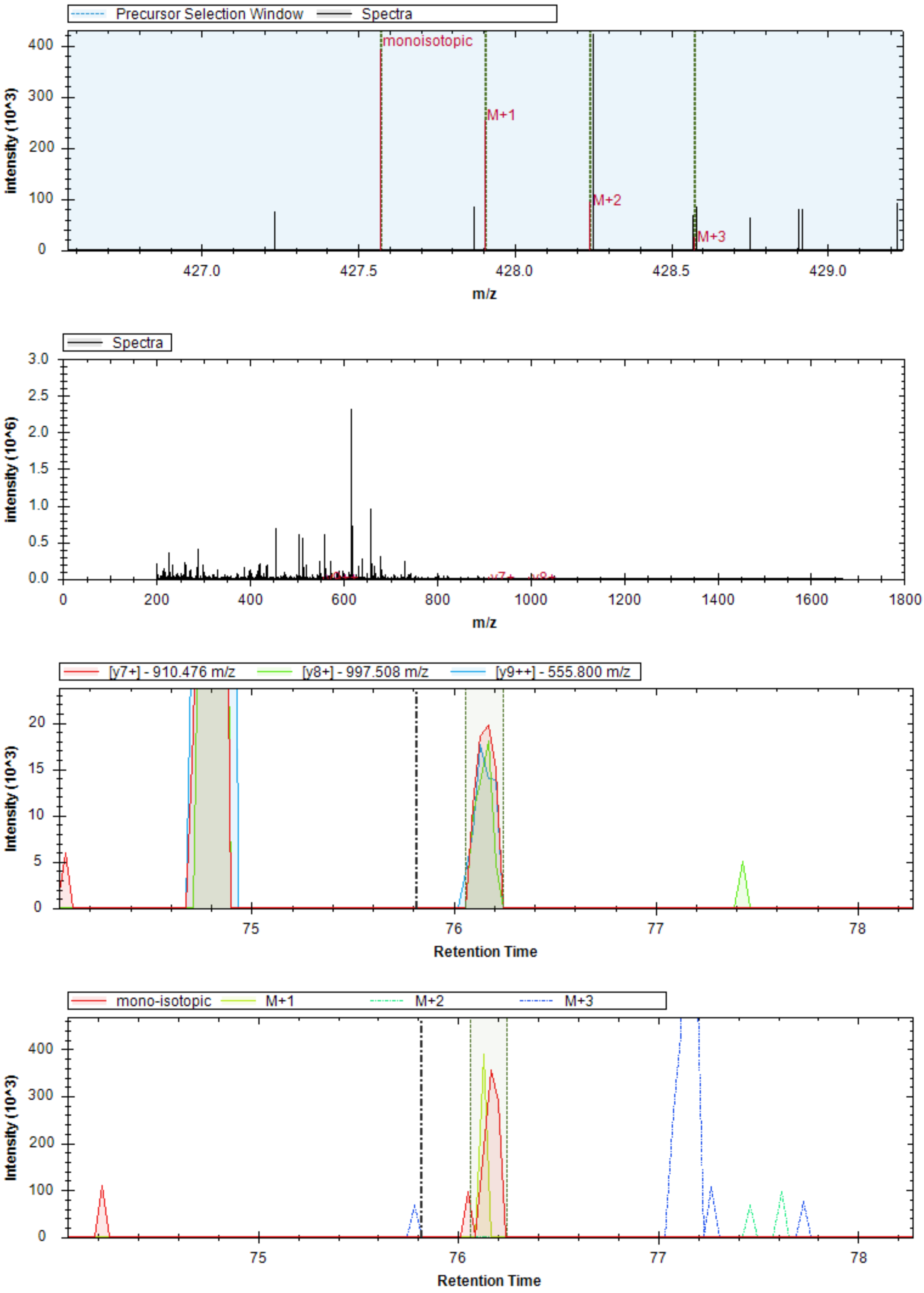

Q52KI8;Q52KI8-2 – _RES[+H+O3+P]PSPAPKPR_.3
Fig4_MP-DIA-barrel-cortex-p9-3_MHRM.htrms

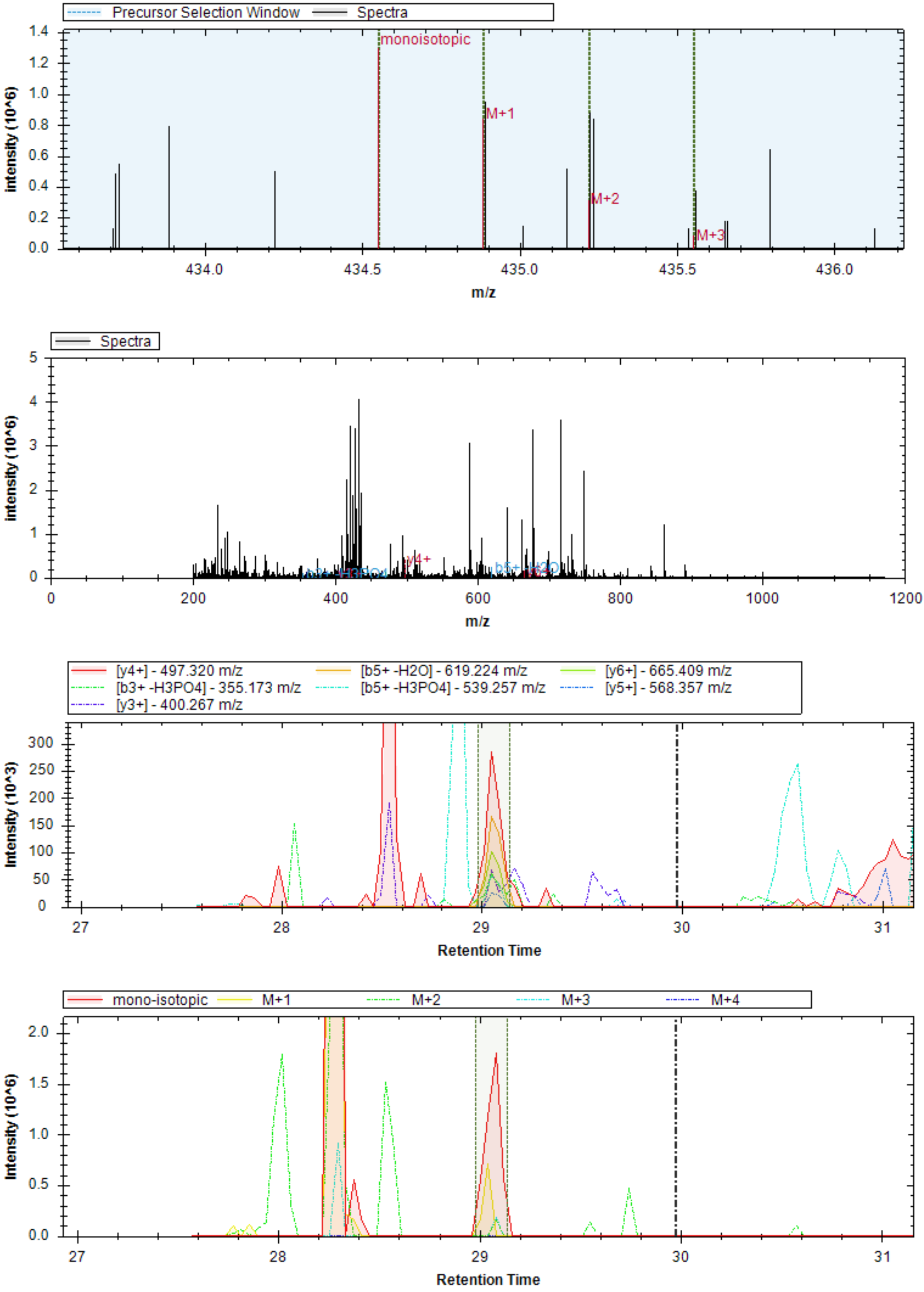

Q62188 – _GS[+H+O3+P]PTRPNPPVR_.3

Fig4_MP-DIA-barrel-cortex-p9-3_MHRM.htrms

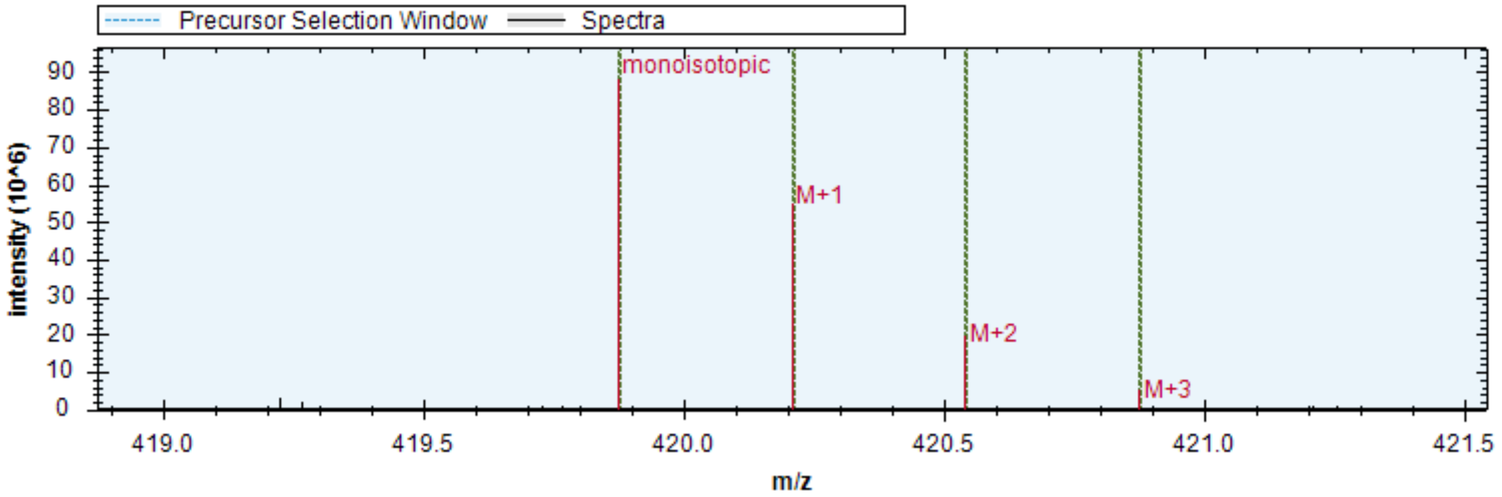

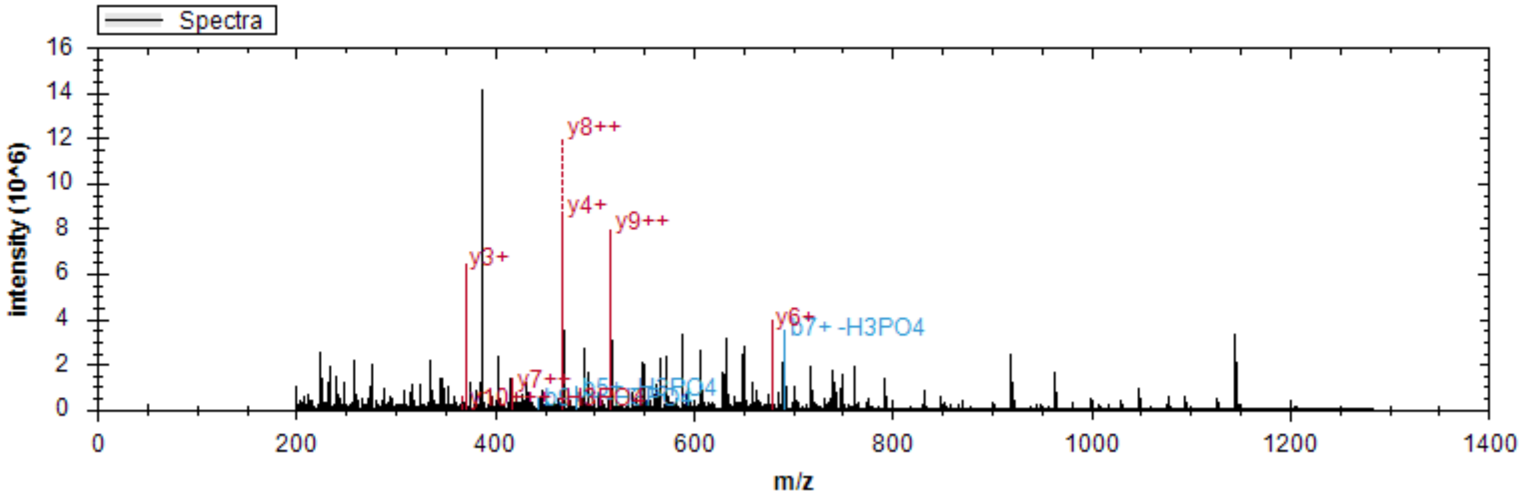

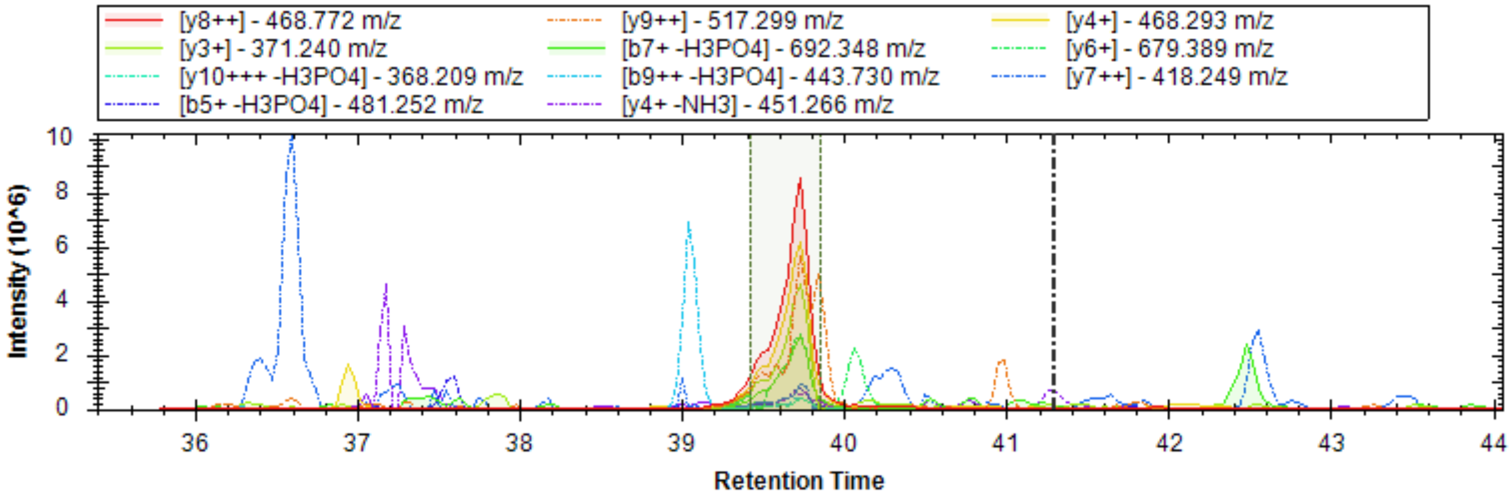

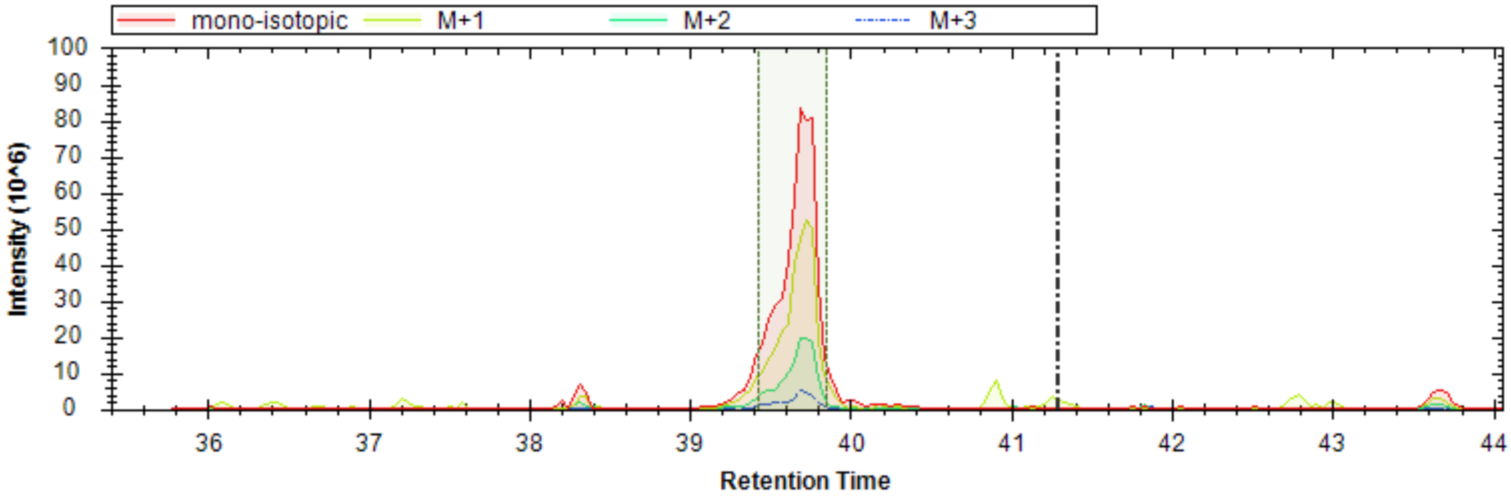

Q93092 – _[+C2+H2+O]SGS[+H+O3+P]PVKR_.2
Fig4_MP-DIA-barrel-cortex-p9-3_MHRM.htrms

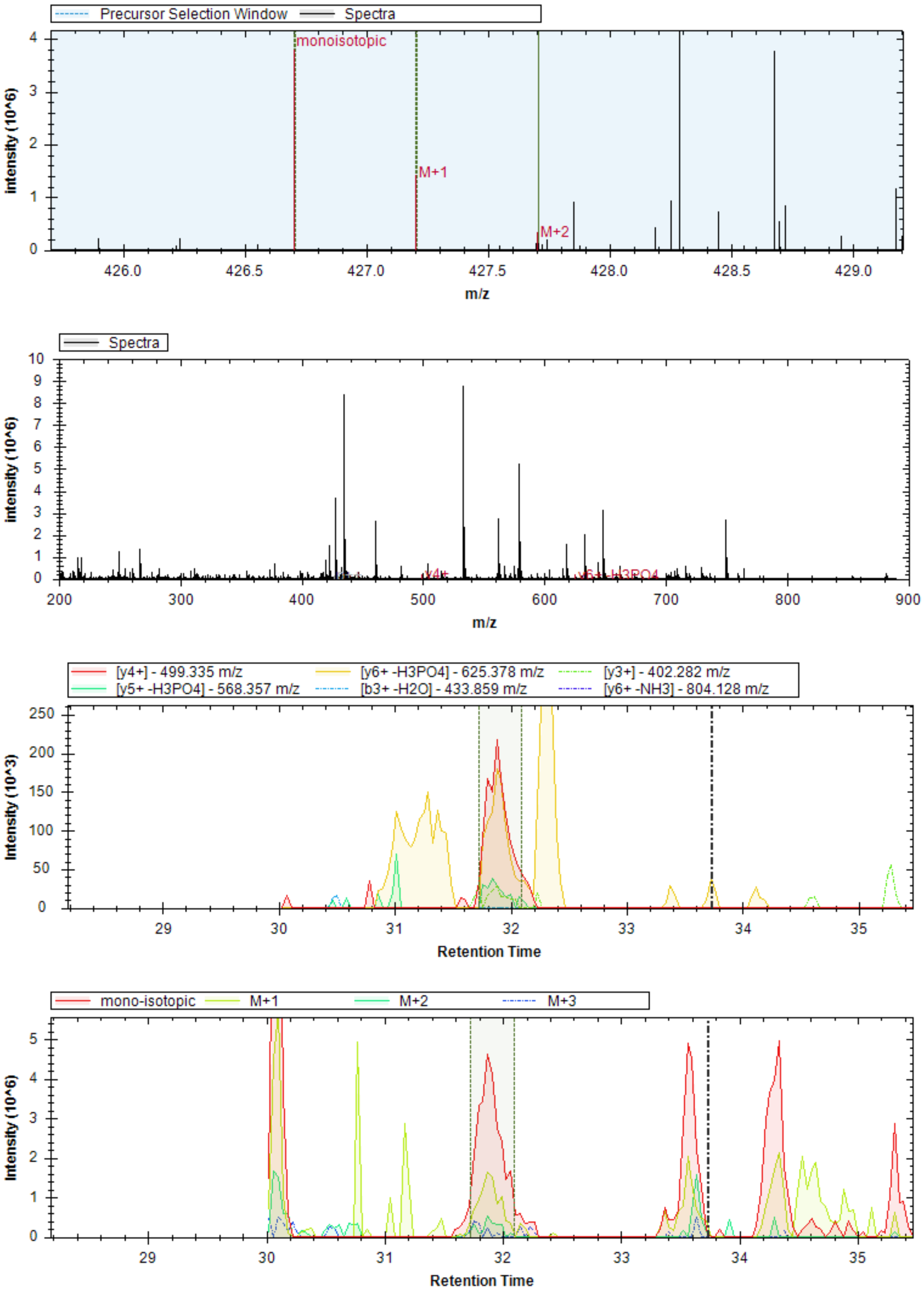

P62484 – _T[+H+O3+P]HKIIAPANLERPVR_.4
Fig4_MP-DIA-barrel-cortex-p9-3_MHRM.htrms

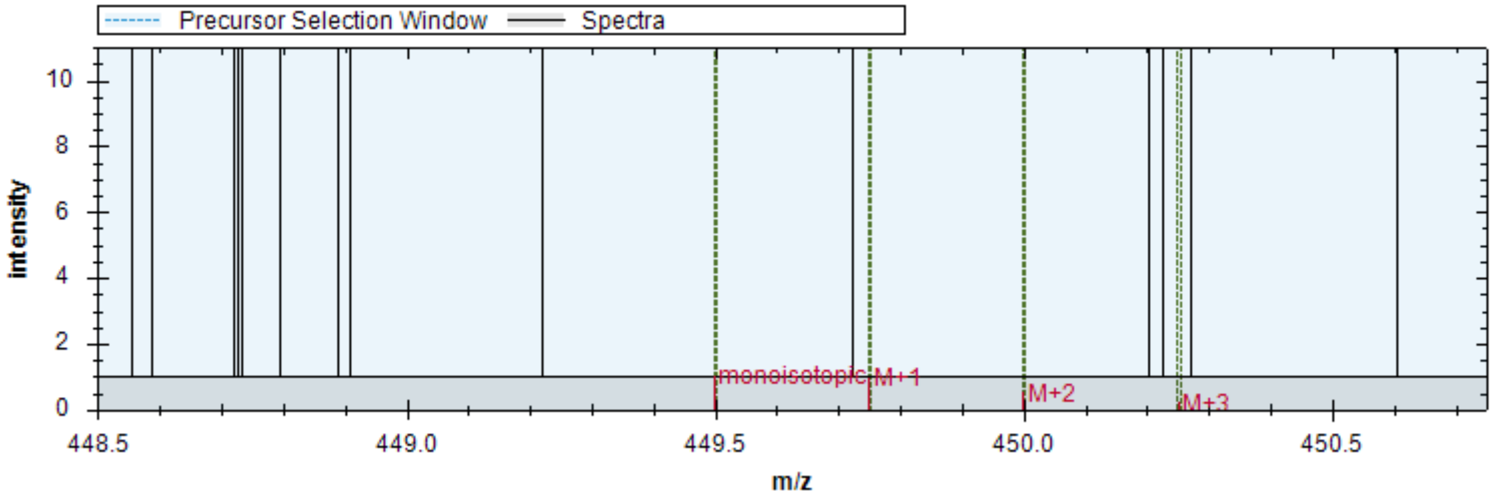

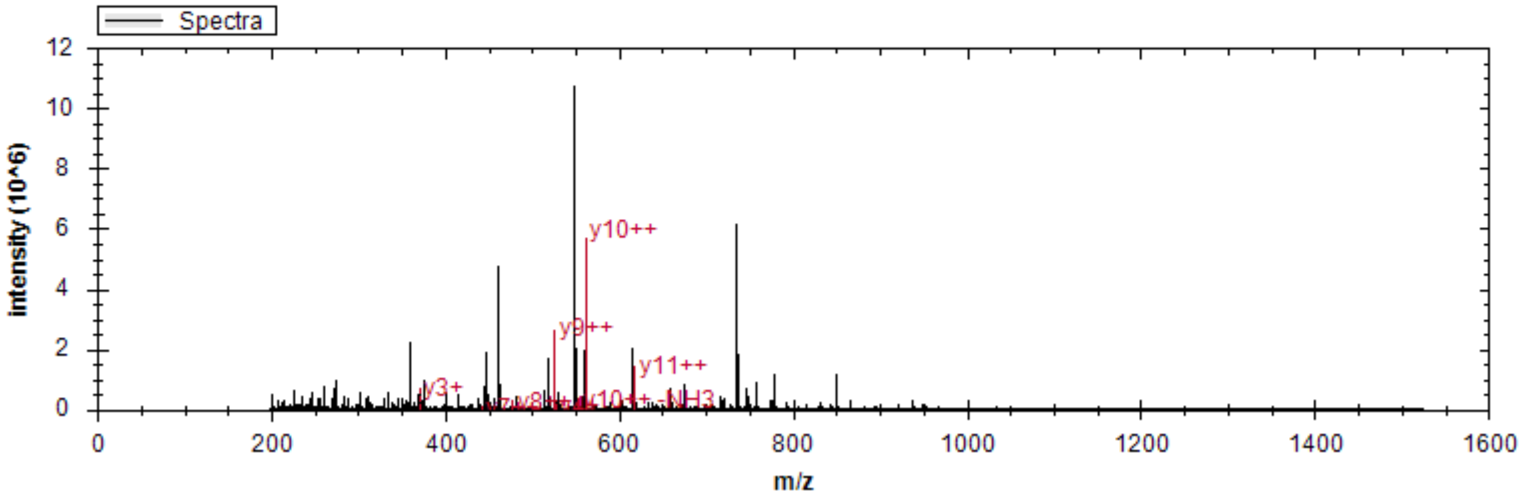

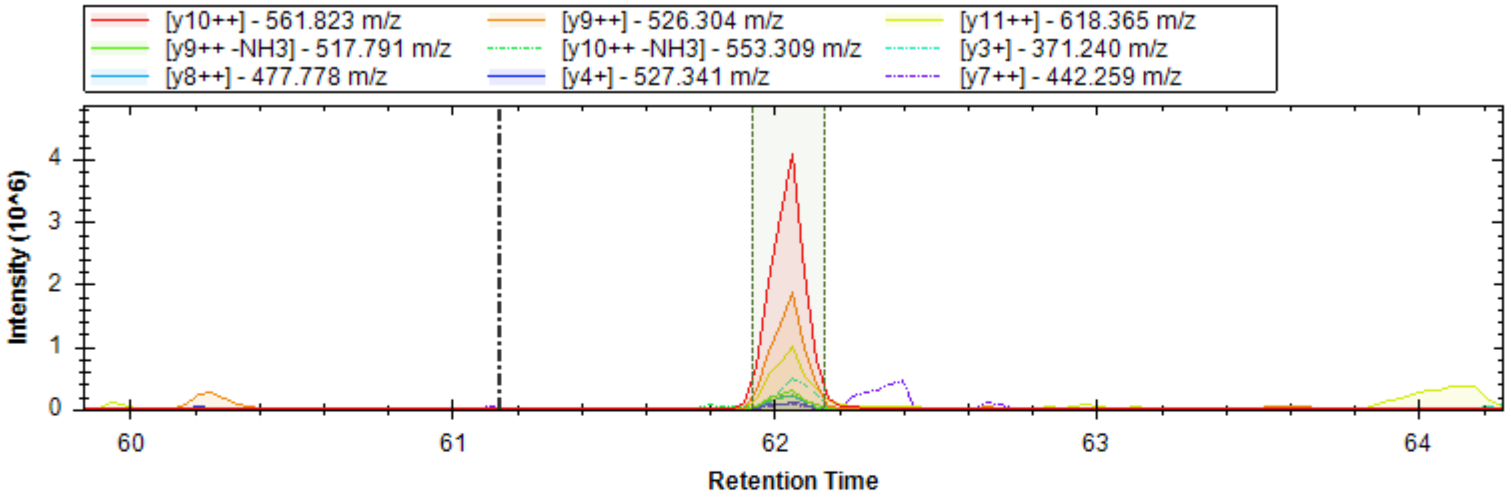

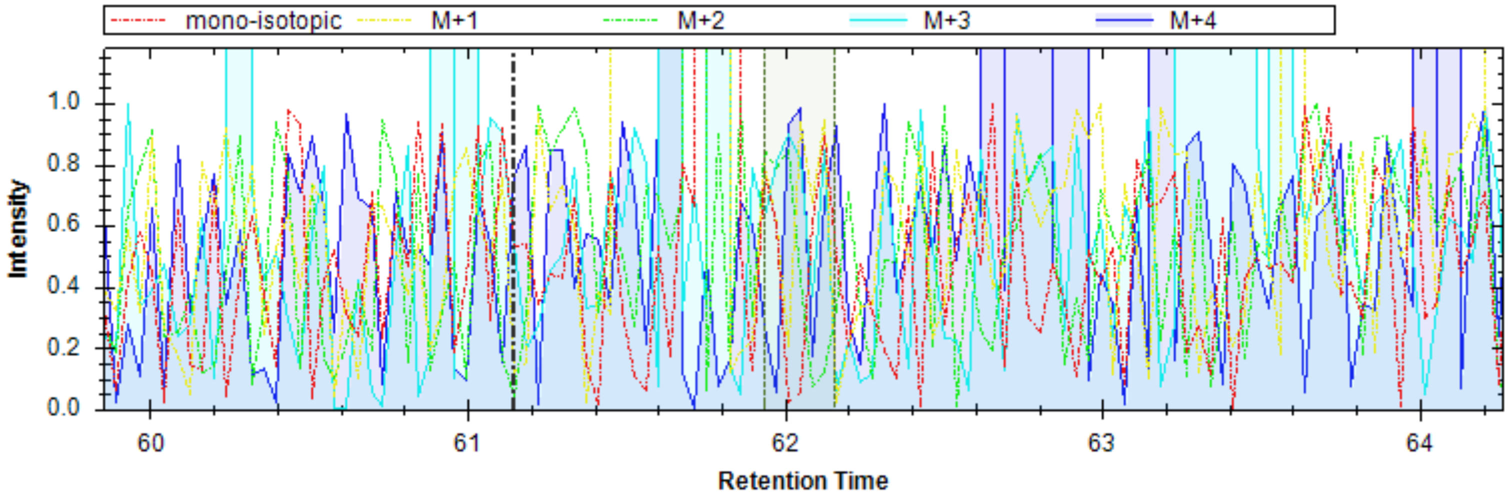

Q811T9;Q811T9-2;Q811[...] – _RQLEVT[+H+O3+P]SLILK_.3

Fig4_MP-DIA-barrel-cortex-p9-3_MHRM.htrms

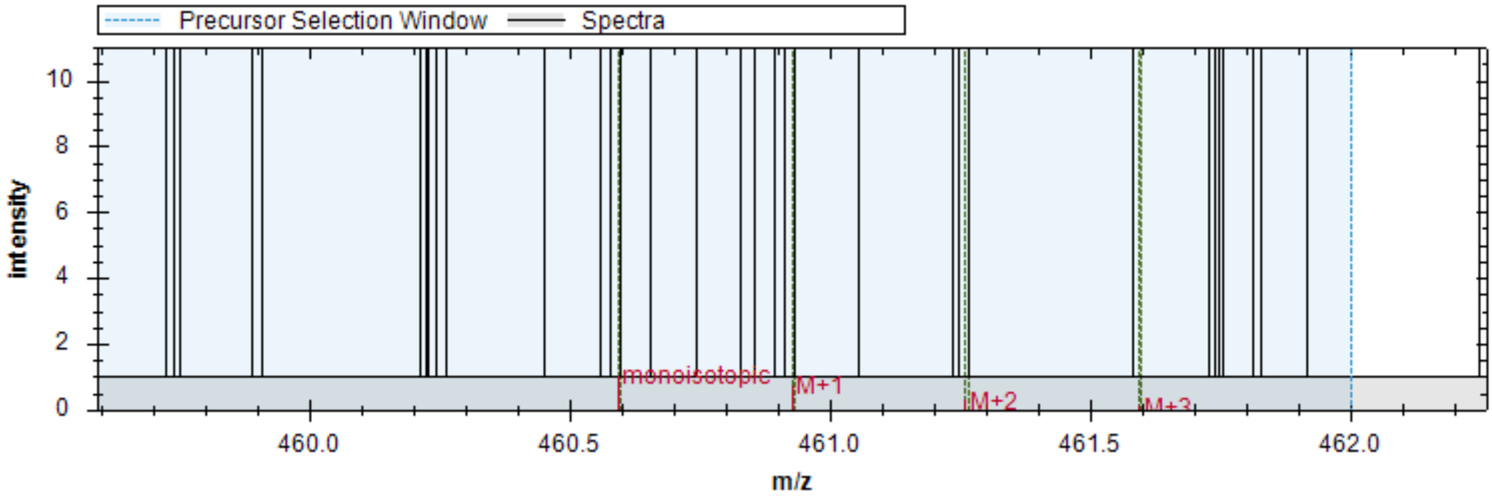

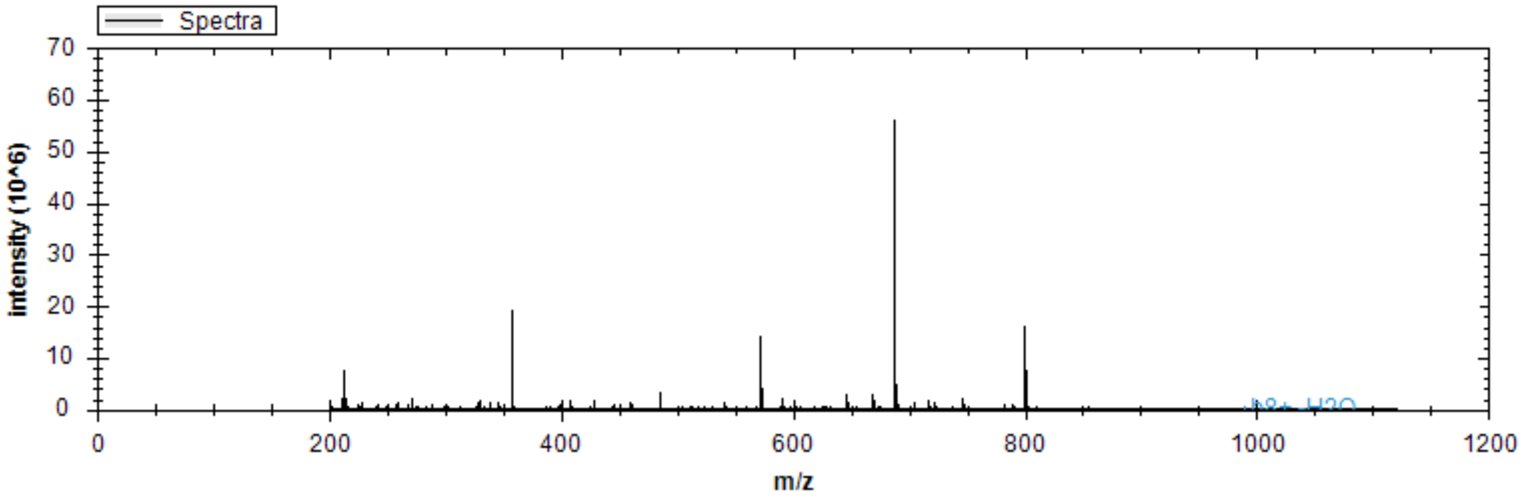

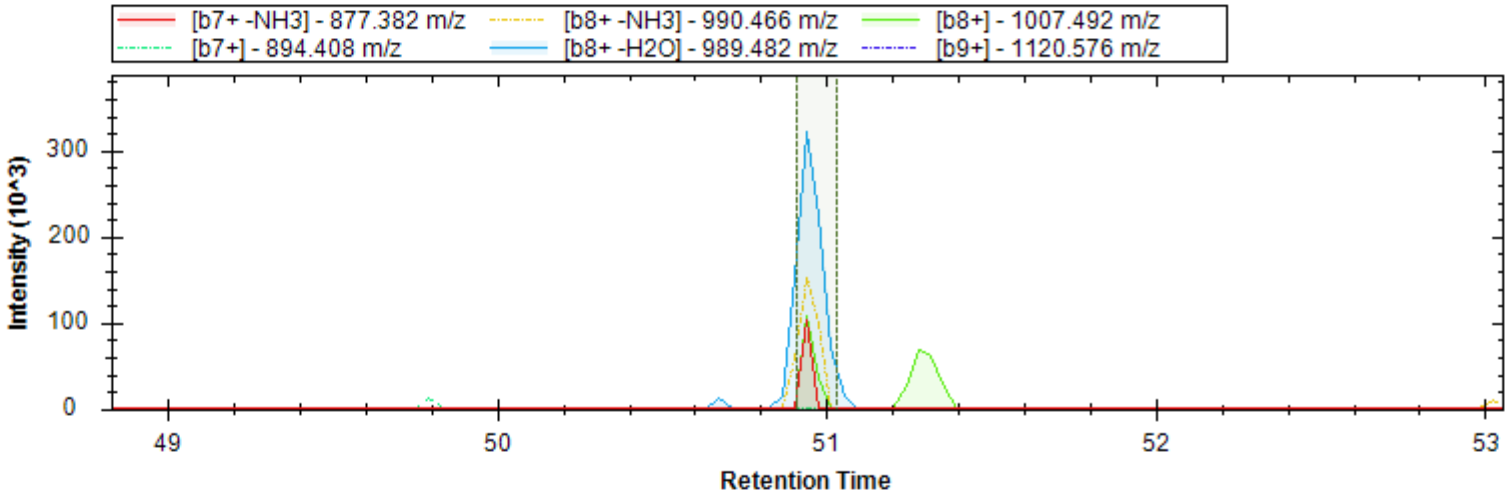

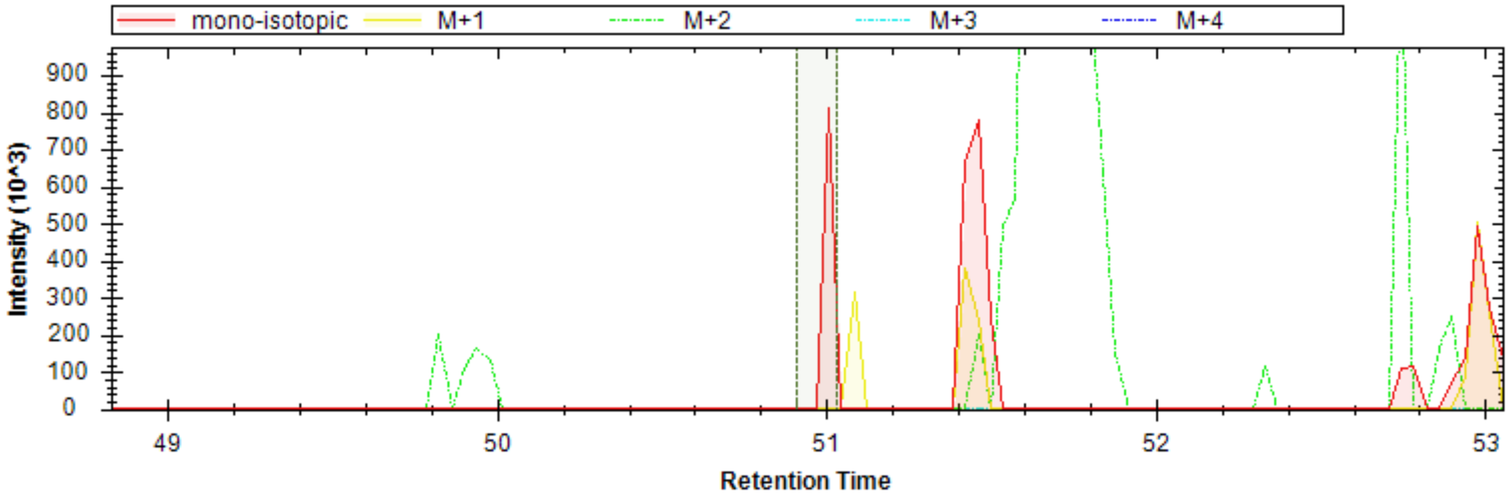

Q8CGI1 – _Y[+H+O3+P]QRS[+H+O3+P]EEELR_.3
Fig4_MP-DIA-barrel-cortex-p9-3_MHRM.htrms

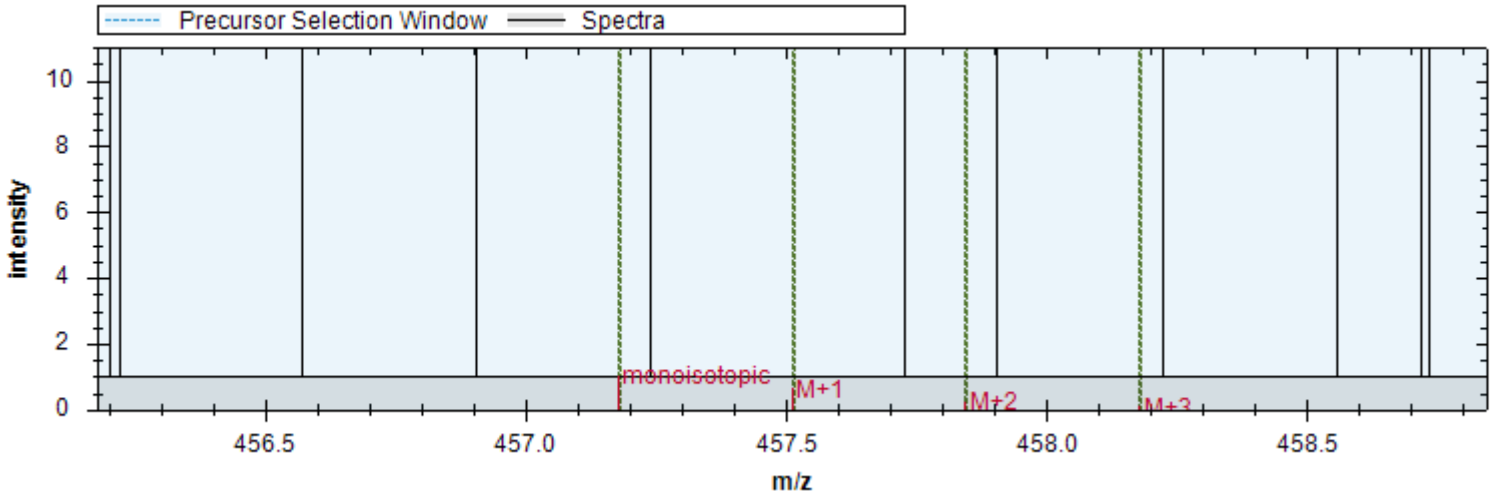

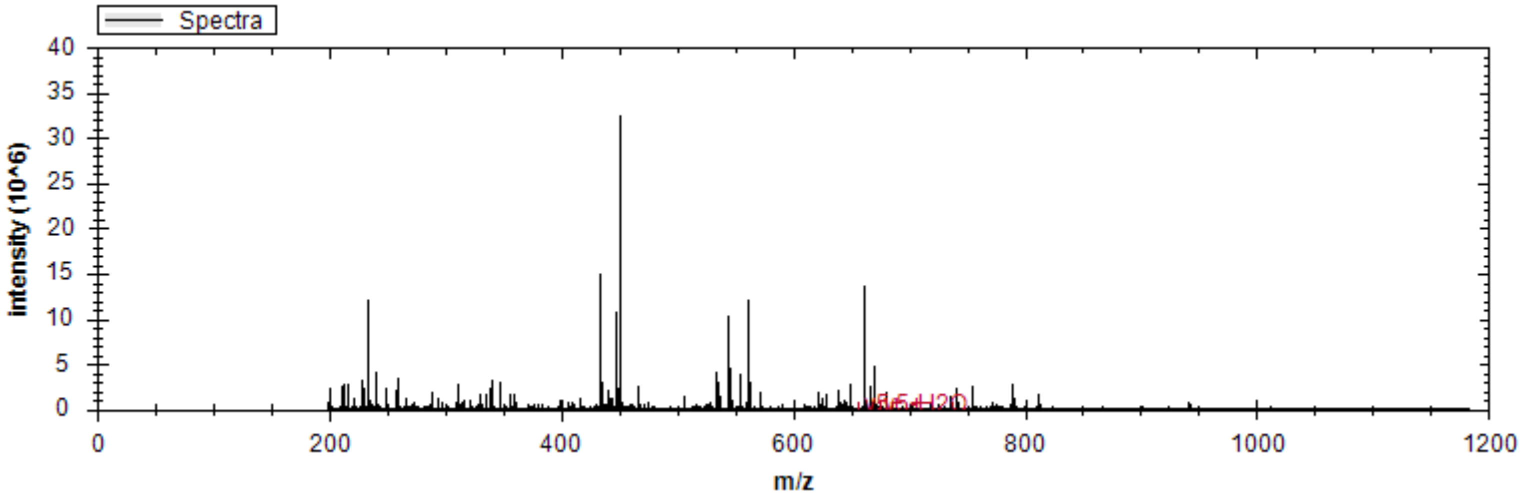

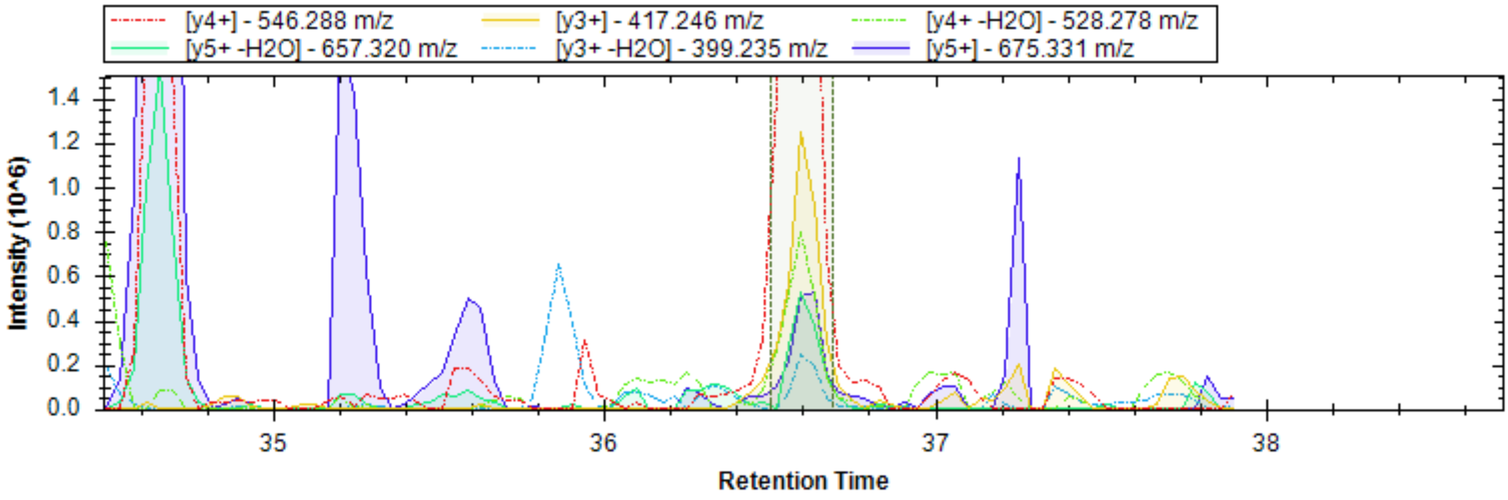

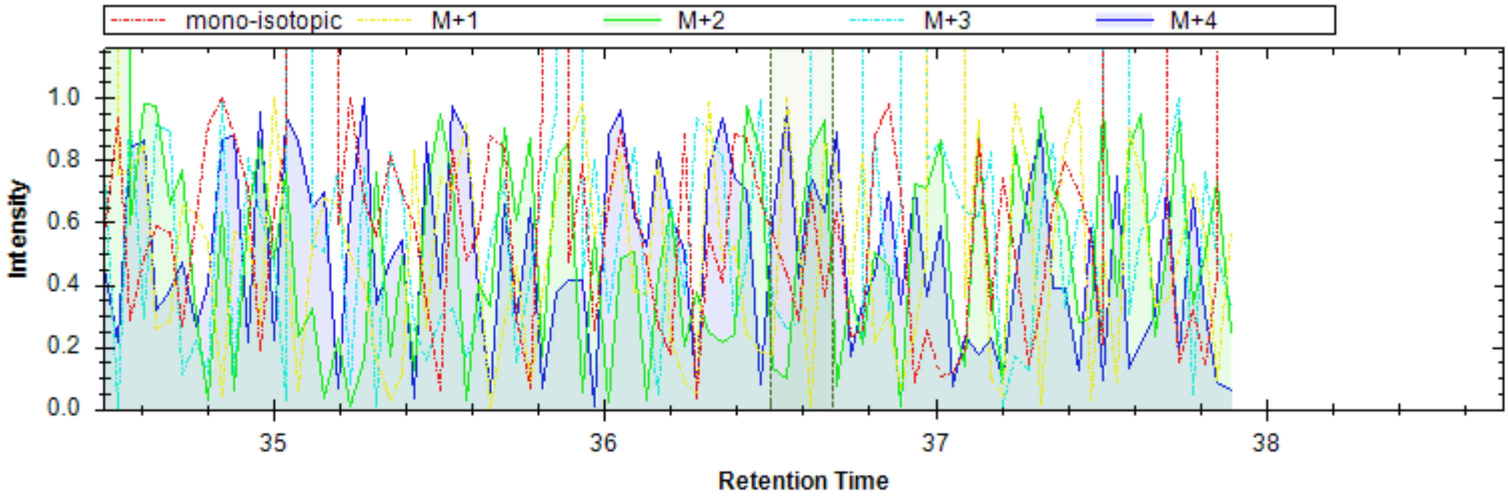

O08553 – _TVT[+H+O3+P]PASSAK_.2

Fig4_MP-DIA-barrel-cortex-p9-3_MHRM.htrms

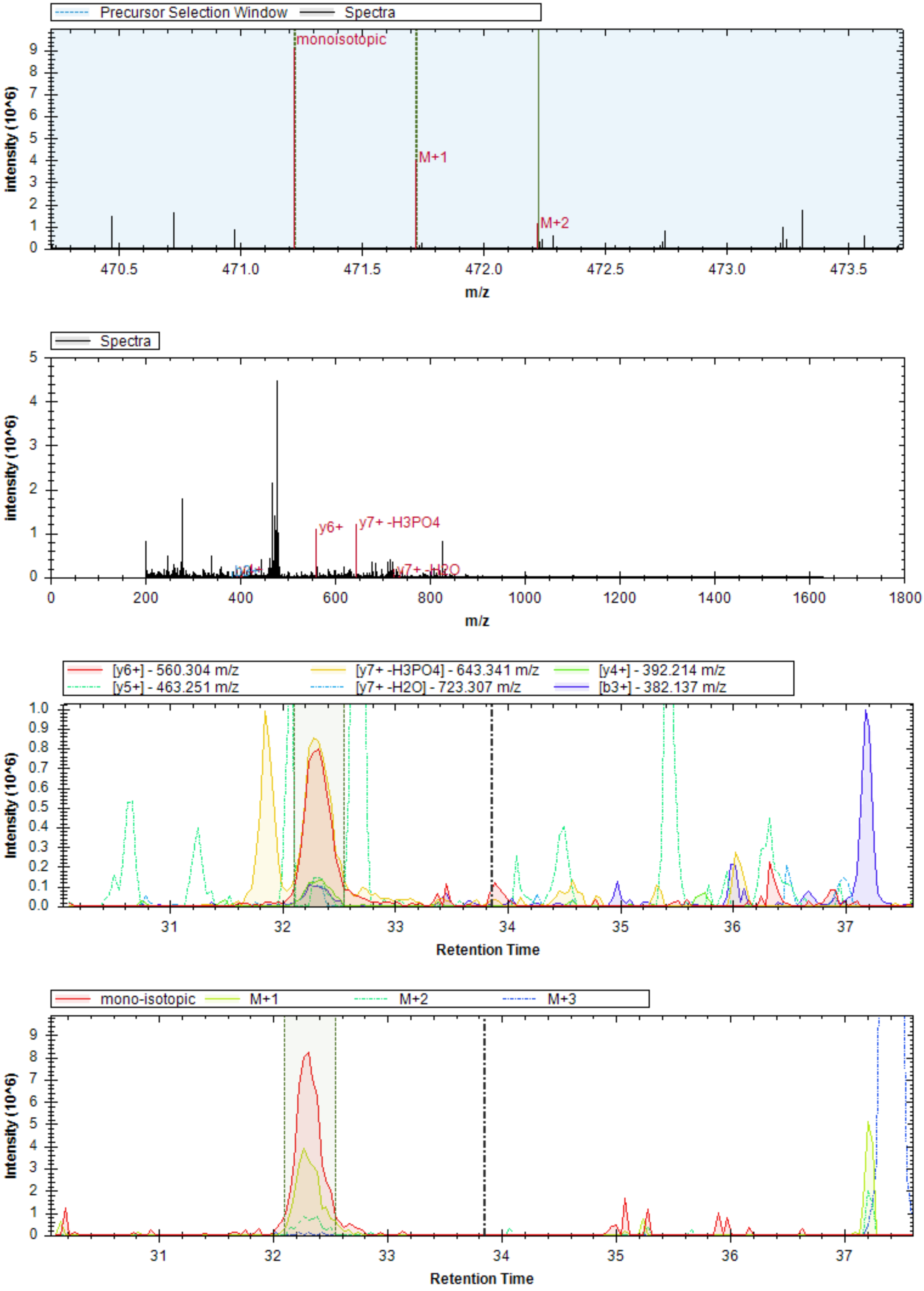

P10637 – _S[+H+O3+P]RTPSLPTPPTR_.3

Fig4_MP-DIA-barrel-cortex-p9-3_MHRM.htrms

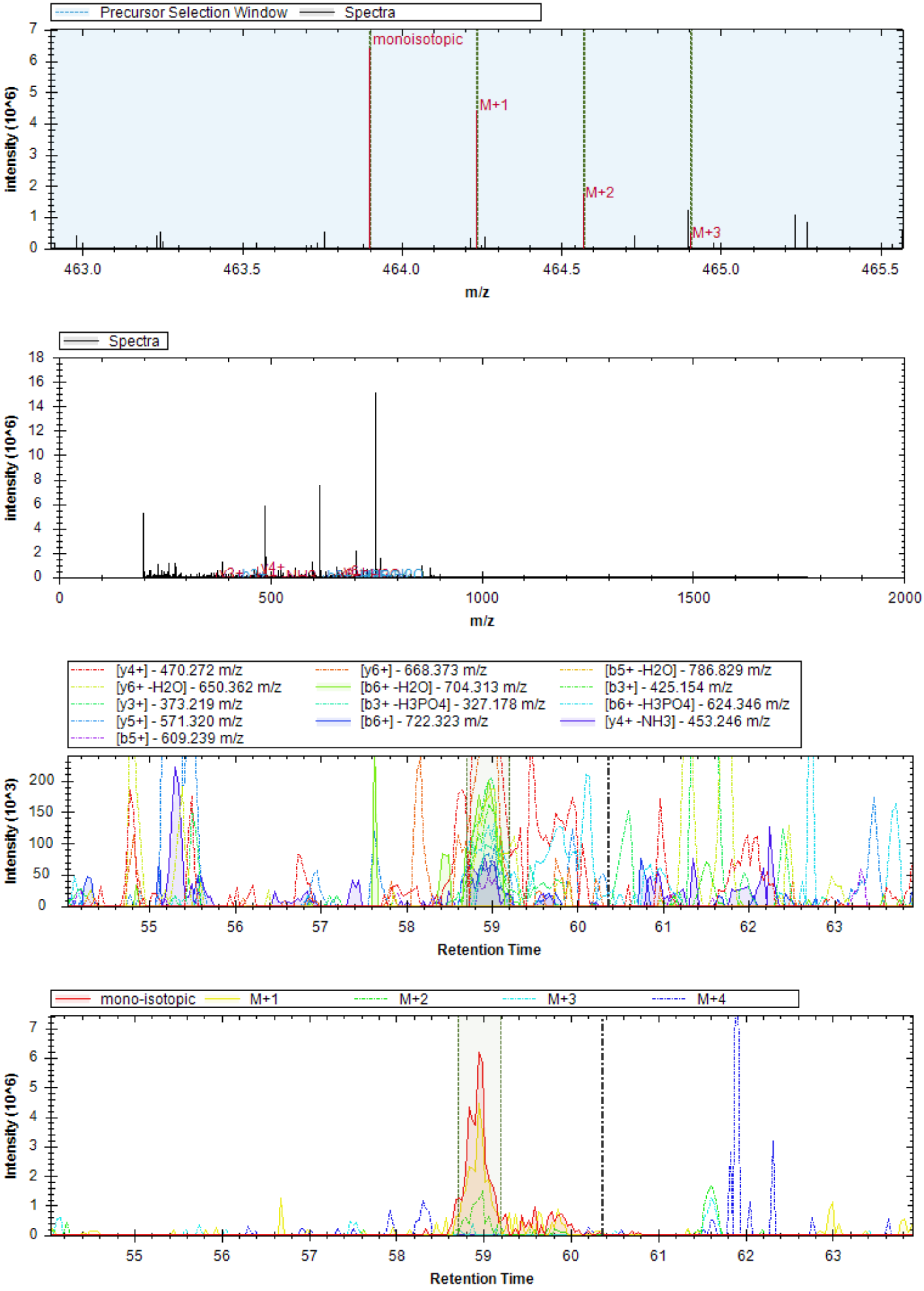

P20357 – _VTDGIS[+H+O3+P]KSPEKR_.3
Fig4_MP-DIA-barrel-cortex-p9-3_MHRM.htrms

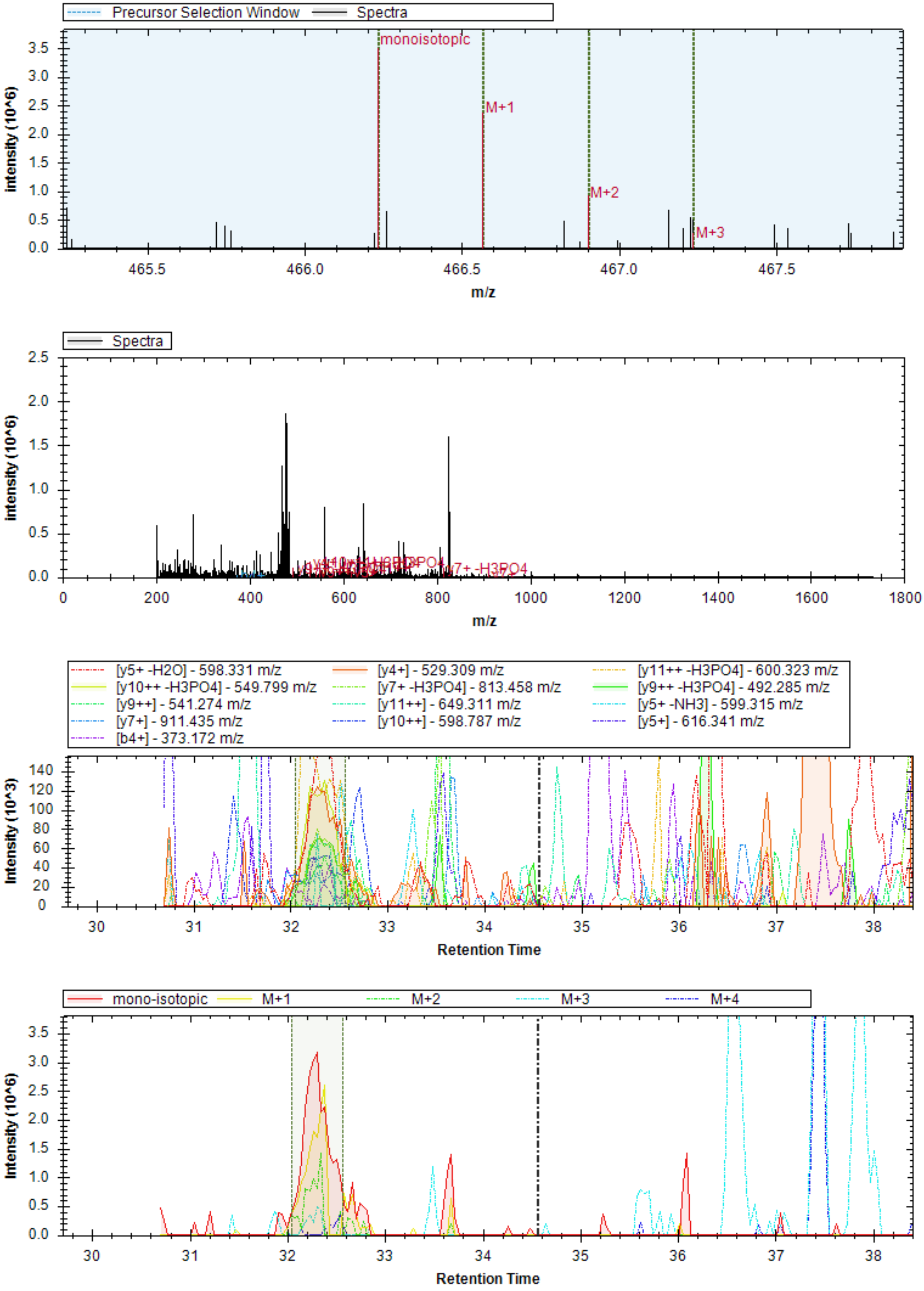

P20357 – _VTDGISKS[+H+O3+P]PEKR_.3

Fig4_MP-DIA-barrel-cortex-p9-3_MHRM.htrms

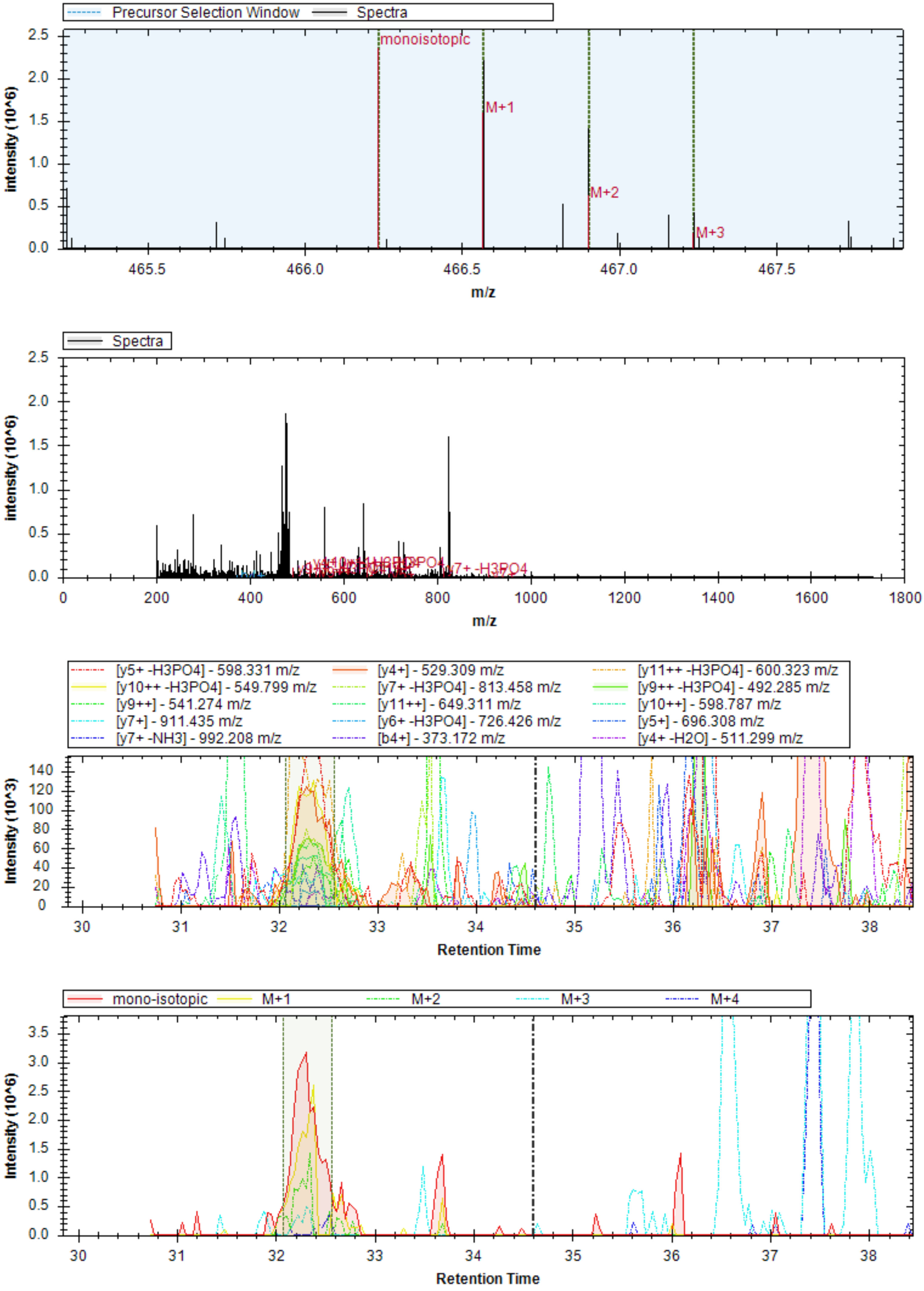

Q9CR81 – _[+C2+H2+O]M[+O]ANHLVKPDS[+H+O3+P]R_.3
Fig4_MP-DIA-barrel-cortex-p9-3_MHRM.htrms

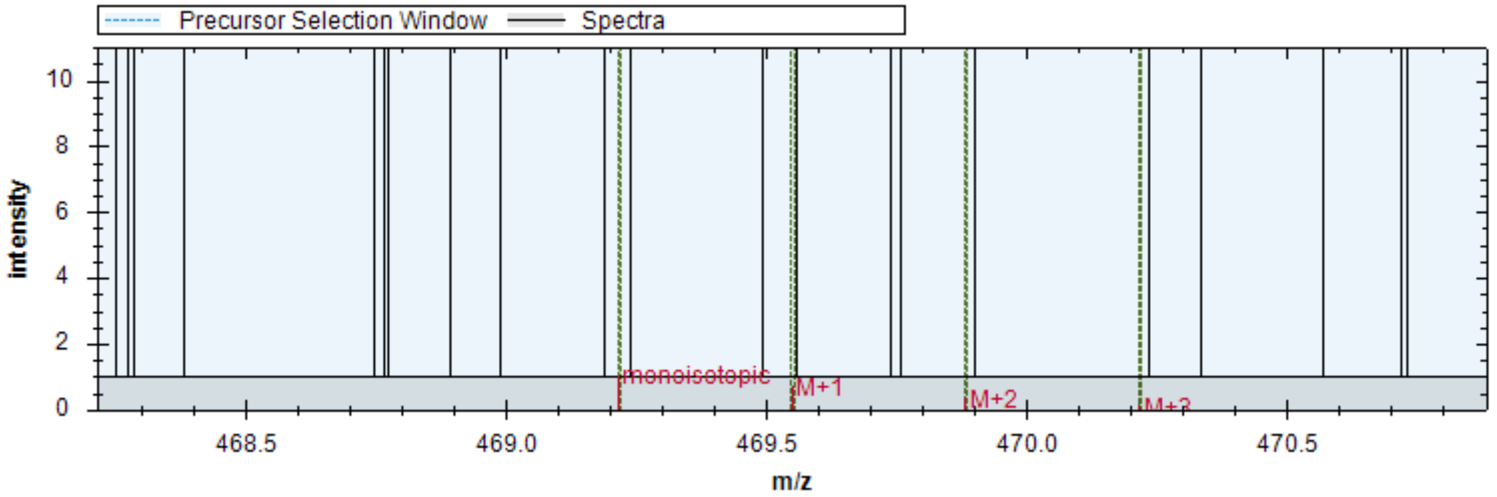

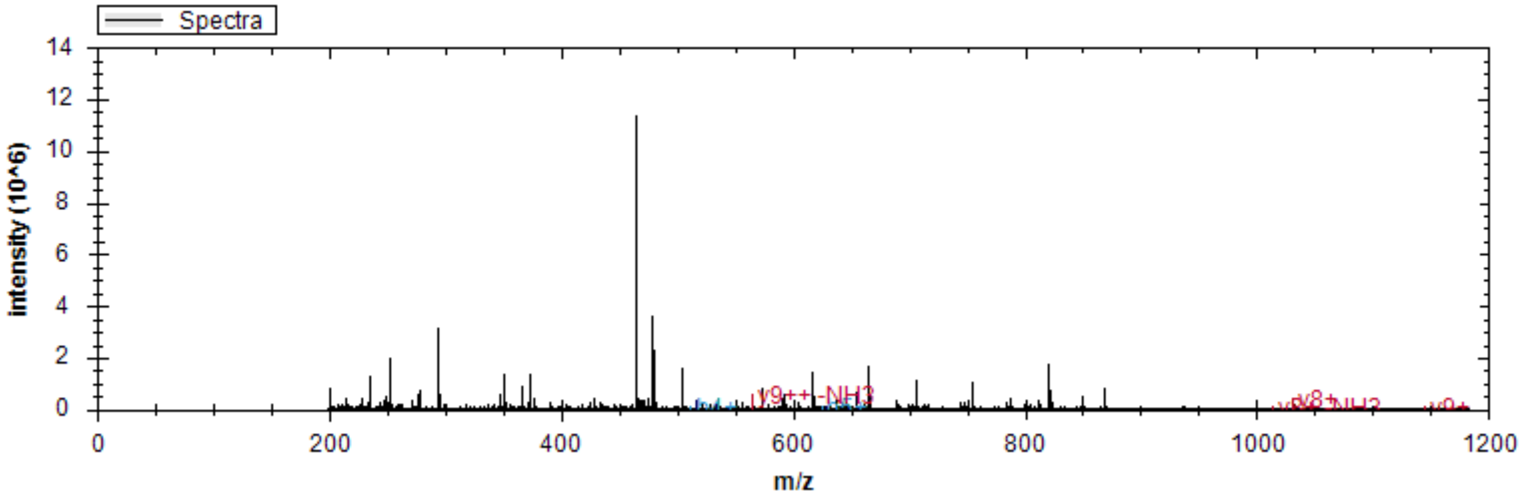

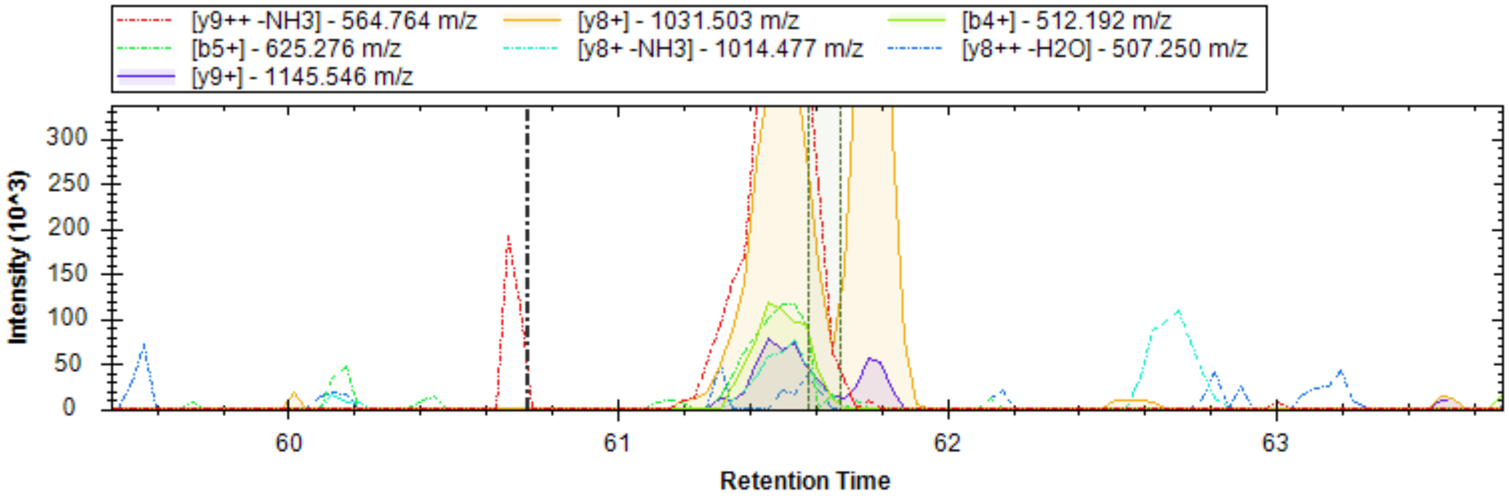

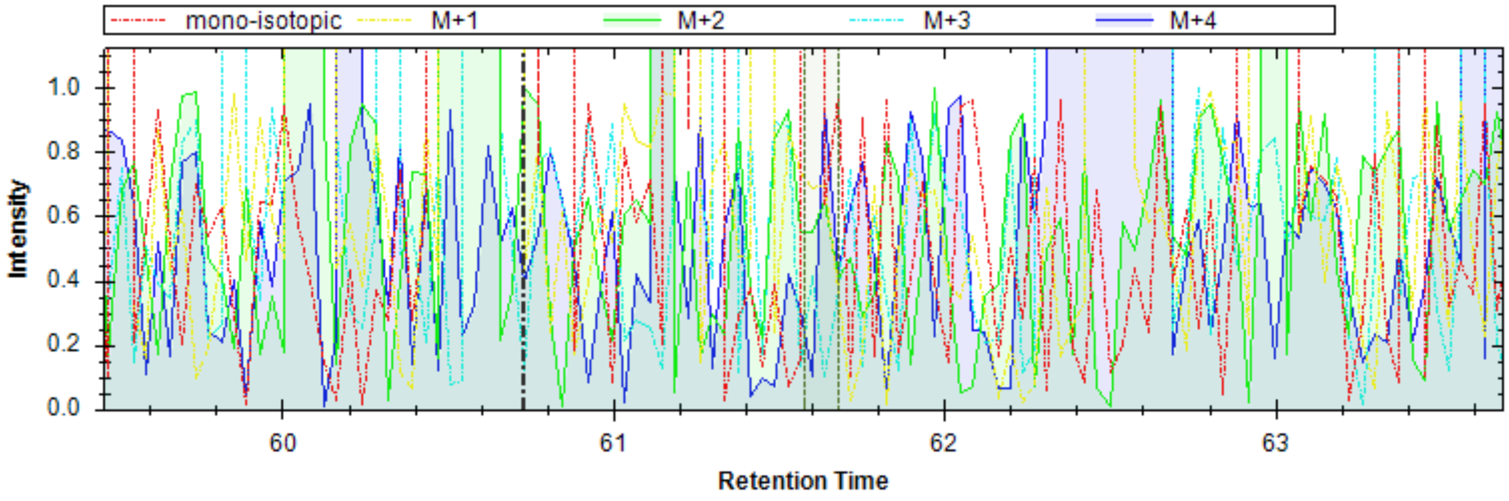

P26645 – _LSGFS[+H+O3+P]FKK_.2
Fig4_MP-DIA-barrel-cortex-p9-3_MHRM.htrms

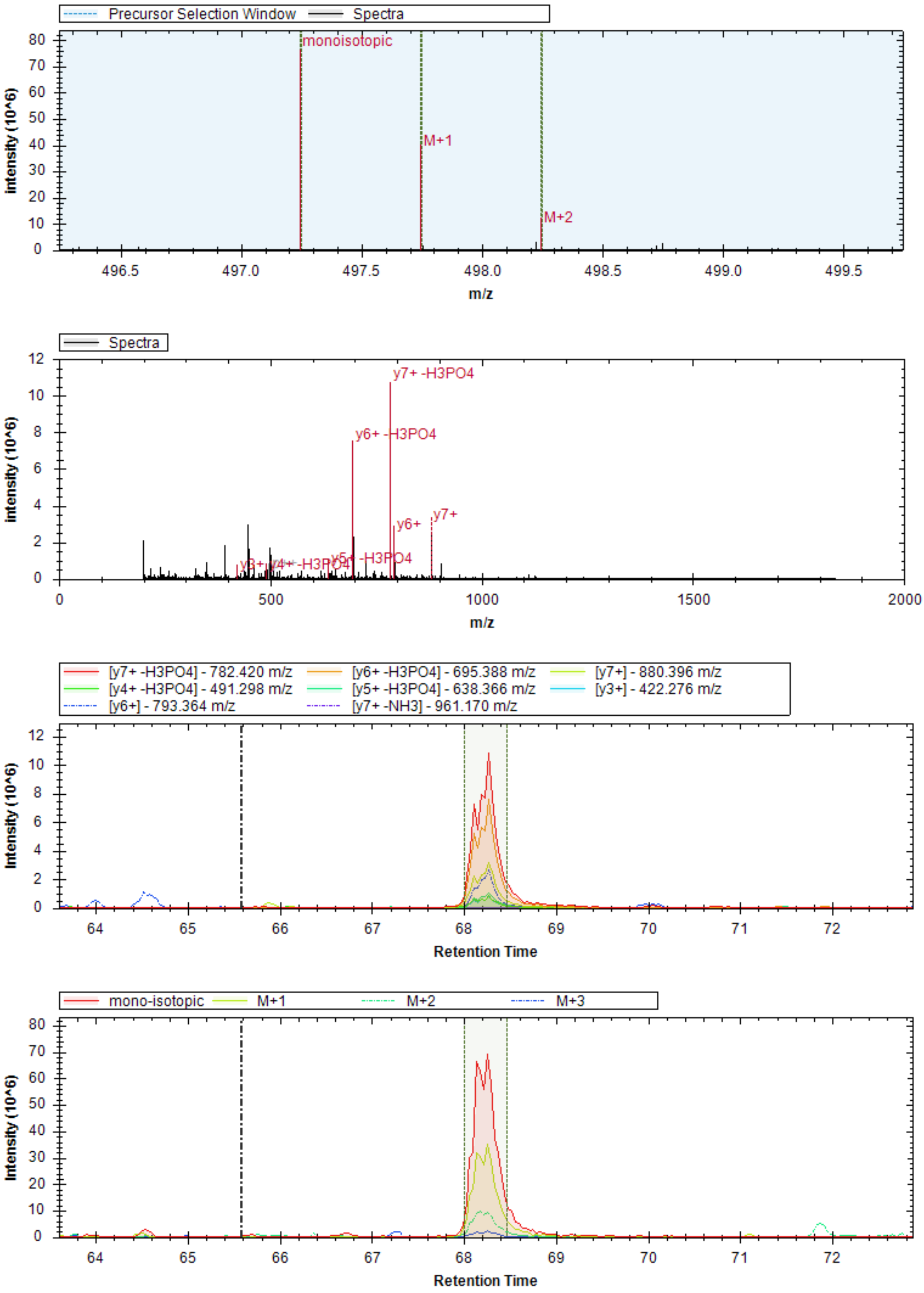

P28667 – _LSGLS[+H+O3+P]FKR_.2
Fig4_MP-DIA-barrel-cortex-p9-3_MHRM.htrms

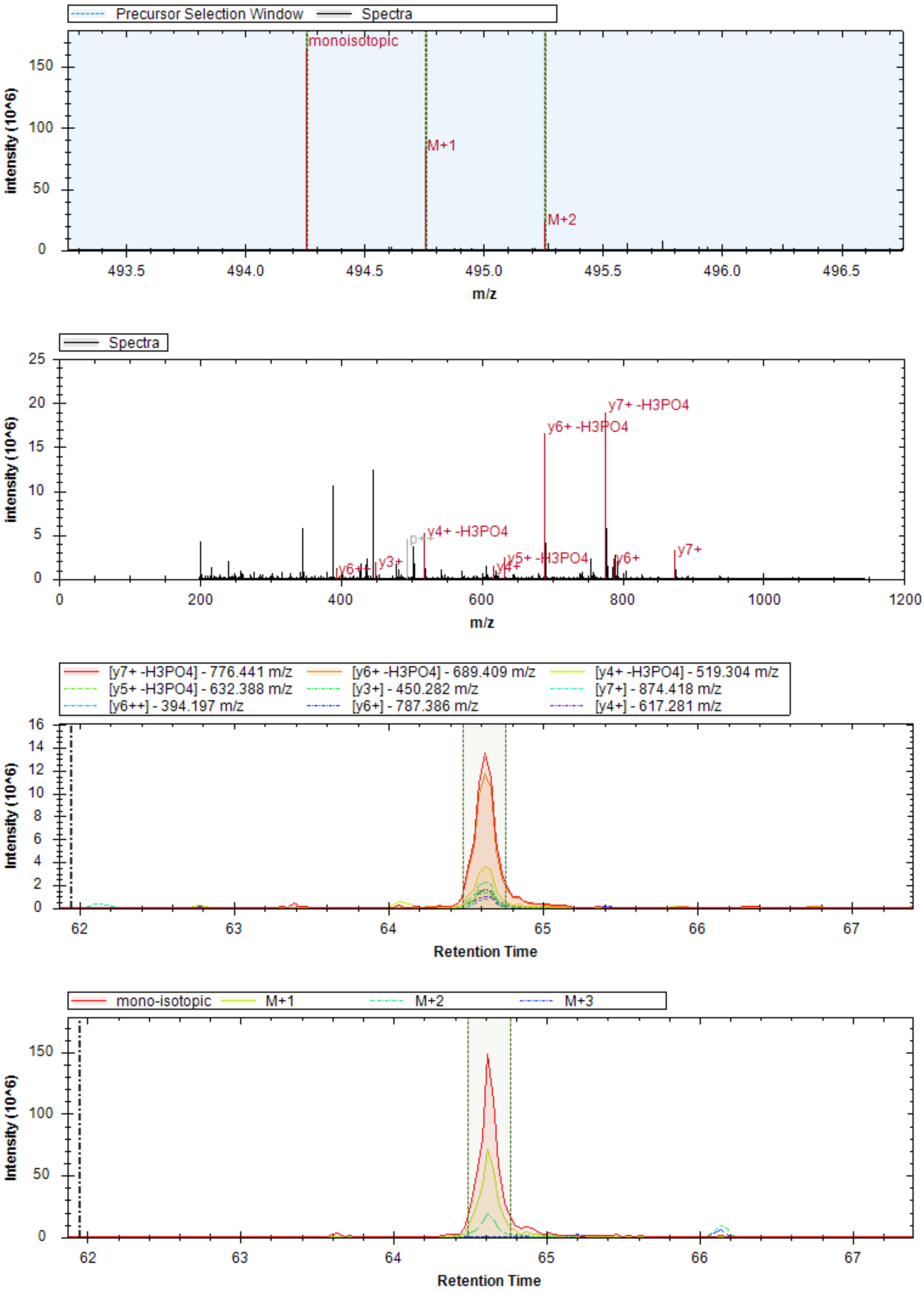

P57776-3 – _ATAPQTQHVS[+H+O3+P]PMR_.3

Fig4_MP-DIA-barrel-cortex-p9-3_MHRM.htrms

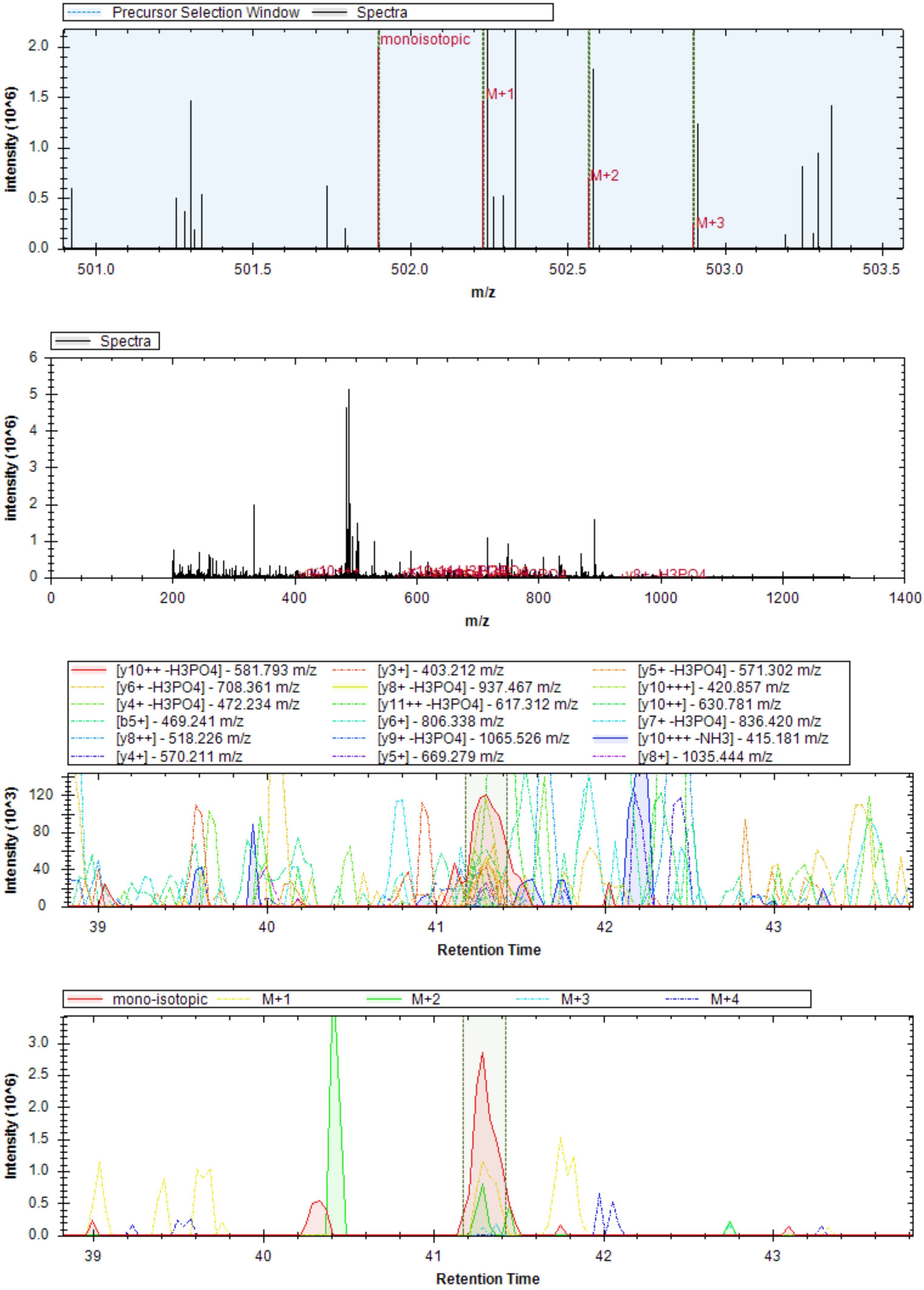

P28738 – _PIRPGHYPAS[+H+O3+P]SPTAVHAVR_.4

Fig4_MP-DIA-barrel-cortex-p9-3_MHRM.htrms

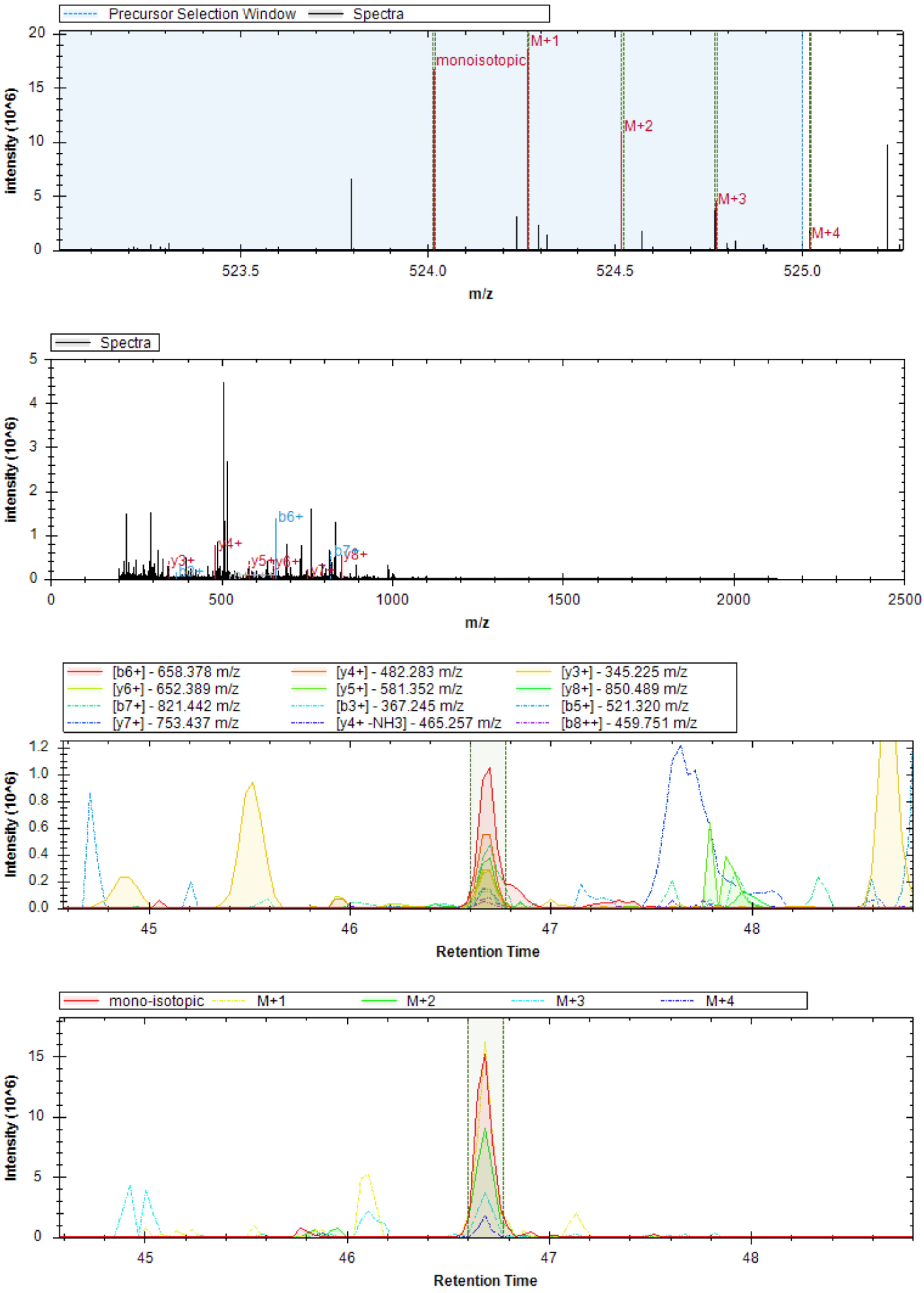

P28738 – _PIRPGHYPASS[+H+O3+P]PTAVHAVR_.4
Fig4_MP-DIA-barrel-cortex-p9-3_MHRM.htrms

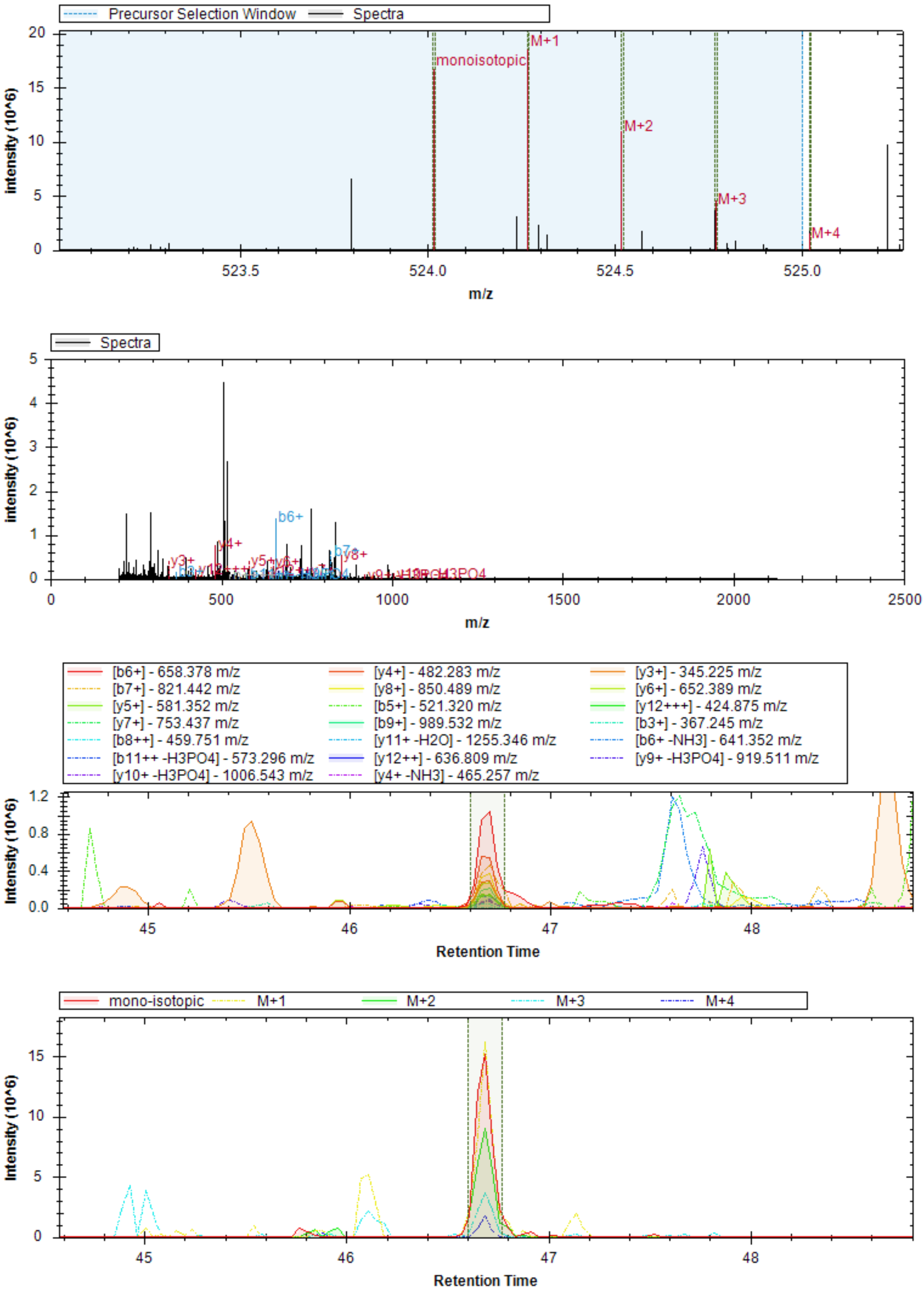

P62996 – _RPHT[+H+O3+P]PTPGIYMGR_.3
Fig4_MP-DIA-barrel-cortex-p9-3_MHRM.htrms

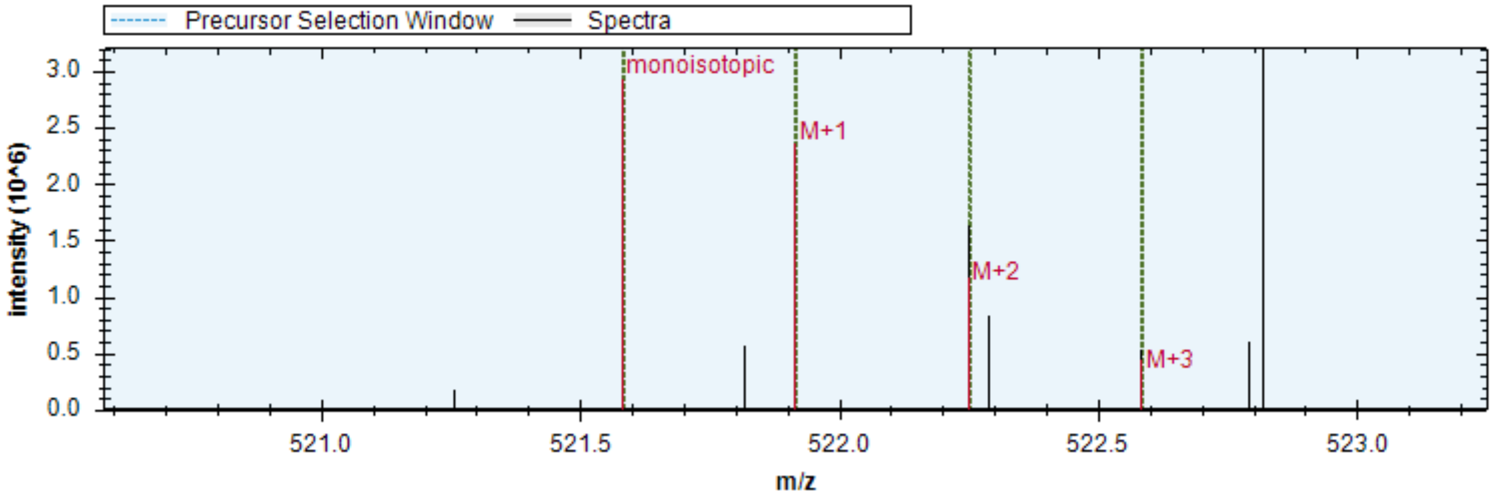

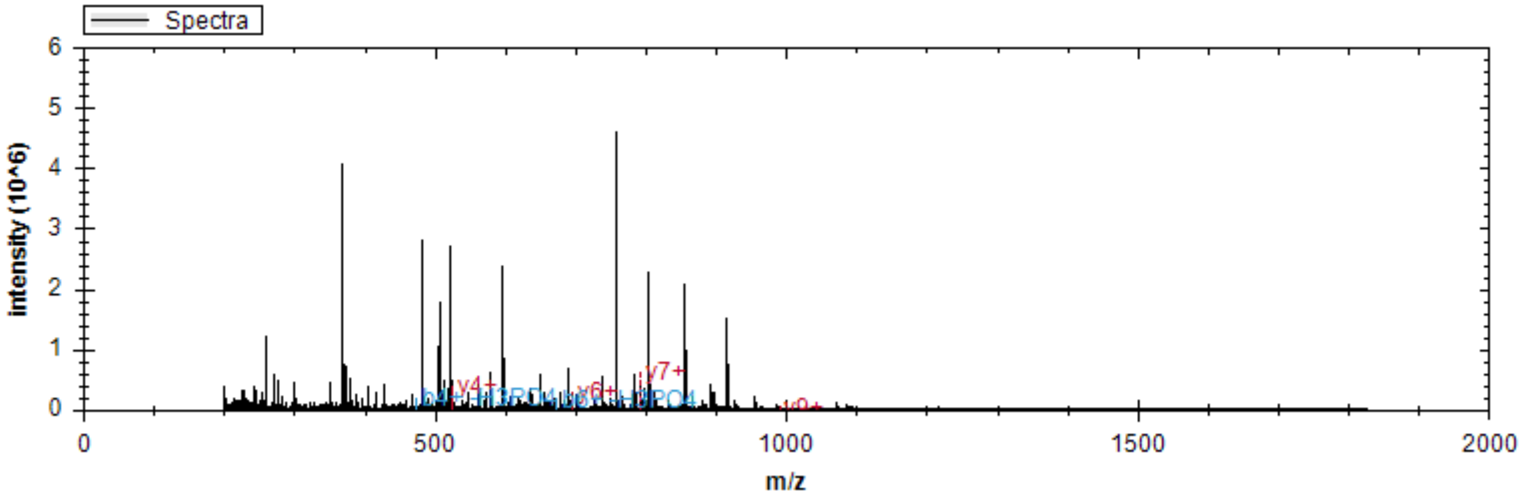

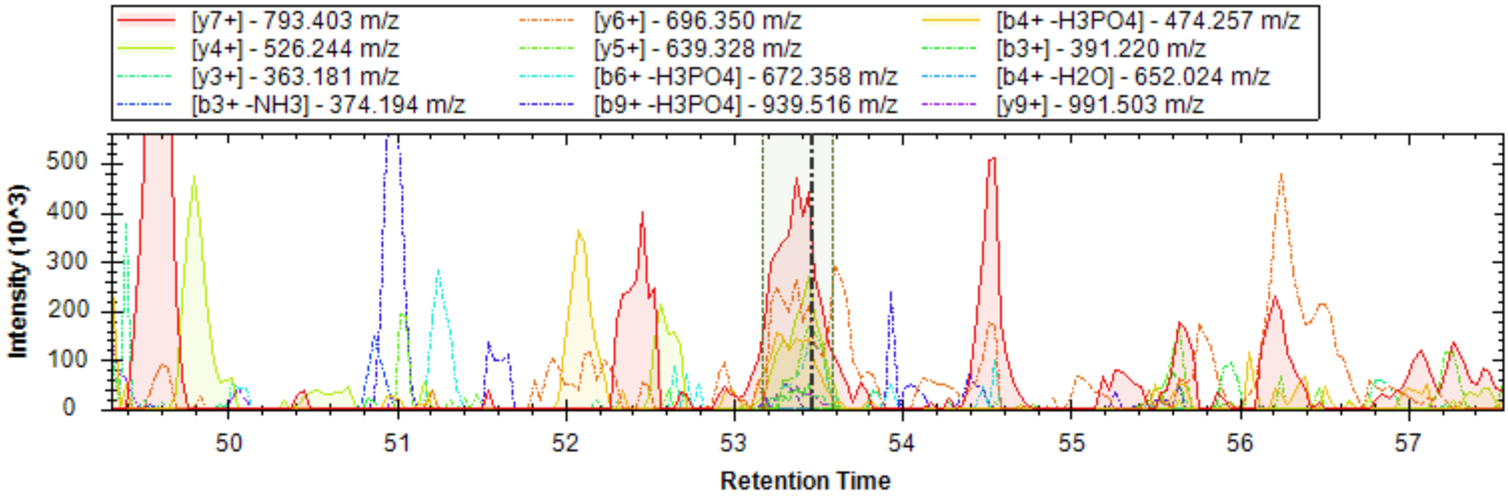

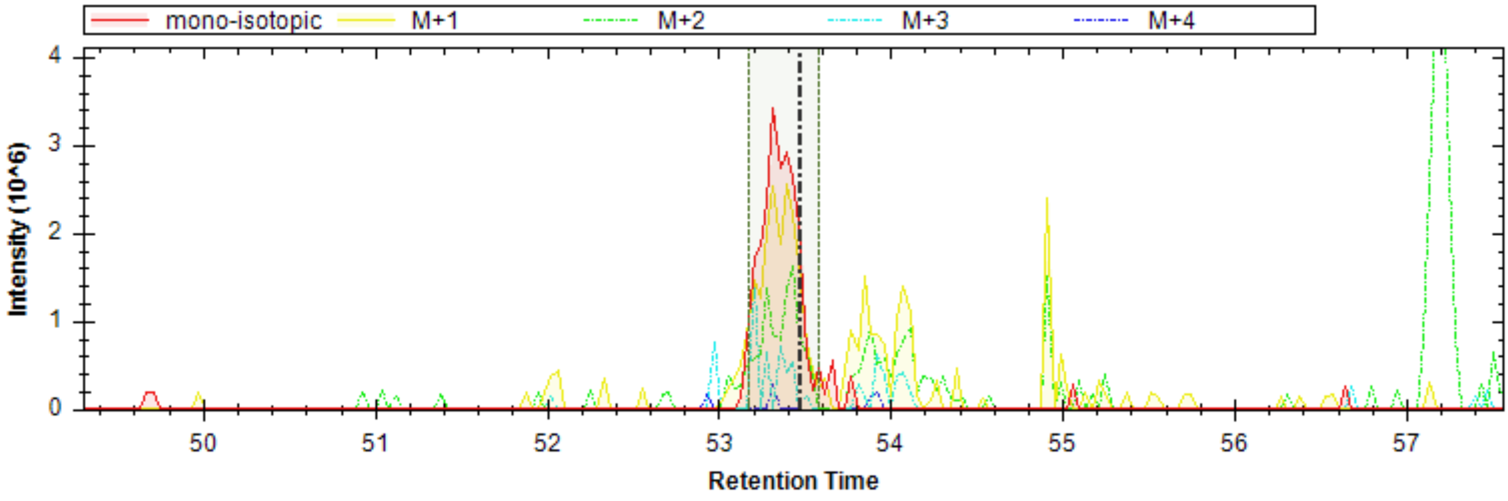

Q8C8R3-7 – _[+C2+H2+O]AHAAAS[+H+O3+P]IKK_.2
Fig4_MP-DIA-barrel-cortex-p9-3_MHRM.htrms

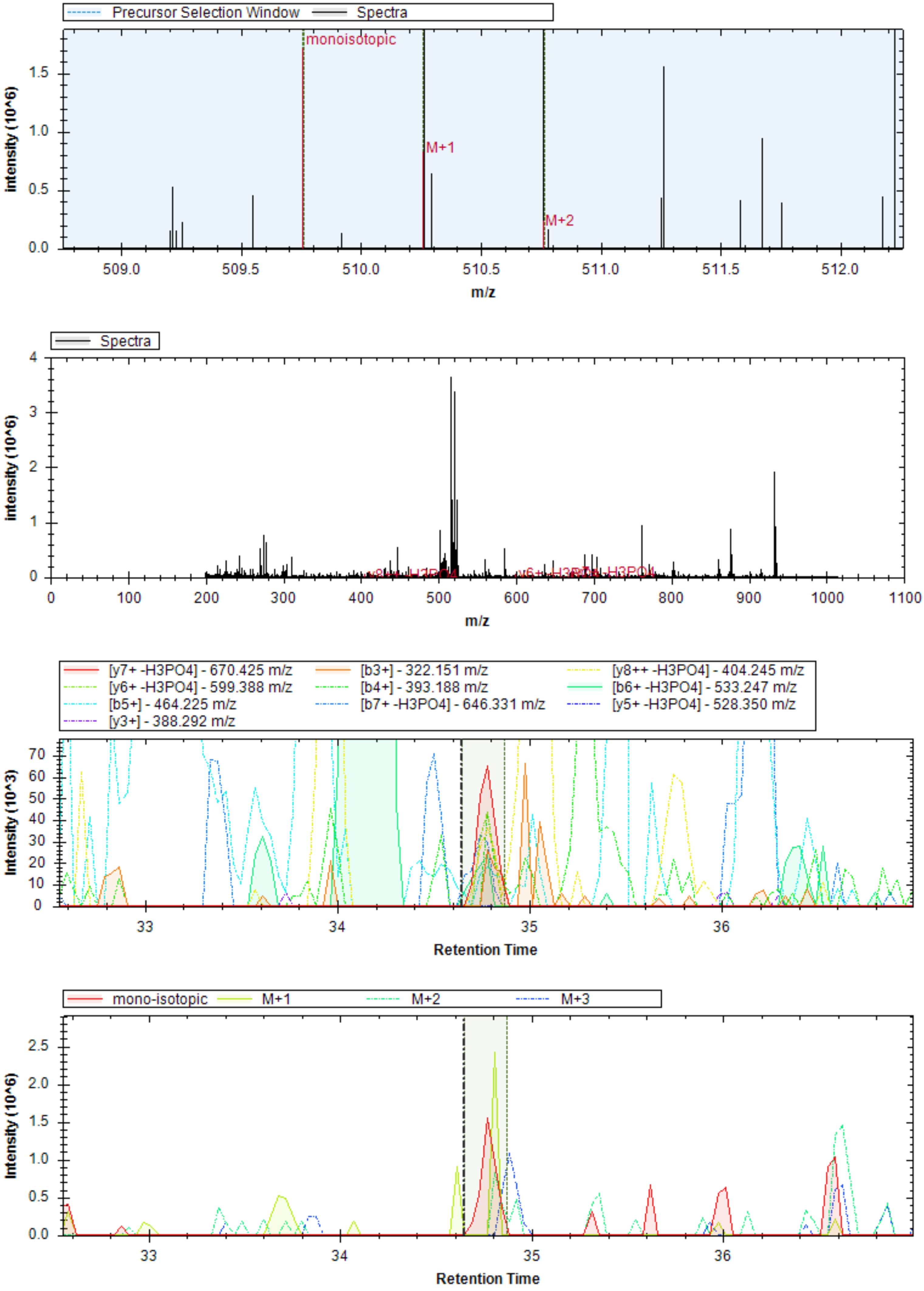

Q8CDY7 – _LVQALC[+C2+H3+N+O]EQIHT[+H+O3+P]R_.3
Fig4_MP-DIA-barrel-cortex-p9-3_MHRM.htrms

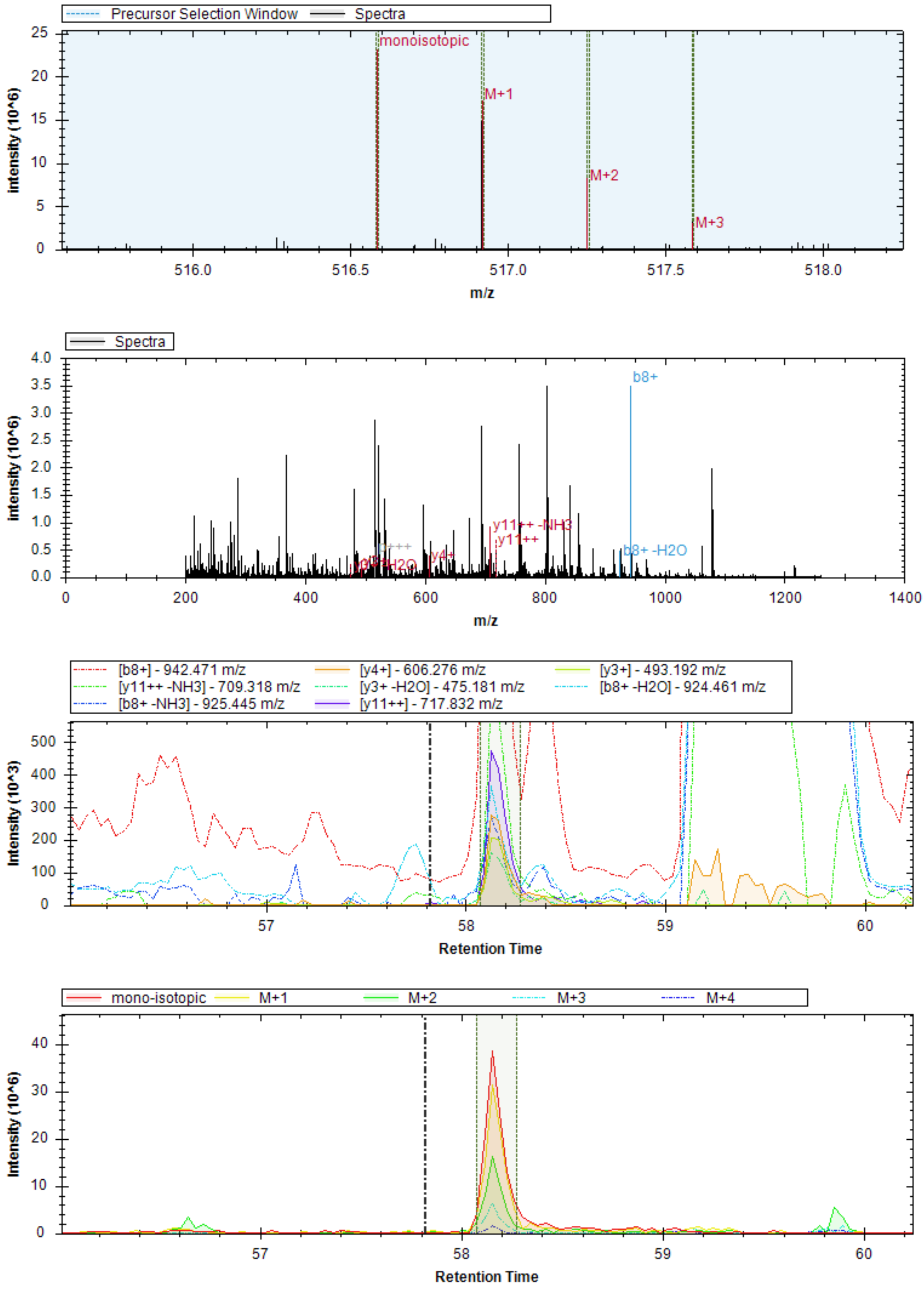

Q9JLM8 – _ISQHGGS[+H+O3+P]STSLSSTK_.3

Fig4_MP-DIA-barrel-cortex-p9-3_MHRM.htrms

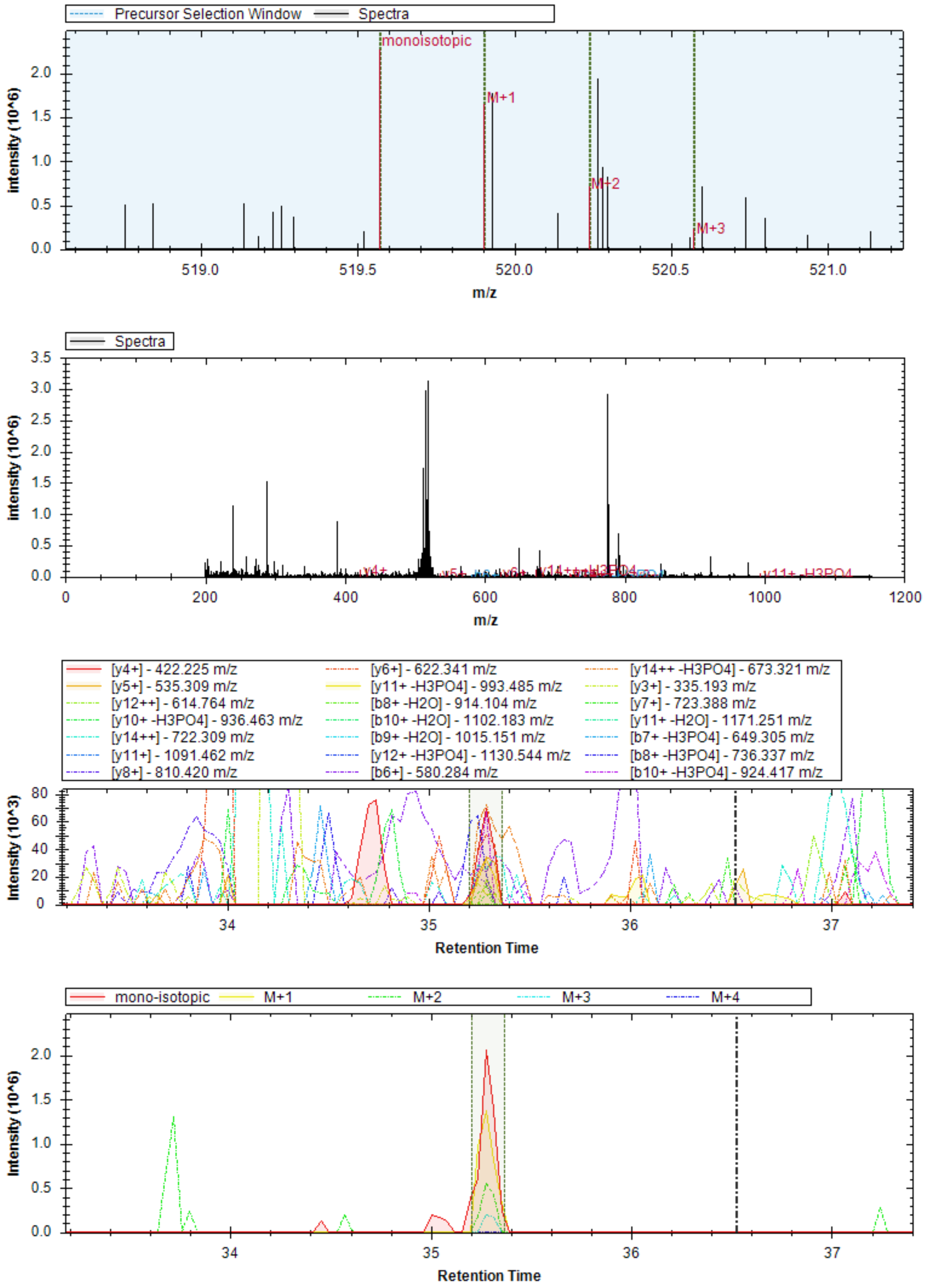

Q9QYC0 – _AAVVT[+H+O3+P]SPPPTTAPHK_.3
Fig4_MP-DIA-barrel-cortex-p9-3_MHRM.htrms

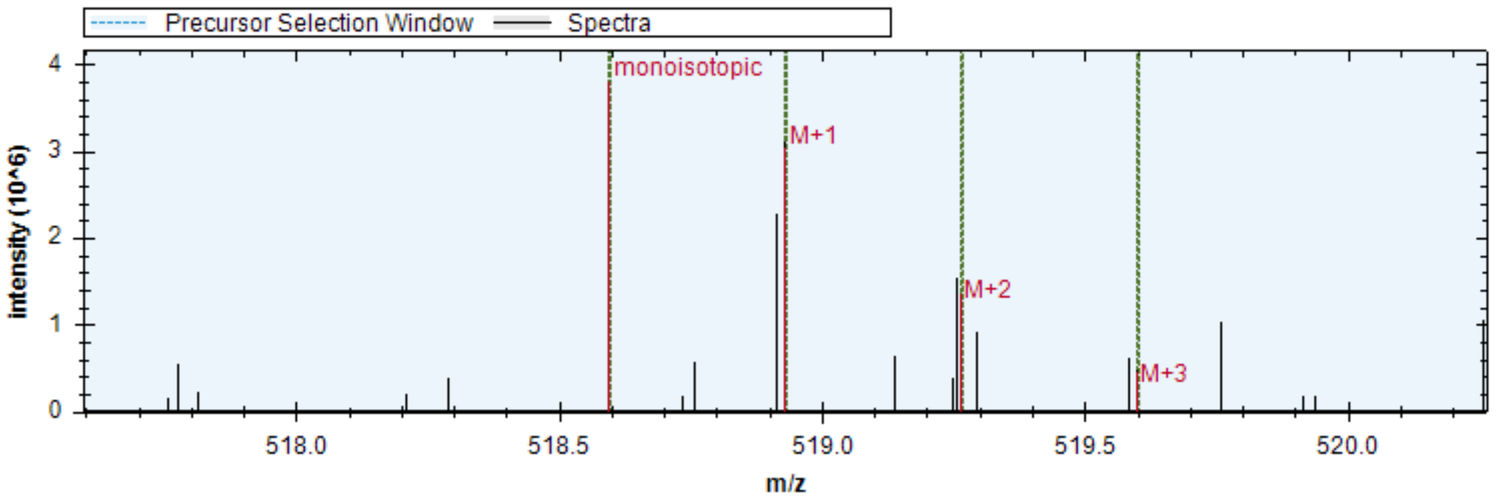

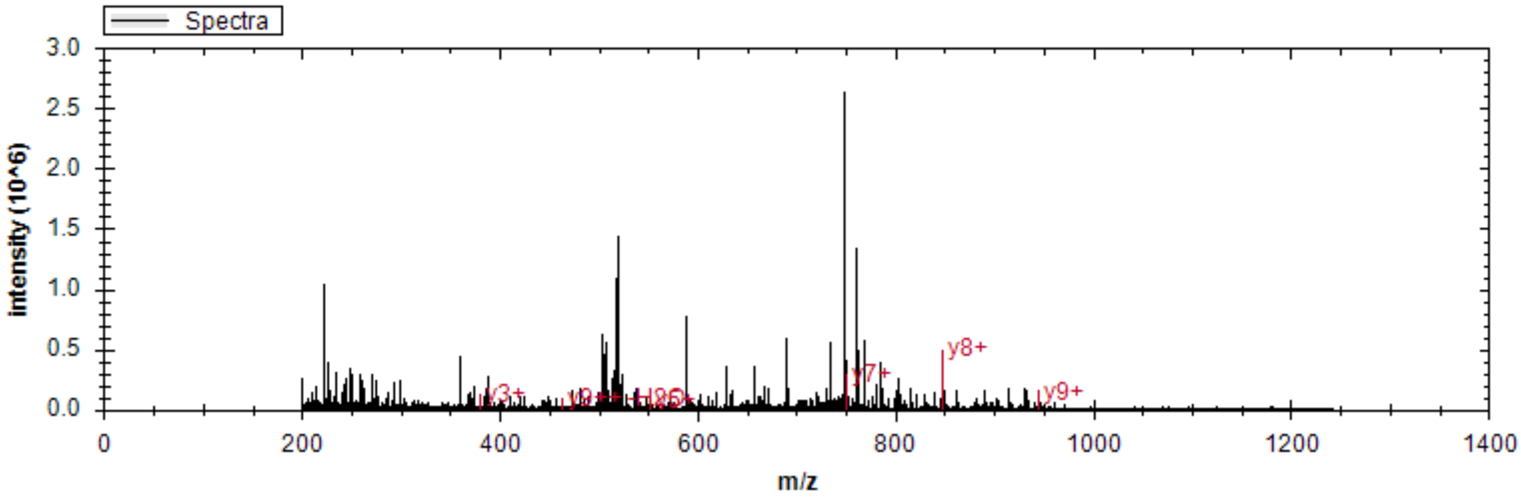

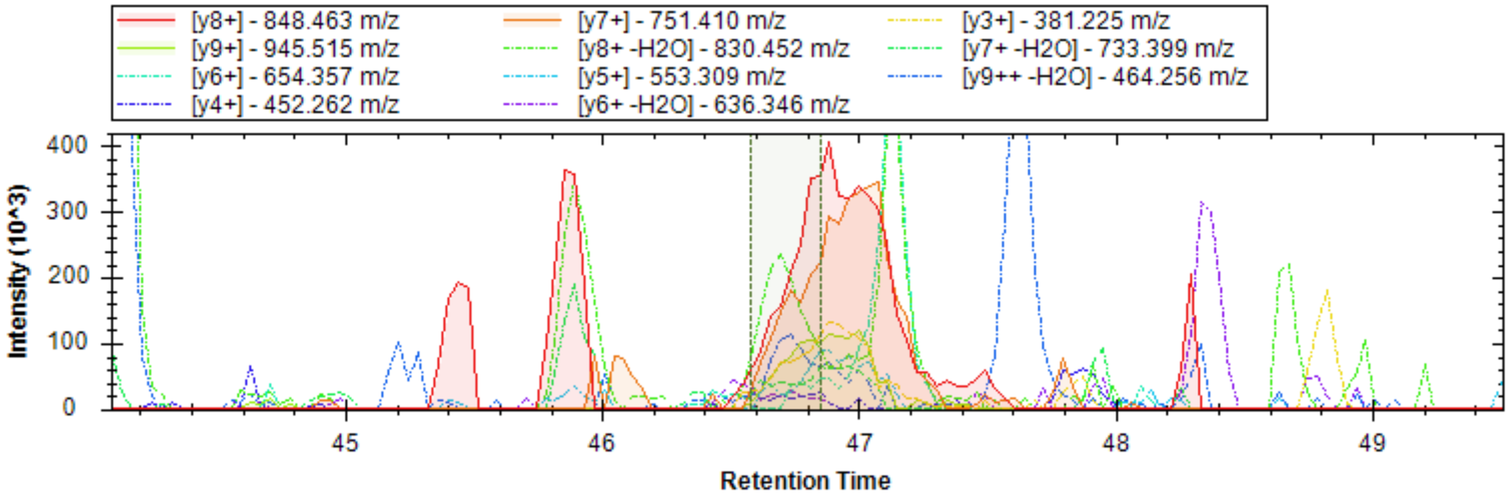

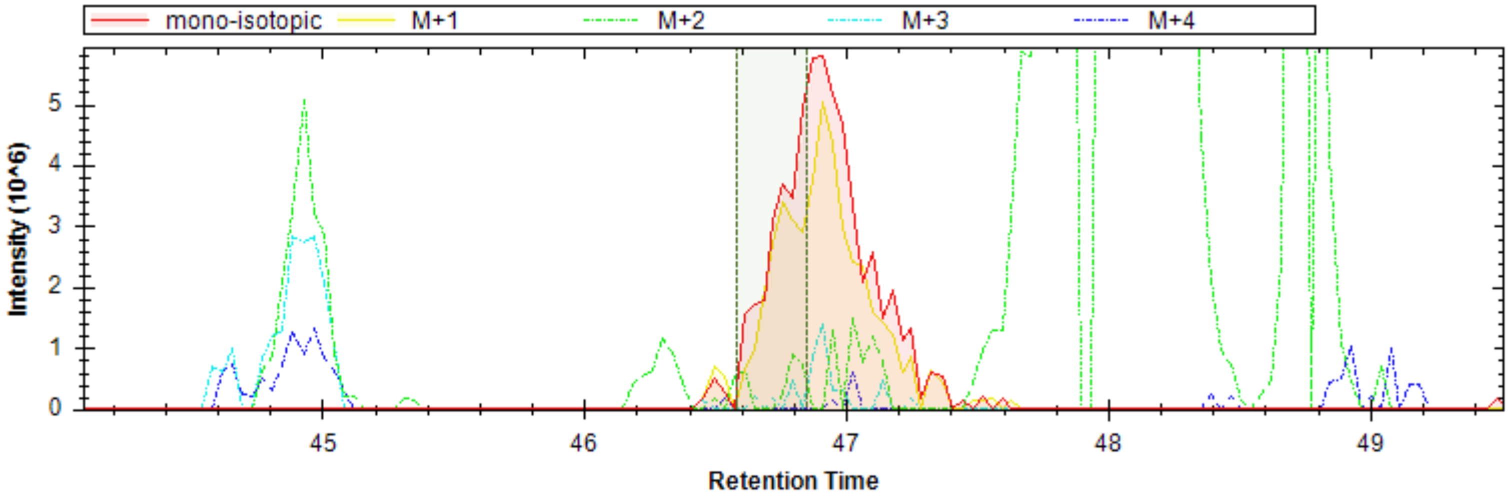

Q9QYC0 – _AAVVTS[+H+O3+P]PPPTTAPHK_.3
Fig4_MP-DIA-barrel-cortex-p9-3_MHRM.htrms

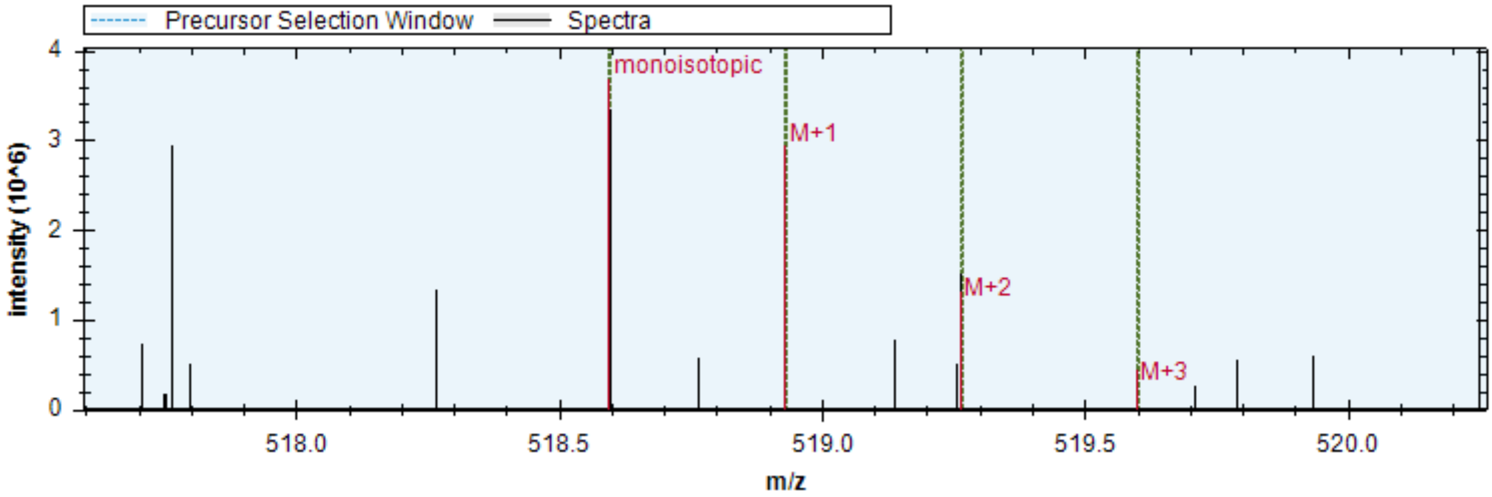

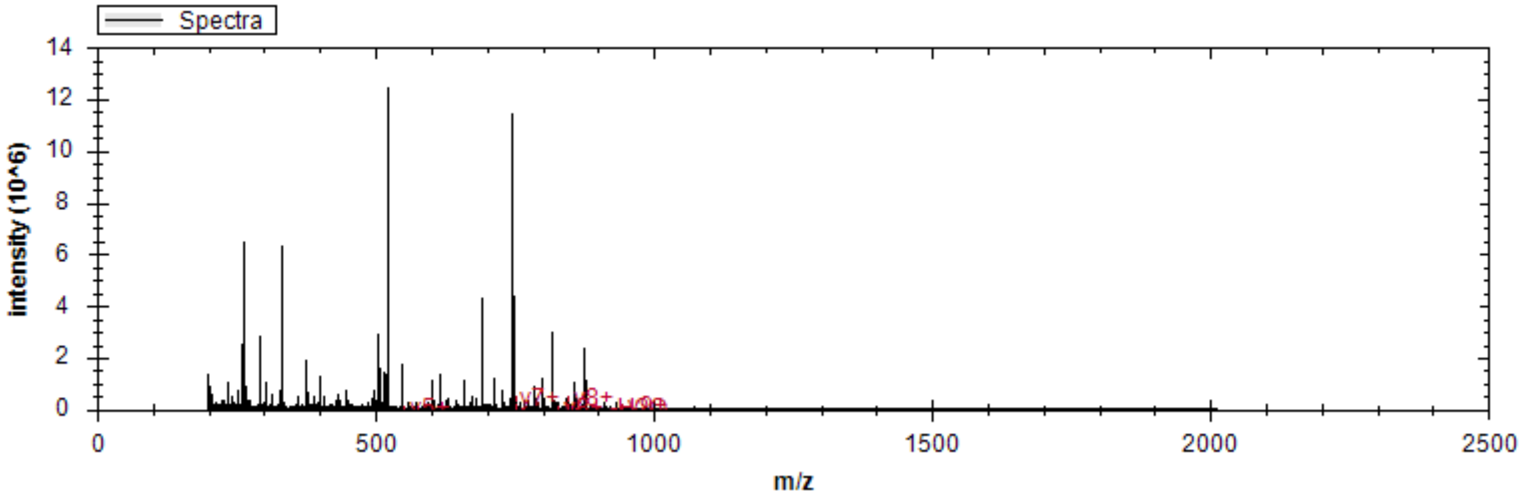

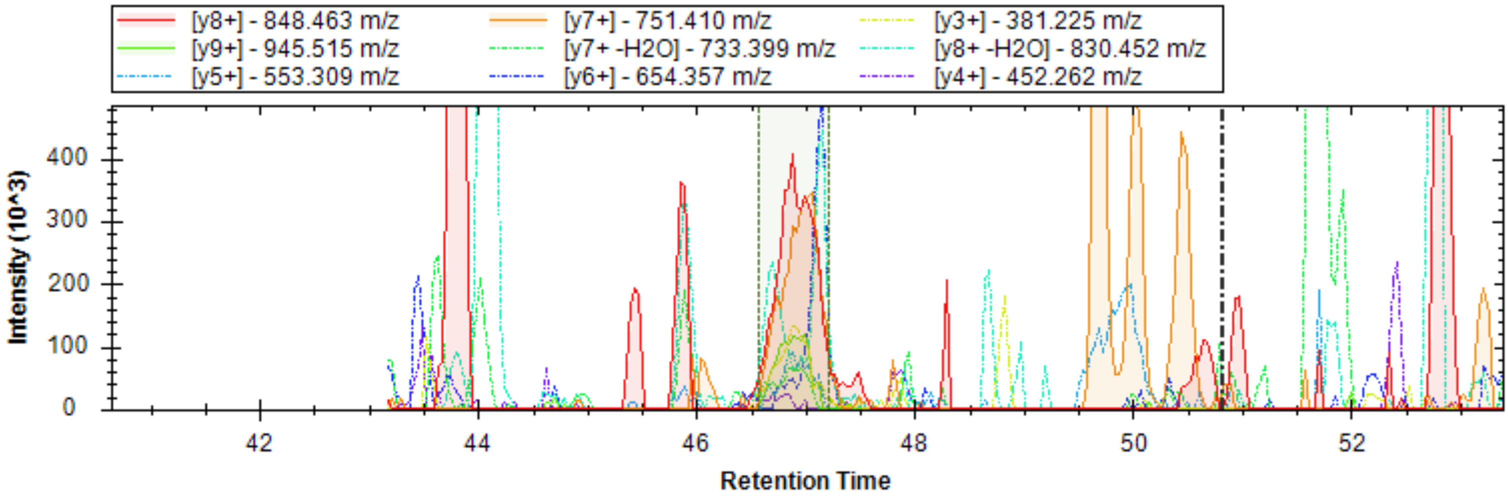

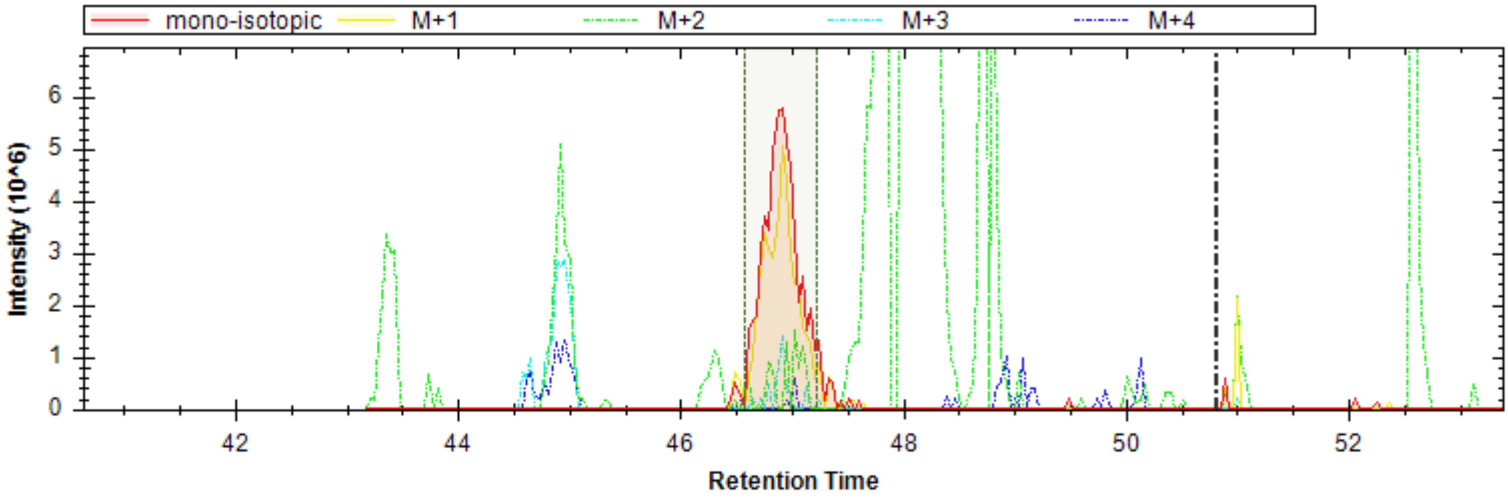

P10637 – _T[+H+O3+P]PPKSPSASK_.2

Fig4_MP-DIA-barrel-cortex-p9-3_MHRM.htrms

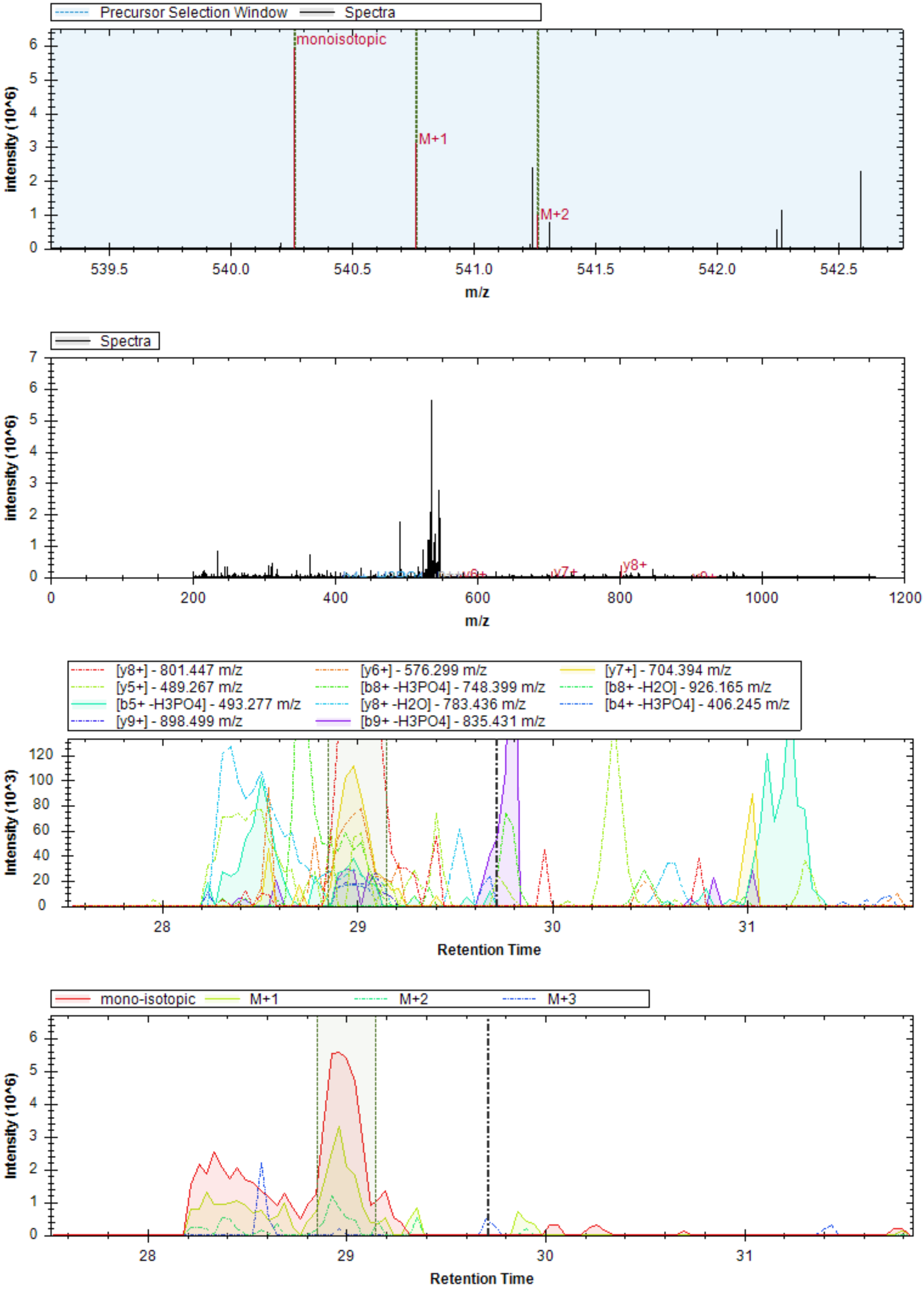

P10637 – _TPPKS[+H+O3+P]PSASK_.2

Fig4_MP-DIA-barrel-cortex-p9-3_MHRM.htrms

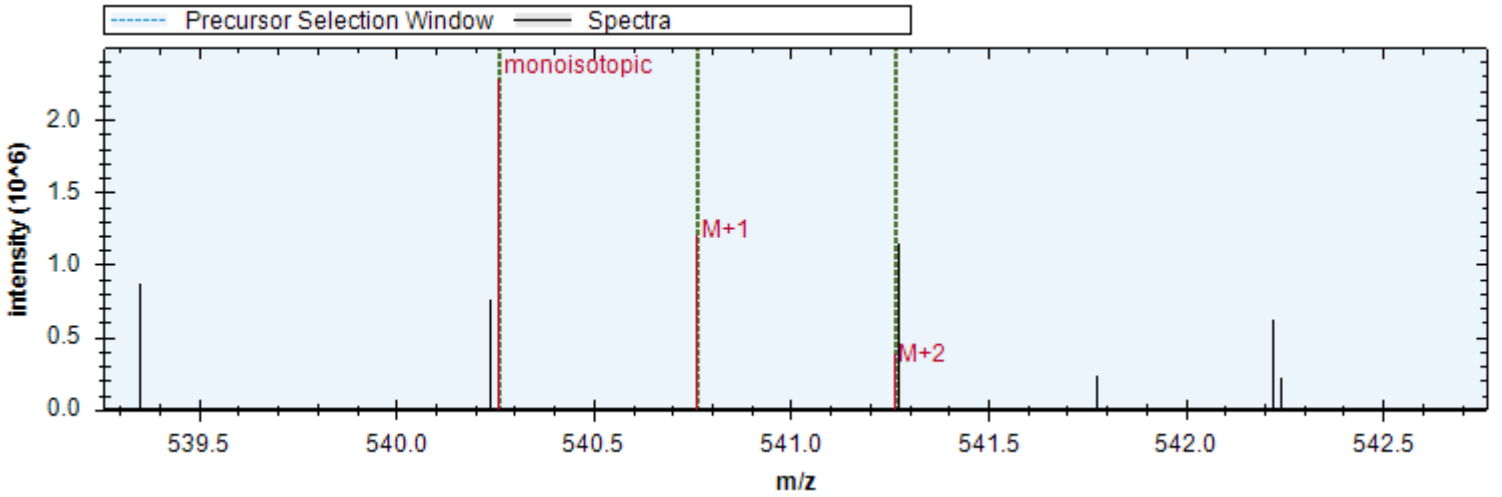

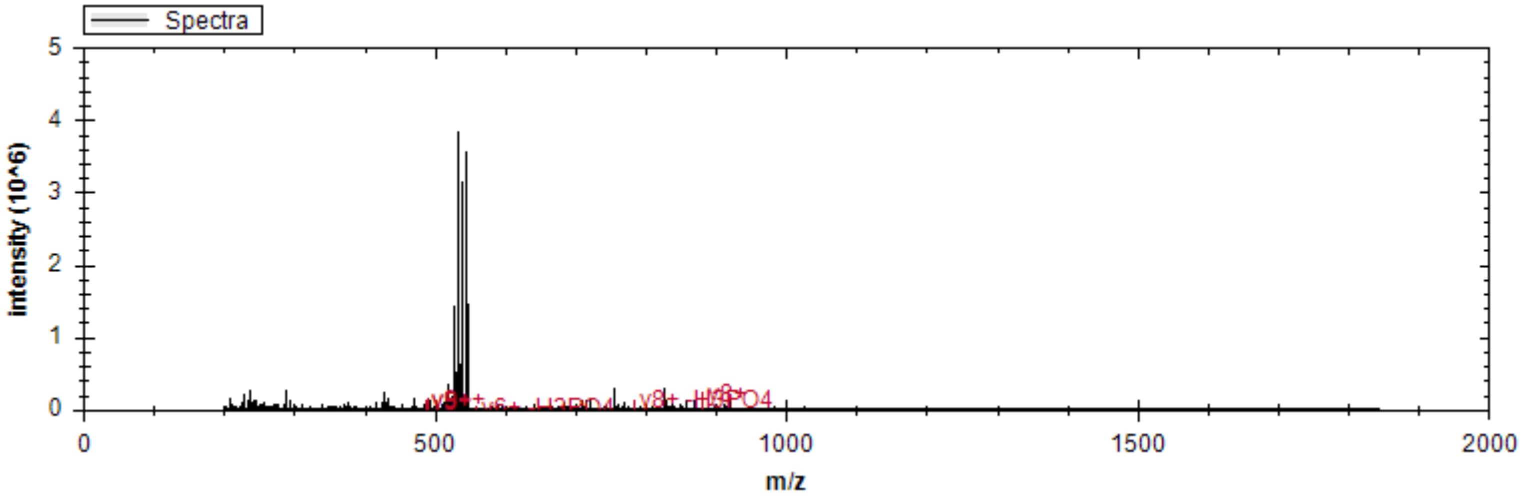

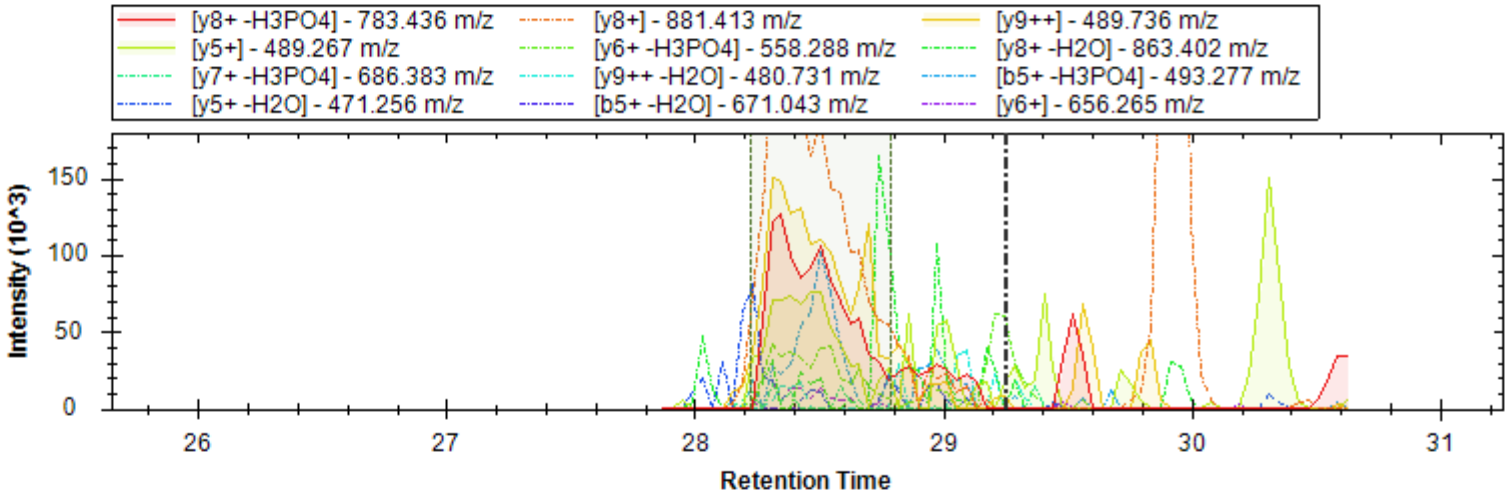

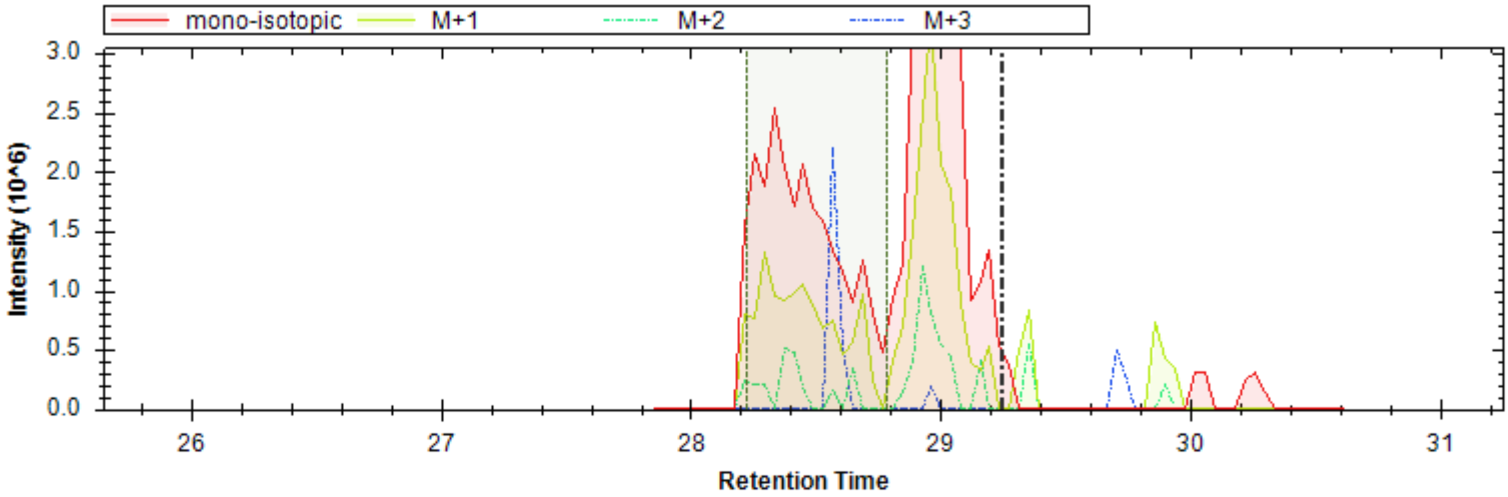

P10637 – _VAVVRT[+H+O3+P]PPKSPSASK_.3

Fig4_MP-DIA-barrel-cortex-p9-3_MHRM.htrms

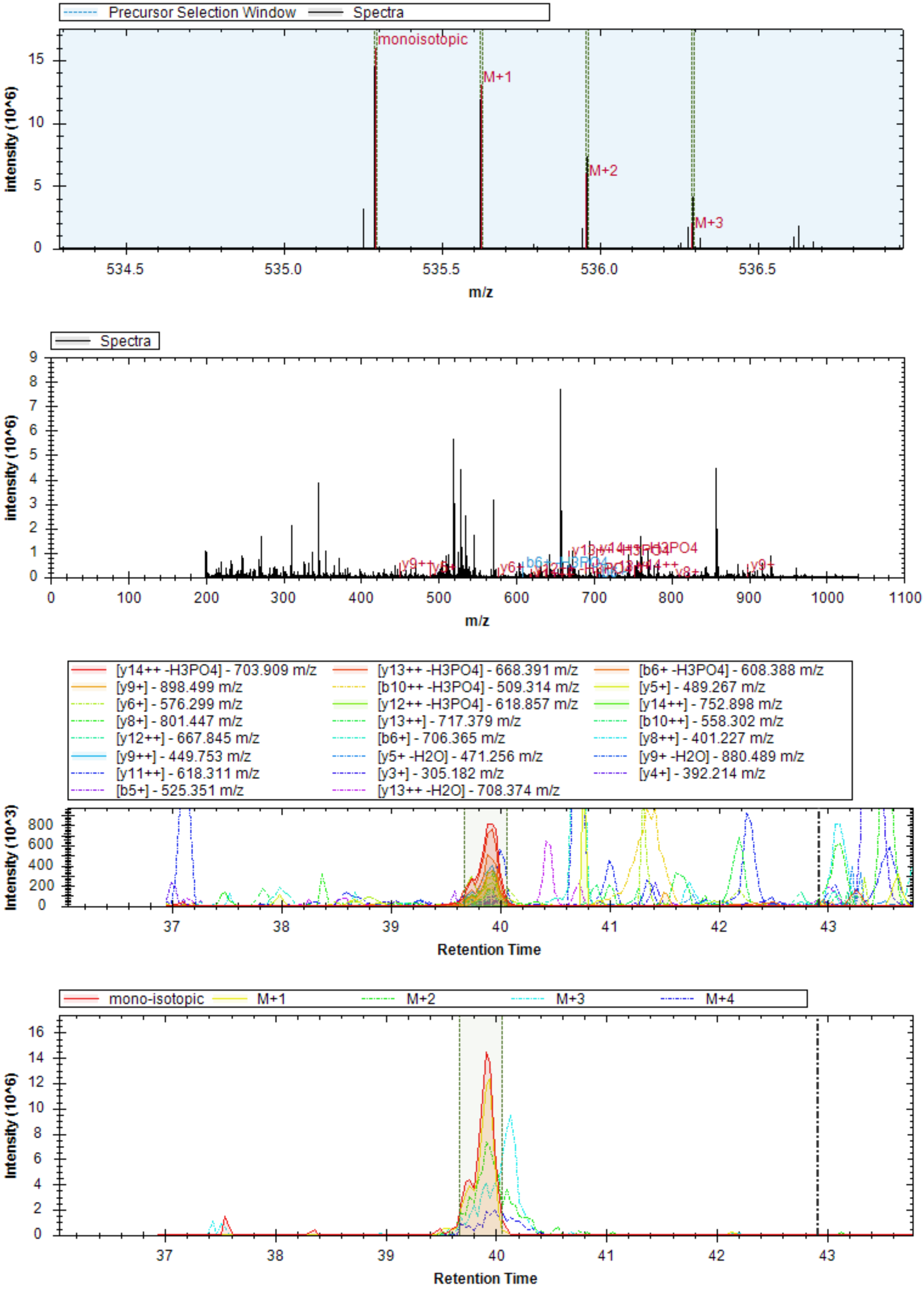

P54227 – _RAS[+H+O3+P]GQAFELILSPR_.3
Fig4_MP-DIA-barrel-cortex-p9-3_MHRM.htrms

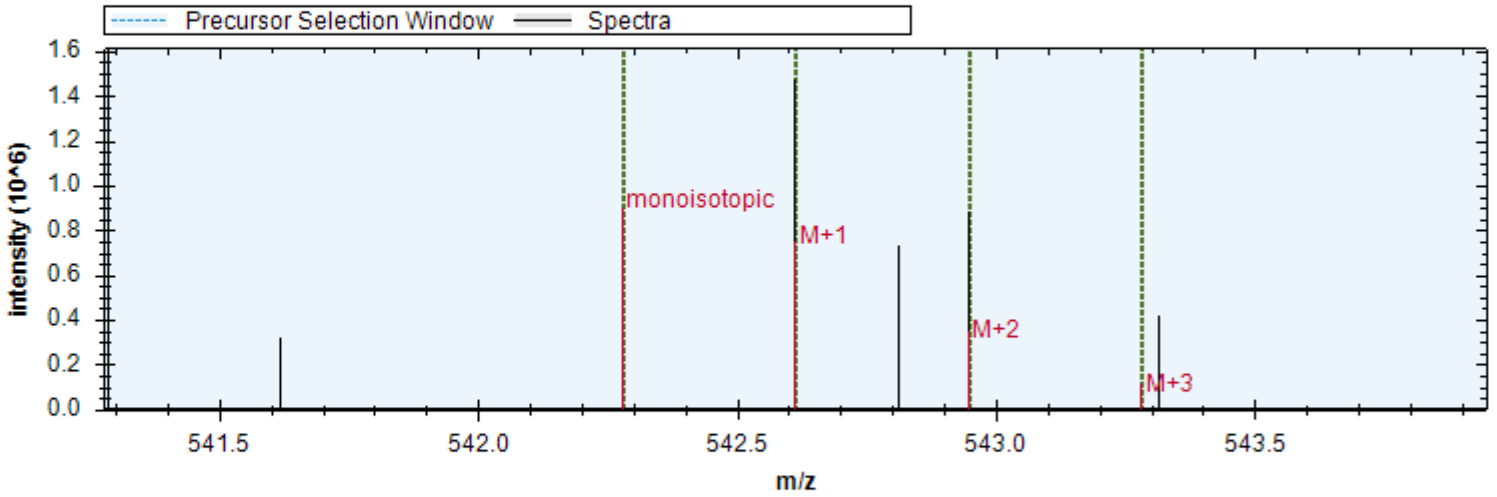

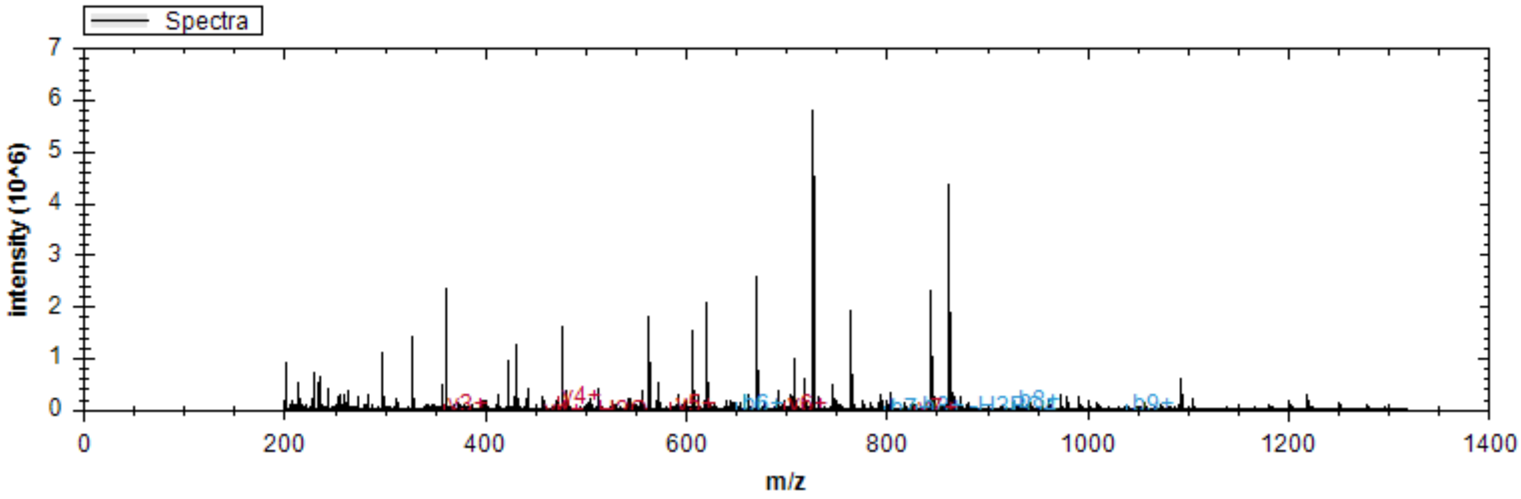

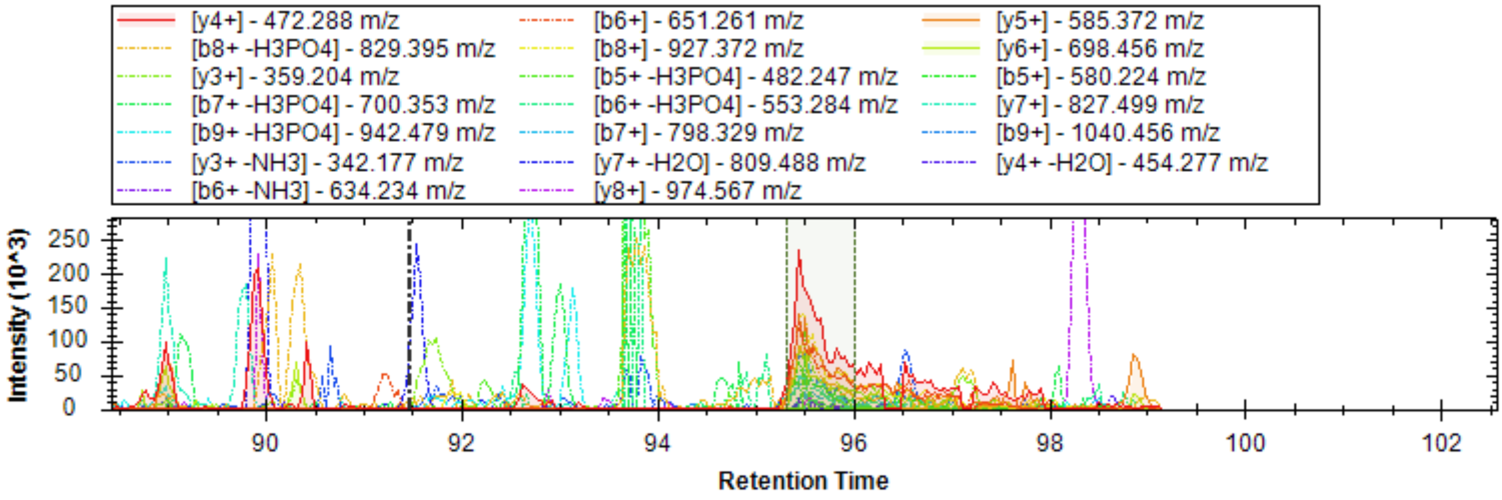

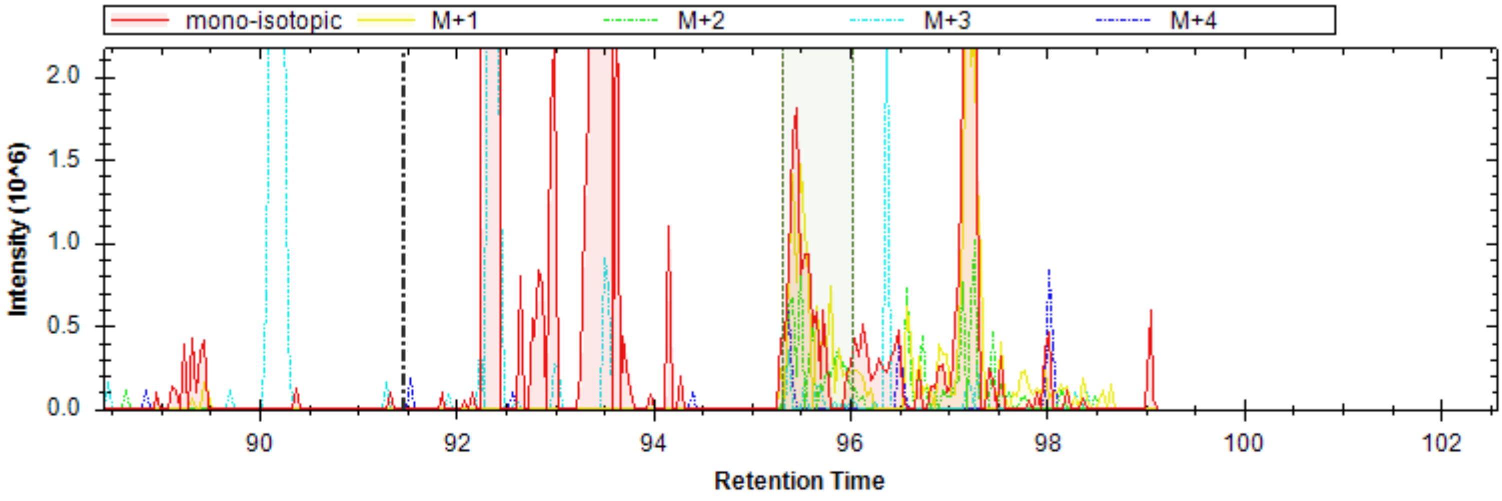

P54227 – _SKESVPDFPLS[+H+O3+P]PPK_.3

Fig4_MP-DIA-barrel-cortex-p9-3_MHRM.htrms

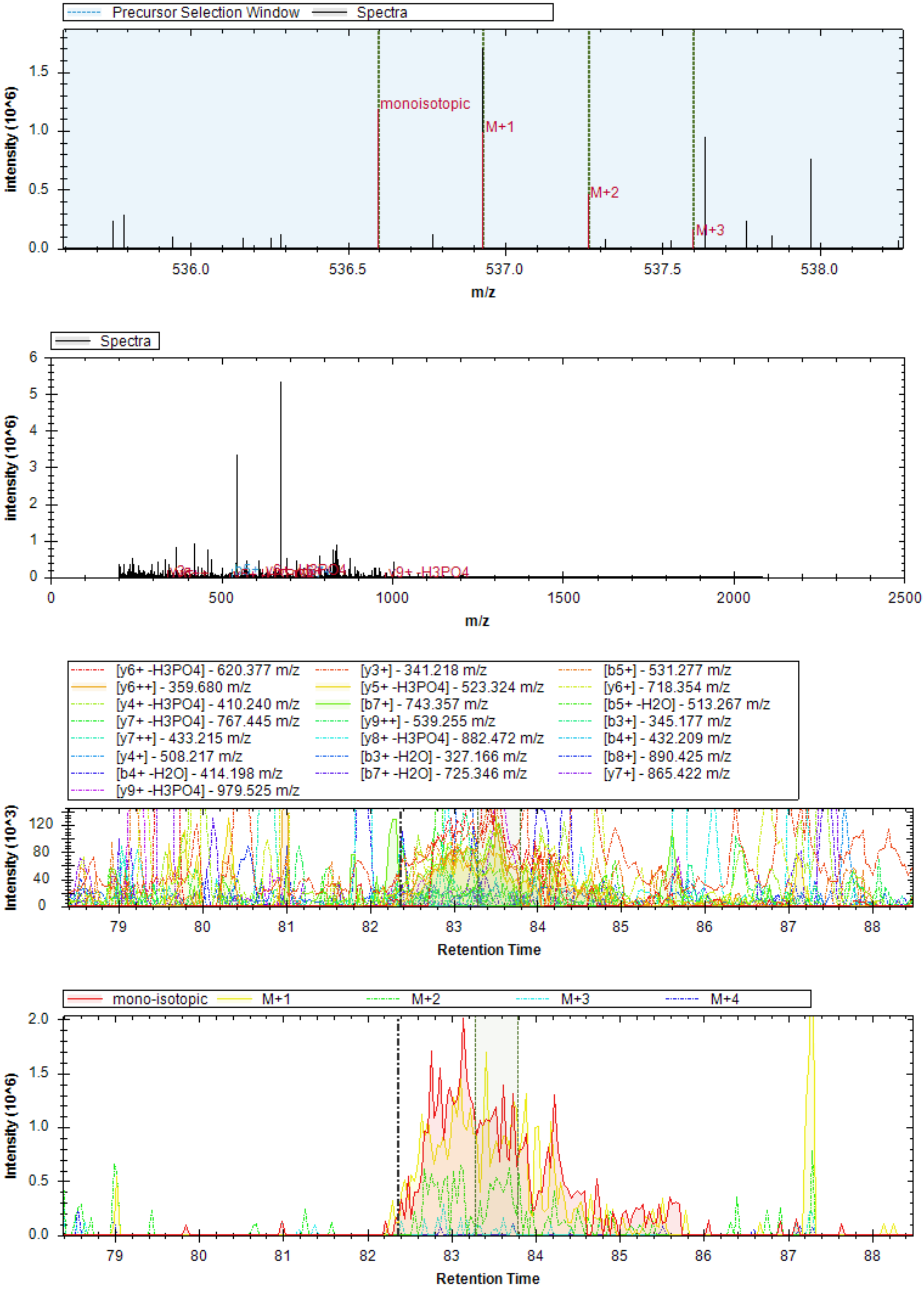

P63318 – _AAPALT[+H+O3+P]PPDR_.2

Fig4_MP-DIA-barrel-cortex-p9-3_MHRM.htrms

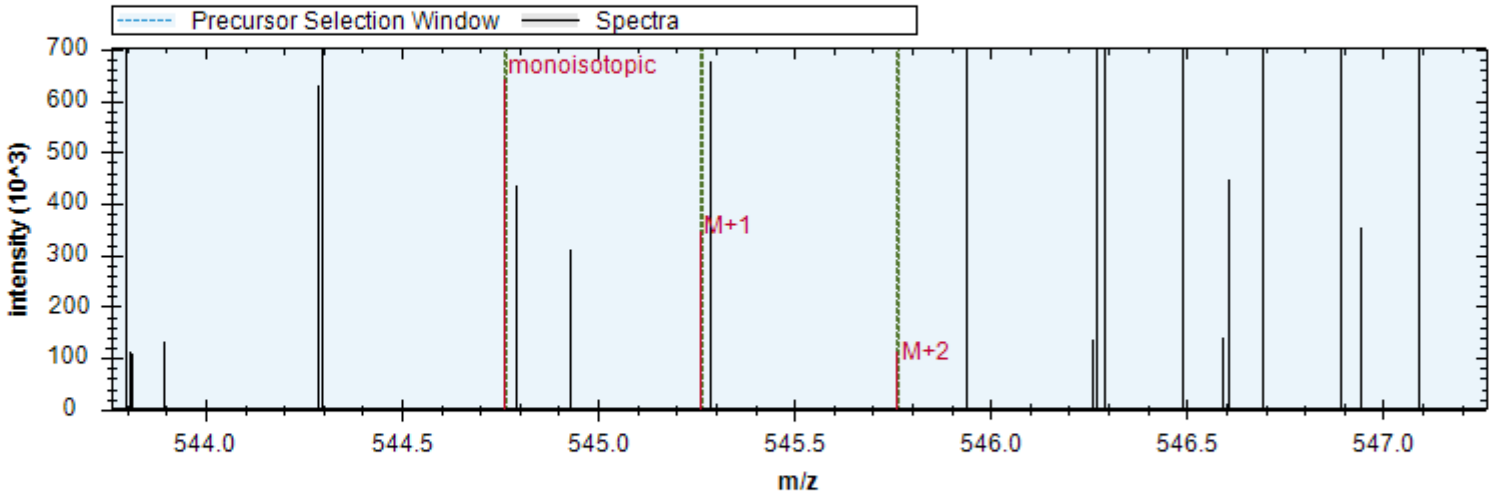

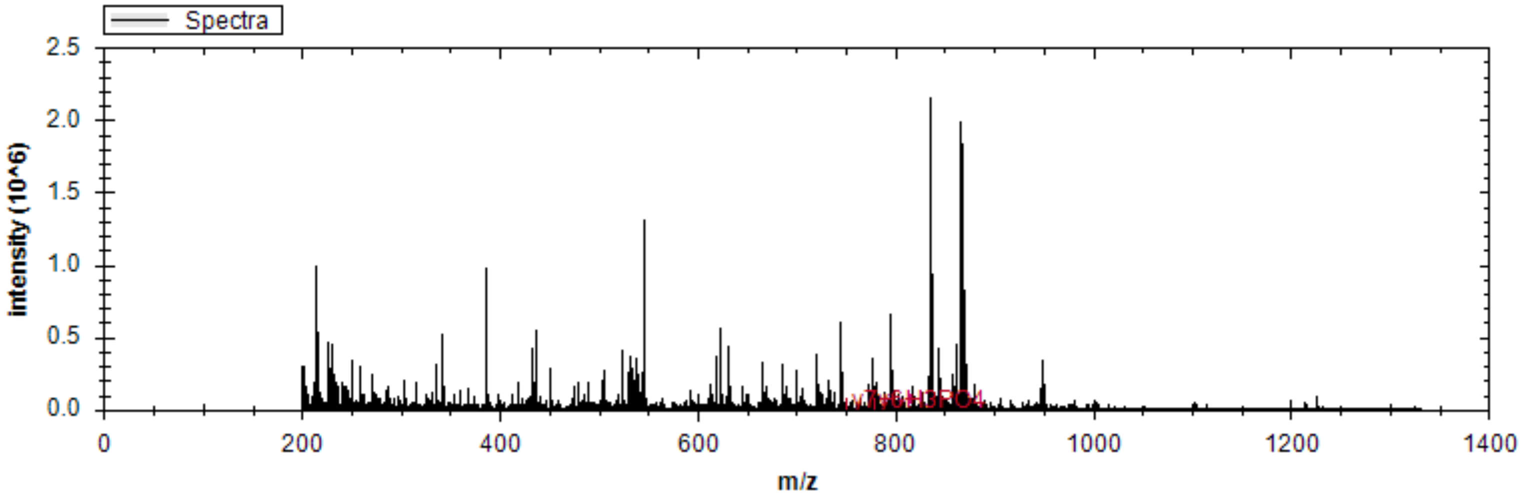

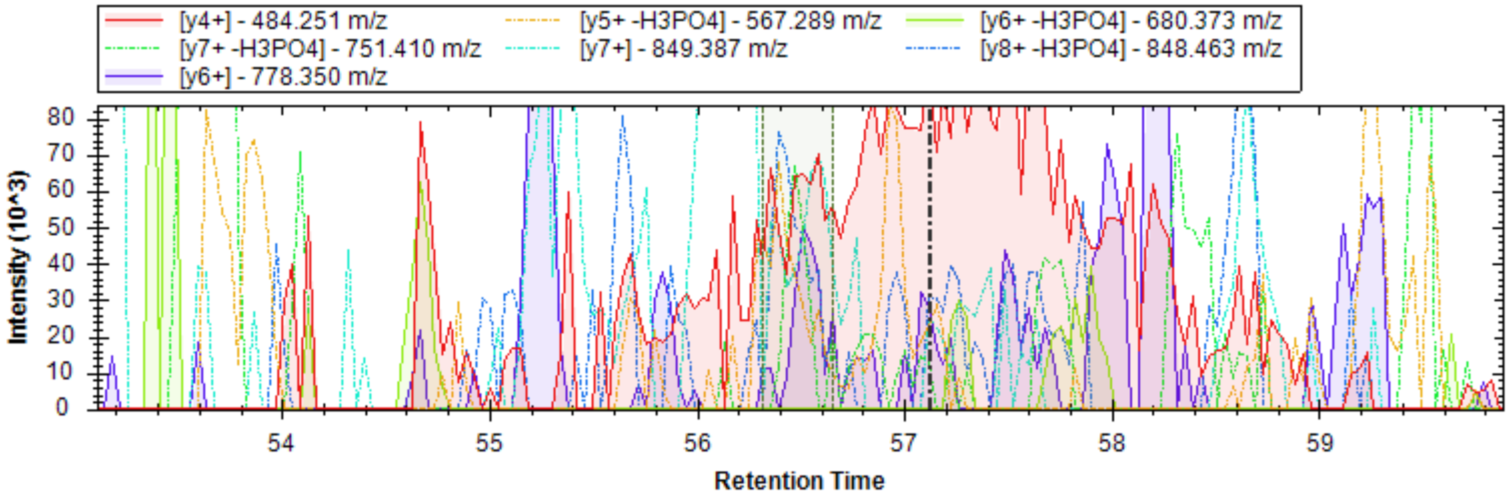

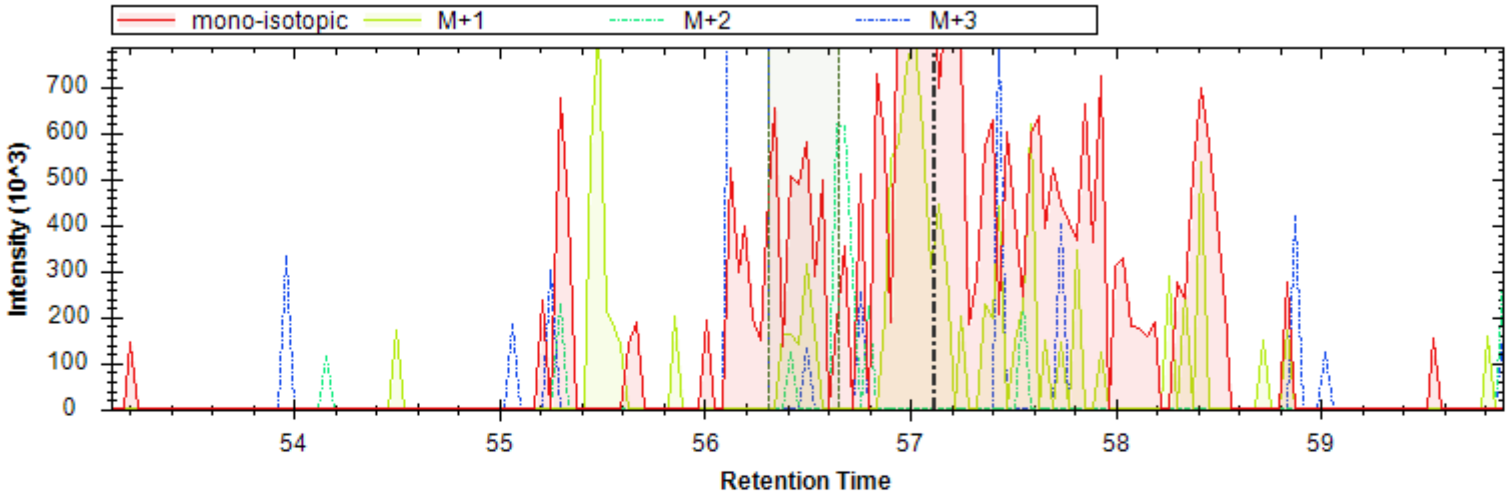

Q7TQD2 – _AANKT[+H+O3+P]PPKSPGDPAR_.3
Fig4_MP-DIA-barrel-cortex-p9-3_MHRM.htrms

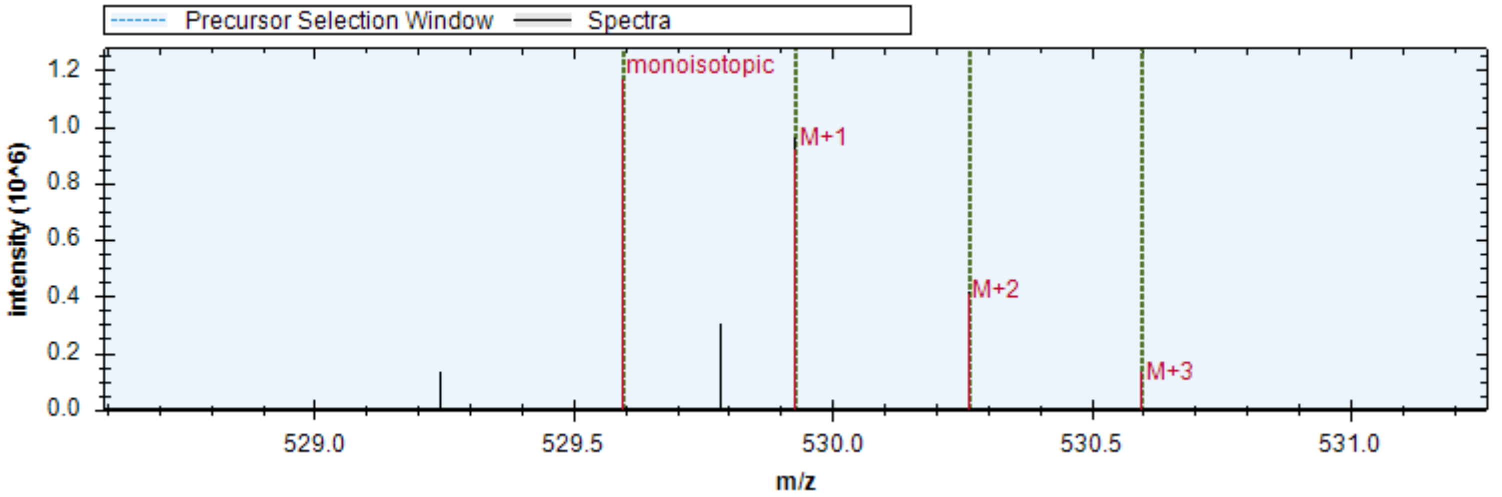

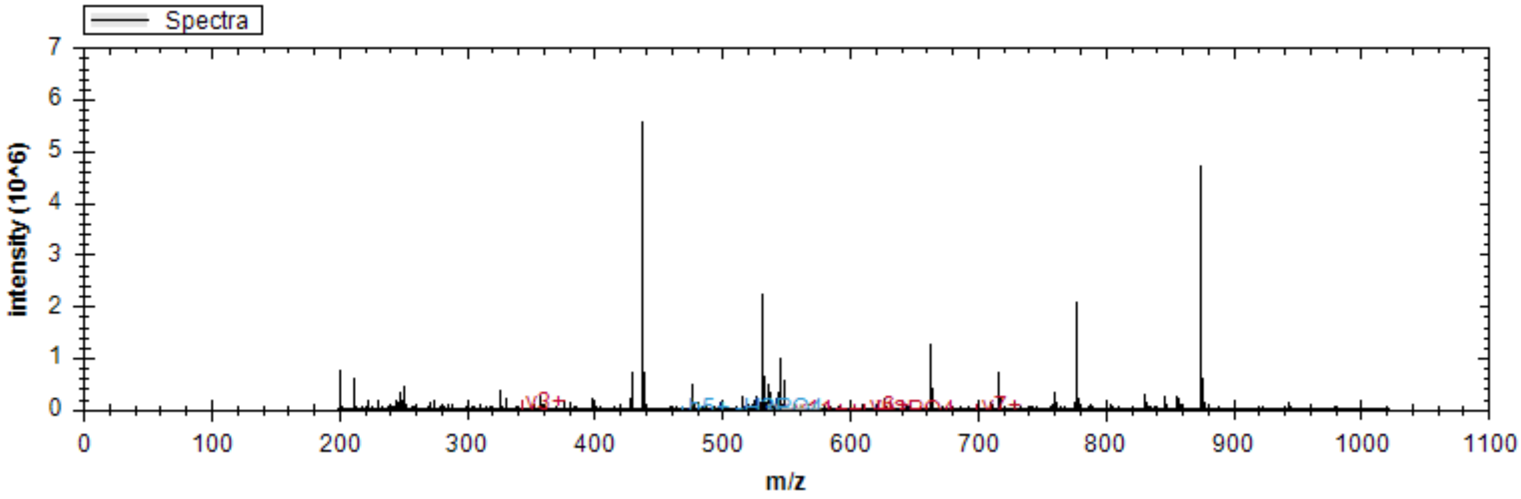

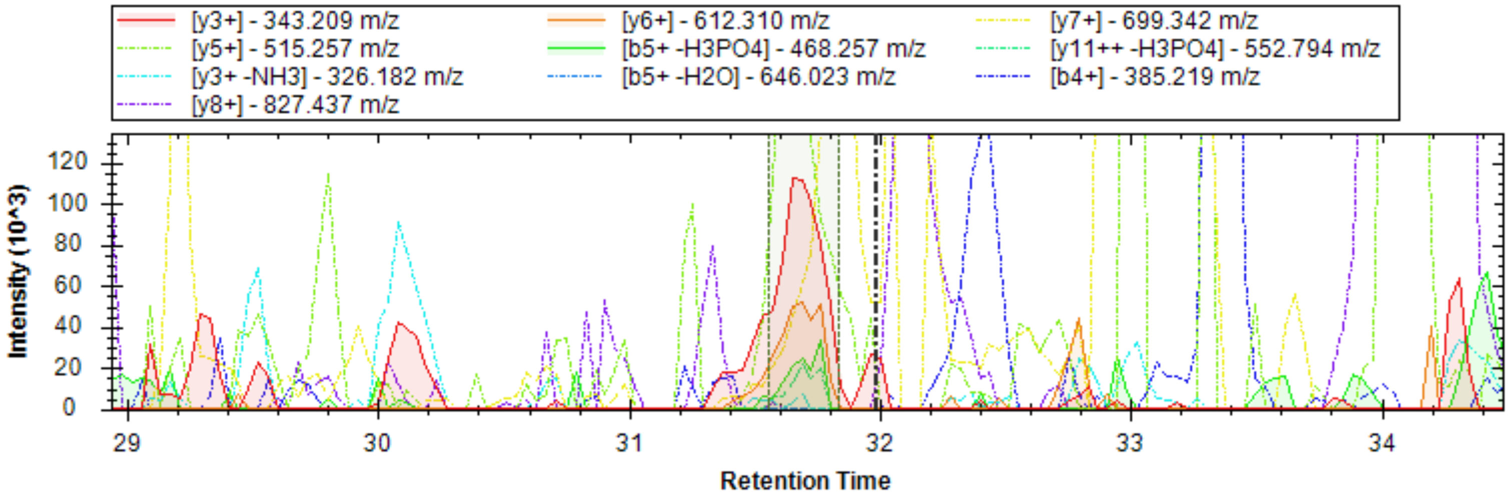

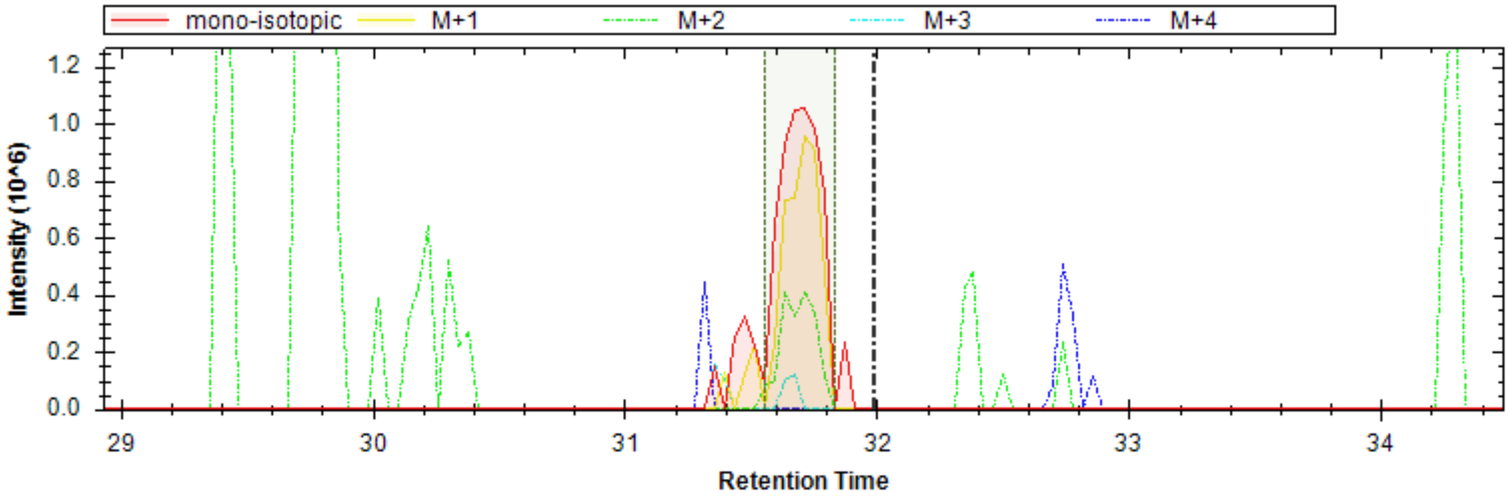

Q9QWI6 – _RFS[+H+O3+P]NVGLVHTSER_.3
Fig4_MP-DIA-barrel-cortex-p9-3_MHRM.htrms

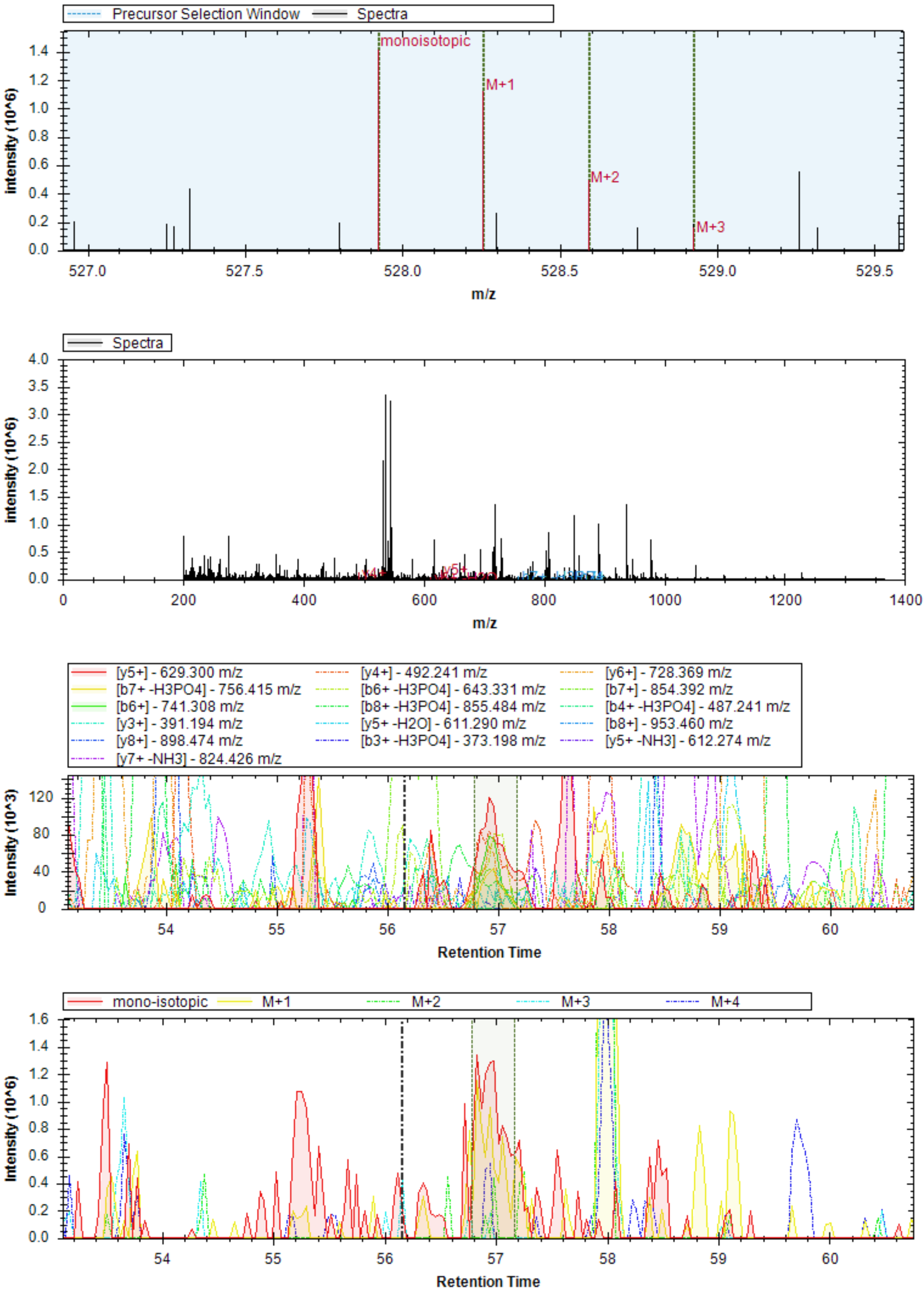

P10637 – _TTPSPKT[+H+O3+P]PPGSGEPPK_.3
Fig4_MP-DIA-barrel-cortex-p9-3_MHRM.htrms

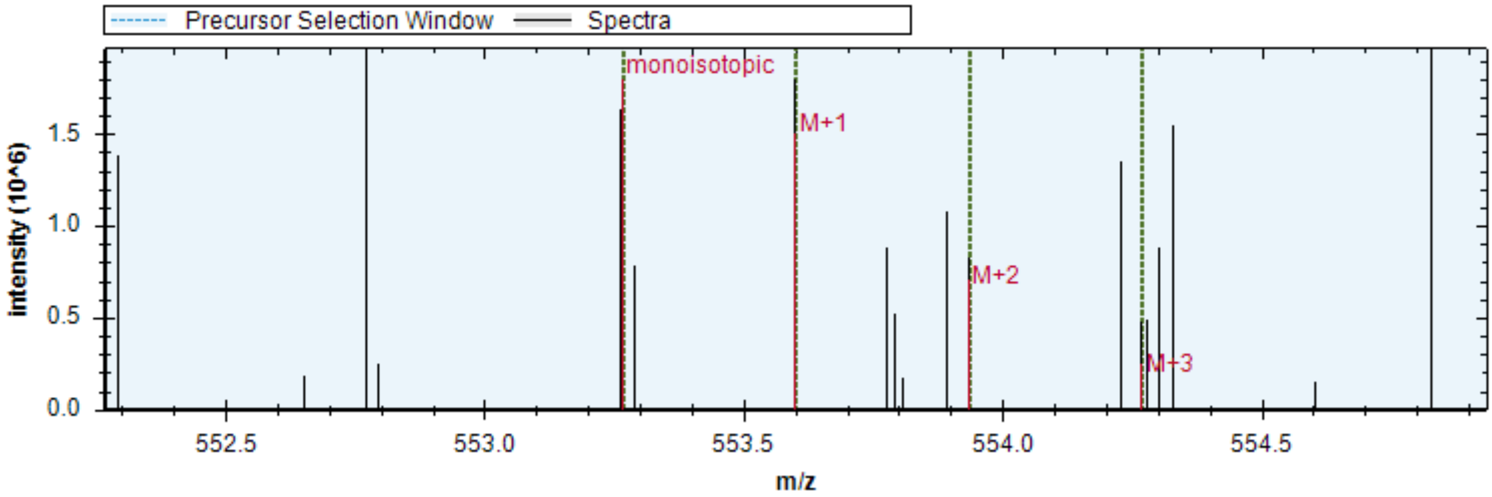

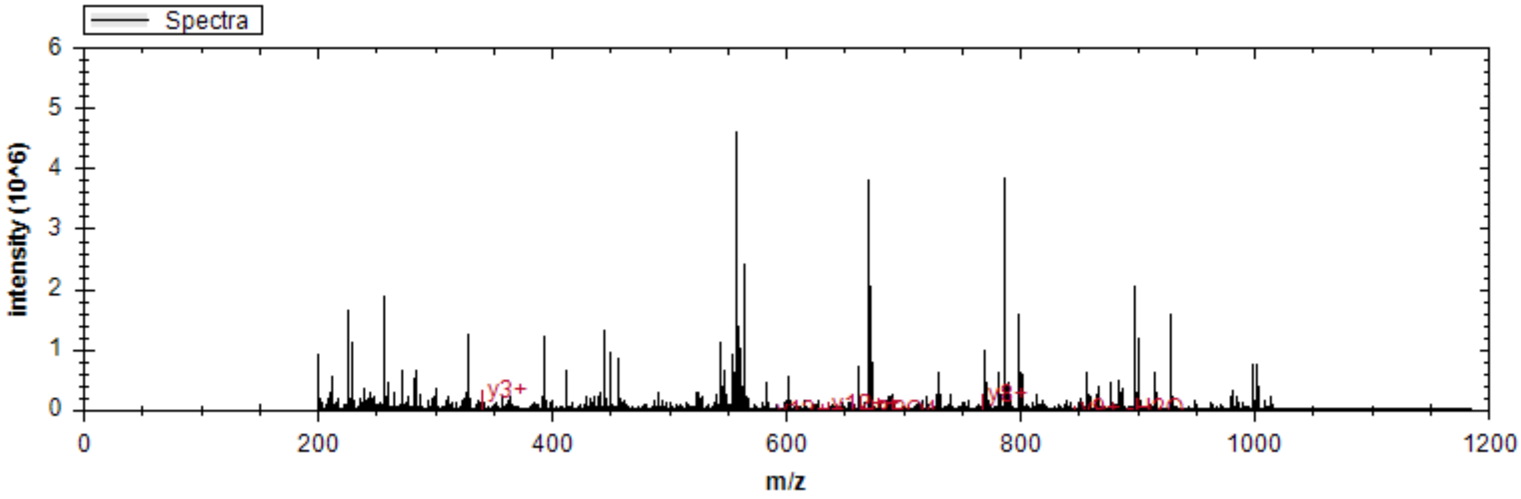

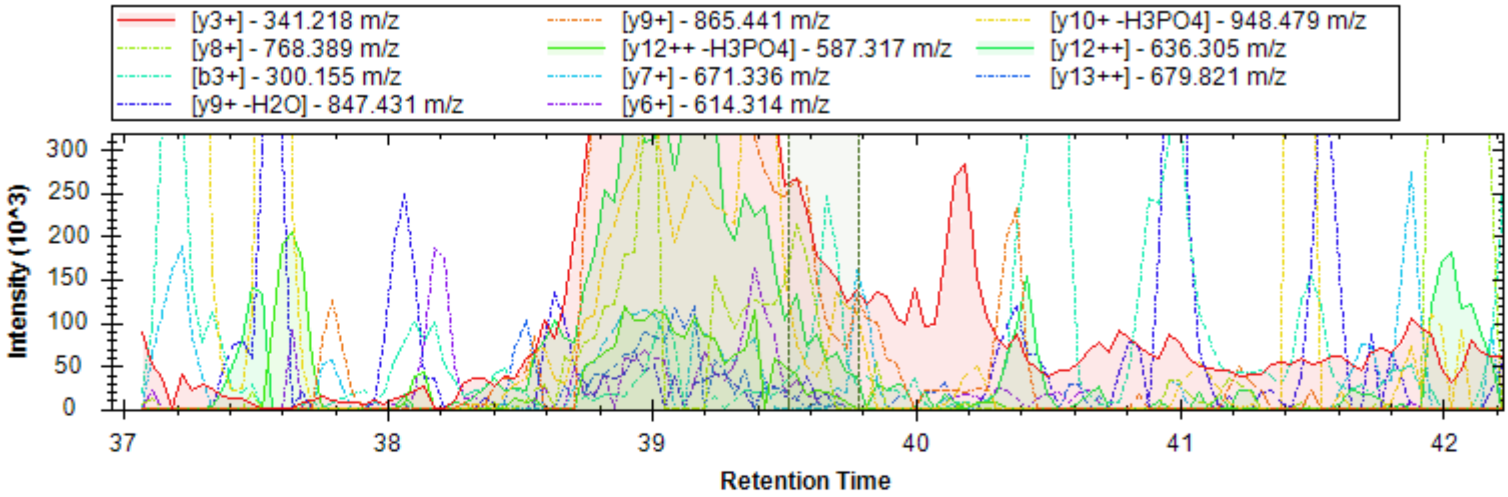

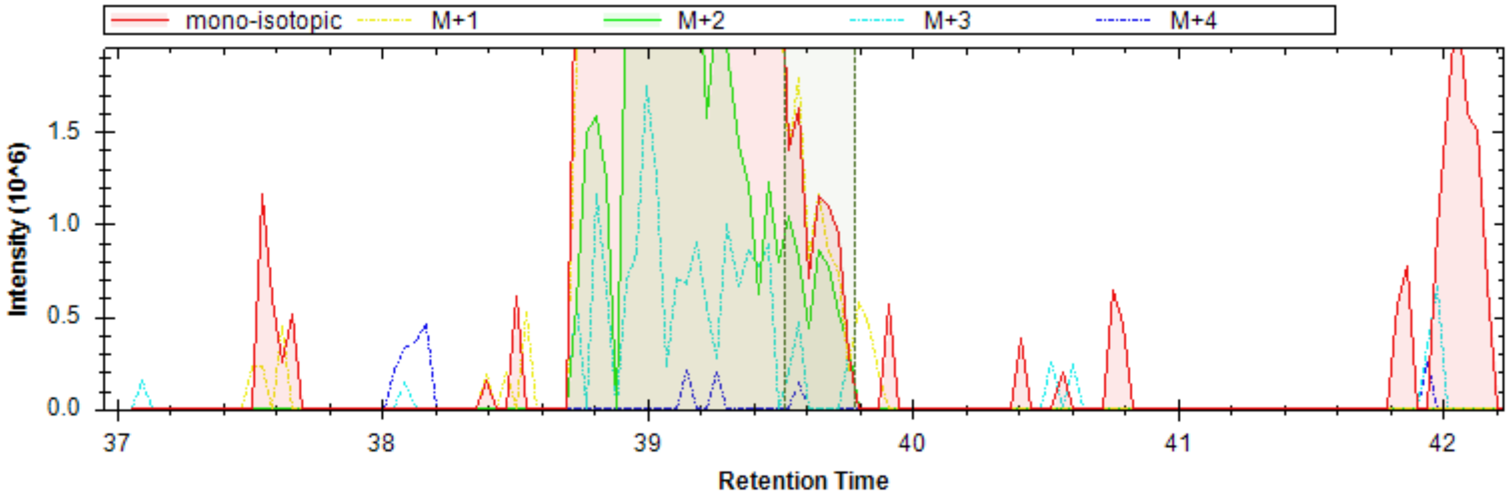

P20357 – _VAIIRT[+H+O3+P]PPKSPATPK_.3
Fig4_MP-DIA-barrel-cortex-p9-3_MHRM.htrms

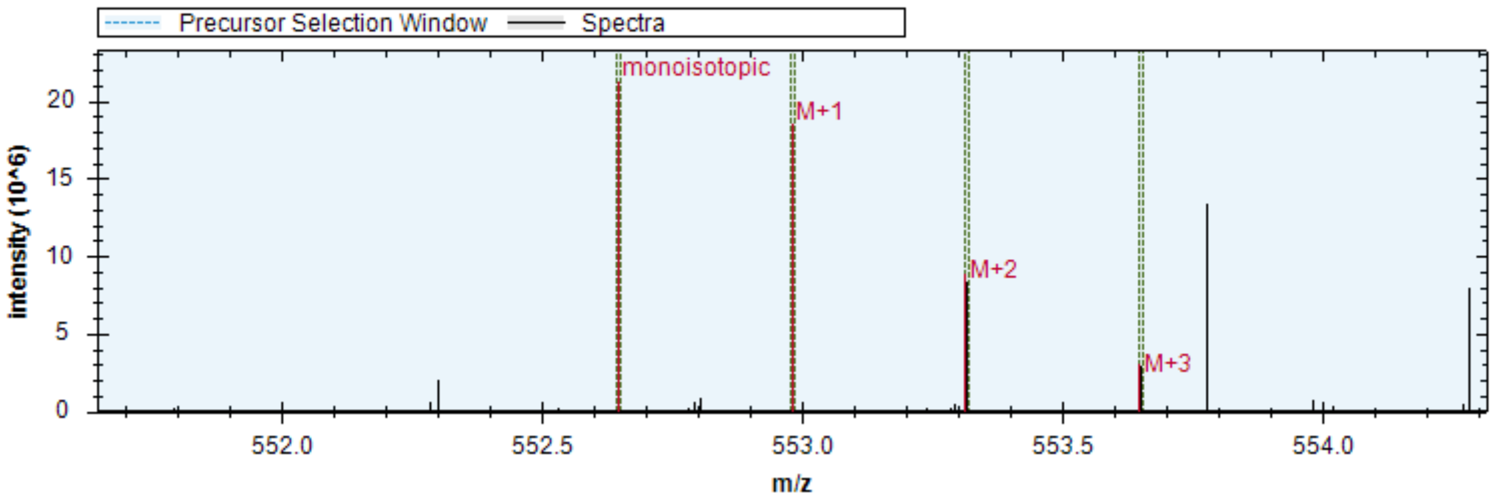

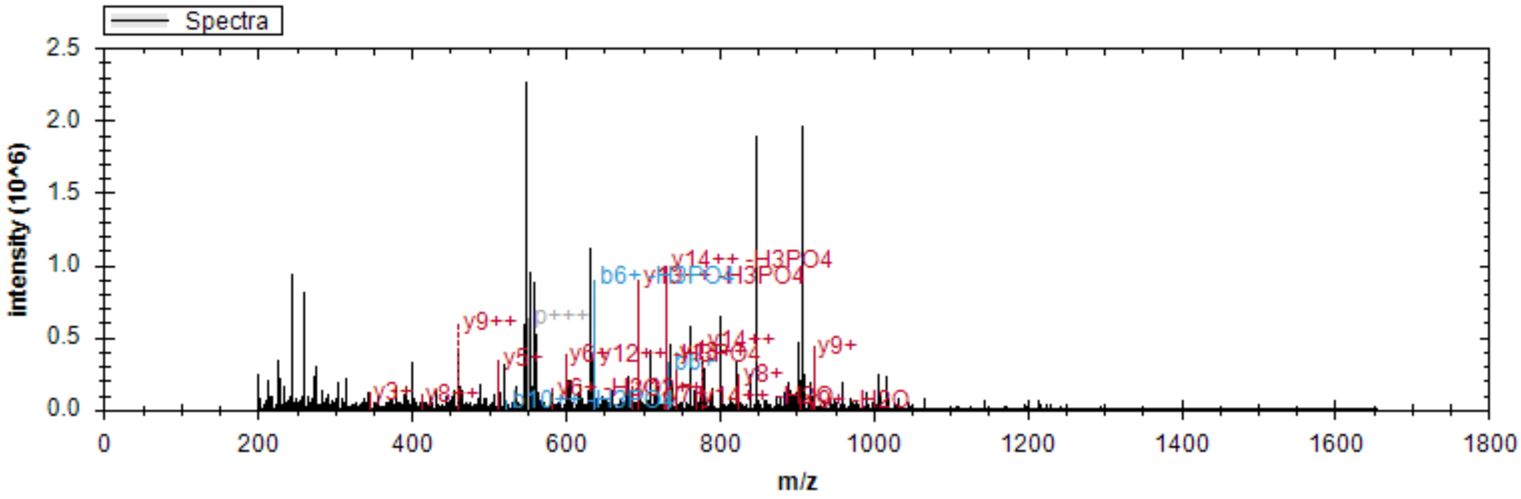

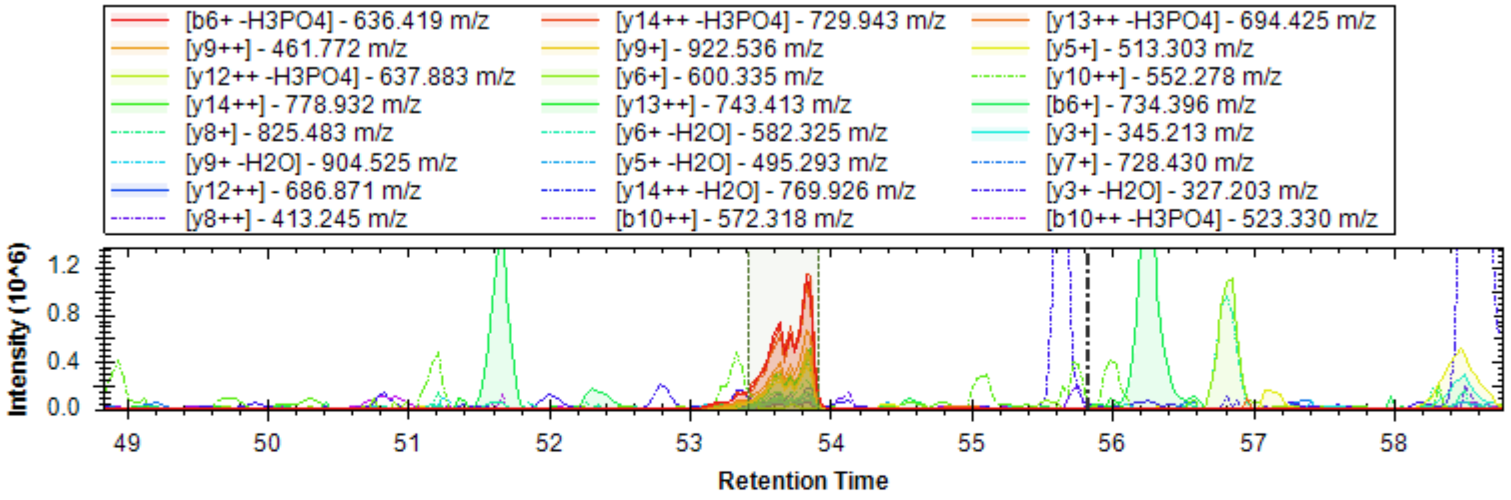

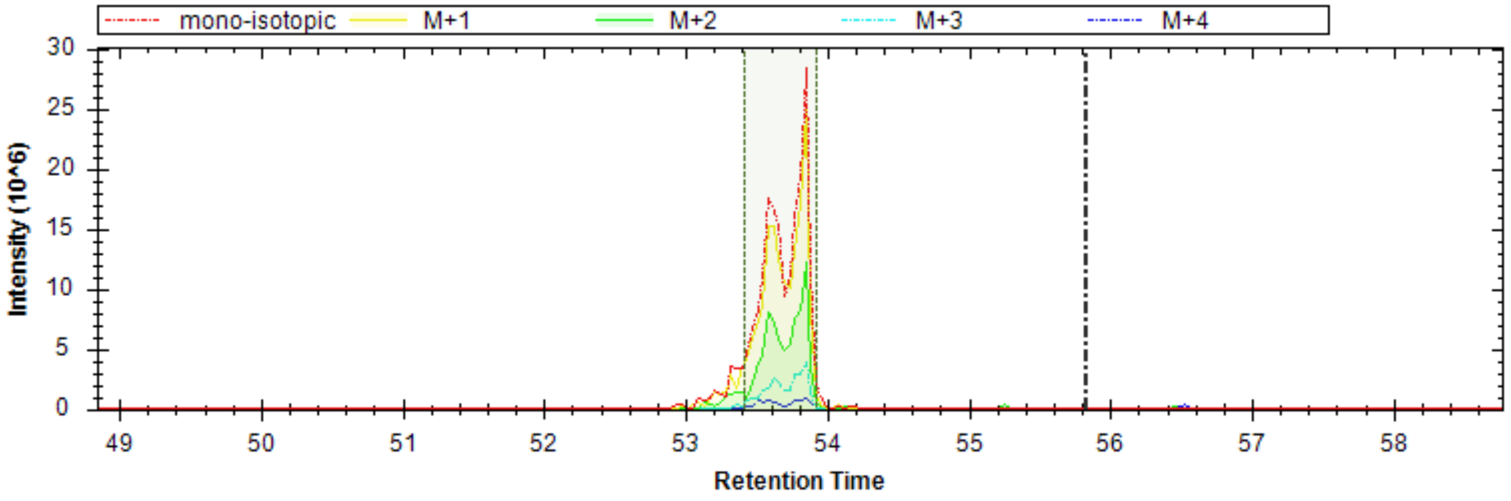

Q9CYT6 – _THTPSPTS[+H+O3+P]PK_.2
Fig4_MP-DIA-barrel-cortex-p9-3_MHRM.htrms

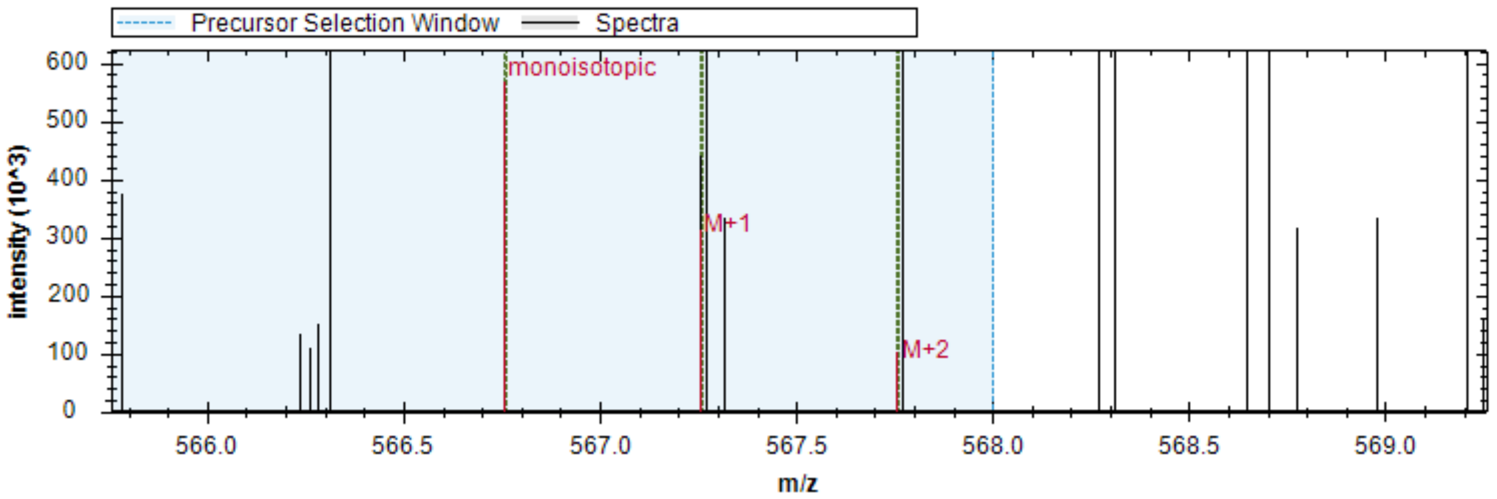

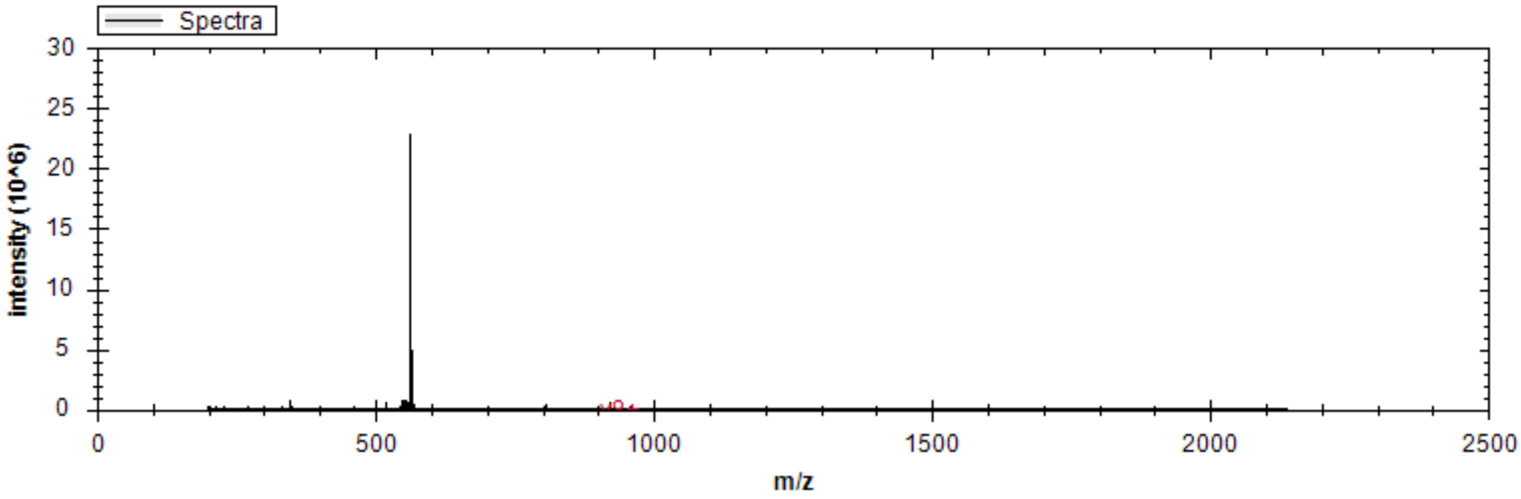

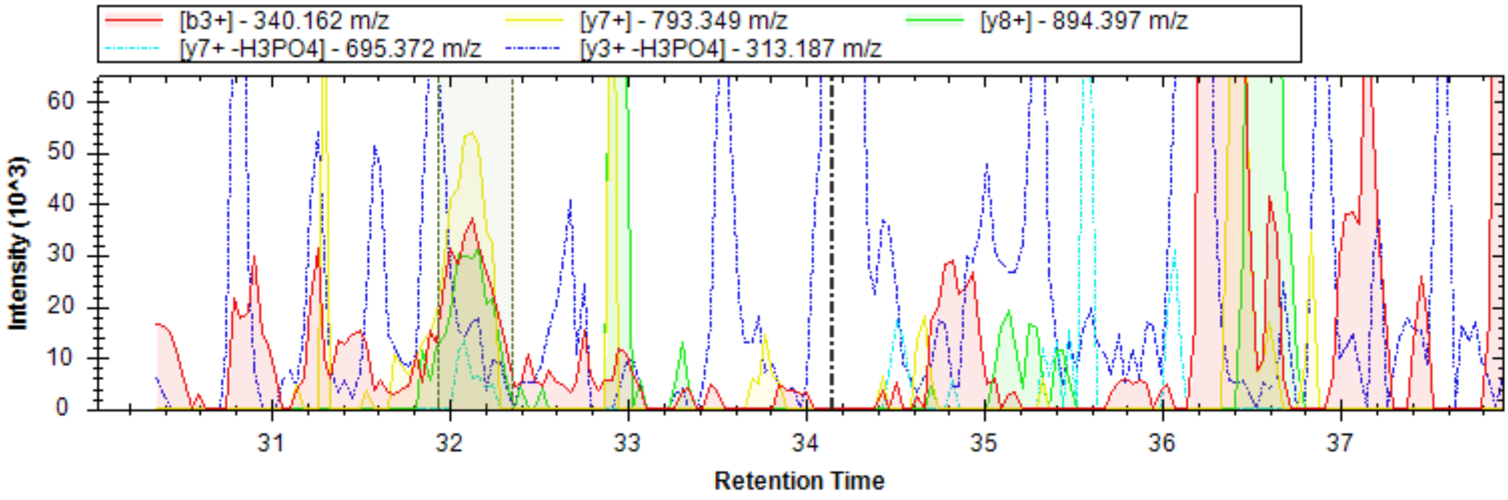

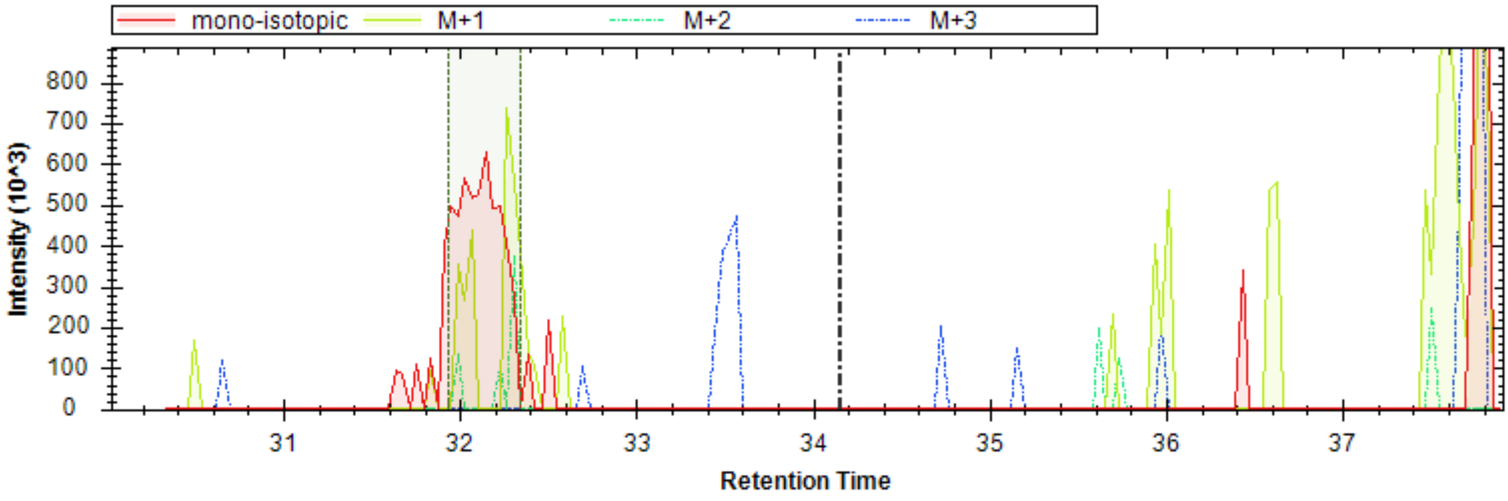

O88935 – _QAS[+H+O3+P]ISGPAPTK_.2

Fig4_MP-DIA-barrel-cortex-p9-3_MHRM.htrms

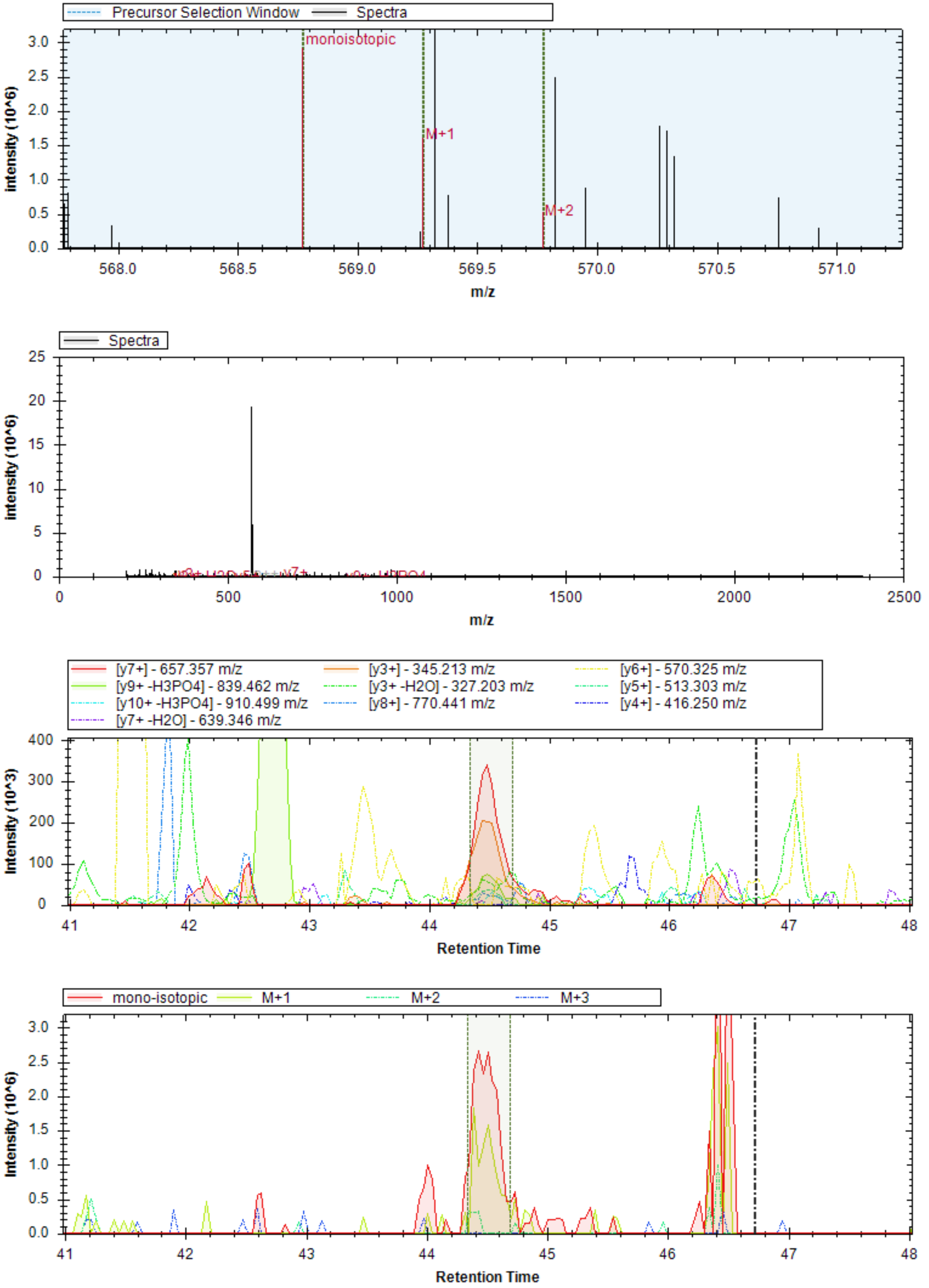

P10637 – _TPS[+H+O3+P]LPTPPTR_.2
Fig4_MP-DIA-barrel-cortex-p9-3_MHRM.htrms

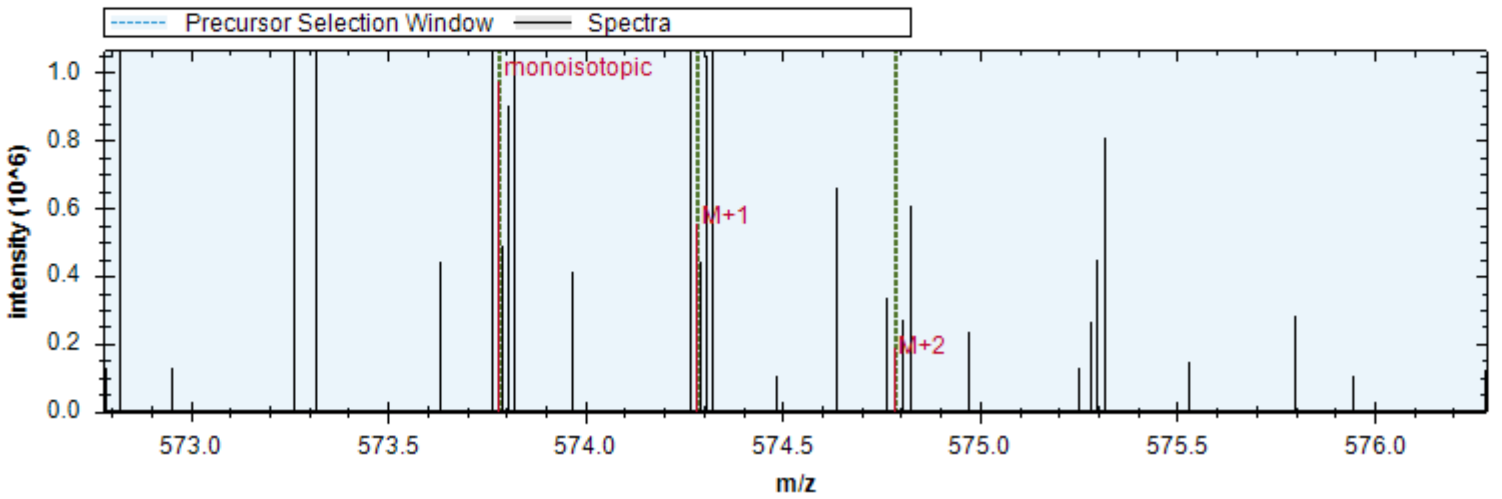

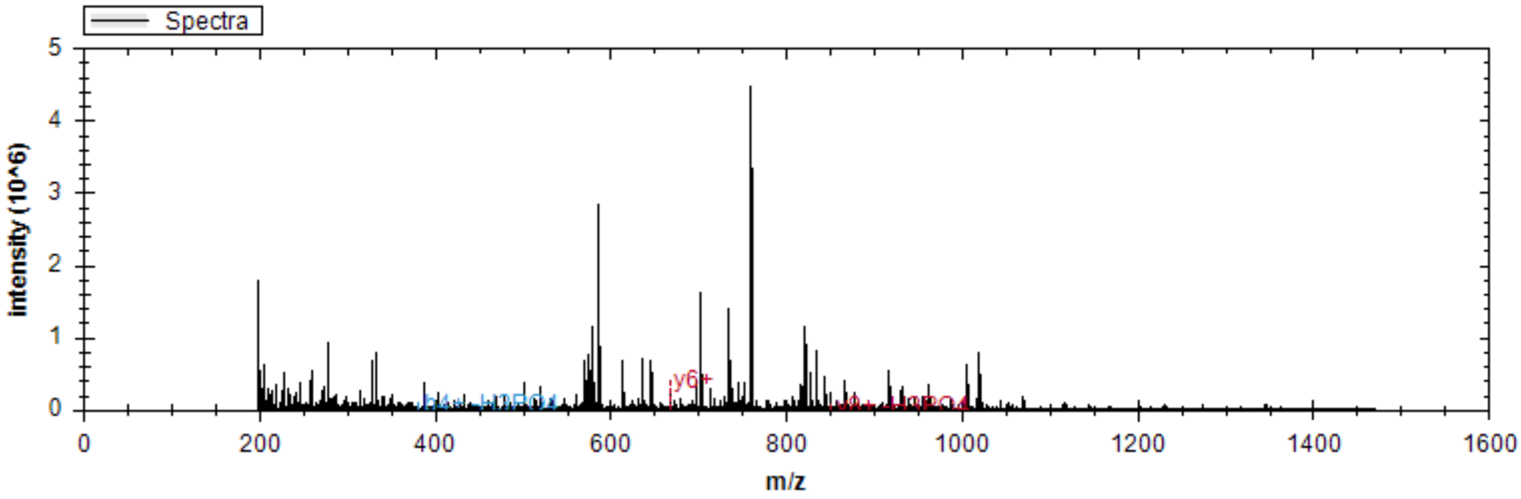

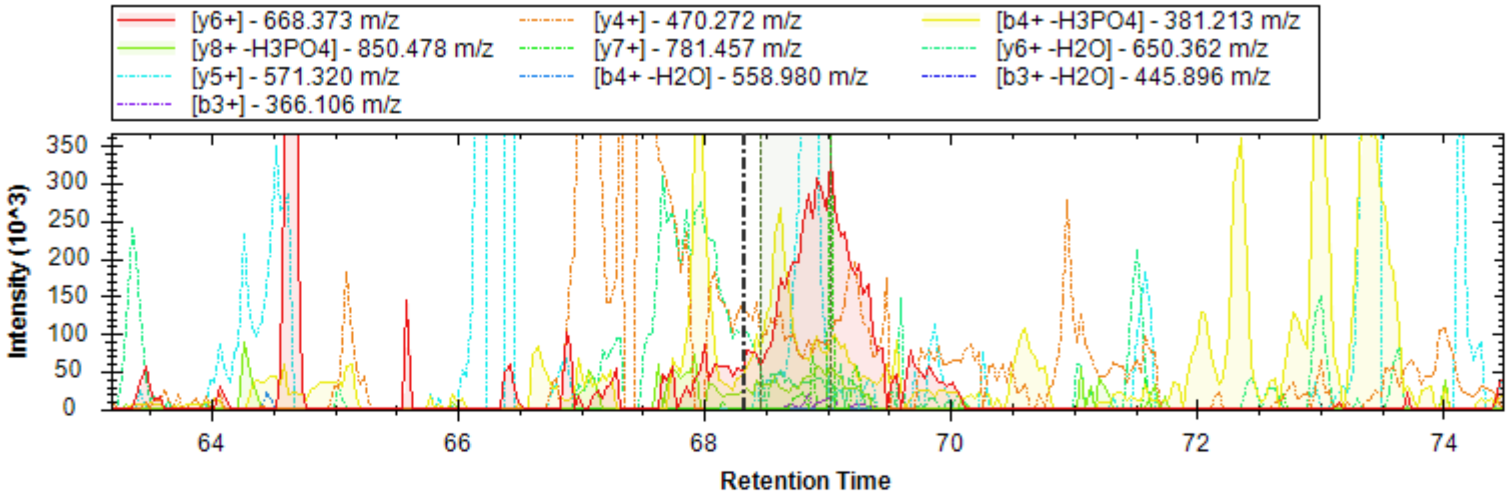

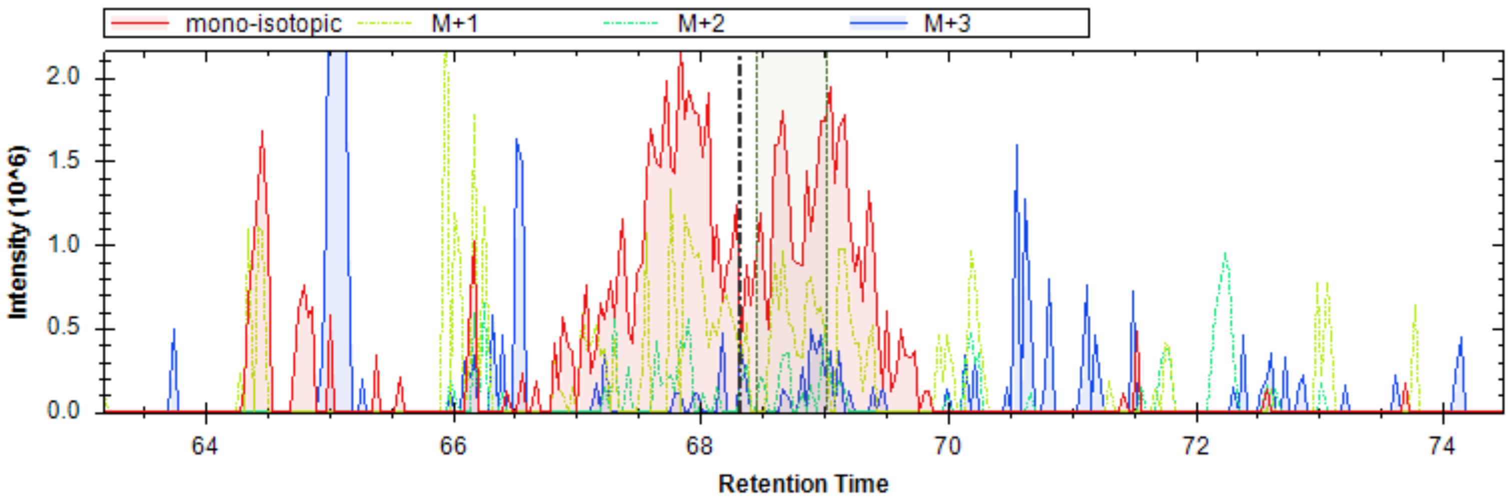

P27546-2 – _T[+H+O3+P]SPSKPSSAPALKPGPK_.3
Fig4_MP-DIA-barrel-cortex-p9-3_MHRM.htrms

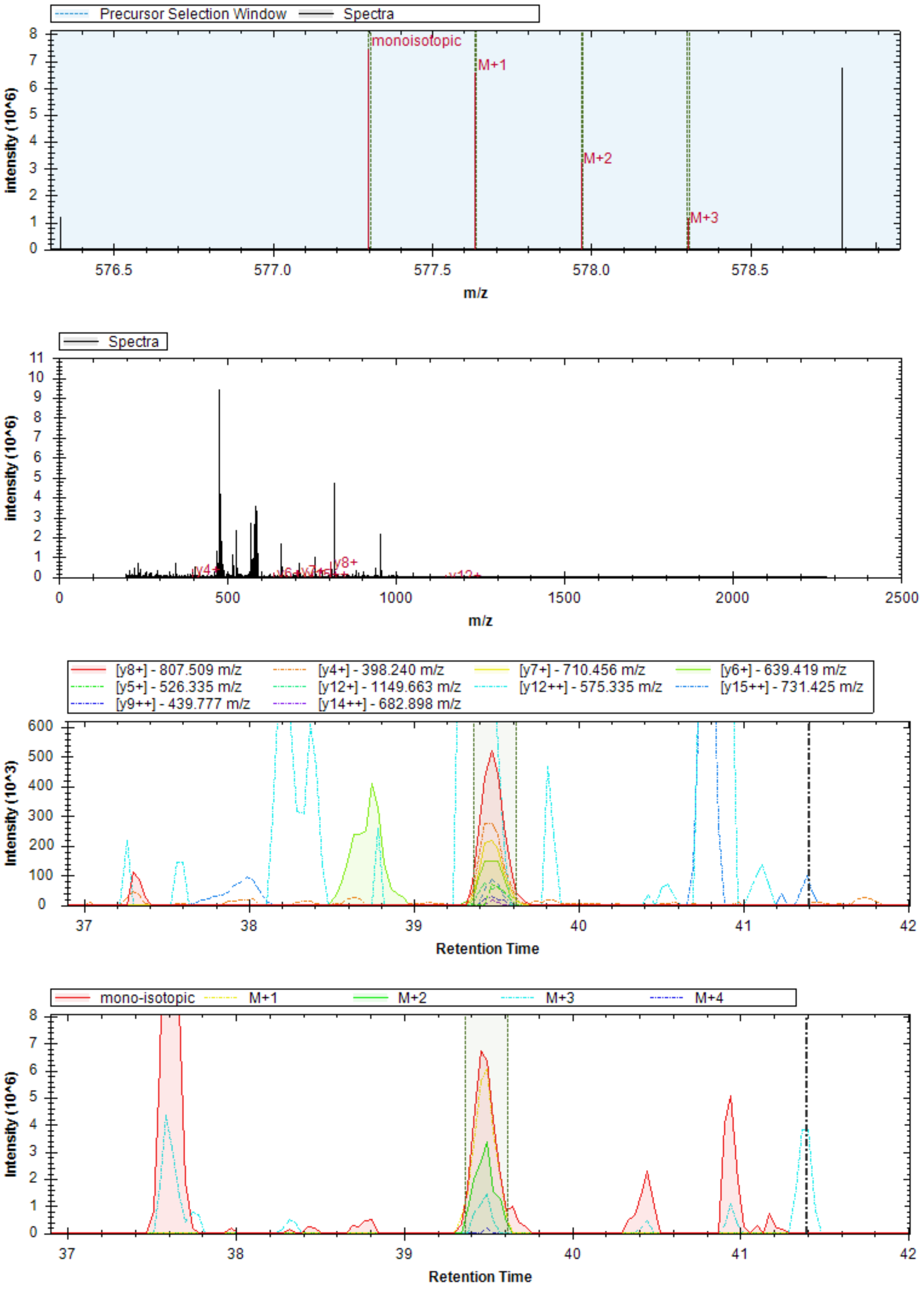

P28667 – _GEVAPKET[+H+O3+P]PK_.2
Fig4_MP-DIA-barrel-cortex-p9-3_MHRM.htrms

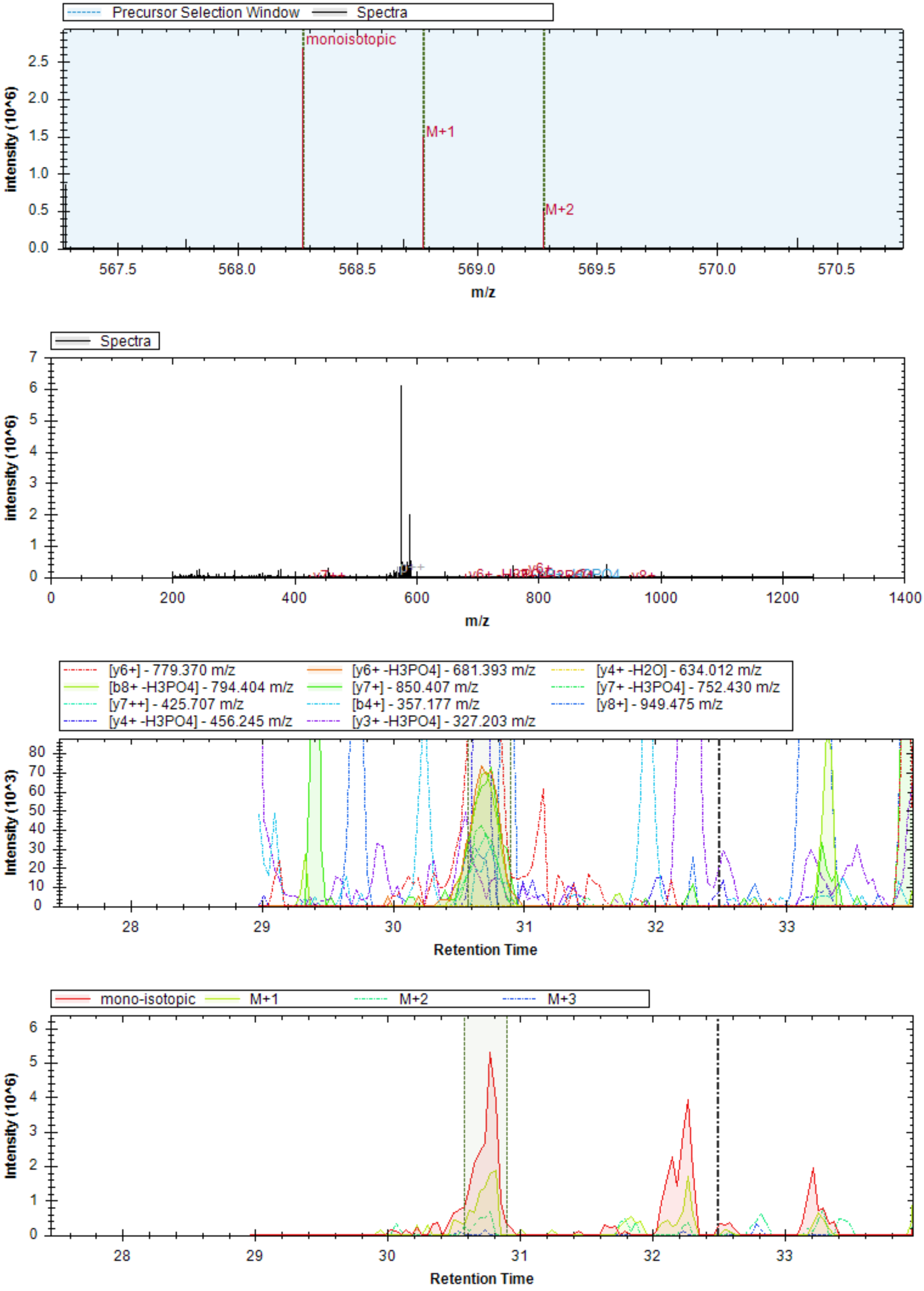

P97792 – _APQS[+H+O3+P]PTLAPAK_.2
Fig4_MP-DIA-barrel-cortex-p9-3_MHRM.htrms

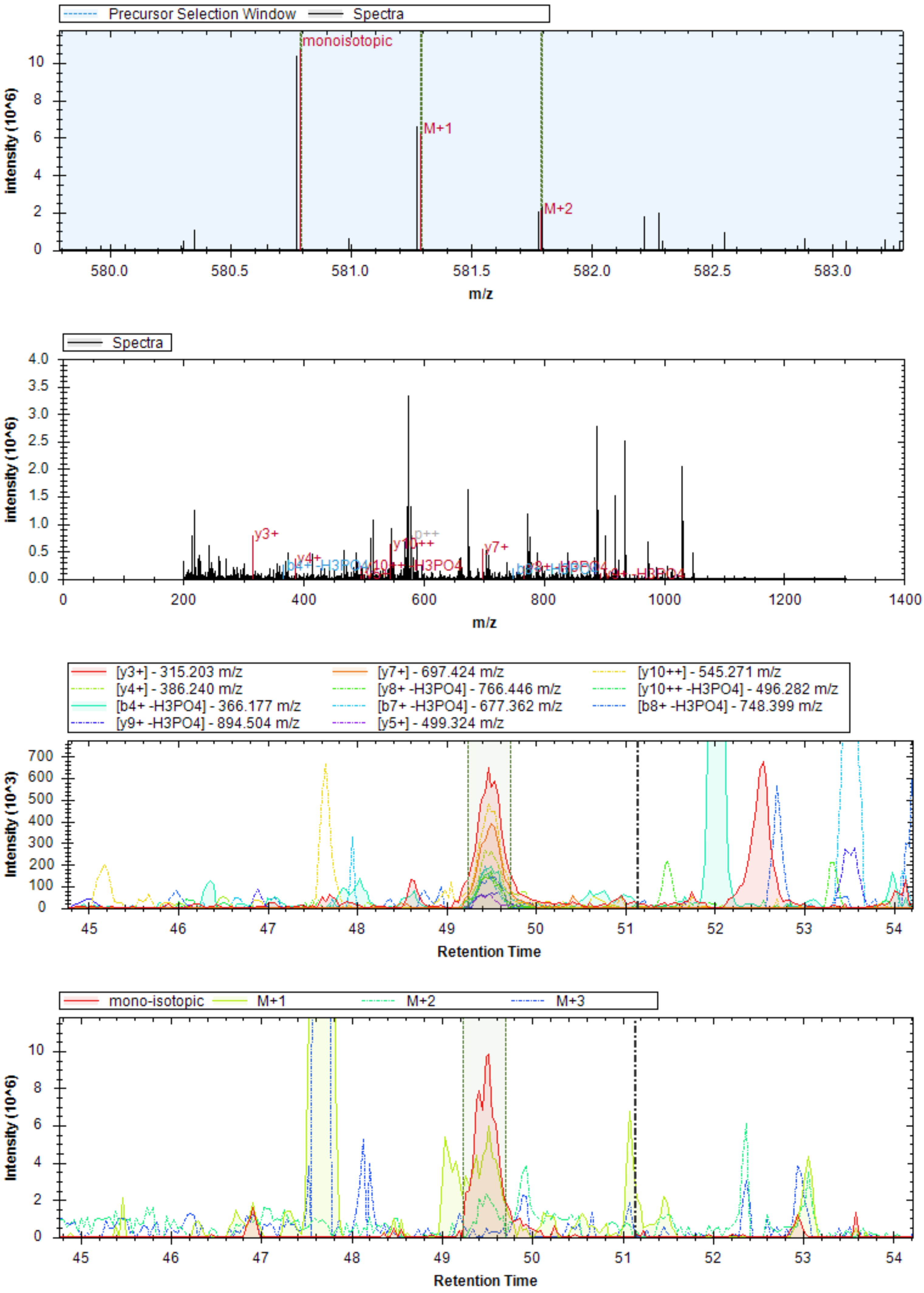

Q64012-2 – _GRLS[+H+O3+P]PVPVPR_.2
Fig4_MP-DIA-barrel-cortex-p9-3_MHRM.htrms

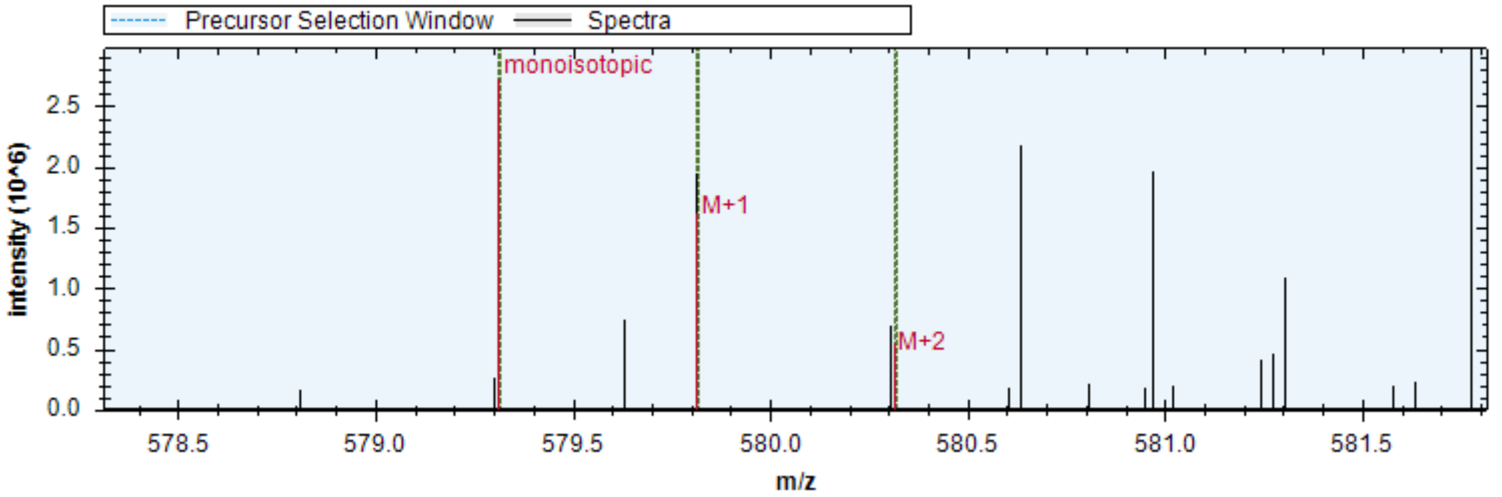

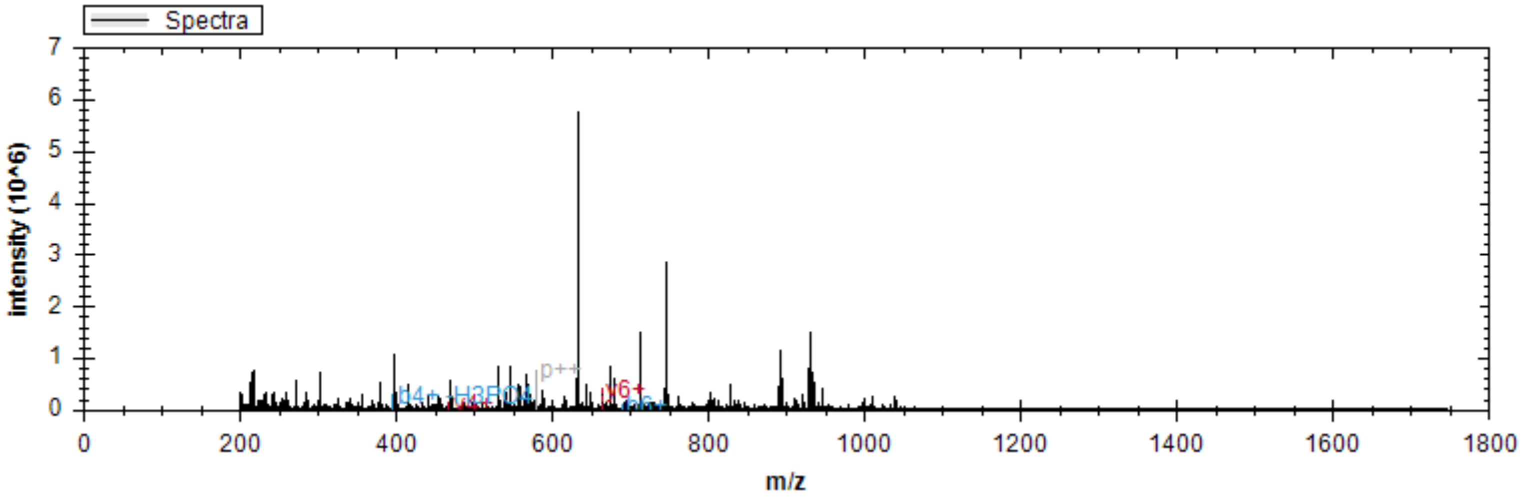

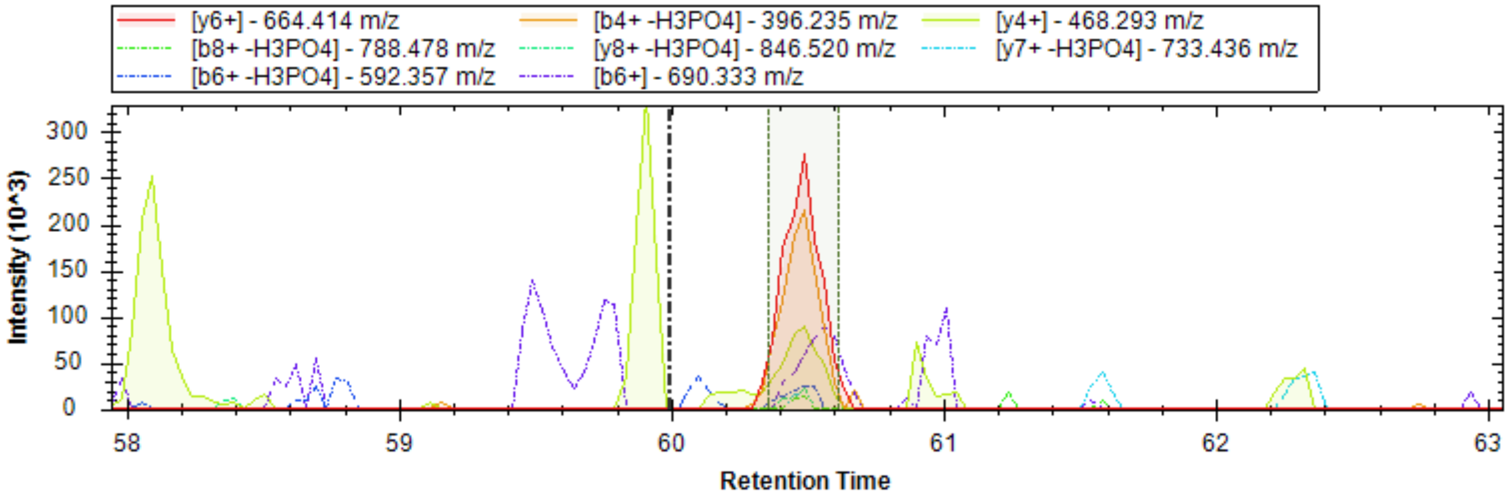

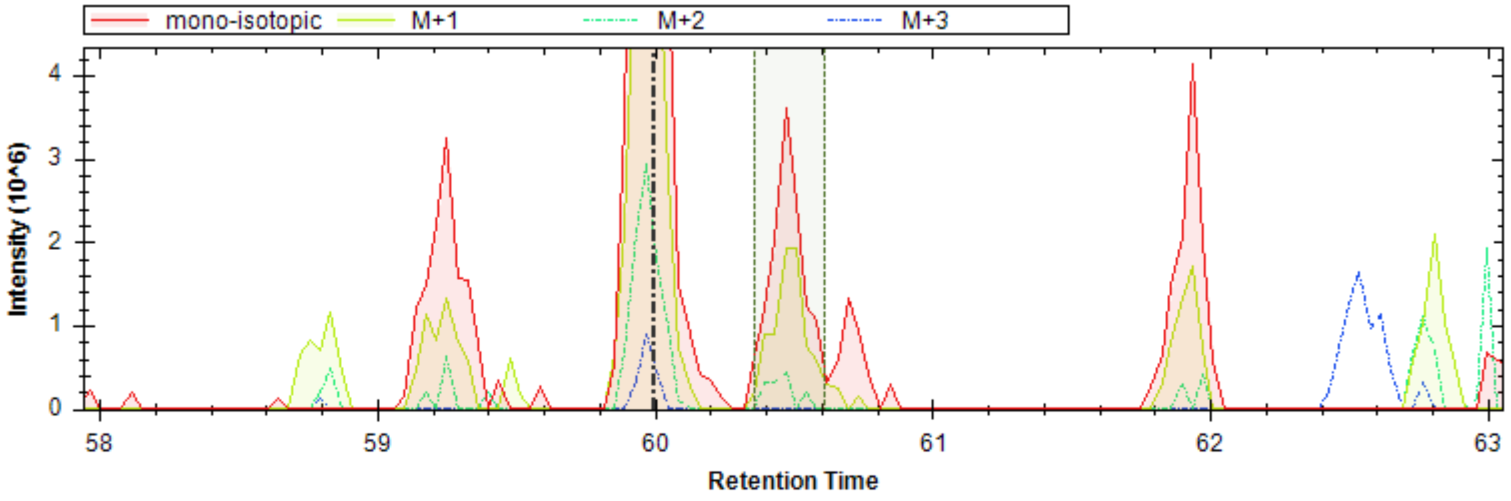

Q8BFU3-4 – _VAS[+H+O3+P]STQVRGASGRELR_.3

Fig4_MP-DIA-barrel-cortex-p9-3_MHRM.htrms

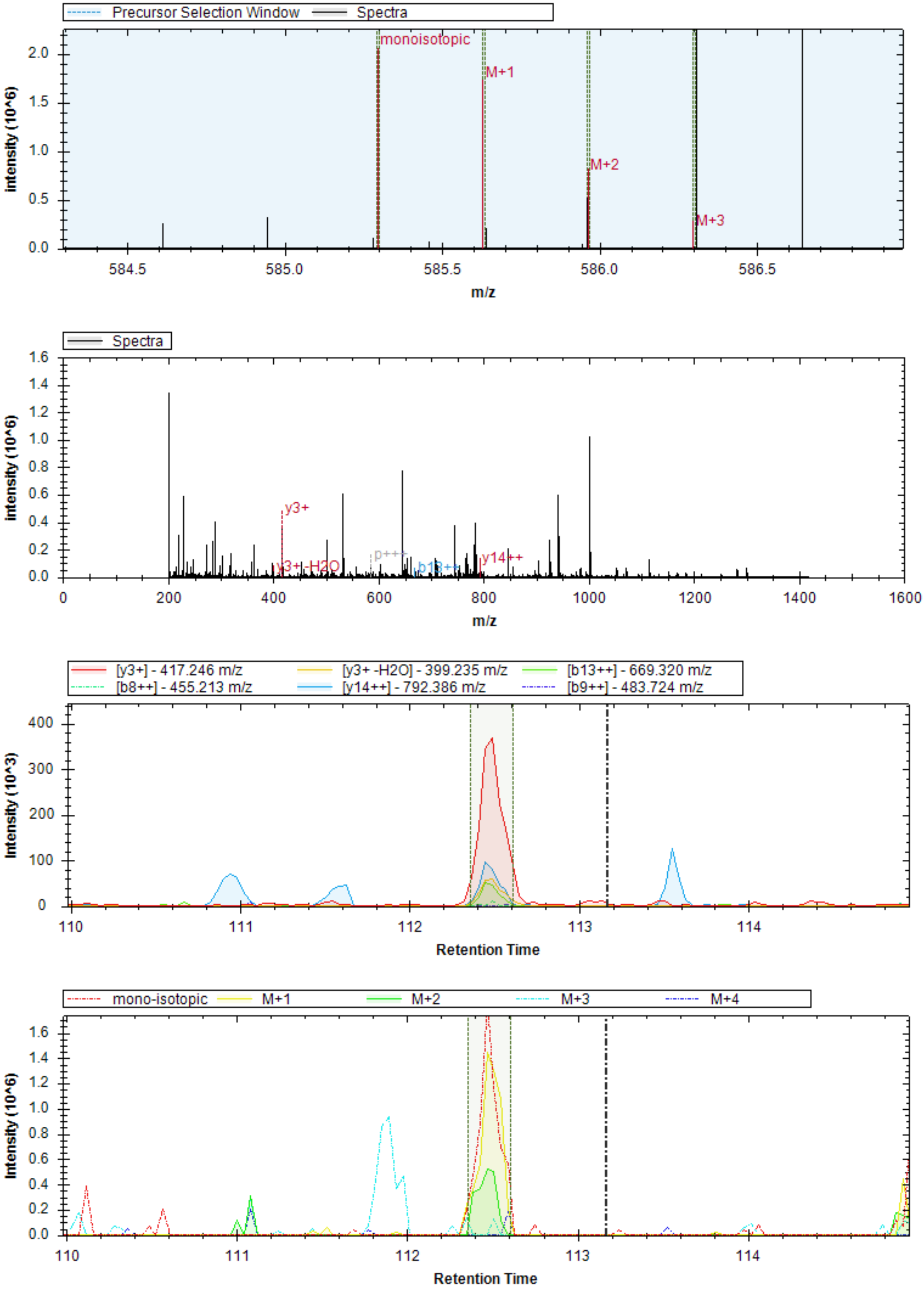

Q8BTI8 – _RVPS[+H+O3+P]PTPVPK_.2

Fig4_MP-DIA-barrel-cortex-p9-3_MHRM.htrms

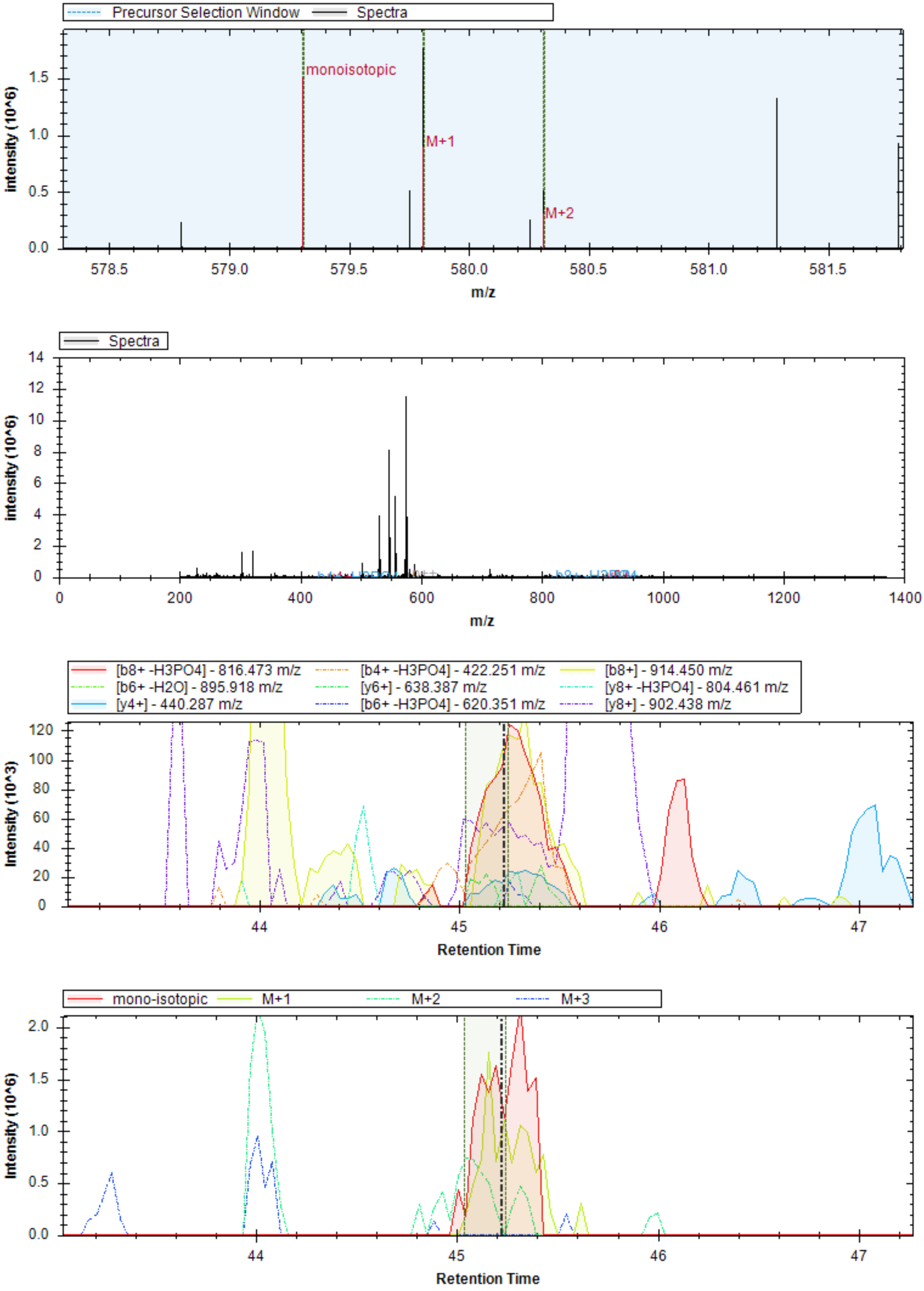

Q9CYT6 – _AQGQIRS[+H+O3+P]PTK_.2
Fig4_MP-DIA-barrel-cortex-p9-3_MHRM.htrms

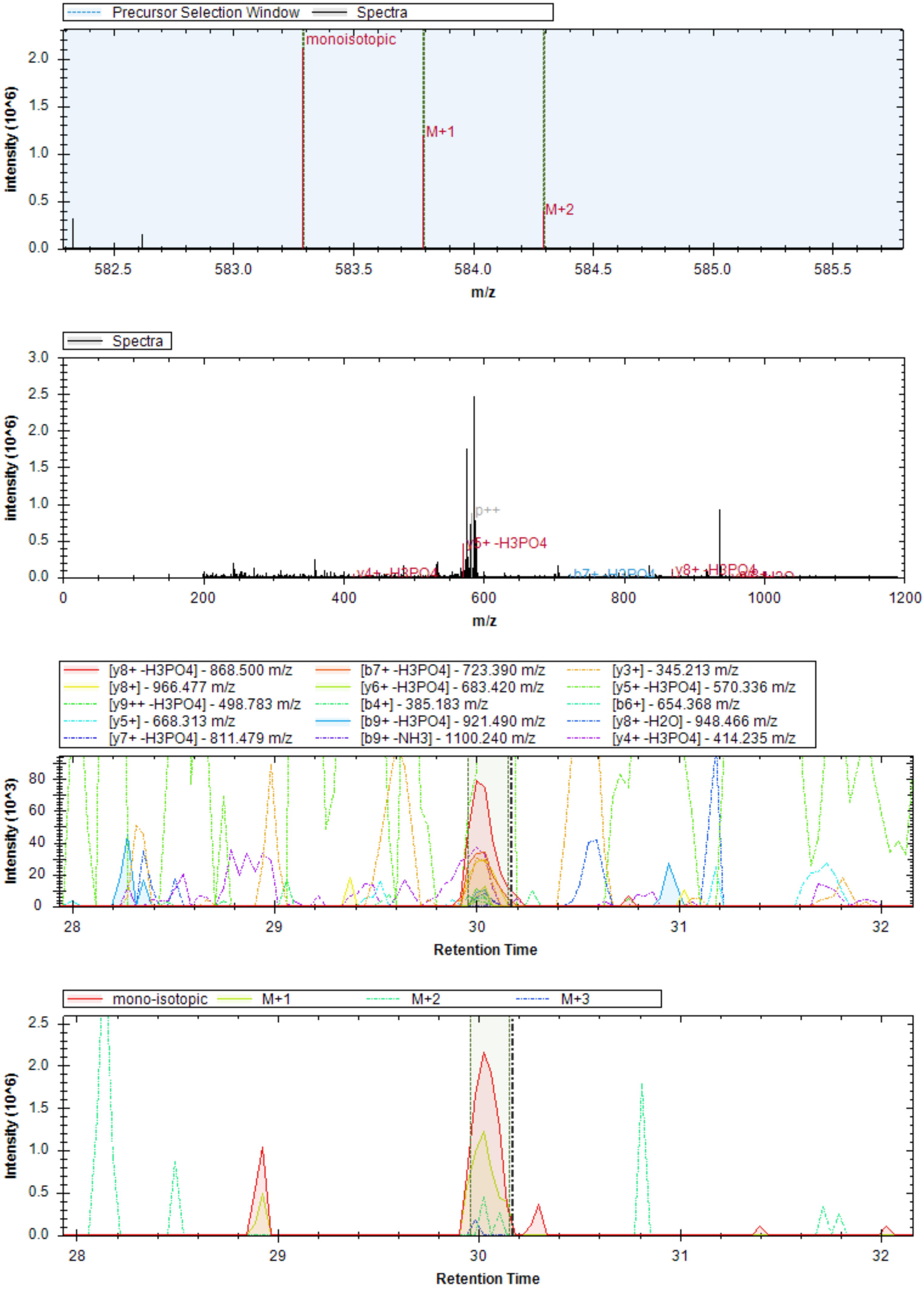

Q9D5Y0 – _ITDT[+H+O3+P]KKTDDLYWR_.3
Fig4_MP-DIA-barrel-cortex-p9-3_MHRM.htrms

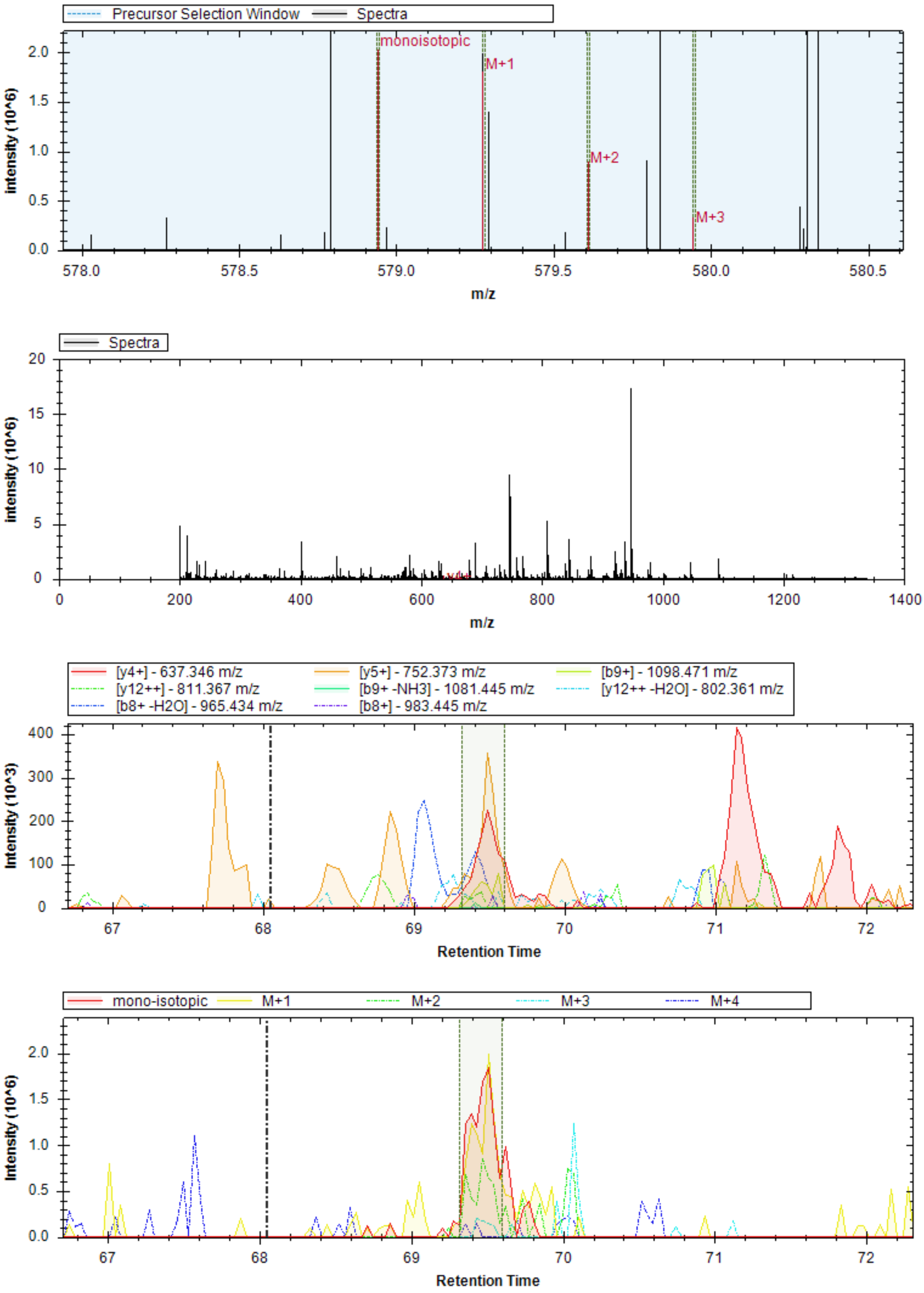

P20357 – _APHWTSASLTEAAAHPHS[+H+O3+P]PEMK_.4

Fig4_MP-DIA-barrel-cortex-p9-3_MHRM.htrms

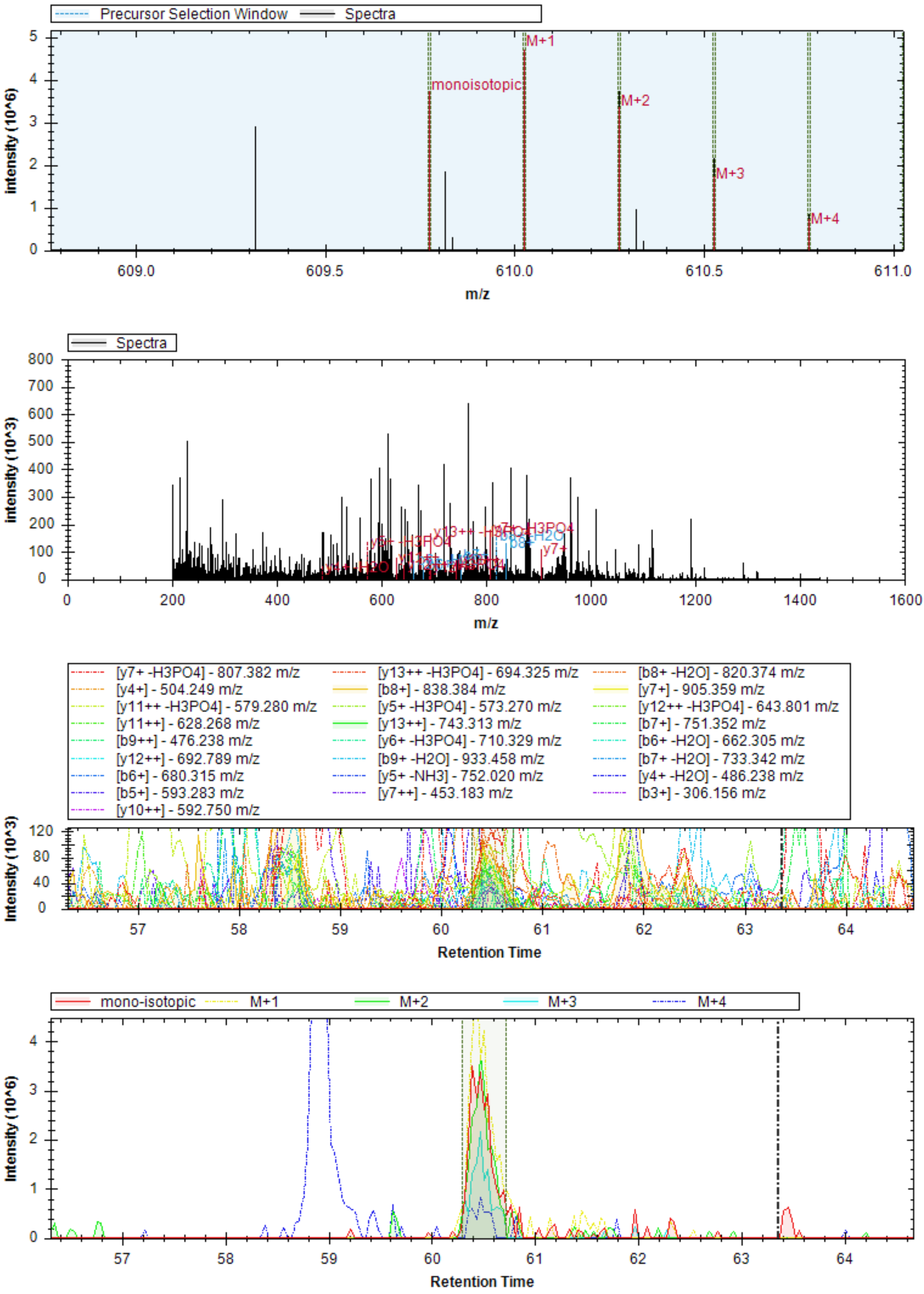

P26645 – _AEDGAAPSPSS[+H+O3+P]ETPKKK_.3
Fig4_MP-DIA-barrel-cortex-p9-3_MHRM.htrms

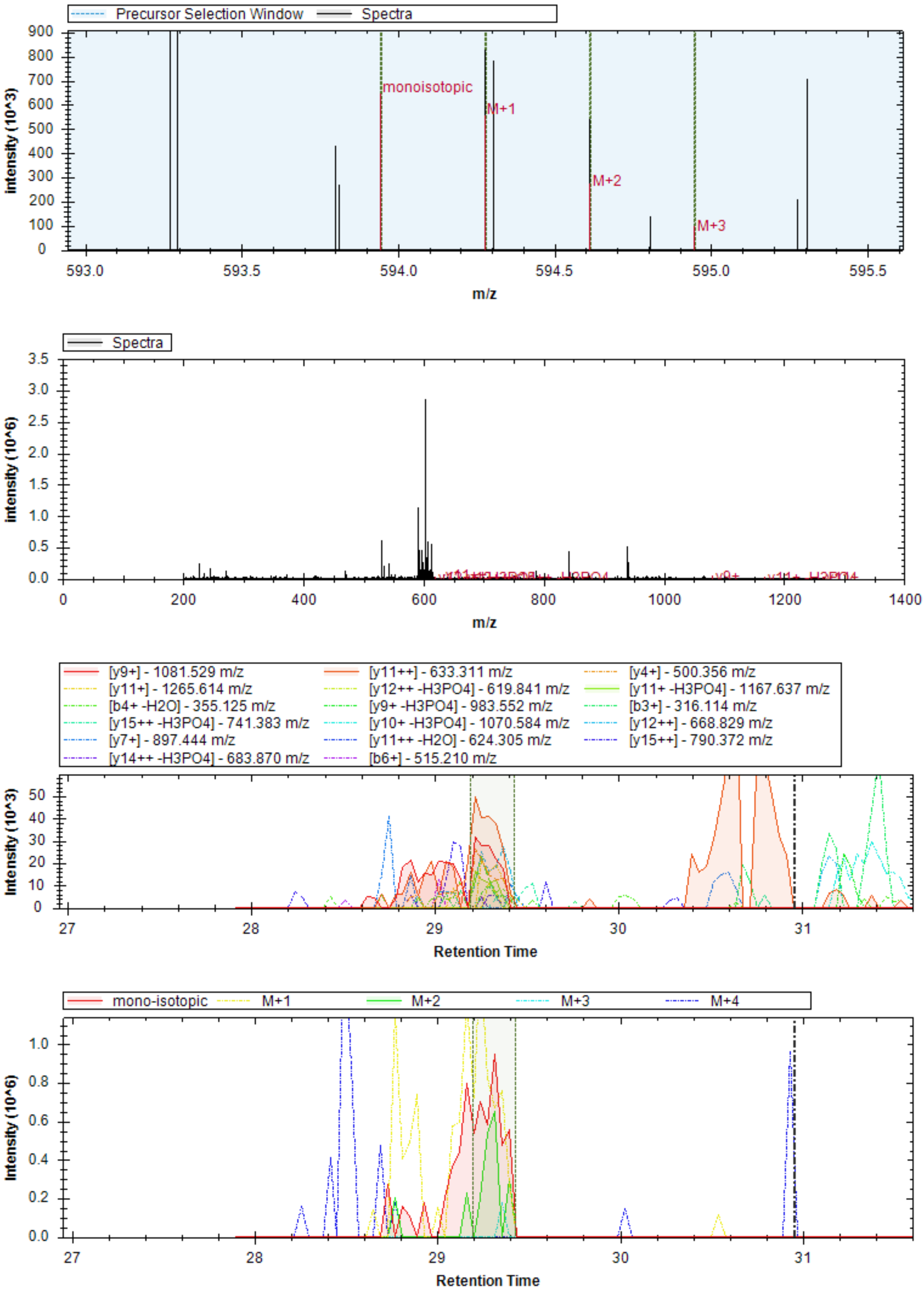

Q62261 – _RPPS[+H+O3+P]PDPNTK_.2
Fig4_MP-DIA-barrel-cortex-p9-3_MHRM.htrms

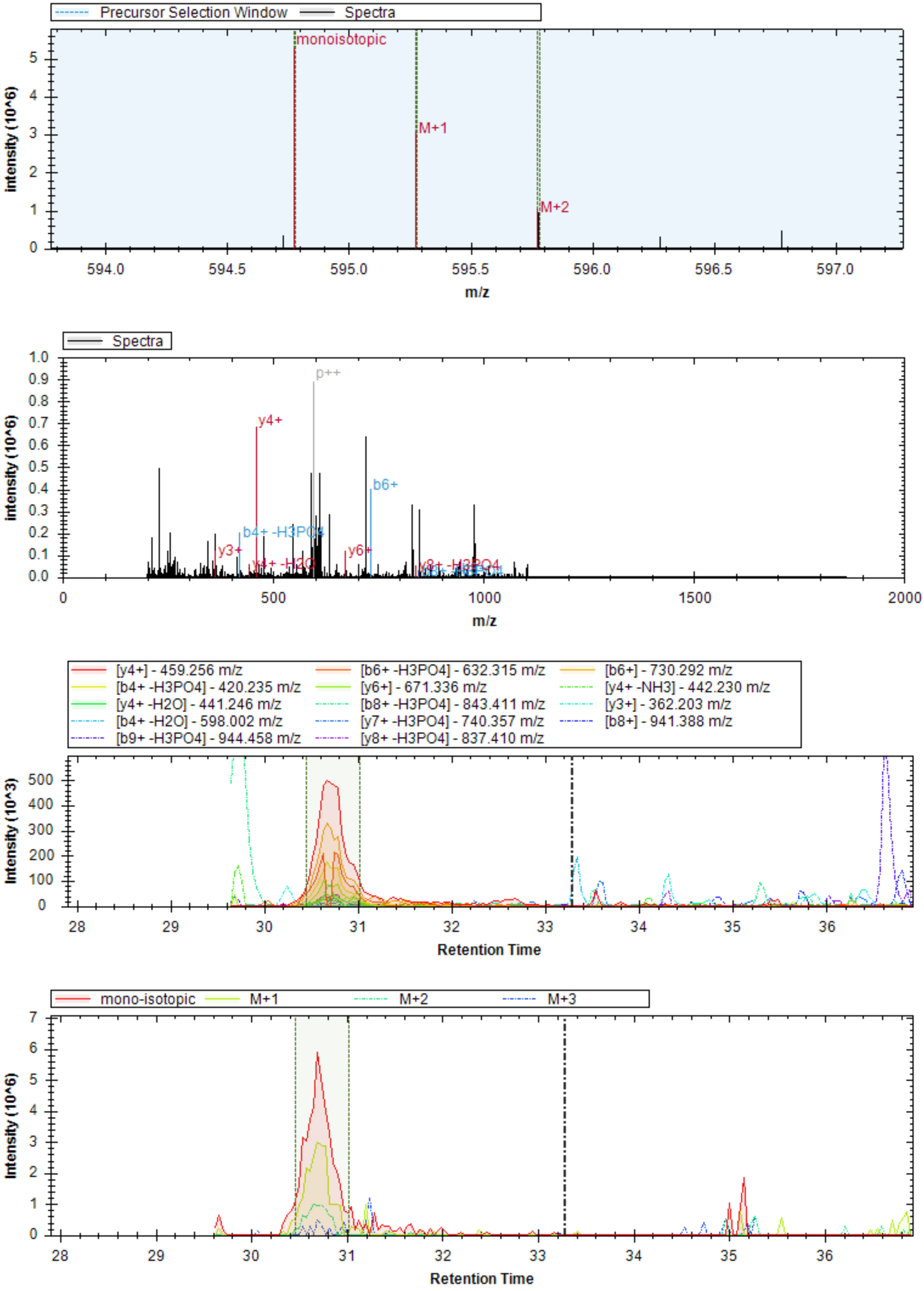

Q80VN0 – _ELTAQIT[+H+O3+P]SIK_.2
Fig4_MP-DIA-barrel-cortex-p9-3_MHRM.htrms

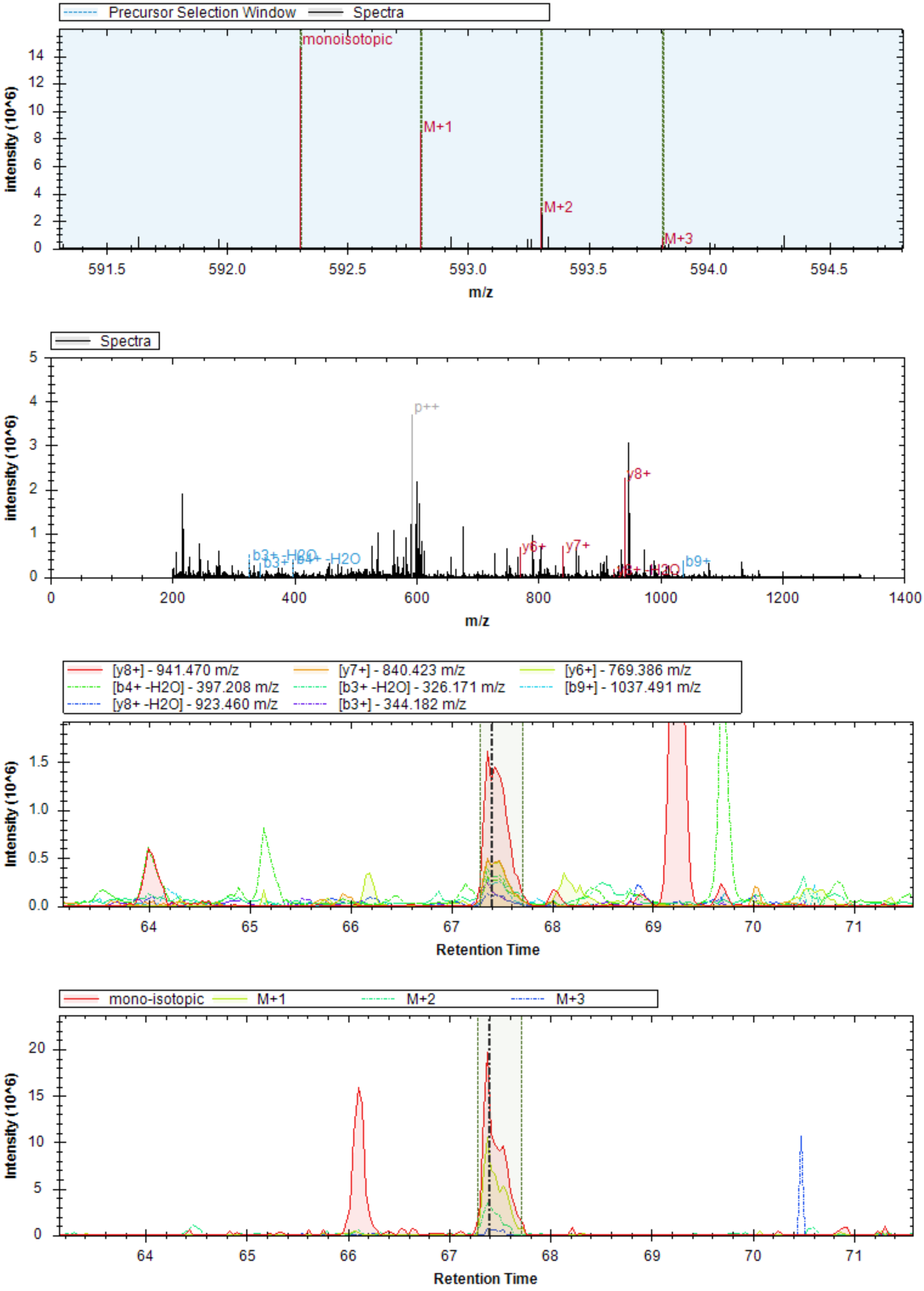

Q99104 – _AIS[+H+O3+P]PTSATSSGR_.2
Fig4_MP-DIA-barrel-cortex-p9-3_MHRM.htrms

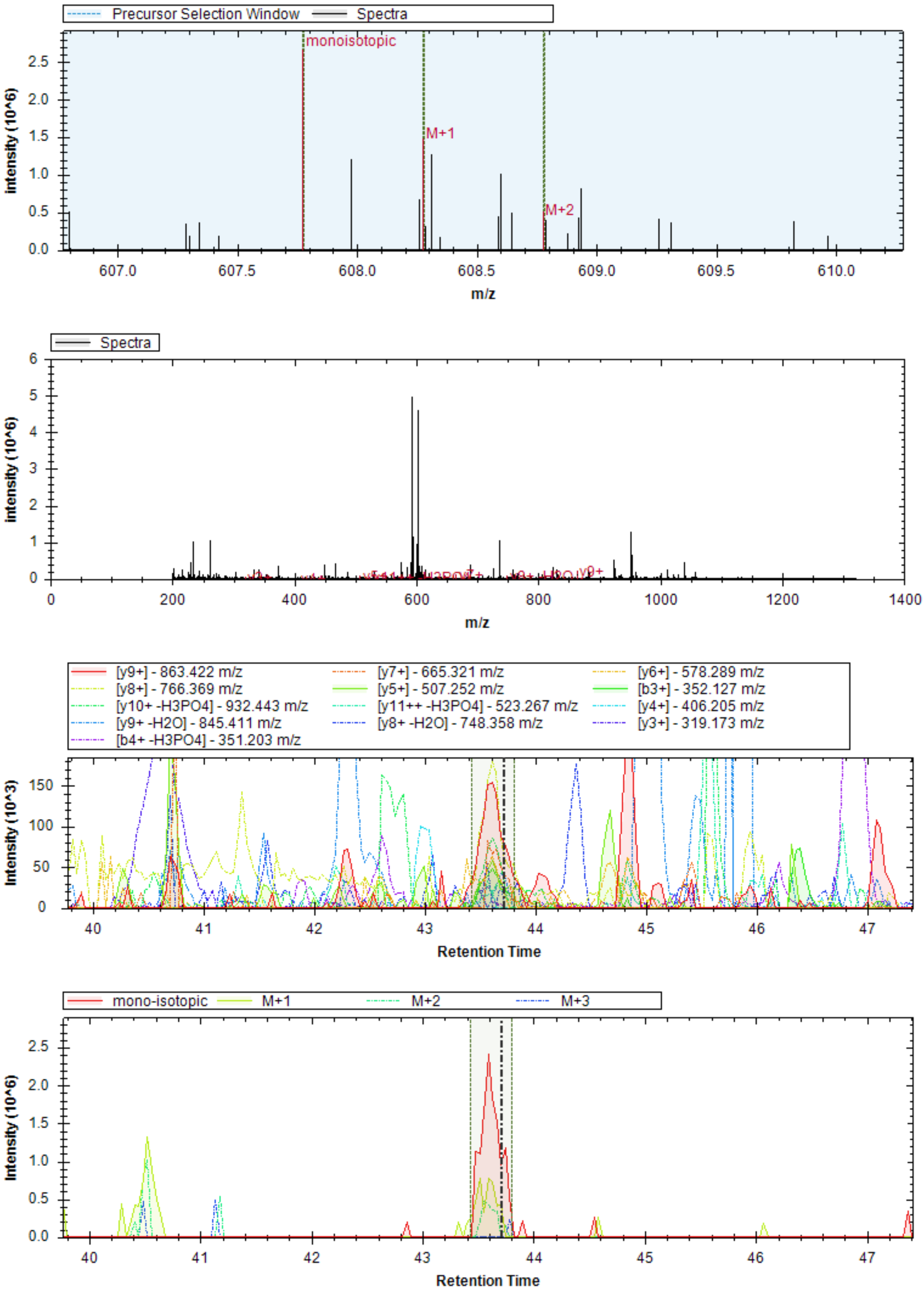

Q9QWI6 – _LSYAGGRPPSYAGS[+H+O3+P]PVHHAAER_.4
Fig4_MP-DIA-barrel-cortex-p9-3_MHRM.htrms

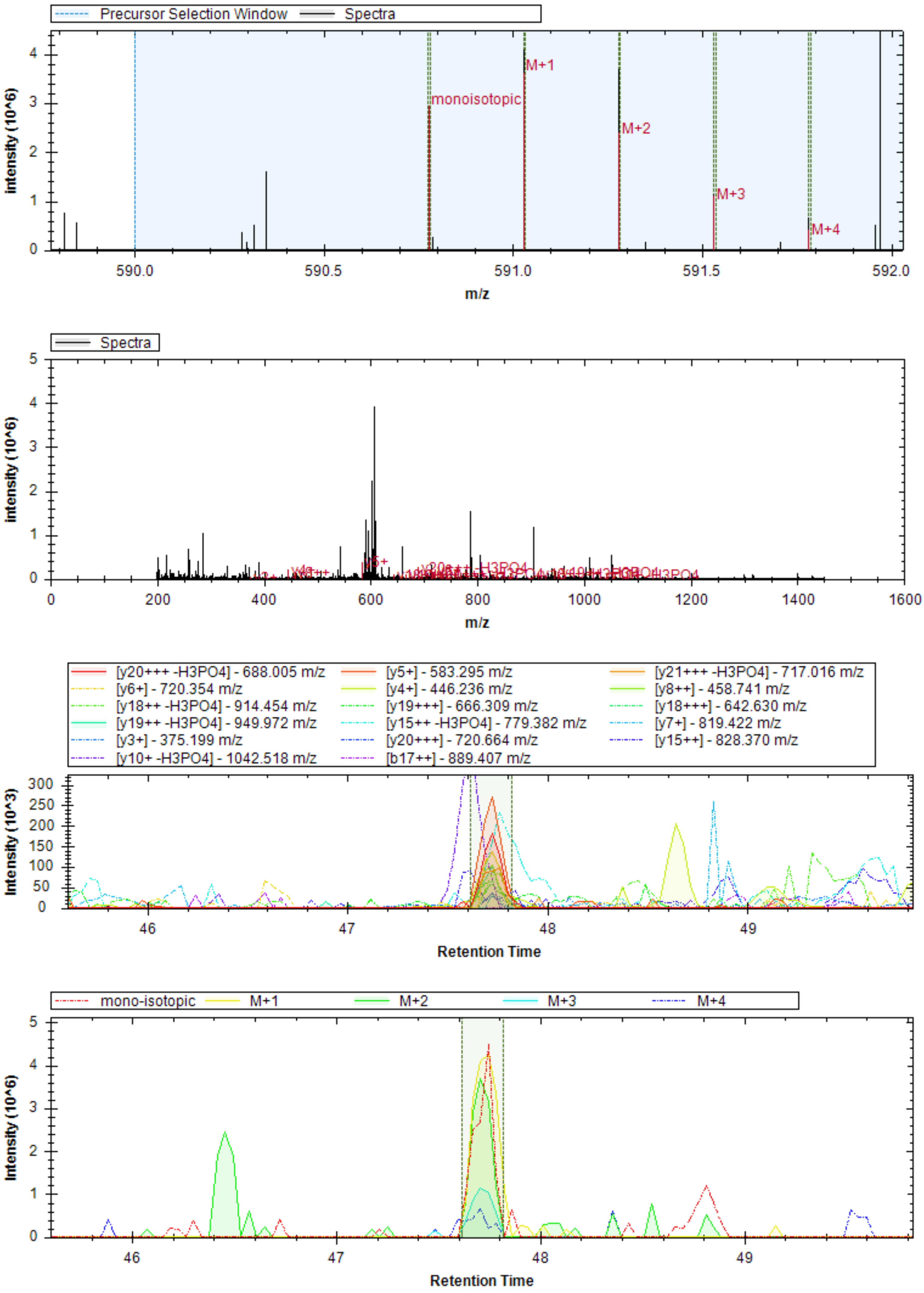

P26645 – _GEATAERPGEAAVASS[+H+O3+P]PSK_.3
Fig4_MP-DIA-barrel-cortex-p9-3_MHRM.htrms

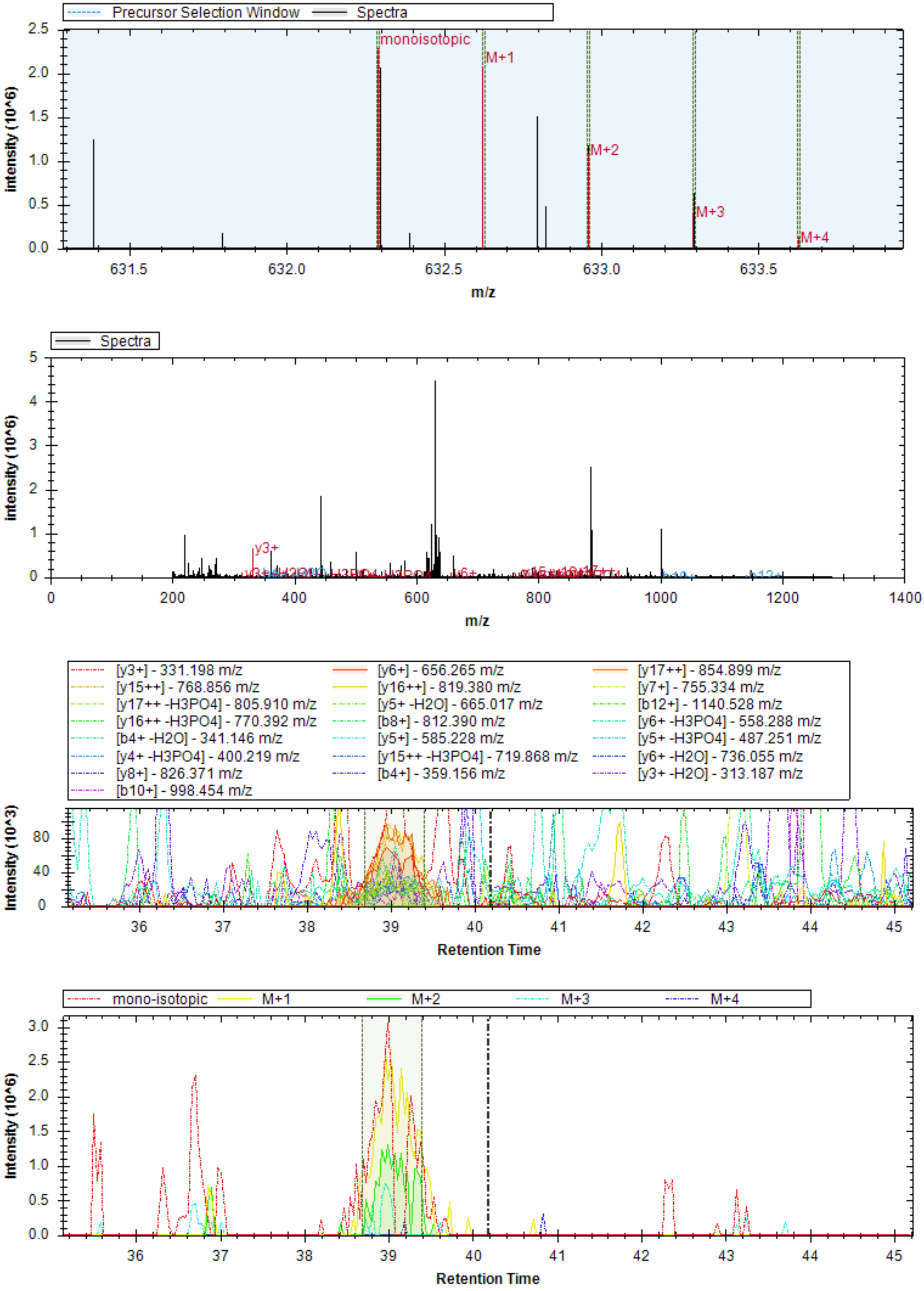

P60904 – _SLS[+H+O3+P]TSGESLYHVLGLDK_.3

Fig4_MP-DIA-barrel-cortex-p9-3_MHRM.htrms

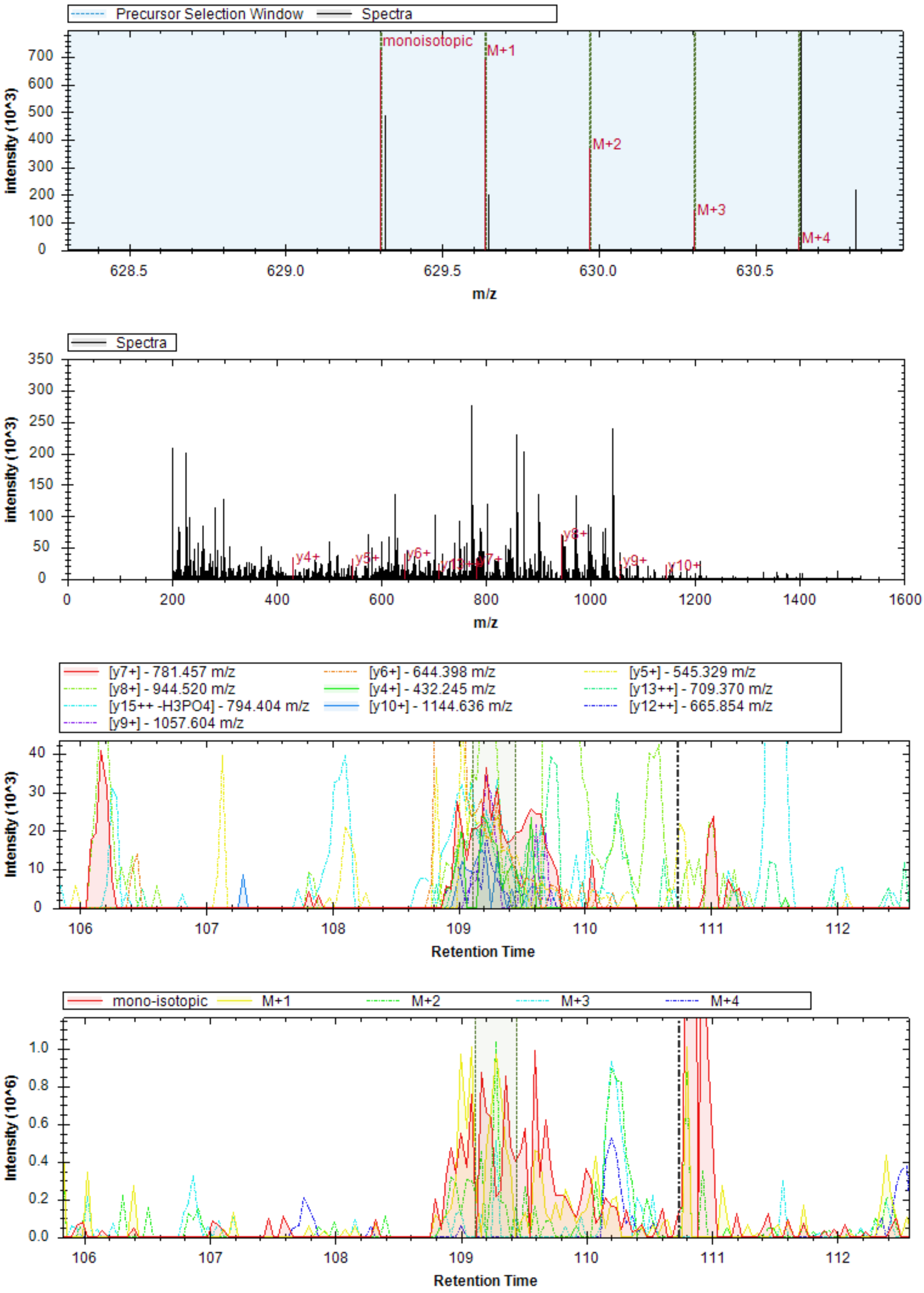

P60904 – _SLST[+H+O3+P]SGESLYHVLGLDK_.3
Fig4_MP-DIA-barrel-cortex-p9-3_MHRM.htrms

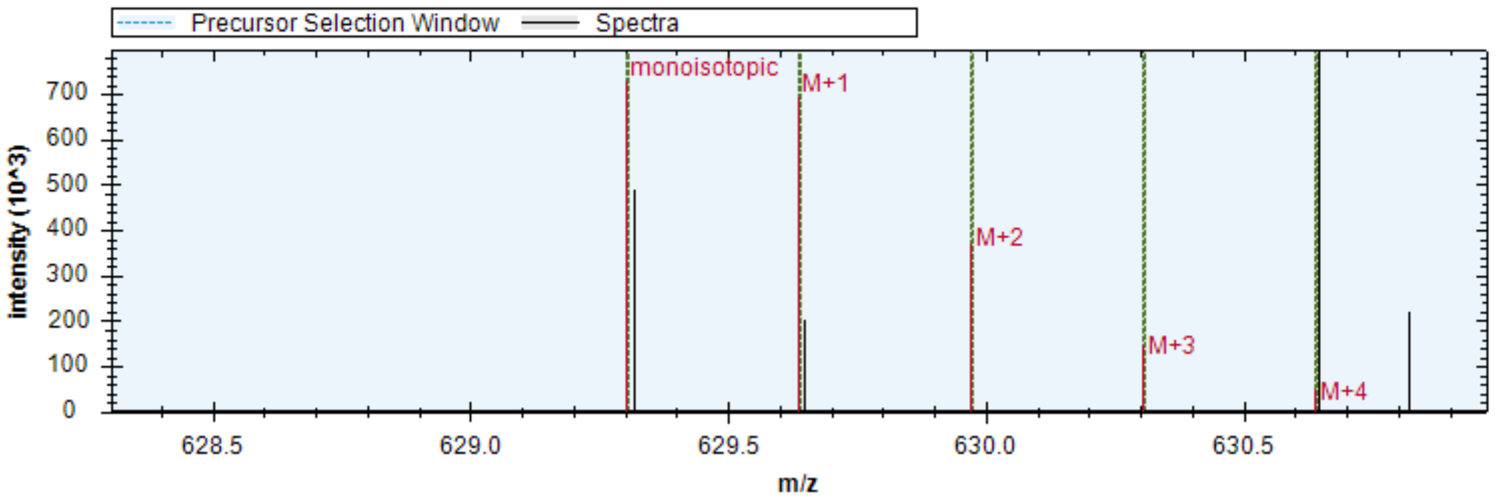

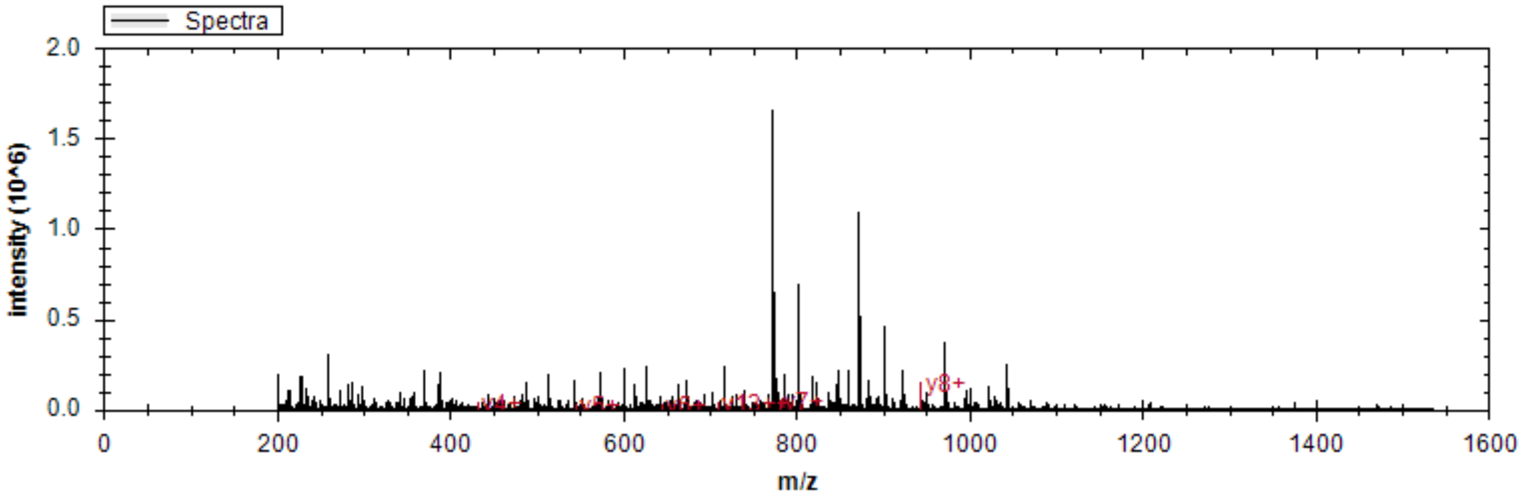

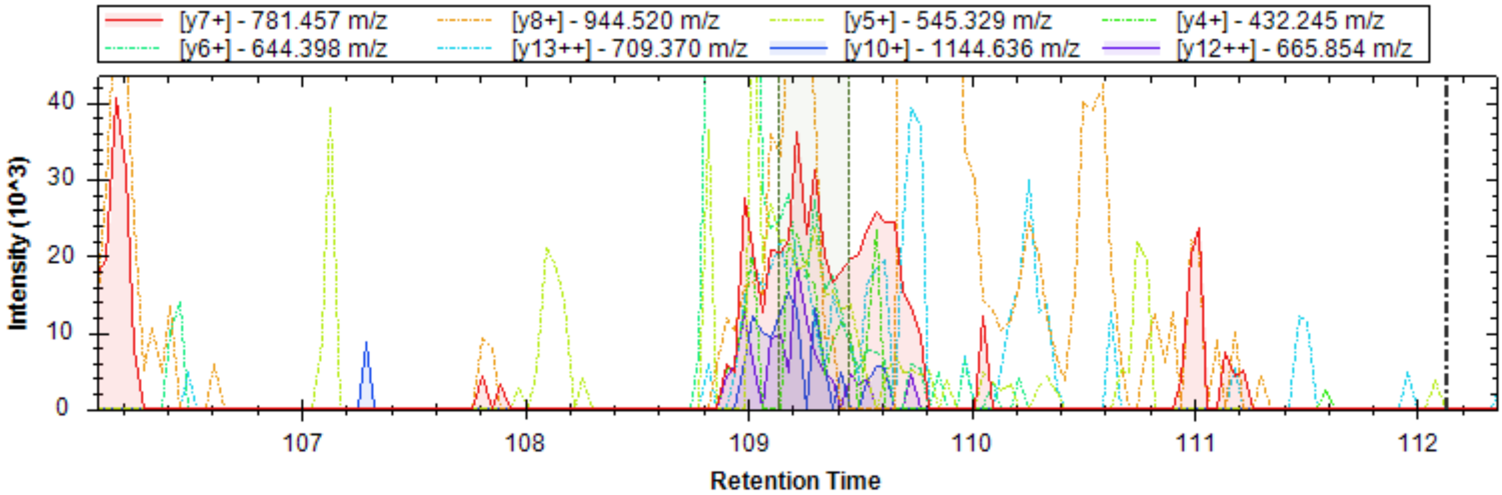

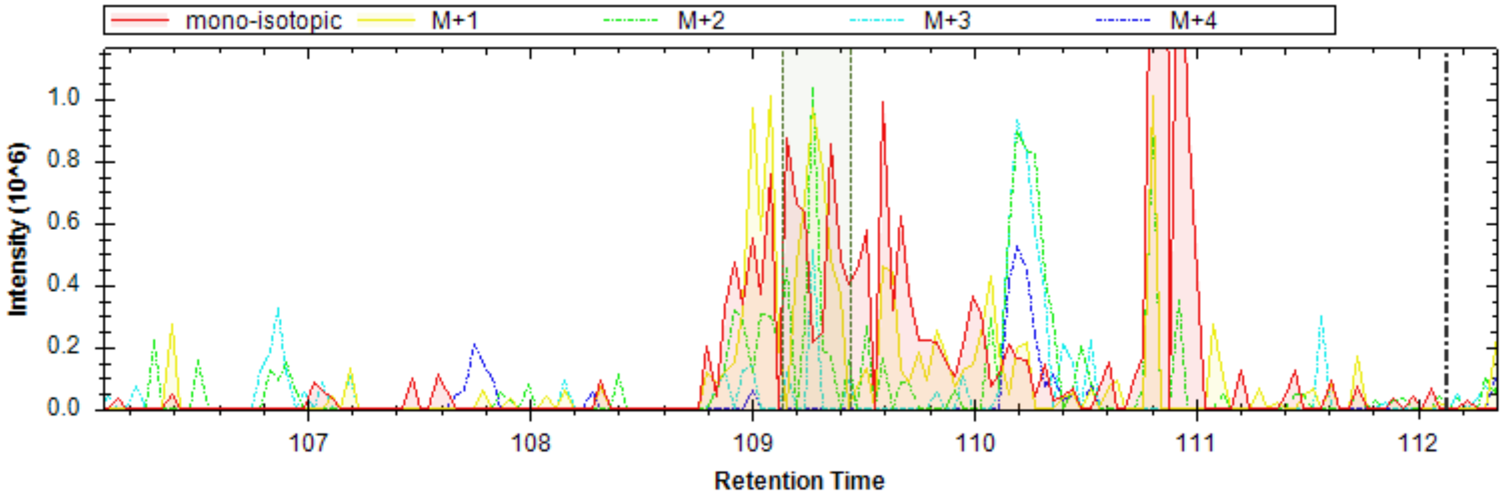

Q9CQS8 – _PGPTPSGTNVGSSGRS[+H+O3+P]PSK_.3
Fig4_MP-DIA-barrel-cortex-p9-3_MHRM.htrms

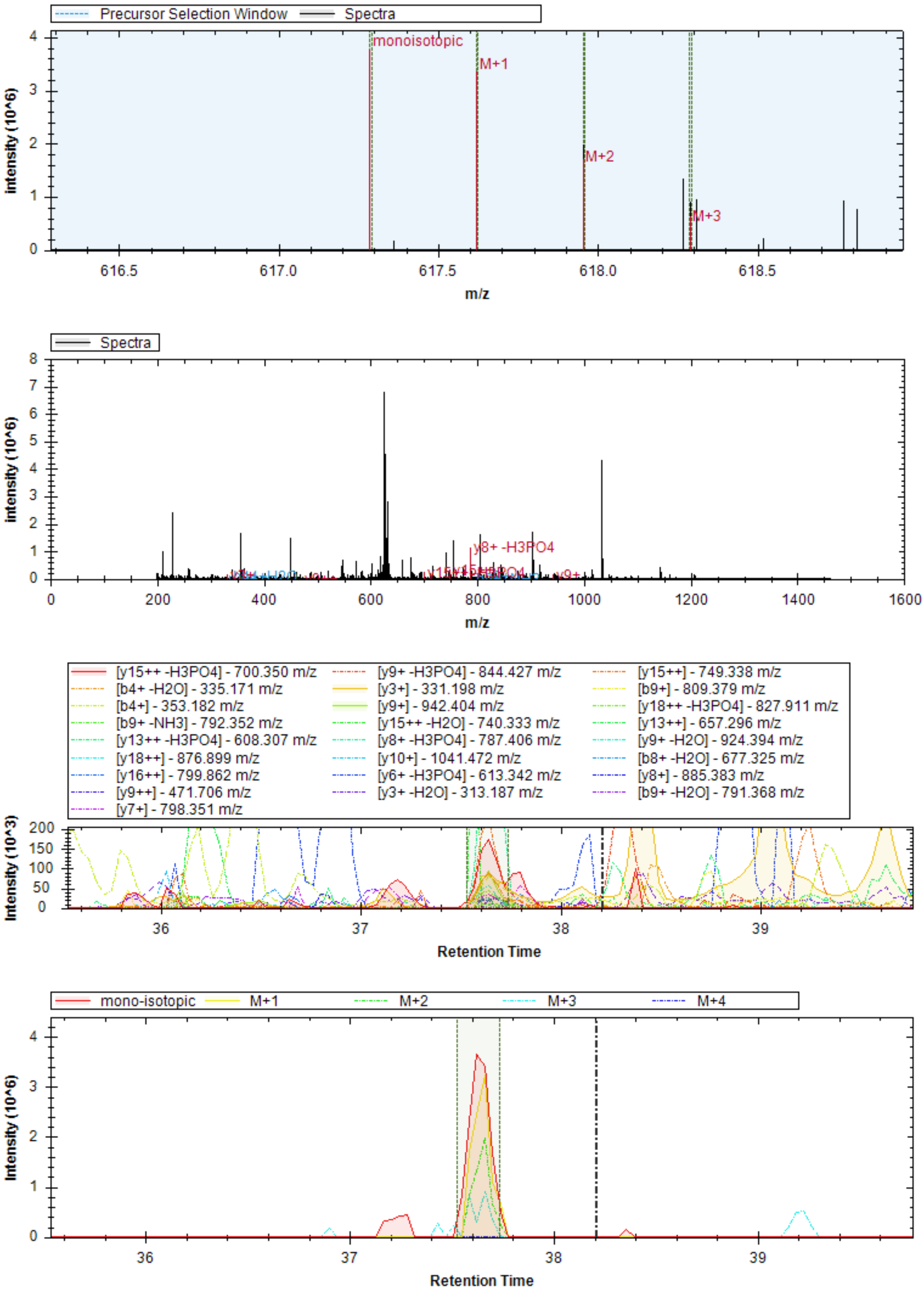

P20357 – _TPGT[+H+O3+P]PGTPSYPR_.2

Fig4_MP-DIA-barrel-cortex-p9-3_MHRM.htrms

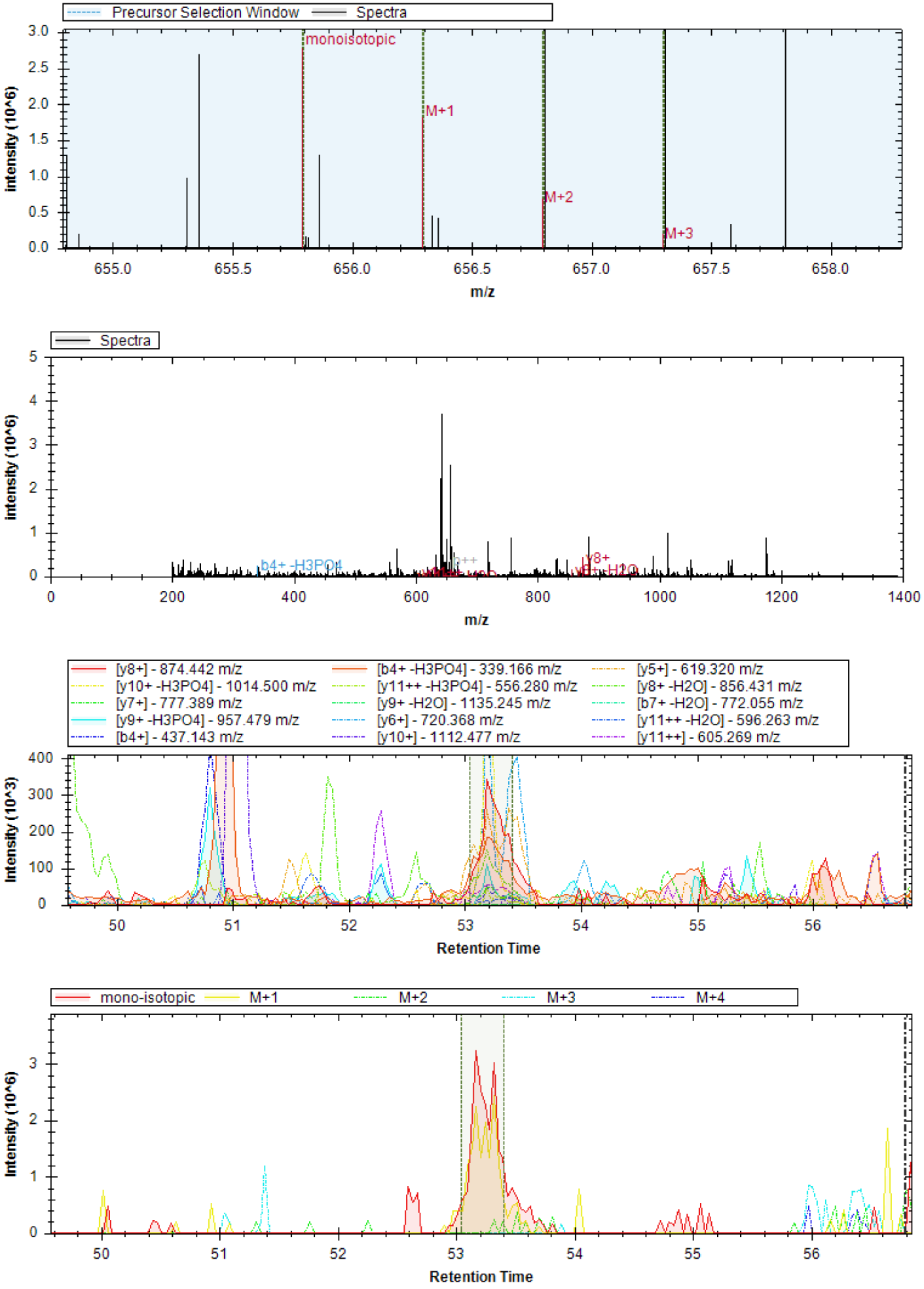

P20357 – _TPGTPGT[+H+O3+P]PSYPR_.2
Fig4_MP-DIA-barrel-cortex-p9-3_MHRM.htrms

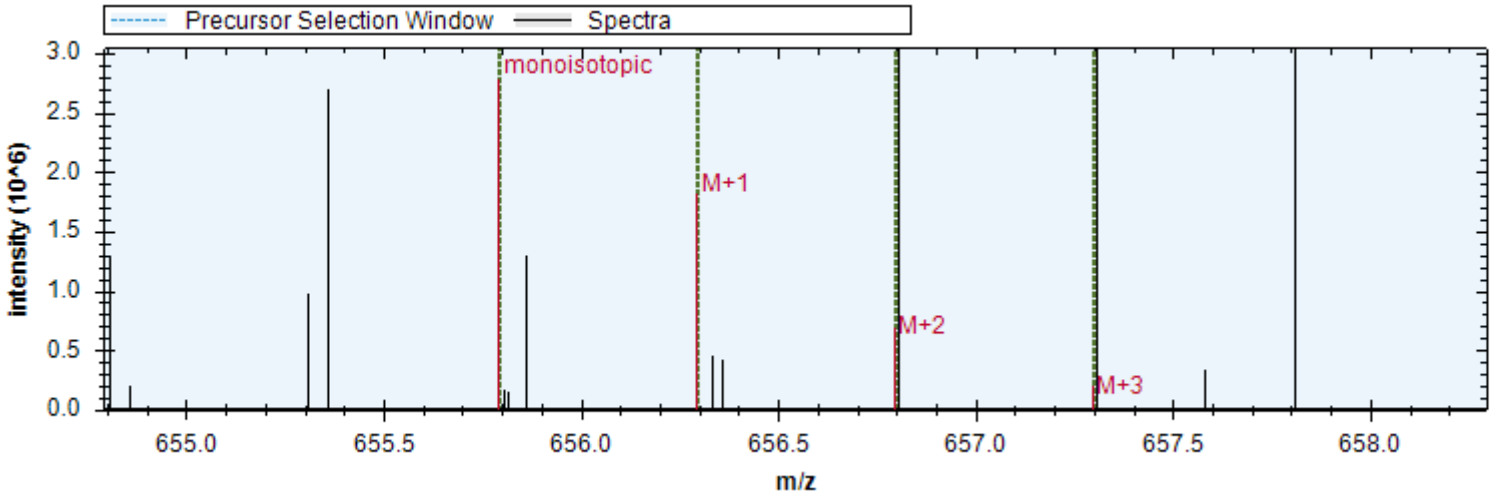

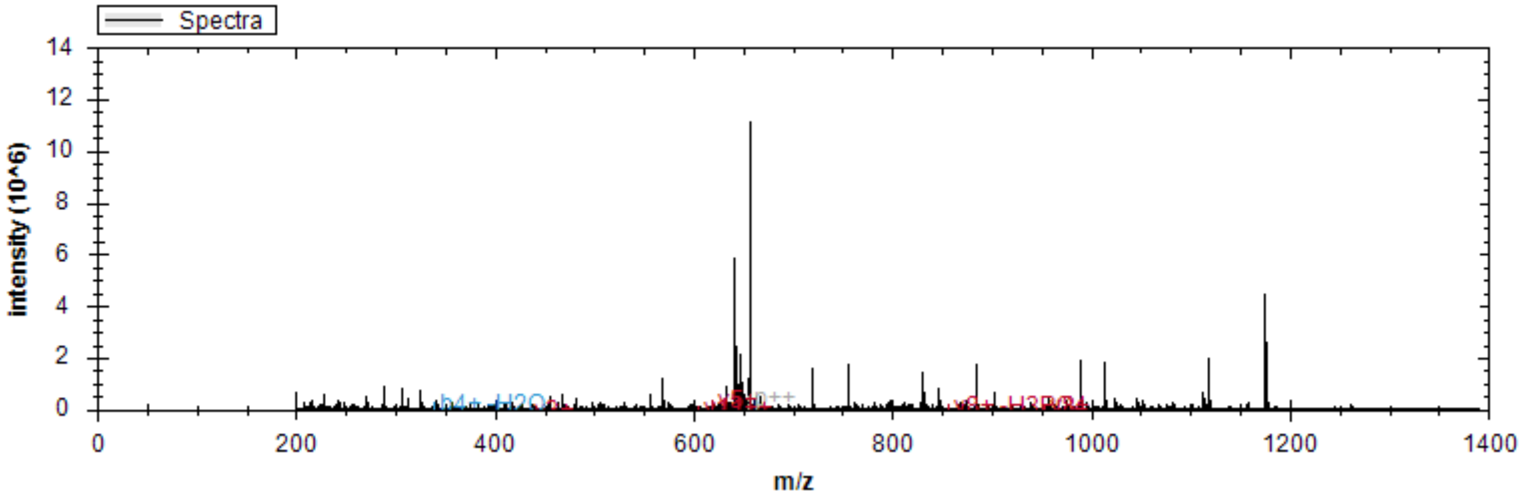

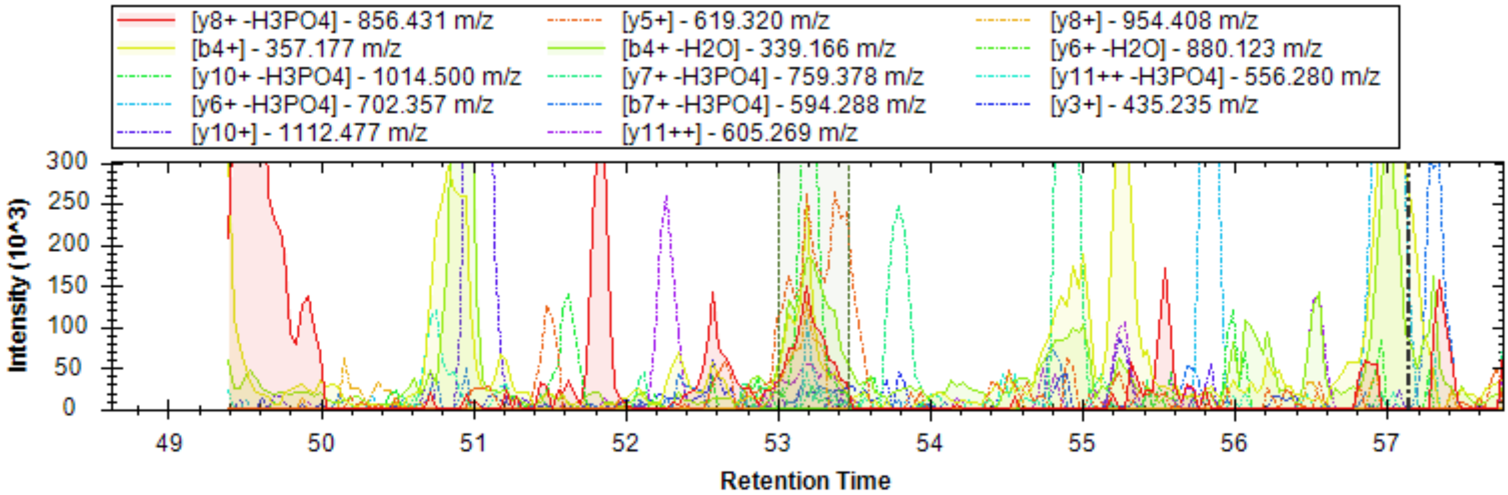

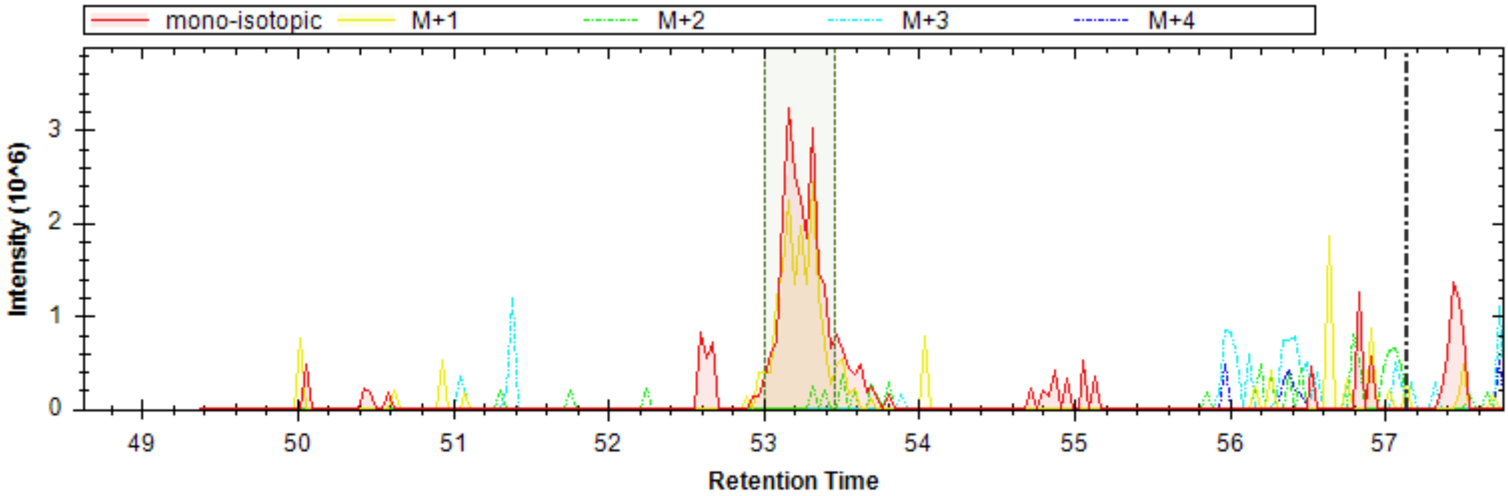

P39447 – _SSGIIRLHT[+H+O3+P]IK_.2
Fig4_MP-DIA-barrel-cortex-p9-3_MHRM.htrms

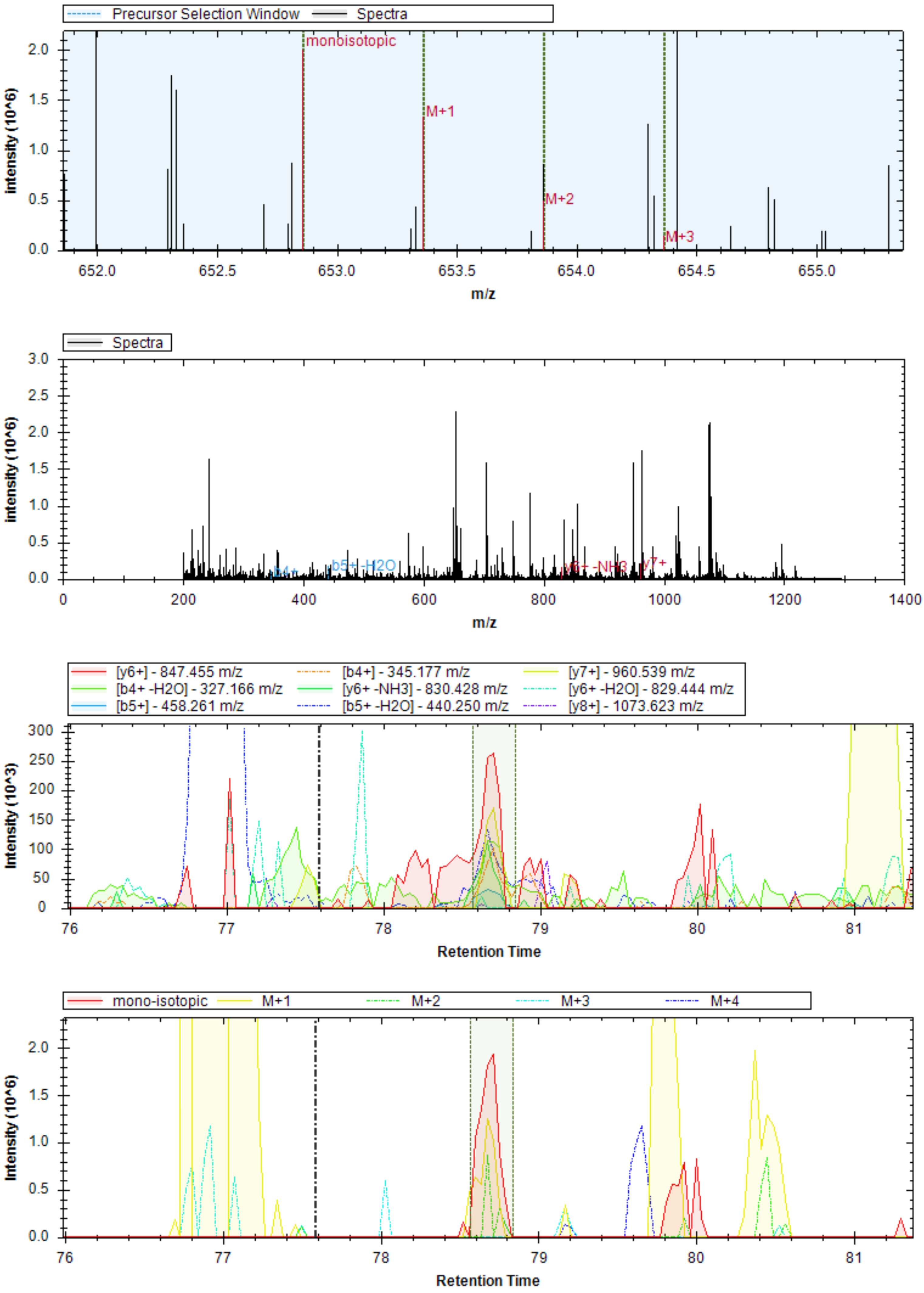

Q8BLJ3 – _M[+O]AS[+H+O3+P]SQGKNELK_.2

Fig4_MP-DIA-barrel-cortex-p9-3_MHRM.htrms

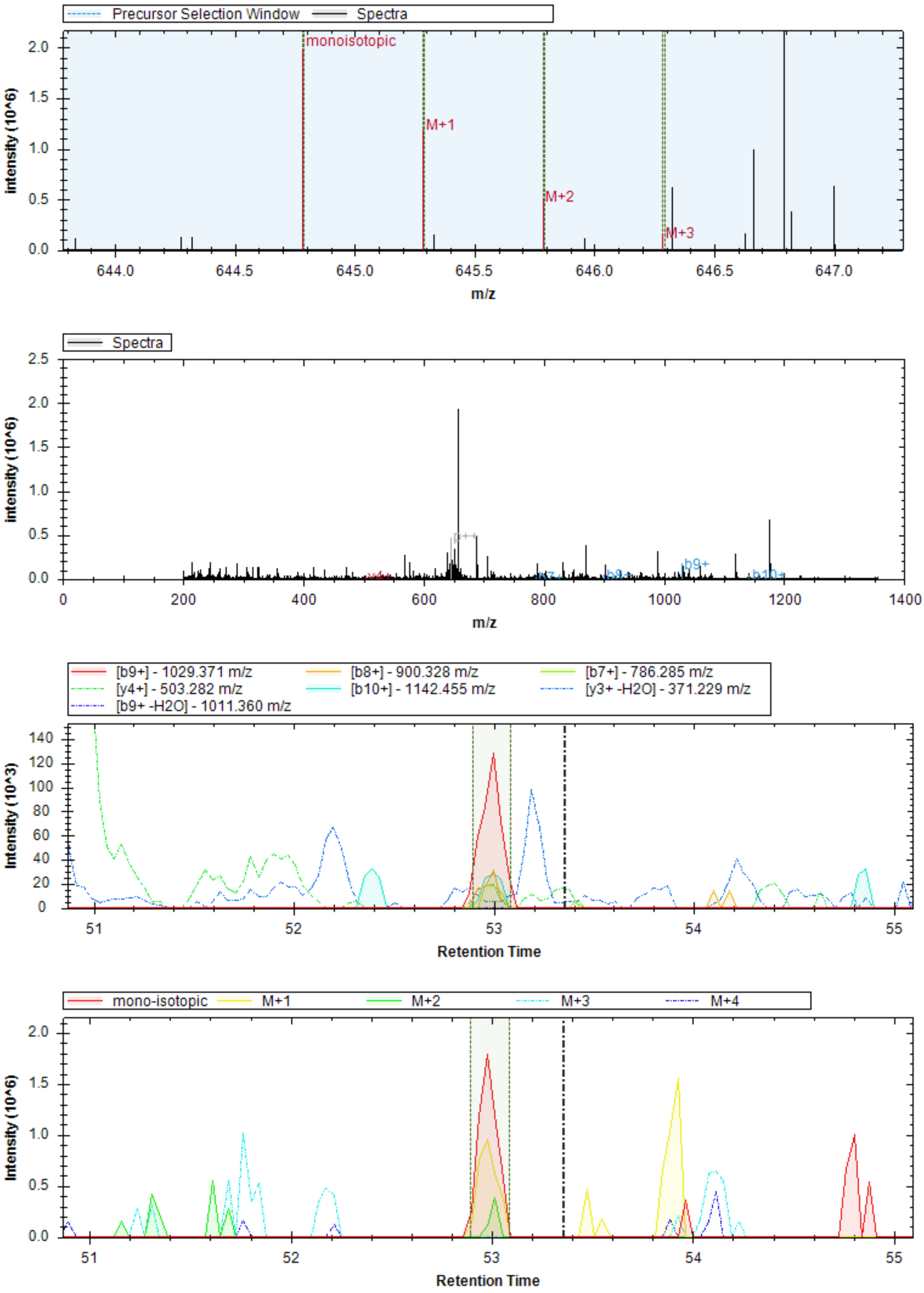

G5E870 – _IIY[+H+O3+P]FADAELLK_.2

Fig4_MP-DIA-barrel-cortex-p9-3_MHRM.htrms

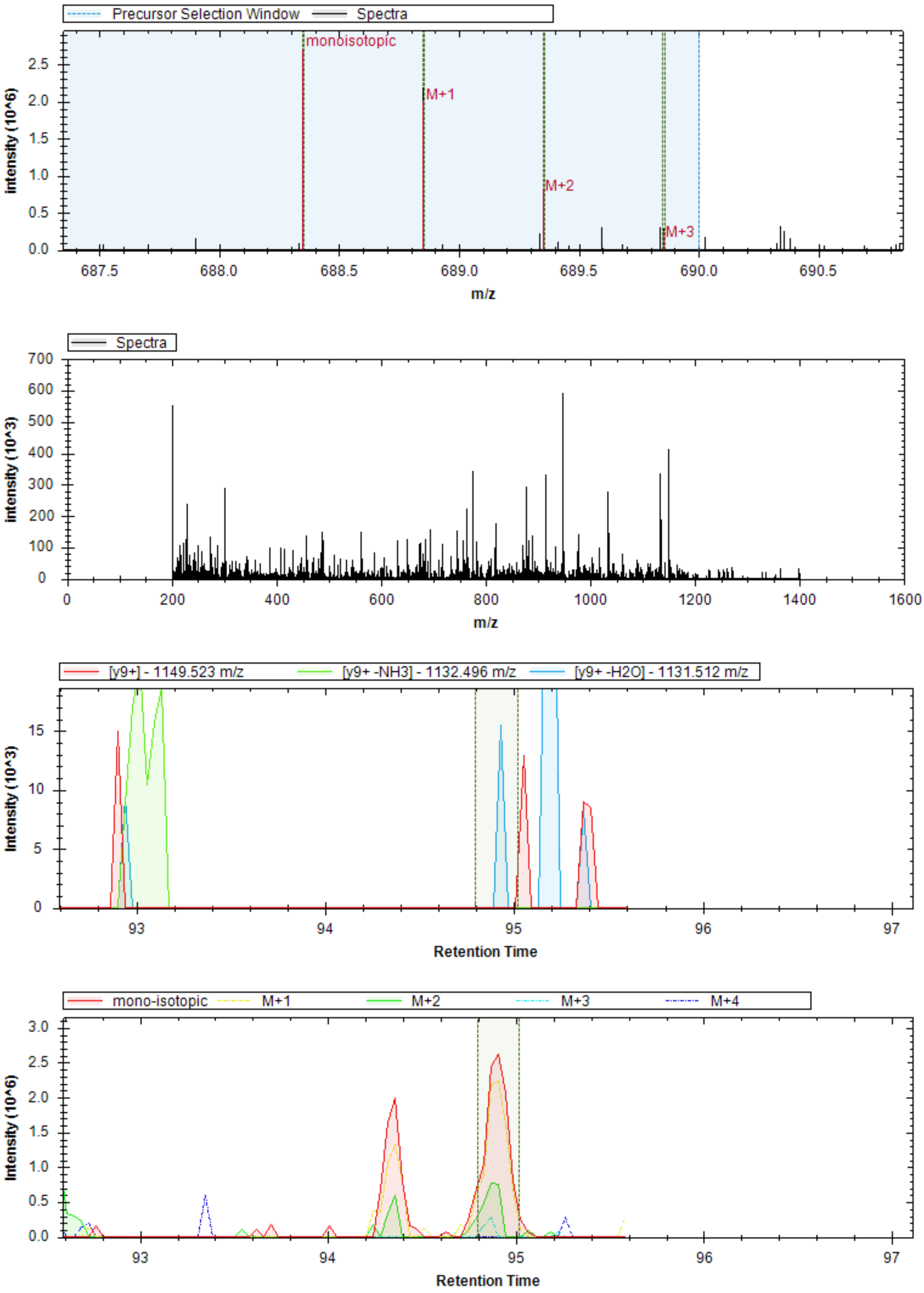

P13595 – _GVTASS[+H+O3+P]SSPASAPK_.2
Fig4_MP-DIA-barrel-cortex-p9-3_MHRM.htrms

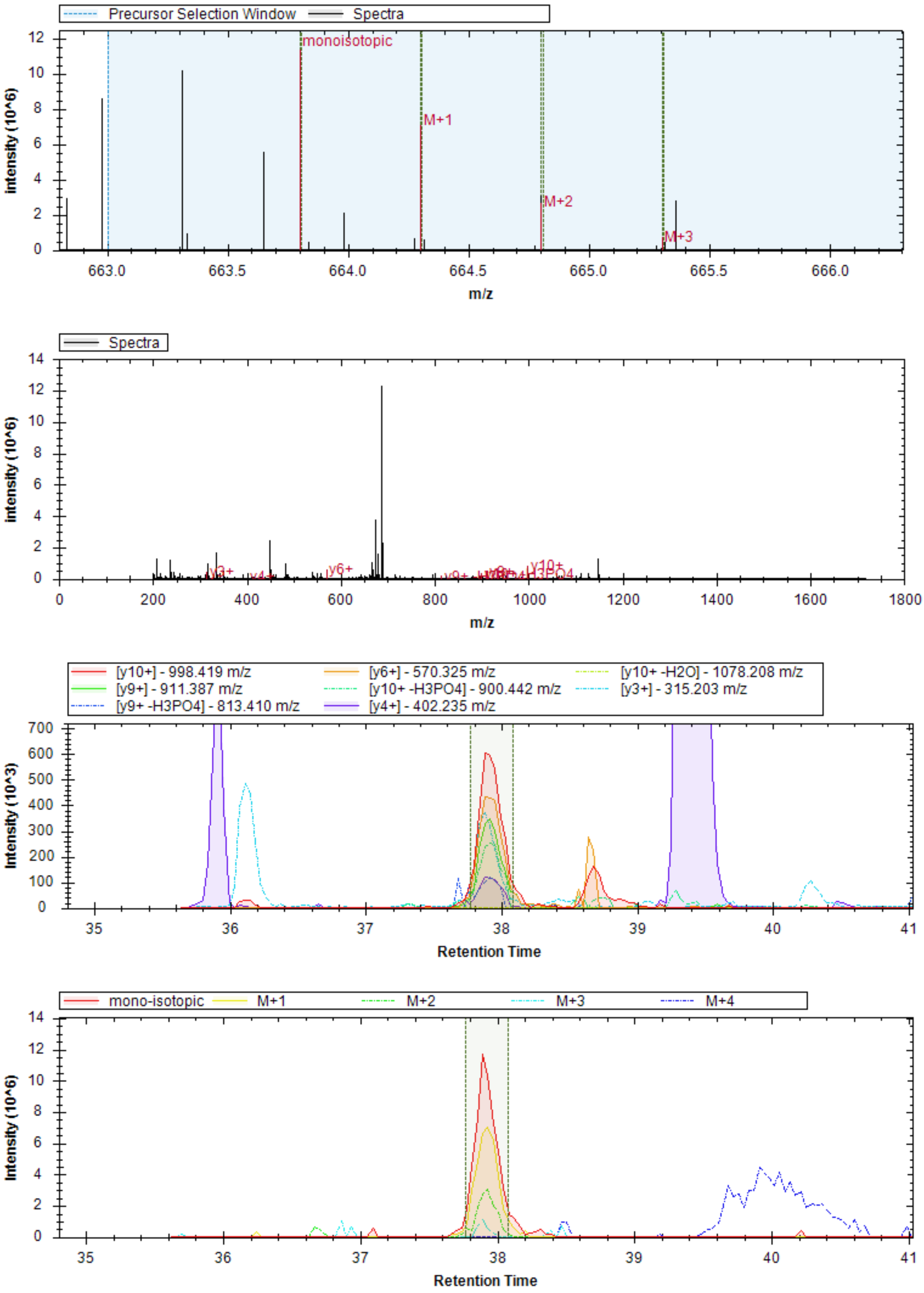

P13595 – _GVTASSS[+H+O3+P]SPASAPK_.2

Fig4_MP-DIA-barrel-cortex-p9-3_MHRM.htrms

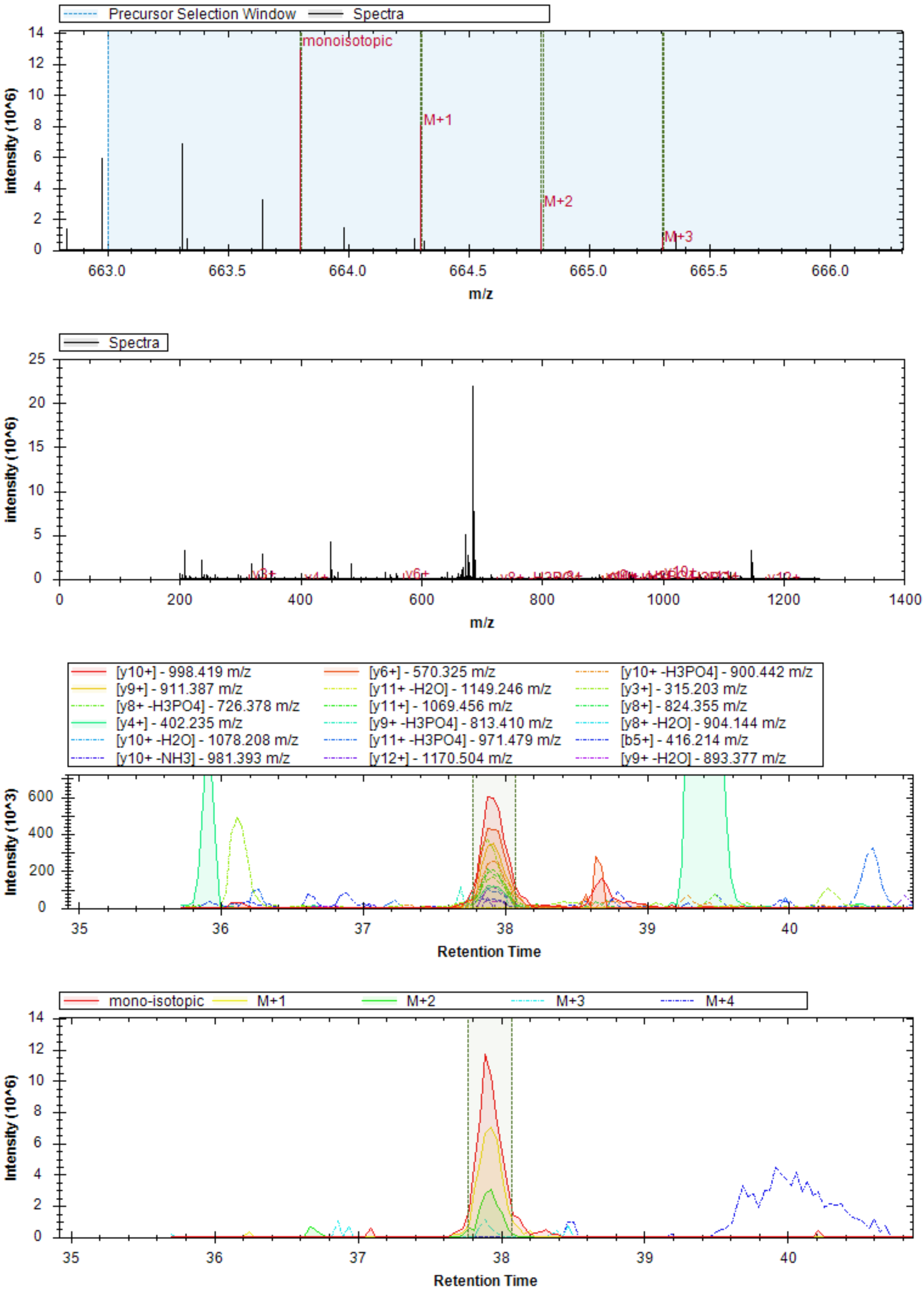

P13595 – _GVTASSSS[+H+O3+P]PASAPK_.2
Fig4_MP-DIA-barrel-cortex-p9-3_MHRM.htrms

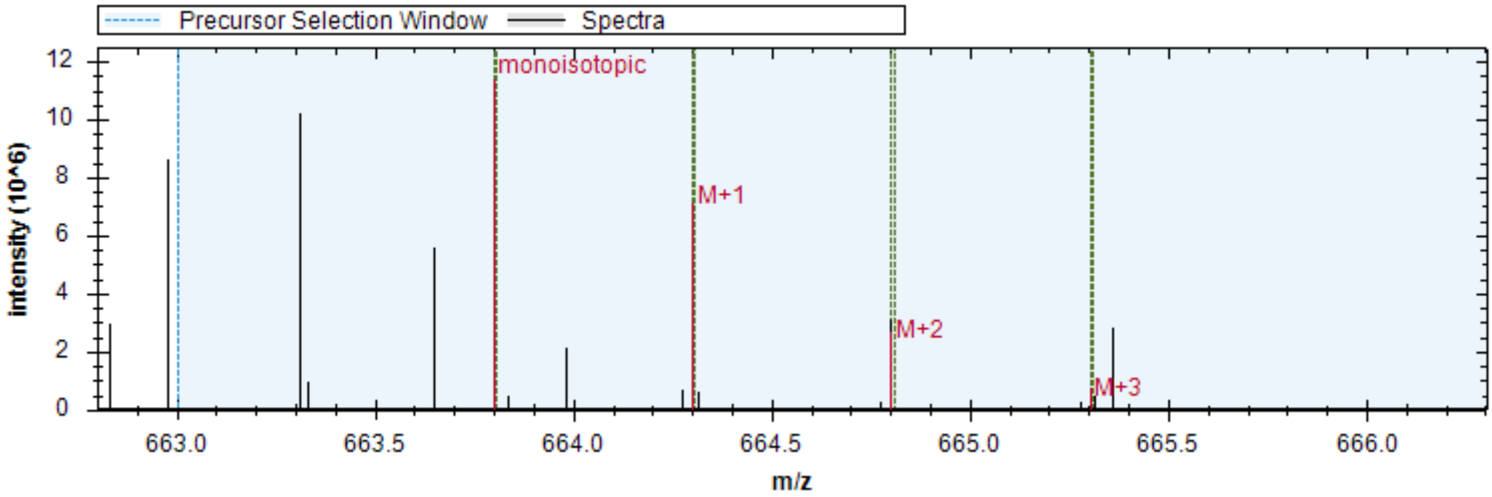

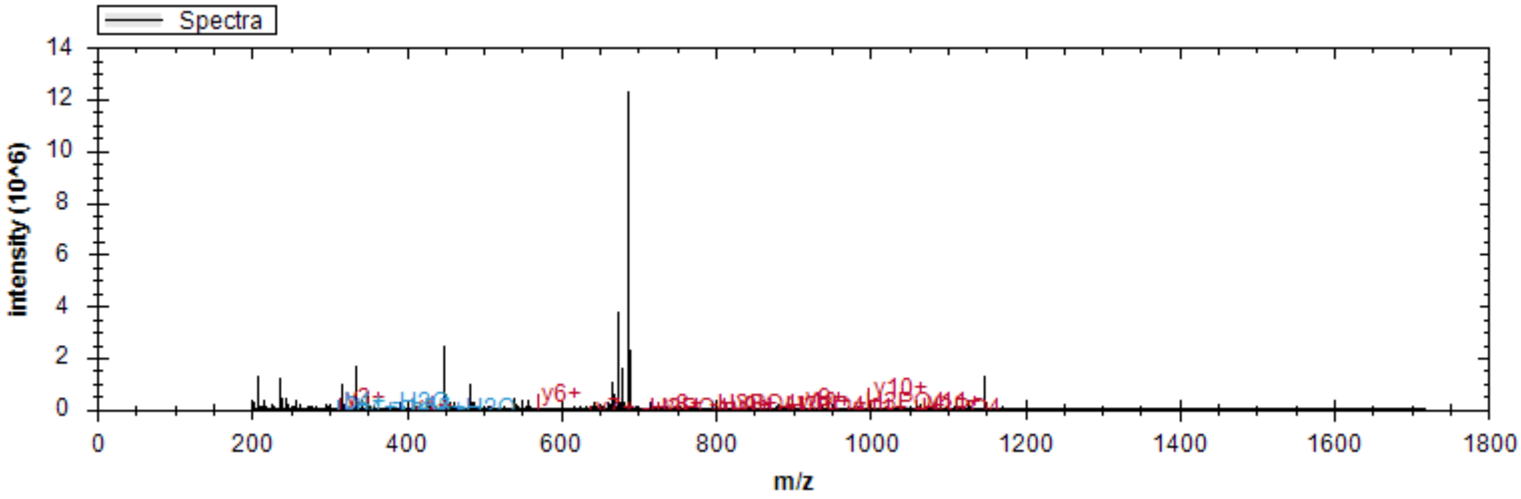

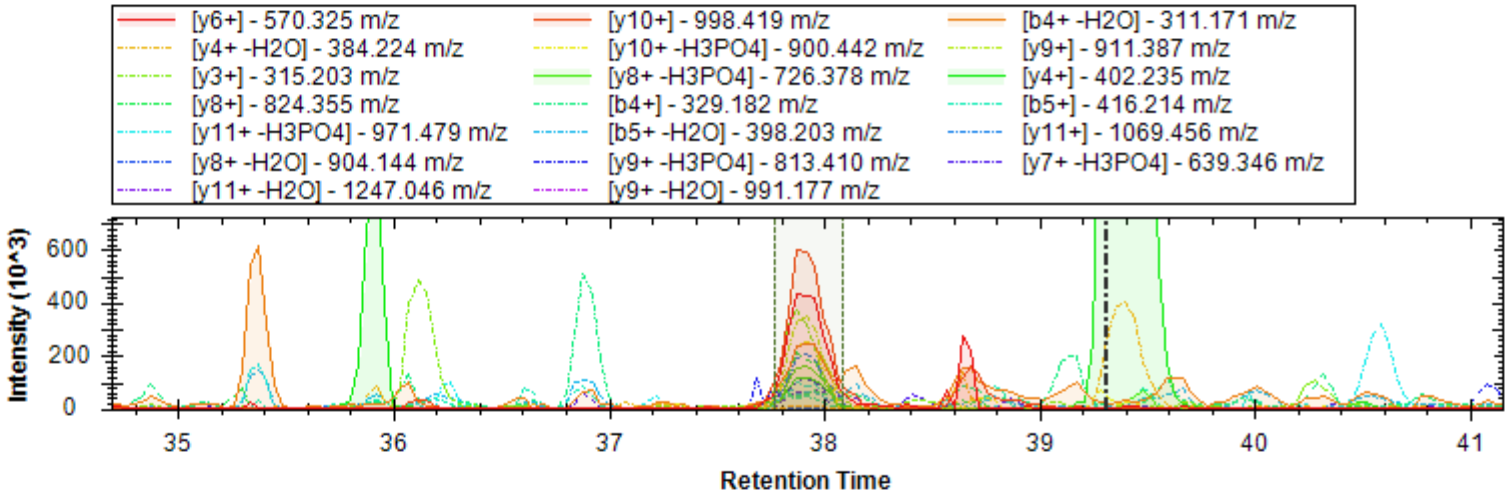

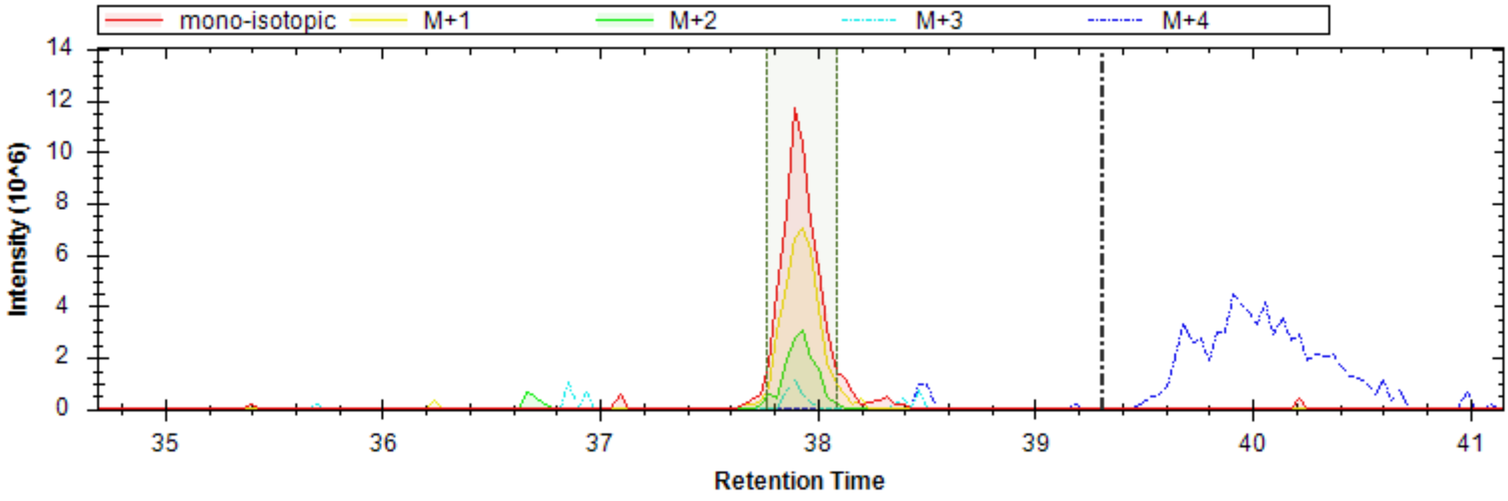

P13595 – _NPPEAATAPAS[+H+O3+P]PK_.2
Fig4_MP-DIA-barrel-cortex-p9-3_MHRM.htrms

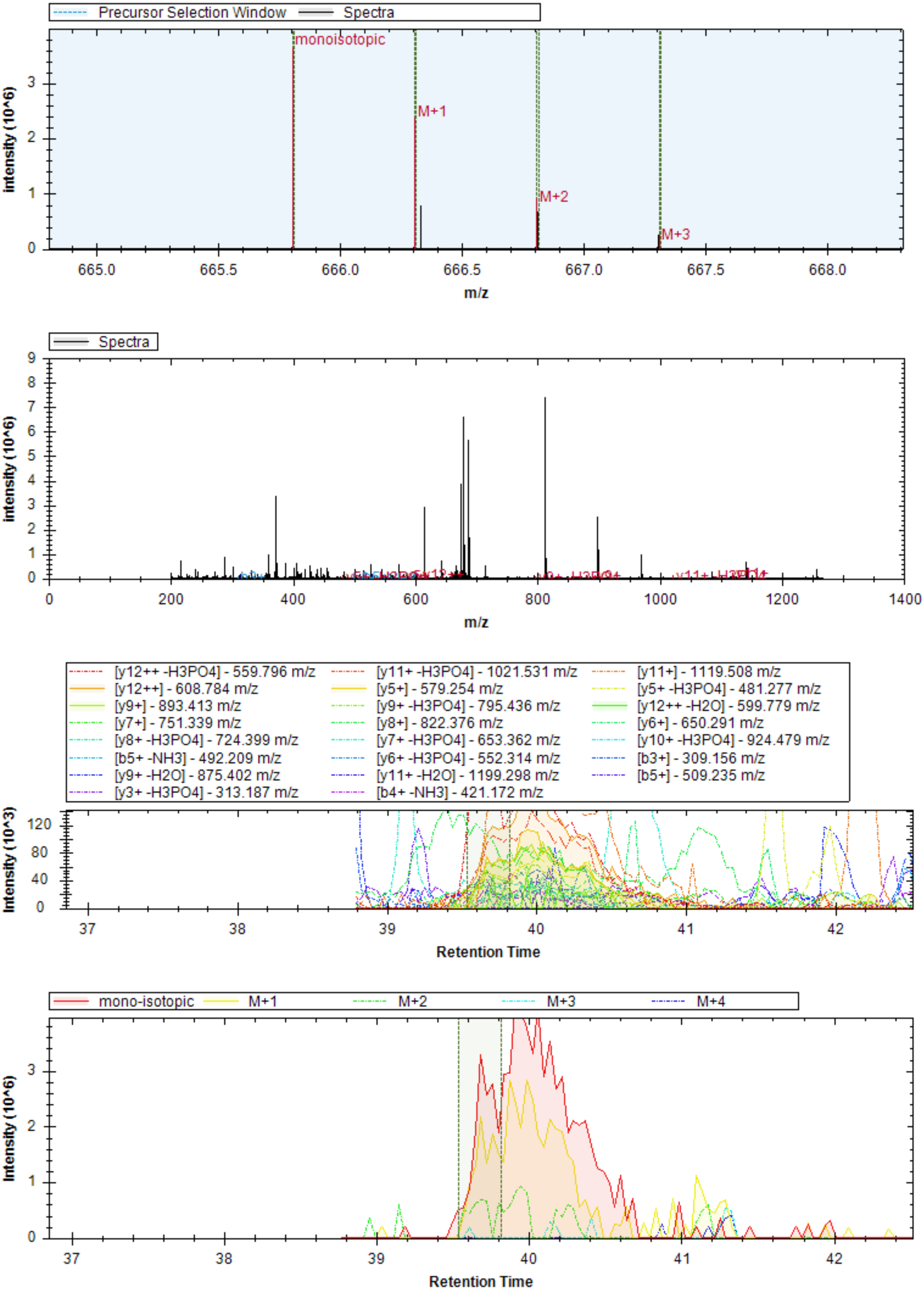

P26361 – _KM[+O]LHS[+H+O3+P]ILHAPMS[+H+O3+P]T[+H+O3+P]ISK_.3
Fig4_MP-DIA-barrel-cortex-p9-3_MHRM.htrms

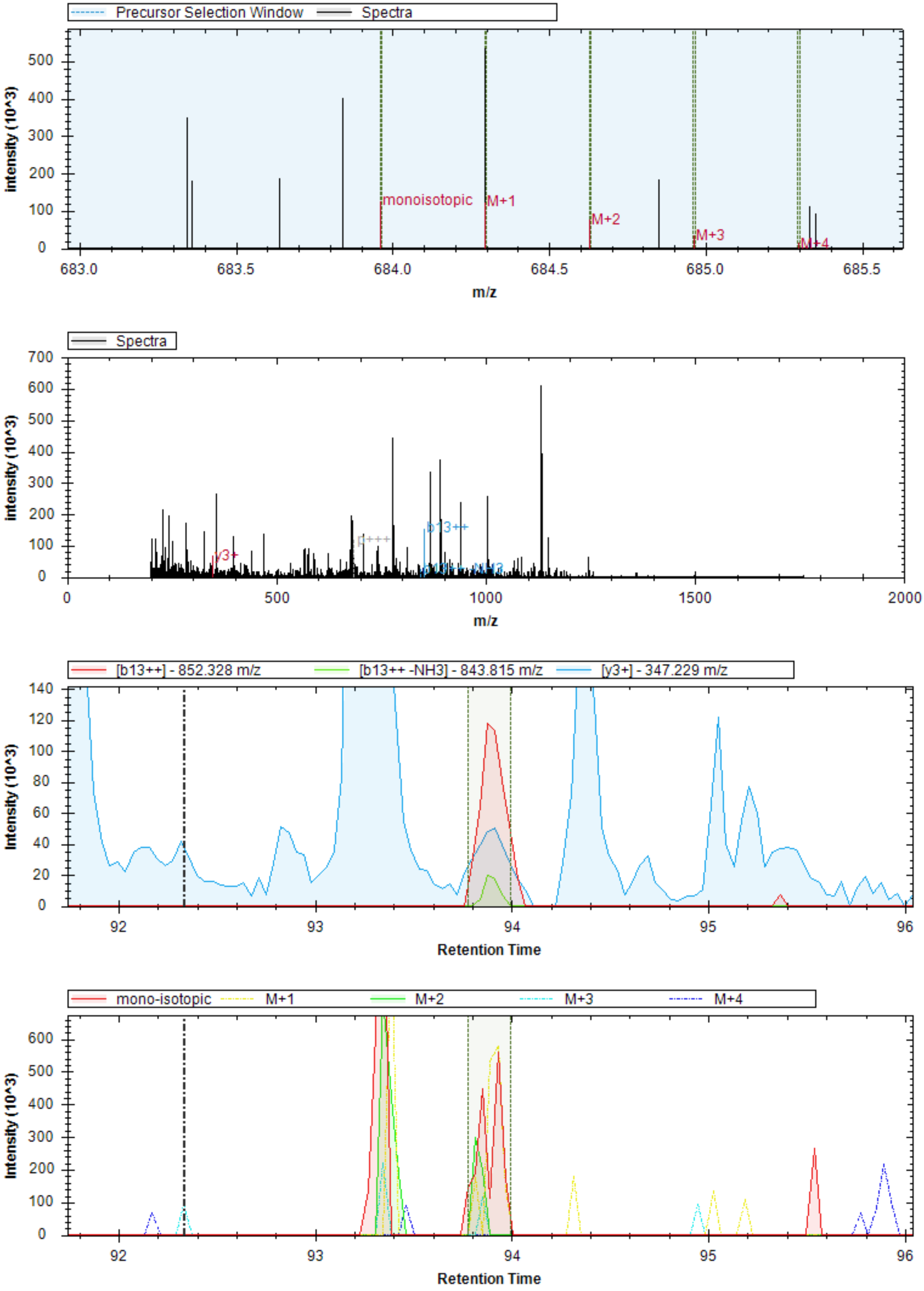

Q03173;Q03173-4;Q03173-5 – _NSRPS[+H+O3+P]SPVNTPSSQPPAAK_.3
Fig4_MP-DIA-barrel-cortex-p9-3_MHRM.htrms

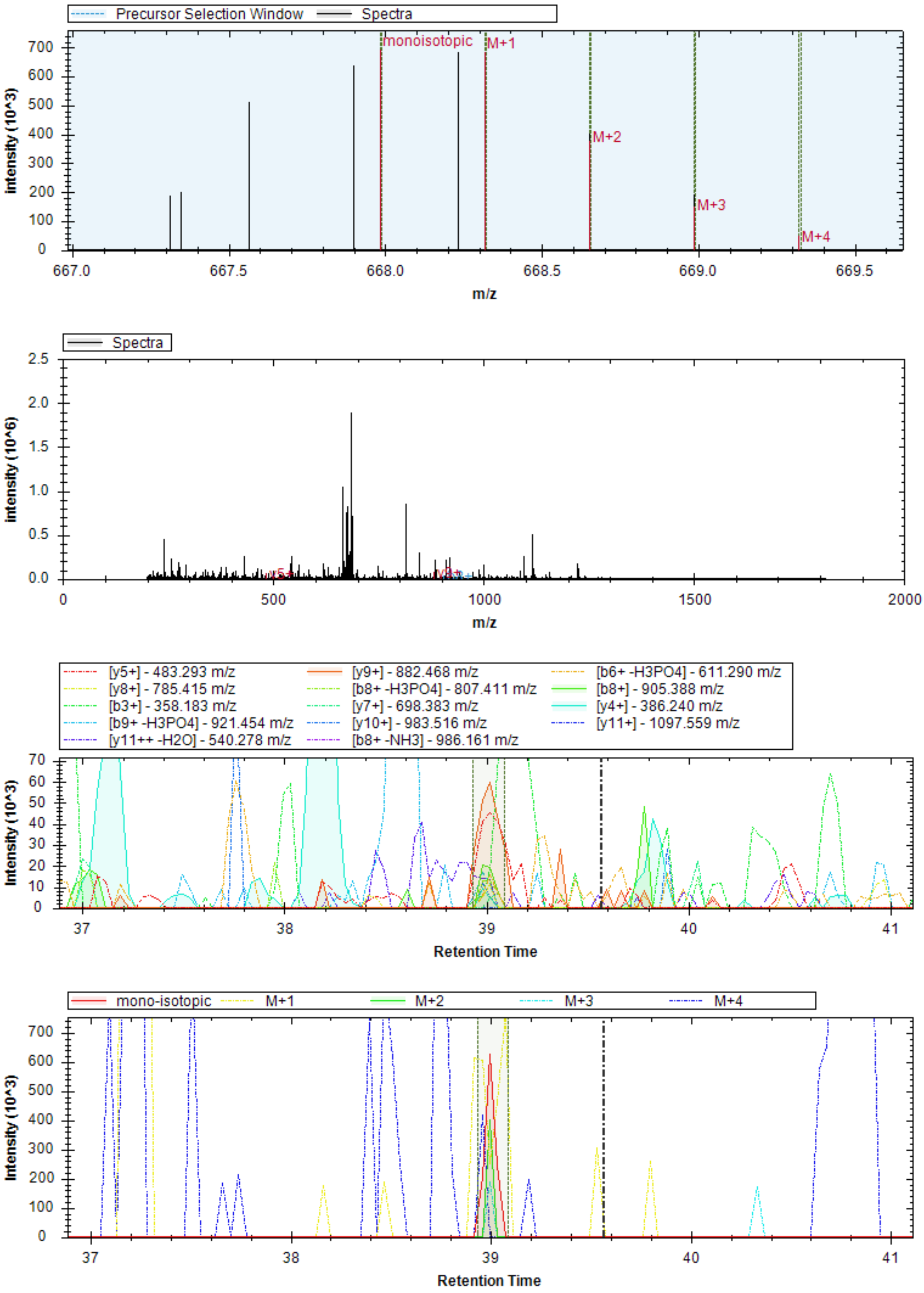

Q3UTZ3 – _SQS[+H+O3+P]FSHQQPSR_.2

Fig4_MP-DIA-barrel-cortex-p9-3_MHRM.htrms

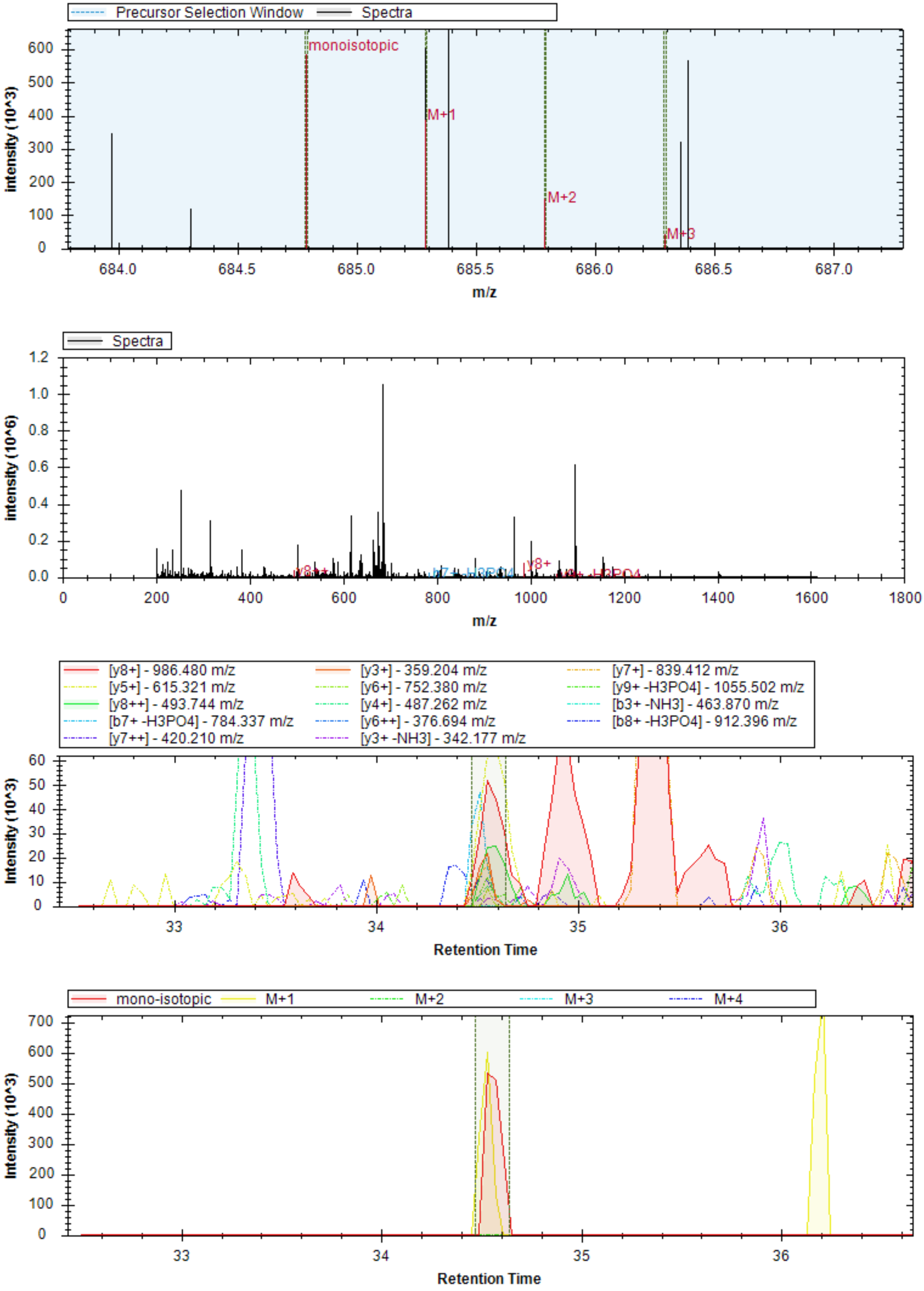

Q6A078 – _T[+H+O3+P]IGLM[+O]KKVVEK_.2
Fig4_MP-DIA-barrel-cortex-p9-3_MHRM.htrms

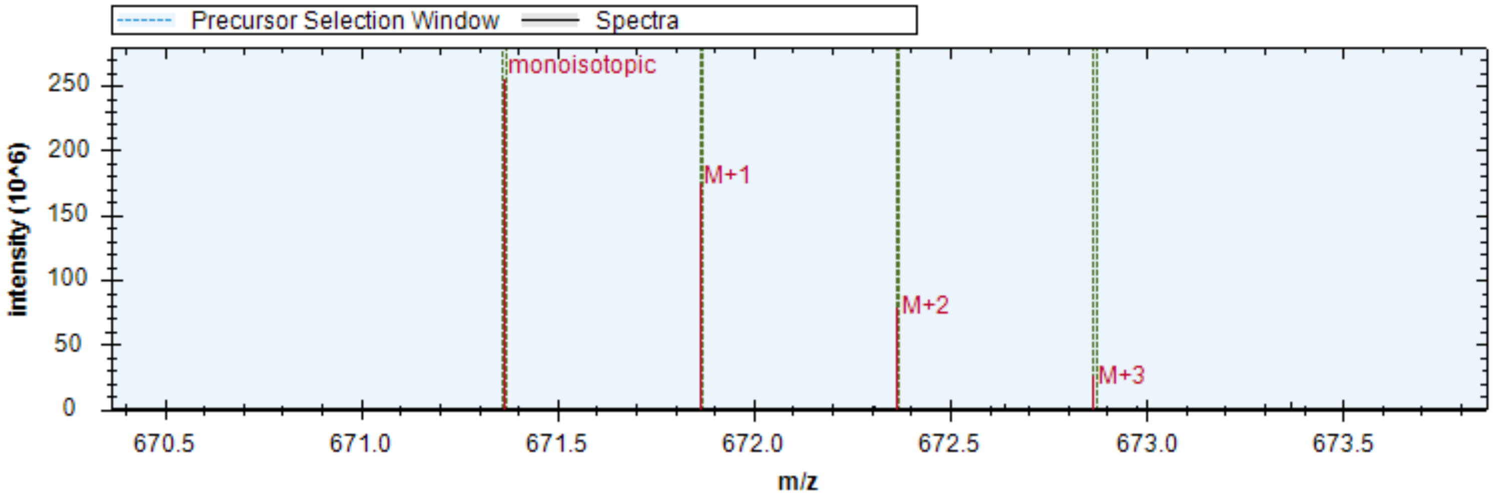

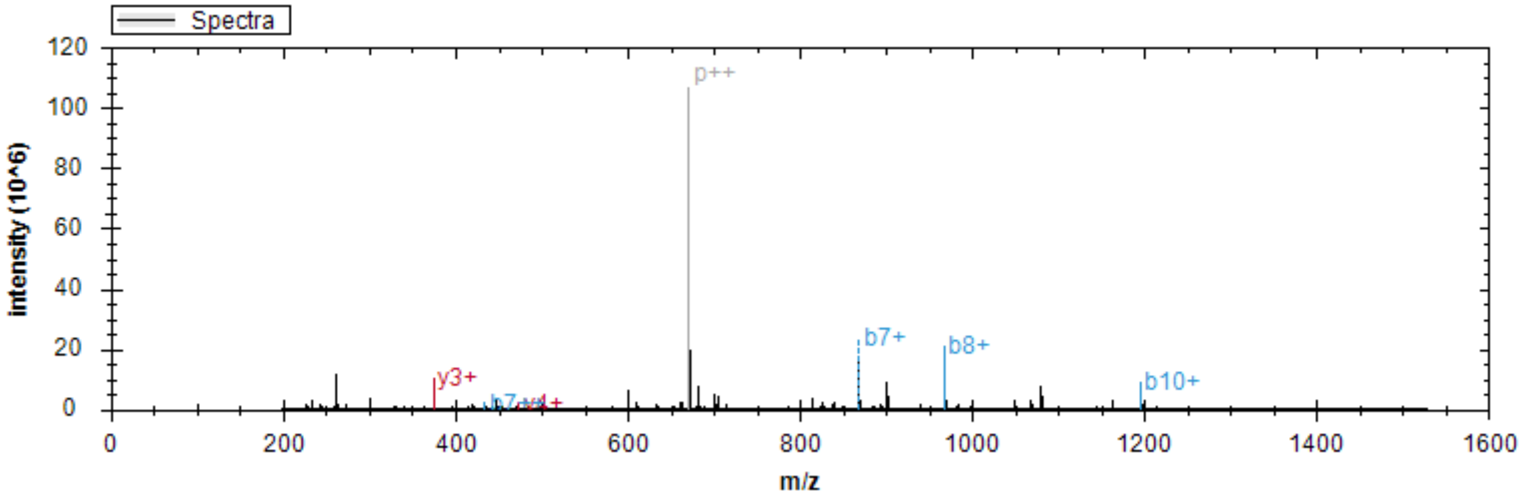

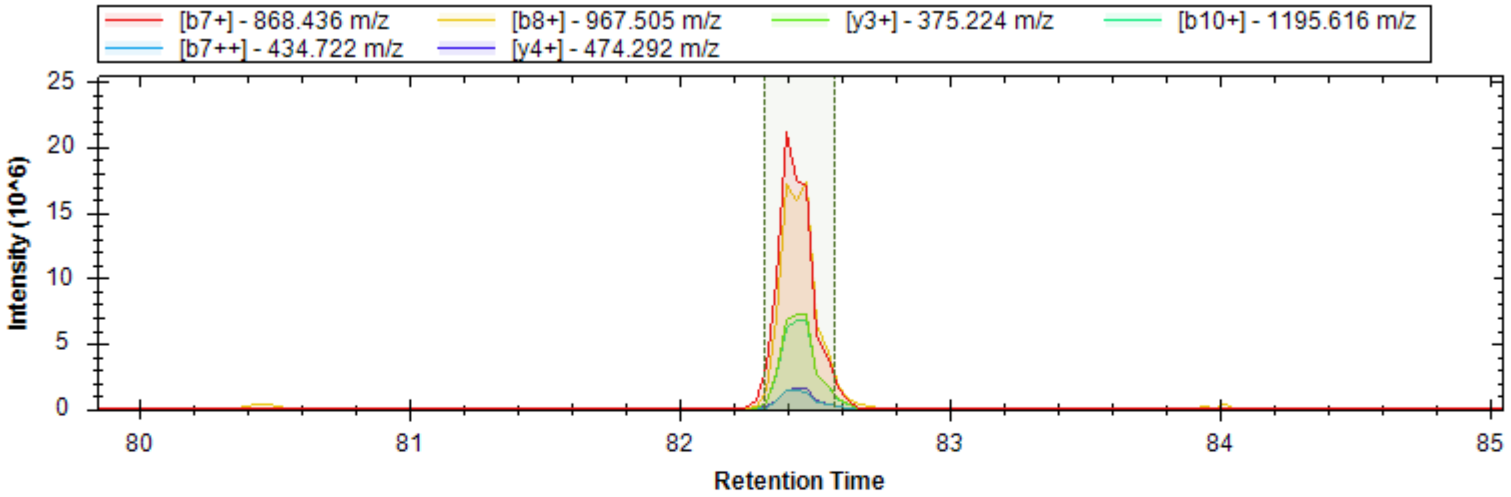

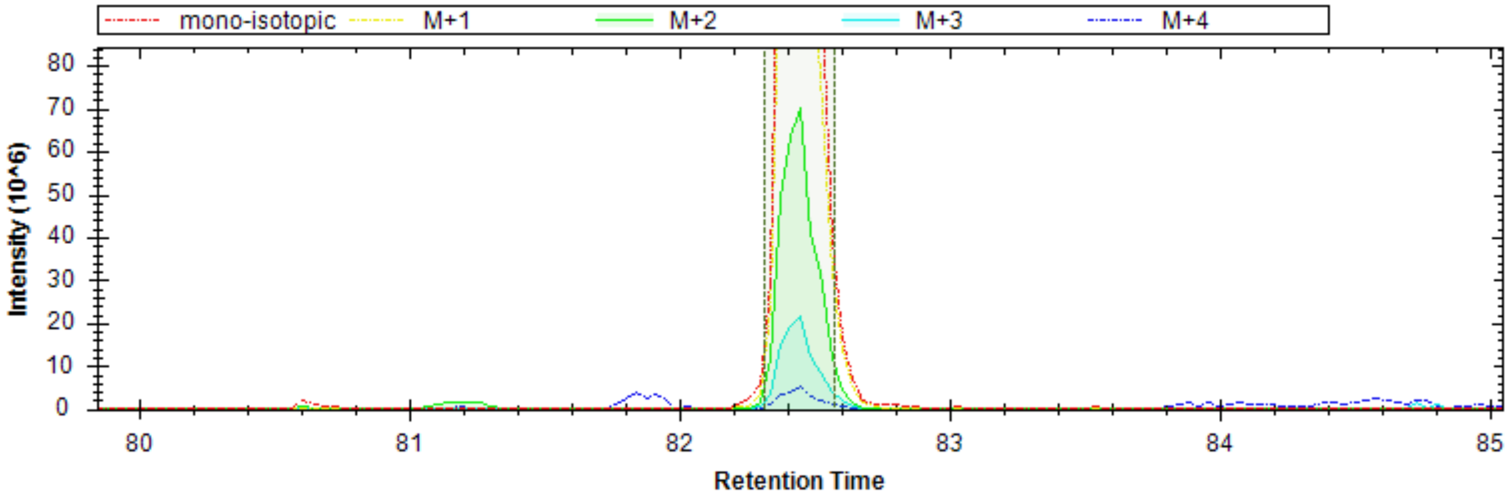

Q8C0E2 – _SMS[+H+O3+P]HQAAIASQR_.2
Fig4_MP-DIA-barrel-cortex-p9-3_MHRM.htrms

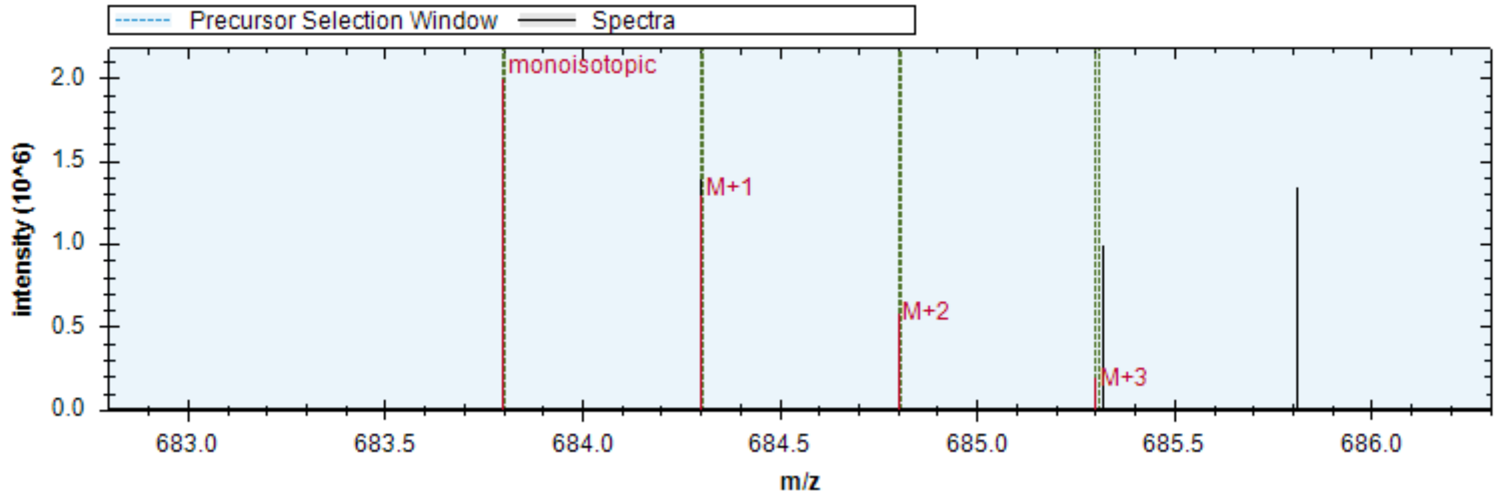

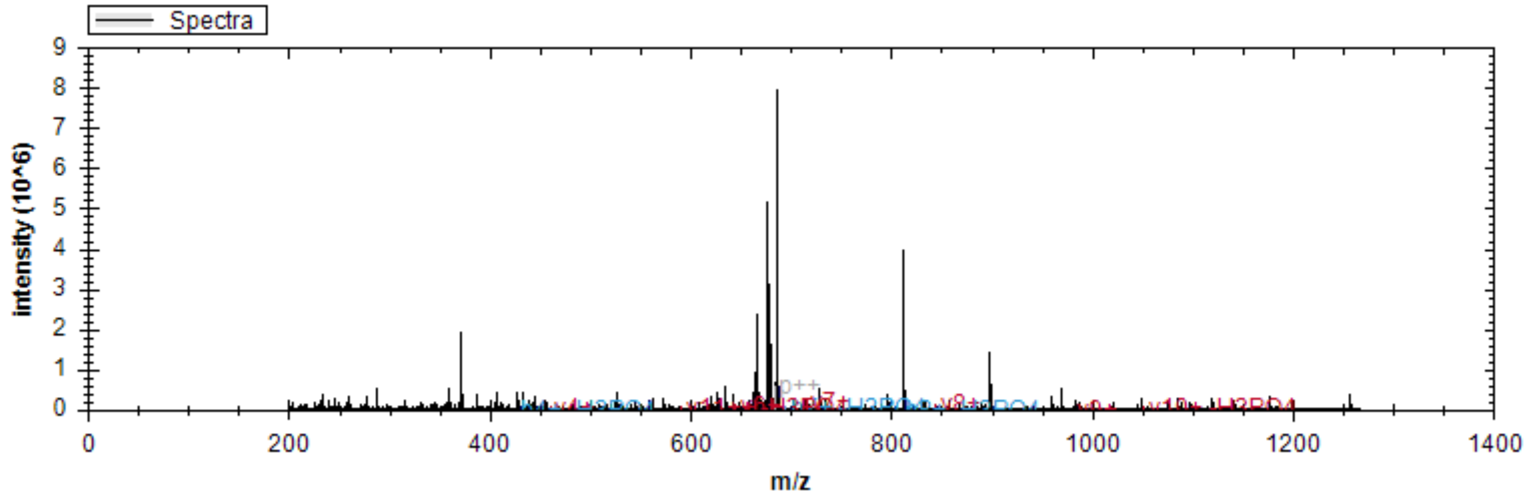

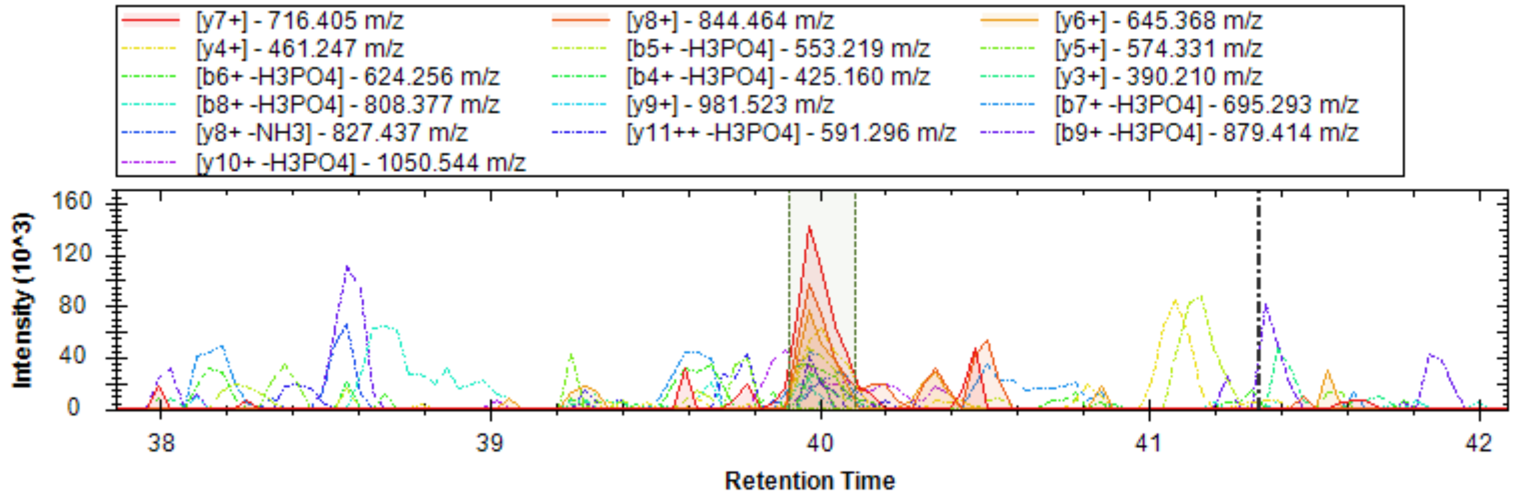

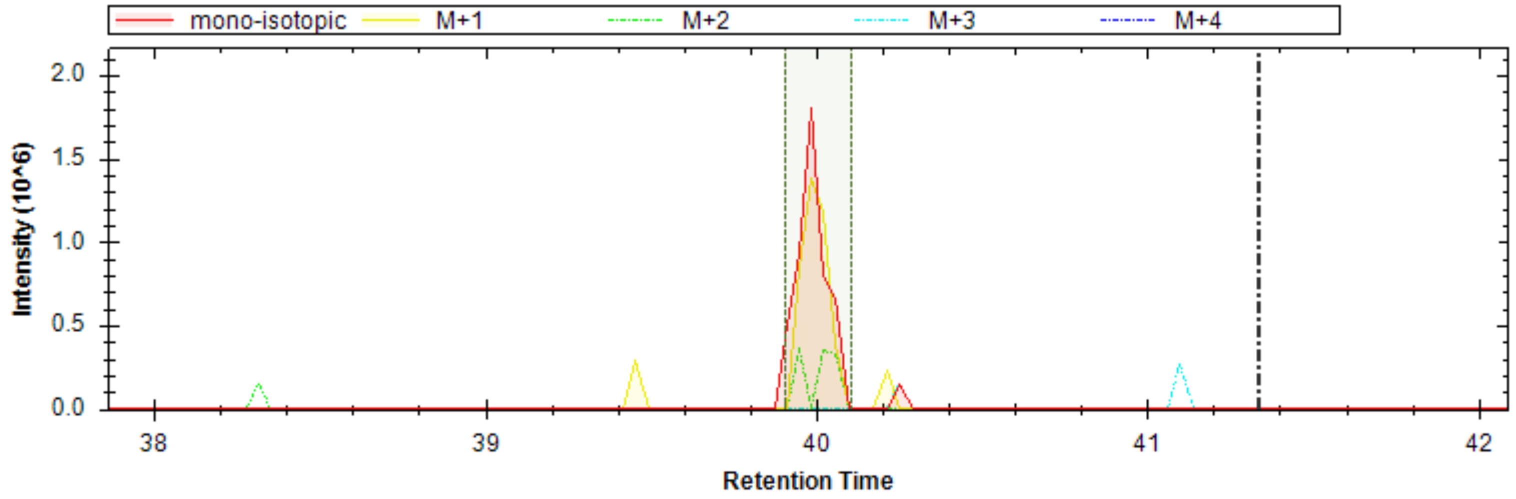

Q8CDU4;Q8CDU4-2 – _[+C2+H2+O]ASLRS[+H+O3+P]ATPRLR_.2

Fig4_MP-DIA-barrel-cortex-p9-3_MHRM.htrms

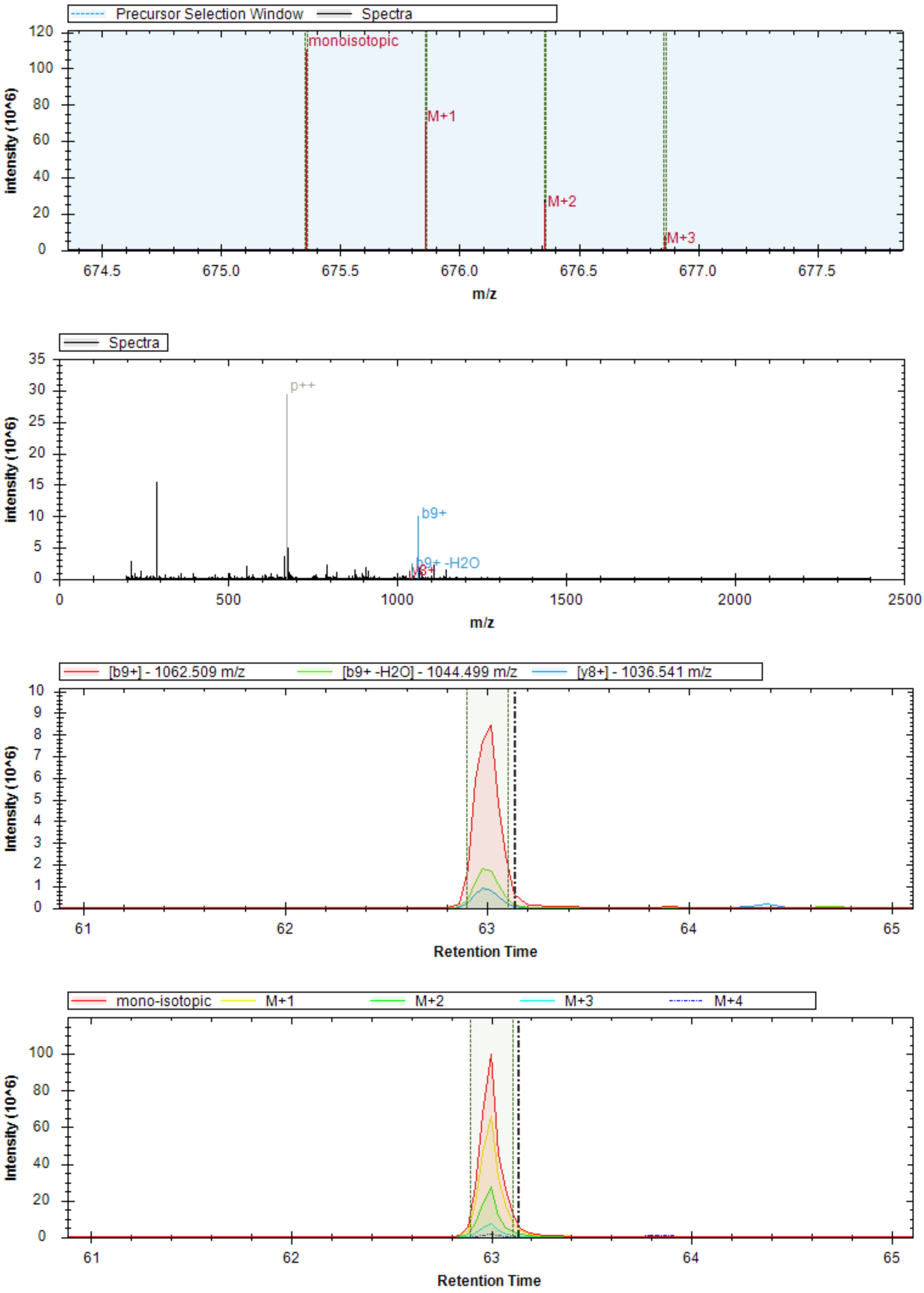

Q8R5L3;Q8R5L3-2 – _LQLY[+H+O3+P]FWKDR_.2
Fig4_MP-DIA-barrel-cortex-p9-3_MHRM.htrms

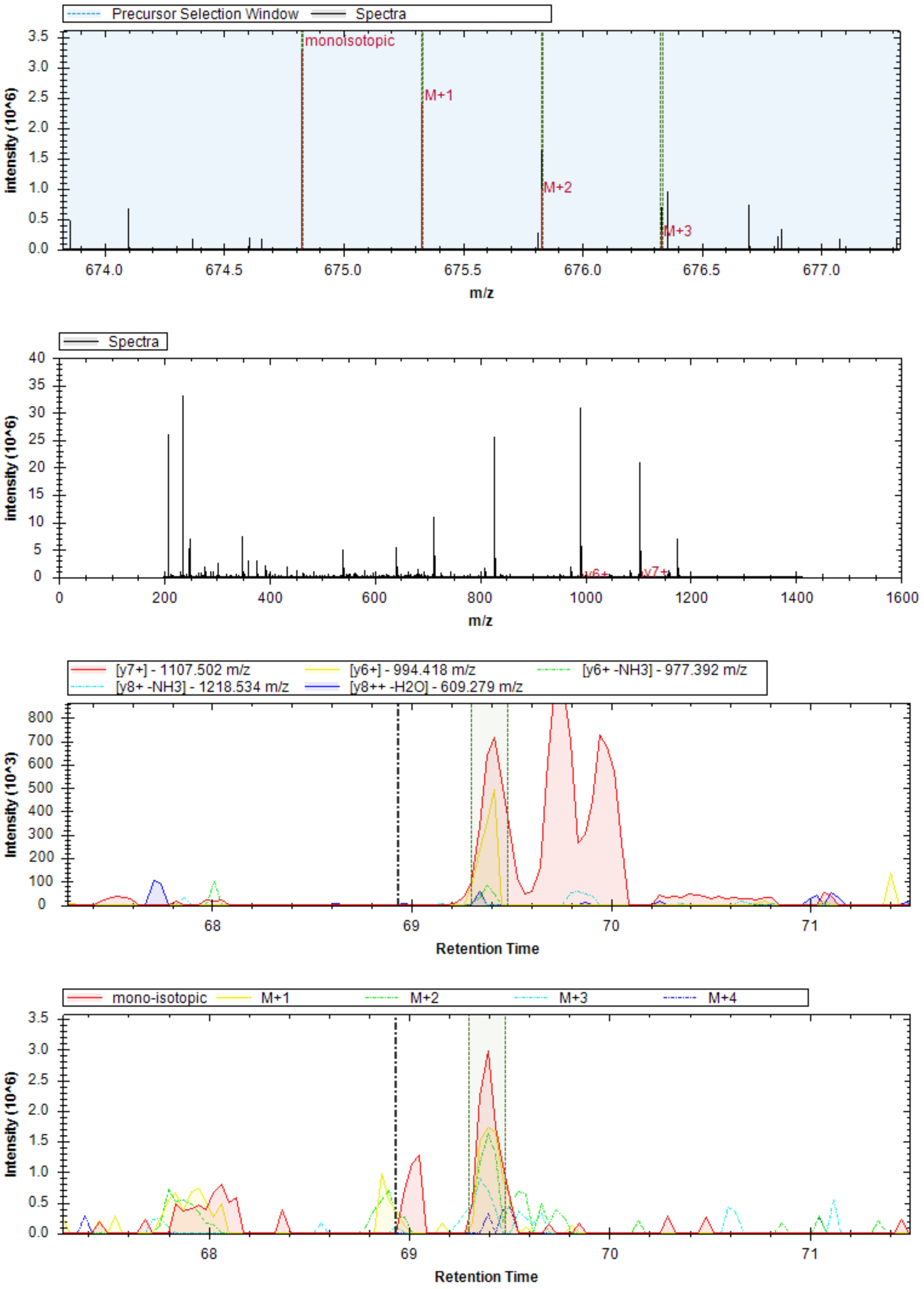

O88935 – _PVAGGPGAPPAARPPAS[+H+O3+P]PSPQR_.3
Fig4_MP-DIA-barrel-cortex-p9-3_MHRM.htrms

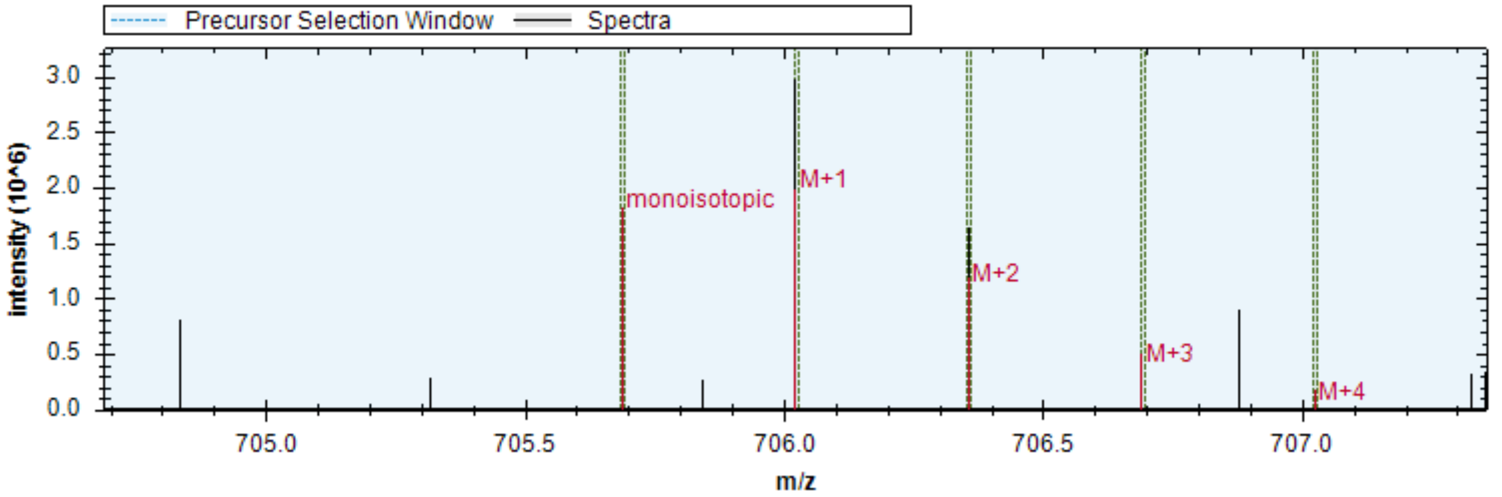

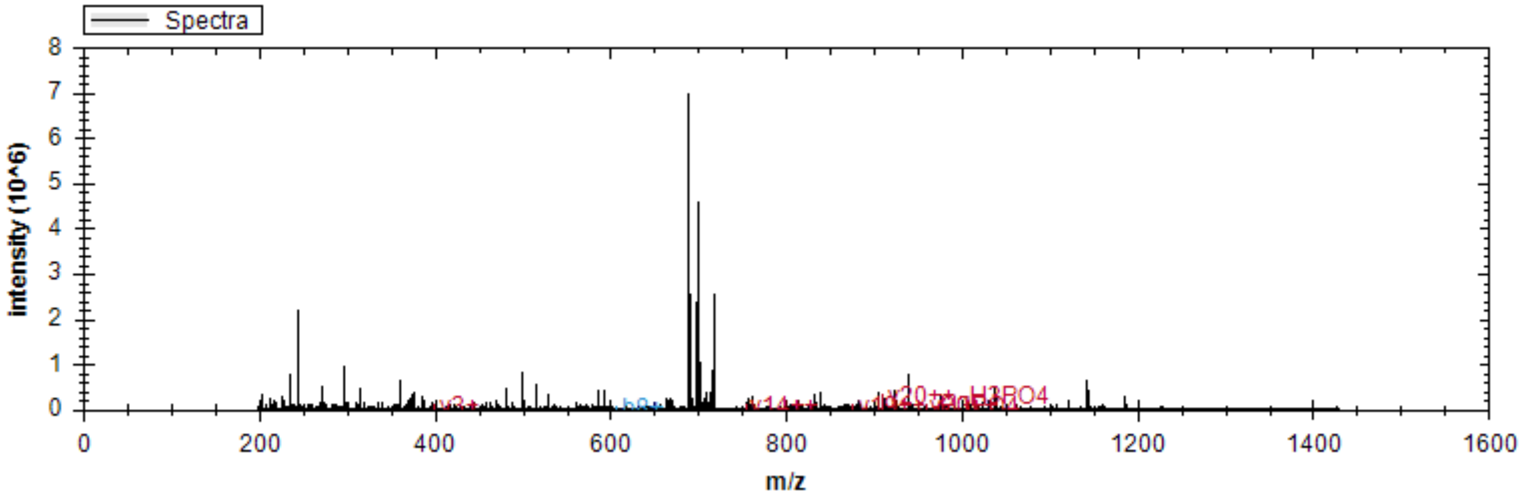

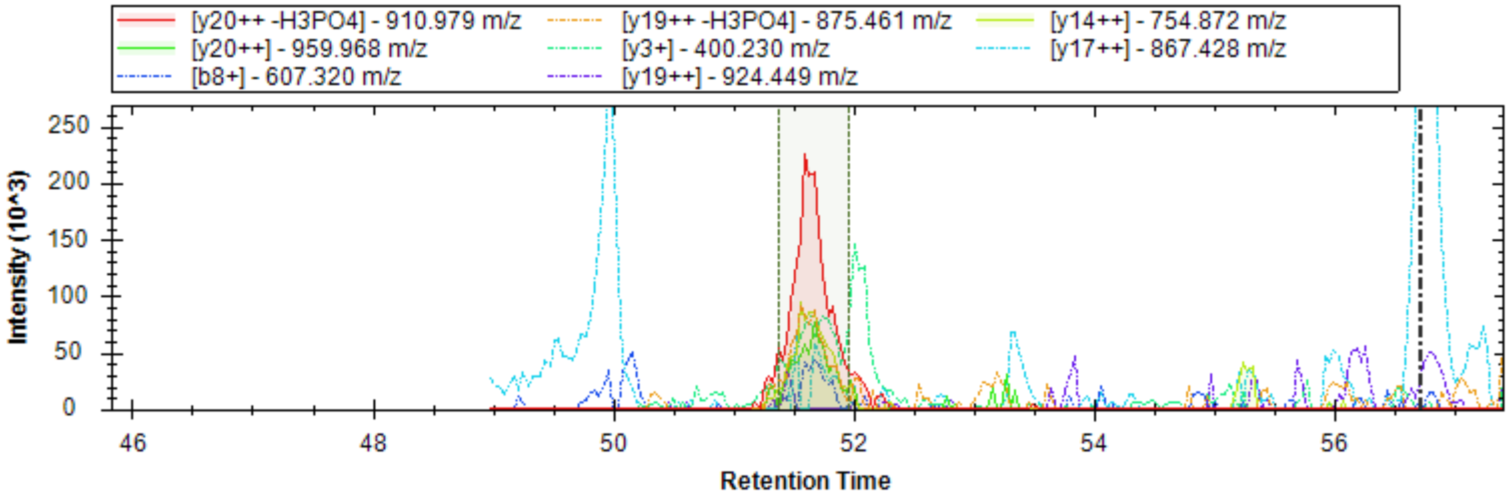

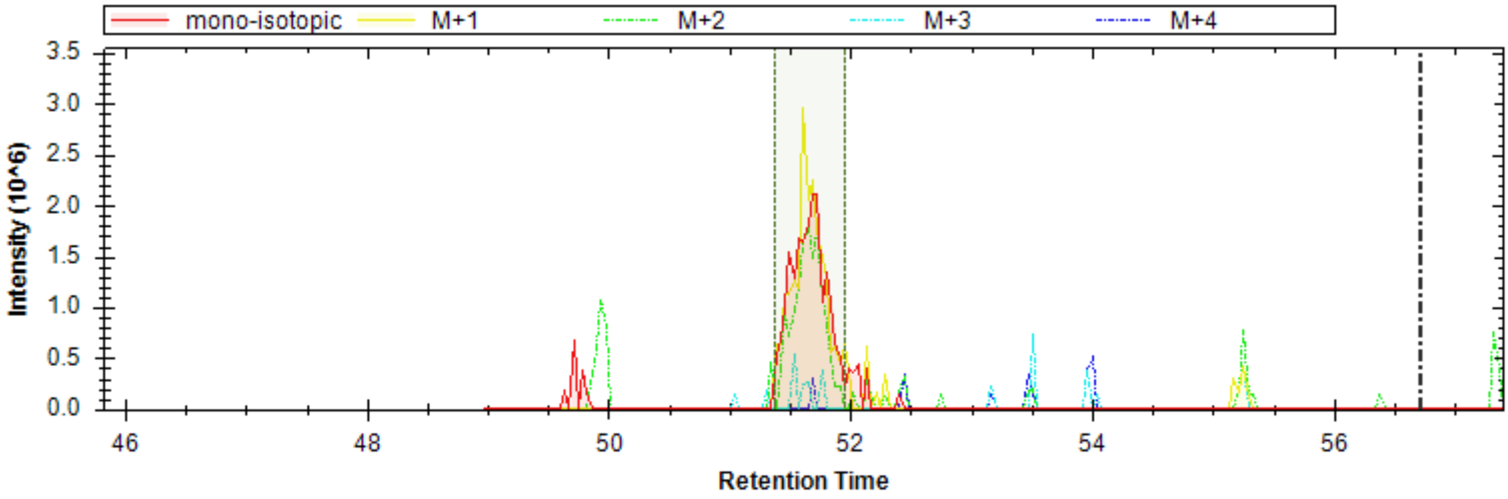

O88935 – _PVAGGPGAPPAARPPASPS[+H+O3+P]PQR_.3
Fig4_MP-DIA-barrel-cortex-p9-3_MHRM.htrms

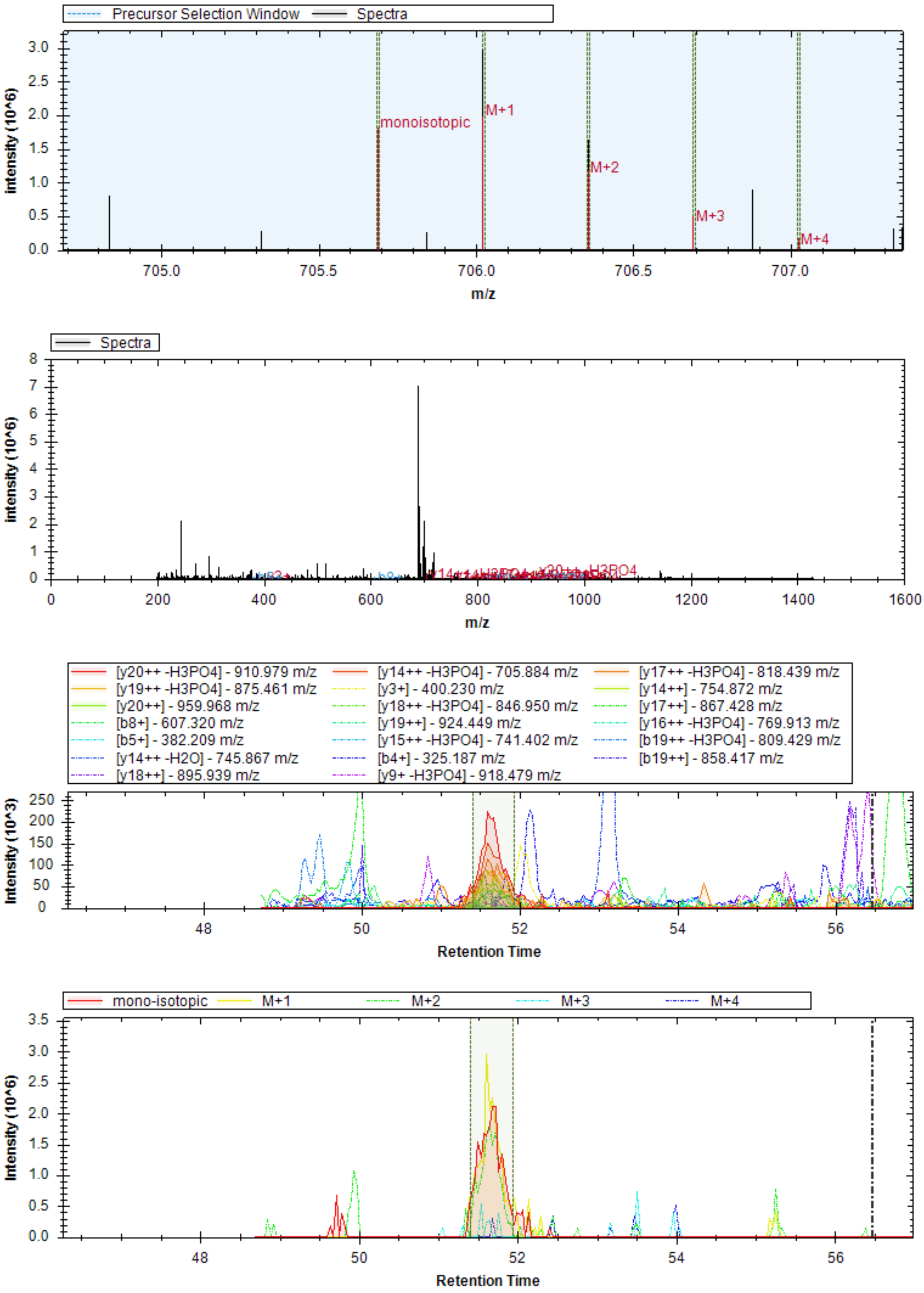

P20357 – _VTDGISKS[+H+O3+P]PEKR_.2
Fig4_MP-DIA-barrel-cortex-p9-3_MHRM.htrms

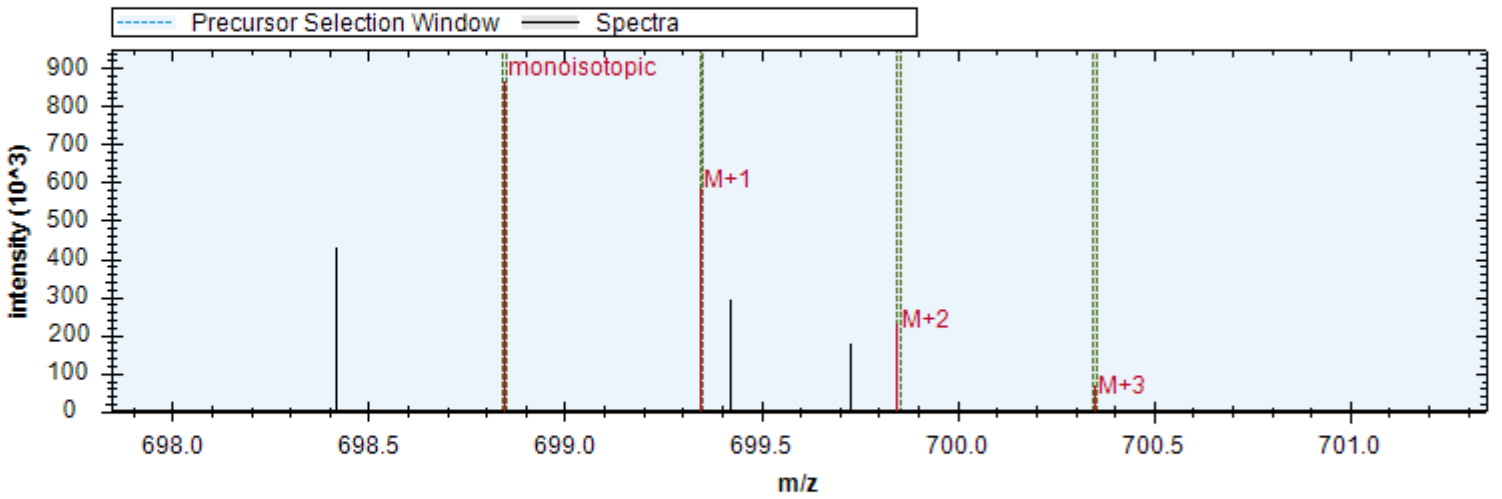

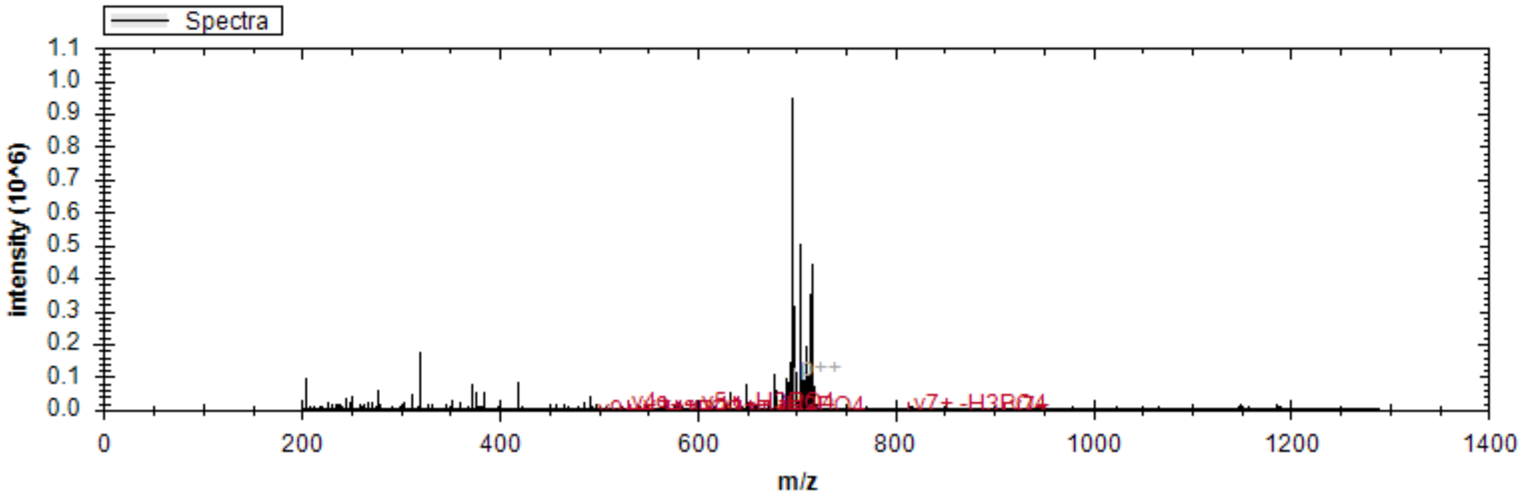

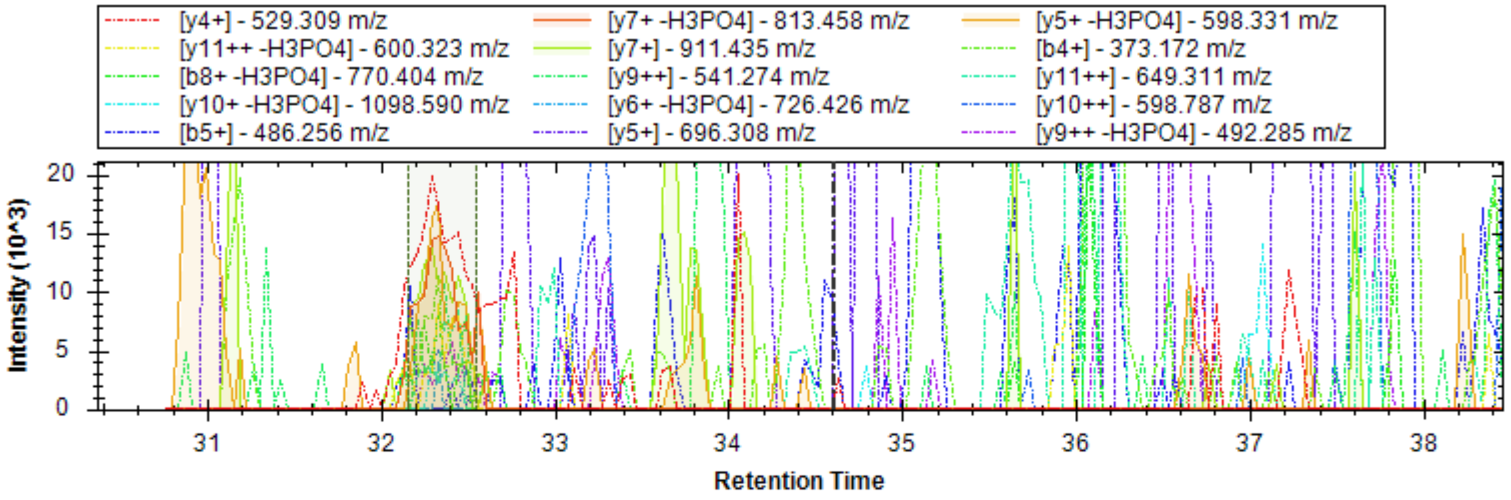

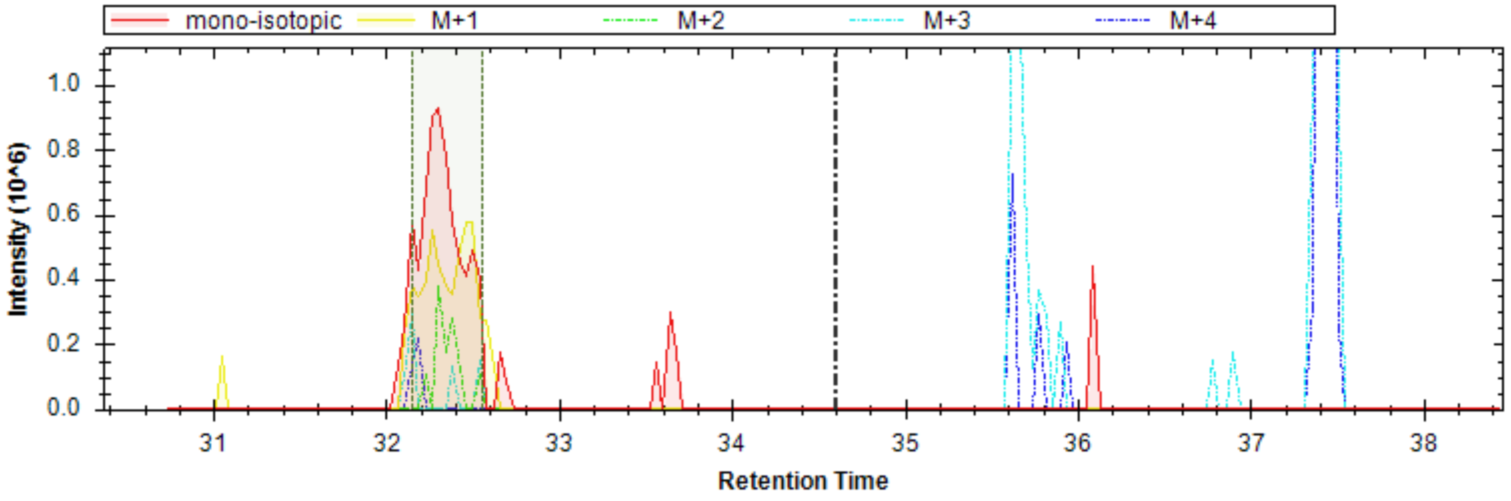

P28738 – _PIRPGHYPAS[+H+O3+P]SPTAVHAVR_.3

Fig4_MP-DIA-barrel-cortex-p9-3_MHRM.htrms

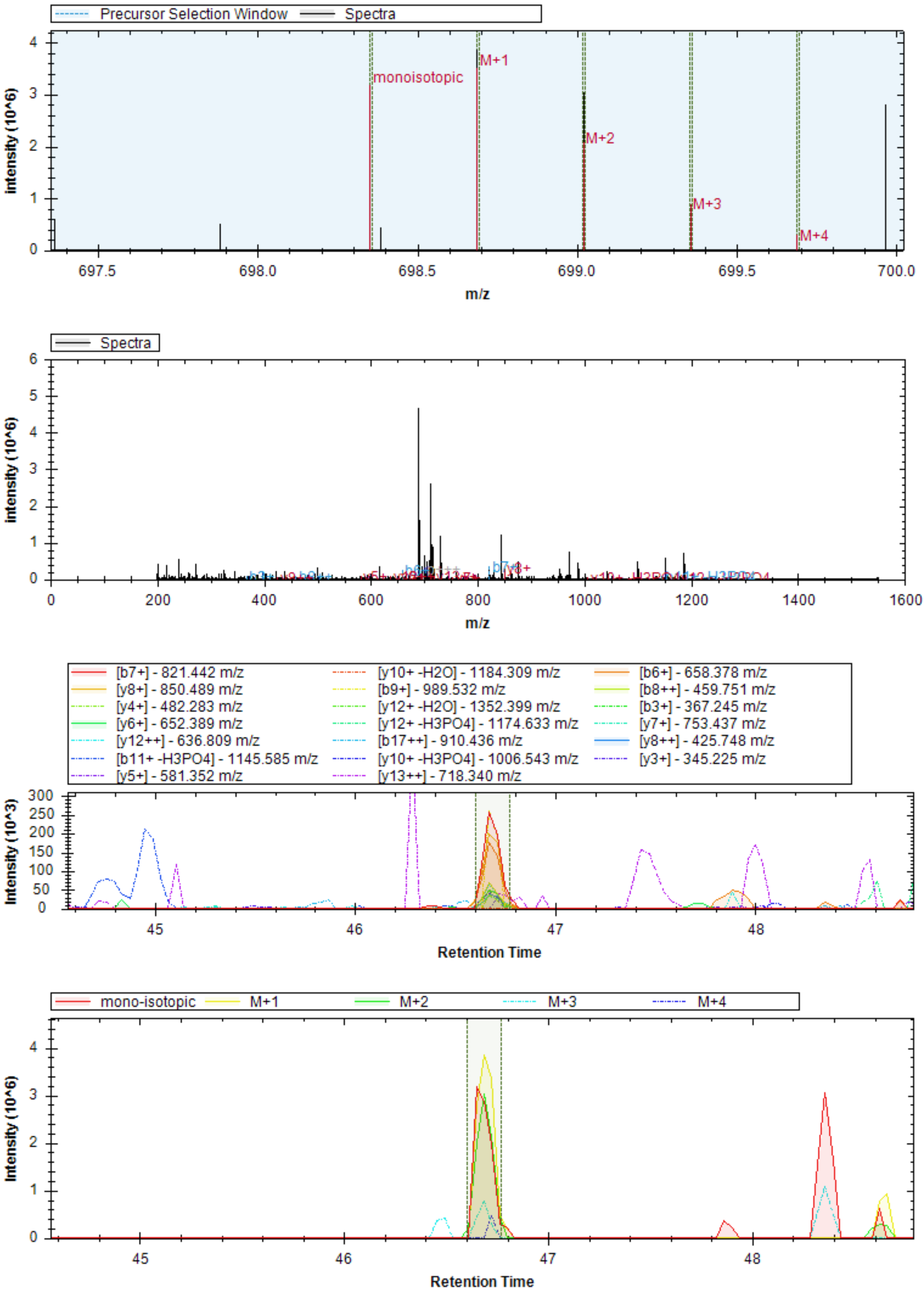

P28738 – _PIRPGHYPASS[+H+O3+P]PTAVHAVR_.3
Fig4_MP-DIA-barrel-cortex-p9-3_MHRM.htrms

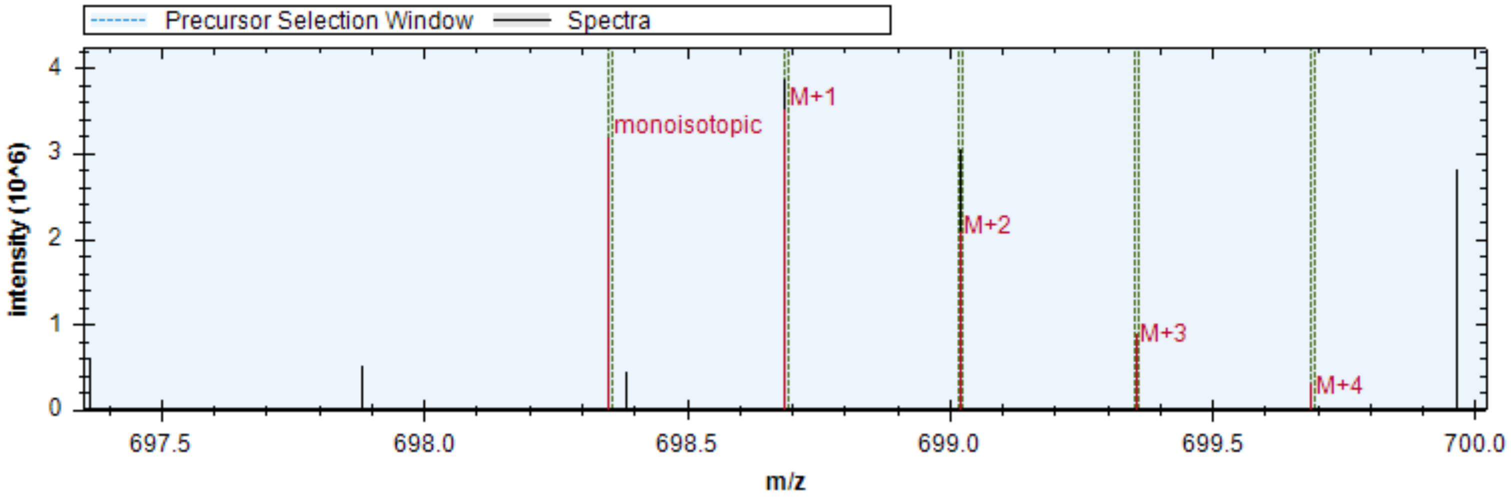

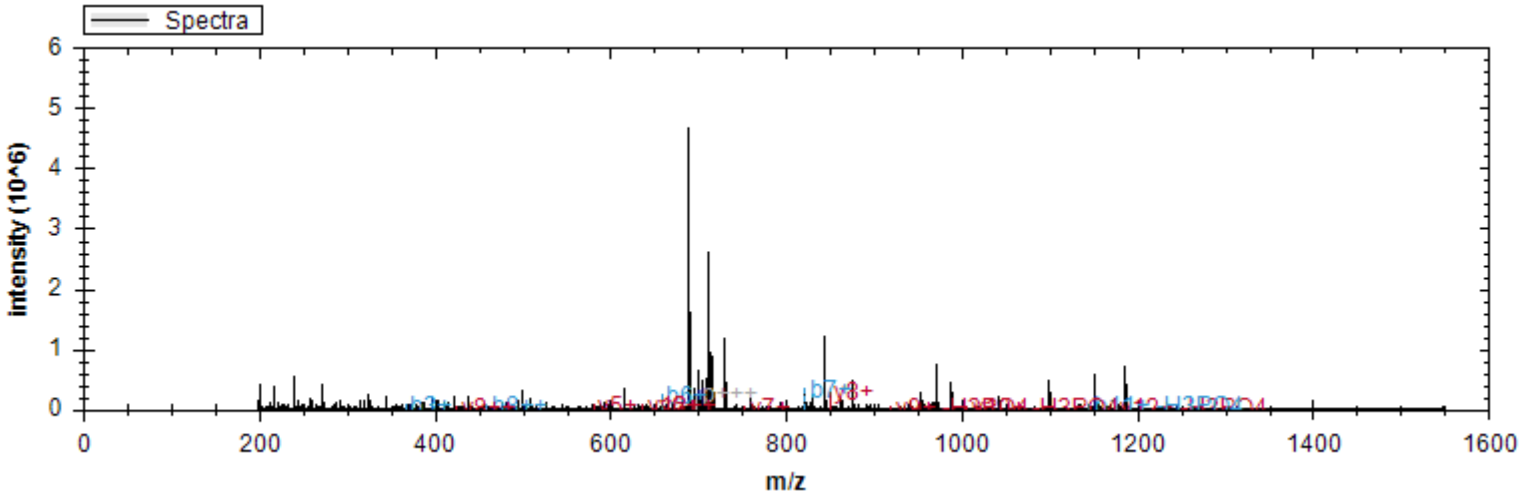

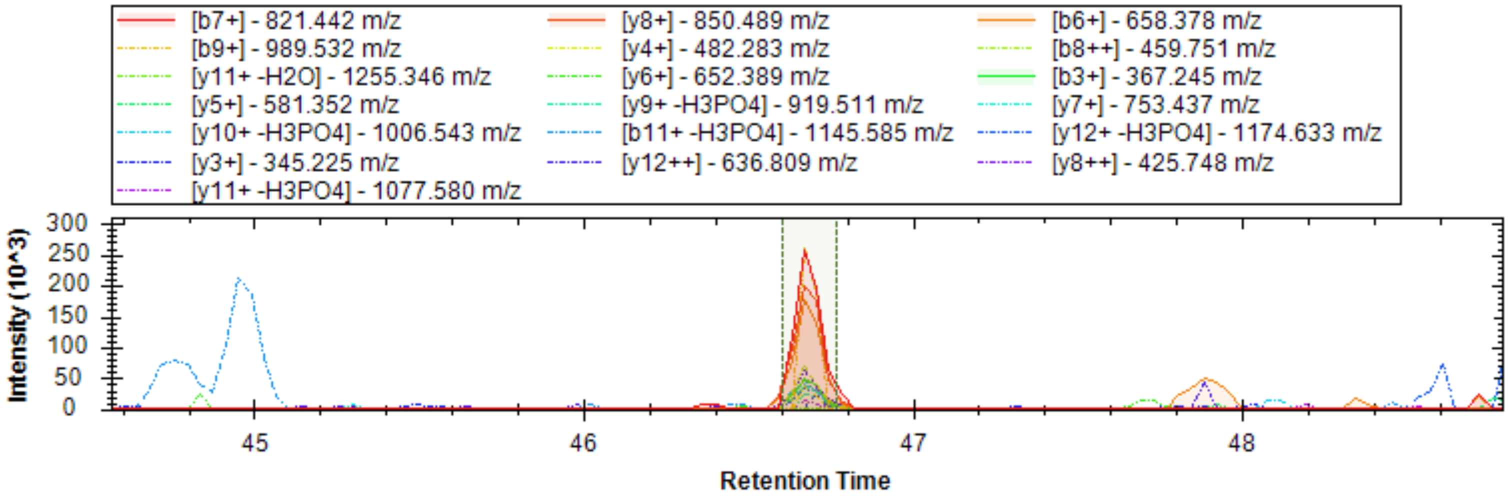

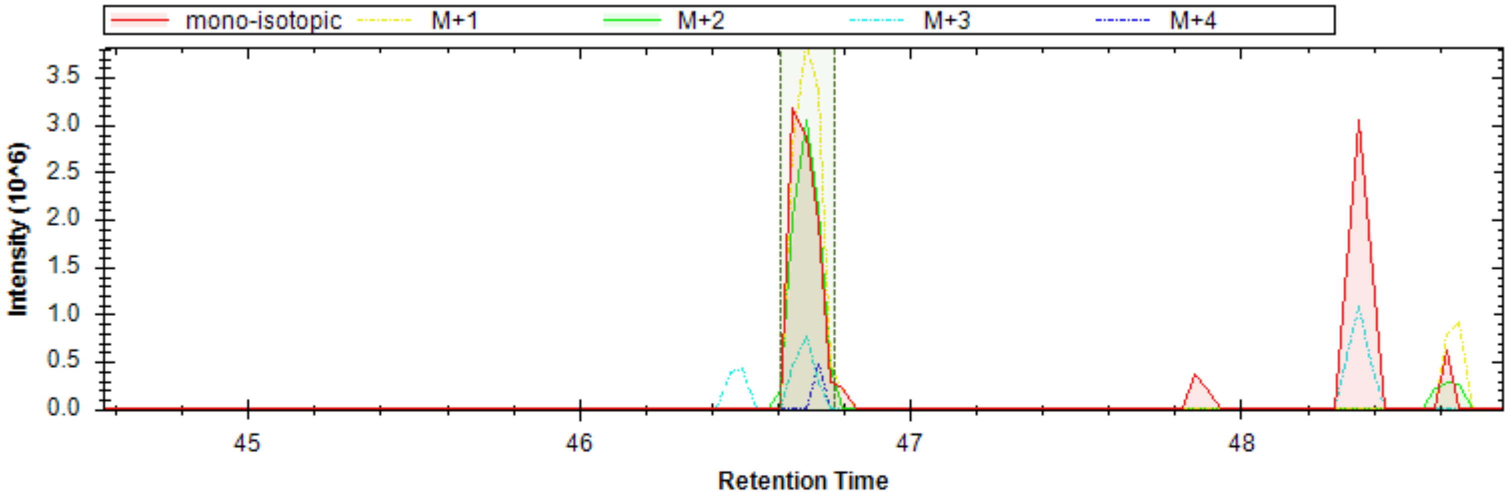

P54227 – _ESVPDFPLS[+H+O3+P]PPK_.2
Fig4_MP-DIA-barrel-cortex-p9-3_MHRM.htrms

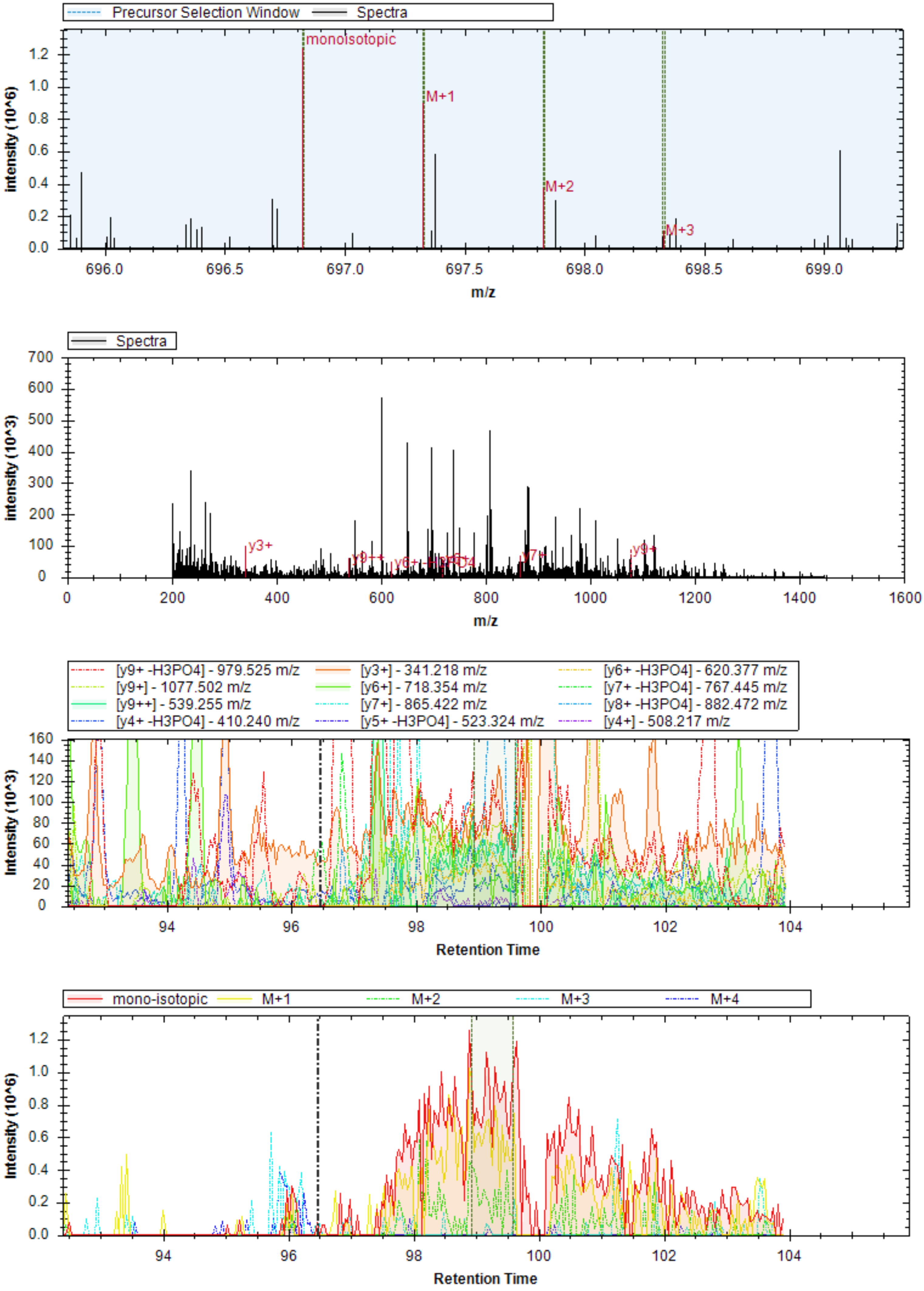

P62751 – _KAY[+H+O3+P]VRLAPDYDALDVANK_.3
Fig4_MP-DIA-barrel-cortex-p9-3_MHRM.htrms

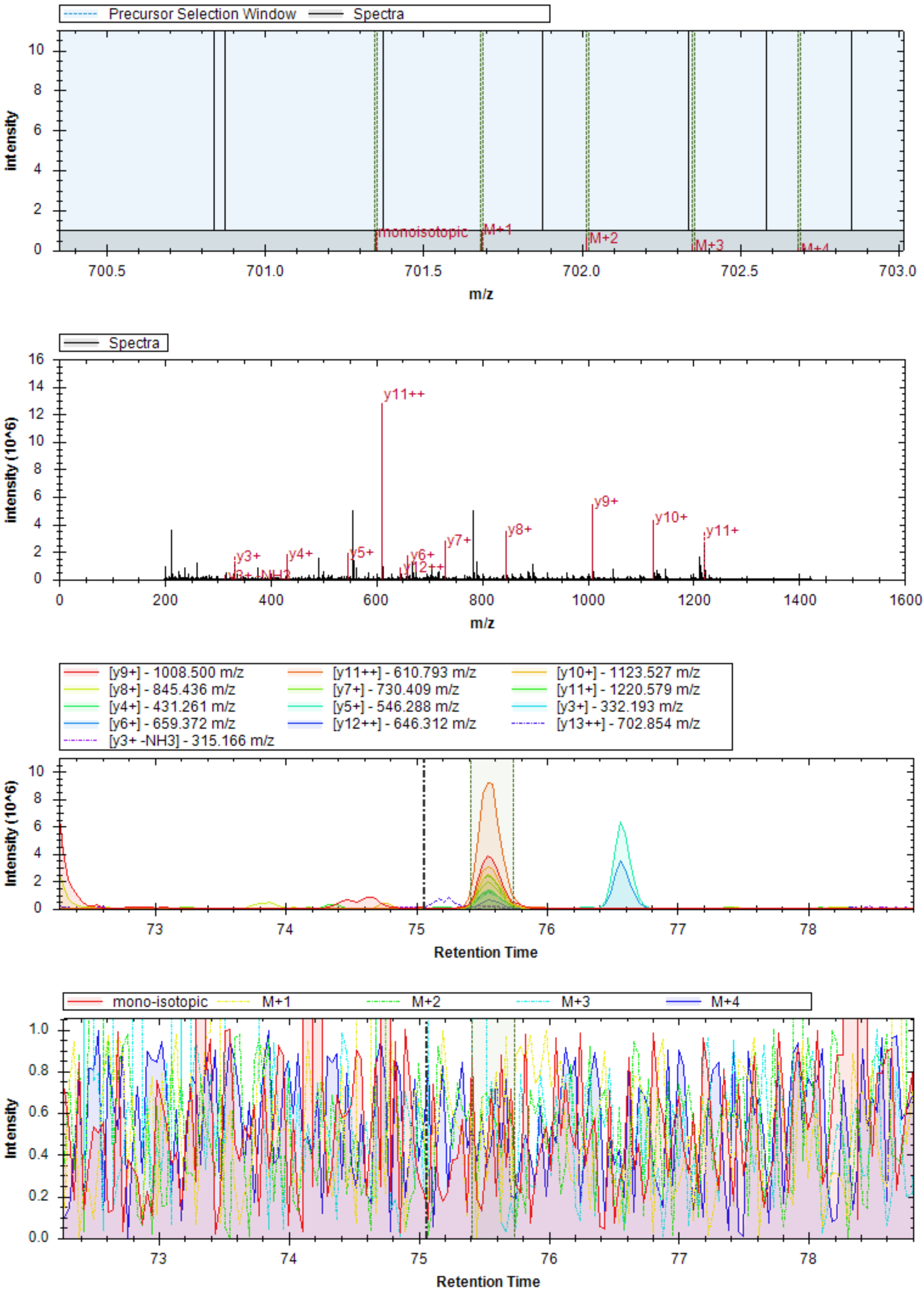

Q3U4B4 – _RHKT[+H+O3+P]LSANEEK_.2
Fig4_MP-DIA-barrel-cortex-p9-3_MHRM.htrms

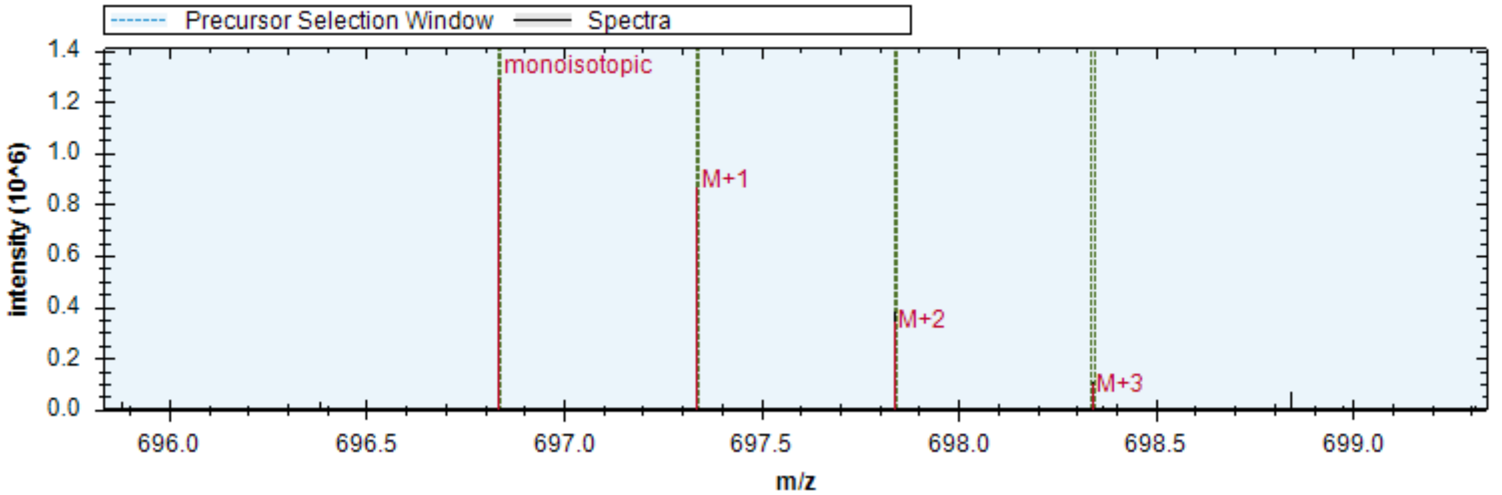

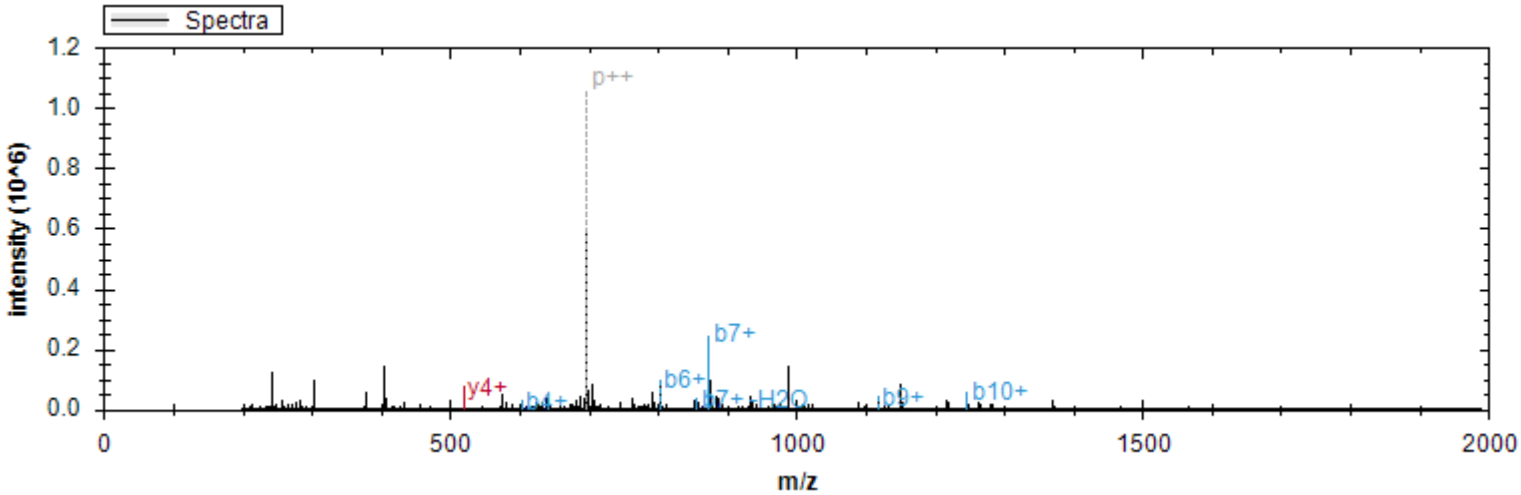

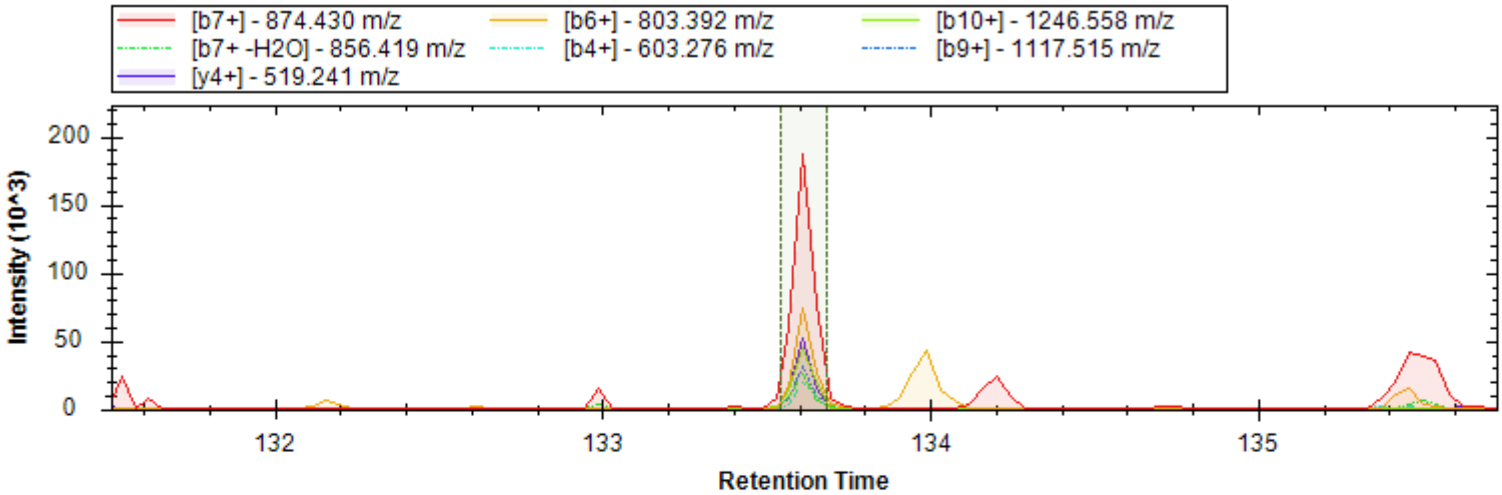

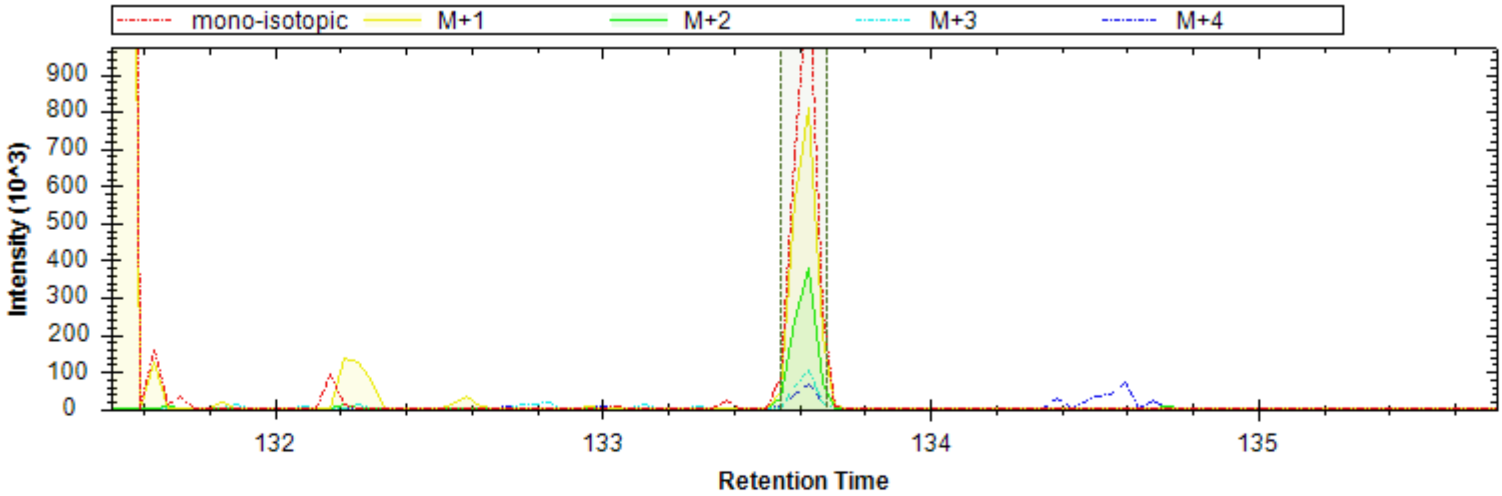

Q3UNH4 – _NSELVS[+H+O3+P]PVKPER_.2

Fig4_MP-DIA-barrel-cortex-p9-3_MHRM.htrms

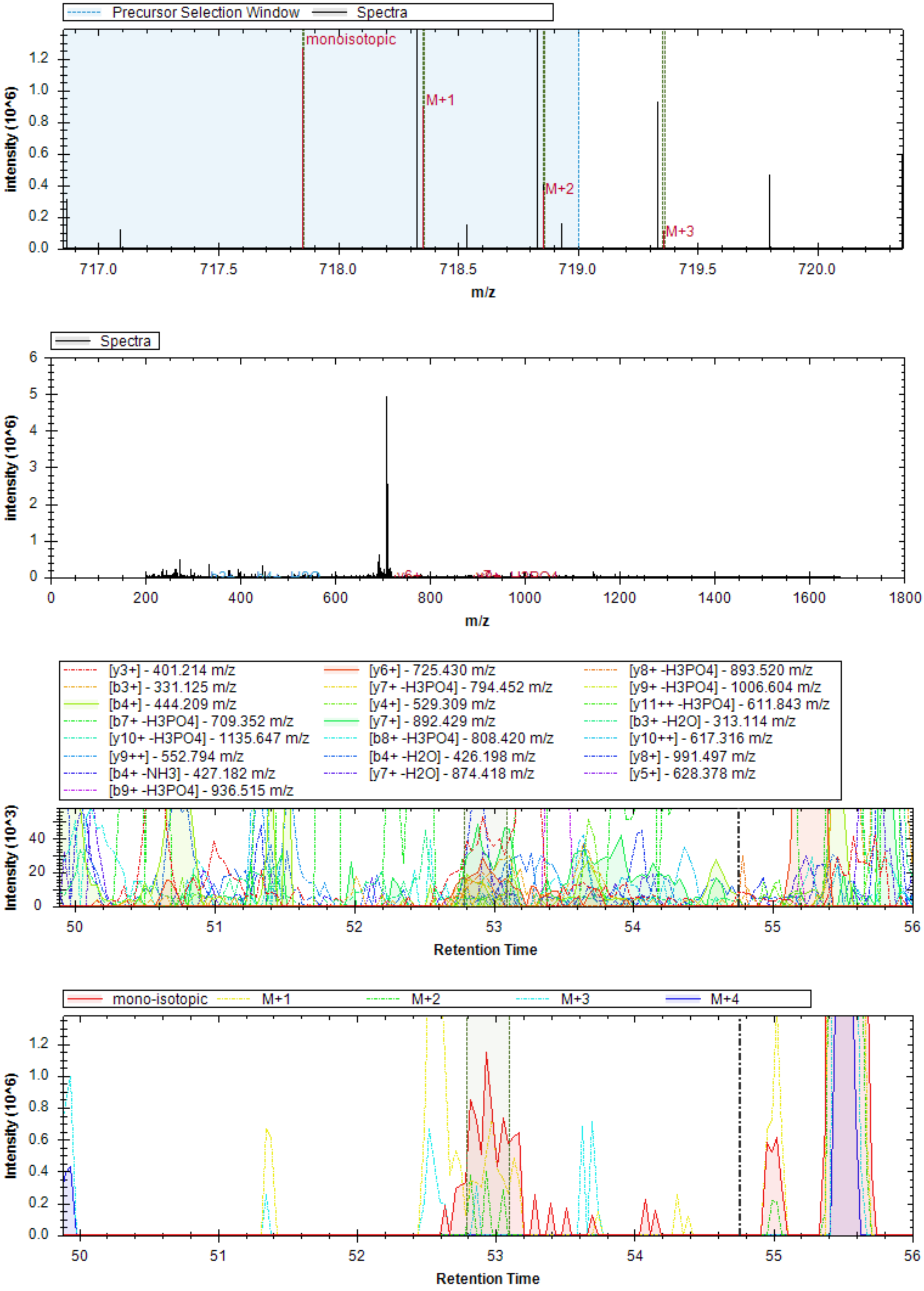

Q8CAA7 – _AVAGVMITAS[+H+O3+P]HNR_.2
Fig4_MP-DIA-barrel-cortex-p9-3_MHRM.htrms

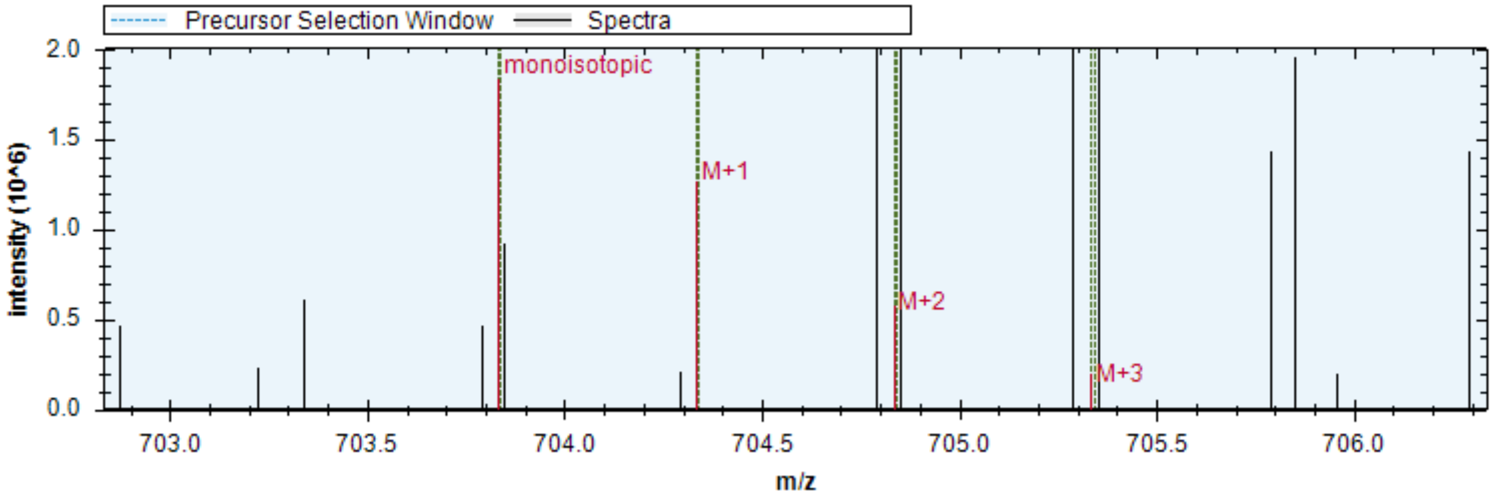

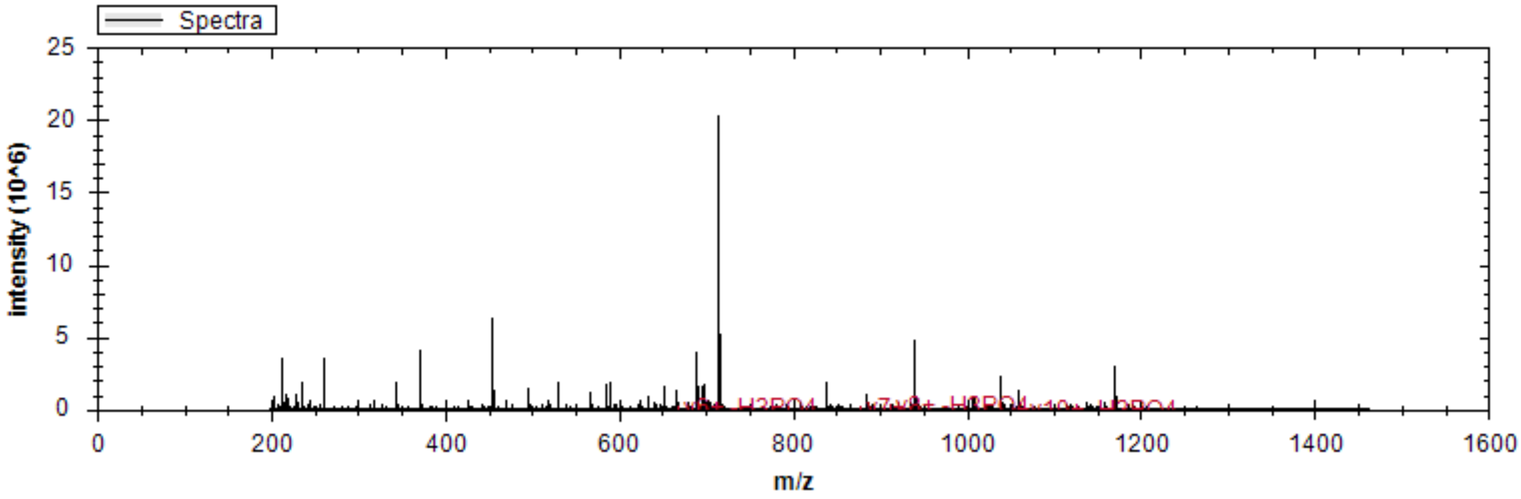

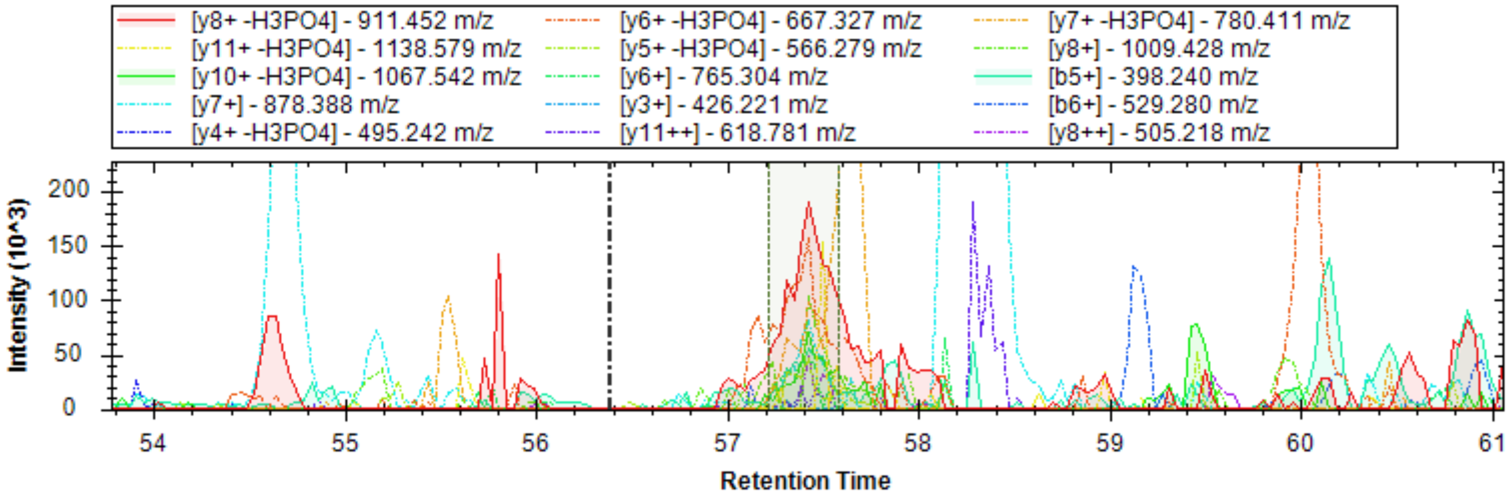

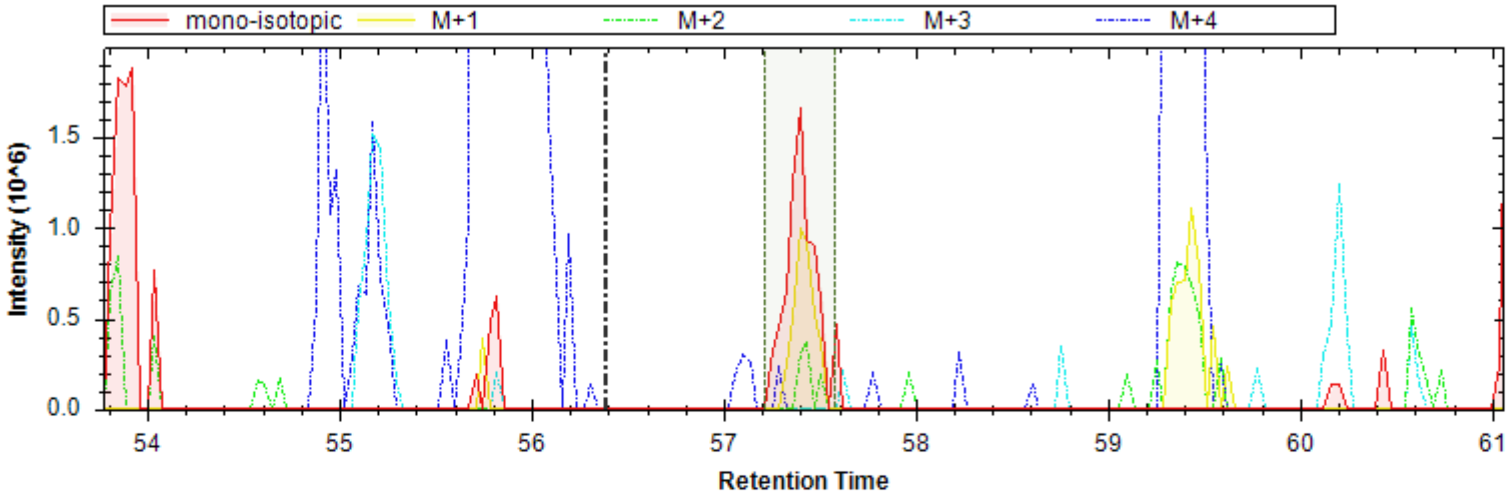

Q9ESE1;Q9ESE1-2 – _VGT[+H+O3+P]VLLIM[+O]HTLK_.2

Fig4_MP-DIA-barrel-cortex-p9-3_MHRM.htrms

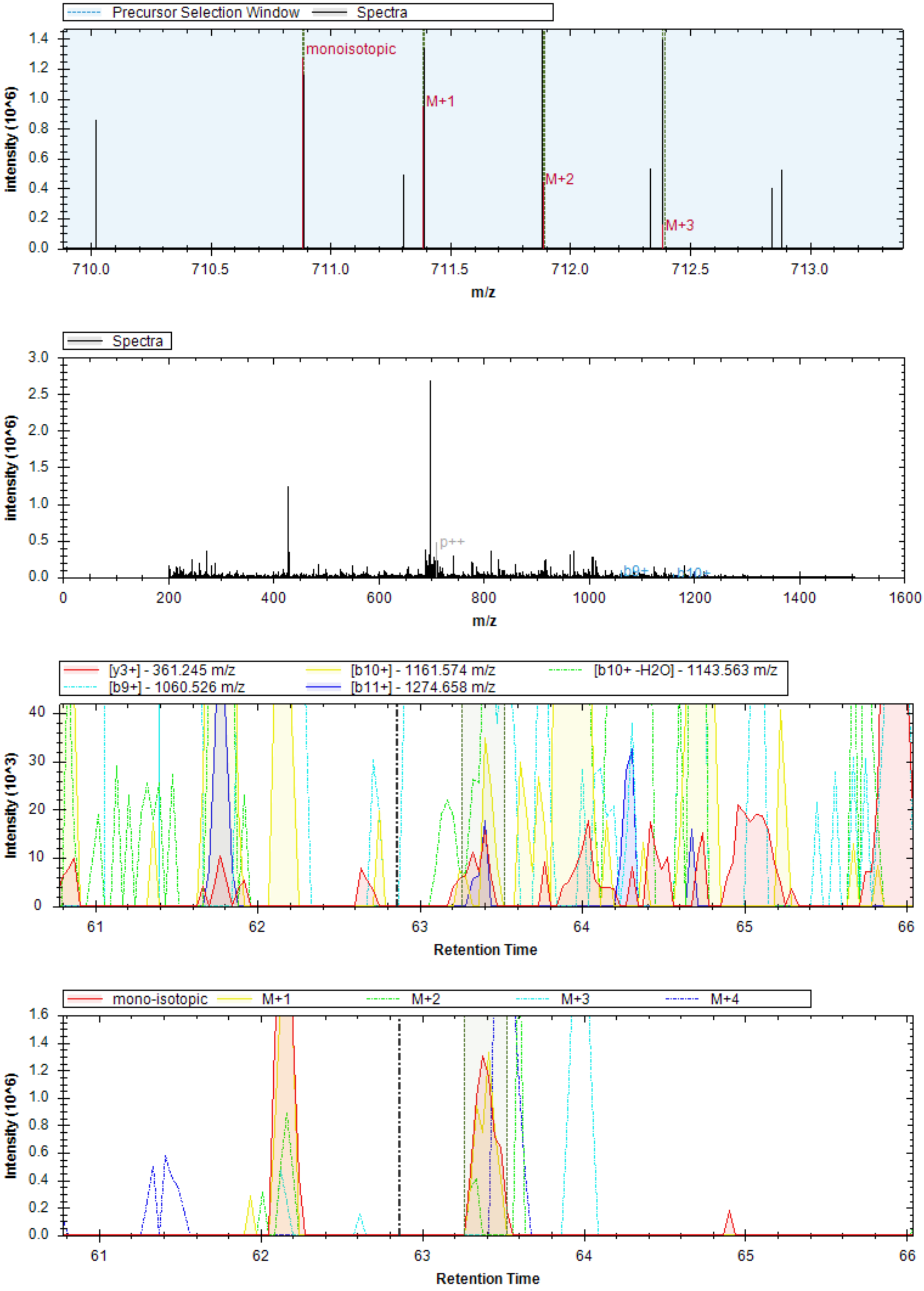

Q9QYC0 – _SPGT[+H+O3+P]PAGEGSGSPPK_.2

Fig4_MP-DIA-barrel-cortex-p9-3_MHRM.htrms

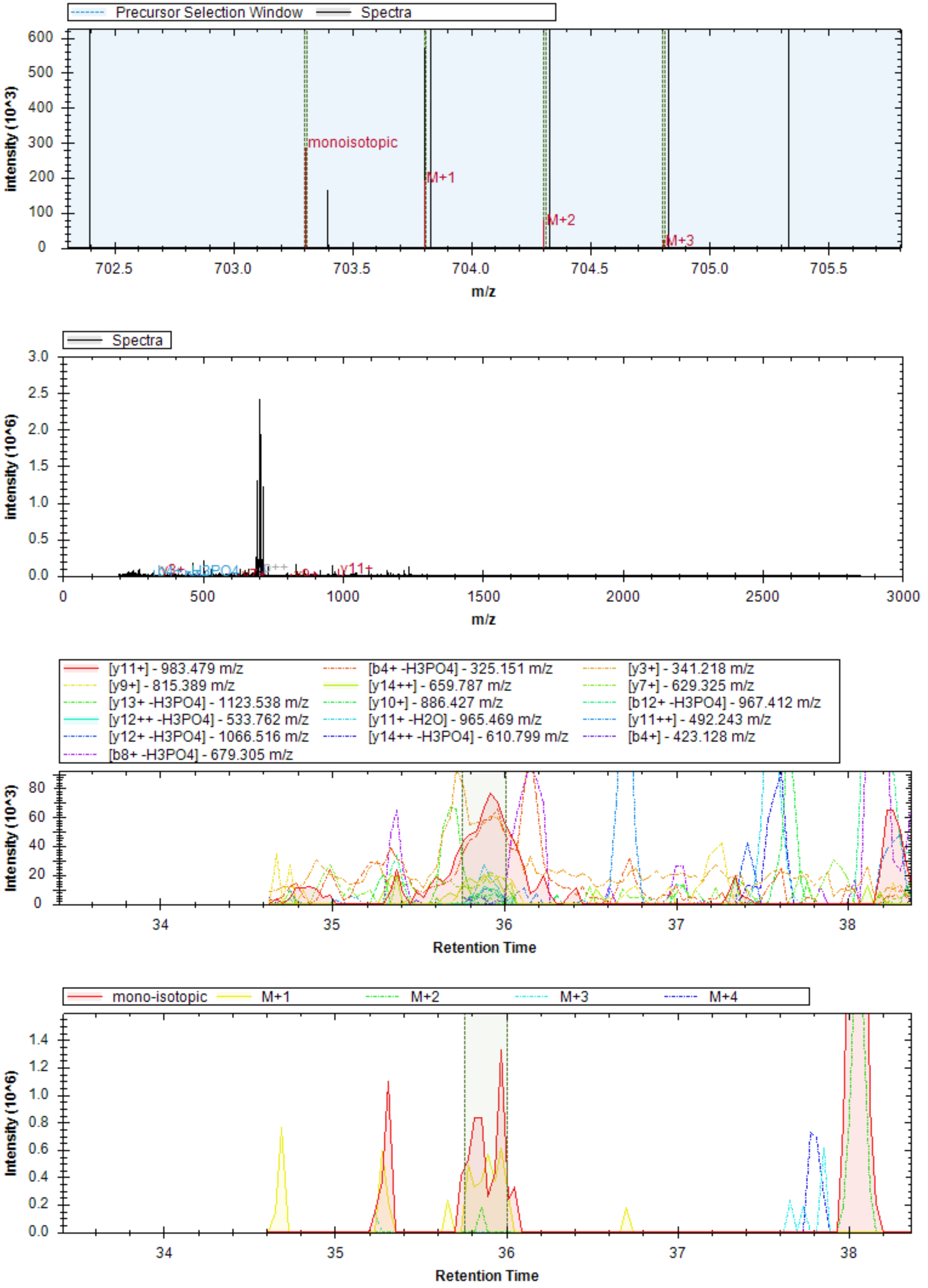

P10637 – _SGYSS[+H+O3+P]PGSPGTPGSR_.2
Fig4_MP-DIA-barrel-cortex-p9-3_MHRM.htrms

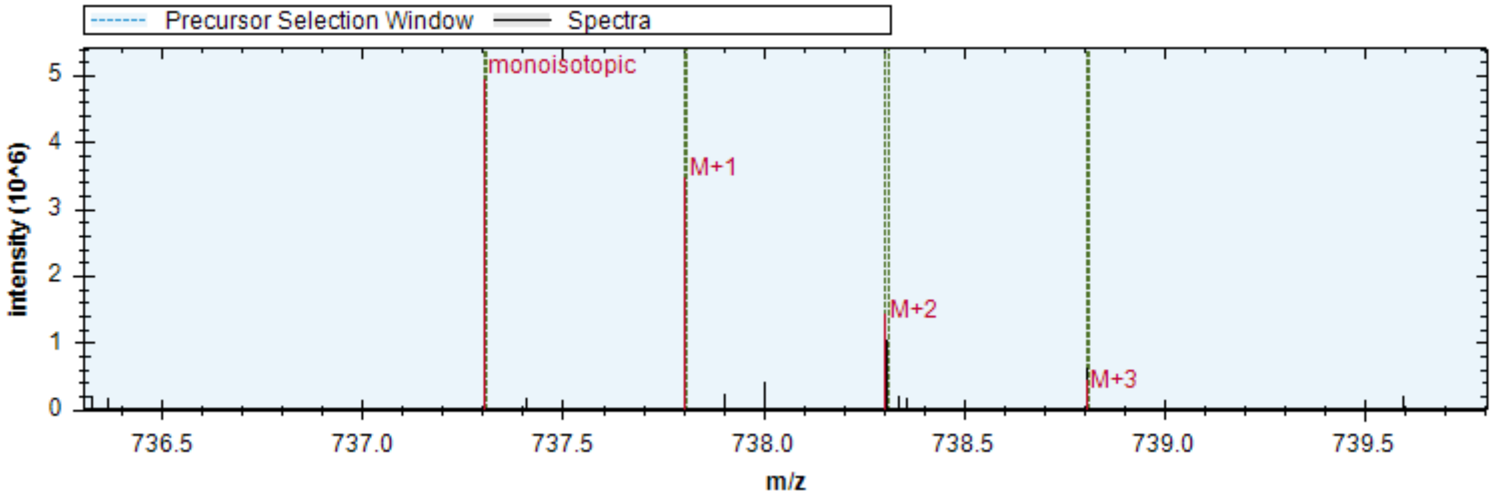

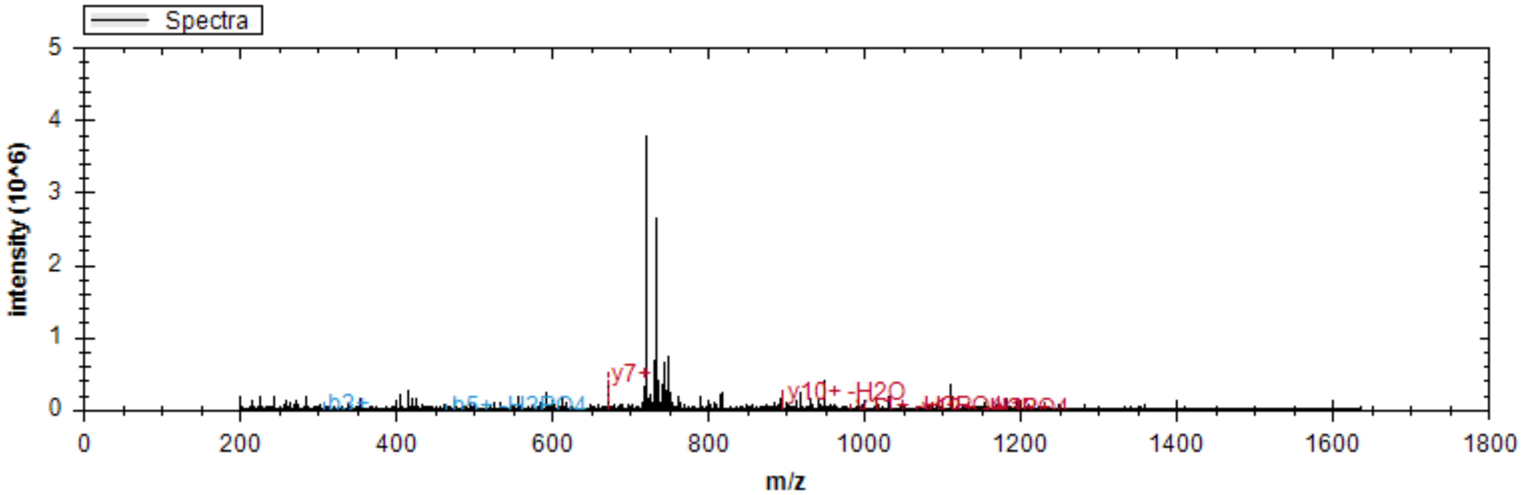

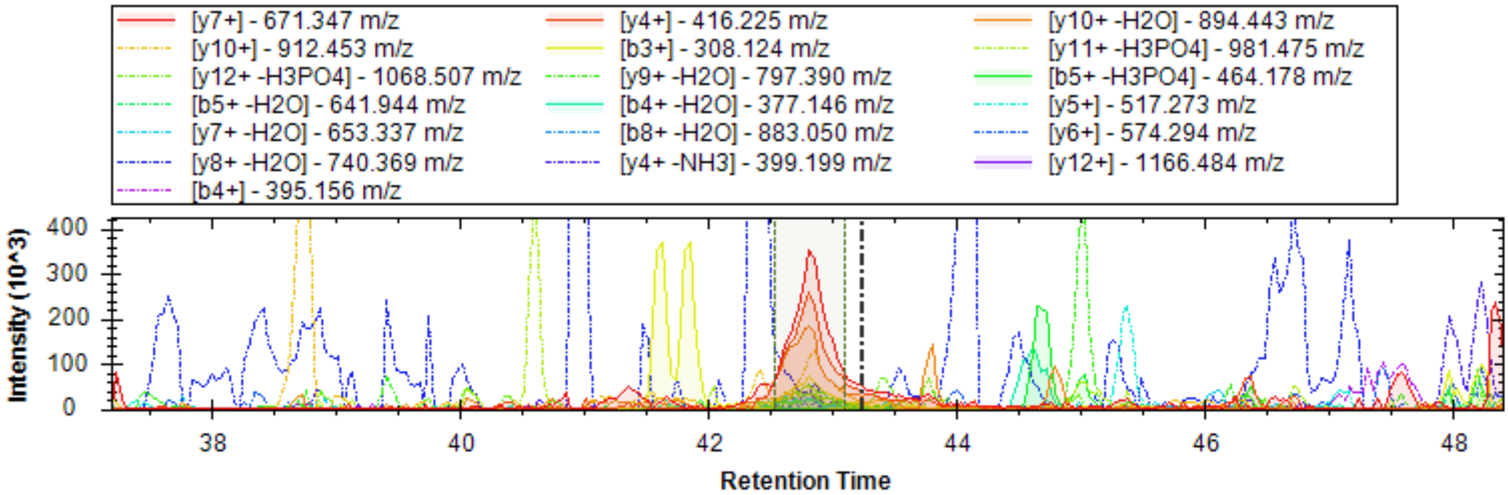

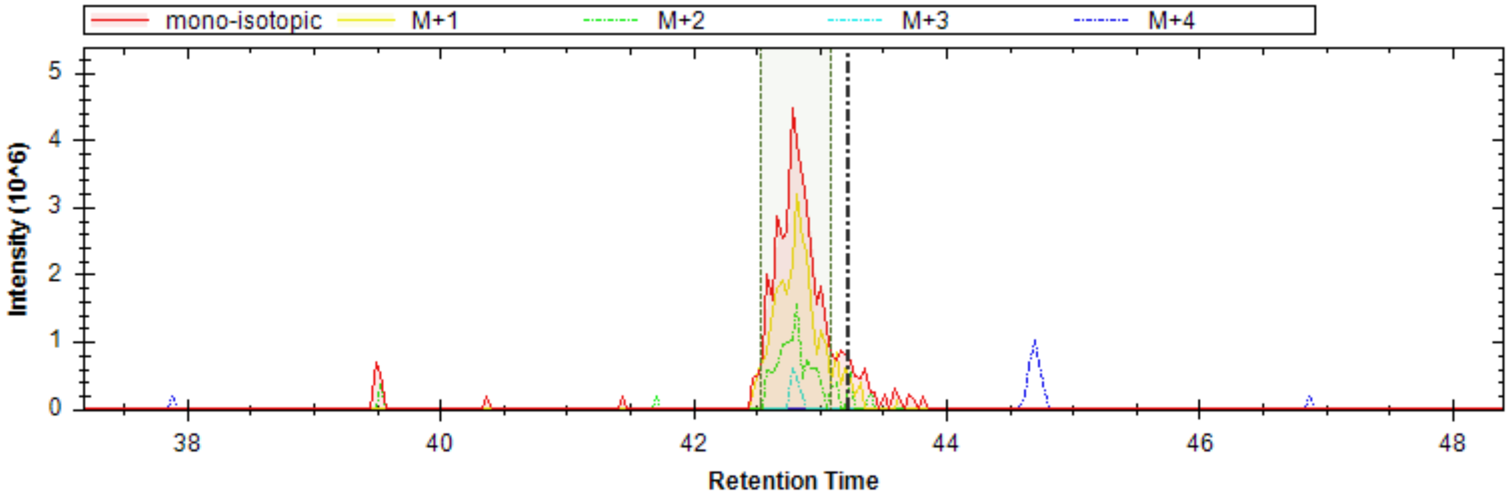

P10637 – _SGYSSPGS[+H+O3+P]PGTPGSR_.2

Fig4_MP-DIA-barrel-cortex-p9-3_MHRM.htrms

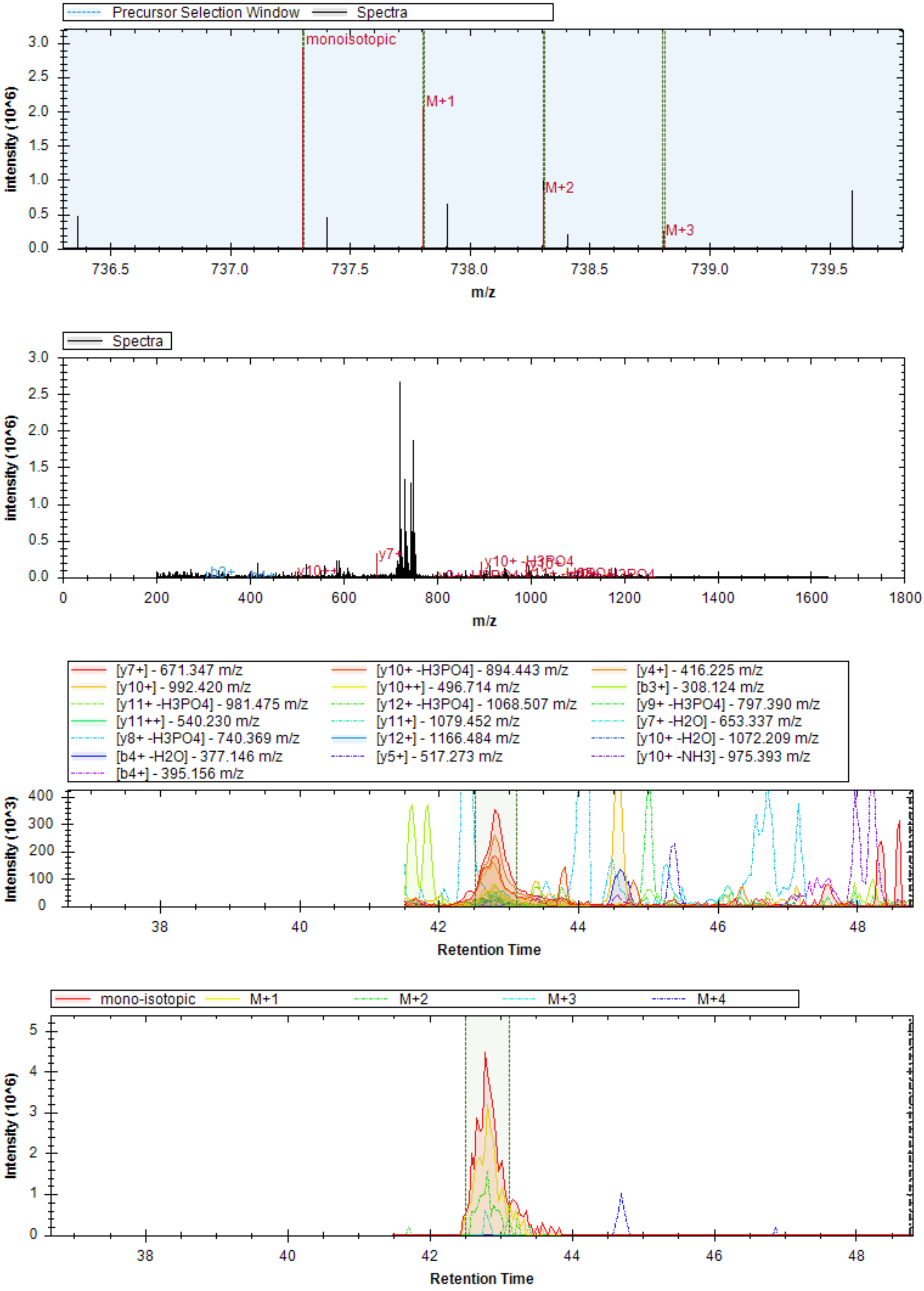

P26645 – _GEATAERPGEAAVAS[+H+O3+P]SPSKANGQENGHVK_.4
Fig4_MP-DIA-barrel-cortex-p9-3_MHRM.htrms

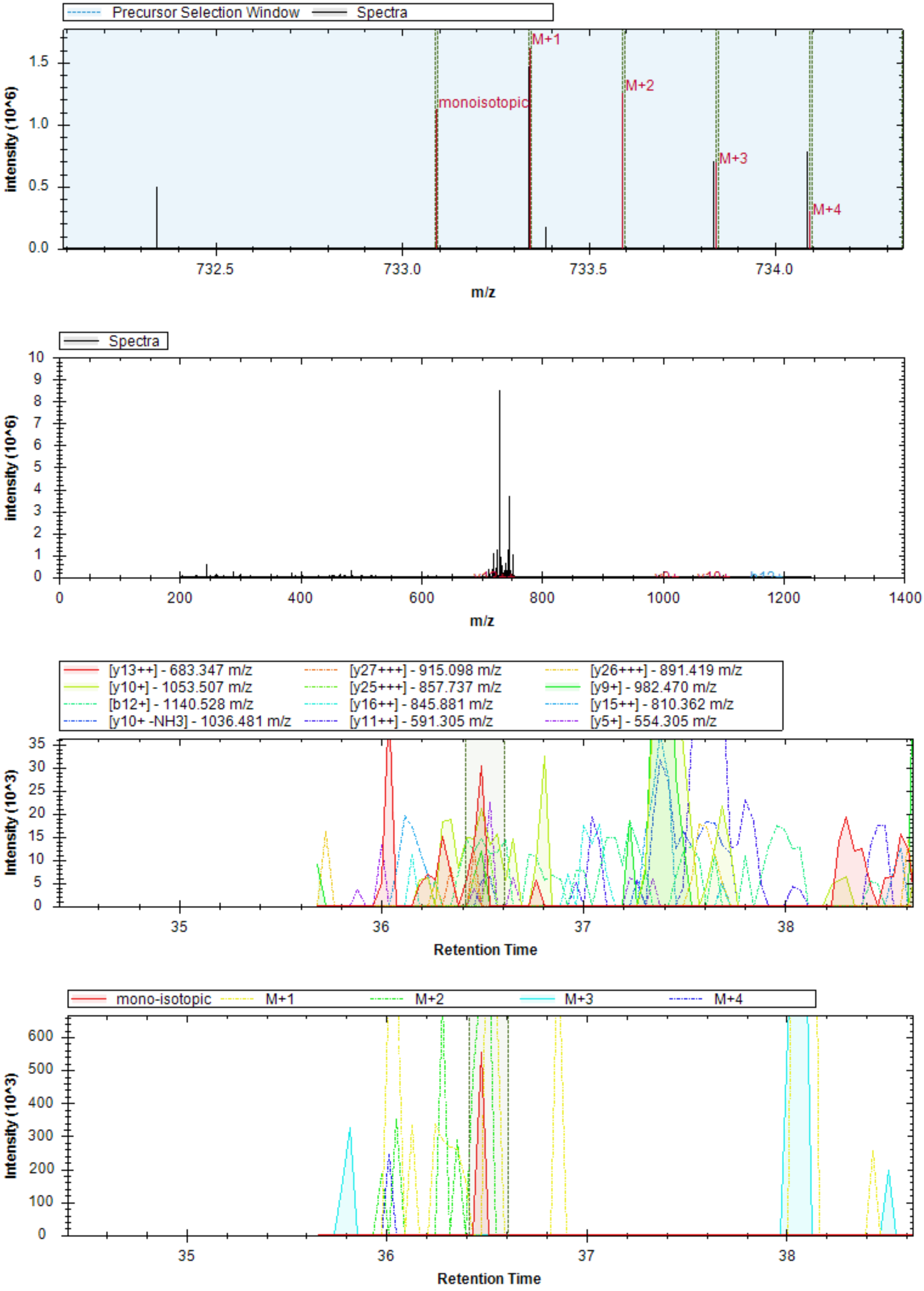

P57776-3 – _ATAPQTQHVS[+H+O3+P]PMR_.2

Fig4_MP-DIA-barrel-cortex-p9-3_MHRM.htrms

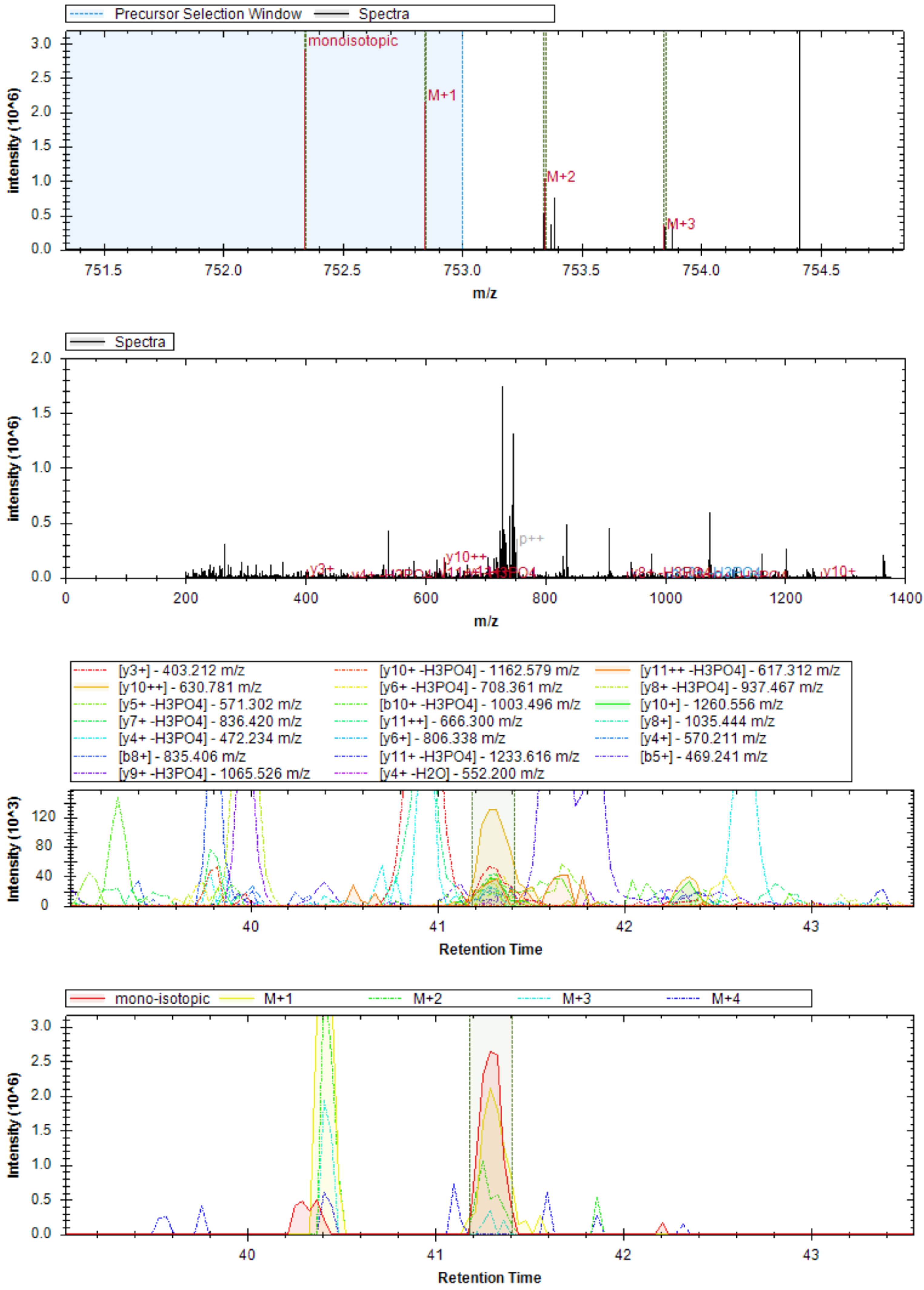

P97798-2 – _NSQDIT[+H+O3+P]PVDNSMDSNIHQR_.3
Fig4_MP-DIA-barrel-cortex-p9-3_MHRM.htrms

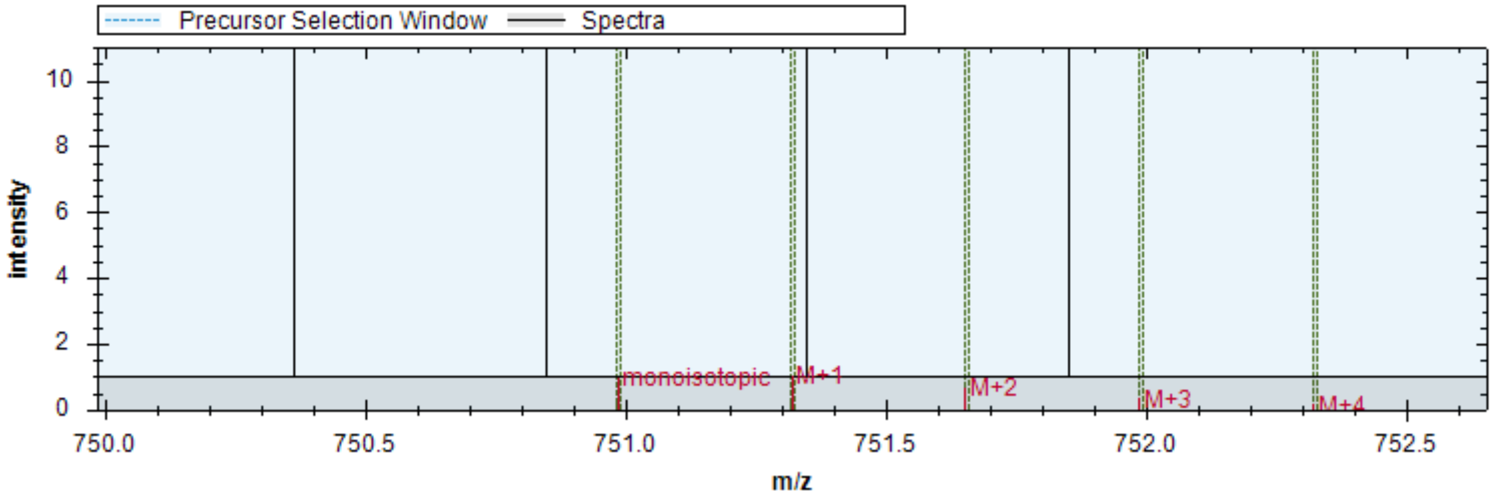

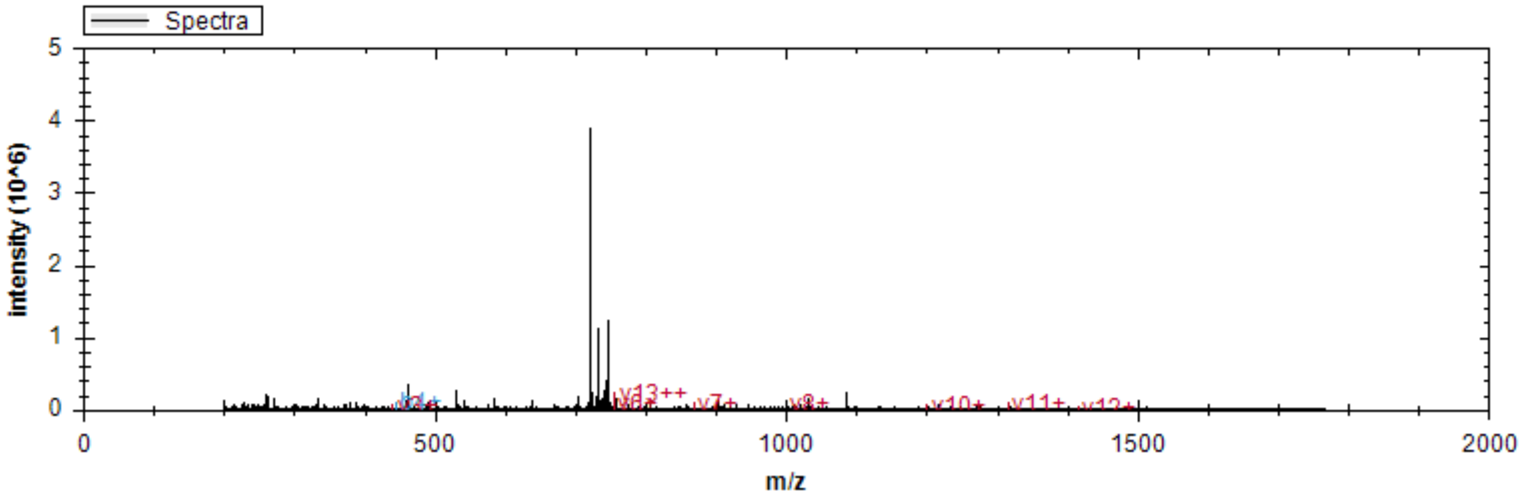

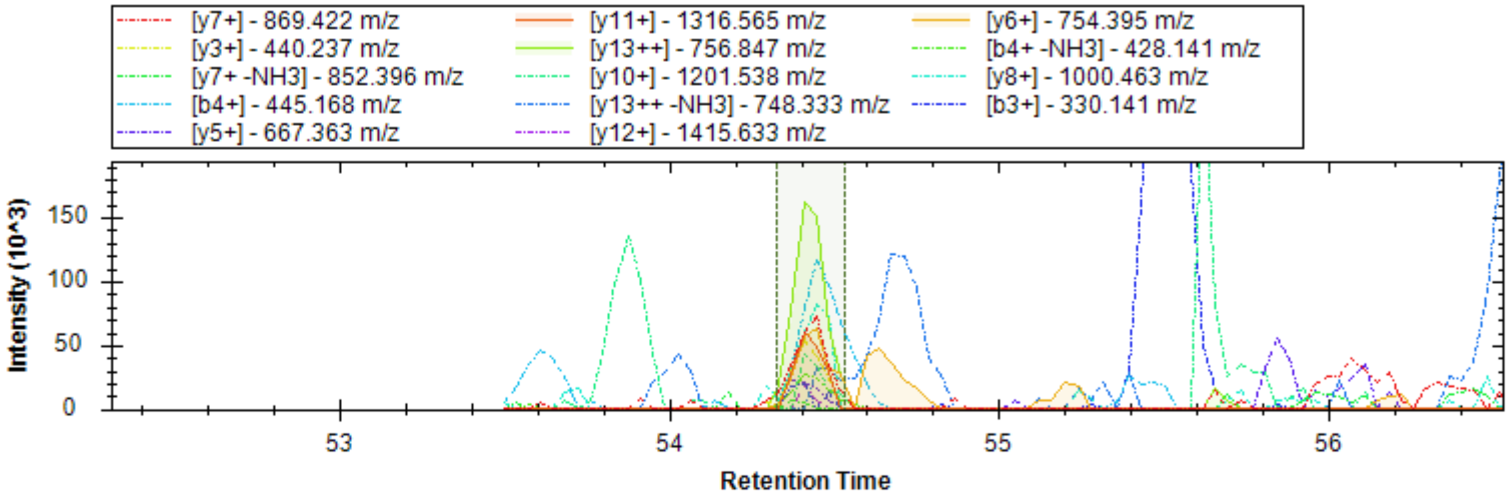

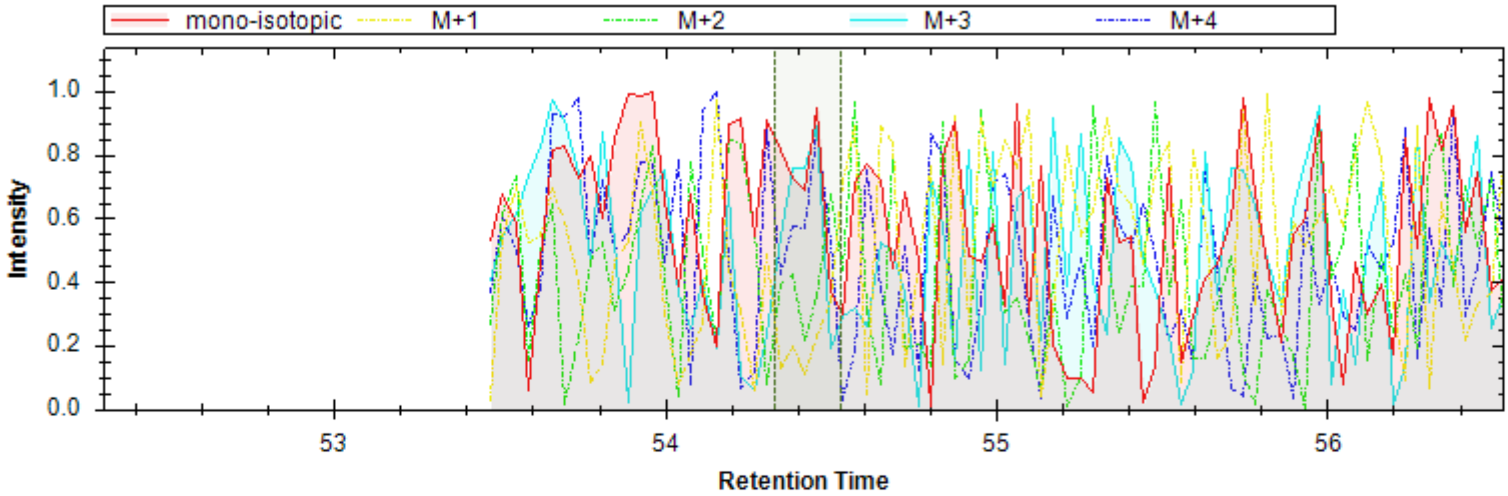

Q3UNH4 – _DLAAAAAQKS[+H+O3+P]PSAEAAAPPPGPR_.3

Fig4_MP-DIA-barrel-cortex-p9-3_MHRM.htrms

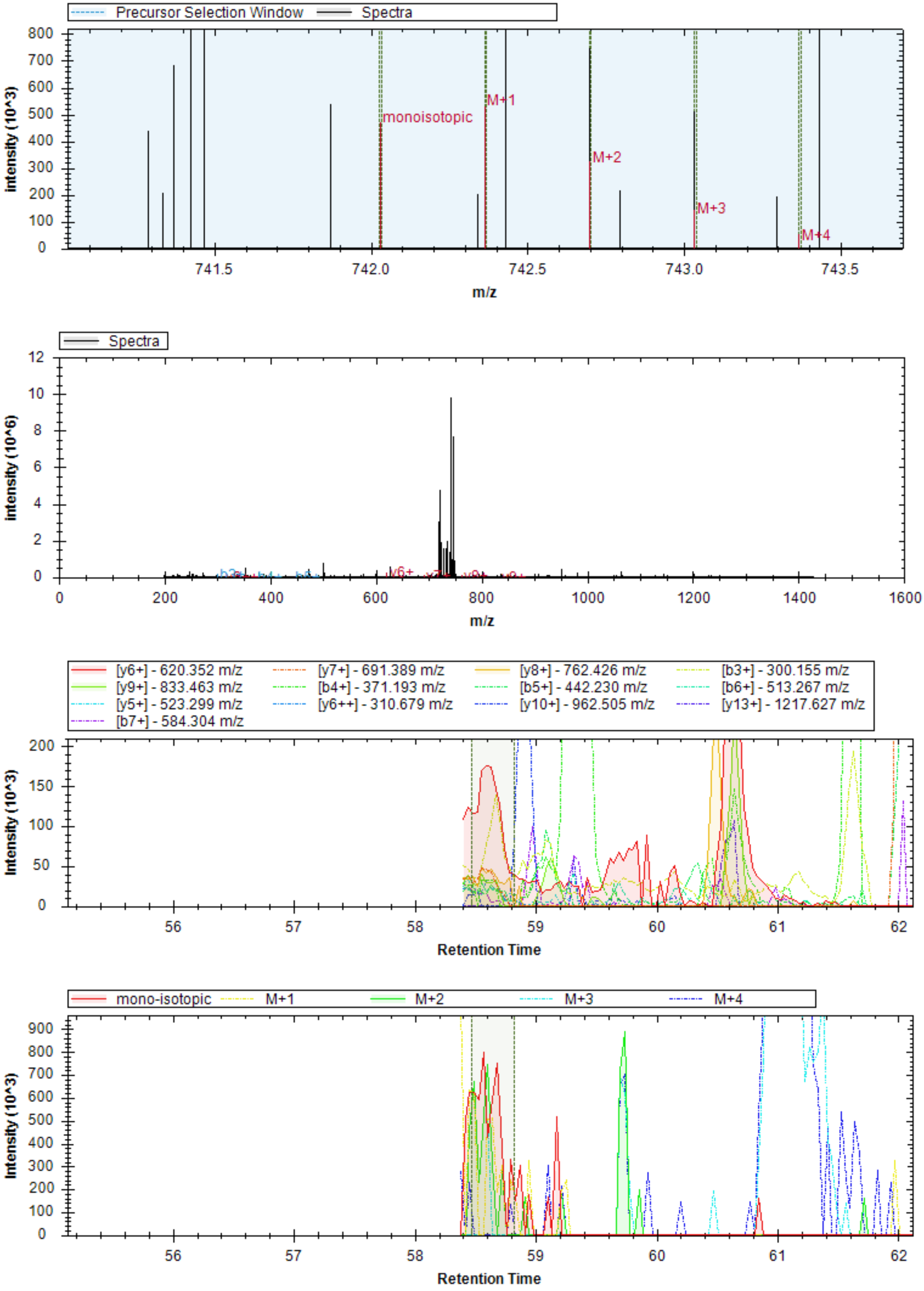

Q3UNH4 – _VITQIPGAT[+H+O3+P]SPGKVETPSLQK_.3
Fig4_MP-DIA-barrel-cortex-p9-3_MHRM.htrms

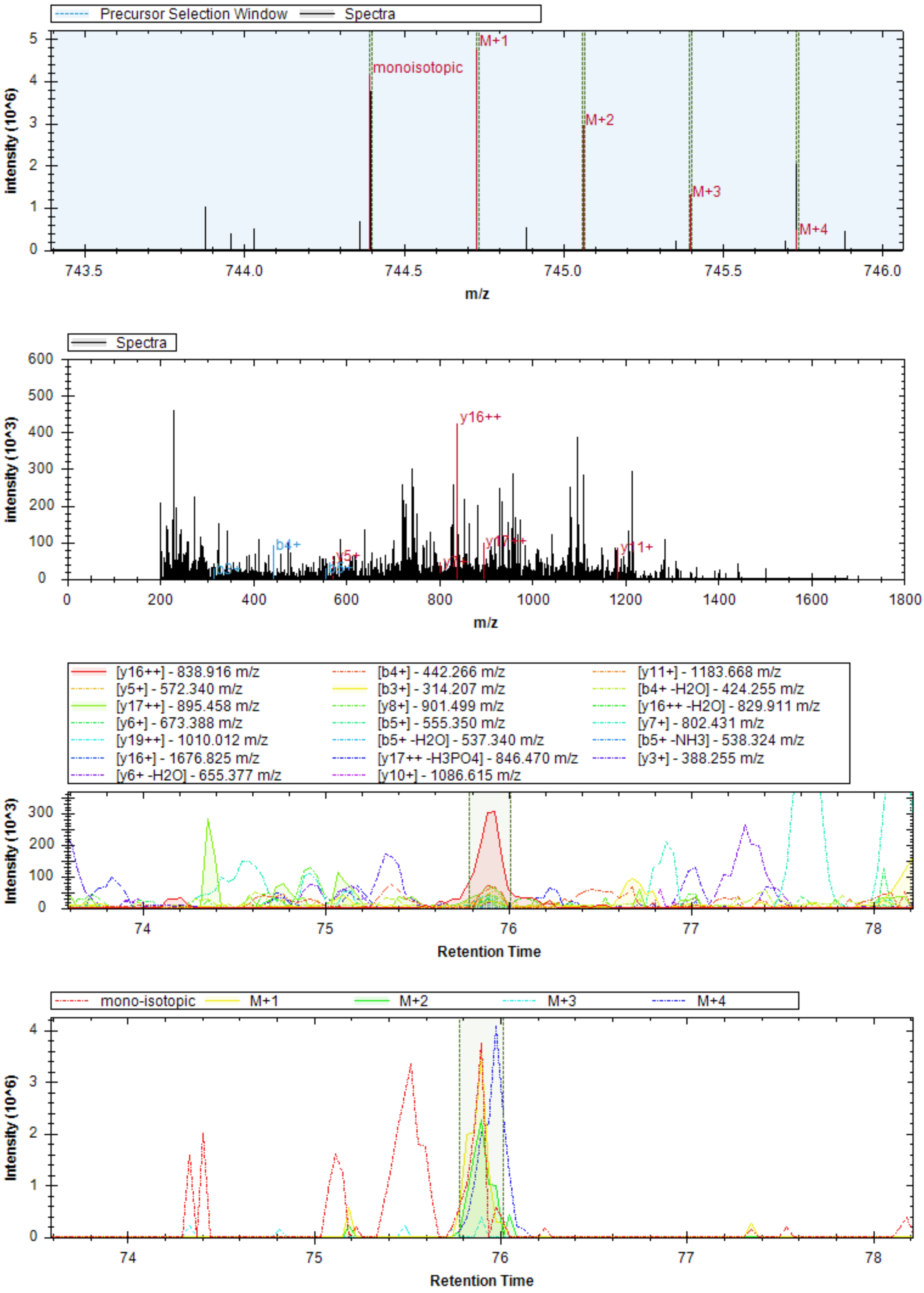

Q3UNH4 – _VITQIPGATS[+H+O3+P]PGKVETPSLQK_.3

Fig4_MP-DIA-barrel-cortex-p9-3_MHRM.htrms

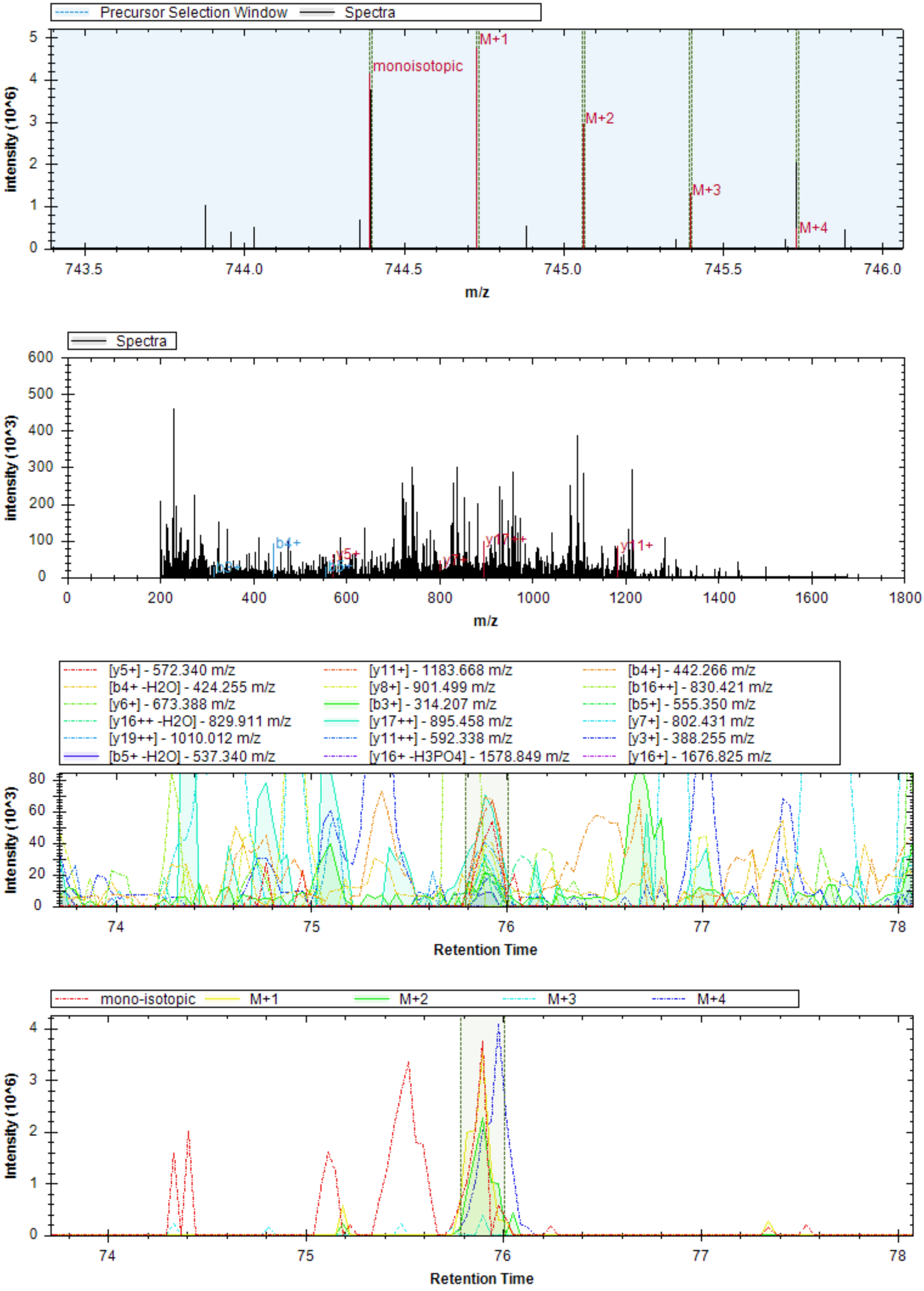

Q8C5R2 – _LAGNEALS[+H+O3+P]PTSPSK_.2

Fig4_MP-DIA-barrel-cortex-p9-3_MHRM.htrms

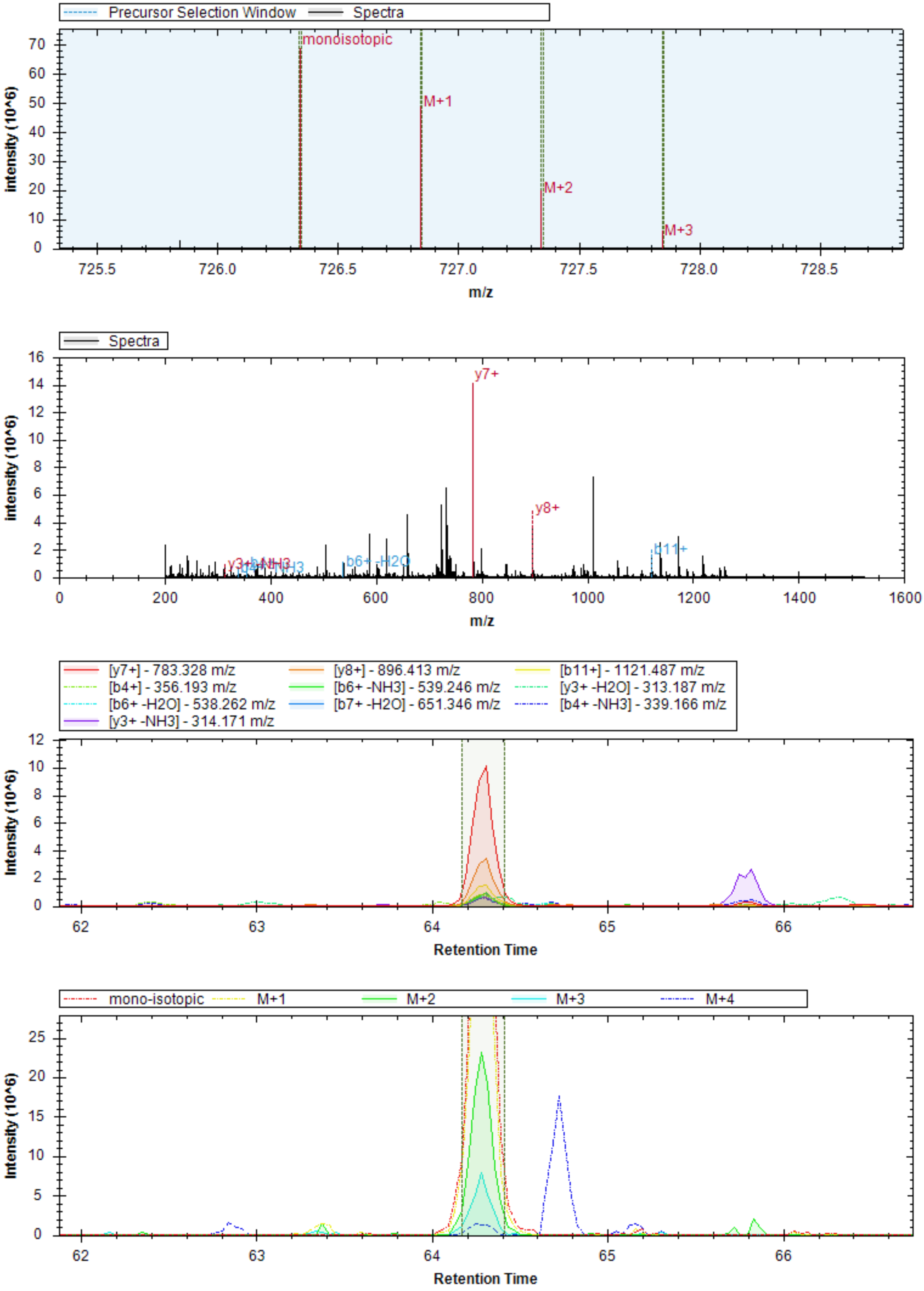

Q8K0U4 – _ETAPTSTYS[+H+O3+P]SPAR_.2
Fig4_MP-DIA-barrel-cortex-p9-3_MHRM.htrms

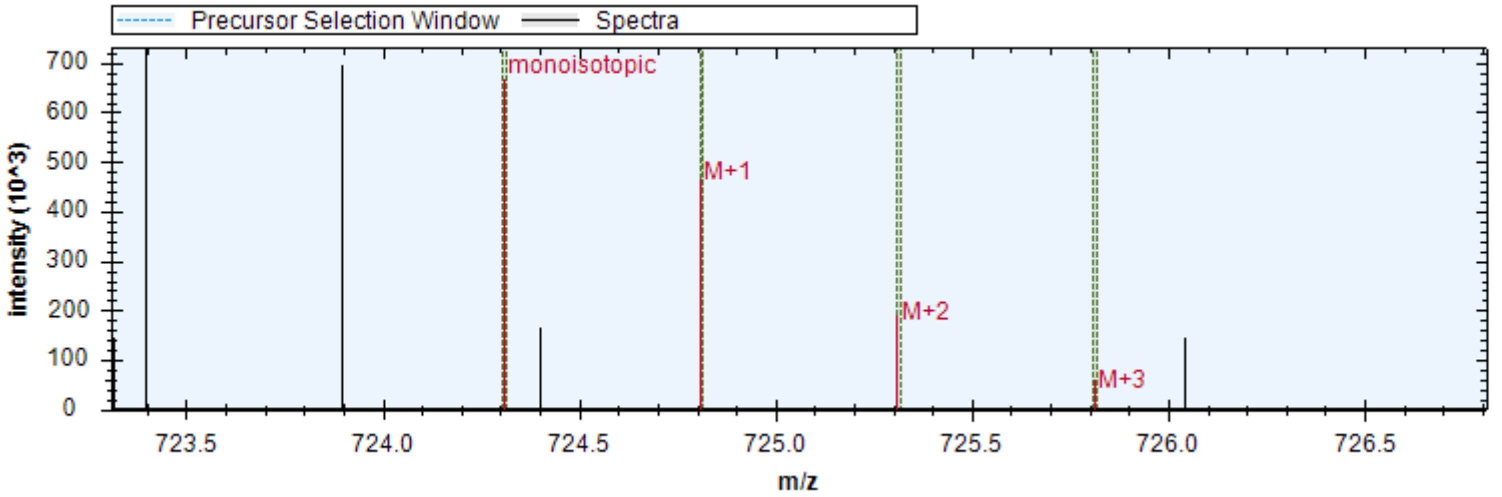

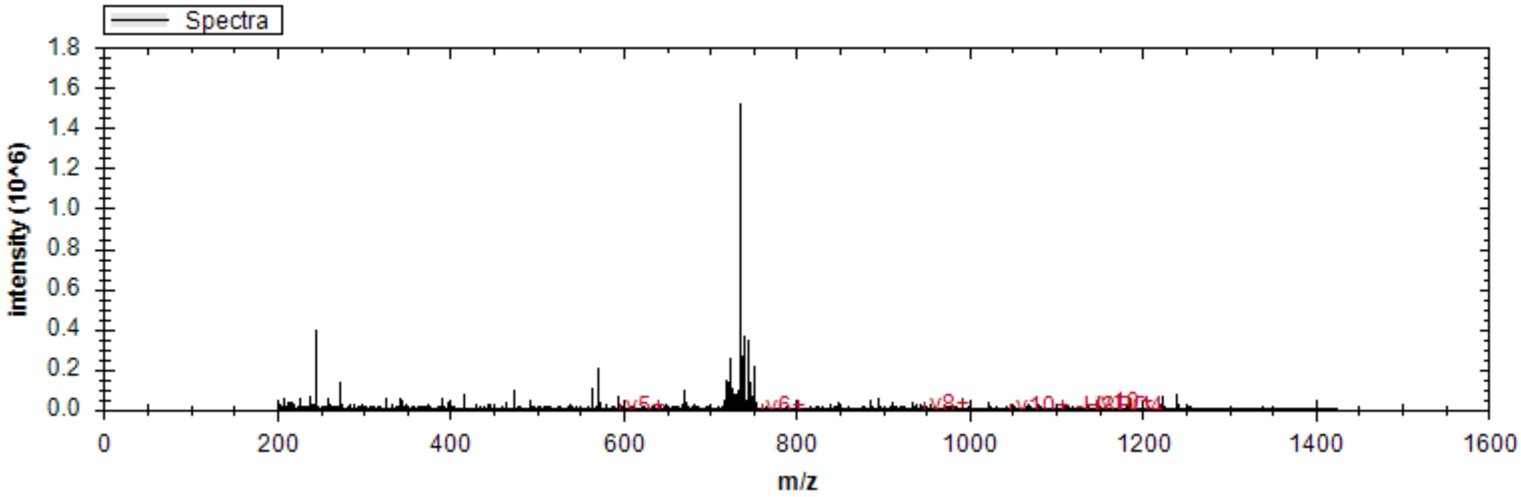

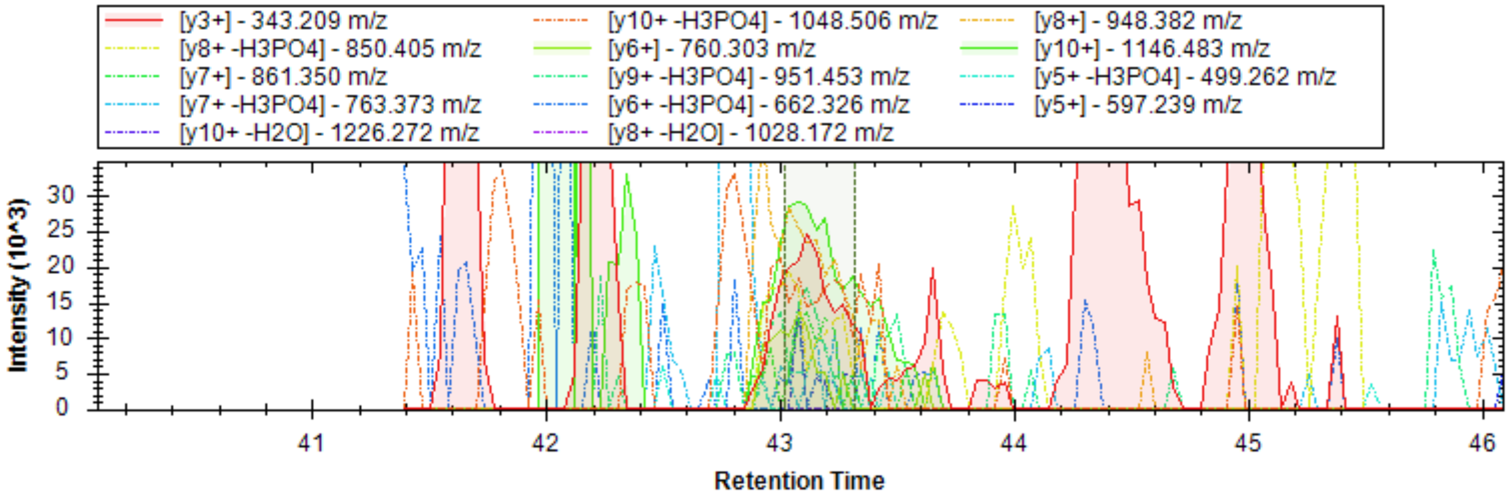

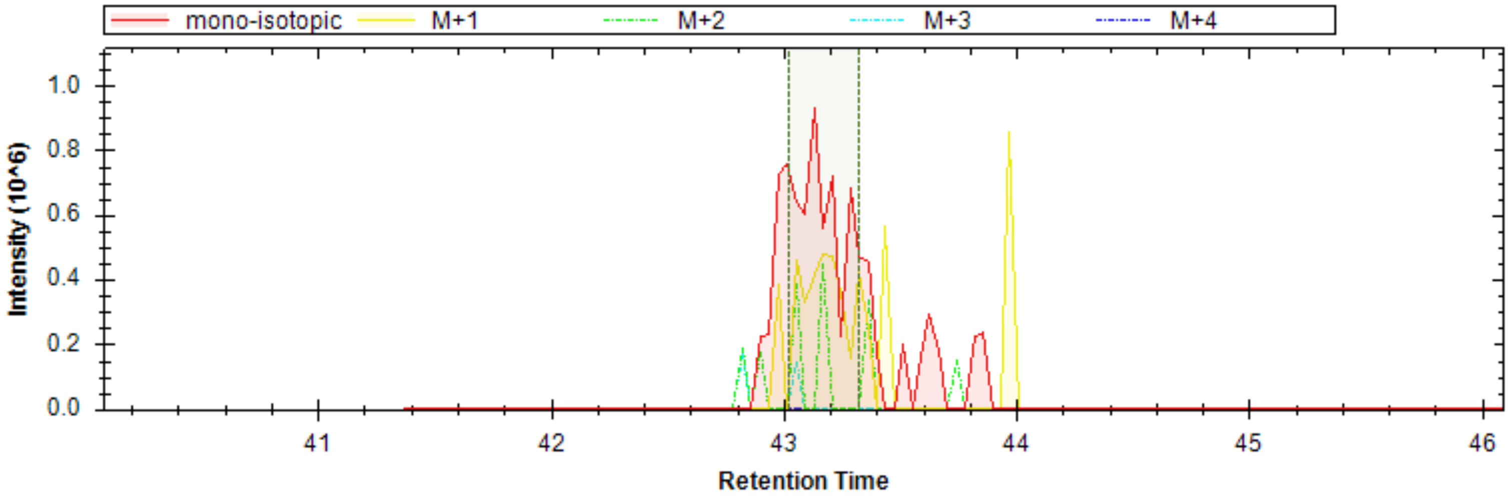

Q8K0U4 – _ETAPTSTYSS[+H+O3+P]PAR_.2
Fig4_MP-DIA-barrel-cortex-p9-3_MHRM.htrms

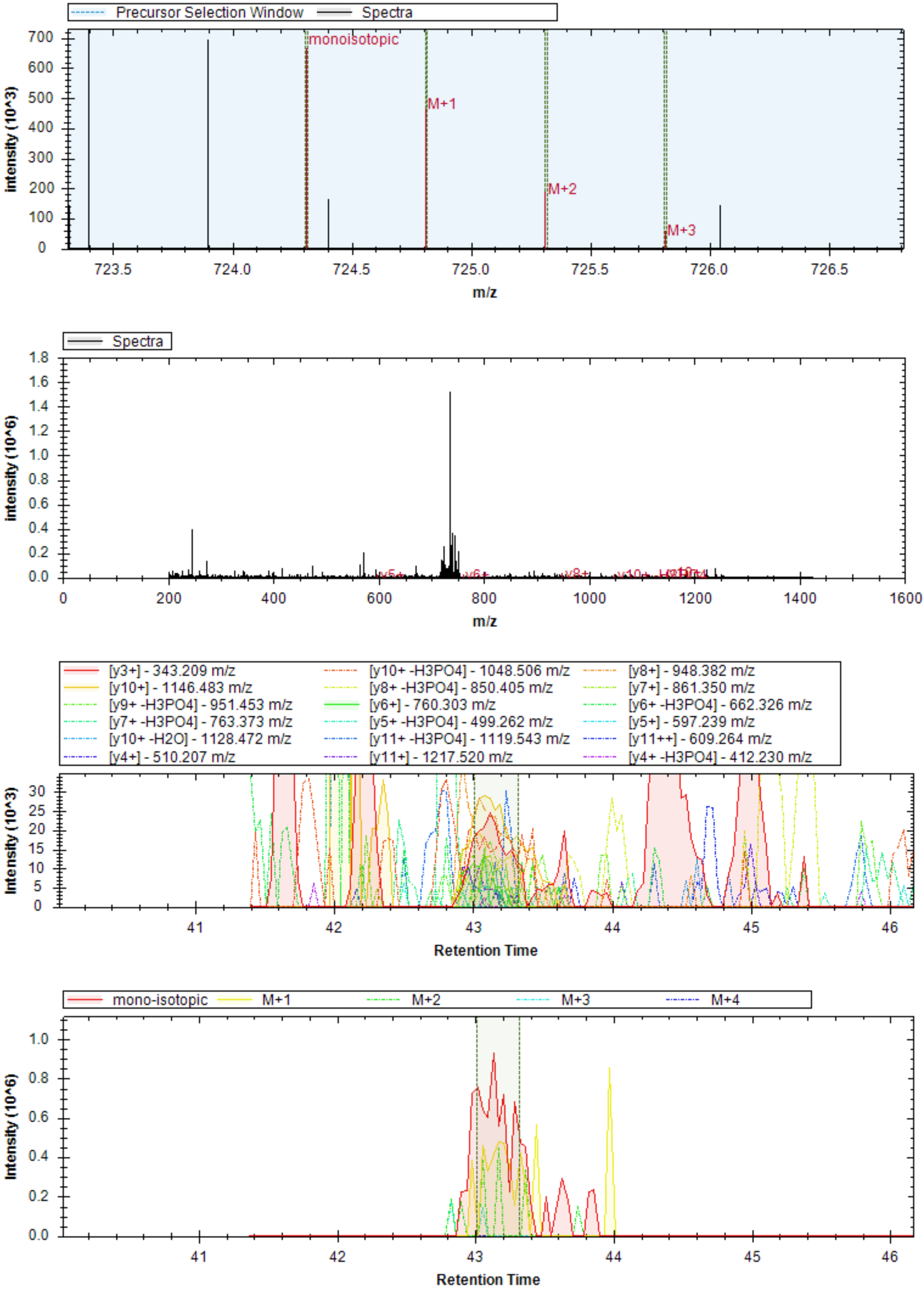

Q8VCI5 – _AKPS[+H+O3+P]PEHAPTISAPDASGPQK_.3
Fig4_MP-DIA-barrel-cortex-p9-3_MHRM.htrms

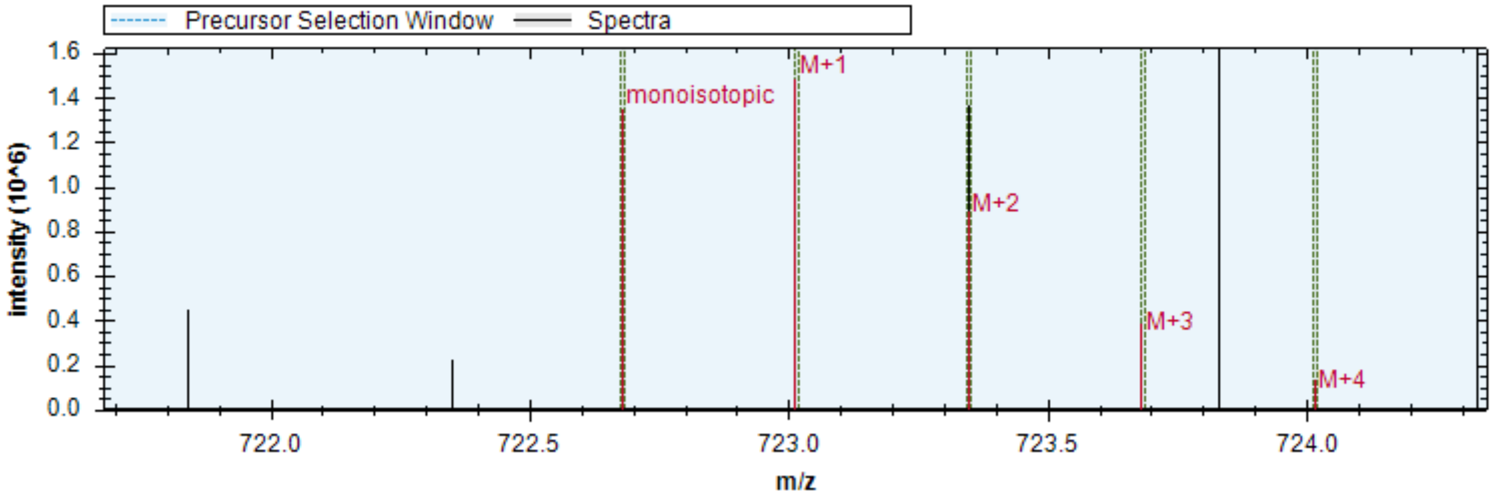

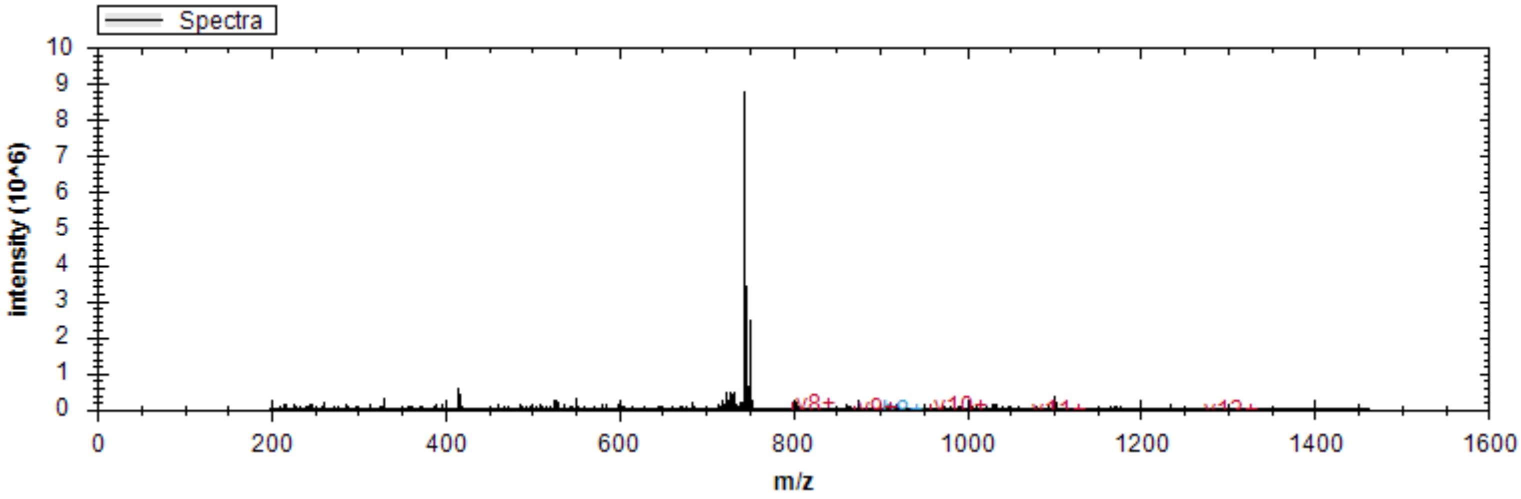

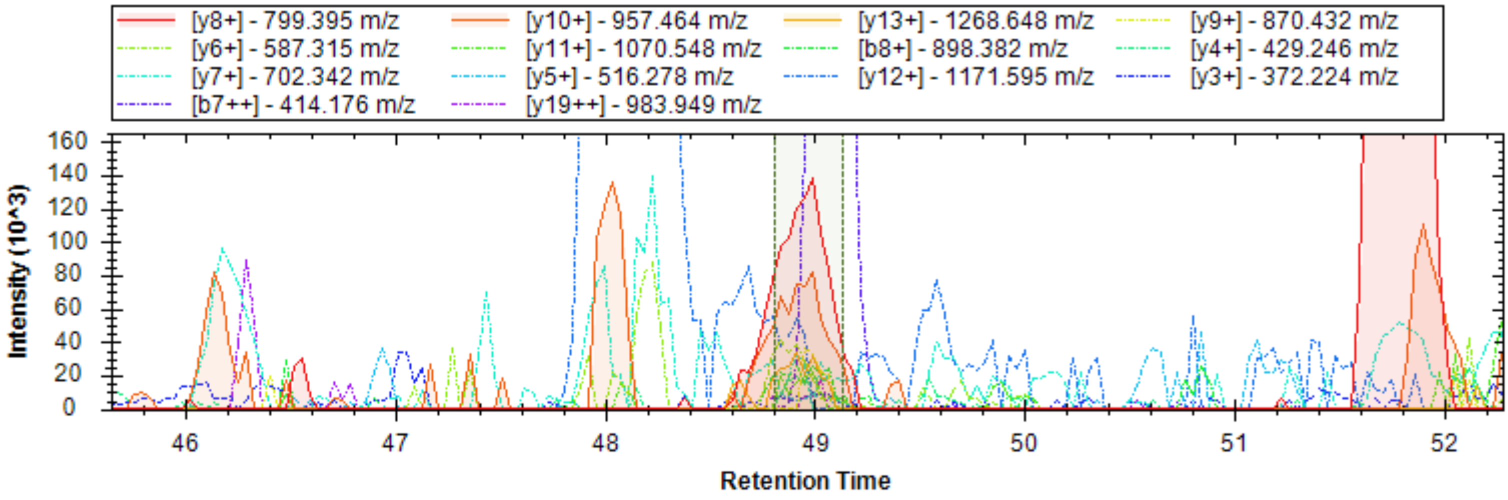

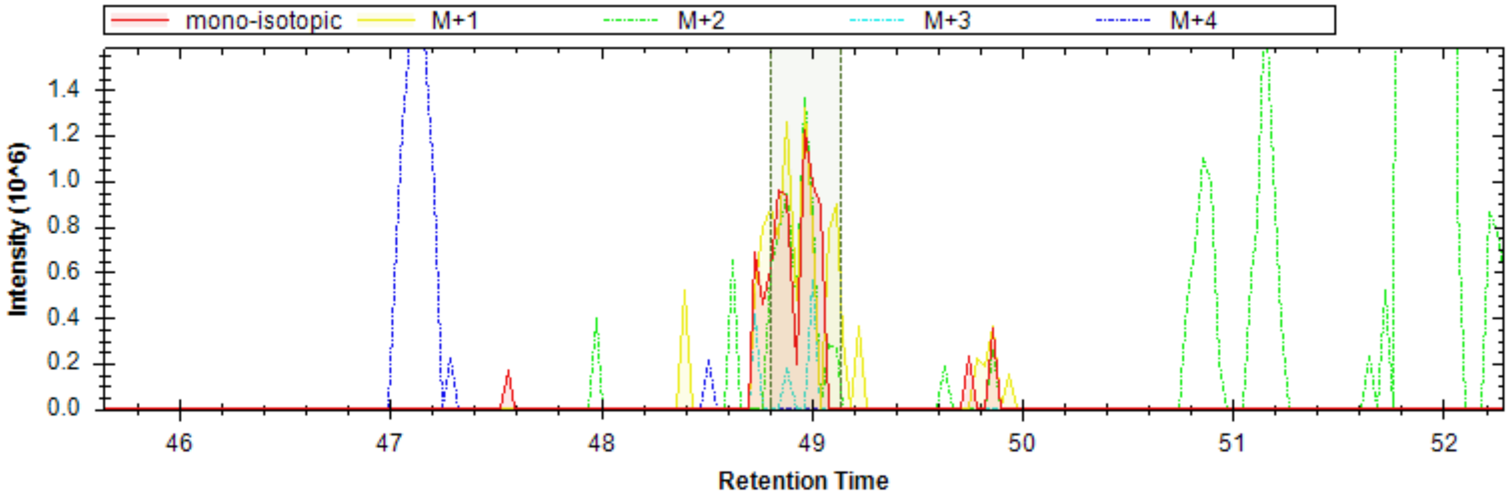

Q9CY18 – _M[+O]GQTVRAVAS[+H+O3+P]SMR_.2
Fig4_MP-DIA-barrel-cortex-p9-3_MHRM.htrms

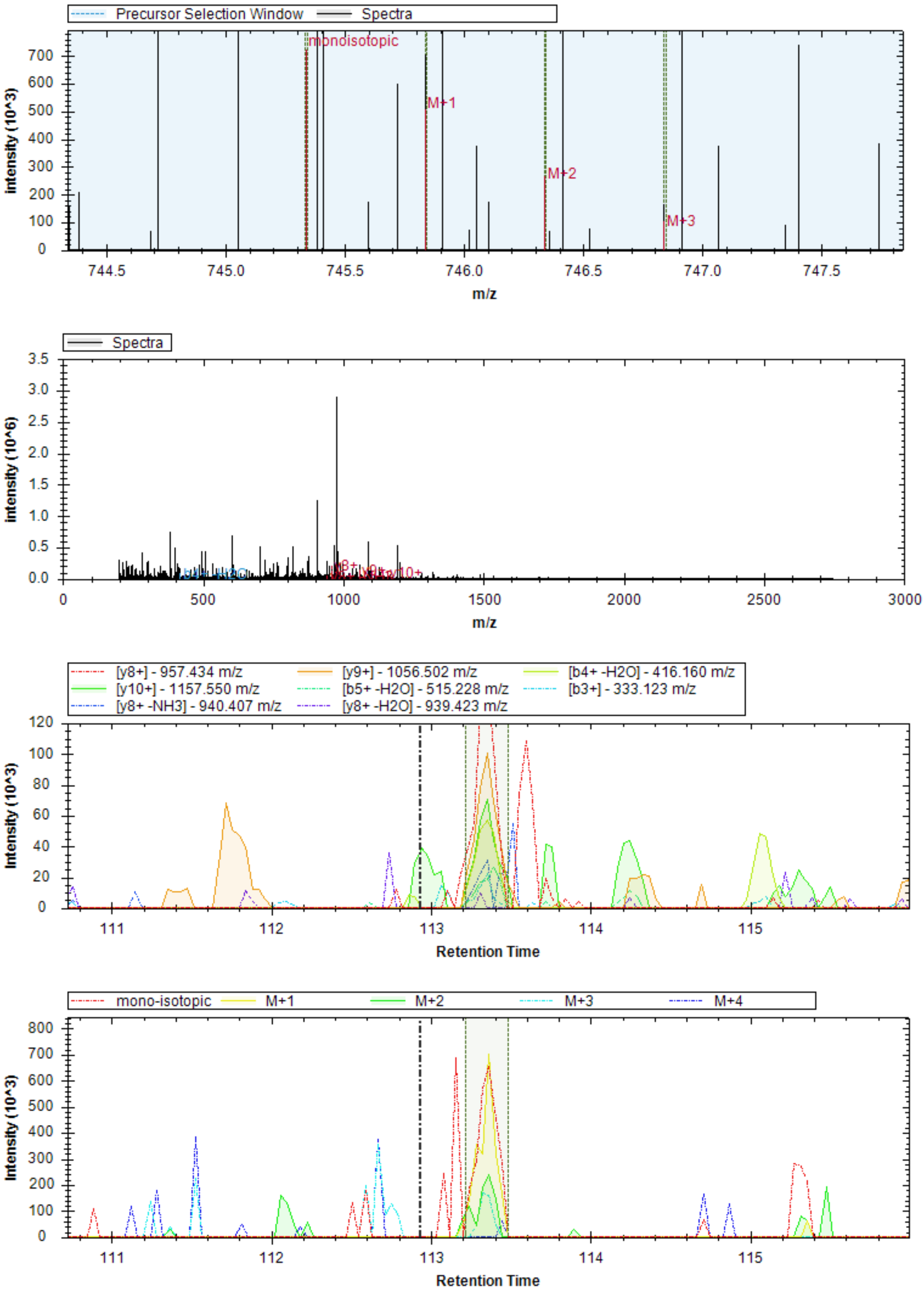

Q9JLM8 – _SKS[+H+O3+P]PASTSSVNGTPGSQLSTPR_.3
Fig4_MP-DIA-barrel-cortex-p9-3_MHRM.htrms

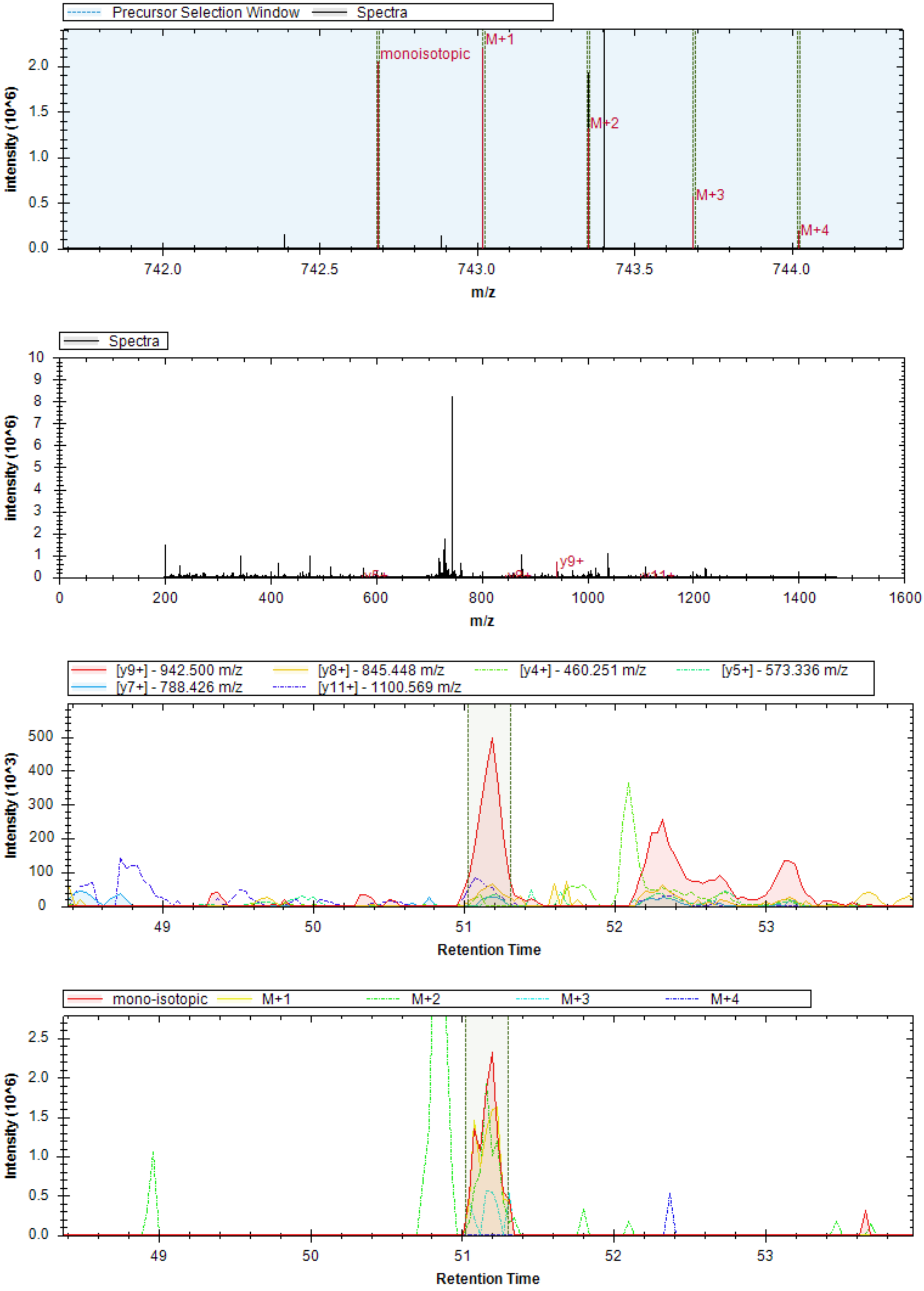

Q9JLM8 – _SPSPSPT[+H+O3+P]SPGSLRK_.2
Fig4_MP-DIA-barrel-cortex-p9-3_MHRM.htrms

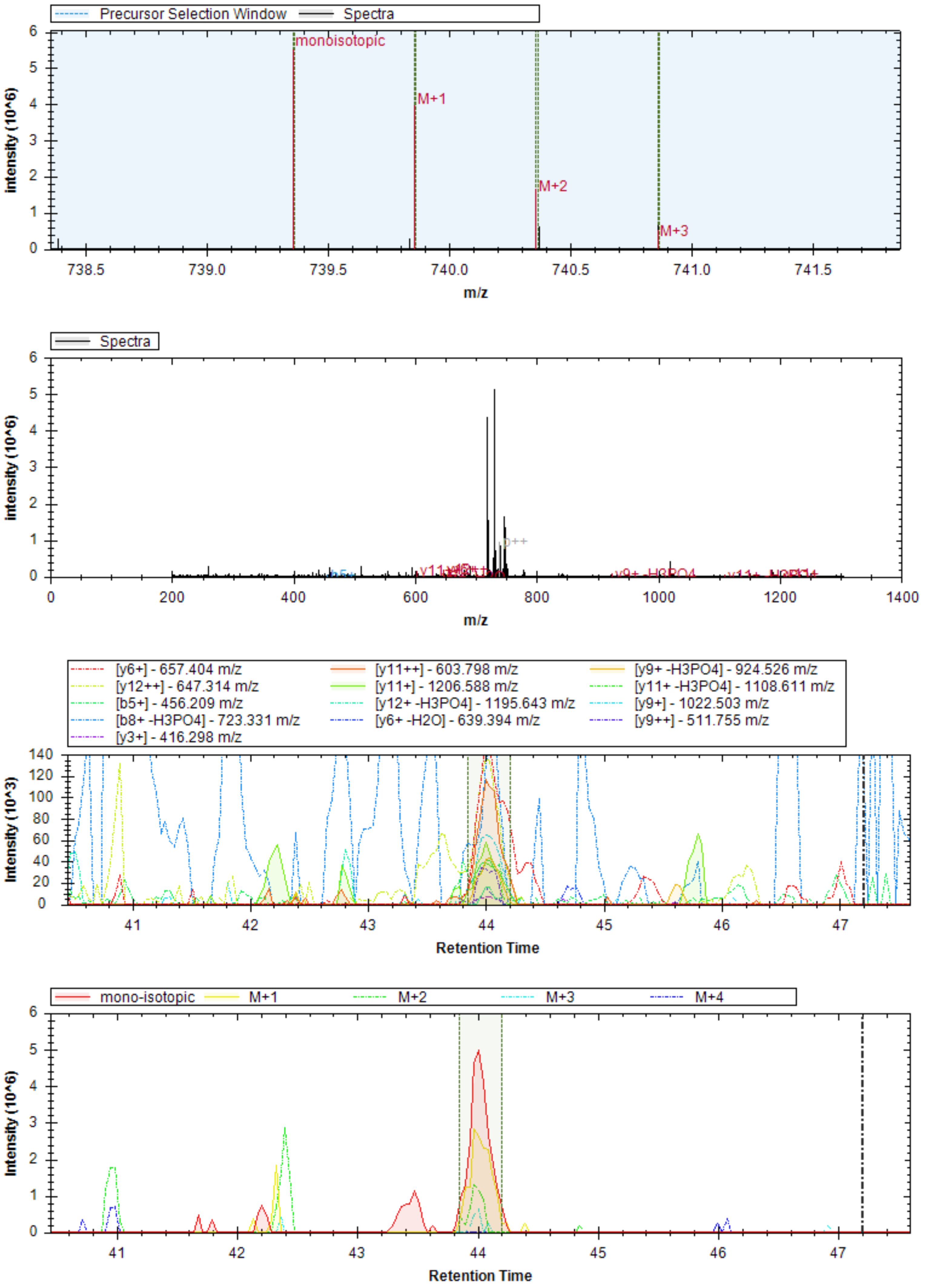

Q9JLM8 – _SPSPSPTS[+H+O3+P]PGSLRK_.2
Fig4_MP-DIA-barrel-cortex-p9-3_MHRM.htrms

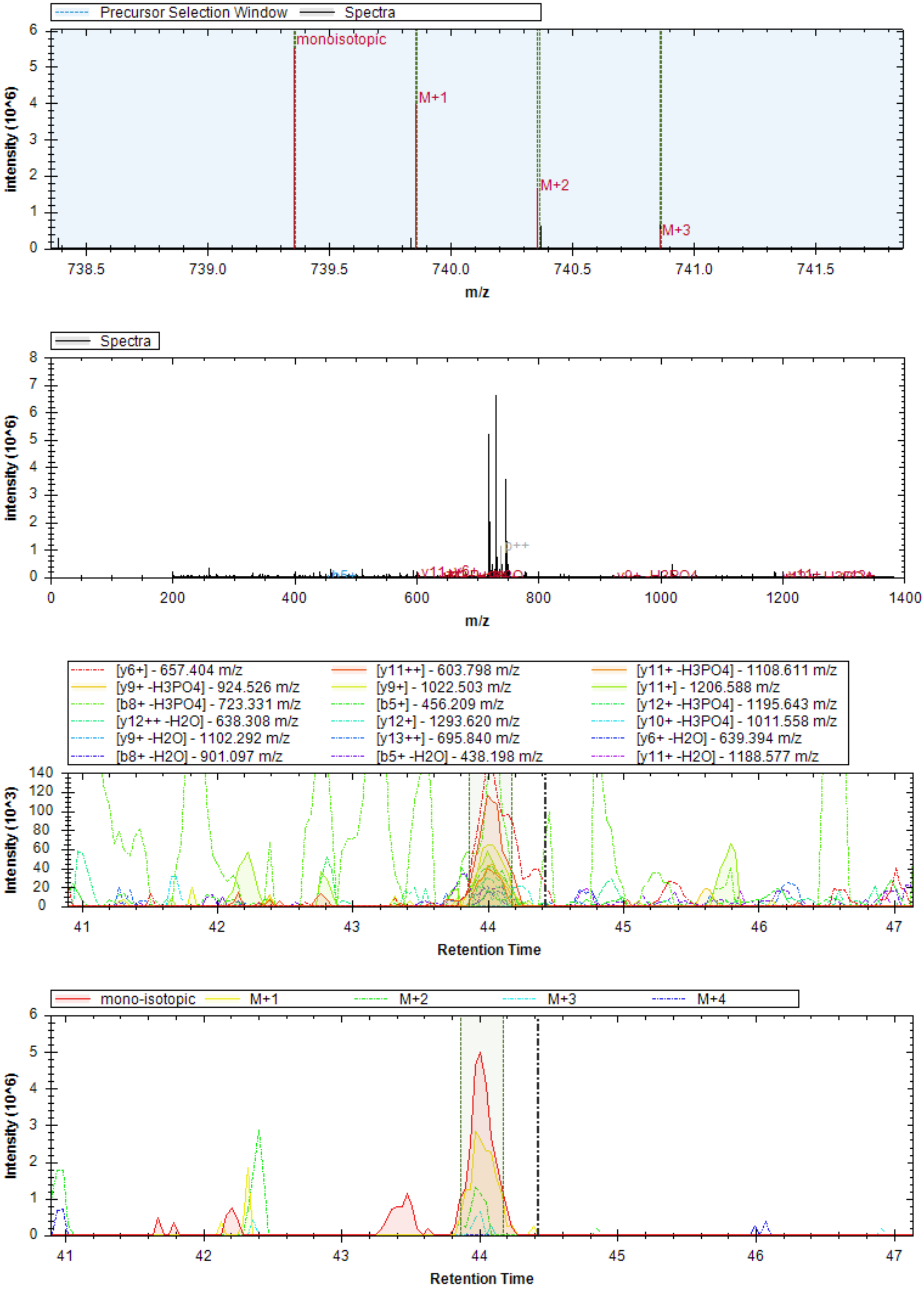

D3Z6S9 – _IPSIEIT[+H+O3+P]ET[+H+O3+P]LPQTQASMLR_.3
Fig4_MP-DIA-barrel-cortex-p9-3_MHRM.htrms

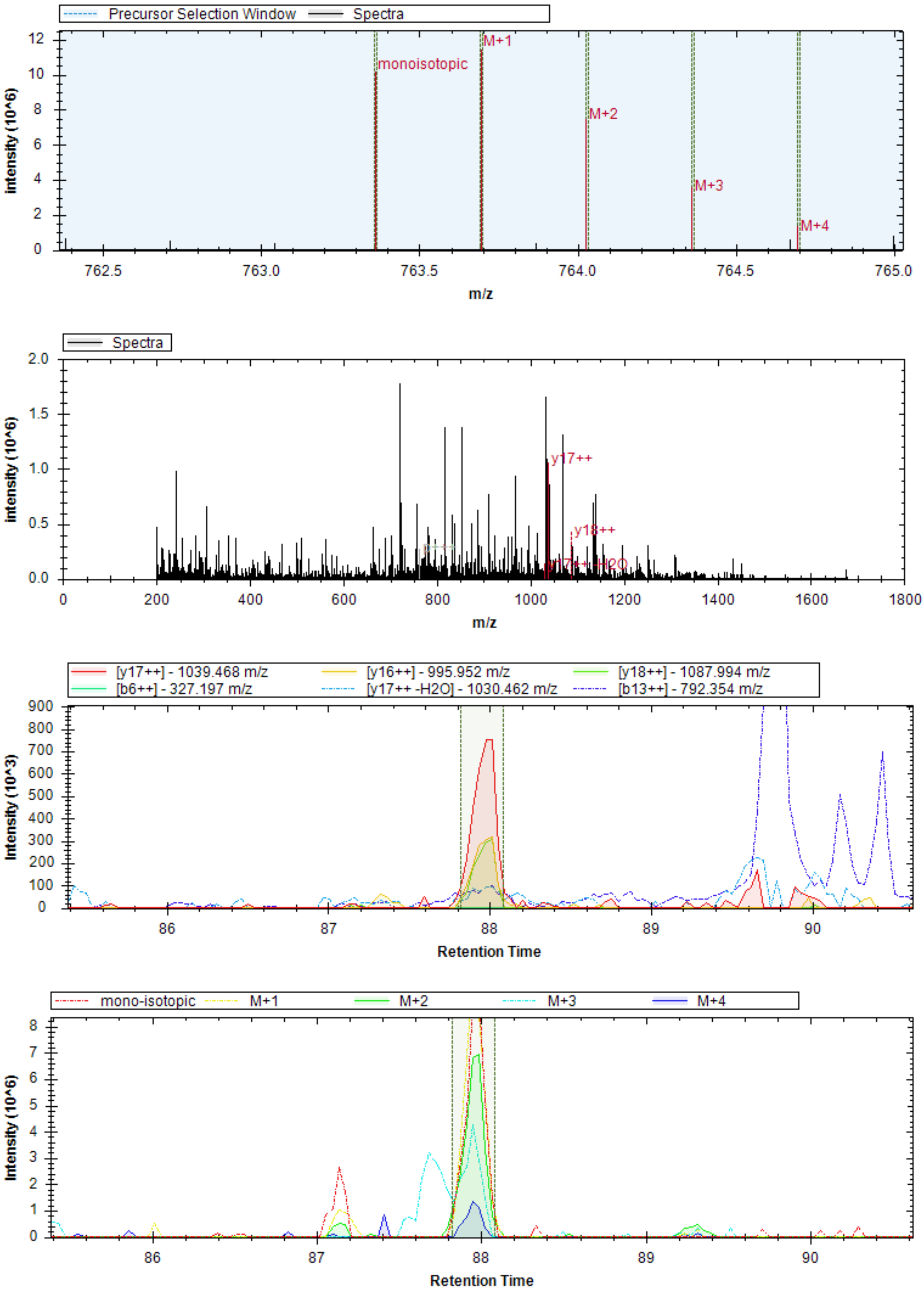

P06837 – _QADVPAAVTDAAAT[+H+O3+P]TPAAEDAATK_.3
Fig4_MP-DIA-barrel-cortex-p9-3_MHRM.htrms

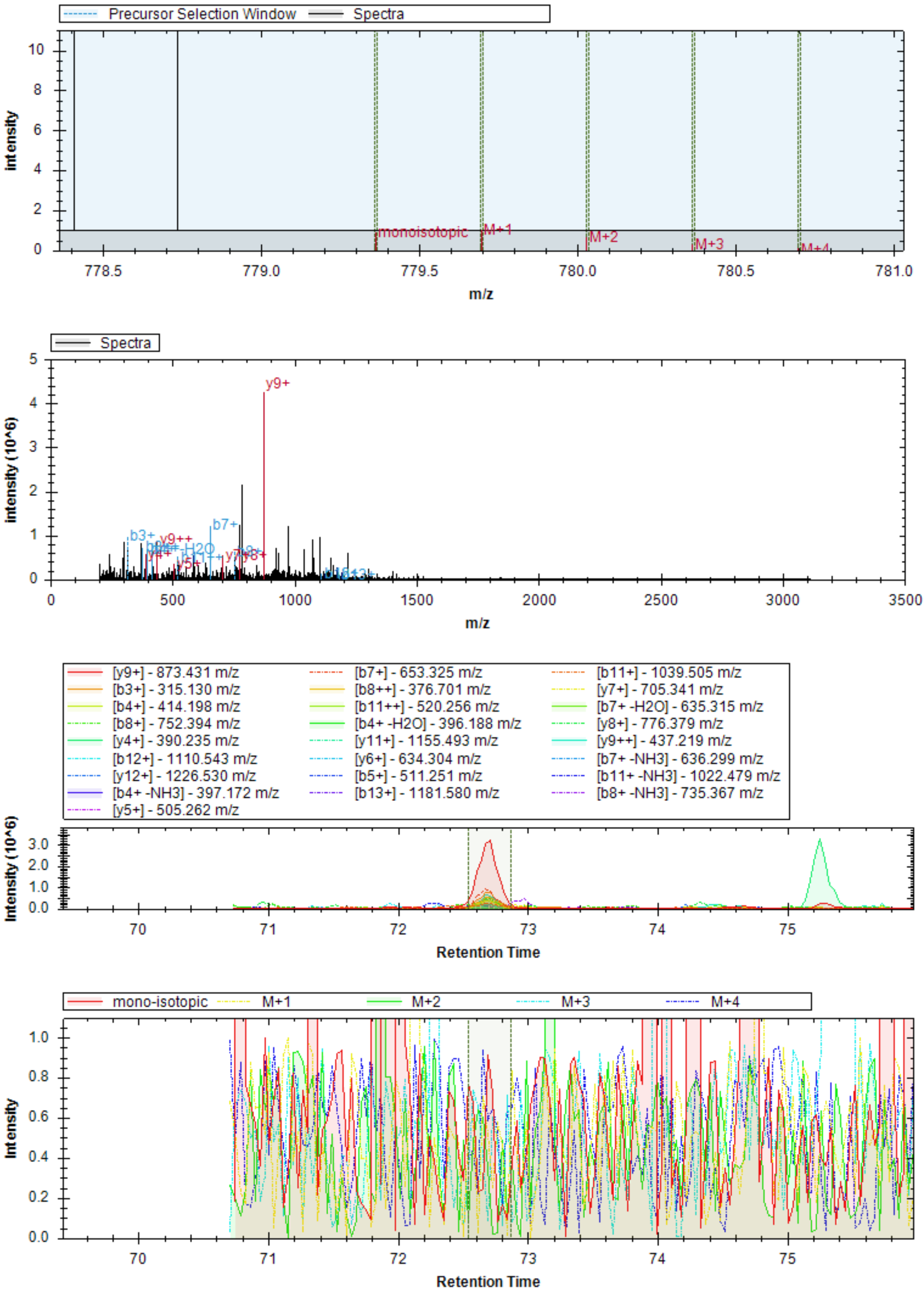

P0C6F1 – _IIDLQM[+O]HDY[+H+O3+P]LR_.2

Fig4_MP-DIA-barrel-cortex-p9-3_MHRM.htrms

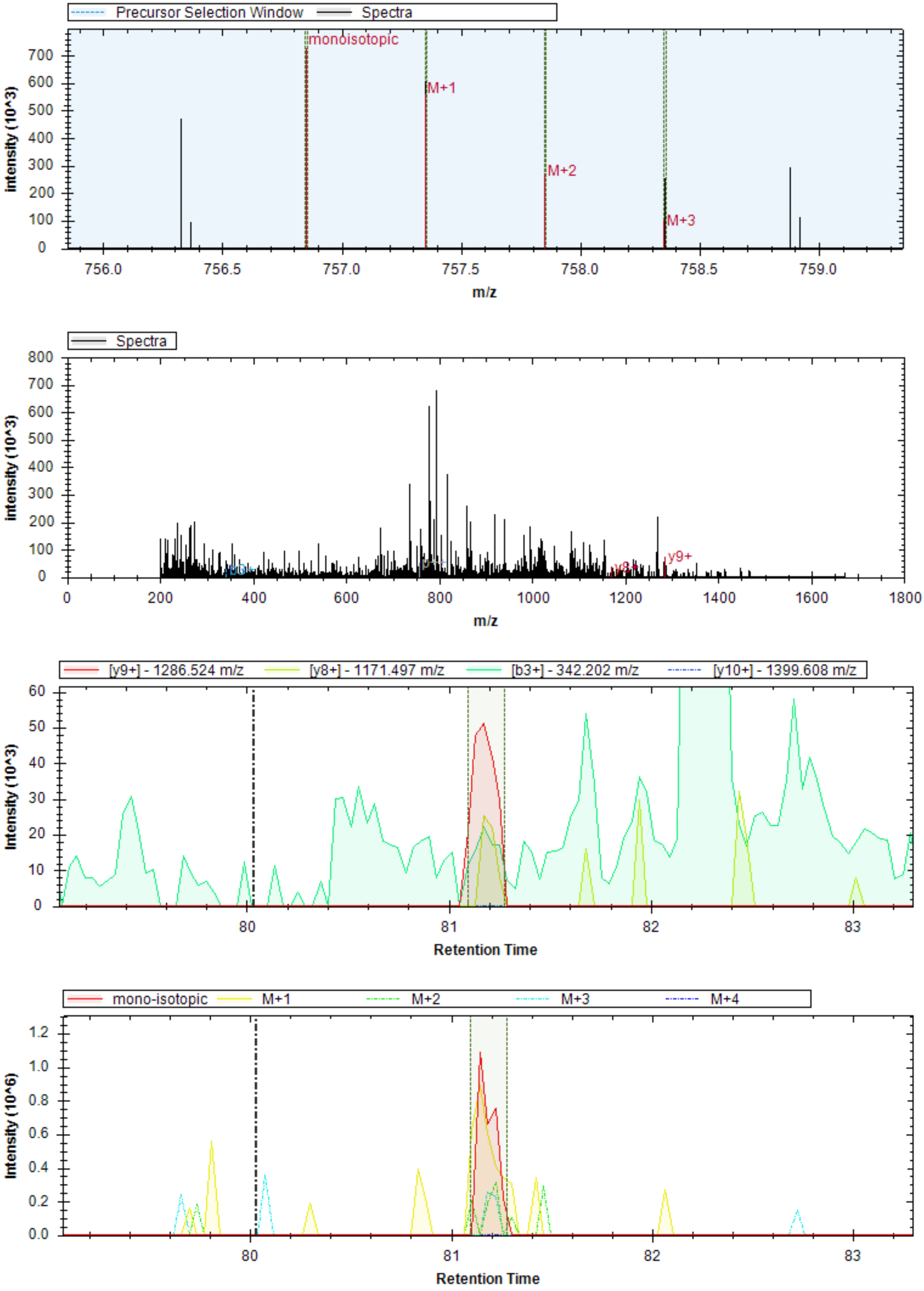

P27546-2 – _AT[+H+O3+P]SPSTLVSTGPSSR_.2

Fig4_MP-DIA-barrel-cortex-p9-3_MHRM.htrms

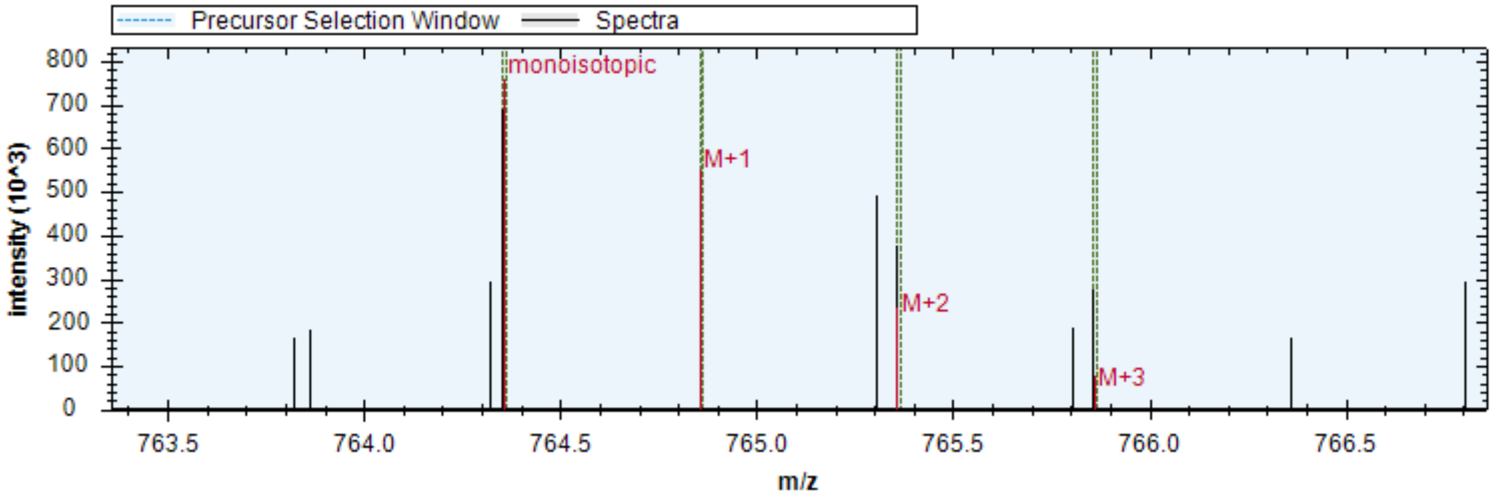

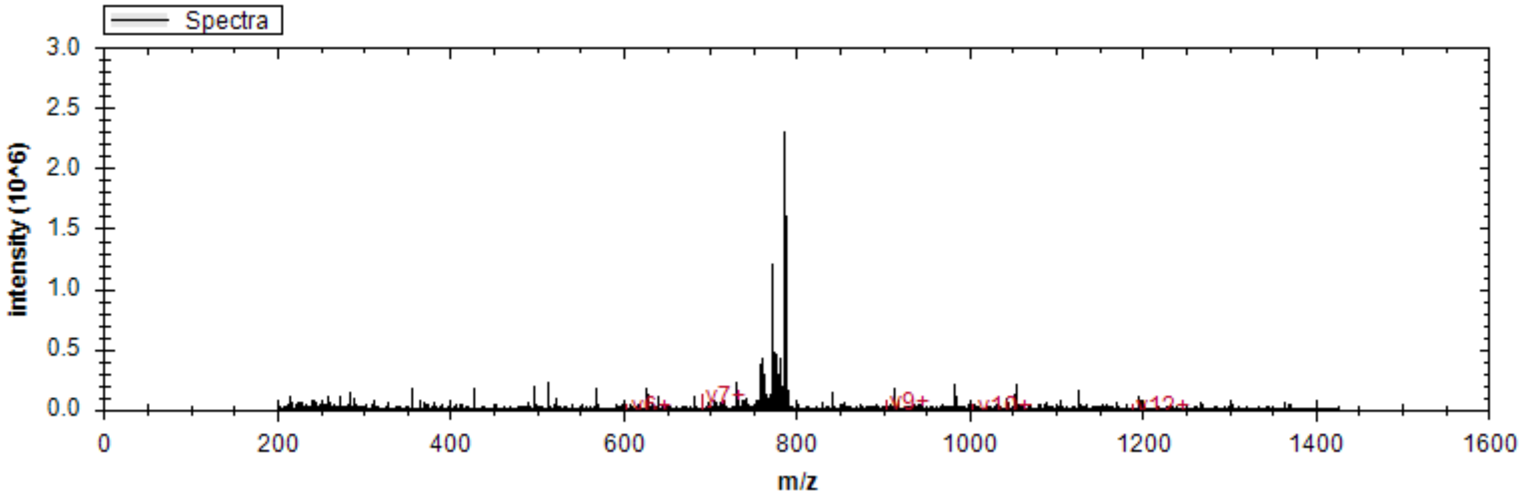

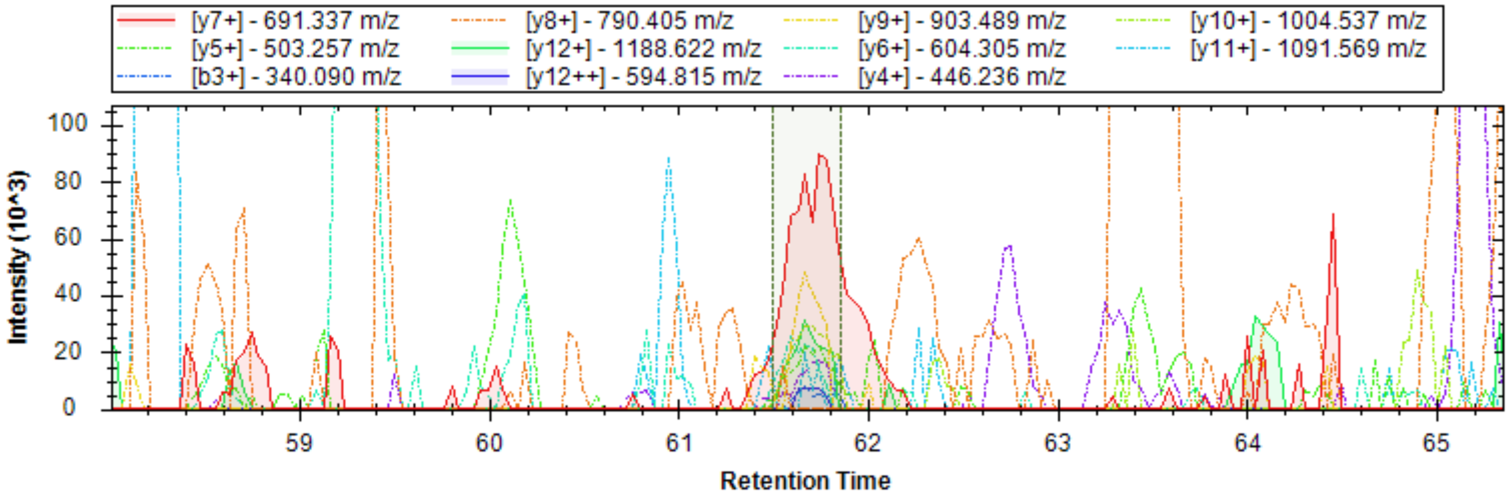

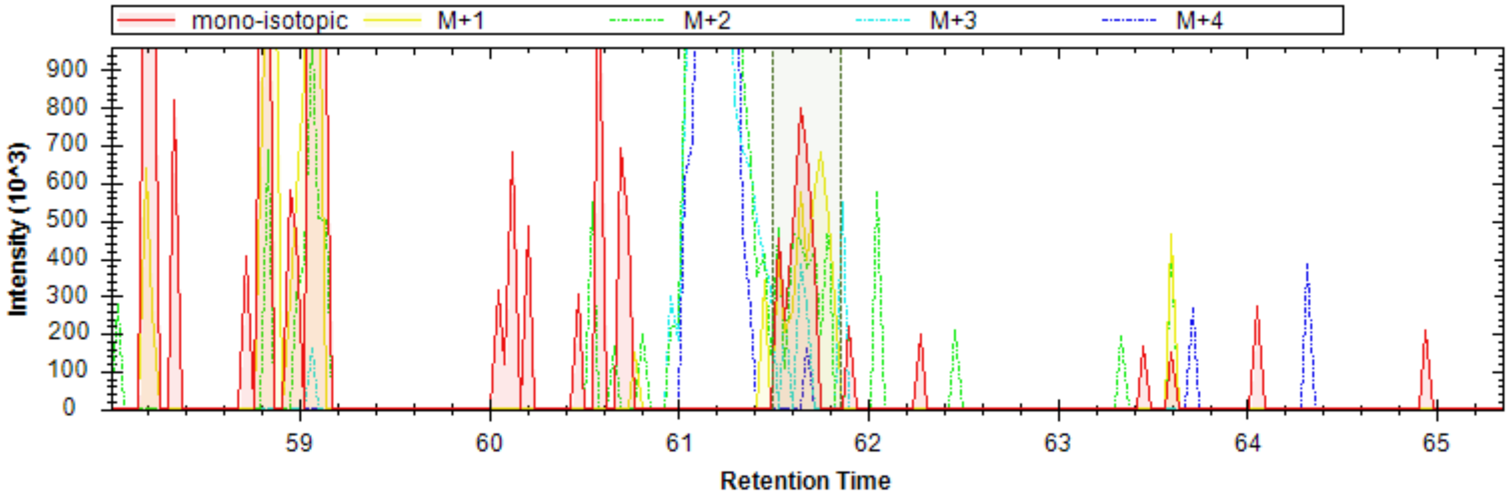

P27546-2 – _ATS[+H+O3+P]PSTLVSTGPSSR_.2
Fig4_MP-DIA-barrel-cortex-p9-3_MHRM.htrms

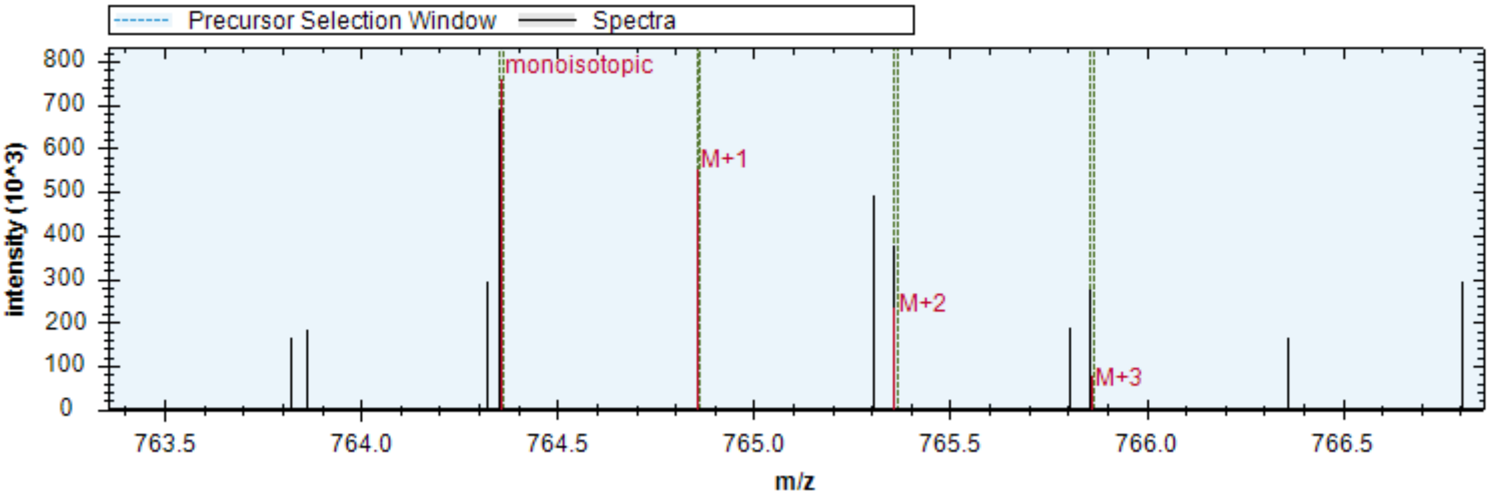

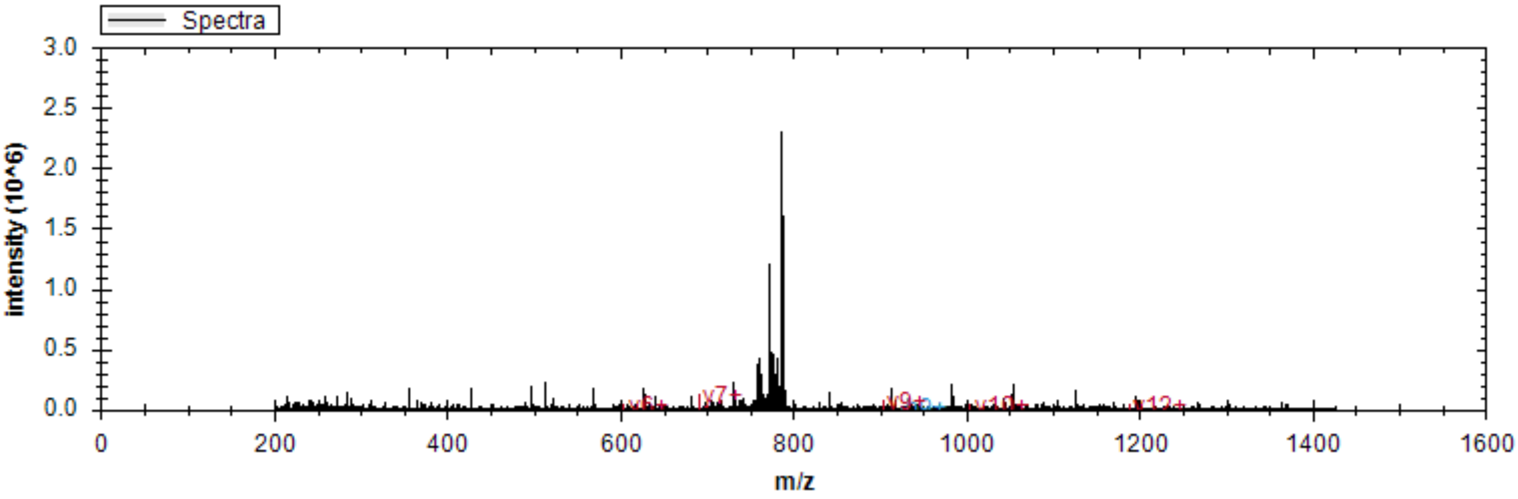

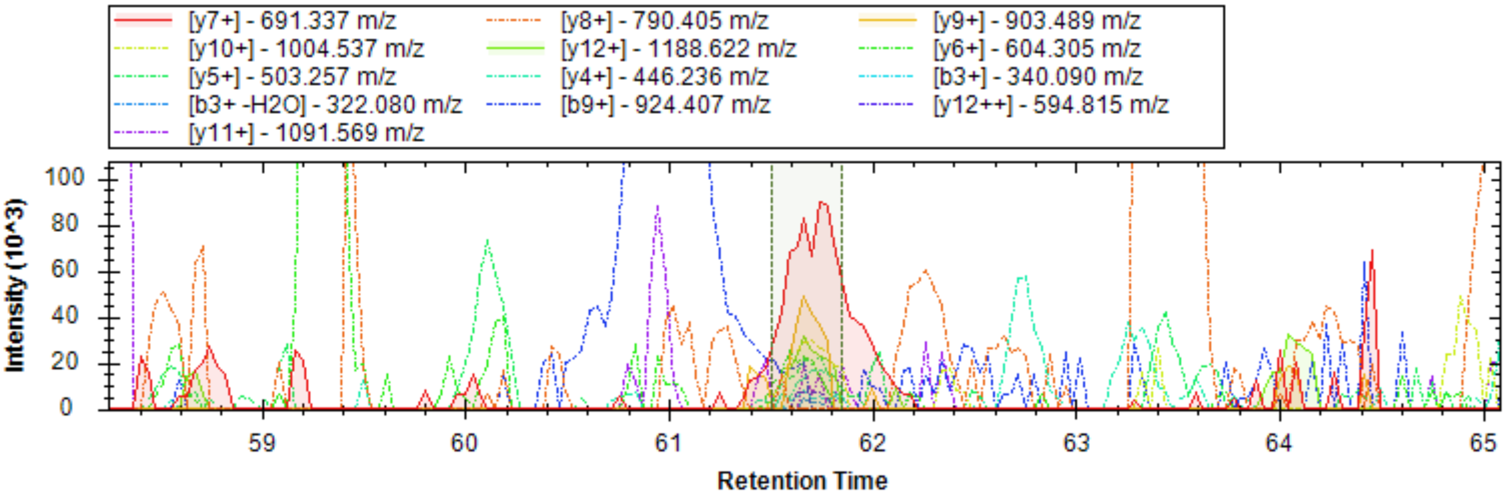

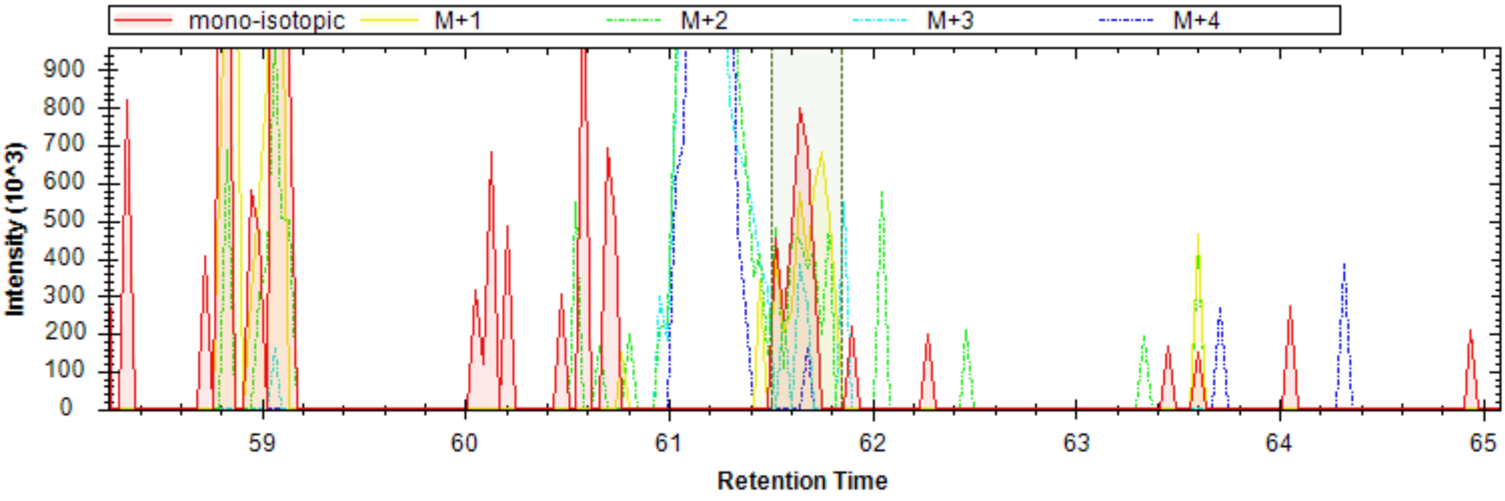

P57776-3 – _ATAPQTQHVS[+H+O3+P]PM[+O]R_.2
Fig4_MP-DIA-barrel-cortex-p9-3_MHRM.htrms

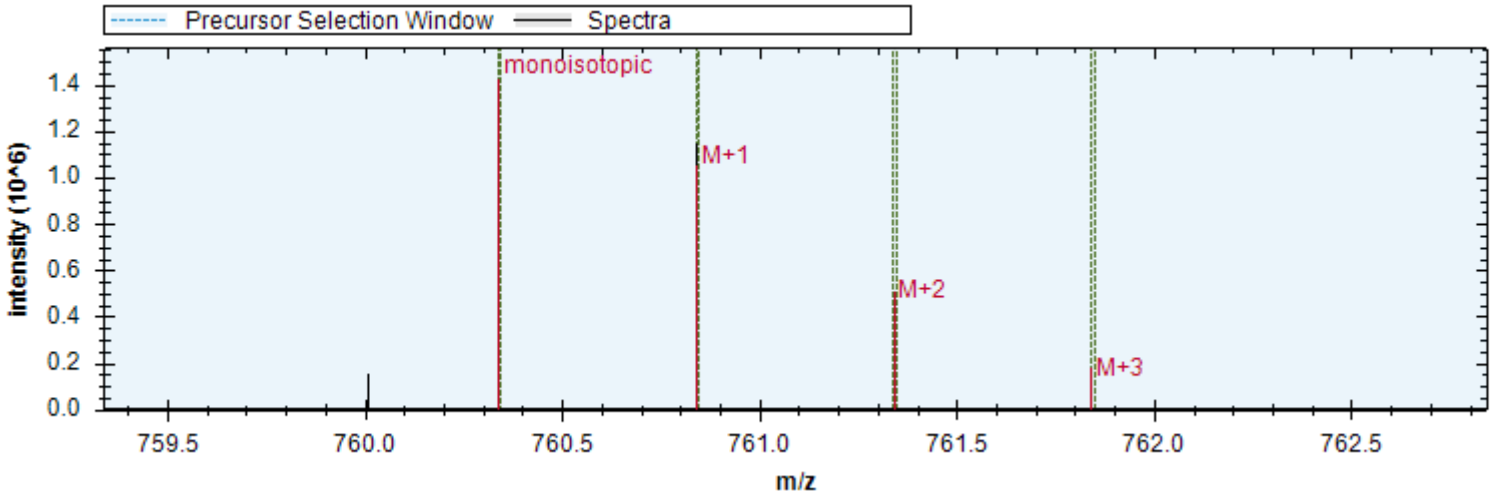

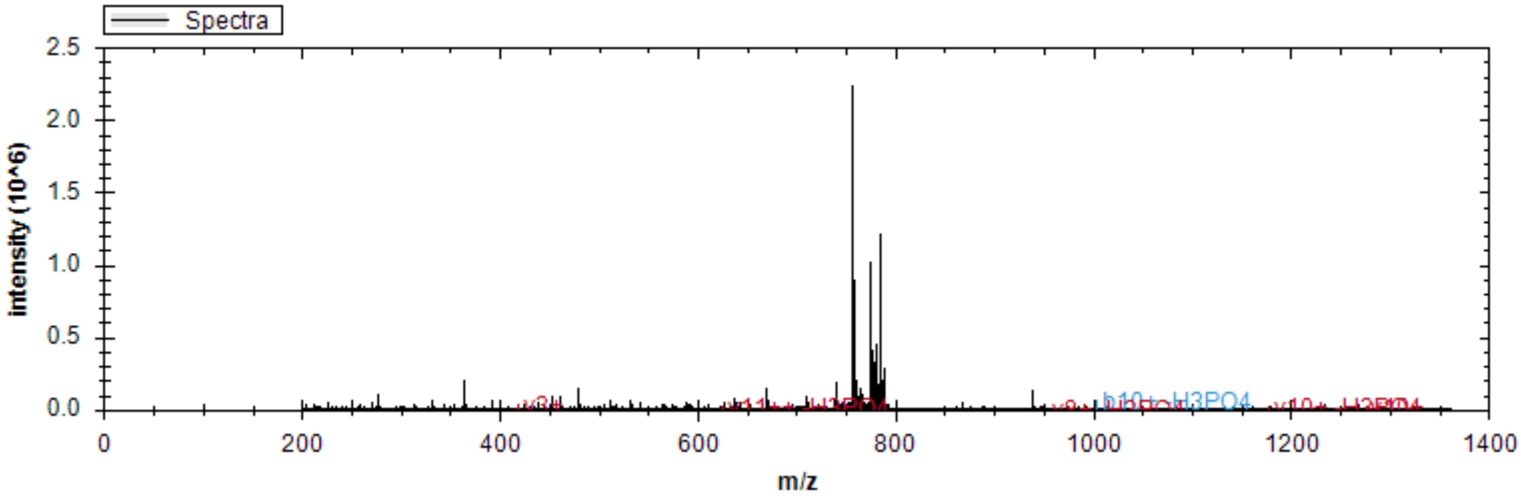

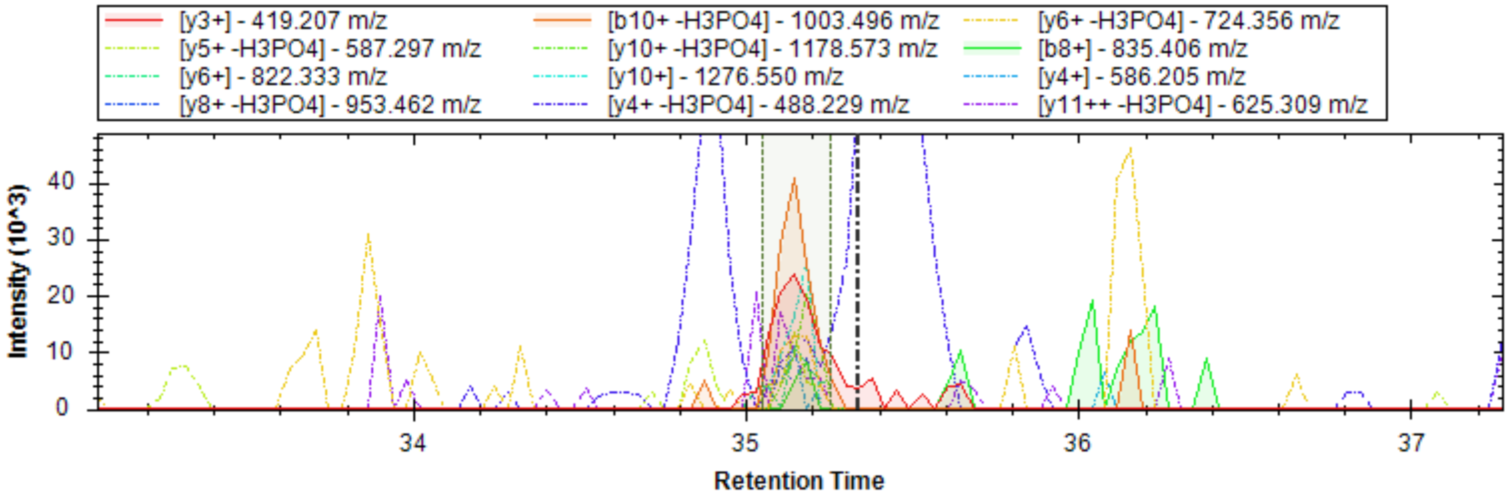

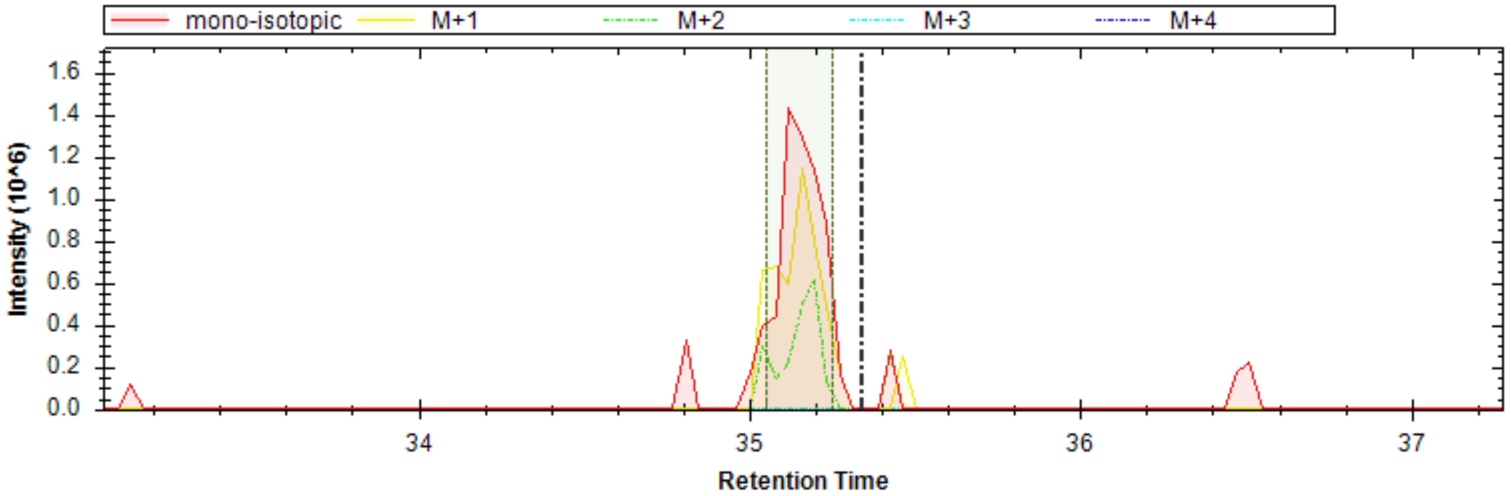

Q3TZM9;Q3TZM9-2;Q3TZM9-3 – _VPDVYIDSM[+O]GYAFT[+H+O3+P]LPLFK_.3
Fig4_MP-DIA-barrel-cortex-p9-3_MHRM.htrms

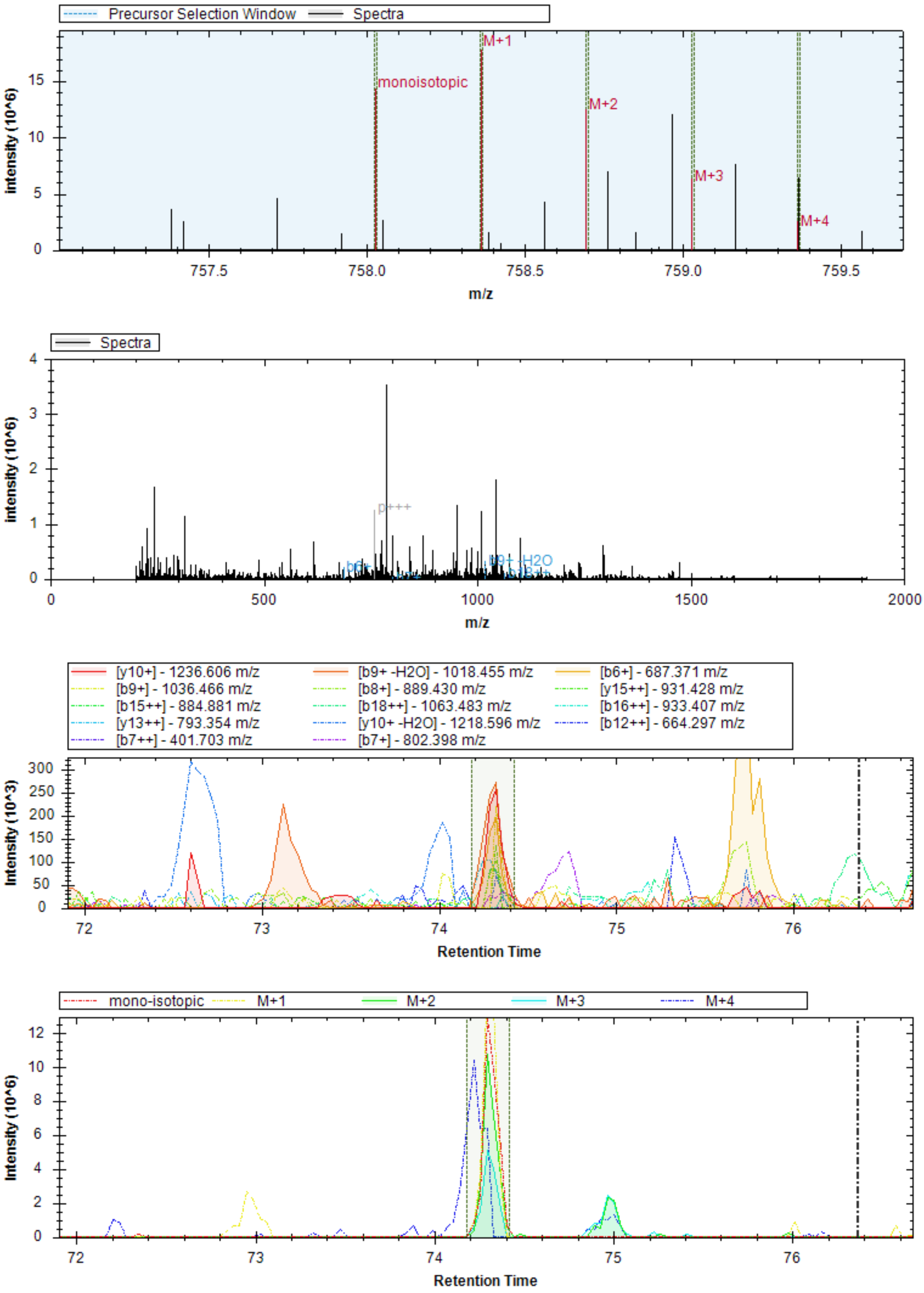

Q68FD9 – _LAQLLS[+H+O3+P]EVPT[+H+O3+P]RR_.2
Fig4_MP-DIA-barrel-cortex-p9-3_MHRM.htrms

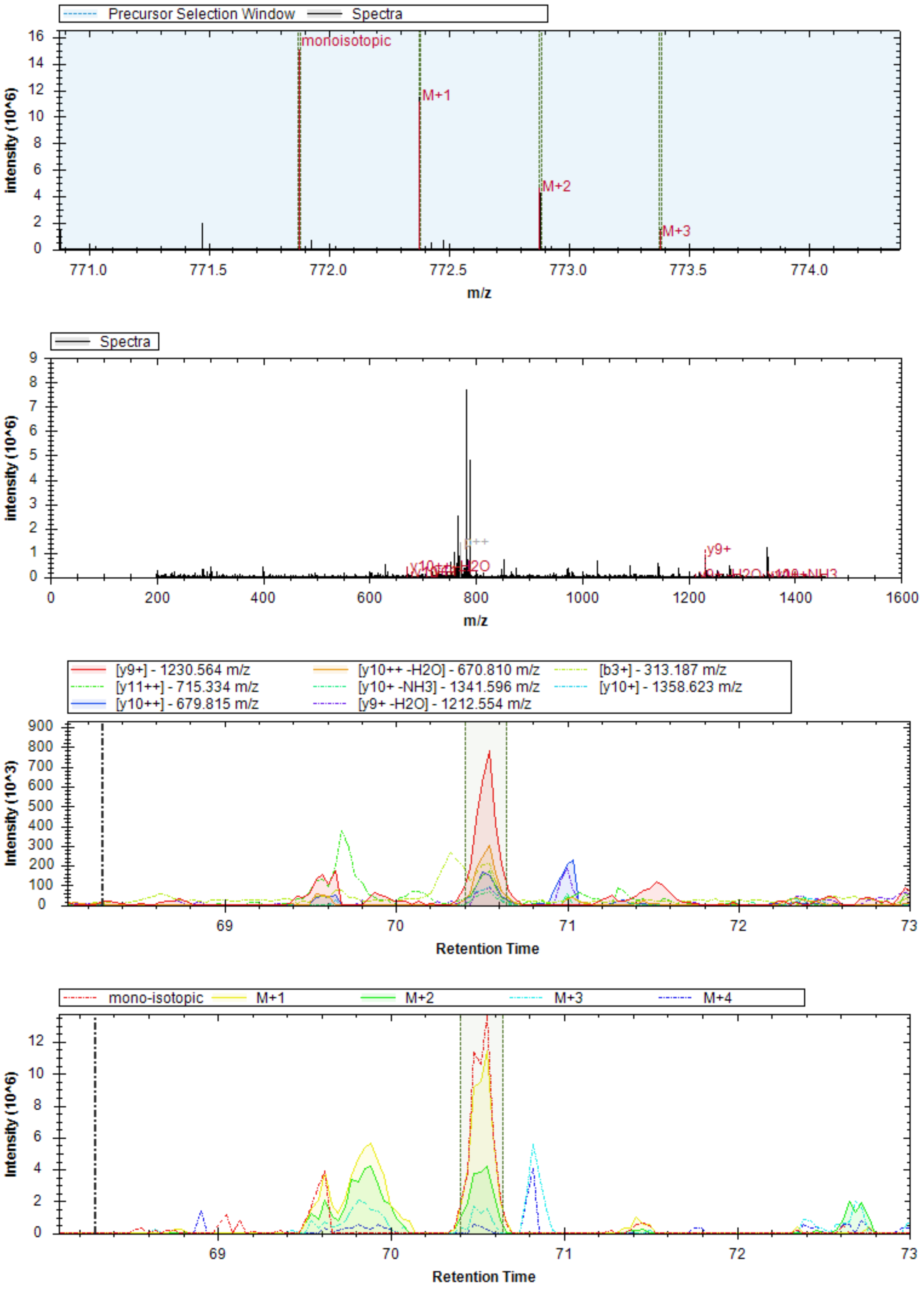

Q6ZQA0;Q6ZQA0-2 – _LHT[+H+O3+P]LLQT[+H+O3+P]AVPTR_.2
Fig4_MP-DIA-barrel-cortex-p9-3_MHRM.htrms

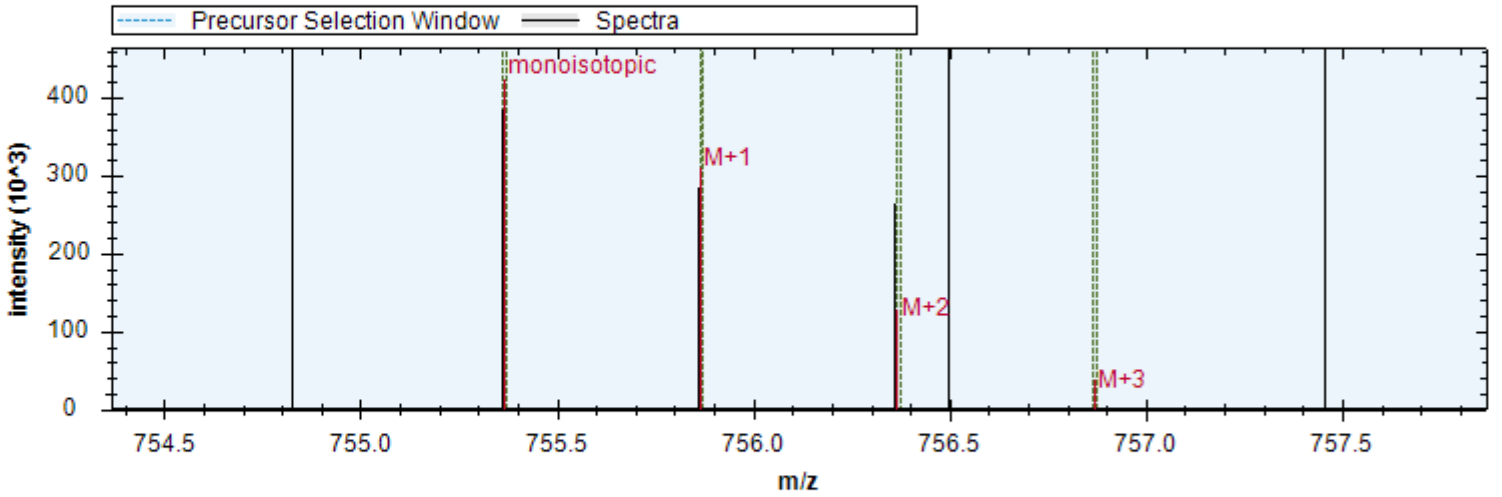

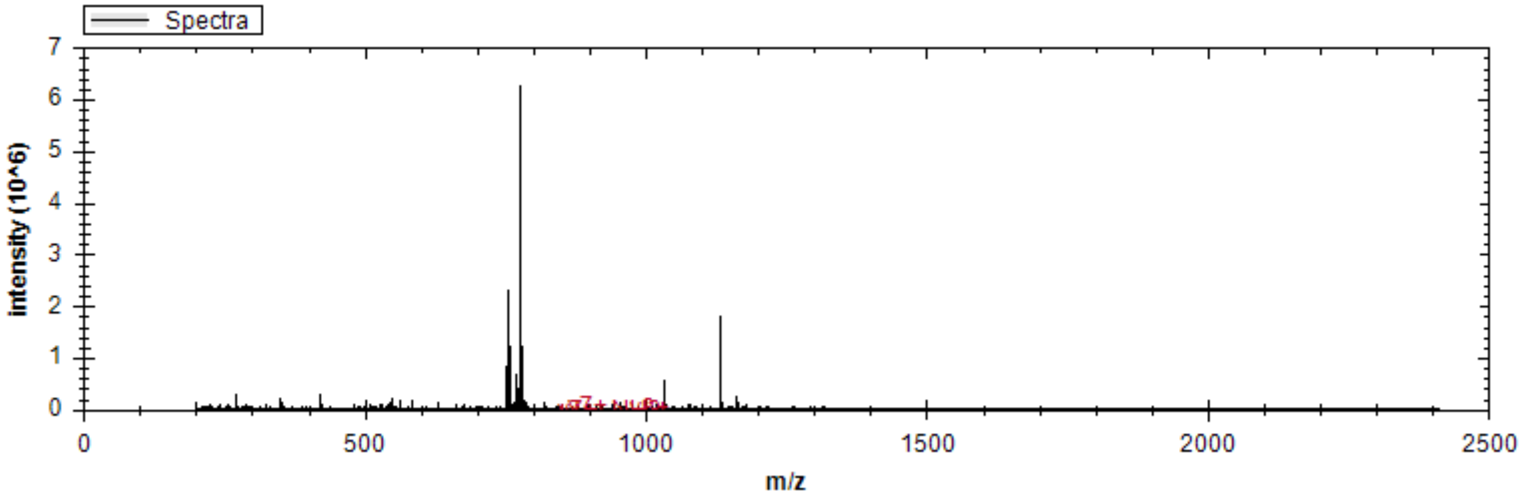

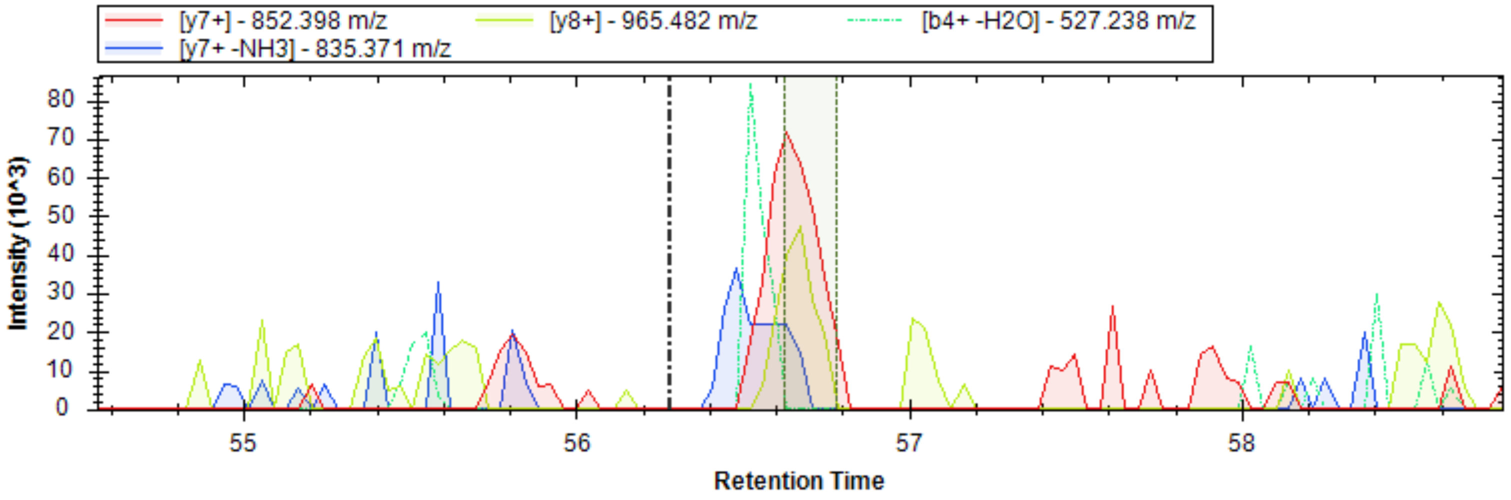

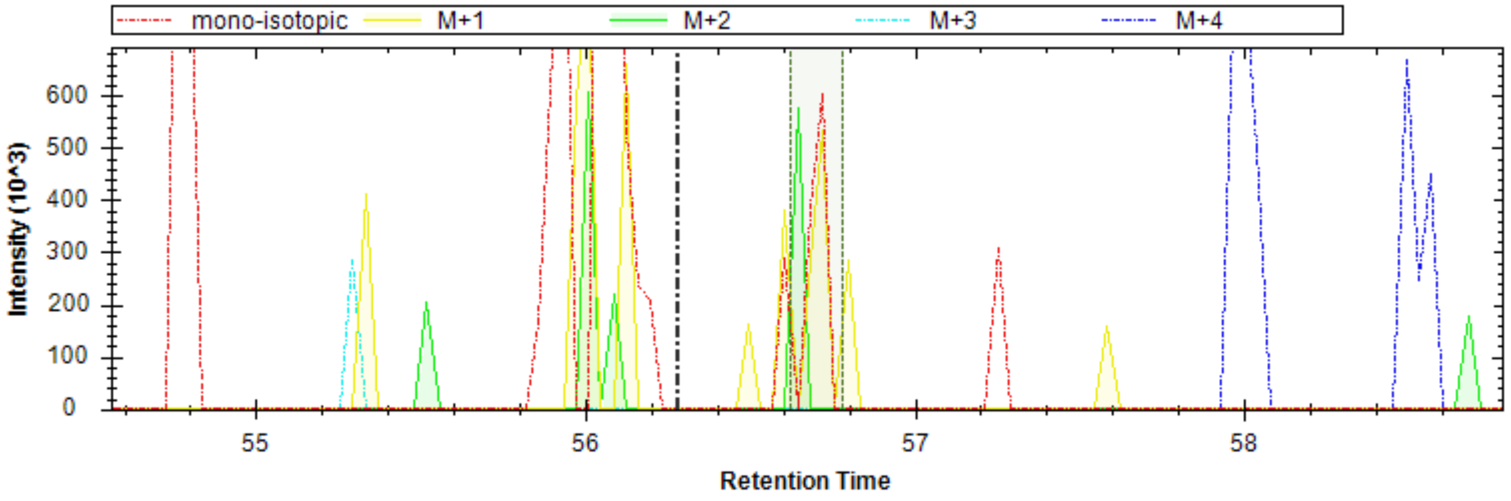

Q8BRT1 – _VLNT[+H+O3+P]GSDVEEAVADALLLGDIR_.3
Fig4_MP-DIA-barrel-cortex-p9-3_MHRM.htrms

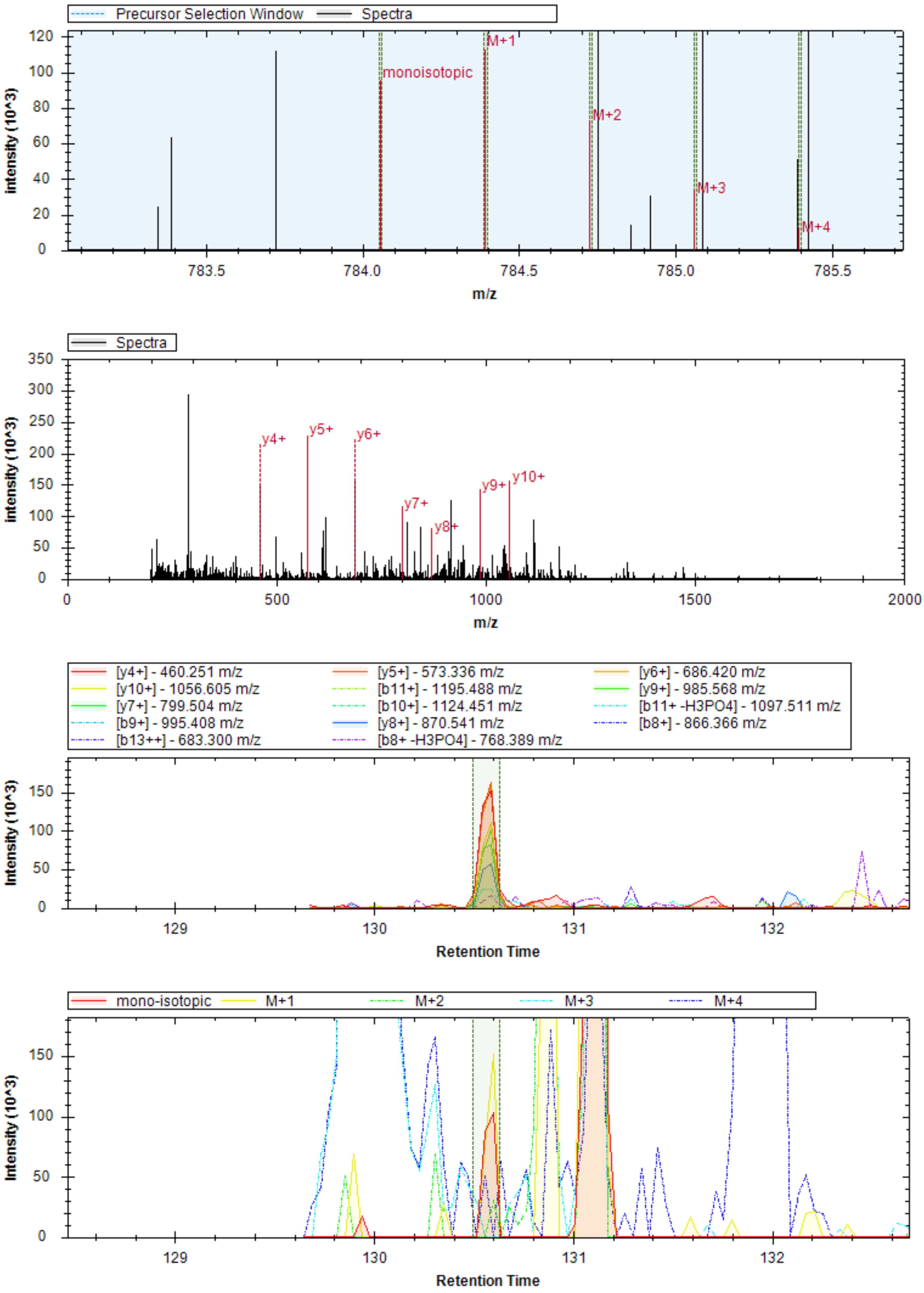

Q8CBW3;Q8CBW3-2;Q8CB[...] – _HNS[+H+O3+P]TTSSTSSGGYR_.2
Fig4_MP-DIA-barrel-cortex-p9-3_MHRM.htrms

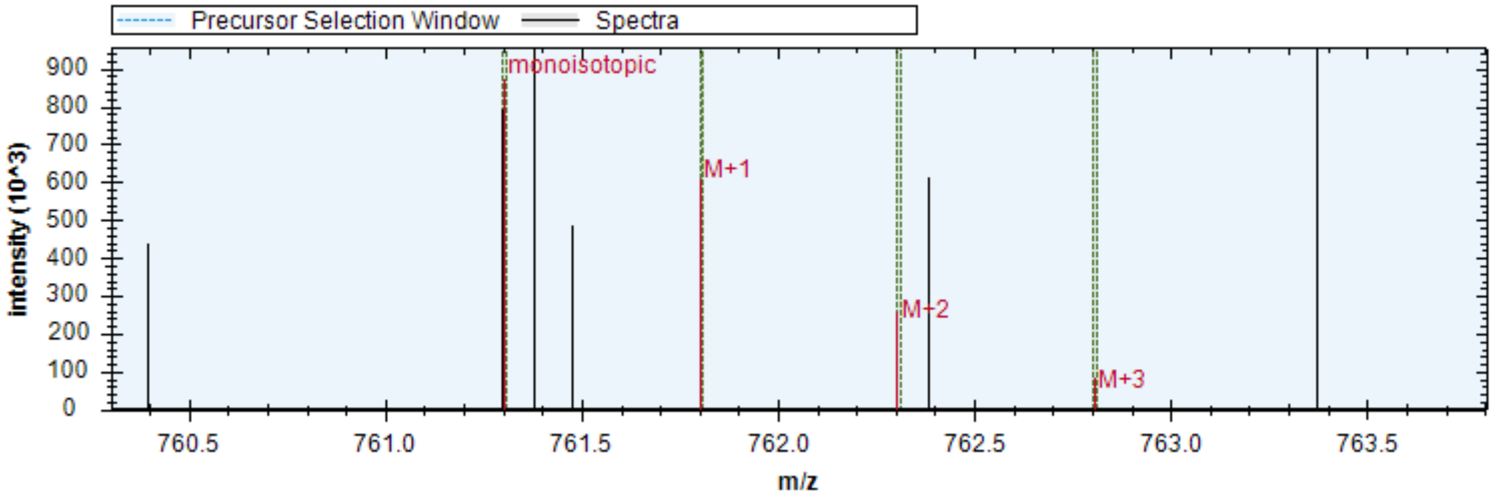

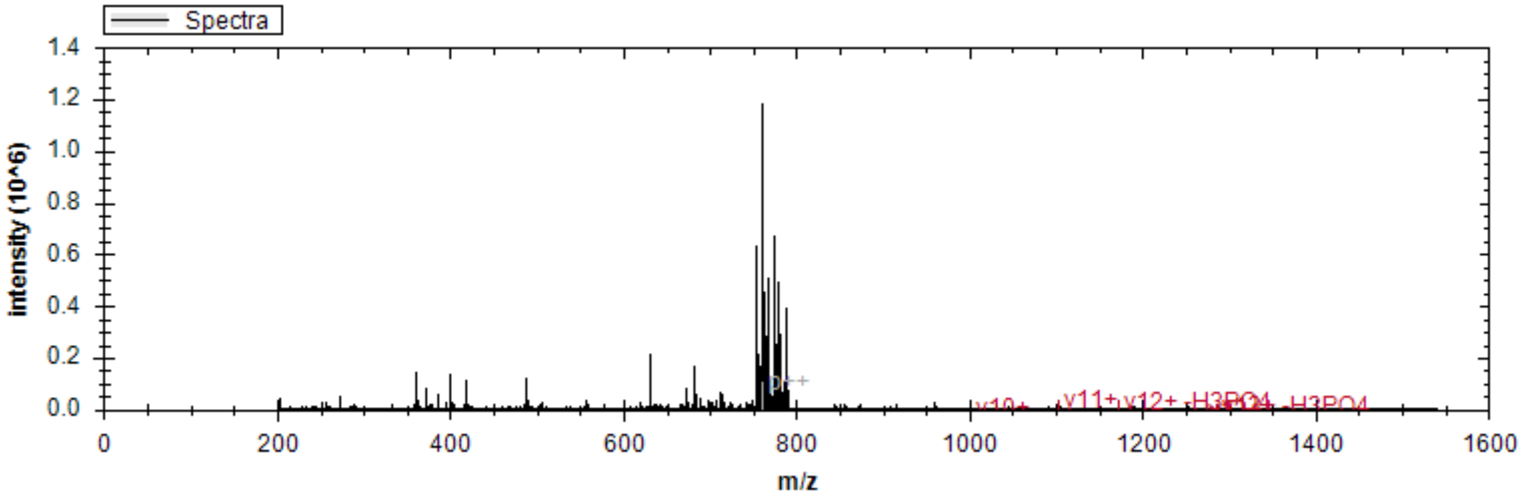

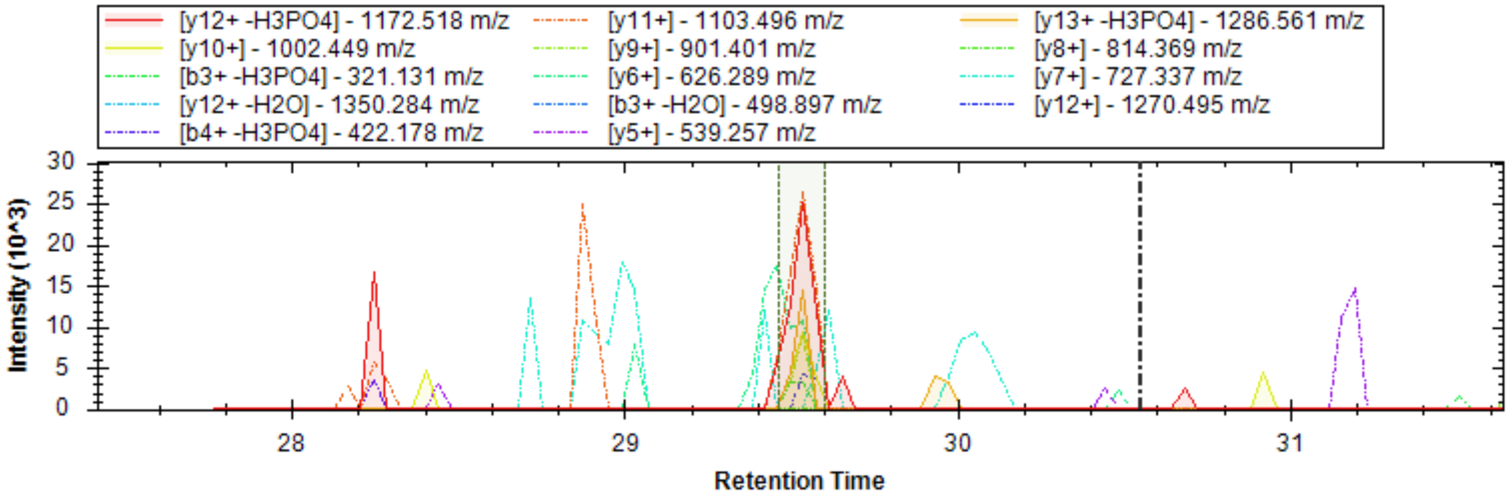

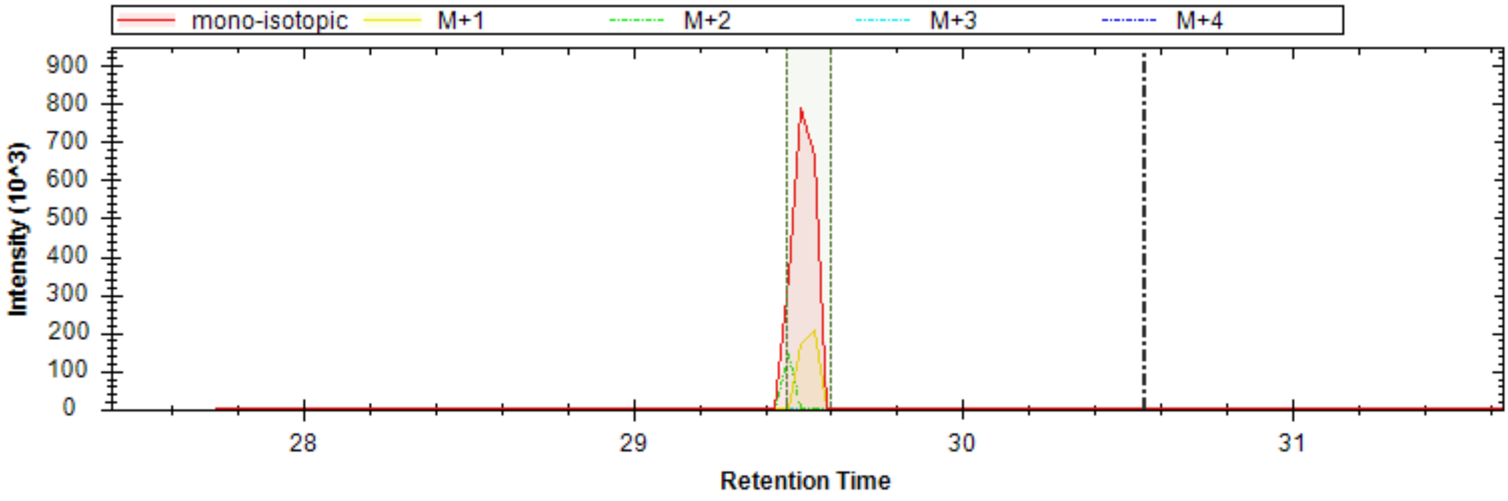

Q9CZX2 – _IHQRY[+H+O3+P]T[+H+O3+P]EIT[+H+O3+P]K_.2
Fig4_MP-DIA-barrel-cortex-p9-3_MHRM.htrms

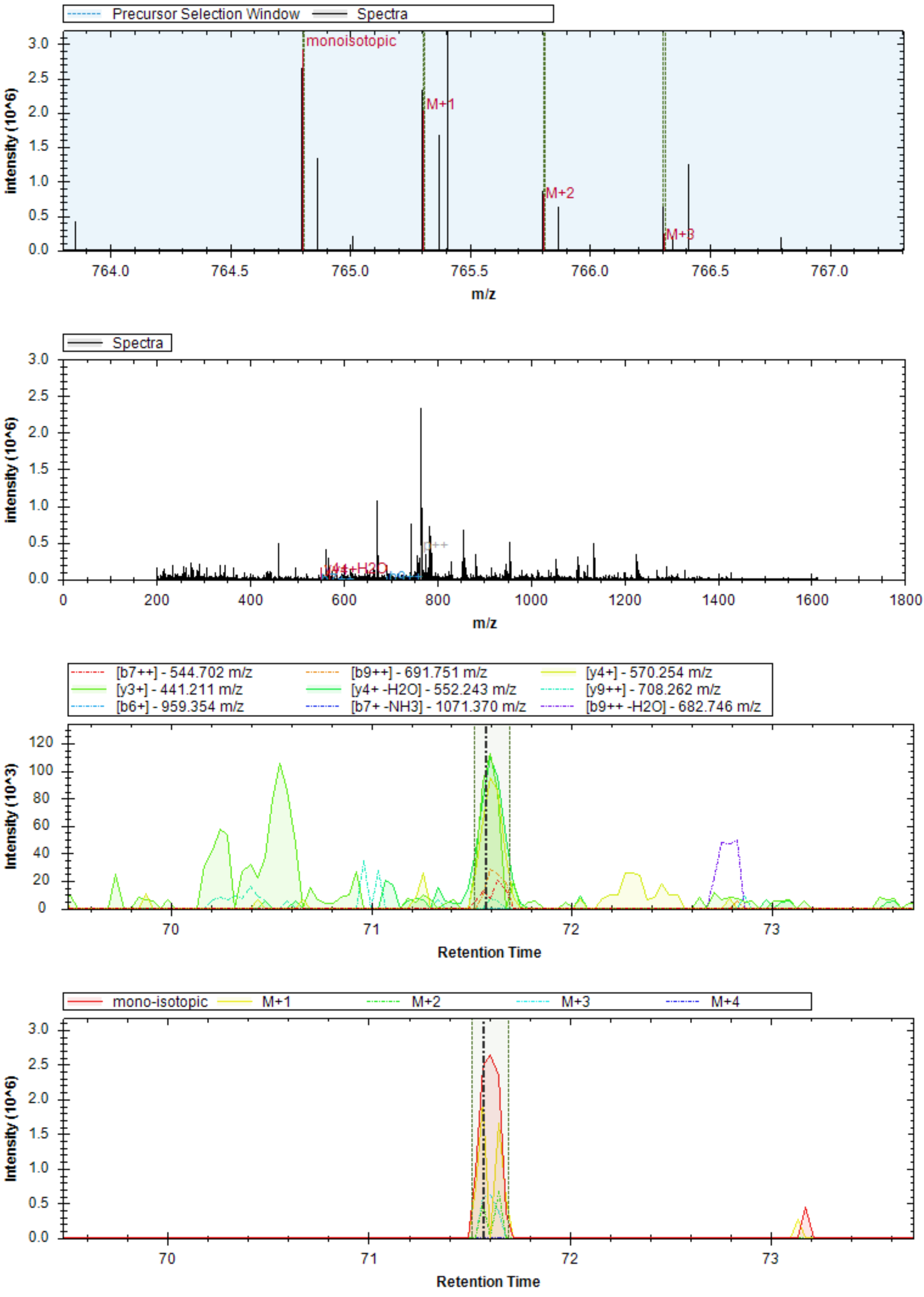

Q9D5R2 – _SNS[+H+O3+P]LPHSAVSNAASK_.2
Fig4_MP-DIA-barrel-cortex-p9-3_MHRM.htrms

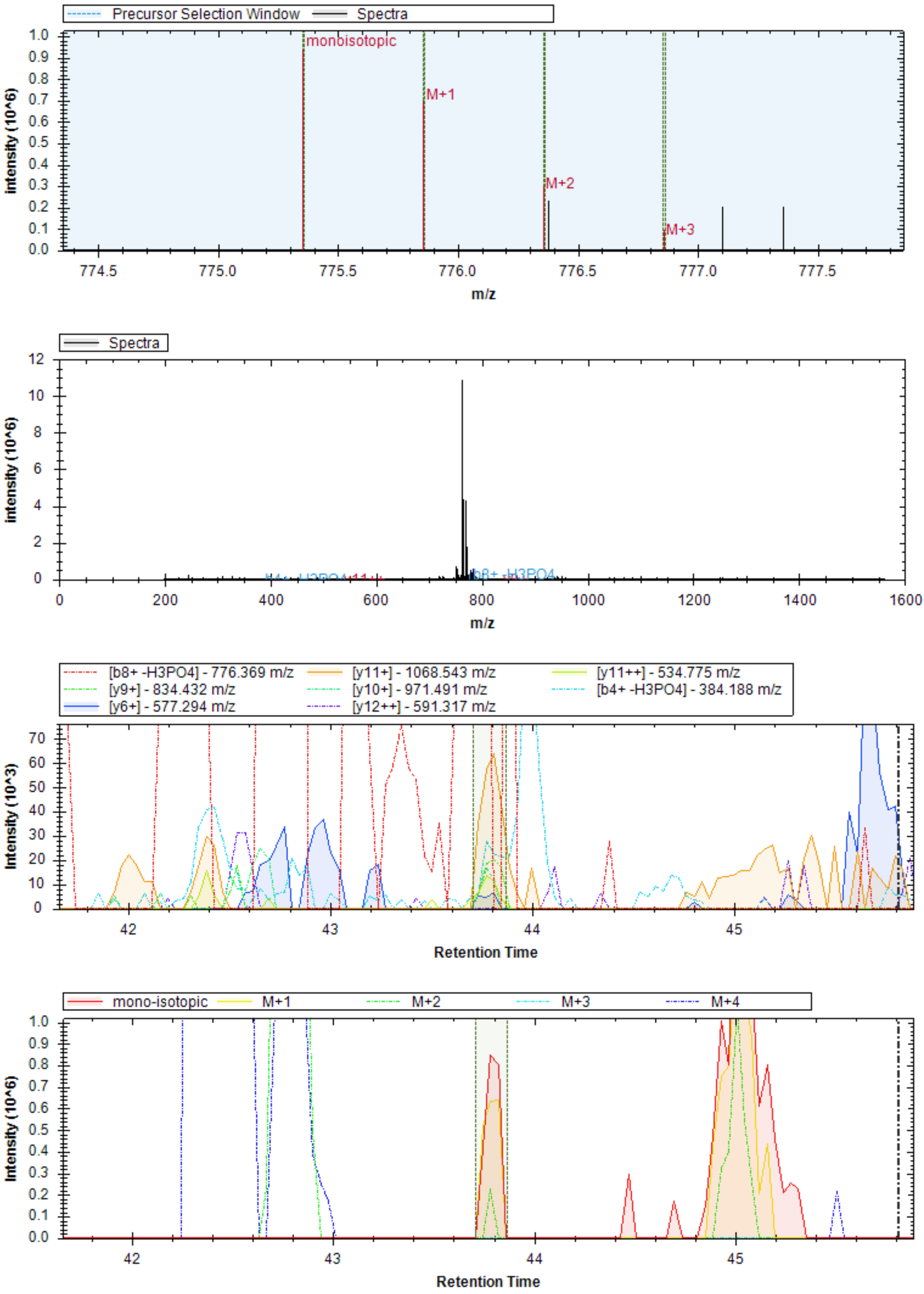

Q9JLM8 – _ISQHGGS[+H+O3+P]STSLSSTK_.2
Fig4_MP-DIA-barrel-cortex-p9-3_MHRM.htrms

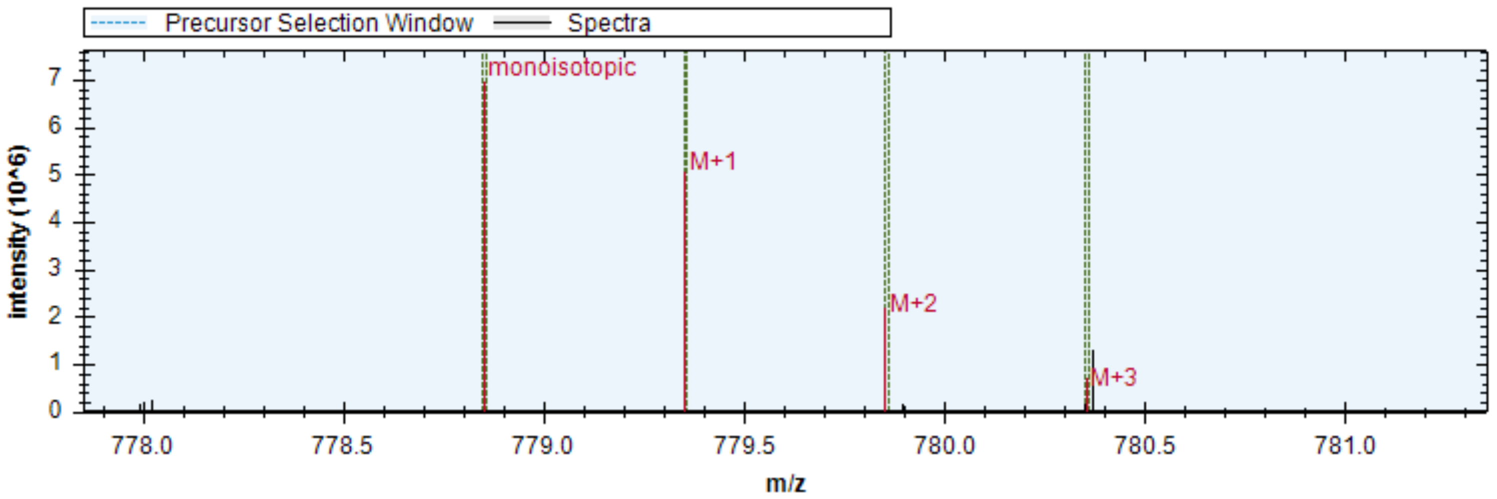

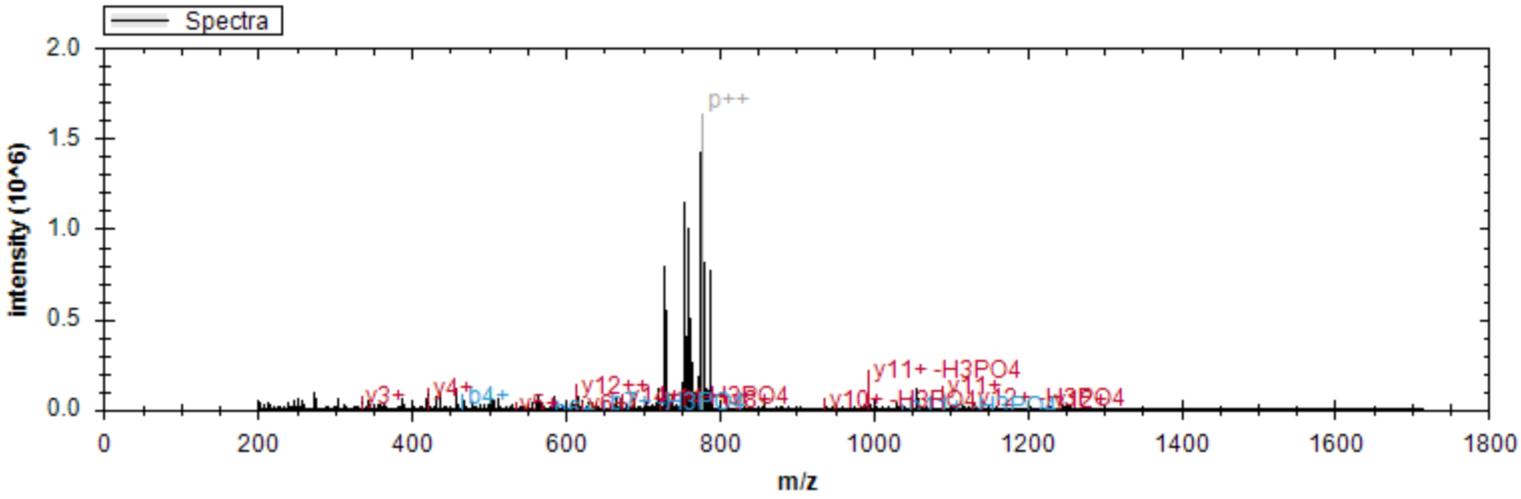

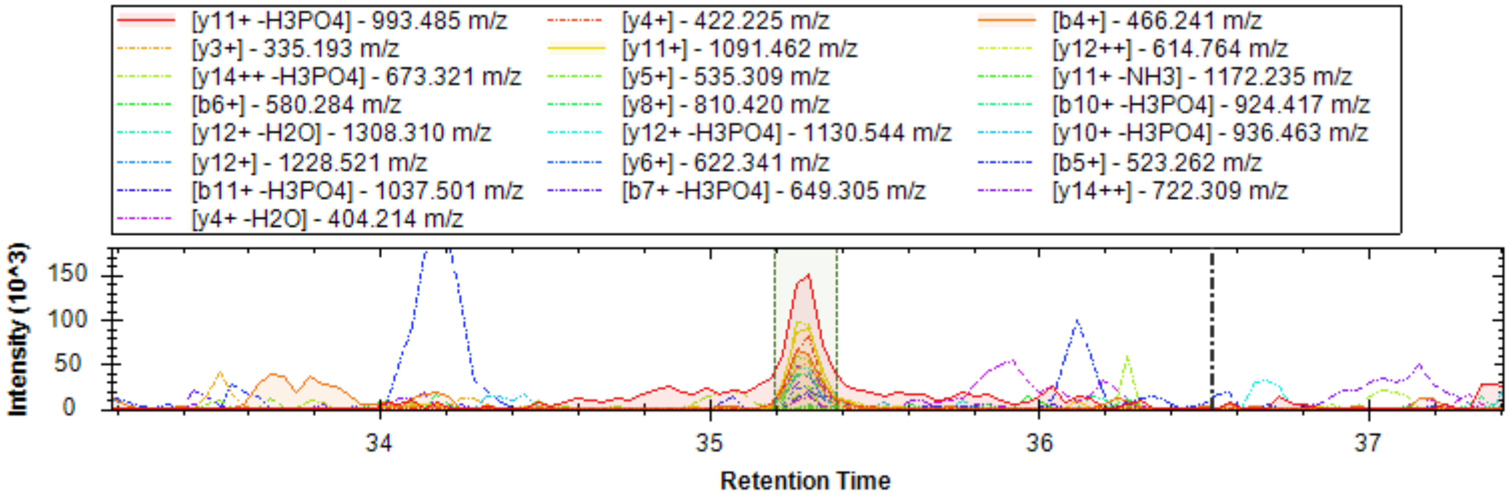

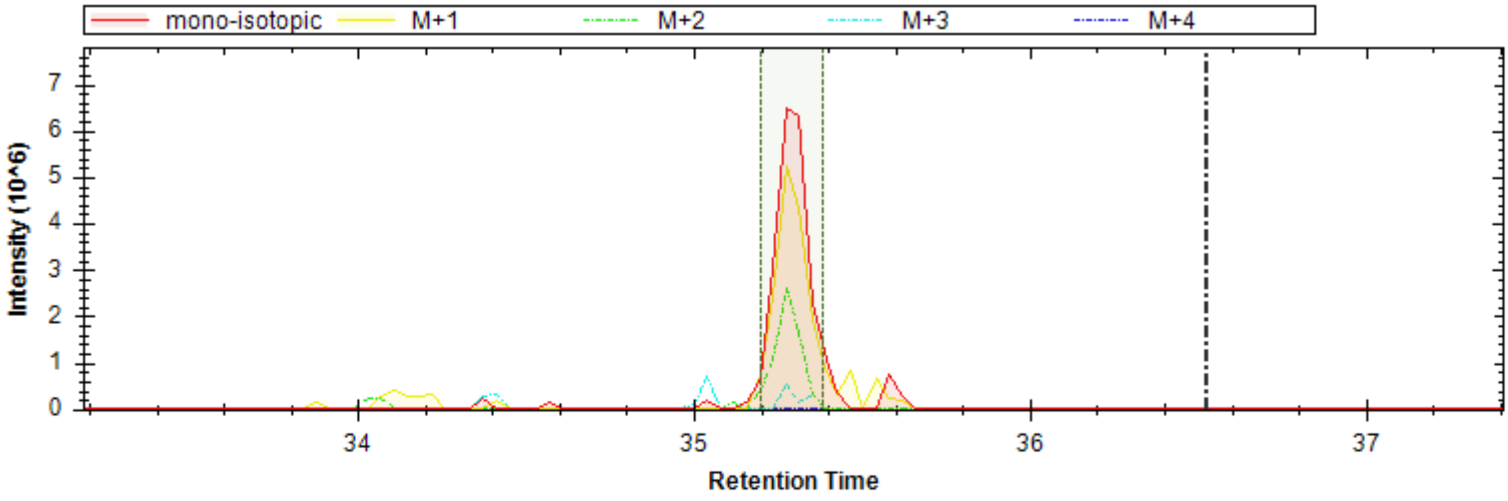

Q9QUG9 – _AQS[+H+O3+P]VSLEGSAPSPSPTHTHHR_.3
Fig4_MP-DIA-barrel-cortex-p9-3_MHRM.htrms

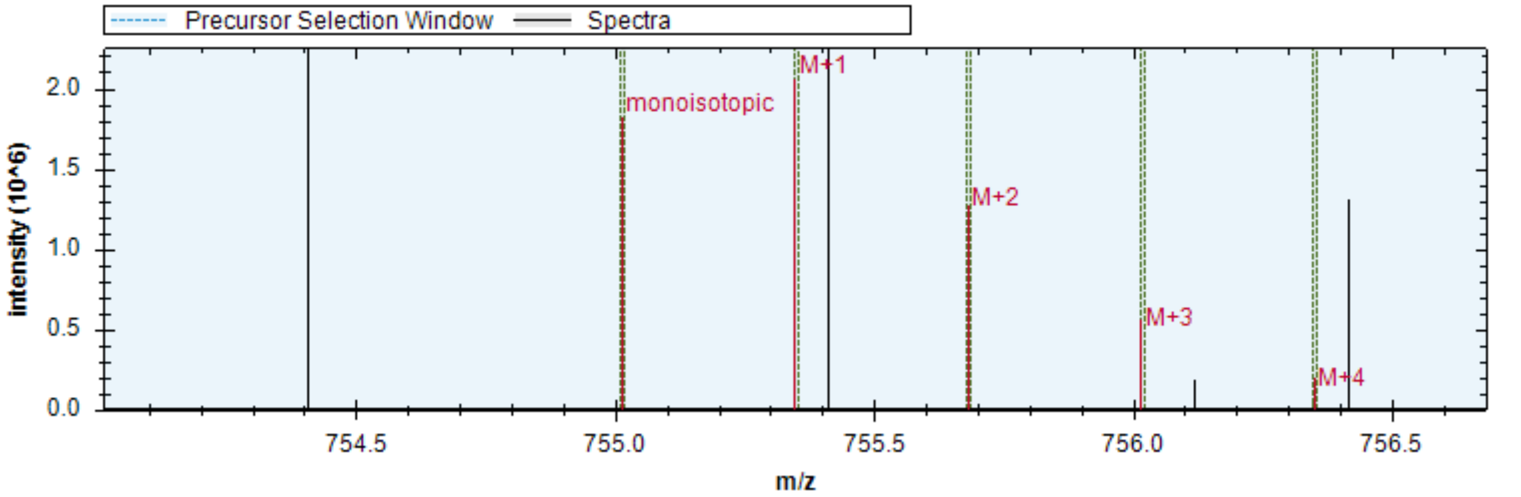

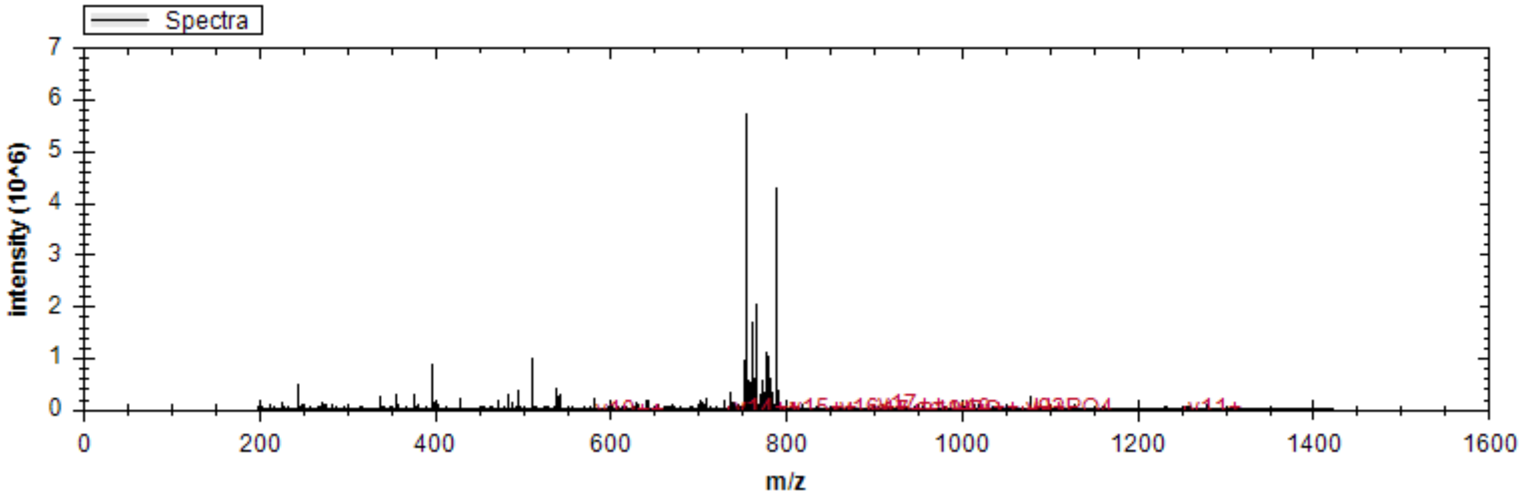

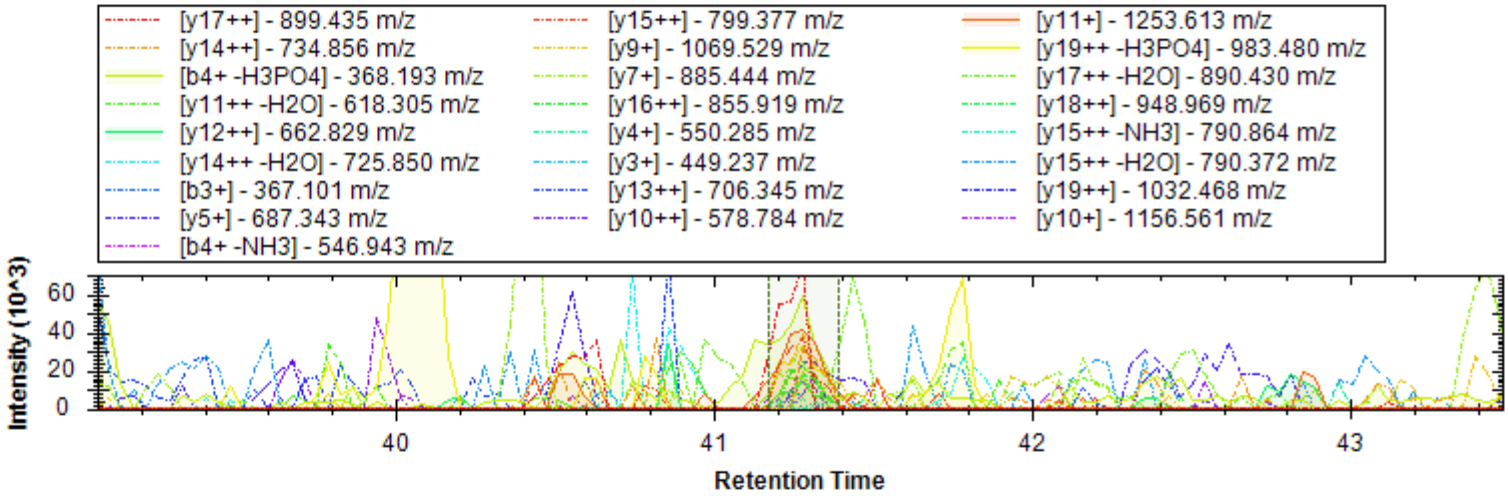

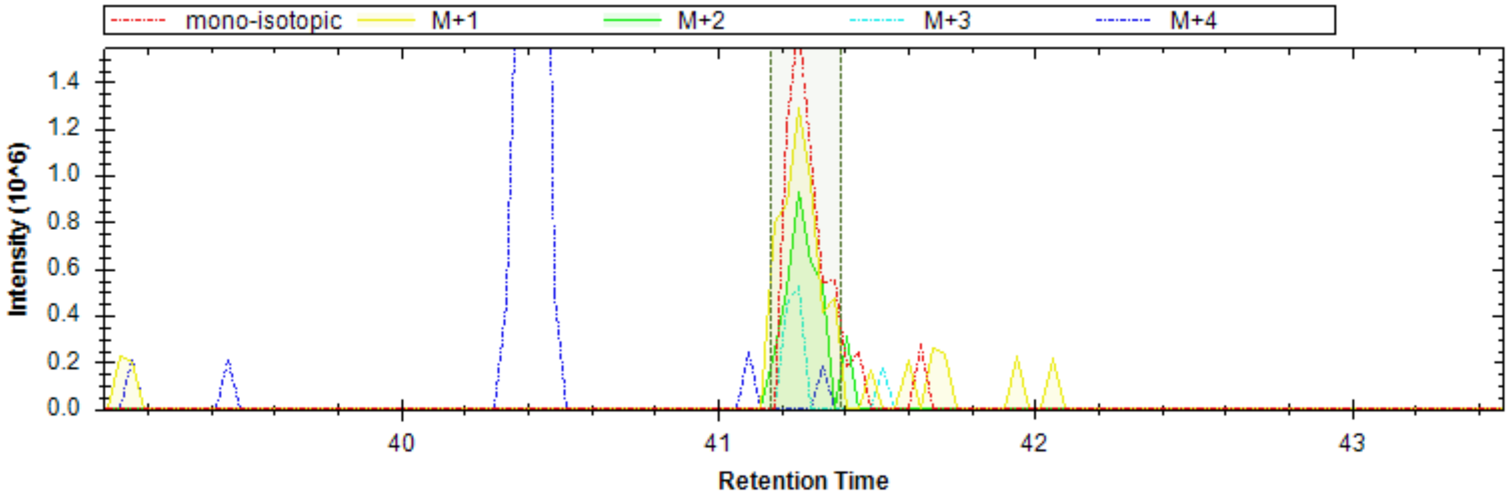

Q9QWI6 – _LSYAGGRPPSYAGS[+H+O3+P]PVHHAAER_.3

Fig4_MP-DIA-barrel-cortex-p9-3_MHRM.htrms

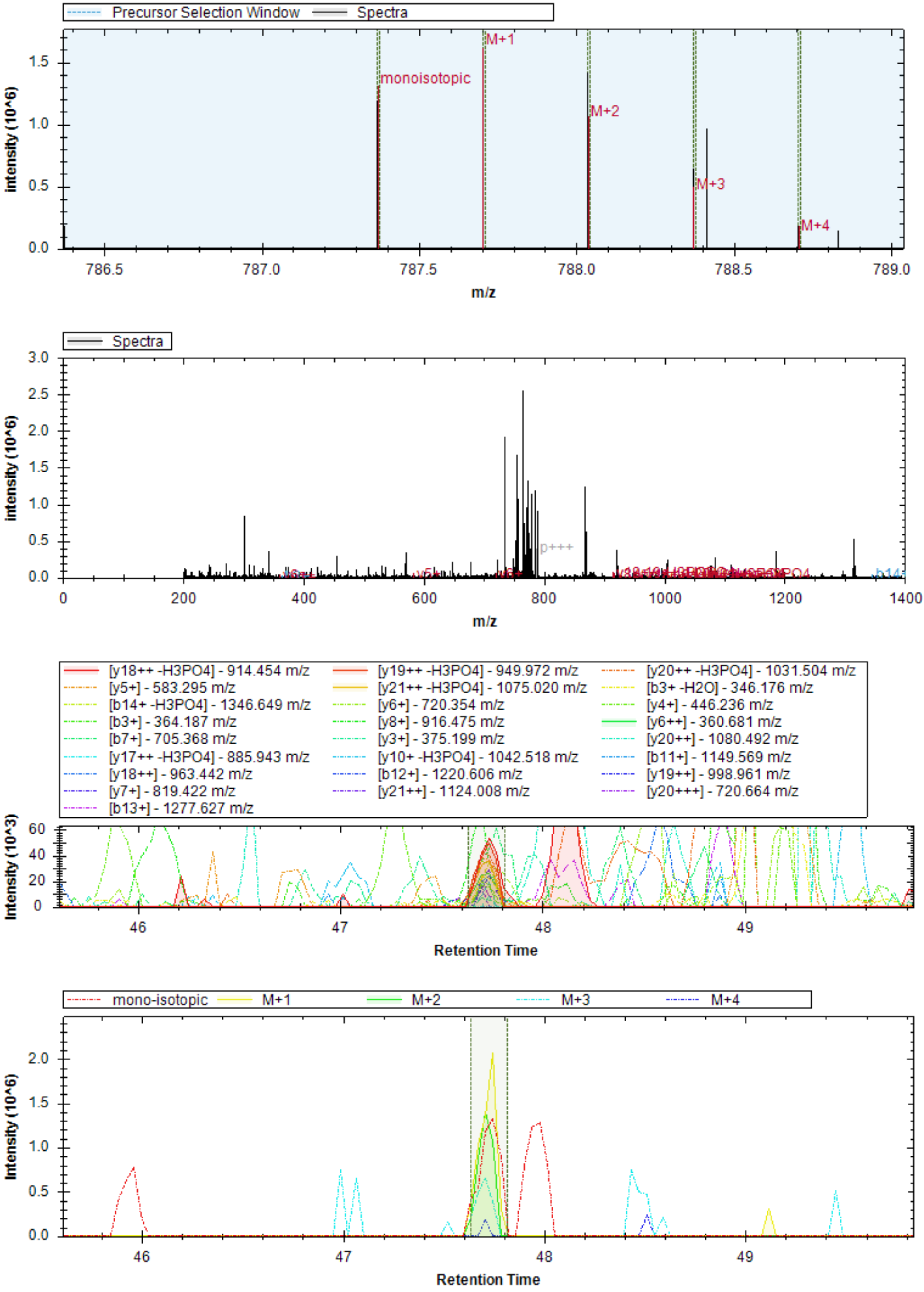

Q9QYC0 – _AAVVTS[+H+O3+P]PPPTTAPHK_.2

Fig4_MP-DIA-barrel-cortex-p9-3_MHRM.htrms

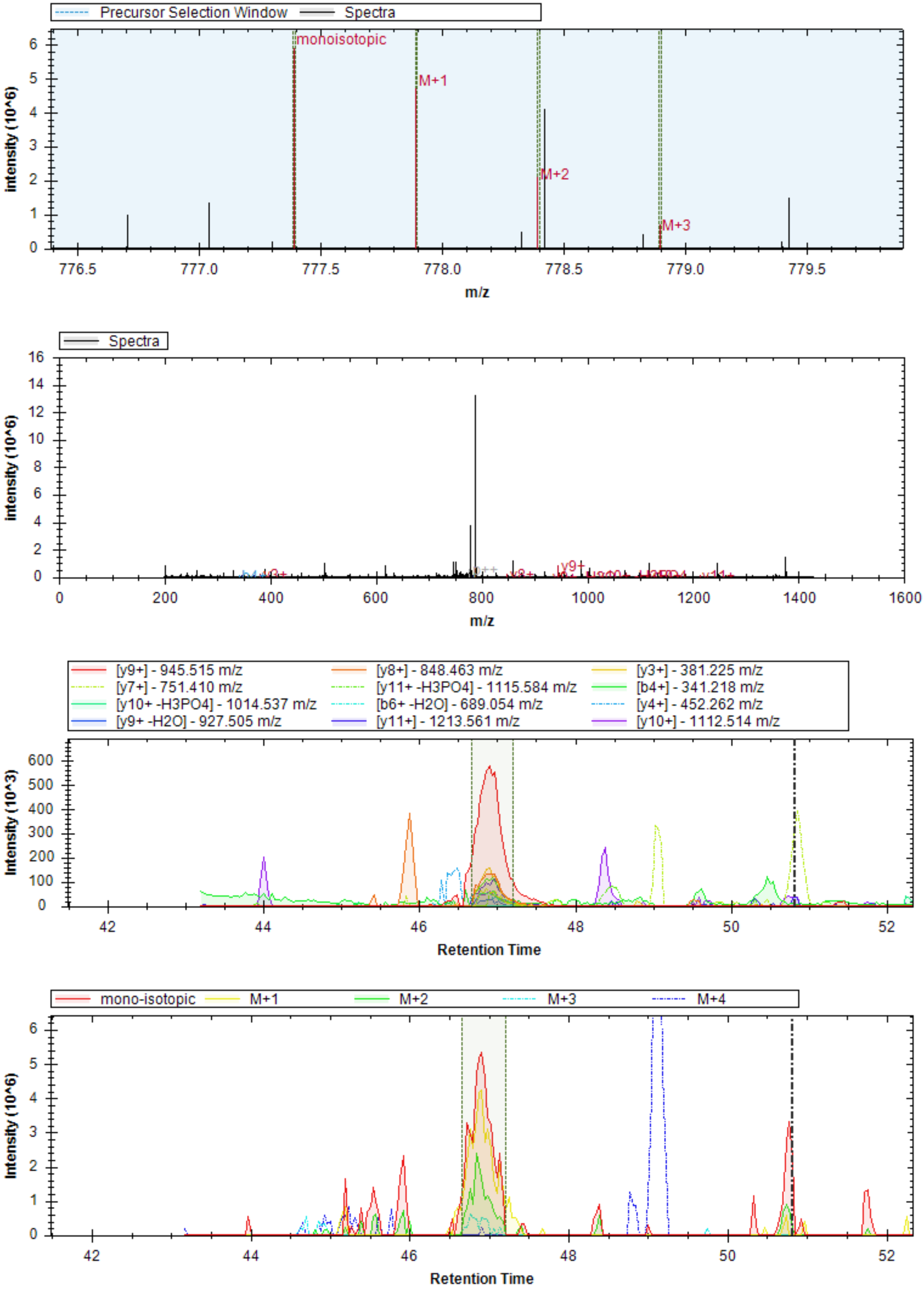

O88935-1 – _PAKPQLAQKPS[+H+O3+P]QDVPPPITAAAGGPPHPQLK_.4
Fig4_MP-DIA-barrel-cortex-p9-3_MHRM.htrms

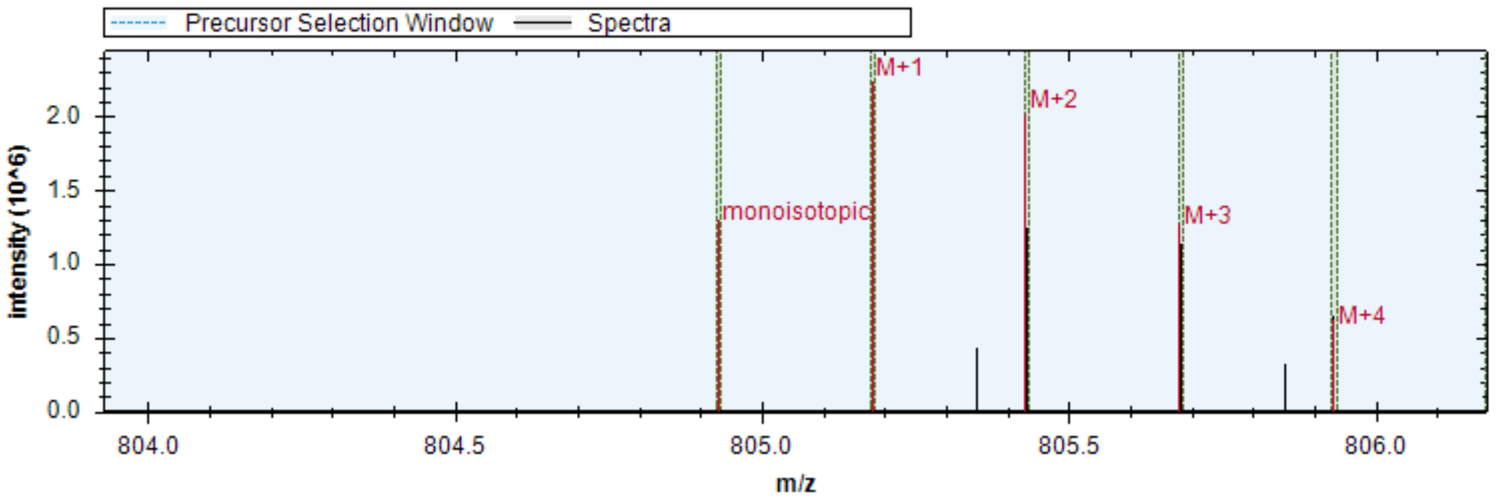

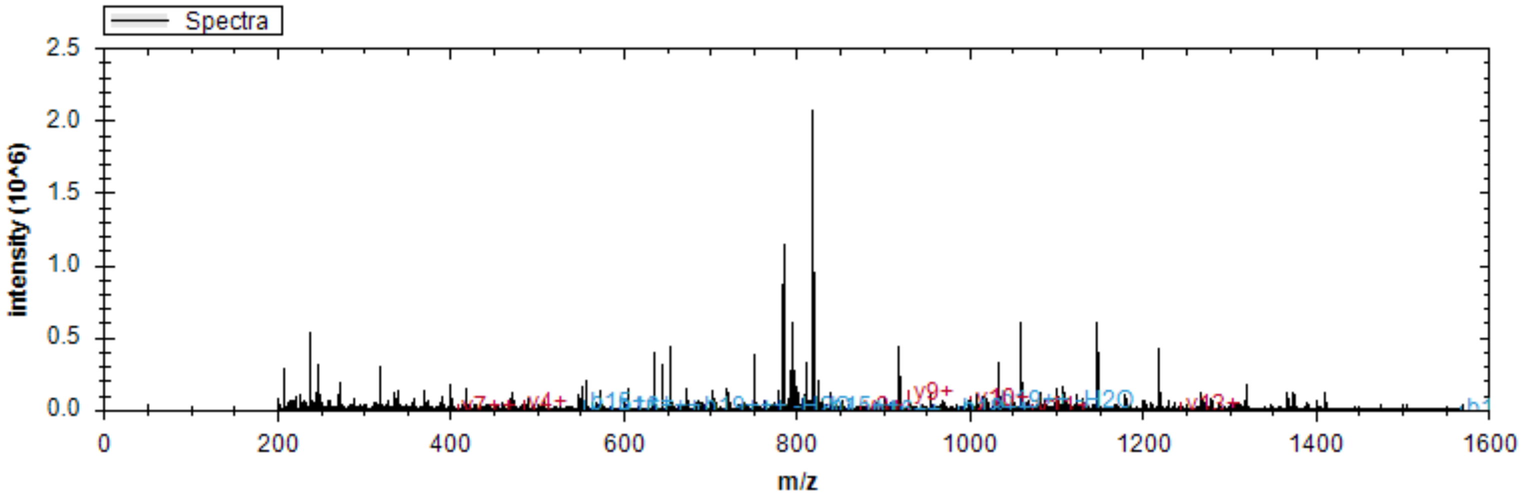

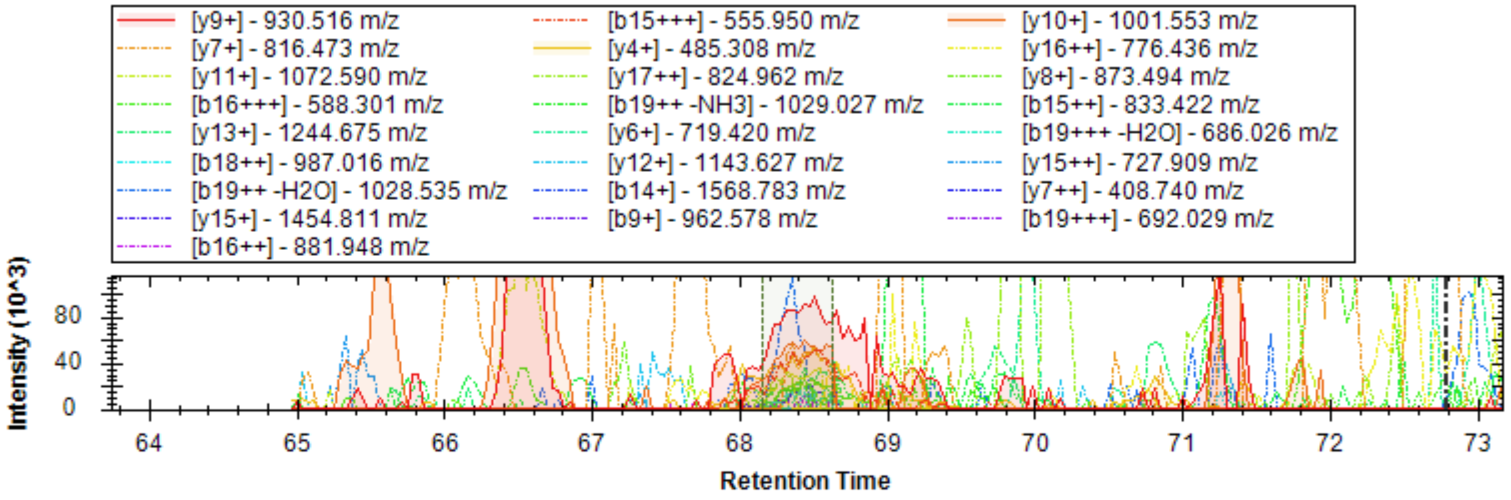

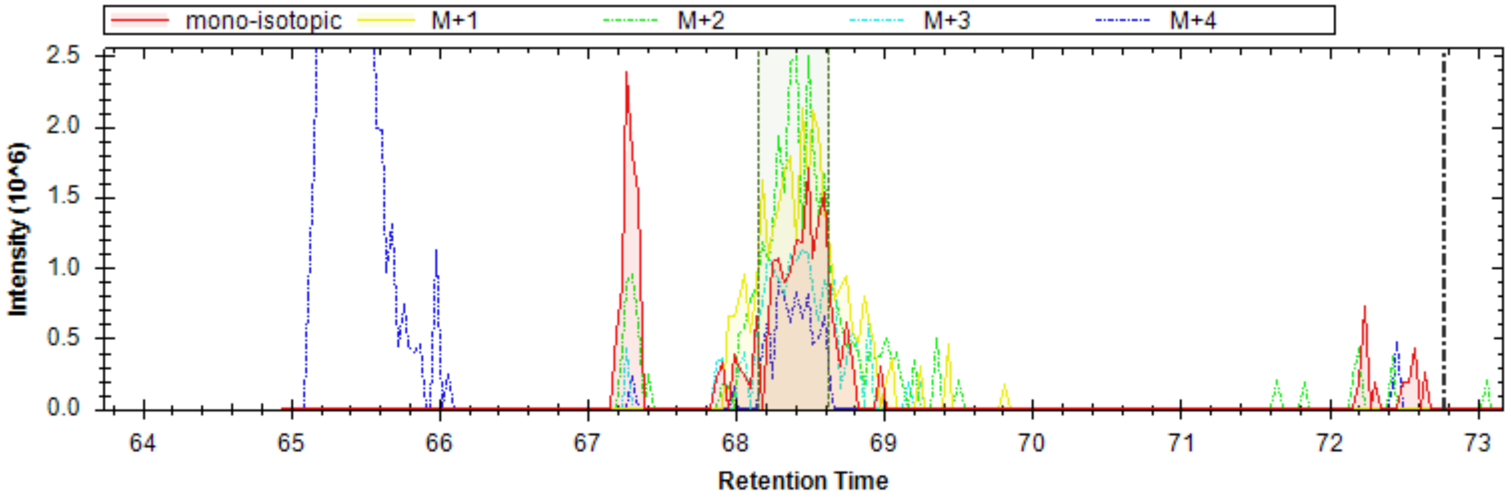

P06837 – _EGDGSATTDAAPAT[+H+O3+P]SPK_.2
Fig4_MP-DIA-barrel-cortex-p9-3_MHRM.htrms

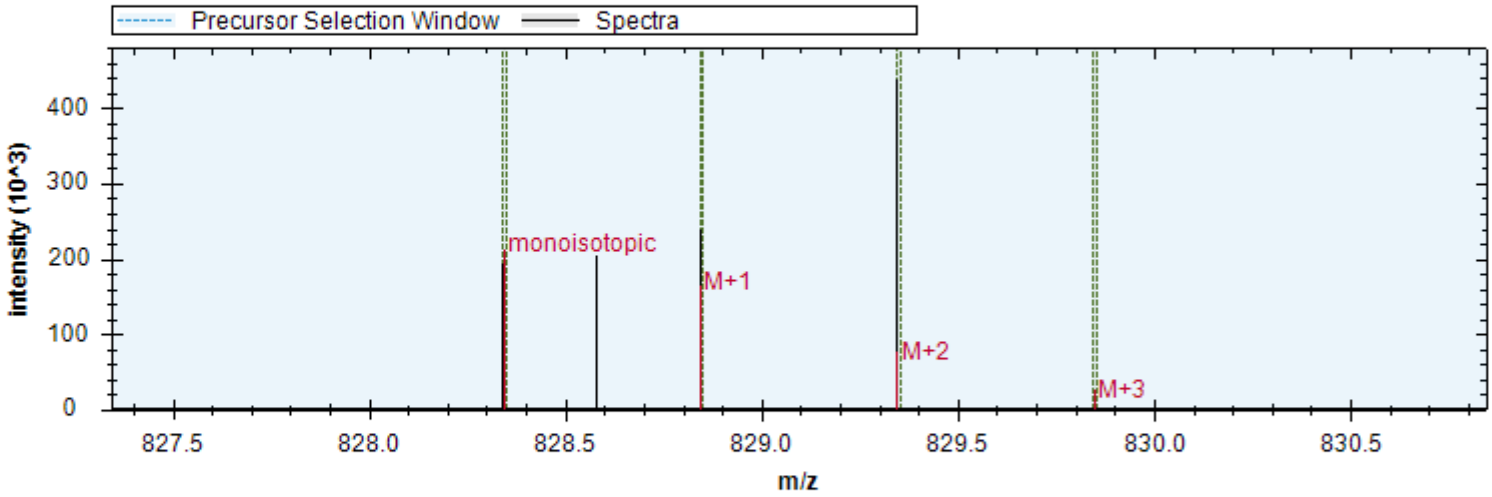

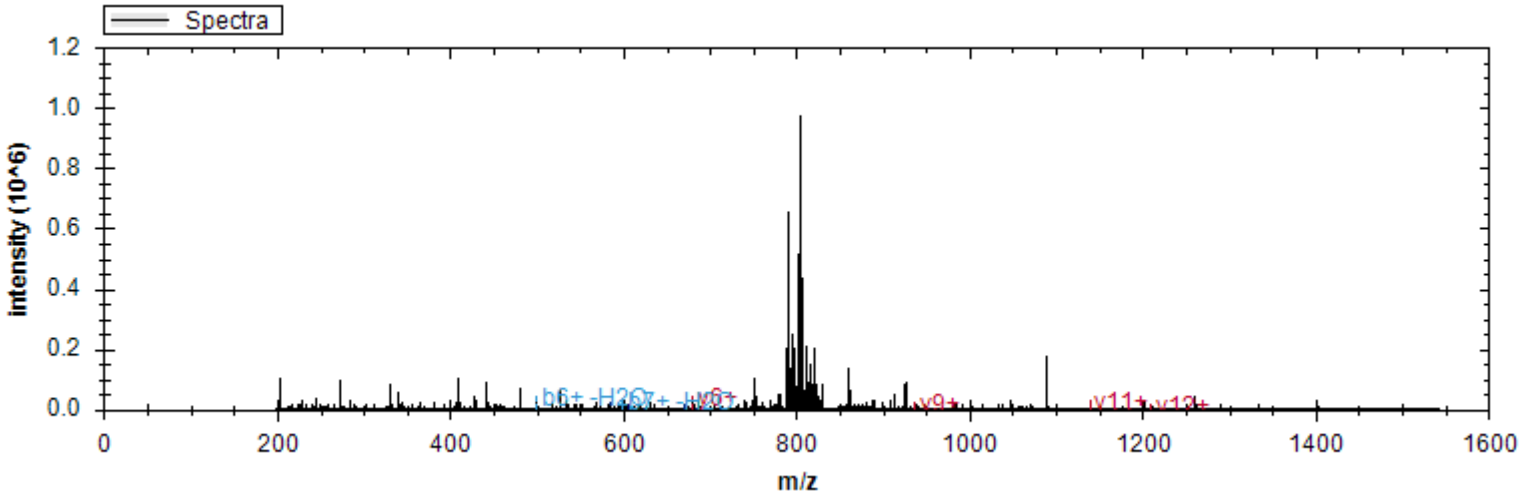

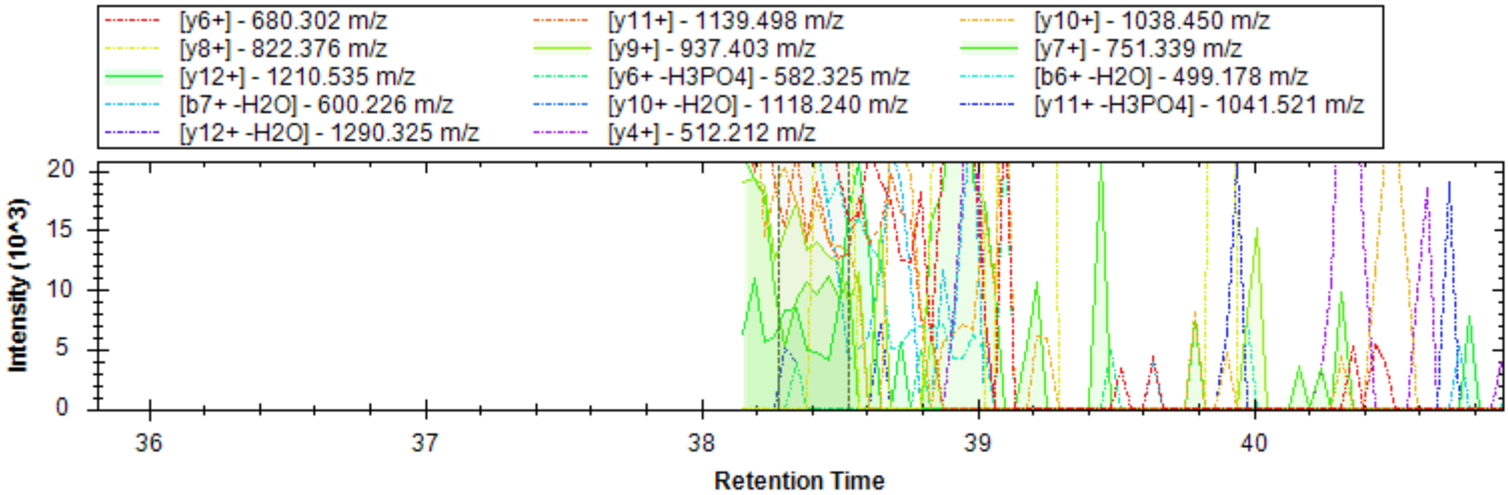

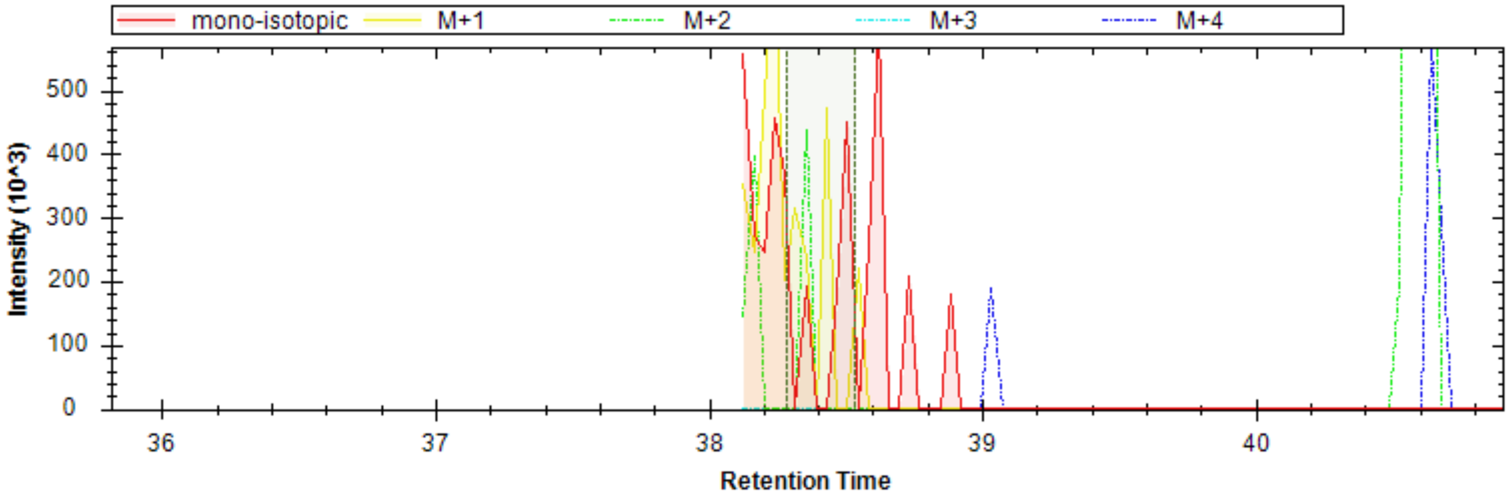

P10637 – _TTPSPKT[+H+O3+P]PPGSGEPPK_.2
Fig4_MP-DIA-barrel-cortex-p9-3_MHRM.htrms

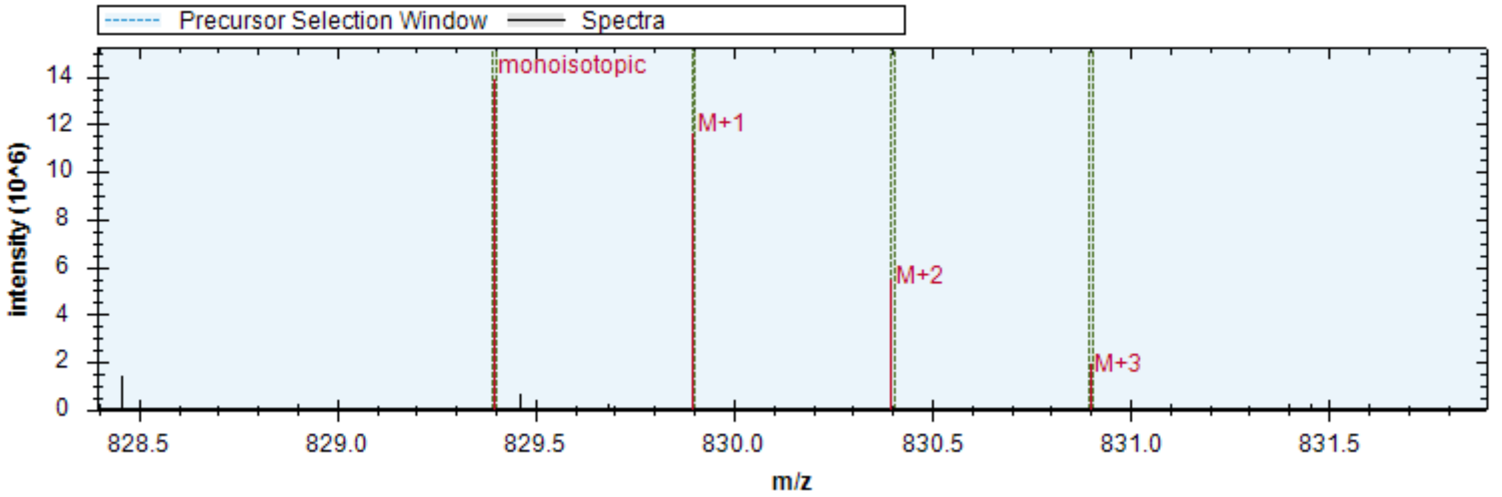

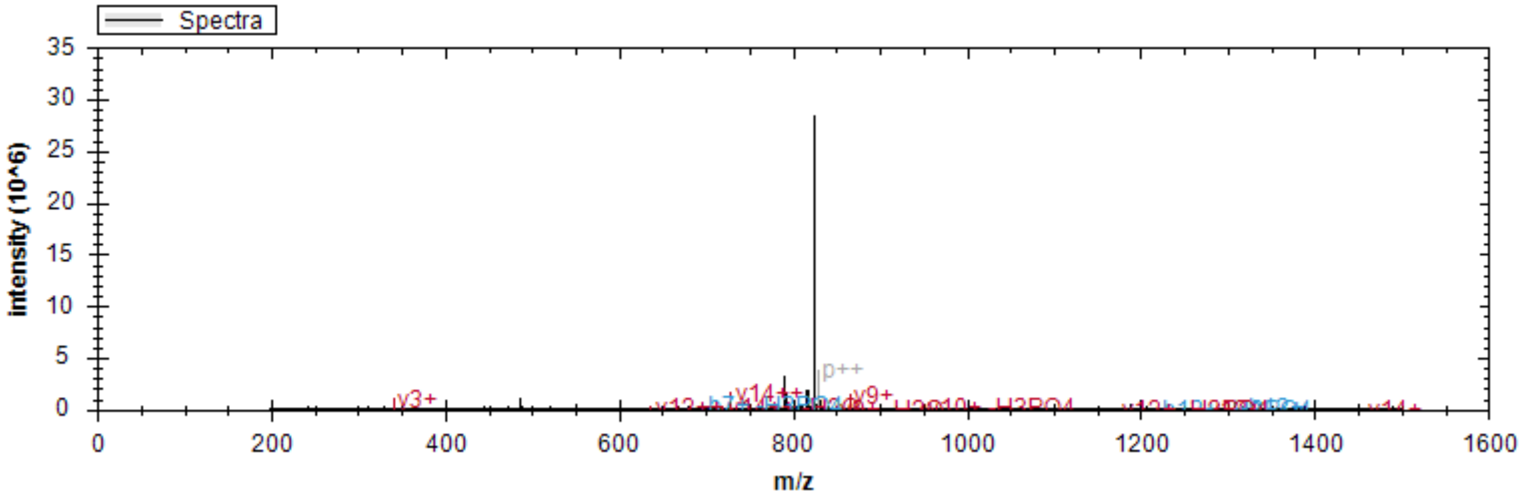

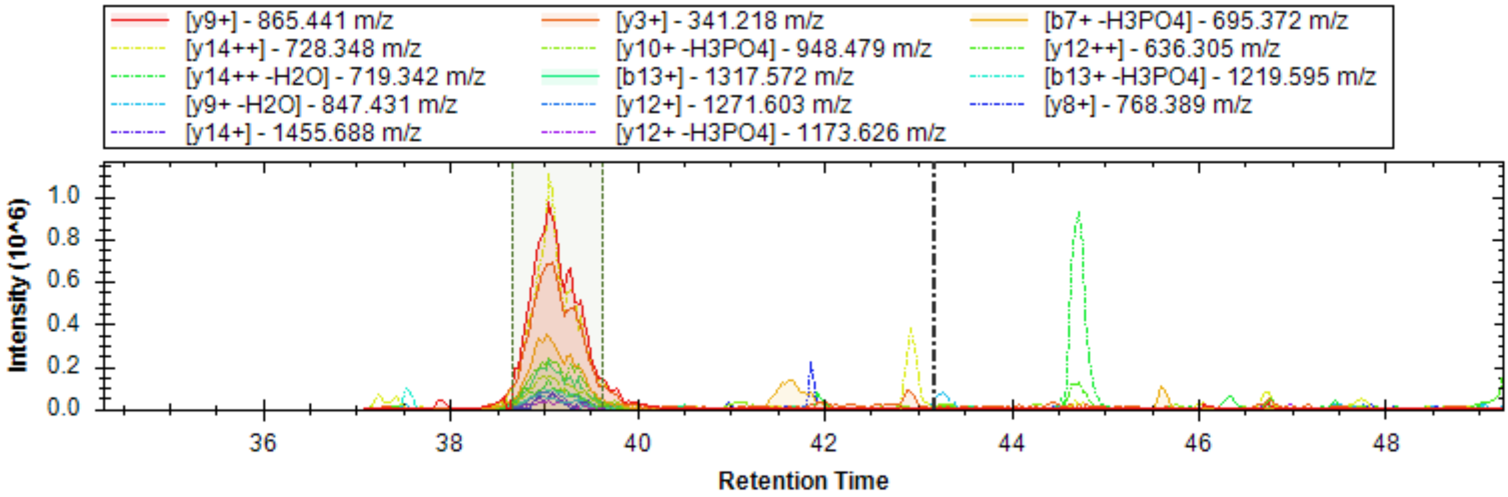

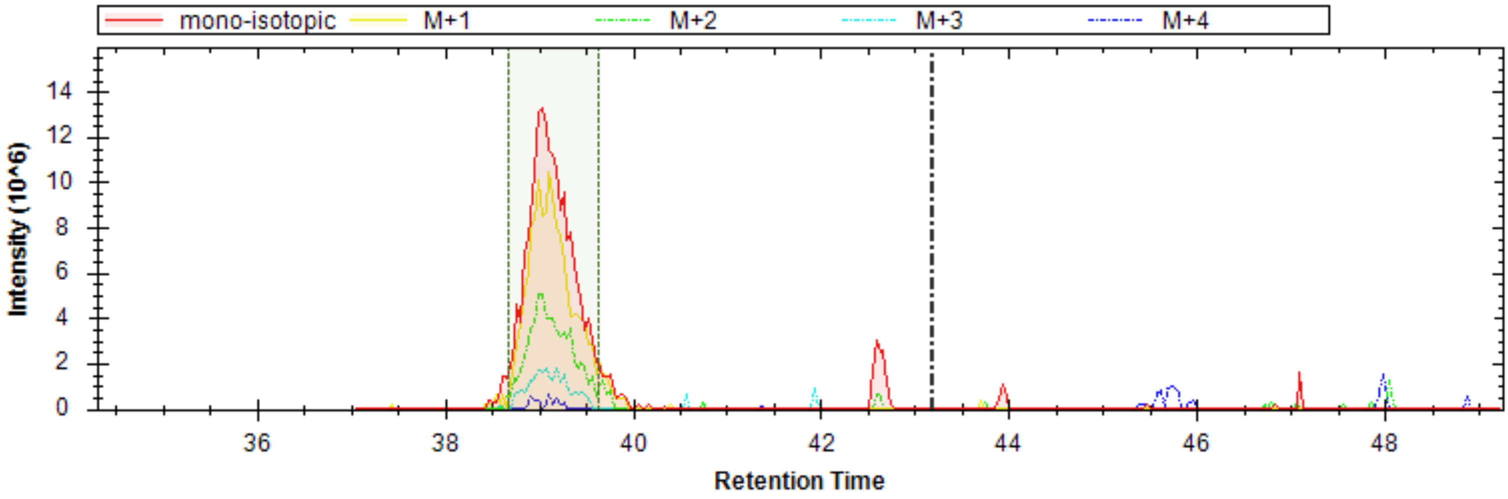

P20357 – _APHWTSASLTEAAAHPHS[+H+O3+P]PEMK_.3

Fig4_MP-DIA-barrel-cortex-p9-3_MHRM.htrms

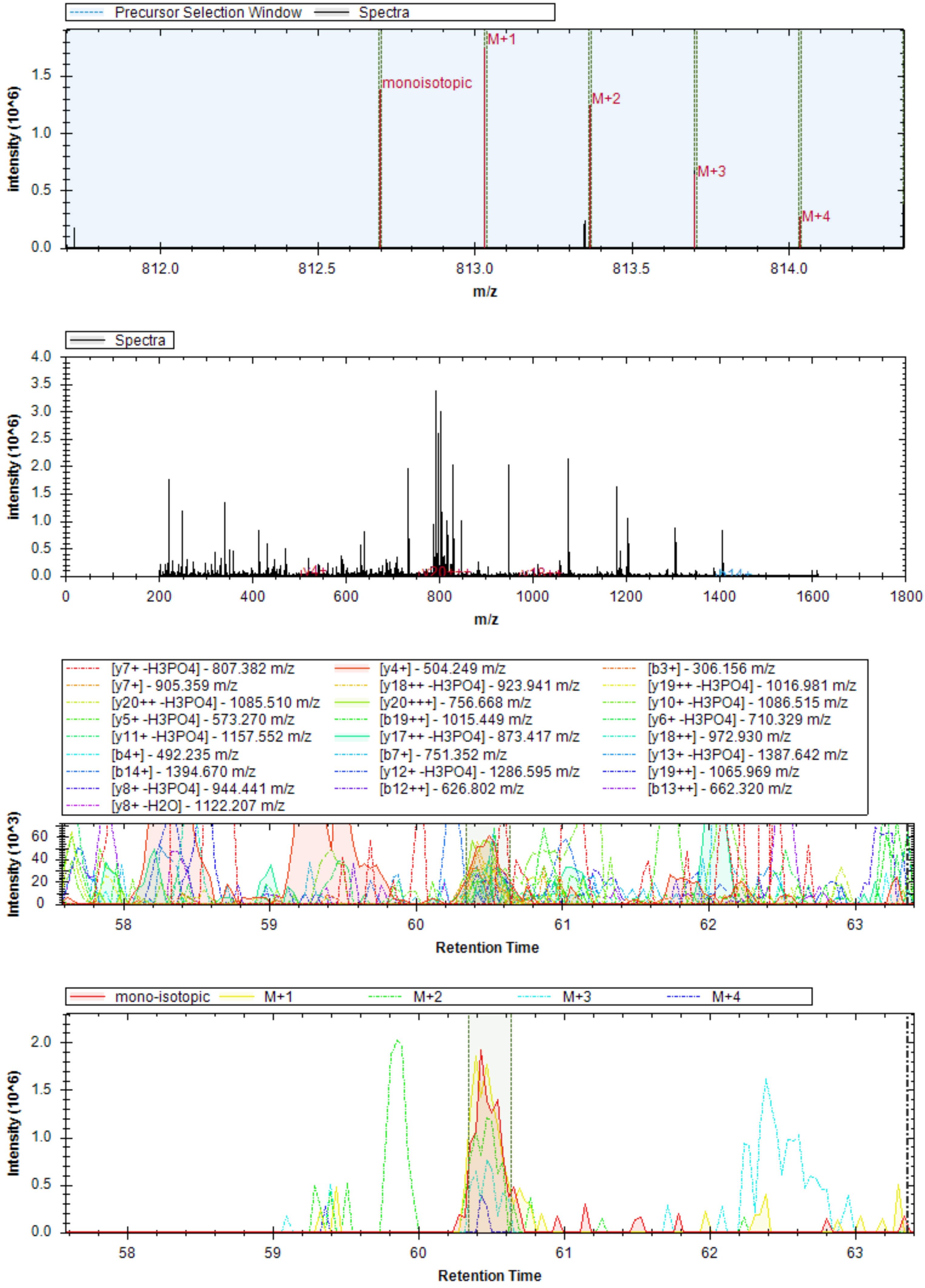

P20357 – _VAIIRT[+H+O3+P]PPKSPATPK_.2
Fig4_MP-DIA-barrel-cortex-p9-3_MHRM.htrms

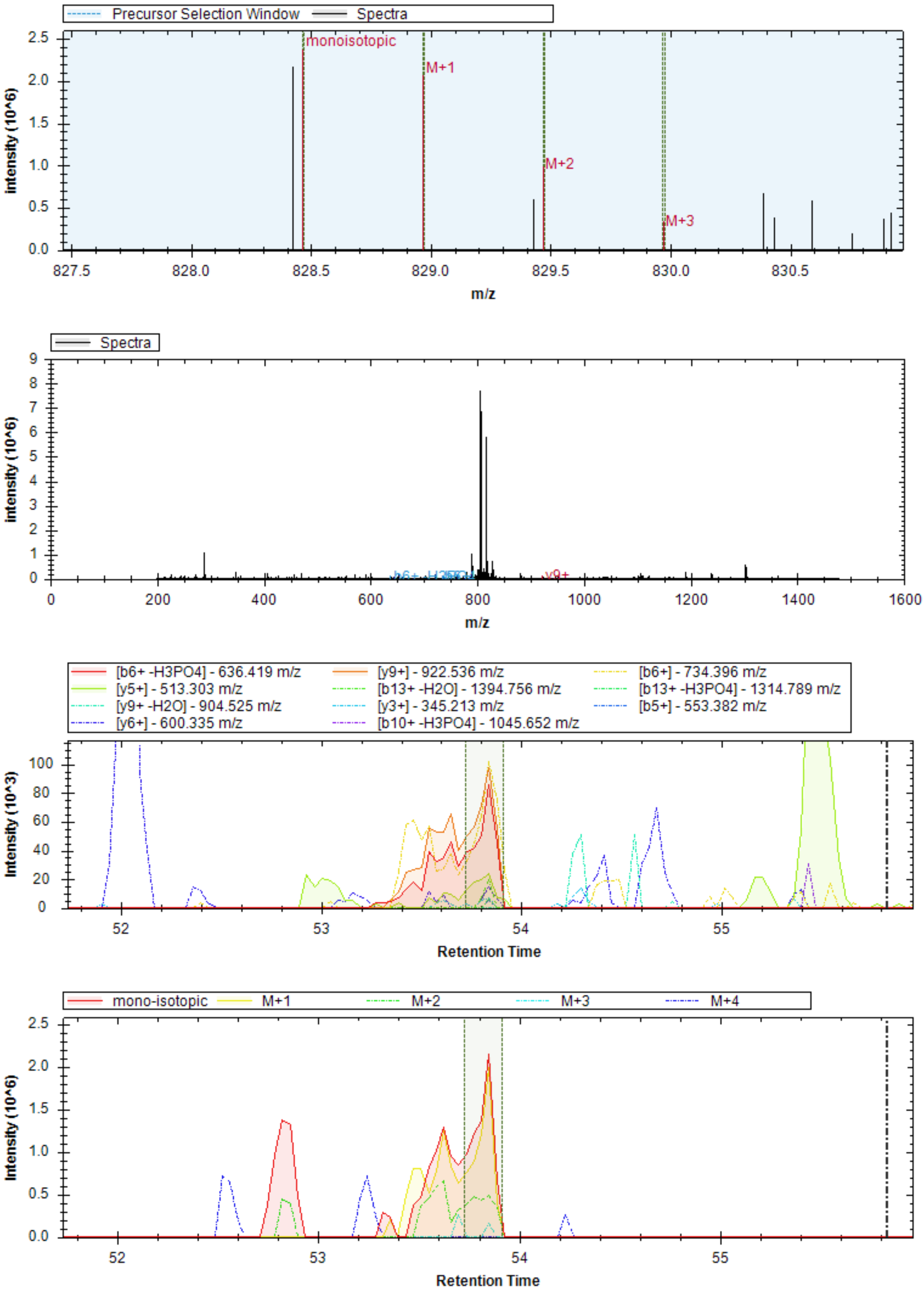

P20357 – _VDHGAEIIT[+H+O3+P]QSPSR_.2

Fig4_MP-DIA-barrel-cortex-p9-3_MHRM.htrms

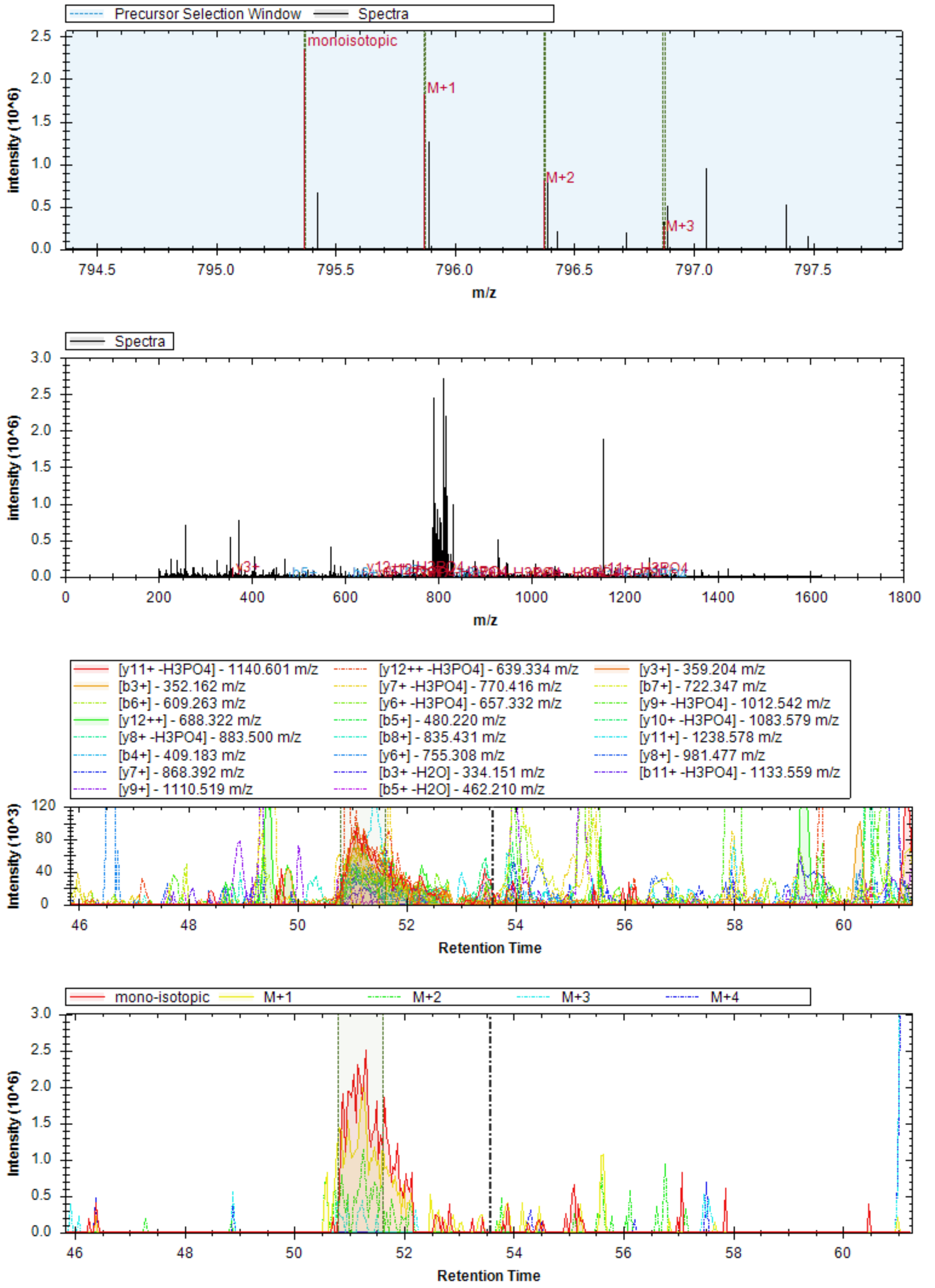

P20357 – _VDHGAEIITQS[+H+O3+P]PSR_.2
Fig4_MP-DIA-barrel-cortex-p9-3_MHRM.htrms

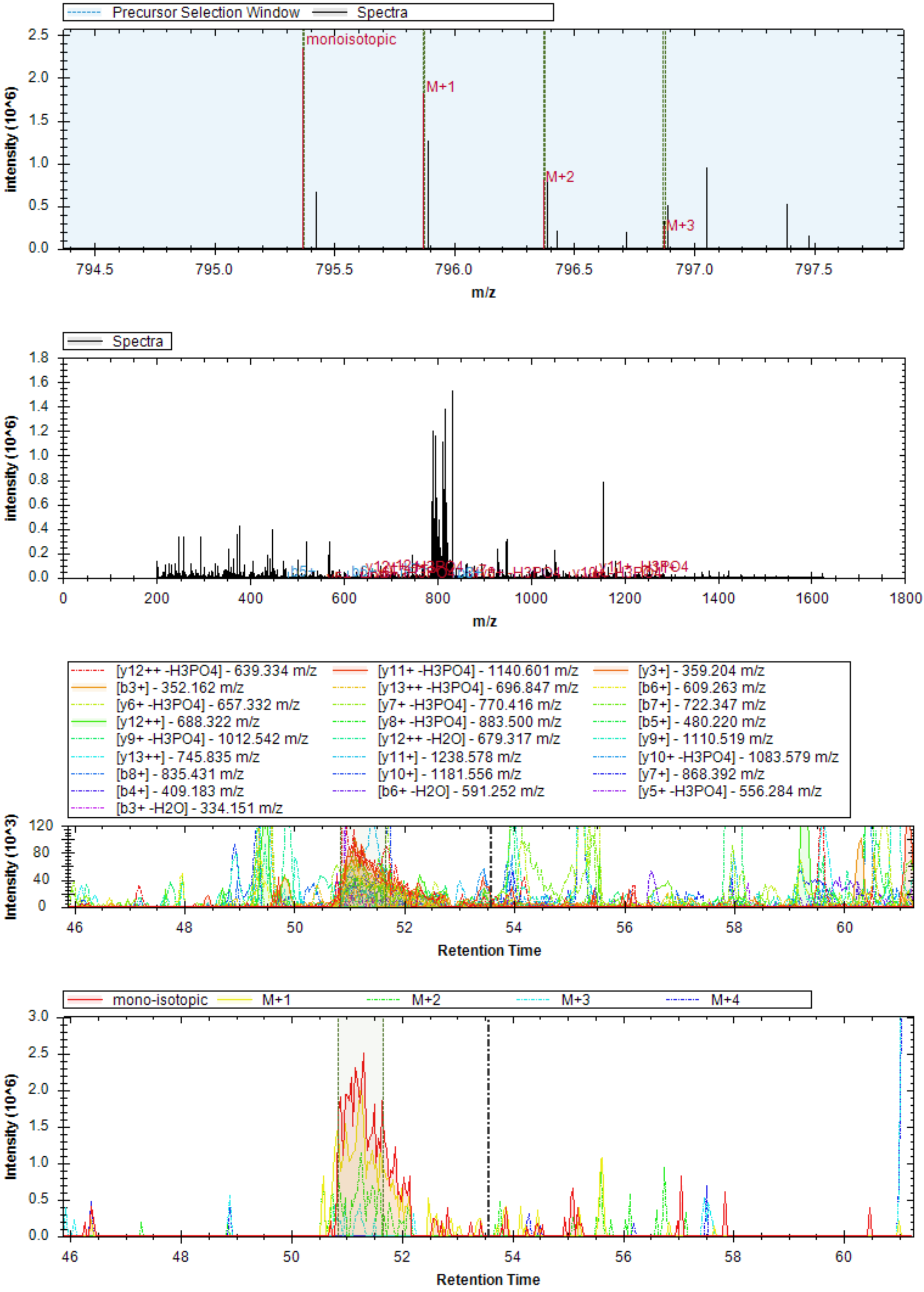

P20357 – _VDHGAEIITQSPS[+H+O3+P]R_.2
Fig4_MP-DIA-barrel-cortex-p9-3_MHRM.htrms

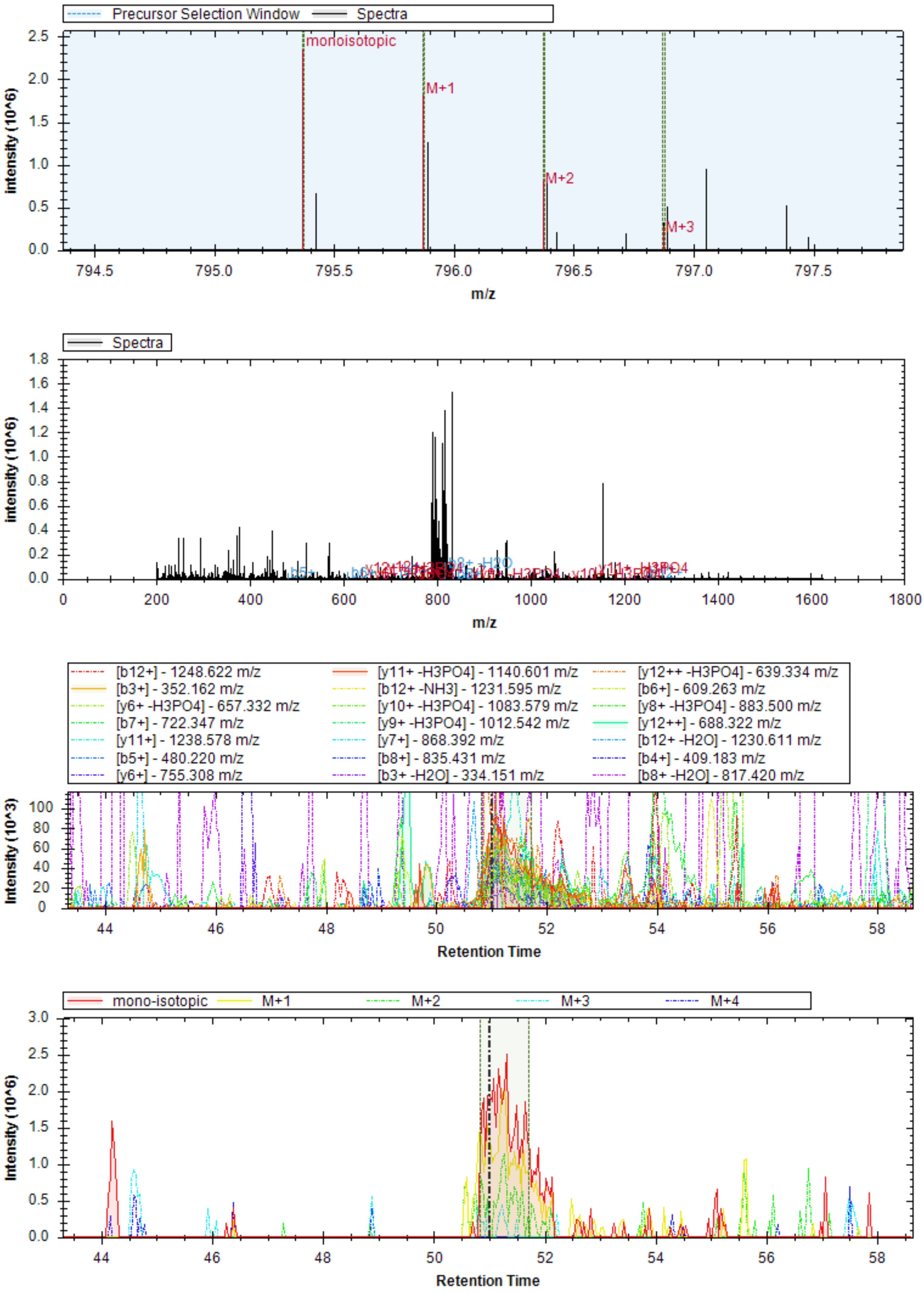

P54227 – _SKESVPDFPLS[+H+O3+P]PPK_.2
Fig4_MP-DIA-barrel-cortex-p9-3_MHRM.htrms

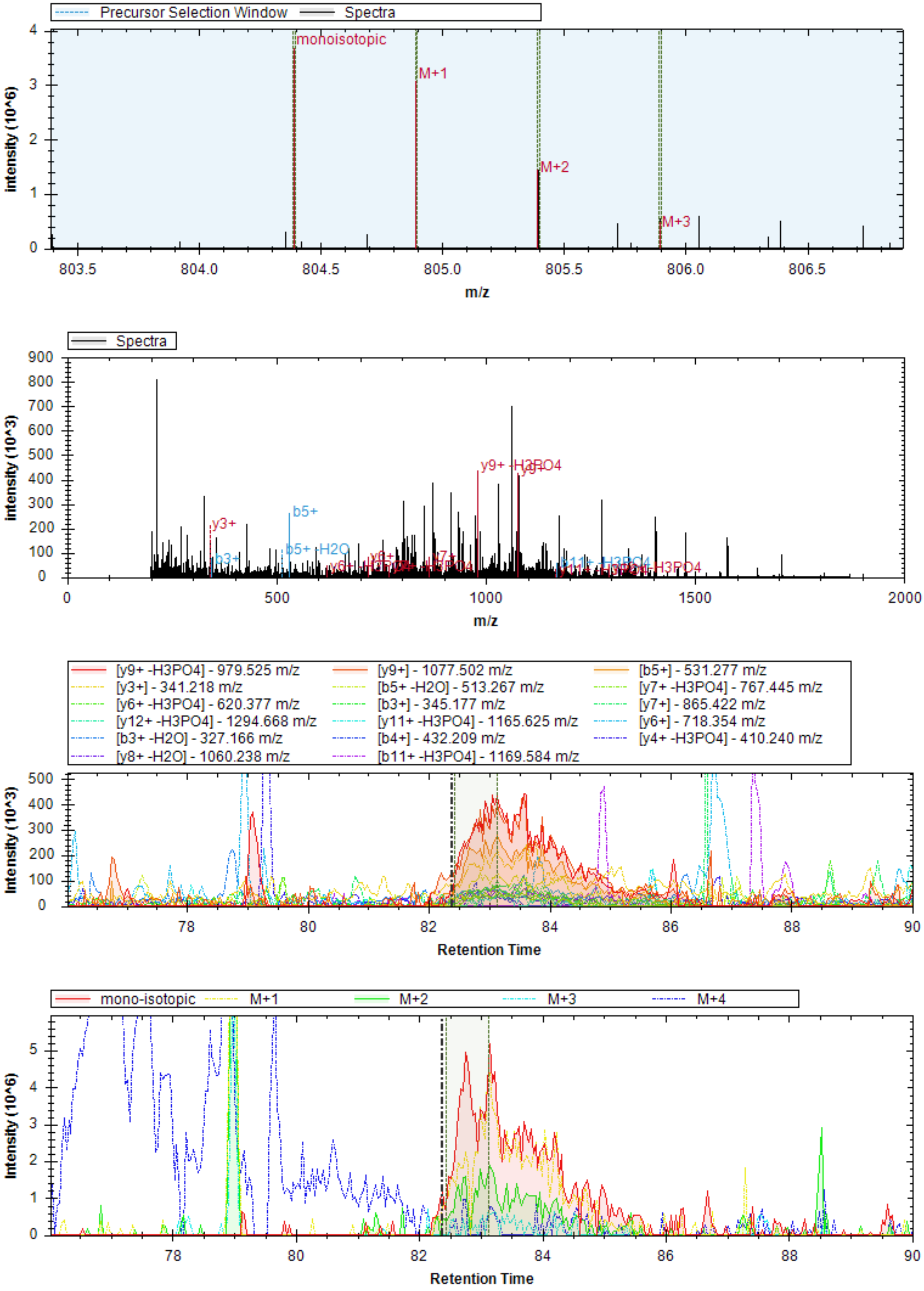

Q45VK7;Q45VK7-2 – _LEEVLAIRT[+H+O3+P]IHEK_.2
Fig4_MP-DIA-barrel-cortex-p9-3_MHRM.htrms

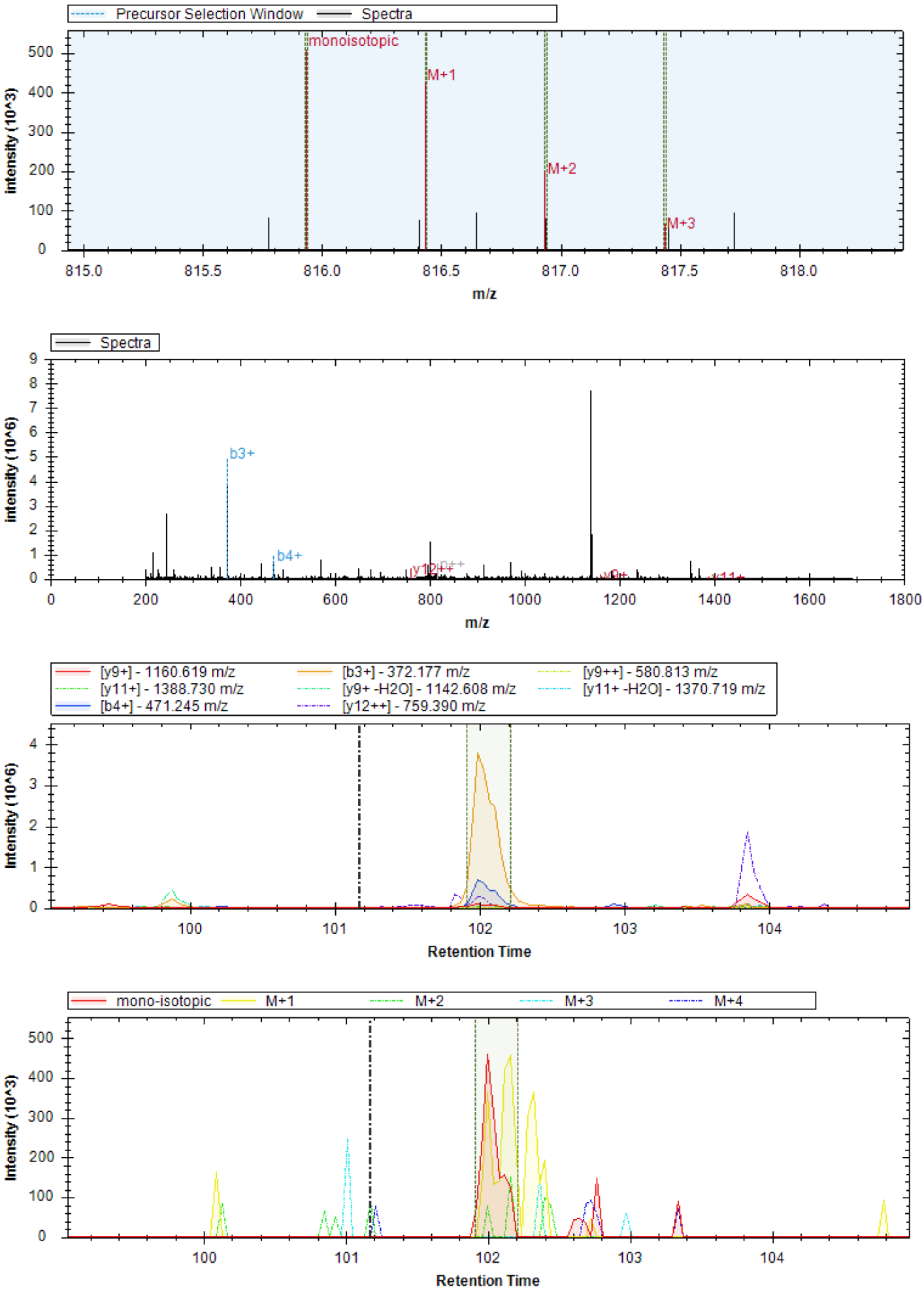

Q61548;Q61548-2;Q61548-3 – _SSPATTVTS[+H+O3+P]PNSTPAK_.2

Fig4_MP-DIA-barrel-cortex-p9-3_MHRM.htrms

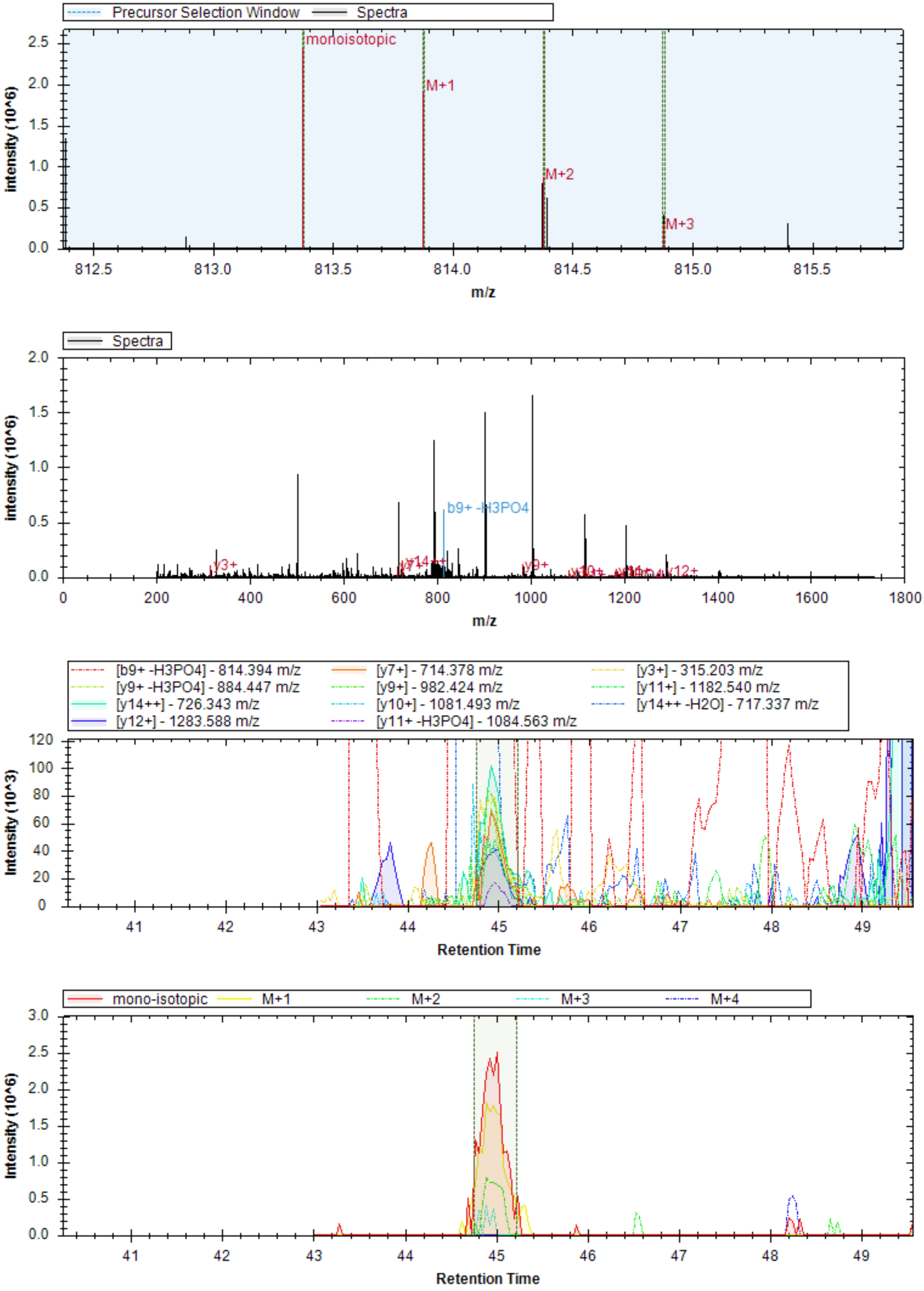

Q64701;Q64701-2 – _LAEILYYKILET[+H+O3+P]IMVQET[+H+O3+P]R_.3
Fig4_MP-DIA-barrel-cortex-p9-3_MHRM.htrms

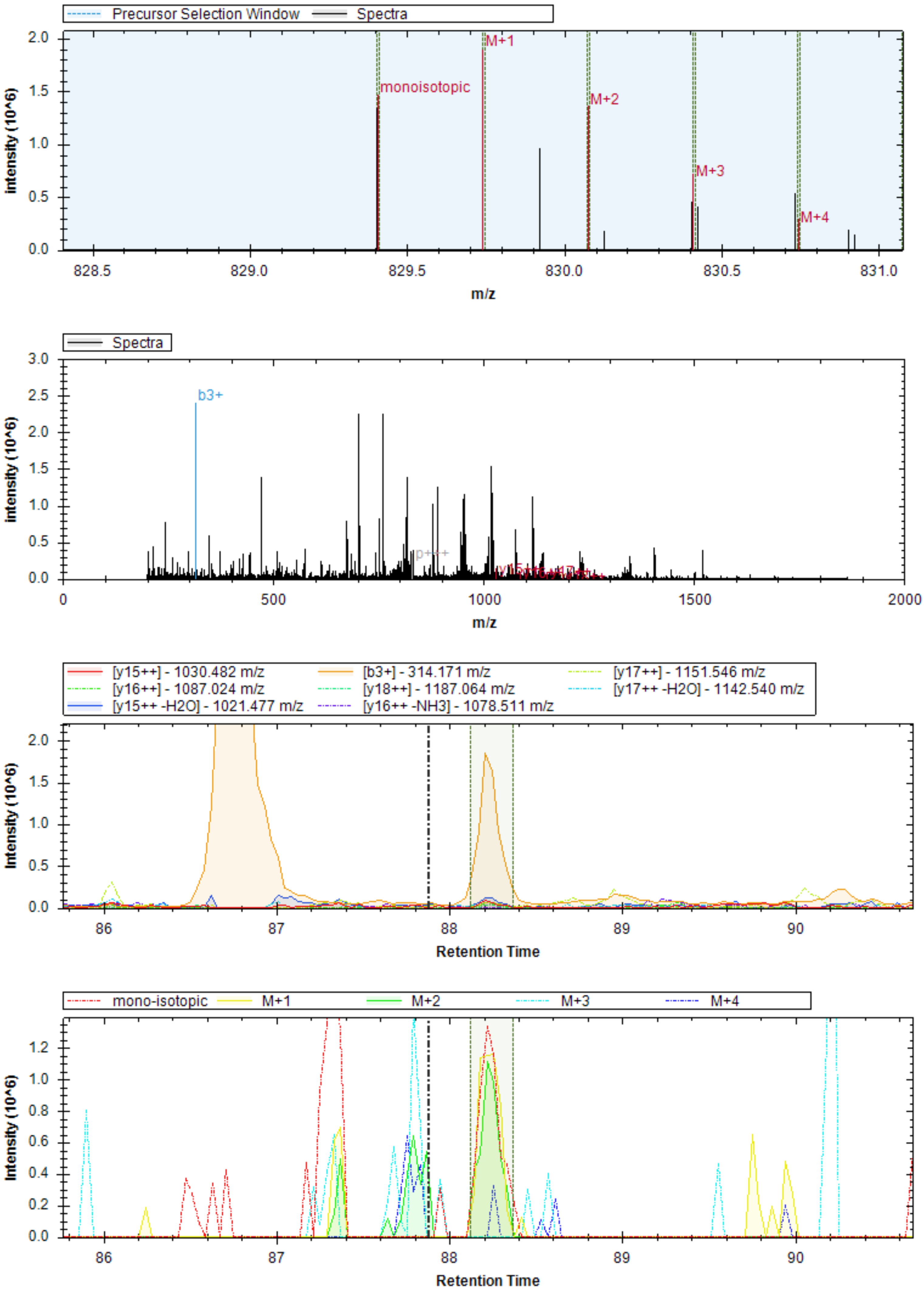

Q91XM9;Q91XM9-2;Q91XM9-7 – _NLSQIENVHGYVLQS[+H+O3+P]HISPLK_.3
Fig4_MP-DIA-barrel-cortex-p9-3_MHRM.htrms

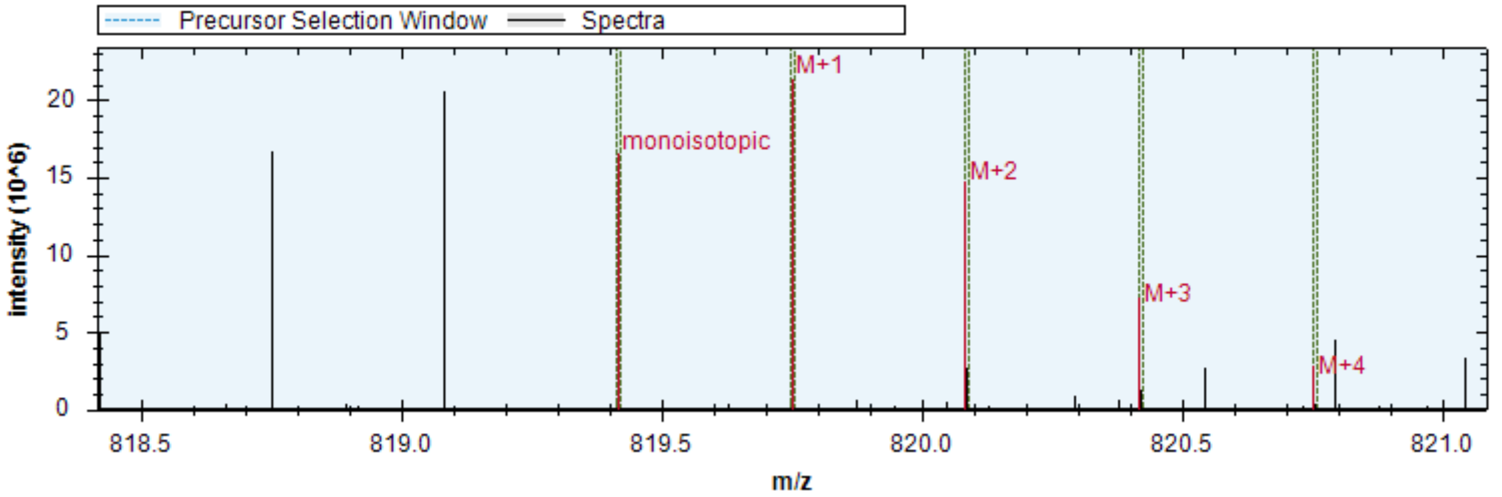

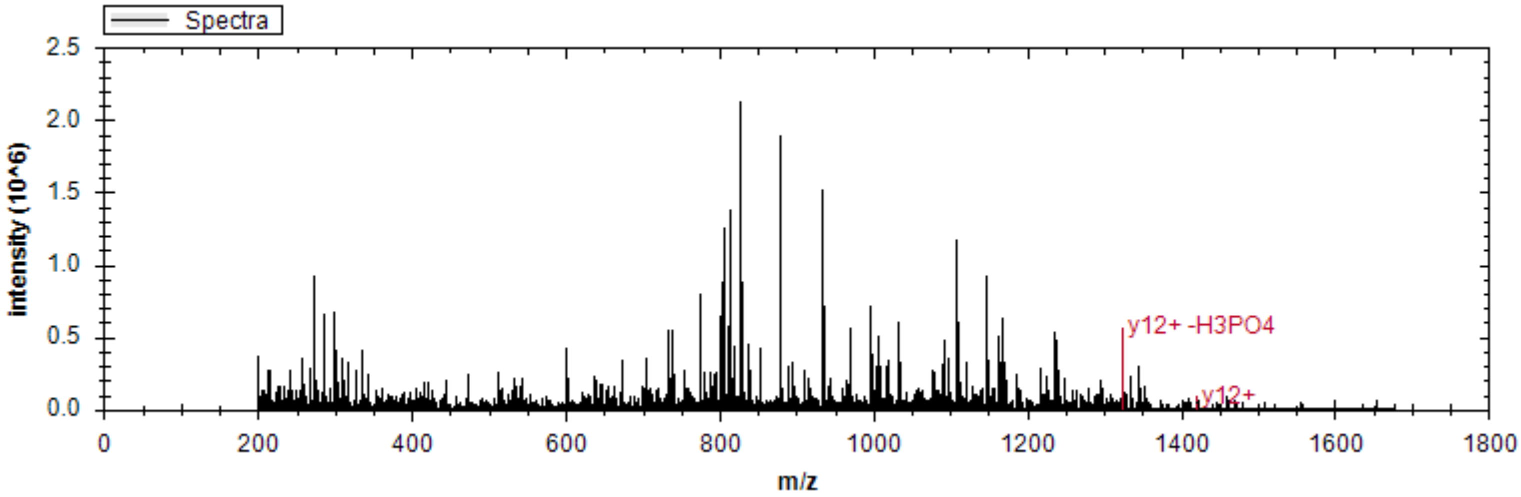

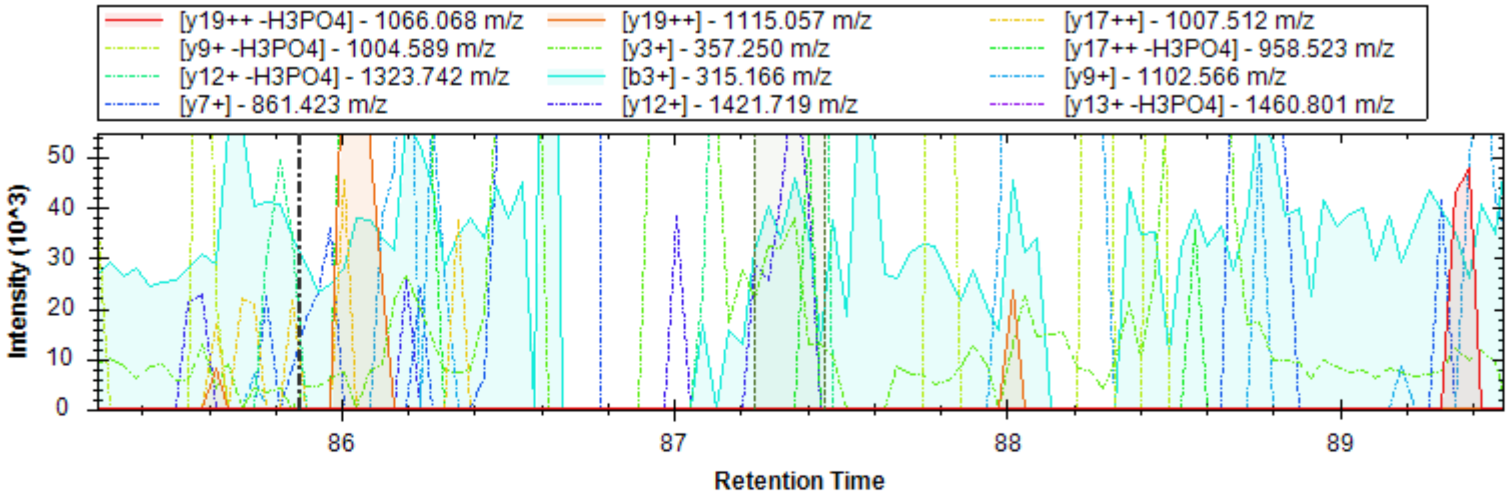

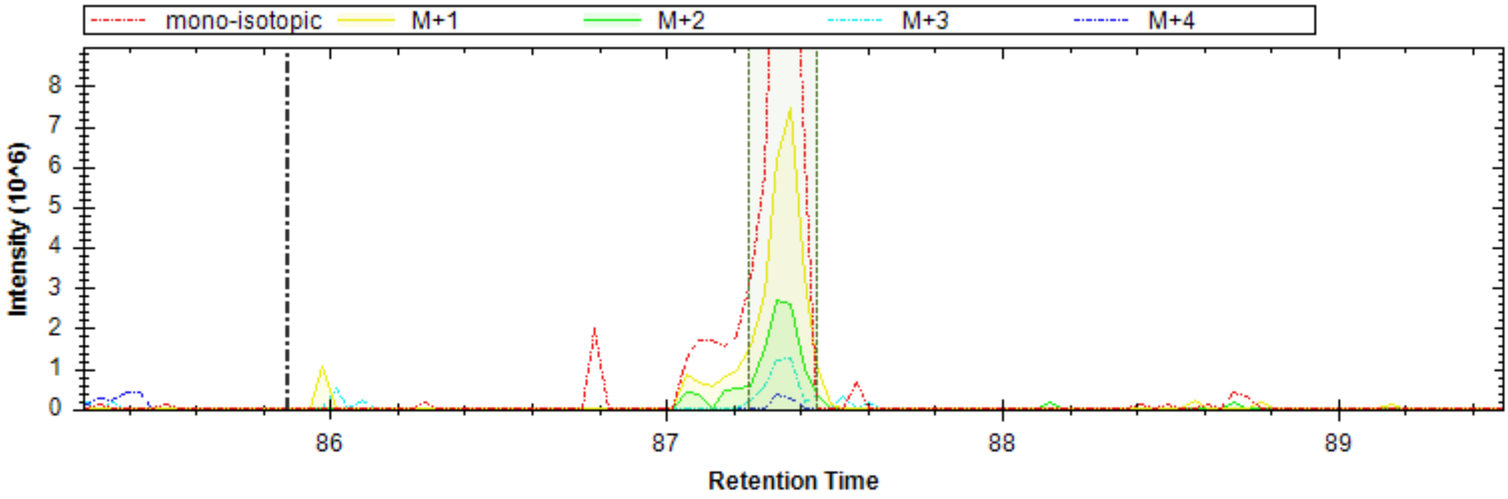

D3YXK2 – _AET[+H+O3+P]LSGLGDASAAGAAAVSSAASETGTR_.3

Fig4_MP-DIA-barrel-cortex-p9-3_MHRM.htrms

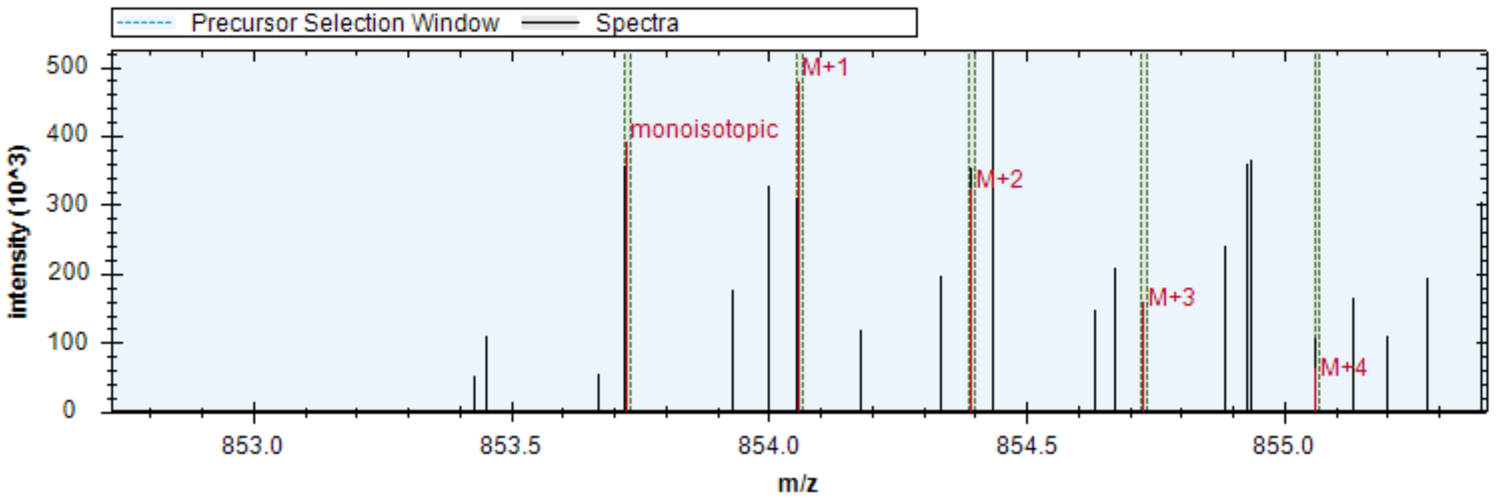

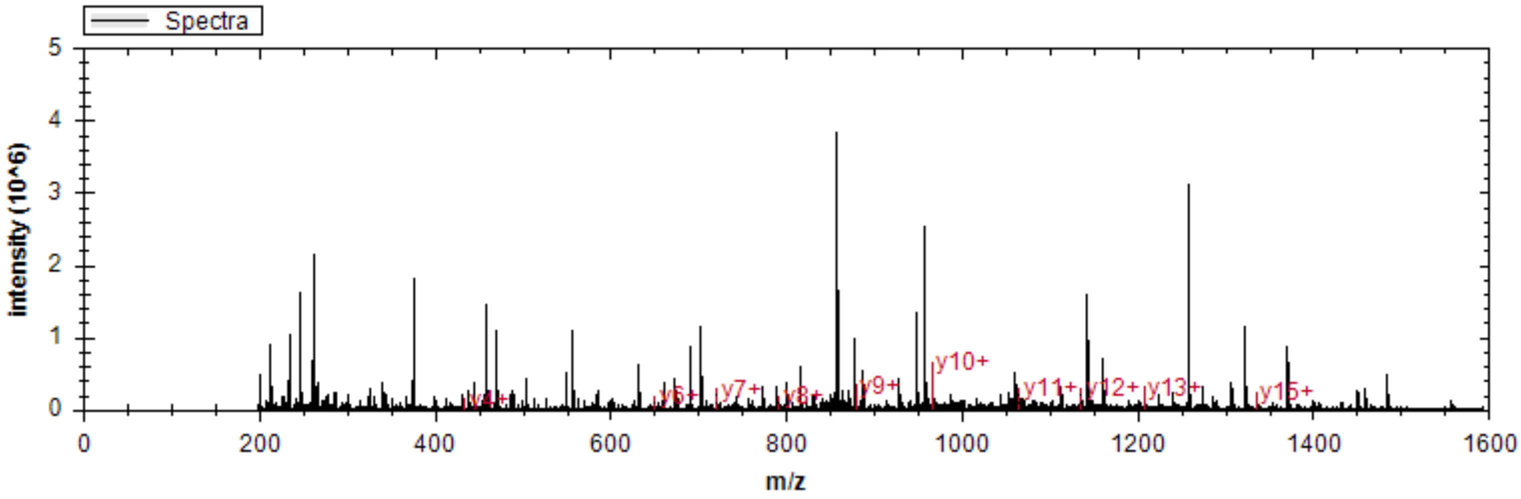

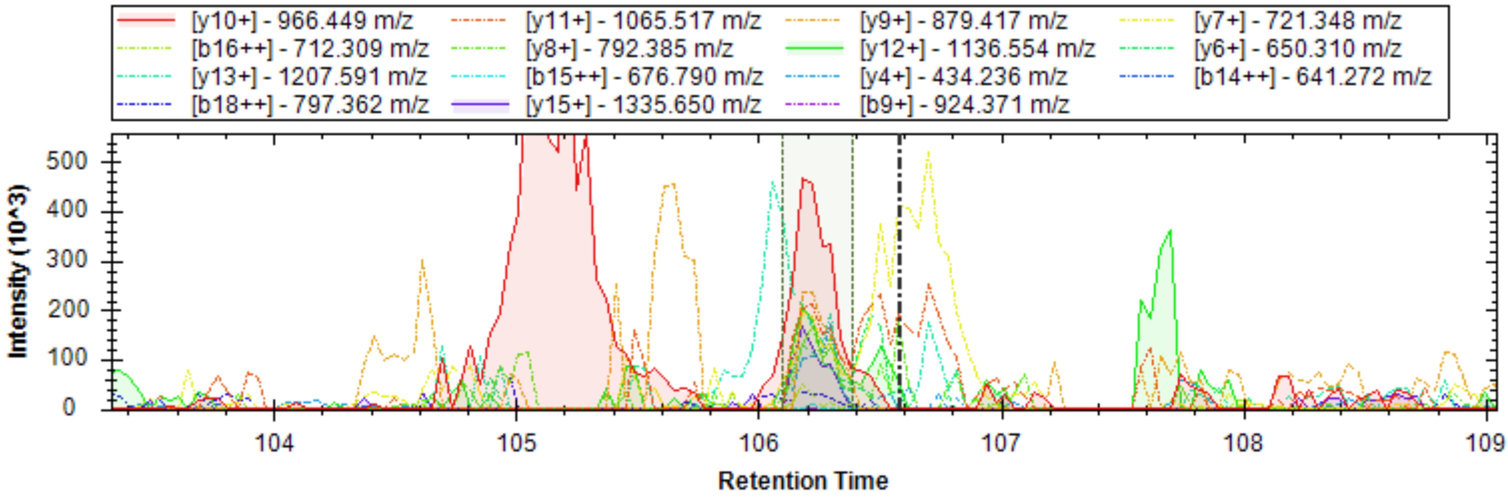

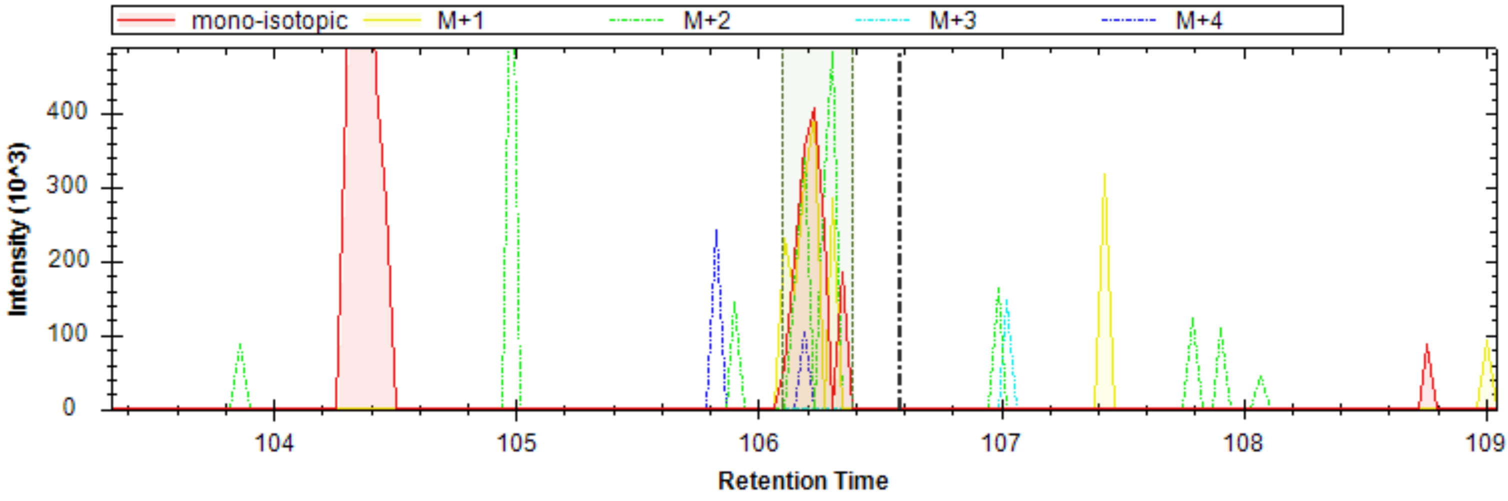

O08784 – _ASAVSPEKAPM[+O]T[+H+O3+P]SKAK_.2
Fig4_MP-DIA-barrel-cortex-p9-3_MHRM.htrms

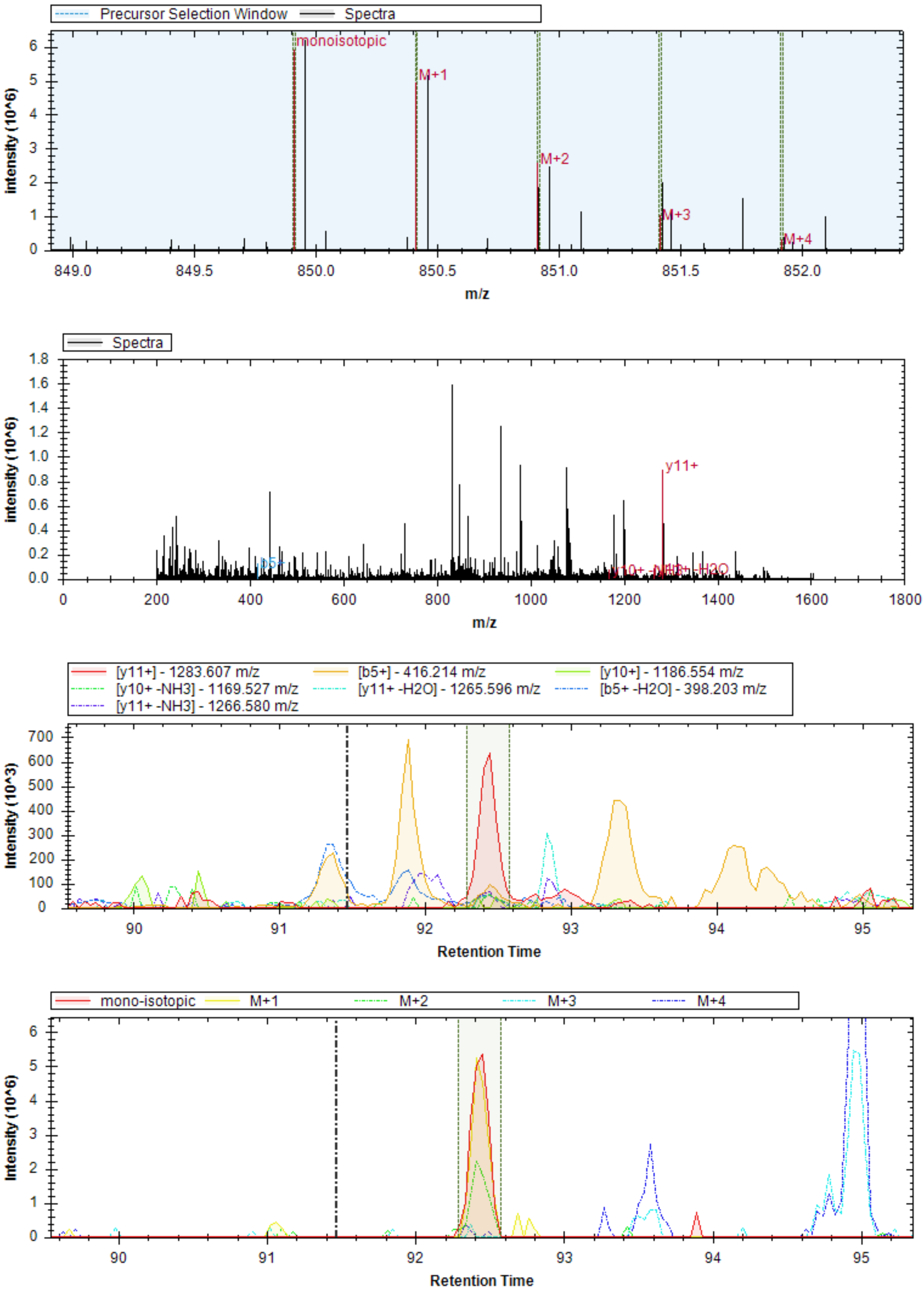

P27546-2 – _AAVGVTGNDITTPPNKEPPPS[+H+O3+P]PEKK_.3

Fig4_MP-DIA-barrel-cortex-p9-3_MHRM.htrms

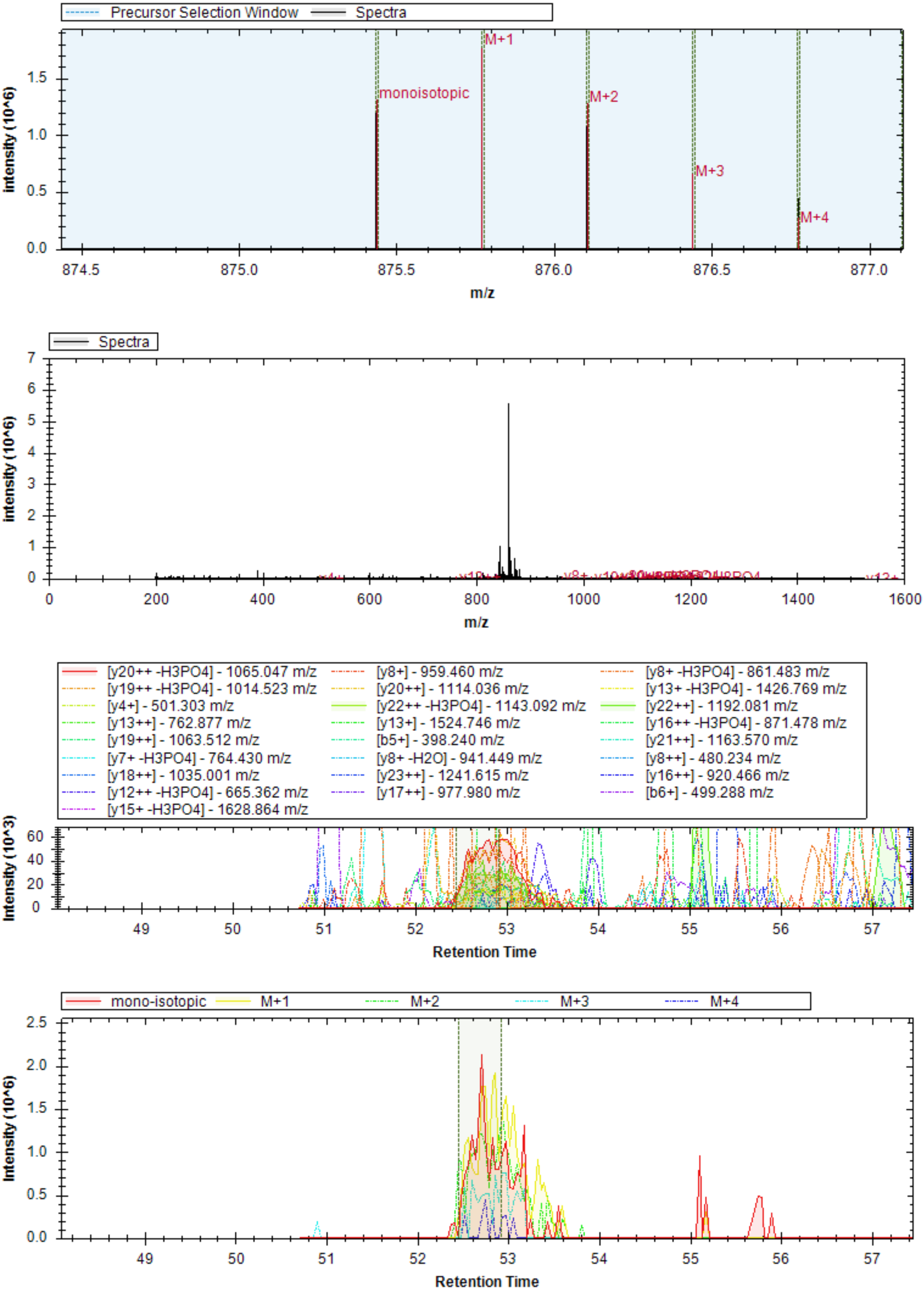

P27546-2 – _NTT[+H+O3+P]PTGAAPPAGMTSTR_.2

Fig4_MP-DIA-barrel-cortex-p9-3_MHRM.htrms

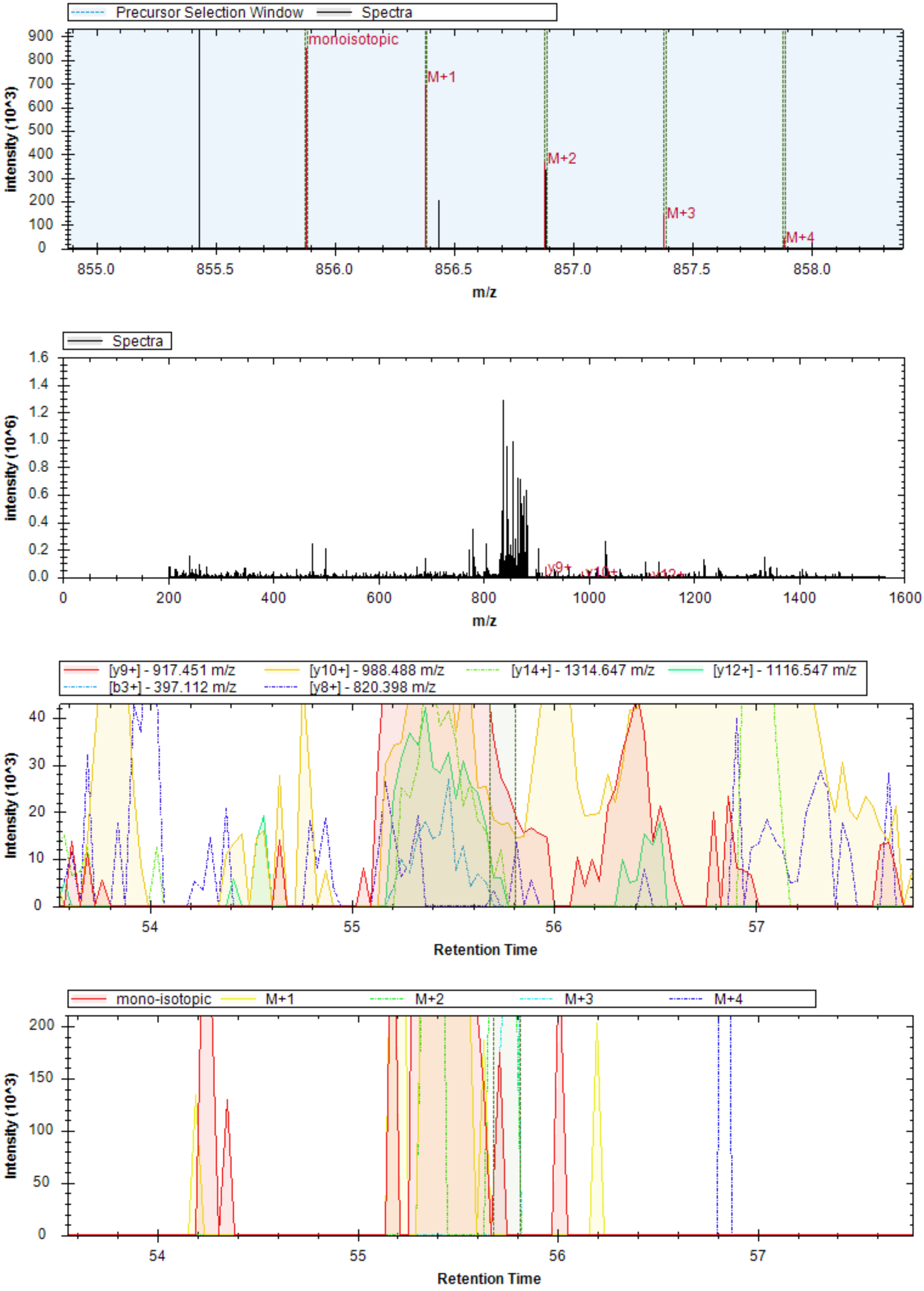

P50538 – _EAEHGYAS[+H+O3+P]M[+O]LPYSK_.2
Fig4_MP-DIA-barrel-cortex-p9-3_MHRM.htrms

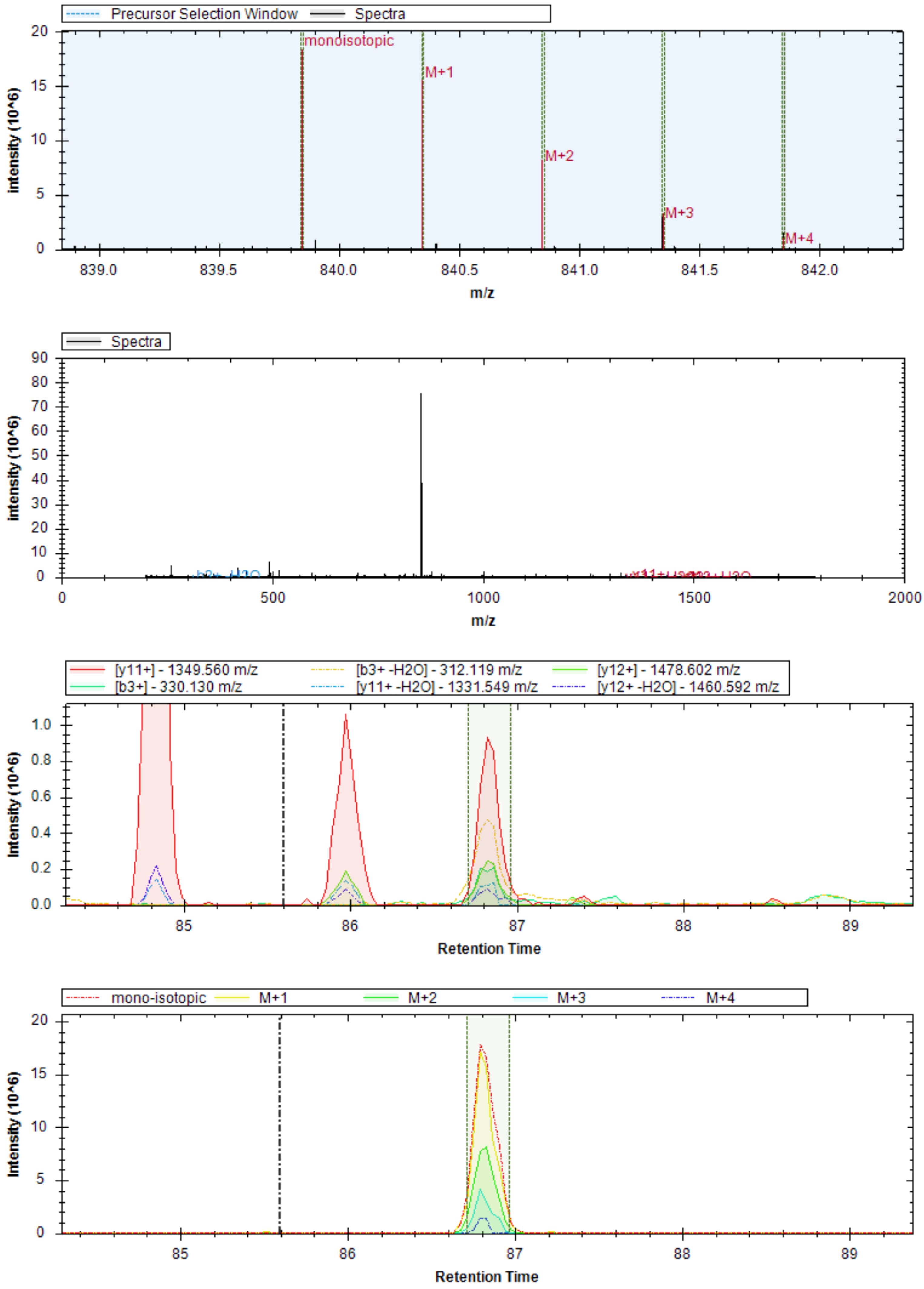

Q61290 – _M[+O]EPS[+H+O3+P]SLPQEIIANAK_.2
Fig4_MP-DIA-barrel-cortex-p9-3_MHRM.htrms

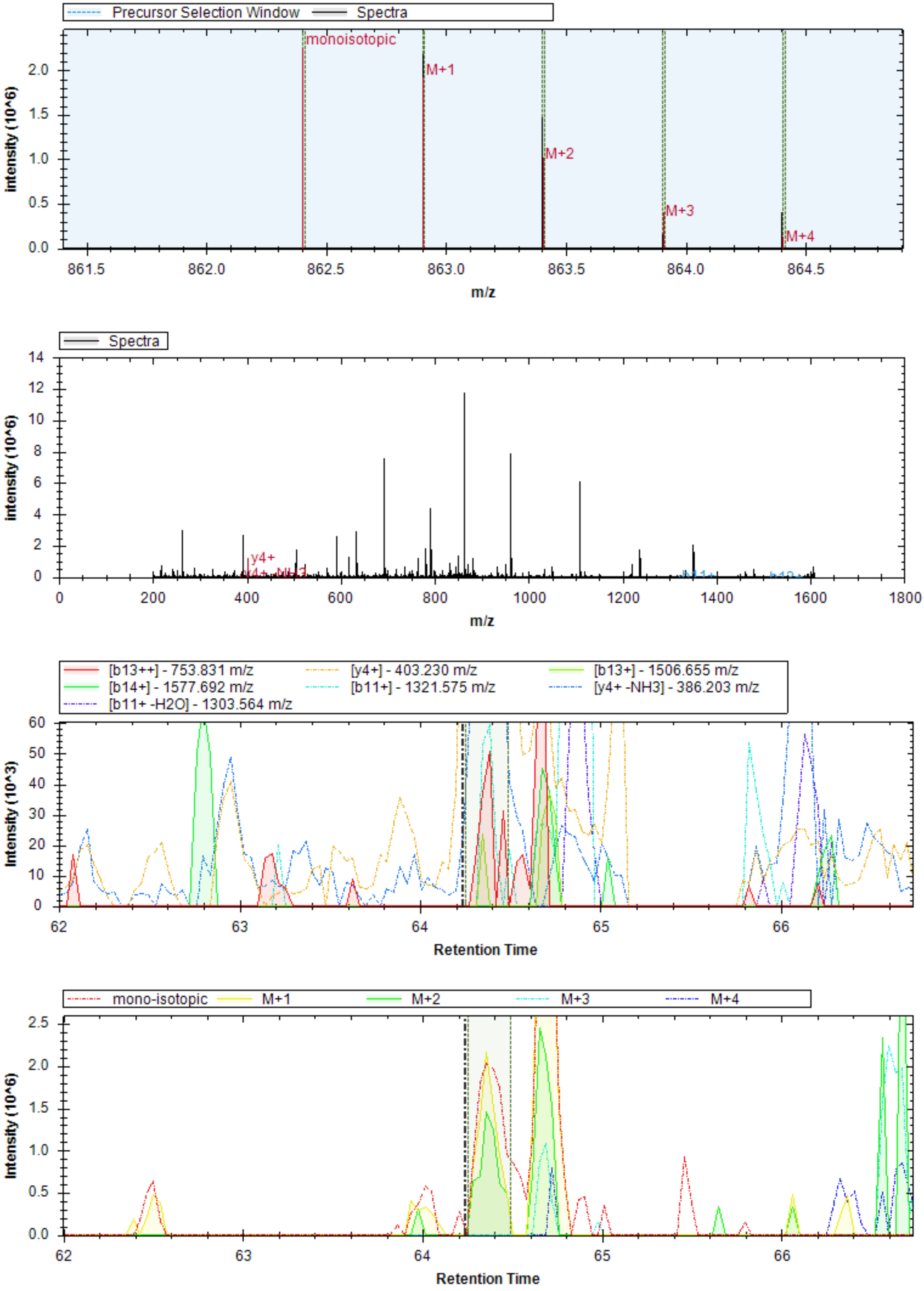

Q62188 – _GM[+O]YDGPVFDLT[+H+O3+P]TTPK_.2

Fig4_MP-DIA-barrel-cortex-p9-3_MHRM.htrms

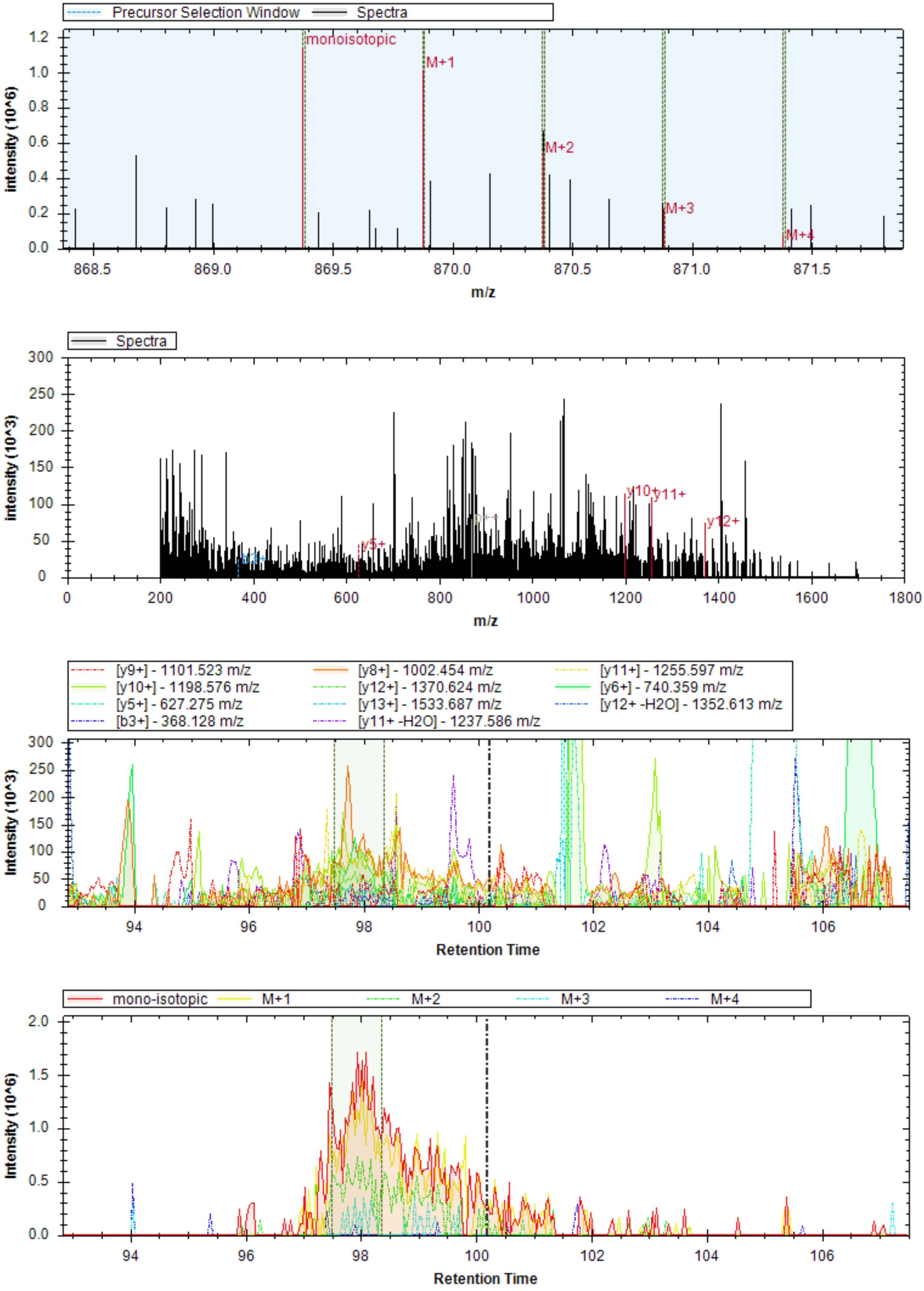

Q62188 – _GM[+O]YDGPVFDLTT[+H+O3+P]TPK_.2

Fig4_MP-DIA-barrel-cortex-p9-3_MHRM.htrms

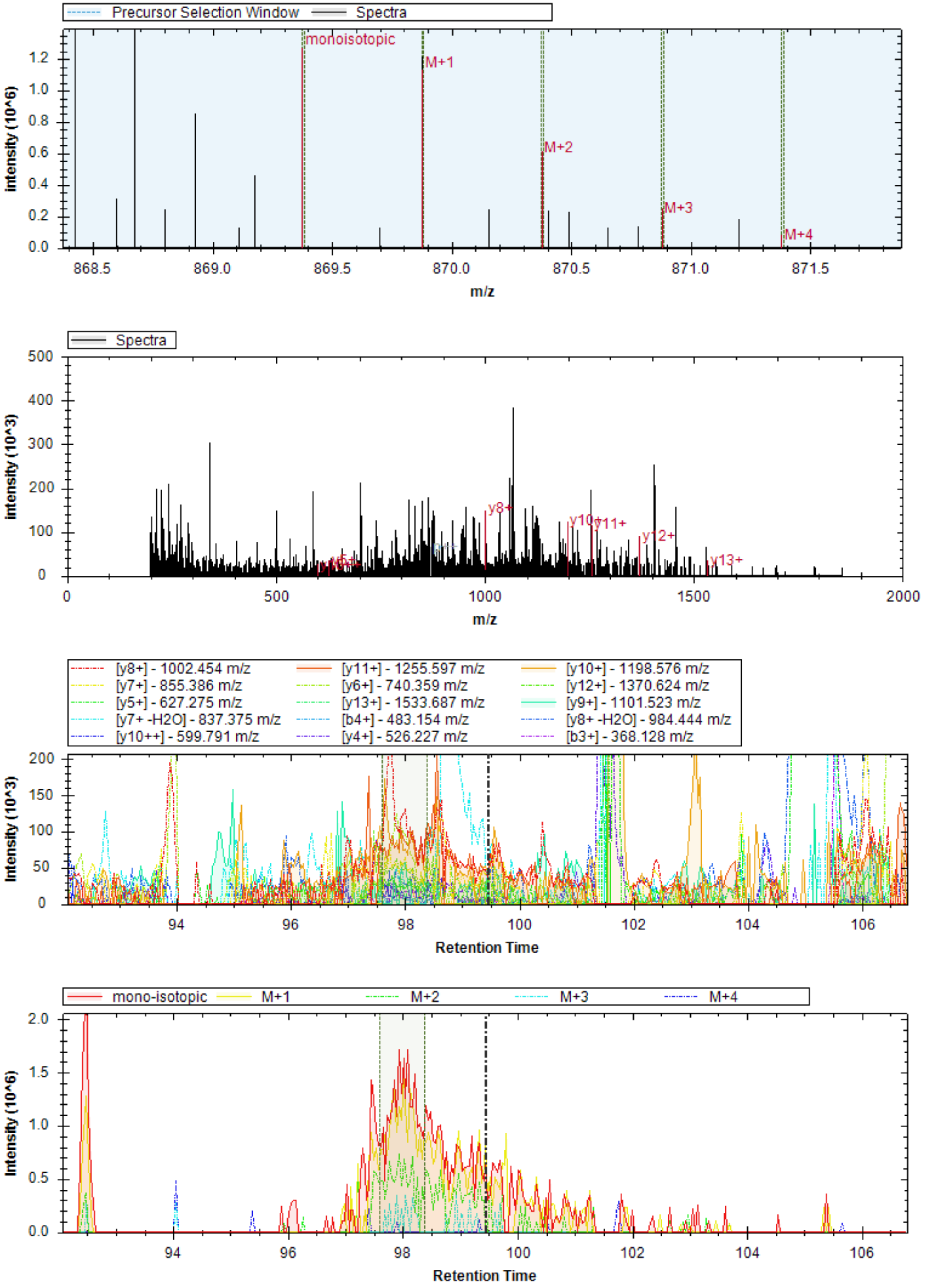

Q62188 – _GM[+O]YDGPVFDLTTT[+H+O3+P]PK_.2
Fig4_MP-DIA-barrel-cortex-p9-3_MHRM.htrms

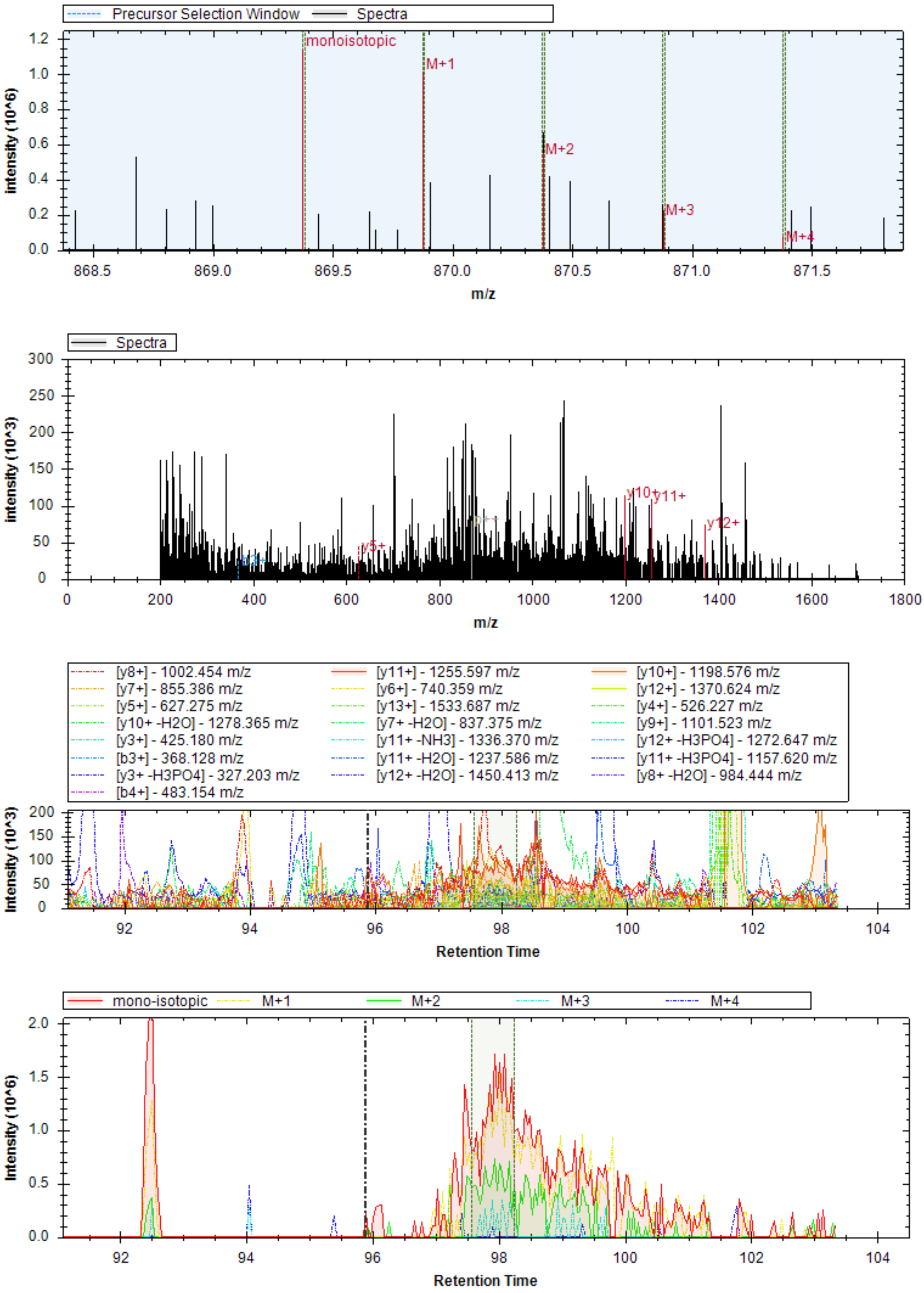

Q9JM96 – _DSSKLPKS[+H+O3+P]LS[+H+O3+P]SSPVK_.2
Fig4_MP-DIA-barrel-cortex-p9-3_MHRM.htrms

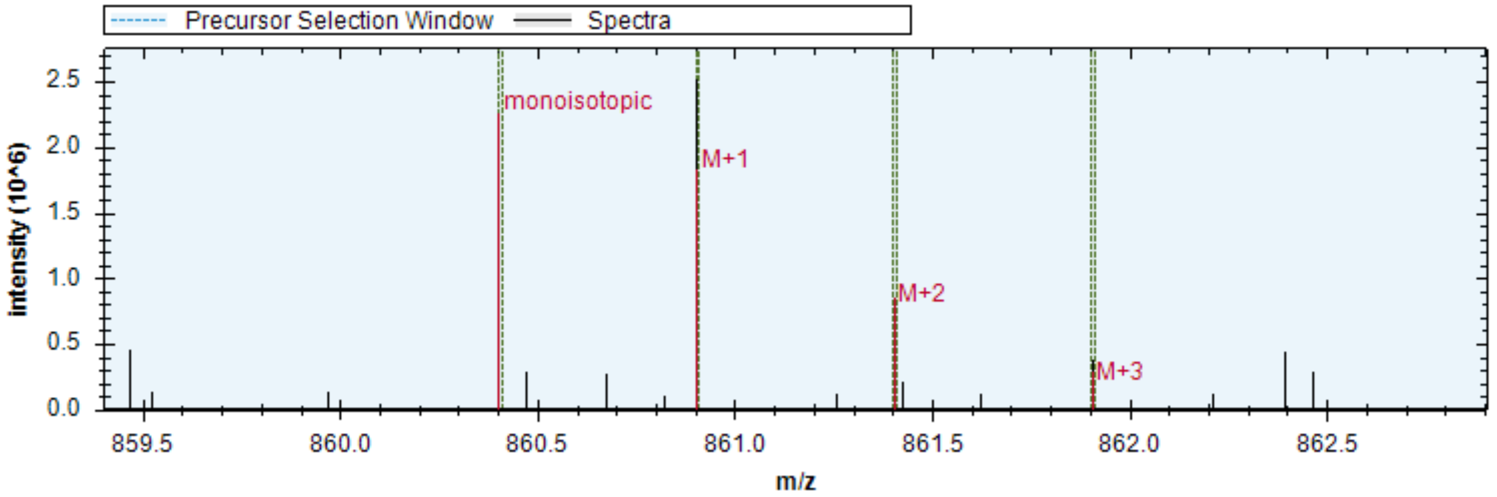

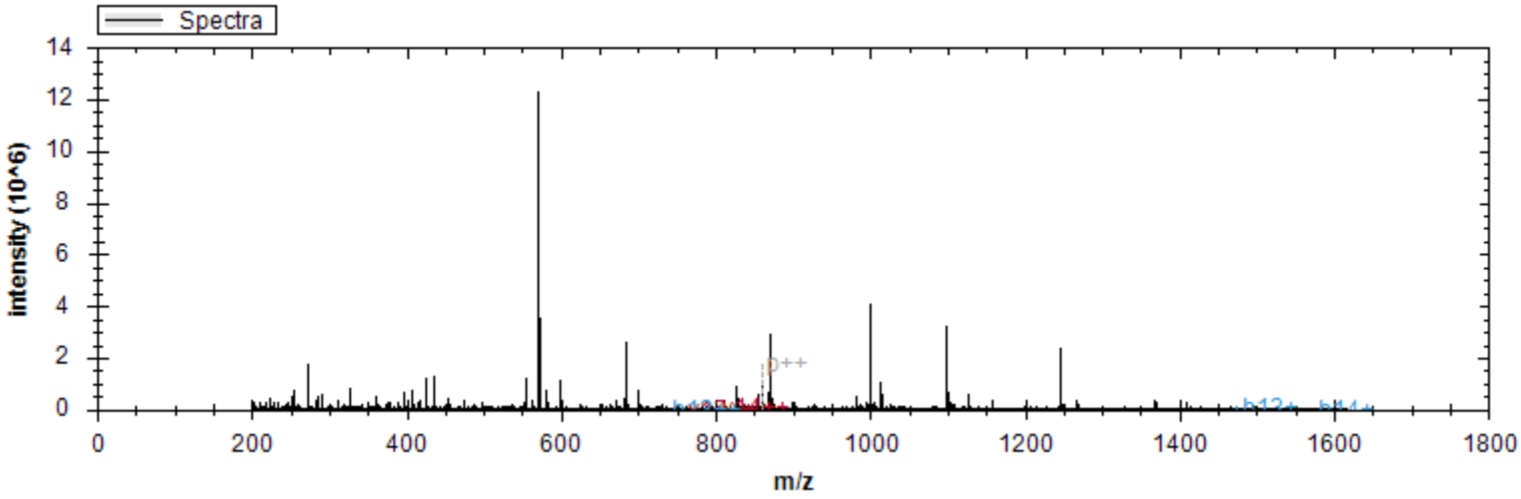

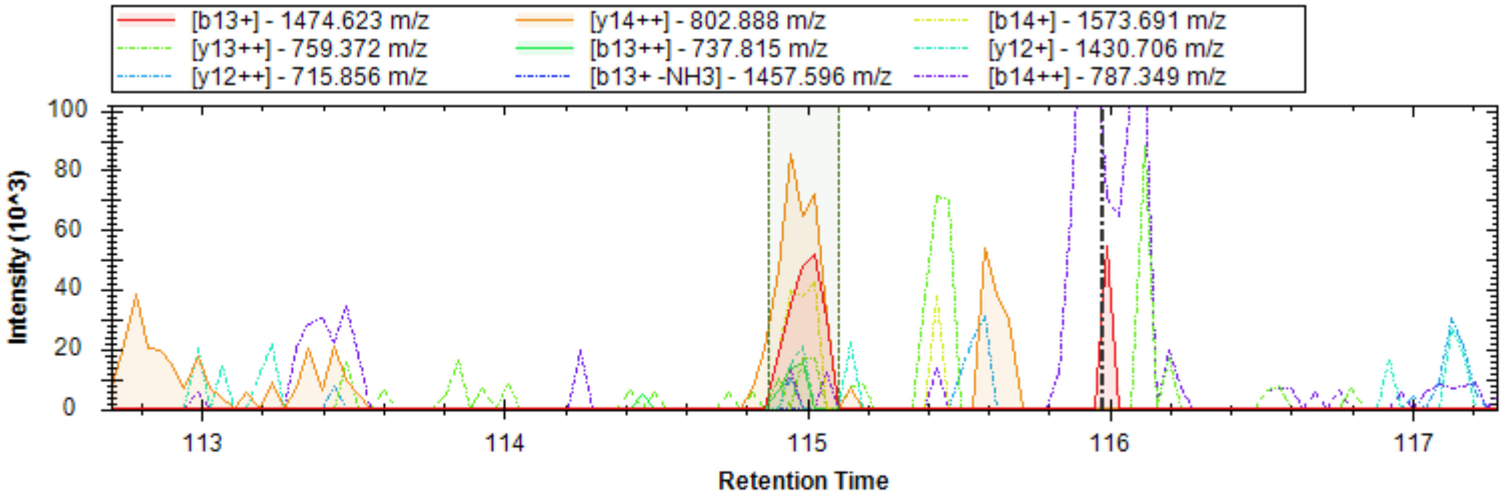

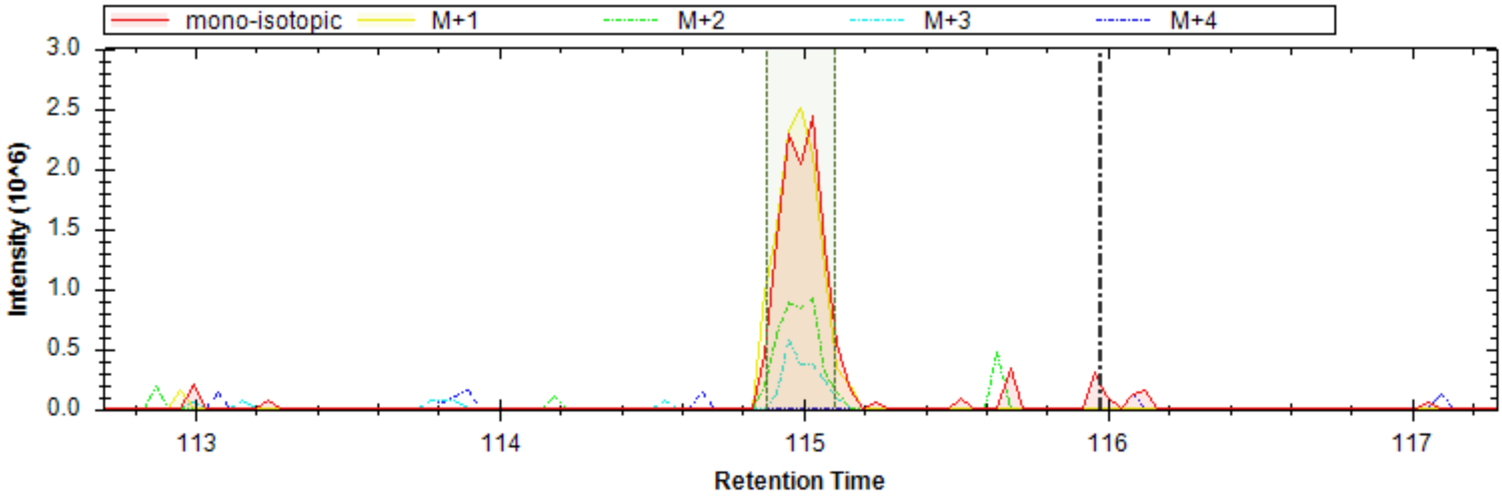

Q9Z0P4 – _SETLVNAQQT[+H+O3+P]PLGTPK_.2
Fig4_MP-DIA-barrel-cortex-p9-3_MHRM.htrms

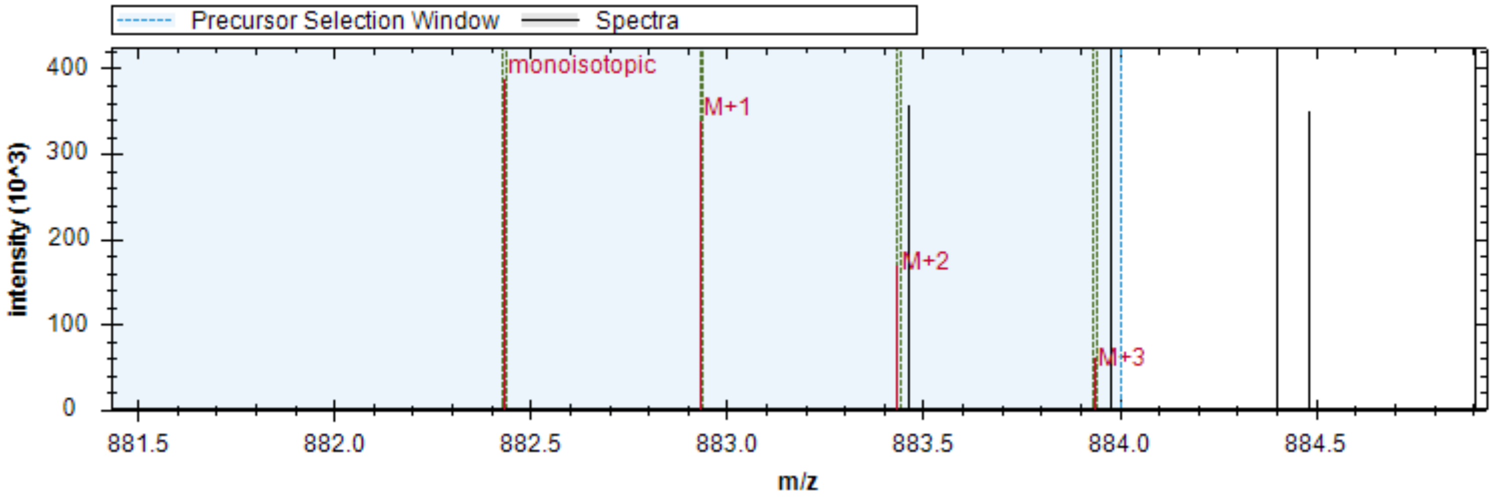

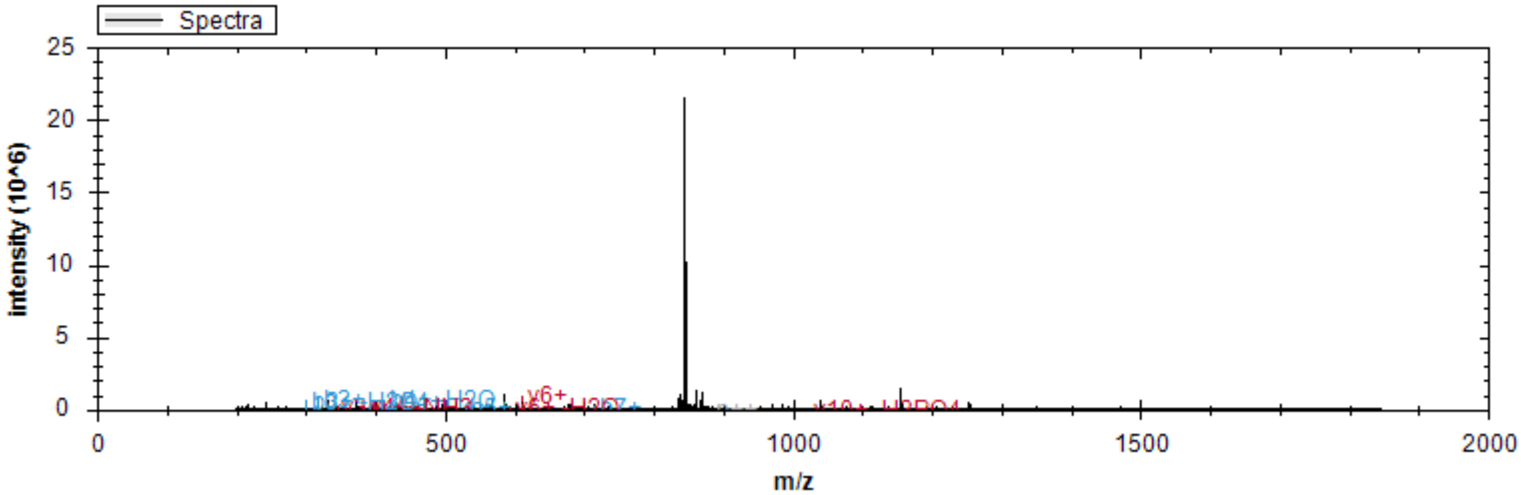

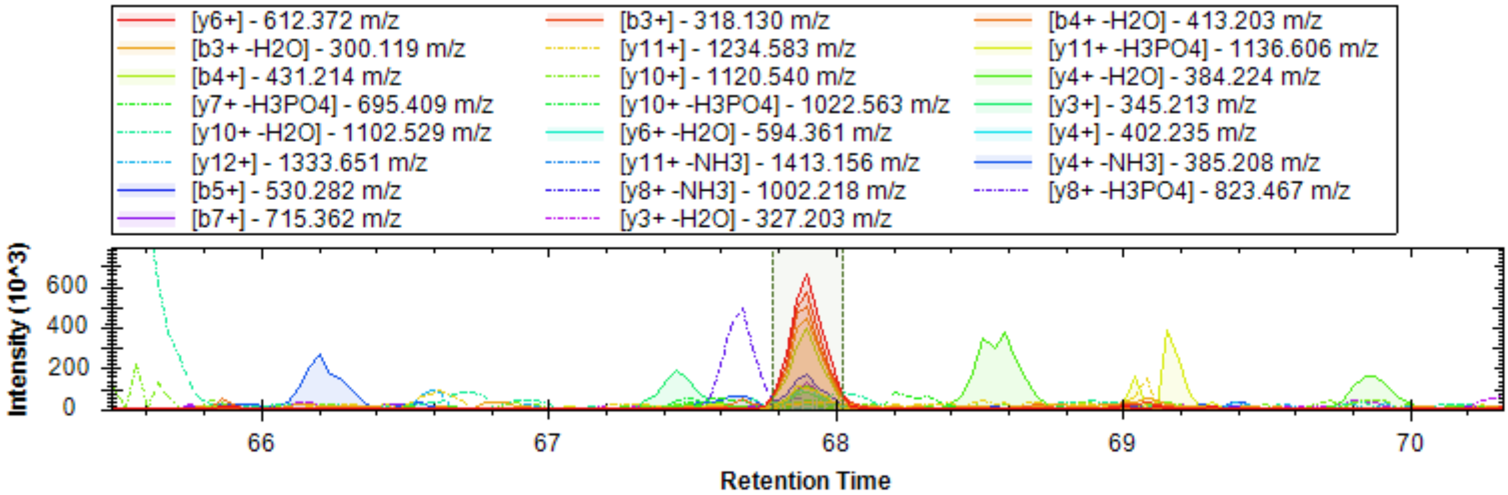

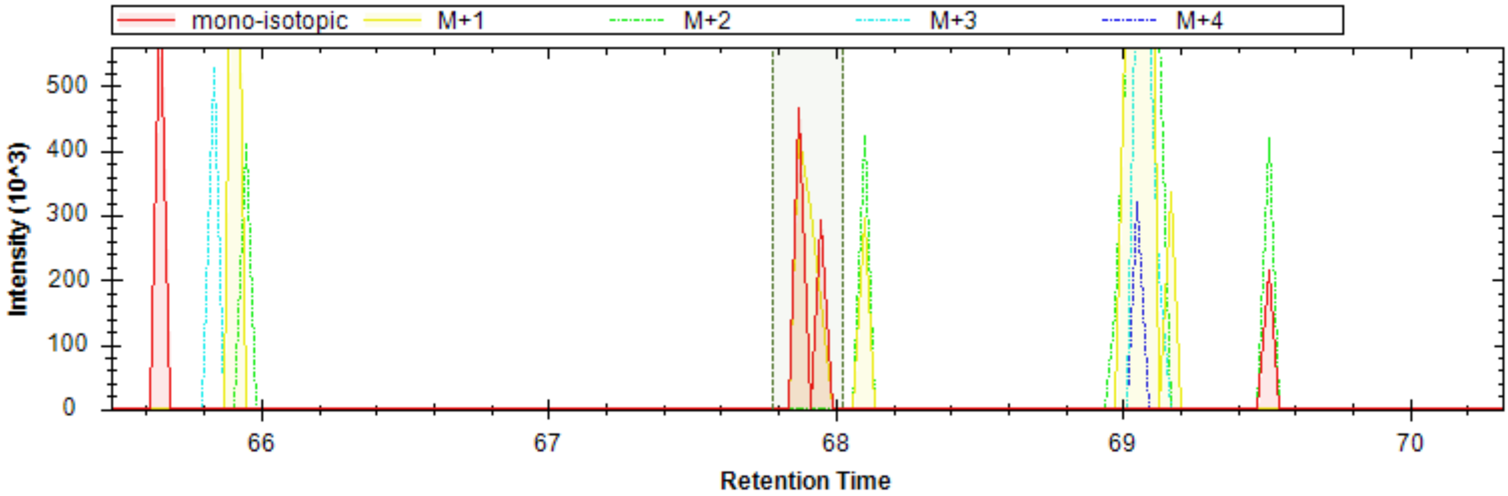

A1A535 – _FLHRGS[+H+O3+P]K_.1
Fig4_MP-DIA-barrel-cortex-p9-3_MHRM.htrms

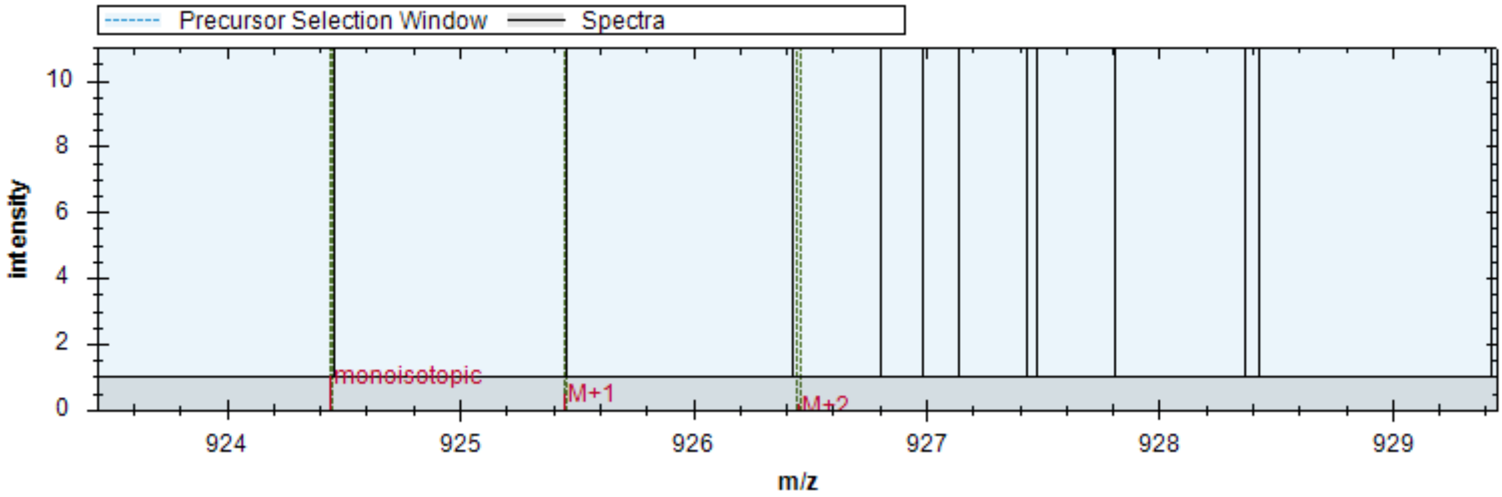

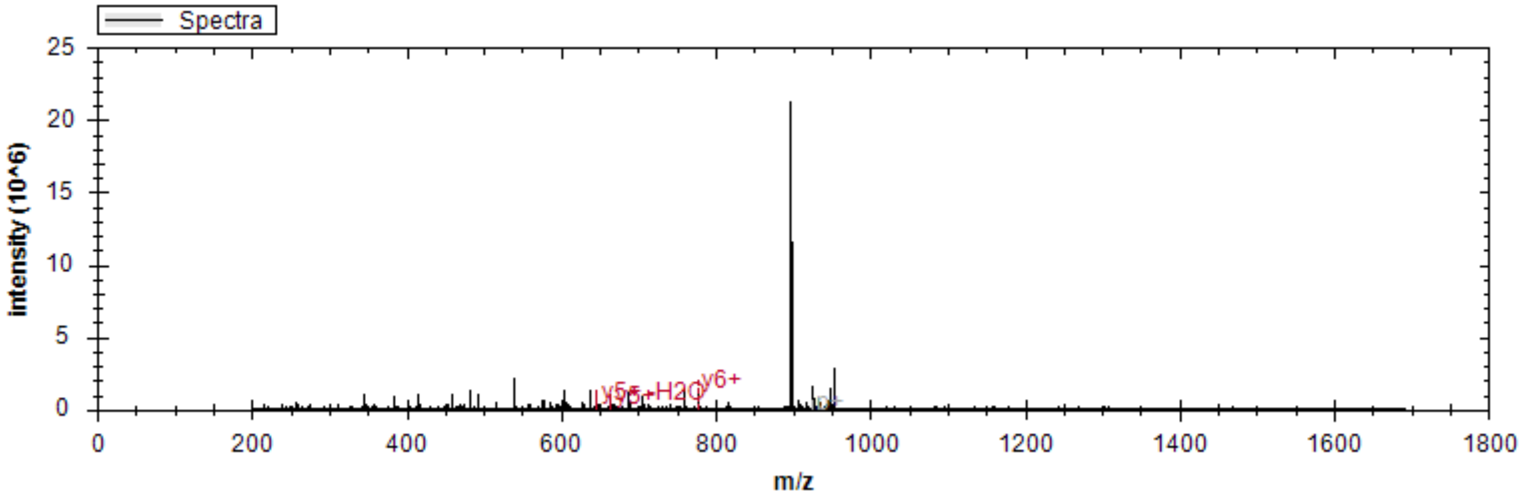

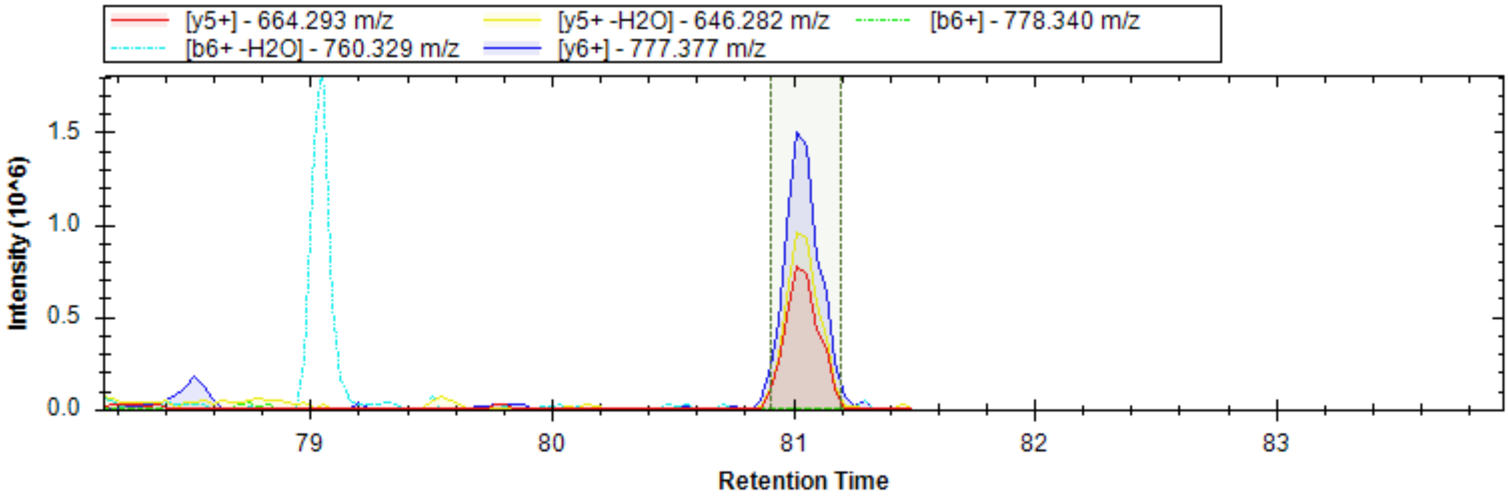

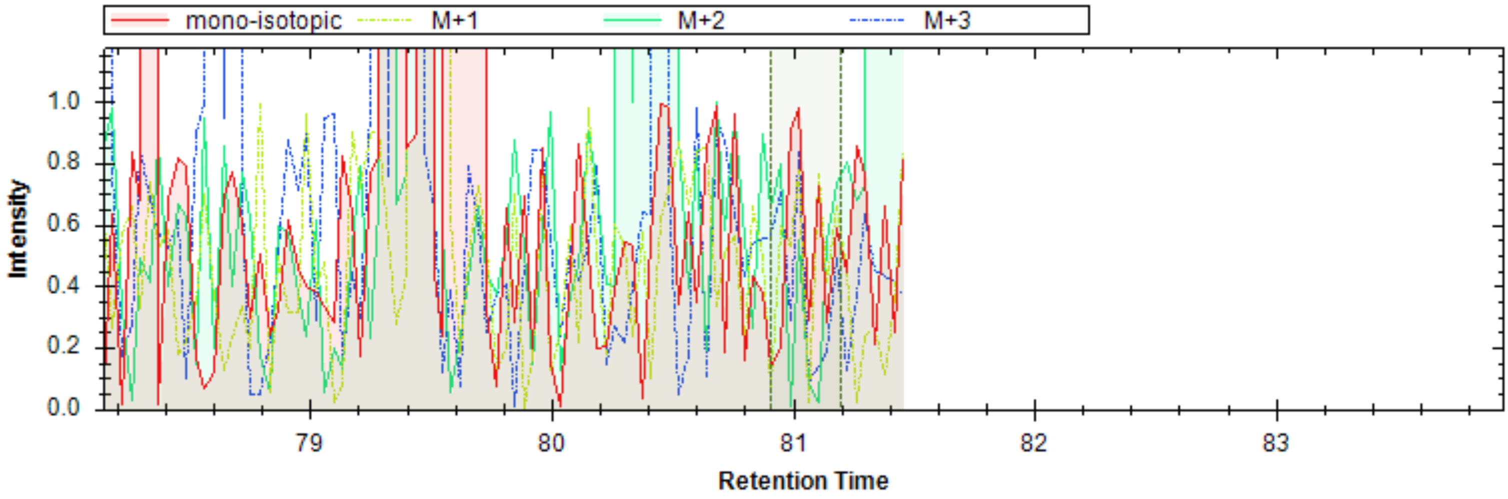

P26645 – _GEATAERPGEAAVASS[+H+O3+P]PSK_.2
Fig4_MP-DIA-barrel-cortex-p9-3_MHRM.htrms

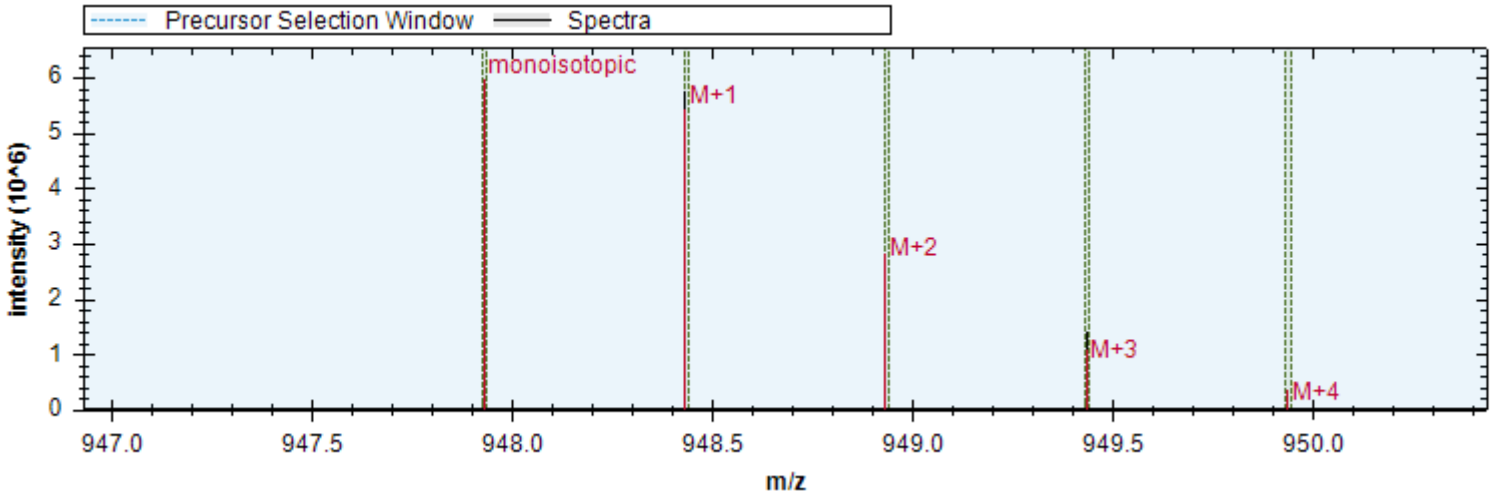

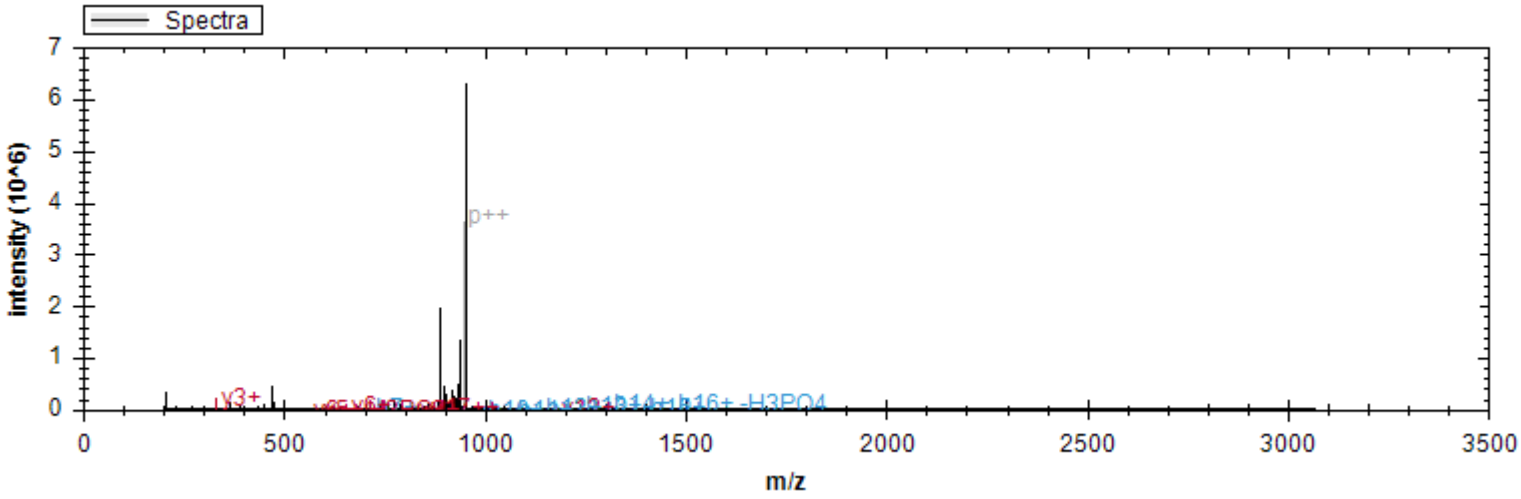

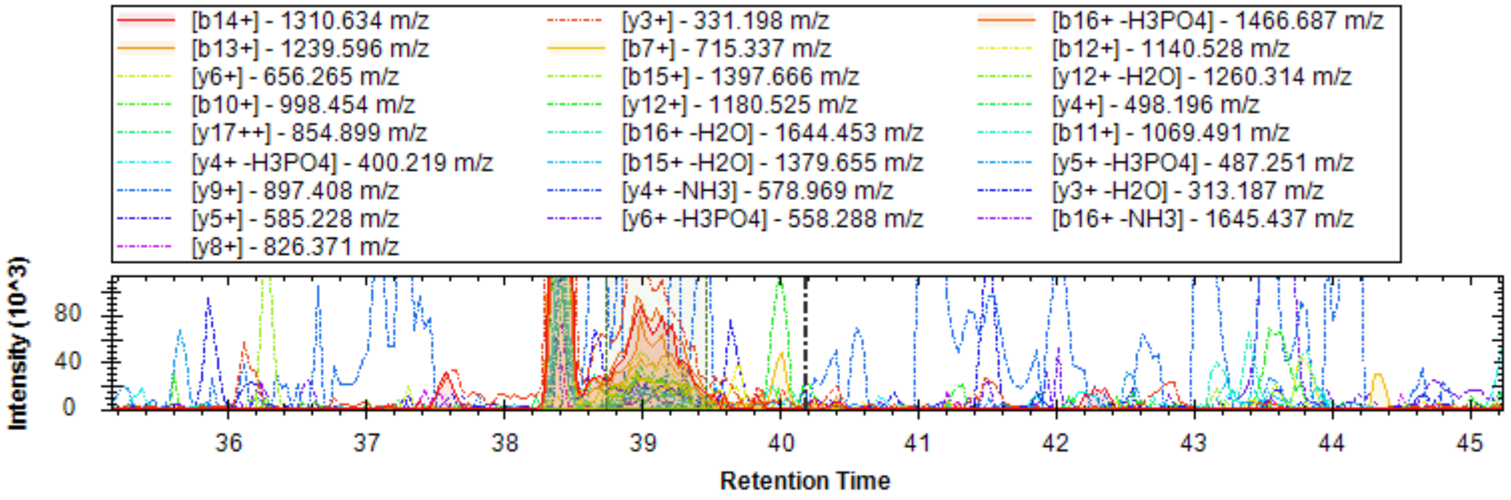

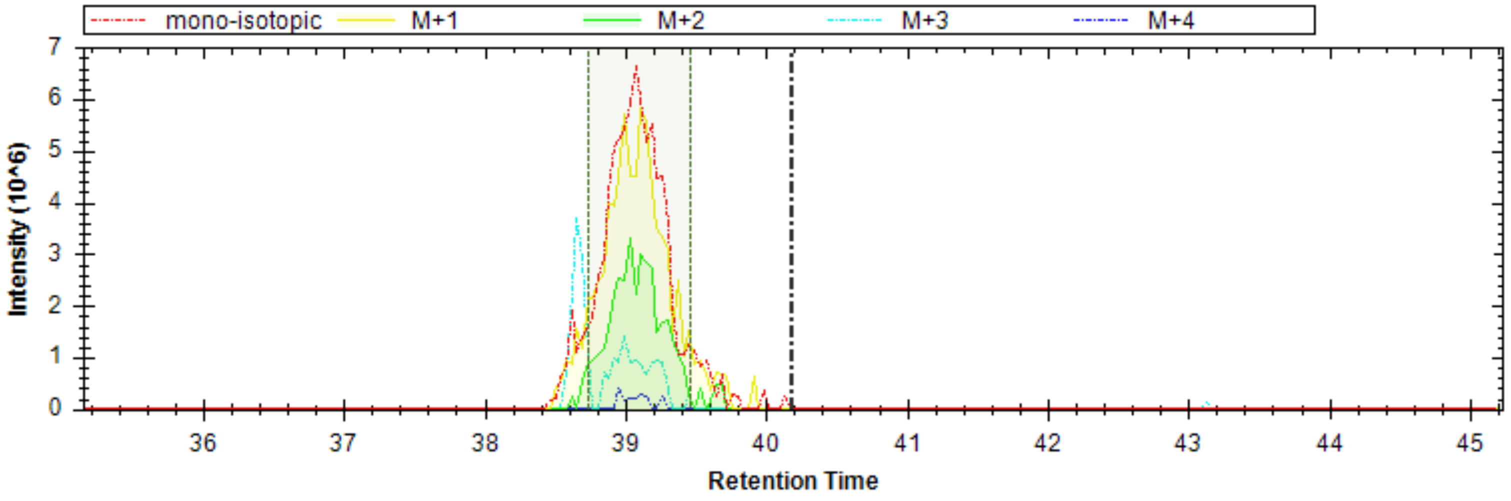

Q8BMA3 – _IIQLC[+C2+H3+N+O]LDLT[+H+O3+P]T[+H+O3+P]AVQK_.2
Fig4_MP-DIA-barrel-cortex-p9-3_MHRM.htrms

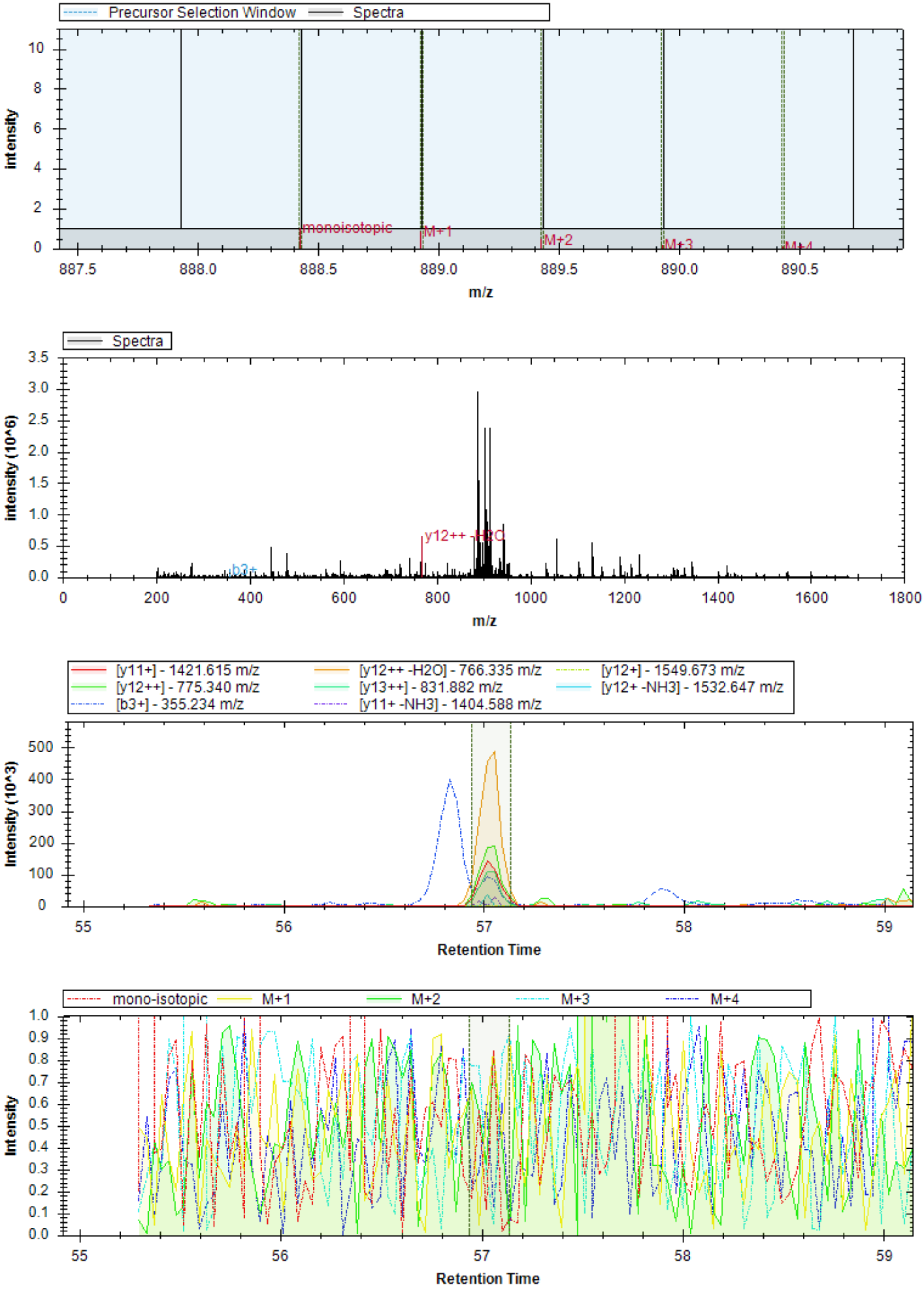

Q8JZP2 – _SQS[+H+O3+P]LTNSLSTSDTSHR_.2
Fig4_MP-DIA-barrel-cortex-p9-3_MHRM.htrms

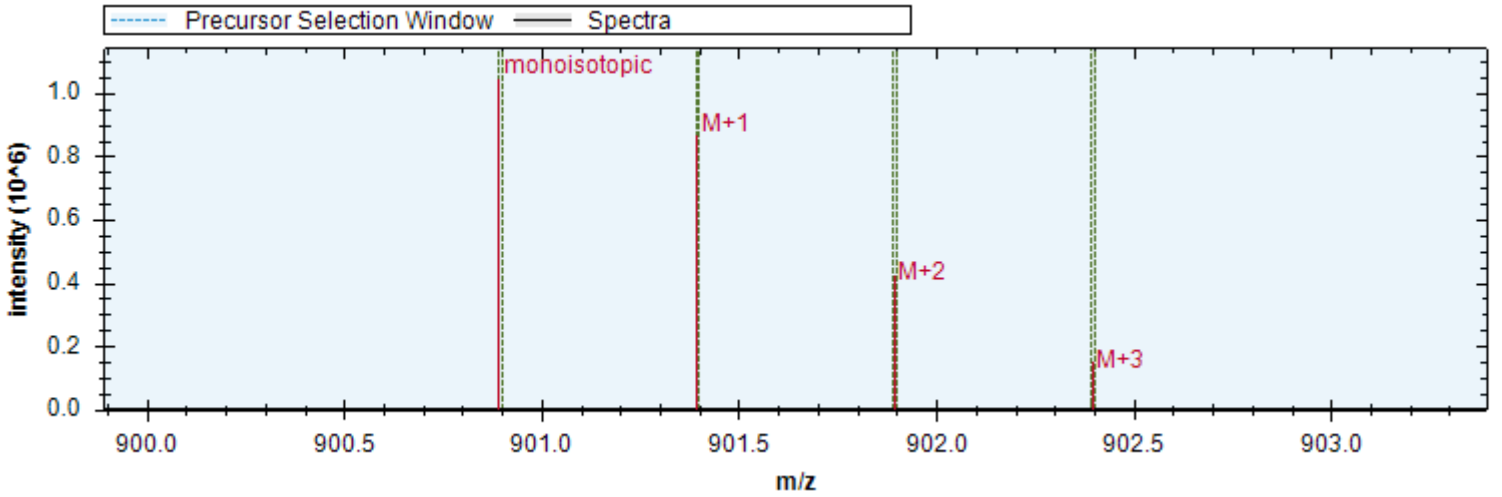

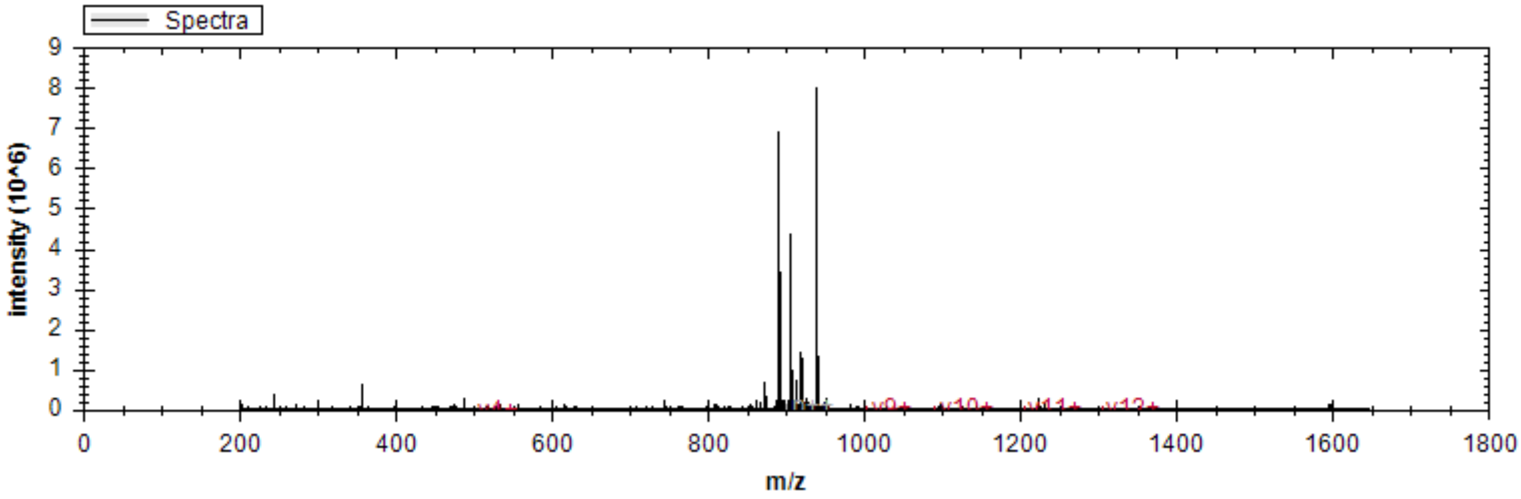

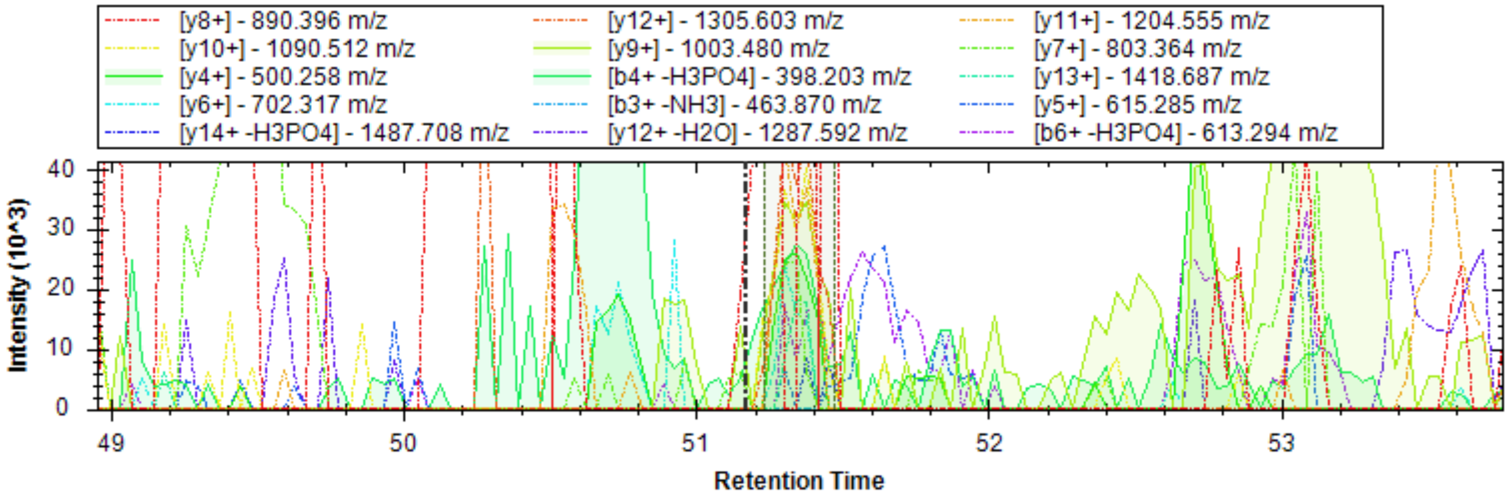

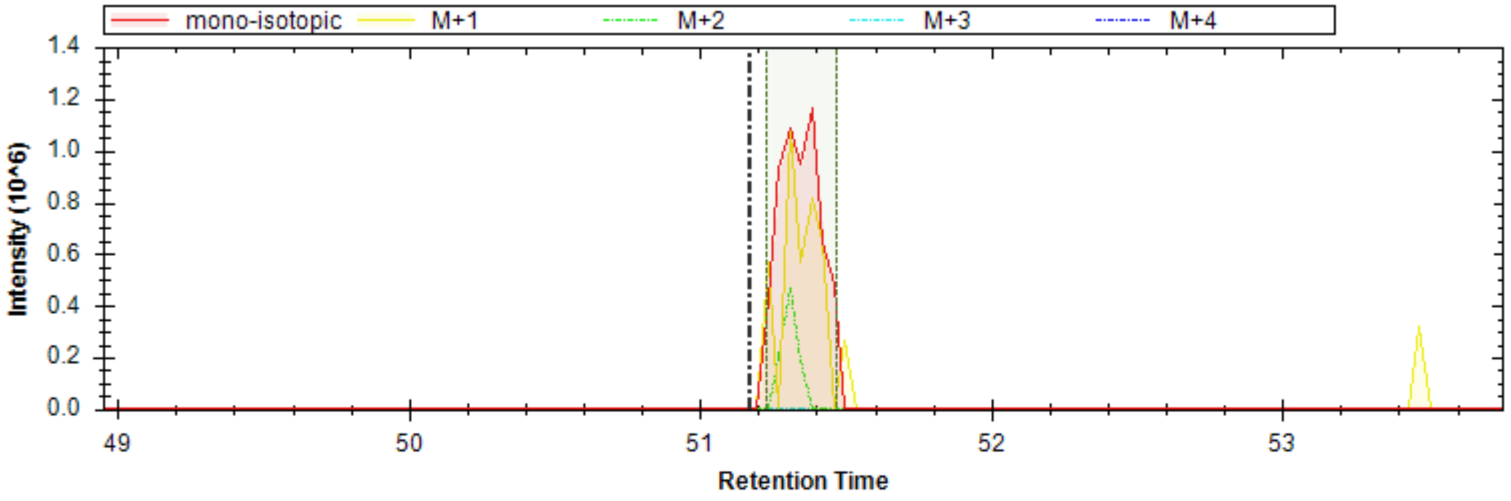

Q91Z31;Q91Z31-2 – _ETSLLAVPGALS[+H+O3+P]PLAIPNAAAAAAAAAAGR_.3
Fig4_MP-DIA-barrel-cortex-p9-3_MHRM.htrms

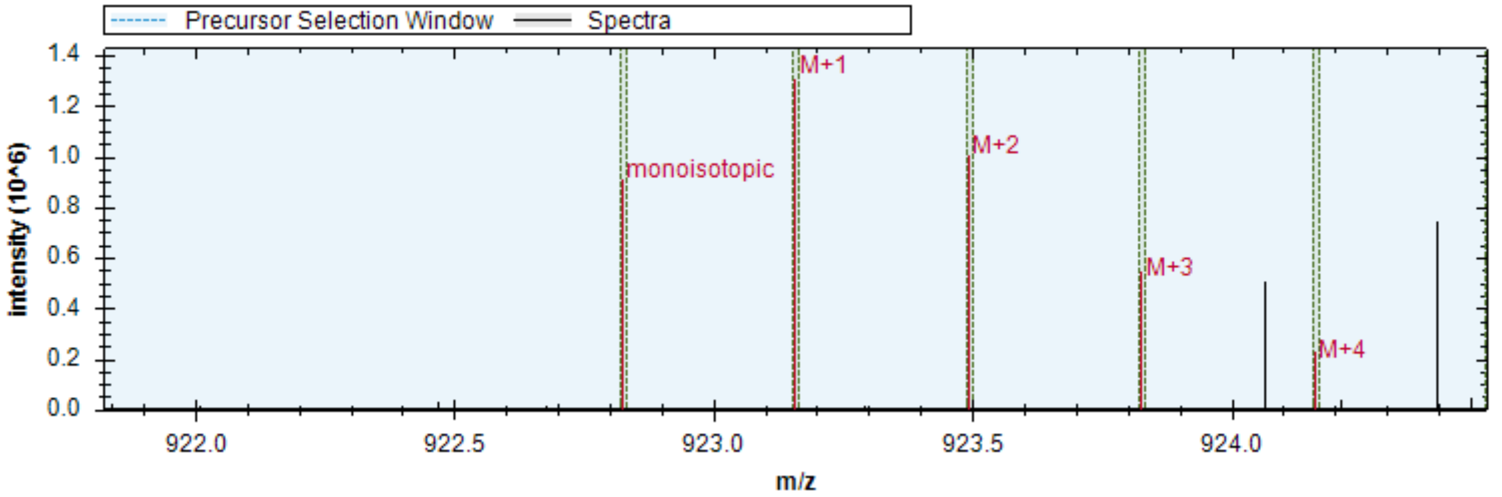

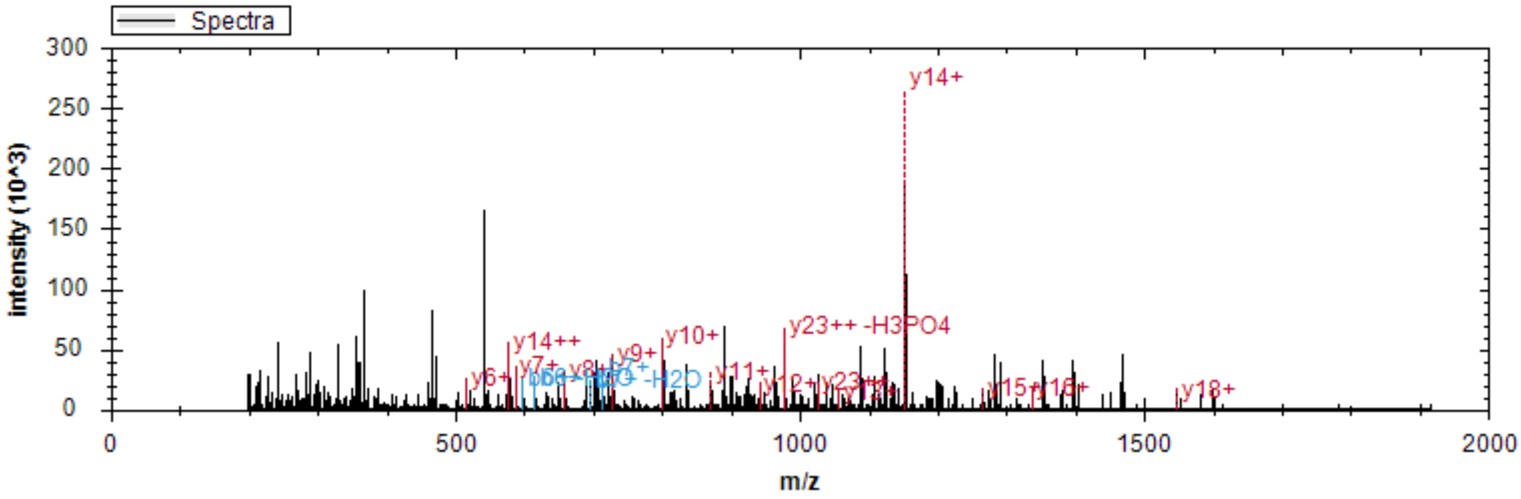

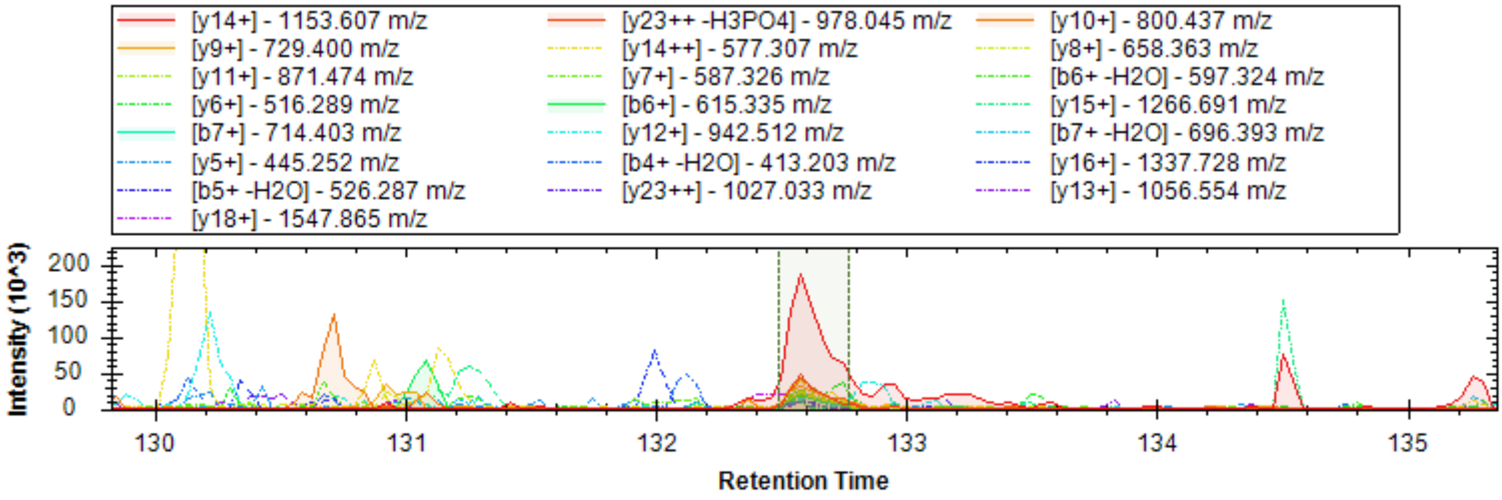

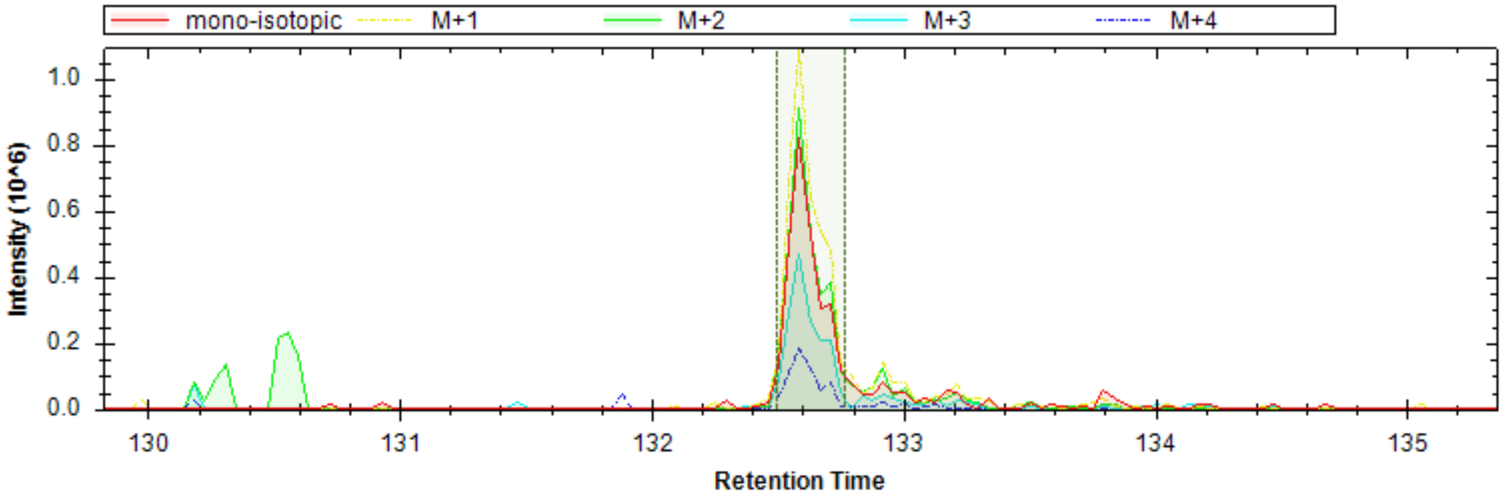

Q99MR1 – _[+C2+H2+O]M[+O]AAET[+H+O3+P]LNFGPEWLR_.2
Fig4_MP-DIA-barrel-cortex-p9-3_MHRM.htrms

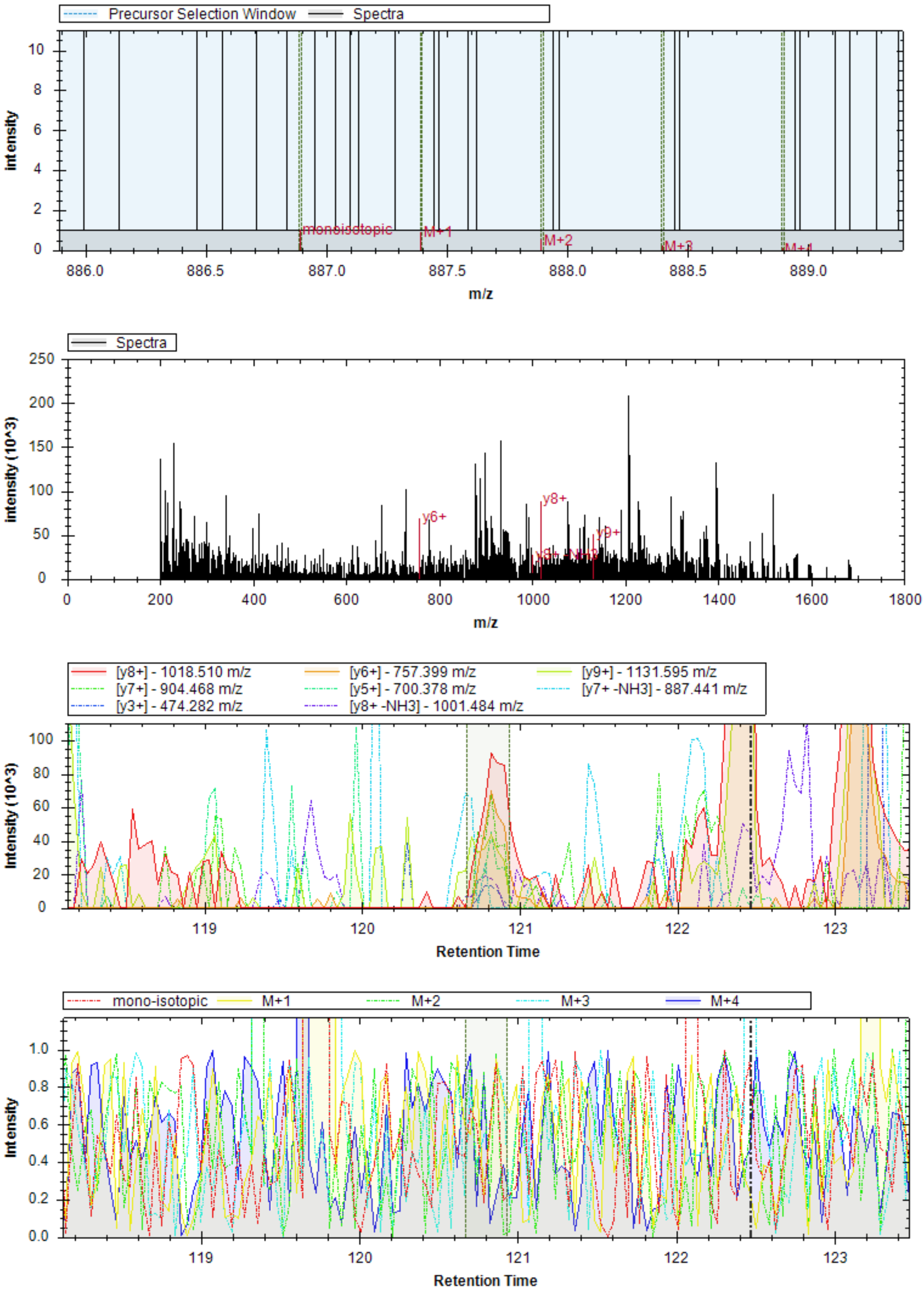

Q9ES97;Q9ES97-2 – _AES[+H+O3+P]SAATQSPSVSSSSSGAEPSALGGGGGSPGAC[+C2+H3+N+O]PALGAK_.4
Fig4_MP-DIA-barrel-cortex-p9-3_MHRM.htrms

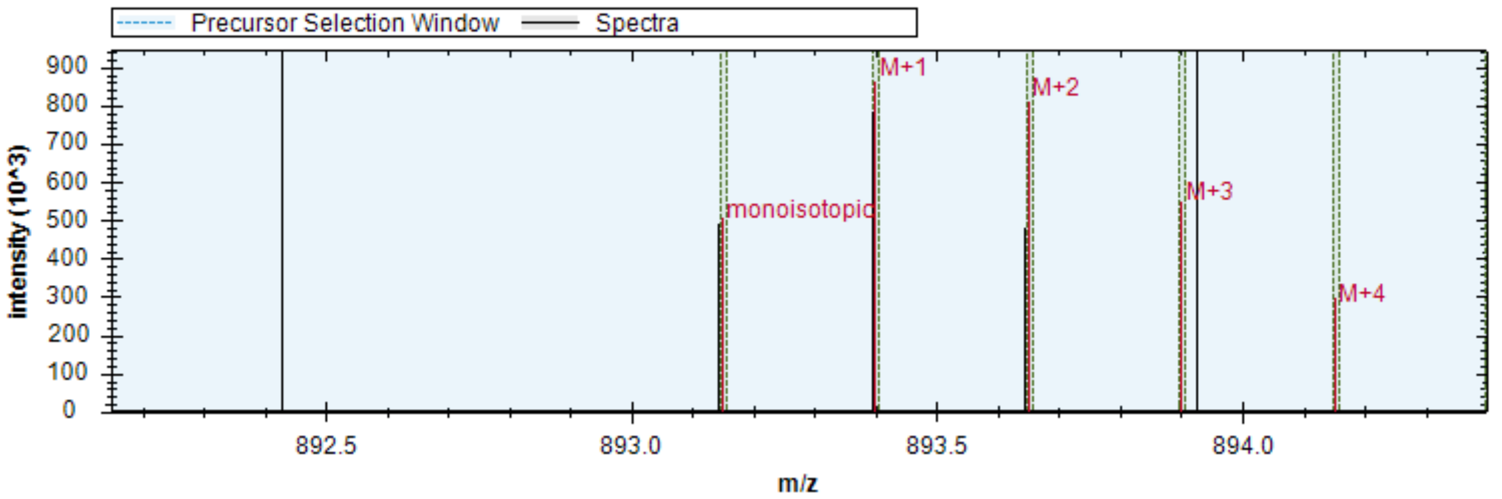

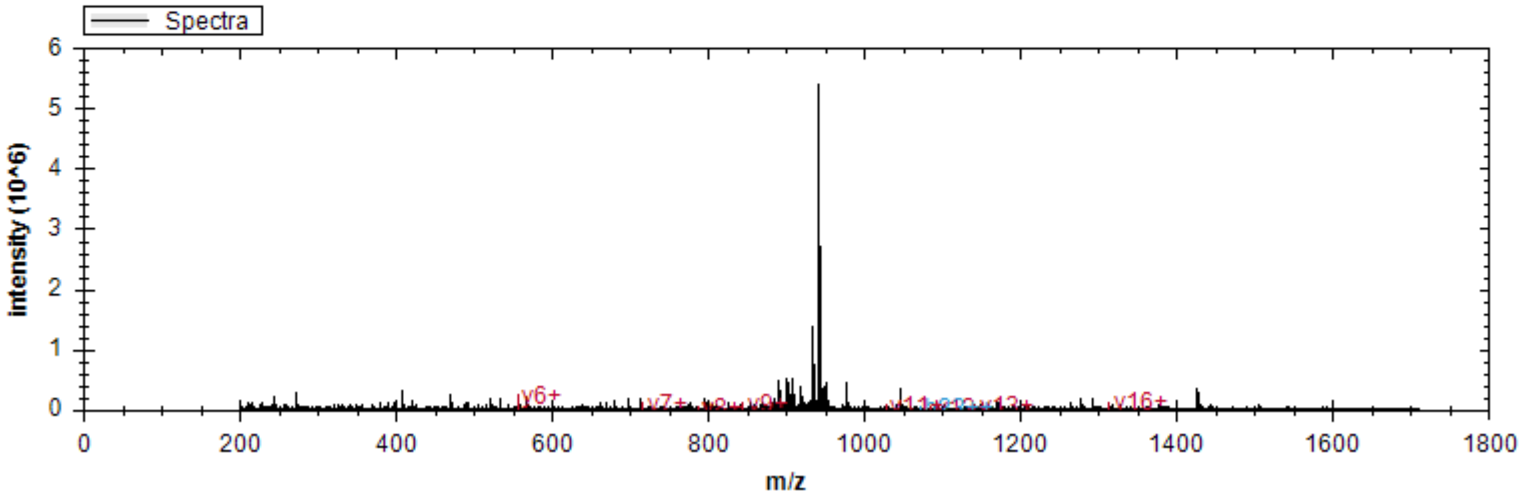

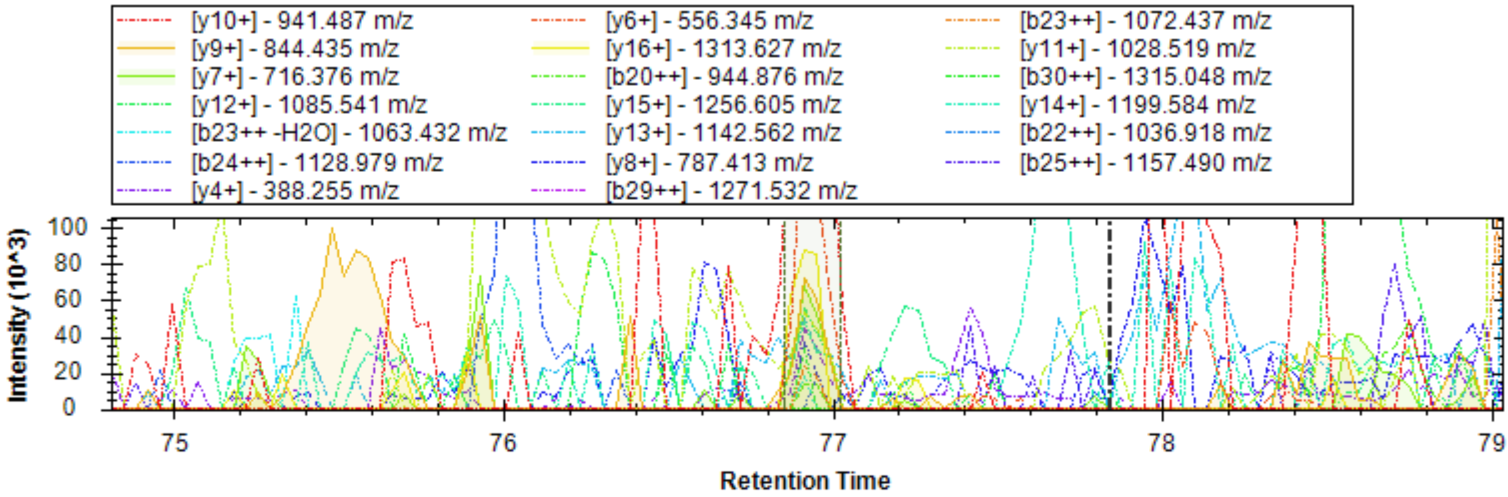

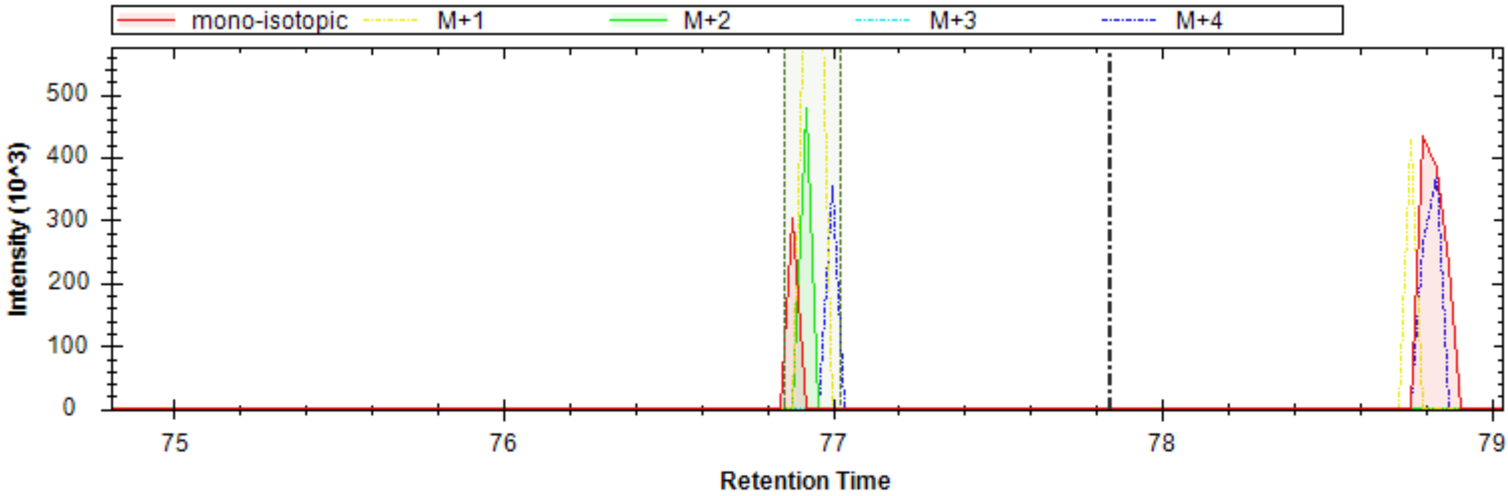

Q9QYR6-2 – _MAS[+H+O3+P]PPPSGPPSAAHTPFHQSPVEEK_.3
Fig4_MP-DIA-barrel-cortex-p9-3_MHRM.htrms

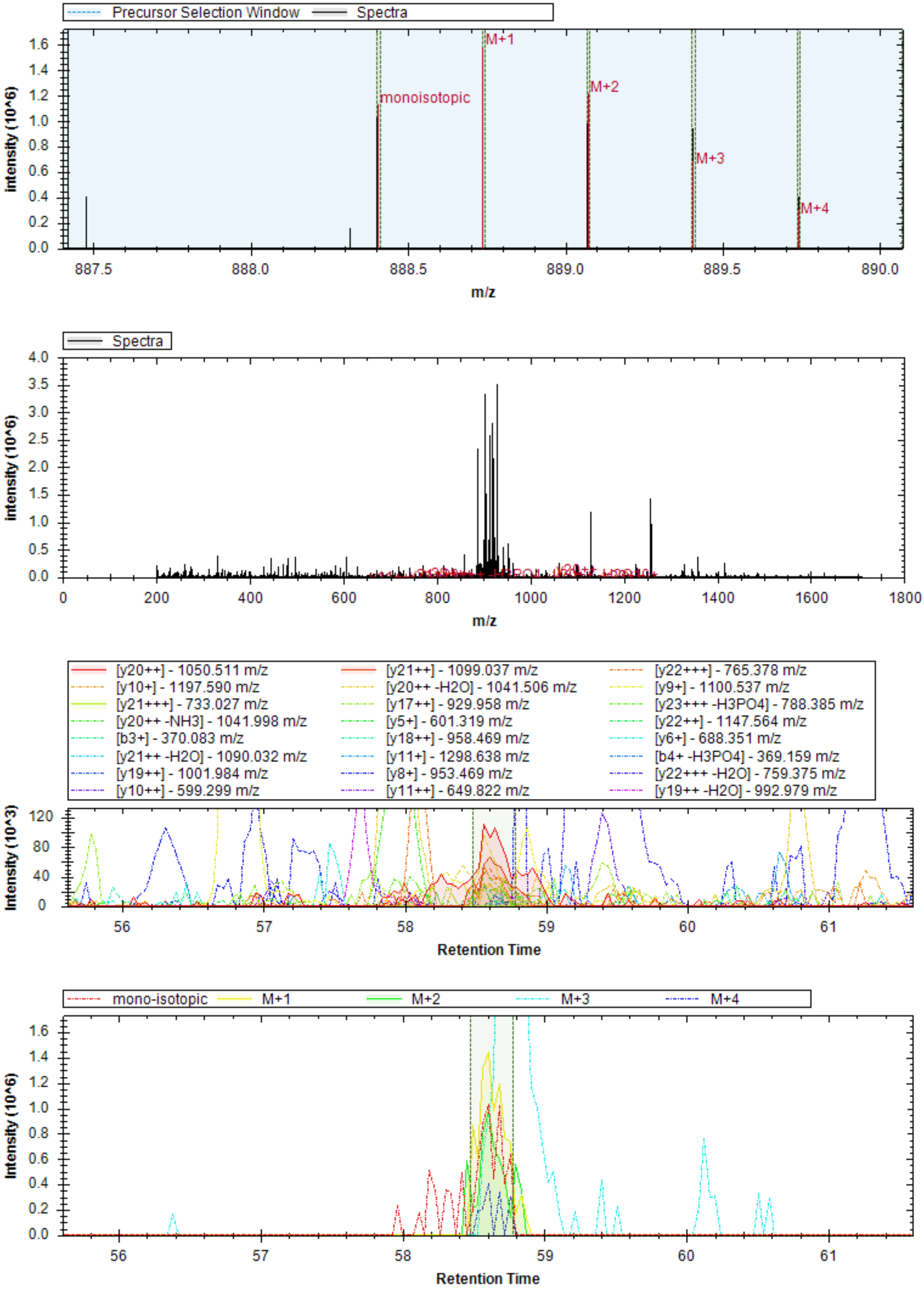

O88485 – _ETQTPLAT[+H+O3+P]HQSEEDEEDEEMVEPK_.3
Fig4_MP-DIA-barrel-cortex-p9-3_MHRM.htrms

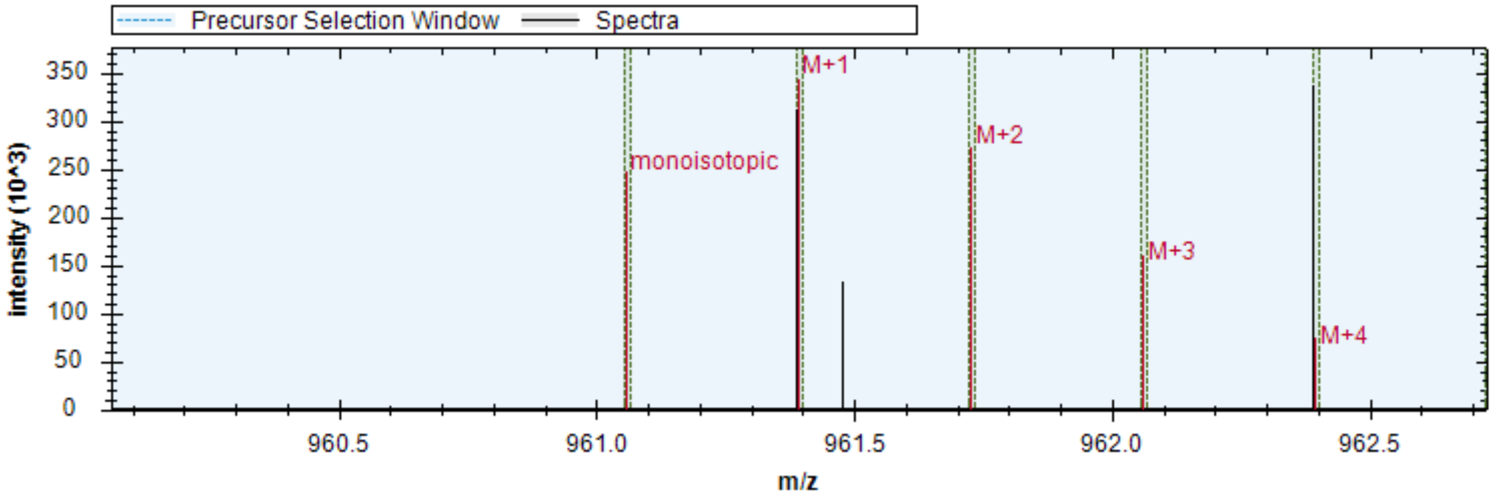

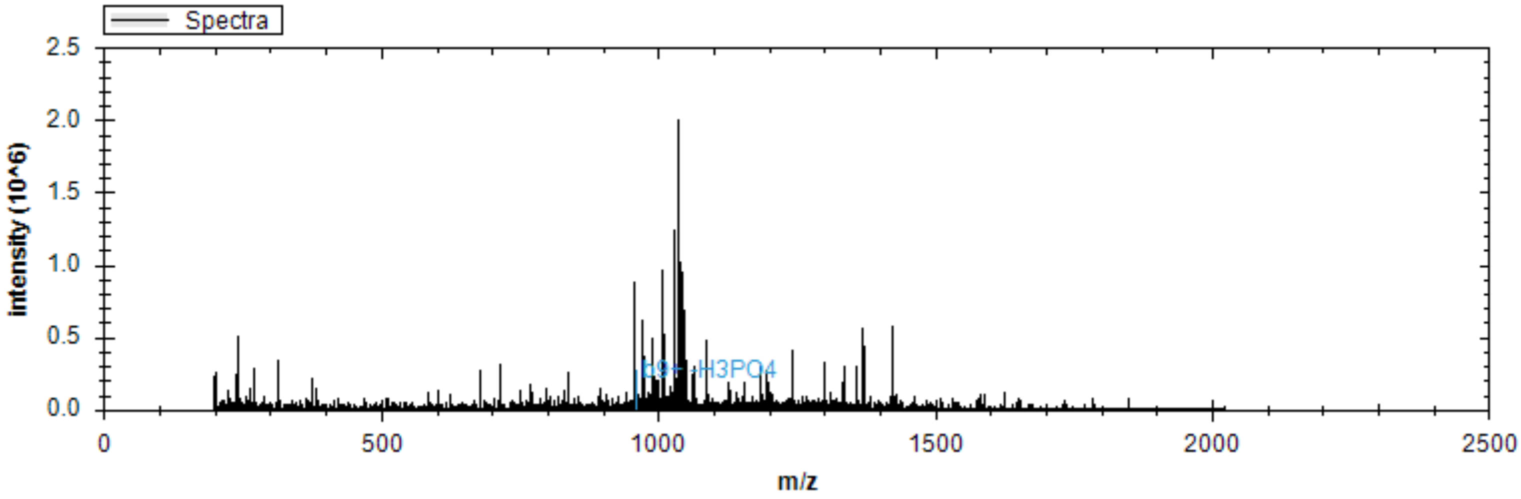

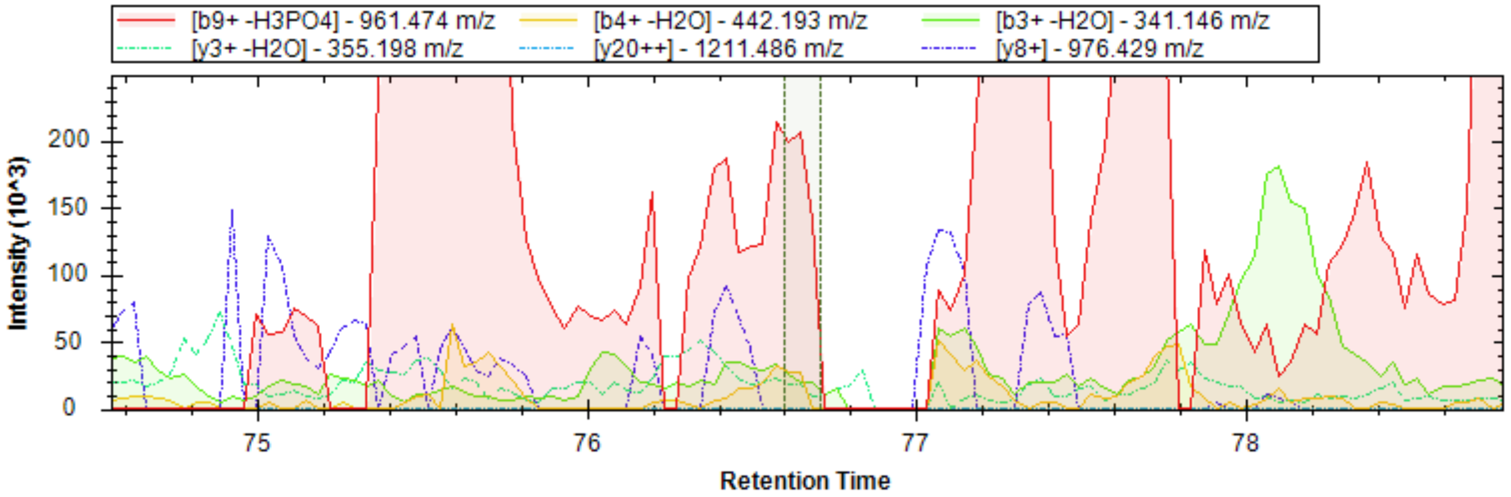

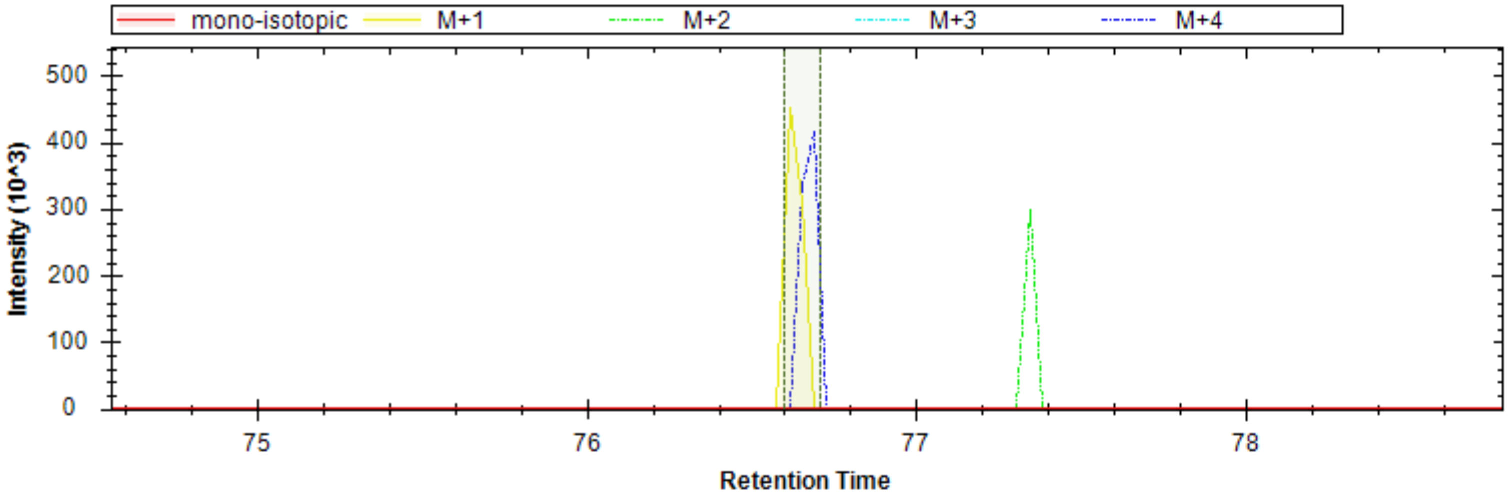

P17742 – _VNPT[+H+O3+P]VFFDITADDEPLGR_.2
Fig4_MP-DIA-barrel-cortex-p9-3_MHRM.htrms

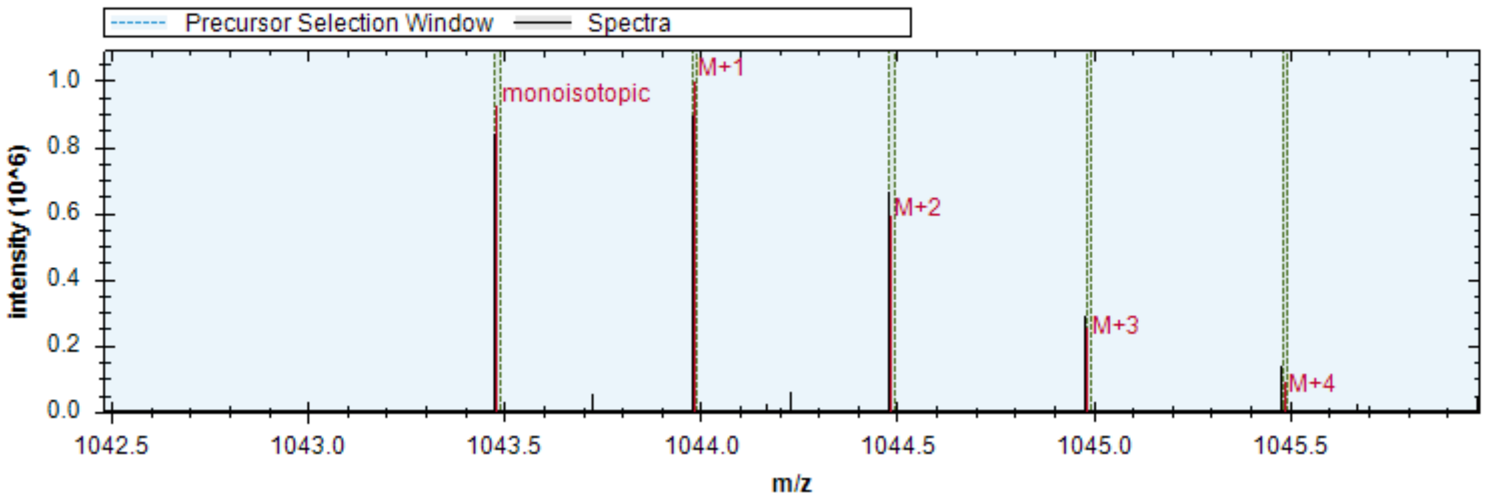

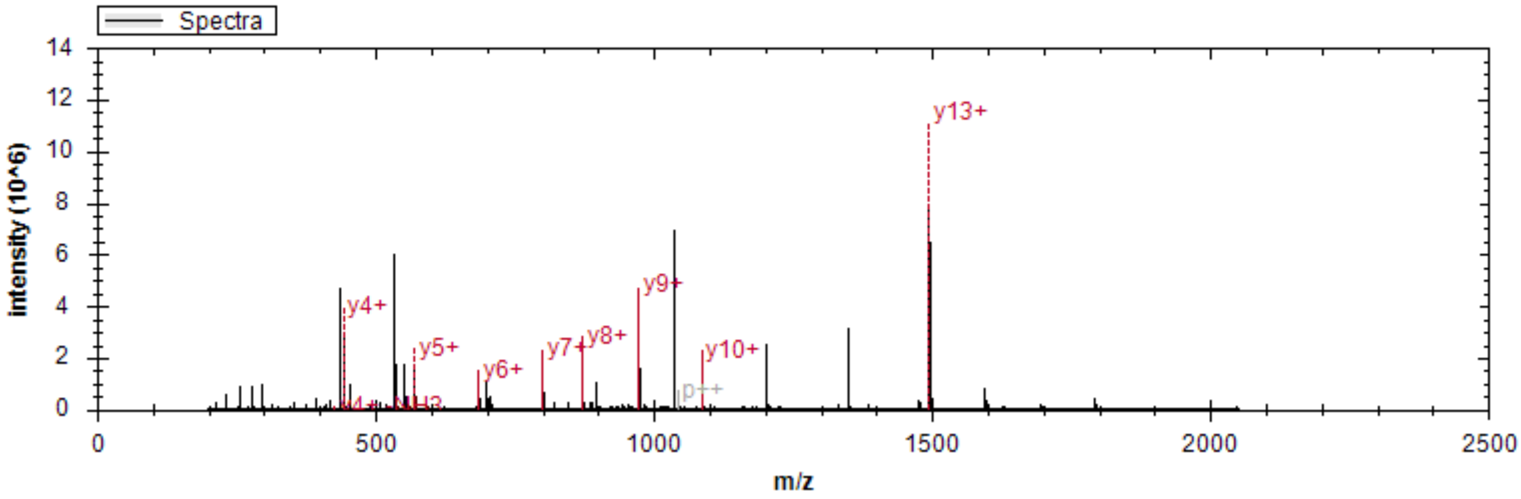

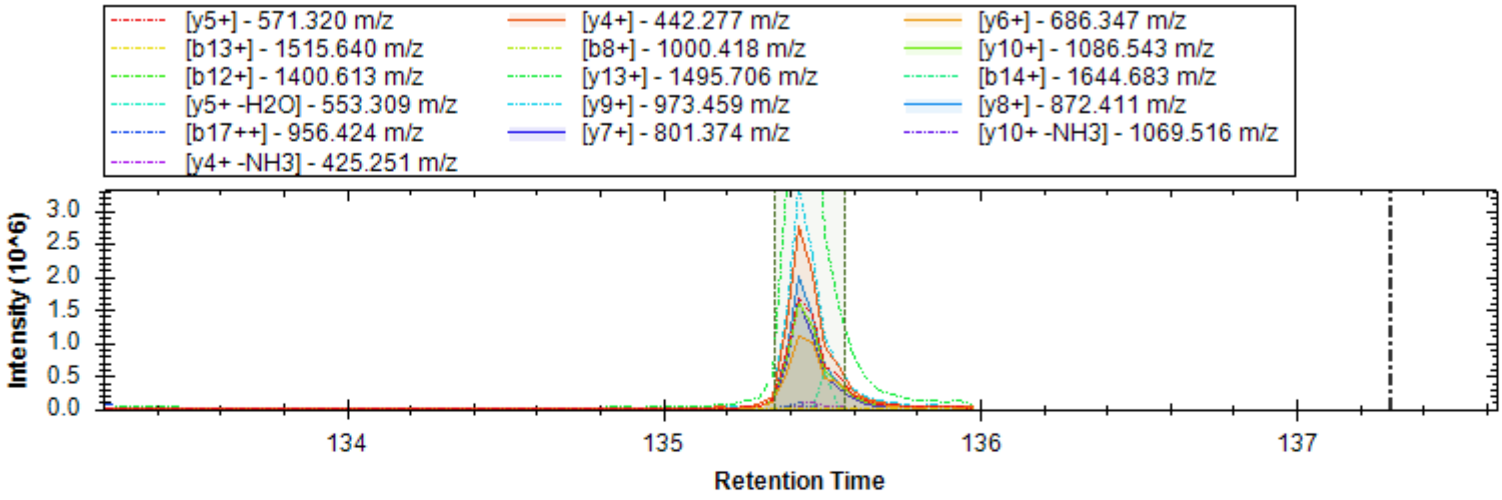

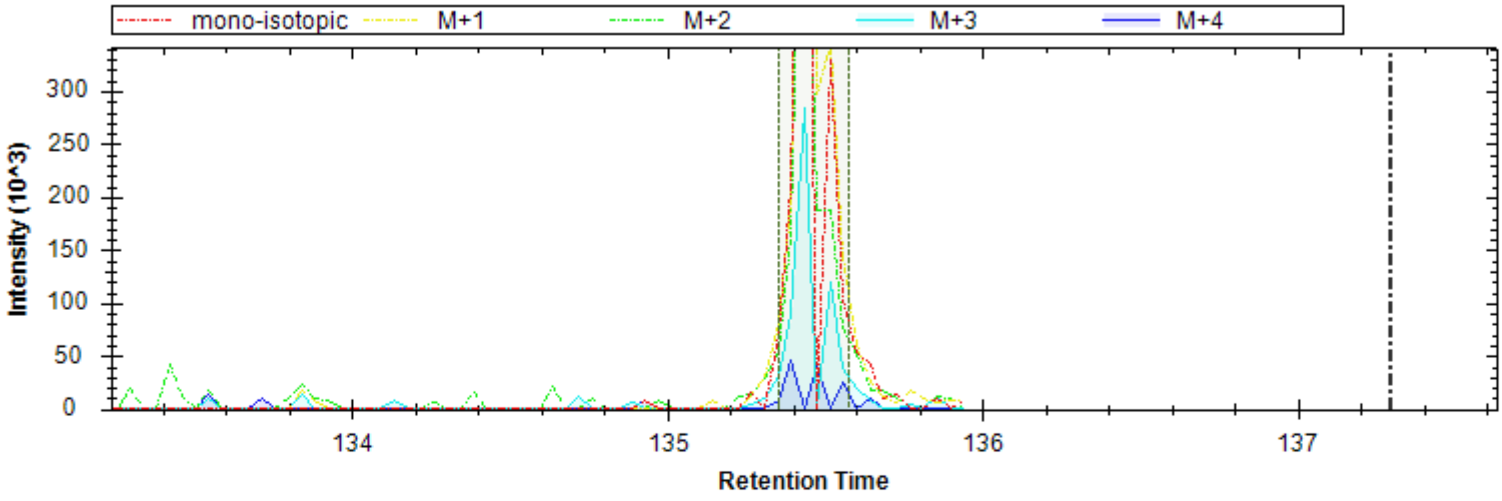

P26645 – _LSGFS[+H+O3+P]FKK_.1
Fig4_MP-DIA-barrel-cortex-p9-3_MHRM.htrms

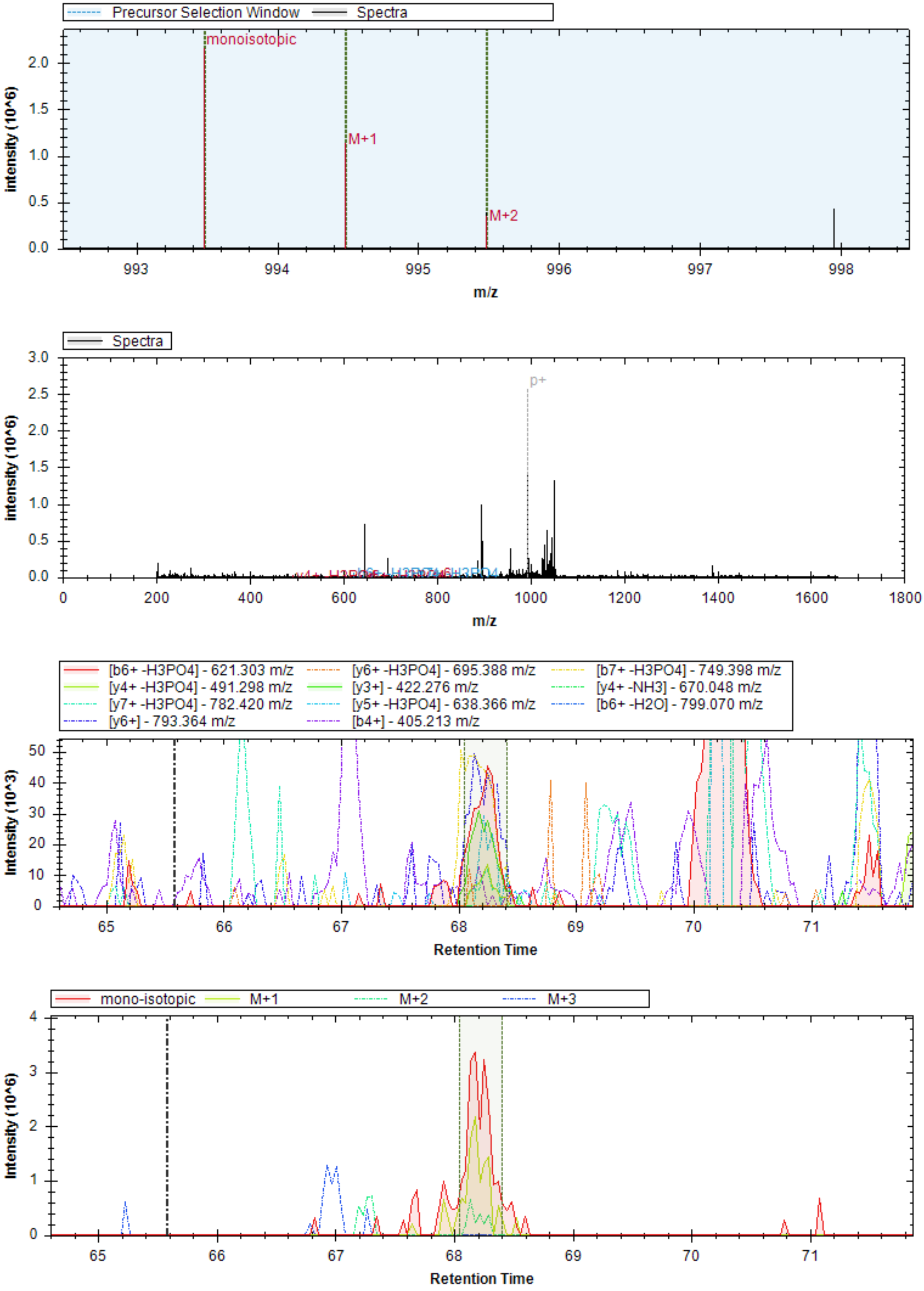

Q8CHK4;Q8CHK4-2;Q8CH[...] – _RGY[+H+O3+P]GKLLIEFS[+H+O3+P]YELSK_.2
Fig4_MP-DIA-barrel-cortex-p9-3_MHRM.htrms

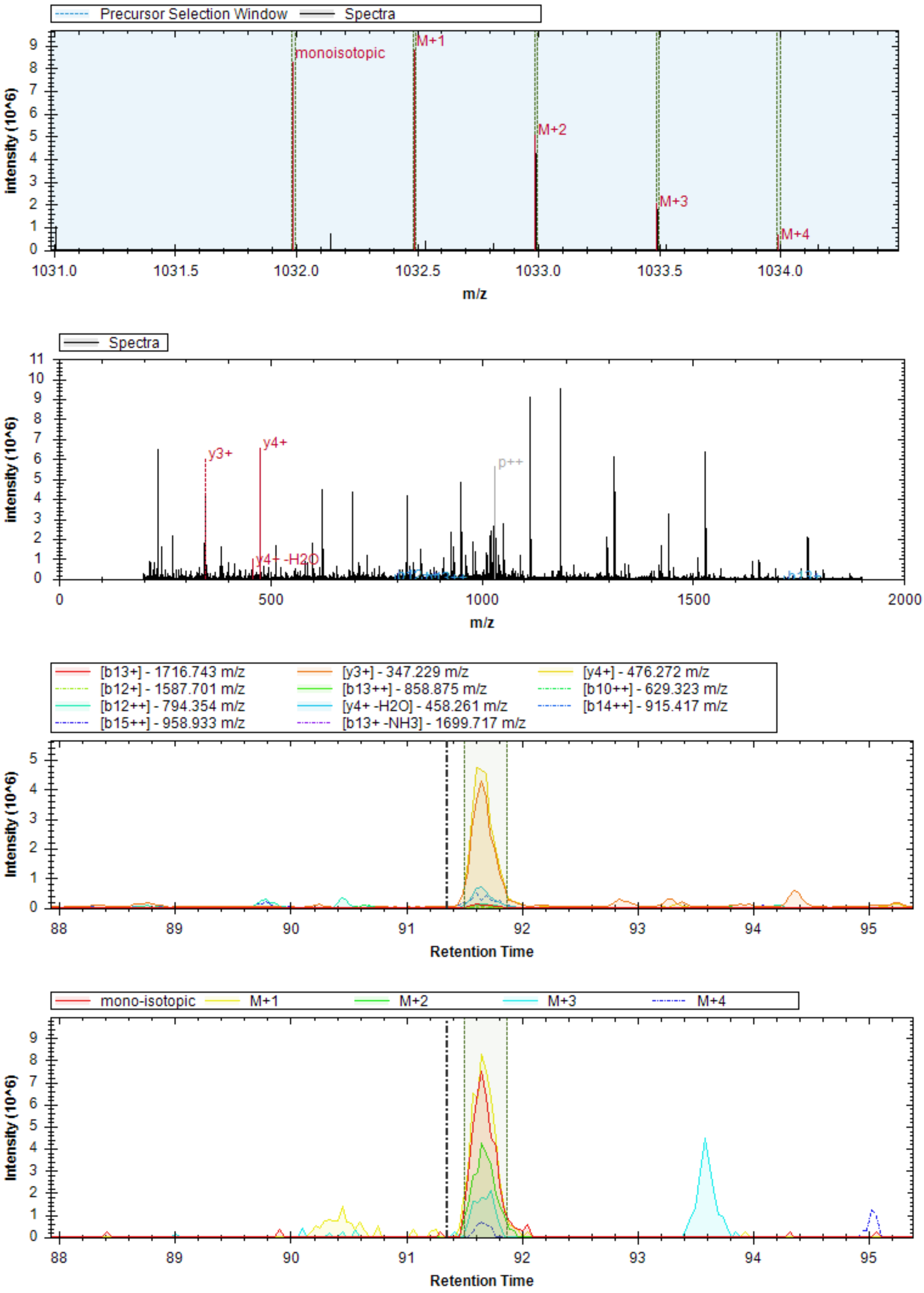

A2ASS6 – _T[+H+O3+P]VS[+H+O3+P]VKVLVLDK_.1
Fig4_MP-DIA-barrel-cortex-p9-3_MHRM.htrms

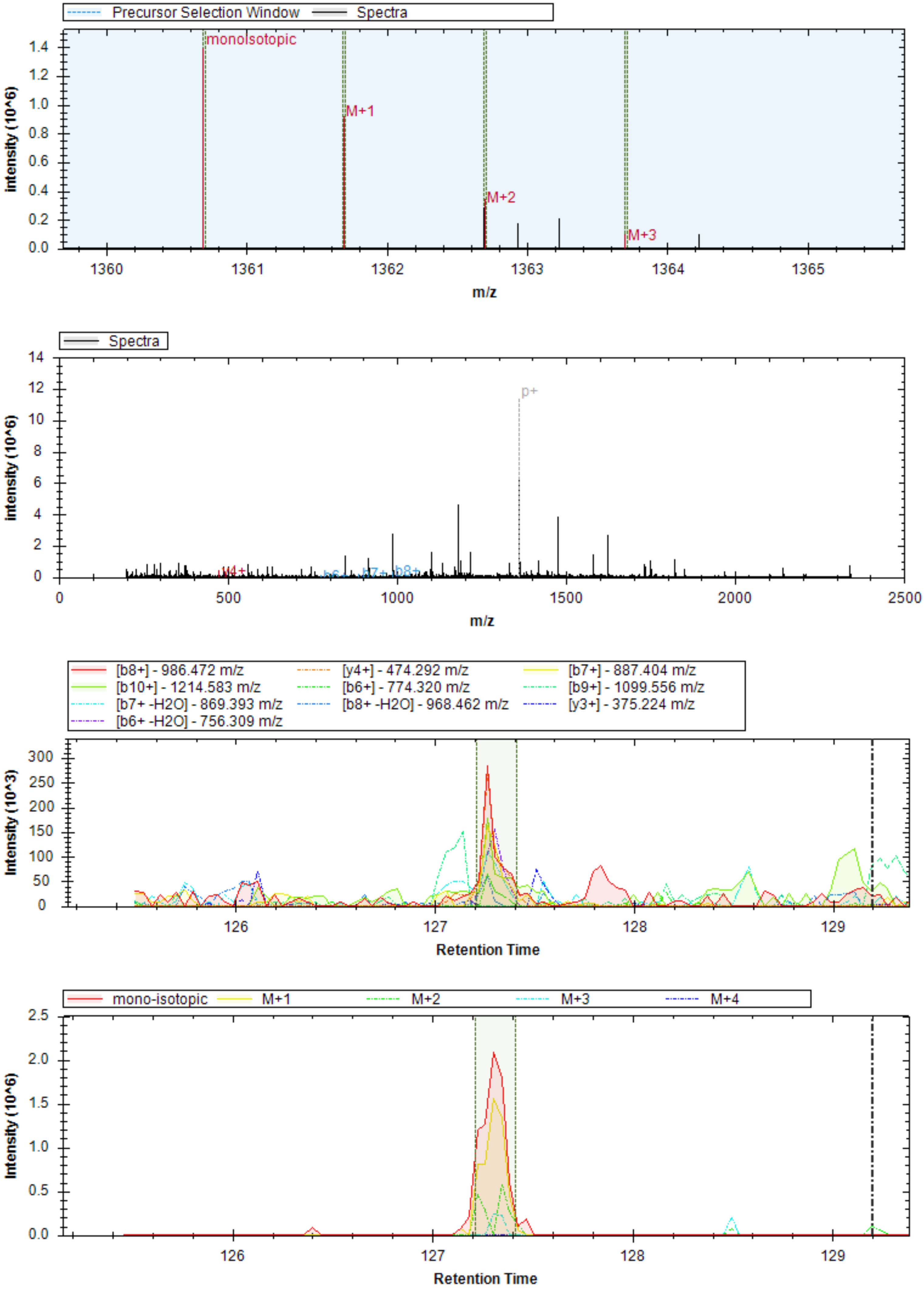

E9Q401 – _MVAEVFIY[+H+O3+P]WSK_.1
Fig4_MP-DIA-barrel-cortex-p9-3_MHRM.htrms

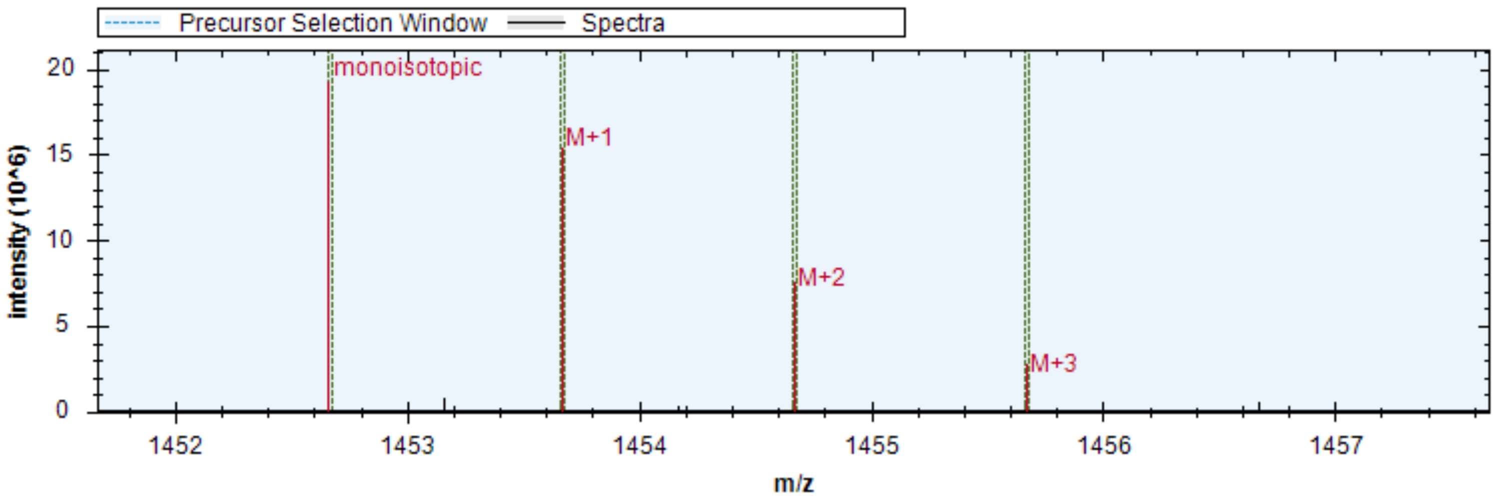

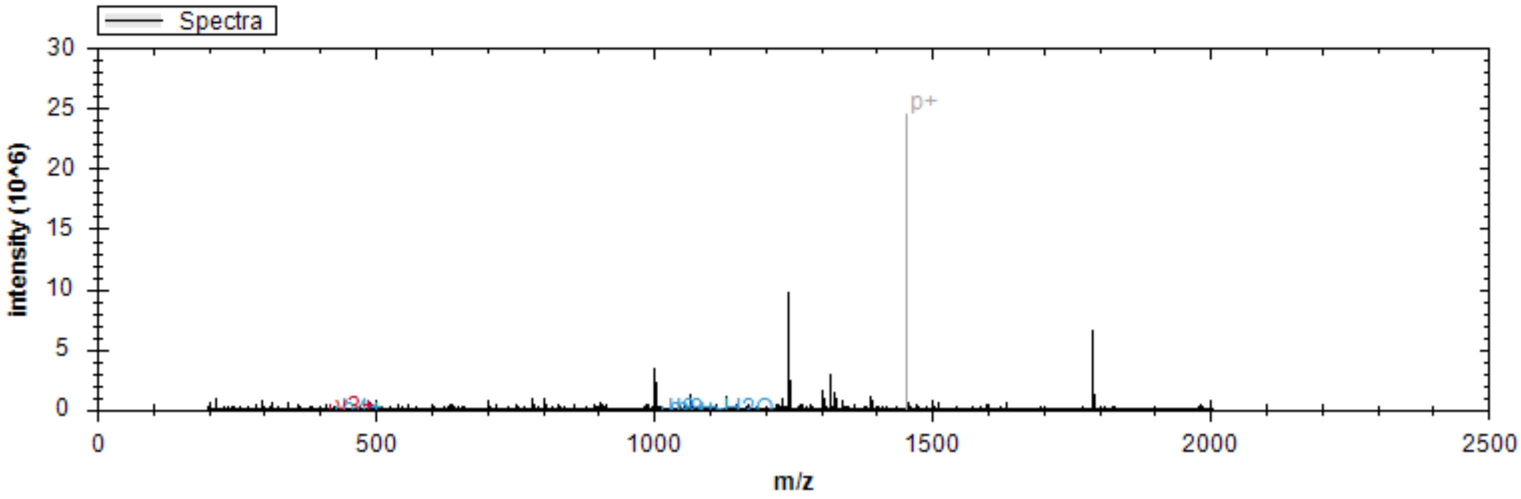

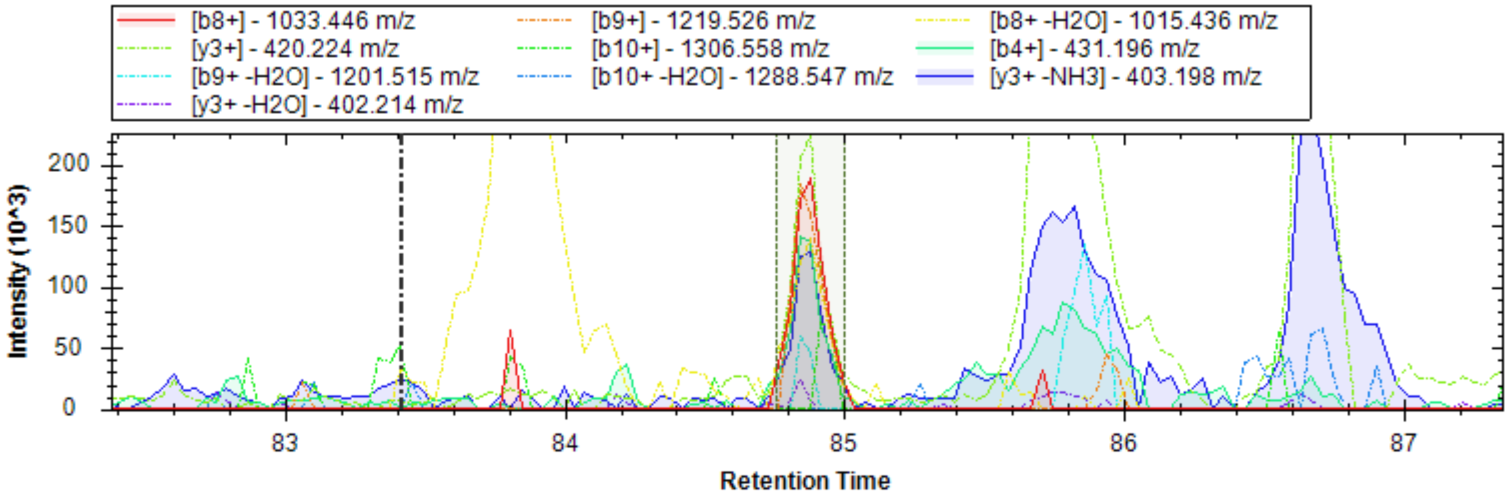

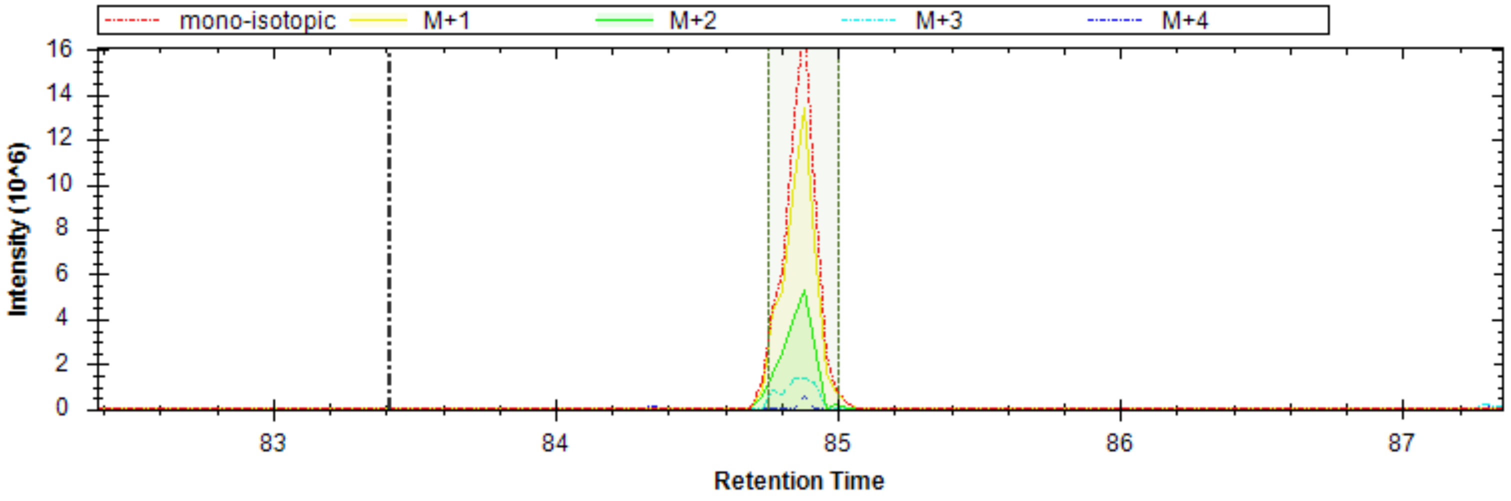

P14873 – _ETAAAHQAS[+H+O3+P]SSPPIDAATAEPYGFR_.2

Fig4_MP-DIA-barrel-cortex-p9-3_MHRM.htrms

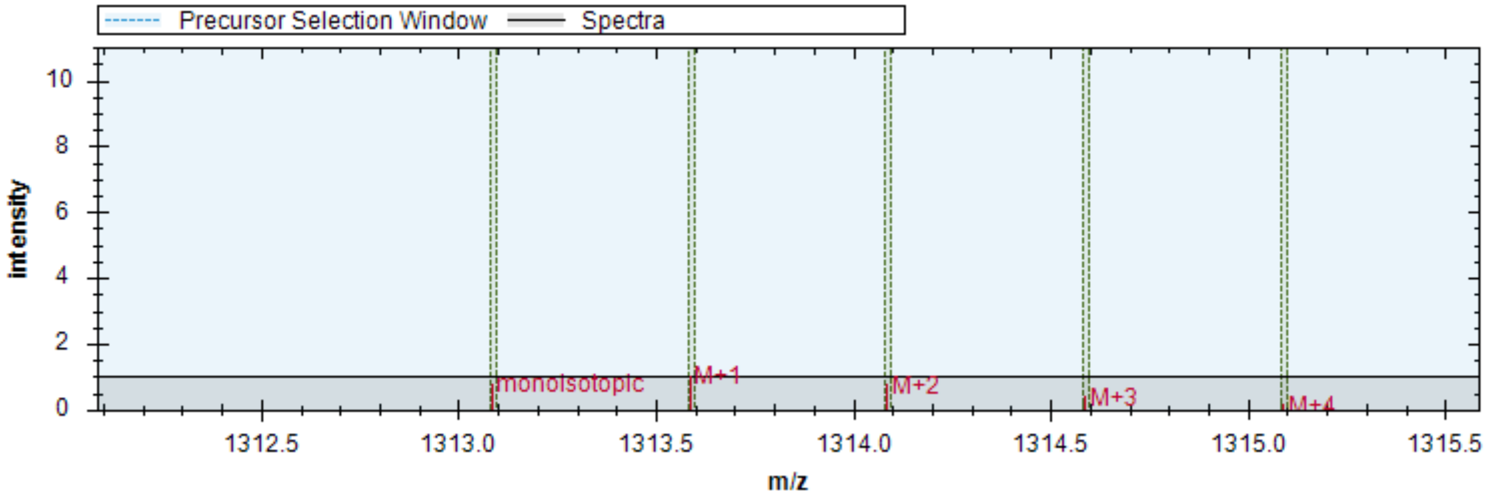

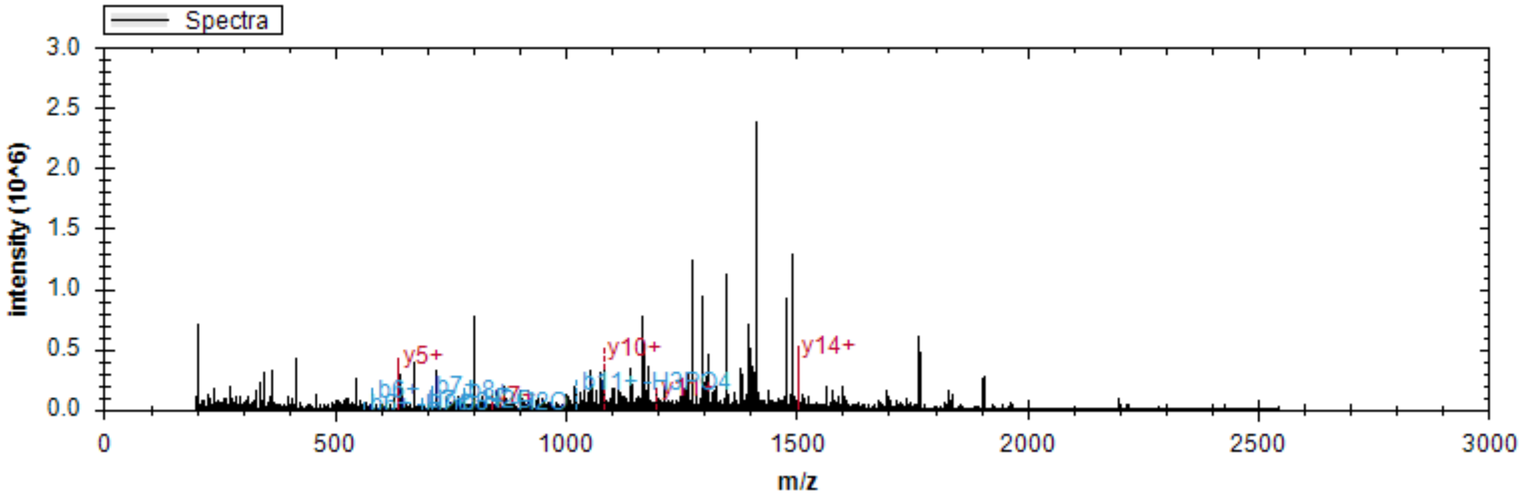

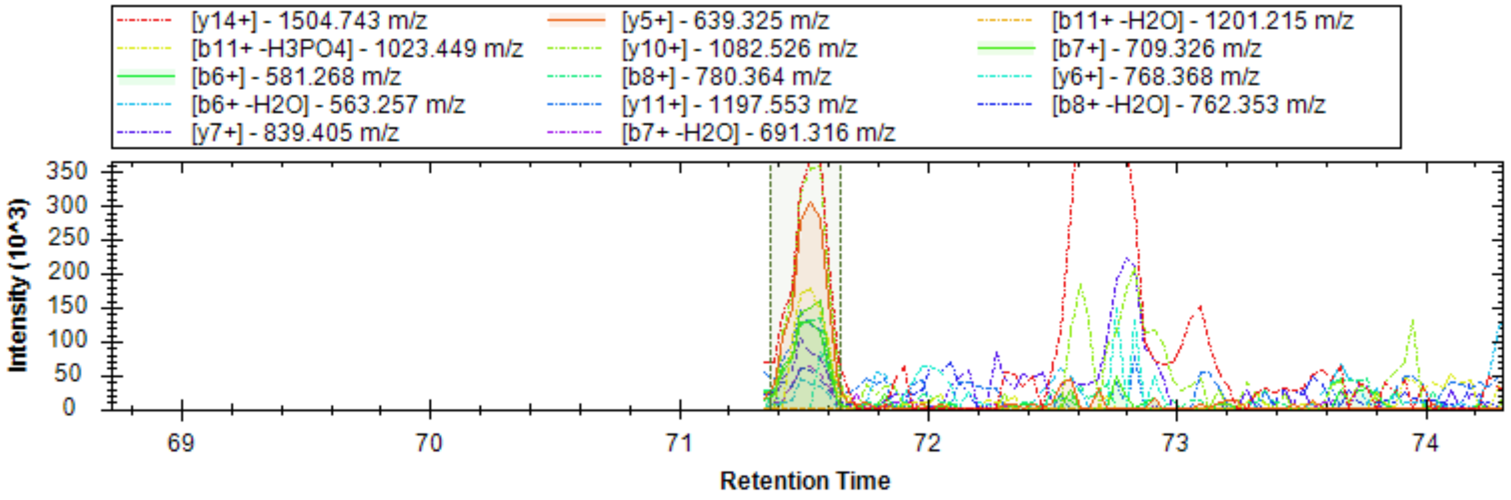

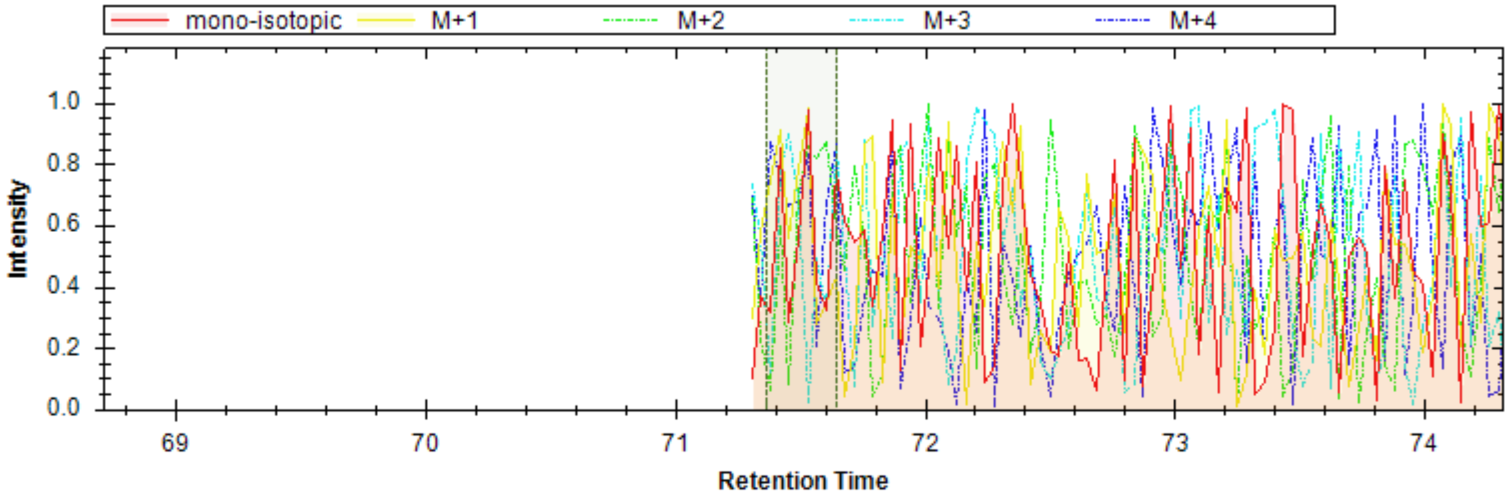

P14873 – _ETAAAHQASS[+H+O3+P]SPPIDAATAEPYGFR_.2
Fig4_MP-DIA-barrel-cortex-p9-3_MHRM.htrms

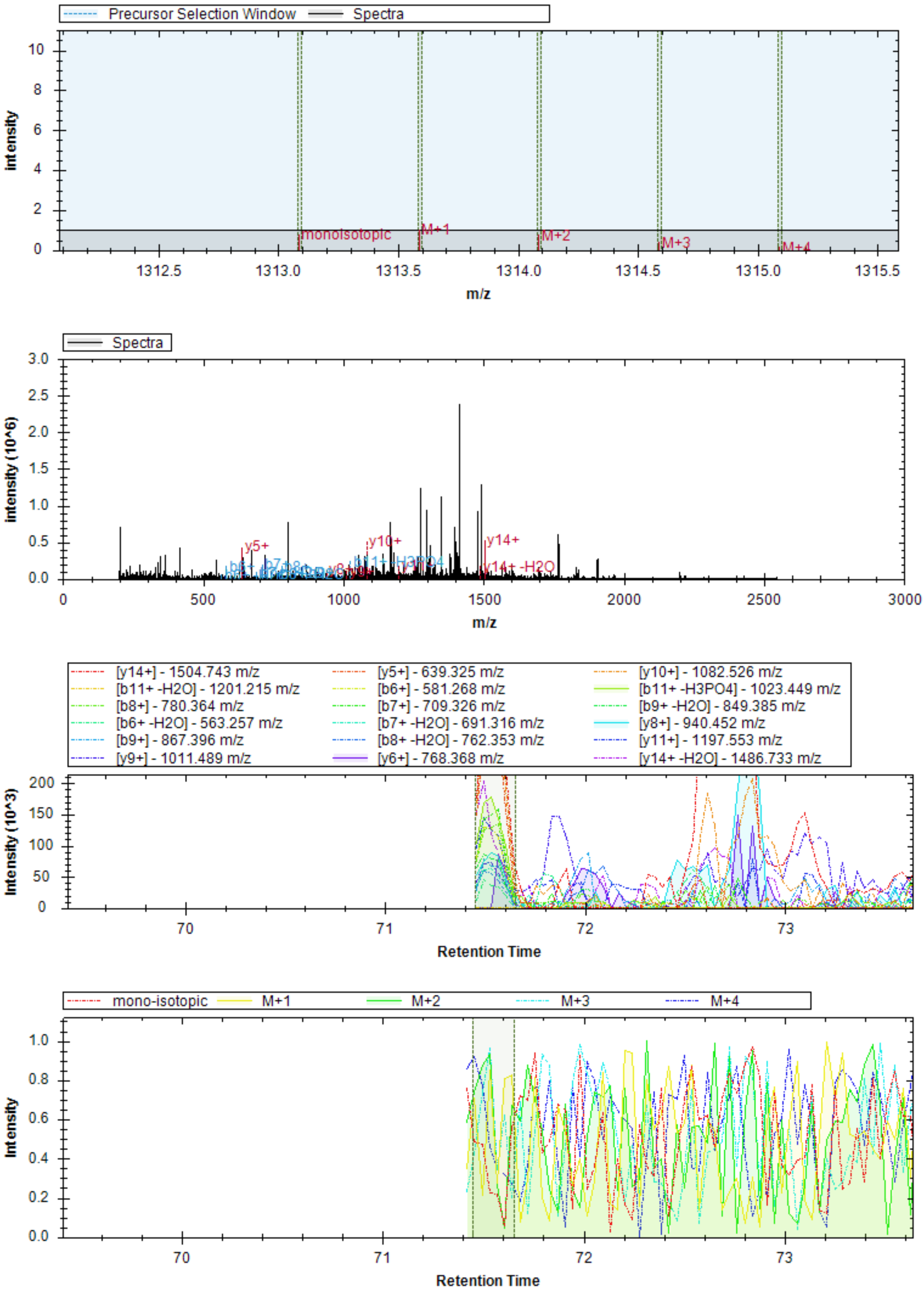

P20357 – _SGTSTPTTPGSTAIT[+H+O3+P]PGTPPSYSSR_.2

Fig4_MP-DIA-barrel-cortex-p9-3_MHRM.htrms

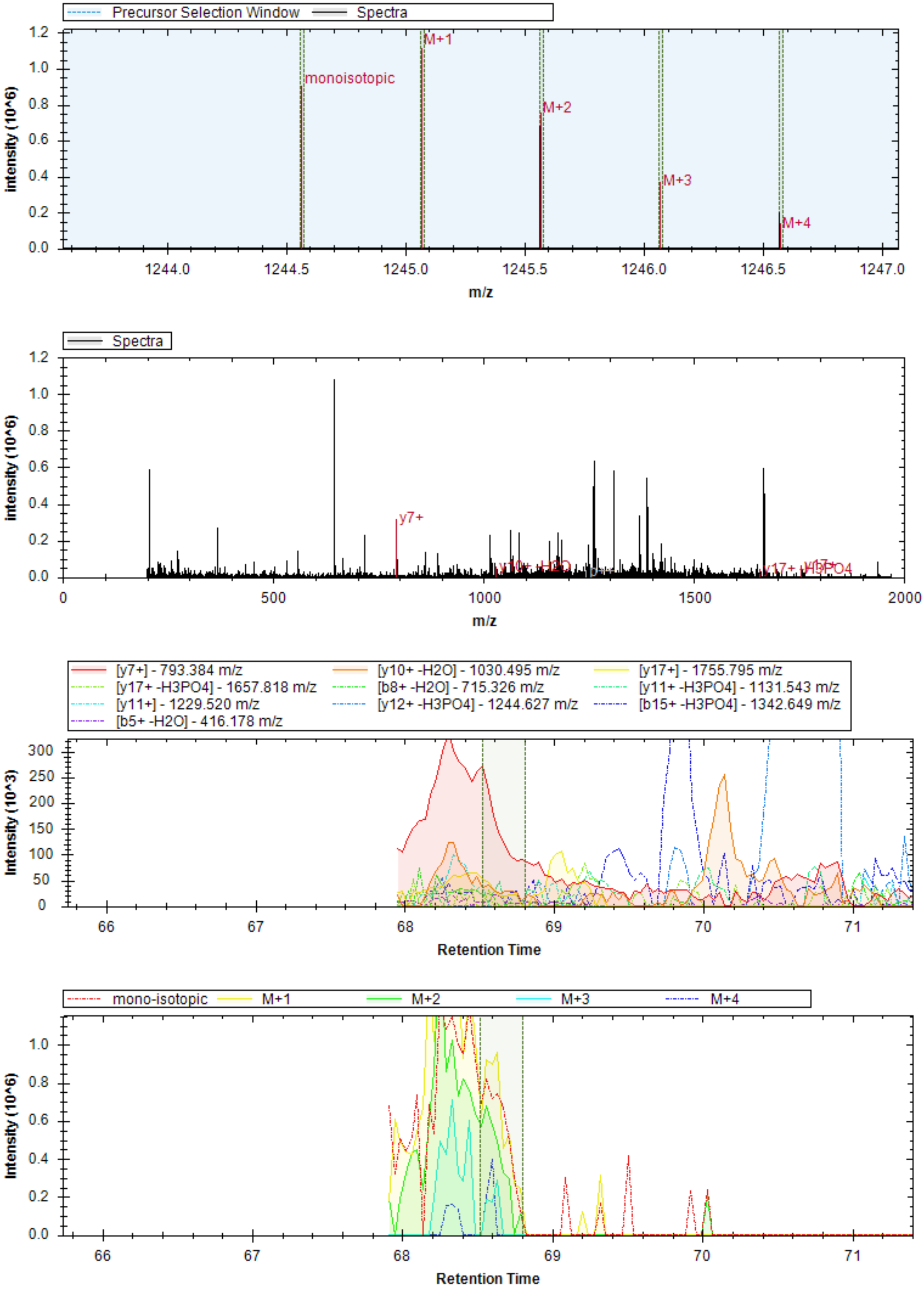

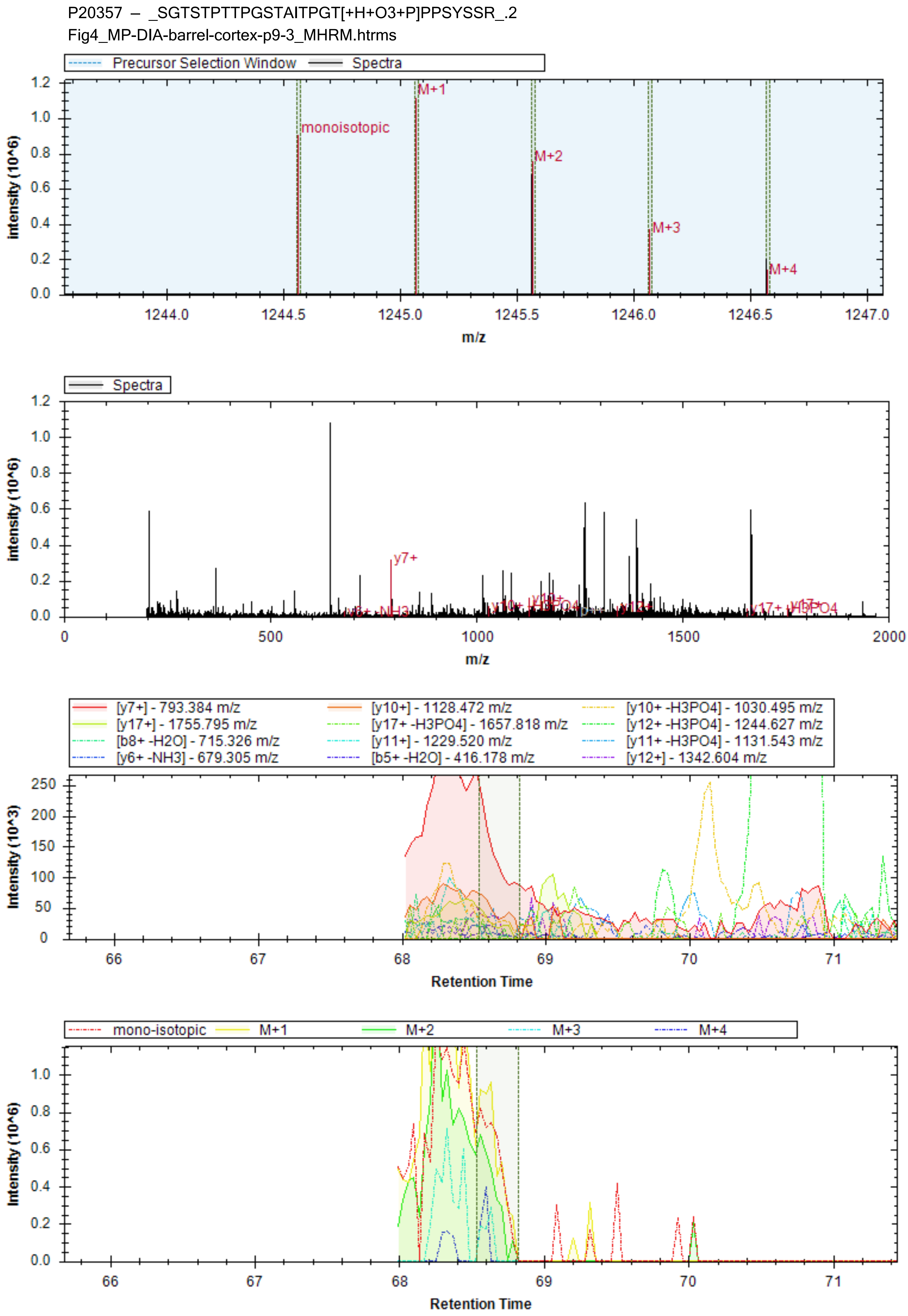

P20357 – _SGTSTPTTPGSTAITPGT[+H+O3+P]PPSYSSR_.2
Fig4_MP-DIA-barrel-cortex-p9-3_MHRM.htrms
Precursor Selection Window
Spectra
monoisotopic
M+1
M+2
M+3
M+4
intensity (10^6)
m/z
Spectra
y7+
intensity (10^6)
m/z
[y7+] - 793.384 m/z
[y10+] - 1128.472 m/z
[y10+ -H3PO4] - 1030.495 m/z
[y17+] - 1755.795 m/z
[y17+ -H3PO4] - 1657.818 m/z
[y12+ -H3PO4] - 1244.627 m/z
[b8+ -H2O] - 715.326 m/z
[y11+] - 1229.520 m/z
[y11+ -H3PO4] - 1131.543 m/z
[y6+ -NH3] - 679.305 m/z
[b5+ -H2O] - 416.178 m/z
[y12+] - 1342.604 m/z
Intensity (10^3)
Retention Time
mono-isotopic
M+1
M+2
M+3
M+4
Intensity (10^6)
Retention Time

Q8BRT1 – _VLNT[+H+O3+P]GSDVEEAVADALLLGDIR_.2
Fig4_MP-DIA-barrel-cortex-p9-3_MHRM.htrms

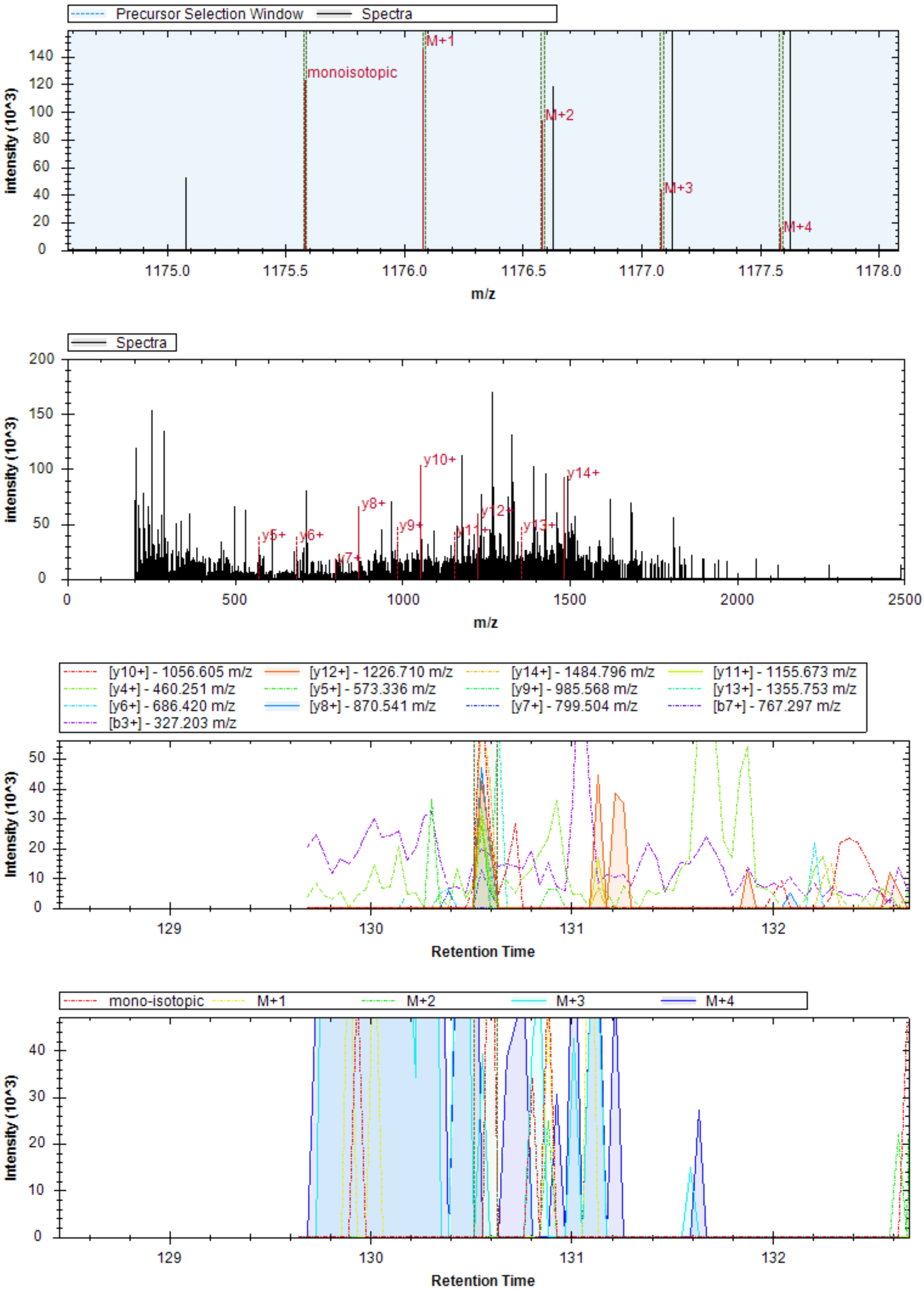

Q8VHY0 – _LLAGPRY[+H+O3+P]GR_.1
Fig4_MP-DIA-barrel-cortex-p9-3_MHRM.htrms

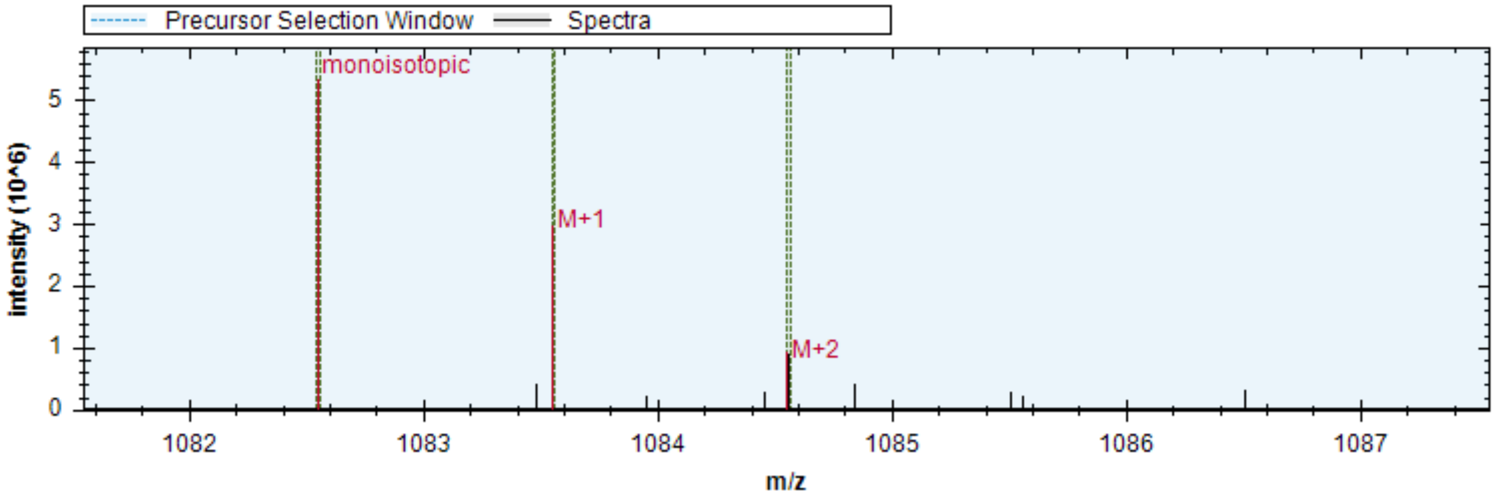

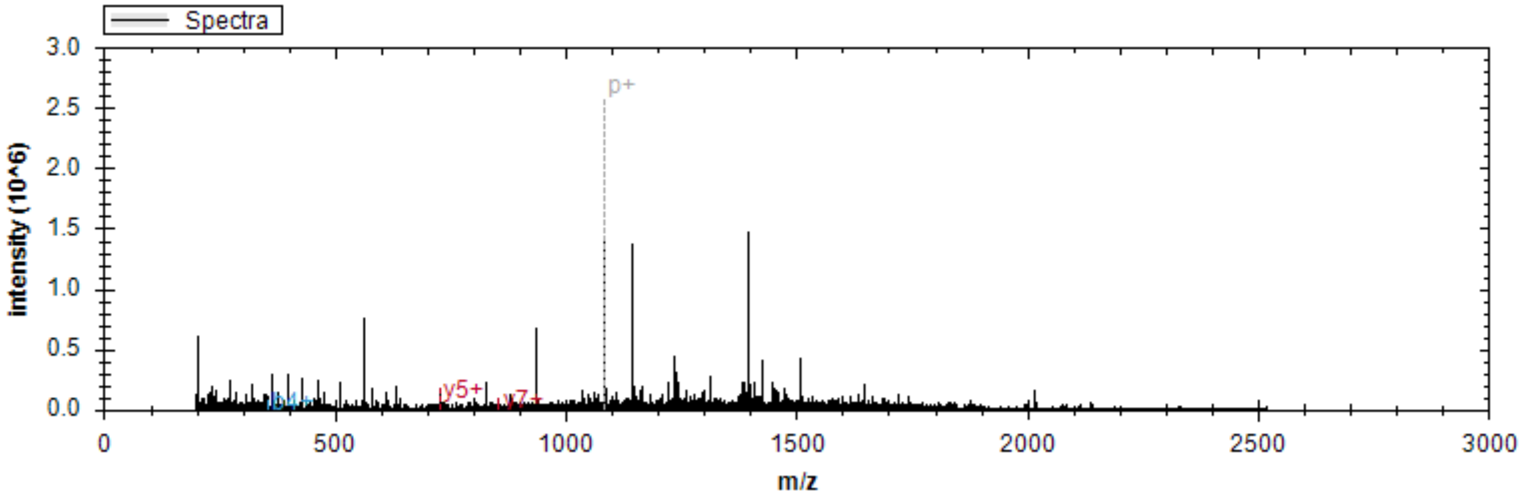

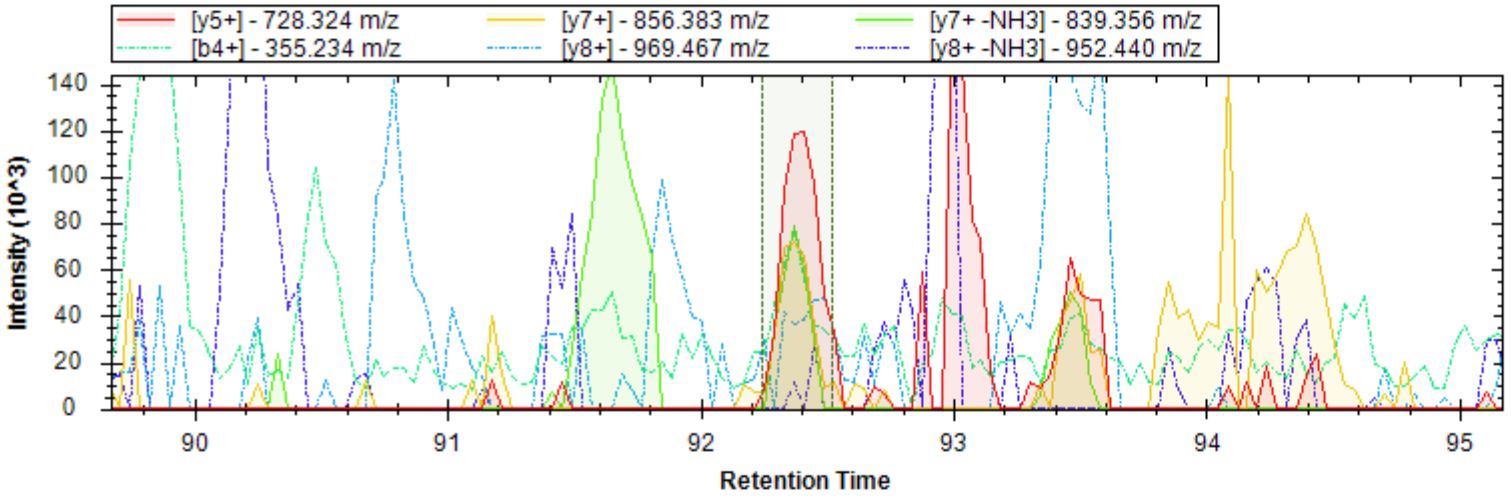

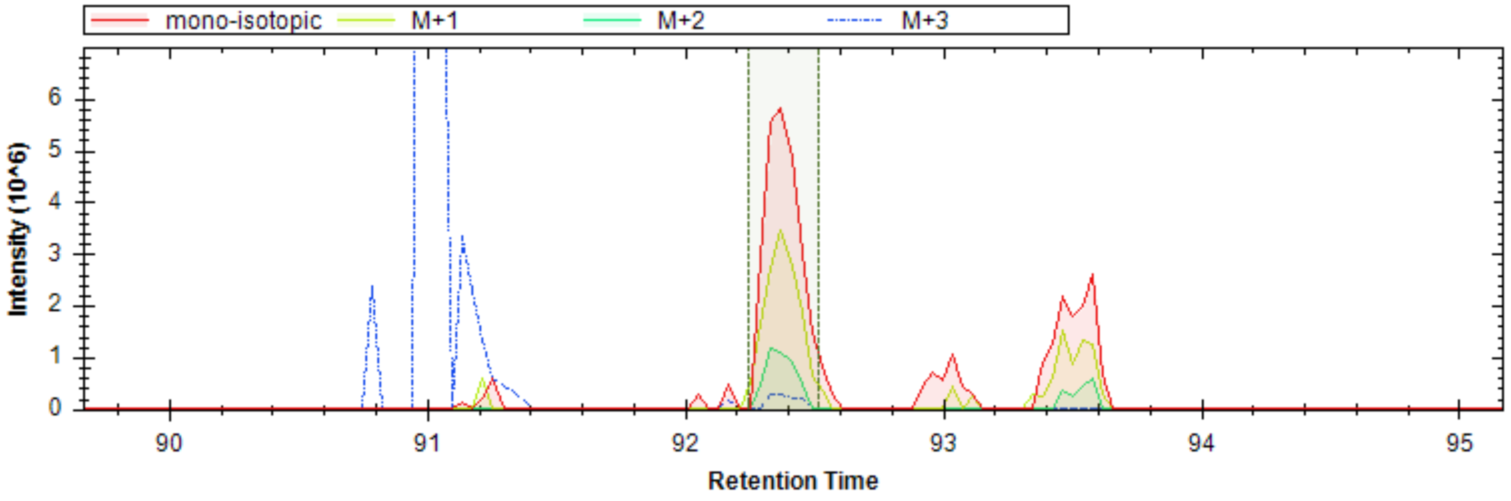

Q921N6;Q921N6-2 – _LIY[+H+O3+P]KPRQAAVTR_.4
Fig4_MP-DIA-barrel-cortex-p15-1_MHRM.htrms

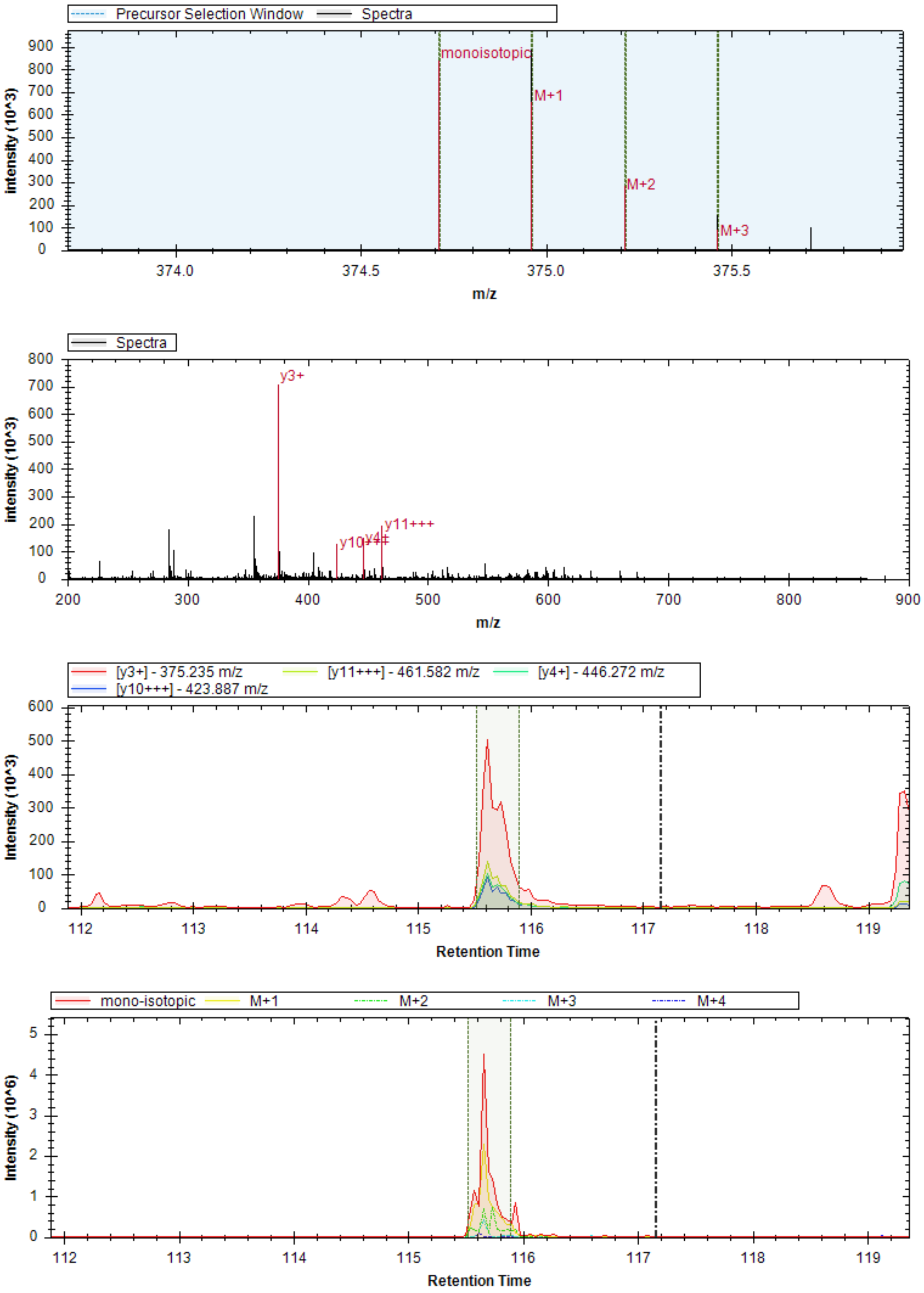

Q9CVB6 – _IIEET[+H+O3+P]LALK_.3
Fig4_MP-DIA-barrel-cortex-p15-1_MHRM.htrms

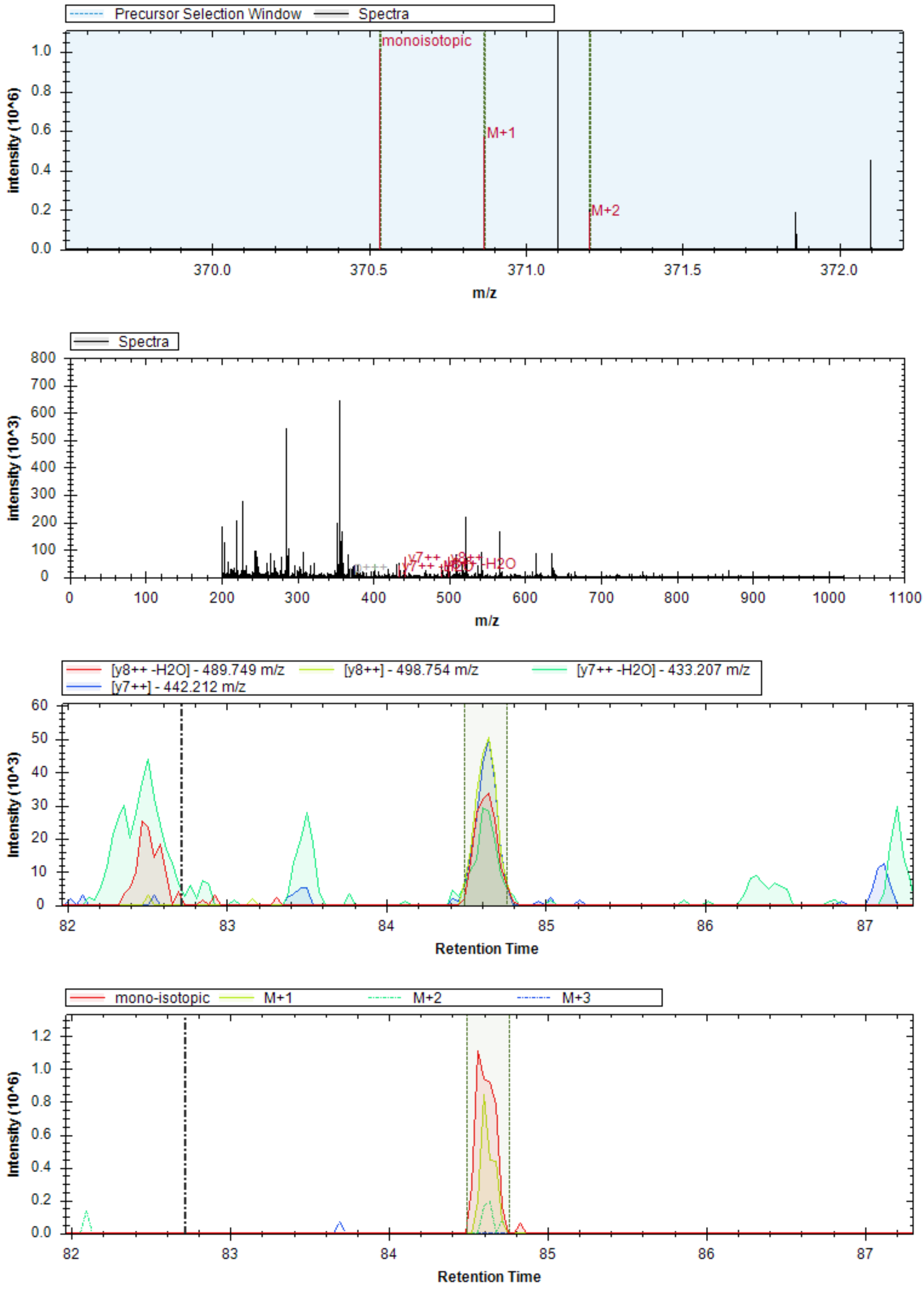

Q9D516;Q9D516-2 – _KLS[+H+O3+P]QGILVIR_.3
Fig4_MP-DIA-barrel-cortex-p15-1_MHRM.htrms

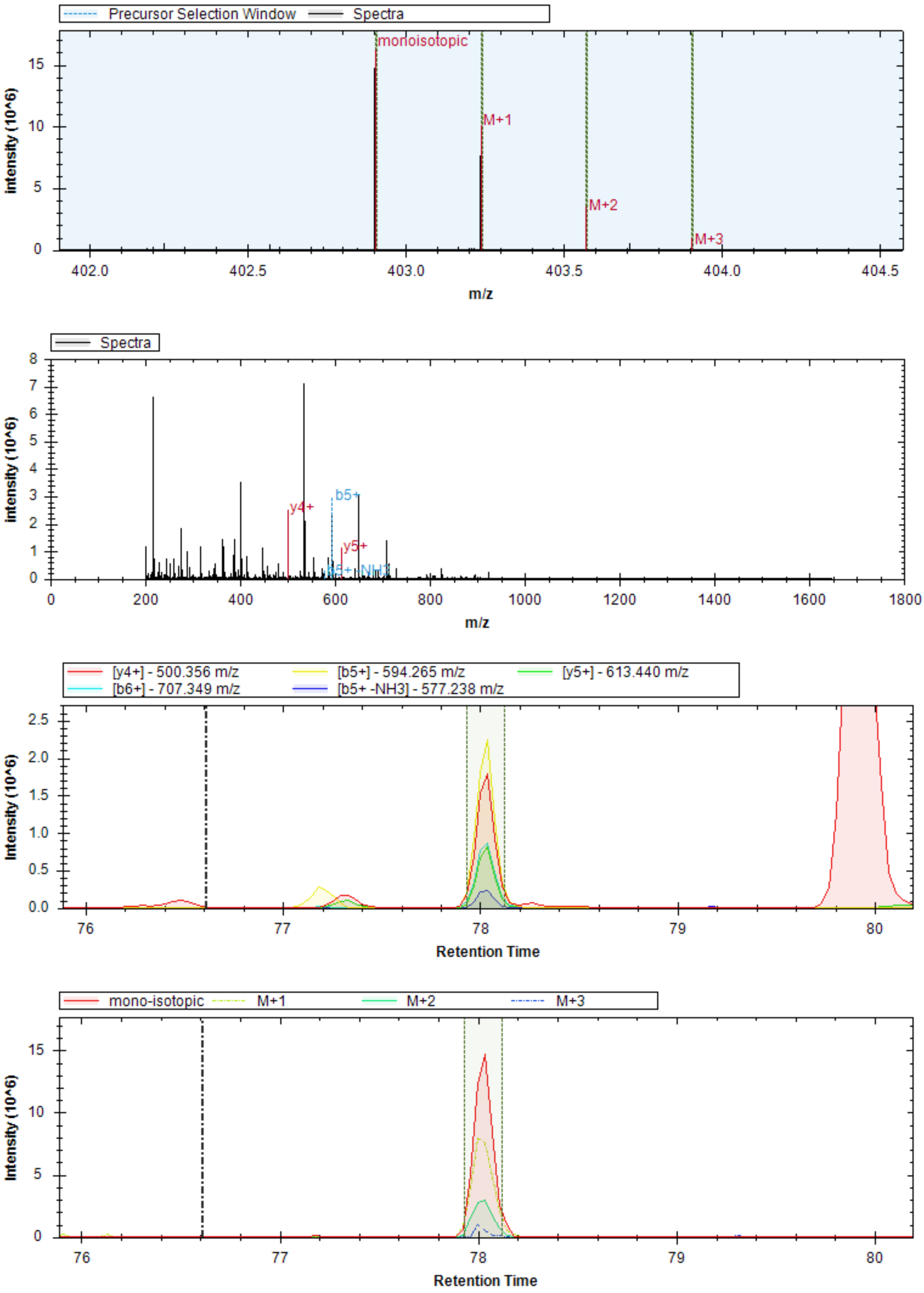

P20357 – _KSEVQAHS[+H+O3+P]PSR_.3
Fig4_MP-DIA-barrel-cortex-p15-1_MHRM.htrms

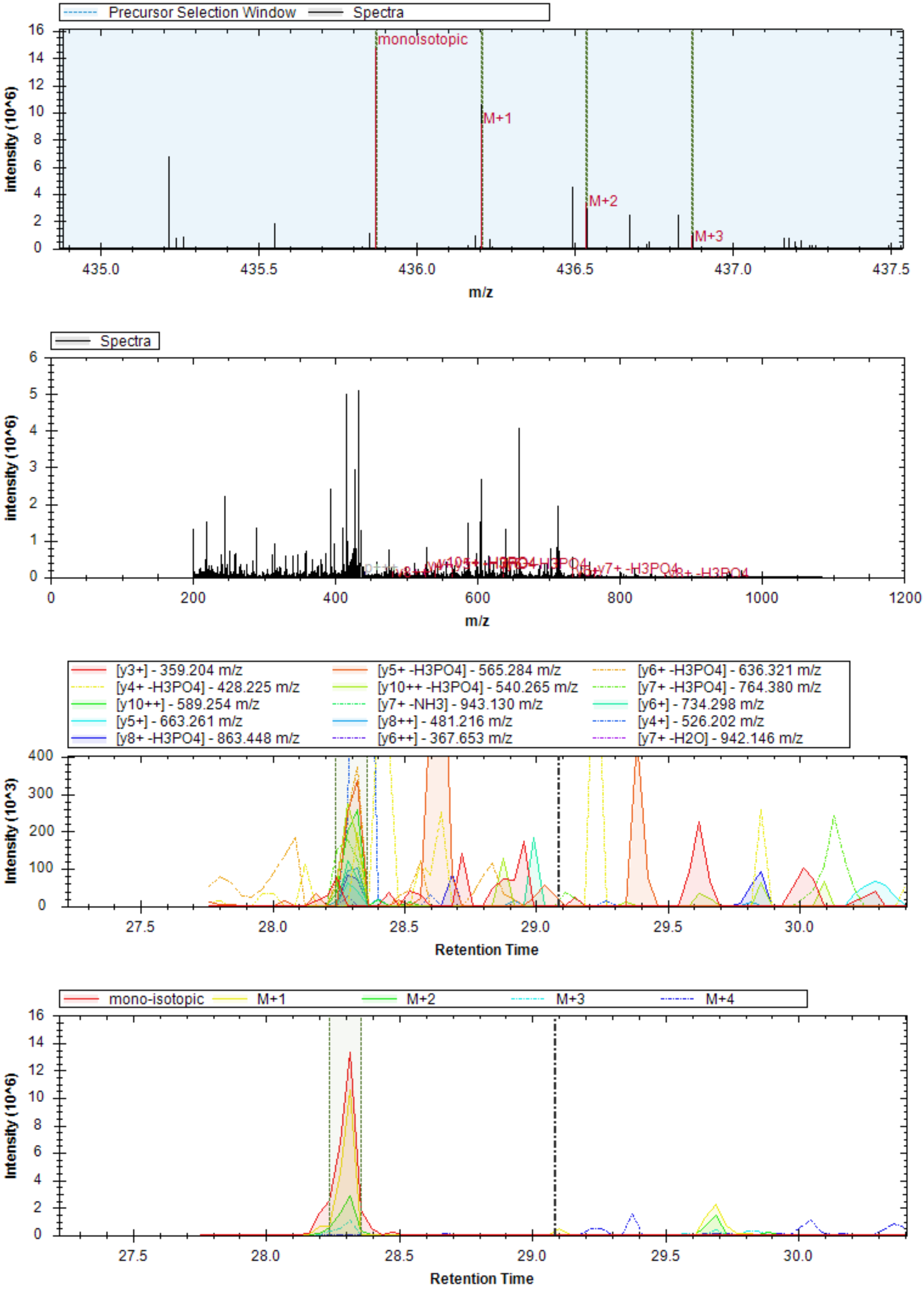

Q03172 – _VALSLLNS[+H+O3+P]KQK_.3

Fig4_MP-DIA-barrel-cortex-p15-1_MHRM.htrms

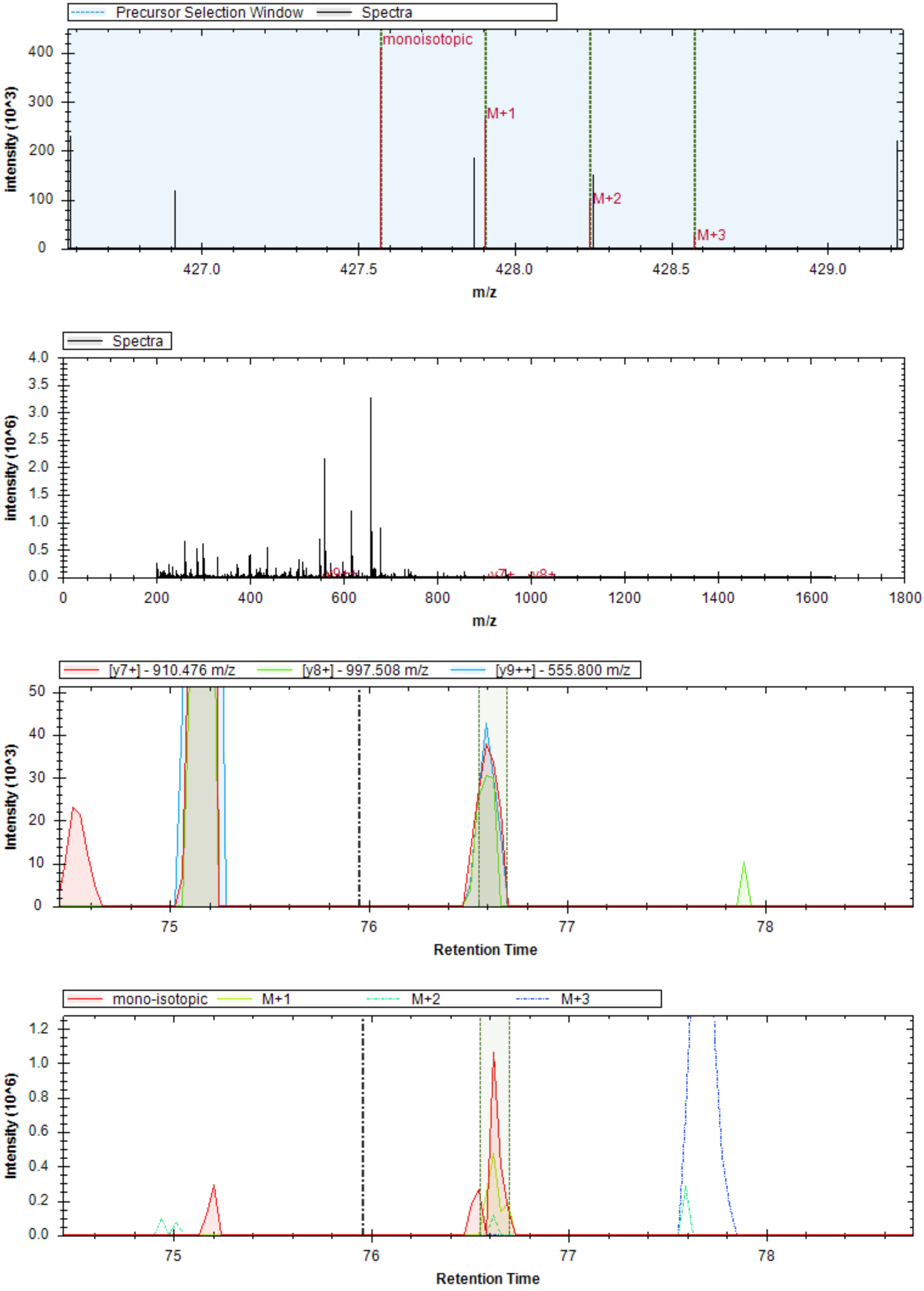

Q52KI8;Q52KI8-2 – _RES[+H+O3+P]PSPAPKPR_.3
Fig4_MP-DIA-barrel-cortex-p15-1_MHRM.htrms

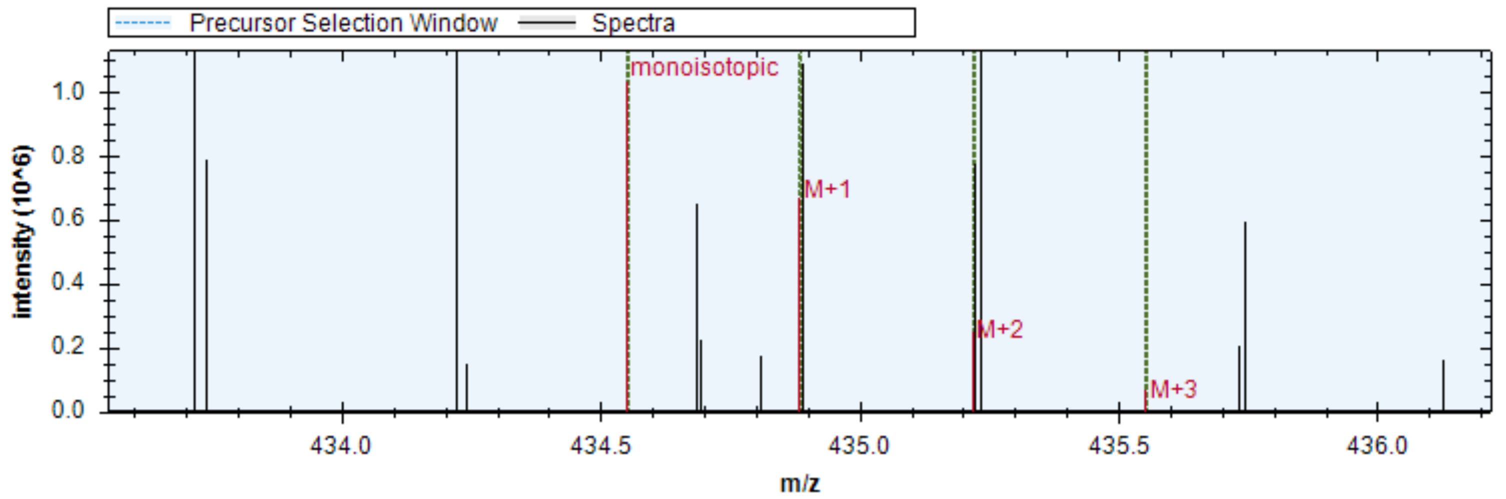

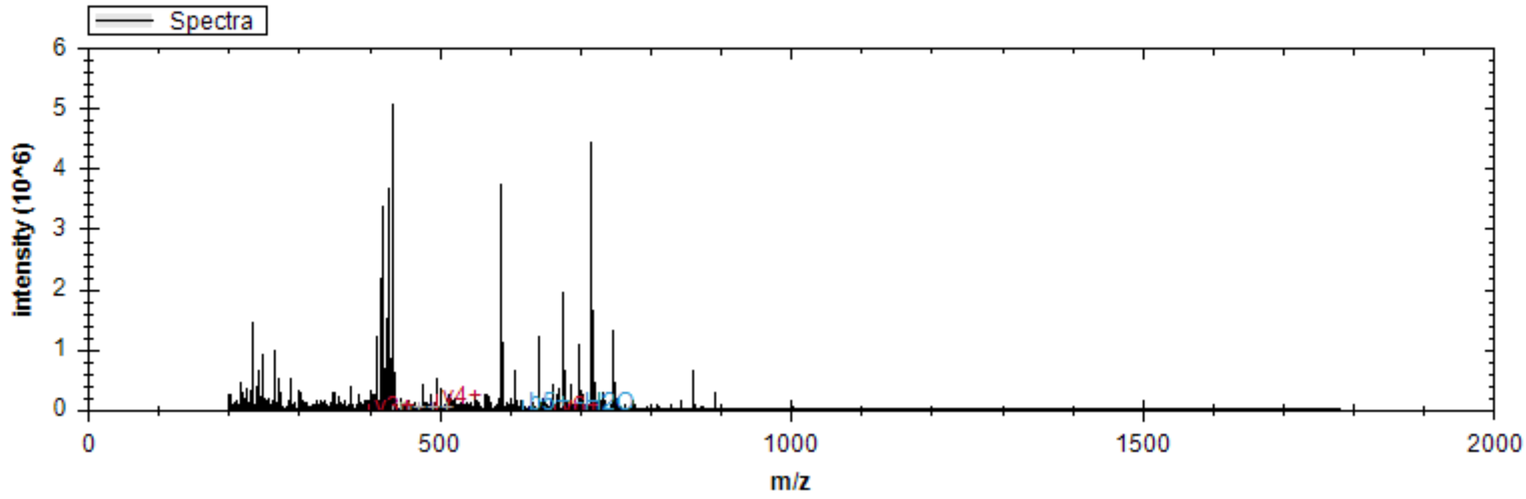

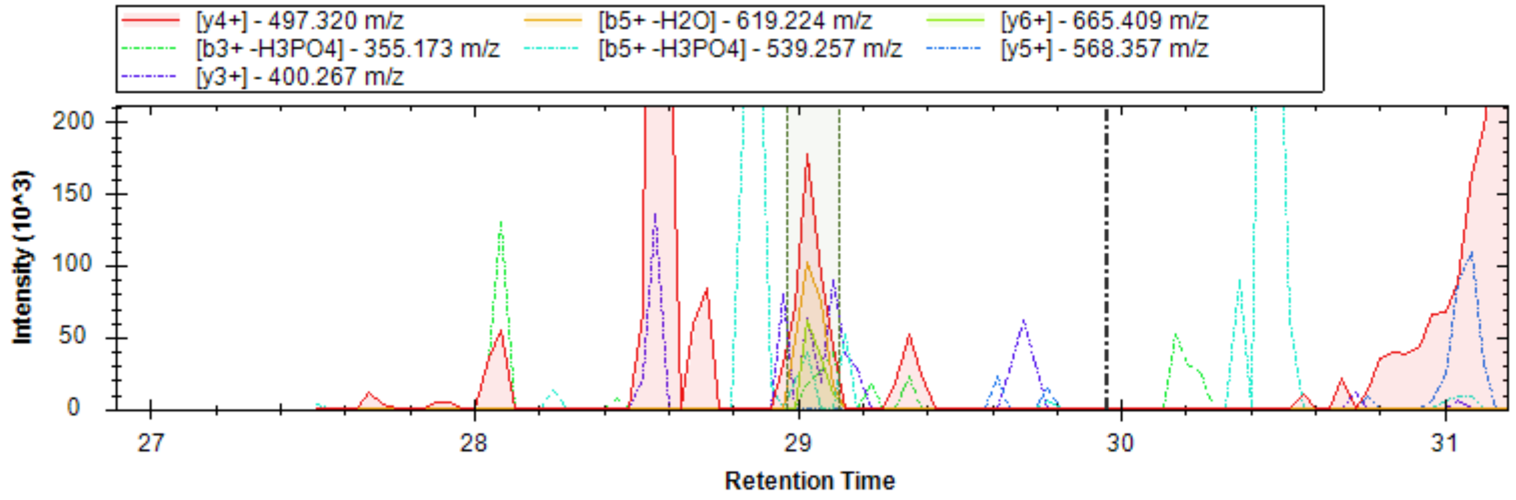

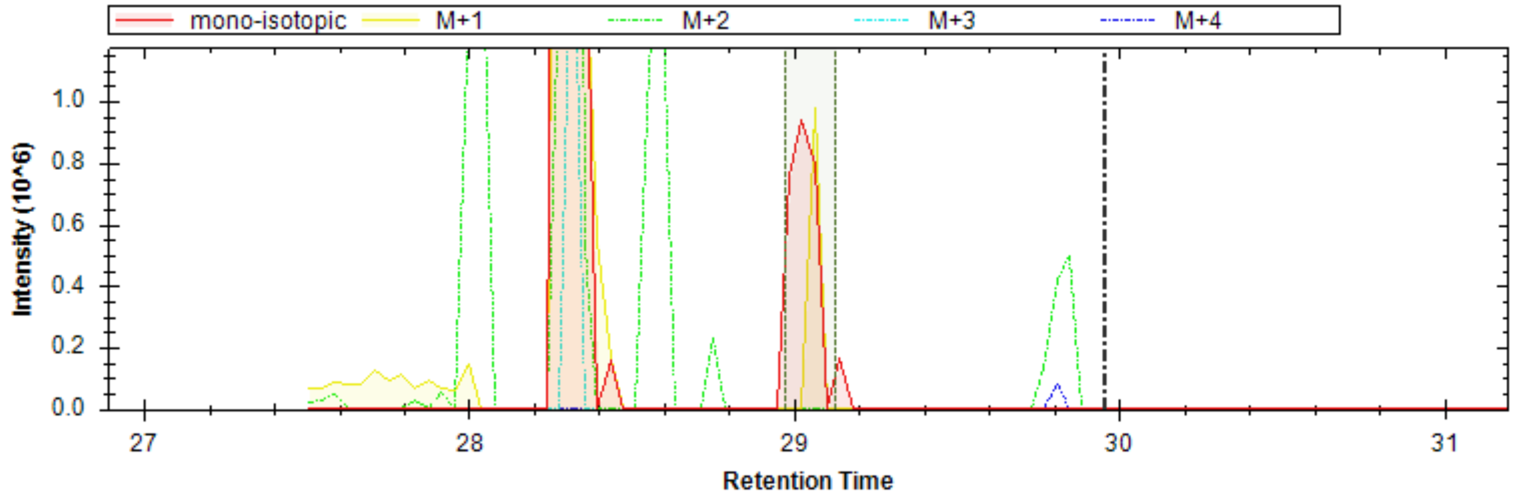

Q62188 – _GS[+H+O3+P]PTRPNPPVR_.3
Fig4_MP-DIA-barrel-cortex-p15-1_MHRM.htrms

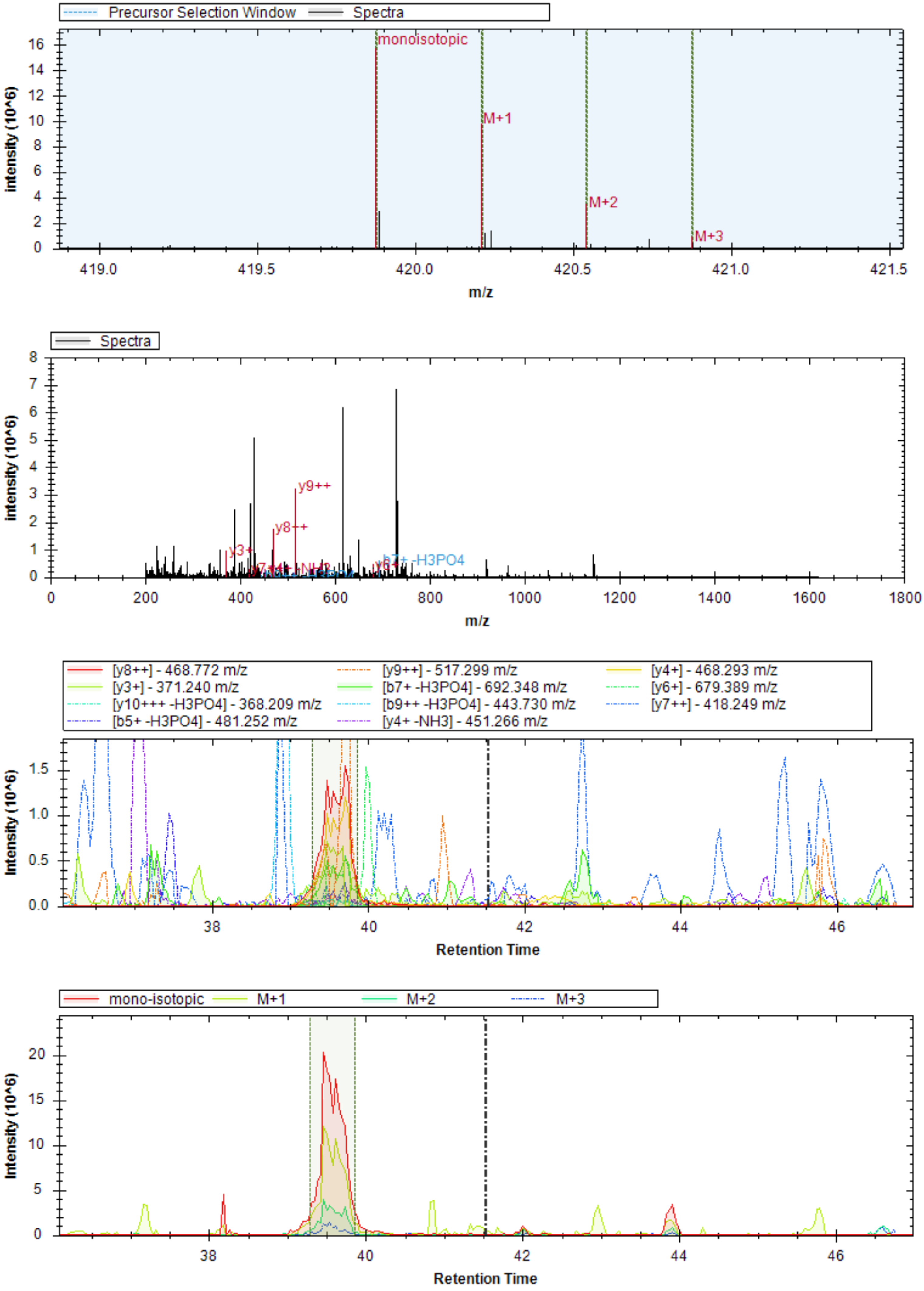

Q93092 – _[+C2+H2+O]SGS[+H+O3+P]PVKR_.2
Fig4_MP-DIA-barrel-cortex-p15-1_MHRM.htrms

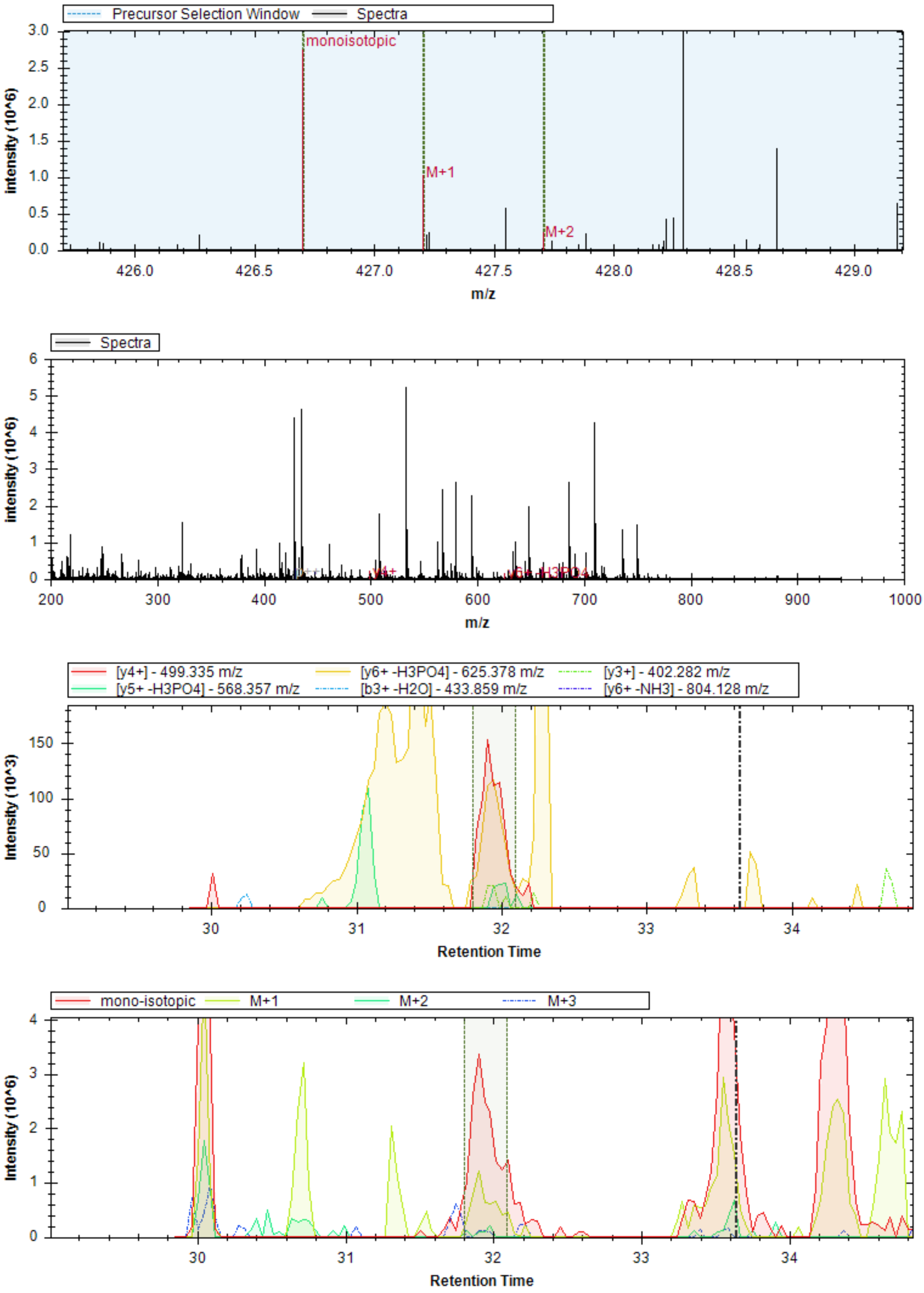

O08553 – _TVT[+H+O3+P]PASSAK_.2
Fig4_MP-DIA-barrel-cortex-p15-1_MHRM.htrms

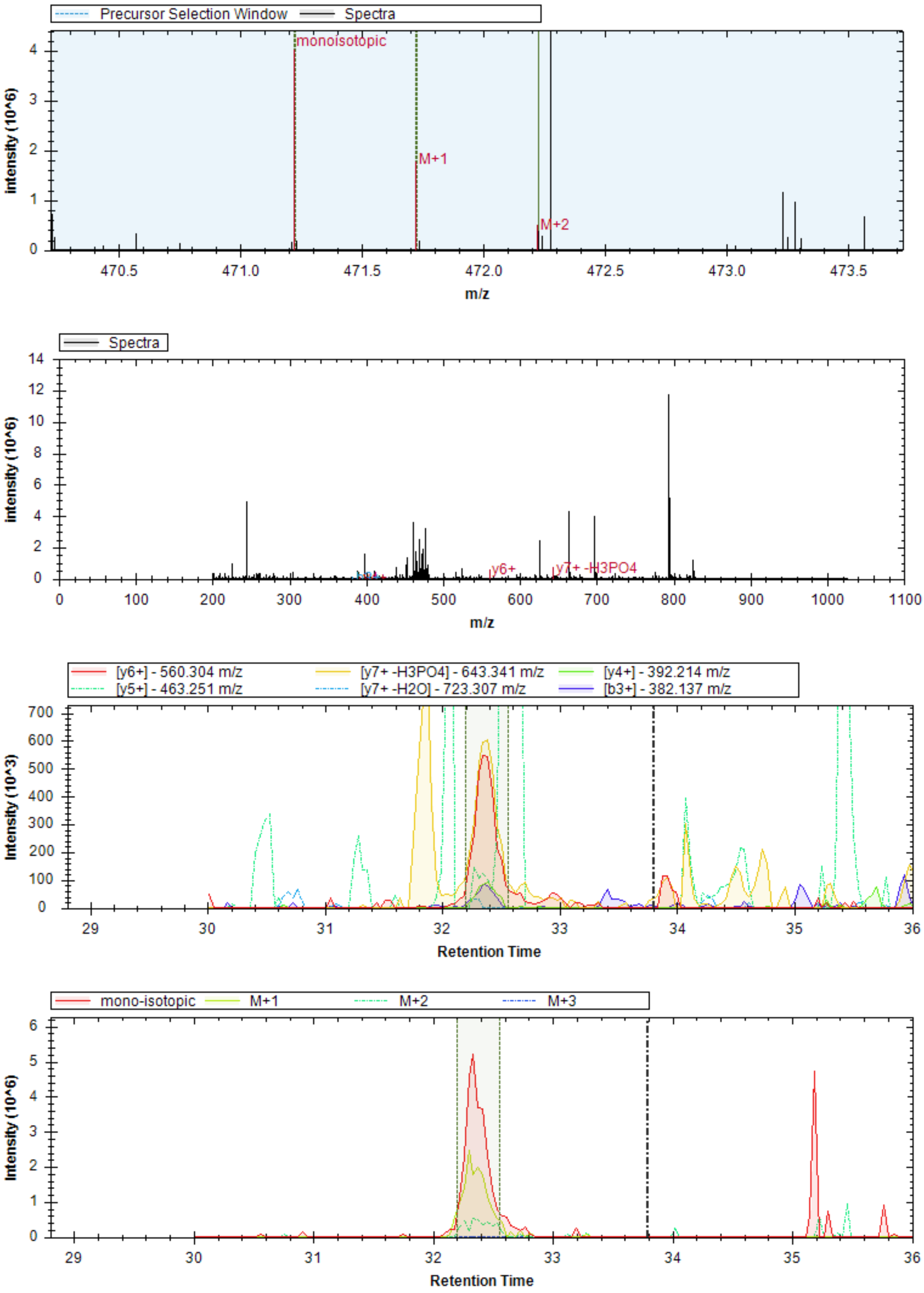

P20357 – _VTDGIS[+H+O3+P]KSPEKR_.3
Fig4_MP-DIA-barrel-cortex-p15-1_MHRM.htrms

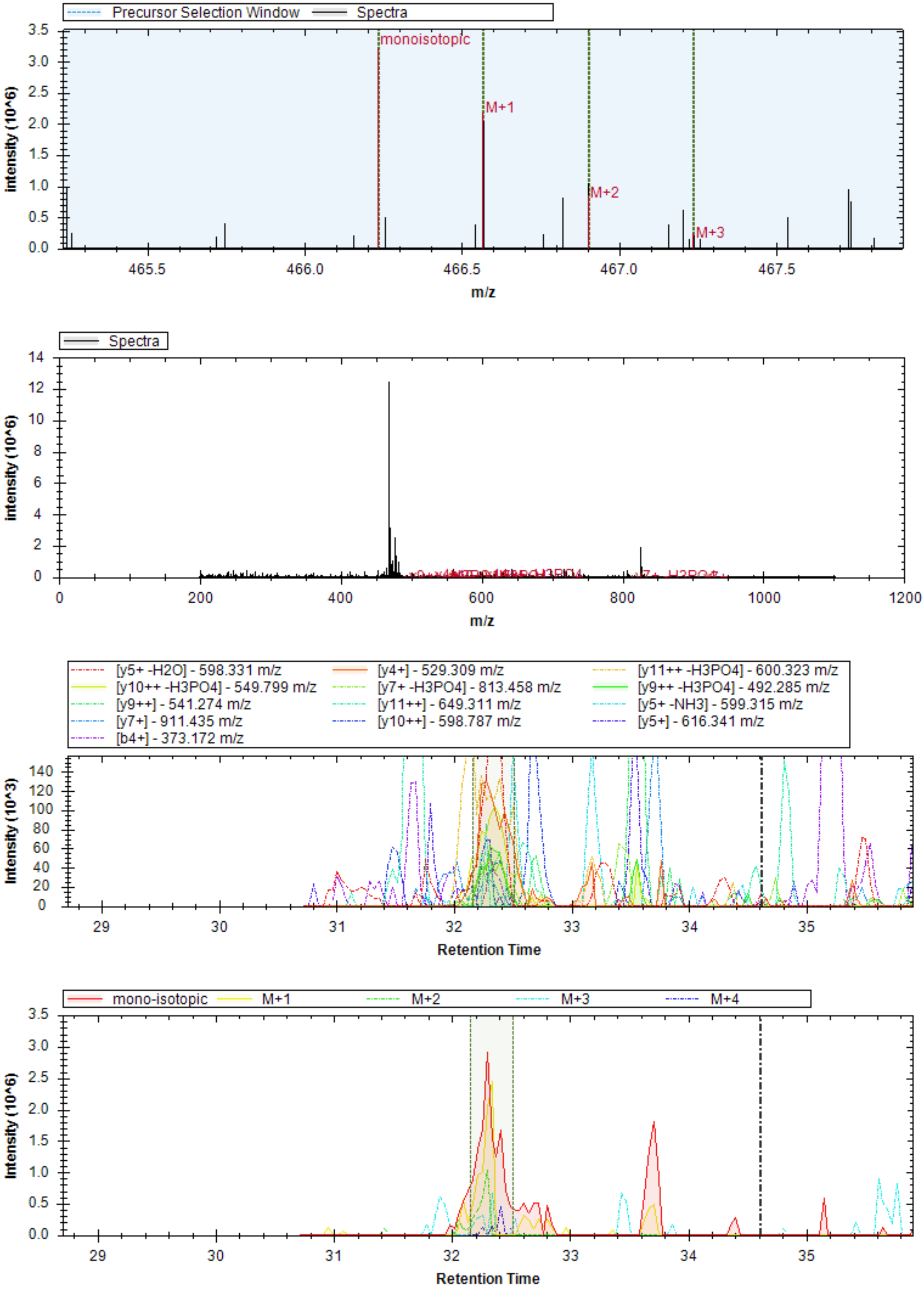

P20357 – _VTDGISKS[+H+O3+P]PEKR_.3

Fig4_MP-DIA-barrel-cortex-p15-1_MHRM.htrms

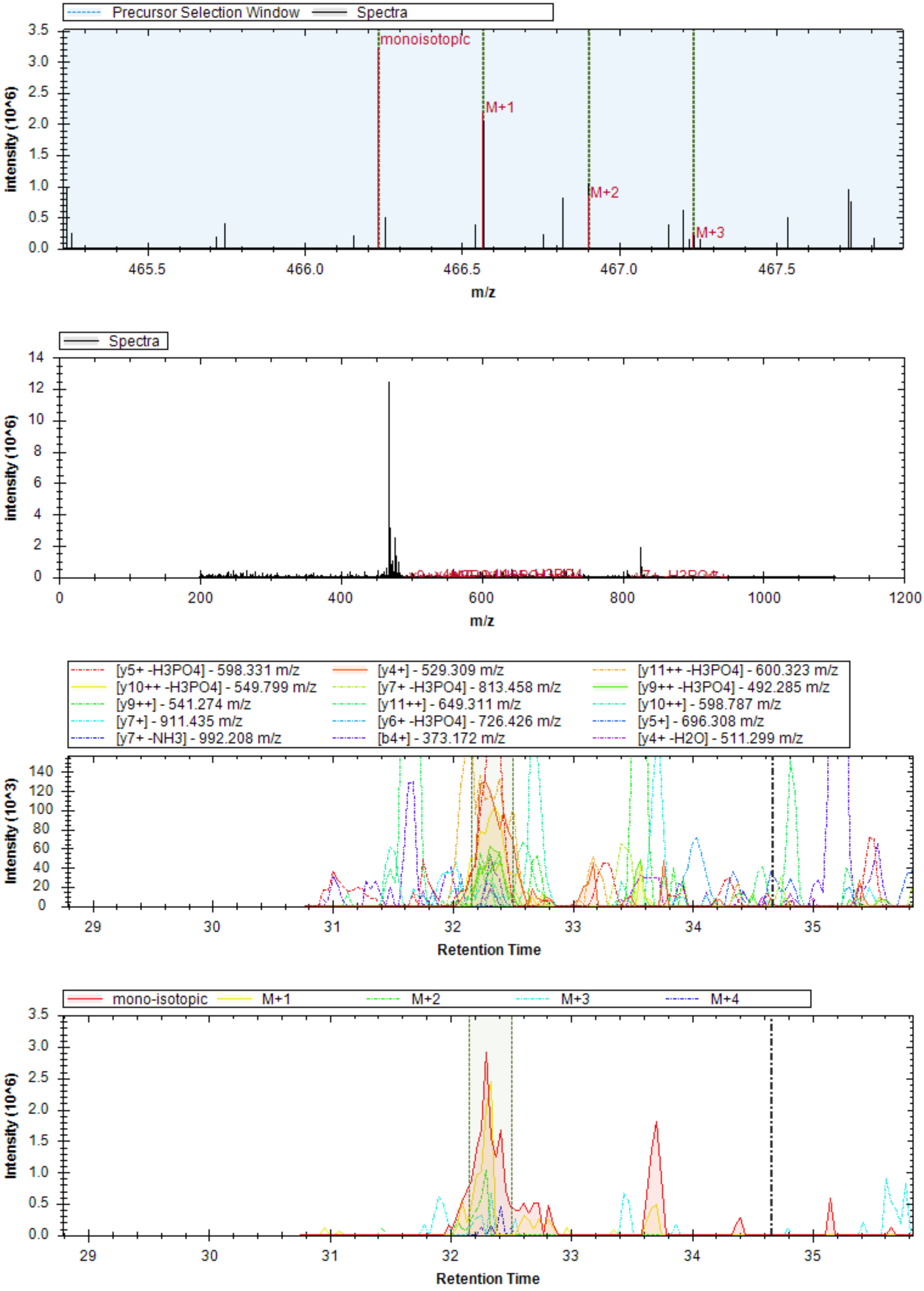

Q9CR81 – _[+C2+H2+O]M[+O]ANHLVKPDS[+H+O3+P]R_.3

Fig4_MP-DIA-barrel-cortex-p15-1_MHRM.htrms

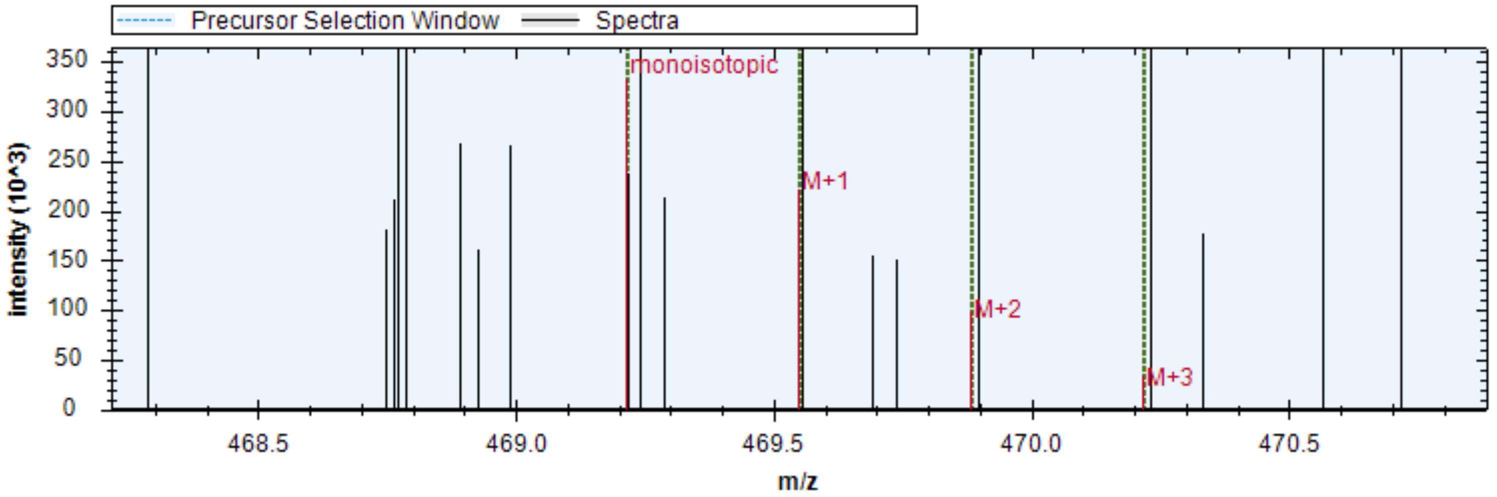

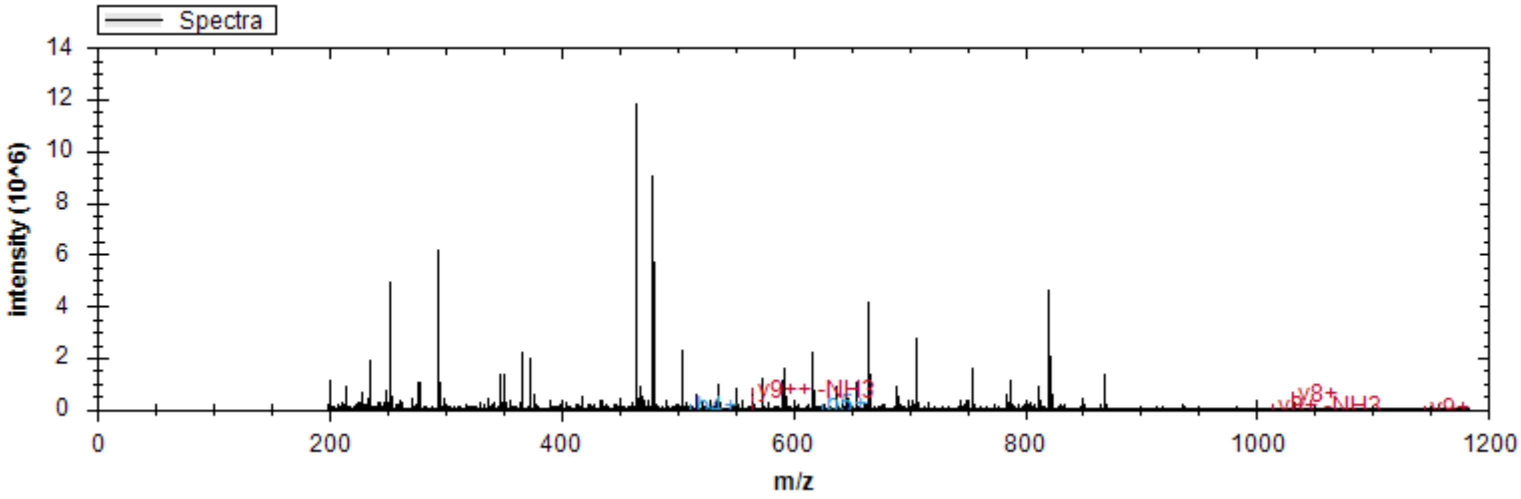

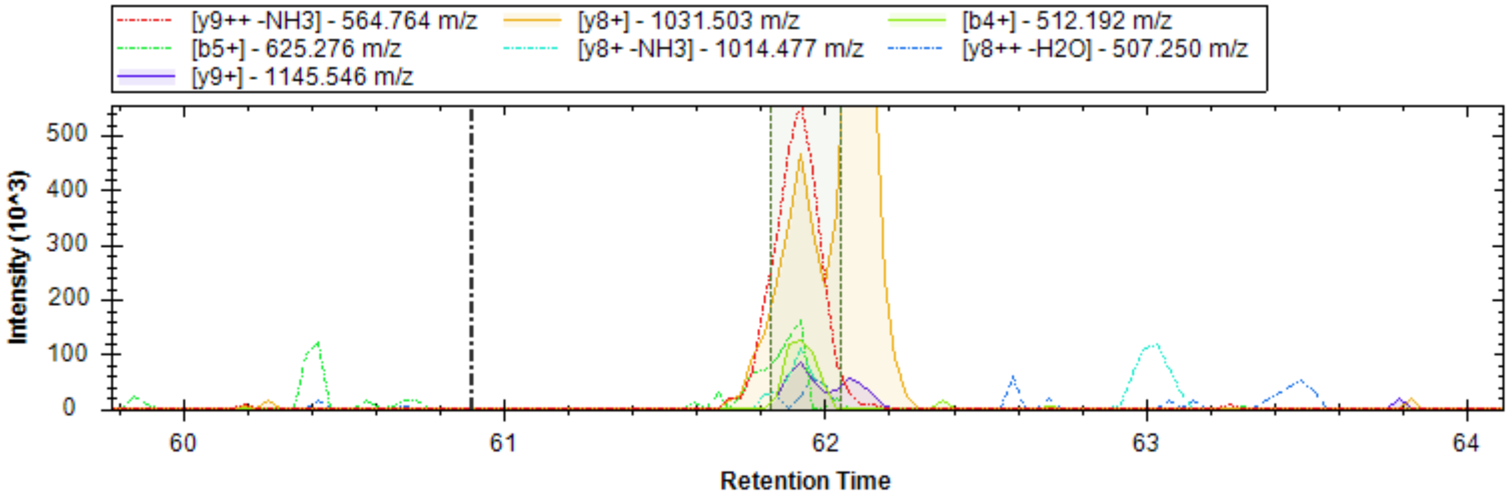

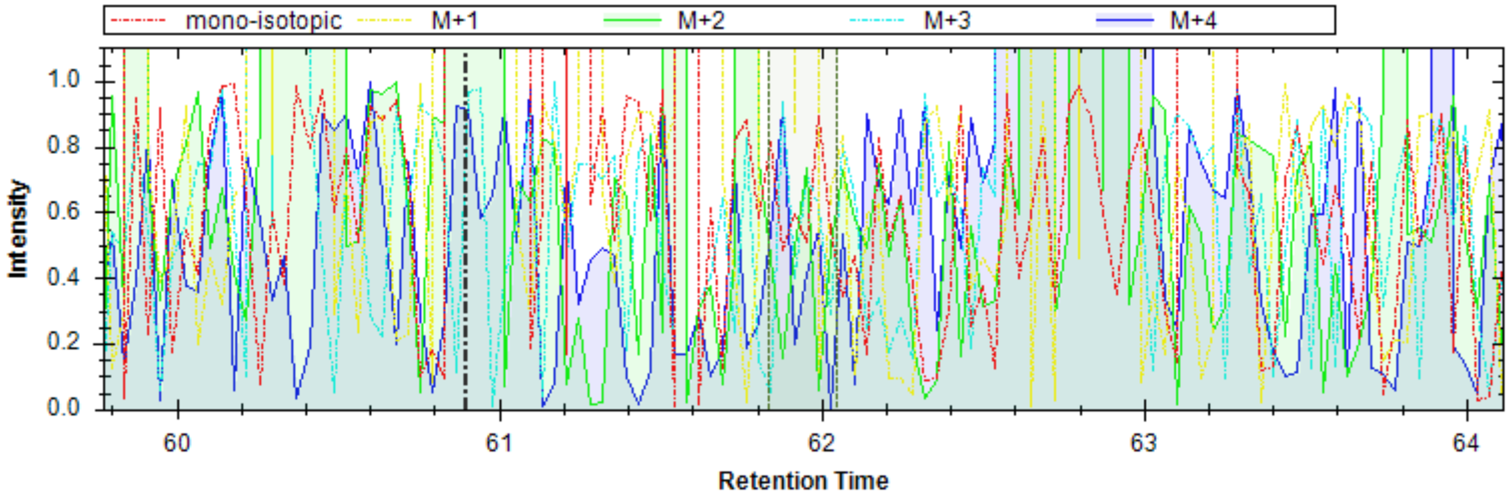

P26645 – _LSGFS[+H+O3+P]FKK_.2

Fig4_MP-DIA-barrel-cortex-p15-1_MHRM.htrms

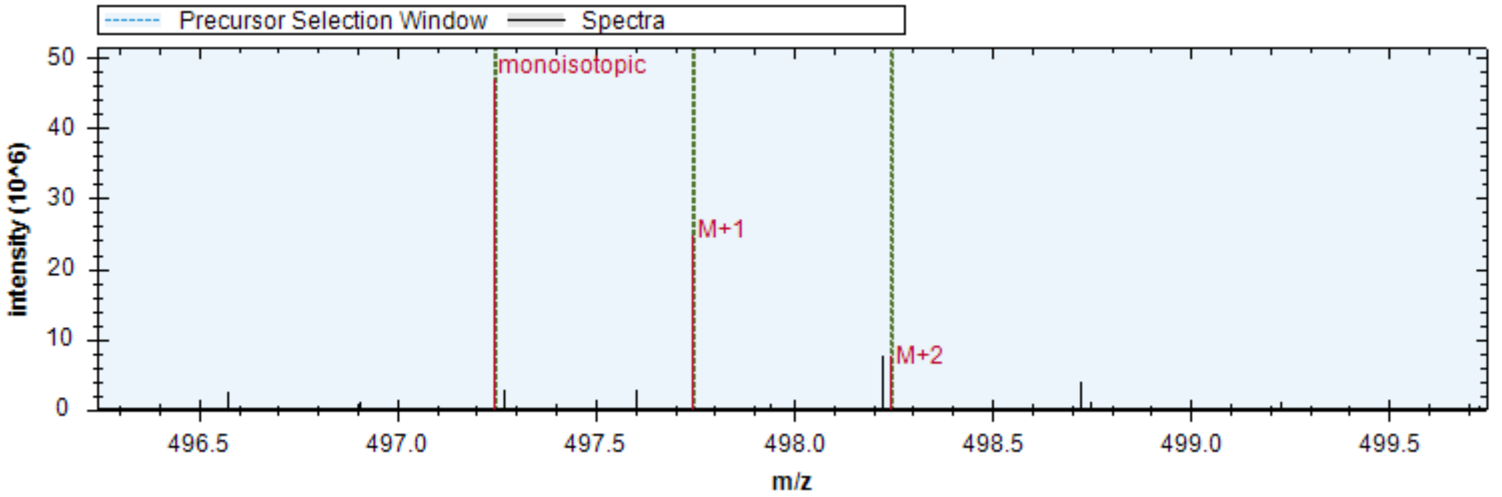

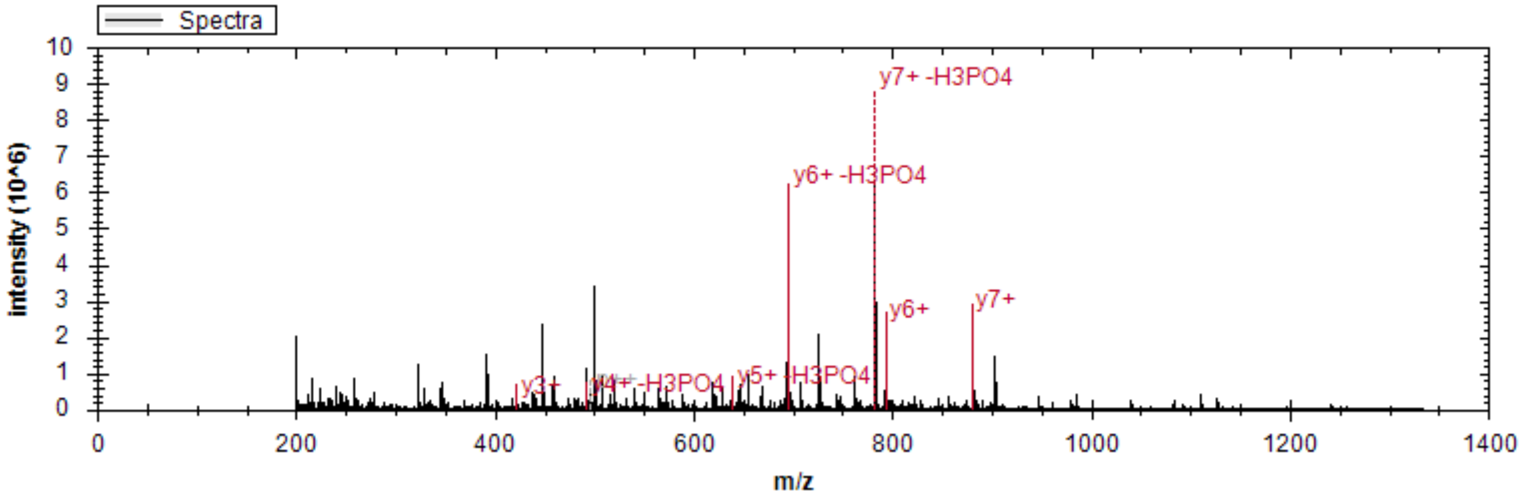

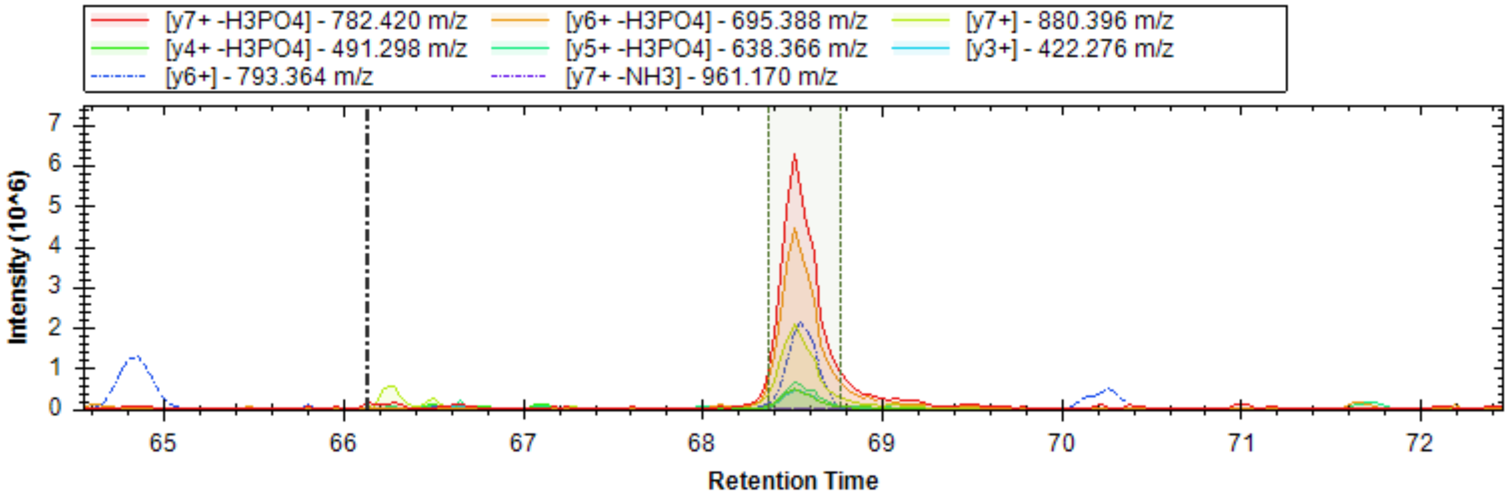

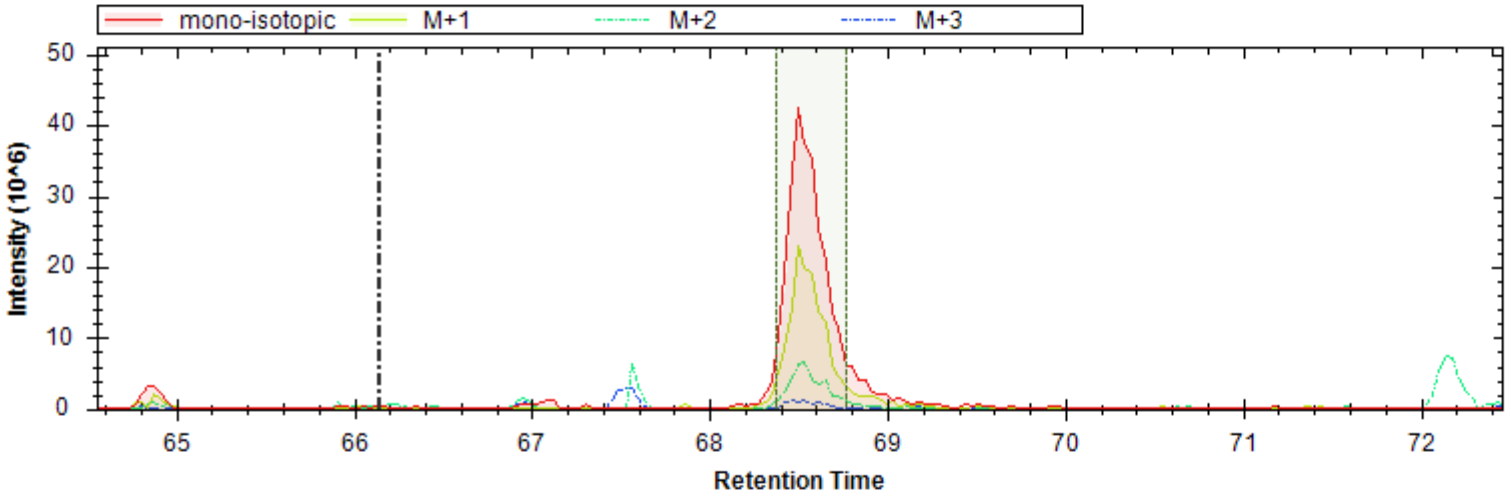

P28667 – _LSGLS[+H+O3+P]FKR_.2
Fig4_MP-DIA-barrel-cortex-p15-1_MHRM.htrms

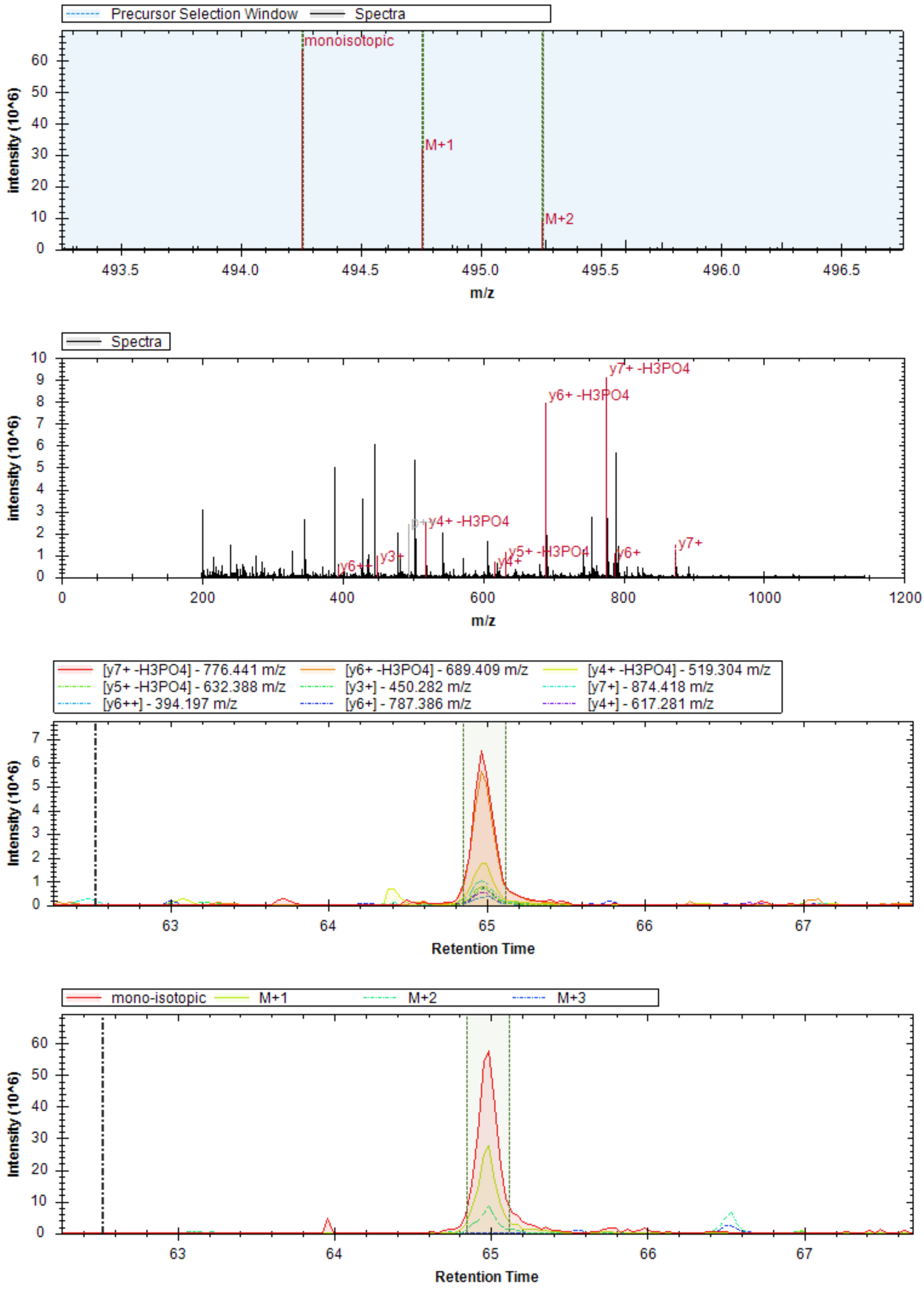

P28738 – _PIRPGHYPAS[+H+O3+P]SPTAVHAVR_.4
Fig4_MP-DIA-barrel-cortex-p15-1_MHRM.htrms

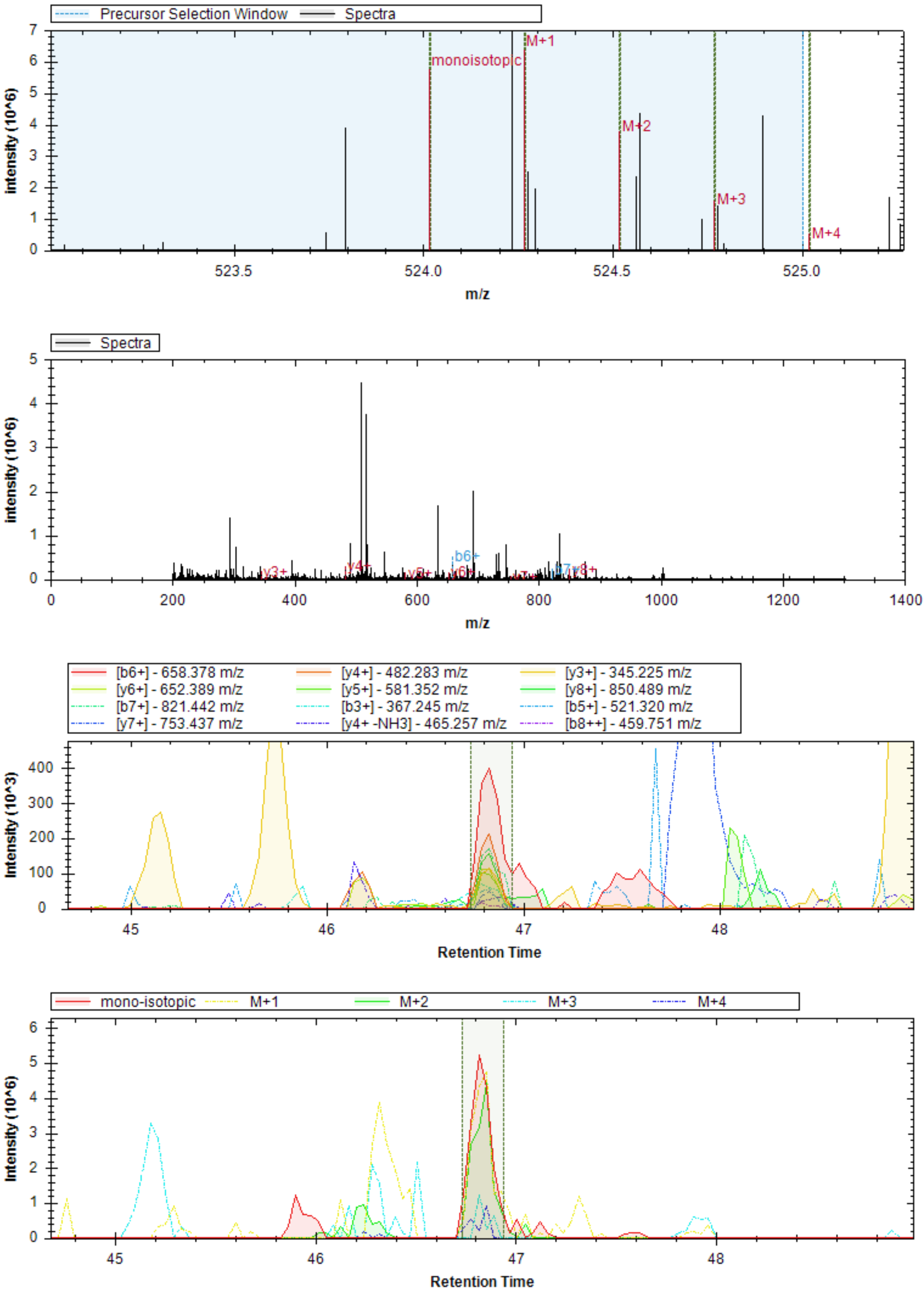

P28738 – _PIRPGHYPASS[+H+O3+P]PTAVHAVR_.4
Fig4_MP-DIA-barrel-cortex-p15-1_MHRM.htrms

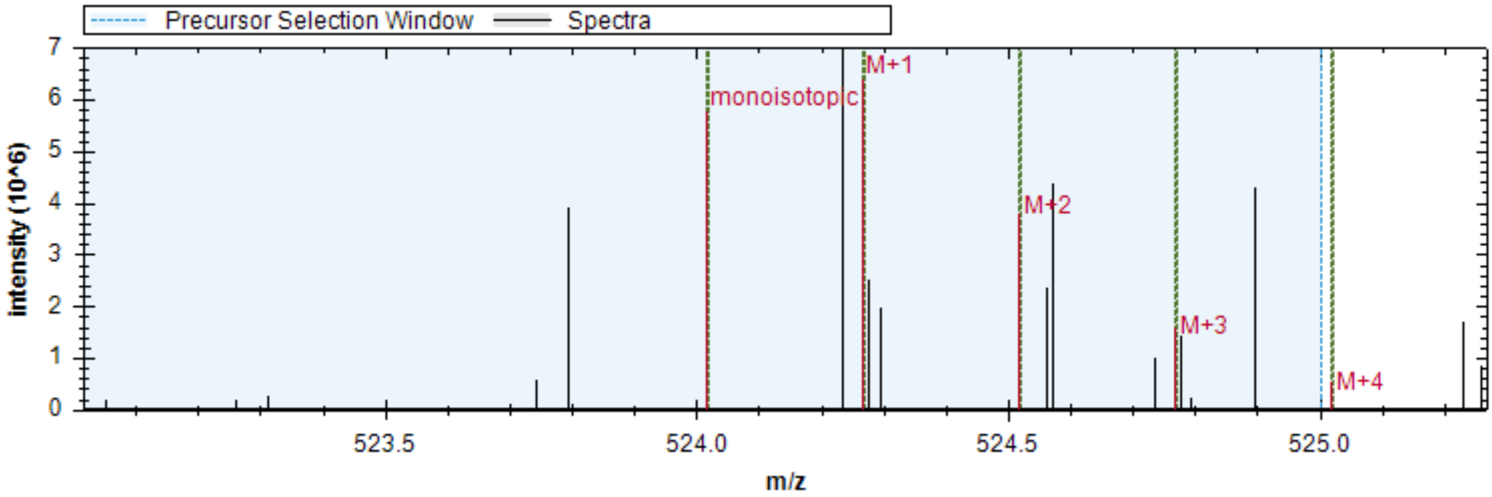

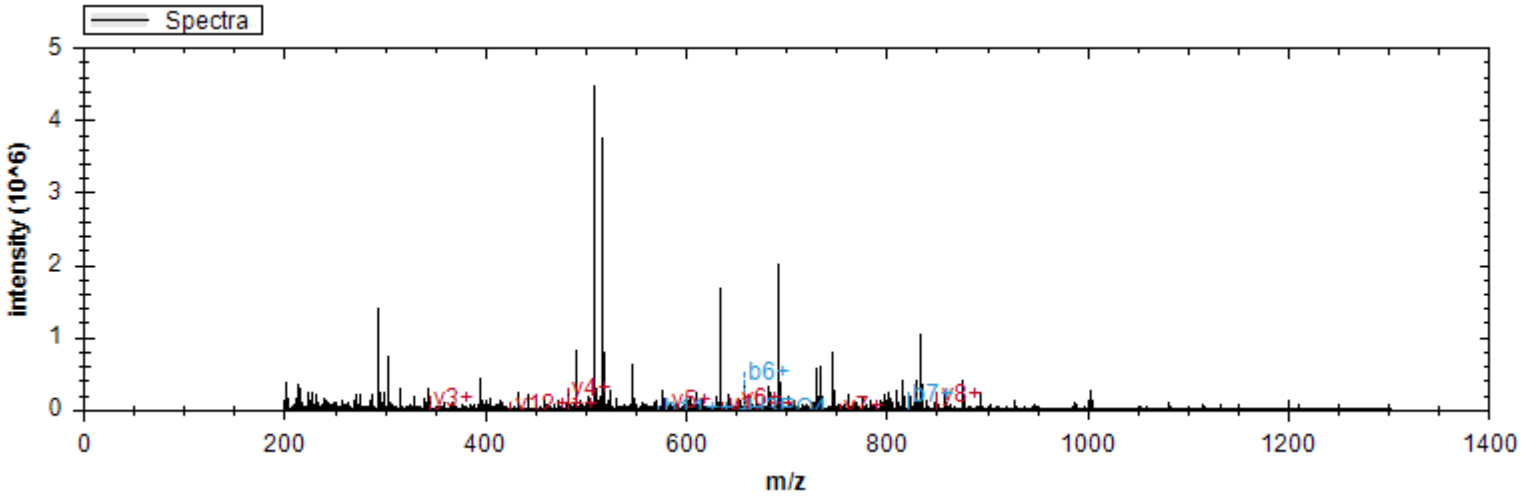

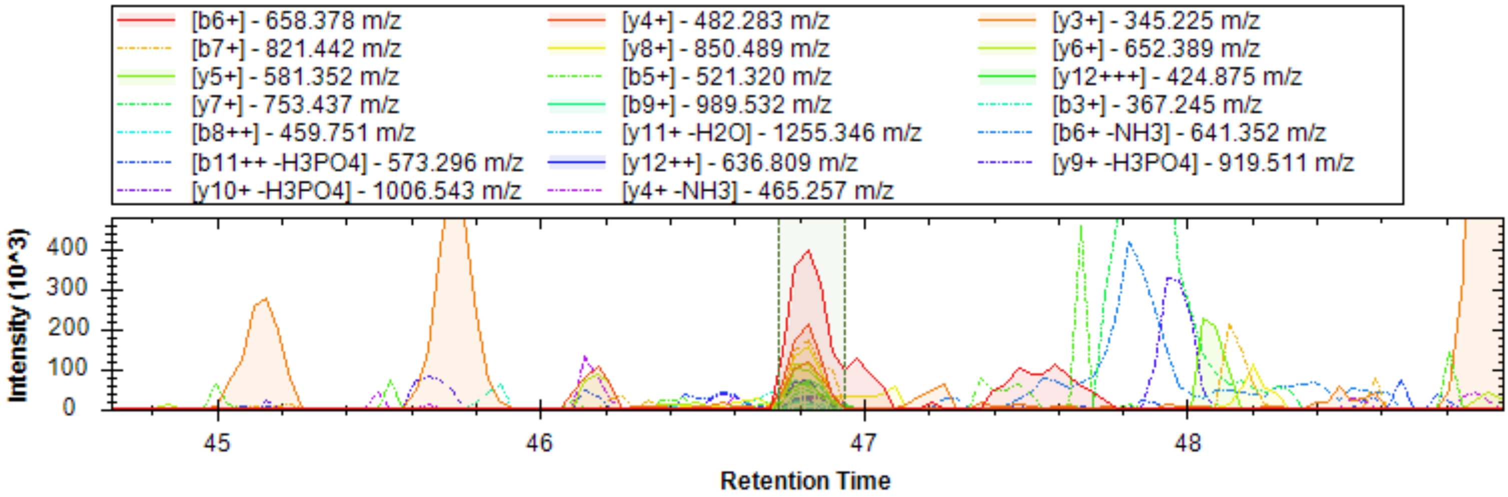

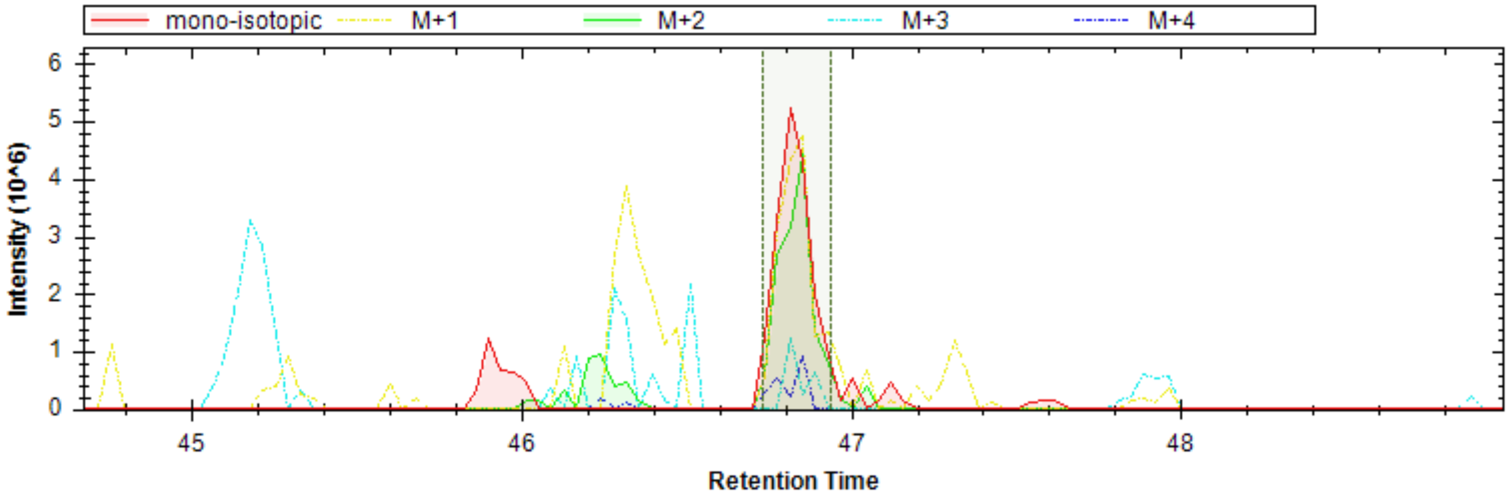

P62996 – _RPHT[+H+O3+P]PTPGIYMGR_.3
Fig4_MP-DIA-barrel-cortex-p15-1_MHRM.htrms

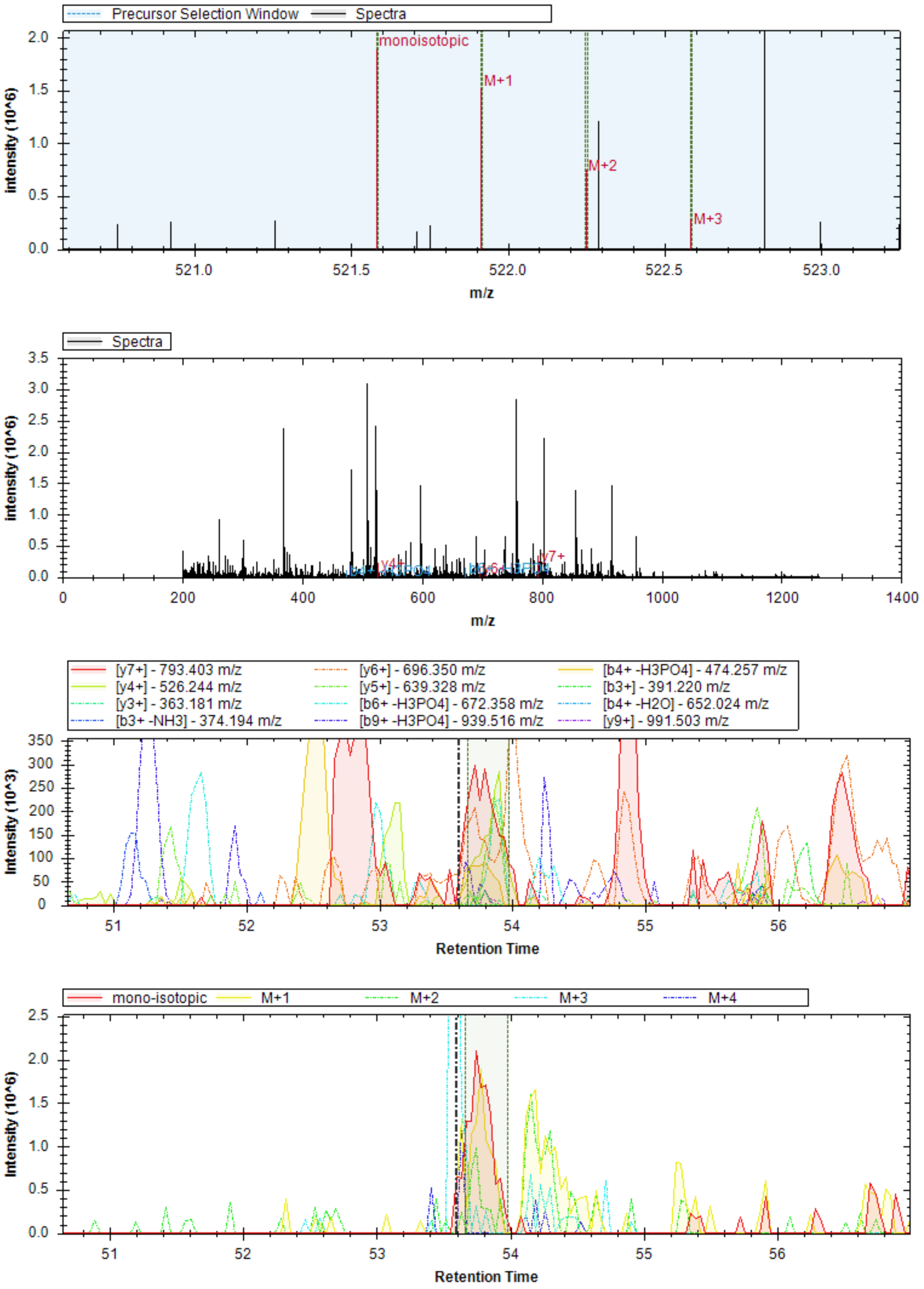

Q8CDY7 – _LVQALC[+C2+H3+N+O]EQIHT[+H+O3+P]R_.3
Fig4_MP-DIA-barrel-cortex-p15-1_MHRM.htrms

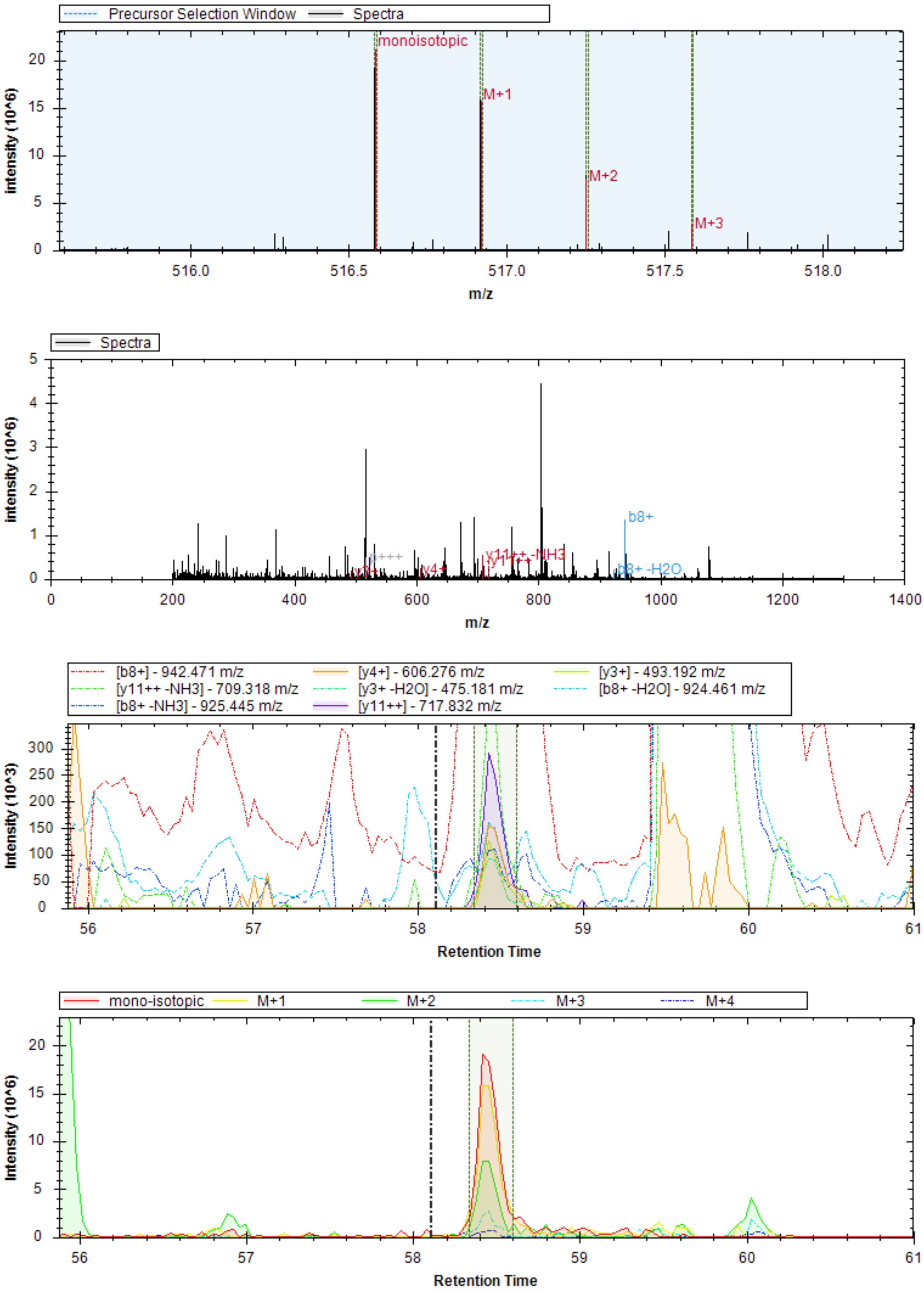

Q9QYC0 – _AAVVT[+H+O3+P]SPPPTTAPHK_.3
Fig4_MP-DIA-barrel-cortex-p15-1_MHRM.htrms

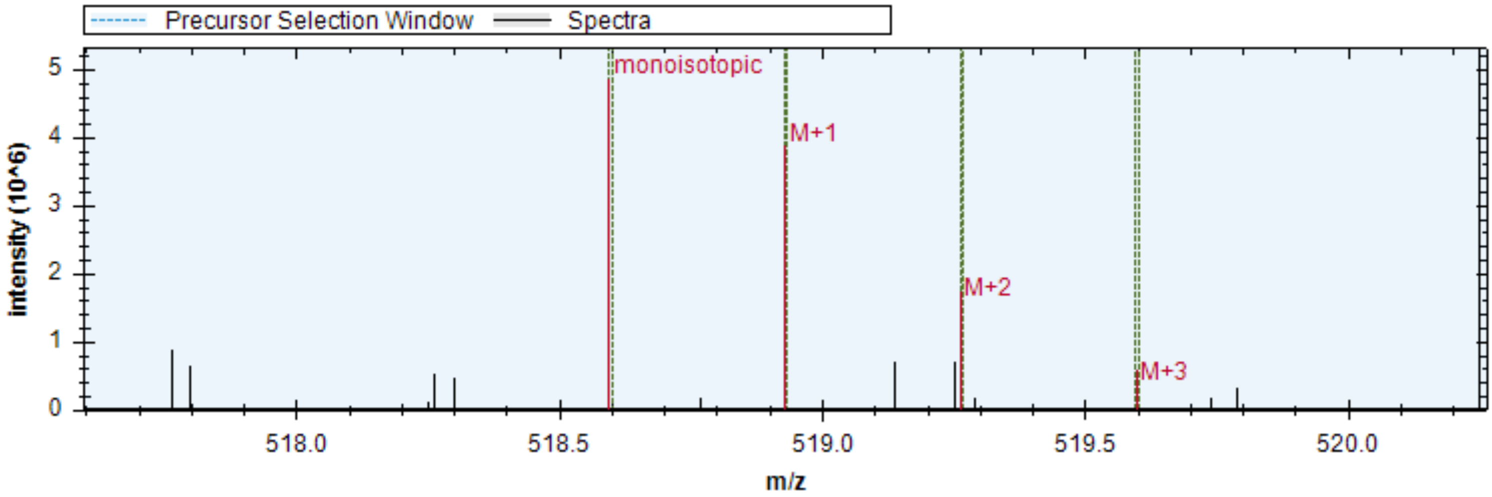

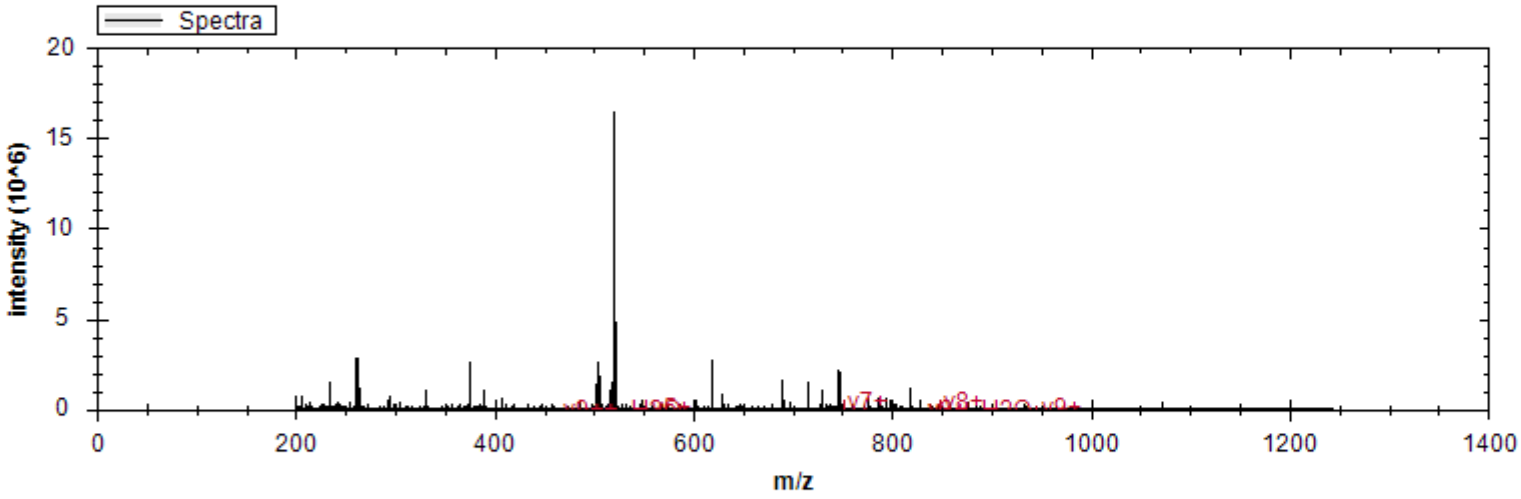

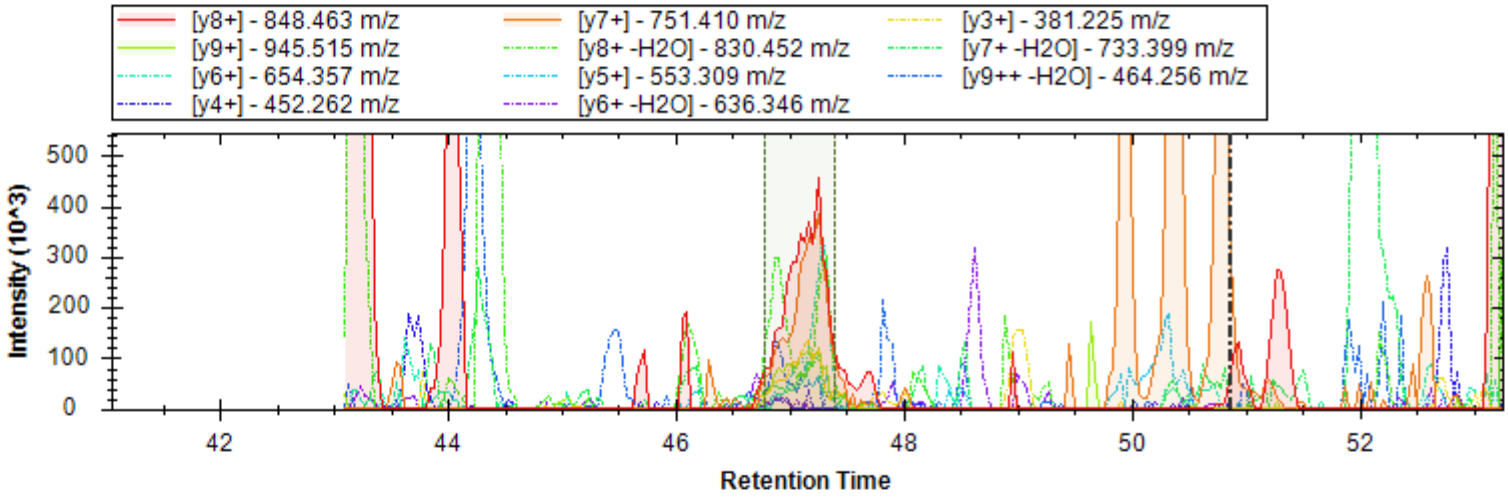

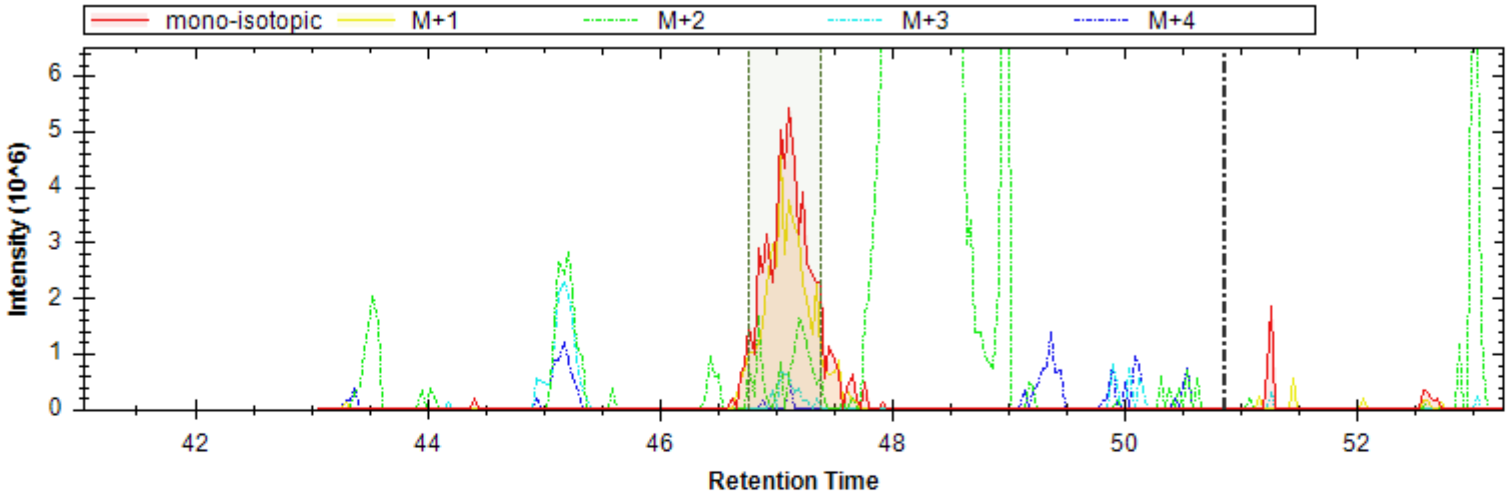

Q9QYC0 – _AAVVTS[+H+O3+P]PPPTTAPHK_.3
Fig4_MP-DIA-barrel-cortex-p15-1_MHRM.htrms

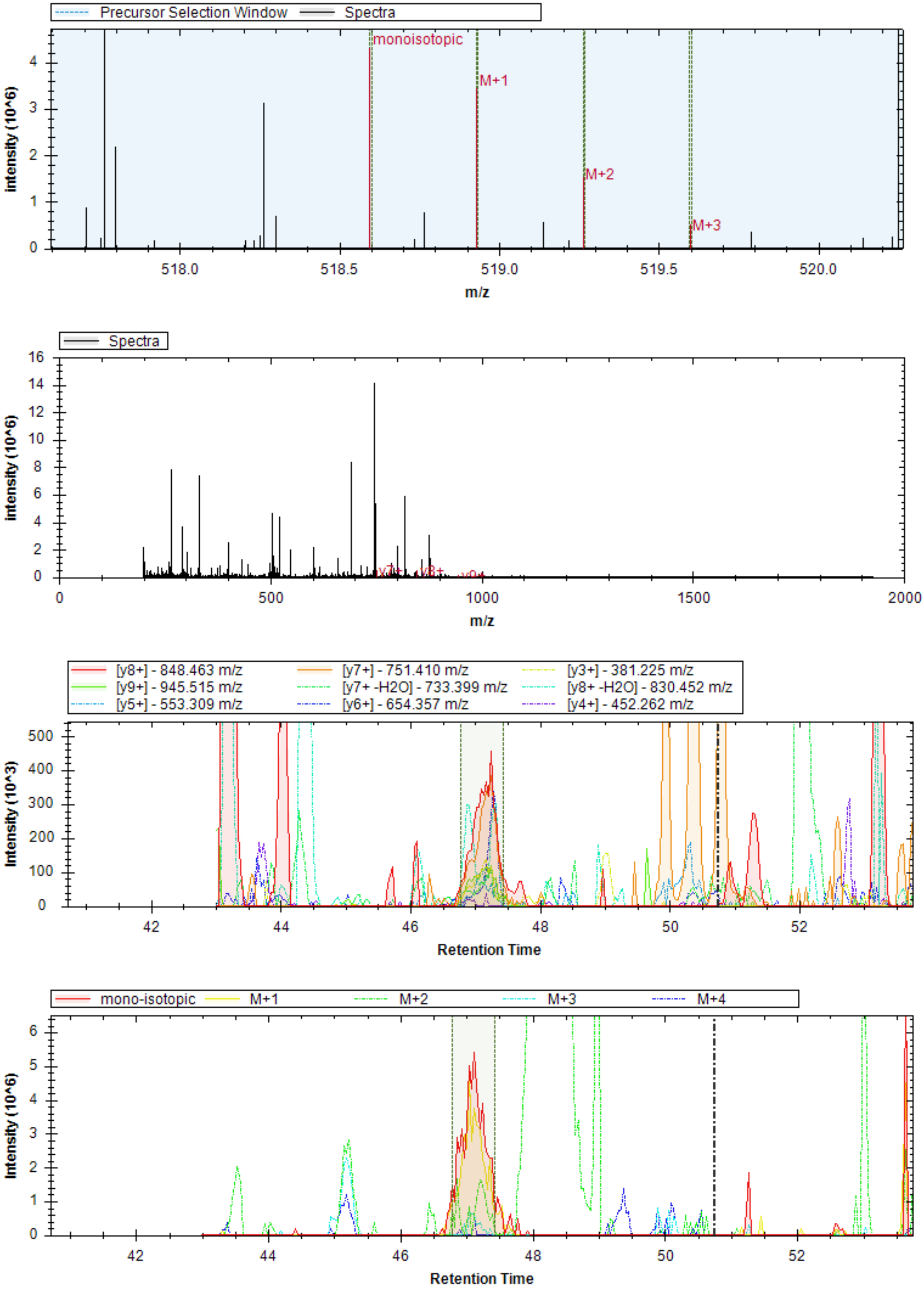

P10637 – _VAVVRT[+H+O3+P]PPKSPSASK_.3
Fig4_MP-DIA-barrel-cortex-p15-1_MHRM.htrms

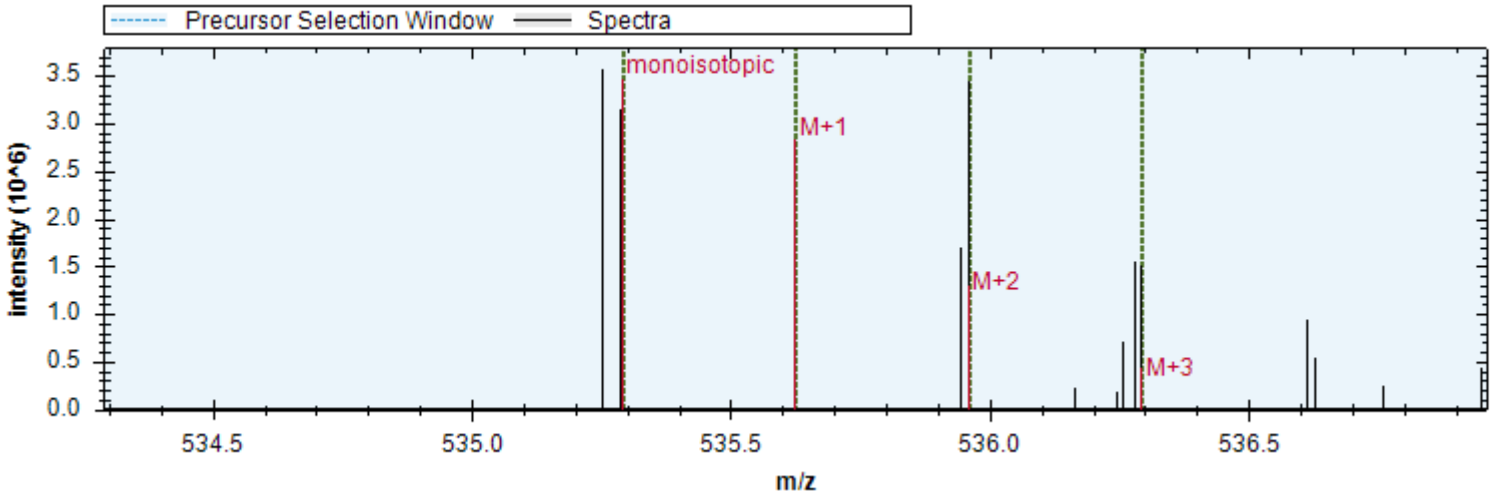

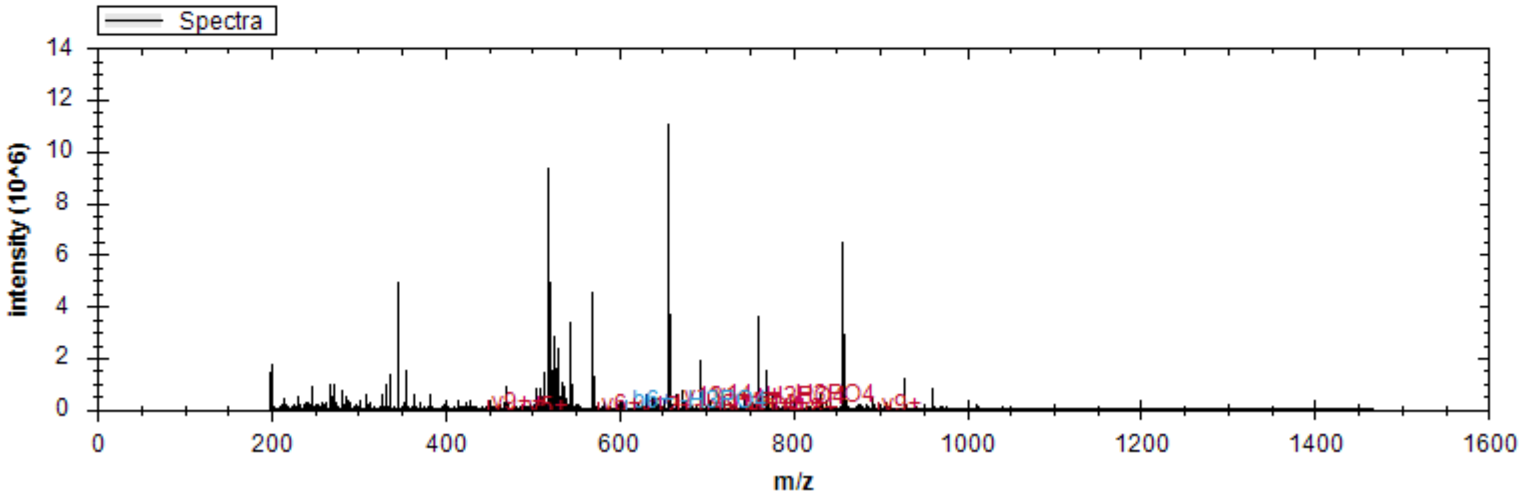

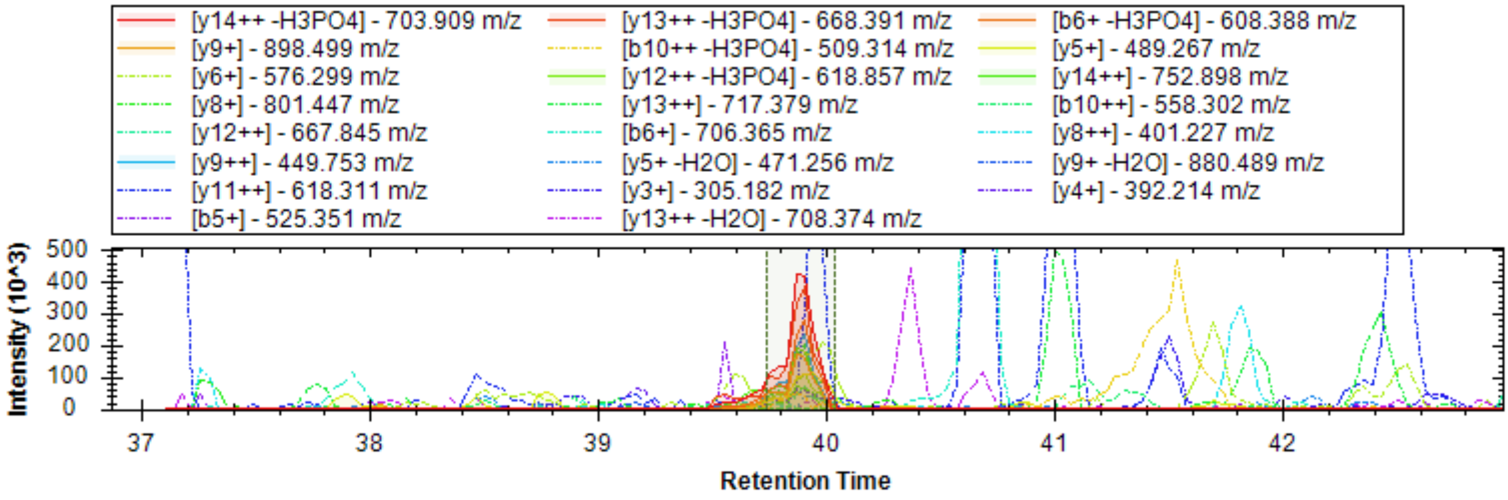

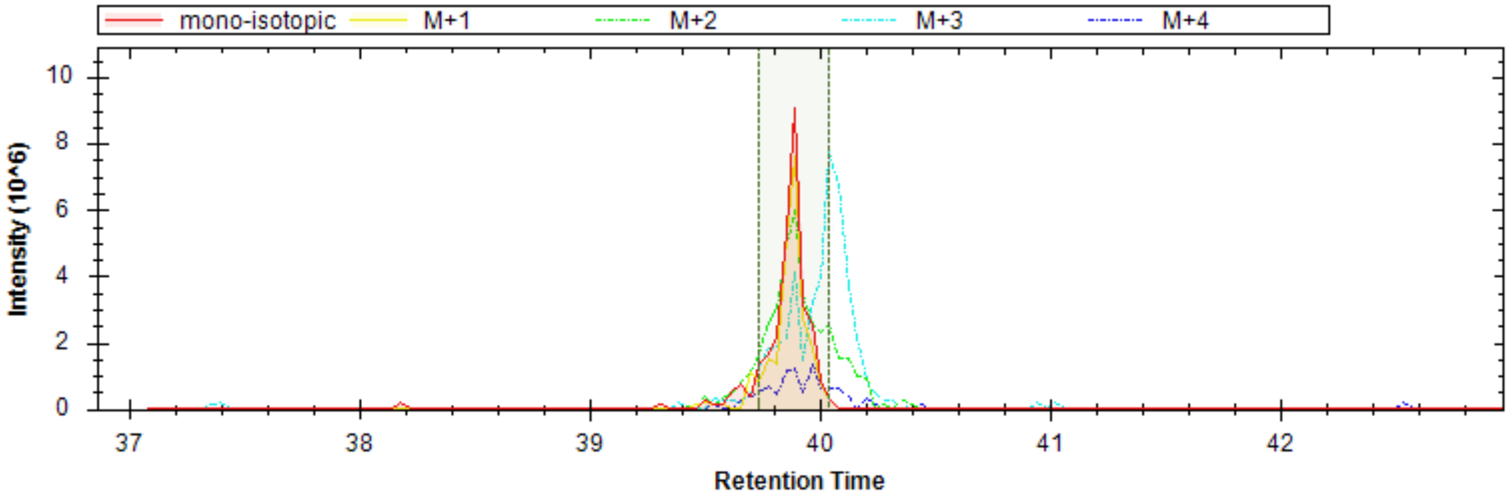

P63318 – _AAPALT[+H+O3+P]PPDR_.2
Fig4_MP-DIA-barrel-cortex-p15-1_MHRM.htrms

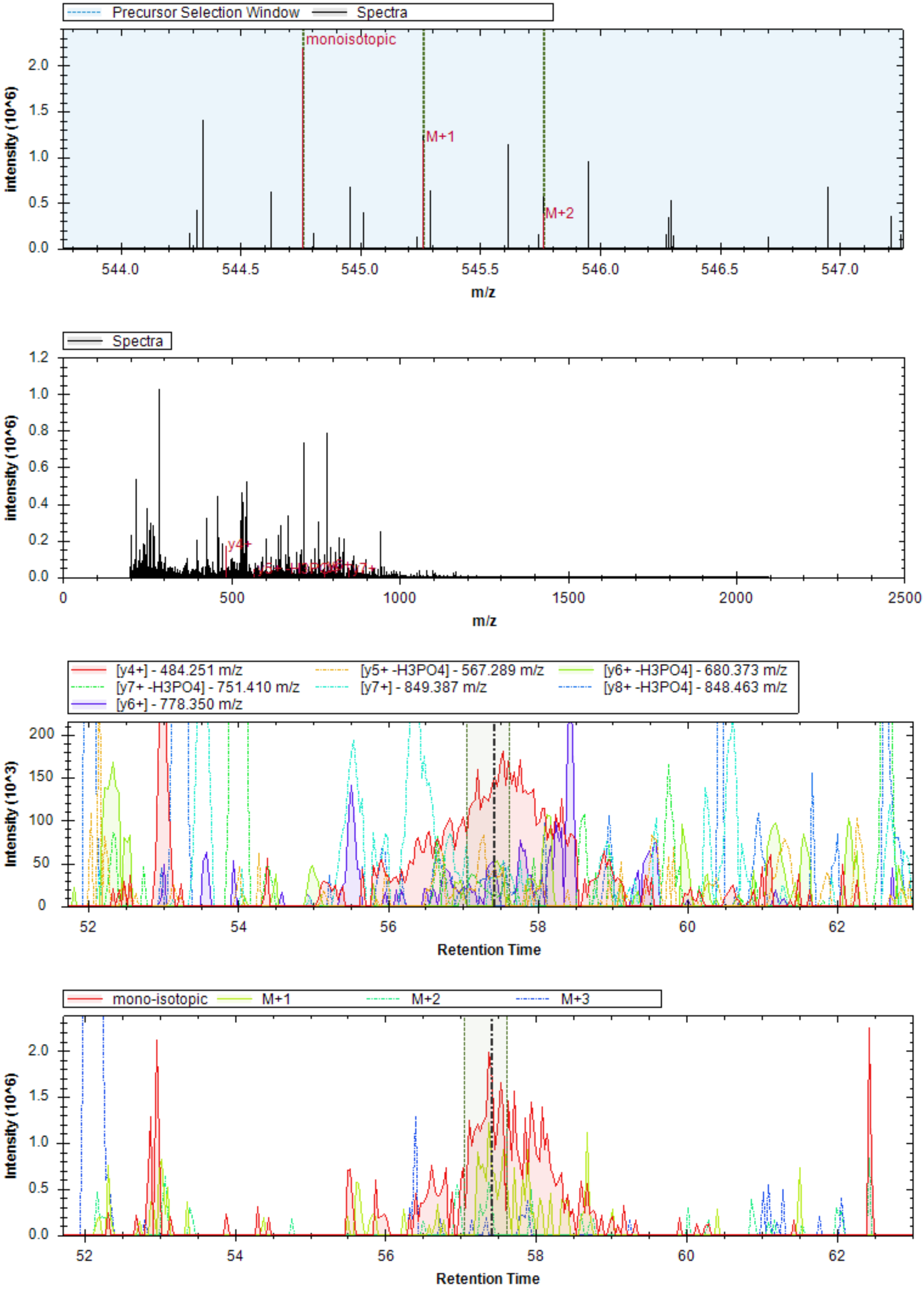

Q7TQD2 – _AANKT[+H+O3+P]PPKSPGDPAR_.3
Fig4_MP-DIA-barrel-cortex-p15-1_MHRM.htrms

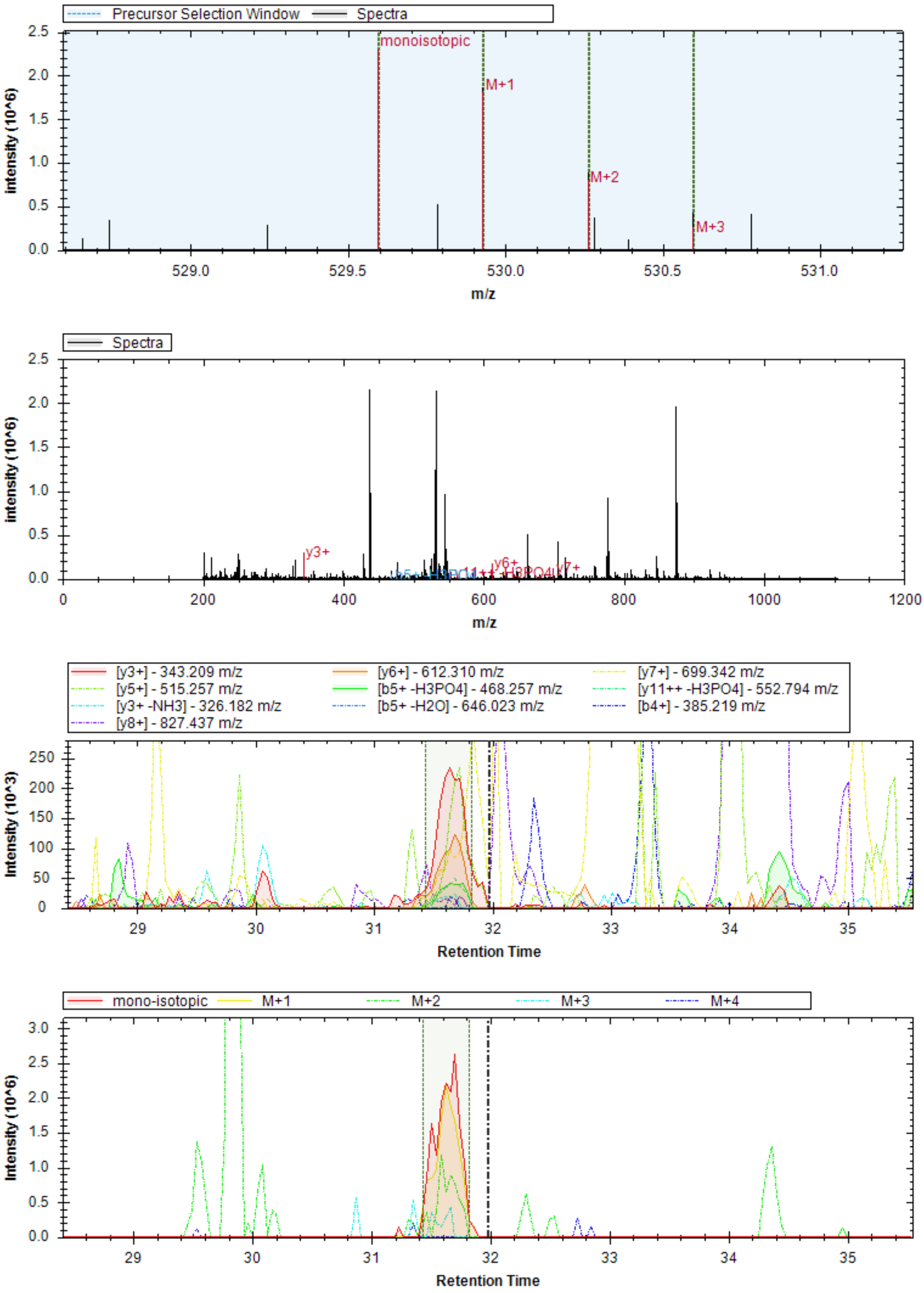

Q9D898 – _AFHAALRNS[+H+O3+P]PINTK_.3

Fig4_MP-DIA-barrel-cortex-p15-1_MHRM.htrms

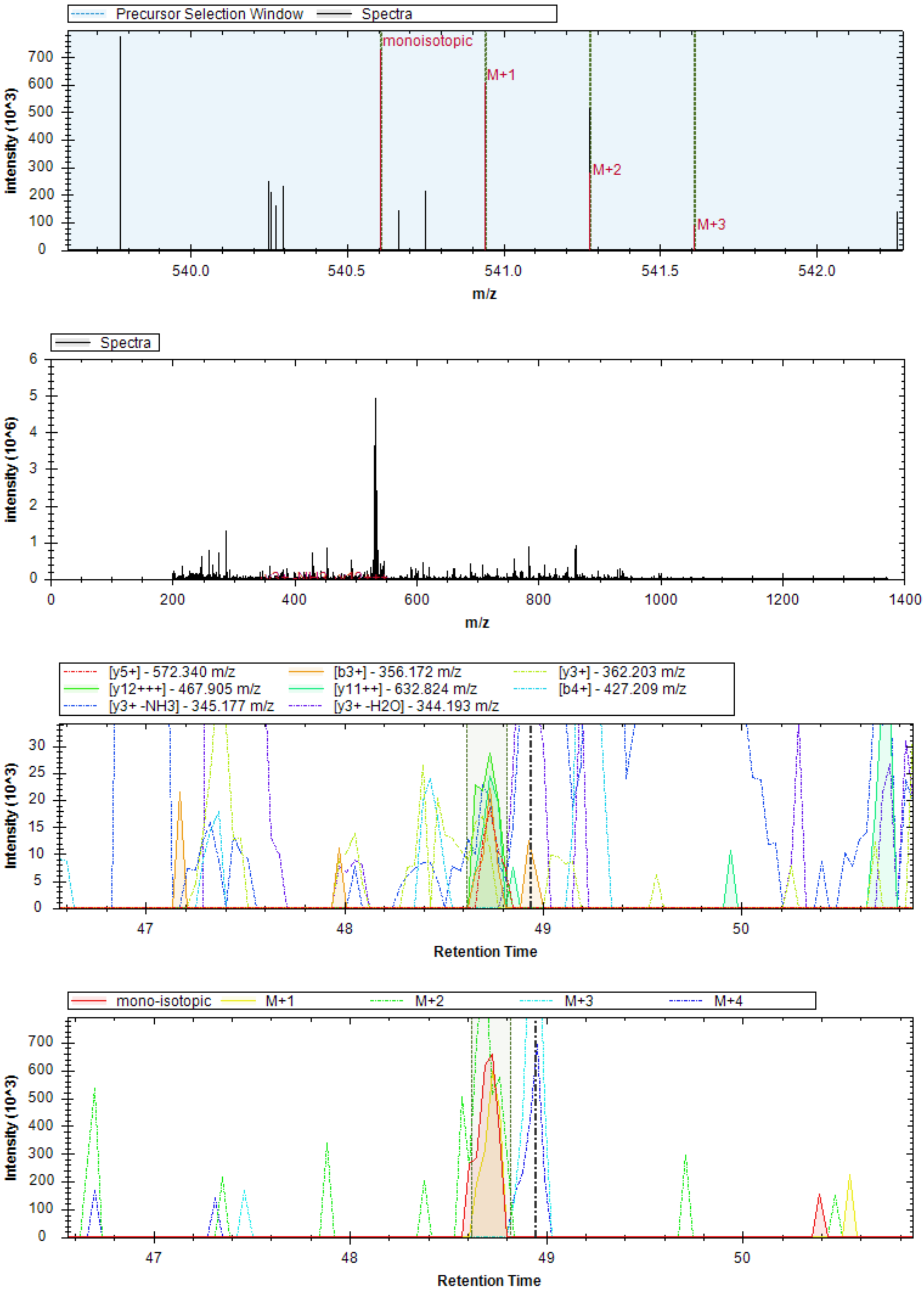

P20357 – _VAIIRT[+H+O3+P]PPKSPATPK_.3
Fig4_MP-DIA-barrel-cortex-p15-1_MHRM.htrms

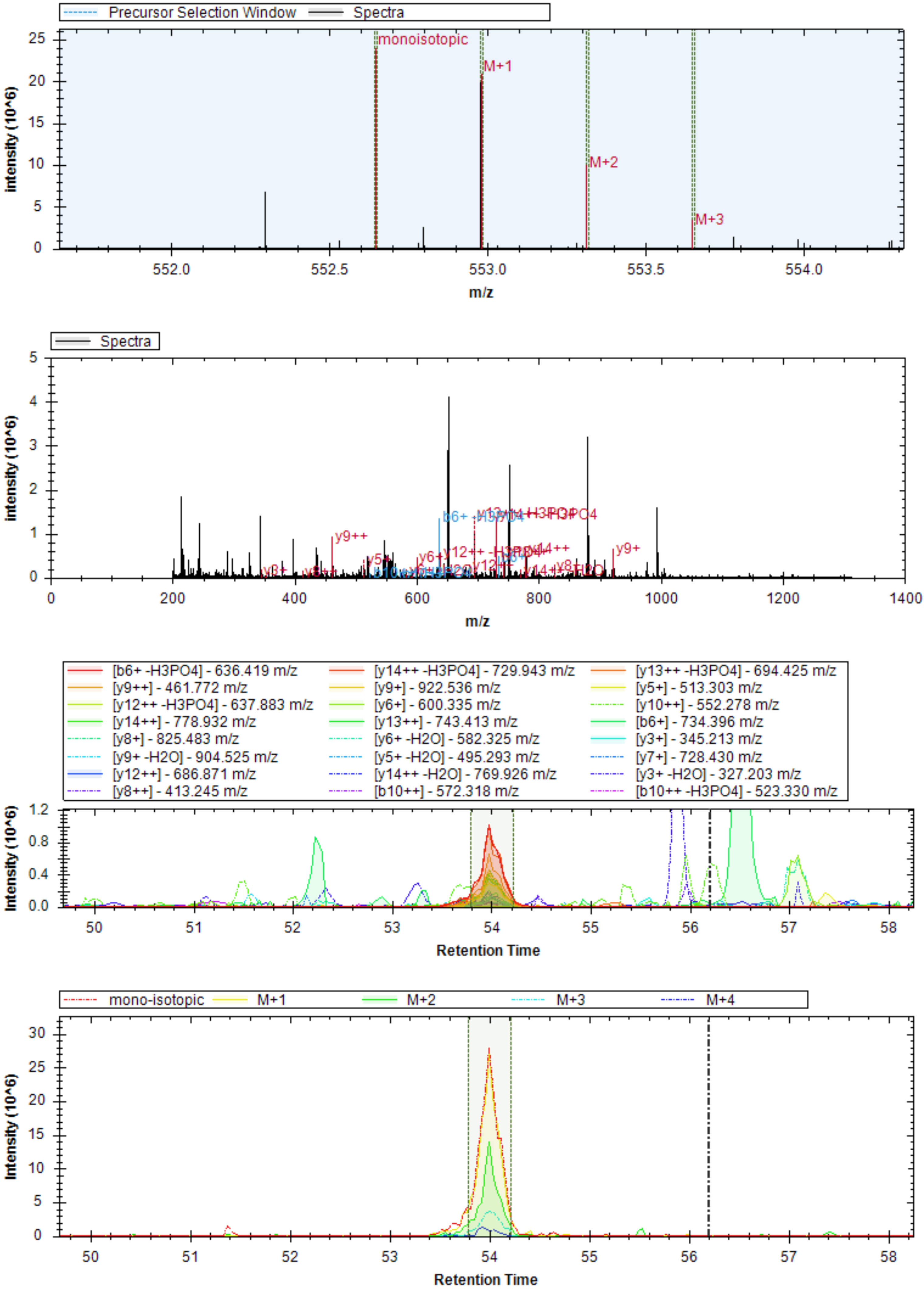

Q9CYT6 – _THTPSPTS[+H+O3+P]PK_.2

Fig4_MP-DIA-barrel-cortex-p15-1_MHRM.htrms

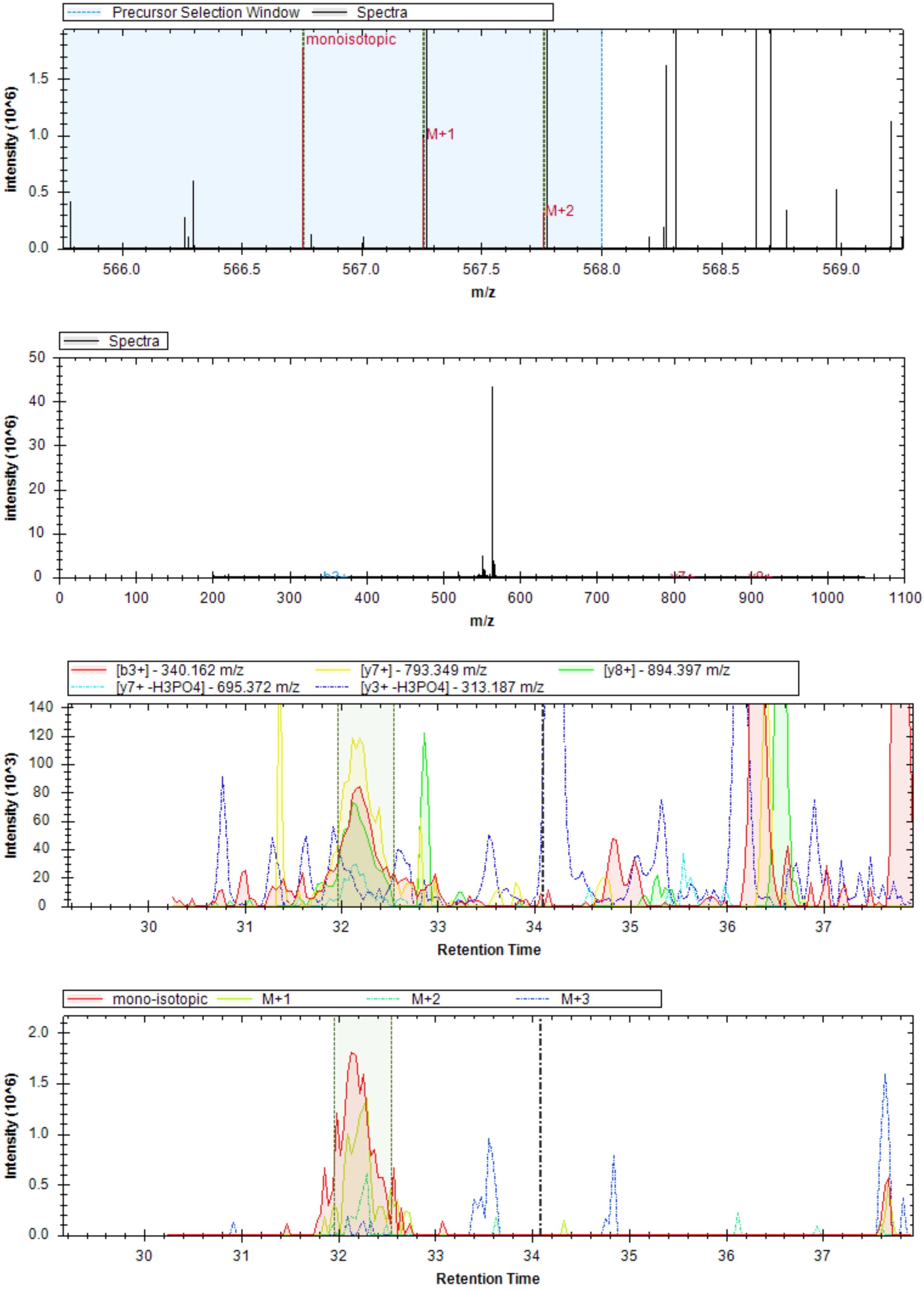

Q9R0K7 – _T[+H+O3+P]SIHNFMAHPEFR_.3
Fig4_MP-DIA-barrel-cortex-p15-1_MHRM.htrms

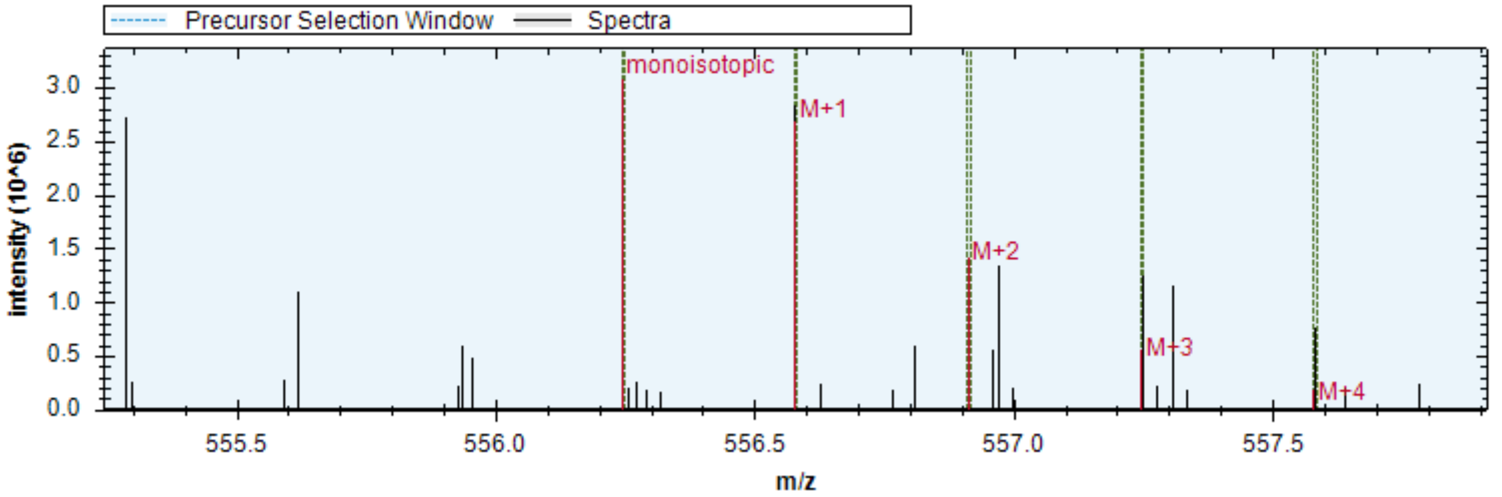

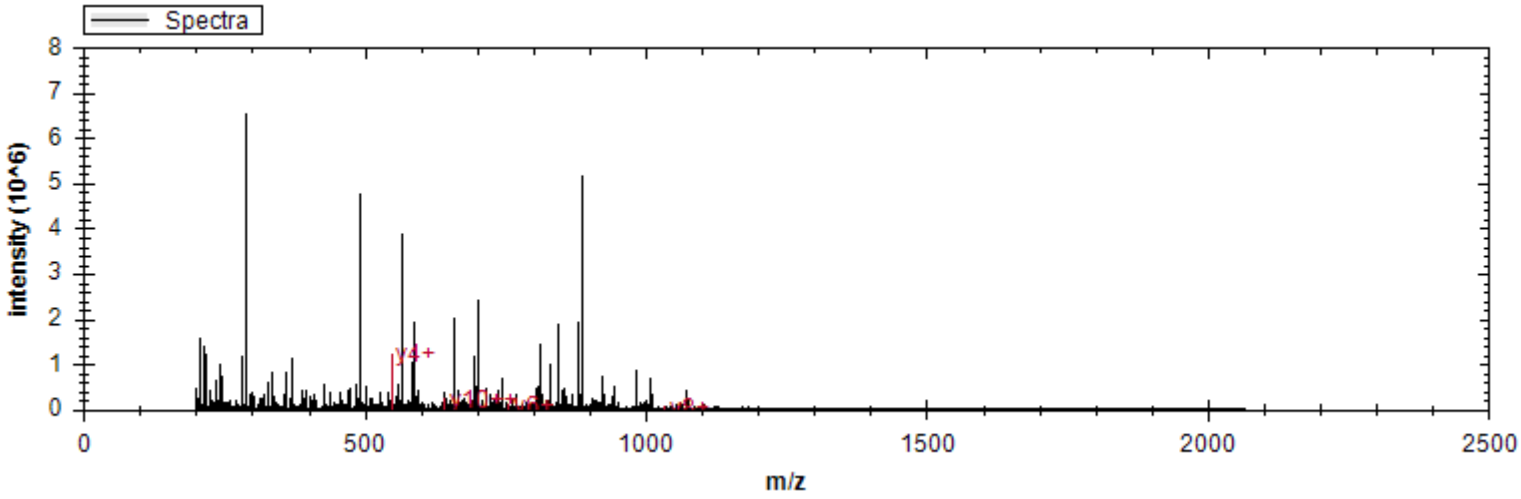

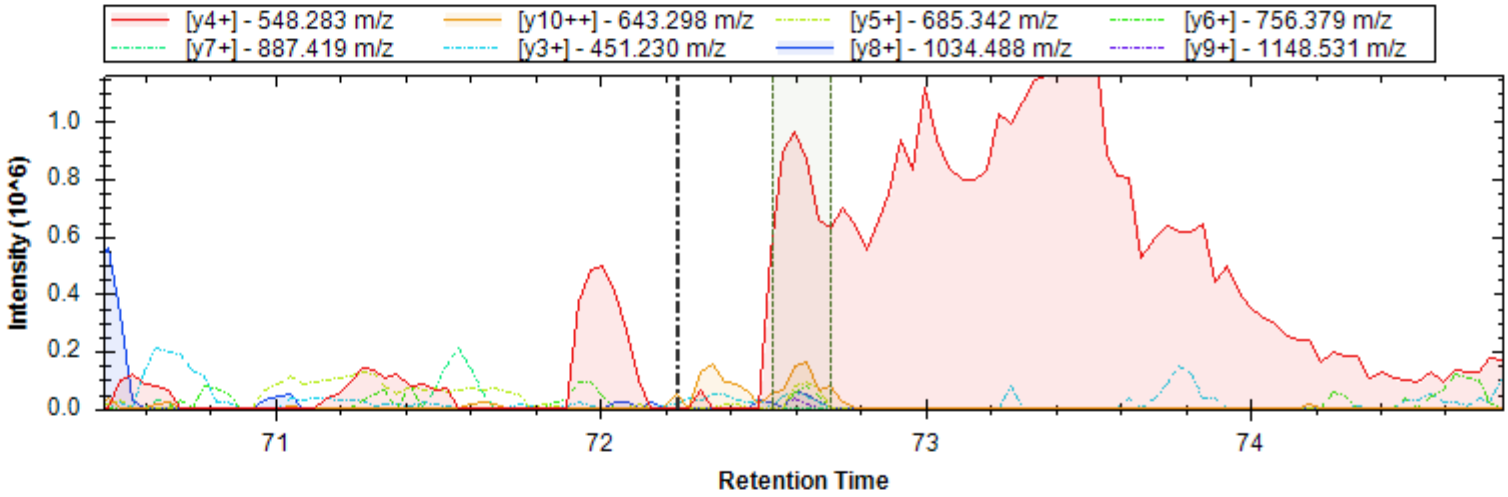

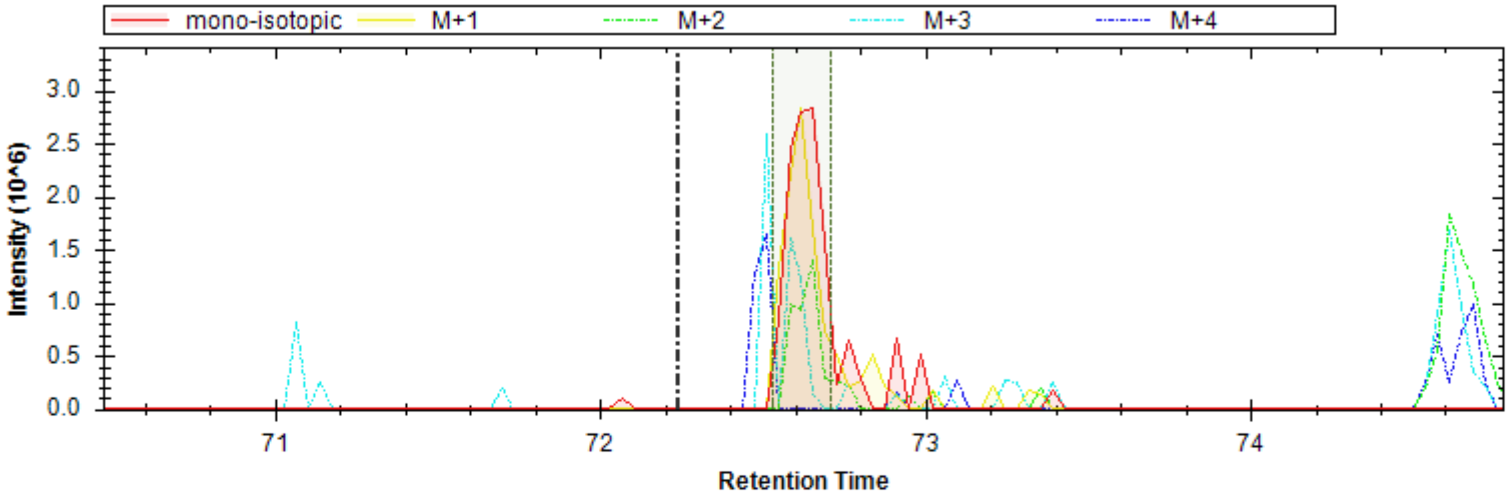

O88935 – _QAS[+H+O3+P]ISGPAPTK_.2
Fig4_MP-DIA-barrel-cortex-p15-1_MHRM.htrms

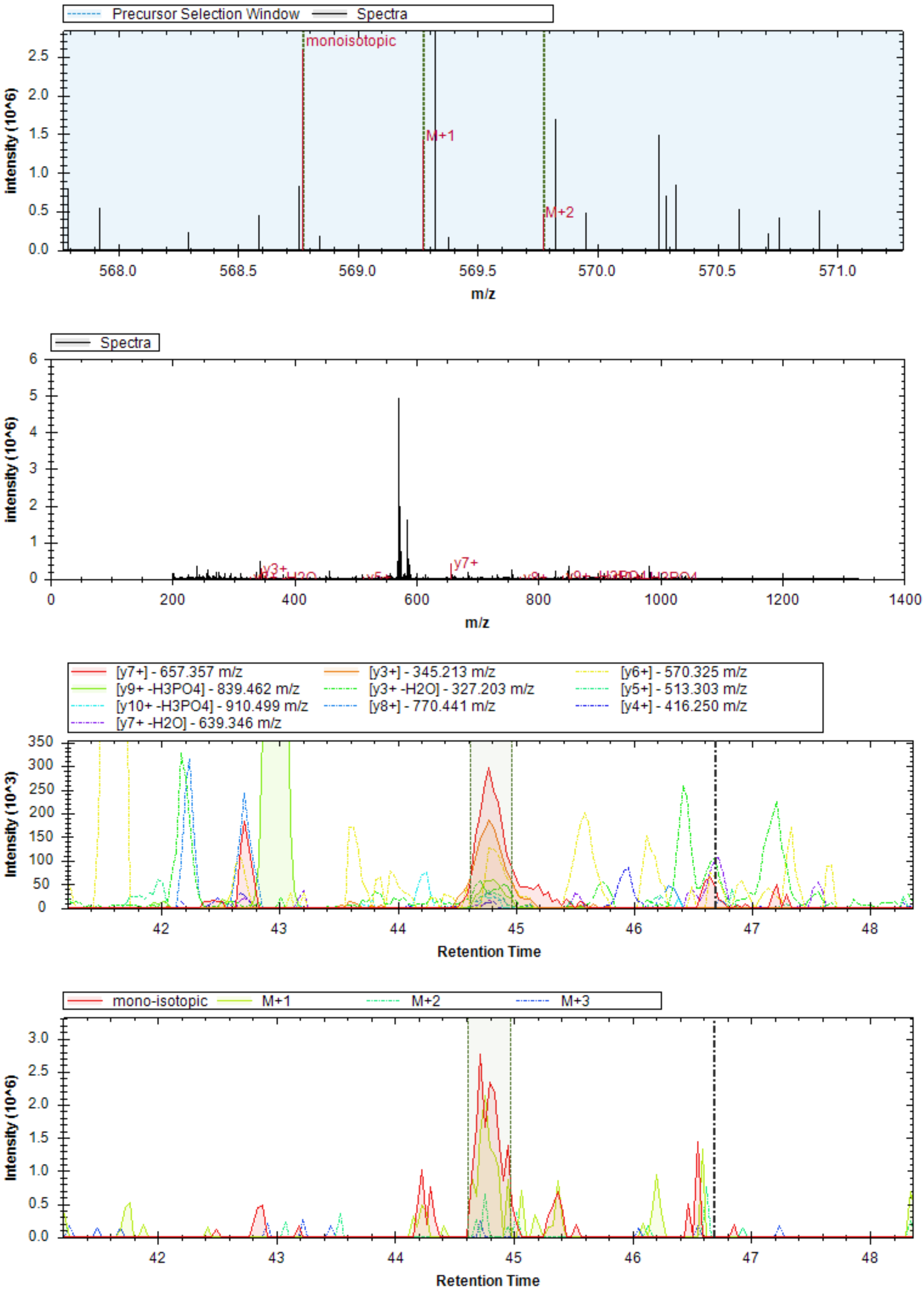

P27546-2 – _T[+H+O3+P]SPSKPSSAPALKPGPK_.3
Fig4_MP-DIA-barrel-cortex-p15-1_MHRM.htrms

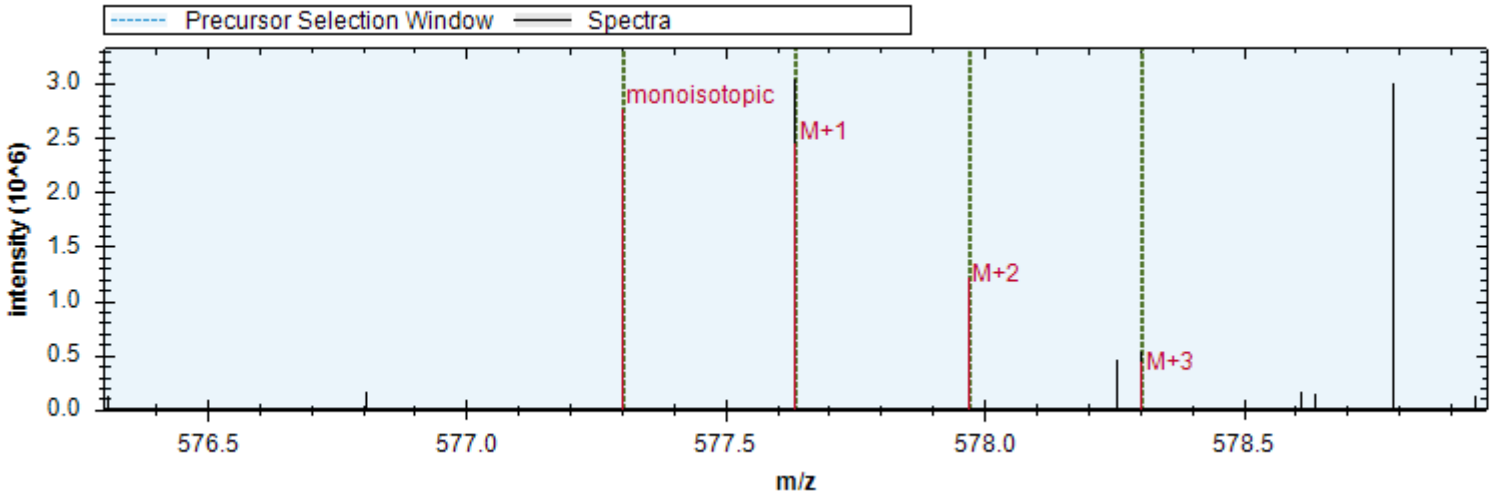

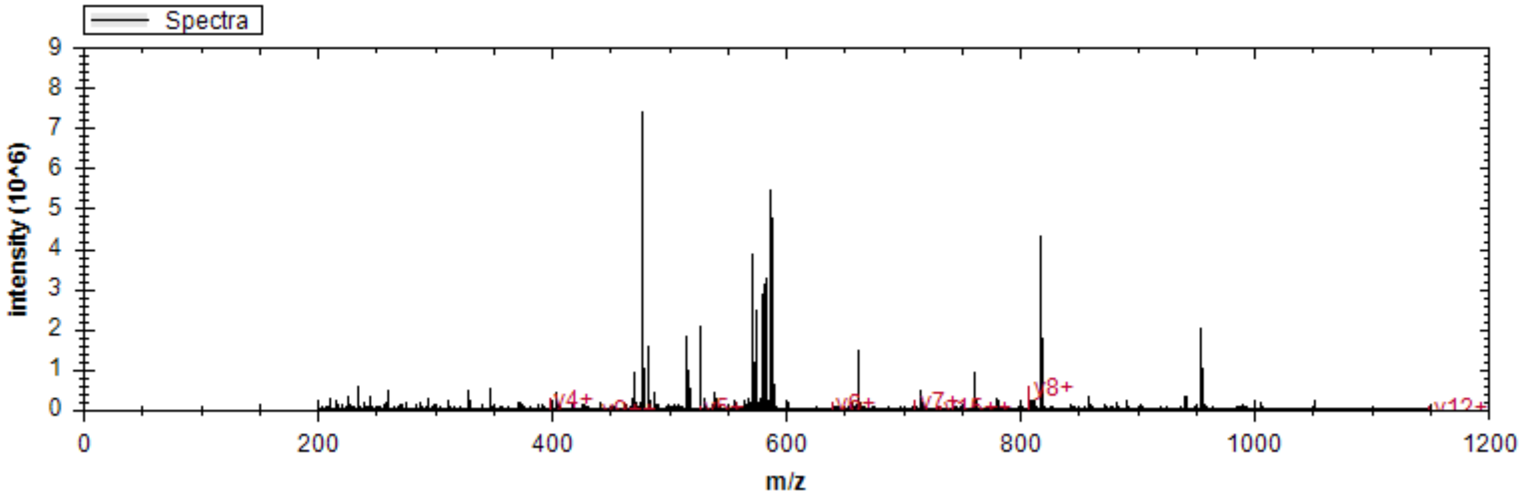

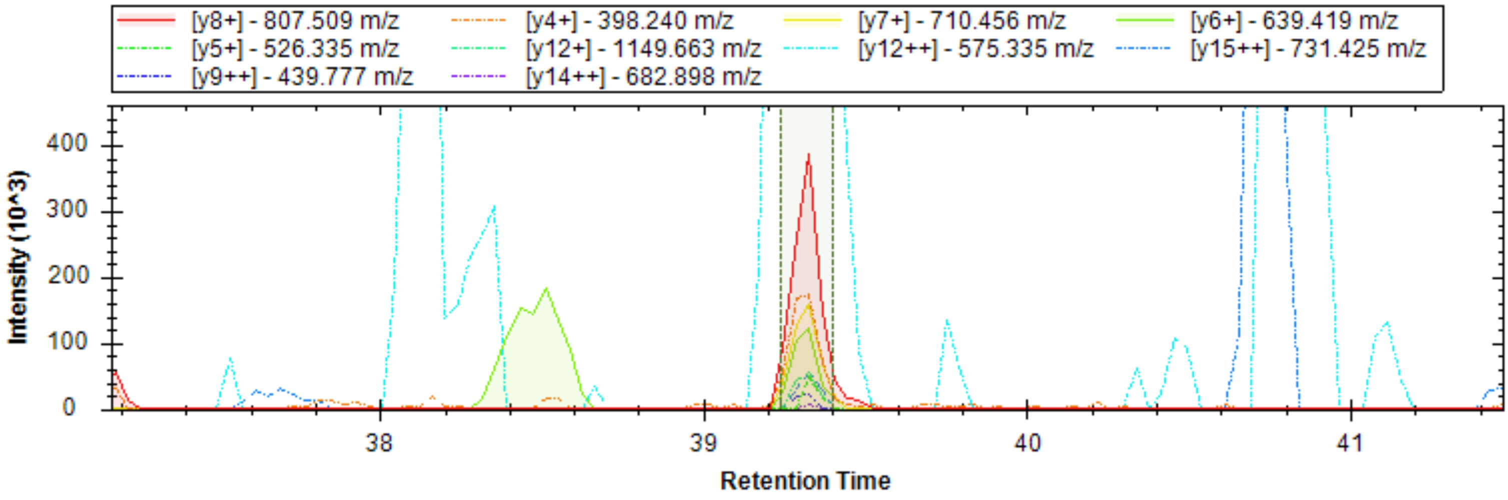

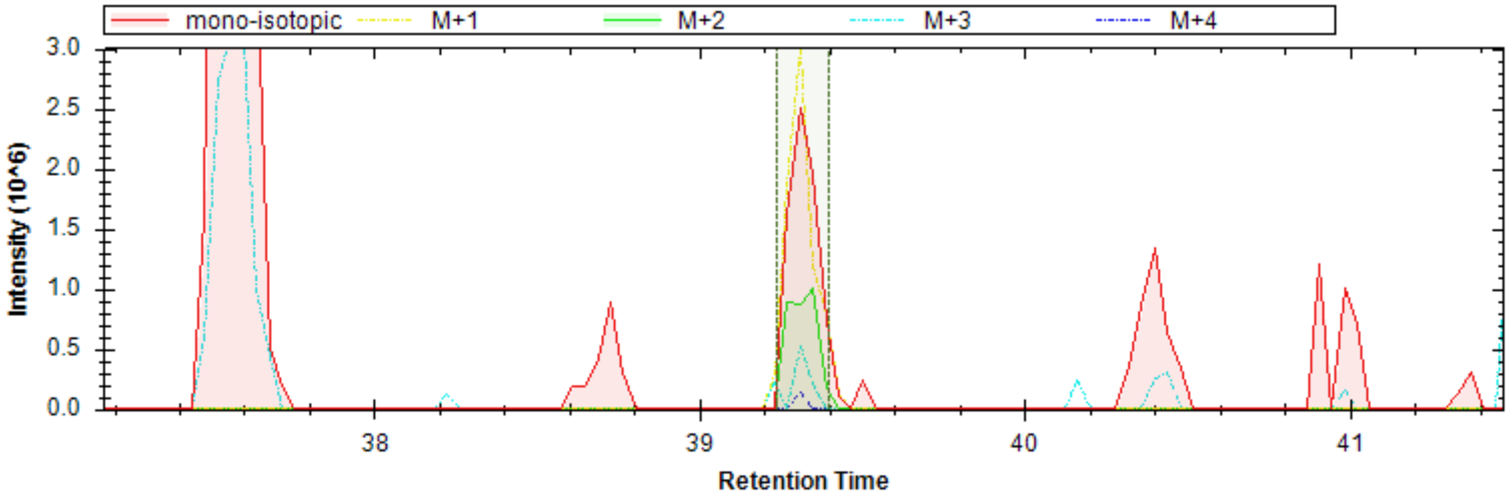

P28667 – _GEVAPKET[+H+O3+P]PK_.2

Fig4_MP-DIA-barrel-cortex-p15-1_MHRM.htrms

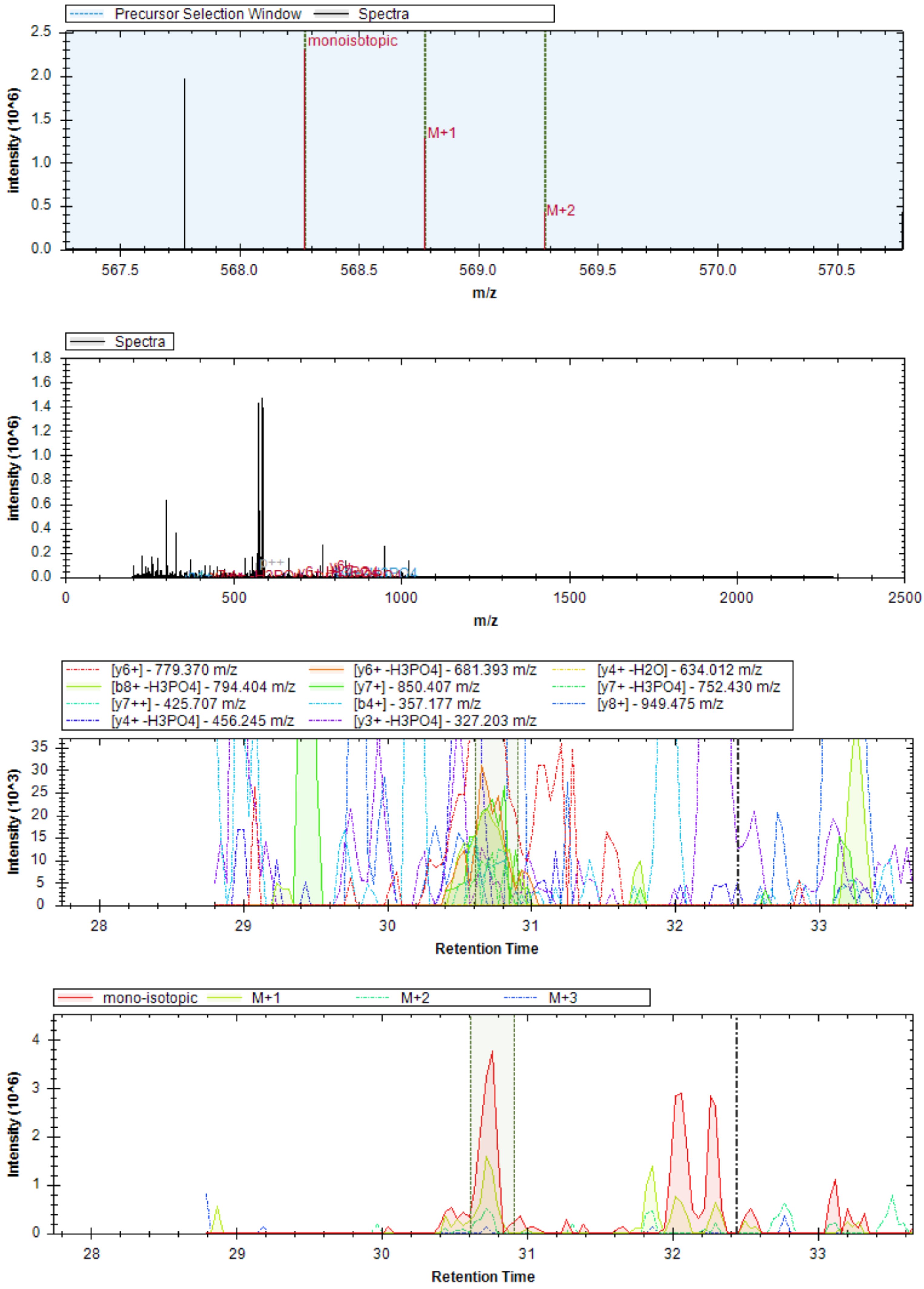

P97792 – _APQS[+H+O3+P]PTLAPAK_.2
Fig4_MP-DIA-barrel-cortex-p15-1_MHRM.htrms

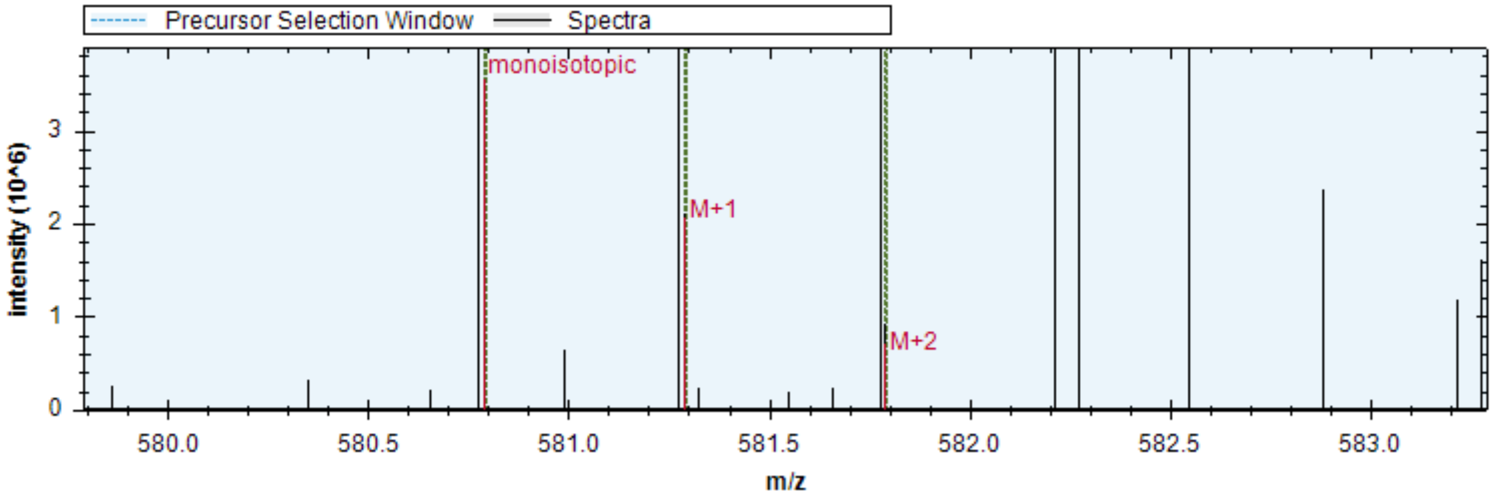

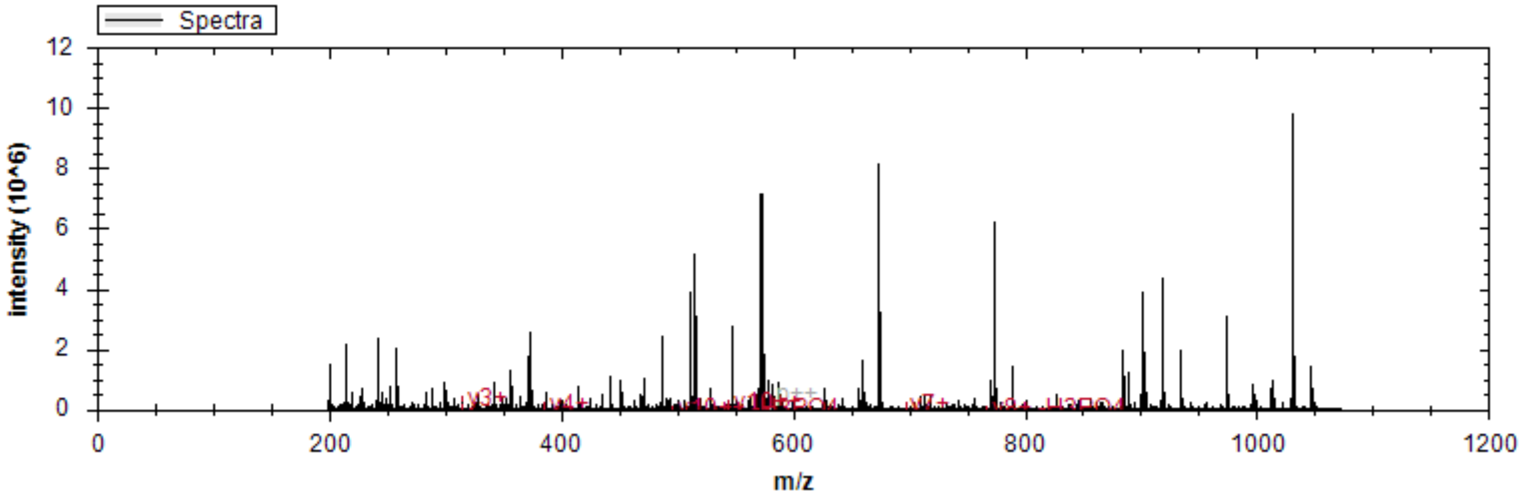

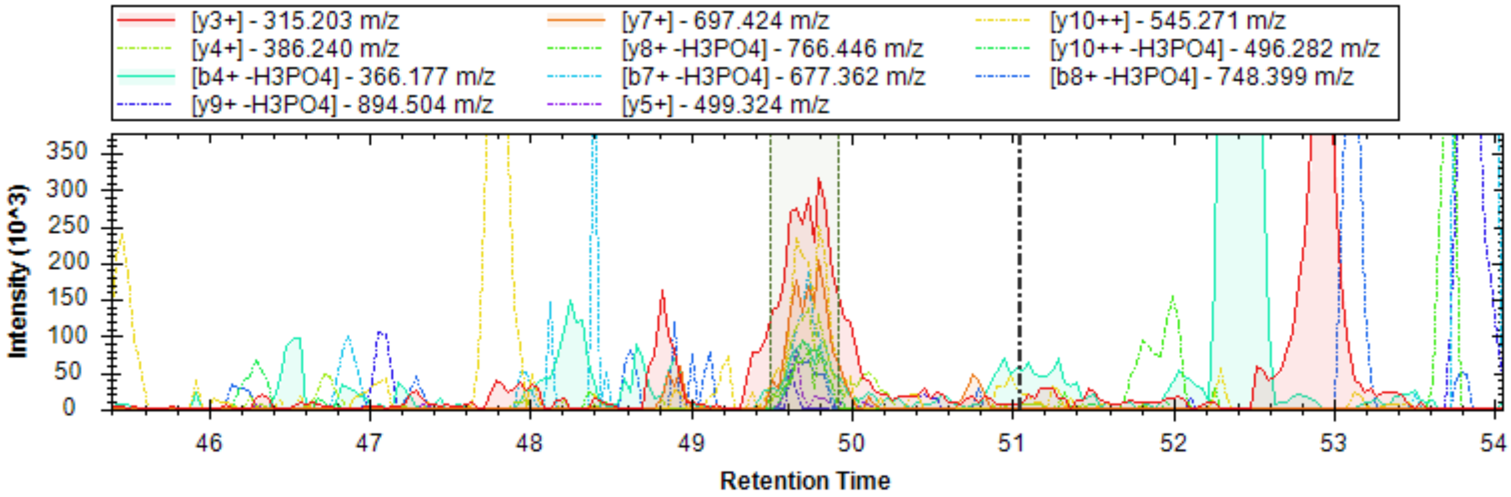

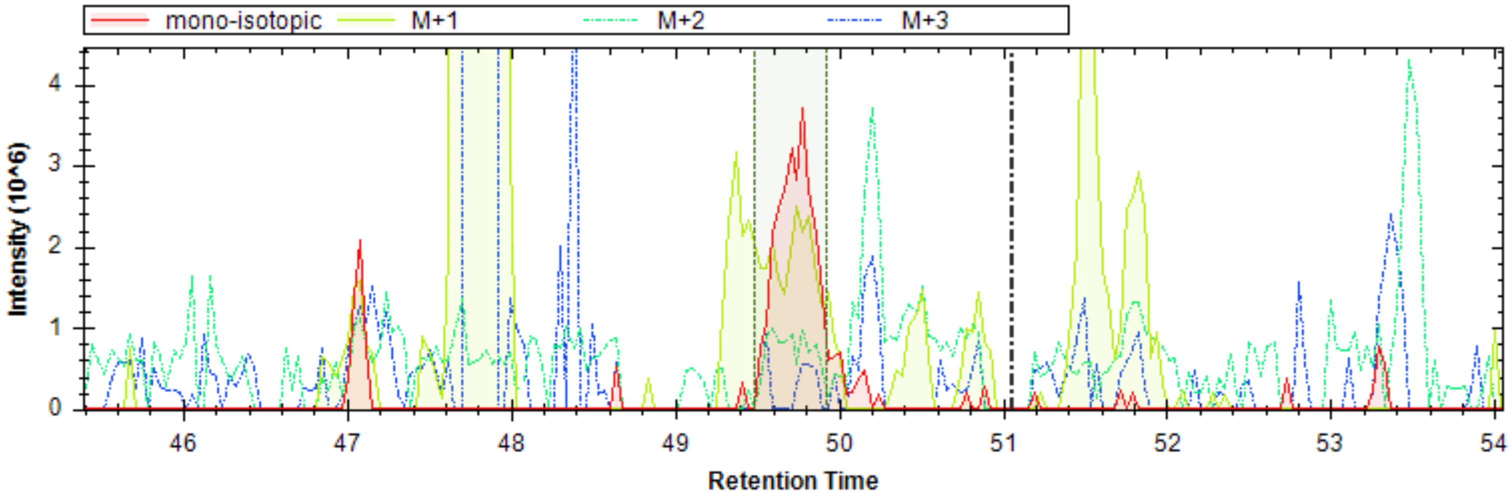

Q8BFU3-4 – _VAS[+H+O3+P]STQVRGASGRELR_.3

Fig4_MP-DIA-barrel-cortex-p15-1_MHRM.htrms

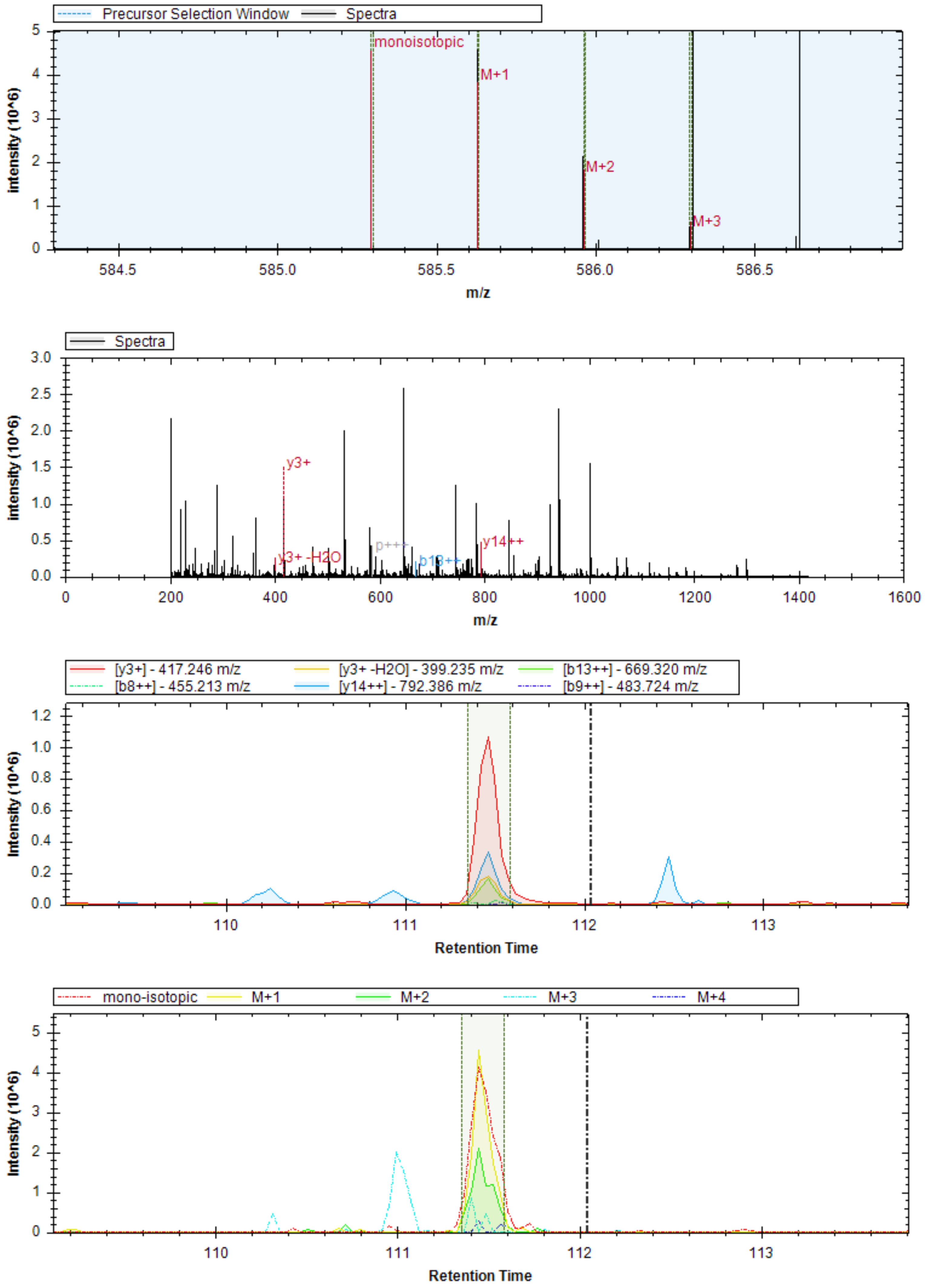

Q9CYT6 – _AQGQIRS[+H+O3+P]PTK_.2
Fig4_MP-DIA-barrel-cortex-p15-1_MHRM.htrms

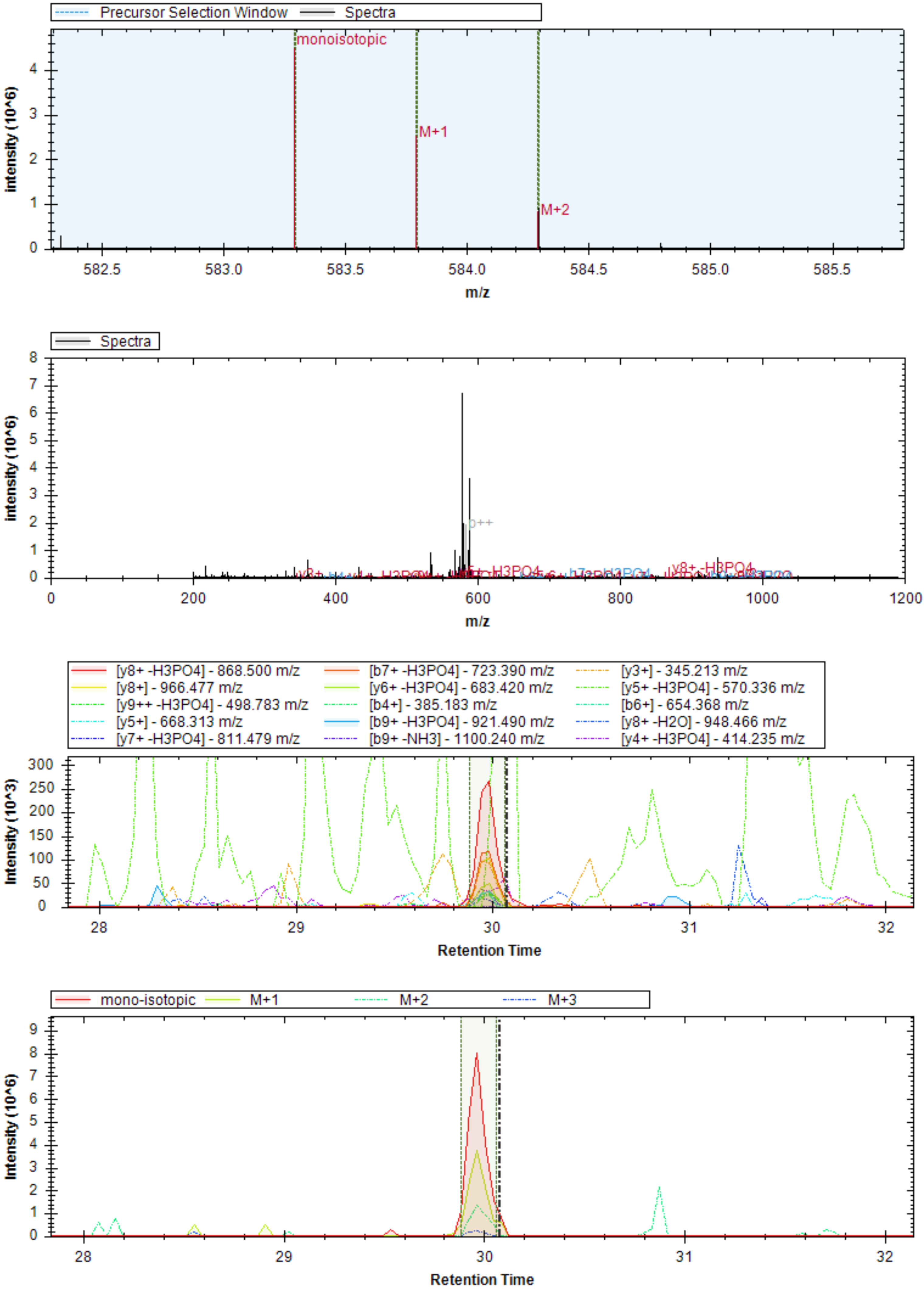

Q9D5Y0 – _ITDT[+H+O3+P]KKTDDLYWR_.3

Fig4_MP-DIA-barrel-cortex-p15-1_MHRM.htrms

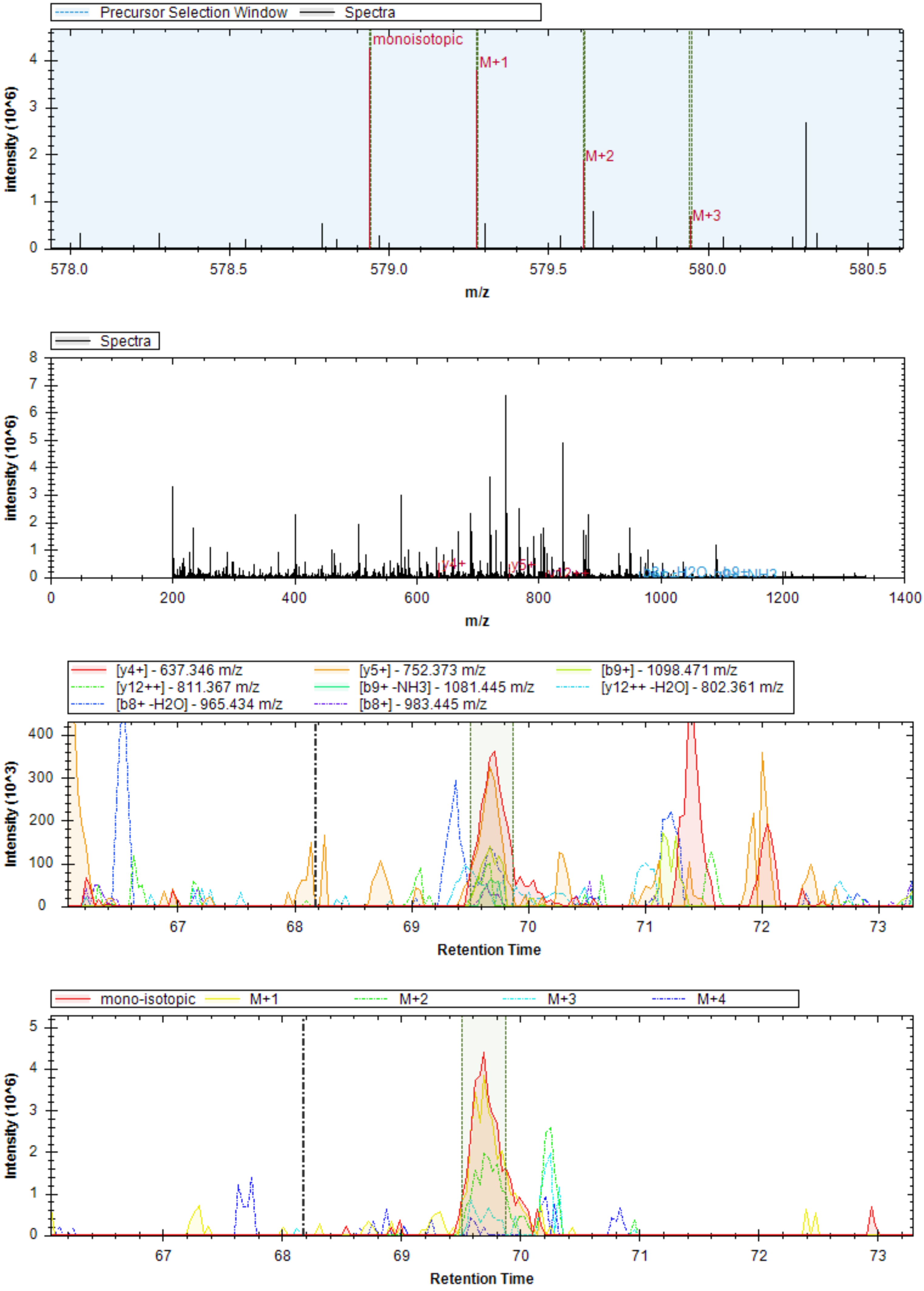

P20357 – _APHWTSASLTEAAAHPHS[+H+O3+P]PEMK_.4

Fig4_MP-DIA-barrel-cortex-p15-1_MHRM.htrms

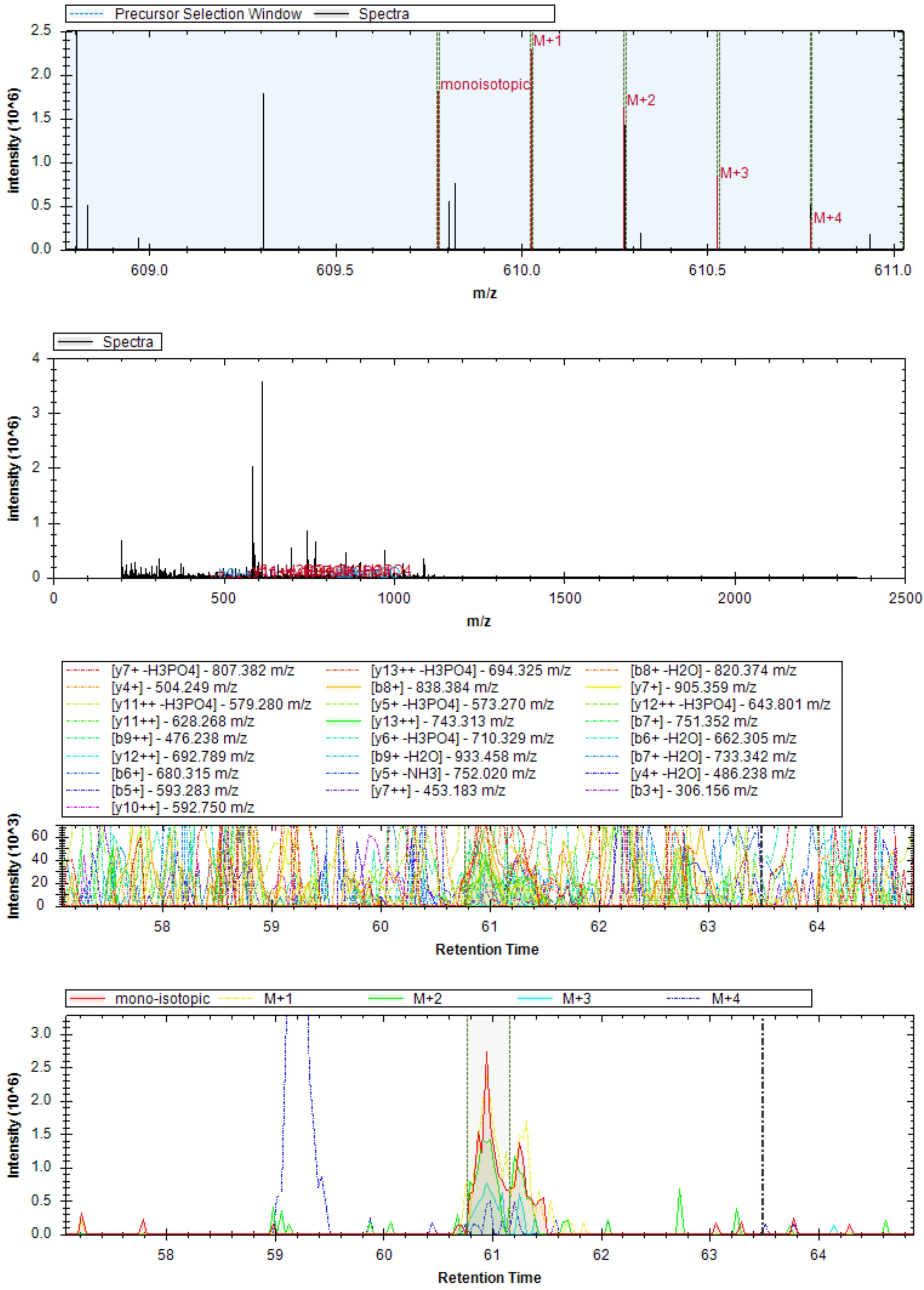

P26645 – _AEDGAAPSPSS[+H+O3+P]ETPKKK_.3
Fig4_MP-DIA-barrel-cortex-p15-1_MHRM.htrms

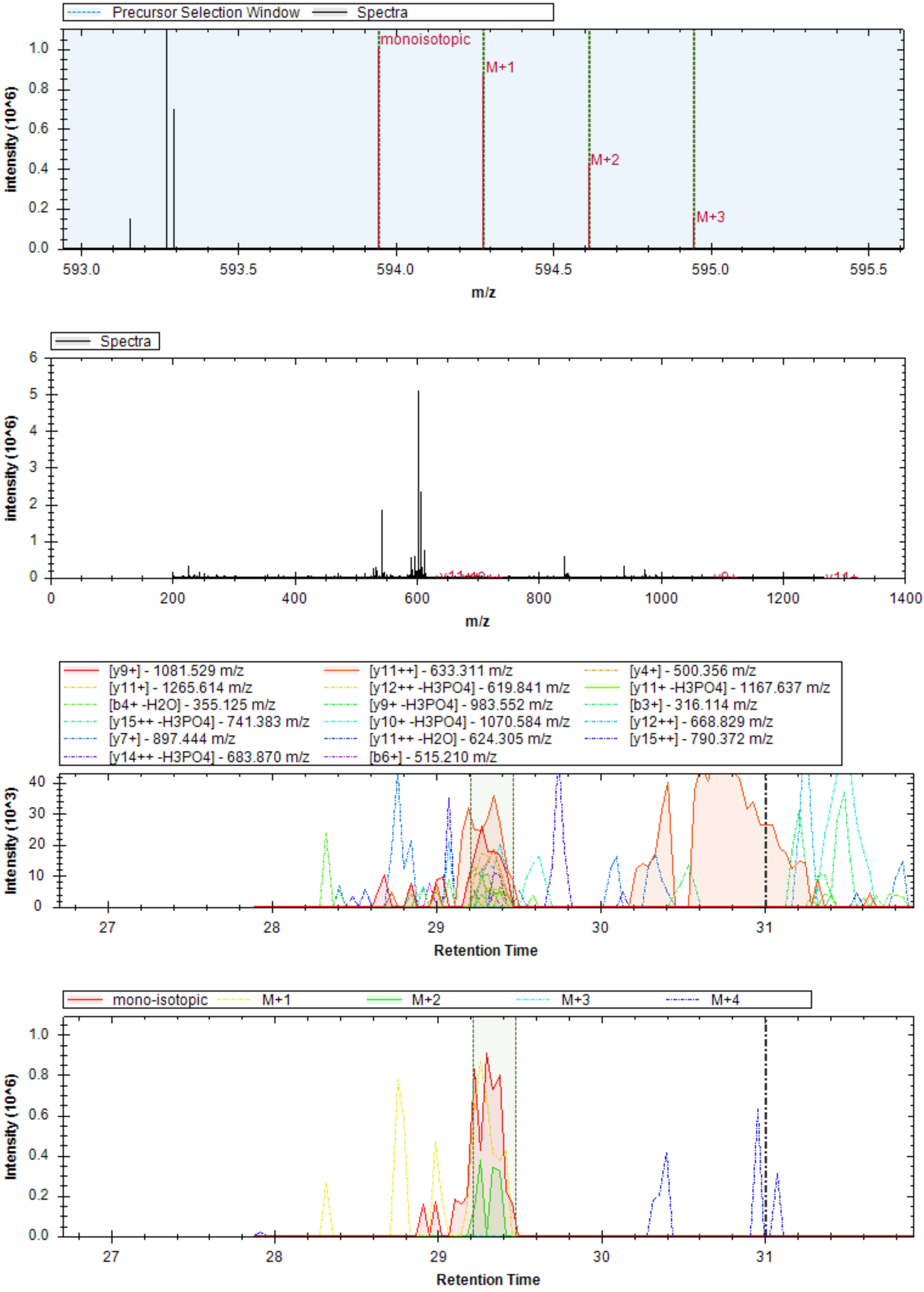

Q62261 – _RPPS[+H+O3+P]PDPNTK_.2

Fig4_MP-DIA-barrel-cortex-p15-1_MHRM.htrms

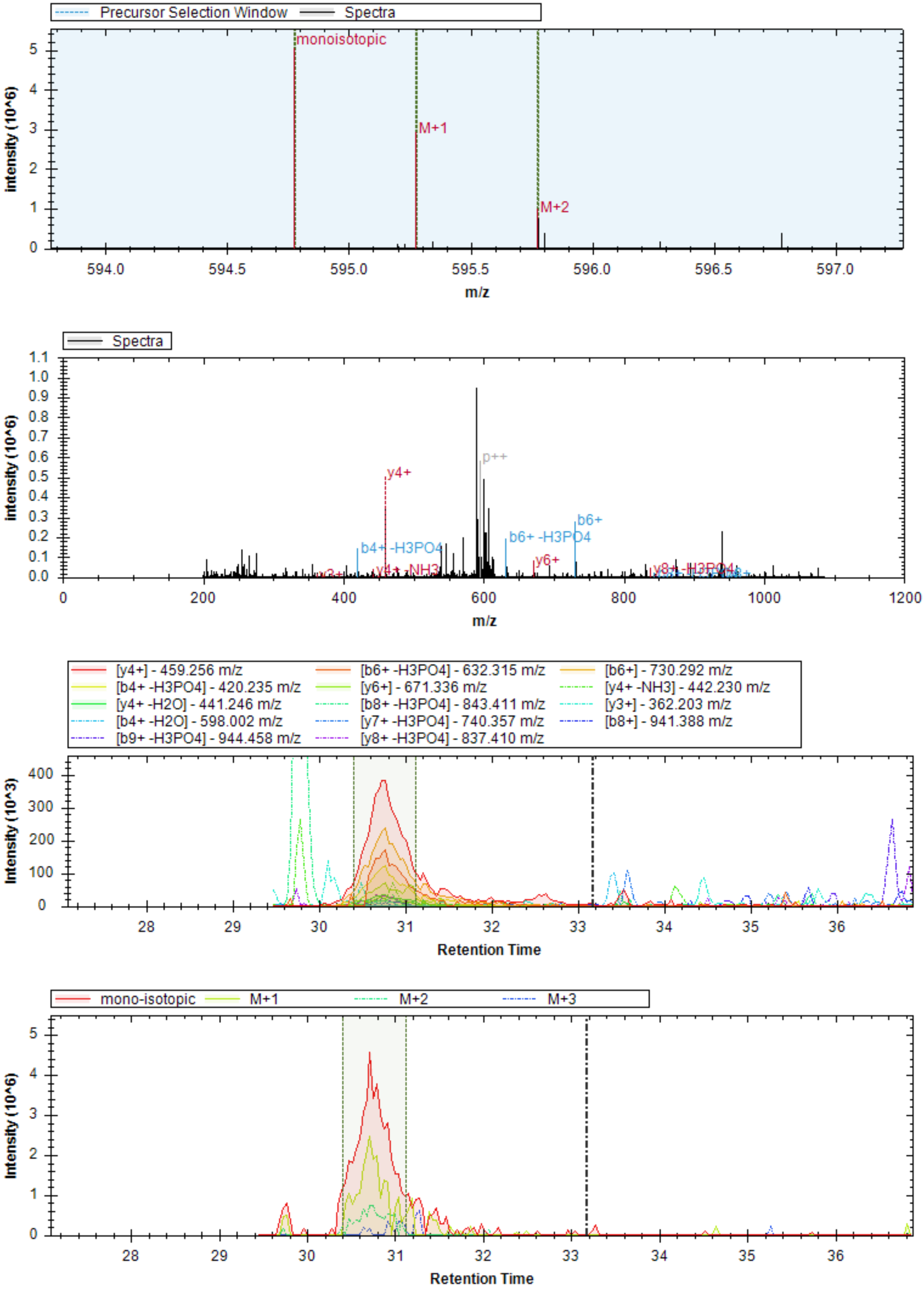

Q80VN0 – _ELTAQIT[+H+O3+P]SIK_.2
Fig4_MP-DIA-barrel-cortex-p15-1_MHRM.htrms

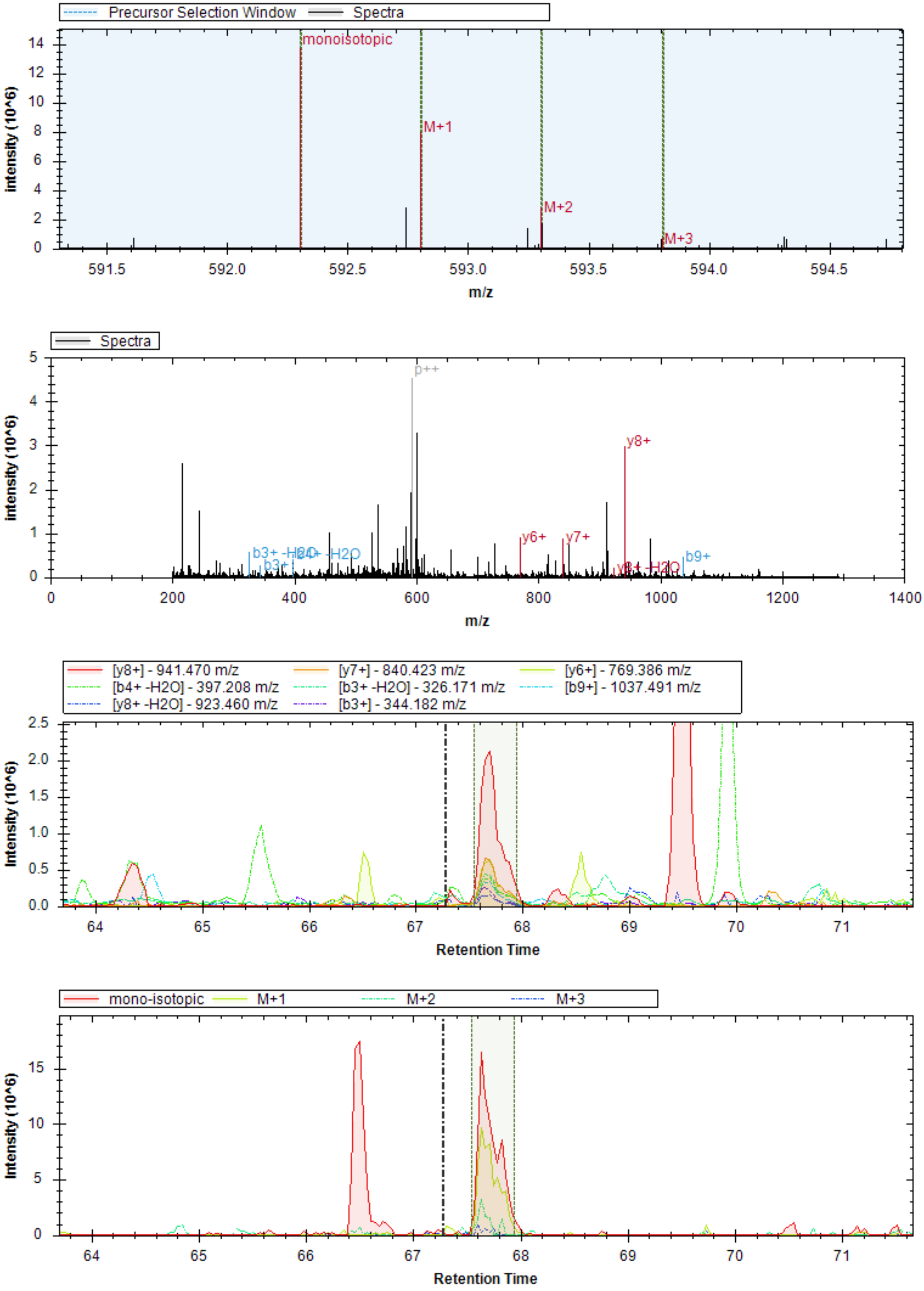

Q8C419 – _SAS[+H+O3+P]AHNLSSEK_.2
Fig4_MP-DIA-barrel-cortex-p15-1_MHRM.htrms

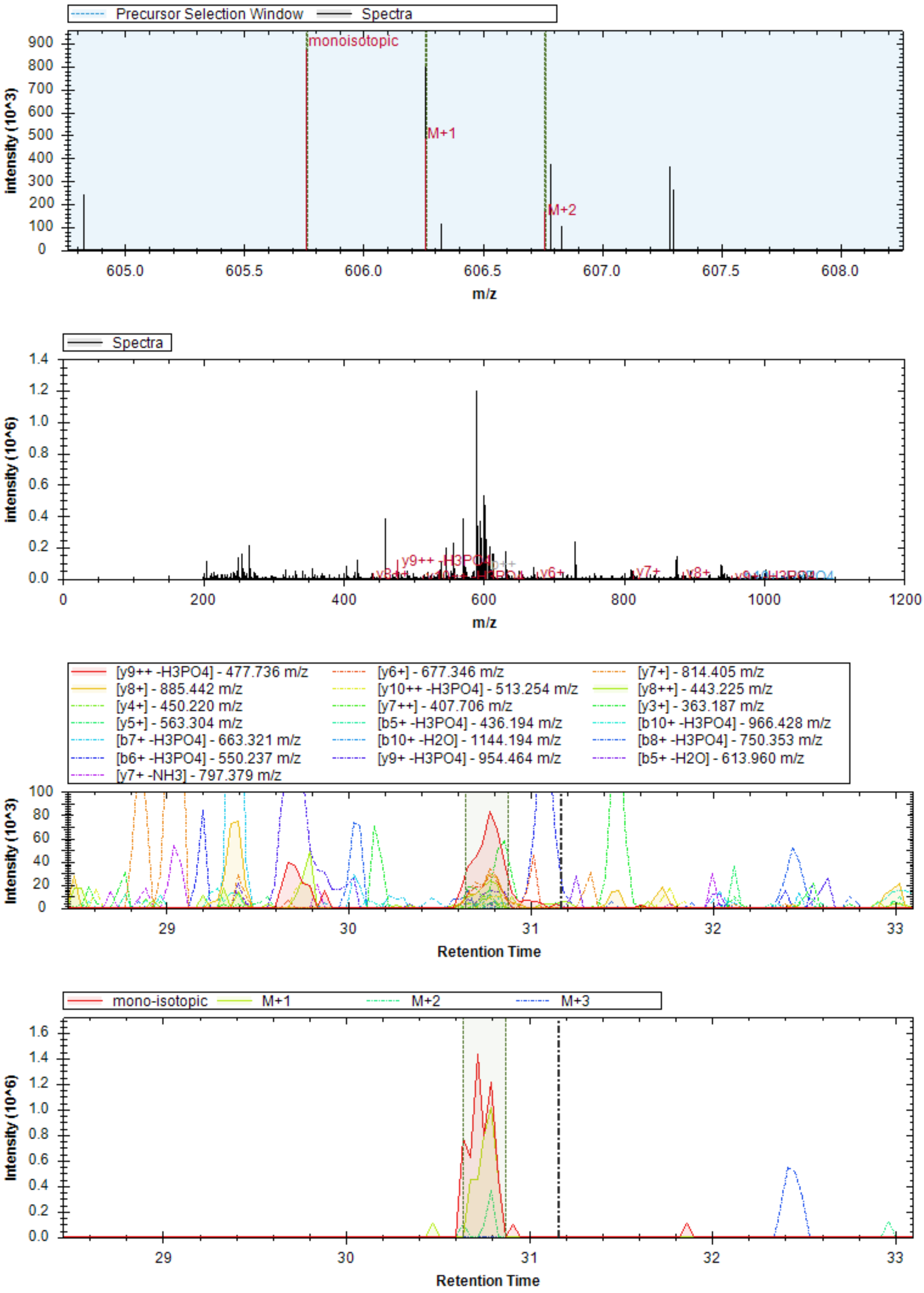

Q99104 – _AIS[+H+O3+P]PTSATSSGR_.2
Fig4_MP-DIA-barrel-cortex-p15-1_MHRM.htrms

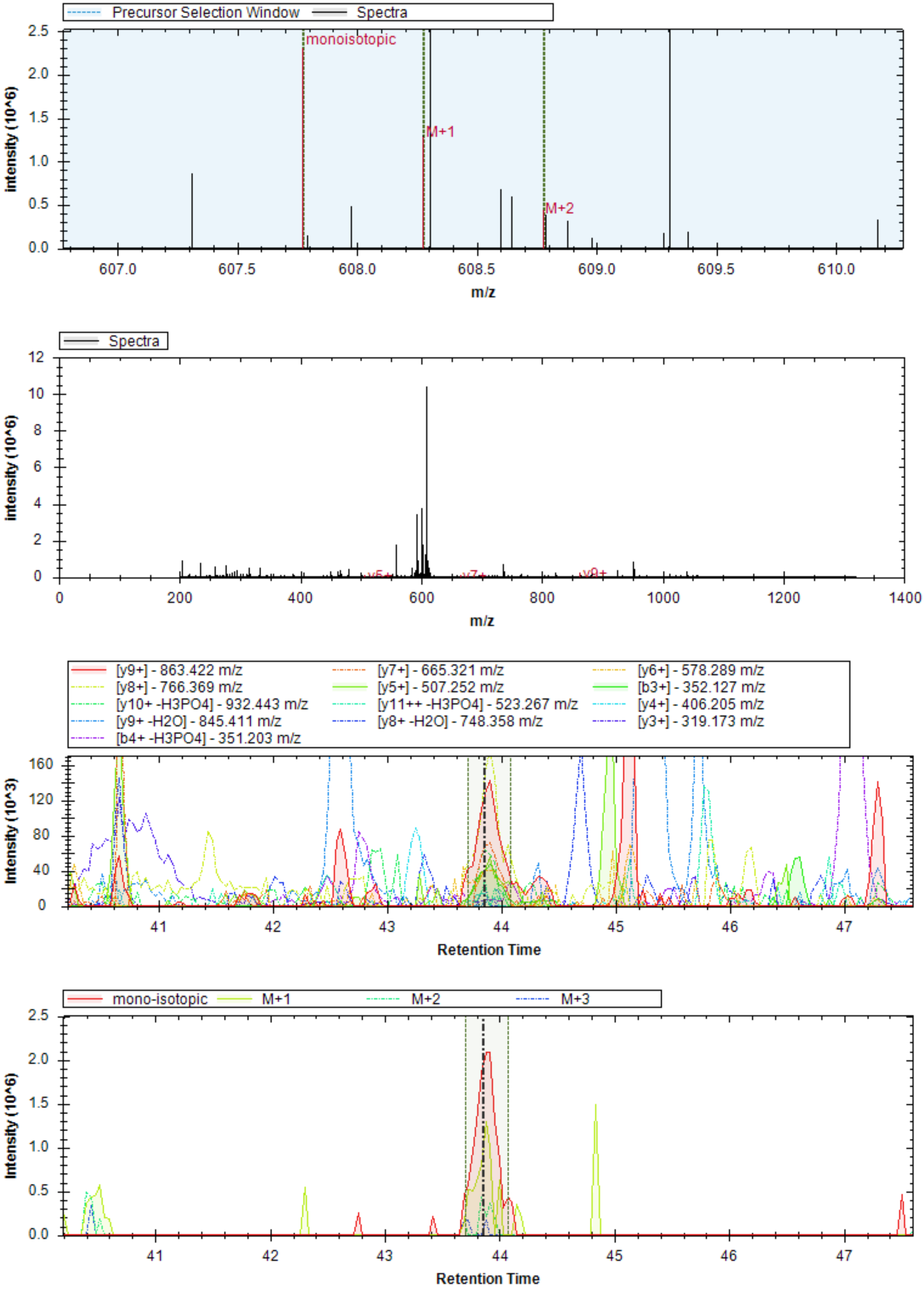

Q9QWI6 – _LSYAGGRPPSYAGS[+H+O3+P]PVHHAAER_.4

Fig4_MP-DIA-barrel-cortex-p15-1_MHRM.htrms

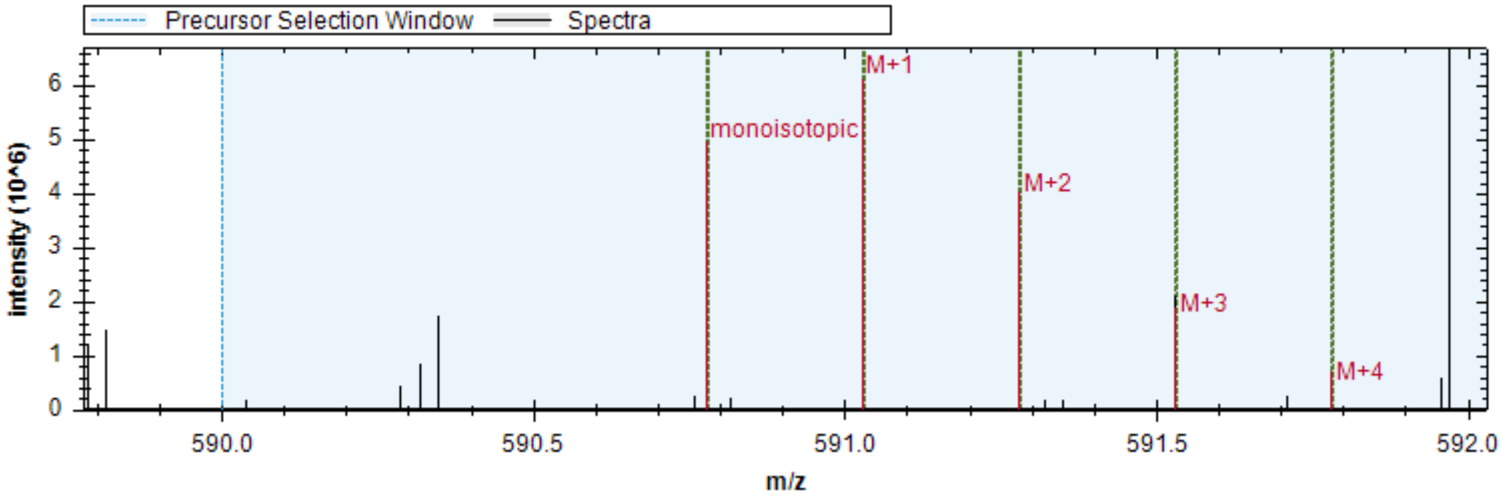

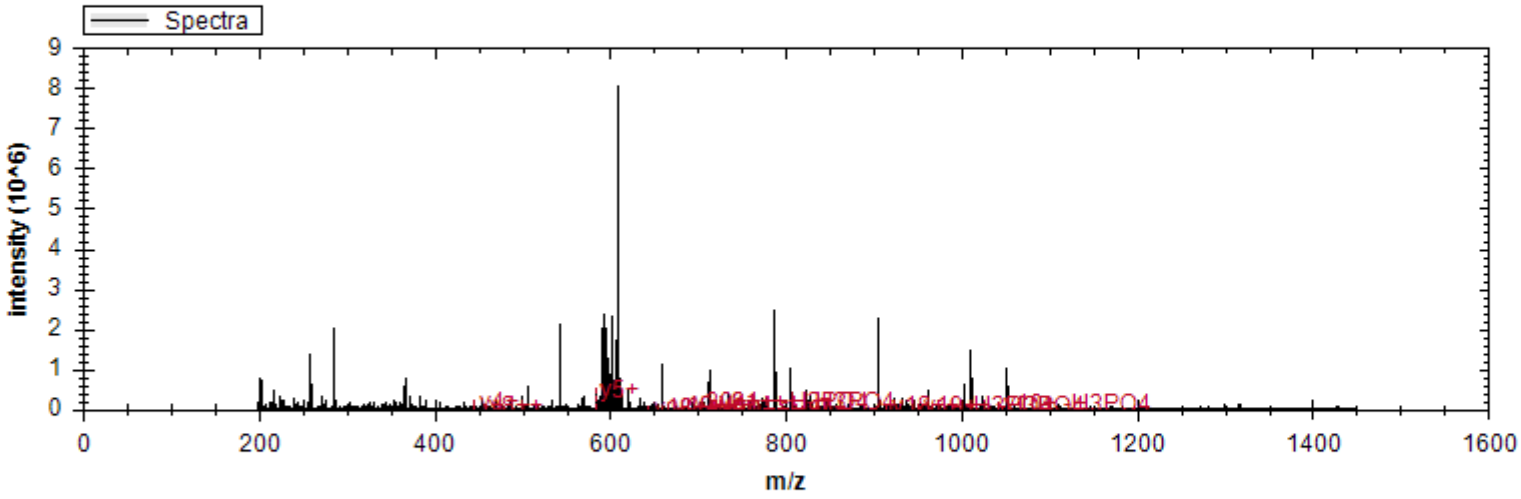

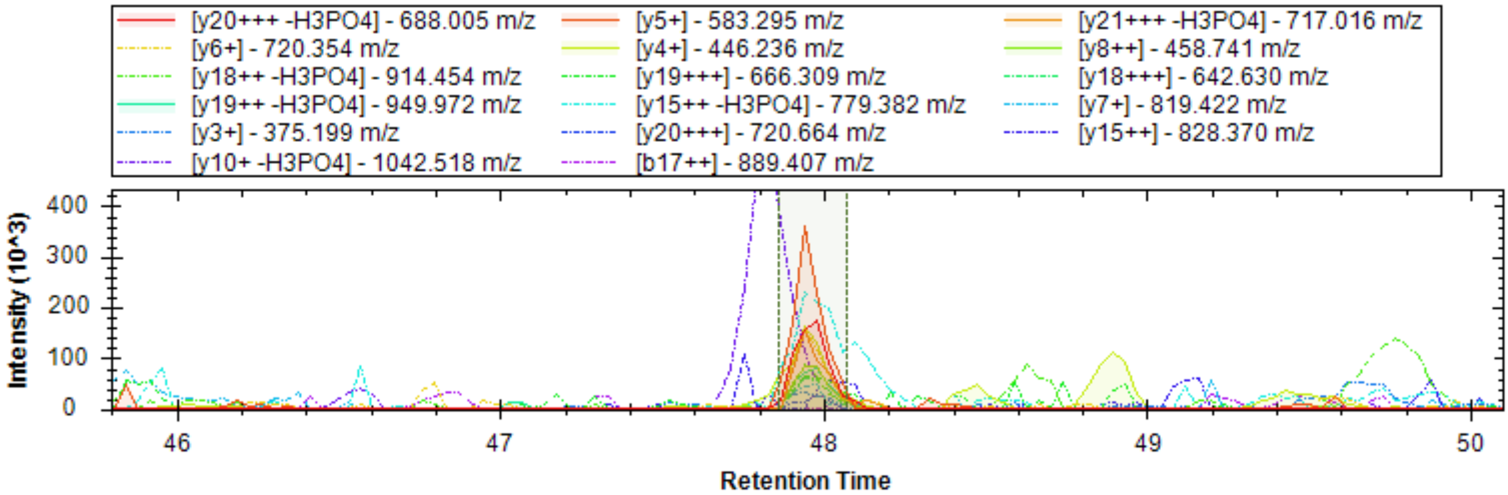

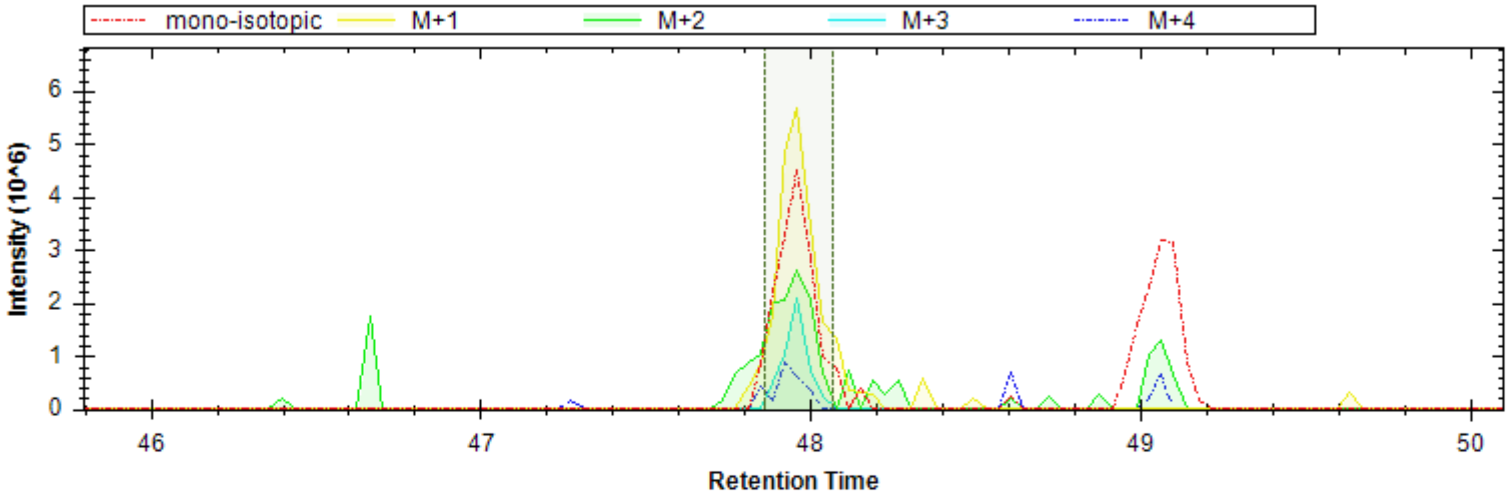

P60904 – _SLS[+H+O3+P]TSGESLYHVLGLDK_.3
Fig4_MP-DIA-barrel-cortex-p15-1_MHRM.htrms

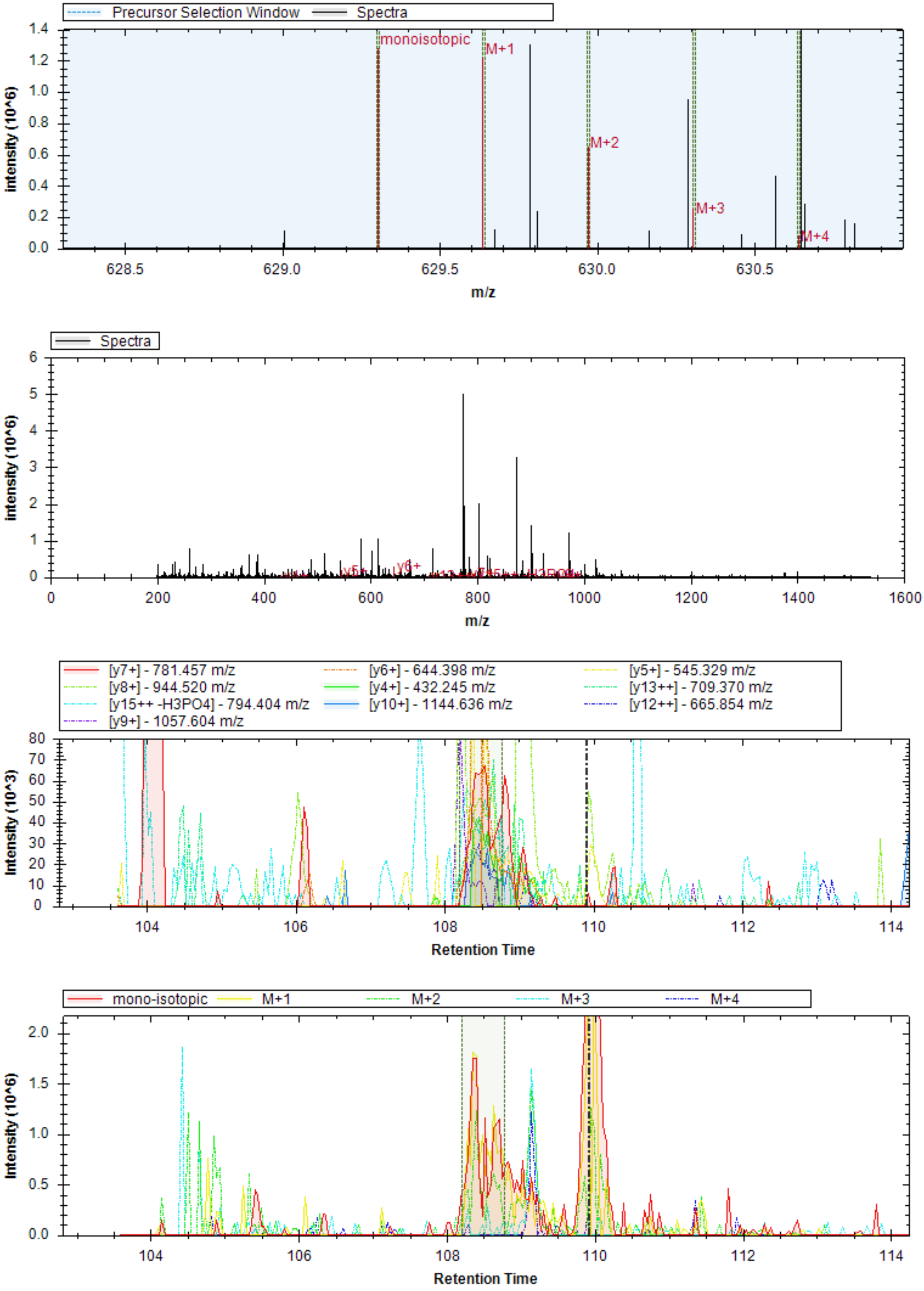

P60904 – _SLST[+H+O3+P]SGESLYHVLGLDK_.3
Fig4_MP-DIA-barrel-cortex-p15-1_MHRM.htrms

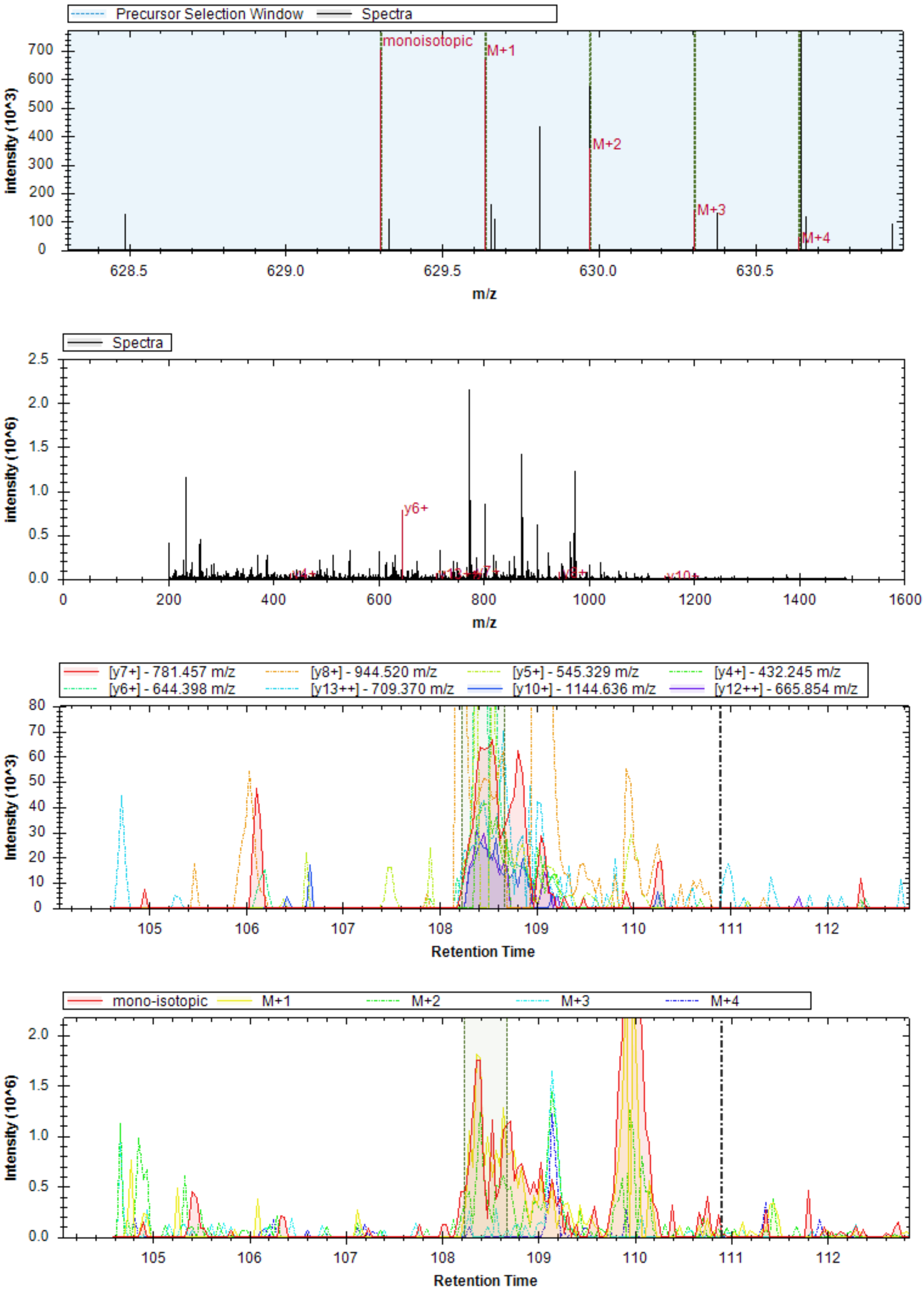

P39447 – _SSGIIRLHT[+H+O3+P]IK_.2
Fig4_MP-DIA-barrel-cortex-p15-1_MHRM.htrms

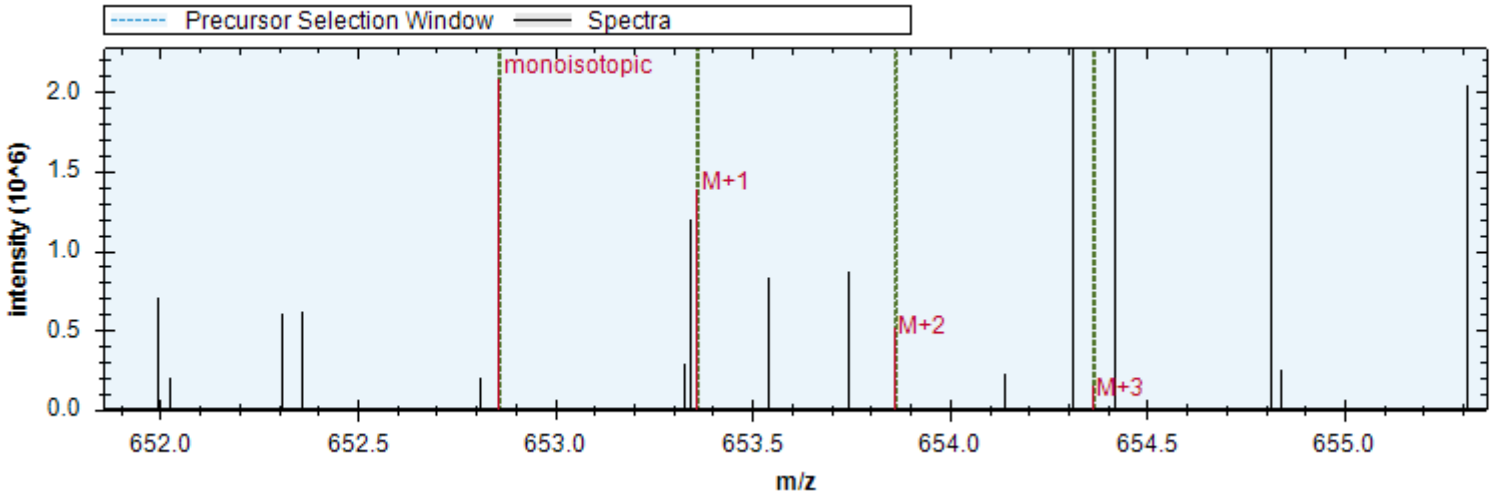

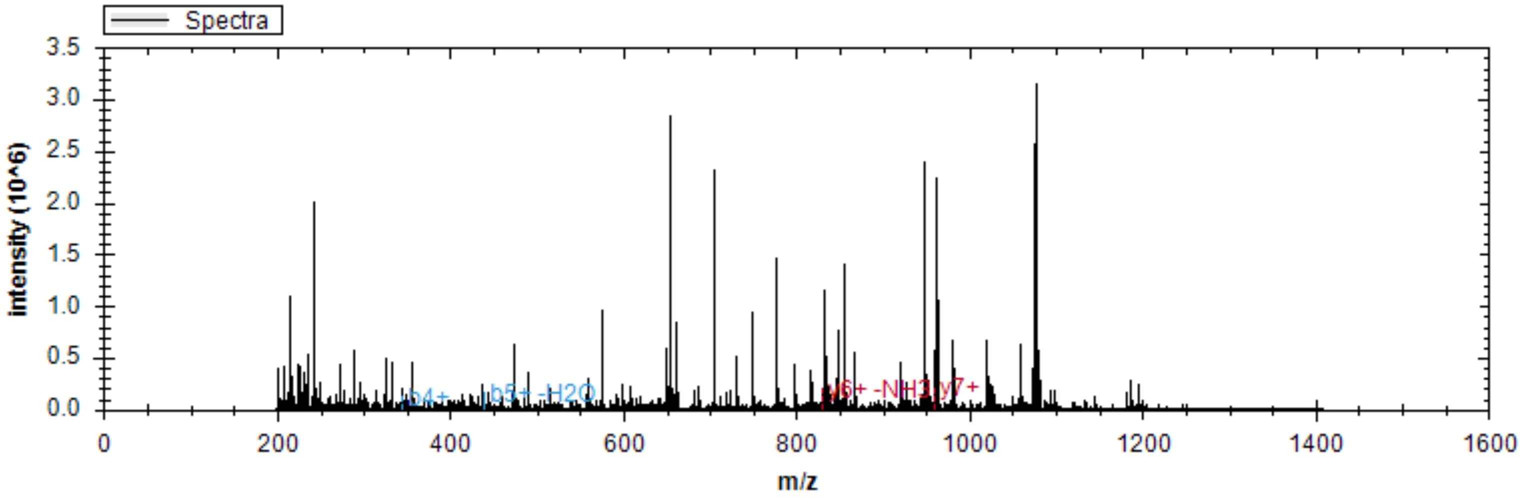

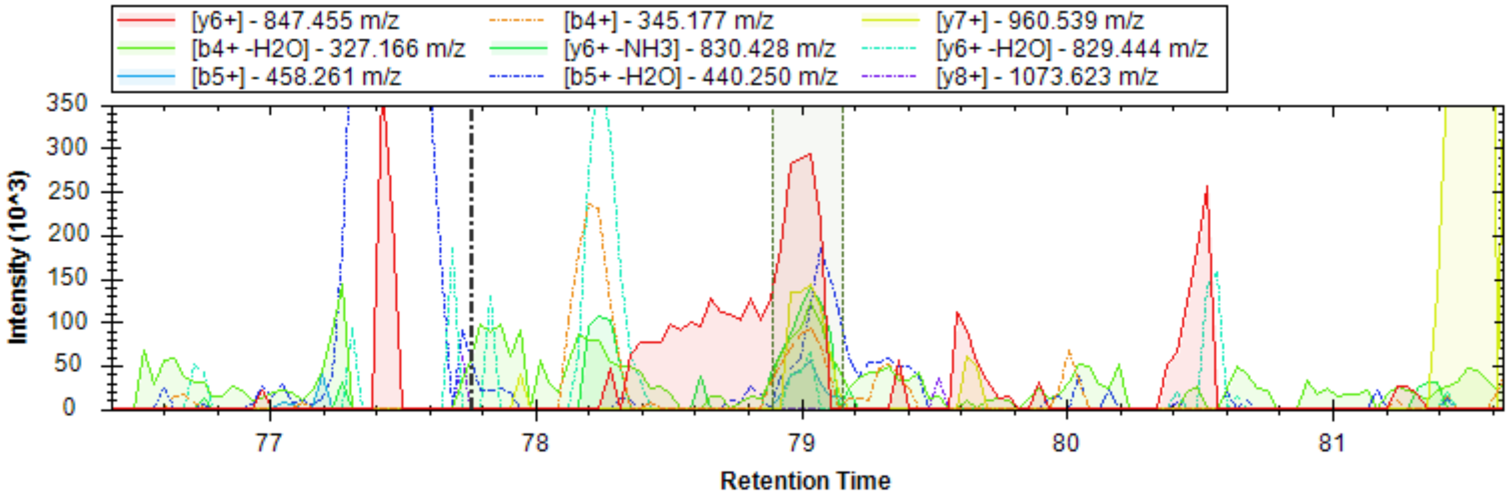

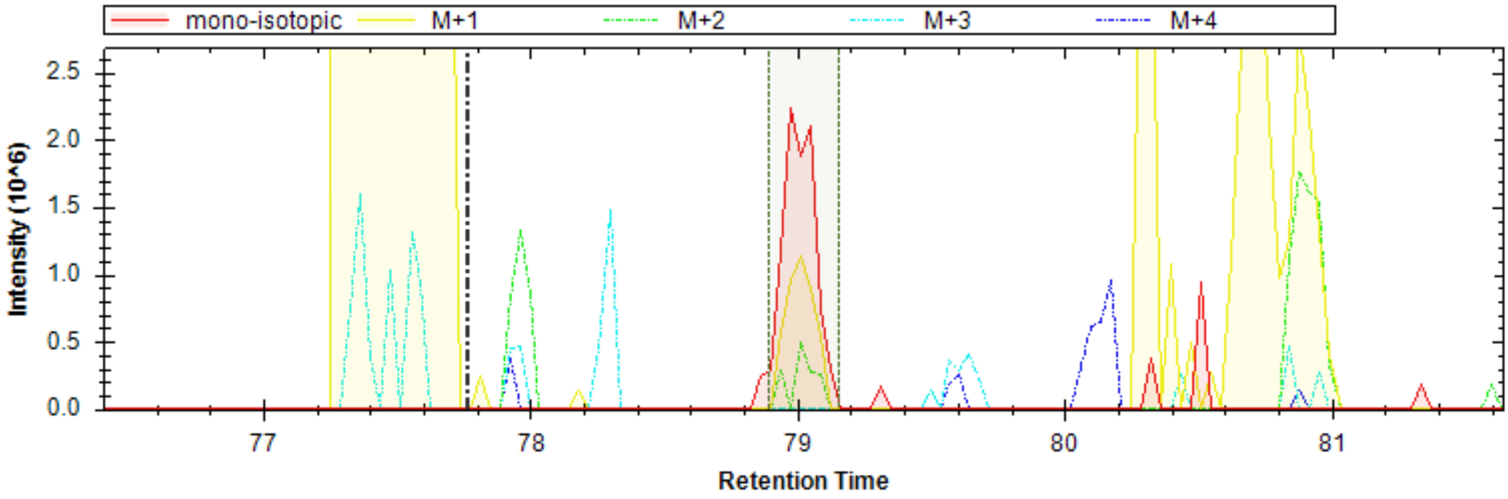

Q8BLJ3 – _M[+O]AS[+H+O3+P]SQGKNELK_.2
Fig4_MP-DIA-barrel-cortex-p15-1_MHRM.htrms

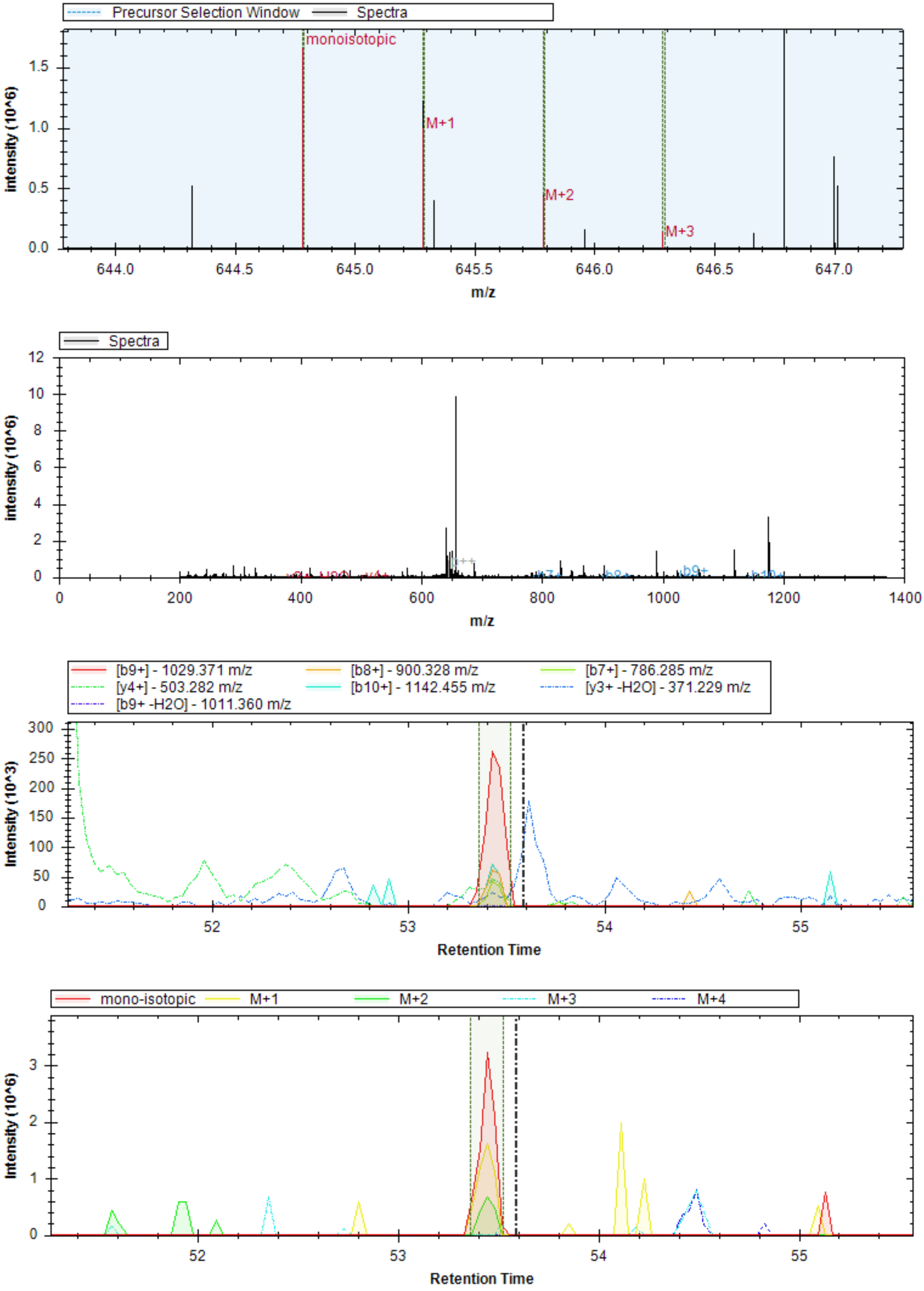

Q8CC35-3 – _AAS[+H+O3+P]PAKPSSLDLVPNLPR_.3
Fig4_MP-DIA-barrel-cortex-p15-1_MHRM.htrms

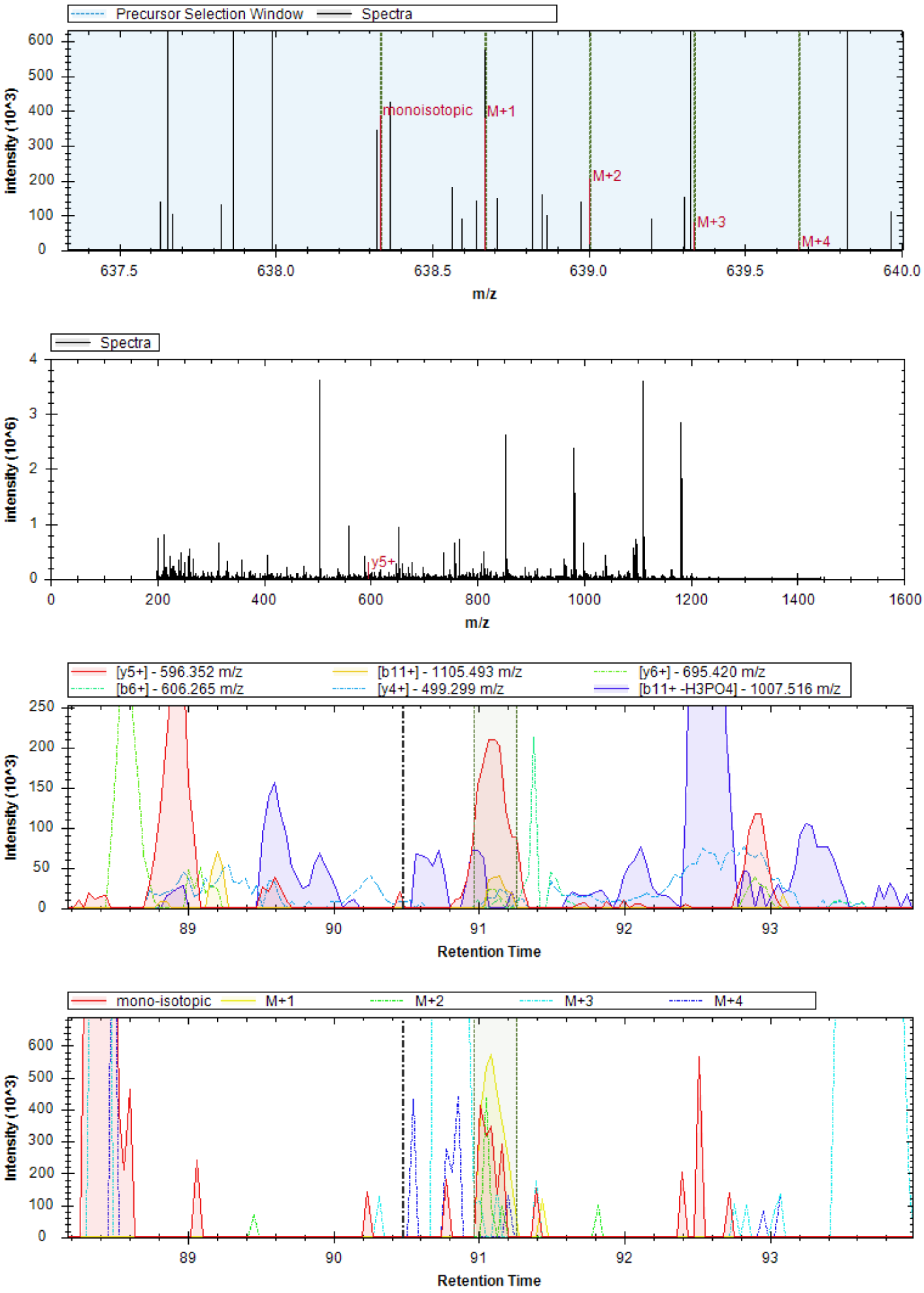

P13595 – _GVTASS[+H+O3+P]SSPASAPK_.2
Fig4_MP-DIA-barrel-cortex-p15-1_MHRM.htrms

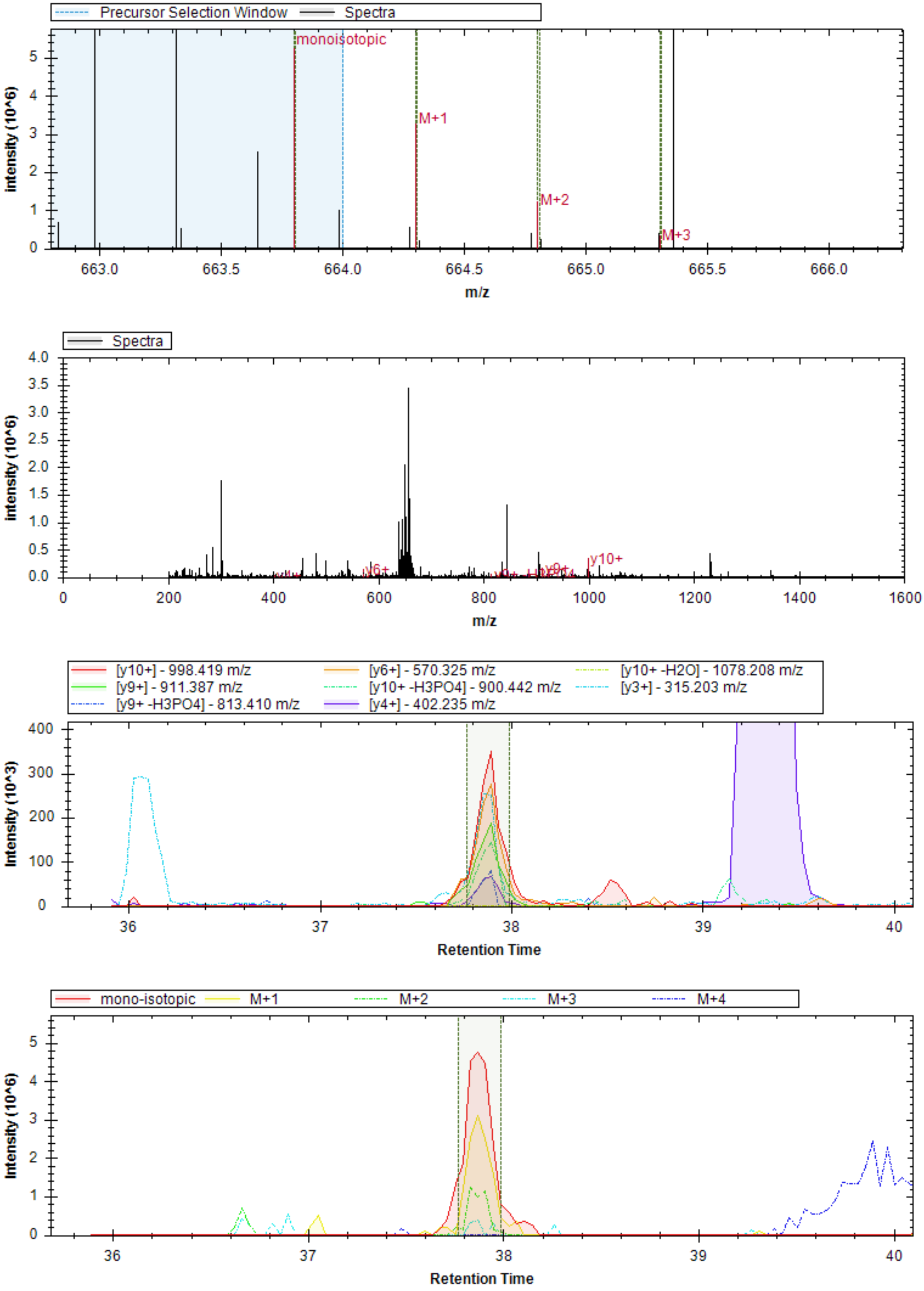

P13595 – _GVTASSS[+H+O3+P]SPASAPK_.2
Fig4_MP-DIA-barrel-cortex-p15-1_MHRM.htrms

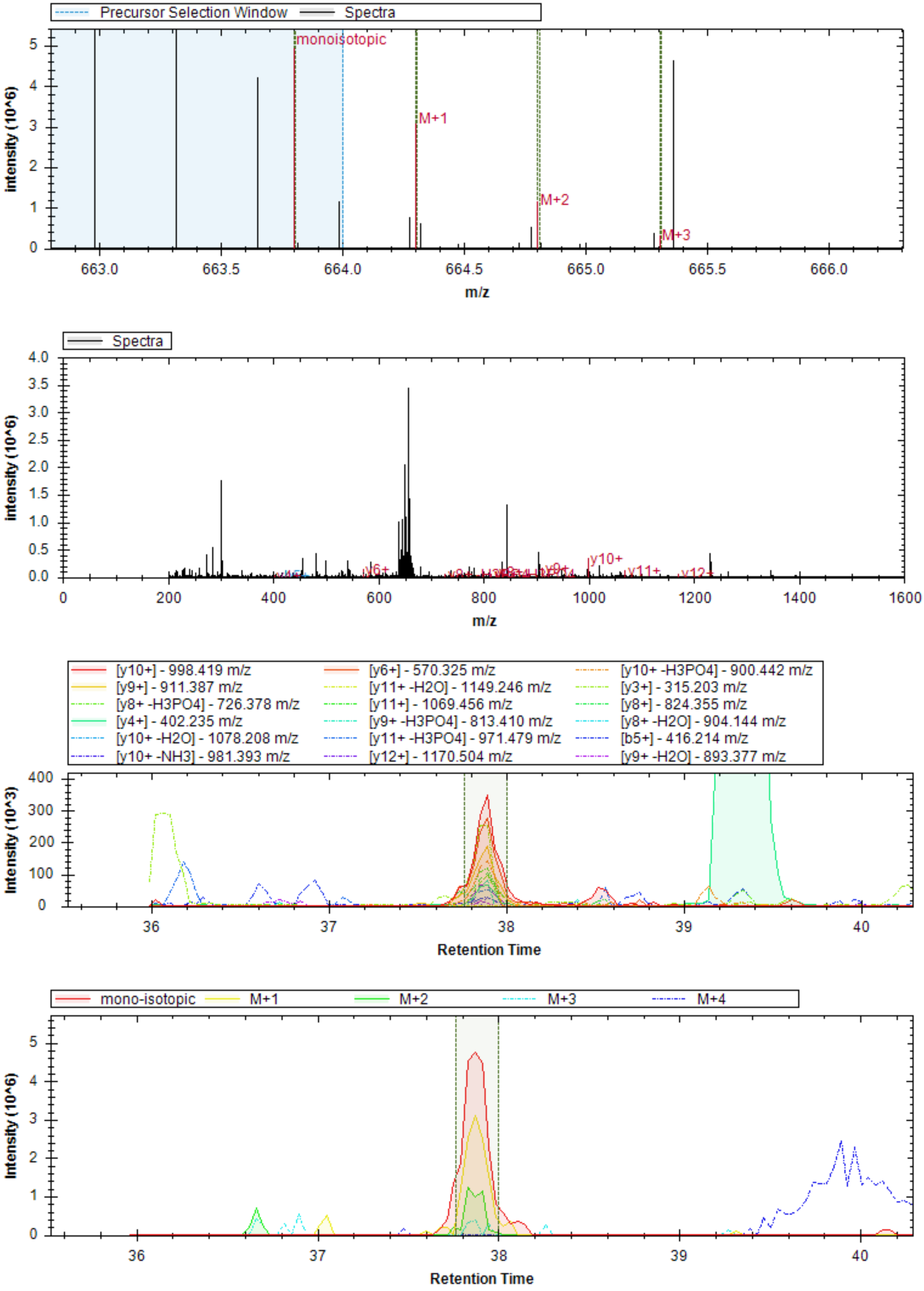

P13595 – _GVTASSSS[+H+O3+P]PASAPK_.2

Fig4_MP-DIA-barrel-cortex-p15-1_MHRM.htrms

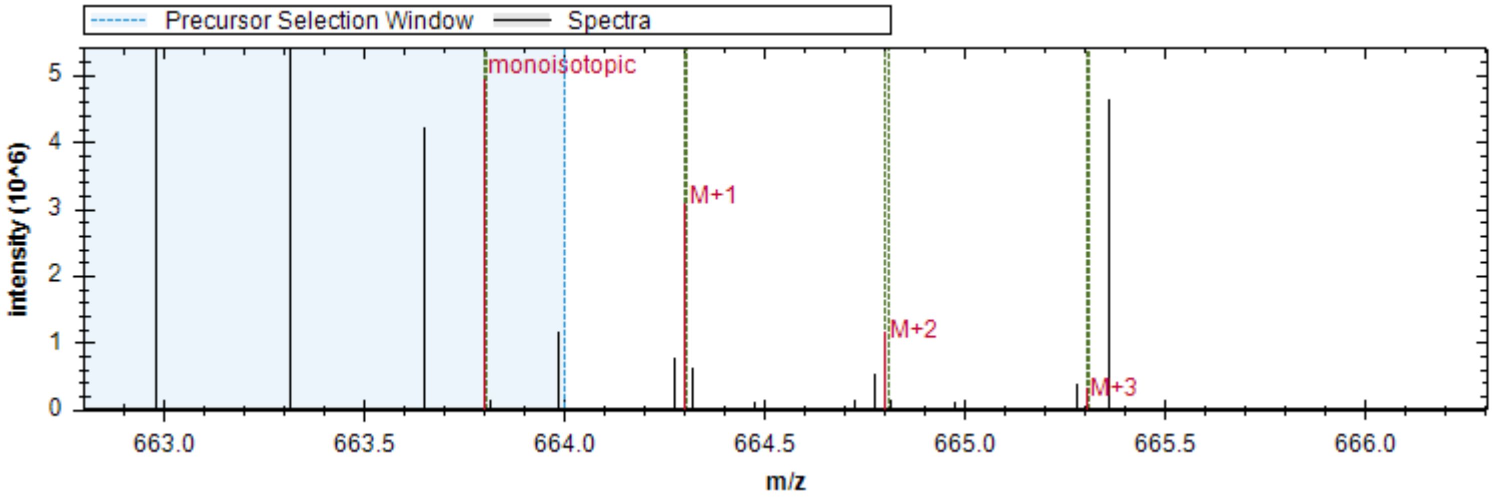

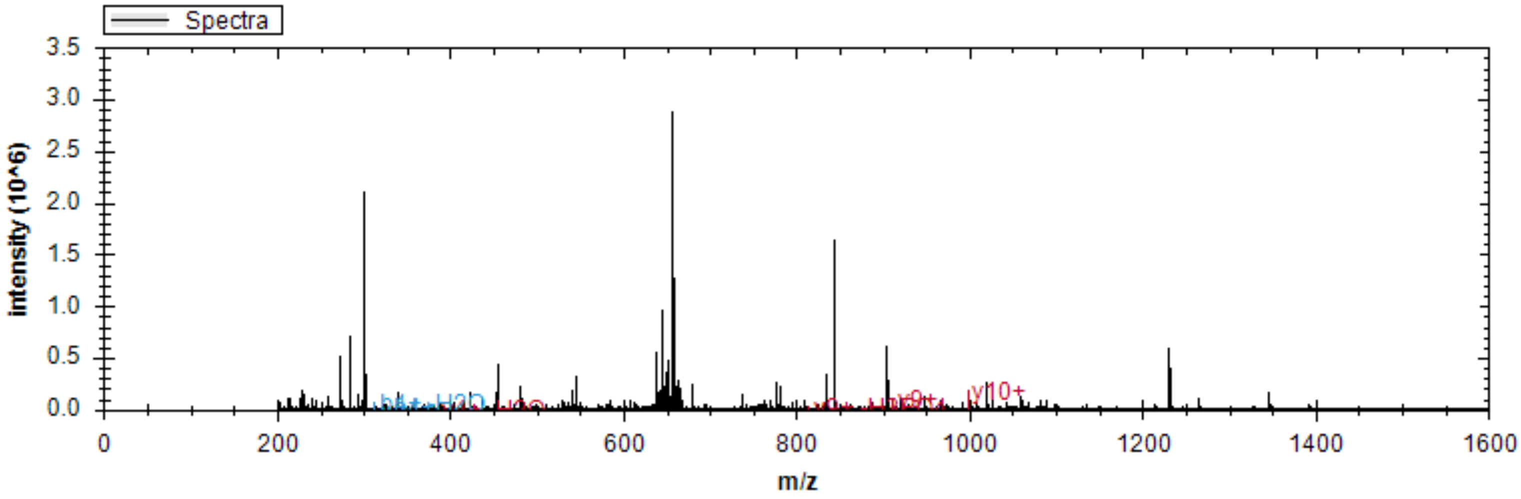

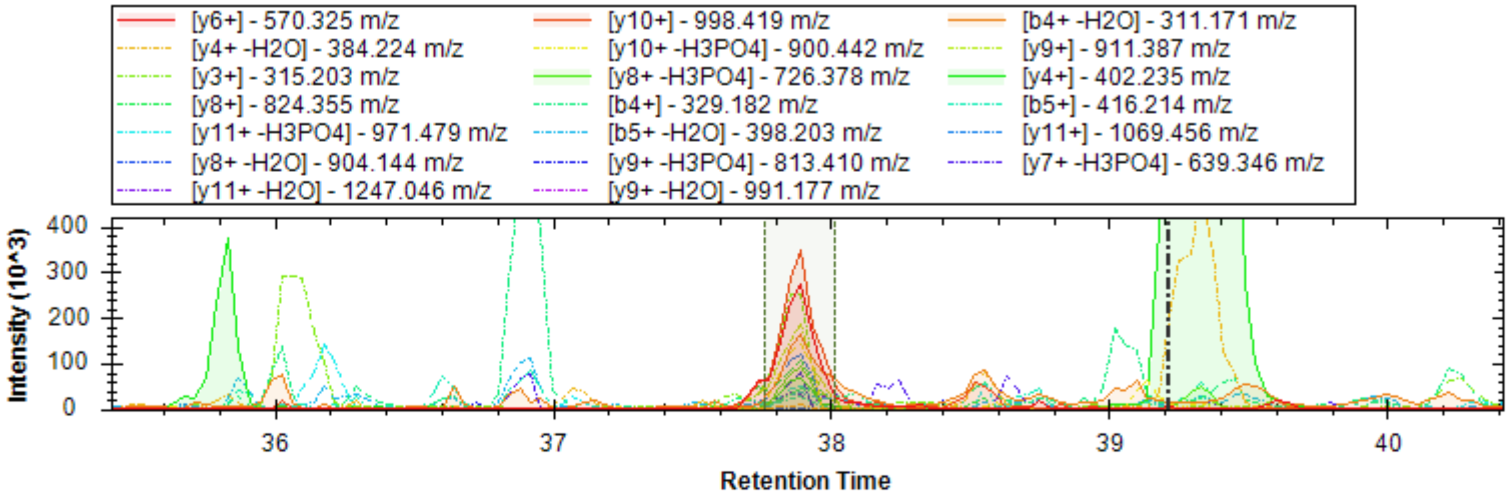

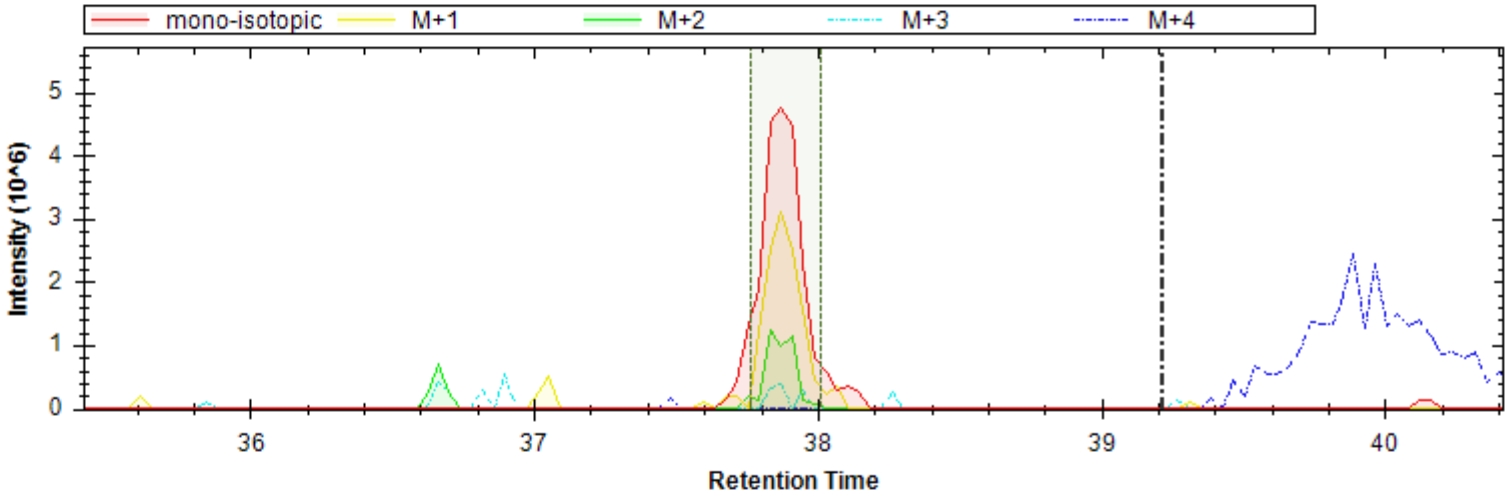

P13595 – _NPPEAATAPAS[+H+O3+P]PK_.2
Fig4_MP-DIA-barrel-cortex-p15-1_MHRM.htrms

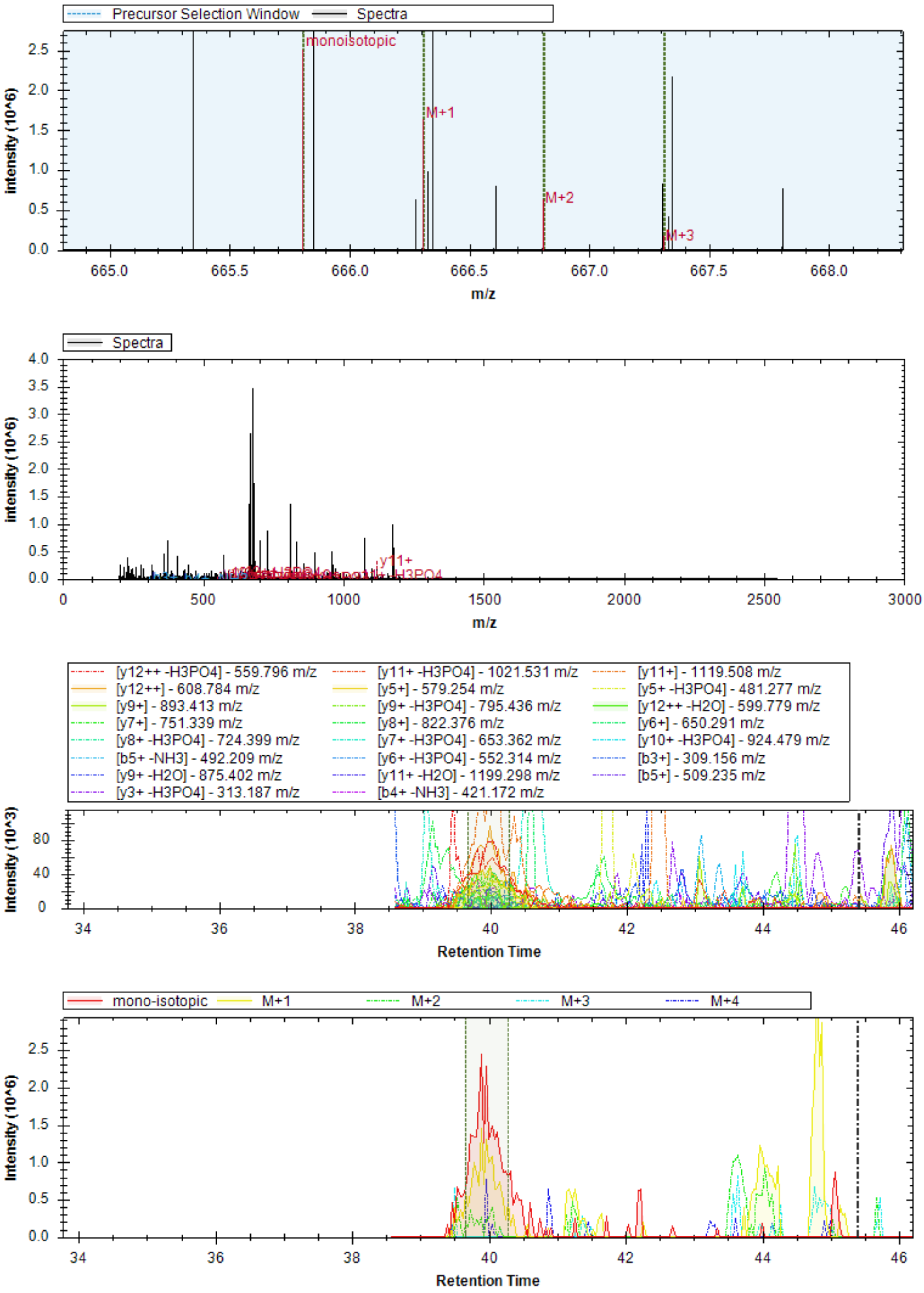

P26361 – _KM[+O]LHS[+H+O3+P]ILHAPMS[+H+O3+P]T[+H+O3+P]ISK_.3
Fig4_MP-DIA-barrel-cortex-p15-1_MHRM.htrms

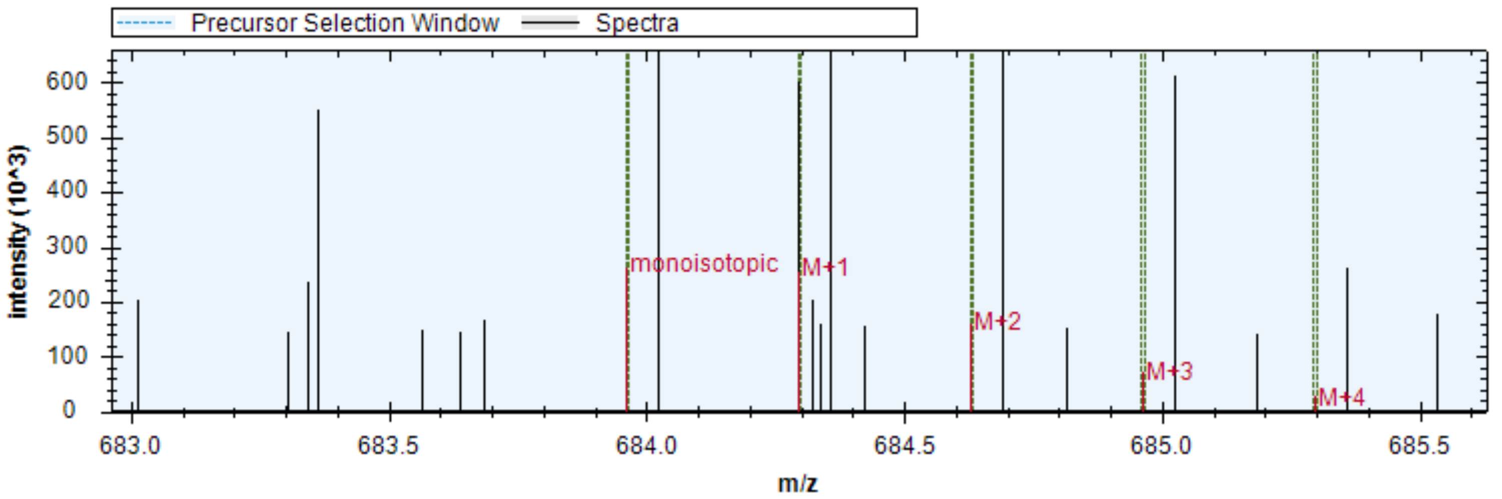

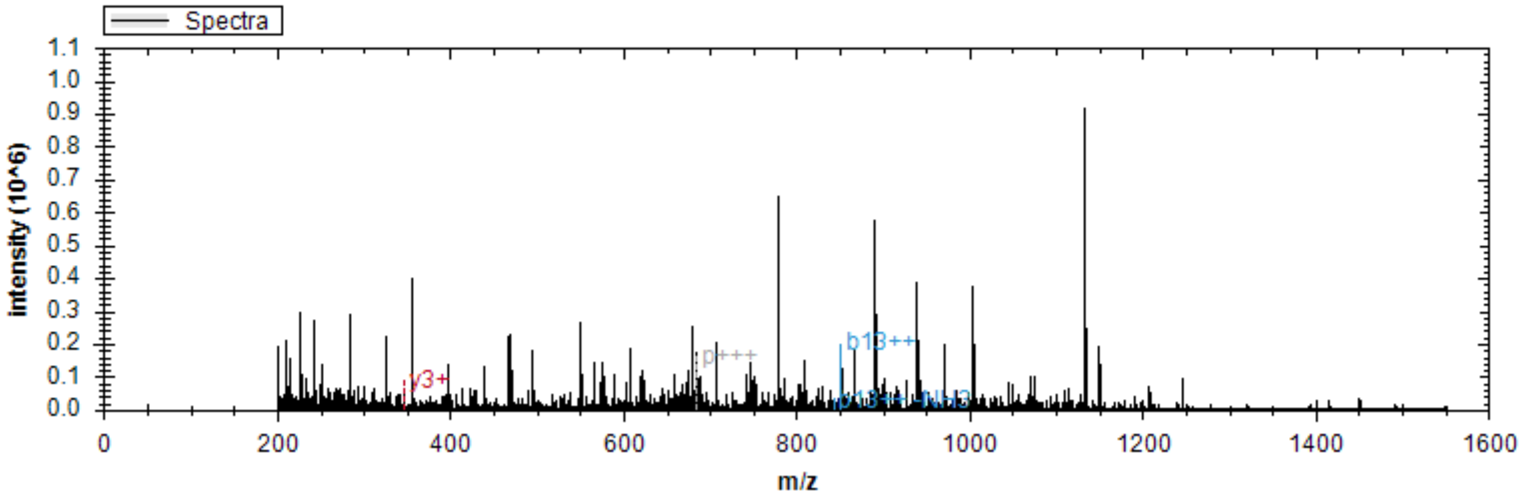

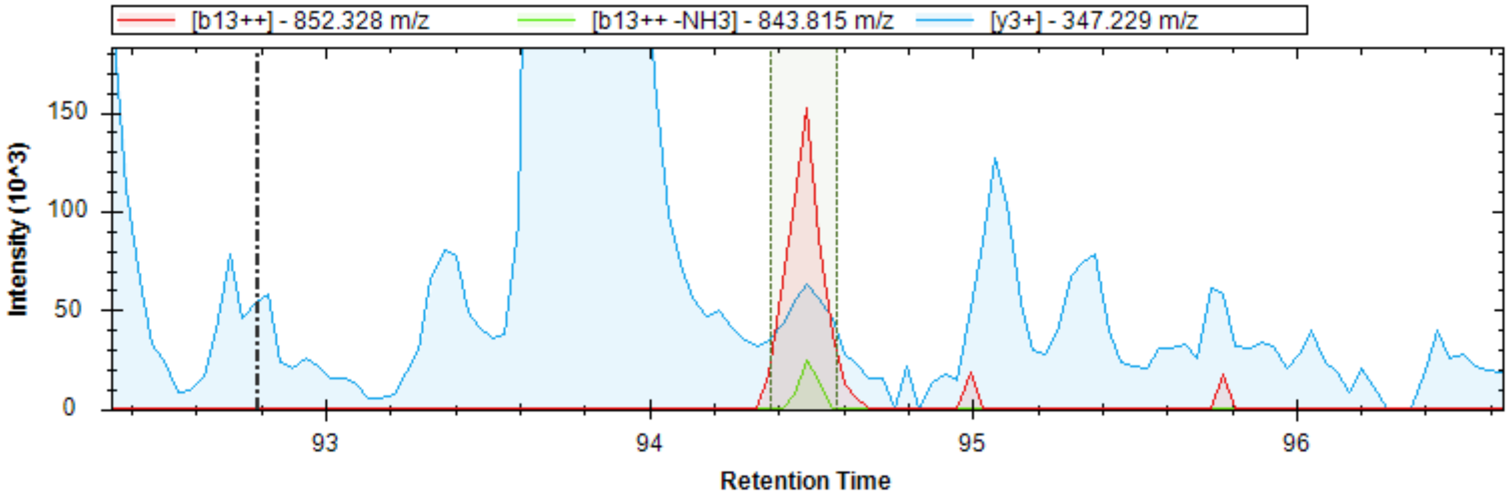

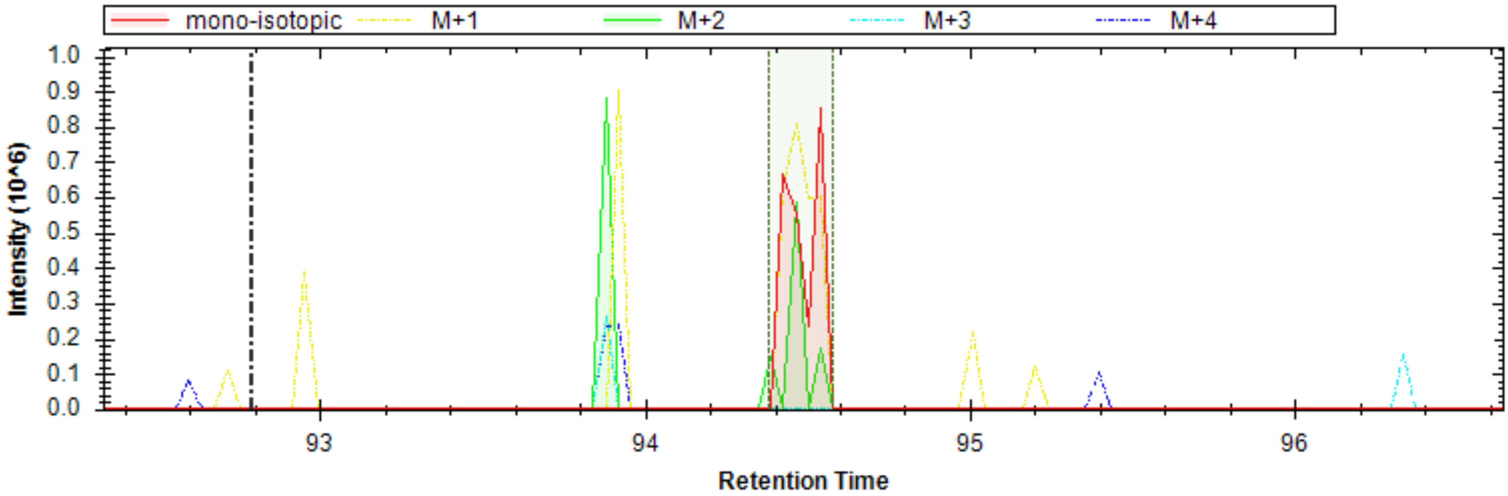

Q6A078 – _T[+H+O3+P]IGLM[+O]KKVVEK_.2
Fig4_MP-DIA-barrel-cortex-p15-1_MHRM.htrms

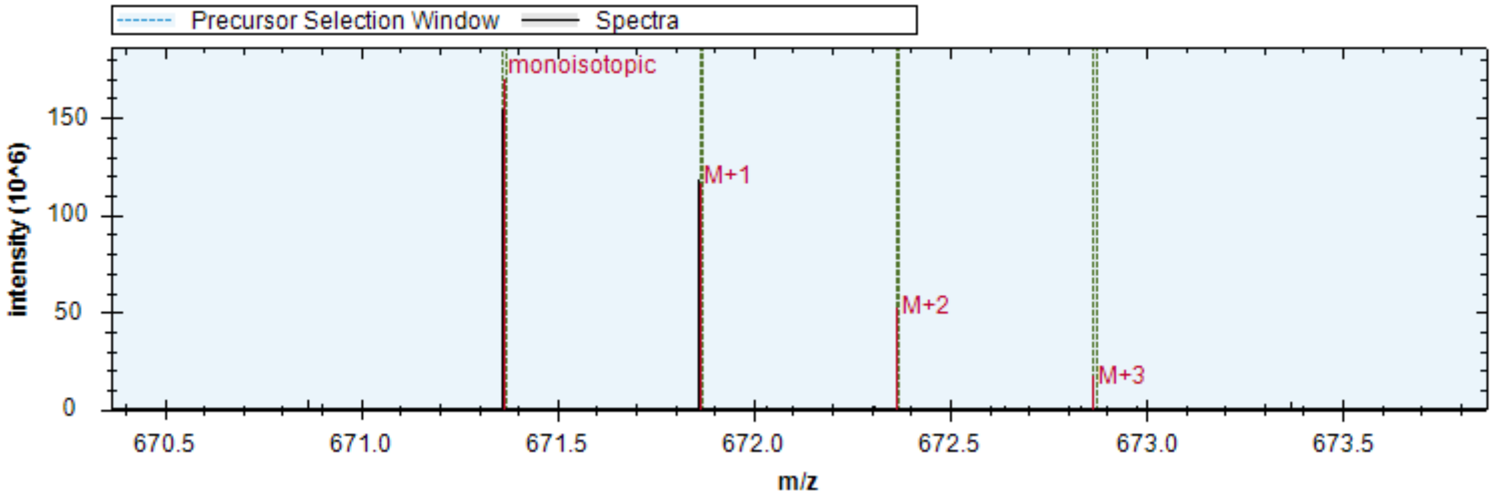

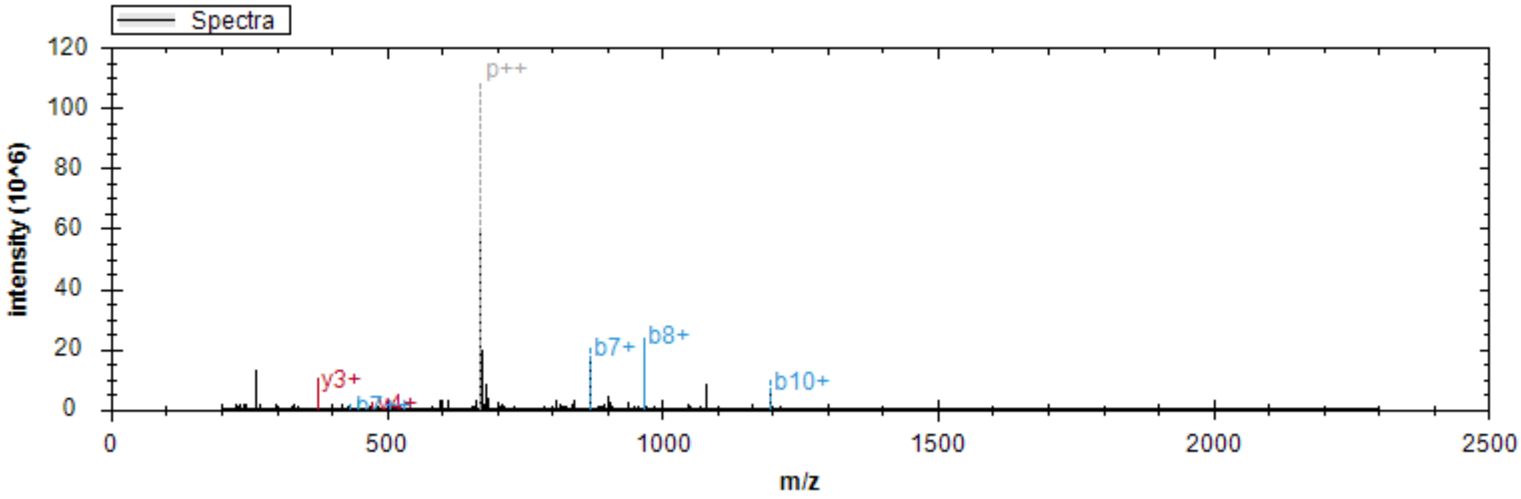

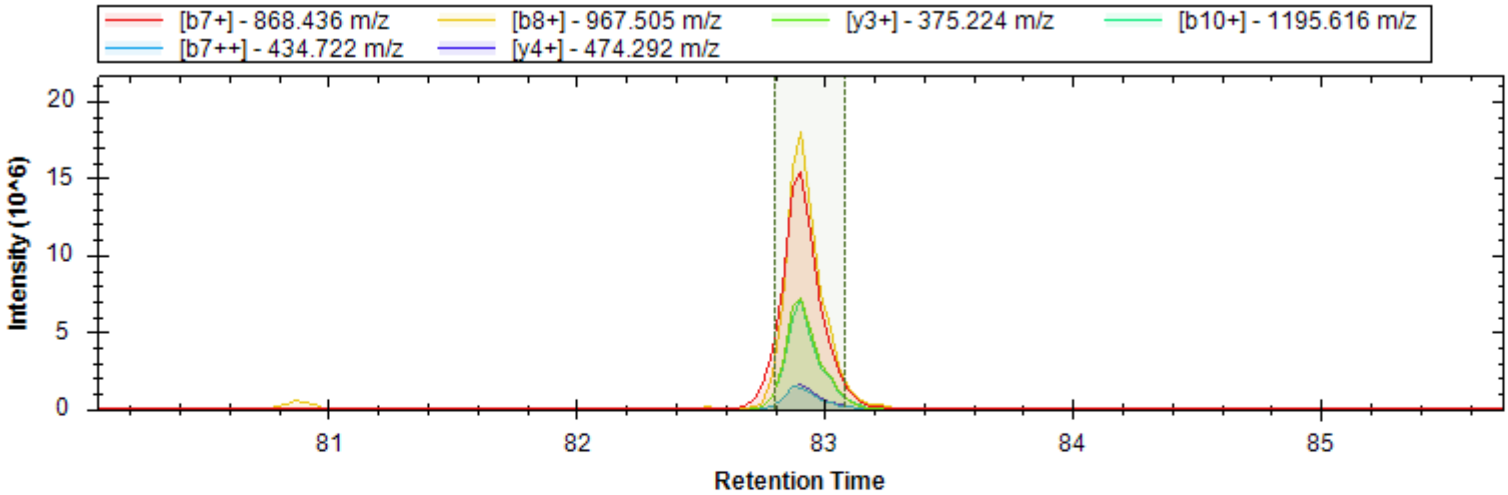

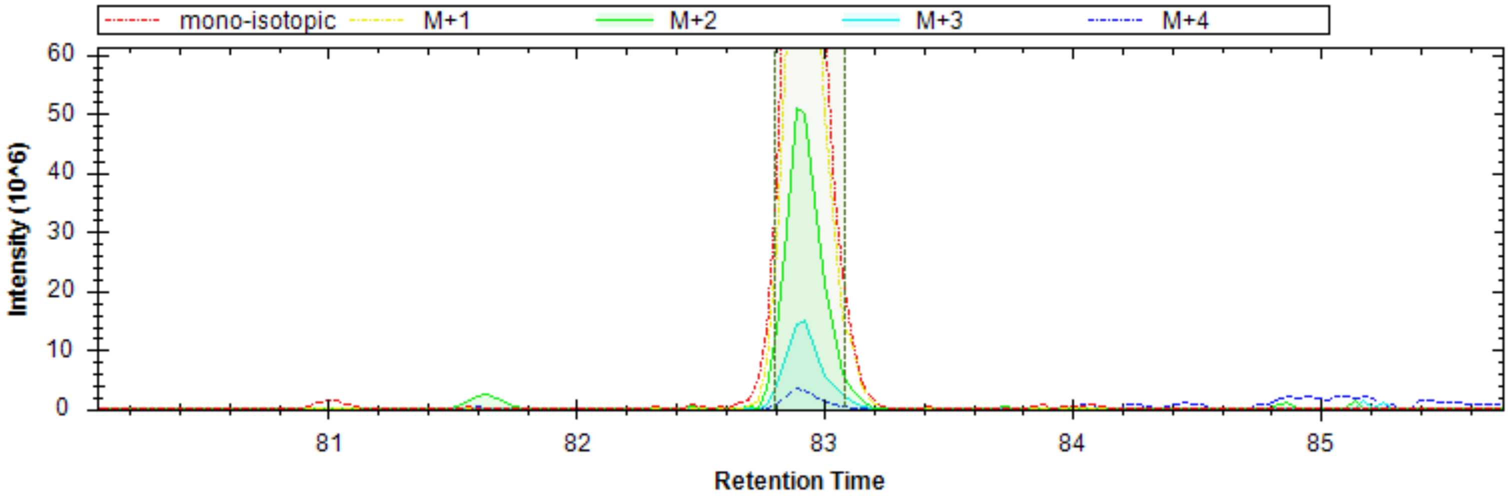

Q8C0E2 – _SMS[+H+O3+P]HQAAIASQR_.2
Fig4_MP-DIA-barrel-cortex-p15-1_MHRM.htrms

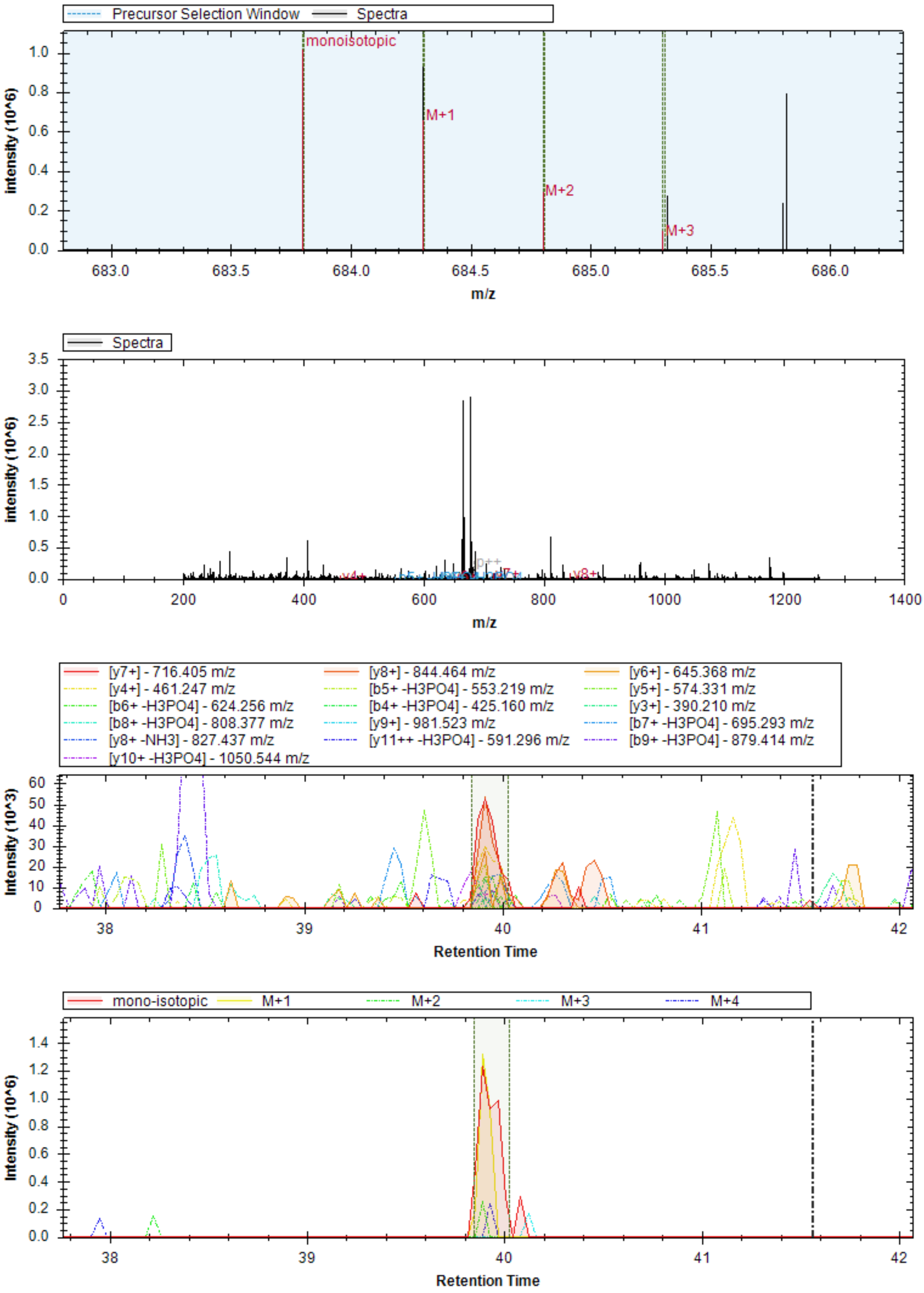

Q8CDU4;Q8CDU4-2 – _[+C2+H2+O]ASLRS[+H+O3+P]ATPRLR_.2
Fig4_MP-DIA-barrel-cortex-p15-1_MHRM.htrms

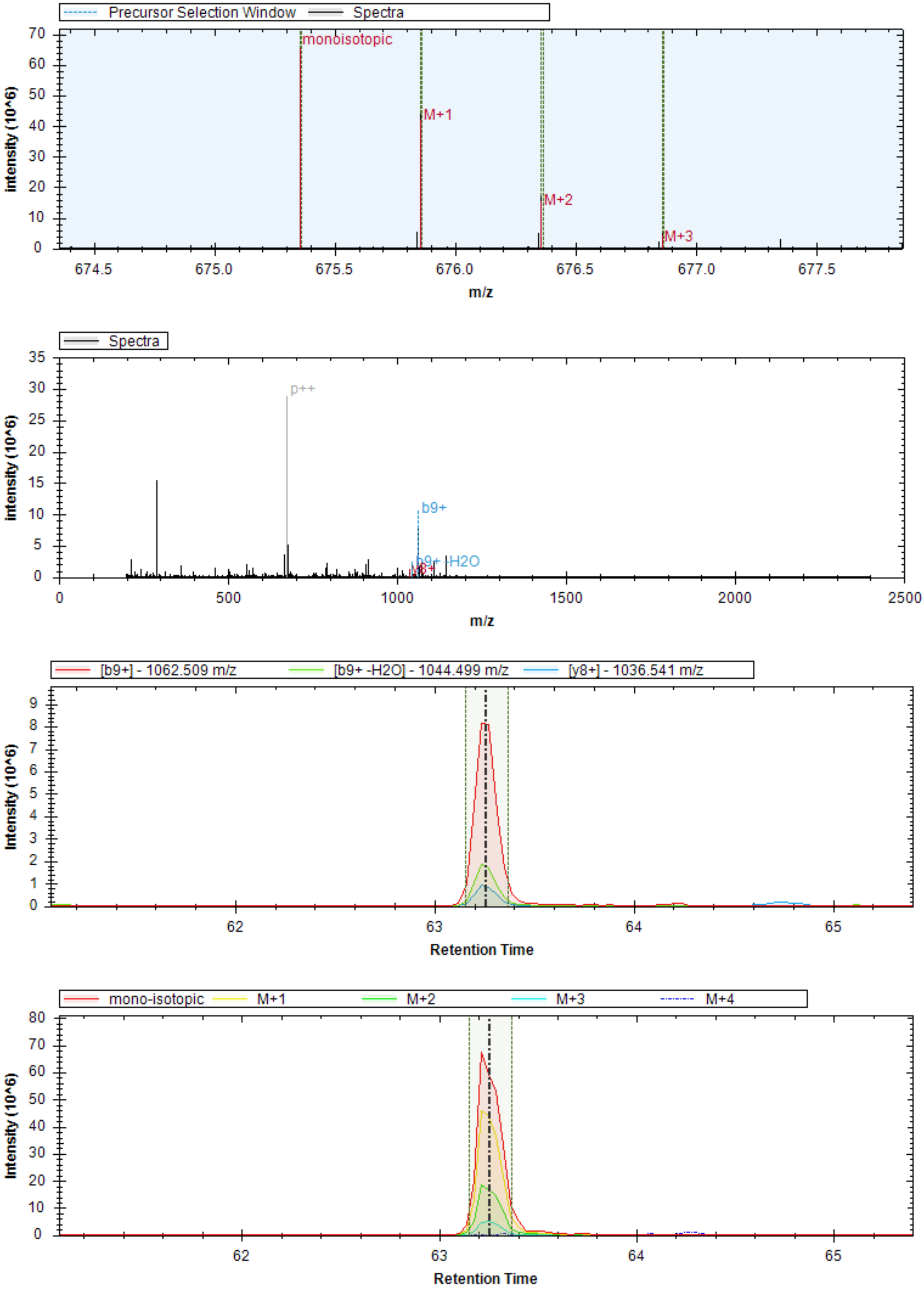

Q9CZU3 – _ESLKNSAT[+H+O3+P]EAAK_.2

Fig4_MP-DIA-barrel-cortex-p15-1_MHRM.htrms

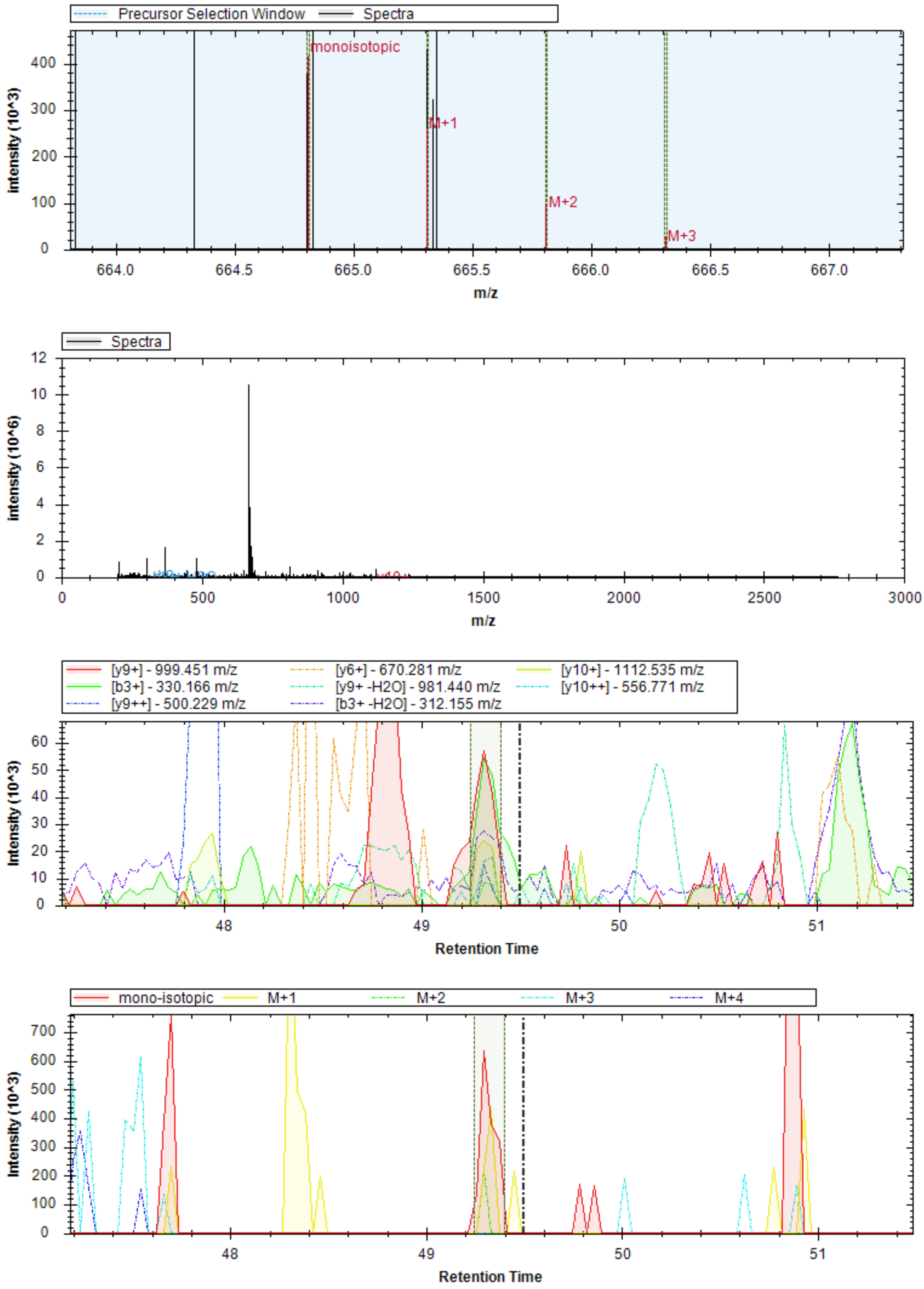

O88935 – _PVAGGPGAPPAARPPAS[+H+O3+P]PSPQR_.3

Fig4_MP-DIA-barrel-cortex-p15-1_MHRM.htrms

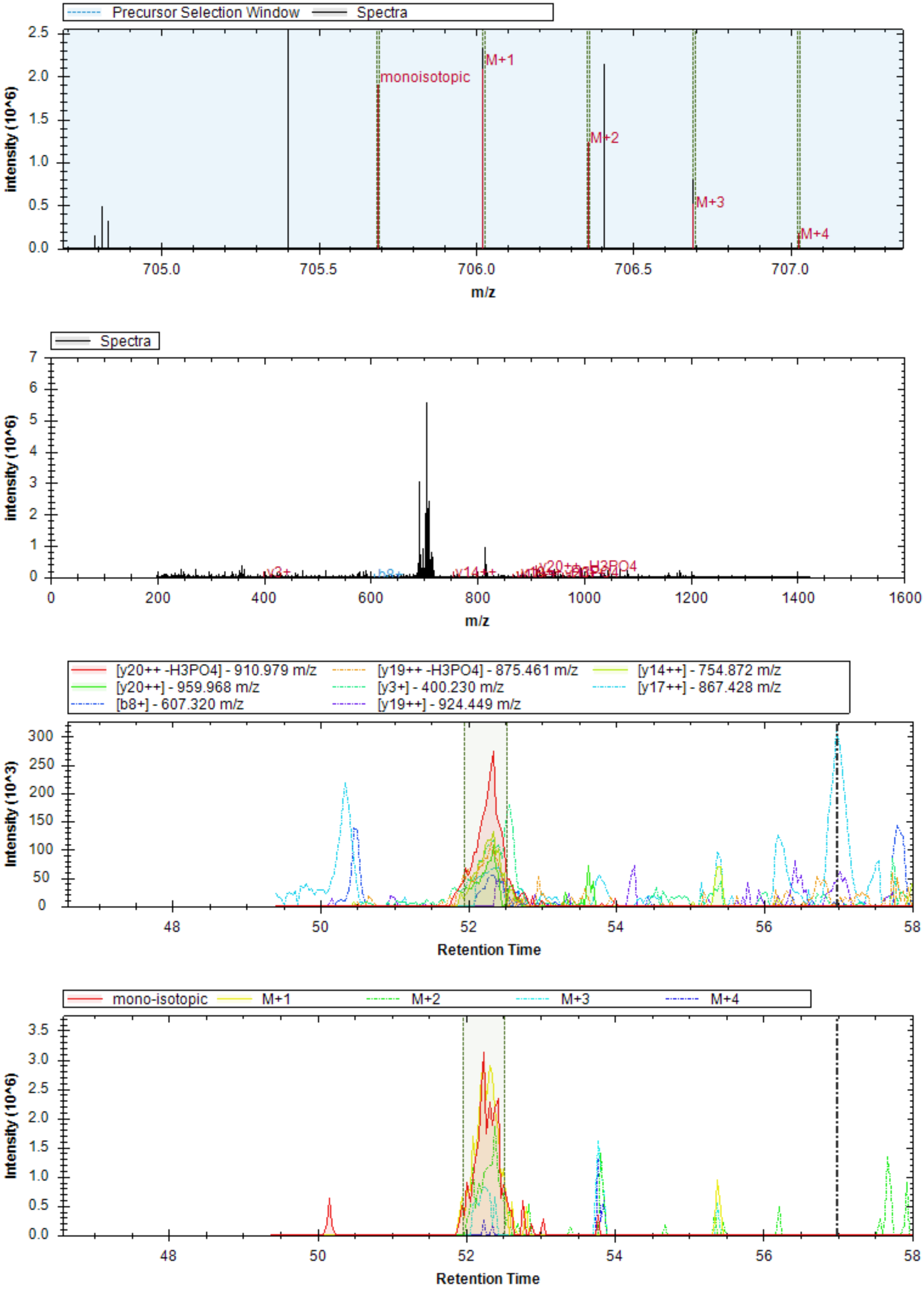

O88935 – _PVAGGPGAPPAARPPASPS[+H+O3+P]PQR_.3
Fig4_MP-DIA-barrel-cortex-p15-1_MHRM.htrms

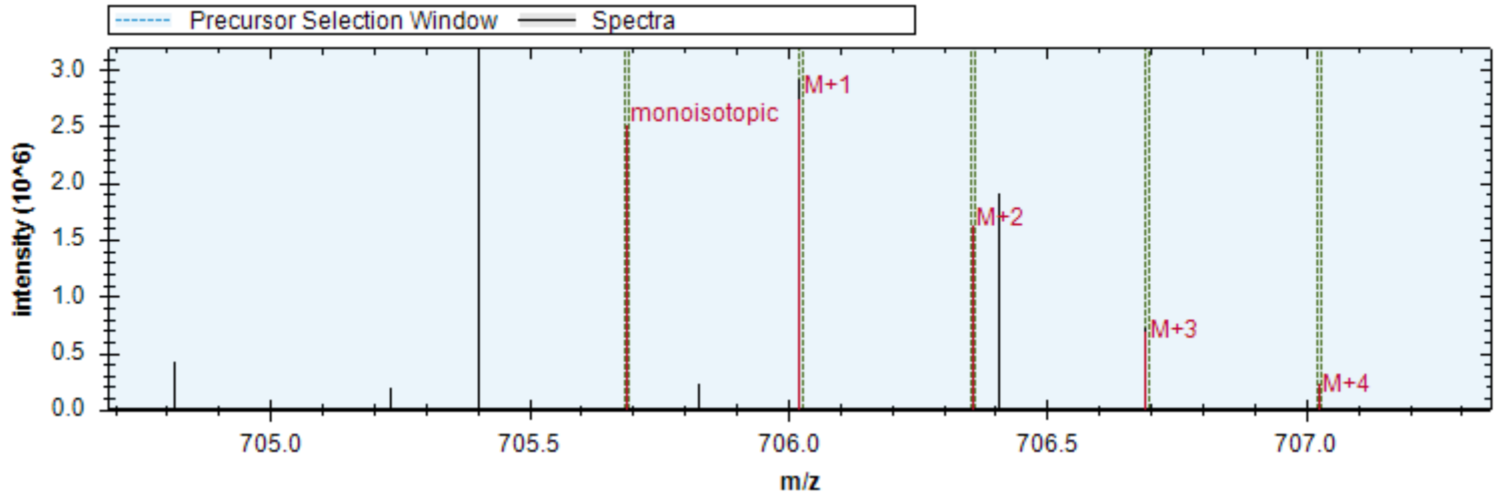

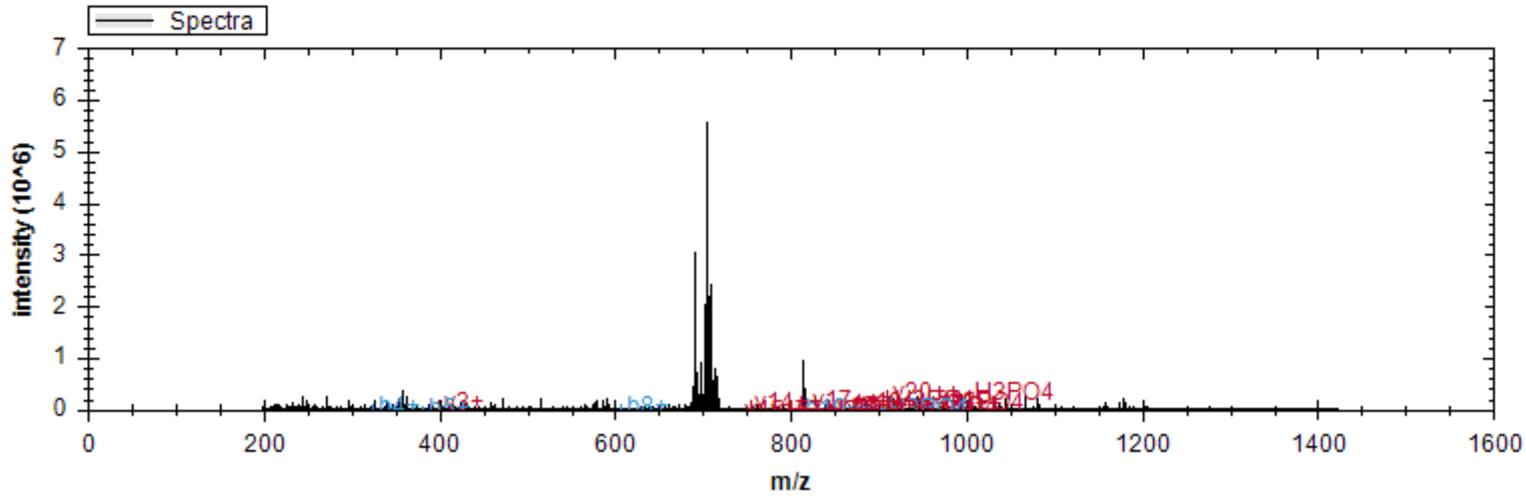

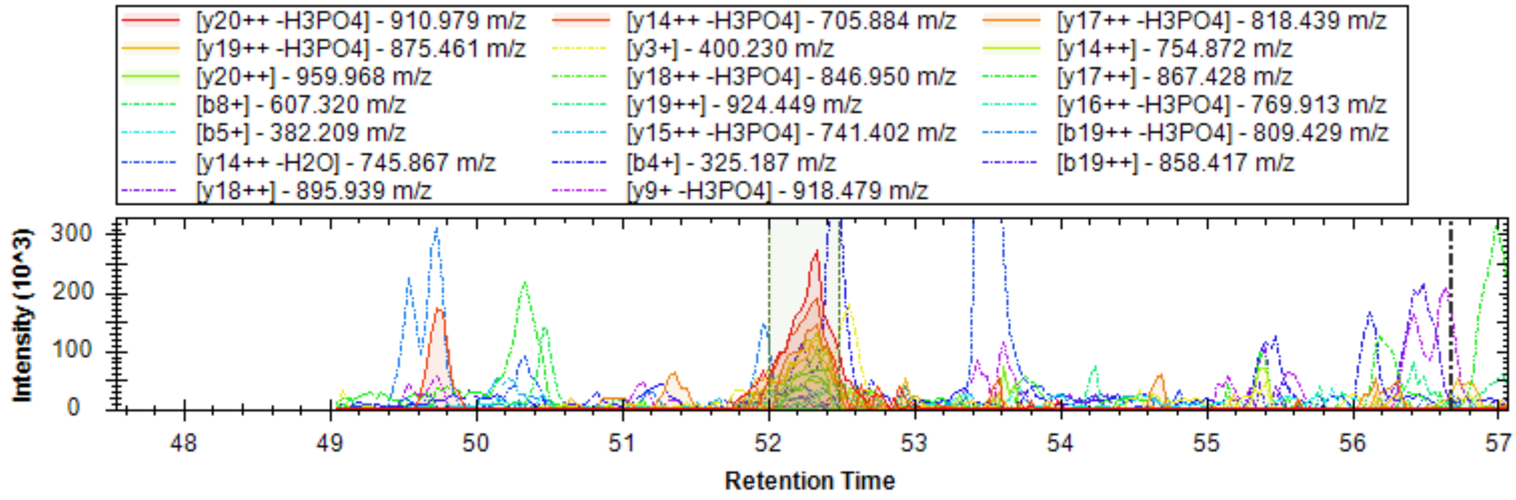

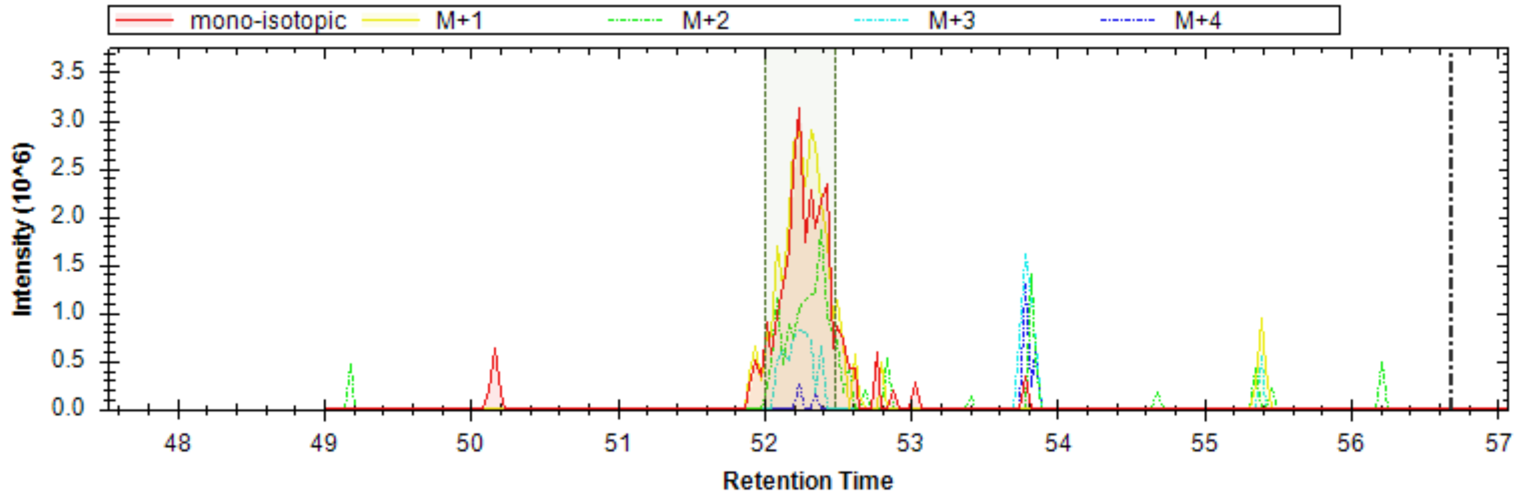

P20357 – _VTDGISKS[+H+O3+P]PEKR_.2
Fig4_MP-DIA-barrel-cortex-p15-1_MHRM.htrms

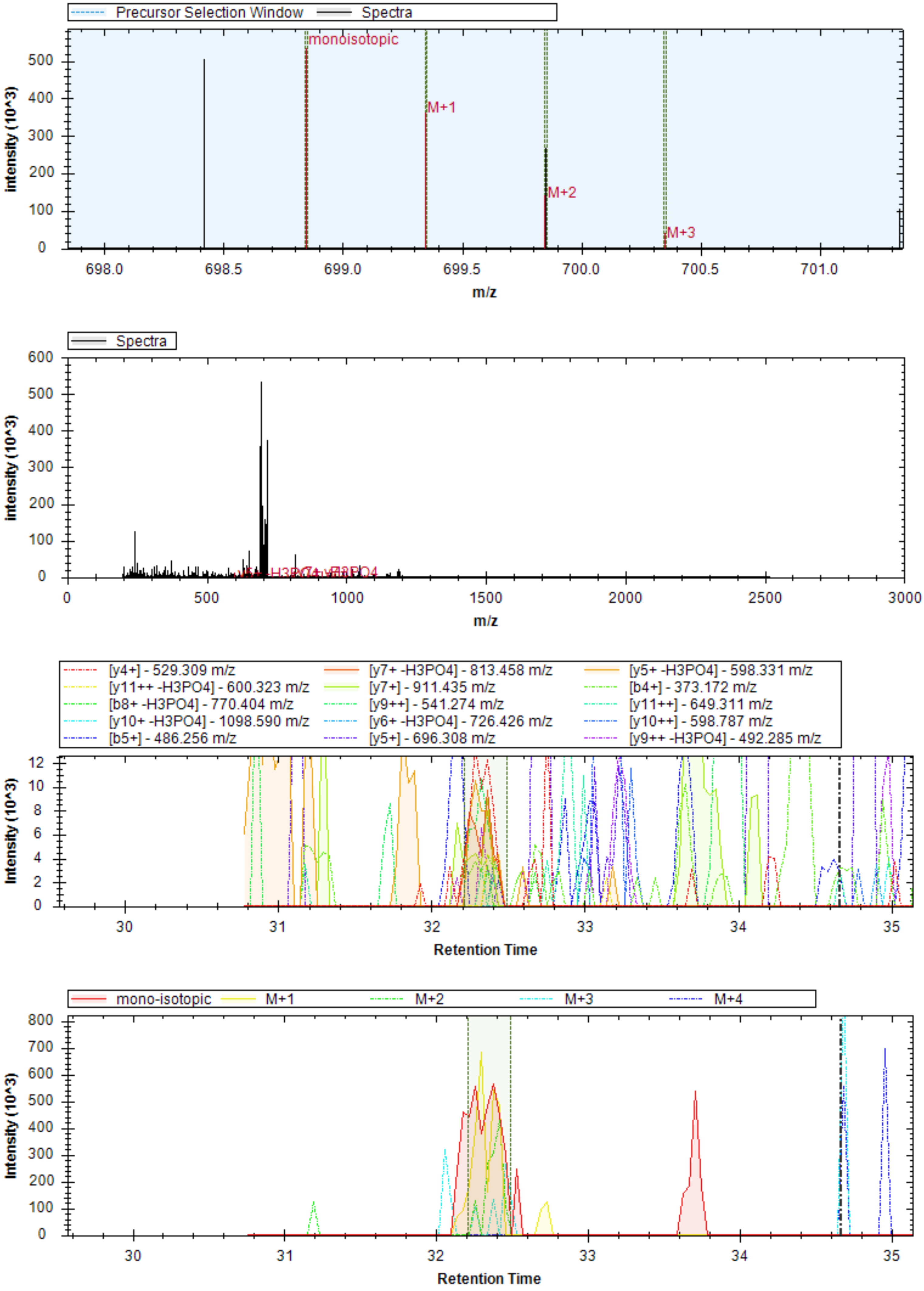

P28738 – _PIRPGHYPAS[+H+O3+P]SPTAVHAVR_.3

Fig4_MP-DIA-barrel-cortex-p15-1_MHRM.htrms

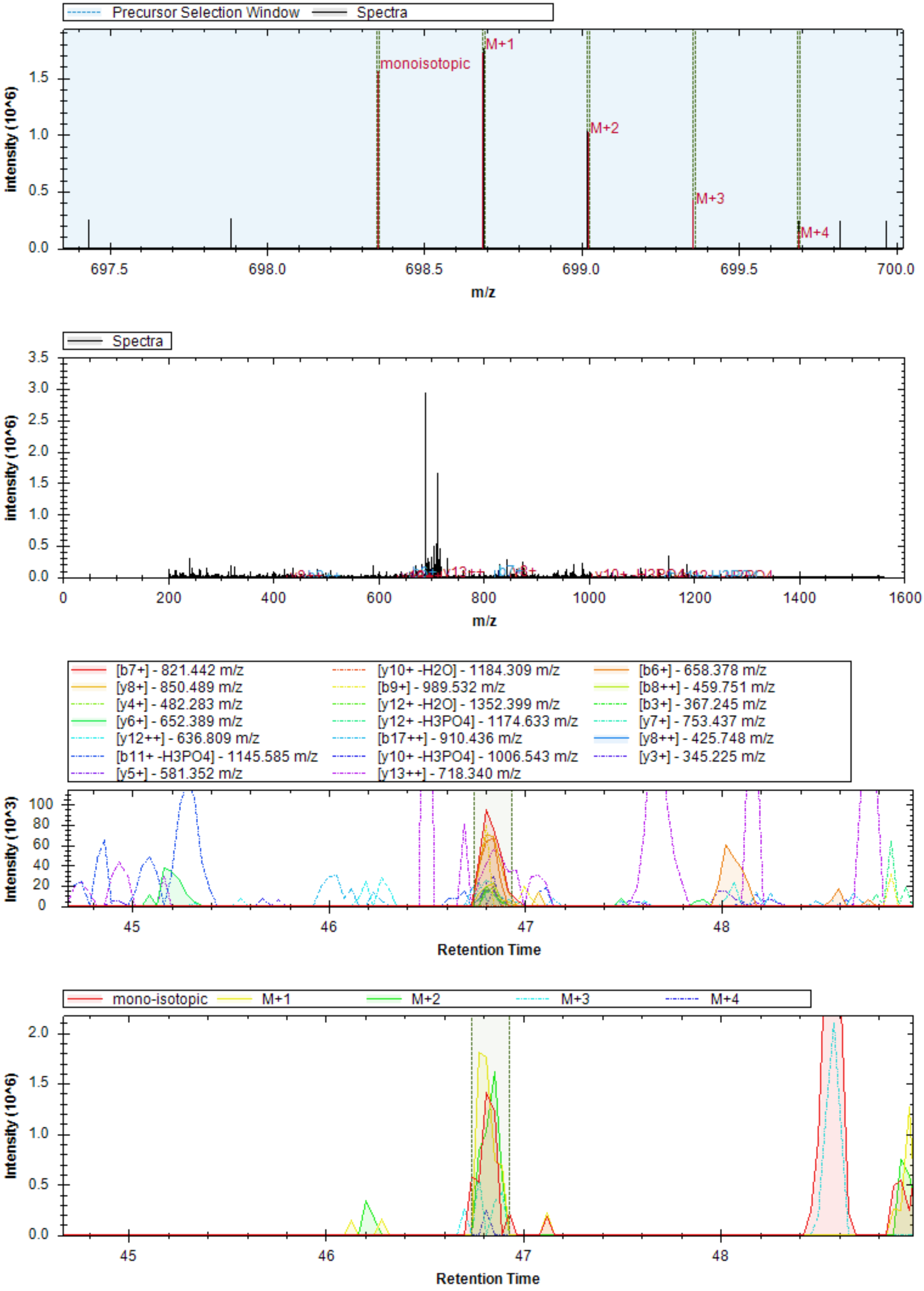

P28738 – _PIRPGHYPASS[+H+O3+P]PTAVHAVR_.3
Fig4_MP-DIA-barrel-cortex-p15-1_MHRM.htrms

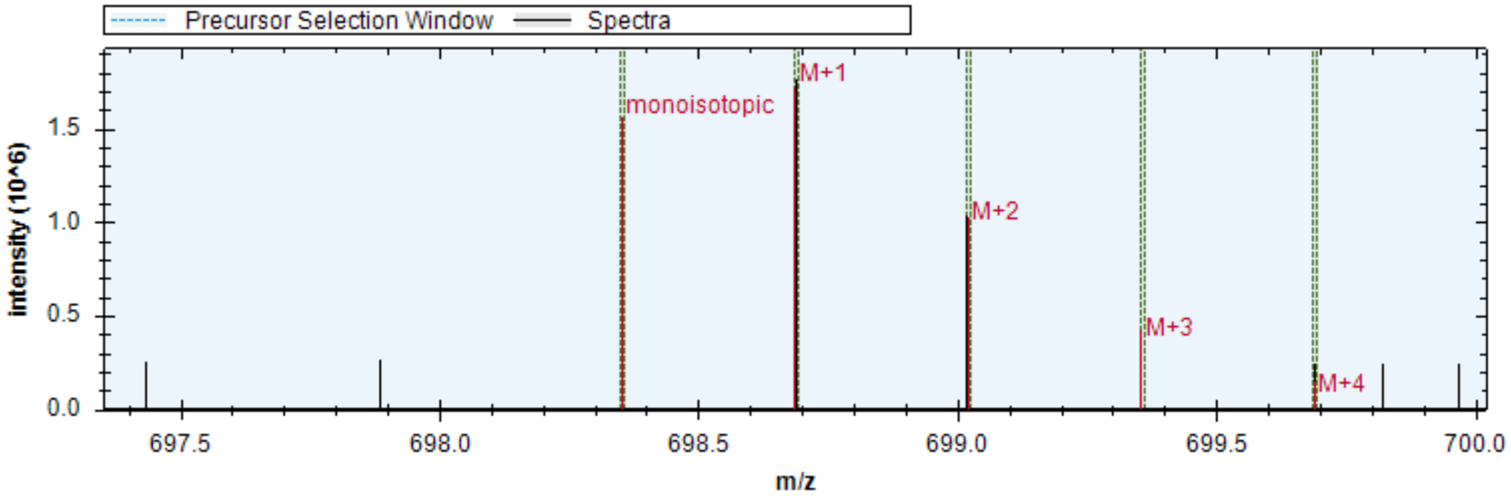

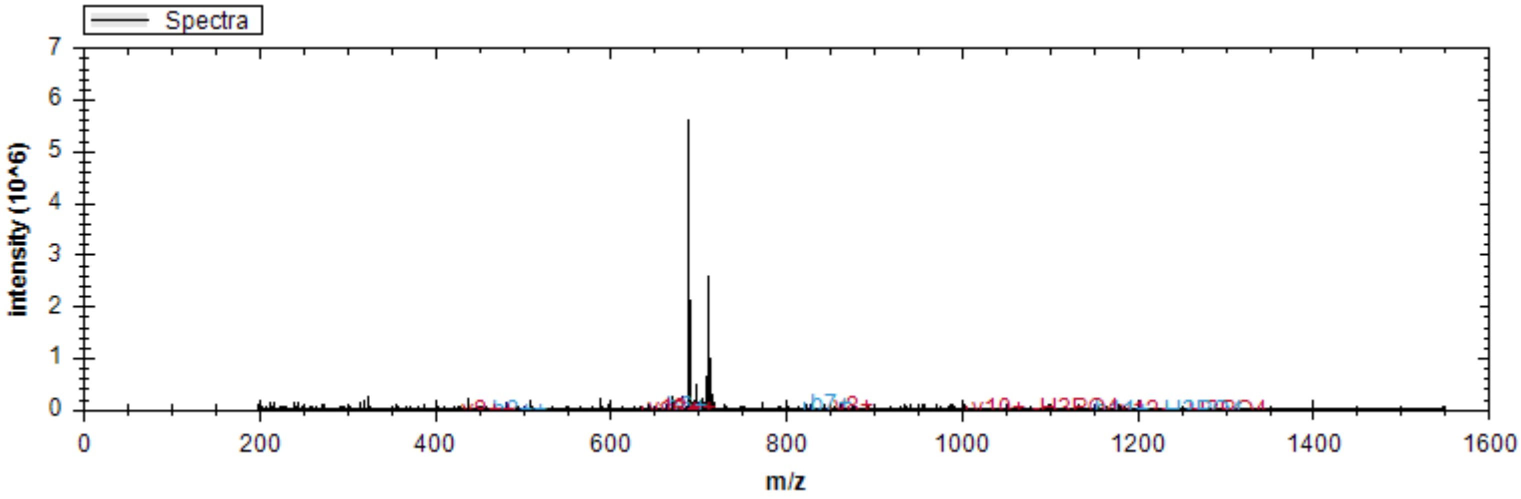

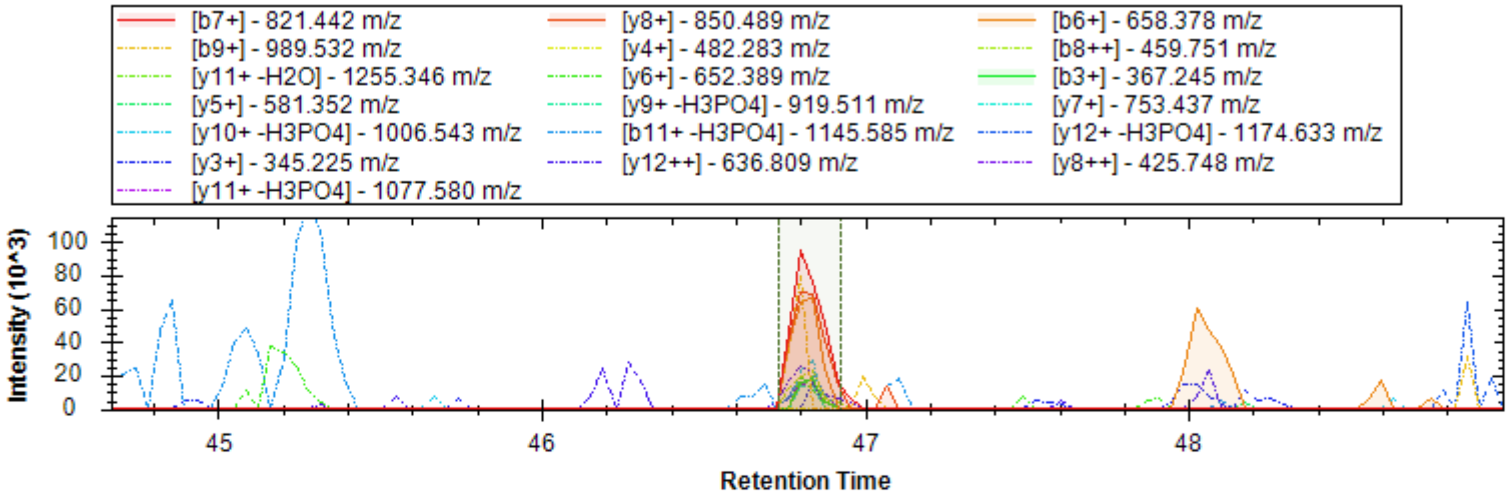

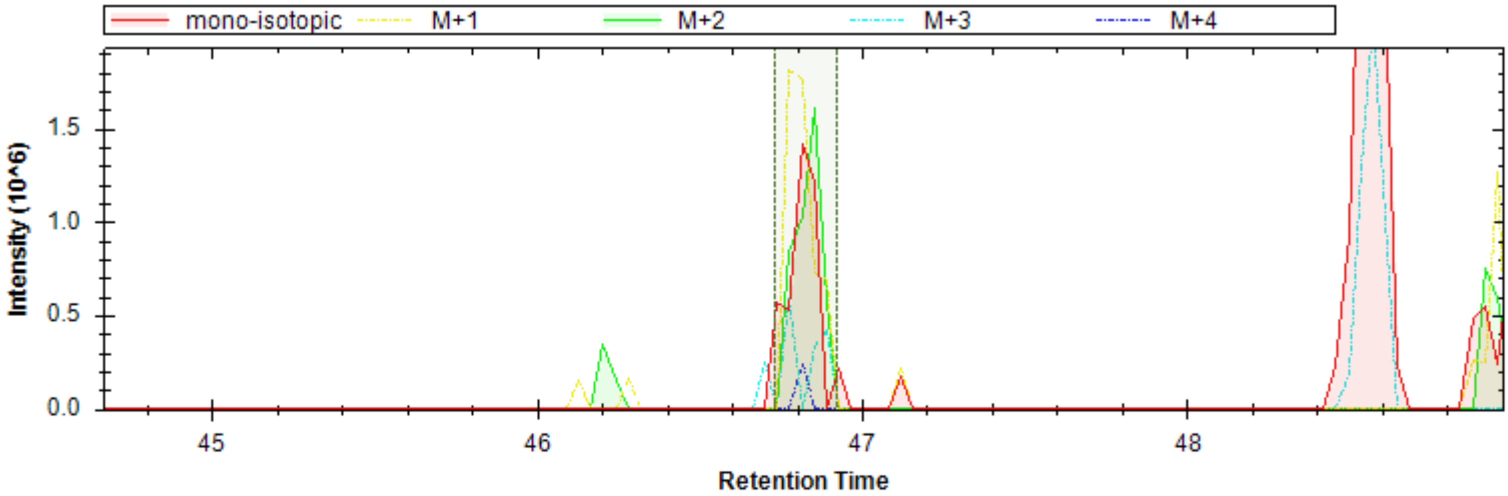

P62751 – _KAY[+H+O3+P]VRLAPDYDALDVANK_.3
Fig4_MP-DIA-barrel-cortex-p15-1_MHRM.htrms

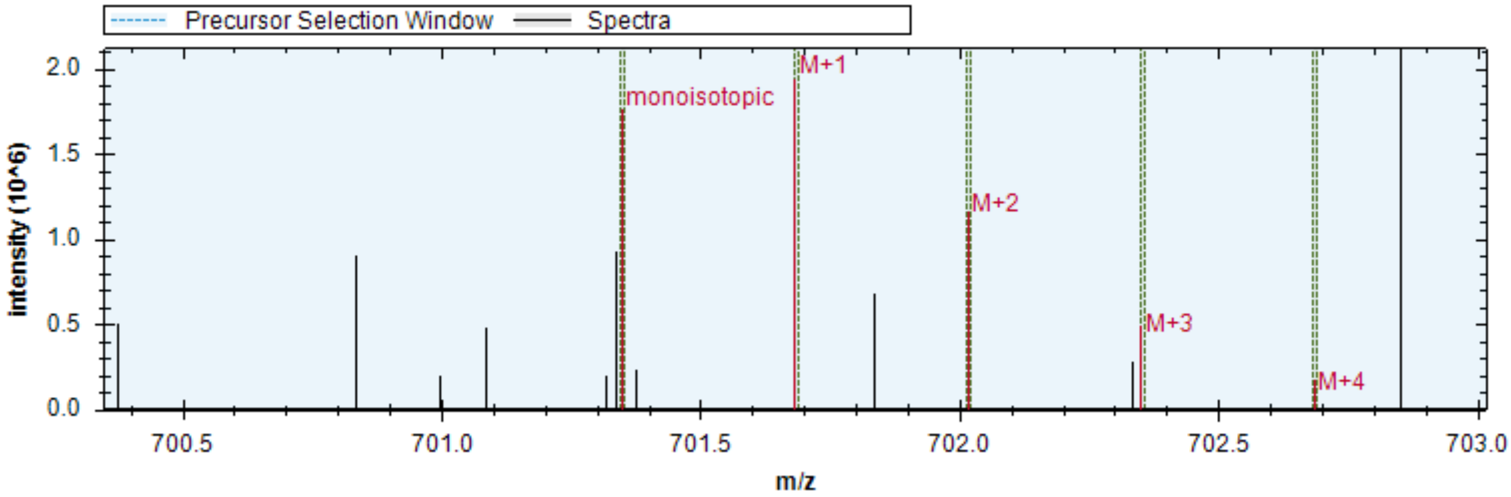

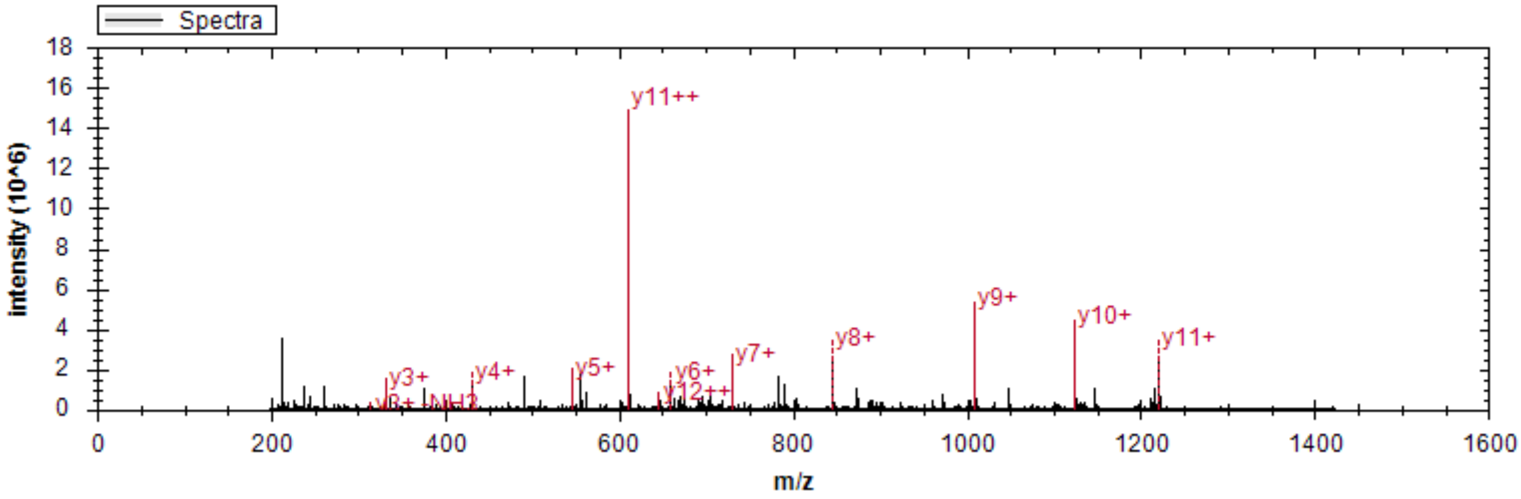

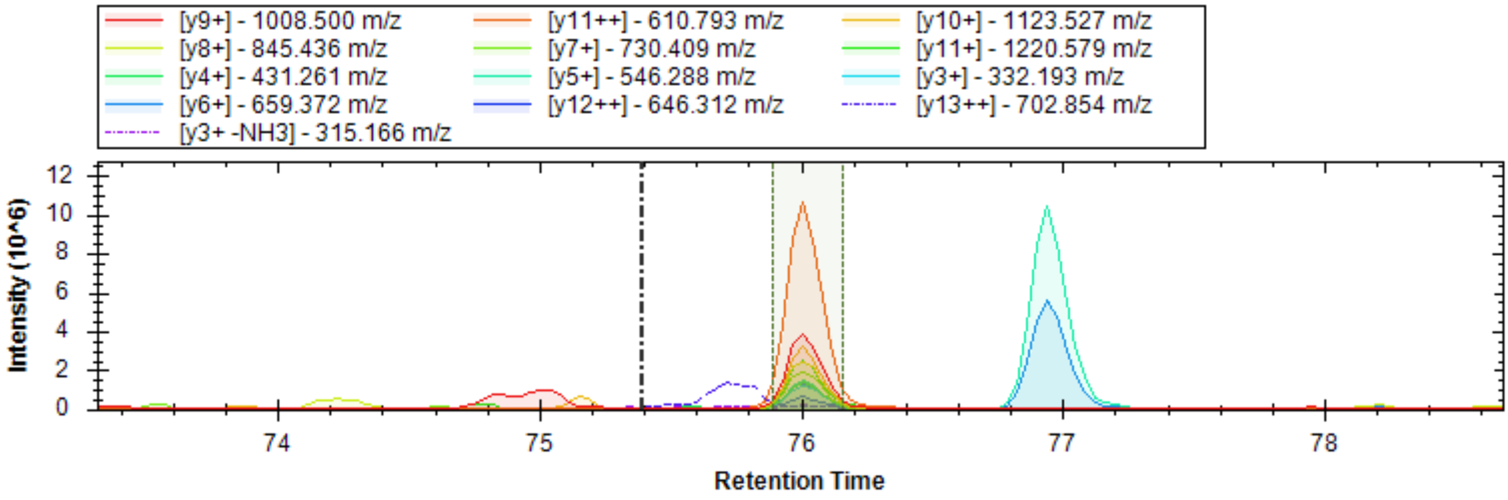

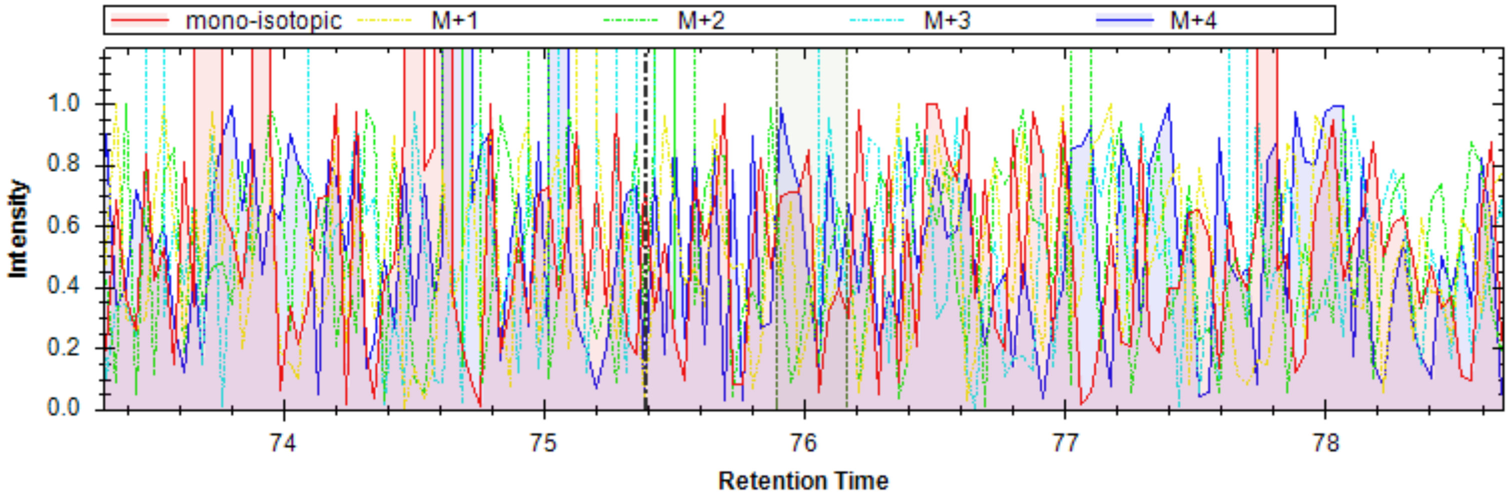

Q3U4B4 – _RHKT[+H+O3+P]LSANEEK_.2
Fig4_MP-DIA-barrel-cortex-p15-1_MHRM.htrms

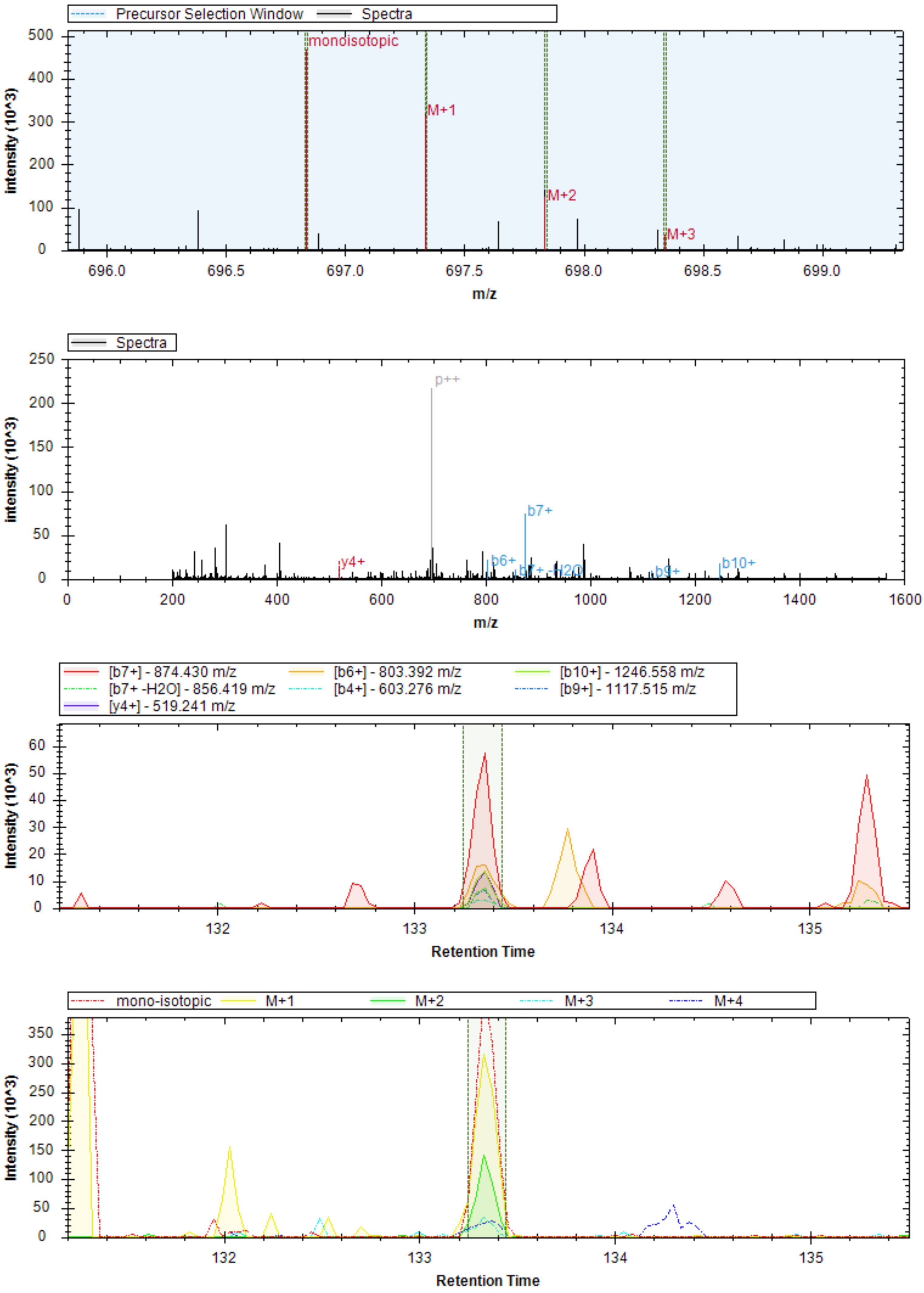

Q99PU8;Q99PU8-2 – _ALGRM[+O]VERS[+H+O3+P]LR_.2
Fig4_MP-DIA-barrel-cortex-p15-1_MHRM.htrms

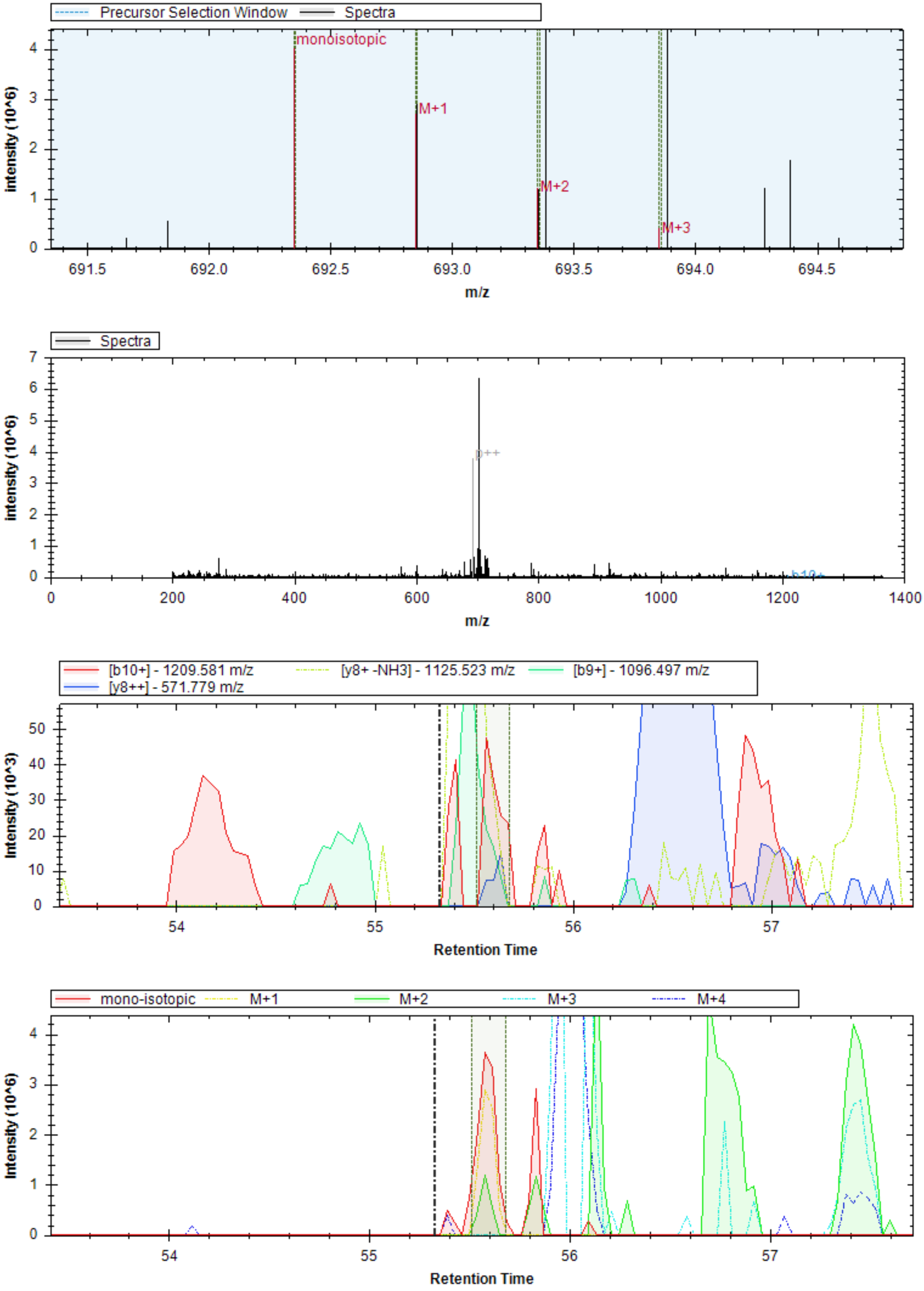

P10637 – _SGYSS[+H+O3+P]PGSPGTPGSR_.2
Fig4_MP-DIA-barrel-cortex-p15-1_MHRM.htrms

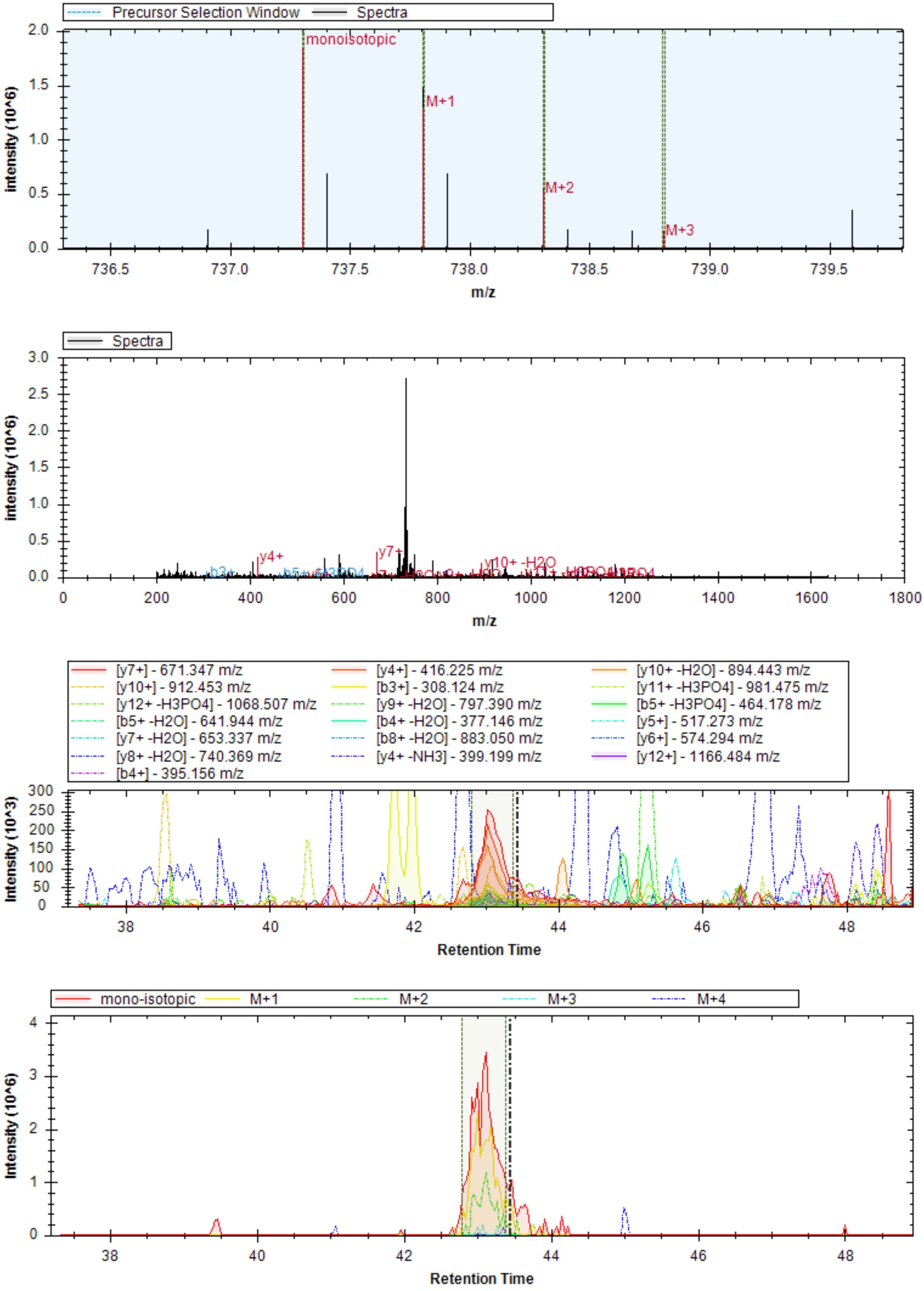

P10637 – _SGYSSPGS[+H+O3+P]PGTPGSR_.2

Fig4_MP-DIA-barrel-cortex-p15-1_MHRM.htrms

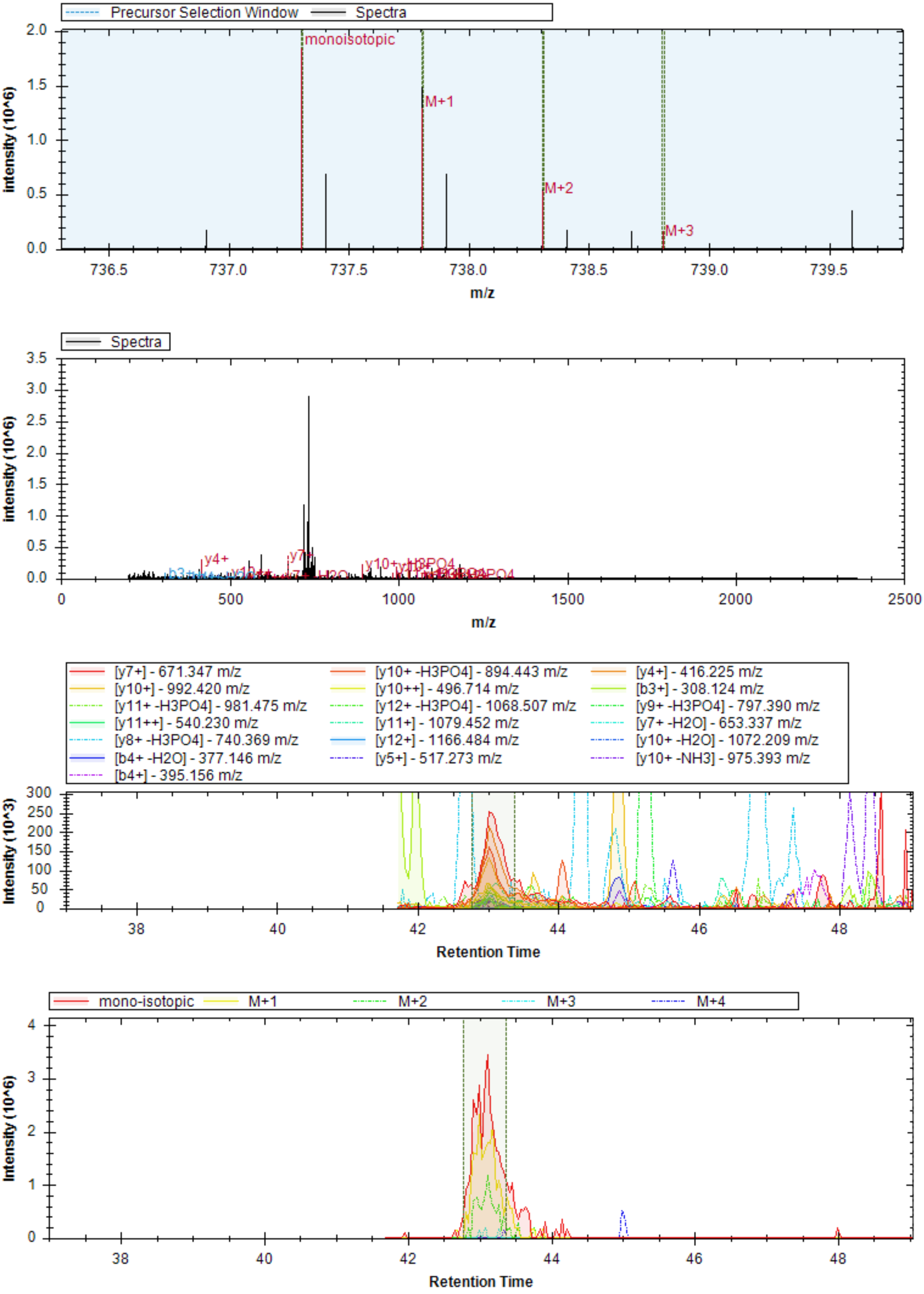

Q8C5R2 – _LAGNEALS[+H+O3+P]PTSPSK_.2
Fig4_MP-DIA-barrel-cortex-p15-1_MHRM.htrms

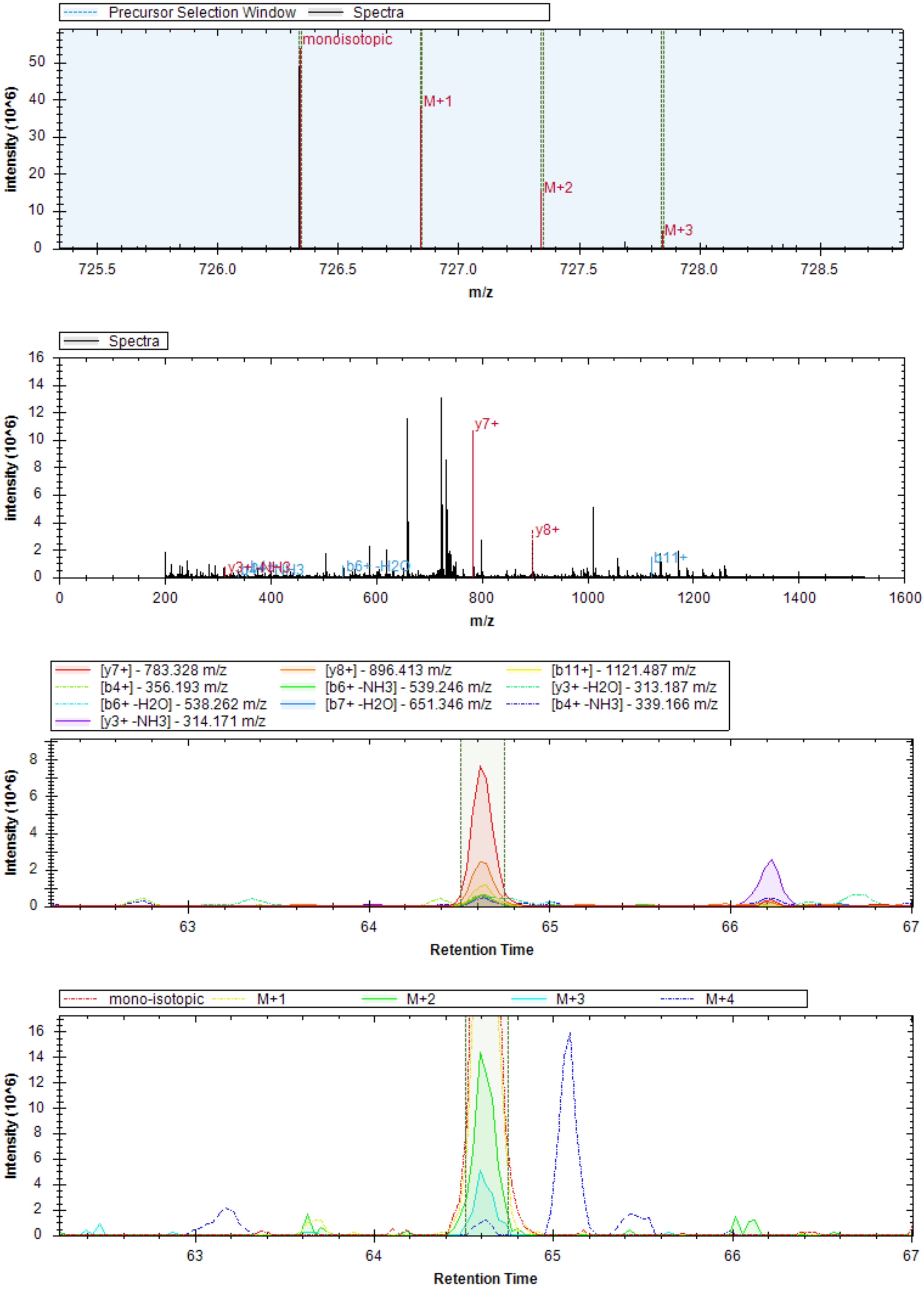

Q8K0U4 – _ETAPTSTYS[+H+O3+P]SPAR_.2
Fig4_MP-DIA-barrel-cortex-p15-1_MHRM.htrms

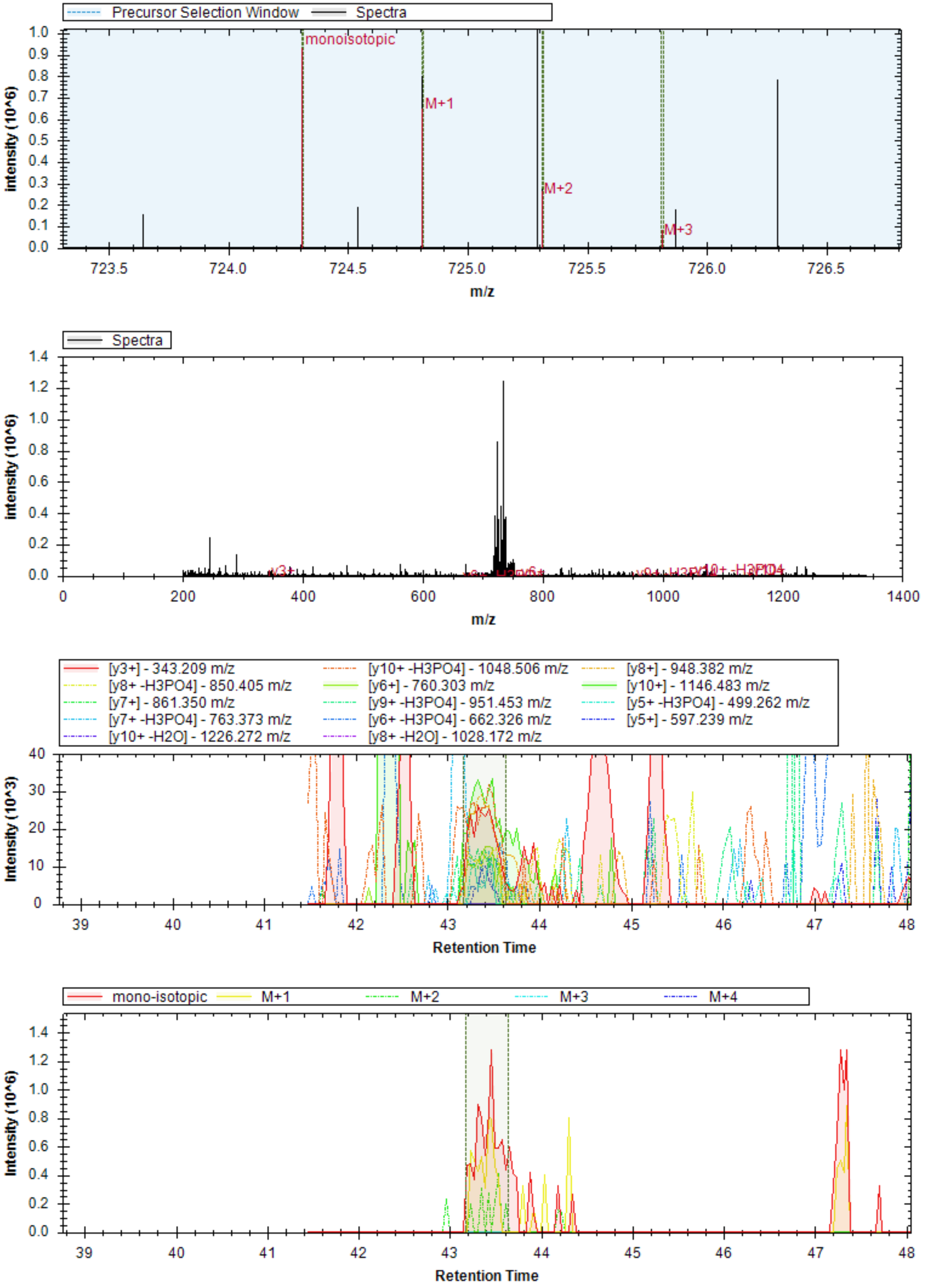

Q8K0U4 – _ETAPTSTYSS[+H+O3+P]PAR_.2
Fig4_MP-DIA-barrel-cortex-p15-1_MHRM.htrms

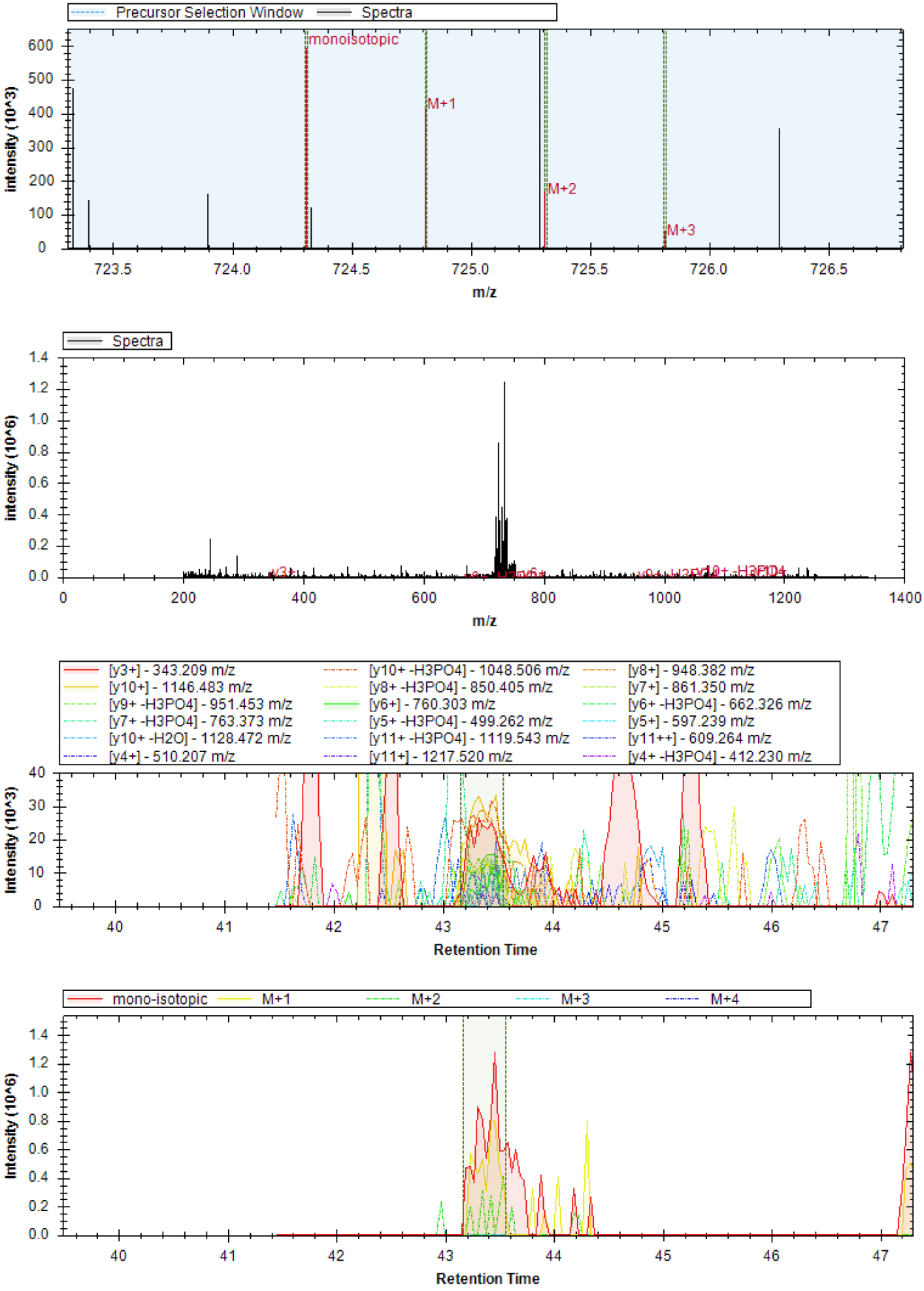

Q9CRB0 – _MEVYIPS[+H+O3+P]FRHEDSDLER_.3
Fig4_MP-DIA-barrel-cortex-p15-1_MHRM.htrms

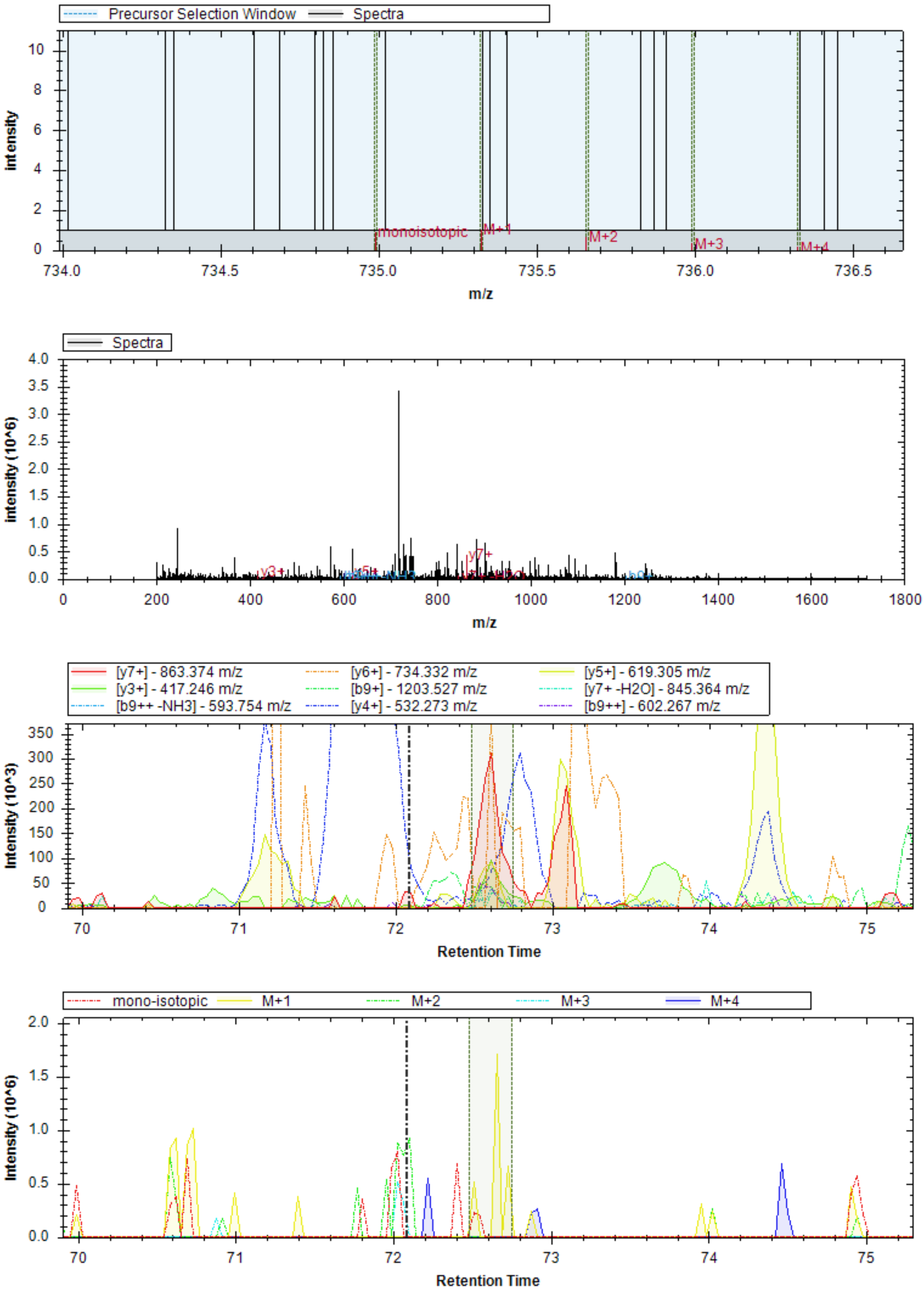

Q9JLM8 – _SKS[+H+O3+P]PASTSSVNGTPGSQLSTPR_.3
Fig4_MP-DIA-barrel-cortex-p15-1_MHRM.htrms

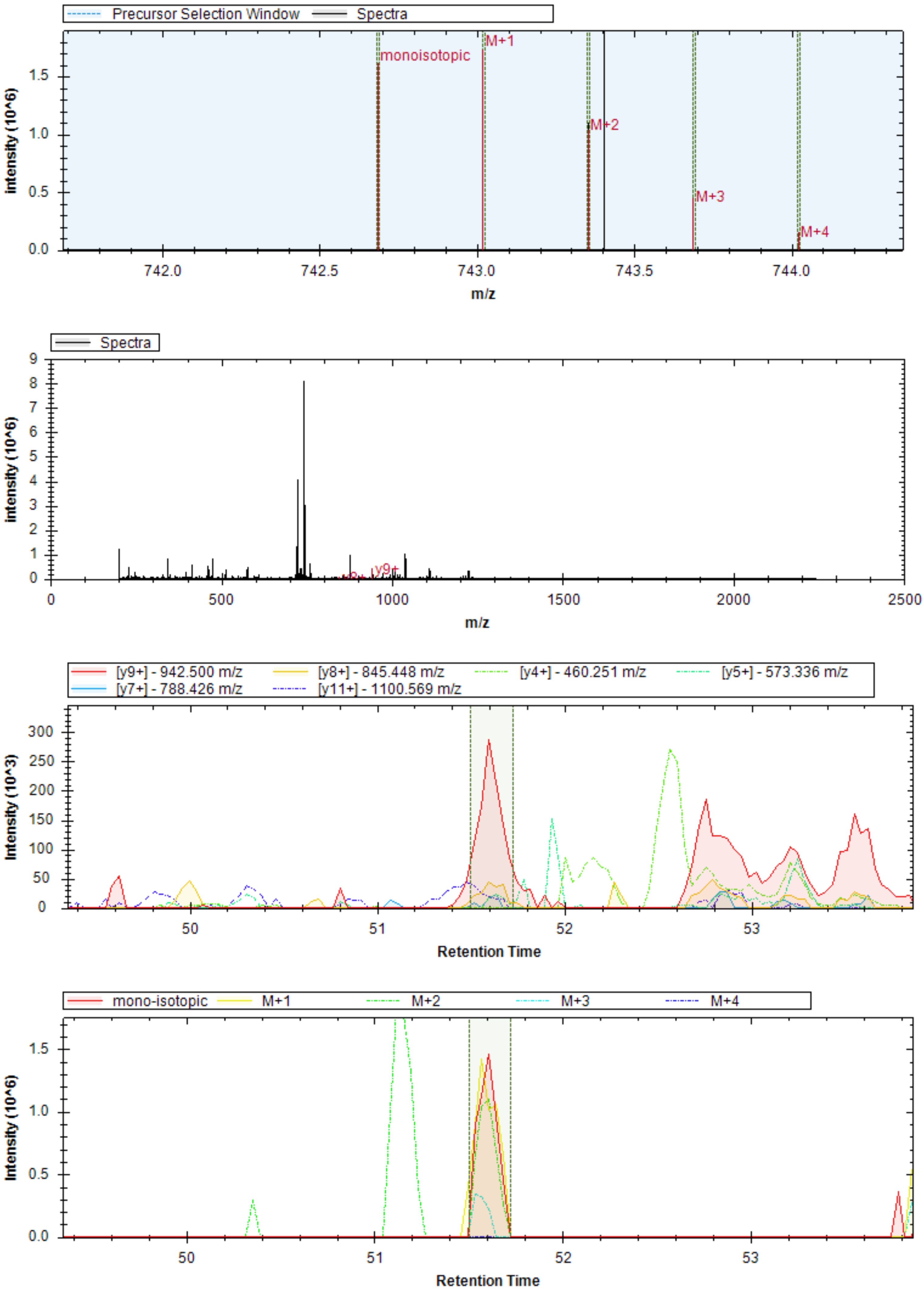

Q9JLM8 – _SPSPSPT[+H+O3+P]SPGSLRK_.2
Fig4_MP-DIA-barrel-cortex-p15-1_MHRM.htrms

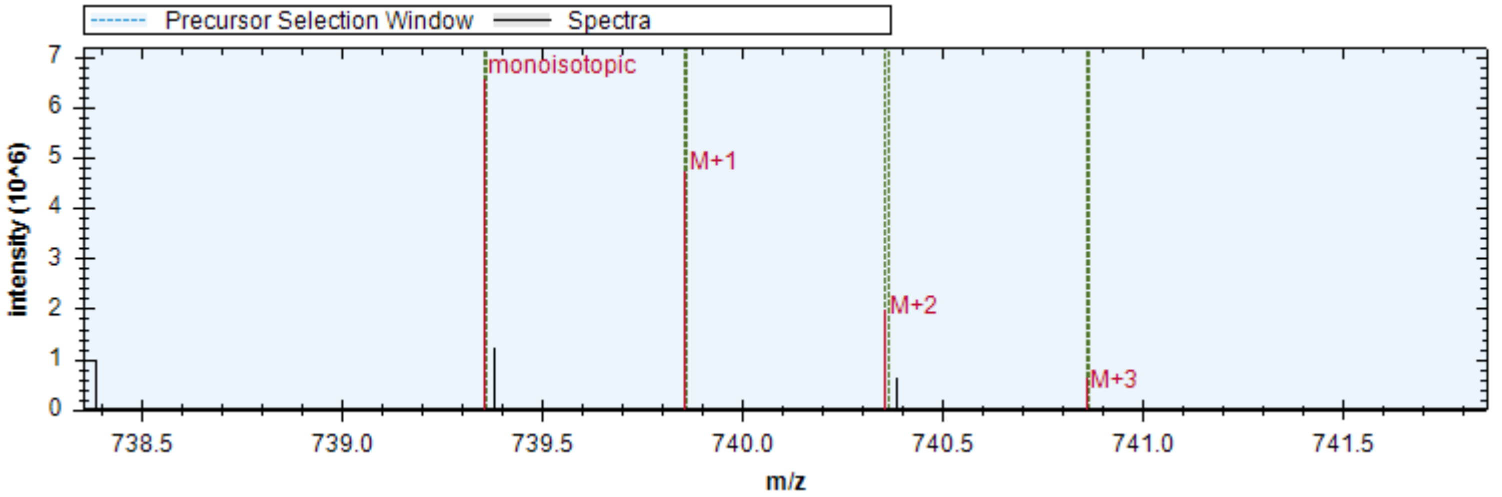

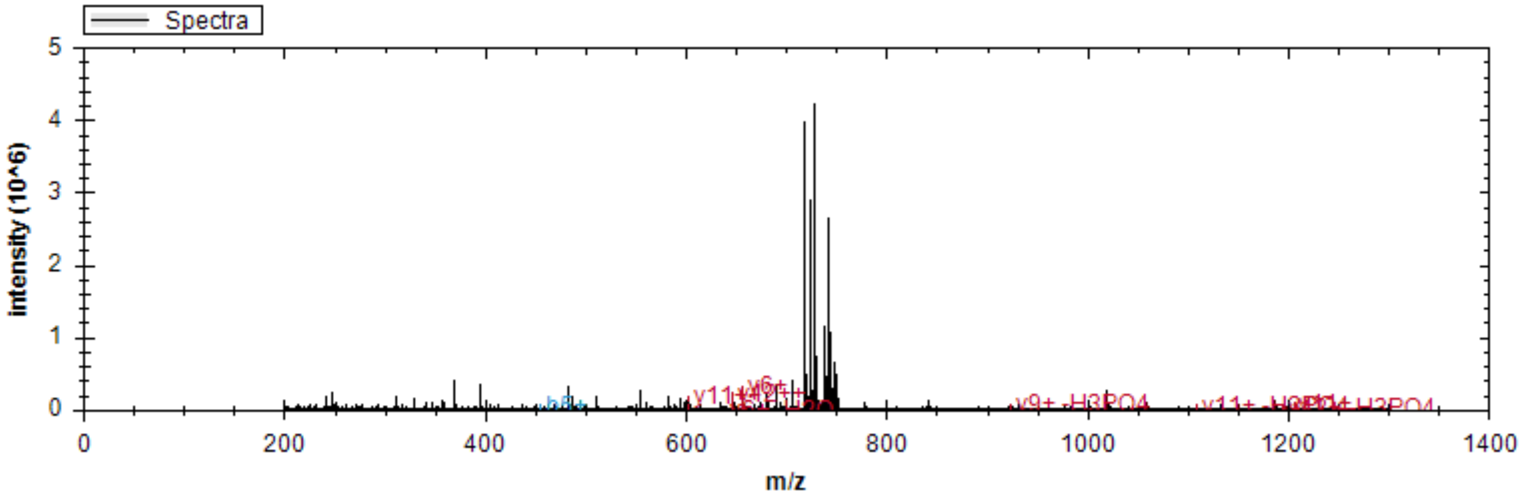

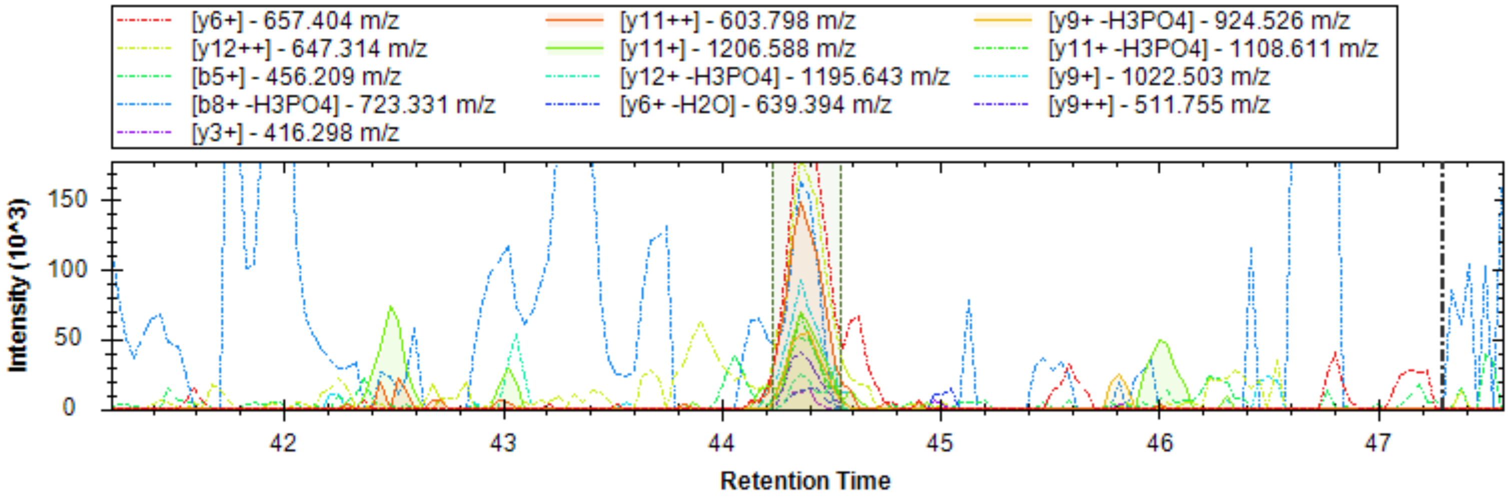

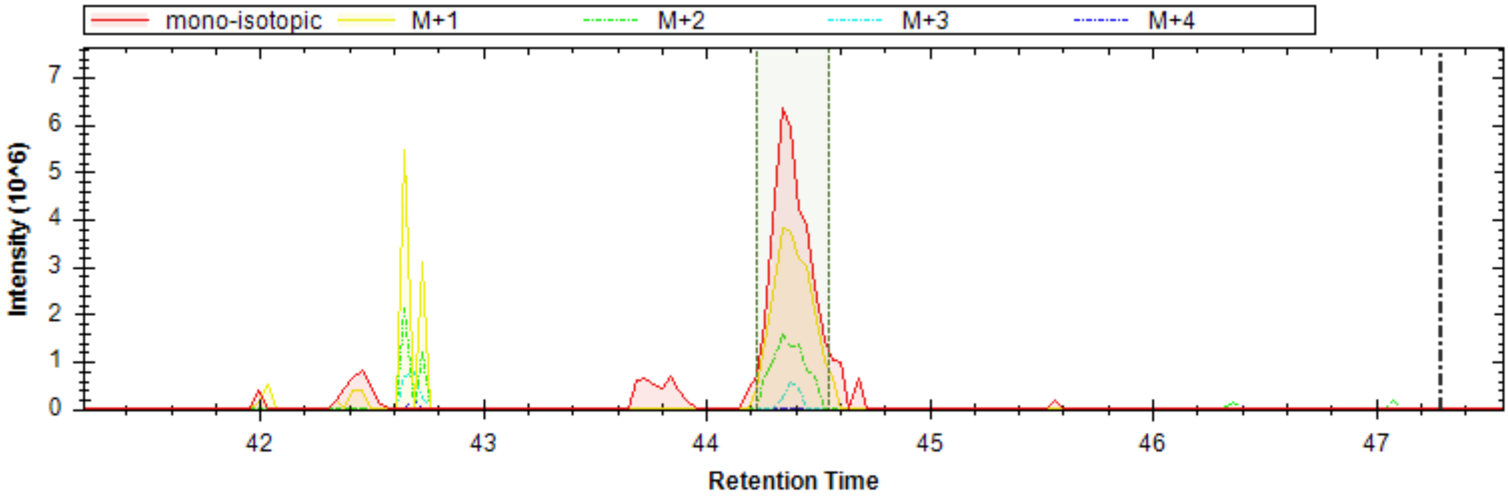

Q9JLM8 – _SPSPSPTS[+H+O3+P]PGSLRK_.2

Fig4_MP-DIA-barrel-cortex-p15-1_MHRM.htrms

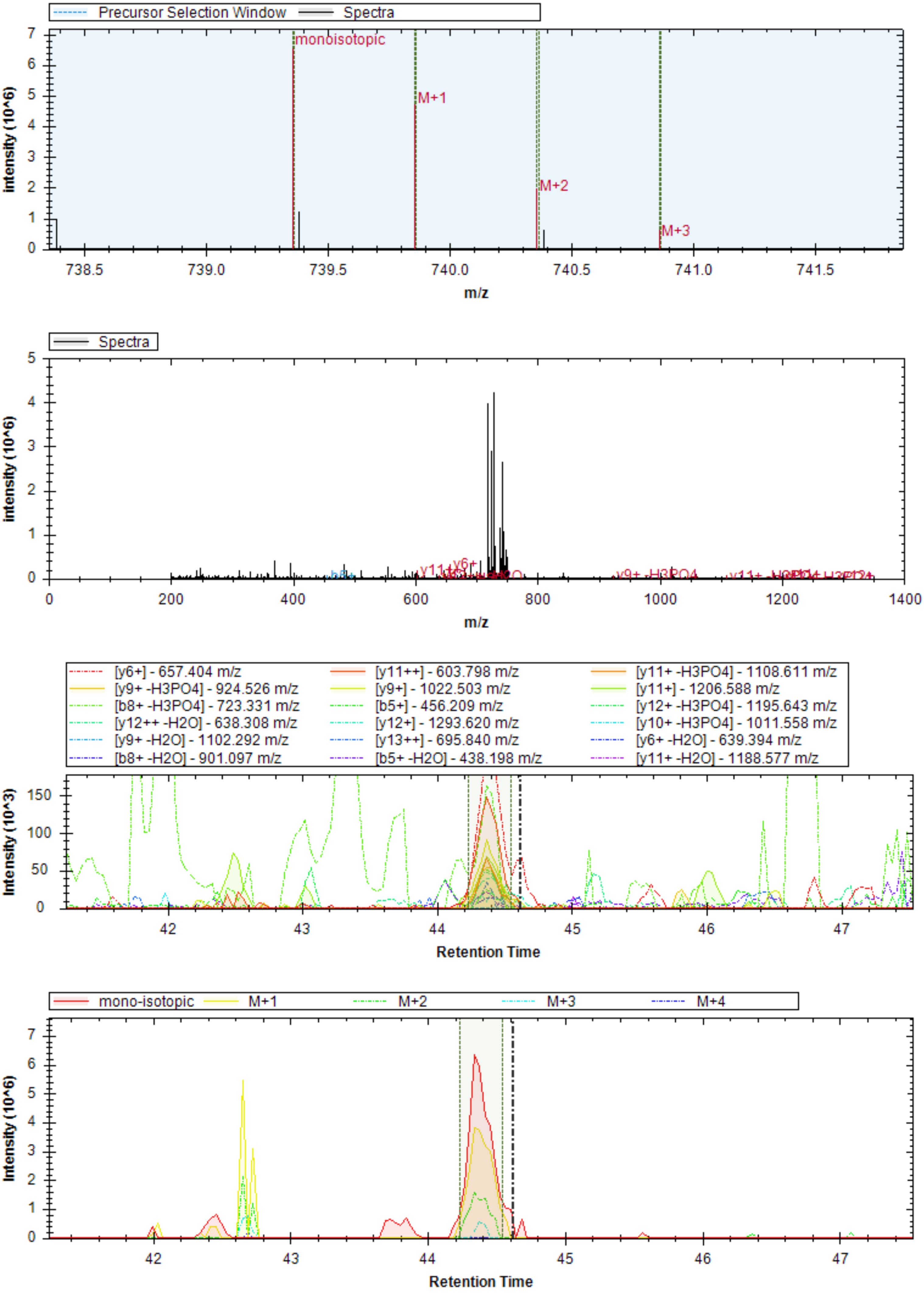

D3Z6S9 – _IPSIEIT[+H+O3+P]ET[+H+O3+P]LPQTQASMLR_.3
Fig4_MP-DIA-barrel-cortex-p15-1_MHRM.htrms

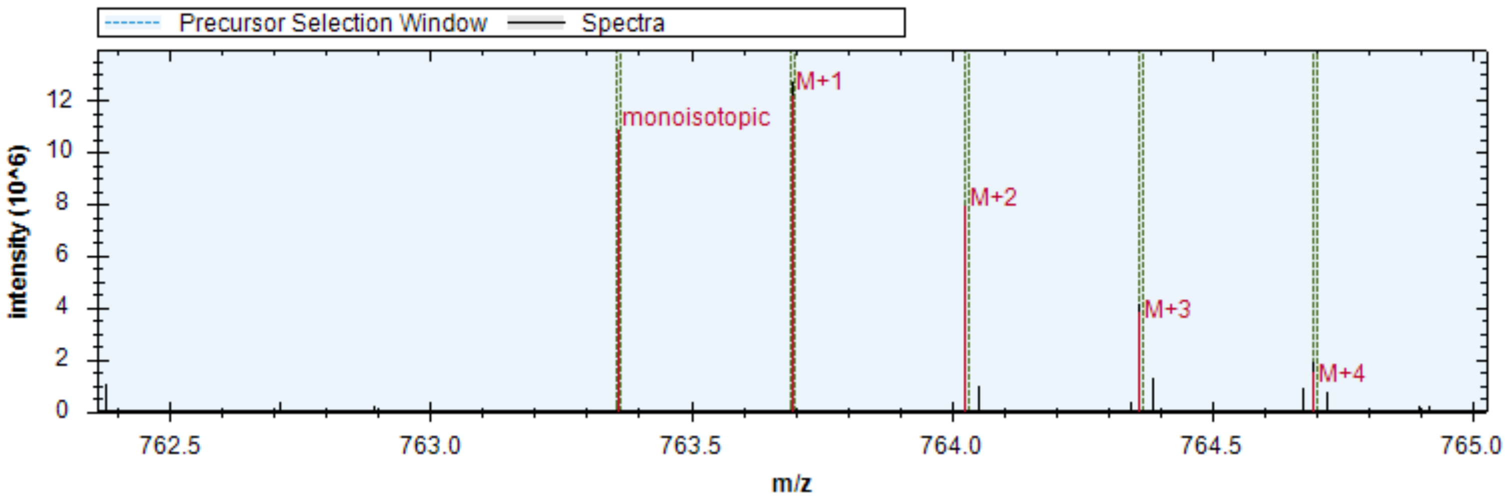

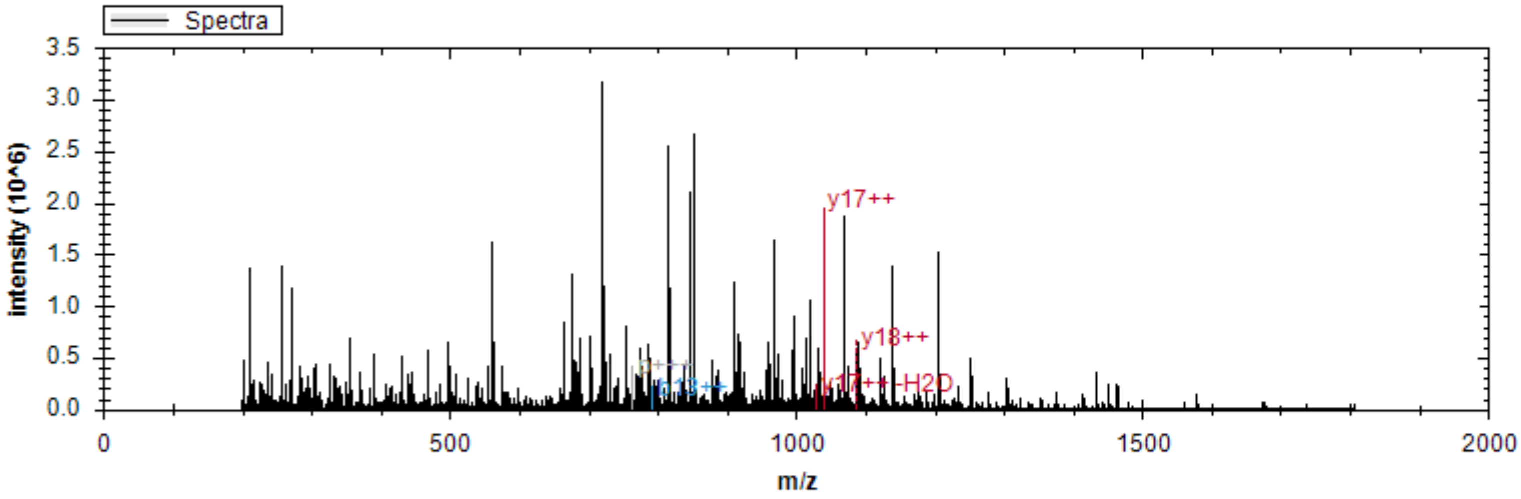

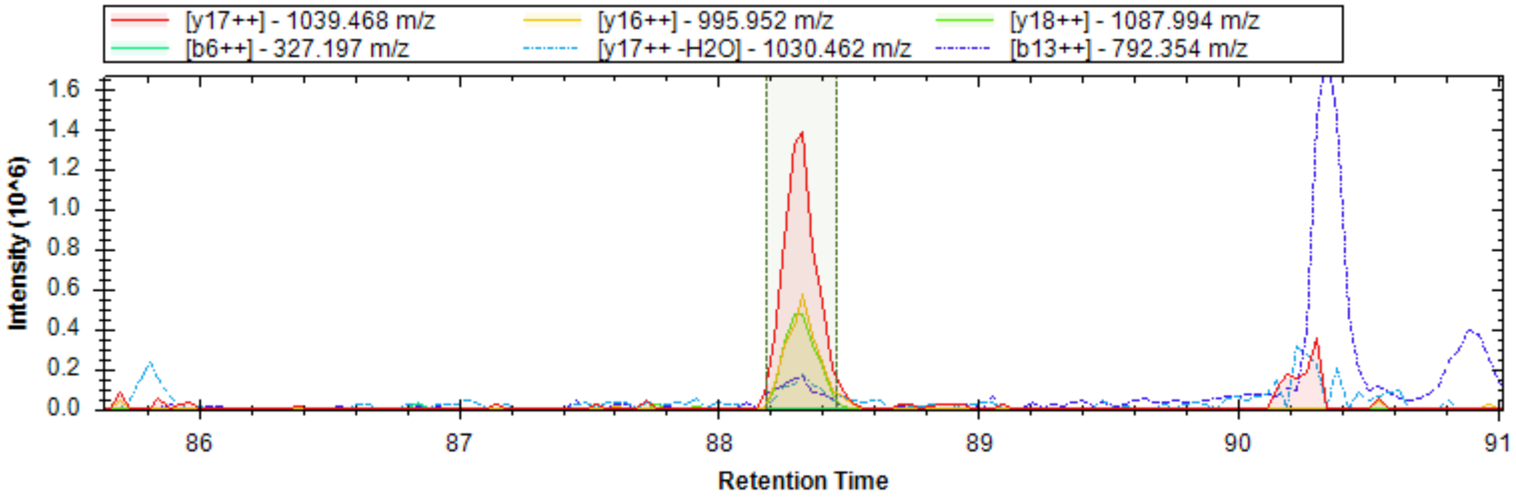

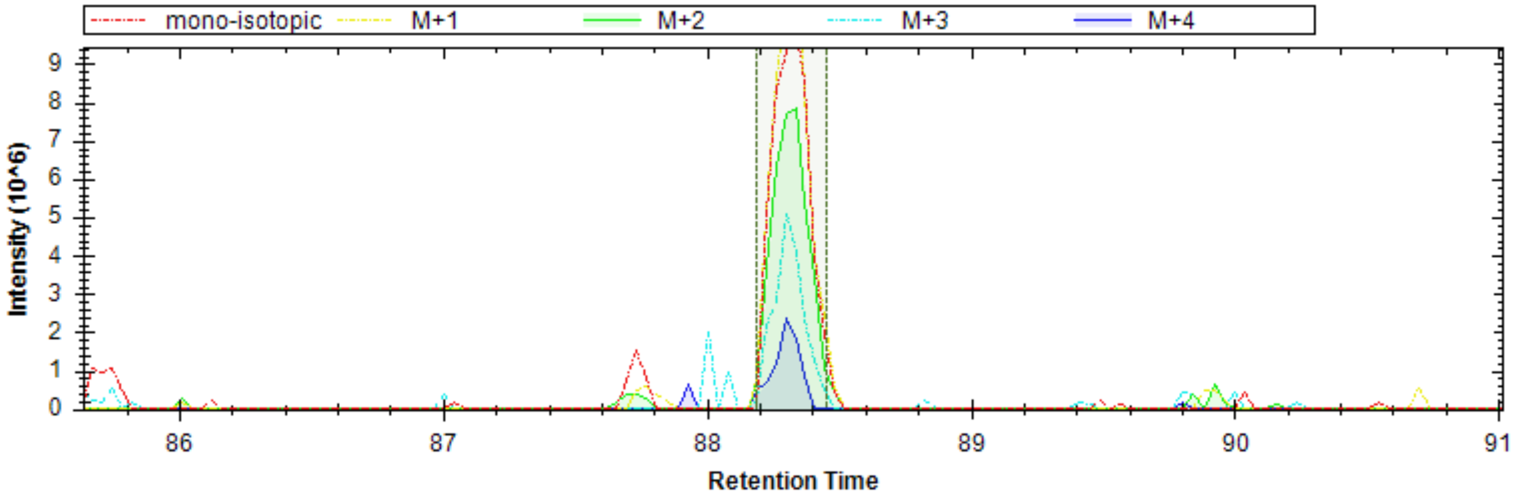

P06837 – _QADVPAAVTDAAAT[+H+O3+P]TPAAEDAATK_.3

Fig4_MP-DIA-barrel-cortex-p15-1_MHRM.htrms

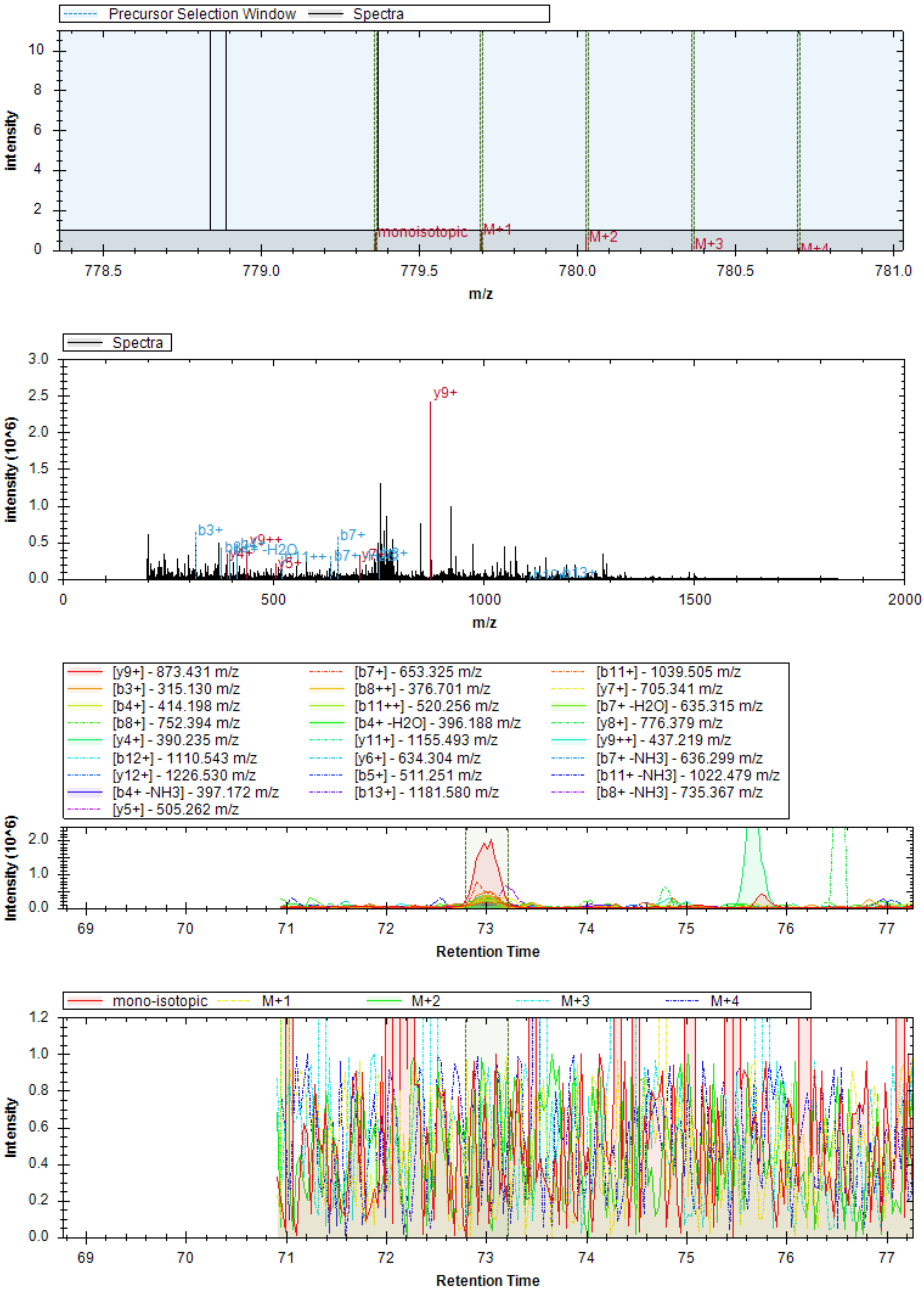

P0C6F1 – _IIDLQM[+O]HDY[+H+O3+P]LR_.2
Fig4_MP-DIA-barrel-cortex-p15-1_MHRM.htrms

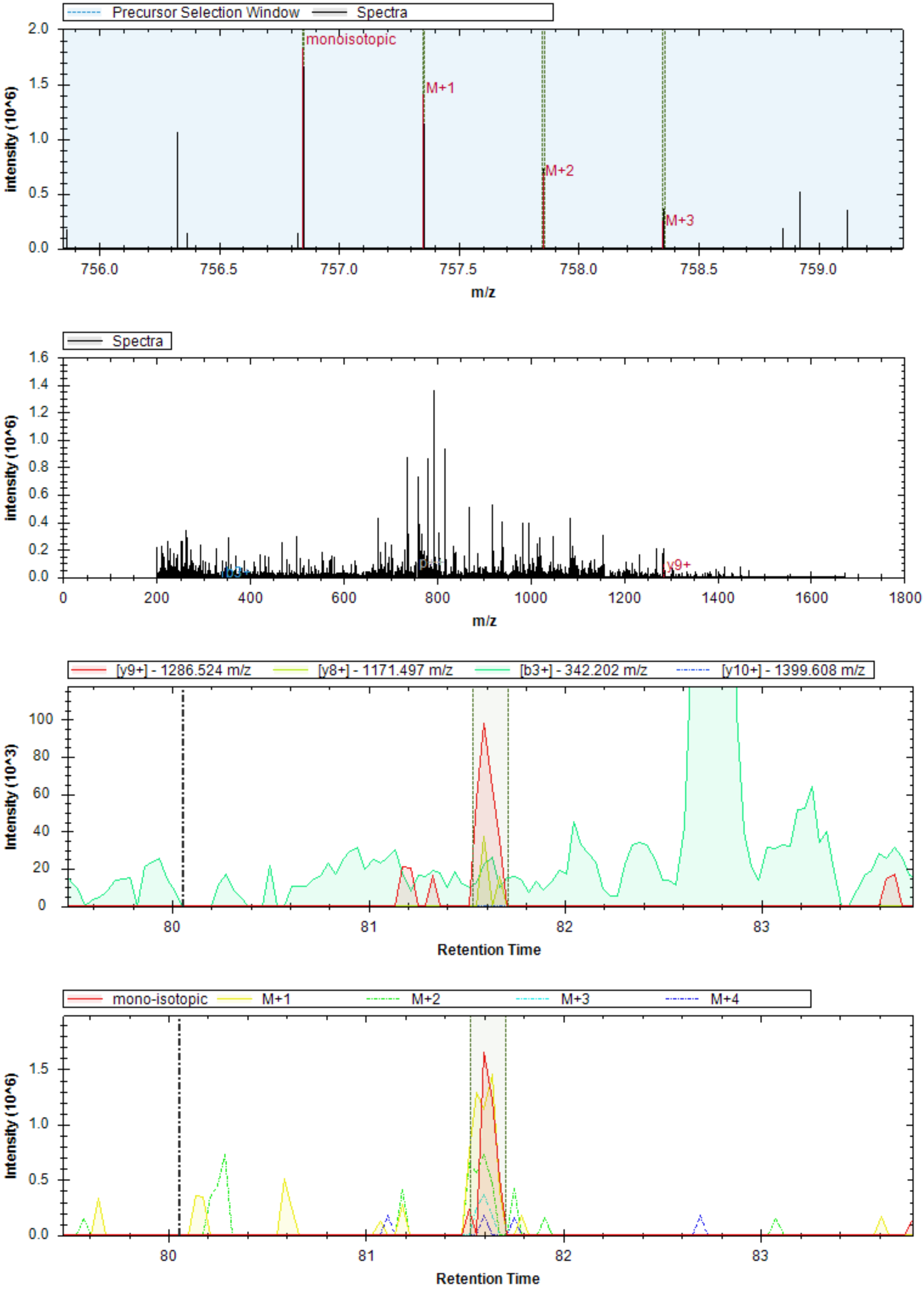

P57776-3 – _ATAPQTQHVS[+H+O3+P]PM[+O]R_.2
Fig4_MP-DIA-barrel-cortex-p15-1_MHRM.htrms

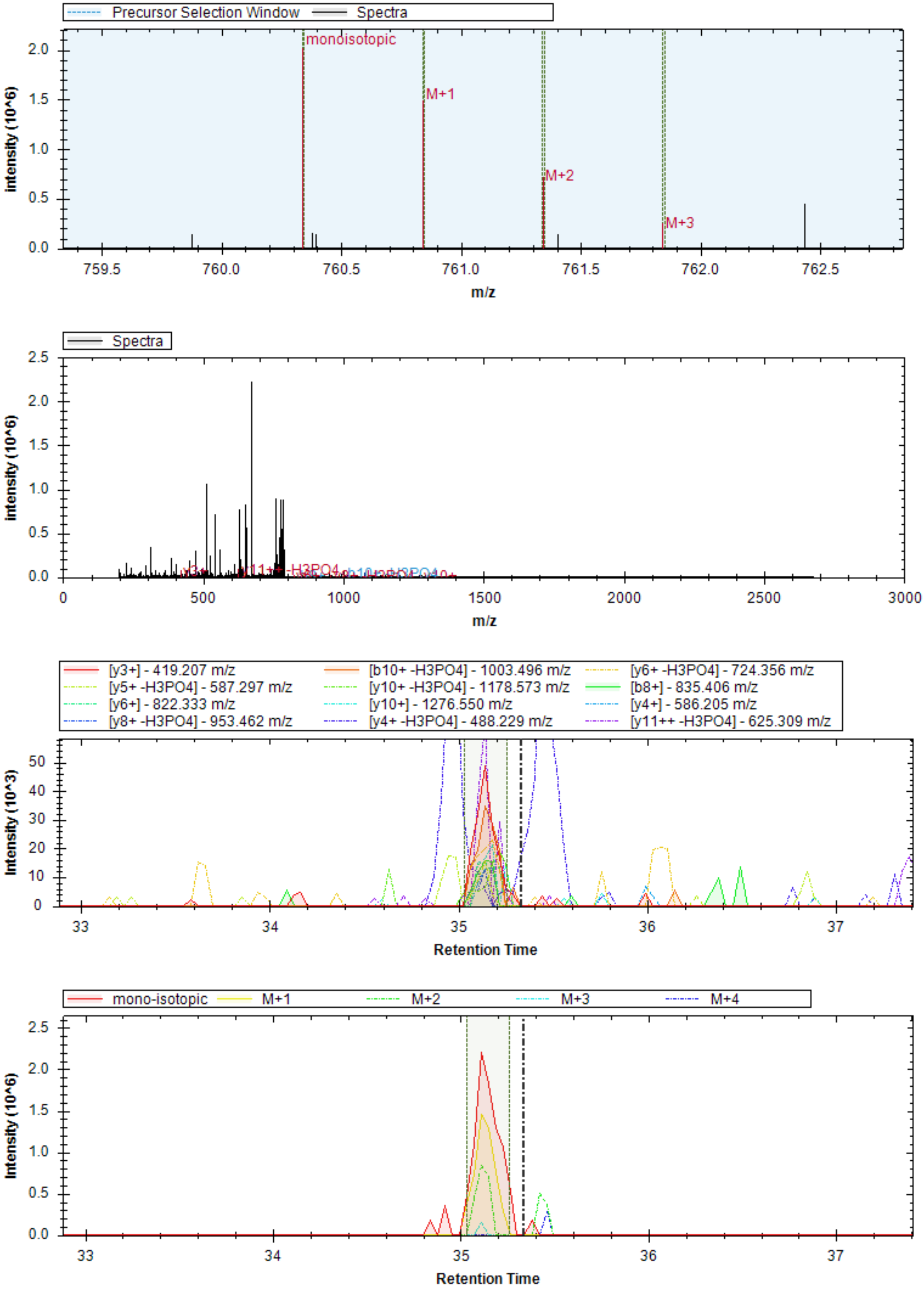

Q3TZM9;Q3TZM9-2;Q3TZM9-3 – _VPDVYIDSM[+O]GYAFT[+H+O3+P]LPLFK_.3
Fig4_MP-DIA-barrel-cortex-p15-1_MHRM.htrms

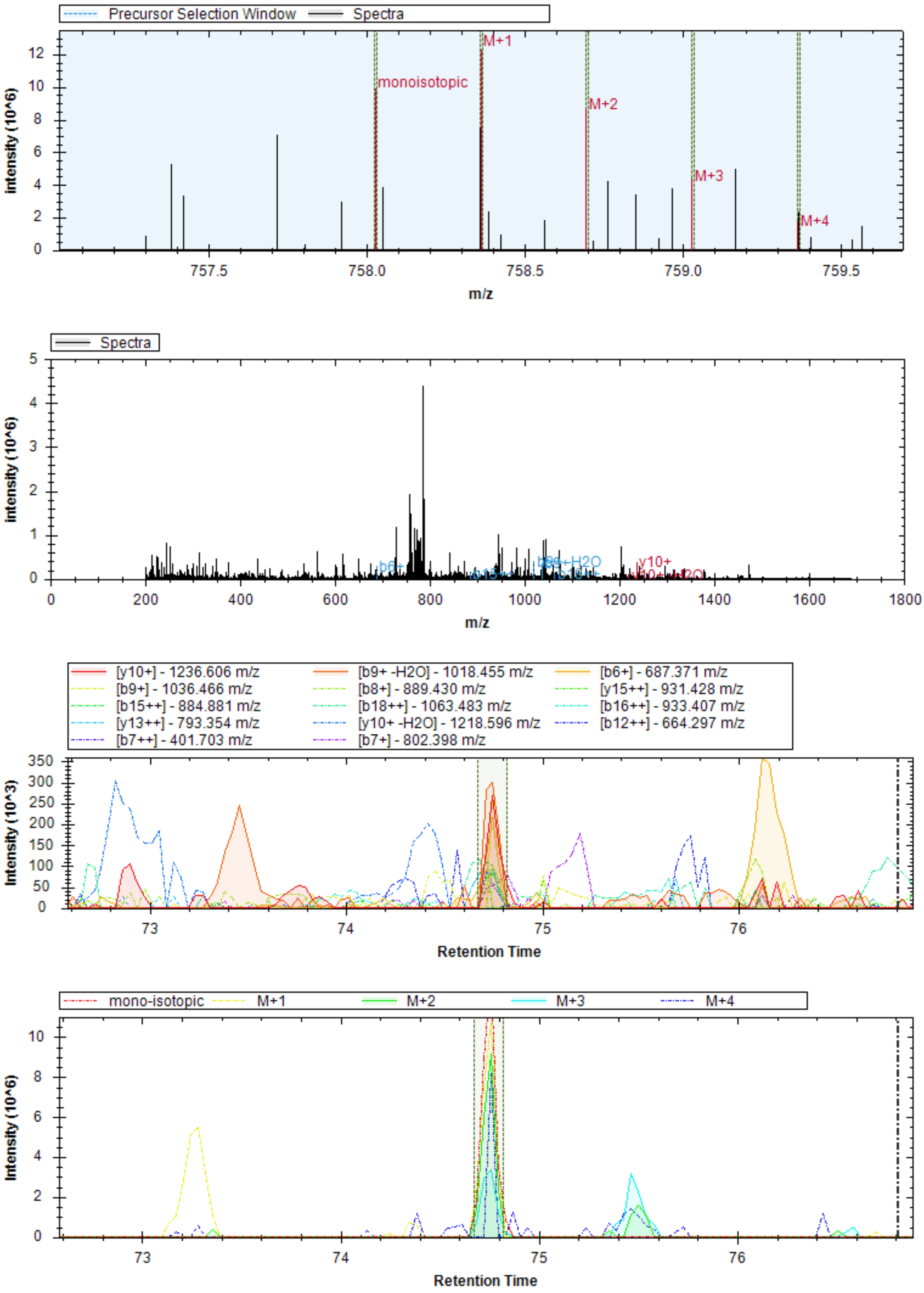

Q68FD9 – _LAQLLS[+H+O3+P]EVPT[+H+O3+P]RR_.2
Fig4_MP-DIA-barrel-cortex-p15-1_MHRM.htrms

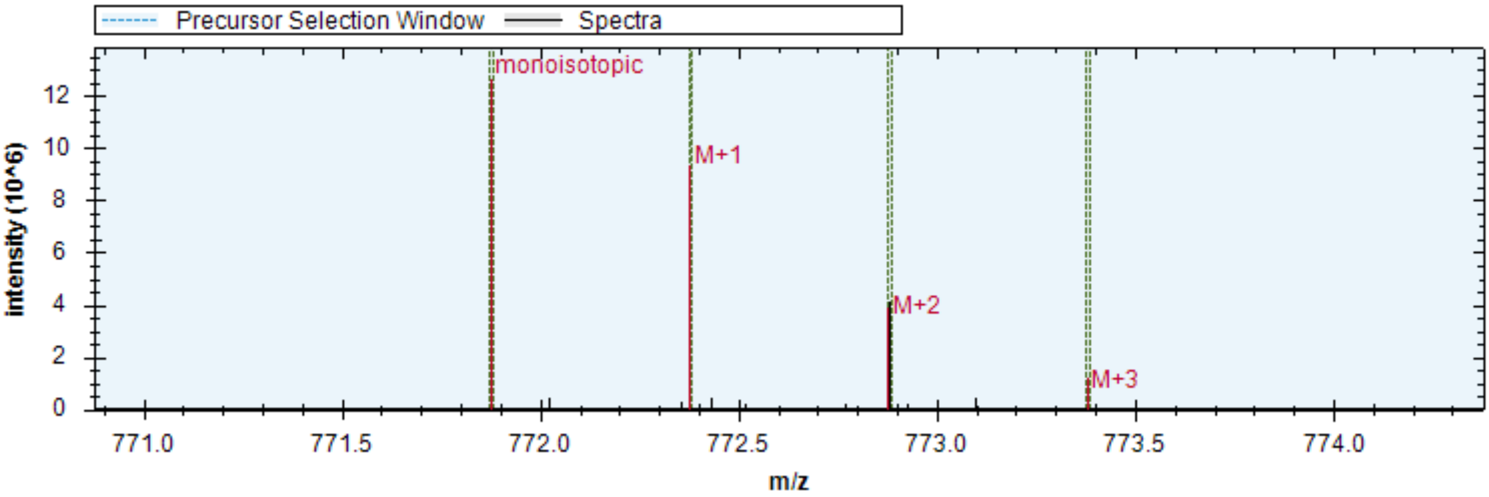

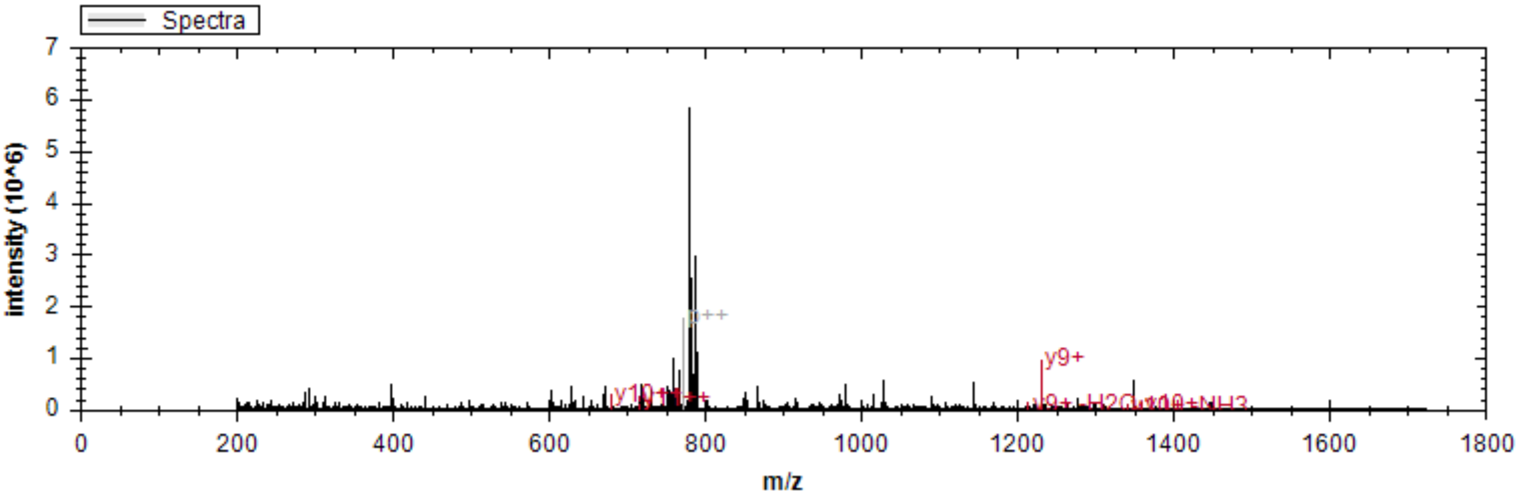

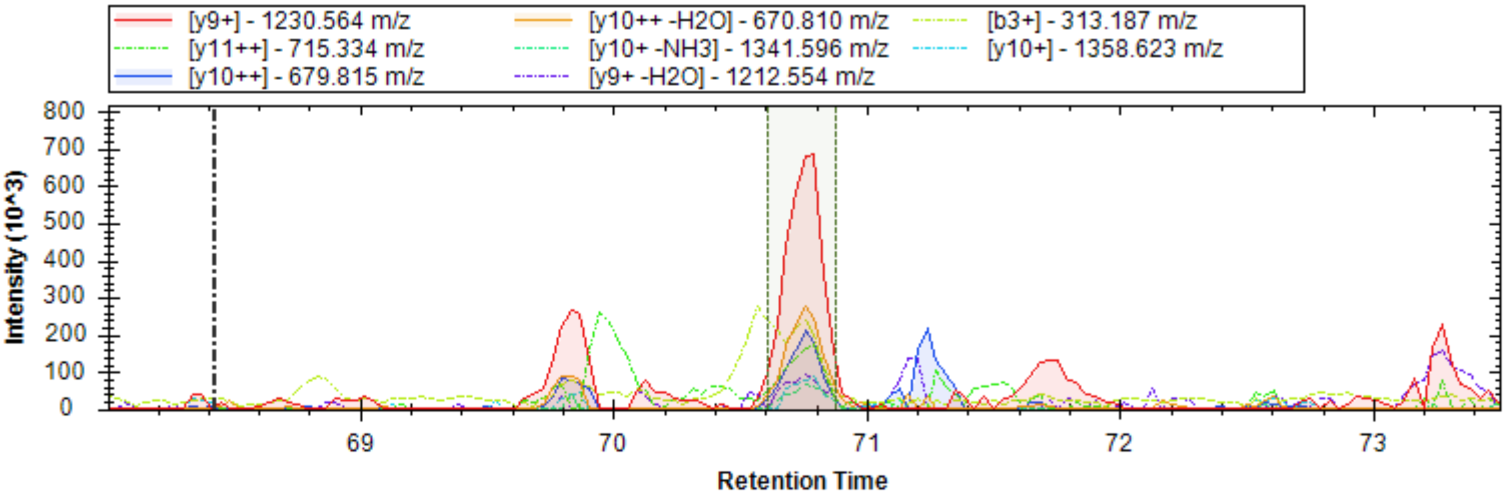

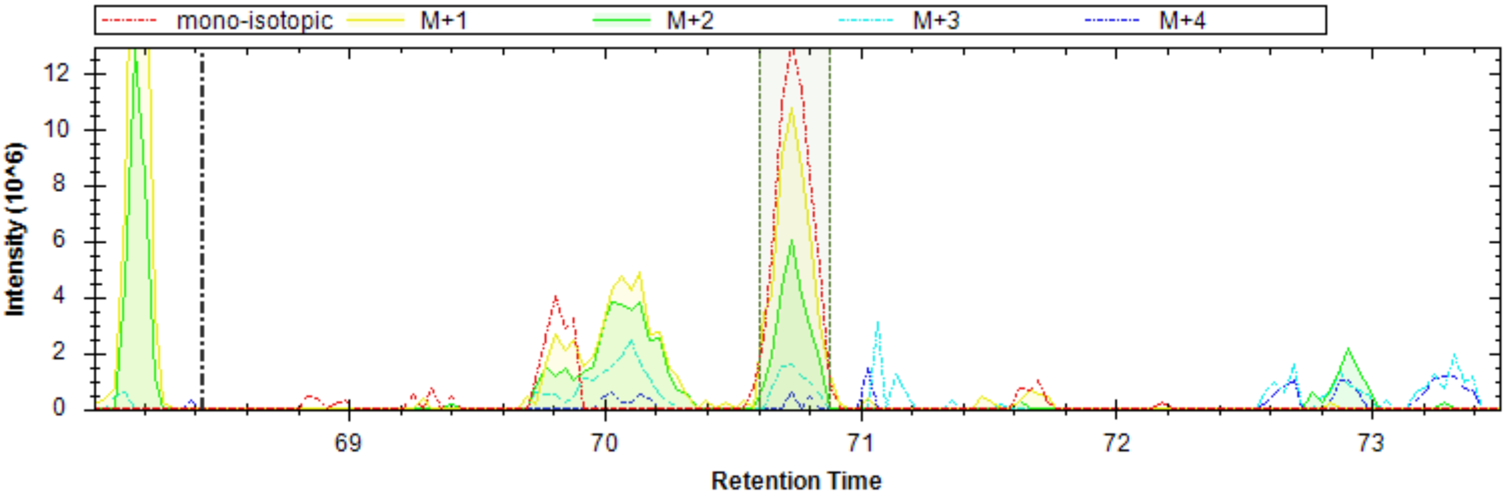

Q6ZQA0;Q6ZQA0-2 – _LHT[+H+O3+P]LLQT[+H+O3+P]AVPTR_.2
Fig4_MP-DIA-barrel-cortex-p15-1_MHRM.htrms

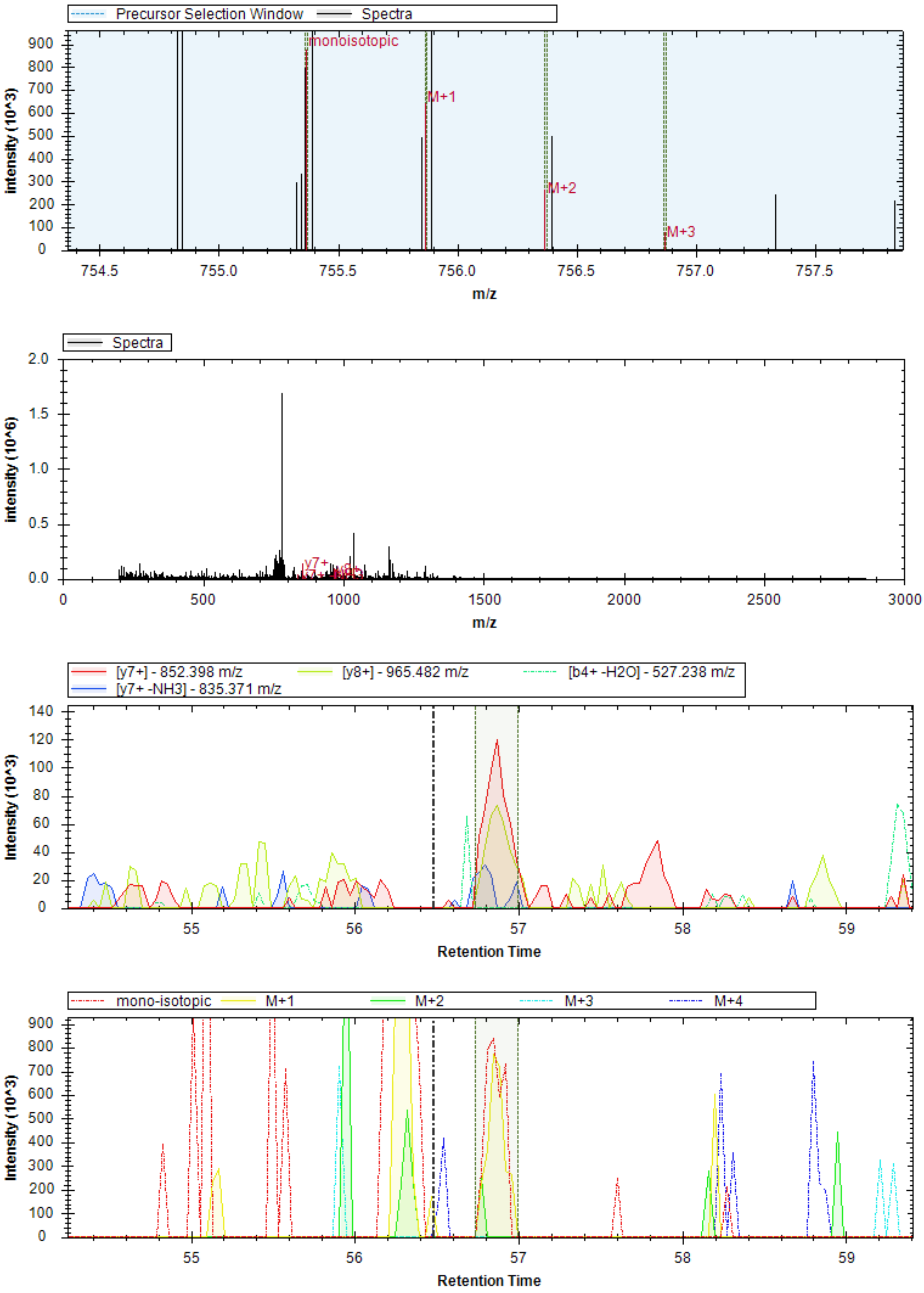

Q9D5R2 – _SNS[+H+O3+P]LPHSAVSNAASK_.2
Fig4_MP-DIA-barrel-cortex-p15-1_MHRM.htrms

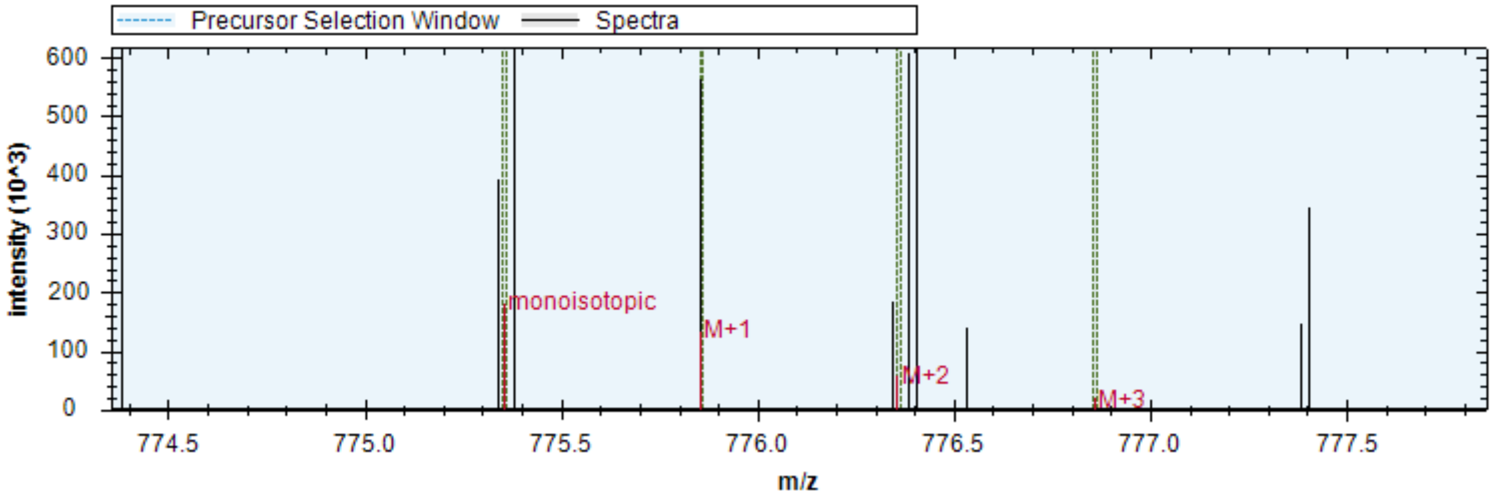

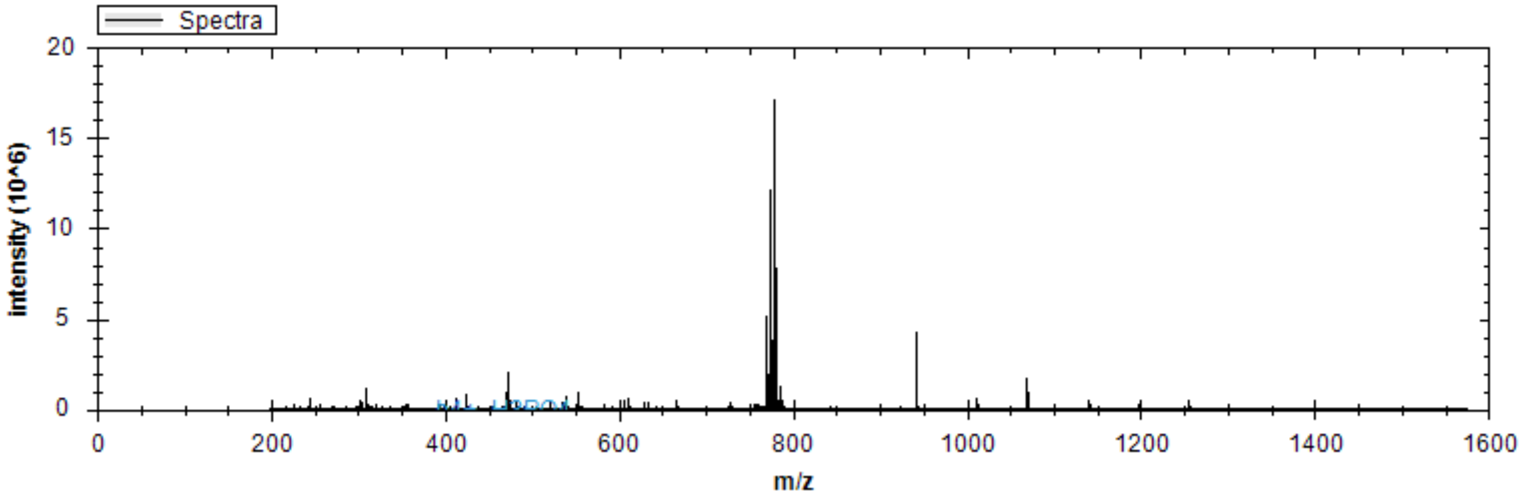

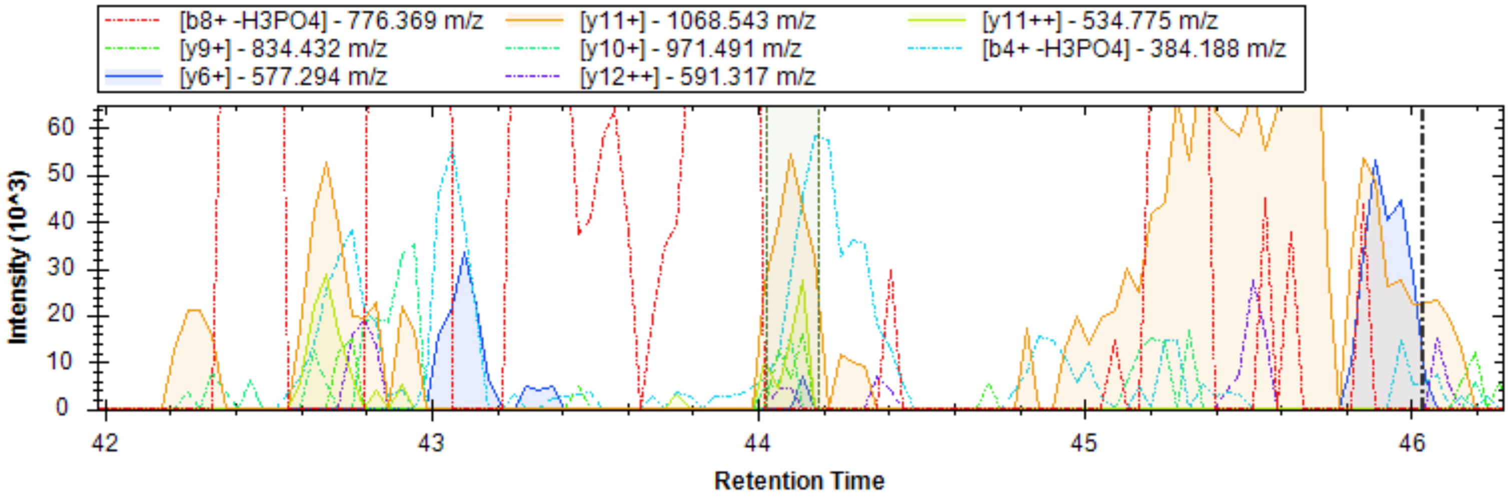

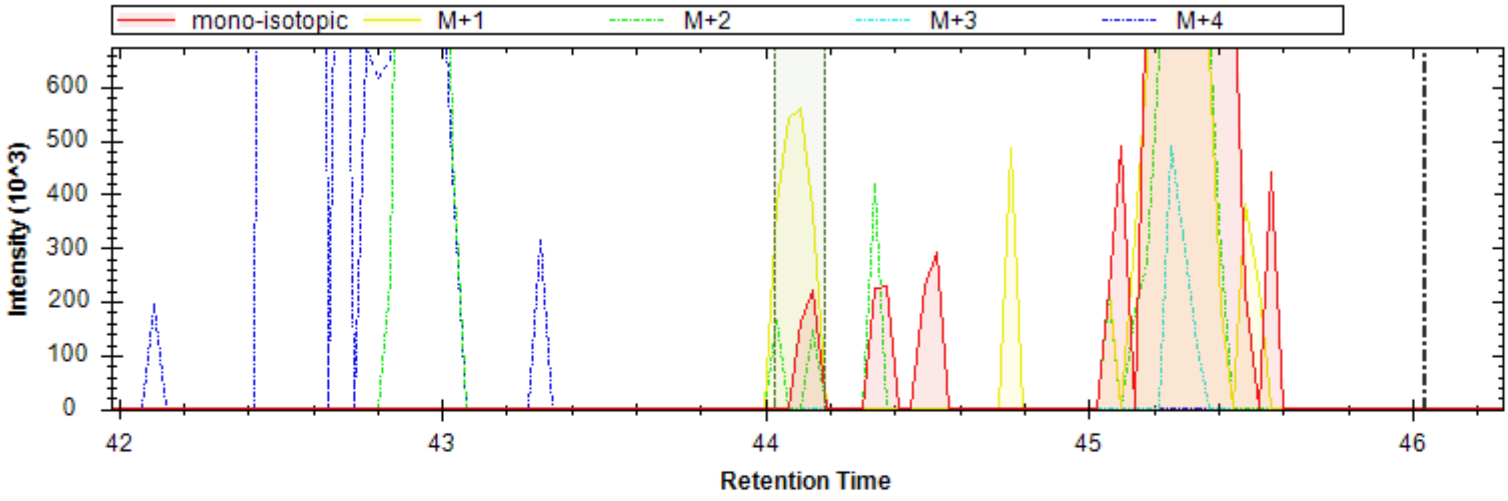

Q9JLM8 – _ISQHGGS[+H+O3+P]STSLSSTK_.2
Fig4_MP-DIA-barrel-cortex-p15-1_MHRM.htrms

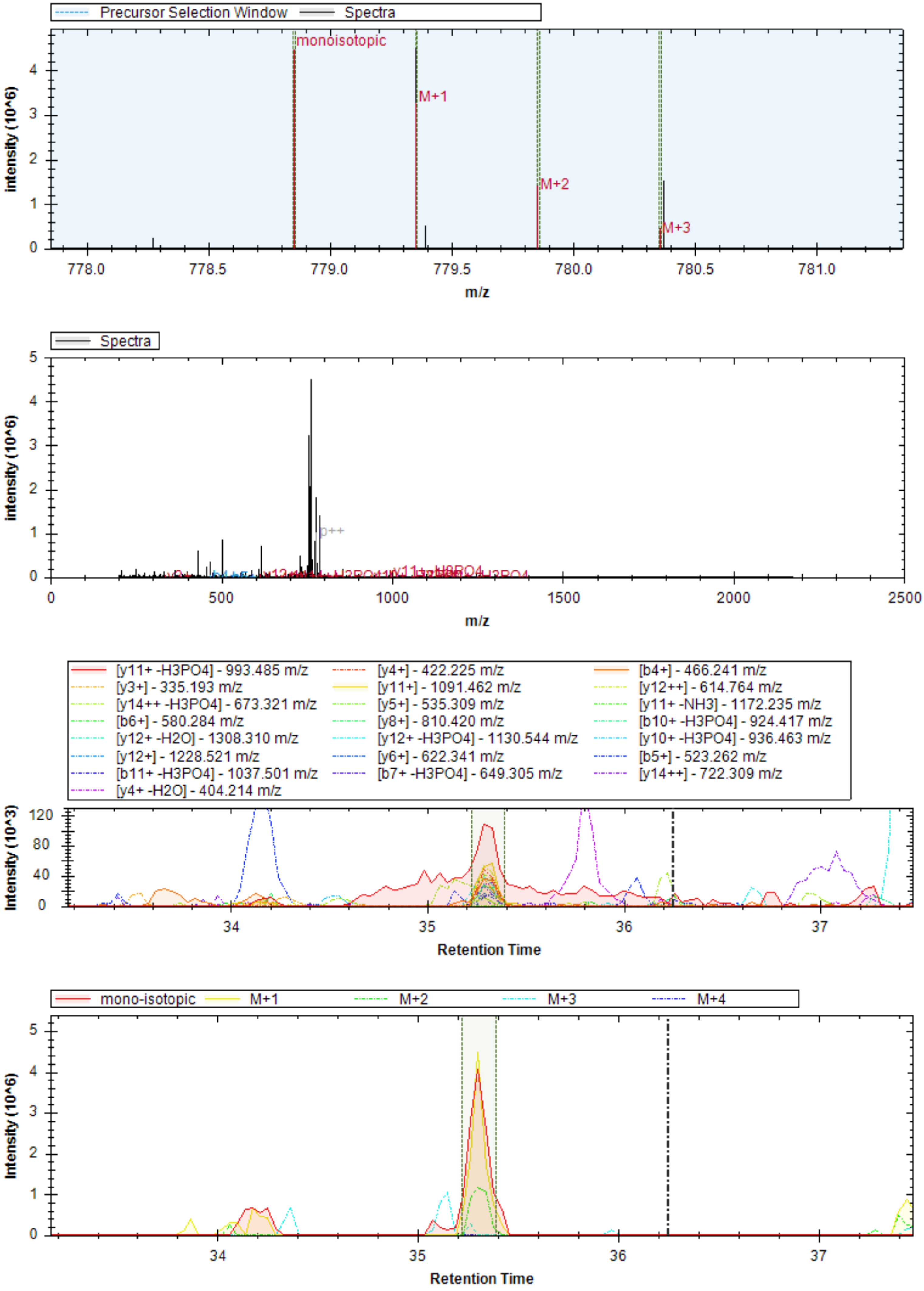

Q9QWI6 – _LSYAGGRPPSYAGS[+H+O3+P]PVHHAAER_.3

Fig4_MP-DIA-barrel-cortex-p15-1_MHRM.htrms

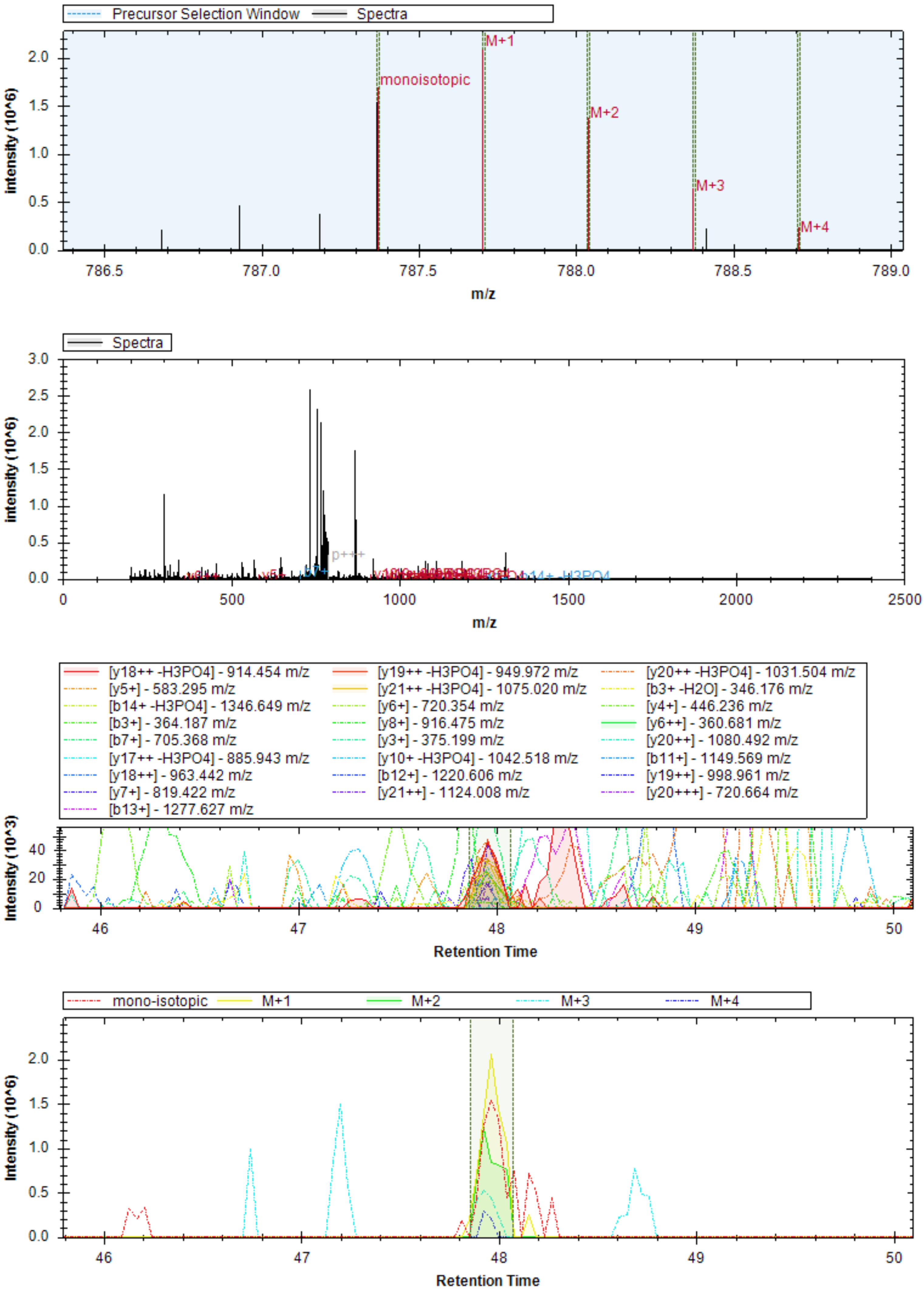

Q9QYC0 – _AAVVTS[+H+O3+P]PPPTTAPHK_.2
Fig4_MP-DIA-barrel-cortex-p15-1_MHRM.htrms

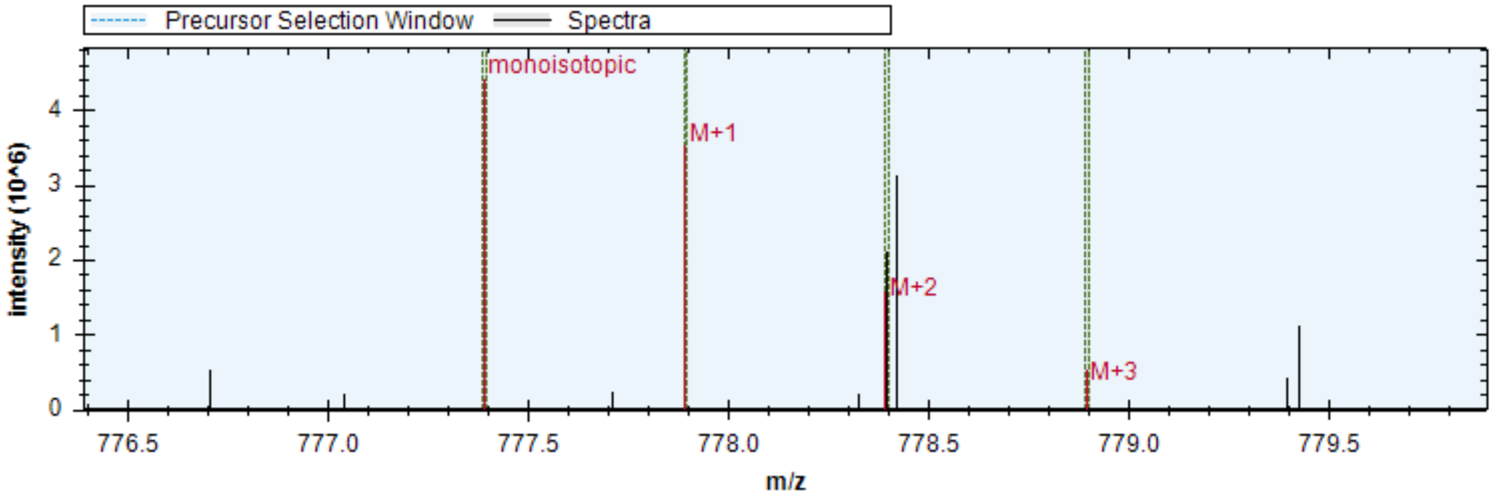

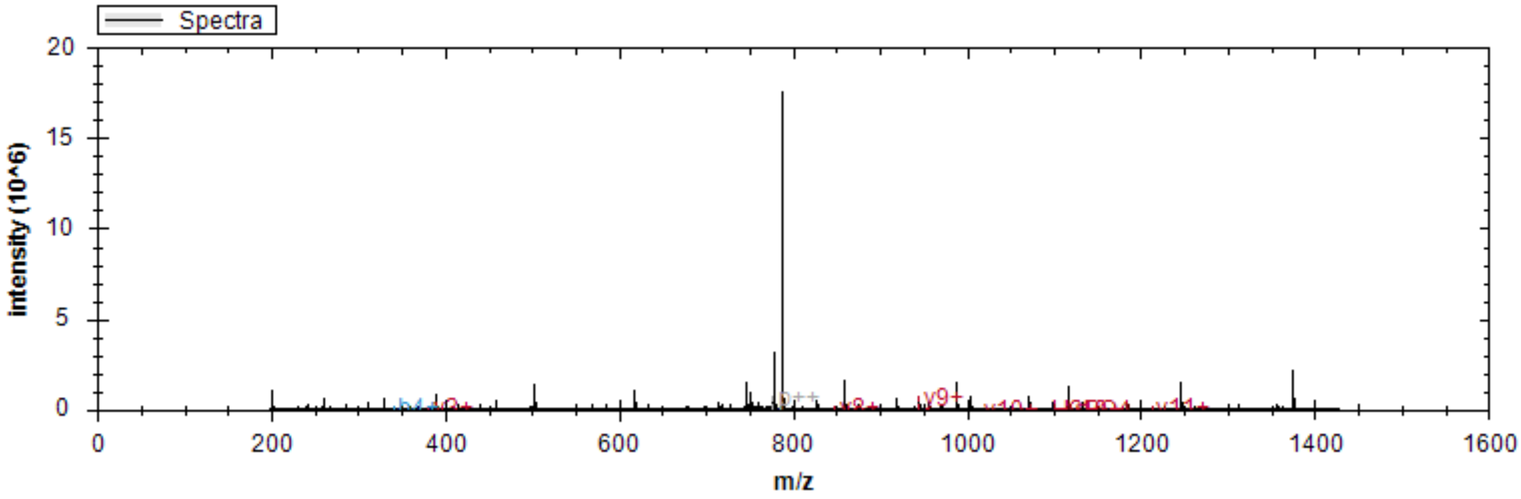

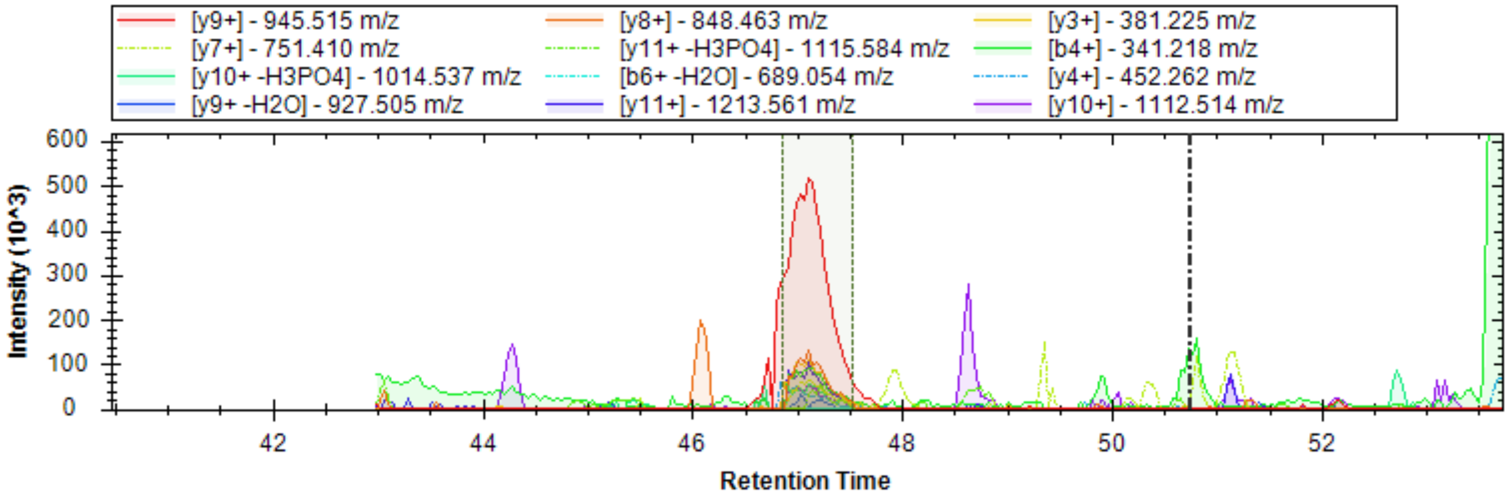

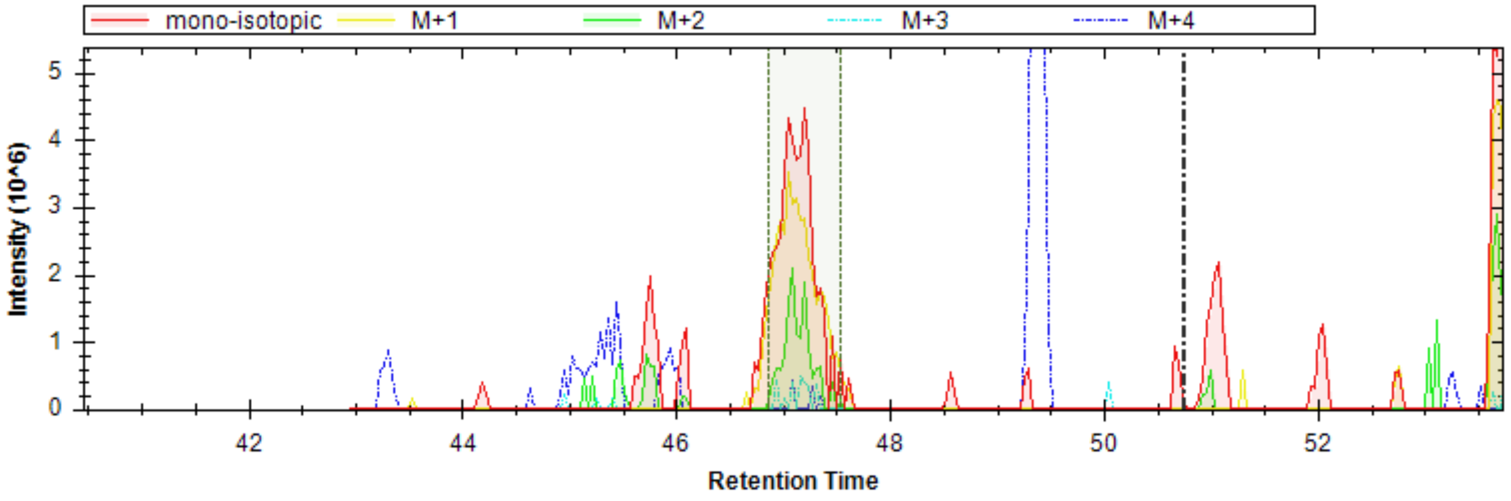

Q9QYR6-2 – _ELSSEPRT[+H+O3+P]PPAQK_.2
Fig4_MP-DIA-barrel-cortex-p15-1_MHRM.htrms

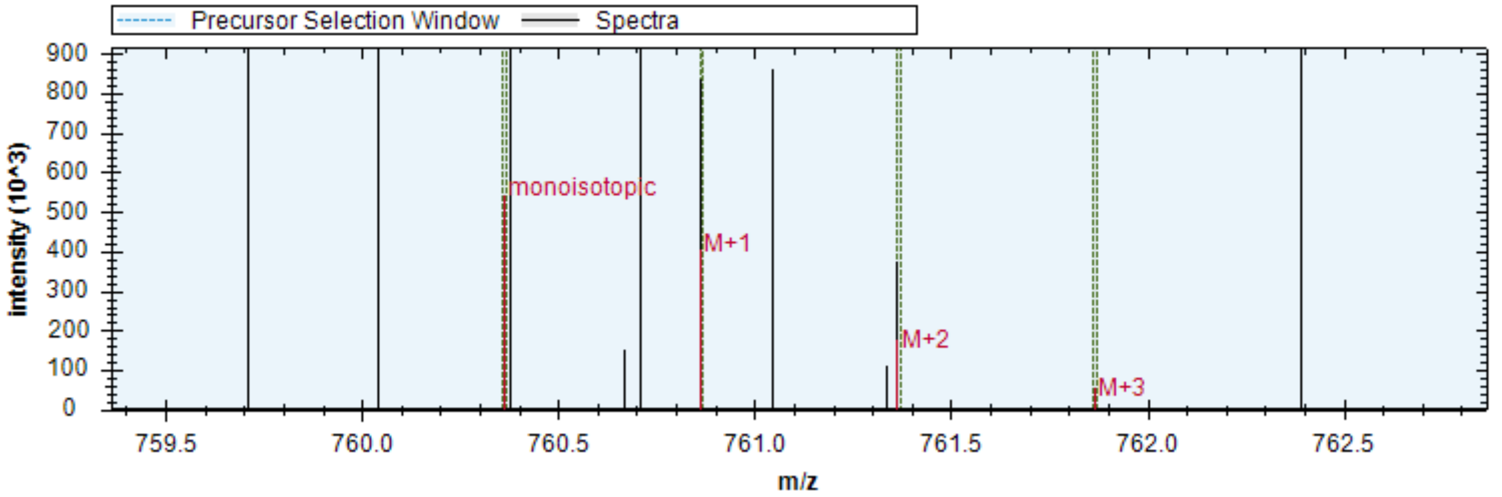

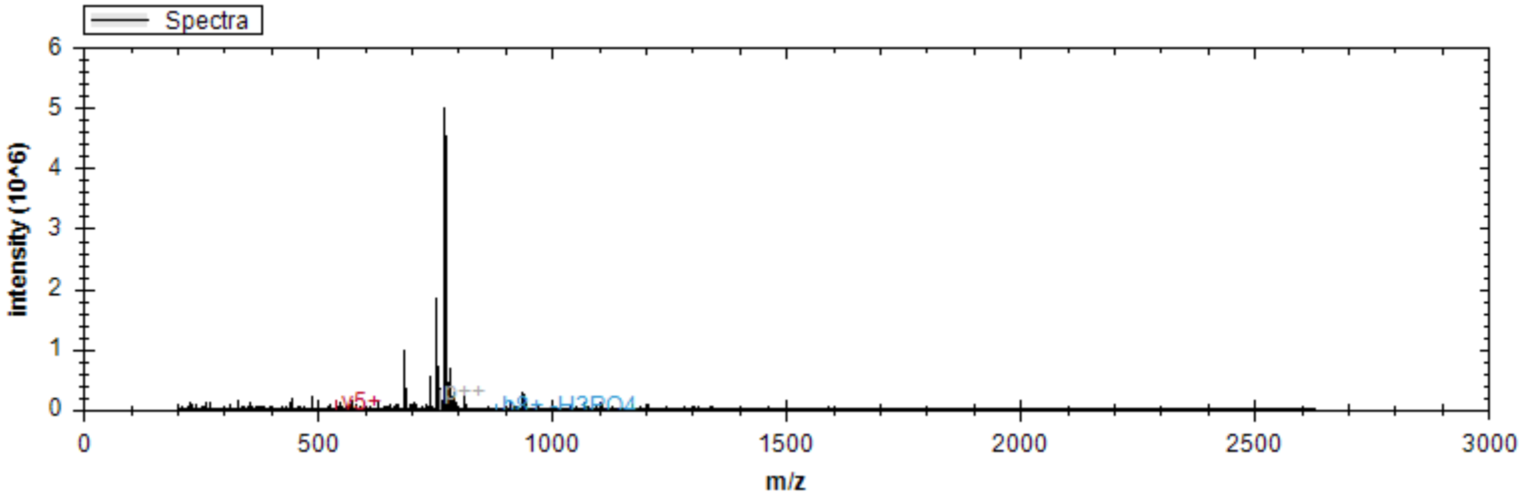

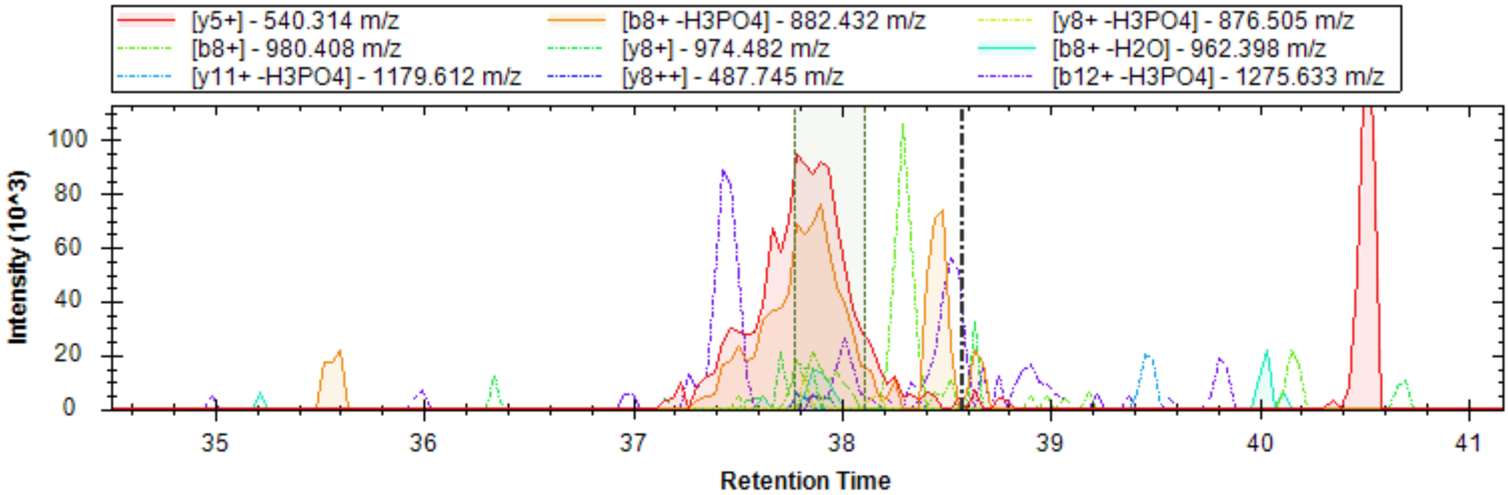

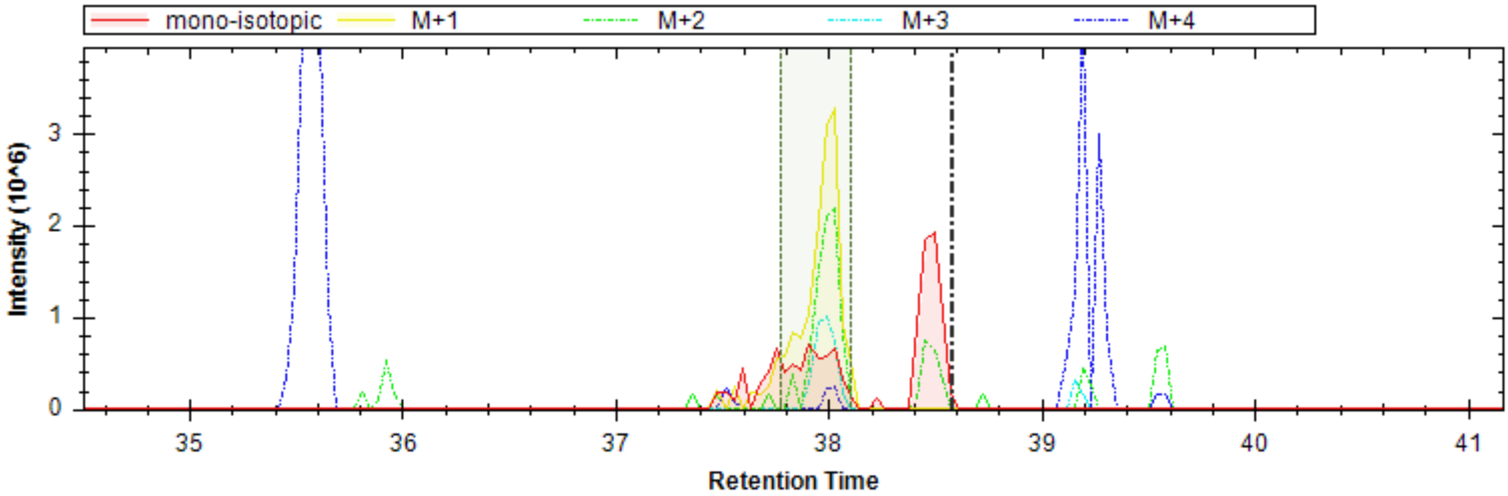

Q9Z0P4 – _ENSAAPSPGRPQS[+H+O3+P]ASPAKEEQK_.3

Fig4_MP-DIA-barrel-cortex-p15-1_MHRM.htrms

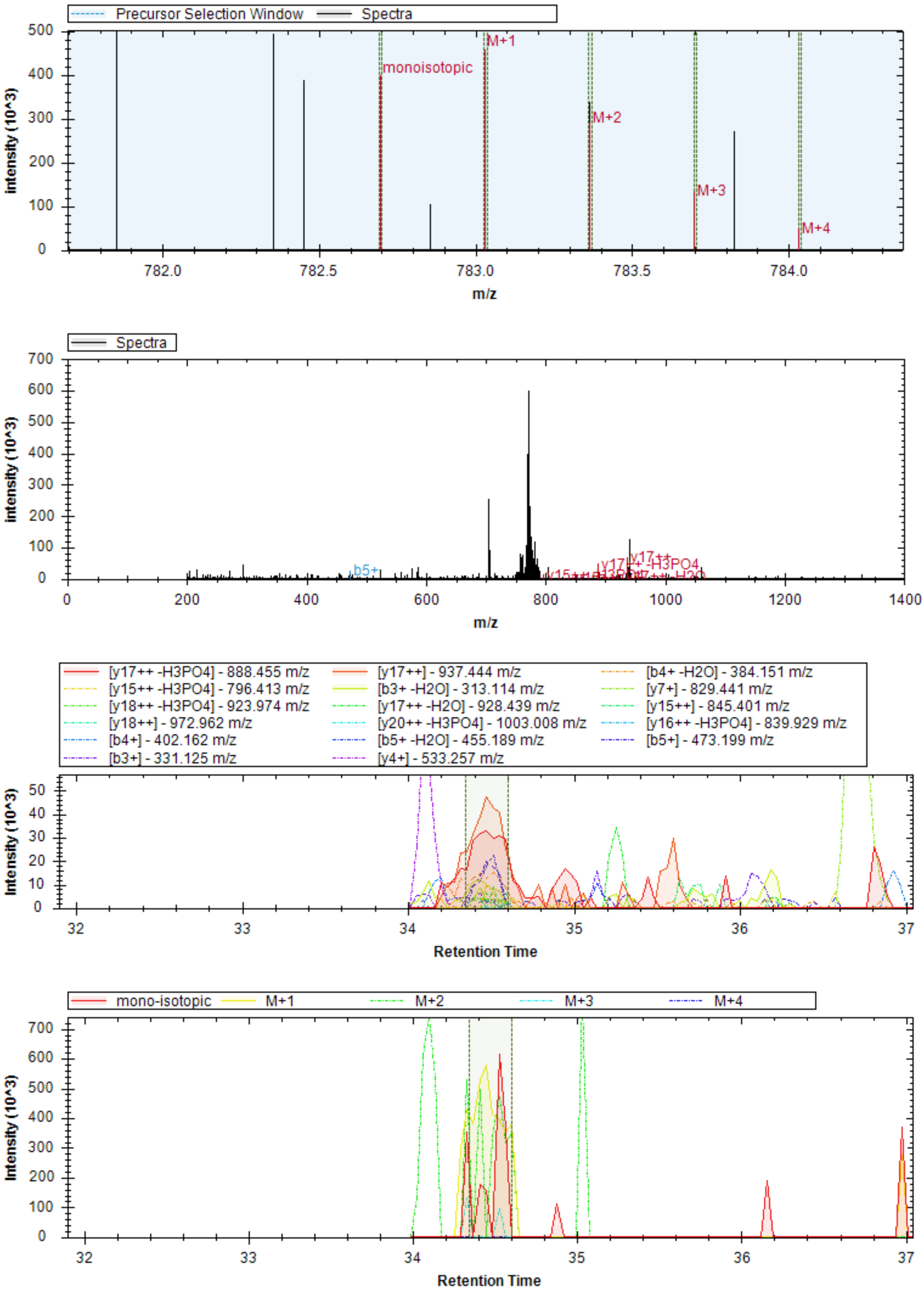

O88935-1 – _PAKPQLAQKPS[+H+O3+P]QDVPPPITAAAGGPPHPQLK_.4
Fig4_MP-DIA-barrel-cortex-p15-1_MHRM.htrms

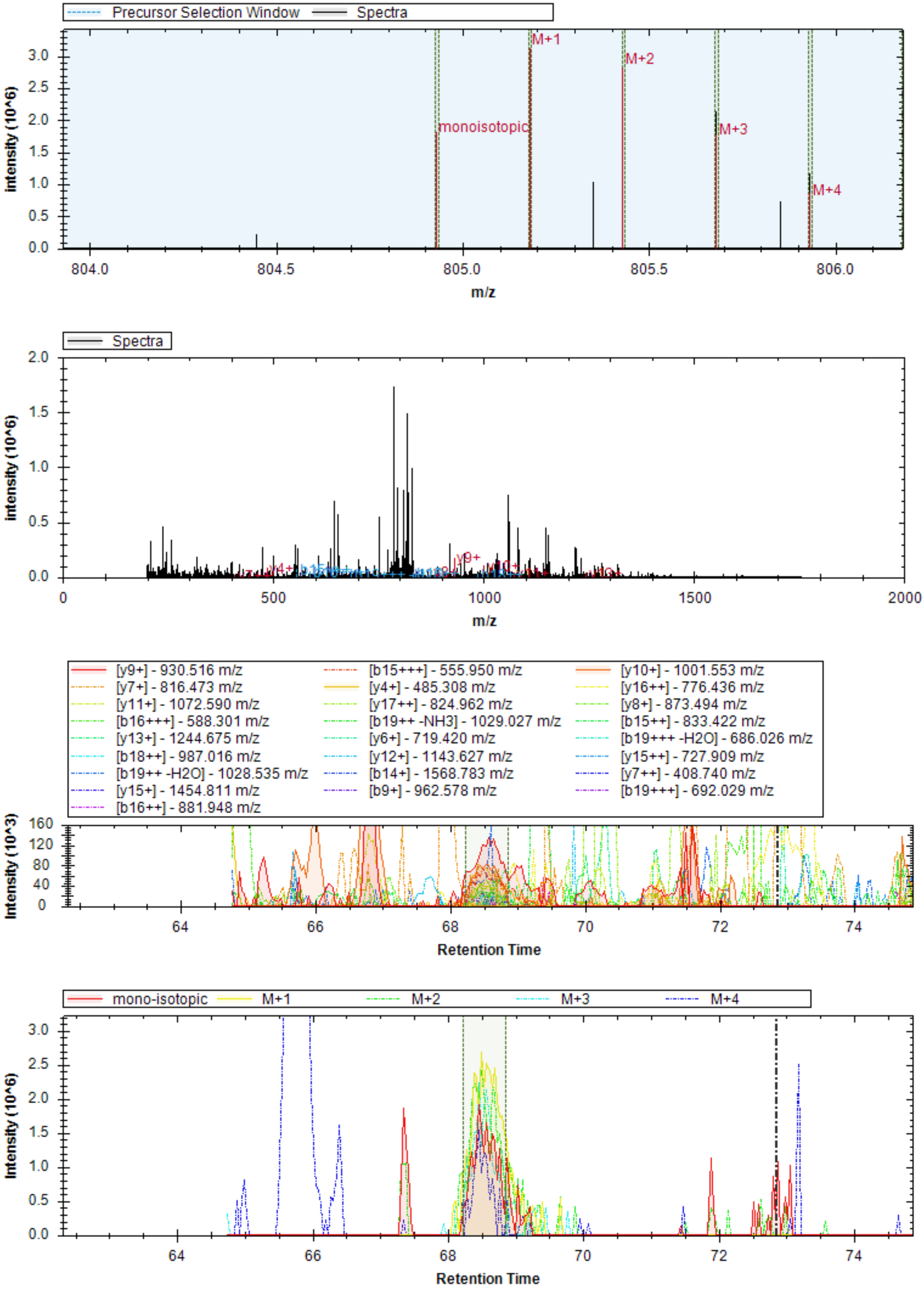

P10637 – _TTPSPKT[+H+O3+P]PPGSGEPPK_.2

Fig4_MP-DIA-barrel-cortex-p15-1_MHRM.htrms

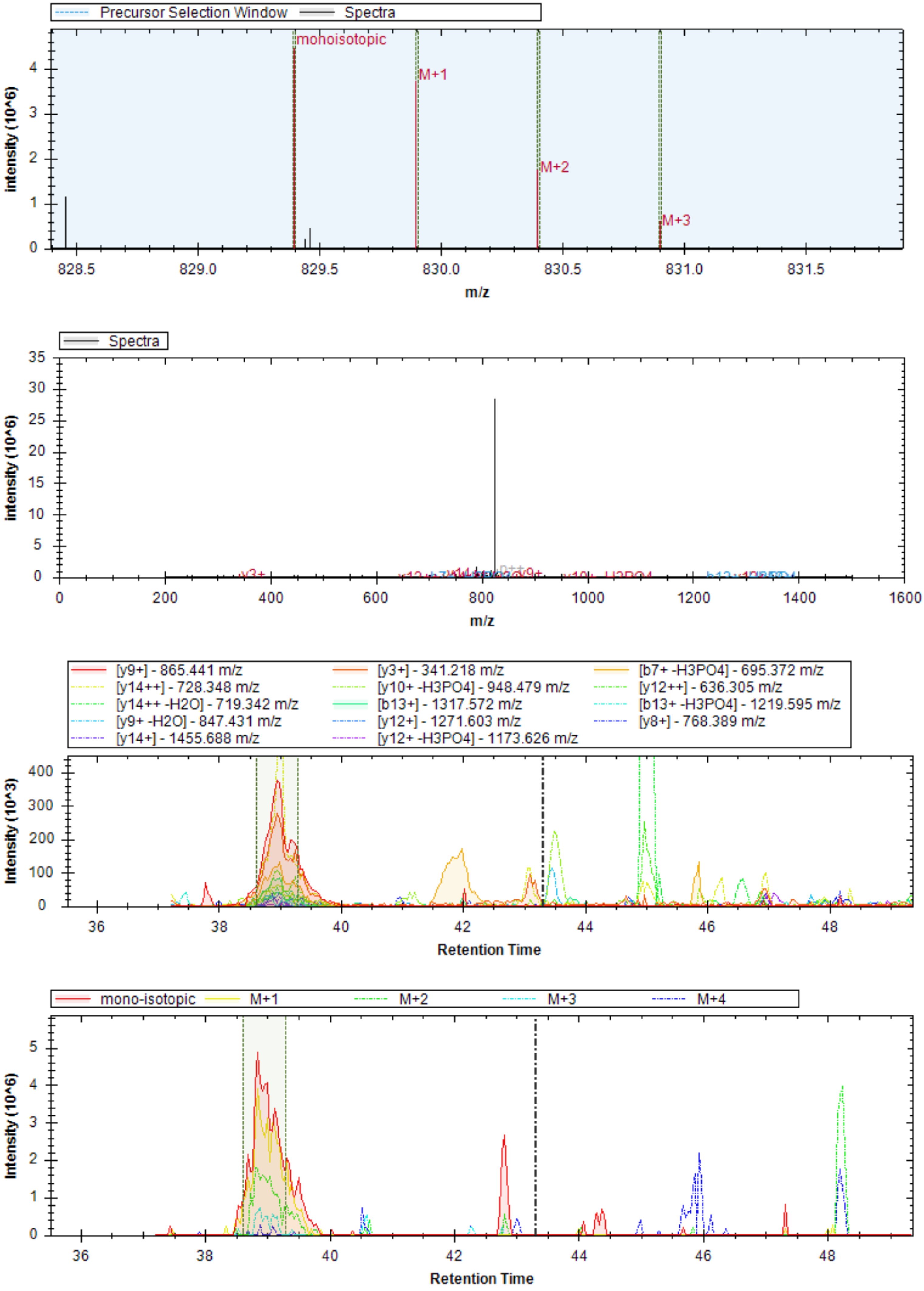

P20357 – _VAIIRT[+H+O3+P]PPKSPATPK_.2

Fig4_MP-DIA-barrel-cortex-p15-1_MHRM.htrms

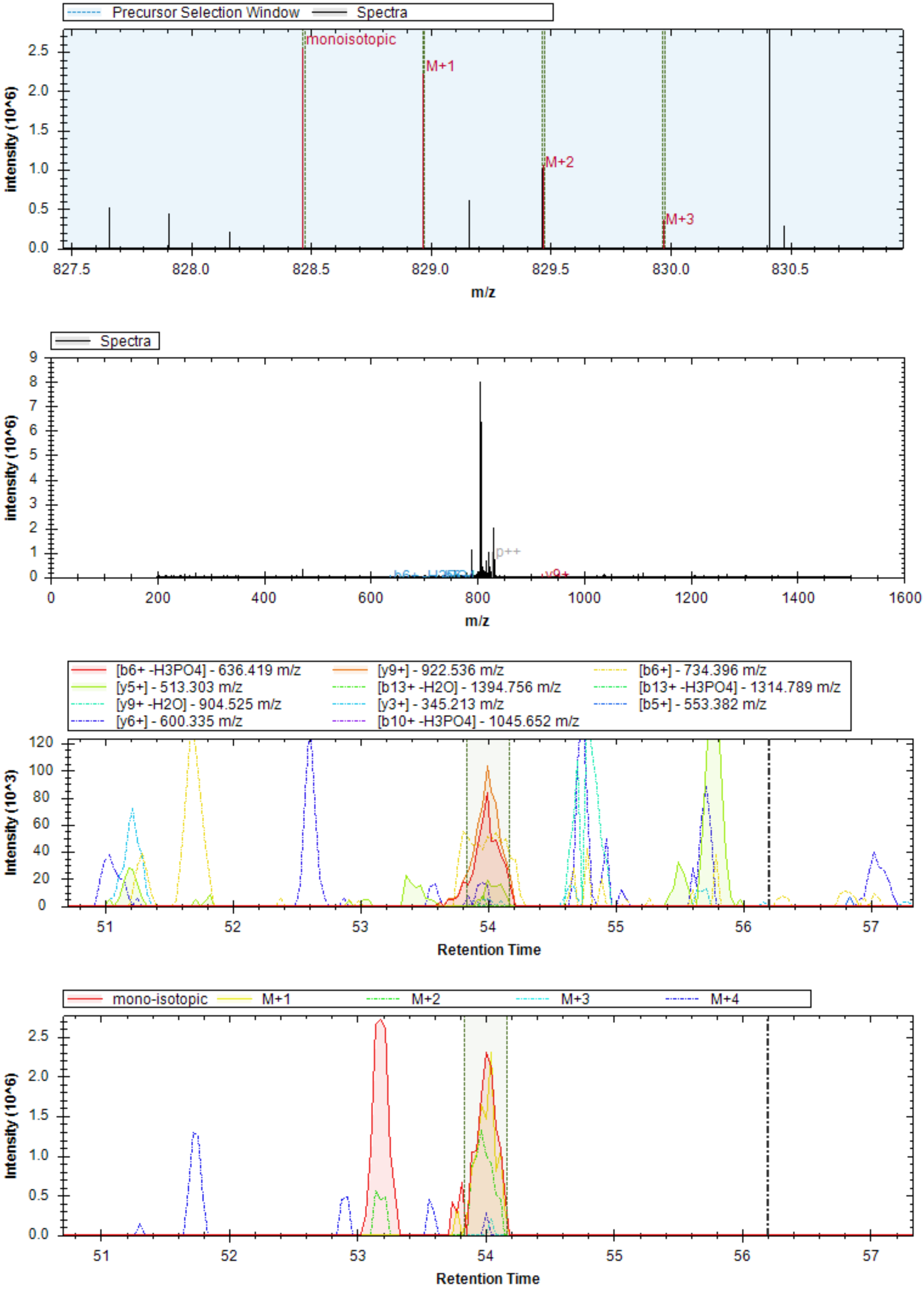

P20357 – _VDHGAEIIT[+H+O3+P]QSPSR_.2
Fig4_MP-DIA-barrel-cortex-p15-1_MHRM.htrms

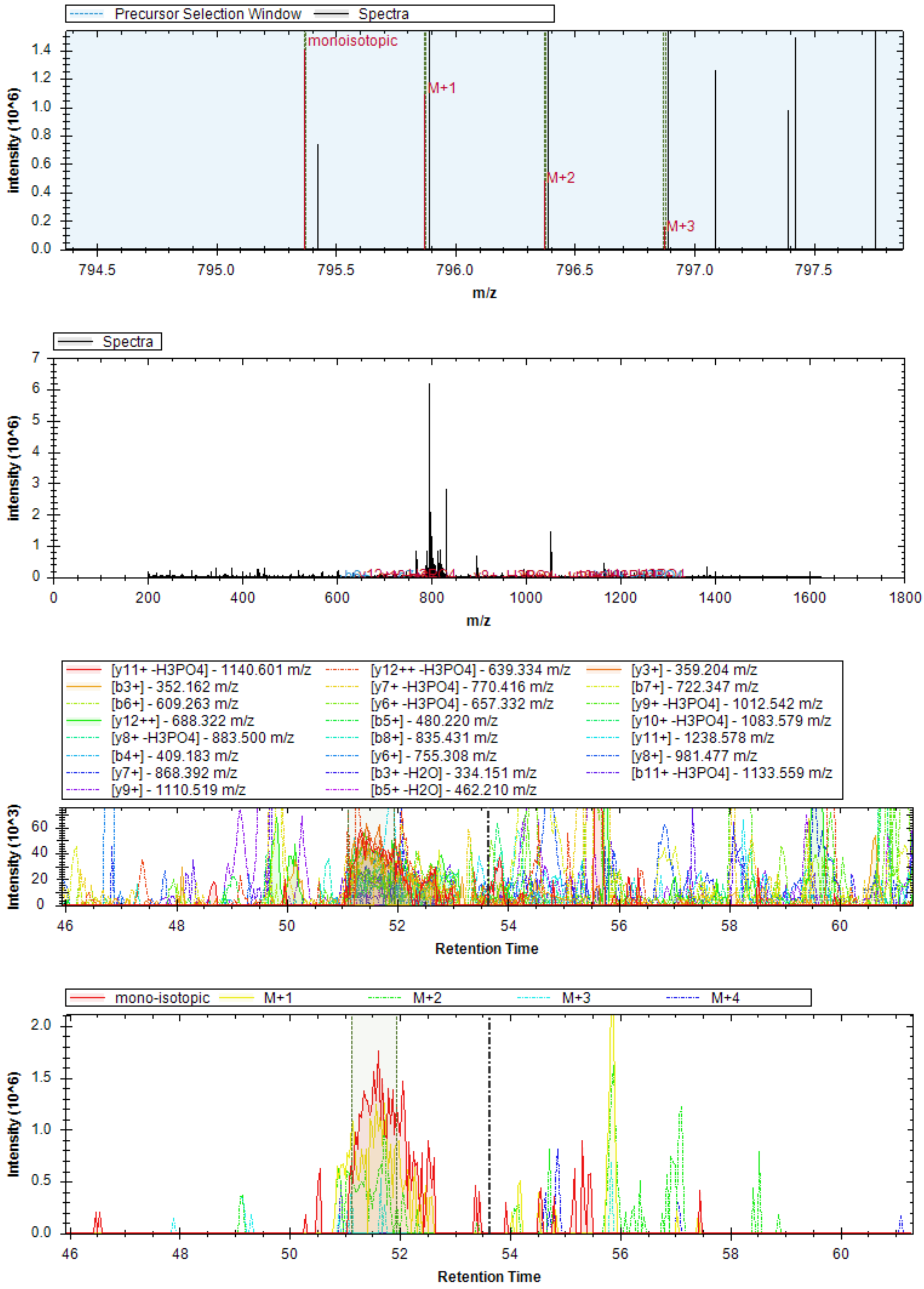

P20357 – _VDHGAEIITQS[+H+O3+P]PSR_.2

Fig4_MP-DIA-barrel-cortex-p15-1_MHRM.htrms

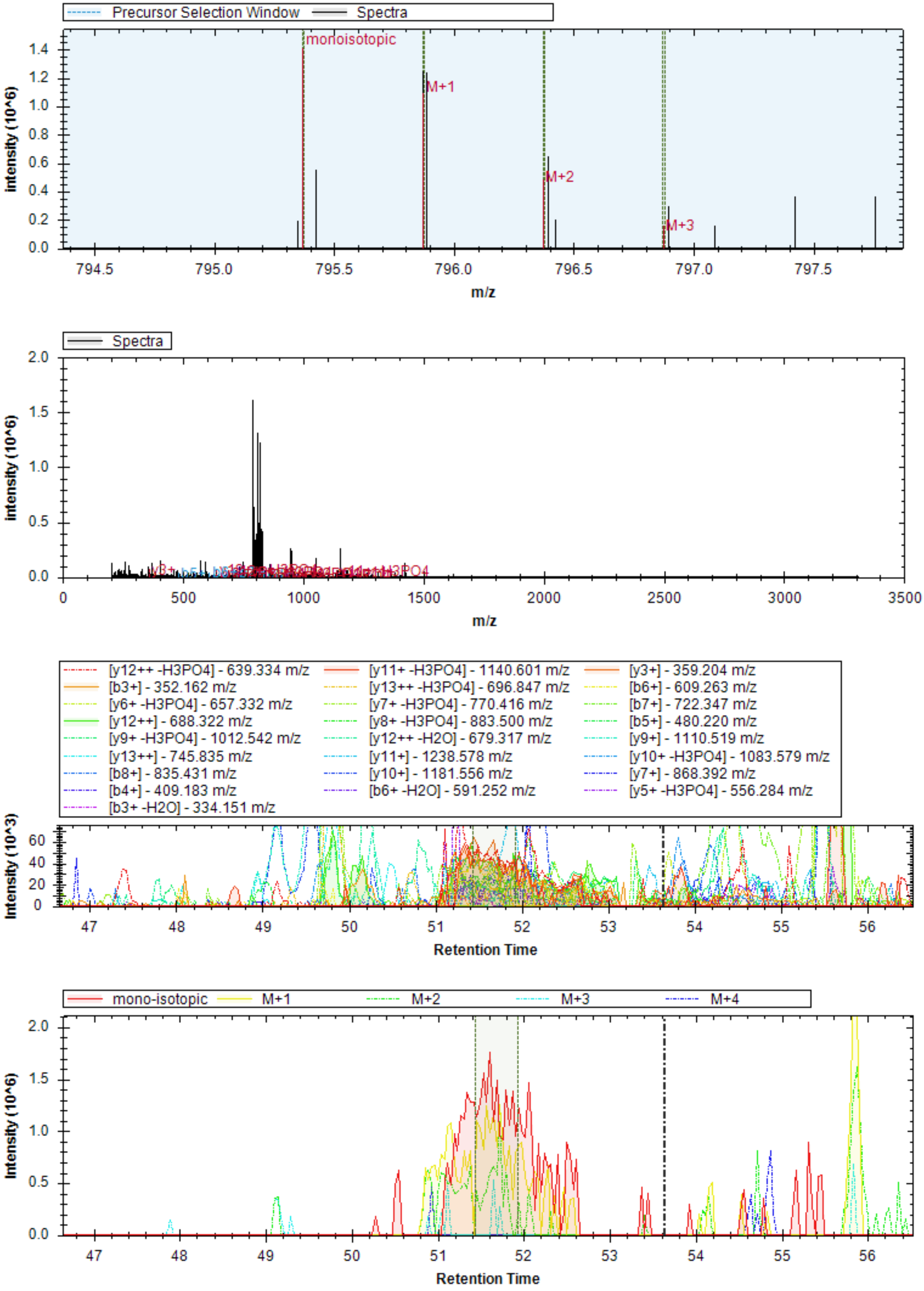

P20357 – _VDHGAEIITQSPS[+H+O3+P]R_.2
Fig4_MP-DIA-barrel-cortex-p15-1_MHRM.htrms

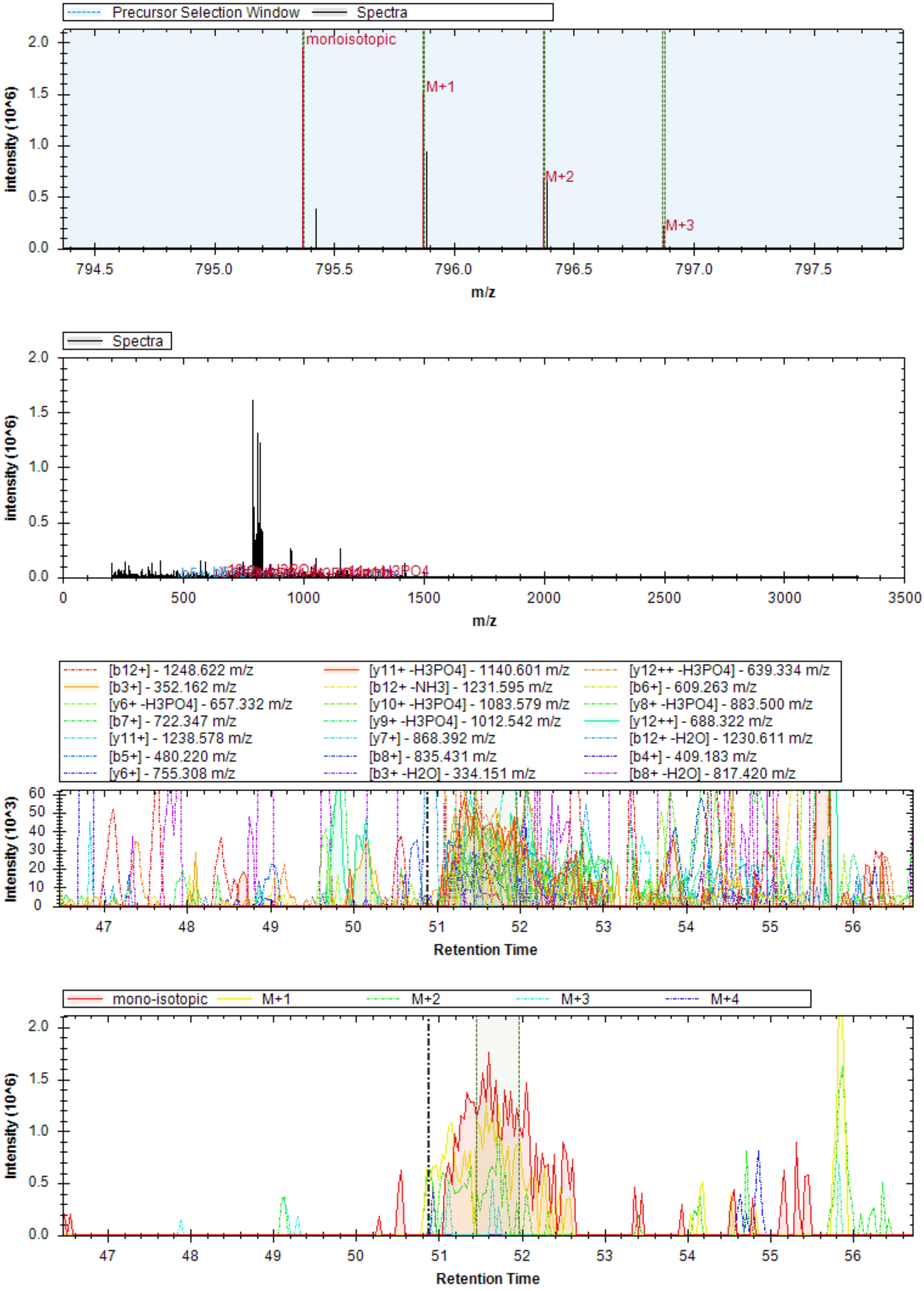

P54227 – _SKESVPDFPLS[+H+O3+P]PPK_.2

Fig4_MP-DIA-barrel-cortex-p15-1_MHRM.htrms

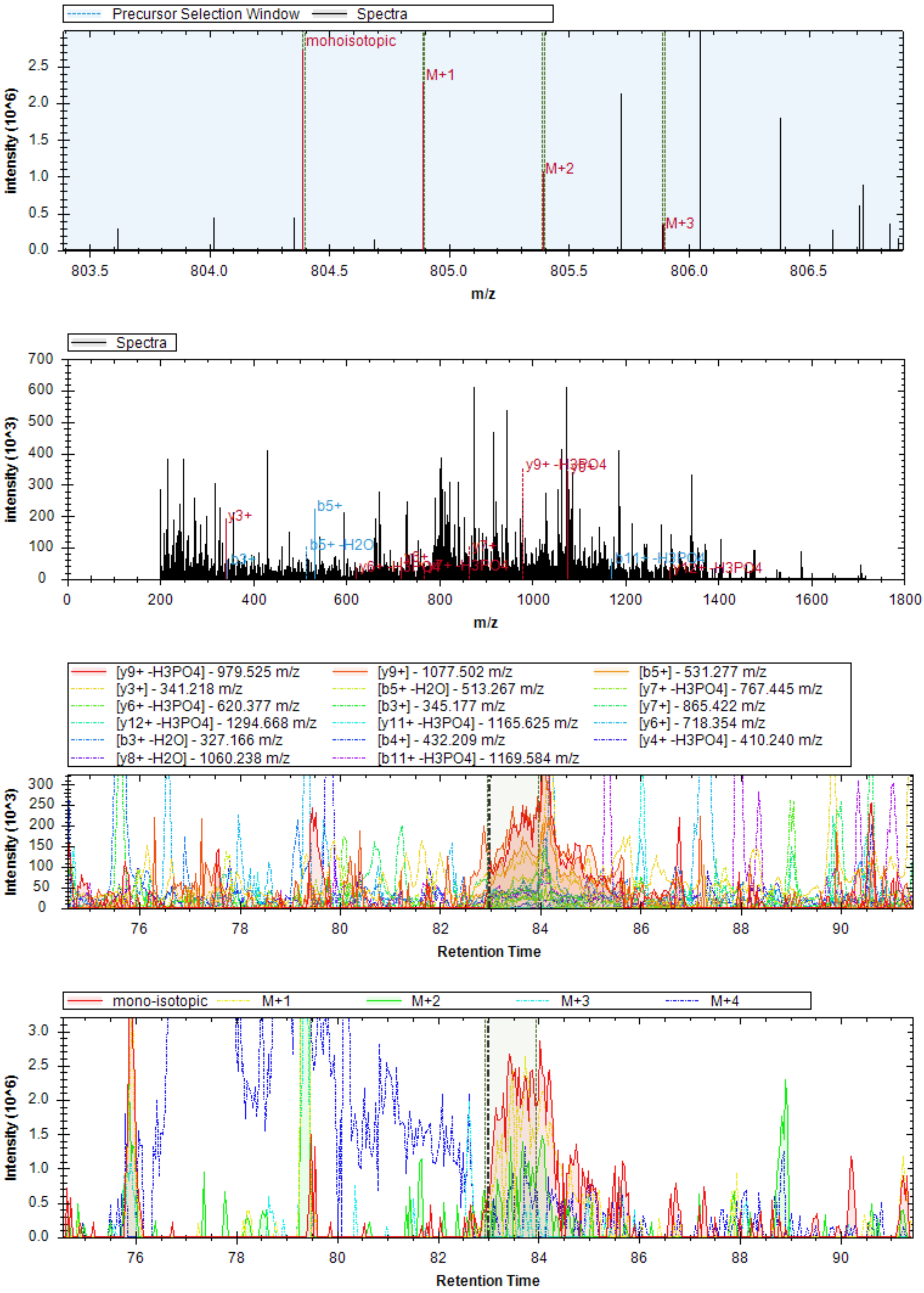

Q60771 – _FYYSSGS[+H+O3+P]SSPTHAK_.2
Fig4_MP-DIA-barrel-cortex-p15-1_MHRM.htrms

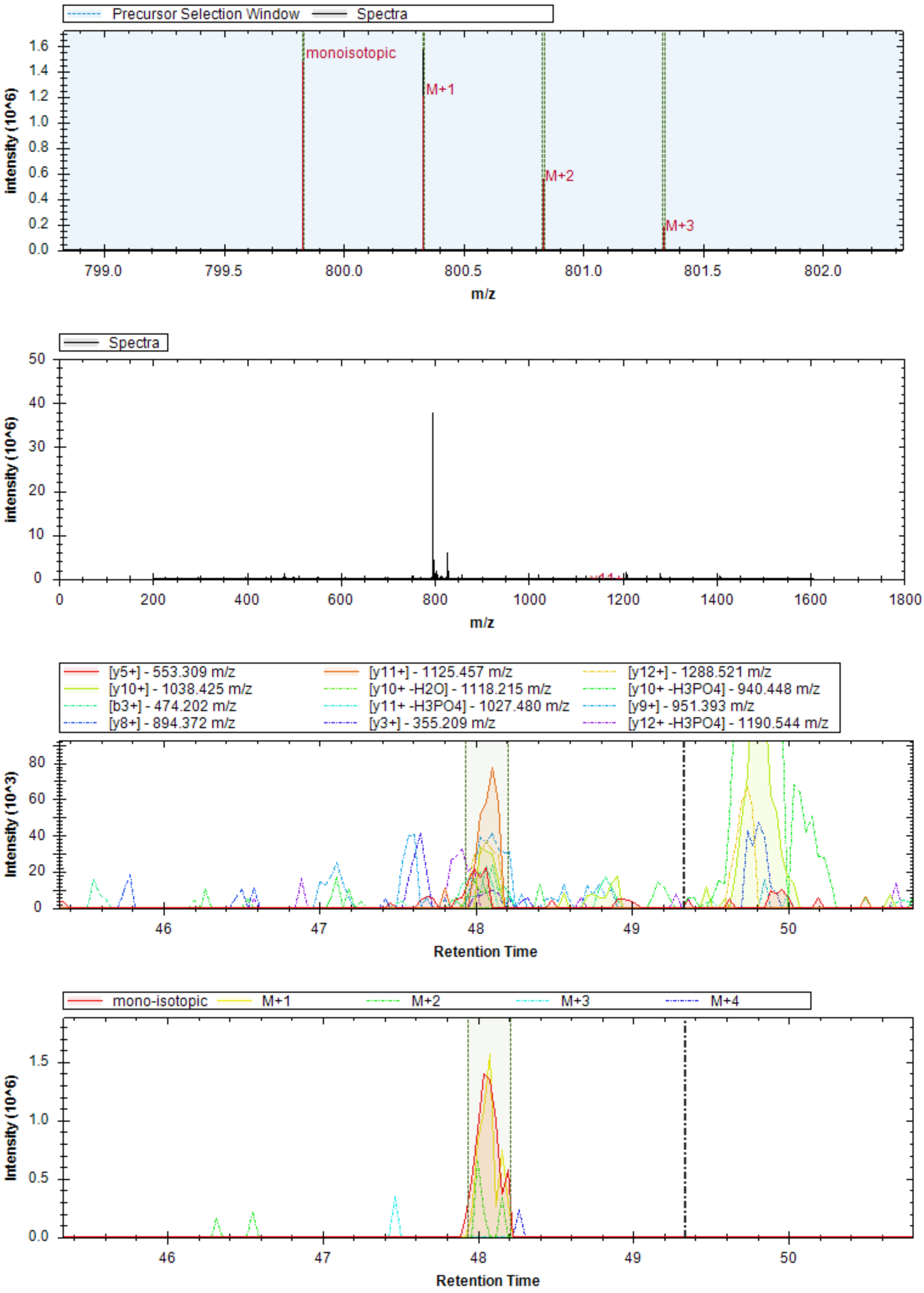

Q60771 – _FYYSSGSSS[+H+O3+P]PTHAK_.2
Fig4_MP-DIA-barrel-cortex-p15-1_MHRM.htrms

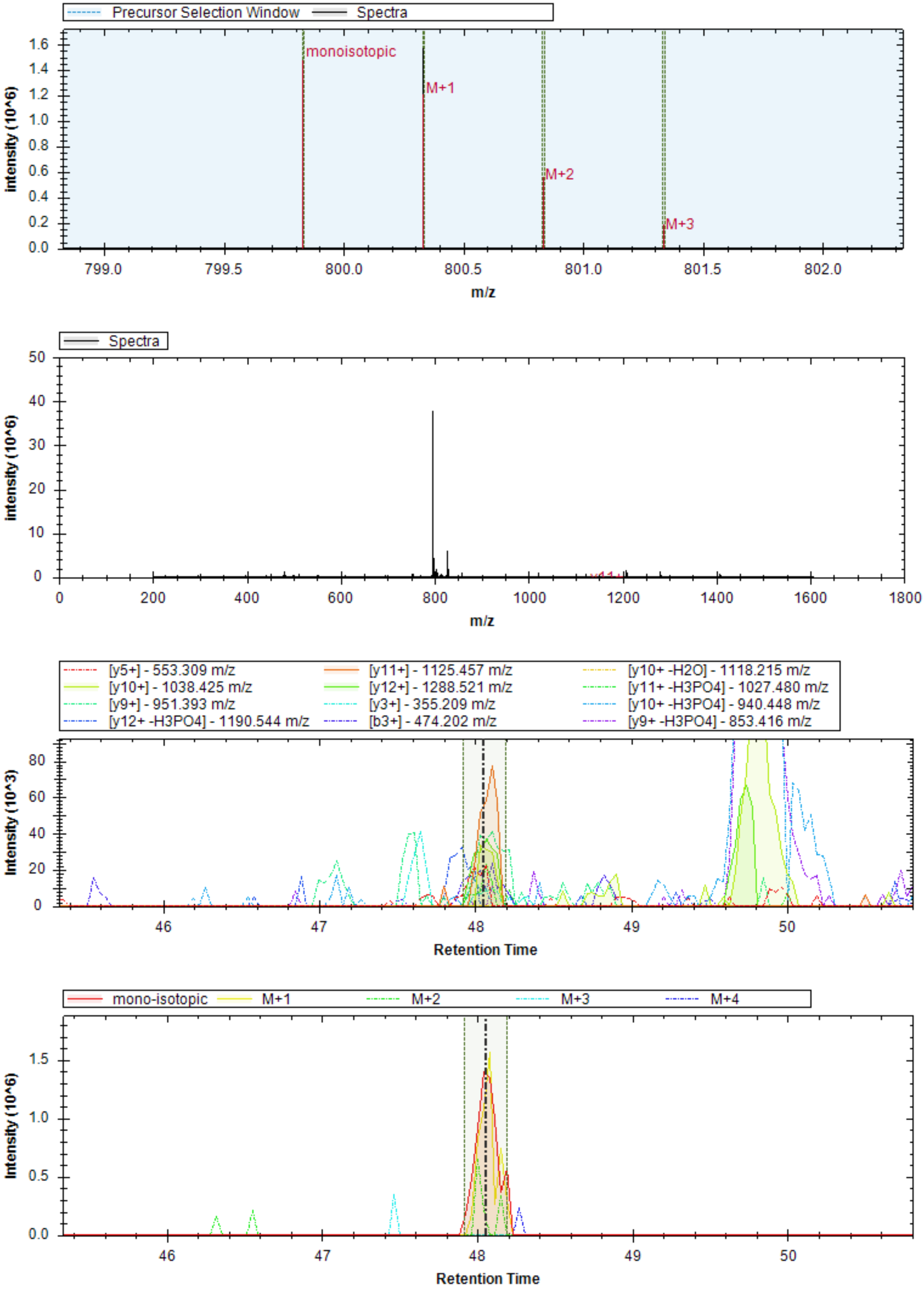

Q61548;Q61548-2;Q61548-3 – _SSPATTVTS[+H+O3+P]PNSTPAK_.2
Fig4_MP-DIA-barrel-cortex-p15-1_MHRM.htrms

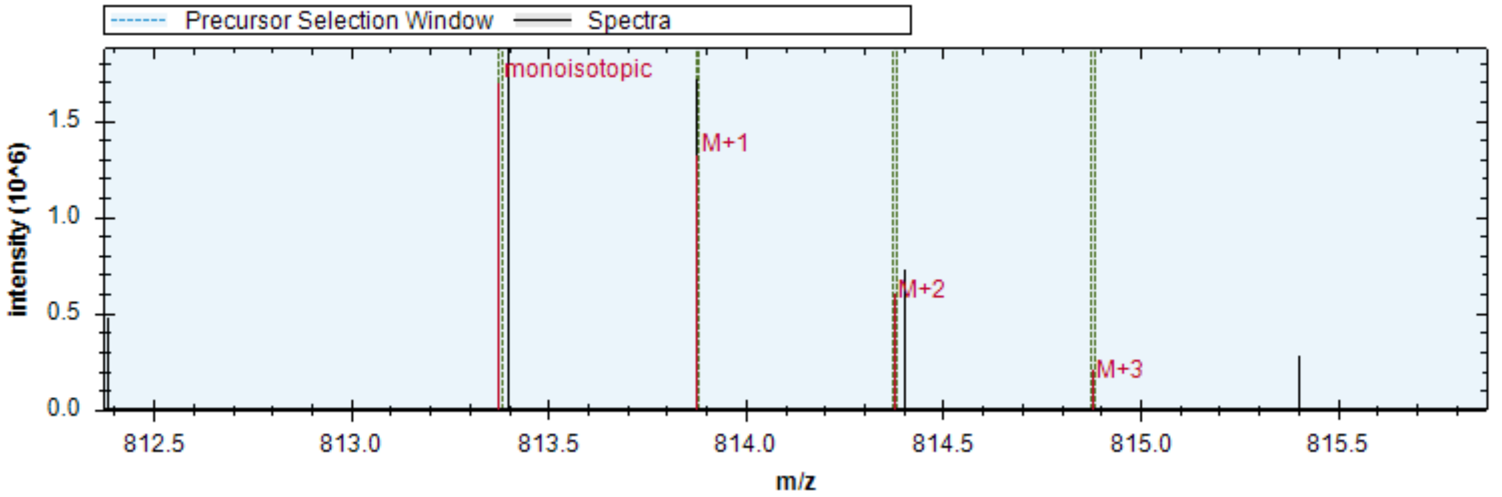

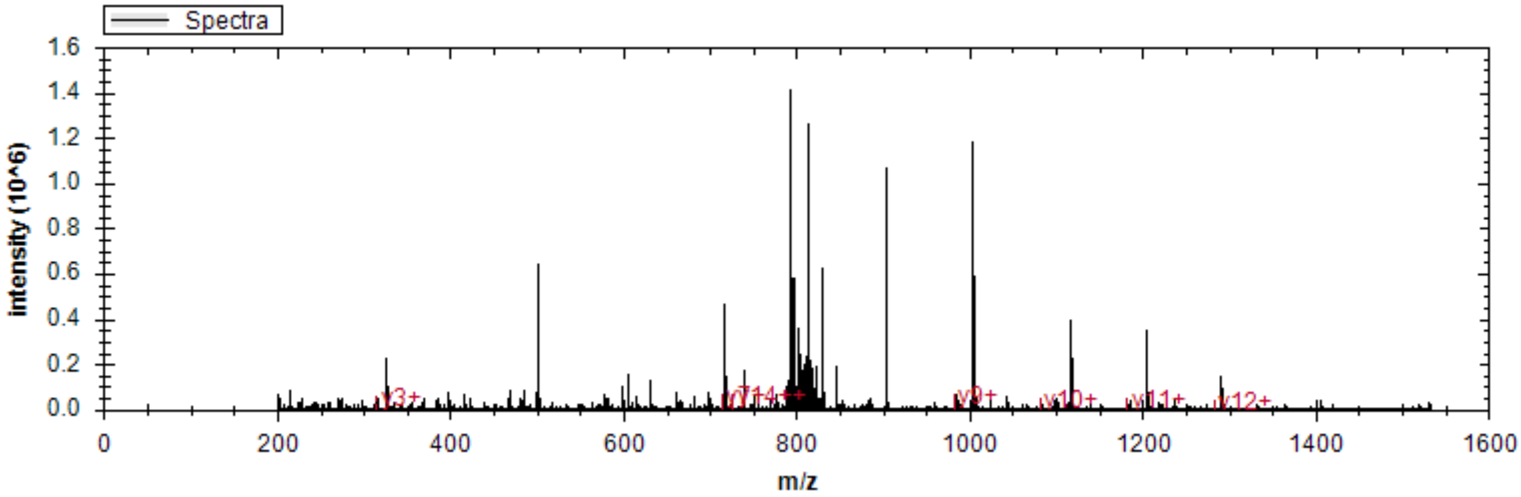

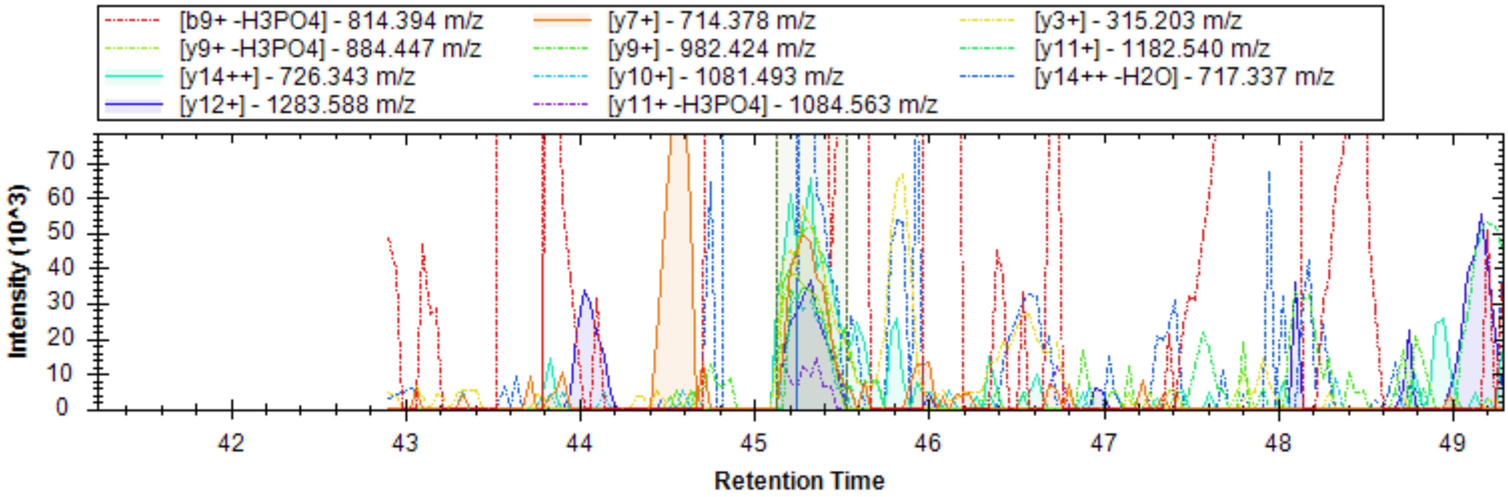

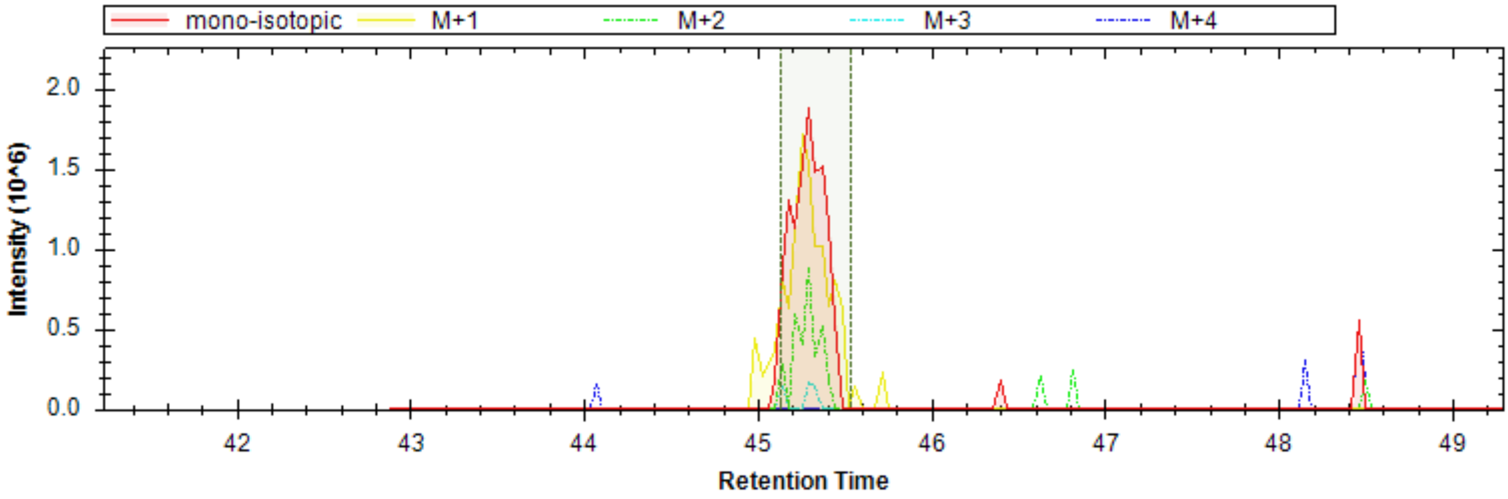

Q64701;Q64701-2 – _LAEILYYKILET[+H+O3+P]IMVQET[+H+O3+P]R_.3
Fig4_MP-DIA-barrel-cortex-p15-1_MHRM.htrms

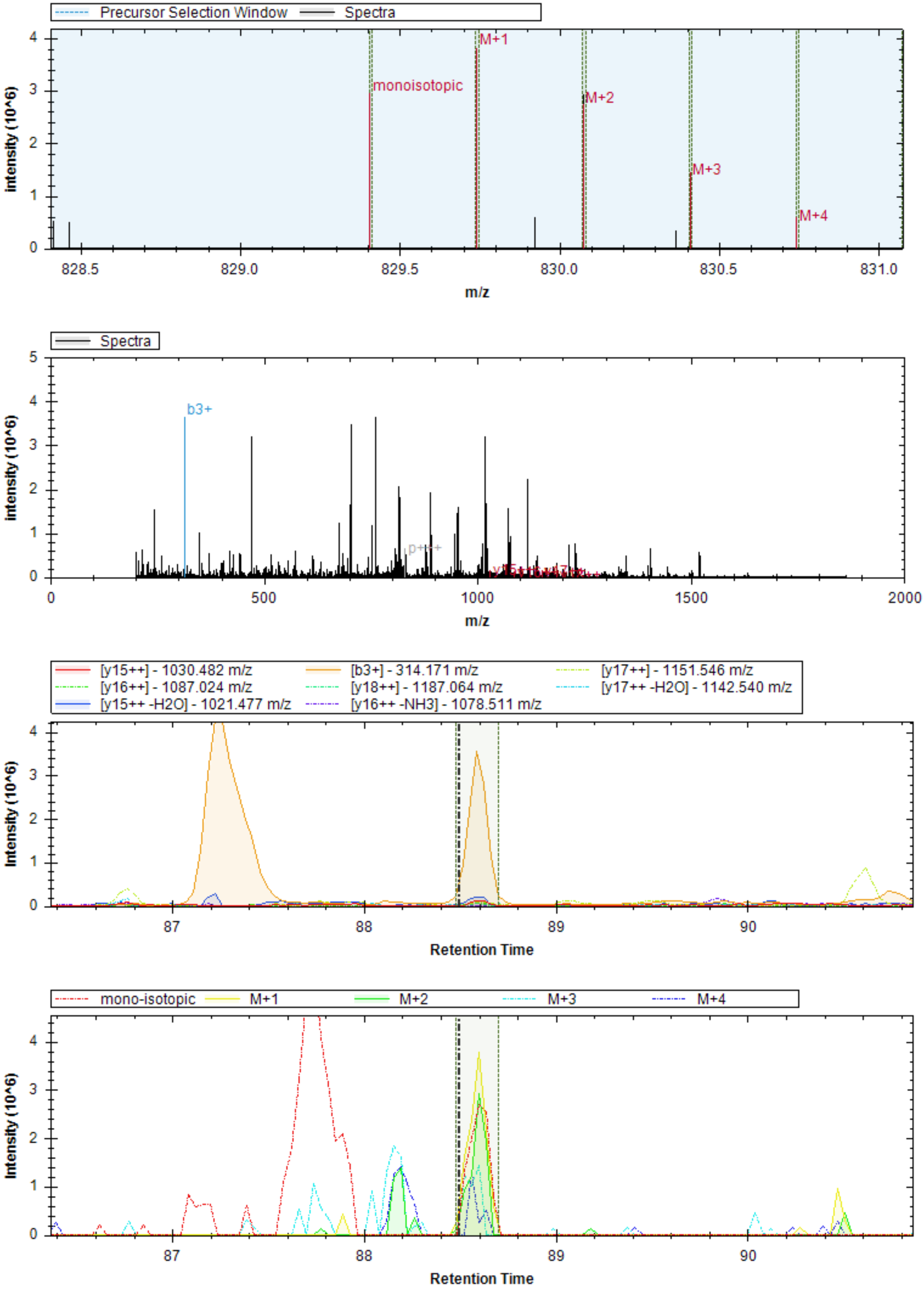

D3YXK2 – _AET[+H+O3+P]LSGLGDASAAGAAAVSSAASETGTR_.3

Fig4_MP-DIA-barrel-cortex-p15-1_MHRM.htrms

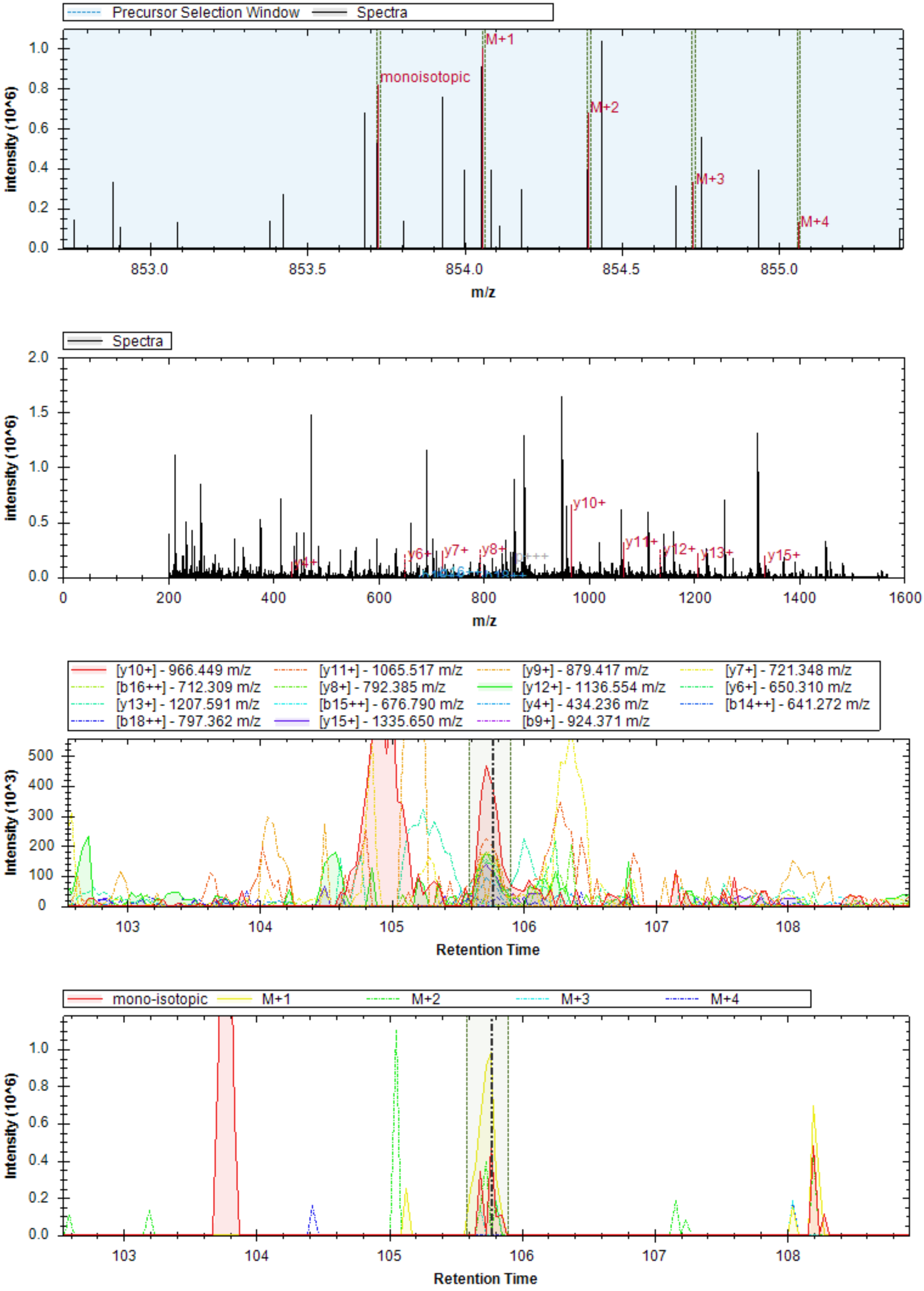

O08784 – _ASAVSPEKAPM[+O]T[+H+O3+P]SKAK_.2

Fig4_MP-DIA-barrel-cortex-p15-1_MHRM.htrms

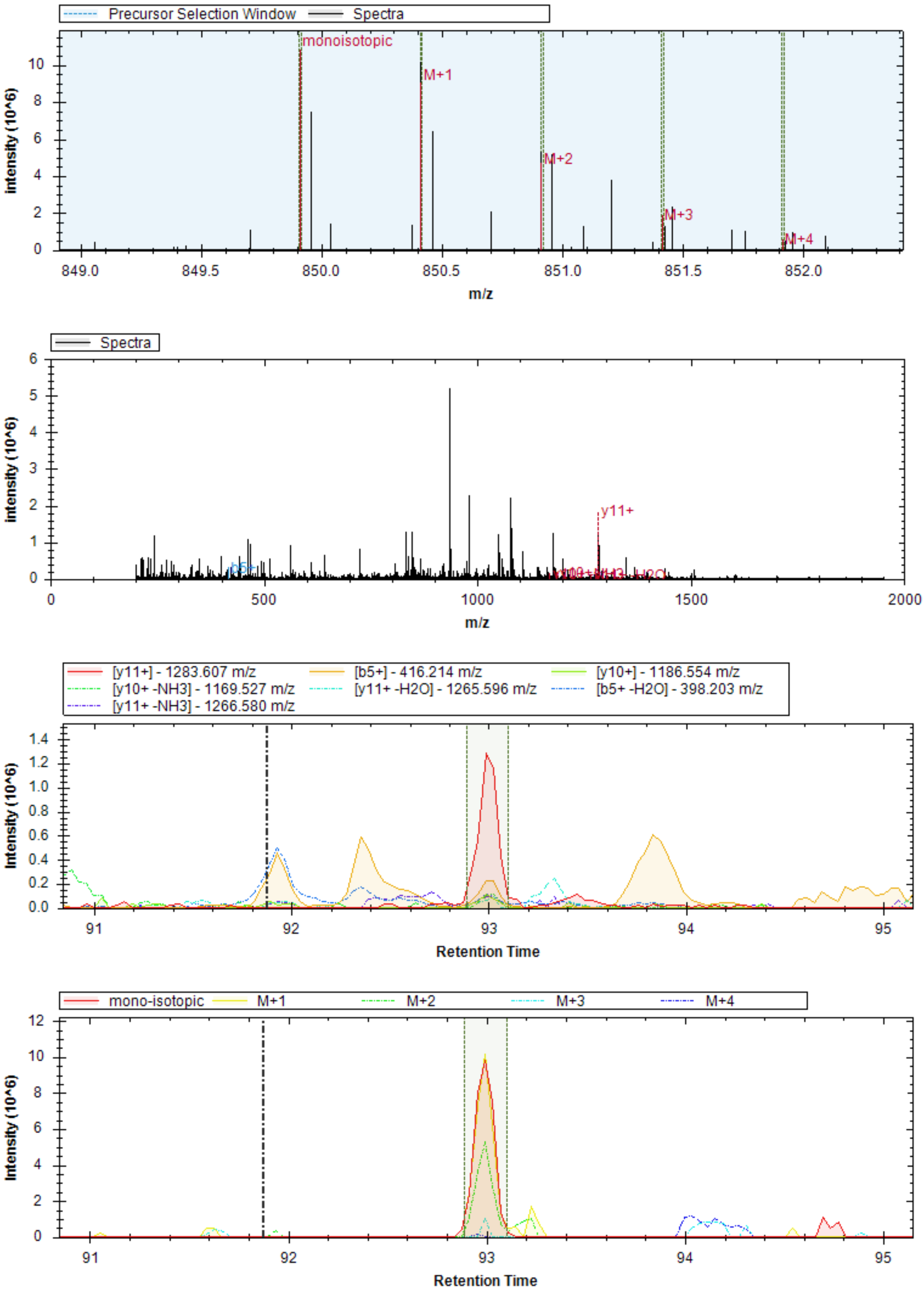

P15533 – _EEM[+O]GIKKNLHS[+H+O3+P]QIK_.2

Fig4_MP-DIA-barrel-cortex-p15-1_MHRM.htrms

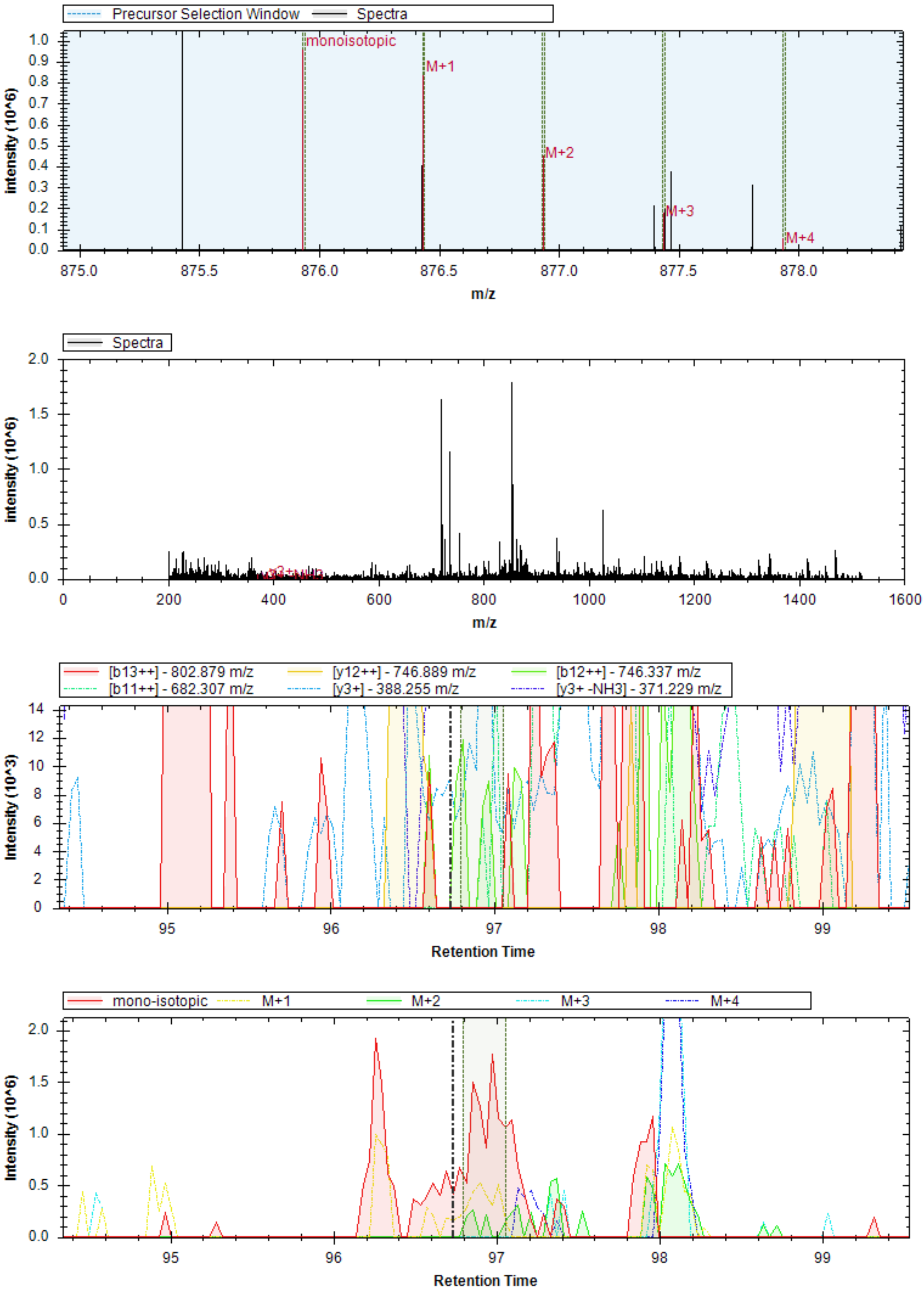

P50538 – _EAEHGYAS[+H+O3+P]M[+O]LPYSK_.2
Fig4_MP-DIA-barrel-cortex-p15-1_MHRM.htrms

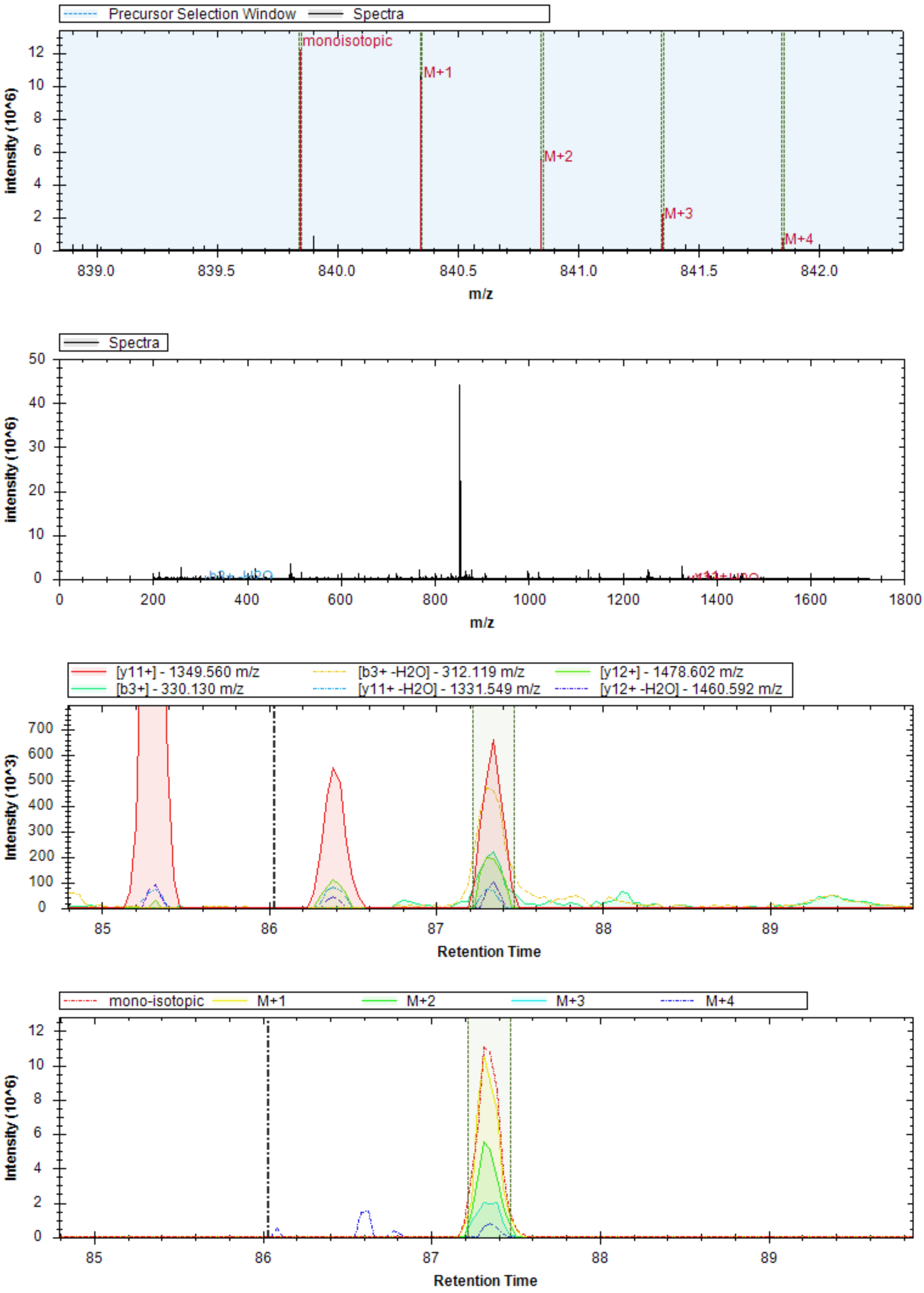

Q61290 – _M[+O]EPS[+H+O3+P]SLPQEIIANAK_.2

Fig4_MP-DIA-barrel-cortex-p15-1_MHRM.htrms

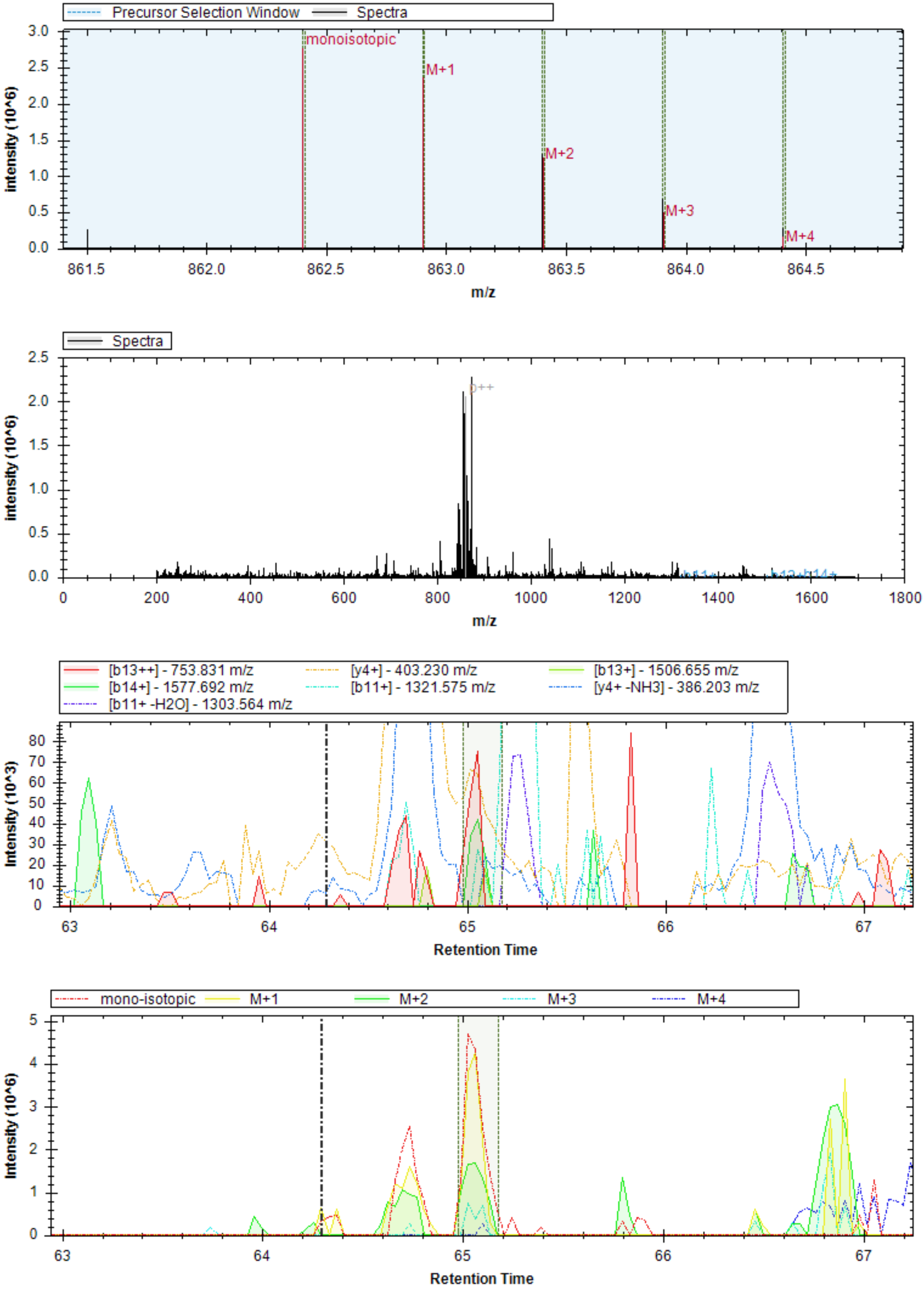

Q62188 – _GM[+O]YDGPVFDLT[+H+O3+P]TTPK_.2
Fig4_MP-DIA-barrel-cortex-p15-1_MHRM.htrms

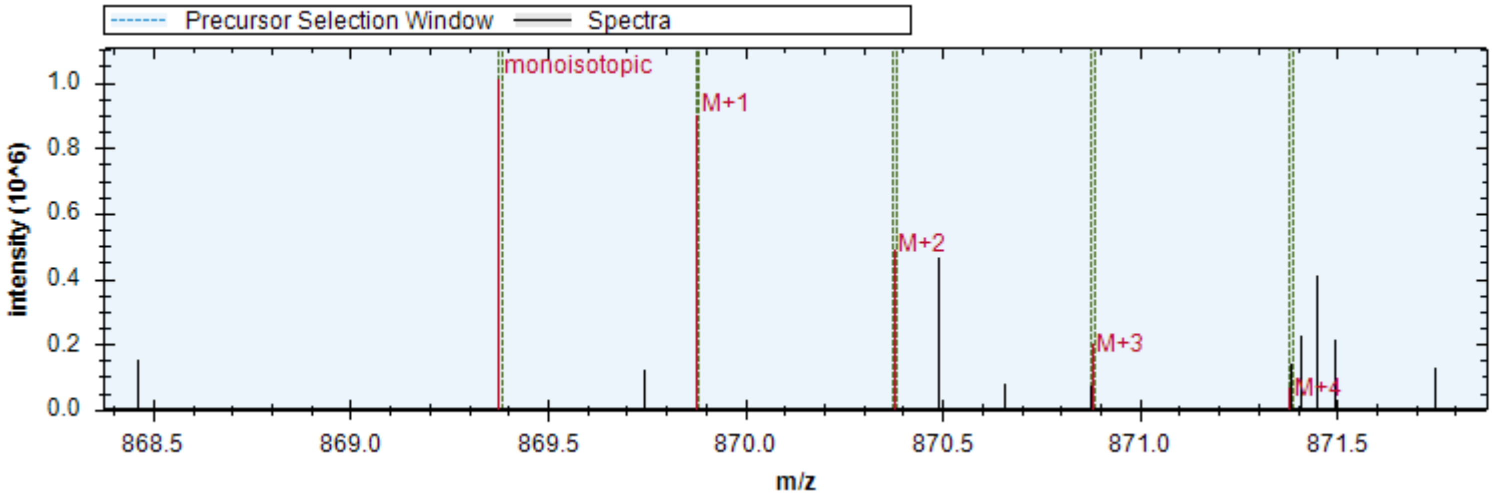

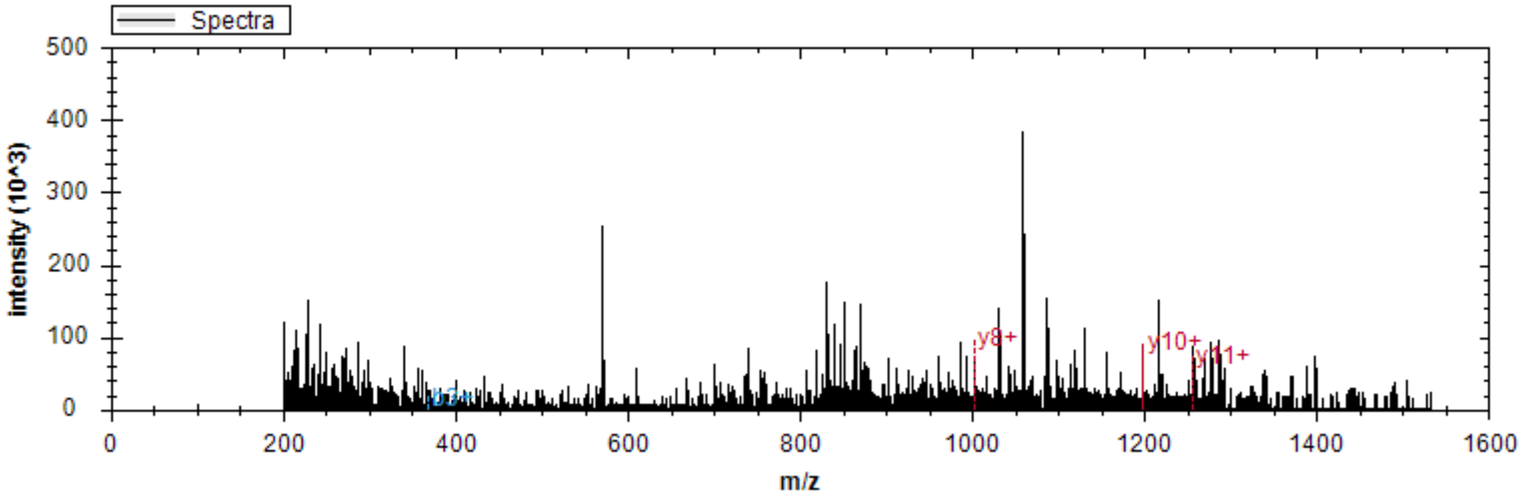

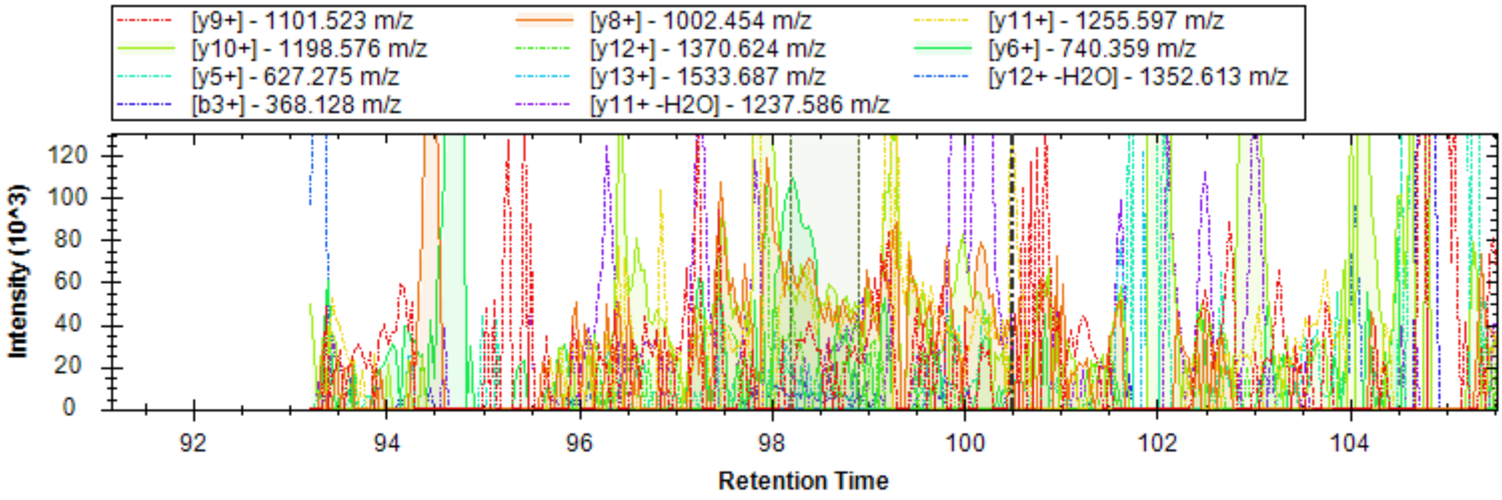

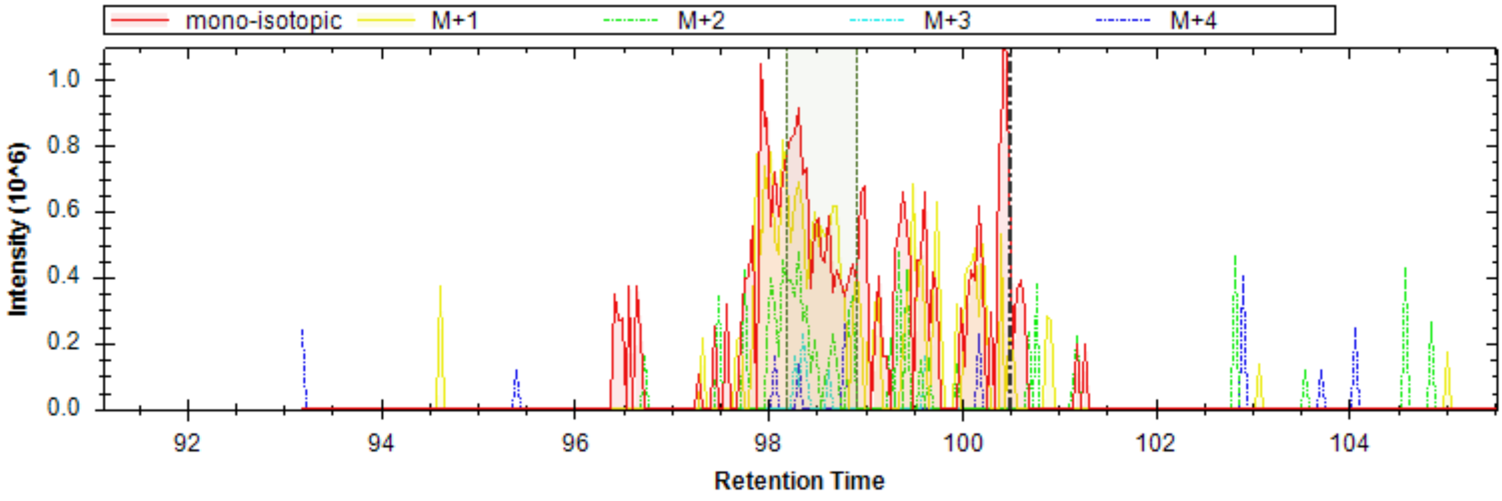

Q9QXS6 – _ASDSGPSSSSSS[+H+O3+P]SSSPPR_.2
Fig4_MP-DIA-barrel-cortex-p15-1_MHRM.htrms

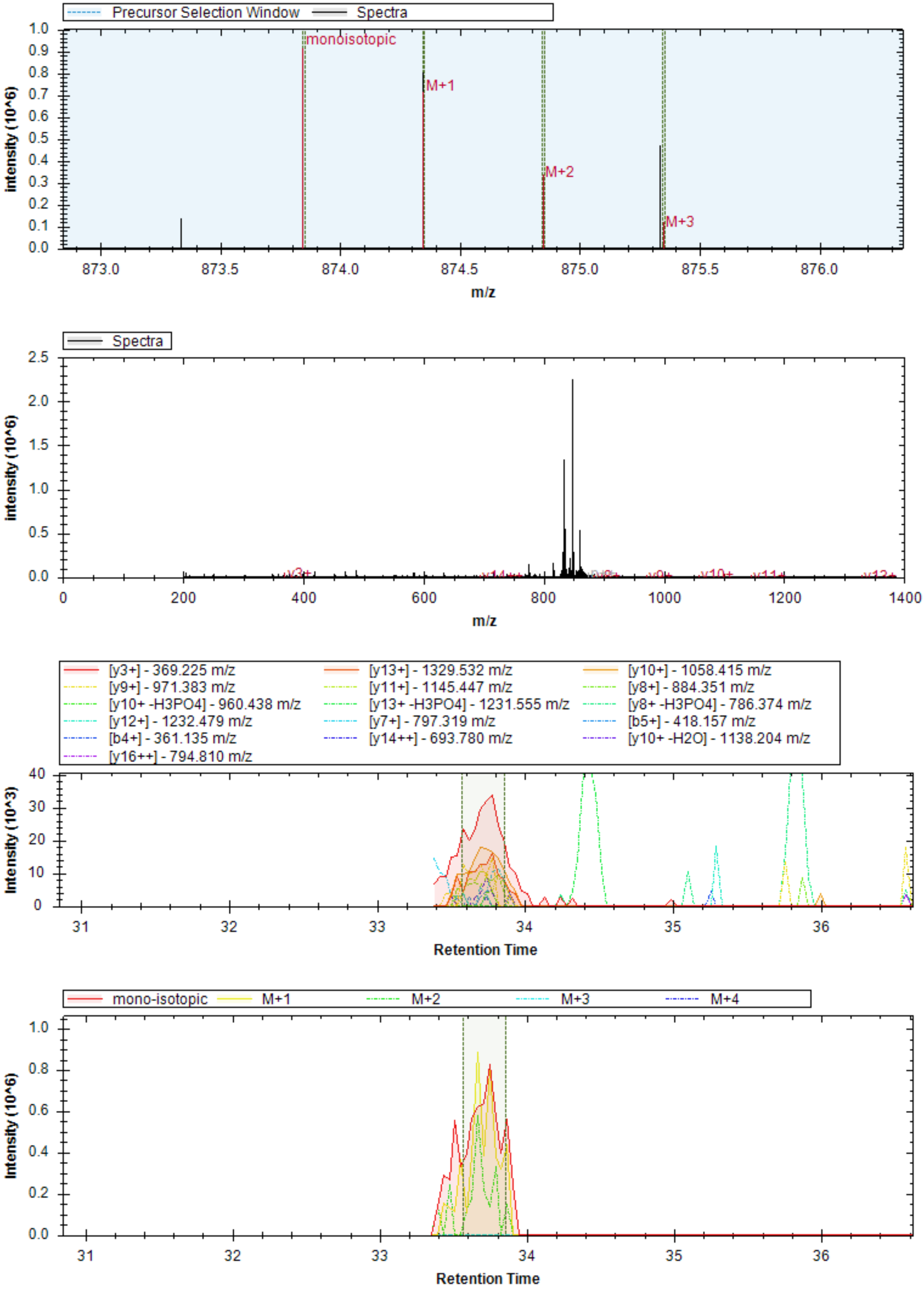

Q9Z0P4 – _SETLVNAQQT[+H+O3+P]PLGTPK_.2
Fig4_MP-DIA-barrel-cortex-p15-1_MHRM.htrms

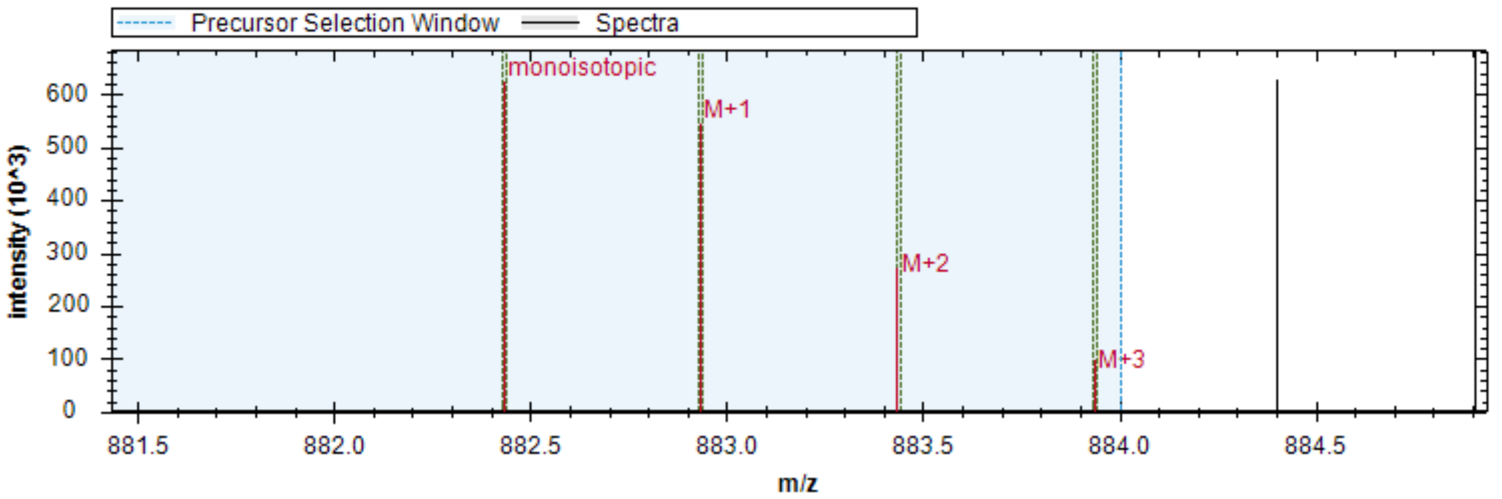

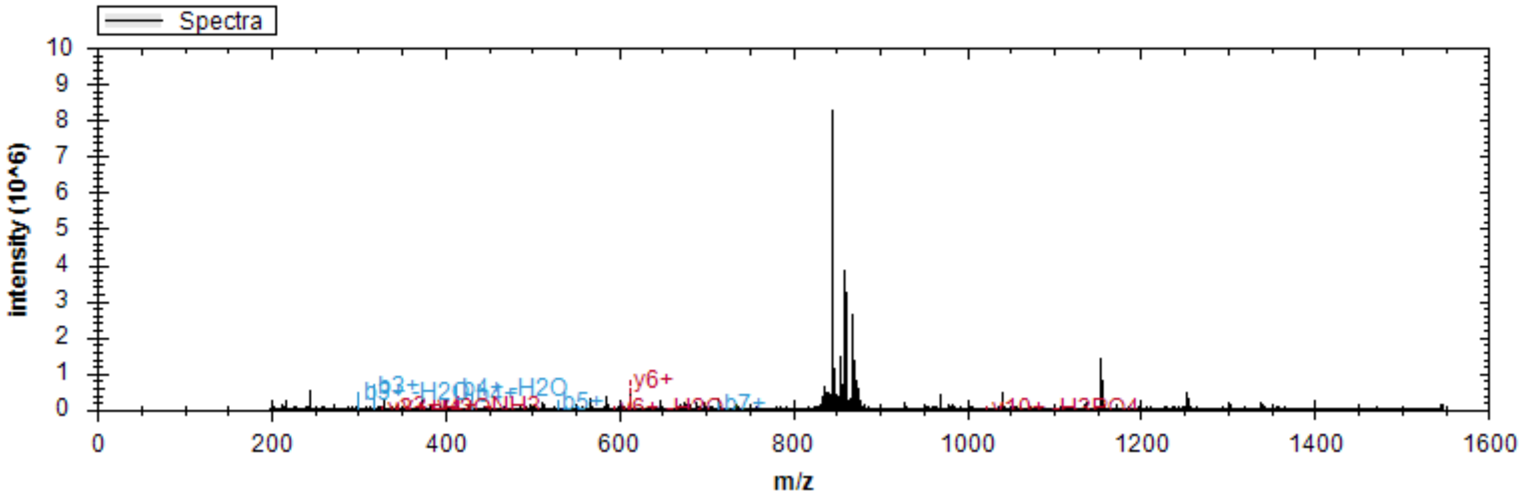

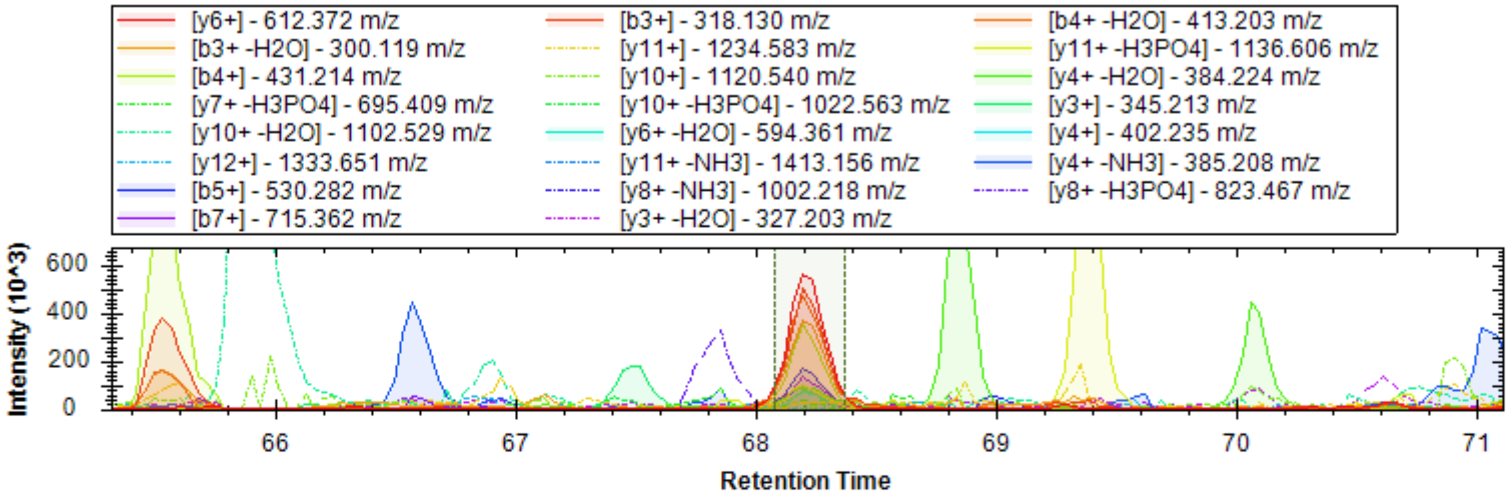

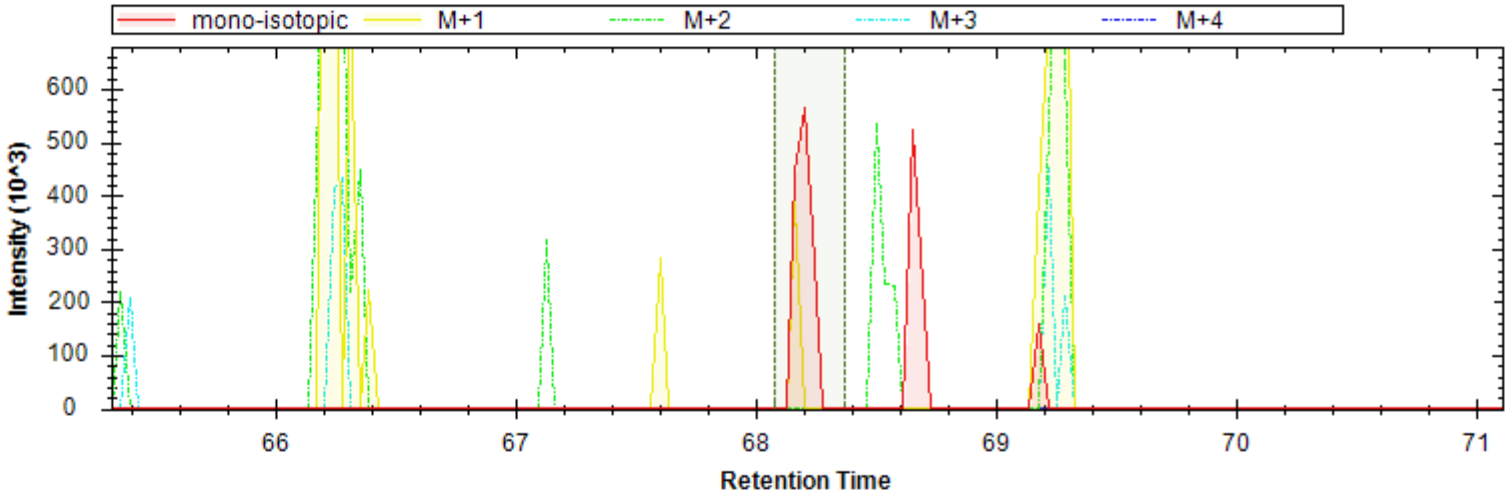

A1A535 – _FLHRGS[+H+O3+P]K_.1
Fig4_MP-DIA-barrel-cortex-p15-1_MHRM.htrms

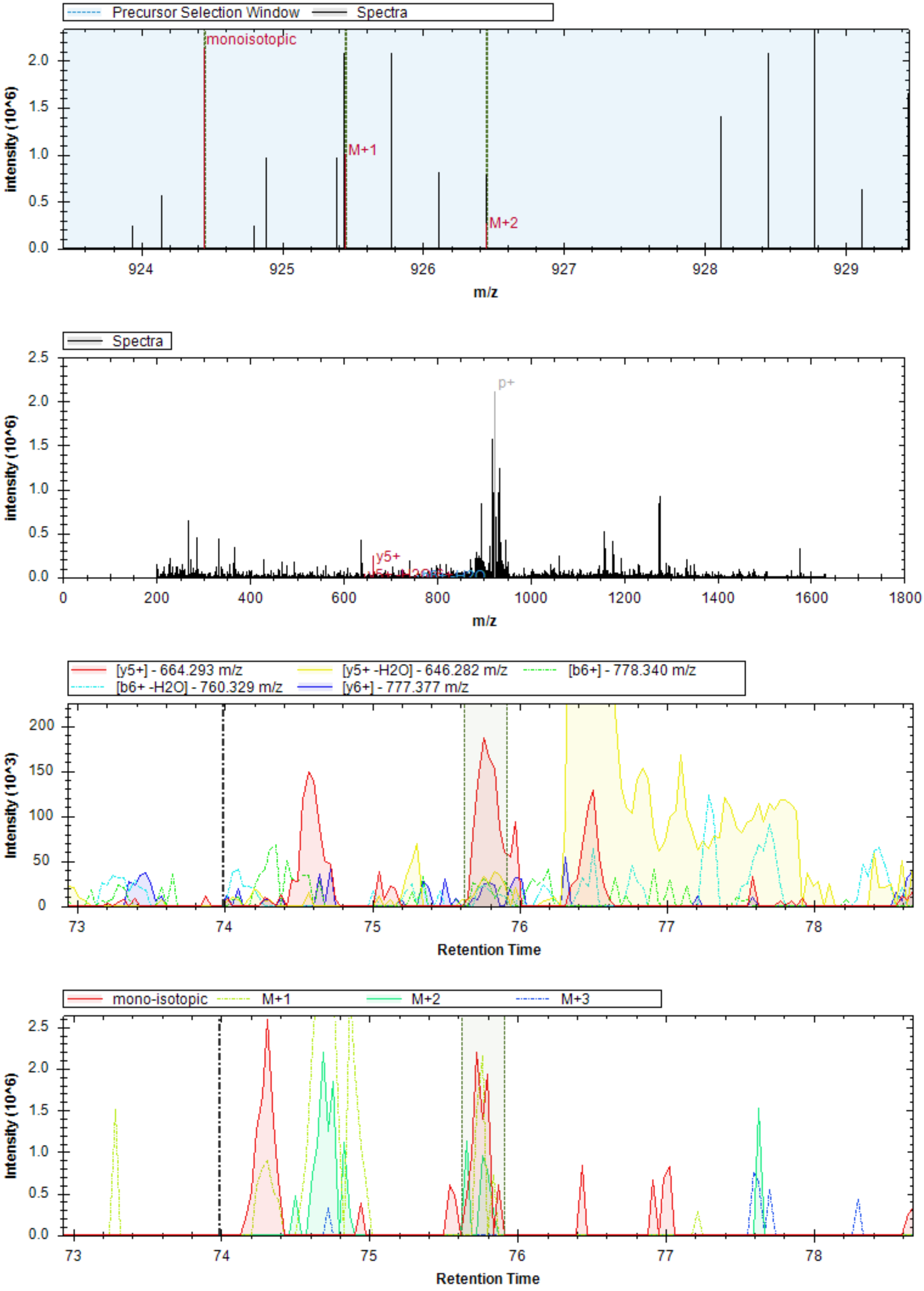

G5E829 – _SATSSSPGS[+H+O3+P]PLHSLETSL_.2

Fig4_MP-DIA-barrel-cortex-p15-1_MHRM.htrms

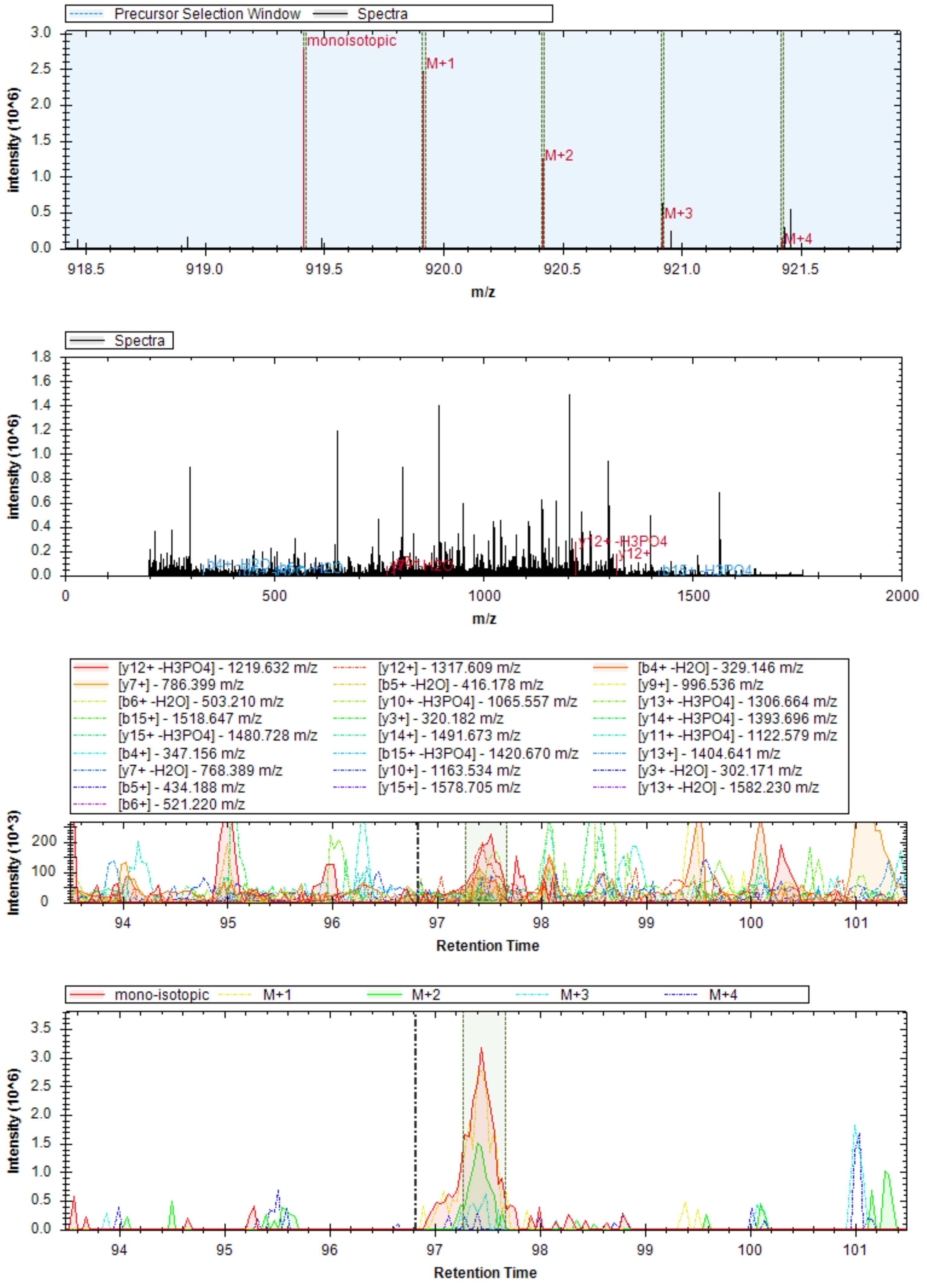

Q8BMA3 – _IIQLC[+C2+H3+N+O]LDLT[+H+O3+P]T[+H+O3+P]AVQK_.2
Fig4_MP-DIA-barrel-cortex-p15-1_MHRM.htrms

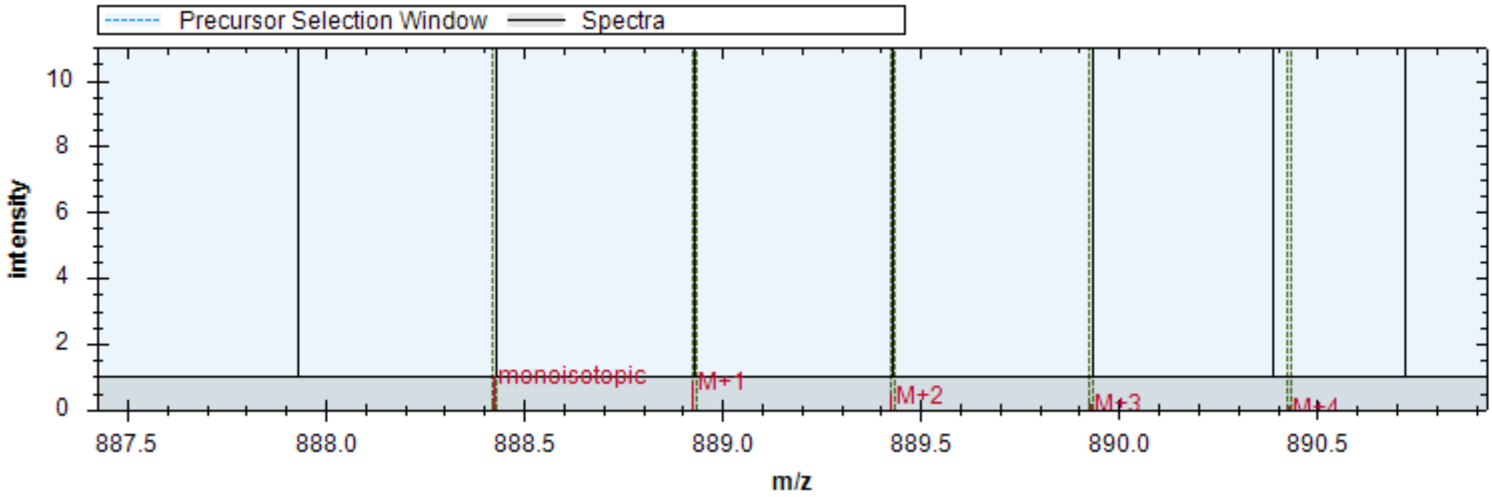

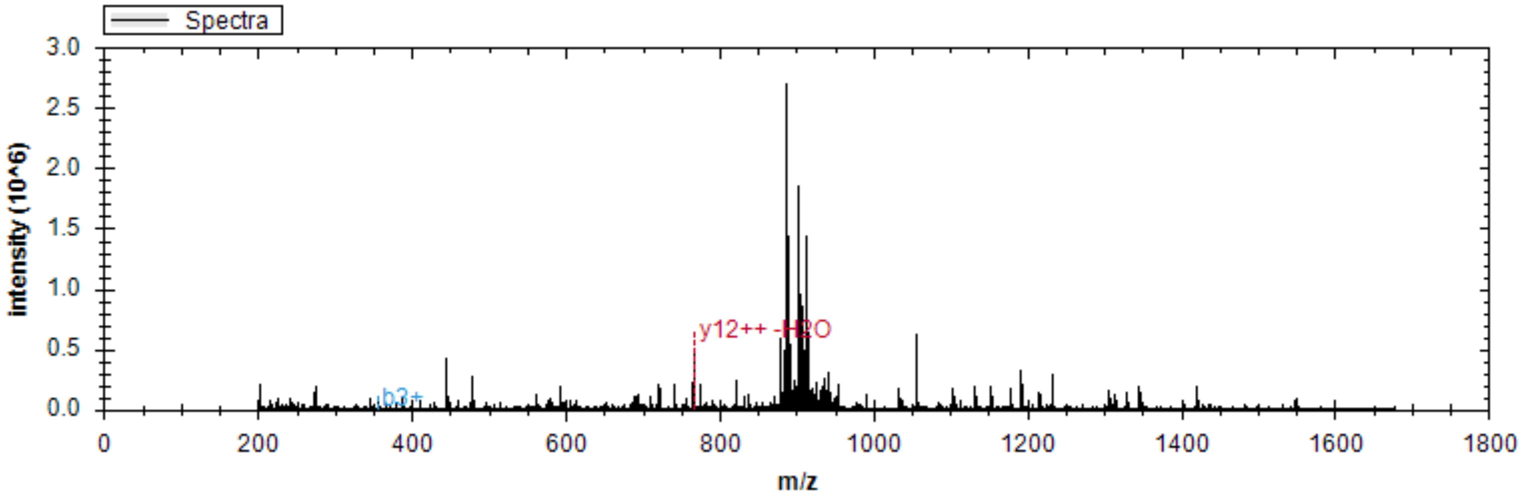

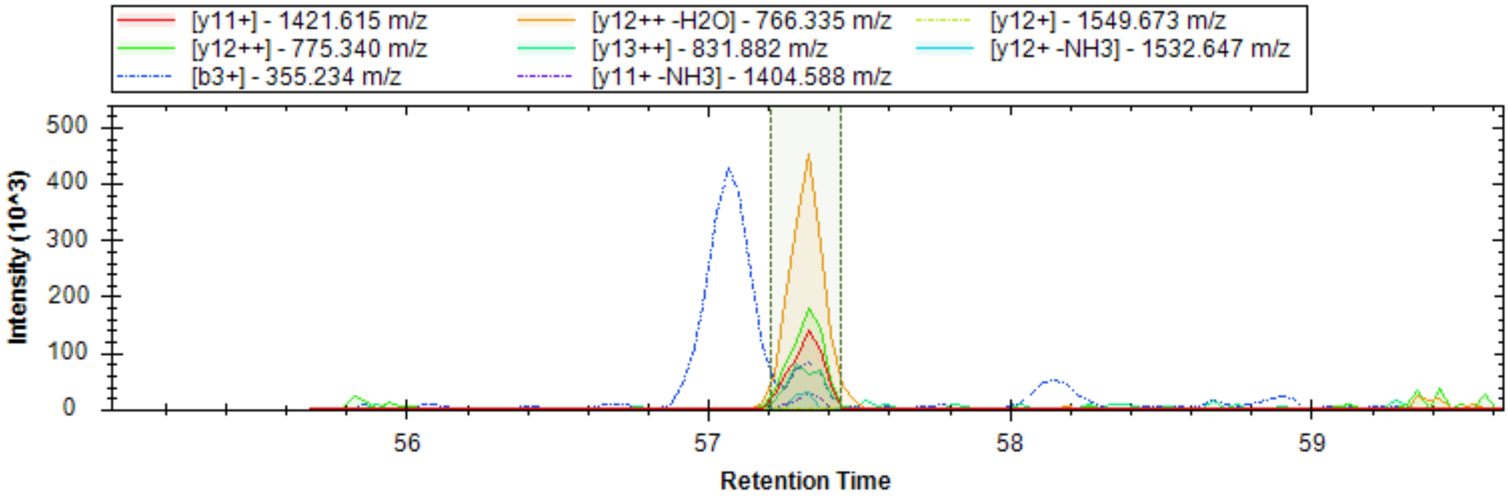

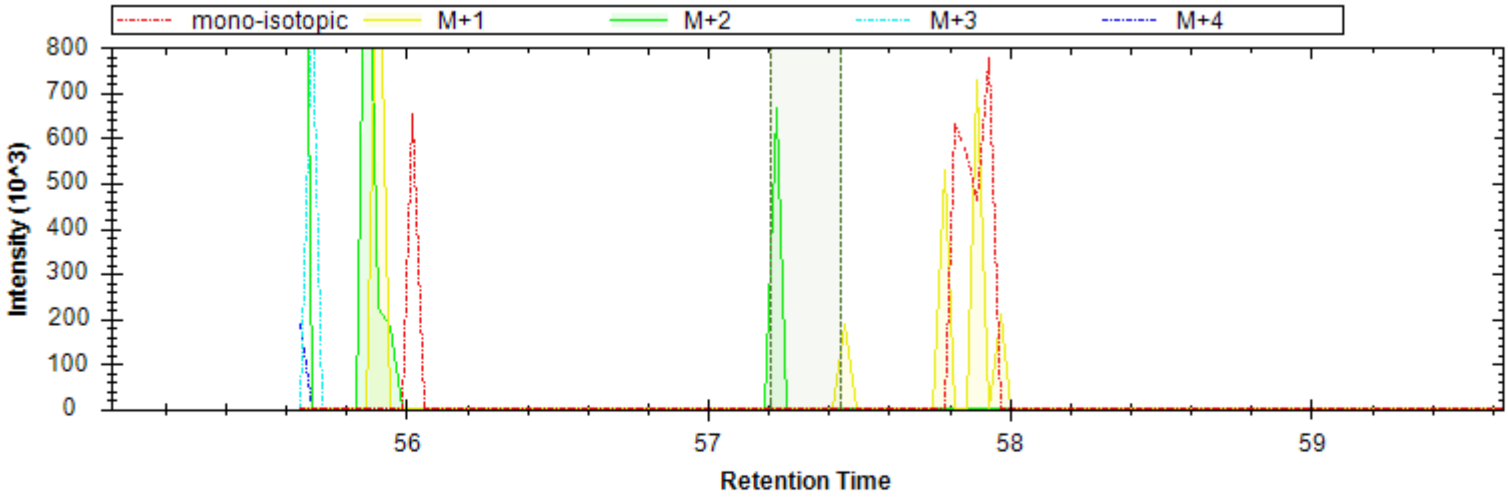

Q91Z31;Q91Z31-2 – _ETSLLAVPGALS[+H+O3+P]PLAIPNAAAAAAAAAAGR_.3
Fig4_MP-DIA-barrel-cortex-p15-1_MHRM.htrms

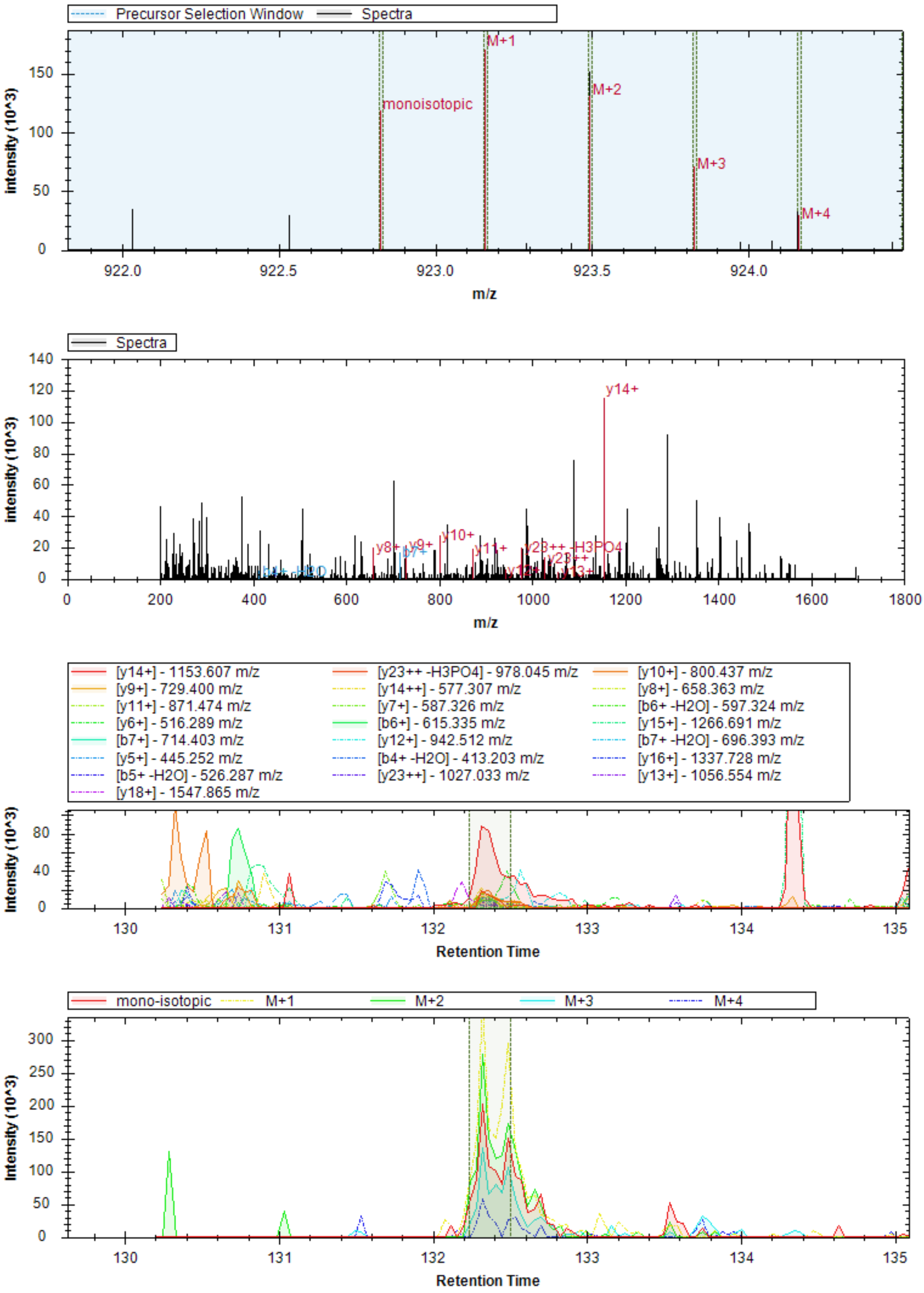

Q9ES97;Q9ES97-2 – _AES[+H+O3+P]SAATQSPSVSSSSSGAEPSALGGGGGSPGAC[+C2+H3+N+O]PALGAK_.4
Fig4_MP-DIA-barrel-cortex-p15-1_MHRM.htrms

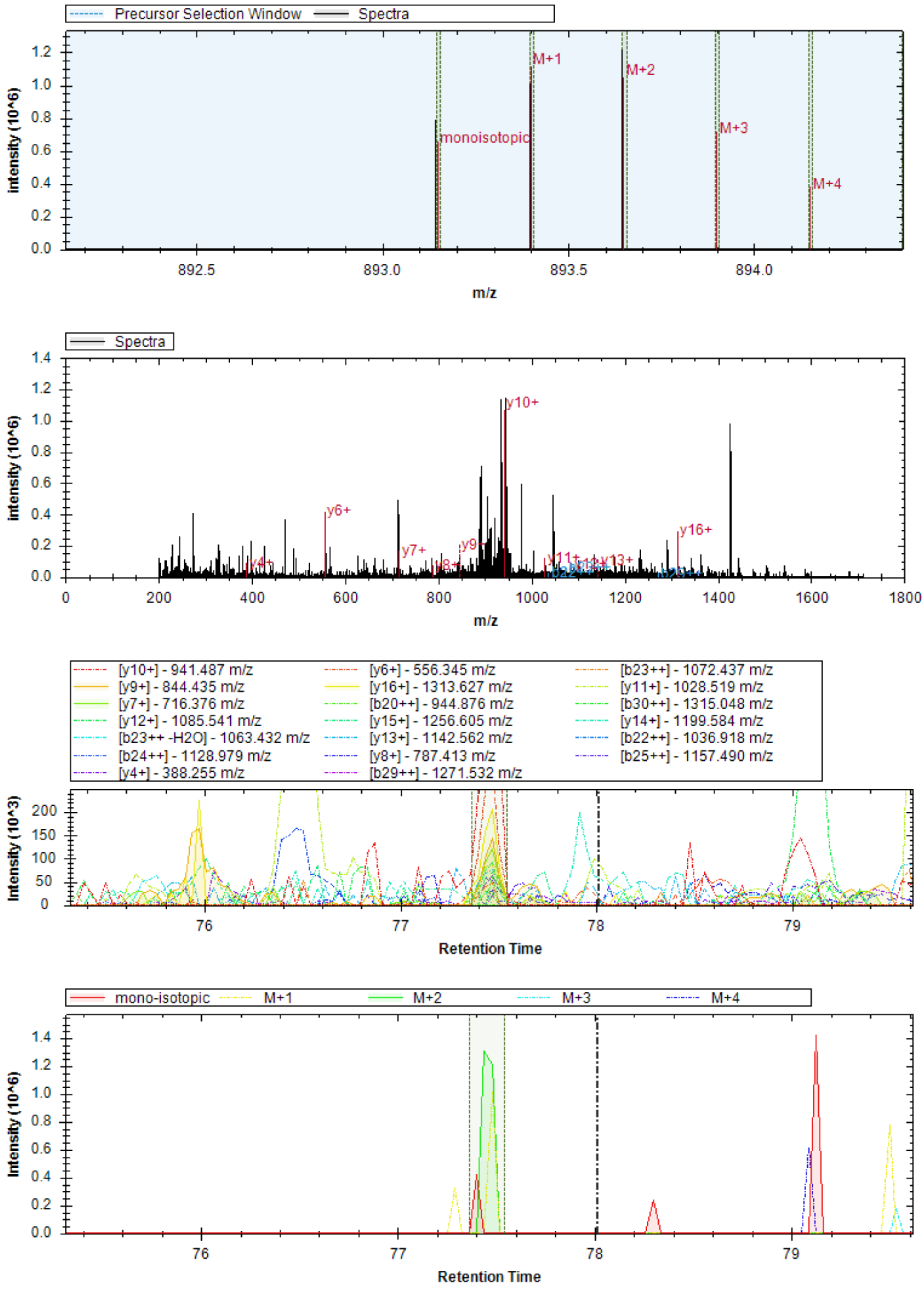

Q9QYR6-2 – _M[+O]AS[+H+O3+P]PPPSGPPSAAHTPFHQSPVEEK_.3

Fig4_MP-DIA-barrel-cortex-p15-1_MHRM.htrms

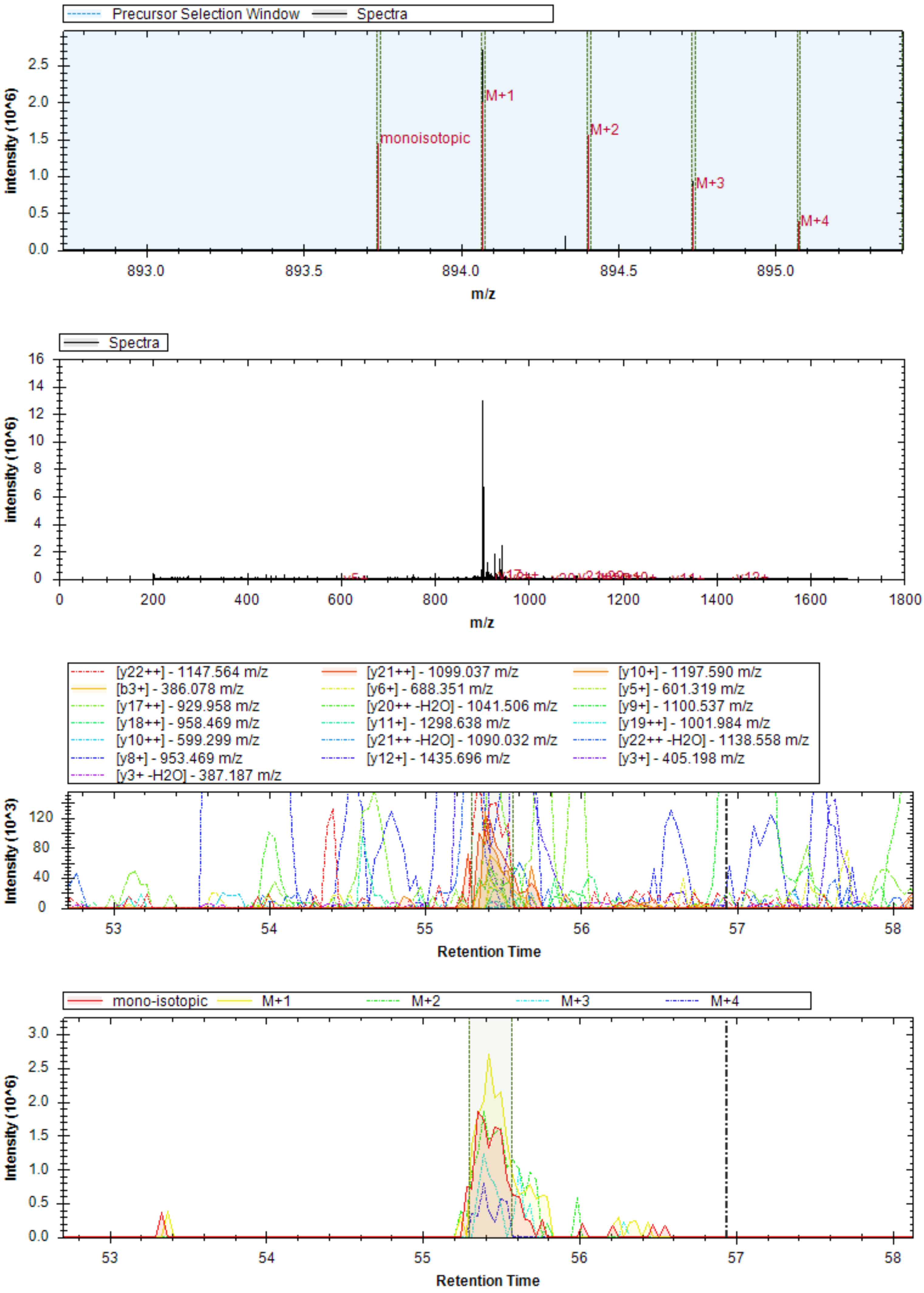

P17742 – _VNPT[+H+O3+P]VFFDITADDEPLGR_.2
Fig4_MP-DIA-barrel-cortex-p15-1_MHRM.htrms

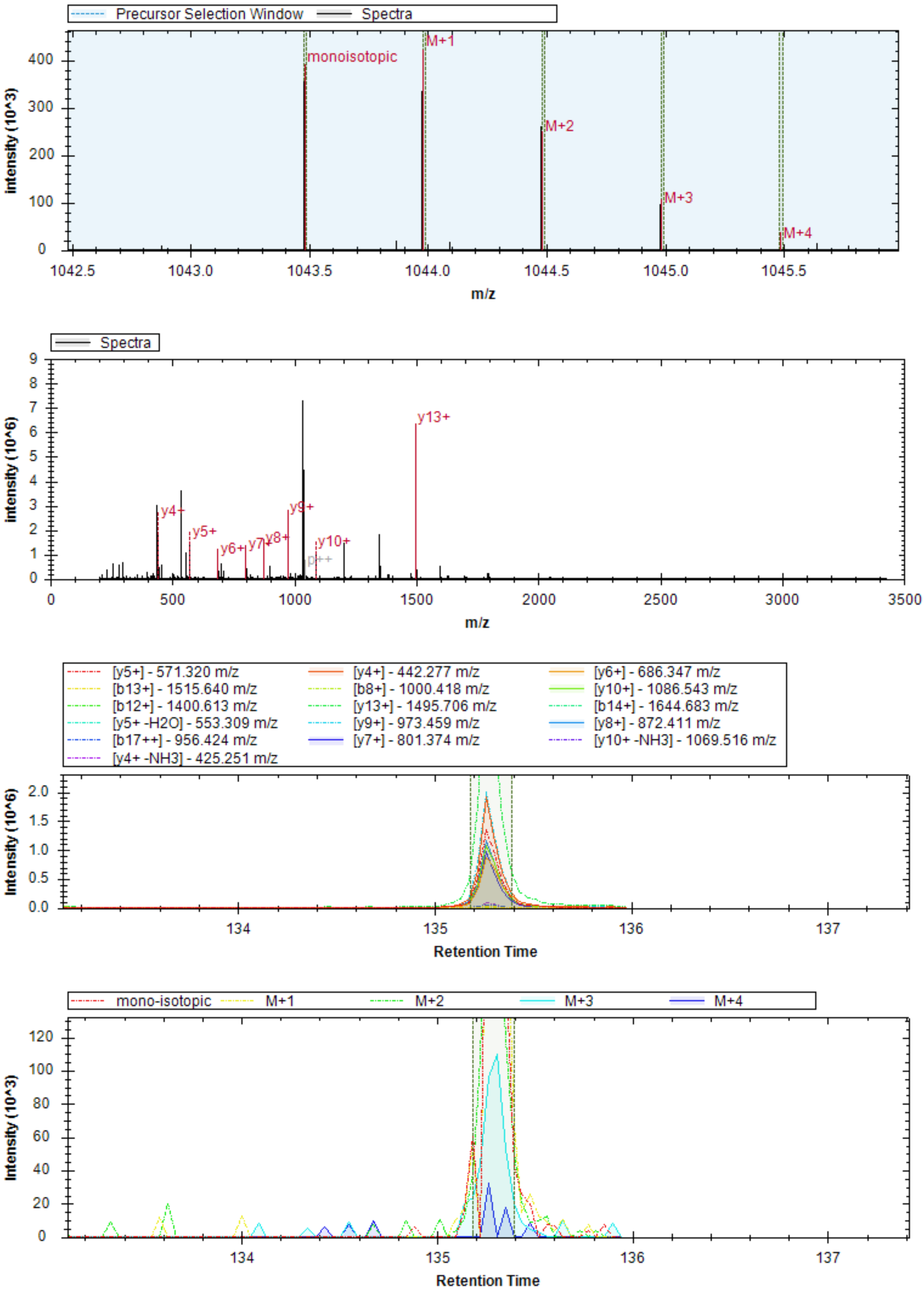

Q64332 – _TPALS[+H+O3+P]PQRPLTTQQPQSGTLKEPDSSK_.3

Fig4_MP-DIA-barrel-cortex-p15-1_MHRM.htrms

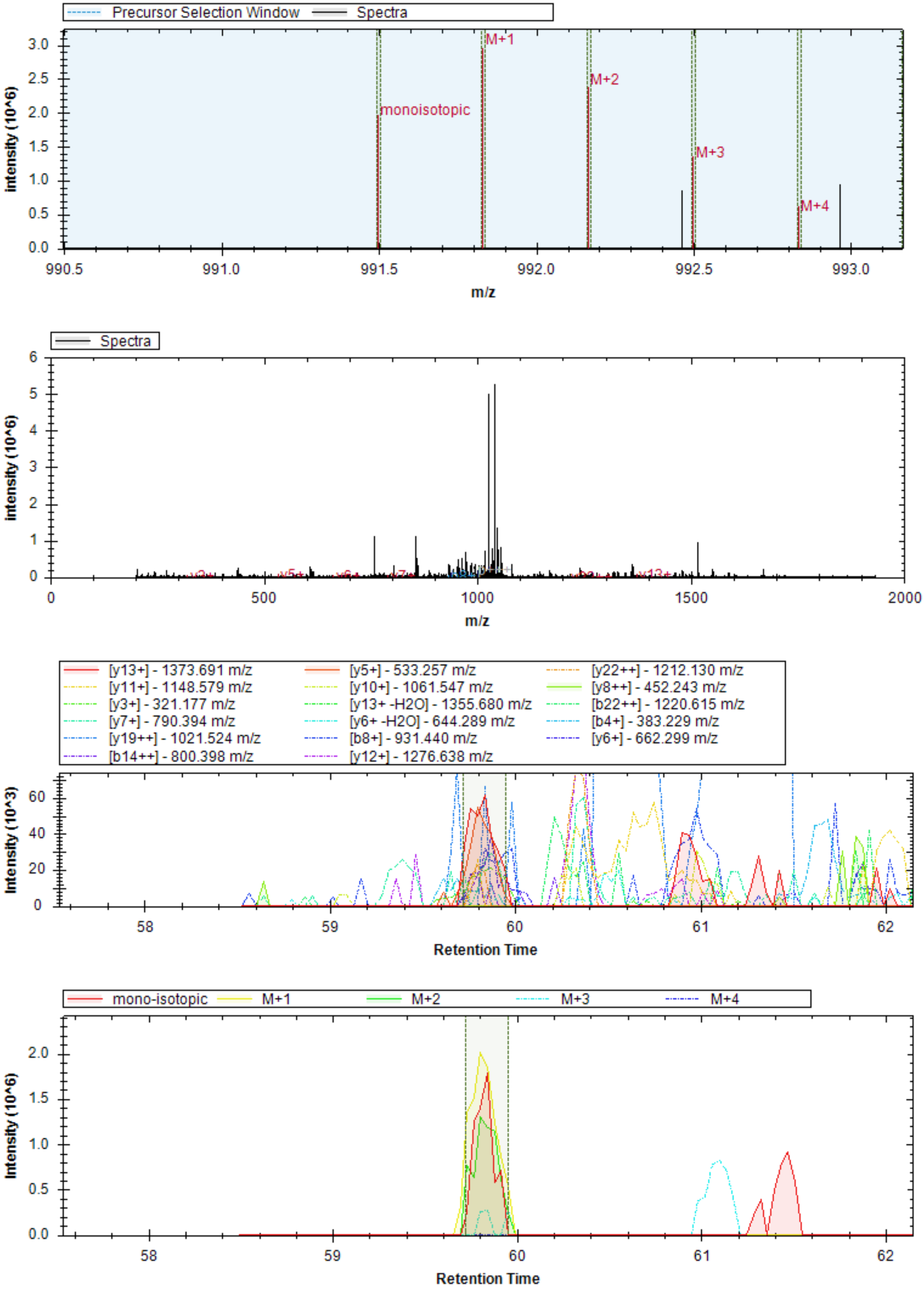

Q8CHK4;Q8CHK4-2;Q8CH[...] – _RGY[+H+O3+P]GKLLIEFS[+H+O3+P]YELSK_.2
Fig4_MP-DIA-barrel-cortex-p15-1_MHRM.htrms

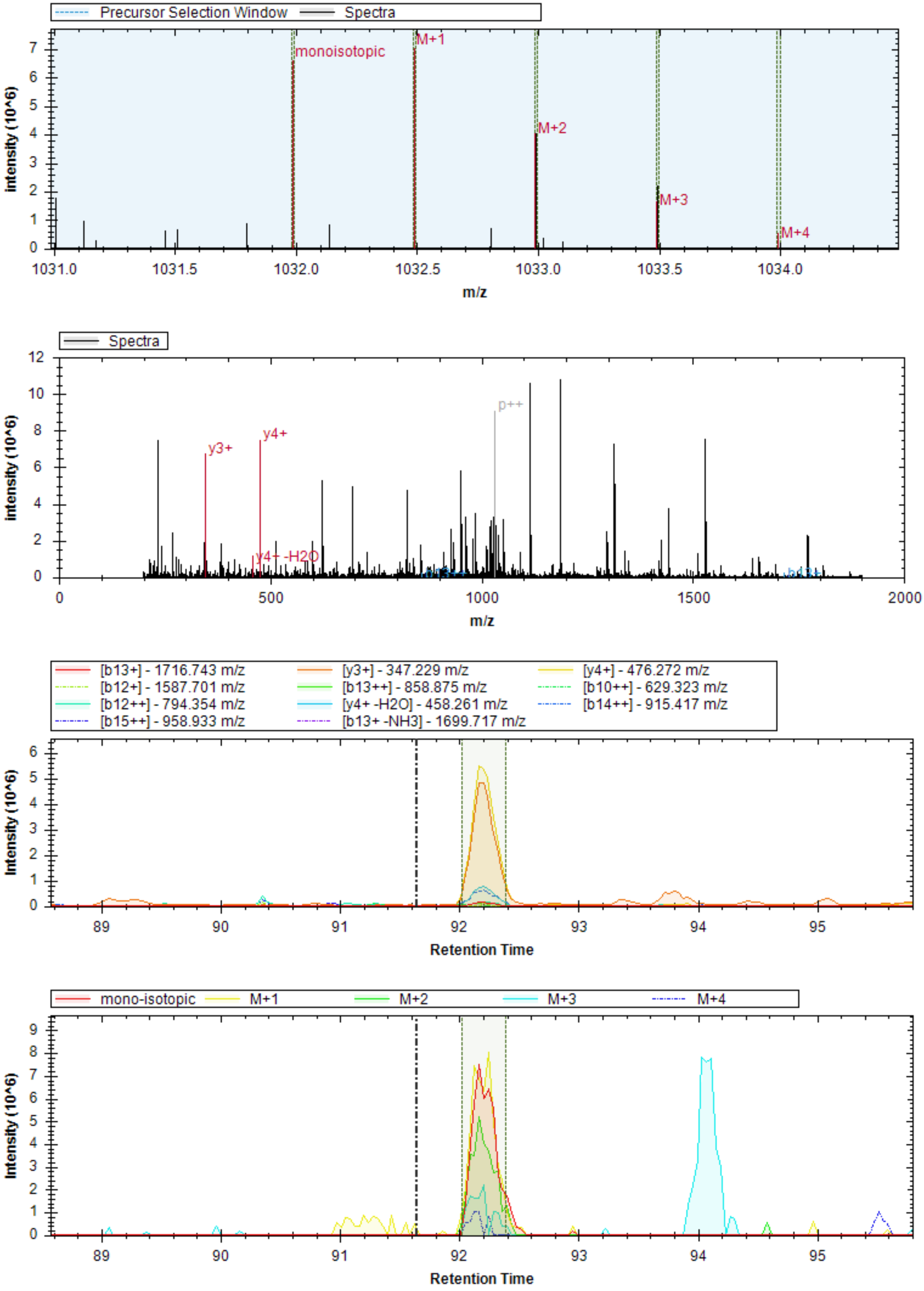

A2ASS6 – _T[+H+O3+P]VS[+H+O3+P]VKVLVLDK_.1
Fig4_MP-DIA-barrel-cortex-p15-1_MHRM.htrms

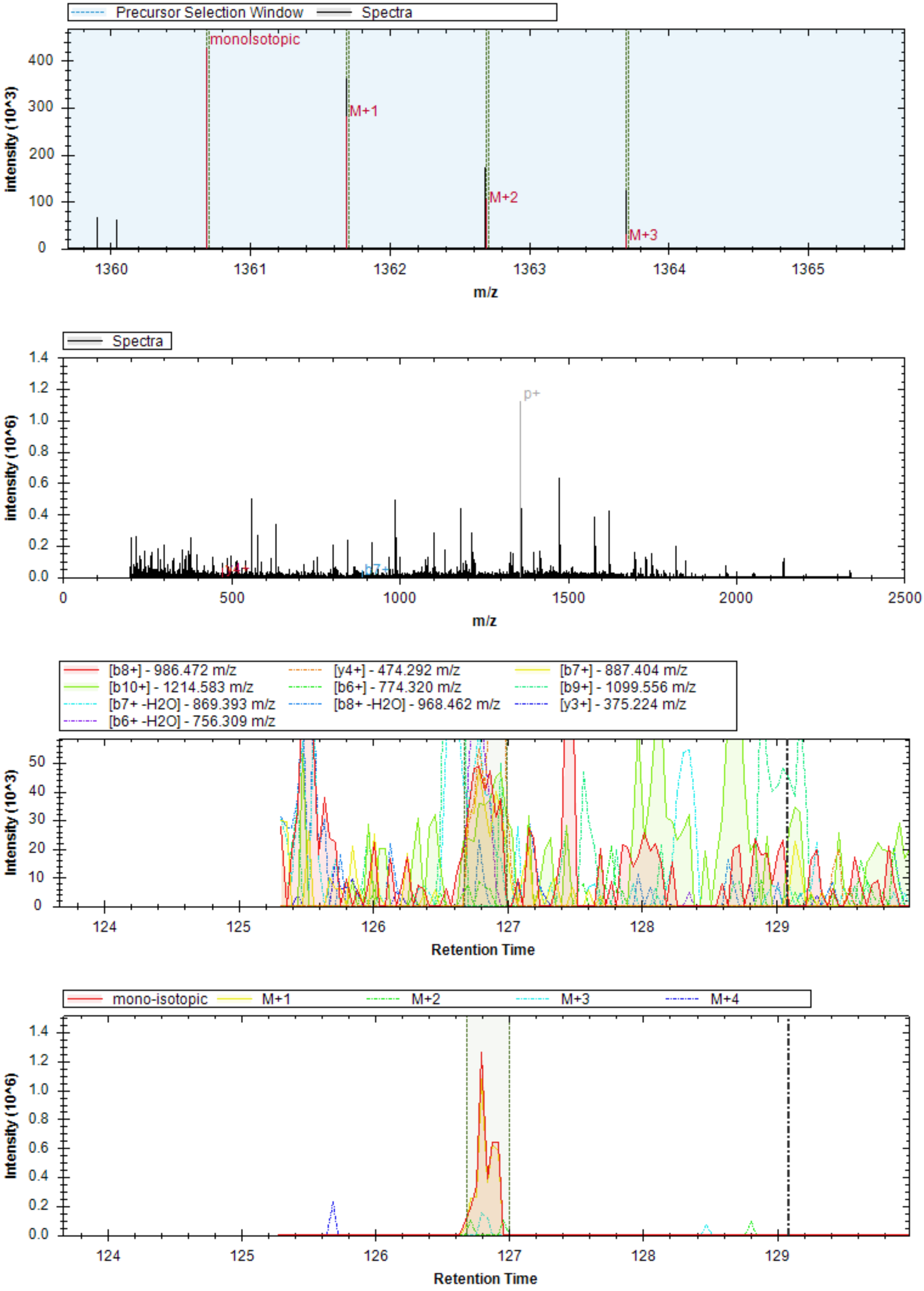

E9Q401 – _MVAEVFIY[+H+O3+P]WSK_.1
Fig4_MP-DIA-barrel-cortex-p15-1_MHRM.htrms

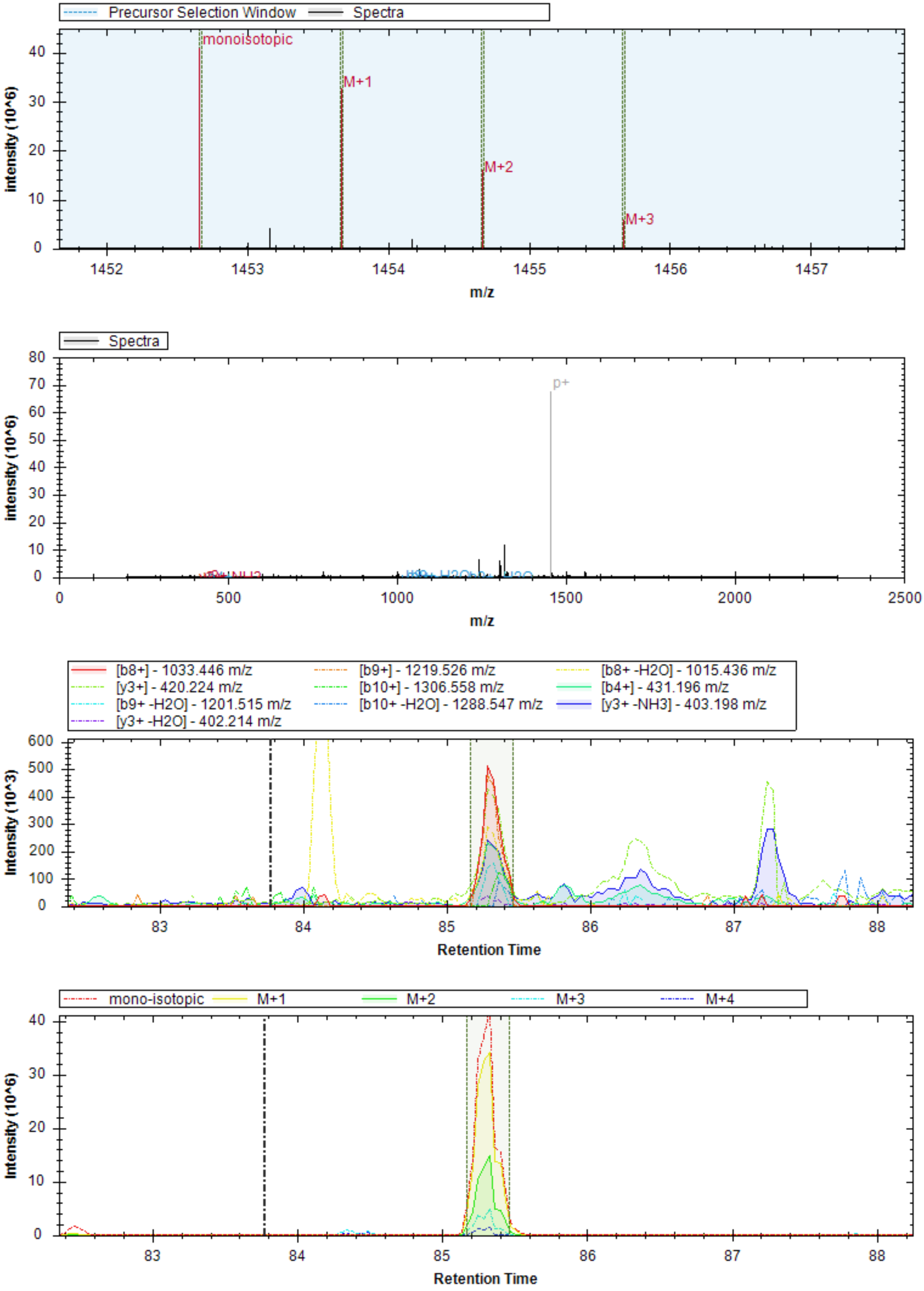

P20357 – _SGTSTPTTPGSTAIT[+H+O3+P]PGTPPSYSSR_.2
Fig4_MP-DIA-barrel-cortex-p15-1_MHRM.htrms

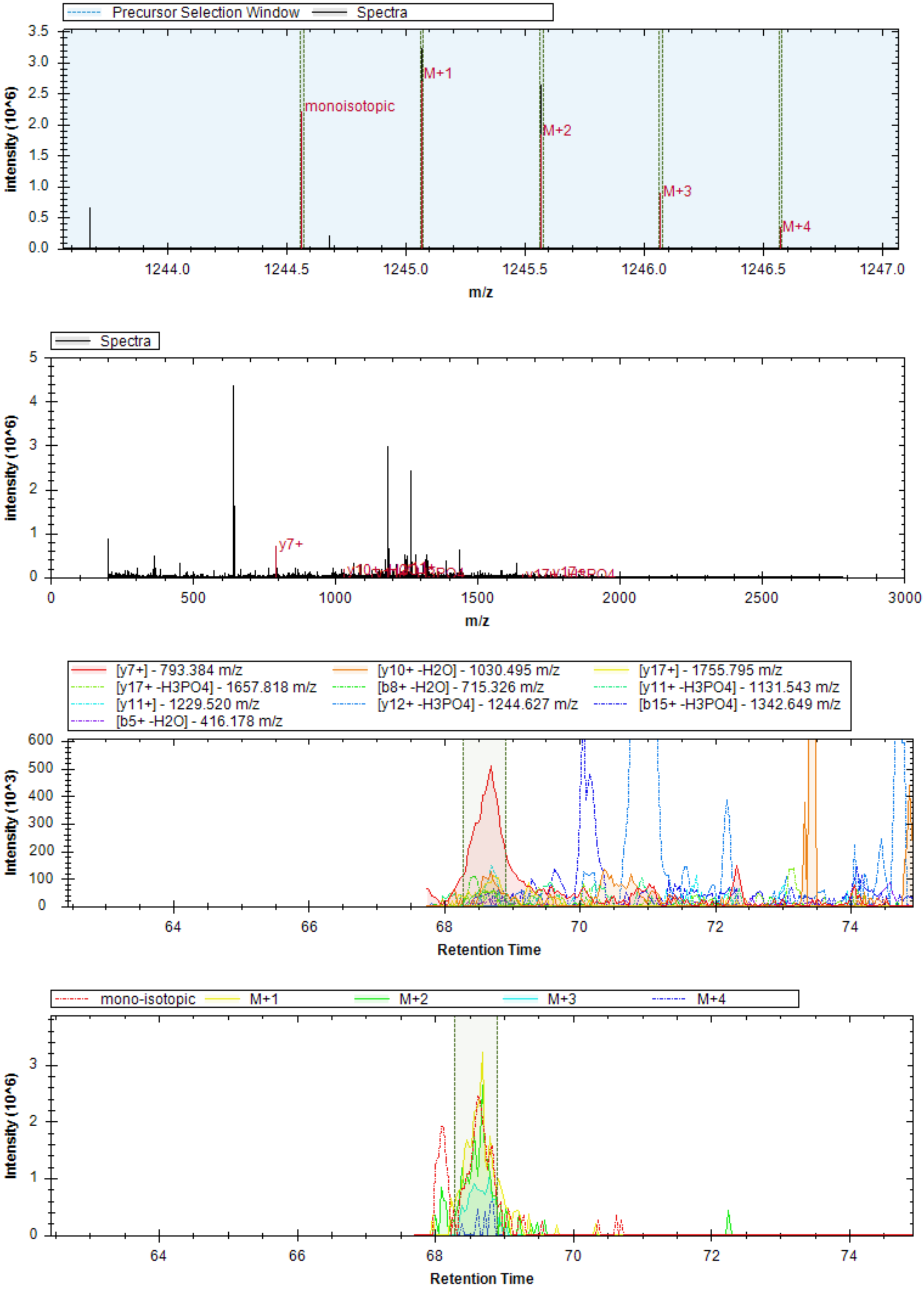

P20357 – _SGTSTPTTPGSTAITPGT[+H+O3+P]PPSYSSR_.2

Fig4_MP-DIA-barrel-cortex-p15-1_MHRM.htrms

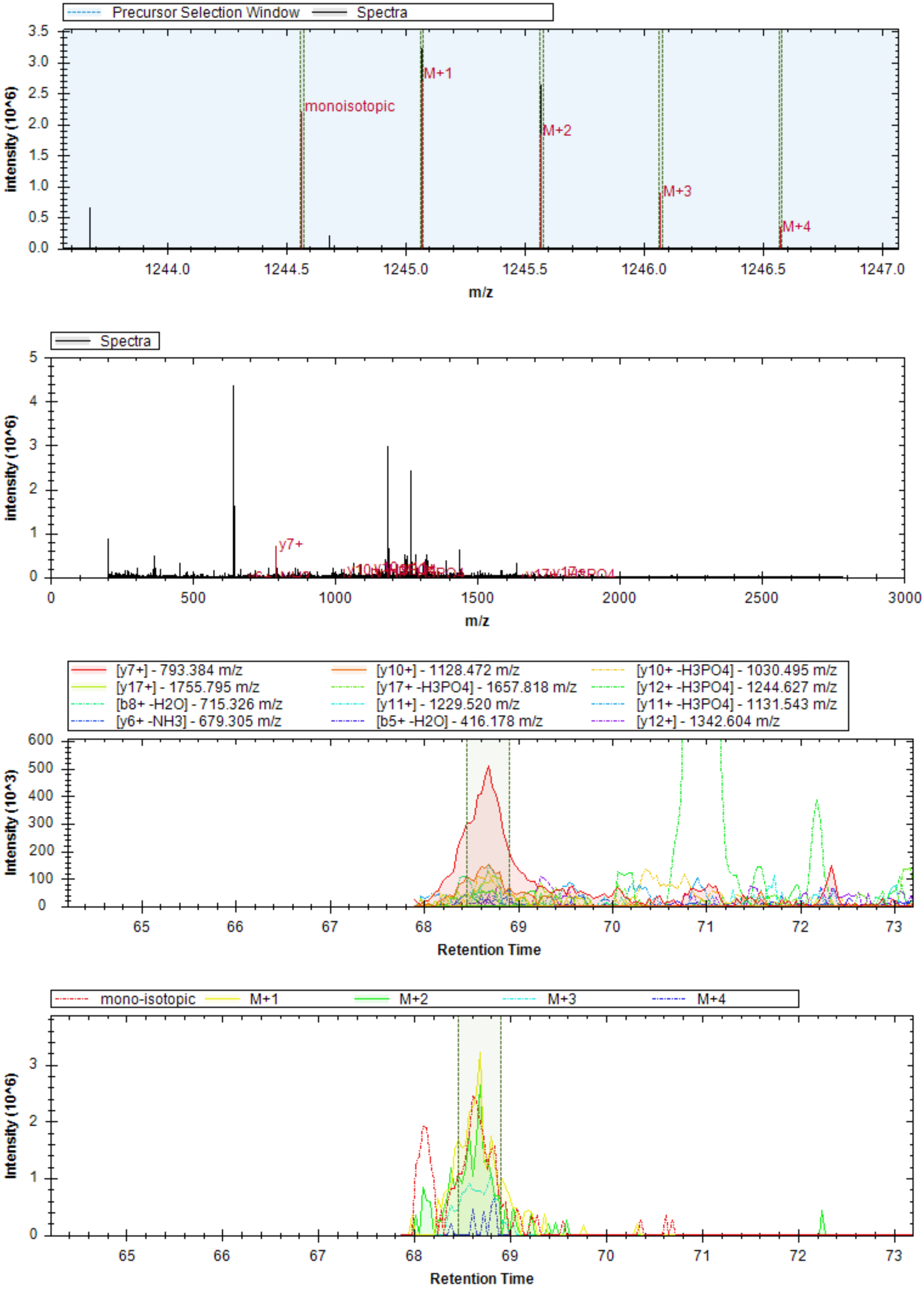

Q8VHY0 – _LLAGPRY[+H+O3+P]GR_.1
Fig4_MP-DIA-barrel-cortex-p15-1_MHRM.htrms

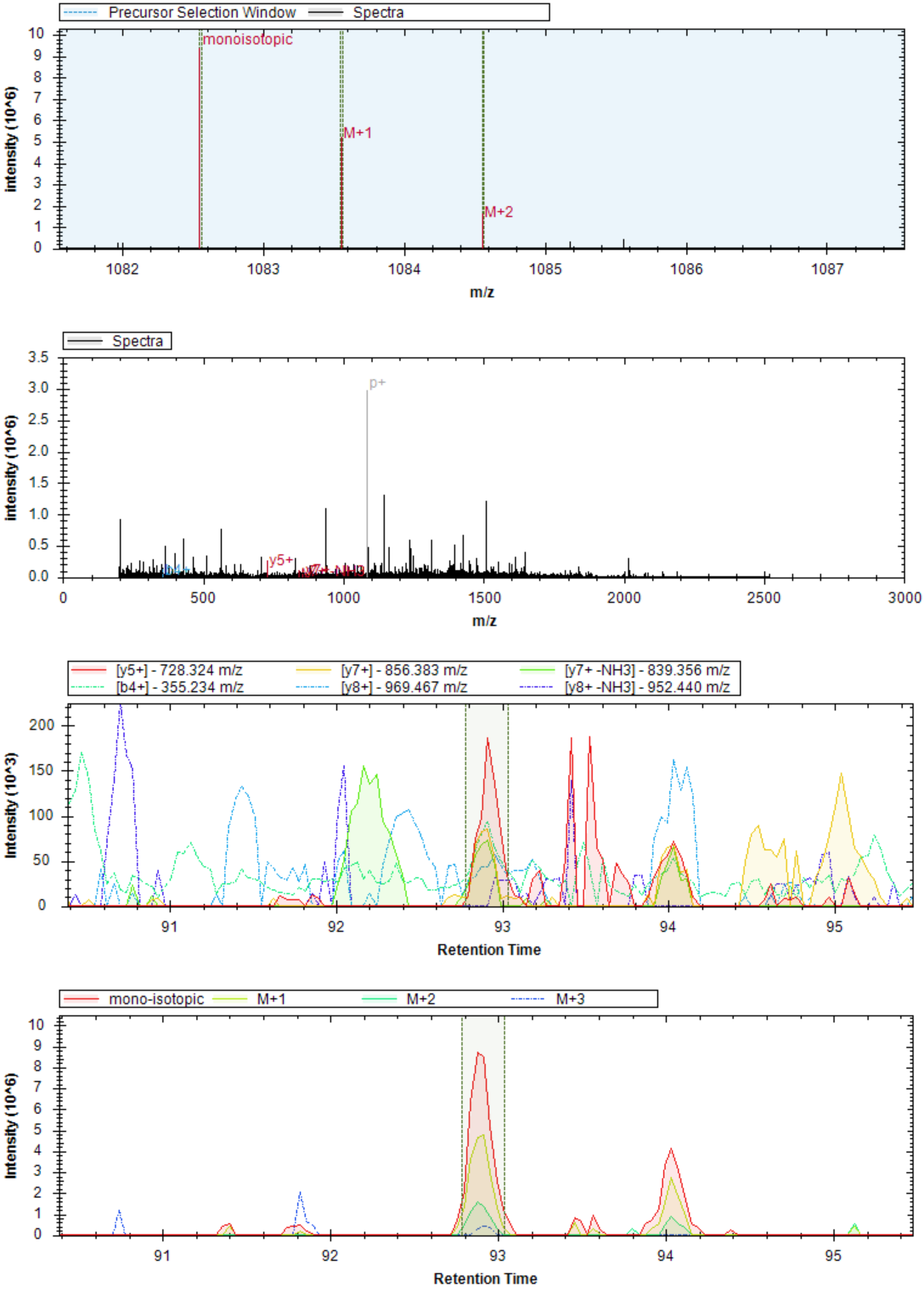

Q921N6;Q921N6-2 – _LIY[+H+O3+P]KPRQAAVTR_.4
Fig4_MP-DIA-barrel-cortex-p15-2_MHRM.htrms

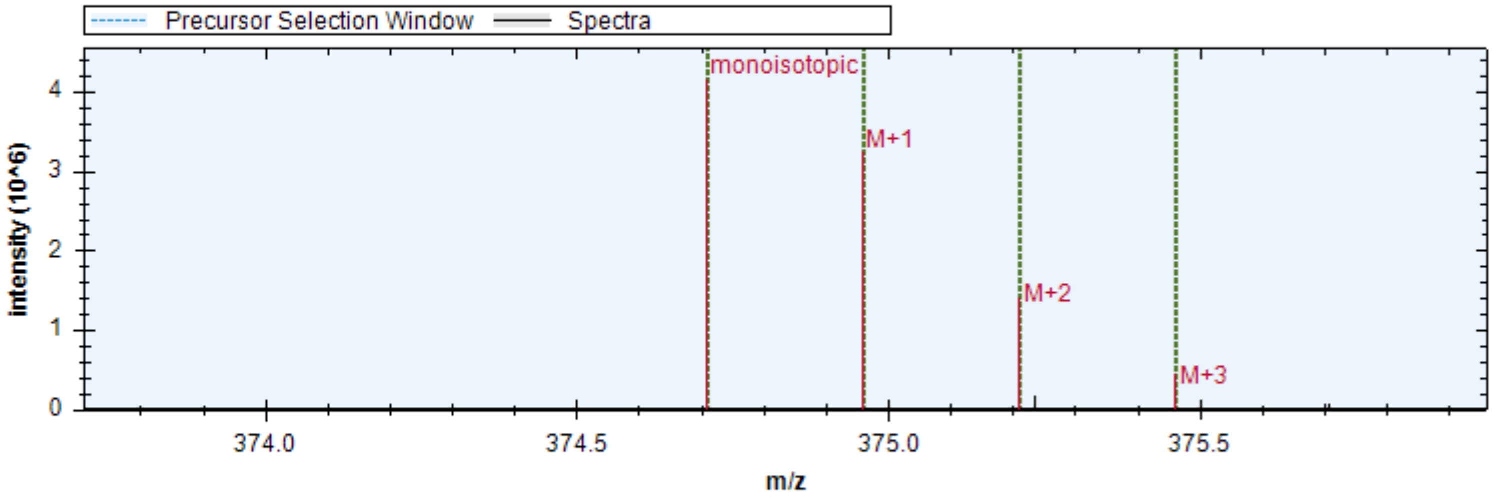

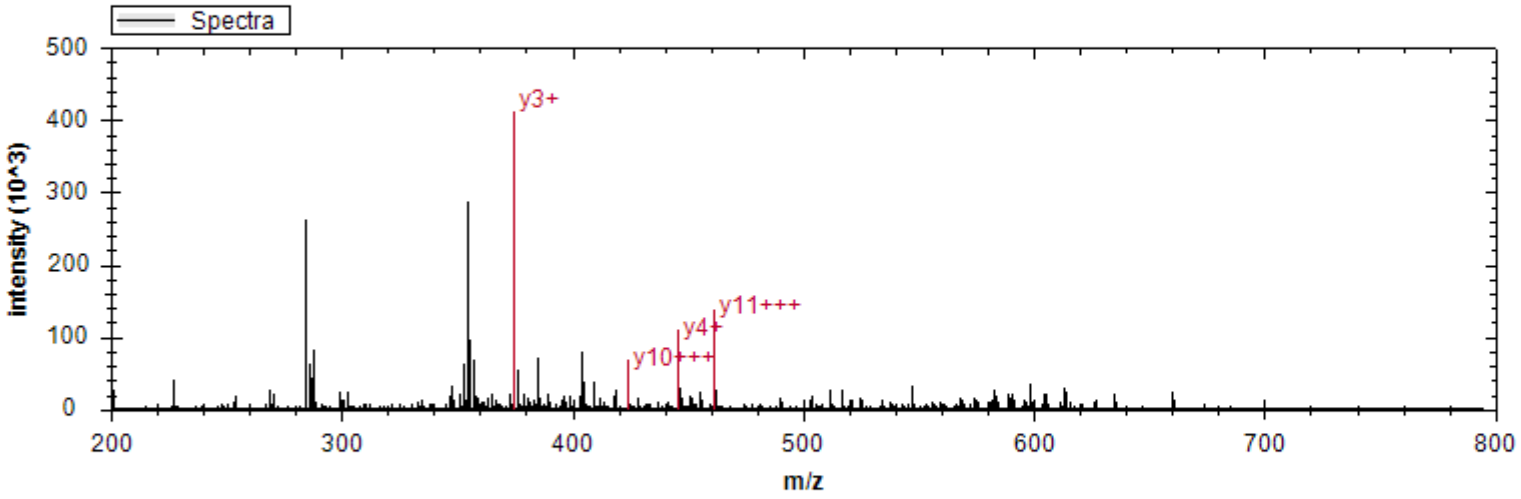

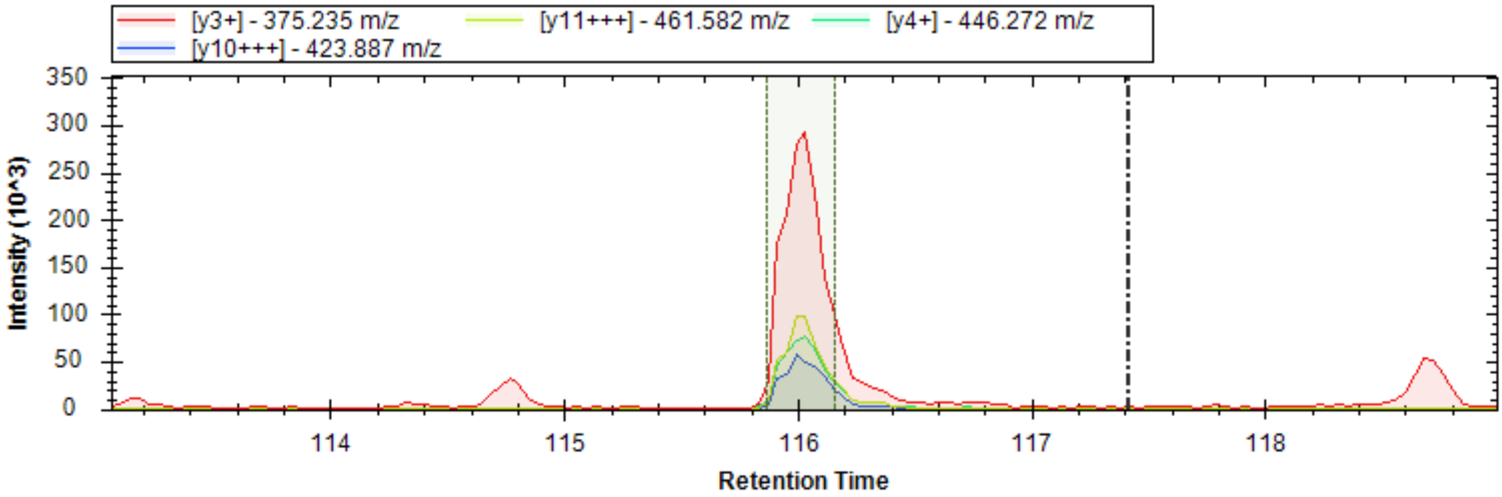

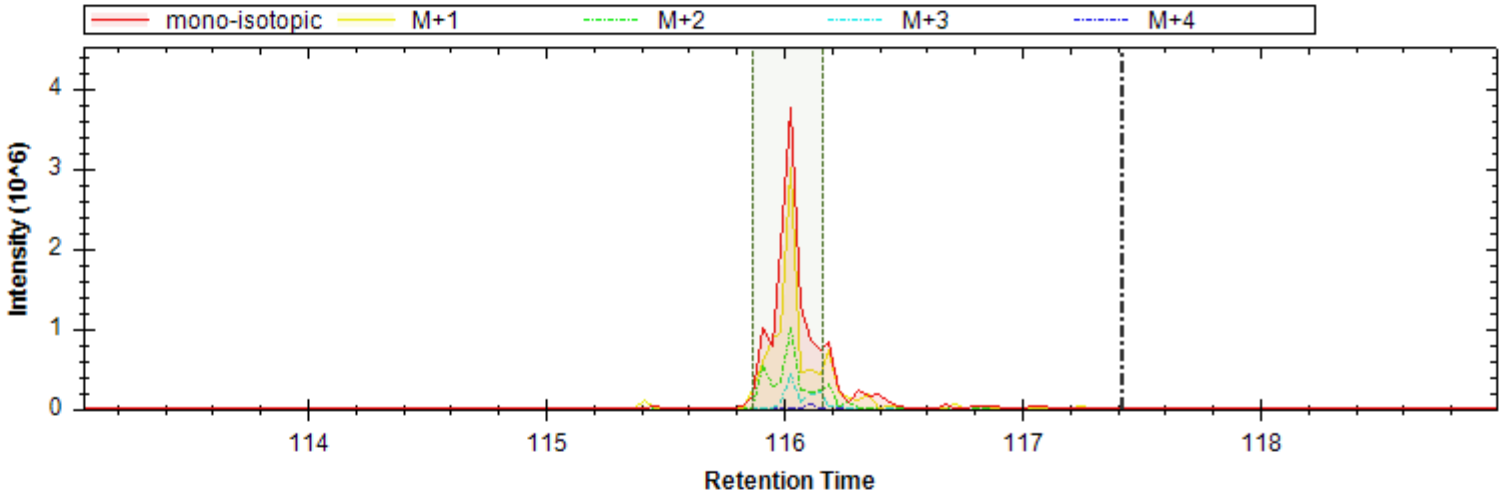

Q9CVB6 – _IIEET[+H+O3+P]LALK_.3
Fig4_MP-DIA-barrel-cortex-p15-2_MHRM.htrms

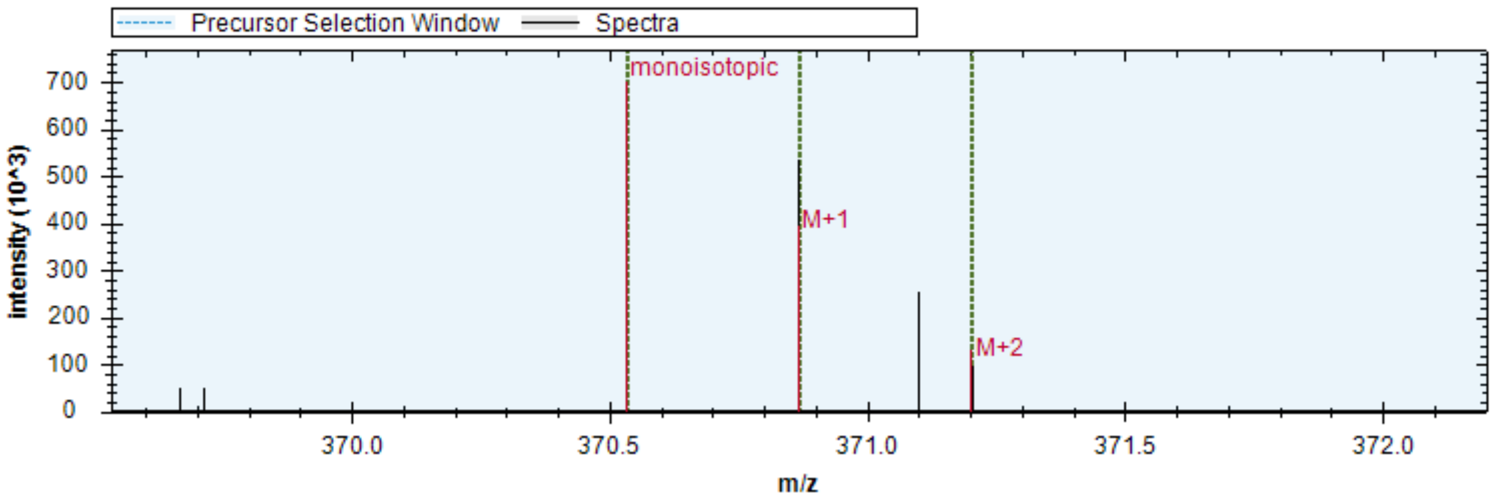

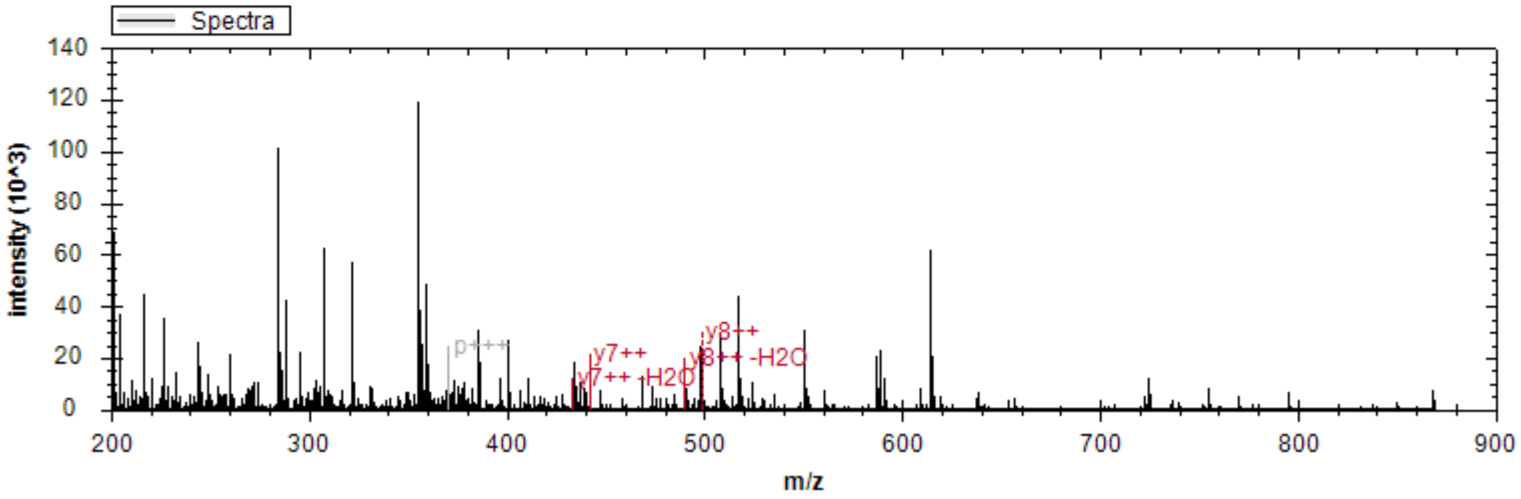

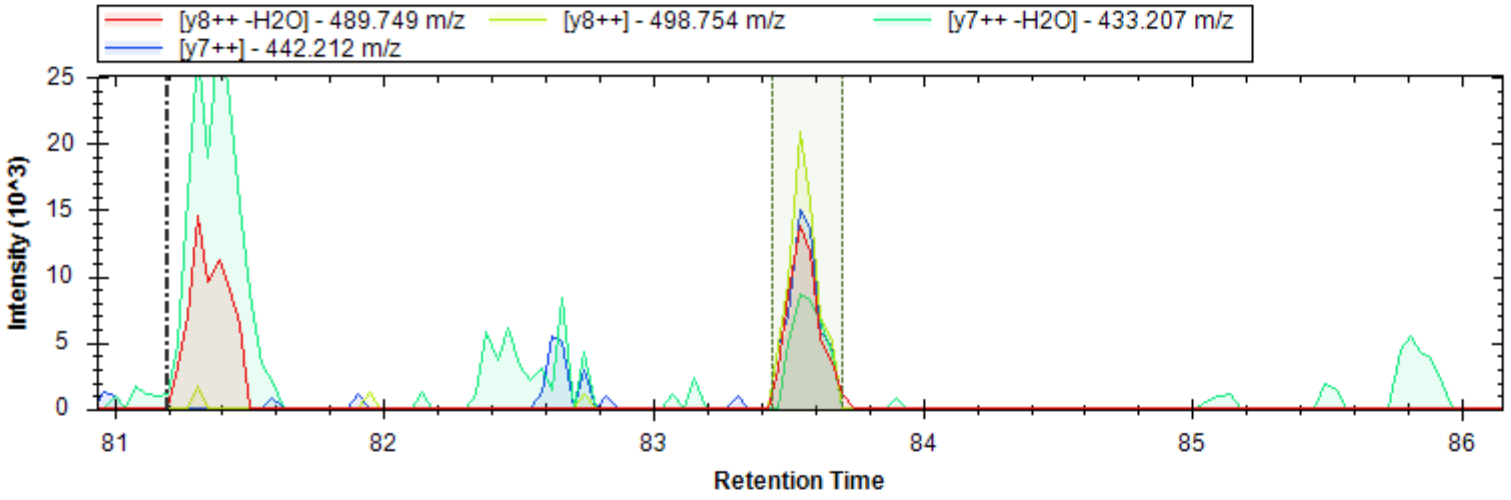

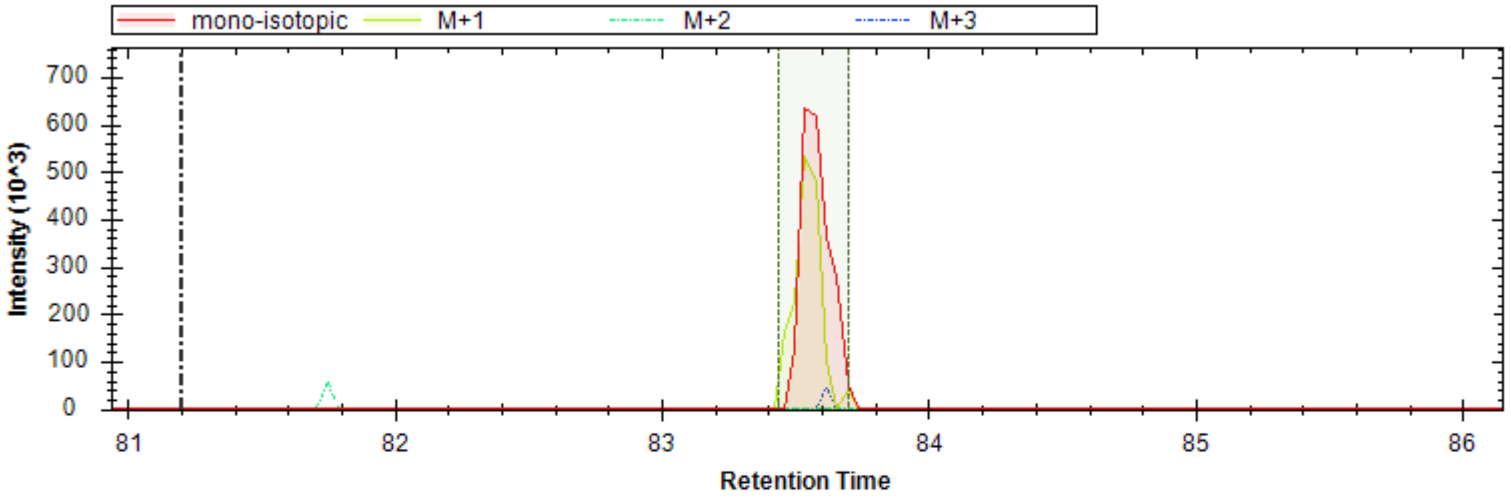

Q9D516;Q9D516-2 – _KLS[+H+O3+P]QGILVIR_.3

Fig4_MP-DIA-barrel-cortex-p15-2_MHRM.htrms

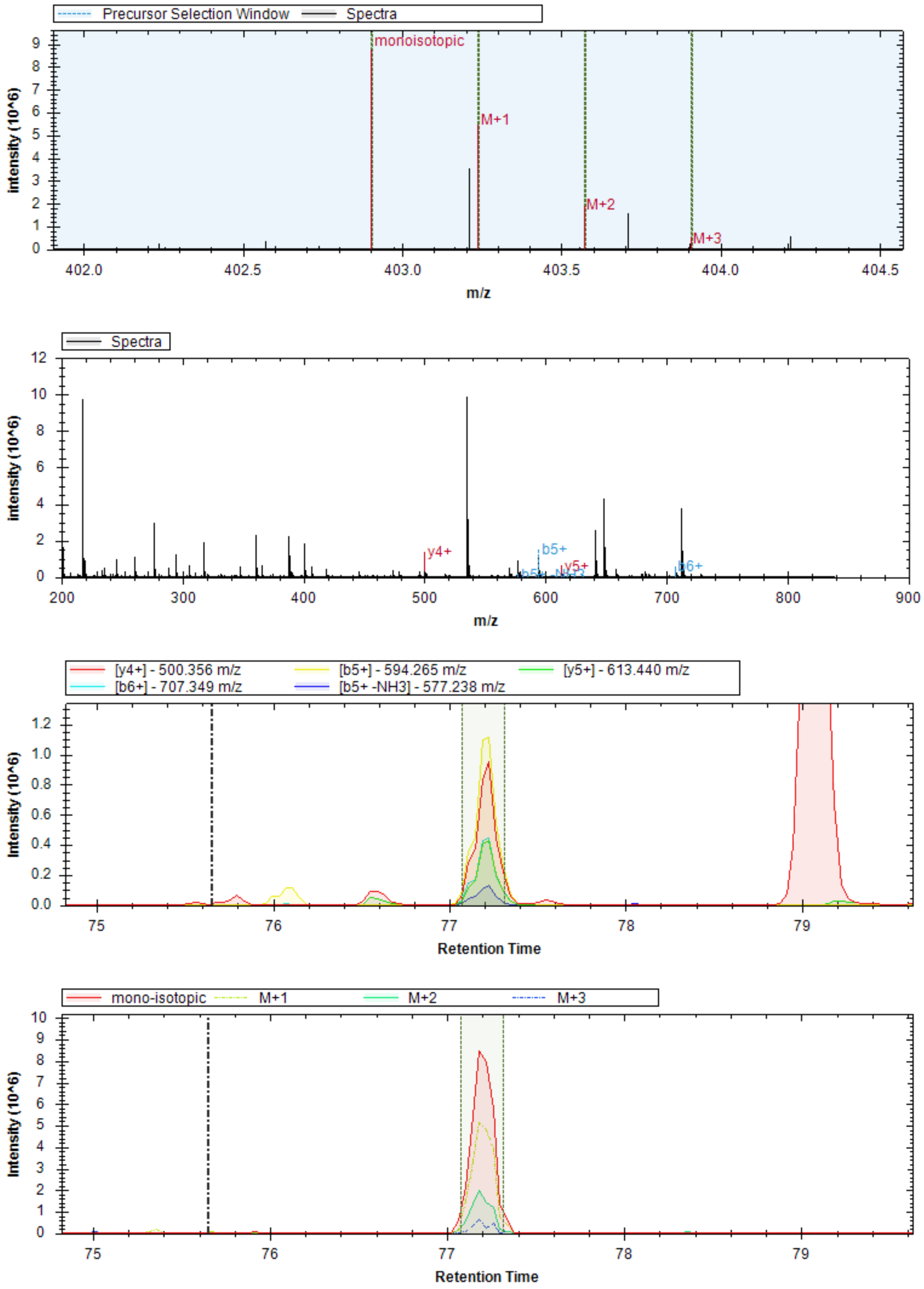

P20357 – _KSEVQAHS[+H+O3+P]PSR_.3
Fig4_MP-DIA-barrel-cortex-p15-2_MHRM.htrms

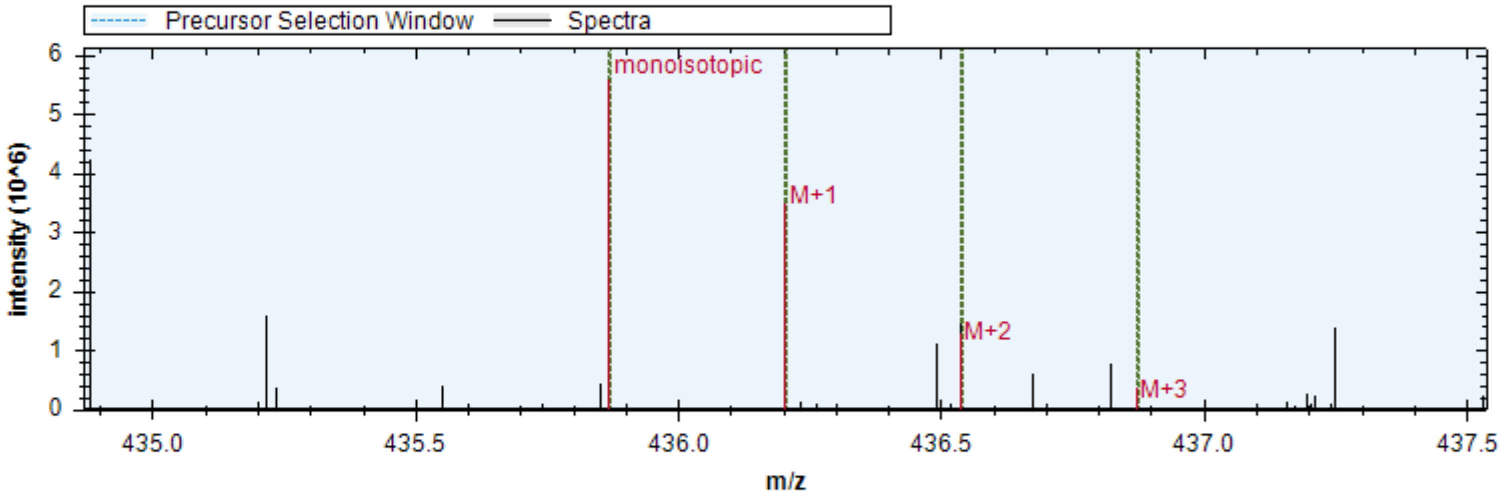

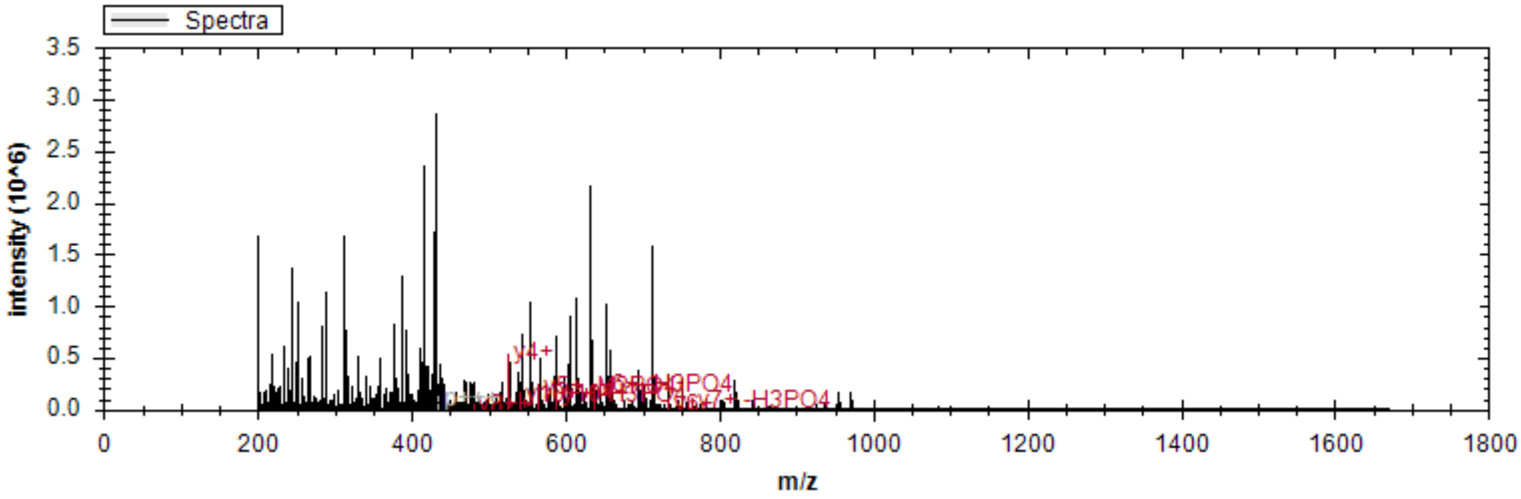

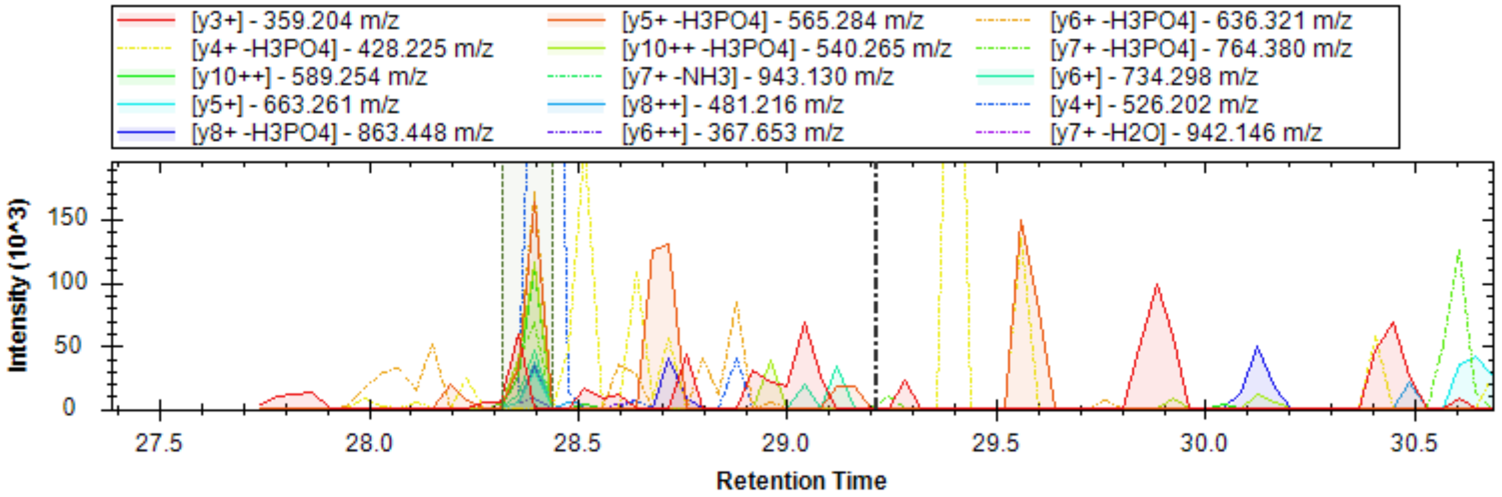

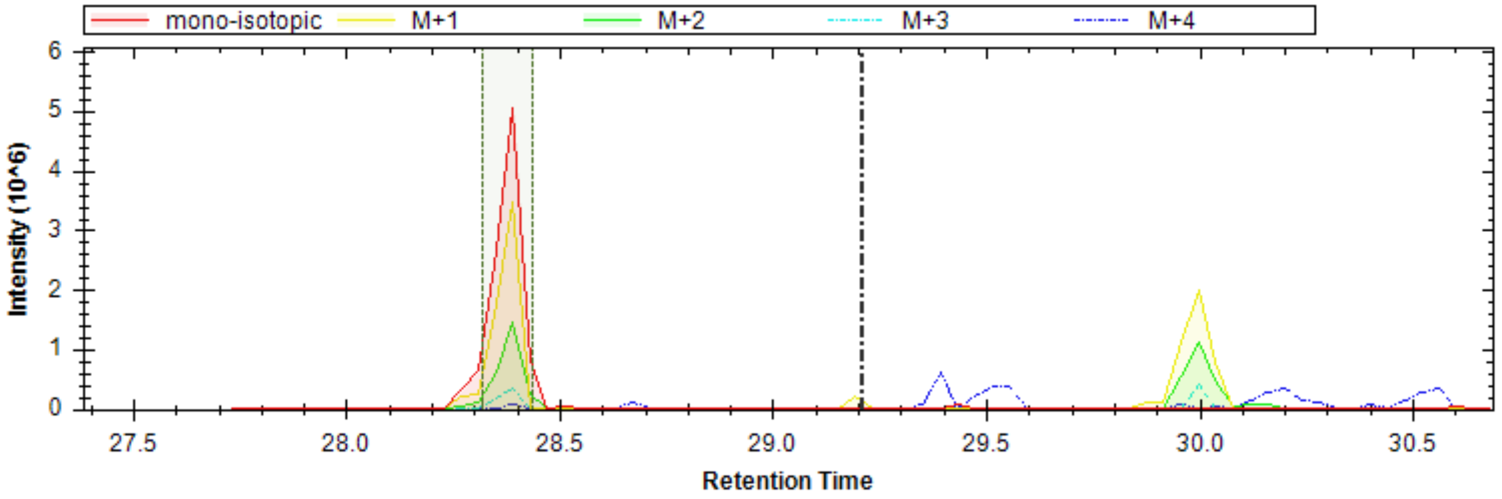

Q03172 – _VALSLLNS[+H+O3+P]KQK_.3
Fig4_MP-DIA-barrel-cortex-p15-2_MHRM.htrms

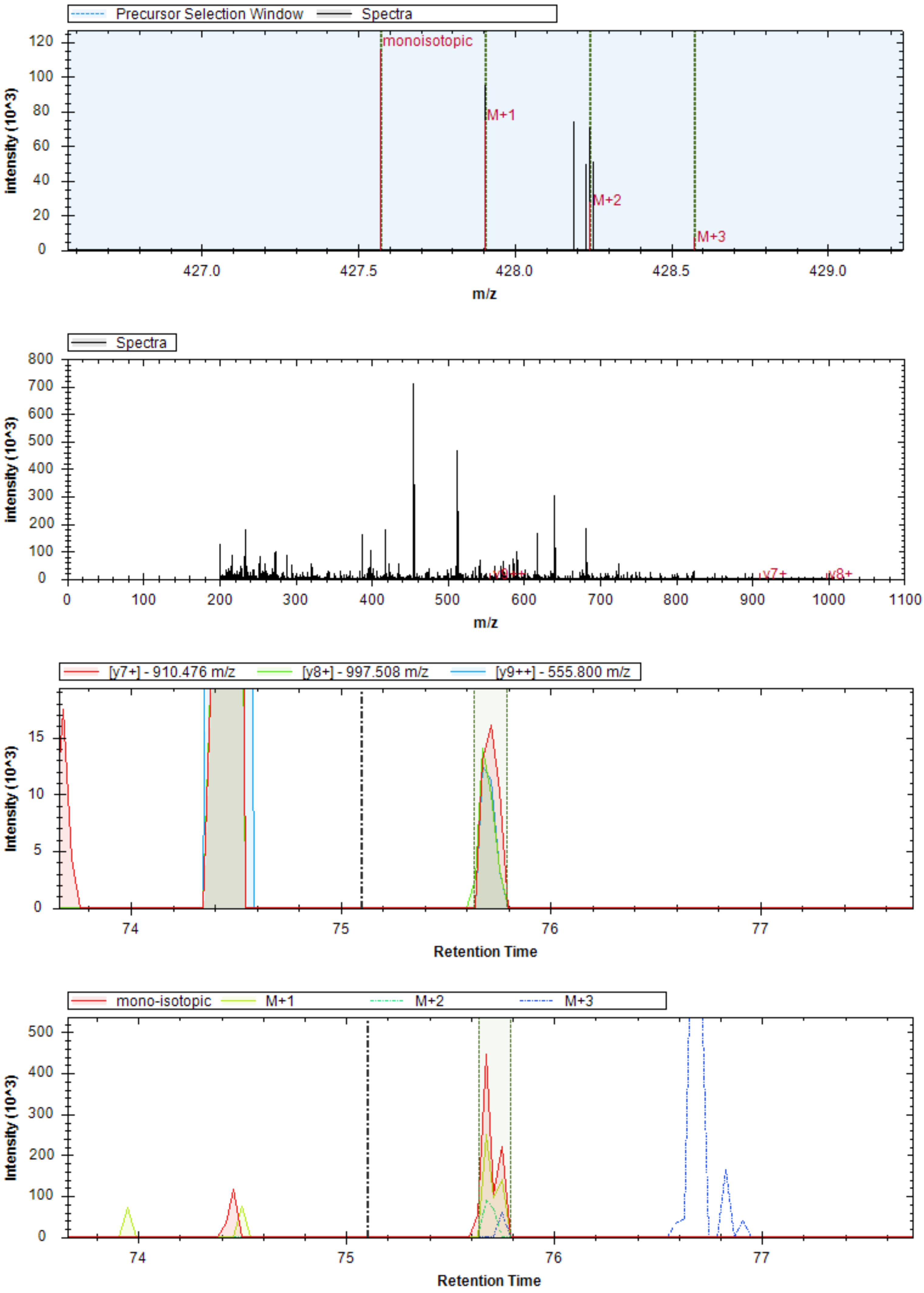

Q52KI8;Q52KI8-2 – _RES[+H+O3+P]PSPAPKPR_.3
Fig4_MP-DIA-barrel-cortex-p15-2_MHRM.htrms

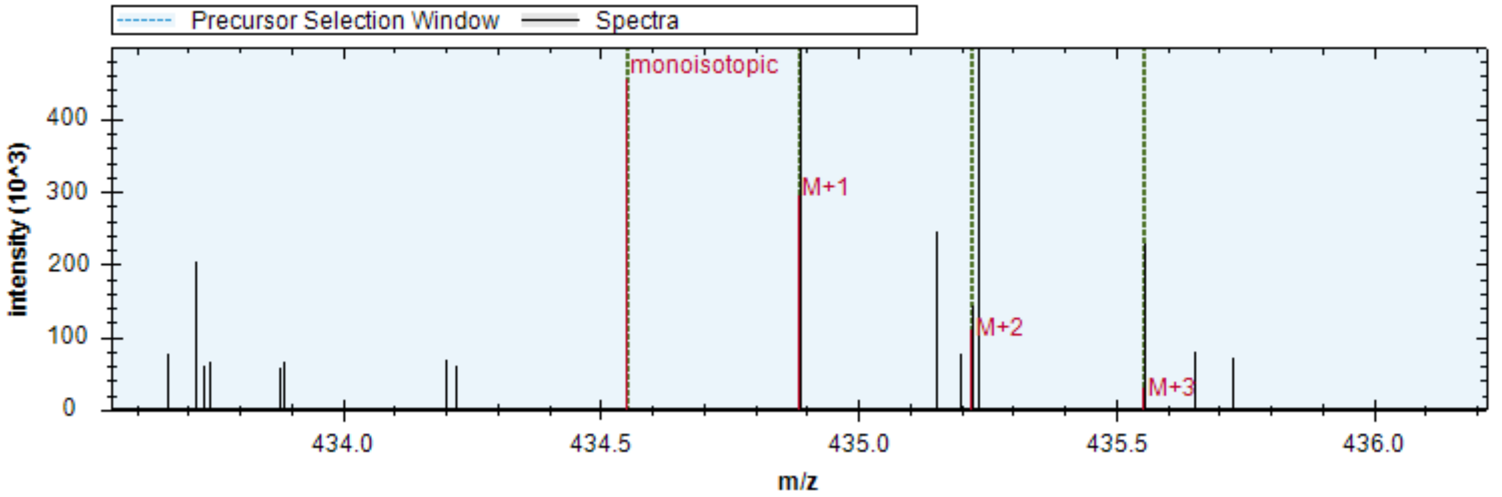

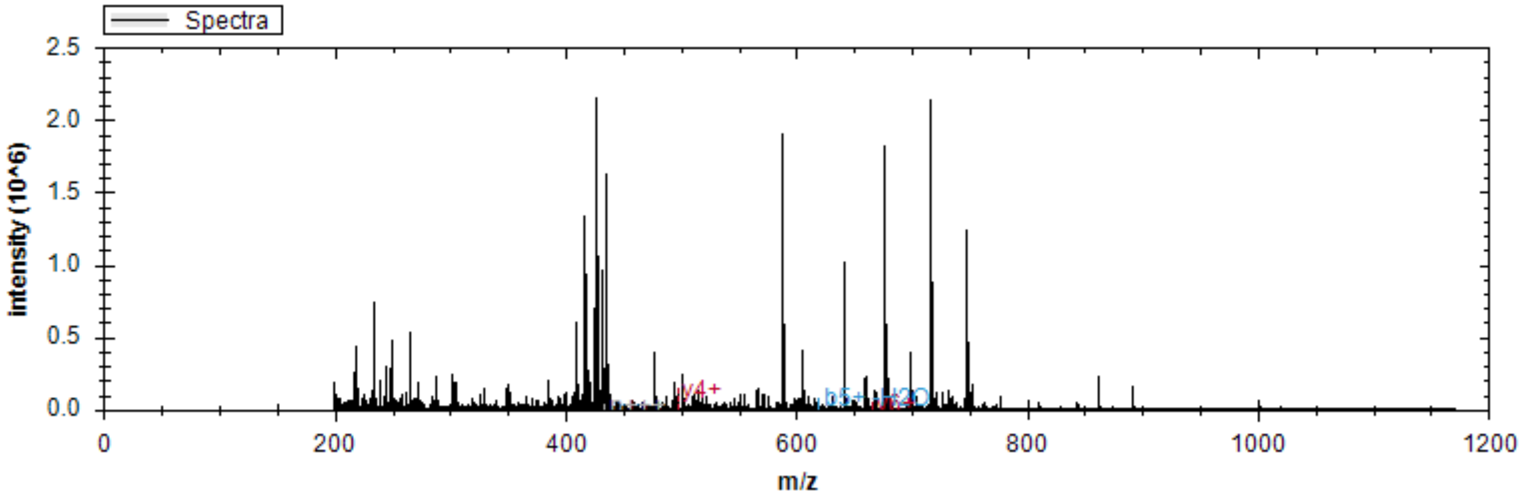

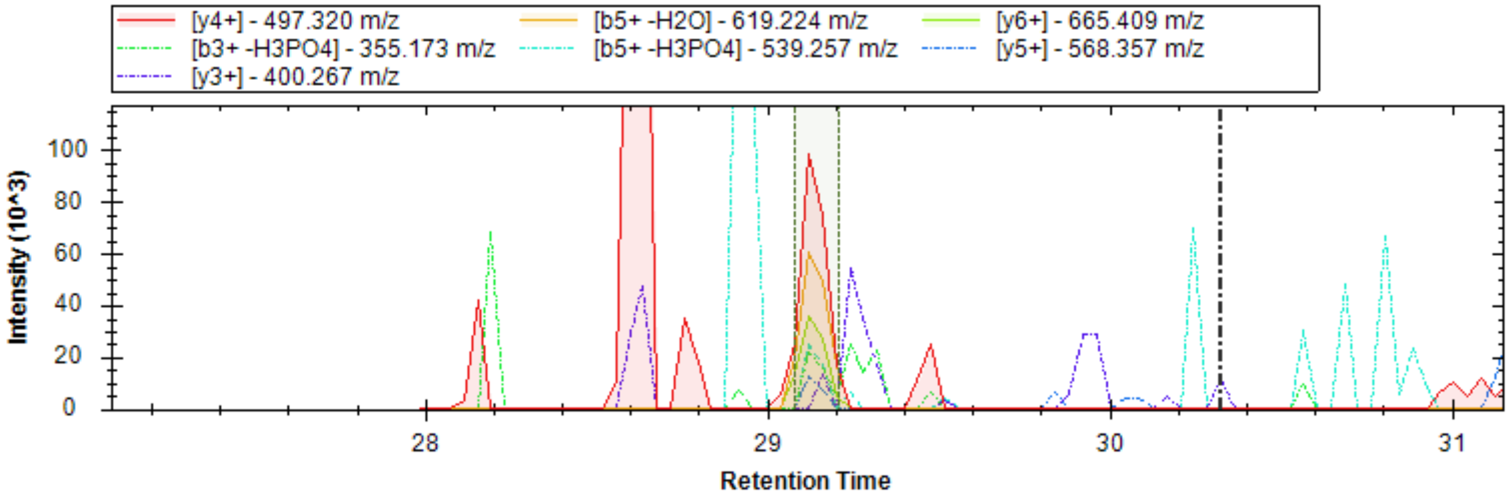

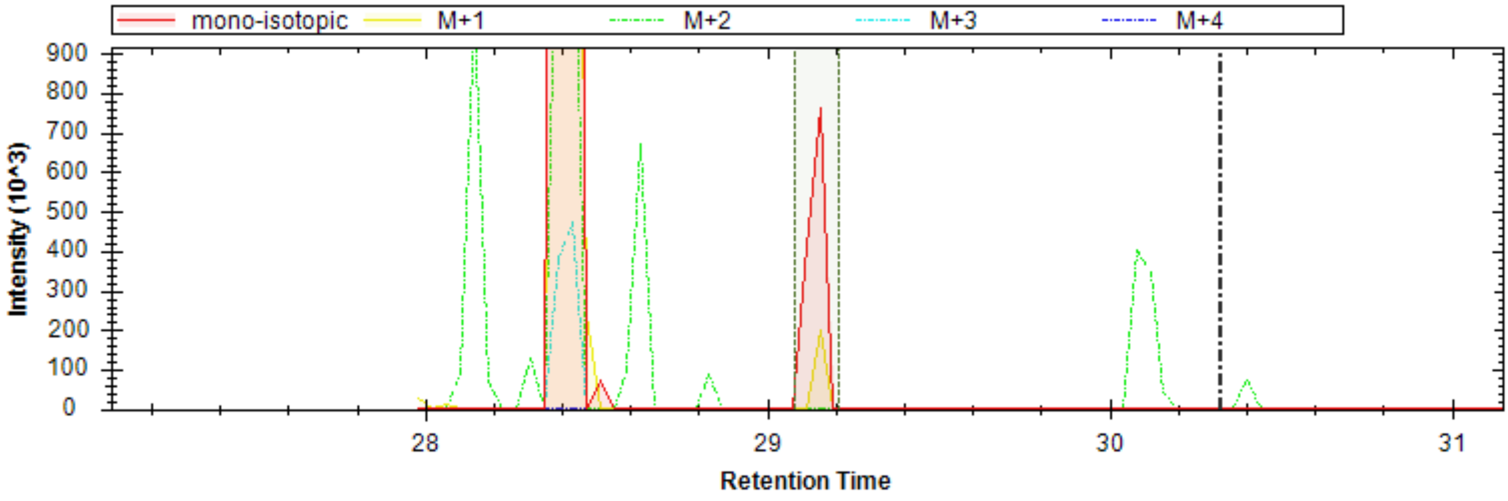

Q62188 – _GS[+H+O3+P]PTRPNPPVR_.3
Fig4_MP-DIA-barrel-cortex-p15-2_MHRM.htrms

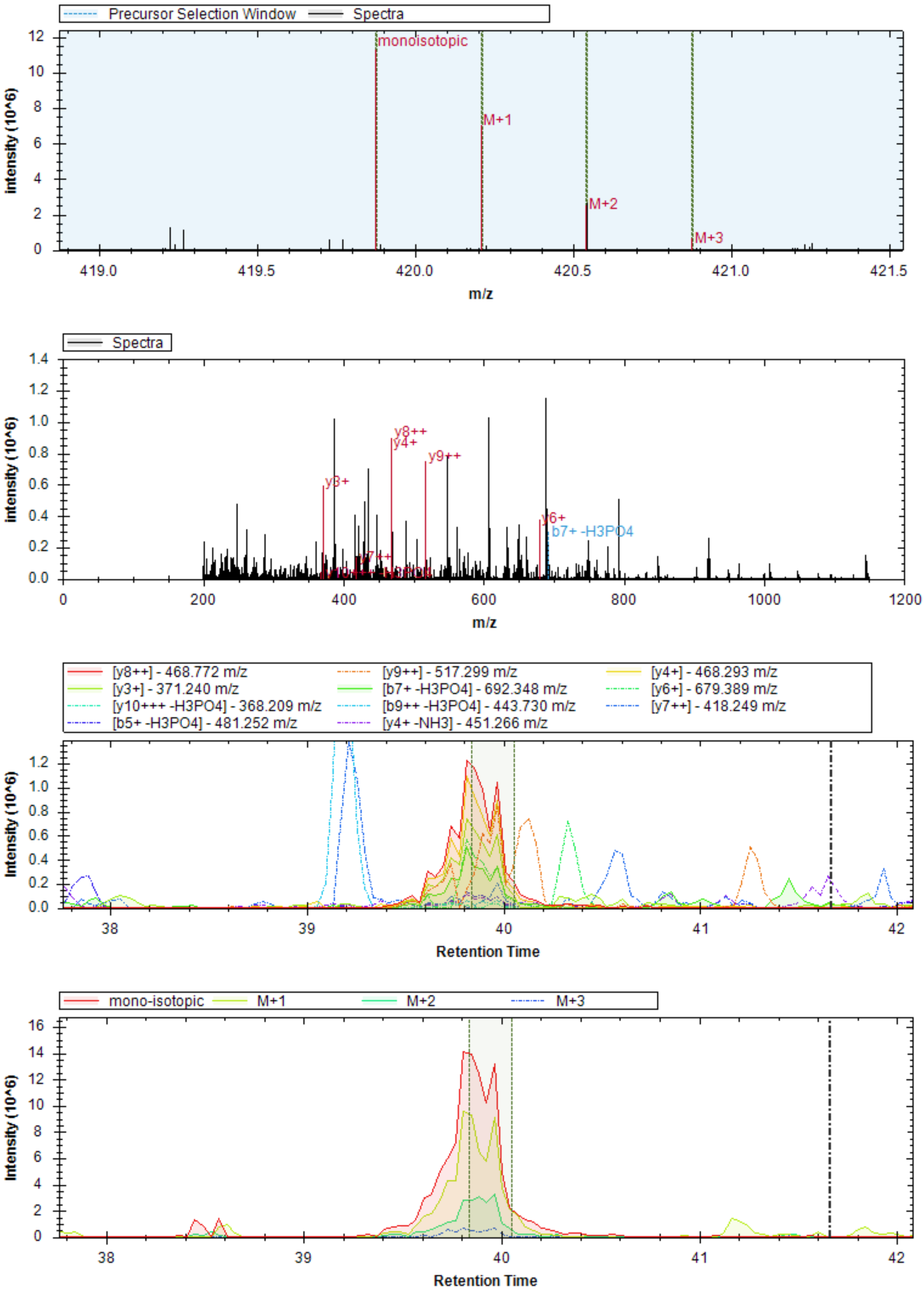

Q8CGI1 – _Y[+H+O3+P]QRS[+H+O3+P]EEELR_.3
Fig4_MP-DIA-barrel-cortex-p15-2_MHRM.htrms

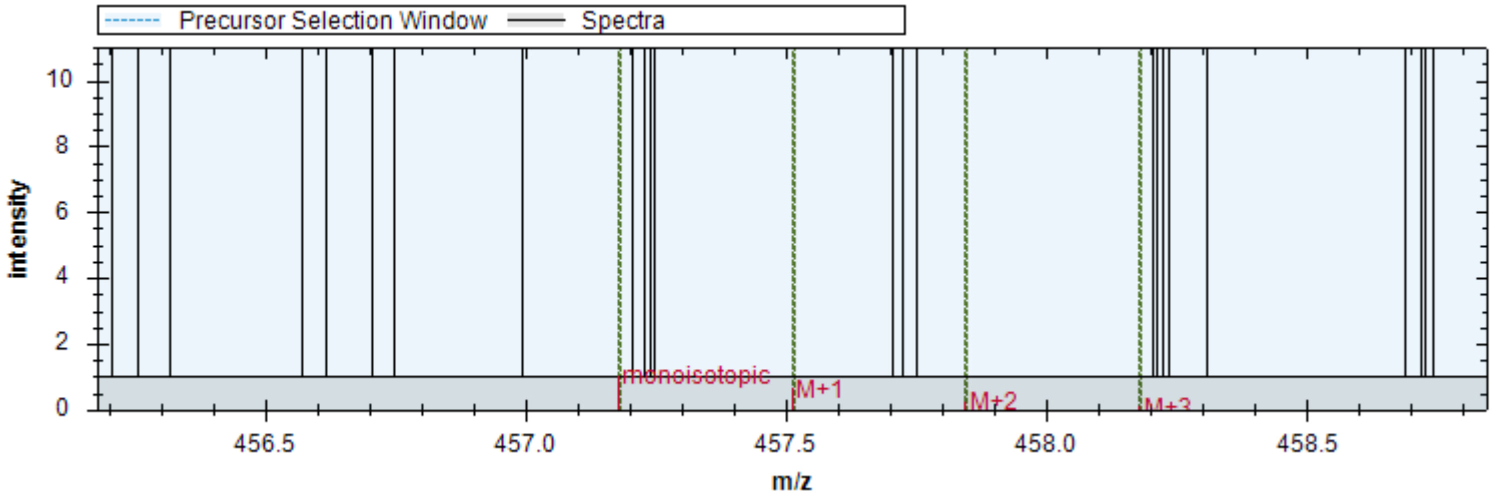

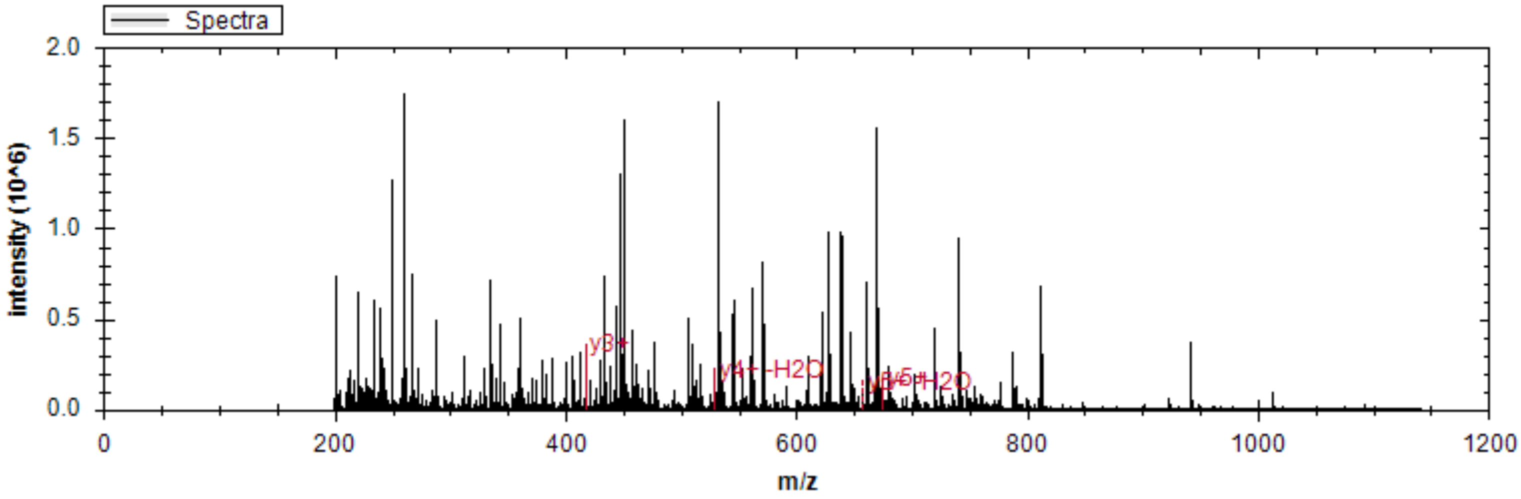

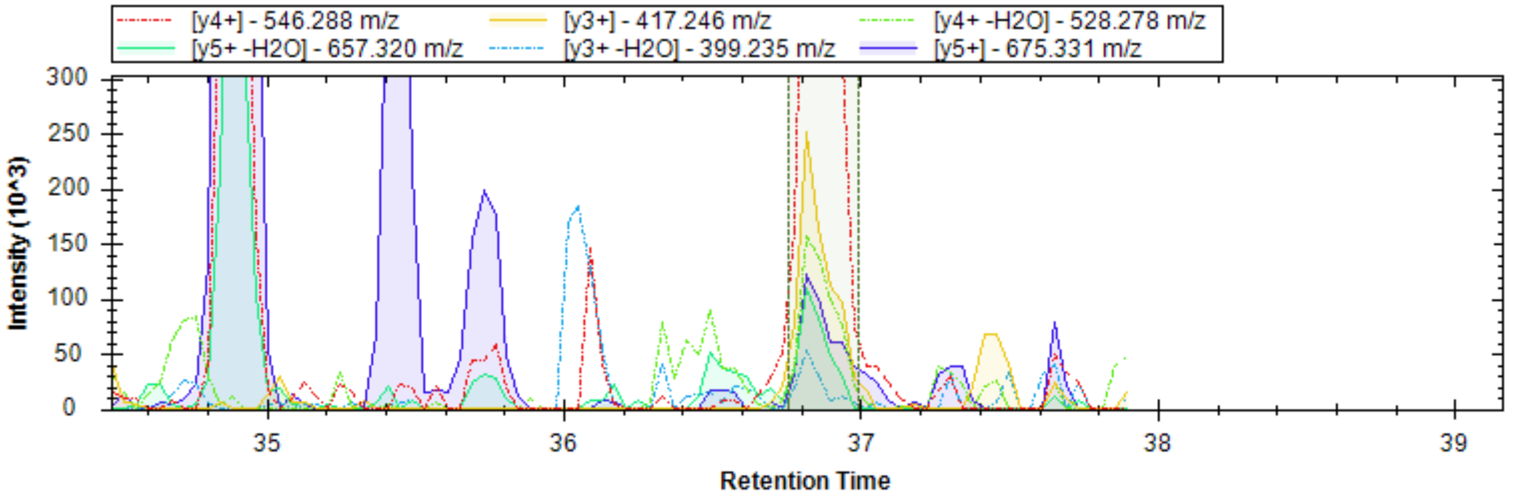

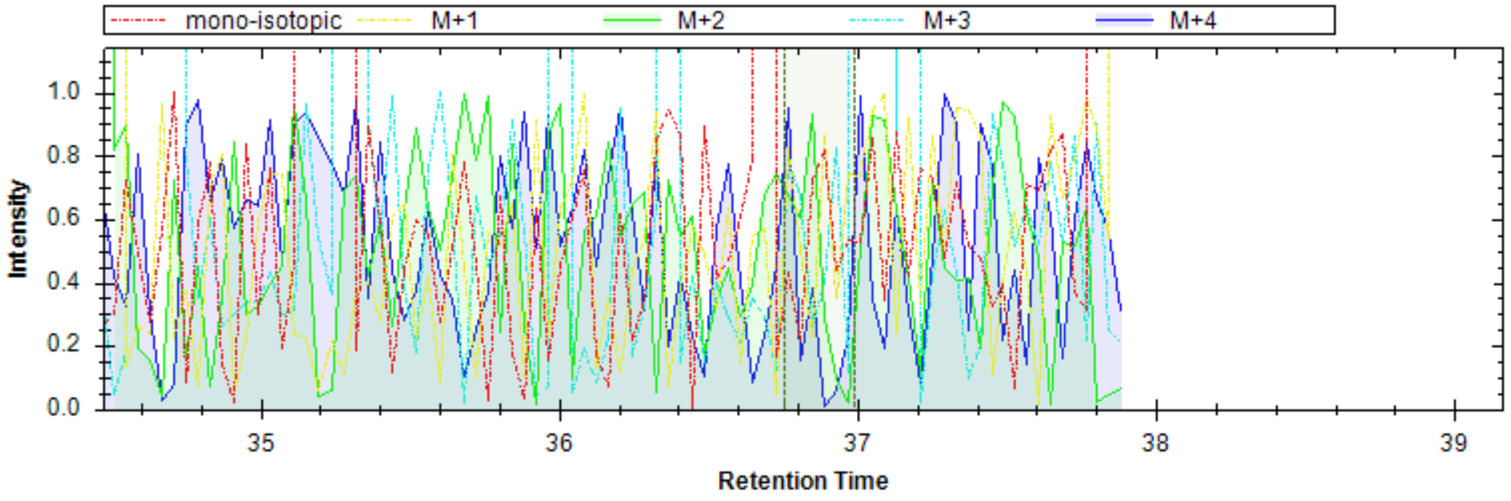

O08553 – _TVT[+H+O3+P]PASSAK_.2
Fig4_MP-DIA-barrel-cortex-p15-2_MHRM.htrms

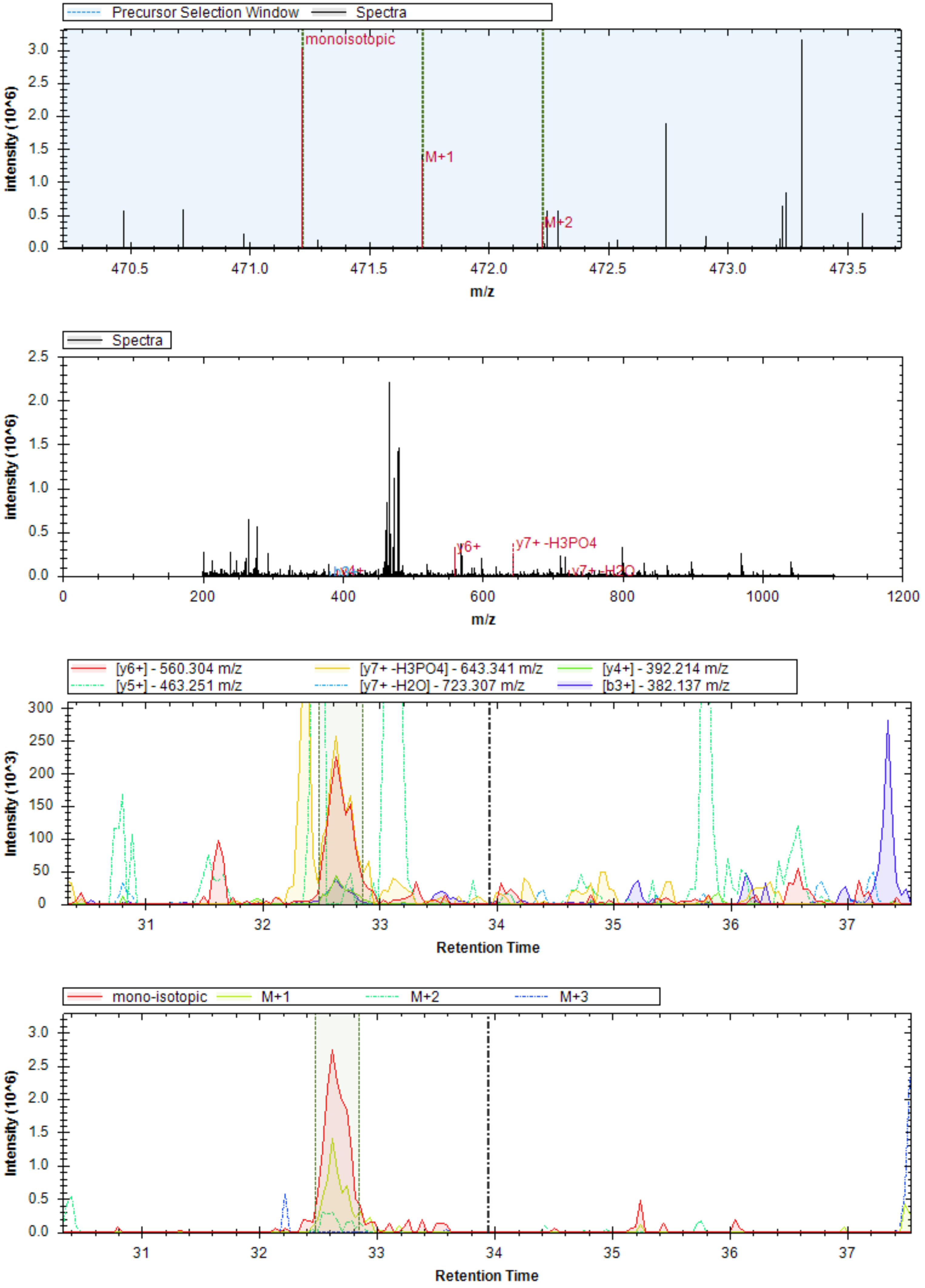

P20357 – _VTDGIS[+H+O3+P]KSPEKR_.3
Fig4_MP-DIA-barrel-cortex-p15-2_MHRM.htrms

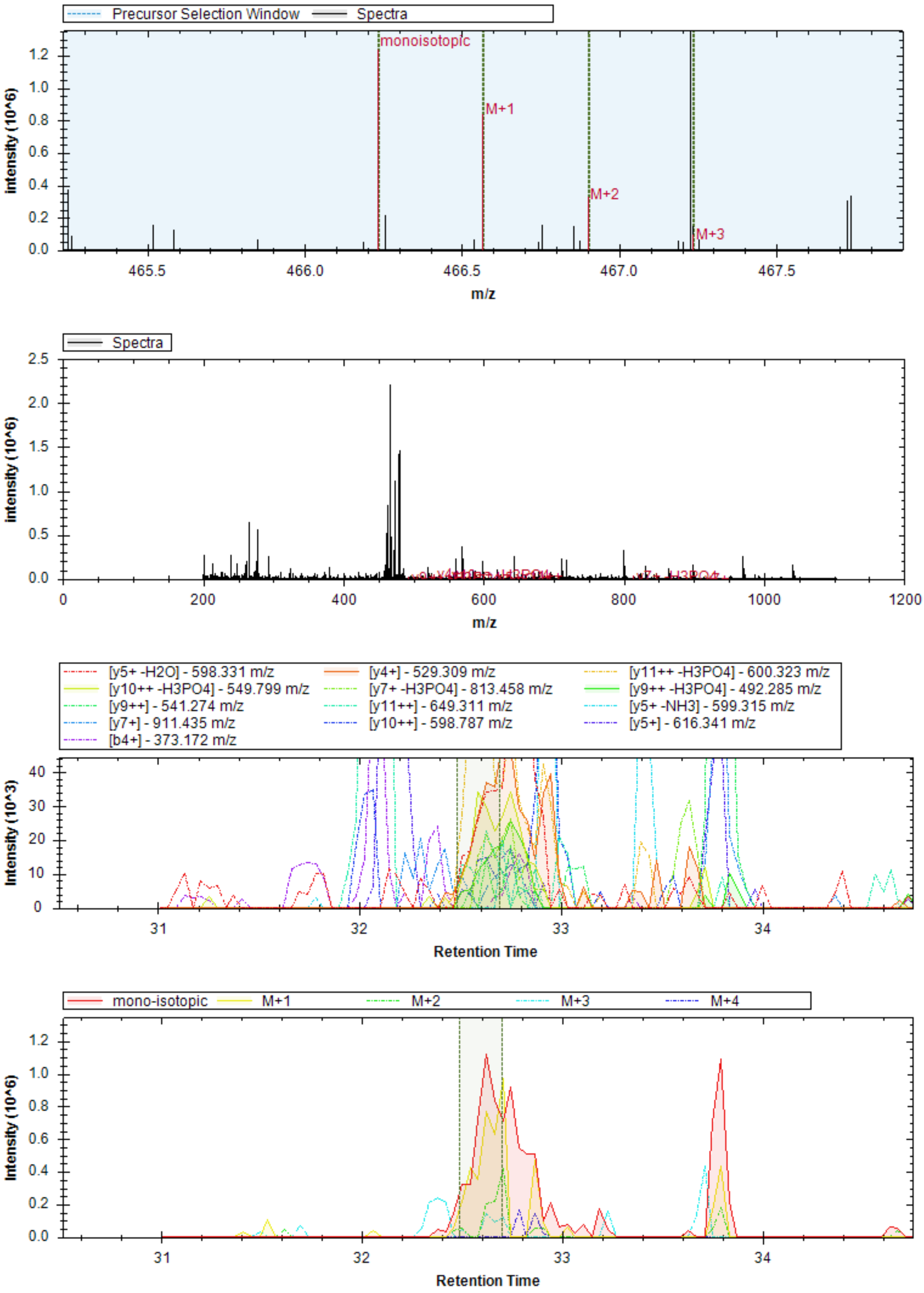

P20357 – _VTDGISKS[+H+O3+P]PEKR_.3
Fig4_MP-DIA-barrel-cortex-p15-2_MHRM.htrms

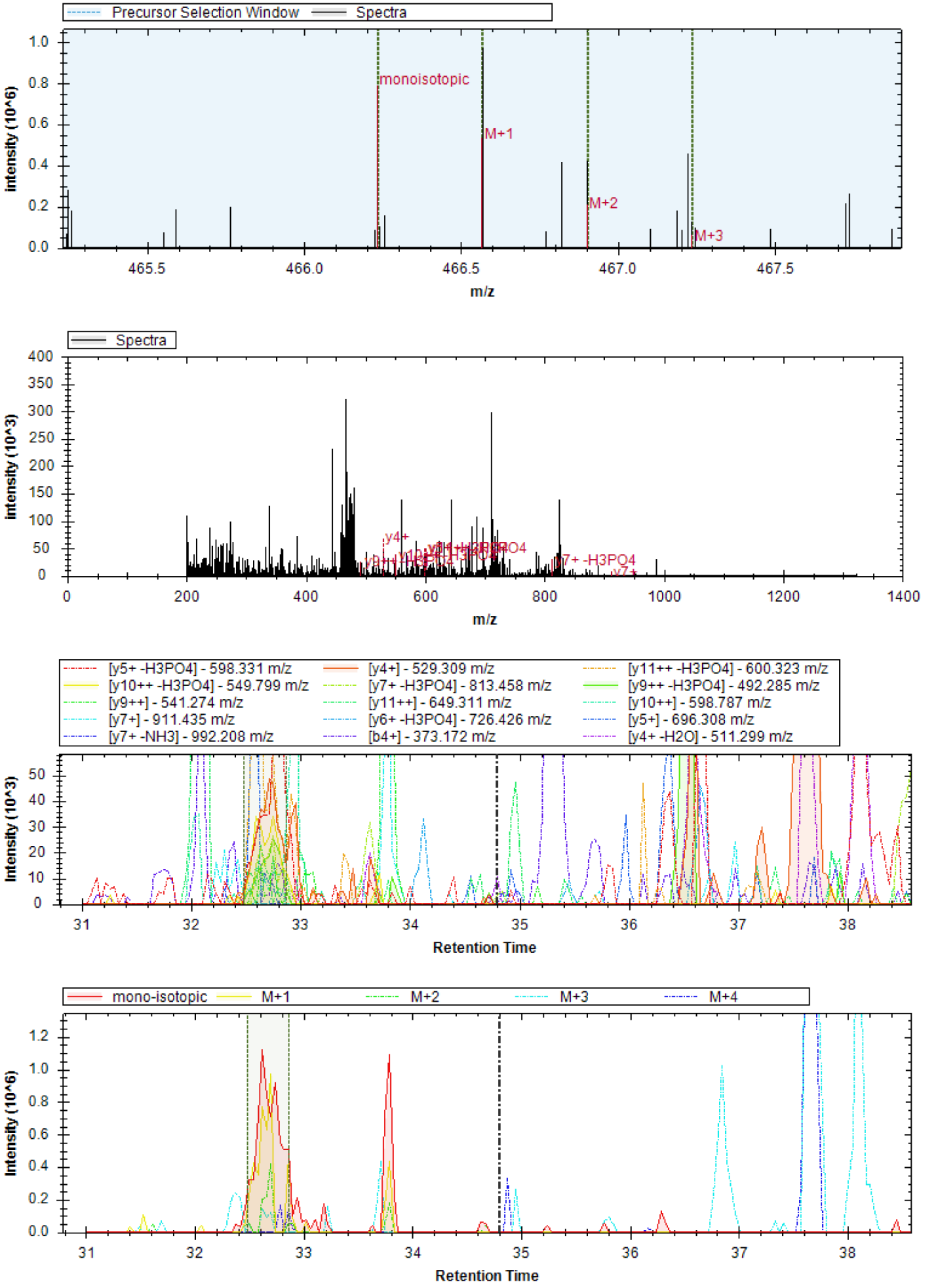

Q8K015;Q8K015-2 – _TVIS[+H+O3+P]EQEVLER_.3

Fig4_MP-DIA-barrel-cortex-p15-2_MHRM.htrms

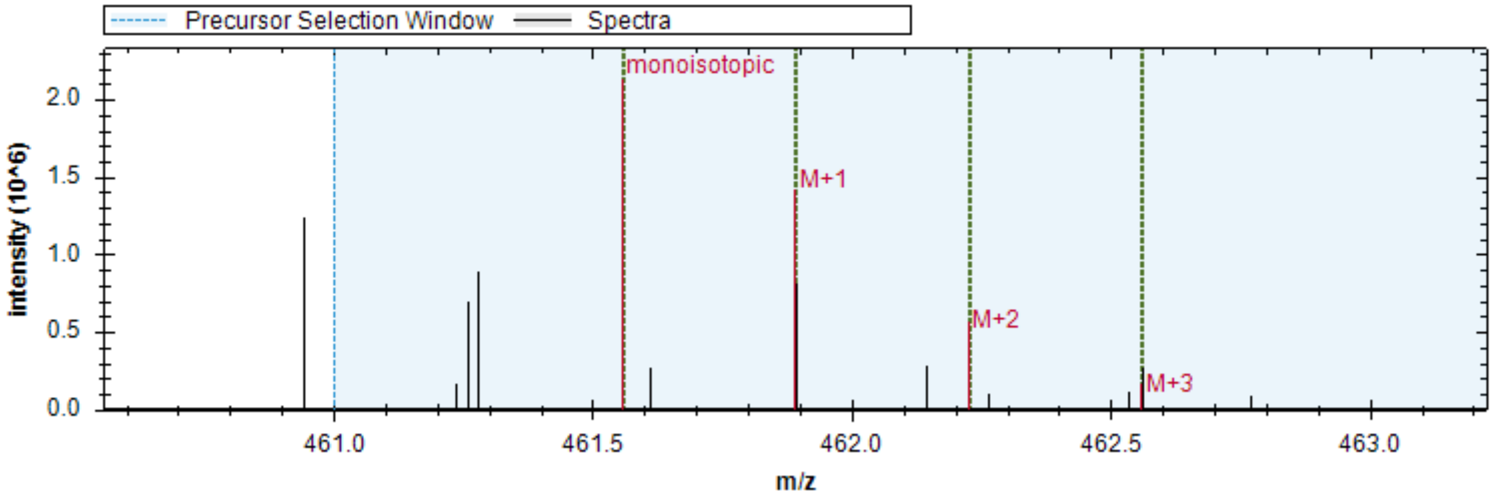

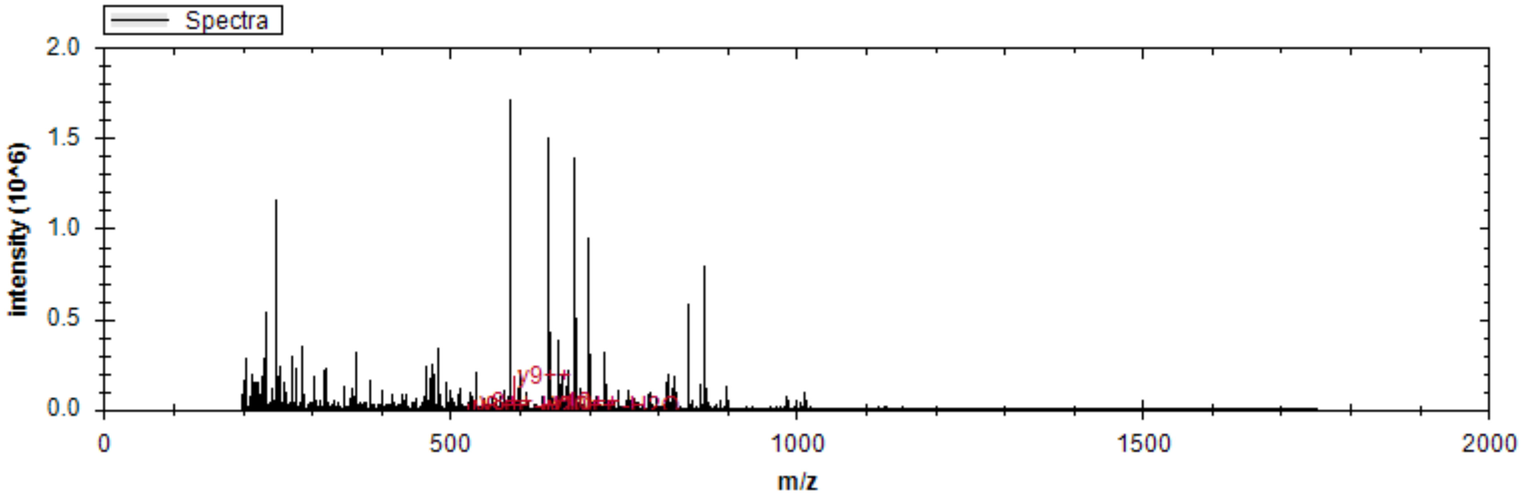

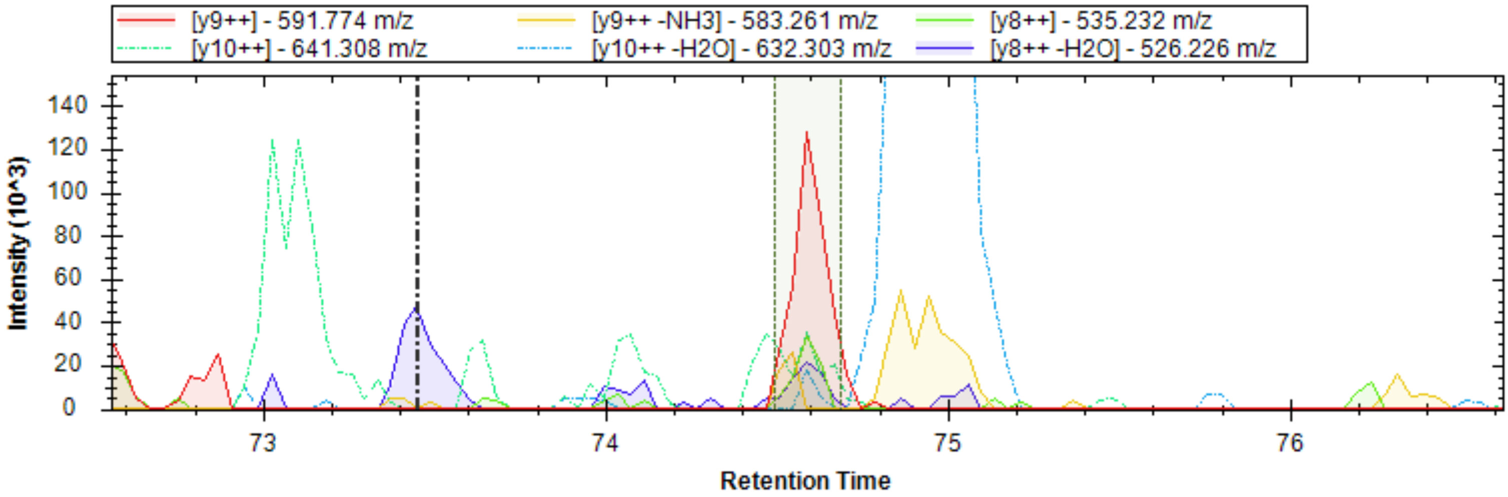

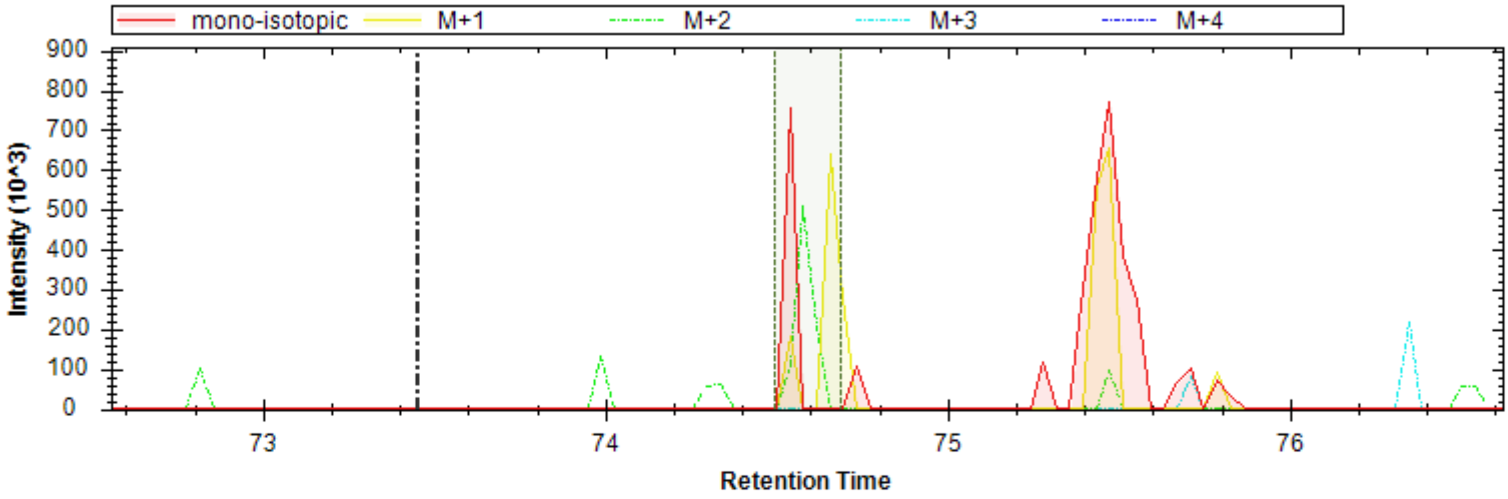

Q9CR81 – _[+C2+H2+O]M[+O]ANHLVKPDS[+H+O3+P]R_.3

Fig4_MP-DIA-barrel-cortex-p15-2_MHRM.htrms

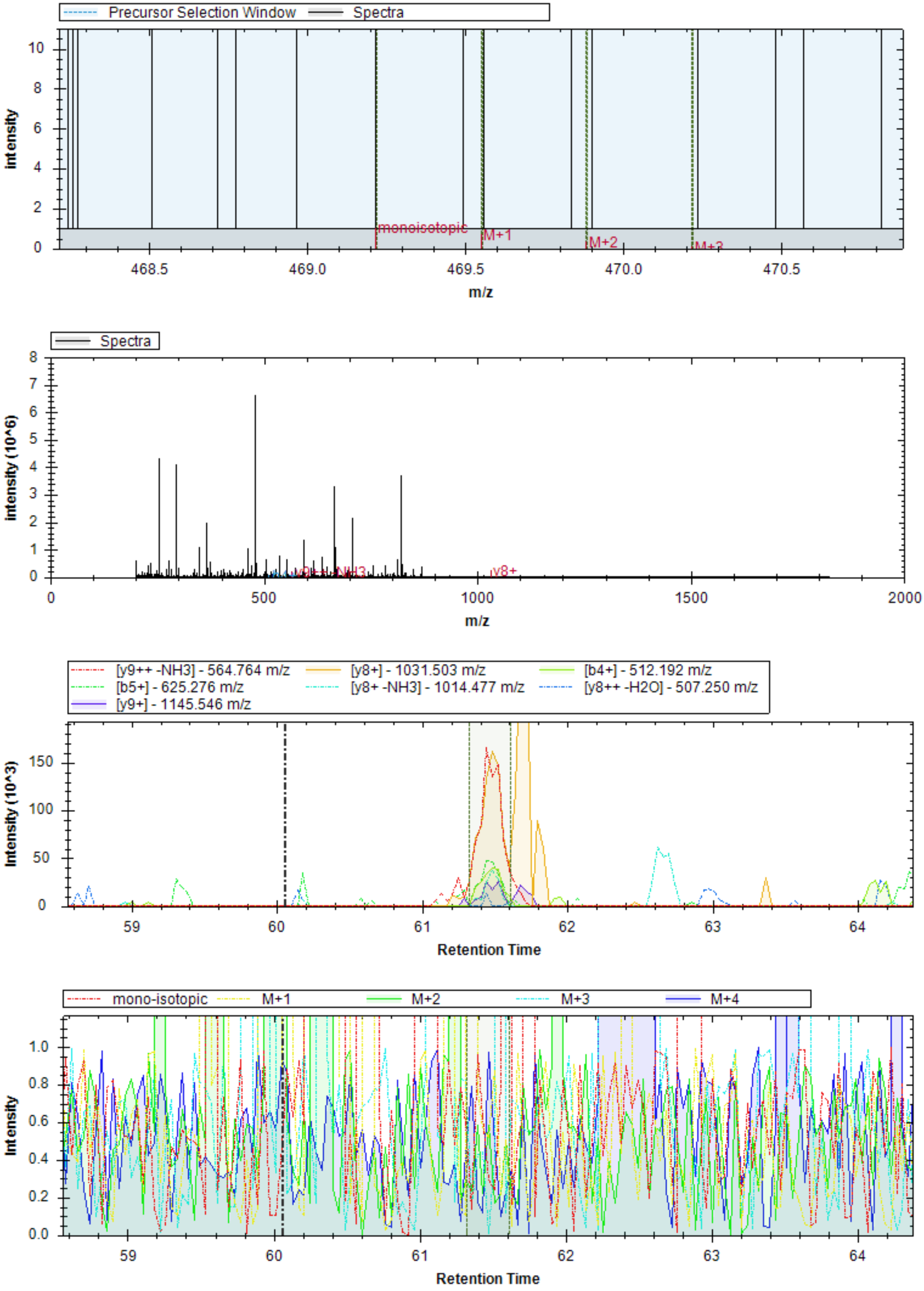

P26645 – _LSGFS[+H+O3+P]FKK_.2
Fig4_MP-DIA-barrel-cortex-p15-2_MHRM.htrms

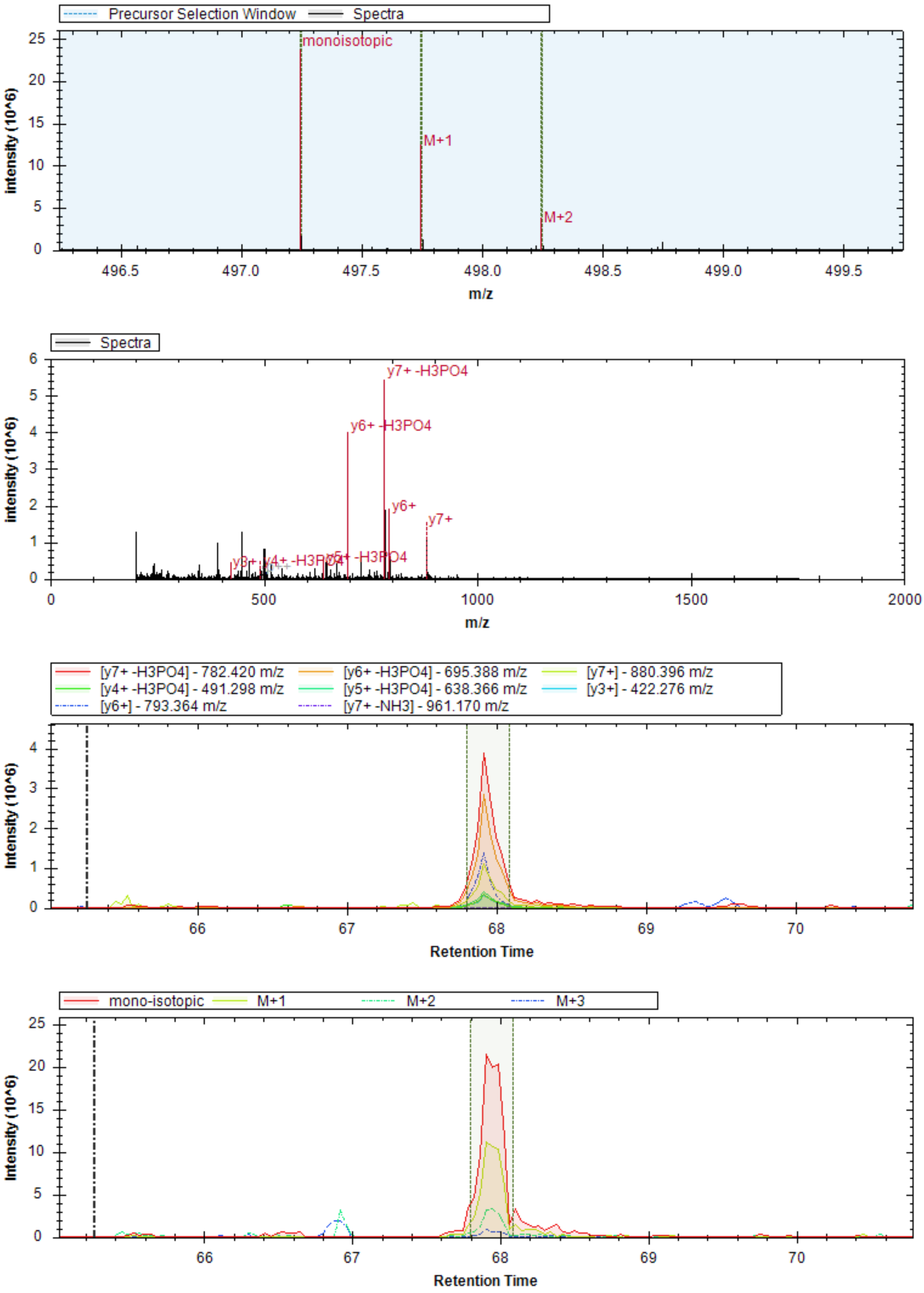

P28667 – _LSGLS[+H+O3+P]FKR_.2
Fig4_MP-DIA-barrel-cortex-p15-2_MHRM.htrms

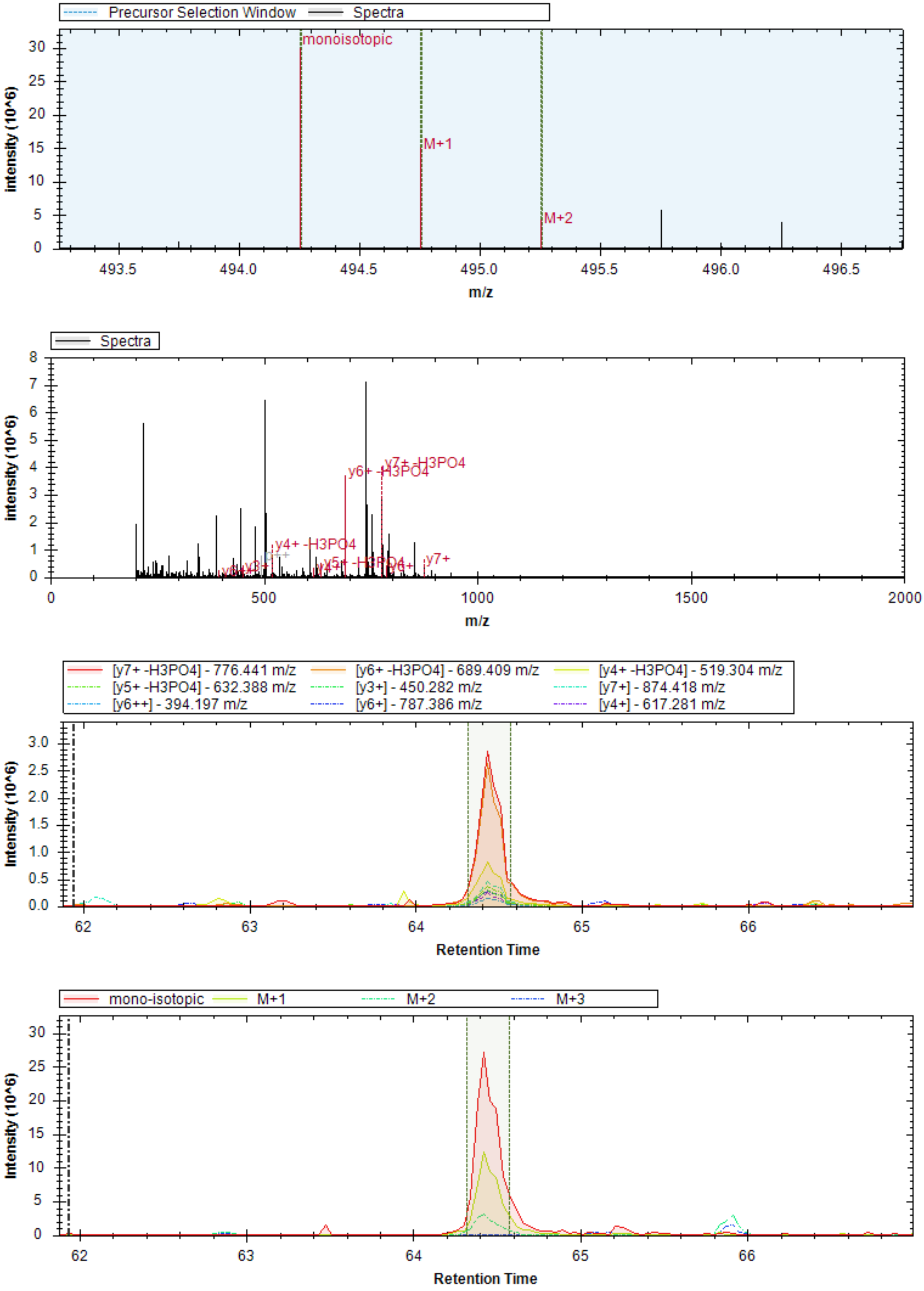

P28738 – _PIRPGHYPAS[+H+O3+P]SPTAVHAVR_.4
Fig4_MP-DIA-barrel-cortex-p15-2_MHRM.htrms

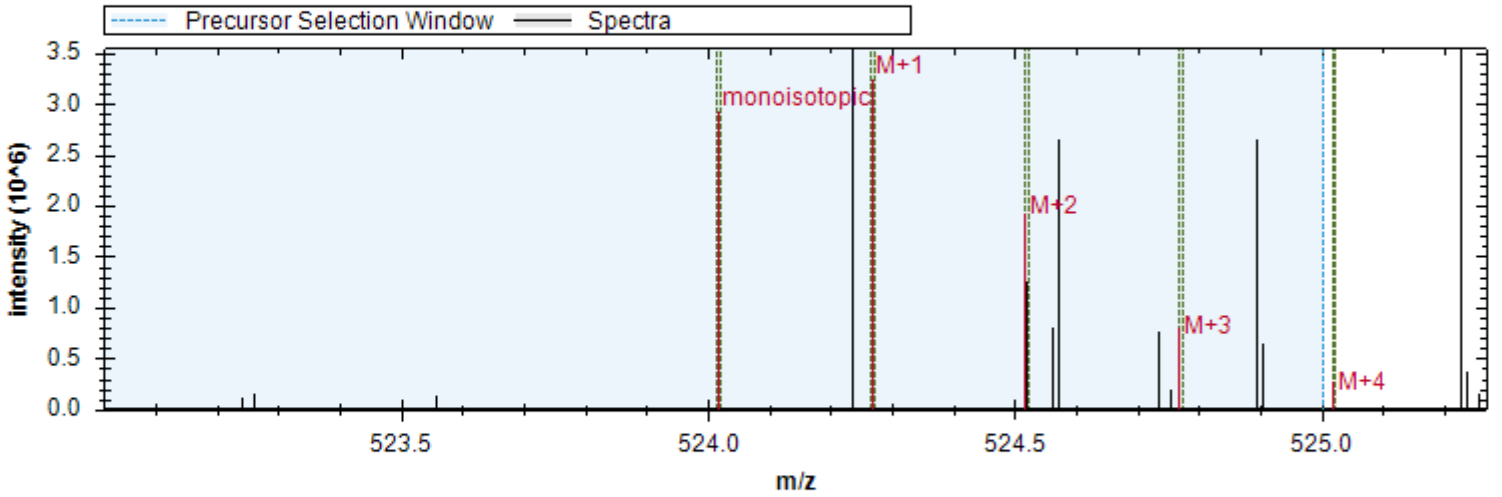

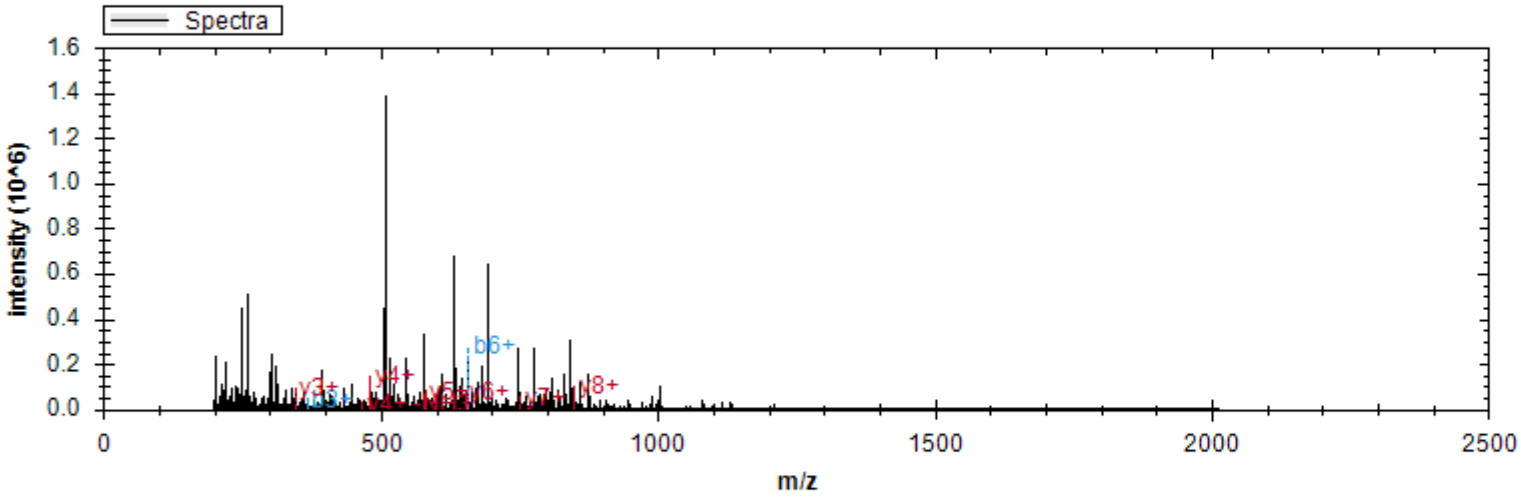

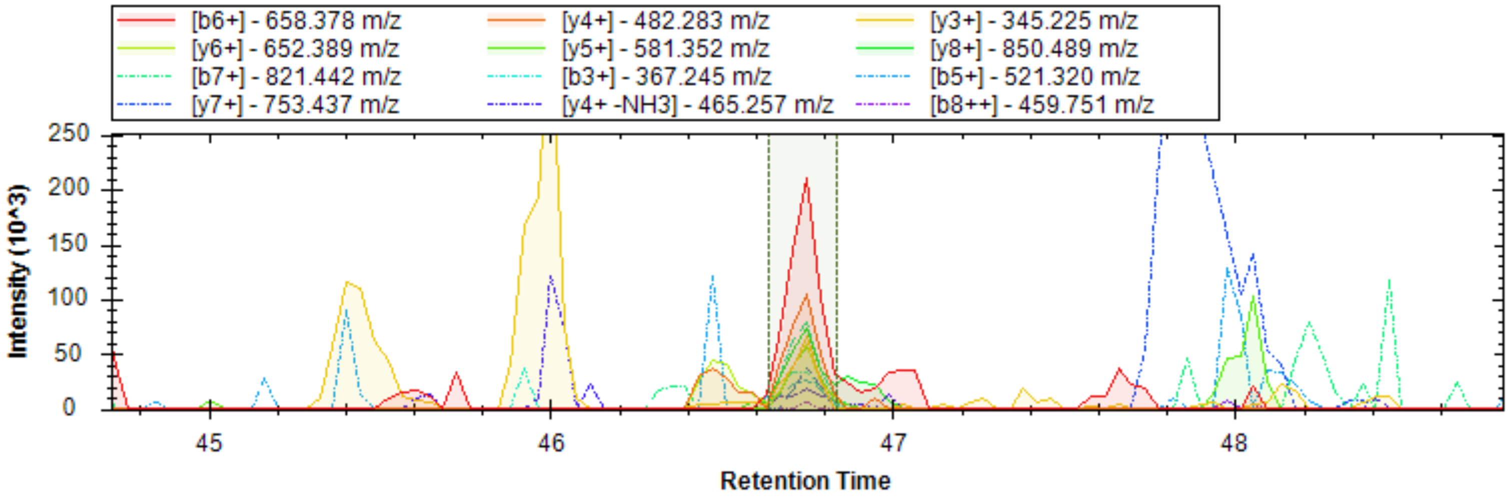

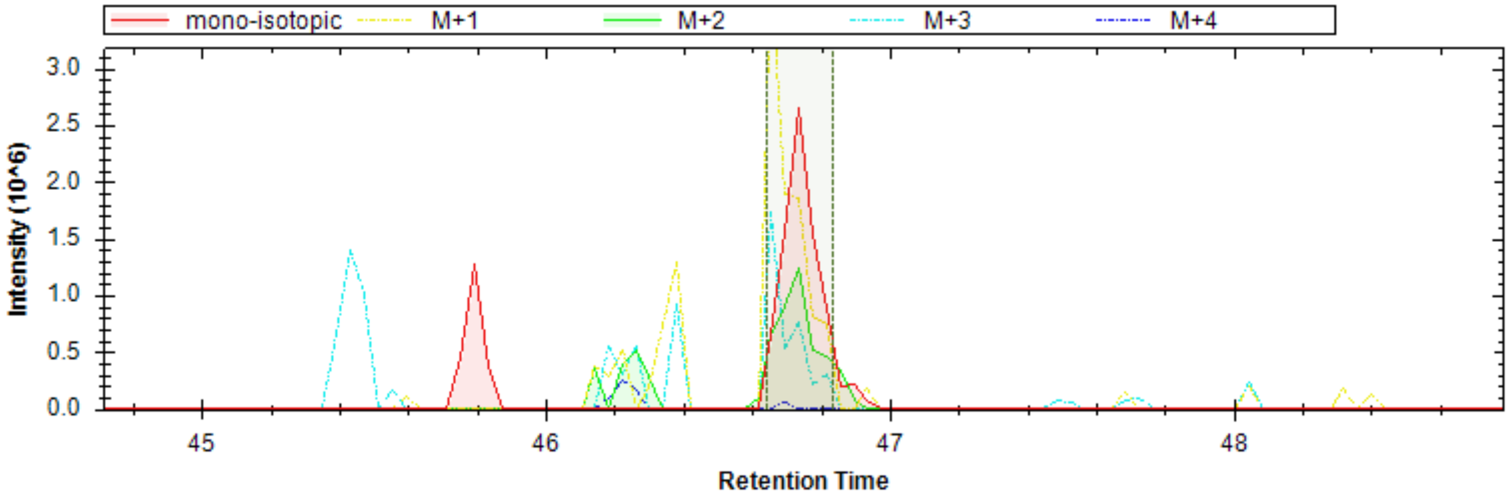

P28738 – _PIRPGHYPASS[+H+O3+P]PTAVHAVR_.4
Fig4_MP-DIA-barrel-cortex-p15-2_MHRM.htrms

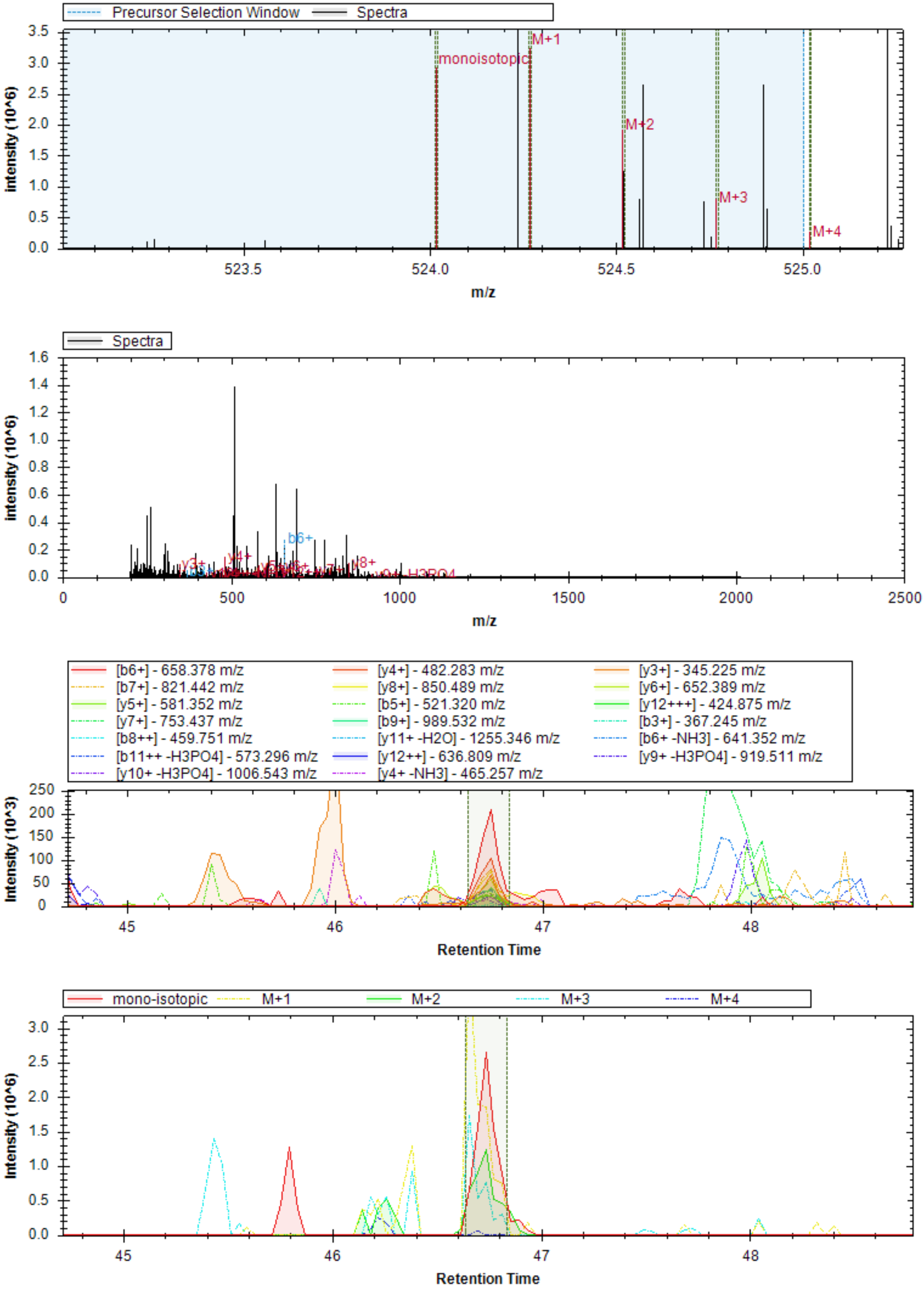

P62996 – _RPHT[+H+O3+P]PTPGIYMGR_.3
Fig4_MP-DIA-barrel-cortex-p15-2_MHRM.htrms

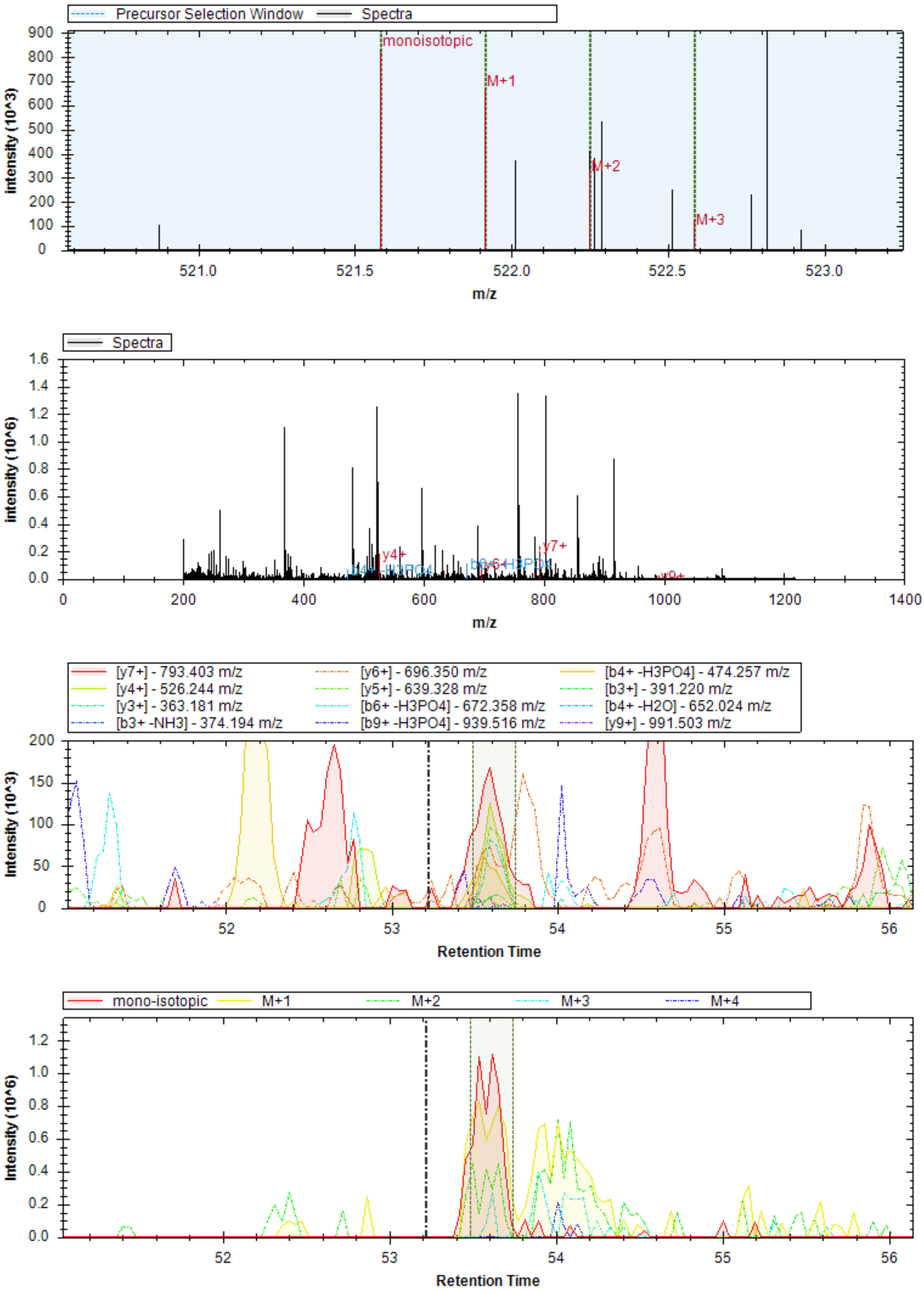

Q8C8R3-7 – _[+C2+H2+O]AHAAAS[+H+O3+P]IKK_.2
Fig4_MP-DIA-barrel-cortex-p15-2_MHRM.htrms

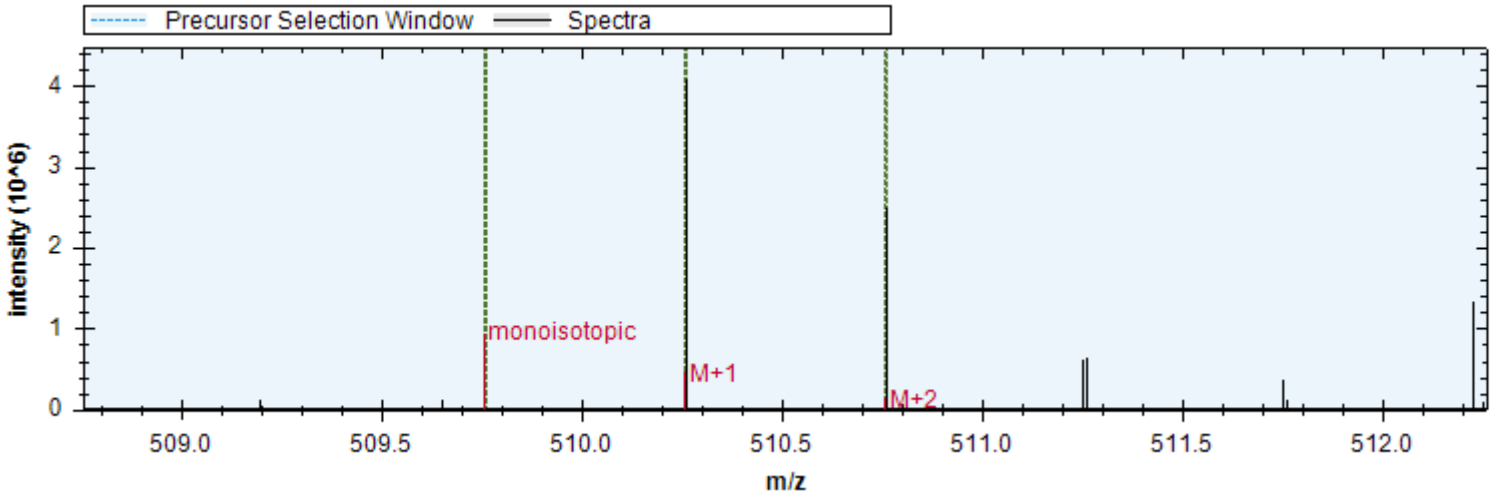

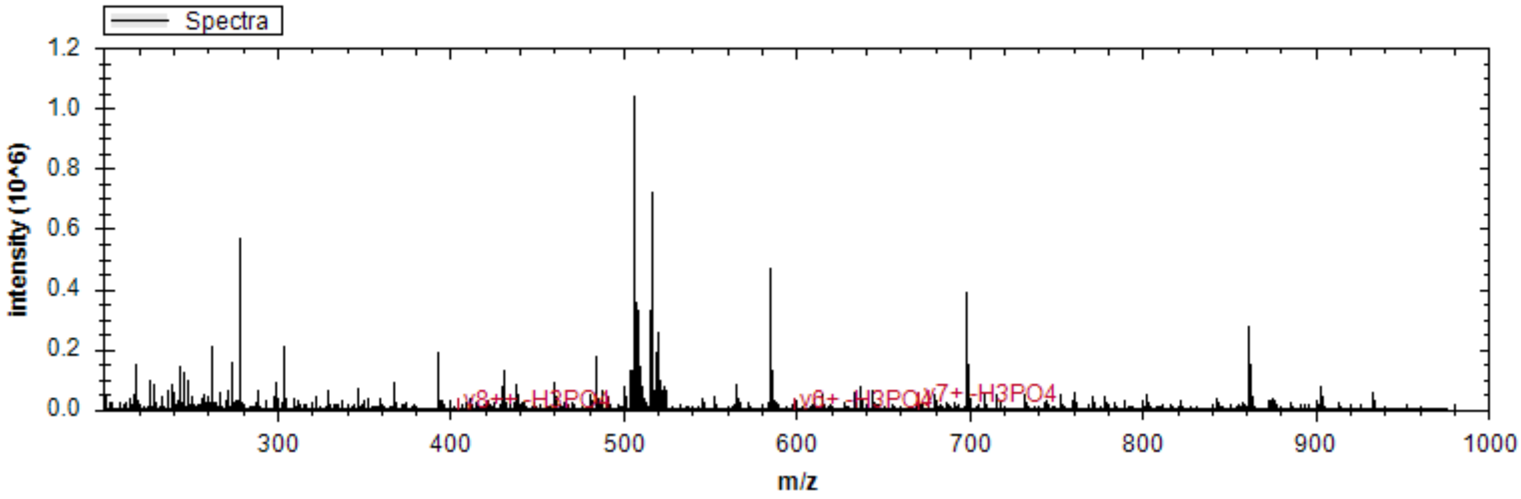

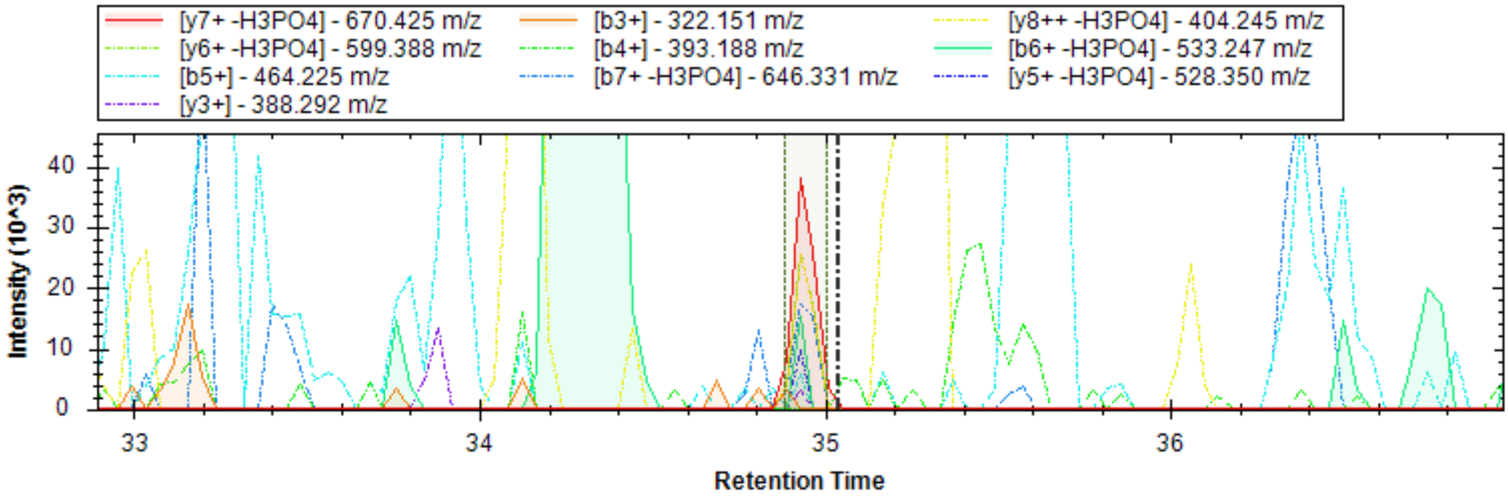

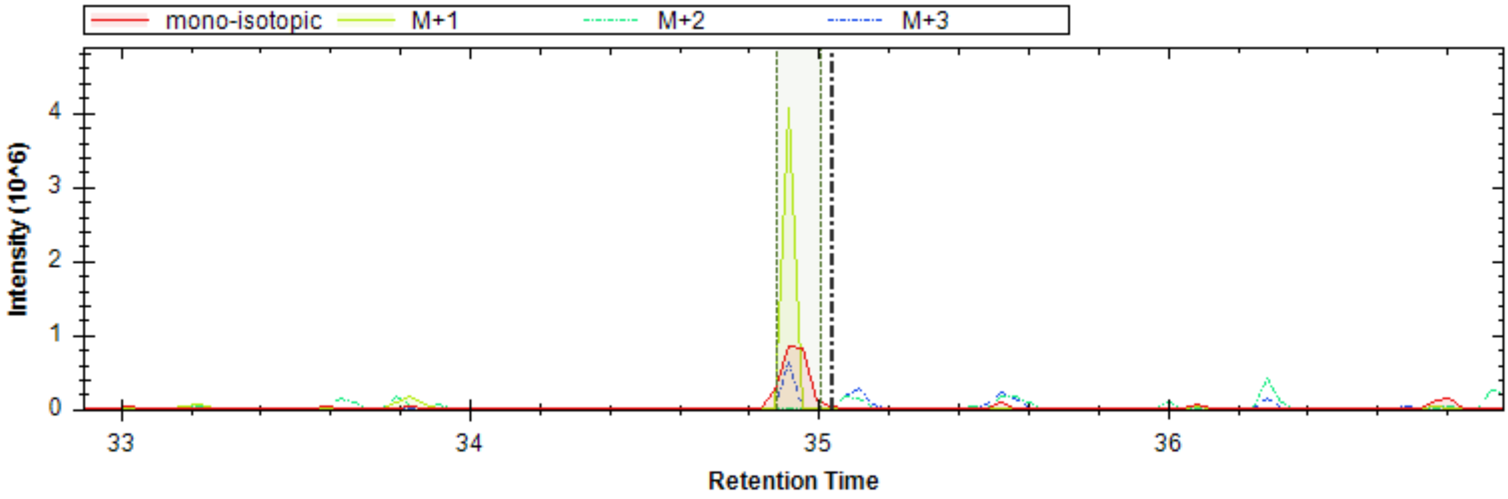

Q8CDY7 – _LVQALC[+C2+H3+N+O]EQIHT[+H+O3+P]R_.3

Fig4_MP-DIA-barrel-cortex-p15-2_MHRM.htrms

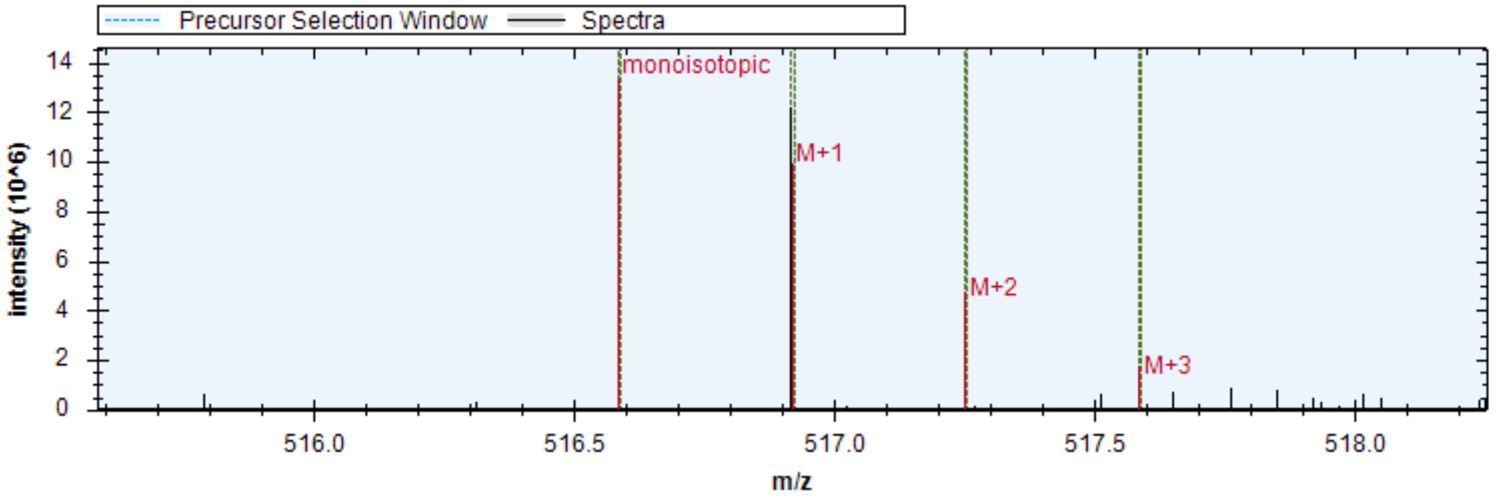

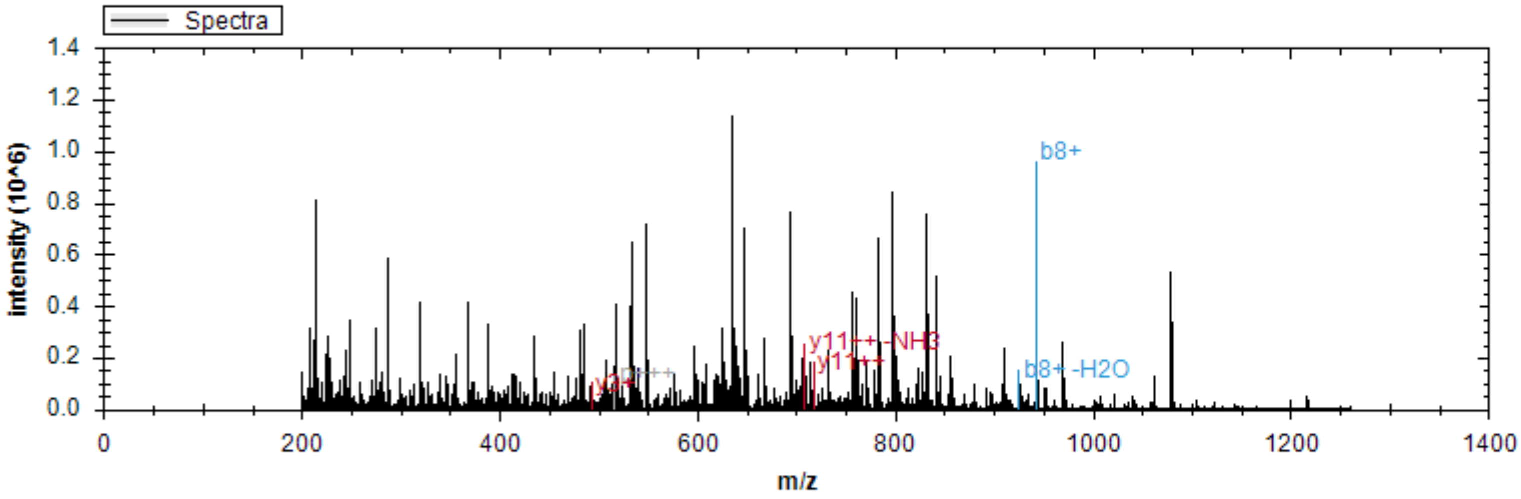

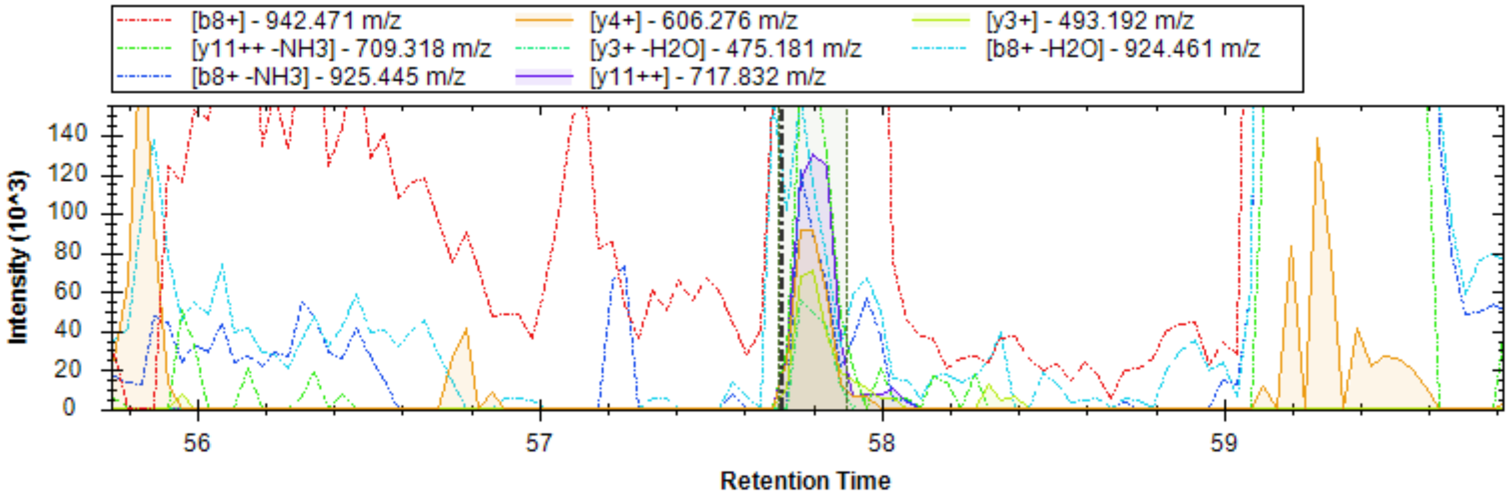

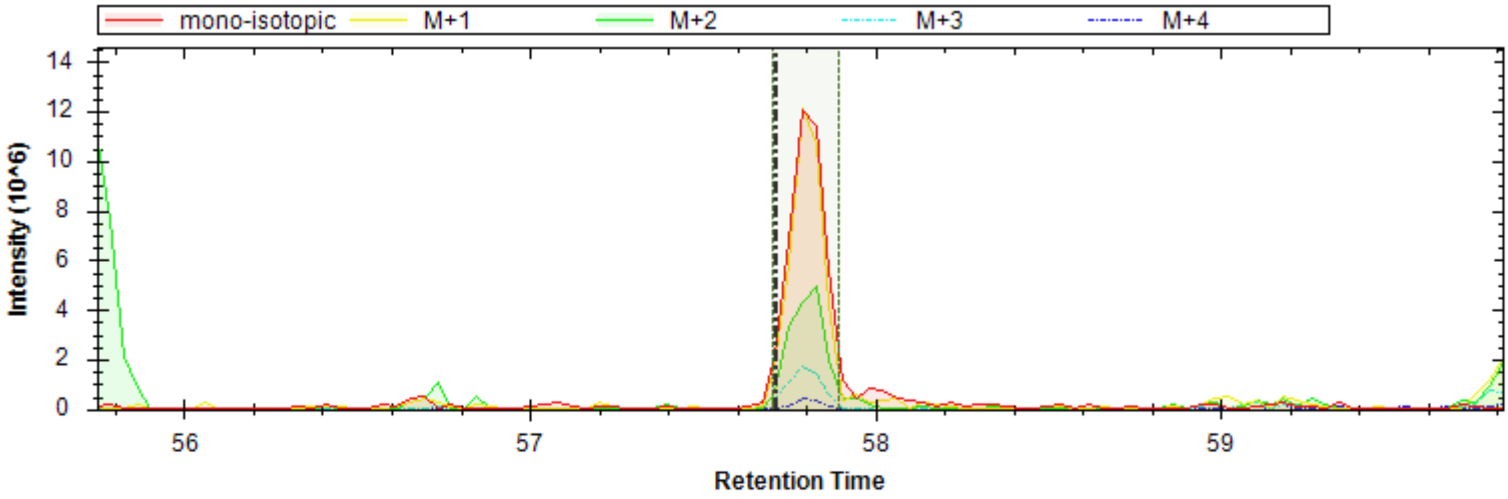

Q9QYC0 – _AAVVT[+H+O3+P]SPPPTTAPHK_.3
Fig4_MP-DIA-barrel-cortex-p15-2_MHRM.htrms

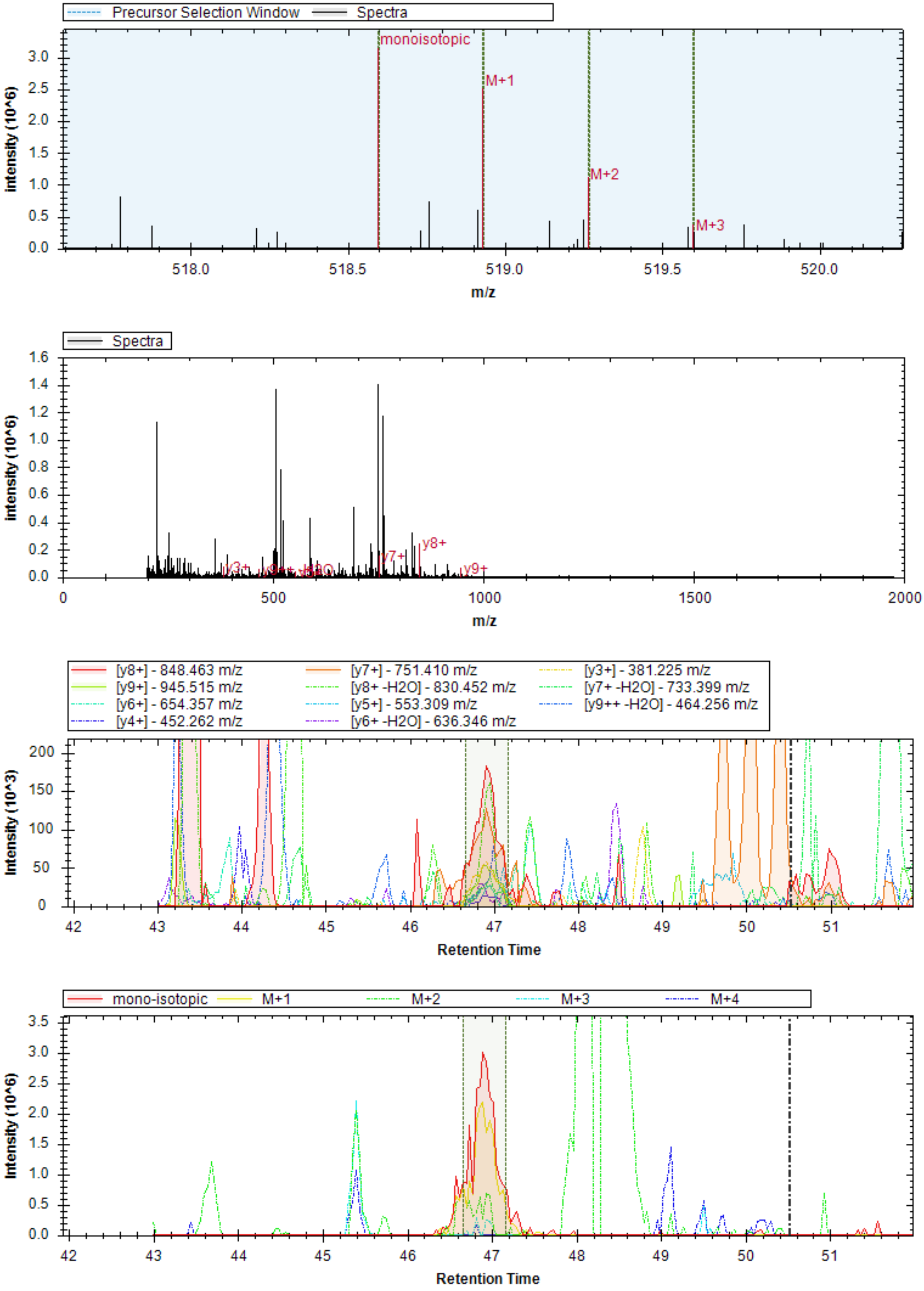

Q9QYC0 – _AAVVTS[+H+O3+P]PPPTTAPHK_.3
Fig4_MP-DIA-barrel-cortex-p15-2_MHRM.htrms

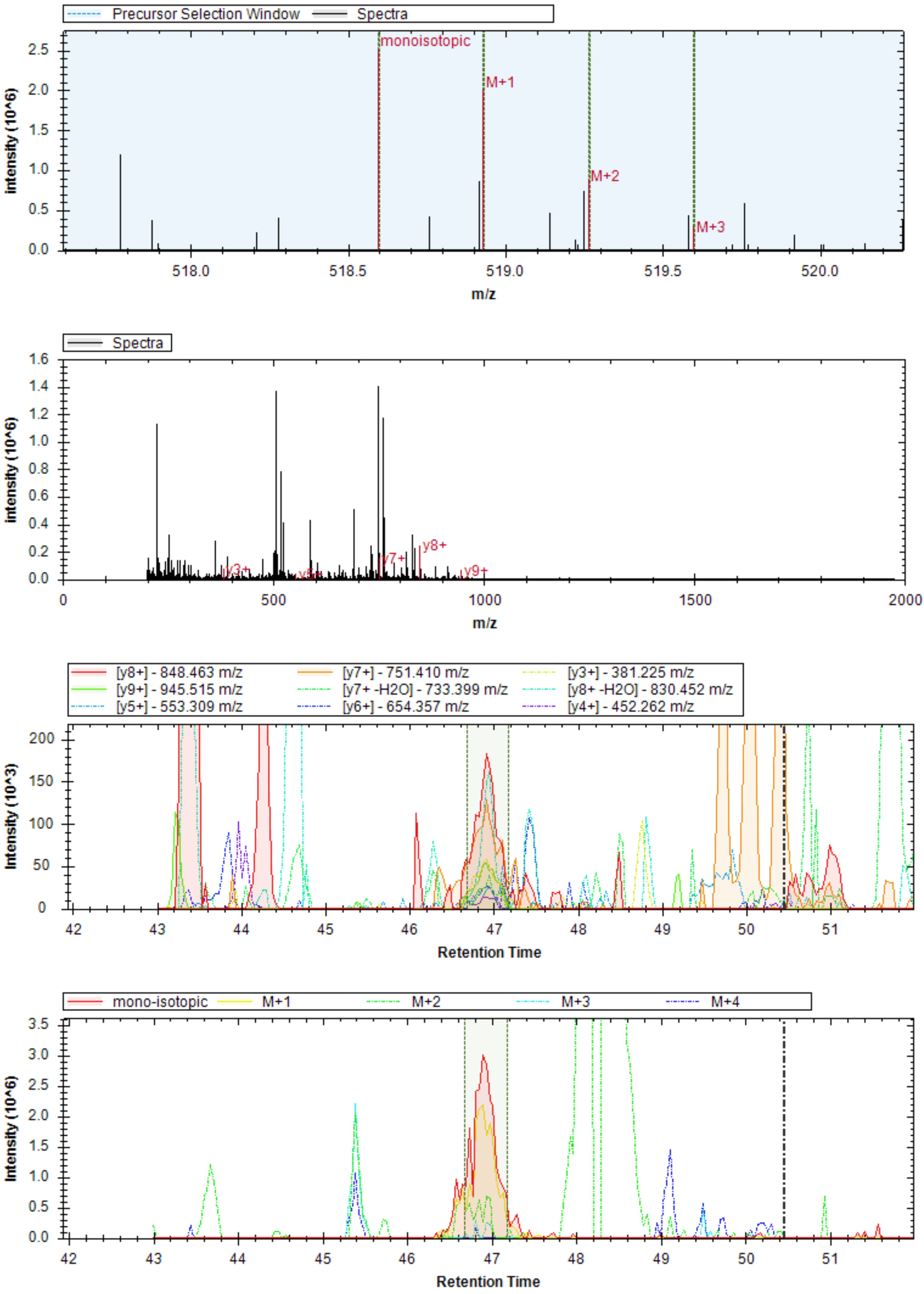

P10637 – _VAVVRT[+H+O3+P]PPKSPSASK_.3

Fig4_MP-DIA-barrel-cortex-p15-2_MHRM.htrms

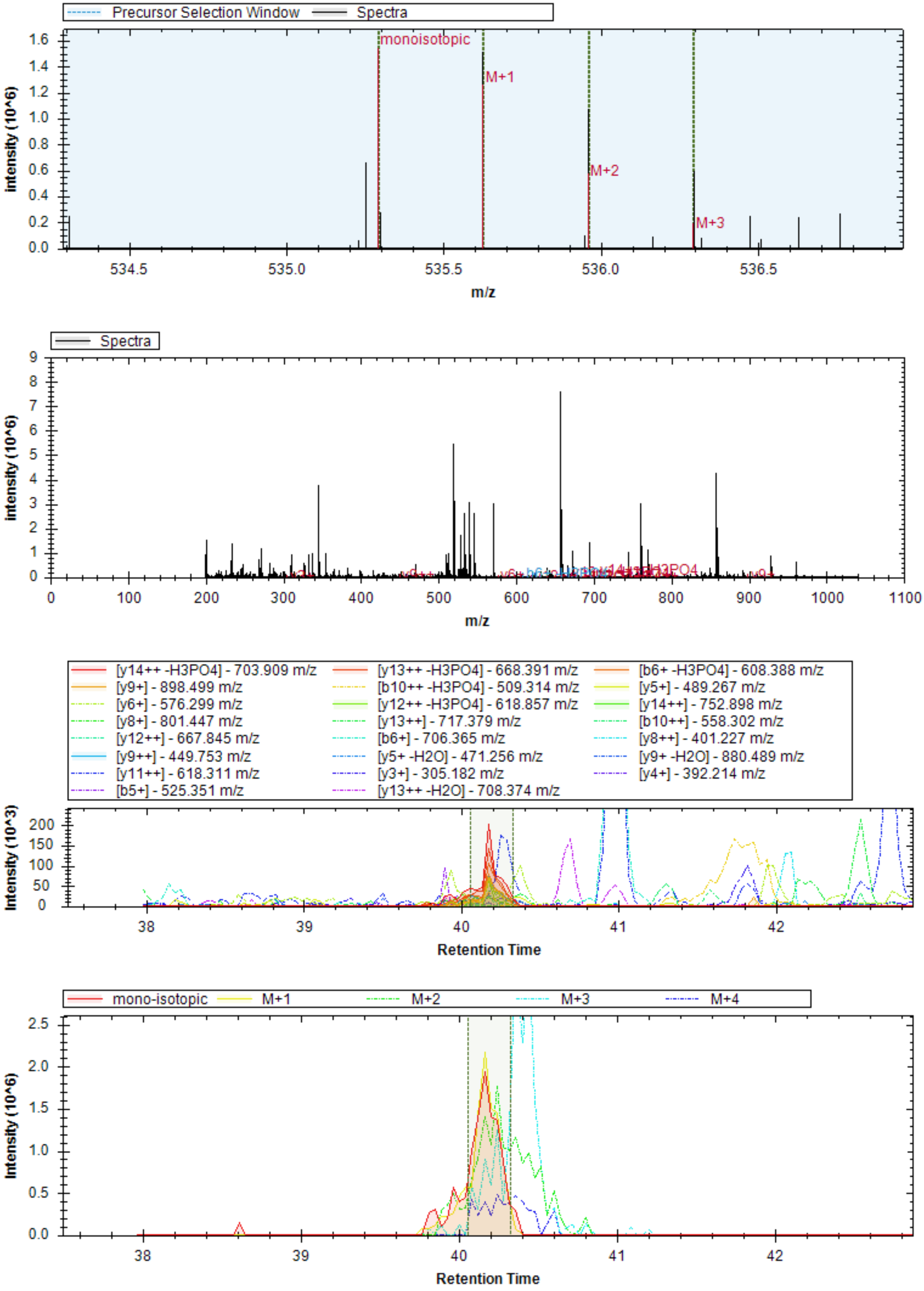

Q7TQD2 – _AANKT[+H+O3+P]PPKSPGDPAR_.3
Fig4_MP-DIA-barrel-cortex-p15-2_MHRM.htrms

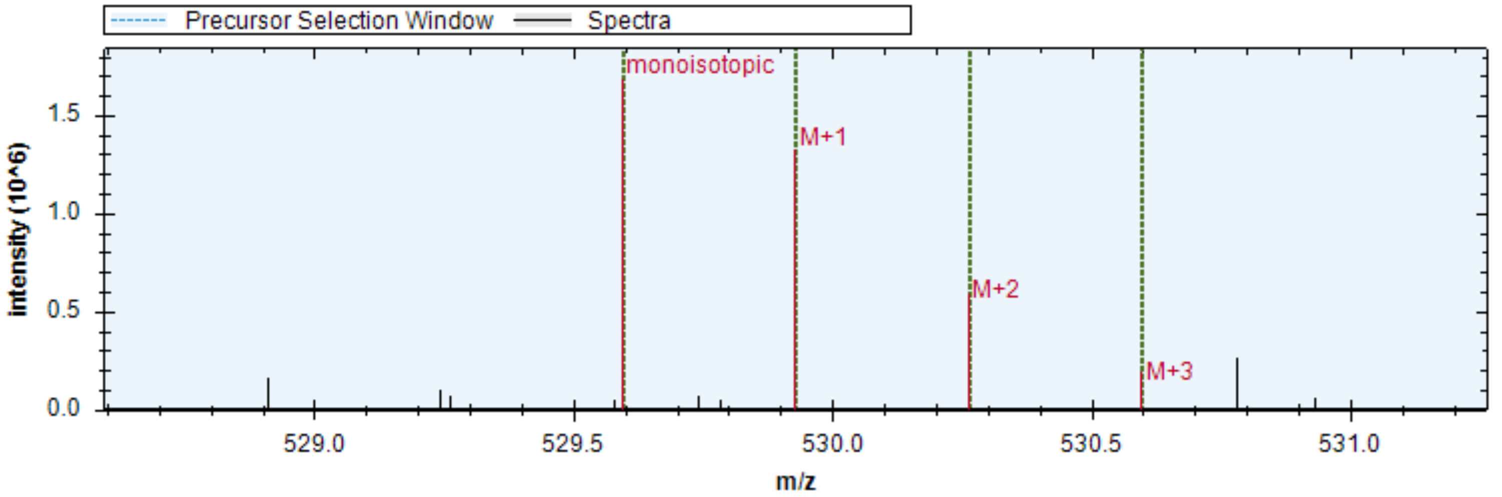

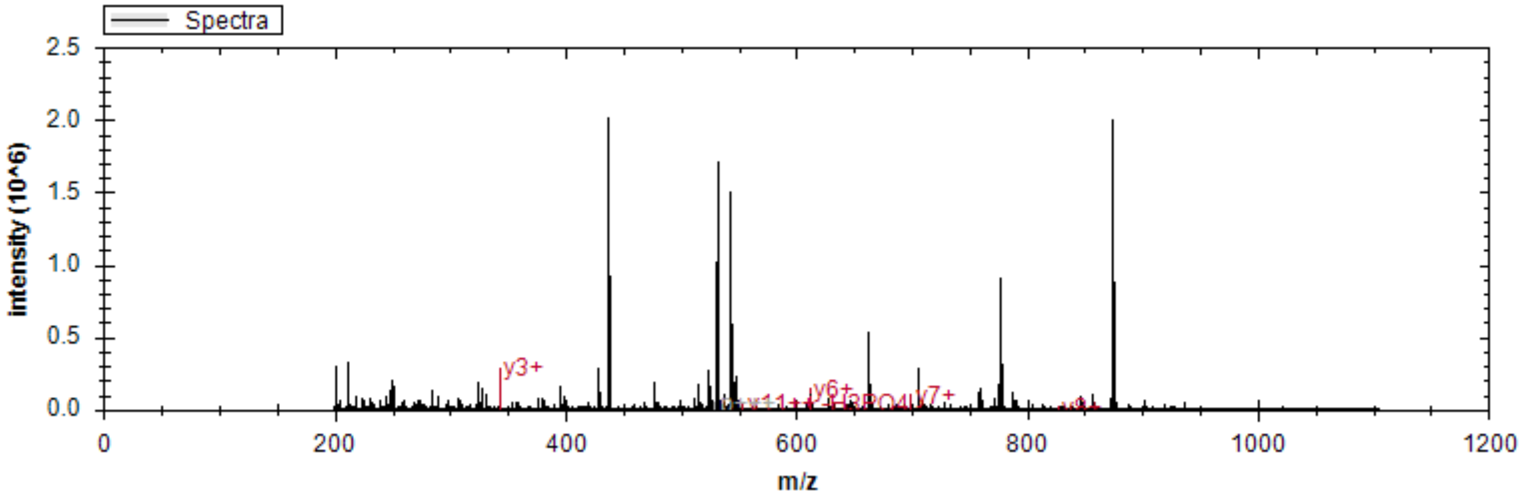

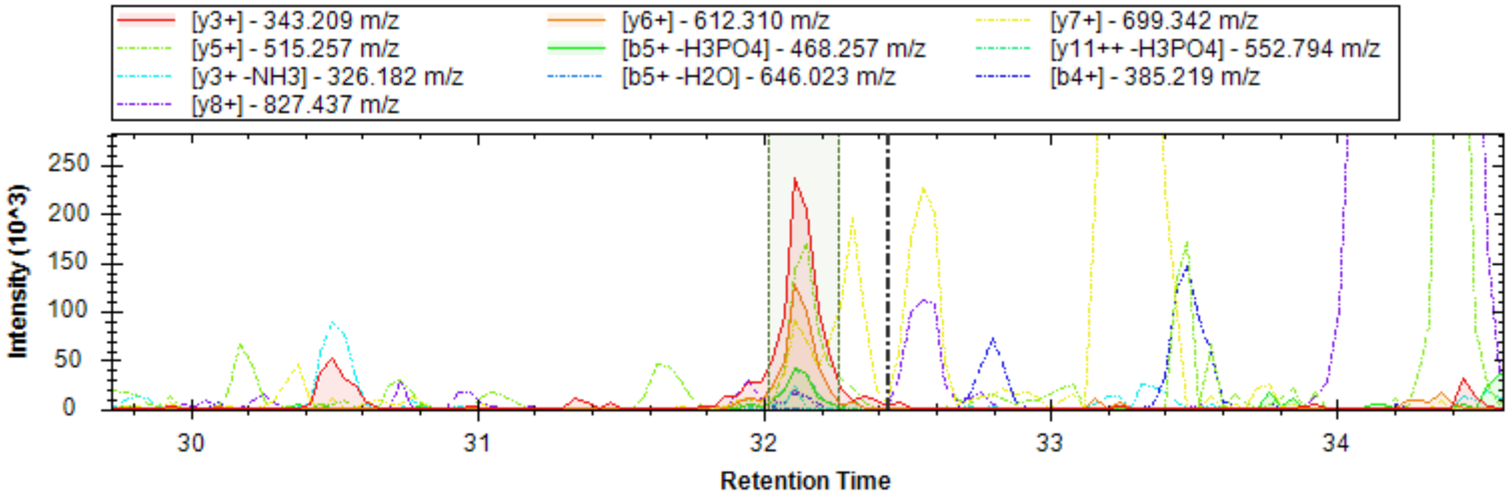

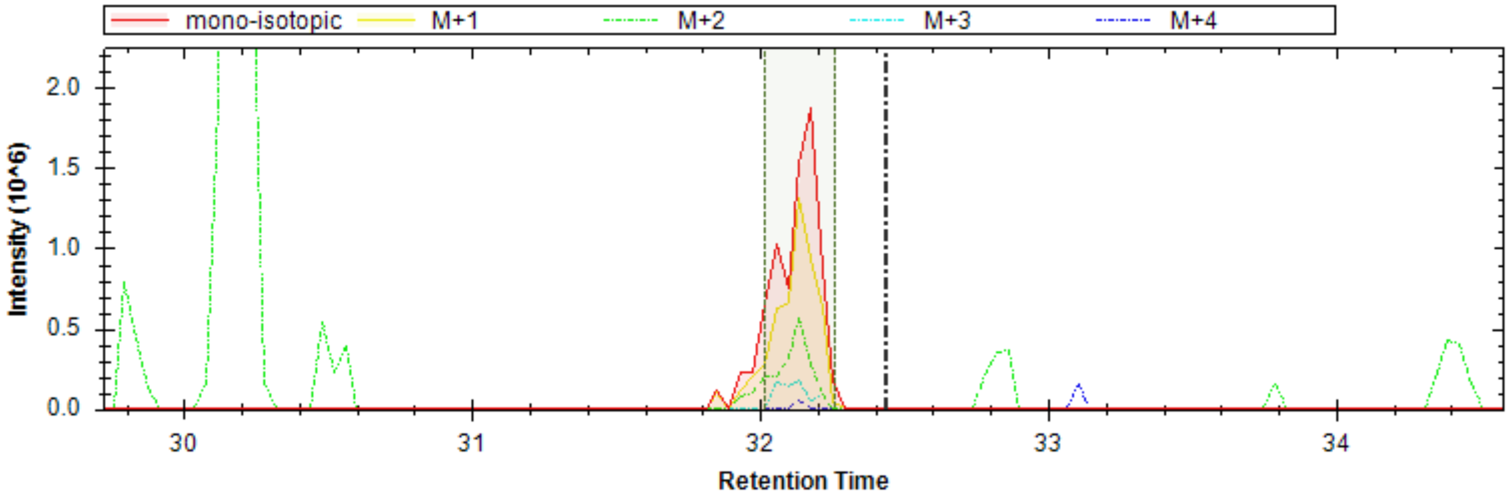

P10637 – _TTPSPKT[+H+O3+P]PPGSGEPPK_.3
Fig4_MP-DIA-barrel-cortex-p15-2_MHRM.htrms

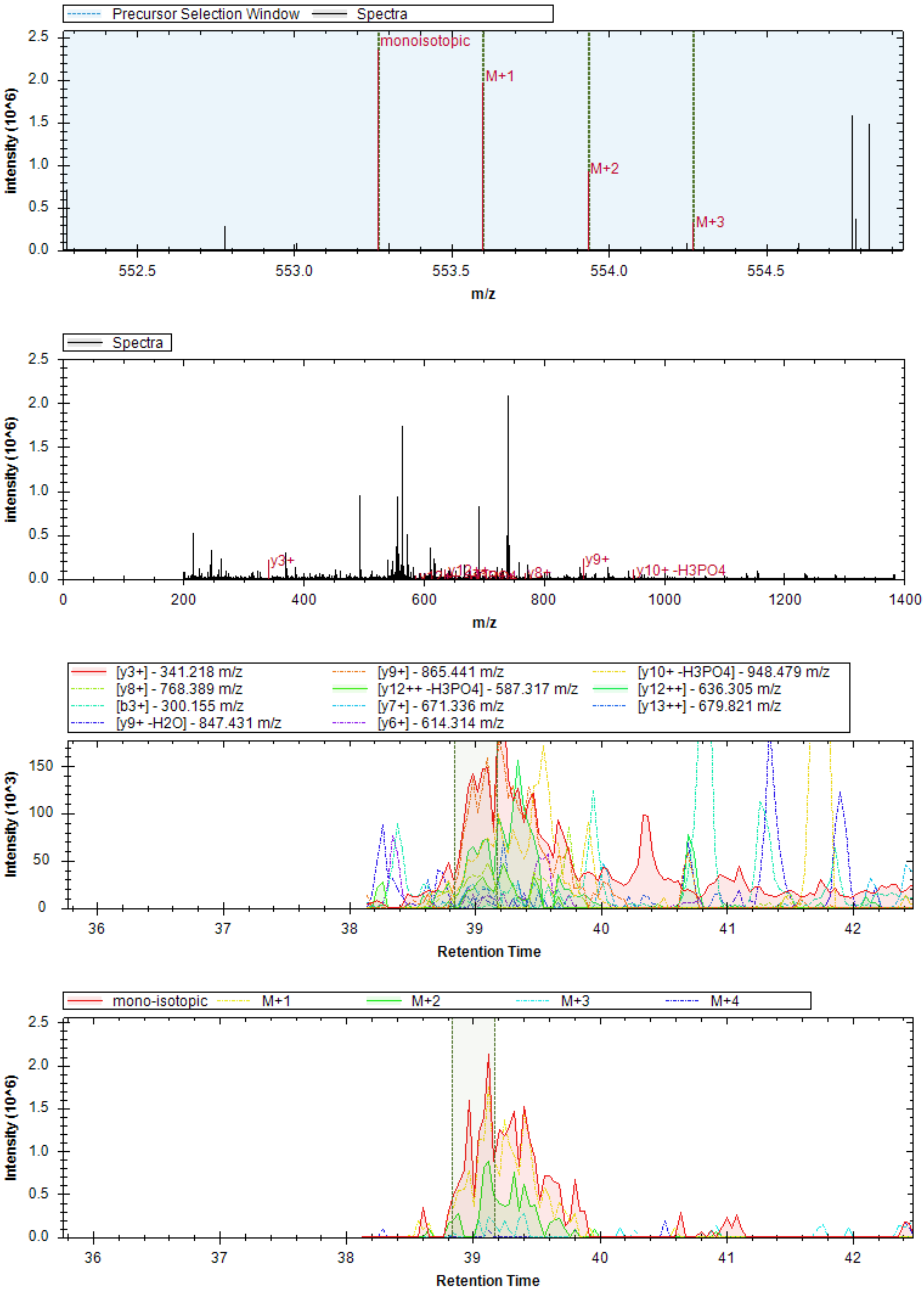

P20357 – _VAIIRT[+H+O3+P]PPKSPATPK_.3
Fig4_MP-DIA-barrel-cortex-p15-2_MHRM.htrms

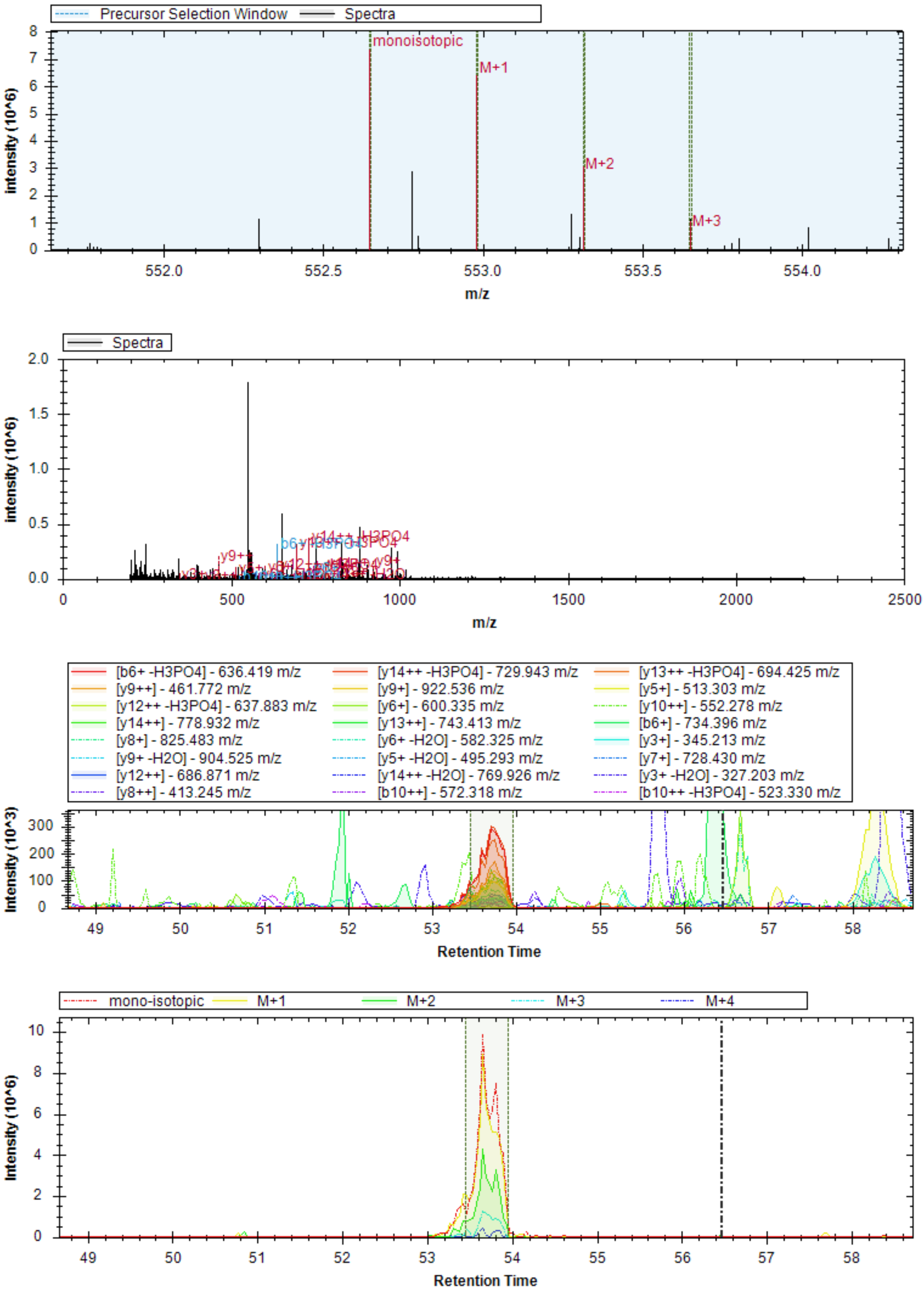

Q9CYT6 – _THTPSPTS[+H+O3+P]PK_.2
Fig4_MP-DIA-barrel-cortex-p15-2_MHRM.htrms

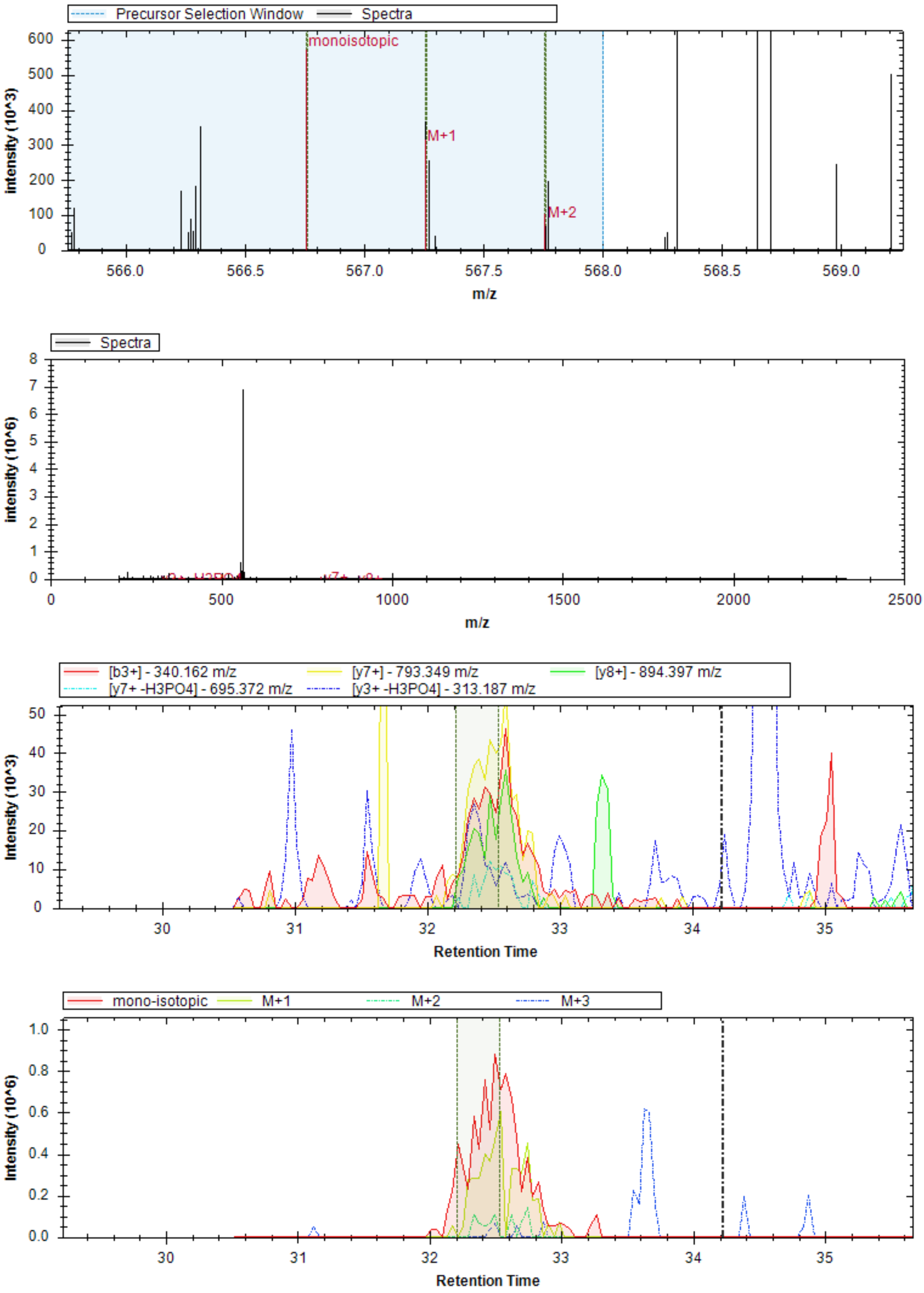

Q9R0K7 – _T[+H+O3+P]SIHNFMAHPEFR_.3
Fig4_MP-DIA-barrel-cortex-p15-2_MHRM.htrms

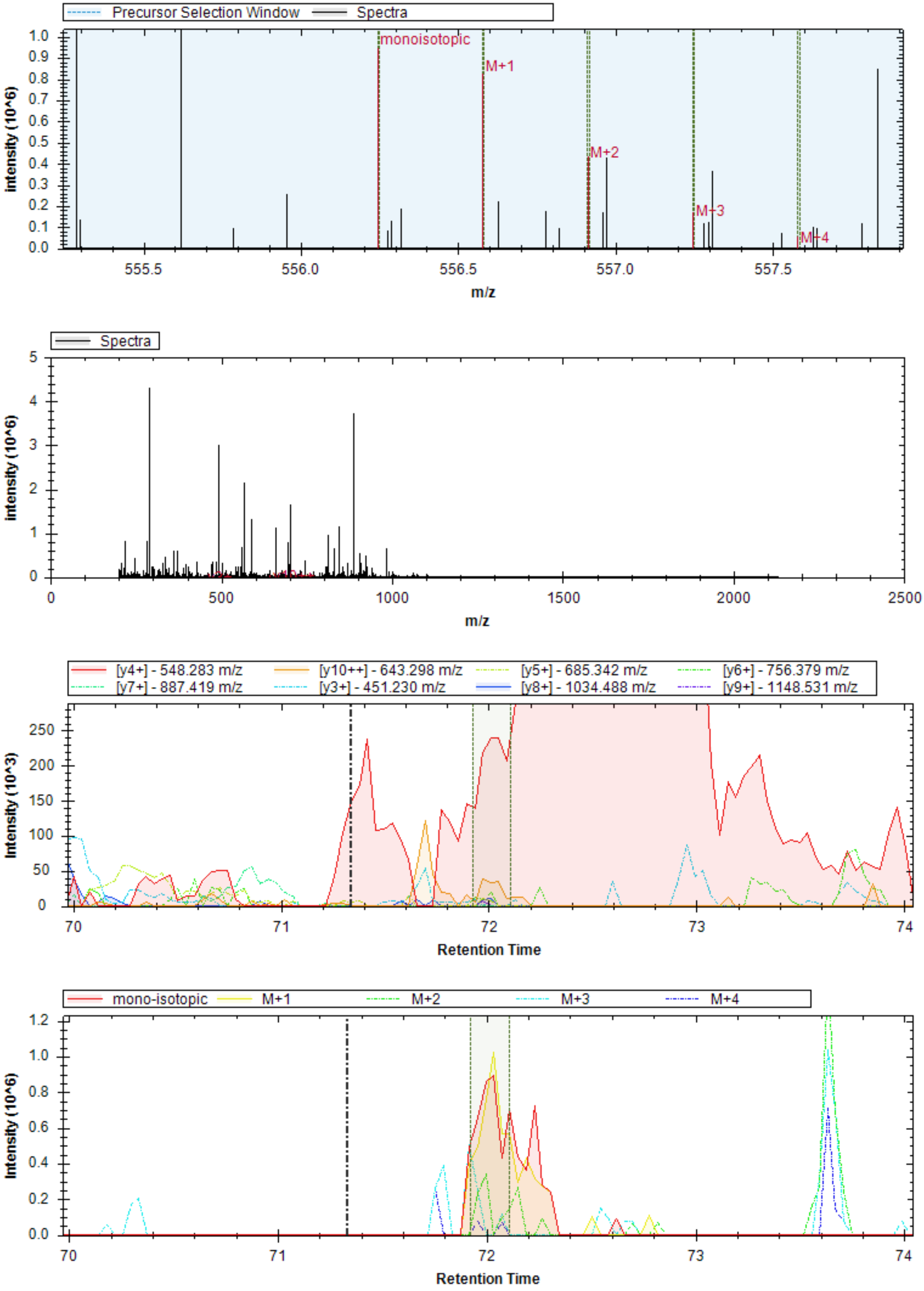

O88935 – _QAS[+H+O3+P]ISGPAPTK_.2
Fig4_MP-DIA-barrel-cortex-p15-2_MHRM.htrms

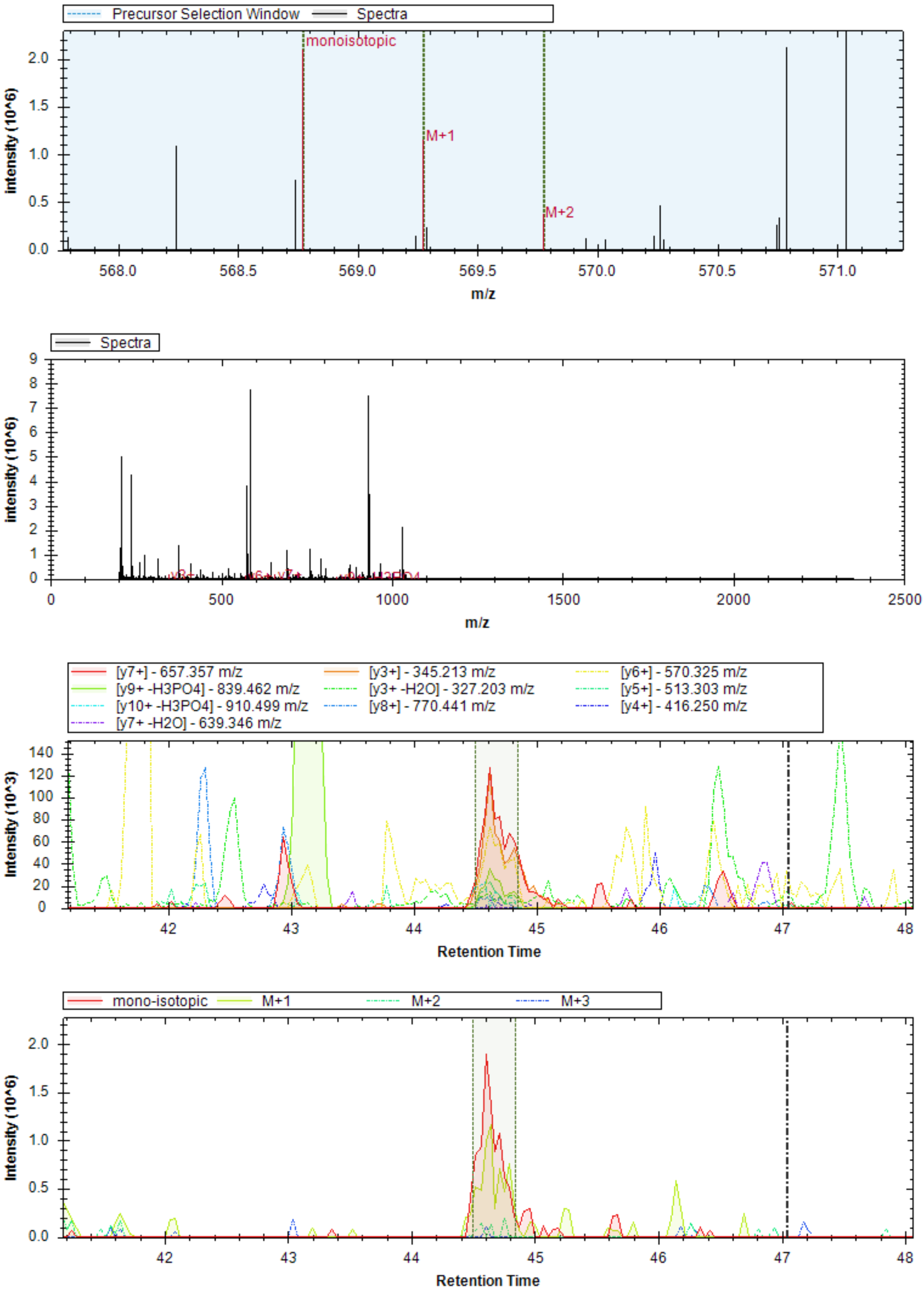

P27546-2 – _T[+H+O3+P]SPSKPSSAPALKPGPK_.3
Fig4_MP-DIA-barrel-cortex-p15-2_MHRM.htrms

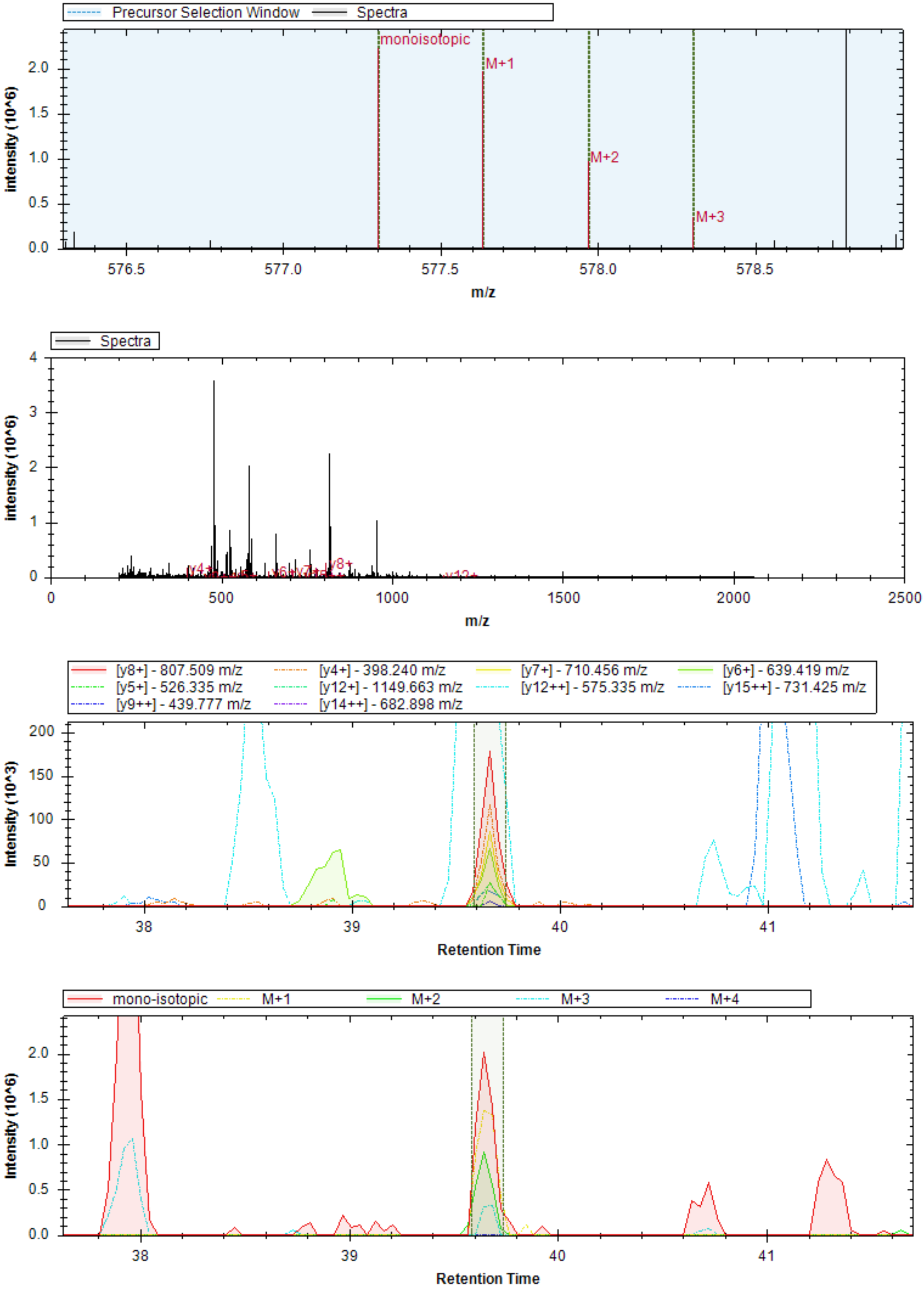

P97792 – _APQS[+H+O3+P]PTLAPAK_.2

Fig4_MP-DIA-barrel-cortex-p15-2_MHRM.htrms

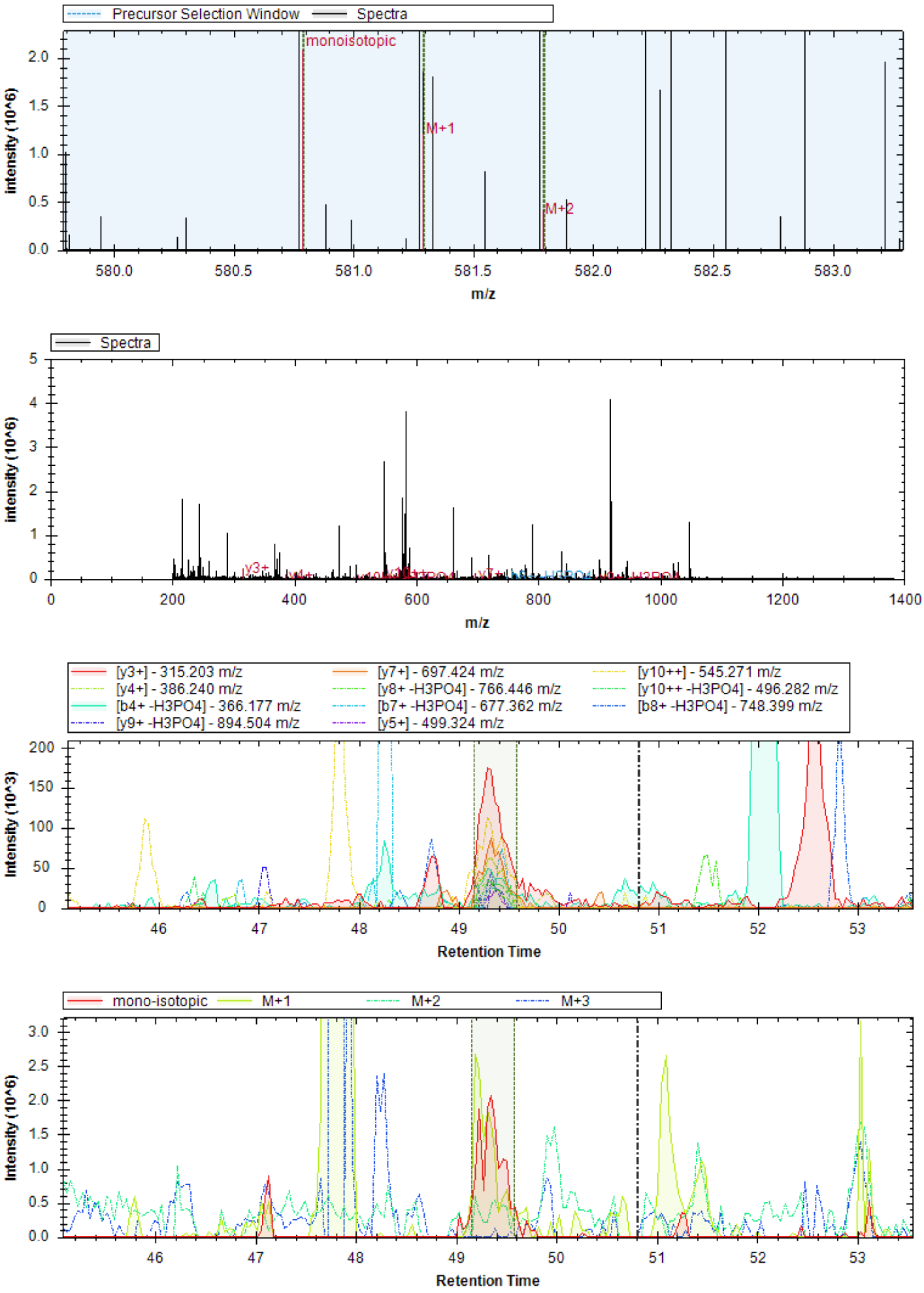

Q64012-2 – _GRLS[+H+O3+P]PVPVPR_.2
Fig4_MP-DIA-barrel-cortex-p15-2_MHRM.htrms

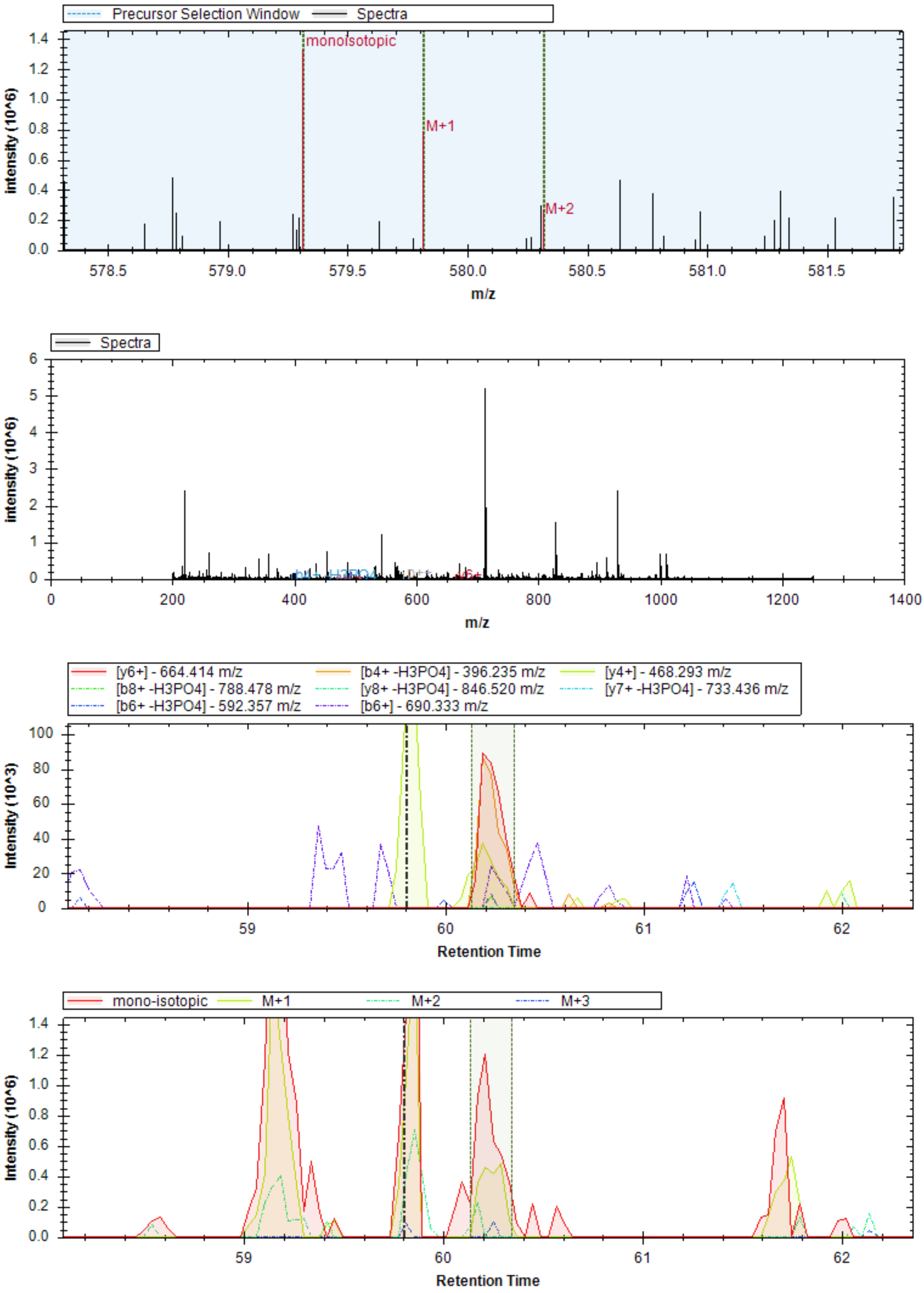

Q8BFU3-4 – _VAS[+H+O3+P]STQVRGASGRELR_.3
Fig4_MP-DIA-barrel-cortex-p15-2_MHRM.htrms

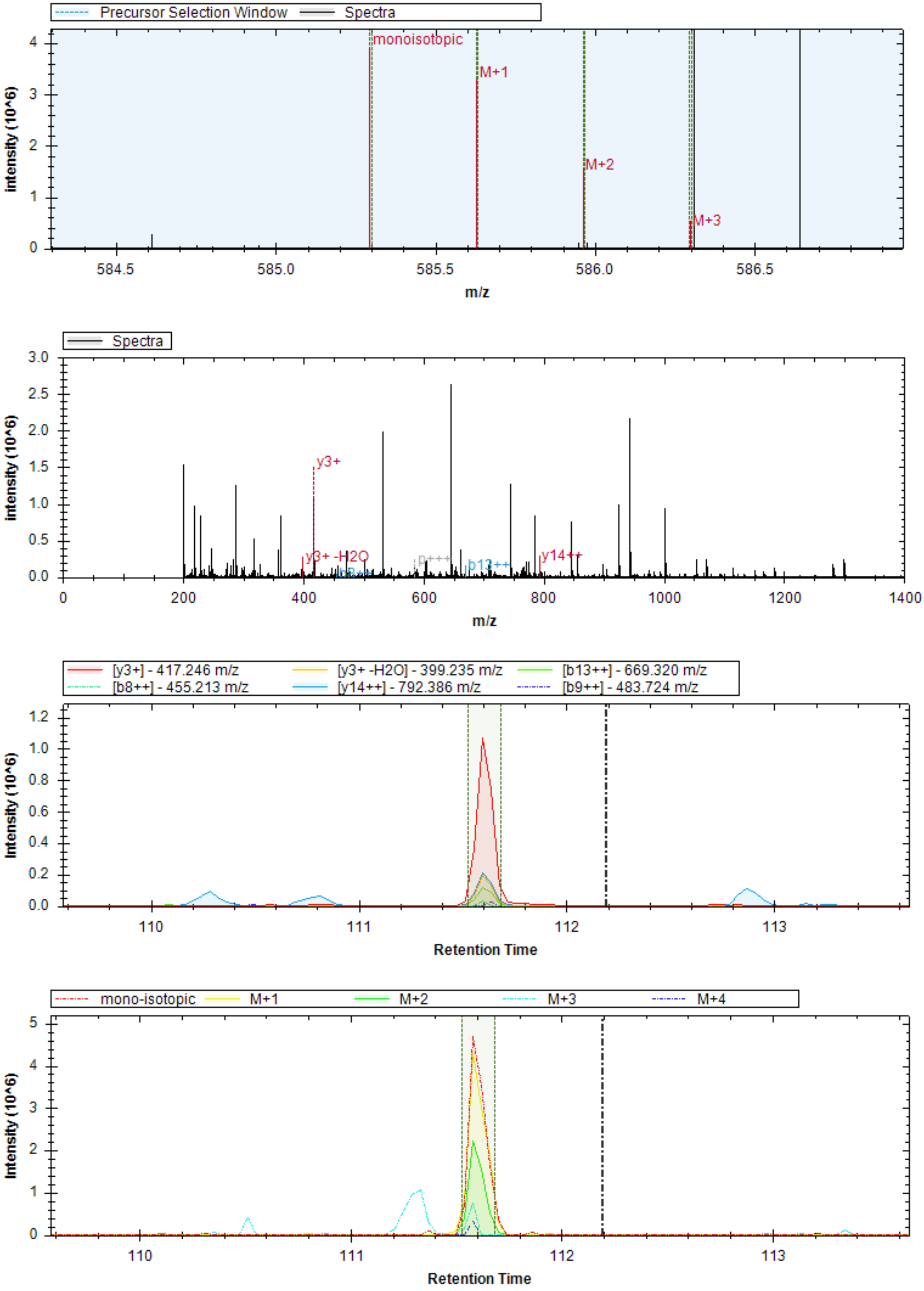

Q9CYT6 – _AQGQIRS[+H+O3+P]PTK_.2

Fig4_MP-DIA-barrel-cortex-p15-2_MHRM.htrms

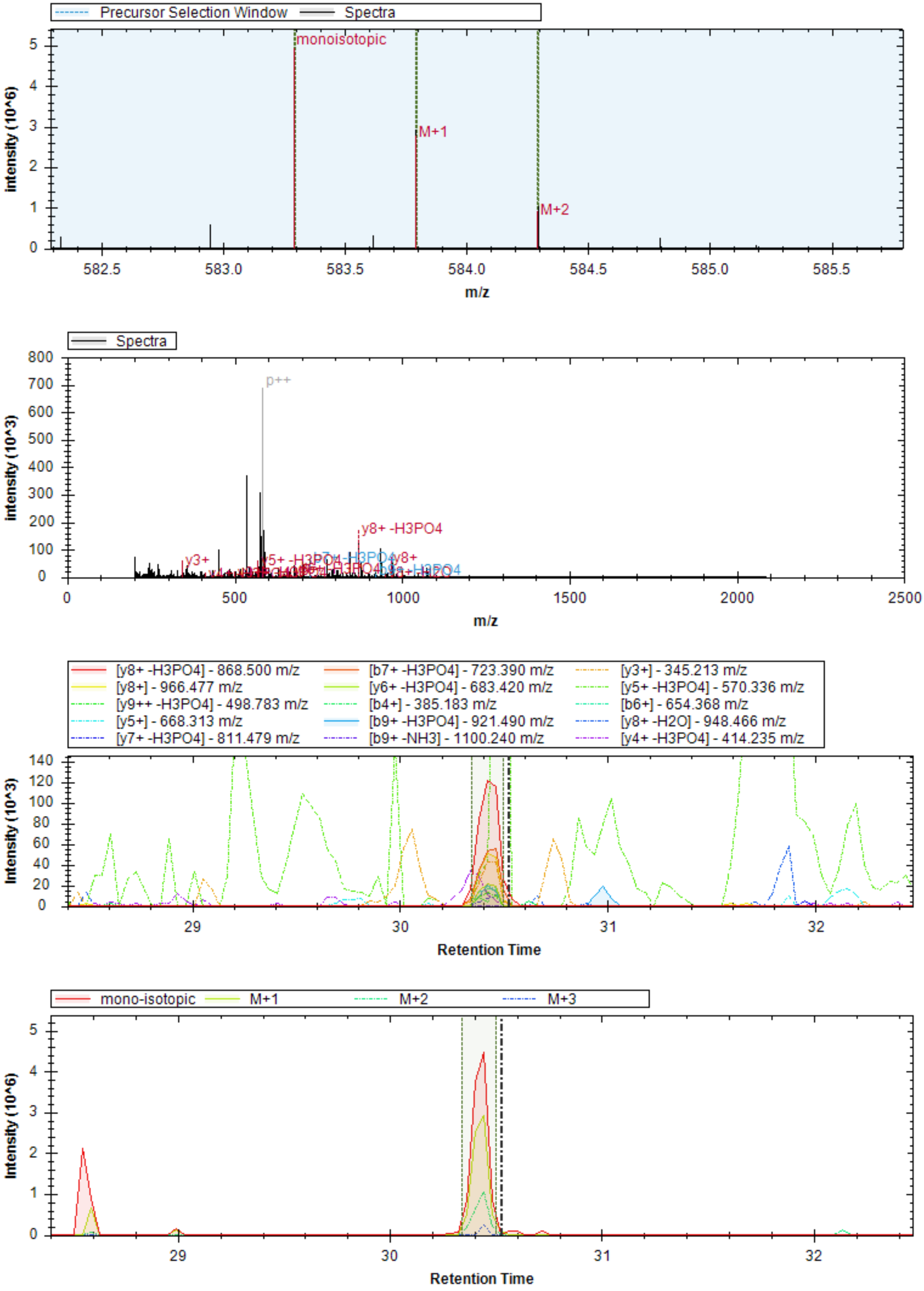

Q9D5Y0 – _ITDT[+H+O3+P]KKTDDLYWR_.3

Fig4_MP-DIA-barrel-cortex-p15-2_MHRM.htrms

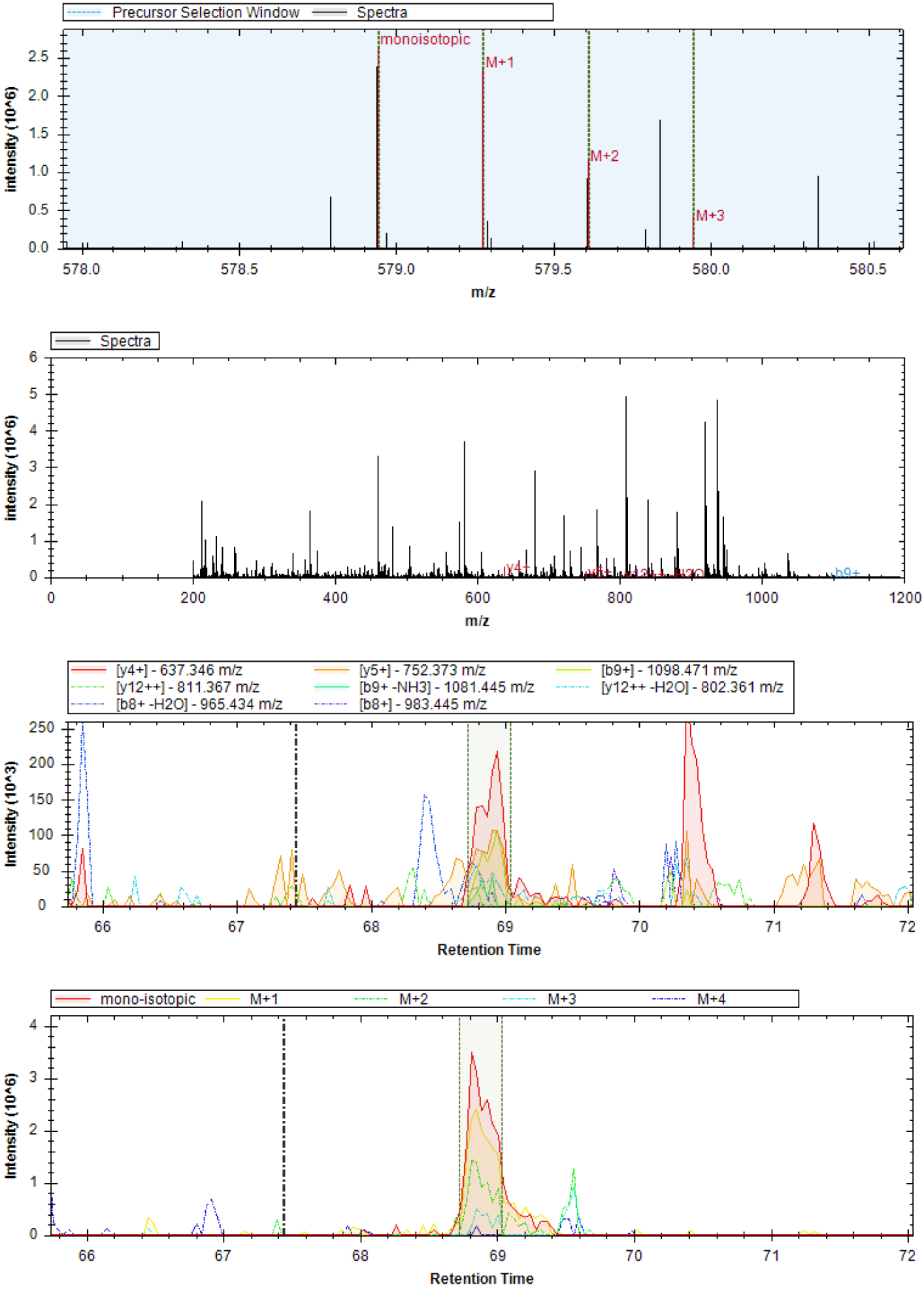

Q9DC63;Q9DC63-2;Q9DC63-3 – _YIDHY[+H+O3+P]AAIK_.2
Fig4_MP-DIA-barrel-cortex-p15-2_MHRM.htrms

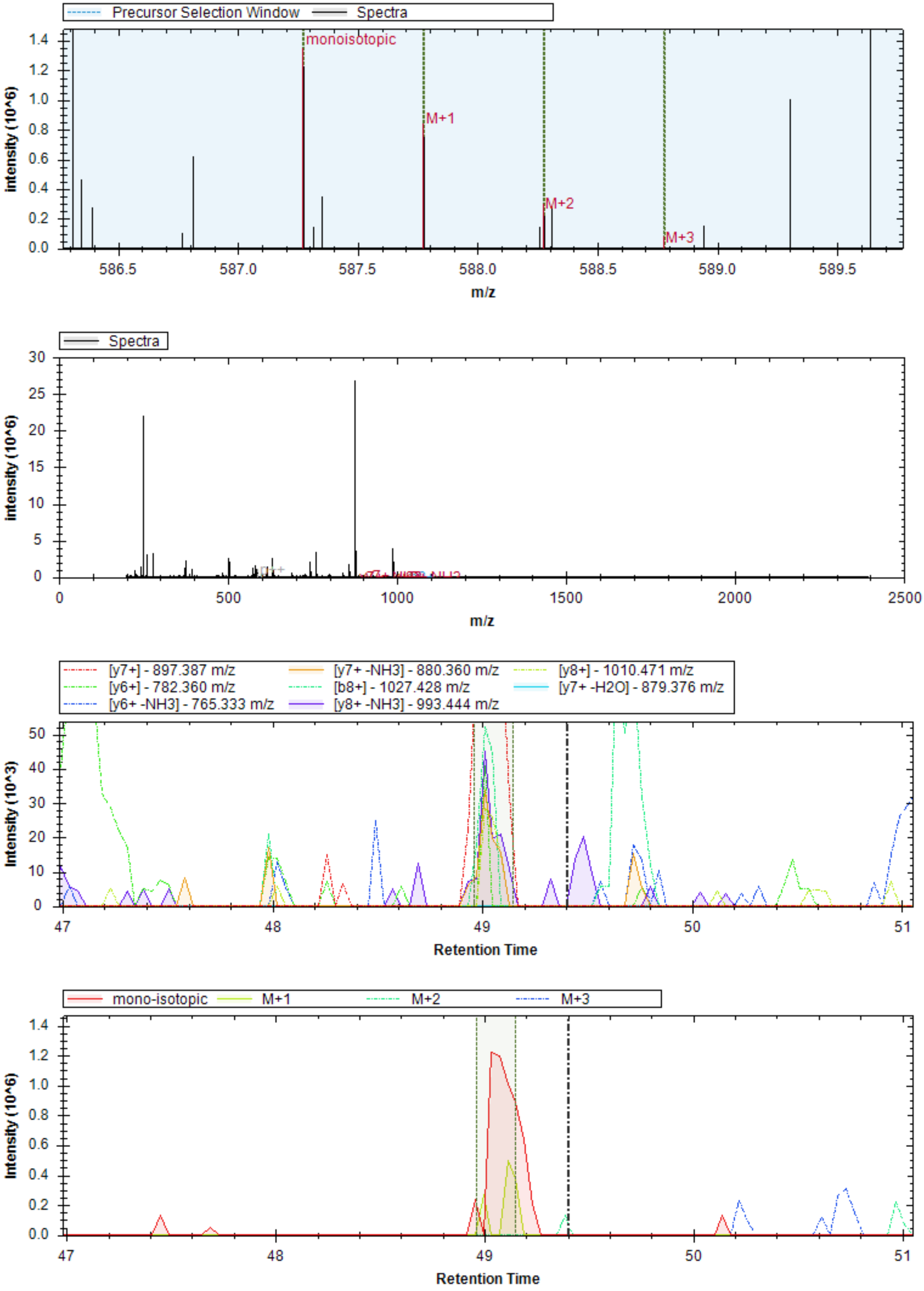

Q62261 – _RPPS[+H+O3+P]PDPNTK_.2
Fig4_MP-DIA-barrel-cortex-p15-2_MHRM.htrms

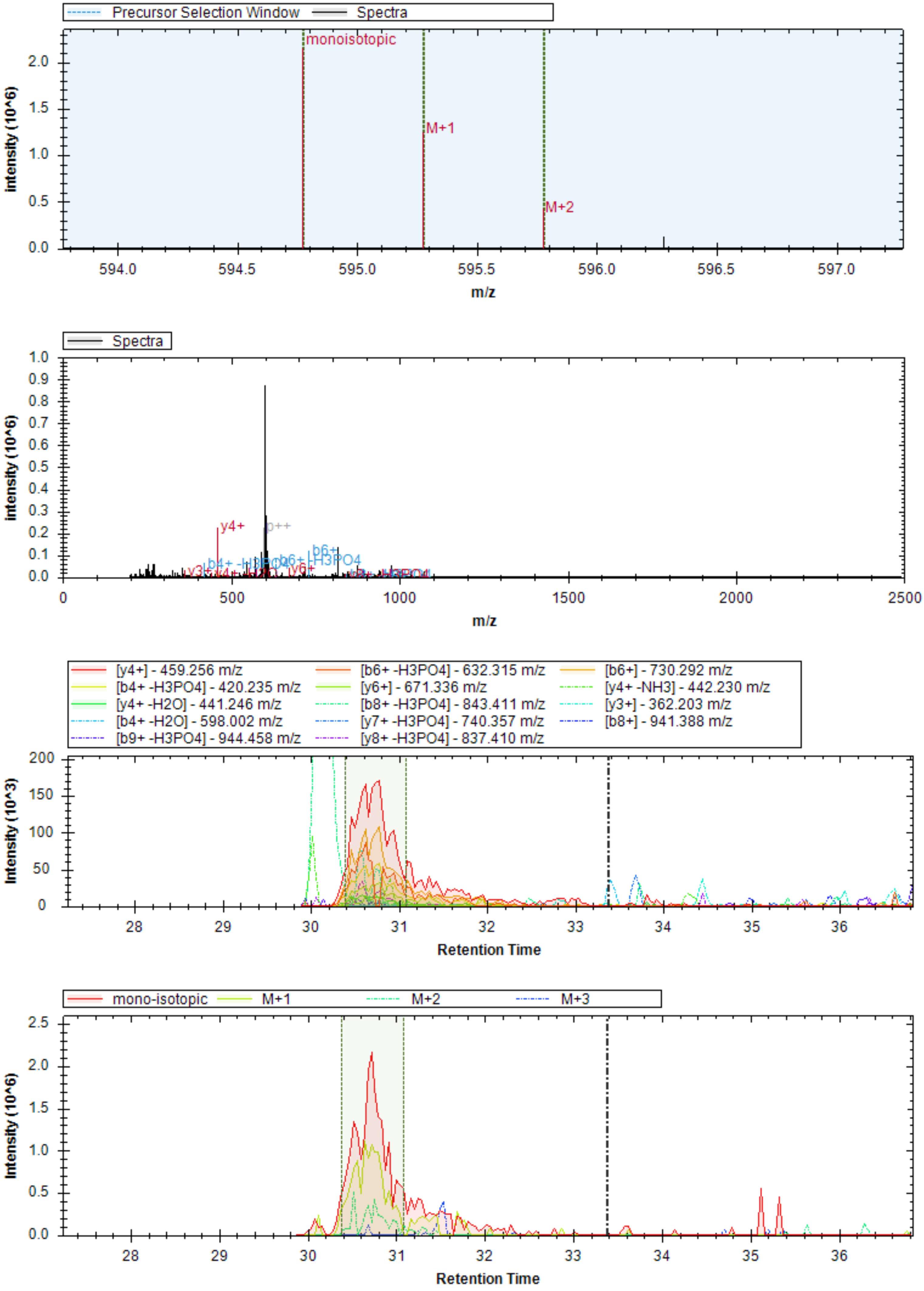

Q80VN0 – _ELTAQIT[+H+O3+P]SIK_.2
Fig4_MP-DIA-barrel-cortex-p15-2_MHRM.htrms

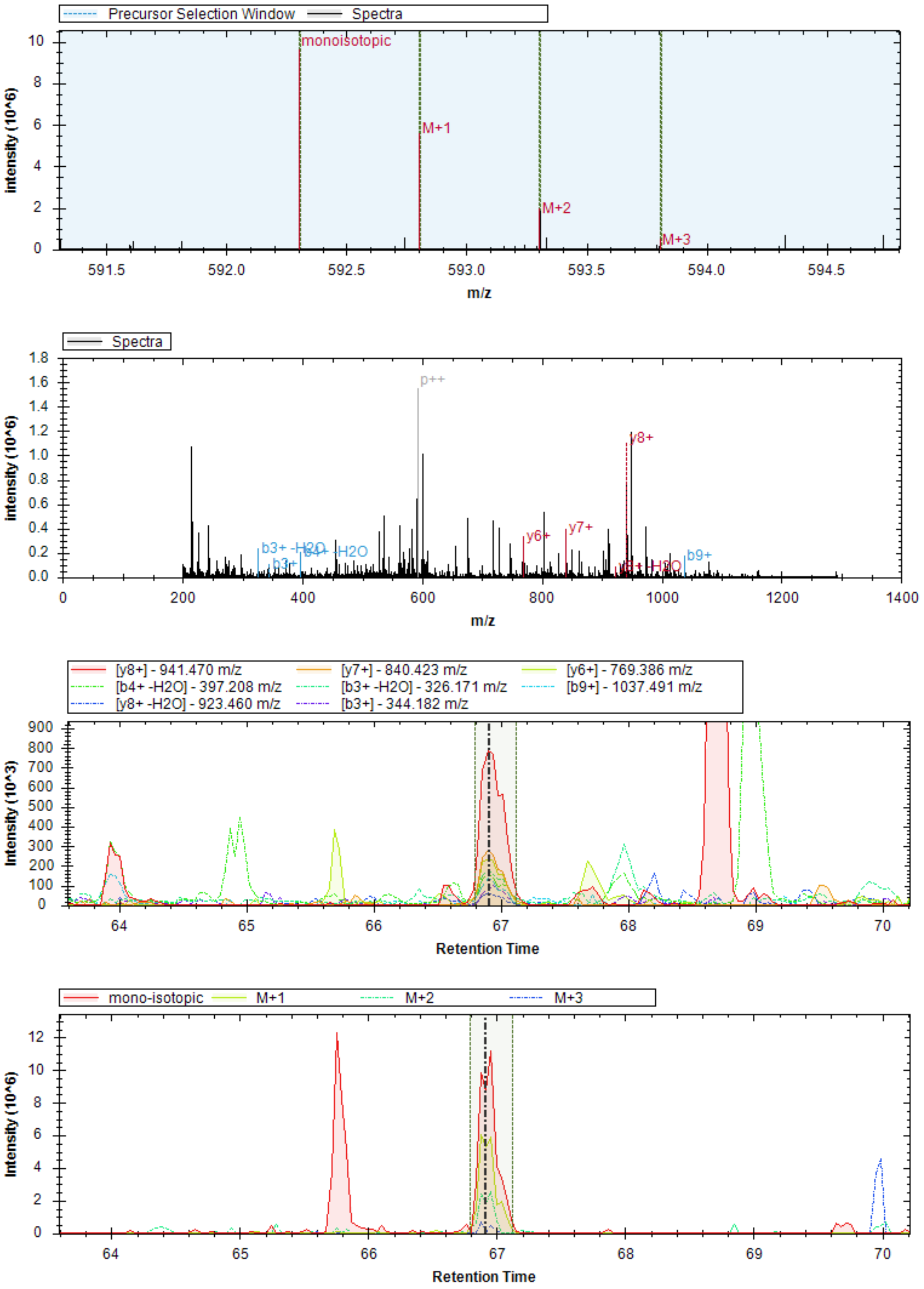

Q8C419 – _SAS[+H+O3+P]AHNLSSEK_.2
Fig4_MP-DIA-barrel-cortex-p15-2_MHRM.htrms

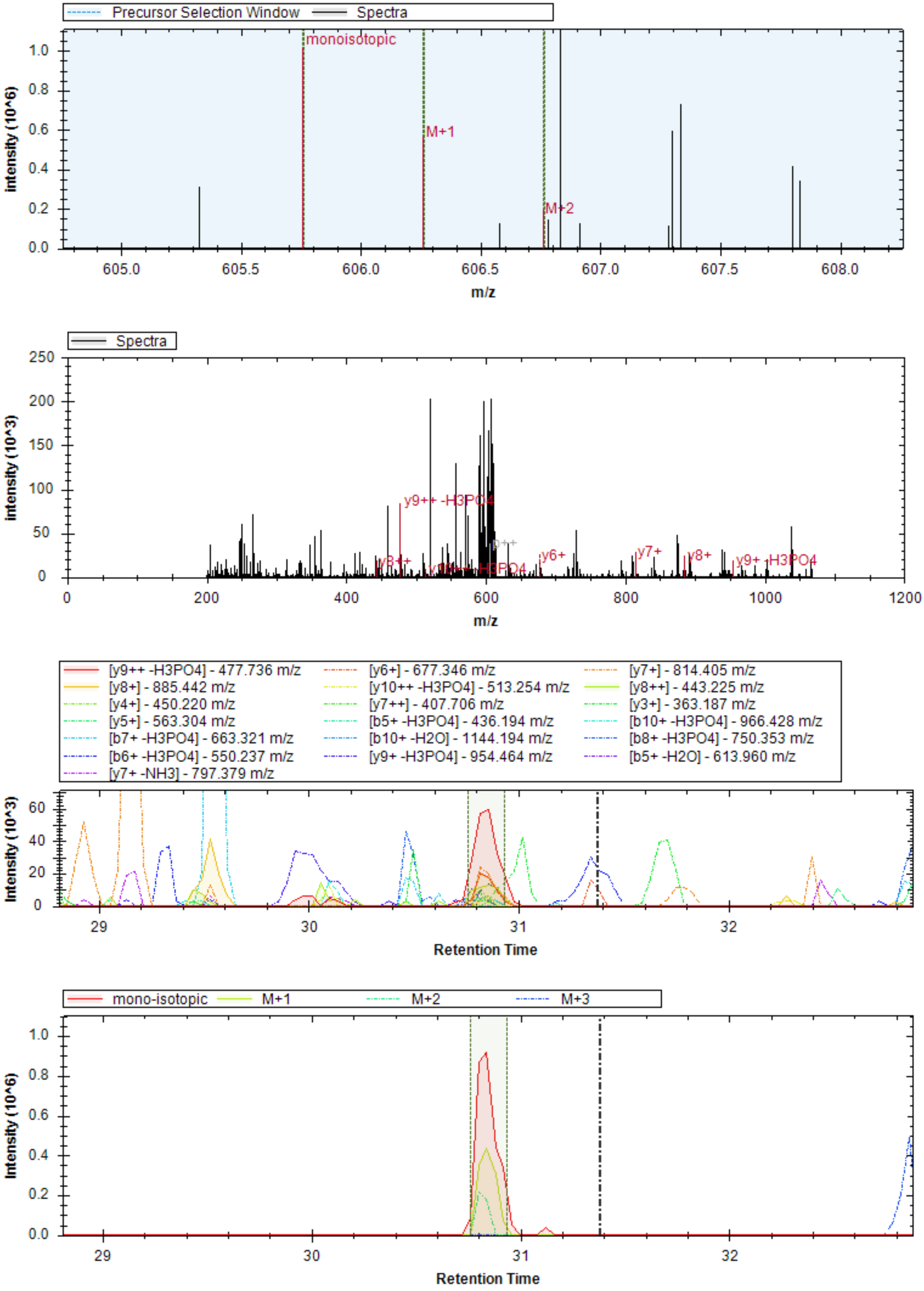

Q99104 – _AIS[+H+O3+P]PTSATSSGR_.2

Fig4_MP-DIA-barrel-cortex-p15-2_MHRM.htrms

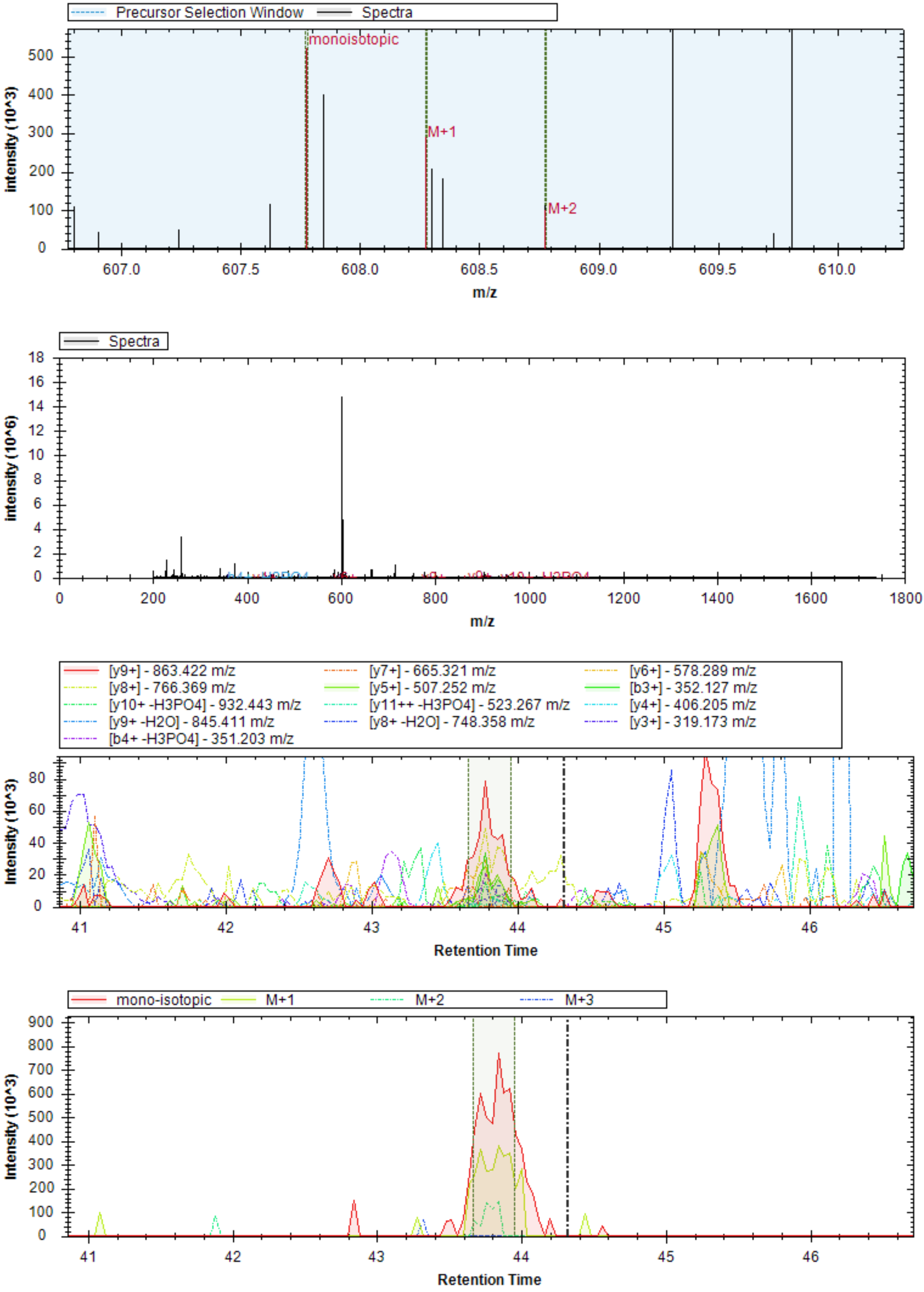

Q9QWI6 – _LSYAGGRPPSYAGS[+H+O3+P]PVHHAAER_.4
Fig4_MP-DIA-barrel-cortex-p15-2_MHRM.htrms

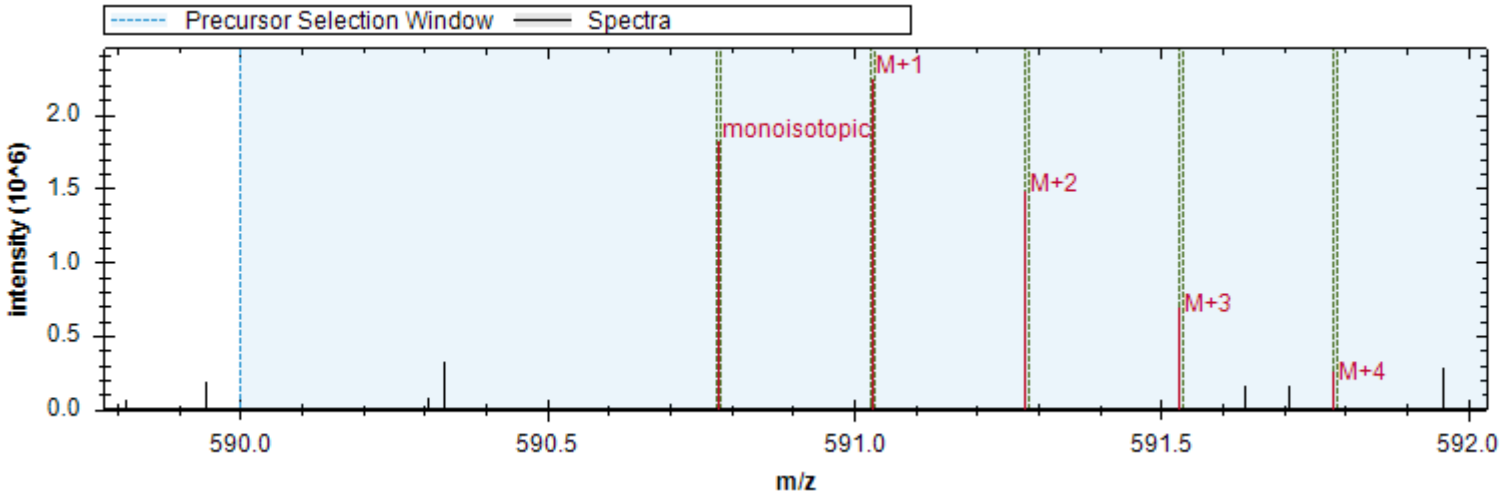

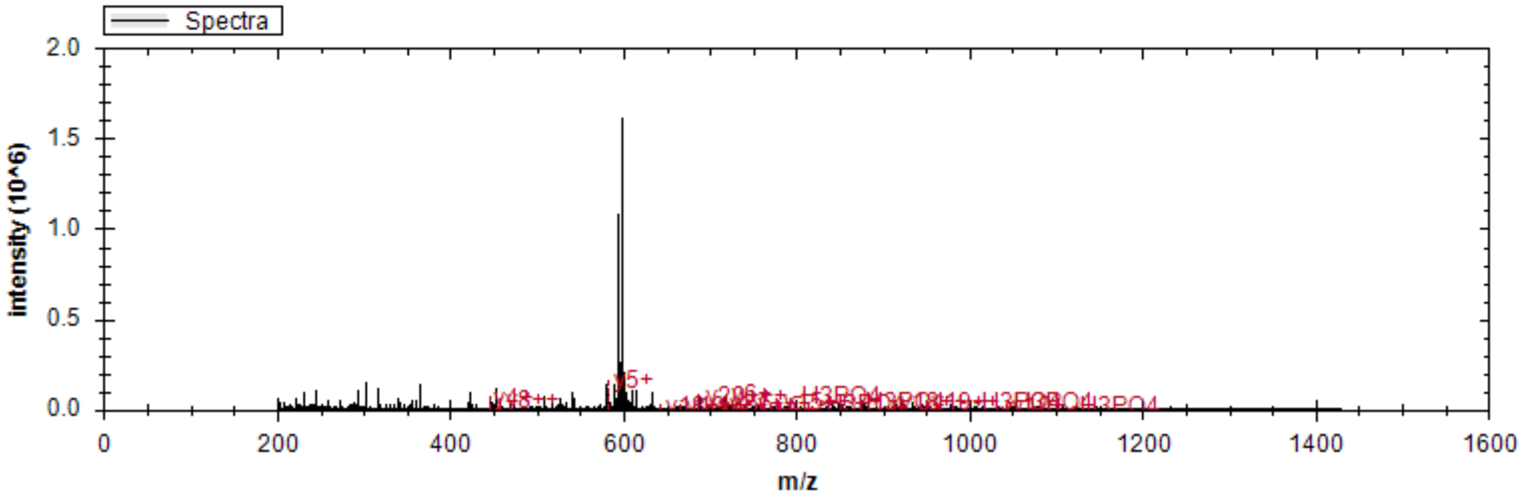

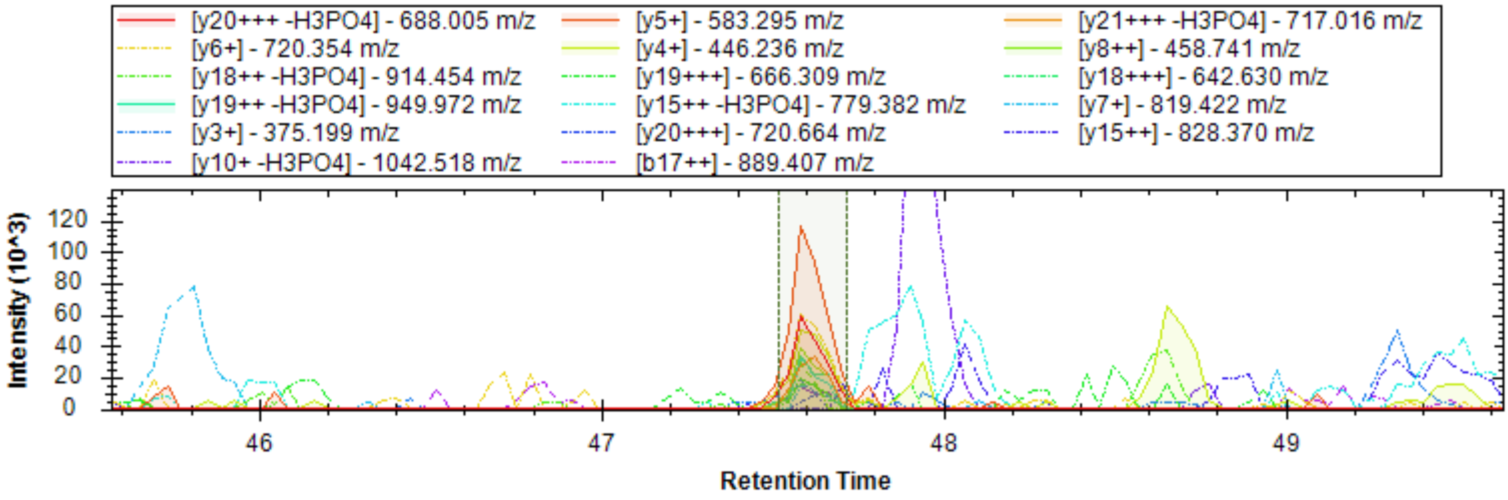

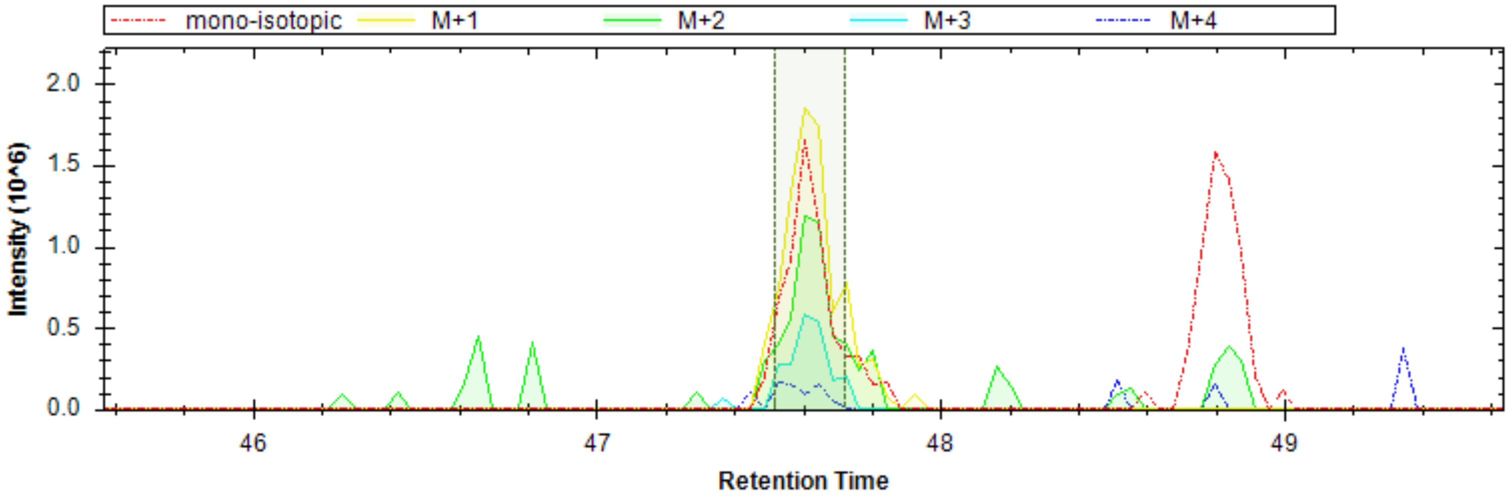

Q9Z2D7 – _KT[+H+O3+P]S[+H+O3+P]LYFSSK_.2
Fig4_MP-DIA-barrel-cortex-p15-2_MHRM.htrms

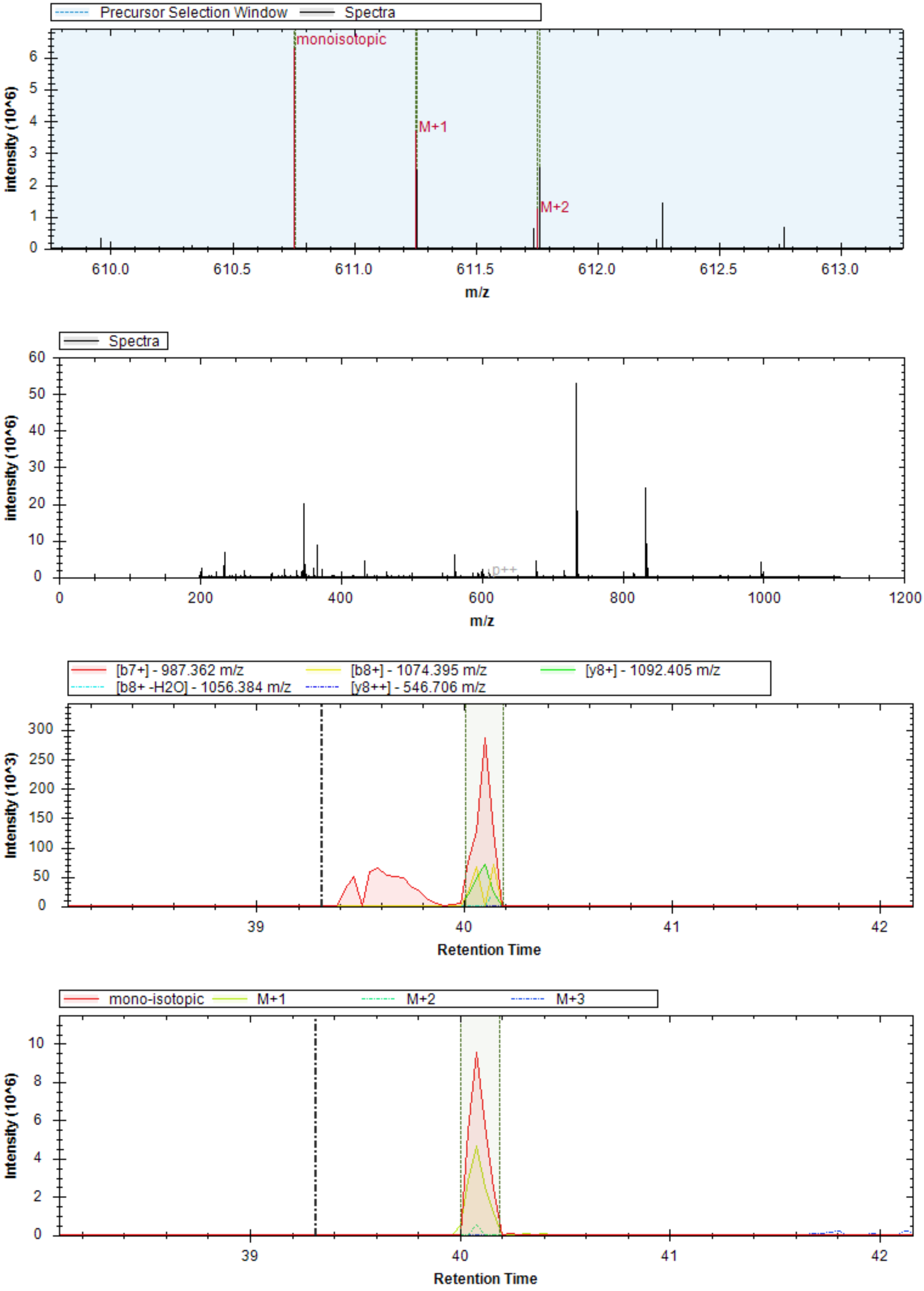

P60904 – _SLS[+H+O3+P]TSGESLYHVLGLDK_.3

Fig4_MP-DIA-barrel-cortex-p15-2_MHRM.htrms

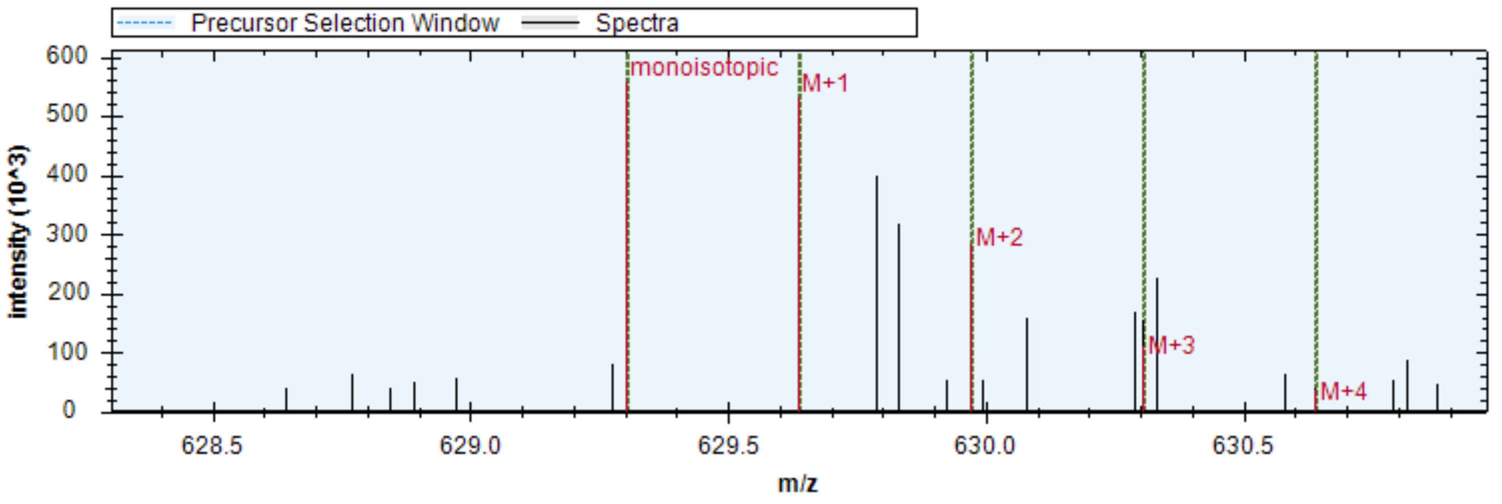

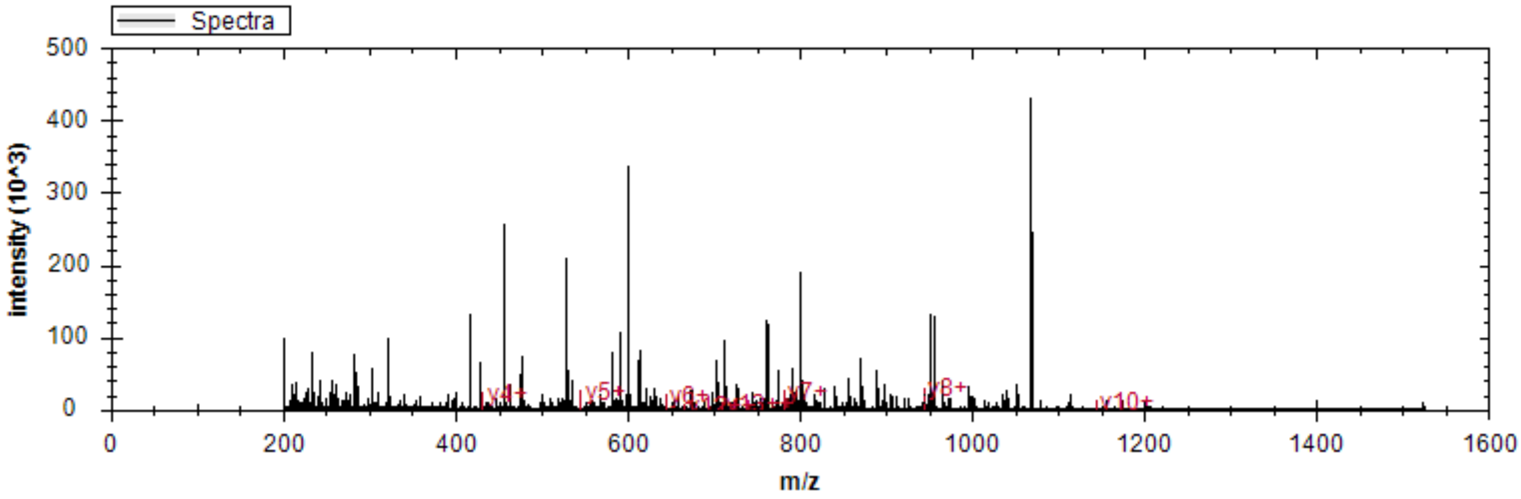

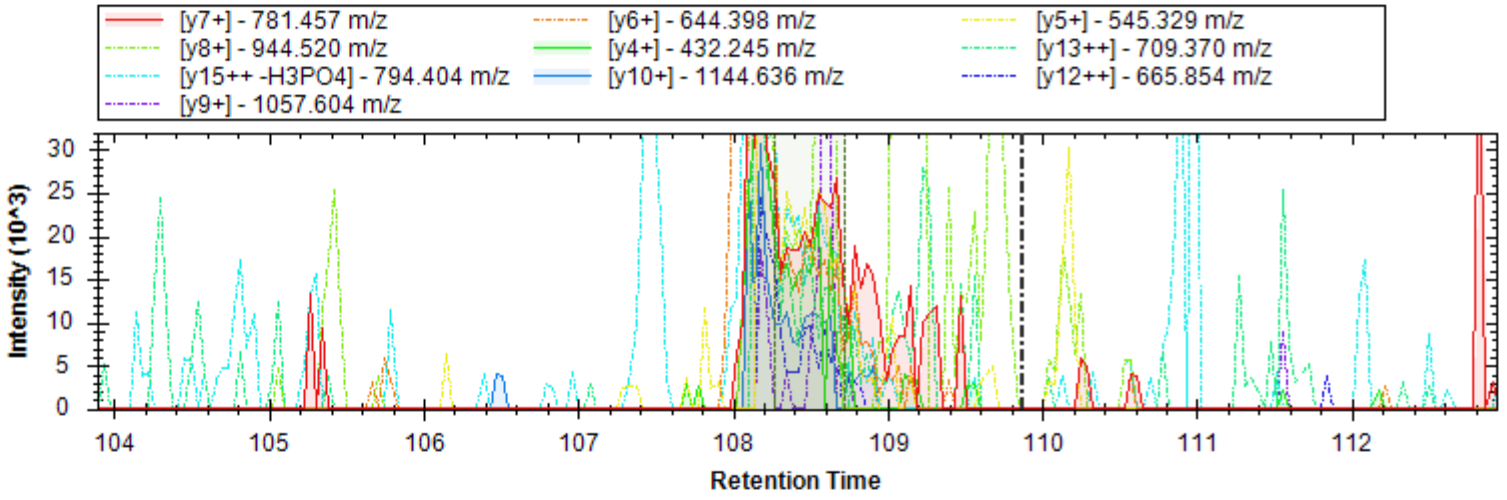

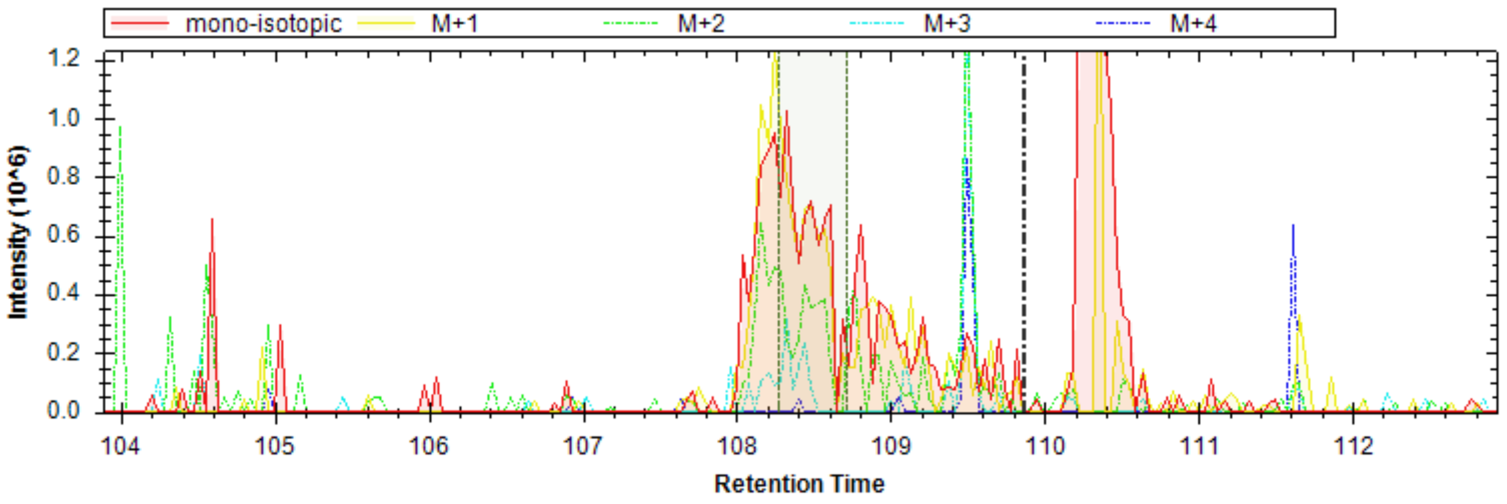

P60904 – _SLST[+H+O3+P]SGESLYHVLGLDK_.3
Fig4_MP-DIA-barrel-cortex-p15-2_MHRM.htrms

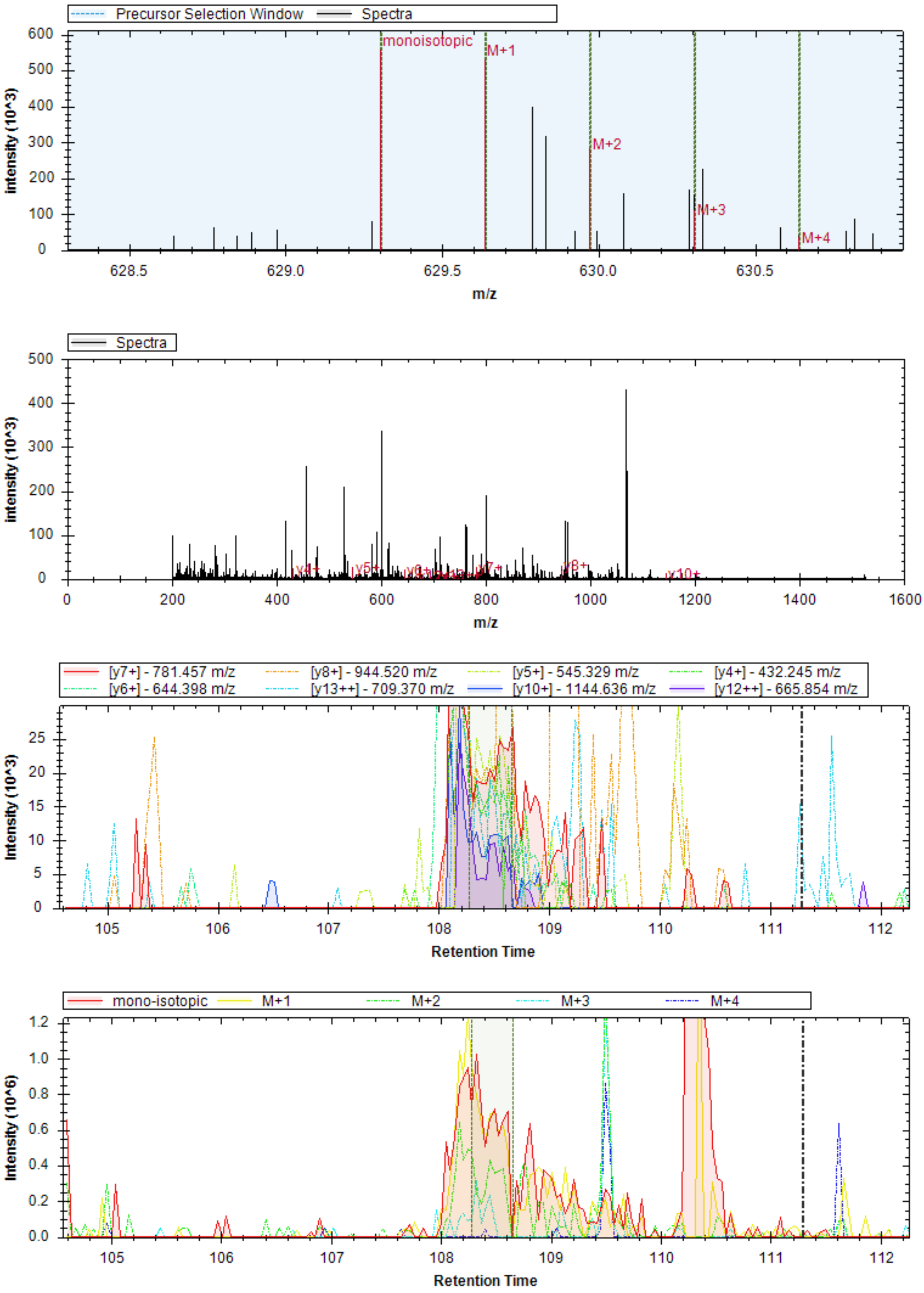

P39447 – _SSGIIRLHT[+H+O3+P]IK_.2

Fig4_MP-DIA-barrel-cortex-p15-2_MHRM.htrms

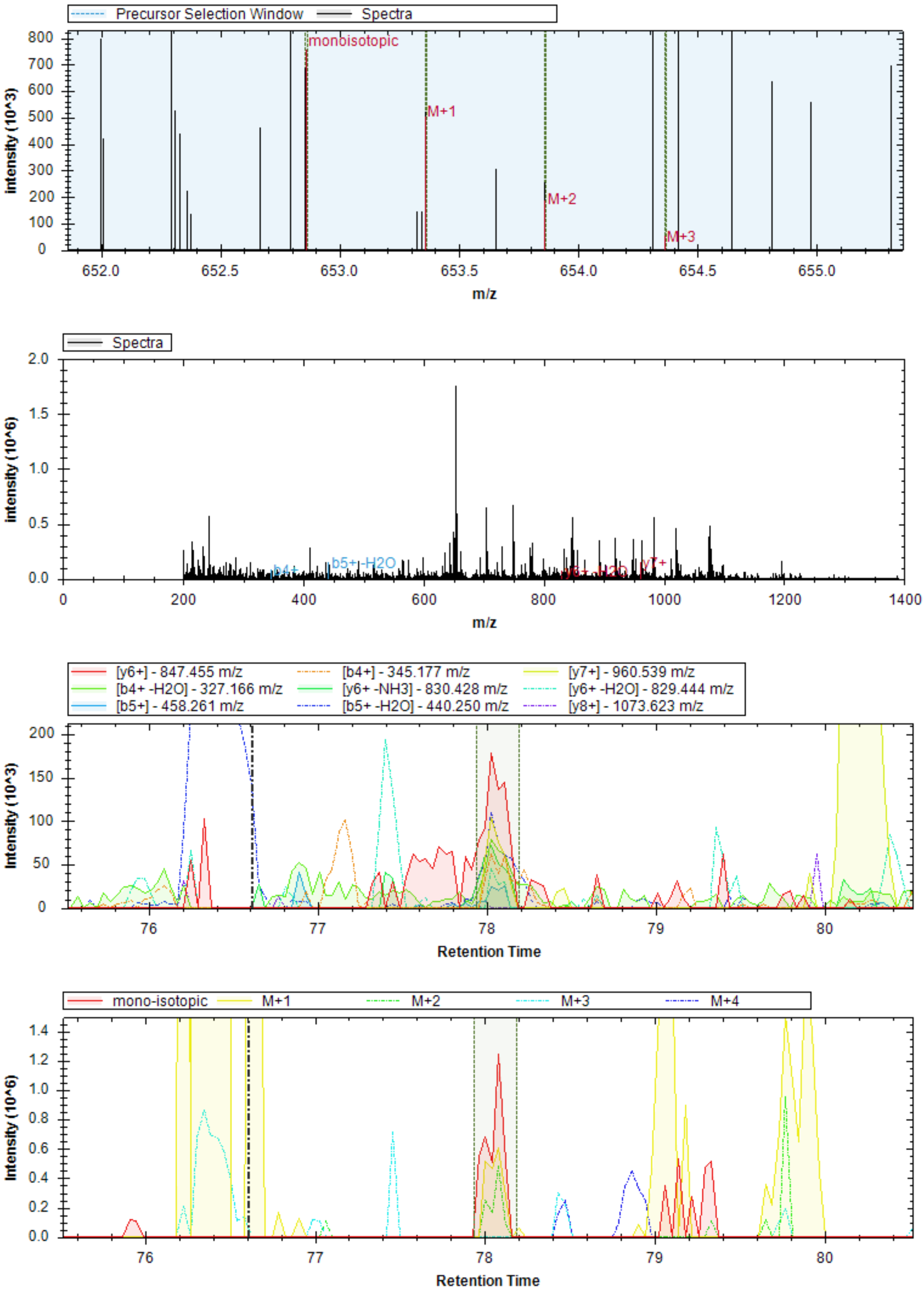

Q8BLJ3 – _M[+O]AS[+H+O3+P]SQGKNELK_.2
Fig4_MP-DIA-barrel-cortex-p15-2_MHRM.htrms

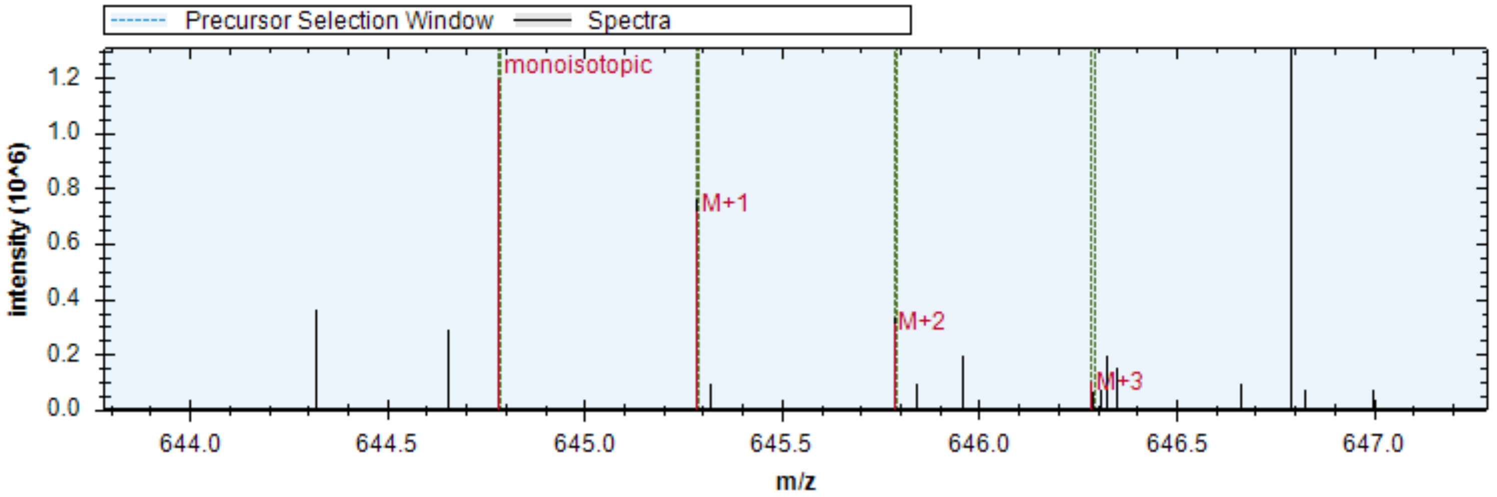

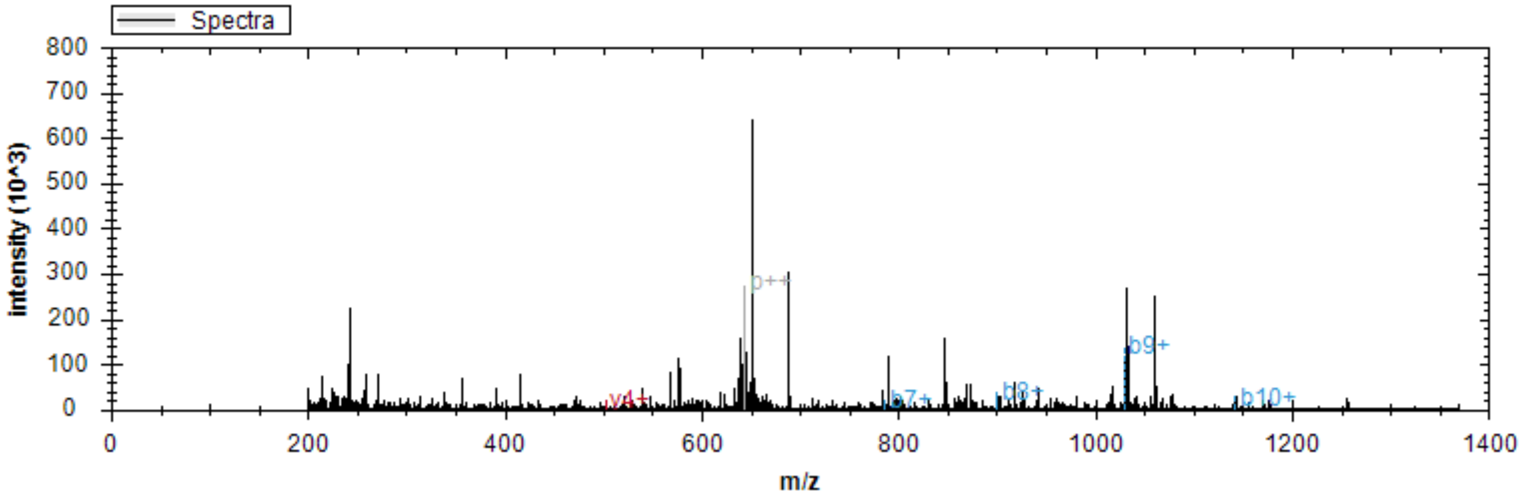

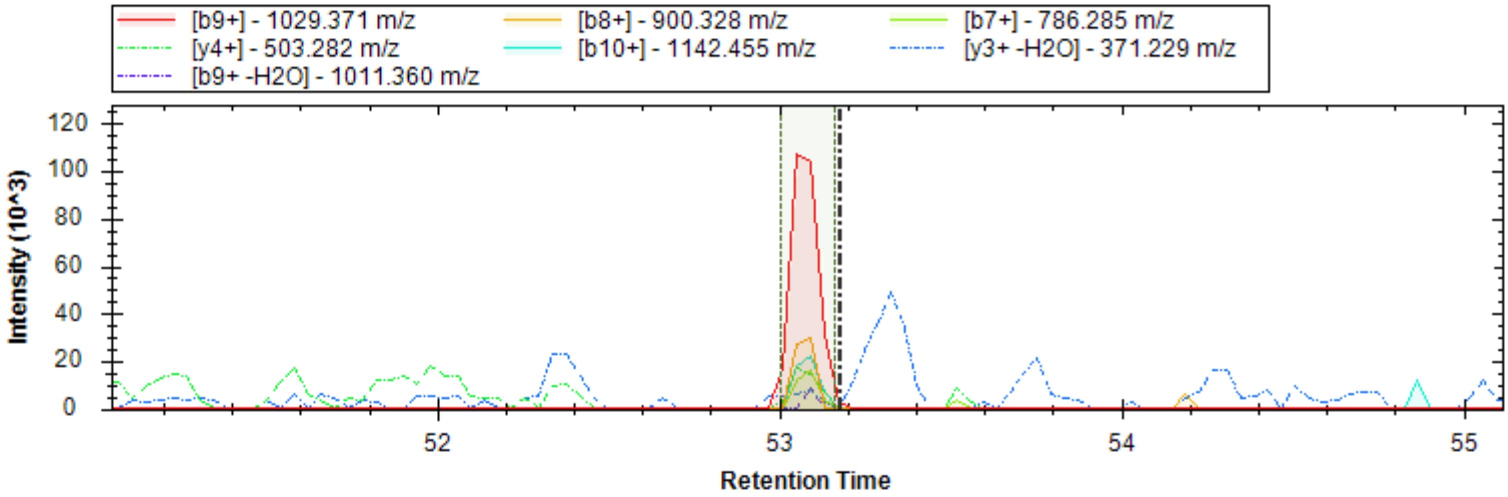

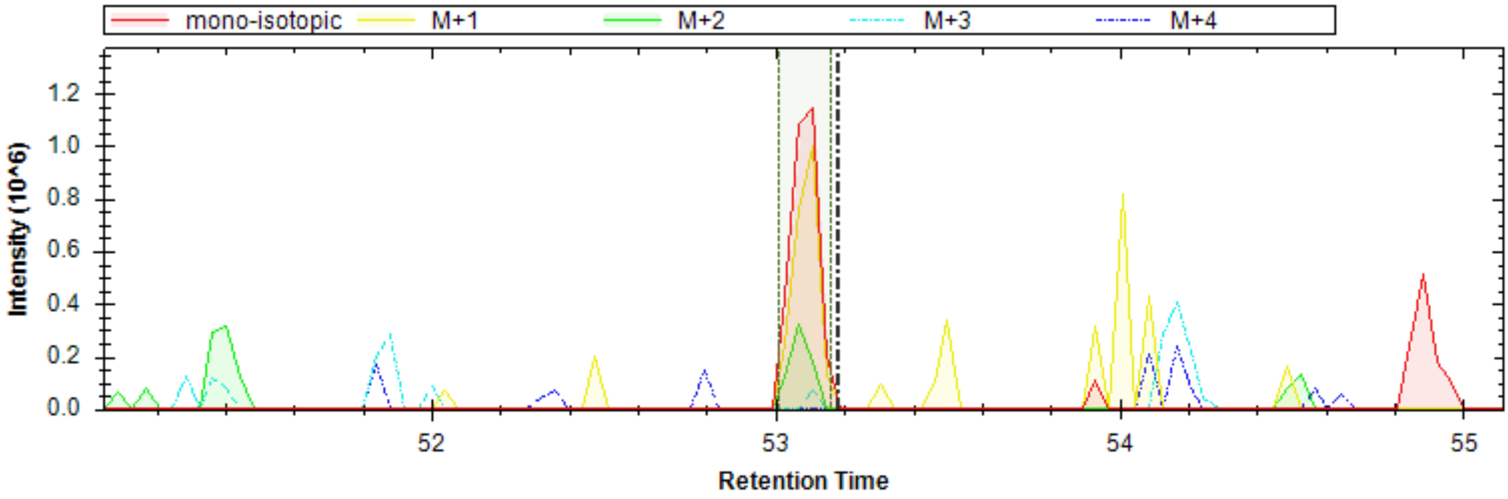

Q8CC35-3 – _AAS[+H+O3+P]PAKPSSLDLVPNLPR_.3

Fig4_MP-DIA-barrel-cortex-p15-2_MHRM.htrms

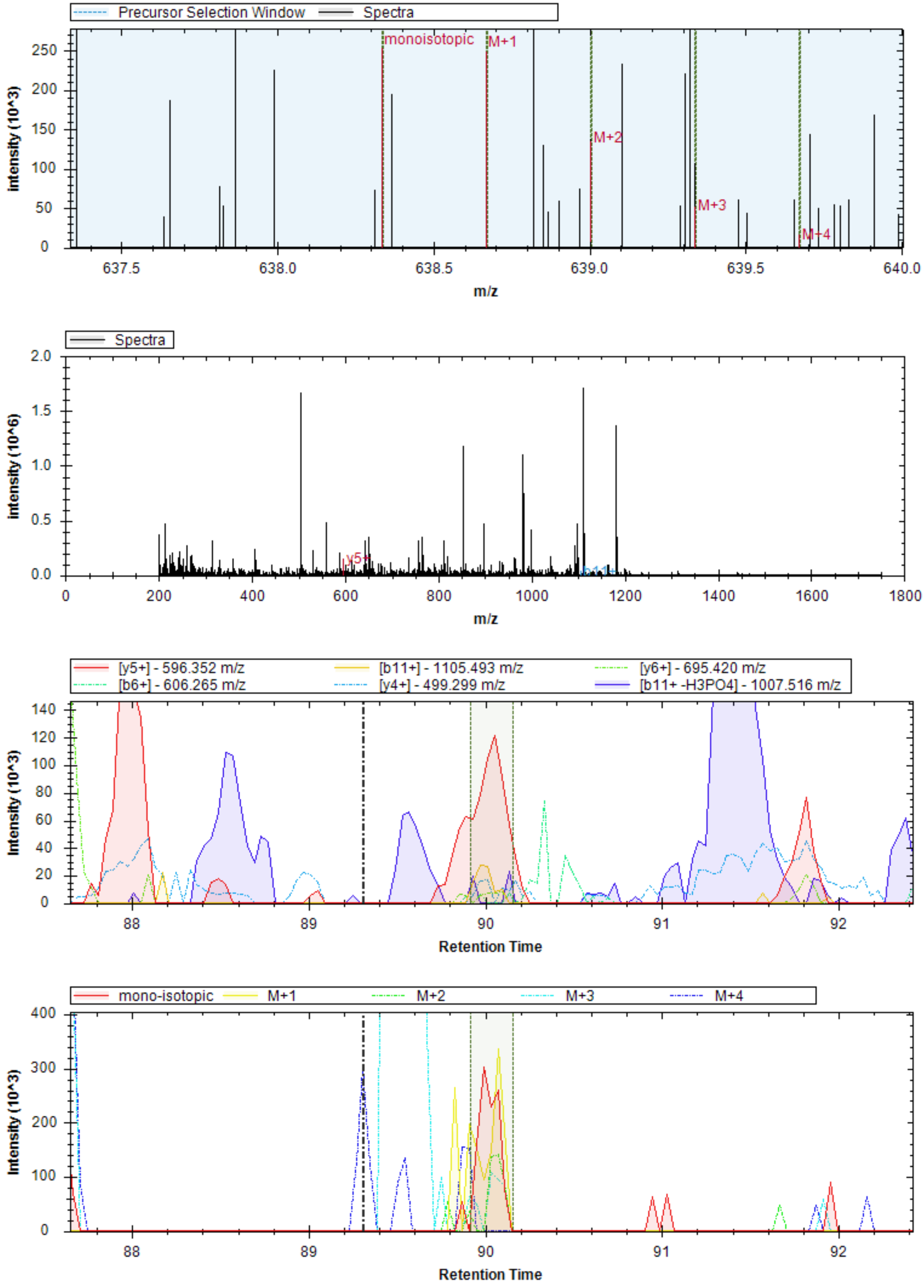

A2AM05;A2AM05-4 – _KDLKMS[+H+O3+P]VLISK_.2
Fig4_MP-DIA-barrel-cortex-p15-2_MHRM.htrms

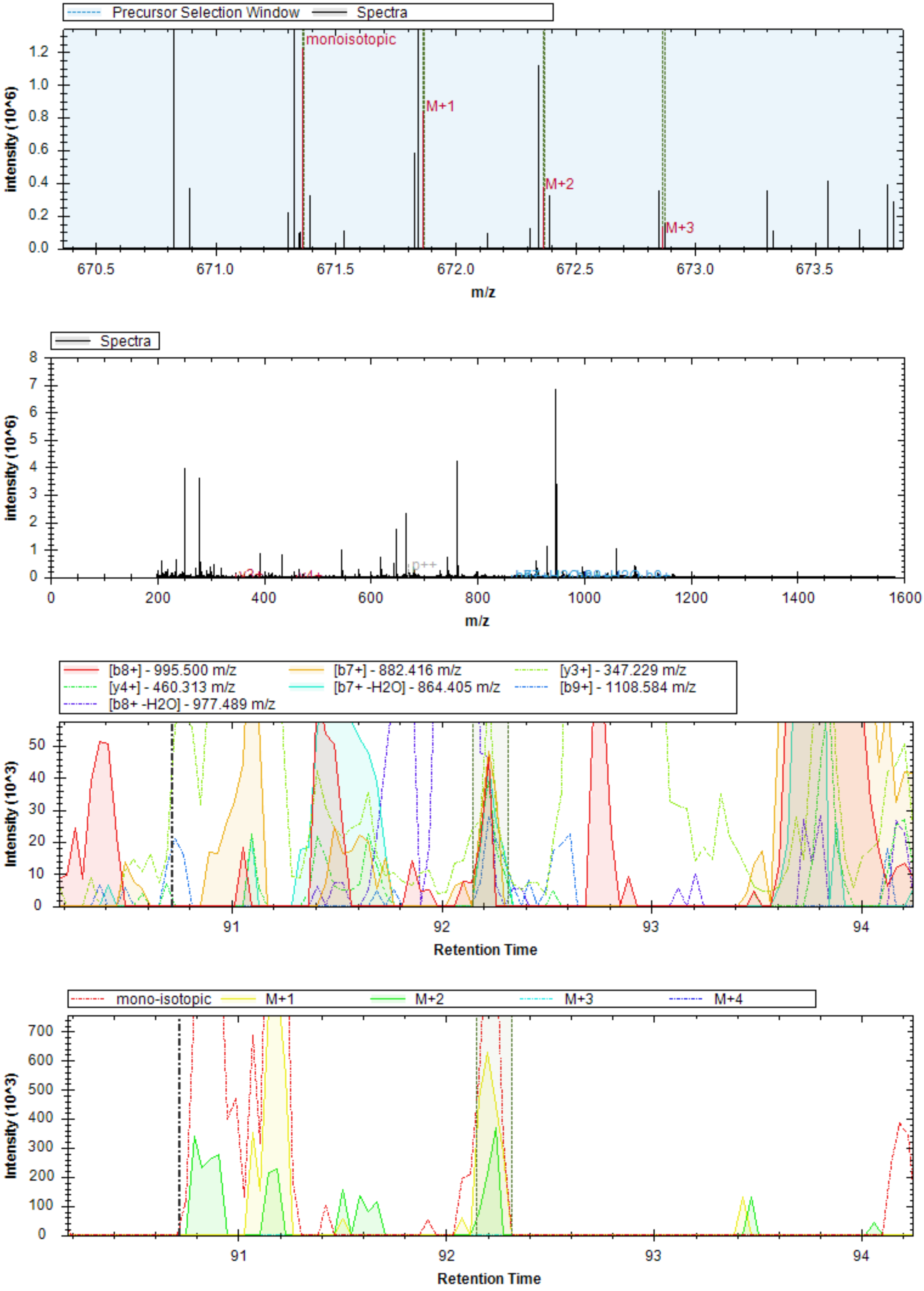

P13595 – _GVTASS[+H+O3+P]SSPASAPK_.2
Fig4_MP-DIA-barrel-cortex-p15-2_MHRM.htrms

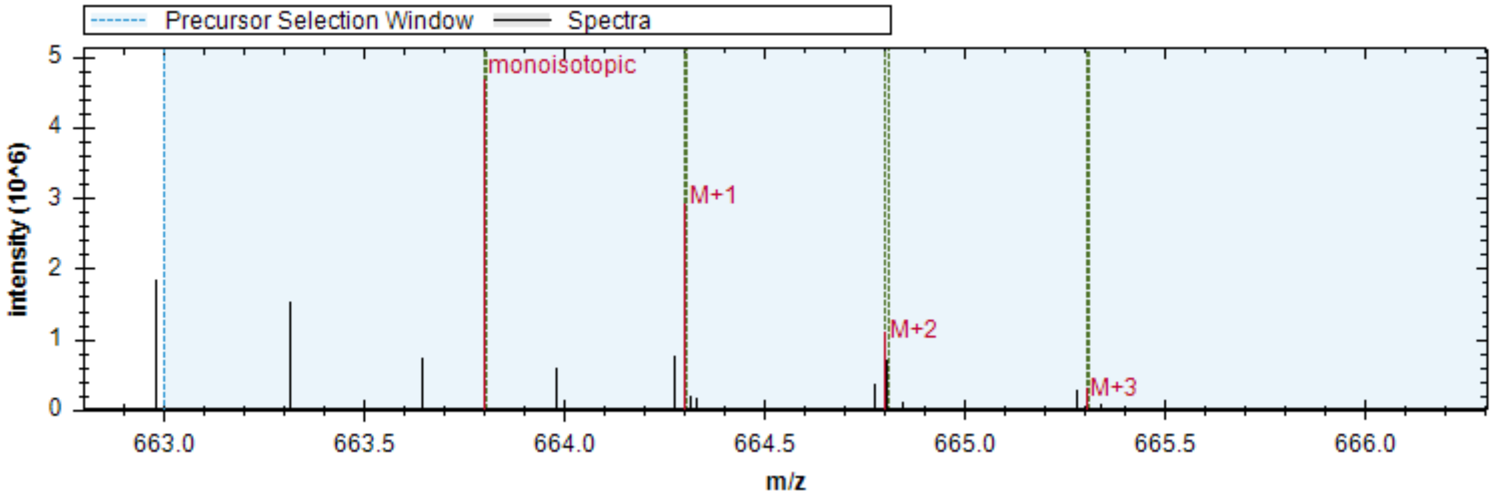

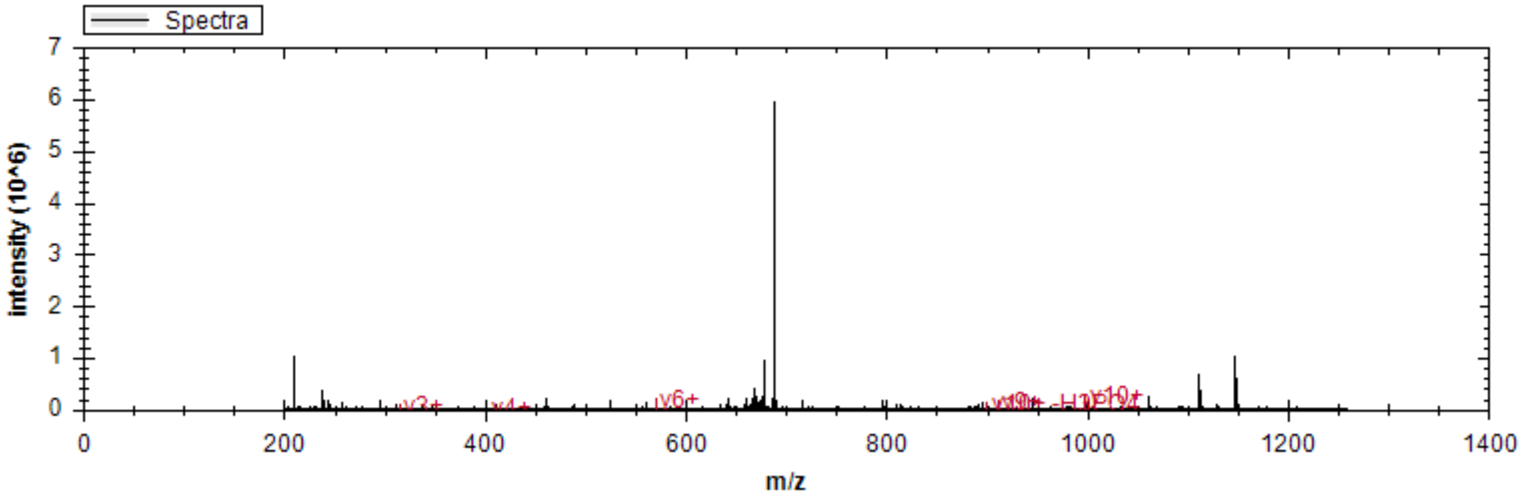

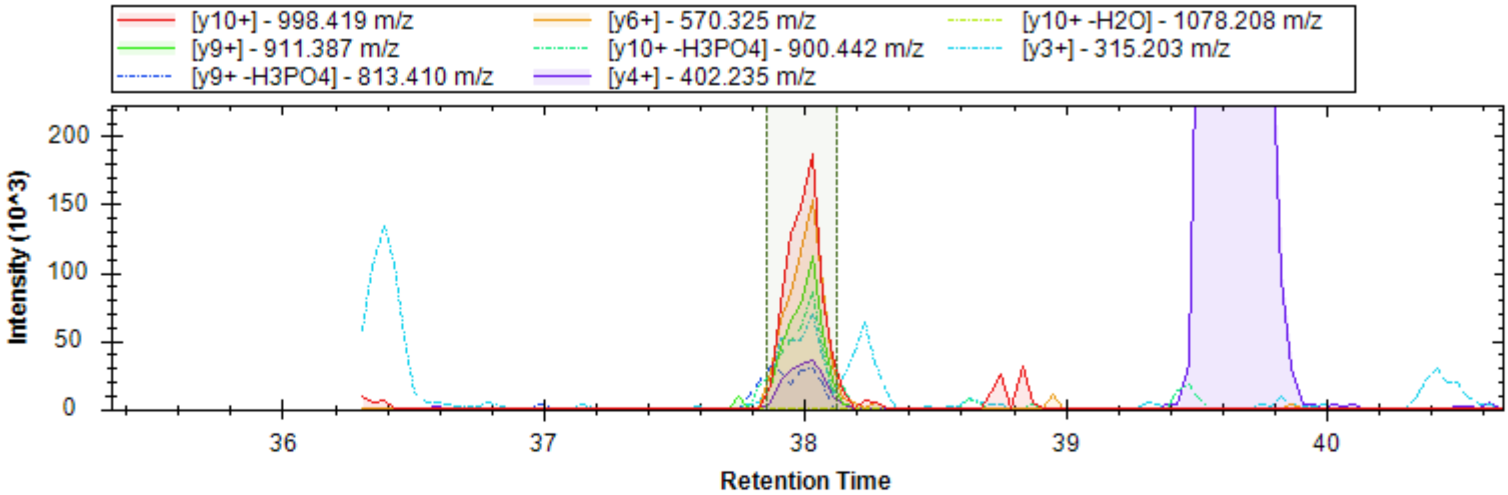

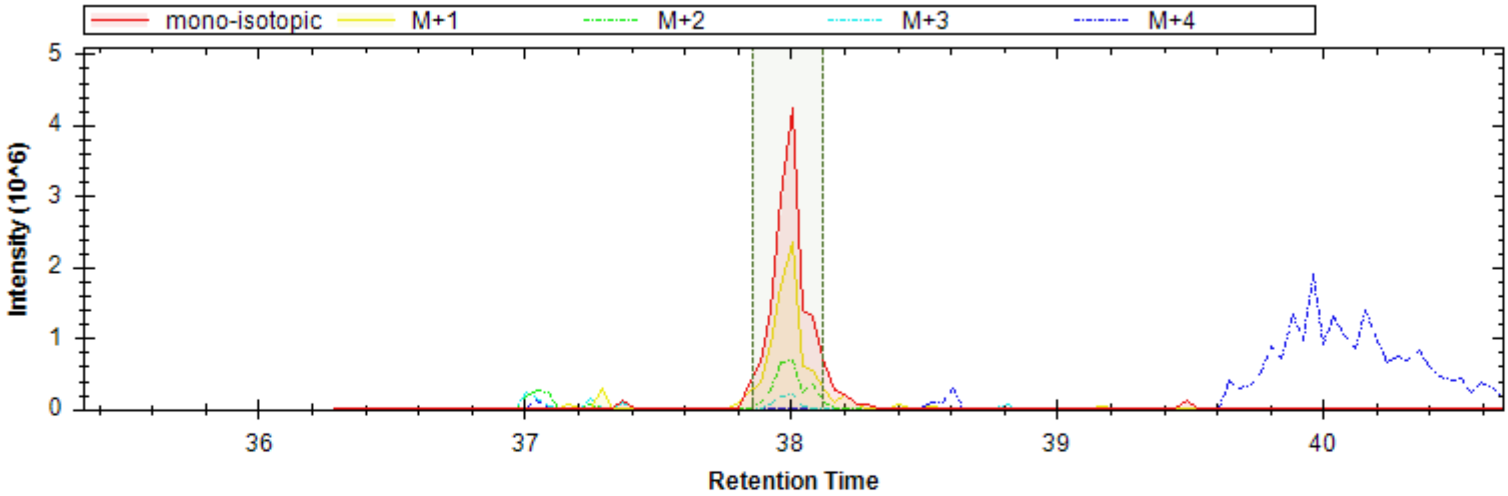

P13595 – _GVTASSS[+H+O3+P]SPASAPK_.2
Fig4_MP-DIA-barrel-cortex-p15-2_MHRM.htrms

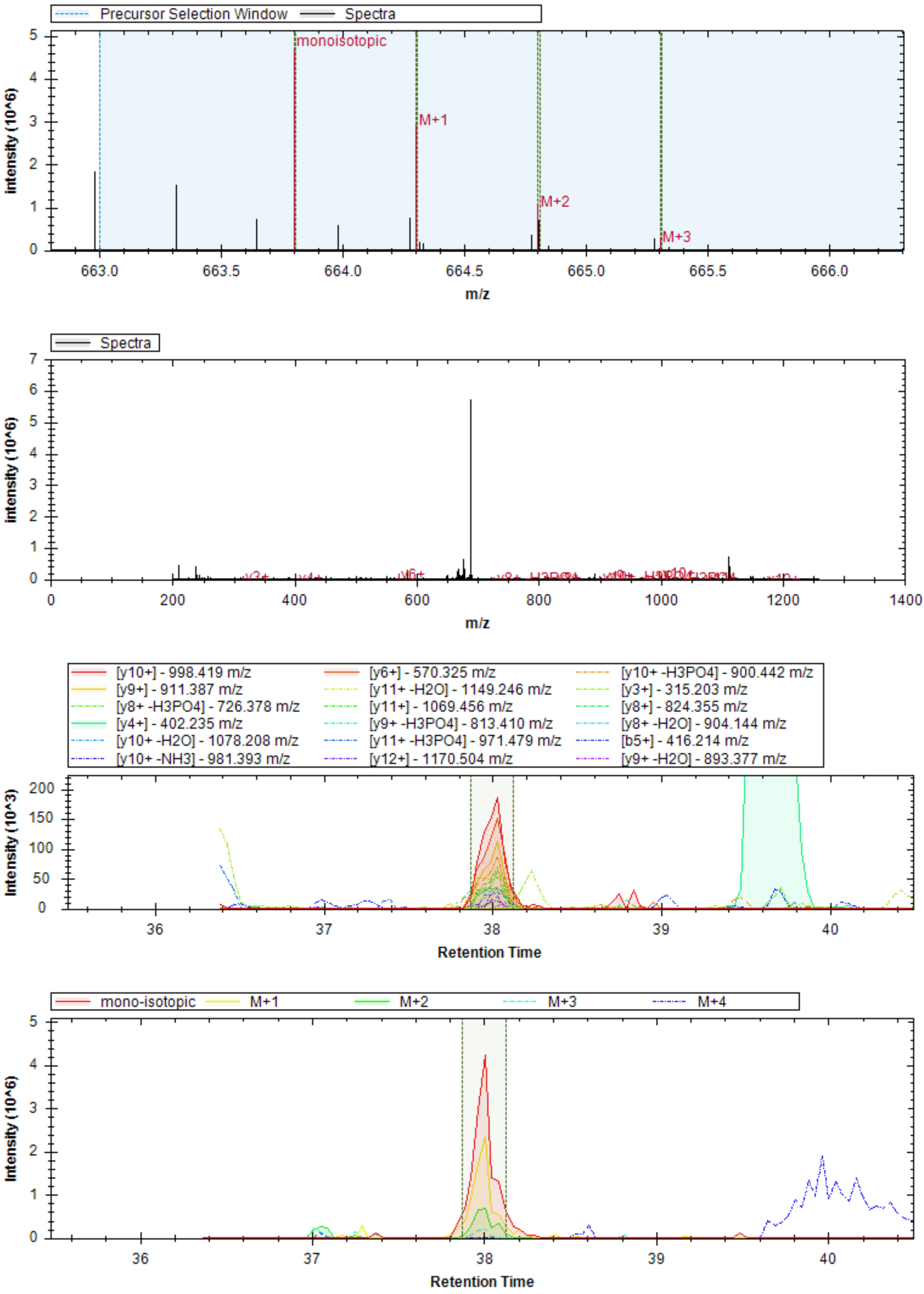

P13595 – _GVTASSSS[+H+O3+P]PASAPK_.2

Fig4_MP-DIA-barrel-cortex-p15-2_MHRM.htrms

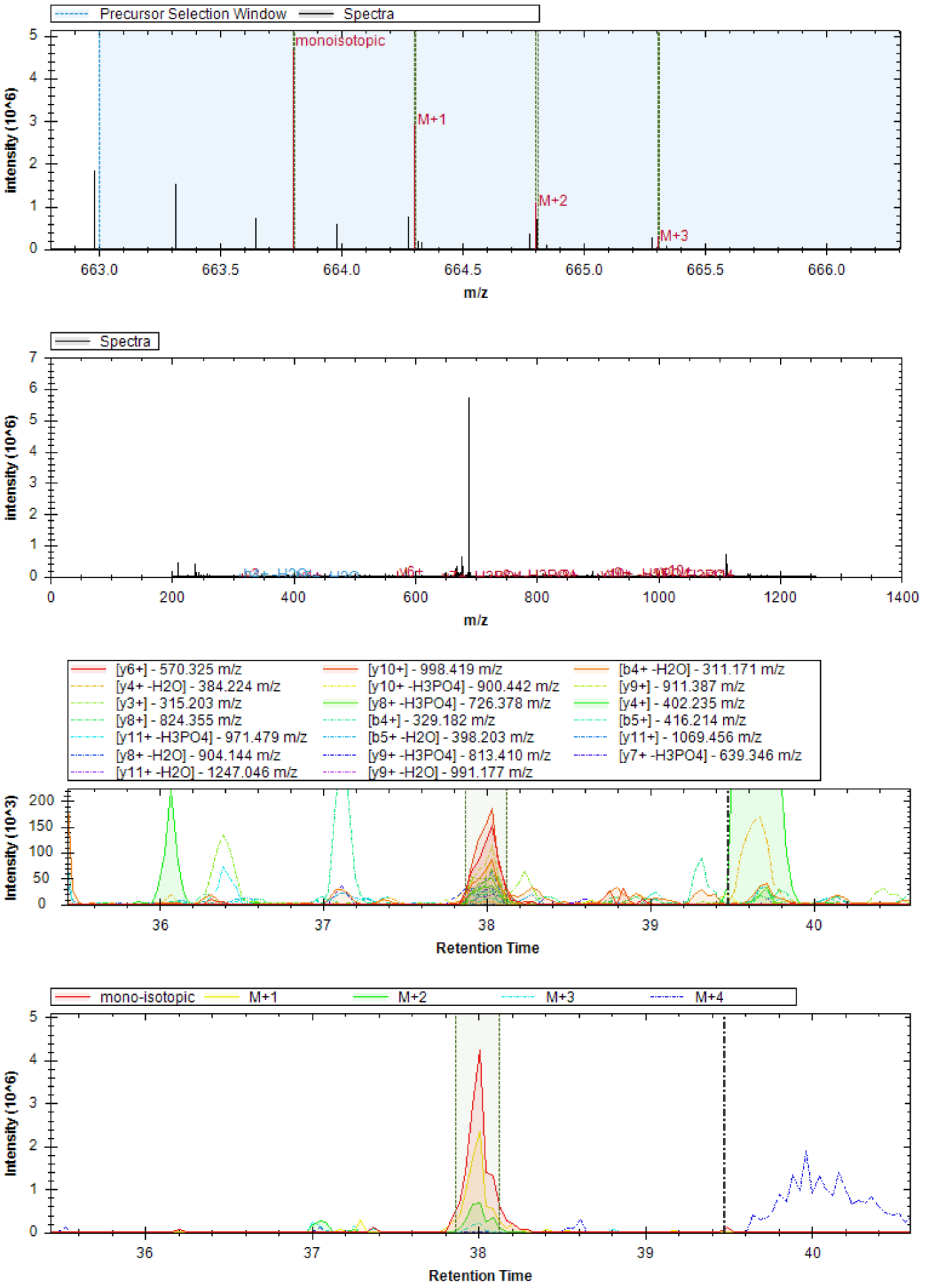

P13595 – _NPPEAATAPAS[+H+O3+P]PK_.2

Fig4_MP-DIA-barrel-cortex-p15-2_MHRM.htrms

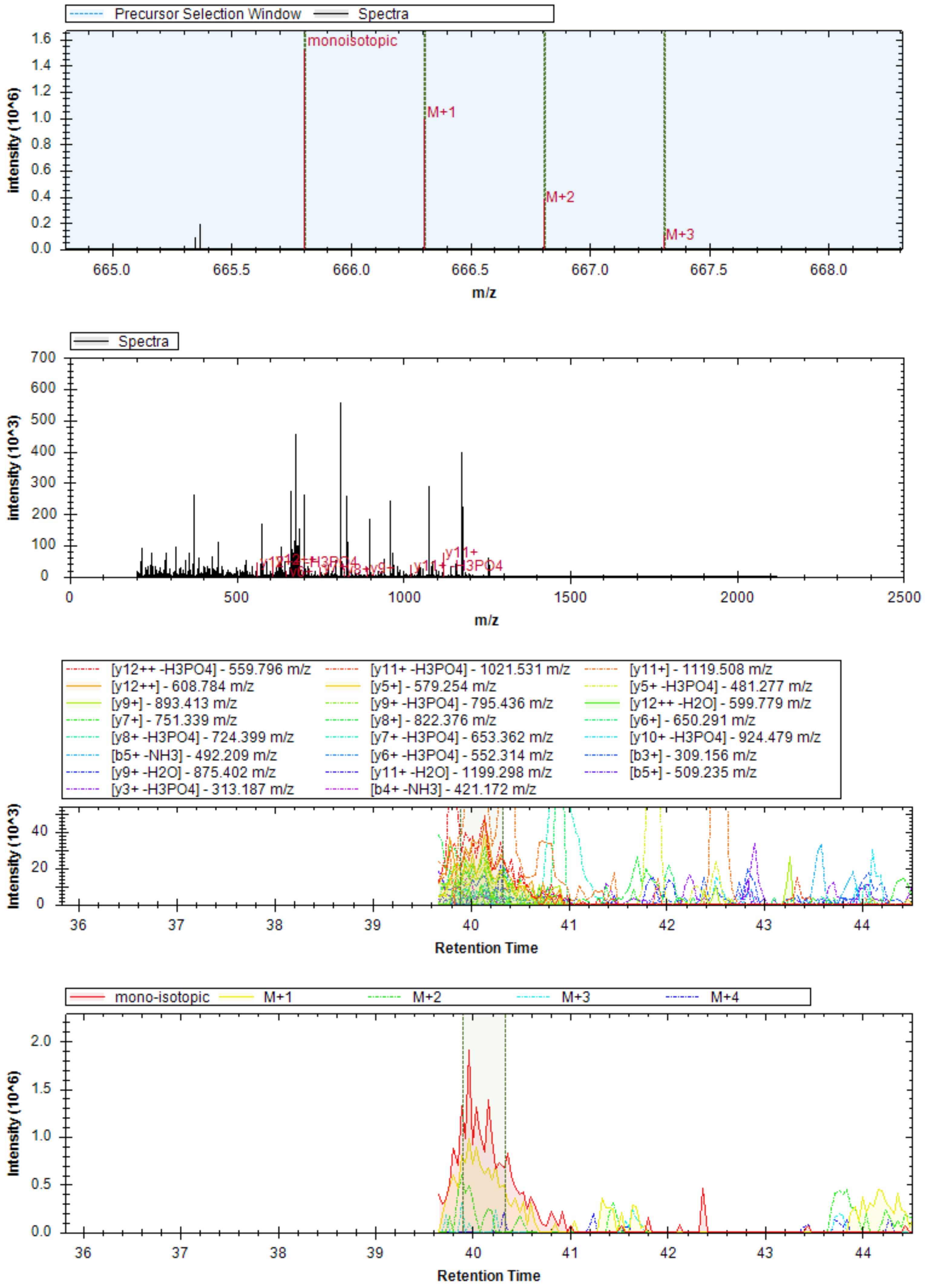

P26361 – _KM[+O]LHS[+H+O3+P]ILHAPMS[+H+O3+P]T[+H+O3+P]ISK_.3
Fig4_MP-DIA-barrel-cortex-p15-2_MHRM.htrms

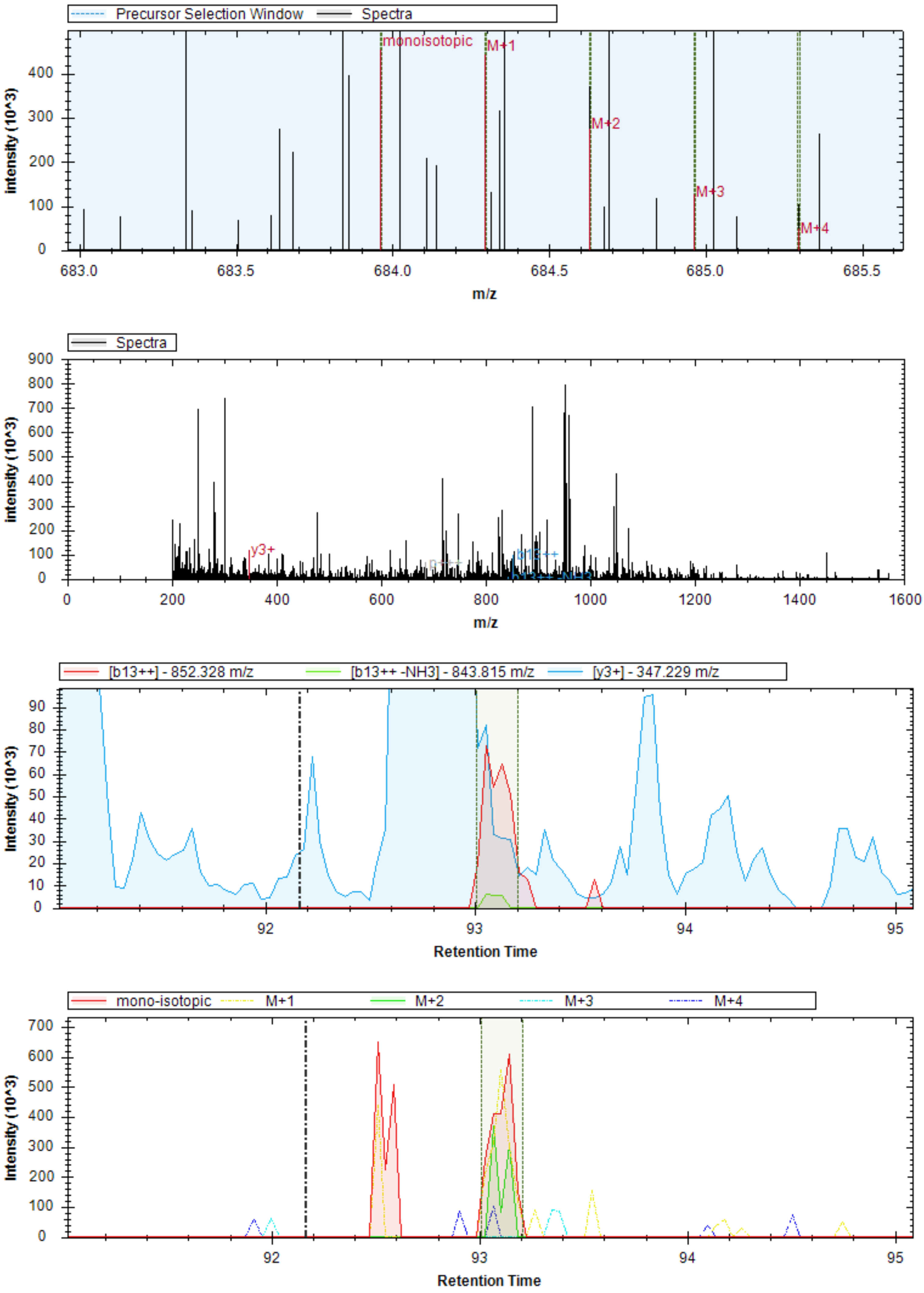

Q6A078 – _T[+H+O3+P]IGLM[+O]KKVVEK_.2
Fig4_MP-DIA-barrel-cortex-p15-2_MHRM.htrms

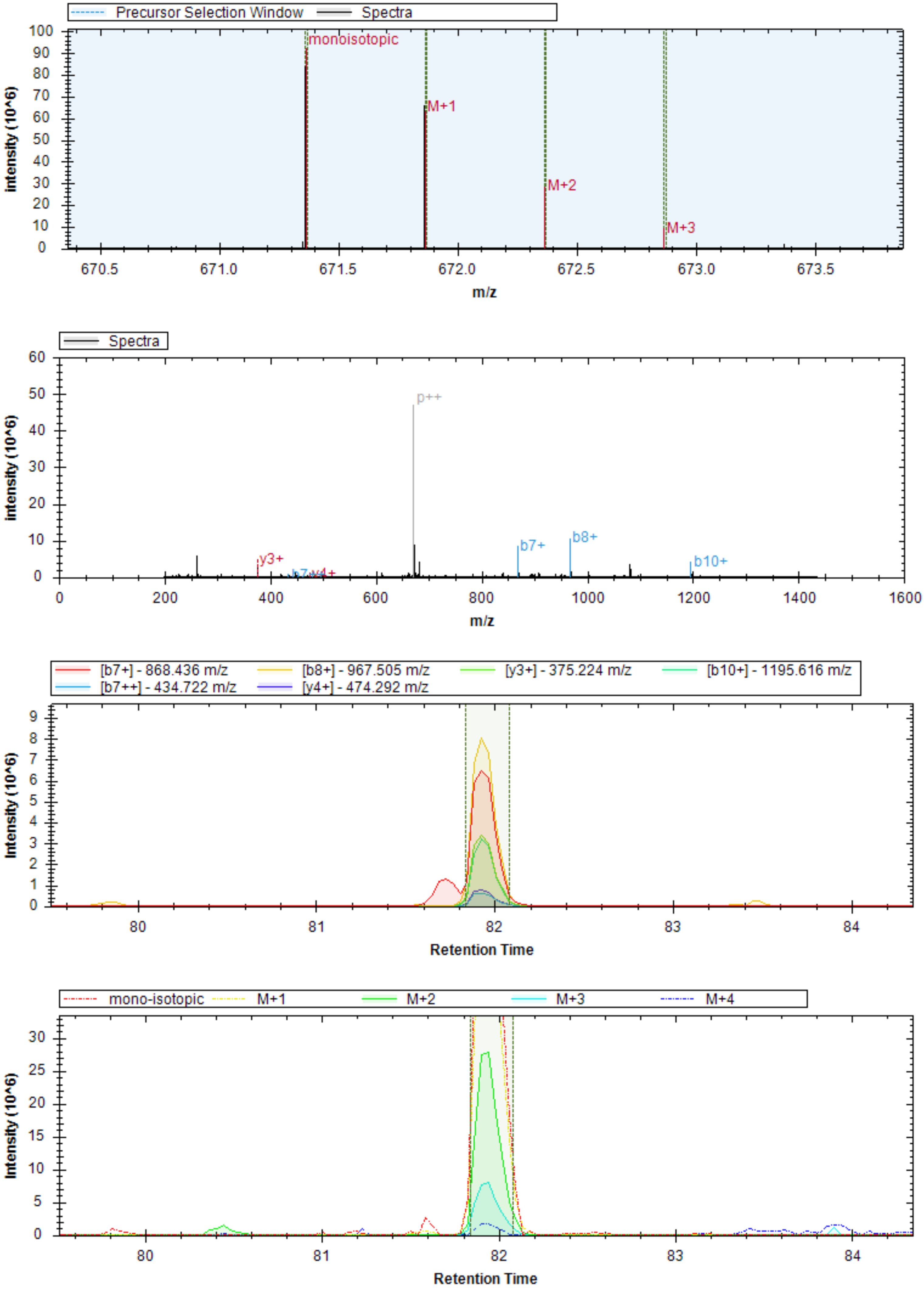

Q8C0E2 – _SMS[+H+O3+P]HQAAIASQR_.2
Fig4_MP-DIA-barrel-cortex-p15-2_MHRM.htrms

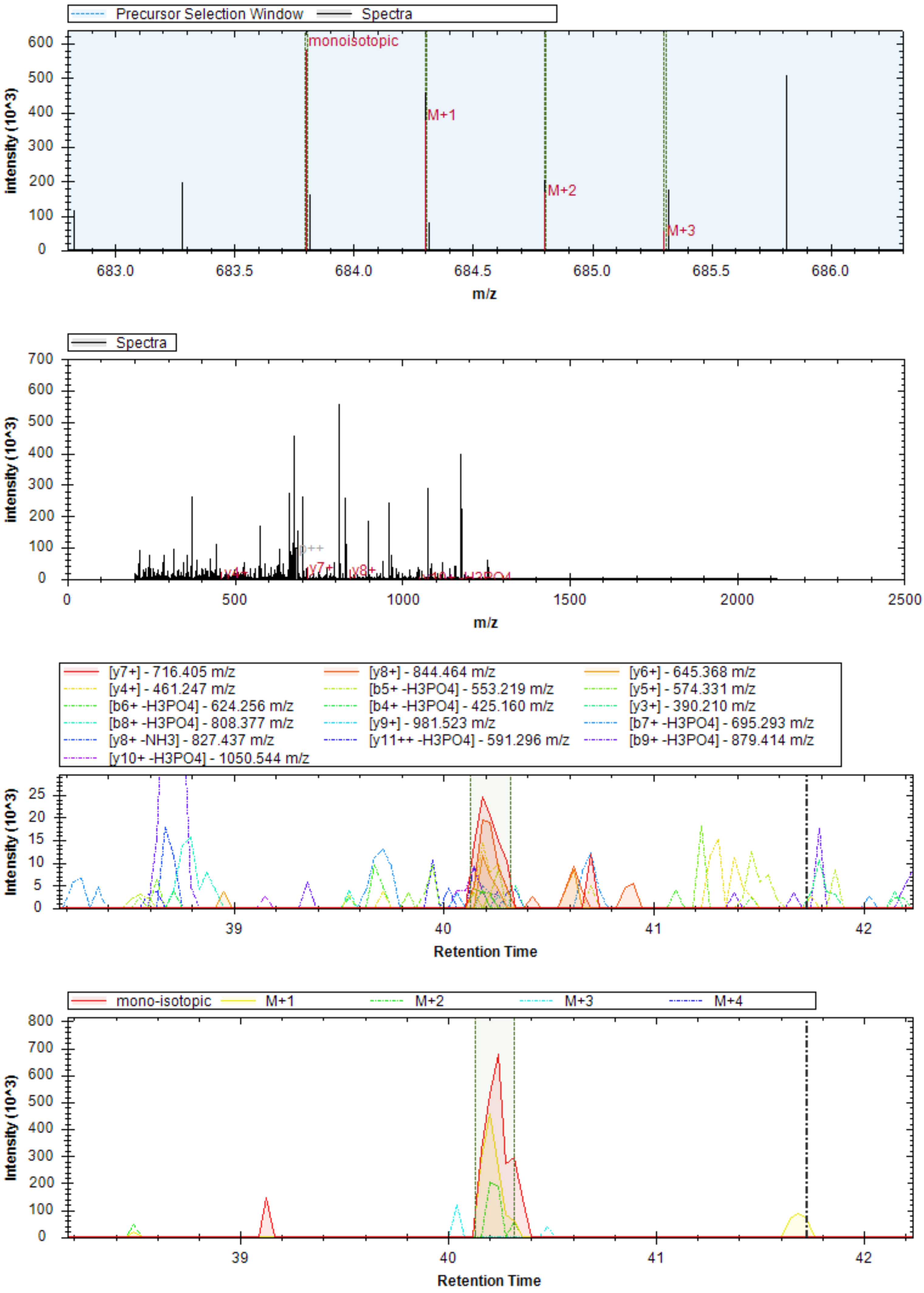

Q8CDU4;Q8CDU4-2 – _[+C2+H2+O]ASLRS[+H+O3+P]ATPRLR_.2
Fig4_MP-DIA-barrel-cortex-p15-2_MHRM.htrms

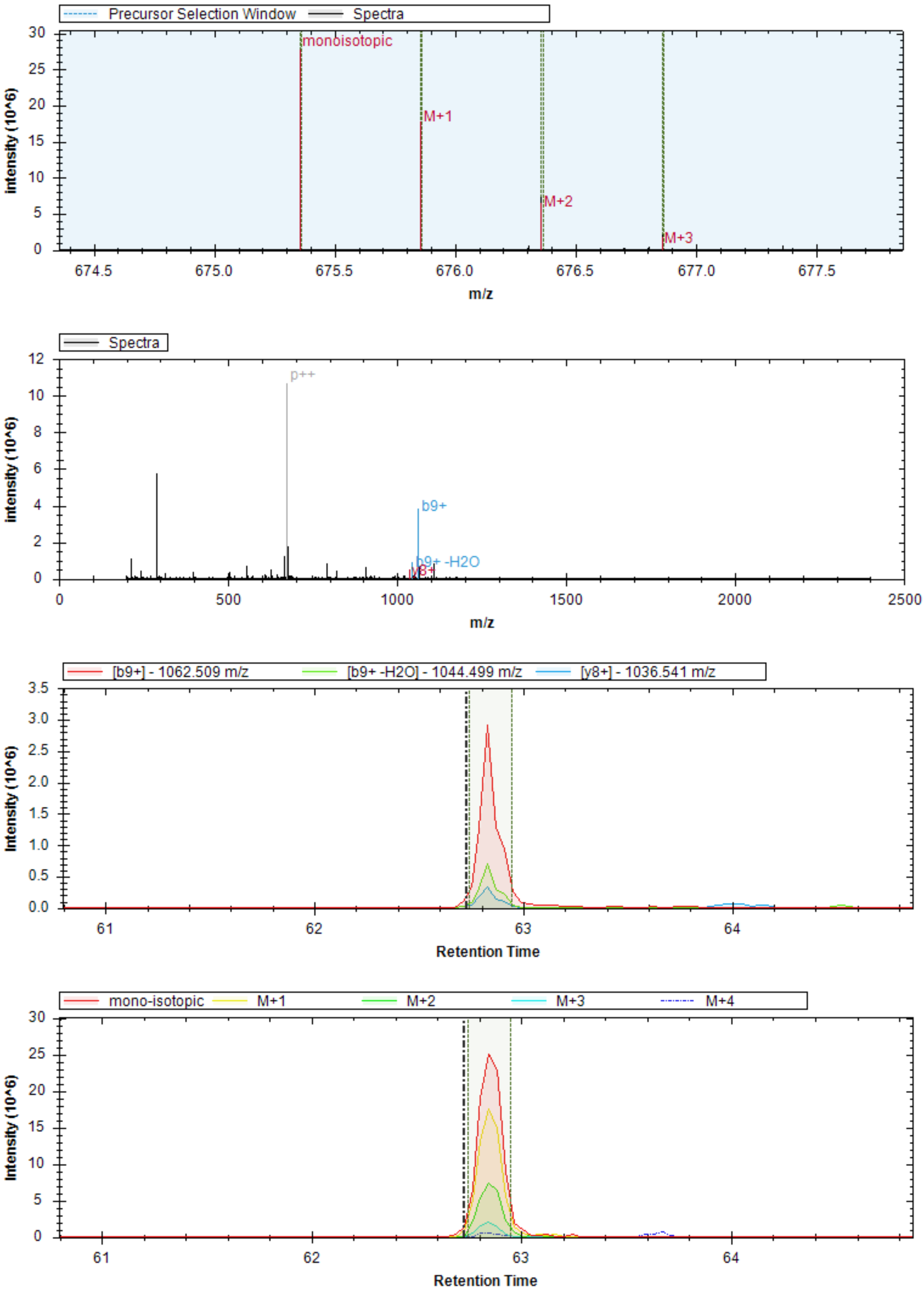

Q9JLM8 – _SPSPSPT[+H+O3+P]SPGSLR_.2
Fig4_MP-DIA-barrel-cortex-p15-2_MHRM.htrms

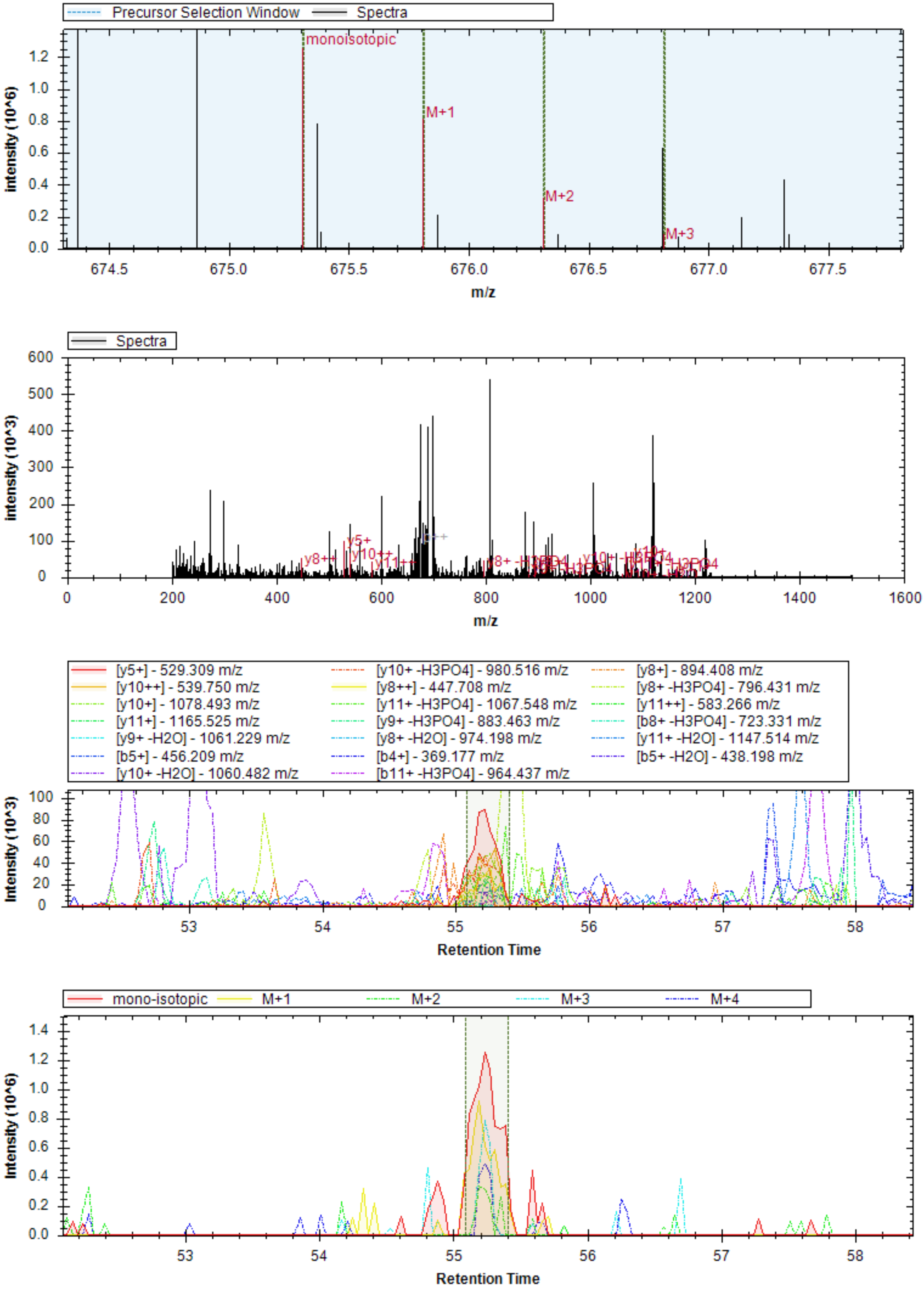

O88935 – _PVAGGPGAPPAARPPAS[+H+O3+P]PSPQR_.3

Fig4_MP-DIA-barrel-cortex-p15-2_MHRM.htrms

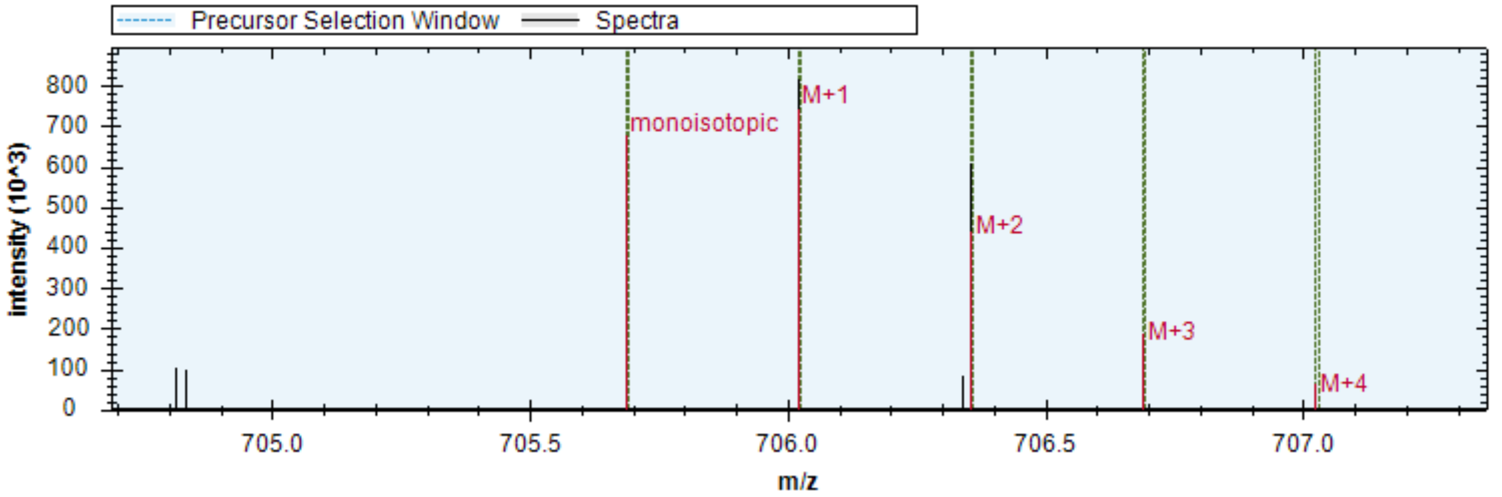

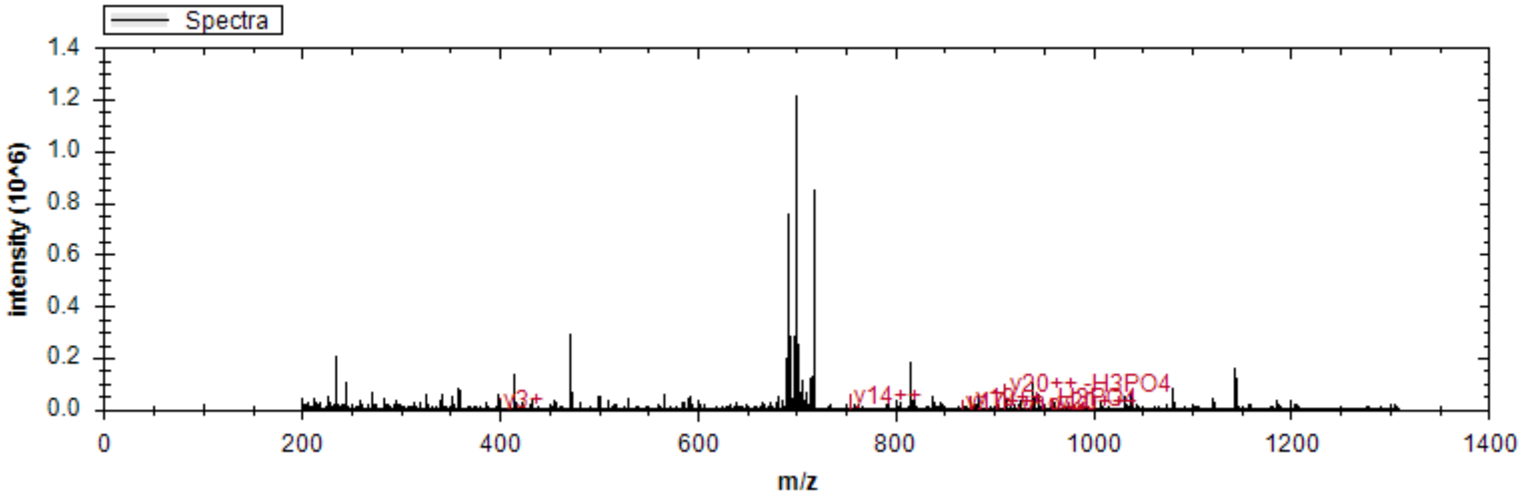

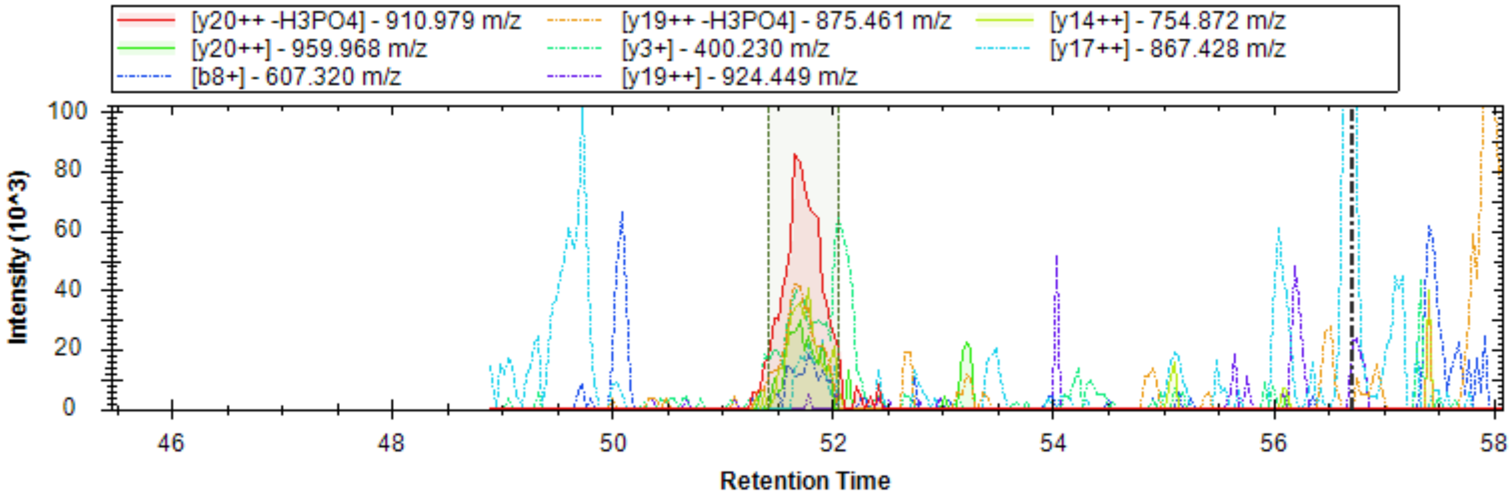

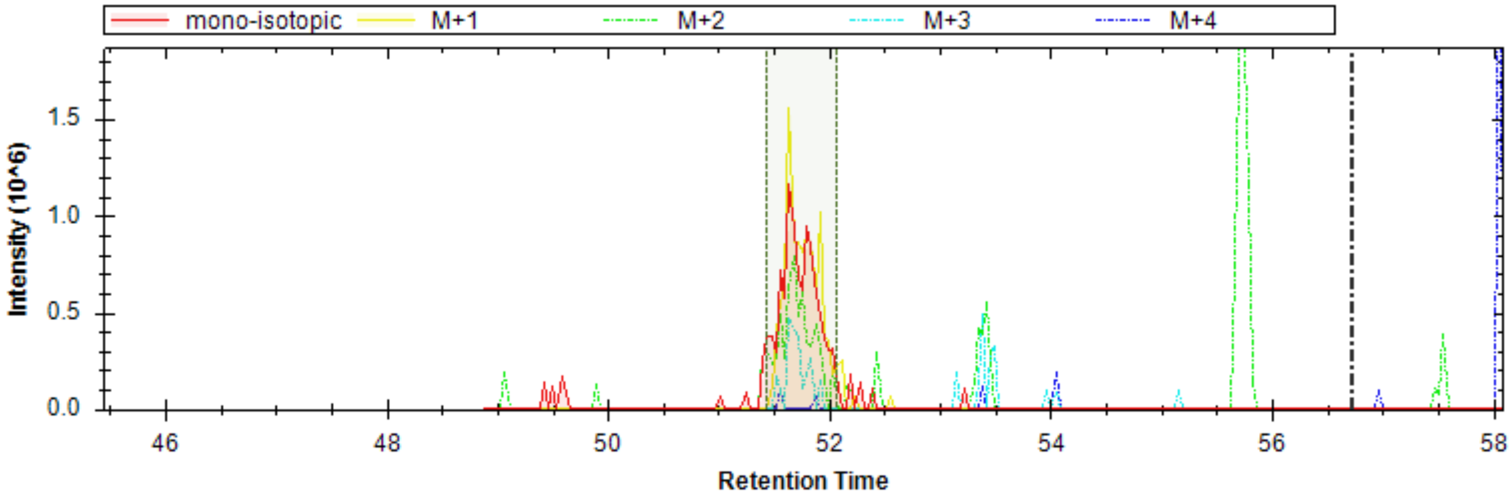

O88935 – _PVAGGPGAPPAARPPASPS[+H+O3+P]PQR_.3

Fig4_MP-DIA-barrel-cortex-p15-2_MHRM.htrms

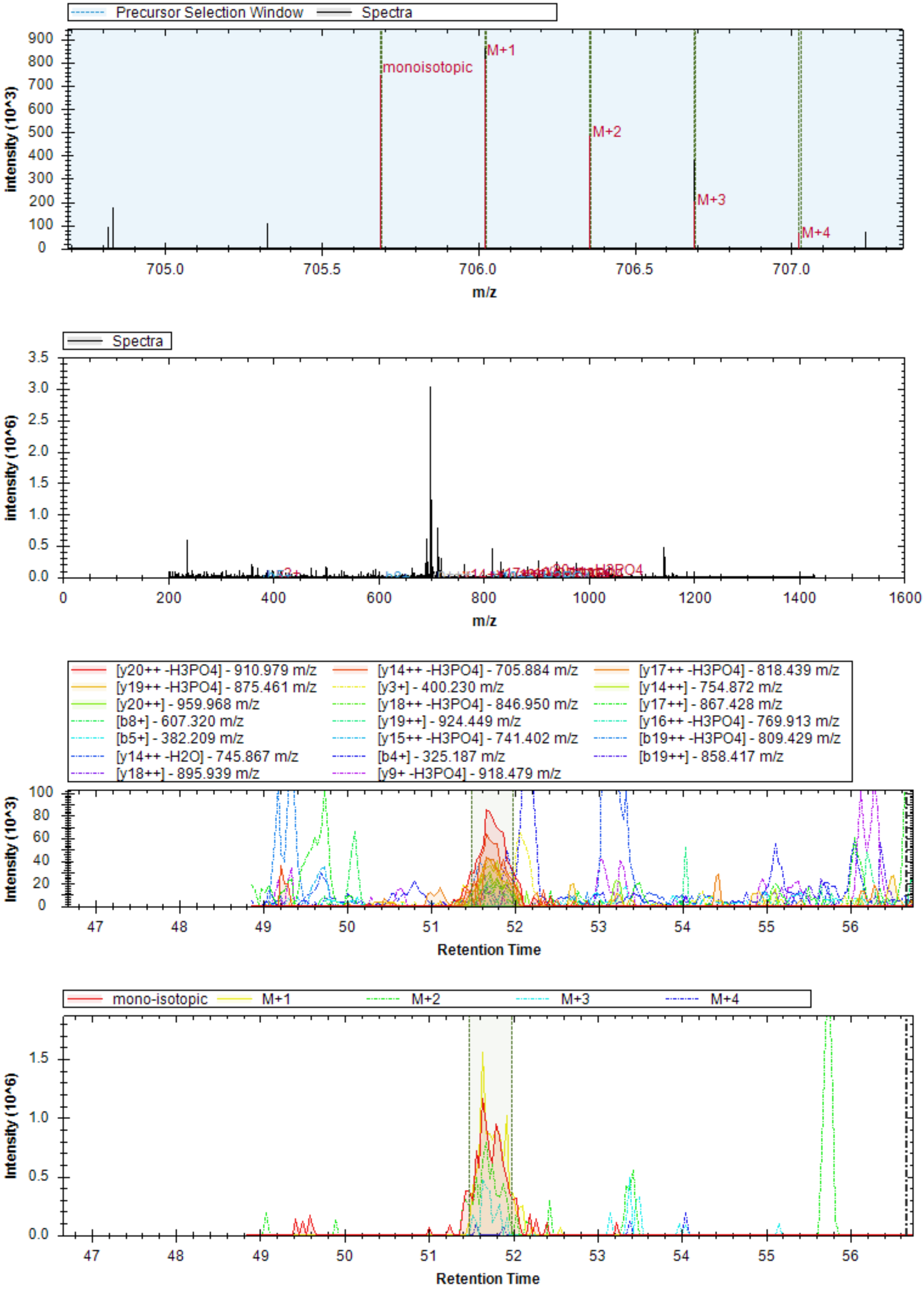

P28738 – _PIRPGHYPAS[+H+O3+P]SPTAVHAVR_.3
Fig4_MP-DIA-barrel-cortex-p15-2_MHRM.htrms

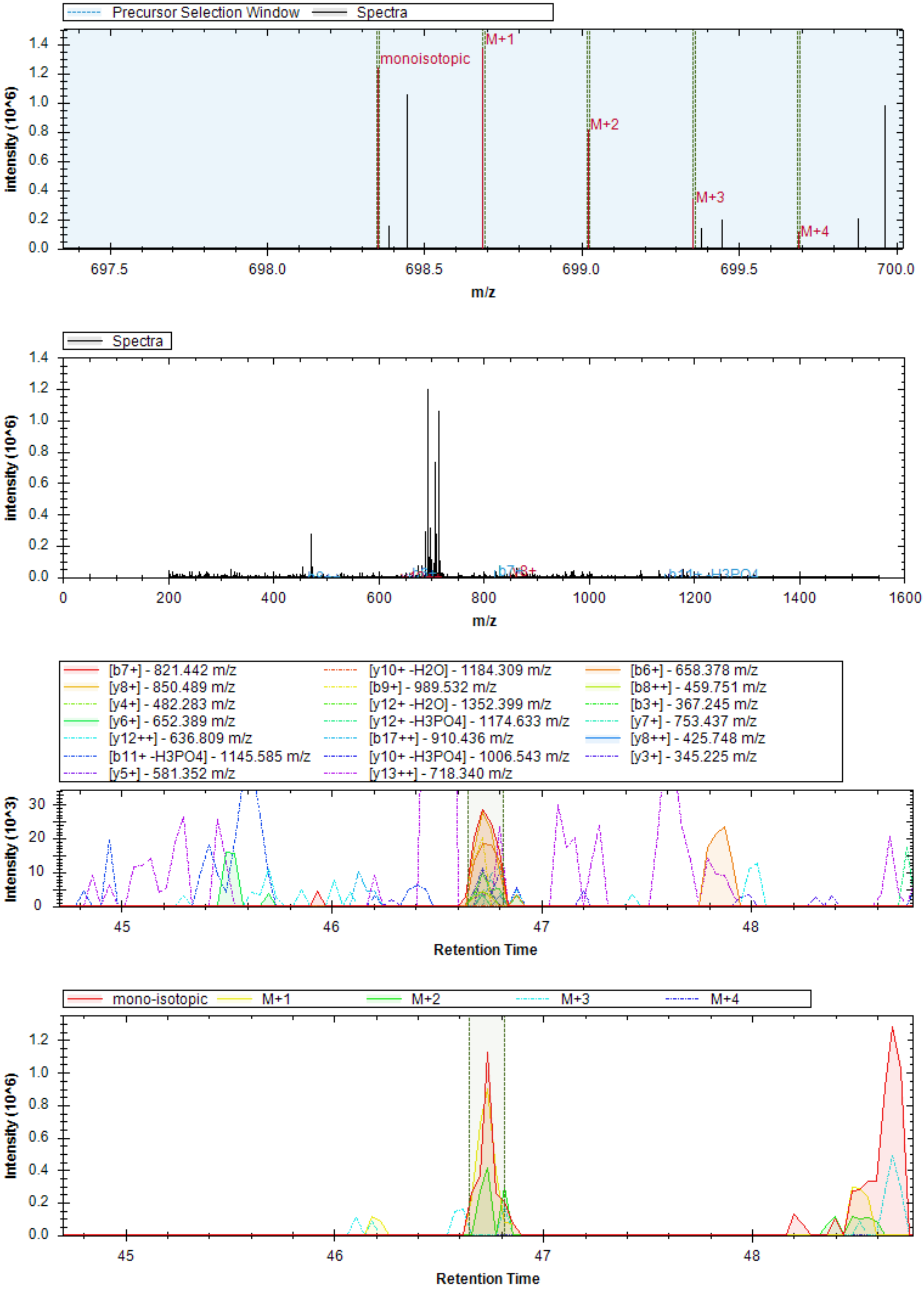

P28738 – _PIRPGHYPASS[+H+O3+P]PTAVHAVR_.3
Fig4_MP-DIA-barrel-cortex-p15-2_MHRM.htrms

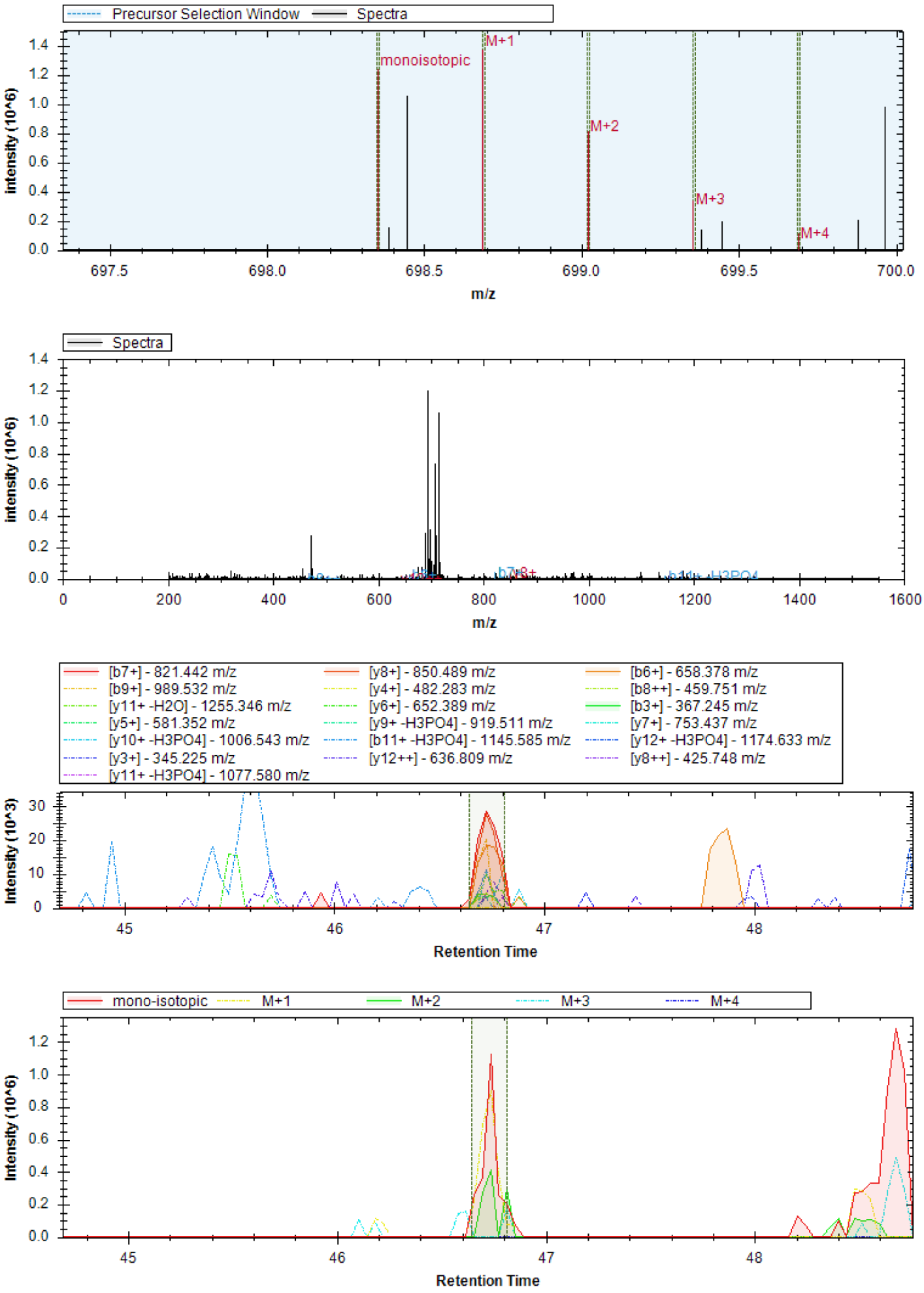

P62751 – _KAY[+H+O3+P]VRLAPDYDALDVANK_.3
Fig4_MP-DIA-barrel-cortex-p15-2_MHRM.htrms

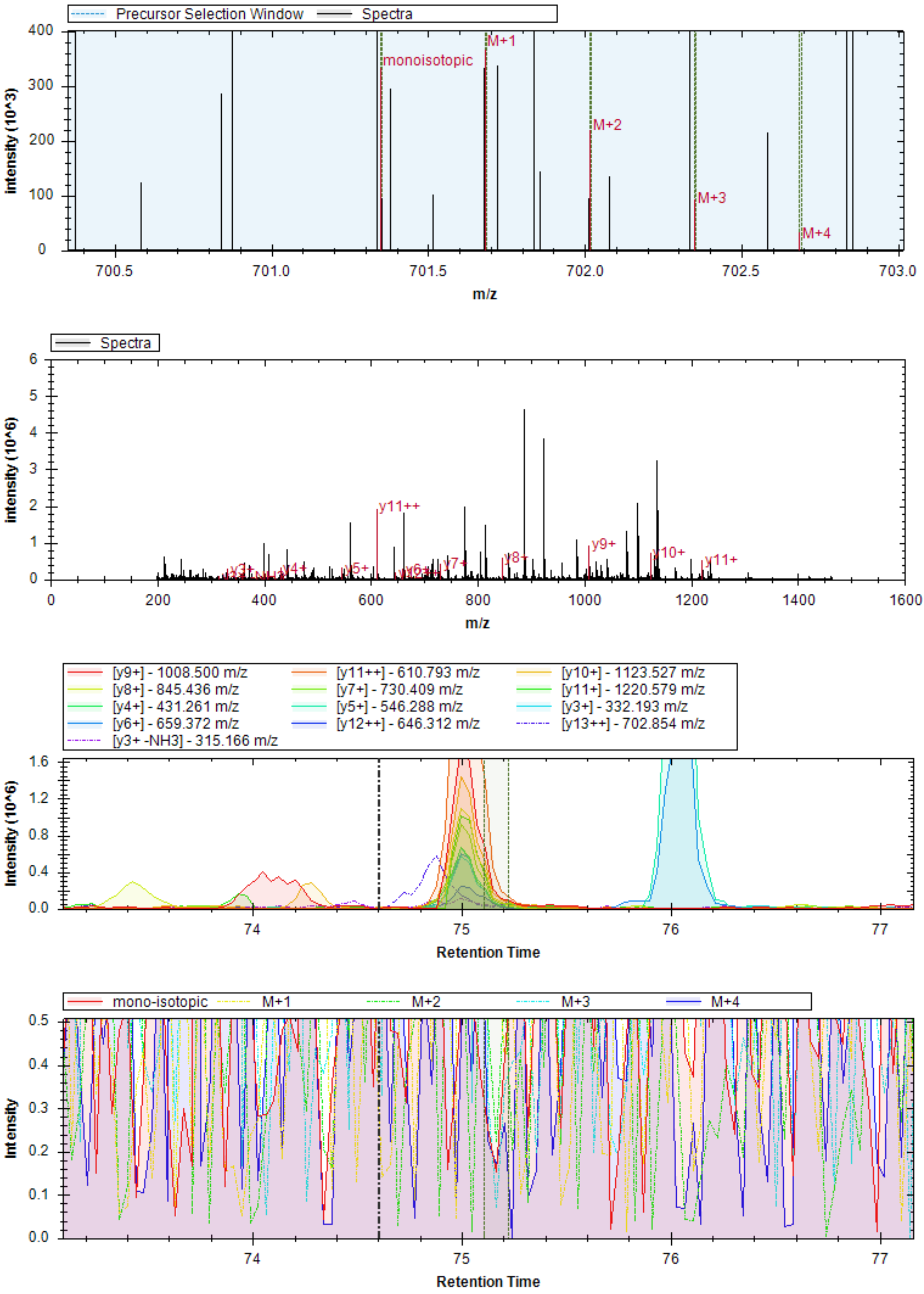

Q3U4B4 – _RHKT[+H+O3+P]LSANEEK_.2

Fig4_MP-DIA-barrel-cortex-p15-2_MHRM.htrms

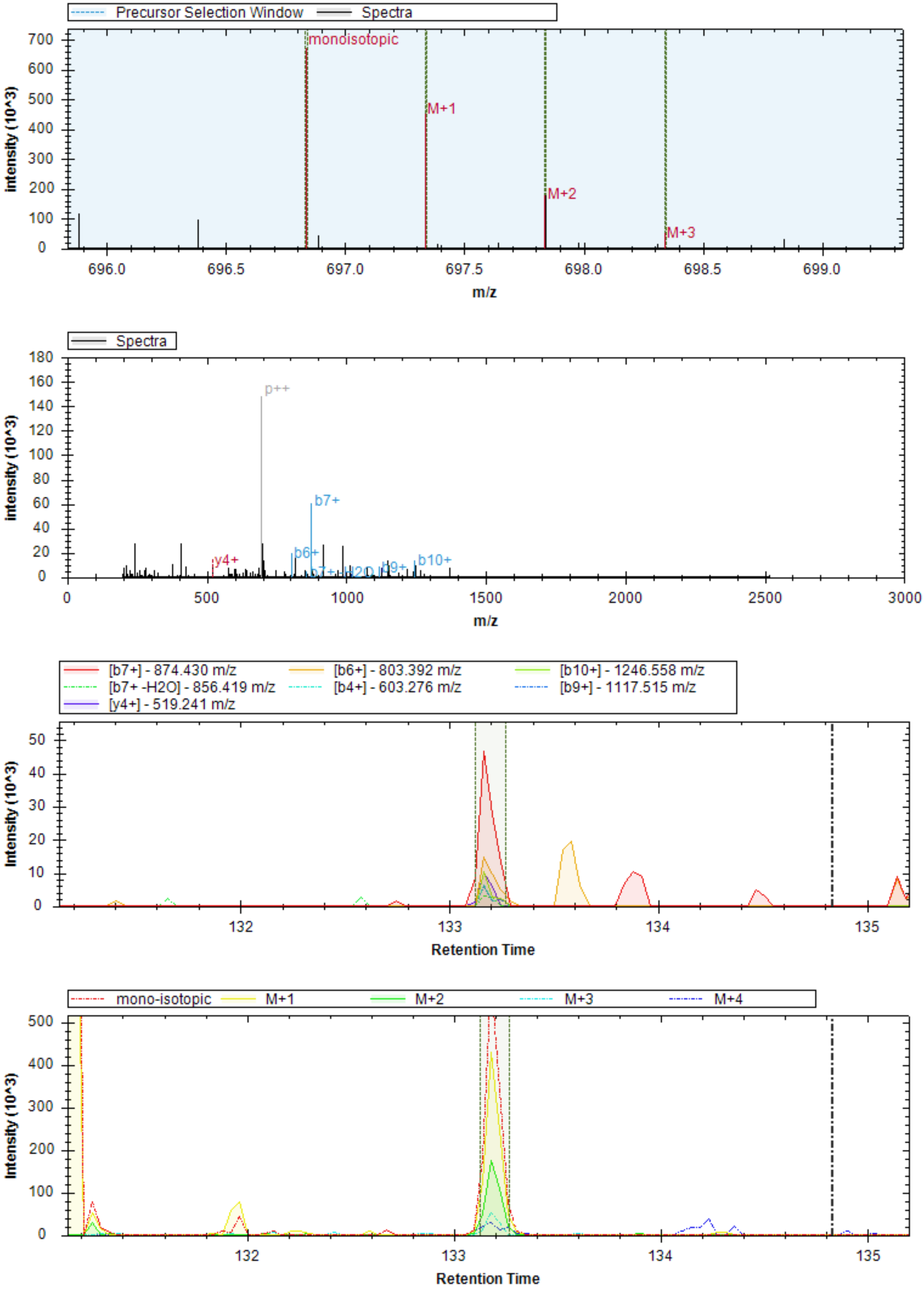

Q8CAA7 – _AVAGVM[+O]ITAS[+H+O3+P]HNR_.2

Fig4_MP-DIA-barrel-cortex-p15-2_MHRM.htrms

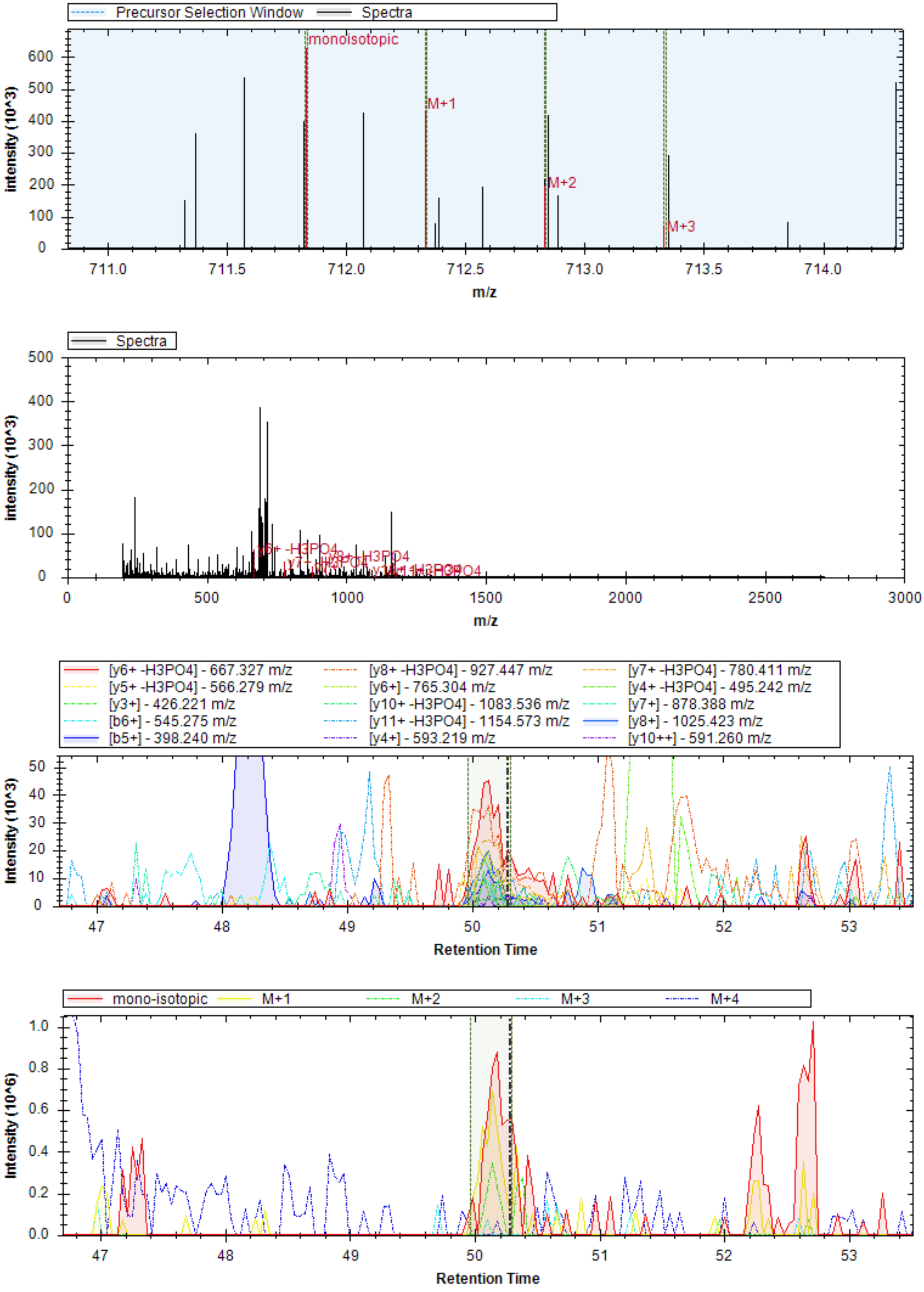

P10637 – _SGYSS[+H+O3+P]PGSPGTPGSR_.2

Fig4_MP-DIA-barrel-cortex-p15-2_MHRM.htrms

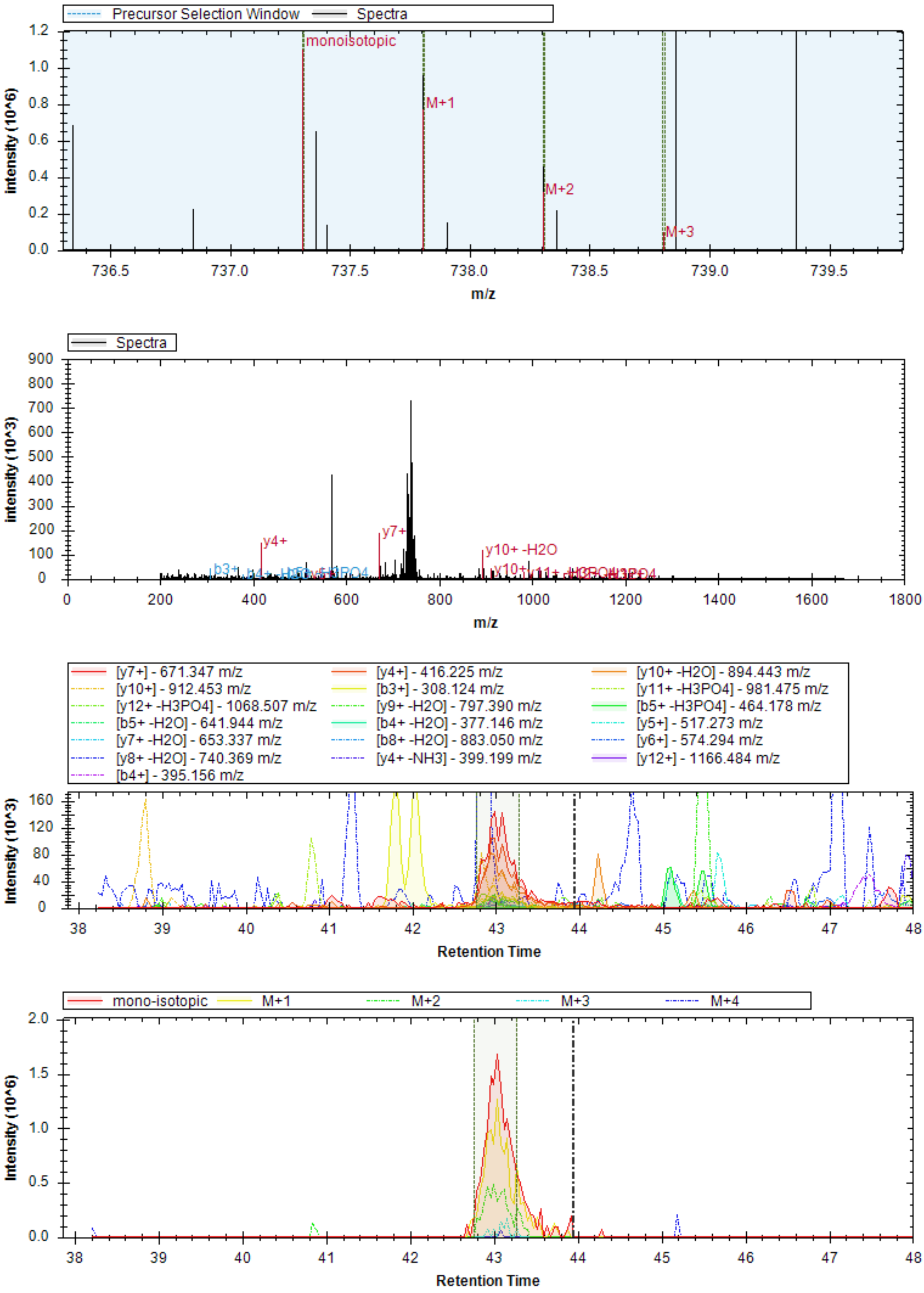

P10637 – _SGYSSPGS[+H+O3+P]PGTPGSR_.2
Fig4_MP-DIA-barrel-cortex-p15-2_MHRM.htrms

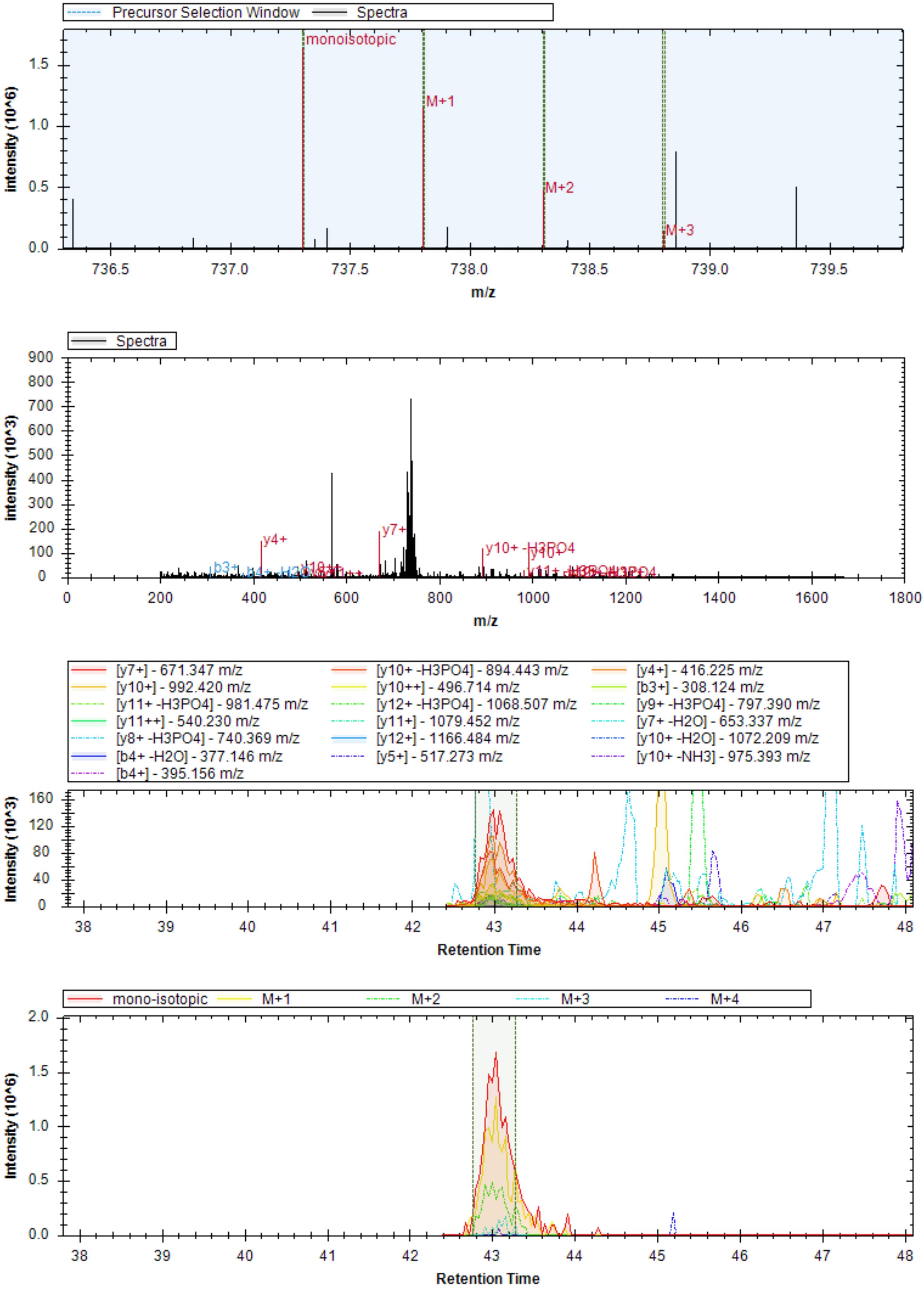

P97798-2 – _NSQDIT[+H+O3+P]PVDNSMDSNIHQR_.3
Fig4_MP-DIA-barrel-cortex-p15-2_MHRM.htrms

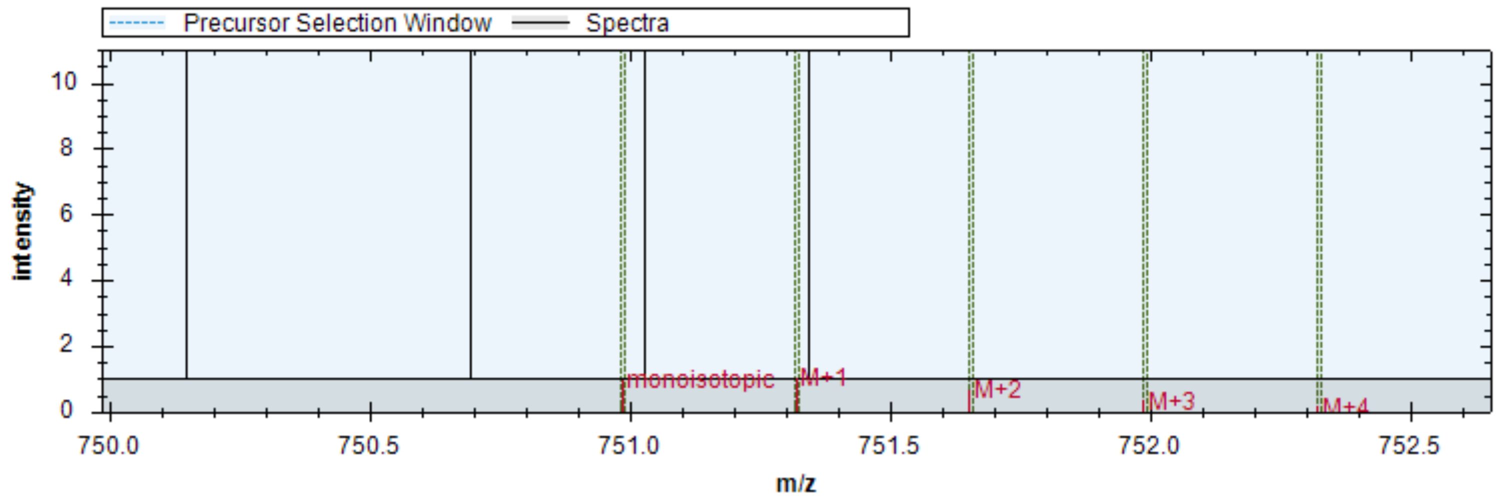

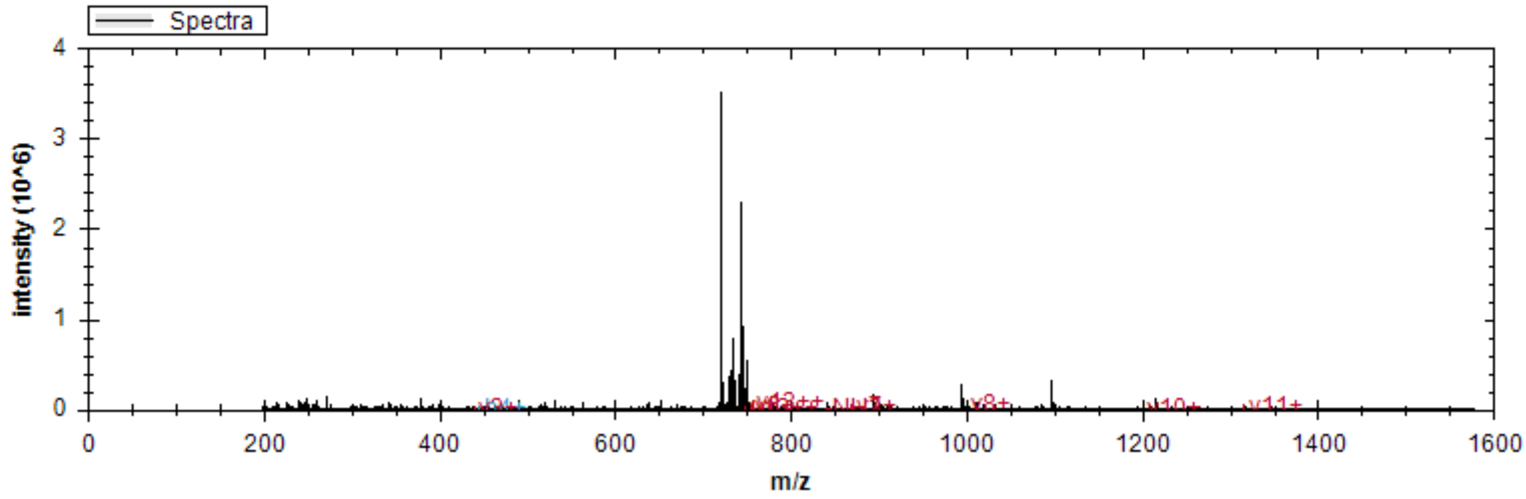

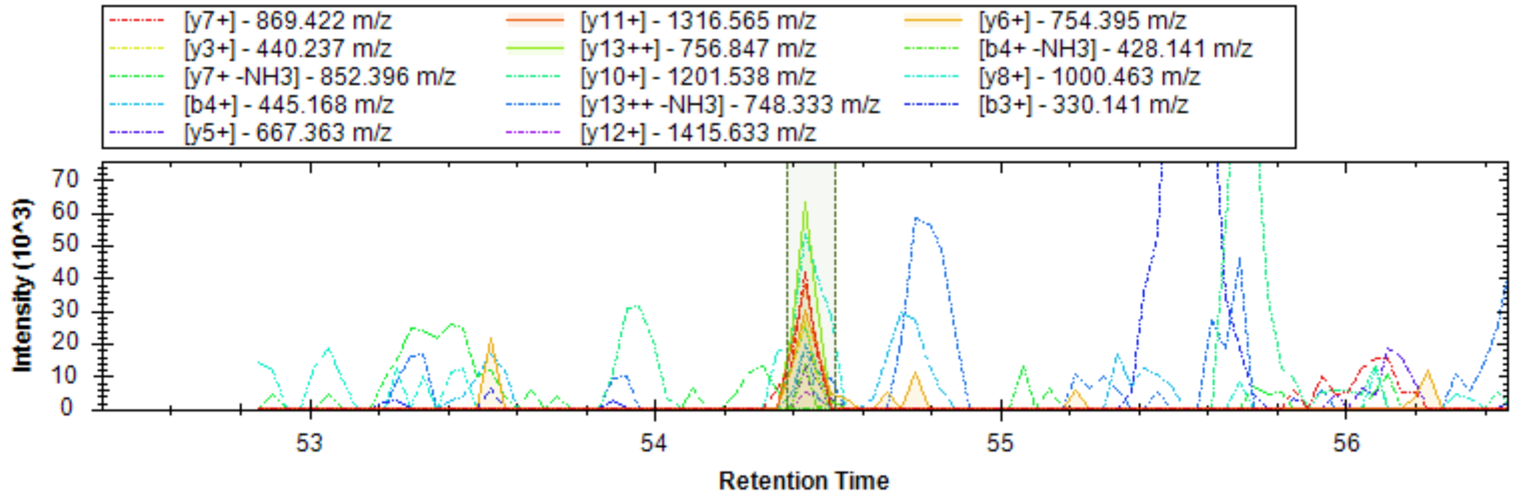

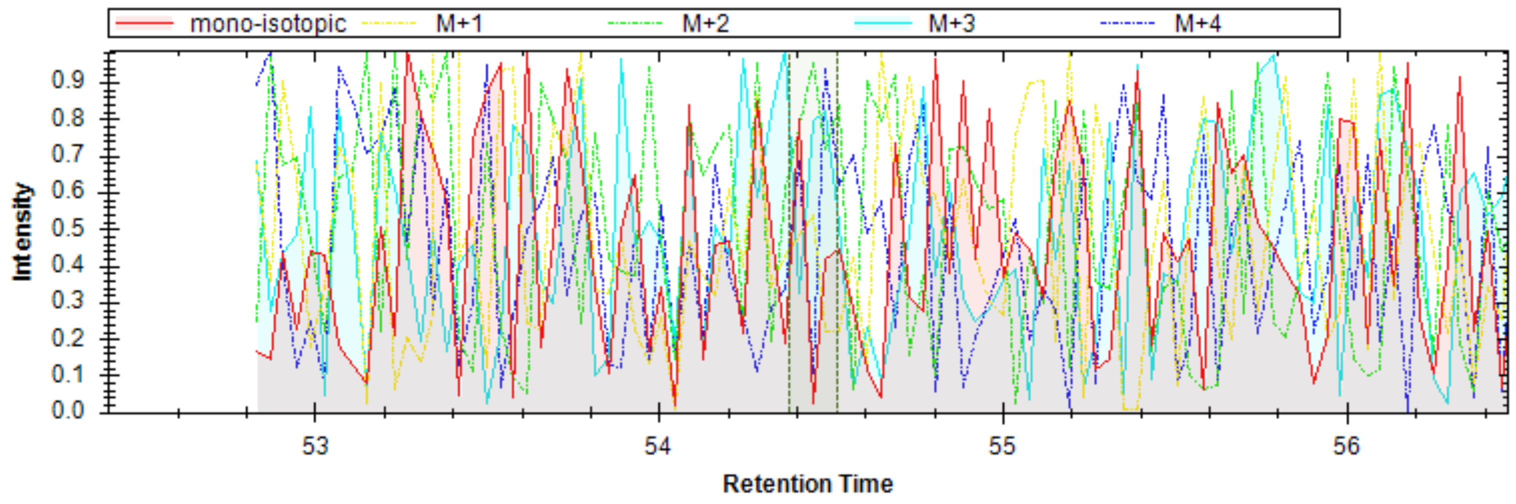

Q3UNH4 – _DLAAAAAQKS[+H+O3+P]PSAEAAAPPPGPR_.3
Fig4_MP-DIA-barrel-cortex-p15-2_MHRM.htrms

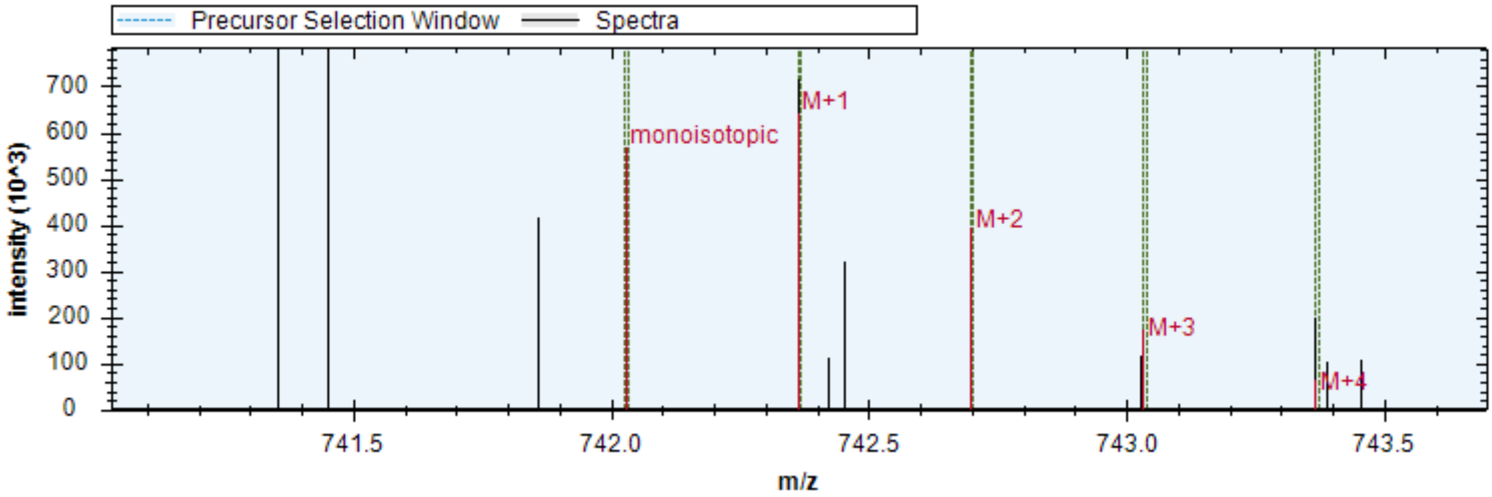

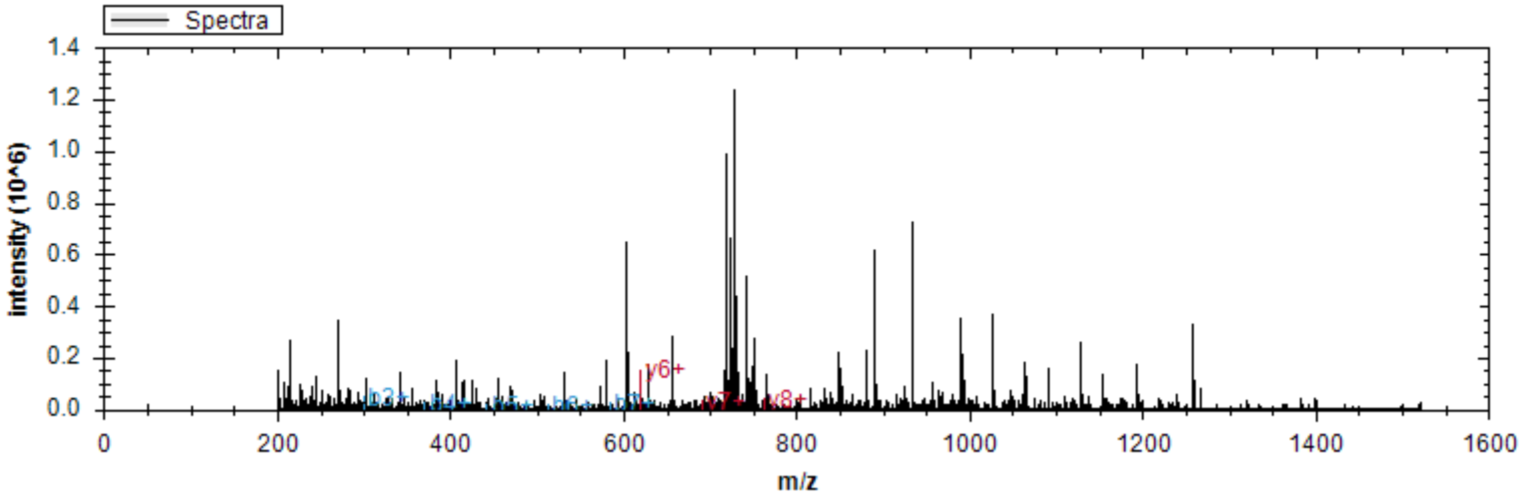

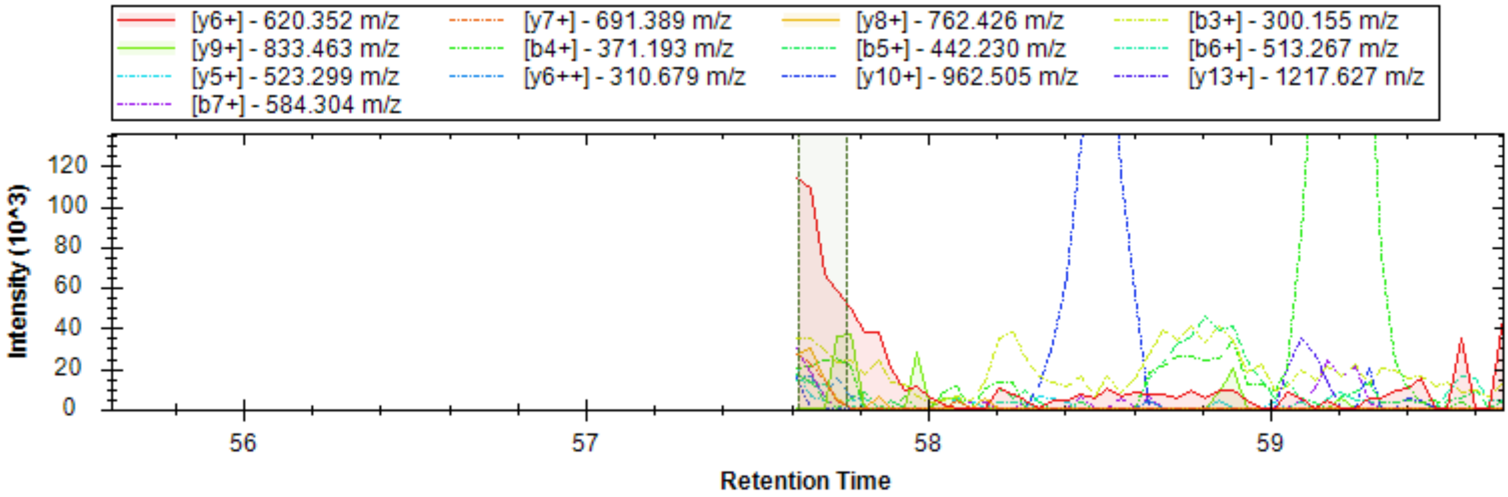

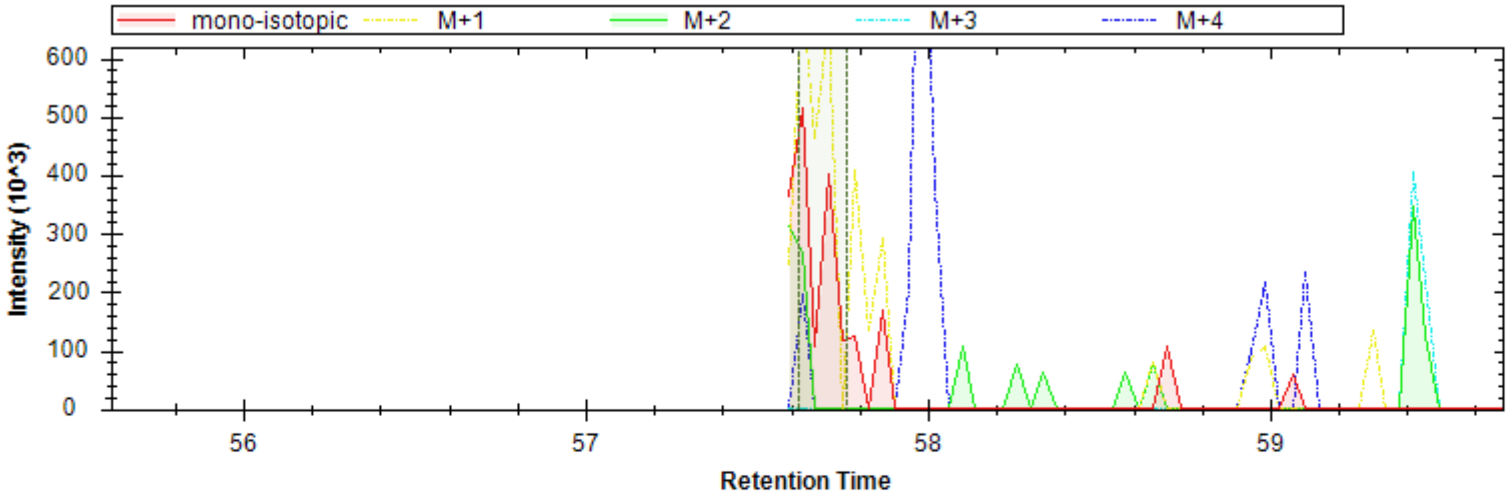

Q8C5R2 – _LAGNEALS[+H+O3+P]PTSPSK_.2
Fig4_MP-DIA-barrel-cortex-p15-2_MHRM.htrms

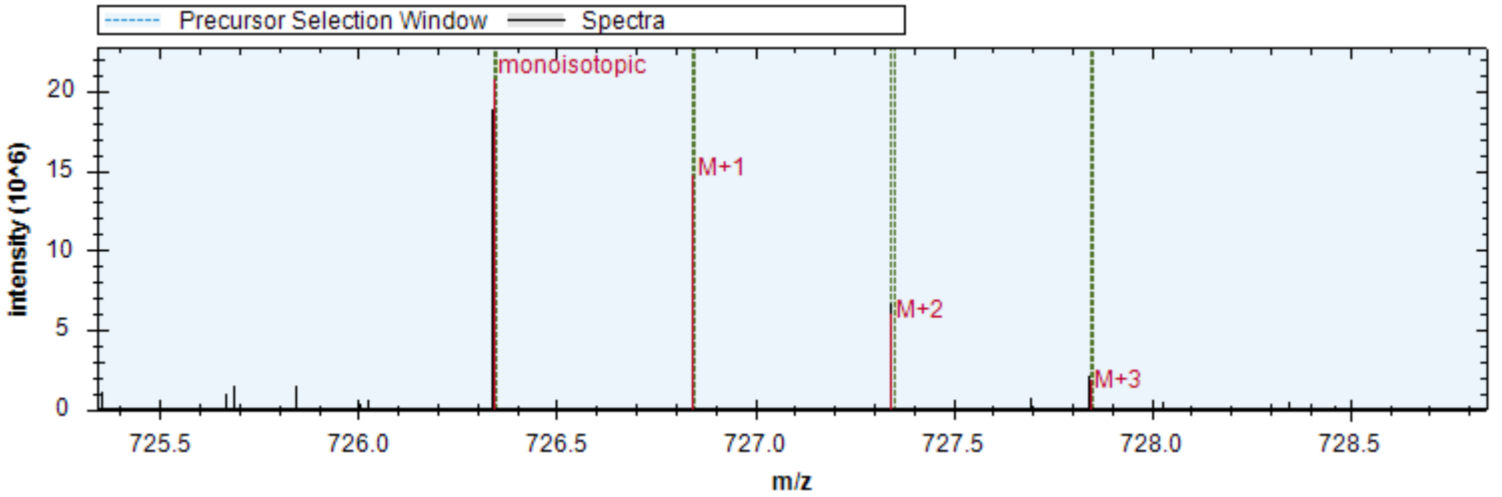

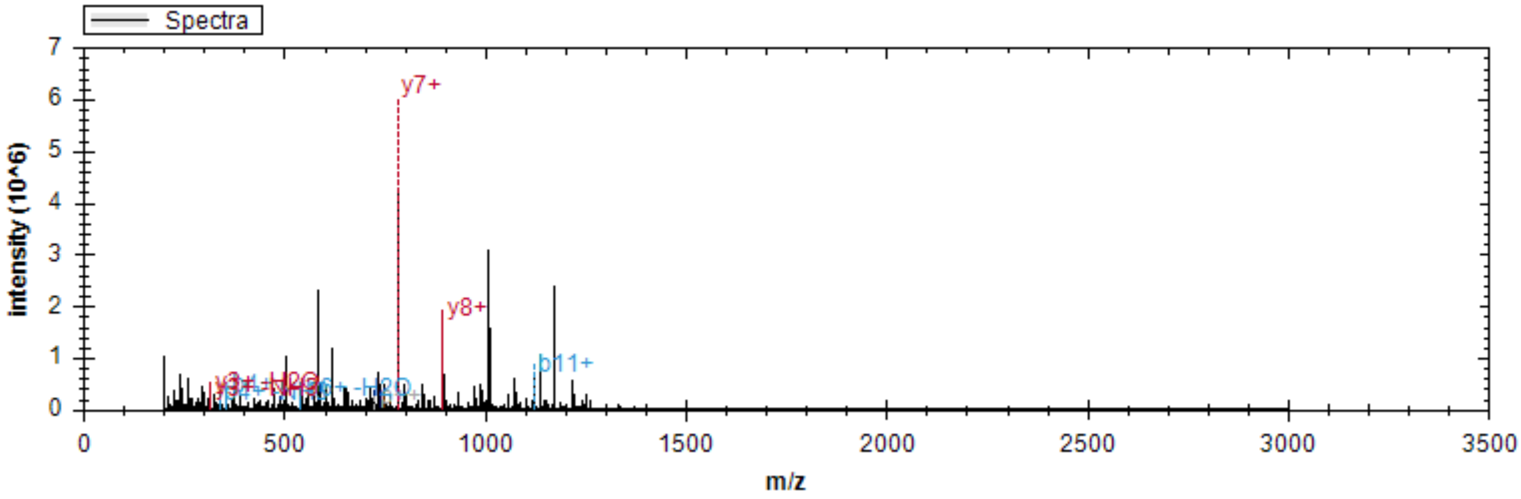

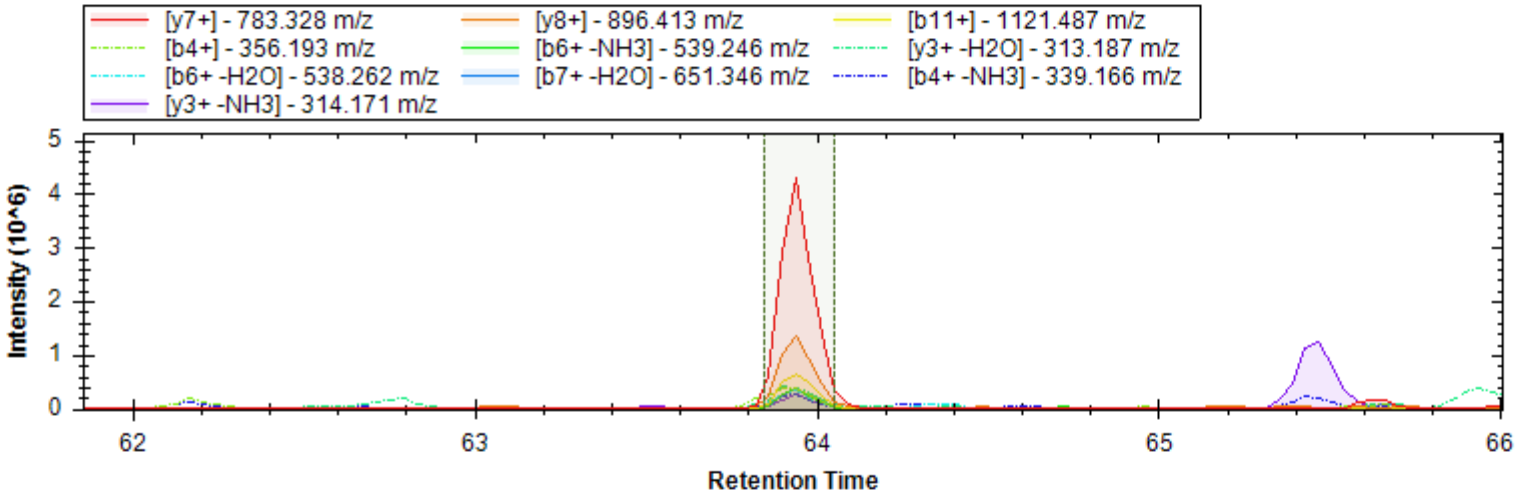

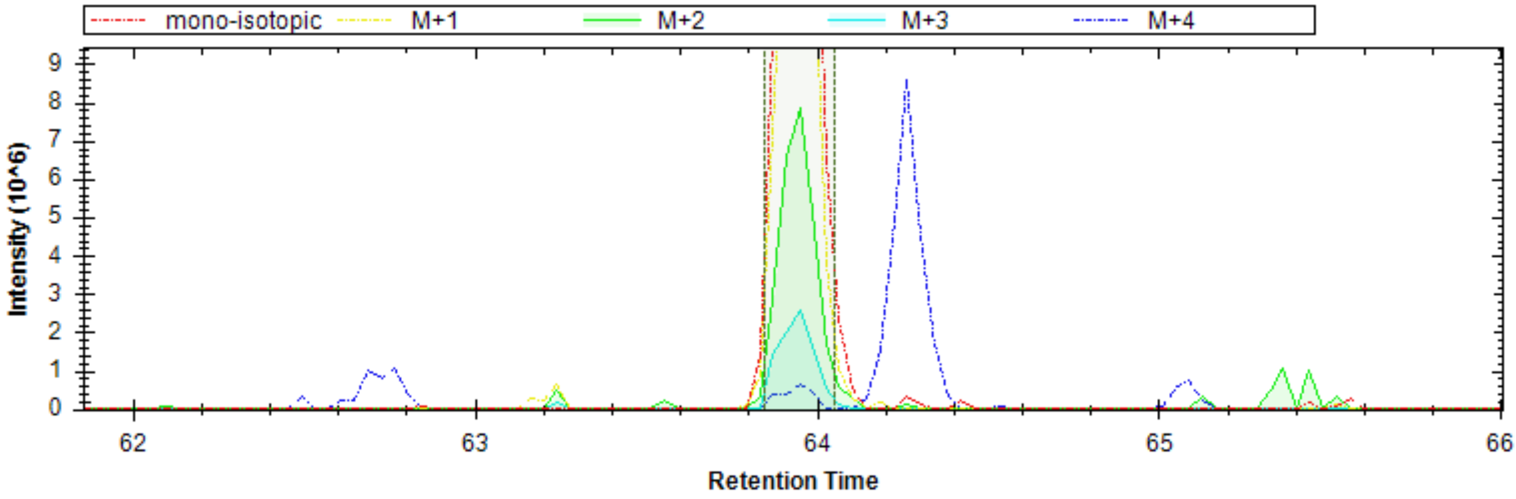

Q8K0U4 – _ETAPTSTYS[+H+O3+P]SPAR_.2
Fig4_MP-DIA-barrel-cortex-p15-2_MHRM.htrms

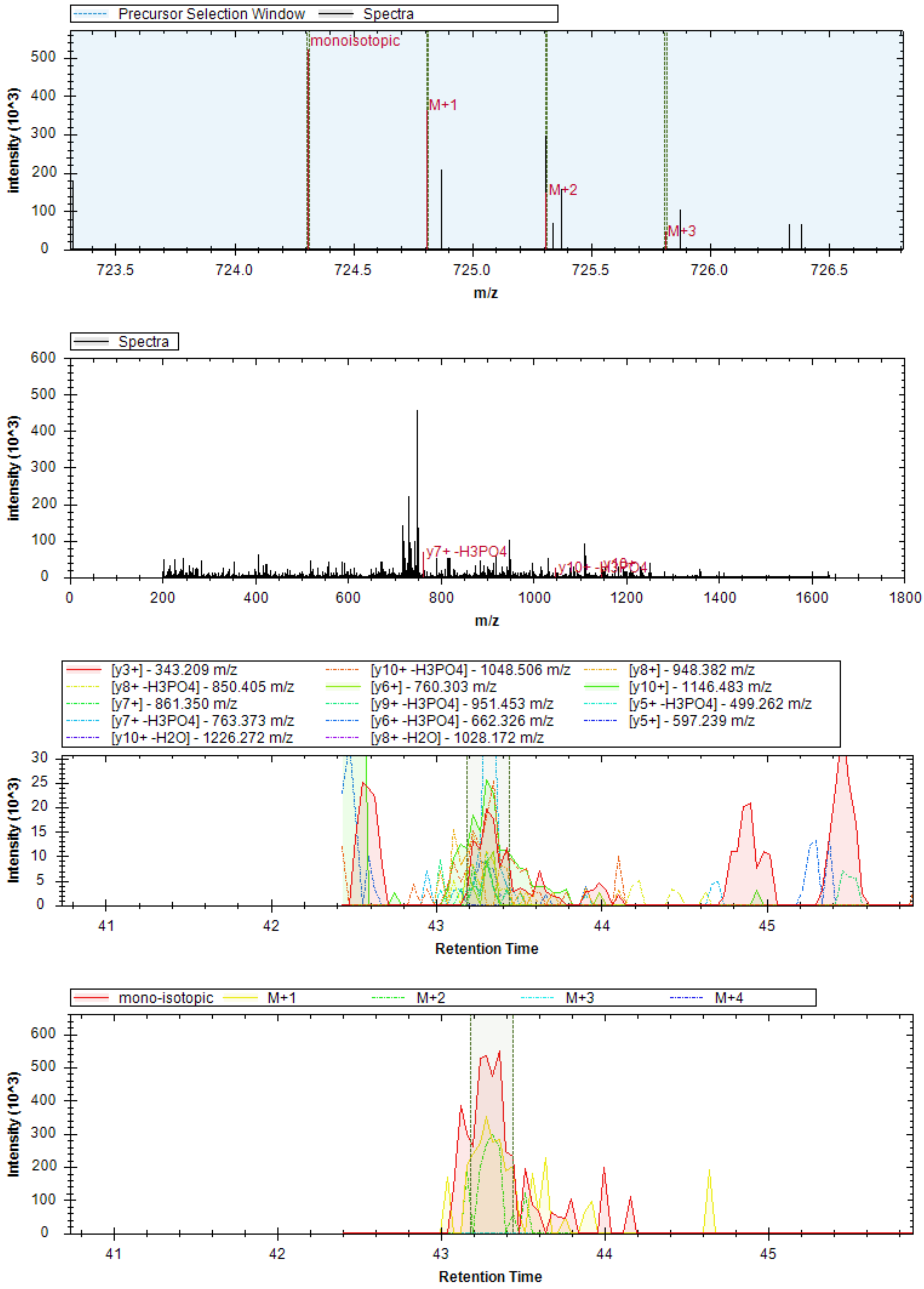

Q9CRB0 – _MEVYIPS[+H+O3+P]FRHEDSDLER_.3

Fig4_MP-DIA-barrel-cortex-p15-2_MHRM.htrms

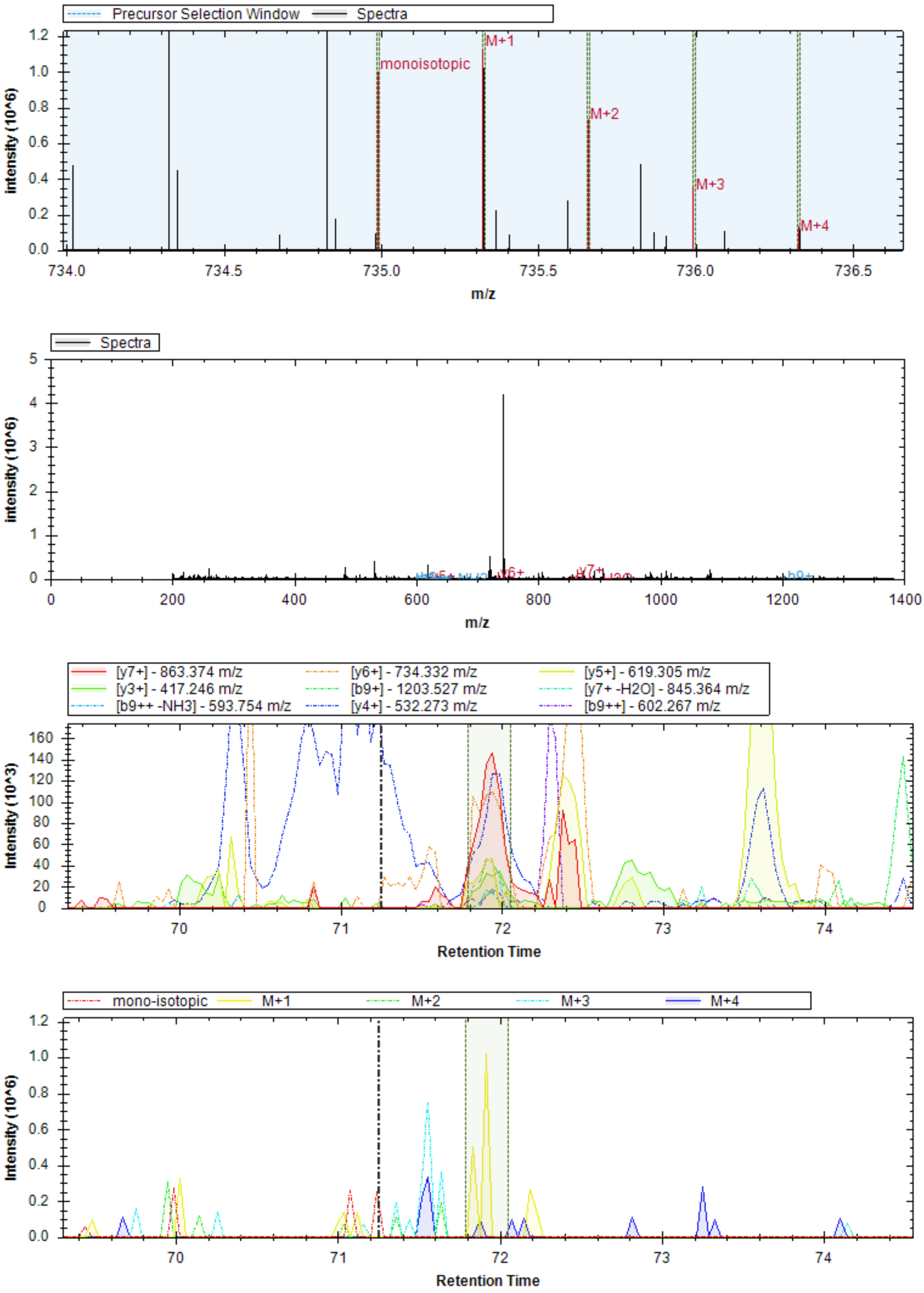

Q9CY18 – _M[+O]GQTVRAVAS[+H+O3+P]SMR_.2

Fig4_MP-DIA-barrel-cortex-p15-2_MHRM.htrms

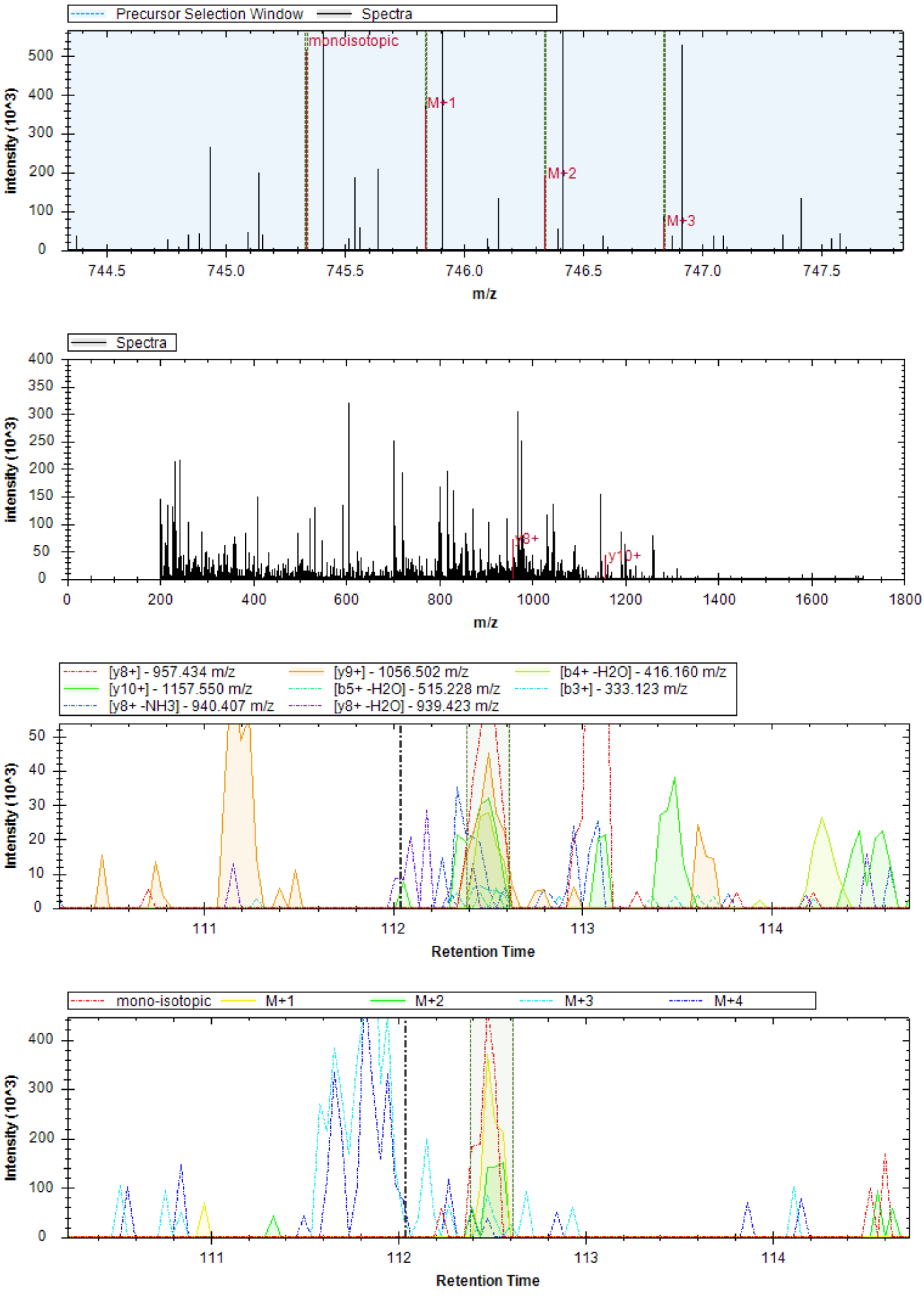

Q9JLM8 – _SKS[+H+O3+P]PASTSSVNGTPGSQLSTPR_.3

Fig4_MP-DIA-barrel-cortex-p15-2_MHRM.htrms

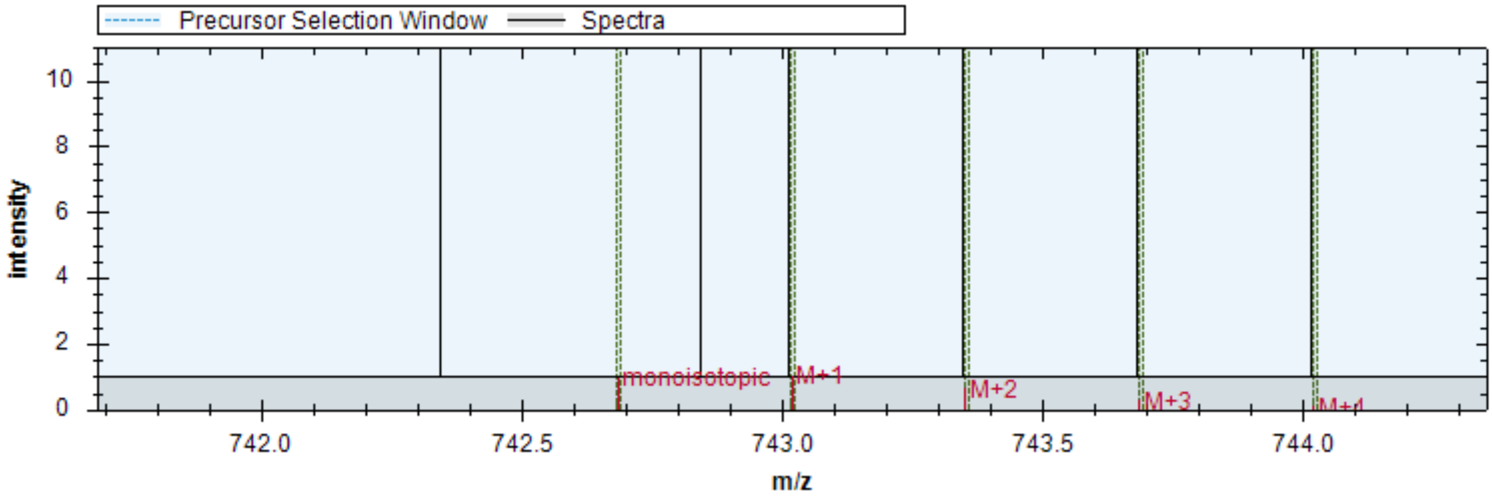

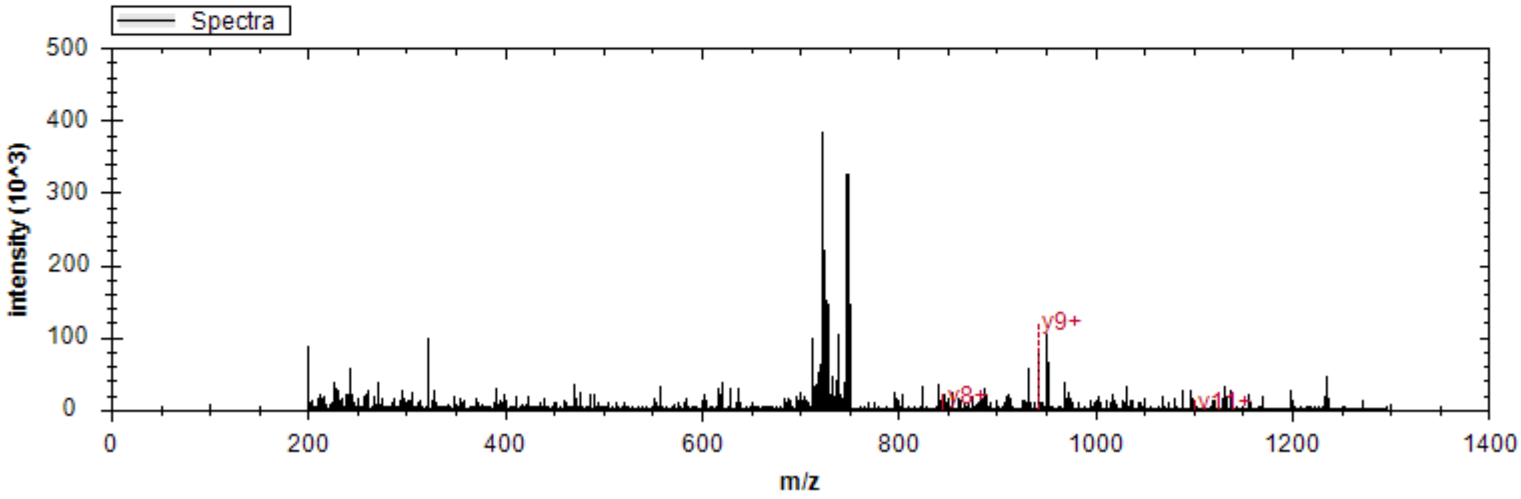

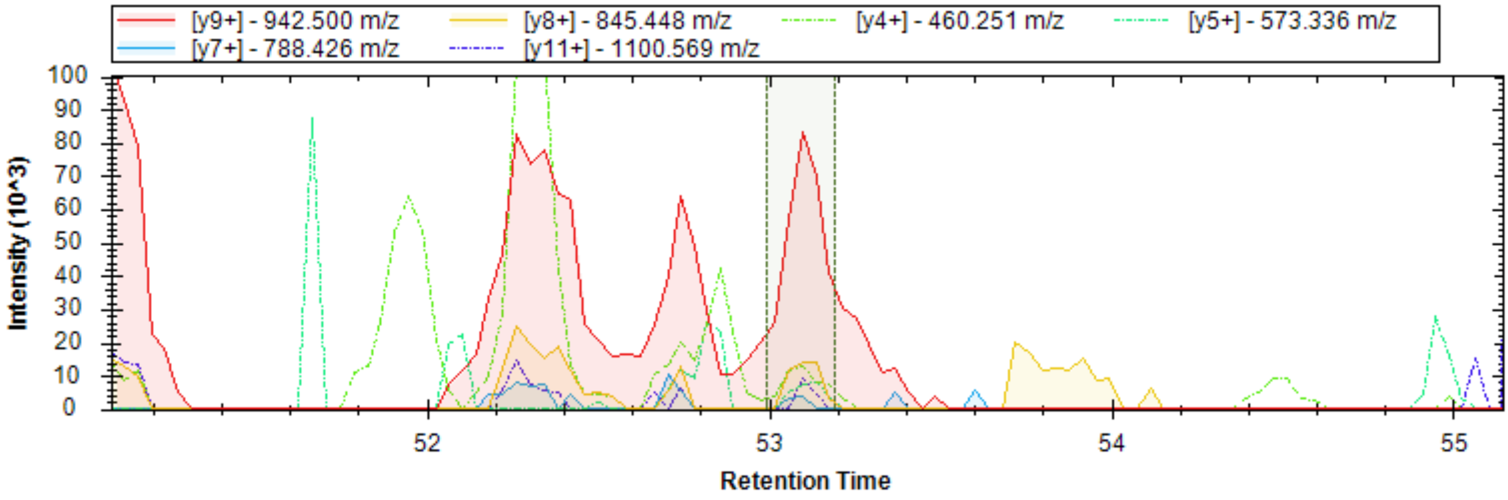

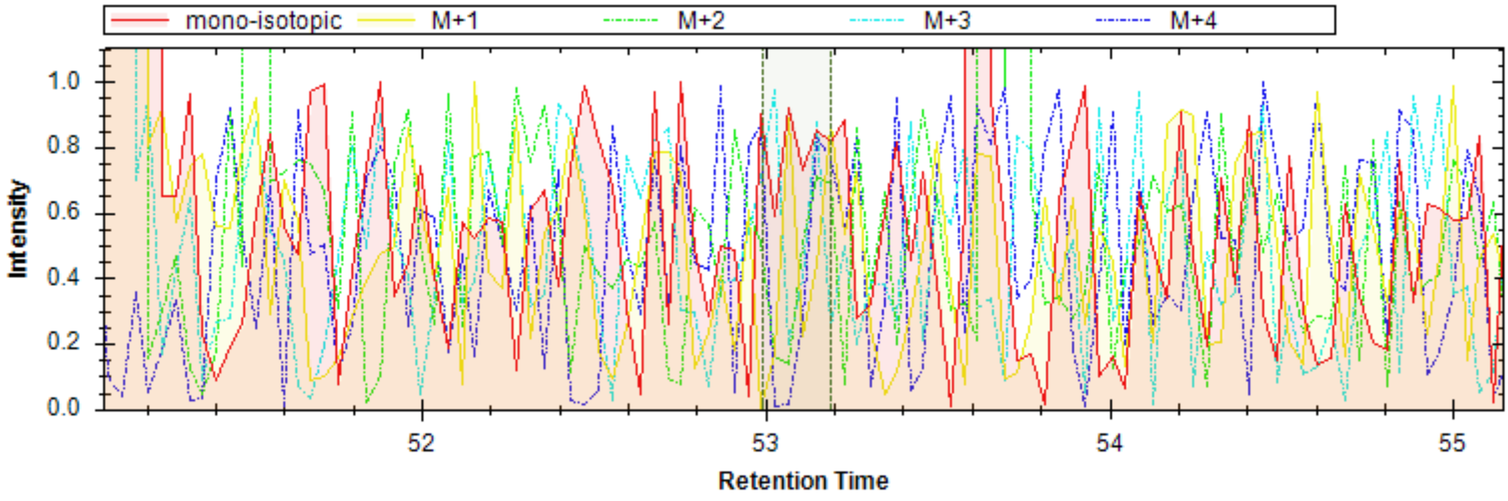

Q9JLM8 – _SPSPSPT[+H+O3+P]SPGSLRK_.2
Fig4_MP-DIA-barrel-cortex-p15-2_MHRM.htrms

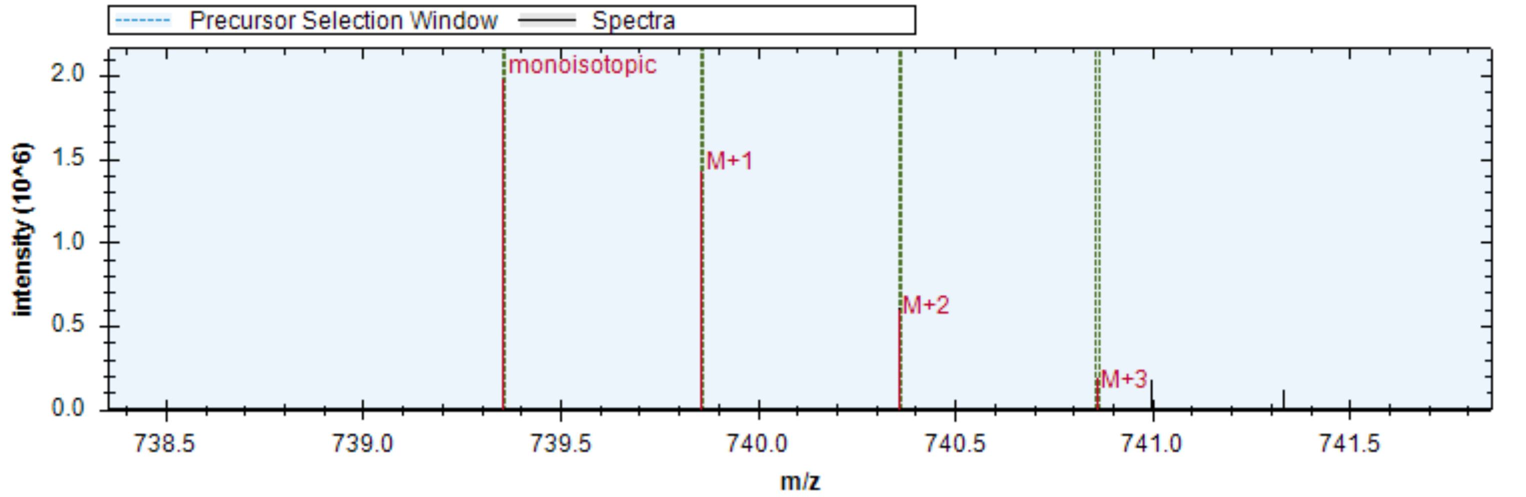

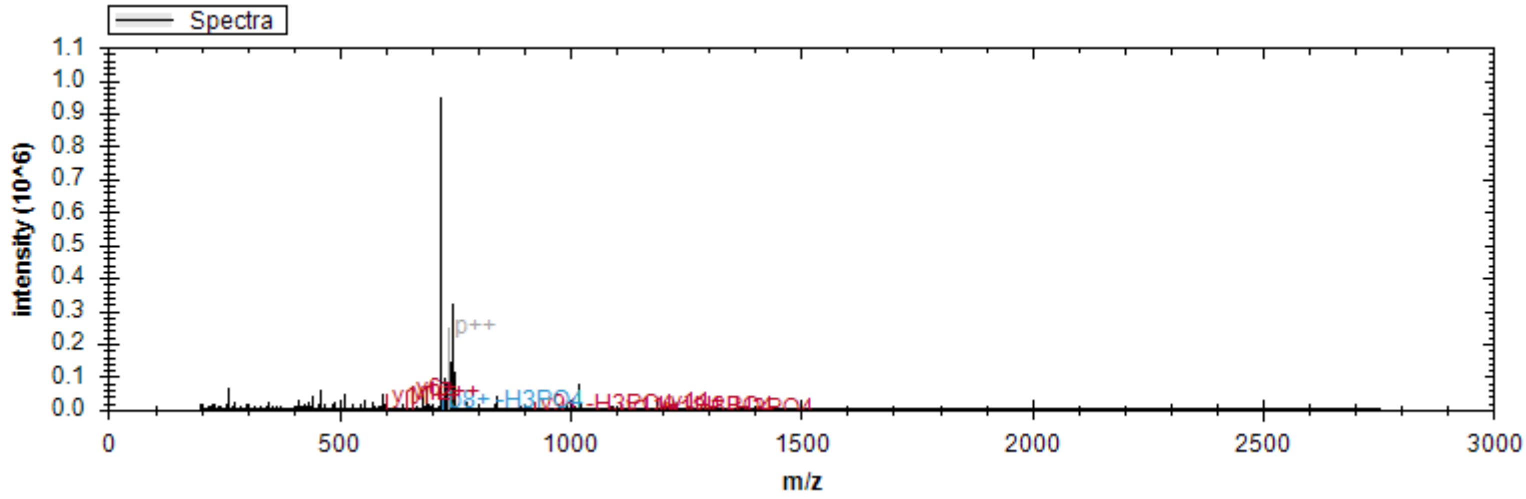

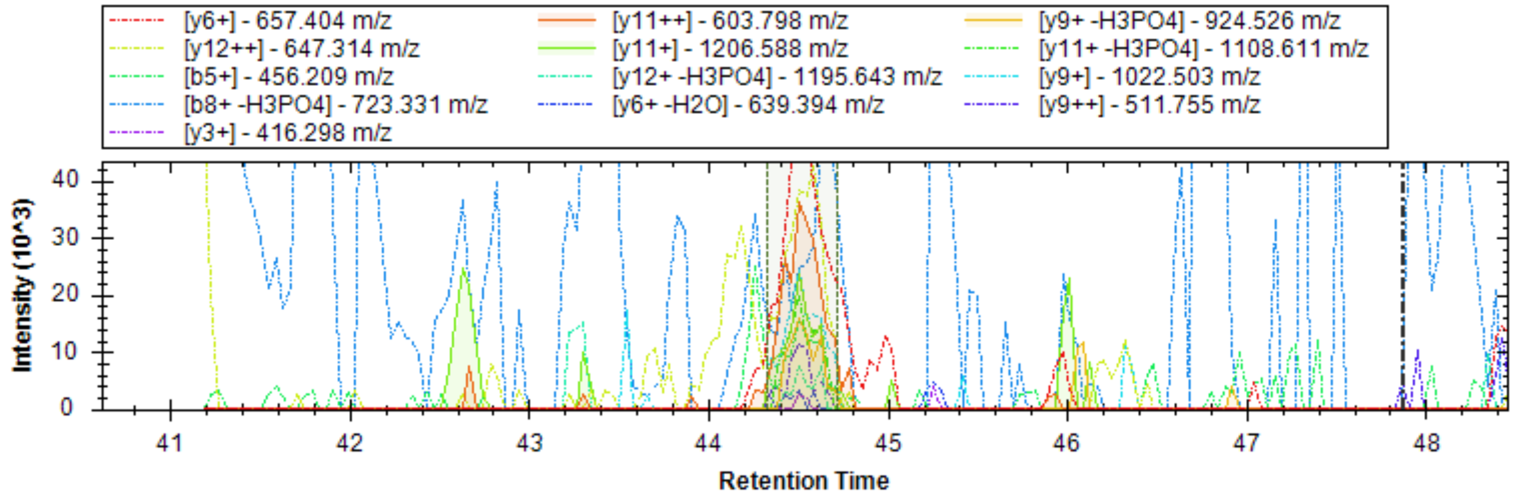

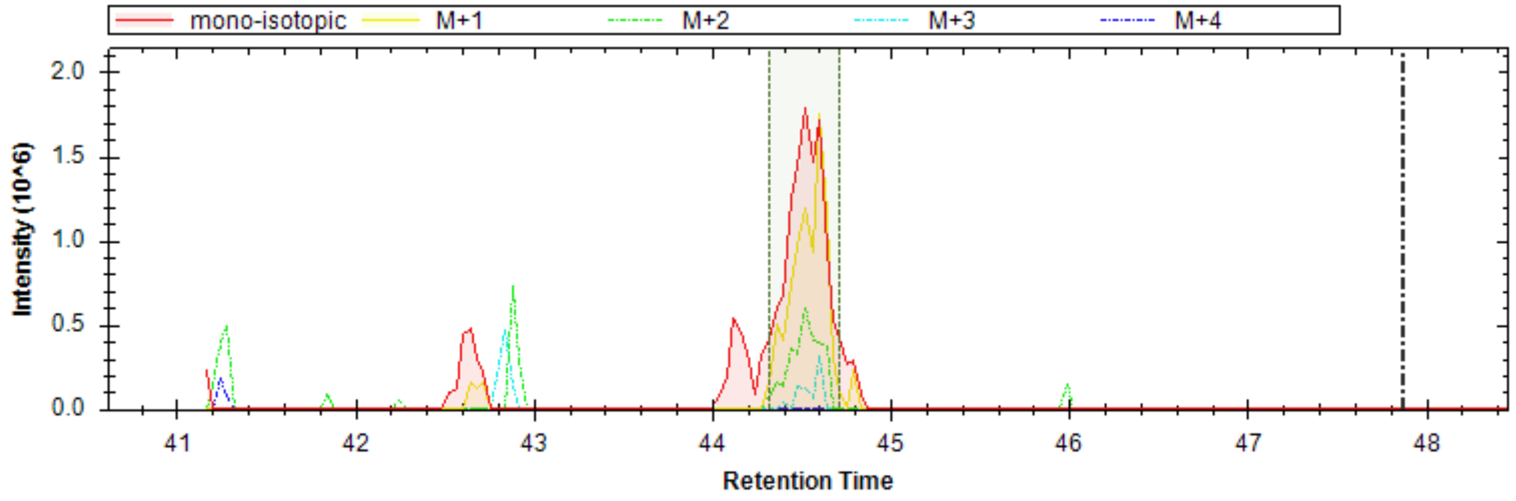

Q9JLM8 – _SPSPSPTS[+H+O3+P]PGSLRK_.2
Fig4_MP-DIA-barrel-cortex-p15-2_MHRM.htrms

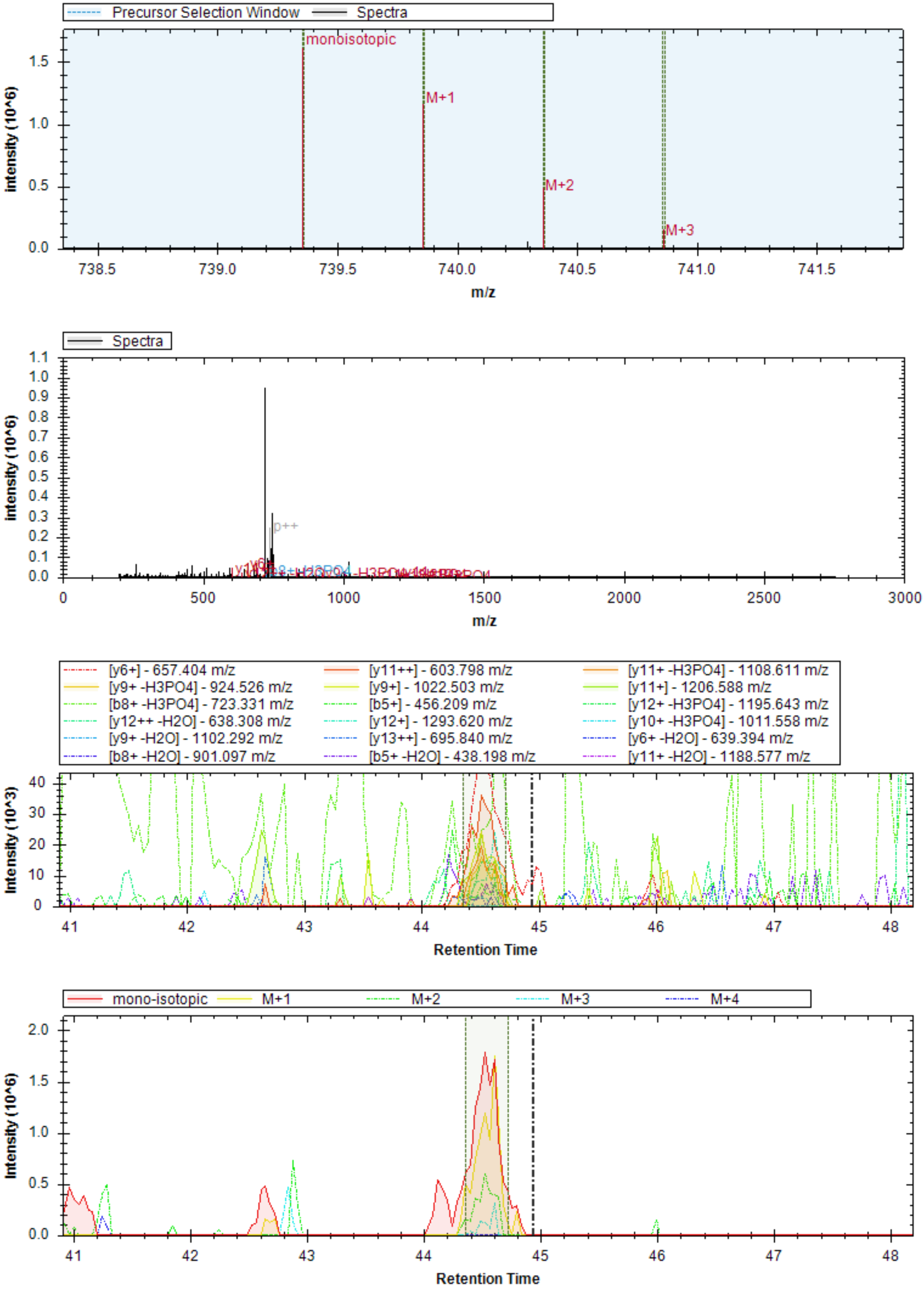

D3Z6S9 – _IPSIEIT[+H+O3+P]ET[+H+O3+P]LPQTQASMLR_.3

Fig4_MP-DIA-barrel-cortex-p15-2_MHRM.htrms

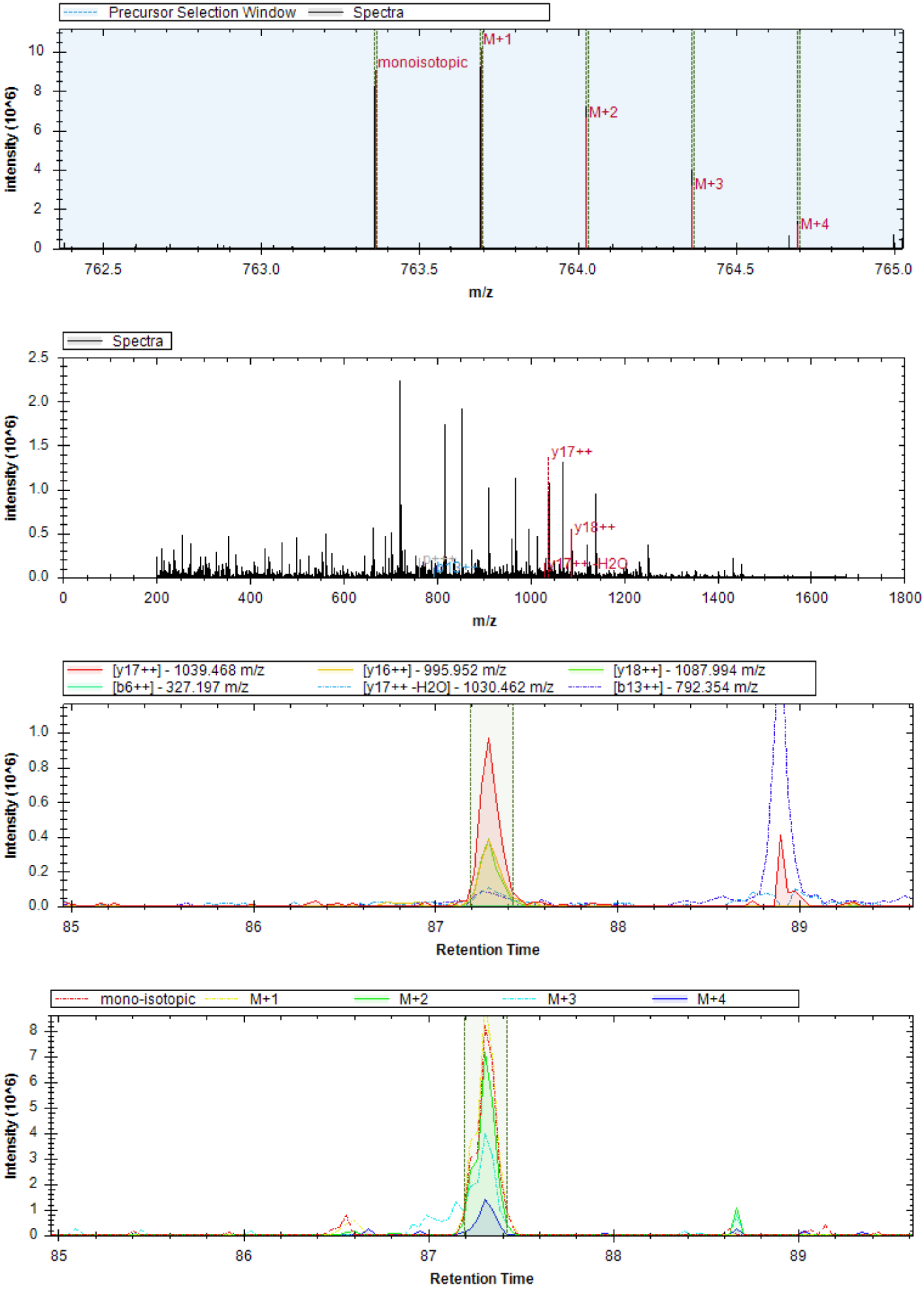

P06837 – _QADVPAAVTDAAAT[+H+O3+P]TPAAEDAATK_.3
Fig4_MP-DIA-barrel-cortex-p15-2_MHRM.htrms

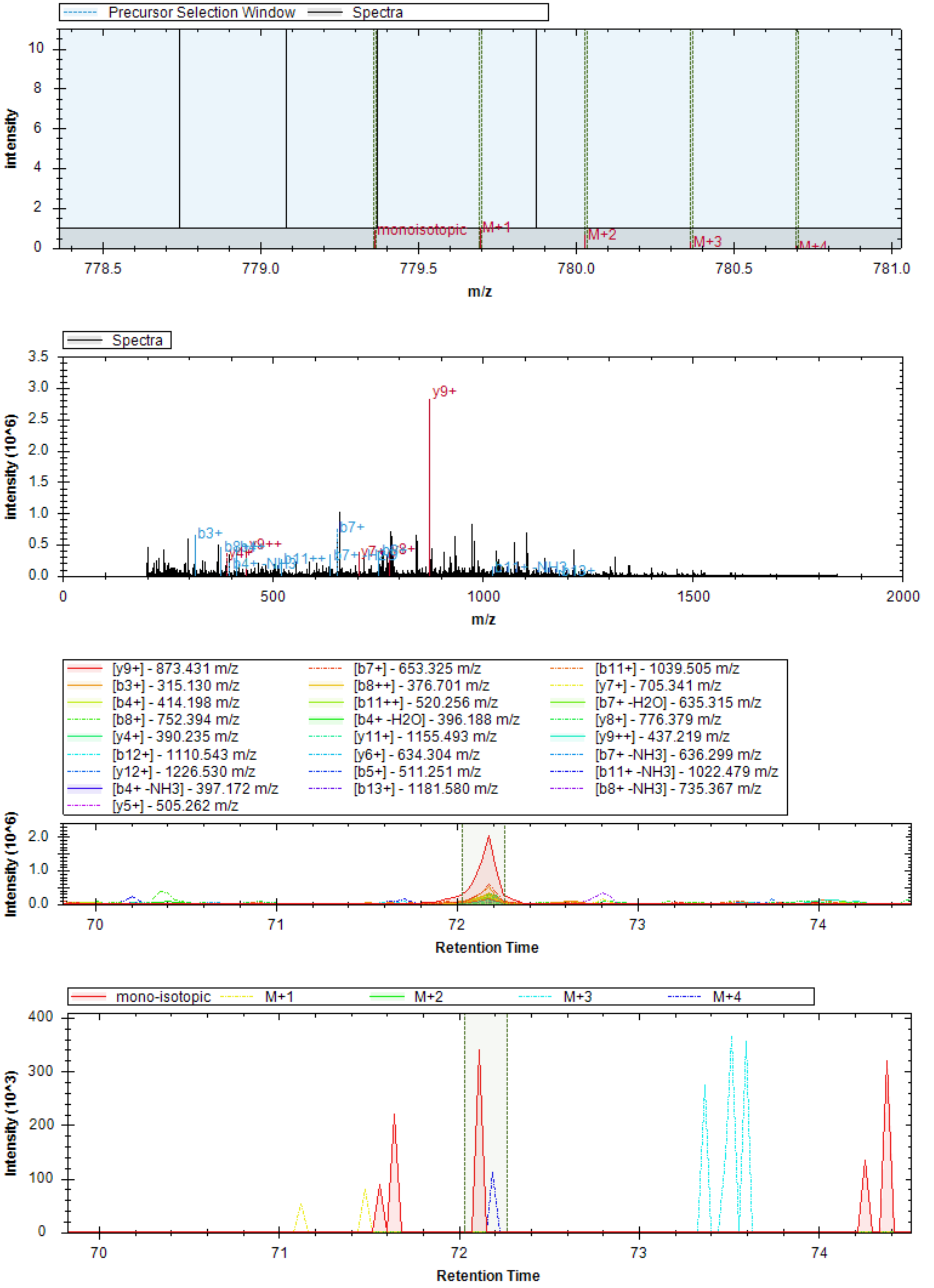

P0C6F1 – _IIDLQM[+O]HDY[+H+O3+P]LR_.2
Fig4_MP-DIA-barrel-cortex-p15-2_MHRM.htrms

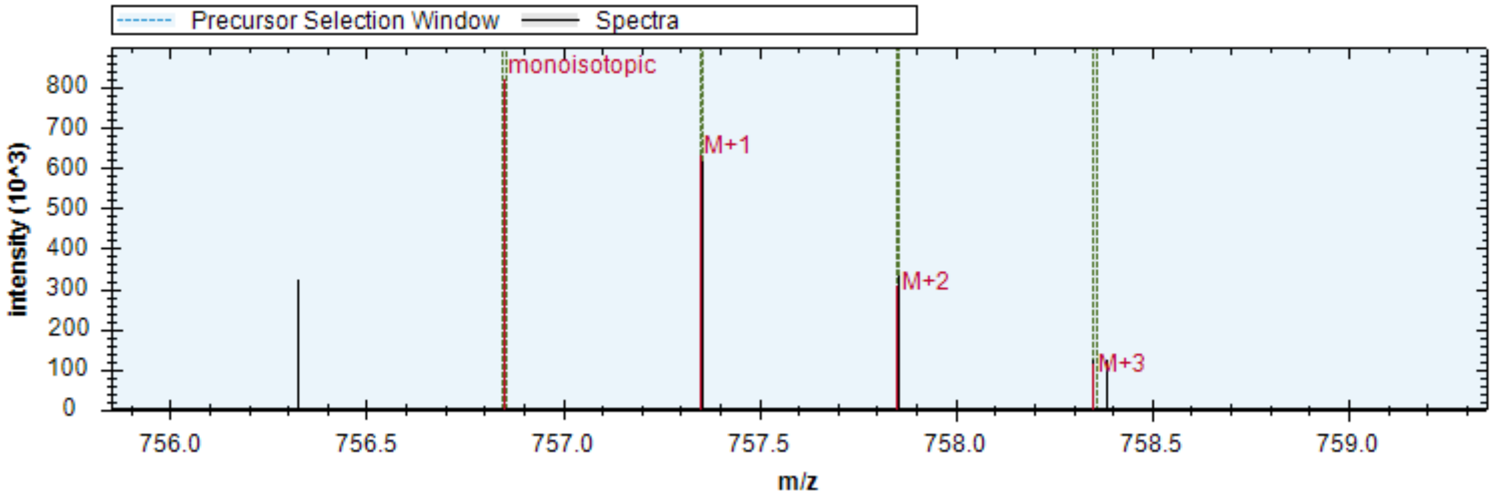

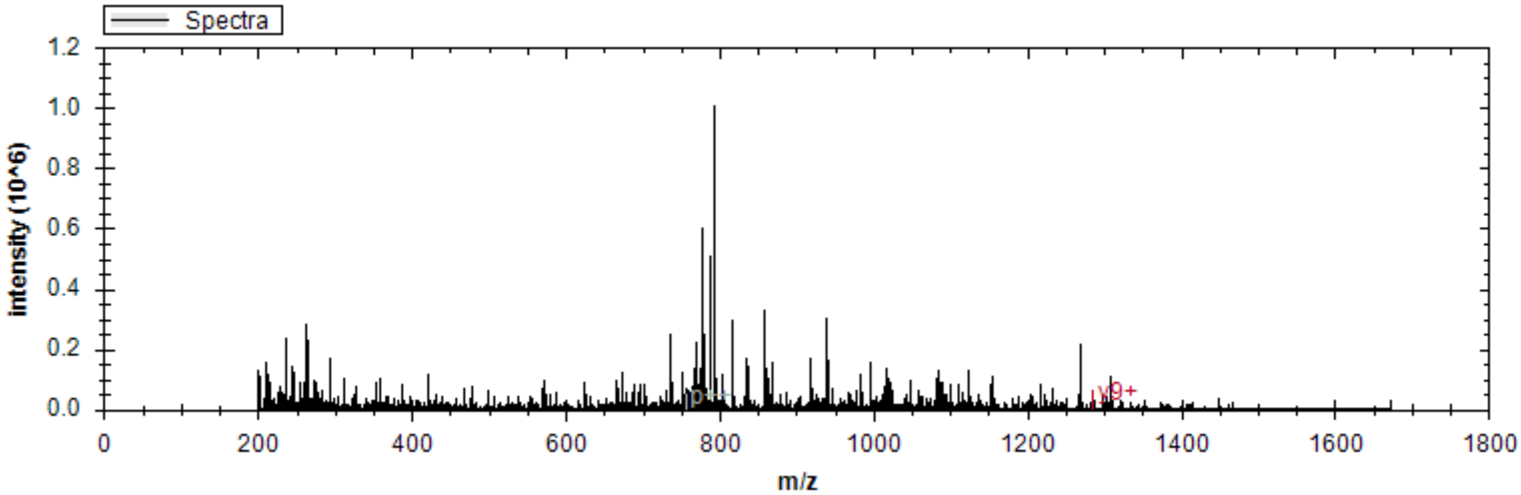

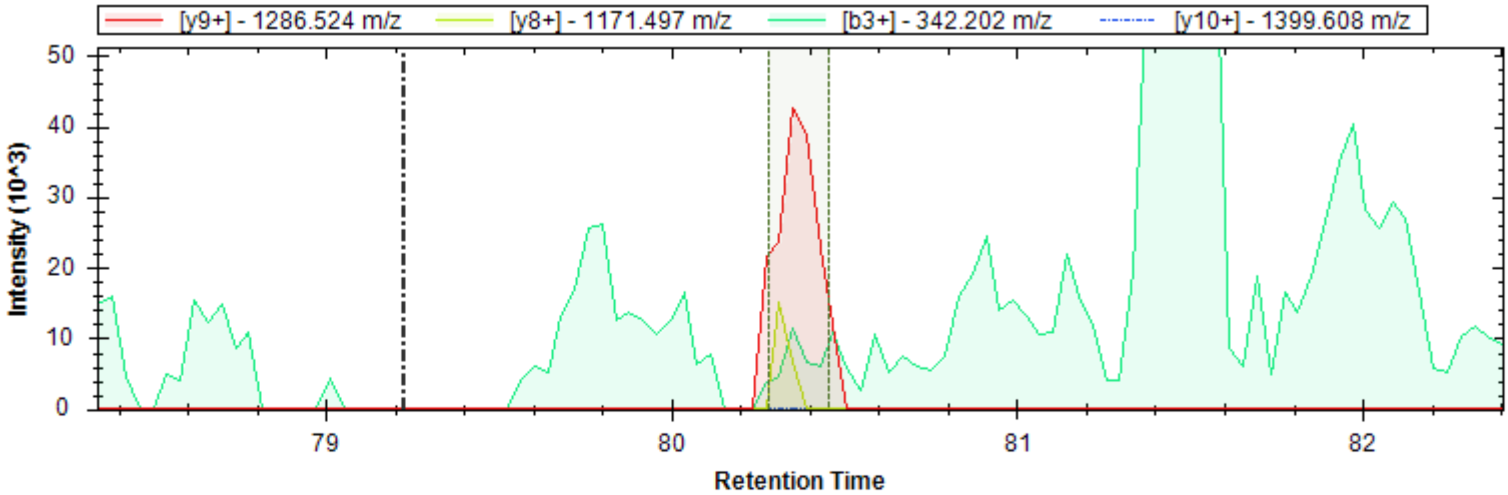

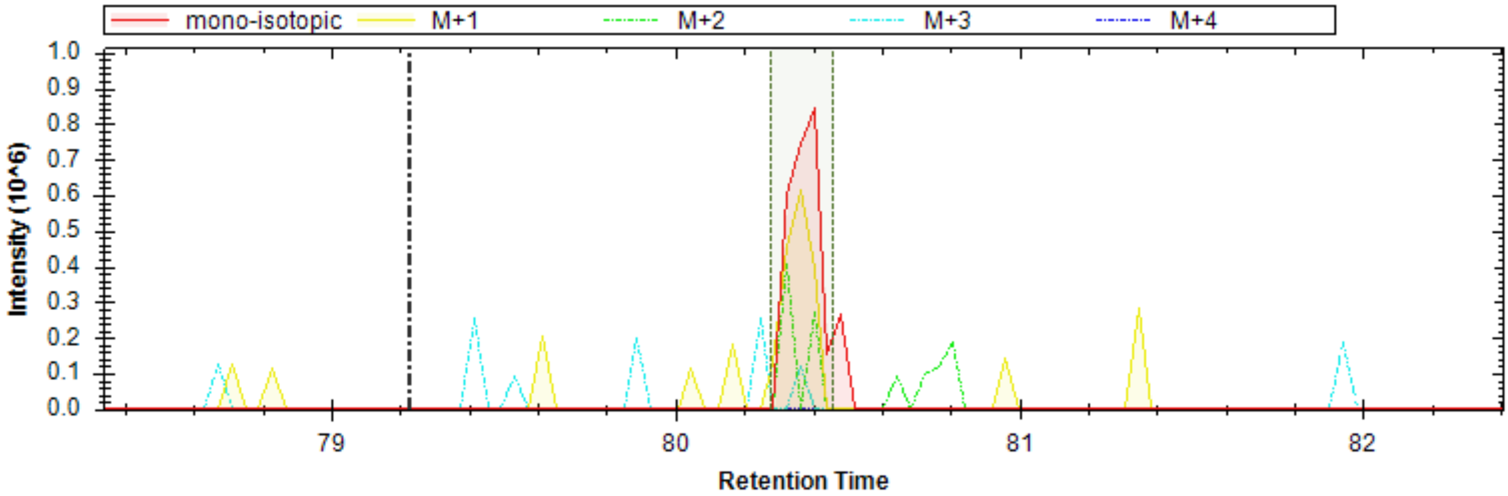

Q68FD9 – _LAQLLS[+H+O3+P]EVPT[+H+O3+P]RR_.2
Fig4_MP-DIA-barrel-cortex-p15-2_MHRM.htrms

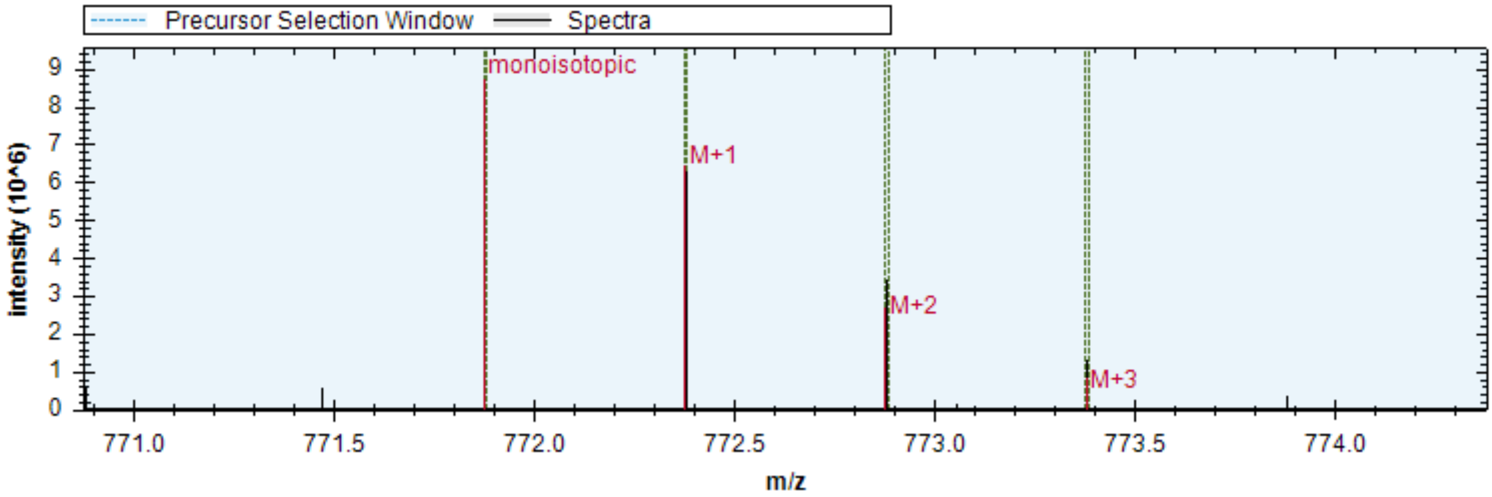

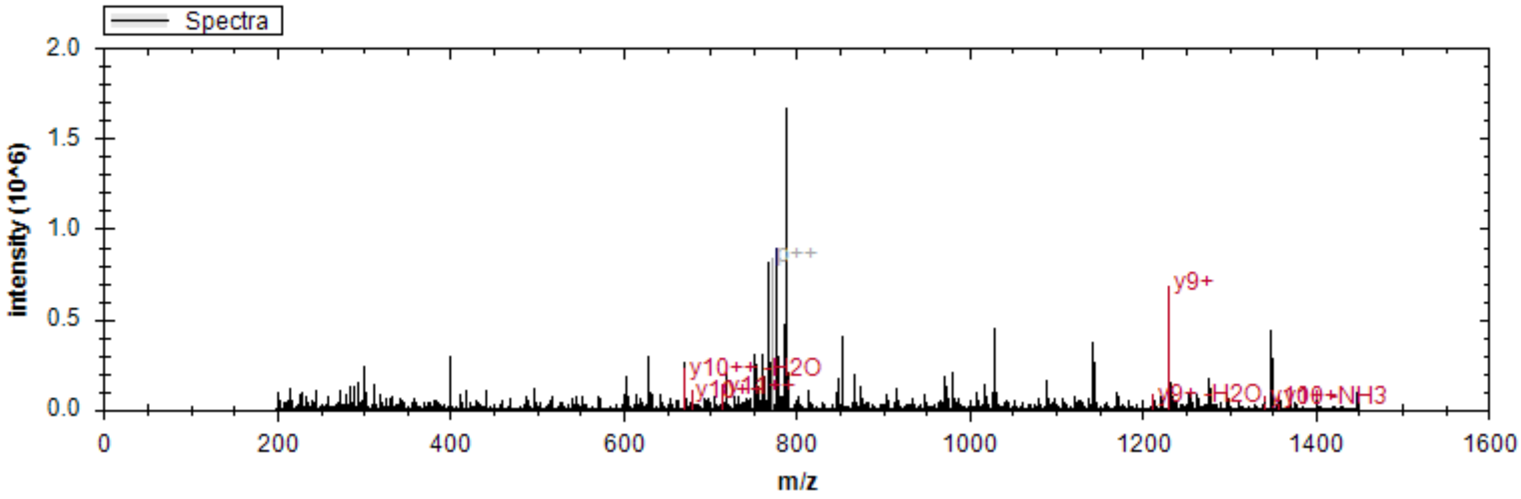

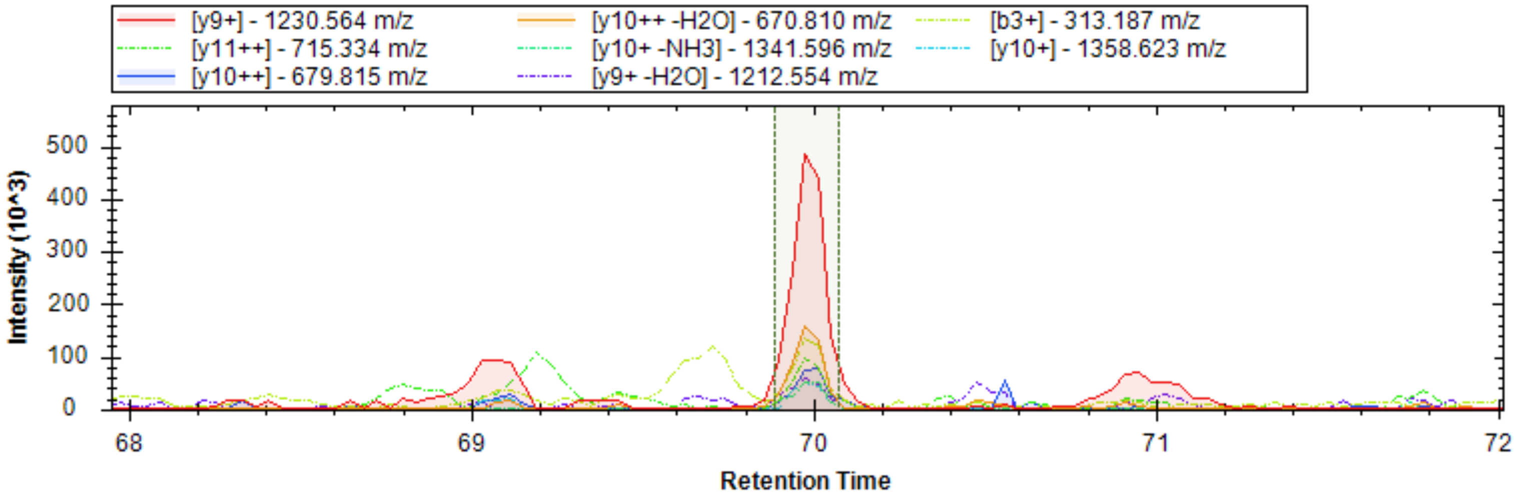

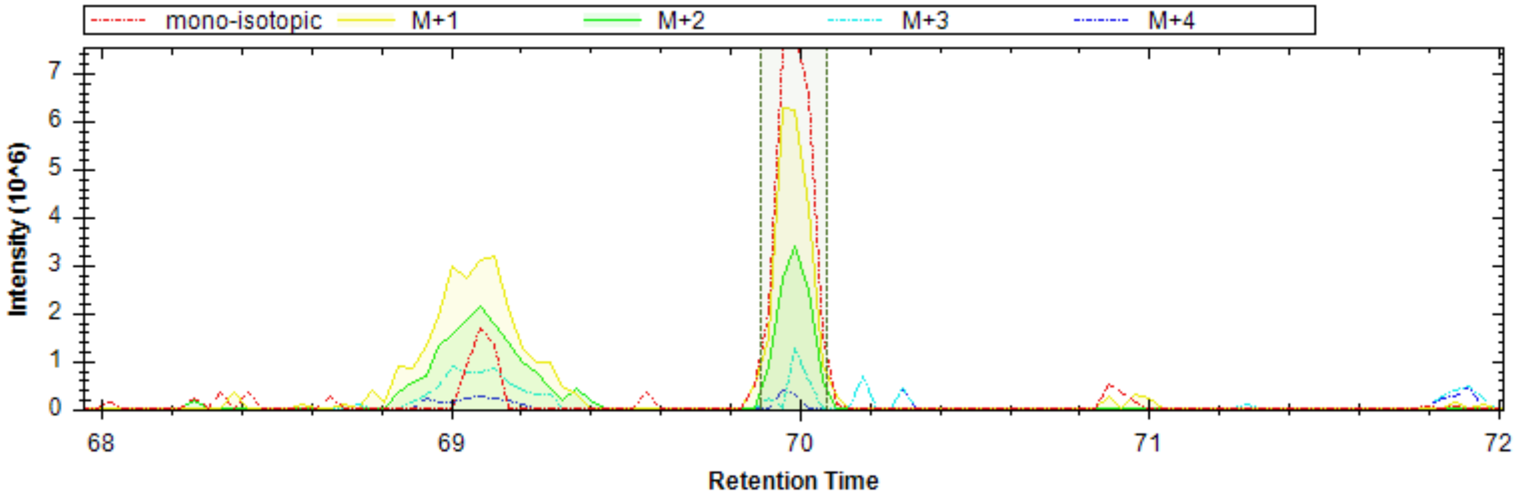

Q8BRT1 – _VLNT[+H+O3+P]GSDVEEAVADALLLGDIR_.3

Fig4_MP-DIA-barrel-cortex-p15-2_MHRM.htrms

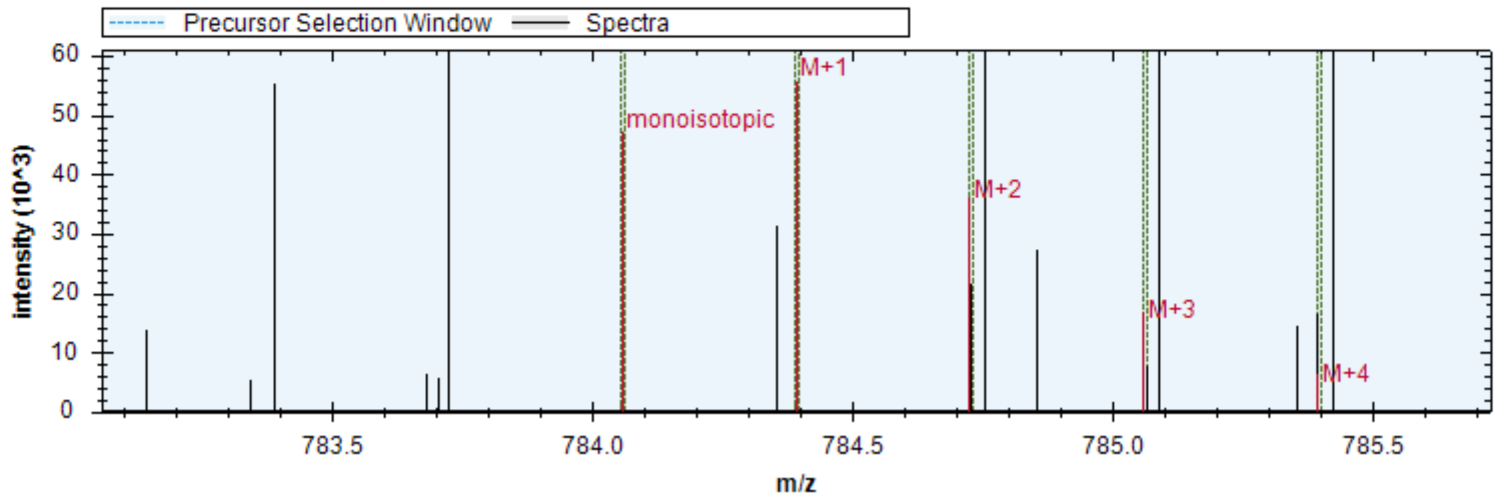

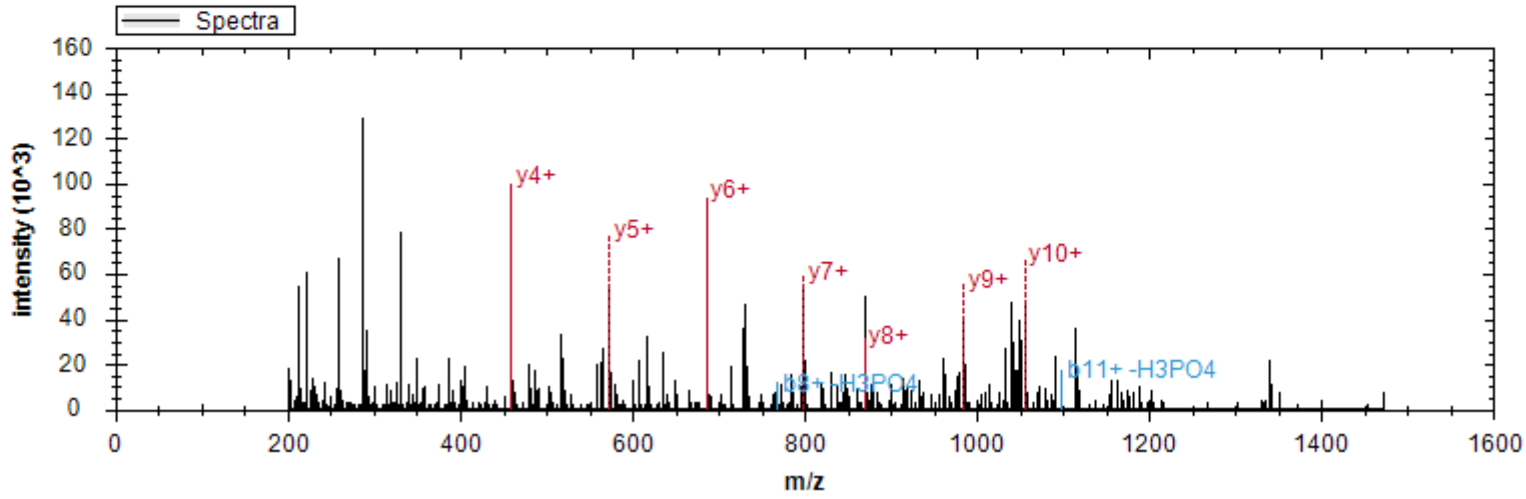

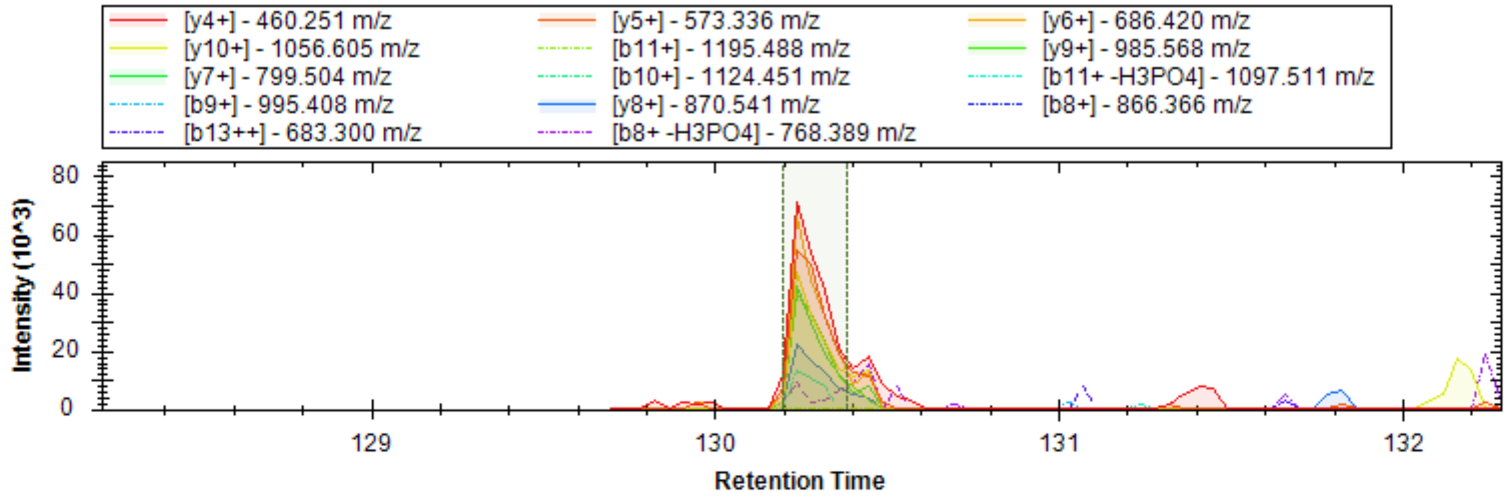

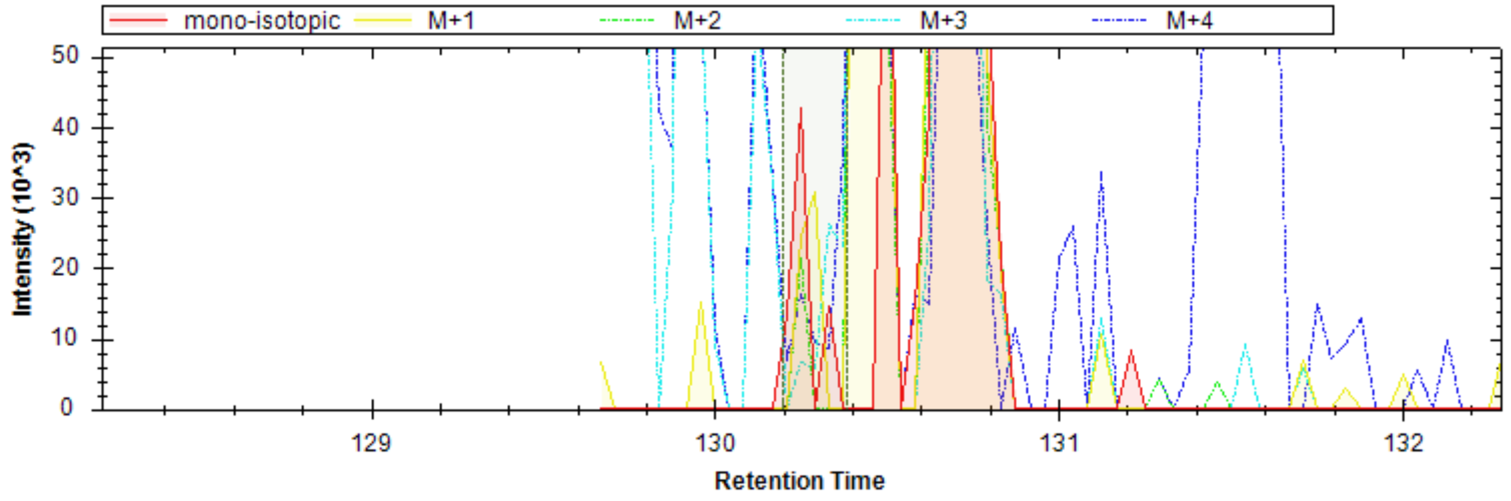

Q9D5R2 – _SNS[+H+O3+P]LPHSAVSNAASK_.2
Fig4_MP-DIA-barrel-cortex-p15-2_MHRM.htrms

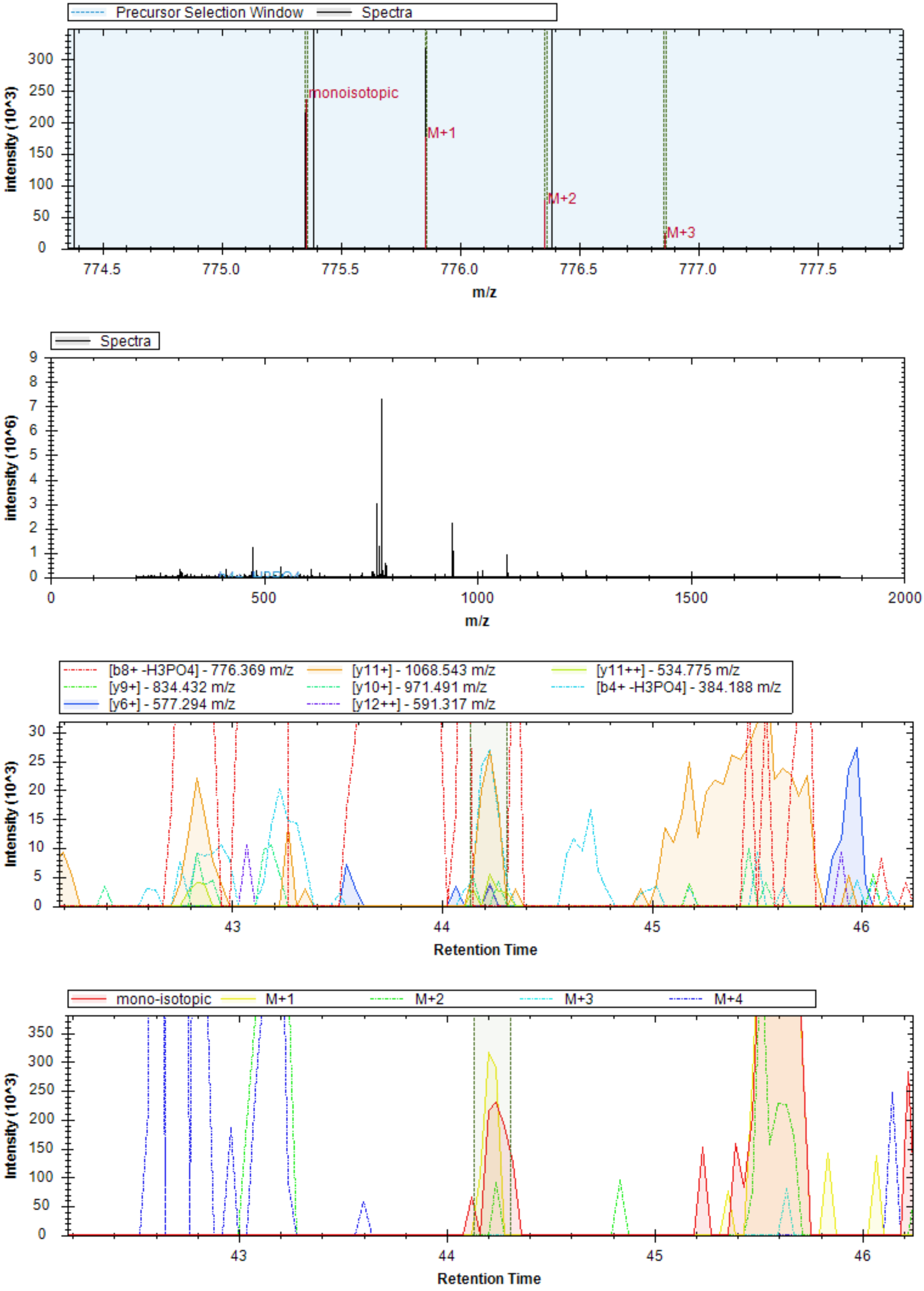

Q9JLM8 – _ISQHGGS[+H+O3+P]STSLSSTK_.2
Fig4_MP-DIA-barrel-cortex-p15-2_MHRM.htrms

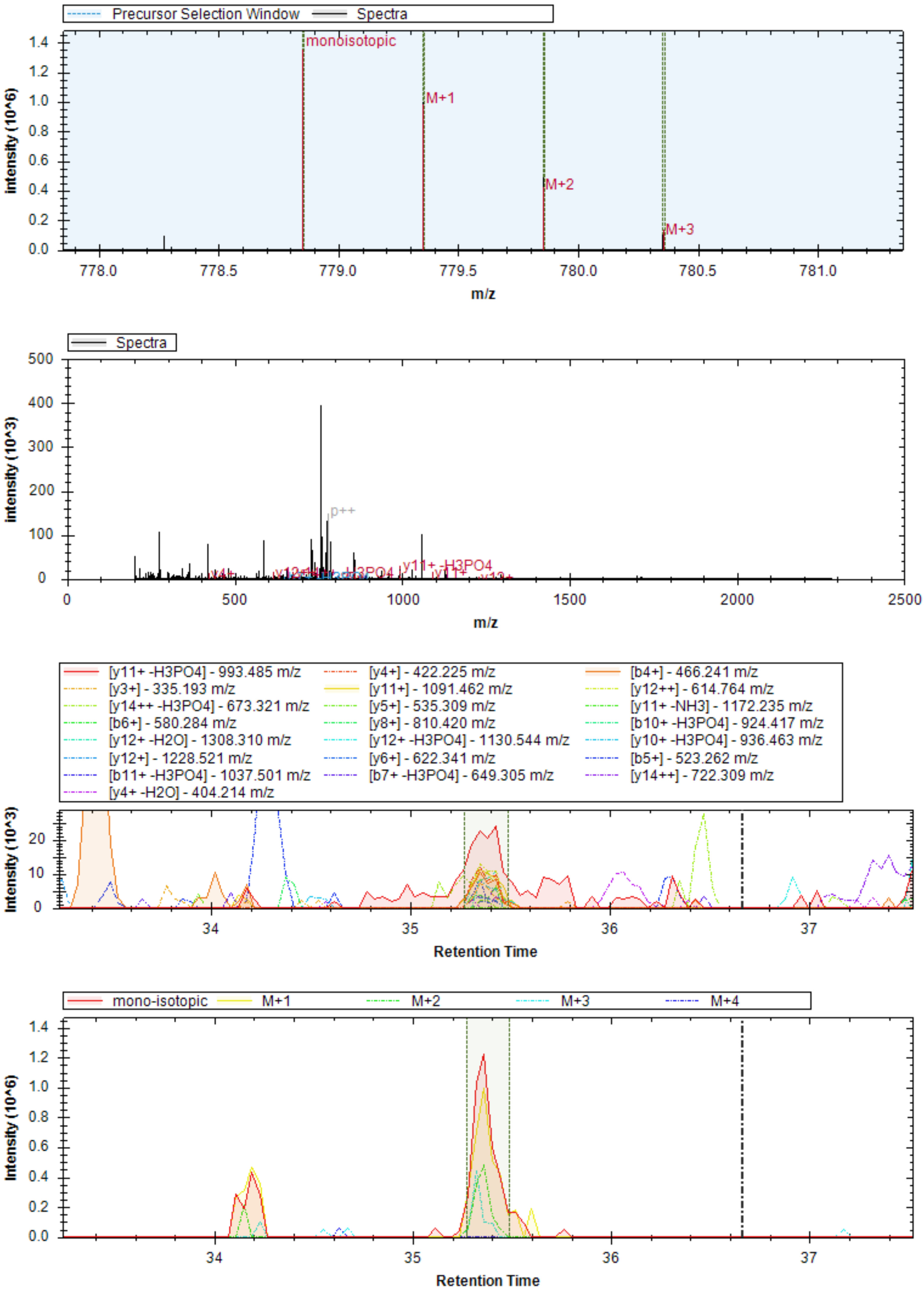

Q9QWI6 – _LSYAGGRPPSYAGS[+H+O3+P]PVHHAAER_.3

Fig4_MP-DIA-barrel-cortex-p15-2_MHRM.htrms

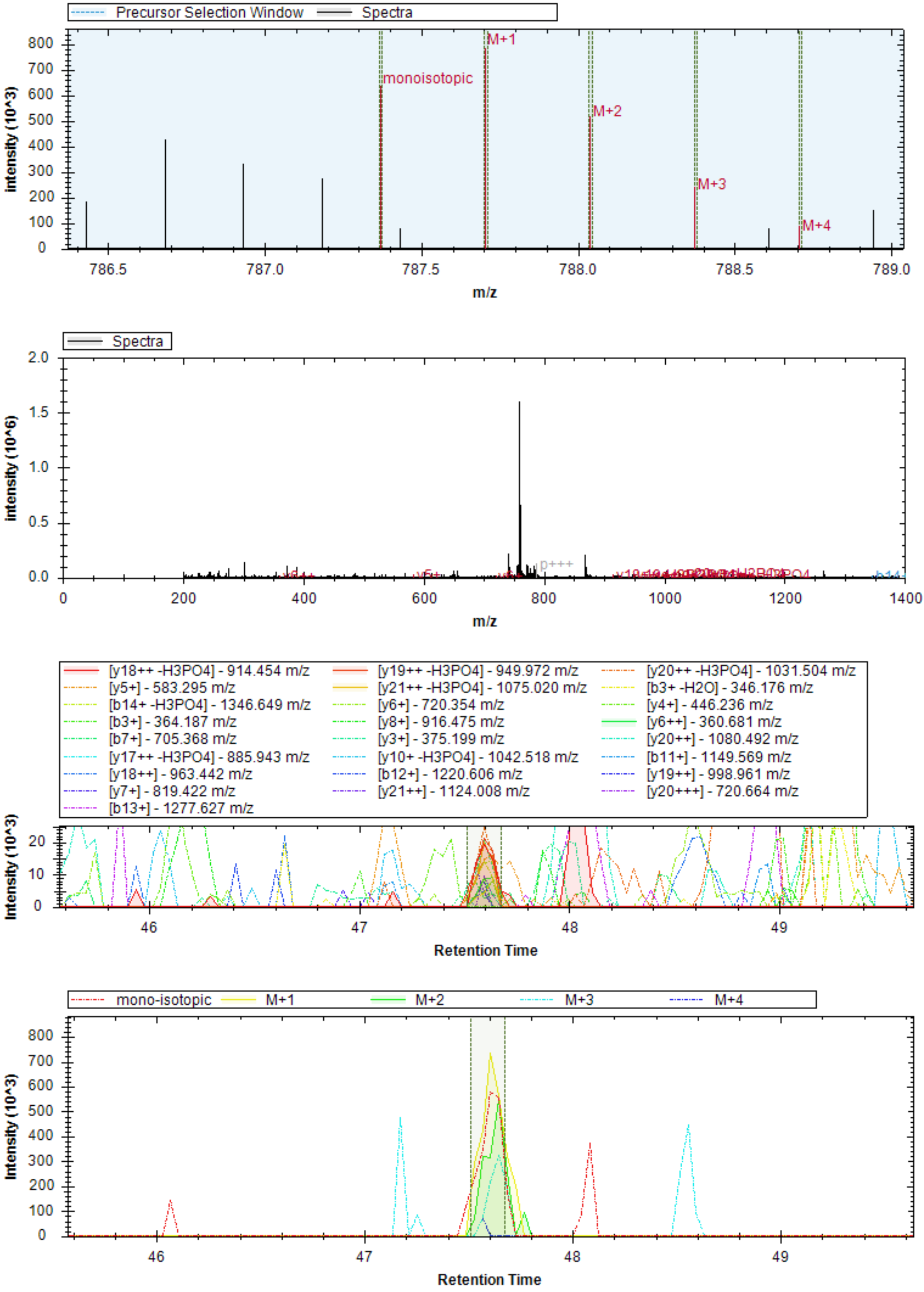

Q9QYC0 – _AAVVTS[+H+O3+P]PPPTTAPHK_.2
Fig4_MP-DIA-barrel-cortex-p15-2_MHRM.htrms

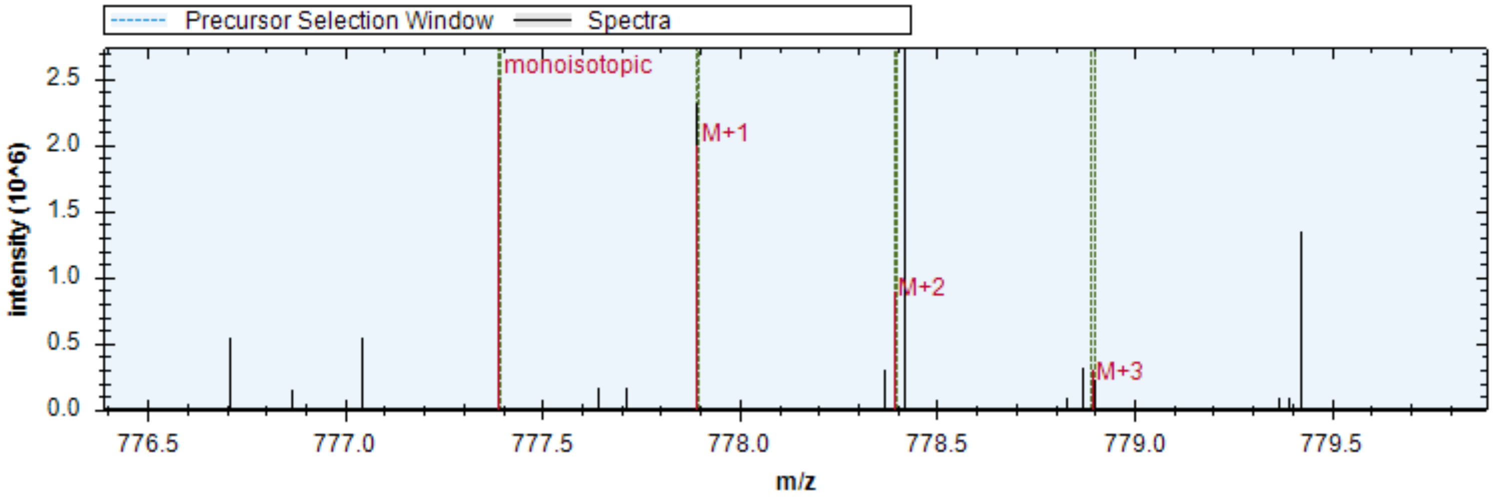

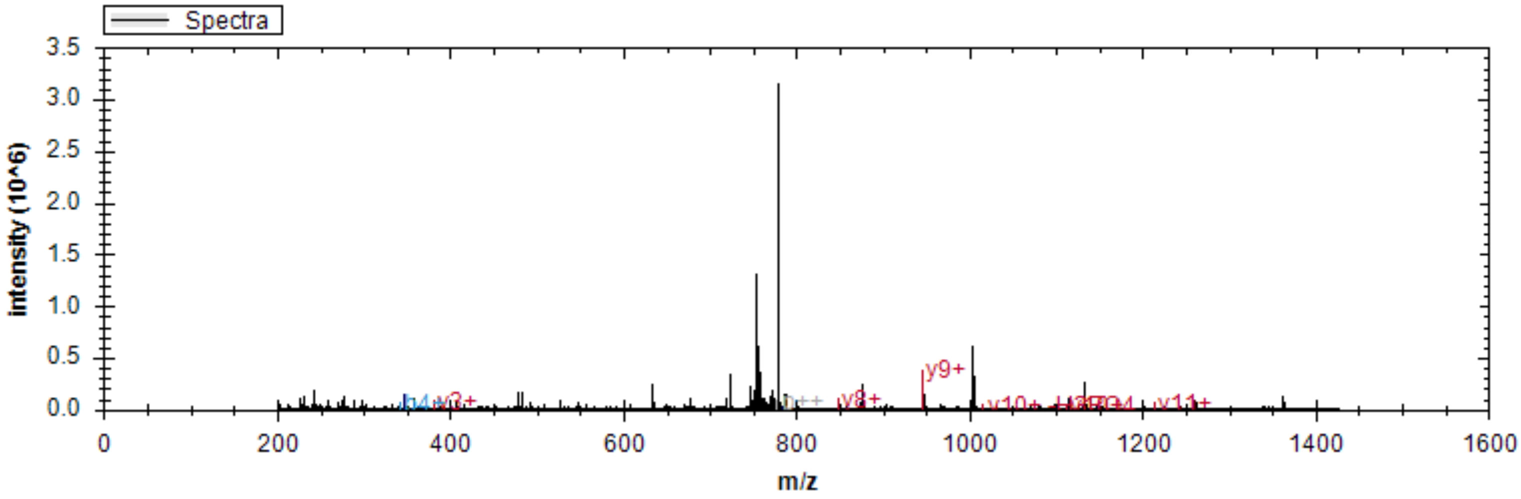

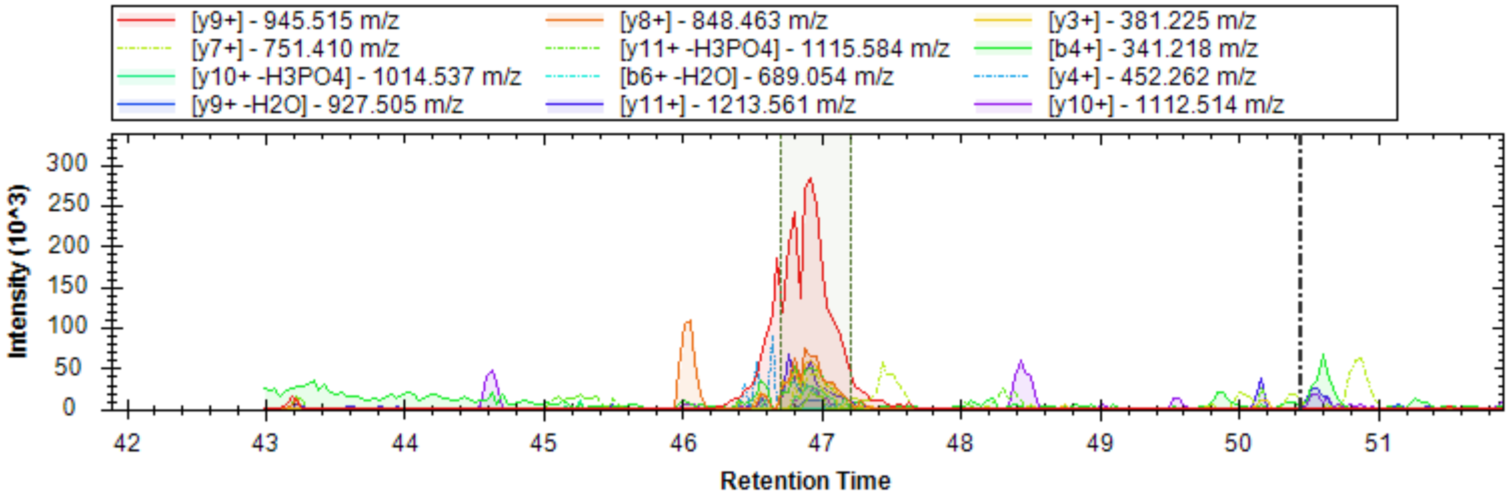

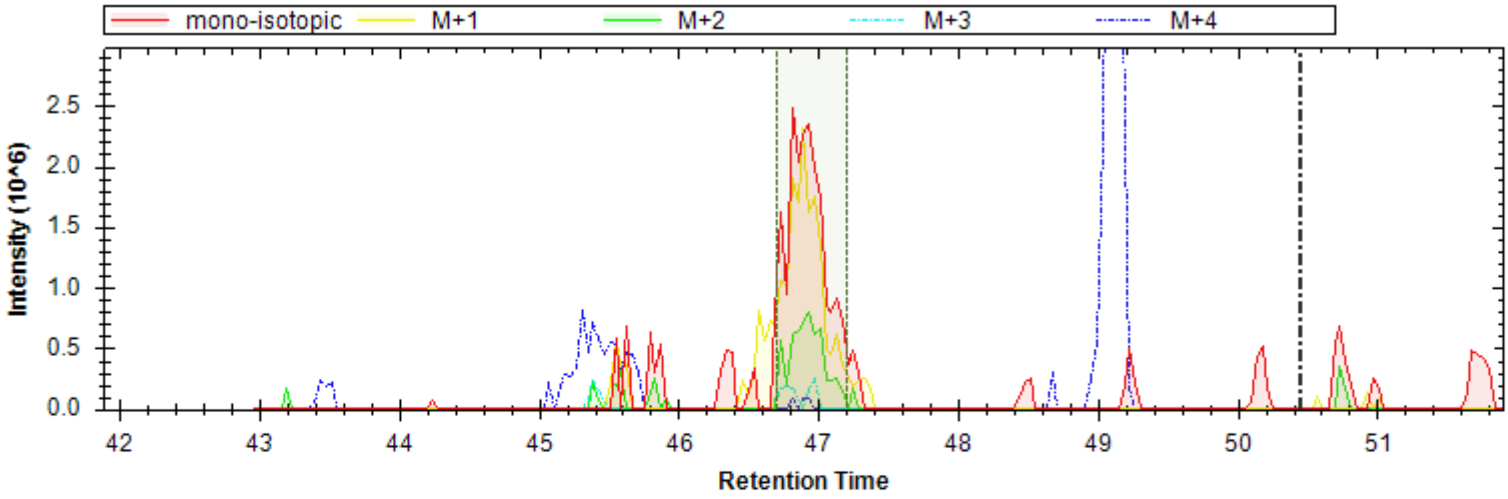

Q9QYR6-2 – _ELSSEPRT[+H+O3+P]PPAQK_.2
Fig4_MP-DIA-barrel-cortex-p15-2_MHRM.htrms

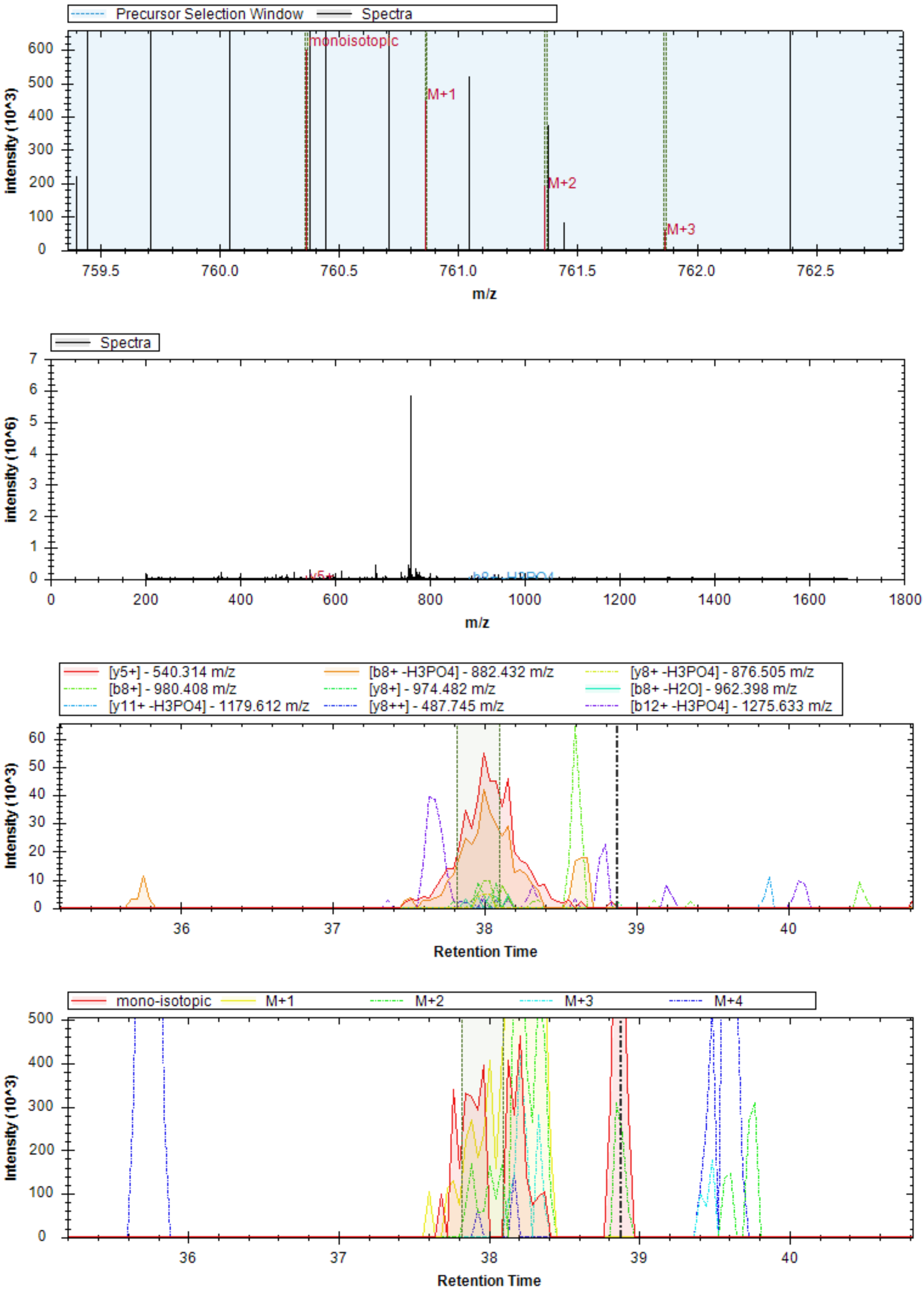

P10637 – _TTPSPKT[+H+O3+P]PPGSGEPPK_.2
Fig4_MP-DIA-barrel-cortex-p15-2_MHRM.htrms

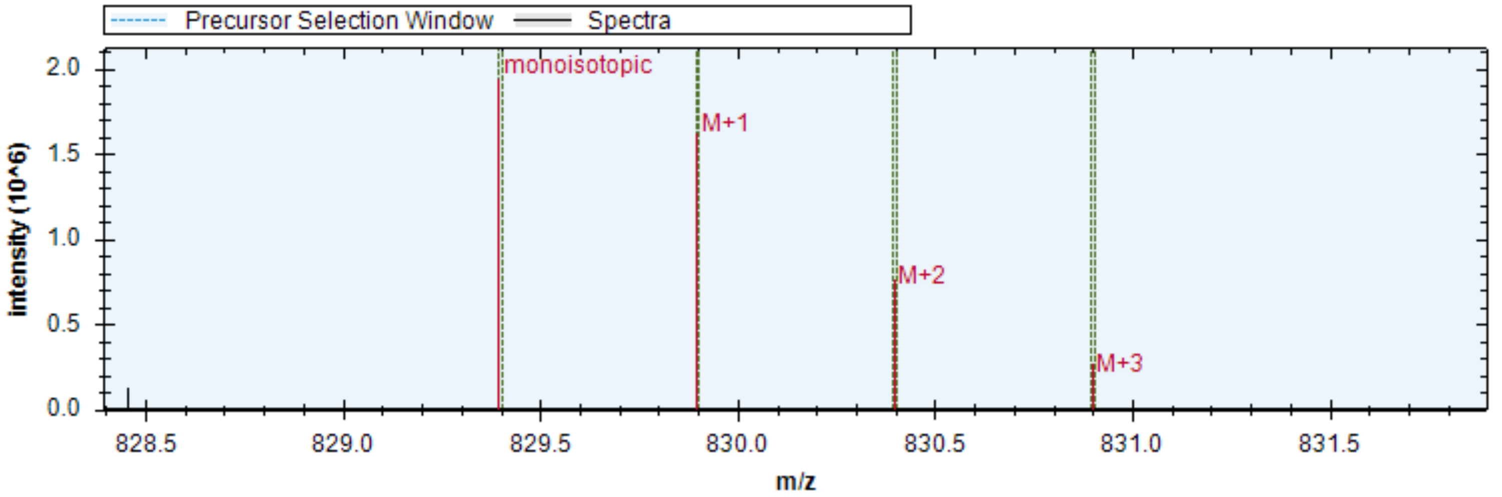

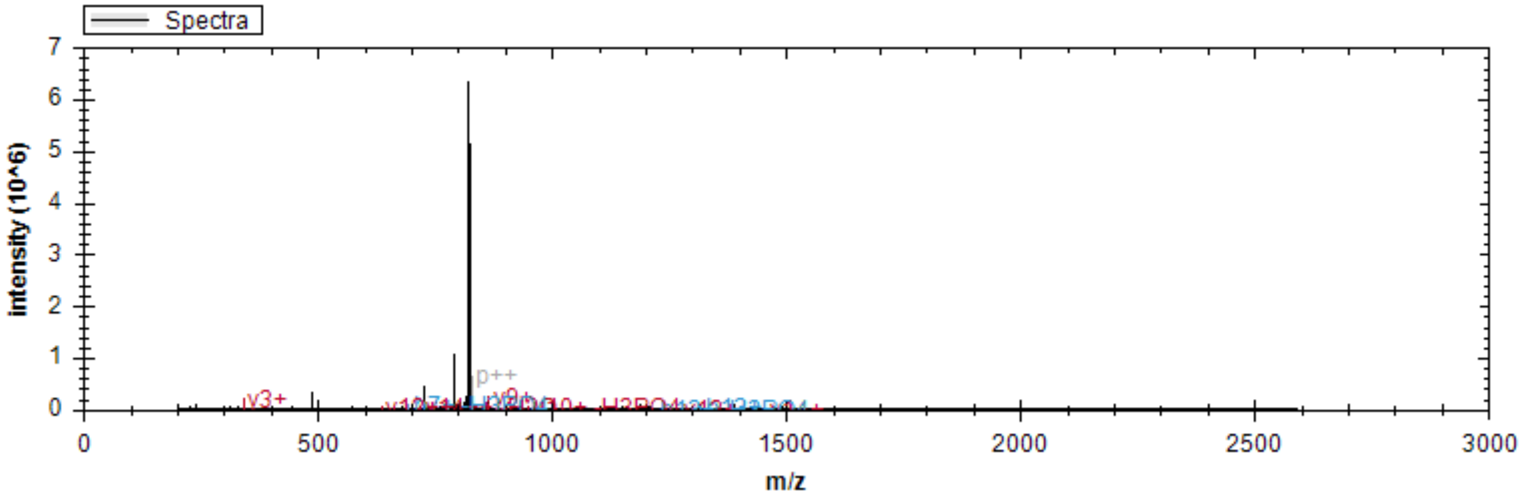

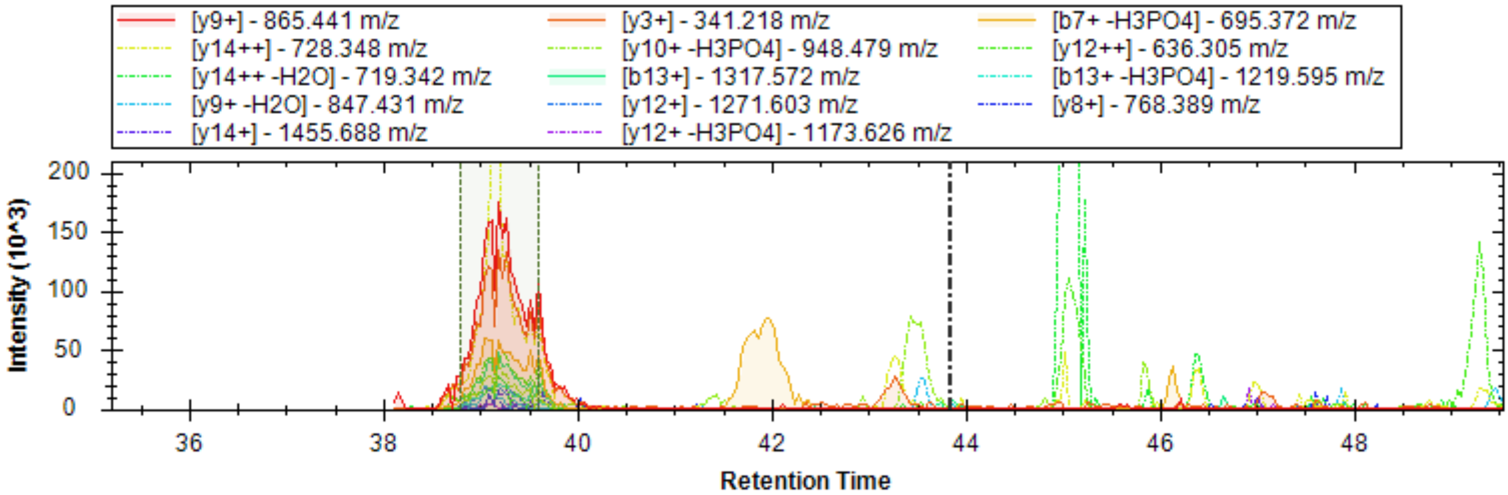

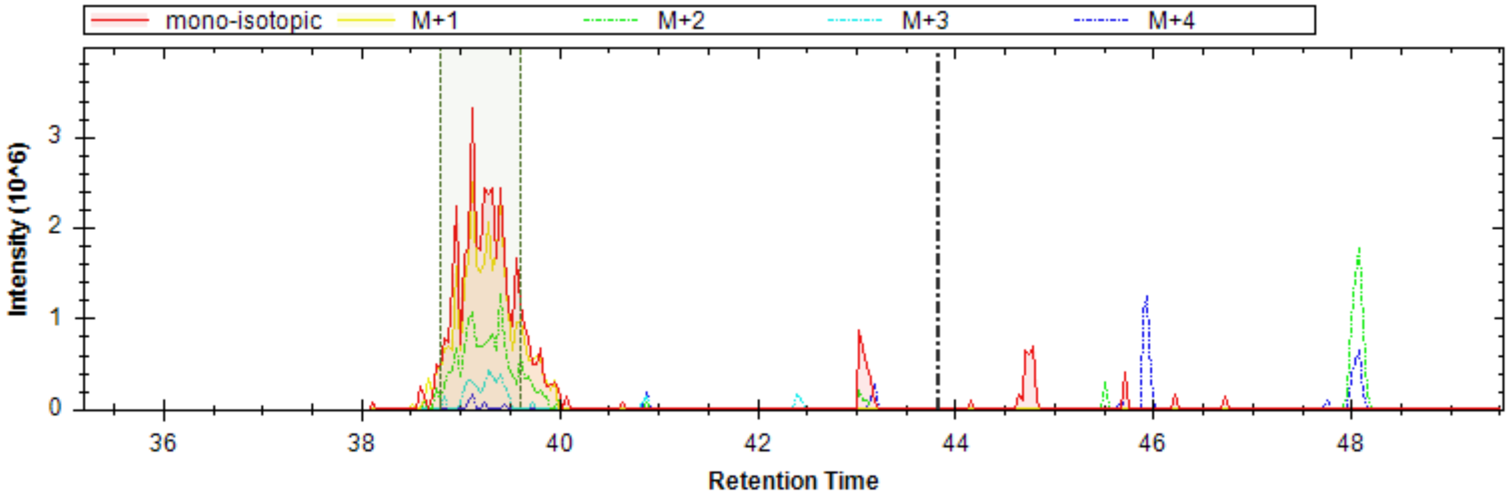

P20357 – _VDHGAEIIT[+H+O3+P]QSPSR_.2
Fig4_MP-DIA-barrel-cortex-p15-2_MHRM.htrms

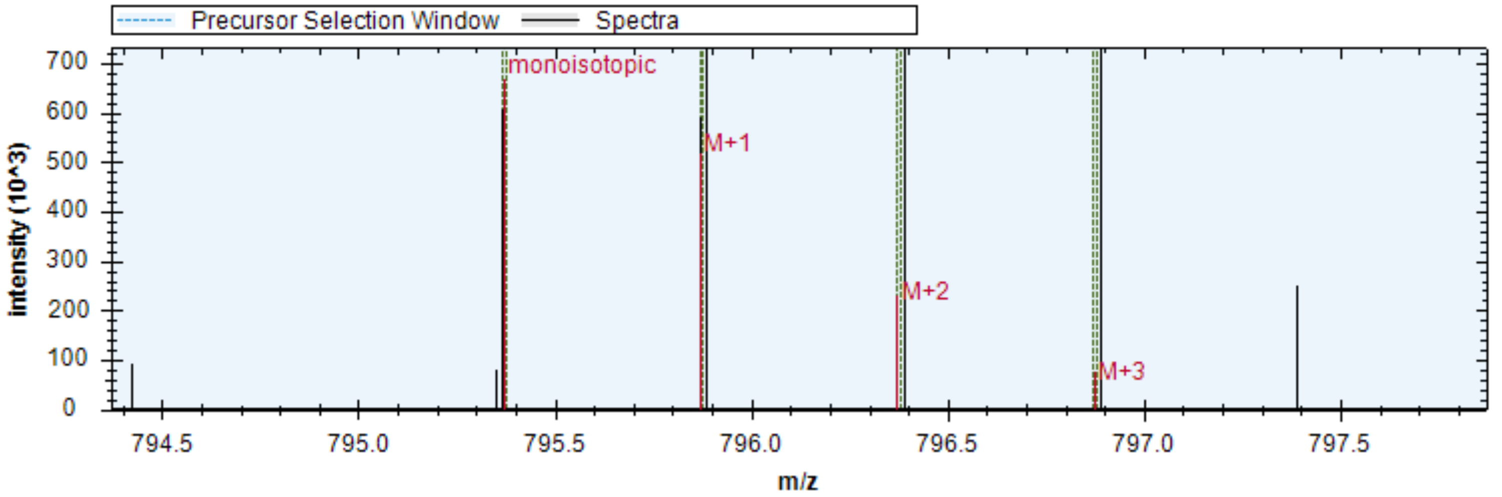

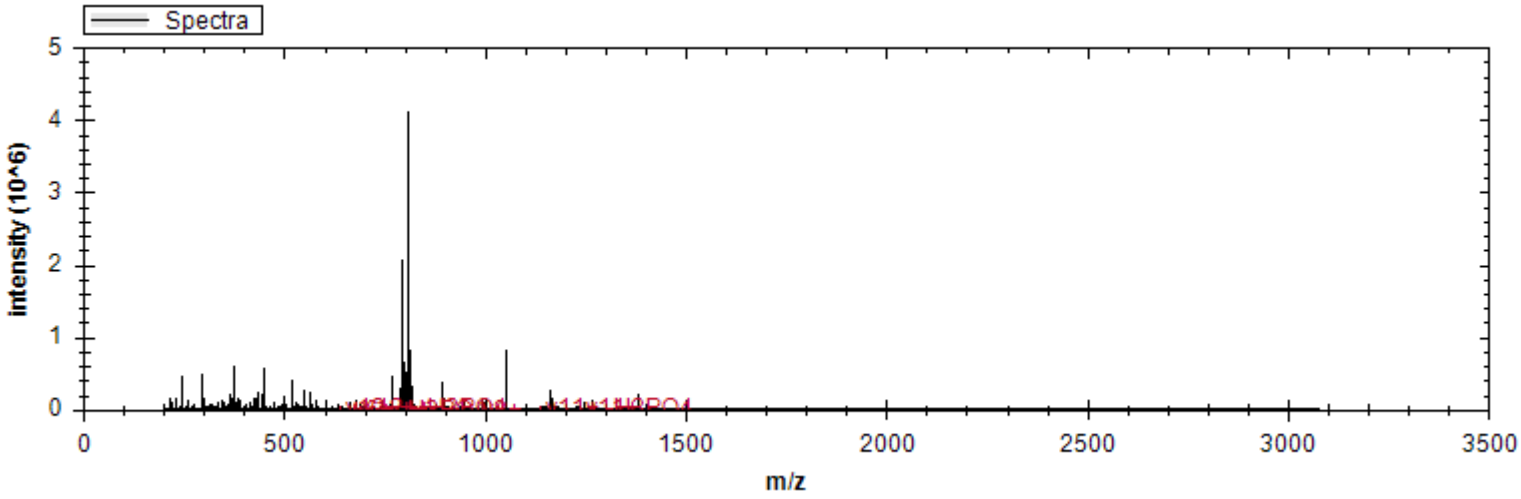

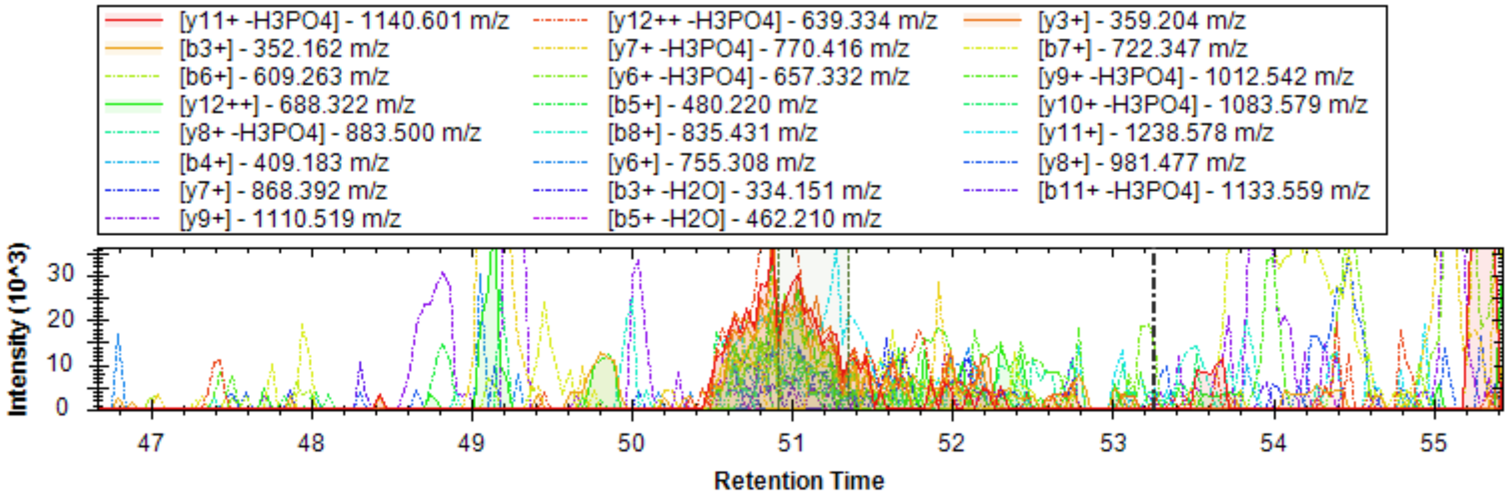

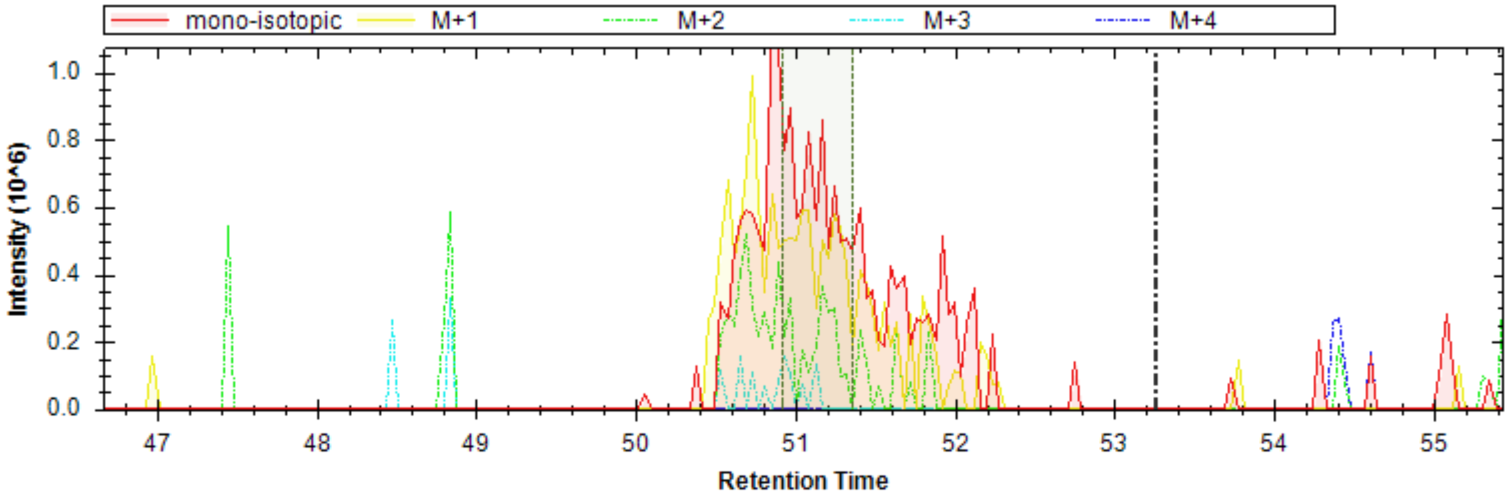

P20357 – _VDHGAEIITQS[+H+O3+P]PSR_.2

Fig4_MP-DIA-barrel-cortex-p15-2_MHRM.htrms

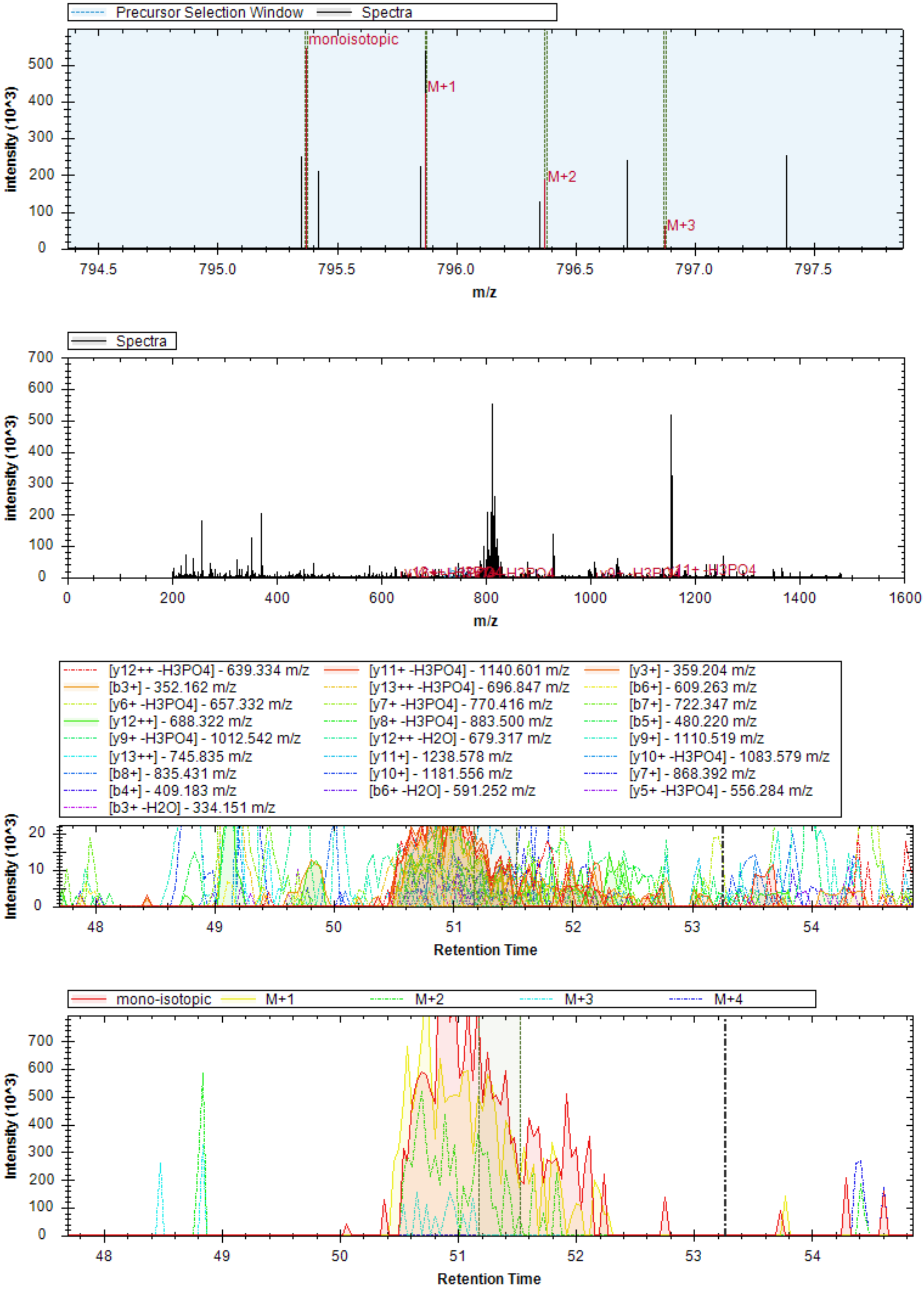

P20357 – _VDHGAEIITQSPS[+H+O3+P]R_.2
Fig4_MP-DIA-barrel-cortex-p15-2_MHRM.htrms

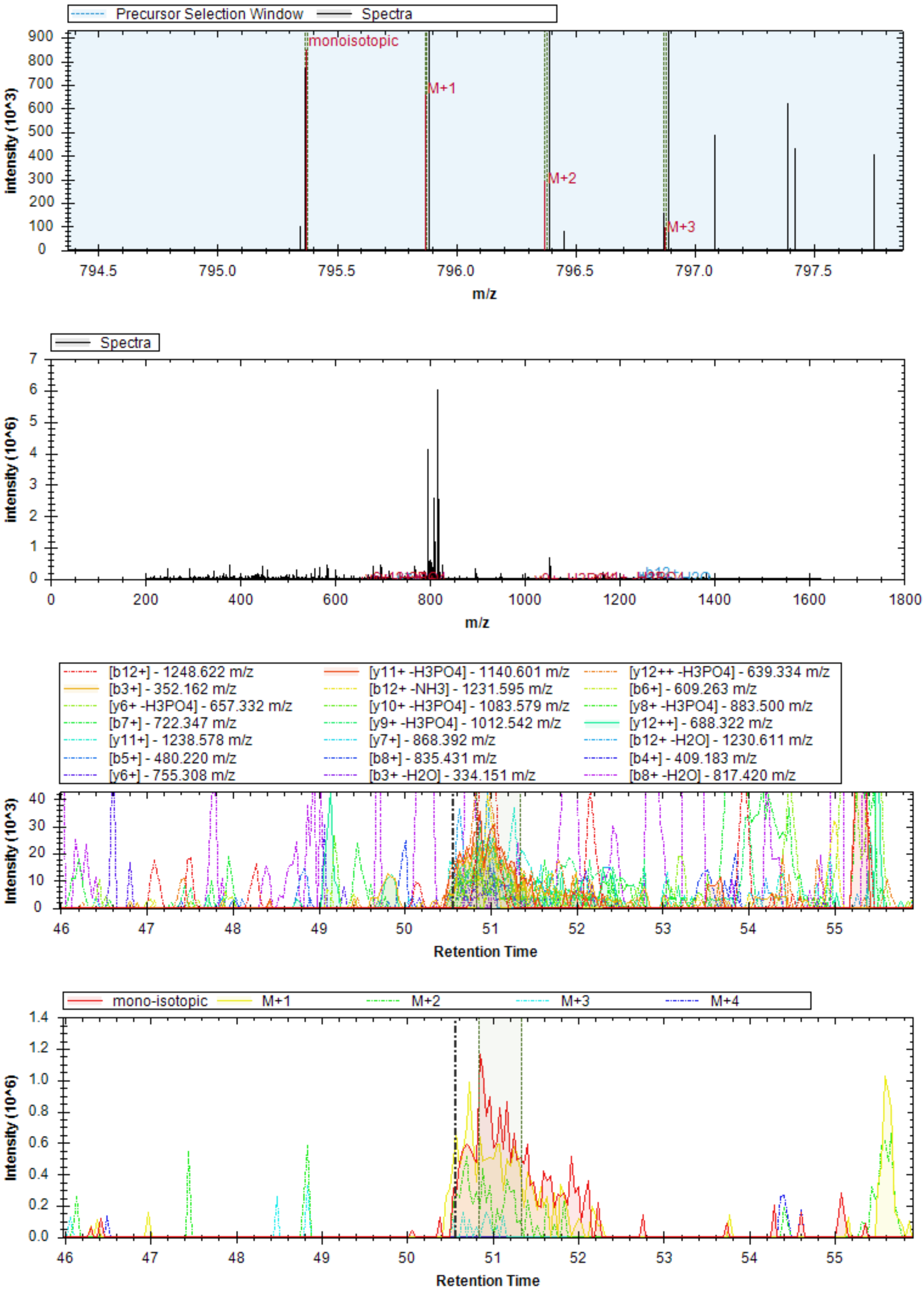

P54227 – _SKESVPDFPLS[+H+O3+P]PPK_.2
Fig4_MP-DIA-barrel-cortex-p15-2_MHRM.htrms

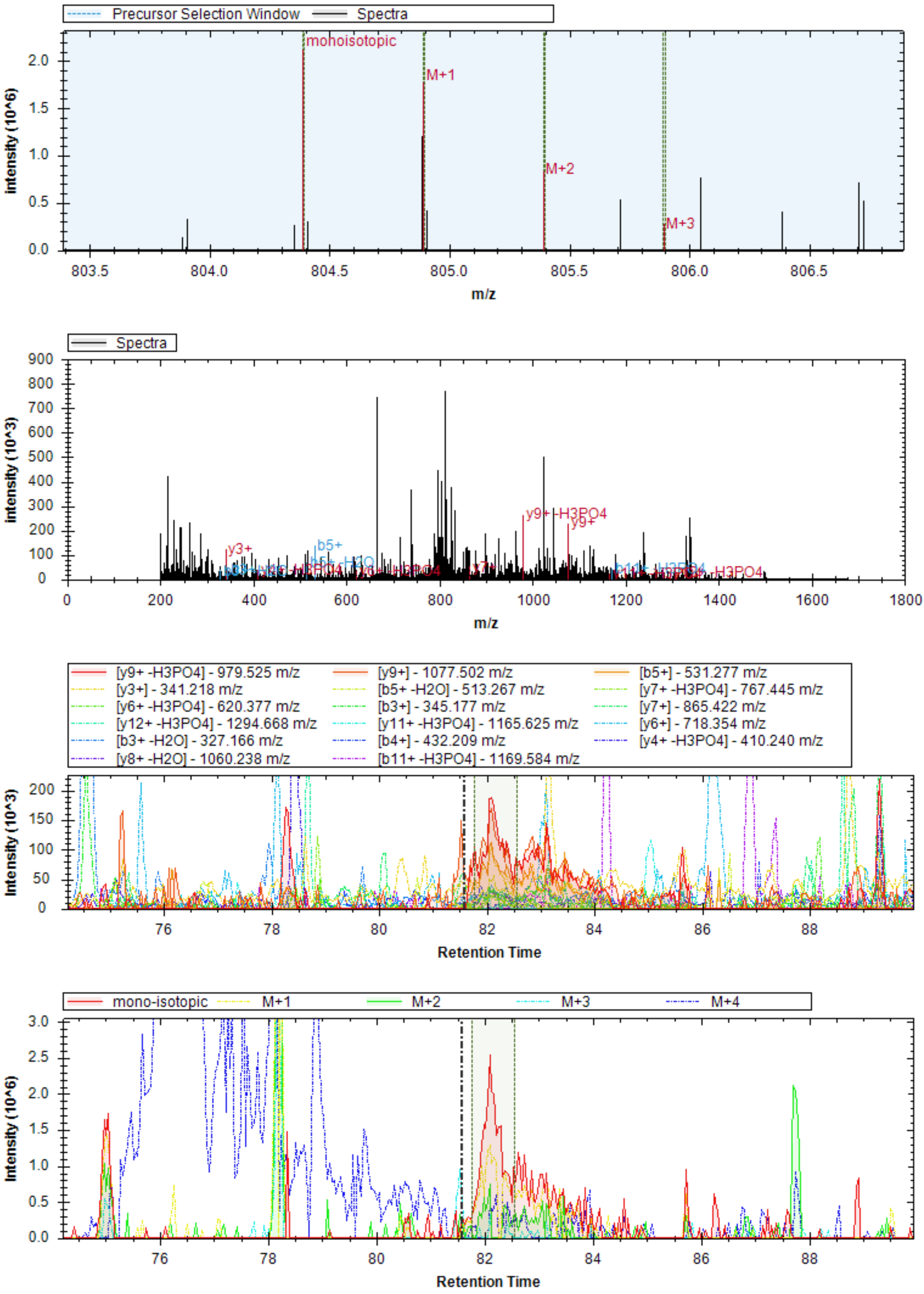

Q60771 – _FYYSSGS[+H+O3+P]SSPTHAK_.2

Fig4_MP-DIA-barrel-cortex-p15-2_MHRM.htrms

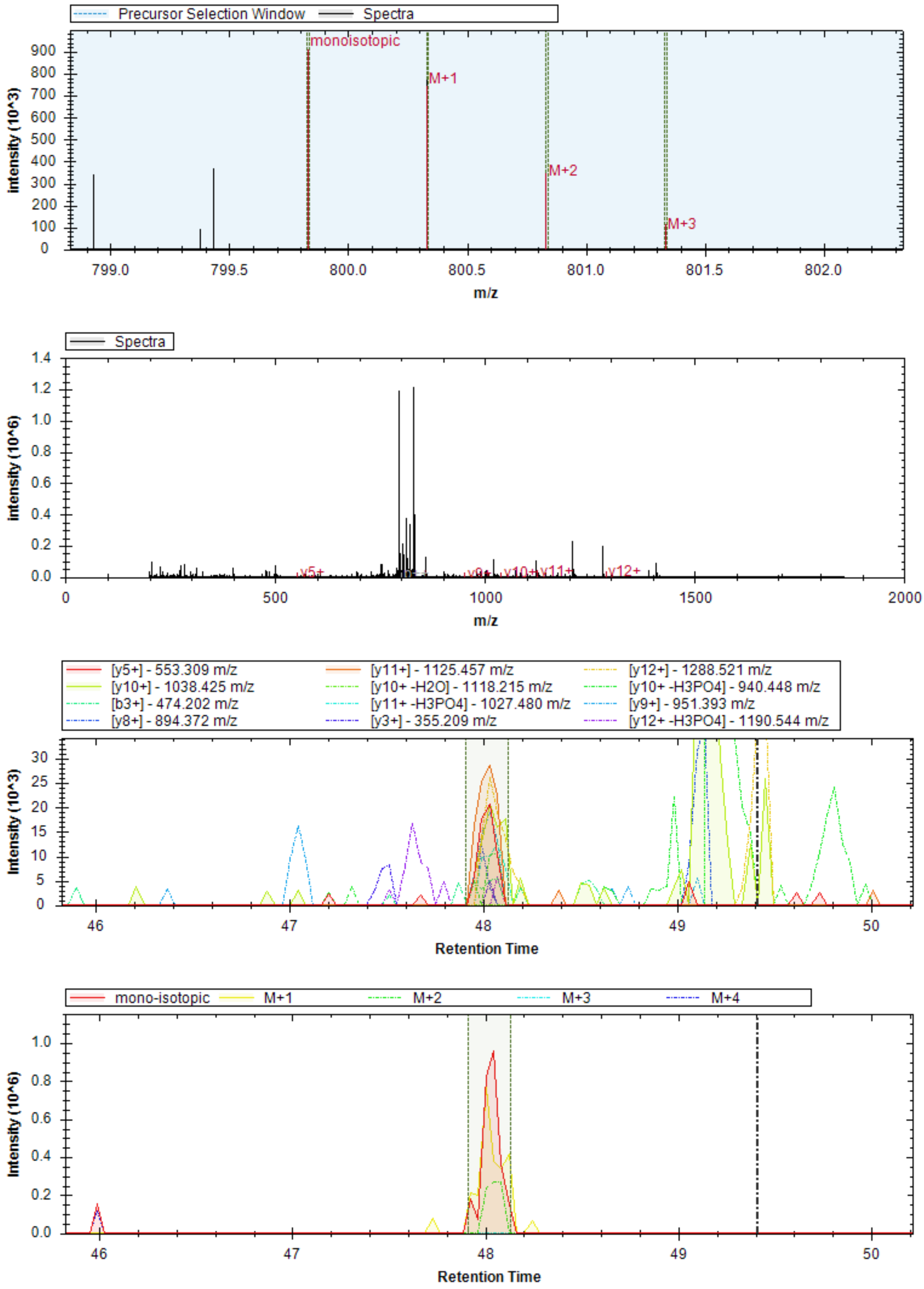

Q60771 – _FYYSSGSSS[+H+O3+P]PTHAK_.2

Fig4_MP-DIA-barrel-cortex-p15-2_MHRM.htrms

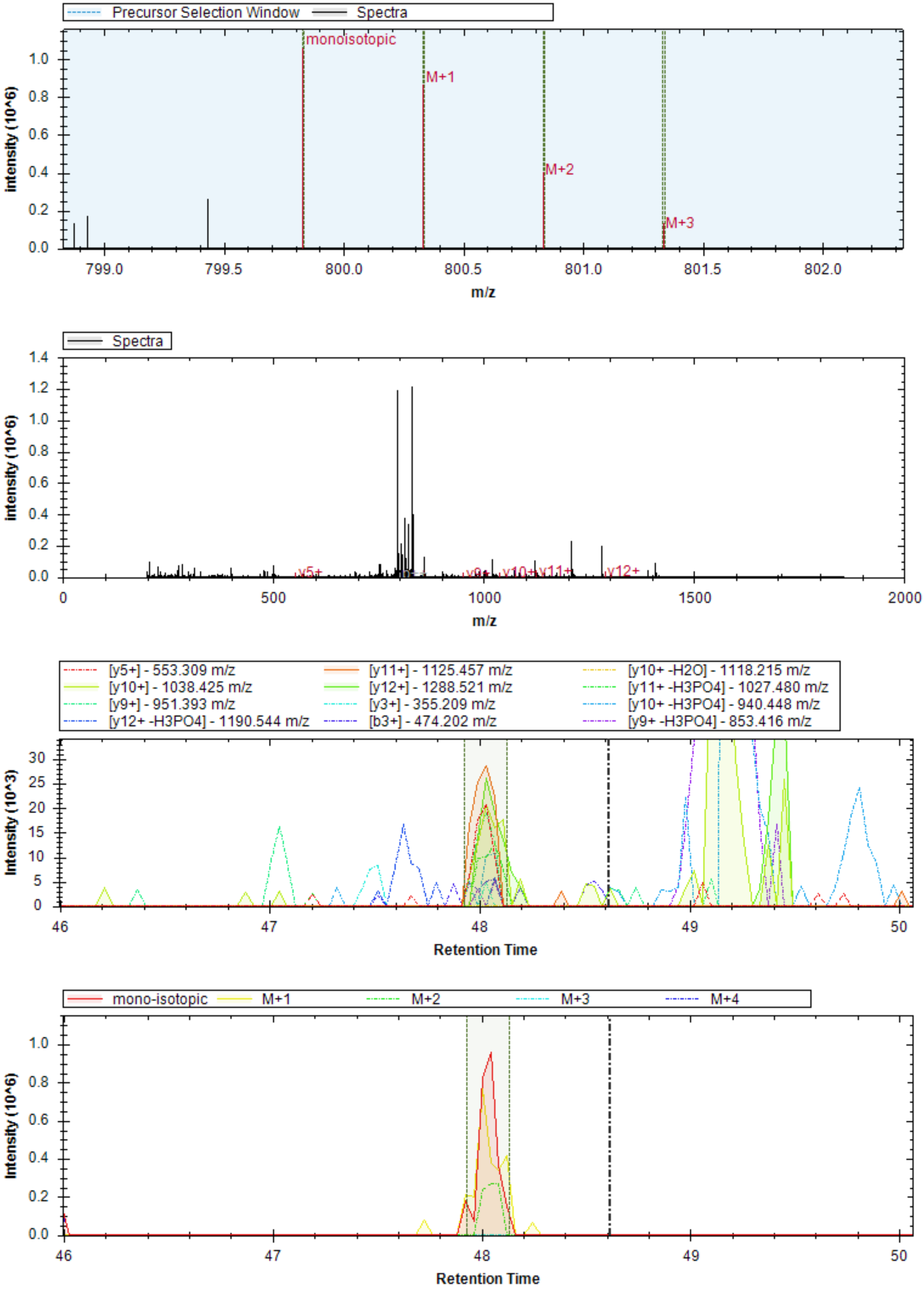

Q91XM9;Q91XM9-2;Q91XM9-7 – _NLSQIENVHGYVLQS[+H+O3+P]HISPLK_.3
Fig4_MP-DIA-barrel-cortex-p15-2_MHRM.htrms

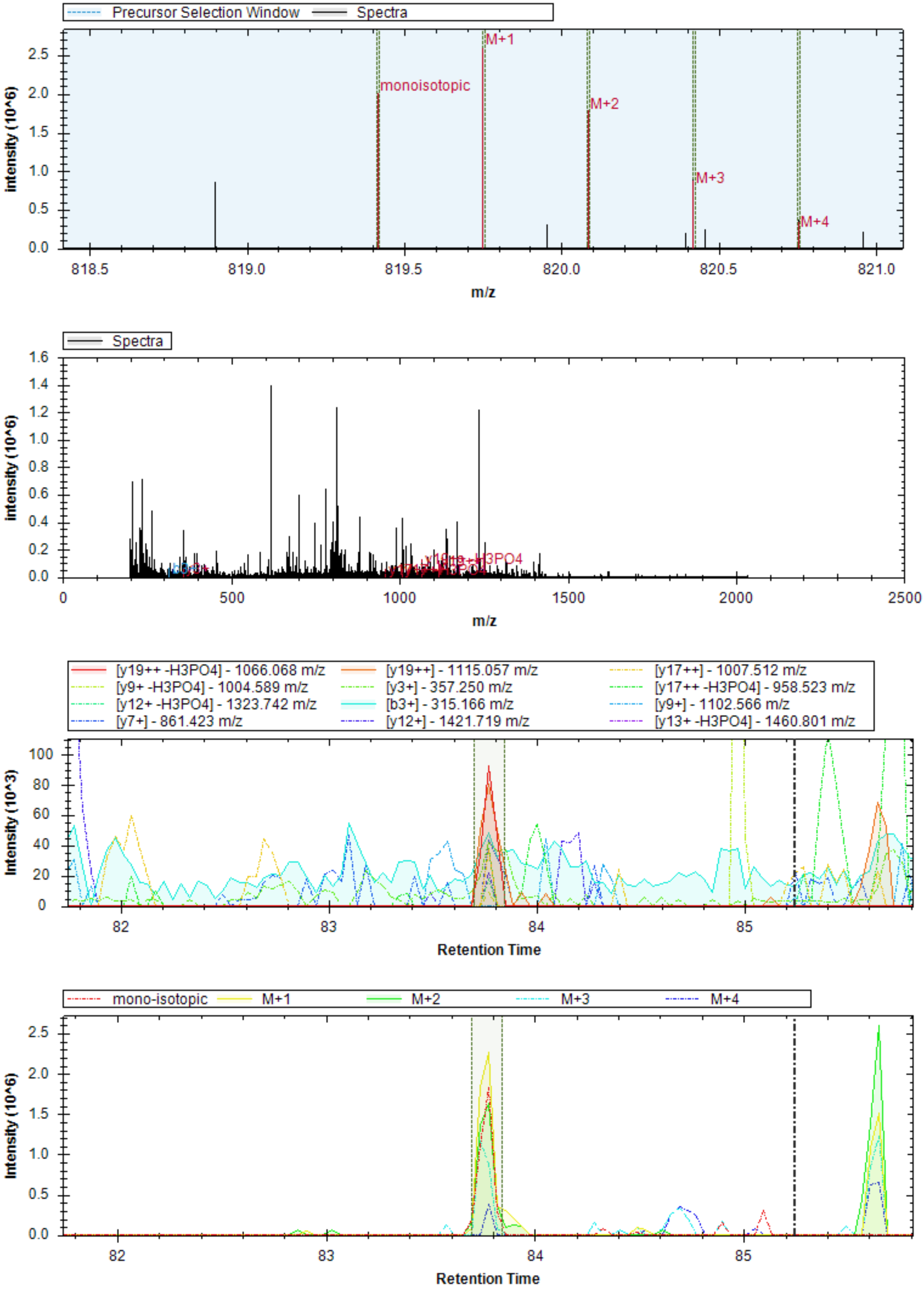

D3YXK2 – _AET[+H+O3+P]LSGLGDASAAGAAAVSSAASETGTR_.3
Fig4_MP-DIA-barrel-cortex-p15-2_MHRM.htrms

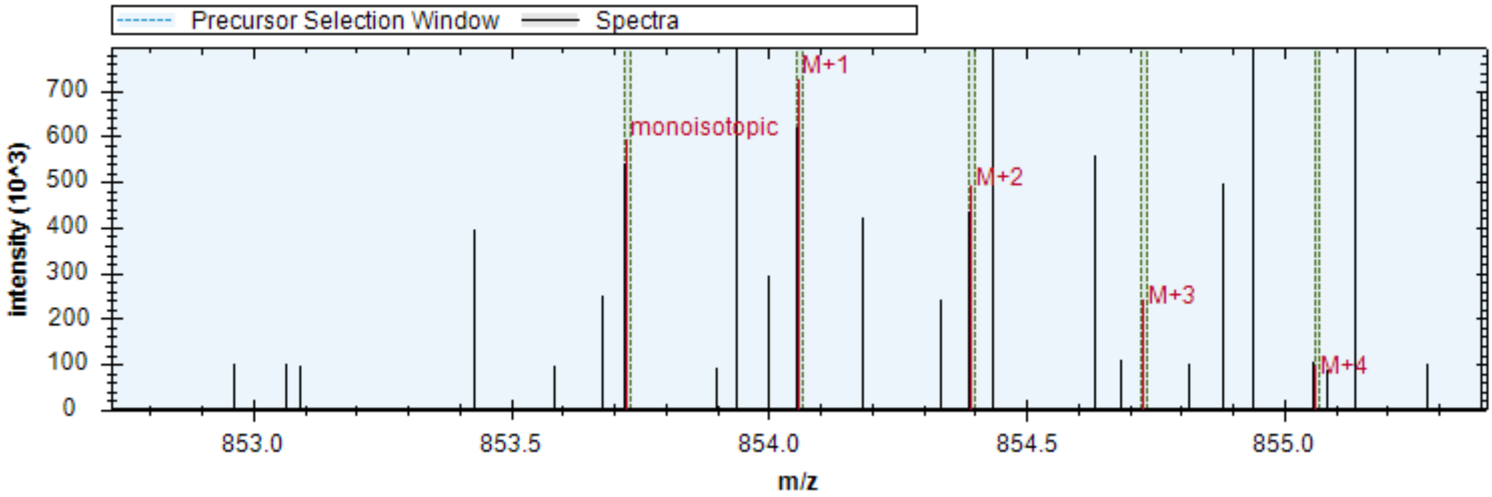

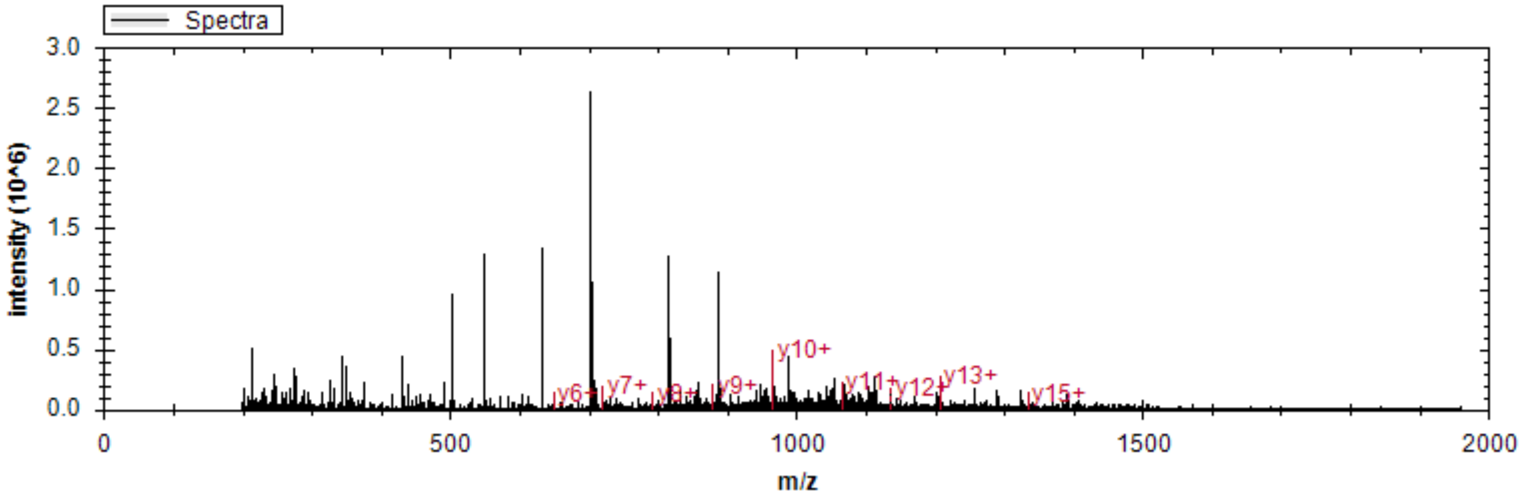

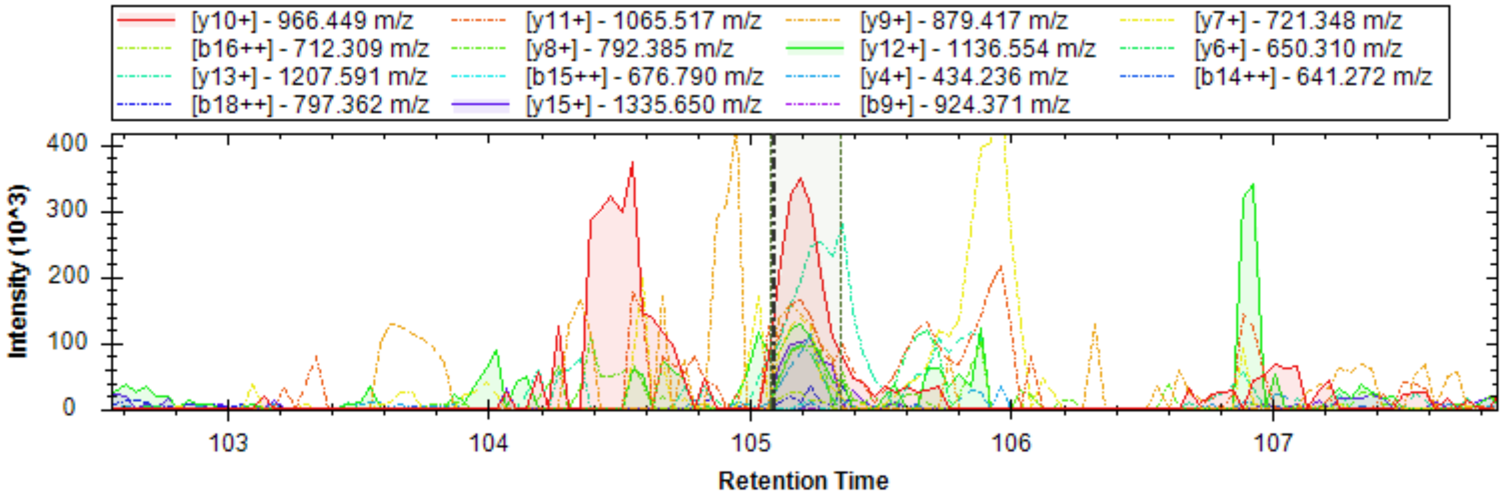

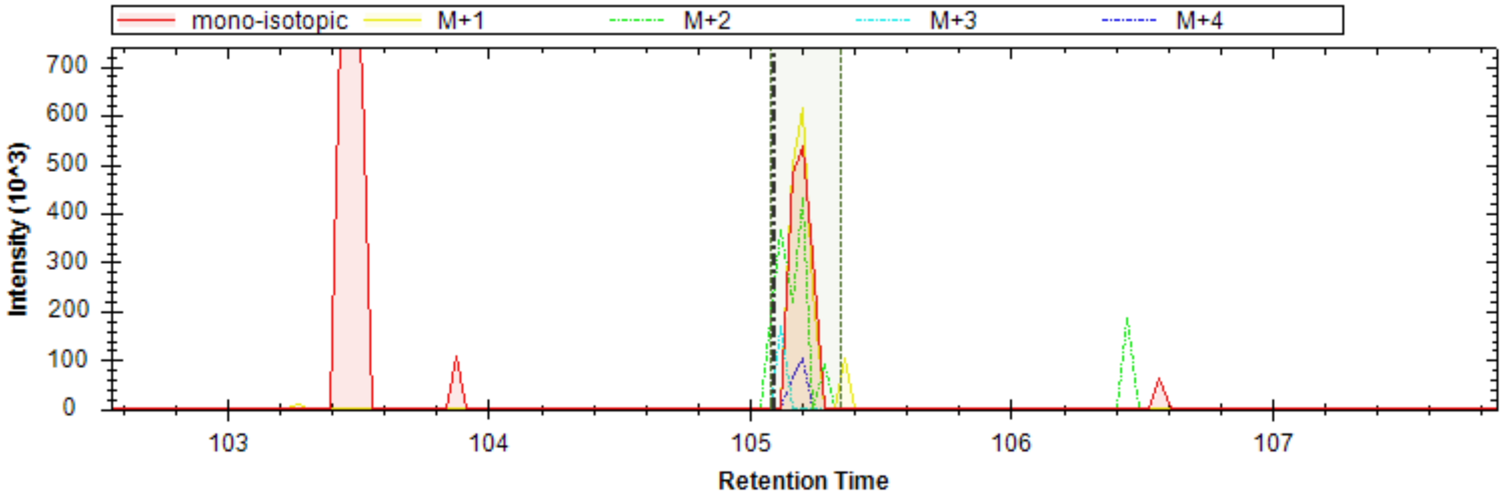

O08784 – _ASAVSPEKAPM[+O]T[+H+O3+P]SKAK_.2
Fig4_MP-DIA-barrel-cortex-p15-2_MHRM.htrms

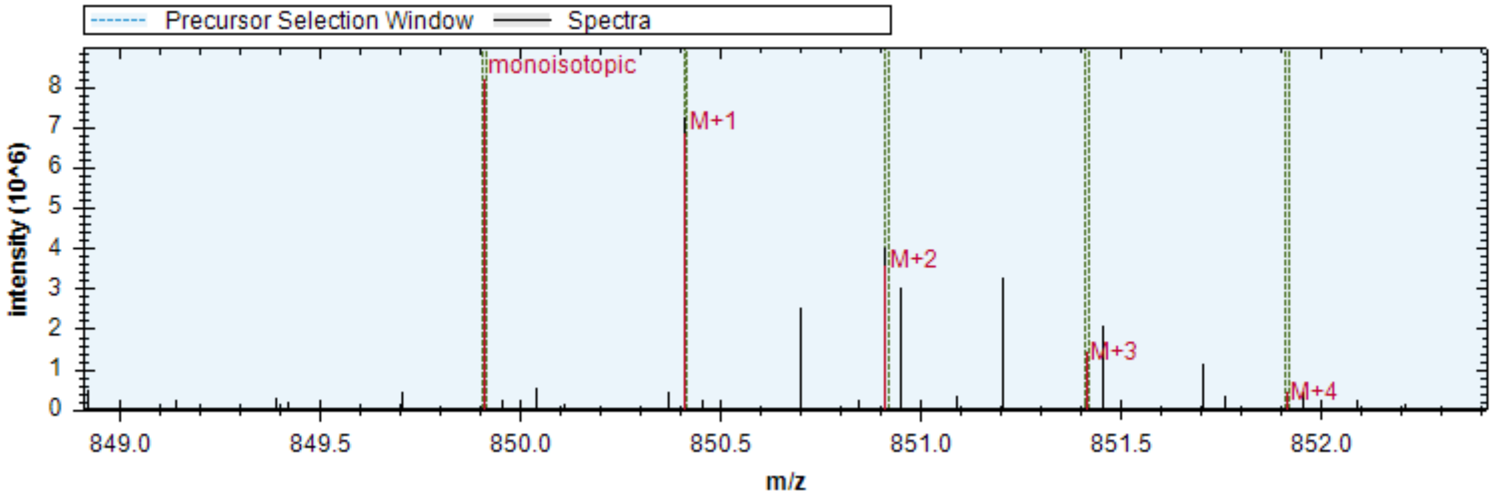

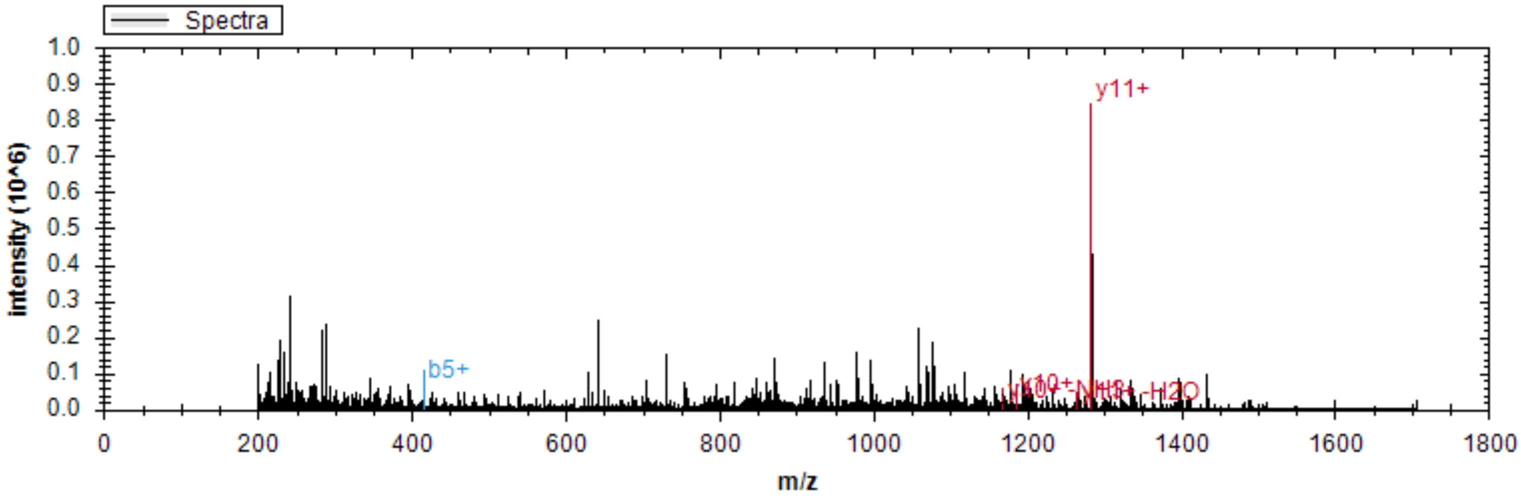

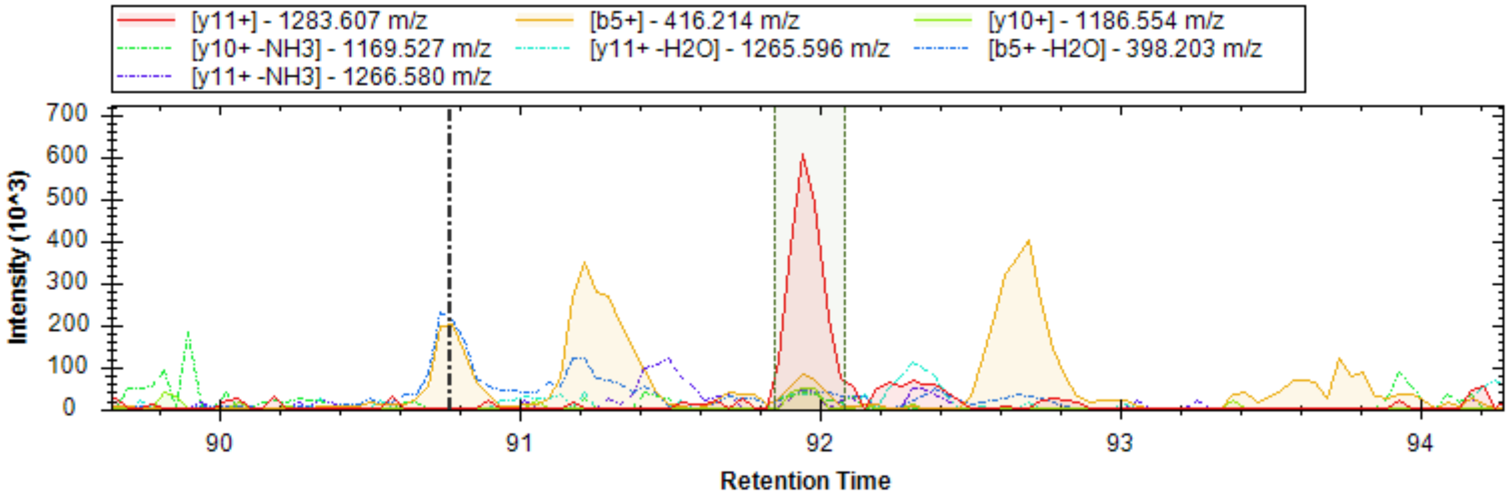

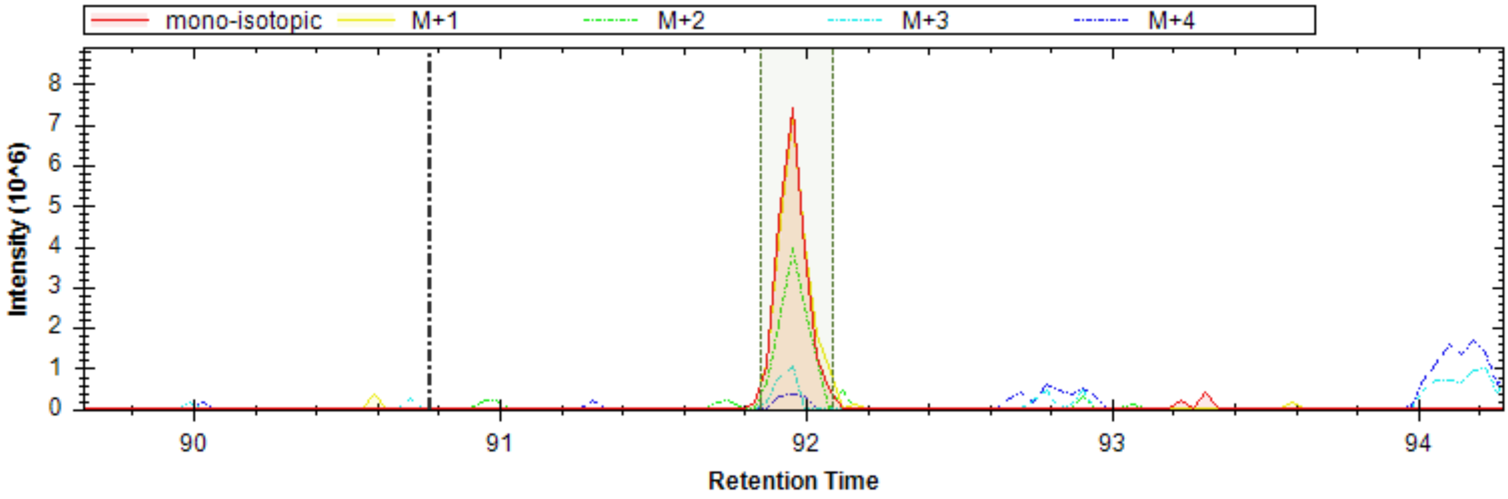

P14873 – _ETAAAHQAS[+H+O3+P]SSPPIDAATAEPYGFR_.3
Fig4_MP-DIA-barrel-cortex-p15-2_MHRM.htrms

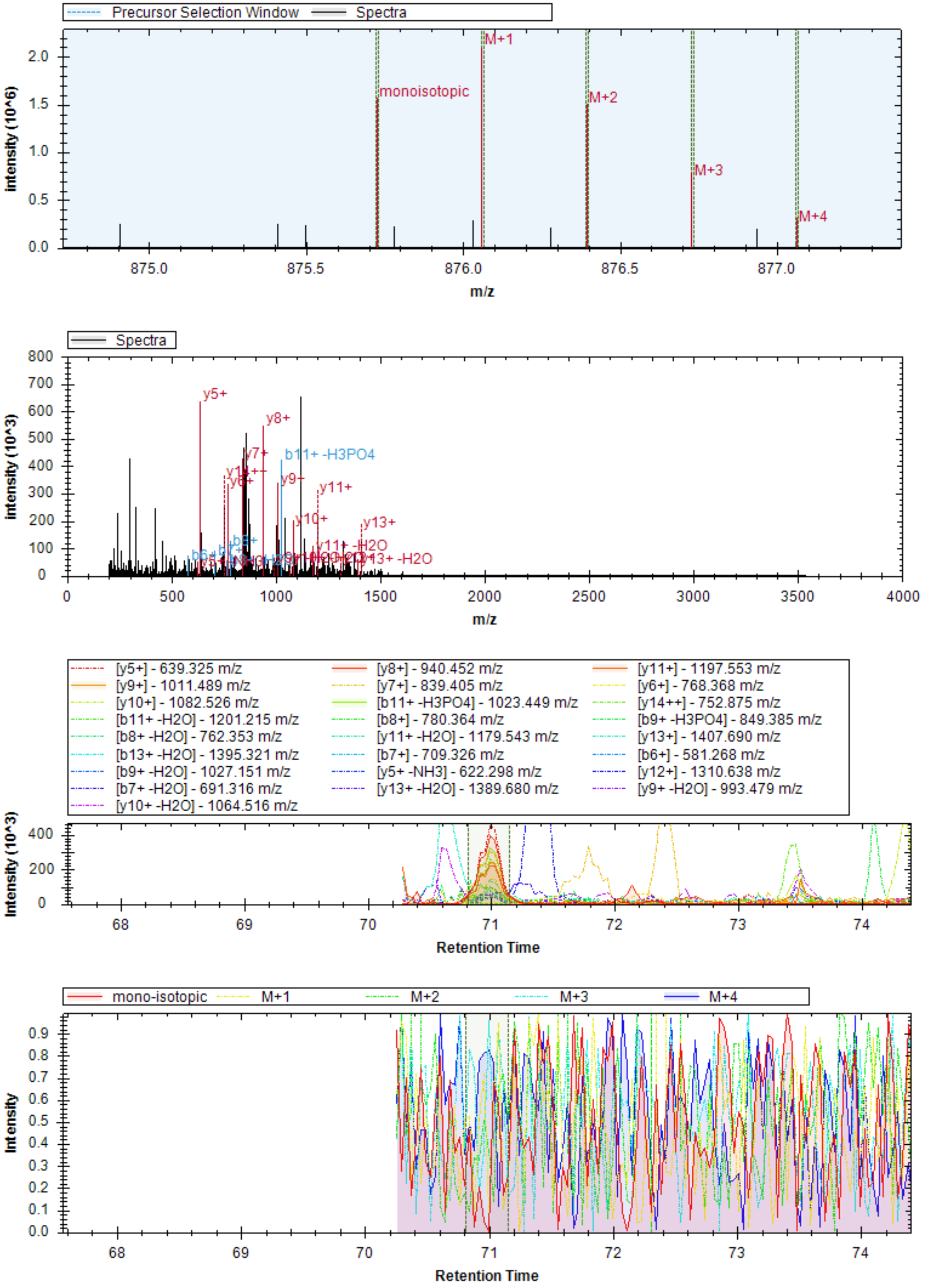

P14873 – _ETAAAHQASS[+H+O3+P]SPPIDAATAEPYGFR_.3
Fig4_MP-DIA-barrel-cortex-p15-2_MHRM.htrms

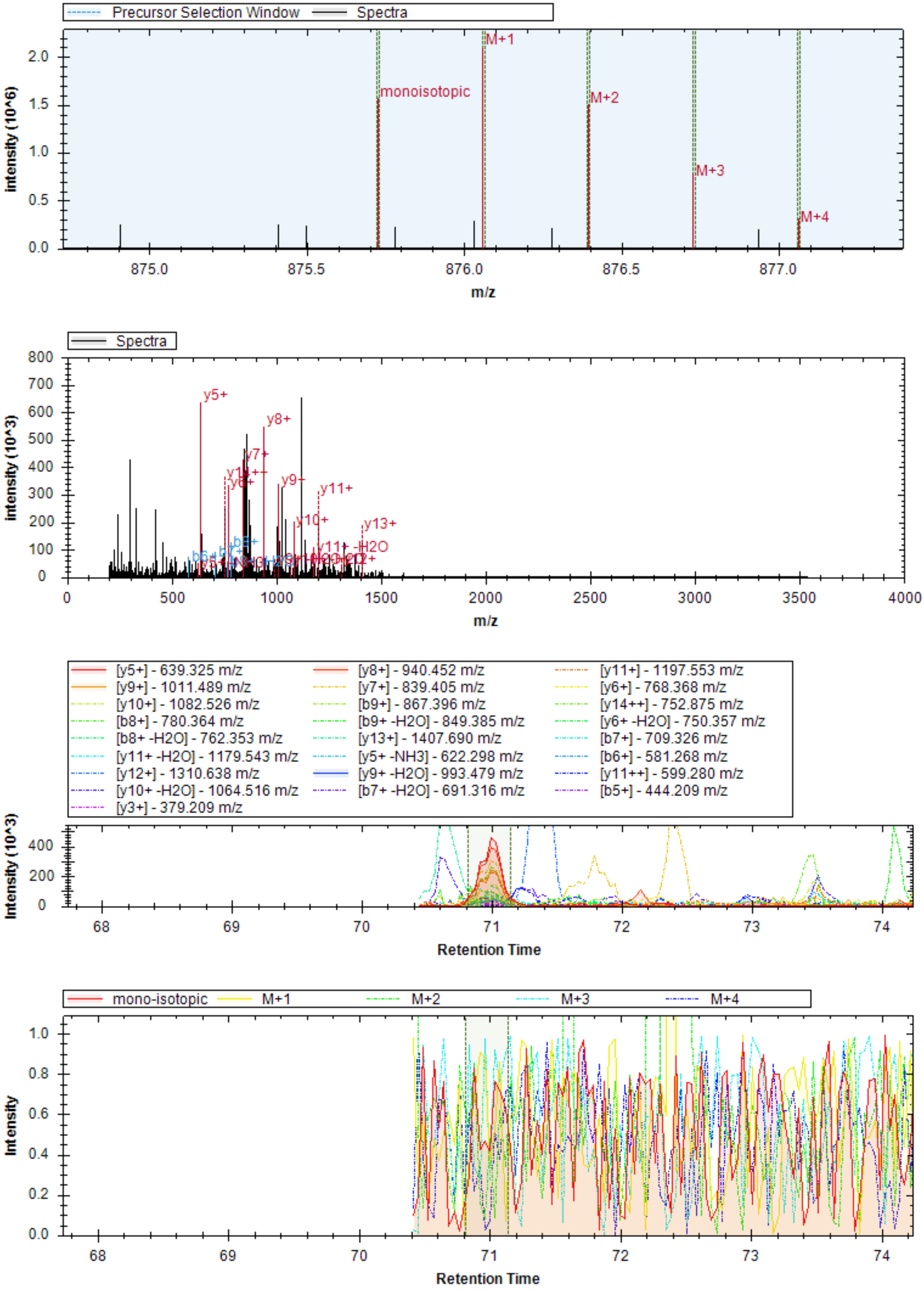

P14873 – _ETAAAHQASSS[+H+O3+P]PPIDAATAEPYGFR_.3

Fig4_MP-DIA-barrel-cortex-p15-2_MHRM.htrms

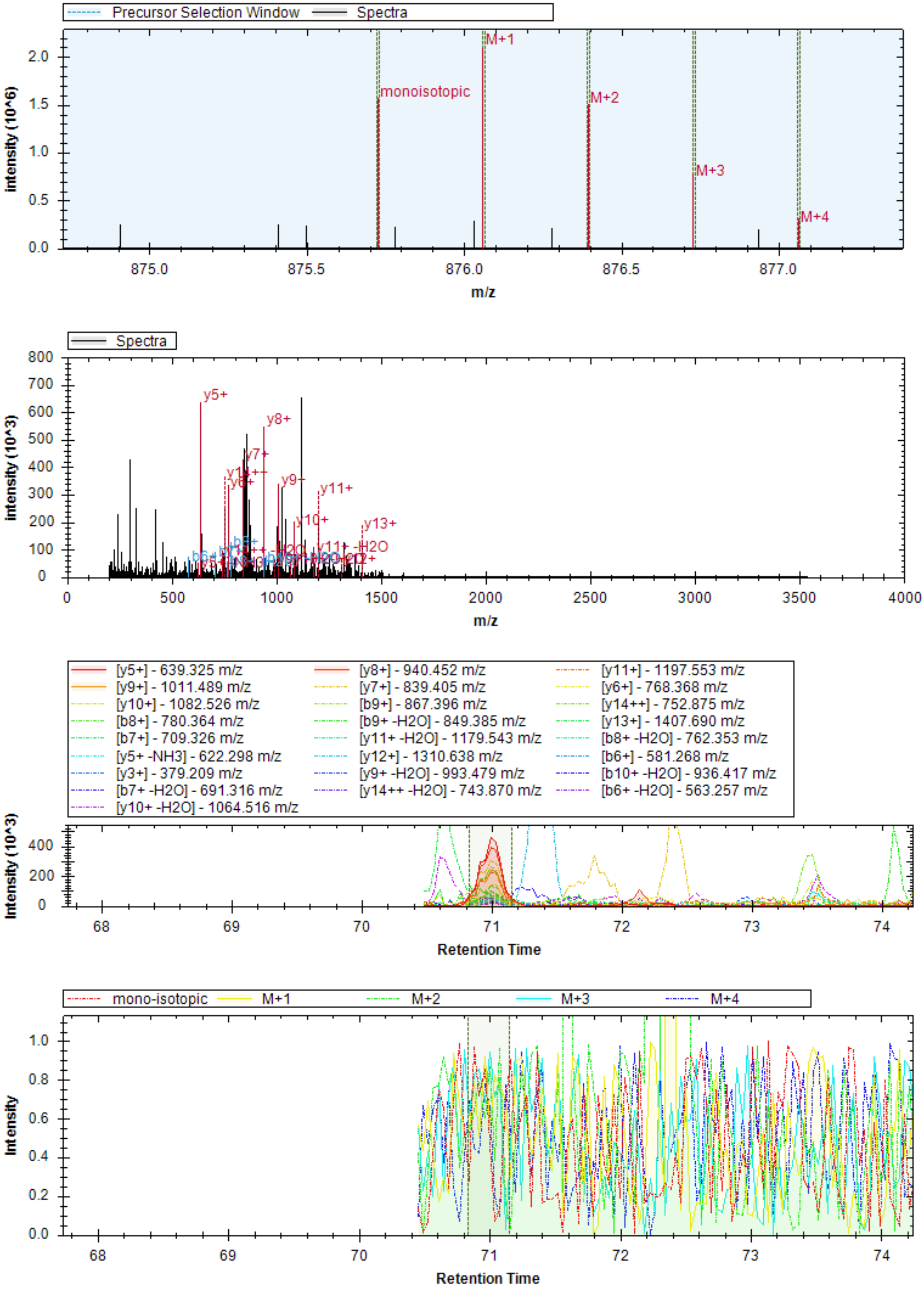

P27546-2 – _NTT[+H+O3+P]PTGAAPPAGMTSTR_.2
Fig4_MP-DIA-barrel-cortex-p15-2_MHRM.htrms

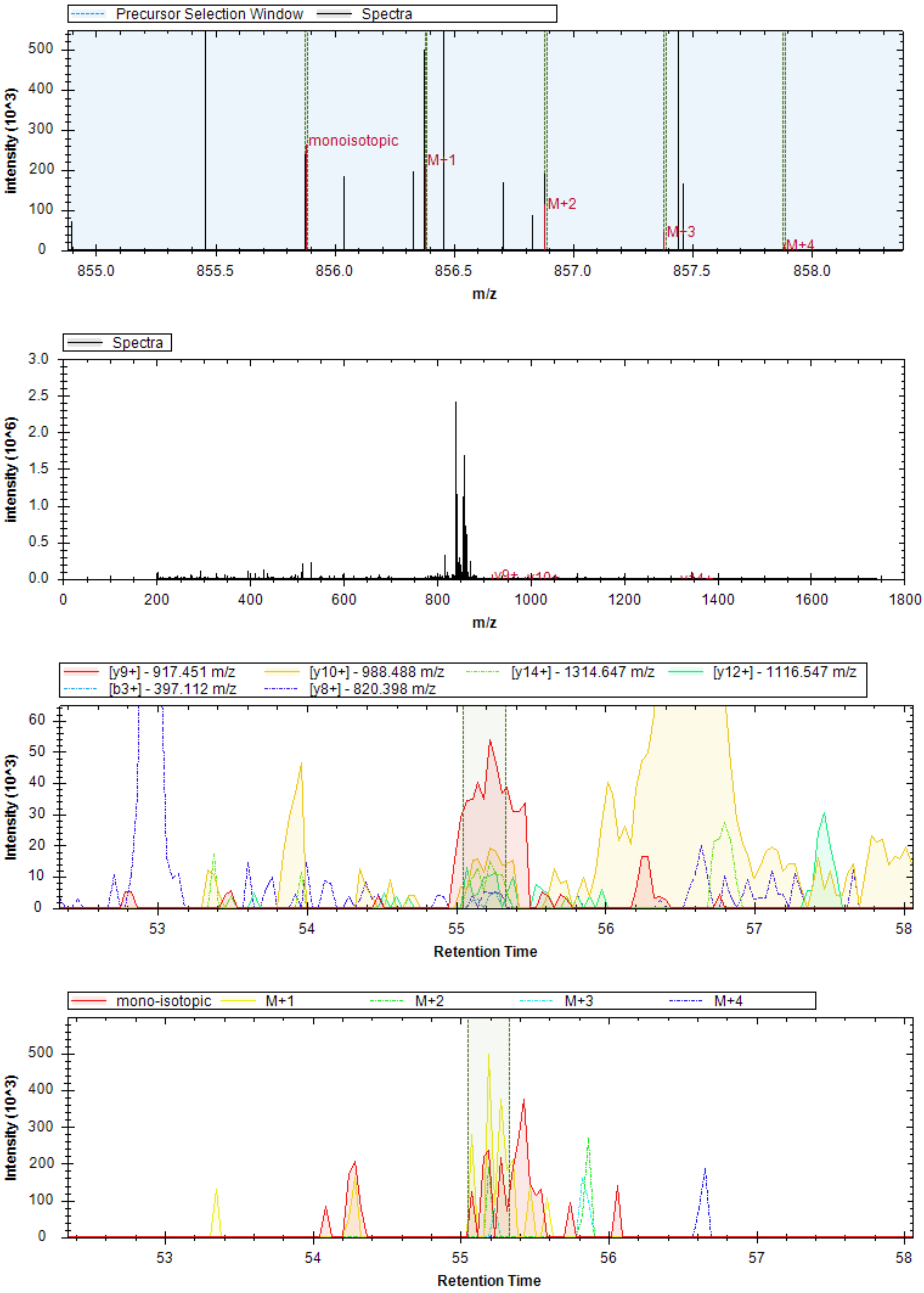

P50538 – _EAEHGYAS[+H+O3+P]M[+O]LPYSK_.2
Fig4_MP-DIA-barrel-cortex-p15-2_MHRM.htrms

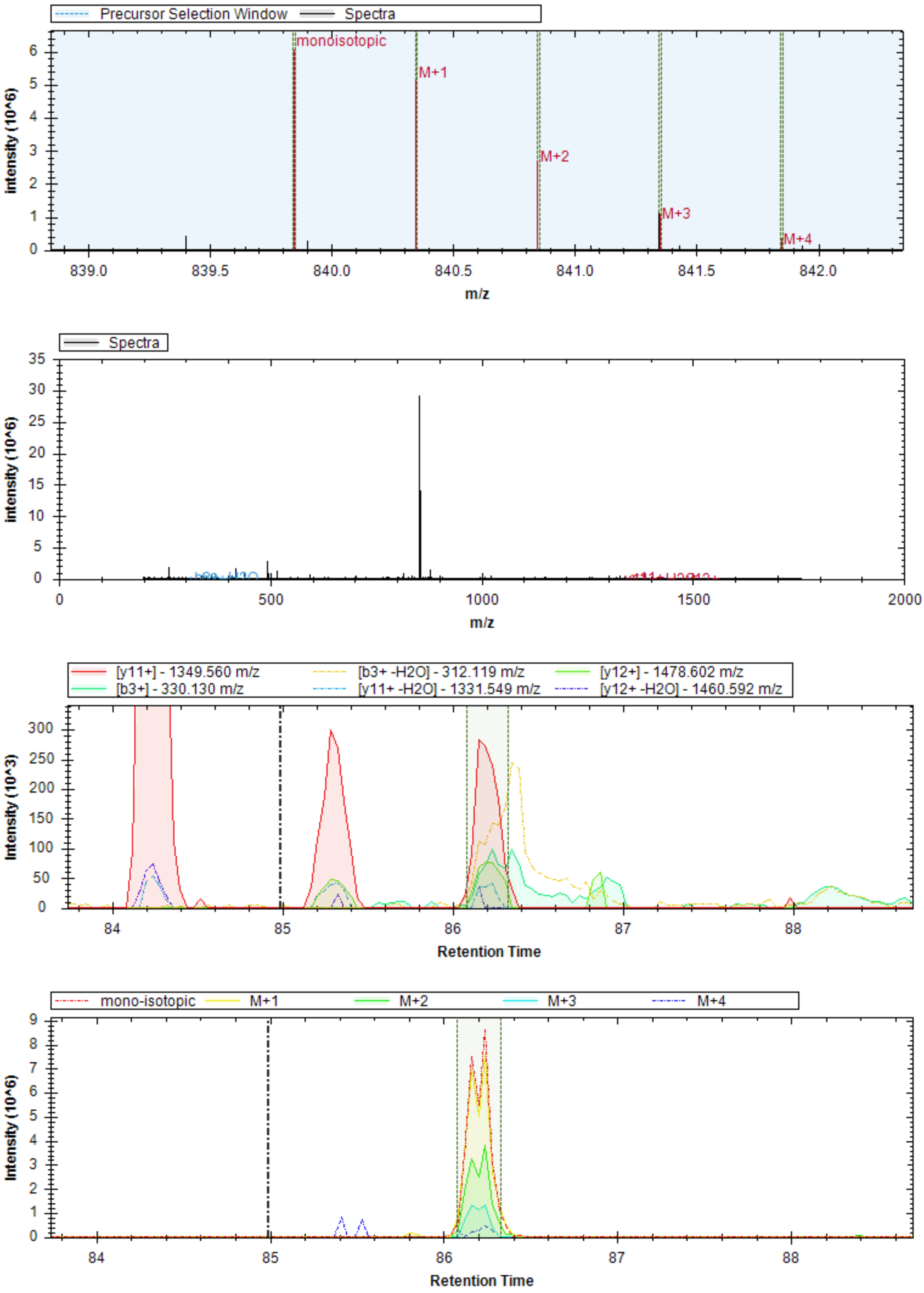

Q62188 – _GM[+O]YDGPVFDLT[+H+O3+P]TTPK_.2
Fig4_MP-DIA-barrel-cortex-p15-2_MHRM.htrms

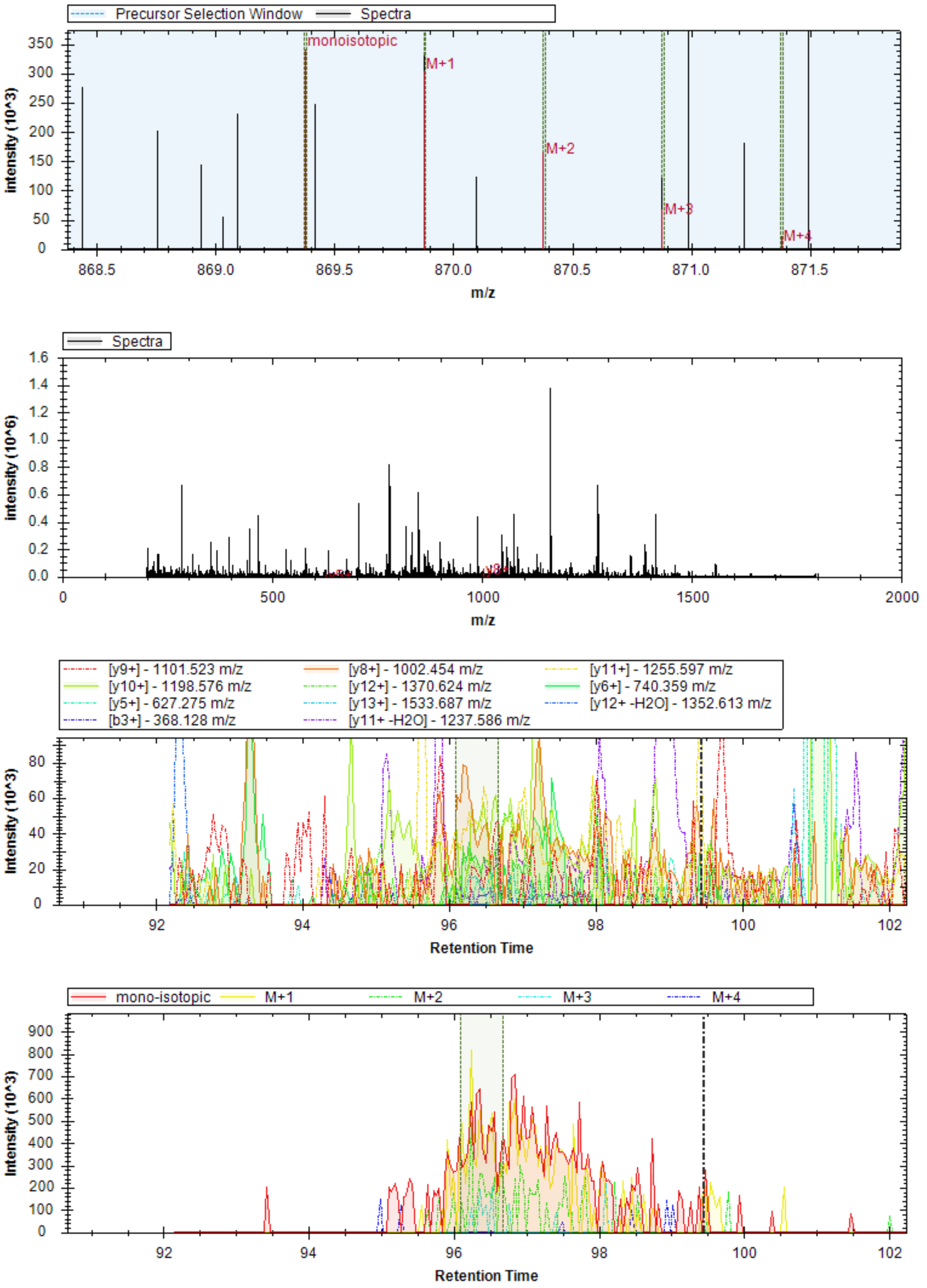

Q9Z0P4 – _SETLVNAQQT[+H+O3+P]PLGTPK_.2
Fig4_MP-DIA-barrel-cortex-p15-2_MHRM.htrms

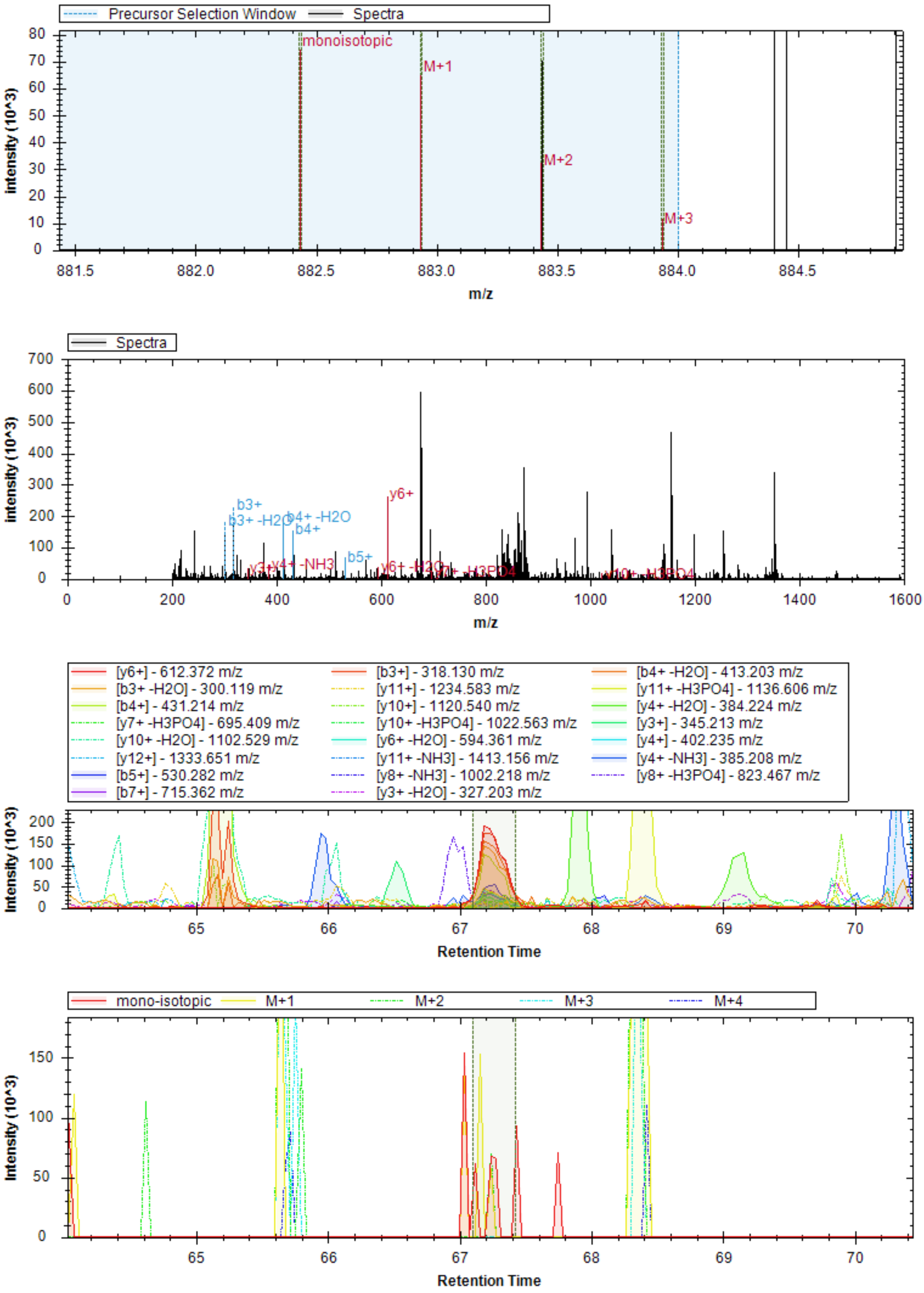

G5E829 – _SATSSSPGS[+H+O3+P]PLHSLETSL_.2
Fig4_MP-DIA-barrel-cortex-p15-2_MHRM.htrms

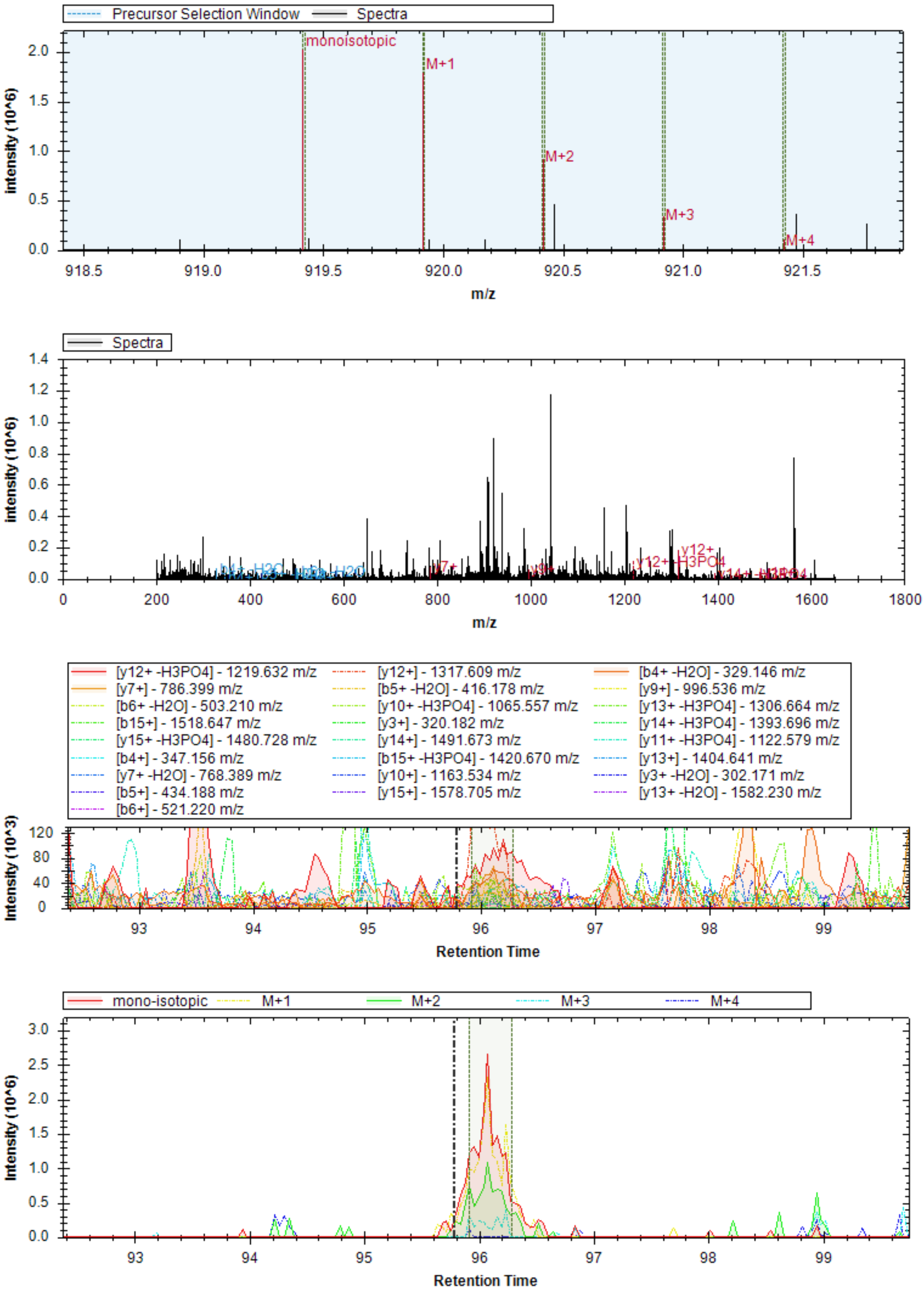

Q8BMA3 – _IIQLC[+C2+H3+N+O]LDLT[+H+O3+P]T[+H+O3+P]AVQK_.2
Fig4_MP-DIA-barrel-cortex-p15-2_MHRM.htrms

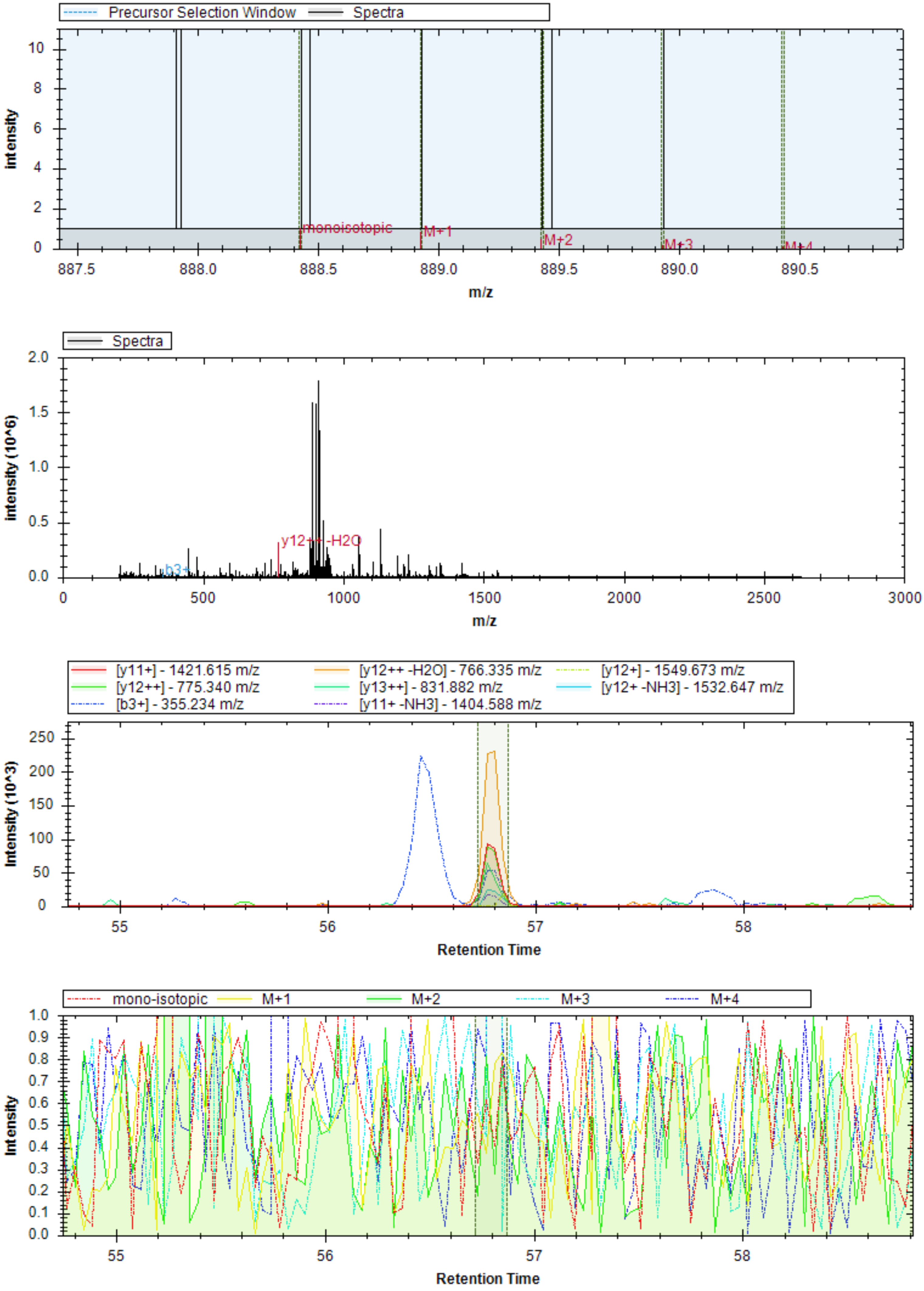

Q91Z31;Q91Z31-2 – _ETSLLAVPGALS[+H+O3+P]PLAIPNAAAAAAAAAAGR_.3
Fig4_MP-DIA-barrel-cortex-p15-2_MHRM.htrms

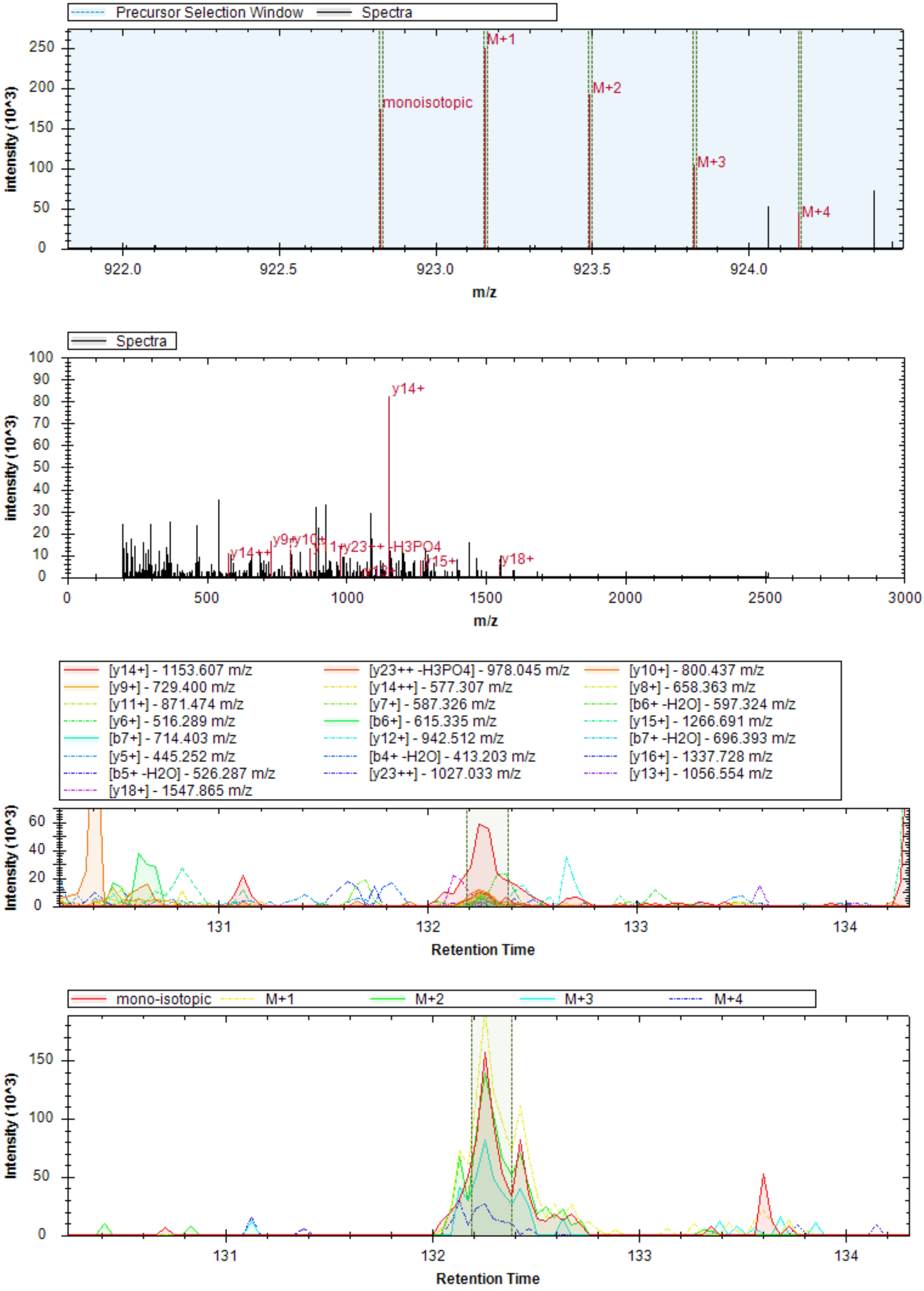

Q9ES97;Q9ES97-2 – _AES[+H+O3+P]SAATQSPSVSSSSSGAEPSALGGGGGSPGAC[+C2+H3+N+O]PALGAK_.4
Fig4_MP-DIA-barrel-cortex-p15-2_MHRM.htrms

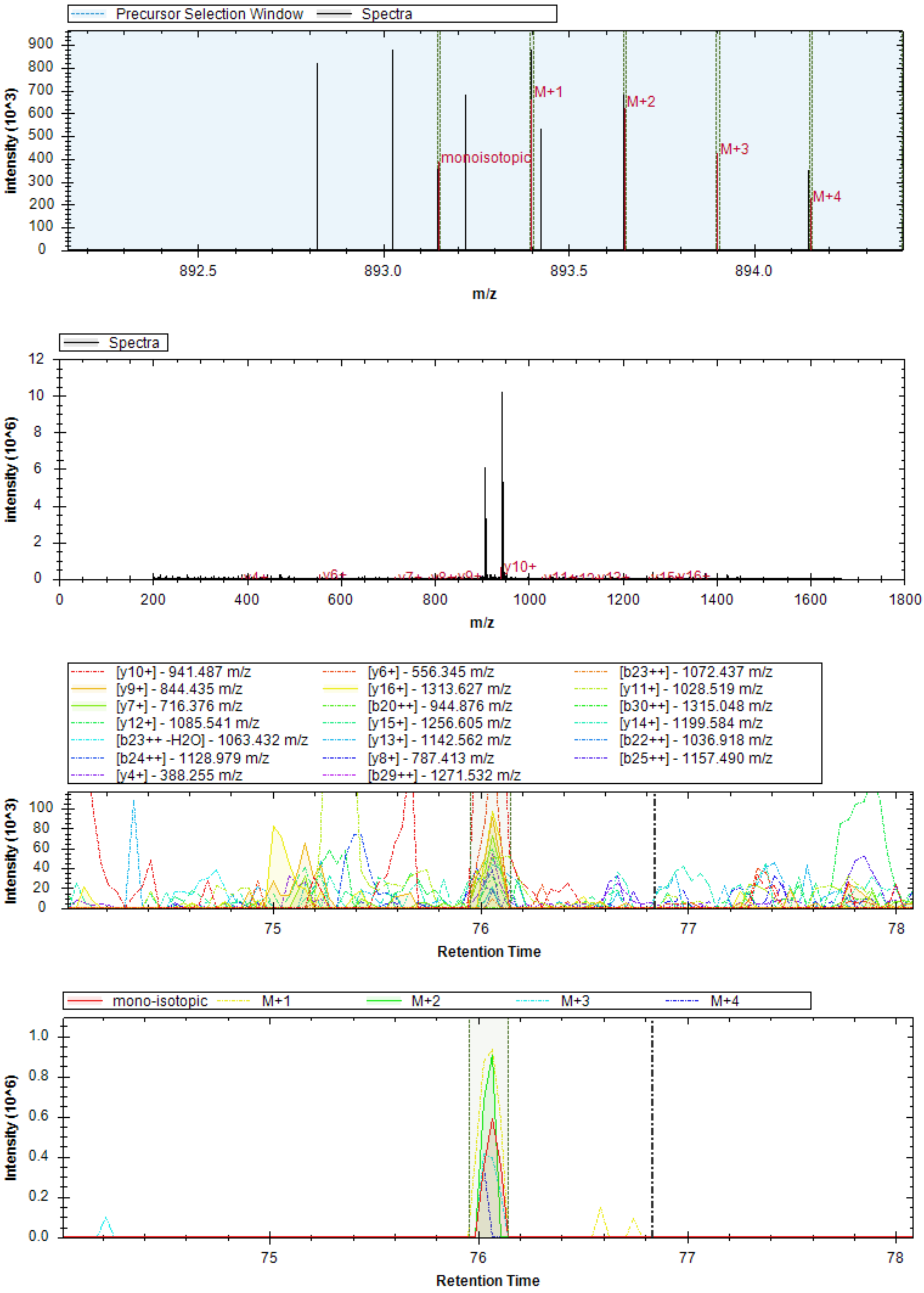

P17742 – _VNPT[+H+O3+P]VFFDITADDEPLGR_.2
Fig4_MP-DIA-barrel-cortex-p15-2_MHRM.htrms

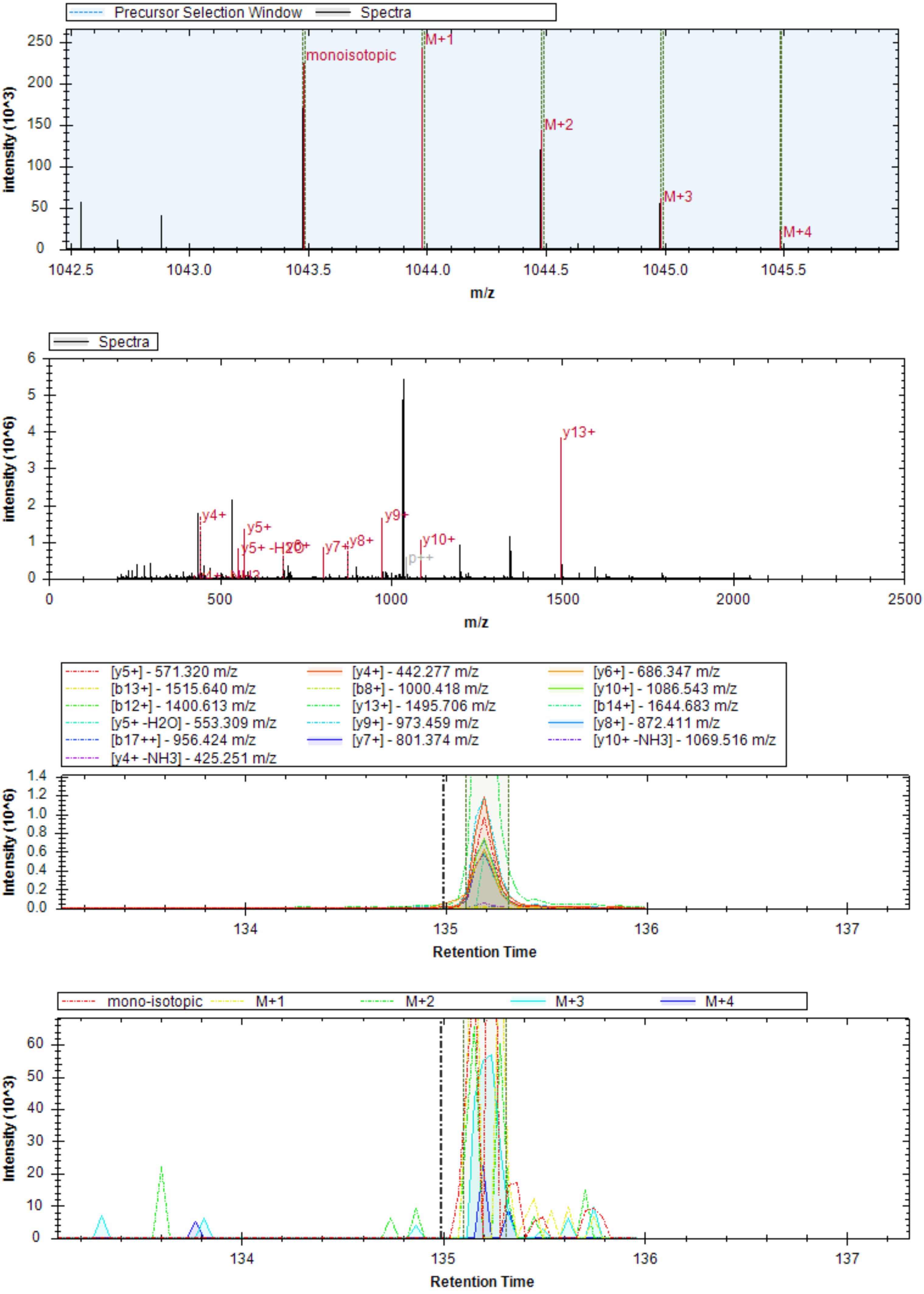

Q8CHK4;Q8CHK4-2;Q8CH[...] – _RGY[+H+O3+P]GKLLIEFS[+H+O3+P]YELSK_.2
Fig4_MP-DIA-barrel-cortex-p15-2_MHRM.htrms

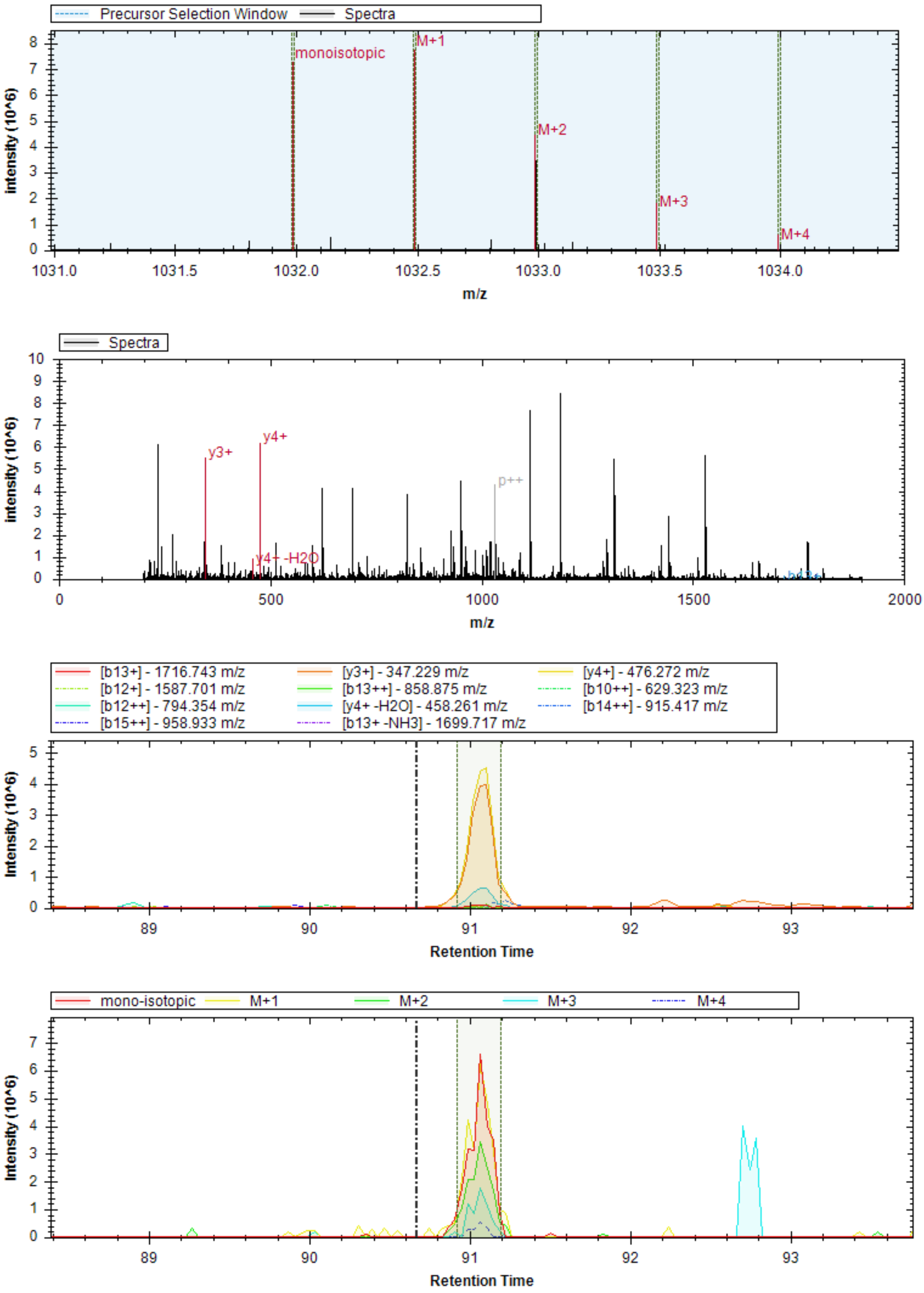

E9Q401 – _MVAEVFIY[+H+O3+P]WSK_.1
Fig4_MP-DIA-barrel-cortex-p15-2_MHRM.htrms

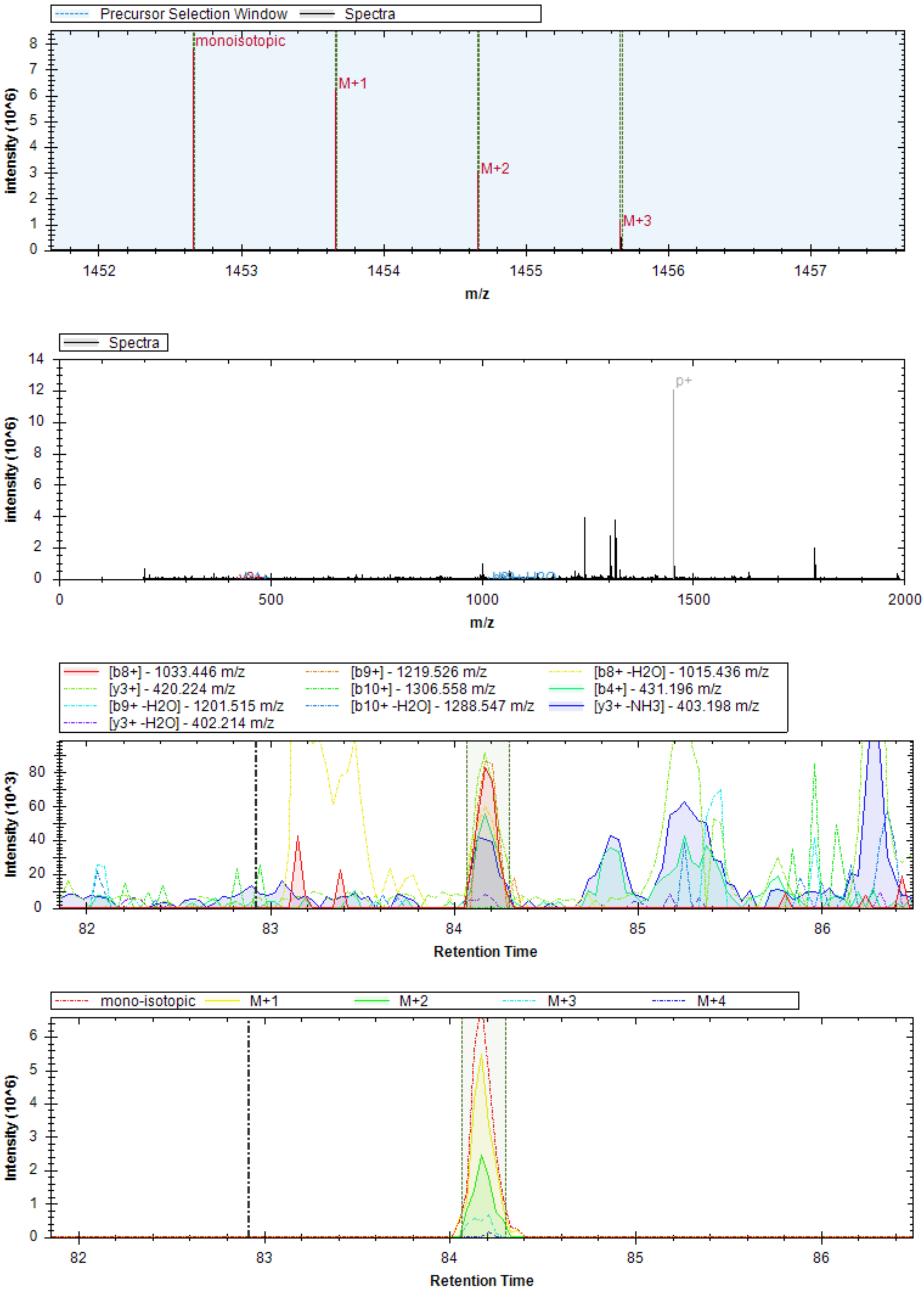

P20357 – _SGTSTPTTPGSTAIT[+H+O3+P]PGTPPSYSSR_.2
Fig4_MP-DIA-barrel-cortex-p15-2_MHRM.htrms

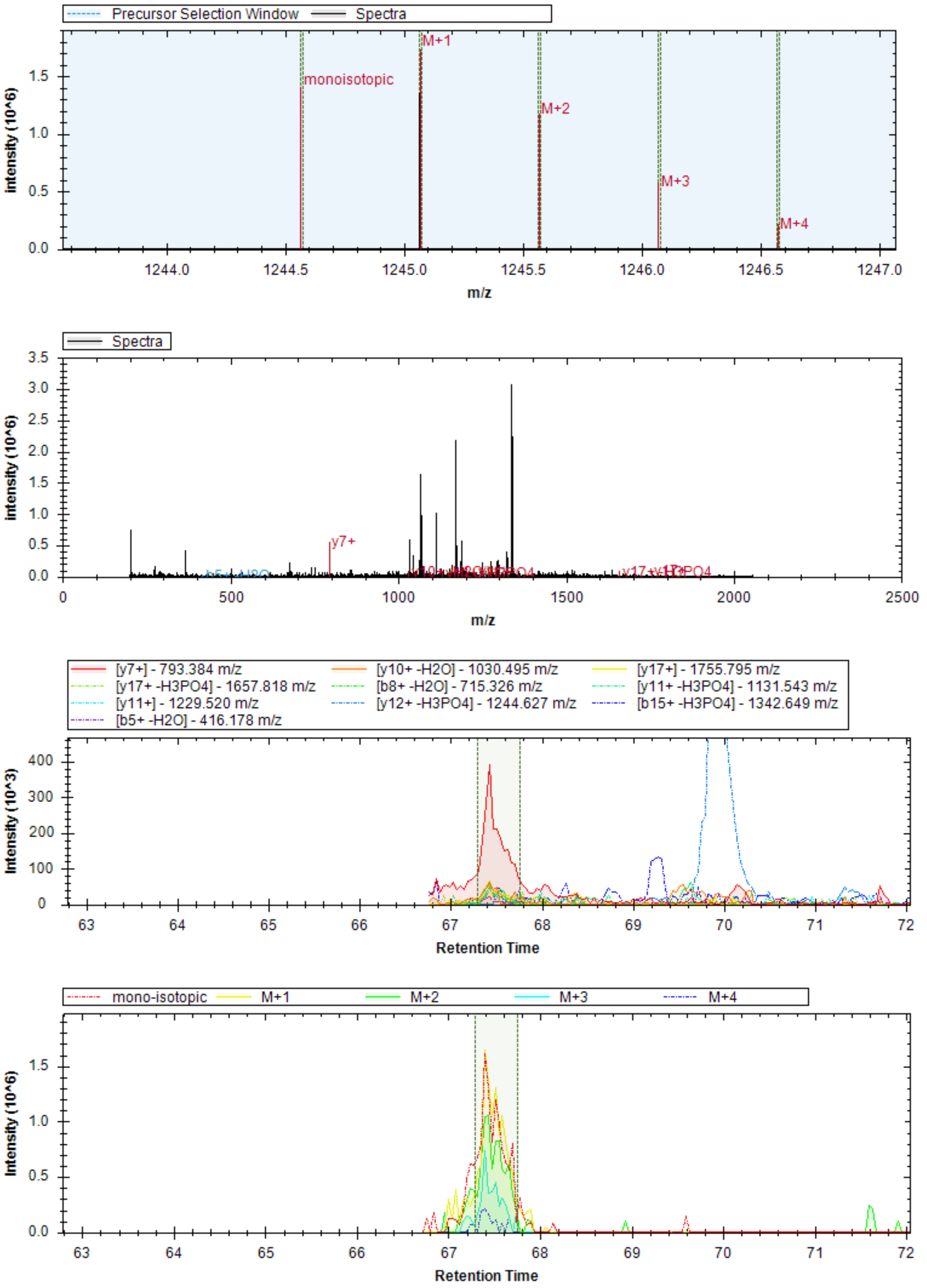

P20357 – _SGTSTPTTPGSTAITPGT[+H+O3+P]PPSYSSR_.2
Fig4_MP-DIA-barrel-cortex-p15-2_MHRM.htrms

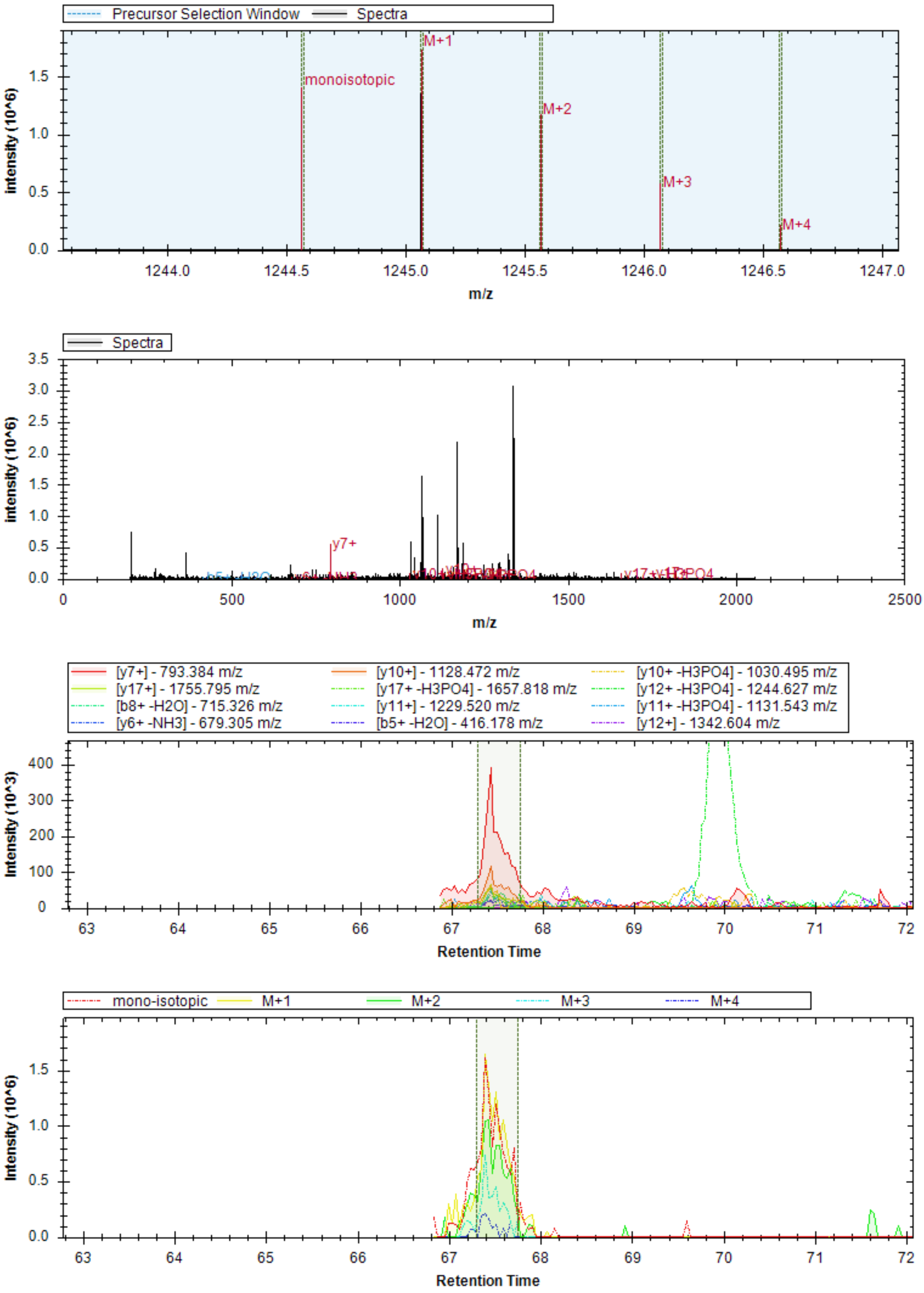

Q8VHY0 – _LLAGPRY[+H+O3+P]GR_.1
Fig4_MP-DIA-barrel-cortex-p15-2_MHRM.htrms

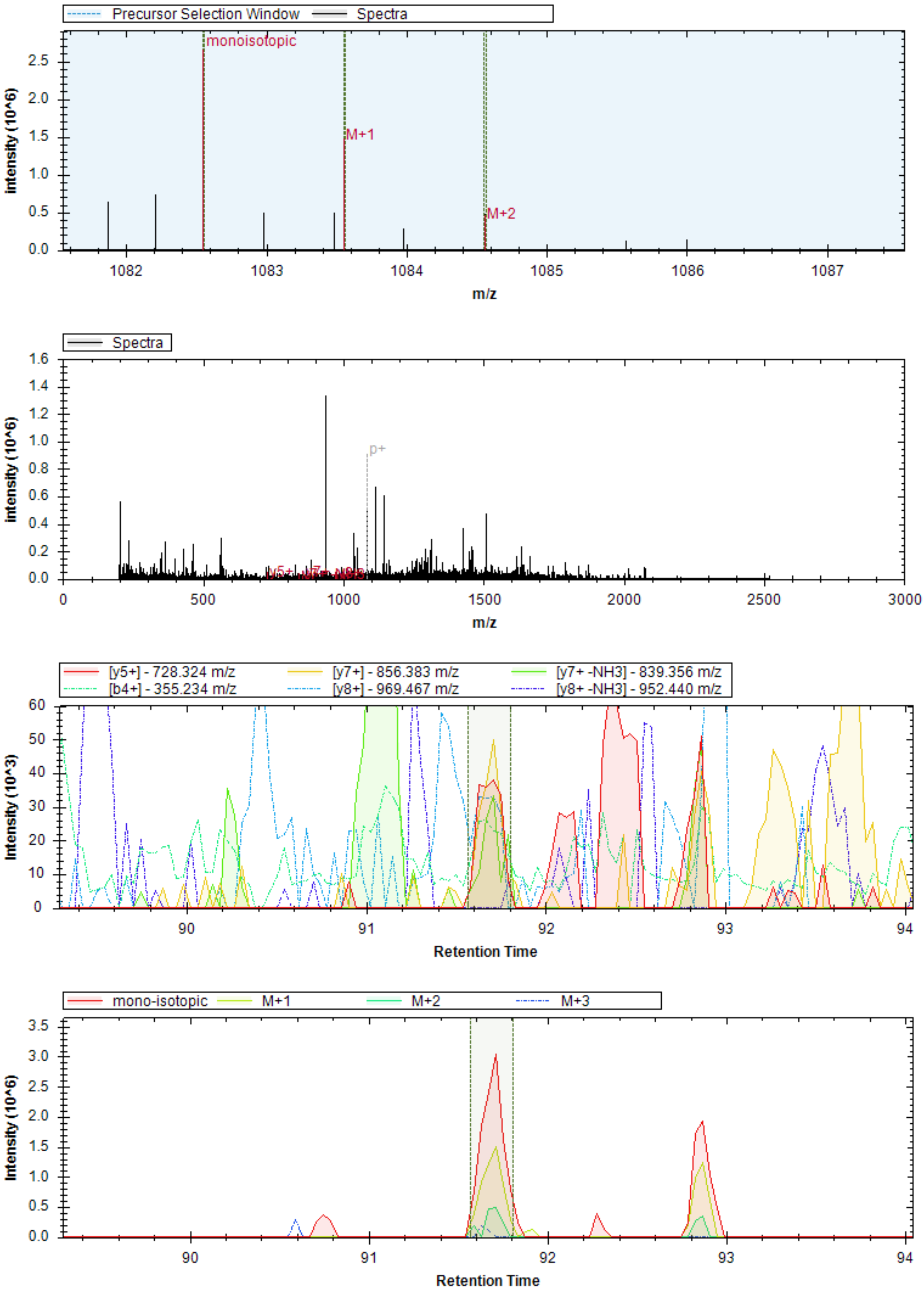

Q921N6;Q921N6-2 – _LIY[+H+O3+P]KPRQAAVTR_.4
Fig4_MP-DIA-barrel-cortex-p15-3_MHRM.htrms

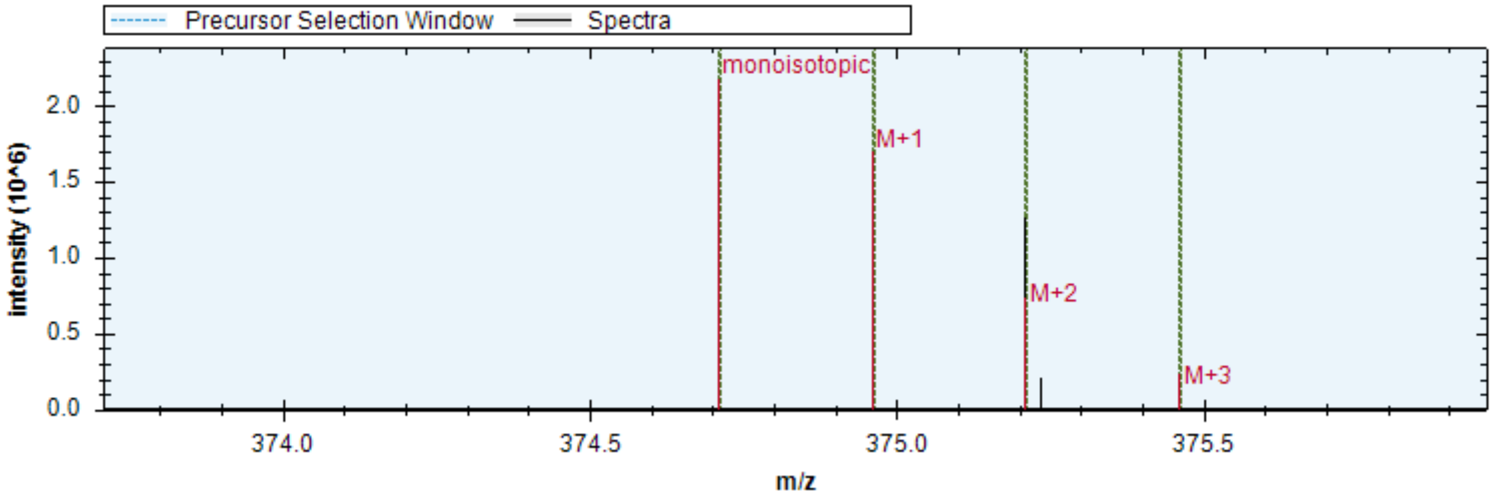

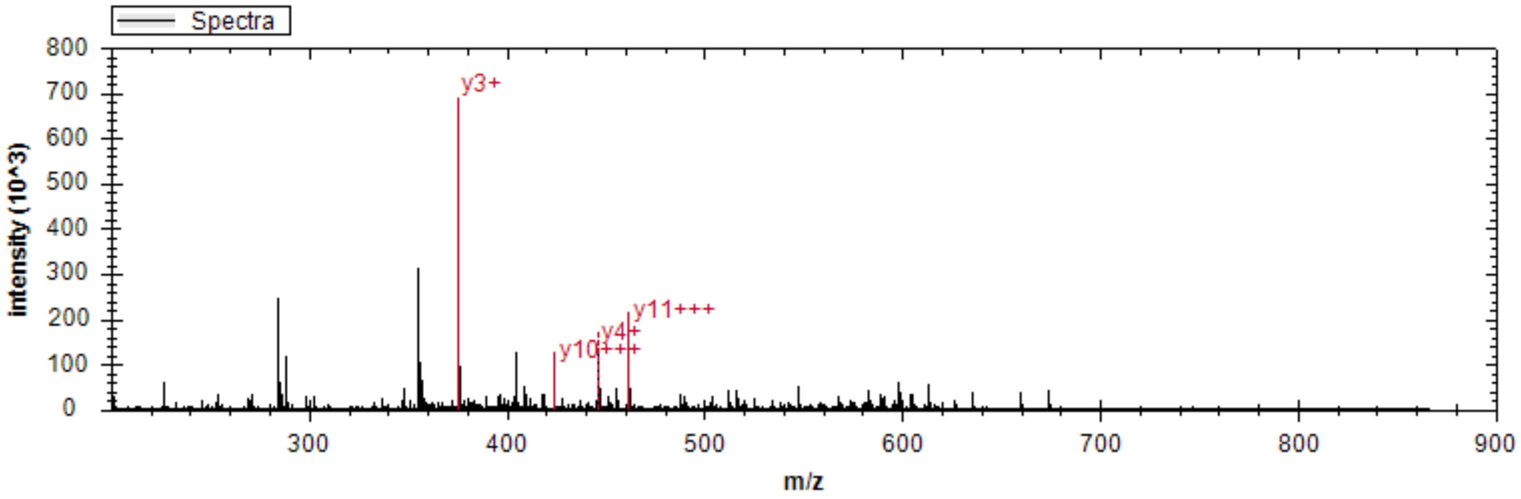

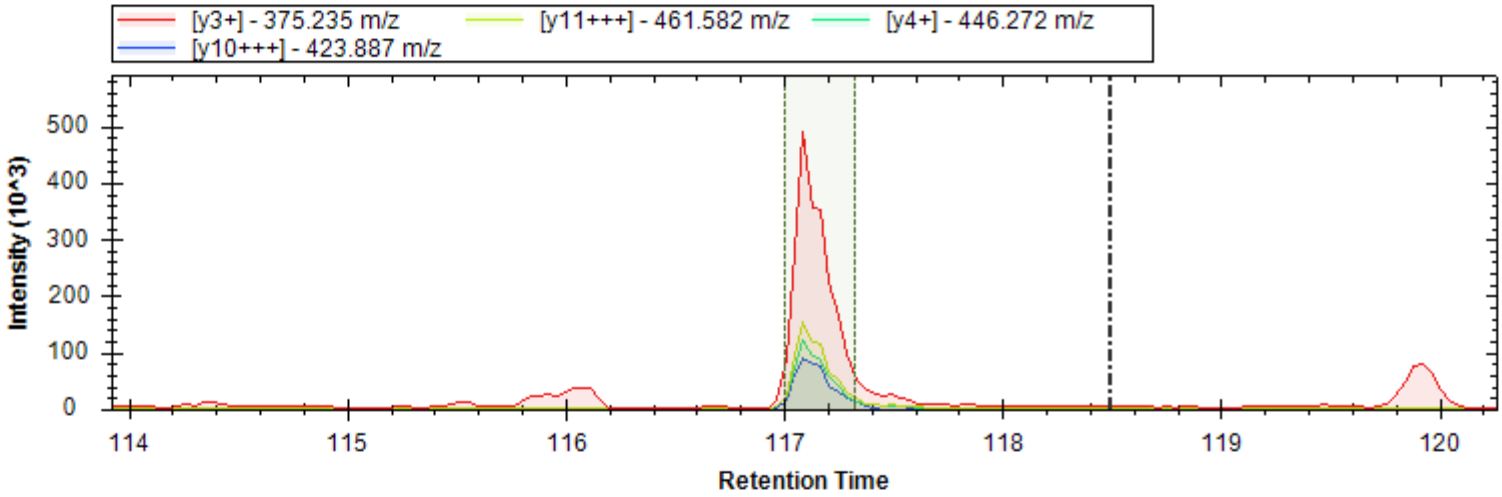

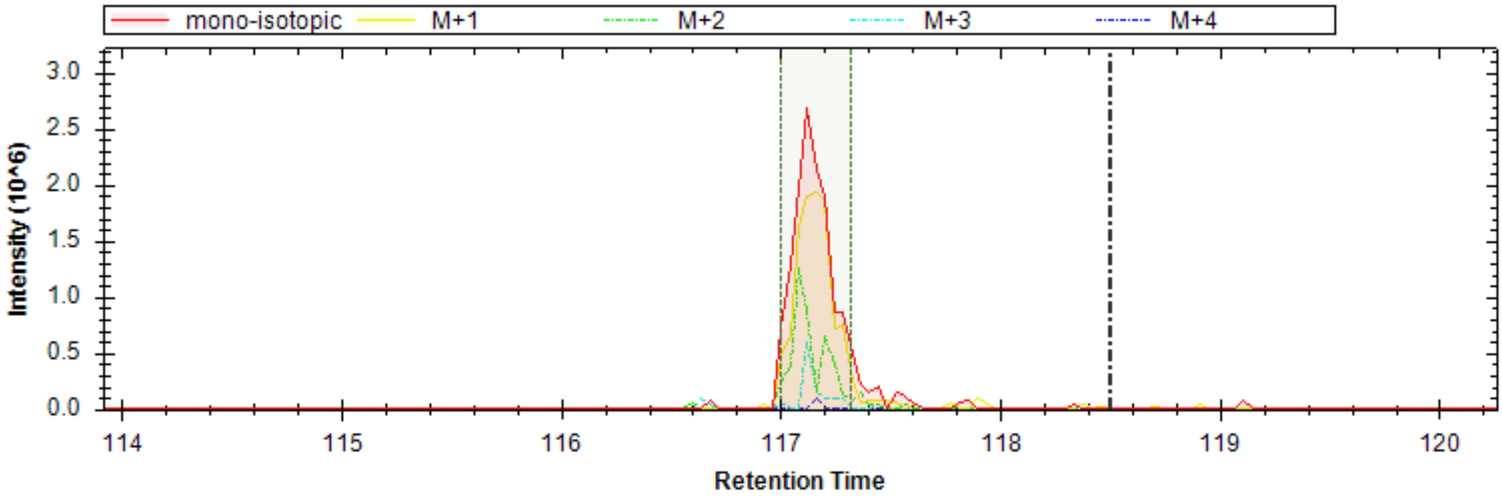

Q9CVB6 – _IIEET[+H+O3+P]LALK_.3
Fig4_MP-DIA-barrel-cortex-p15-3_MHRM.htrms

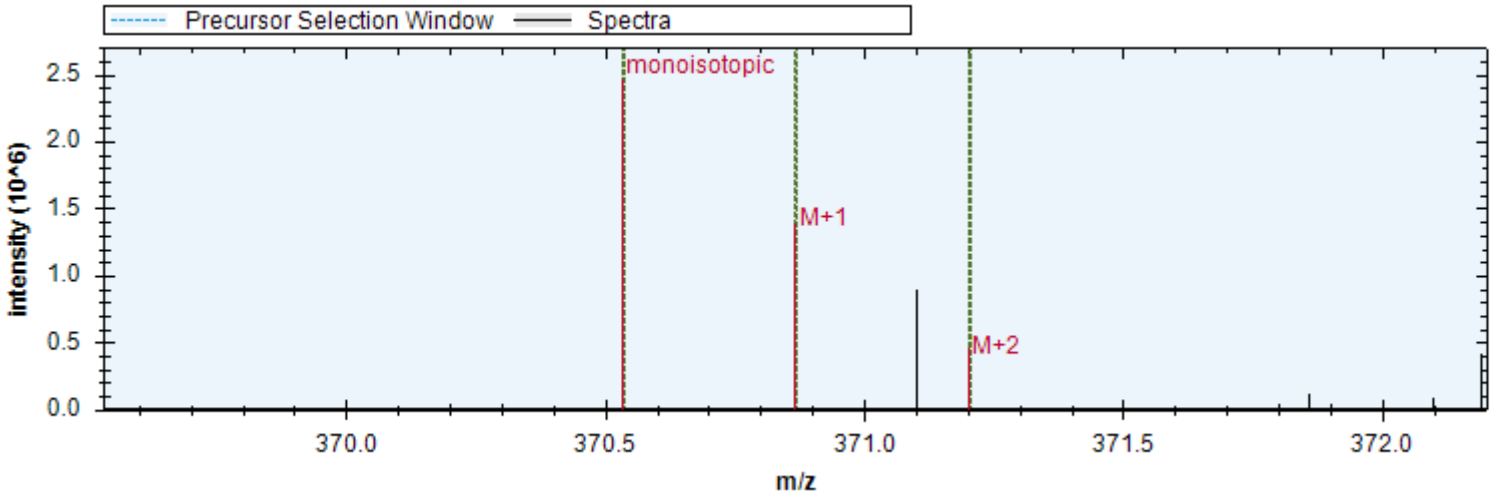

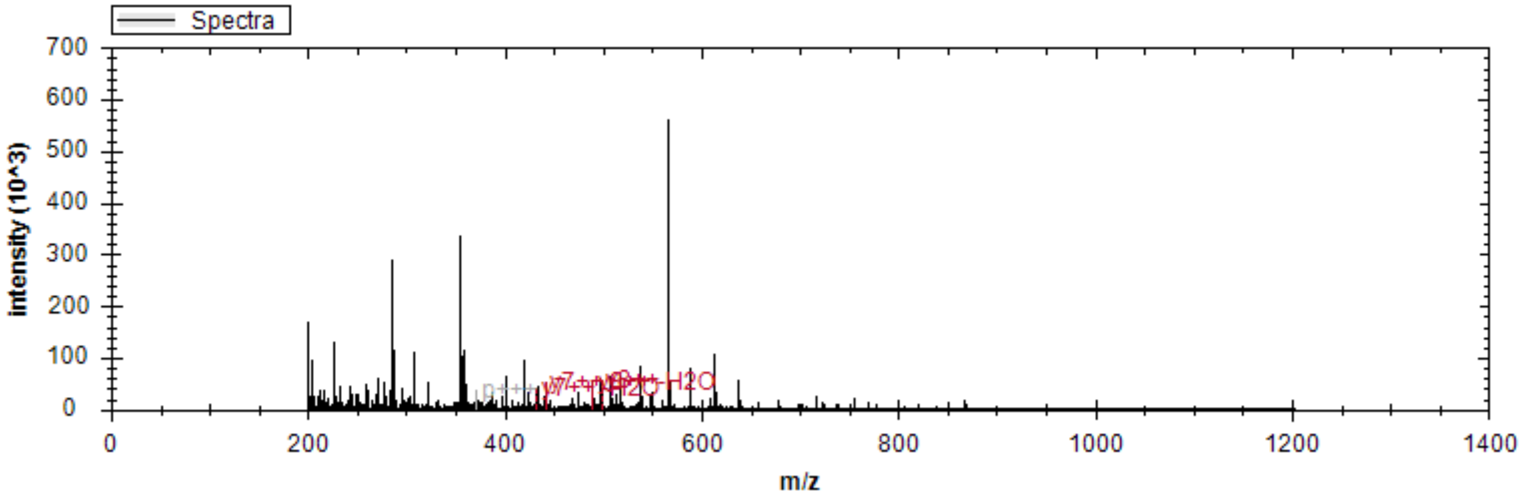

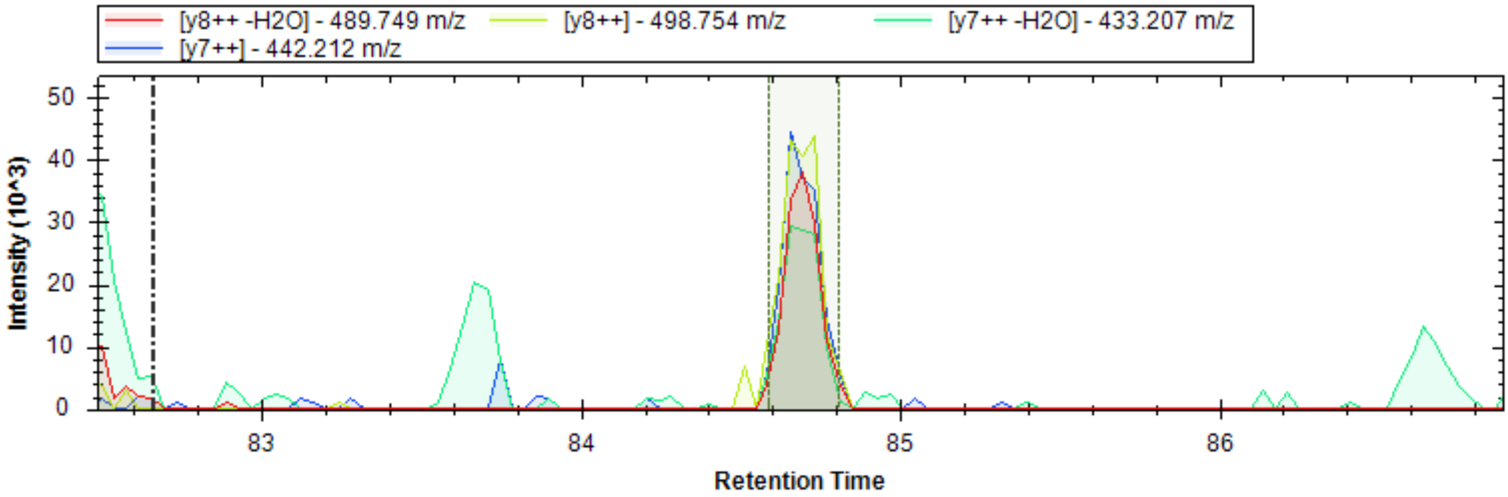

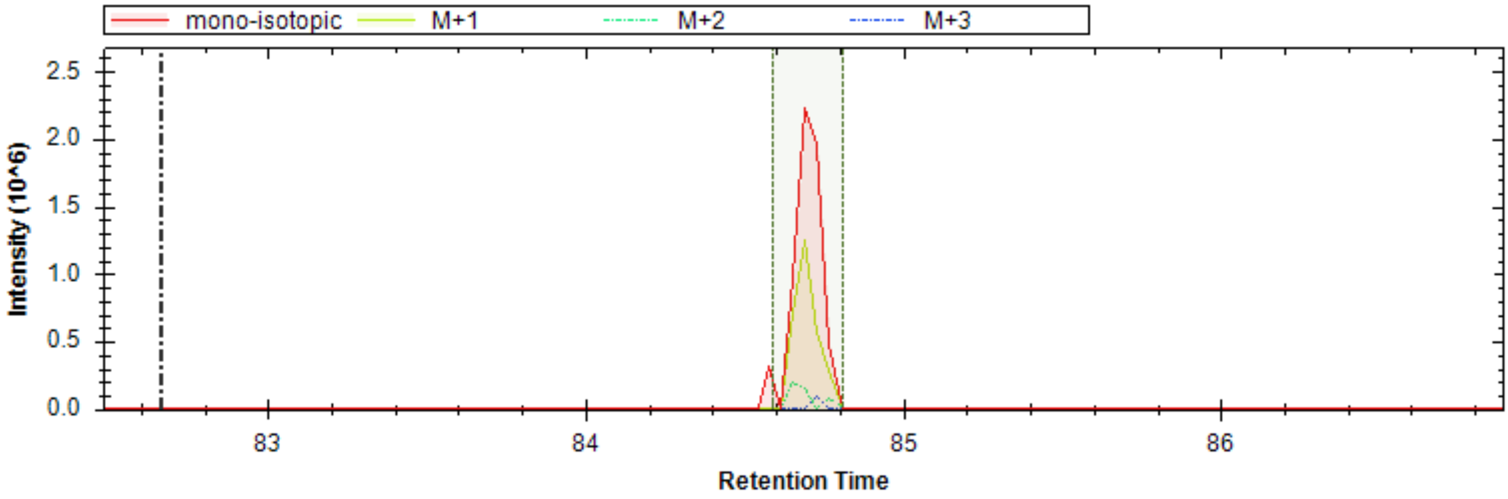

Q9D516;Q9D516-2 – _KLS[+H+O3+P]QGILVIR_.3
Fig4_MP-DIA-barrel-cortex-p15-3_MHRM.htrms

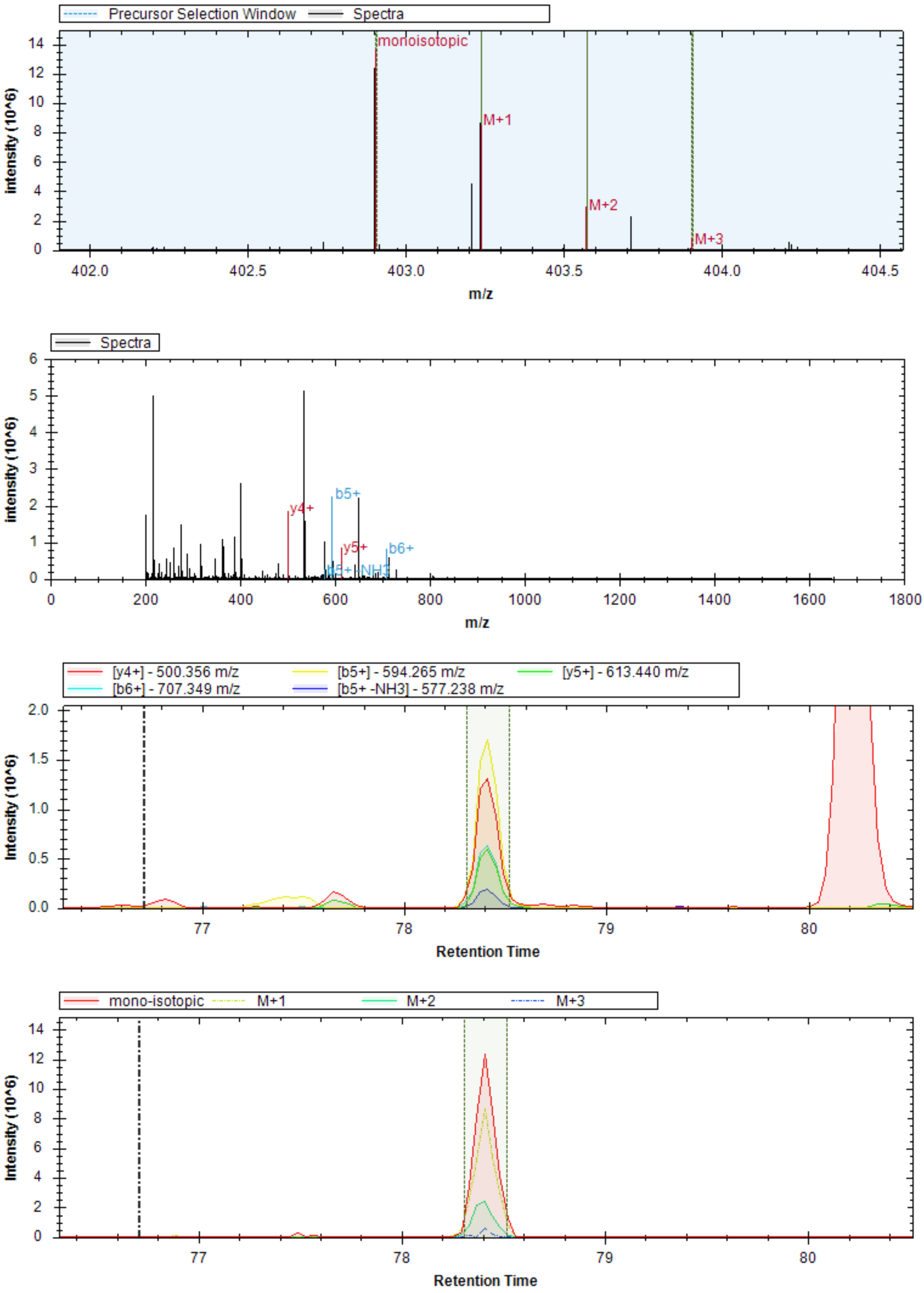

P20357 – _KSEVQAHS[+H+O3+P]PSR_.3

Fig4_MP-DIA-barrel-cortex-p15-3_MHRM.htrms

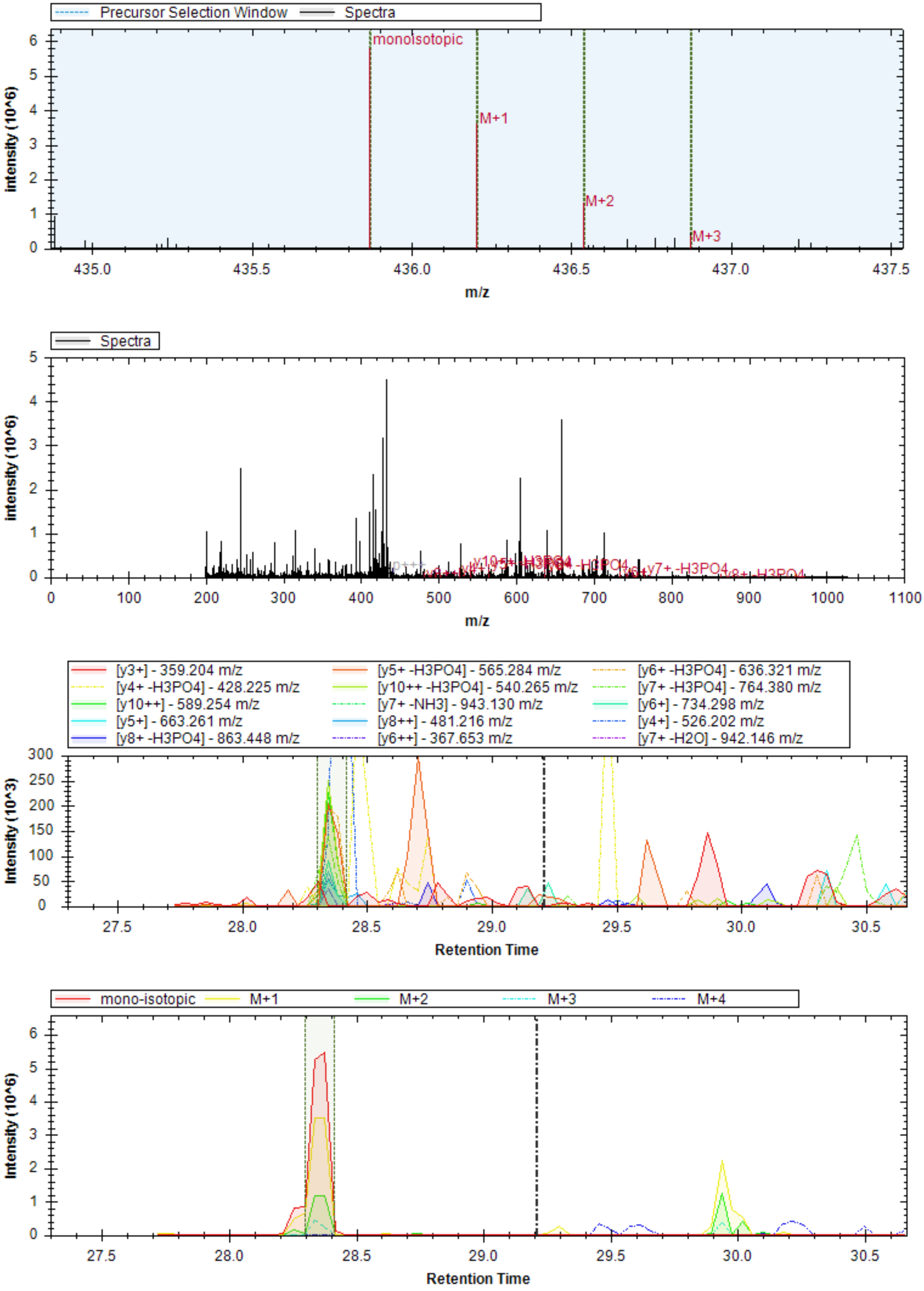

Q03172 – _VALSLLNS[+H+O3+P]KQK_.3

Fig4_MP-DIA-barrel-cortex-p15-3_MHRM.htrms

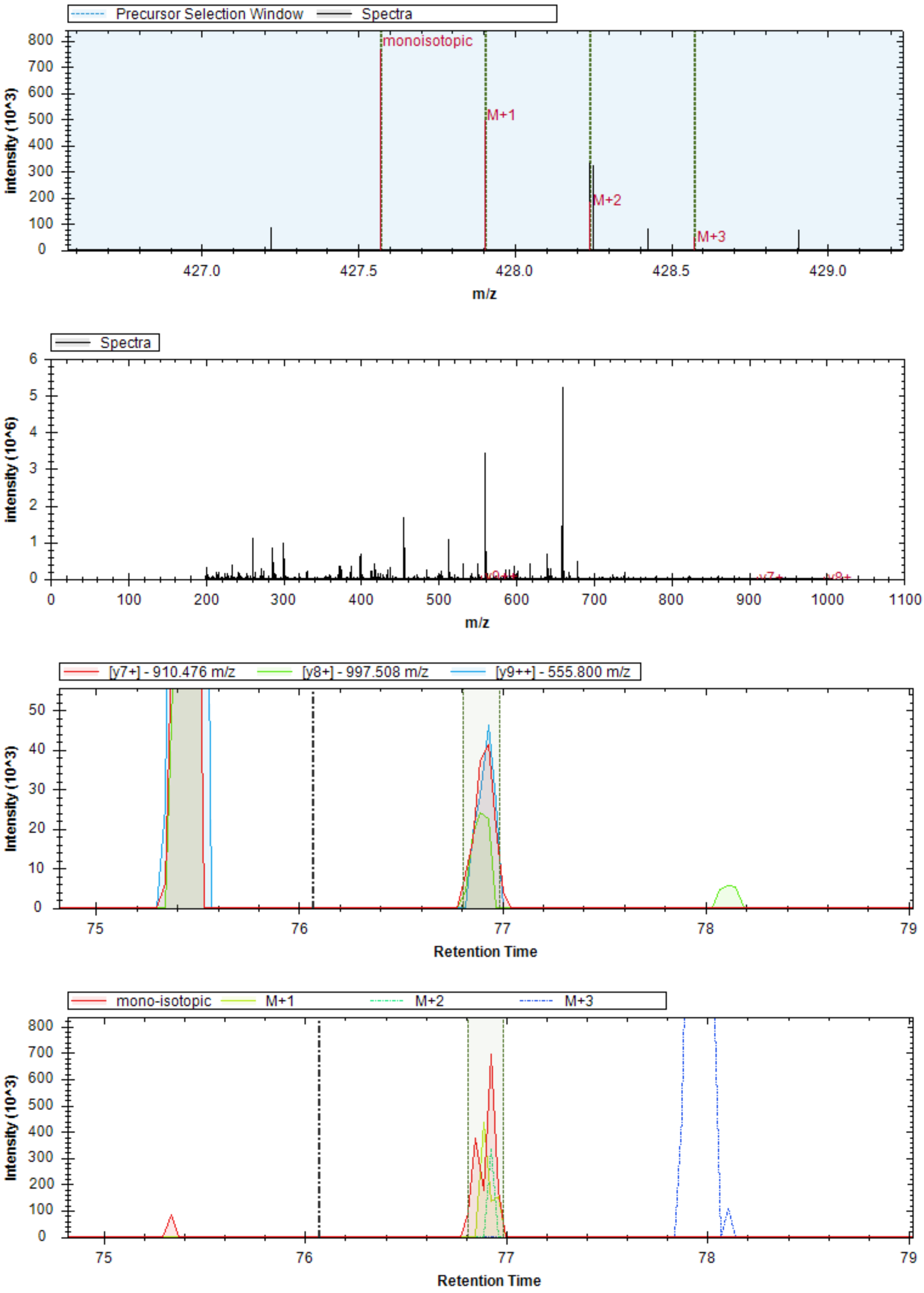

Q52KI8;Q52KI8-2 – _RES[+H+O3+P]PSPAPKPR_.3

Fig4_MP-DIA-barrel-cortex-p15-3_MHRM.htrms

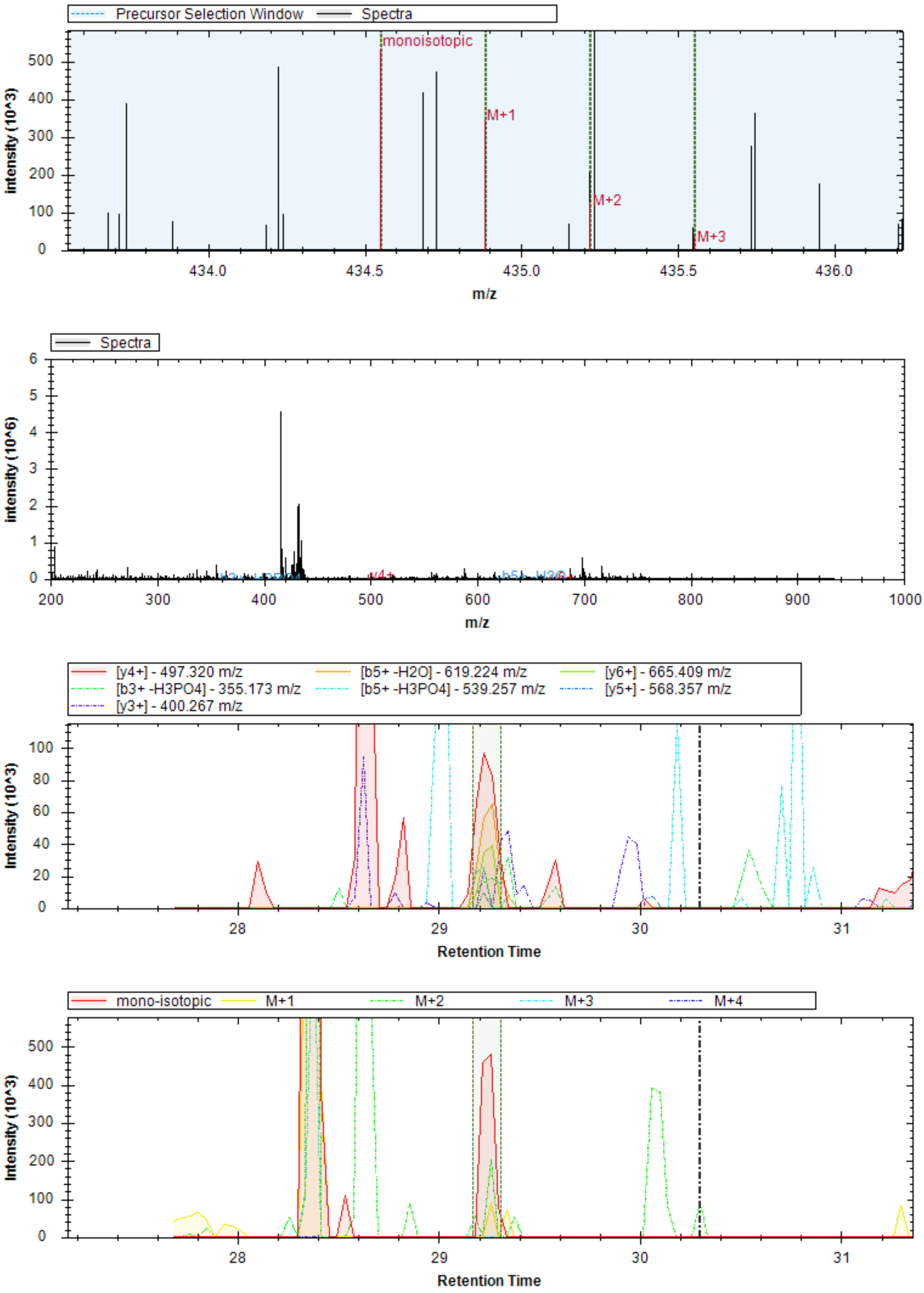

Q62188 – _GS[+H+O3+P]PTRPNPPVR_.3
Fig4_MP-DIA-barrel-cortex-p15-3_MHRM.htrms

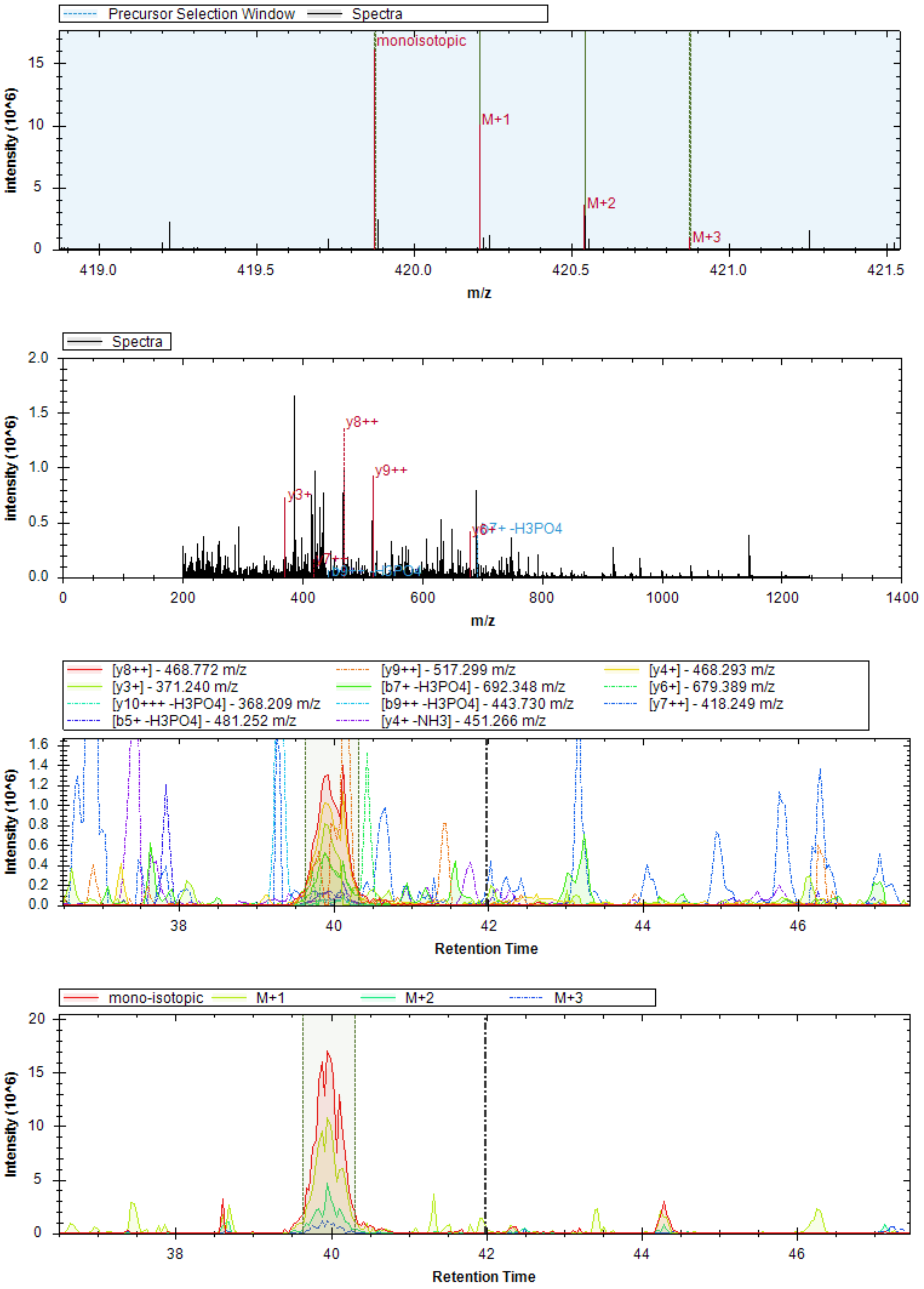

Q93092 – _[+C2+H2+O]SGS[+H+O3+P]PVKR_.2

Fig4_MP-DIA-barrel-cortex-p15-3_MHRM.htrms

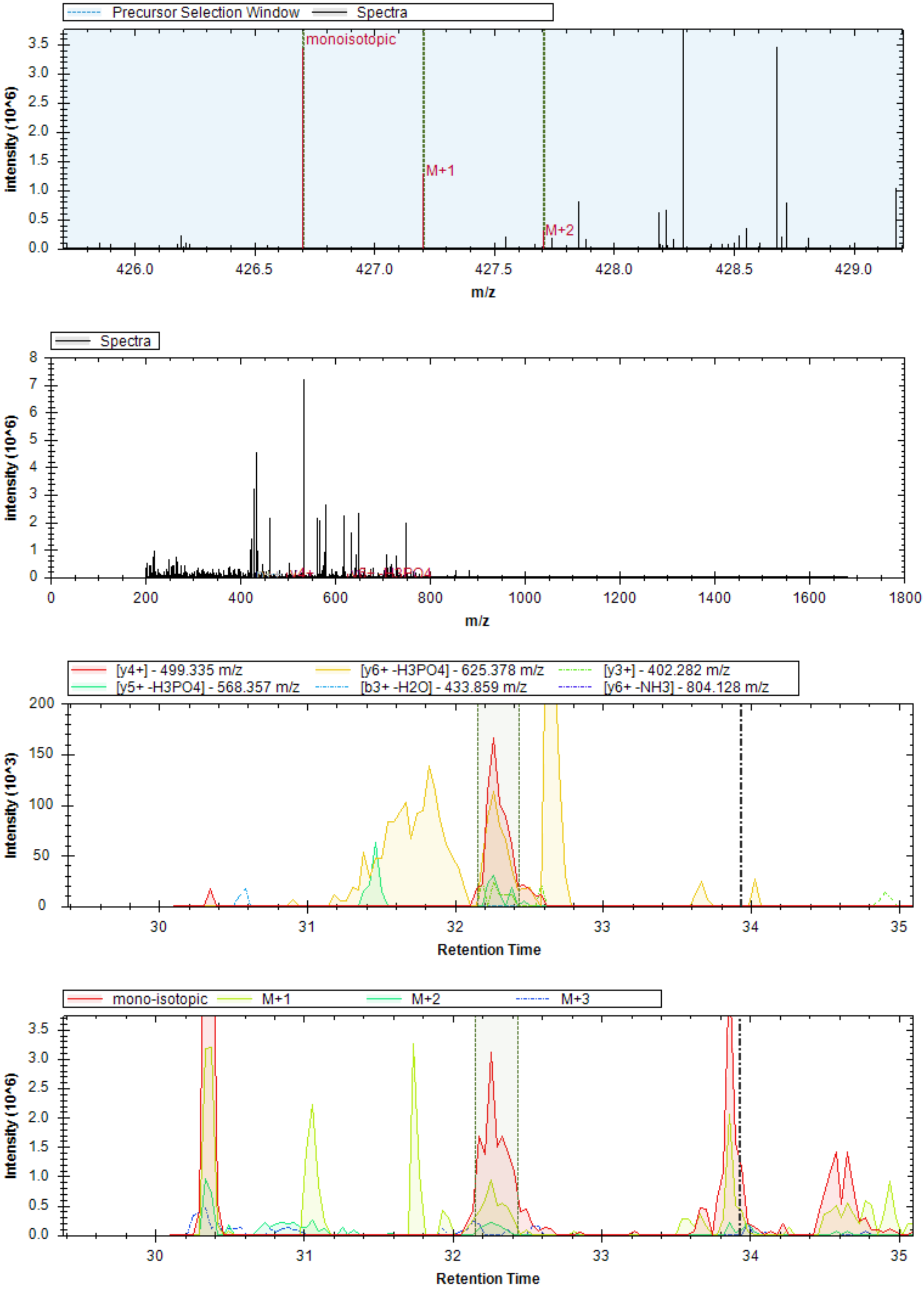

P62484 – _T[+H+O3+P]HKIIAPANLERPVR_.4
Fig4_MP-DIA-barrel-cortex-p15-3_MHRM.htrms

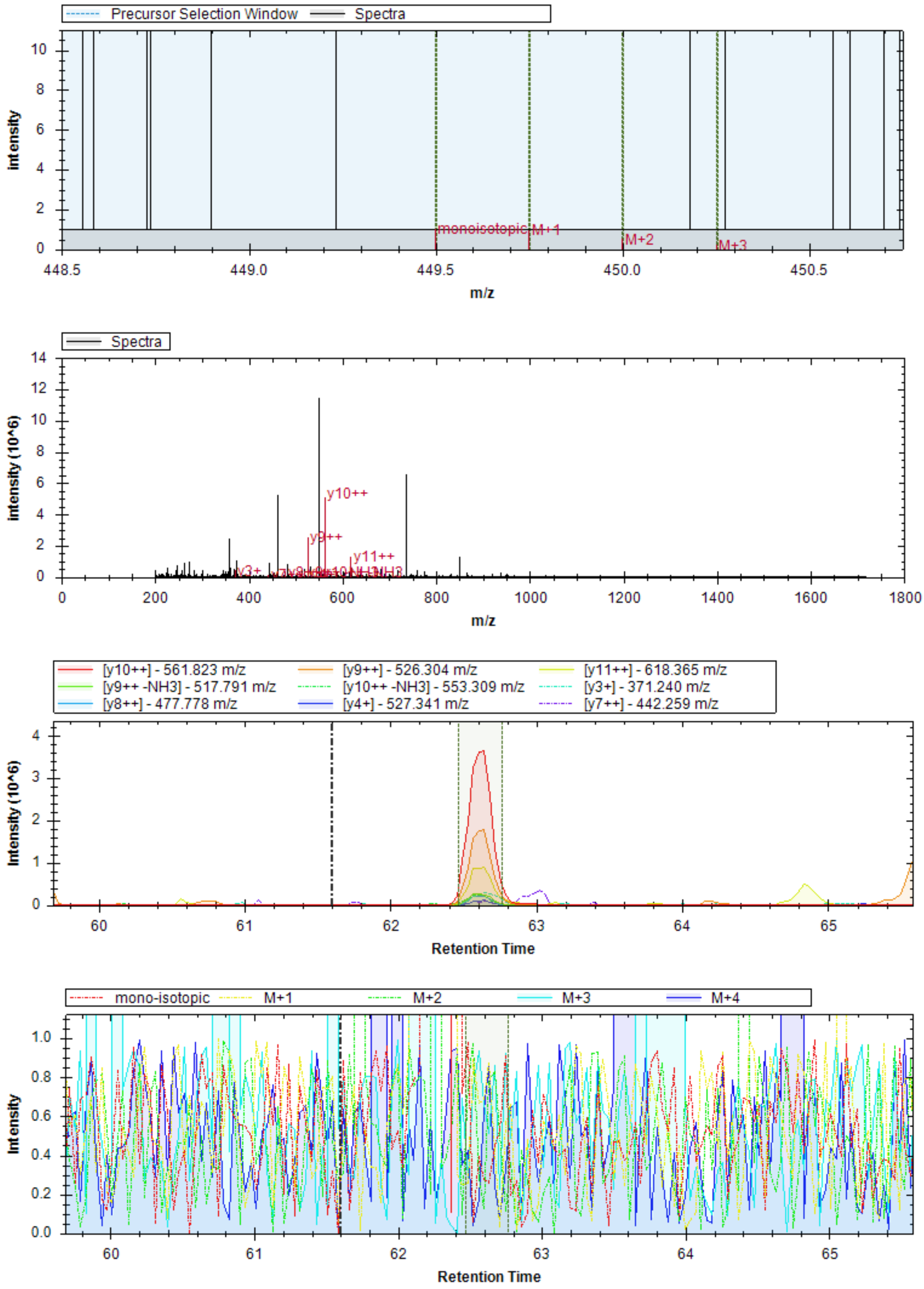

Q811T9;Q811T9-2;Q811[...] – _RQLEVT[+H+O3+P]SLILK_.3
Fig4_MP-DIA-barrel-cortex-p15-3_MHRM.htrms

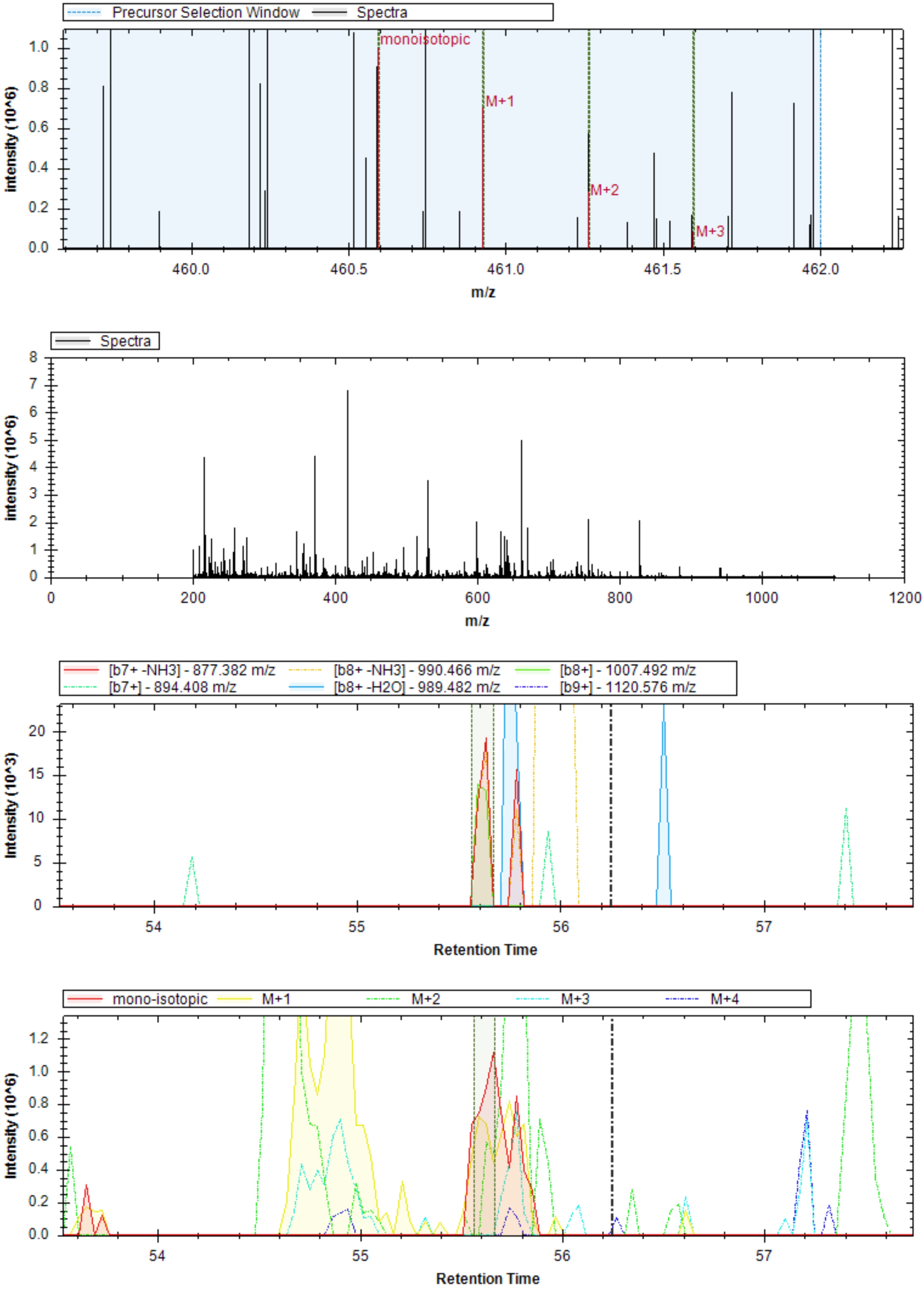

Q8CGI1 – _Y[+H+O3+P]QRS[+H+O3+P]EEELR_.3

Fig4_MP-DIA-barrel-cortex-p15-3_MHRM.htrms

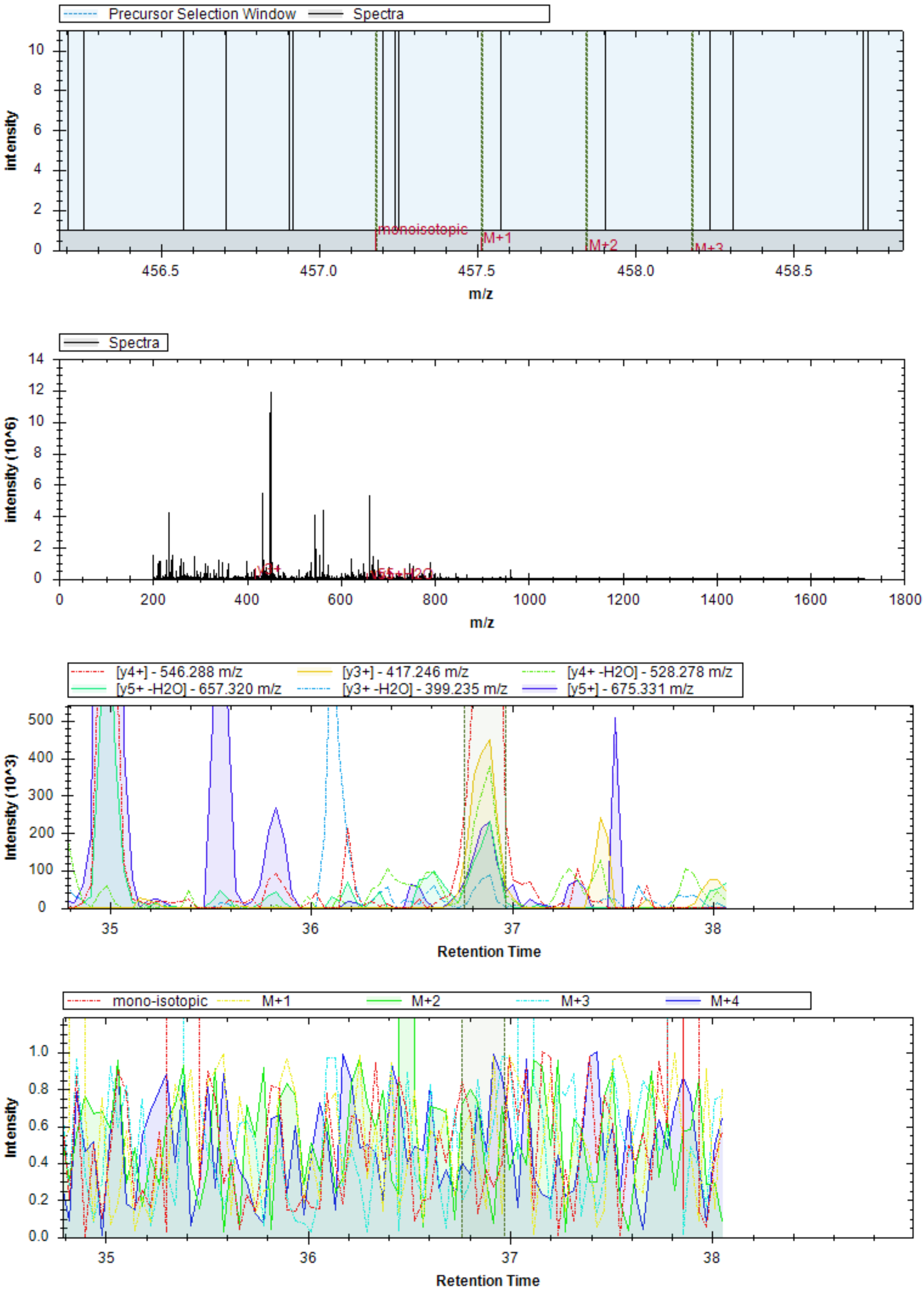

O08553 – _TVT[+H+O3+P]PASSAK_.2
Fig4_MP-DIA-barrel-cortex-p15-3_MHRM.htrms

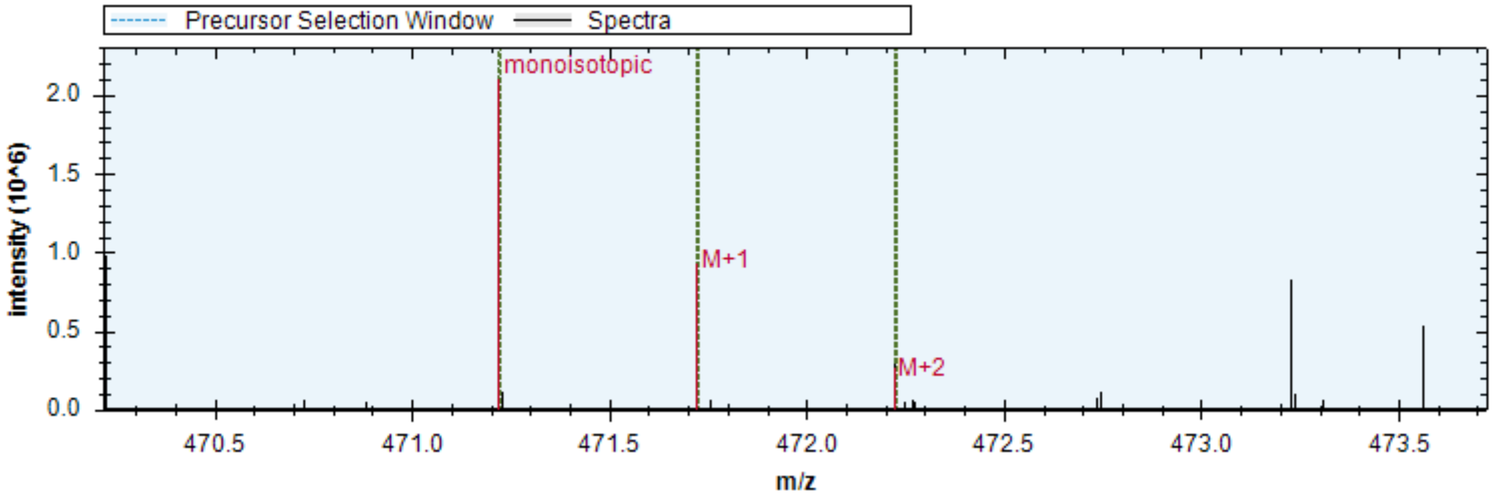

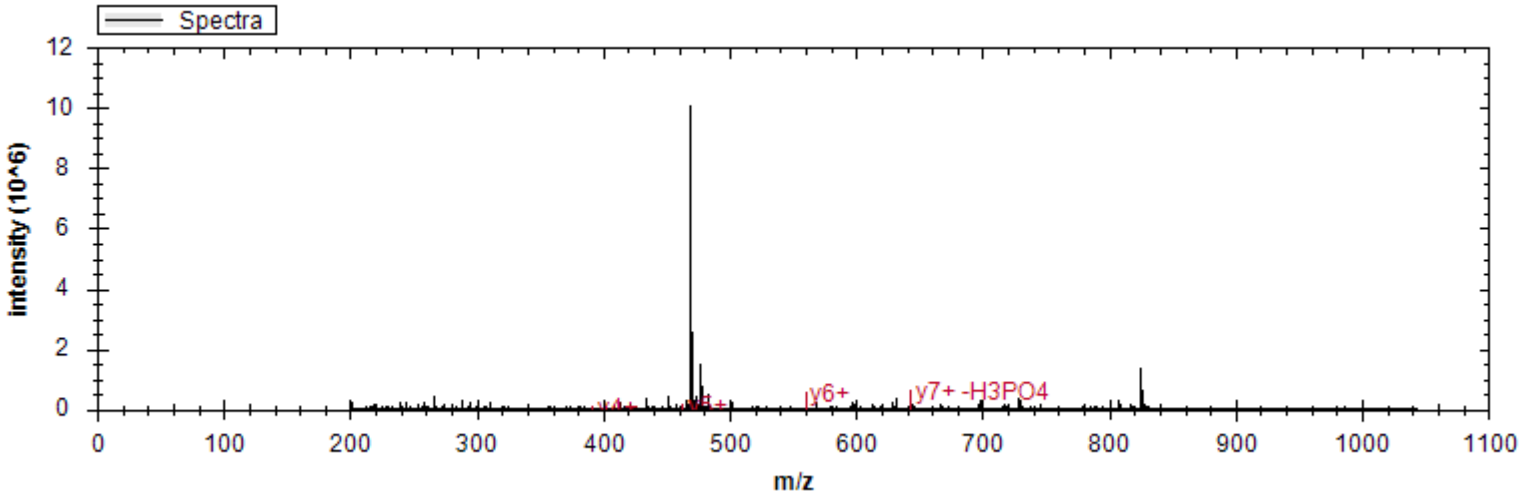

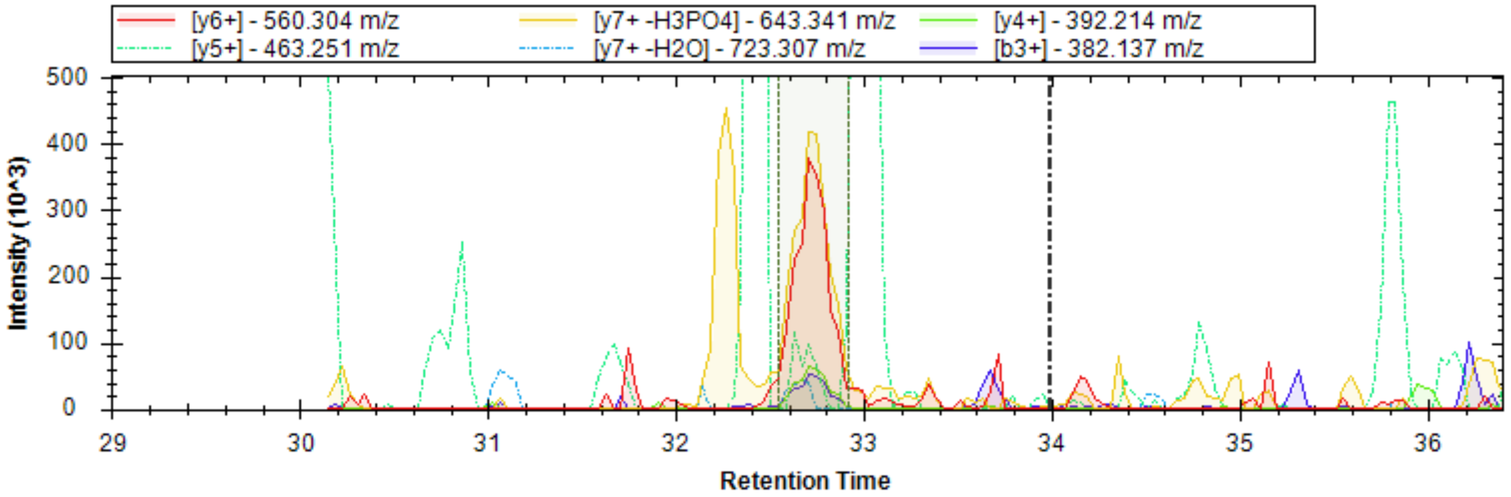

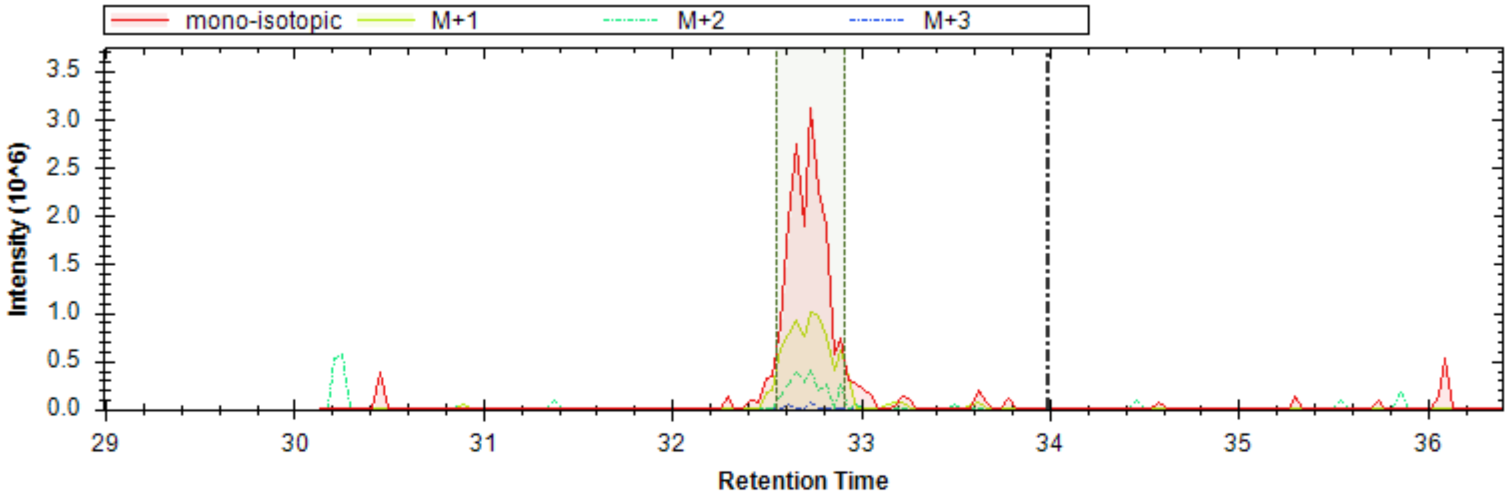

P20357 – _VTDGIS[+H+O3+P]KSPEKR_.3

Fig4_MP-DIA-barrel-cortex-p15-3_MHRM.htrms

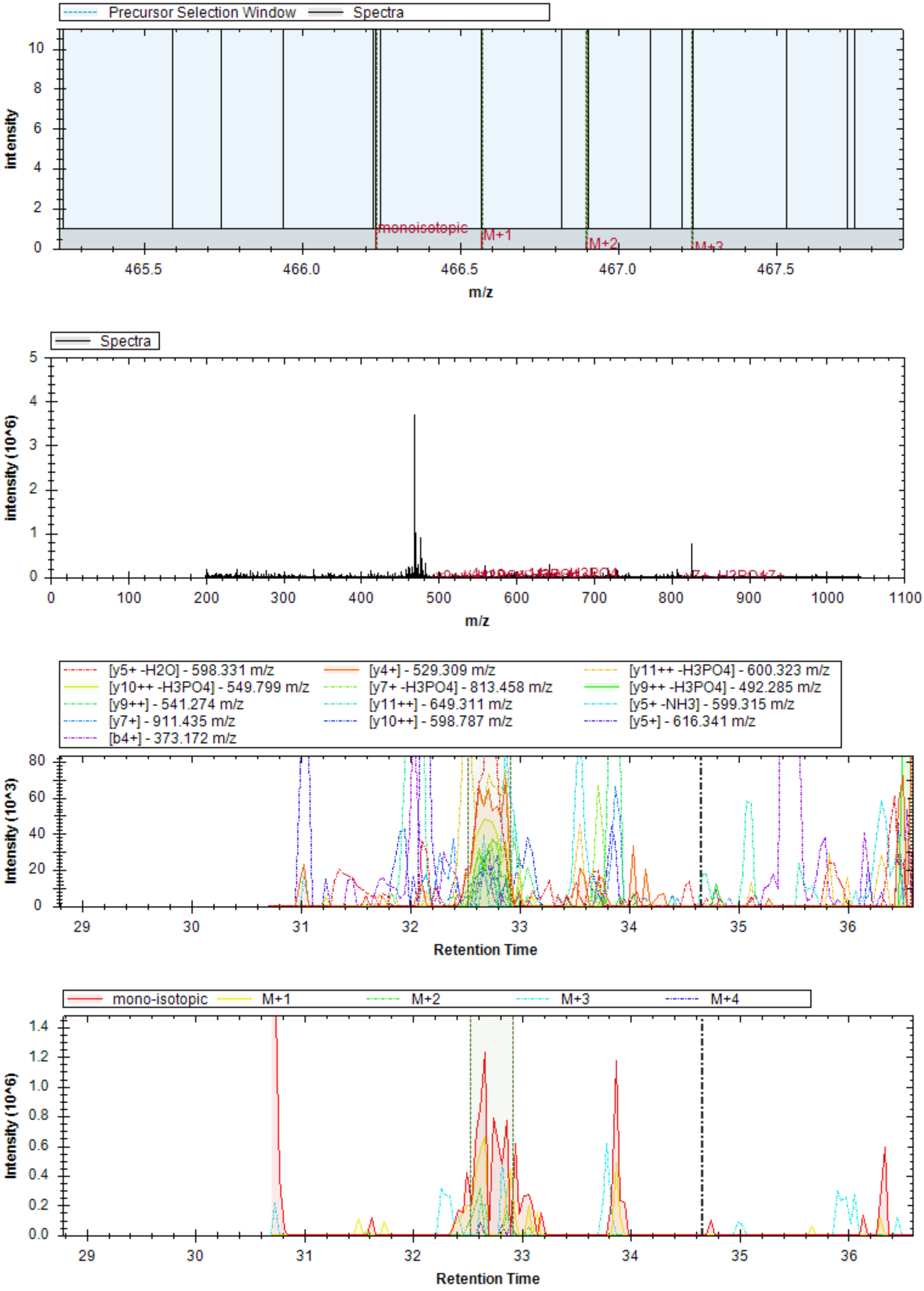

P20357 – _VTDGISKS[+H+O3+P]PEKR_.3

Fig4_MP-DIA-barrel-cortex-p15-3_MHRM.htrms

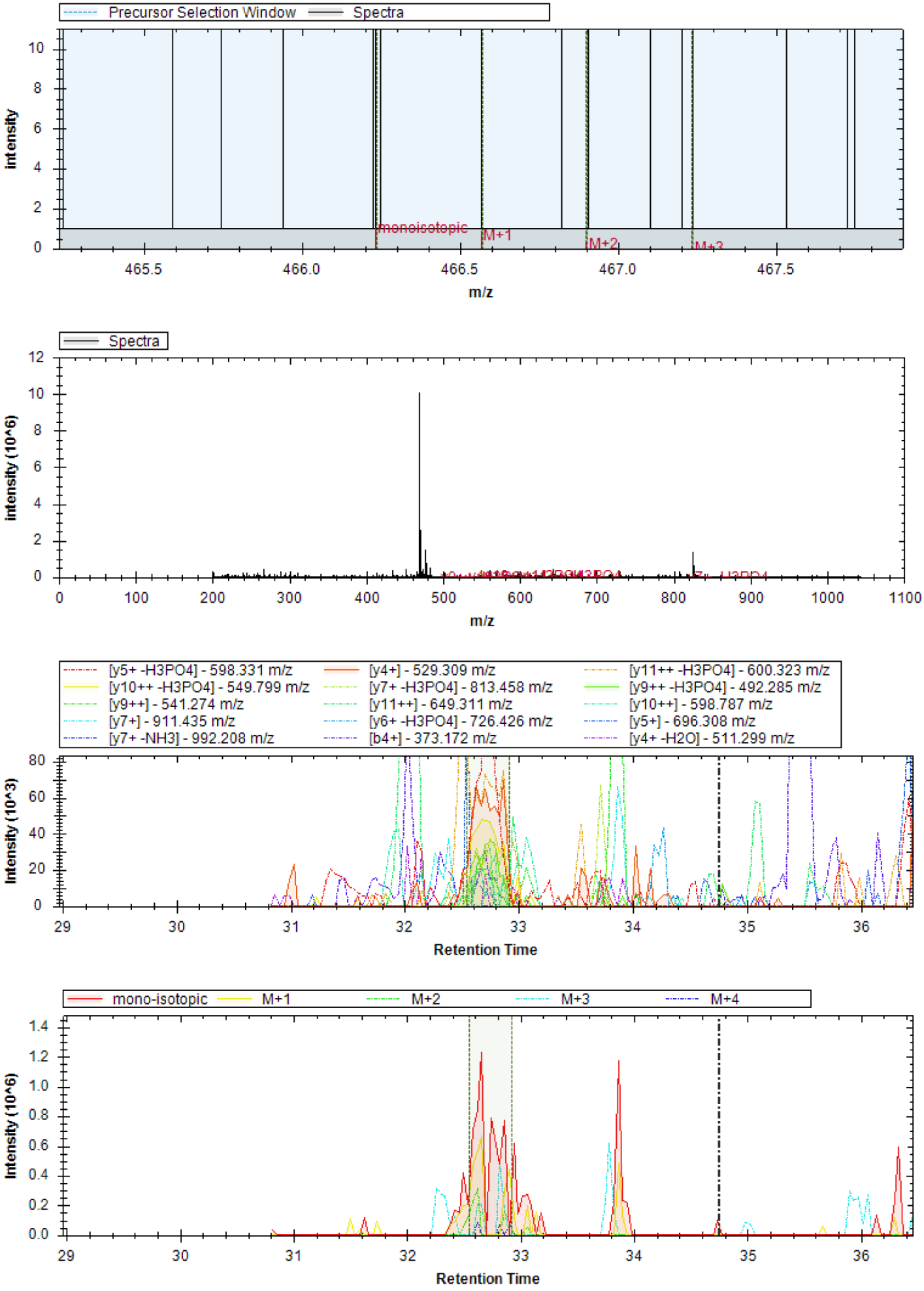

Q3UTJ2-2 – _RGS[+H+O3+P]LPDNSILHR_.3
Fig4_MP-DIA-barrel-cortex-p15-3_MHRM.htrms

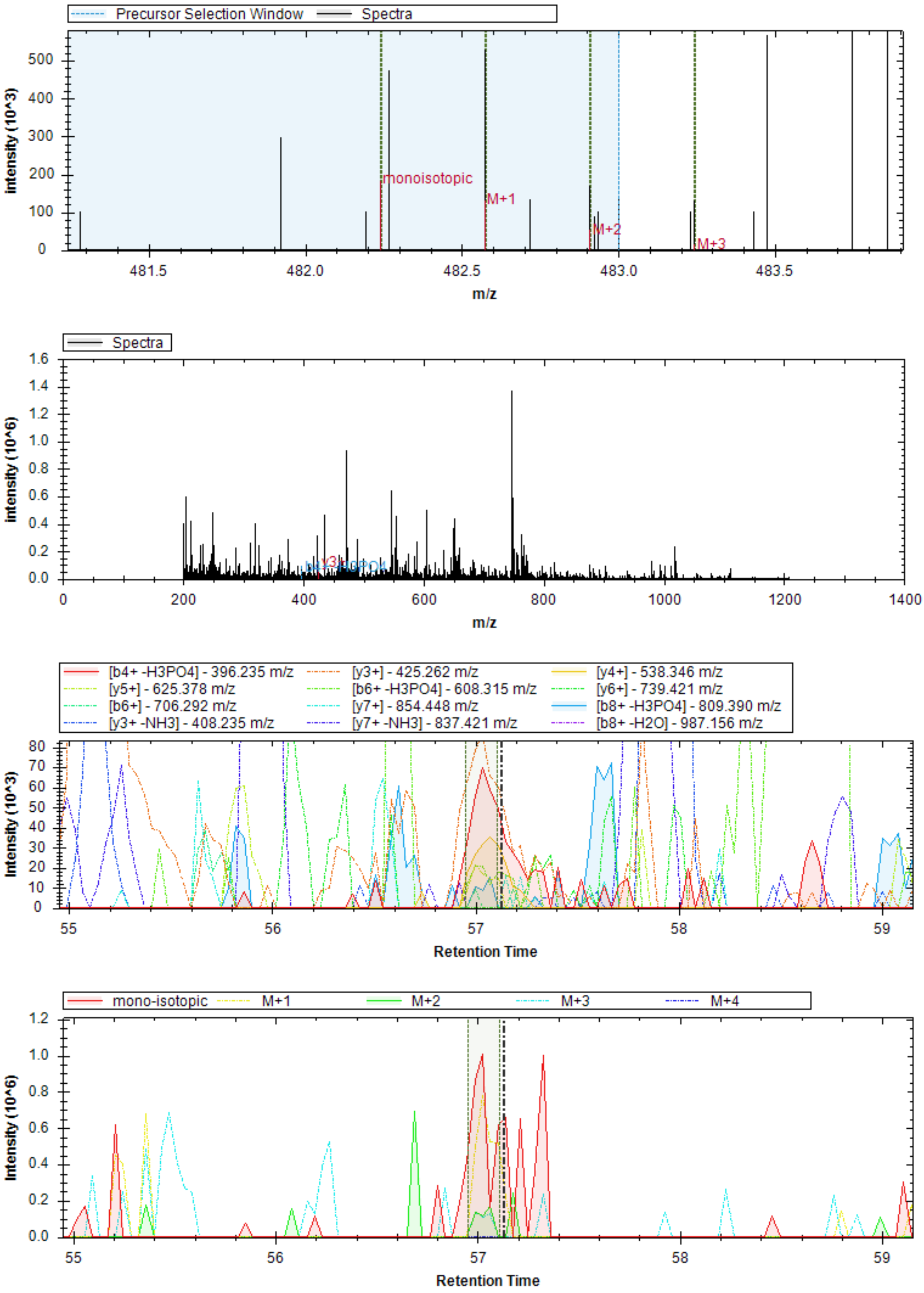

Q8K015;Q8K015-2 – _TVIS[+H+O3+P]EQEVLER_.3
Fig4_MP-DIA-barrel-cortex-p15-3_MHRM.htrms

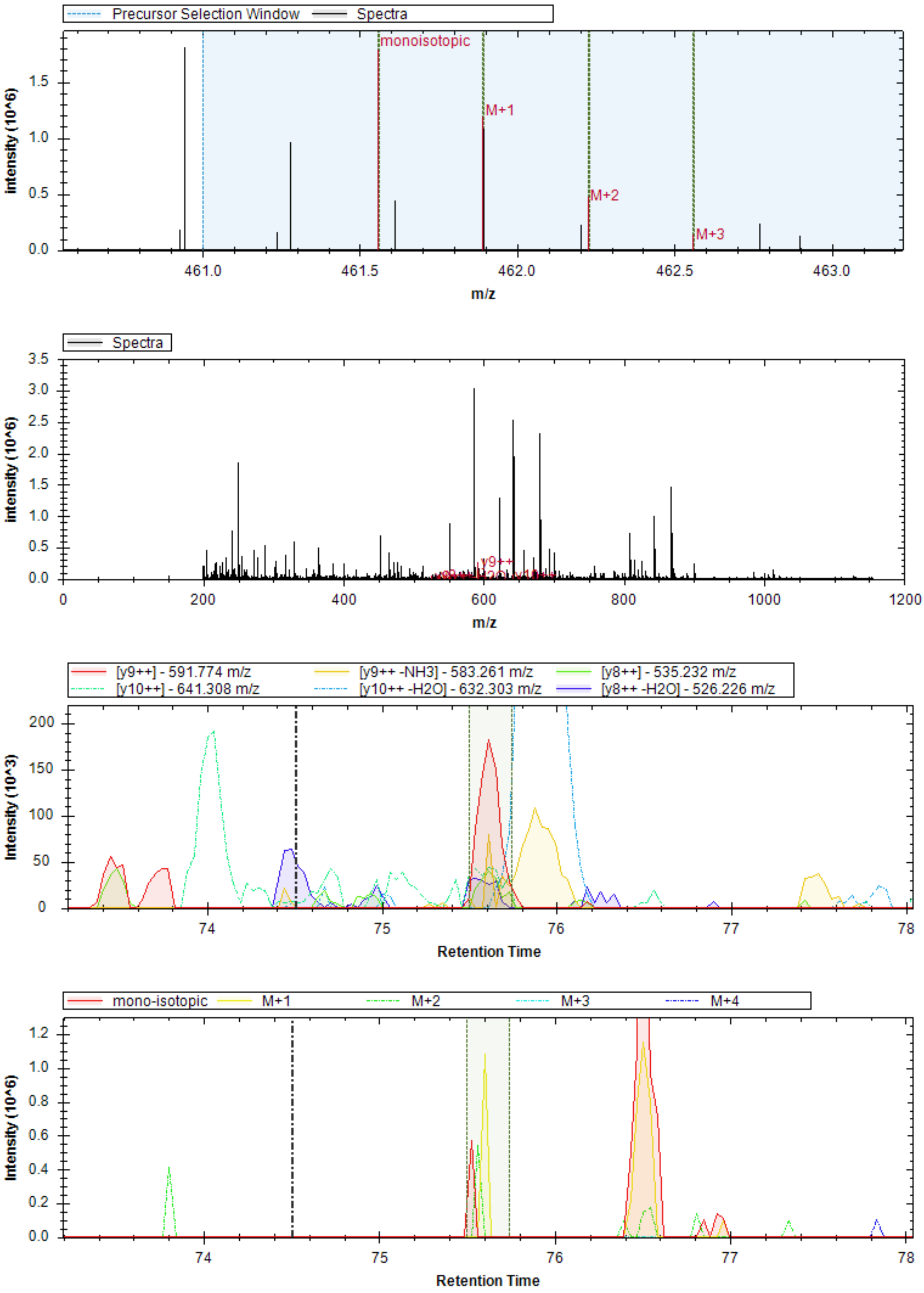

Q9CR81 – _[+C2+H2+O]M[+O]ANHLVKPDS[+H+O3+P]R_.3
Fig4_MP-DIA-barrel-cortex-p15-3_MHRM.htrms

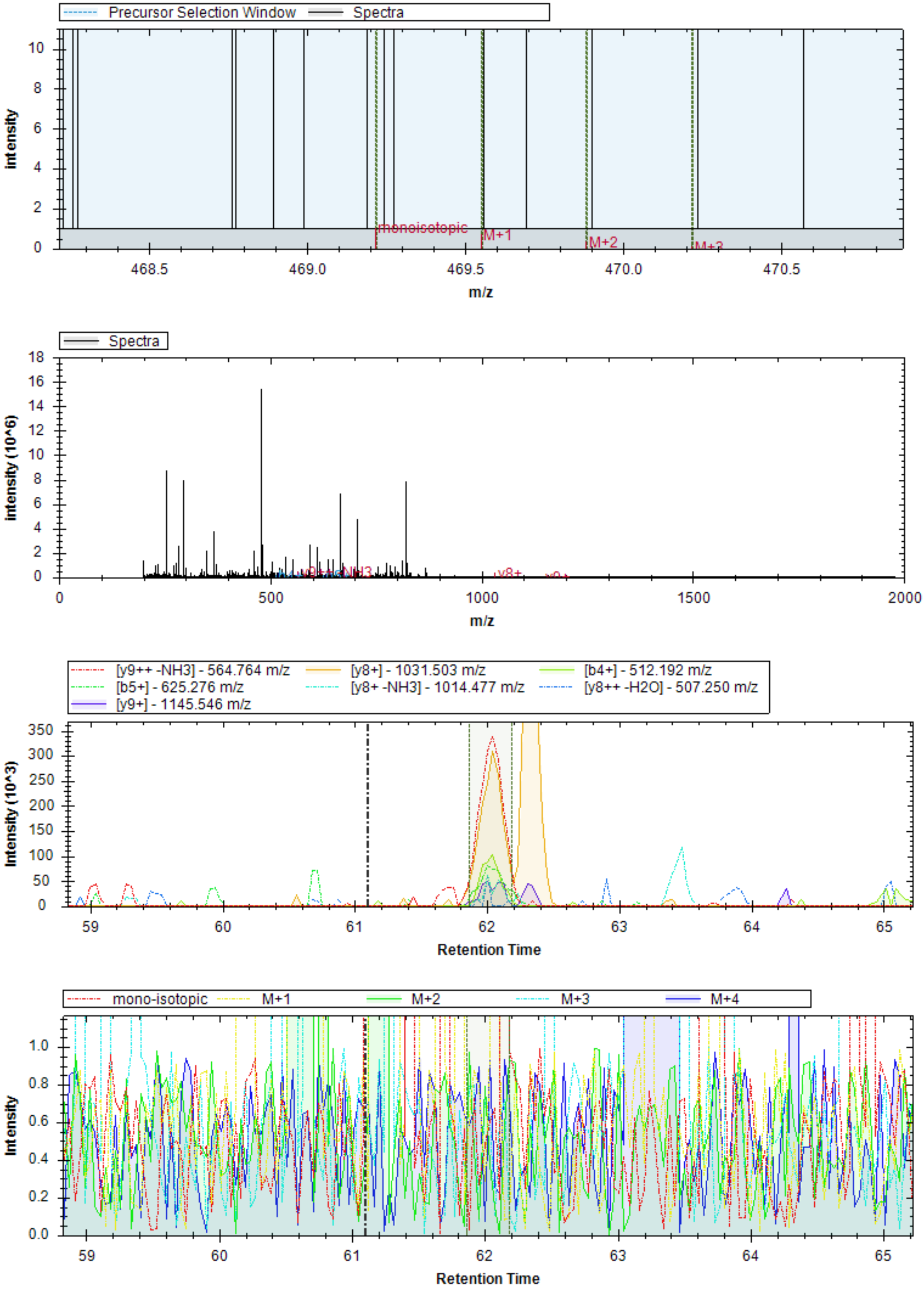

P26645 – _LSGFS[+H+O3+P]FKK_.2
Fig4_MP-DIA-barrel-cortex-p15-3_MHRM.htrms

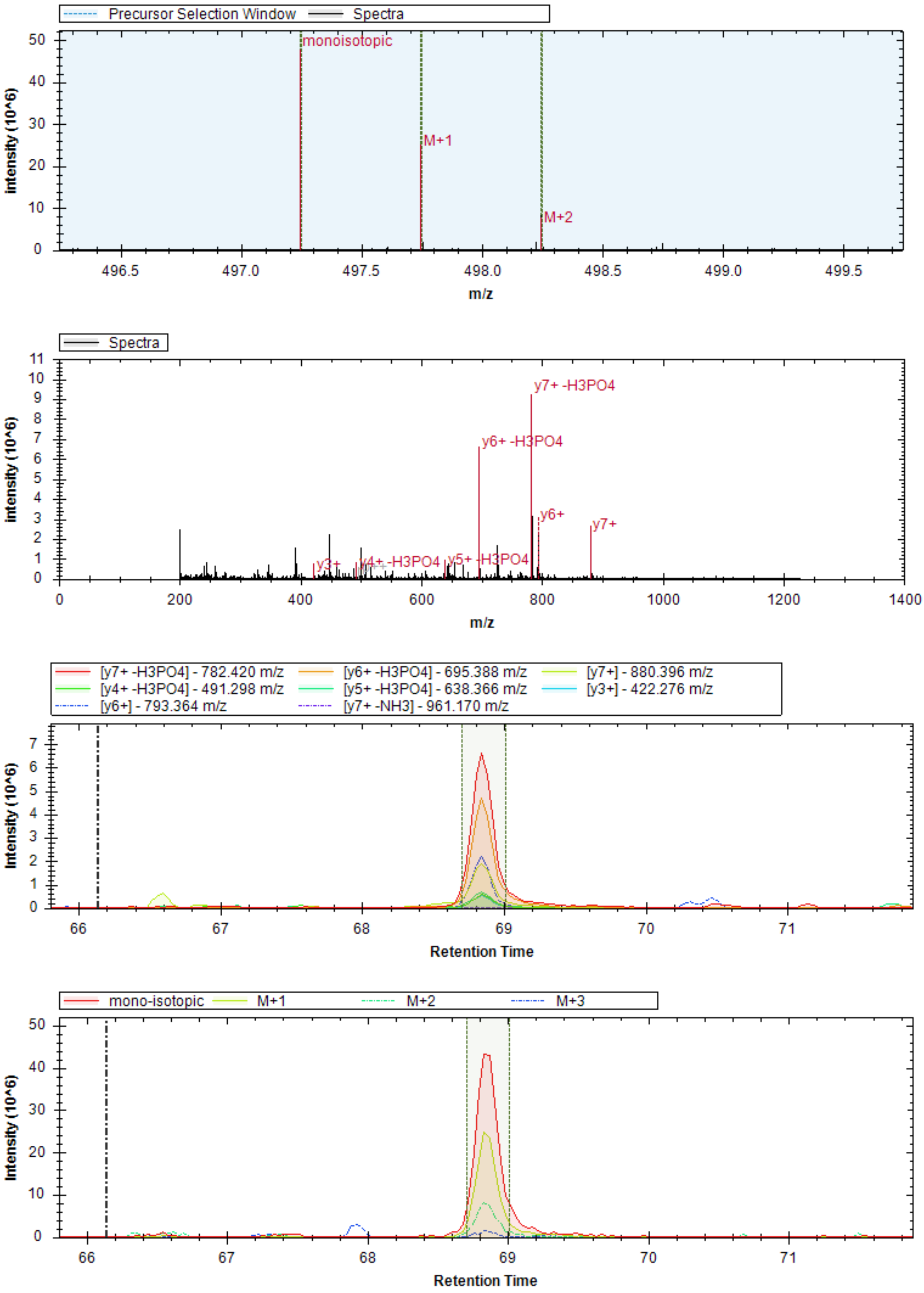

P28652 – _NSSAITS[+H+O3+P]PK_.2

Fig4_MP-DIA-barrel-cortex-p15-3_MHRM.htrms

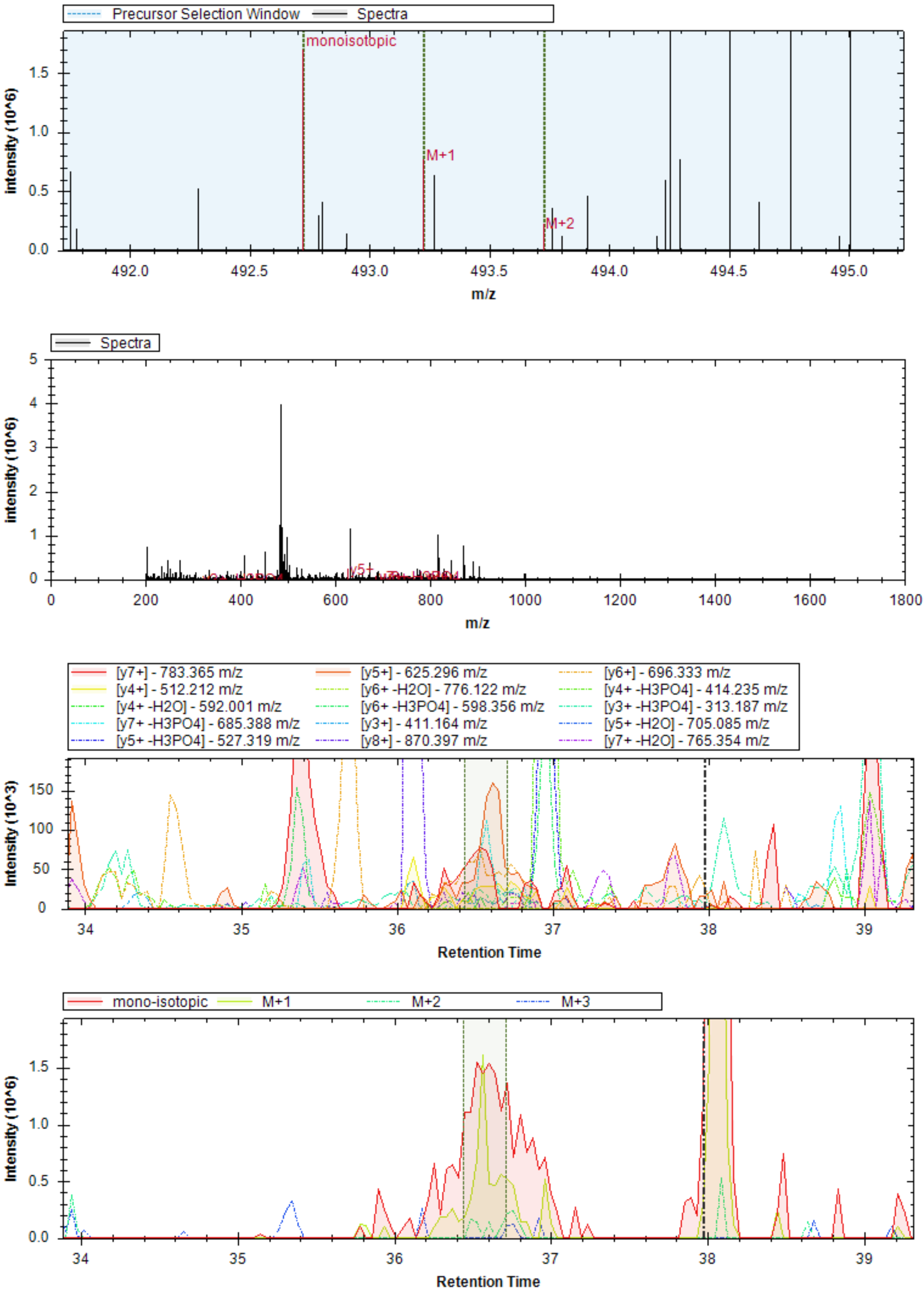

P28667 – _LSGLS[+H+O3+P]FKR_.2
Fig4_MP-DIA-barrel-cortex-p15-3_MHRM.htrms

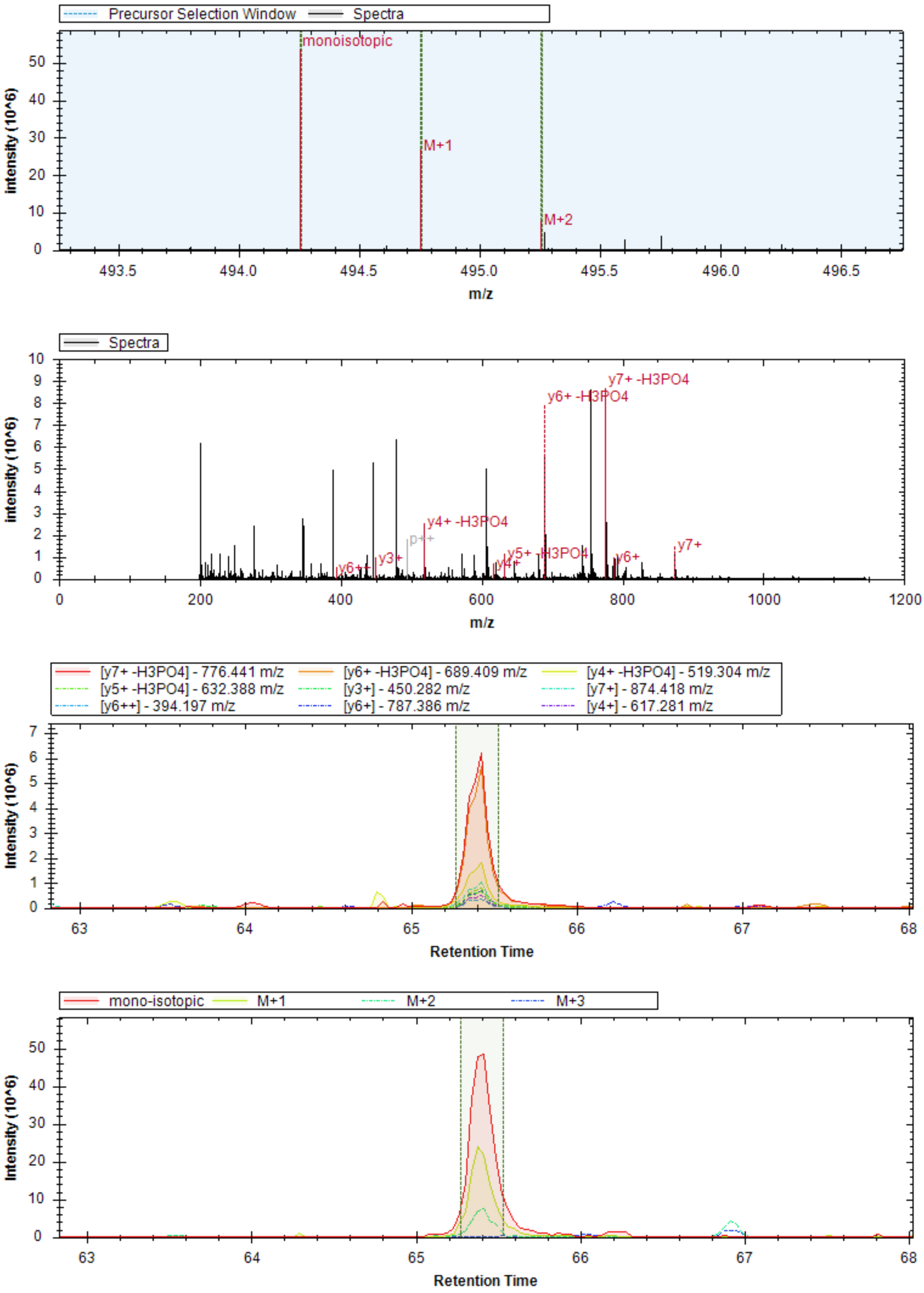

P28738 – _PIRPGHYPAS[+H+O3+P]SPTAVHAVR_.4
Fig4_MP-DIA-barrel-cortex-p15-3_MHRM.htrms

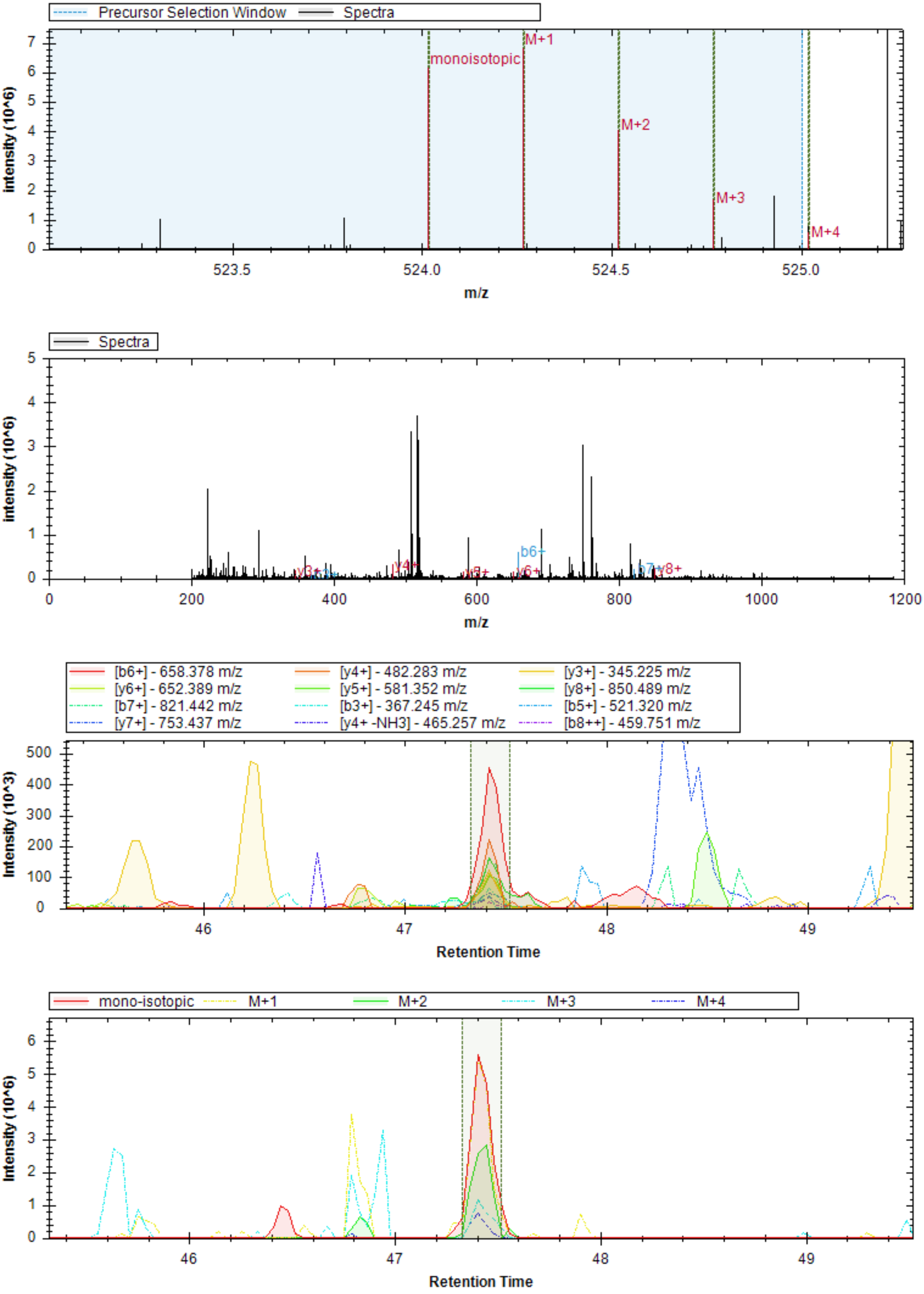

P28738 – _PIRPGHYPASS[+H+O3+P]PTAVHAVR_.4
Fig4_MP-DIA-barrel-cortex-p15-3_MHRM.htrms

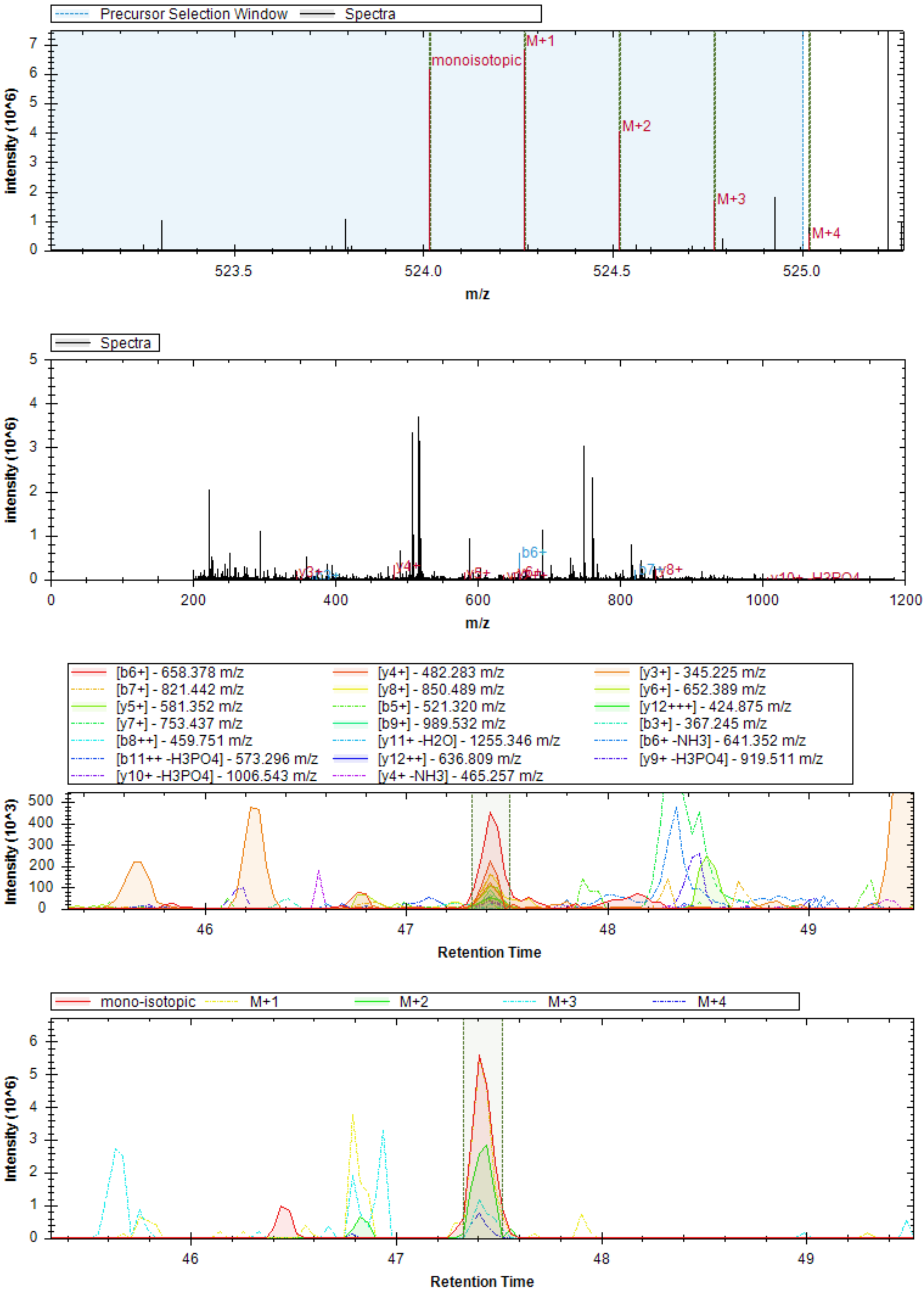

P62996 – _RPHT[+H+O3+P]PTPGIYMGR_.3
Fig4_MP-DIA-barrel-cortex-p15-3_MHRM.htrms

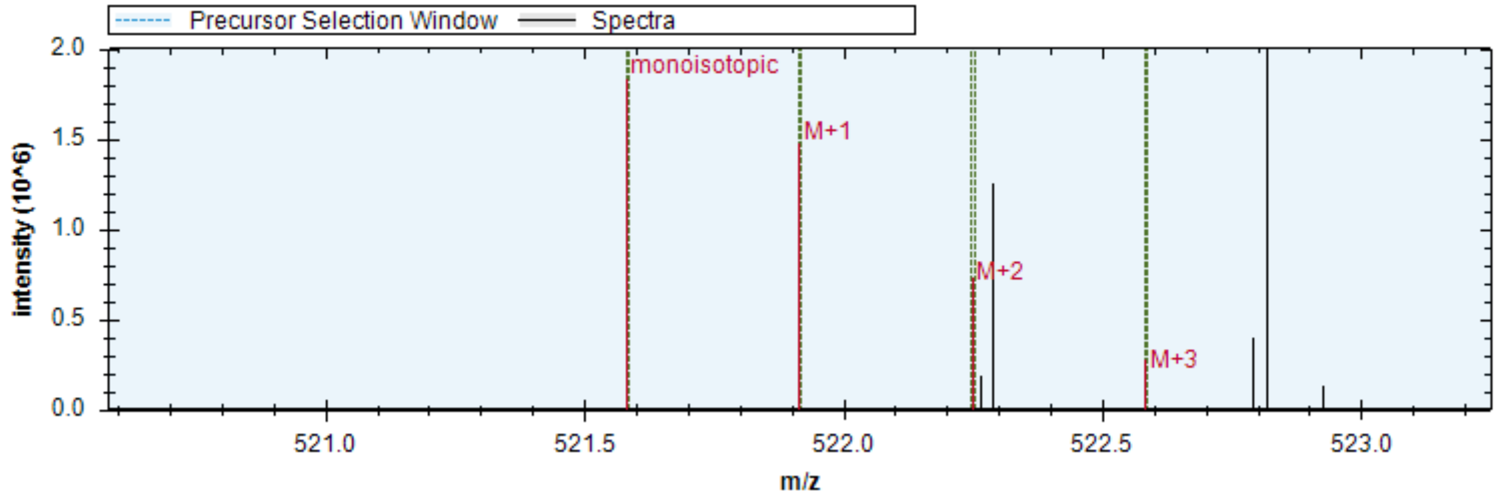

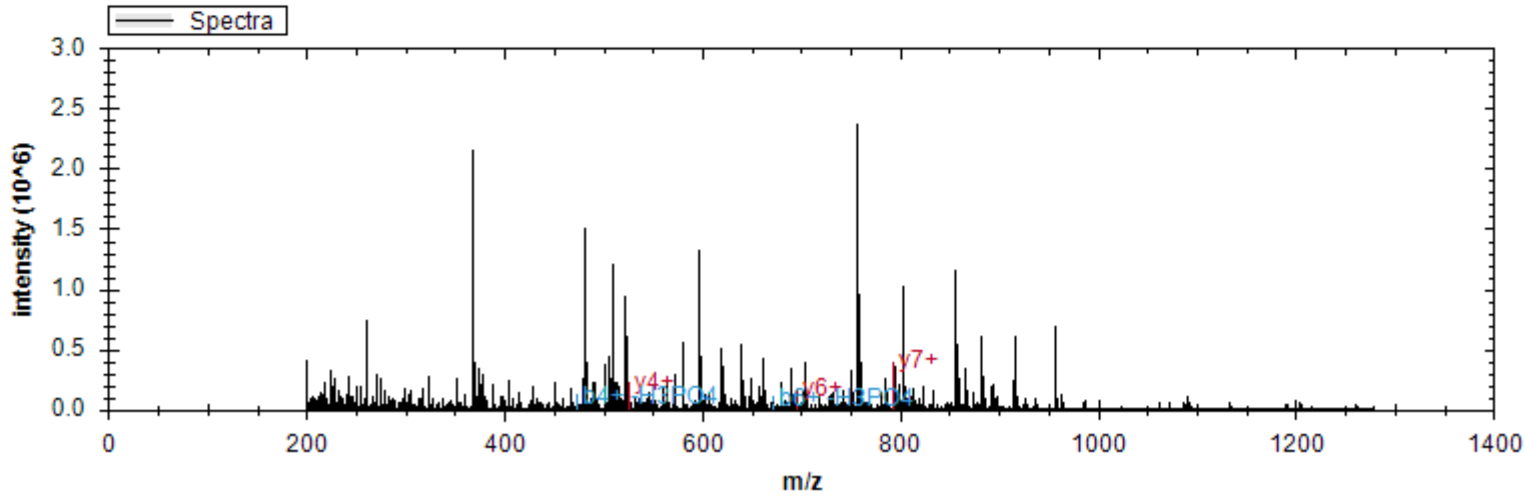

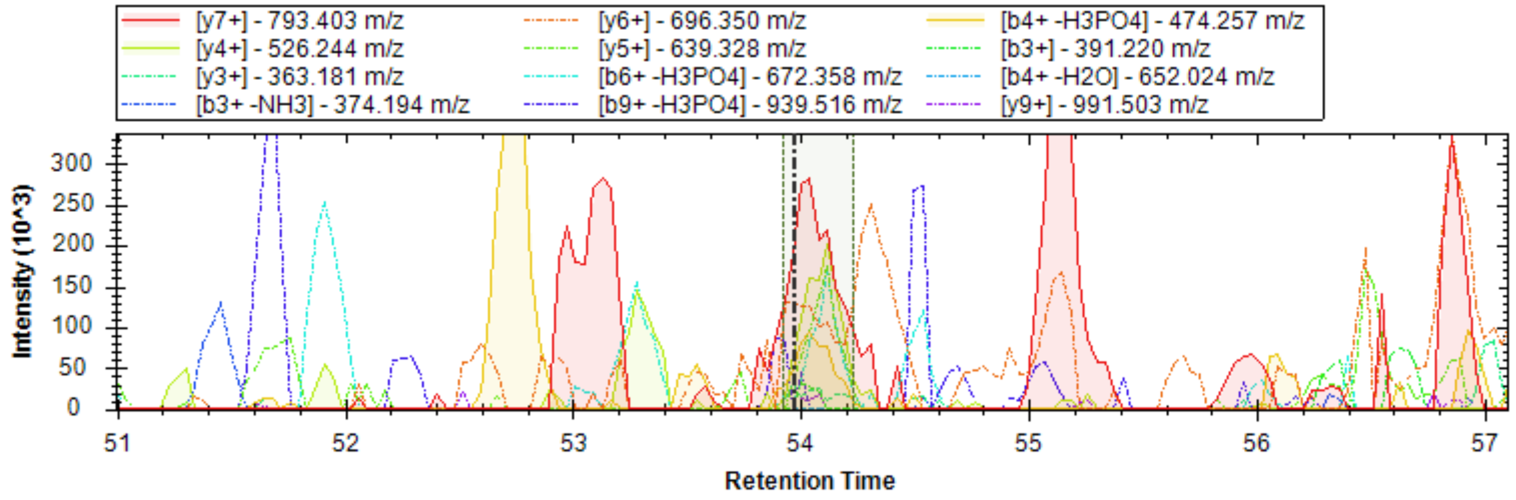

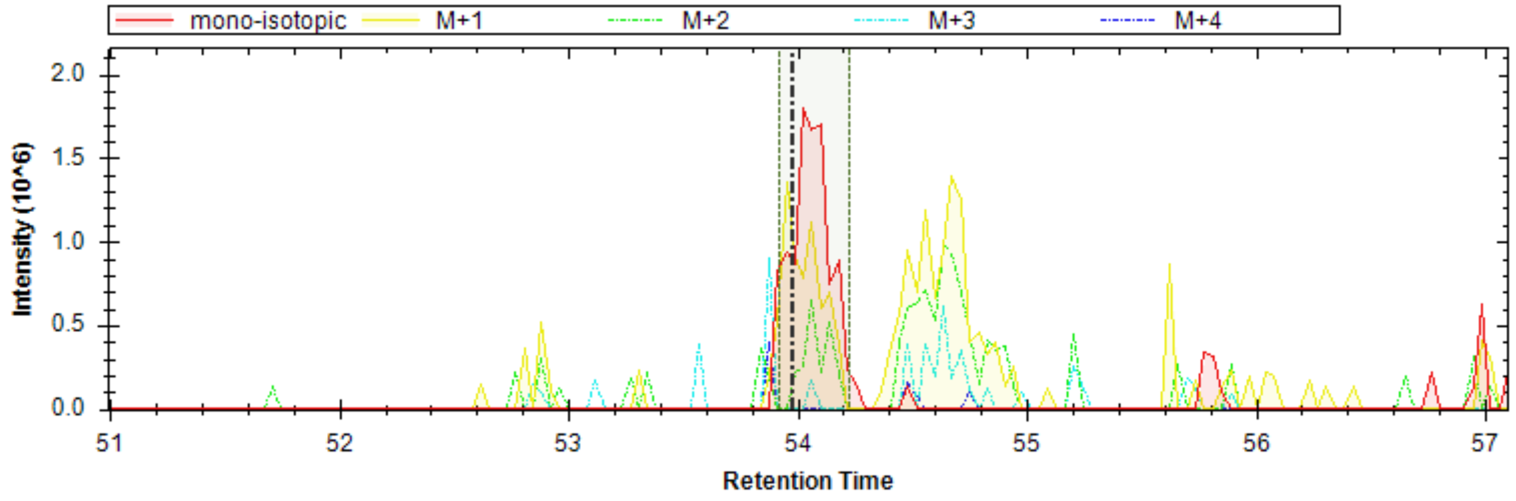

Q8C8R3-7 – _[+C2+H2+O]AHAAAS[+H+O3+P]IKK_.2
Fig4_MP-DIA-barrel-cortex-p15-3_MHRM.htrms

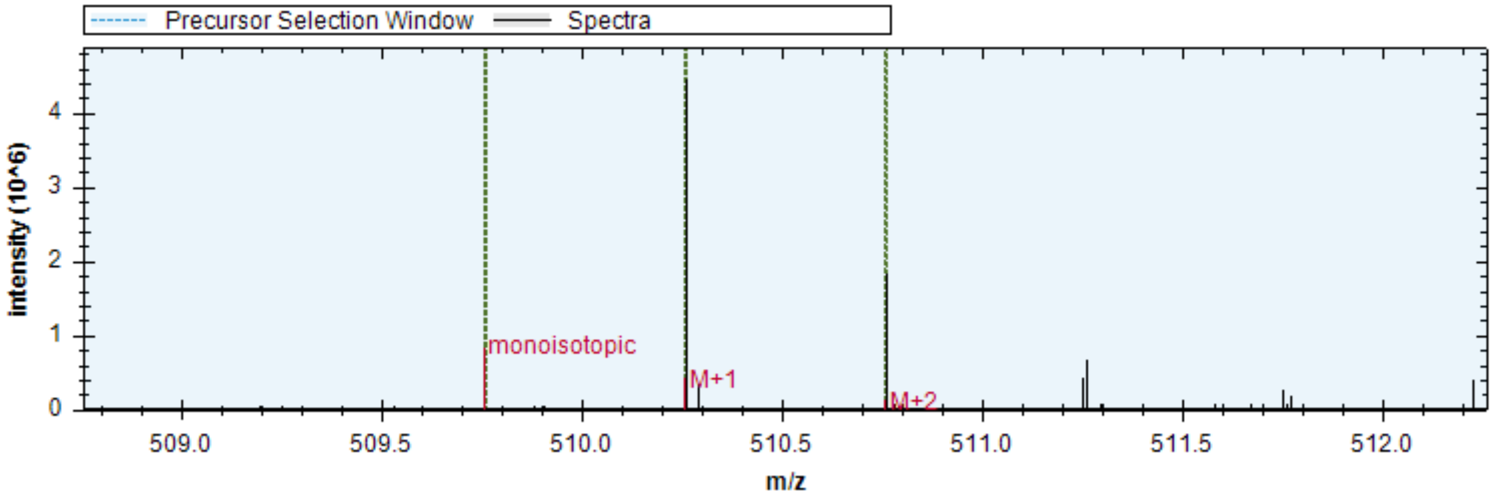

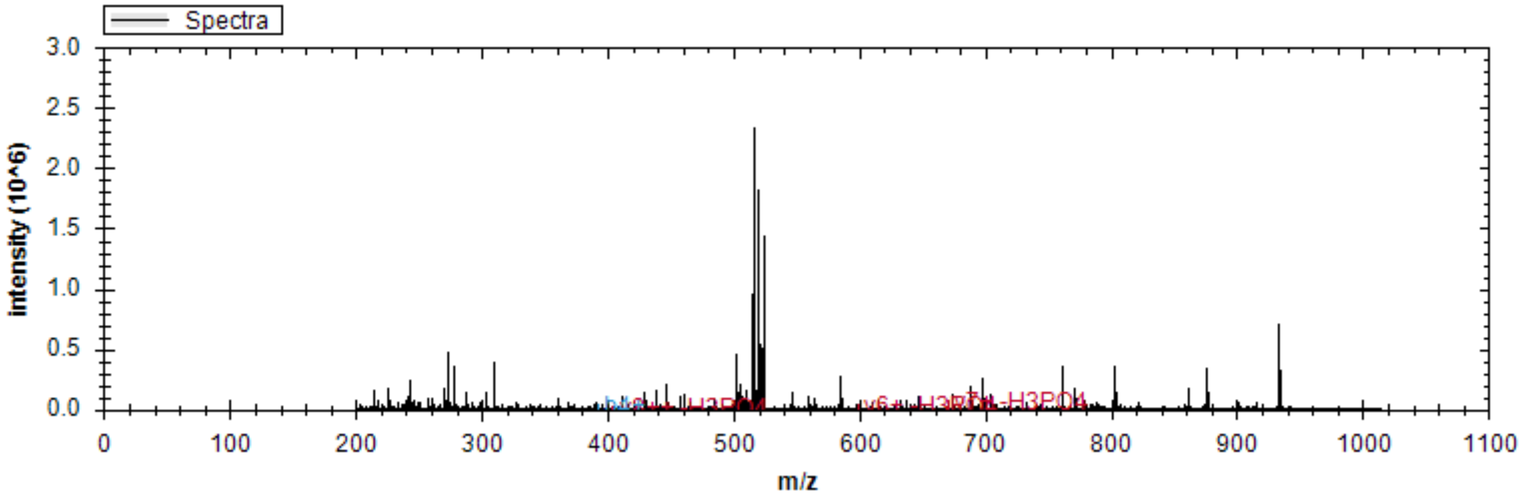

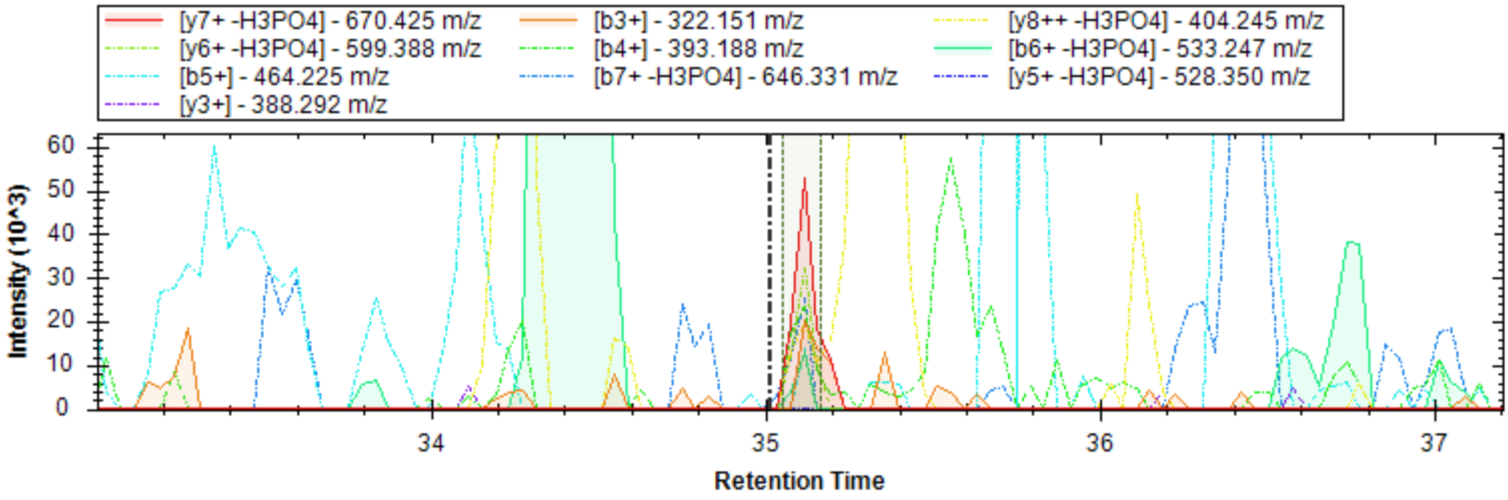

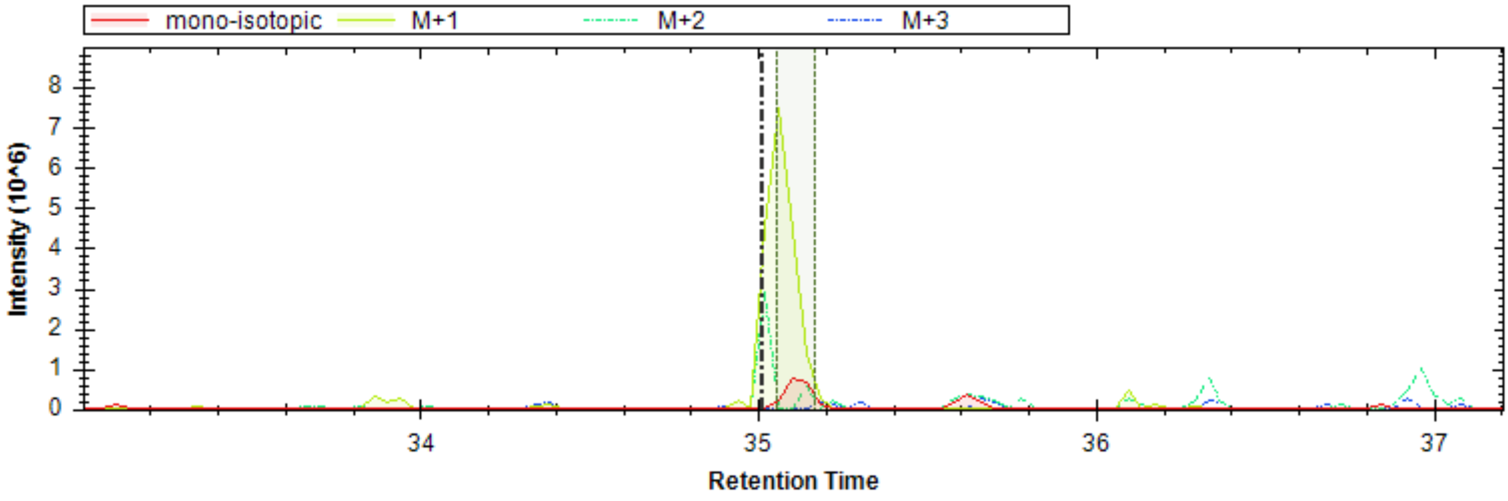

Q8CDY7 – _LVQALC[+C2+H3+N+O]EQIHT[+H+O3+P]R_.3
Fig4_MP-DIA-barrel-cortex-p15-3_MHRM.htrms

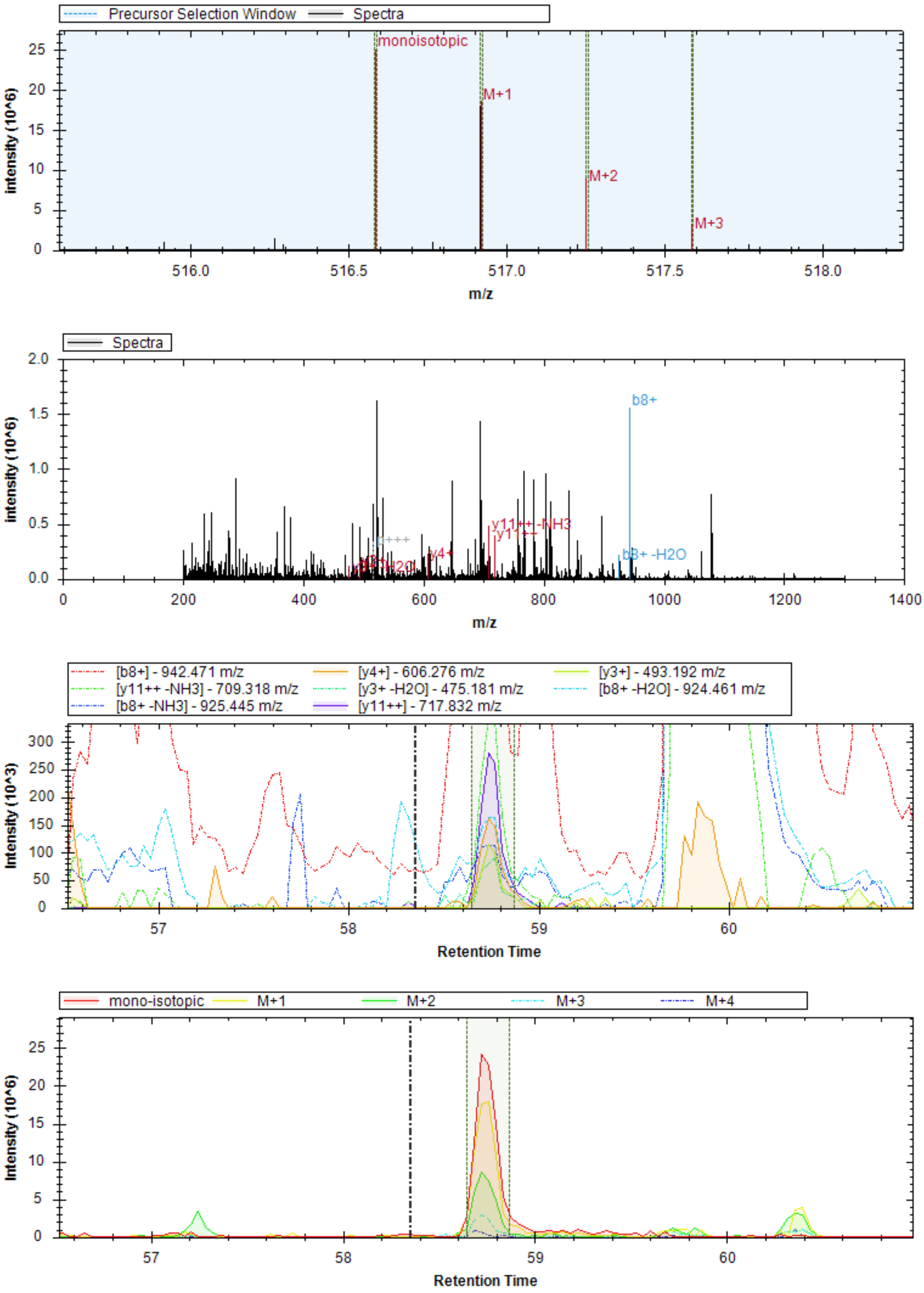

Q8JZP2 – _SPGS[+H+O3+P]PQLSR_.2
Fig4_MP-DIA-barrel-cortex-p15-3_MHRM.htrms

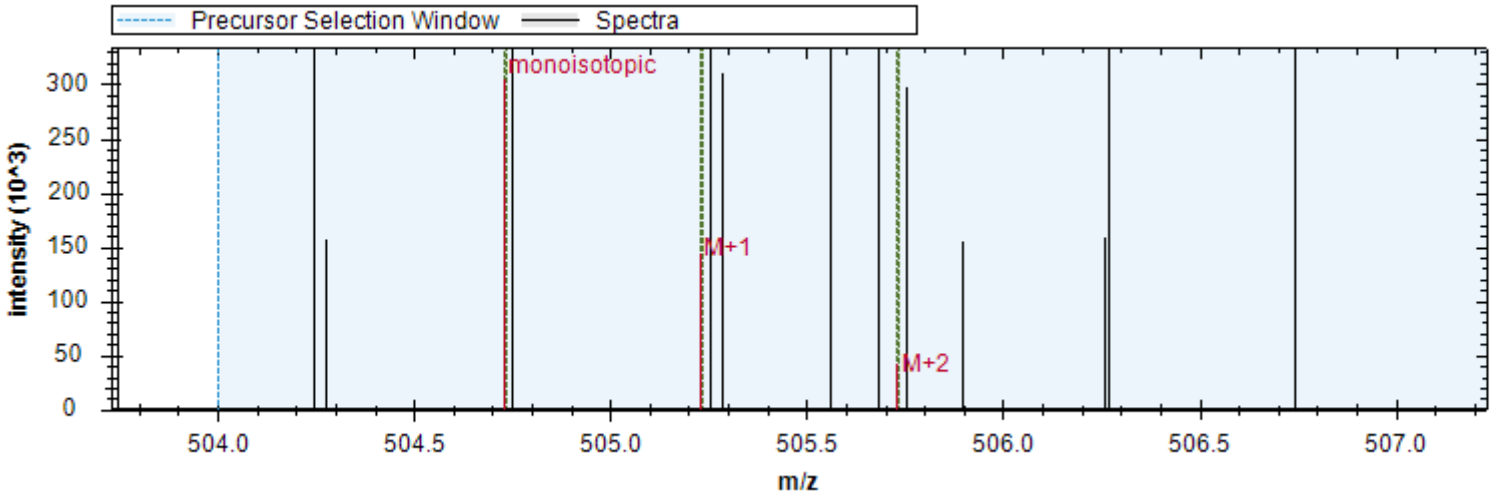

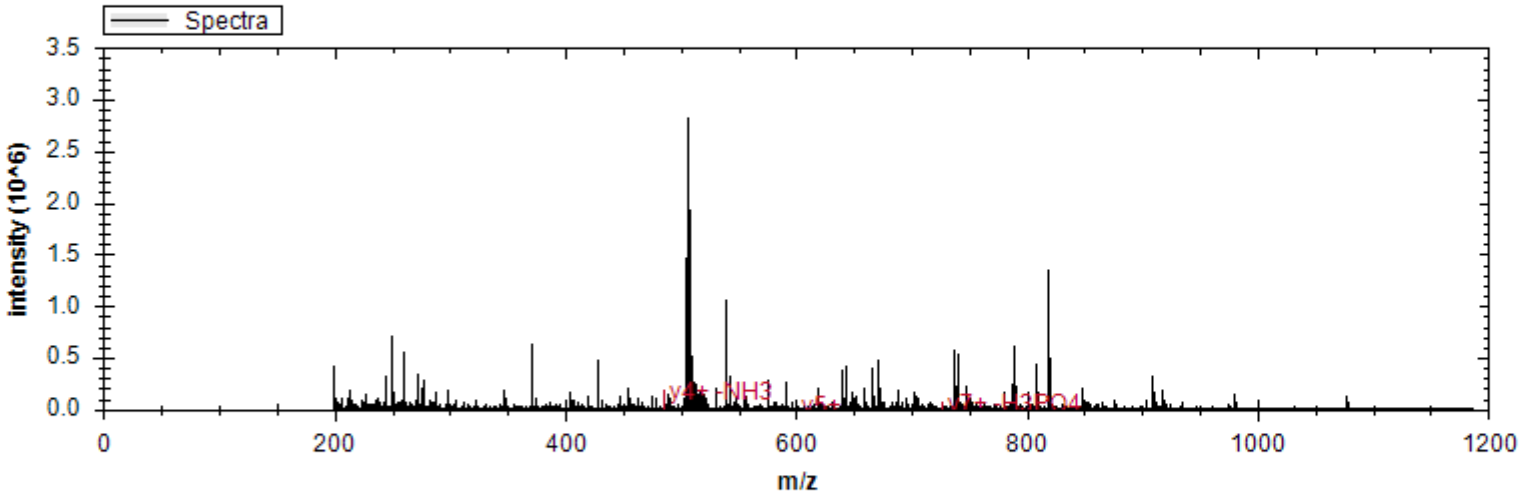

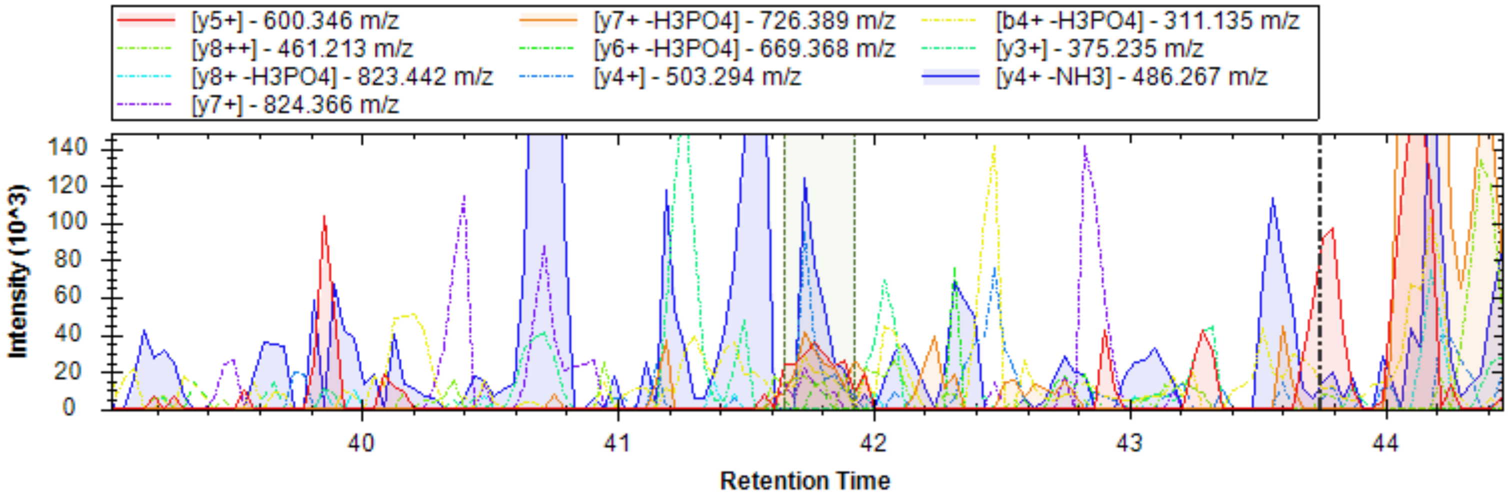

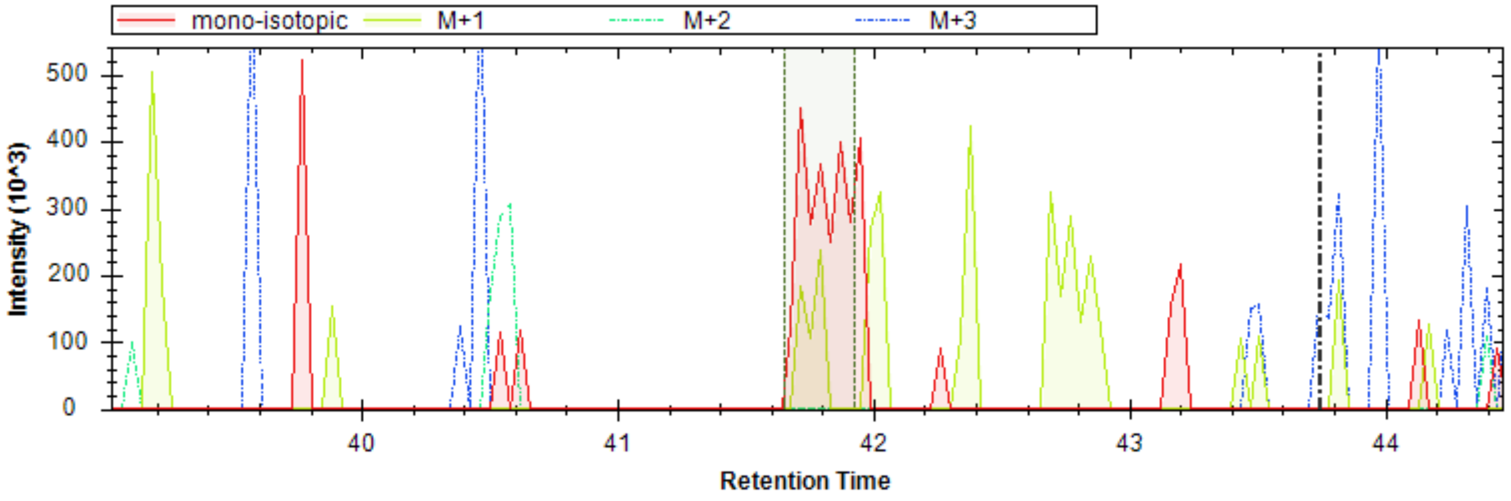

Q9QYC0 – _AAVVT[+H+O3+P]SPPPTTAPHK_.3

Fig4_MP-DIA-barrel-cortex-p15-3_MHRM.htrms

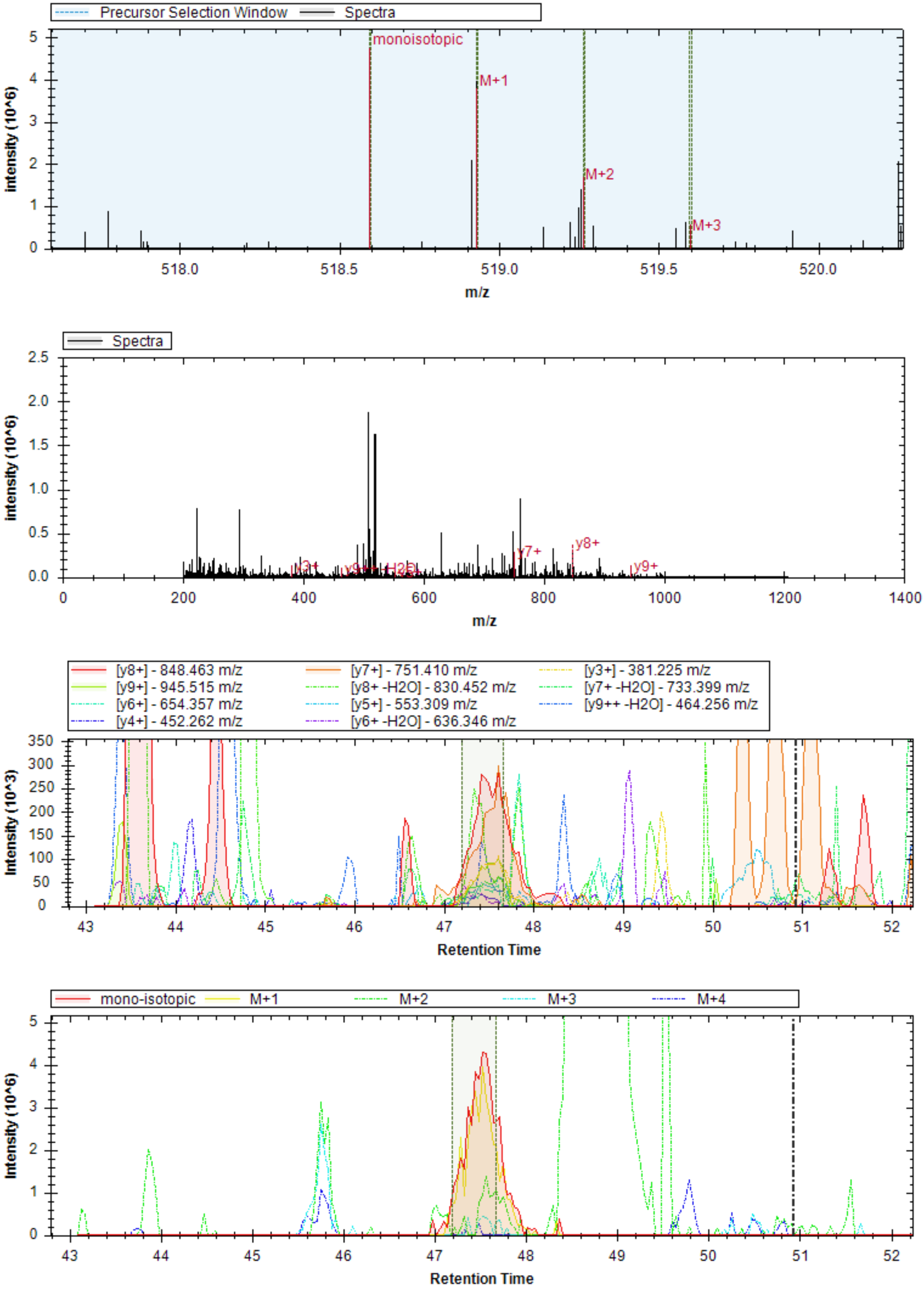

Q9QYC0 – _AAVVTS[+H+O3+P]PPPTTAPHK_.3

Fig4_MP-DIA-barrel-cortex-p15-3_MHRM.htrms

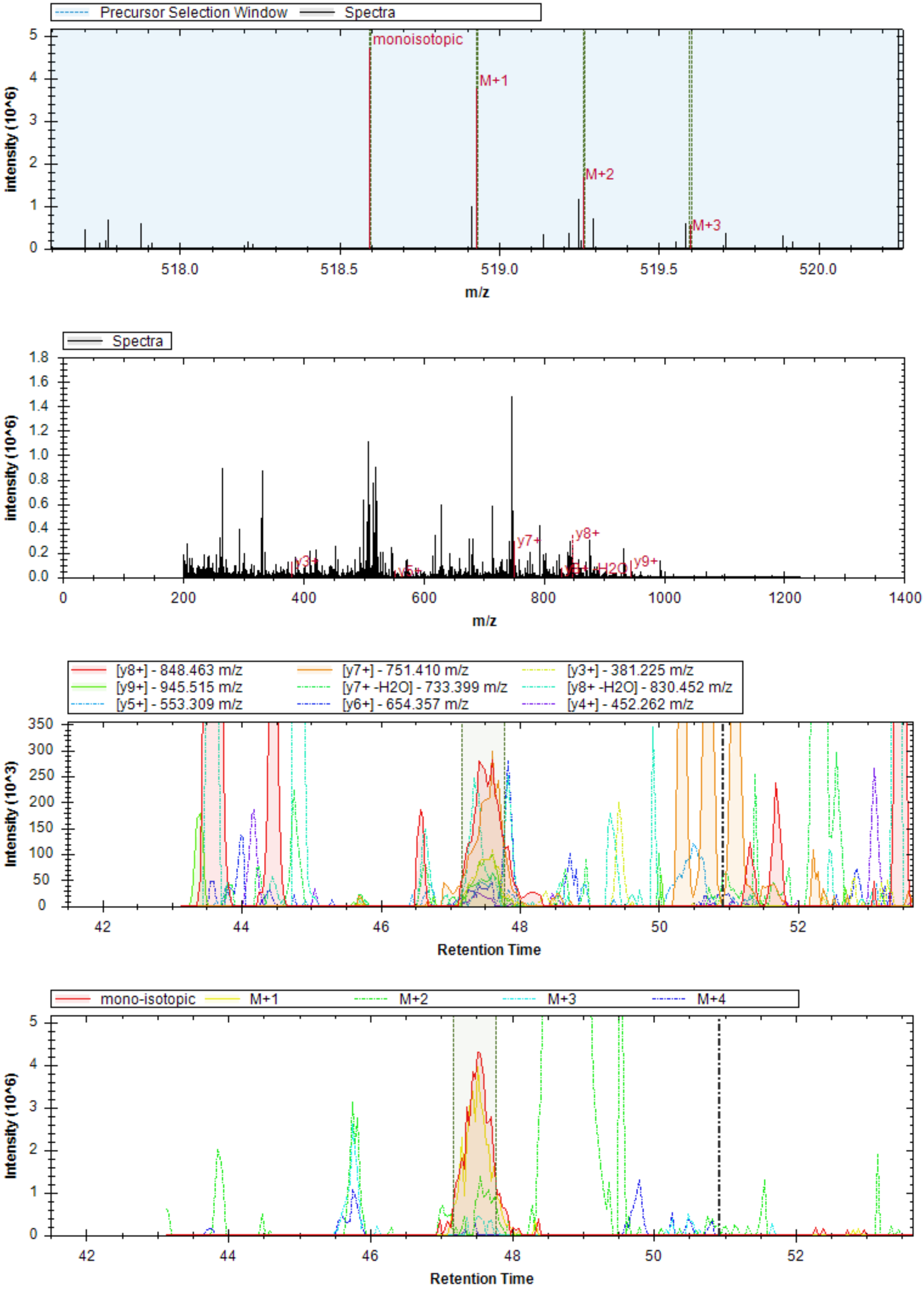

P10637 – _TPPKS[+H+O3+P]PSASK_.2
Fig4_MP-DIA-barrel-cortex-p15-3_MHRM.htrms

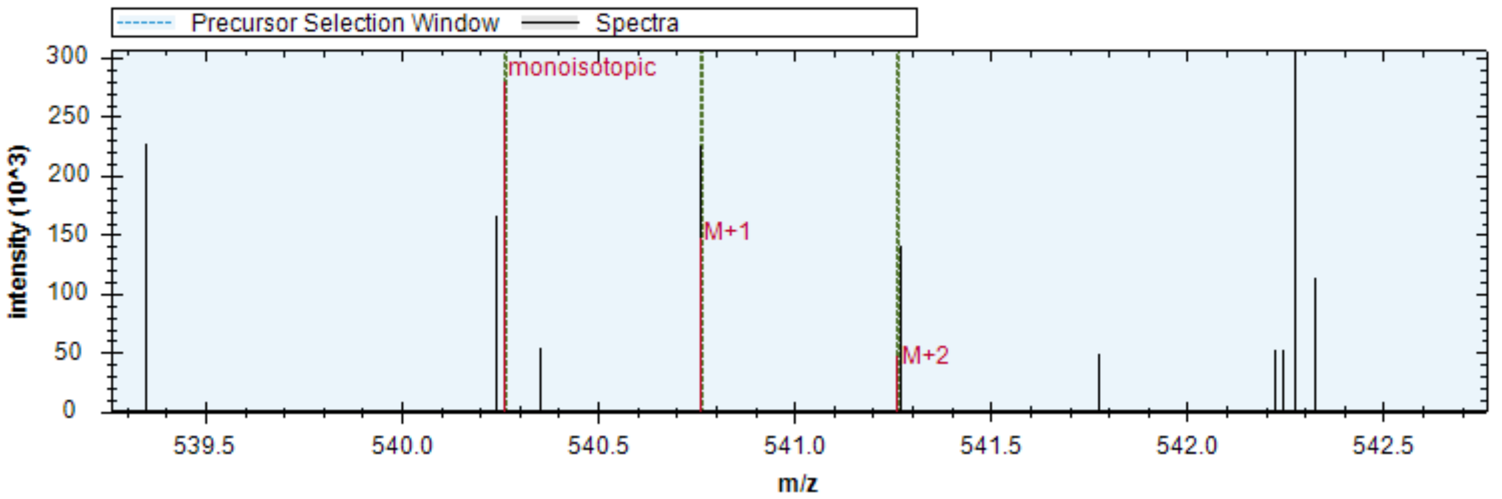

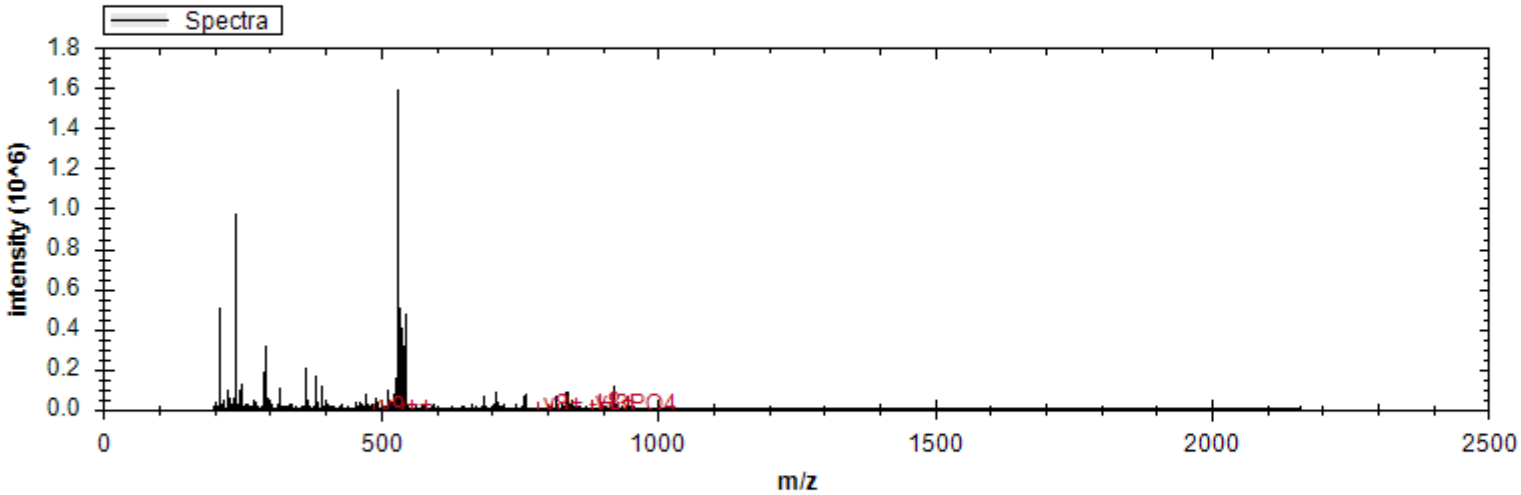

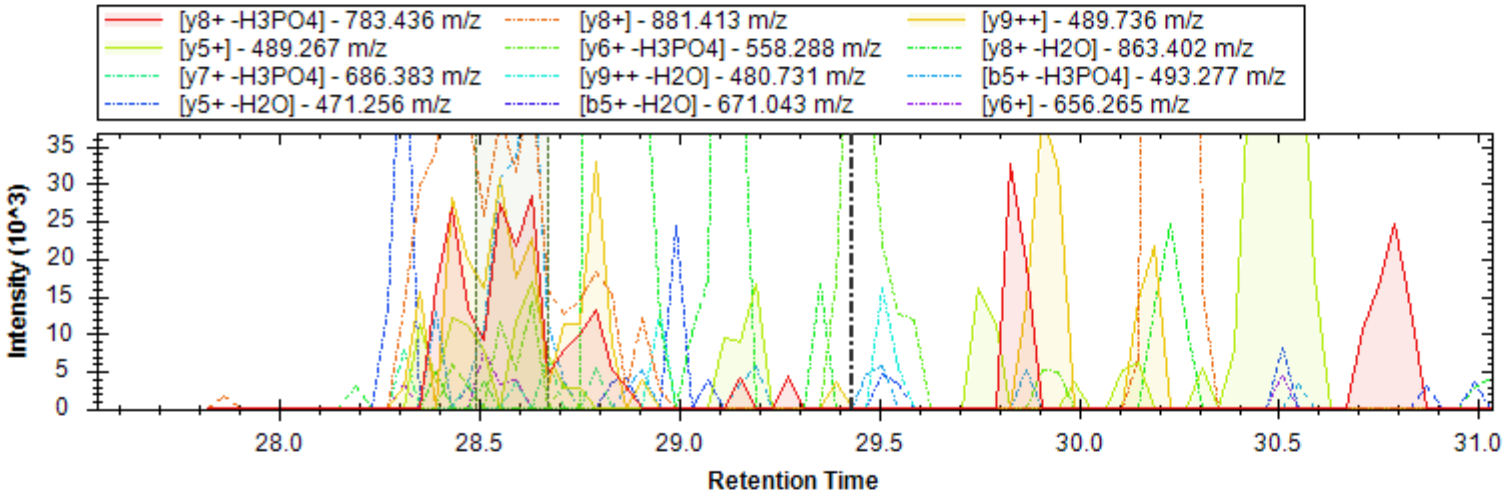

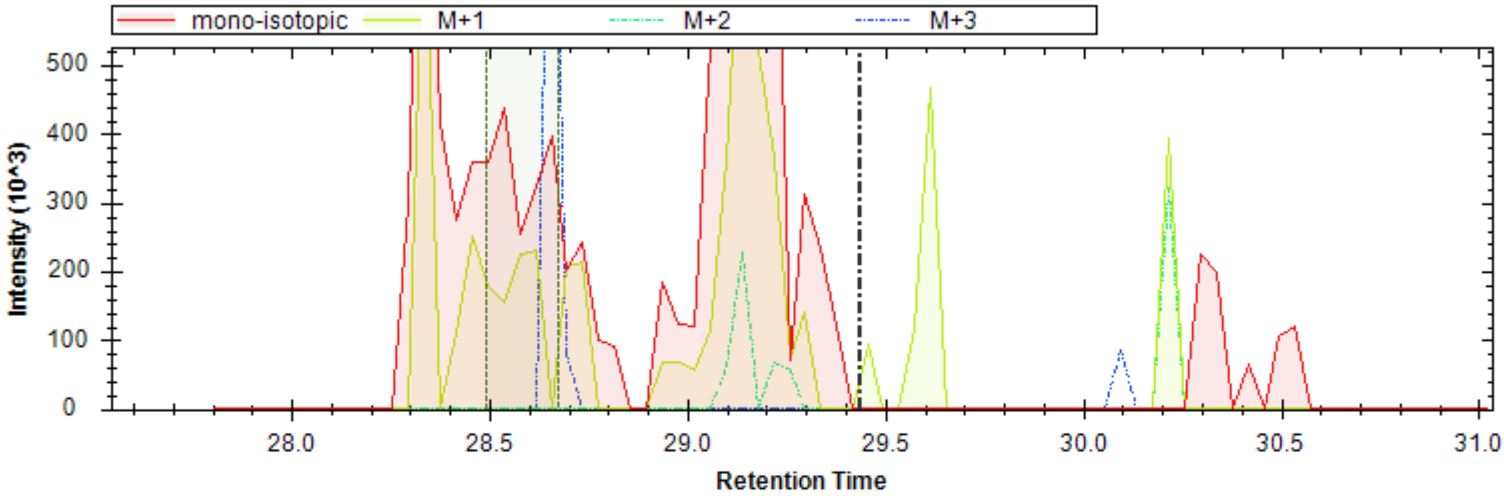

P10637 – _VAVVRT[+H+O3+P]PPKSPSASK_.3
Fig4_MP-DIA-barrel-cortex-p15-3_MHRM.htrms

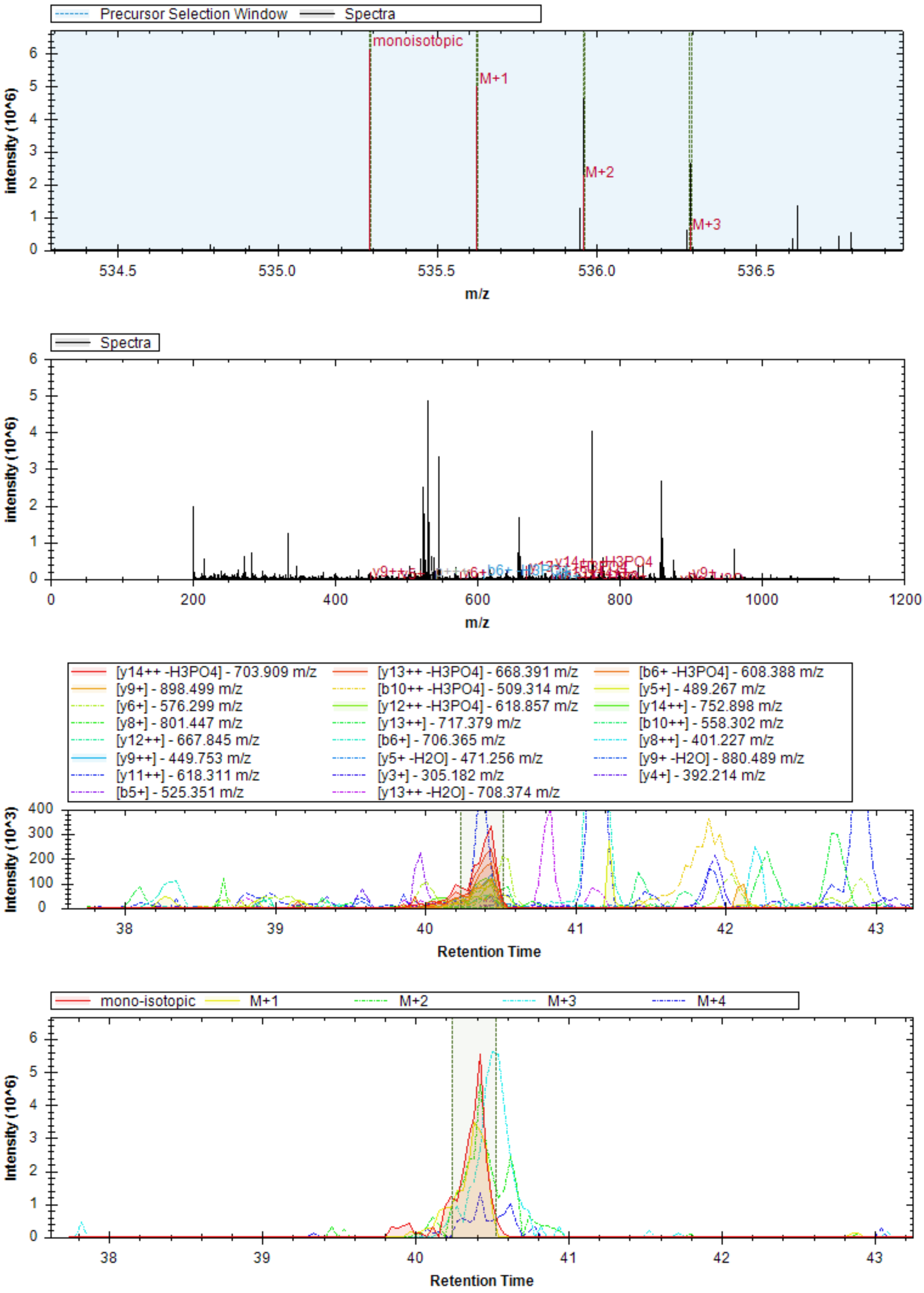

P54227 – _SKESVPDFPLS[+H+O3+P]PPK_.3
Fig4_MP-DIA-barrel-cortex-p15-3_MHRM.htrms

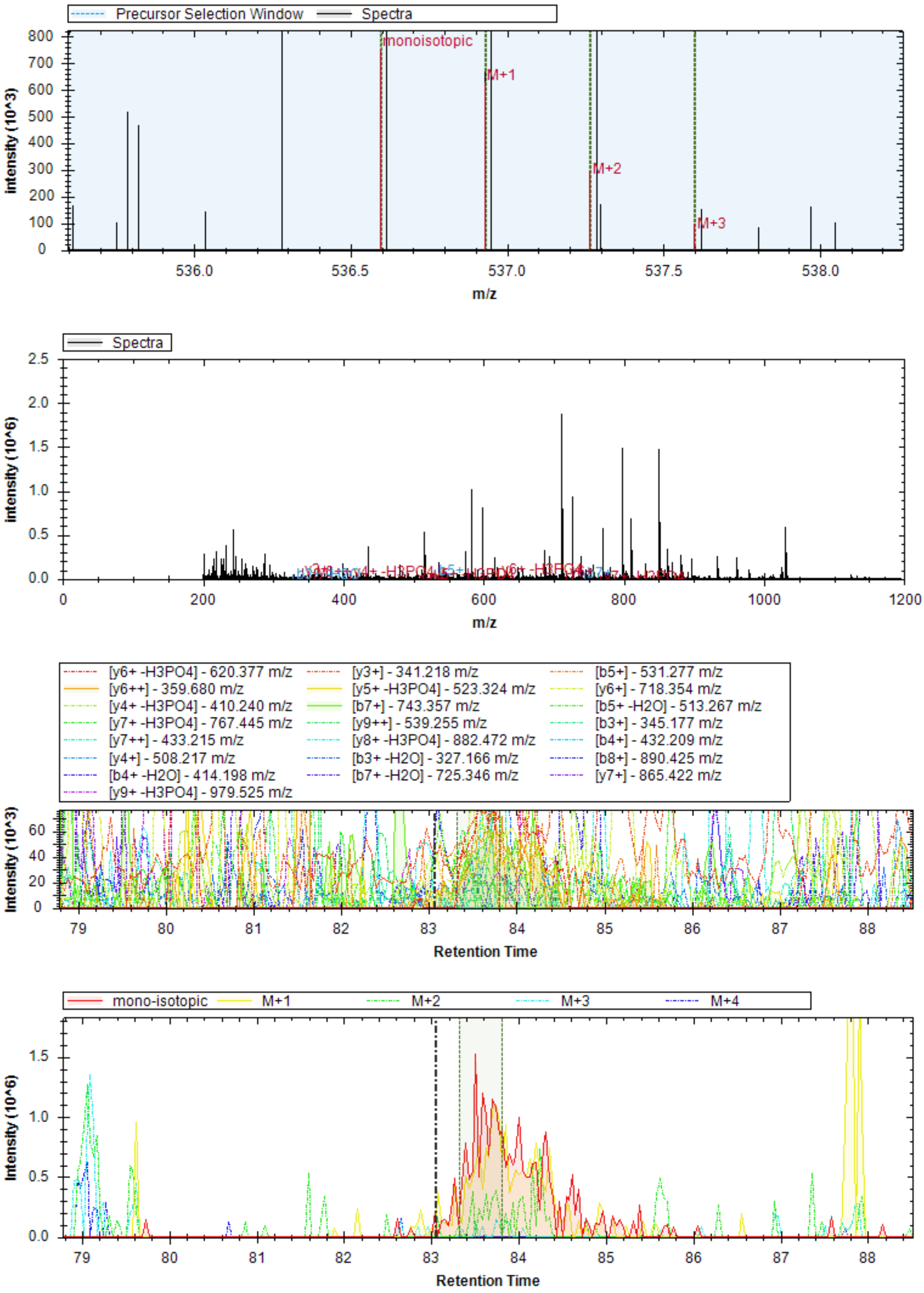

P63318 – _AAPALT[+H+O3+P]PPDR_.2
Fig4_MP-DIA-barrel-cortex-p15-3_MHRM.htrms

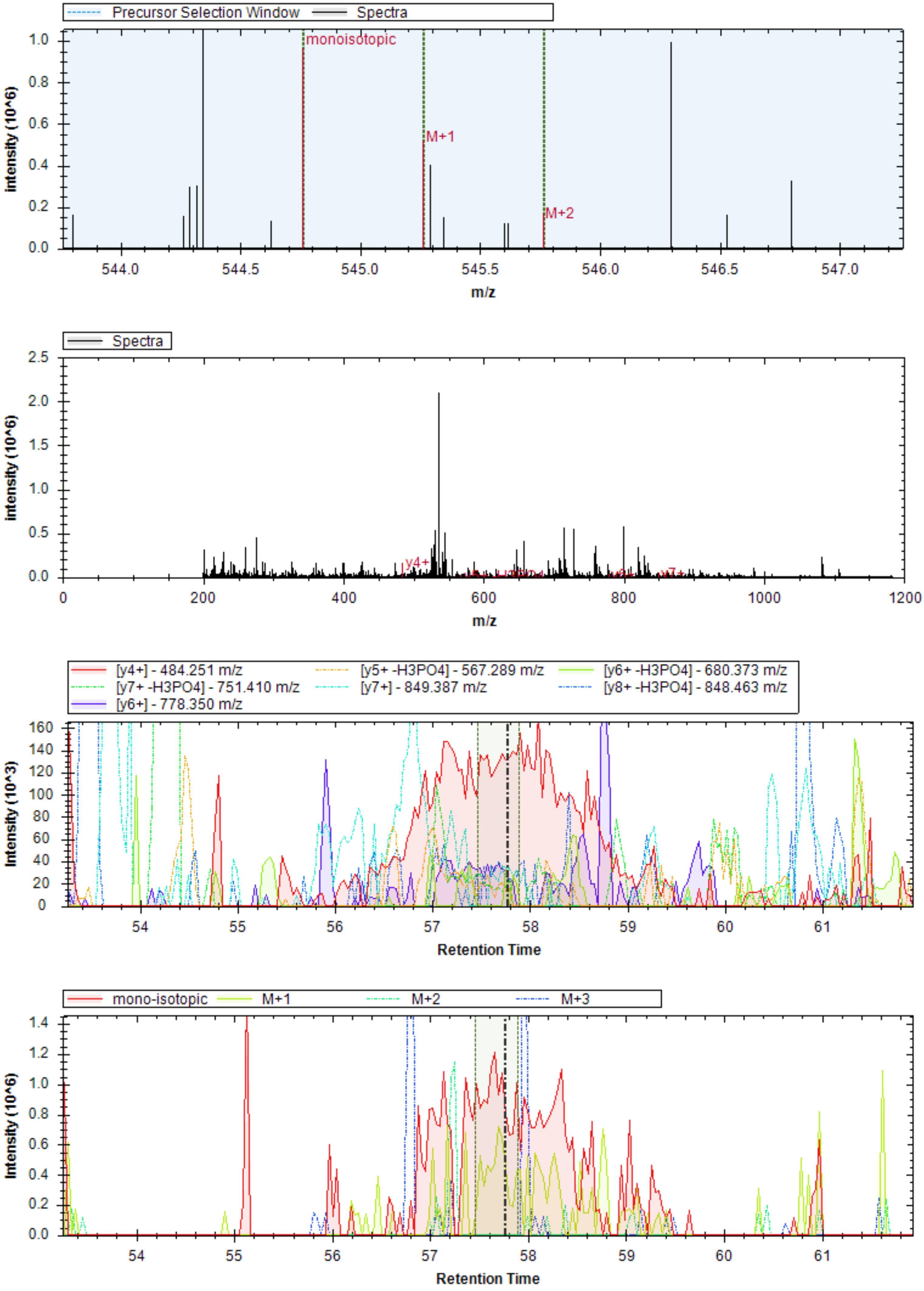

Q7TQD2 – _AANKT[+H+O3+P]PPKSPGDPAR_.3
Fig4_MP-DIA-barrel-cortex-p15-3_MHRM.htrms

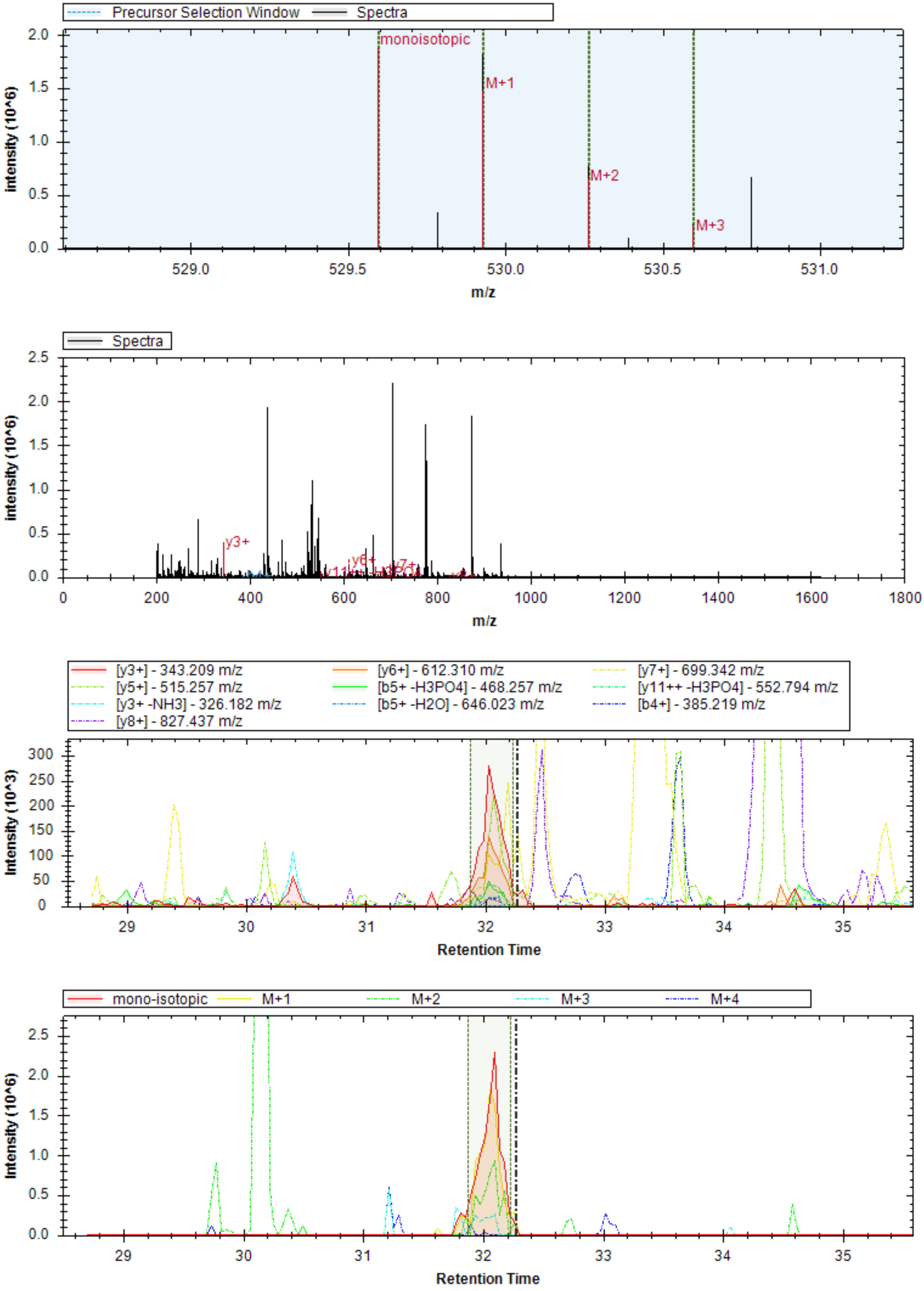

Q9D898 – _AFHAALRNS[+H+O3+P]PINTK_.3
Fig4_MP-DIA-barrel-cortex-p15-3_MHRM.htrms

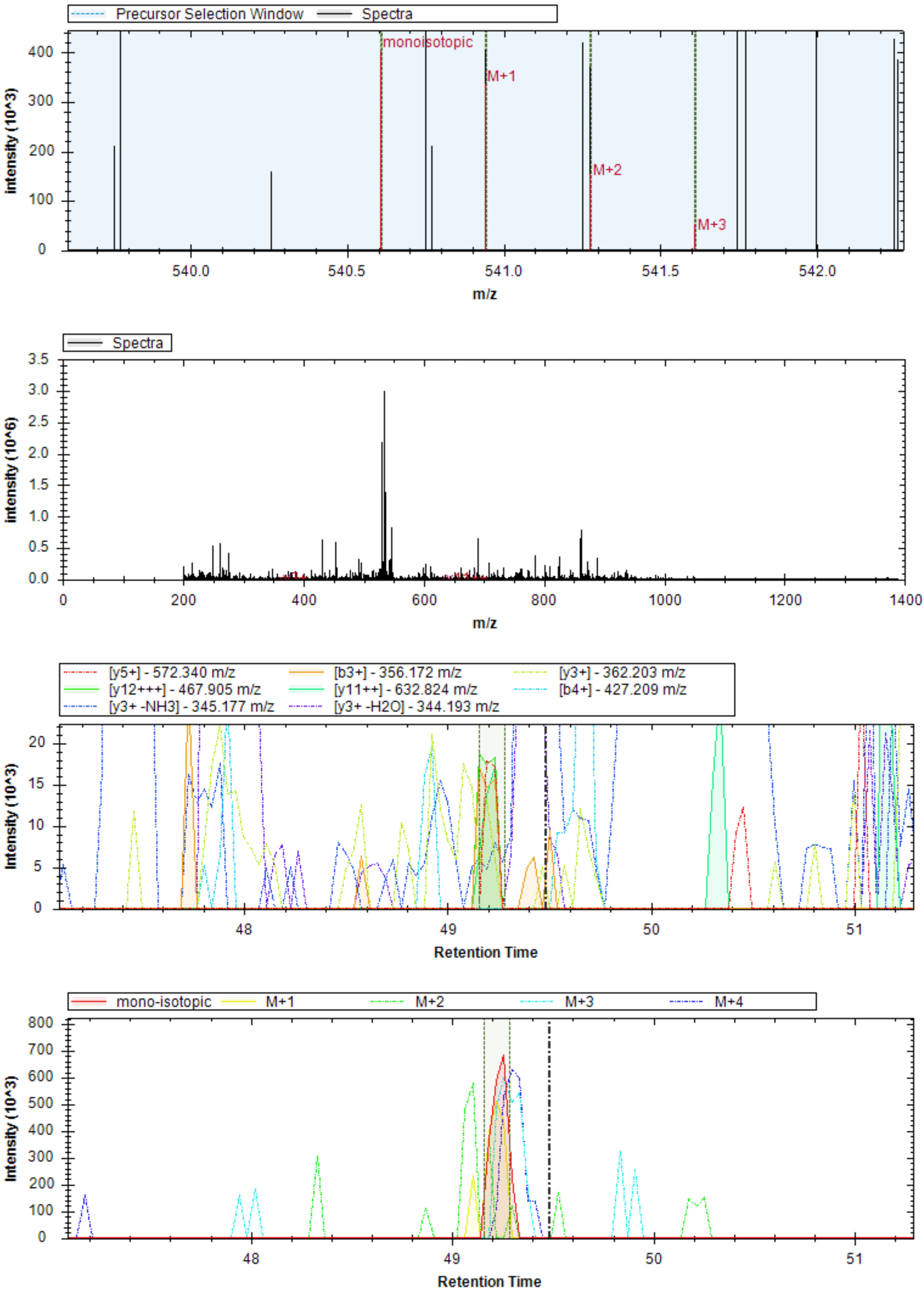

P10637 – _TTPSPKT[+H+O3+P]PPGSGEPPK_.3
Fig4_MP-DIA-barrel-cortex-p15-3_MHRM.htrms

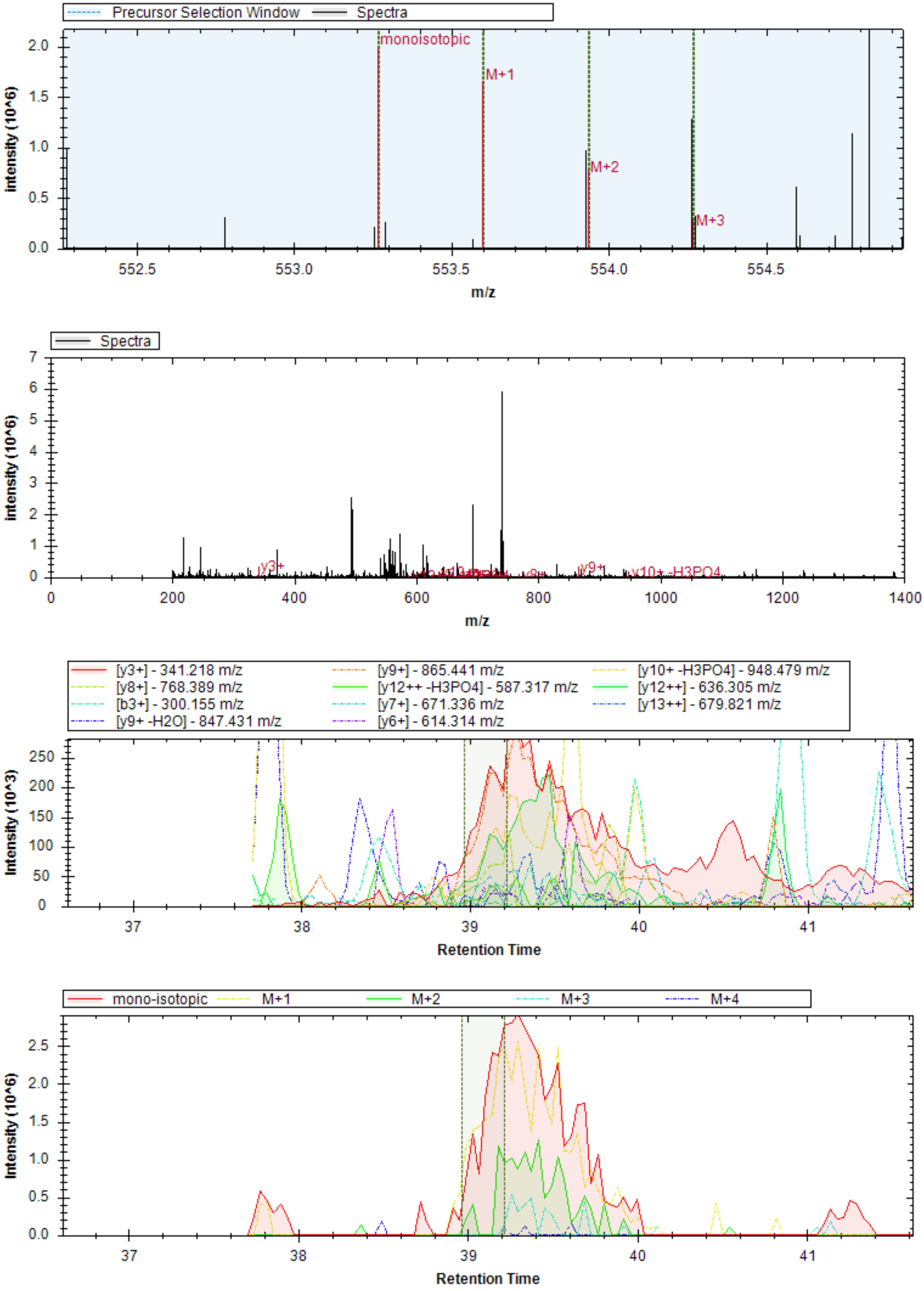

P20357 – _VAIIRT[+H+O3+P]PPKSPATPK_.3
Fig4_MP-DIA-barrel-cortex-p15-3_MHRM.htrms

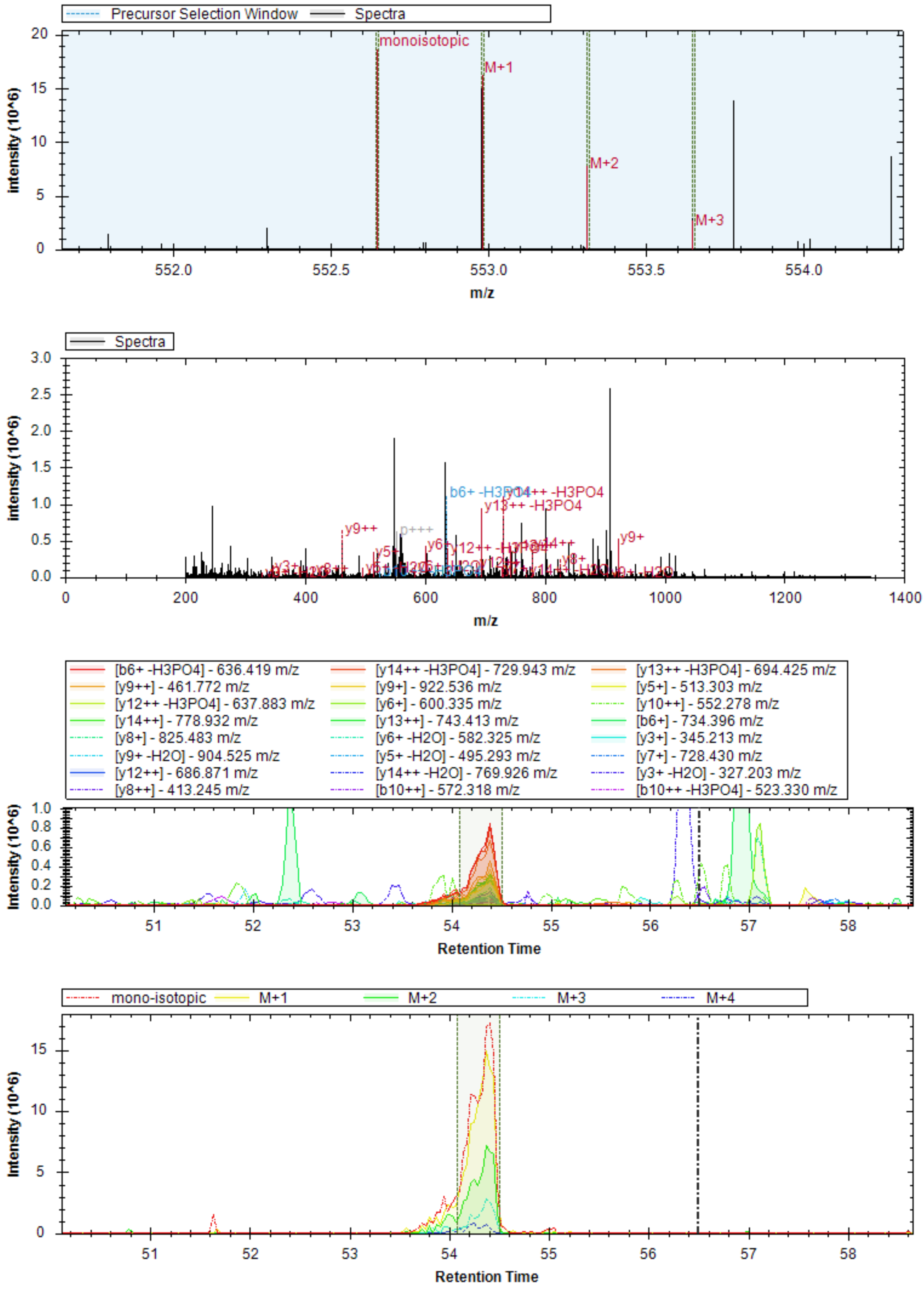

Q9CYT6 – _THTPSPTS[+H+O3+P]PK_.2
Fig4_MP-DIA-barrel-cortex-p15-3_MHRM.htrms

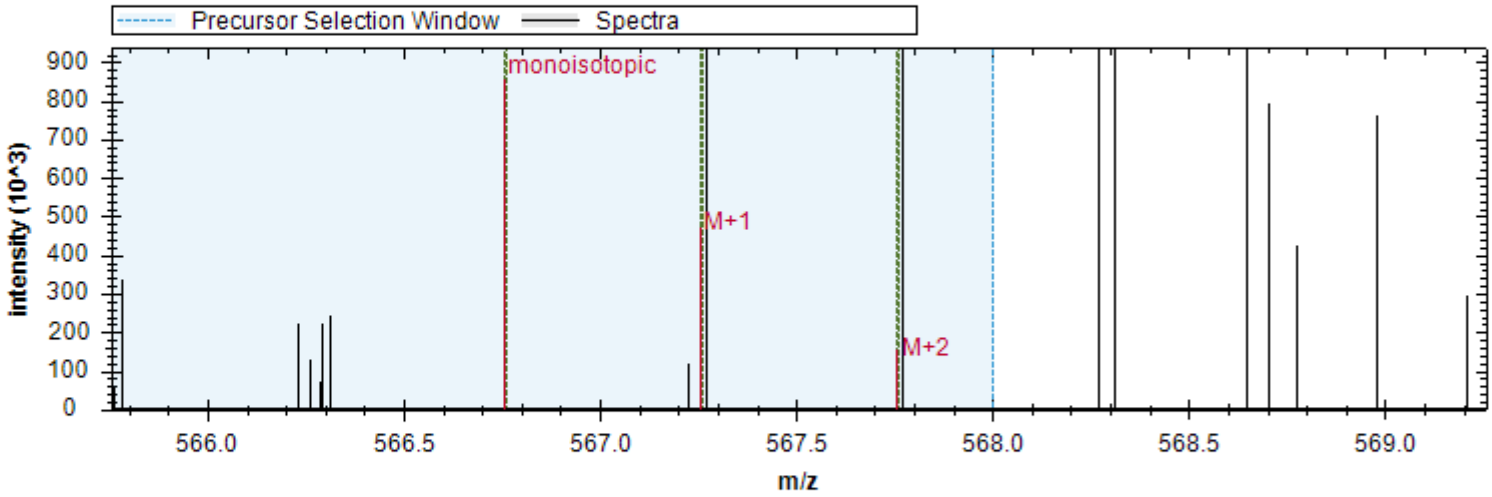

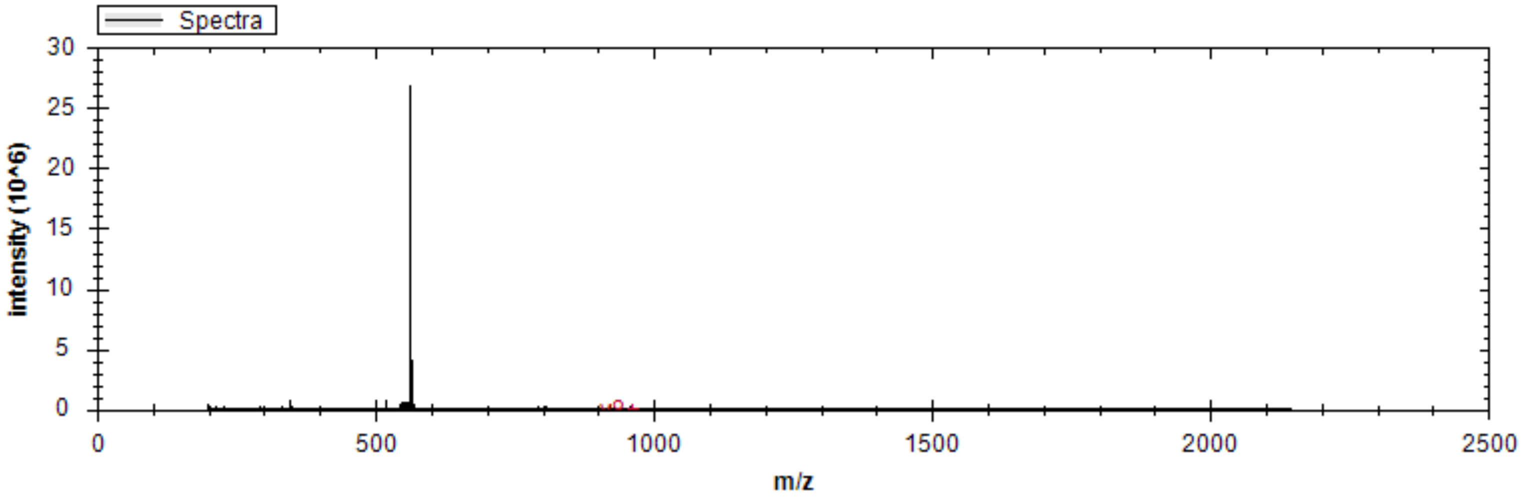

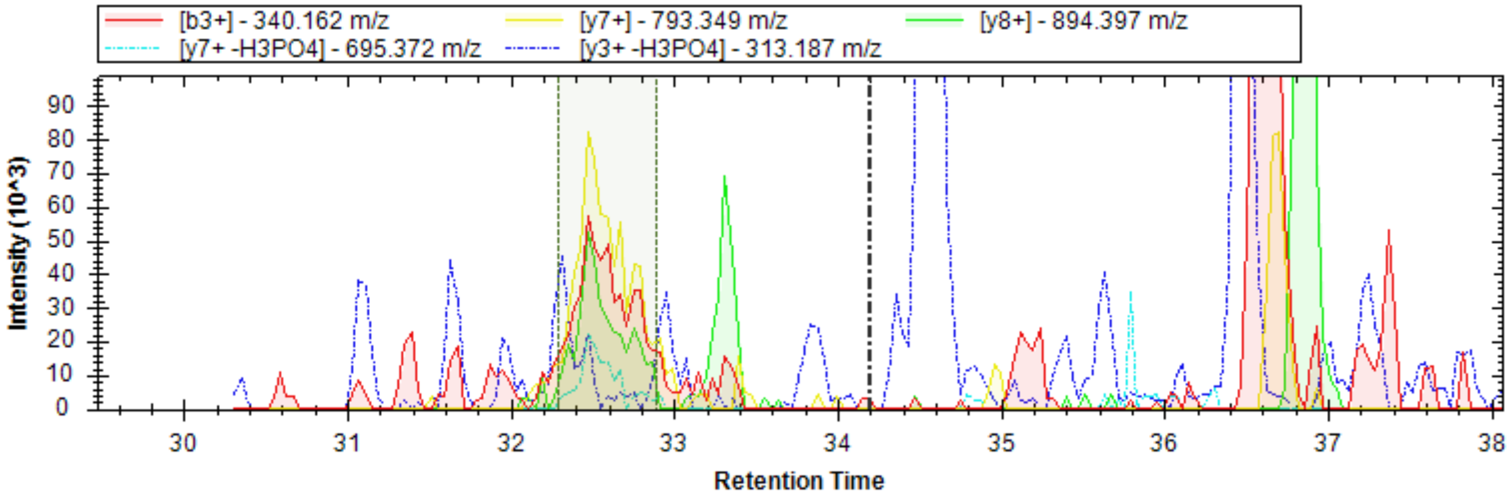

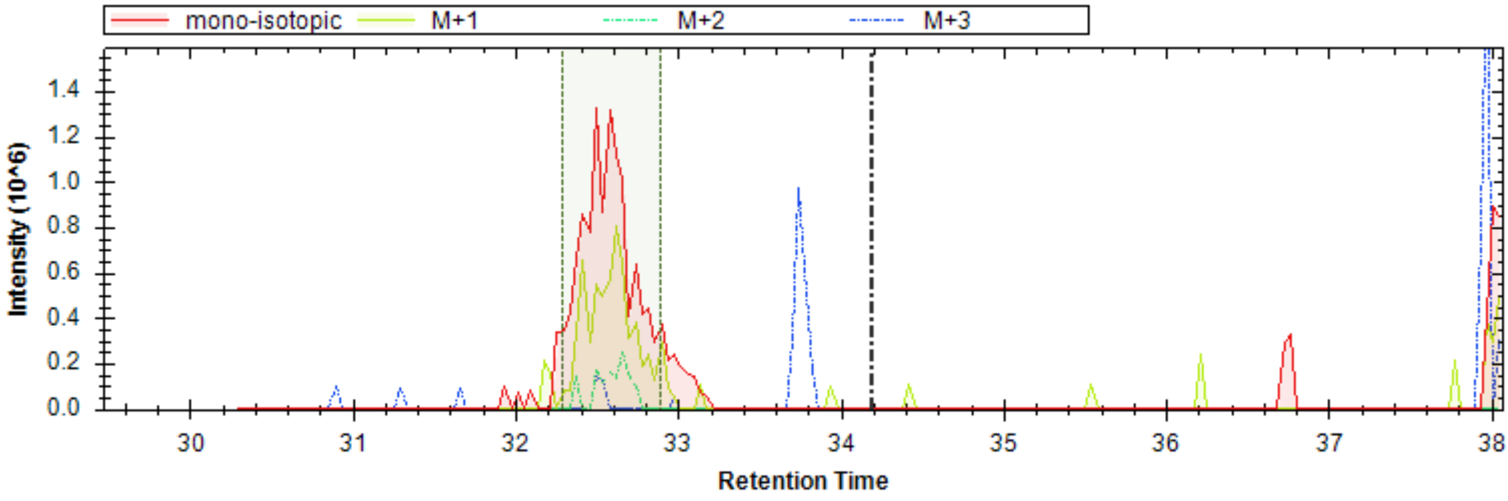

Q9R0K7 – _T[+H+O3+P]SIHNFMAHPEFR_.3

Fig4_MP-DIA-barrel-cortex-p15-3_MHRM.htrms

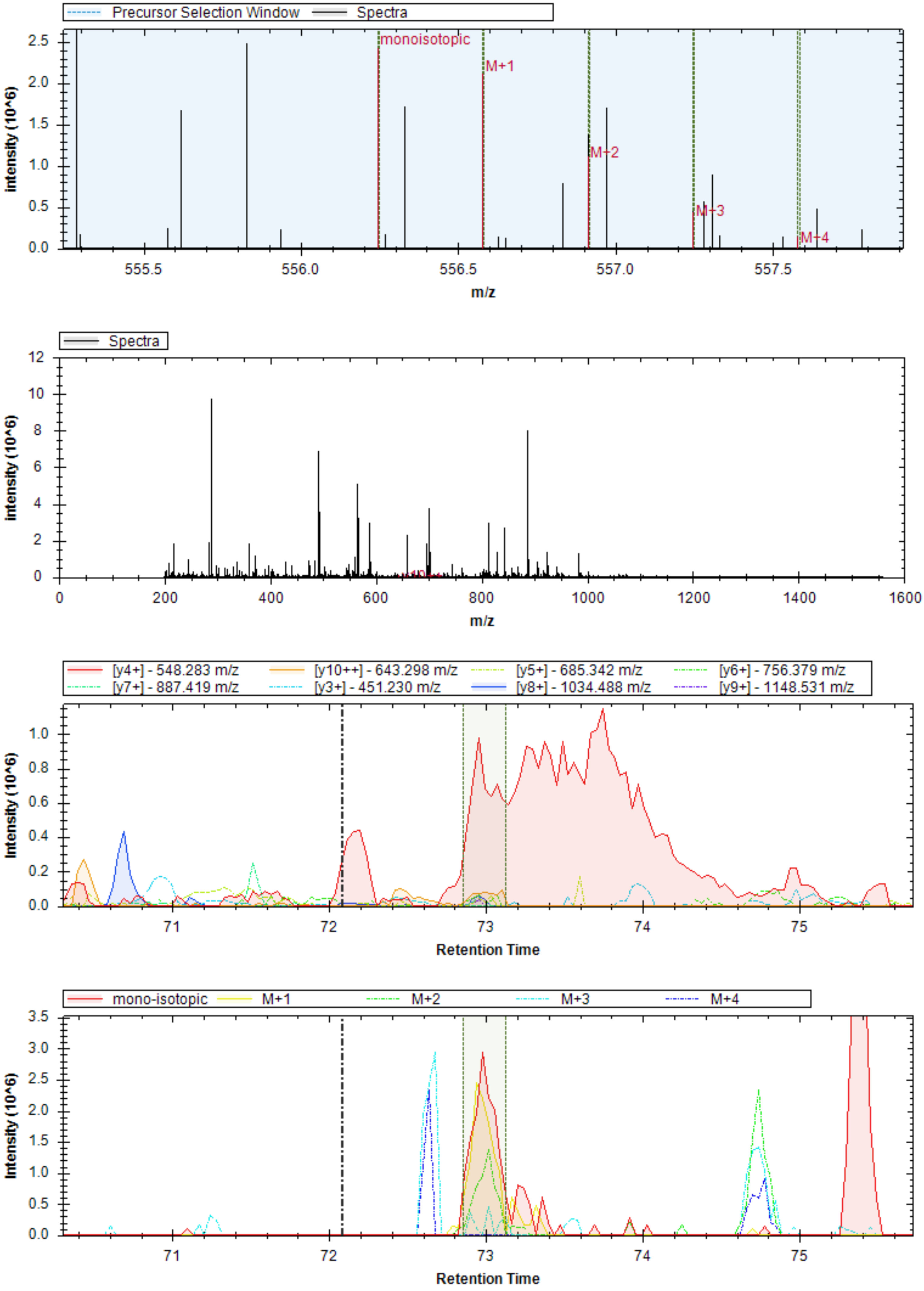

O88935 – _QAS[+H+O3+P]ISGPAPTK_.2
Fig4_MP-DIA-barrel-cortex-p15-3_MHRM.htrms

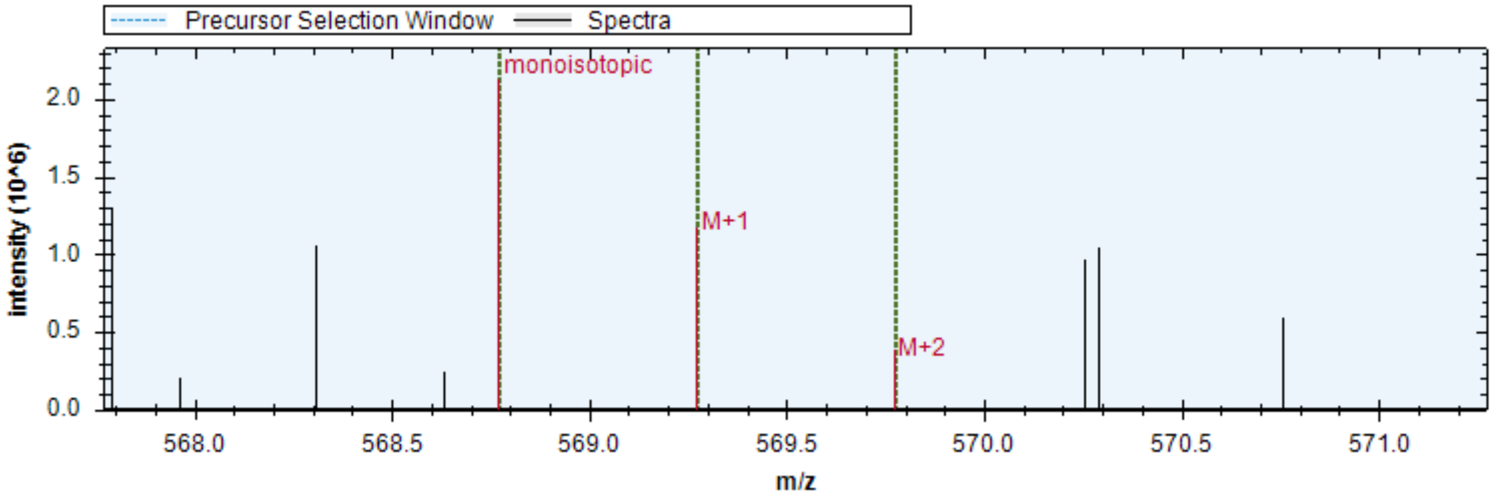

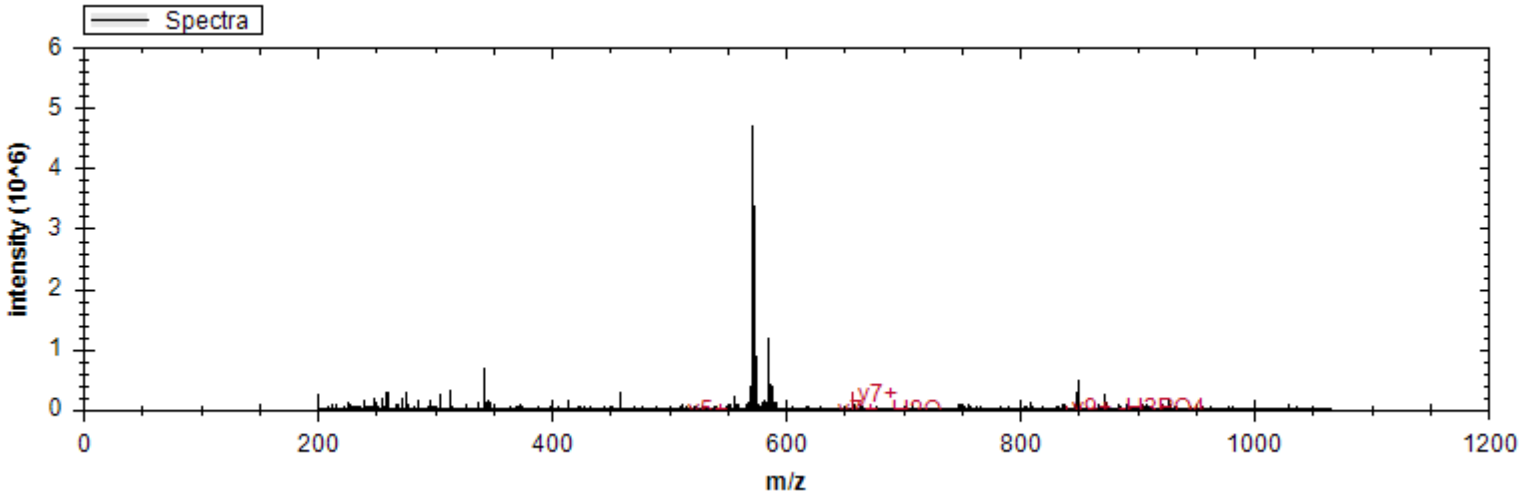

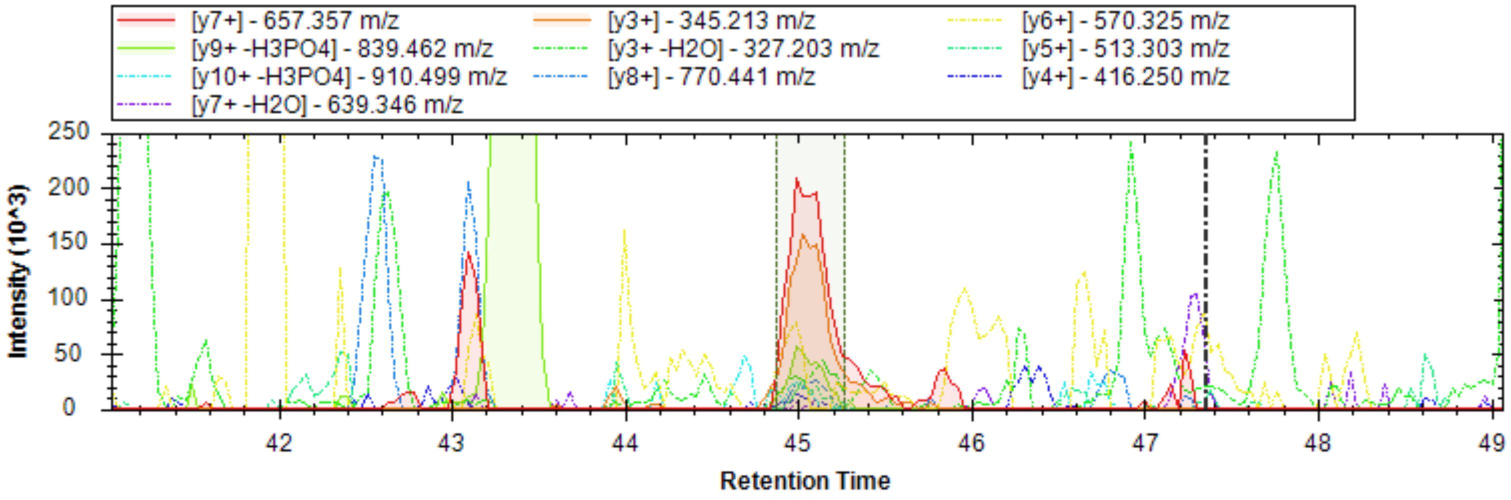

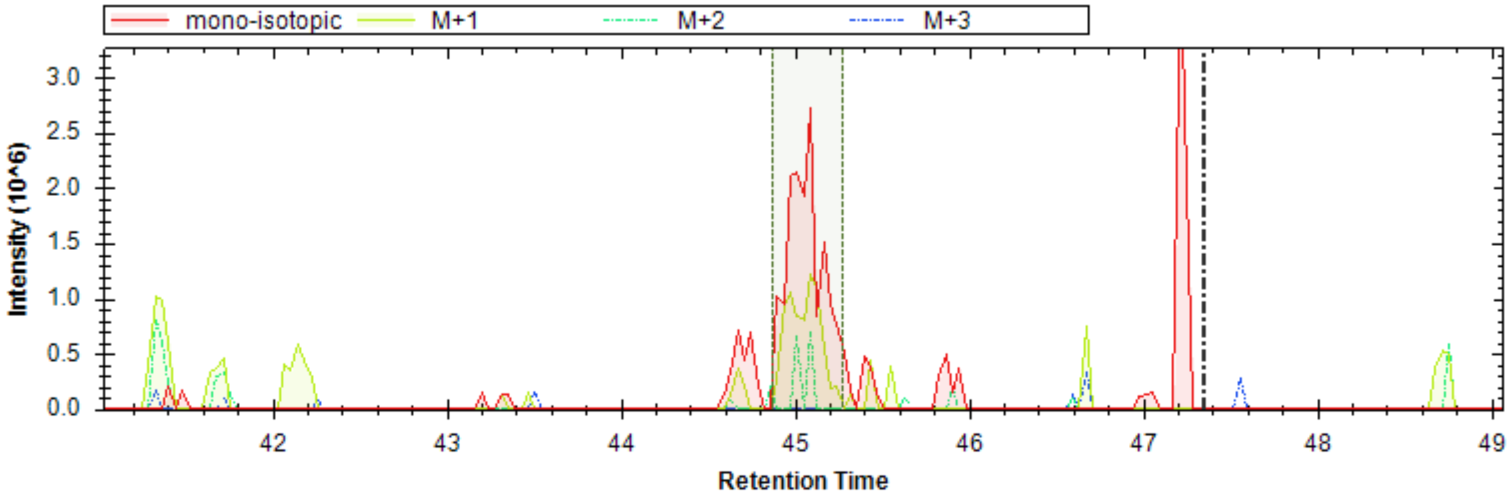

P27546-2 – _T[+H+O3+P]SPSKPSSAPALKPGPK_.3

Fig4_MP-DIA-barrel-cortex-p15-3_MHRM.htrms

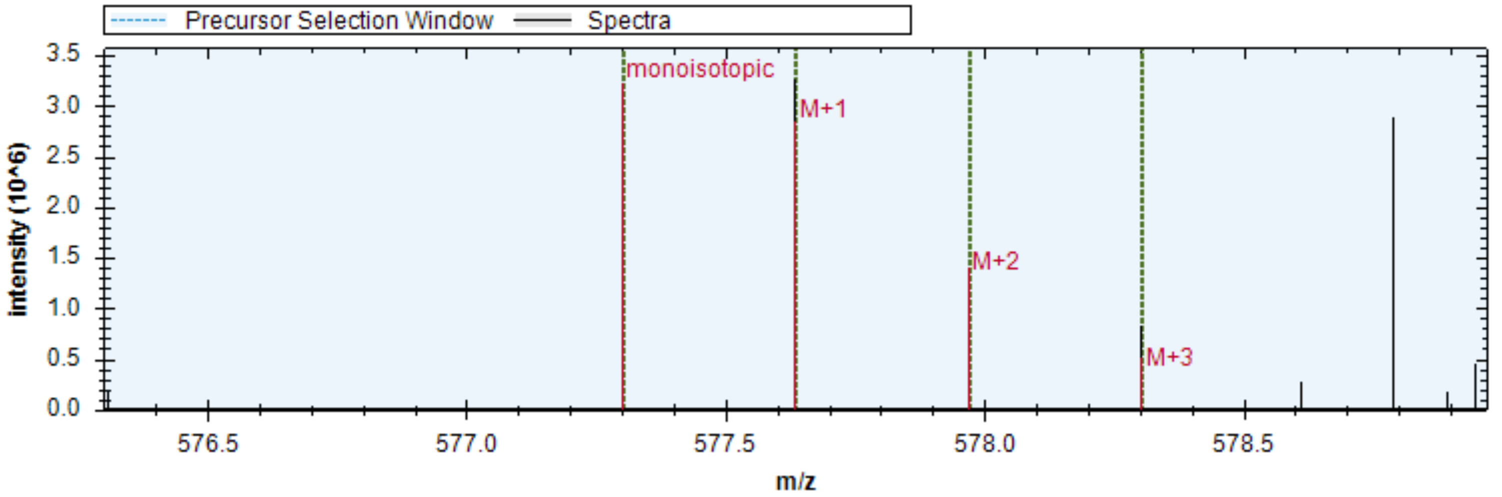

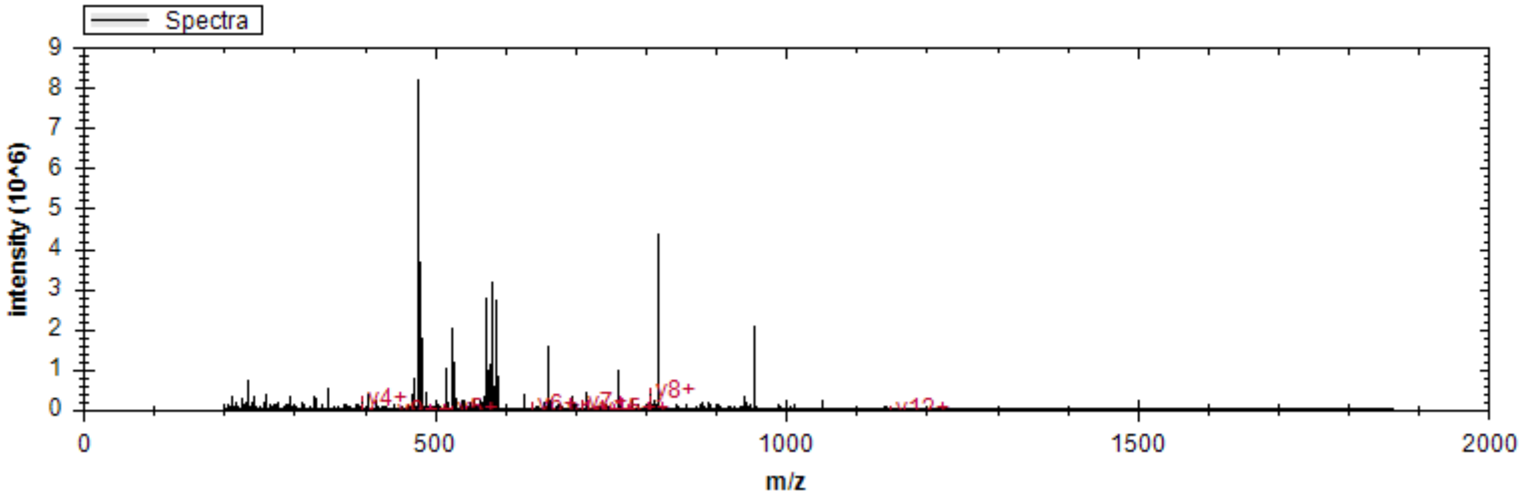

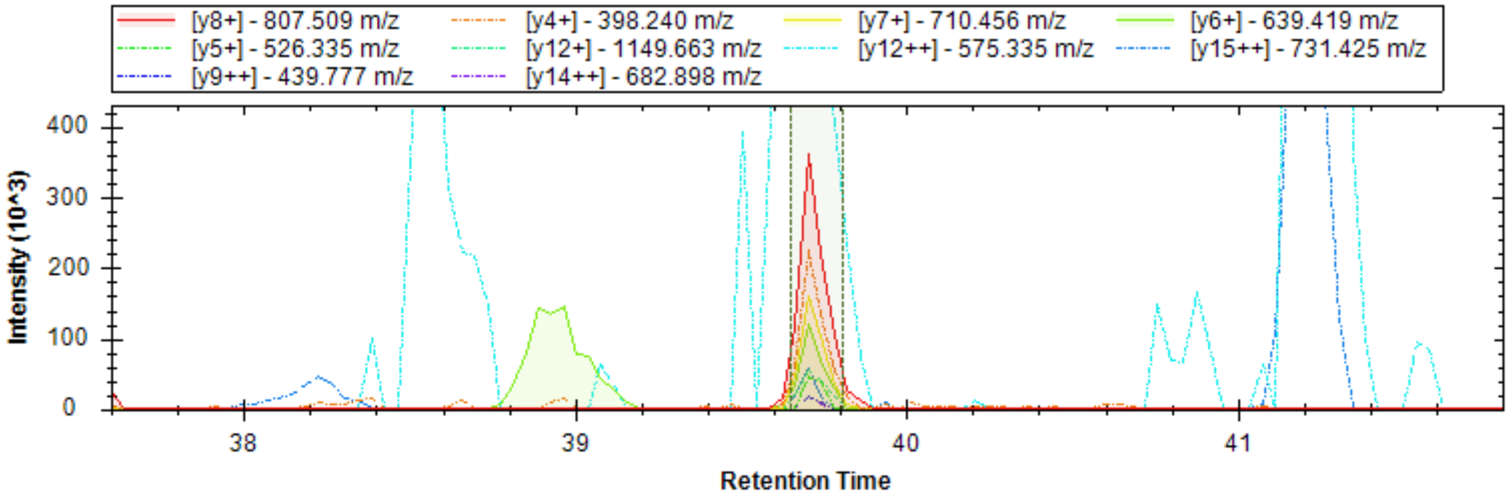

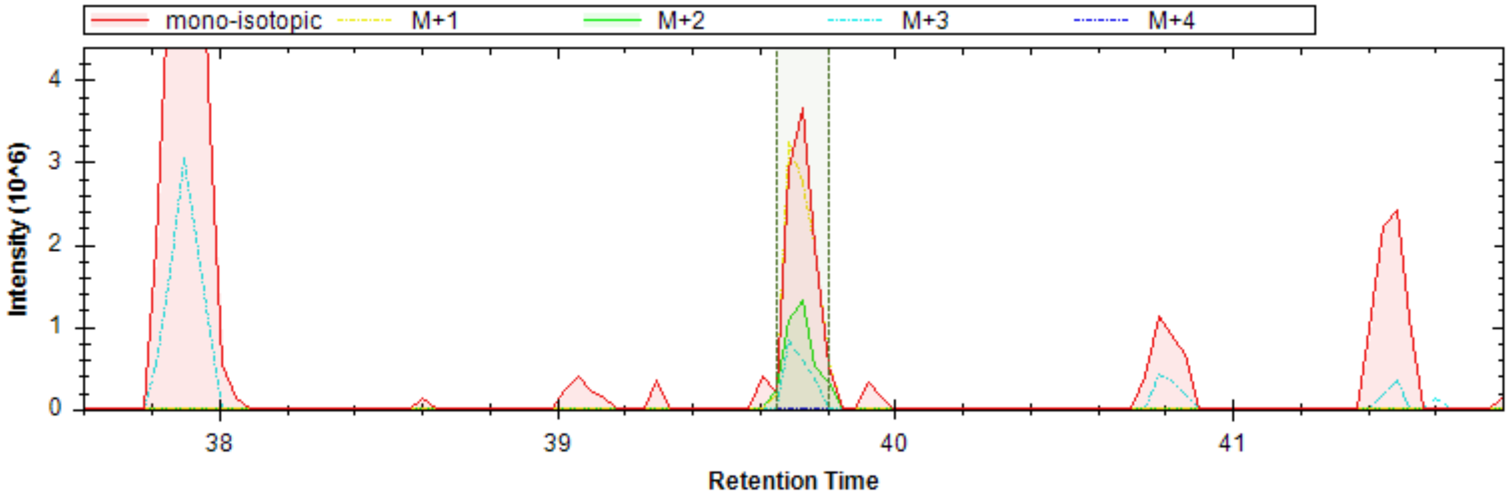

P28667 – _GEVAPKET[+H+O3+P]PK_.2
Fig4_MP-DIA-barrel-cortex-p15-3_MHRM.htrms

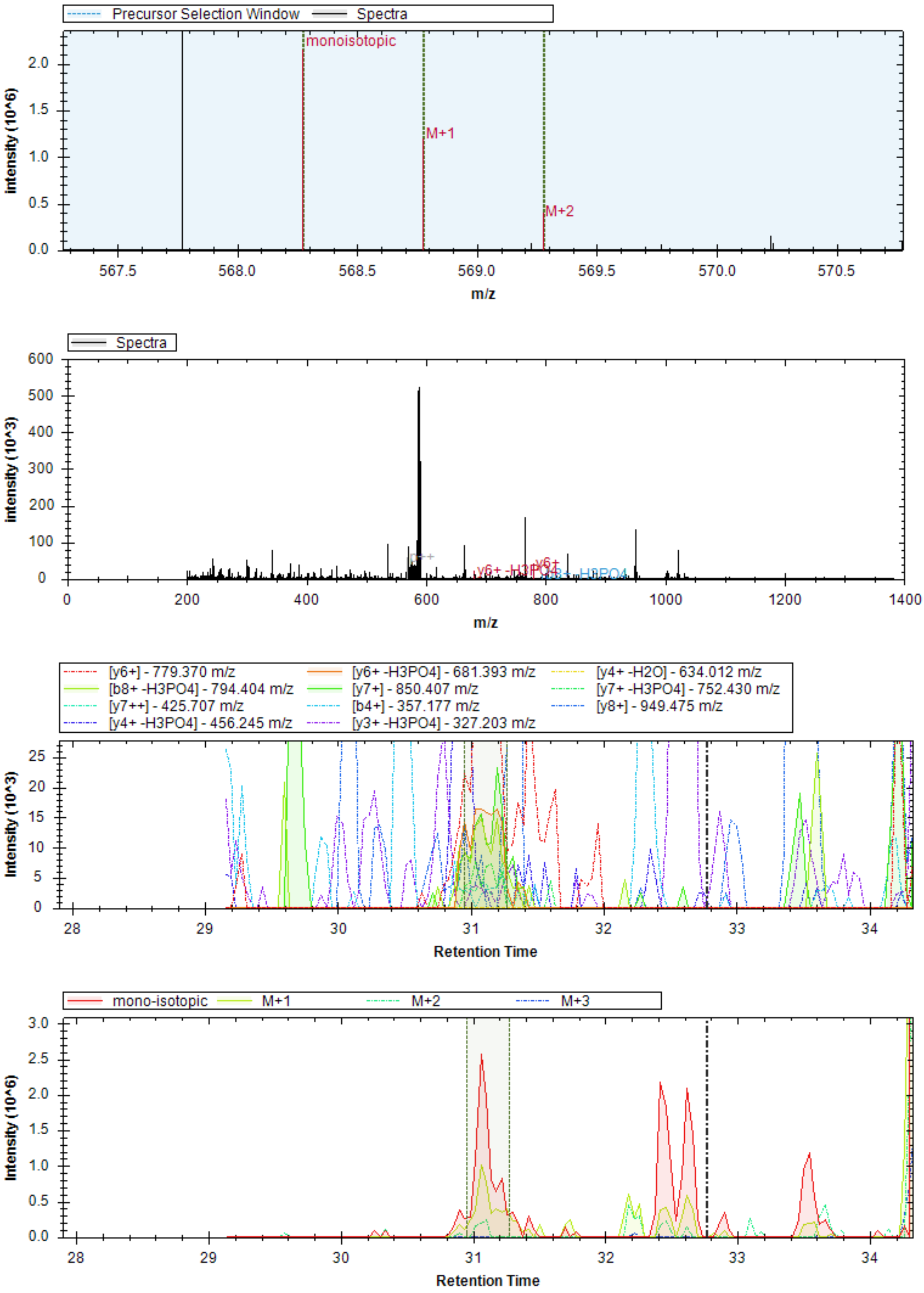

P97792 – _APQS[+H+O3+P]PTLAPAK_.2
Fig4_MP-DIA-barrel-cortex-p15-3_MHRM.htrms

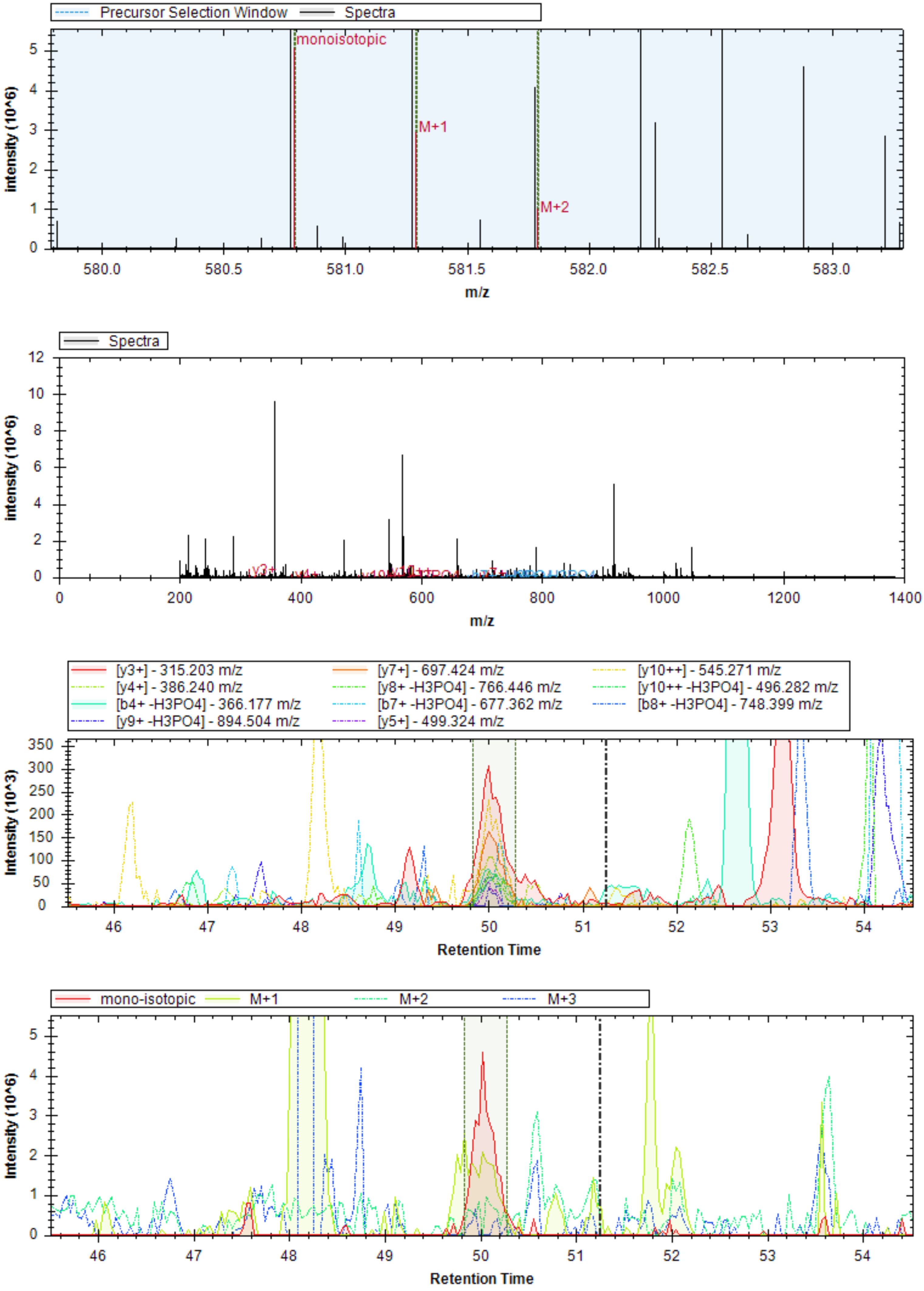

Q64012-2 – _GRLS[+H+O3+P]PVPVPR_.2

Fig4_MP-DIA-barrel-cortex-p15-3_MHRM.htrms

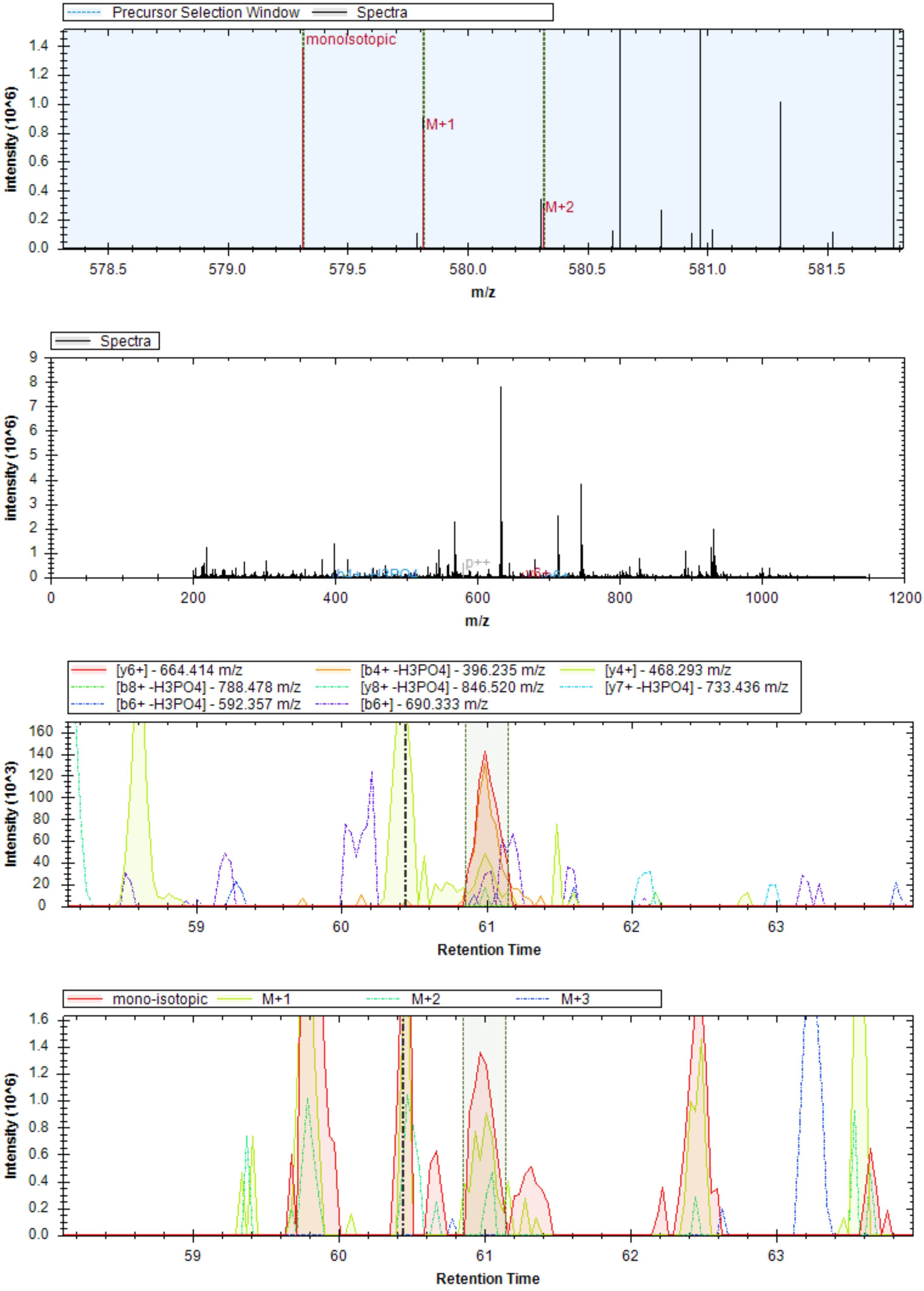

Q8BFU3-4 – _VAS[+H+O3+P]STQVRGASGRELR_.3
Fig4_MP-DIA-barrel-cortex-p15-3_MHRM.htrms

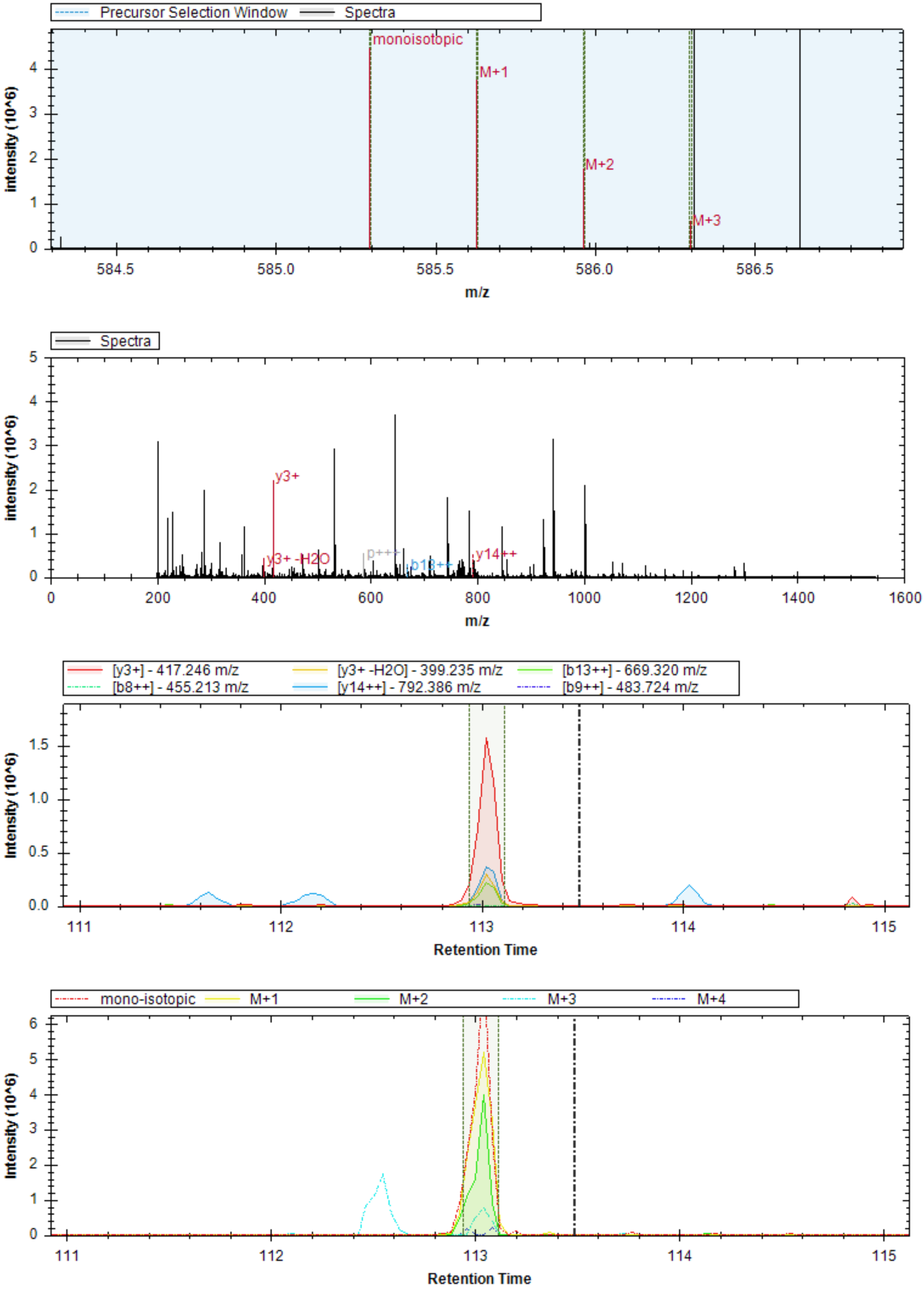

Q8BTI8 – _RVPS[+H+O3+P]PTPVPK_.2
Fig4_MP-DIA-barrel-cortex-p15-3_MHRM.htrms

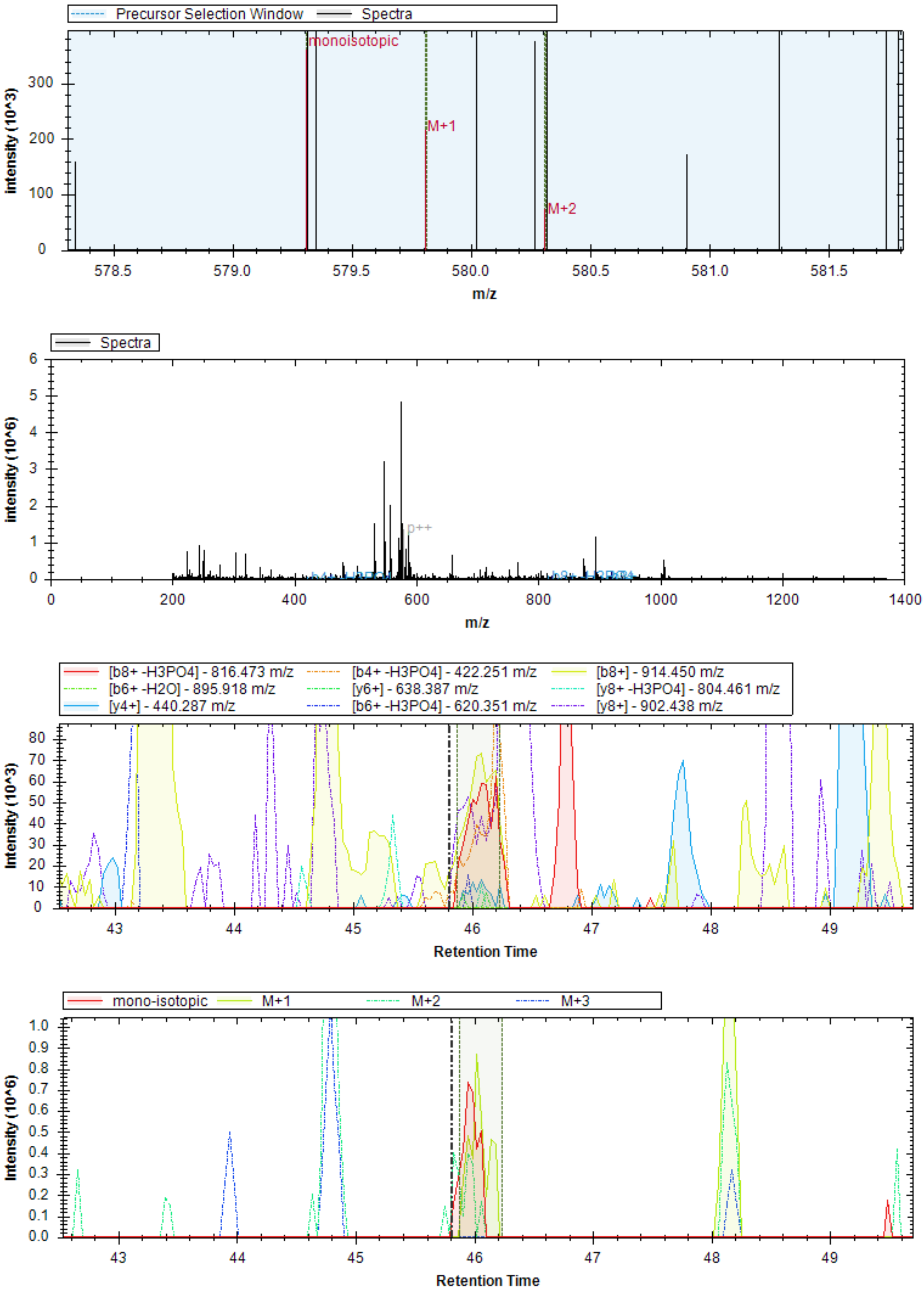

Q9CYT6 – _AQGQIRS[+H+O3+P]PTK_.2
Fig4_MP-DIA-barrel-cortex-p15-3_MHRM.htrms

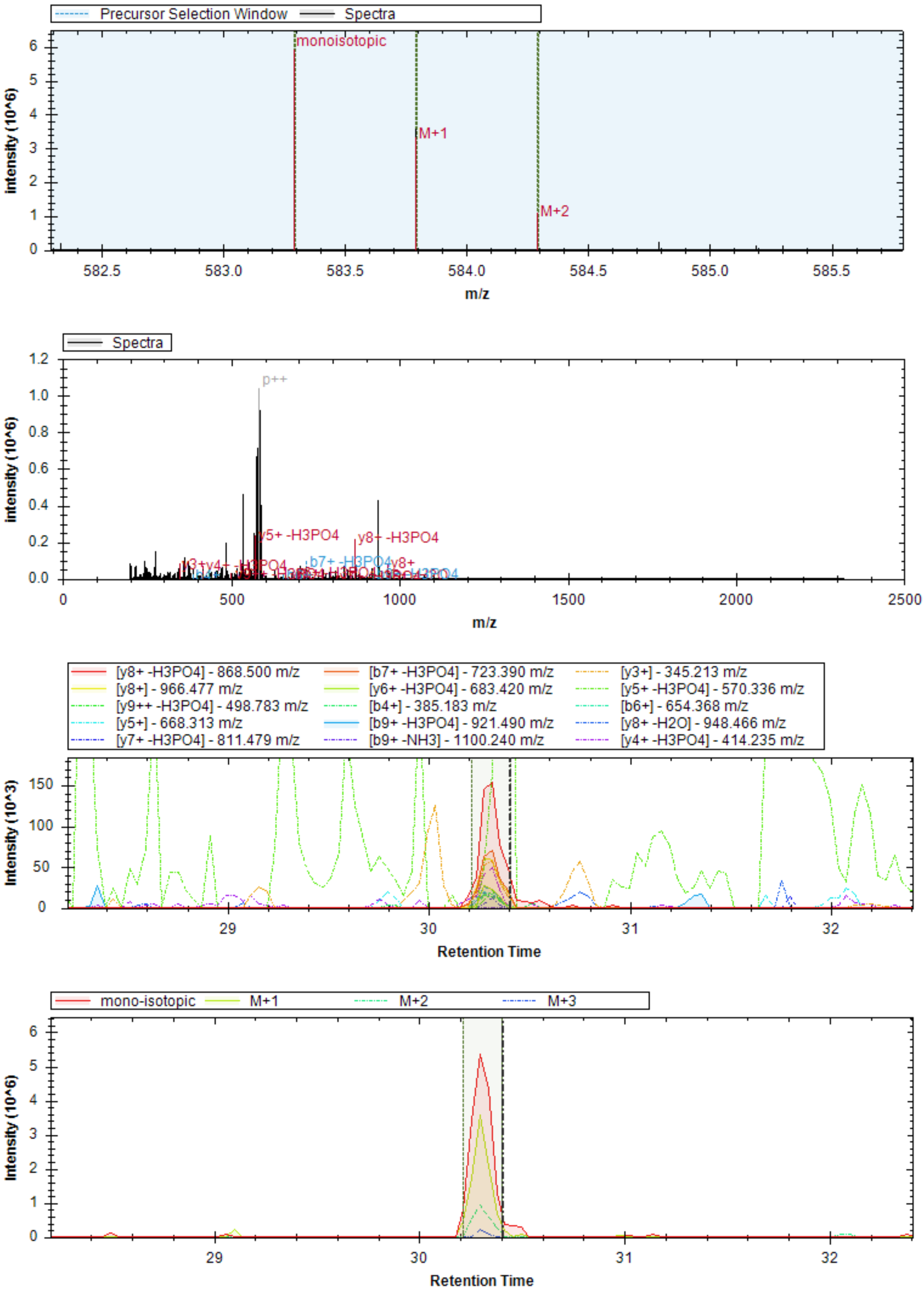

Q9D5Y0 – _ITDT[+H+O3+P]KKTDDLYWR_.3
Fig4_MP-DIA-barrel-cortex-p15-3_MHRM.htrms

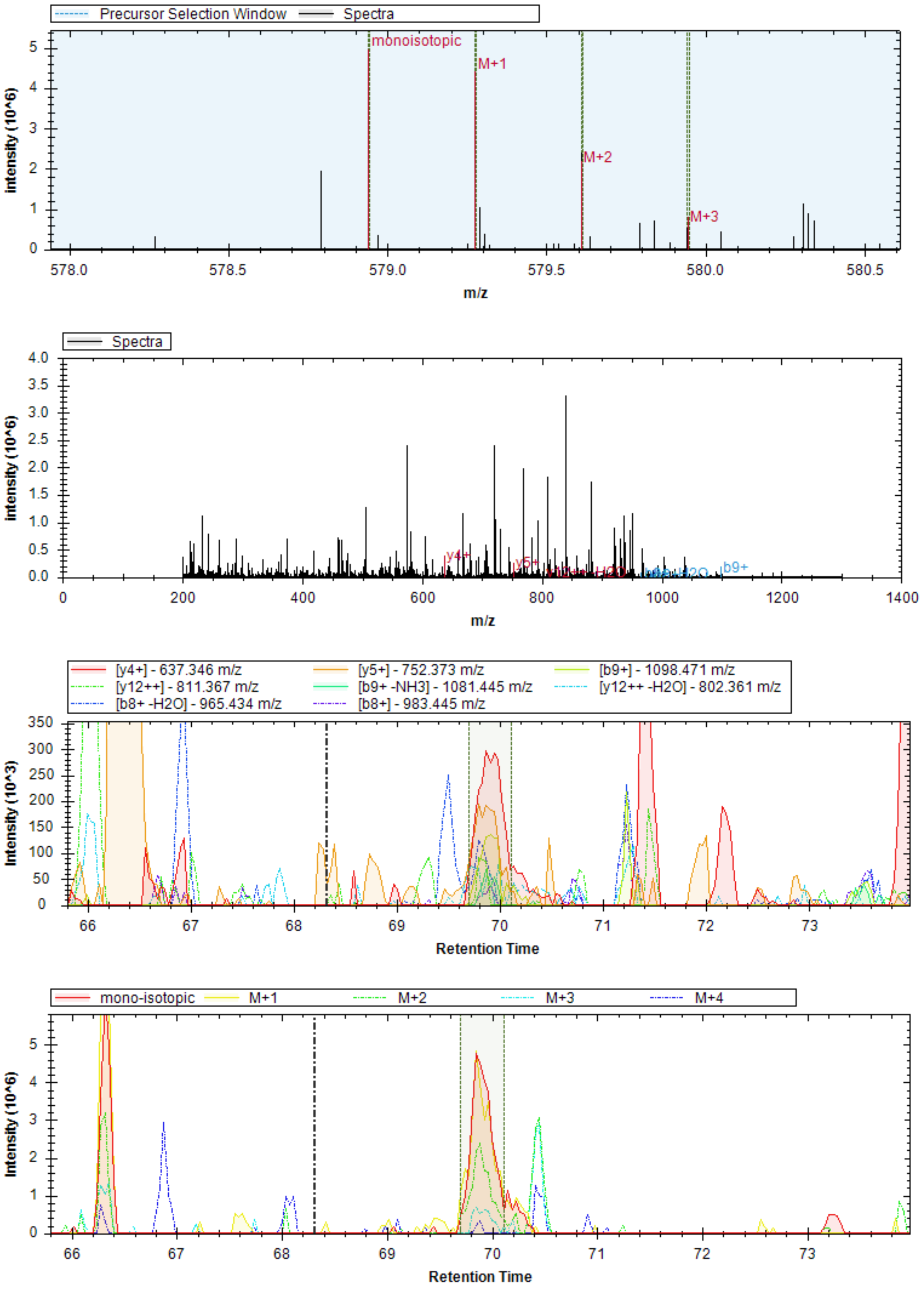

Q9DC63;Q9DC63-2;Q9DC63-3 – _YIDHY[+H+O3+P]AAIK_.2
Fig4_MP-DIA-barrel-cortex-p15-3_MHRM.htrms

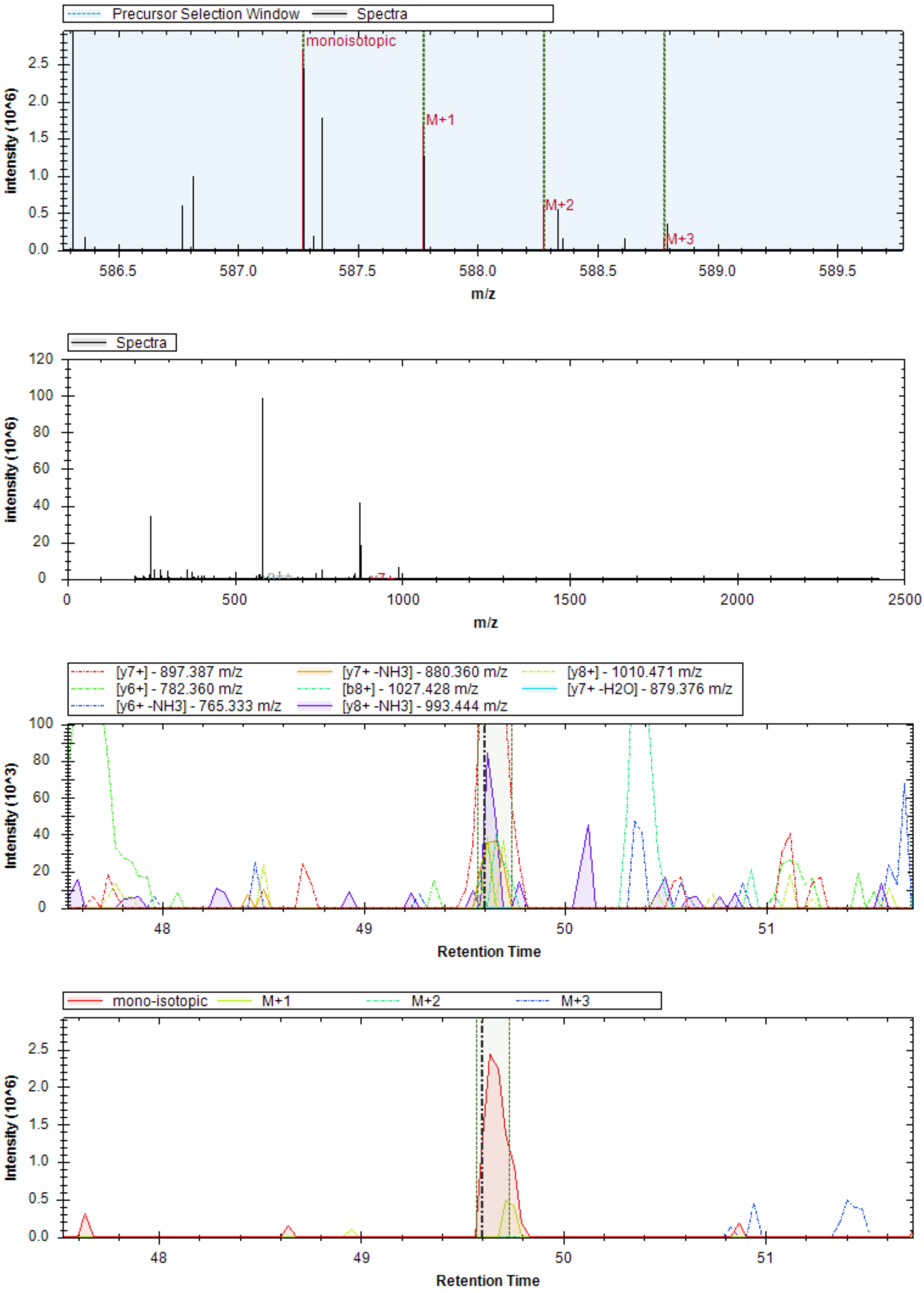

P20357 – _APHWTSASLTEAAAHPHS[+H+O3+P]PEMK_.4
Fig4_MP-DIA-barrel-cortex-p15-3_MHRM.htrms

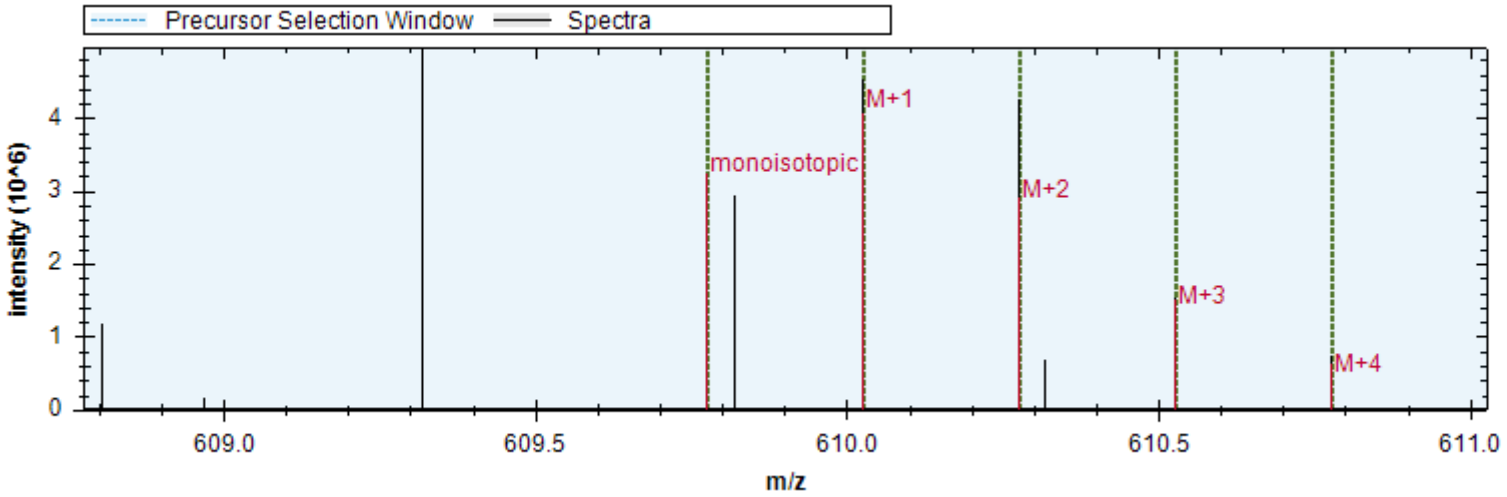

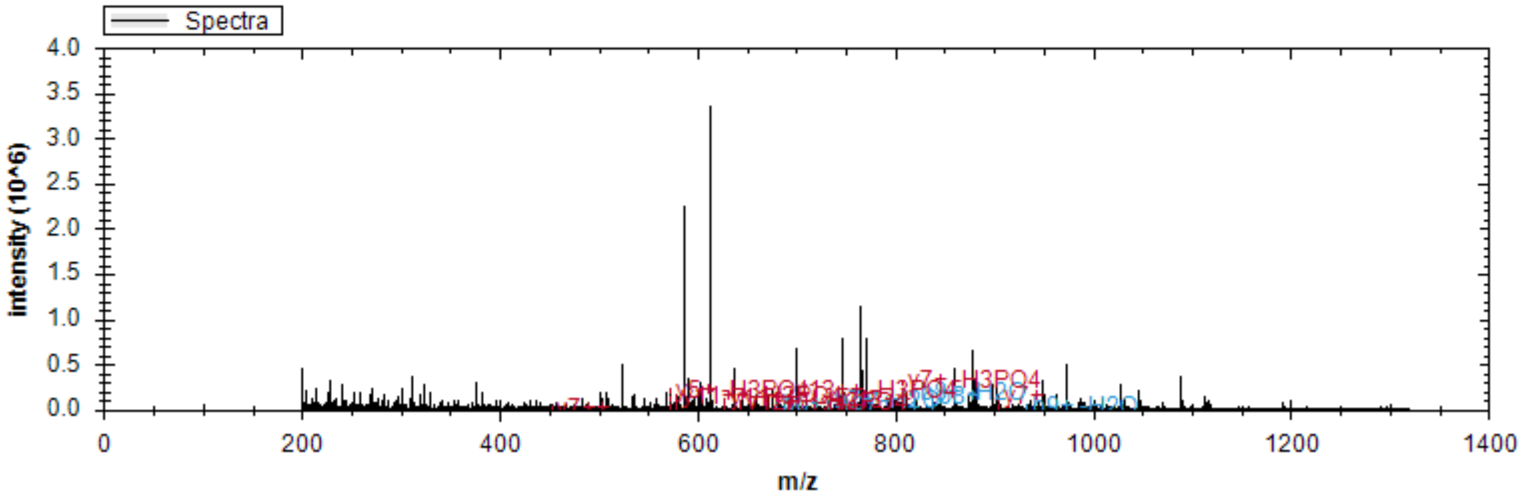

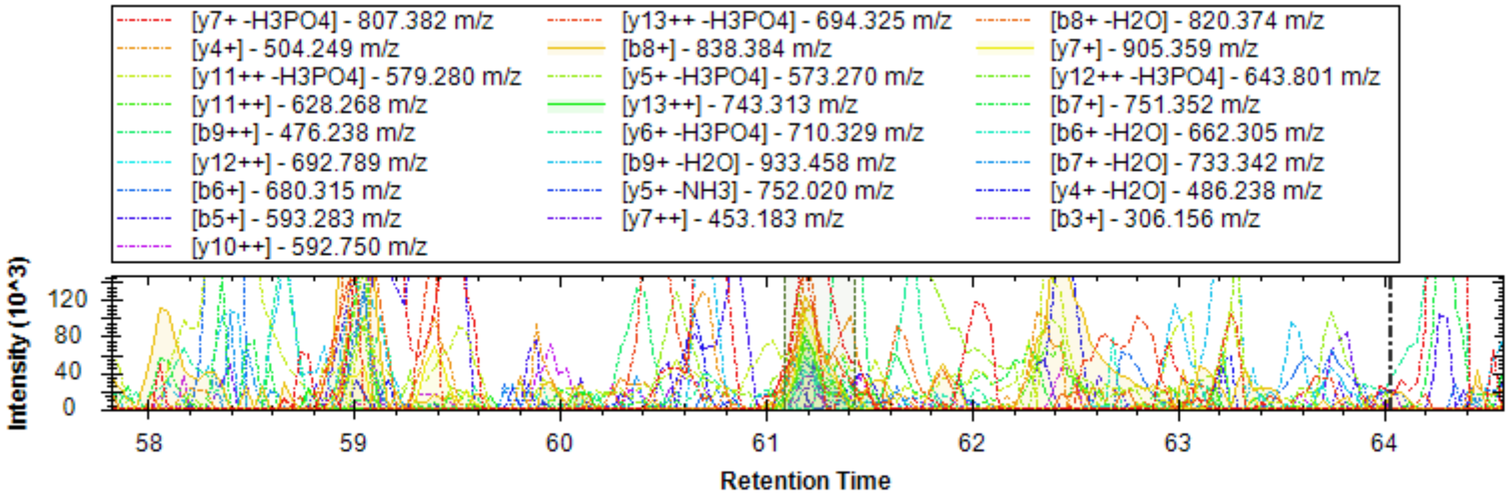

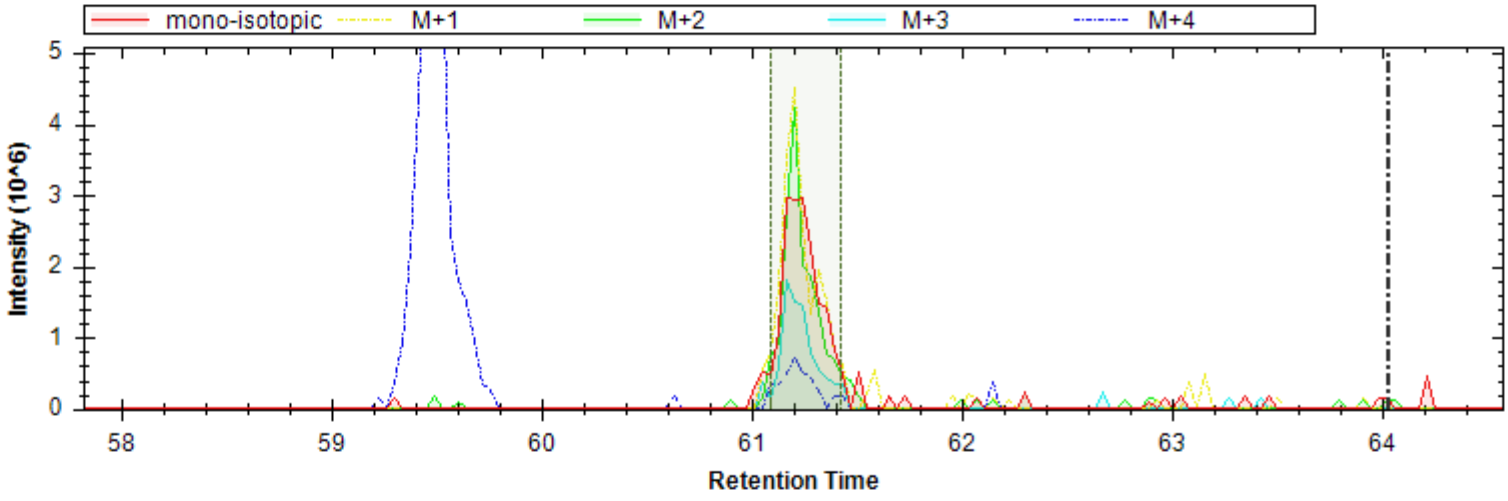

P26645 – _AEDGAAPSPSS[+H+O3+P]ETPKKK_.3
Fig4_MP-DIA-barrel-cortex-p15-3_MHRM.htrms

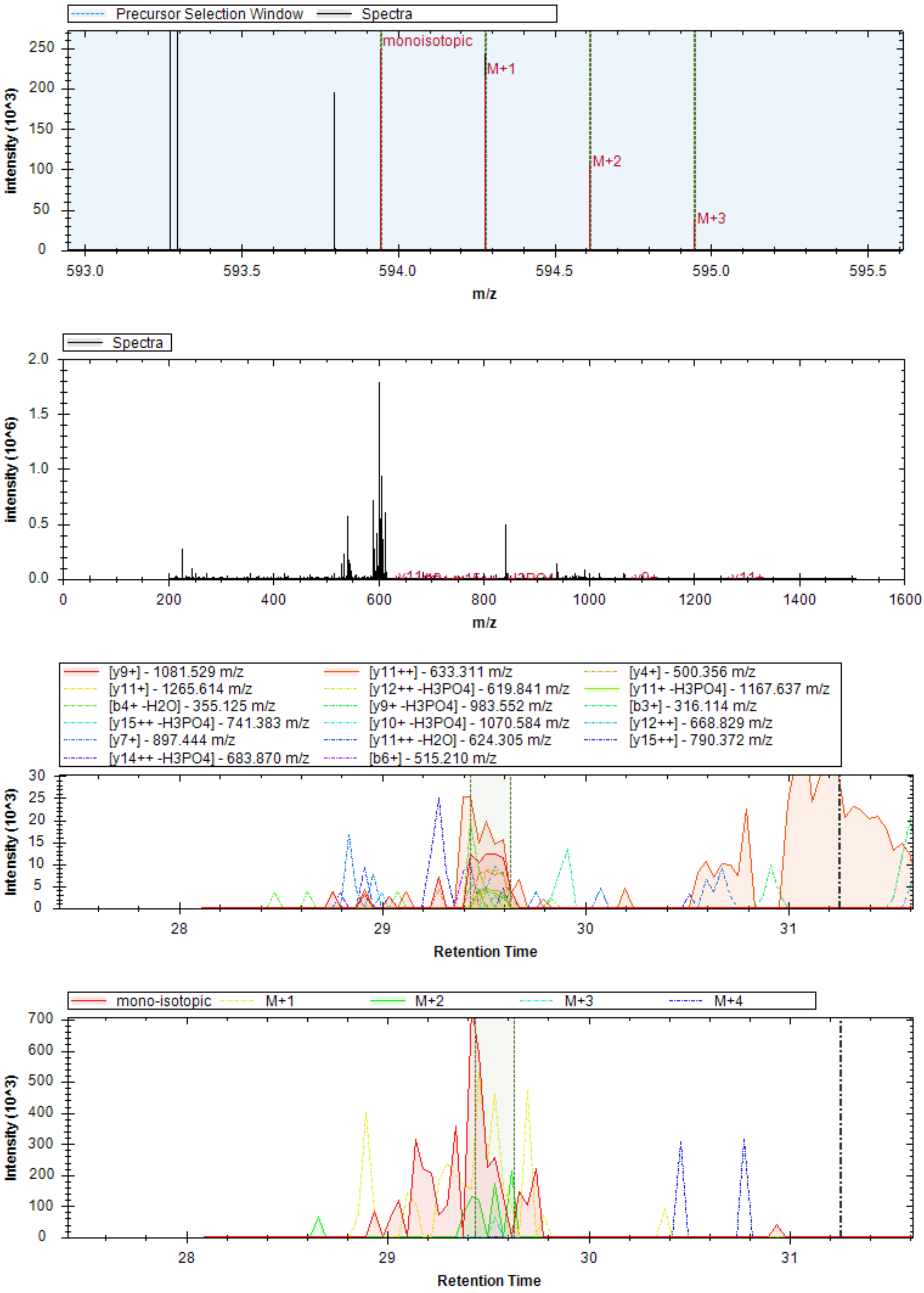

Q62261 – _RPPS[+H+O3+P]PDPNTK_.2

Fig4_MP-DIA-barrel-cortex-p15-3_MHRM.htrms

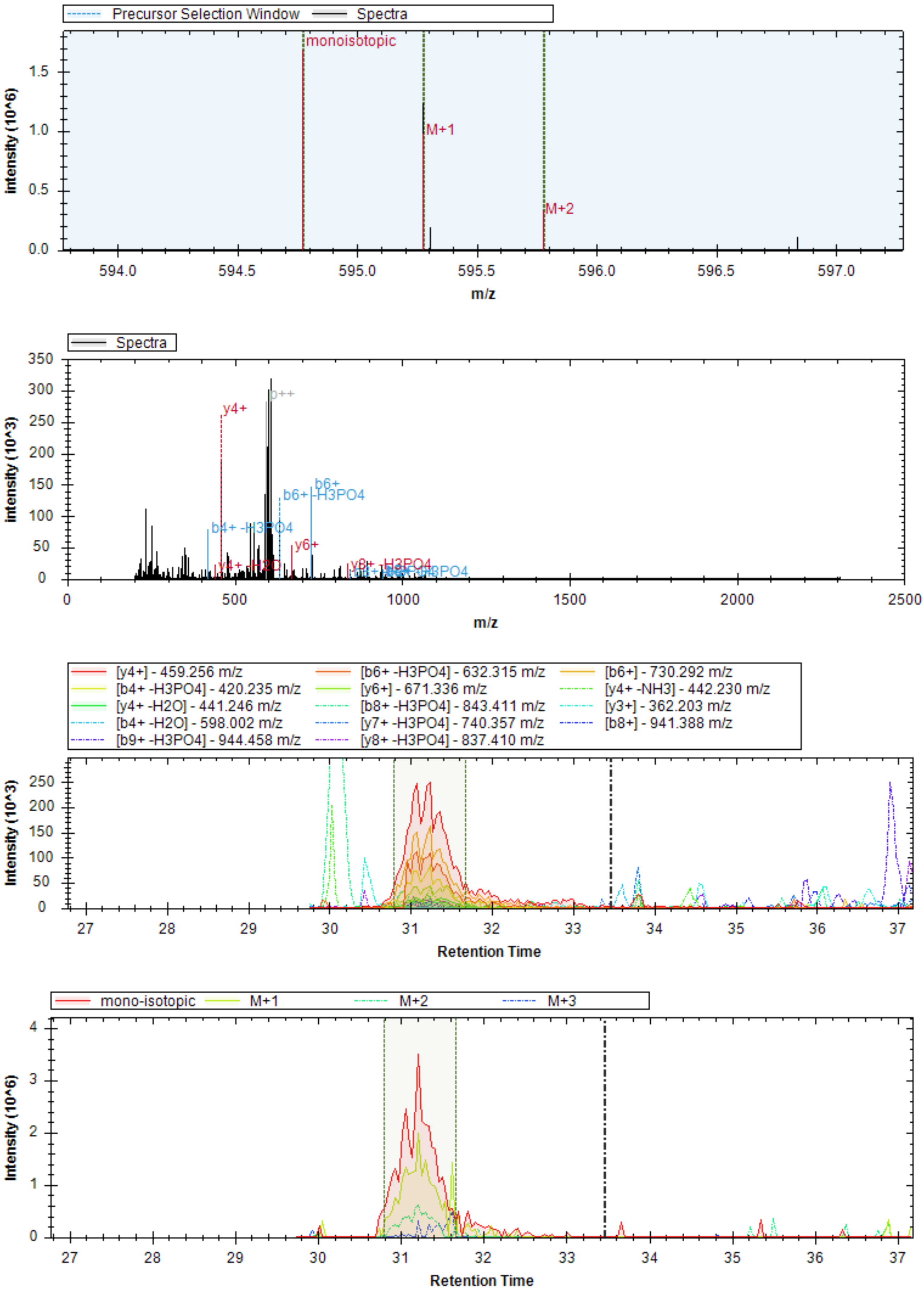

Q80VN0 – _ELTAQIT[+H+O3+P]SIK_.2

Fig4_MP-DIA-barrel-cortex-p15-3_MHRM.htrms

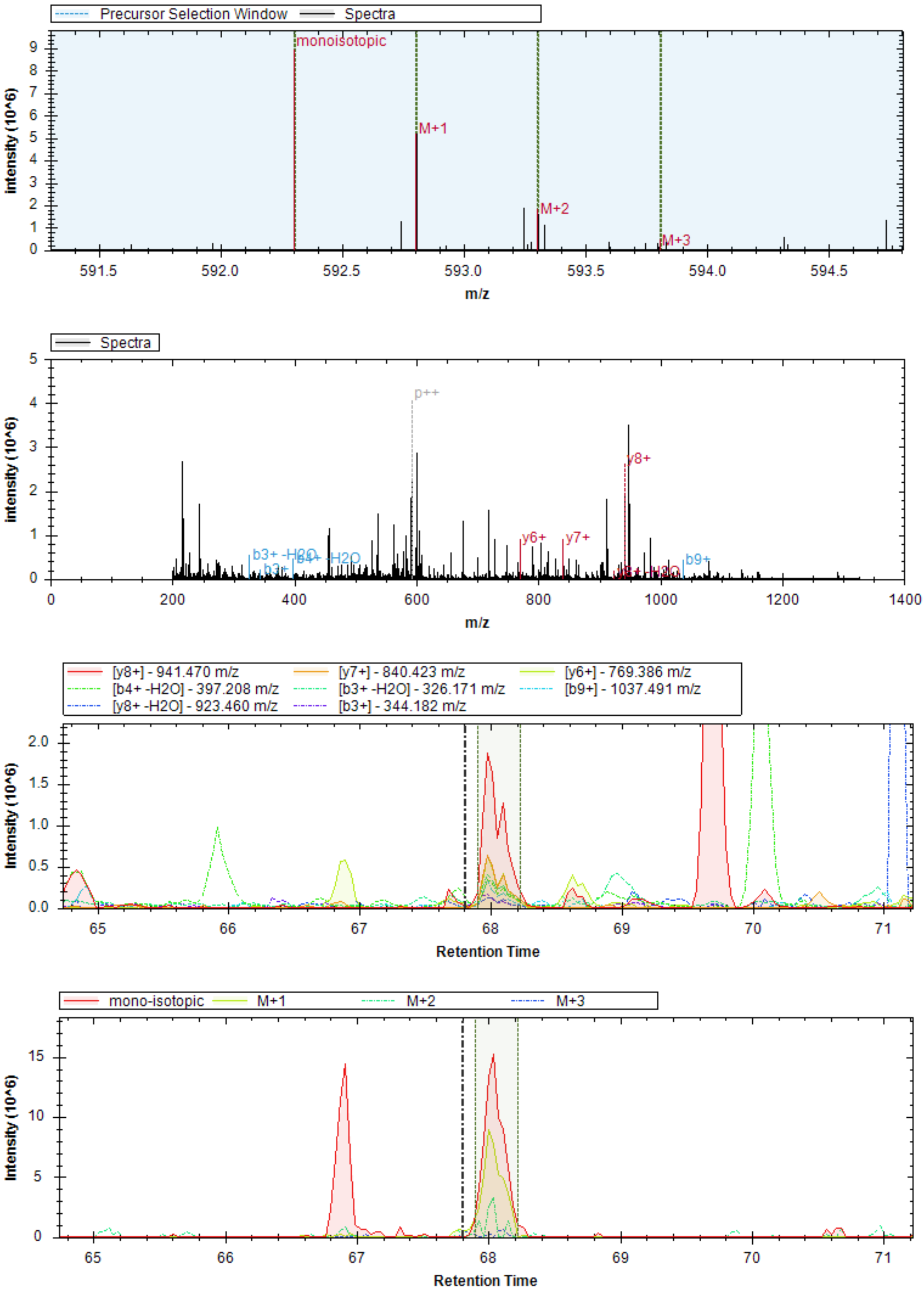

Q8C419 – _SAS[+H+O3+P]AHNLSSEK_.2
Fig4_MP-DIA-barrel-cortex-p15-3_MHRM.htrms

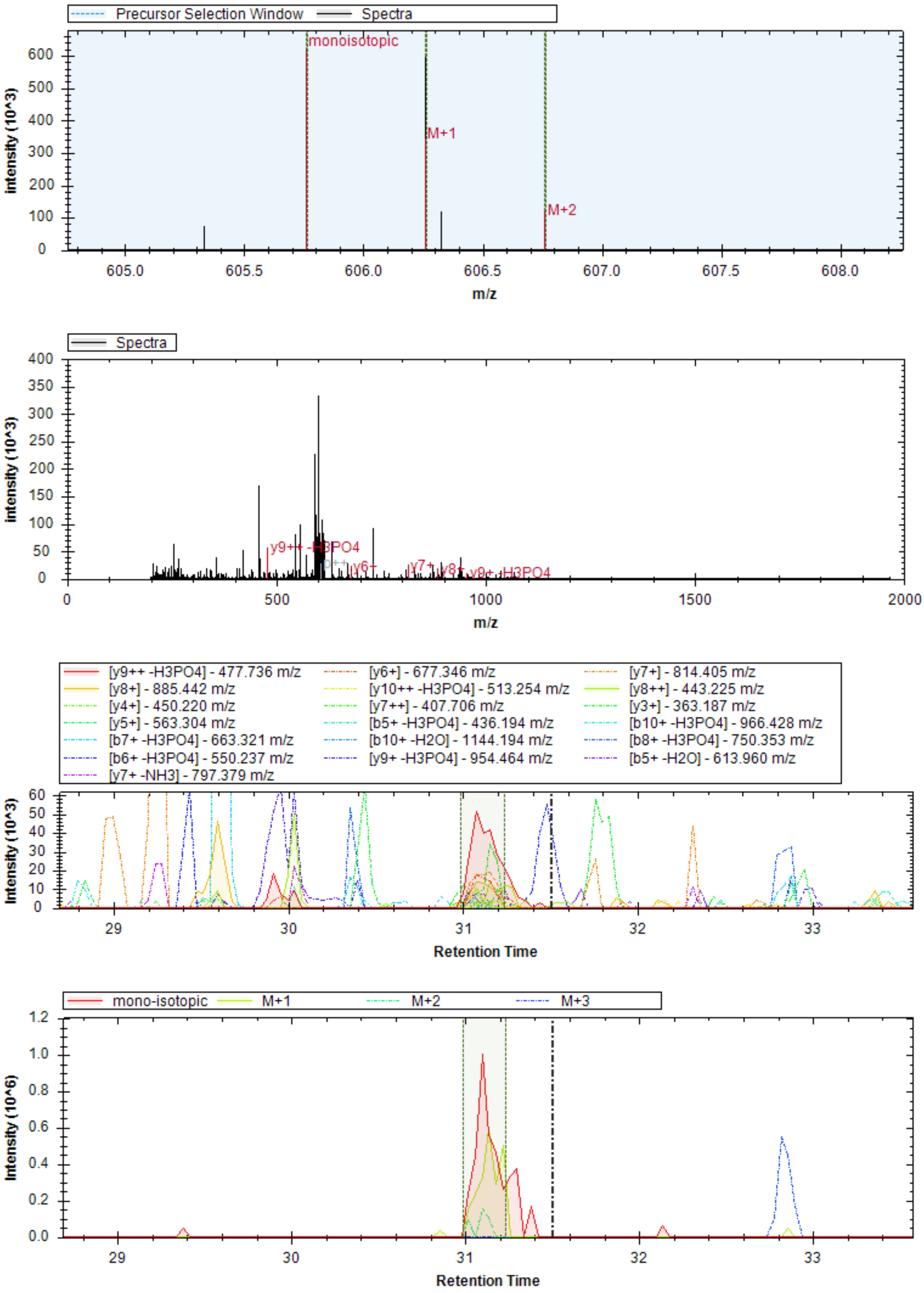

Q99104 – _AIS[+H+O3+P]PTSATSSGR_.2
Fig4_MP-DIA-barrel-cortex-p15-3_MHRM.htrms

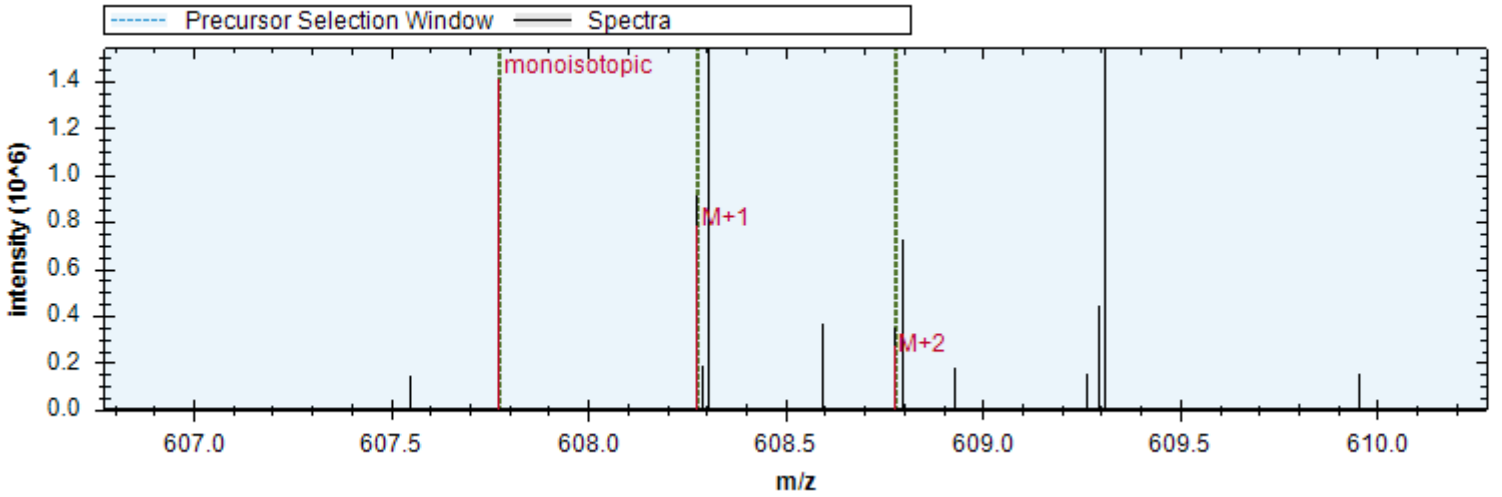

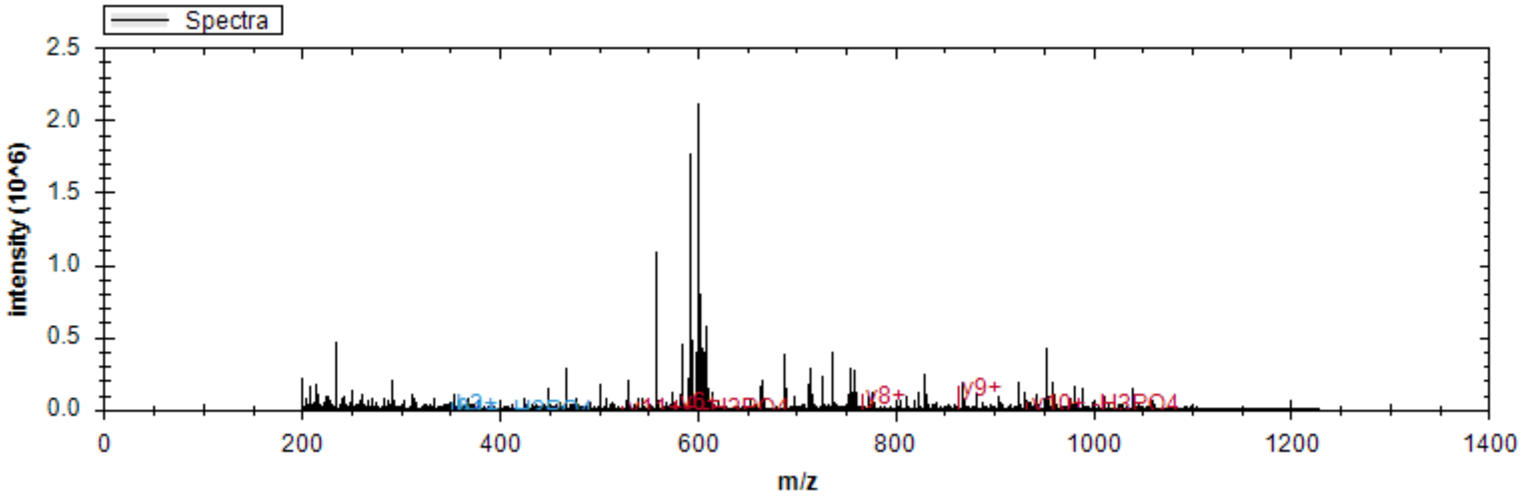

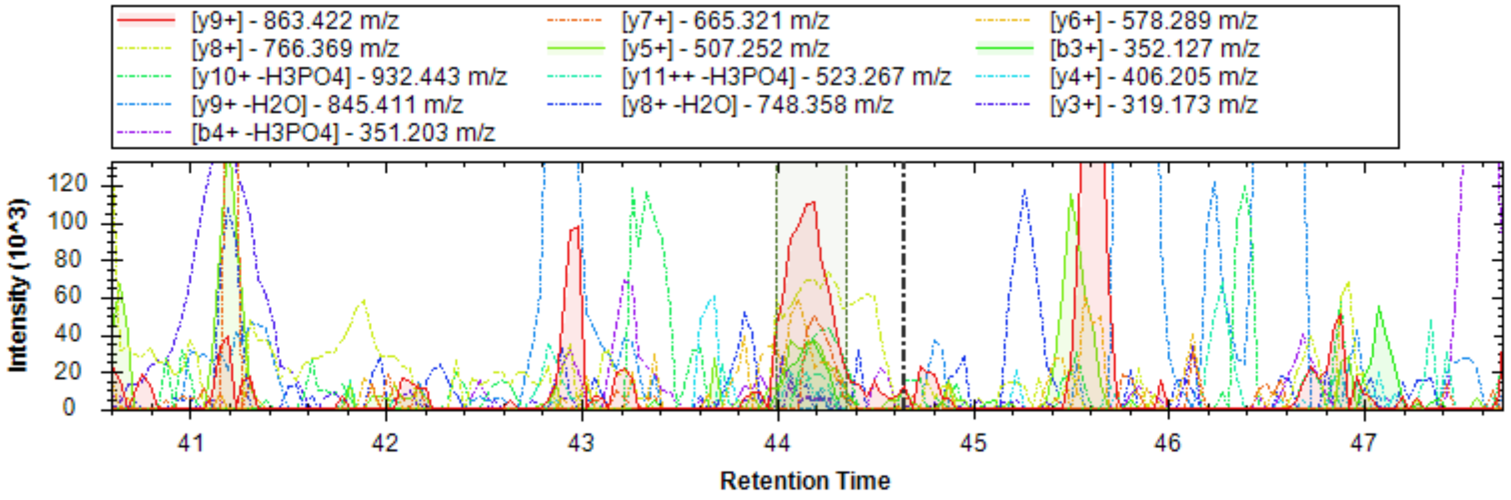

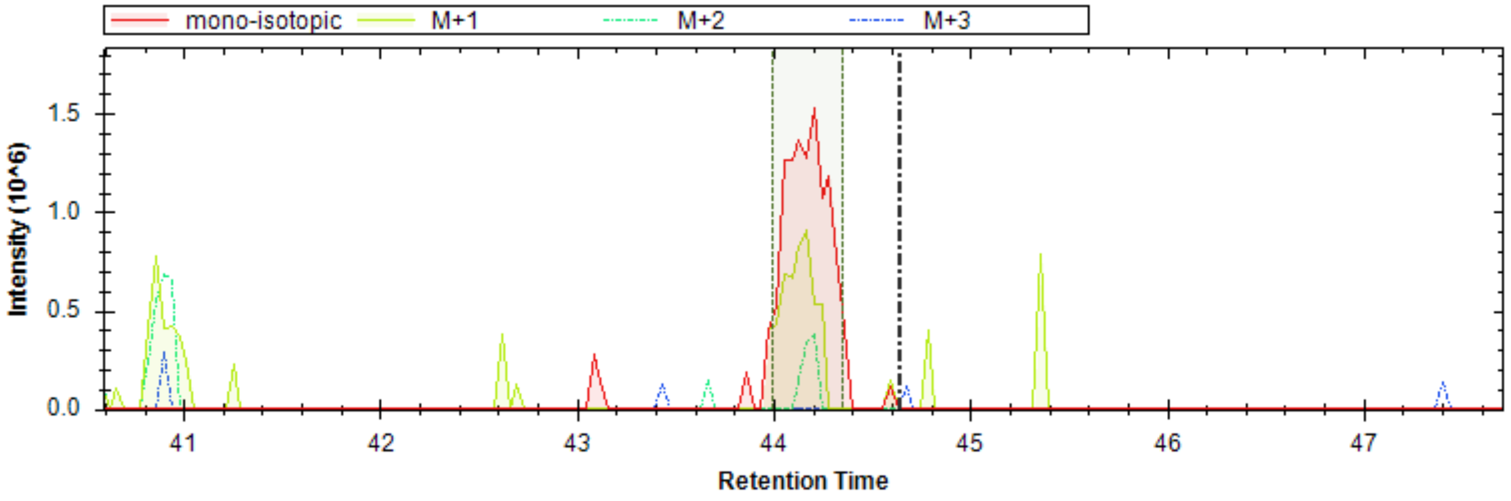

Q9EQF6 – _DLHES[+H+O3+P]SFSLSGSQIDDHVPKR_.4

Fig4_MP-DIA-barrel-cortex-p15-3_MHRM.htrms

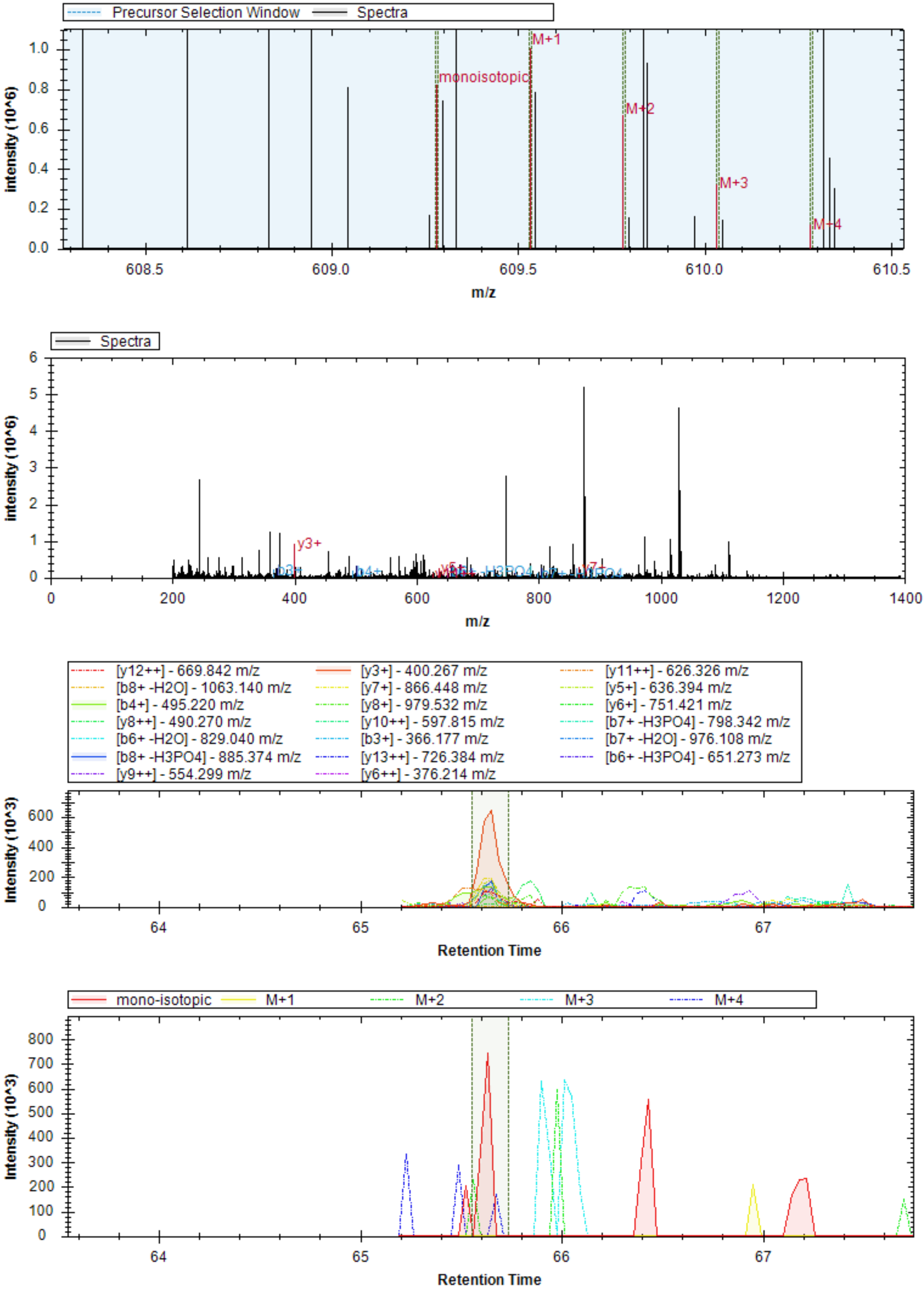

Q9EQF6 – _DLHESS[+H+O3+P]FSLSGSQIDDHVPKR_.4
Fig4_MP-DIA-barrel-cortex-p15-3_MHRM.htrms

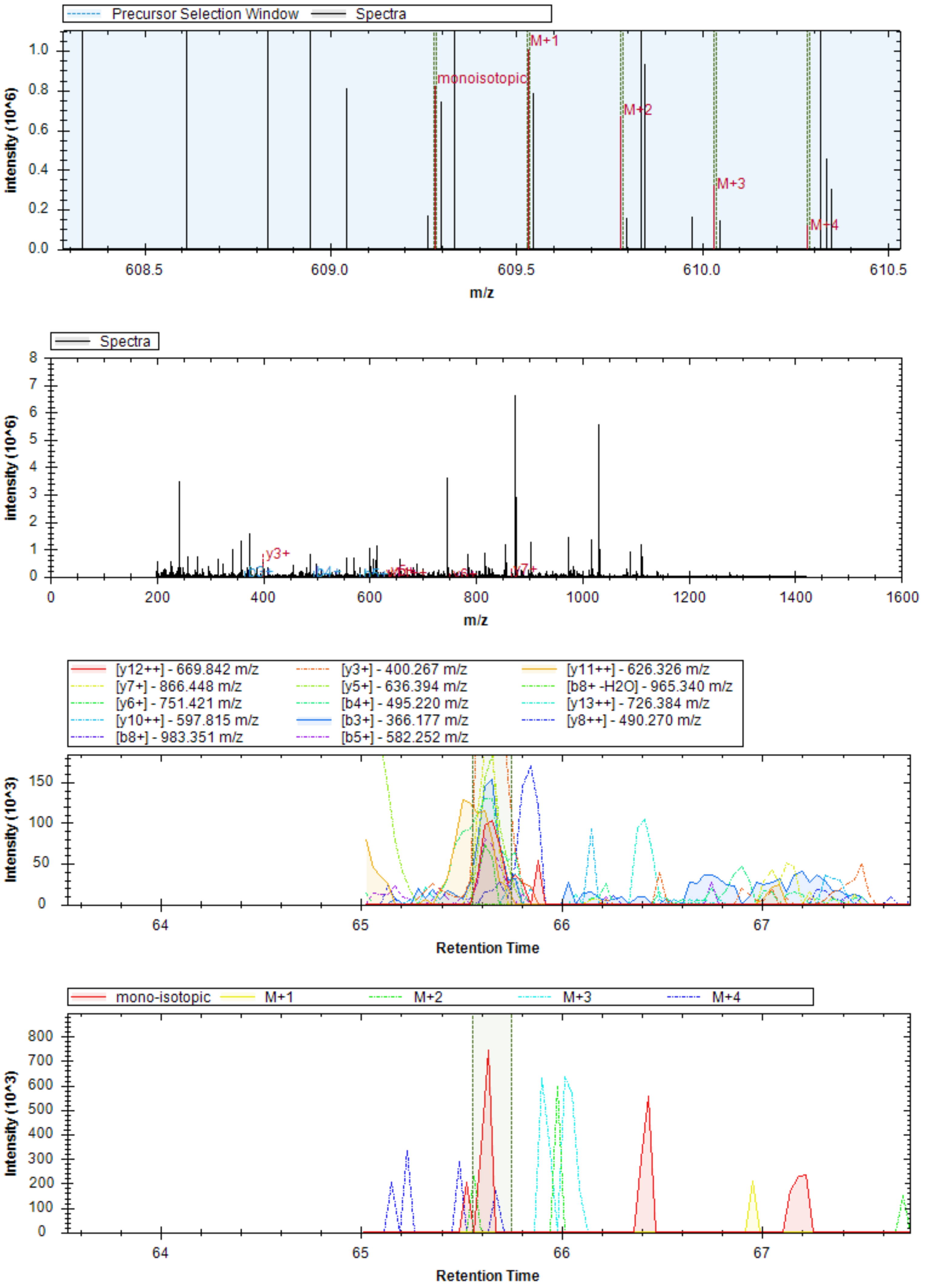

Q9QWI6 – _LSYAGGRPPSYAGS[+H+O3+P]PVHHAAER_.4

Fig4_MP-DIA-barrel-cortex-p15-3_MHRM.htrms

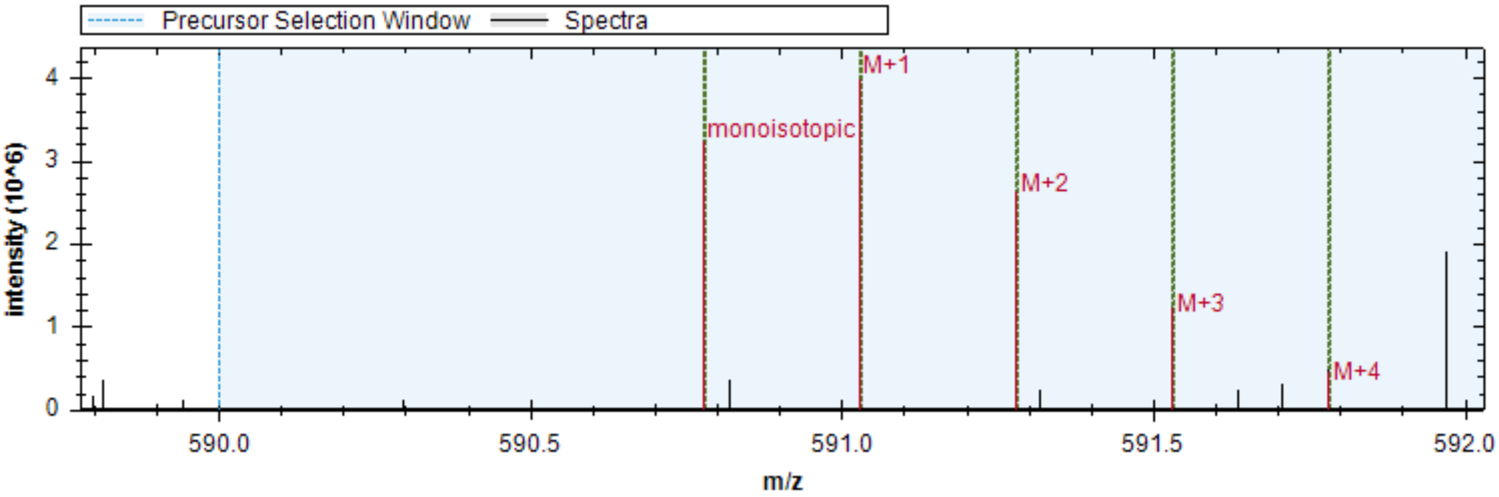

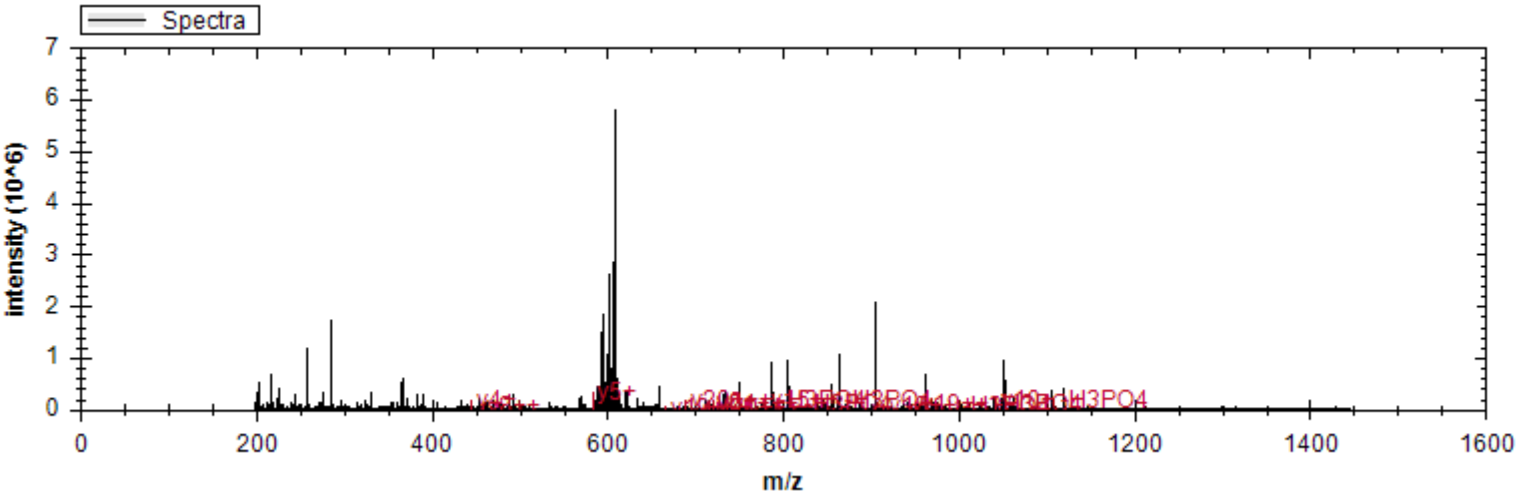

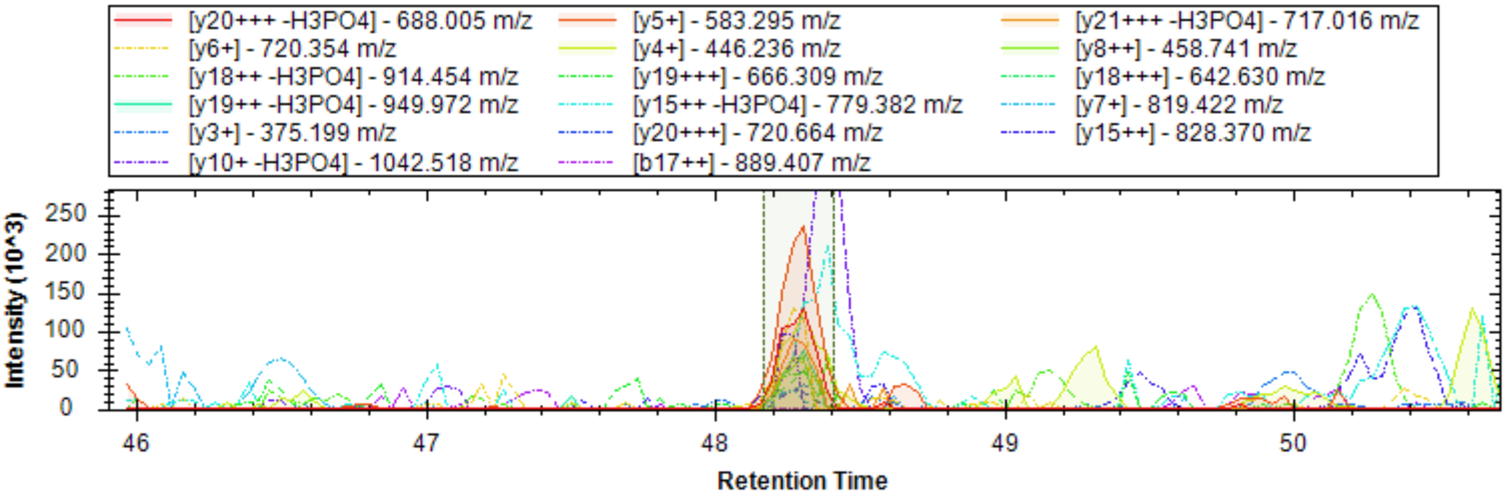

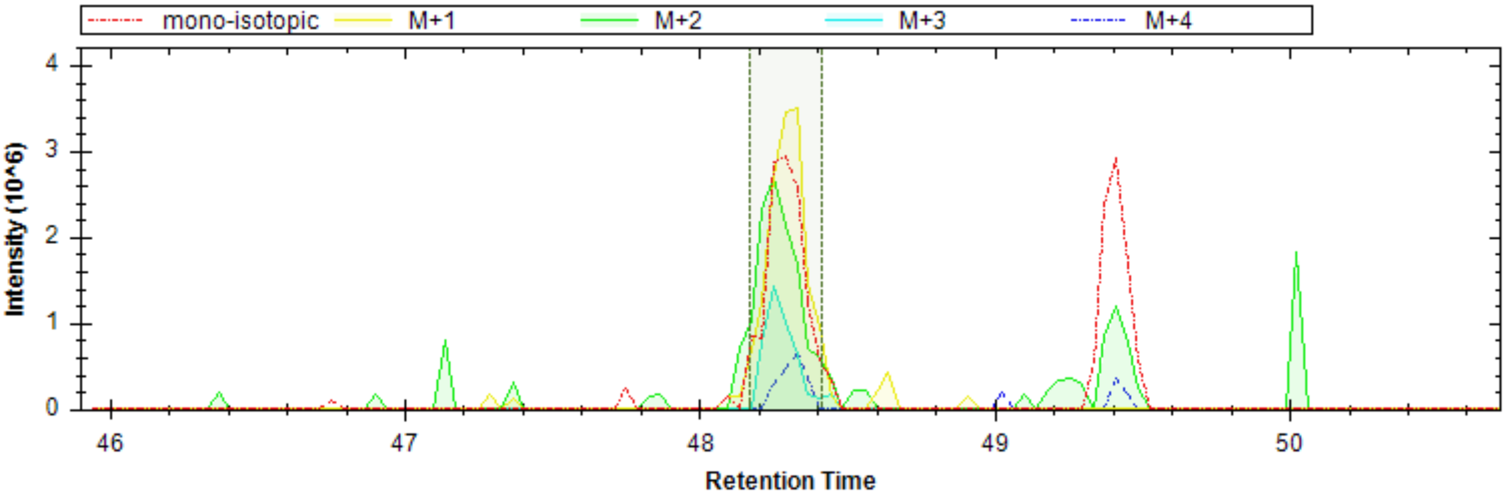

Q9Z2D7 – _KT[+H+O3+P]S[+H+O3+P]LYFSSK_.2

Fig4_MP-DIA-barrel-cortex-p15-3_MHRM.htrms

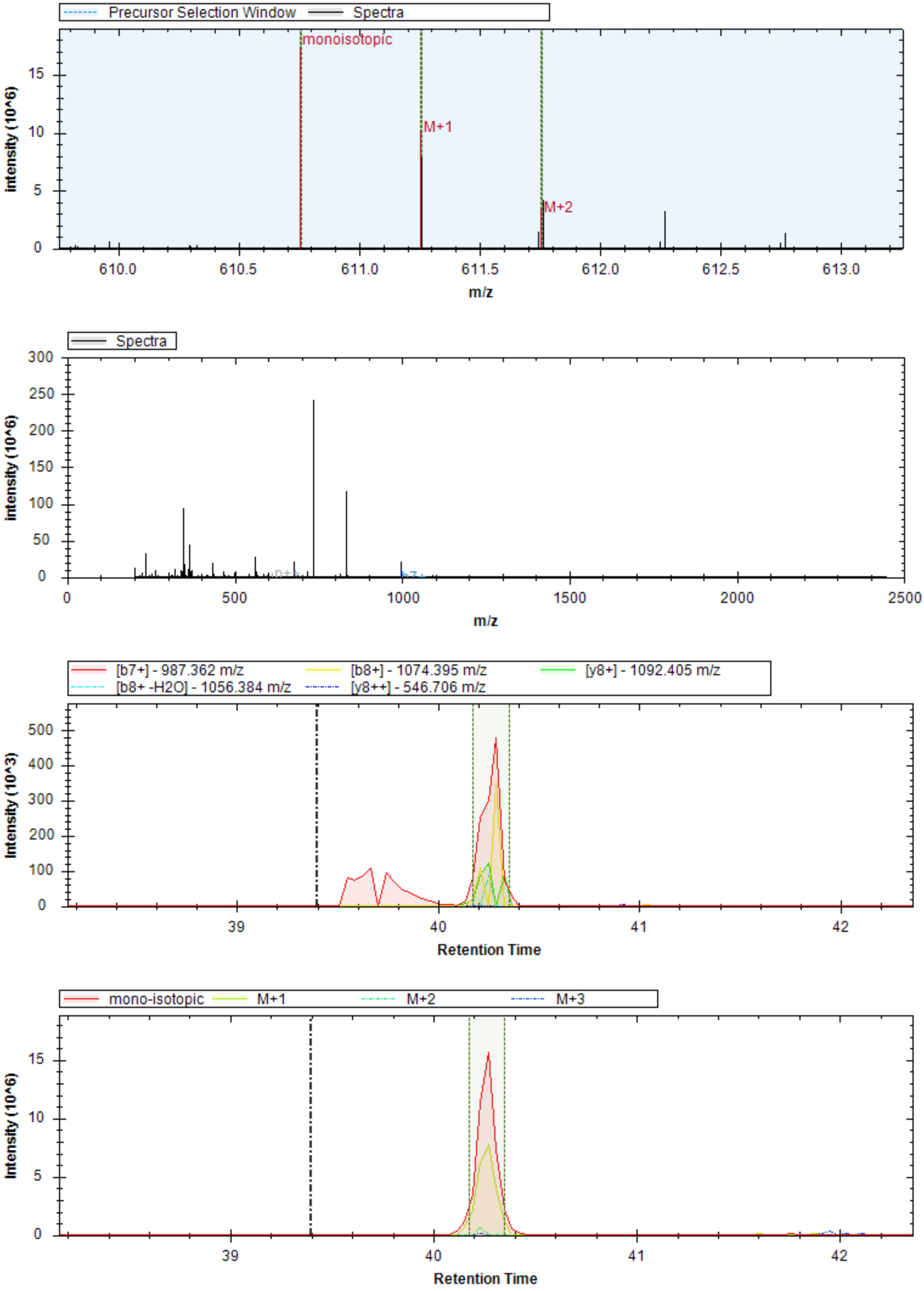

P26645 – _GEATAERPGEAAVASS[+H+O3+P]PSK_.3
Fig4_MP-DIA-barrel-cortex-p15-3_MHRM.htrms

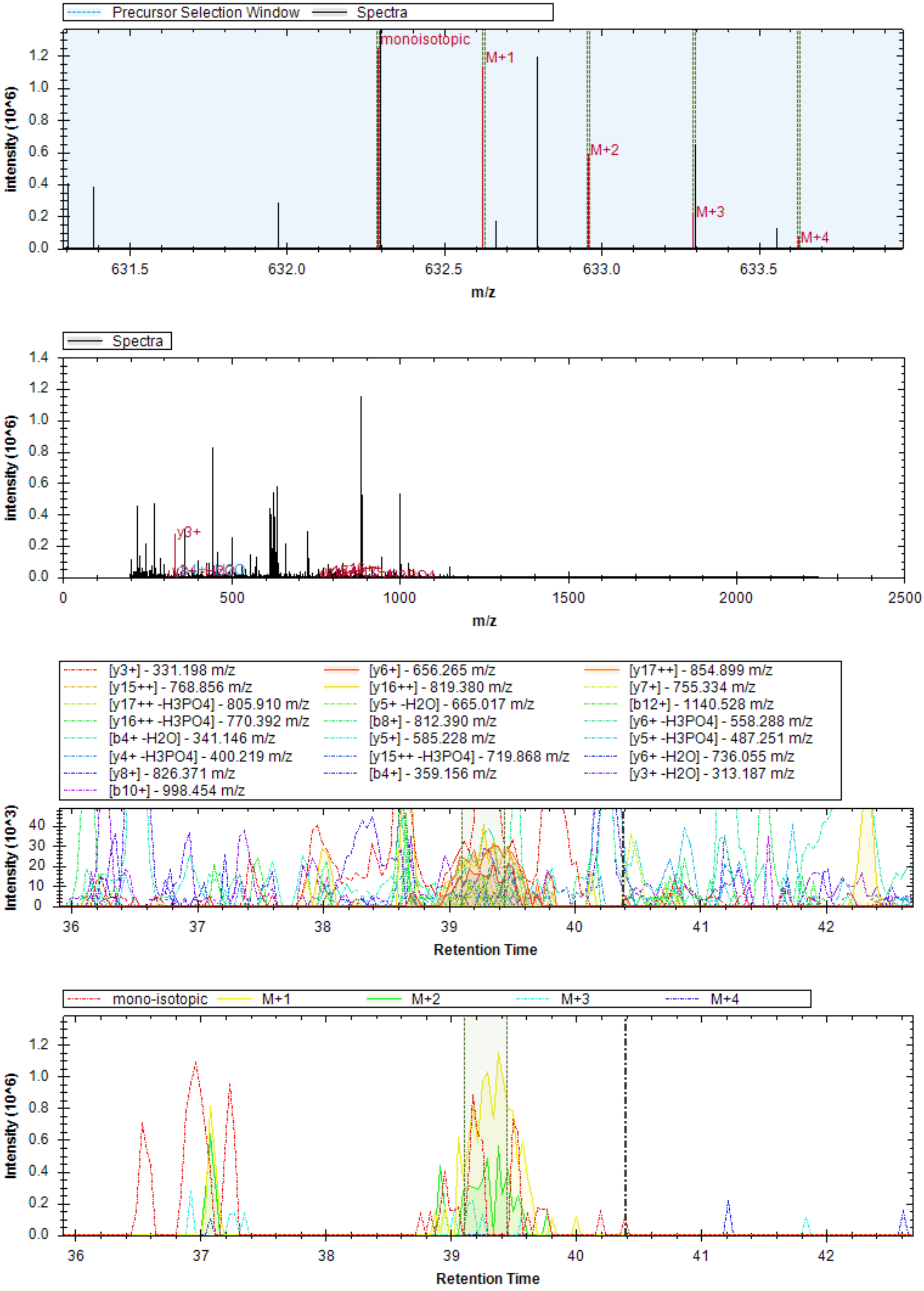

P60904 – _SLS[+H+O3+P]TSGESLYHVLGLDK_.3
Fig4_MP-DIA-barrel-cortex-p15-3_MHRM.htrms

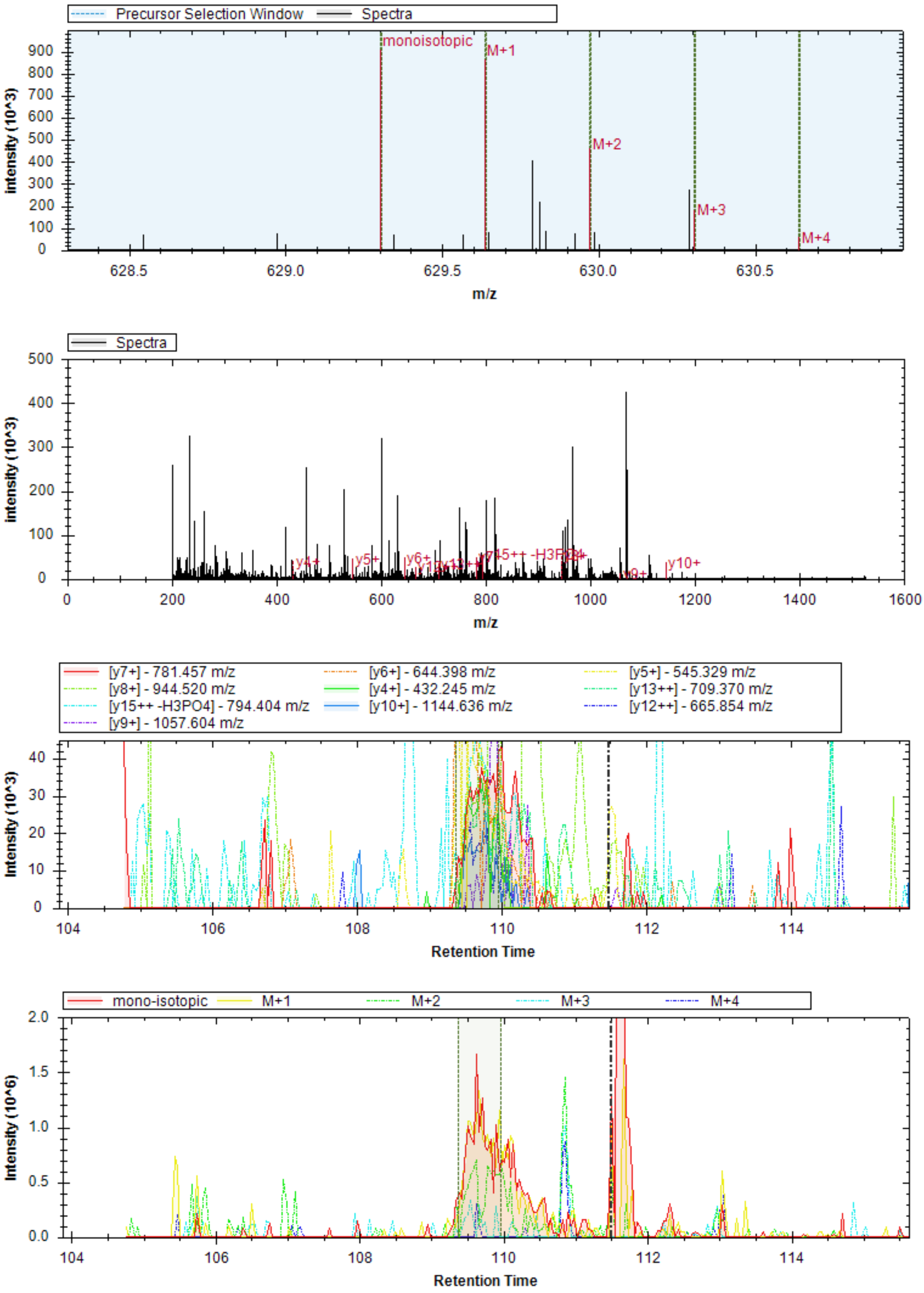

P60904 – _SLST[+H+O3+P]SGESLYHVLGLDK_.3

Fig4_MP-DIA-barrel-cortex-p15-3_MHRM.htrms

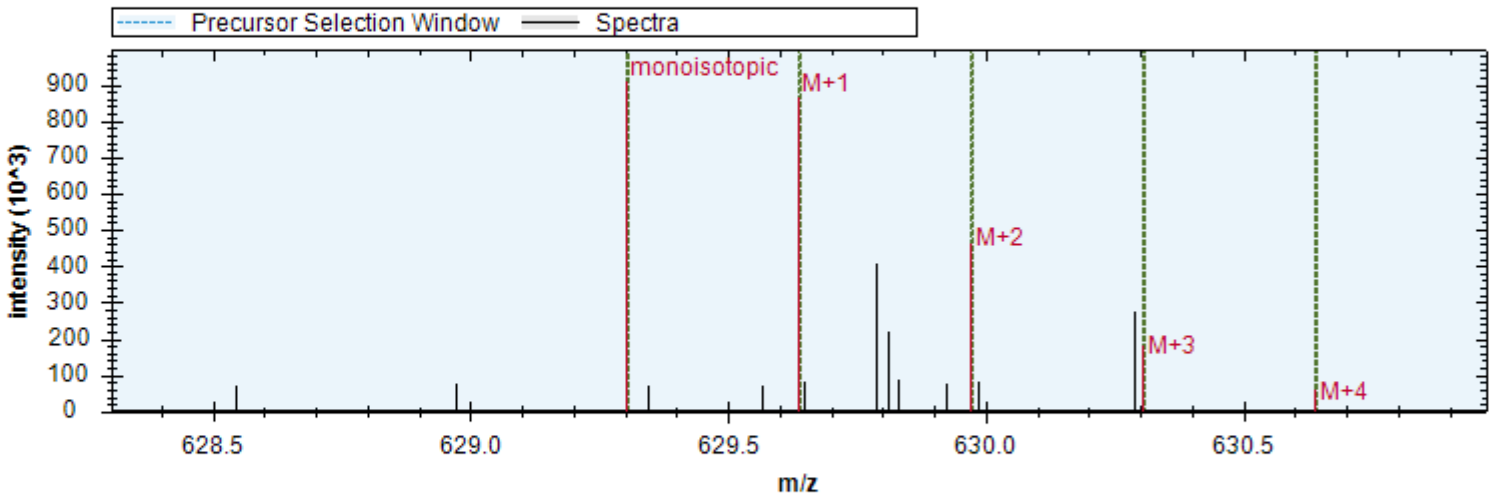

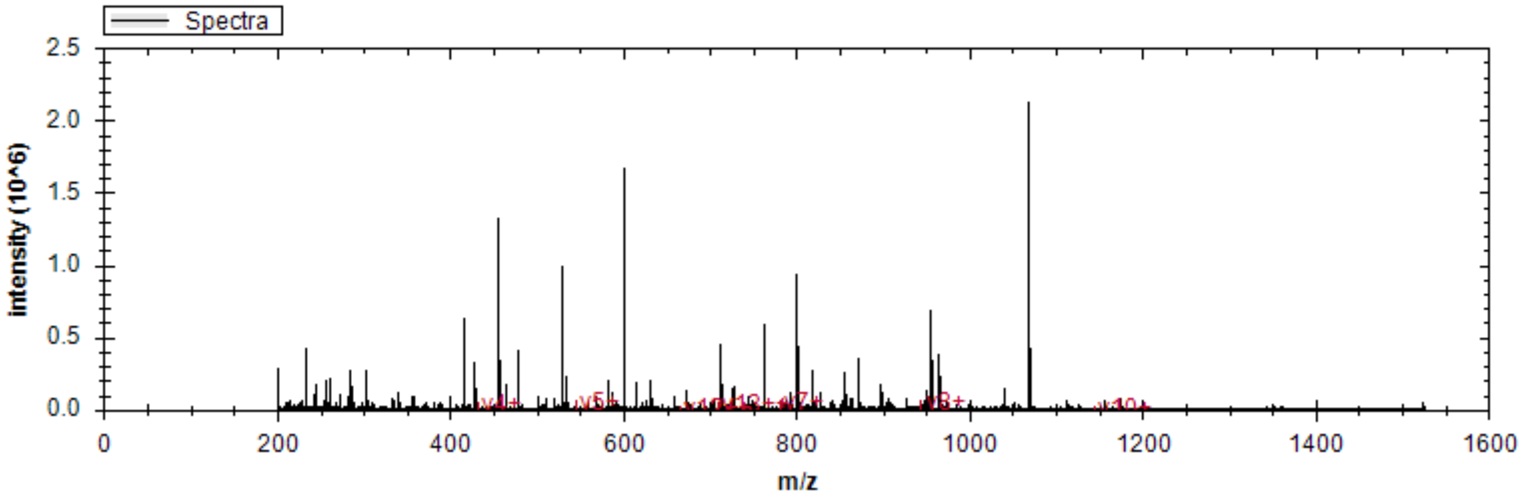

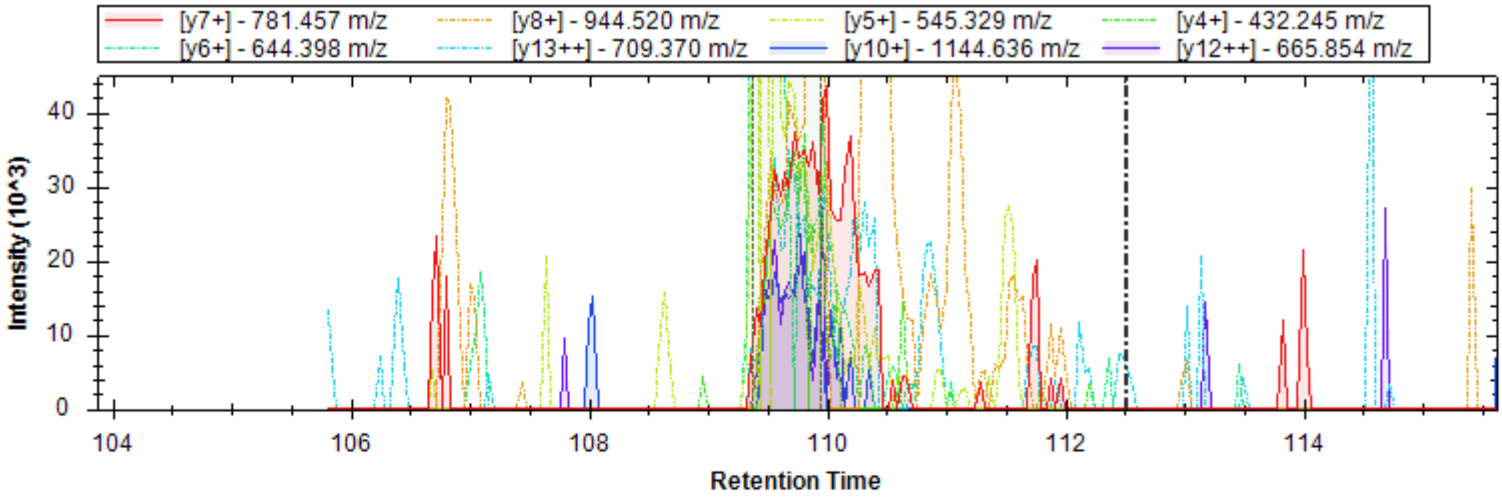

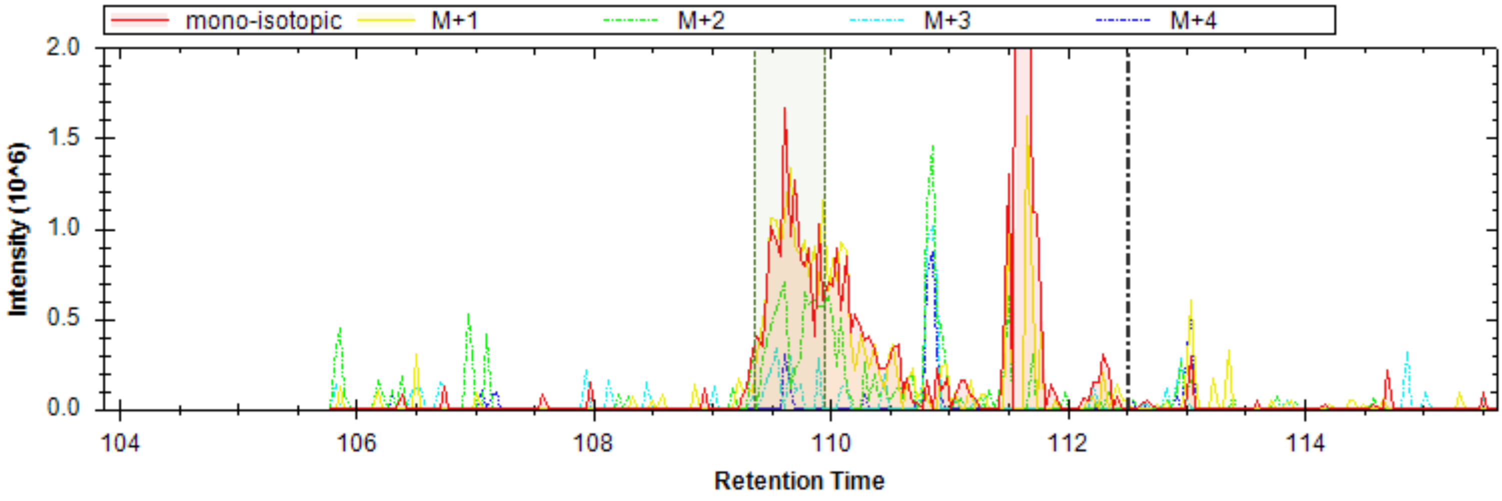

Q9CQS8 – _PGPTPSGTNVGSSGRS[+H+O3+P]PSK_.3
Fig4_MP-DIA-barrel-cortex-p15-3_MHRM.htrms

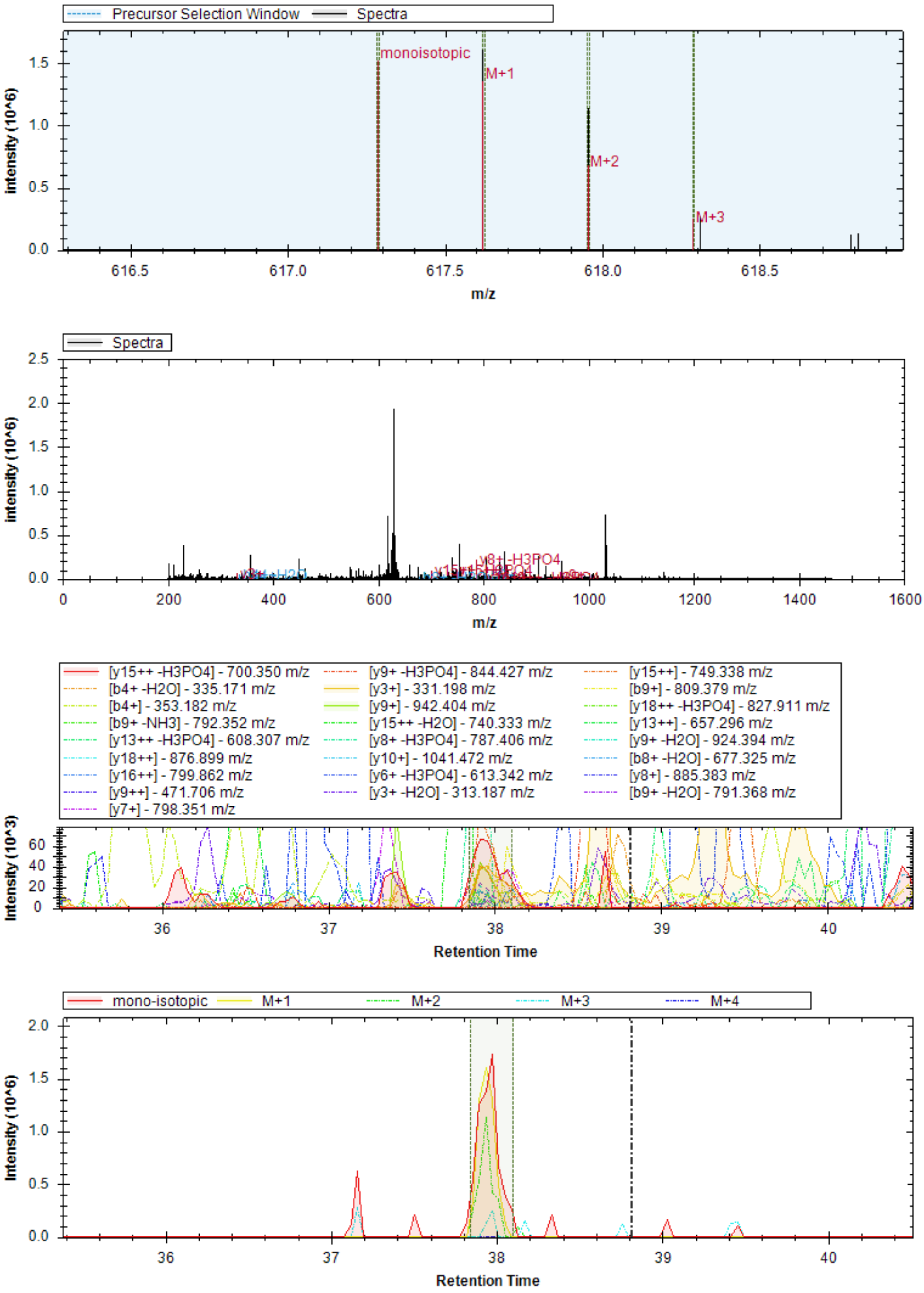

P20357 – _TPGT[+H+O3+P]PGTPSYPR_.2
Fig4_MP-DIA-barrel-cortex-p15-3_MHRM.htrms

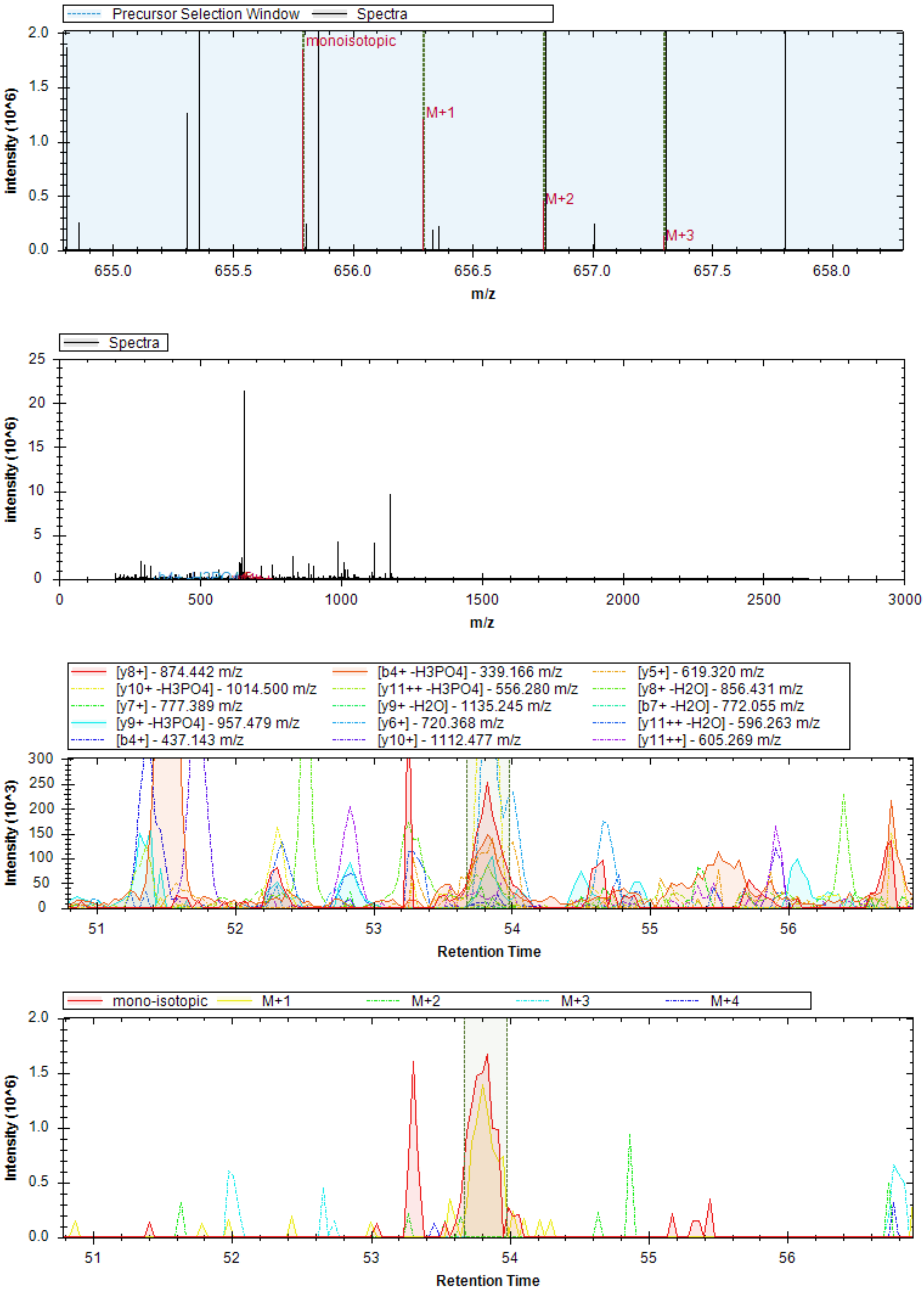

P39447 – _SSGIIRLHT[+H+O3+P]IK_.2
Fig4_MP-DIA-barrel-cortex-p15-3_MHRM.htrms

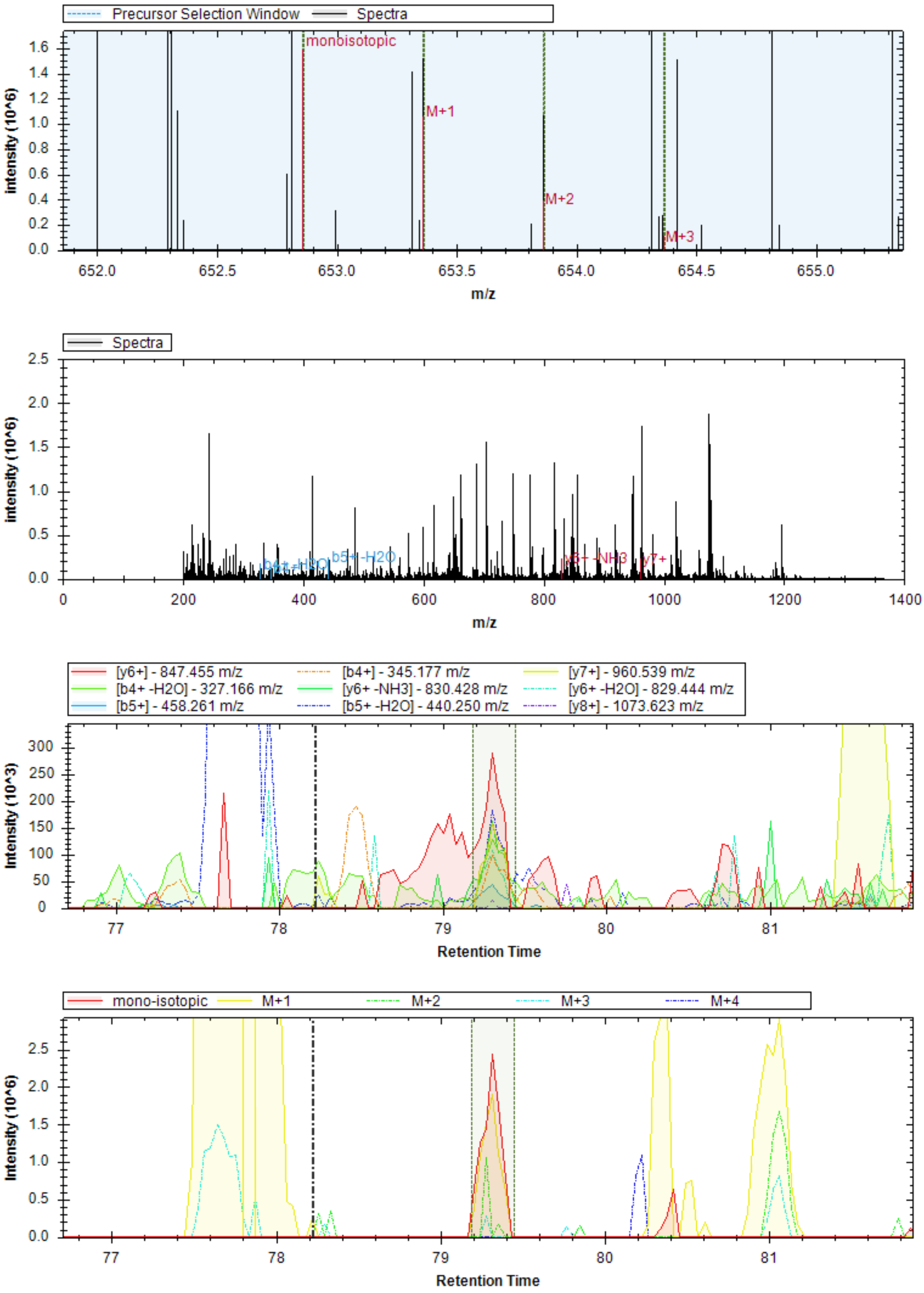

Q80XI3;Q80XI3-3 – _TSS[+H+O3+P]PTSLPPLAR_.2
Fig4_MP-DIA-barrel-cortex-p15-3_MHRM.htrms

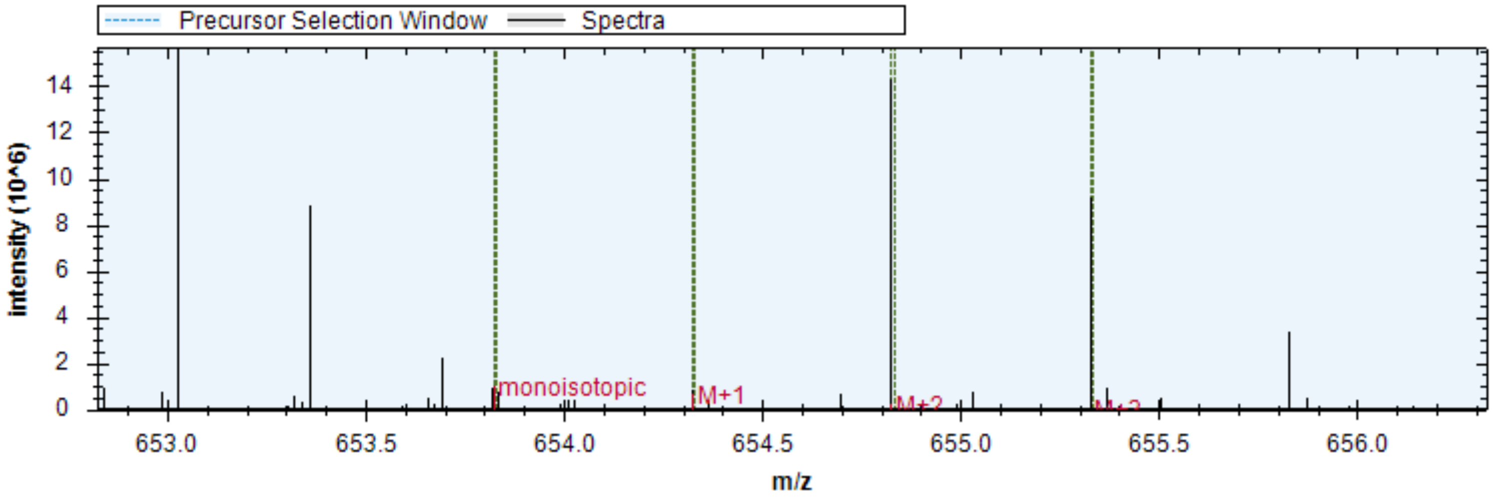

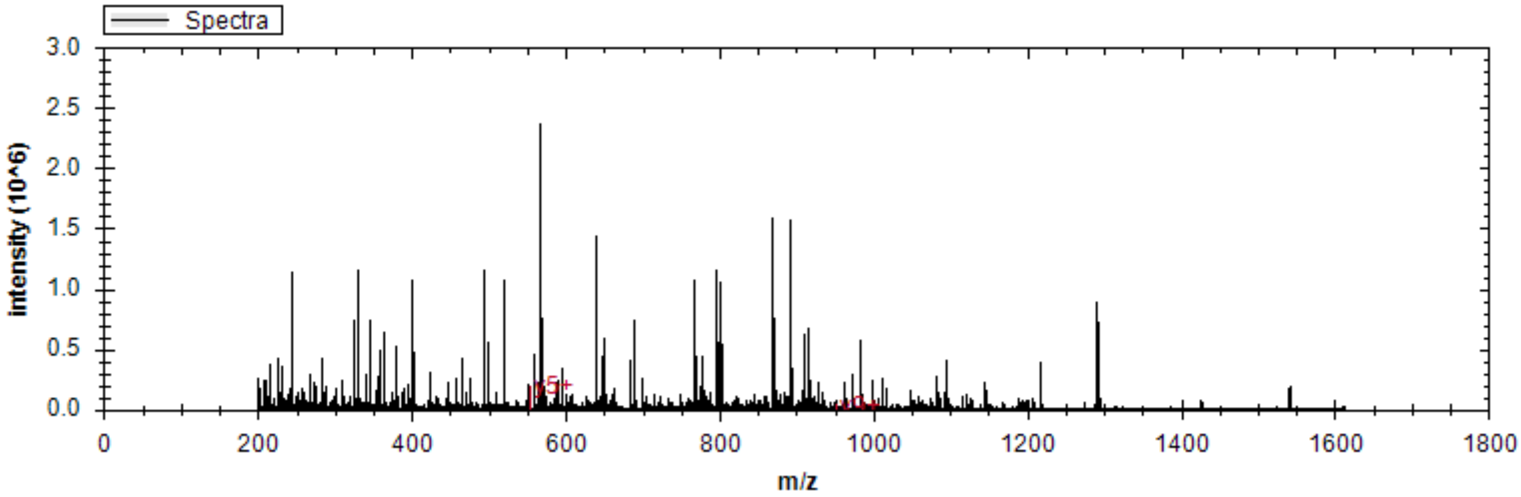

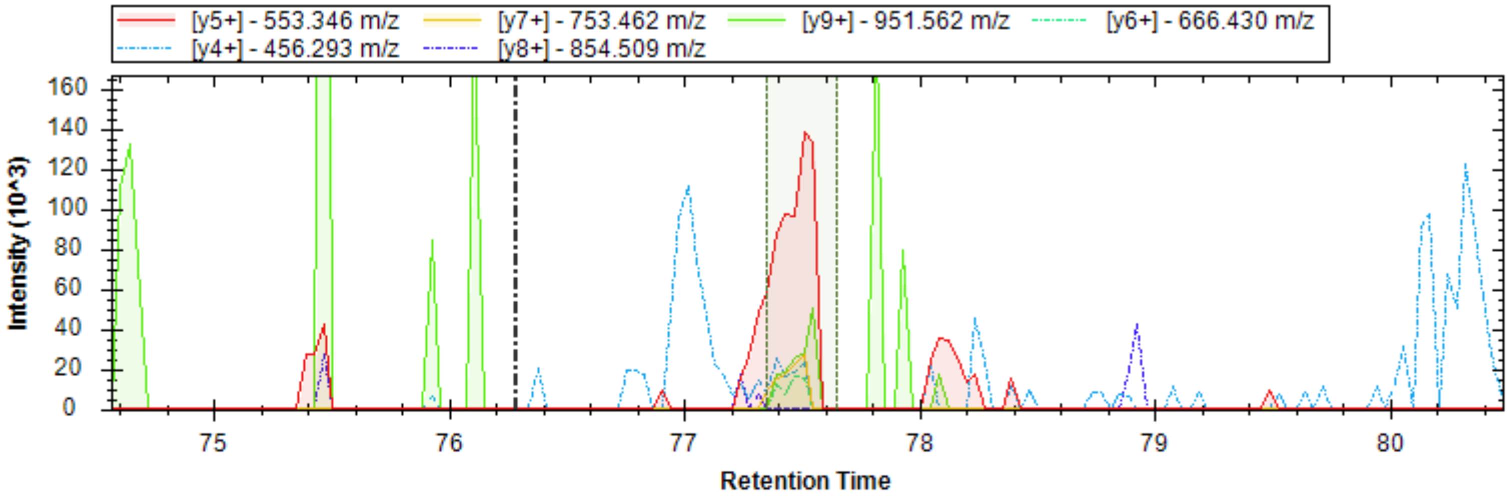

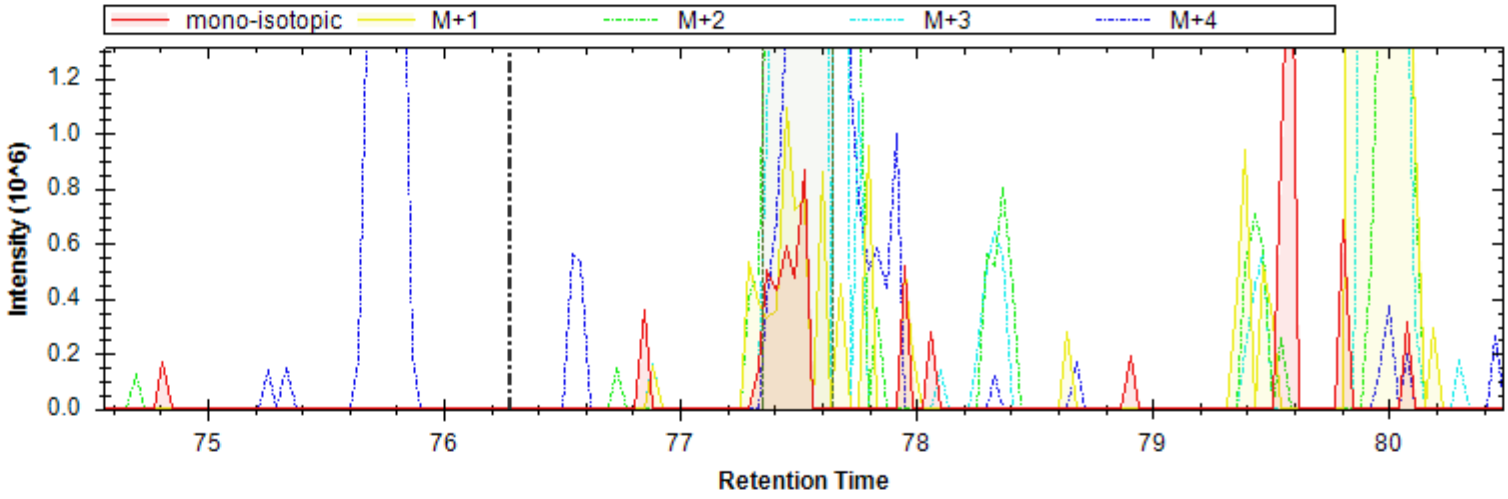

Q8BLJ3 – _M[+O]AS[+H+O3+P]SQGKNELK_.2
Fig4_MP-DIA-barrel-cortex-p15-3_MHRM.htrms

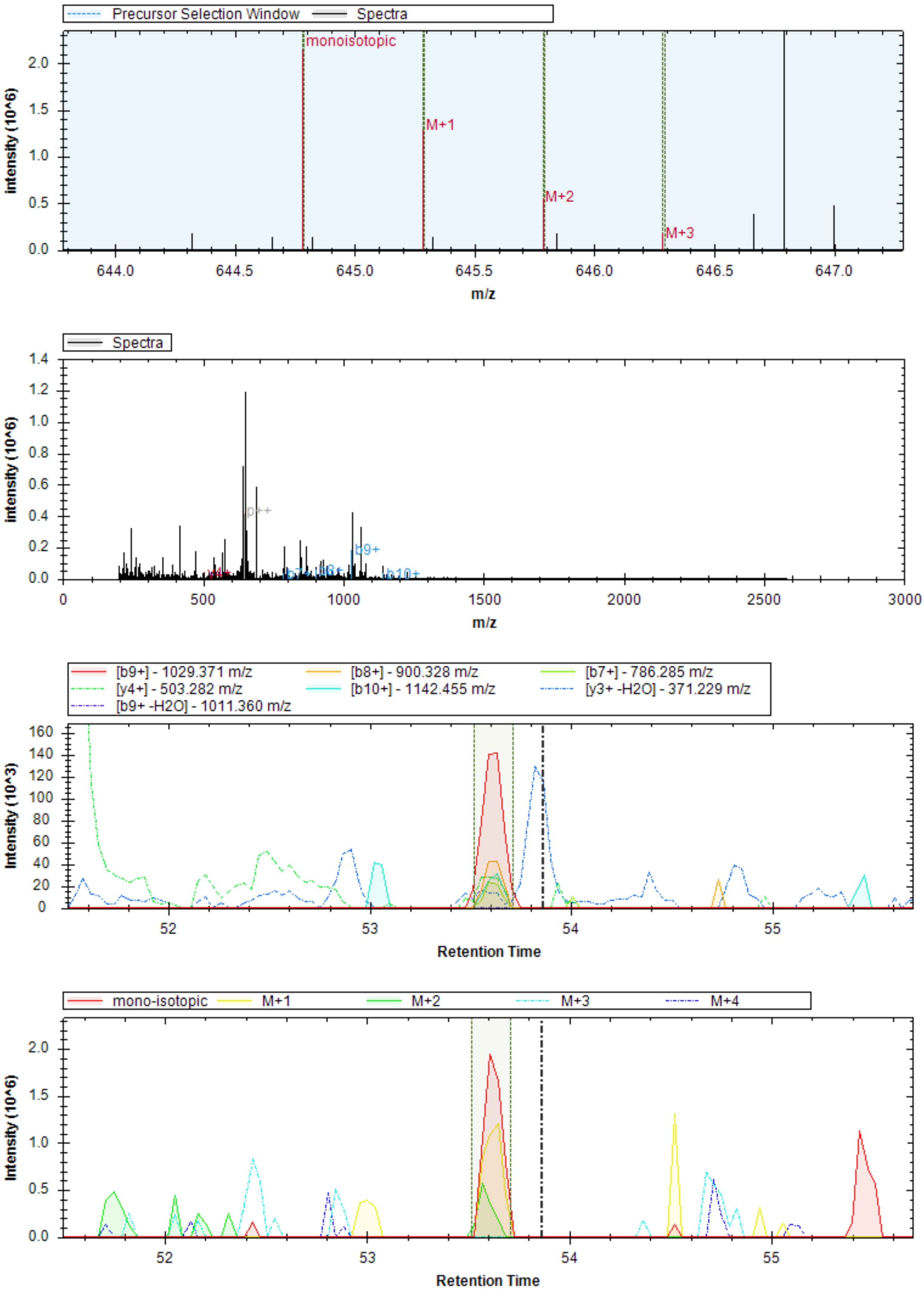

Q8CC35-3 – _AAS[+H+O3+P]PAKPSSLDLVPNLPR_.3
Fig4_MP-DIA-barrel-cortex-p15-3_MHRM.htrms

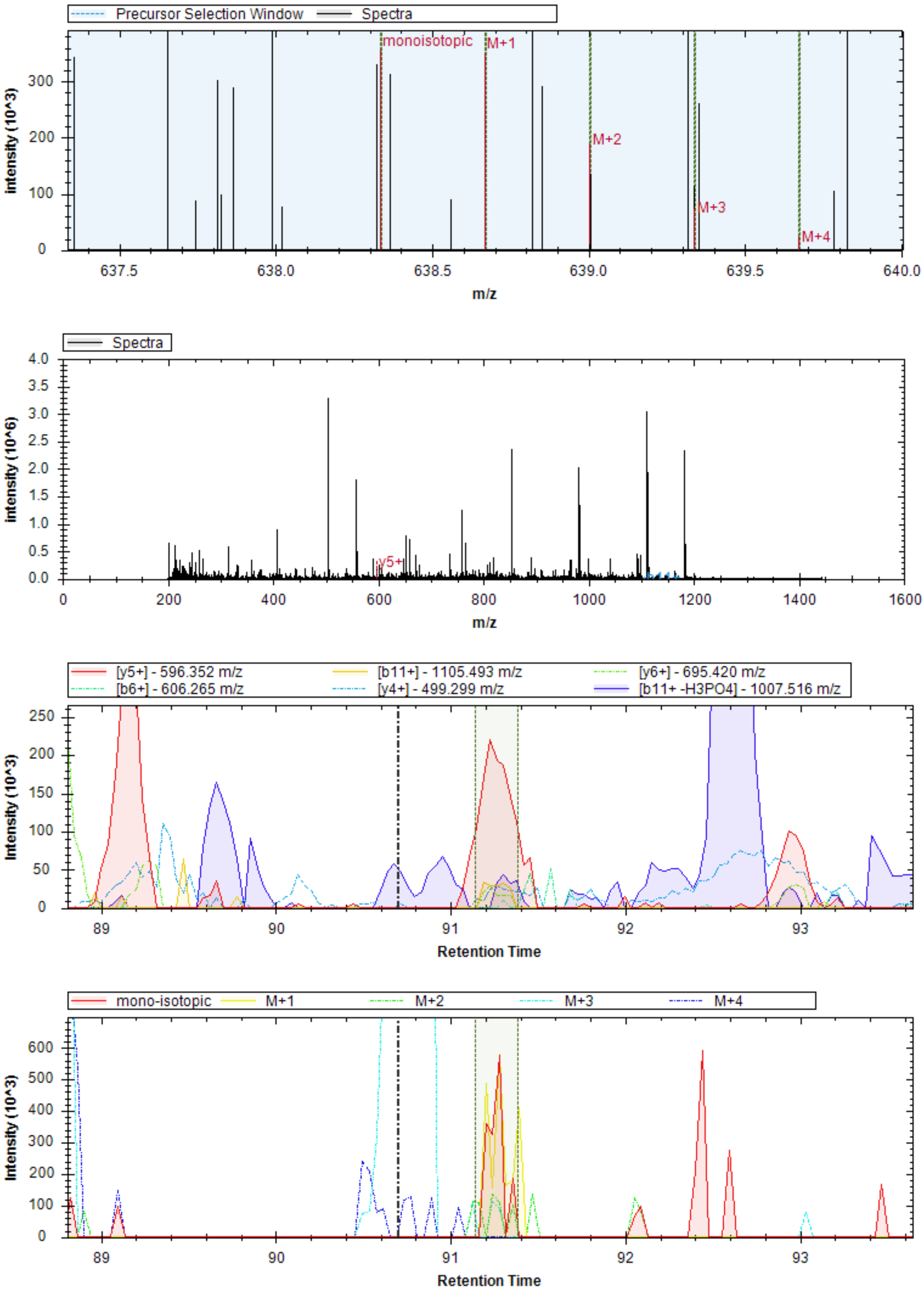

Q9DC63;Q9DC63-2;Q9DC63-3 – _YIDHY[+H+O3+P]AAIKK_.2
Fig4_MP-DIA-barrel-cortex-p15-3_MHRM.htrms

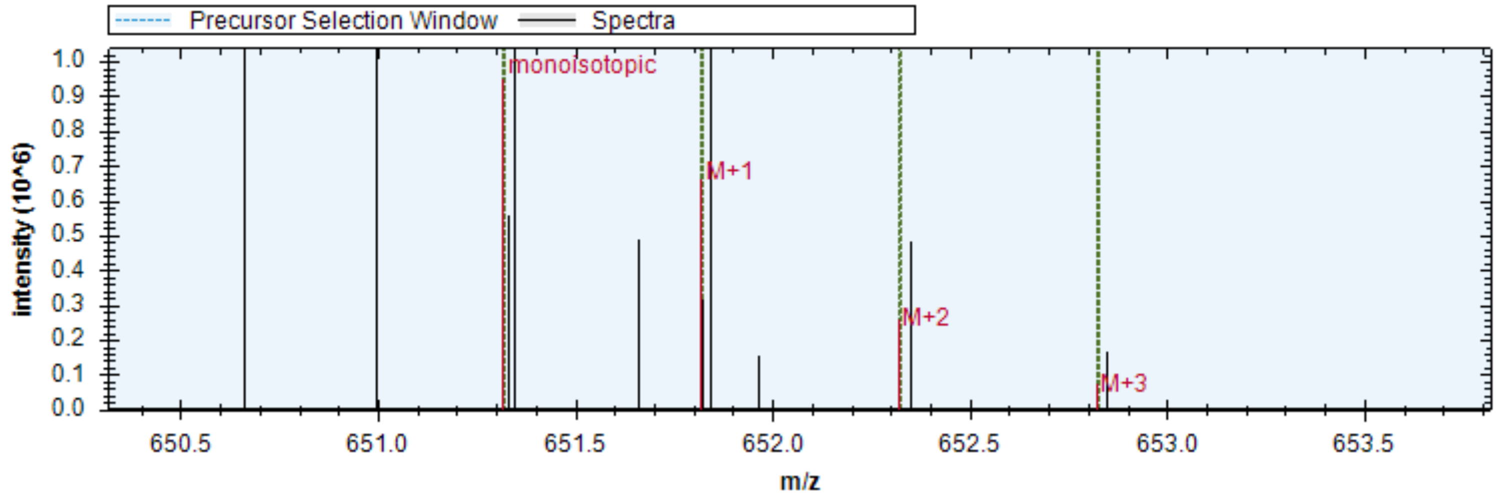

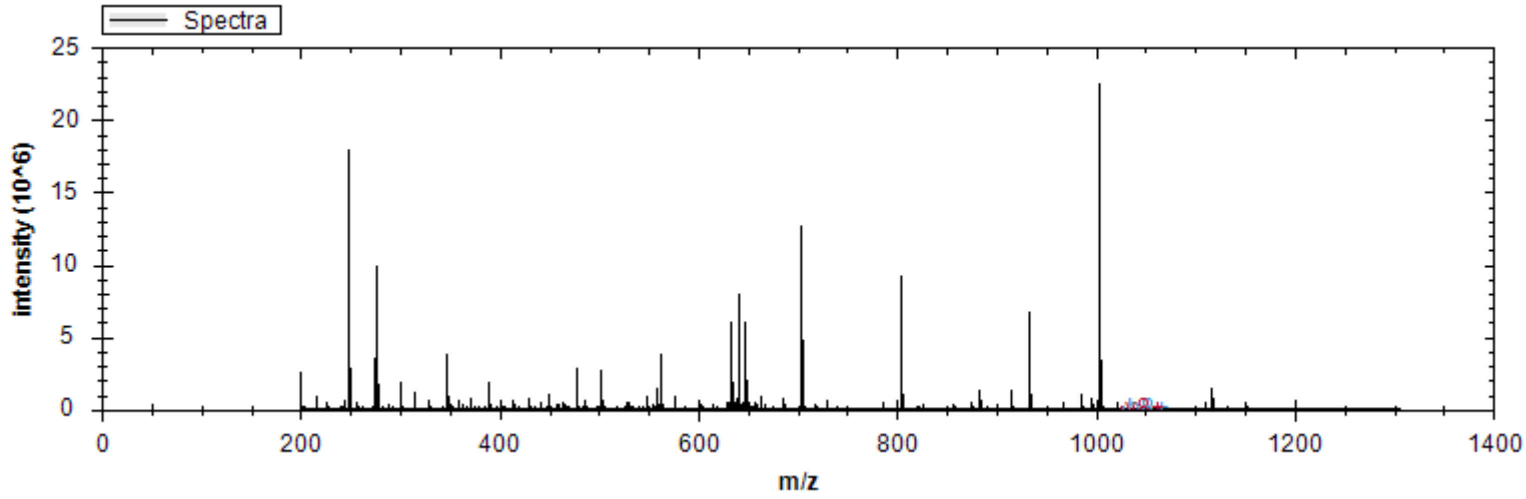

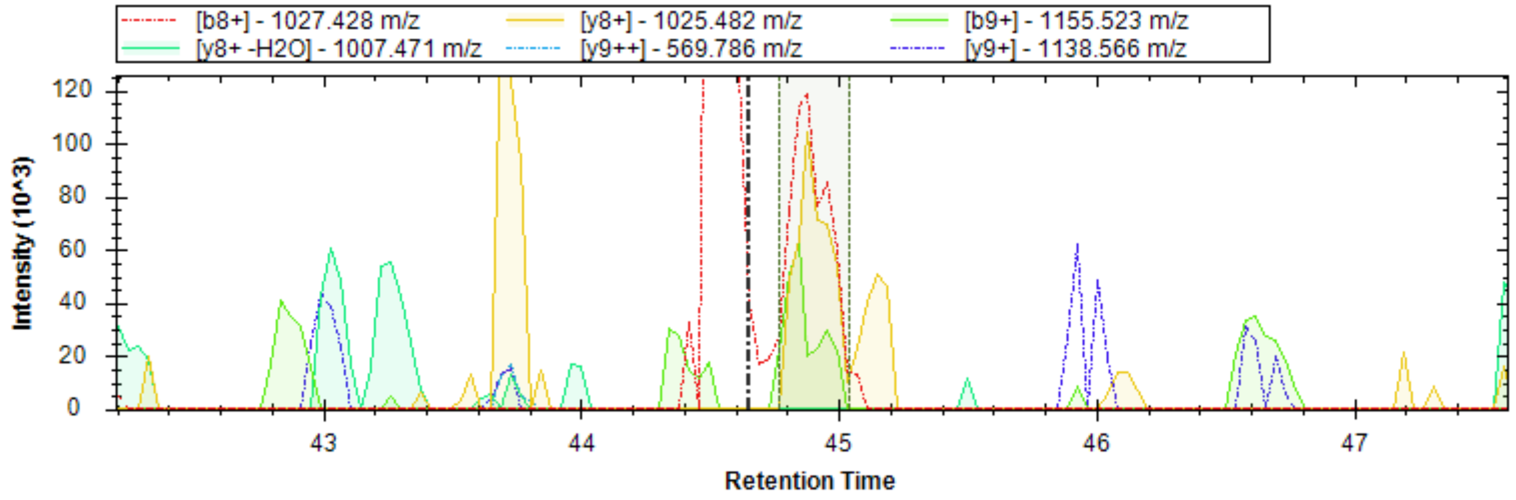

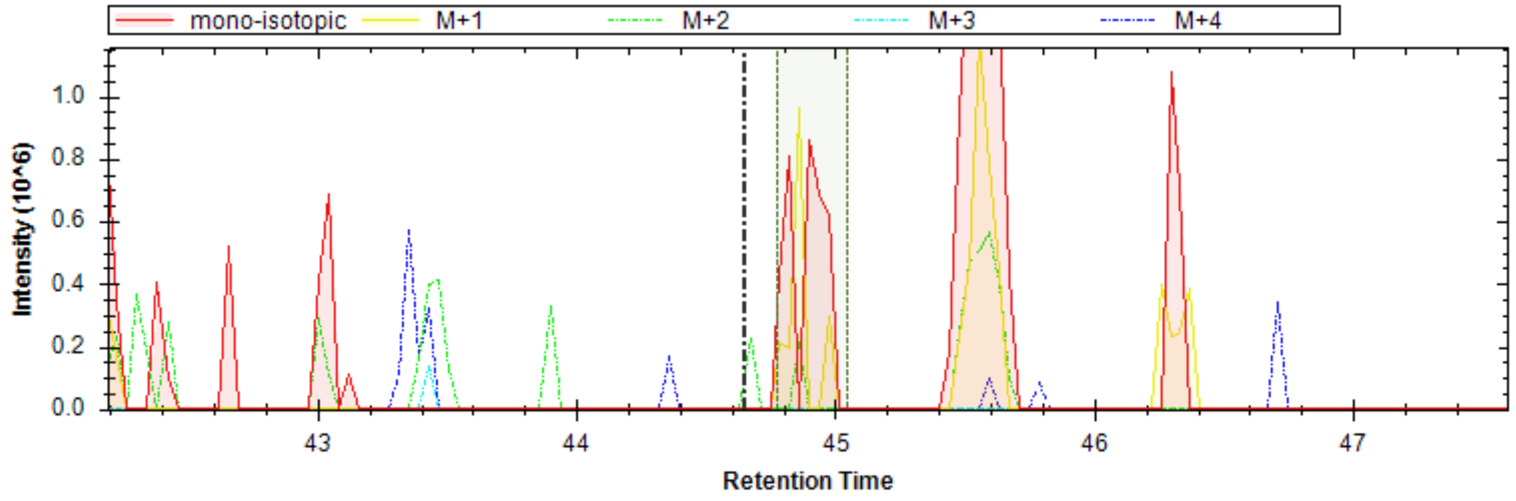

A2AM05;A2AM05-4 – _KDLKMS[+H+O3+P]VLISK_.2

Fig4_MP-DIA-barrel-cortex-p15-3_MHRM.htrms

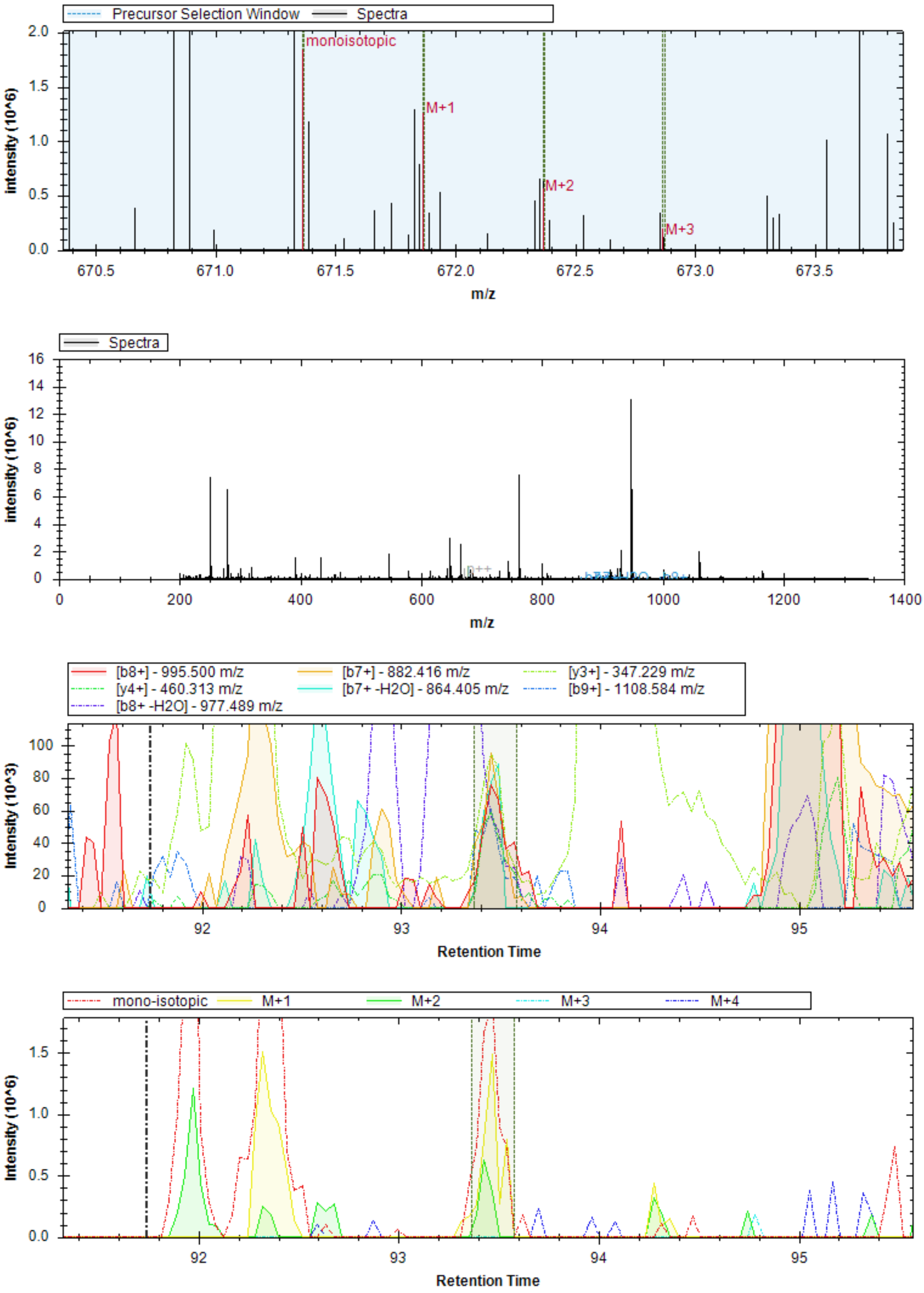

P13595 – _GVTASS[+H+O3+P]SSPASAPK_.2
Fig4_MP-DIA-barrel-cortex-p15-3_MHRM.htrms

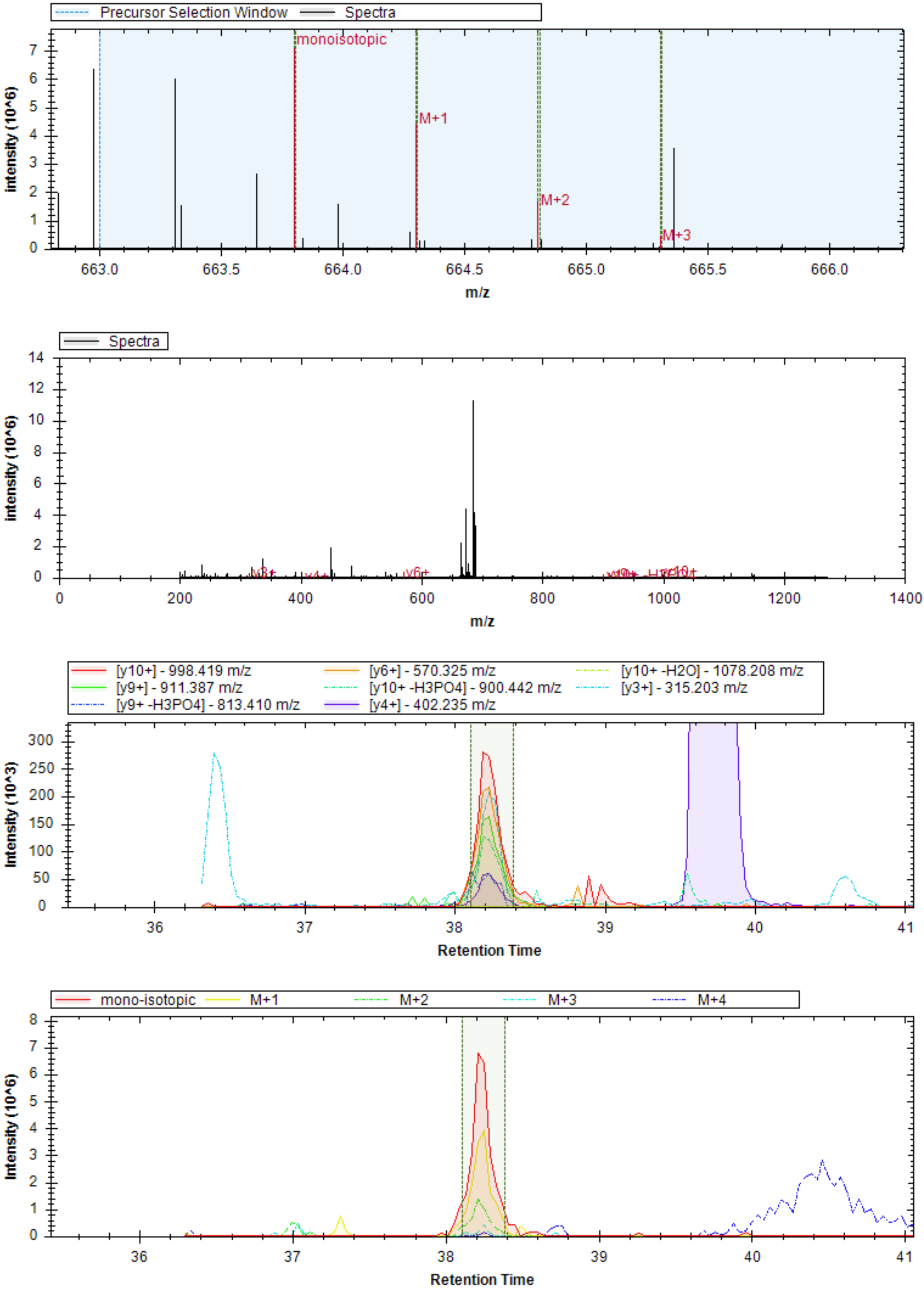

P13595 – _GVTASSS[+H+O3+P]SPASAPK_.2
Fig4_MP-DIA-barrel-cortex-p15-3_MHRM.htrms

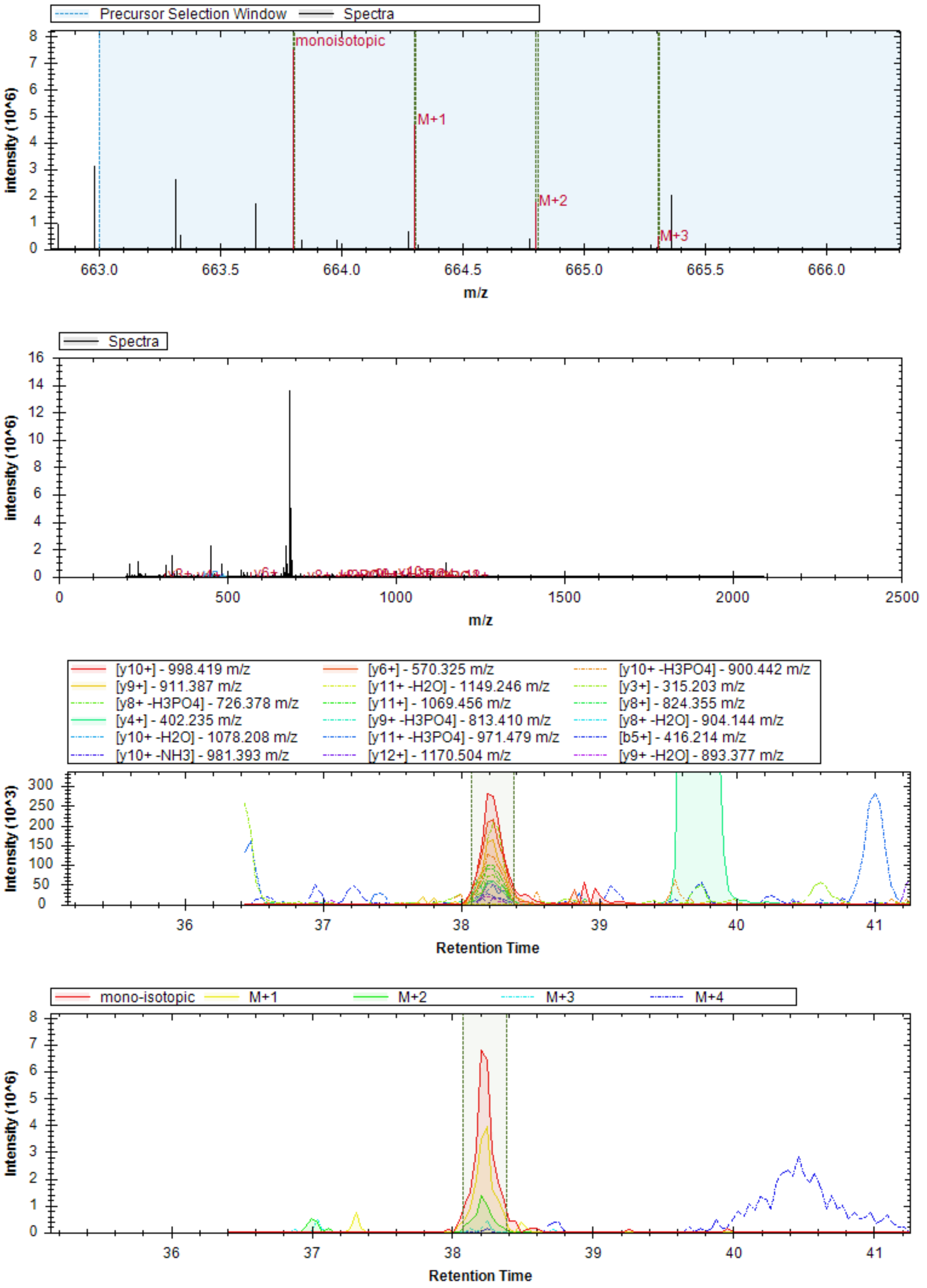

P13595 – _GVTASSSS[+H+O3+P]PASAPK_.2

Fig4_MP-DIA-barrel-cortex-p15-3_MHRM.htrms

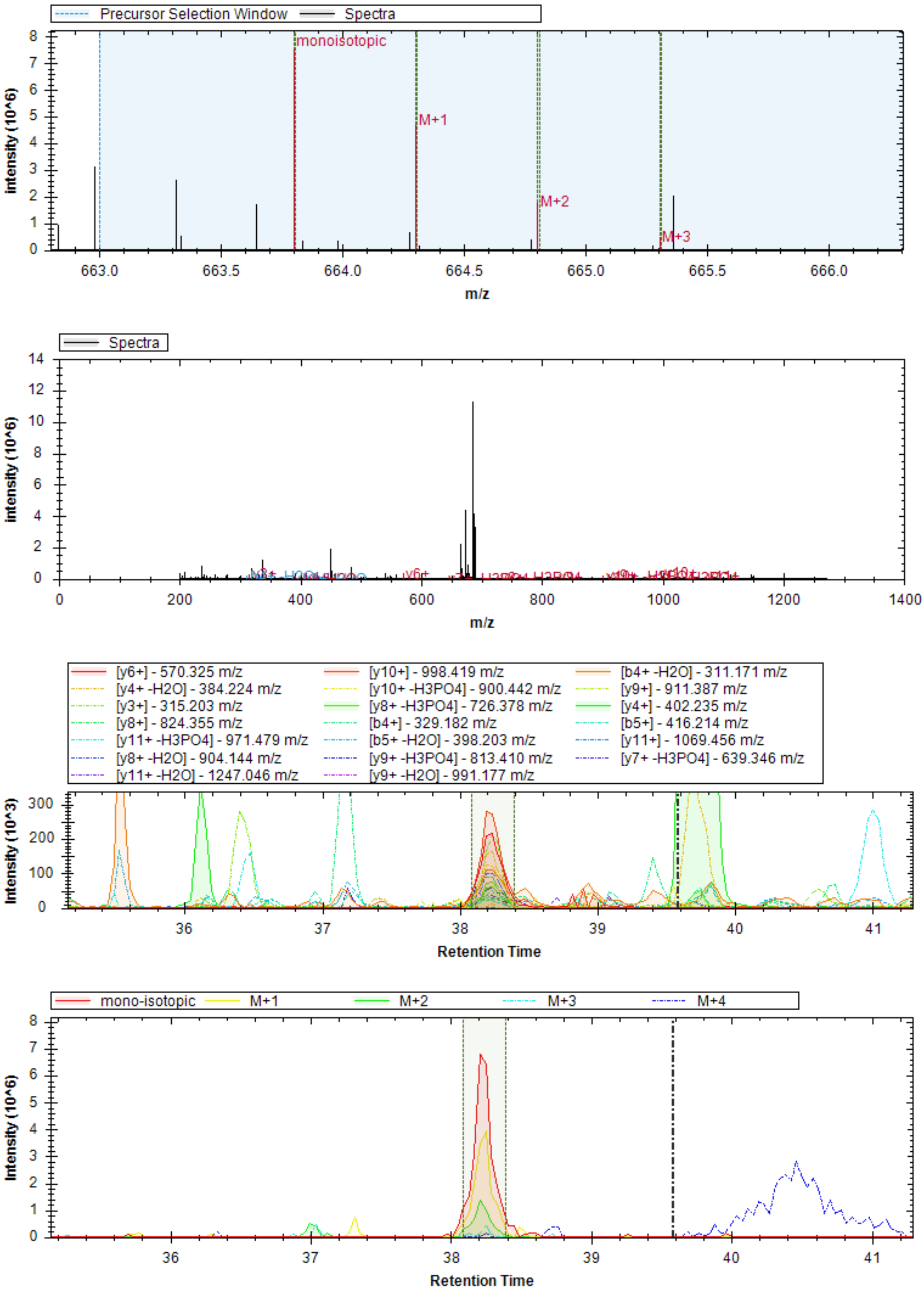

P13595 – _NPPEAATAPAS[+H+O3+P]PK_.2
Fig4_MP-DIA-barrel-cortex-p15-3_MHRM.htrms

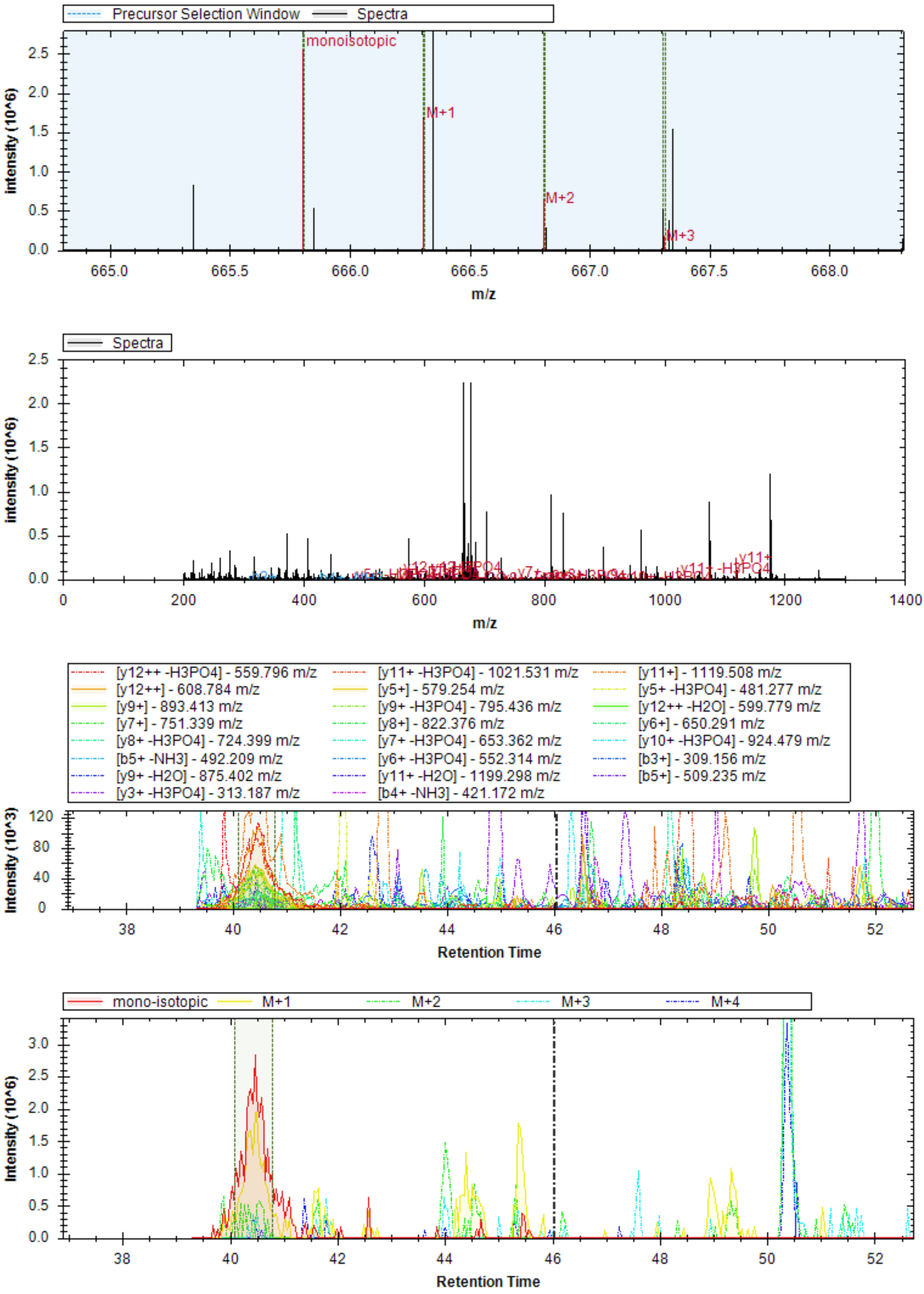

P26361 – _KM[+O]LHS[+H+O3+P]ILHAPMS[+H+O3+P]T[+H+O3+P]ISK_.3

Fig4_MP-DIA-barrel-cortex-p15-3_MHRM.htrms

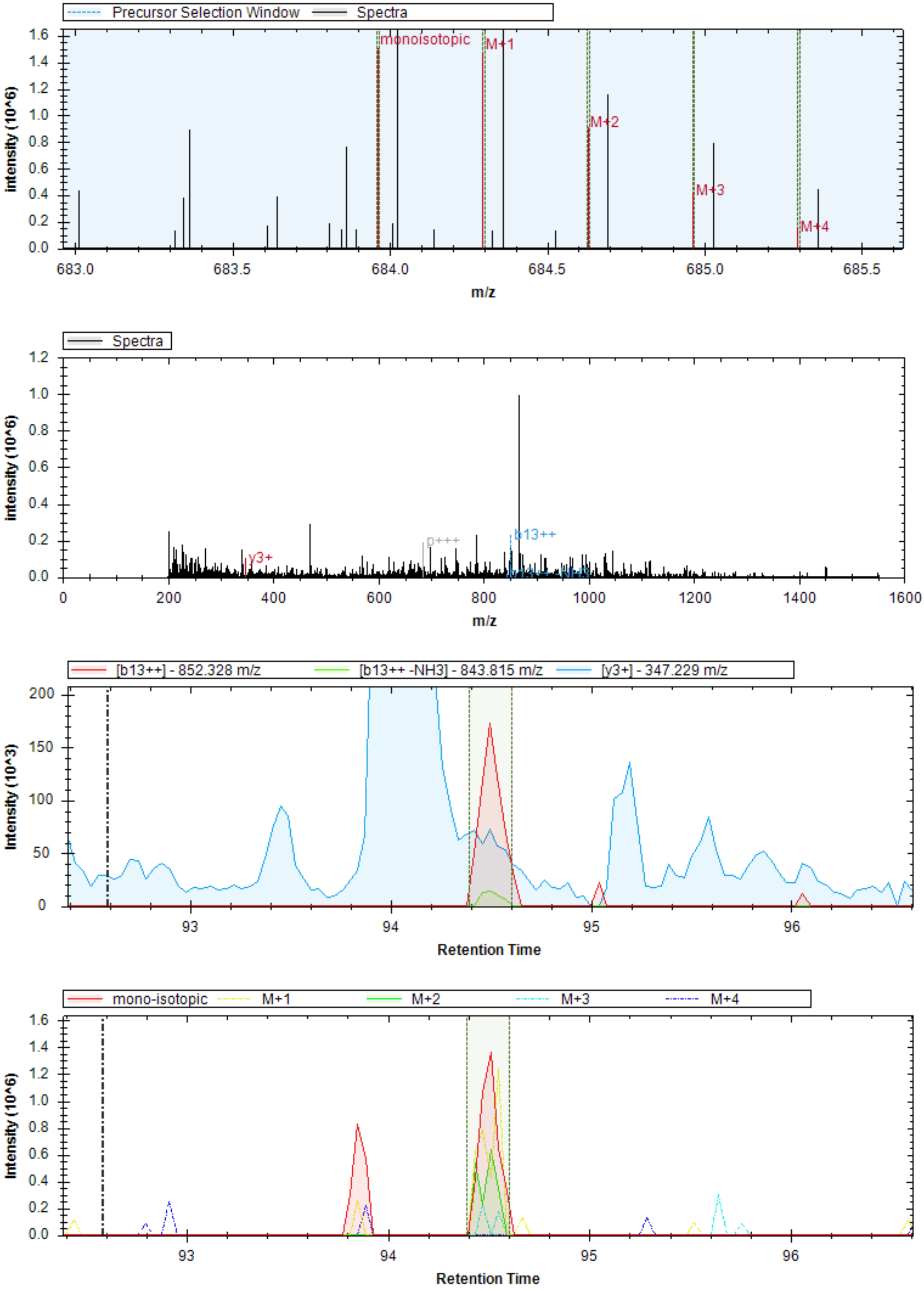

Q03173;Q03173-4;Q03173-5 – _NSRPS[+H+O3+P]SPVNTPSSQPPAAK_.3
Fig4_MP-DIA-barrel-cortex-p15-3_MHRM.htrms

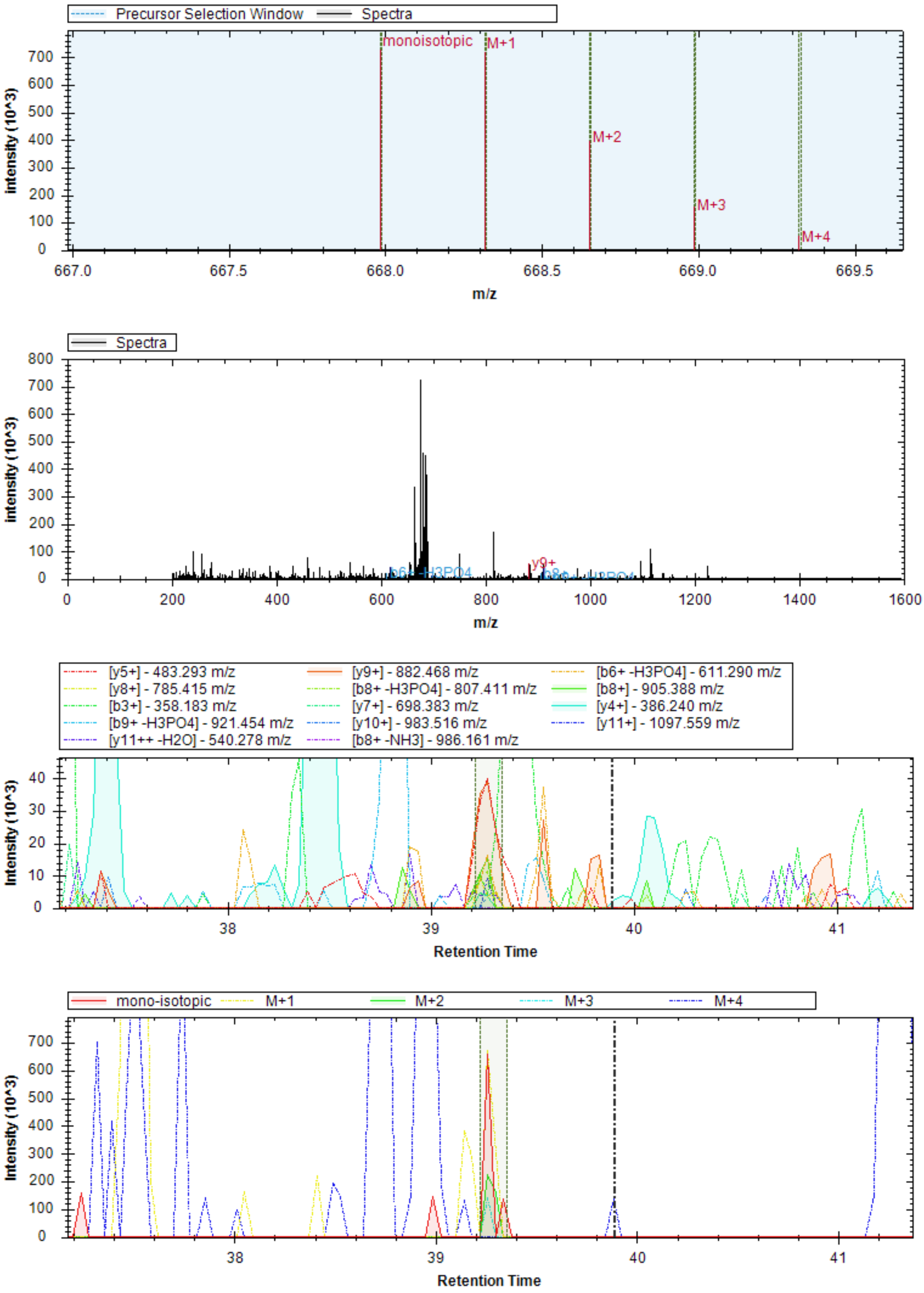

Q6A078 – _T[+H+O3+P]IGLM[+O]KKVVEK_.2
Fig4_MP-DIA-barrel-cortex-p15-3_MHRM.htrms

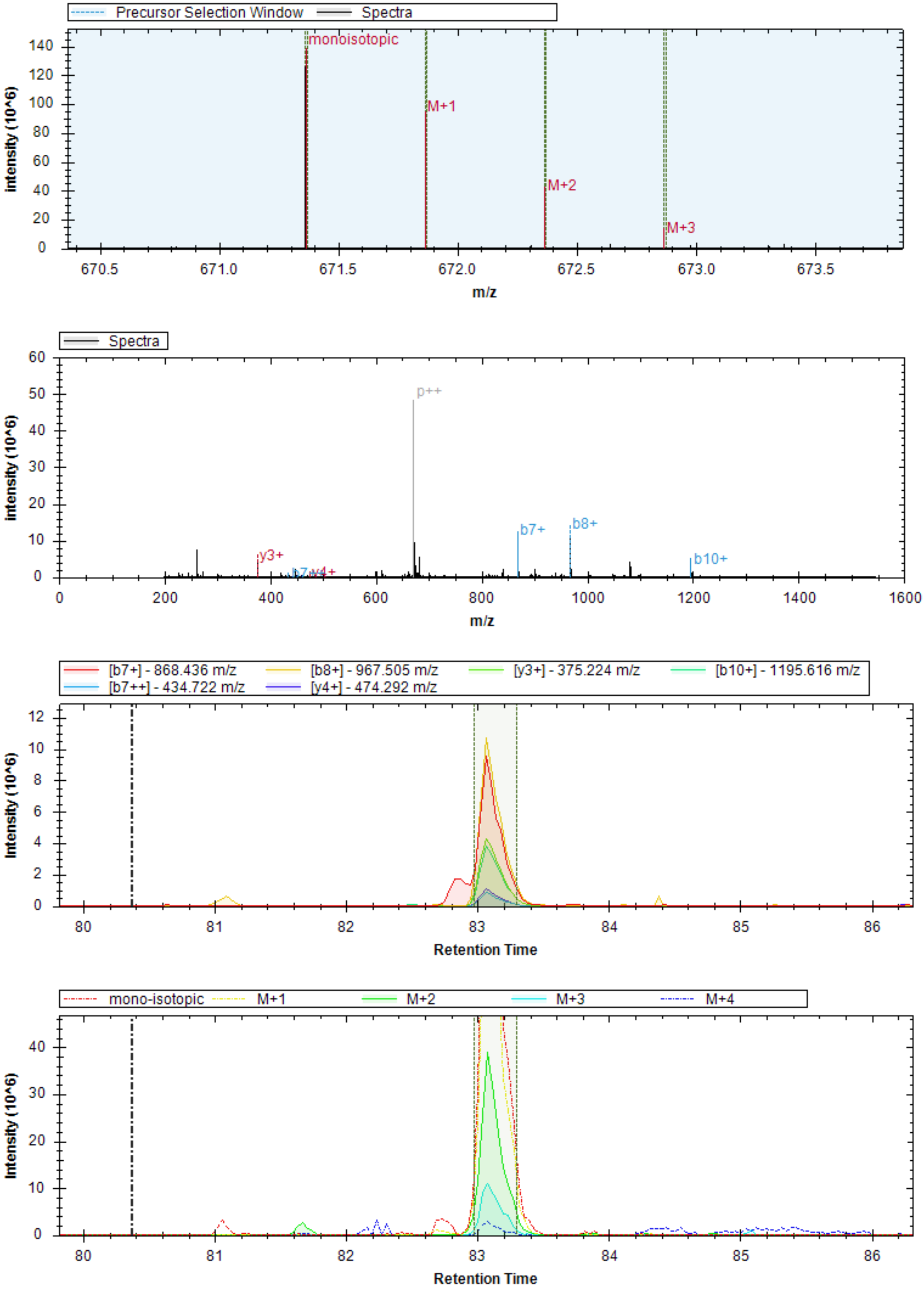

Q8C0E2 – _SMS[+H+O3+P]HQAAIASQR_.2
Fig4_MP-DIA-barrel-cortex-p15-3_MHRM.htrms

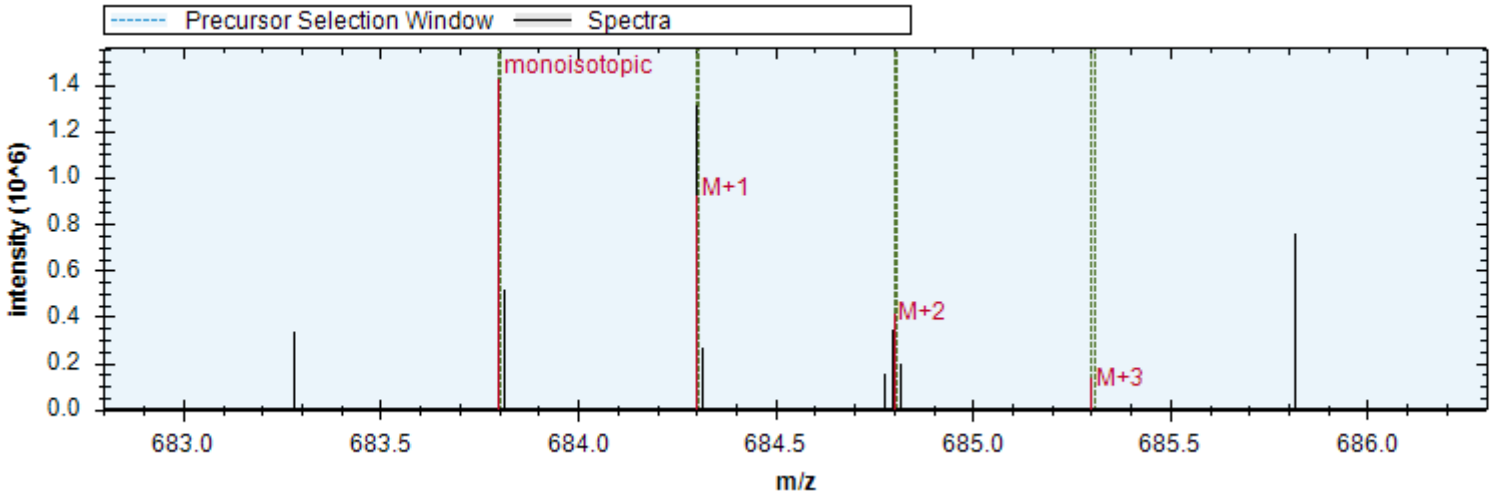

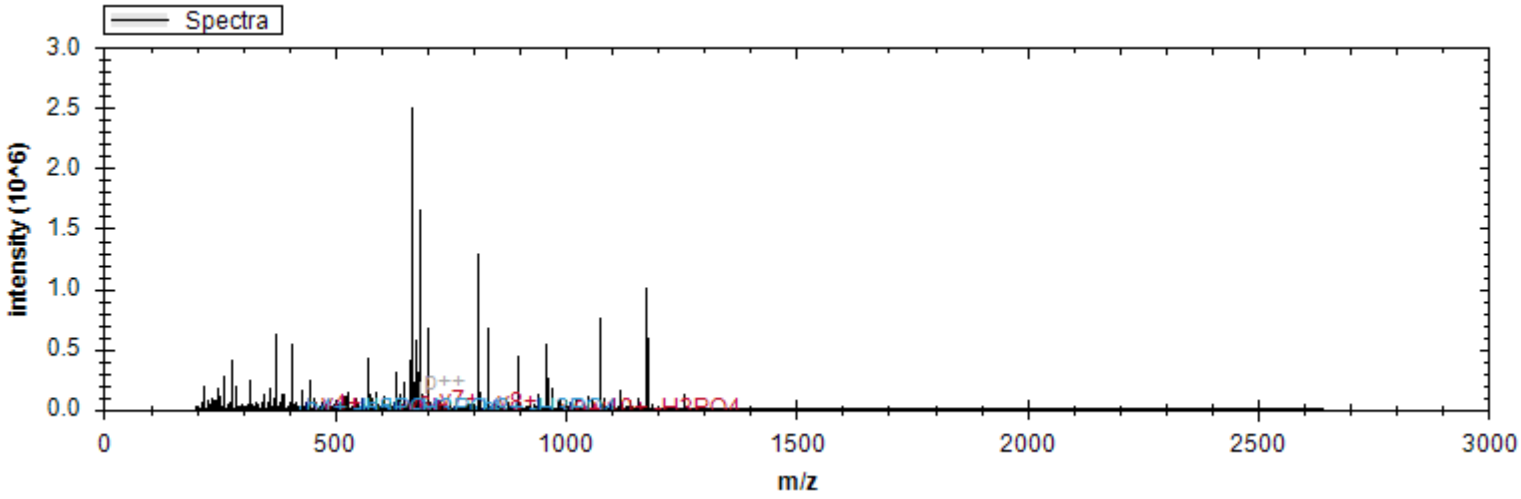

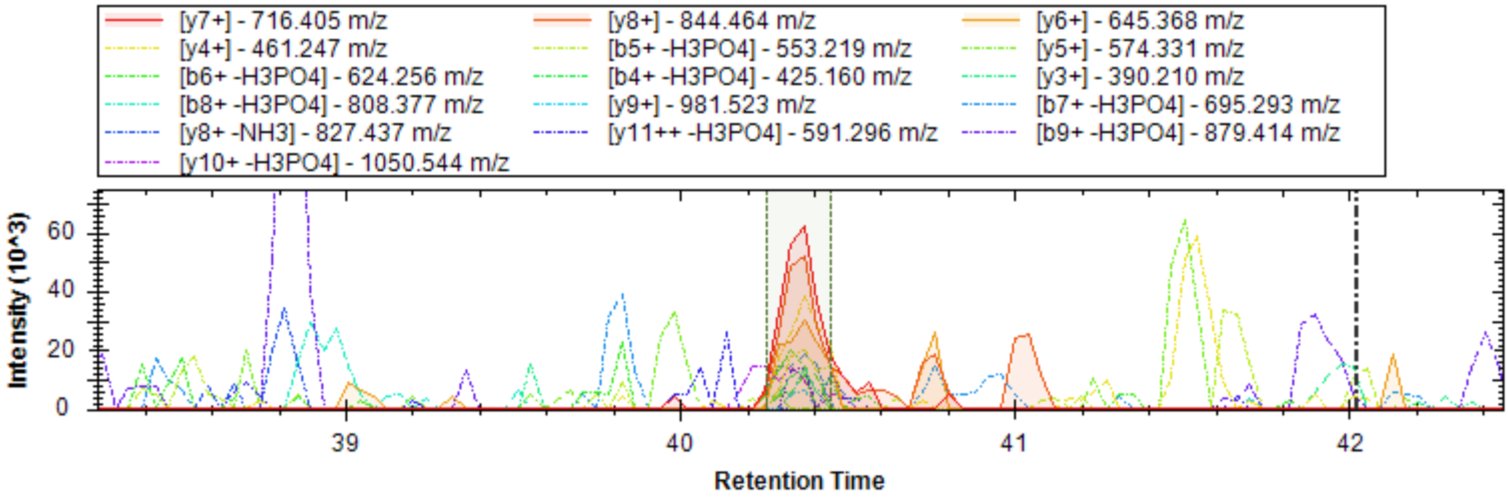

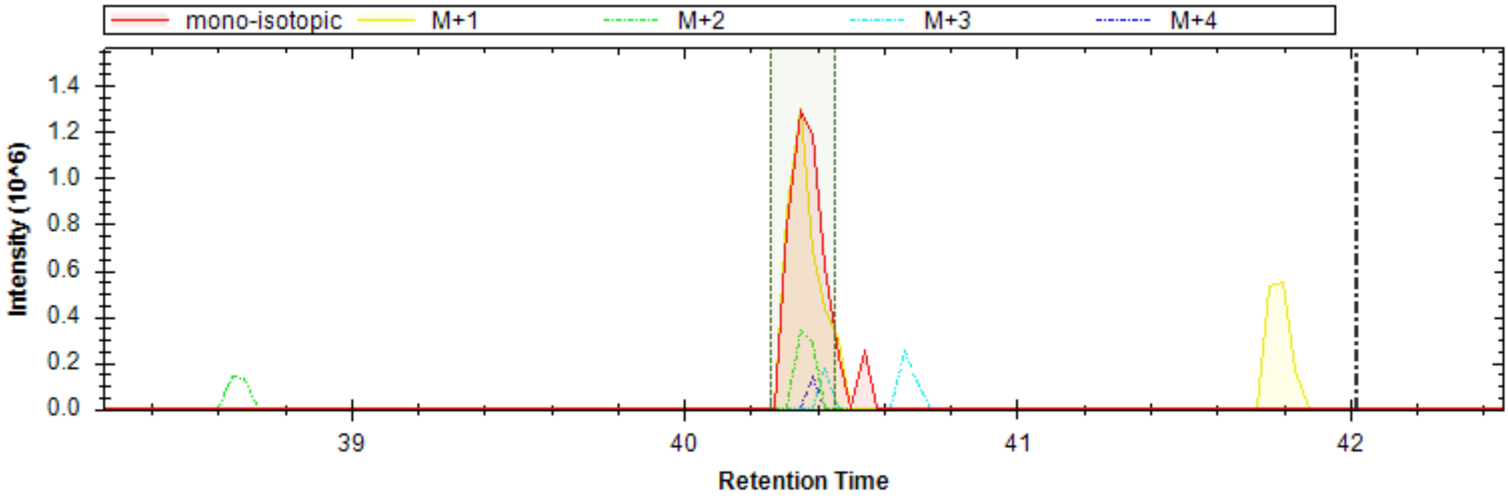

Q8CDU4;Q8CDU4-2 – _[+C2+H2+O]ASLRS[+H+O3+P]ATPRLR_.2

Fig4_MP-DIA-barrel-cortex-p15-3_MHRM.htrms

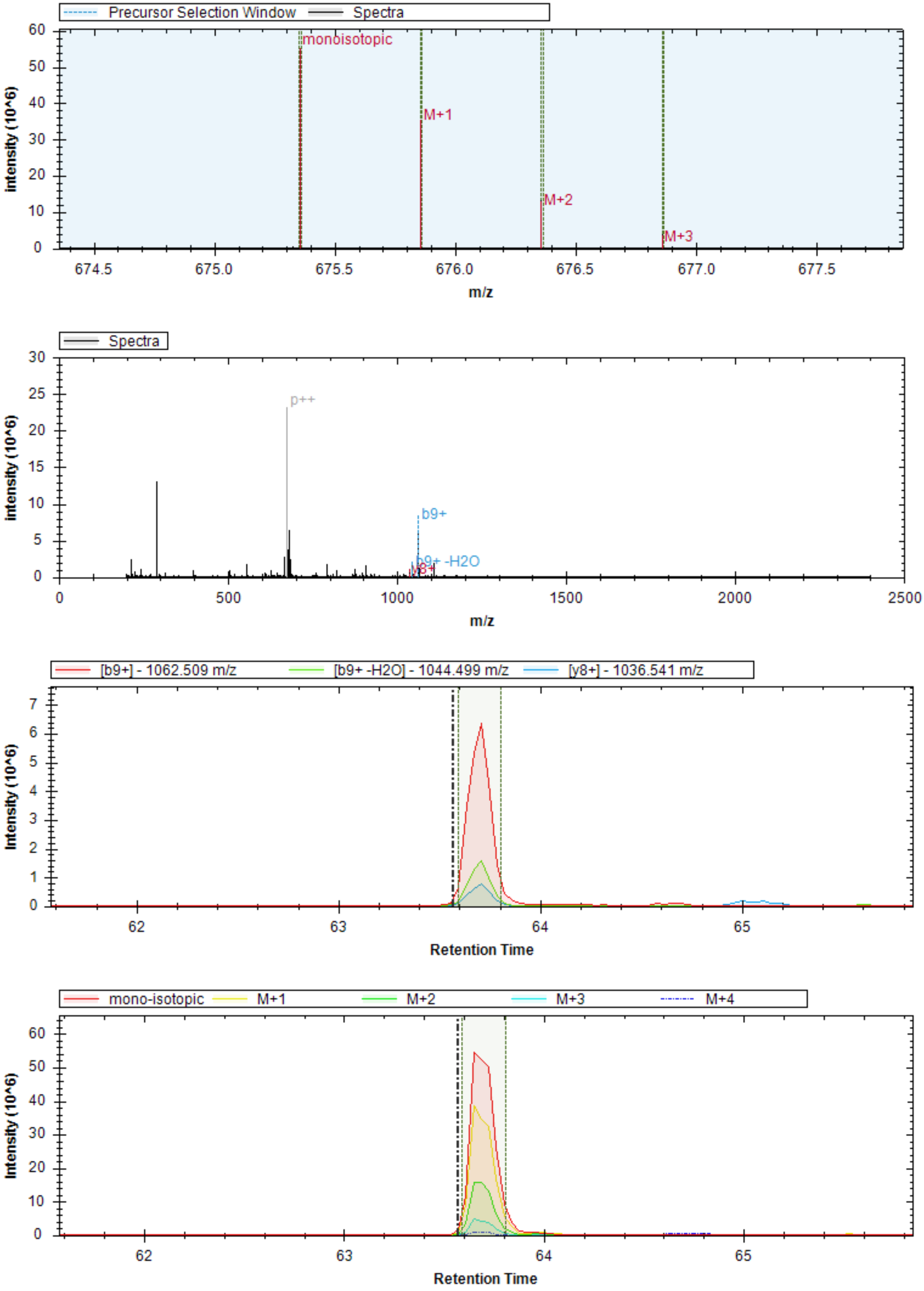

Q8R5L3;Q8R5L3-2 – _LQLY[+H+O3+P]FWKDR_.2
Fig4_MP-DIA-barrel-cortex-p15-3_MHRM.htrms

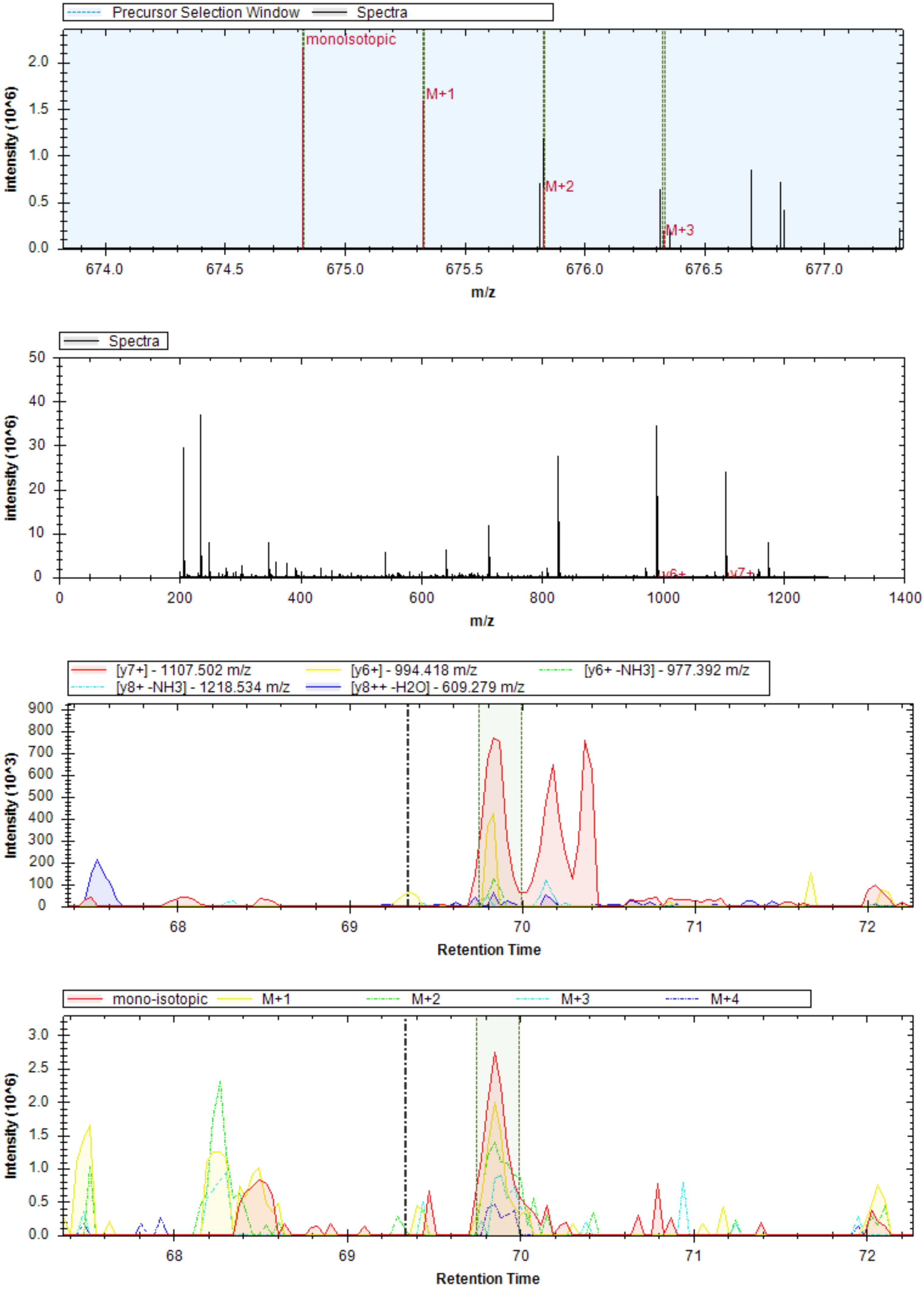

Q9JLM8 – _SPSPSPT[+H+O3+P]SPGSLR_.2
Fig4_MP-DIA-barrel-cortex-p15-3_MHRM.htrms

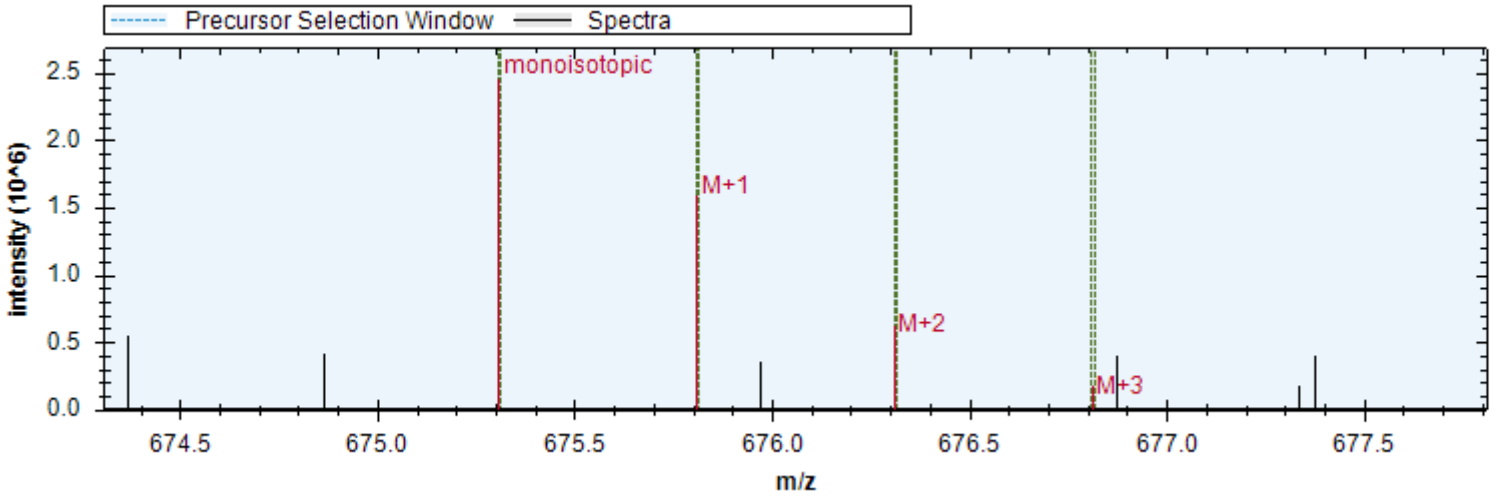

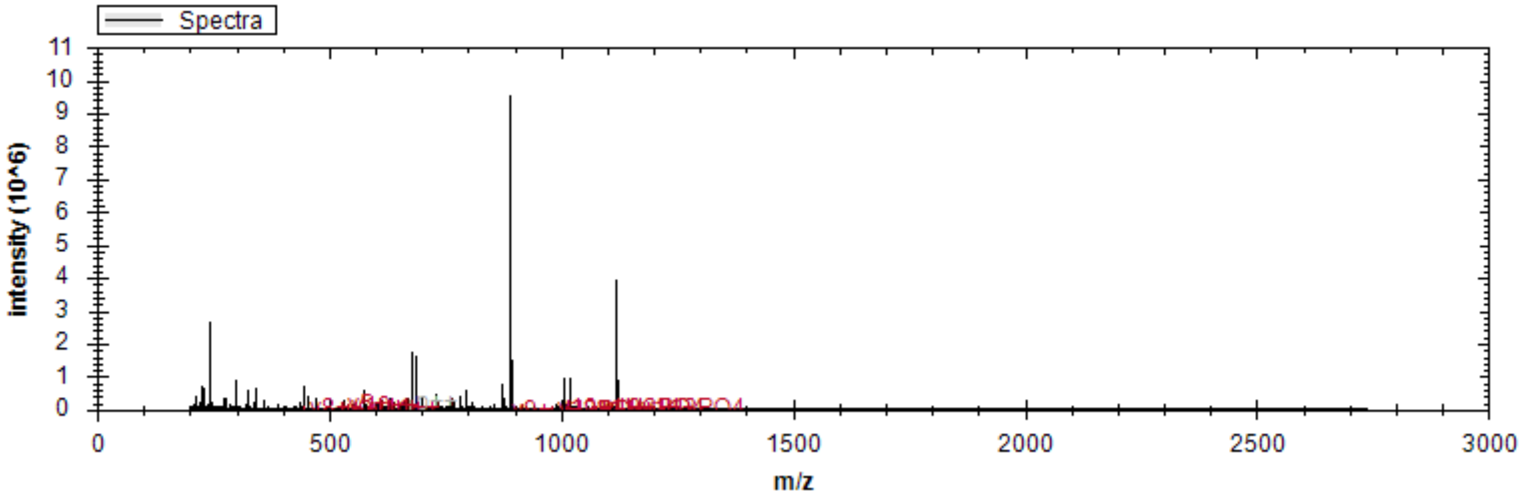

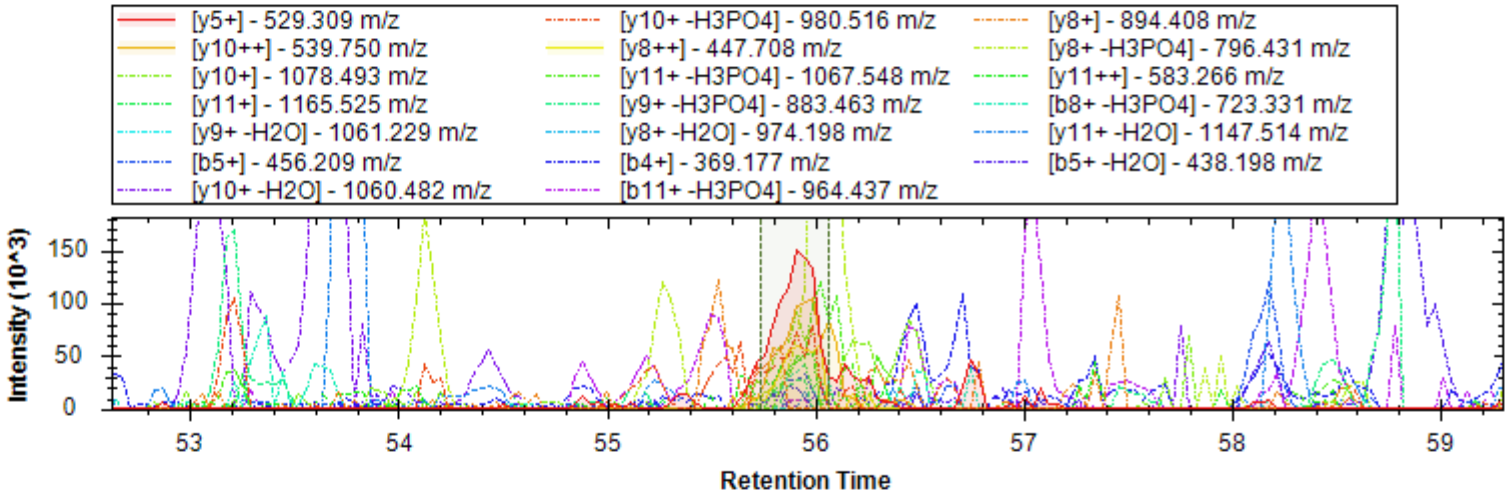

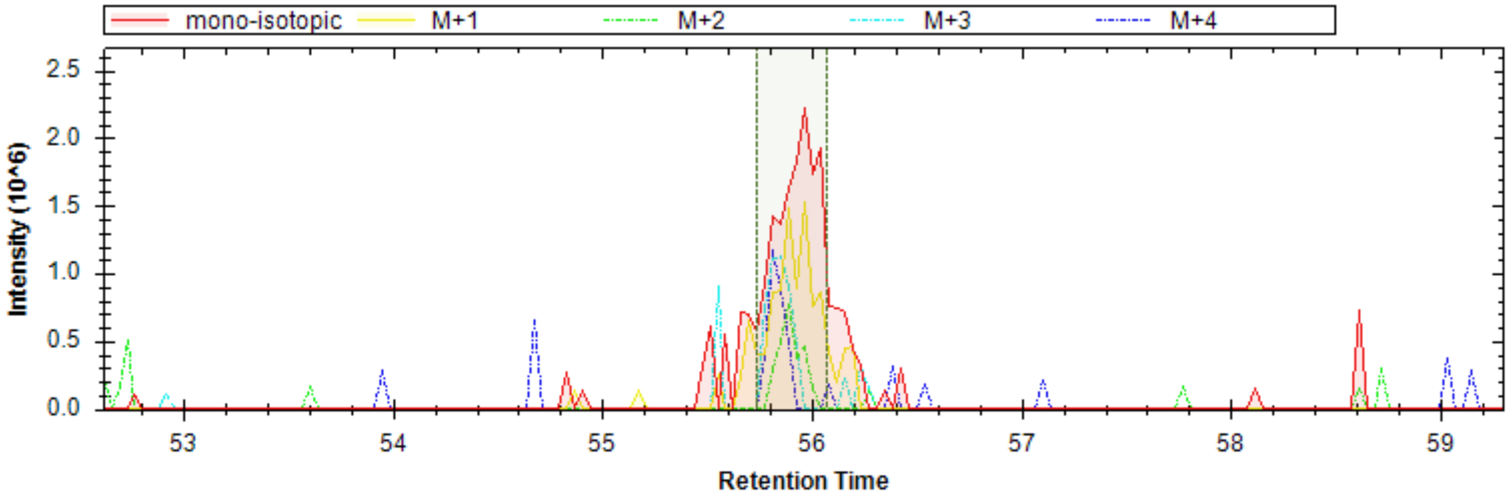

Q9JLM8 – _SPSPSPTS[+H+O3+P]PGSLR_.2
Fig4_MP-DIA-barrel-cortex-p15-3_MHRM.htrms

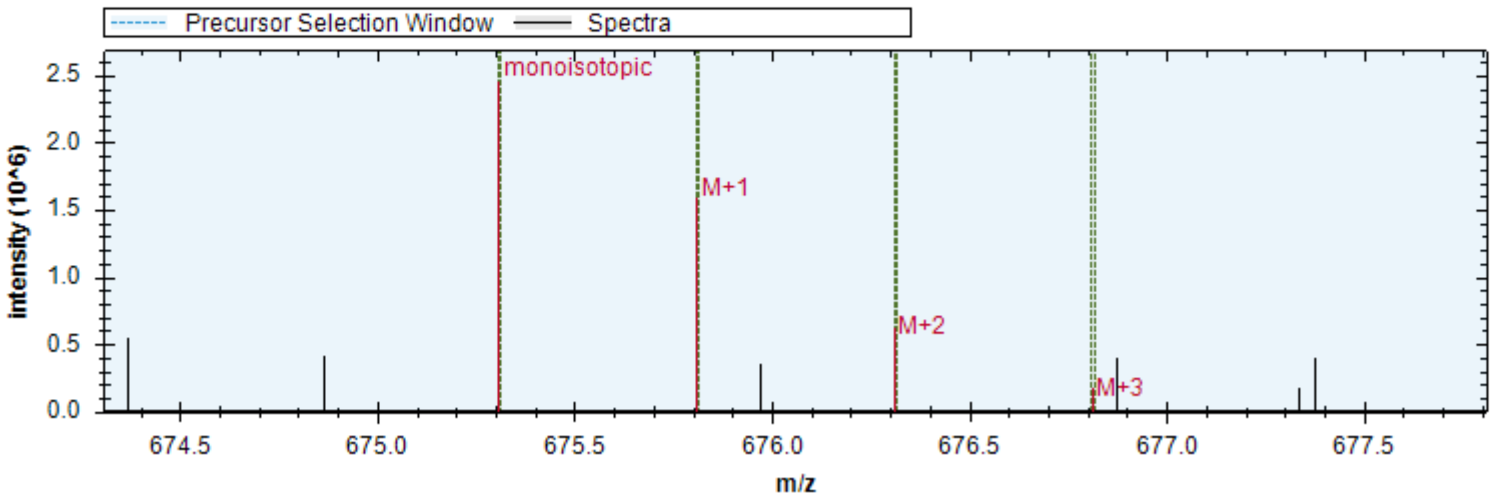

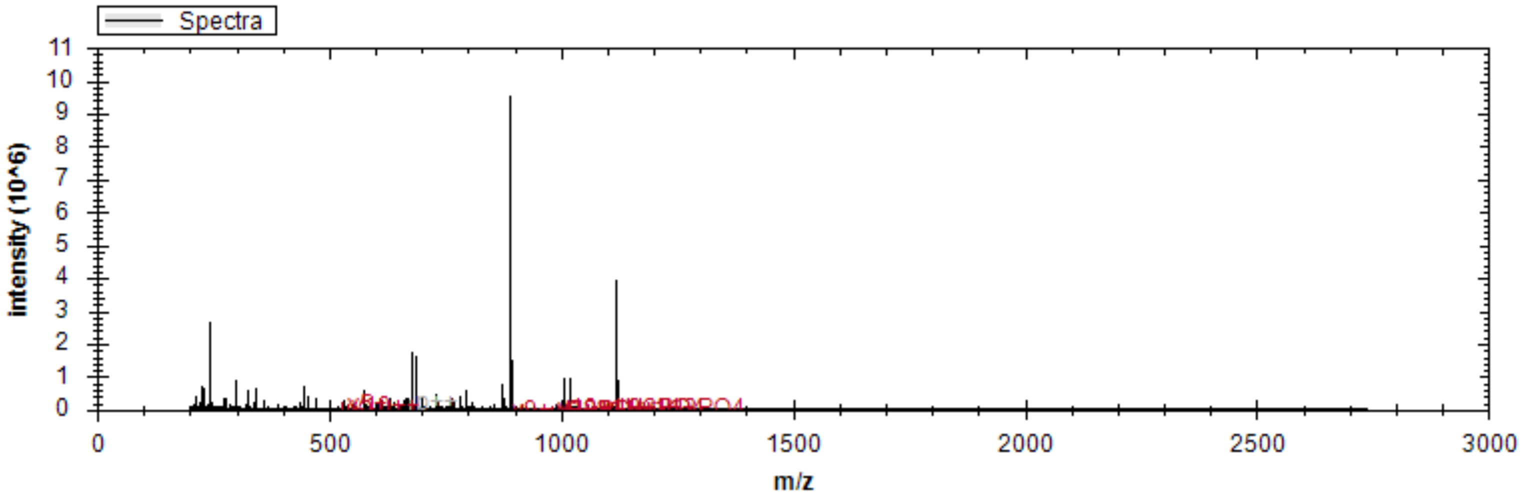

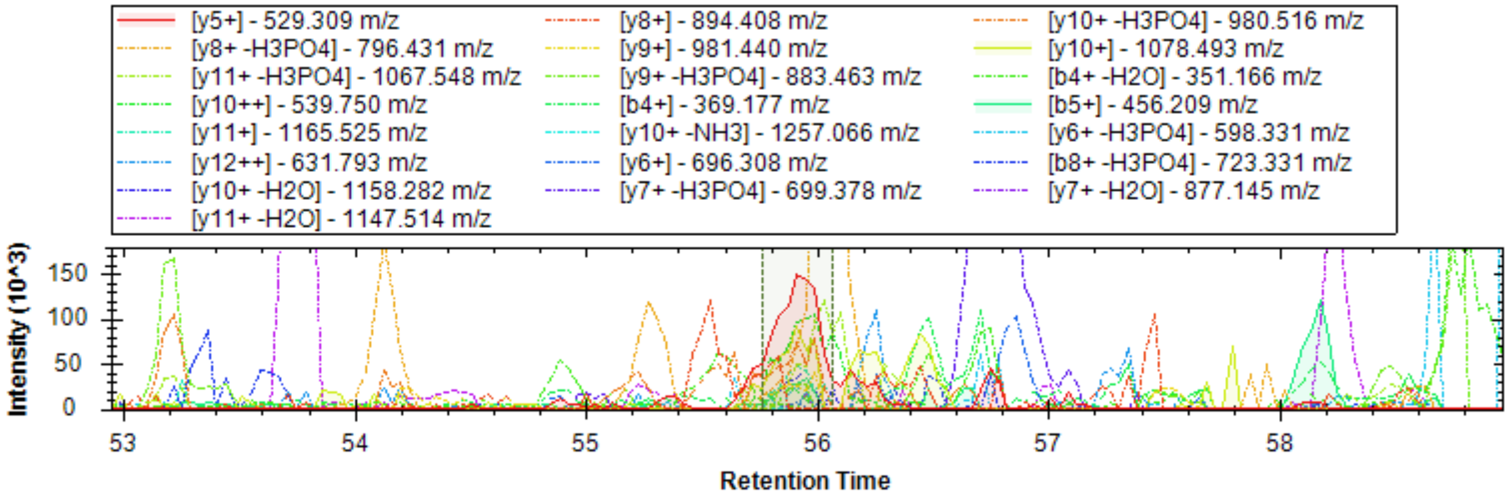

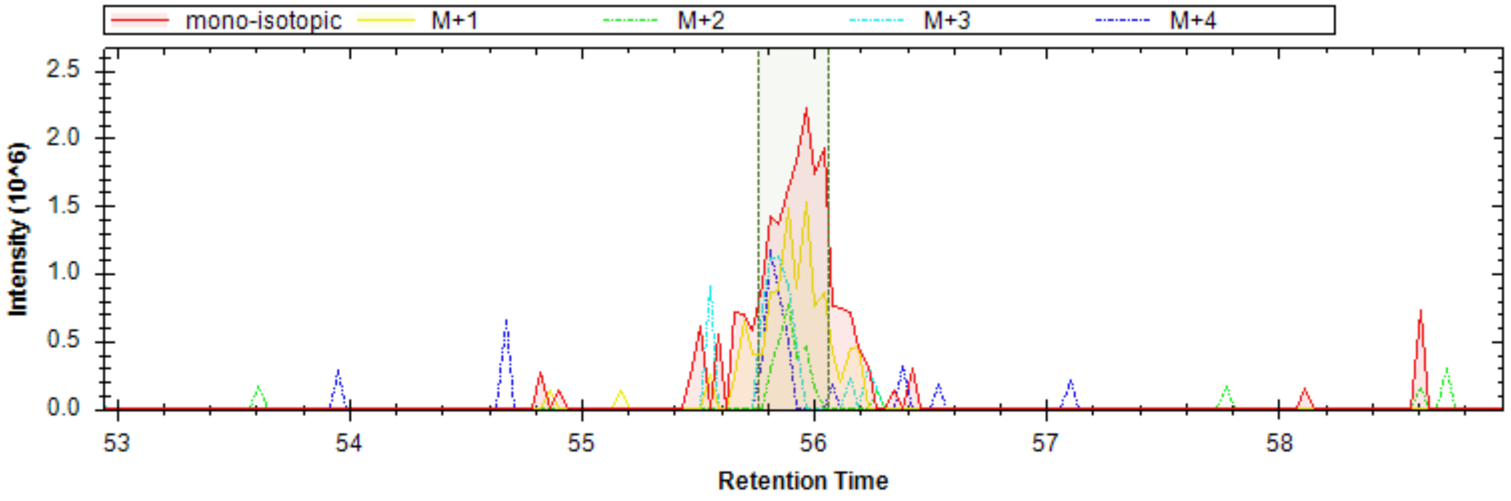

O88935 – _PVAGGPGAPPAARPPAS[+H+O3+P]PSPQR_.3
Fig4_MP-DIA-barrel-cortex-p15-3_MHRM.htrms

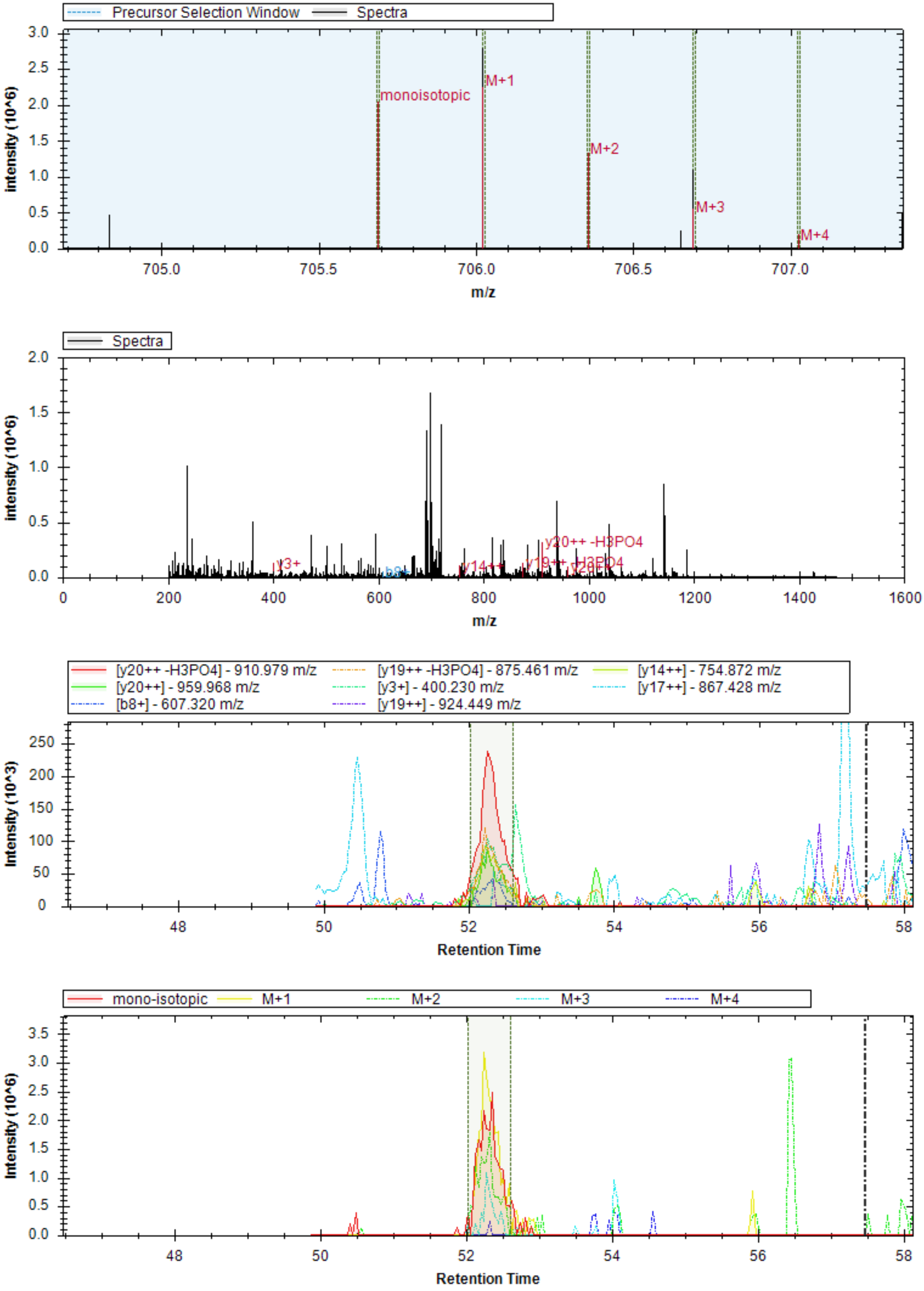

O88935 – _PVAGGPGAPPAARPPASPS[+H+O3+P]PQR_.3
Fig4_MP-DIA-barrel-cortex-p15-3_MHRM.htrms

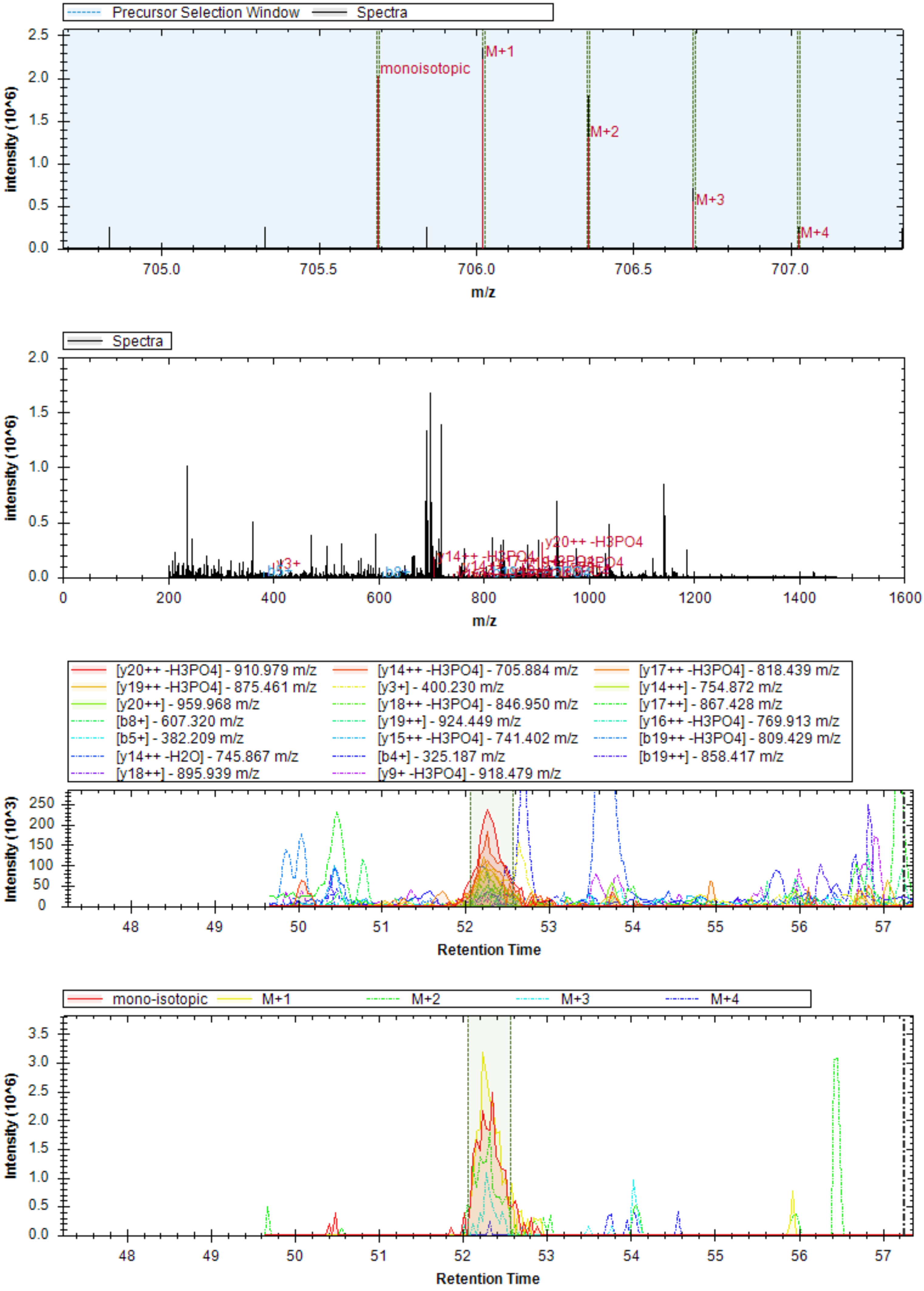

P28738 – _PIRPGHYPAS[+H+O3+P]SPTAVHAVR_.3

Fig4_MP-DIA-barrel-cortex-p15-3_MHRM.htrms

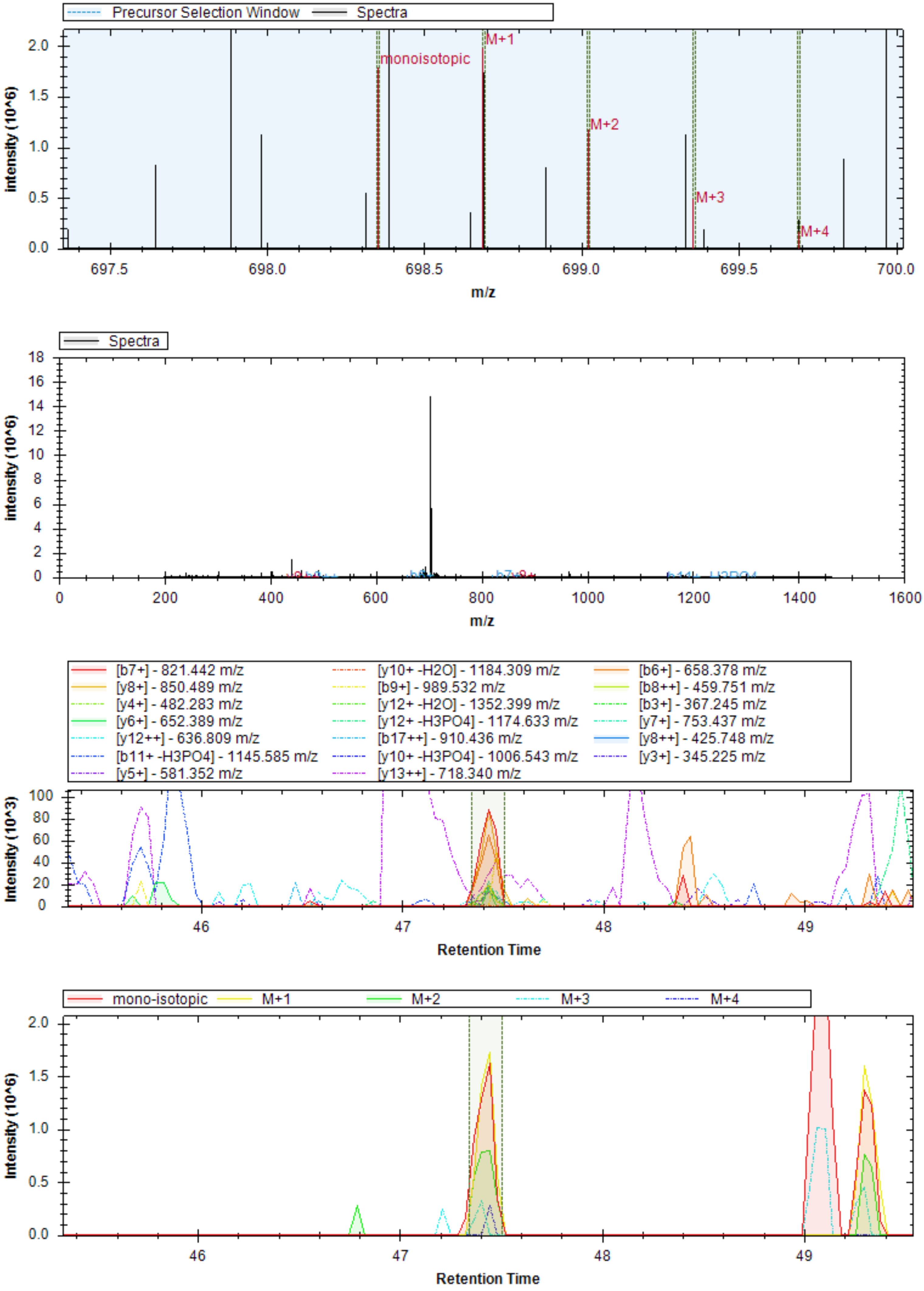

P28738 – _PIRPGHYPASS[+H+O3+P]PTAVHAVR_.3
Fig4_MP-DIA-barrel-cortex-p15-3_MHRM.htrms

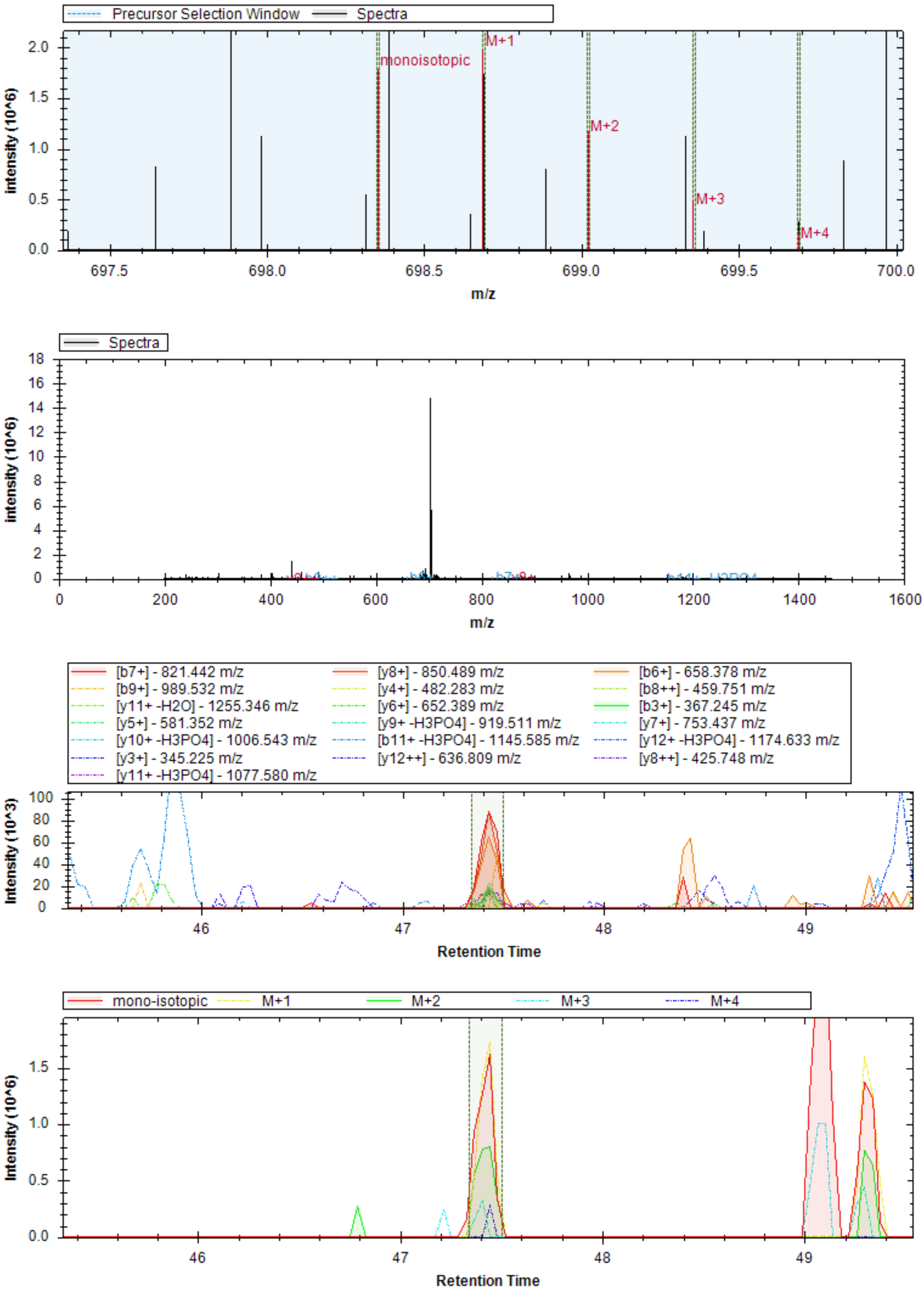

P62751 – _KAY[+H+O3+P]VRLAPDYDALDVANK_.3
Fig4_MP-DIA-barrel-cortex-p15-3_MHRM.htrms

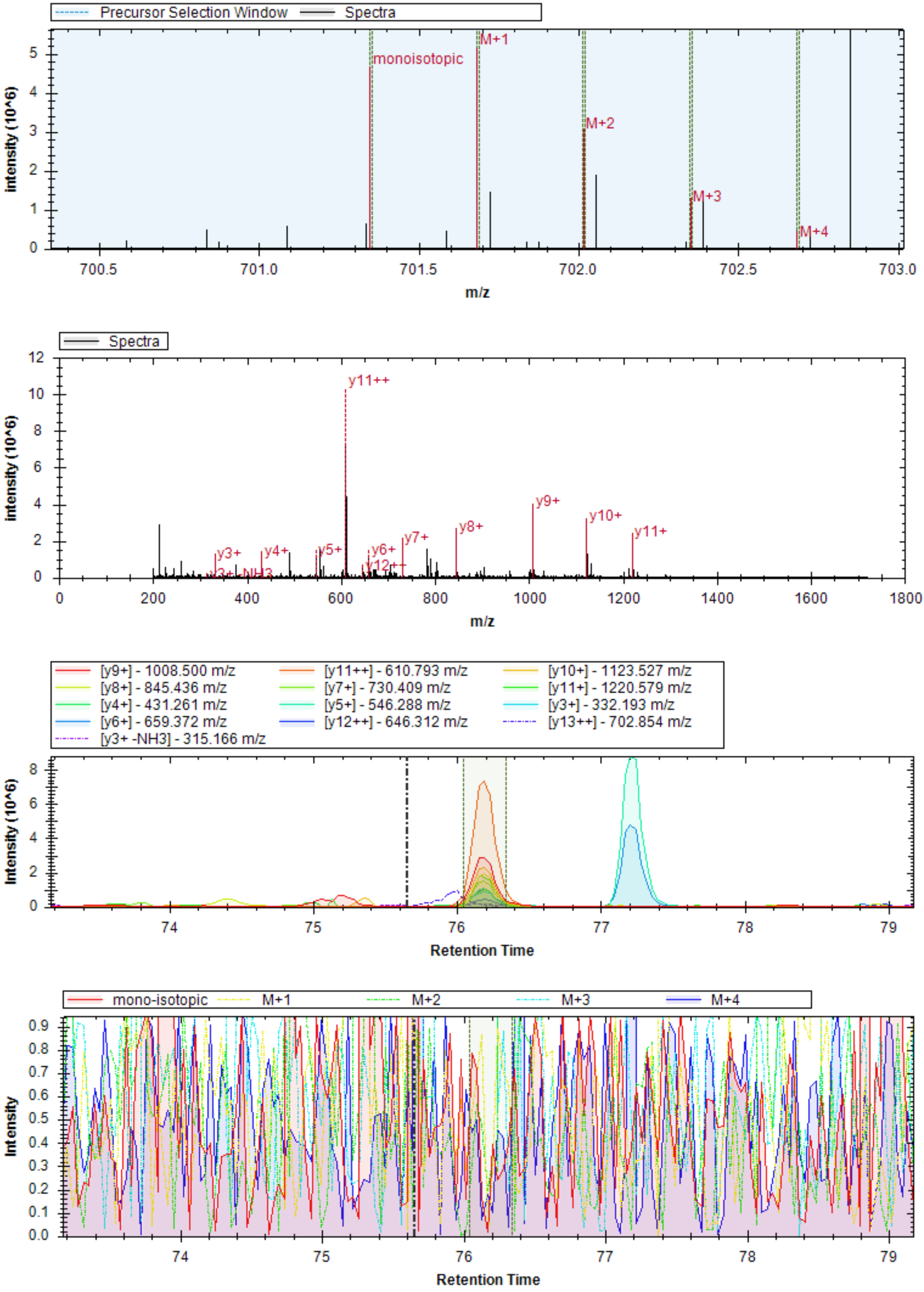

Q3U4B4 – _RHKT[+H+O3+P]LSANEEK_.2
Fig4_MP-DIA-barrel-cortex-p15-3_MHRM.htrms

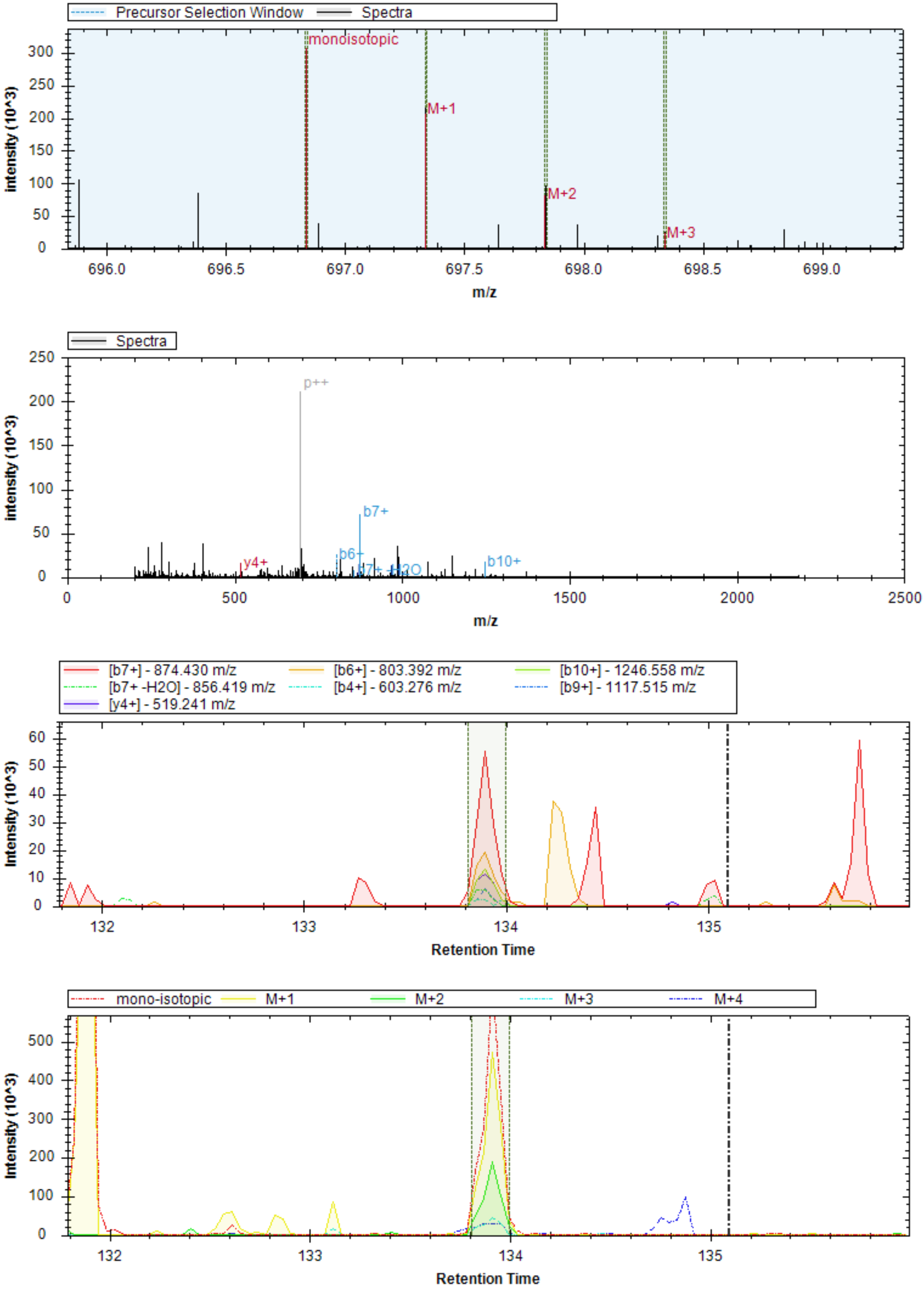

Q8CAA7 – _AVAGVM[+O]ITAS[+H+O3+P]HNR_.2
Fig4_MP-DIA-barrel-cortex-p15-3_MHRM.htrms

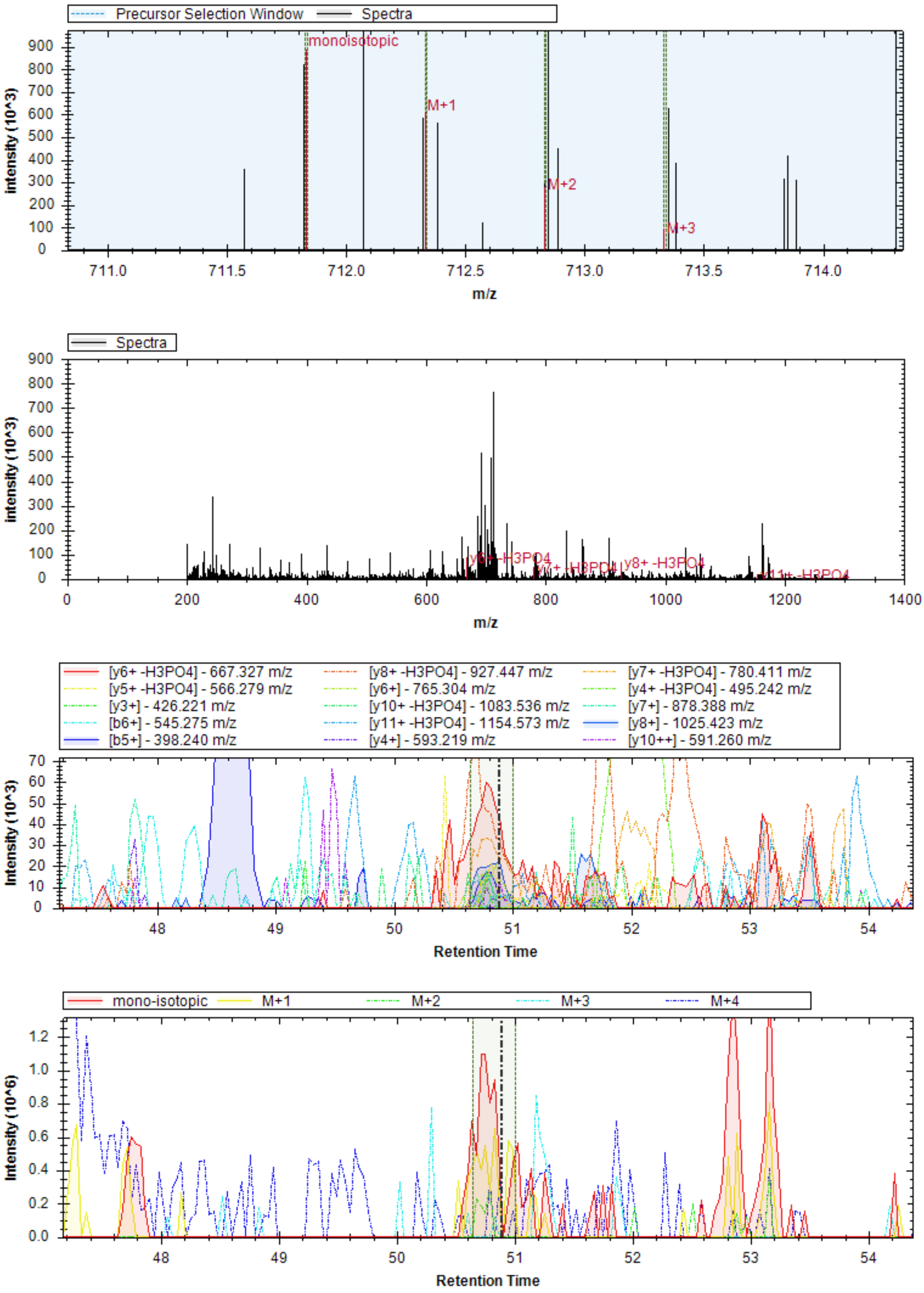

Q8CAA7 – _AVAGVMIT[+H+O3+P]ASHNR_.2
Fig4_MP-DIA-barrel-cortex-p15-3_MHRM.htrms

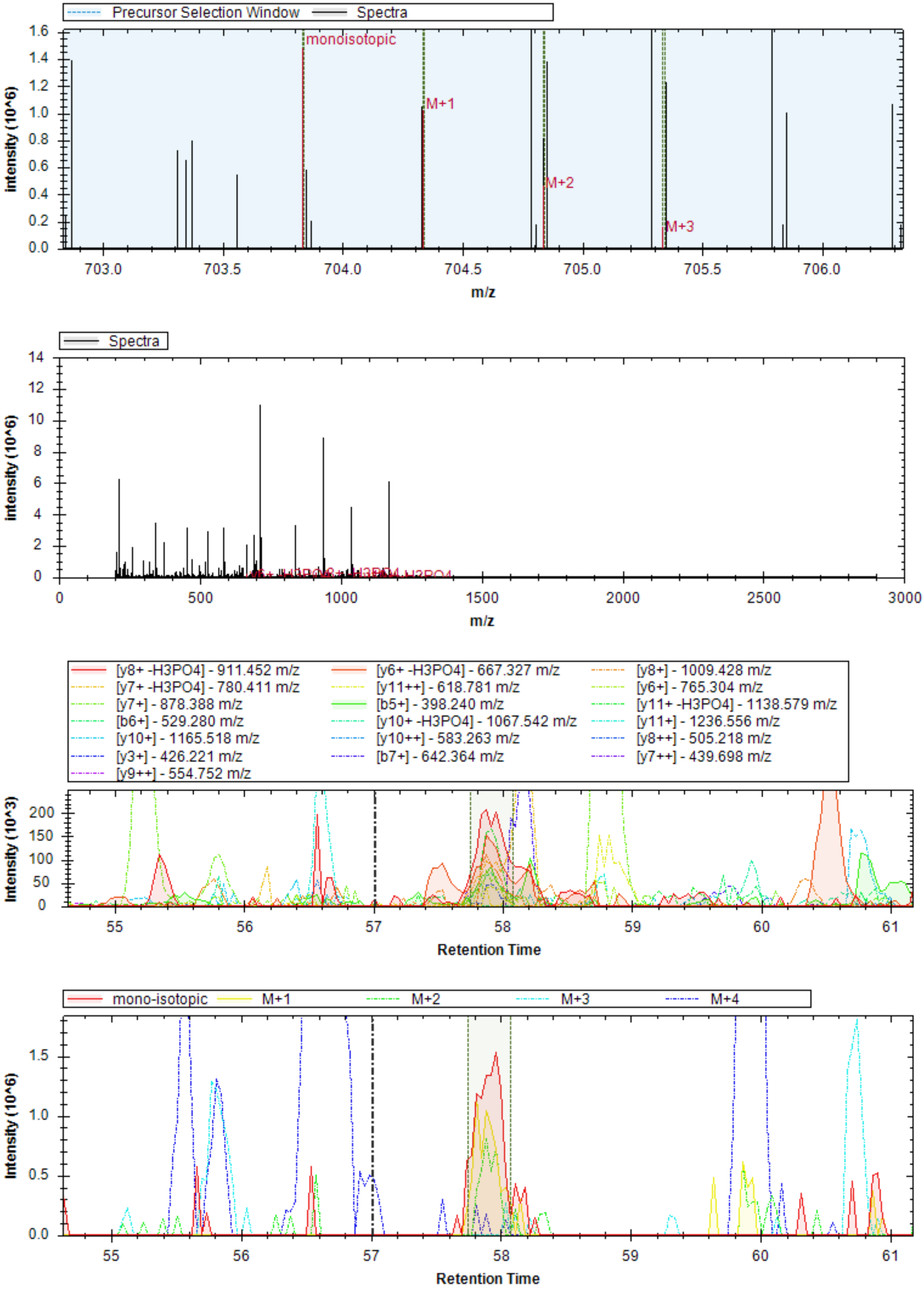

Q8CAA7 – _AVAGVMITAS[+H+O3+P]HNR_.2
Fig4_MP-DIA-barrel-cortex-p15-3_MHRM.htrms

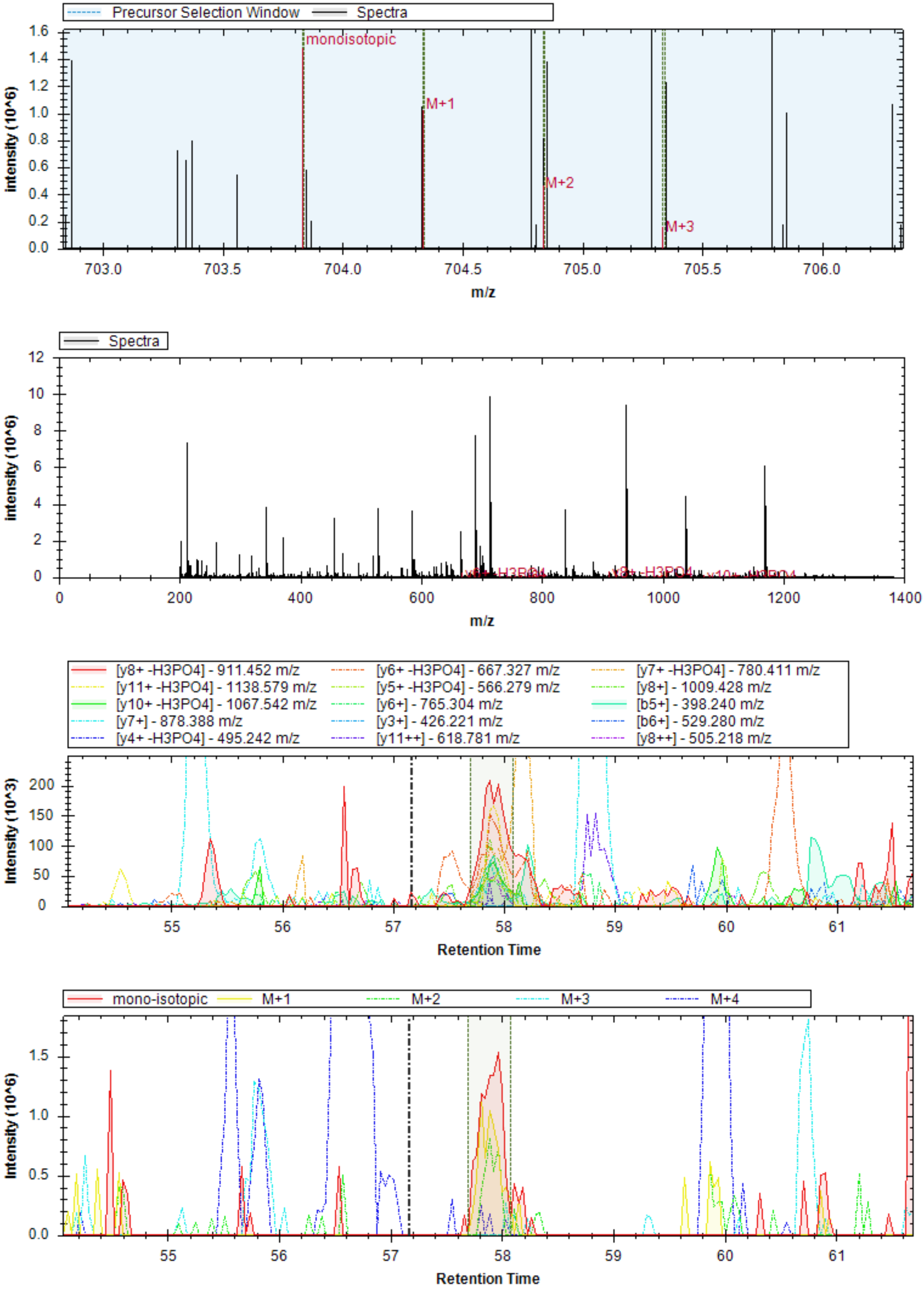

Q99PU8;Q99PU8-2 – _ALGRM[+O]VERS[+H+O3+P]LR_.2

Fig4_MP-DIA-barrel-cortex-p15-3_MHRM.htrms

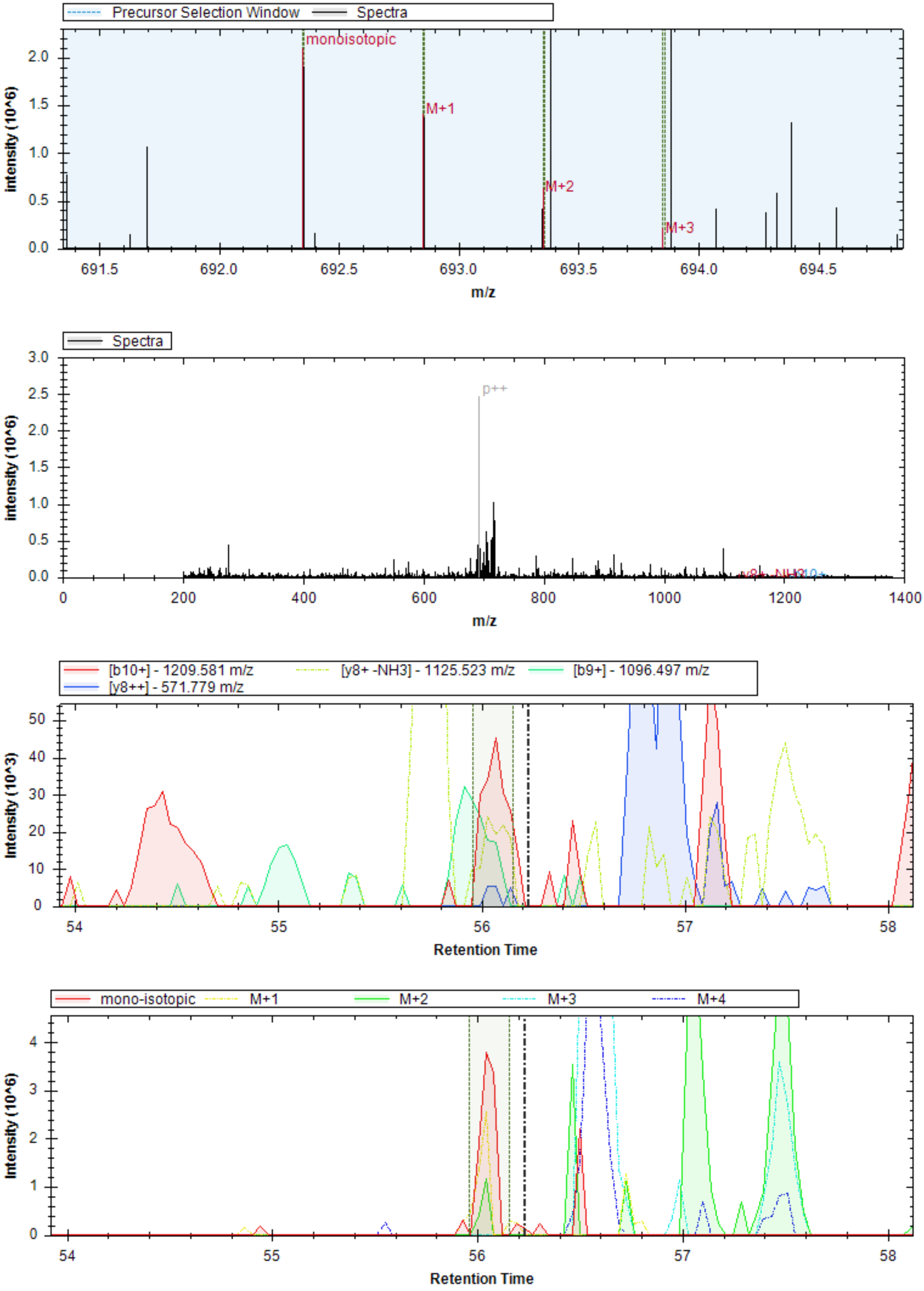

Q9QYC0 – _SPGT[+H+O3+P]PAGEGSGSPPK_.2
Fig4_MP-DIA-barrel-cortex-p15-3_MHRM.htrms

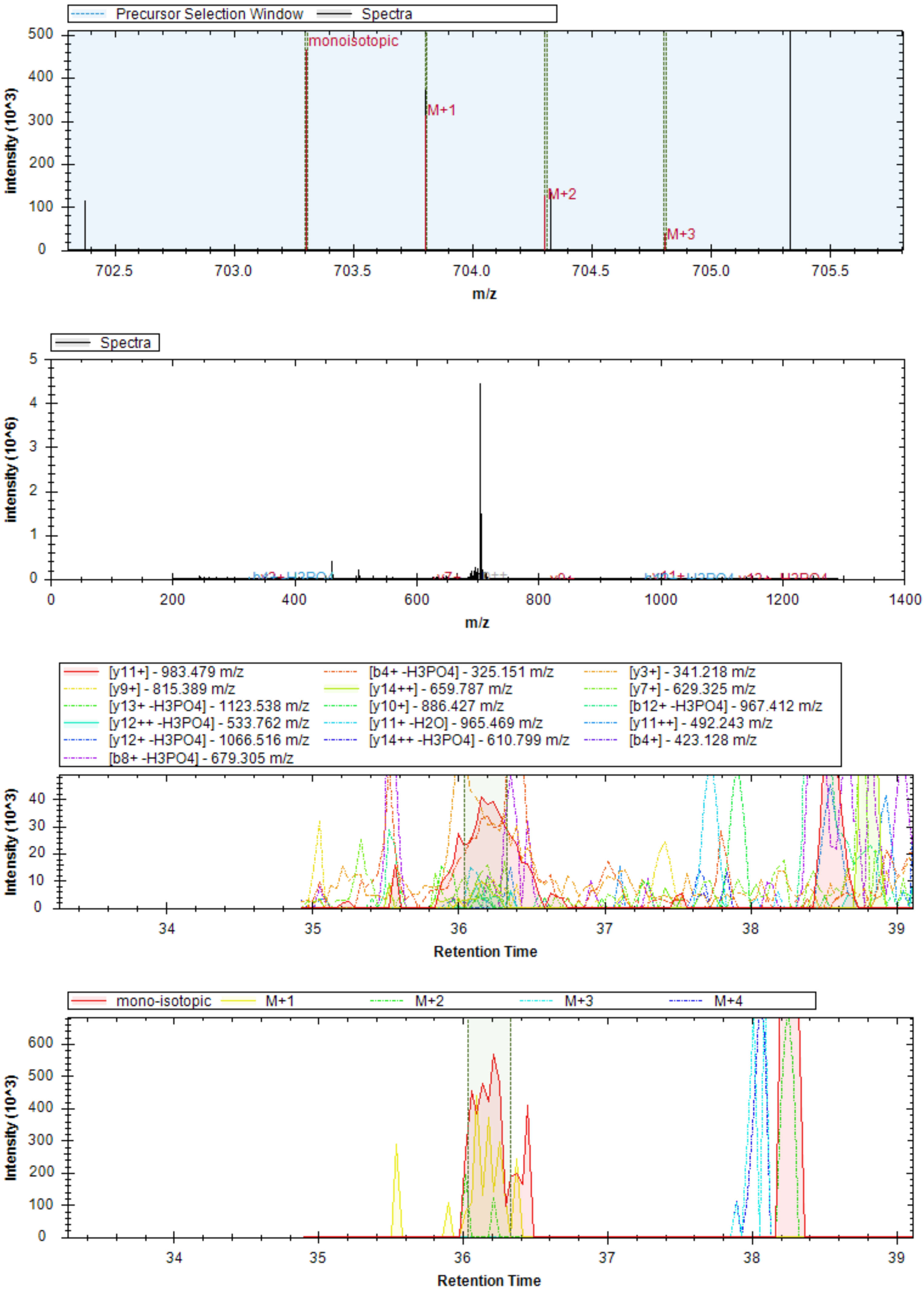

P10637 – _SGYSS[+H+O3+P]PGSPGTPGSR_.2
Fig4_MP-DIA-barrel-cortex-p15-3_MHRM.htrms

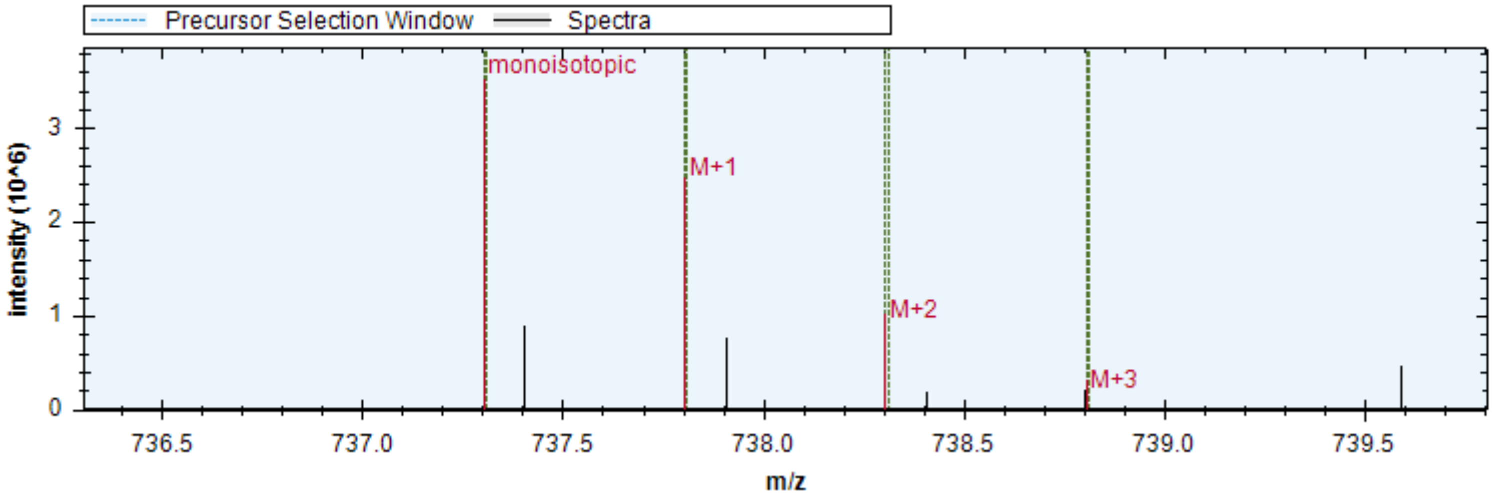

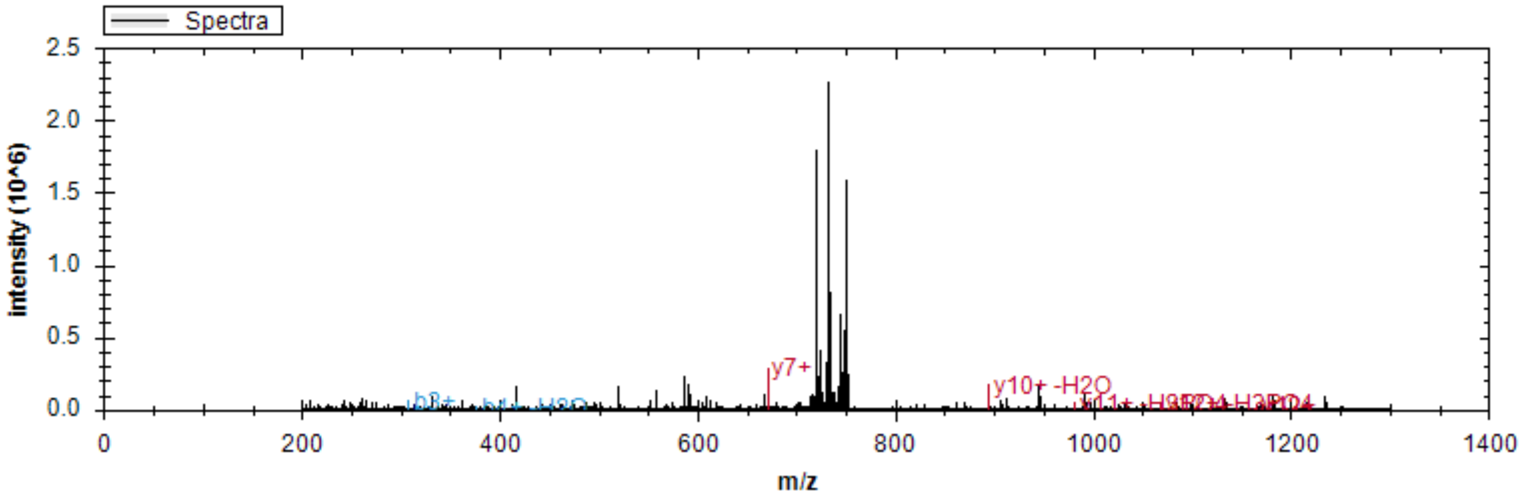

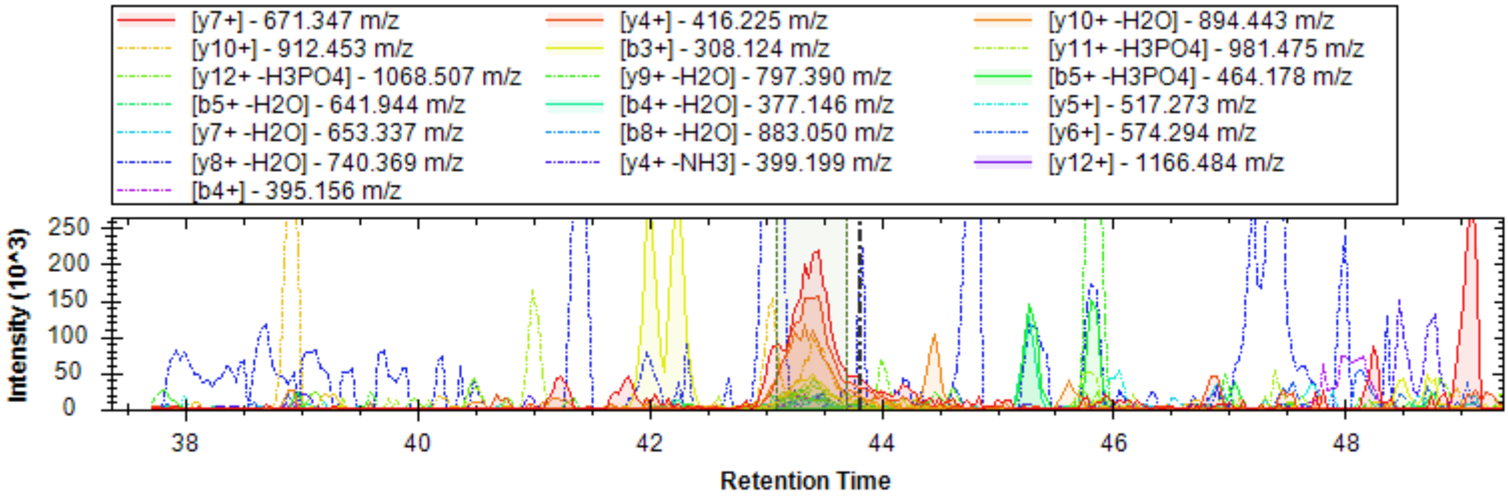

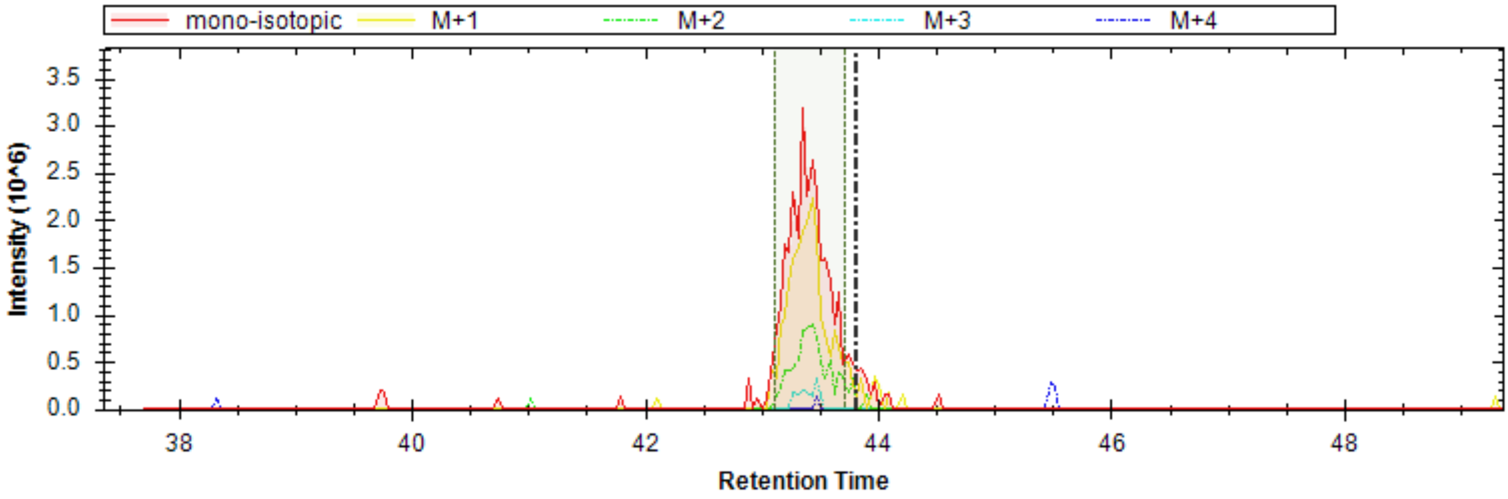

P10637 – _SGYSSPGS[+H+O3+P]PGTPGSR_.2
Fig4_MP-DIA-barrel-cortex-p15-3_MHRM.htrms

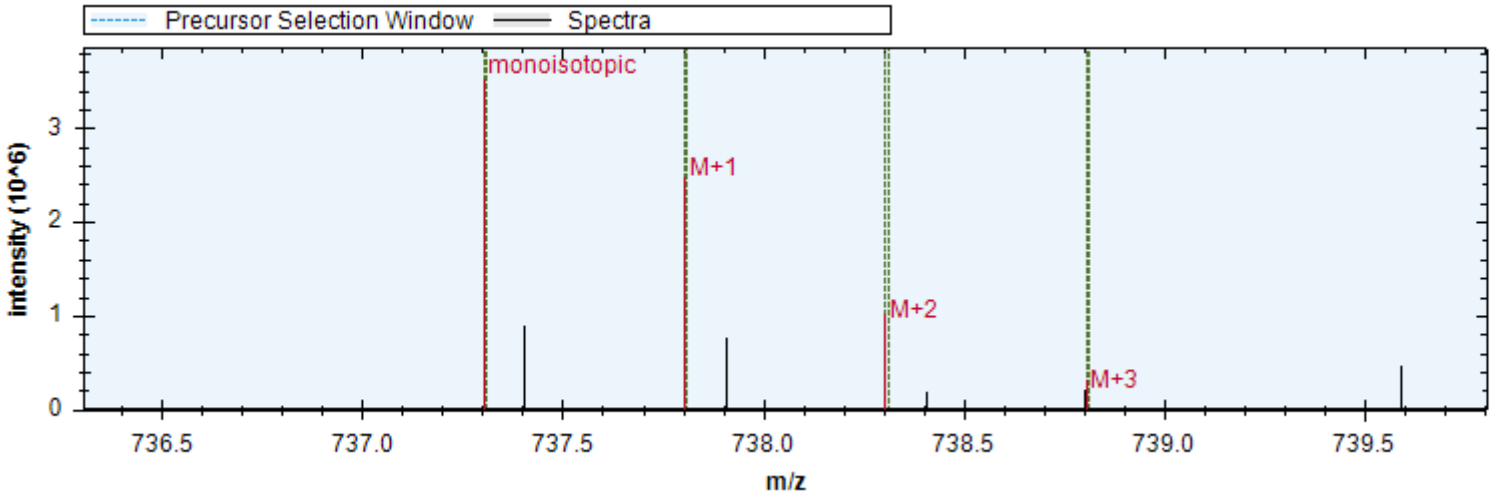

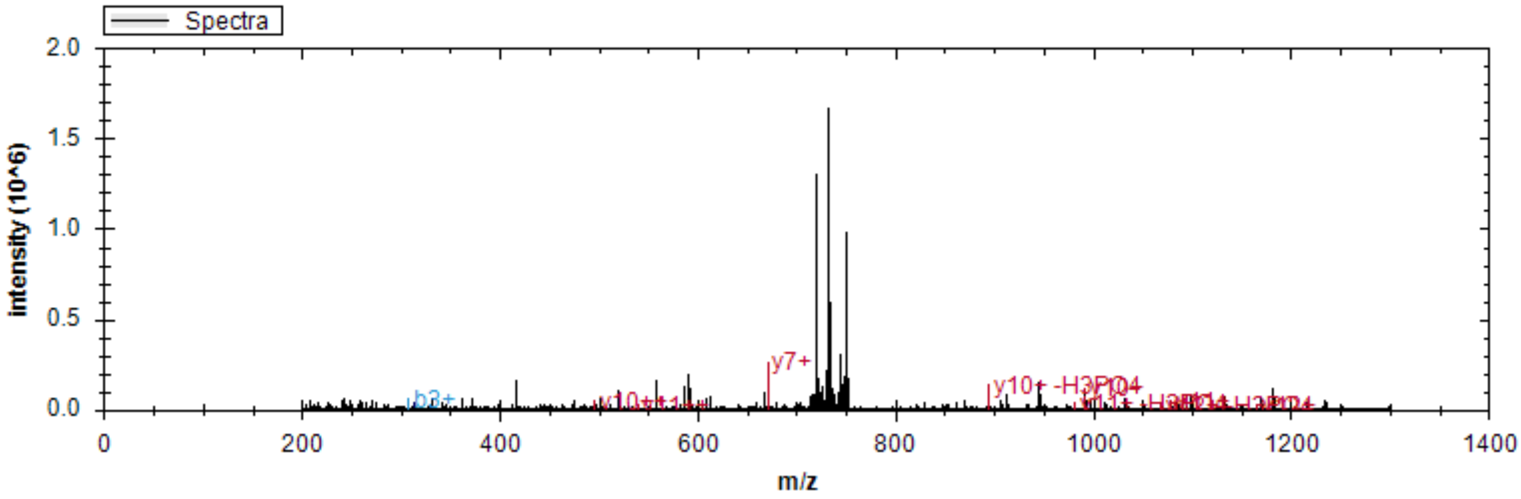

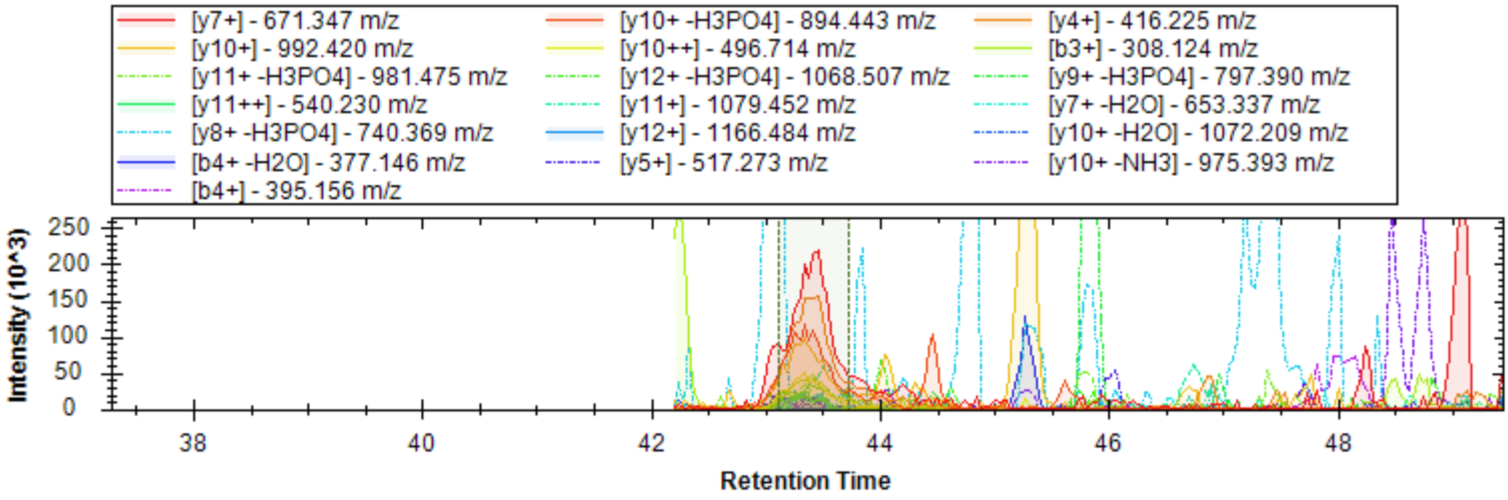

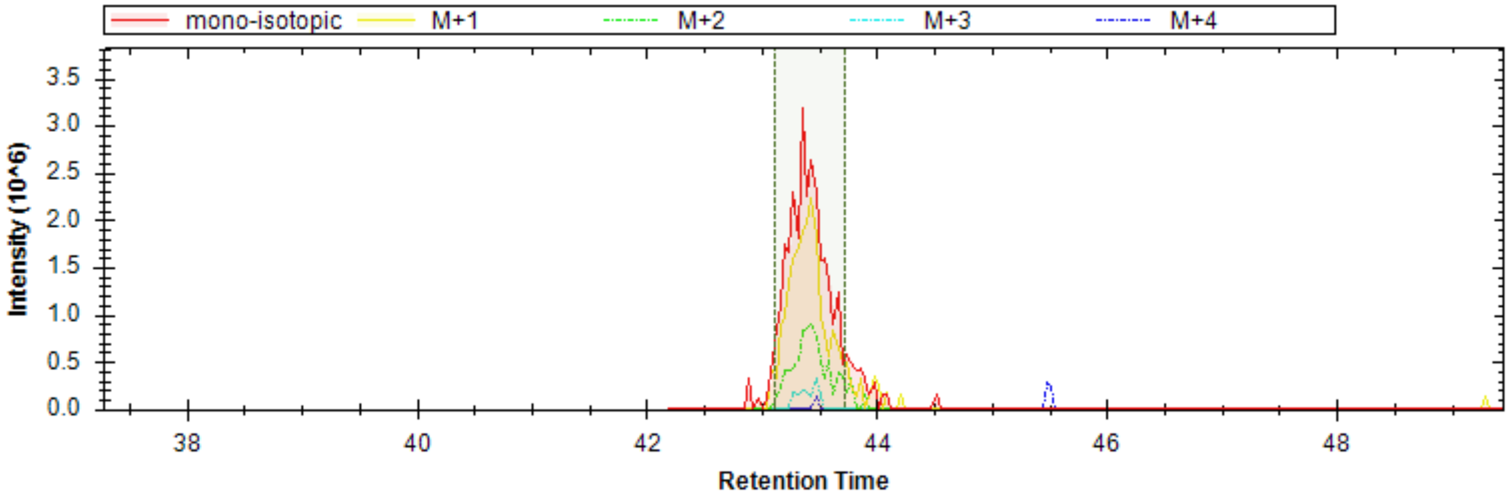

P57776-3 – _ATAPQTQHVS[+H+O3+P]PMR_.2
Fig4_MP-DIA-barrel-cortex-p15-3_MHRM.htrms

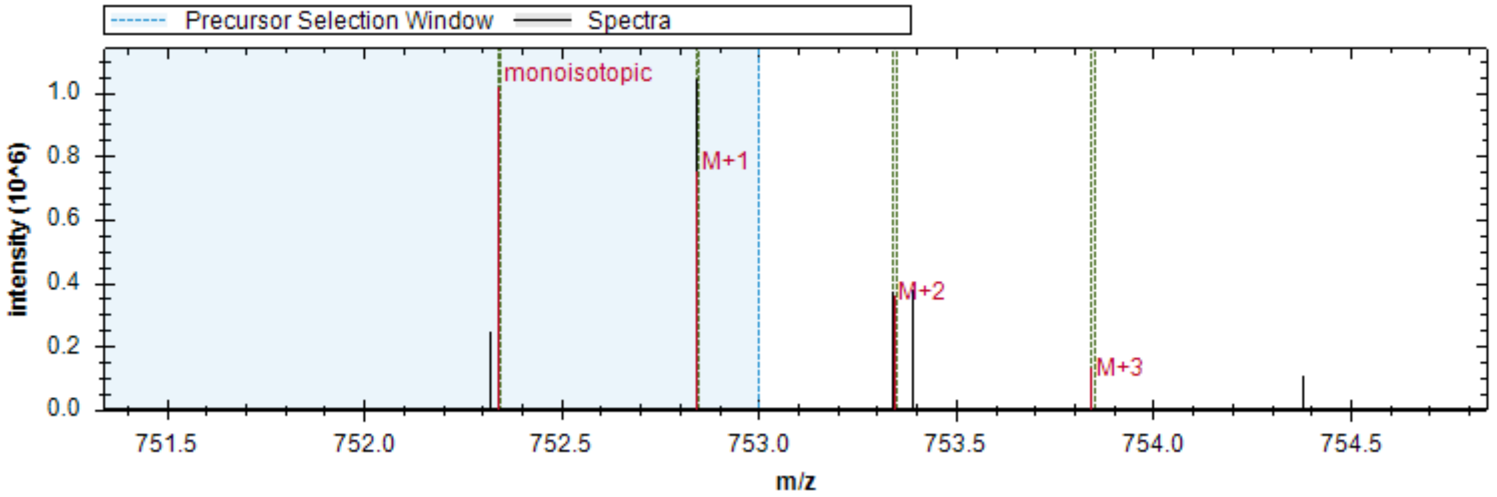

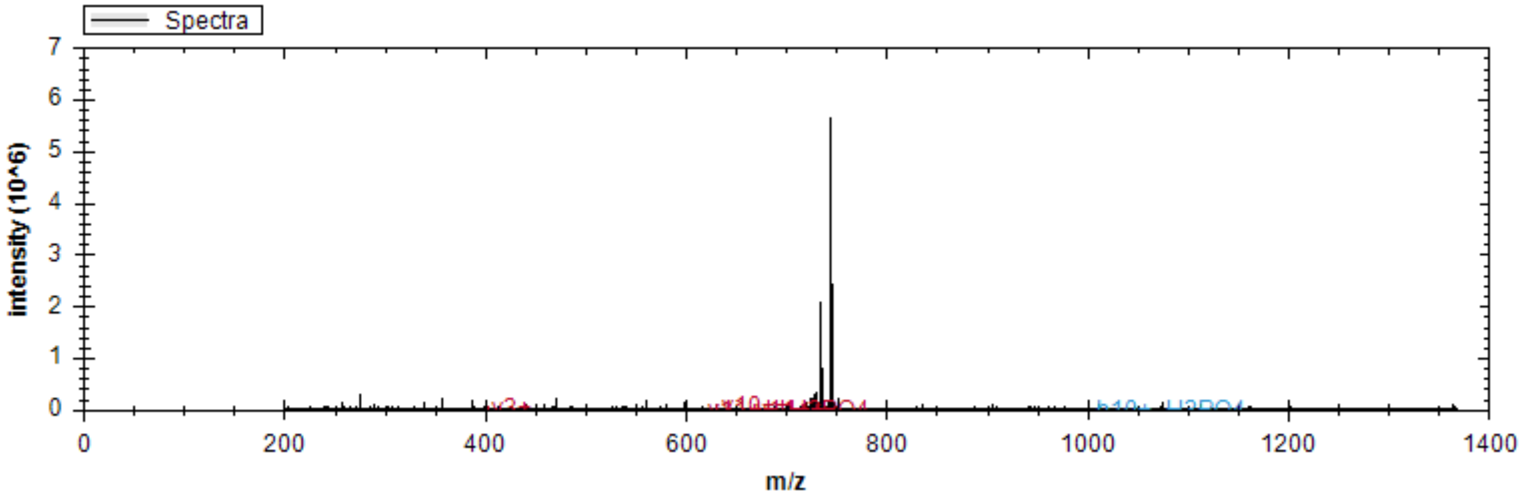

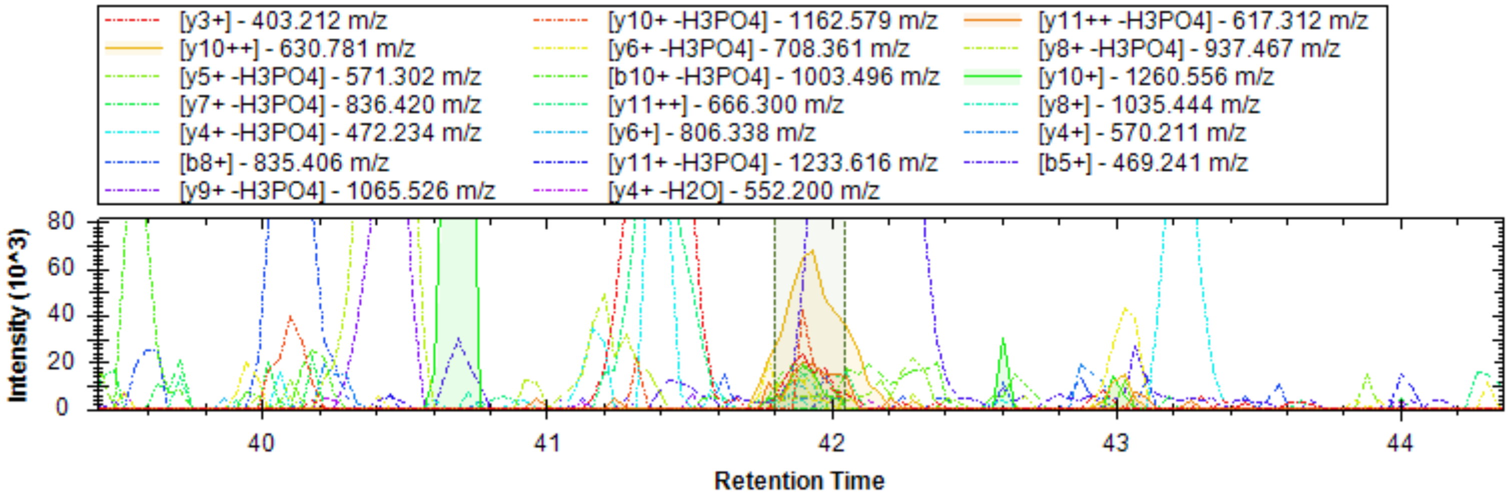

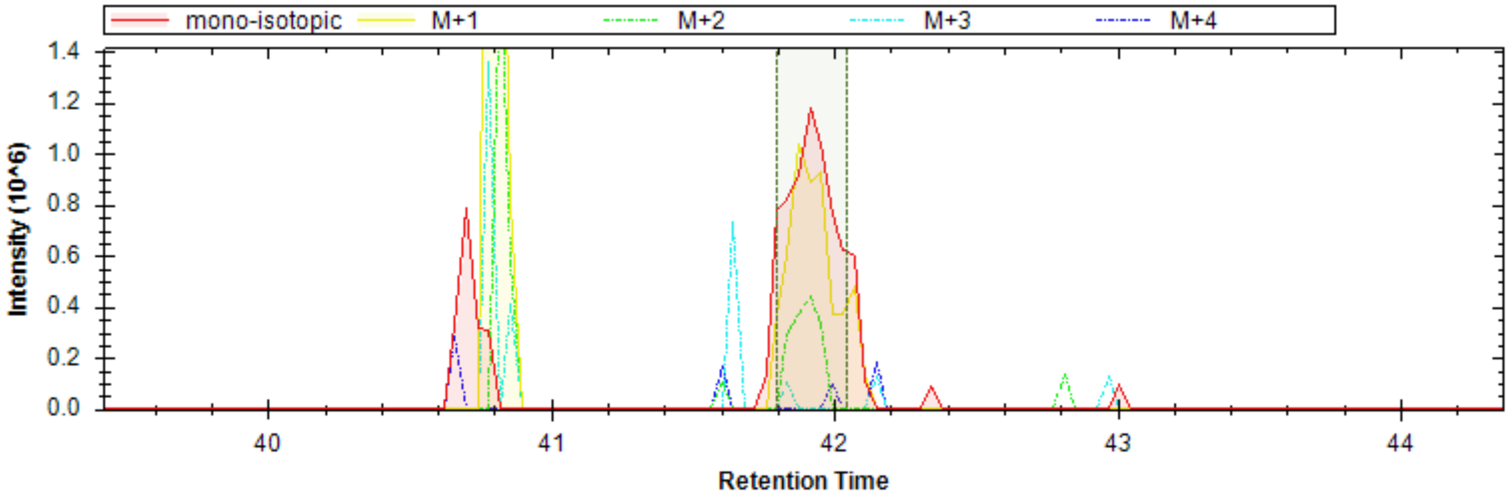

P97798-2 – _NSQDIT[+H+O3+P]PVDNSMDSNIHQR_.3
Fig4_MP-DIA-barrel-cortex-p15-3_MHRM.htrms

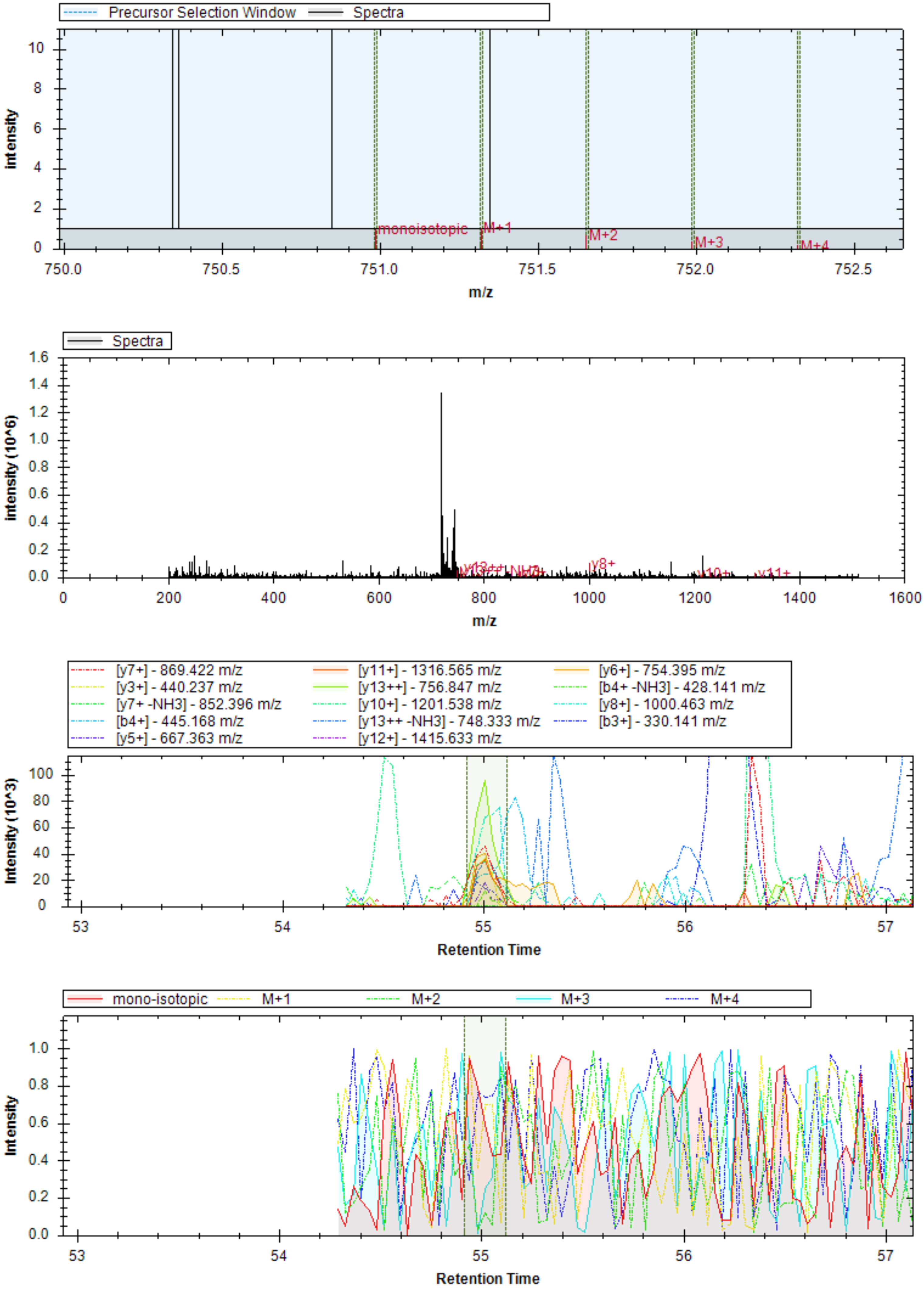

Q8C5R2 – _LAGNEALS[+H+O3+P]PTSPSK_.2
Fig4_MP-DIA-barrel-cortex-p15-3_MHRM.htrms

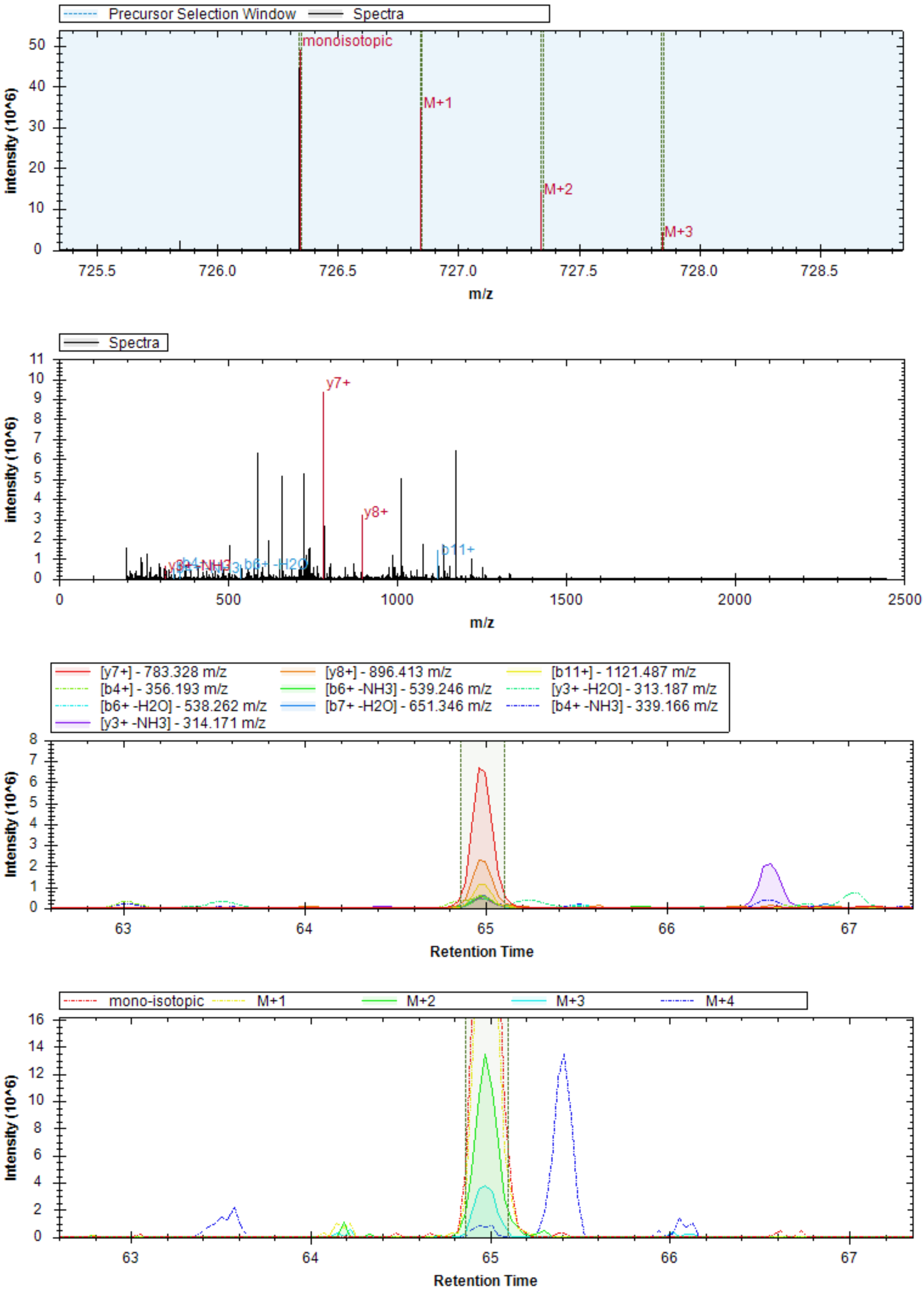

Q8K0U4 – _ETAPTSTYS[+H+O3+P]SPAR_.2

Fig4_MP-DIA-barrel-cortex-p15-3_MHRM.htrms

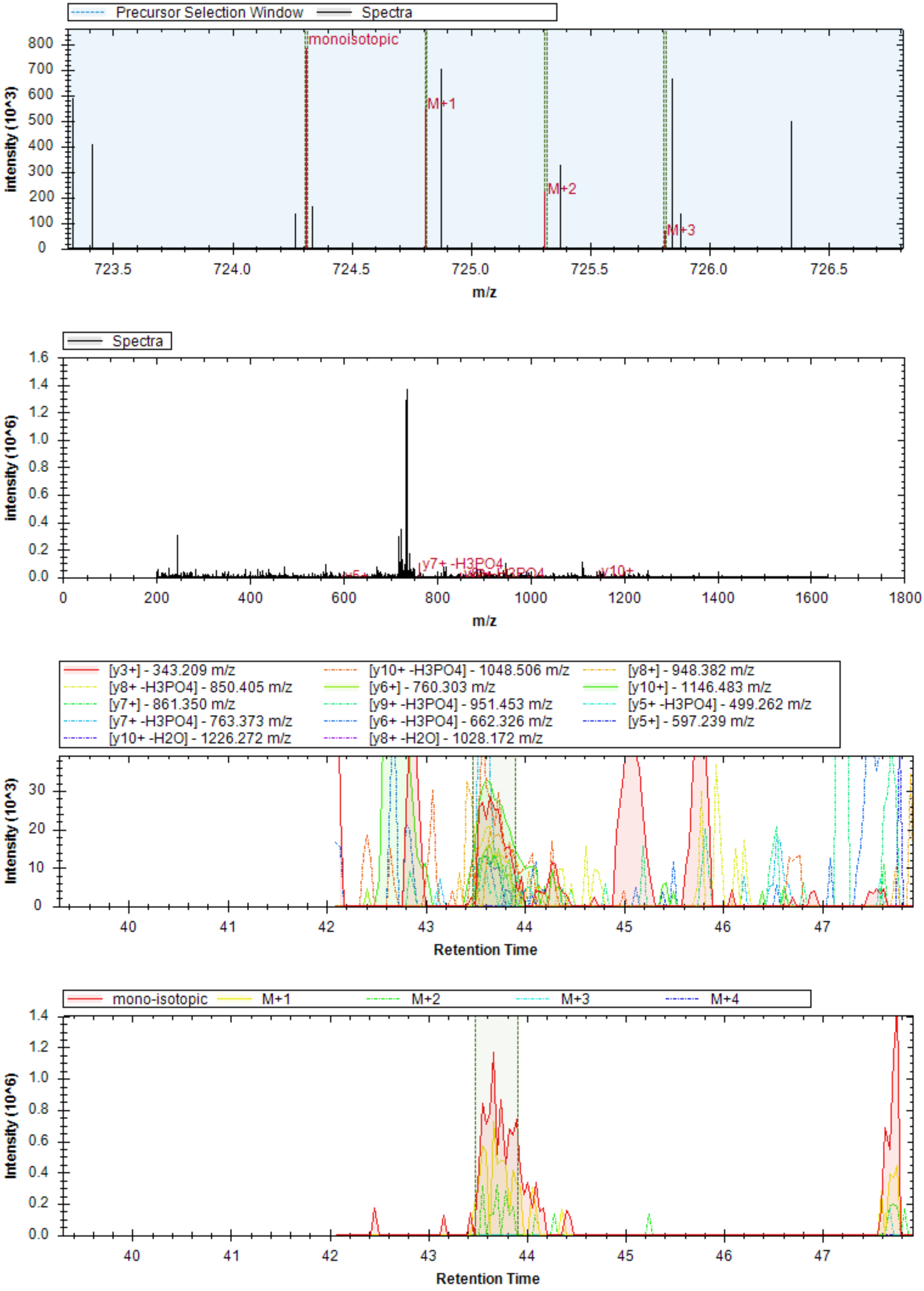

Q8K0U4 – _ETAPTSTYSS[+H+O3+P]PAR_.2
Fig4_MP-DIA-barrel-cortex-p15-3_MHRM.htrms

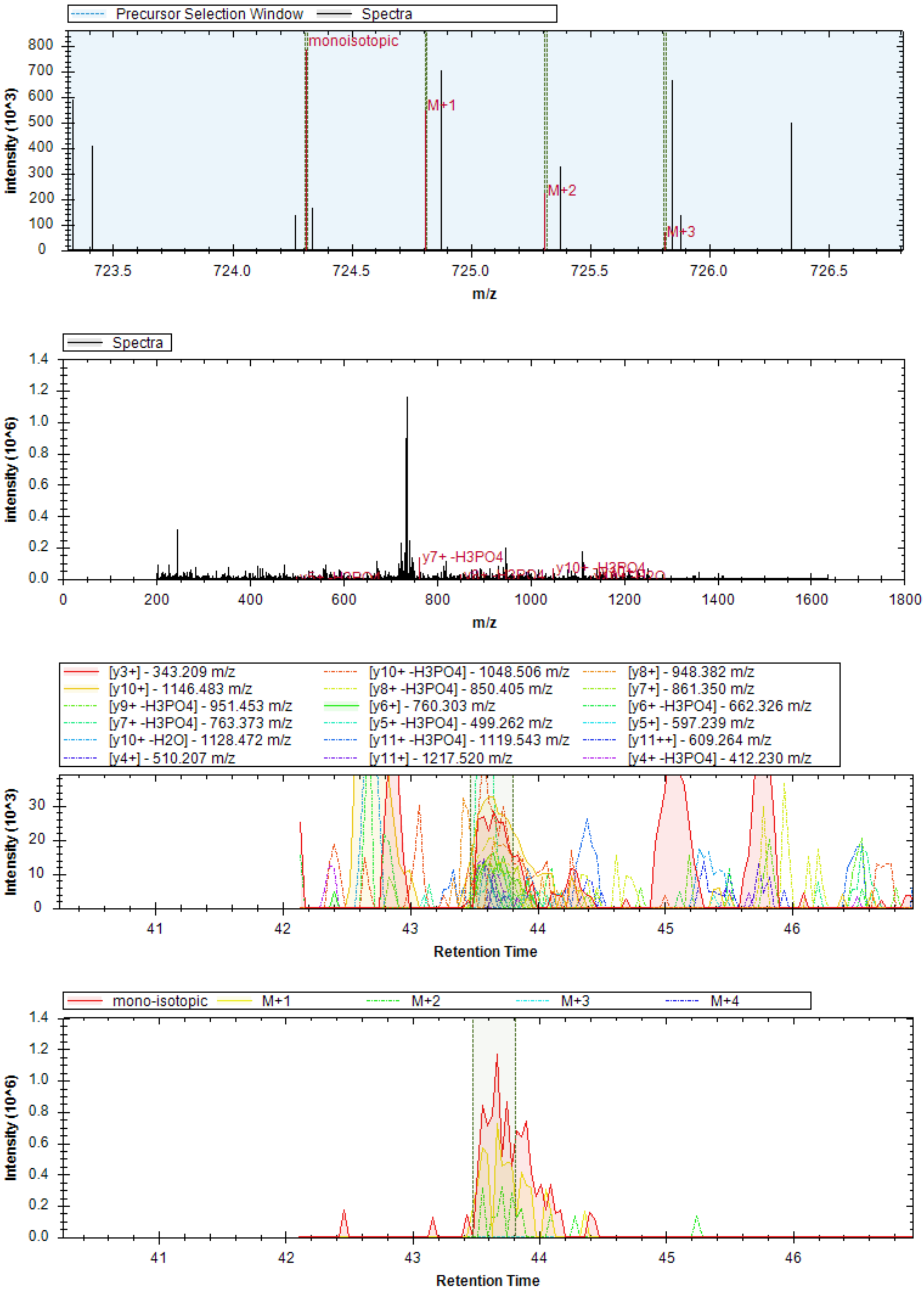

Q8VCI5 – _AKPS[+H+O3+P]PEHAPTISAPDASGPQK_.3
Fig4_MP-DIA-barrel-cortex-p15-3_MHRM.htrms

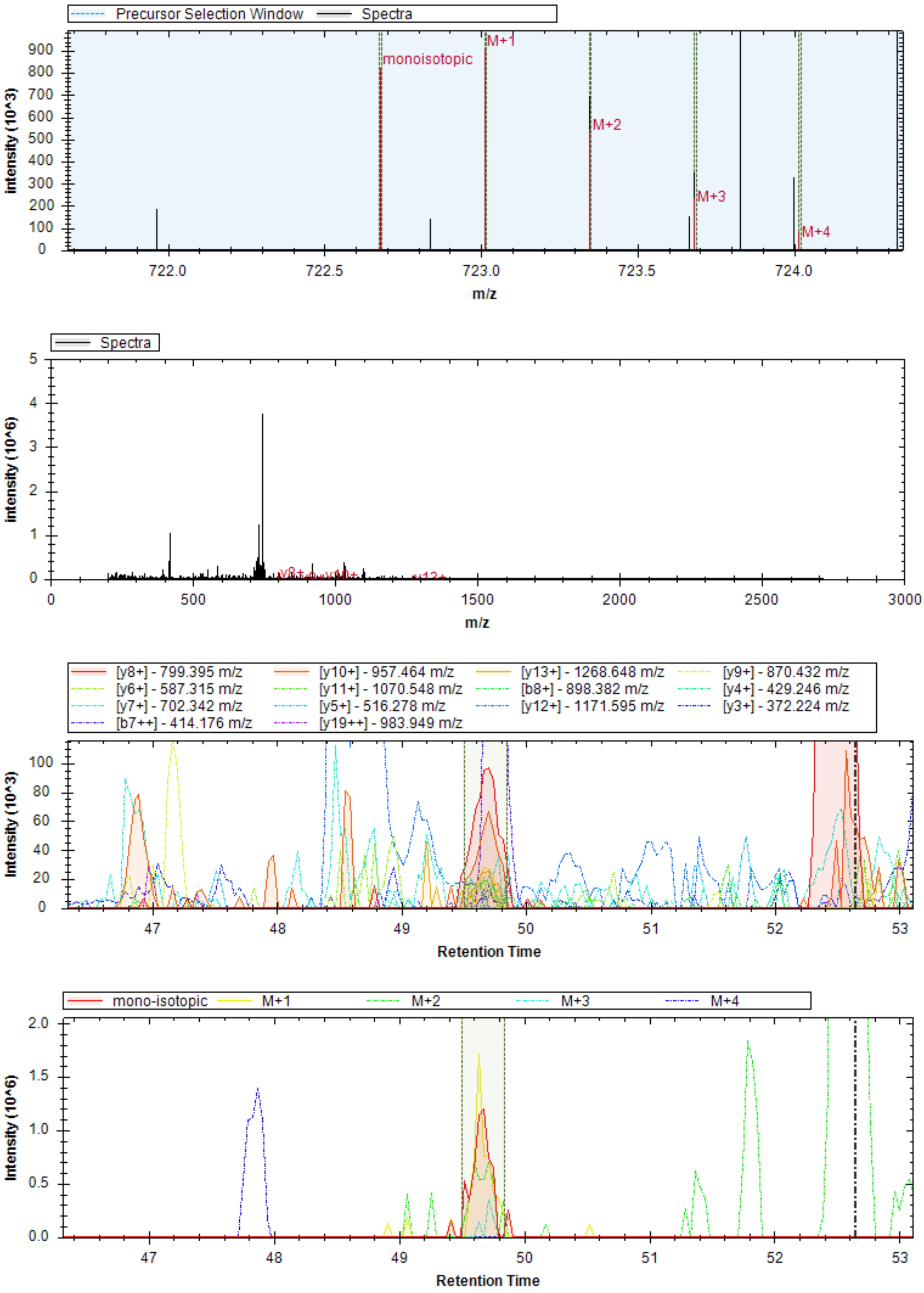

Q9CRB0 – _MEVYIPS[+H+O3+P]FRHEDSDLER_.3

Fig4_MP-DIA-barrel-cortex-p15-3_MHRM.htrms

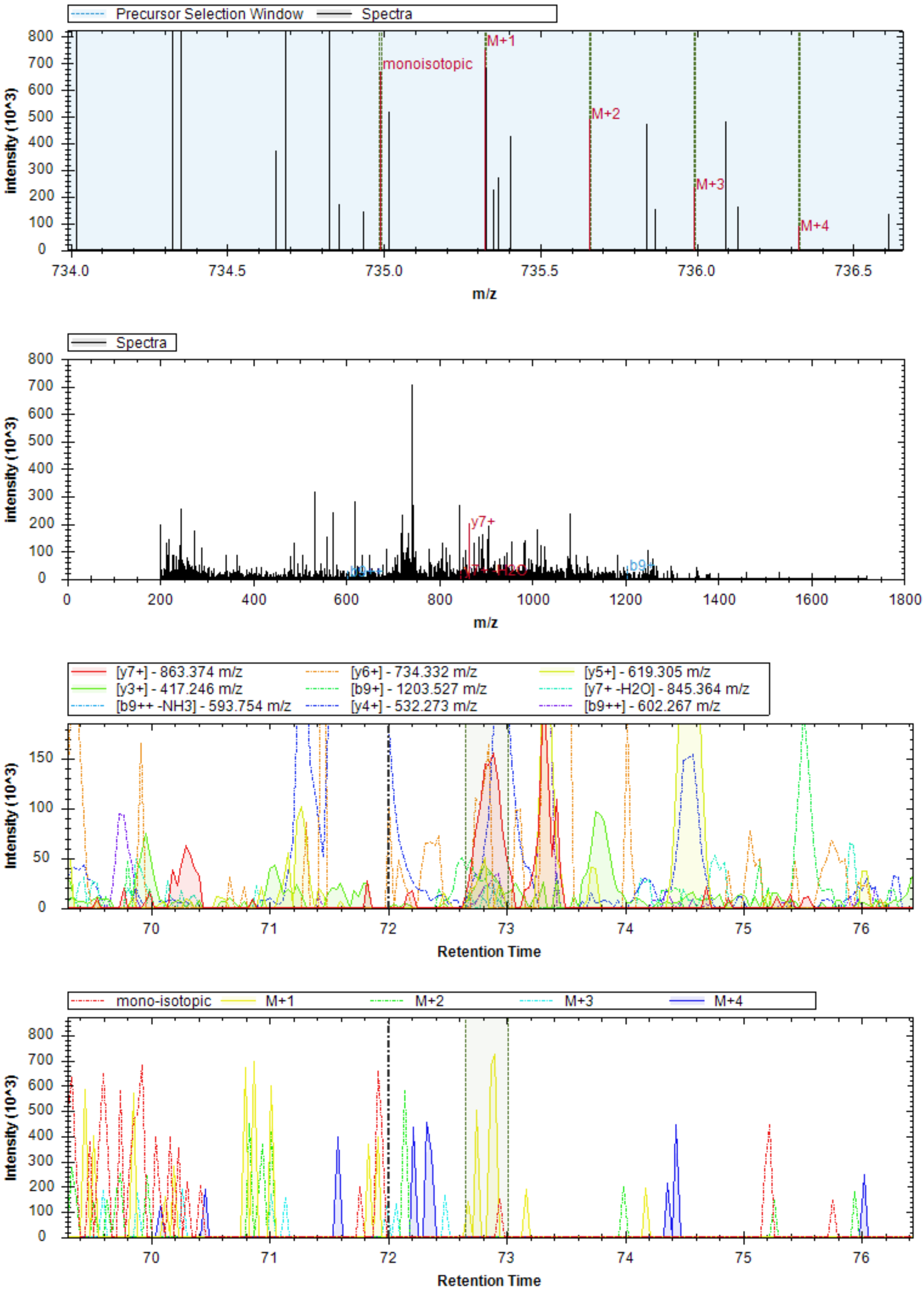

Q9CY18 – _M[+O]GQTVRAVAS[+H+O3+P]SMR_.2

Fig4_MP-DIA-barrel-cortex-p15-3_MHRM.htrms

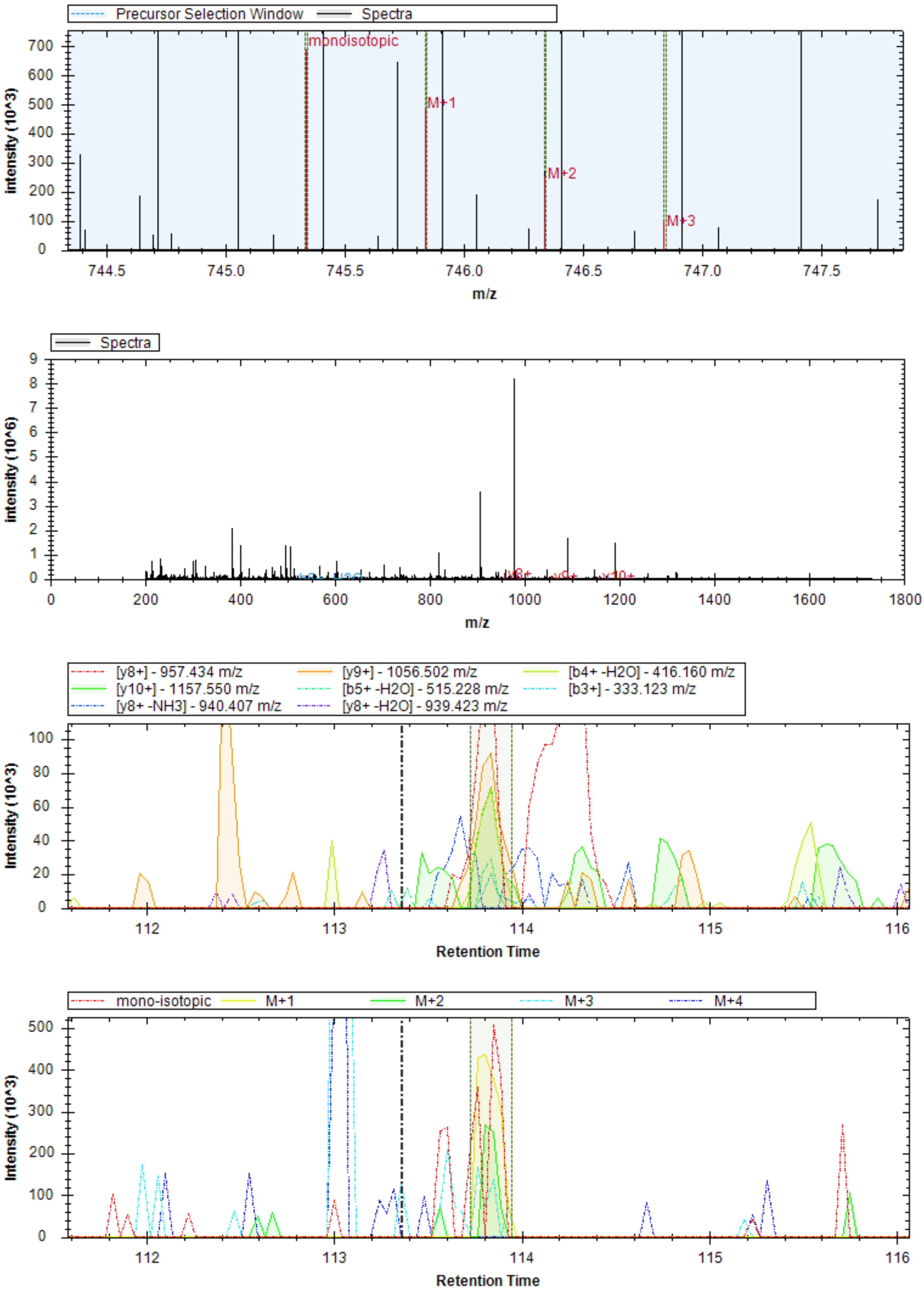

Q9JLM8 – _SKS[+H+O3+P]PASTSSVNGTPGSQLSTPR_.3
Fig4_MP-DIA-barrel-cortex-p15-3_MHRM.htrms

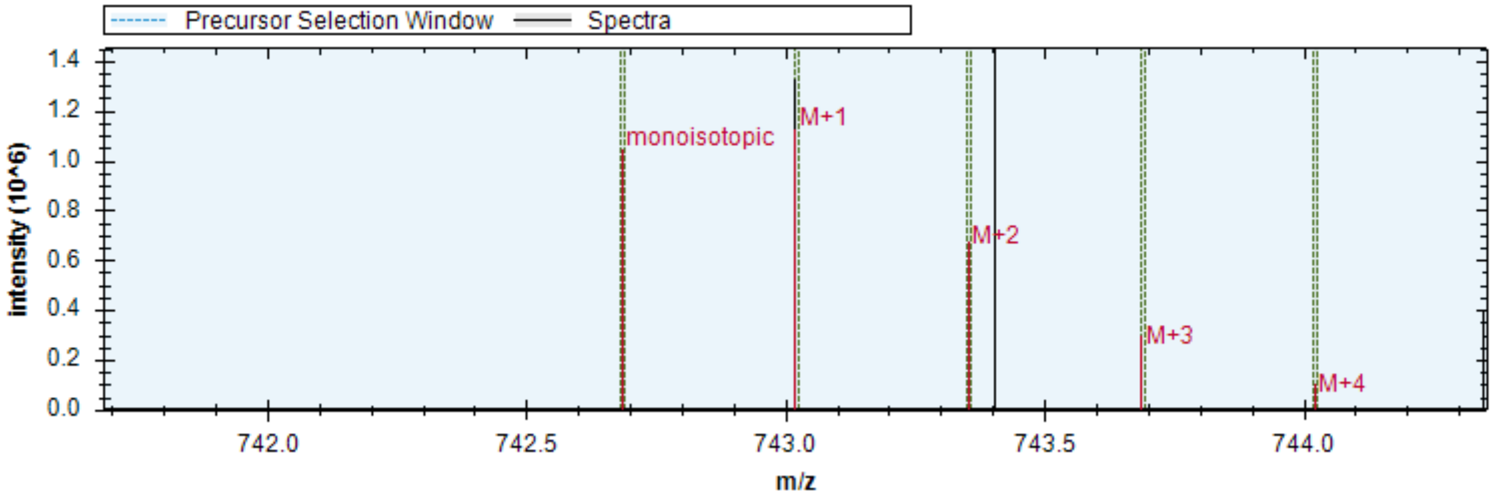

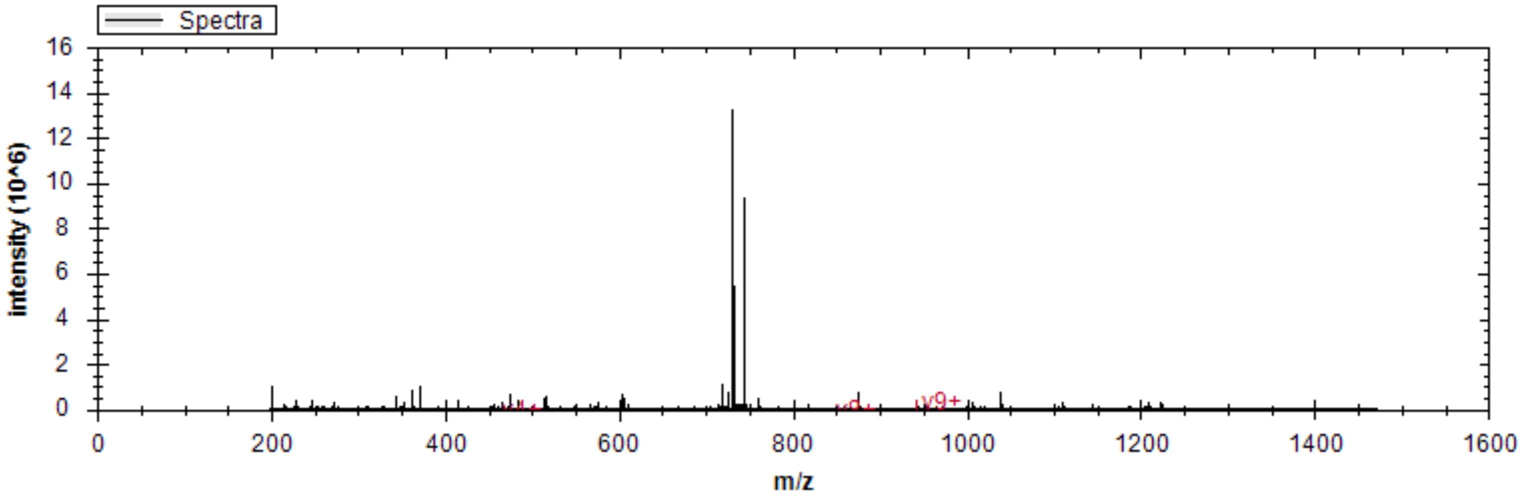

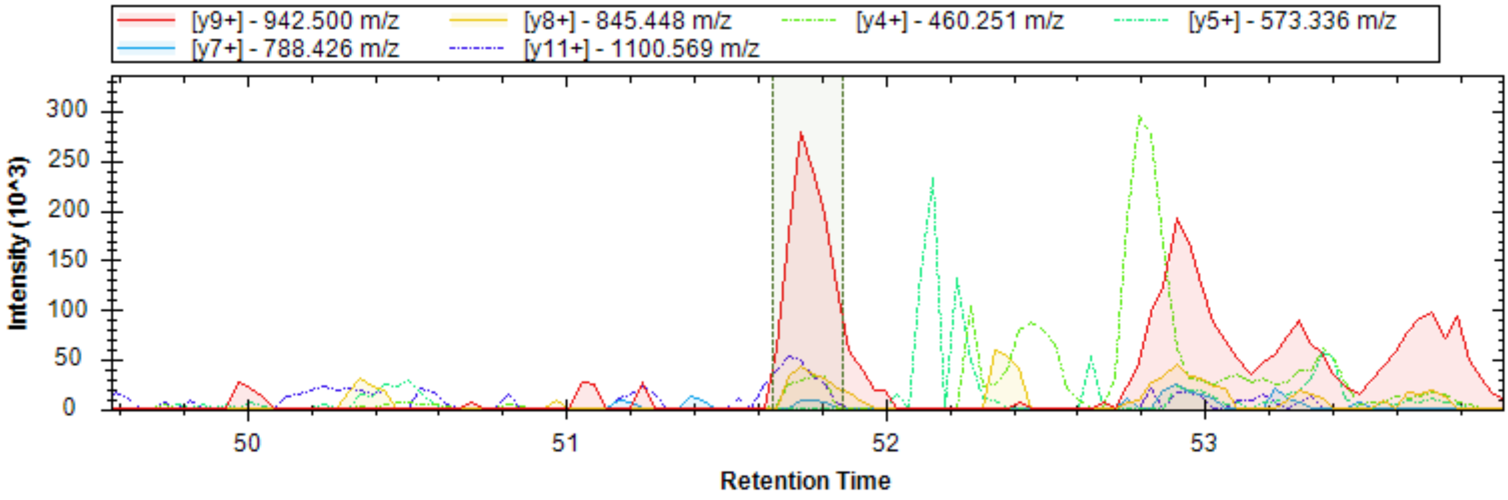

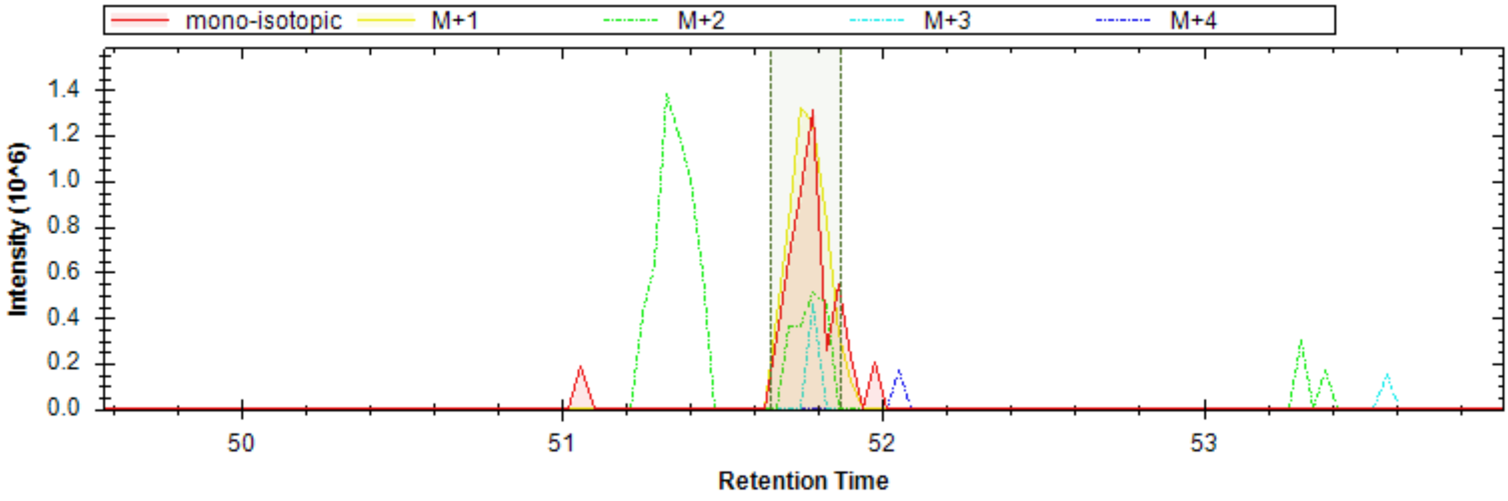

Q9JLM8 – _SPSPSPT[+H+O3+P]SPGSLRK_.2
Fig4_MP-DIA-barrel-cortex-p15-3_MHRM.htrms

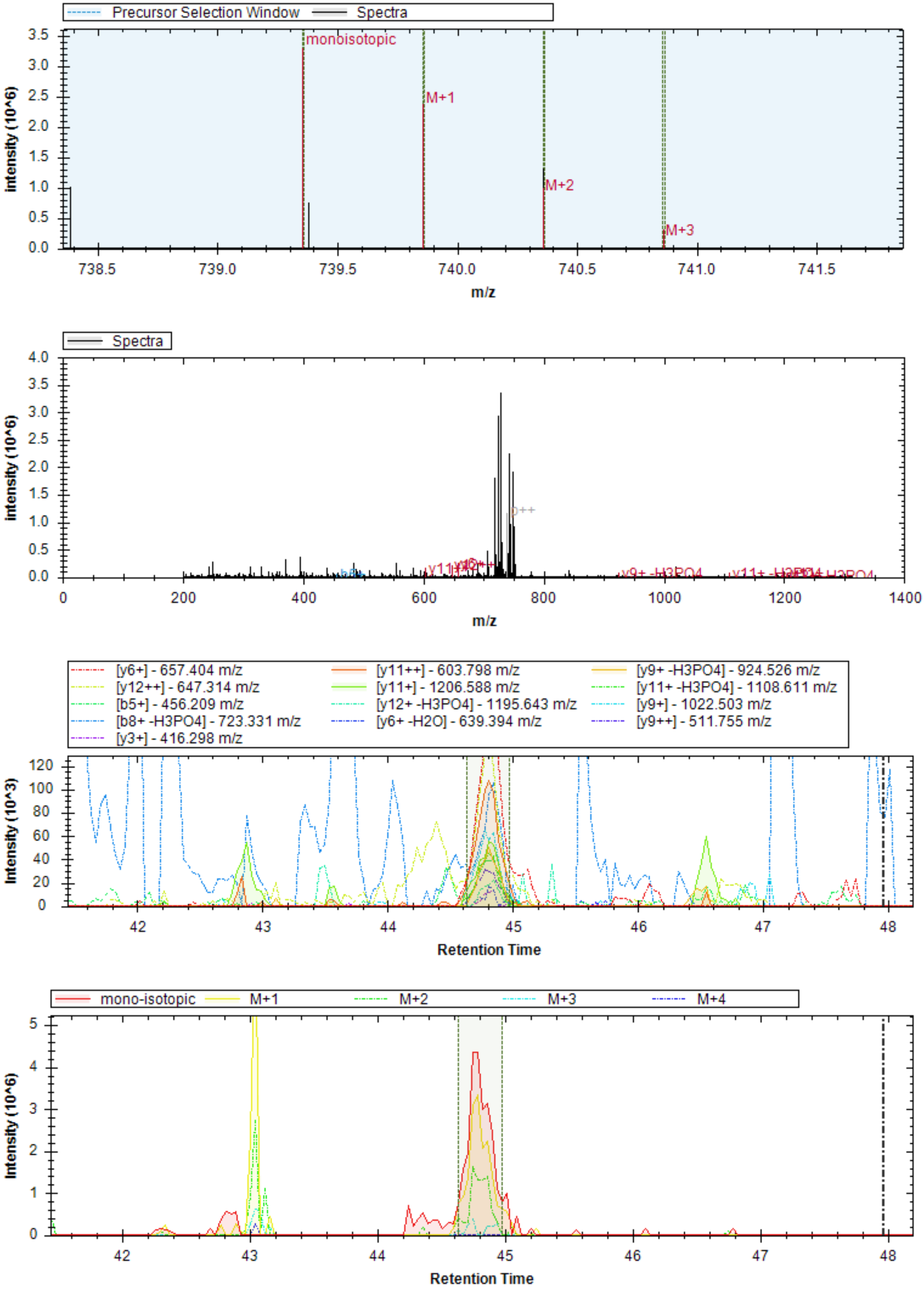

Q9JLM8 – _SPSPSPTS[+H+O3+P]PGSLRK_.2
Fig4_MP-DIA-barrel-cortex-p15-3_MHRM.htrms

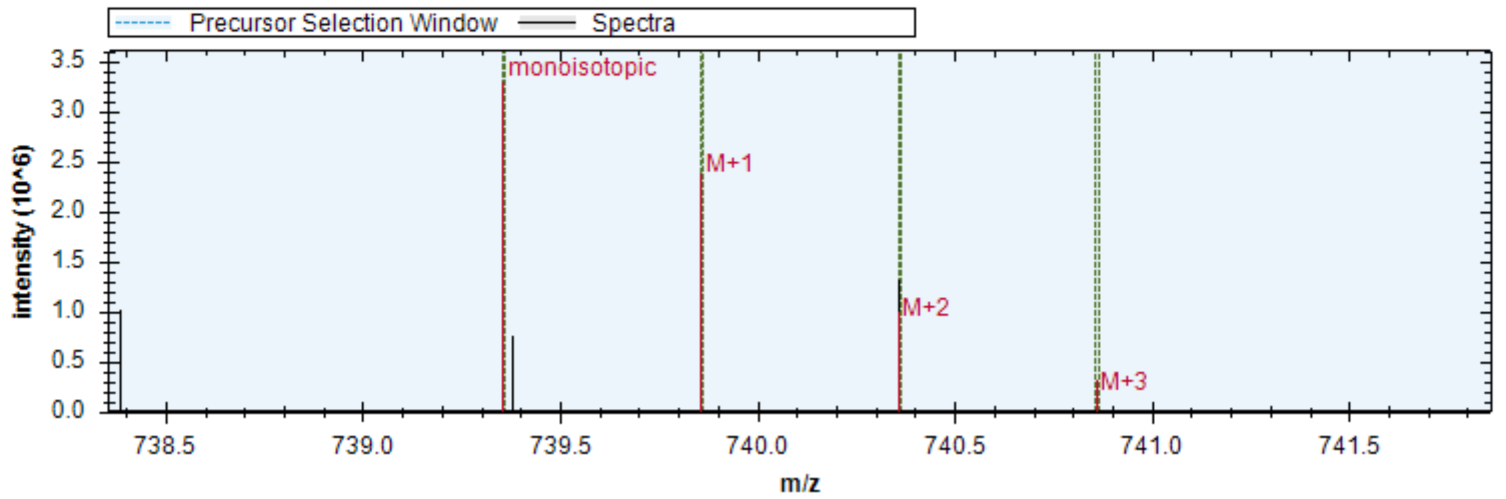

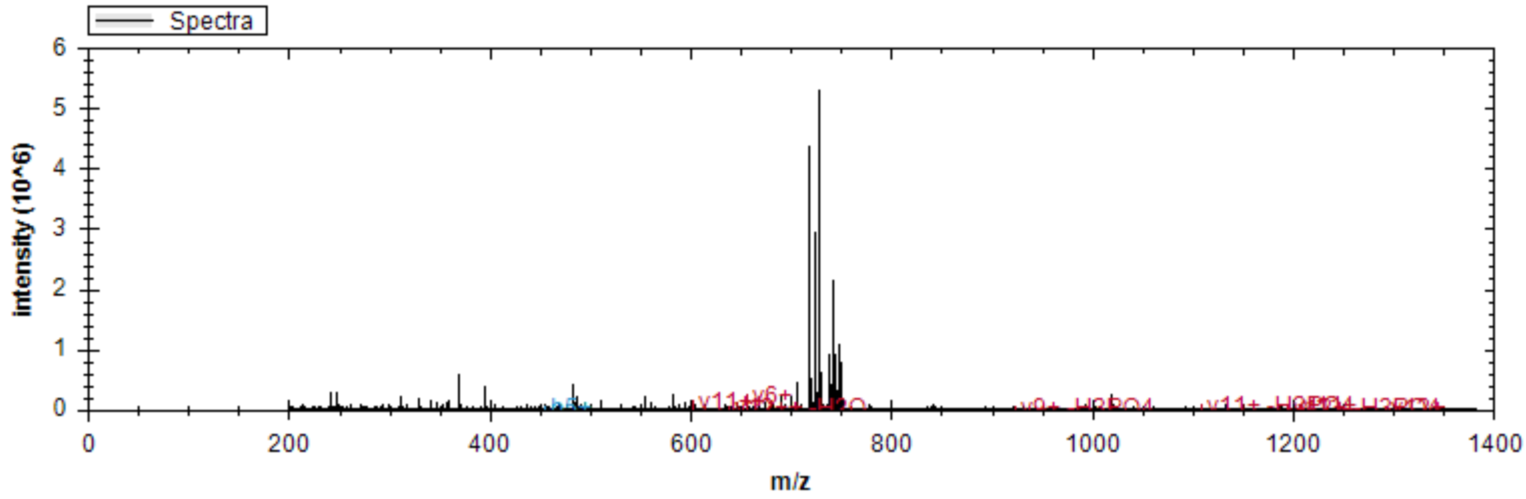

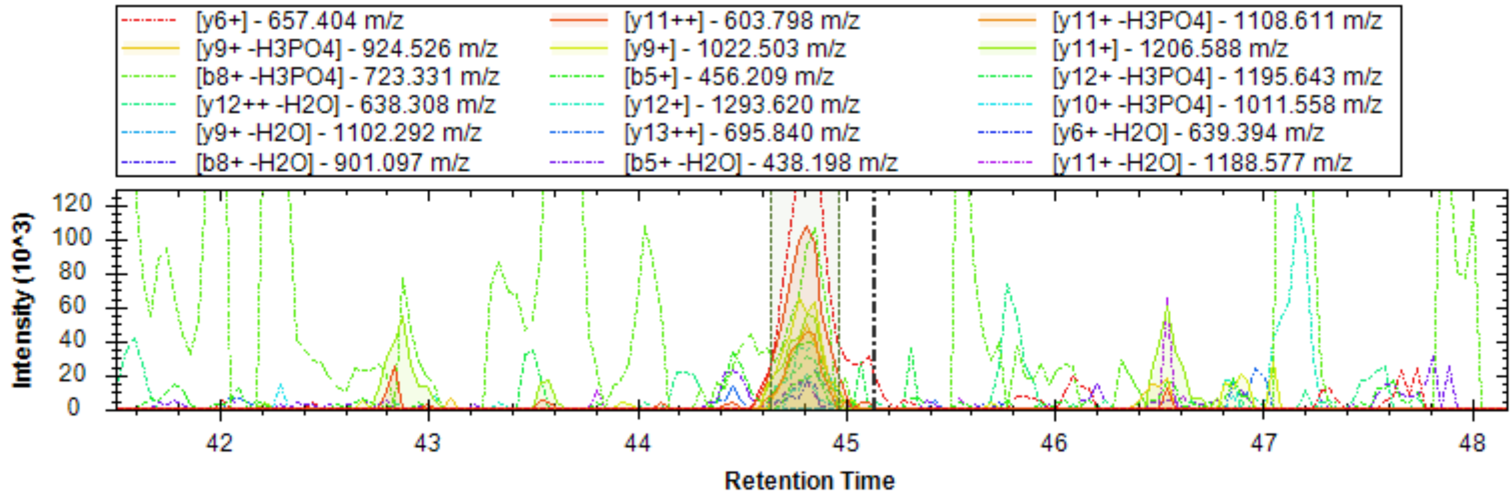

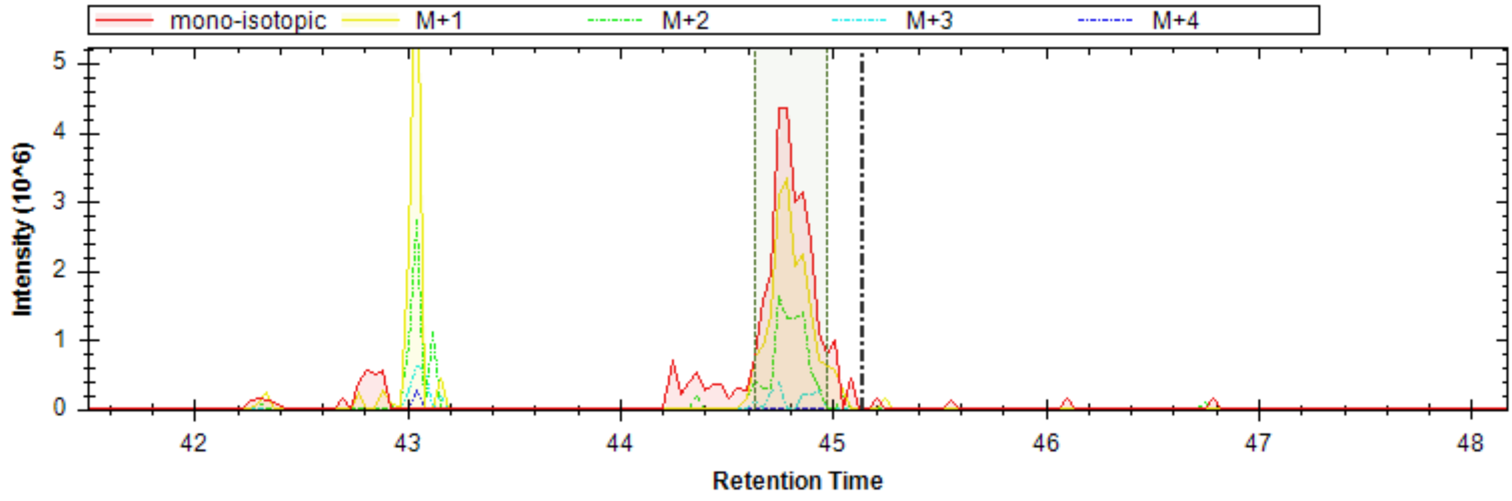

D3Z6S9 – _IPSIEIT[+H+O3+P]ET[+H+O3+P]LPQTQASMLR_.3
Fig4_MP-DIA-barrel-cortex-p15-3_MHRM.htrms

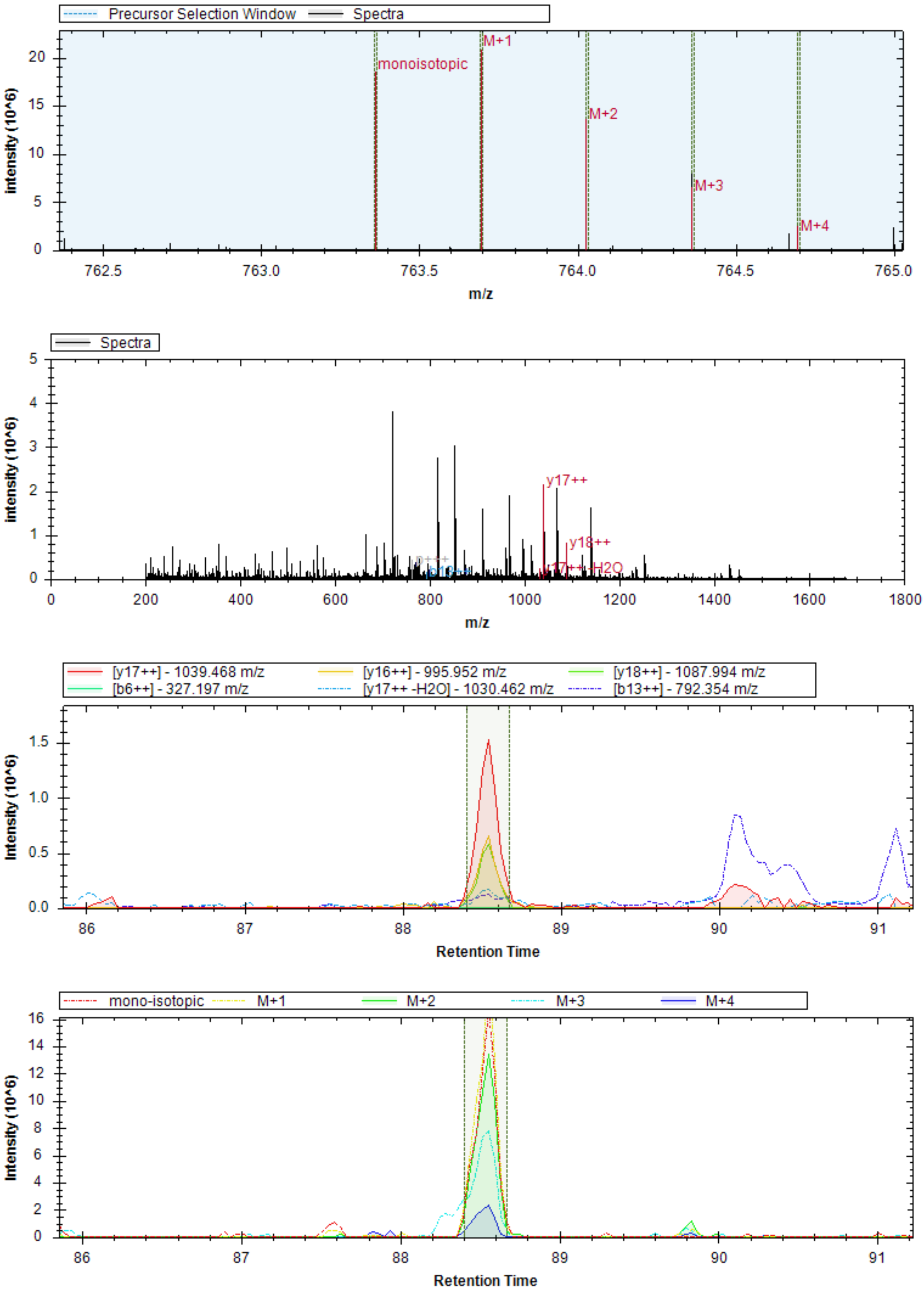

P06837 – _QADVPAAVTDAAAT[+H+O3+P]TPAAEDAATK_.3

Fig4_MP-DIA-barrel-cortex-p15-3_MHRM.htrms

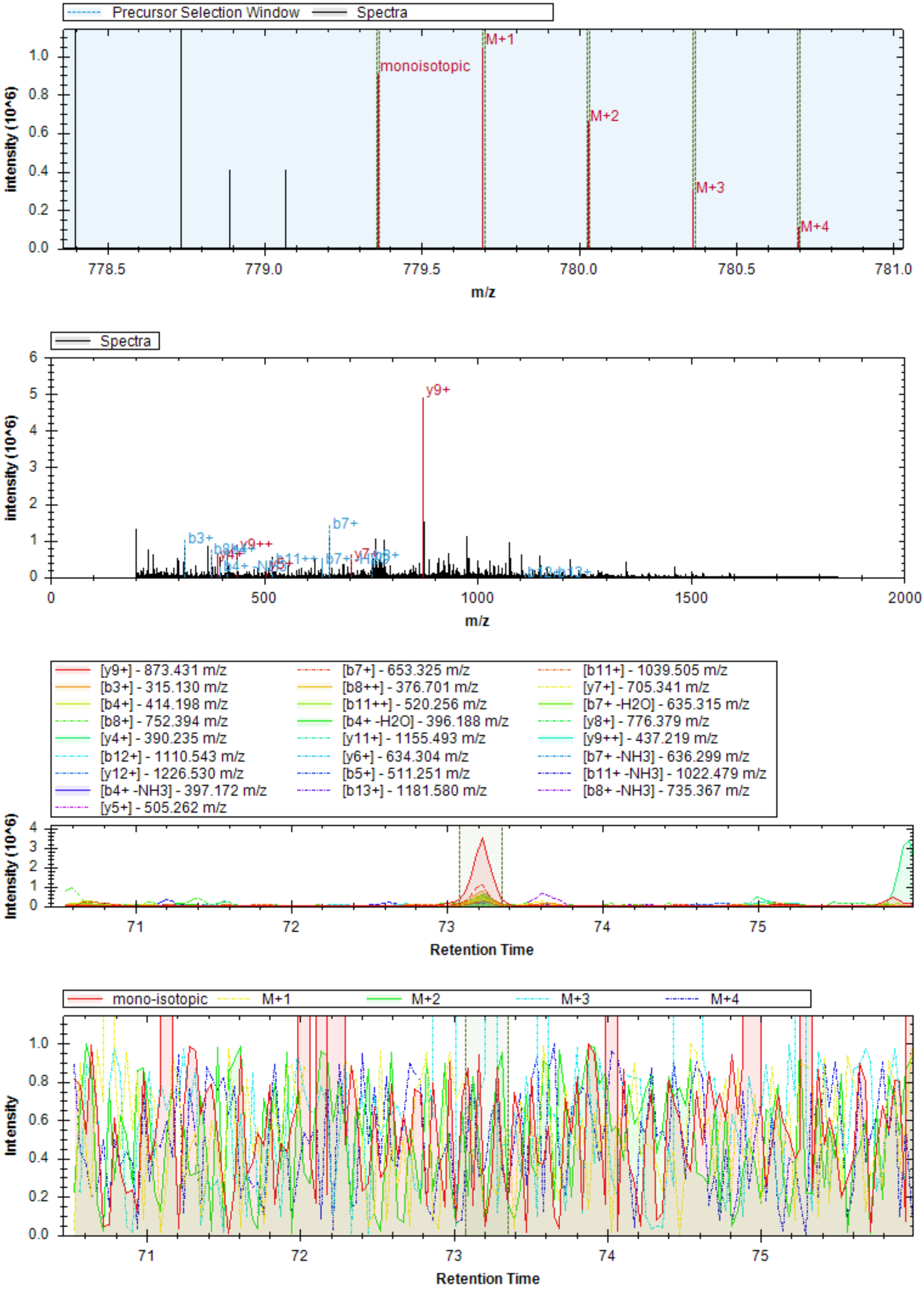

P0C6F1 – _IIDLQM[+O]HDY[+H+O3+P]LR_.2
Fig4_MP-DIA-barrel-cortex-p15-3_MHRM.htrms

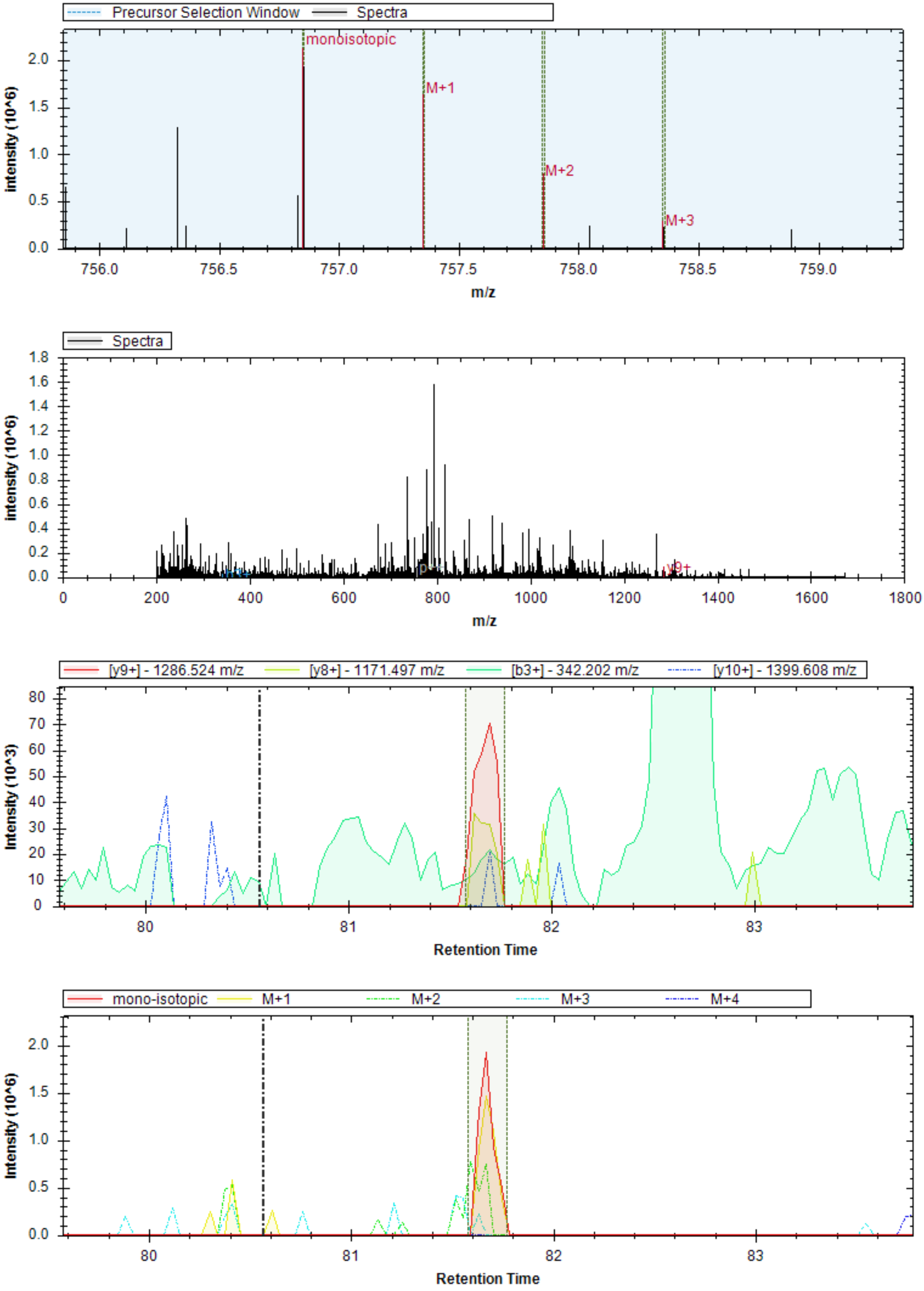

P57776-3 – _ATAPQTQHVS[+H+O3+P]PM[+O]R_.2
Fig4_MP-DIA-barrel-cortex-p15-3_MHRM.htrms

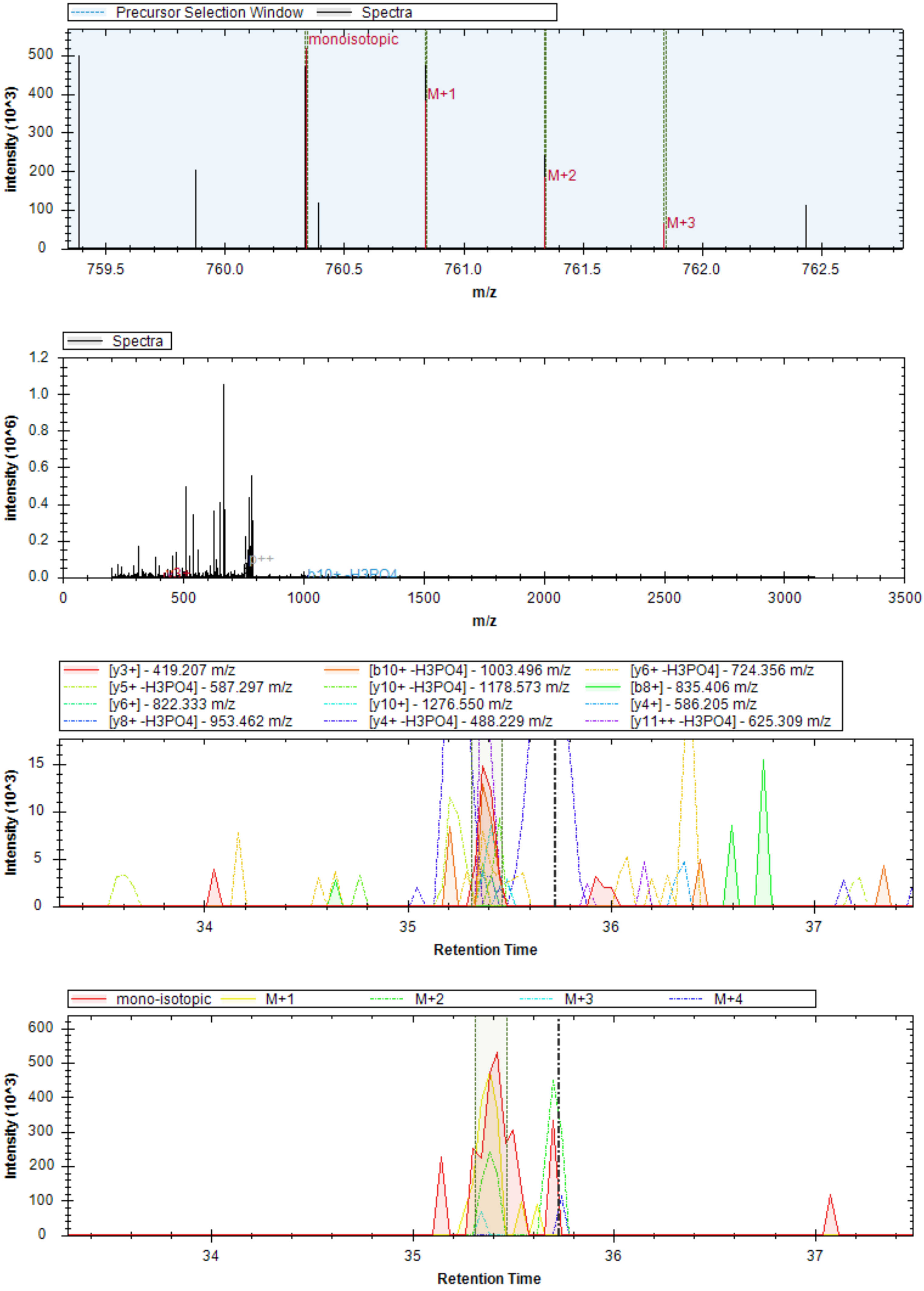

Q3TZM9;Q3TZM9-2;Q3TZM9-3 – _VPDVYIDSM[+O]GYAFT[+H+O3+P]LPLFK_.3
Fig4_MP-DIA-barrel-cortex-p15-3_MHRM.htrms

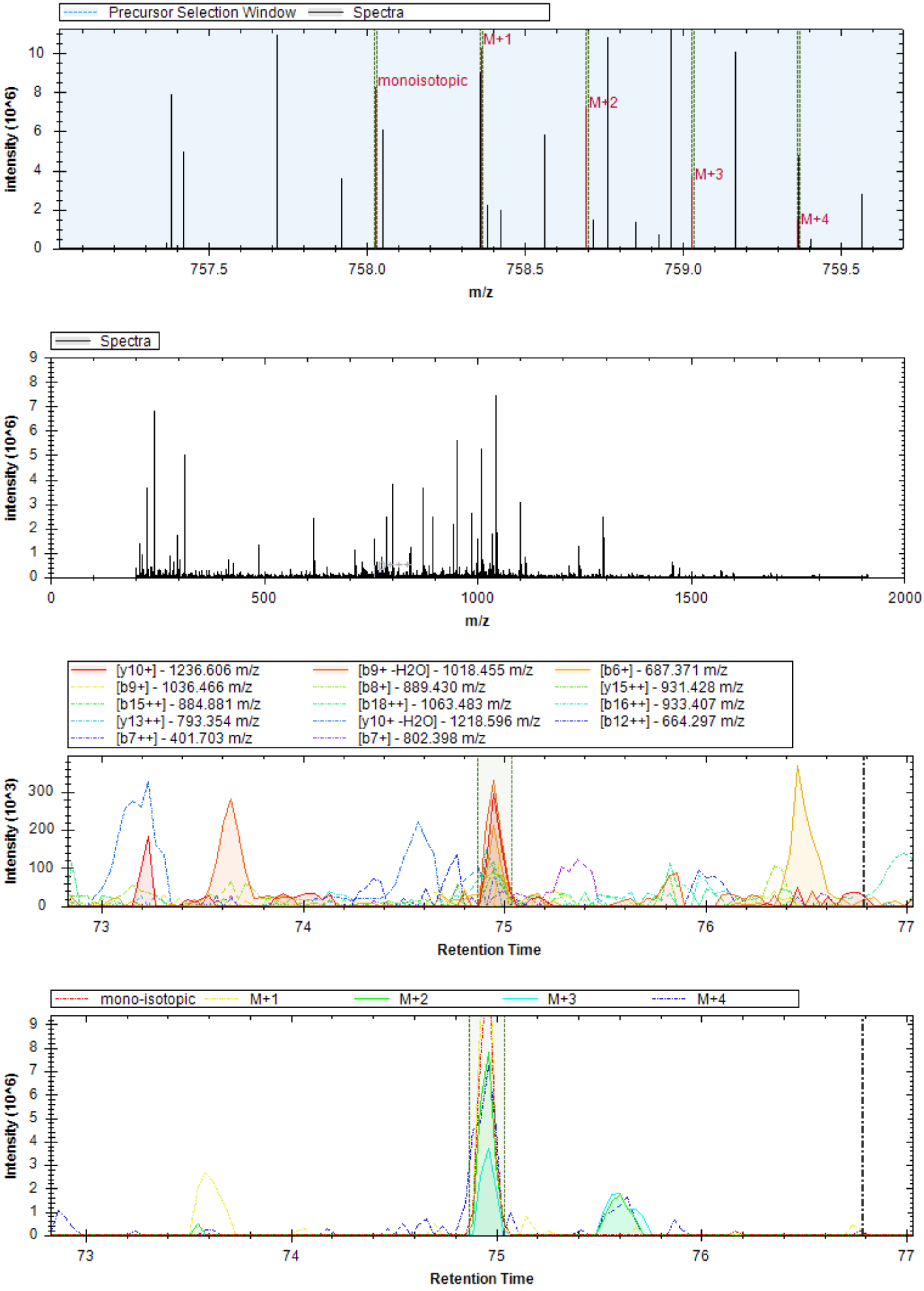

Q68FD9 – _LAQLLS[+H+O3+P]EVPT[+H+O3+P]RR_.2
Fig4_MP-DIA-barrel-cortex-p15-3_MHRM.htrms

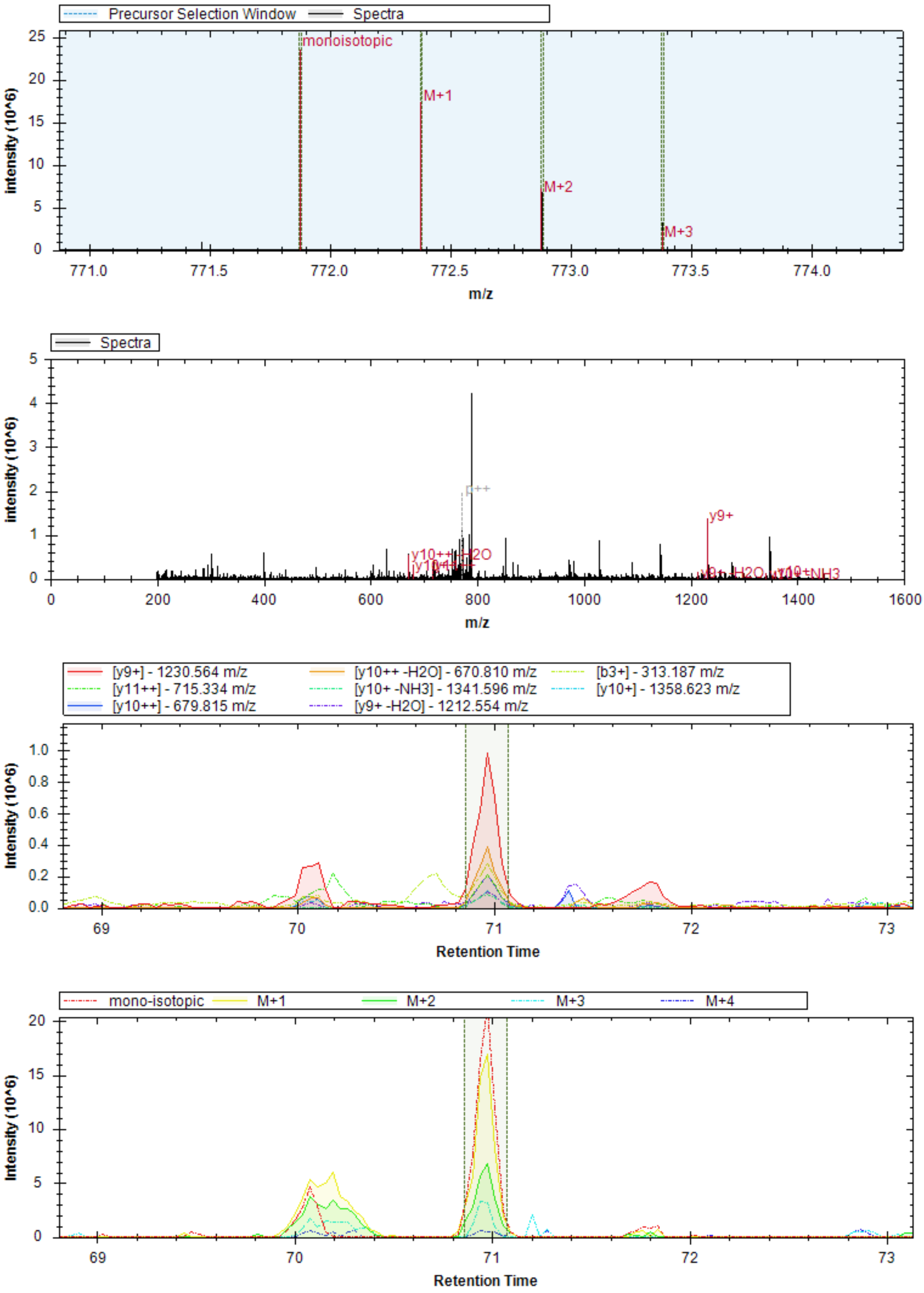

Q6ZQA0;Q6ZQA0-2 – _LHT[+H+O3+P]LLQT[+H+O3+P]AVPTR_.2
Fig4_MP-DIA-barrel-cortex-p15-3_MHRM.htrms

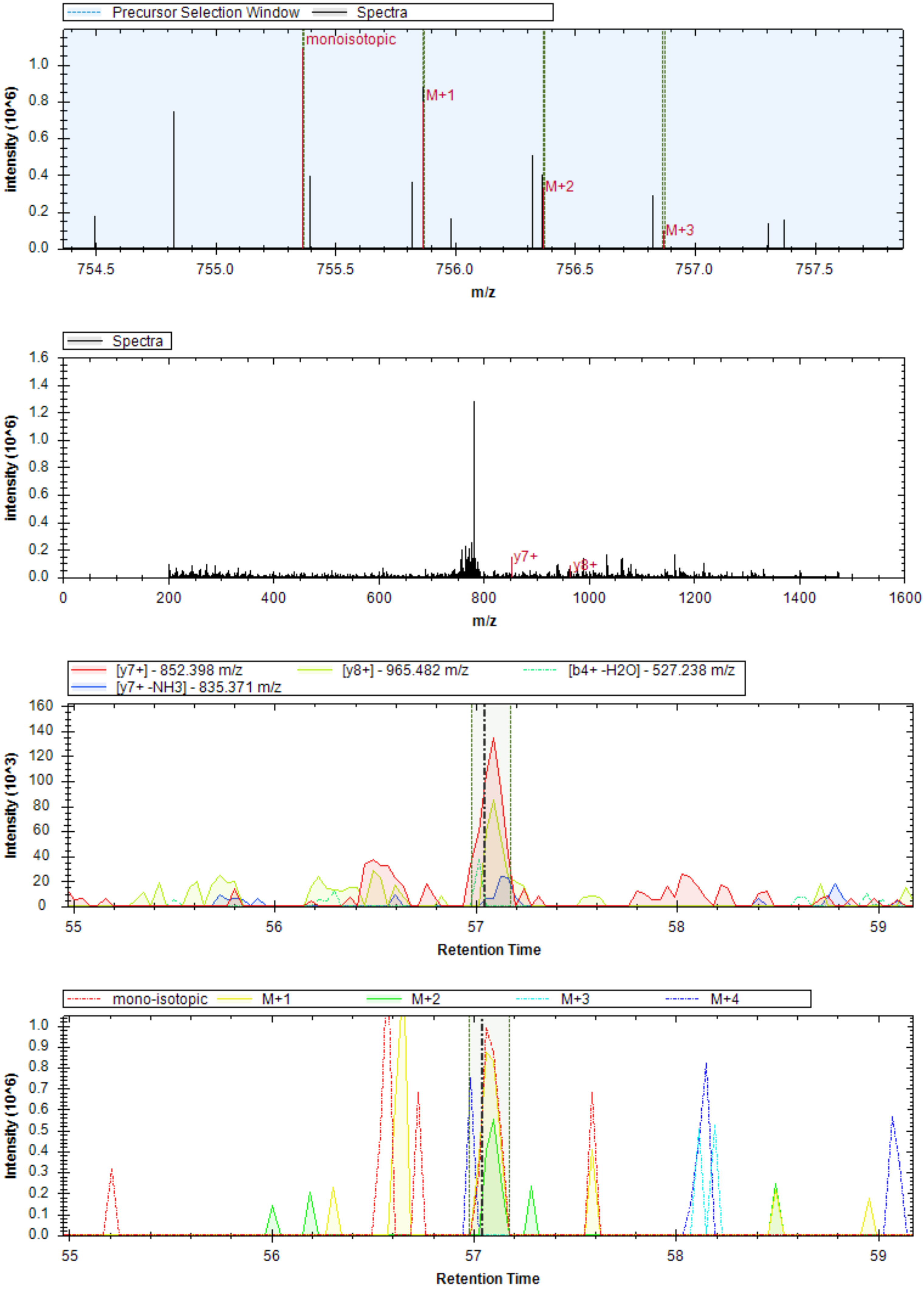

Q8BRT1 – _VLNT[+H+O3+P]GSDVEEAVADALLLGDIR_.3
Fig4_MP-DIA-barrel-cortex-p15-3_MHRM.htrms

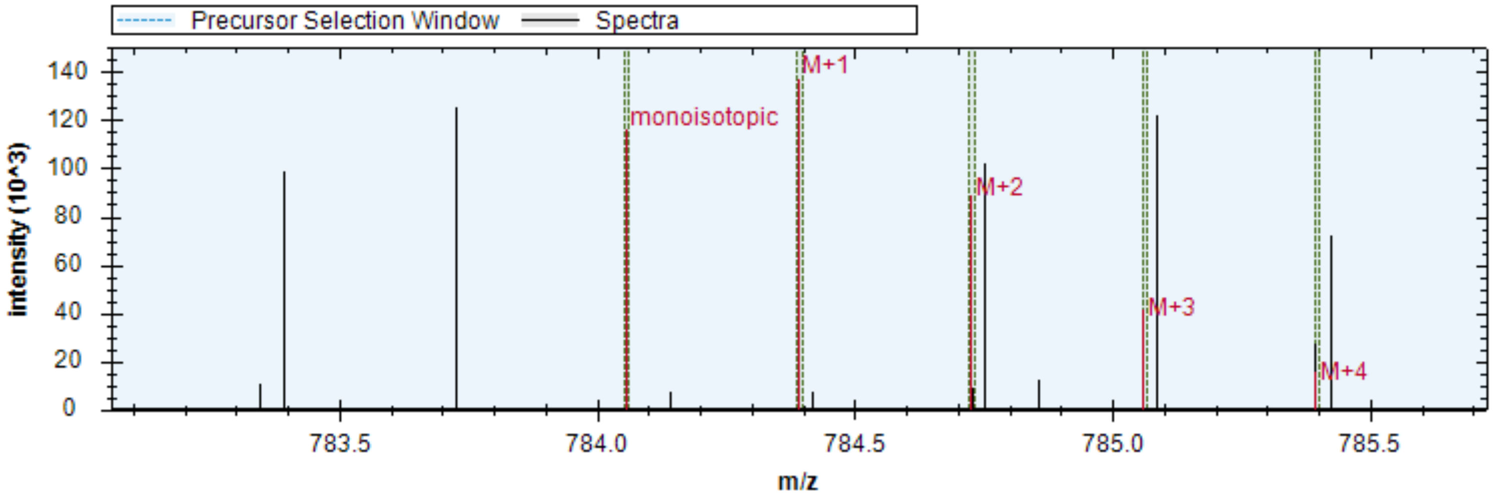

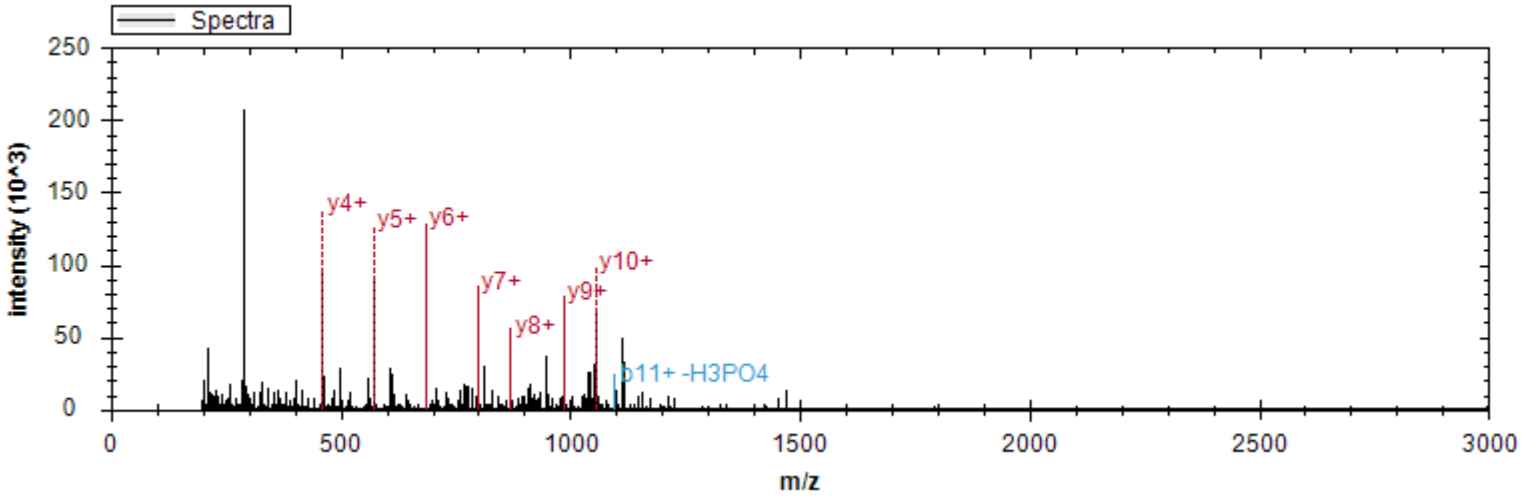

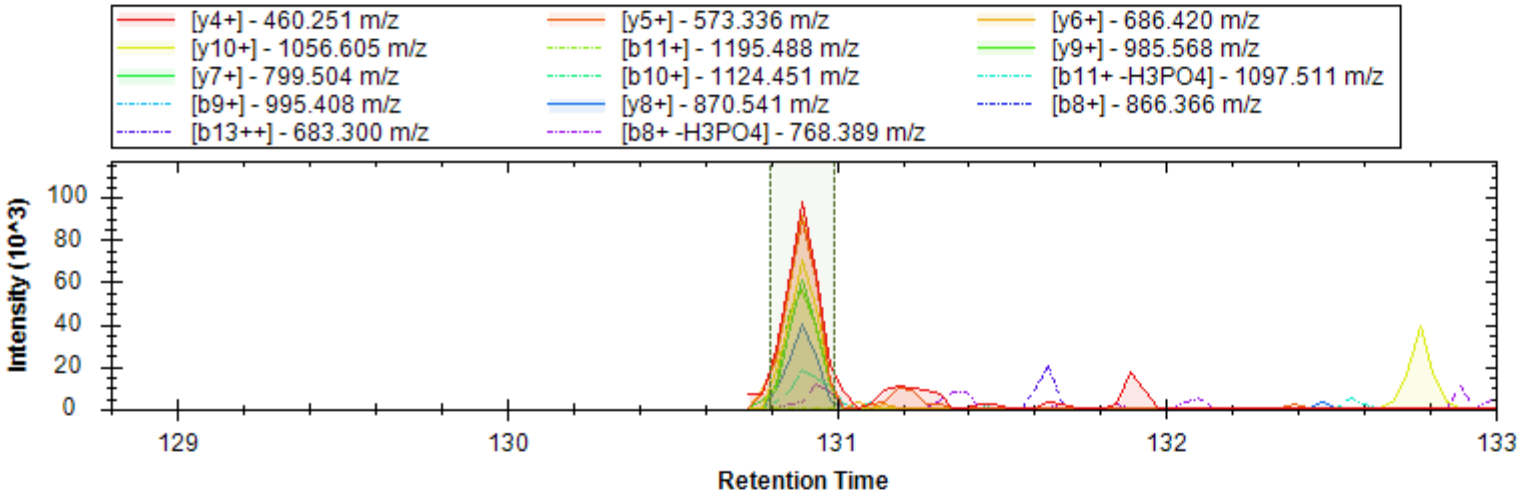

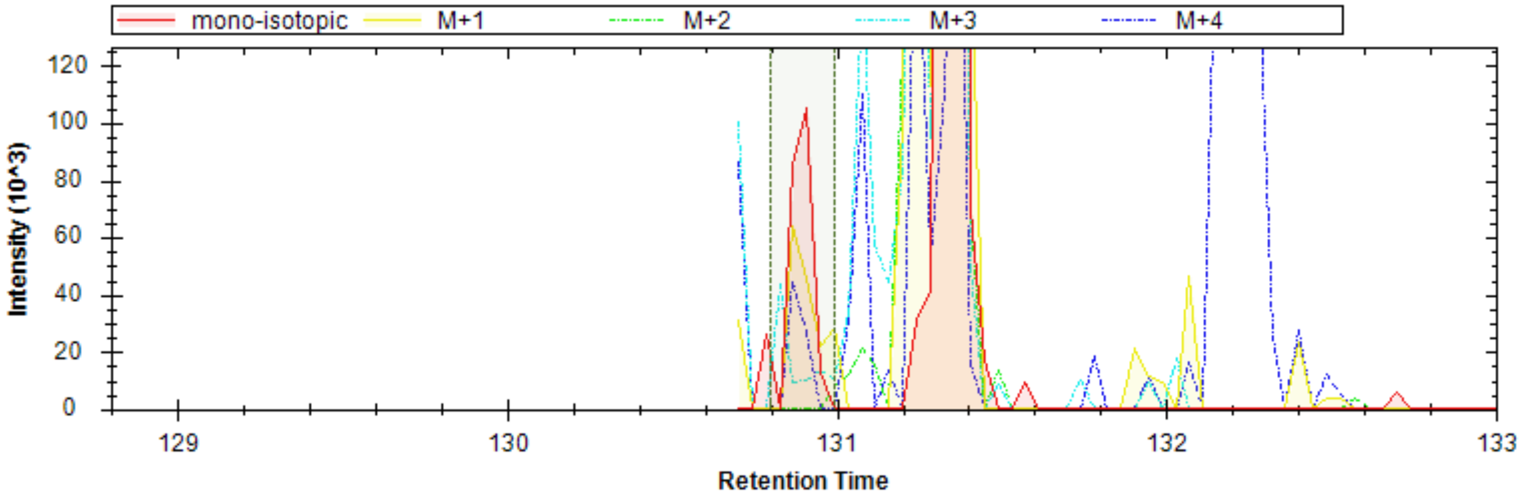

Q8CBW3;Q8CBW3-2;Q8CB[...] – _HNS[+H+O3+P]TTSSTSSGGYR_.2

Fig4_MP-DIA-barrel-cortex-p15-3_MHRM.htrms

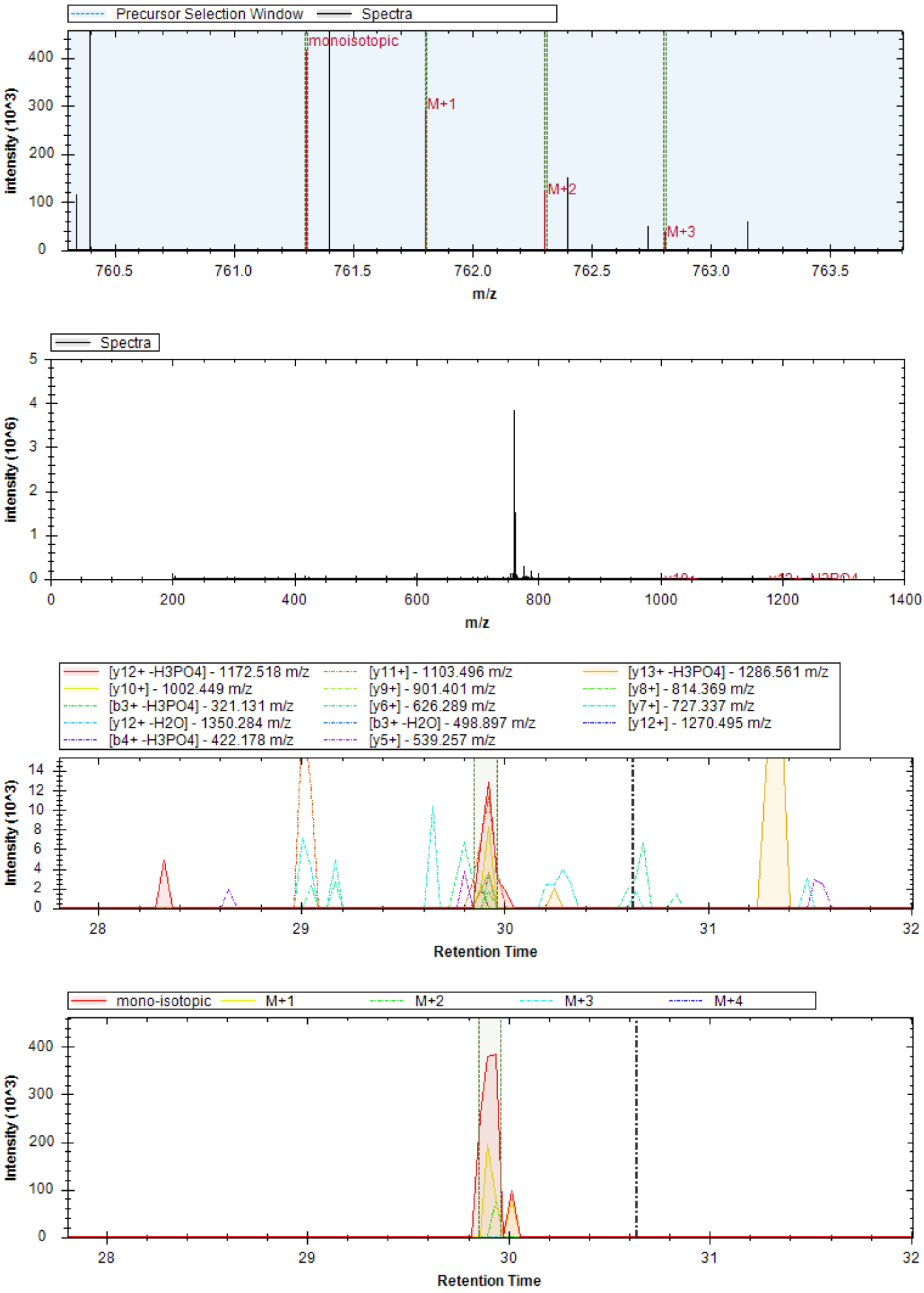

Q925N0 – _ADT[+H+O3+P]ATTASAASAAASAPNASTDAPPFQLGKPR_.4
Fig4_MP-DIA-barrel-cortex-p15-3_MHRM.htrms

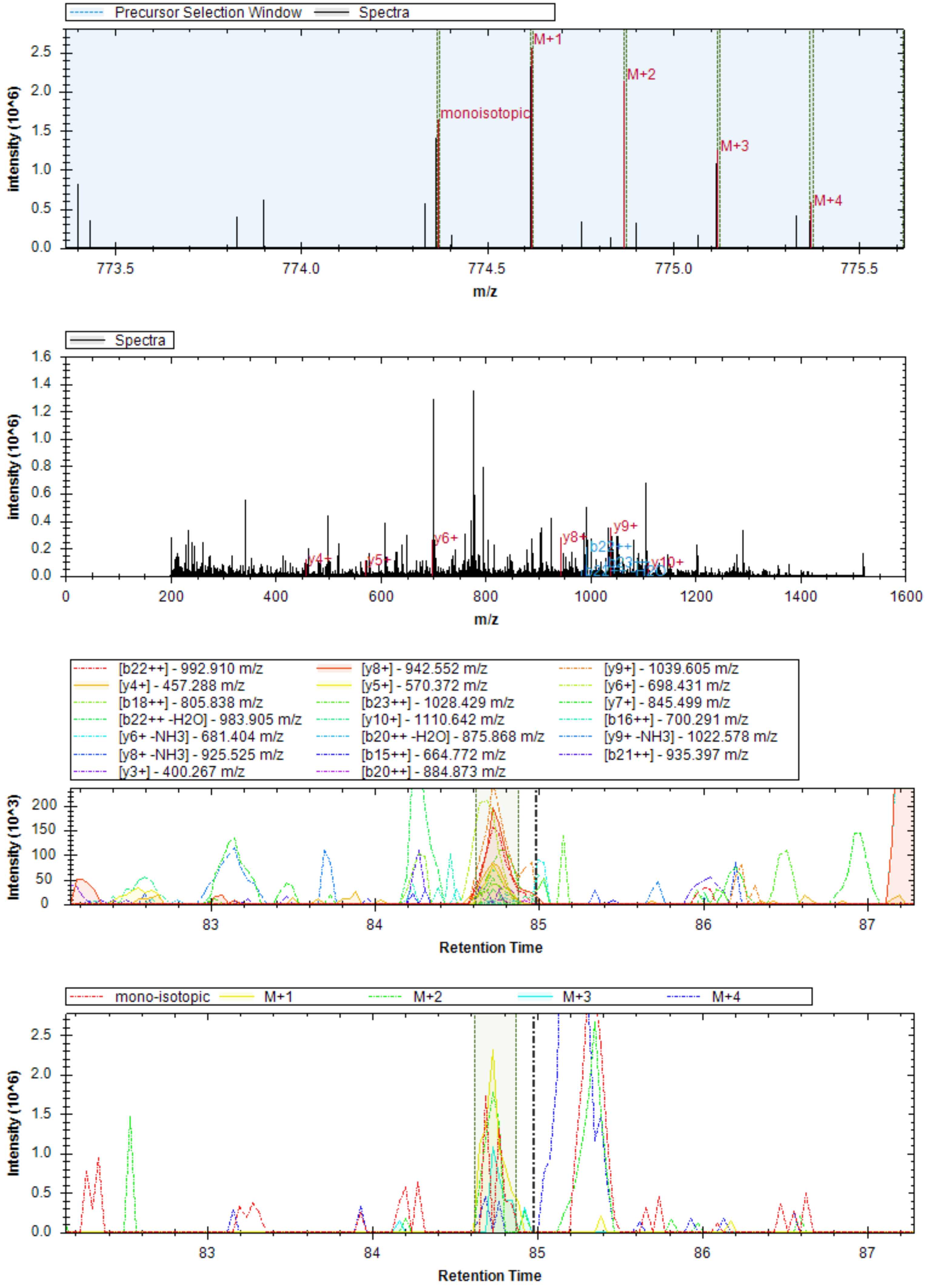

Q9CZX2 – _IHQRY[+H+O3+P]T[+H+O3+P]EIT[+H+O3+P]K_.2

Fig4_MP-DIA-barrel-cortex-p15-3_MHRM.htrms

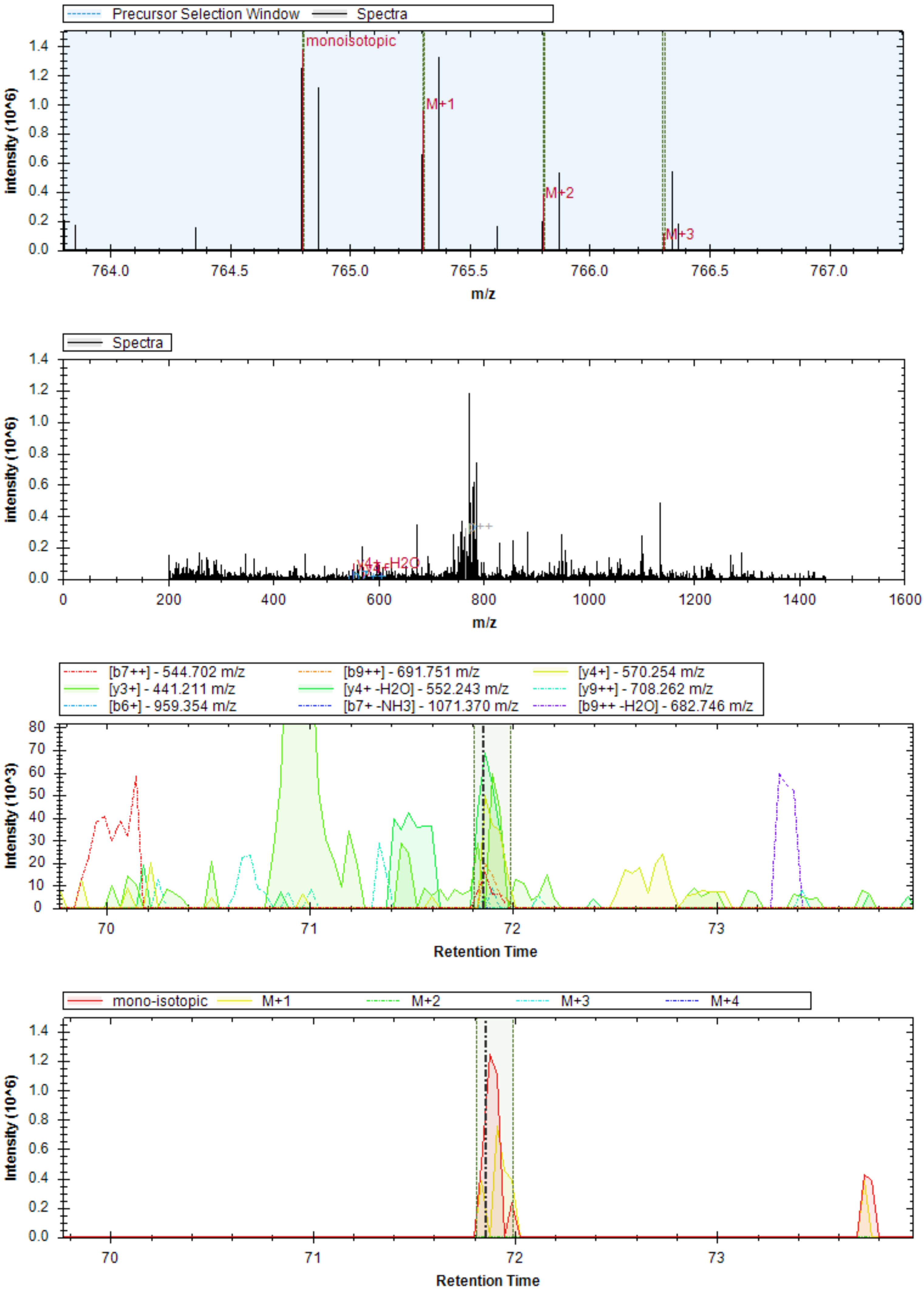

Q9D5R2 – _SNS[+H+O3+P]LPHSAVSNAASK_.2
Fig4_MP-DIA-barrel-cortex-p15-3_MHRM.htrms

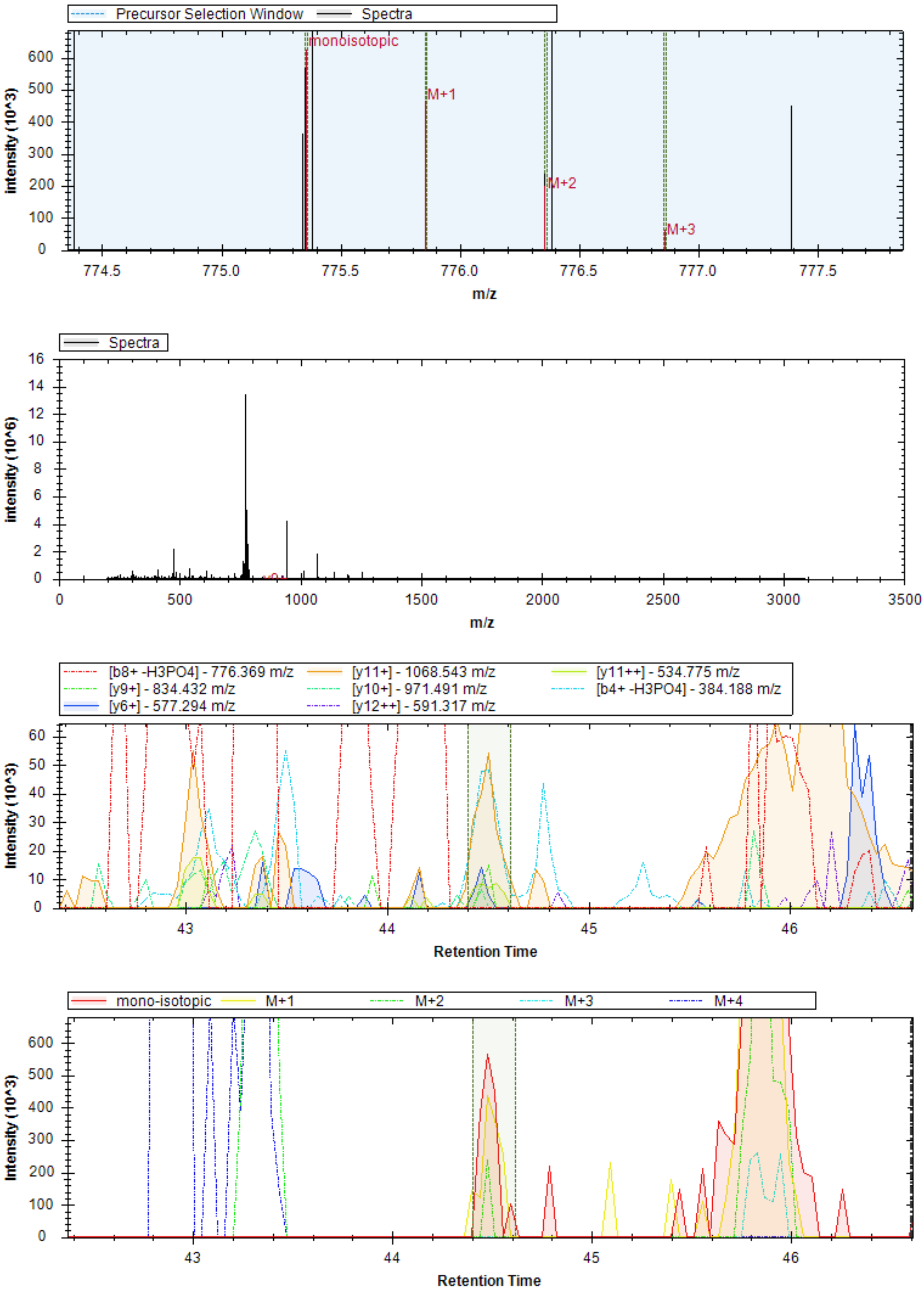

Q9JLM8 – _ISQHGGS[+H+O3+P]STSLSSTK_.2
Fig4_MP-DIA-barrel-cortex-p15-3_MHRM.htrms

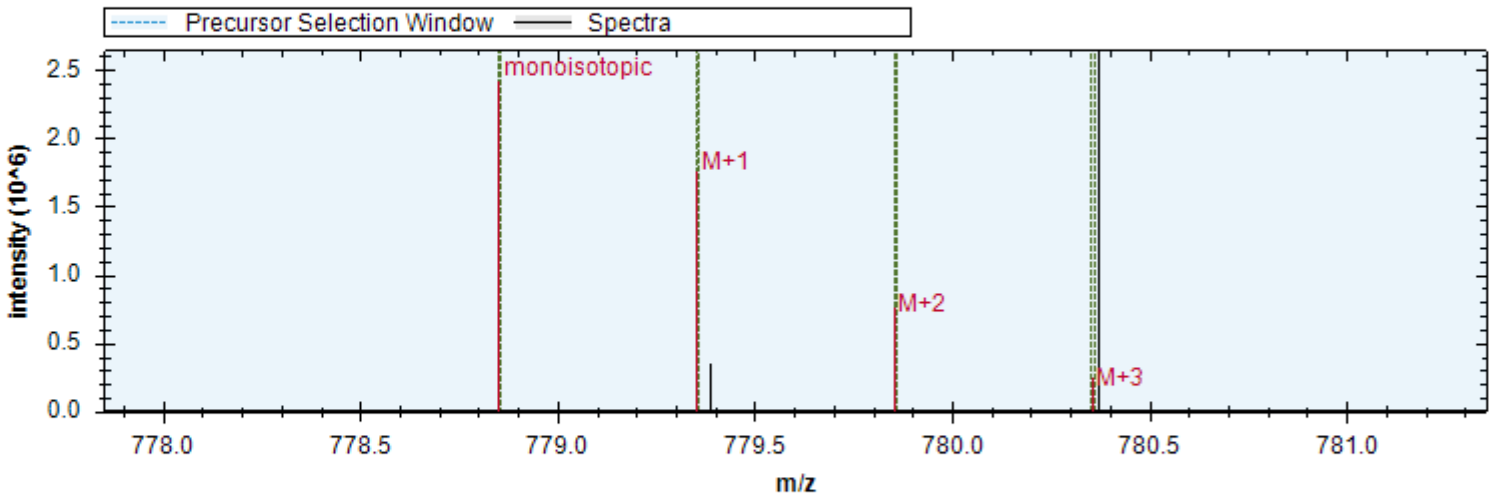

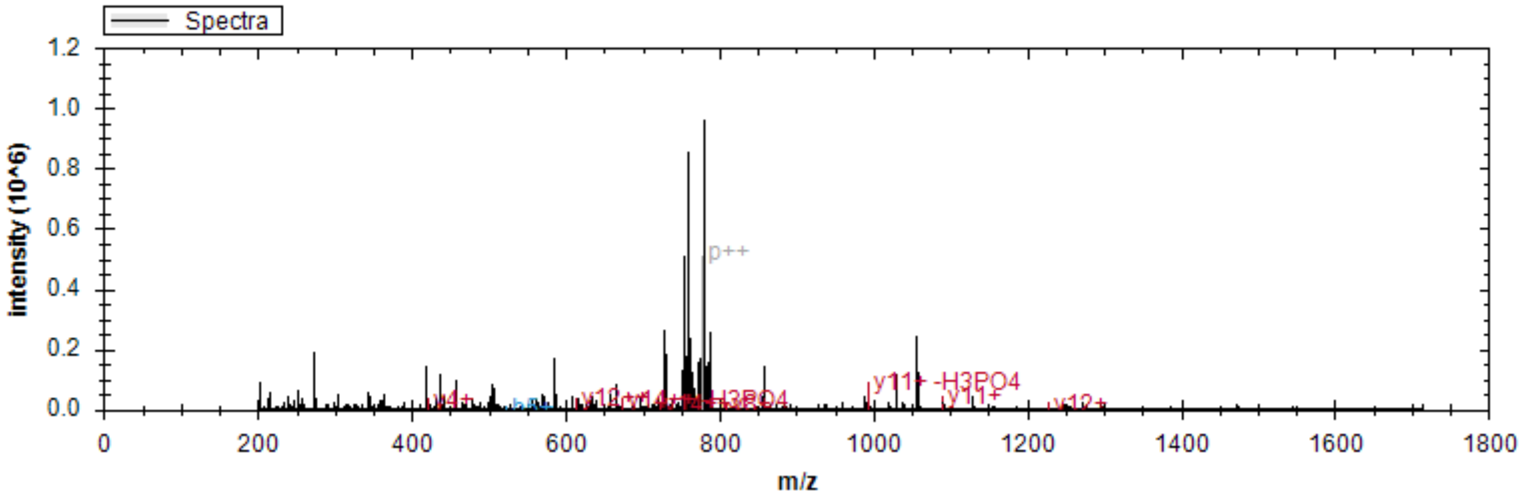

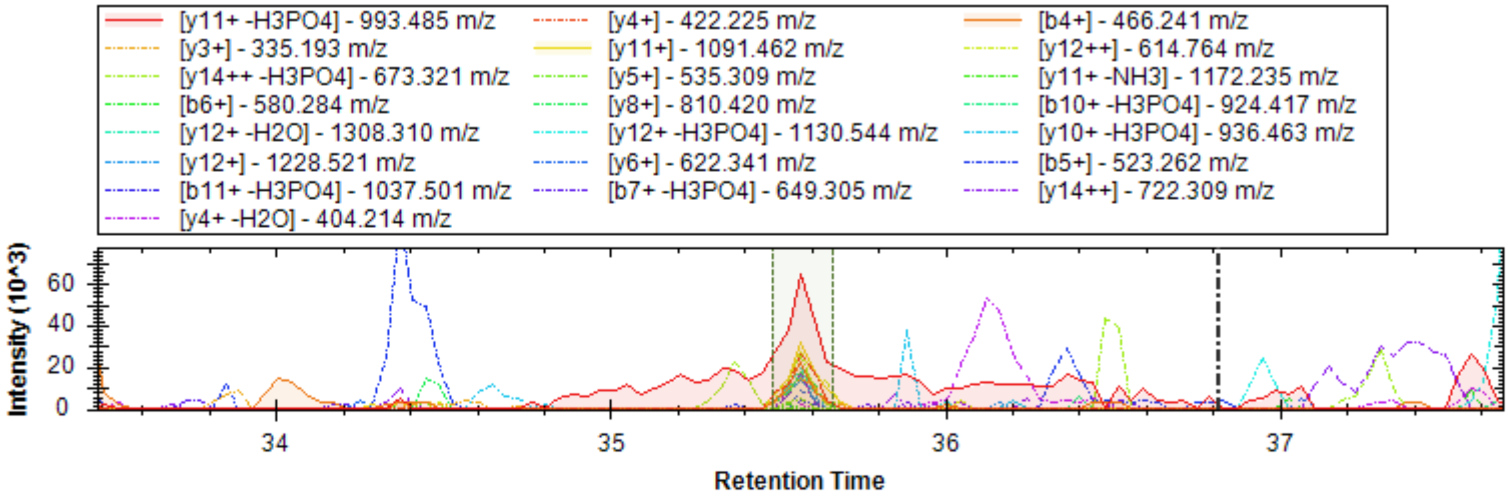

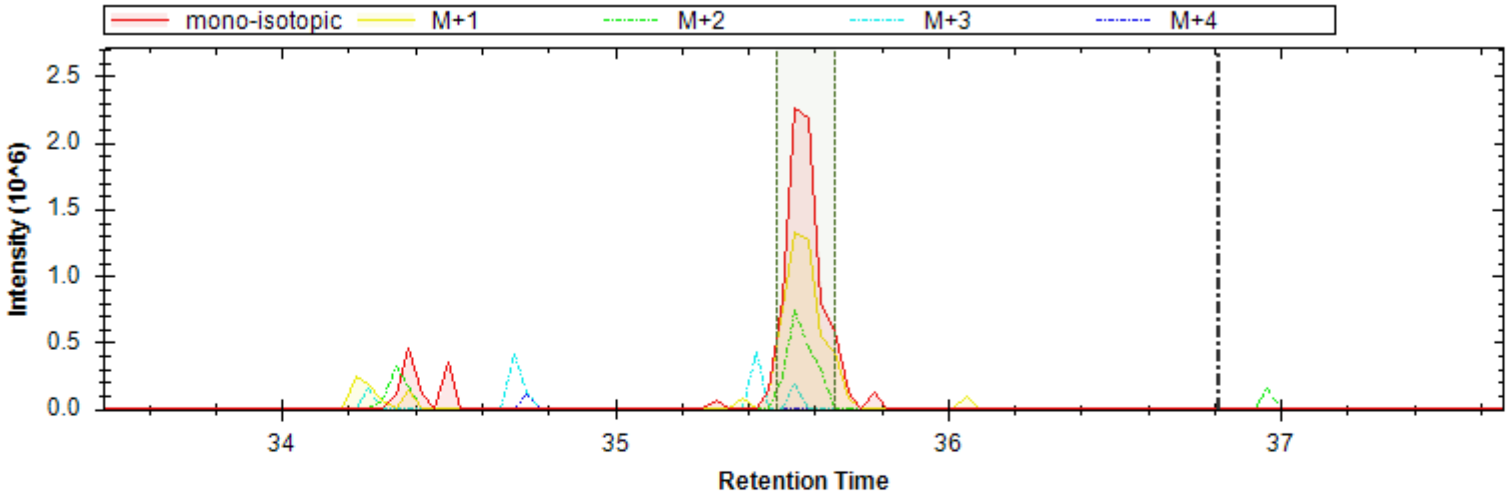

Q9QWI6 – _LSYAGGRPPSYAGS[+H+O3+P]PVHHAAER_.3
Fig4_MP-DIA-barrel-cortex-p15-3_MHRM.htrms

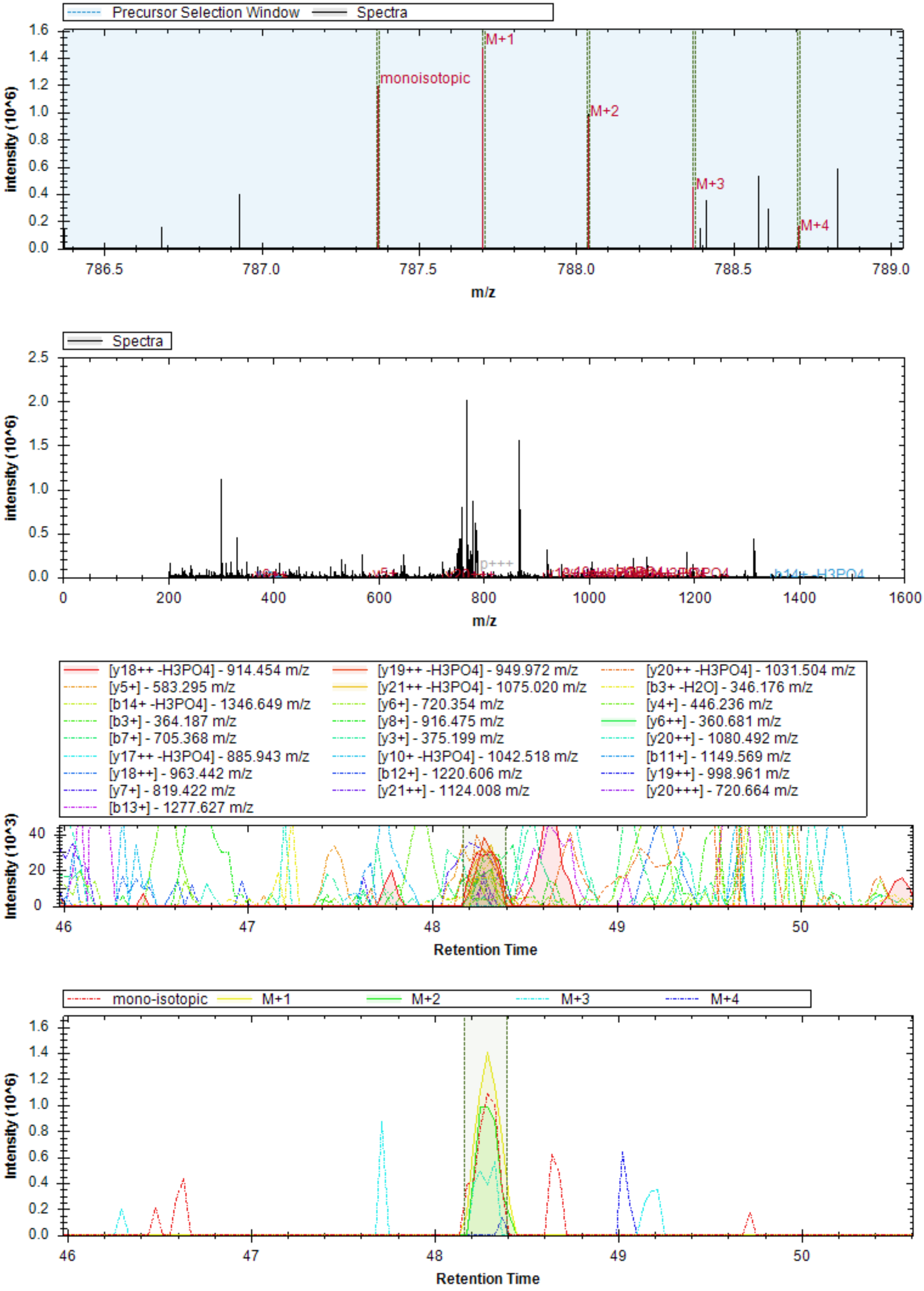

Q9QYC0 – _AAVVTS[+H+O3+P]PPPTTAPHK_.2
Fig4_MP-DIA-barrel-cortex-p15-3_MHRM.htrms

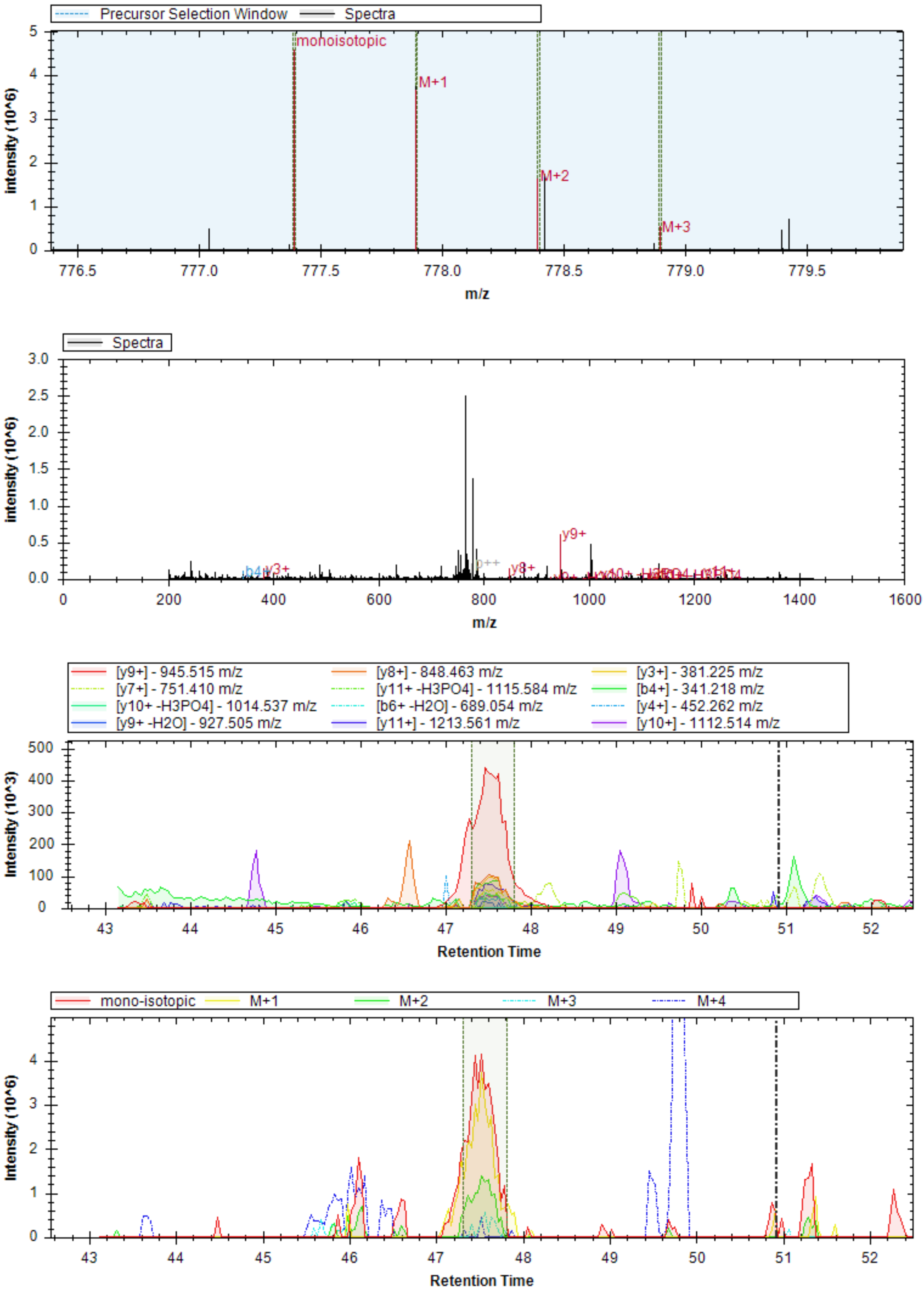

Q9QYR6-2 – _ELSSEPRT[+H+O3+P]PPAQK_.2

Fig4_MP-DIA-barrel-cortex-p15-3_MHRM.htrms

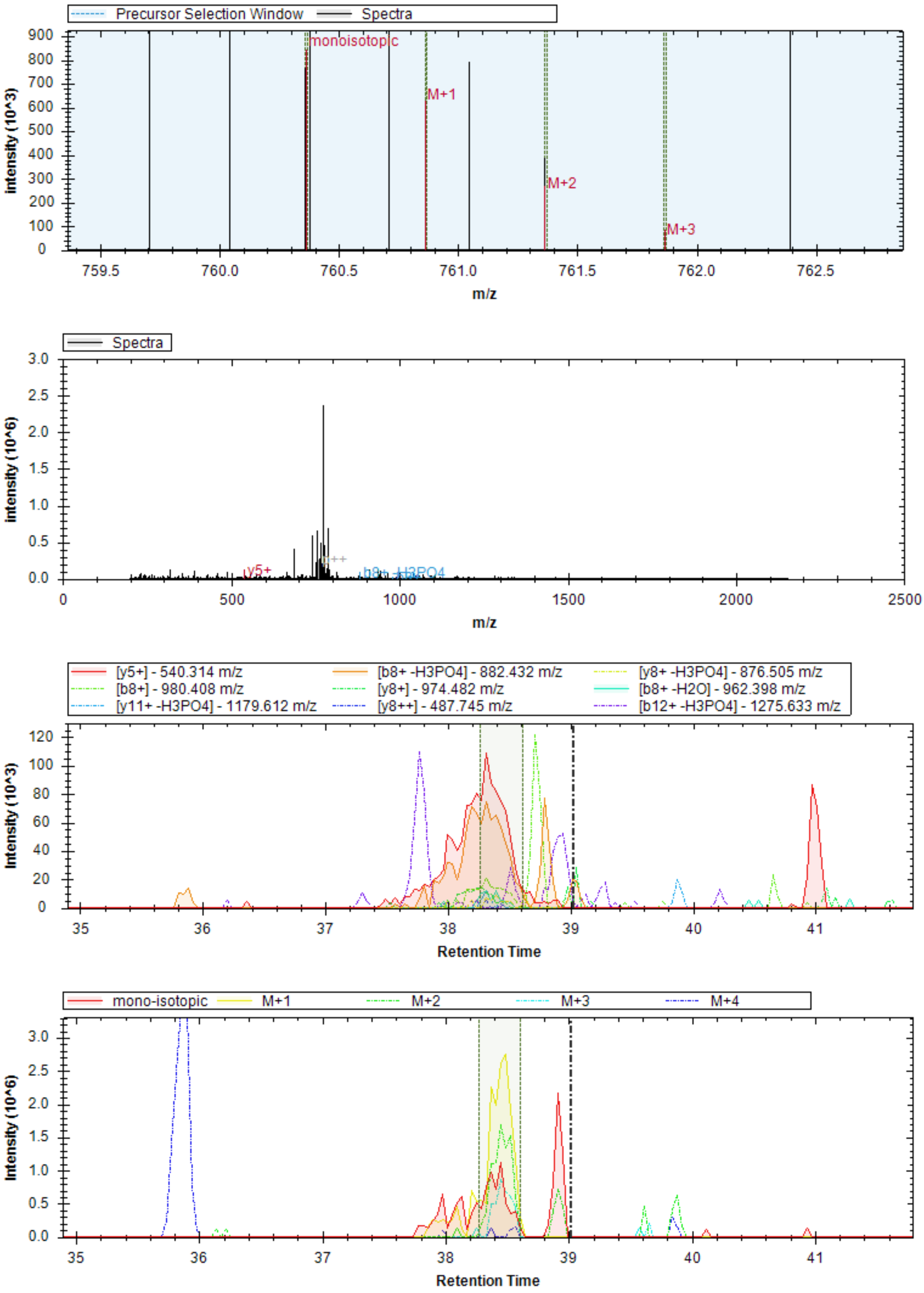

Q9Z0P4 – _ENSAAPSPGRPQS[+H+O3+P]ASPAKEEQK_.3
Fig4_MP-DIA-barrel-cortex-p15-3_MHRM.htrms

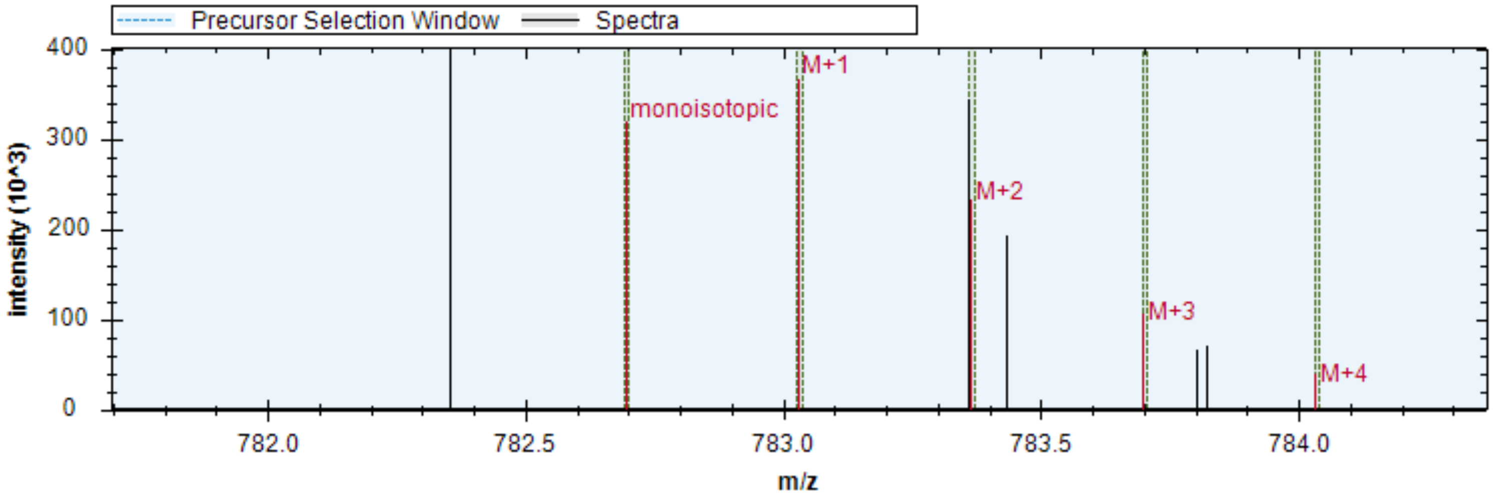

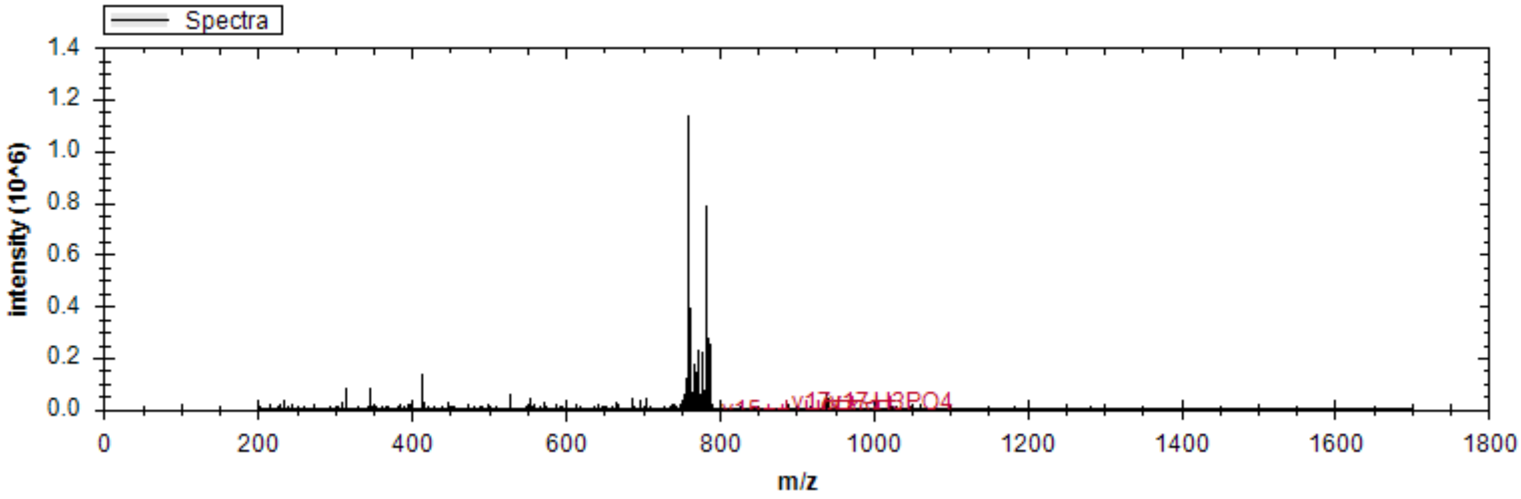

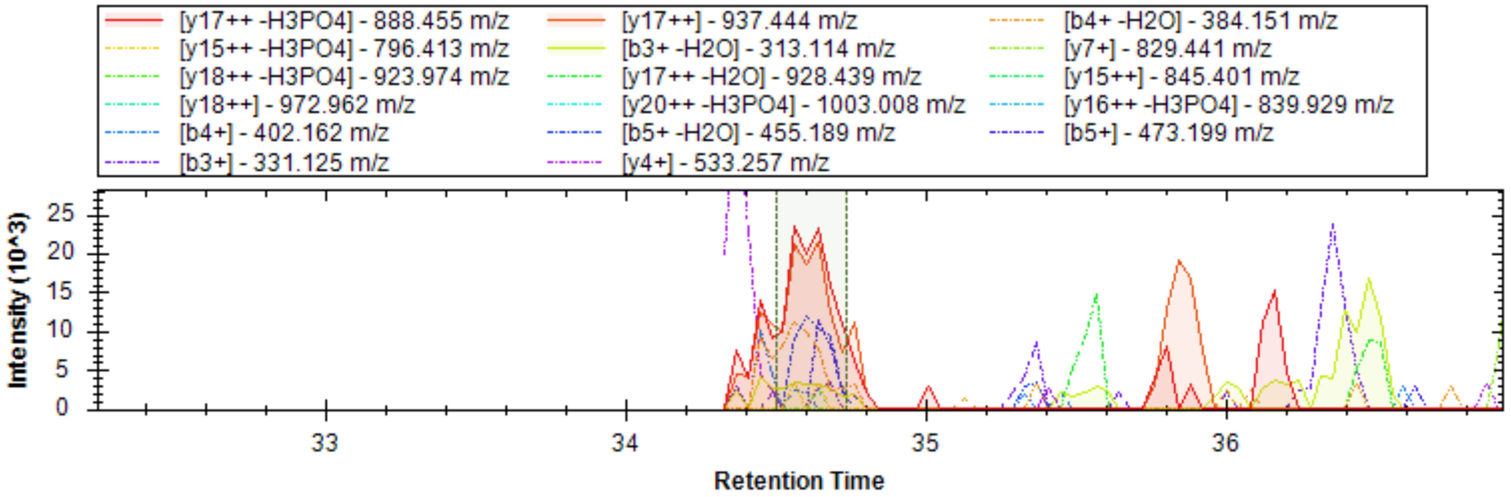

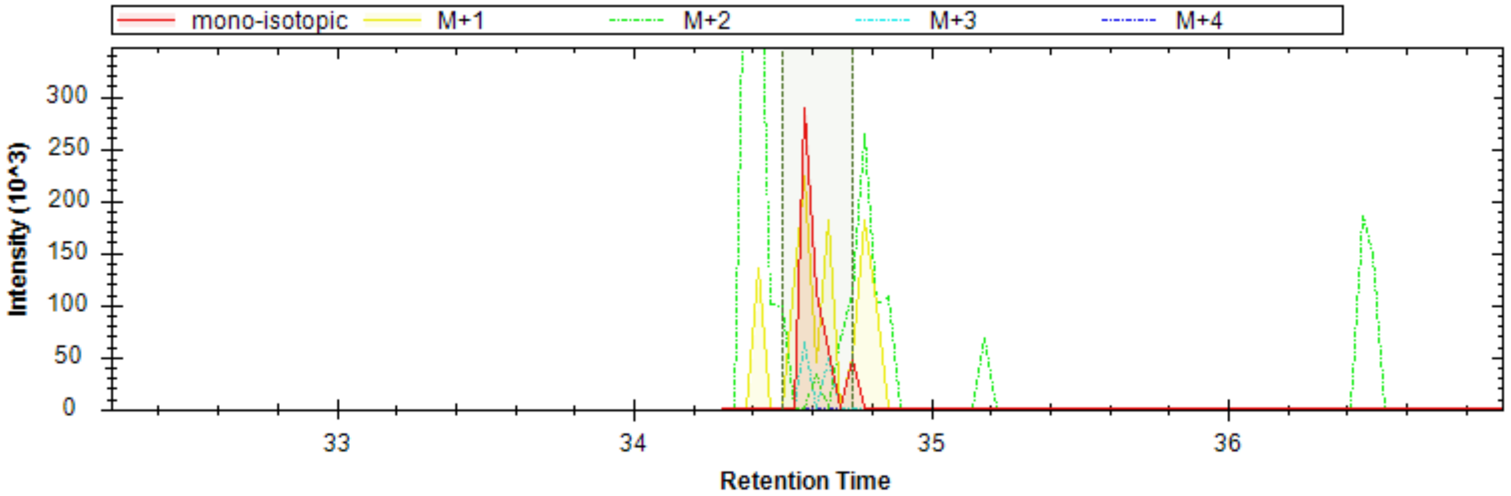

Q9Z2C4 – _GSS[+H+O3+P]PTHSATPVHTSV_.2
Fig4_MP-DIA-barrel-cortex-p15-3_MHRM.htrms

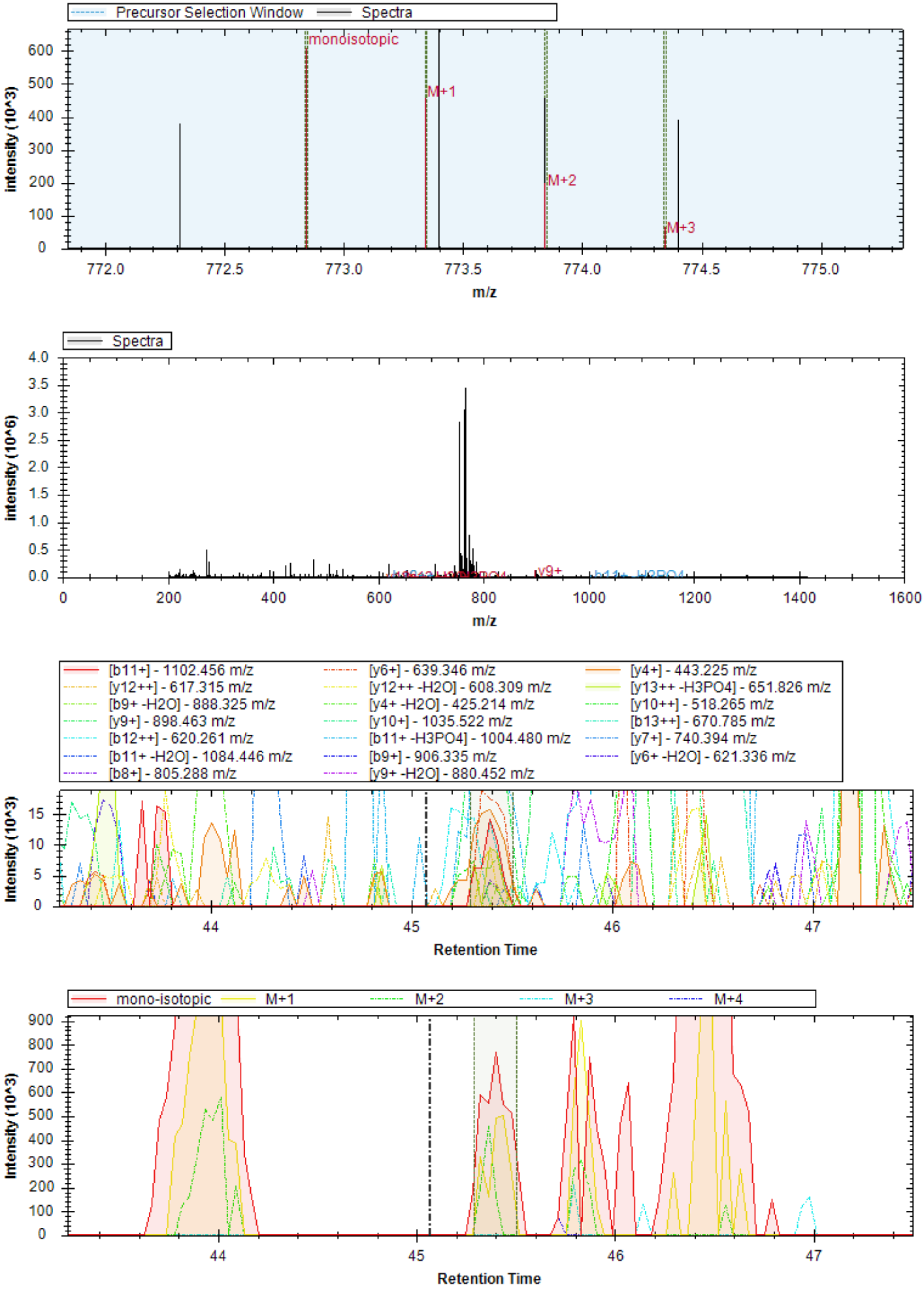

O88935 – _LPS[+H+O3+P]PTAAPQQSASQATPVTQGQGR_.3

Fig4_MP-DIA-barrel-cortex-p15-3_MHRM.htrms

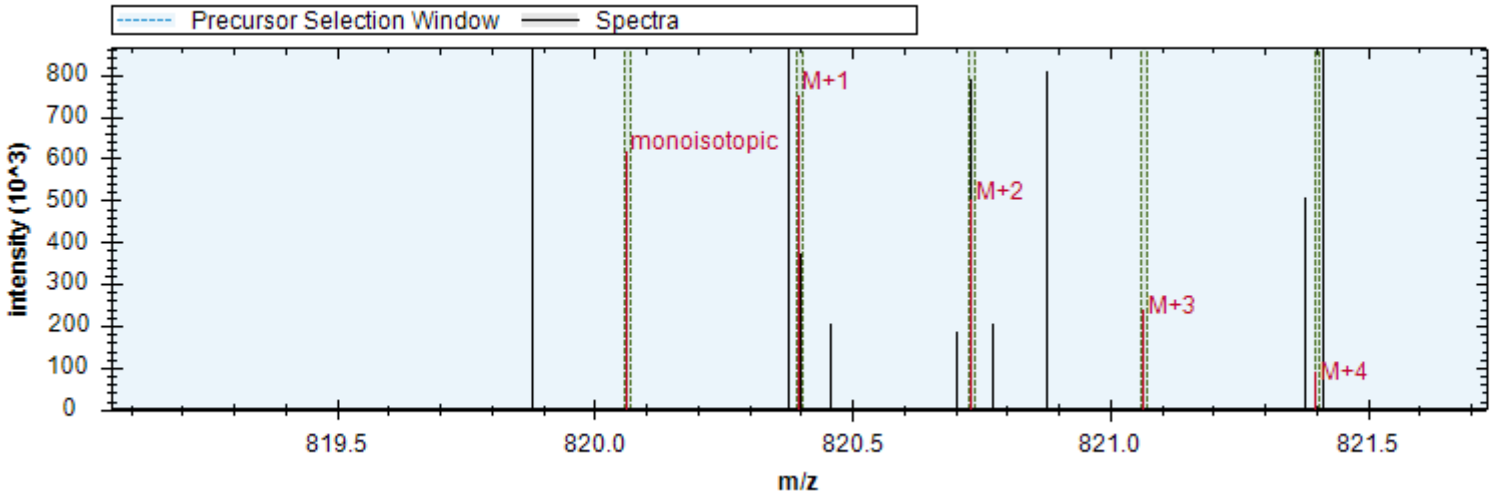

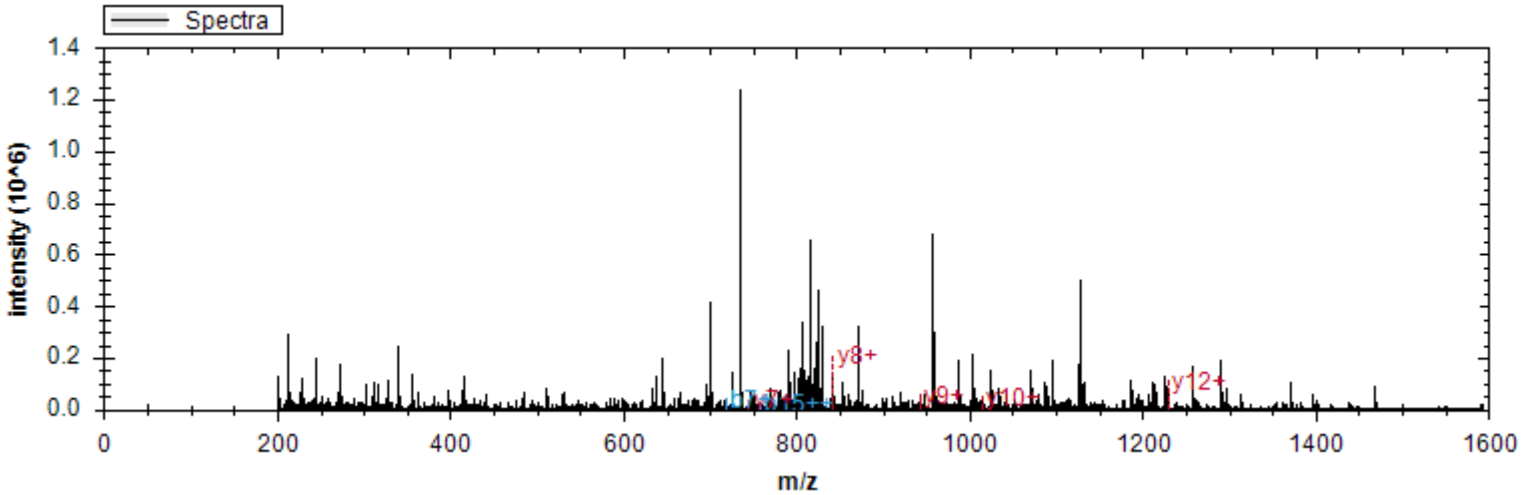

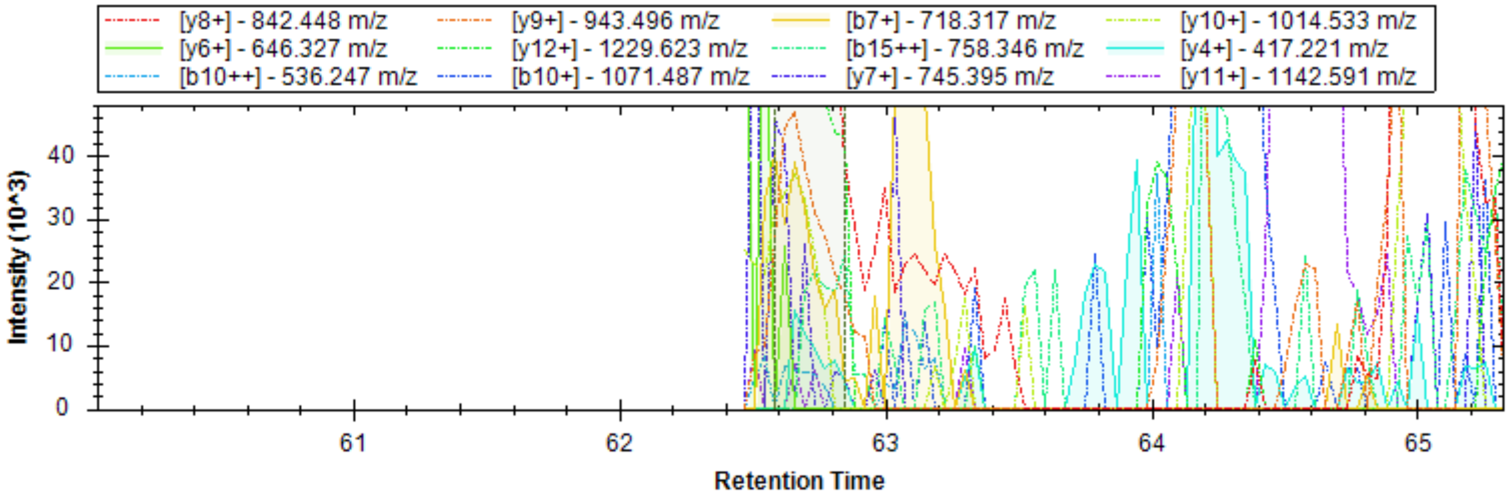

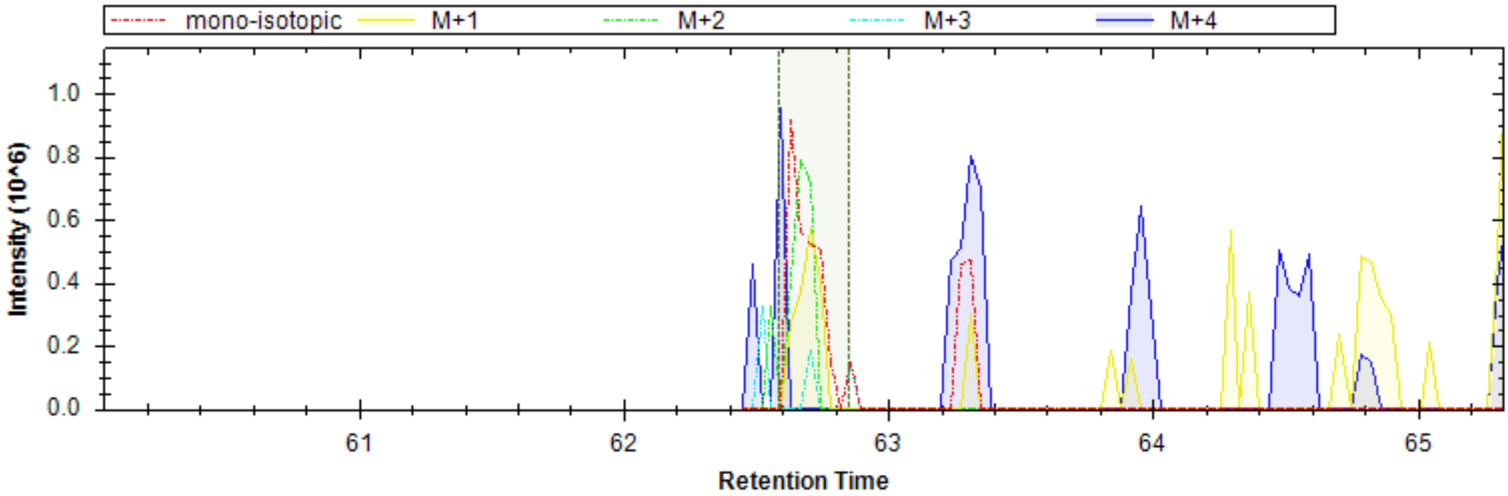

O88935-1 – _PAKPQLAQKPS[+H+O3+P]QDVPPPITAAAGGPPHPQLK_.4
Fig4_MP-DIA-barrel-cortex-p15-3_MHRM.htrms

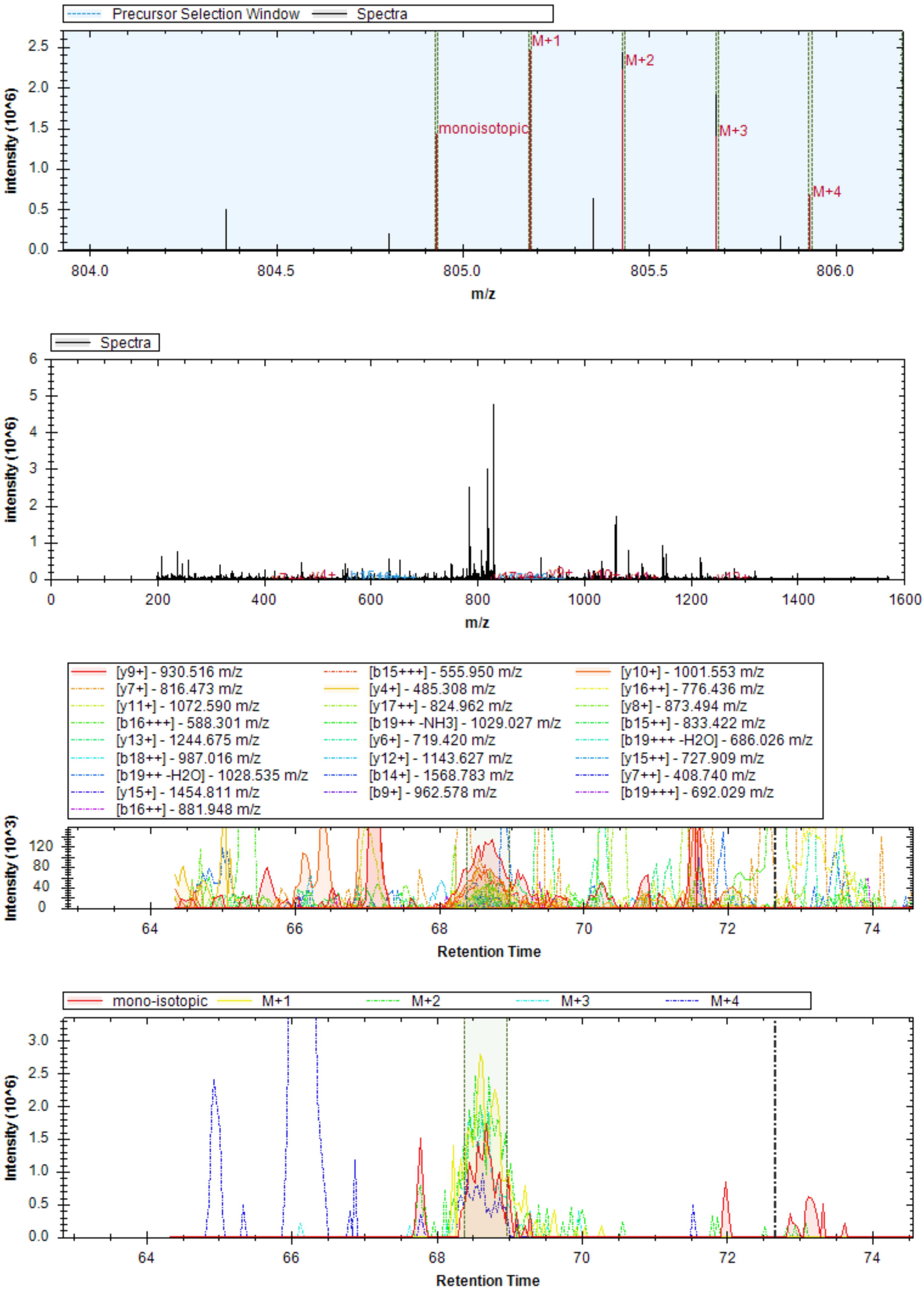

P10637 – _TTPSPKT[+H+O3+P]PPGSGEPPK_.2

Fig4_MP-DIA-barrel-cortex-p15-3_MHRM.htrms

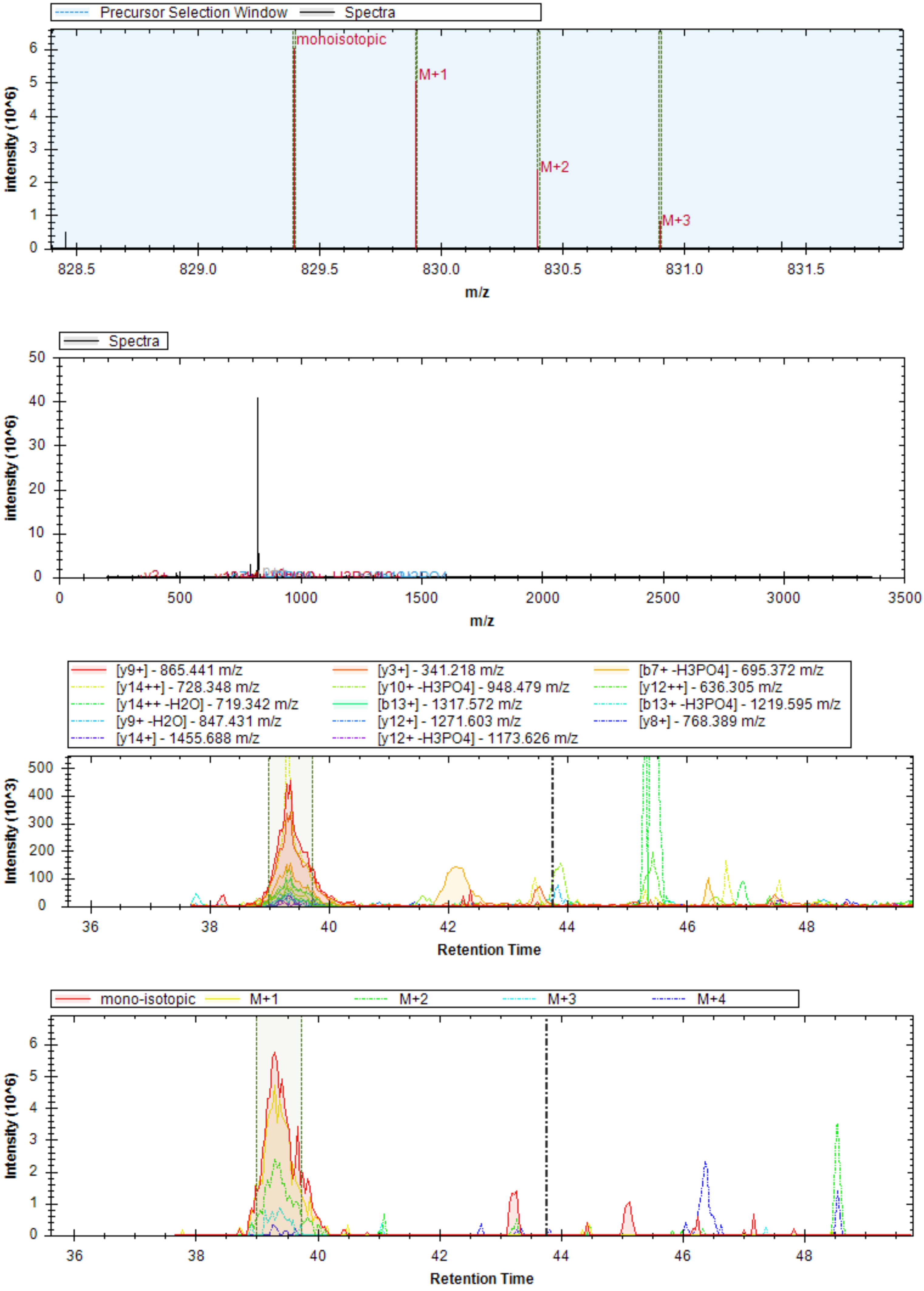

P20357 – _APHWTSASLTEAAAHPHS[+H+O3+P]PEMK_.3

Fig4_MP-DIA-barrel-cortex-p15-3_MHRM.htrms

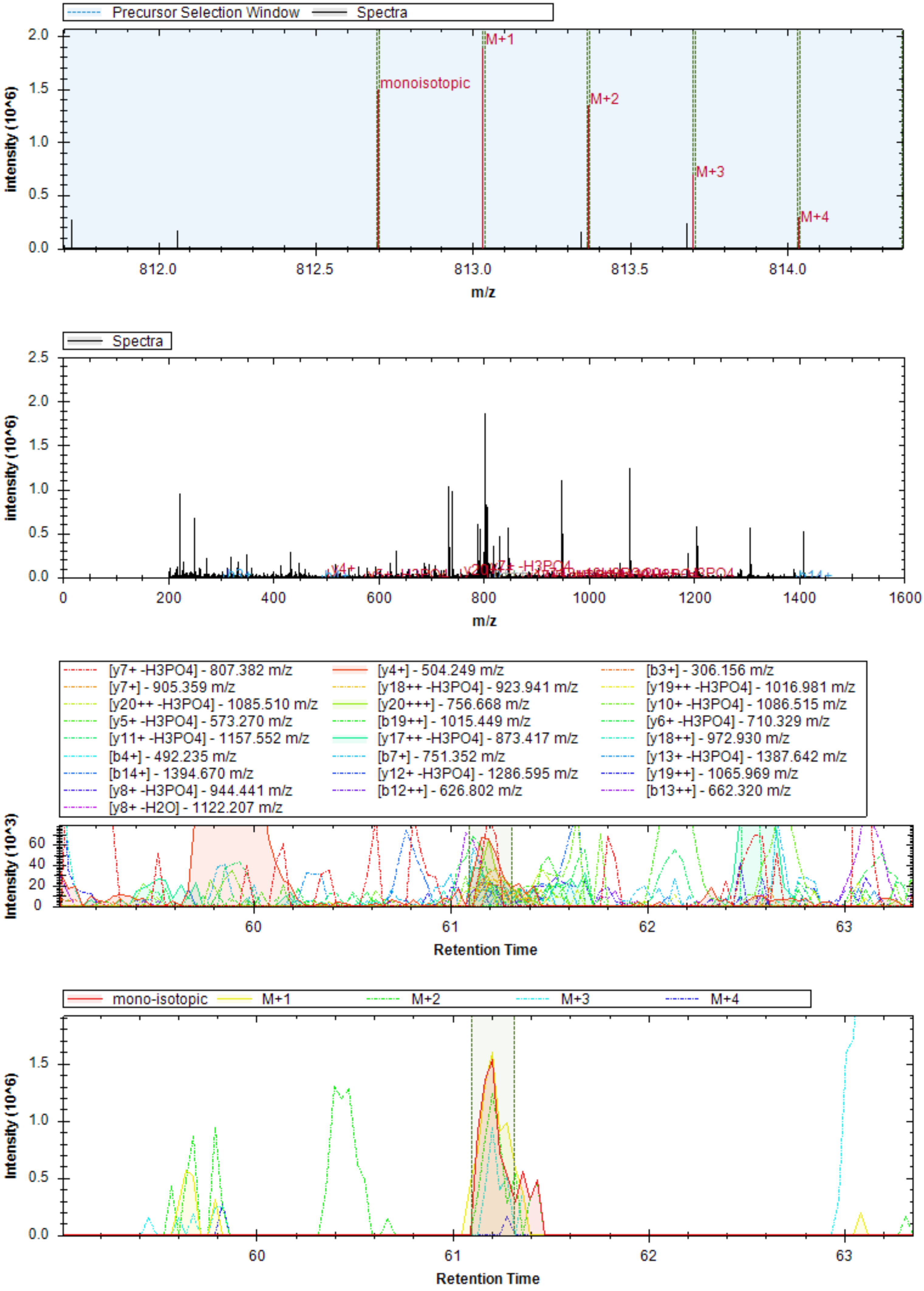

P20357 – _VDHGAEIIT[+H+O3+P]QSPSR_.2
Fig4_MP-DIA-barrel-cortex-p15-3_MHRM.htrms

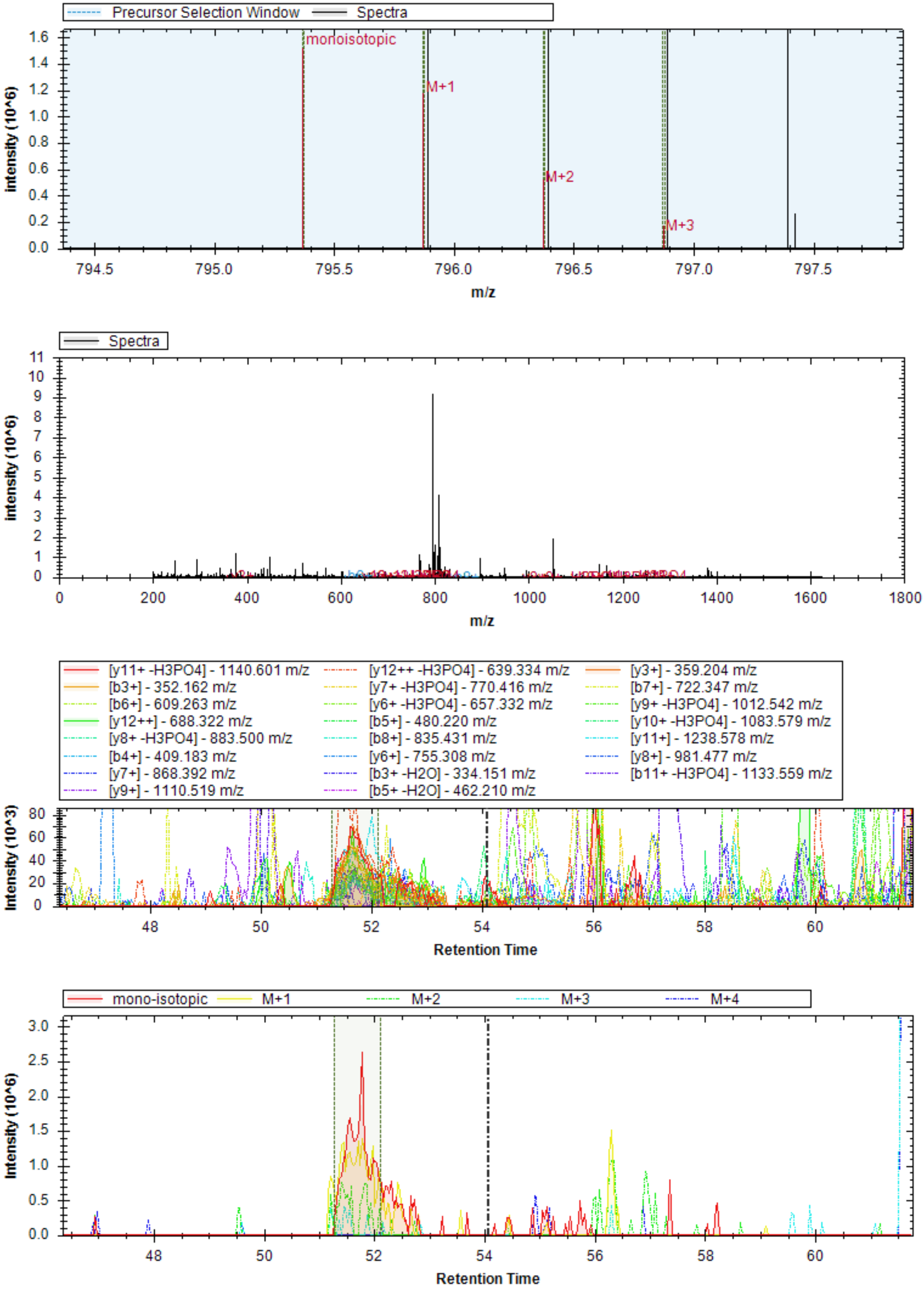

P20357 – _VDHGAEIITQS[+H+O3+P]PSR_.2
Fig4_MP-DIA-barrel-cortex-p15-3_MHRM.htrms

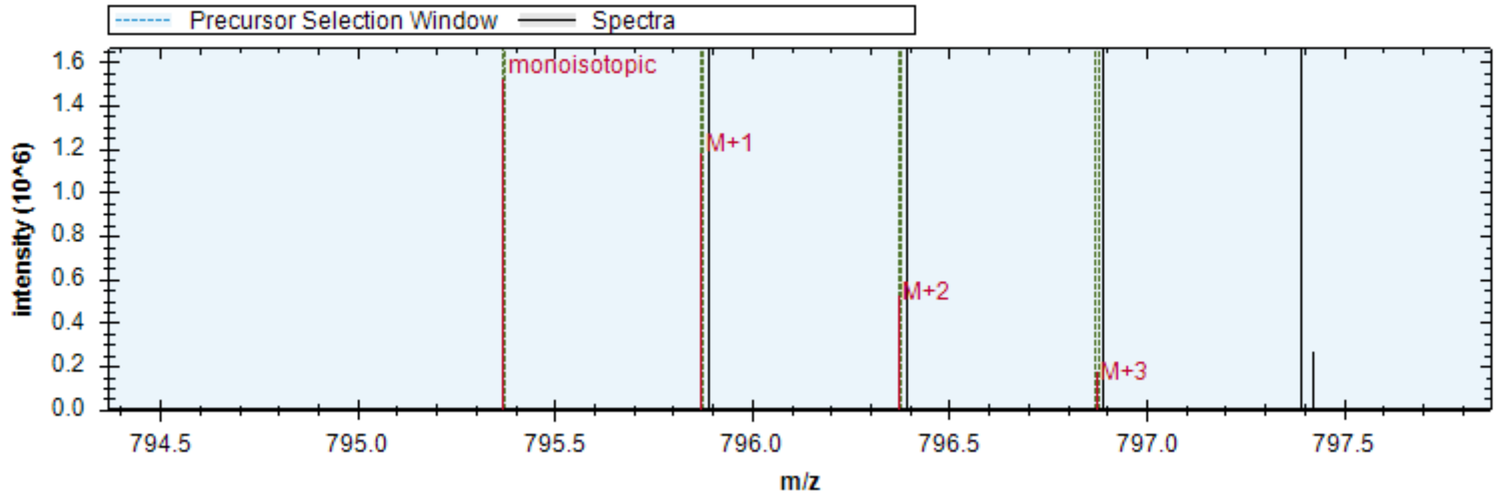

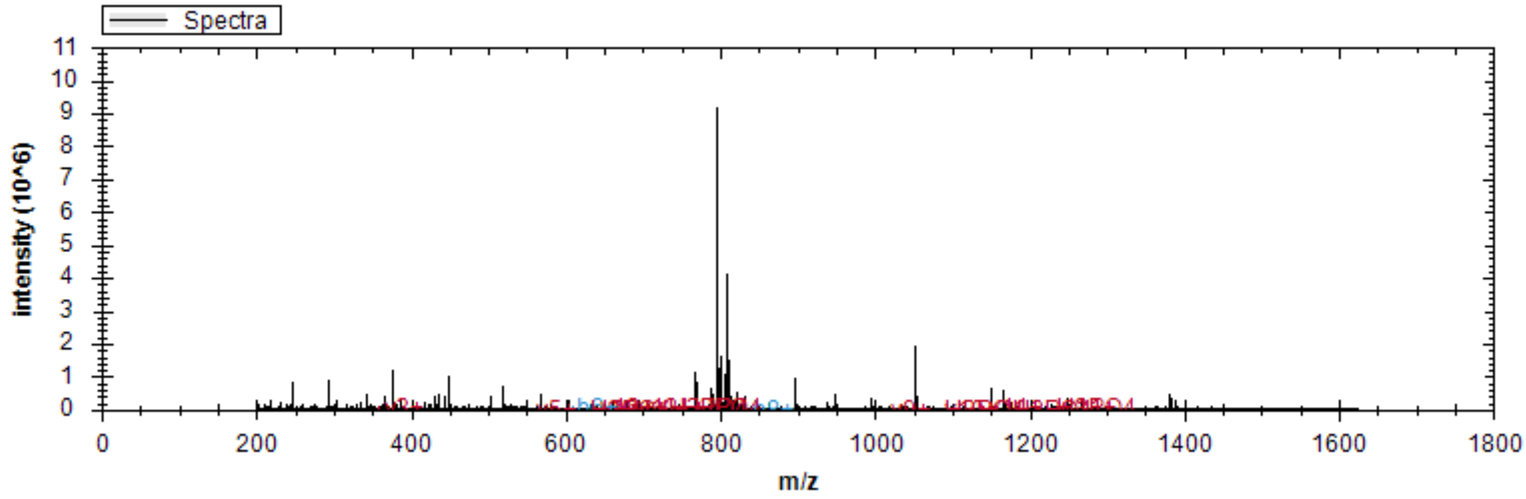

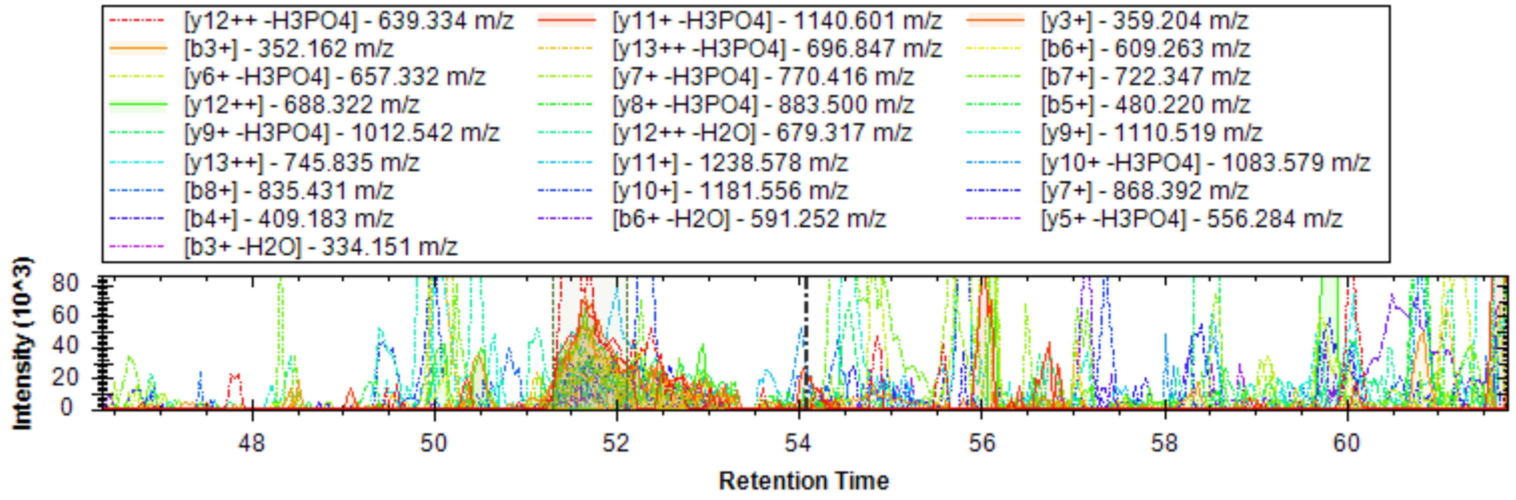

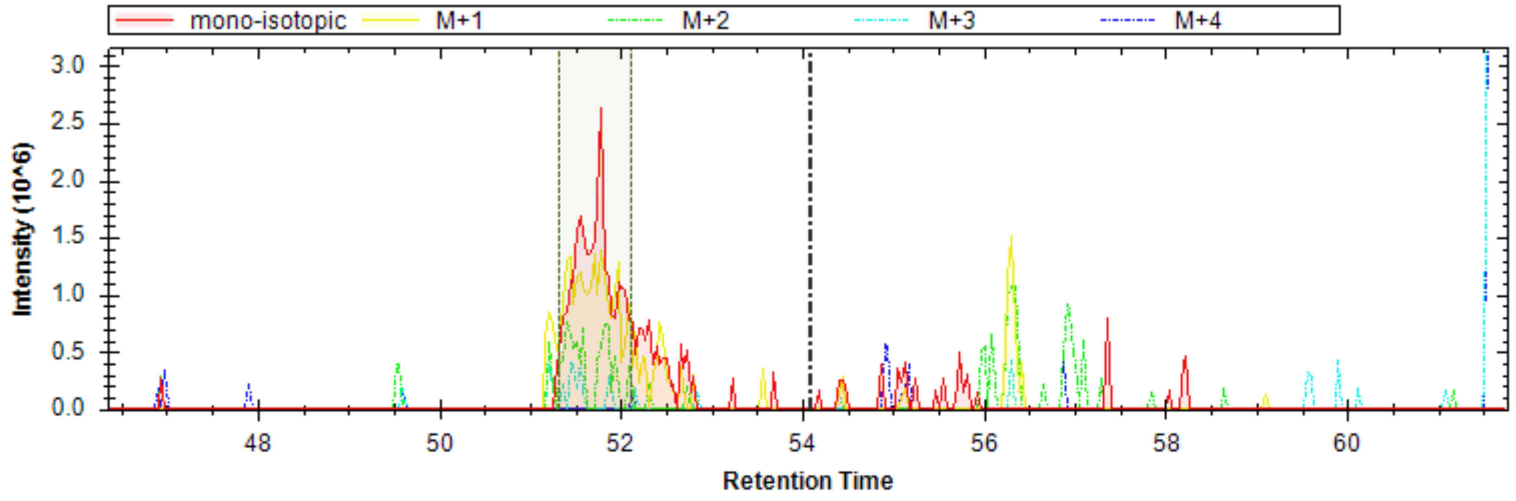

P20357 – _VDHGAEIITQSPS[+H+O3+P]R_.2
Fig4_MP-DIA-barrel-cortex-p15-3_MHRM.htrms

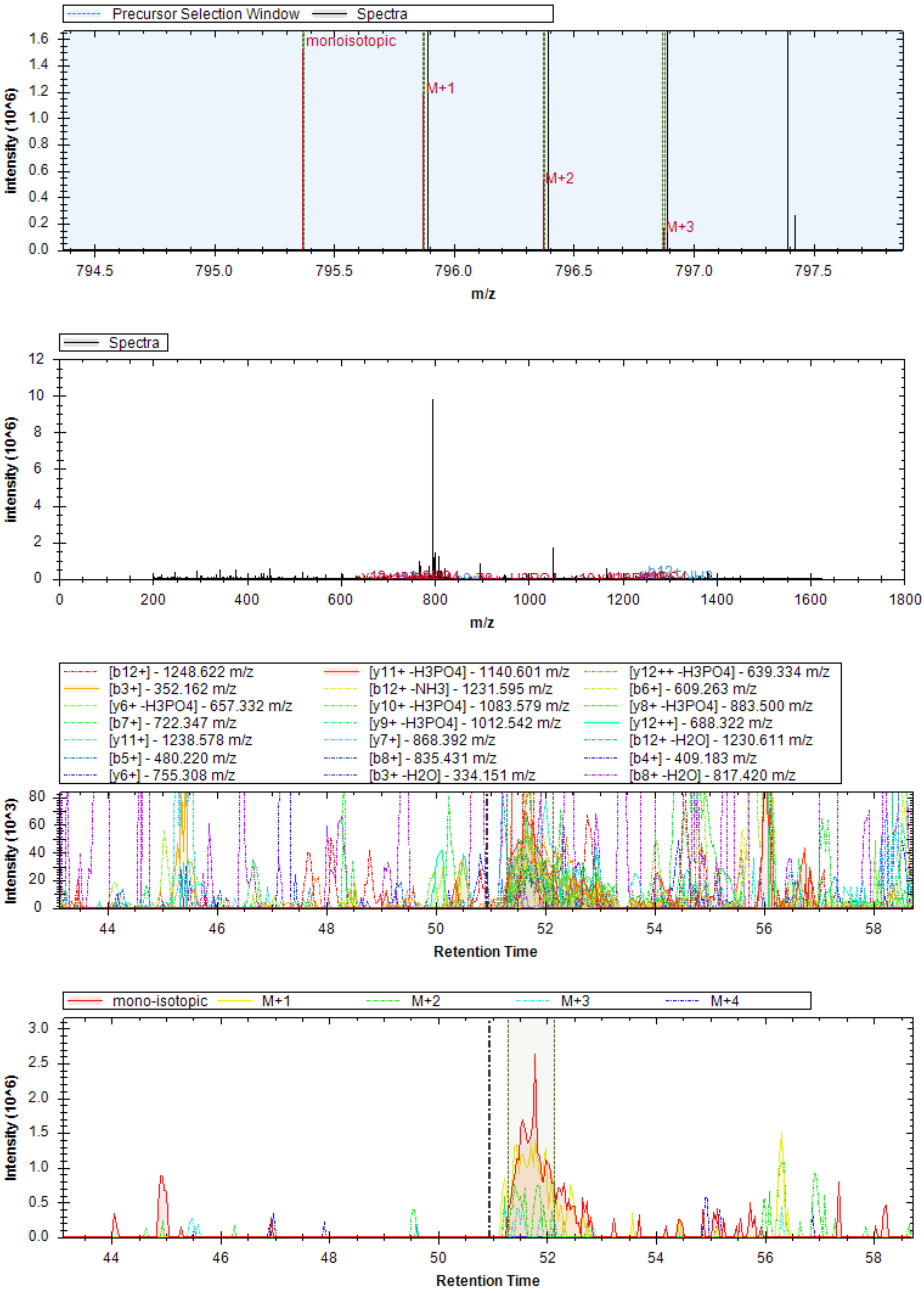

P54227 – _SKESVPDFPLS[+H+O3+P]PPK_.2

Fig4_MP-DIA-barrel-cortex-p15-3_MHRM.htrms

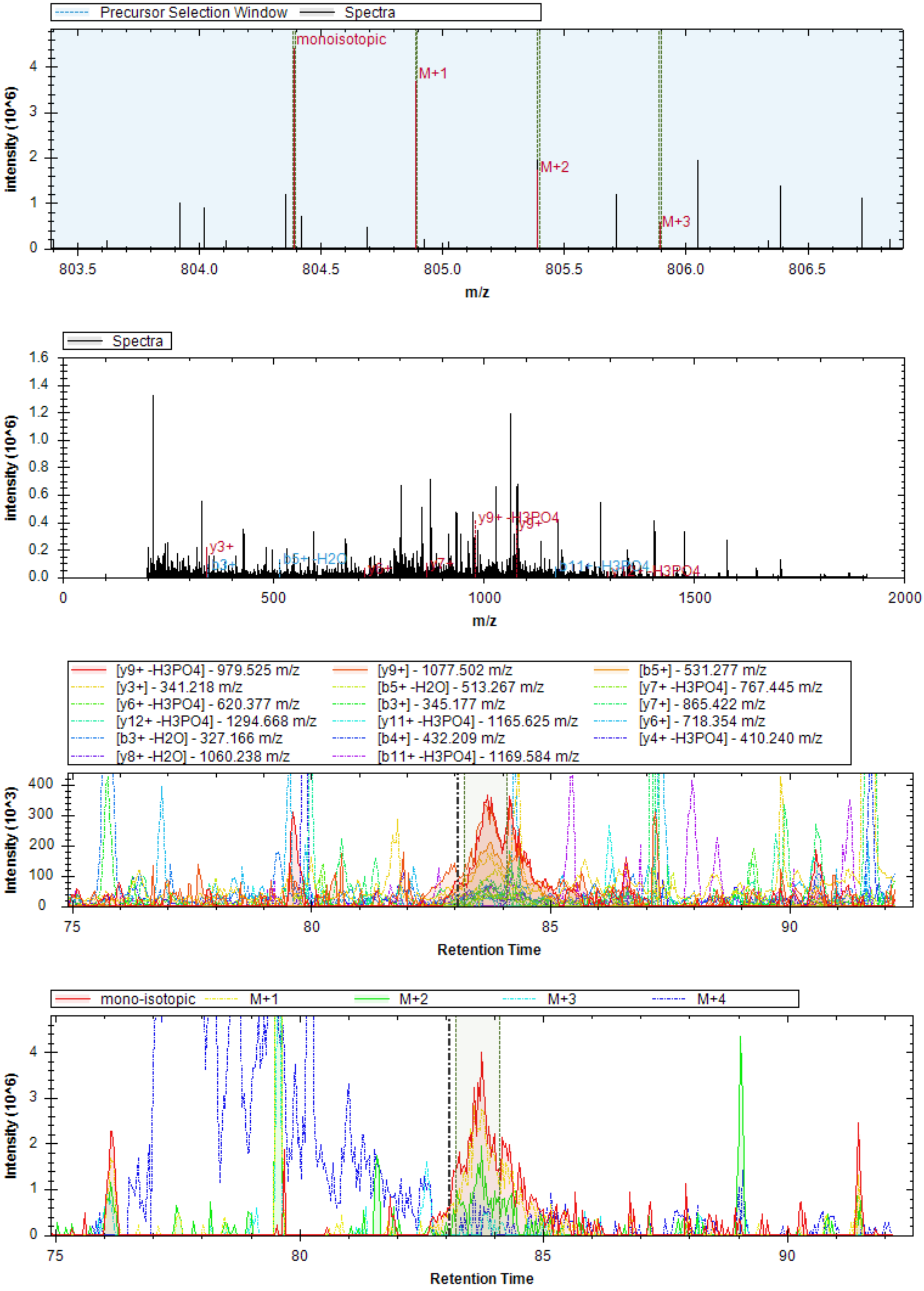

Q60771 – _FYYSSGS[+H+O3+P]SSPTHAK_.2
Fig4_MP-DIA-barrel-cortex-p15-3_MHRM.htrms

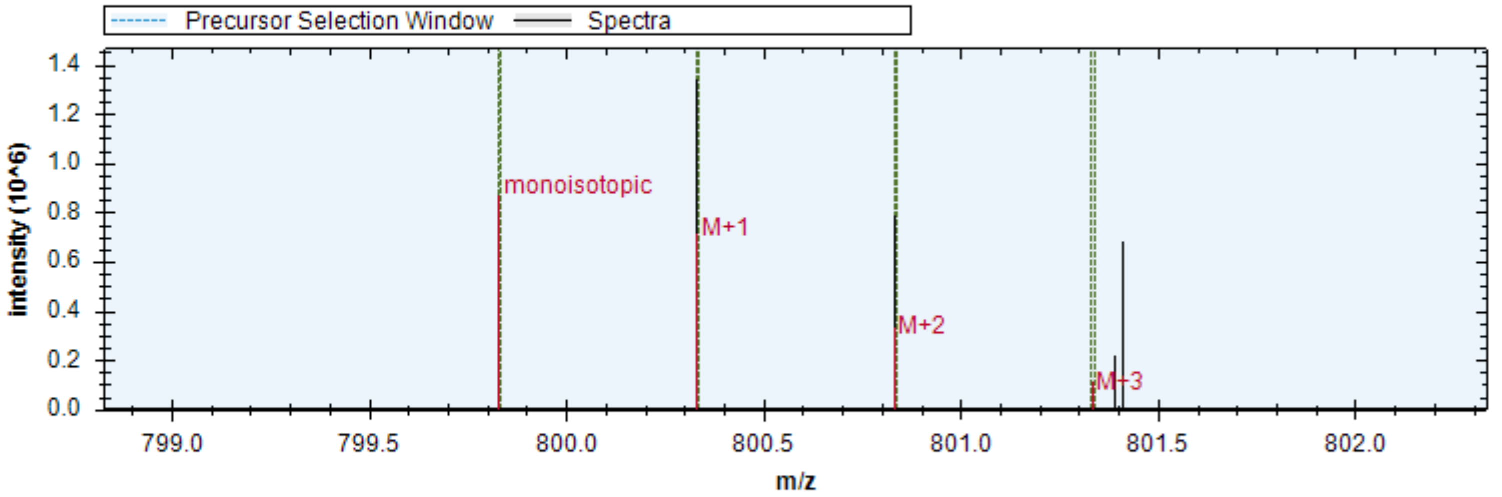

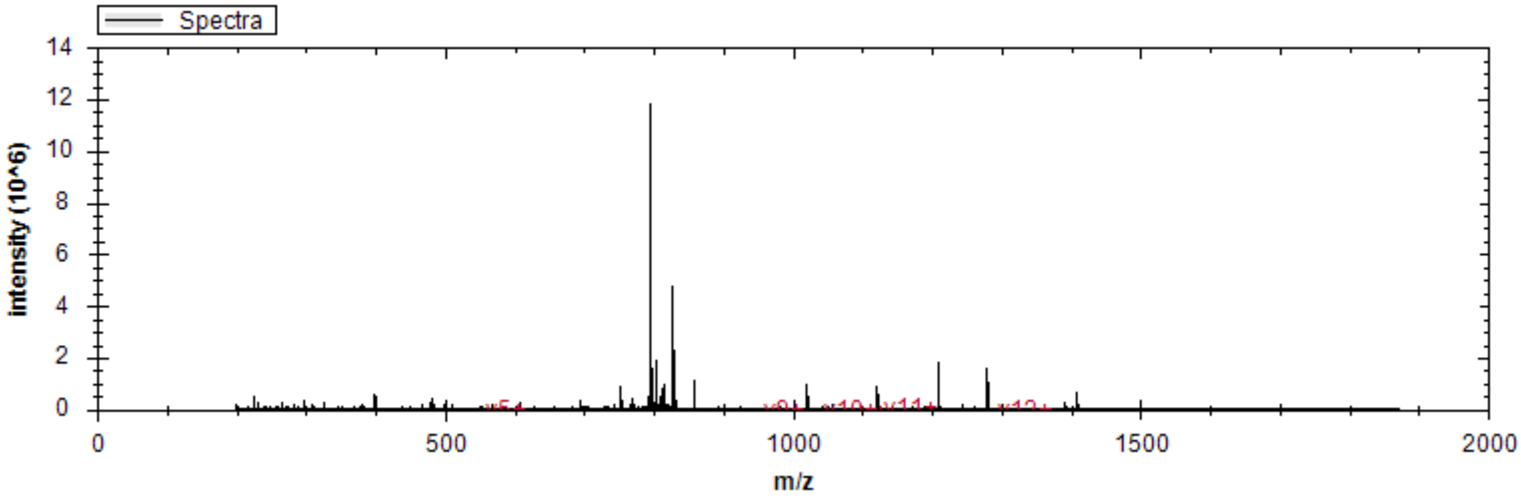

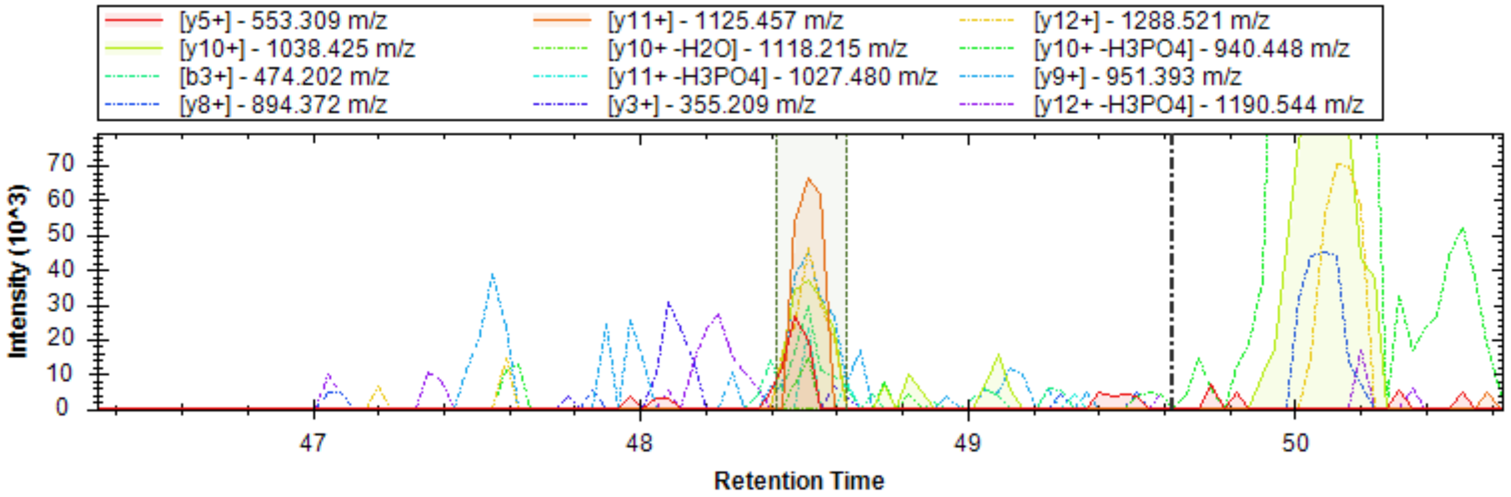

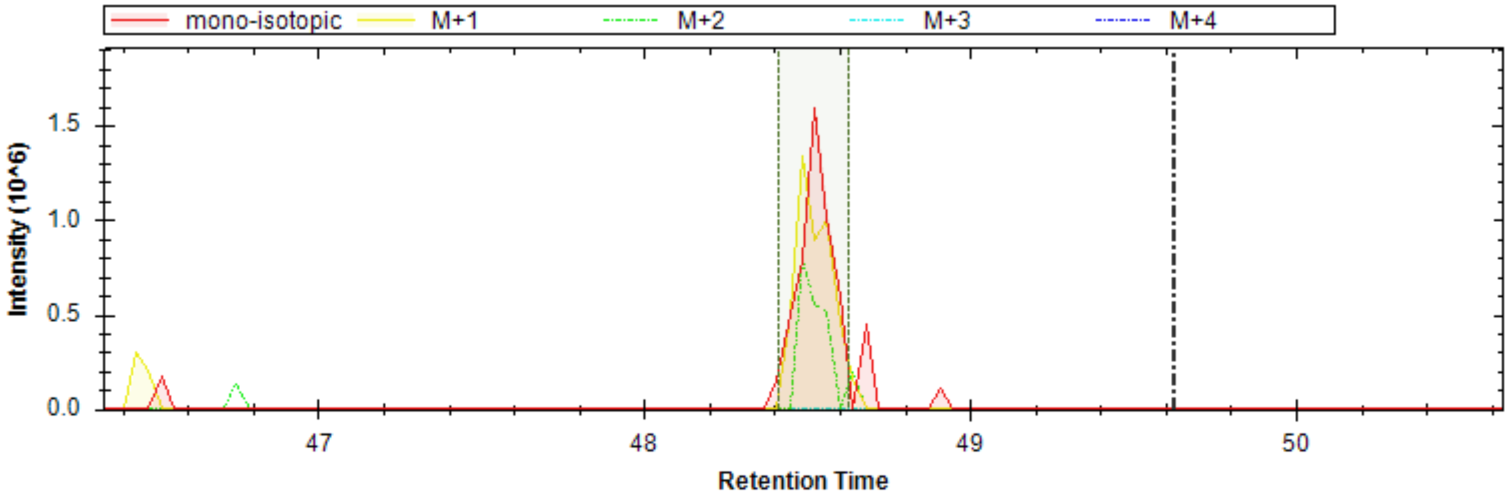

Q60771 – _FYYSSGSSS[+H+O3+P]PTHAK_.2

Fig4_MP-DIA-barrel-cortex-p15-3_MHRM.htrms

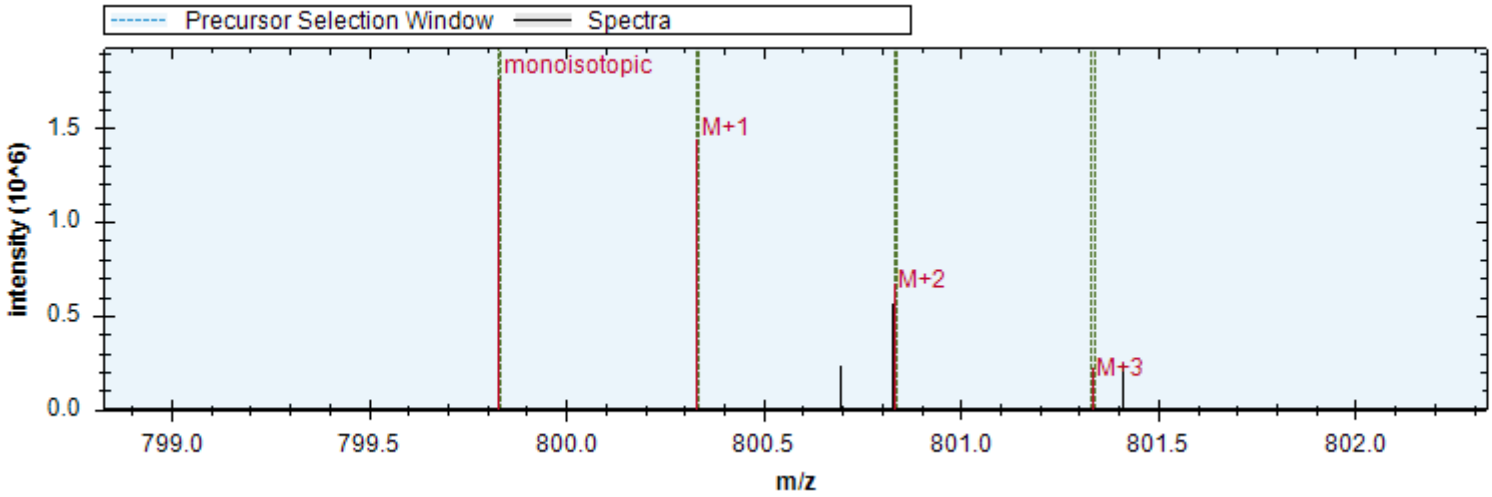

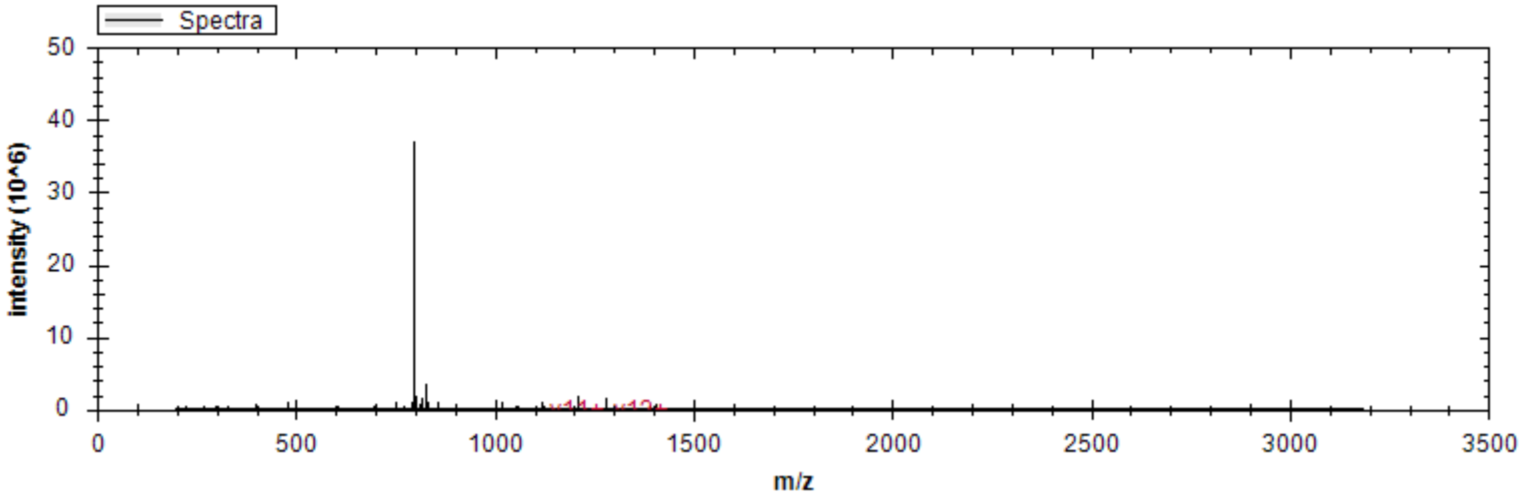

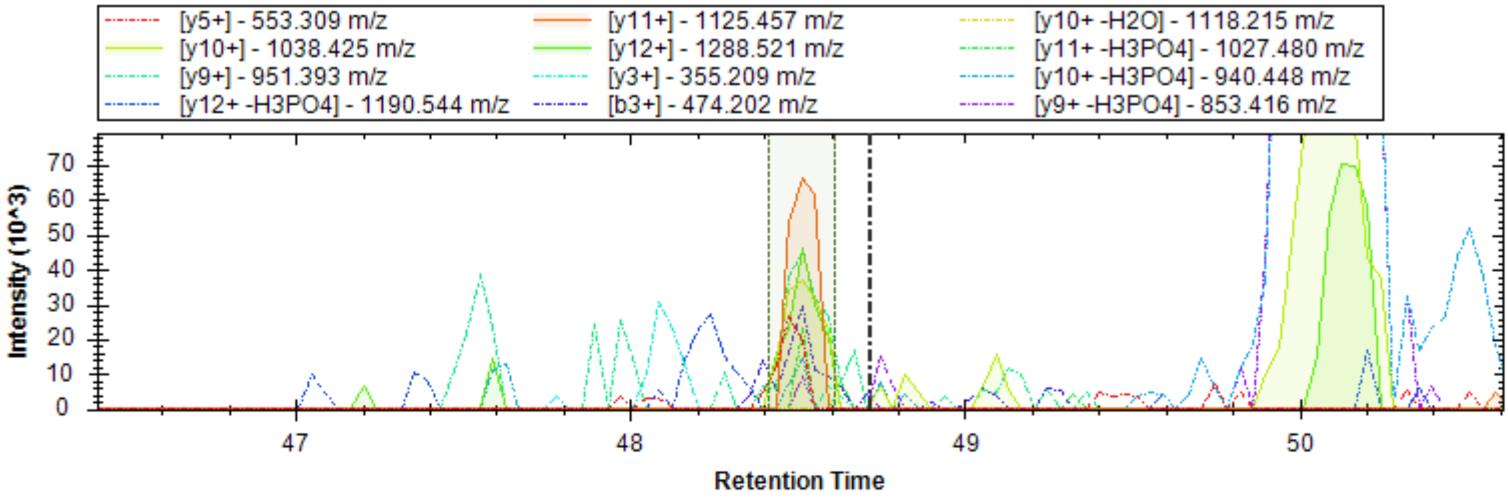

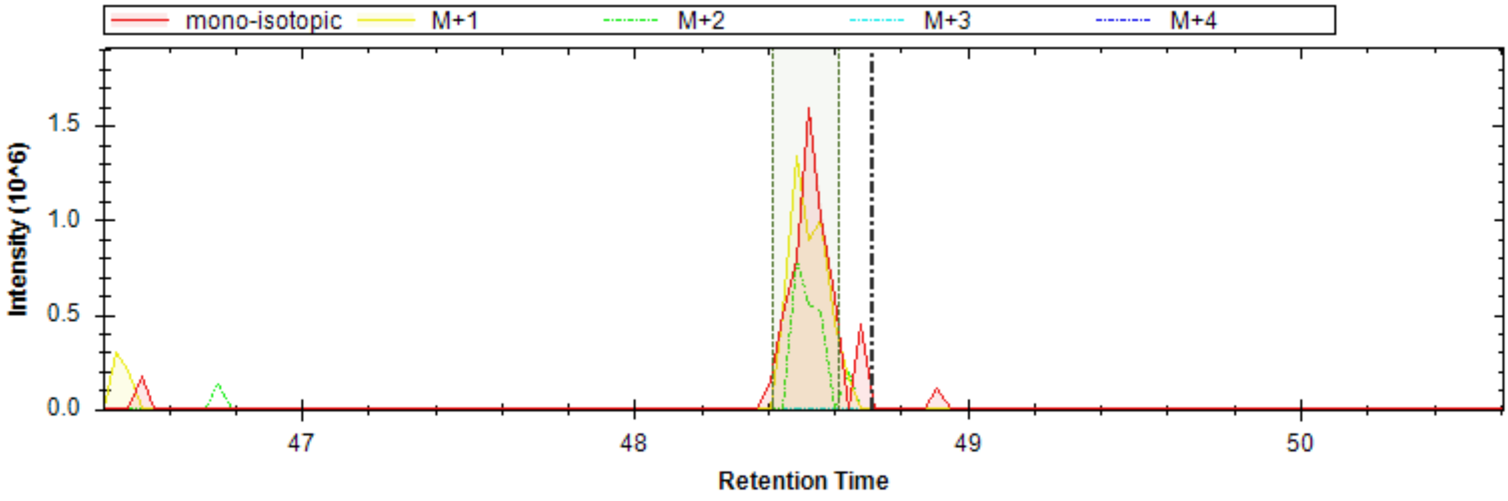

Q61548;Q61548-2;Q61548-3 – _SSPATTVTS[+H+O3+P]PNSTPAK_.2
Fig4_MP-DIA-barrel-cortex-p15-3_MHRM.htrms

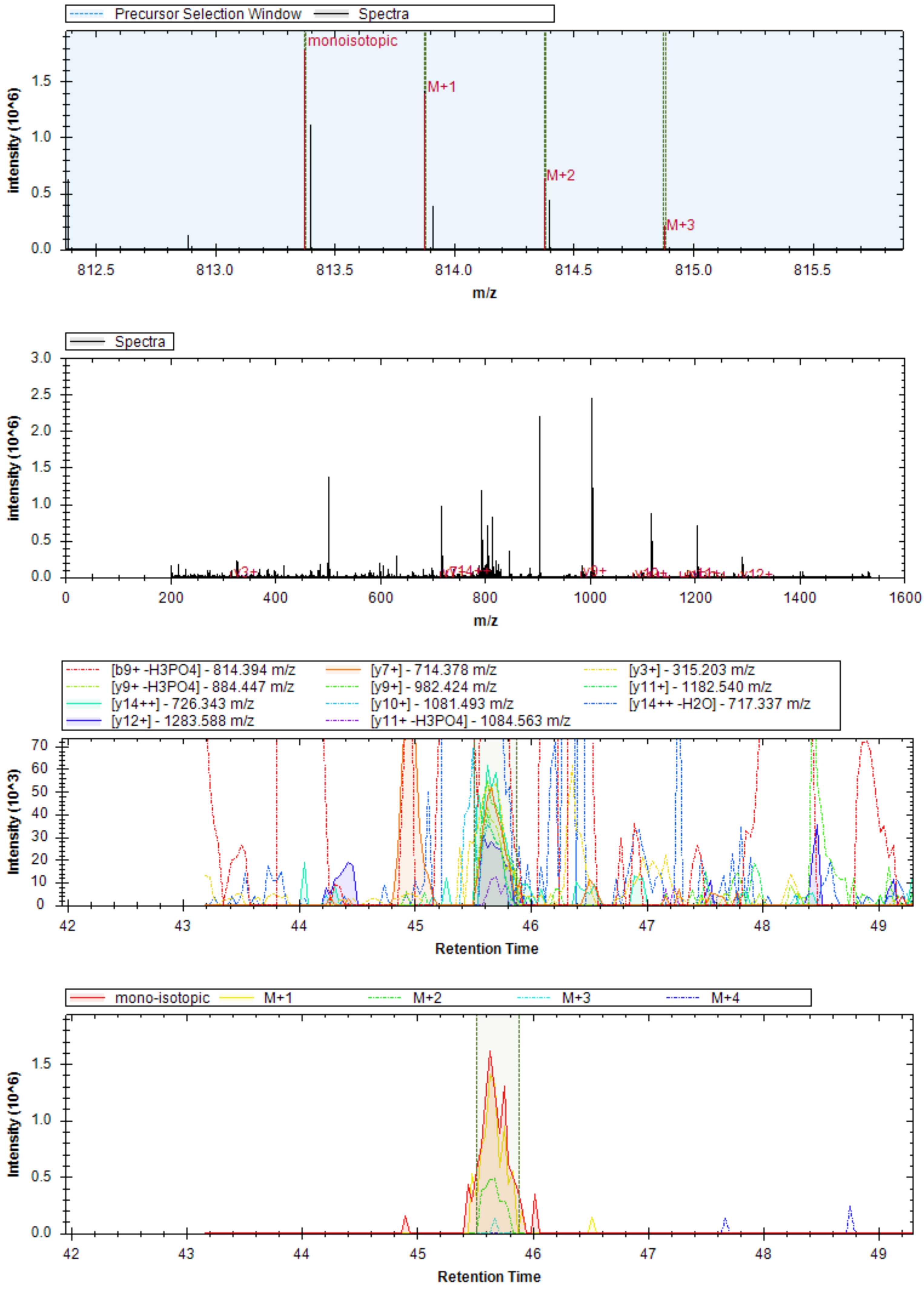

Q64701;Q64701-2 – _LAEILYYKILET[+H+O3+P]IMVQET[+H+O3+P]R_.3
Fig4_MP-DIA-barrel-cortex-p15-3_MHRM.htrms

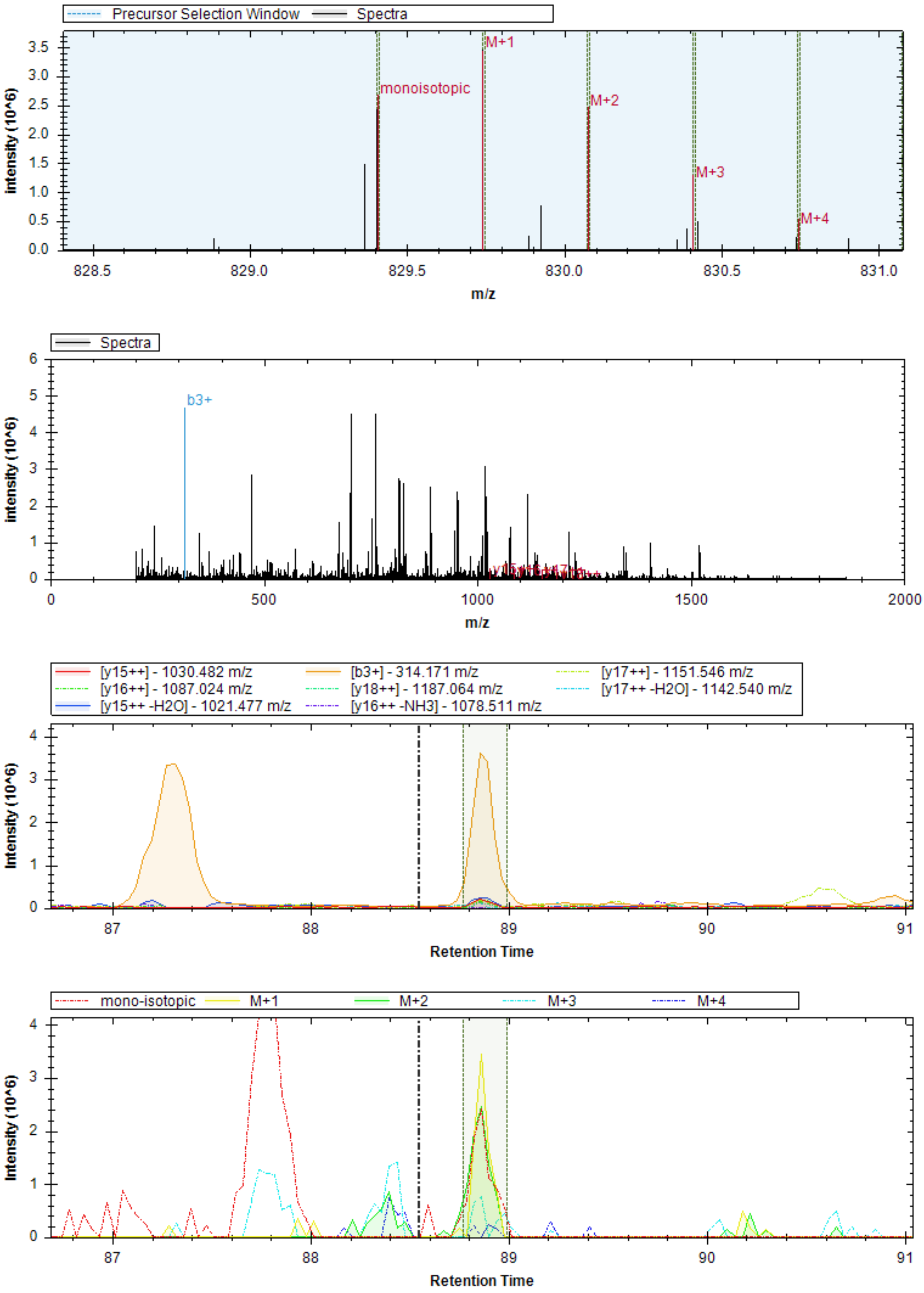

Q7TQD2 – _AANKT[+H+O3+P]PPKSPGDPAR_.2

Fig4_MP-DIA-barrel-cortex-p15-3_MHRM.htrms

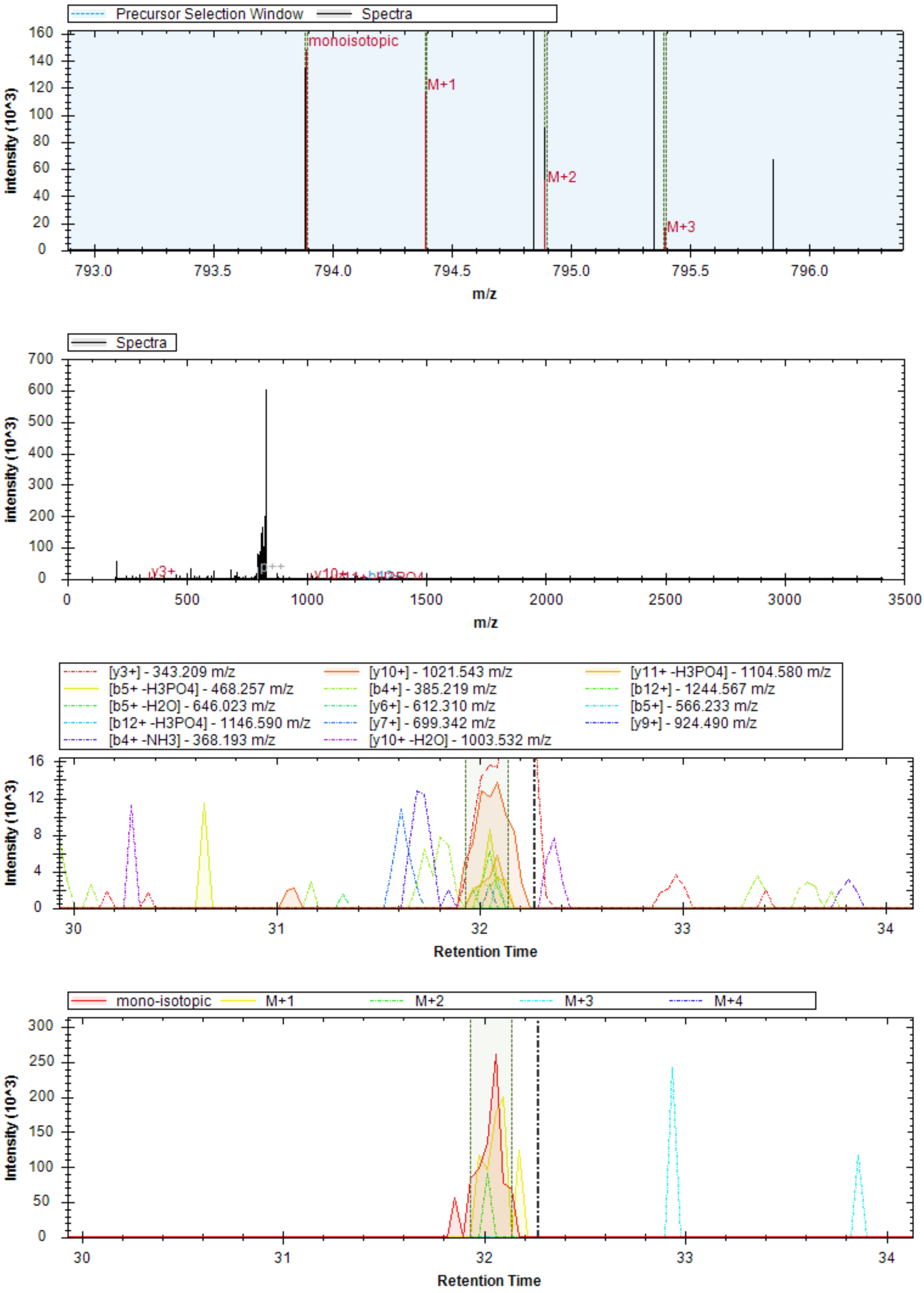

Q91XM9;Q91XM9-2;Q91XM9-7 – _NLSQIENVHGYVLQS[+H+O3+P]HISPLK_.3

Fig4_MP-DIA-barrel-cortex-p15-3_MHRM.htrms

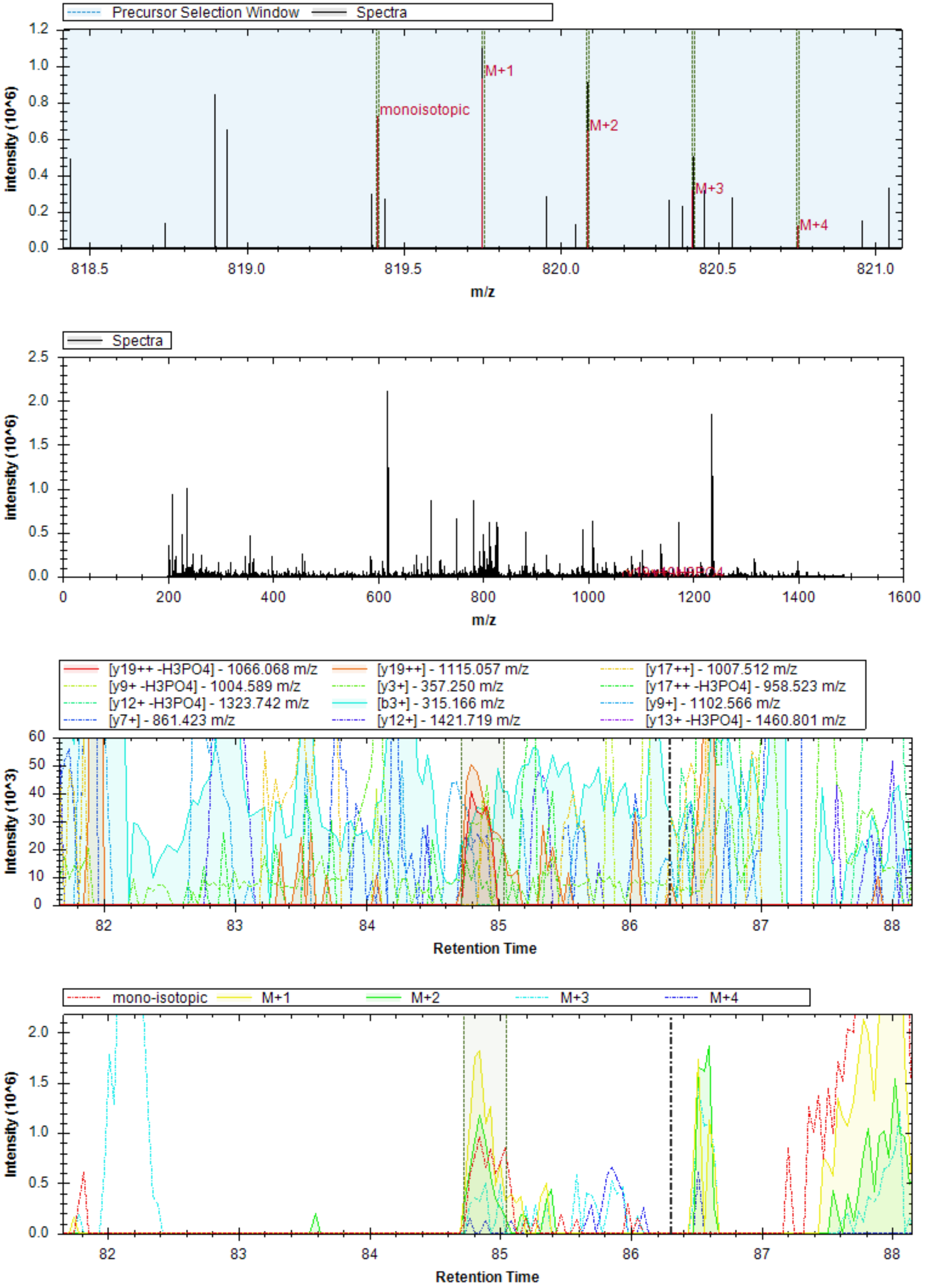

D3YXK2 – _AET[+H+O3+P]LSGLGDASAAGAAAVSSAASETGTR_.3
Fig4_MP-DIA-barrel-cortex-p15-3_MHRM.htrms

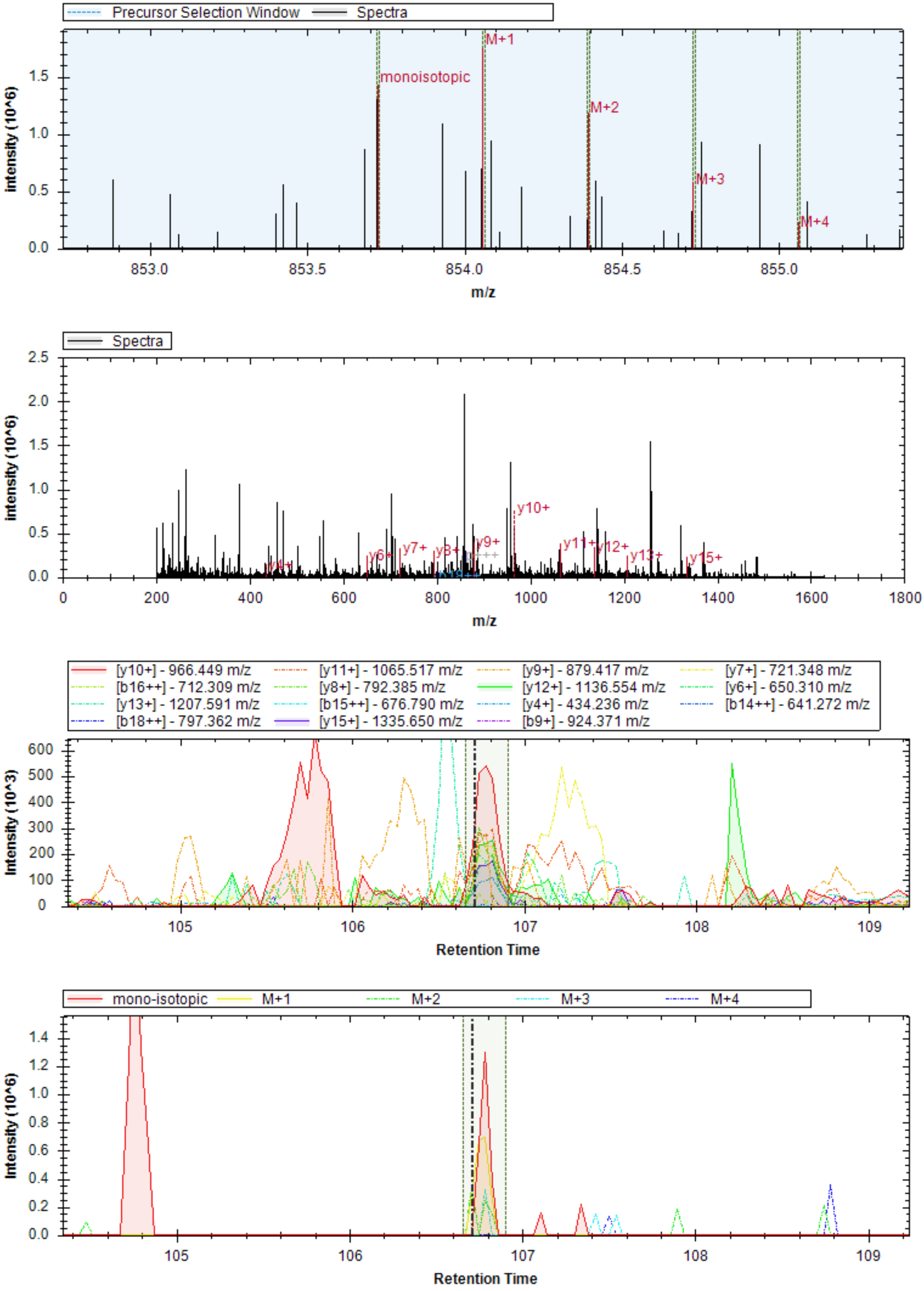

O08784 – _ASAVSPEKAPM[+O]T[+H+O3+P]SKAK_.2
Fig4_MP-DIA-barrel-cortex-p15-3_MHRM.htrms

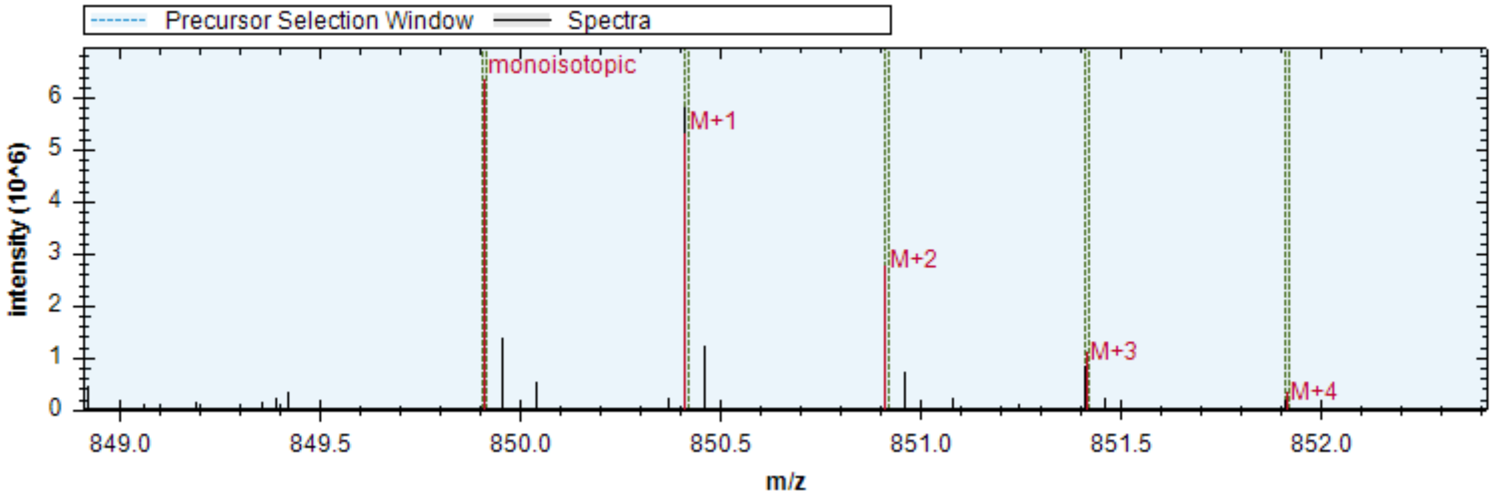

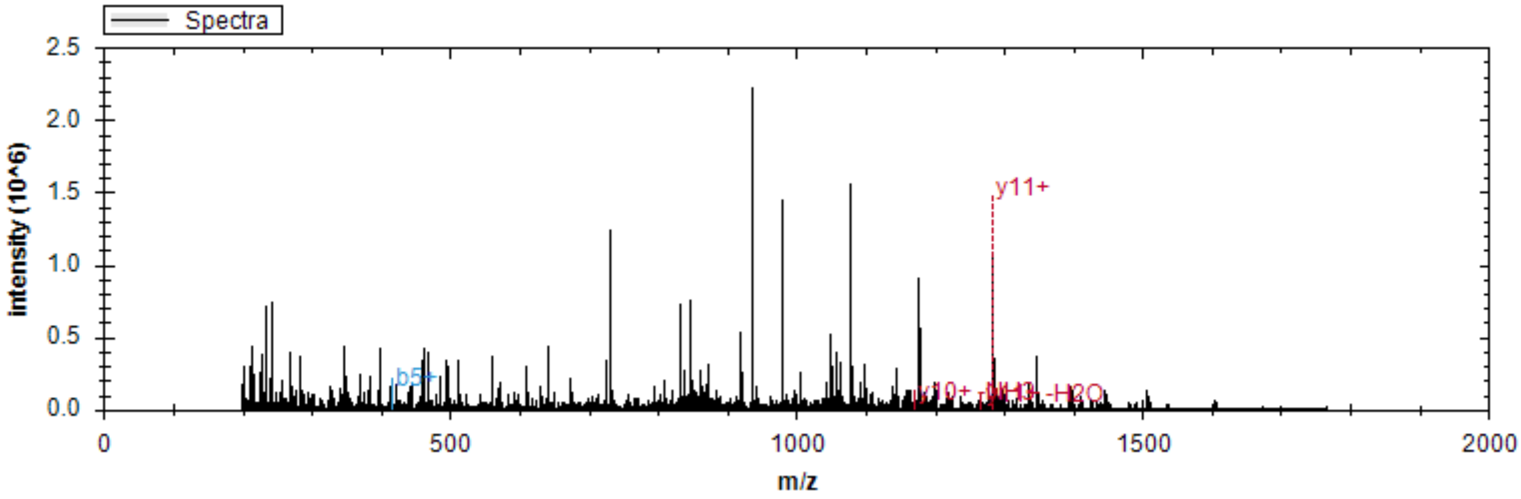

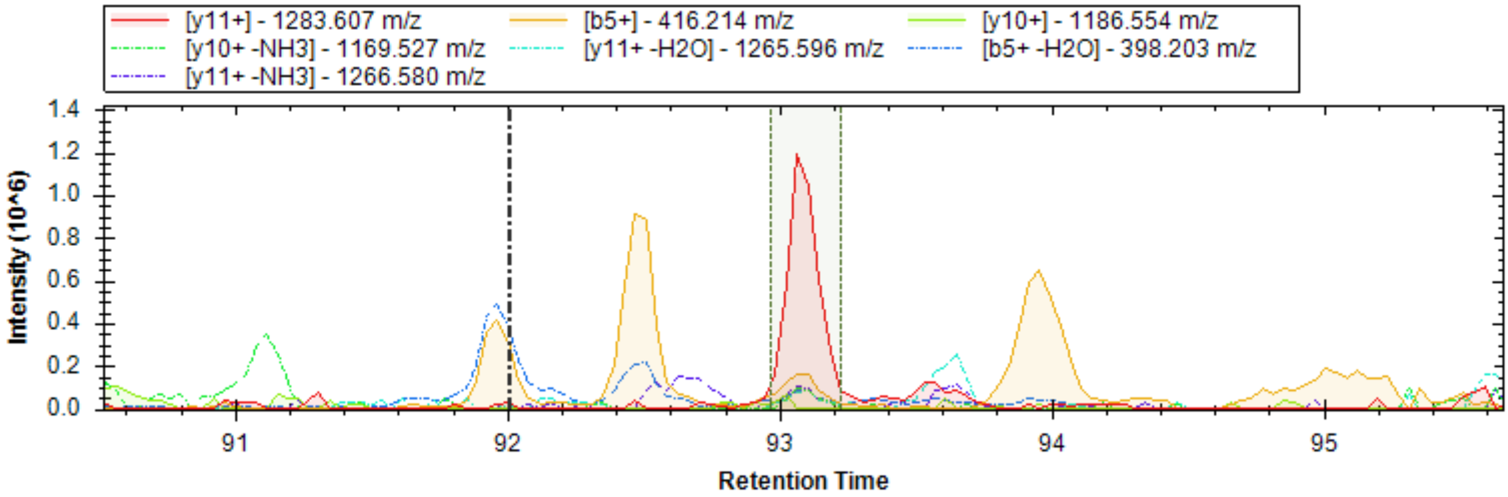

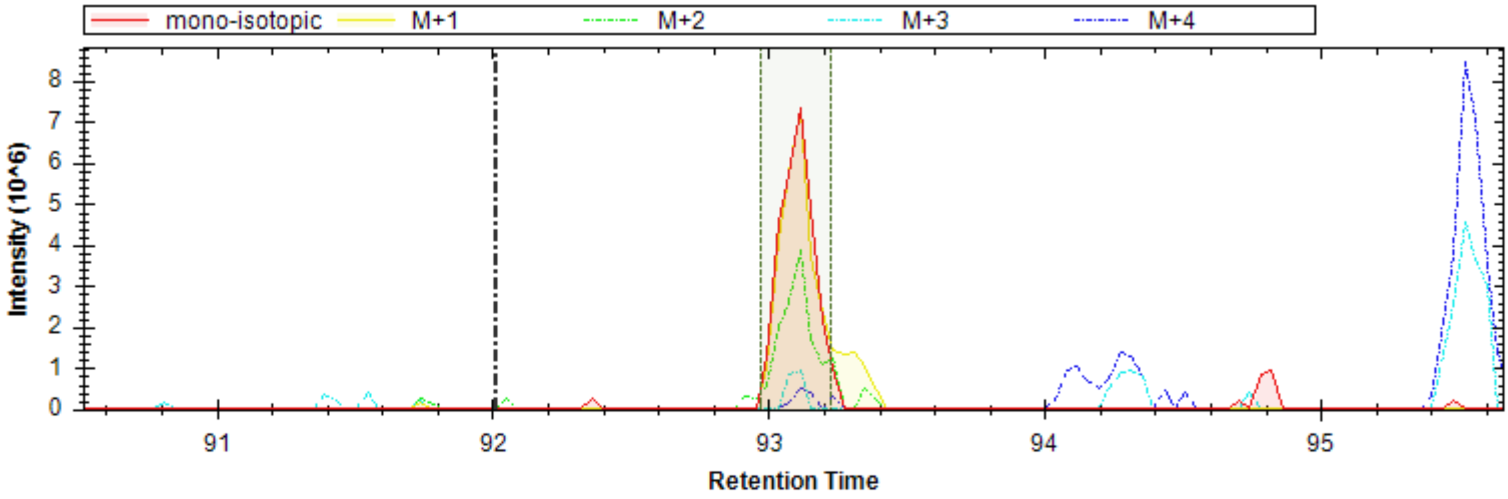

P14873 – _ETAAAHQAS[+H+O3+P]SSPPIDAATAEPYGFR_.3
Fig4_MP-DIA-barrel-cortex-p15-3_MHRM.htrms

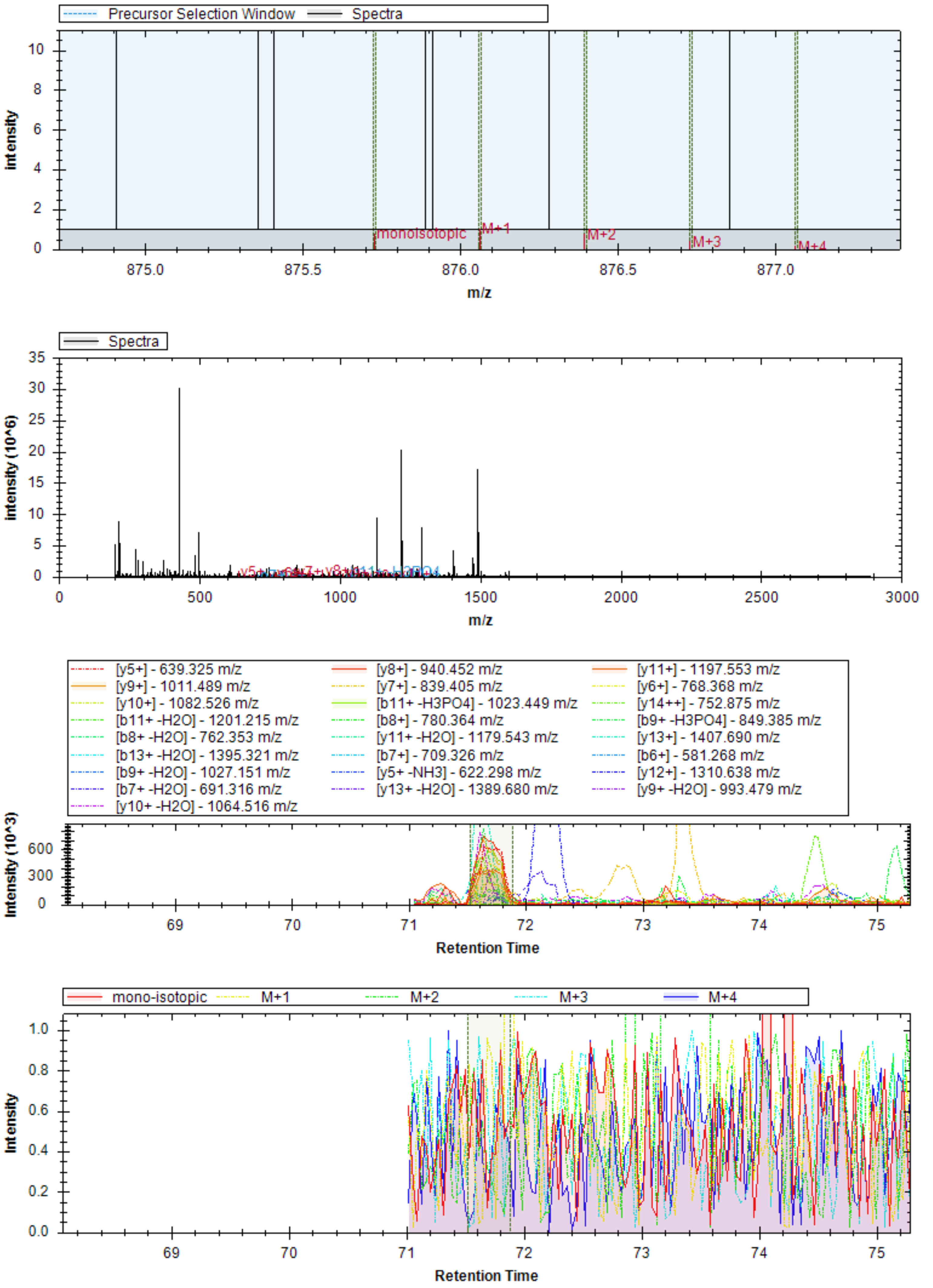

P14873 – _ETAAAHQASSS[+H+O3+P]PPIDAATAEPYGFR_.3
Fig4_MP-DIA-barrel-cortex-p15-3_MHRM.htrms

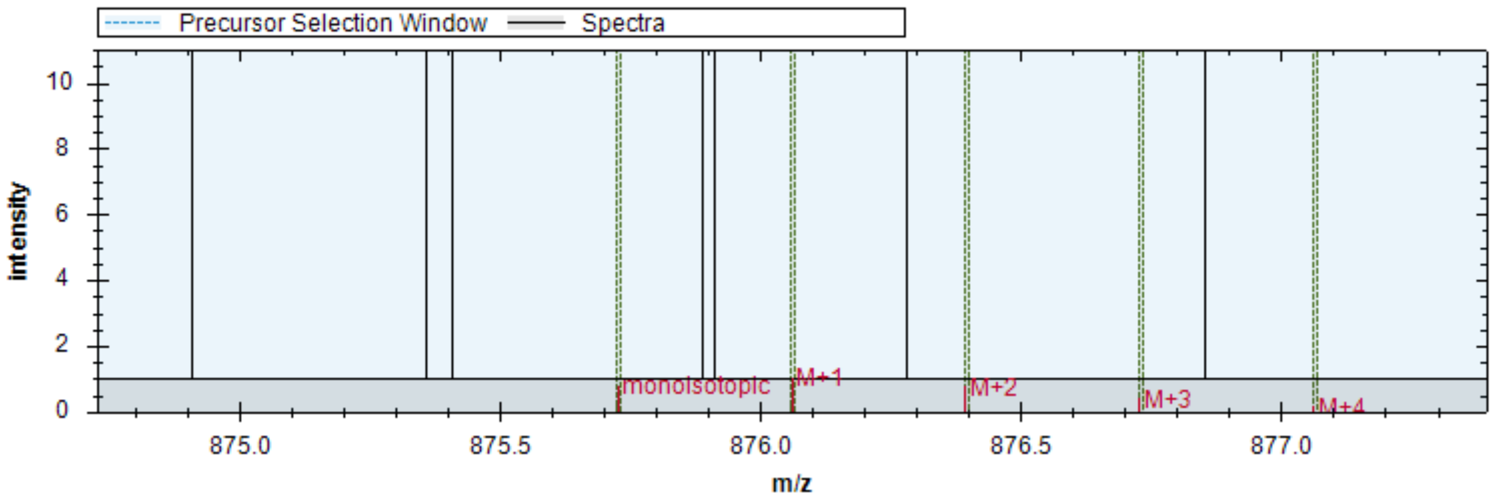

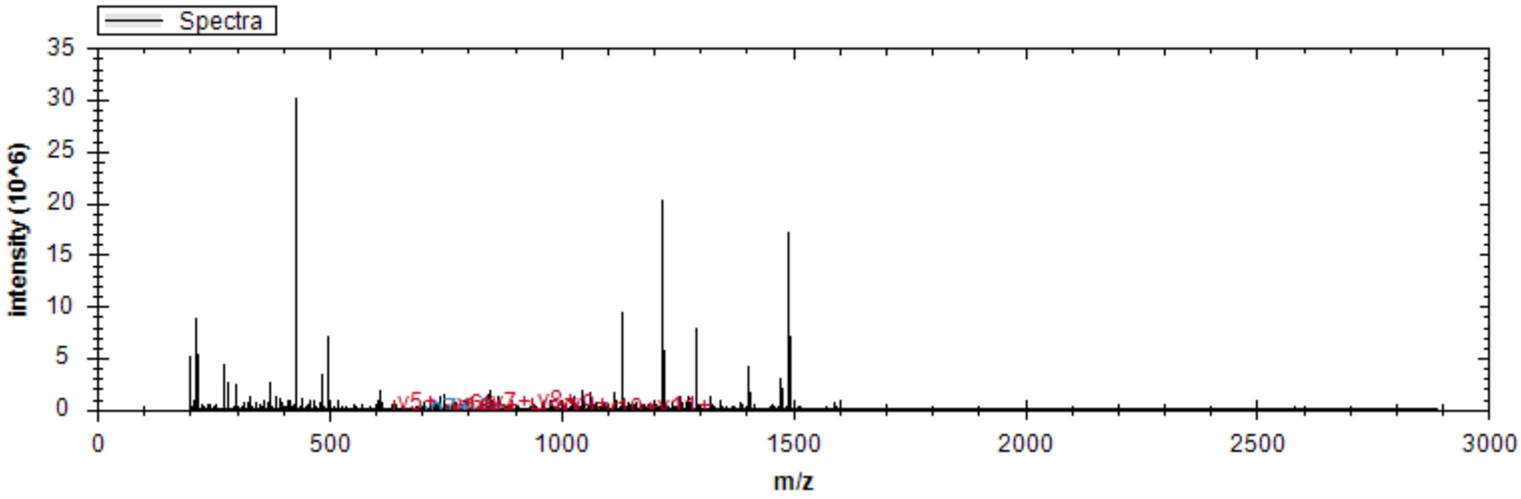

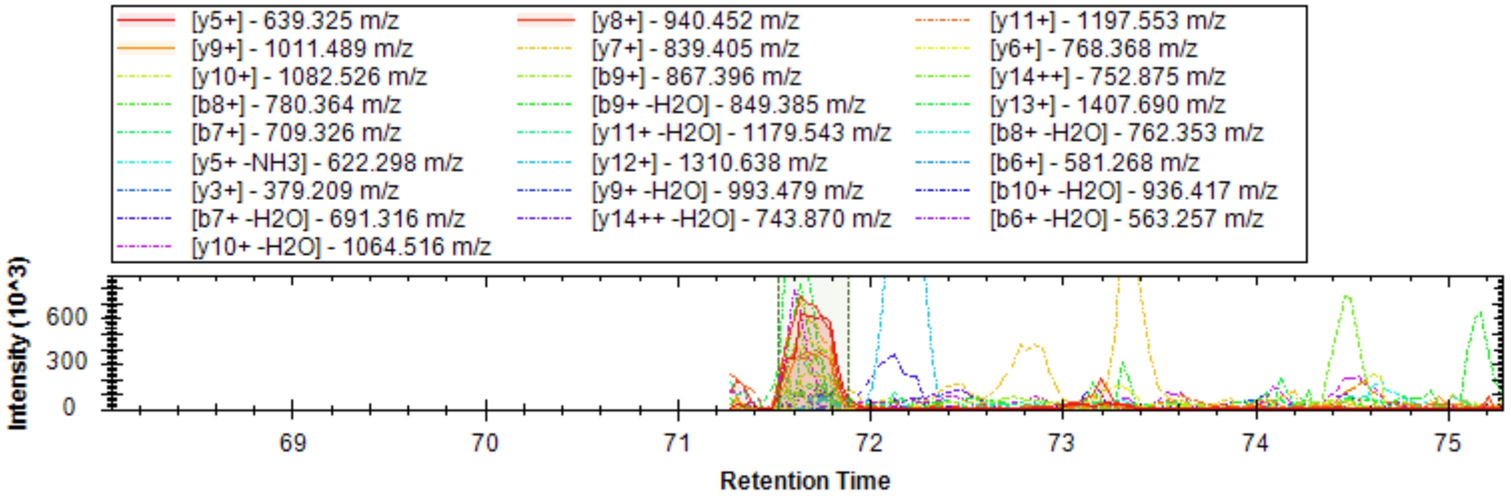

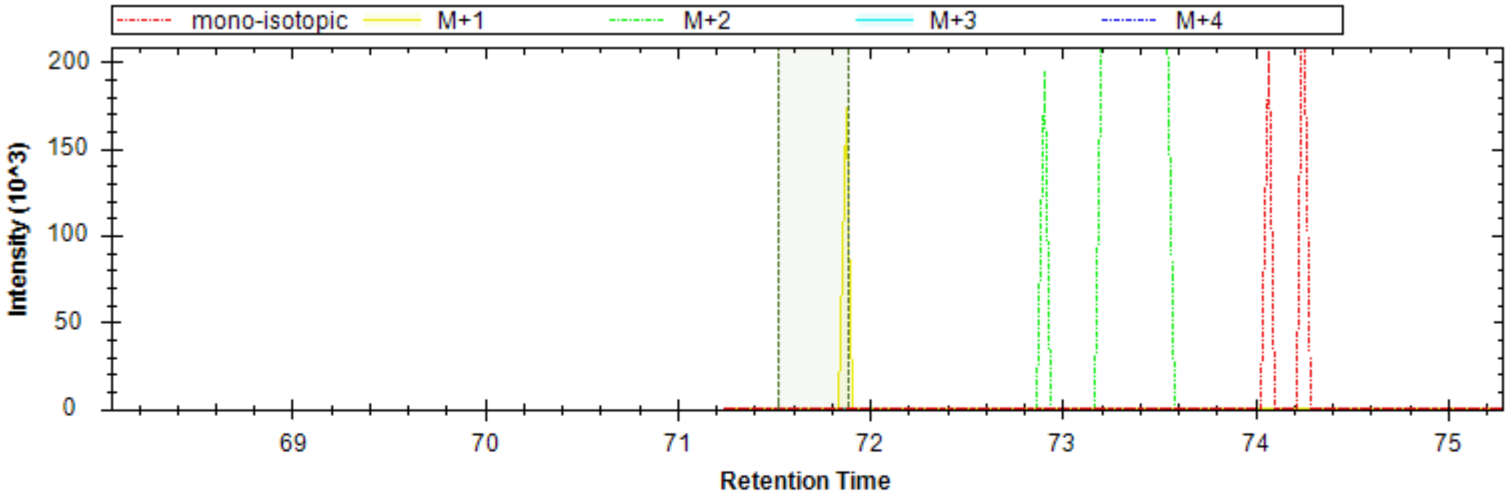

P20917 – _NVTES[+H+O3+P]SSFSGGDNPHVLYSPEFR_.3
Fig4_MP-DIA-barrel-cortex-p15-3_MHRM.htrms

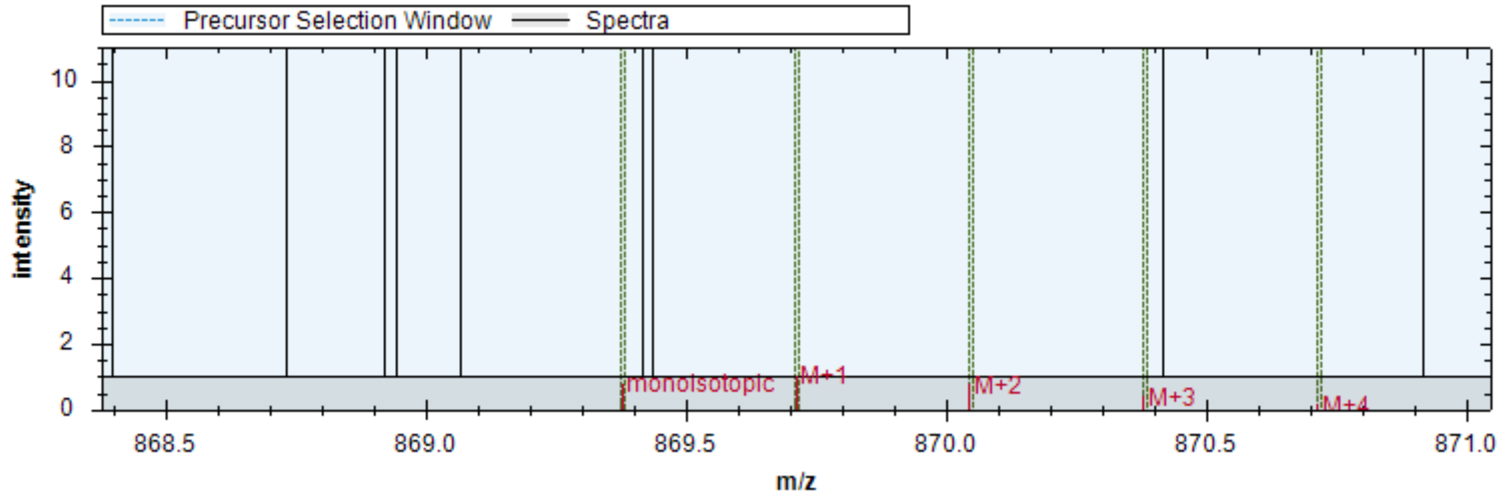

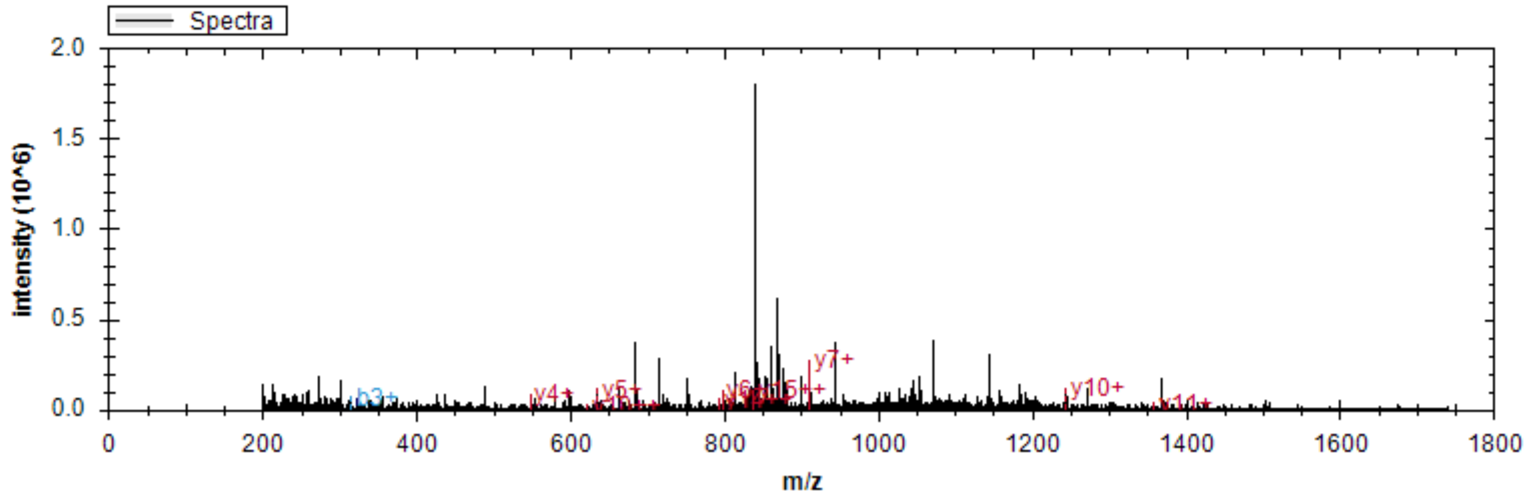

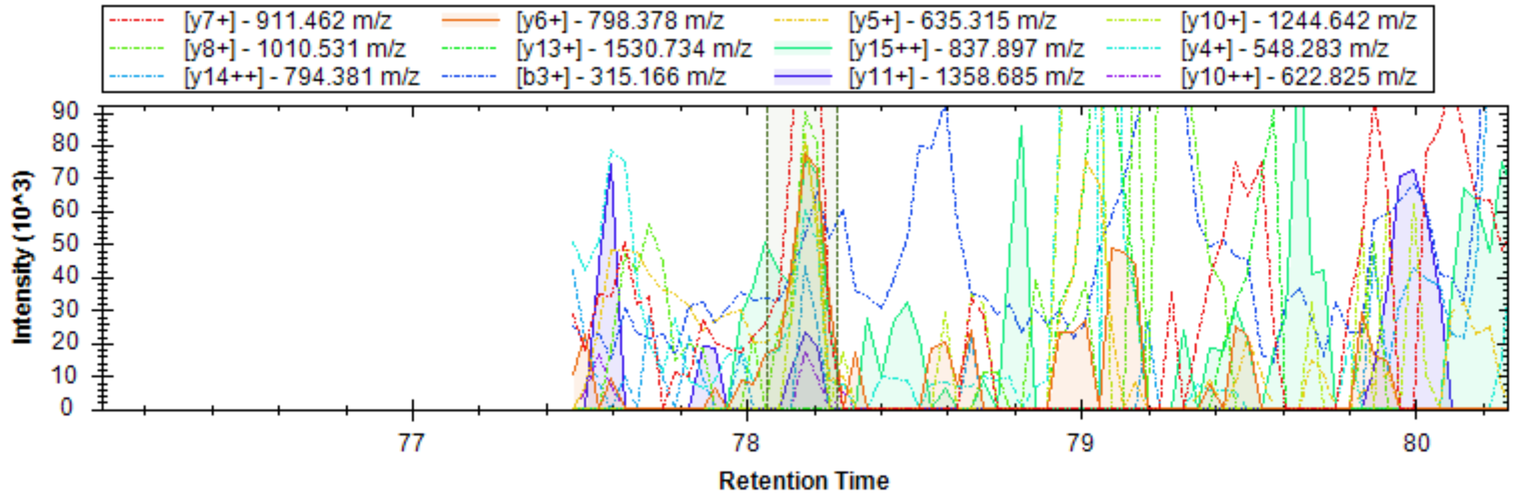

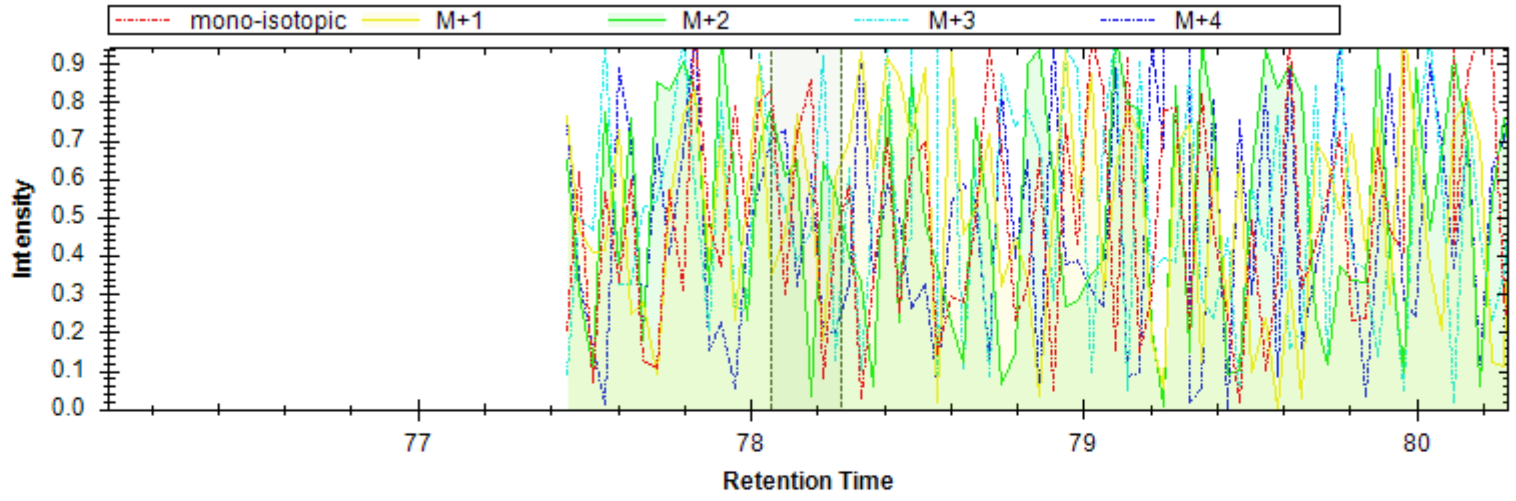

P20917 – _NVTESSS[+H+O3+P]FSGGDNPHVLYSPEFR_.3
Fig4_MP-DIA-barrel-cortex-p15-3_MHRM.htrms

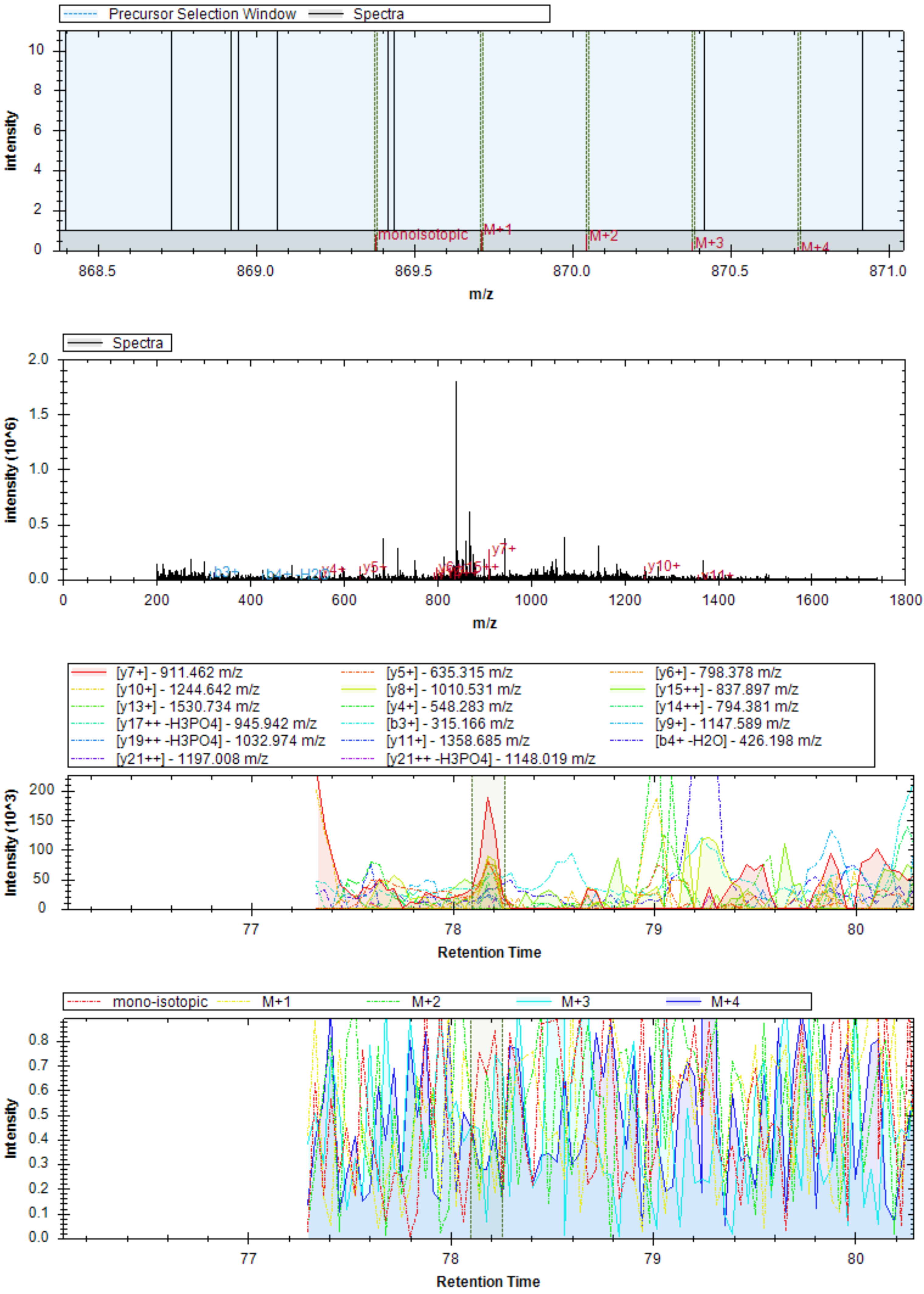

P20917 – _NVTESSSFS[+H+O3+P]GGDNPHVLYSPEFR_.3
Fig4_MP-DIA-barrel-cortex-p15-3_MHRM.htrms

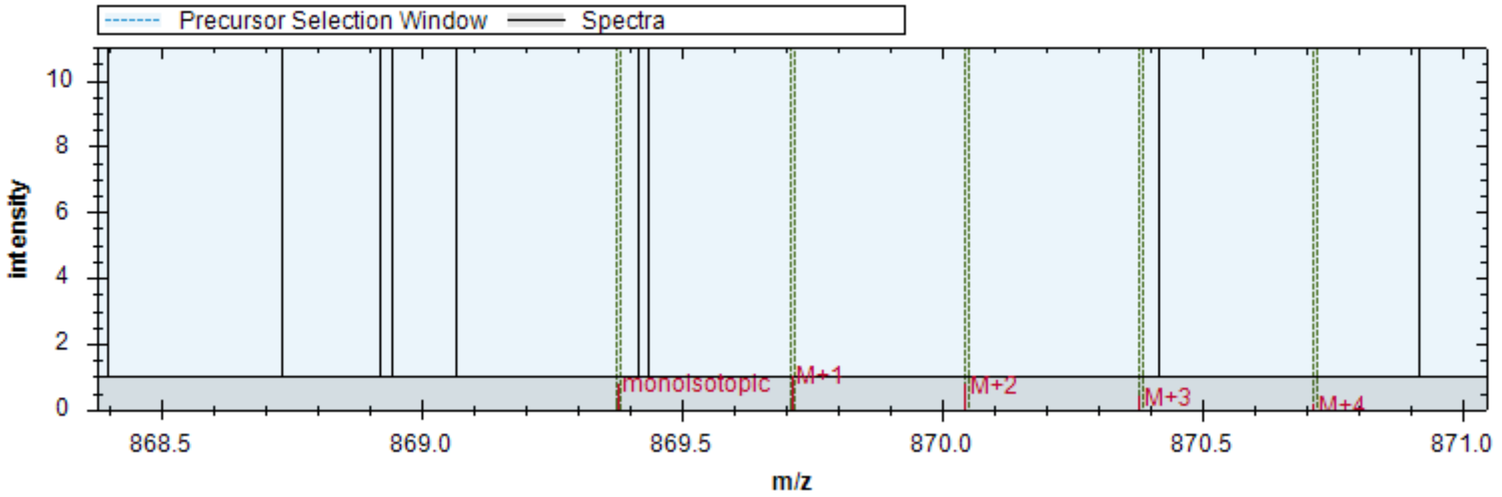

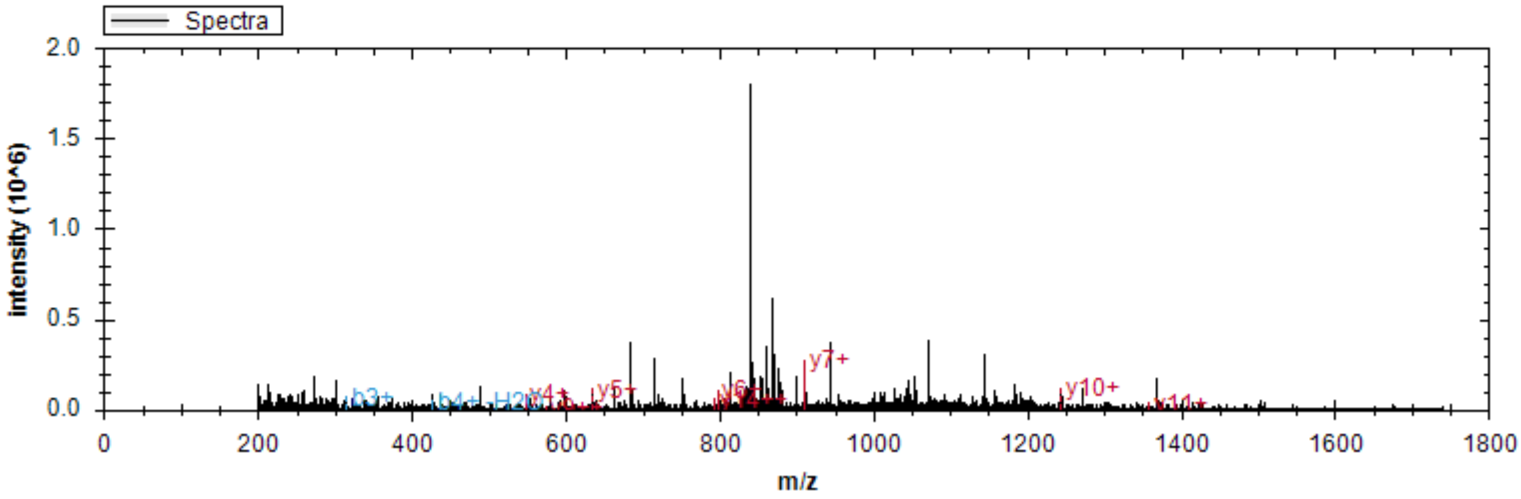

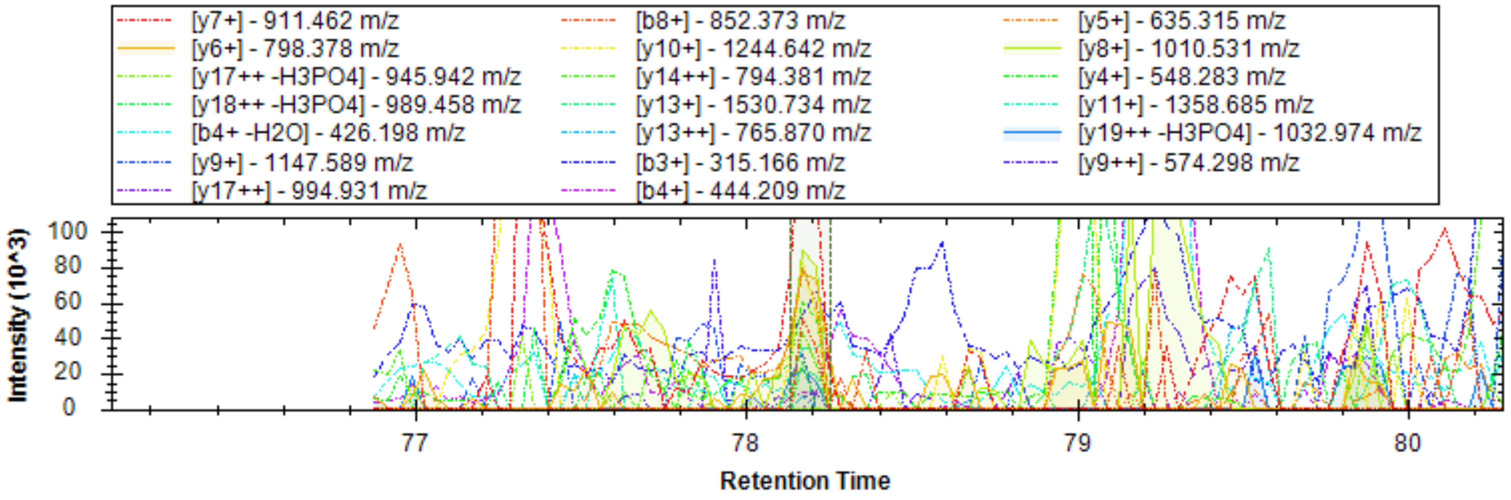

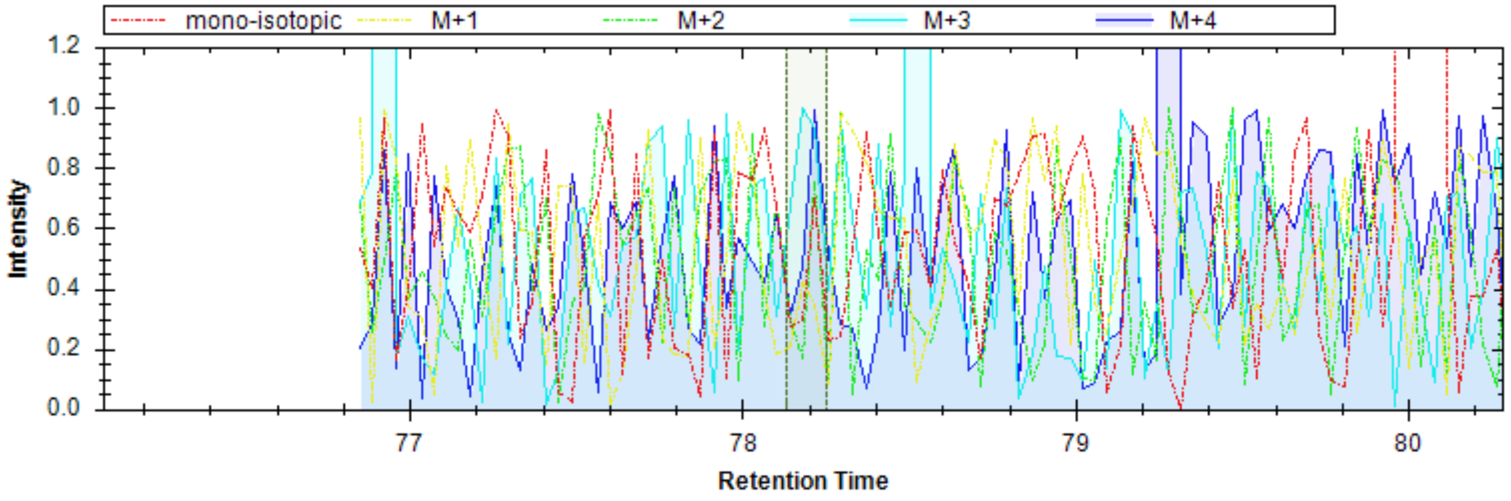

P50538 – _EAEHGYAS[+H+O3+P]M[+O]LPYSK_.2
Fig4_MP-DIA-barrel-cortex-p15-3_MHRM.htrms

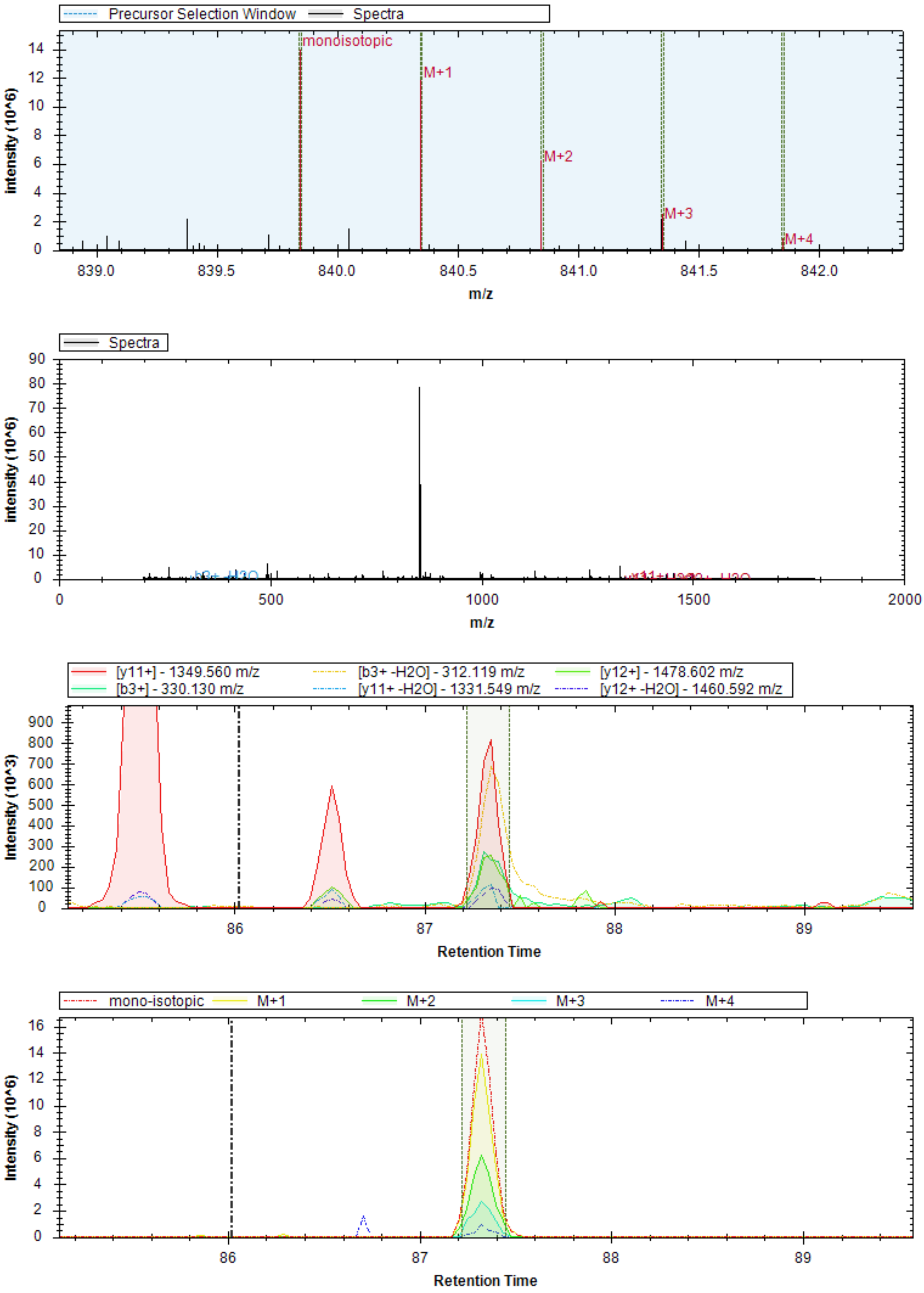

Q3UNH4 – _S[+H+O3+P]VATGPMTPQAAAPPAVPPVFPEVR_.3

Fig4_MP-DIA-barrel-cortex-p15-3_MHRM.htrms

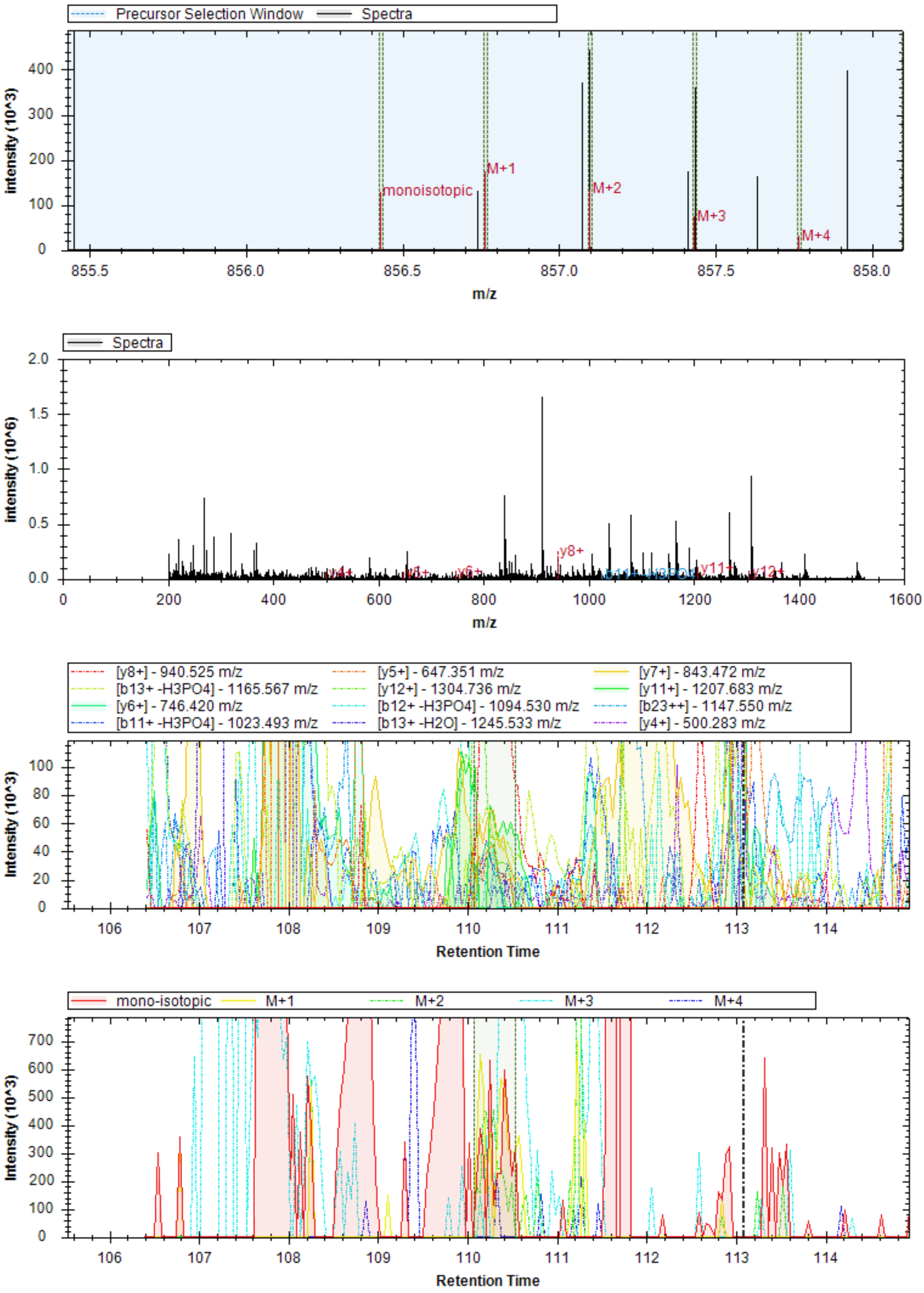

Q61290 – _M[+O]EPS[+H+O3+P]SLPQEIIANAK_.2
Fig4_MP-DIA-barrel-cortex-p15-3_MHRM.htrms

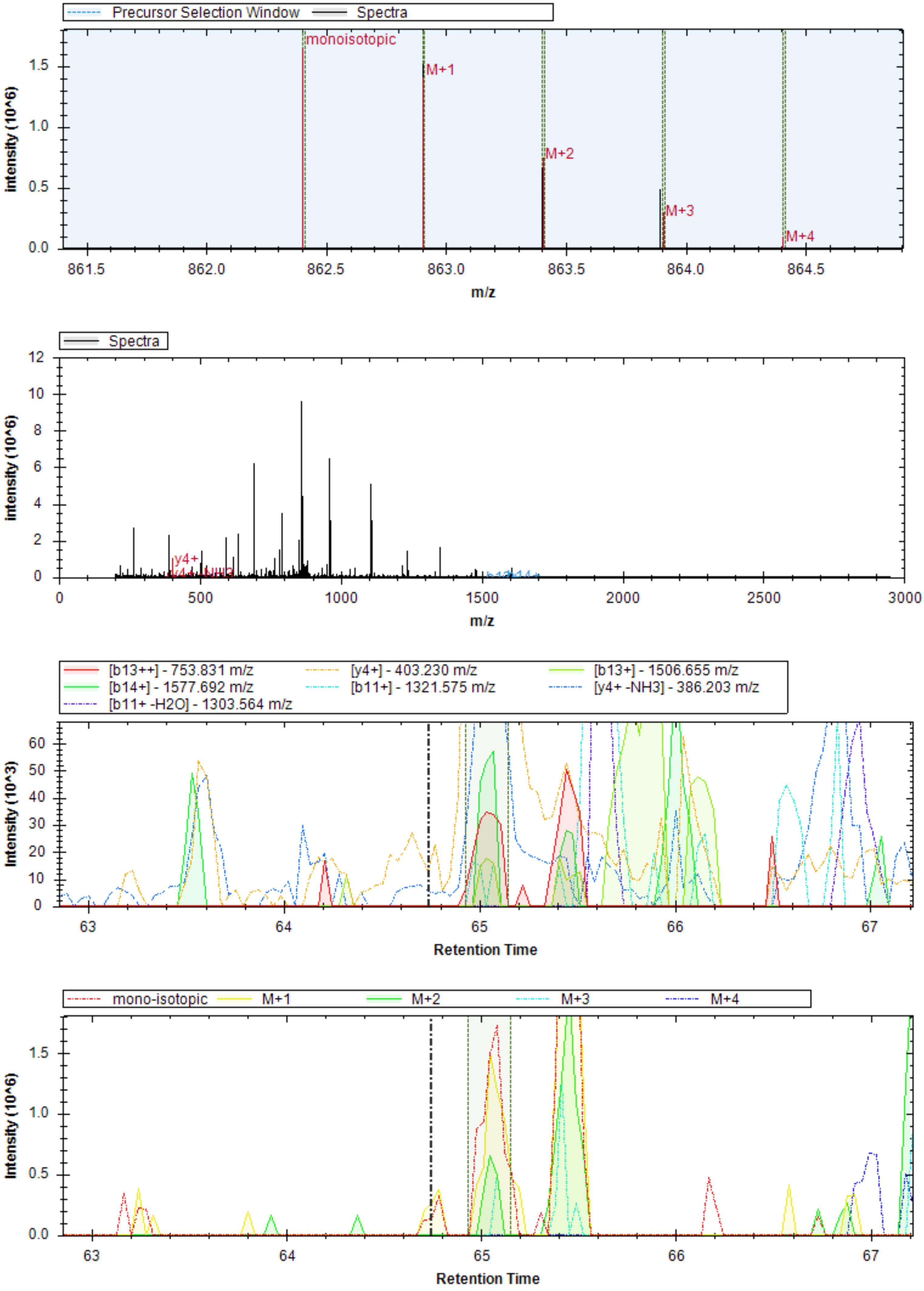

Q920B9 – _KSNVSYKNPS[+H+O3+P]LM[+O]PK_.2
Fig4_MP-DIA-barrel-cortex-p15-3_MHRM.htrms

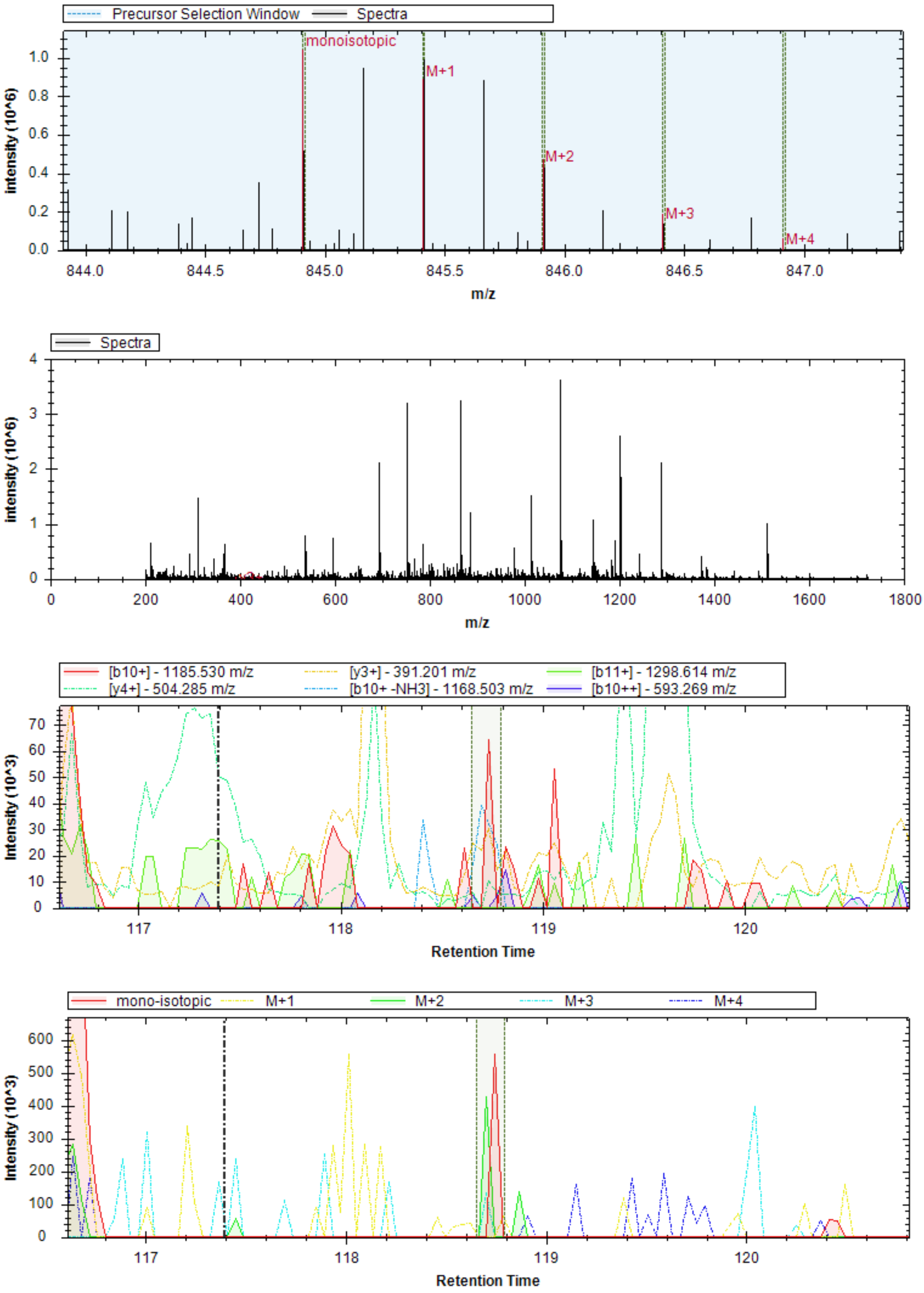

Q9JM96 – _DSSKLPKS[+H+O3+P]LS[+H+O3+P]SSPVK_.2
Fig4_MP-DIA-barrel-cortex-p15-3_MHRM.htrms

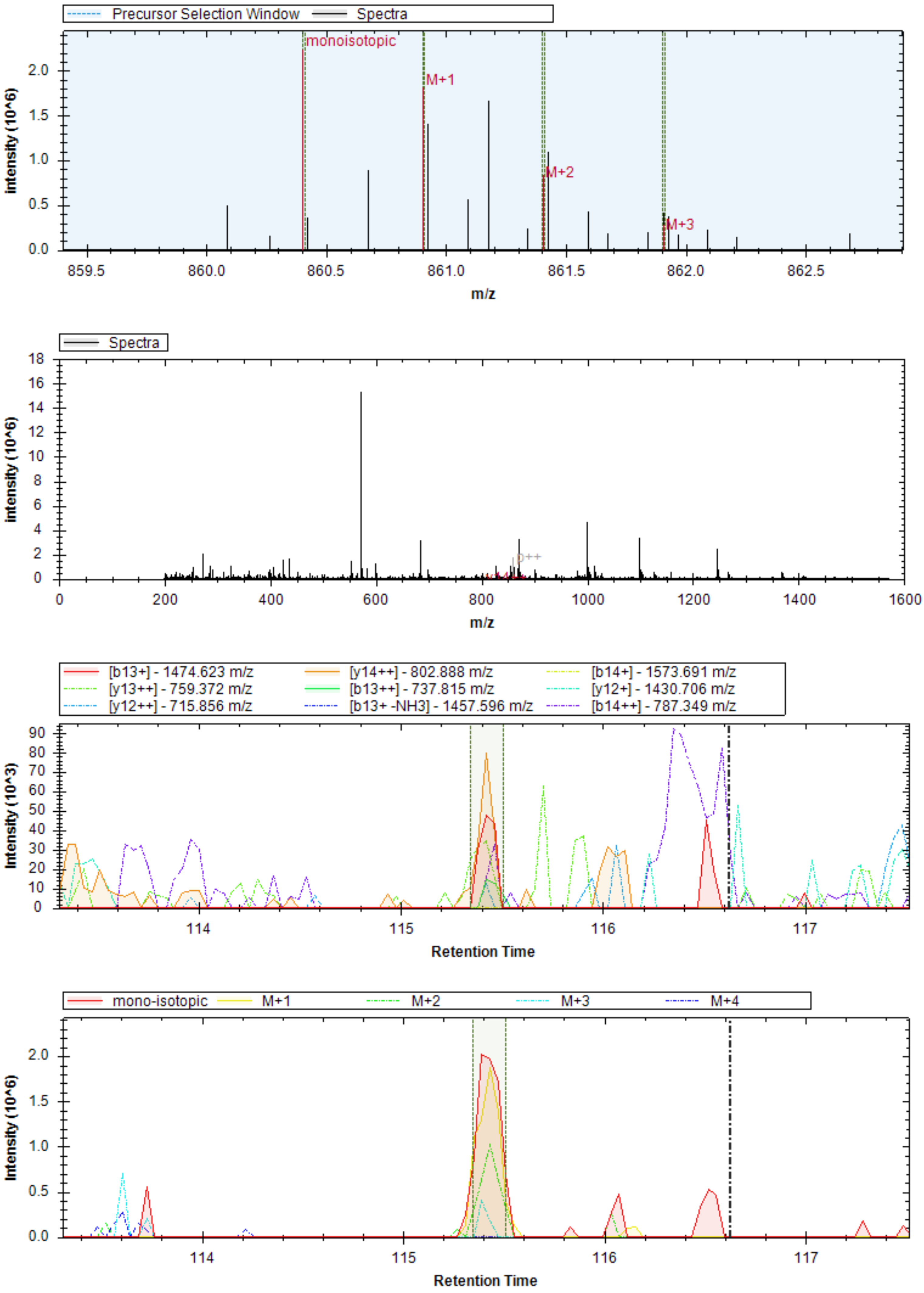

Q9QXS6 – _ASDSGPSSSSSS[+H+O3+P]SSSPPR_.2

Fig4_MP-DIA-barrel-cortex-p15-3_MHRM.htrms

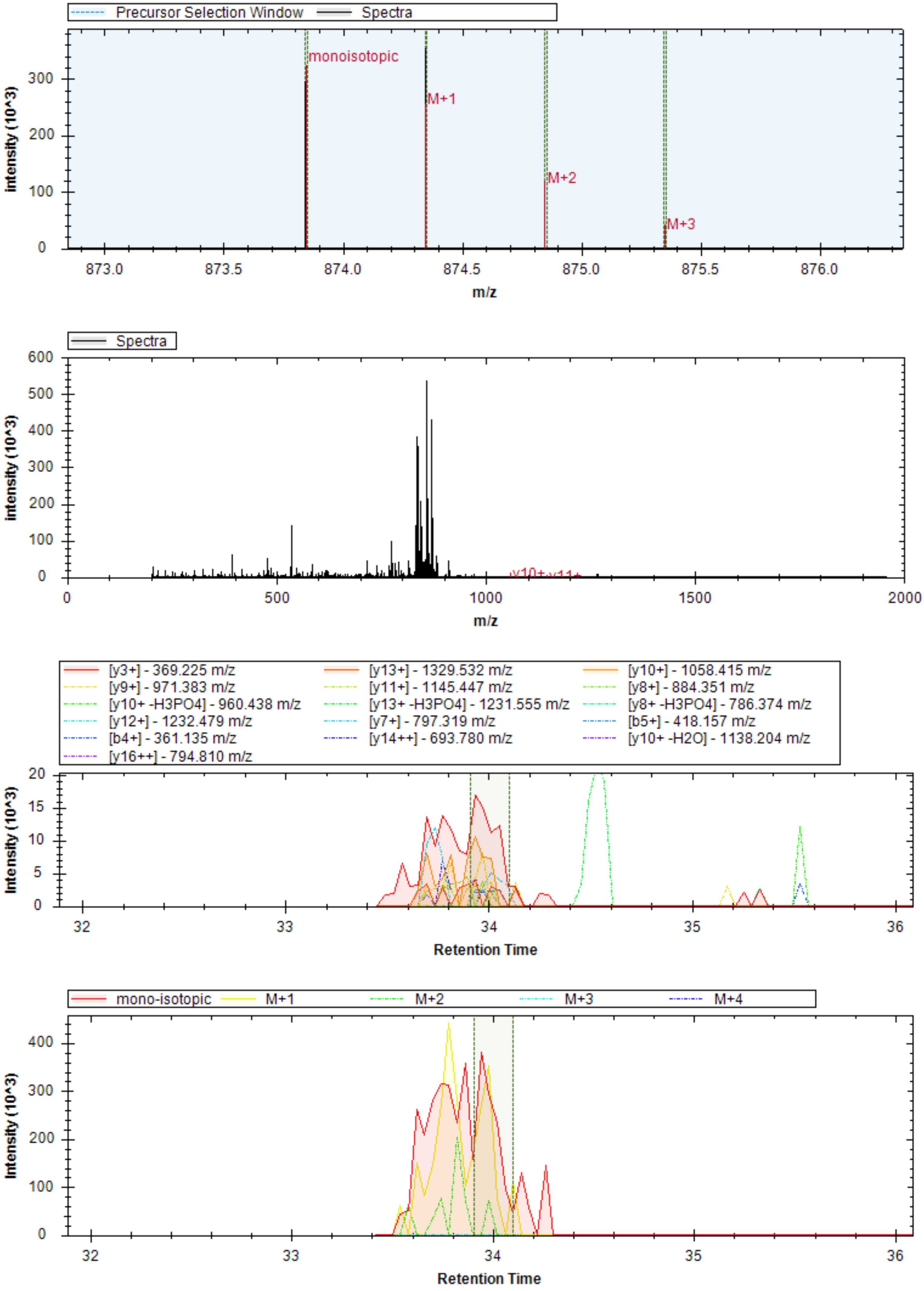

Q9Z0P4 – _SETLVNAQQT[+H+O3+P]PLGTPK_.2
Fig4_MP-DIA-barrel-cortex-p15-3_MHRM.htrms

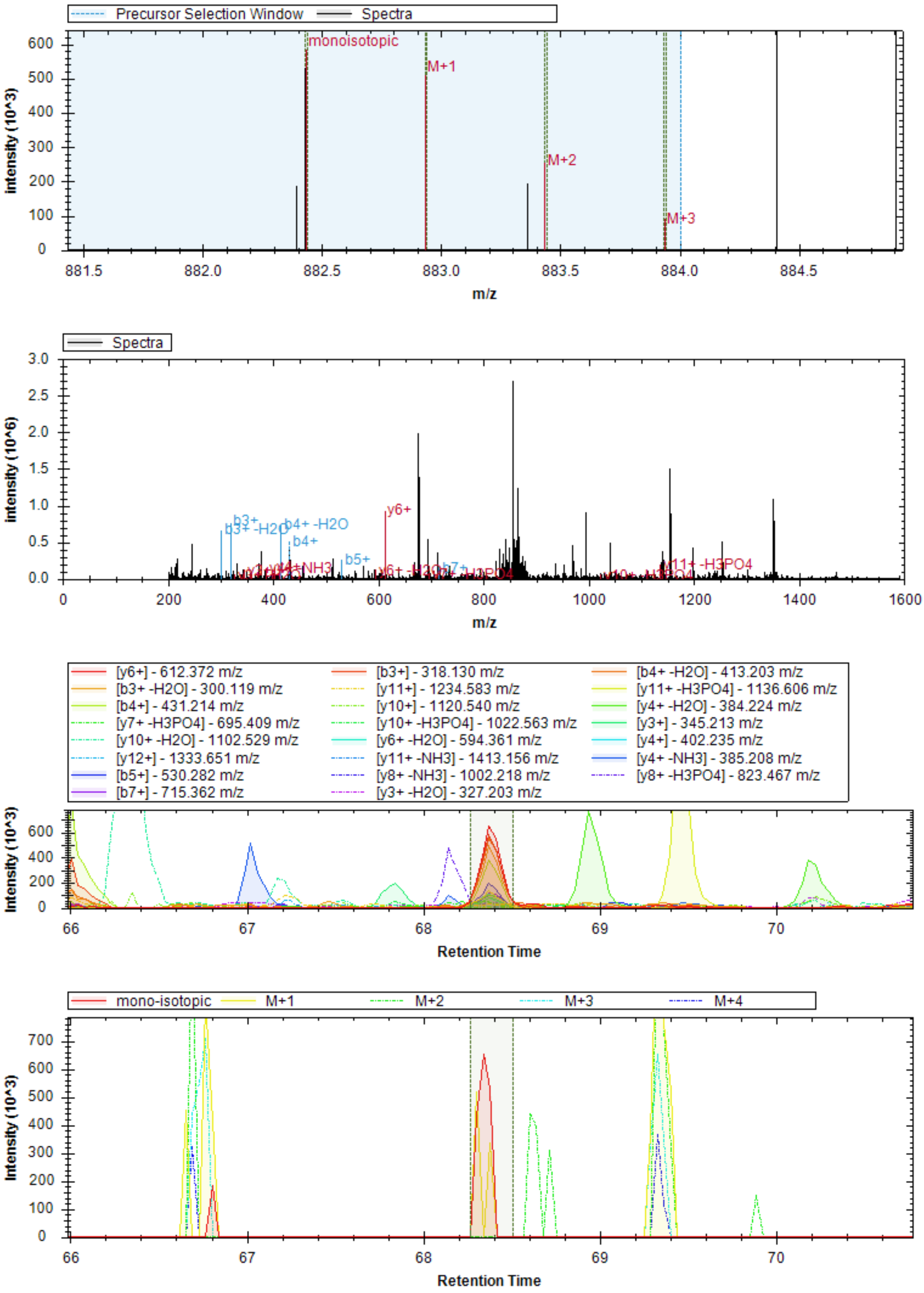

Q9Z0P4 – _SETLVNAQQTPLGT[+H+O3+P]PK_.2
Fig4_MP-DIA-barrel-cortex-p15-3_MHRM.htrms

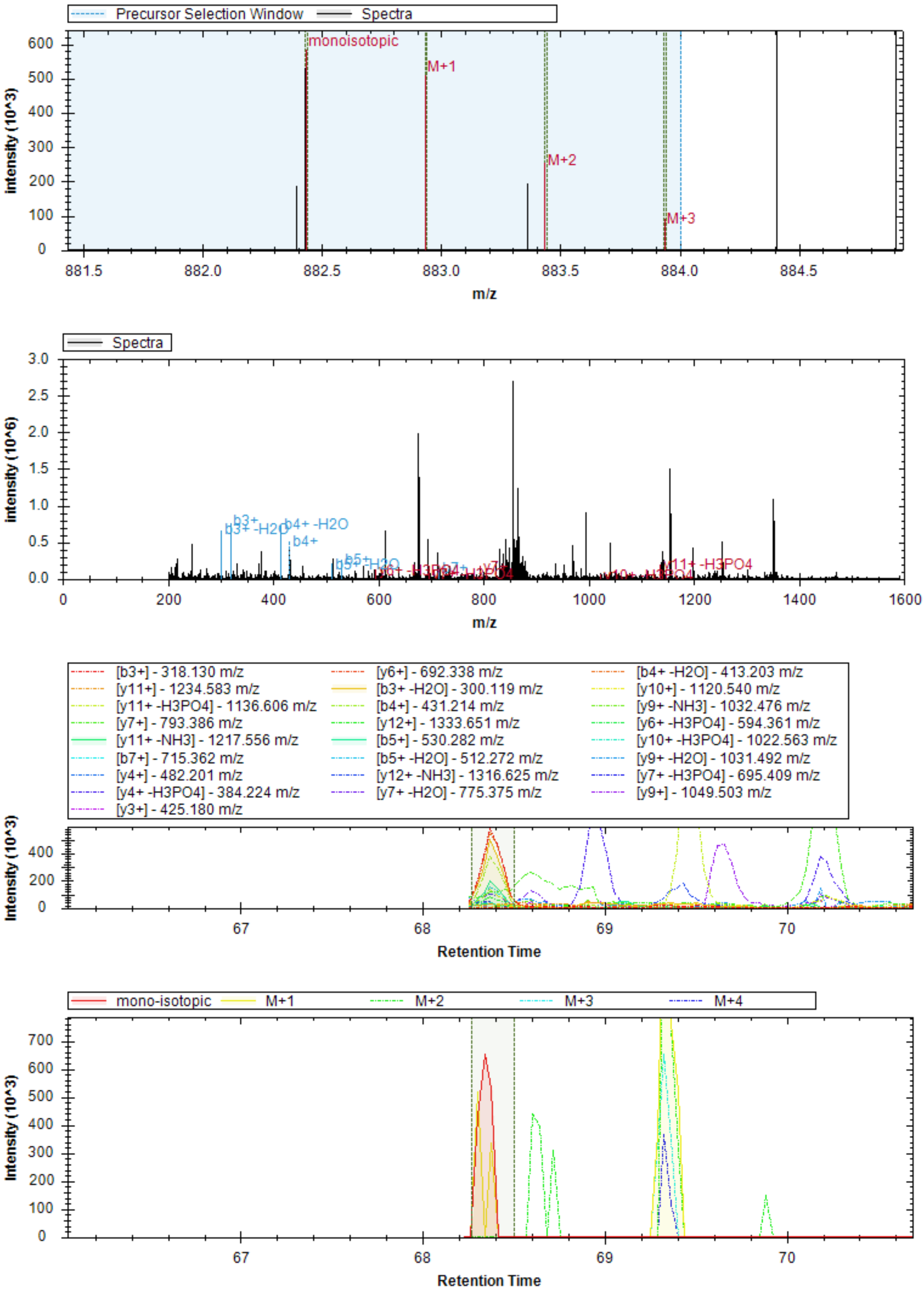

A1A535 – _FLHRGS[+H+O3+P]K_.1
Fig4_MP-DIA-barrel-cortex-p15-3_MHRM.htrms

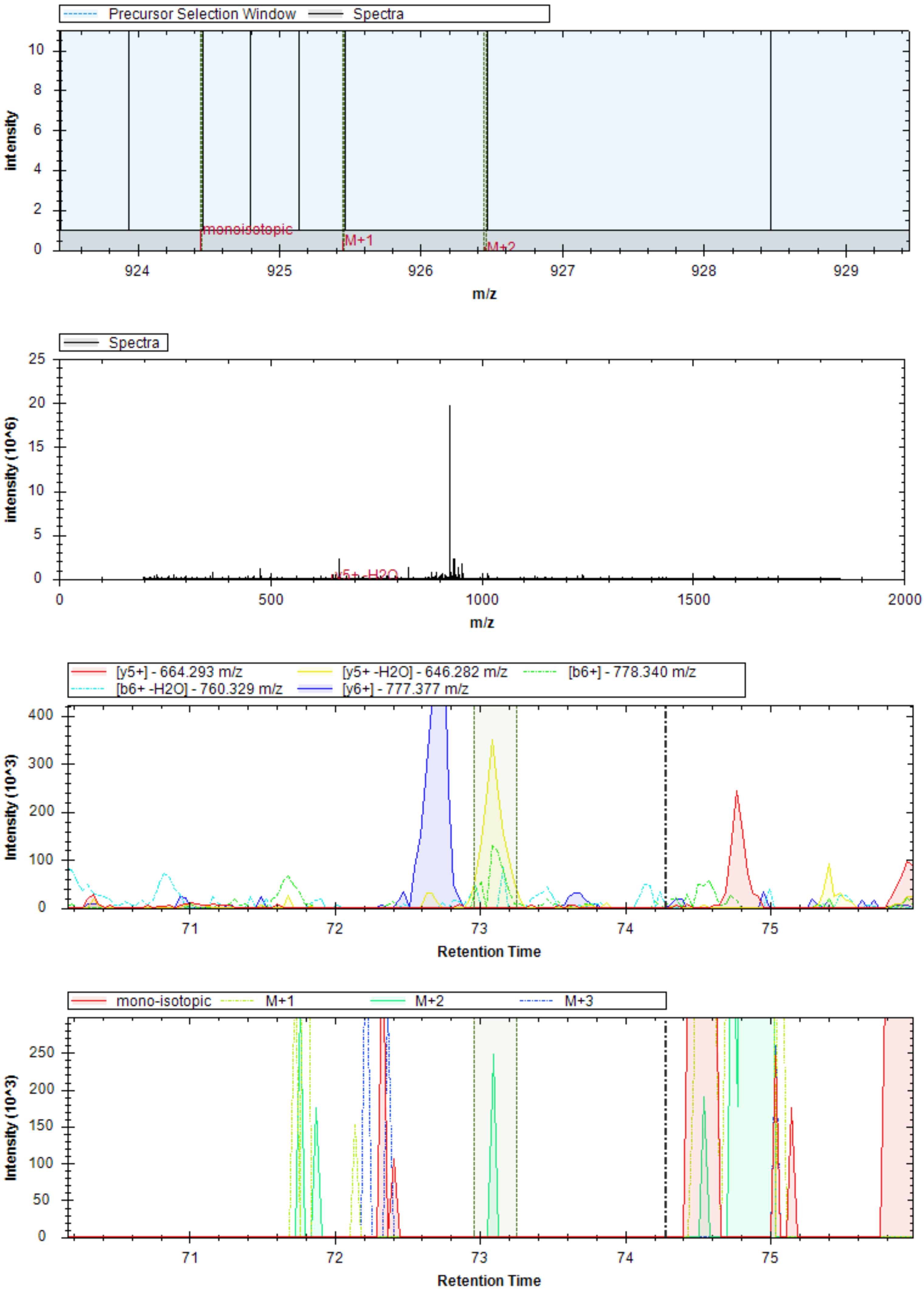

G5E829 – _SATSSSPGS[+H+O3+P]PLHSLETSL_.2

Fig4_MP-DIA-barrel-cortex-p15-3_MHRM.htrms

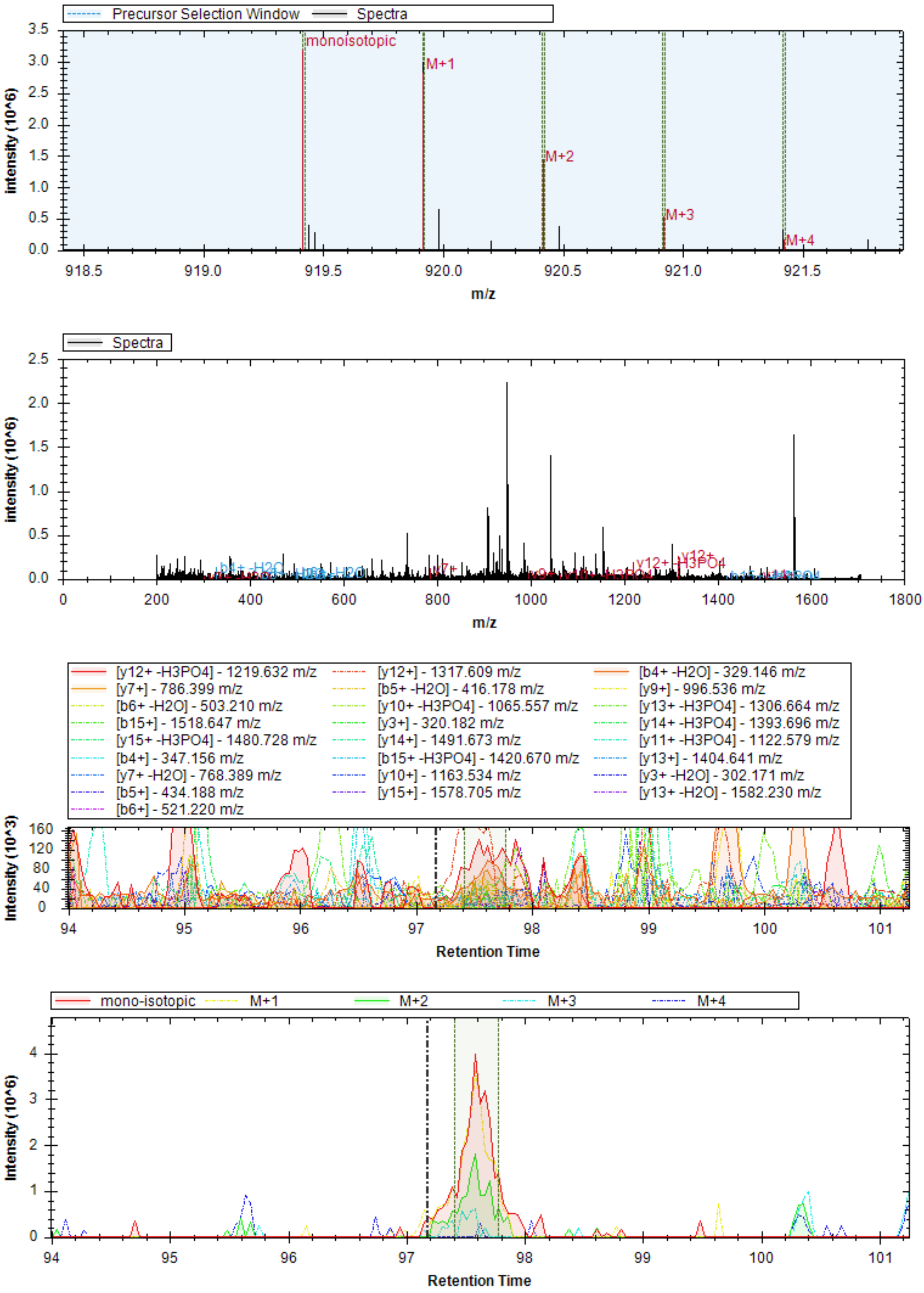

P26645 – _GEATAERPGEAAVASS[+H+O3+P]PSK_.2

Fig4_MP-DIA-barrel-cortex-p15-3_MHRM.htrms

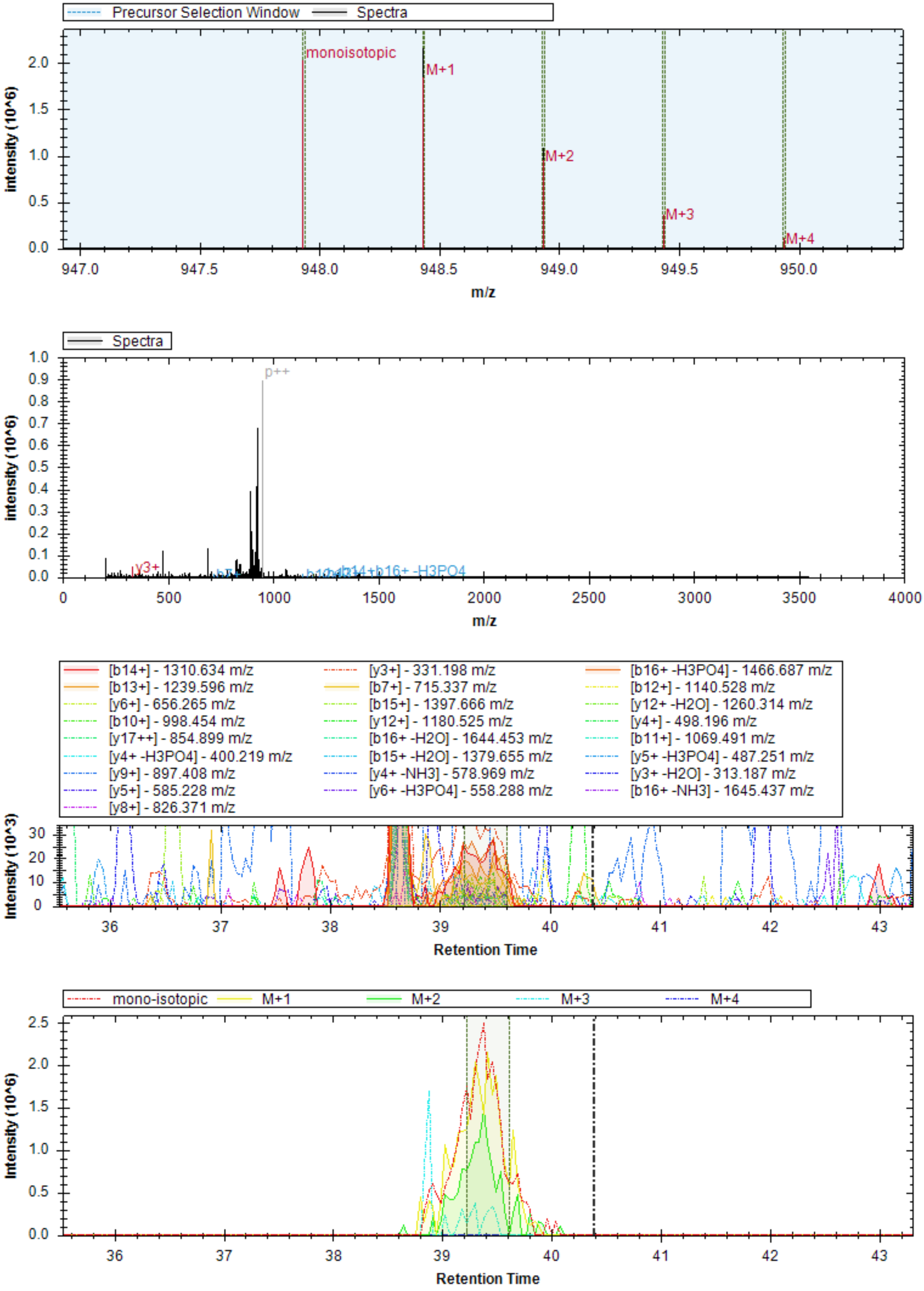

Q8BMA3 – _IIQLC[+C2+H3+N+O]LDLT[+H+O3+P]T[+H+O3+P]AVQK_.2
Fig4_MP-DIA-barrel-cortex-p15-3_MHRM.htrms

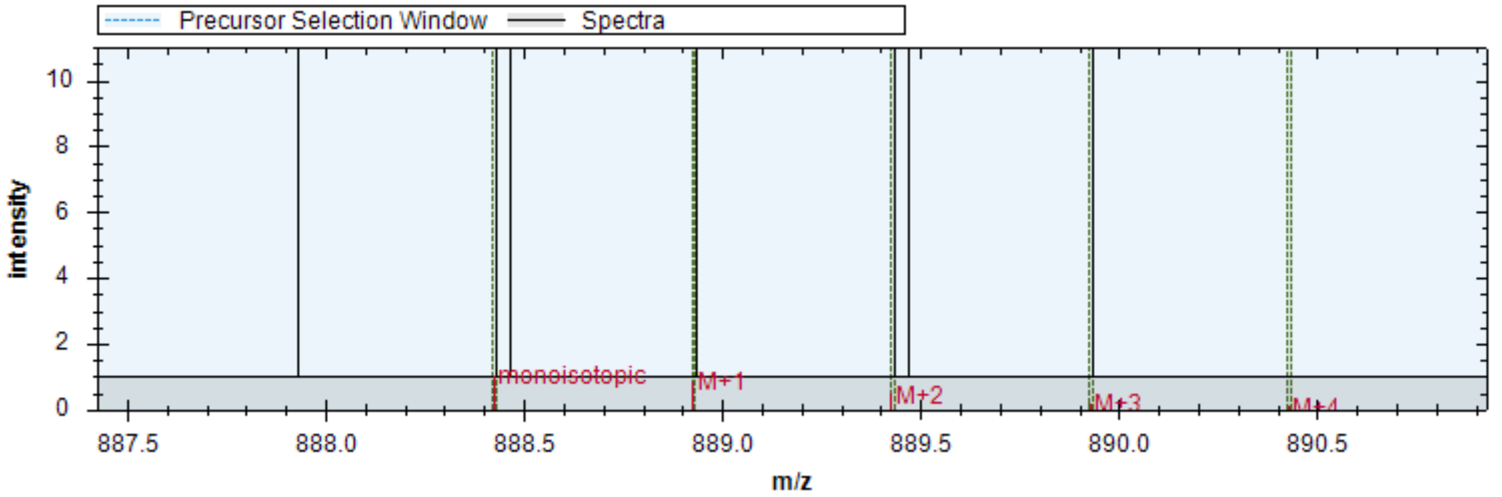

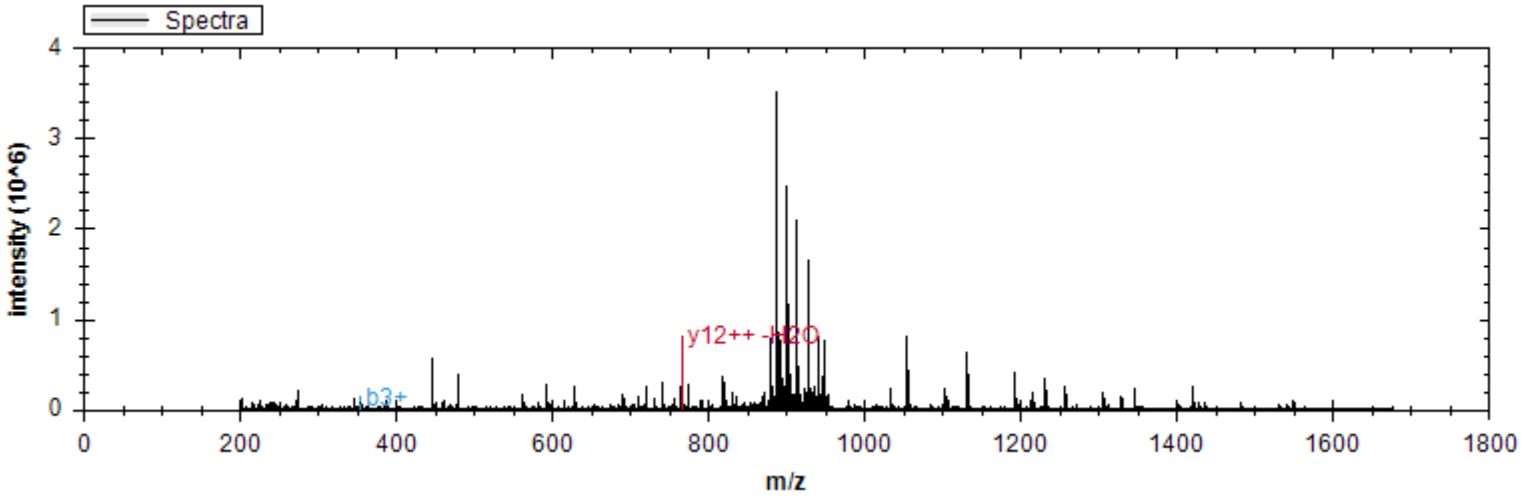

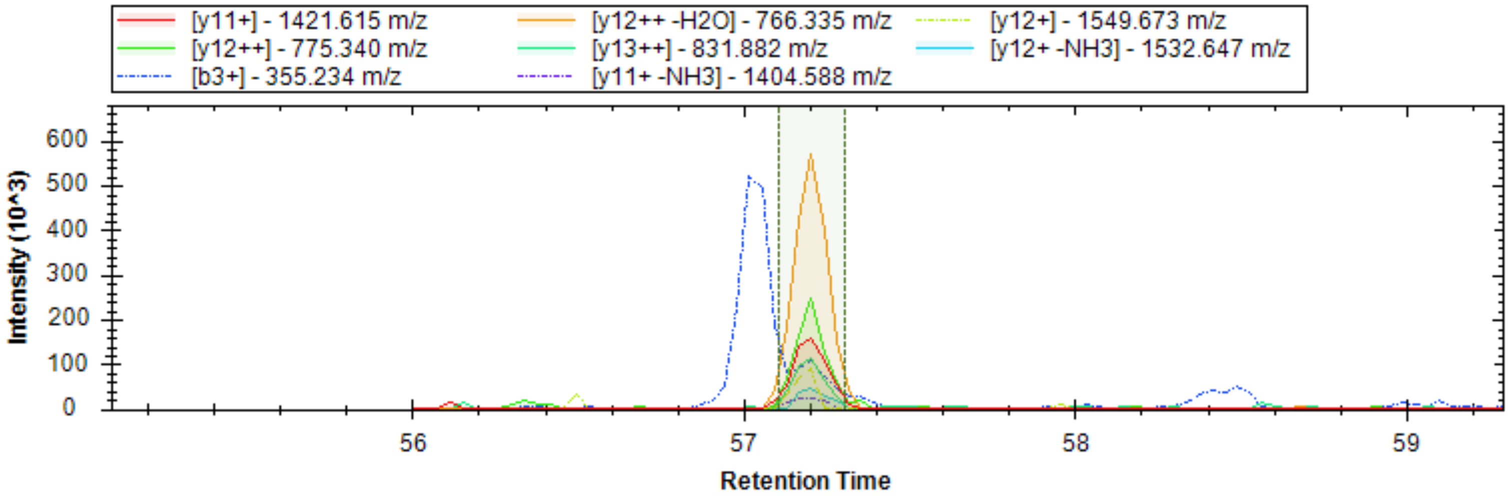

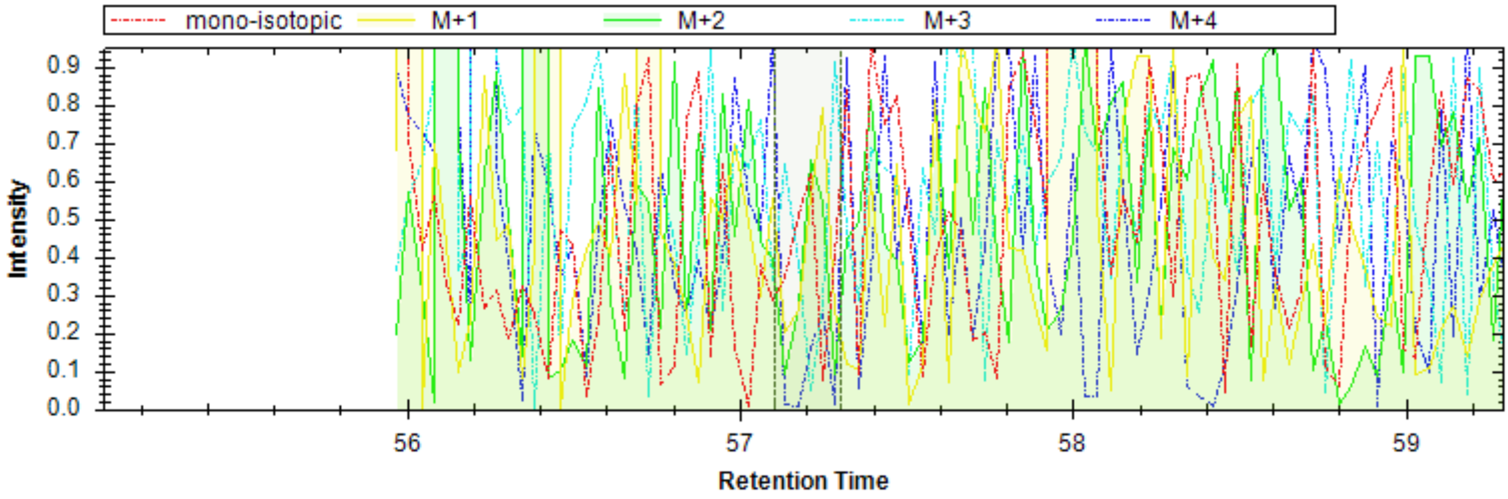

Q8BX90 – _IQALNSLGAGPFS[+H+O3+P]HTIK_.2
Fig4_MP-DIA-barrel-cortex-p15-3_MHRM.htrms

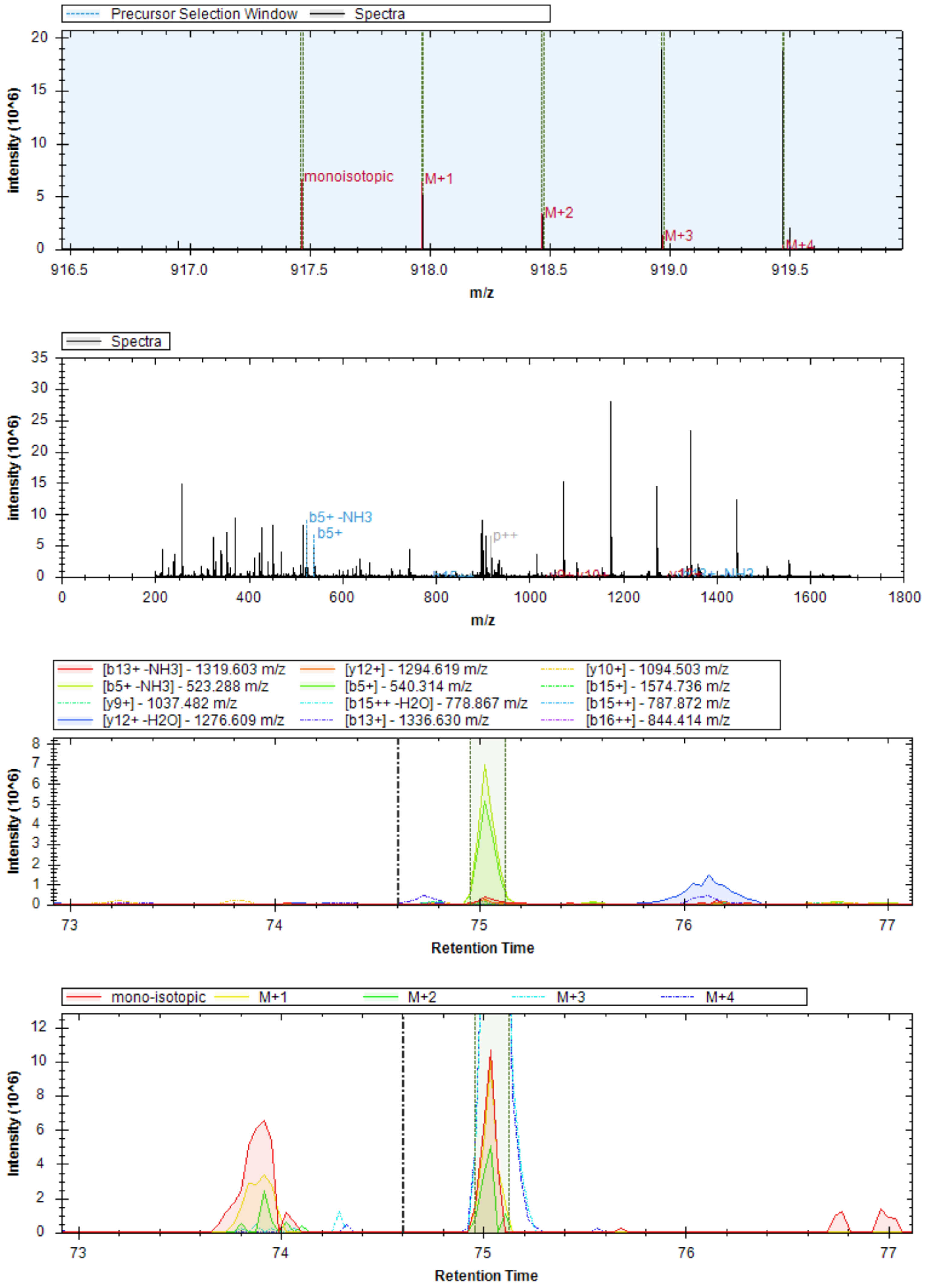

Q8JZP2 – _SQS[+H+O3+P]LTNSLSTSDTSHR_.2
Fig4_MP-DIA-barrel-cortex-p15-3_MHRM.htrms

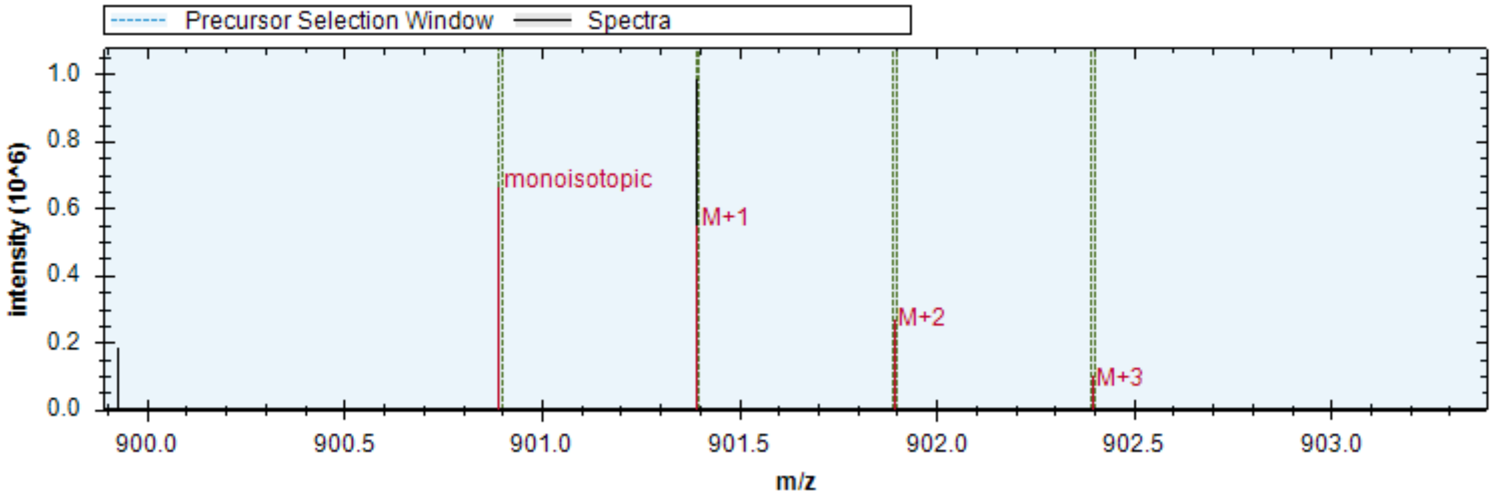

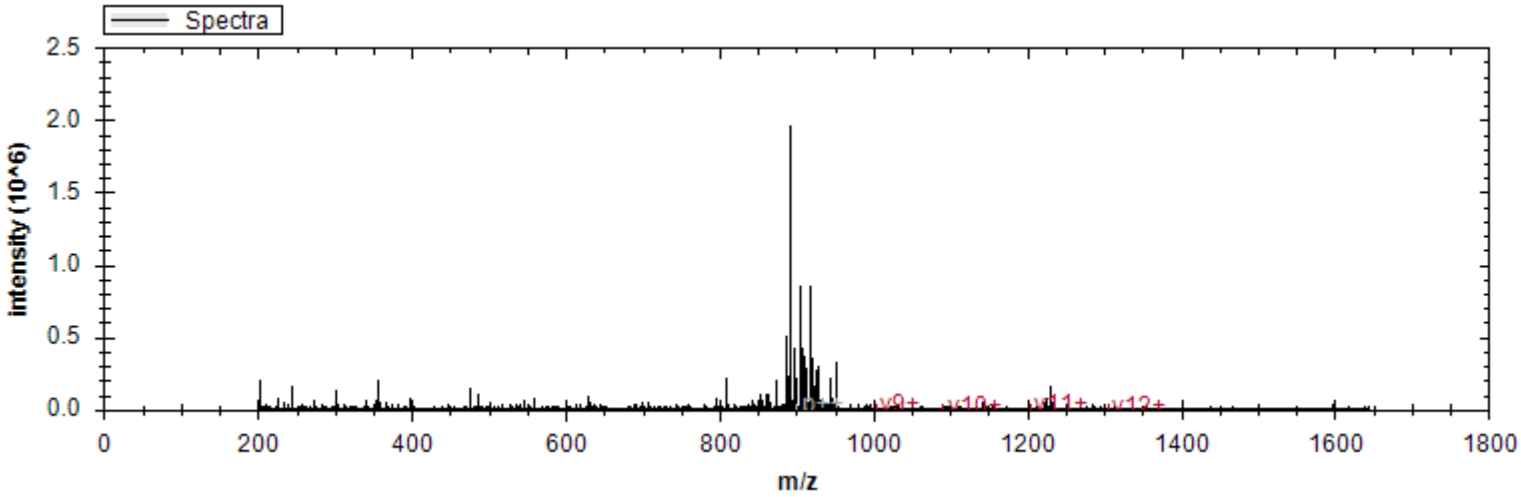

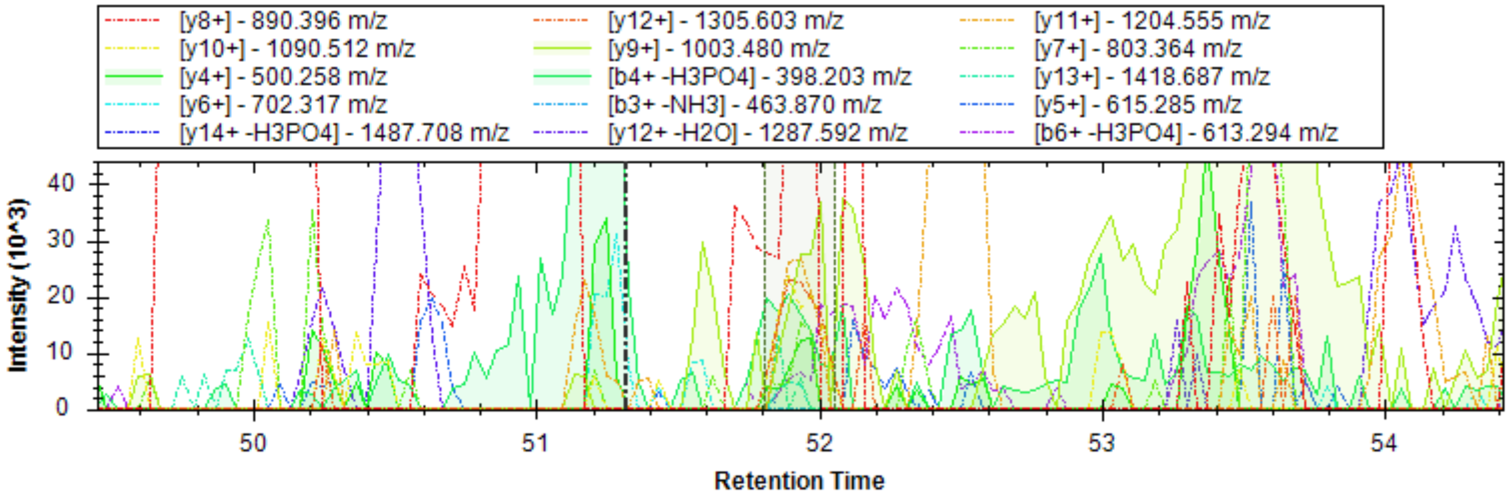

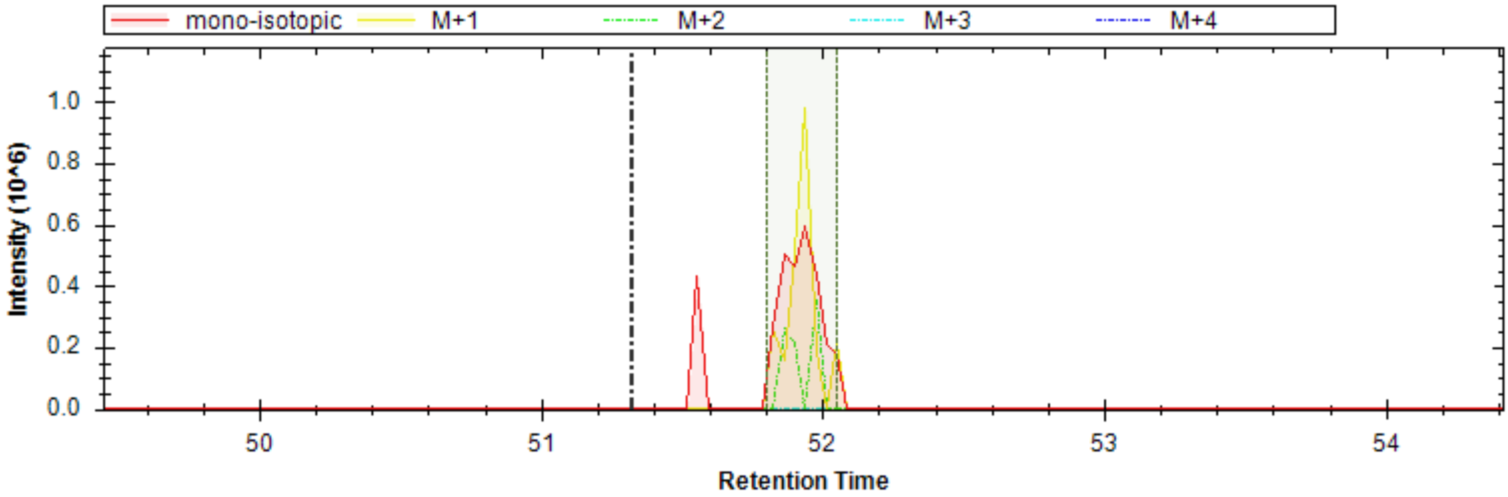

Q91Z31;Q91Z31-2 – _ETSLLAVPGALS[+H+O3+P]PLAIPNAAAAAAAAAAGR_.3
Fig4_MP-DIA-barrel-cortex-p15-3_MHRM.htrms

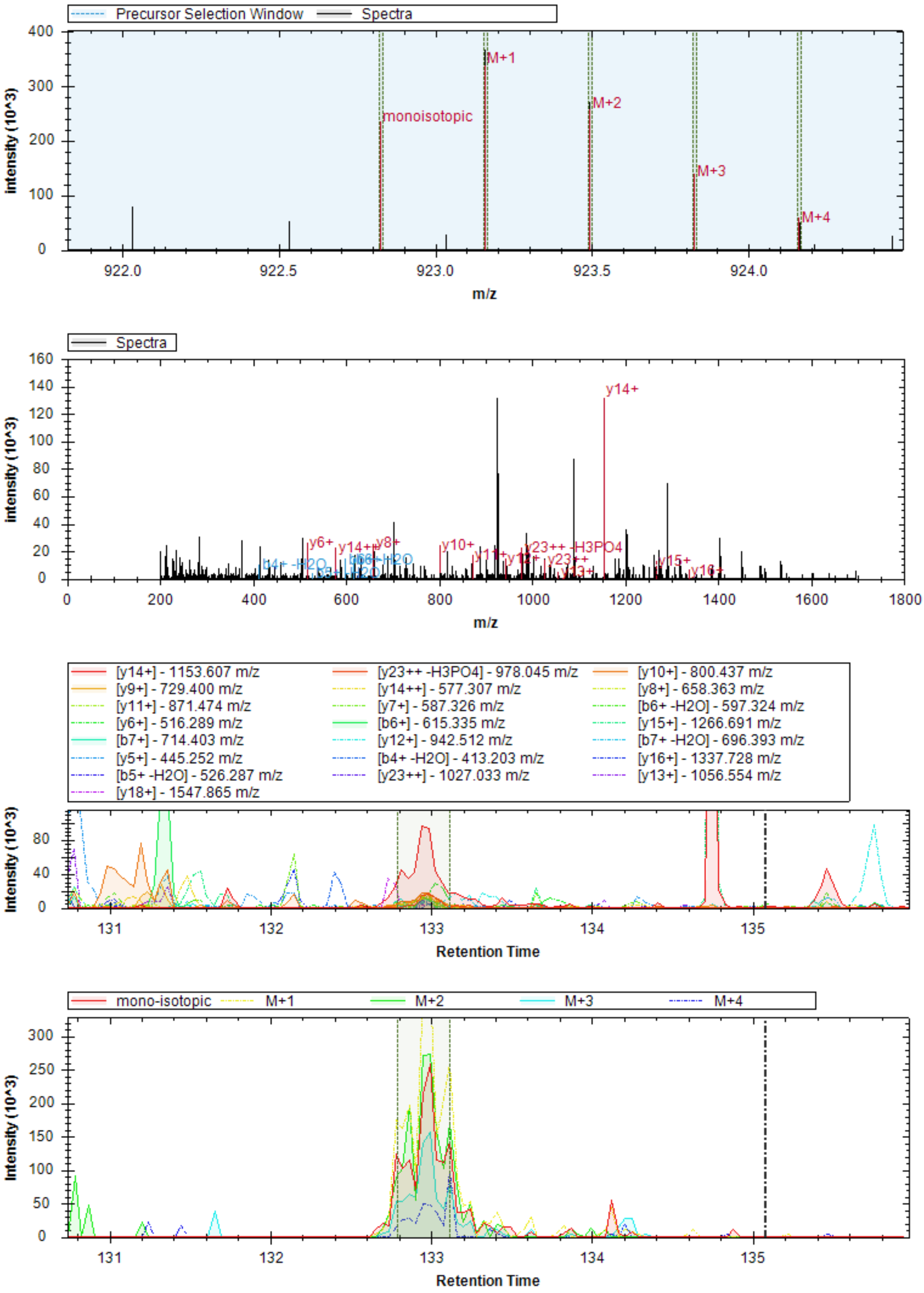

Q9CZT4;Q9CZT4-2 – _KEQM[+O]RAATVLPS[+H+O3+P]VQIK_.2
Fig4_MP-DIA-barrel-cortex-p15-3_MHRM.htrms

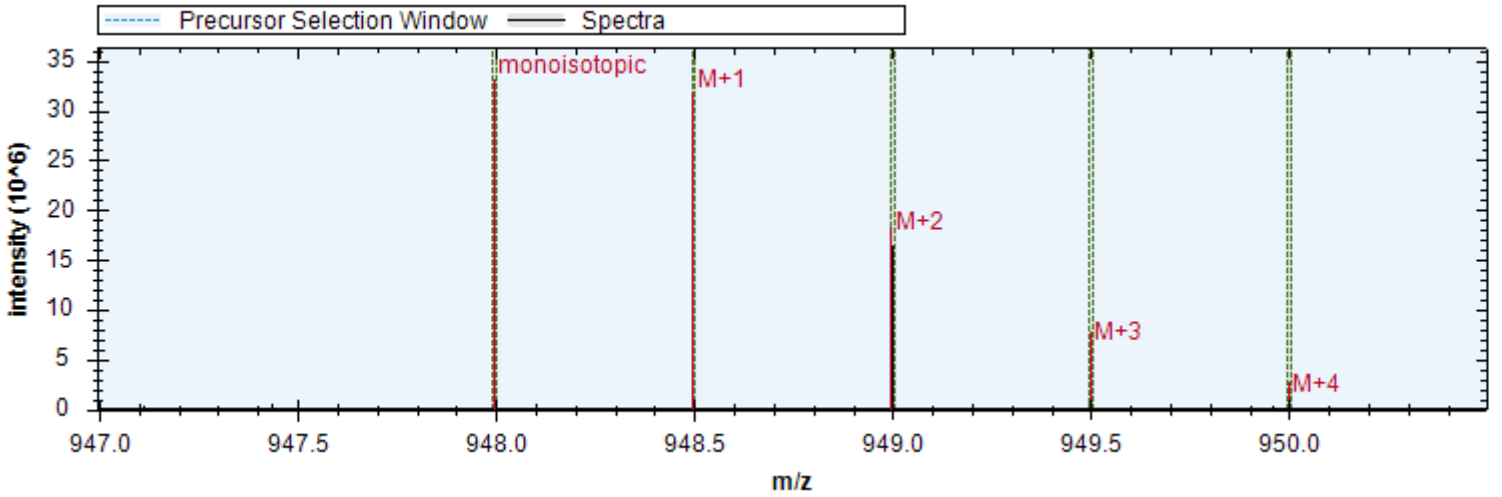

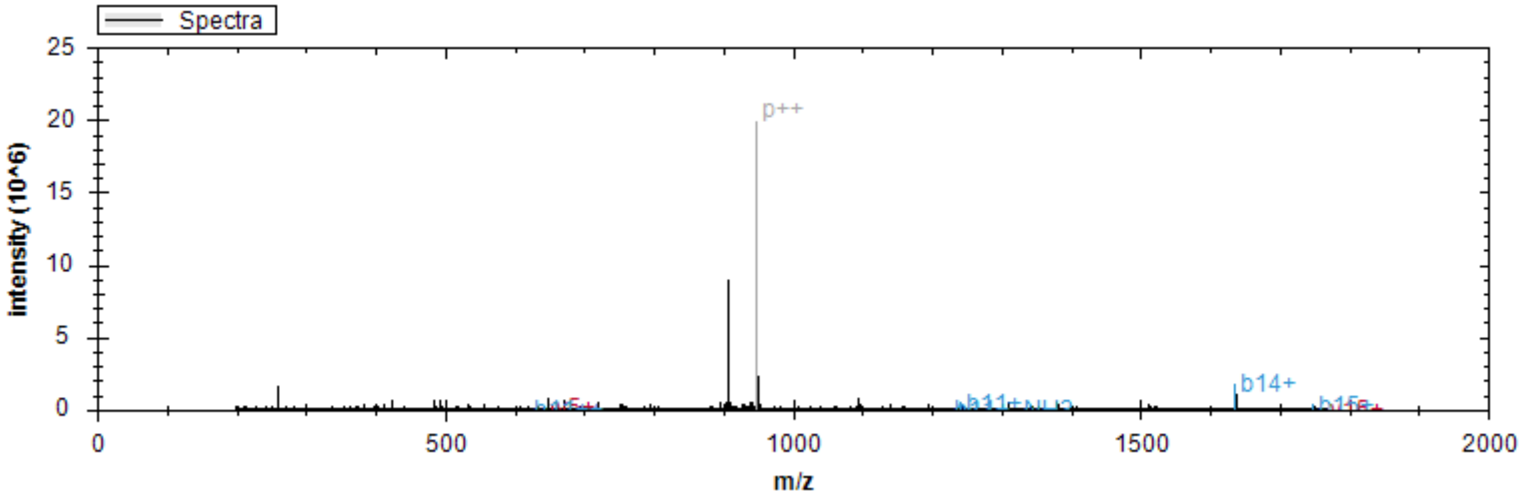

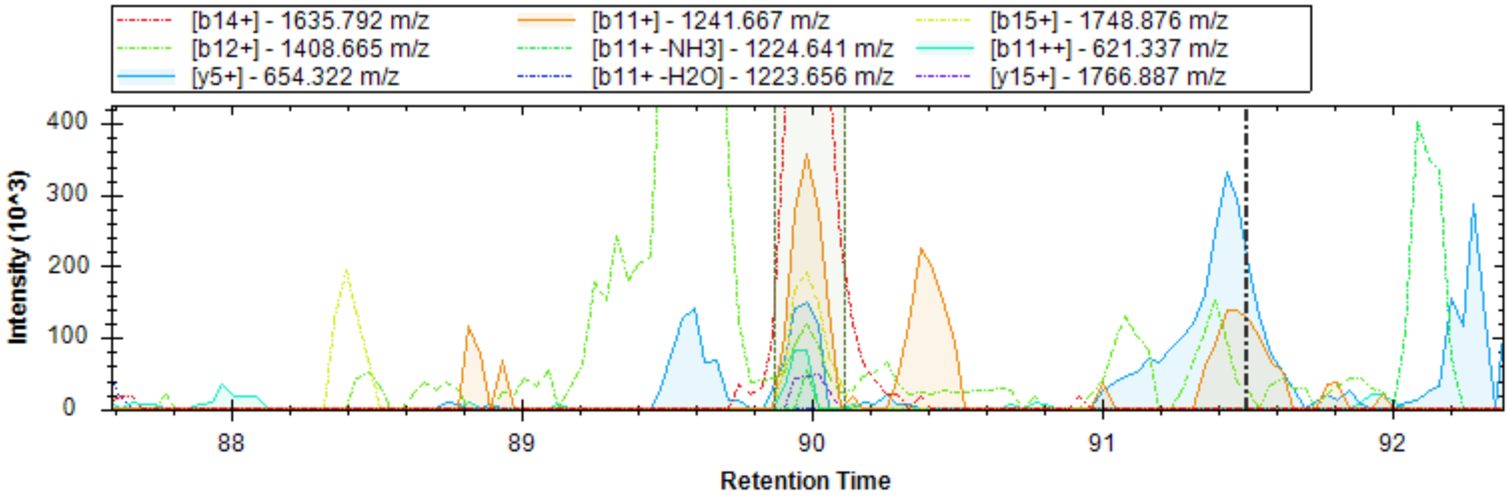

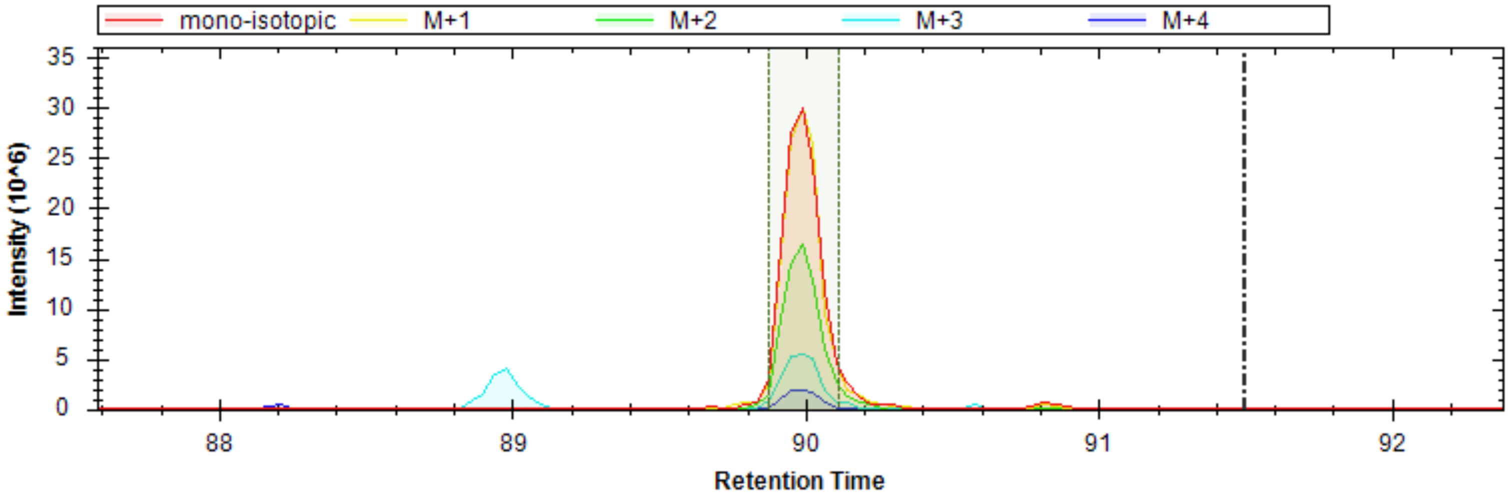

Q9ES97;Q9ES97-2 – _AES[+H+O3+P]SAATQSPSVSSSSSGAEPSALGGGGGSPGAC[+C2+H3+N+O]PALGAK_.4
Fig4_MP-DIA-barrel-cortex-p15-3_MHRM.htrms

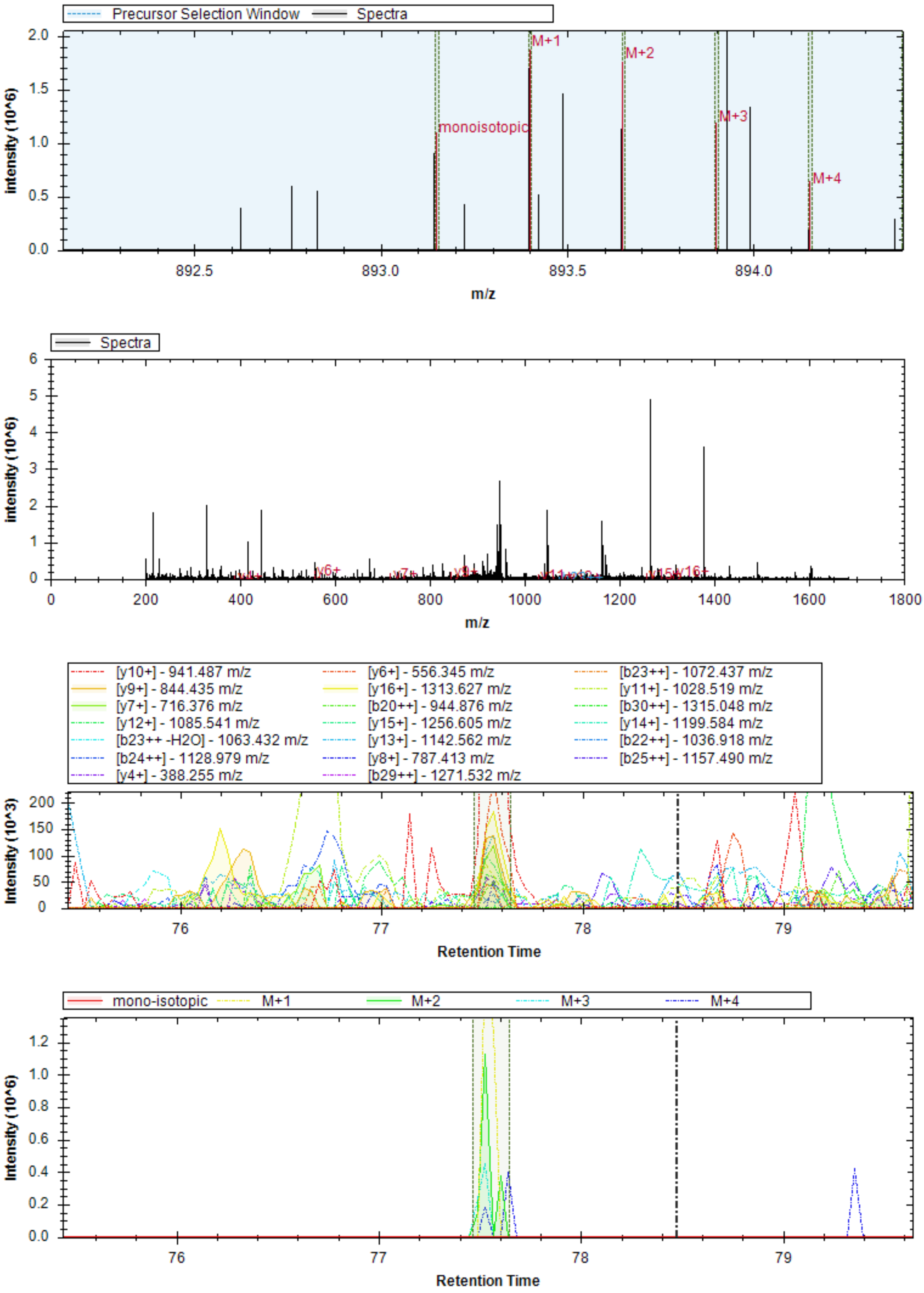

Q9QYR6-2 – _MAS[+H+O3+P]PPPSGPPSAAHTPFHQSPVEEK_.3

Fig4_MP-DIA-barrel-cortex-p15-3_MHRM.htrms

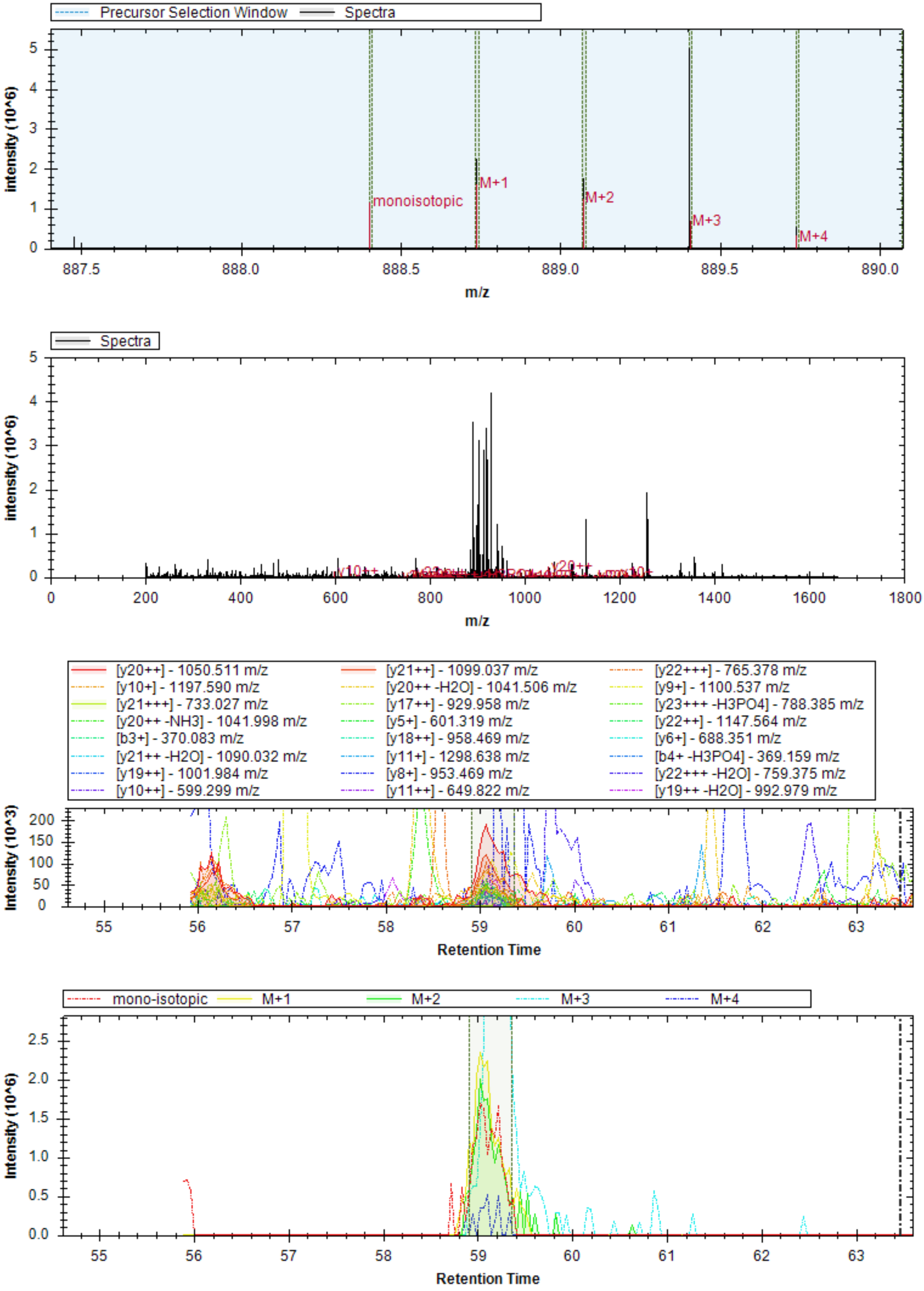

A2A891;A2A891-2;A2A8[...] – _TAVIVQQKLRS[+H+O3+P]S[+H+O3+P]LLT[+H+O3+P]K_.2
Fig4_MP-DIA-barrel-cortex-p15-3_MHRM.htrms

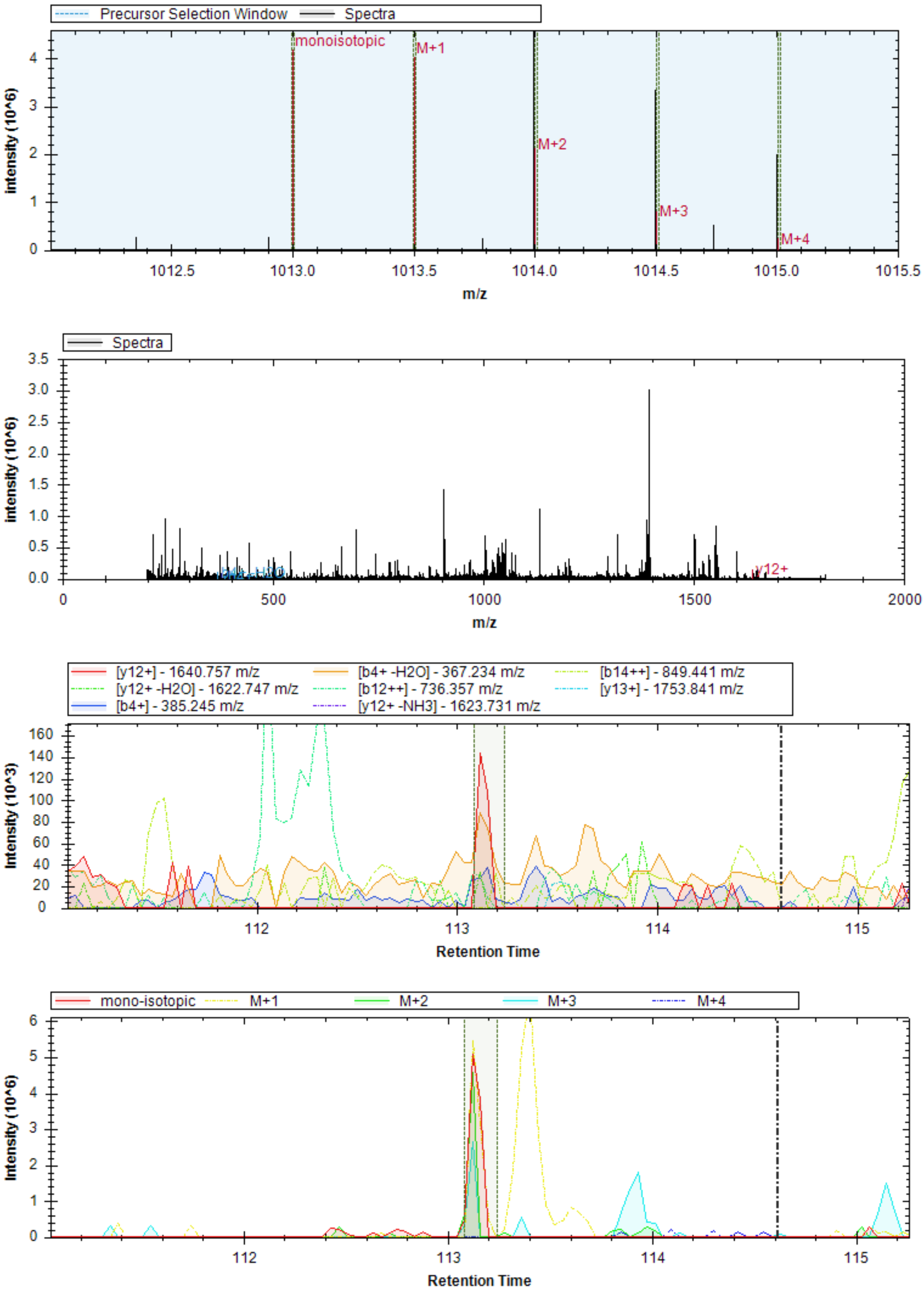

P17742 – _VNPT[+H+O3+P]VFFDITADDEPLGR_.2
Fig4_MP-DIA-barrel-cortex-p15-3_MHRM.htrms

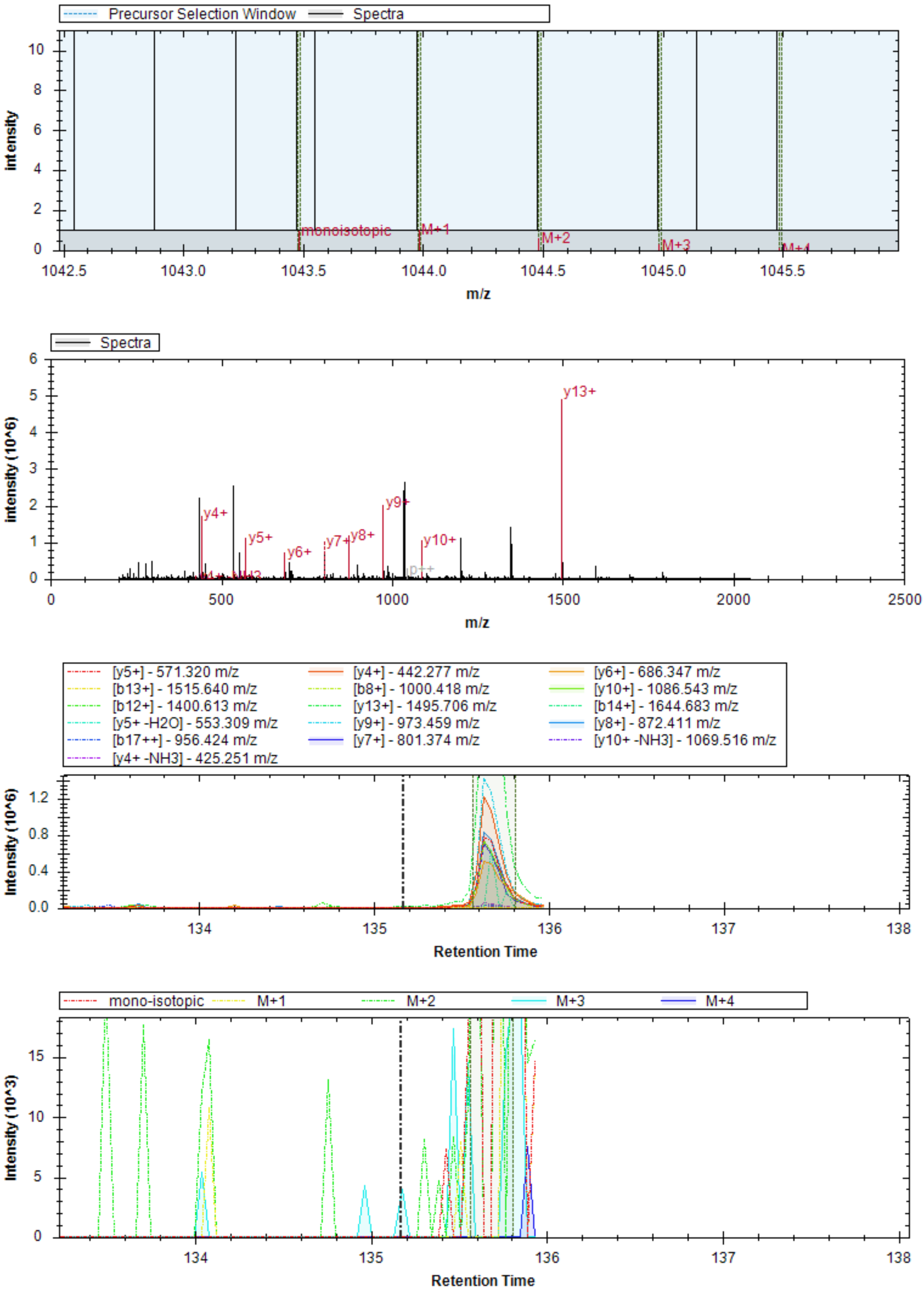

Q8CHK4;Q8CHK4-2;Q8CH[...] – _RGY[+H+O3+P]GKLLIEFS[+H+O3+P]YELSK_.2
Fig4_MP-DIA-barrel-cortex-p15-3_MHRM.htrms

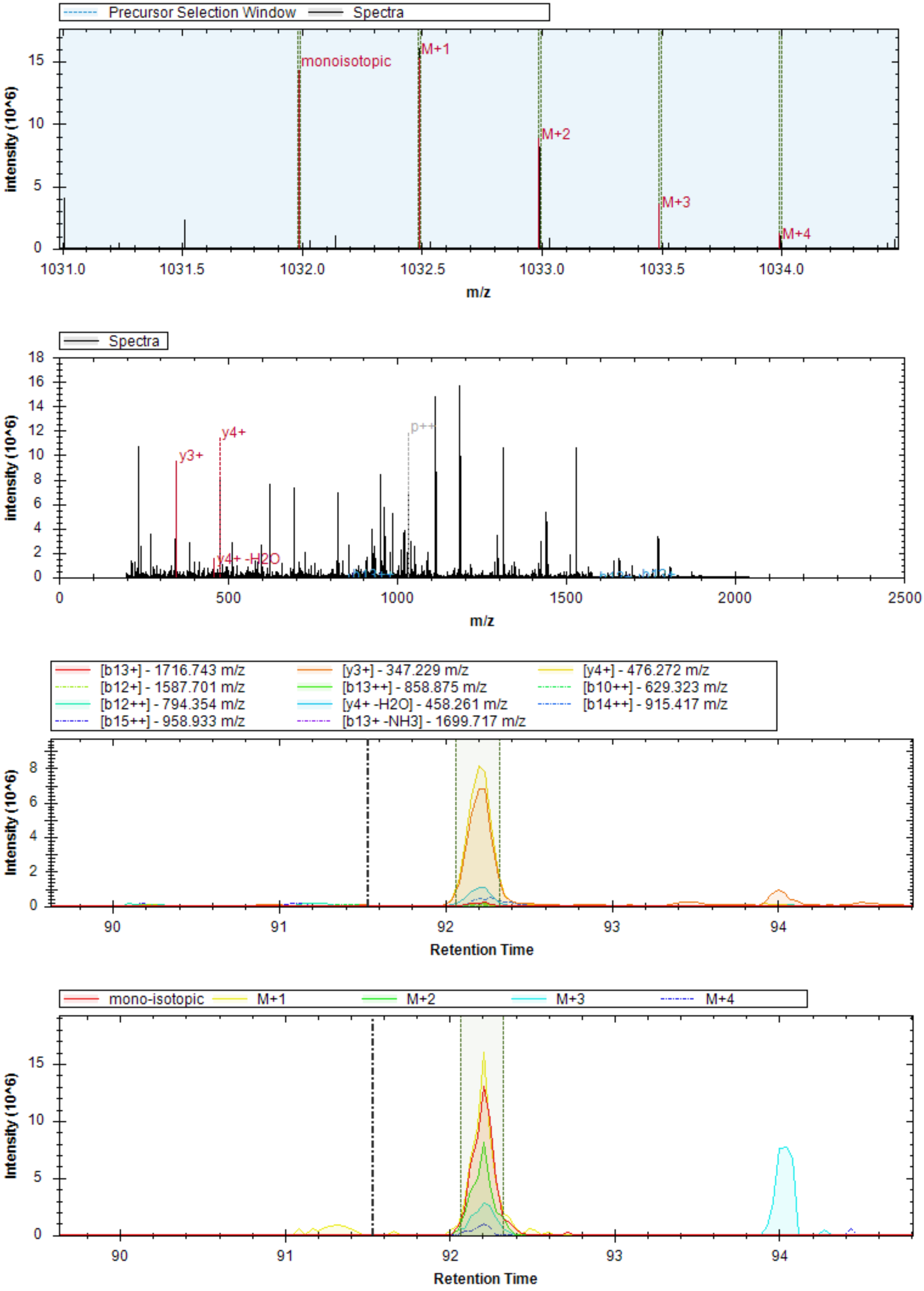

A2ASS6 – _T[+H+O3+P]VS[+H+O3+P]VKVLVLDK_.1
Fig4_MP-DIA-barrel-cortex-p15-3_MHRM.htrms

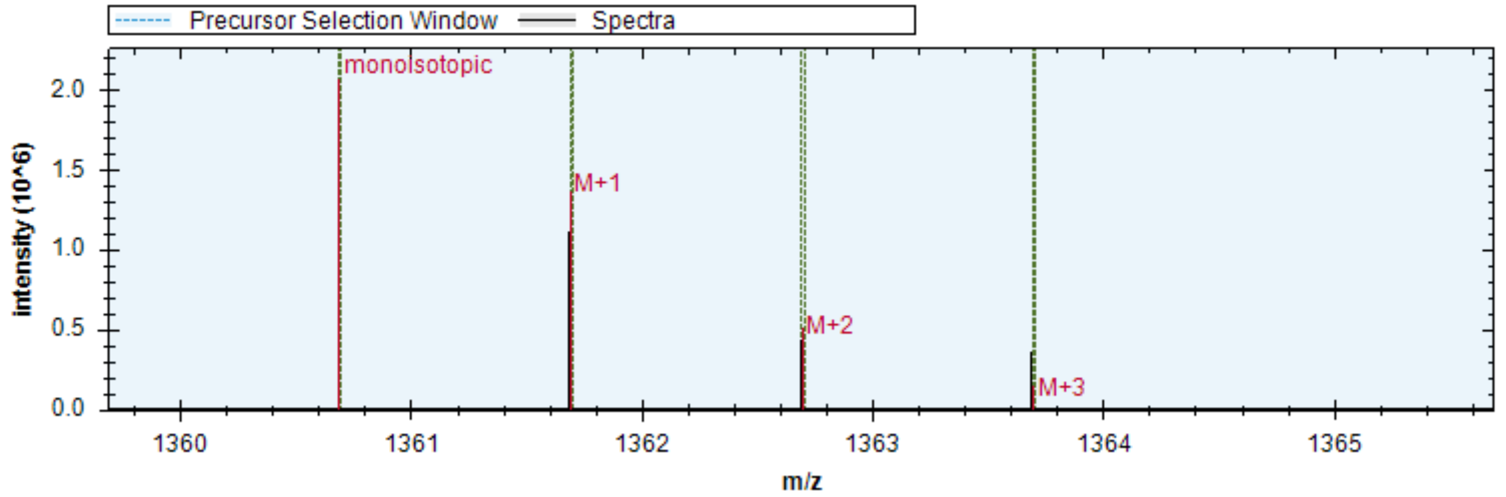

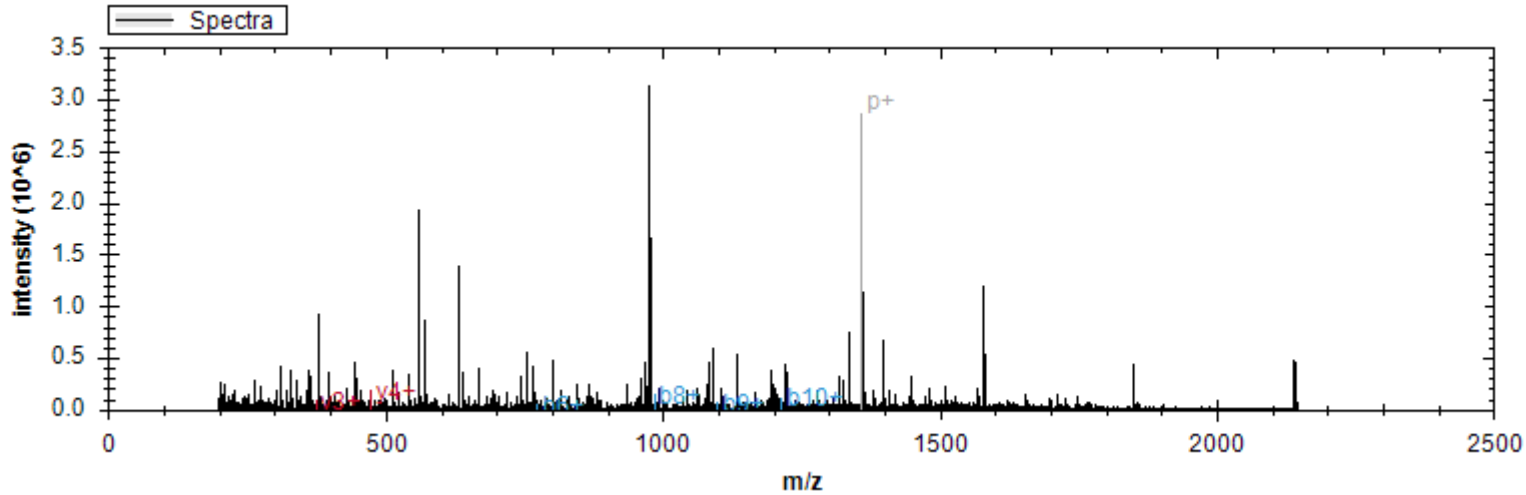

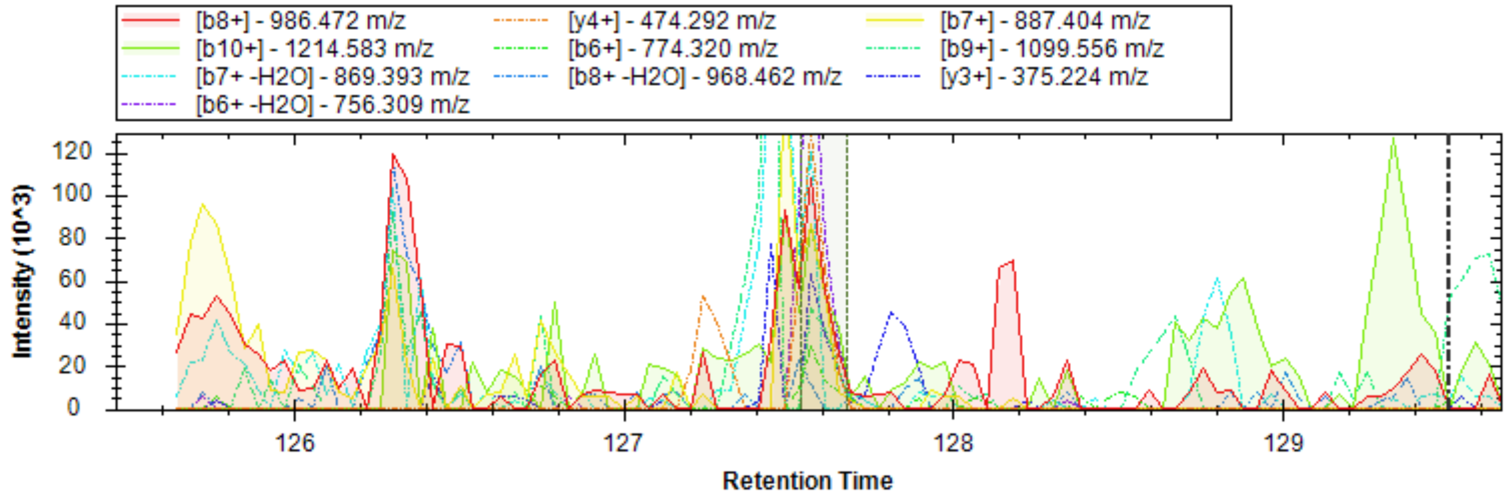

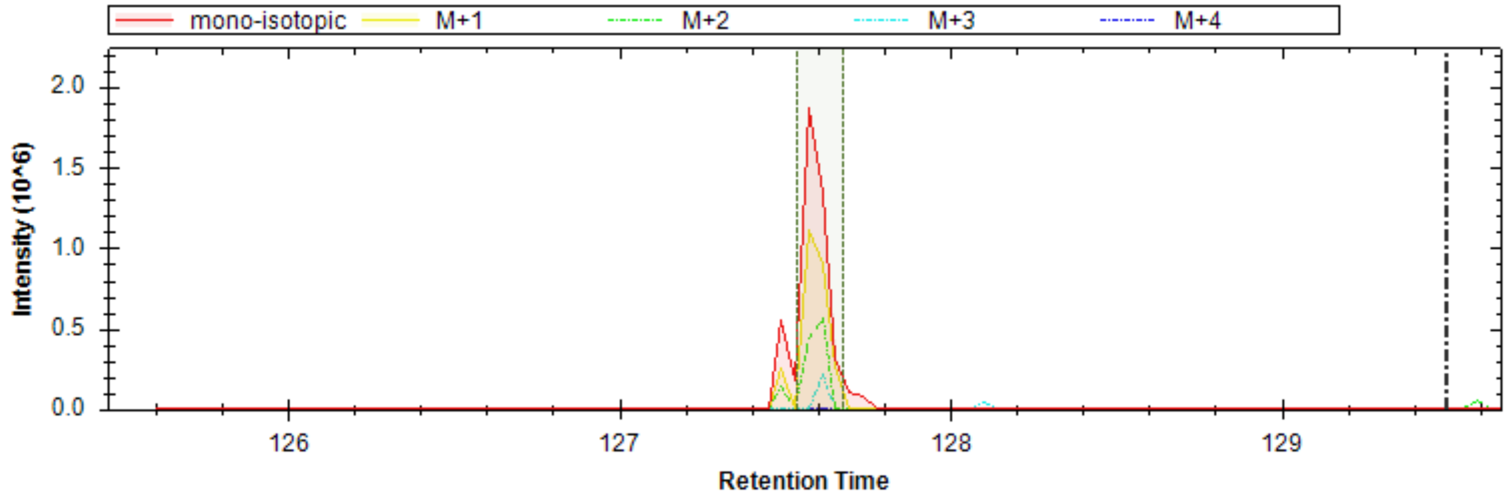

E9Q401 – _MVAEVFIY[+H+O3+P]WSK_.1

Fig4_MP-DIA-barrel-cortex-p15-3_MHRM.htrms

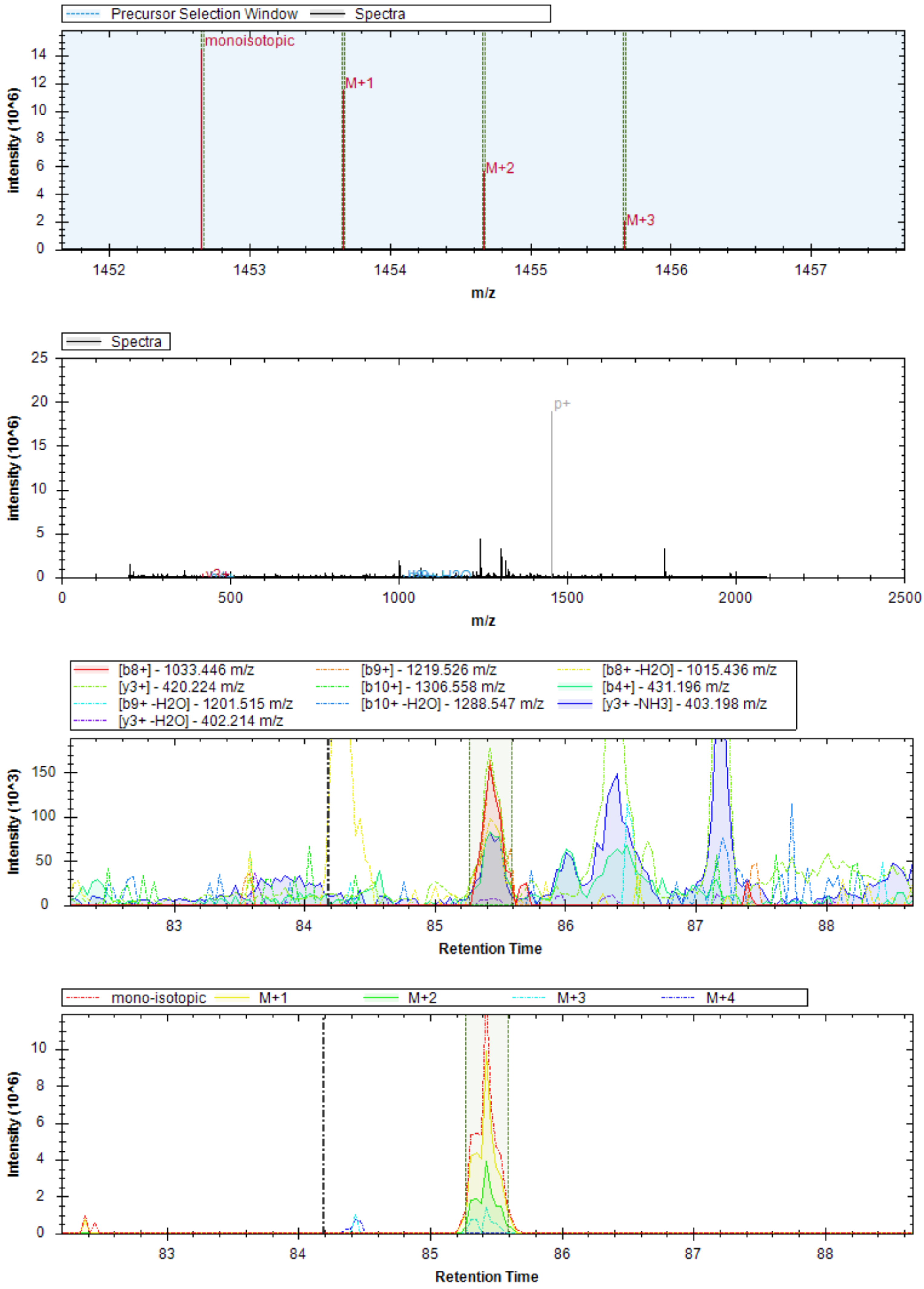

O35526 – _T[+H+O3+P]TTSEELEDMLESGNPAIFASGIIMDSSISK_.3

Fig4_MP-DIA-barrel-cortex-p15-3_MHRM.htrms

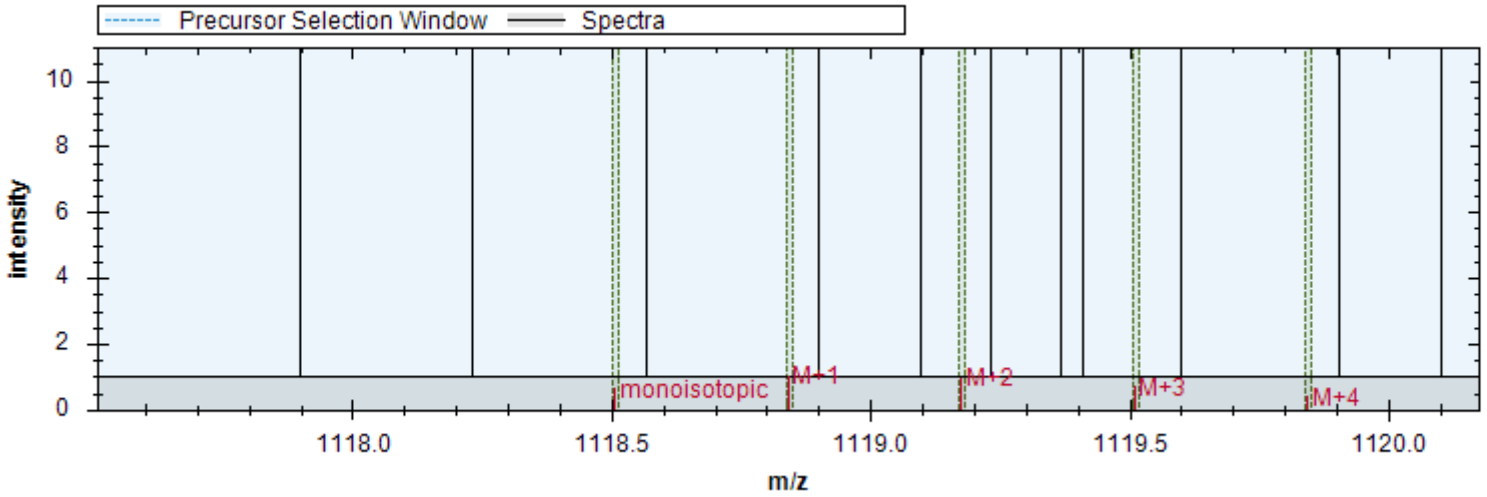

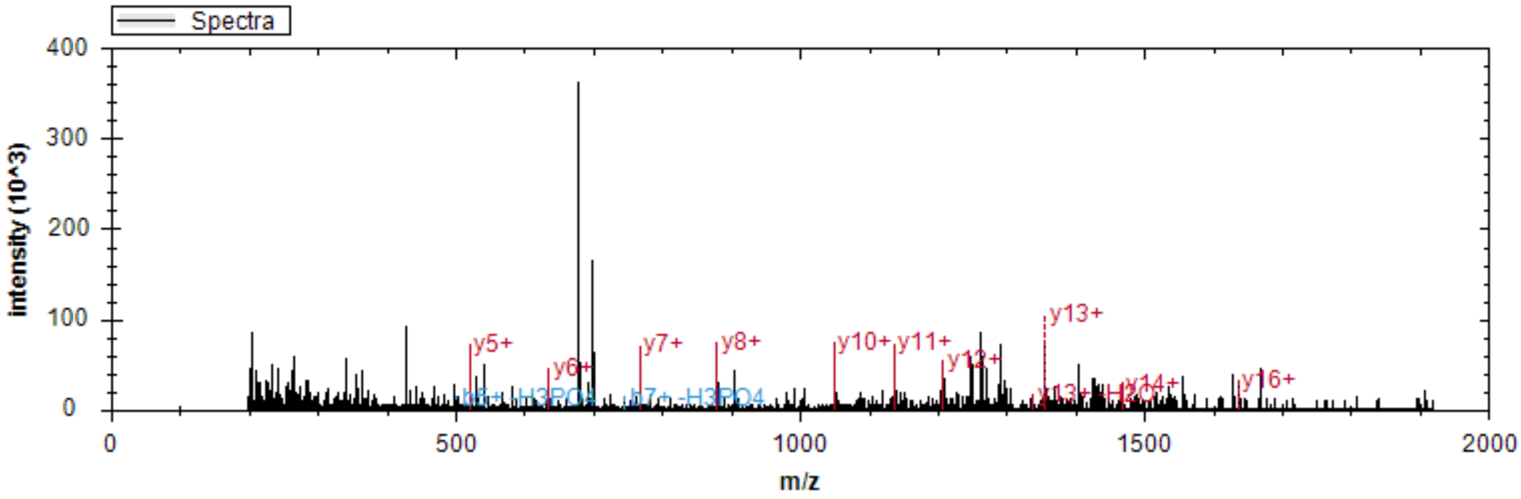

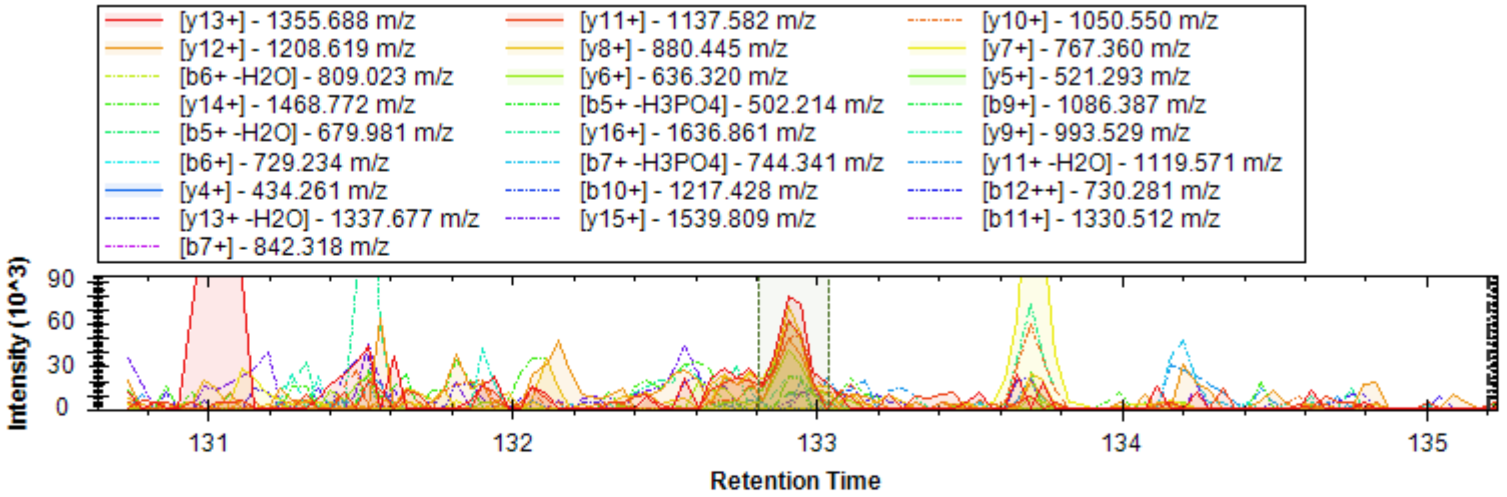

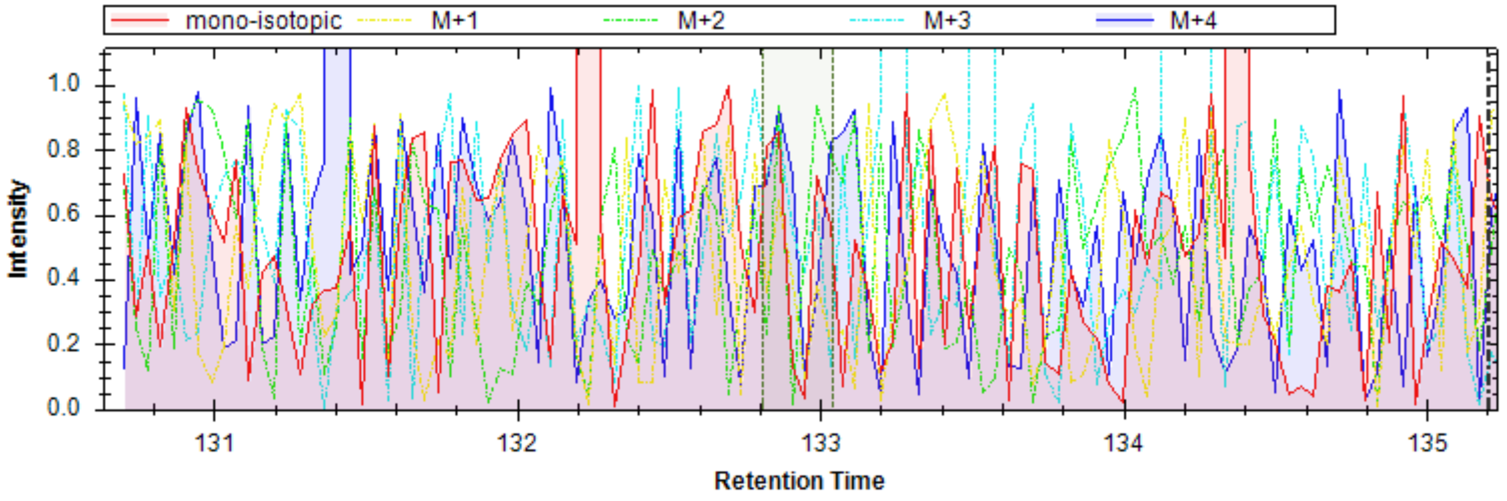

O35526 – _TTTS[+H+O3+P]EELEDMLESGNPAIFASGIIMDSSISK_.3
Fig4_MP-DIA-barrel-cortex-p15-3_MHRM.htrms

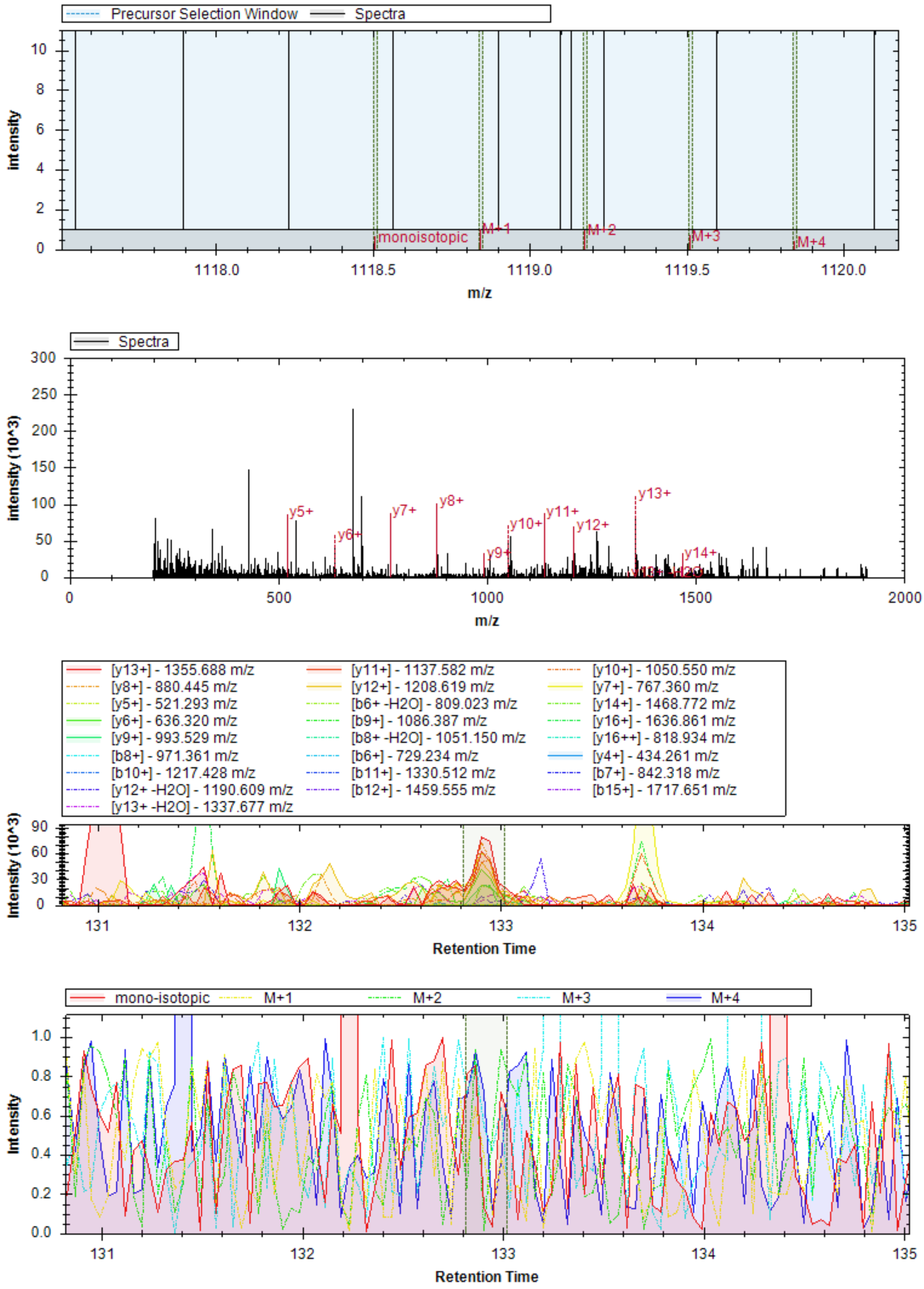

P20357 – _SGTSTPTTPGSTAIT[+H+O3+P]PGTPPSYSSR_.2
Fig4_MP-DIA-barrel-cortex-p15-3_MHRM.htrms

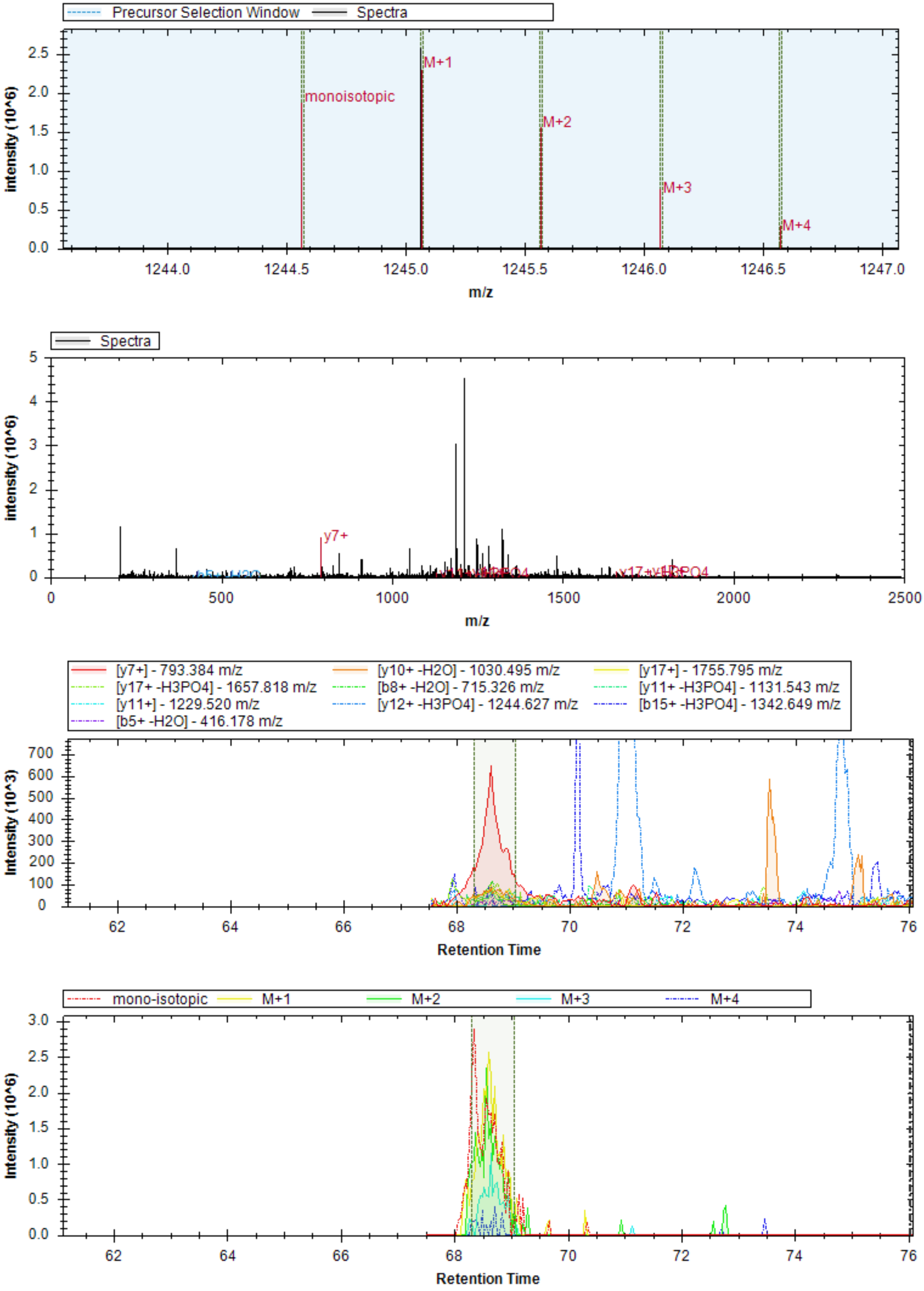

P20357 – _SGTSTPTTPGSTAITPGT[+H+O3+P]PPSYSSR_.2
Fig4_MP-DIA-barrel-cortex-p15-3_MHRM.htrms

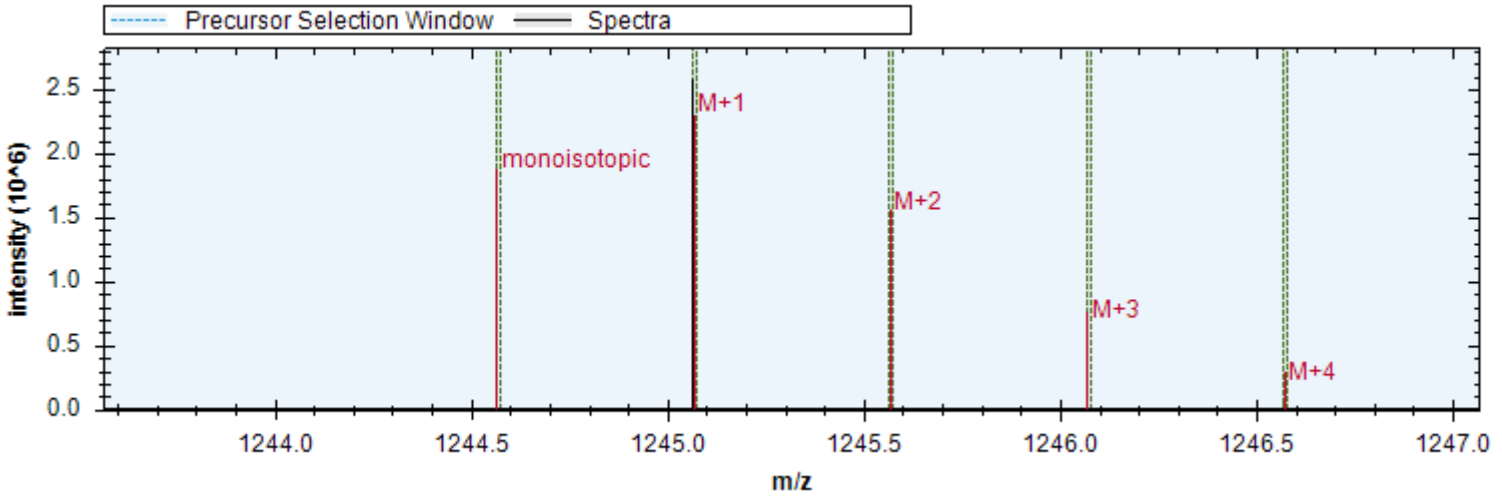

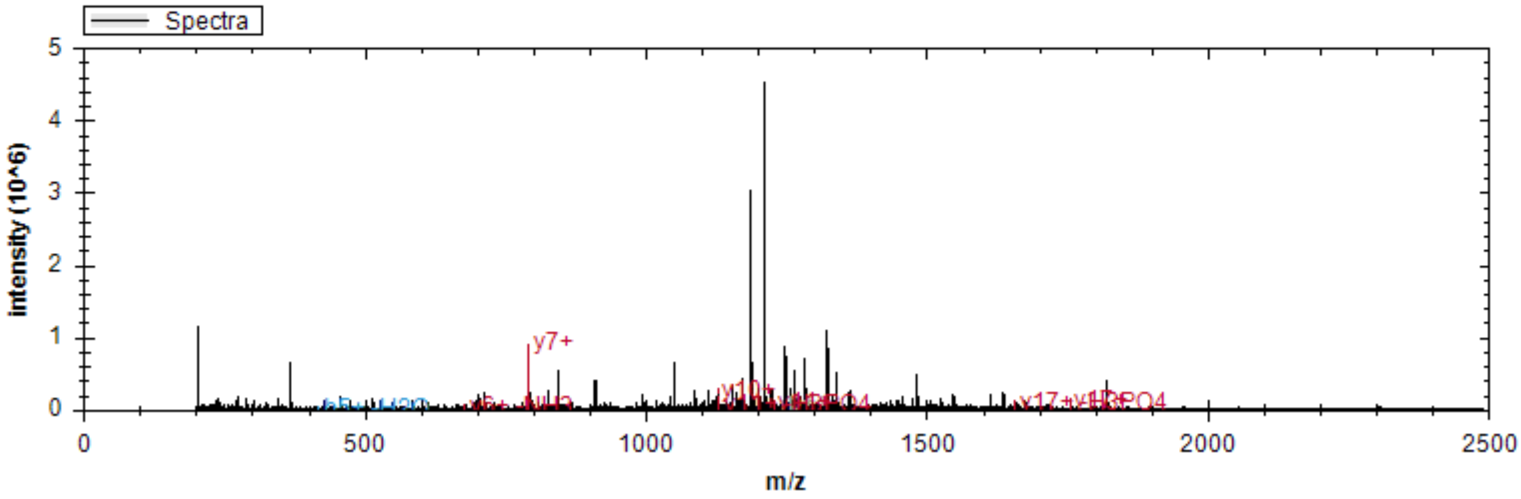

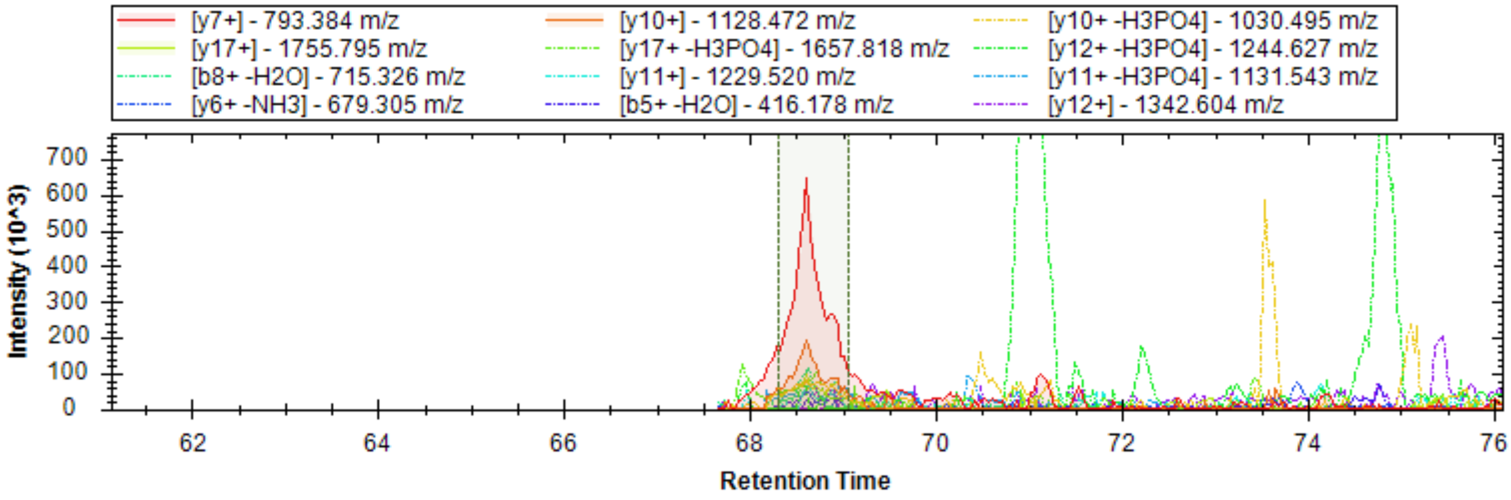

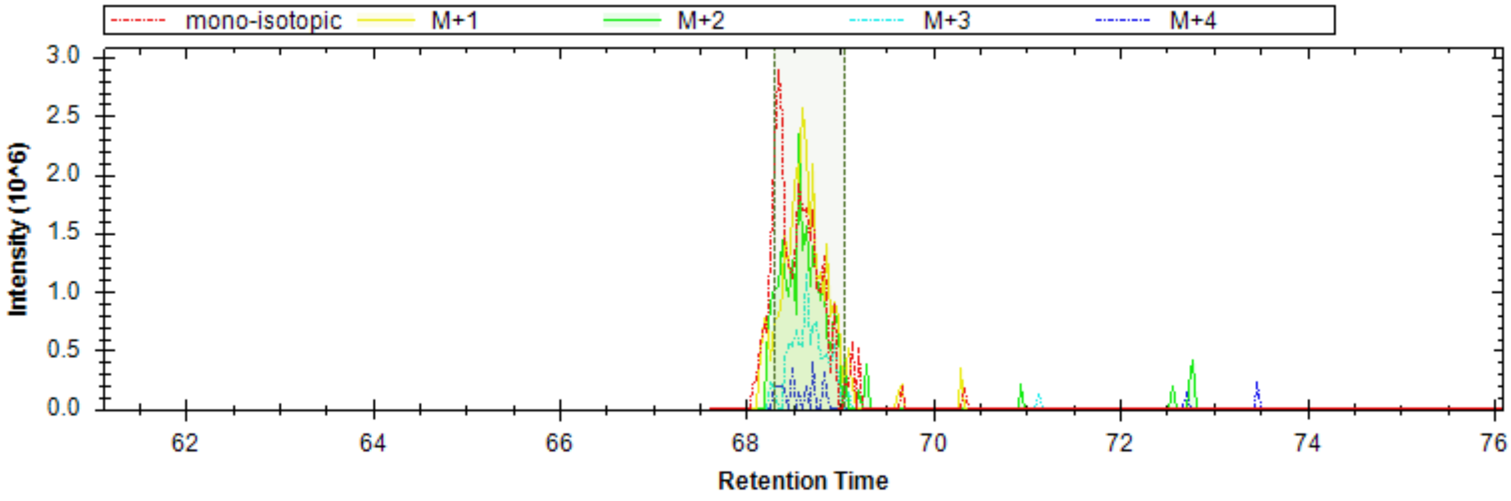

Q61329 – _ES[+H+O3+P]PKPEEQK_.1
Fig4_MP-DIA-barrel-cortex-p15-3_MHRM.htrms

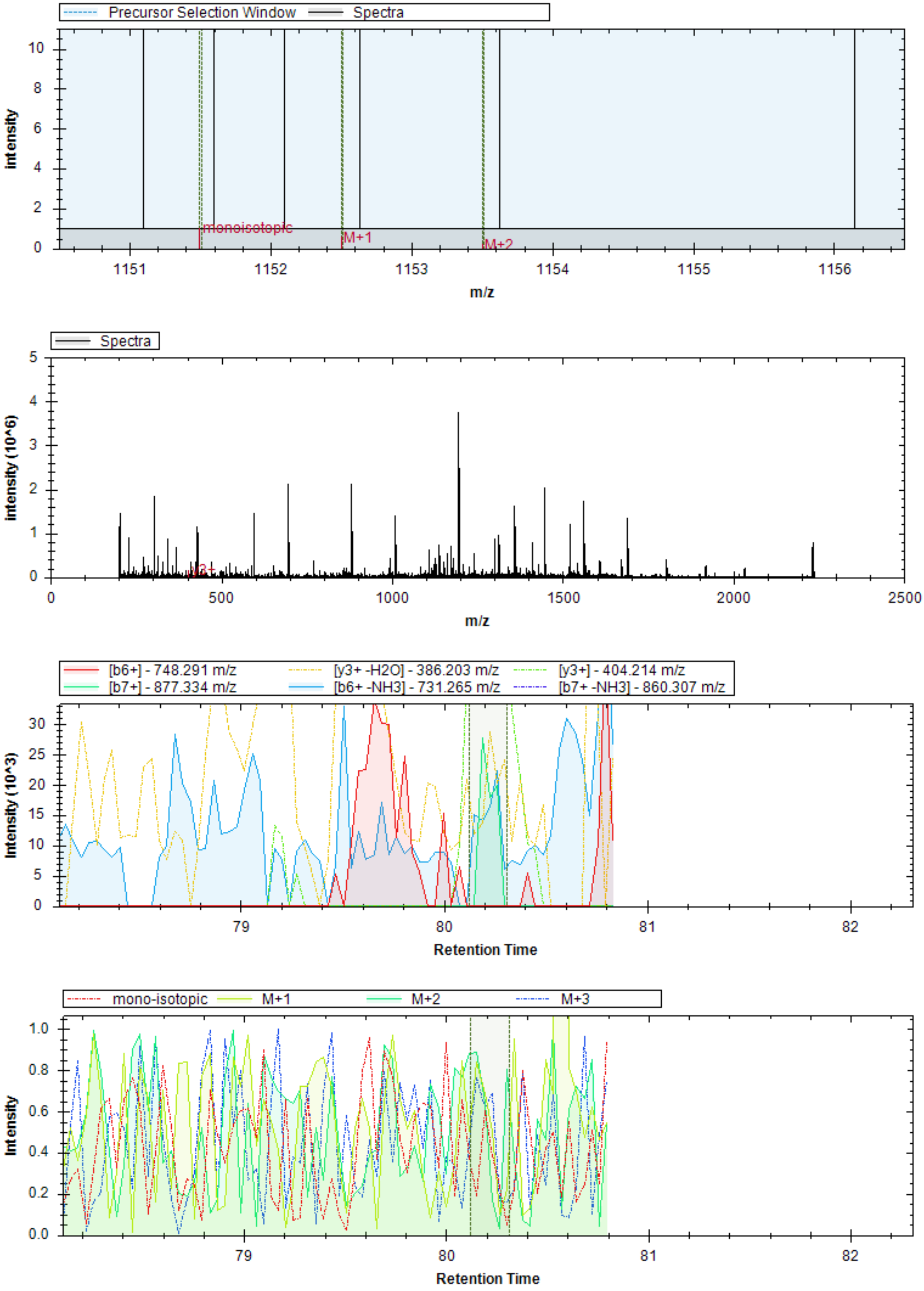

Q8BRT1 – _VLNT[+H+O3+P]GSDVEEAVADALLLGDIR_.2
Fig4_MP-DIA-barrel-cortex-p15-3_MHRM.htrms

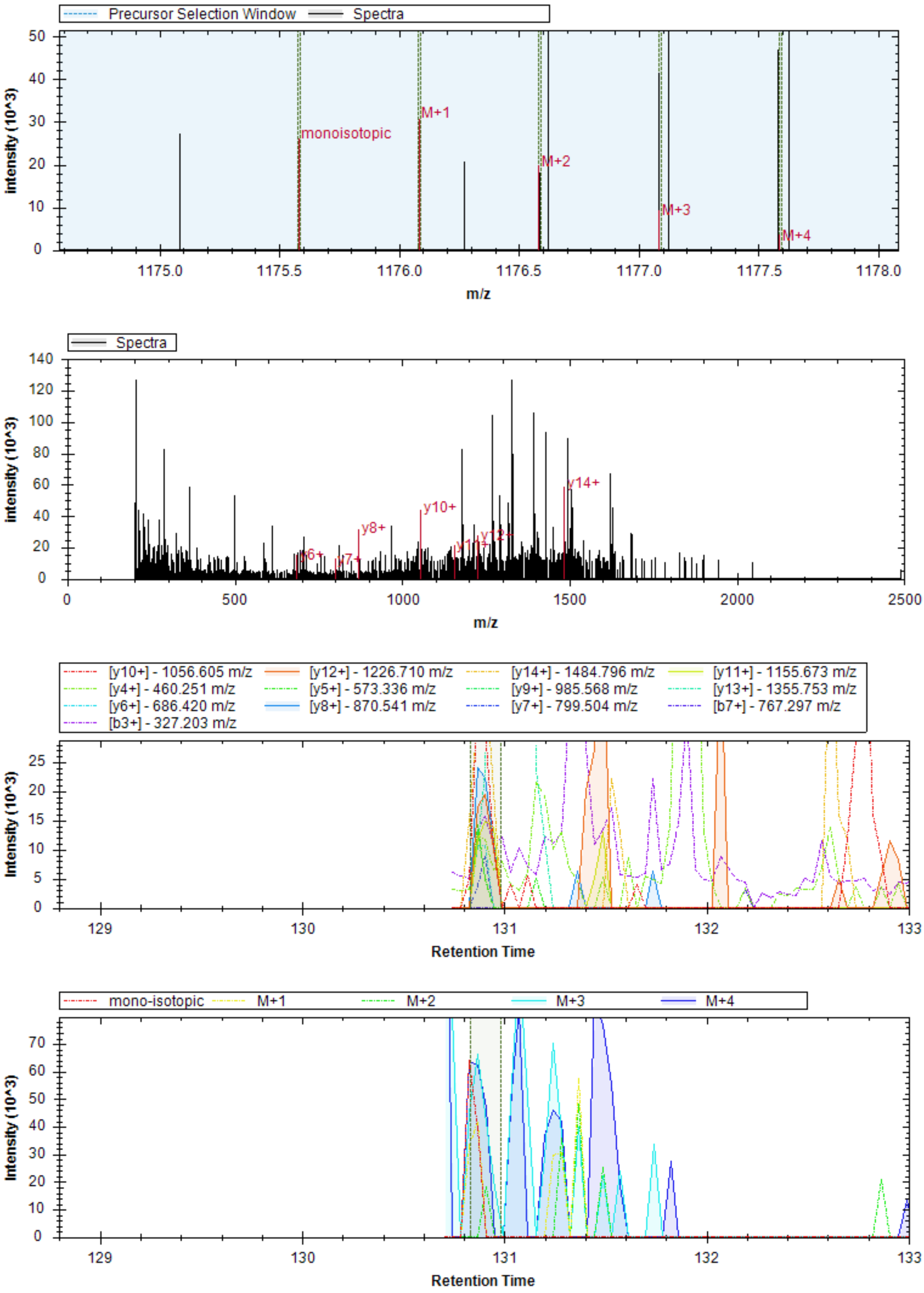

Q8BRT1 – _VLNTGS[+H+O3+P]DVEEAVADALLLGDIR_.2
Fig4_MP-DIA-barrel-cortex-p15-3_MHRM.htrms

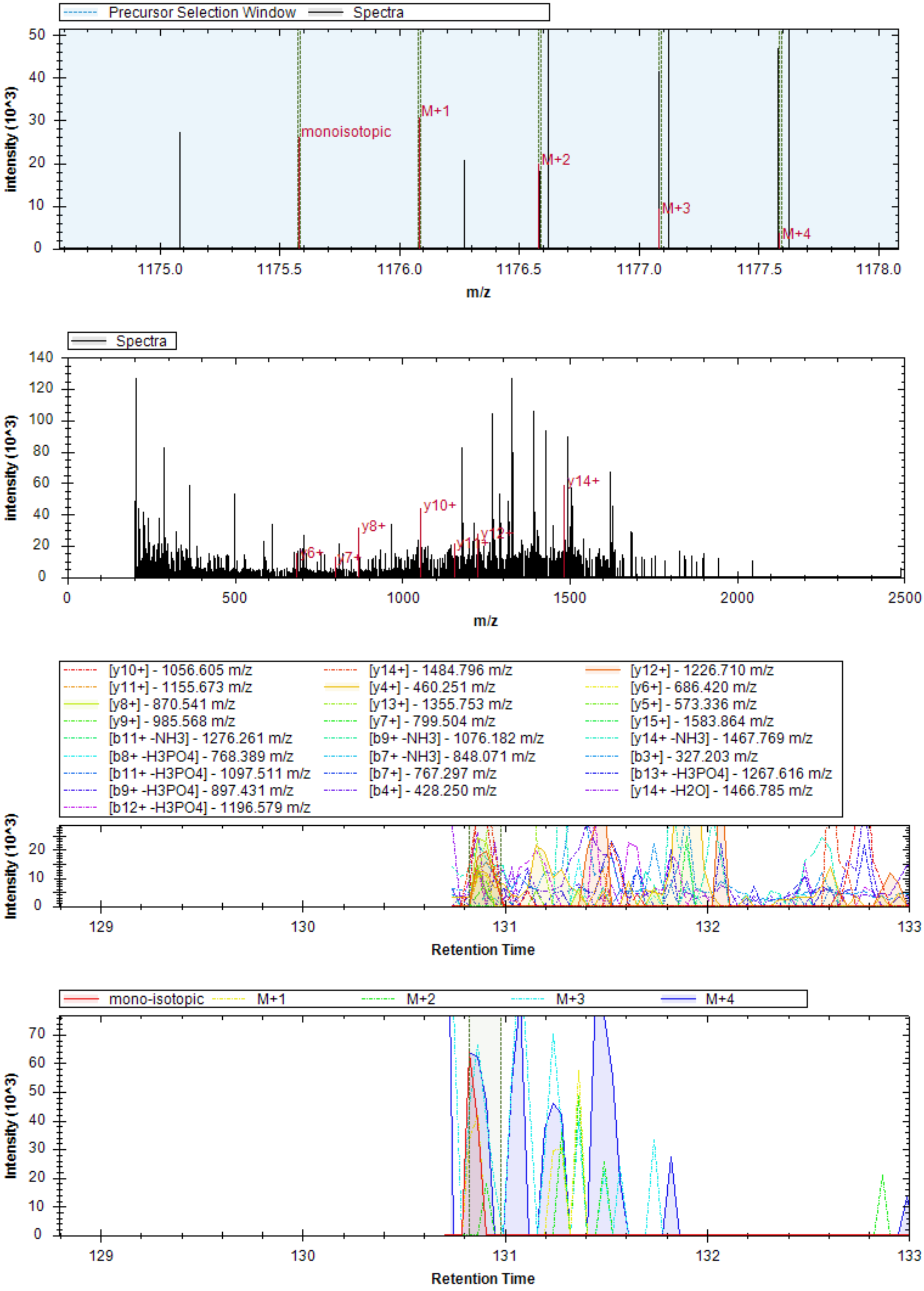

Q8VHY0 – _LLAGPRY[+H+O3+P]GR_.1
Fig4_MP-DIA-barrel-cortex-p15-3_MHRM.htrms

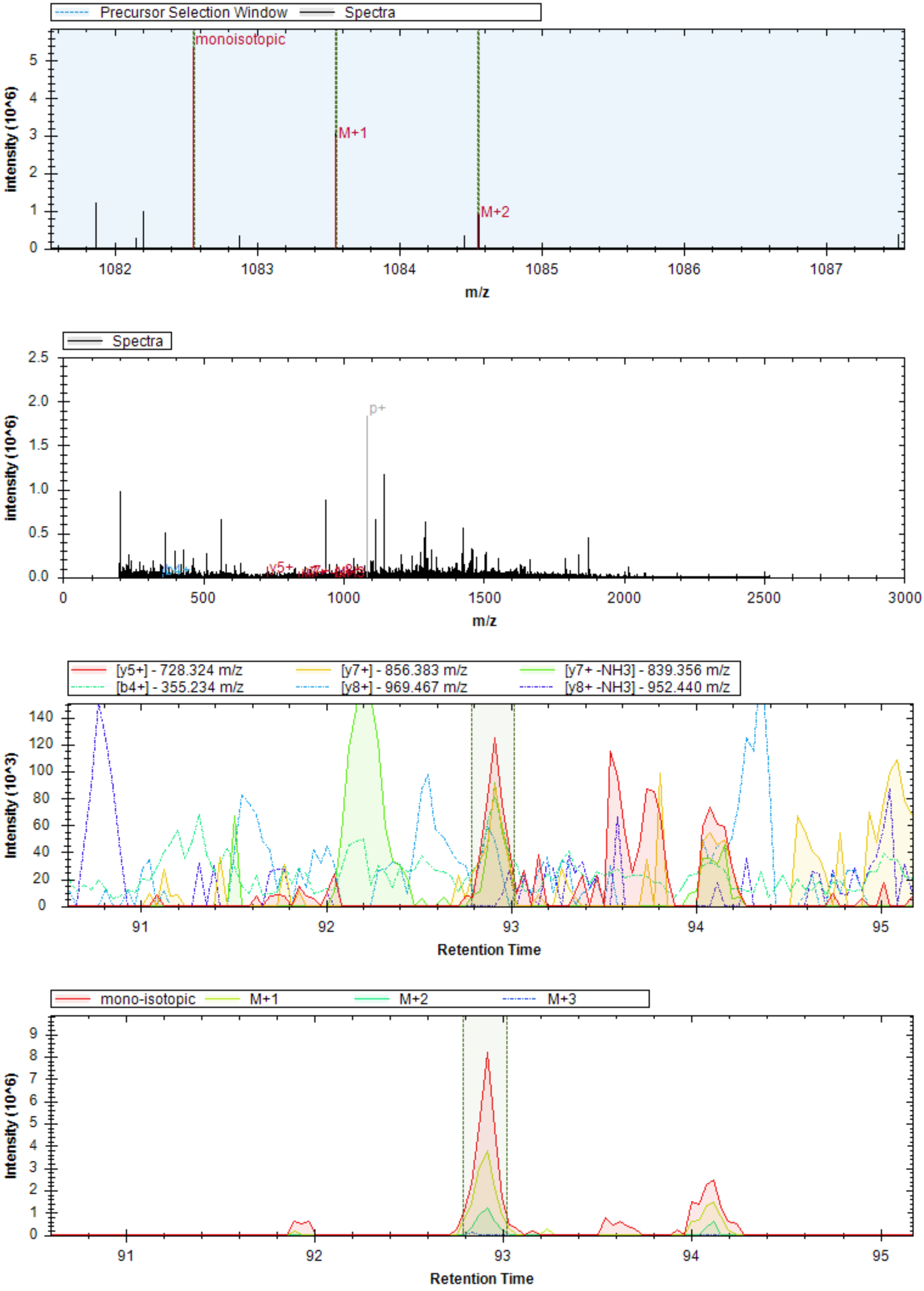

Q9EQF6 – _DLHESS[+H+O3+P]FSLSGSQIDDHVPK_.2
Fig4_MP-DIA-barrel-cortex-p15-3_MHRM.htrms

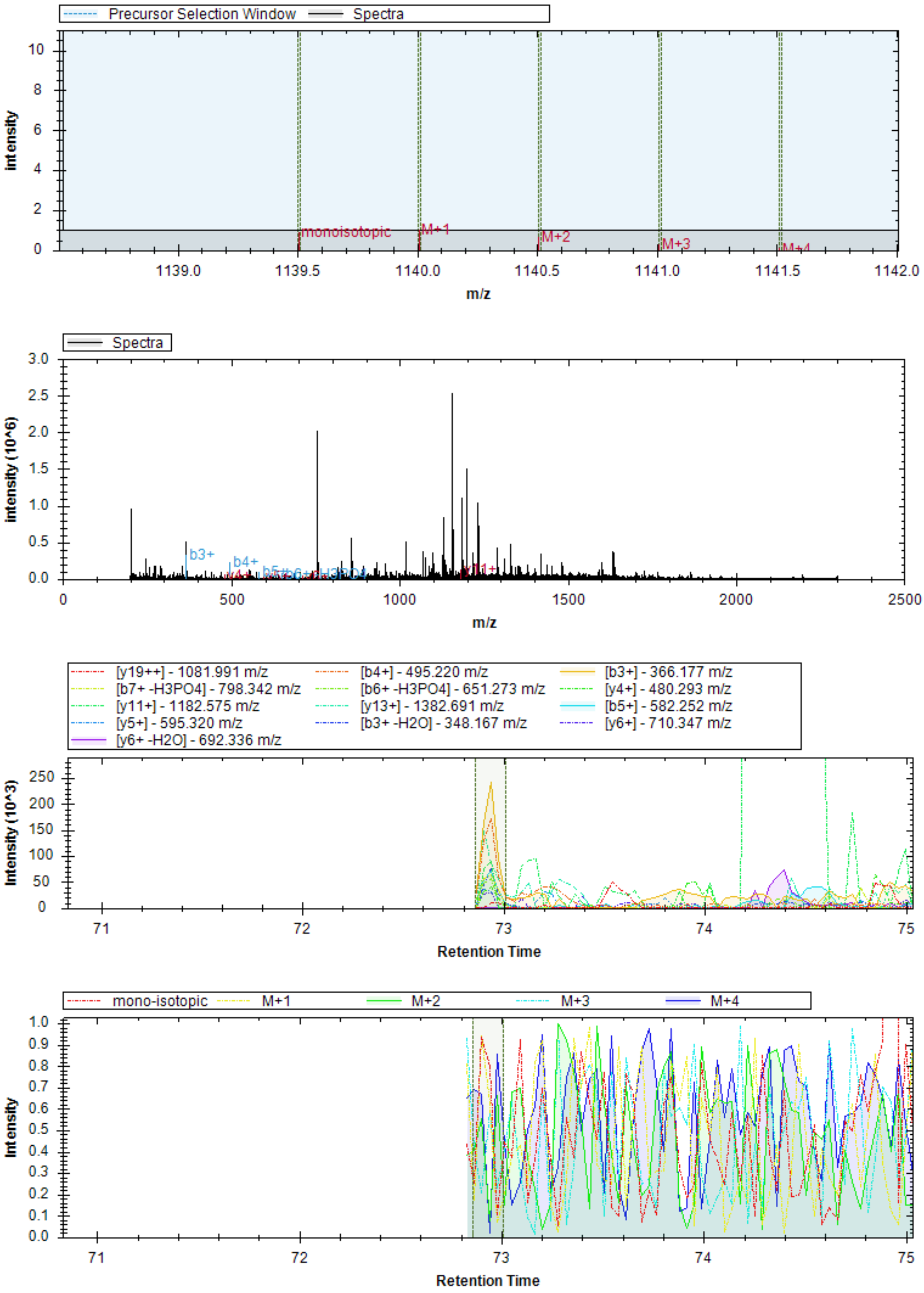

Q921N6;Q921N6-2 – _LIY[+H+O3+P]KPRQAAVTR_.4
Fig4_MP-DIA-barrel-cortex-p30-1_MHRM.htrms

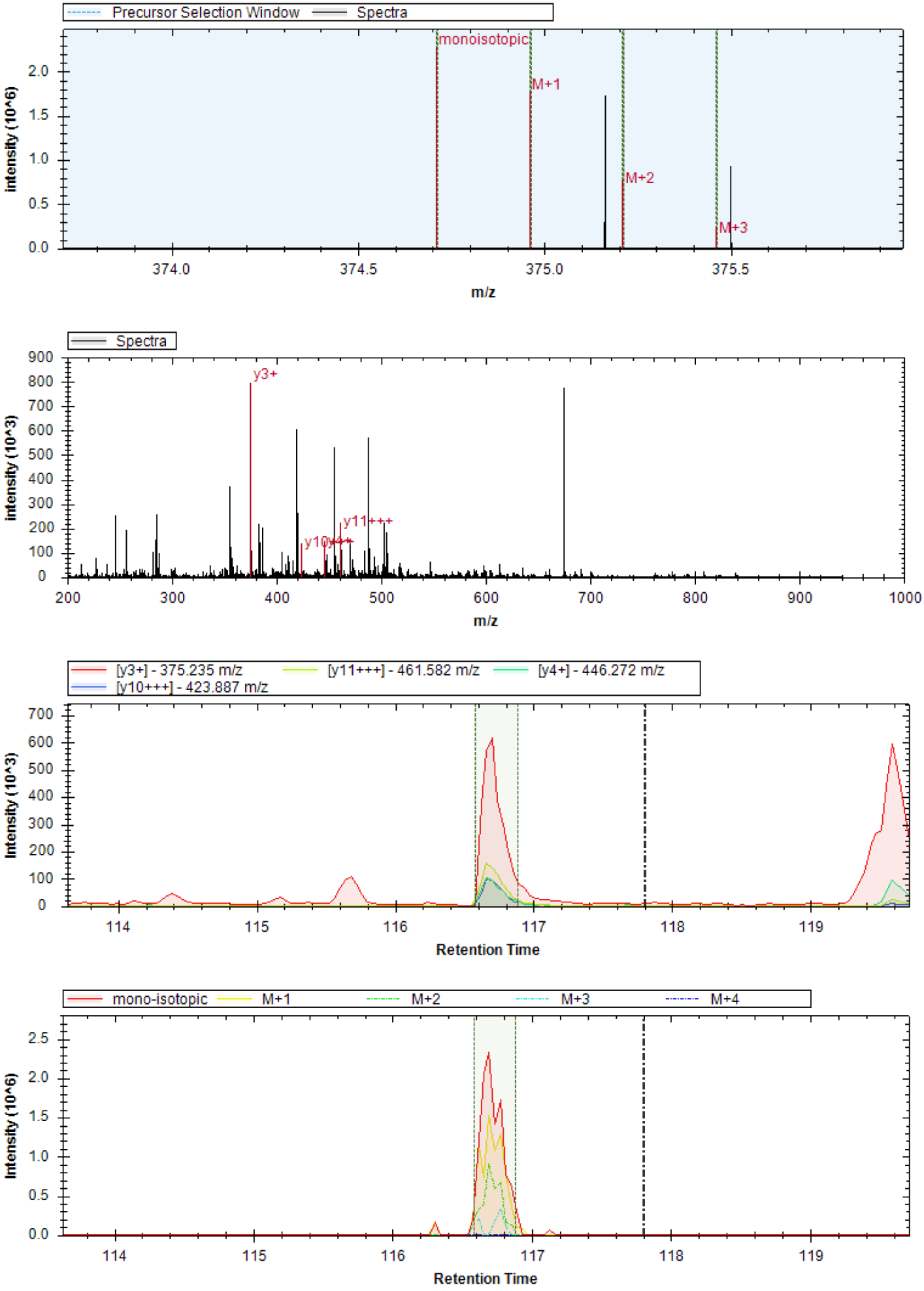

Q9CVB6 – _IIEET[+H+O3+P]LALK_.3

Fig4_MP-DIA-barrel-cortex-p30-1_MHRM.htrms

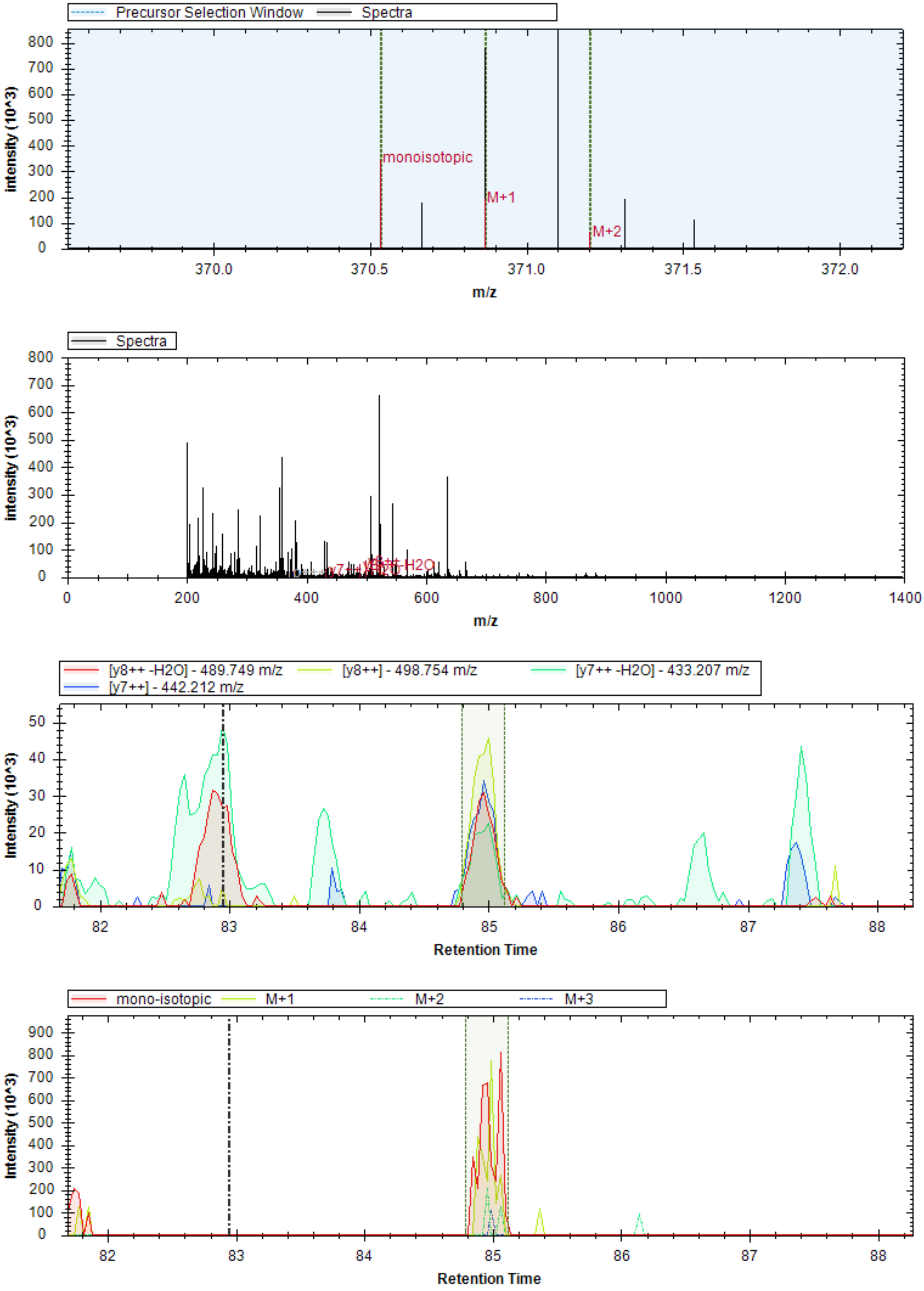

Q9D516;Q9D516-2 – _KLS[+H+O3+P]QGILVIR_.3
Fig4_MP-DIA-barrel-cortex-p30-1_MHRM.htrms

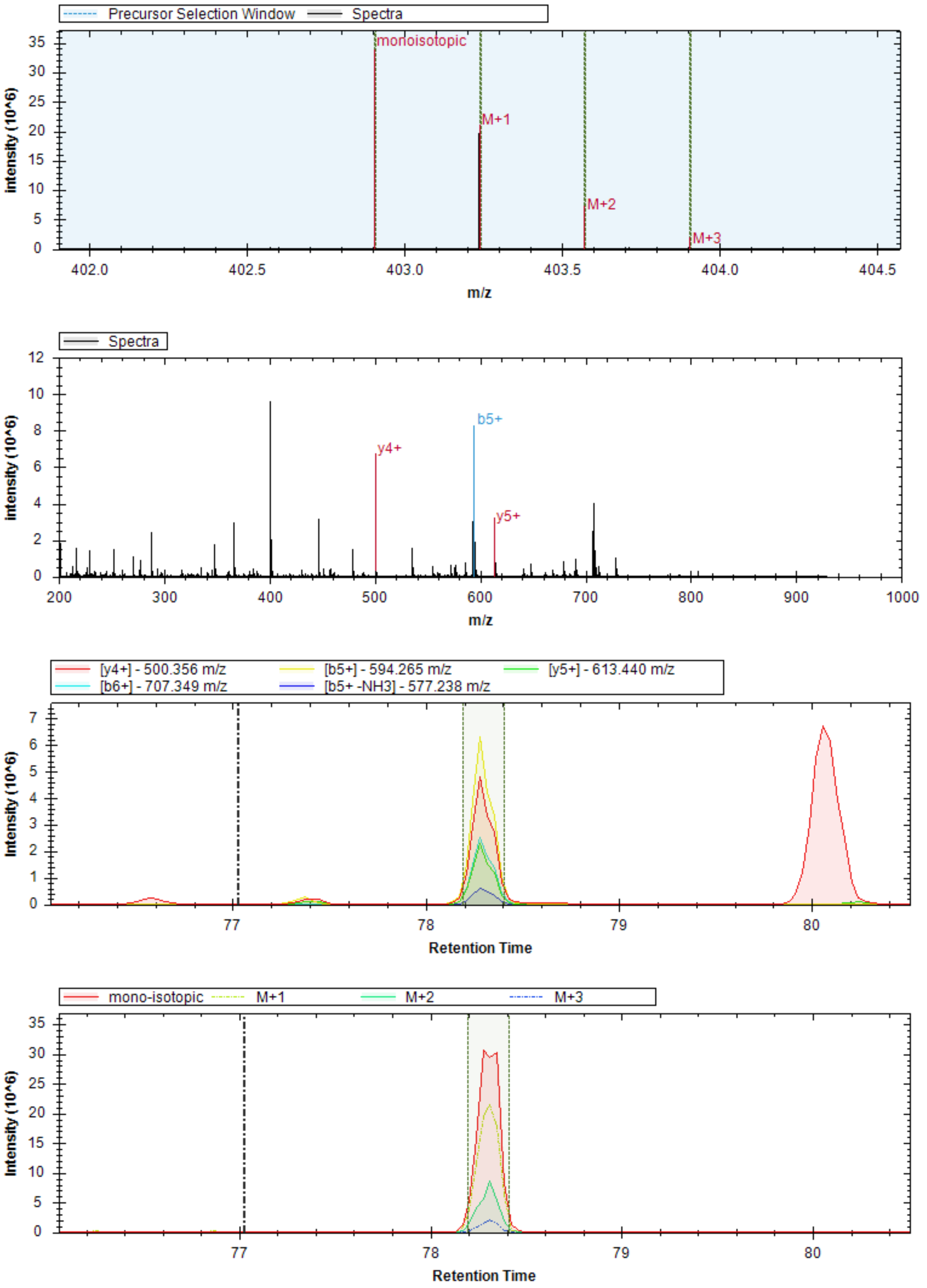

P20357 – _KSEVQAHS[+H+O3+P]PSR_.3
Fig4_MP-DIA-barrel-cortex-p30-1_MHRM.htrms

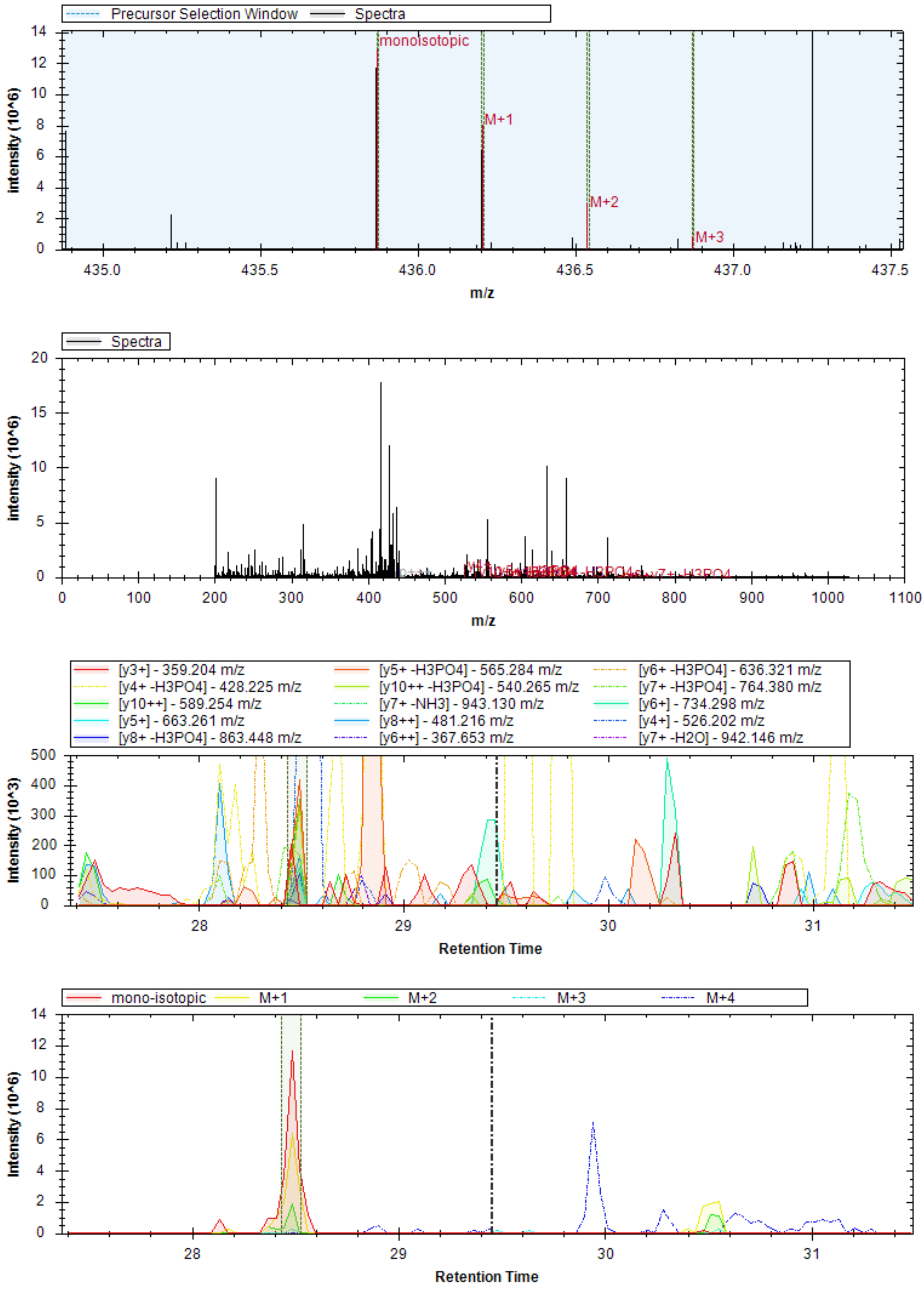

Q03172 – _VALSLLNS[+H+O3+P]KQK_.3
Fig4_MP-DIA-barrel-cortex-p30-1_MHRM.htrms

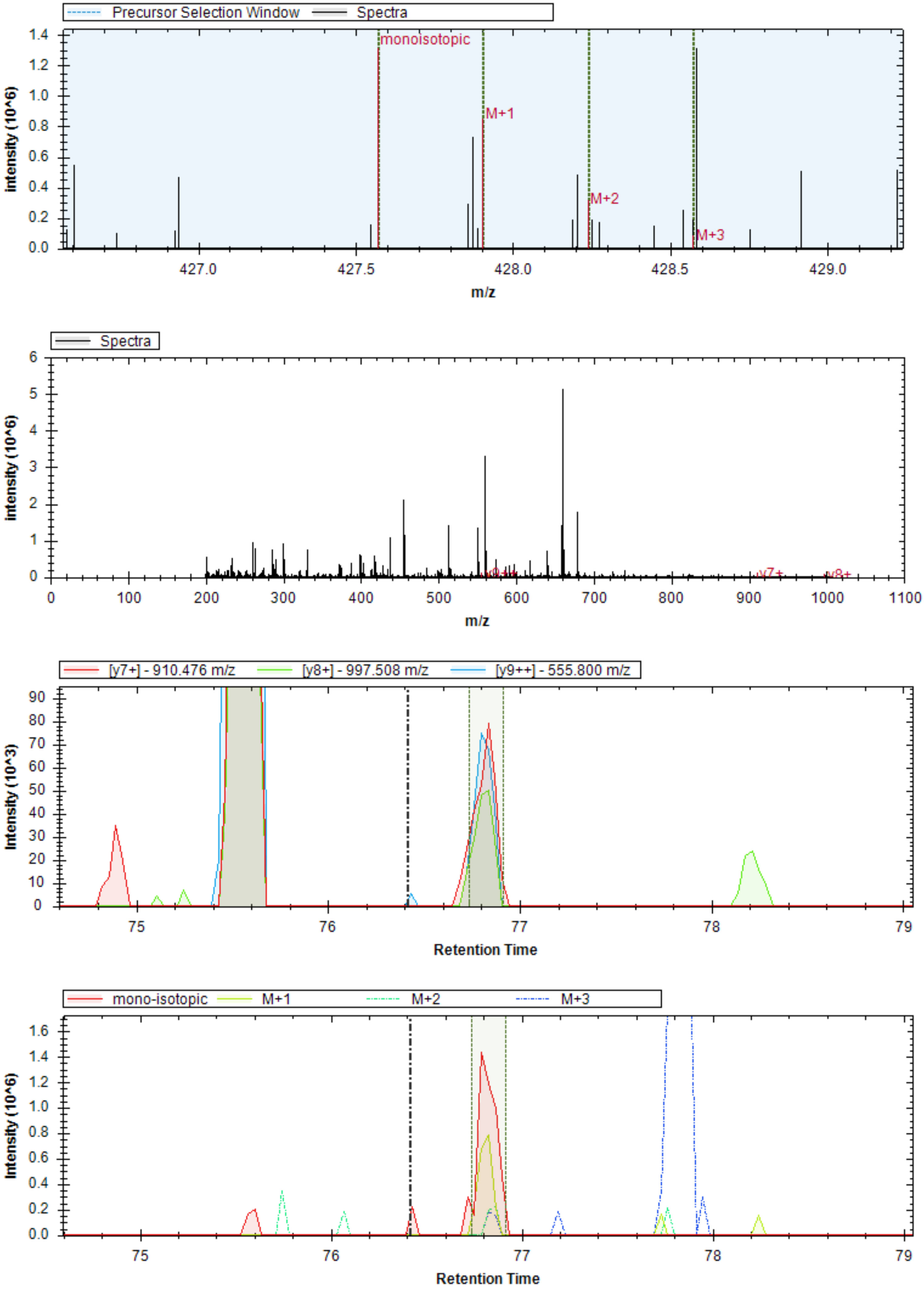

Q52KI8;Q52KI8-2 – _RES[+H+O3+P]PSPAPKPR_.3
Fig4_MP-DIA-barrel-cortex-p30-1_MHRM.htrms

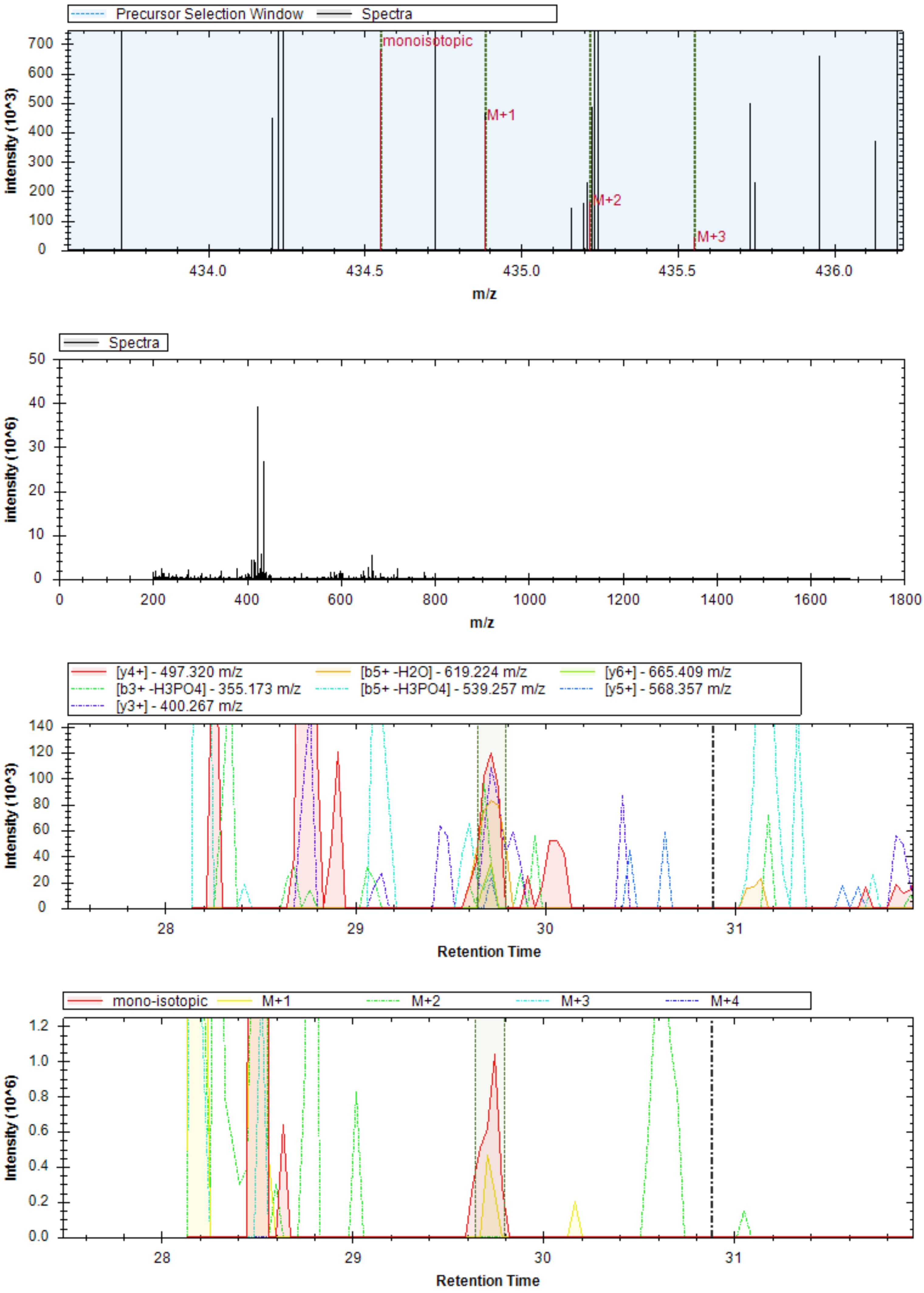

Q62188 – _GS[+H+O3+P]PTRPNPPVR_.3
Fig4_MP-DIA-barrel-cortex-p30-1_MHRM.htrms

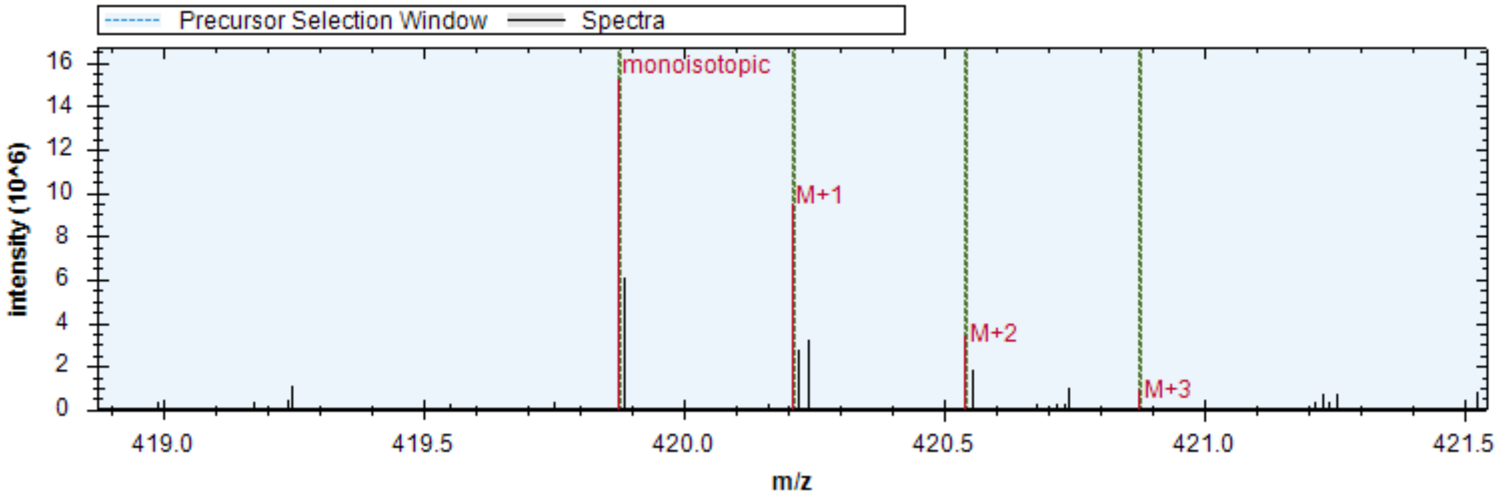

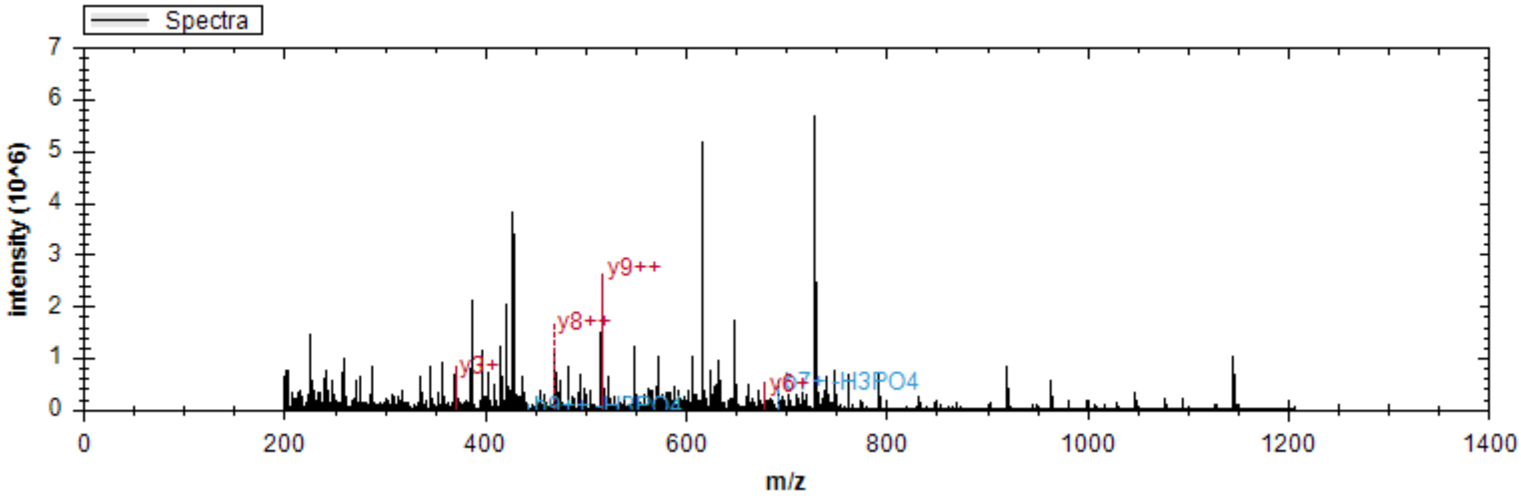

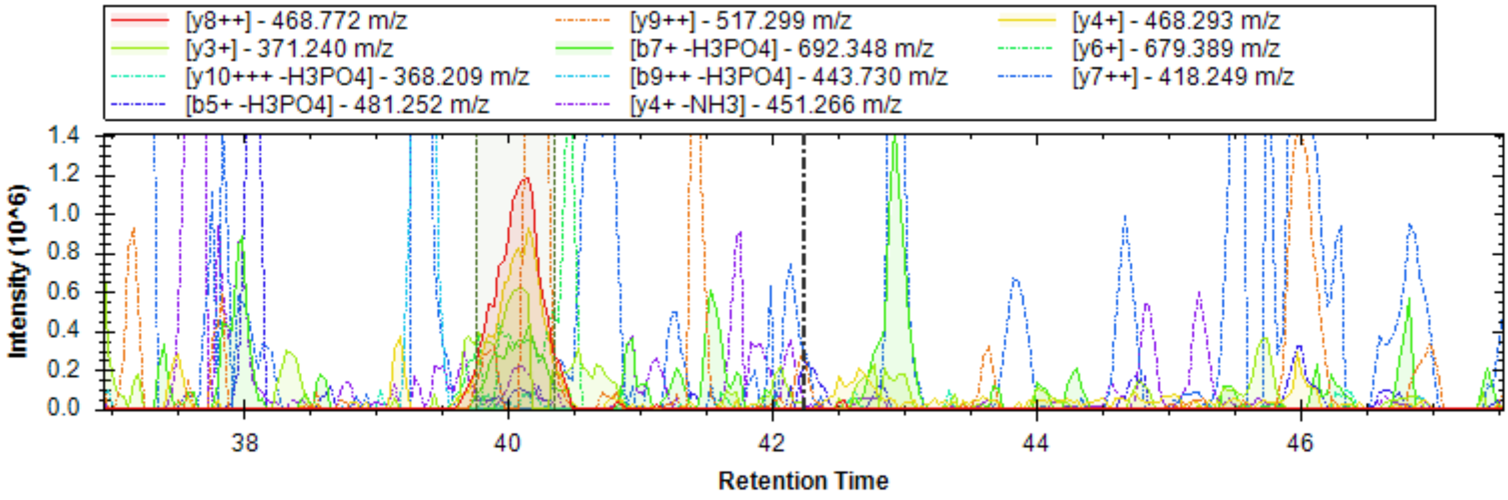

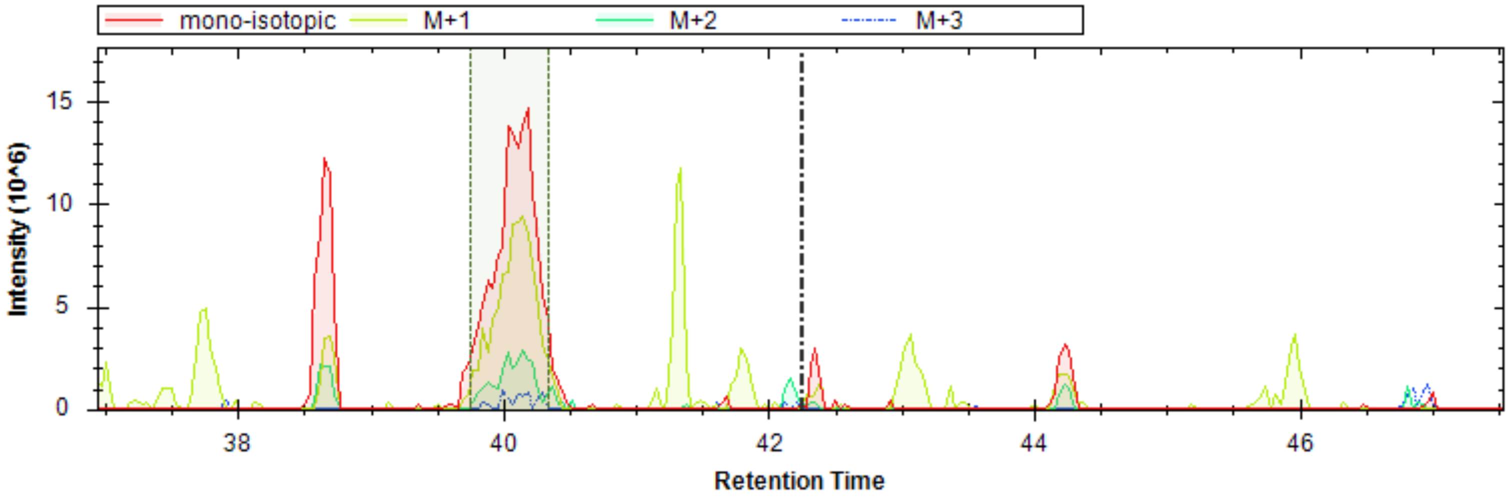

P62484 – _T[+H+O3+P]HKIIAPANLERPVR_.4
Fig4_MP-DIA-barrel-cortex-p30-1_MHRM.htrms

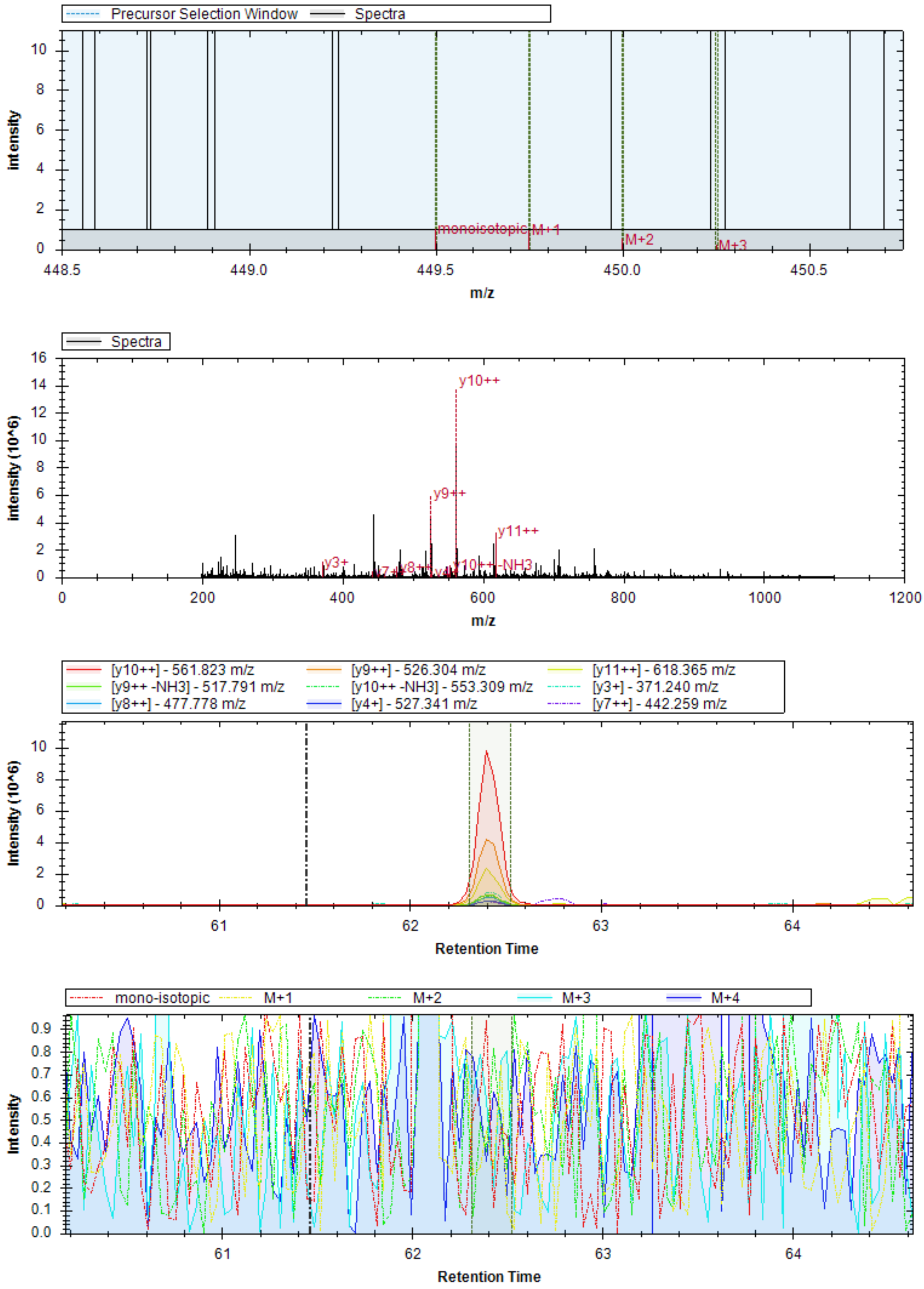

O08553 – _TVT[+H+O3+P]PASSAK_.2
Fig4_MP-DIA-barrel-cortex-p30-1_MHRM.htrms

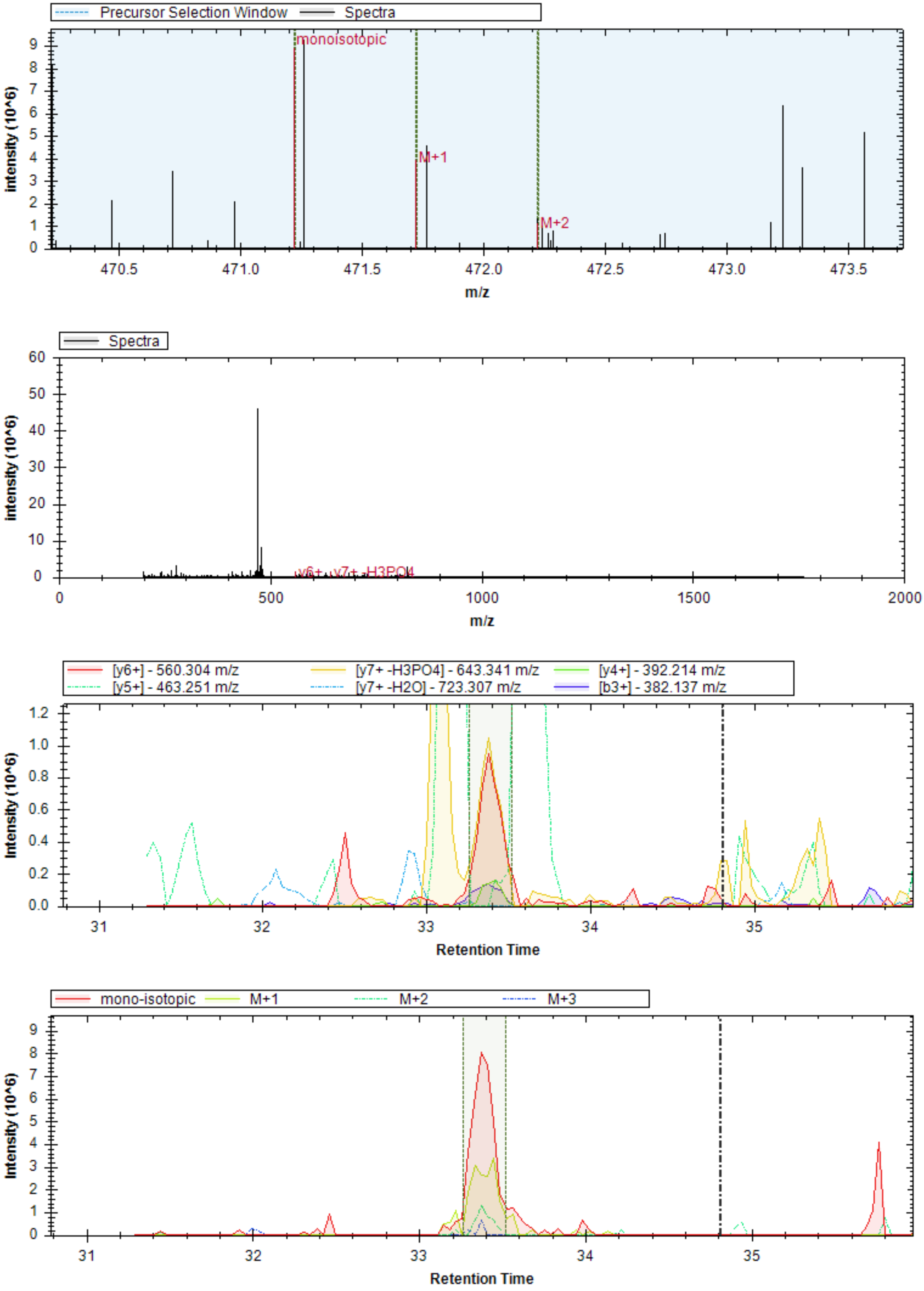

P26645 – _LSGFS[+H+O3+P]FKK_.2
Fig4_MP-DIA-barrel-cortex-p30-1_MHRM.htrms

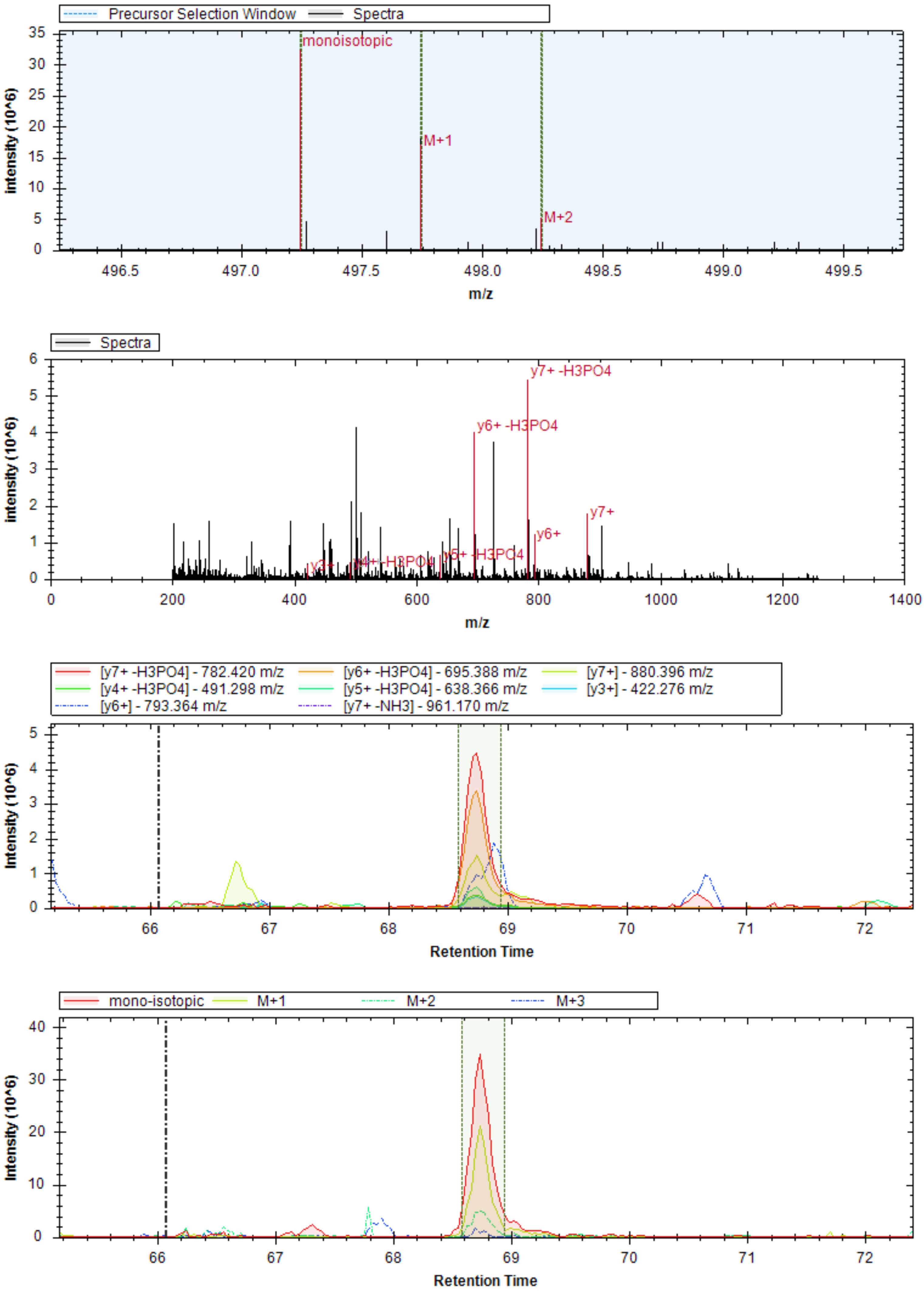

P28667 – _LSGLS[+H+O3+P]FKR_.2
Fig4_MP-DIA-barrel-cortex-p30-1_MHRM.htrms

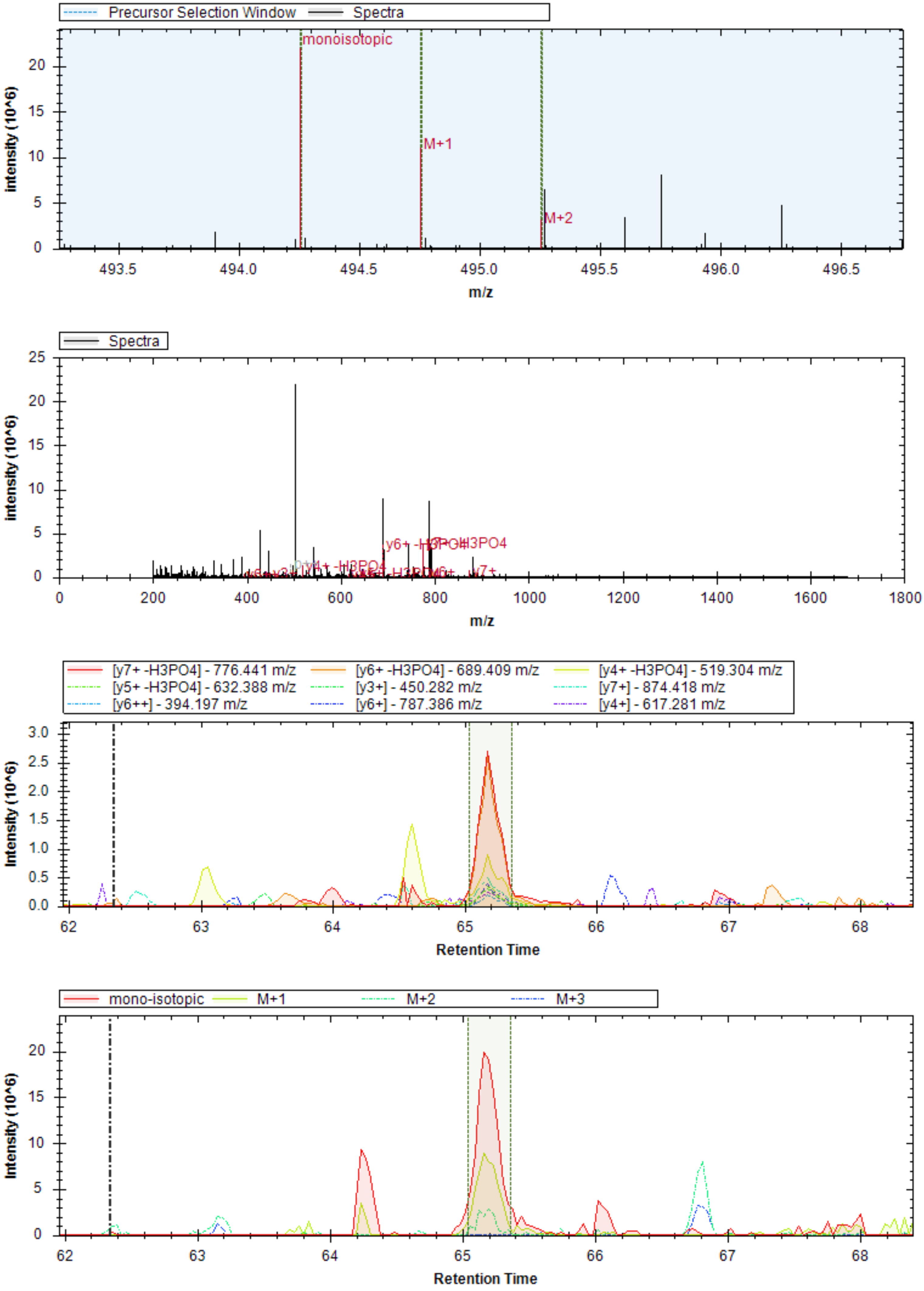

P08553 – _S[+H+O3+P]PVEEVKPKPEAK_.3
Fig4_MP-DIA-barrel-cortex-p30-1_MHRM.htrms

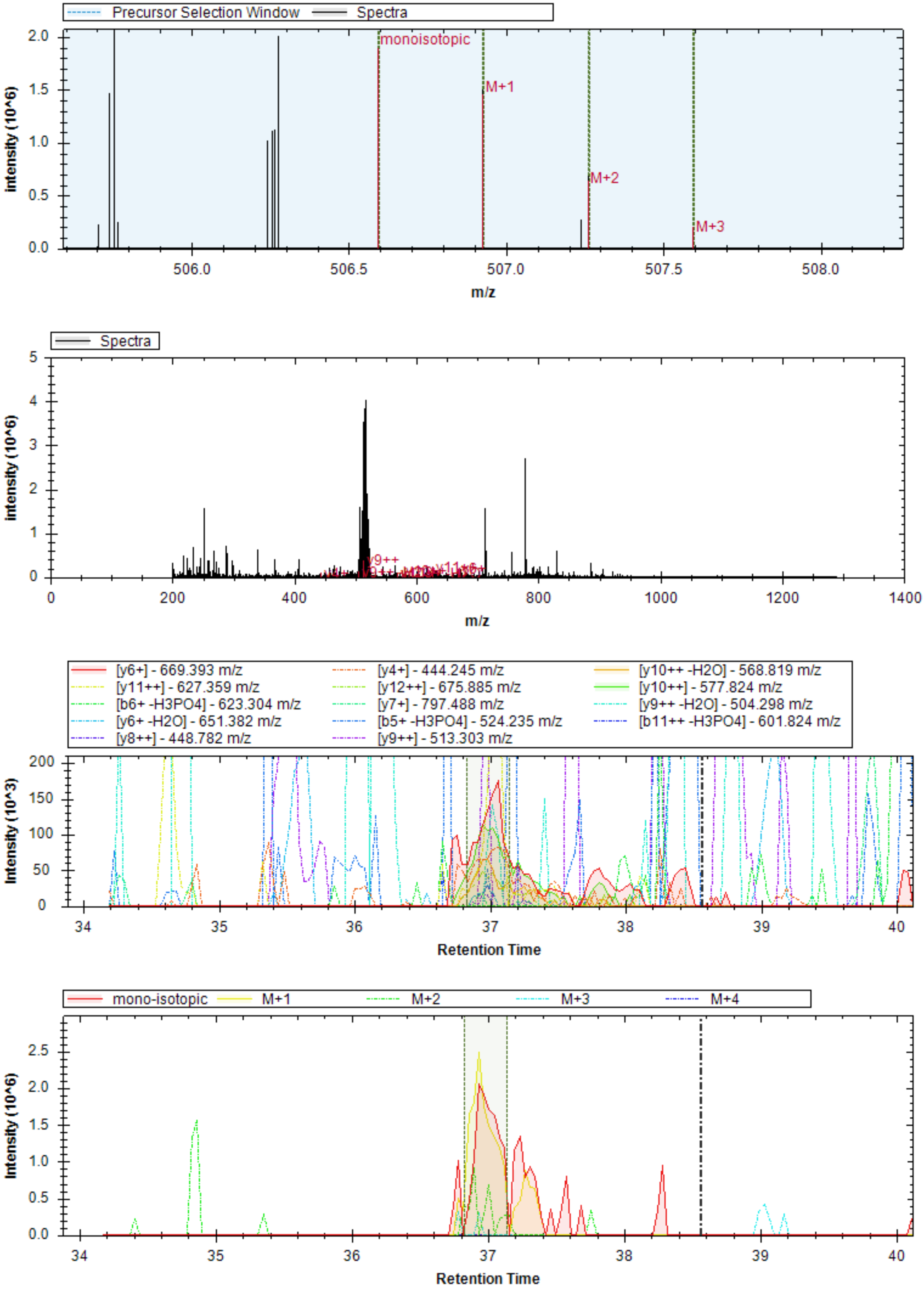

P28738 – _PIRPGHYPAS[+H+O3+P]SPTAVHAVR_.4
Fig4_MP-DIA-barrel-cortex-p30-1_MHRM.htrms

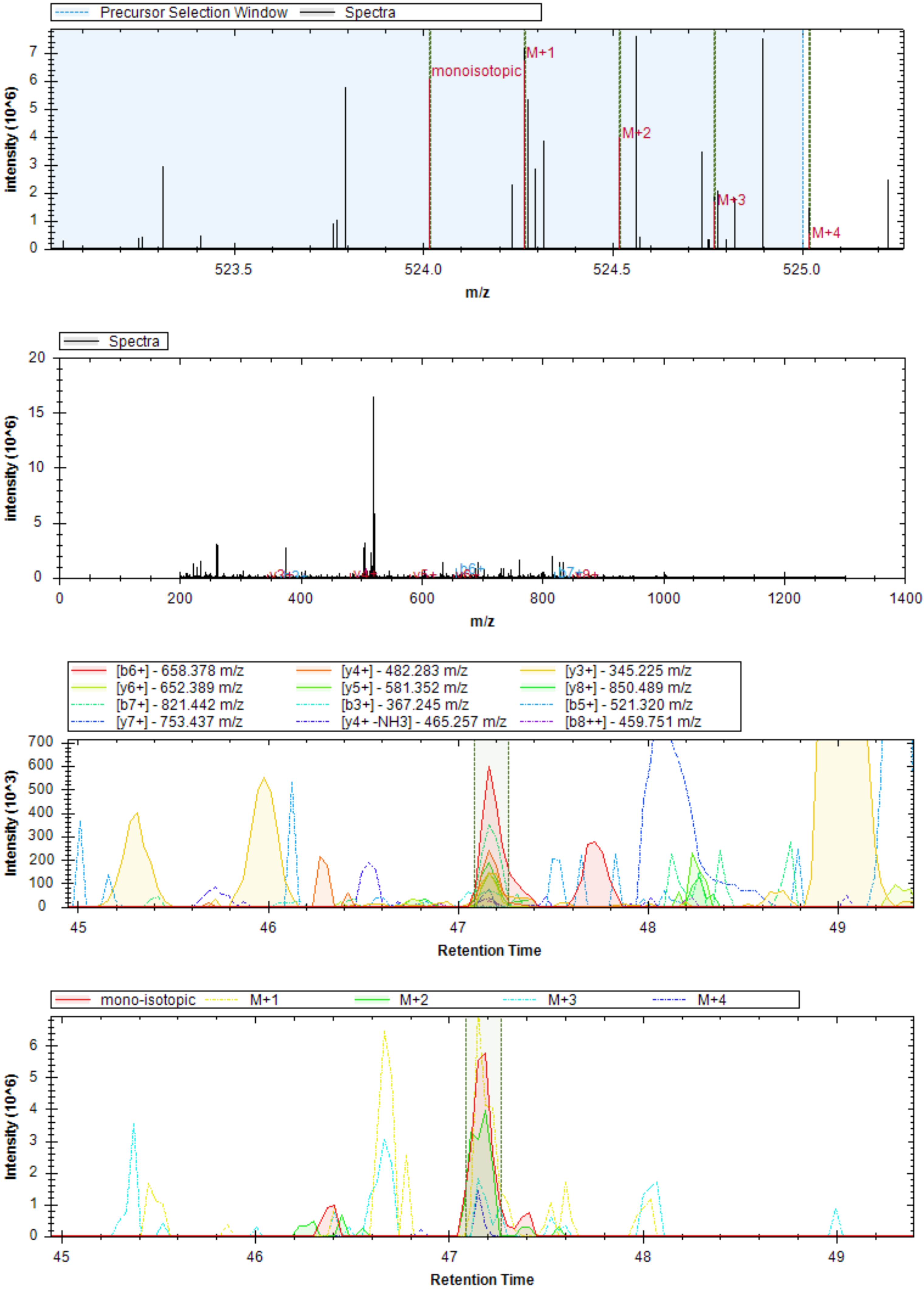

P28738 – _PIRPGHYPASS[+H+O3+P]PTAVHAVR_.4
Fig4_MP-DIA-barrel-cortex-p30-1_MHRM.htrms

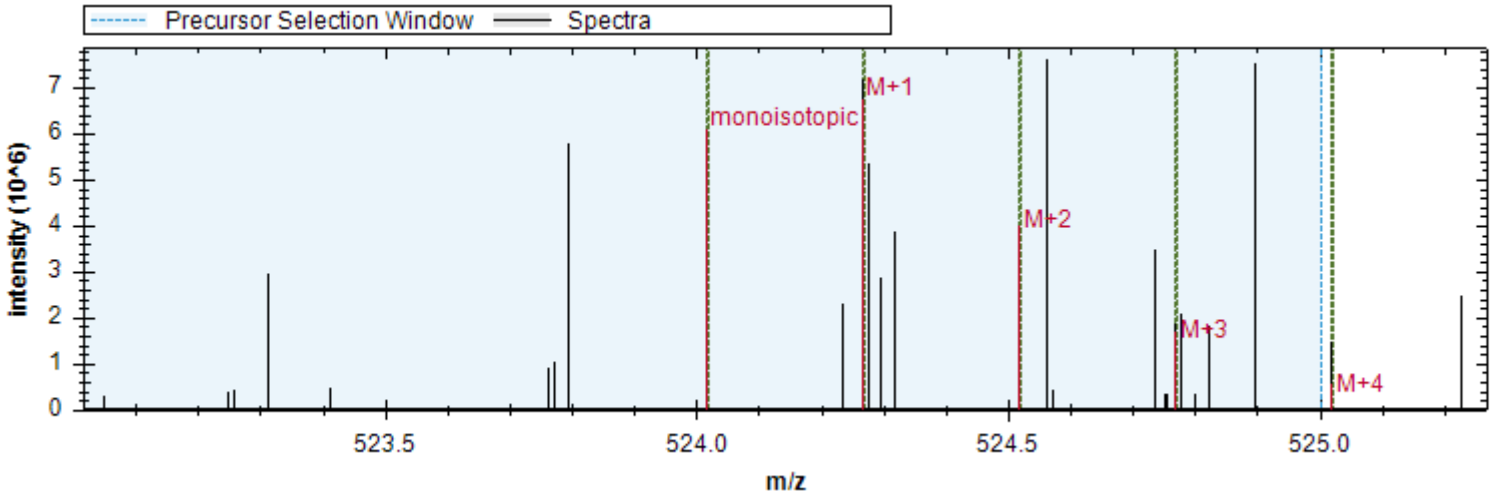

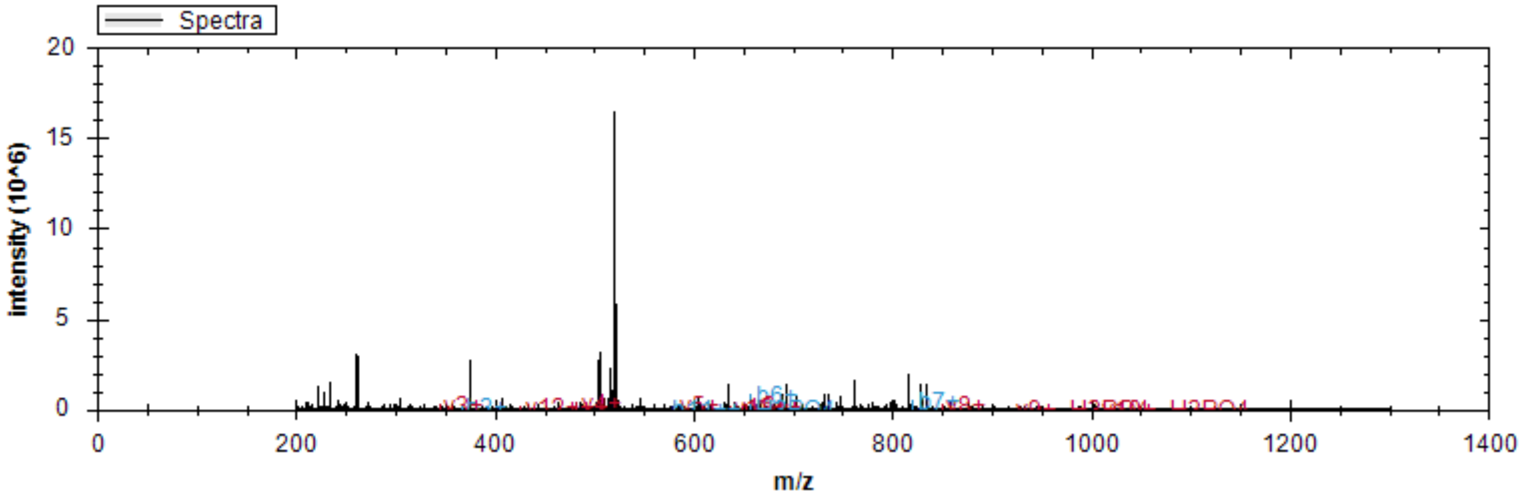

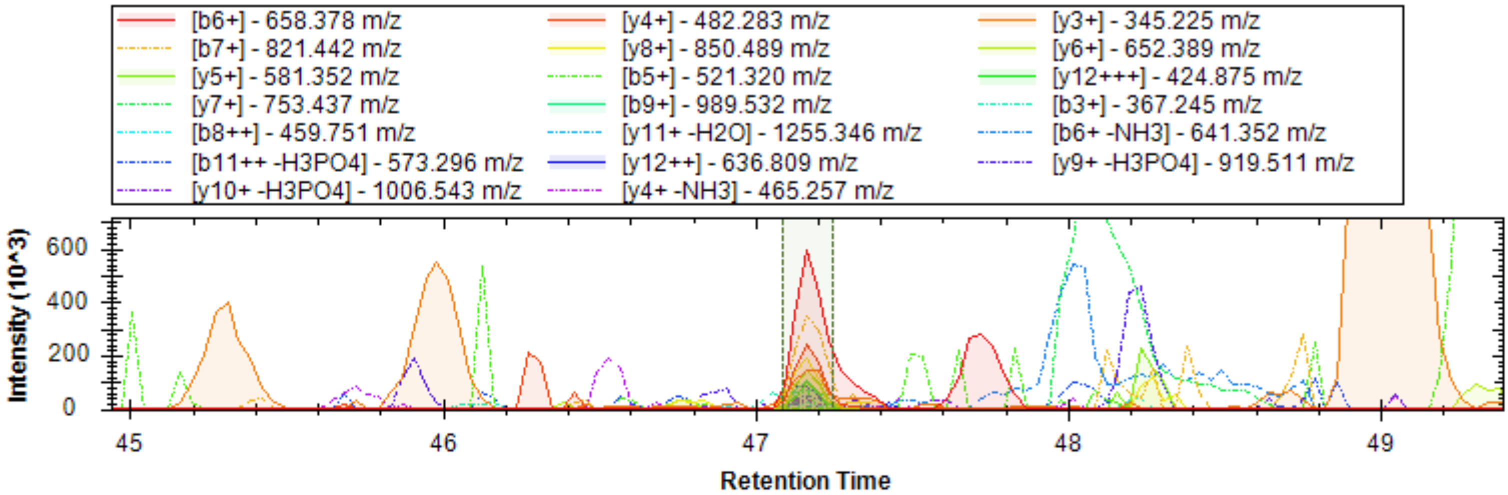

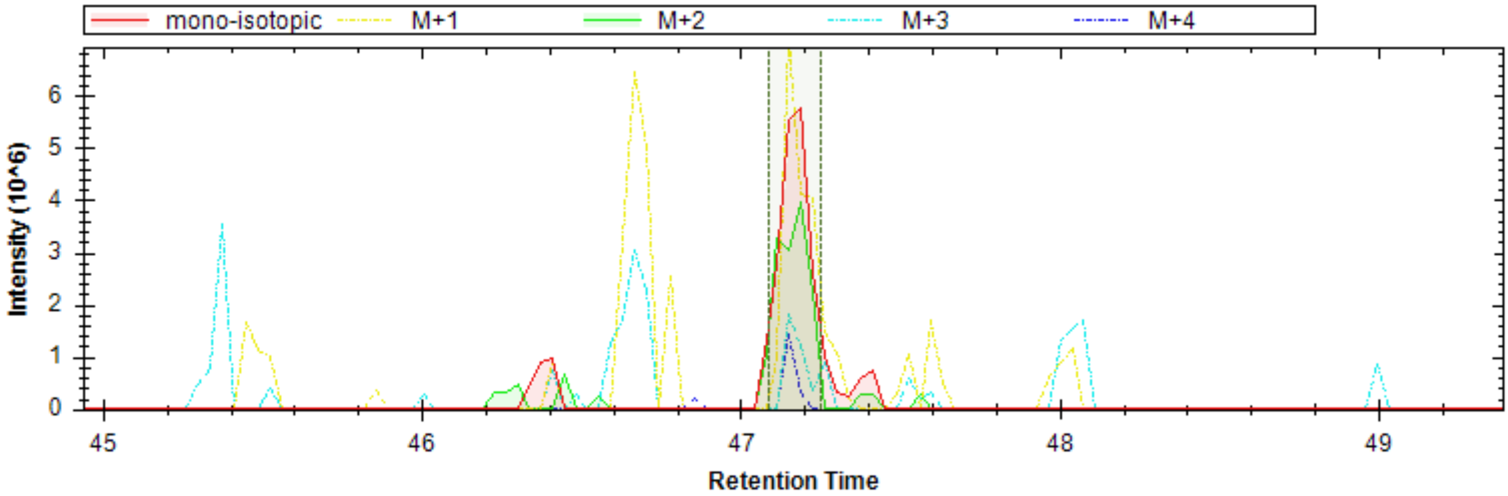

Q8CDY7 – _LVQALC[+C2+H3+N+O]EQIHT[+H+O3+P]R_.3
Fig4_MP-DIA-barrel-cortex-p30-1_MHRM.htrms

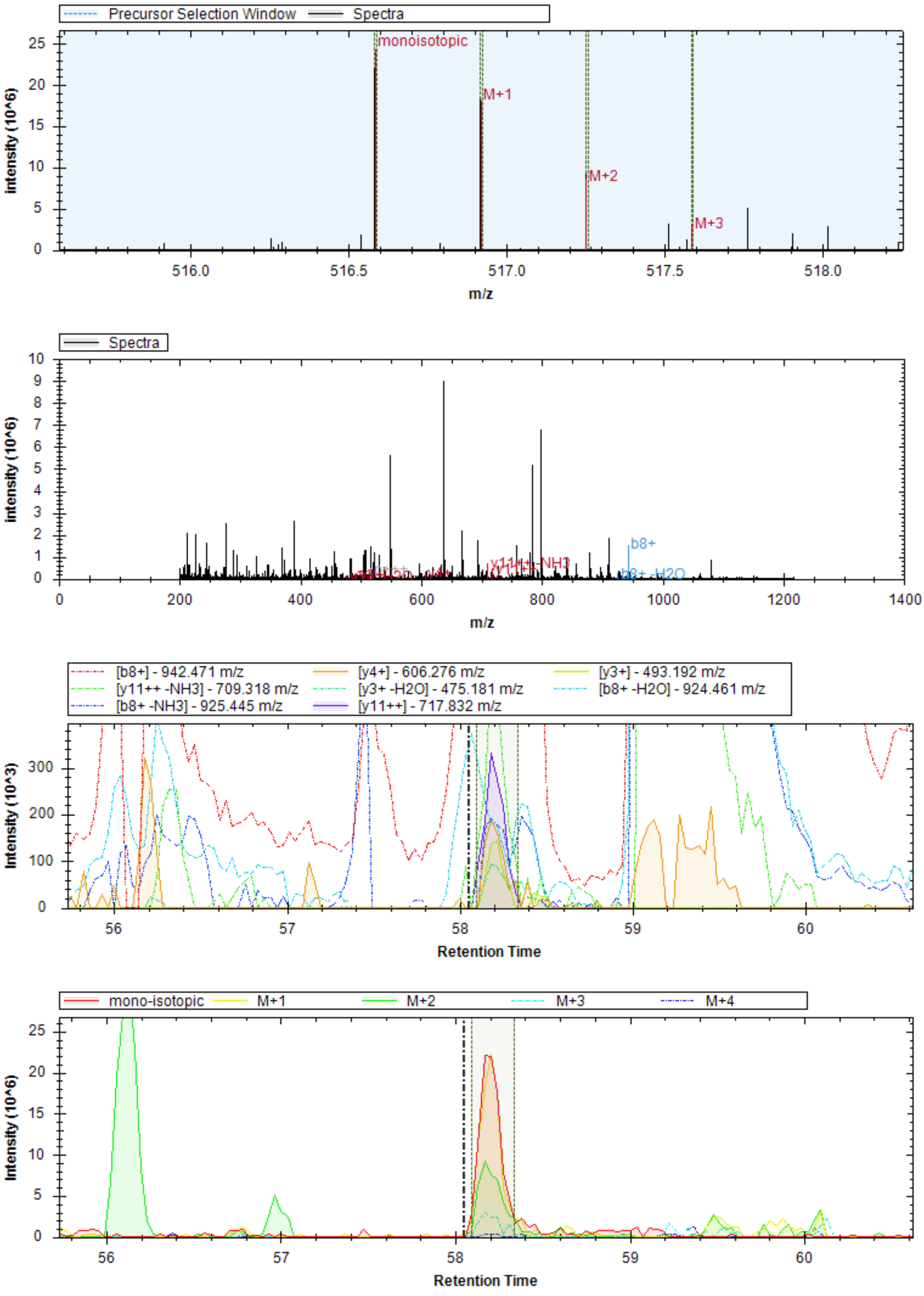

Q9QYC0 – _AAVVT[+H+O3+P]SPPPTTAPHK_.3
Fig4_MP-DIA-barrel-cortex-p30-1_MHRM.htrms

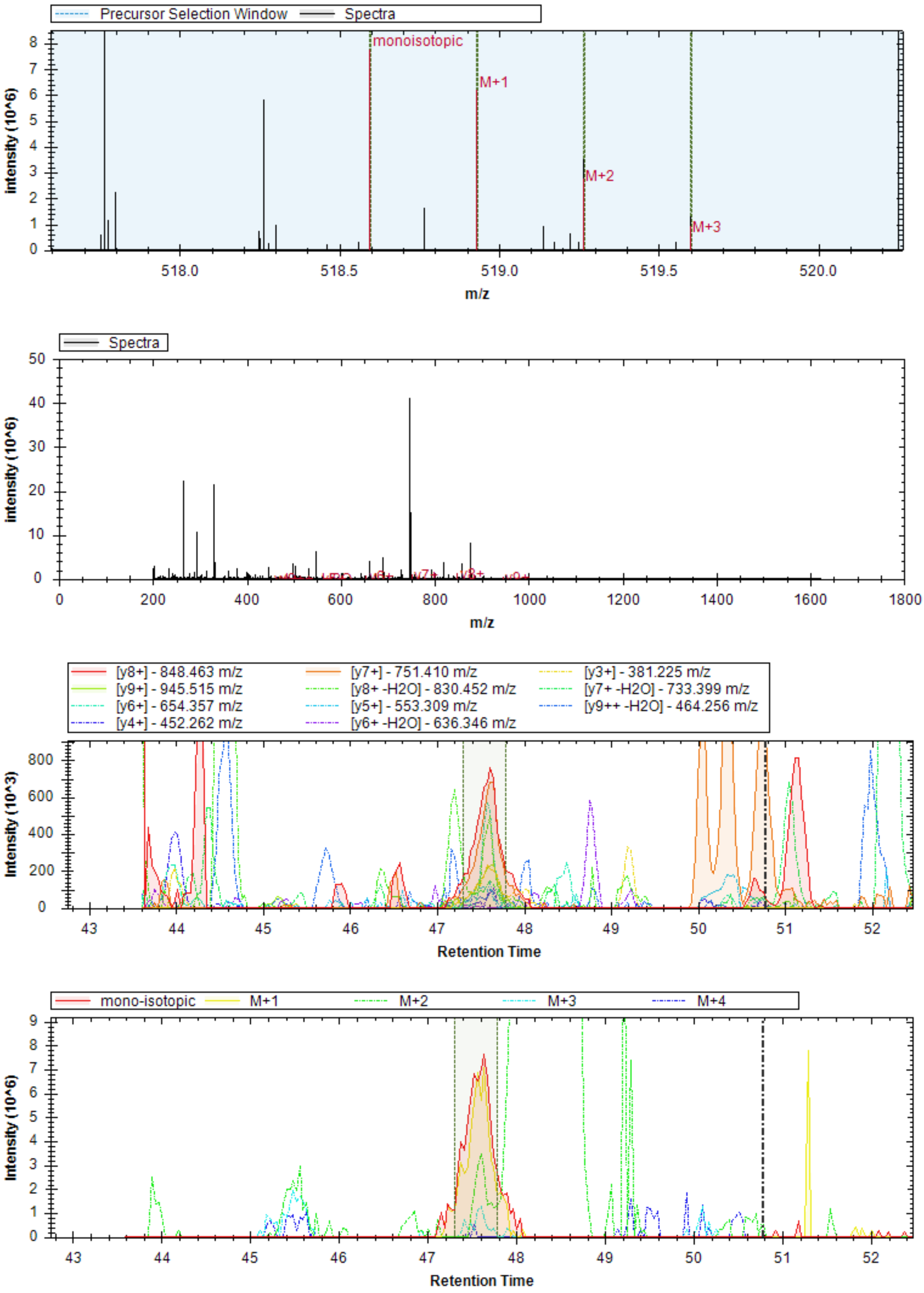

Q9QYC0 – _AAVVTS[+H+O3+P]PPPTTAPHK_.3
Fig4_MP-DIA-barrel-cortex-p30-1_MHRM.htrms

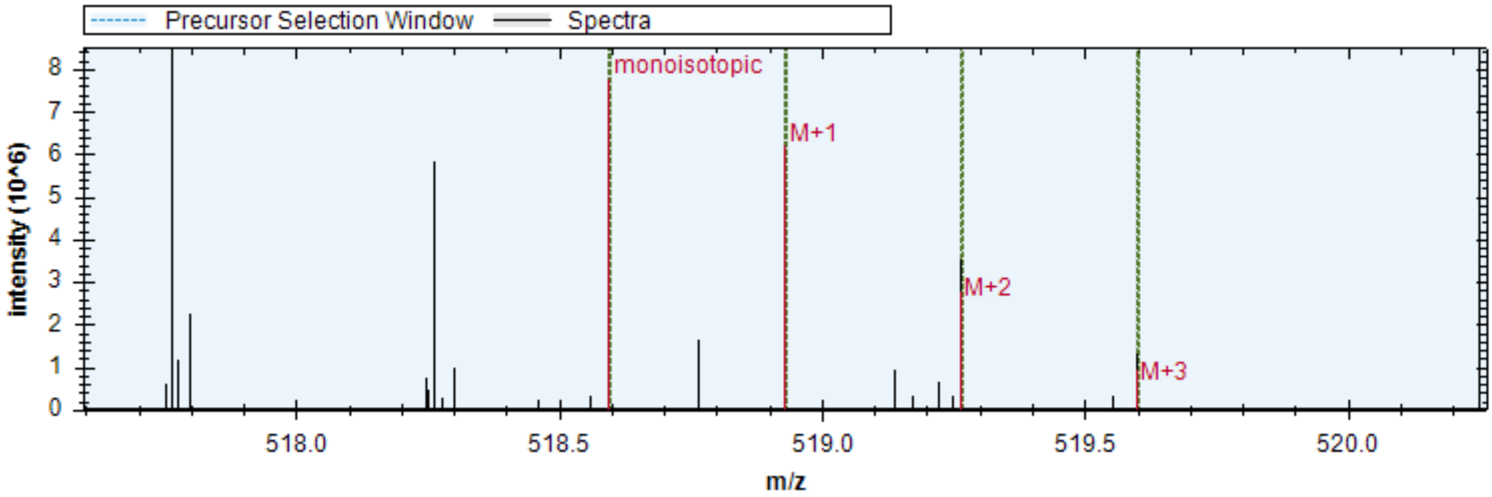

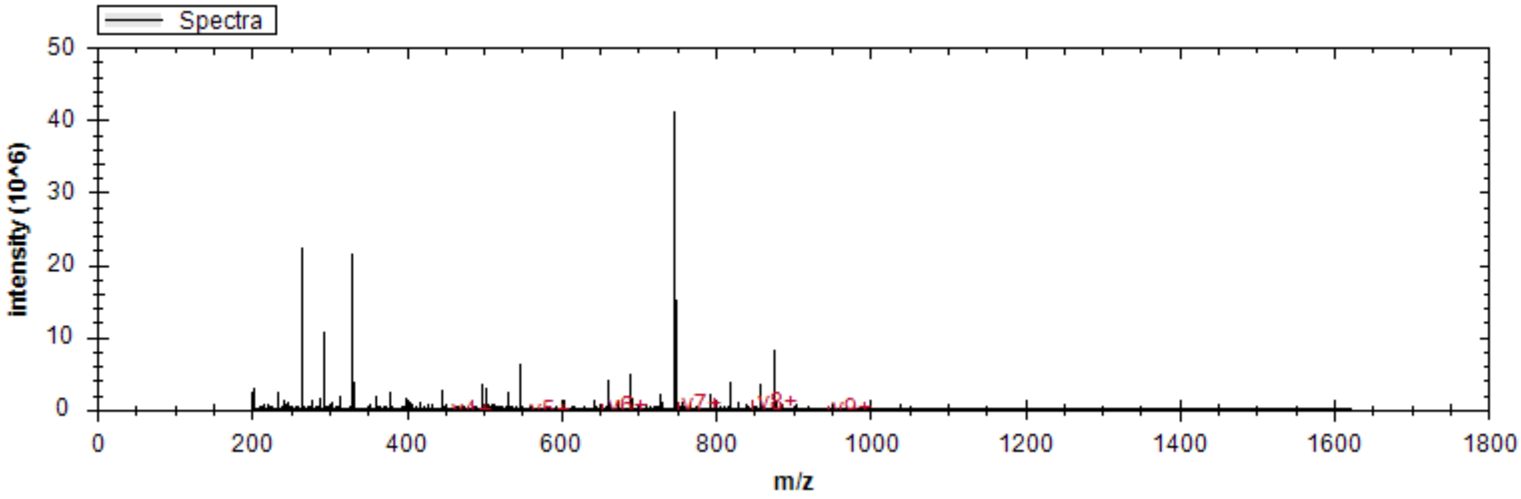

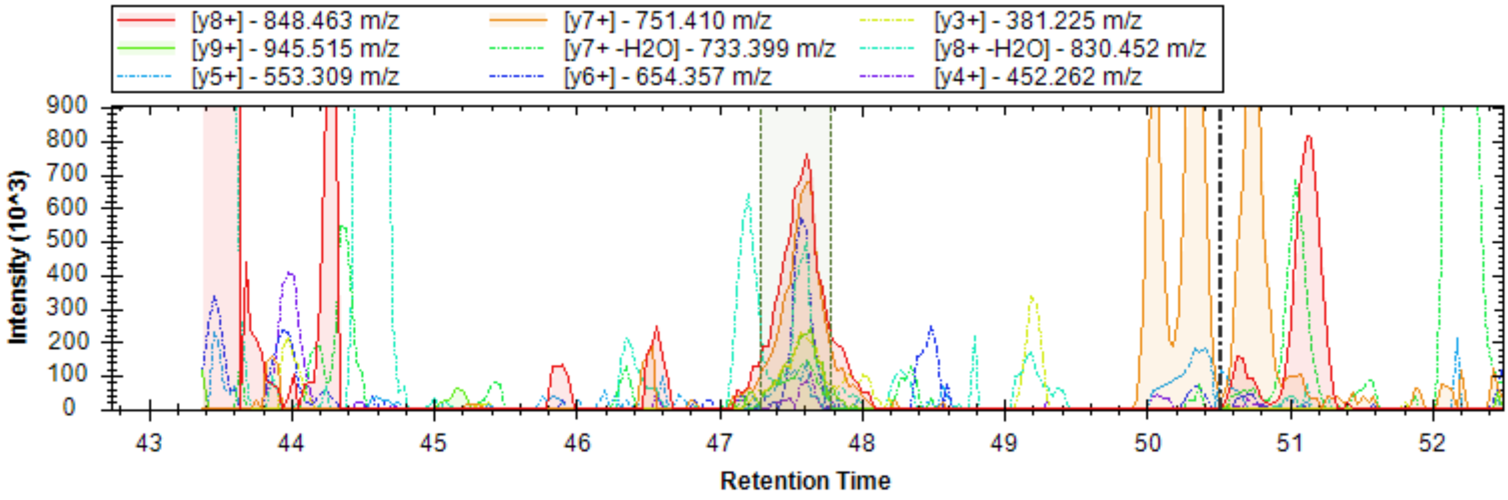

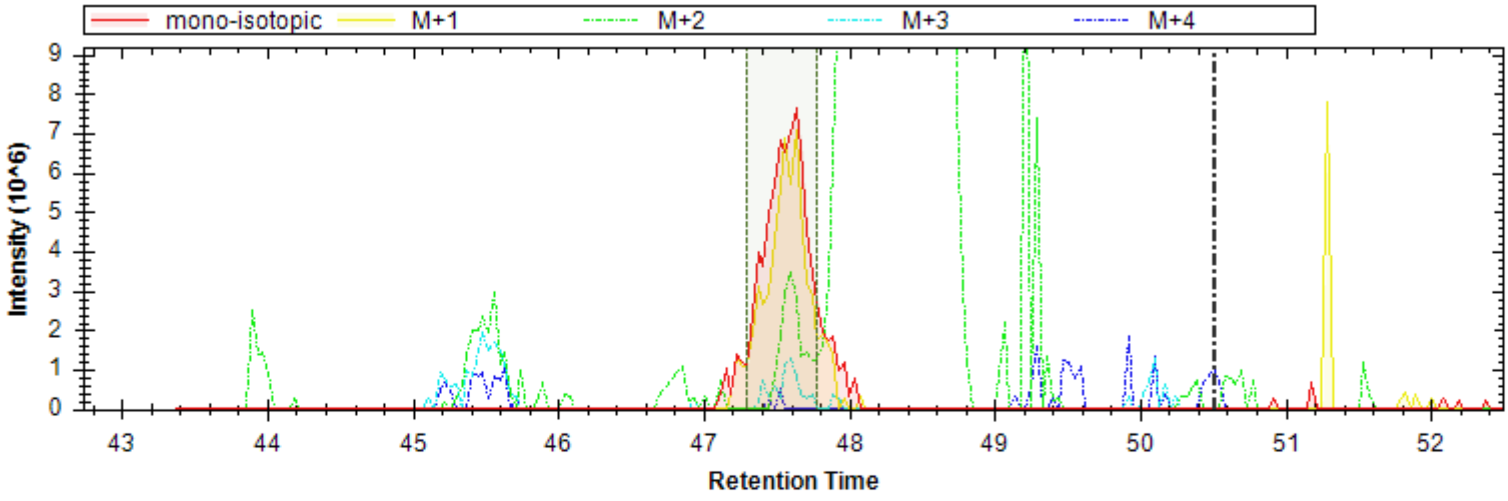

P10637 – _VAVVRT[+H+O3+P]PPKSPSASK_.3

Fig4_MP-DIA-barrel-cortex-p30-1_MHRM.htrms

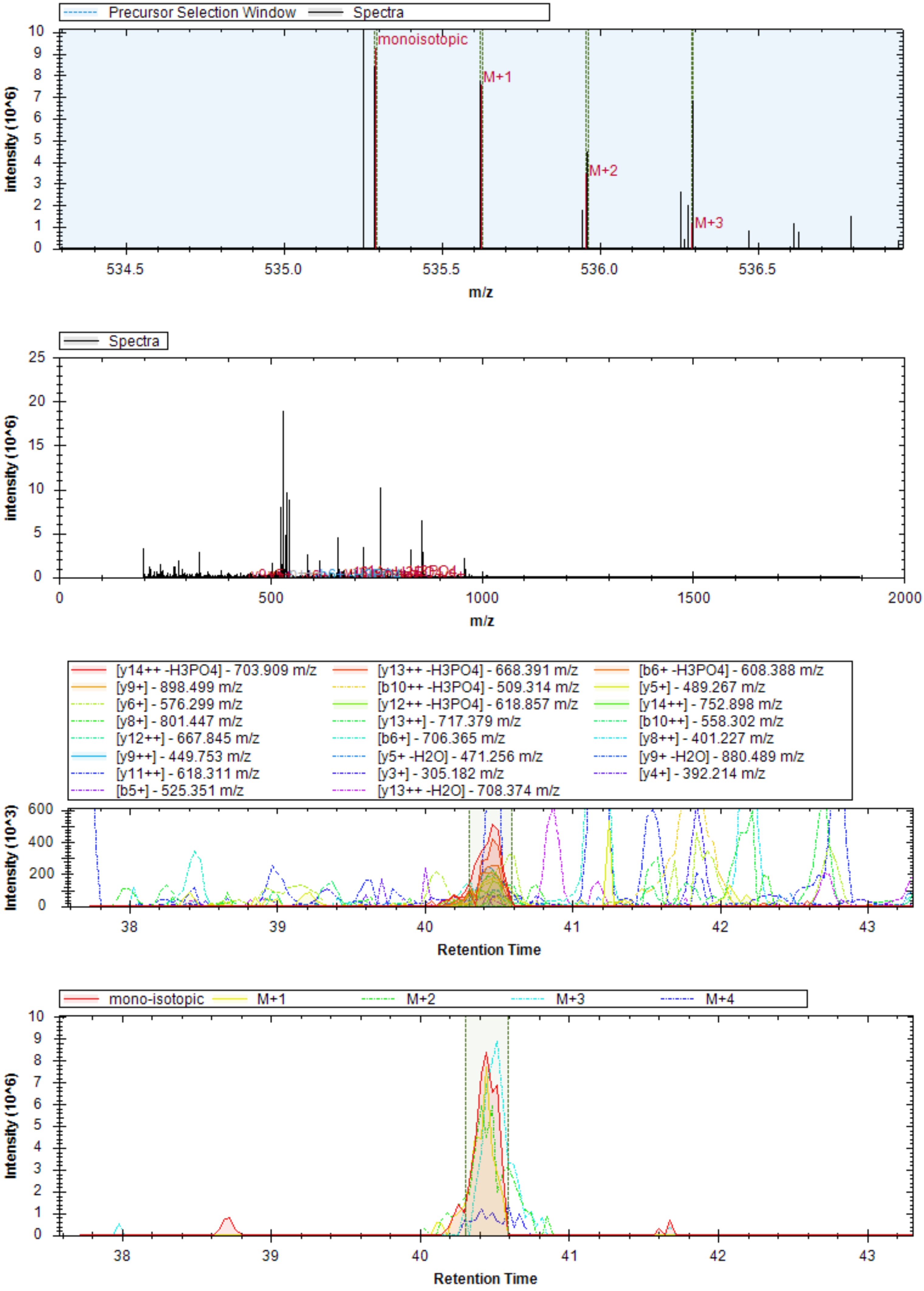

Q7TQD2 – _AANKT[+H+O3+P]PPKSPGDPAR_.3
Fig4_MP-DIA-barrel-cortex-p30-1_MHRM.htrms

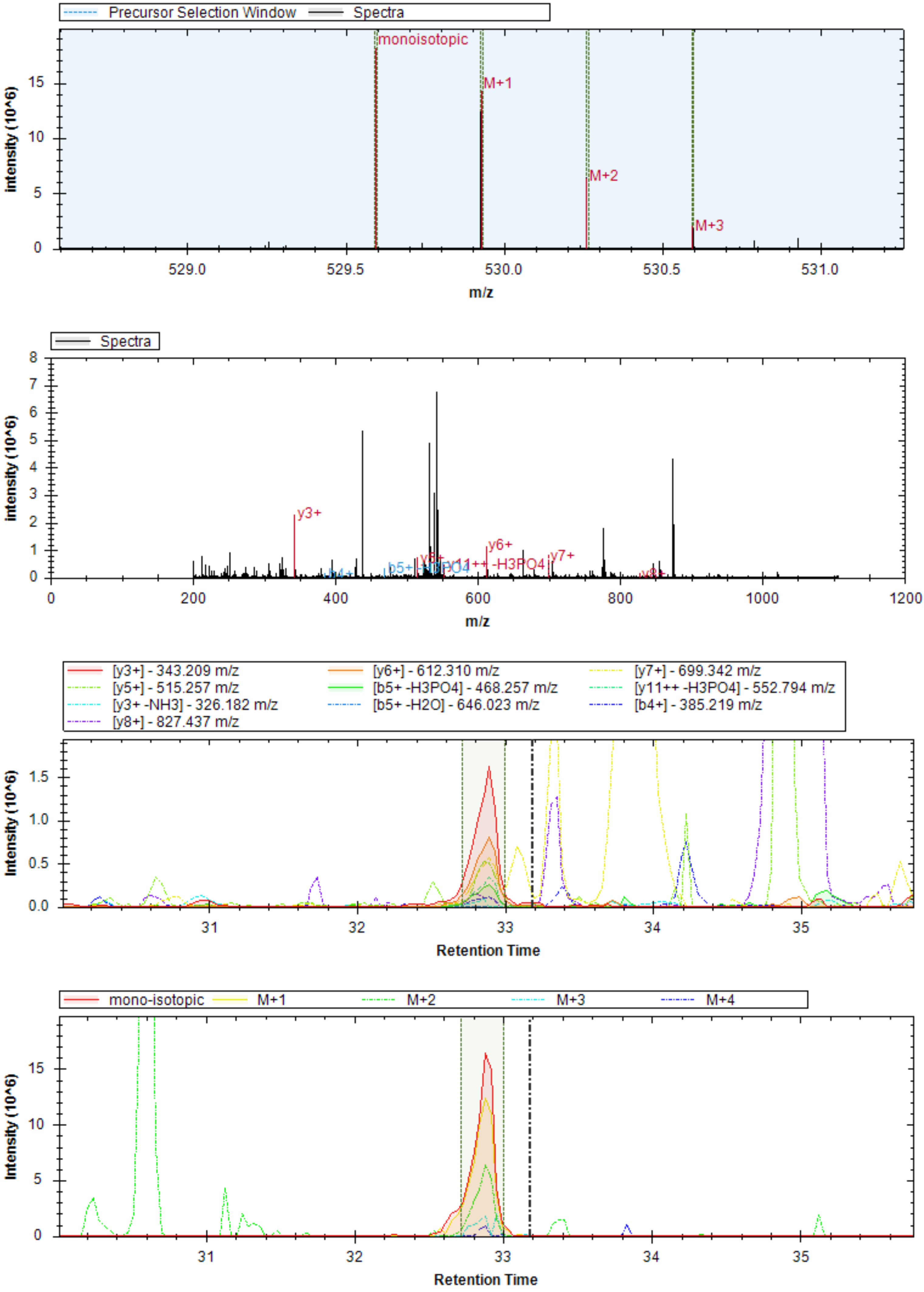

P20357 – _VAIIRT[+H+O3+P]PPKSPATPK_.3
Fig4_MP-DIA-barrel-cortex-p30-1_MHRM.htrms

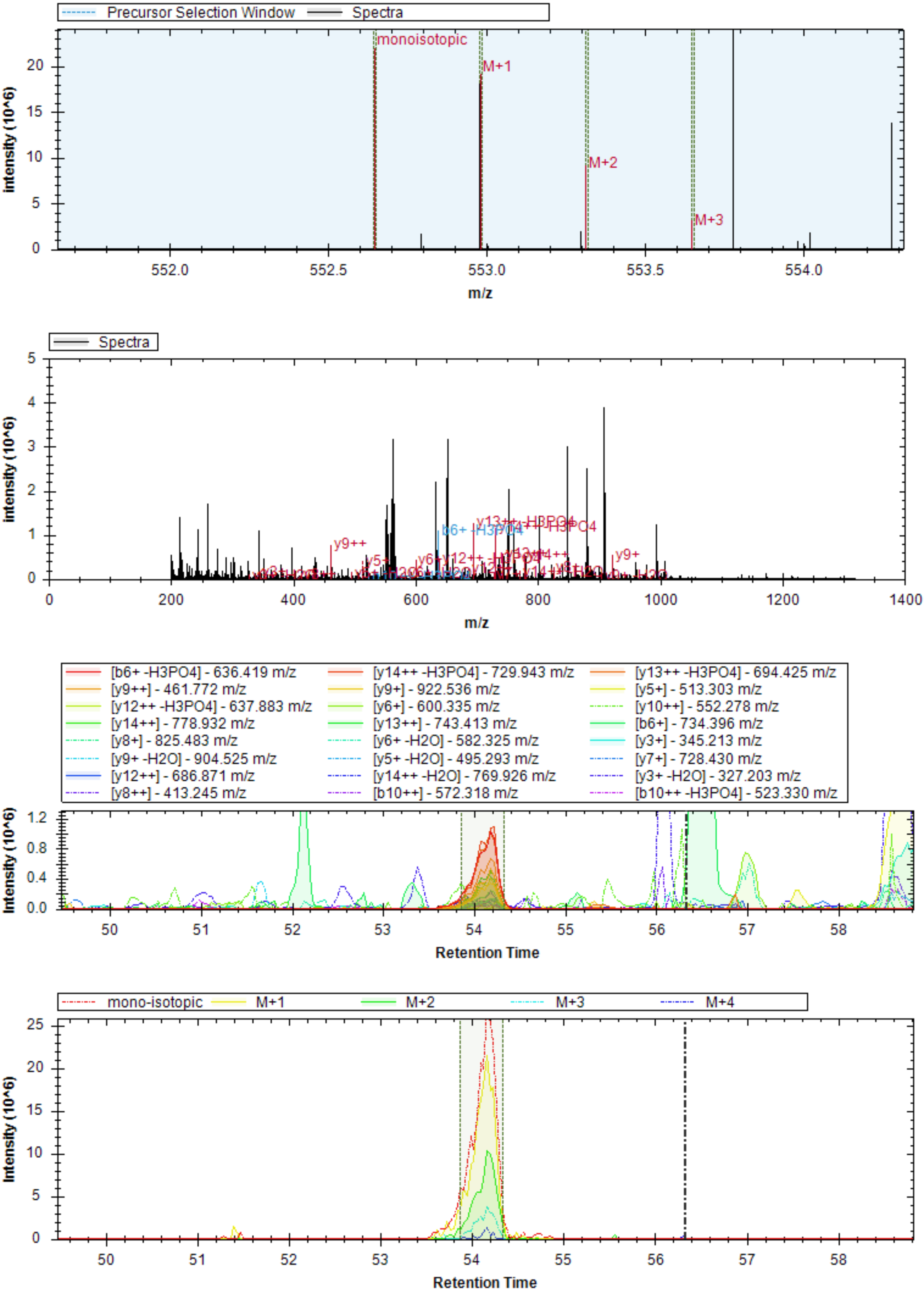

Q9CYT6 – _THTPSPTS[+H+O3+P]PK_.2

Fig4_MP-DIA-barrel-cortex-p30-1_MHRM.htrms

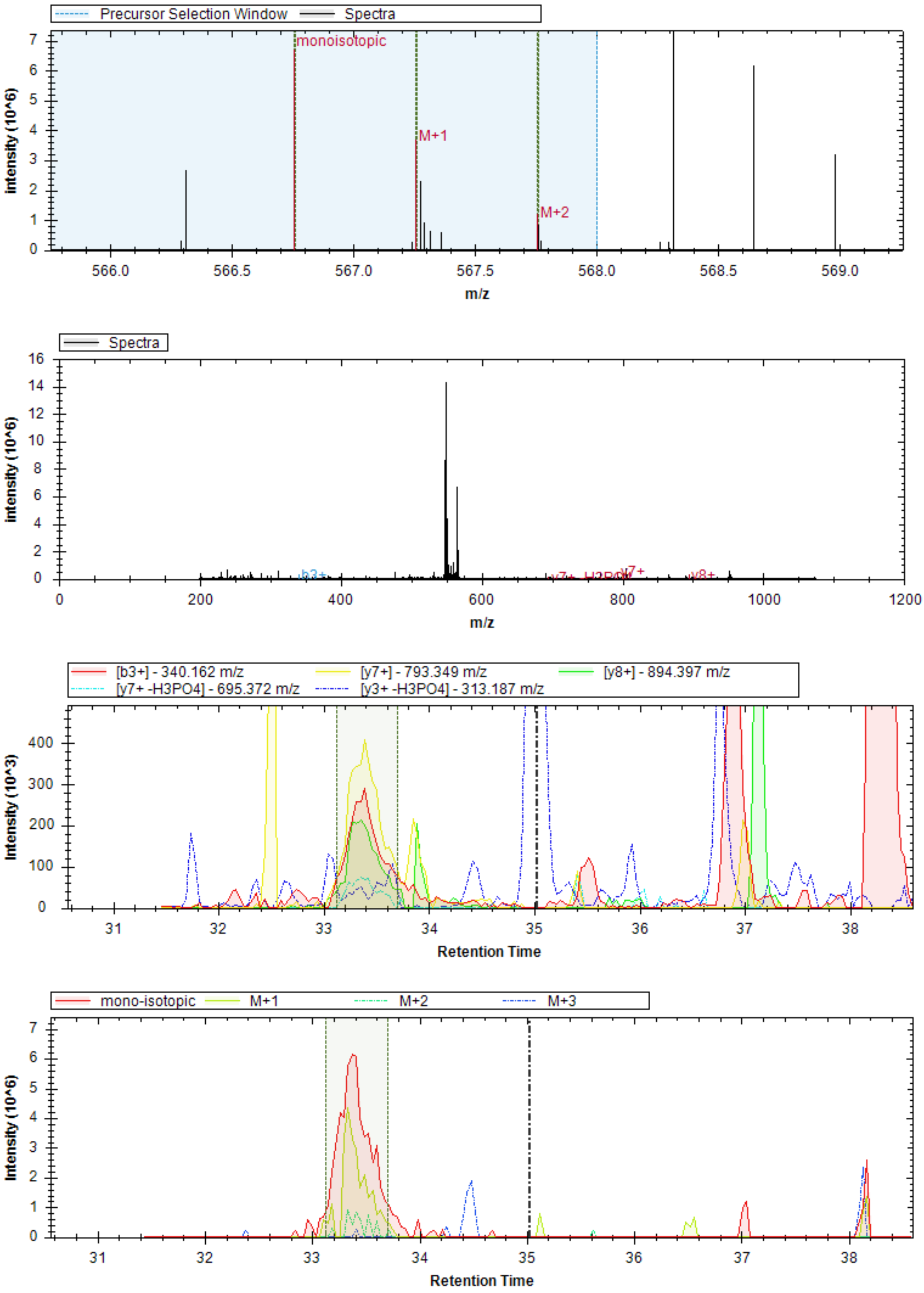

Q9R0K7 – _T[+H+O3+P]SIHNFMAHPEFR_.3
Fig4_MP-DIA-barrel-cortex-p30-1_MHRM.htrms

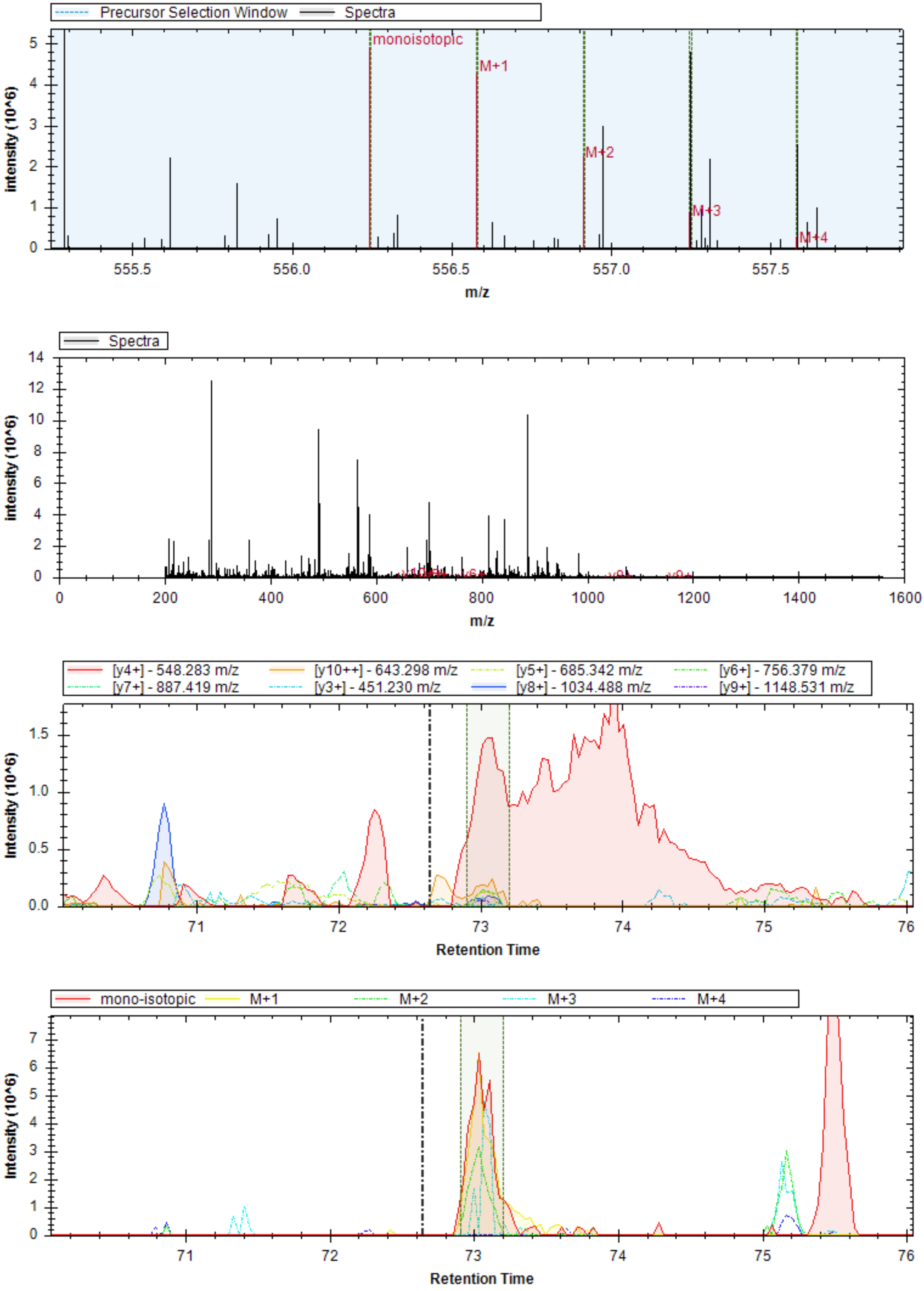

O88935 – _QAS[+H+O3+P]ISGPAPTK_.2
Fig4_MP-DIA-barrel-cortex-p30-1_MHRM.htrms

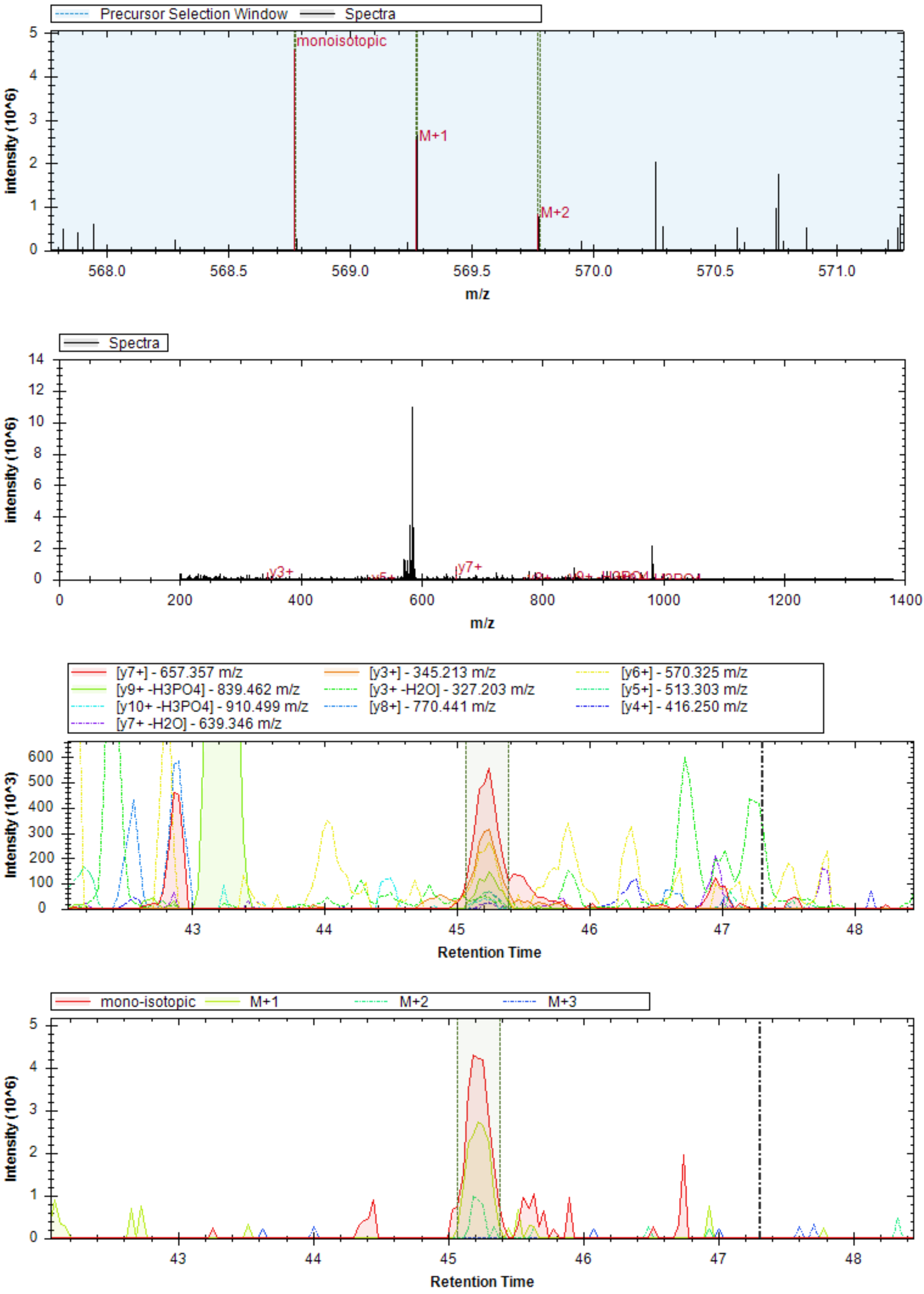

P27546-2 – _T[+H+O3+P]SPSKPSSAPALKPGPK_.3
Fig4_MP-DIA-barrel-cortex-p30-1_MHRM.htrms

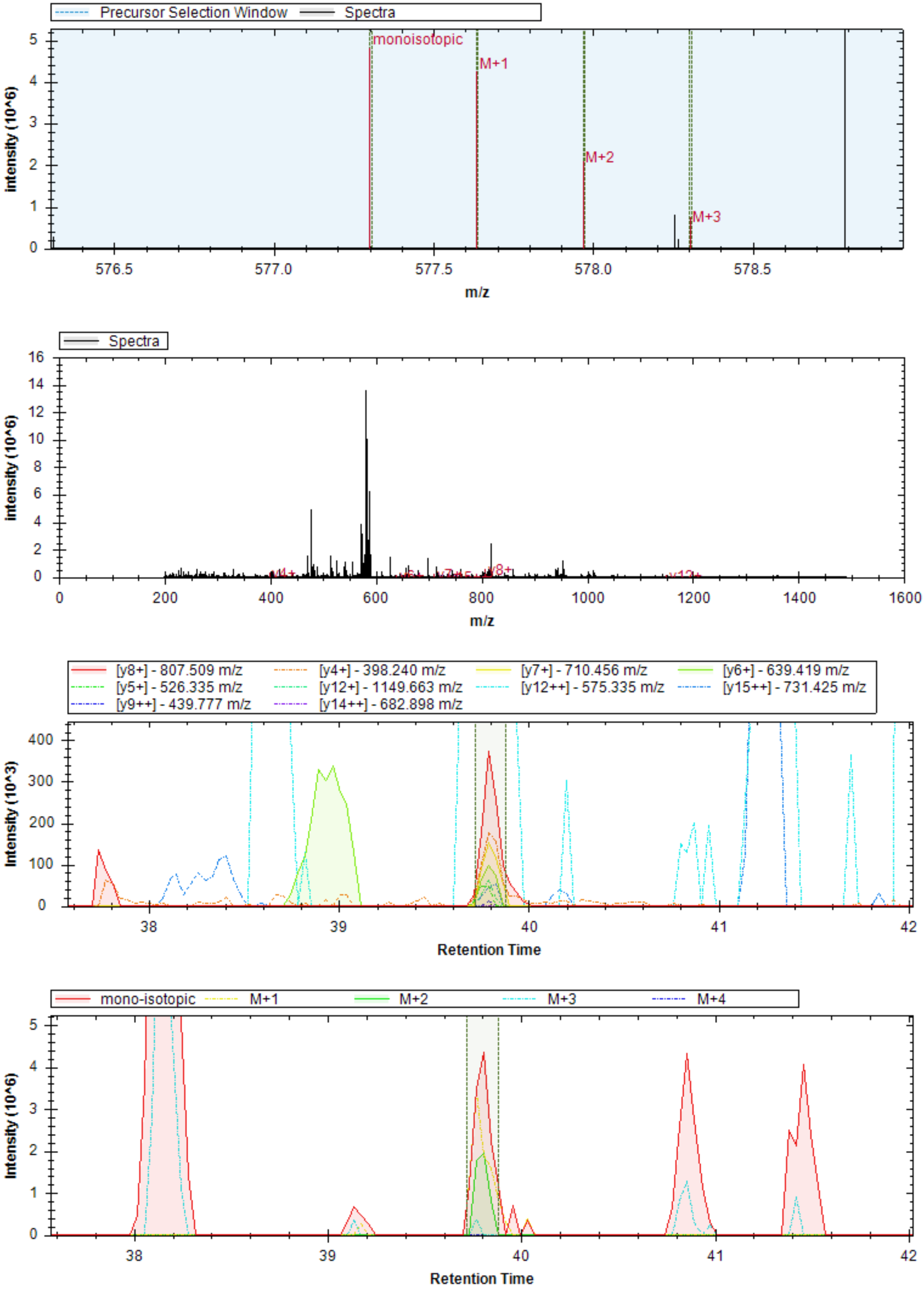

Q8BFU3-4 – _VAS[+H+O3+P]STQVRGASGRELR_.3

Fig4_MP-DIA-barrel-cortex-p30-1_MHRM.htrms

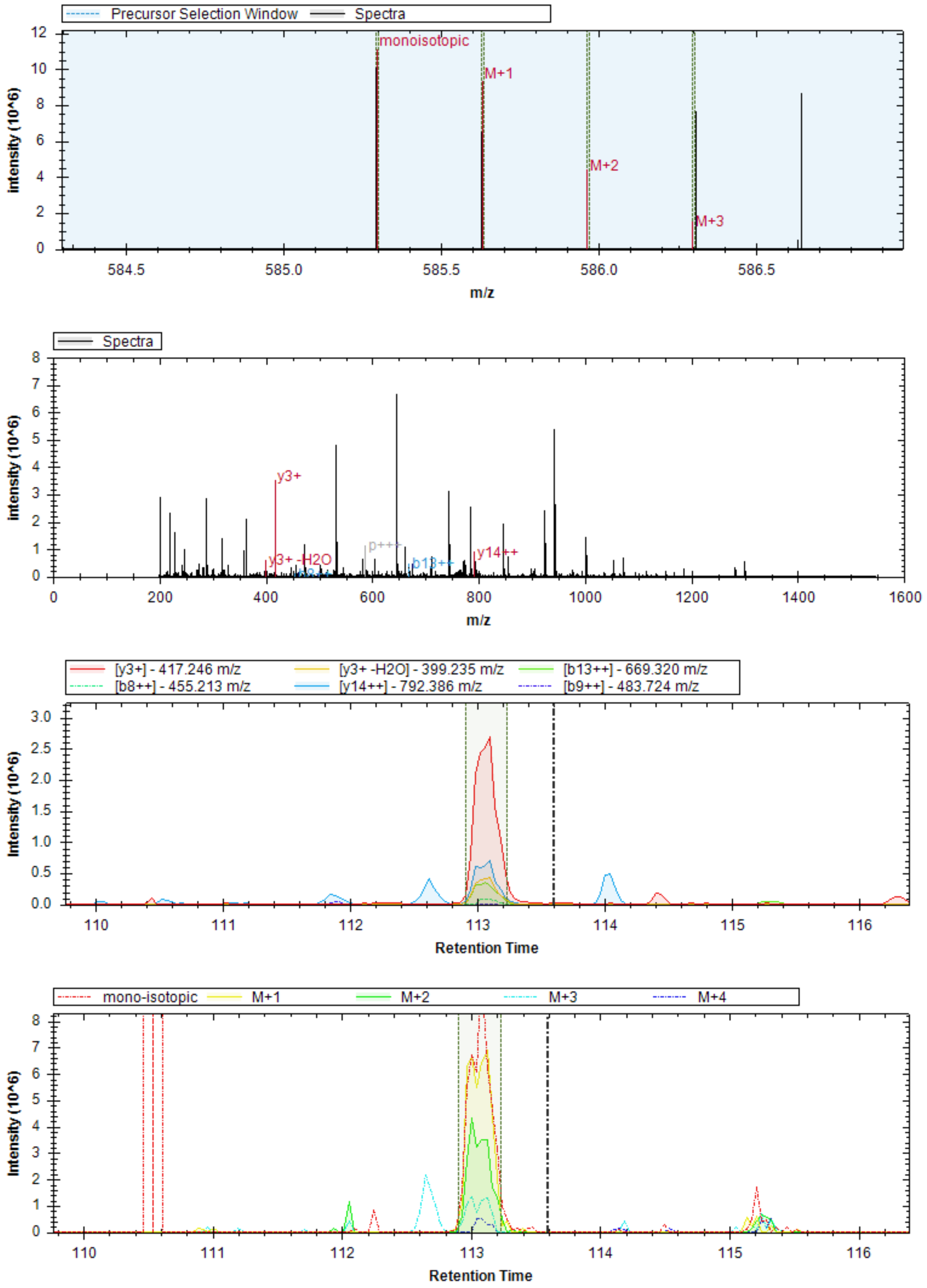

Q8BTI8 – _RVPS[+H+O3+P]PTPVPK_.2
Fig4_MP-DIA-barrel-cortex-p30-1_MHRM.htrms

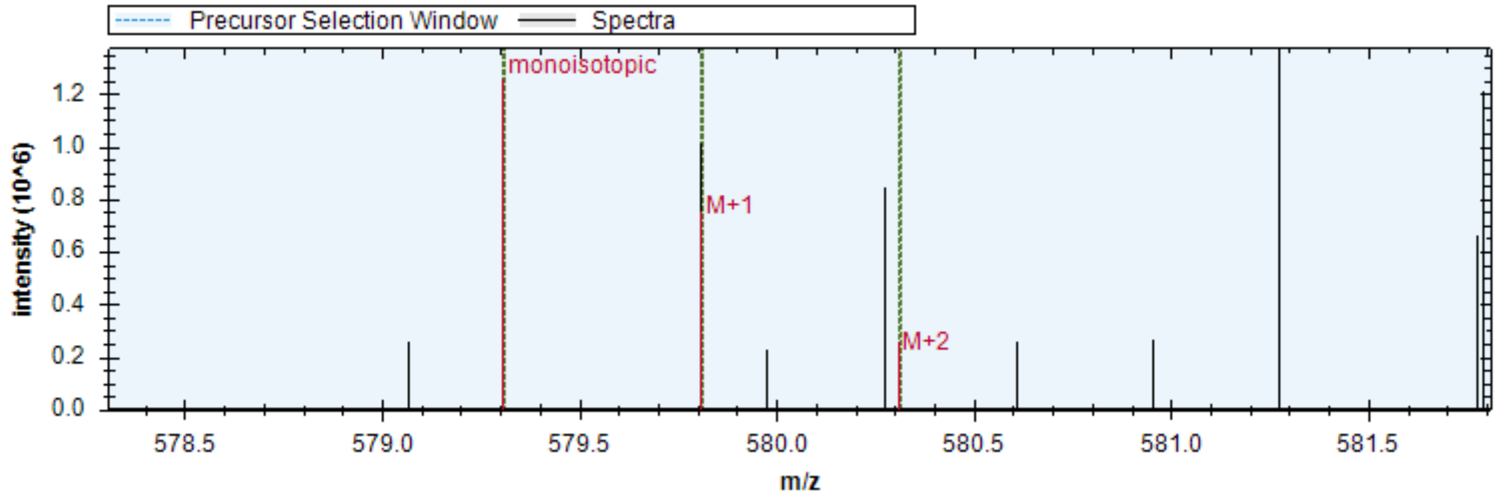

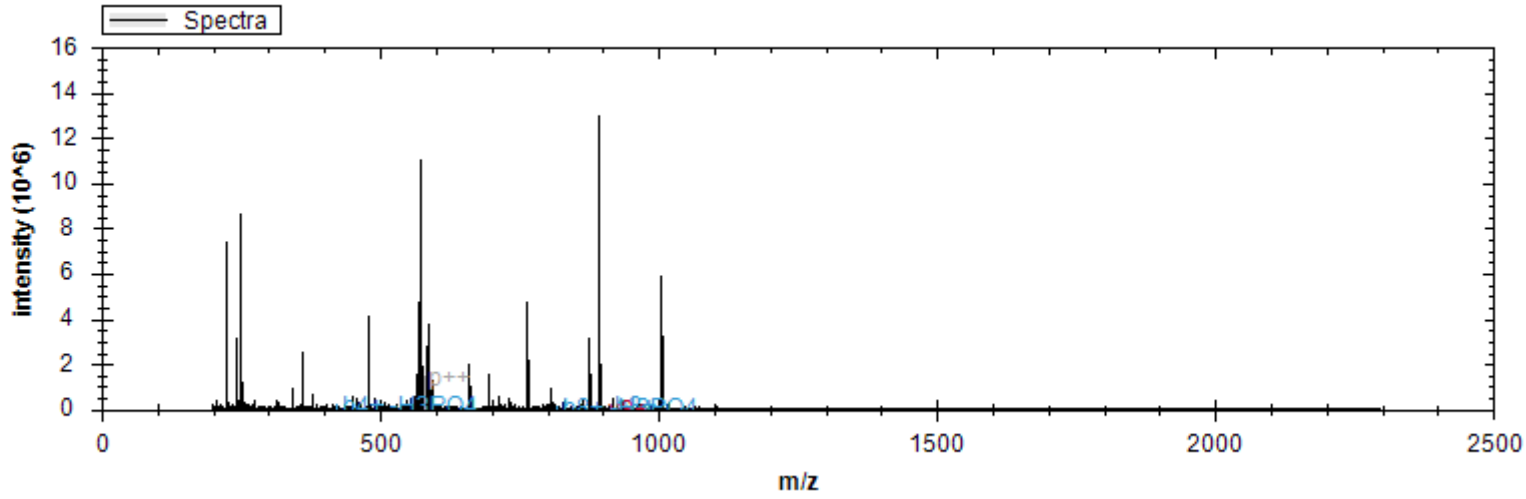

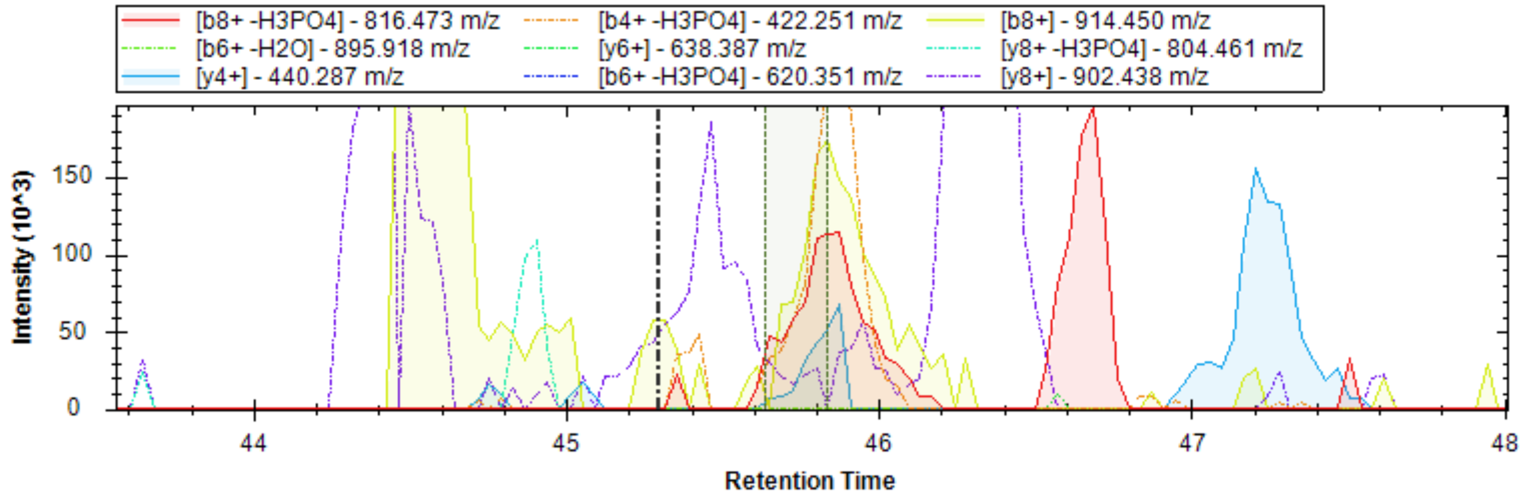

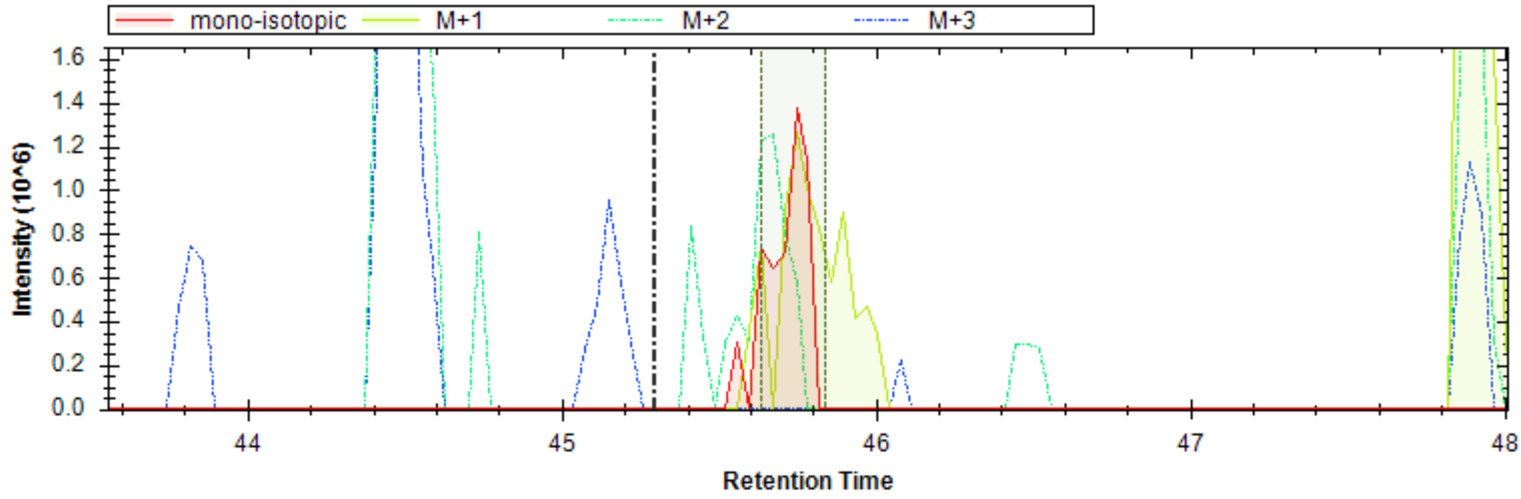

Q9CYT6 – _AQGQIRS[+H+O3+P]PTK_.2

Fig4_MP-DIA-barrel-cortex-p30-1_MHRM.htrms

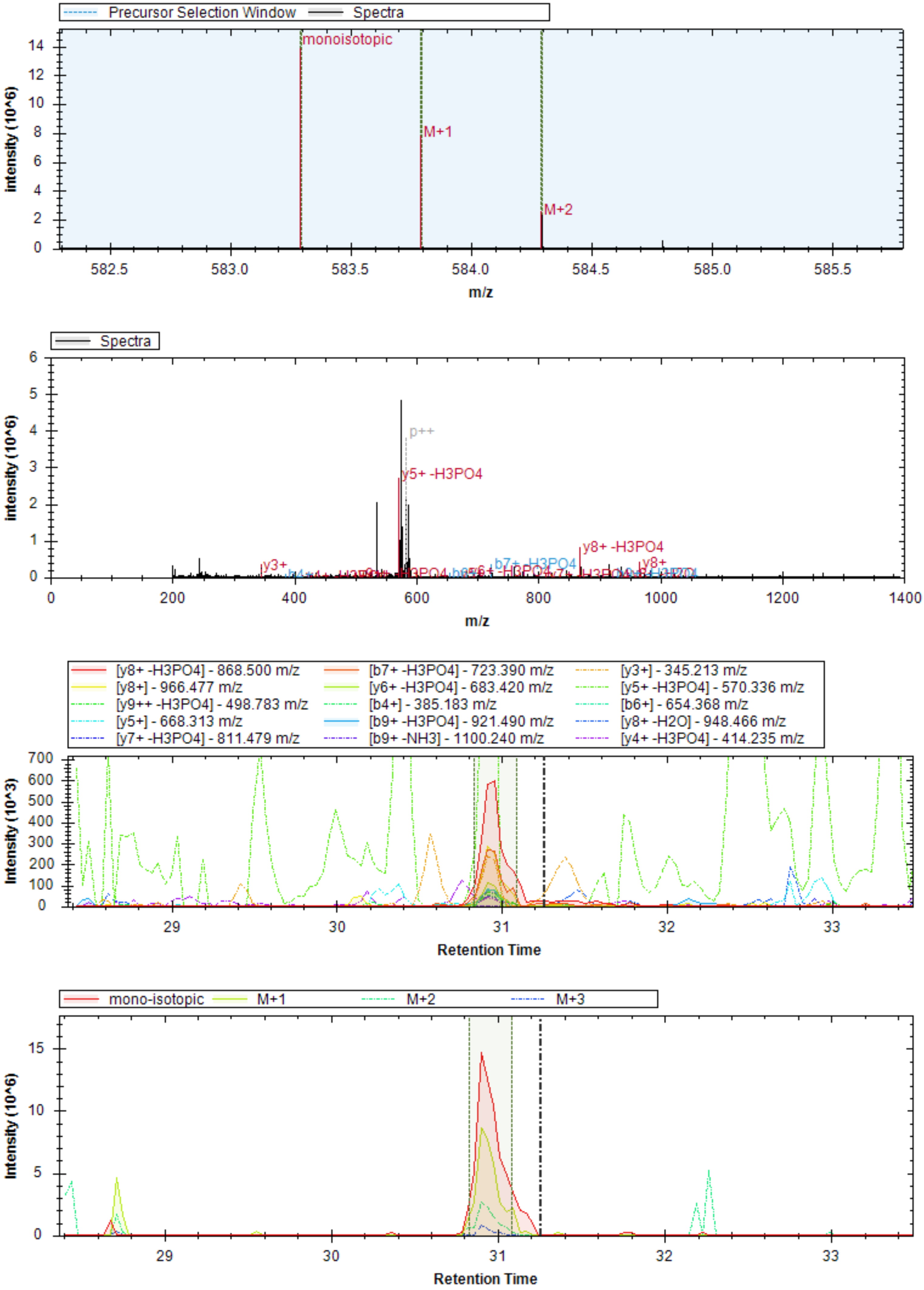

Q9D5Y0 – _ITDT[+H+O3+P]KKTDDLYWR_.3
Fig4_MP-DIA-barrel-cortex-p30-1_MHRM.htrms

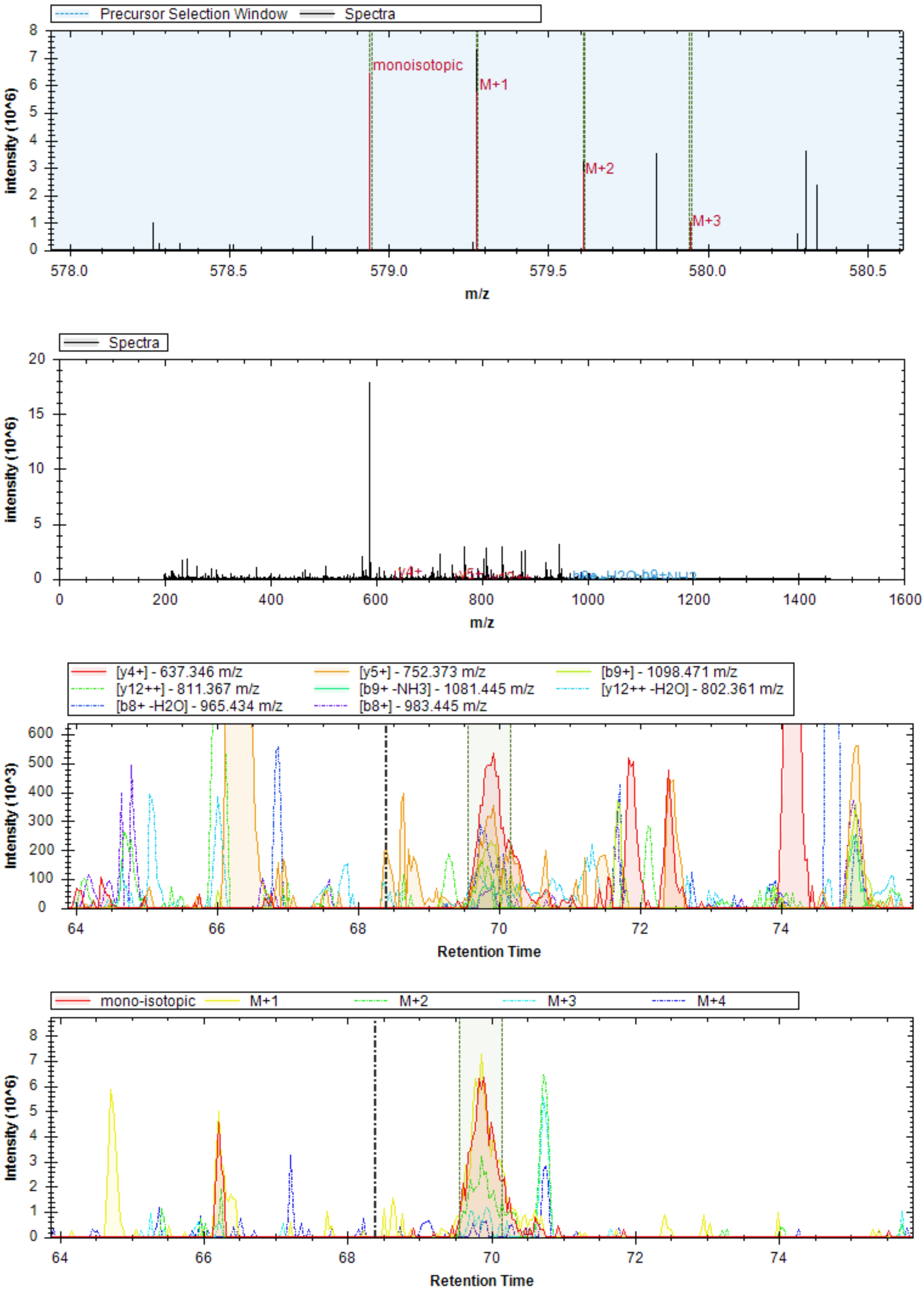

P04370-4 – _TTHYGS[+H+O3+P]LPQK_.2
Fig4_MP-DIA-barrel-cortex-p30-1_MHRM.htrms

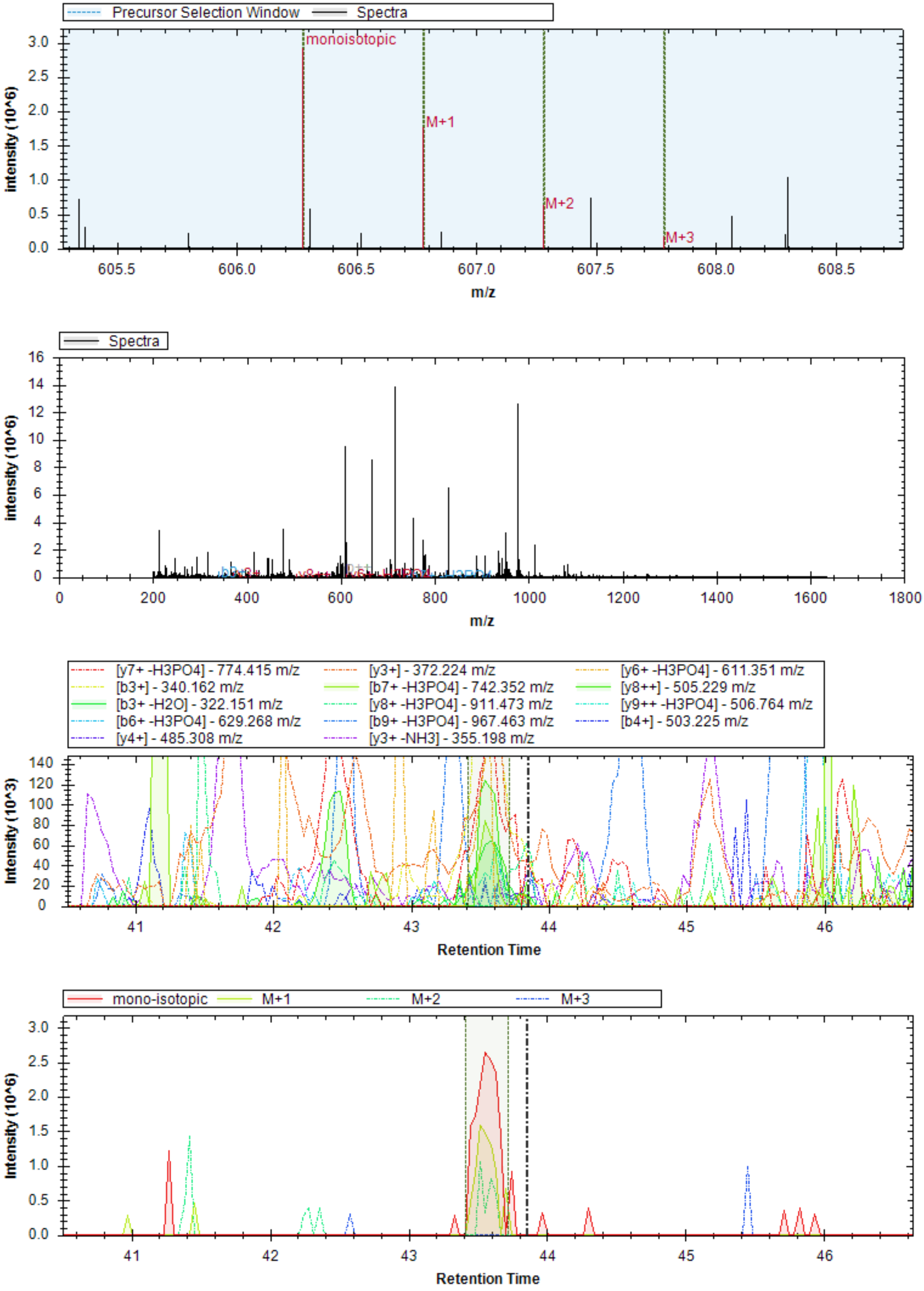

Q62261 – _RPPS[+H+O3+P]PDPNTK_.2
Fig4_MP-DIA-barrel-cortex-p30-1_MHRM.htrms

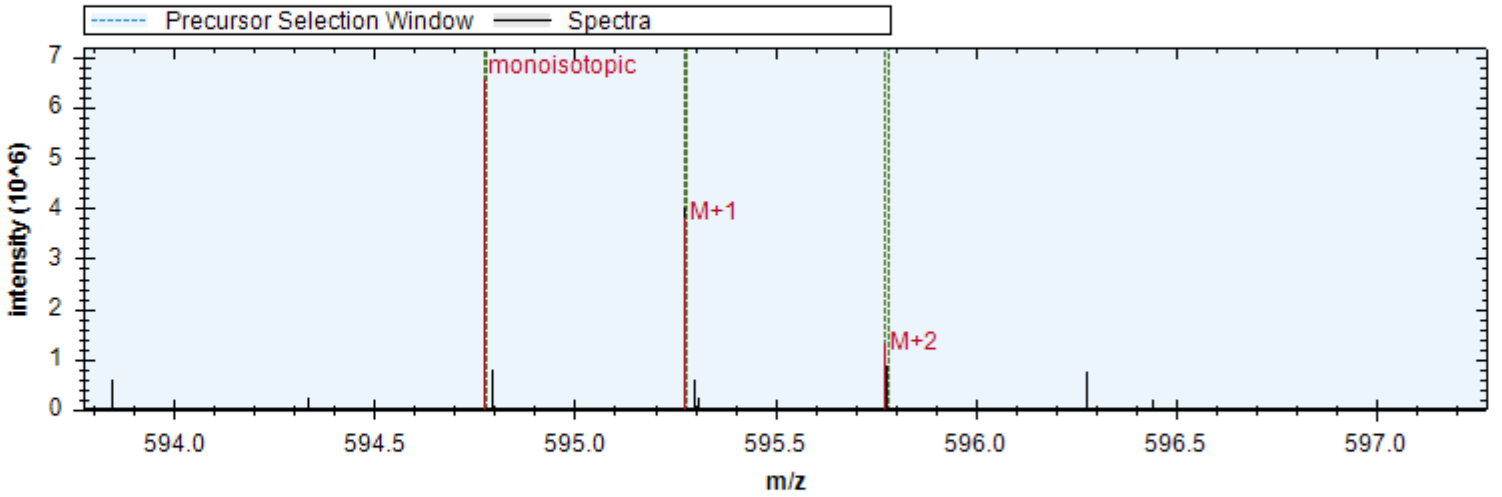

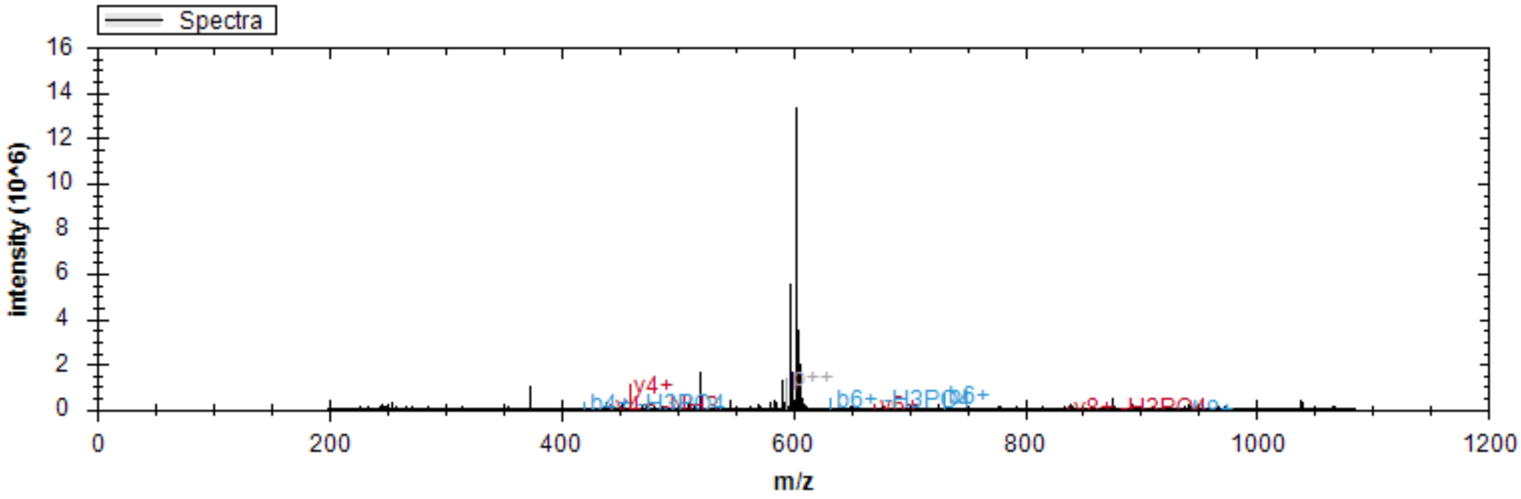

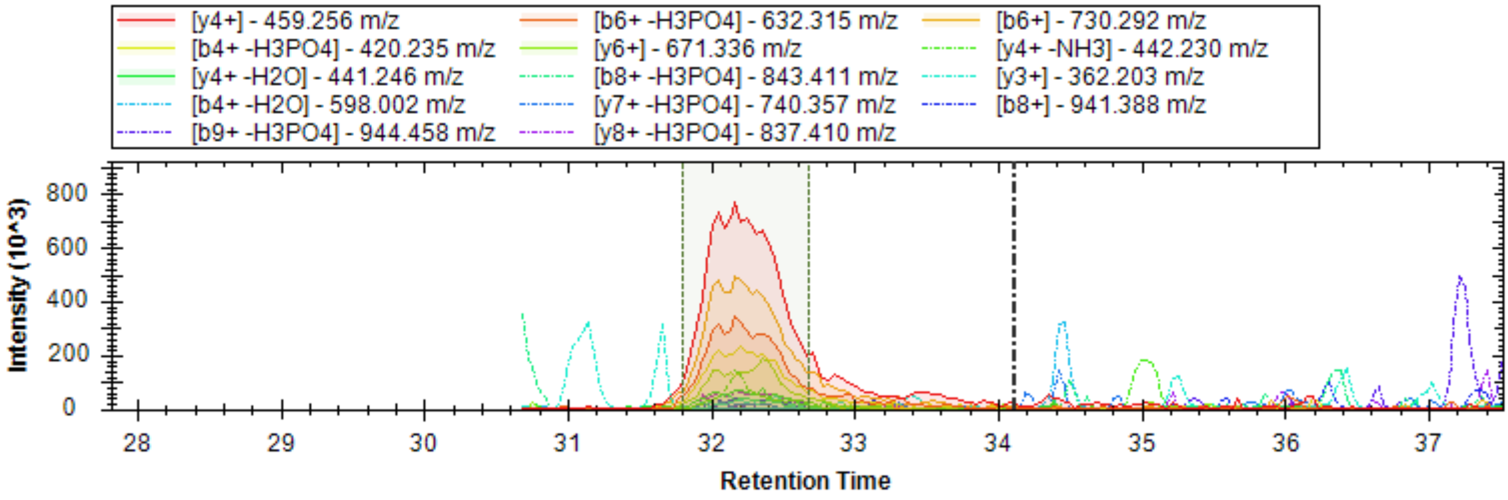

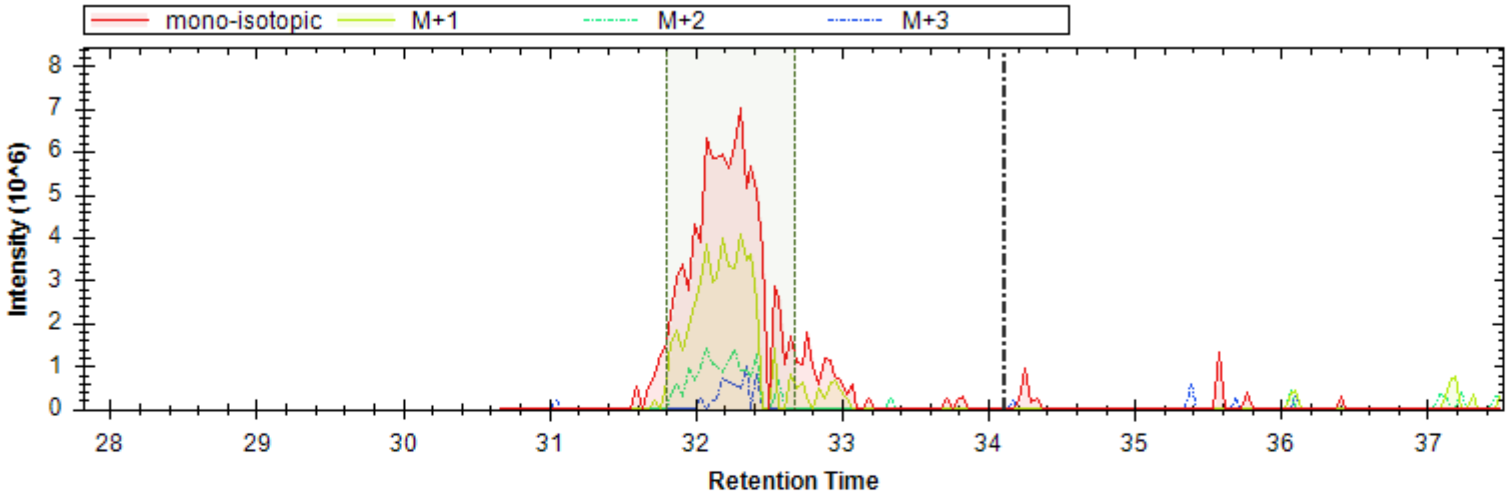

Q80VN0 – _ELTAQIT[+H+O3+P]SIK_.2
Fig4_MP-DIA-barrel-cortex-p30-1_MHRM.htrms

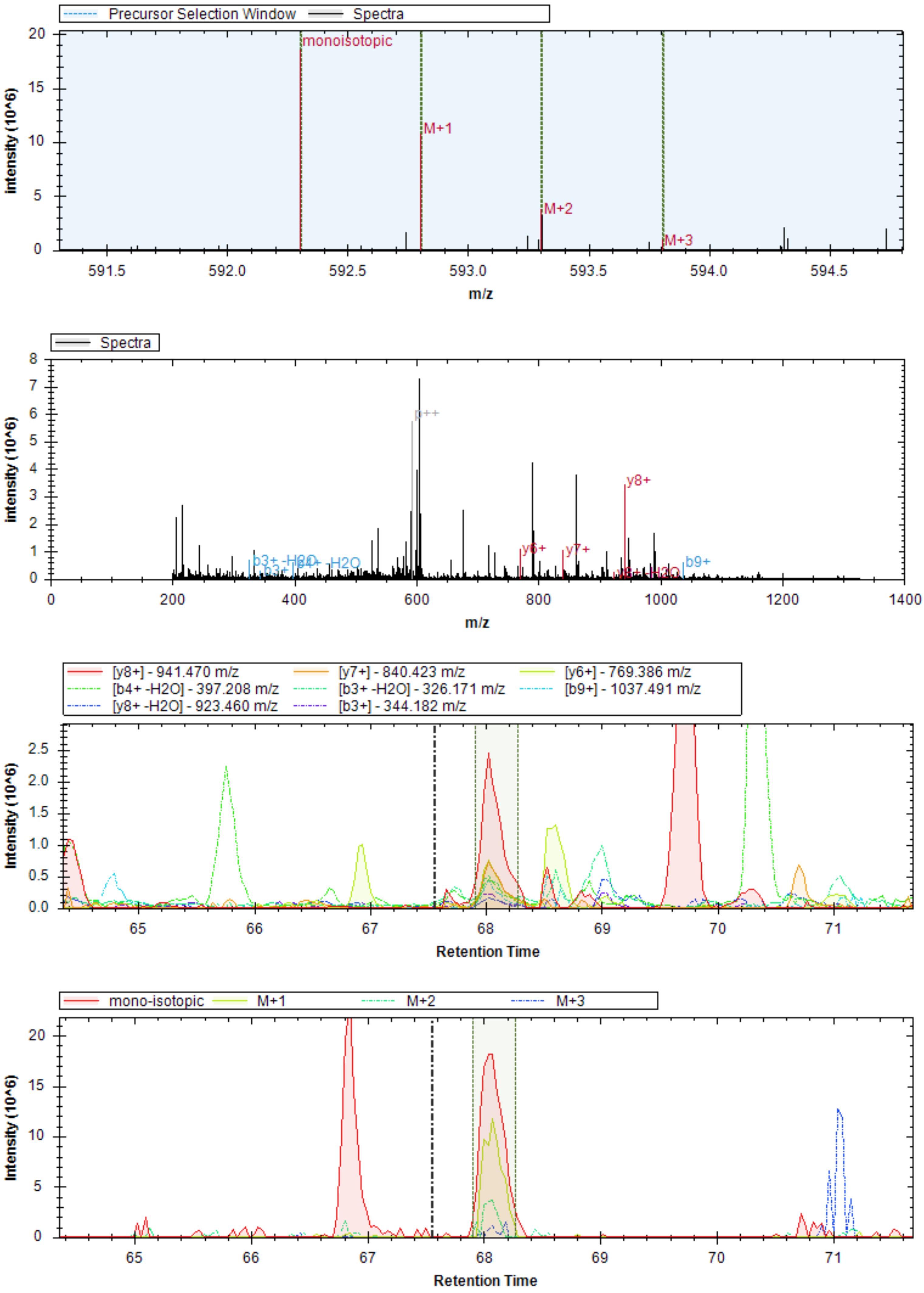

Q99104 – _AIS[+H+O3+P]PTSATSSGR_.2
Fig4_MP-DIA-barrel-cortex-p30-1_MHRM.htrms

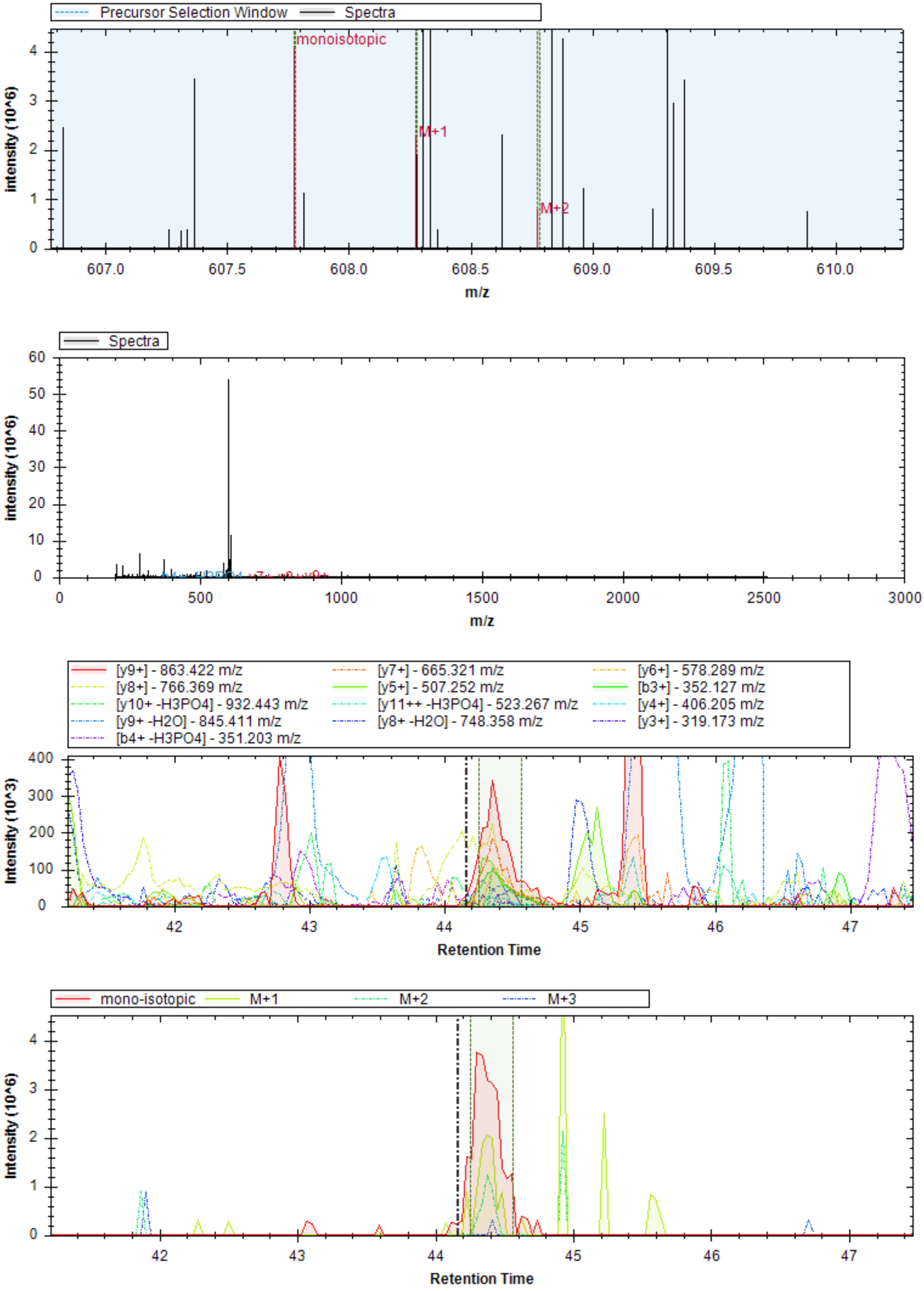

Q9QWI6 – _LSYAGGRPPSYAGS[+H+O3+P]PVHHAAER_.4

Fig4_MP-DIA-barrel-cortex-p30-1_MHRM.htrms

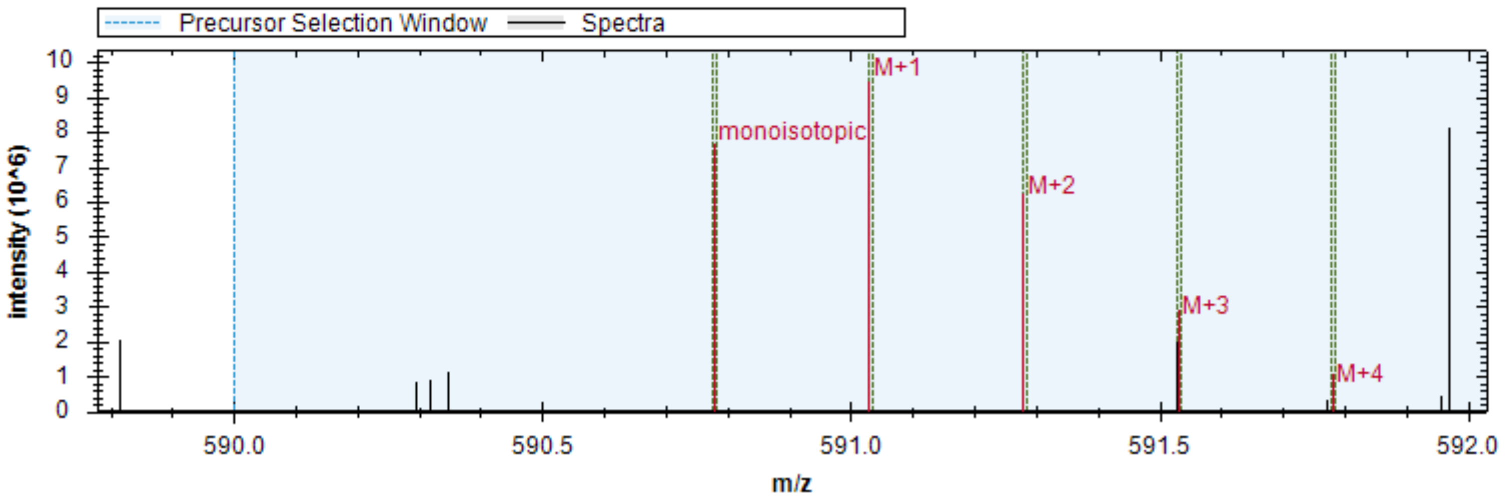

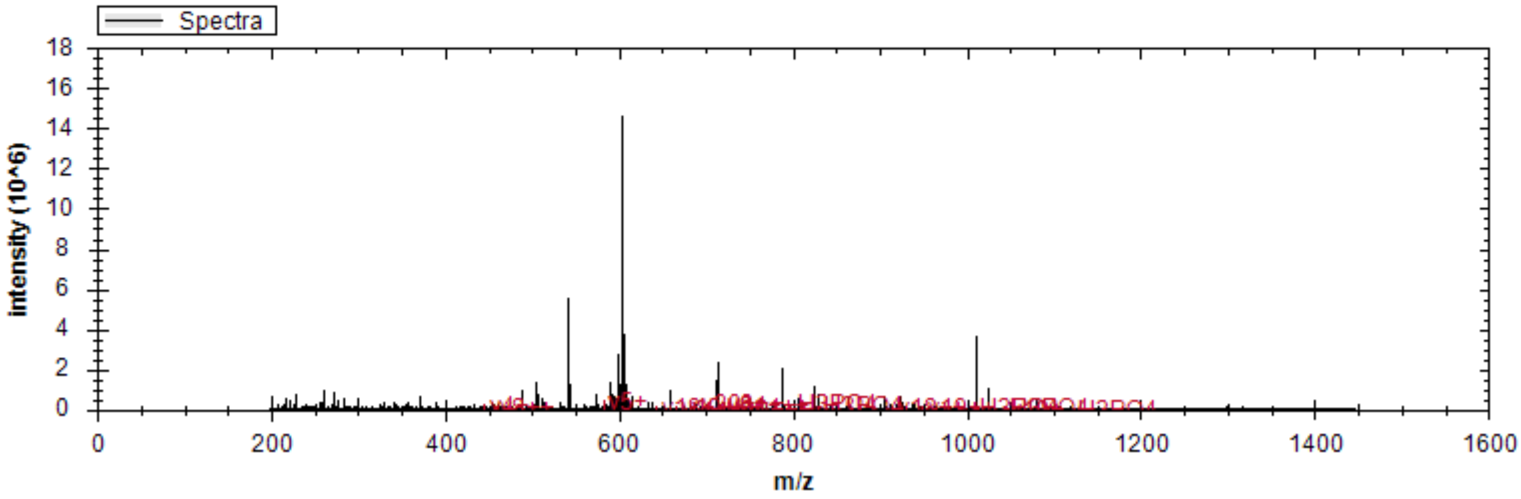

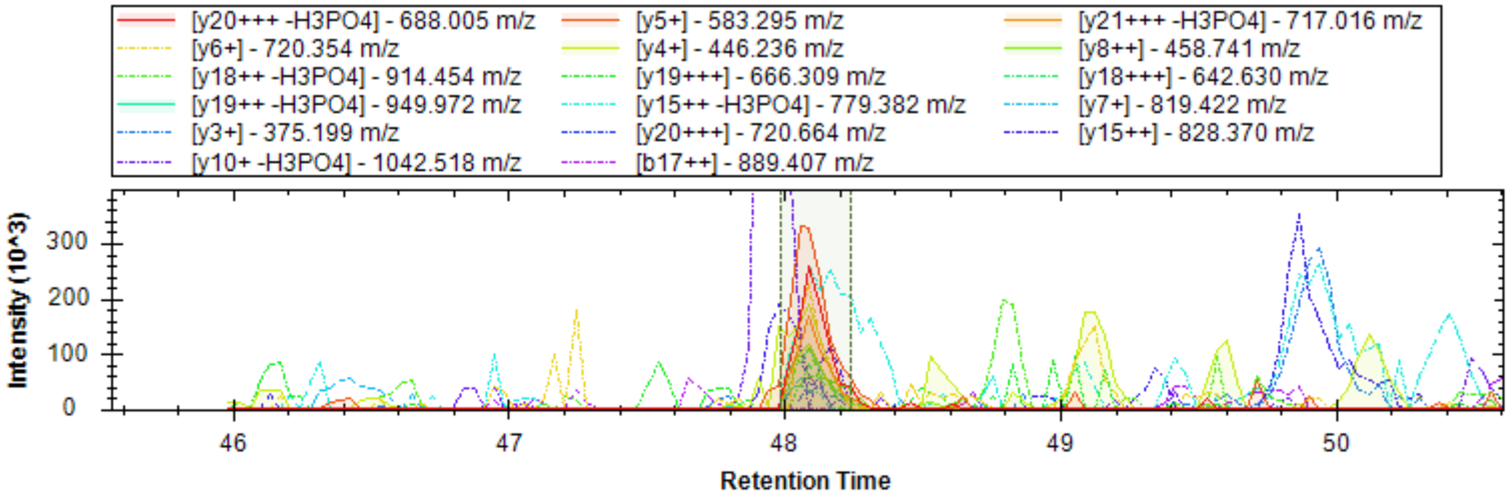

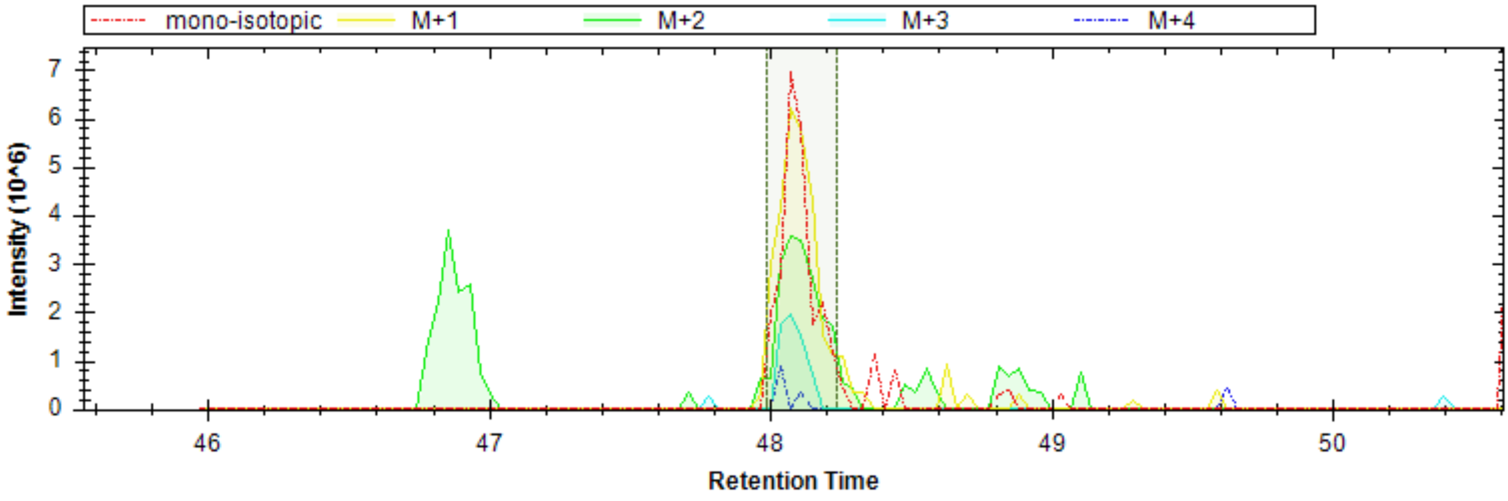

P60904 – _SLS[+H+O3+P]TSGESLYHVLGLDK_.3
Fig4_MP-DIA-barrel-cortex-p30-1_MHRM.htrms

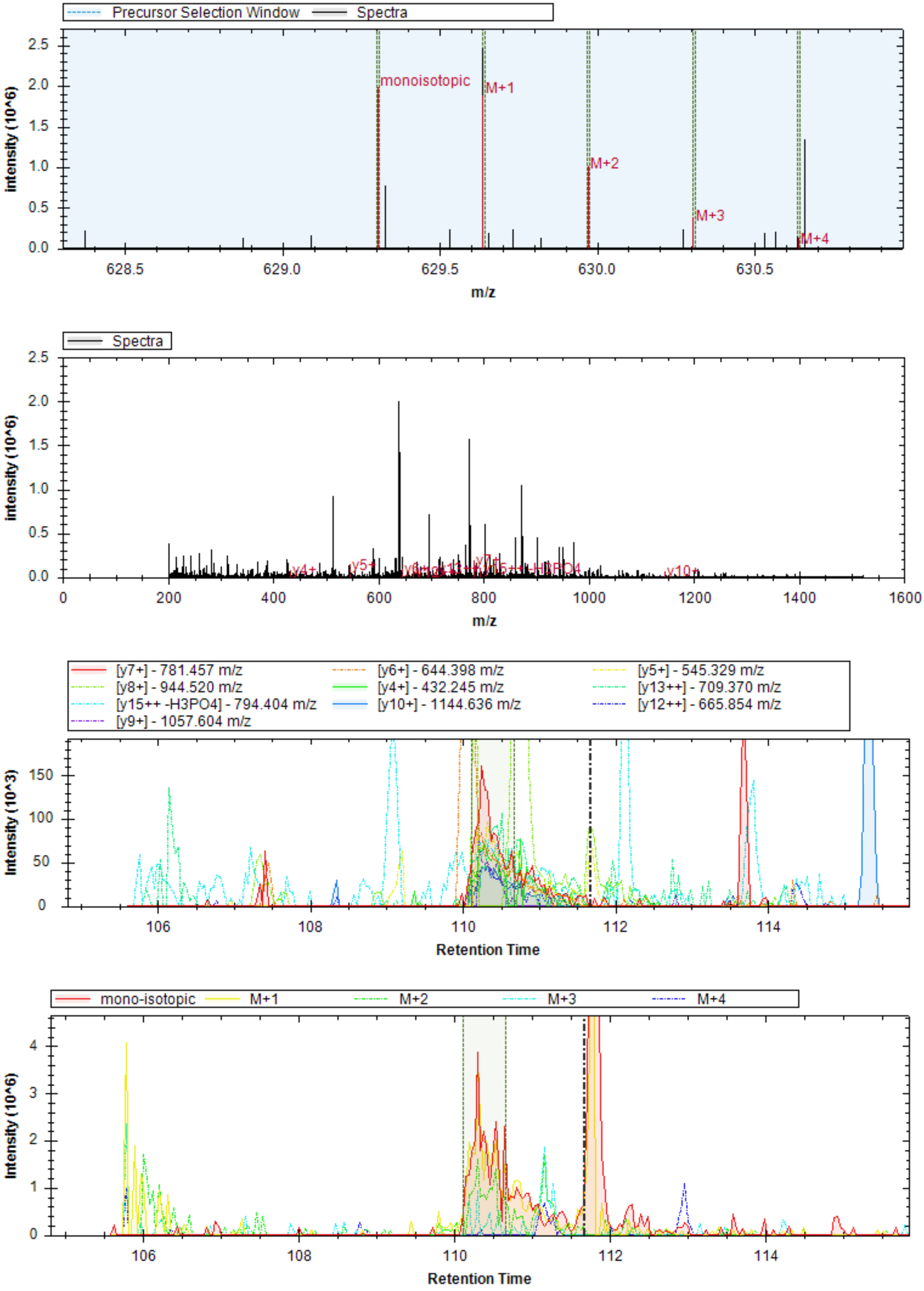

P60904 – _SLST[+H+O3+P]SGESLYHVLGLDK_.3

Fig4_MP-DIA-barrel-cortex-p30-1_MHRM.htrms

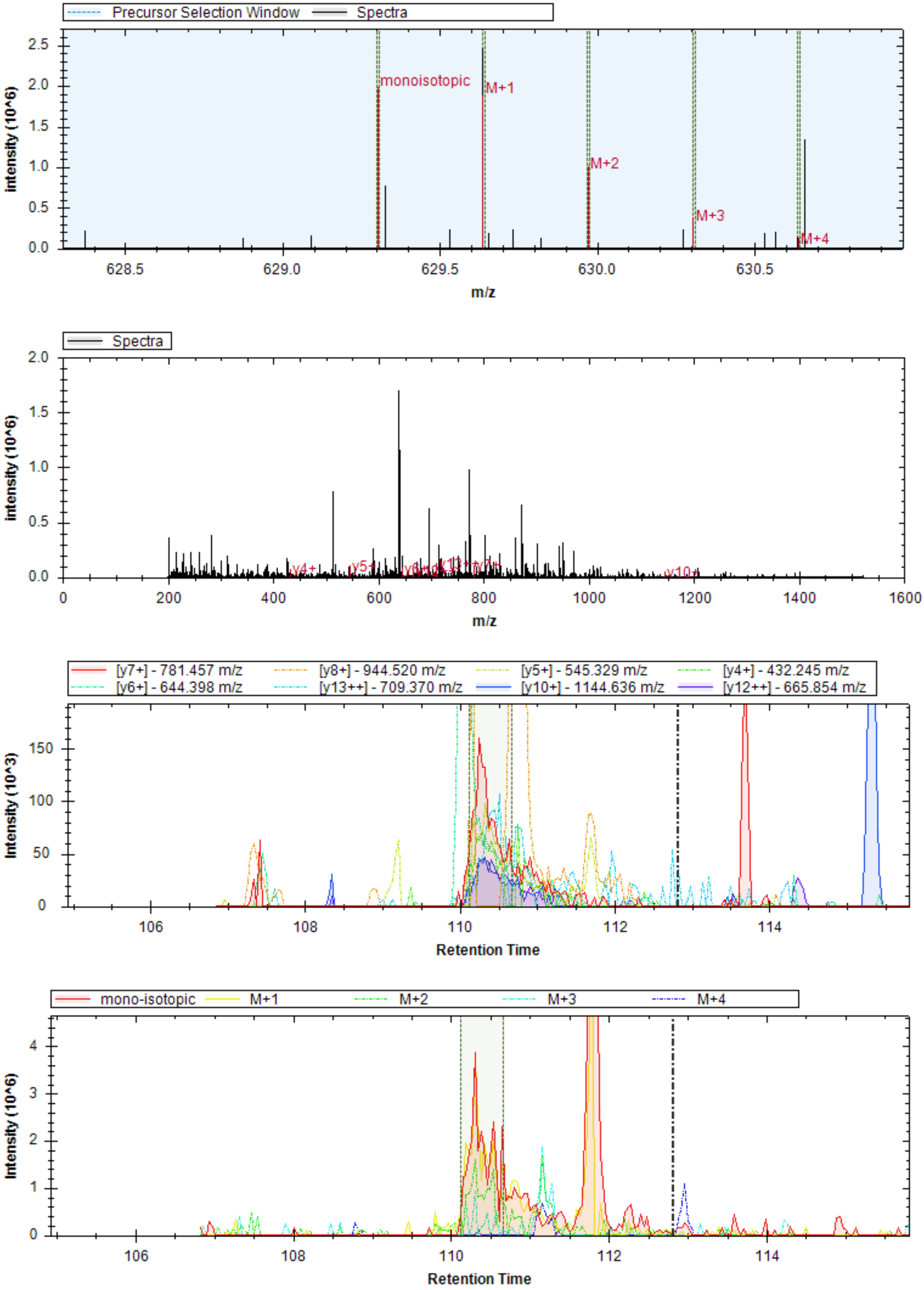

P39447 – _SSGIIRLHT[+H+O3+P]IK_.2
Fig4_MP-DIA-barrel-cortex-p30-1_MHRM.htrms

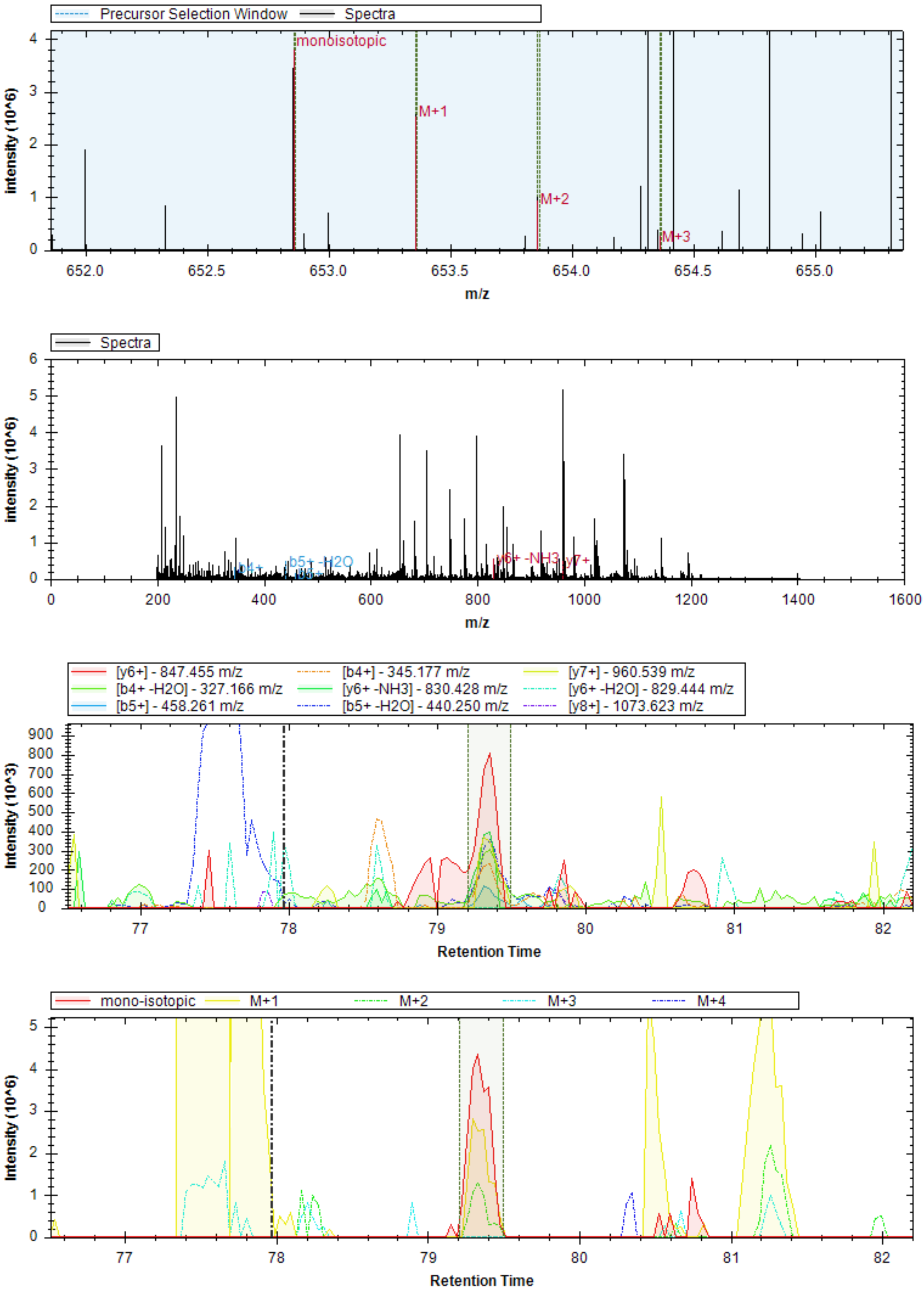

Q8BLJ3 – _M[+O]AS[+H+O3+P]SQGKNELK_.2
Fig4_MP-DIA-barrel-cortex-p30-1_MHRM.htrms

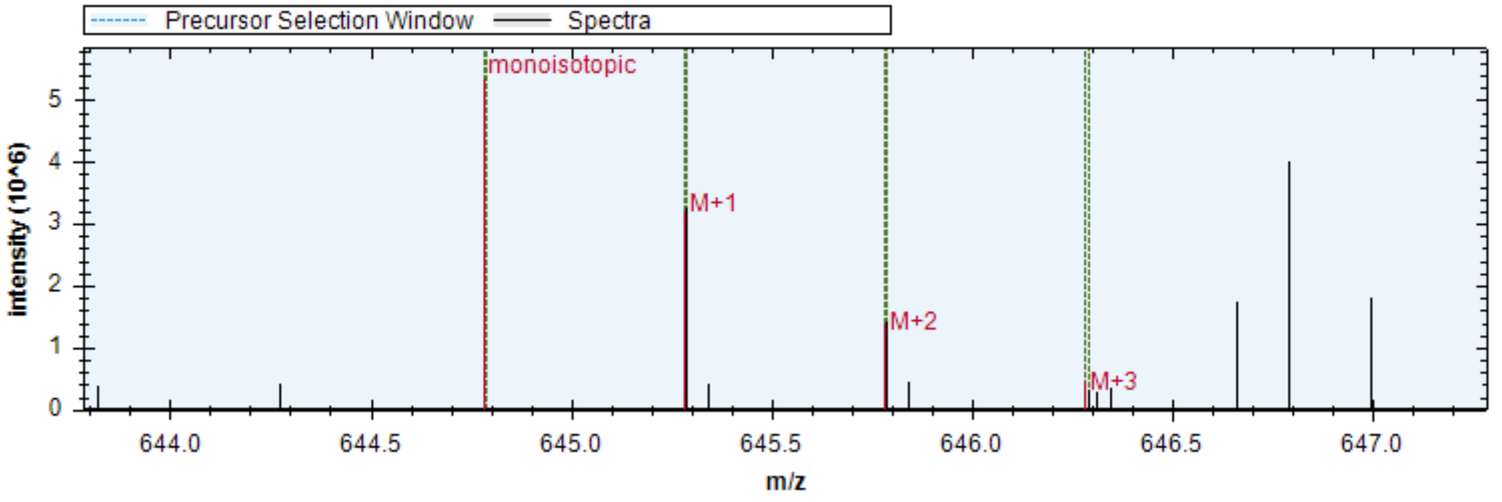

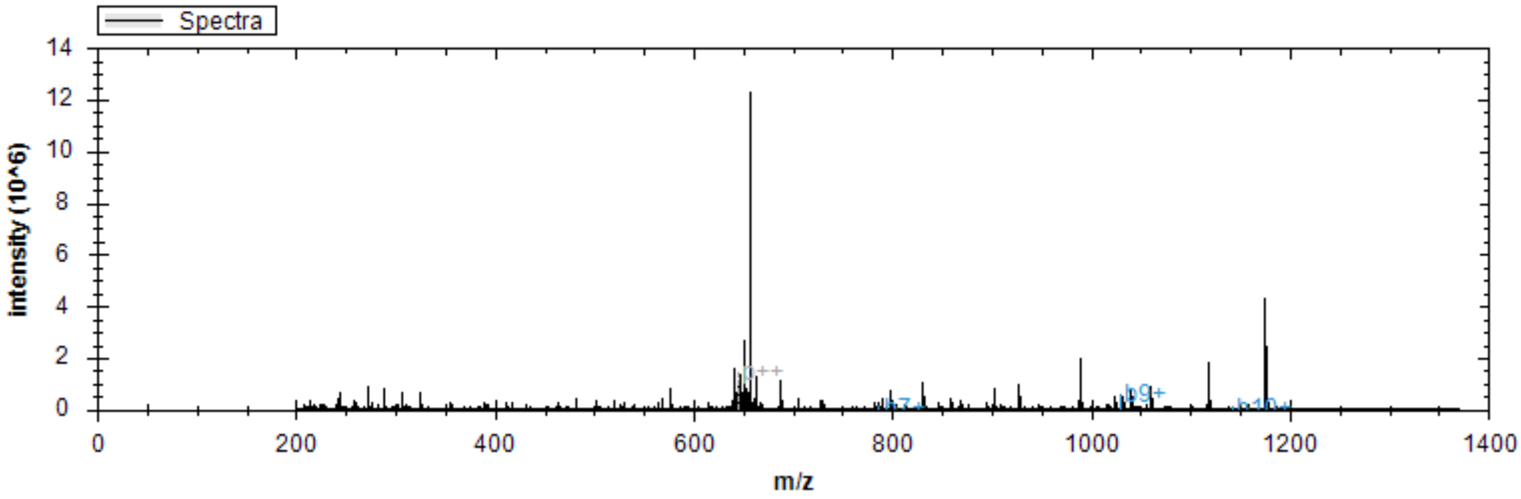

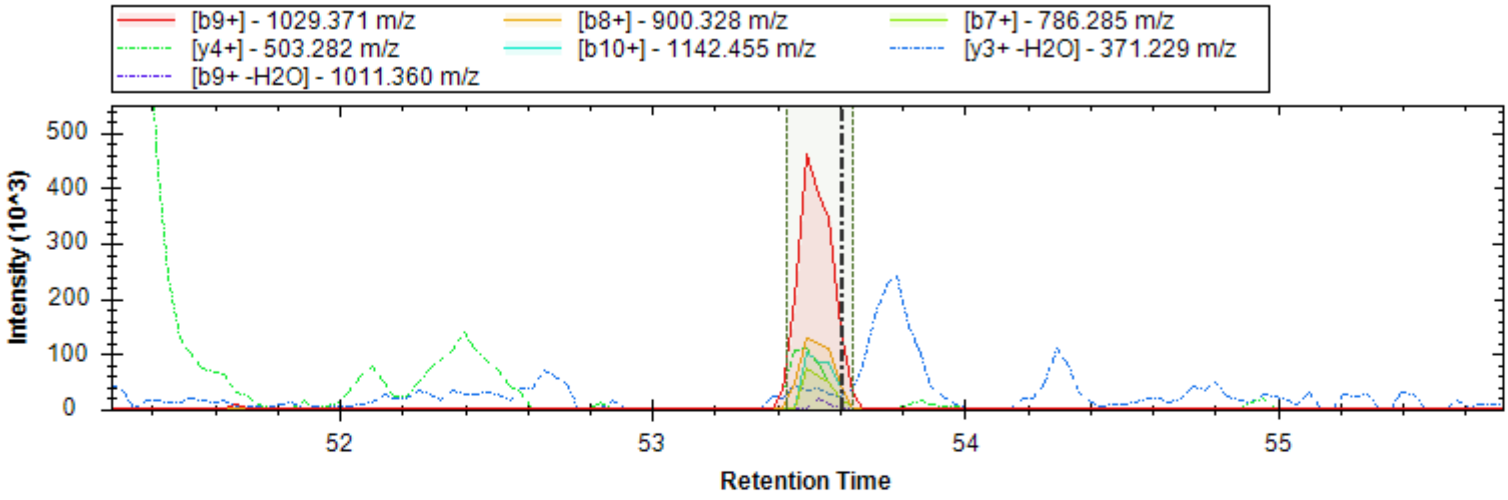

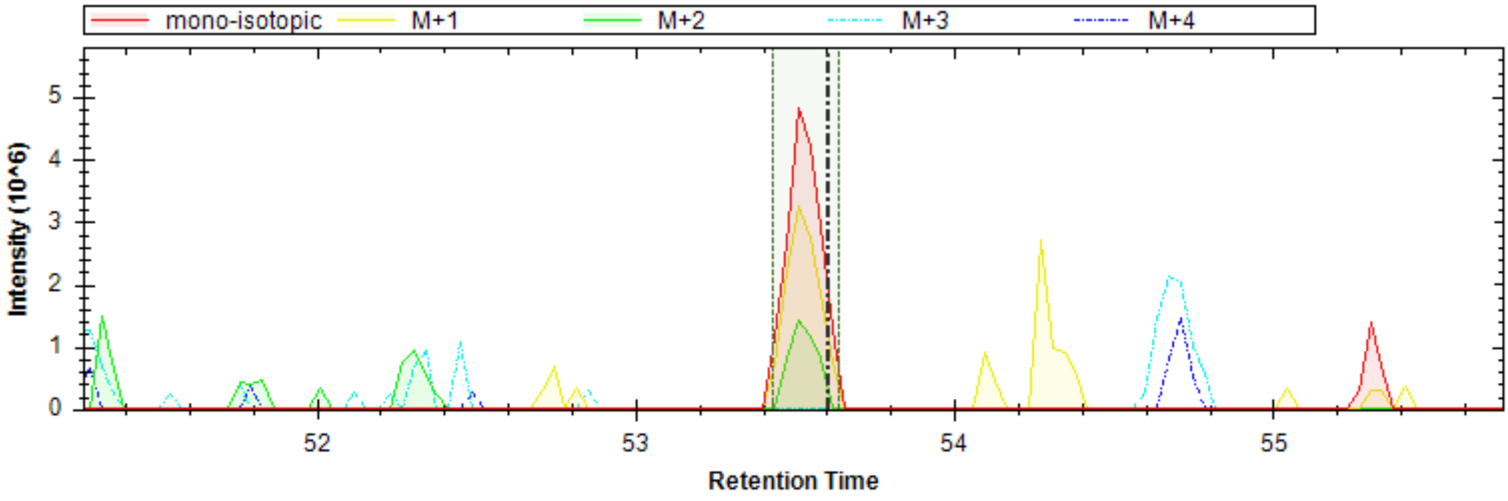

G5E870 – _IIY[+H+O3+P]FADAELLK_.2

Fig4_MP-DIA-barrel-cortex-p30-1_MHRM.htrms

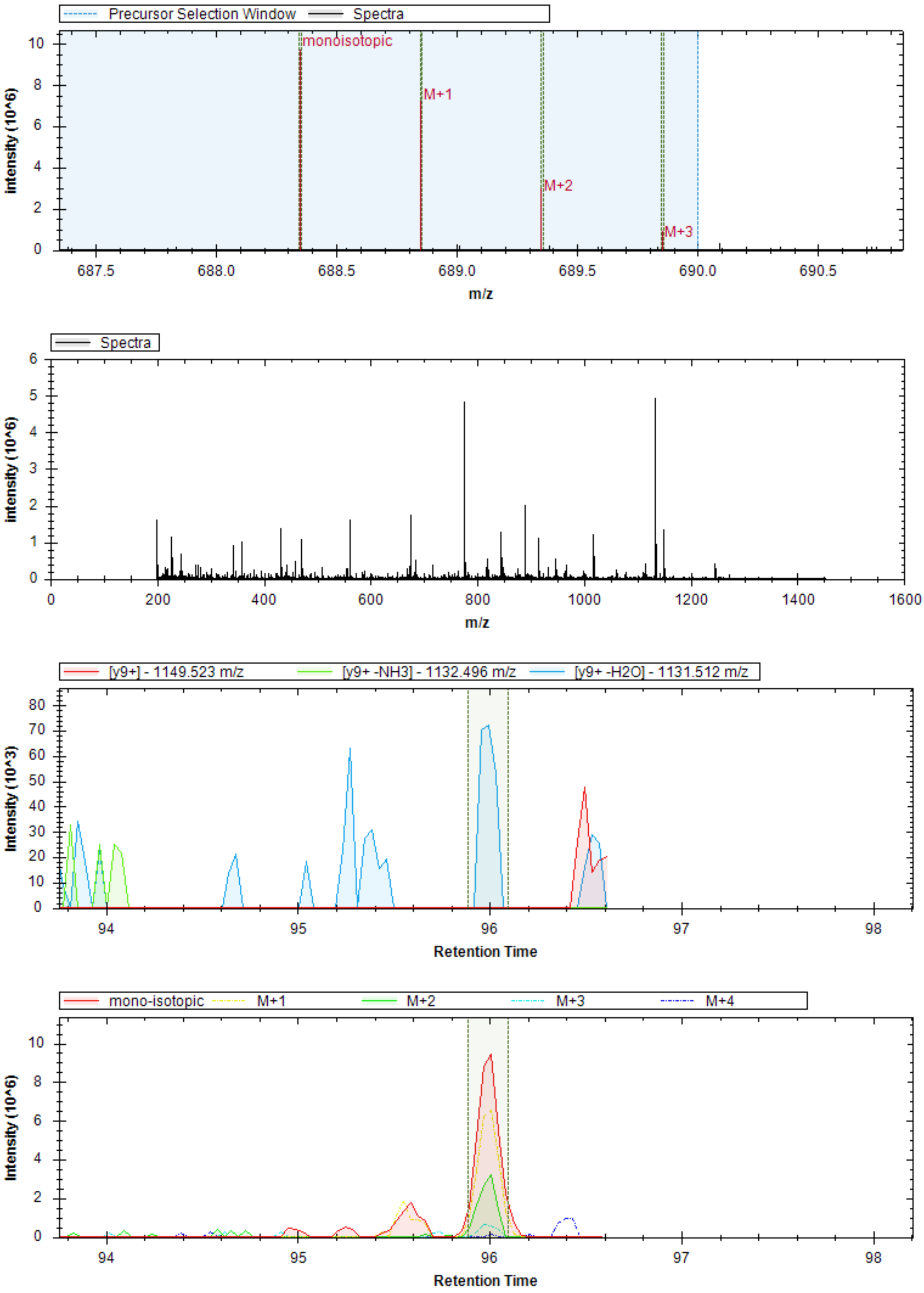

P13595 – _GVTASS[+H+O3+P]SSPASAPK_.2
Fig4_MP-DIA-barrel-cortex-p30-1_MHRM.htrms

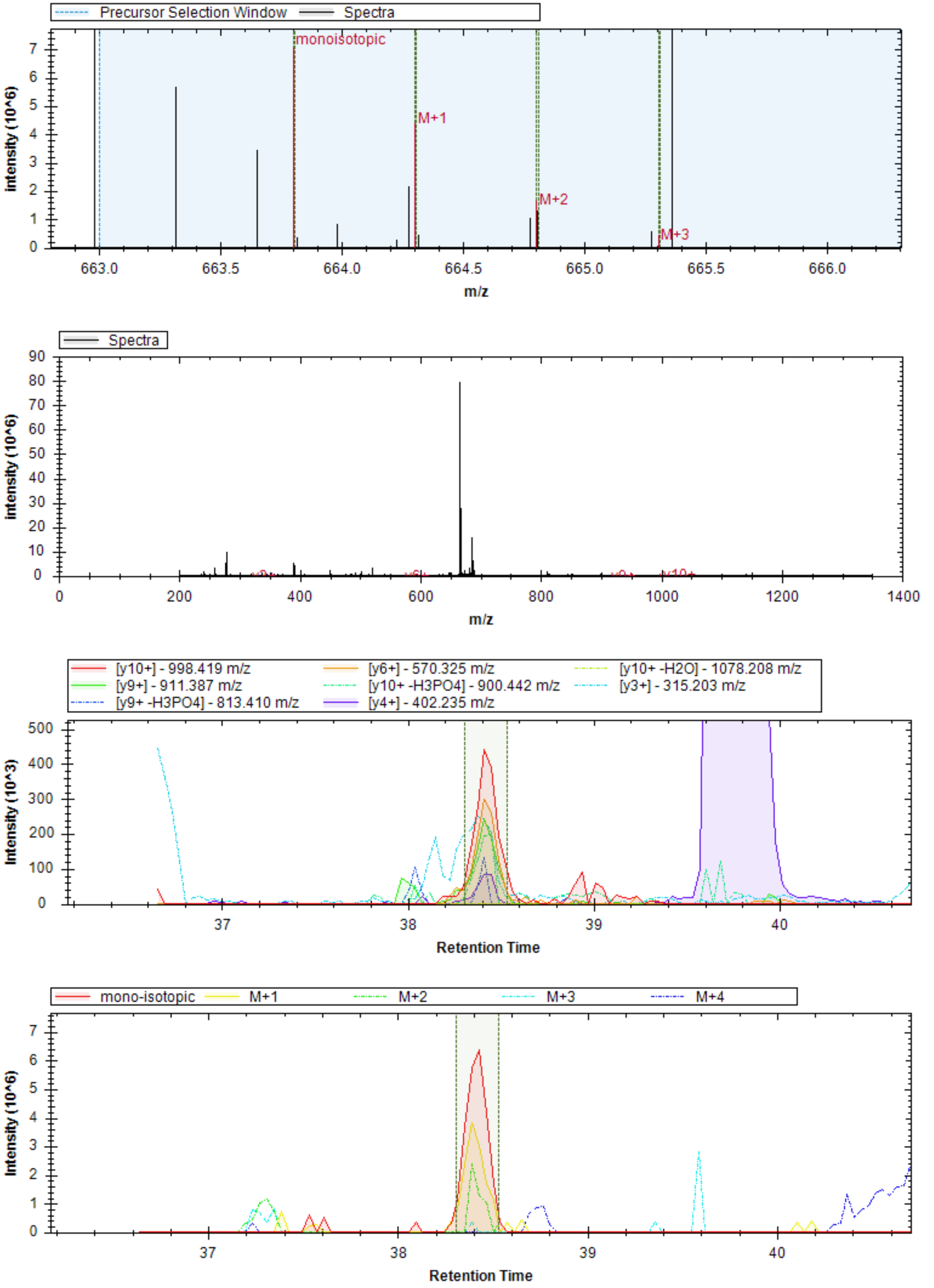

P13595 – _GVTASSS[+H+O3+P]SPASAPK_.2
Fig4_MP-DIA-barrel-cortex-p30-1_MHRM.htrms

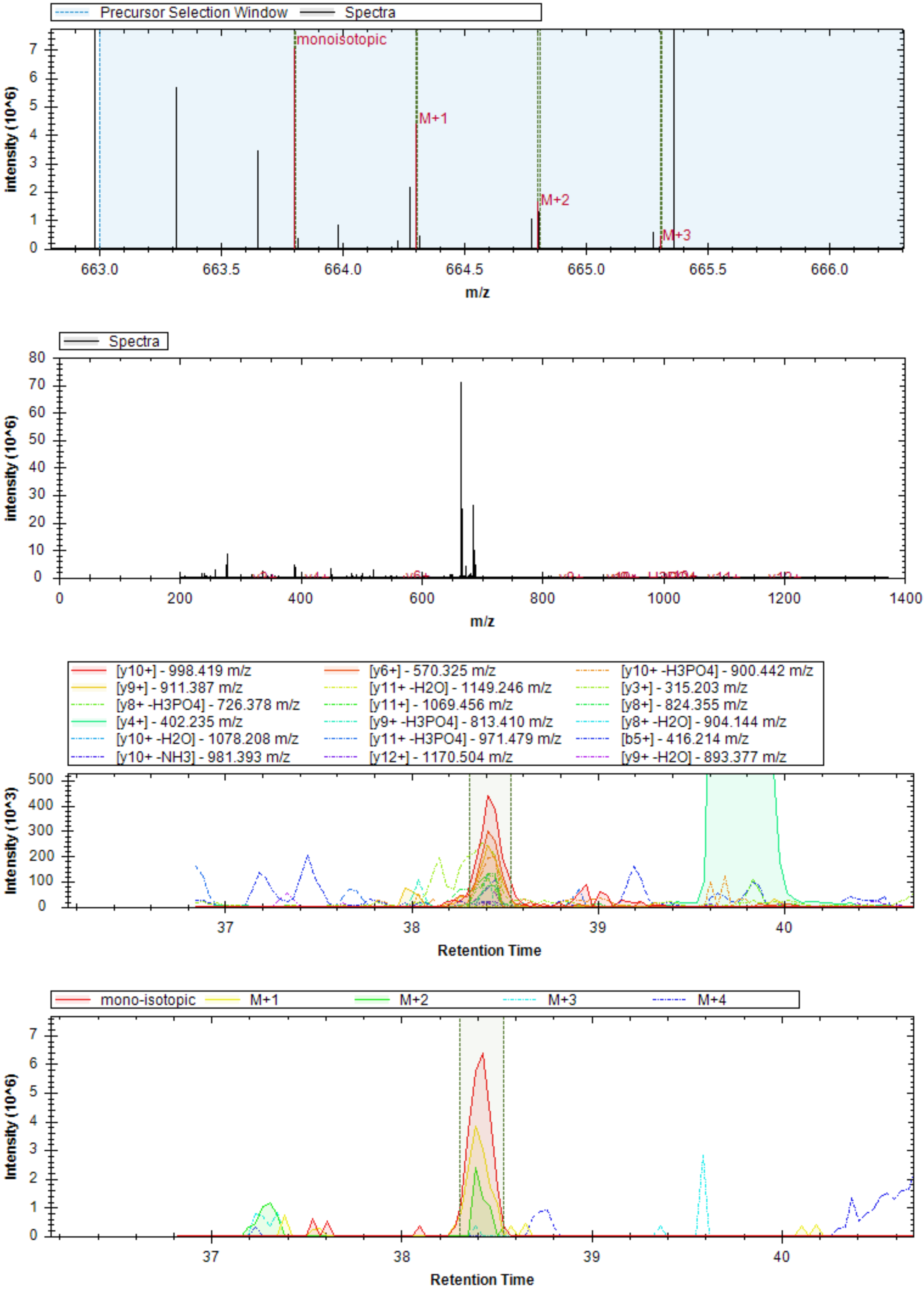

P13595 – _GVTASSSS[+H+O3+P]PASAPK_.2
Fig4_MP-DIA-barrel-cortex-p30-1_MHRM.htrms

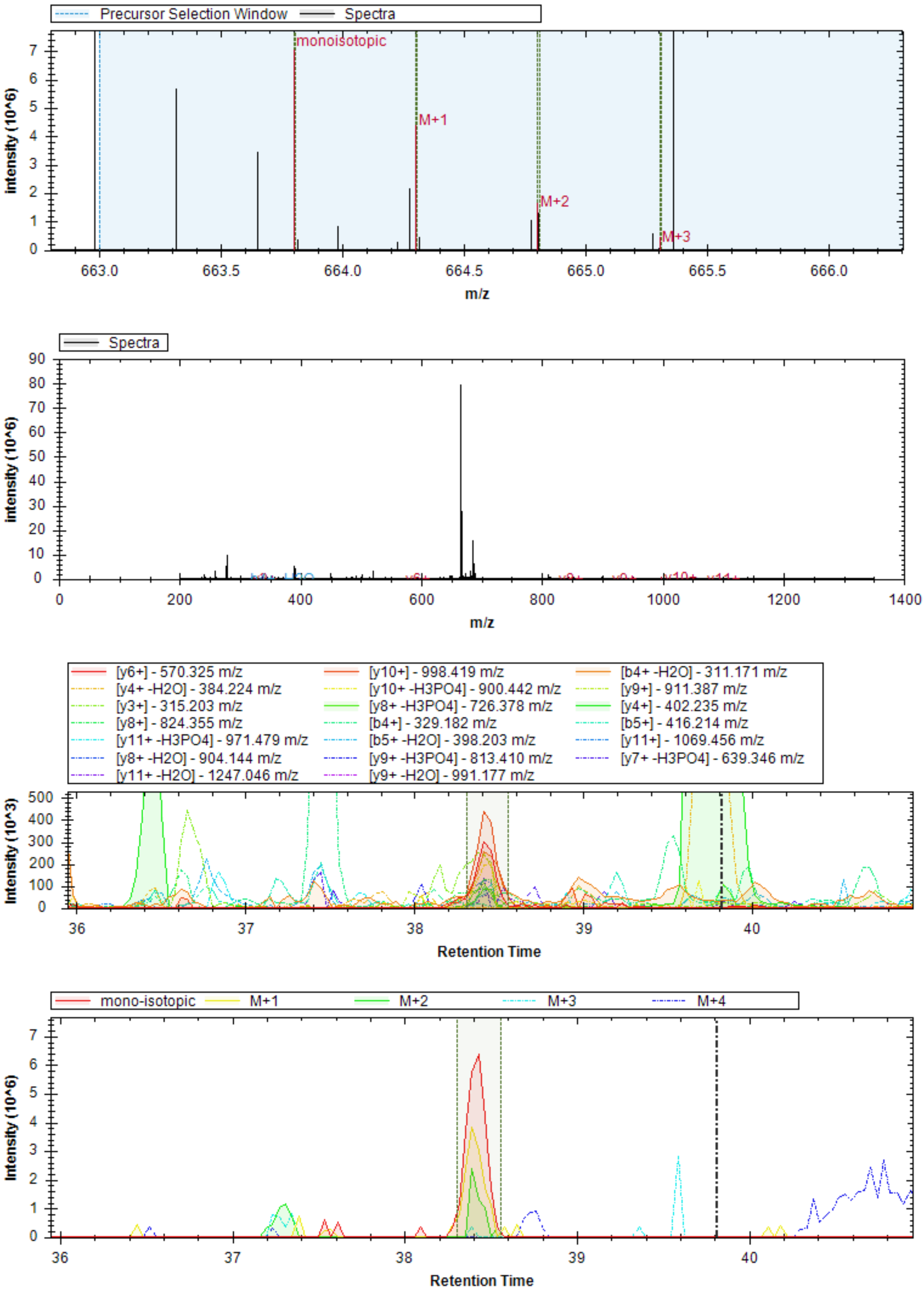

P13595 – _NPPEAATAPAS[+H+O3+P]PK_.2
Fig4_MP-DIA-barrel-cortex-p30-1_MHRM.htrms

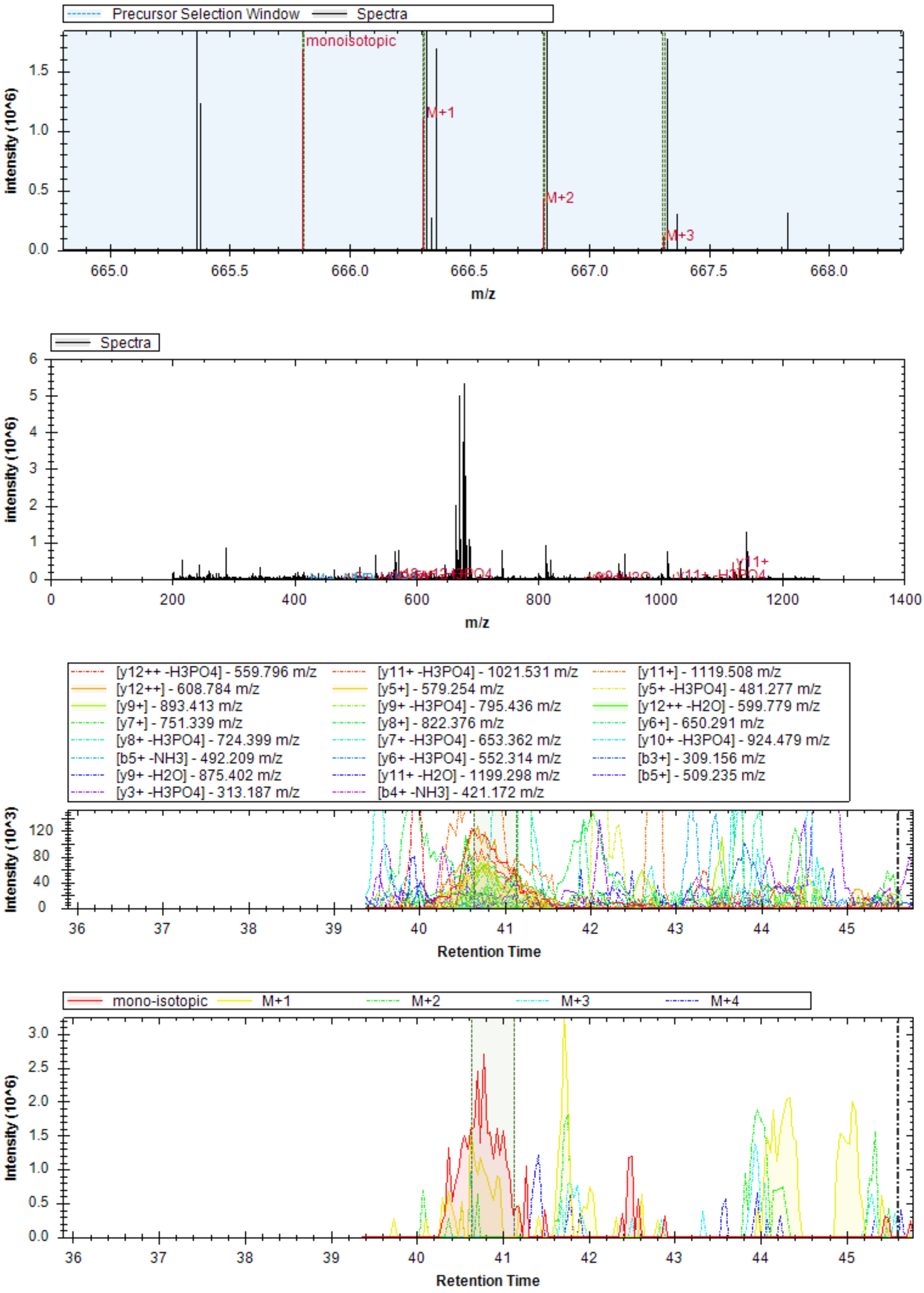

Q6A078 – _T[+H+O3+P]IGLM[+O]KKVVEK_.2
Fig4_MP-DIA-barrel-cortex-p30-1_MHRM.htrms

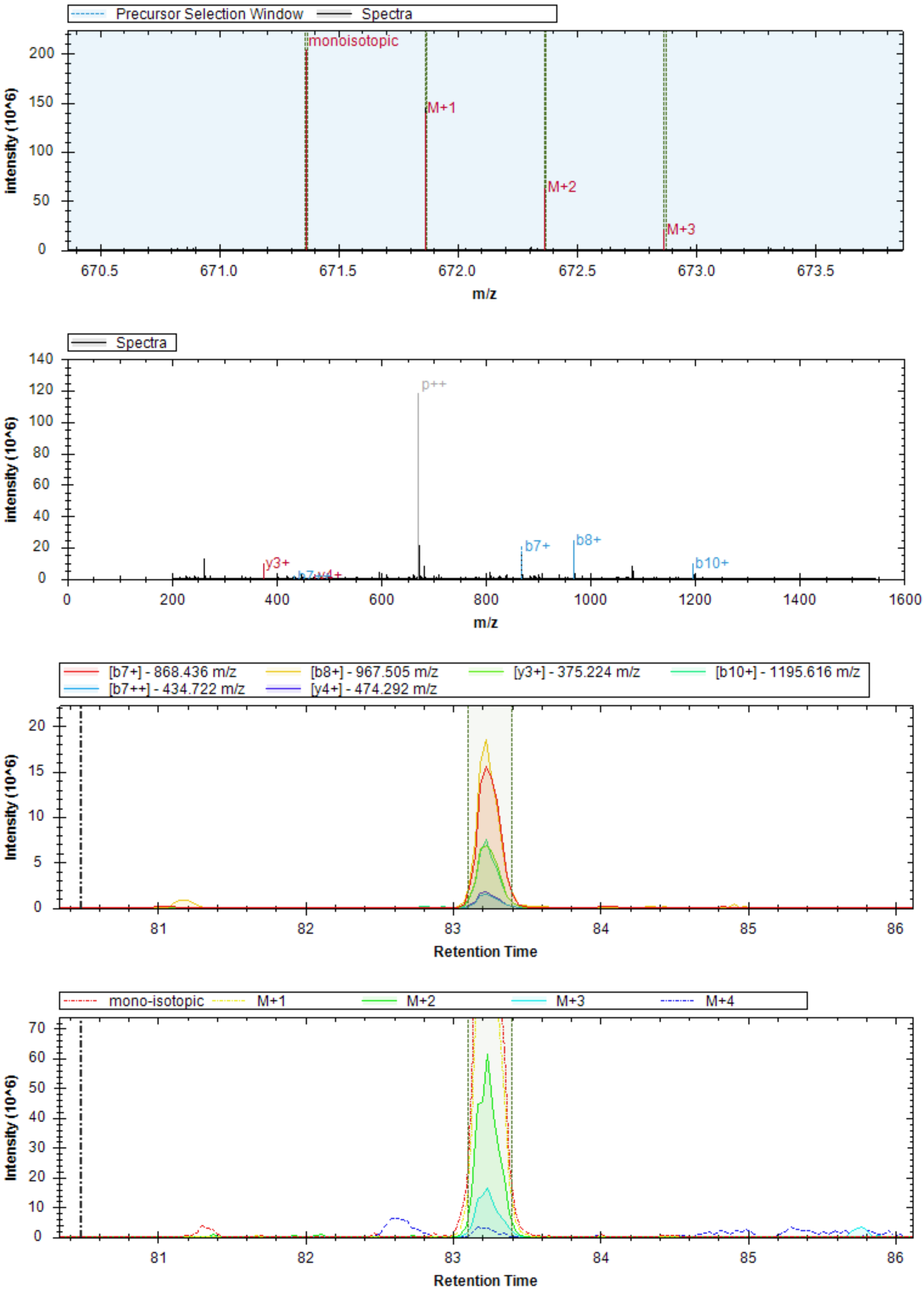

Q8C0E2 – _SMS[+H+O3+P]HQAAIASQR_.2
Fig4_MP-DIA-barrel-cortex-p30-1_MHRM.htrms

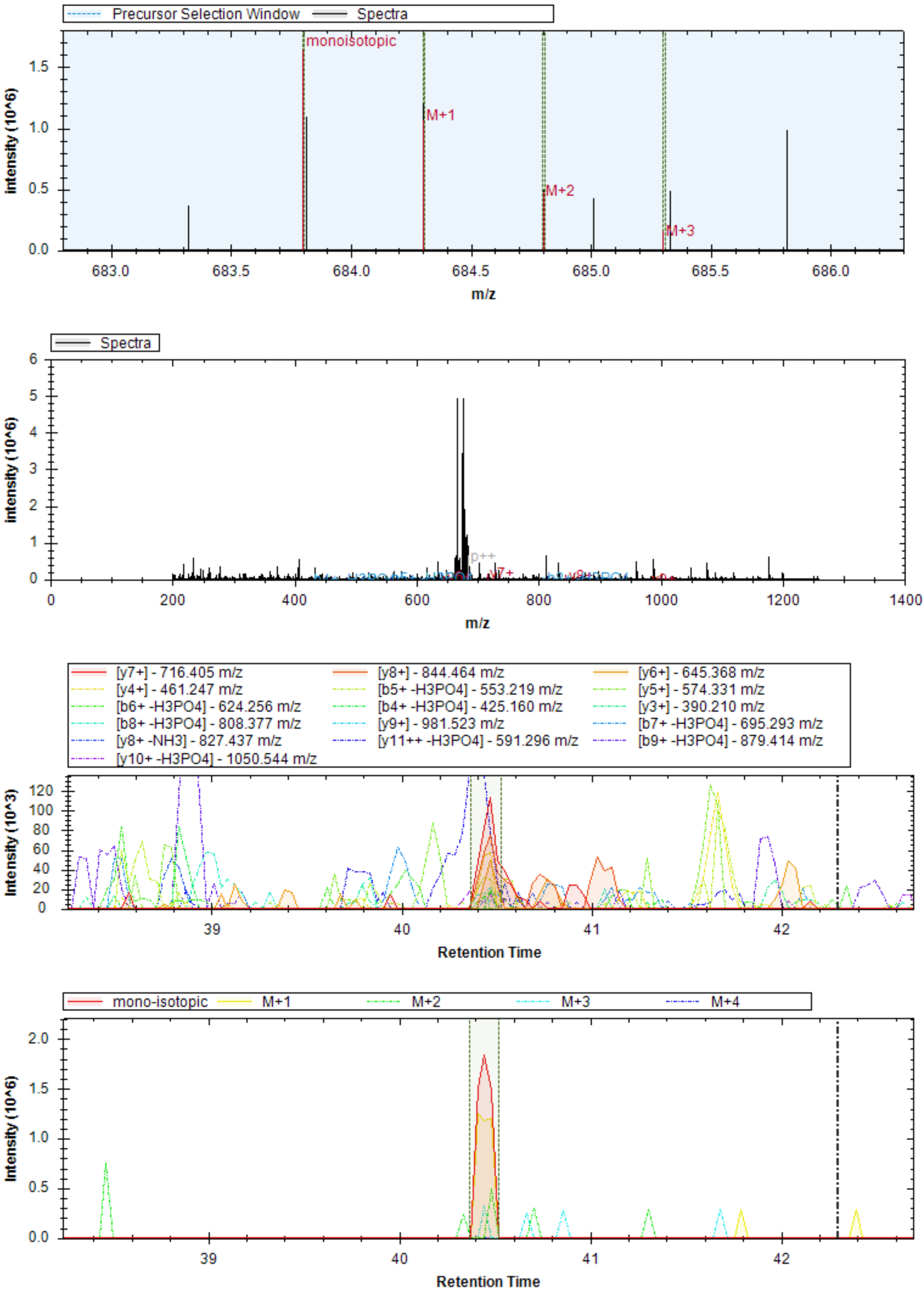

Q8CDU4;Q8CDU4-2 – _[+C2+H2+O]ASLRS[+H+O3+P]ATPRLR_.2
Fig4_MP-DIA-barrel-cortex-p30-1_MHRM.htrms

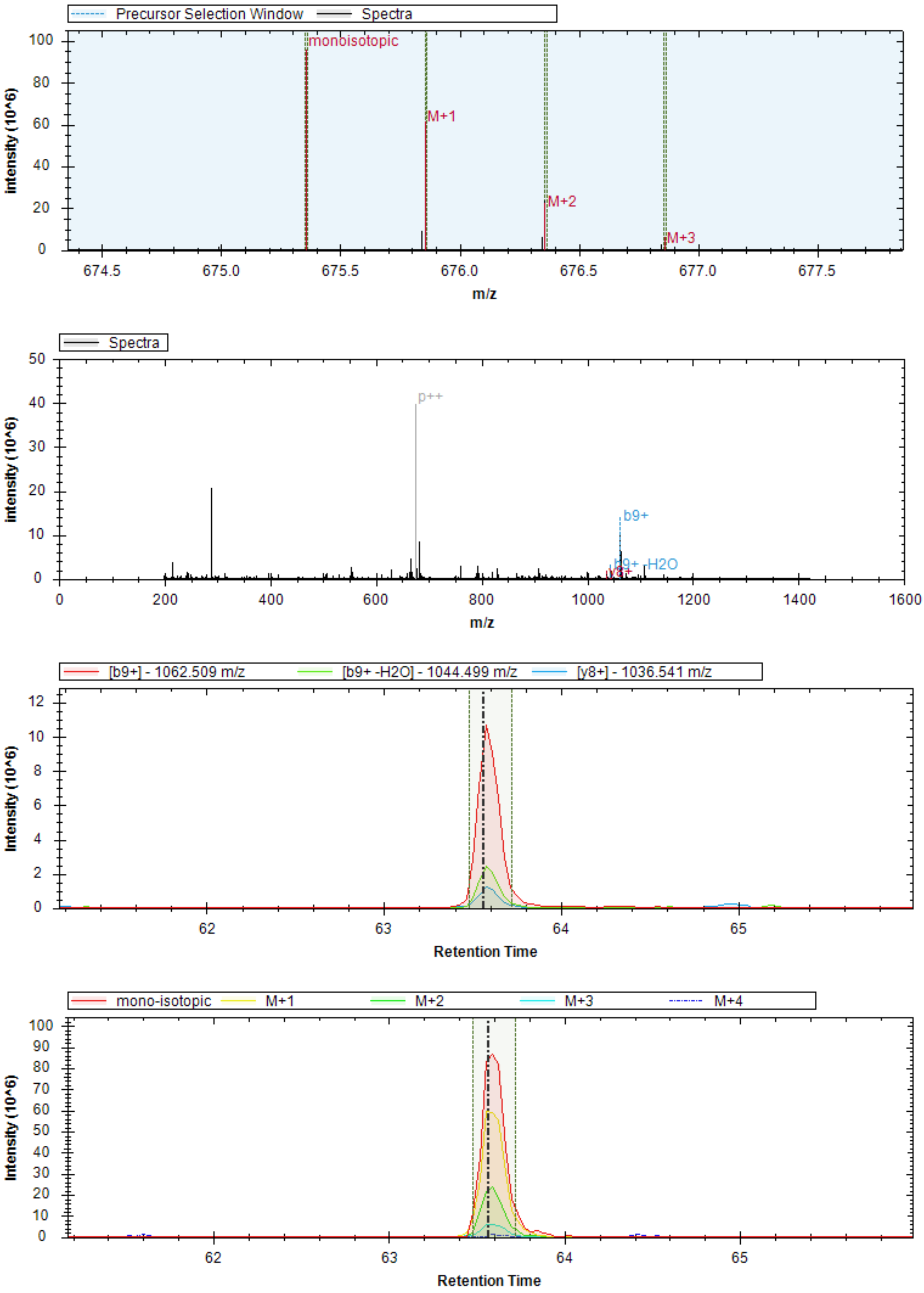

Q9CZU3 – _ESLKNSAT[+H+O3+P]EAAK_.2
Fig4_MP-DIA-barrel-cortex-p30-1_MHRM.htrms

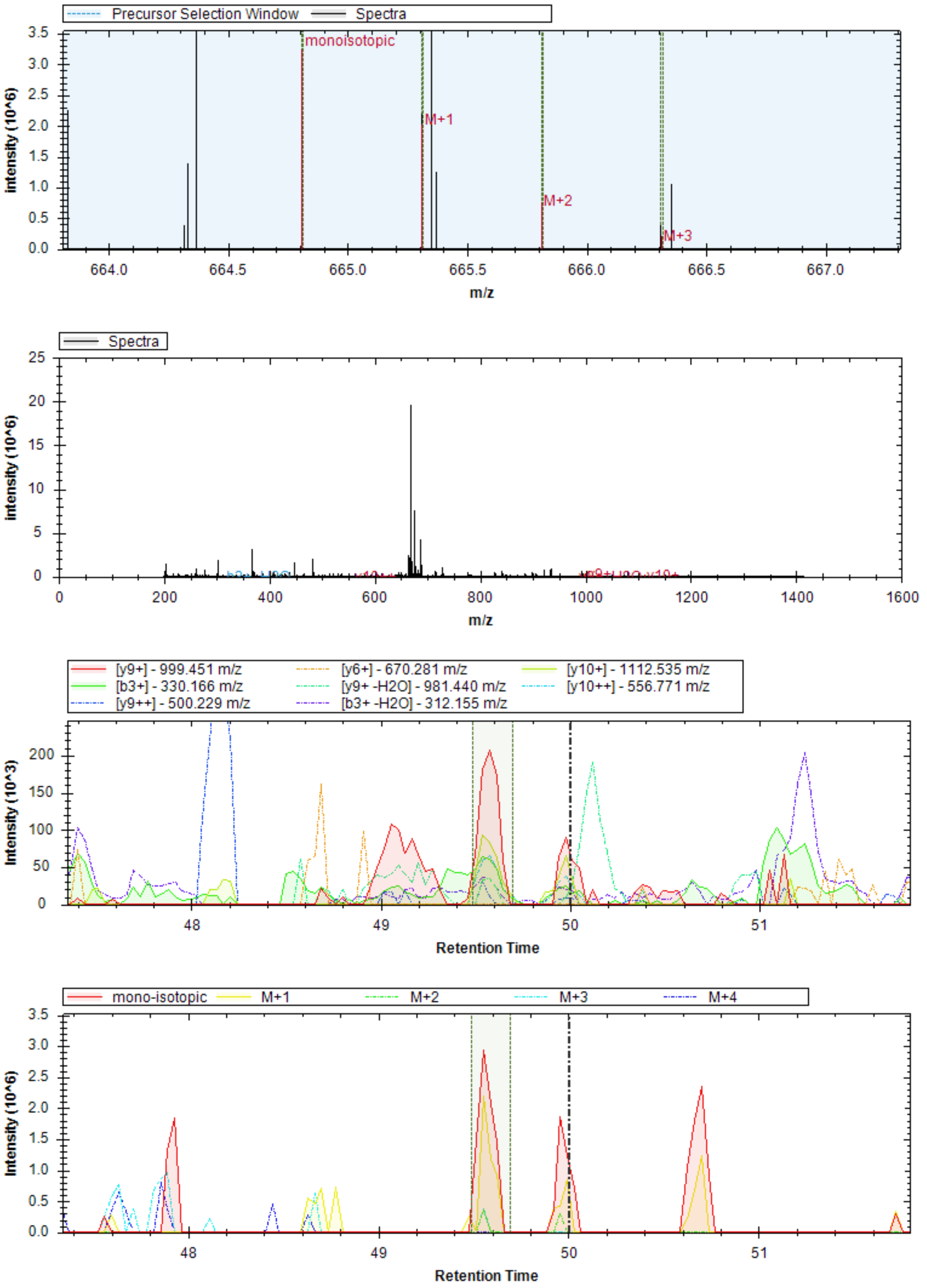

O88935 – _PVAGGPGAPPAARPPAS[+H+O3+P]PSPQR_.3

Fig4_MP-DIA-barrel-cortex-p30-1_MHRM.htrms

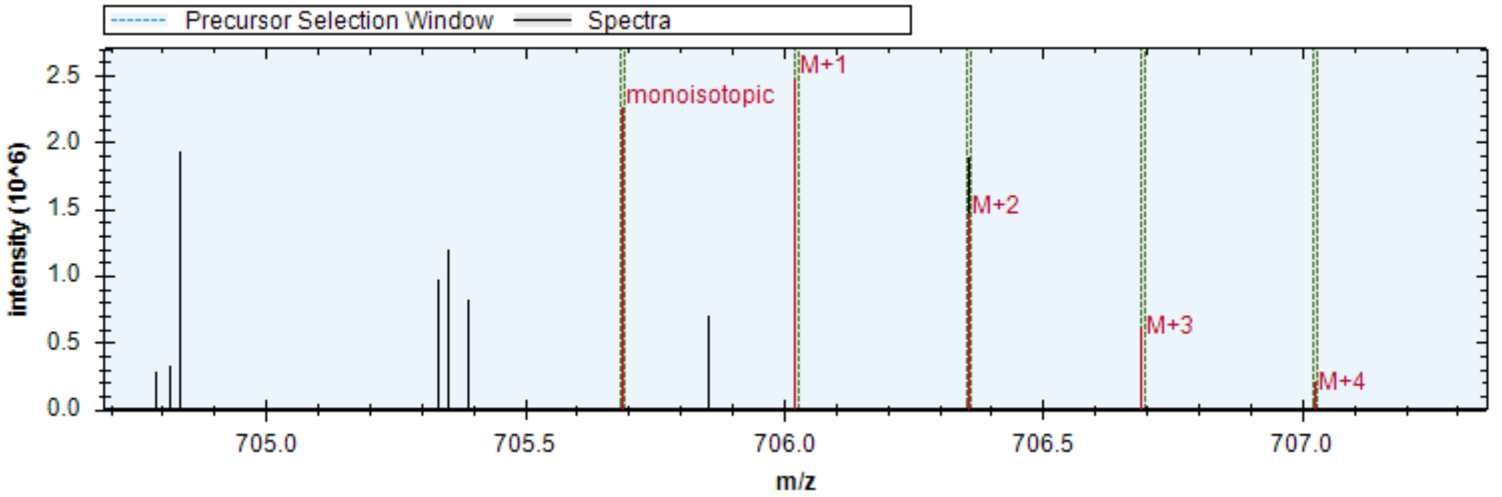

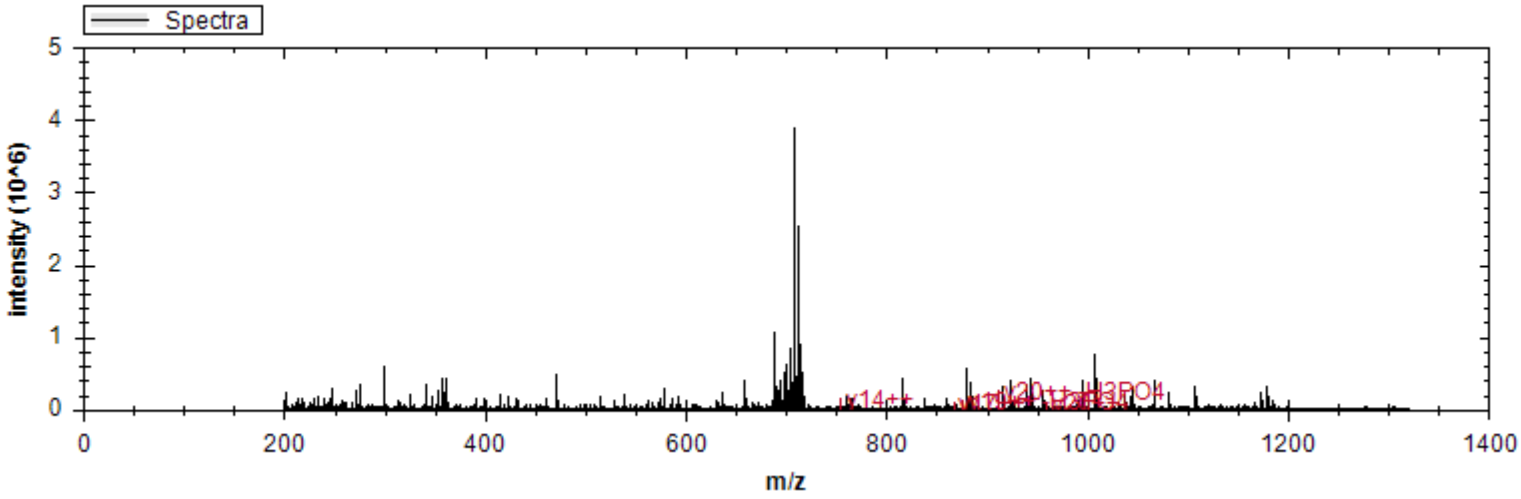

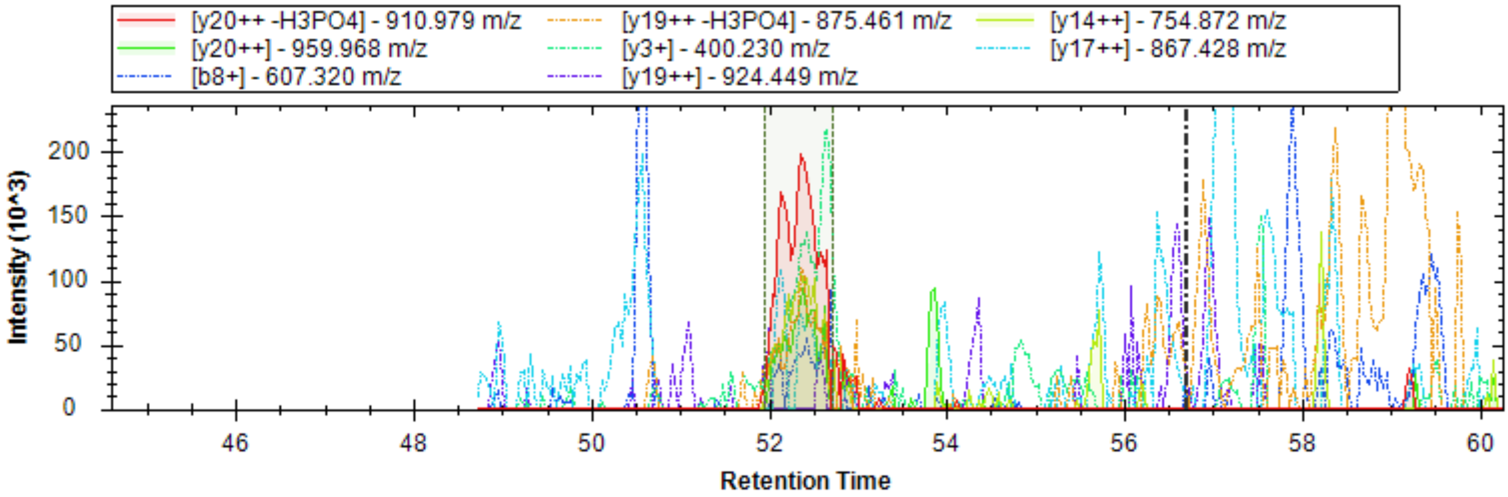

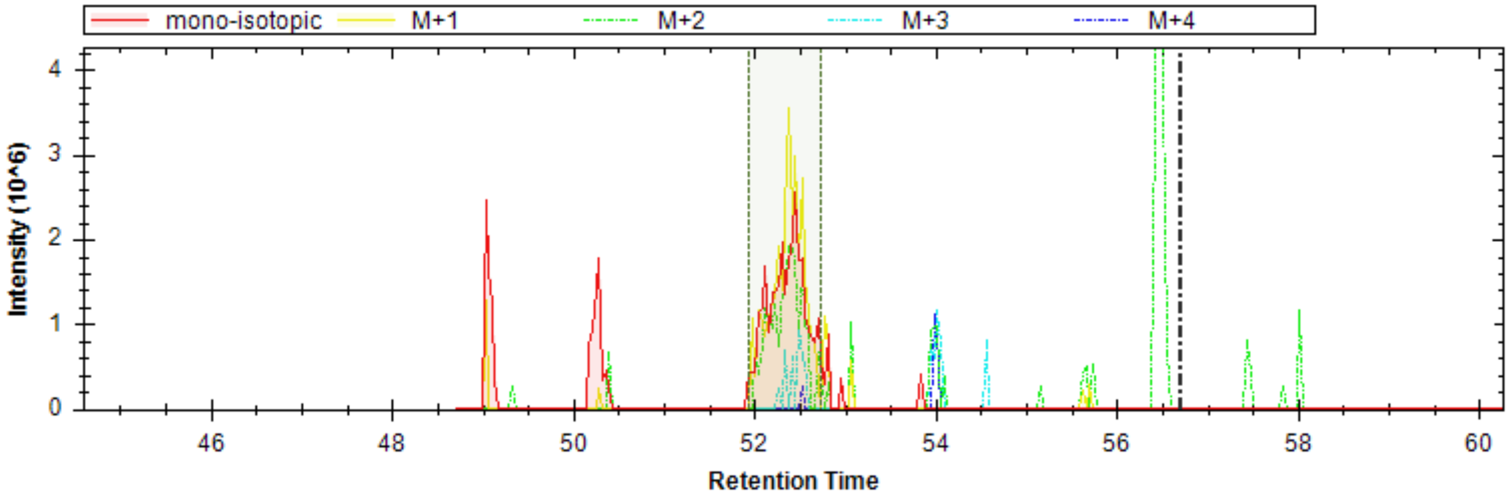

O88935 – _PVAGGPGAPPAARPPASPS[+H+O3+P]PQR_.3

Fig4_MP-DIA-barrel-cortex-p30-1_MHRM.htrms

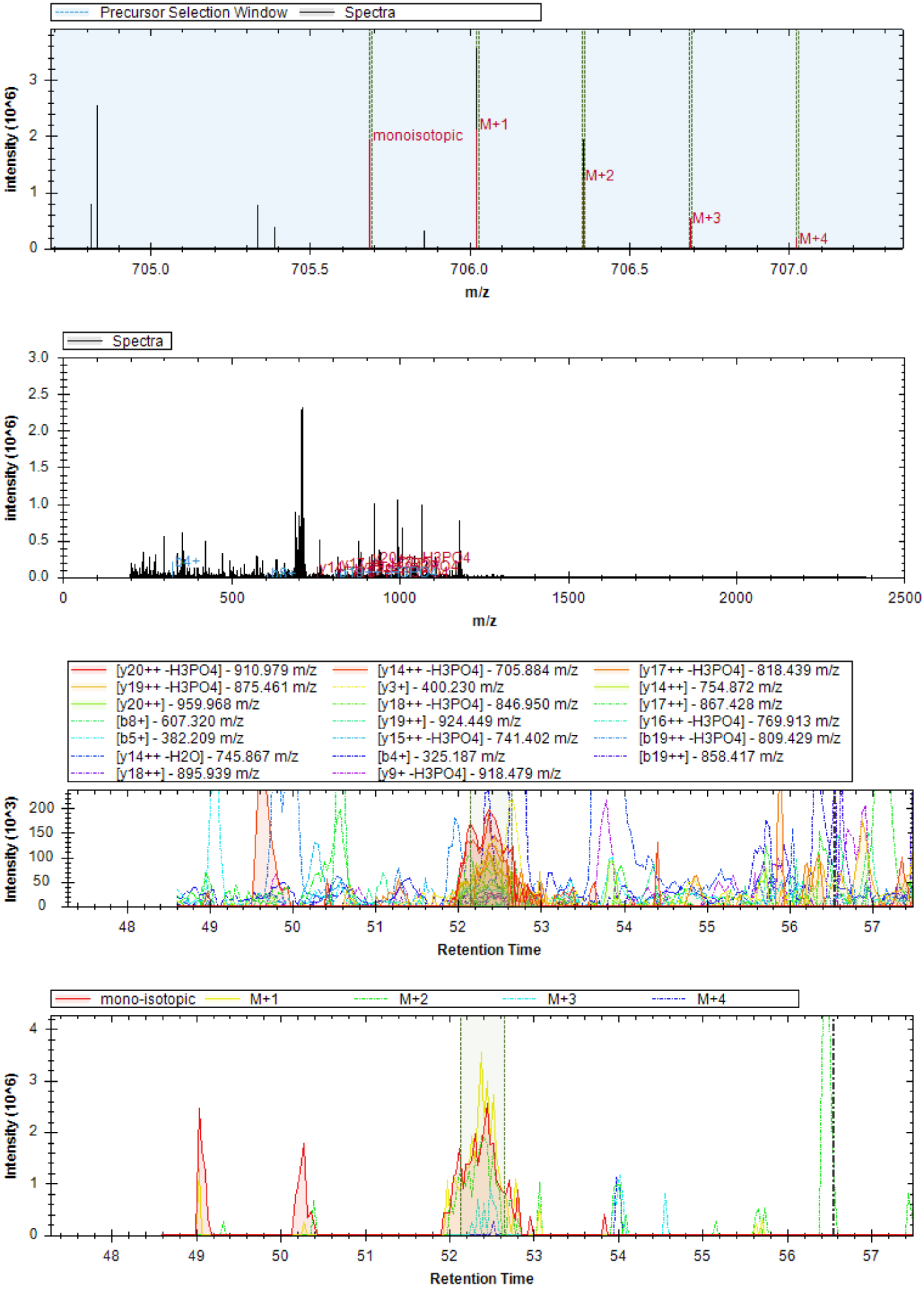

P28738 – _PIRPGHYPAS[+H+O3+P]SPTAVHAVR_.3
Fig4_MP-DIA-barrel-cortex-p30-1_MHRM.htrms

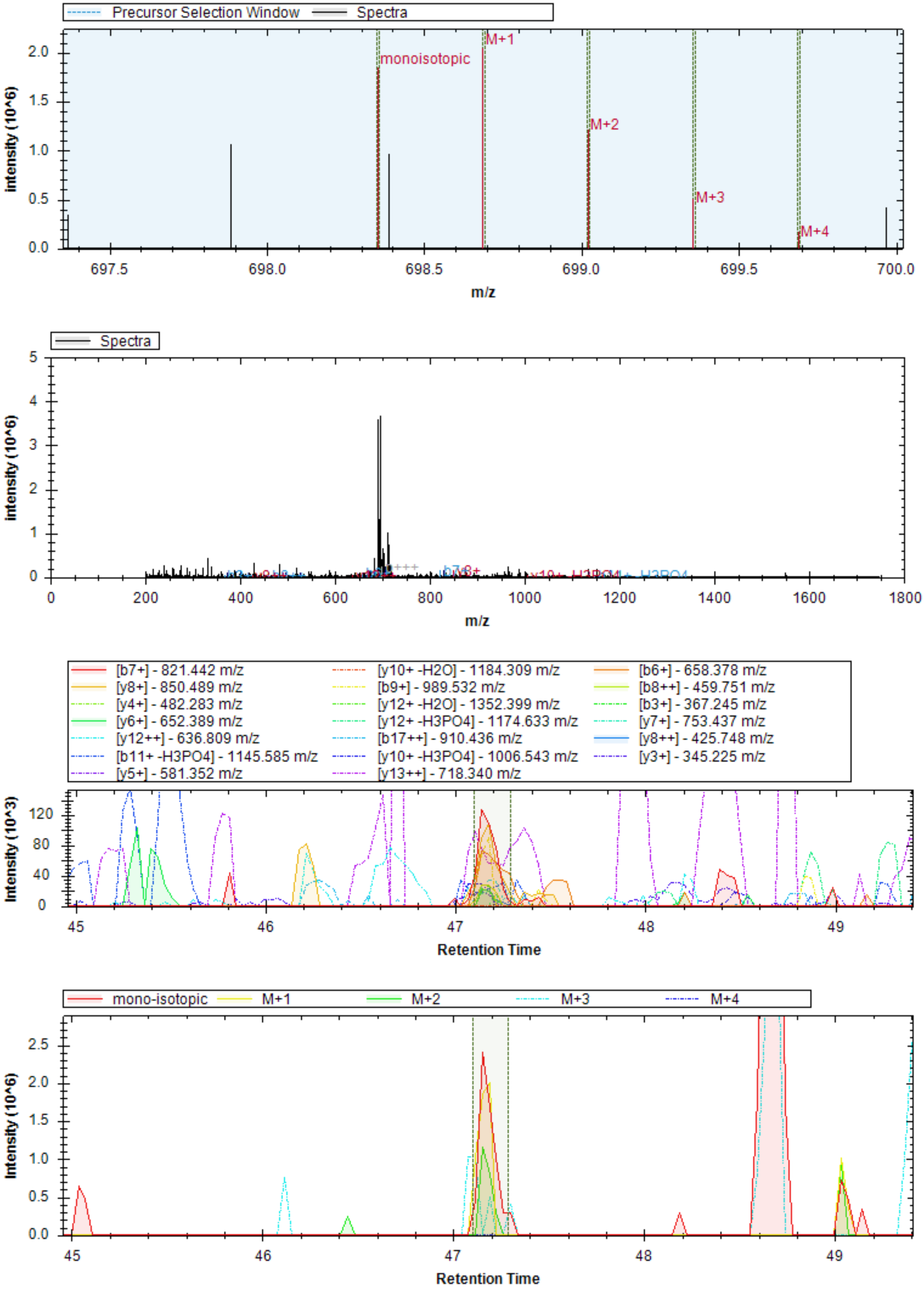

P28738 – _PIRPGHYPASS[+H+O3+P]PTAVHAVR_.3
Fig4_MP-DIA-barrel-cortex-p30-1_MHRM.htrms

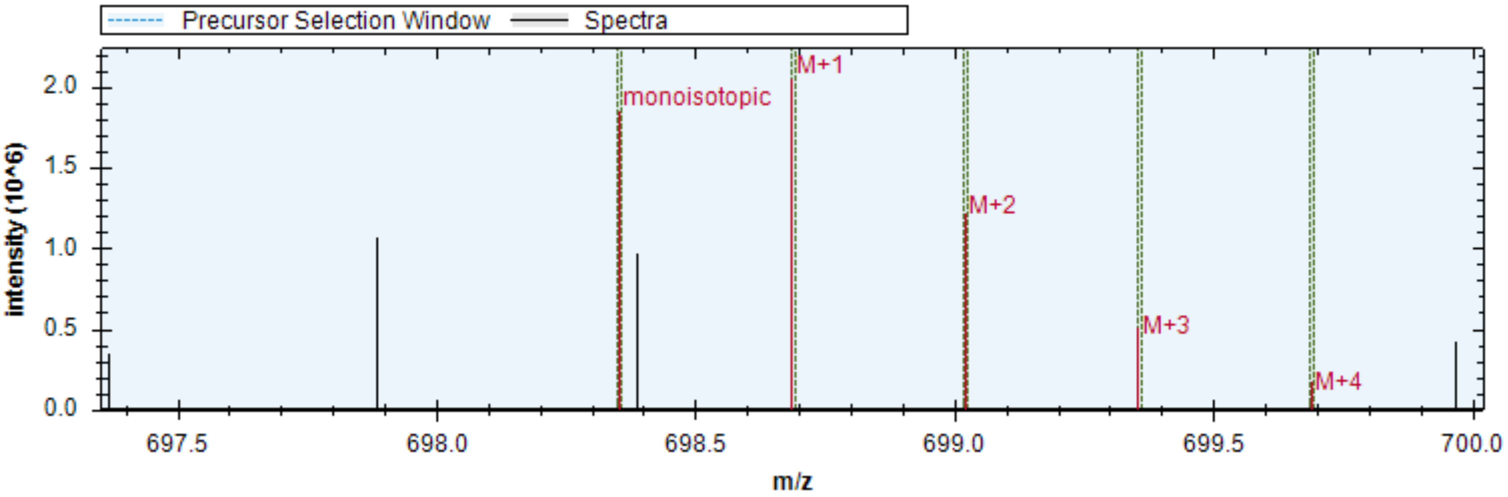

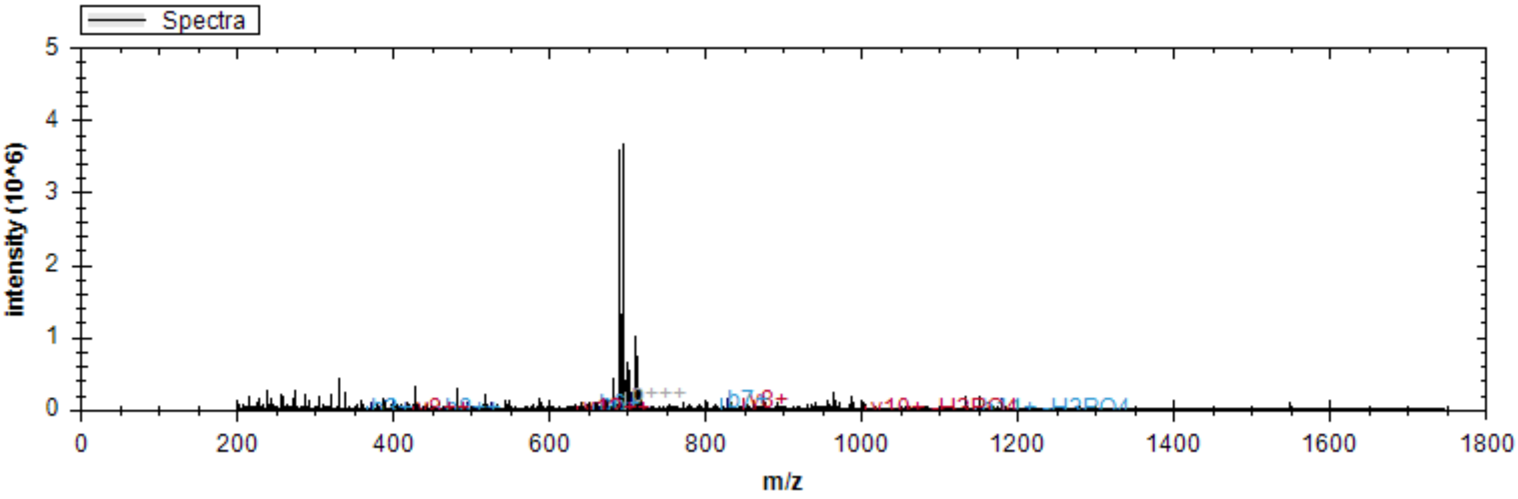

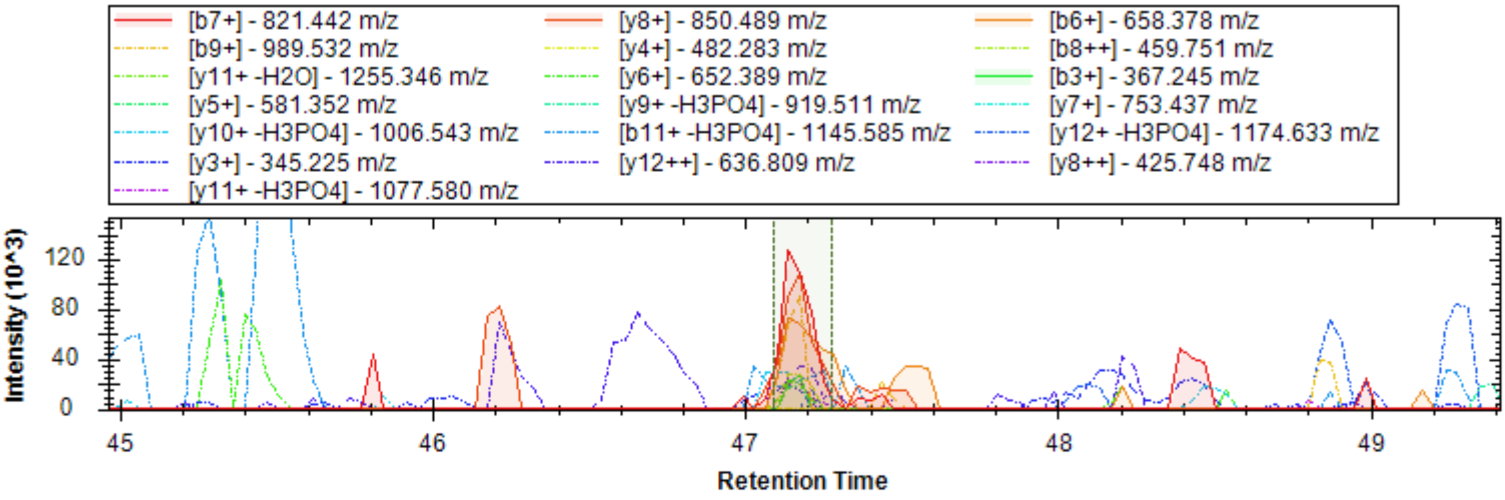

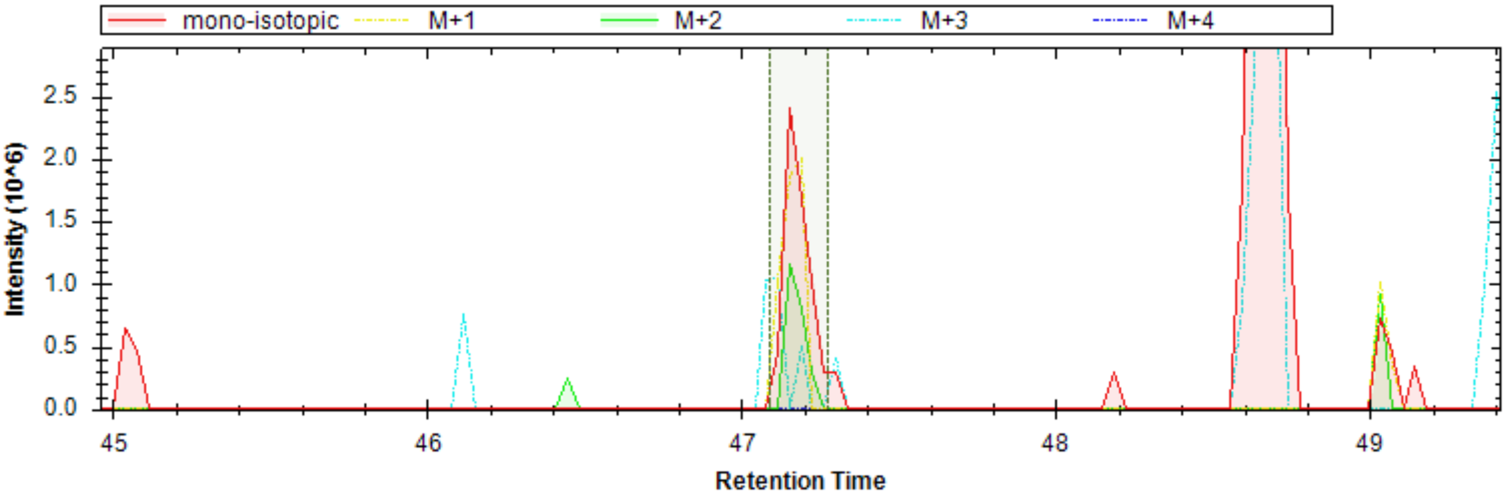

P62751 – _KAY[+H+O3+P]VRLAPDYDALDVANK_.3

Fig4_MP-DIA-barrel-cortex-p30-1_MHRM.htrms

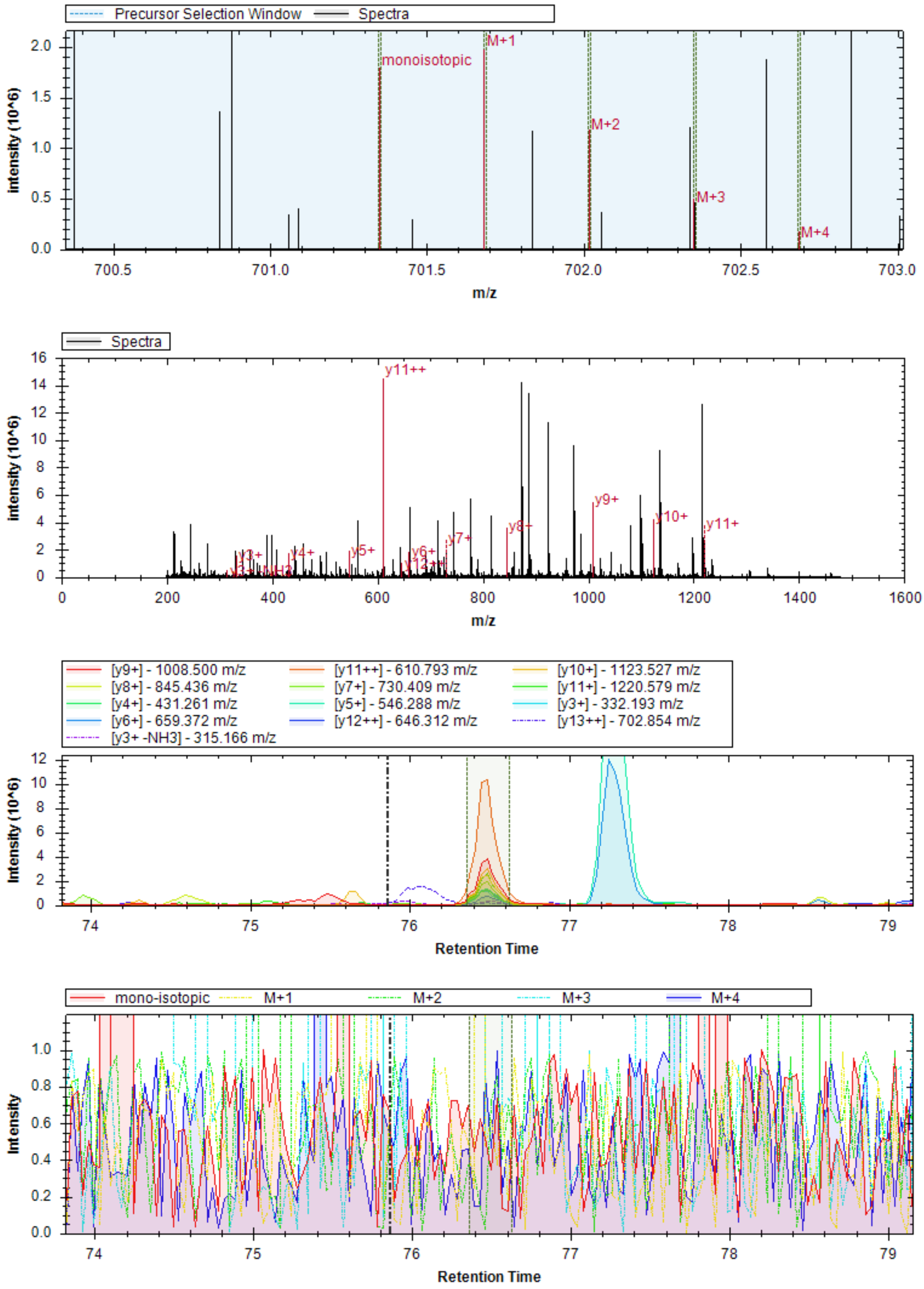

Q3U4B4 – _RHKT[+H+O3+P]LSANEEK_.2
Fig4_MP-DIA-barrel-cortex-p30-1_MHRM.htrms

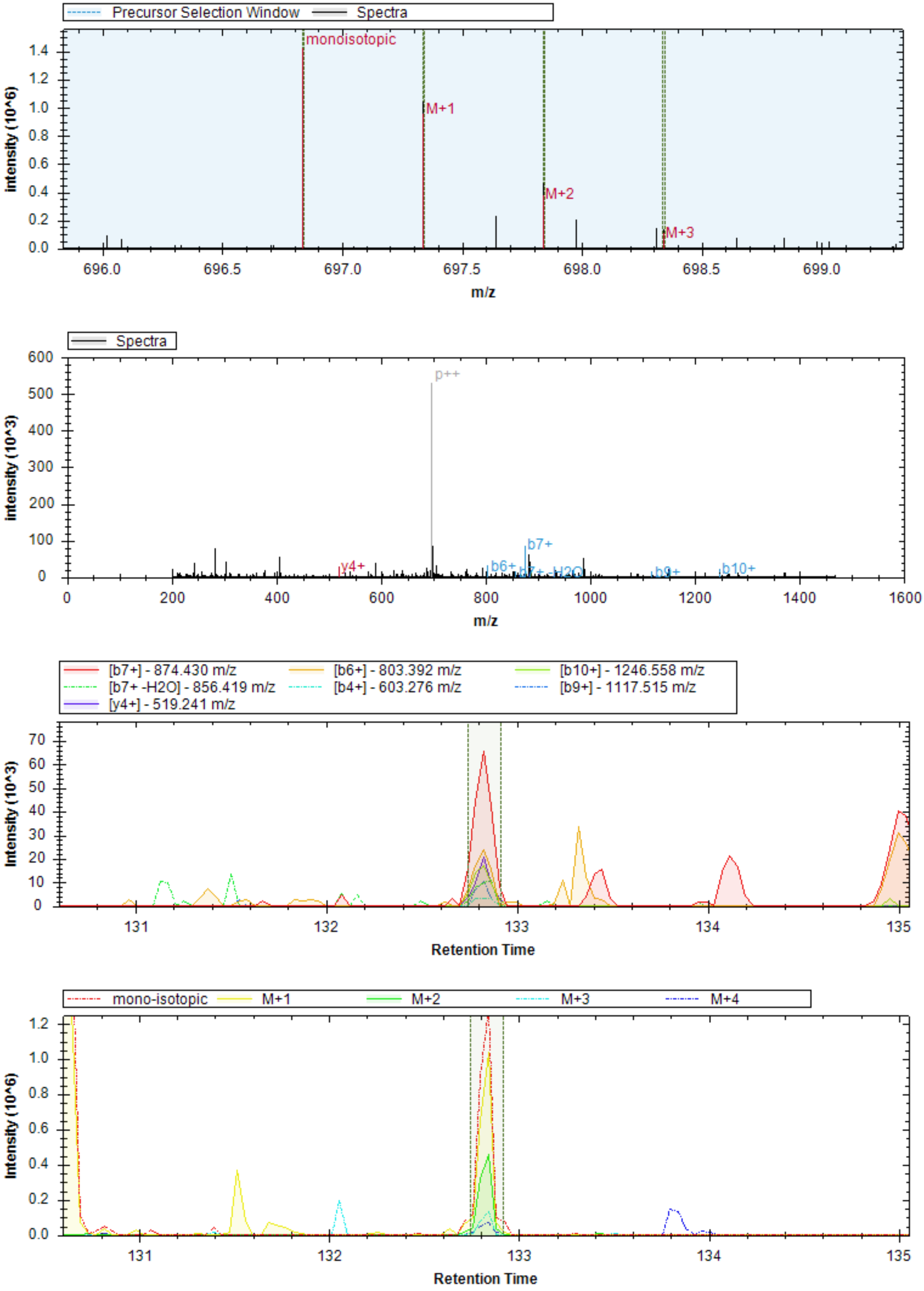

P10637 – _SGYSS[+H+O3+P]PGSPGTPGSR_.2

Fig4_MP-DIA-barrel-cortex-p30-1_MHRM.htrms

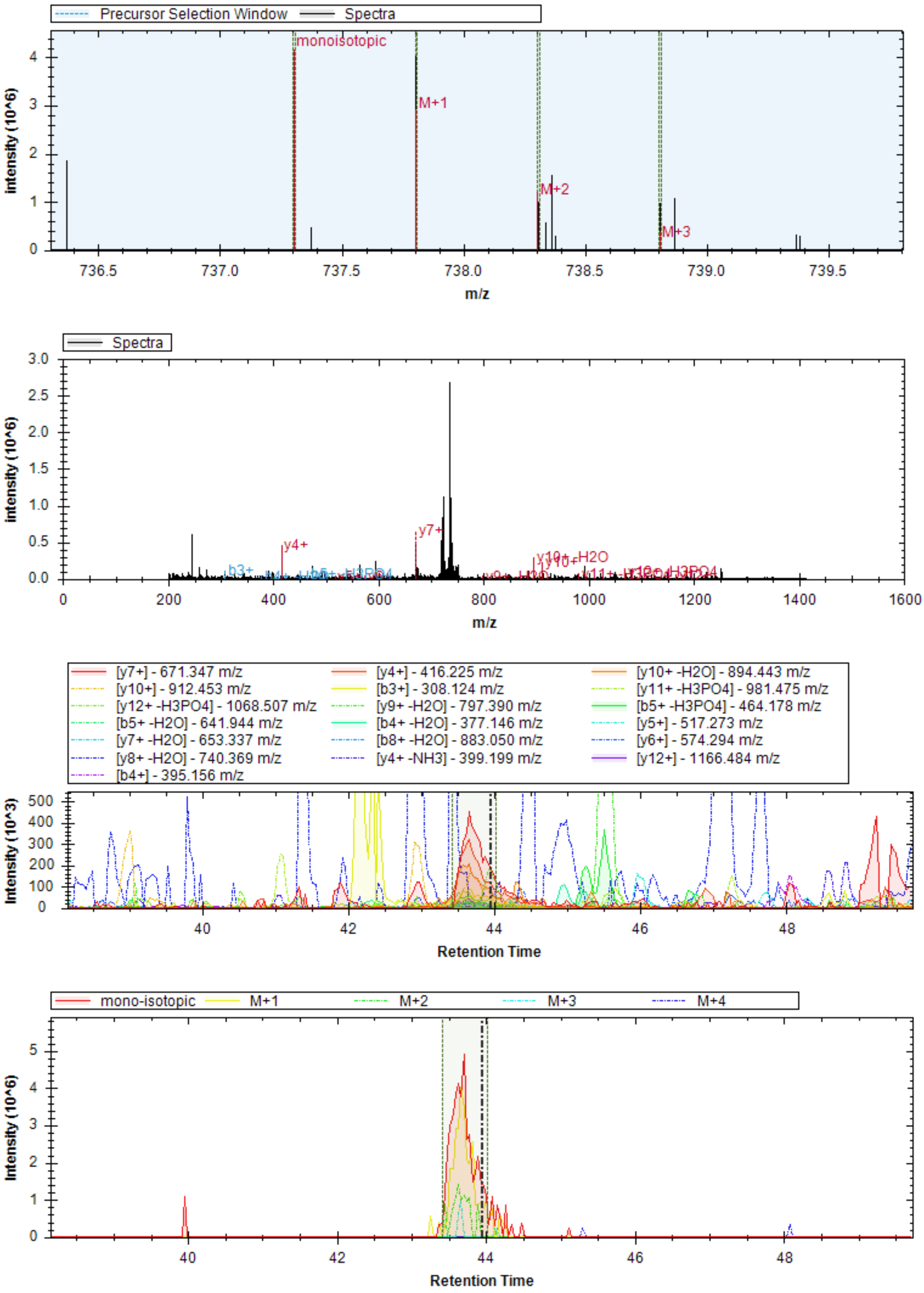

P10637 – _SGYSSPGS[+H+O3+P]PGTPGSR_.2
Fig4_MP-DIA-barrel-cortex-p30-1_MHRM.htrms

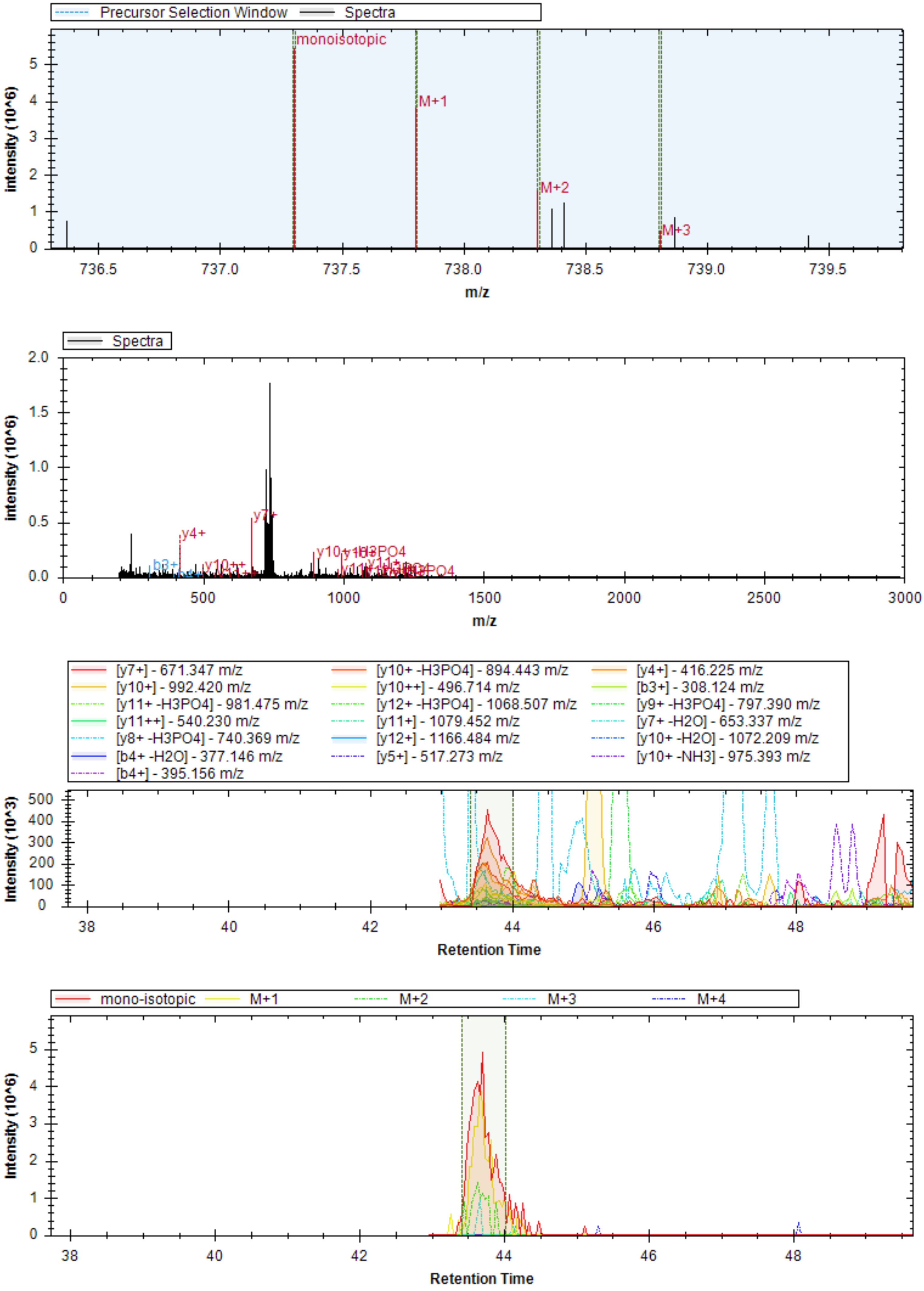

Q8C5R2 – _LAGNEALS[+H+O3+P]PTSPSK_.2
Fig4_MP-DIA-barrel-cortex-p30-1_MHRM.htrms

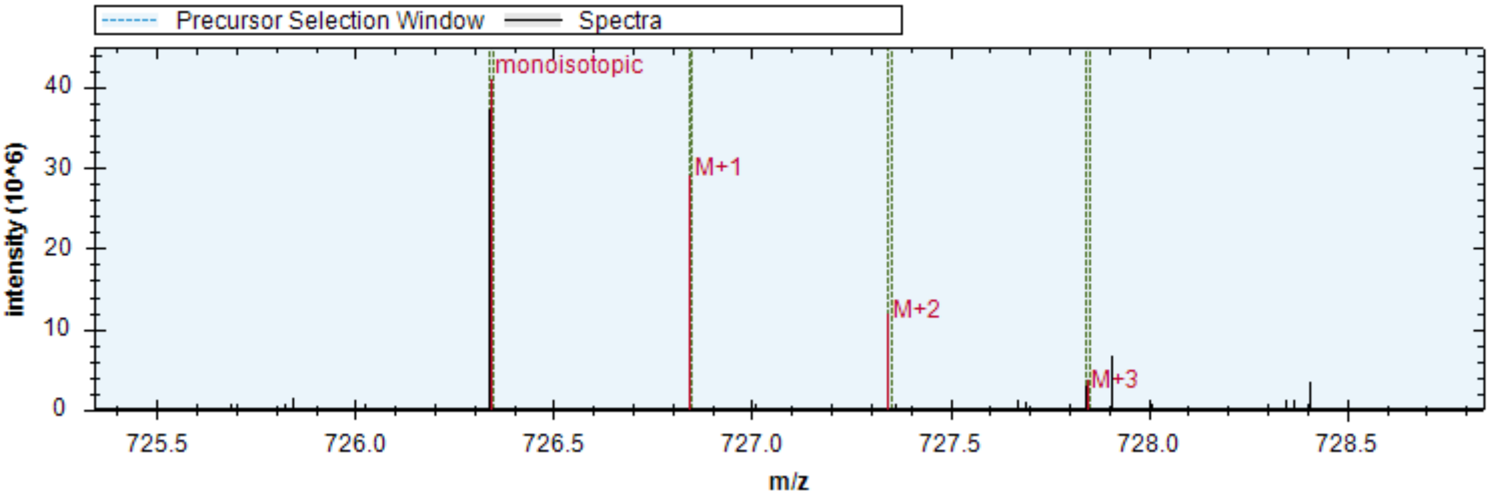

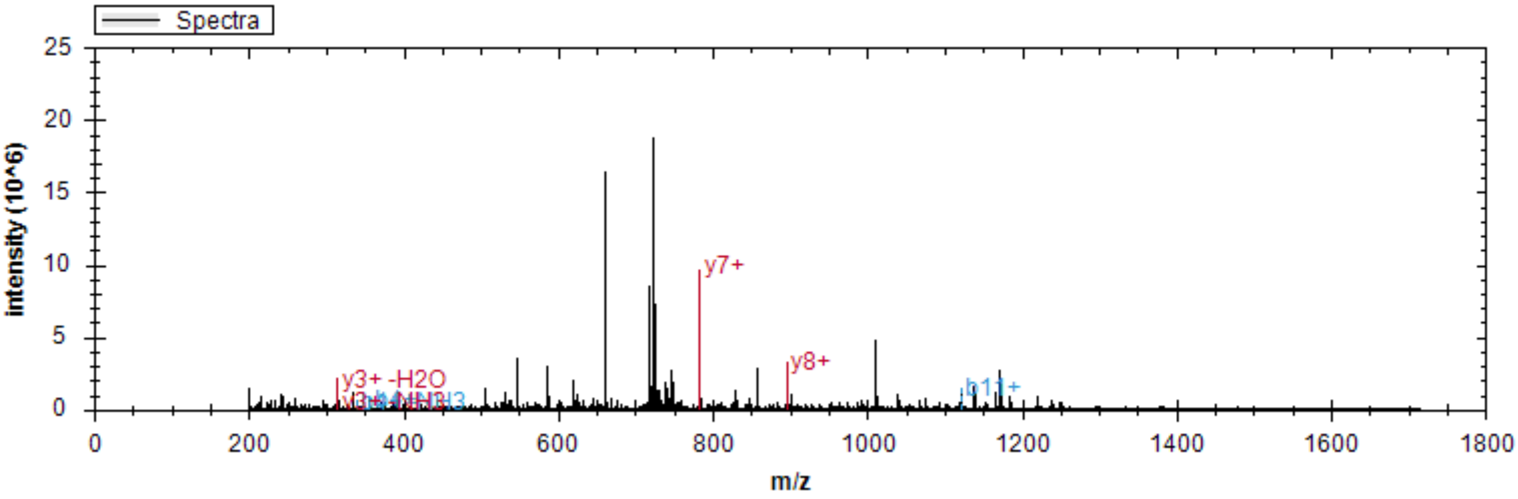

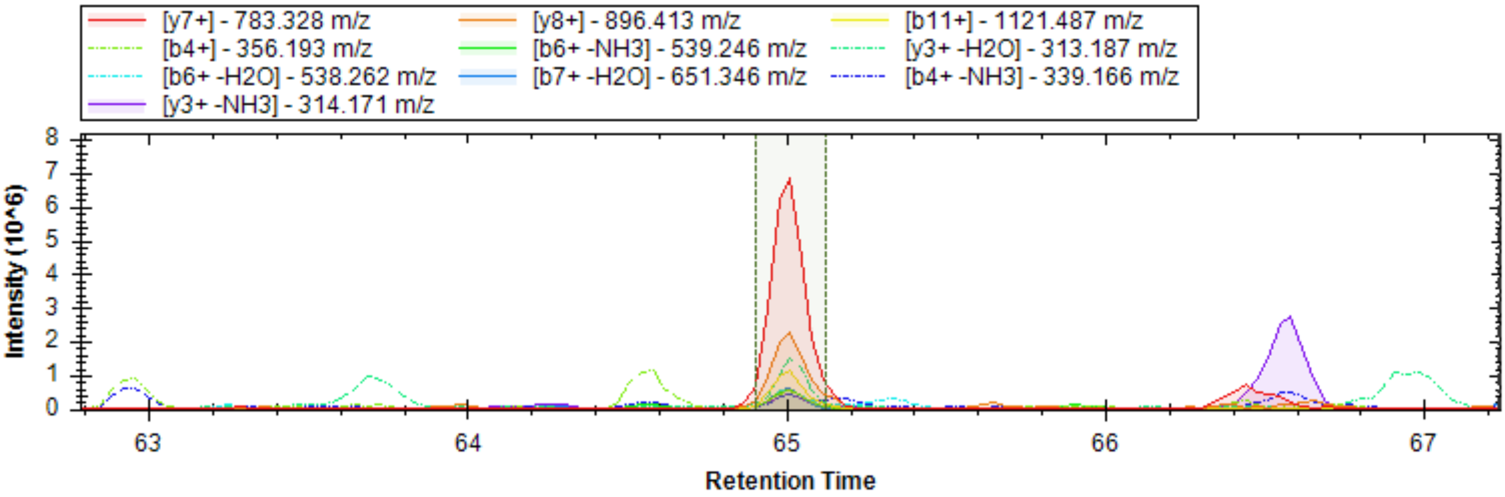

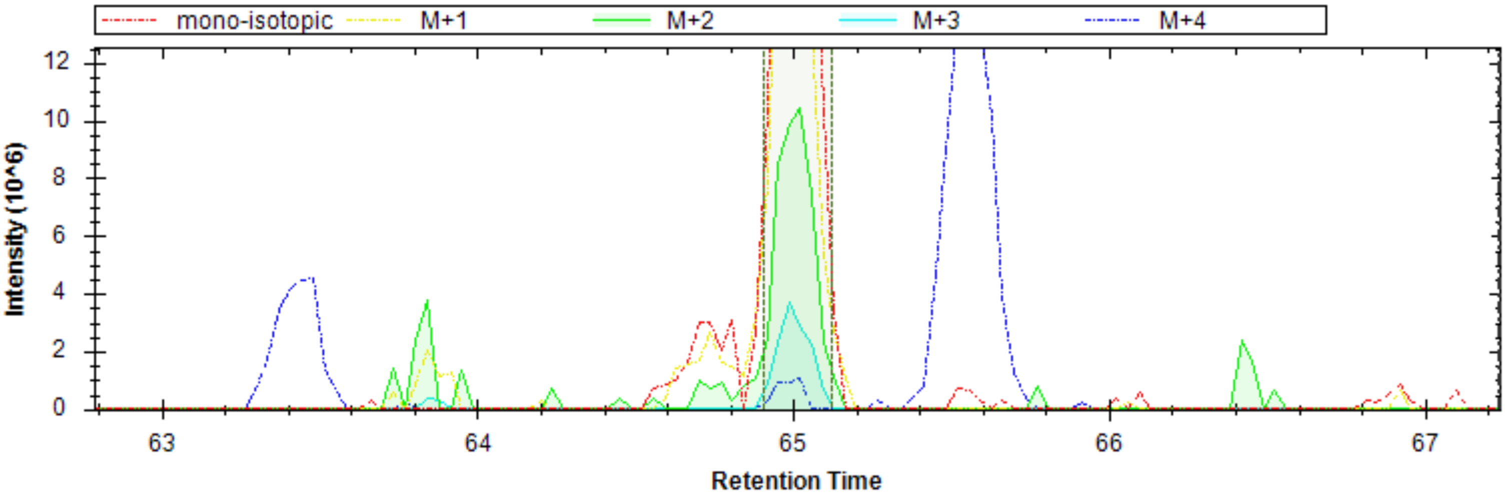

Q8K0U4 – _ETAPTSTYS[+H+O3+P]SPAR_.2
Fig4_MP-DIA-barrel-cortex-p30-1_MHRM.htrms

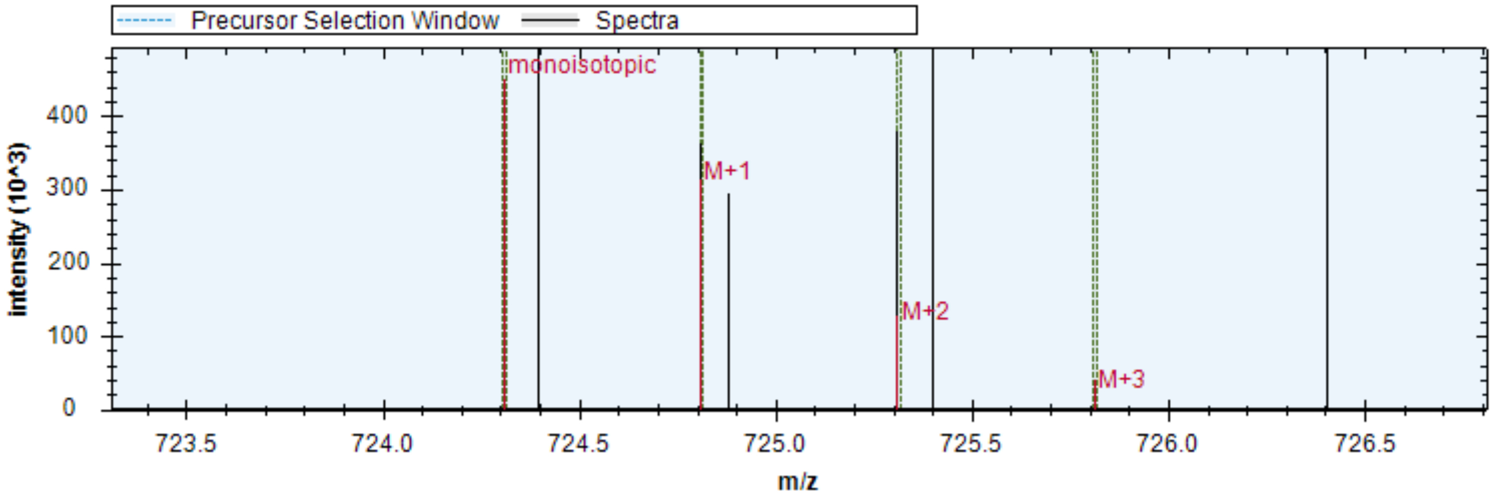

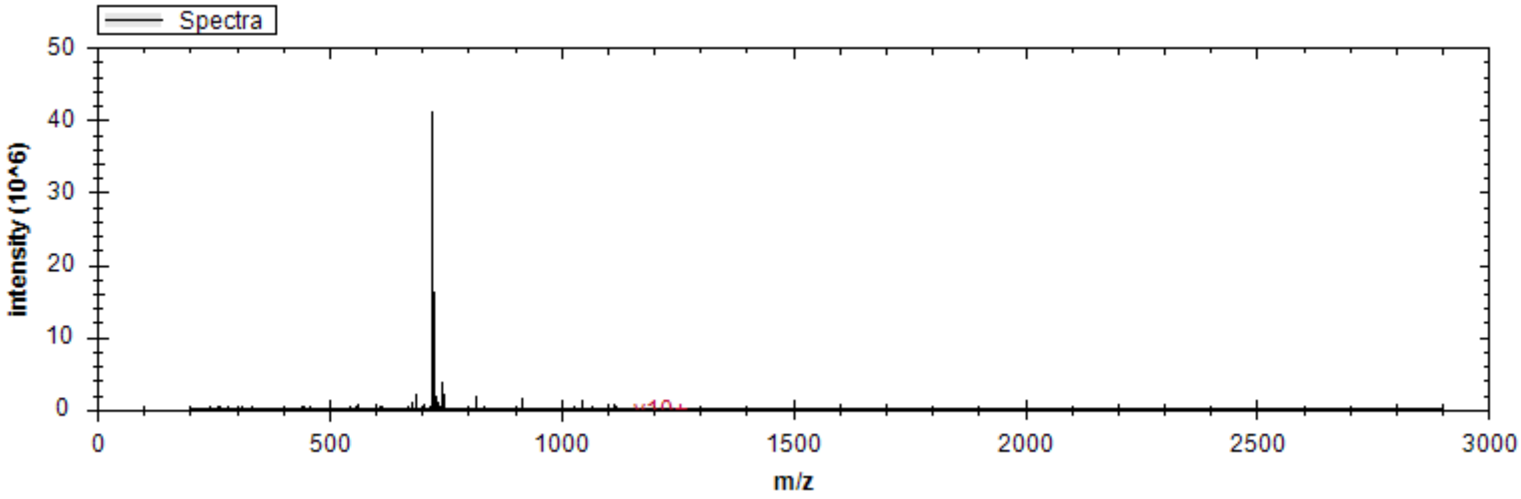

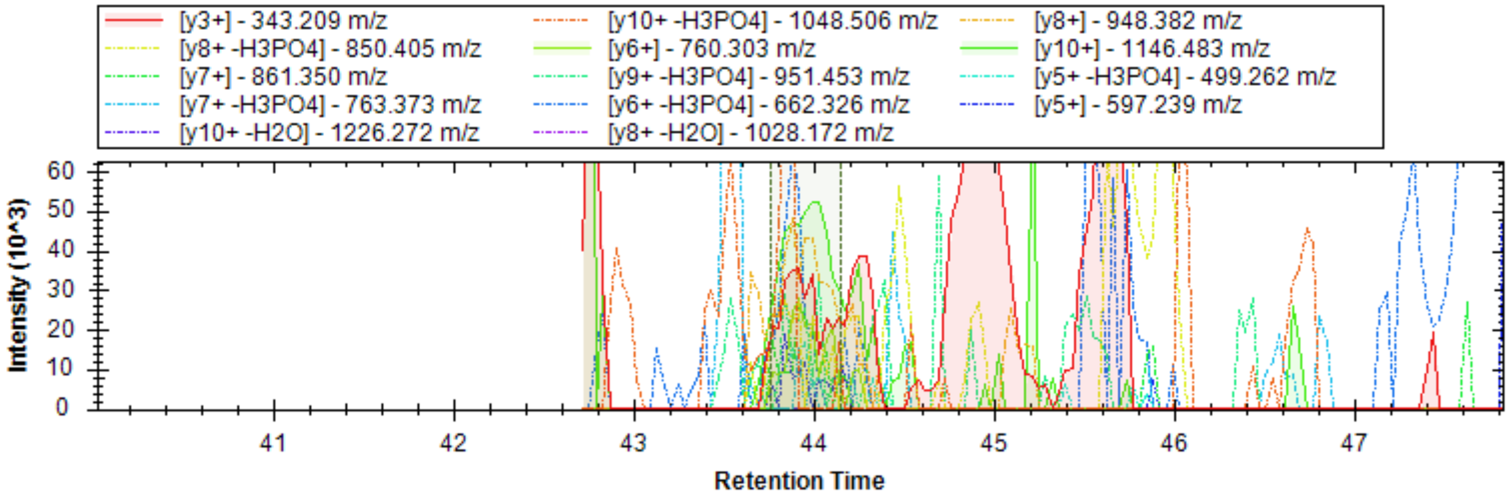

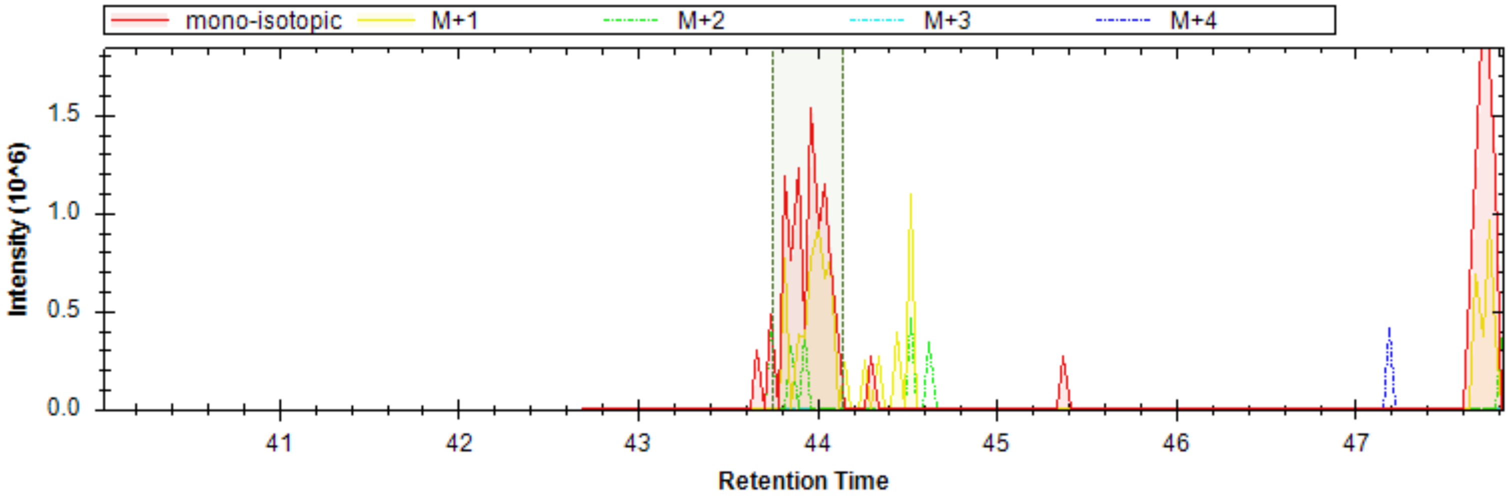

Q9CRB0 – _MEVYIPS[+H+O3+P]FRHEDSDLER_.3
Fig4_MP-DIA-barrel-cortex-p30-1_MHRM.htrms

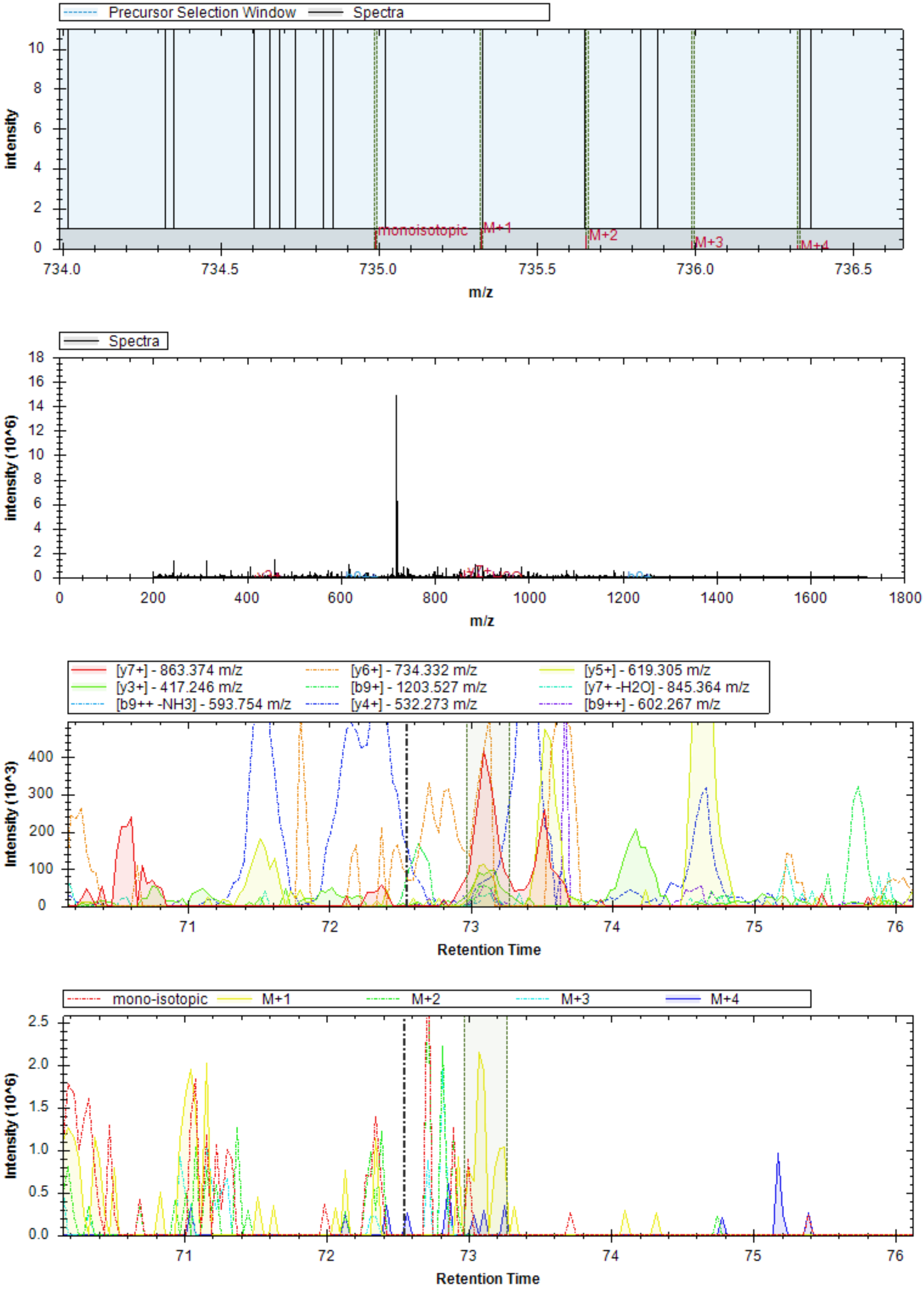

Q9JLM8 – _SPSPSPT[+H+O3+P]SPGSLRK_.2
Fig4_MP-DIA-barrel-cortex-p30-1_MHRM.htrms

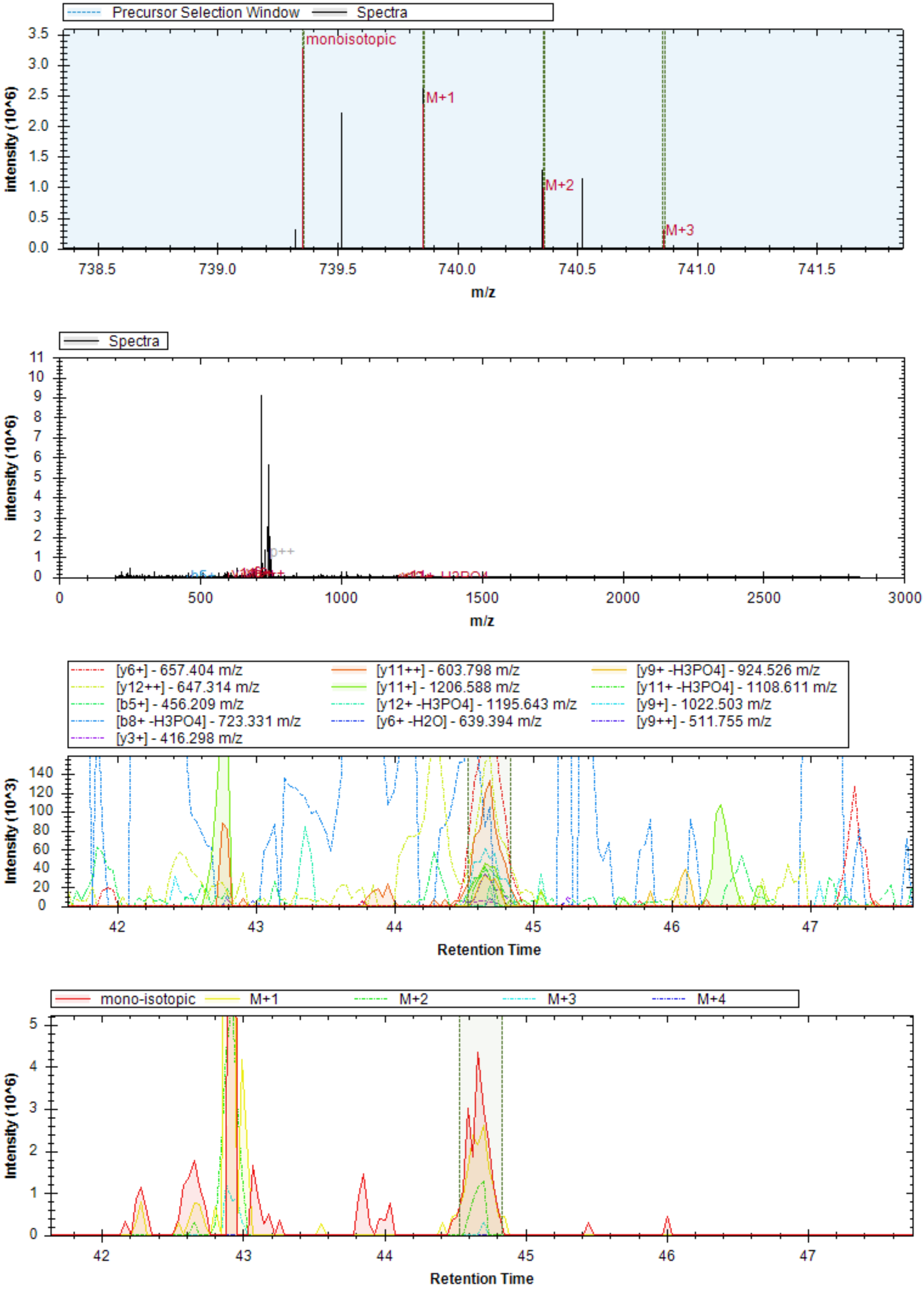

Q9JLM8 – _SPSPSPTS[+H+O3+P]PGSLRK_.2
Fig4_MP-DIA-barrel-cortex-p30-1_MHRM.htrms

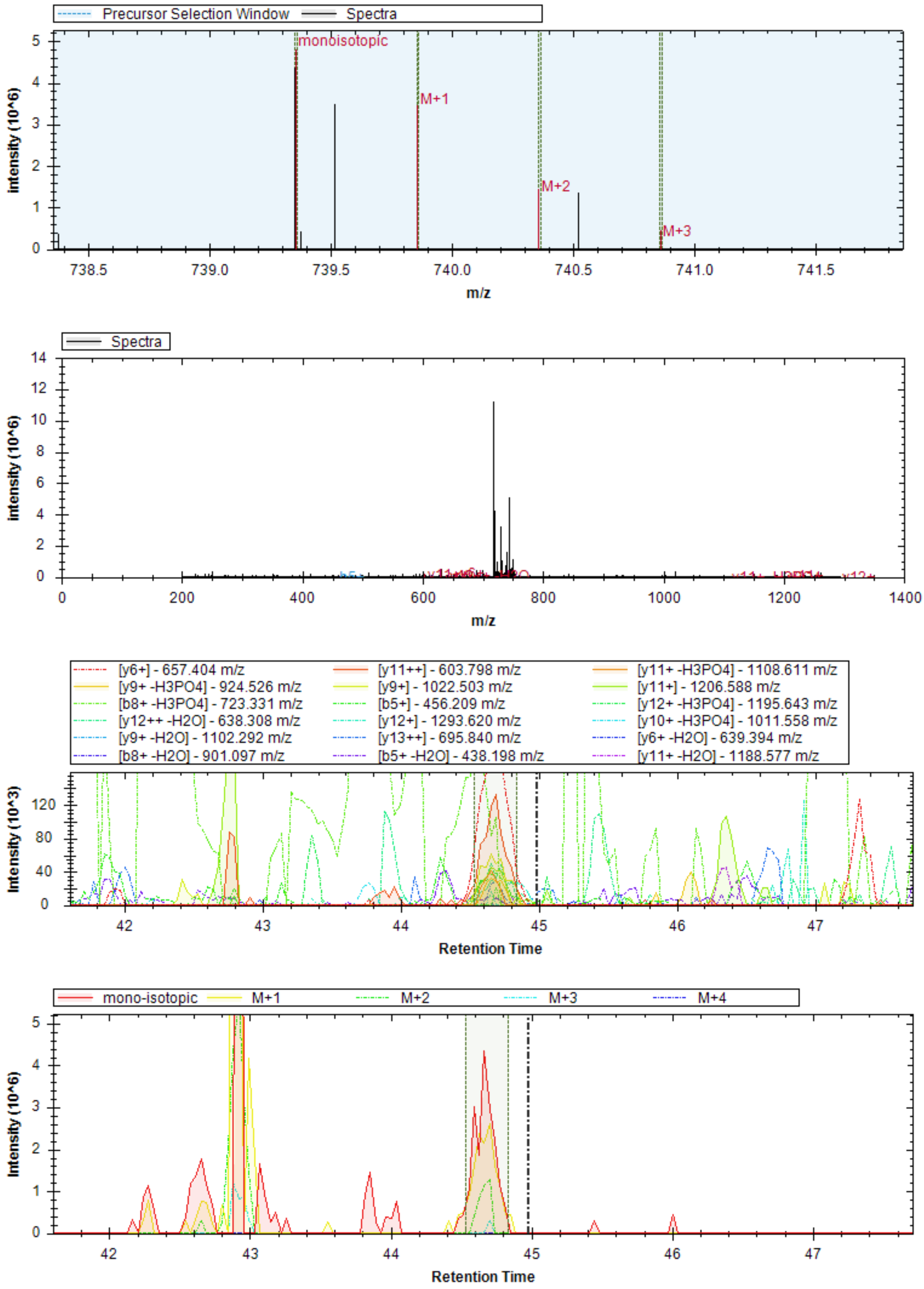

D3Z6S9 – _IPSIEIT[+H+O3+P]ET[+H+O3+P]LPQTQASMLR_.3
Fig4_MP-DIA-barrel-cortex-p30-1_MHRM.htrms

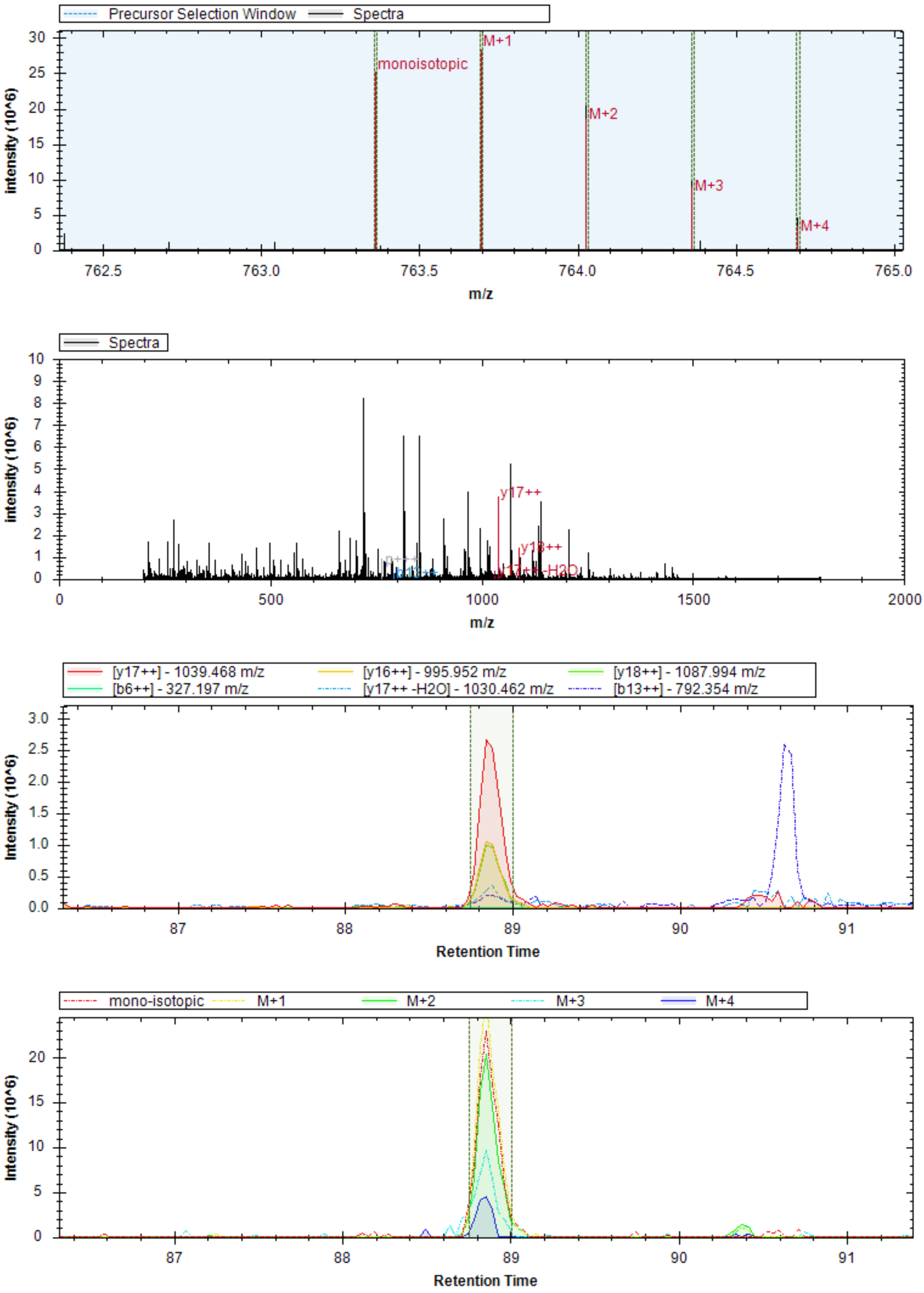

P0C6F1 – _IIDLQM[+O]HDY[+H+O3+P]LR_.2
Fig4_MP-DIA-barrel-cortex-p30-1_MHRM.htrms

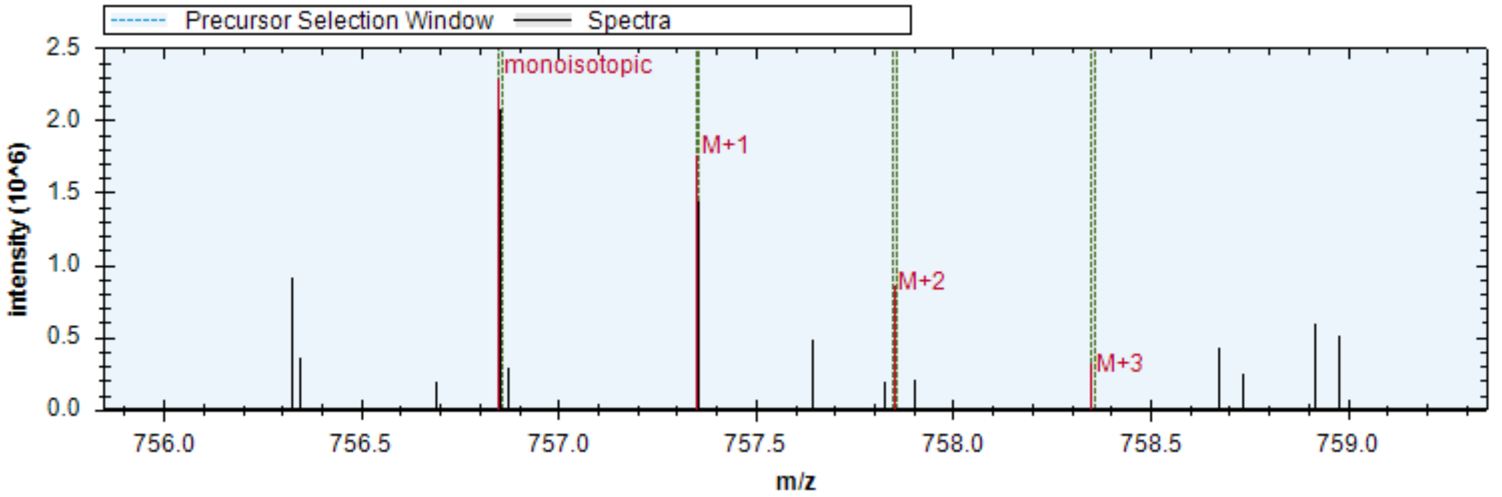

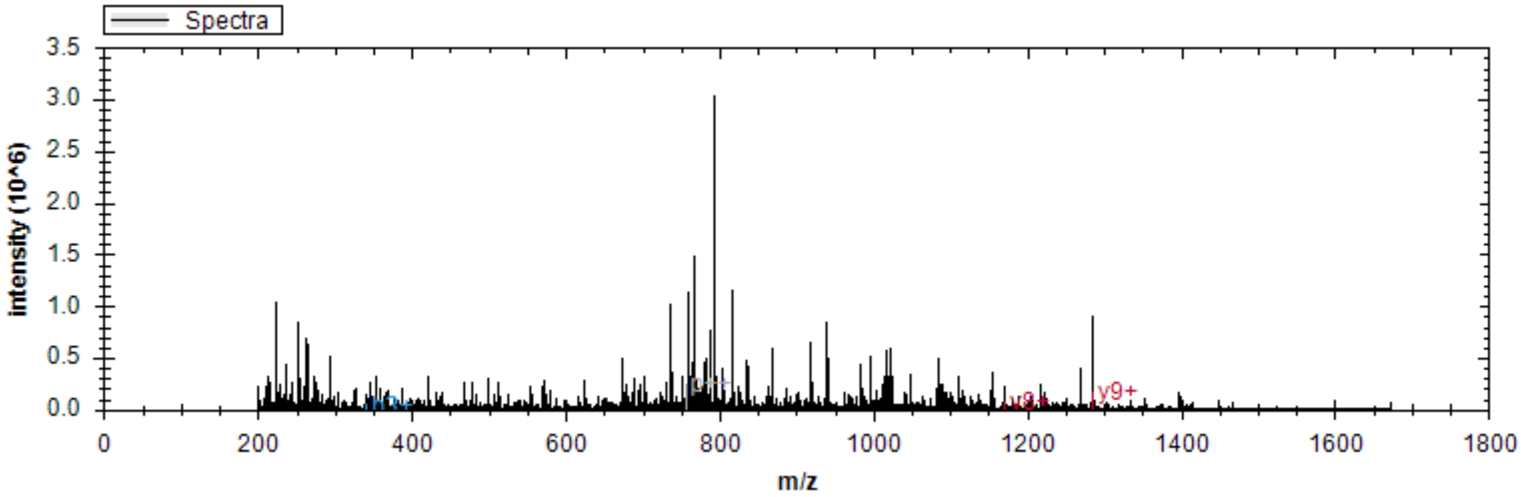

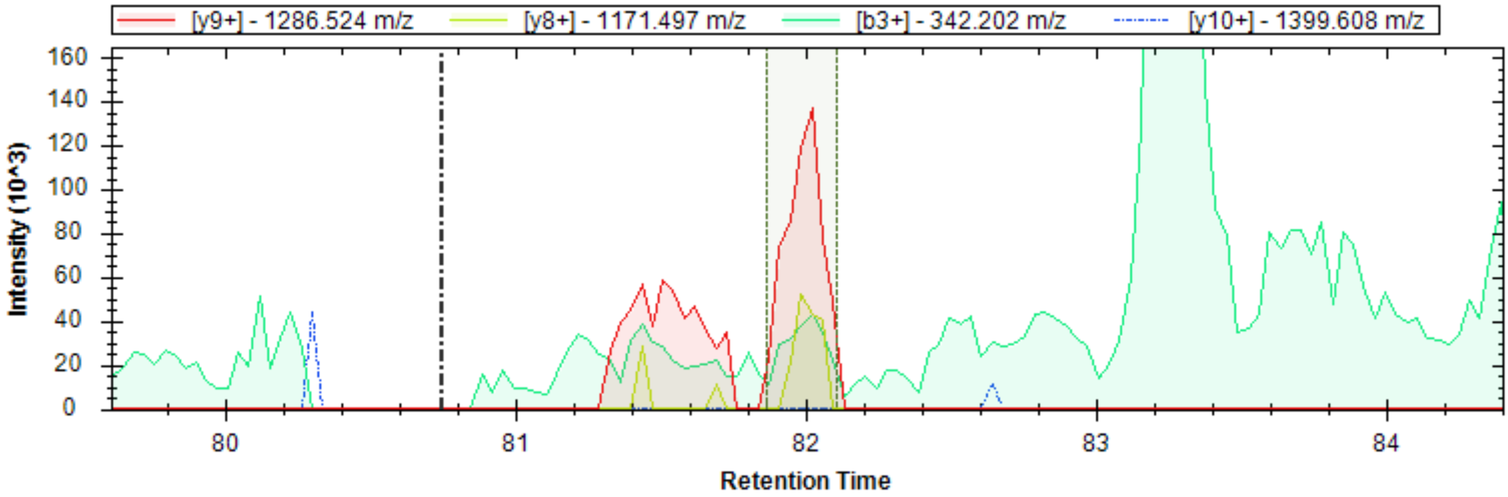

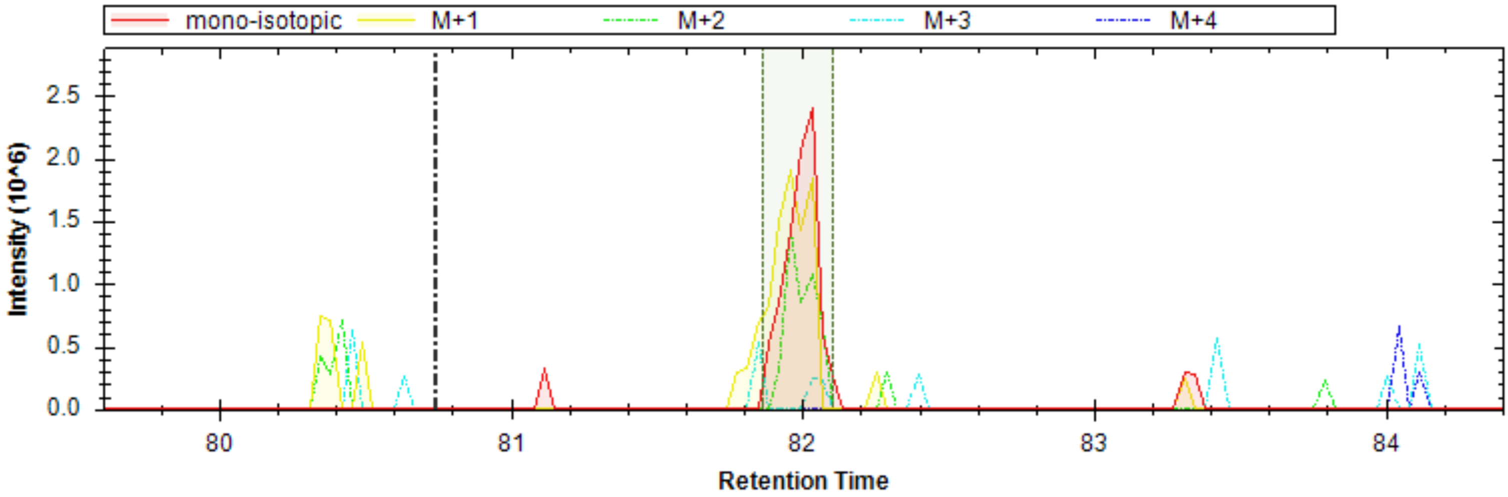

Q68FD9 – _LAQLLS[+H+O3+P]EVPT[+H+O3+P]RR_.2

Fig4_MP-DIA-barrel-cortex-p30-1_MHRM.htrms

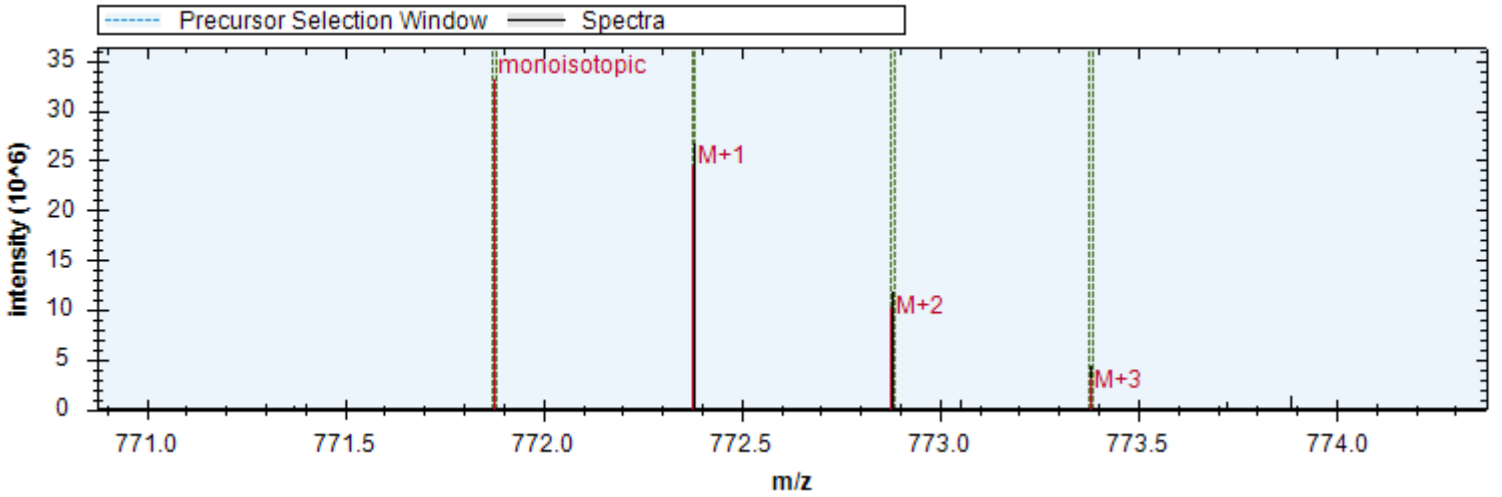

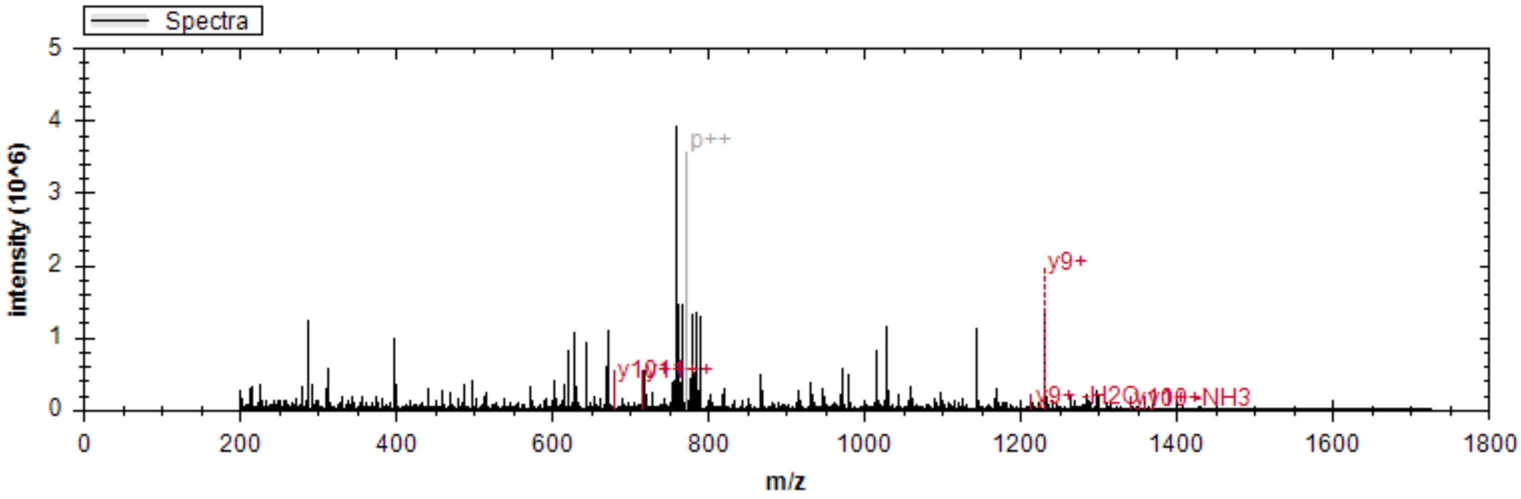

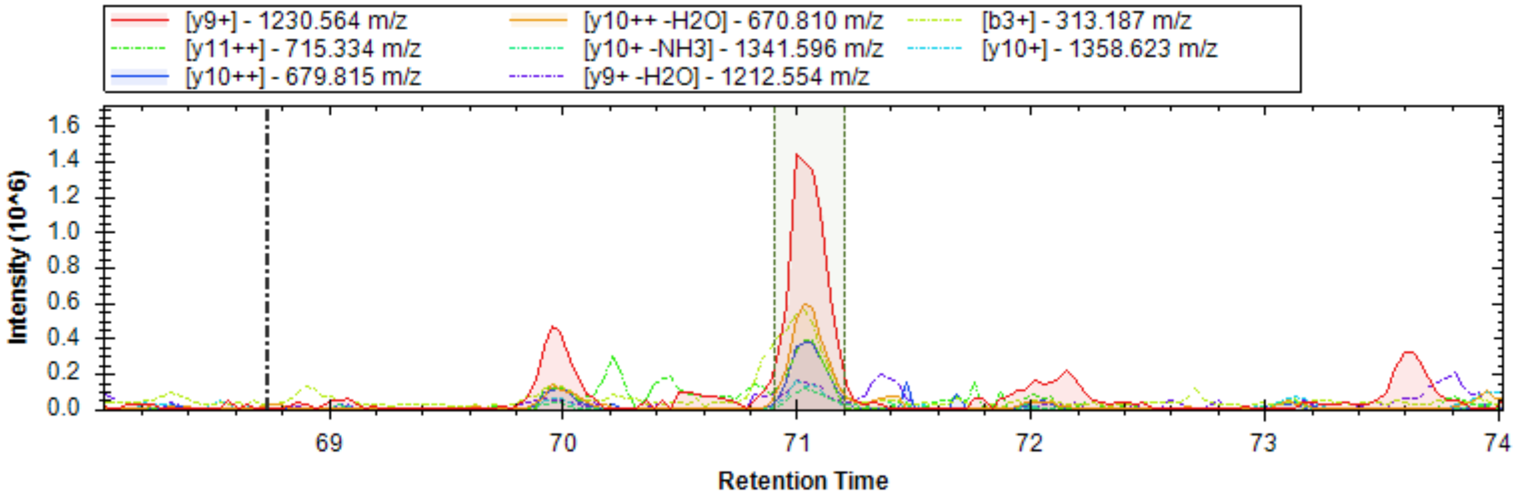

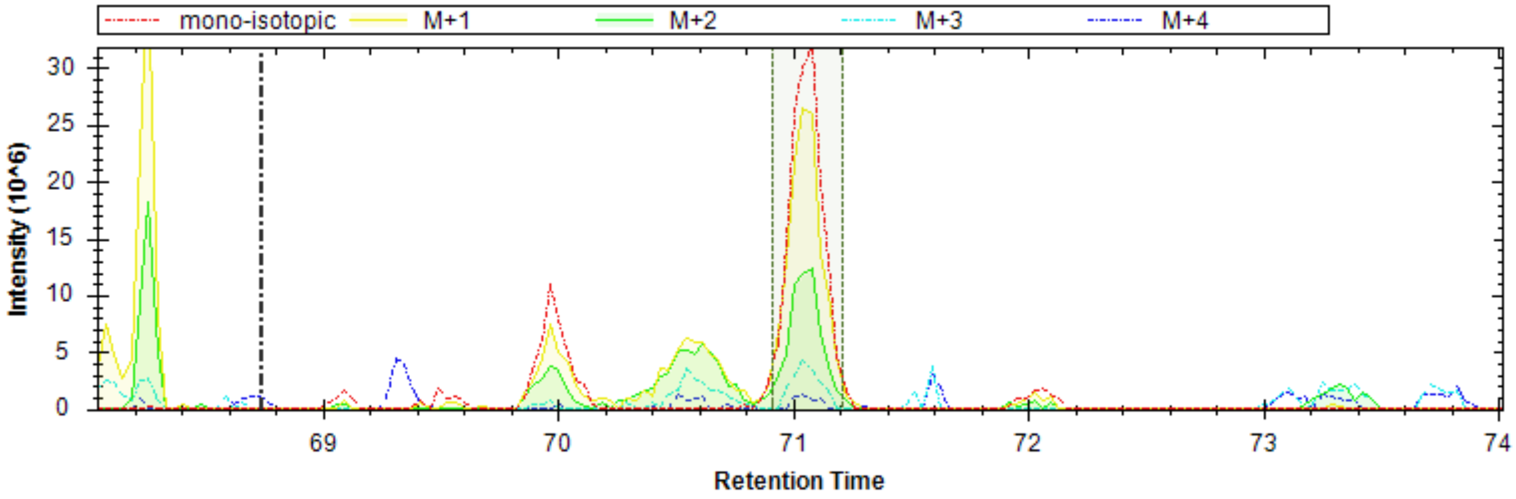

Q8BRT1 – _VLNT[+H+O3+P]GSDVEEAVADALLLGDIR_.3
Fig4_MP-DIA-barrel-cortex-p30-1_MHRM.htrms

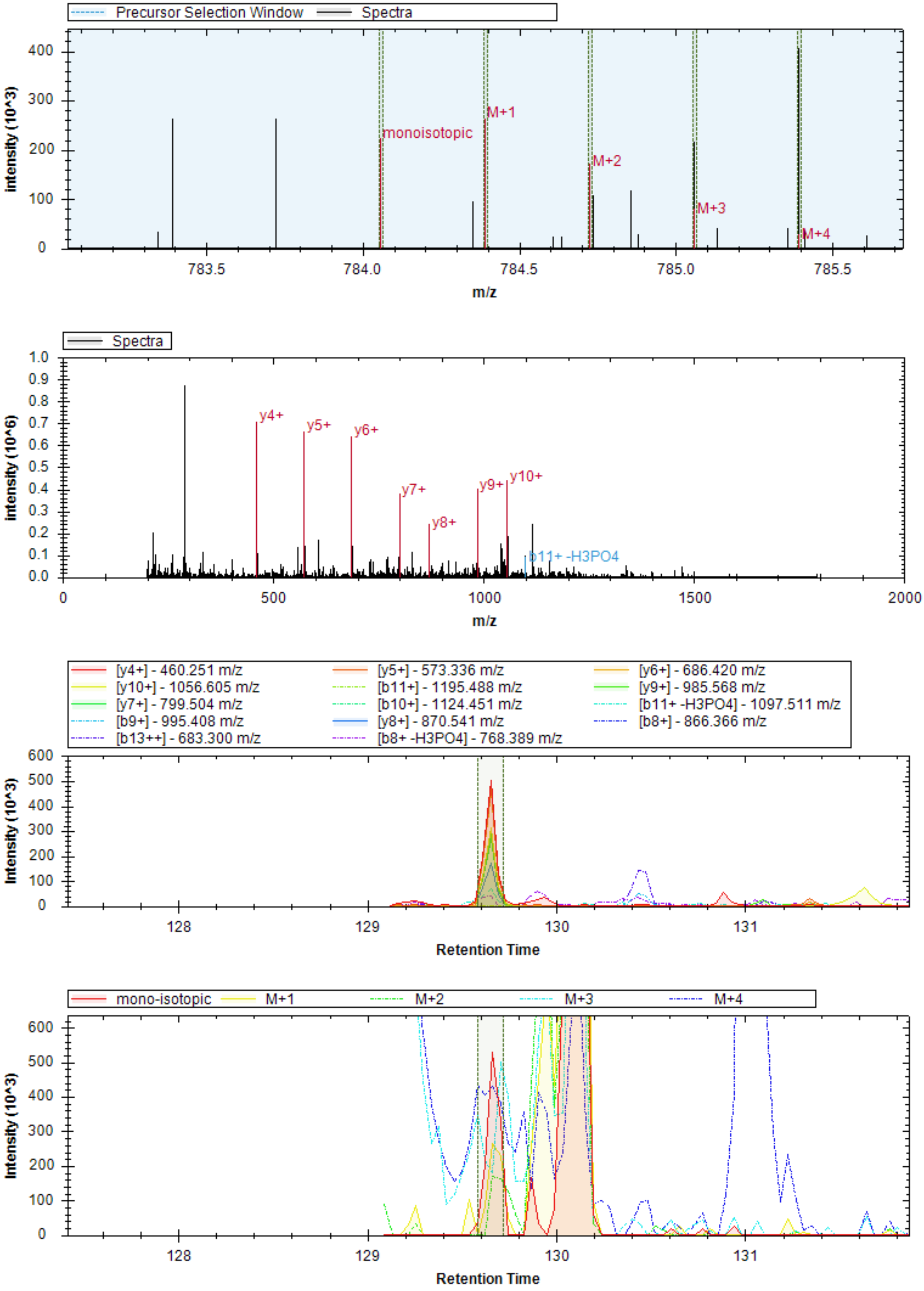

Q8CBW3;Q8CBW3-2;Q8CB[...] – _HNS[+H+O3+P]TTSSTSSGGYR_.2
Fig4_MP-DIA-barrel-cortex-p30-1_MHRM.htrms

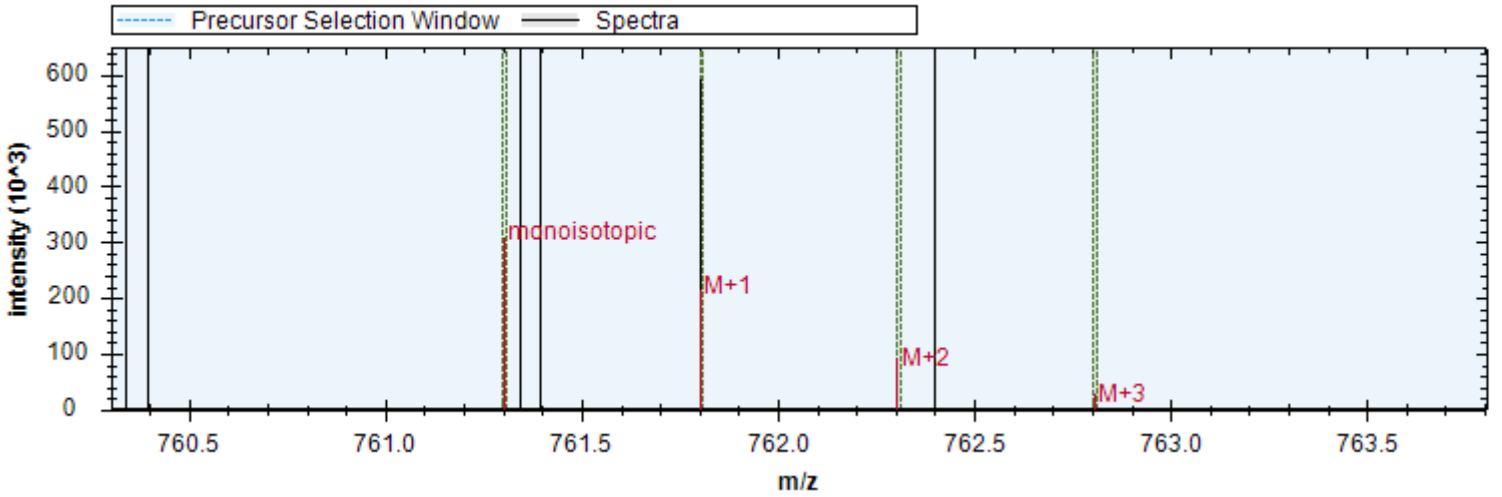

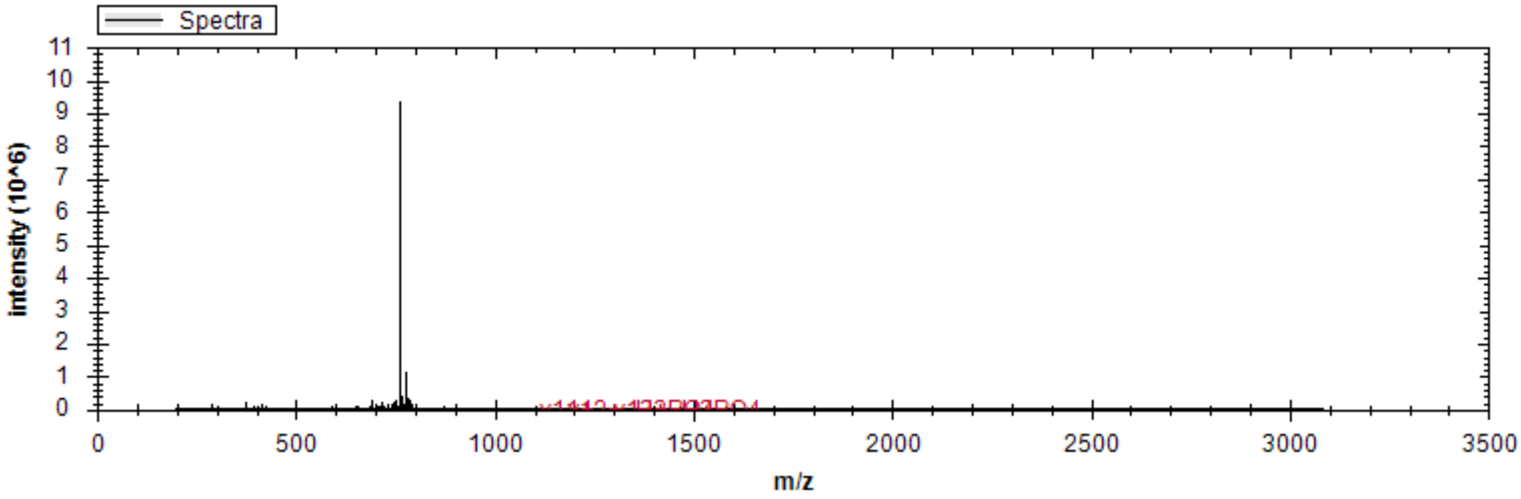

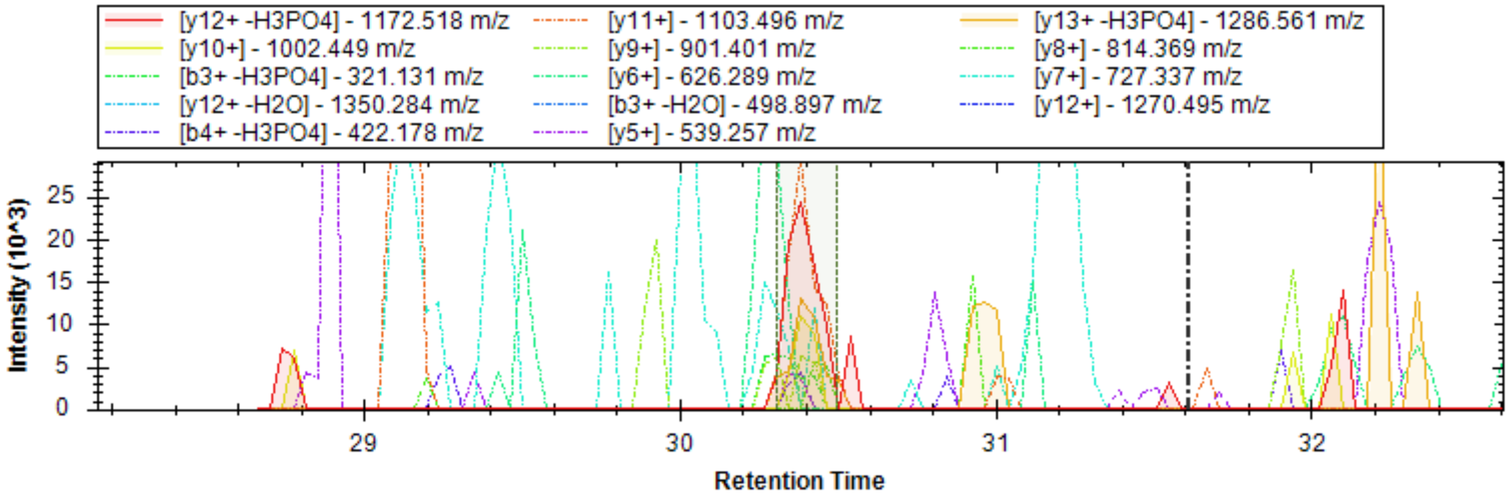

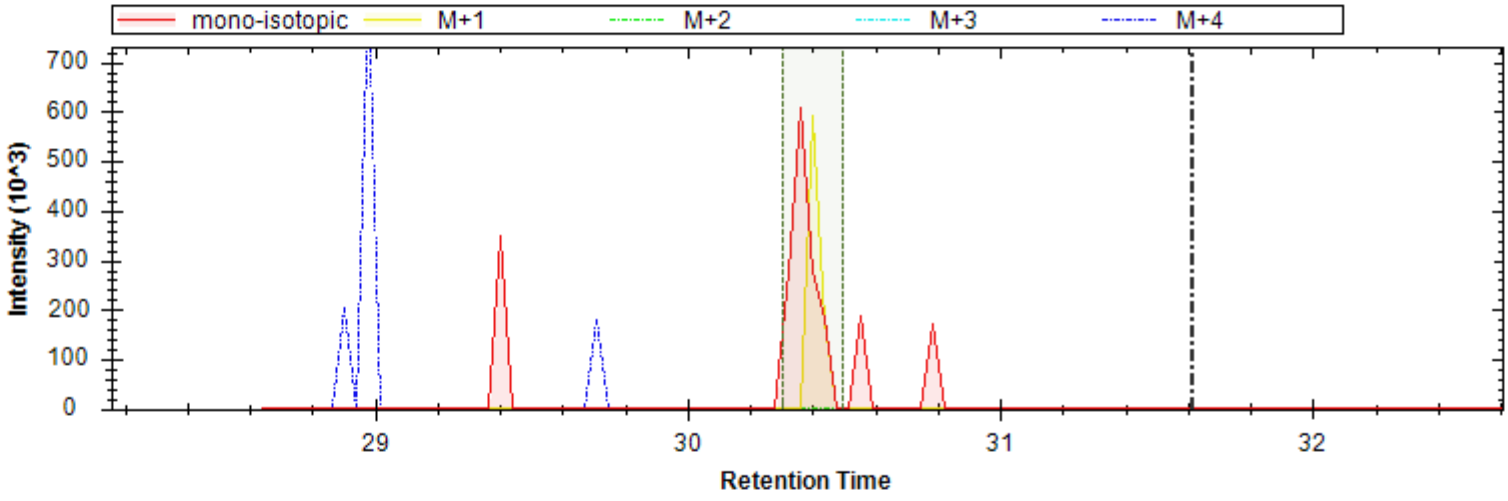

Q9QWI6 – _LSYAGGRPPSYAGS[+H+O3+P]PVHHAAER_.3
Fig4_MP-DIA-barrel-cortex-p30-1_MHRM.htrms

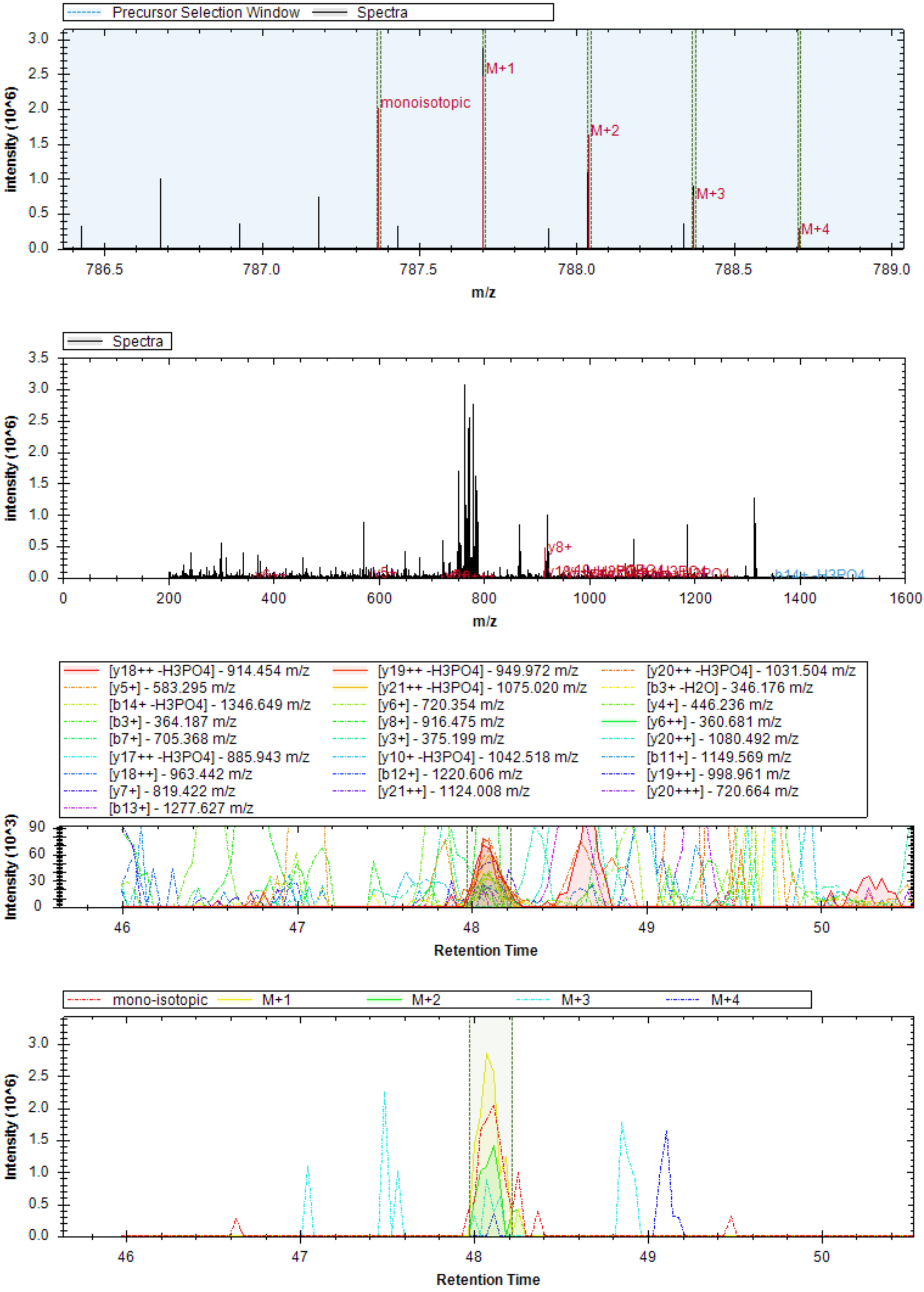

Q9QYC0 – _AAVVTS[+H+O3+P]PPPTTAPHK_.2
Fig4_MP-DIA-barrel-cortex-p30-1_MHRM.htrms

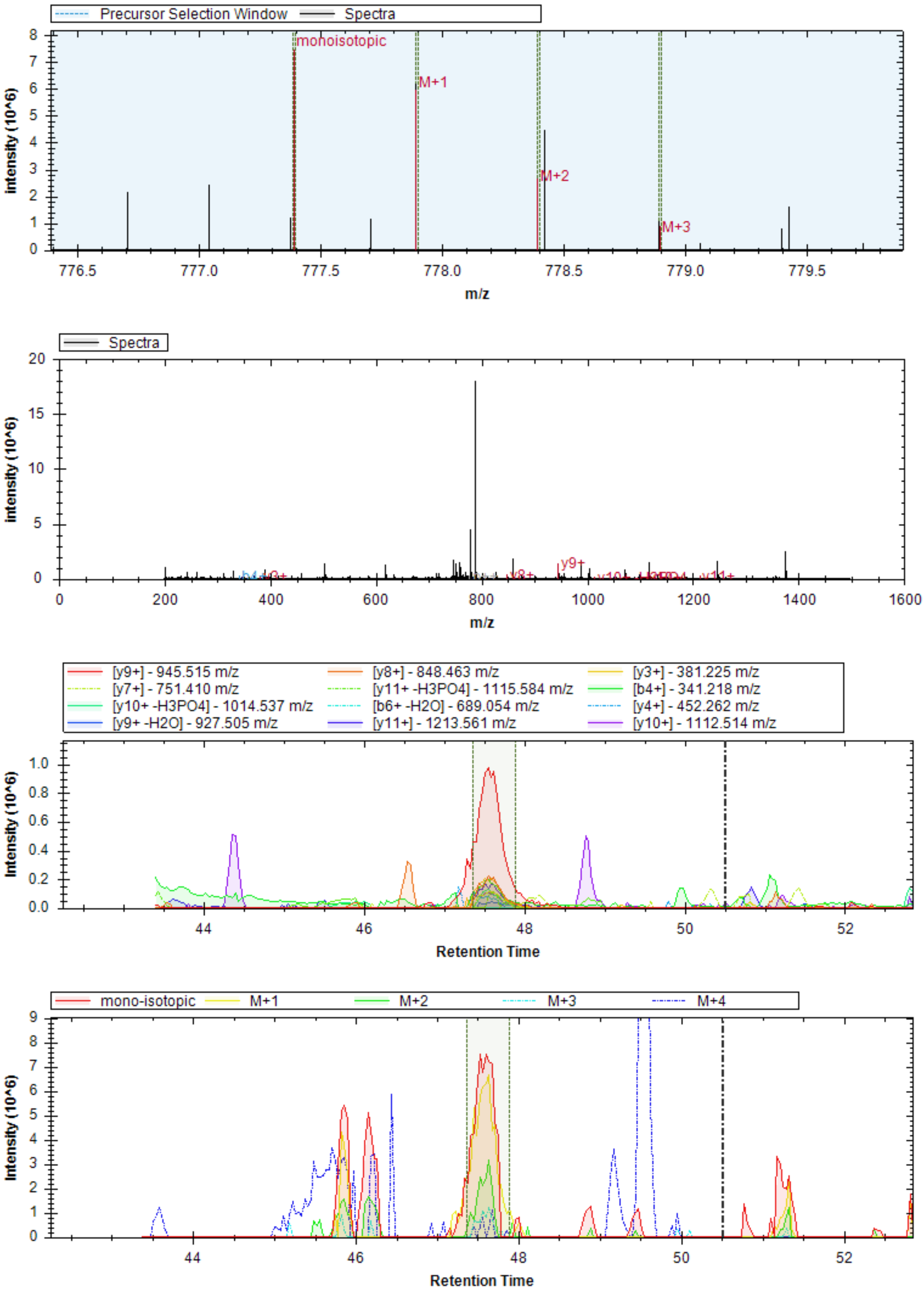

Q9QYR6-2 – _ELSSEPRT[+H+O3+P]PPAQK_.2
Fig4_MP-DIA-barrel-cortex-p30-1_MHRM.htrms

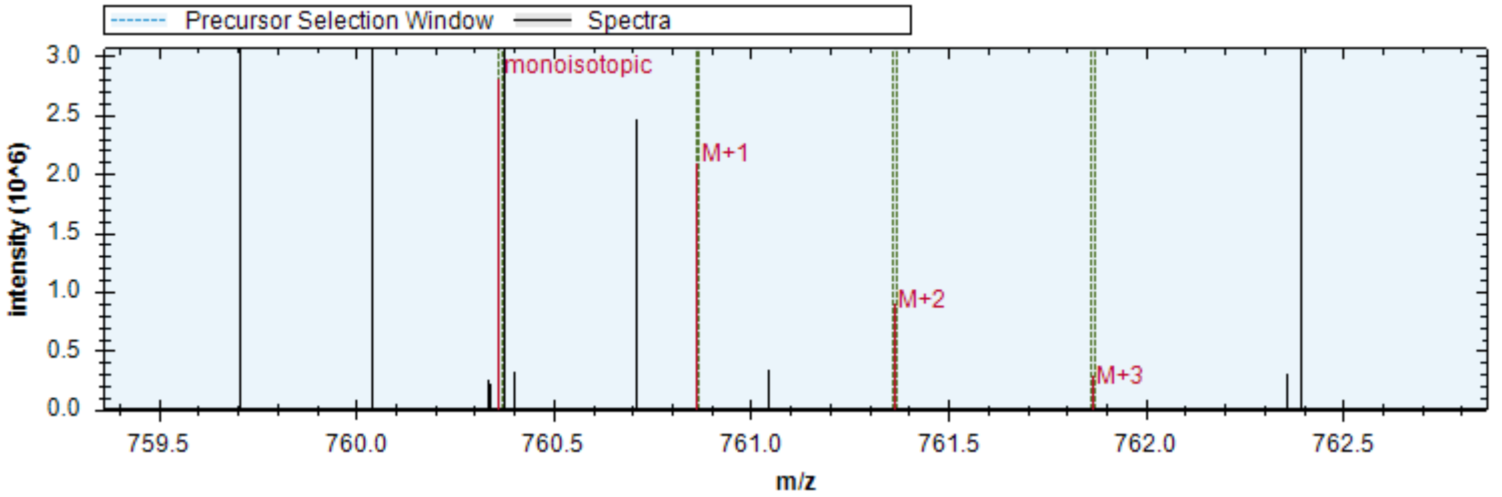

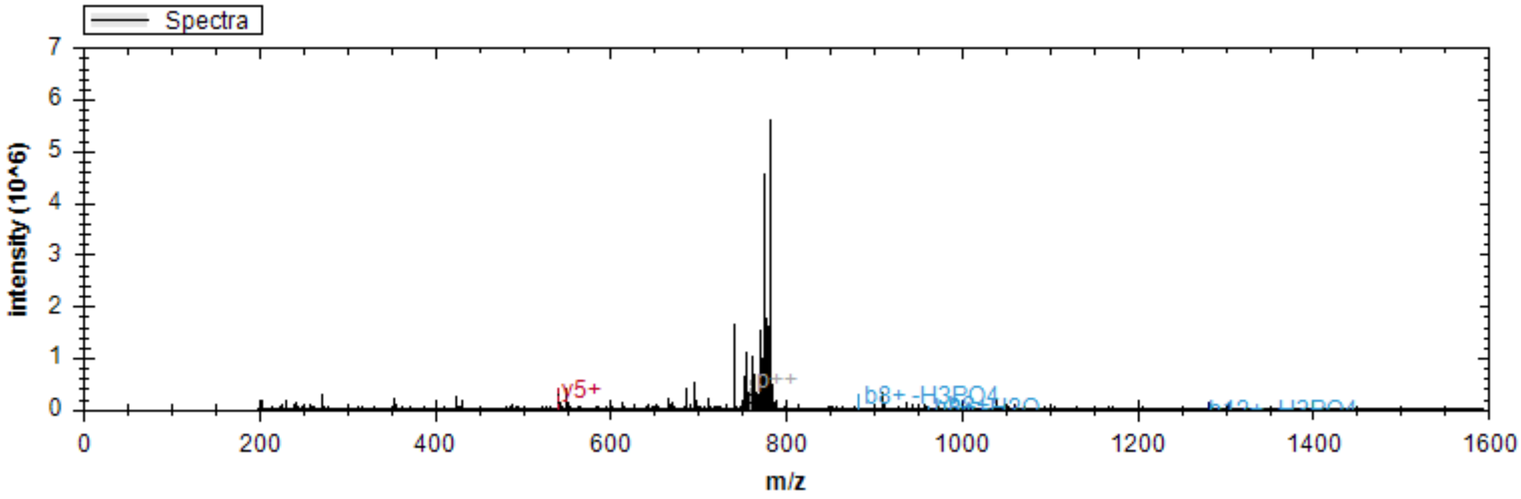

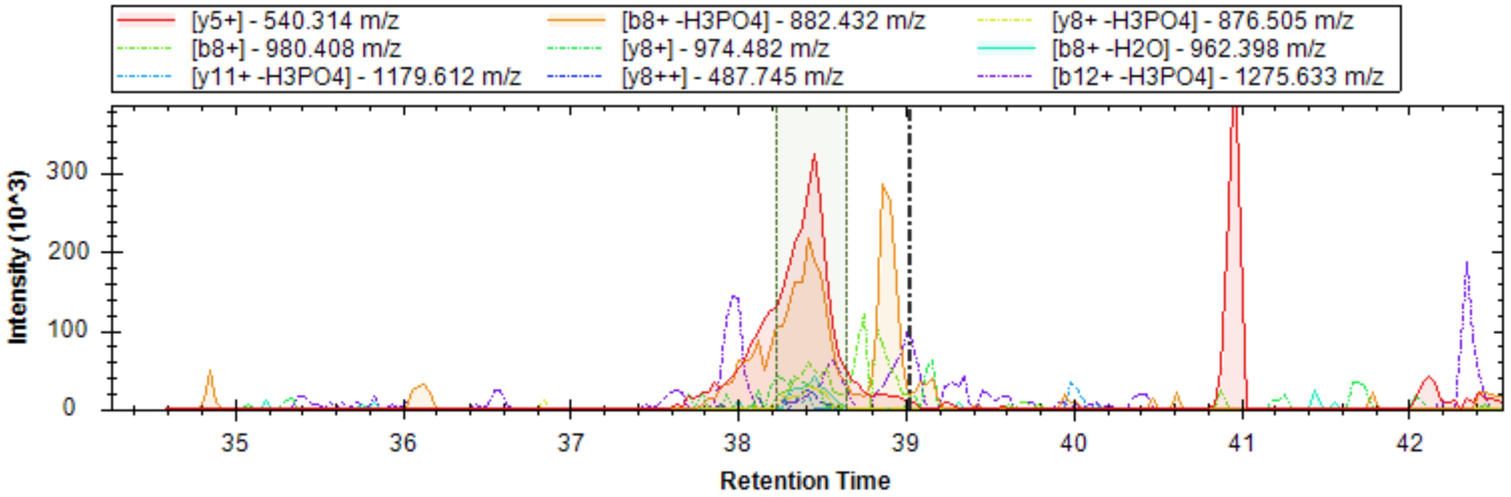

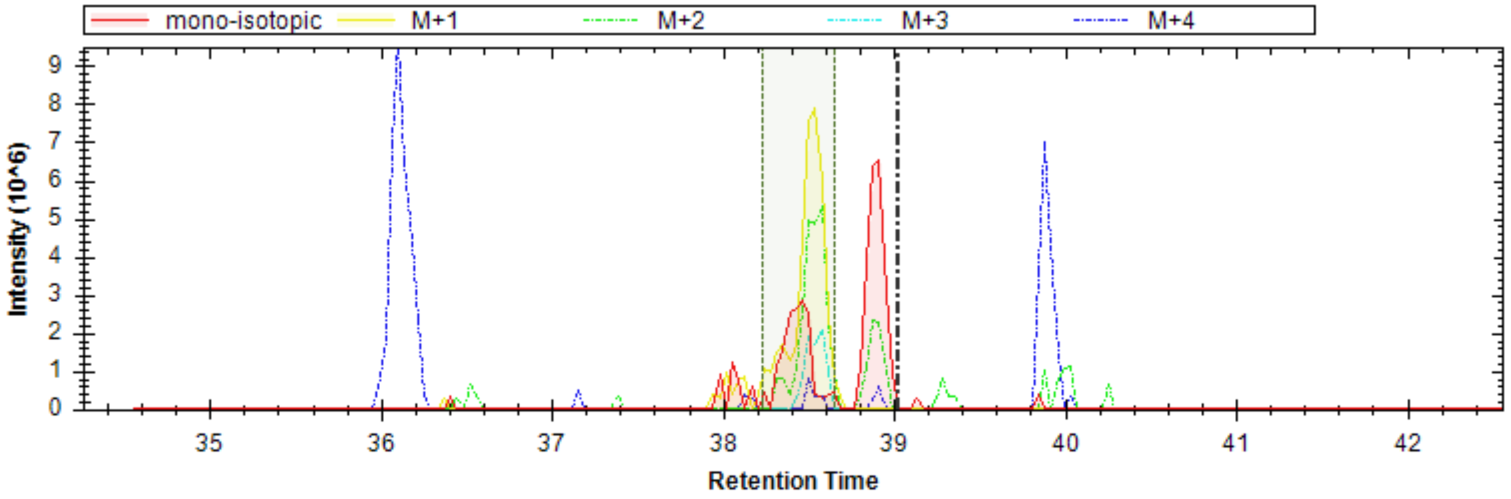

Q9Z0P4 – _ENSAAPSPGRPQS[+H+O3+P]ASPAKEEQK_.3
Fig4_MP-DIA-barrel-cortex-p30-1_MHRM.htrms

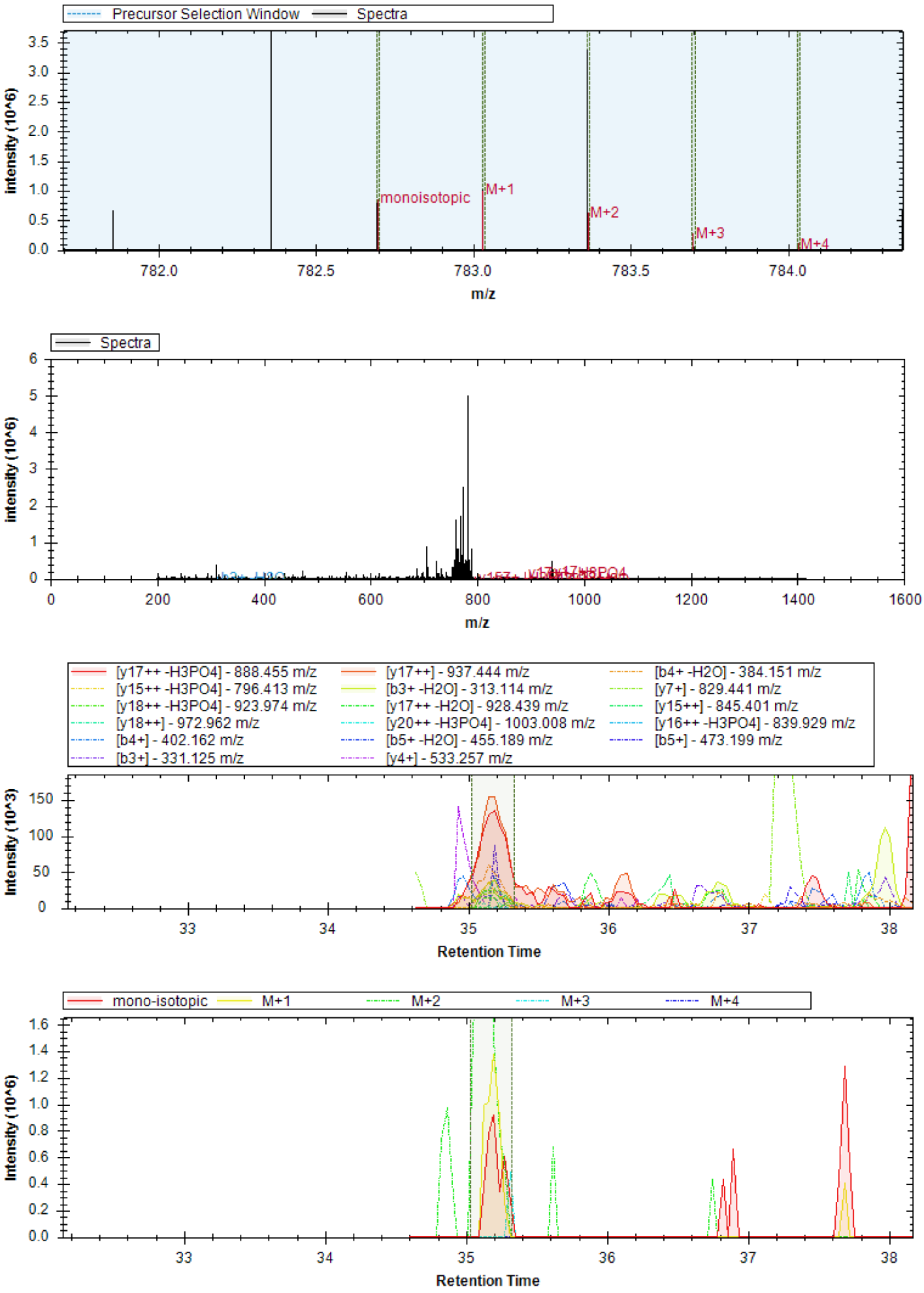

Q9Z2C4 – _GSS[+H+O3+P]PTHSATPVHTSV_.2
Fig4_MP-DIA-barrel-cortex-p30-1_MHRM.htrms

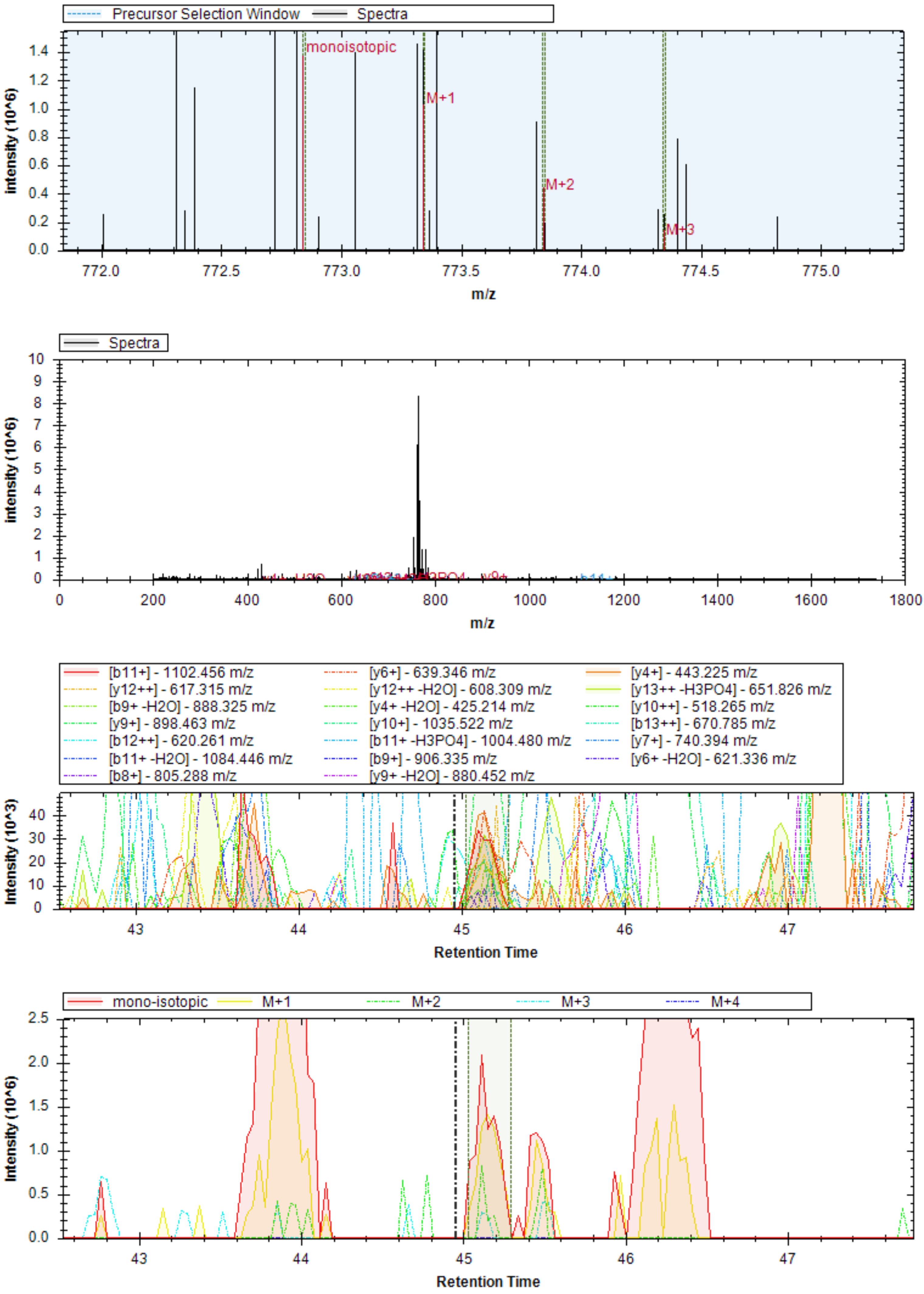

P08249 – _NS[+H+O3+P]PLVSRLTLYDIAHTPGVAADLSHIETR_.4
Fig4_MP-DIA-barrel-cortex-p30-1_MHRM.htrms

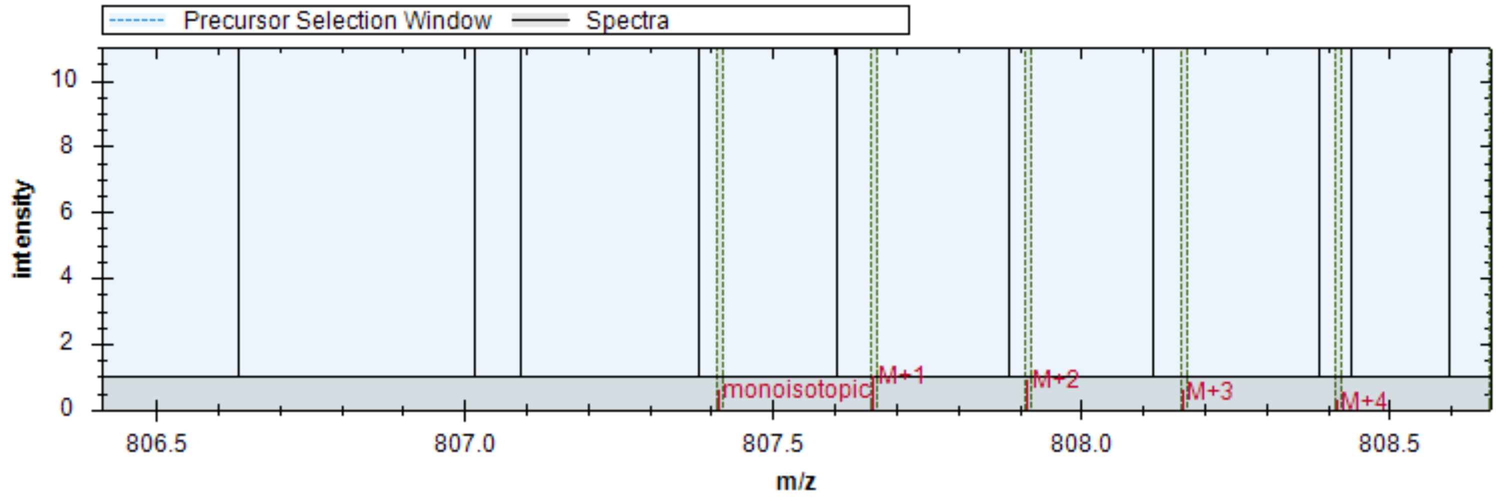

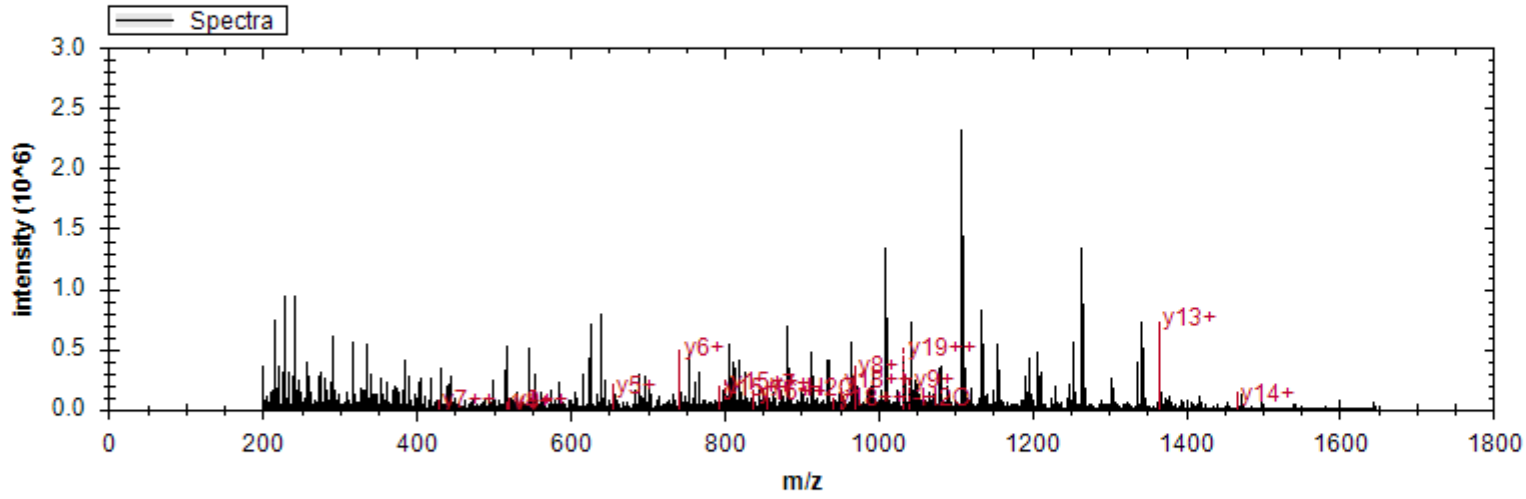

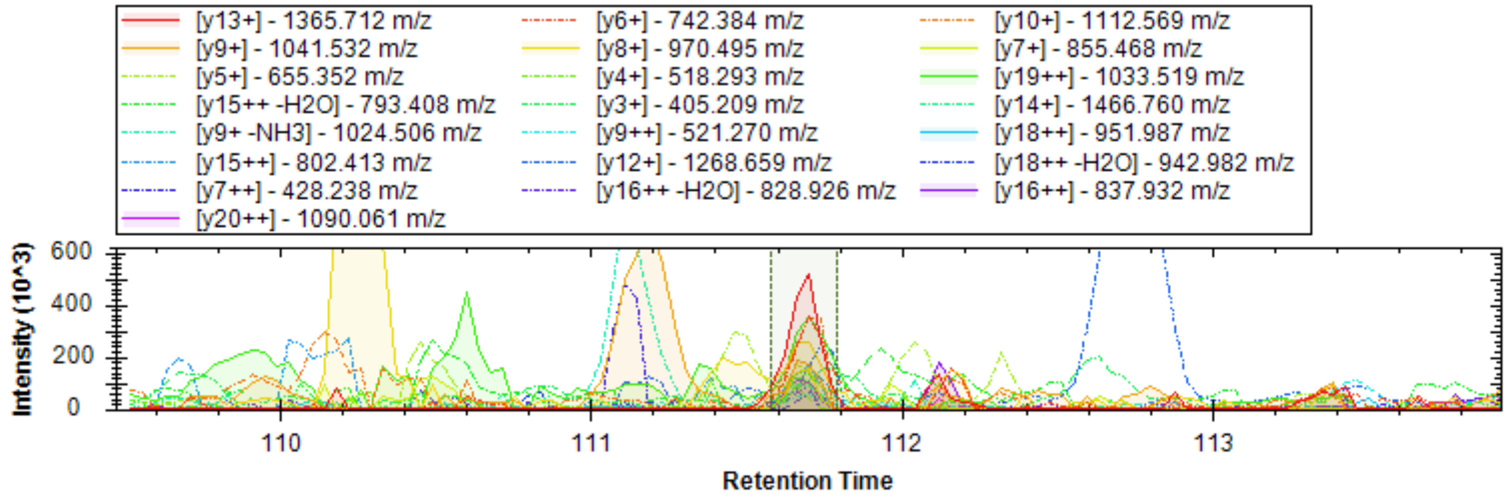

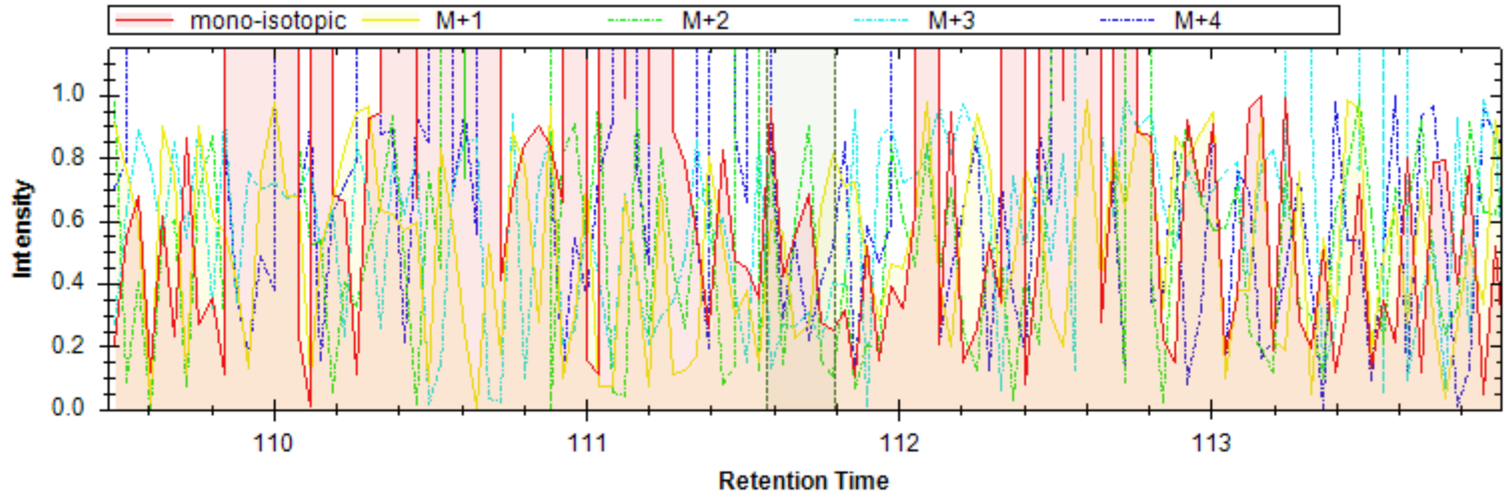

P10637 – _TTPSPKT[+H+O3+P]PPGSGEPPK_.2
Fig4_MP-DIA-barrel-cortex-p30-1_MHRM.htrms

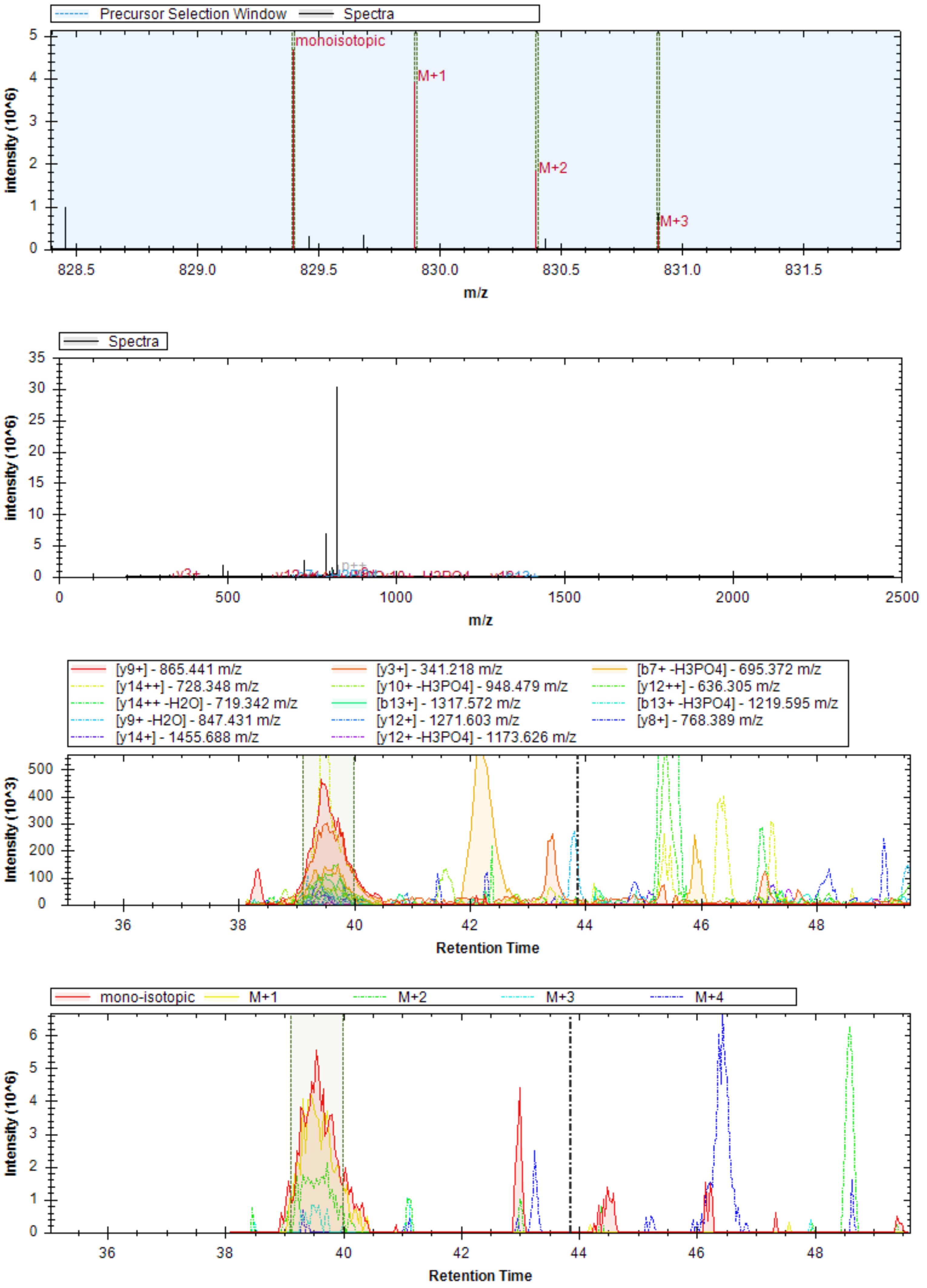

P20357 – _VDHGAEIIT[+H+O3+P]QSPSR_.2

Fig4_MP-DIA-barrel-cortex-p30-1_MHRM.htrms

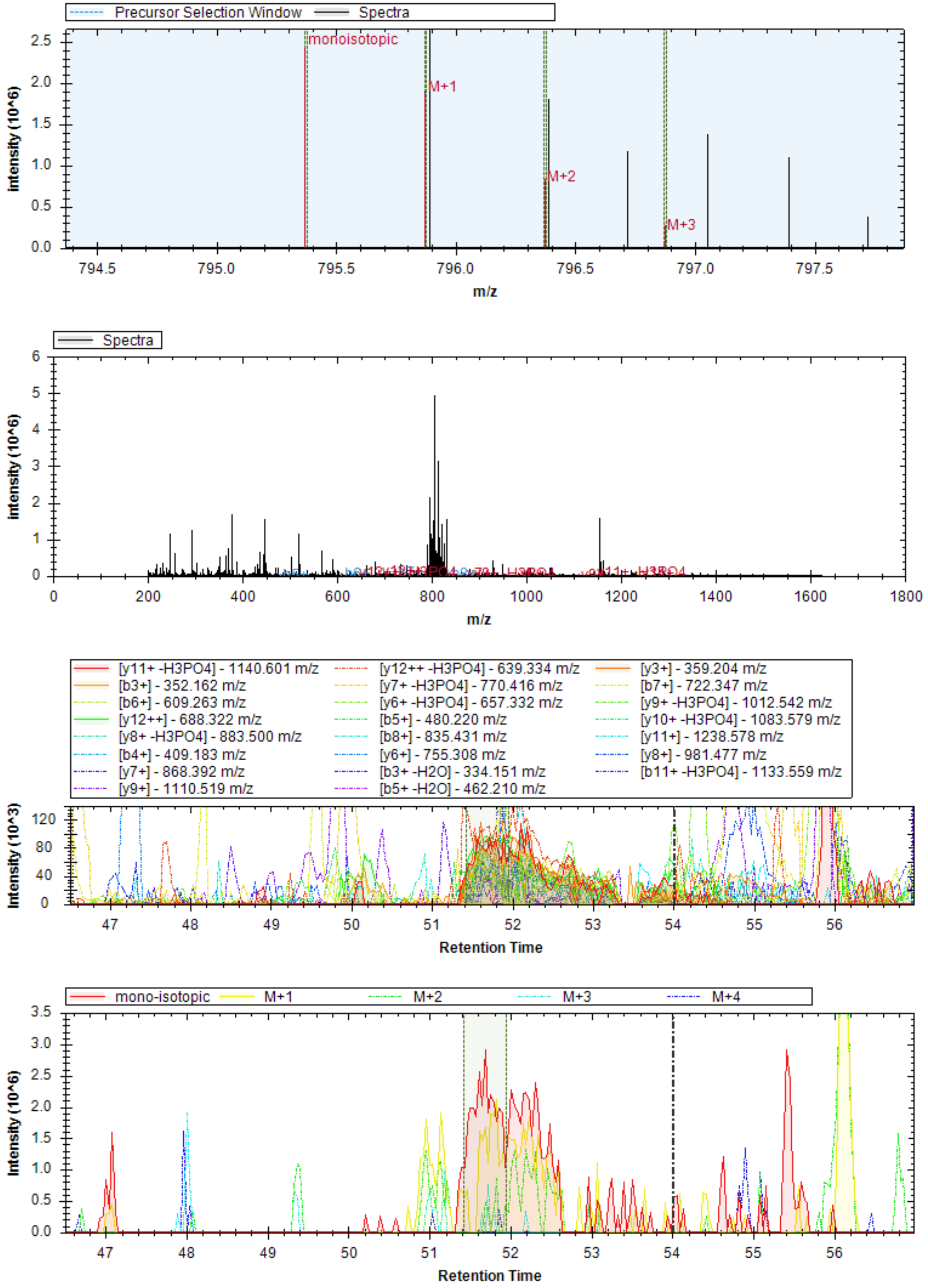

P20357 – _VDHGAEIITQS[+H+O3+P]PSR_.2

Fig4_MP-DIA-barrel-cortex-p30-1_MHRM.htrms

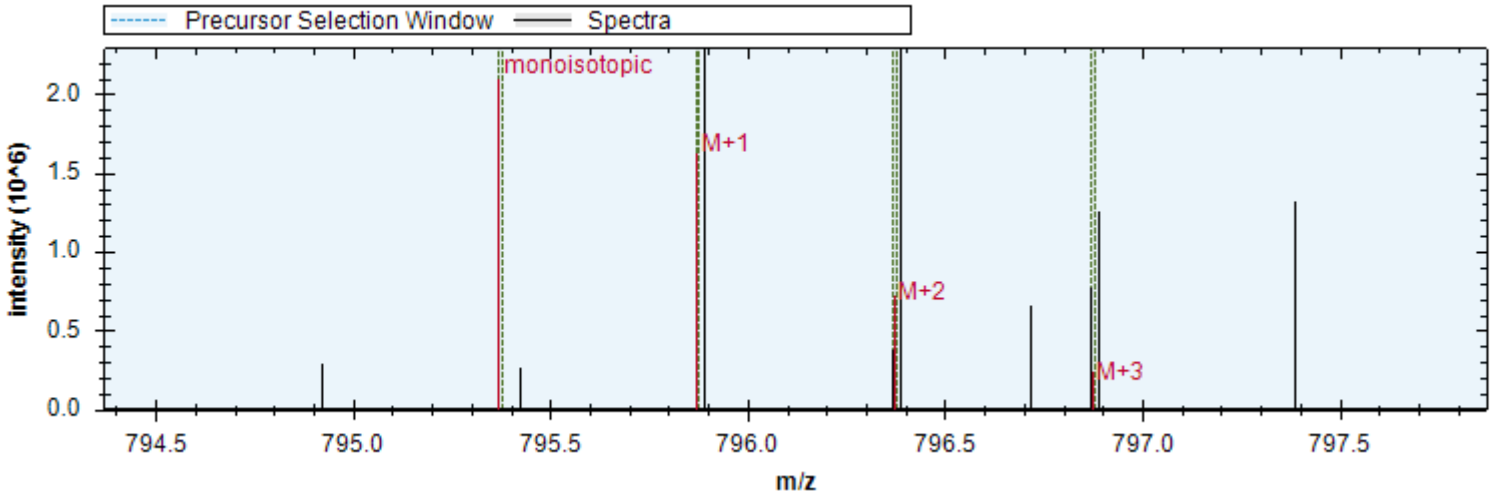

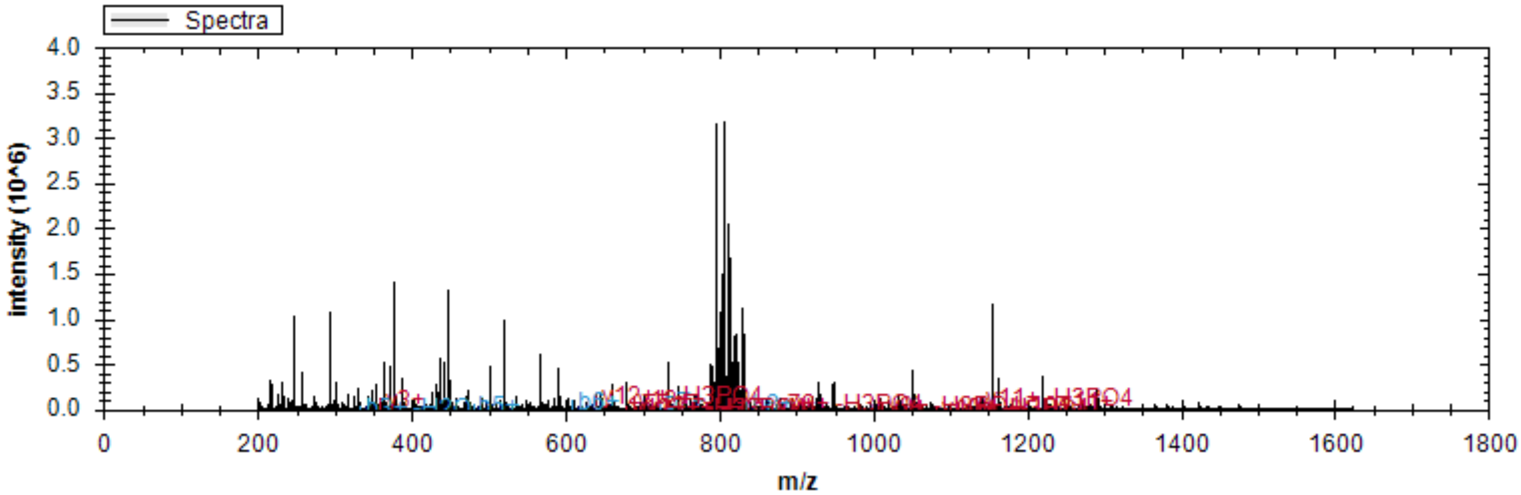

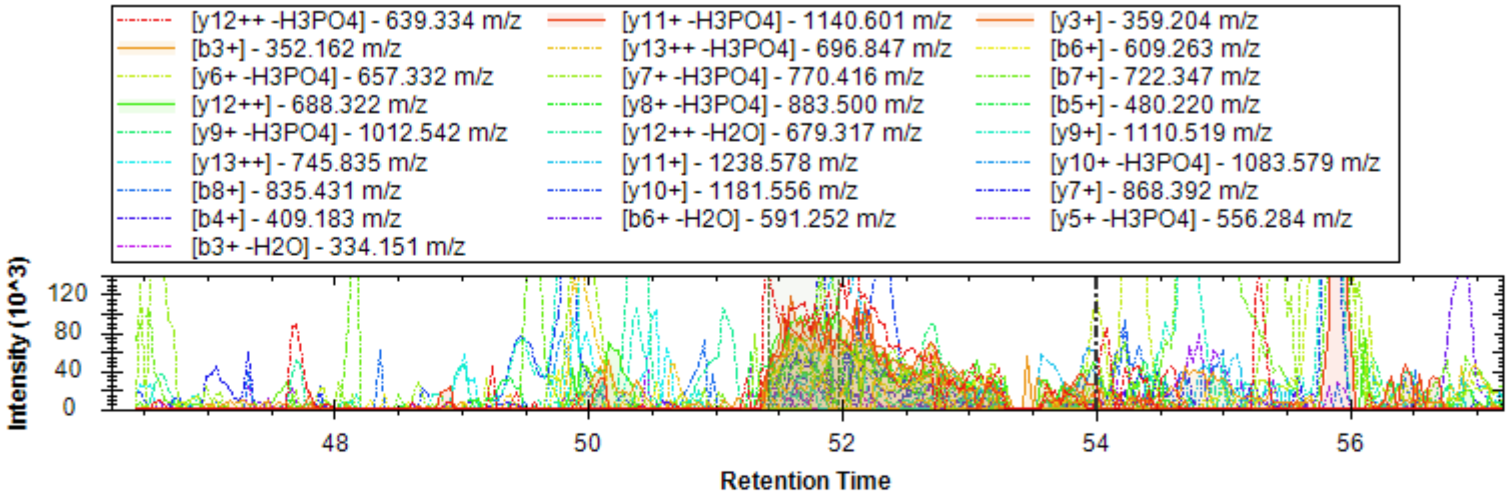

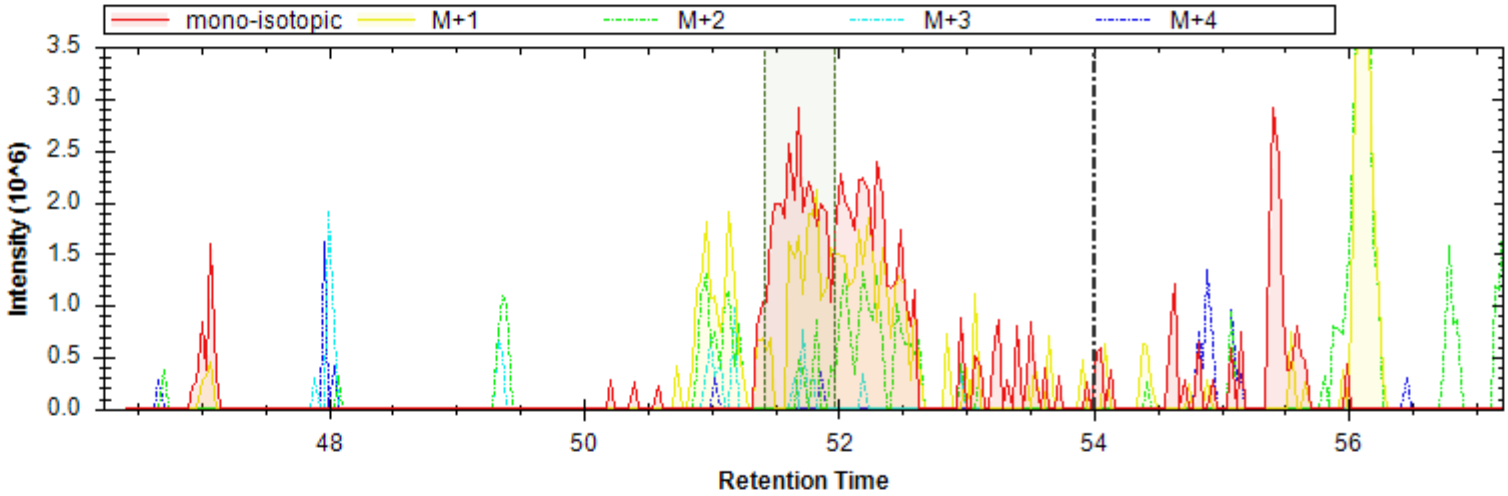

P20357 – _VDHGAEIITQSPS[+H+O3+P]R_.2
Fig4_MP-DIA-barrel-cortex-p30-1_MHRM.htrms

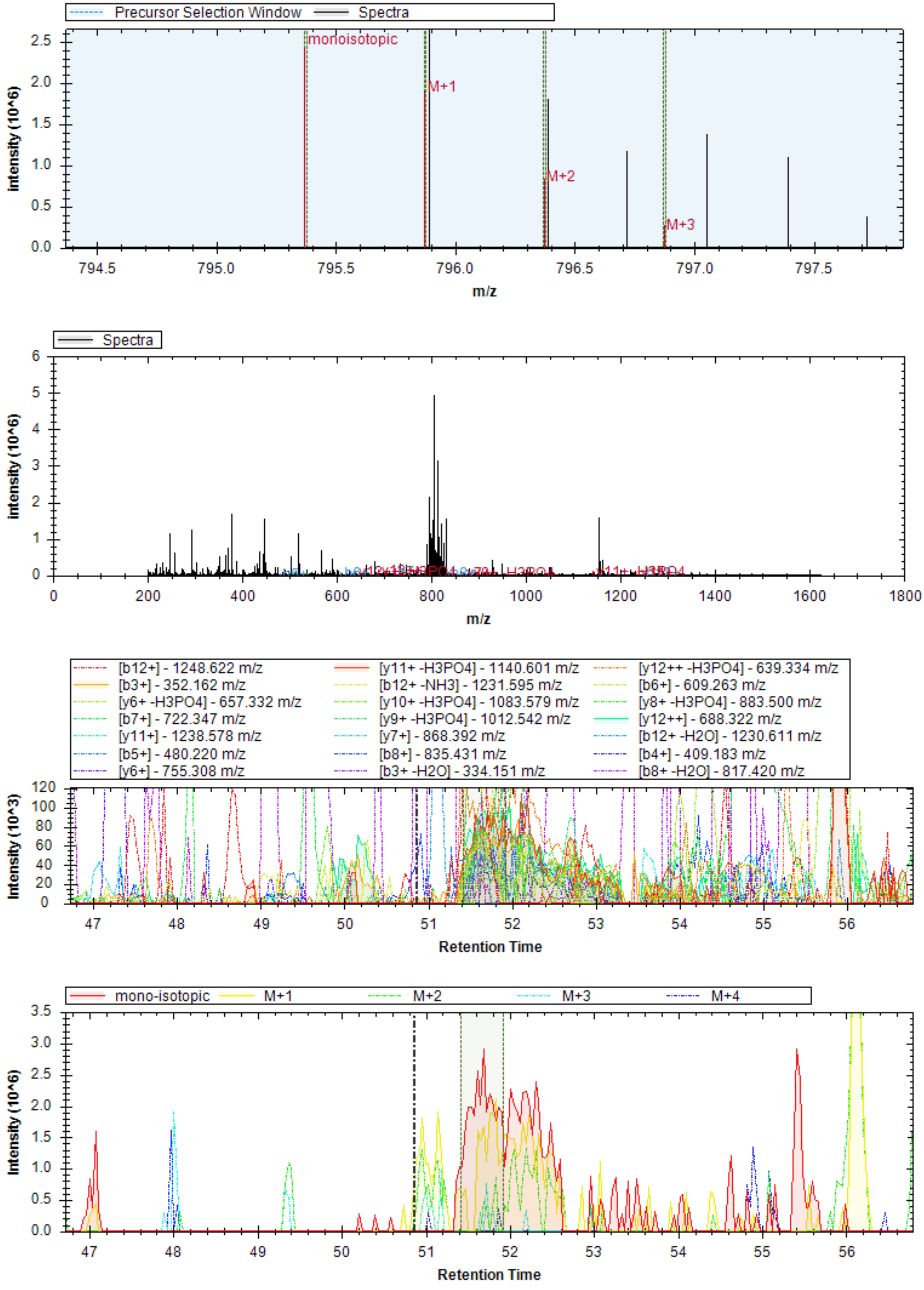

Q60771 – _FYYSS[+H+O3+P]GSSSPTHAK_.2

Fig4_MP-DIA-barrel-cortex-p30-1_MHRM.htrms

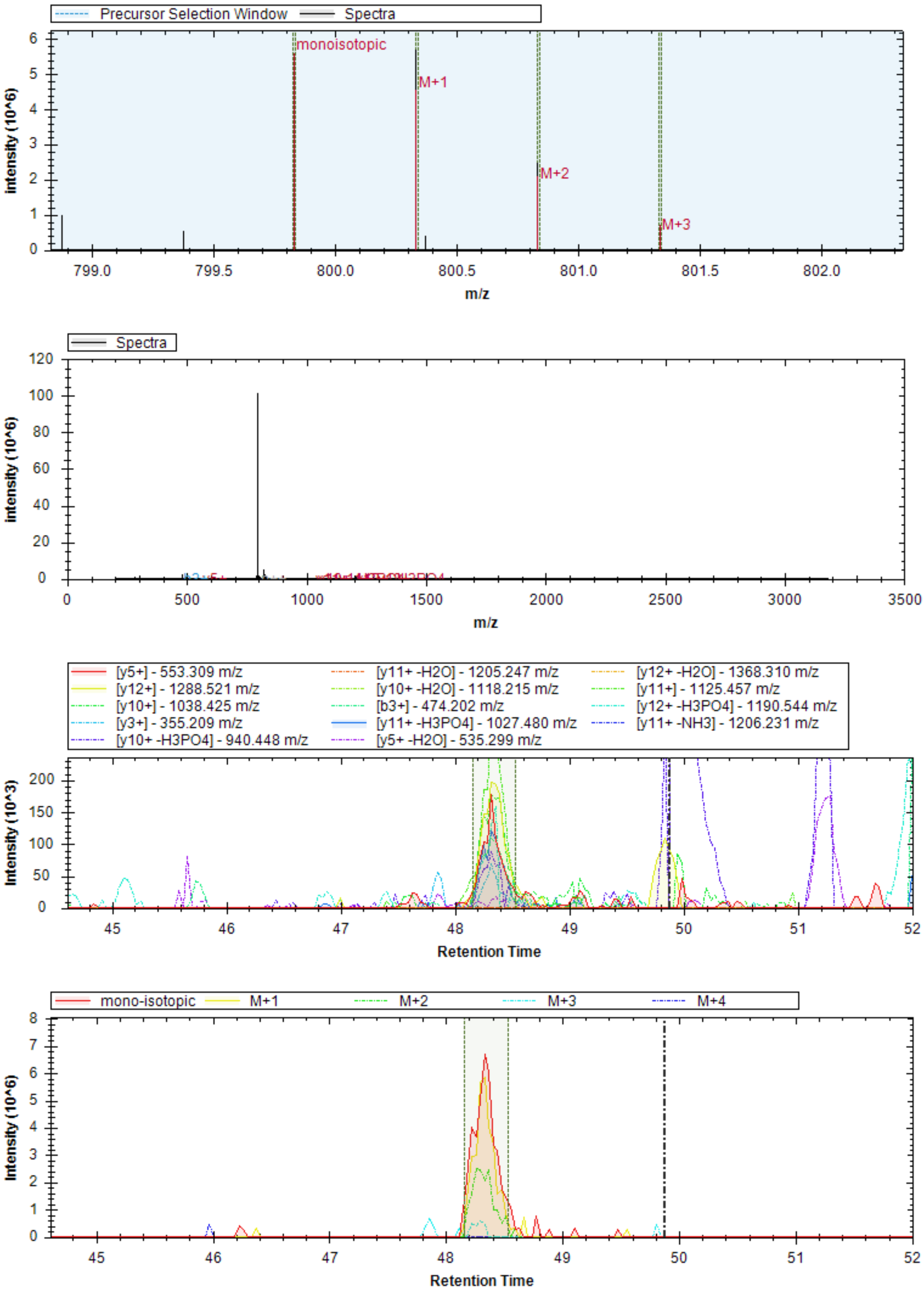

Q60771 – _FYYSSGS[+H+O3+P]SSPTHAK_.2
Fig4_MP-DIA-barrel-cortex-p30-1_MHRM.htrms

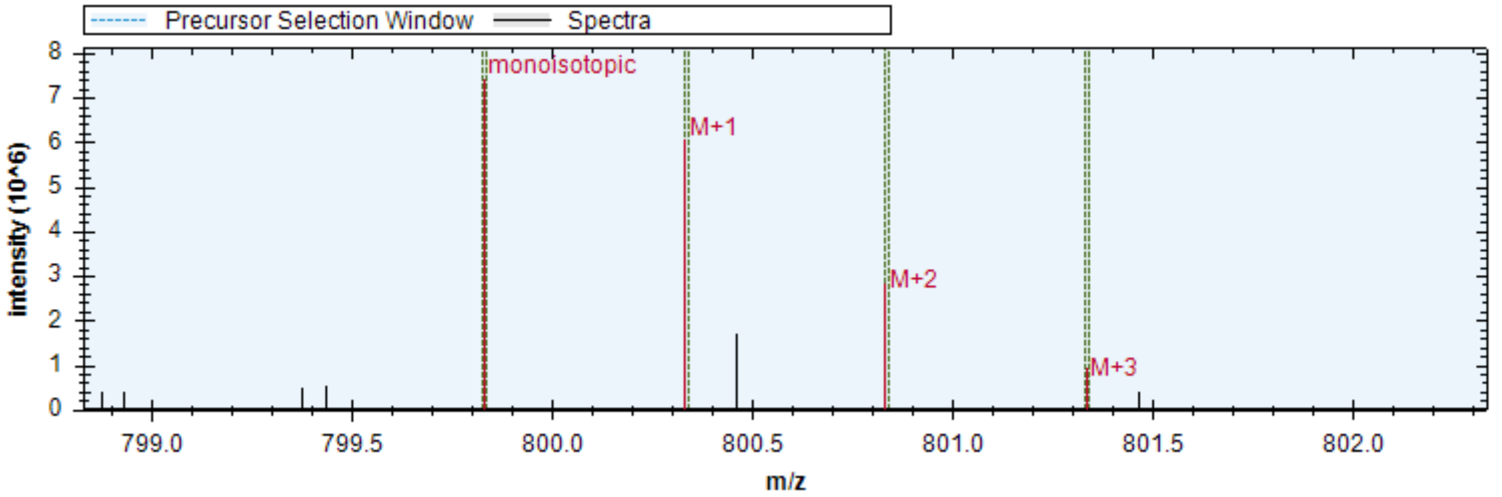

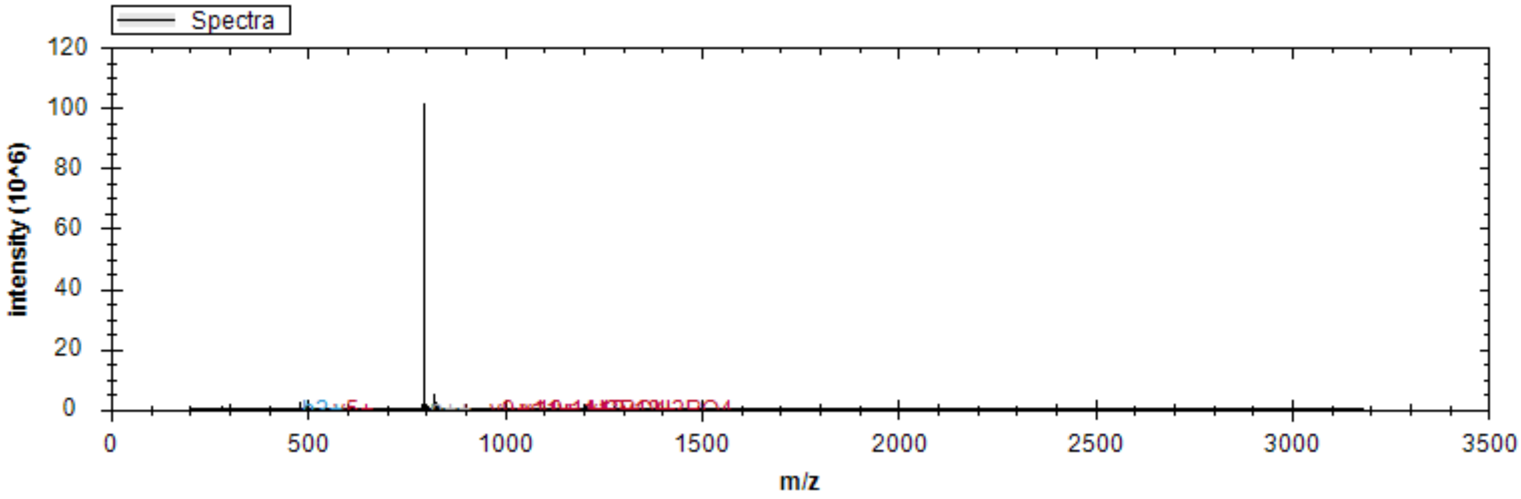

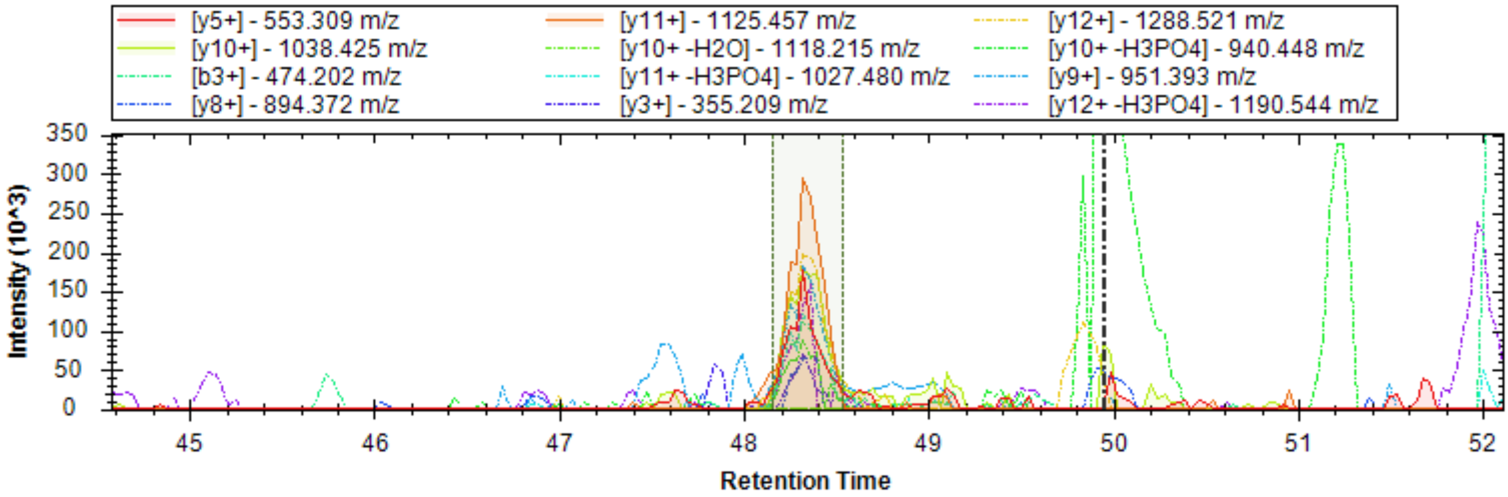

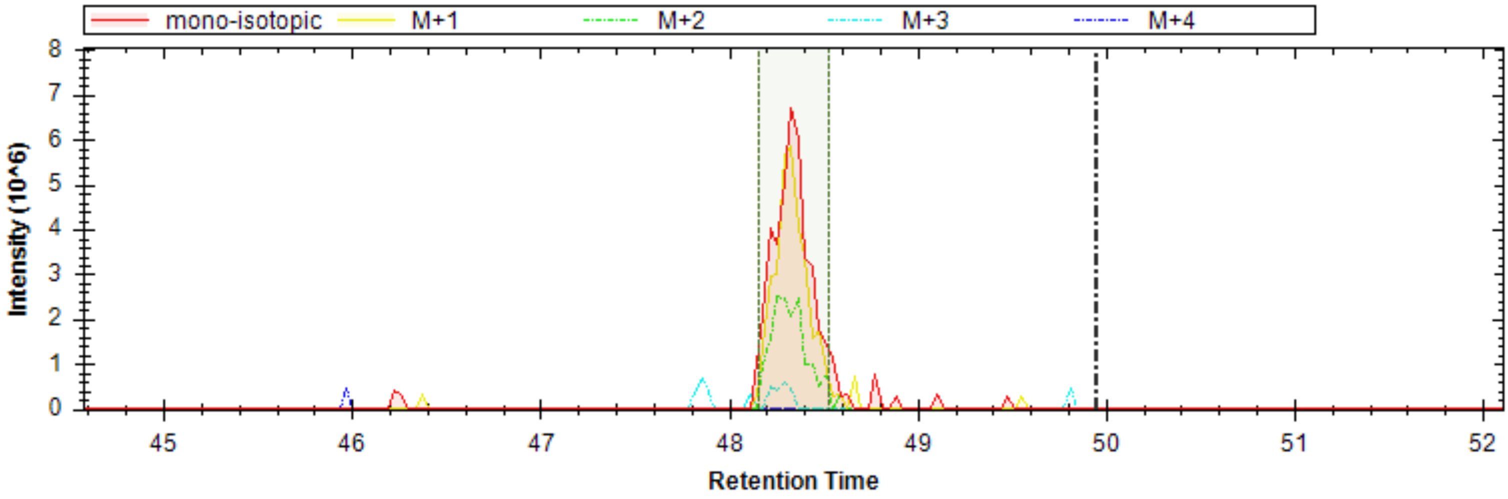

Q60771 – _FYYSSGSSS[+H+O3+P]PTHAK_.2
Fig4_MP-DIA-barrel-cortex-p30-1_MHRM.htrms

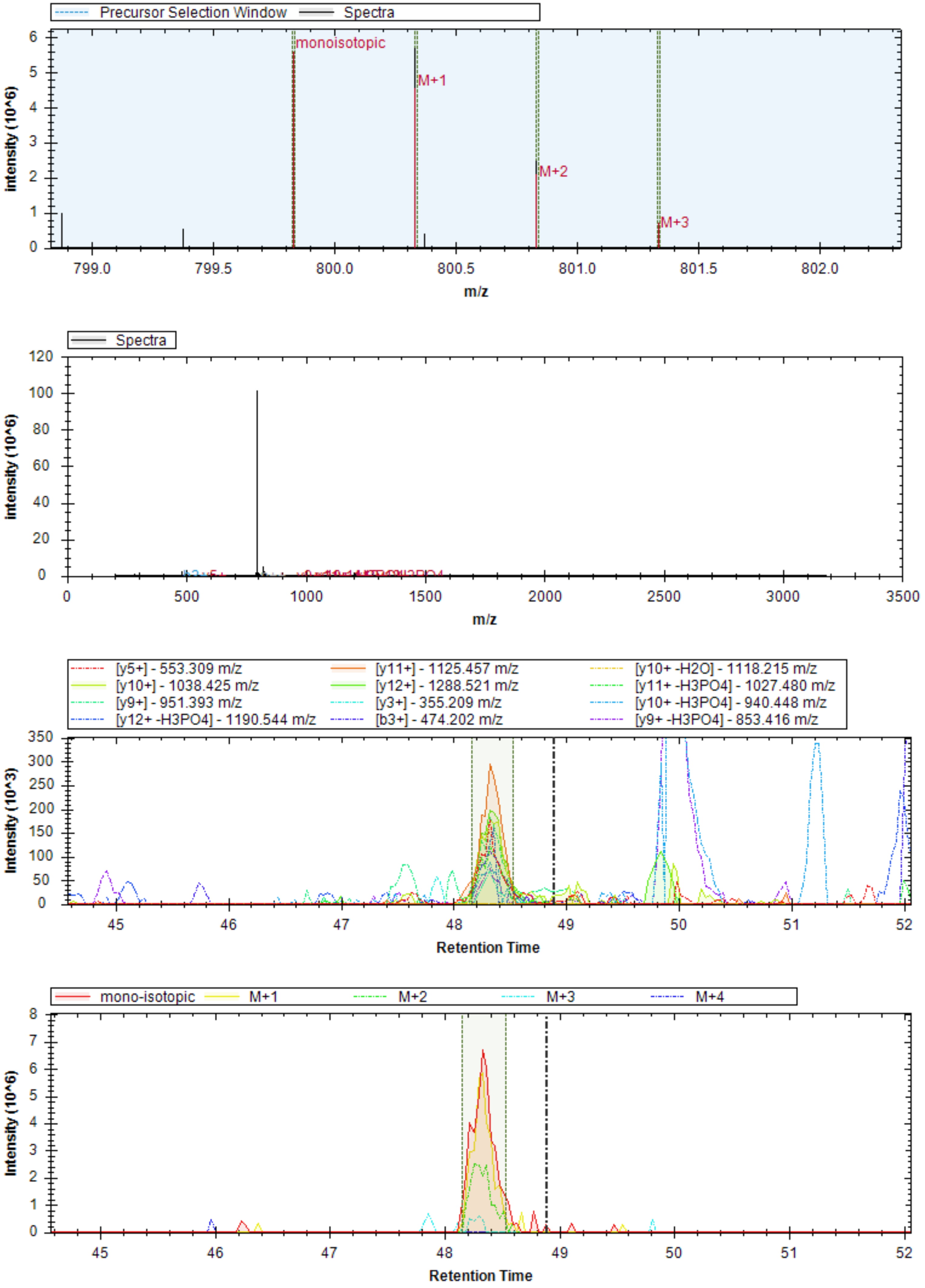

Q64701;Q64701-2 – _LAEILYYKILET[+H+O3+P]IMVQET[+H+O3+P]R_.3
Fig4_MP-DIA-barrel-cortex-p30-1_MHRM.htrms

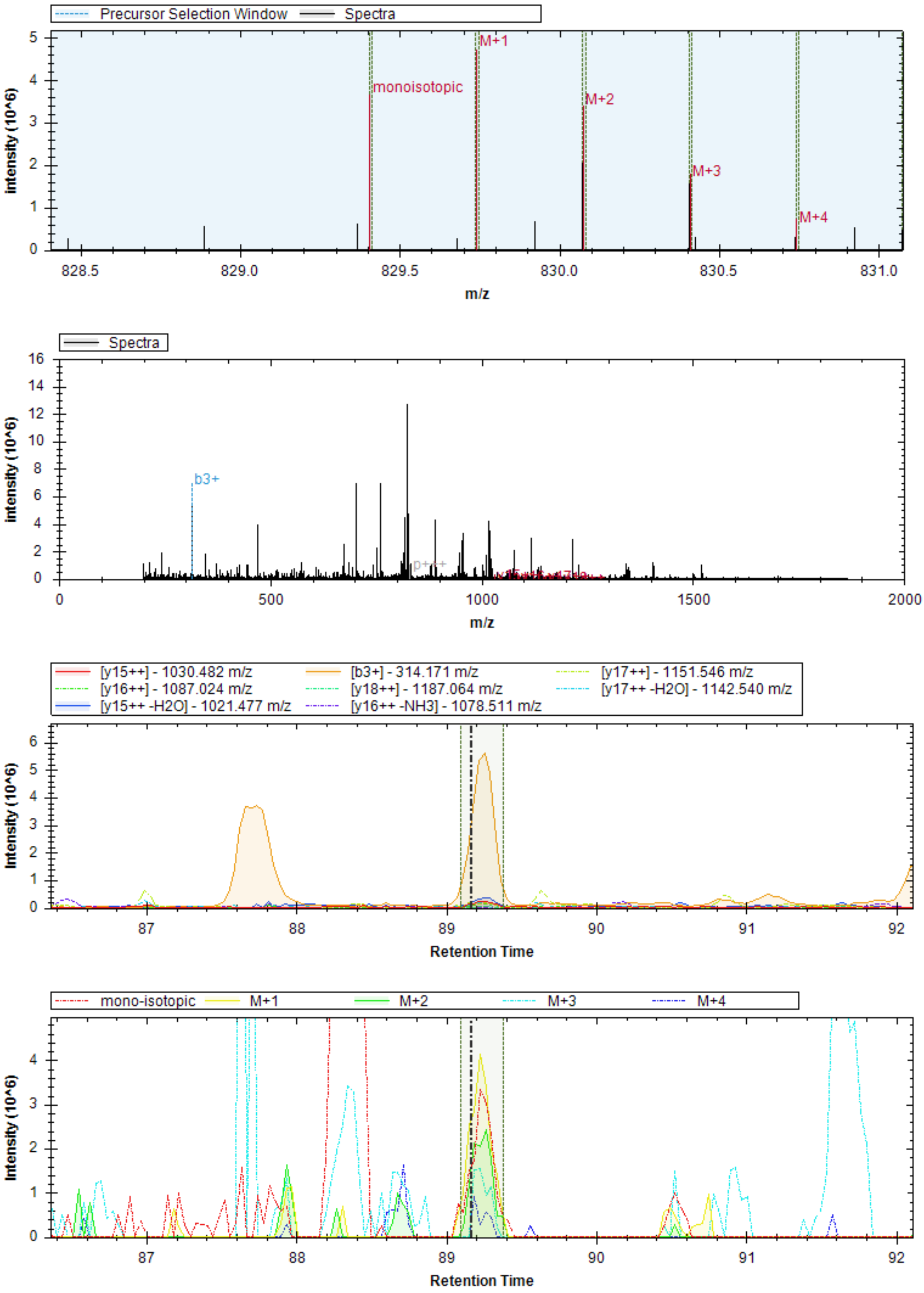

Q7TQD2 – _AANKT[+H+O3+P]PPKSPGDPAR_.2
Fig4_MP-DIA-barrel-cortex-p30-1_MHRM.htrms

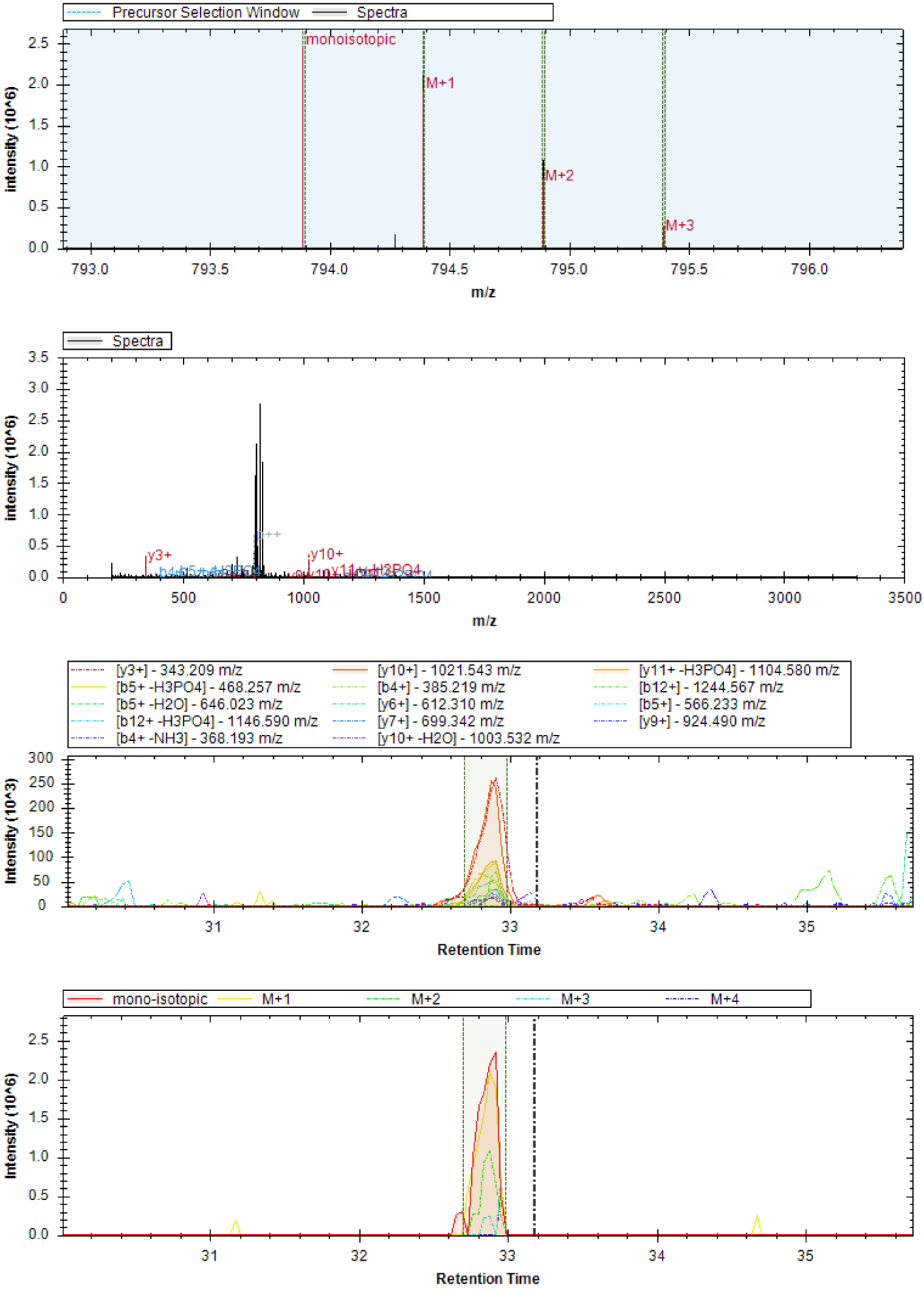

Q91XM9;Q91XM9-2;Q91XM9-7 – _NLSQIENVHGYVLQS[+H+O3+P]HISPLK_.3
Fig4_MP-DIA-barrel-cortex-p30-1_MHRM.htrms

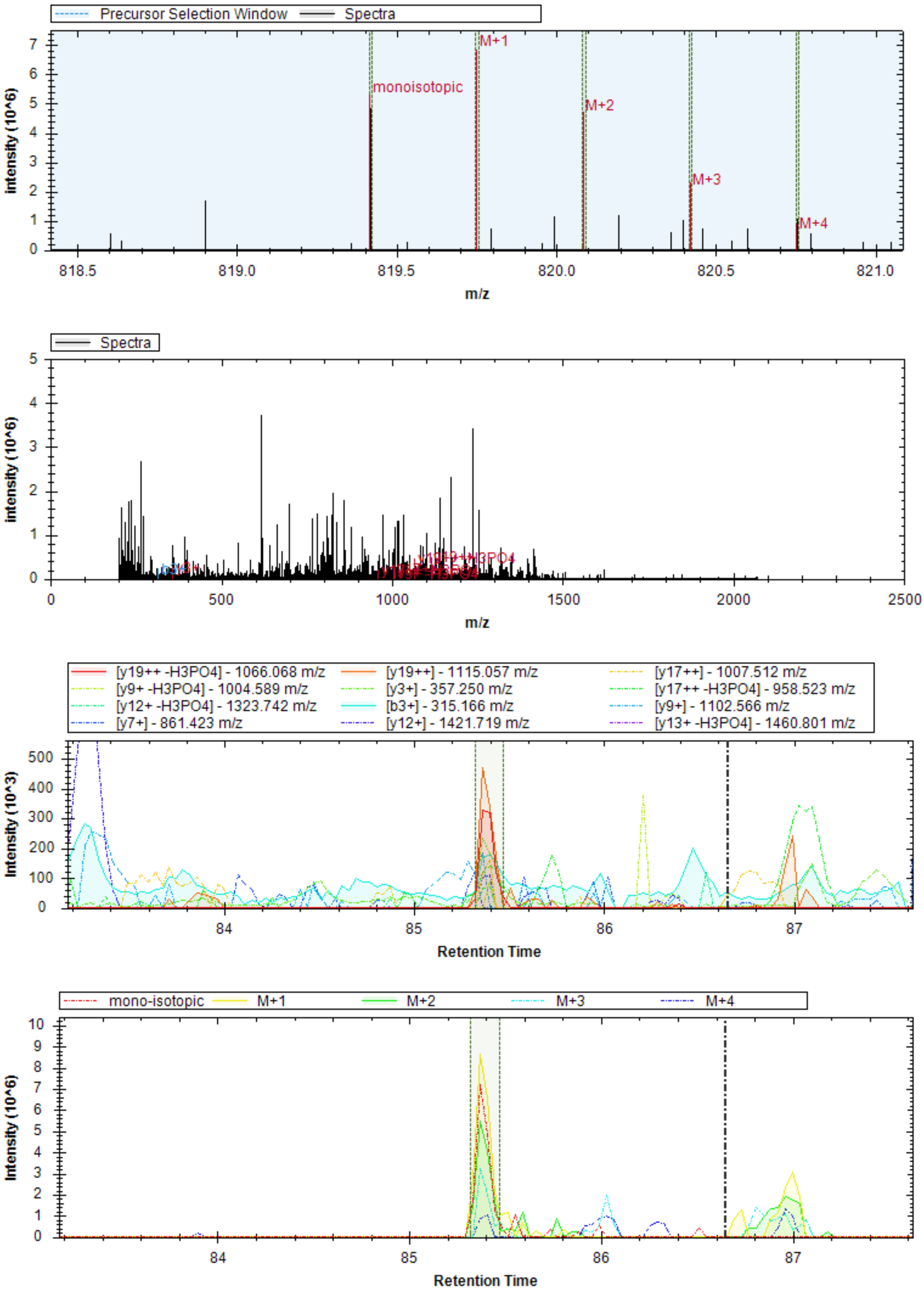

Q91XM9;Q91XM9-2;Q91XM9-7 – _NLSQIENVHGYVLQSHIS[+H+O3+P]PLK_.3
Fig4_MP-DIA-barrel-cortex-p30-1_MHRM.htrms

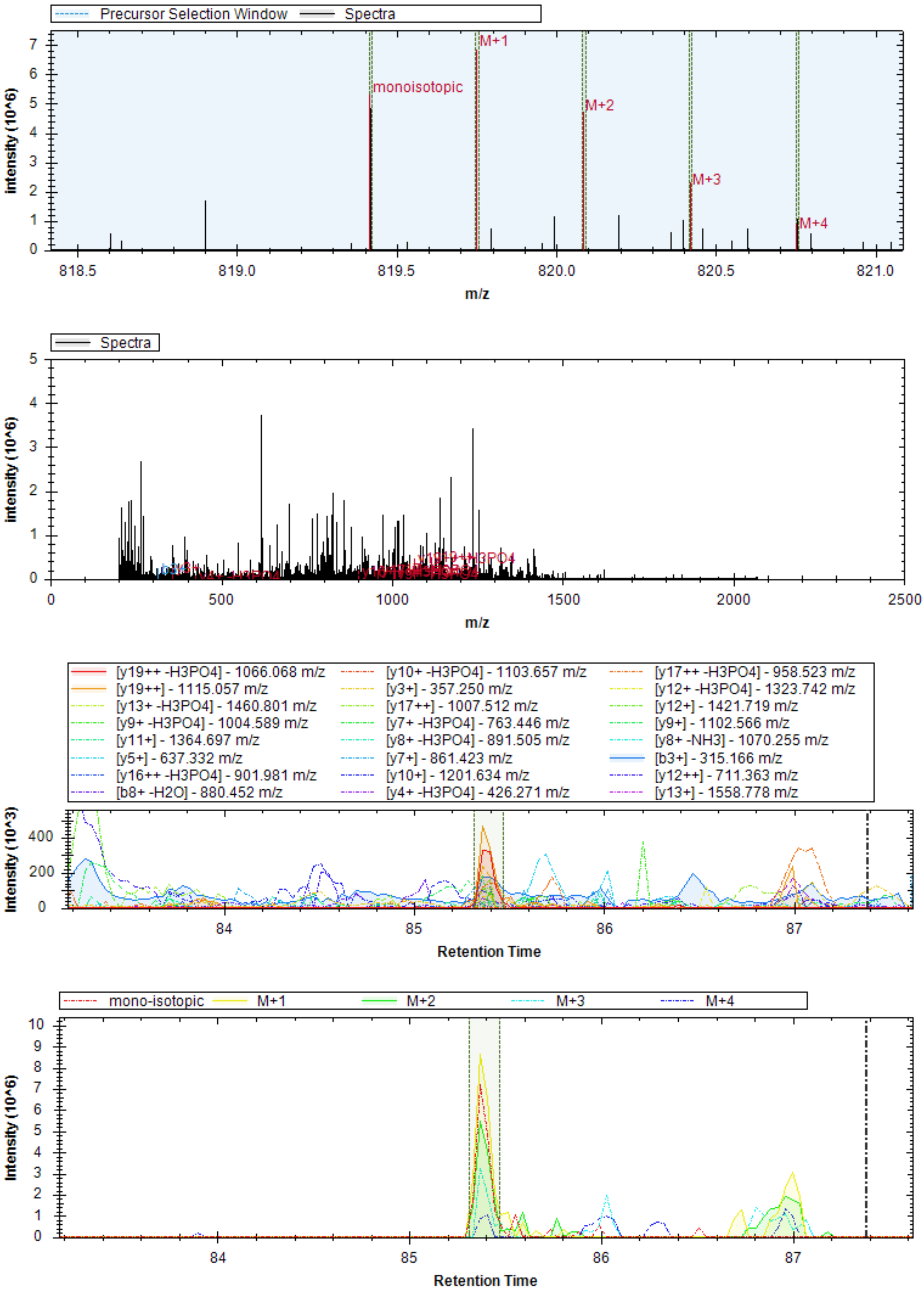

O08784 – _ASAVSPEKAPM[+O]T[+H+O3+P]SKAK_.2
Fig4_MP-DIA-barrel-cortex-p30-1_MHRM.htrms

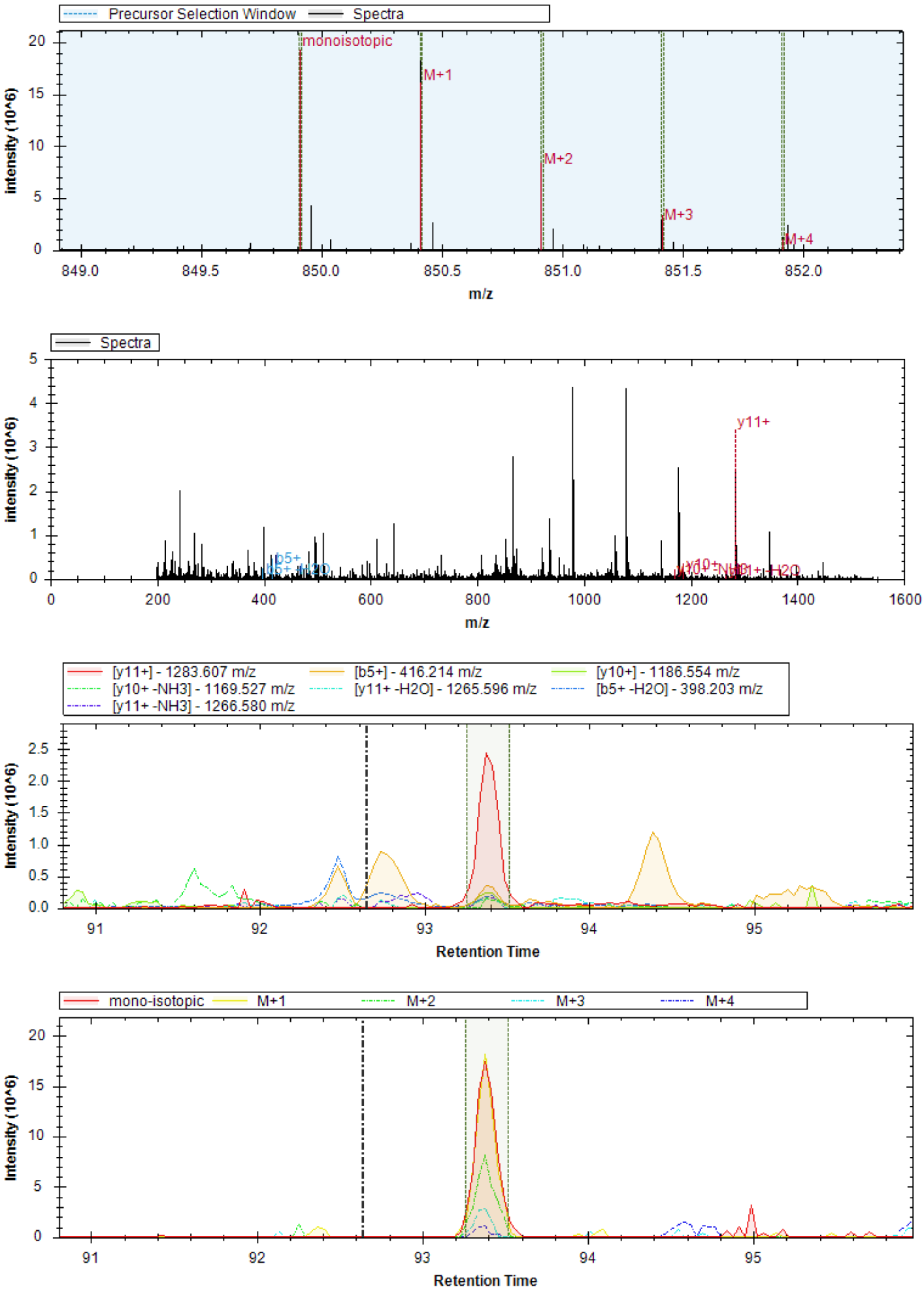

P14873 – _ETAAAHQAS[+H+O3+P]SSPPIDAATAEPYGFR_.3
Fig4_MP-DIA-barrel-cortex-p30-1_MHRM.htrms

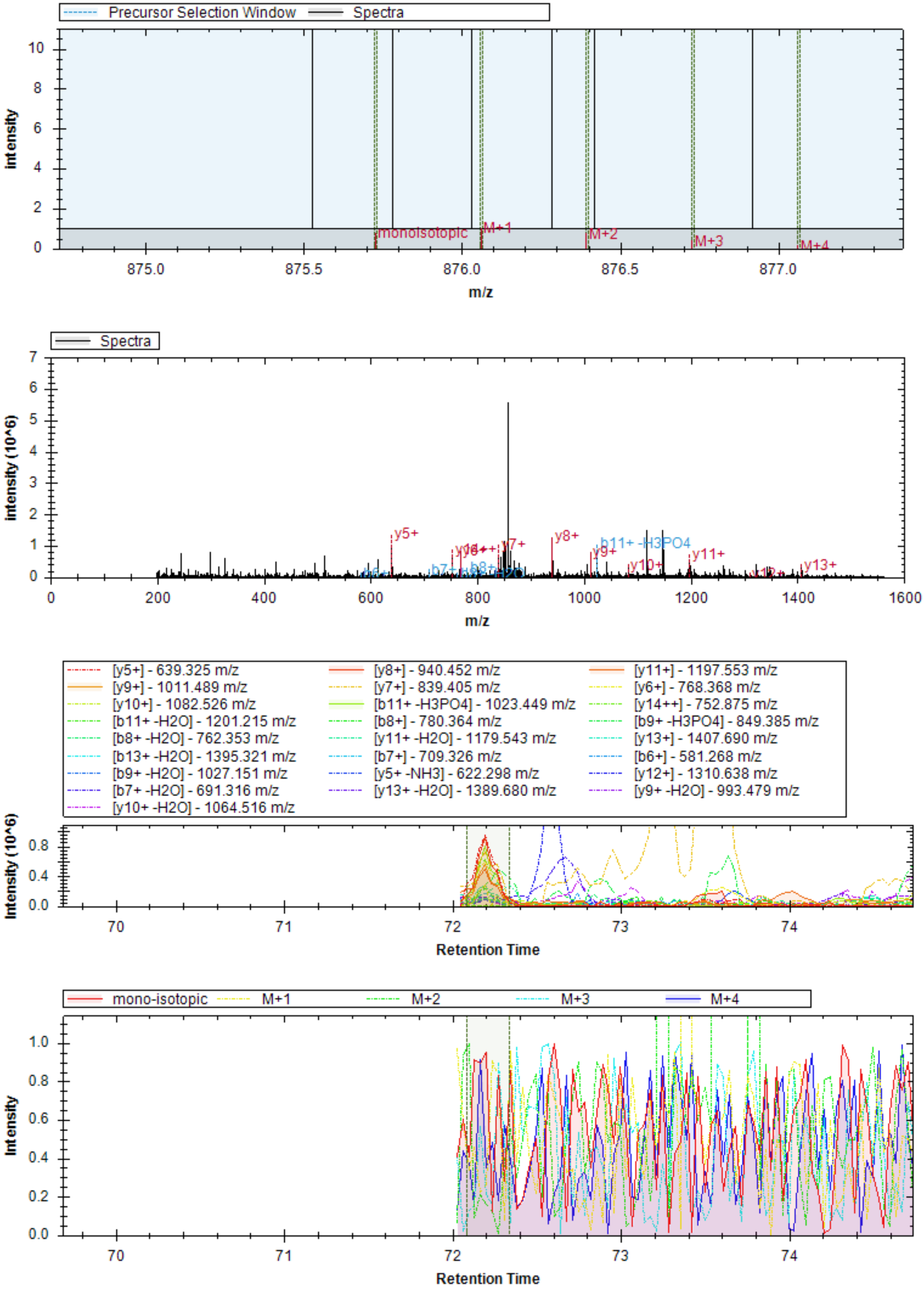

P50538 – _EAEHGYAS[+H+O3+P]M[+O]LPYSK_.2
Fig4_MP-DIA-barrel-cortex-p30-1_MHRM.htrms

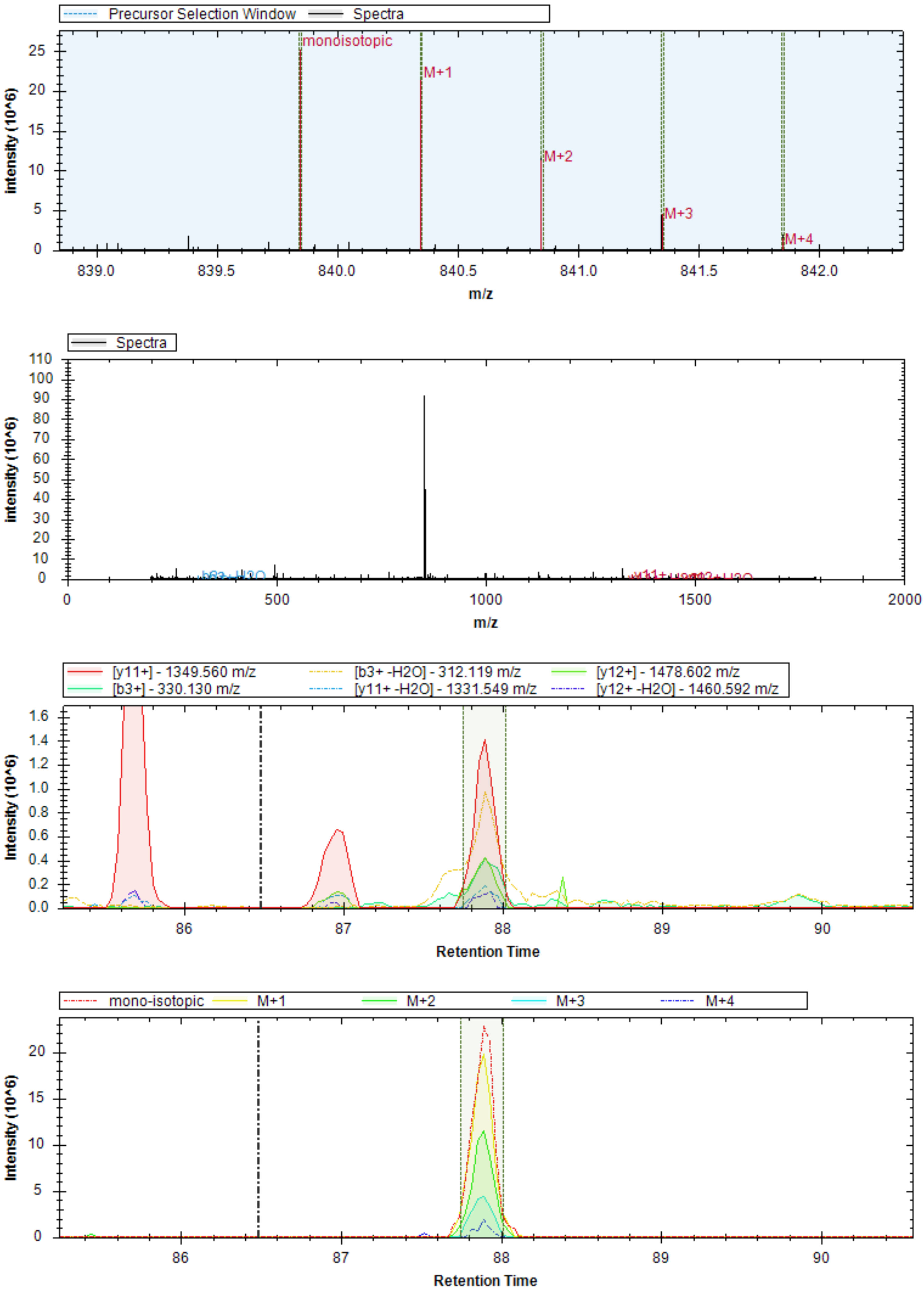

Q61290 – _M[+O]EPS[+H+O3+P]SLPQEIIANAK_.2
Fig4_MP-DIA-barrel-cortex-p30-1_MHRM.htrms

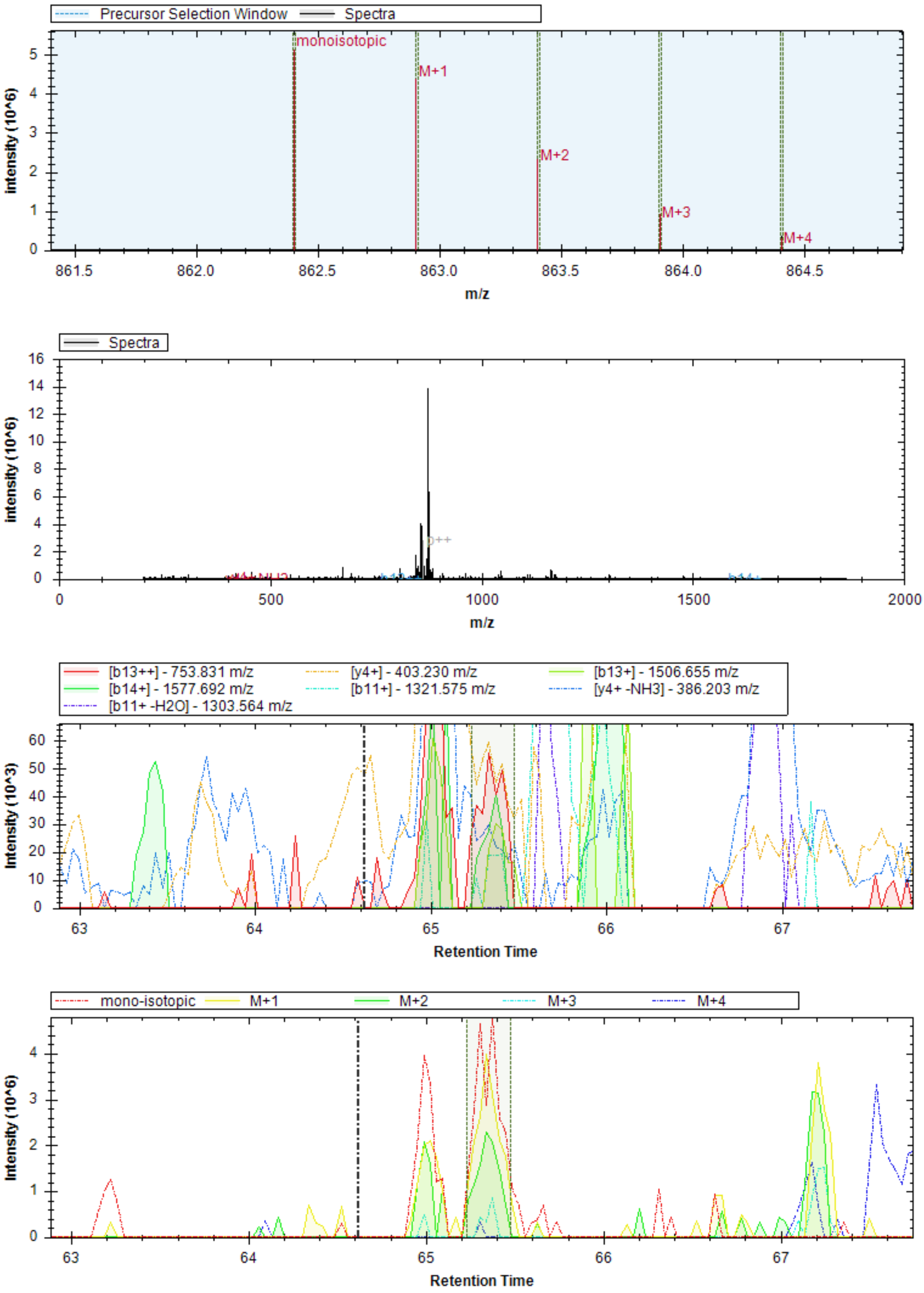

Q9QXS6 – _ASDSGPSSSSSS[+H+O3+P]SSSPPR_.2
Fig4_MP-DIA-barrel-cortex-p30-1_MHRM.htrms

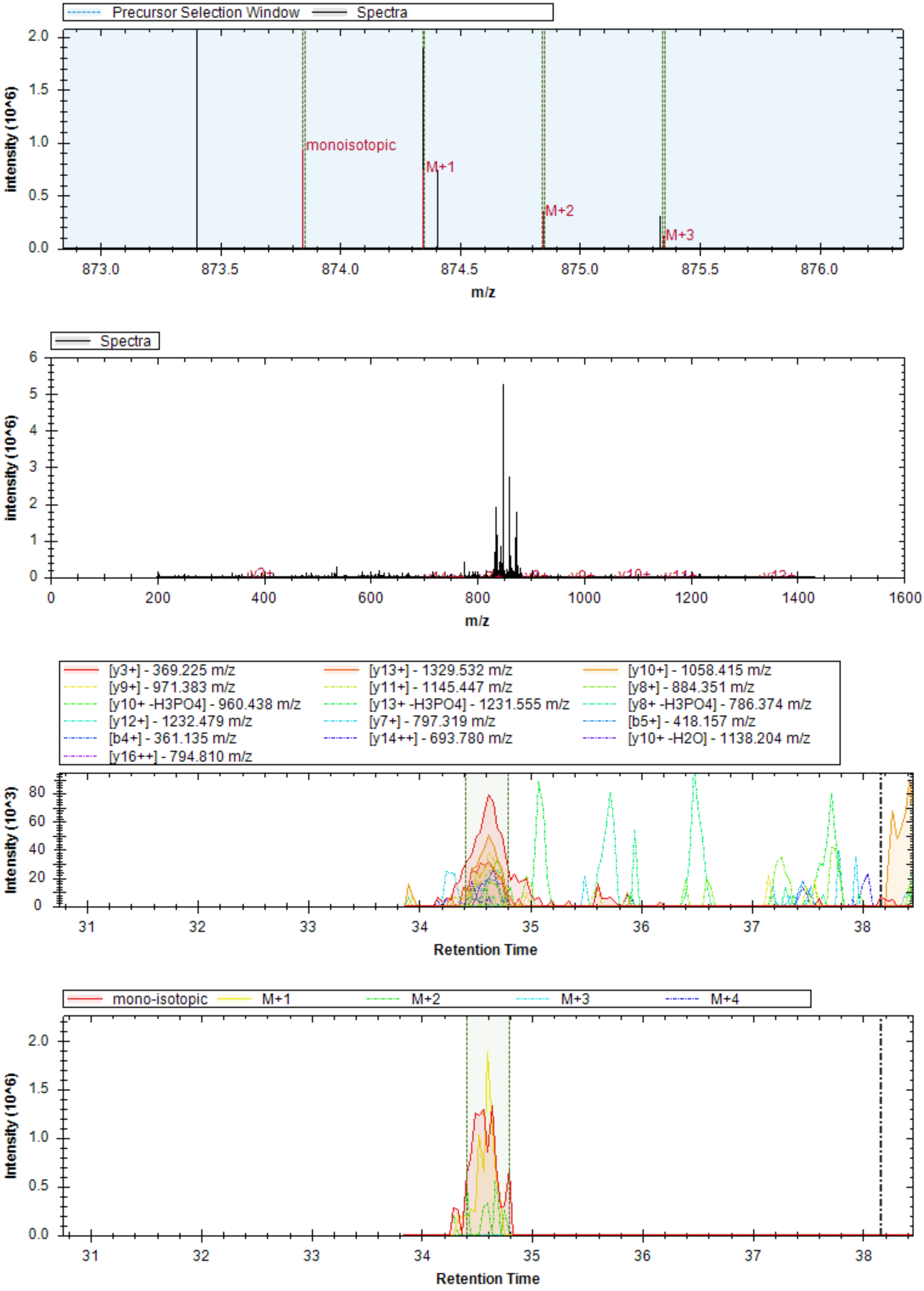

A1A535 – _FLHRGS[+H+O3+P]K_.1
Fig4_MP-DIA-barrel-cortex-p30-1_MHRM.htrms

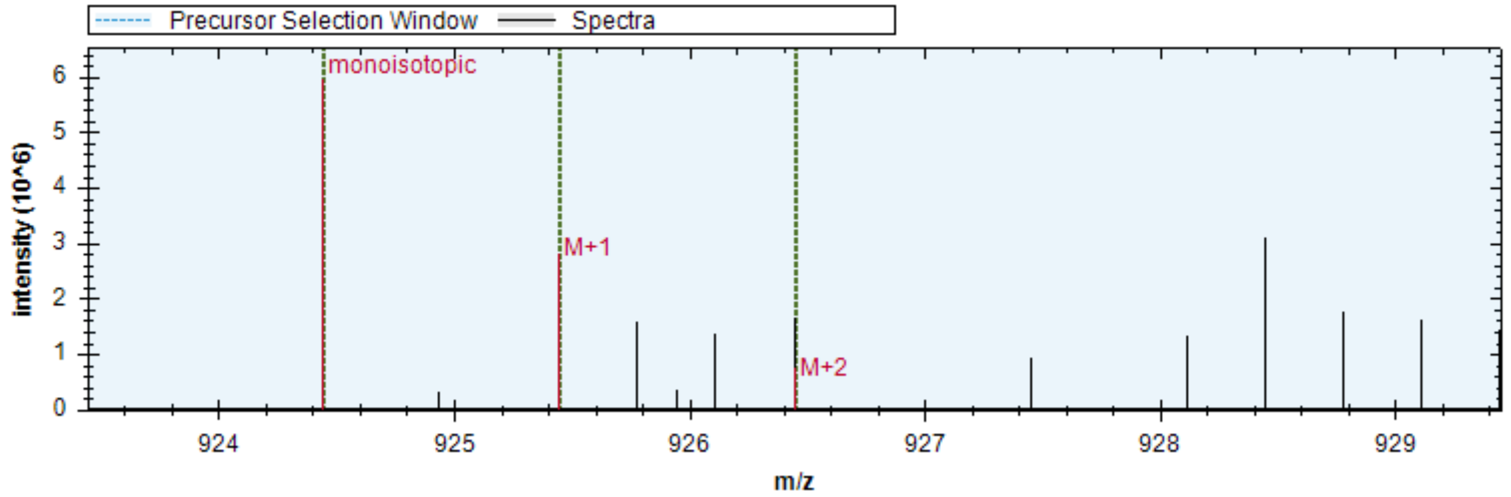

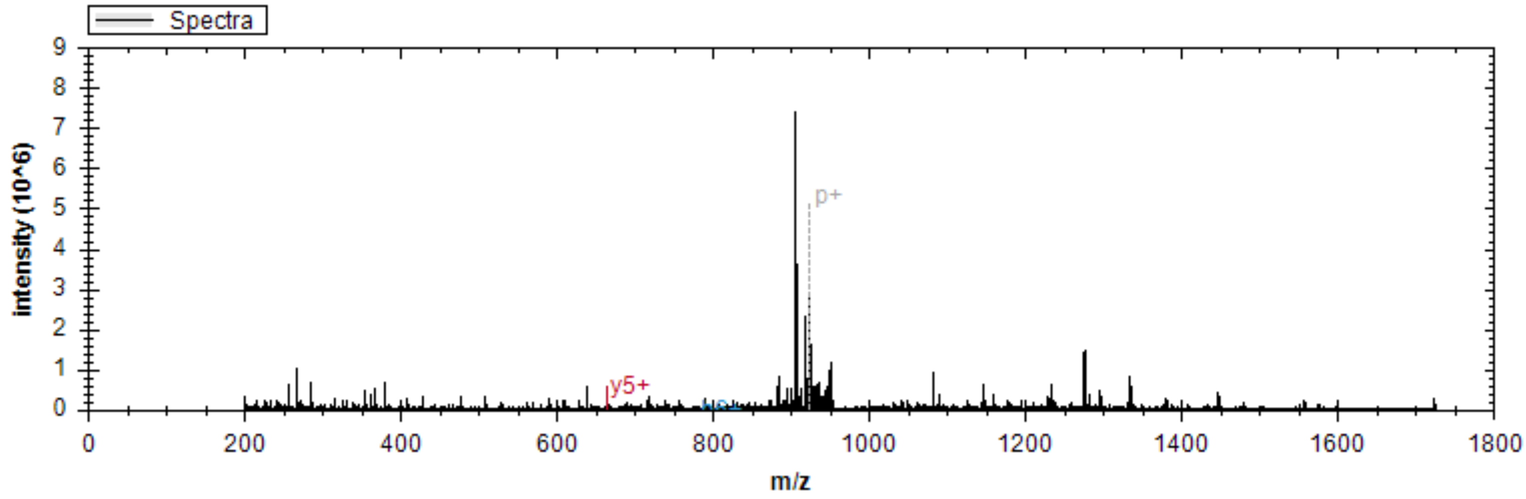

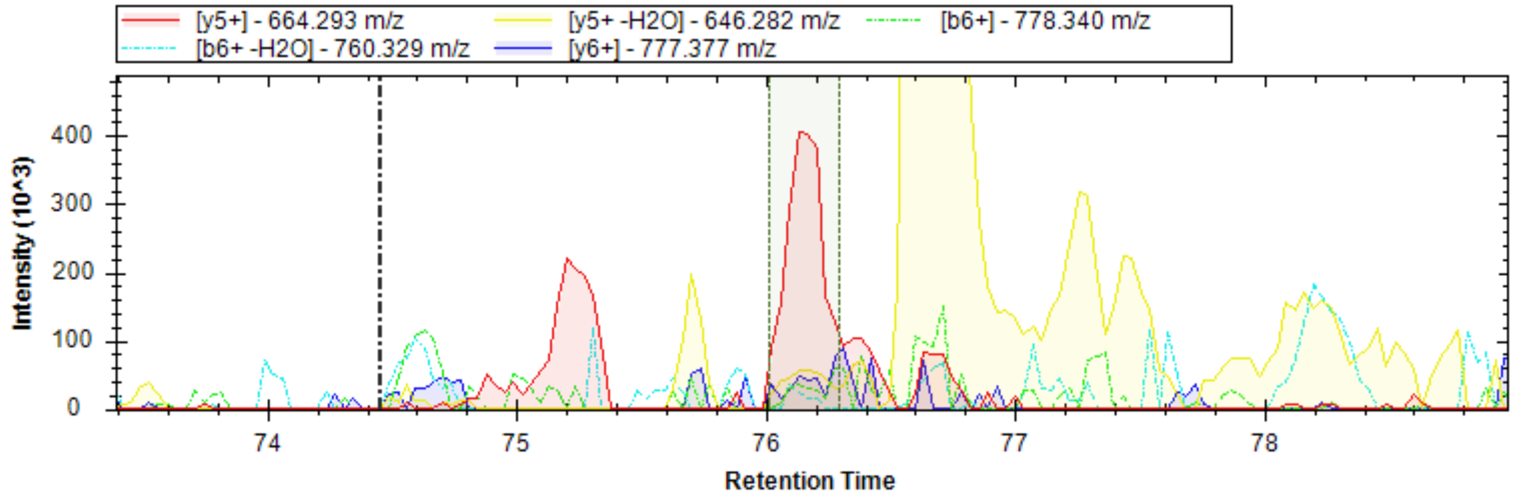

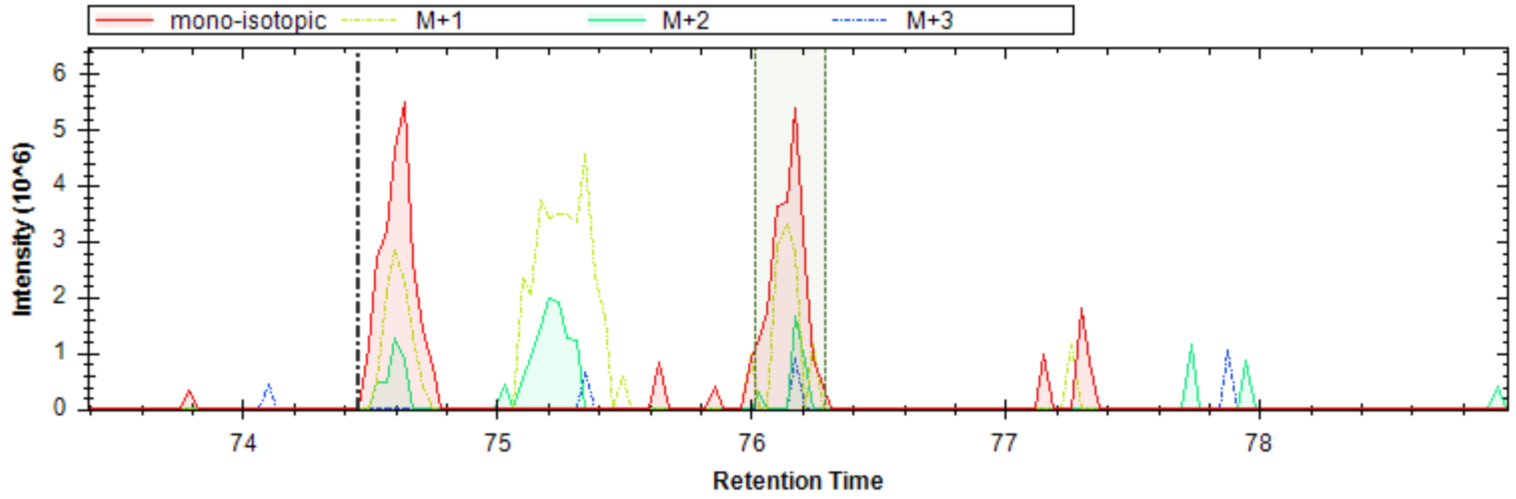

G5E829 – _SATSSSPGS[+H+O3+P]PLHSLETSL_.2
Fig4_MP-DIA-barrel-cortex-p30-1_MHRM.htrms

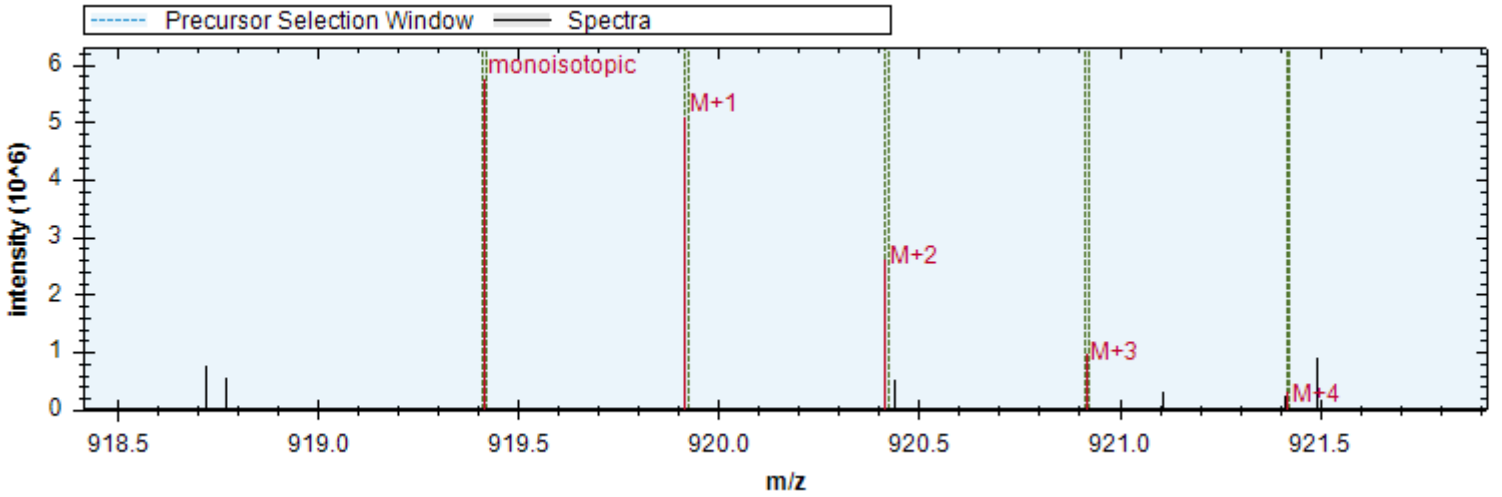

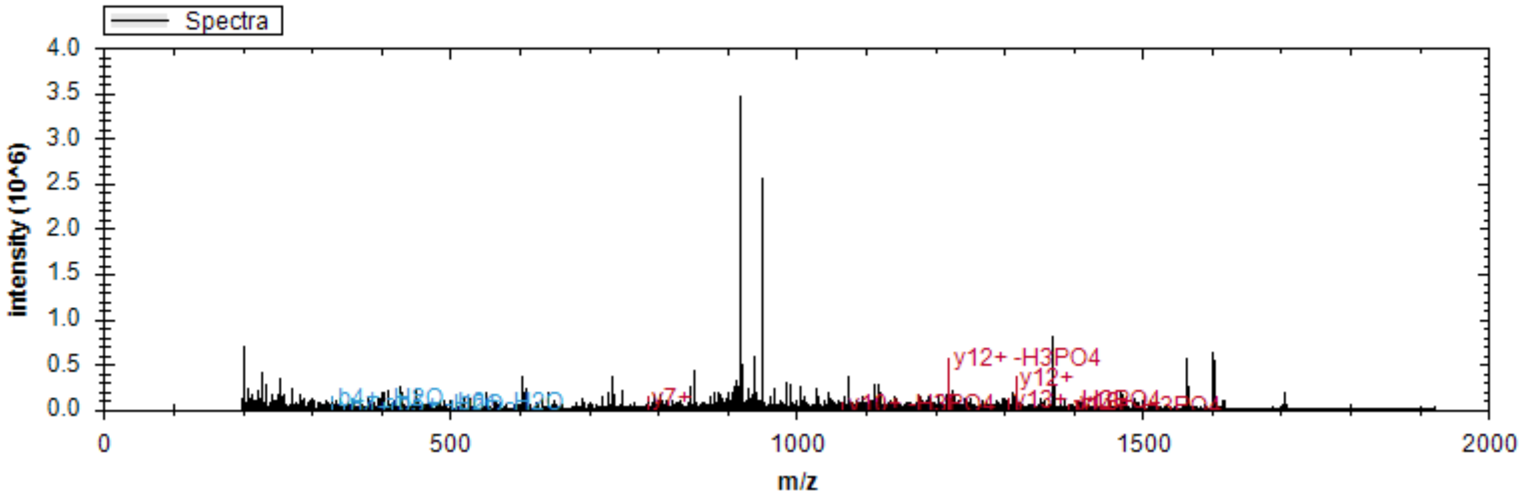

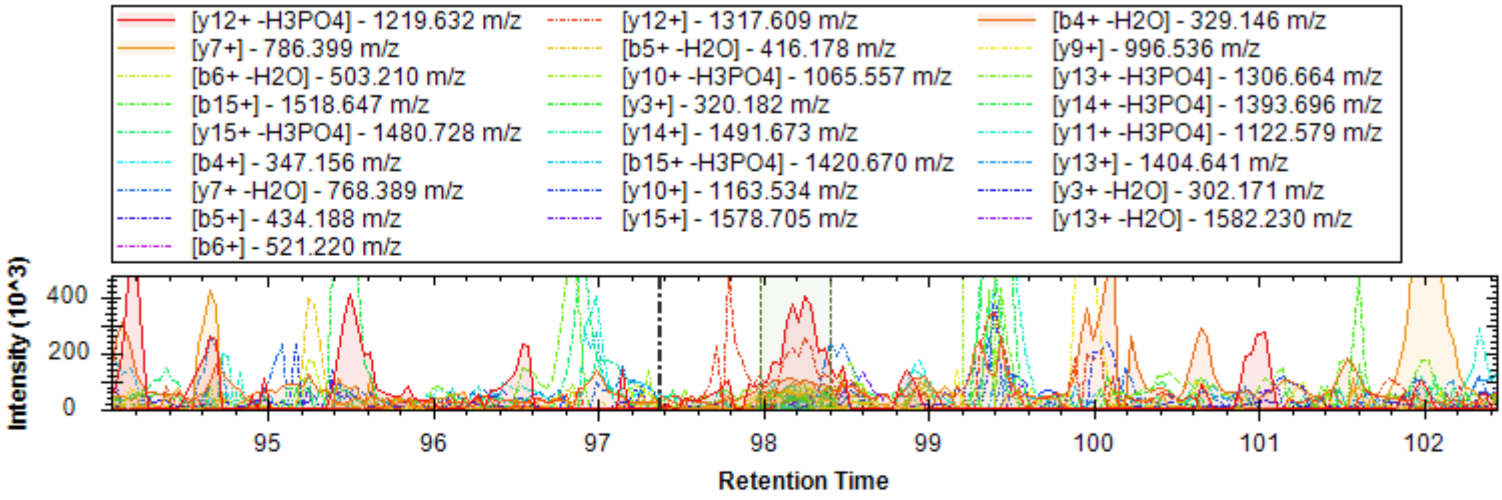

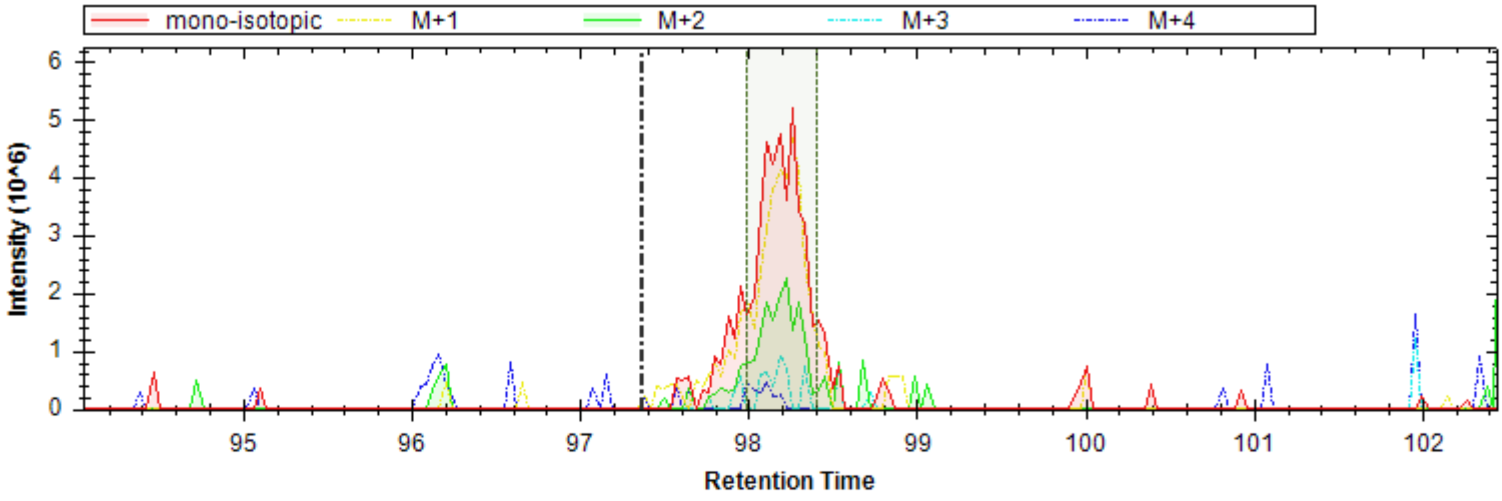

Q8BMA3 – _IIQLC[+C2+H3+N+O]LDLT[+H+O3+P]T[+H+O3+P]AVQK_.2

Fig4_MP-DIA-barrel-cortex-p30-1_MHRM.htrms

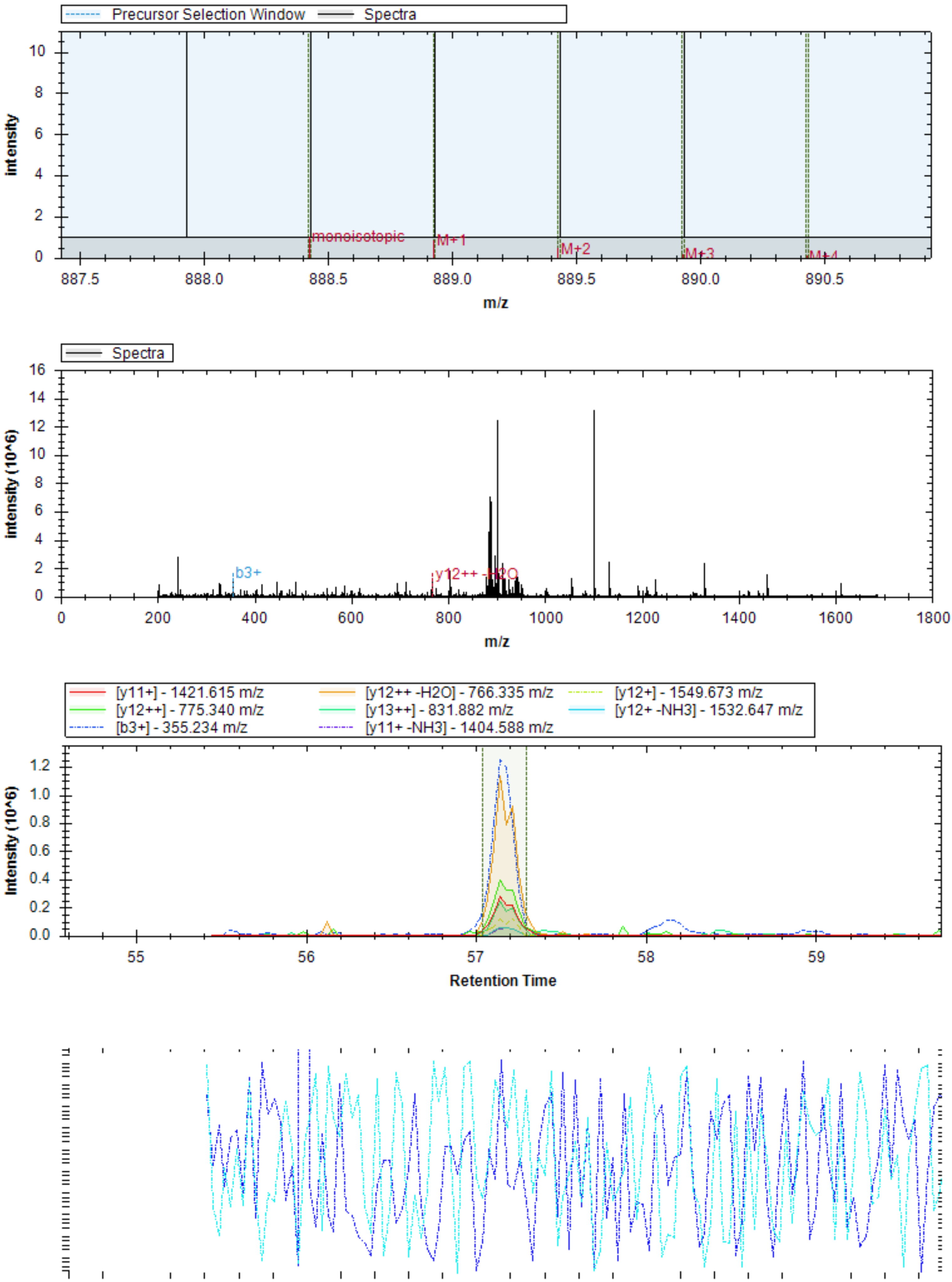

Q91Z31;Q91Z31-2 – _ETSLLAVPGALS[+H+O3+P]PLAIPNAAAAAAAAAAGR_.3
Fig4_MP-DIA-barrel-cortex-p30-1_MHRM.htrms

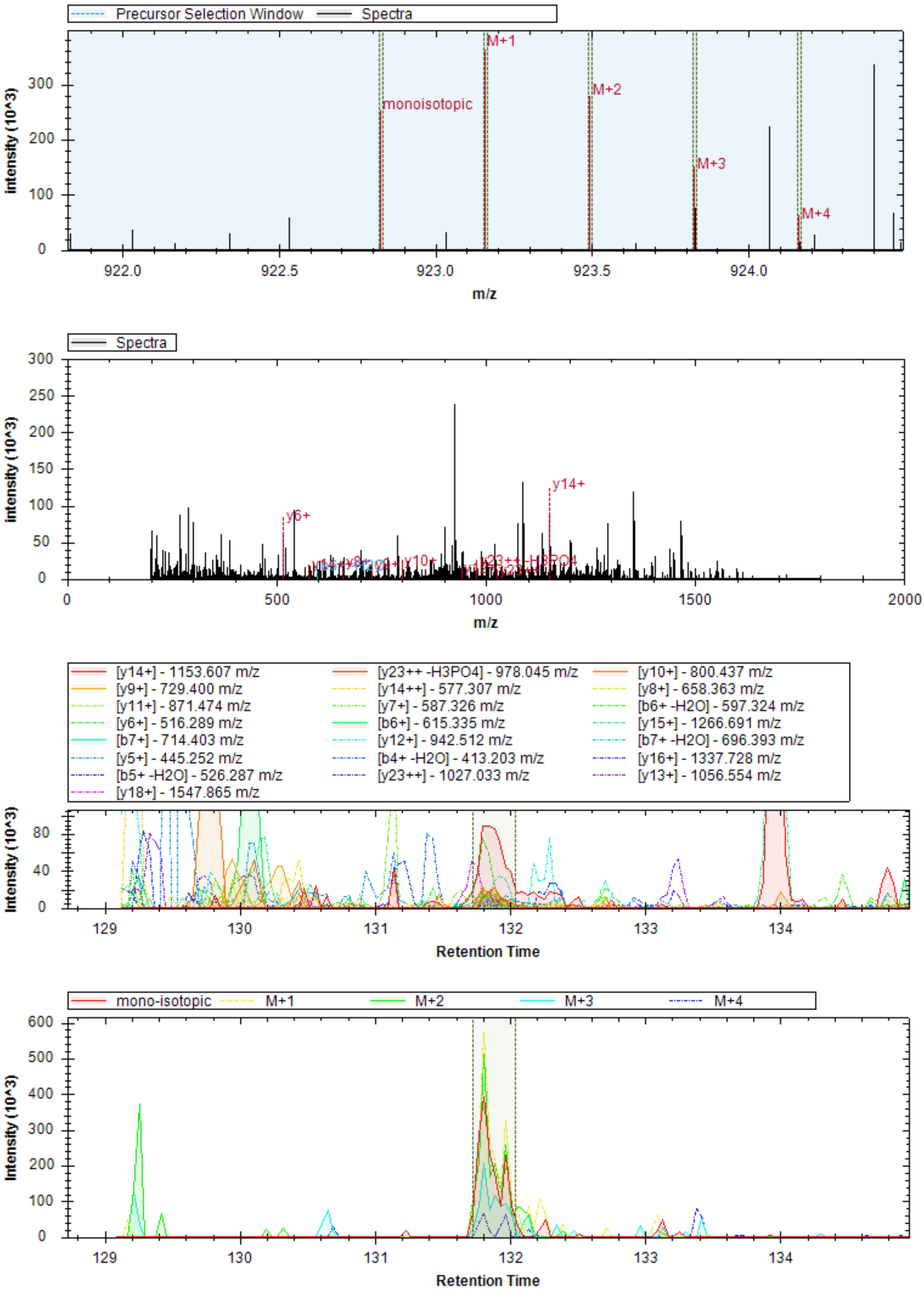

Q9CZT4;Q9CZT4-2 – _KEQM[+O]RAATVLPS[+H+O3+P]VQIK_.2
Fig4_MP-DIA-barrel-cortex-p30-1_MHRM.htrms

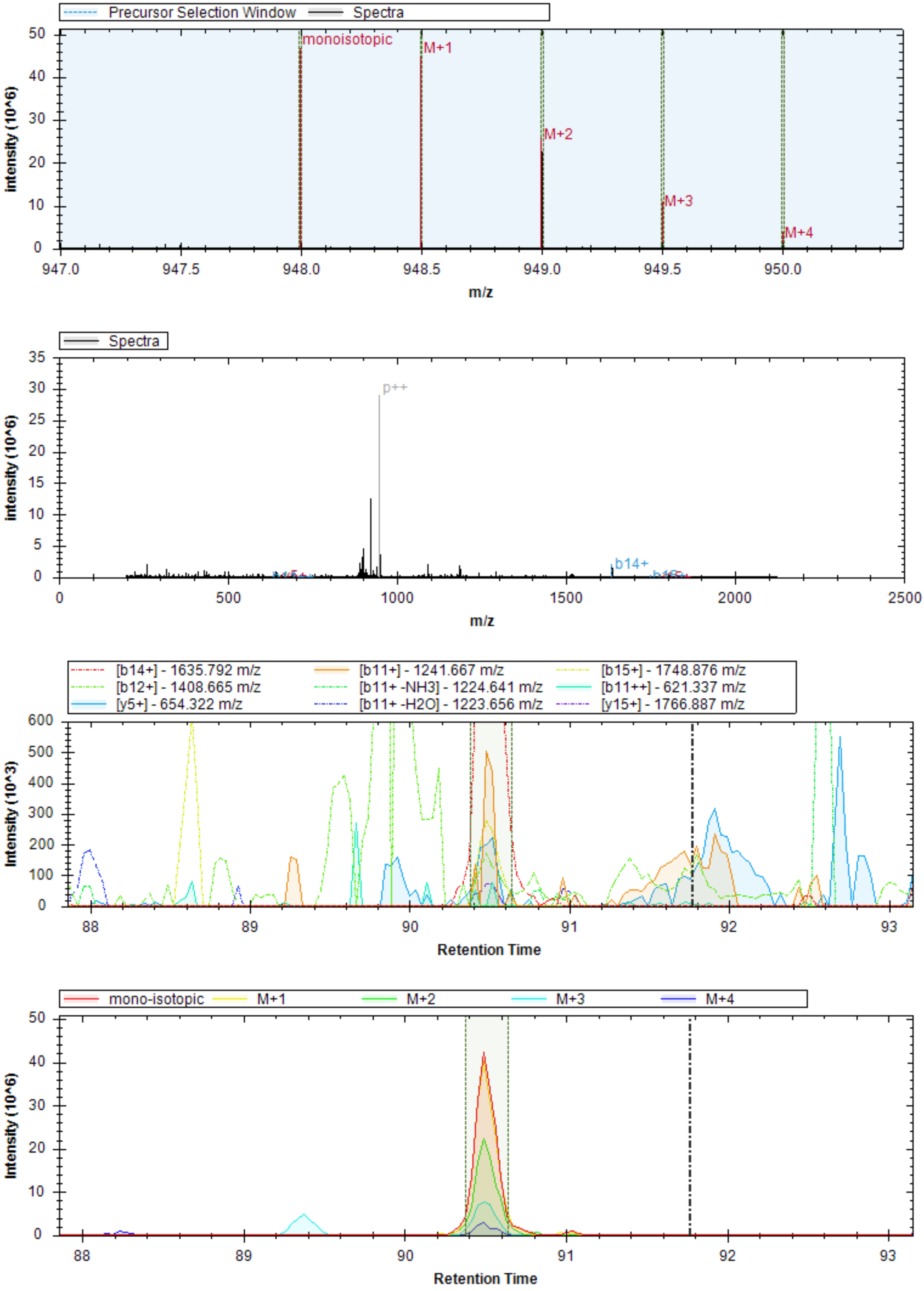

Q9ES97;Q9ES97-2 – _AES[+H+O3+P]SAATQSPSVSSSSSGAEPSALGGGGGSPGAC[+C2+H3+N+O]PALGAK_.4
Fig4_MP-DIA-barrel-cortex-p30-1_MHRM.htrms

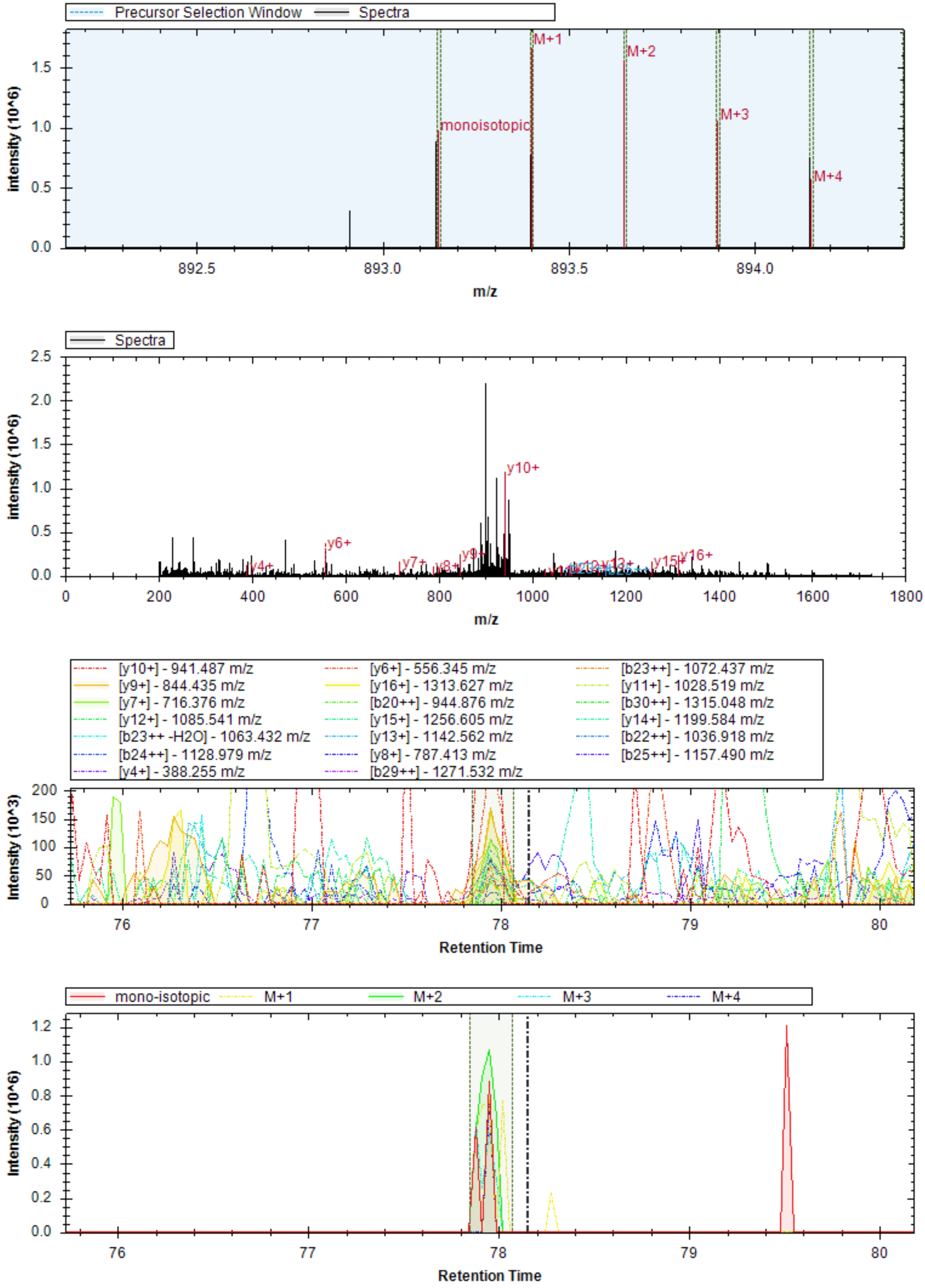

Q9QYR6-2 – _M[+O]AS[+H+O3+P]PPPSGPPSAAHTPFHQSPVEEK_.3

Fig4_MP-DIA-barrel-cortex-p30-1_MHRM.htrms

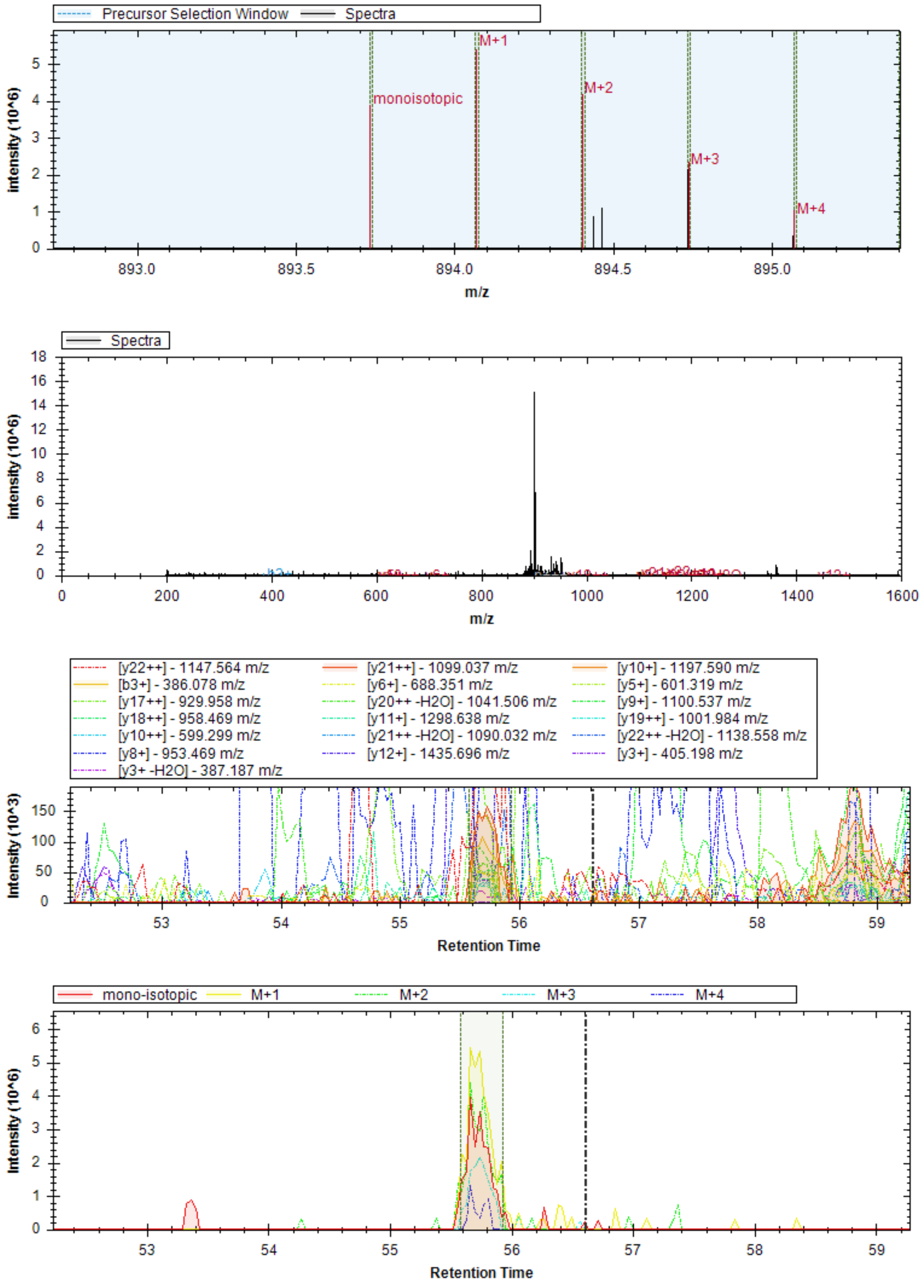

Q9QYR6-2 – _MAS[+H+O3+P]PPPSGPPSAAHTPFHQSPVEEK_.3
Fig4_MP-DIA-barrel-cortex-p30-1_MHRM.htrms

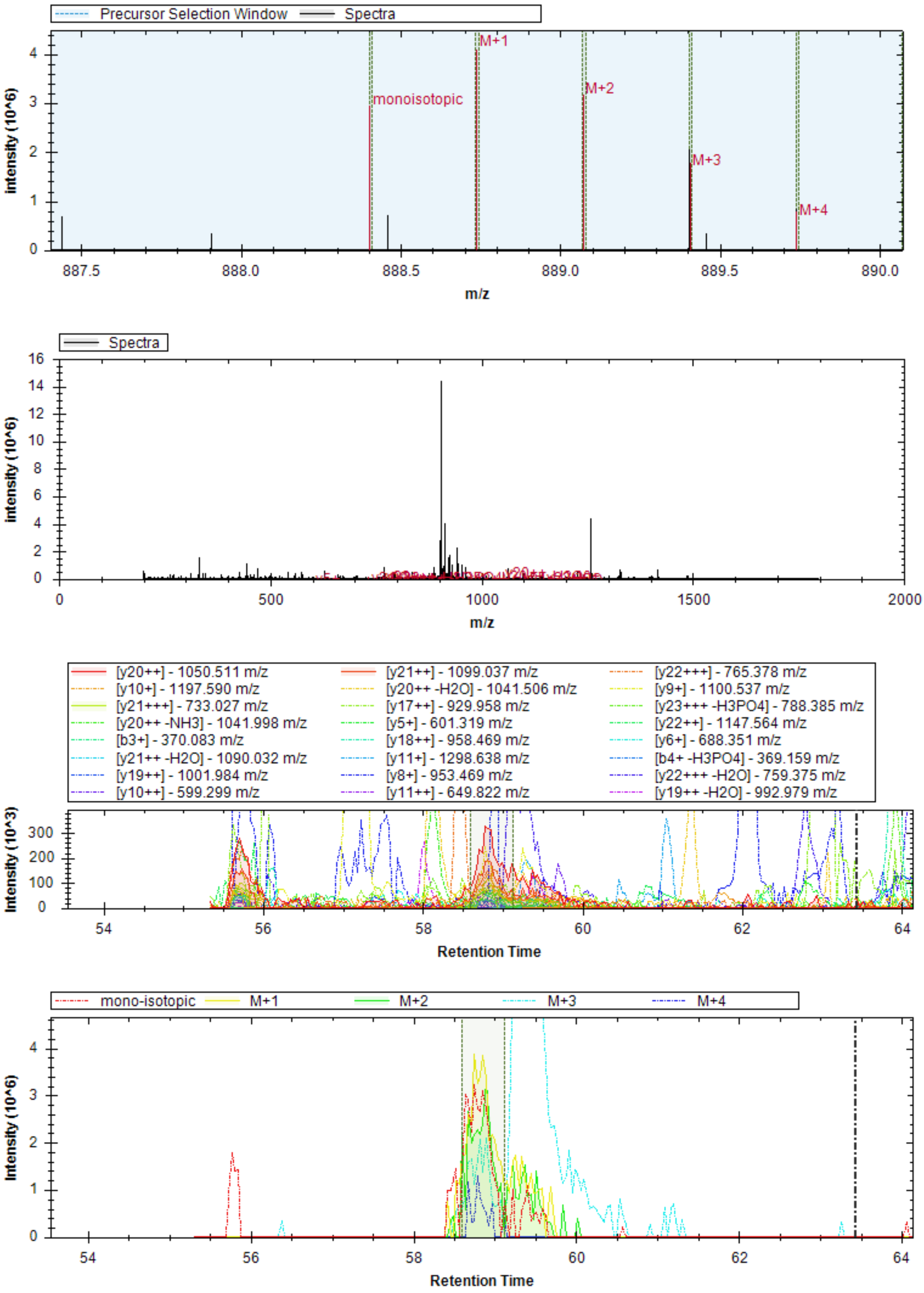

A2A891;A2A891-2;A2A8[...] – _TAVIVQQKLRS[+H+O3+P]S[+H+O3+P]LLT[+H+O3+P]K_.2
Fig4_MP-DIA-barrel-cortex-p30-1_MHRM.htrms

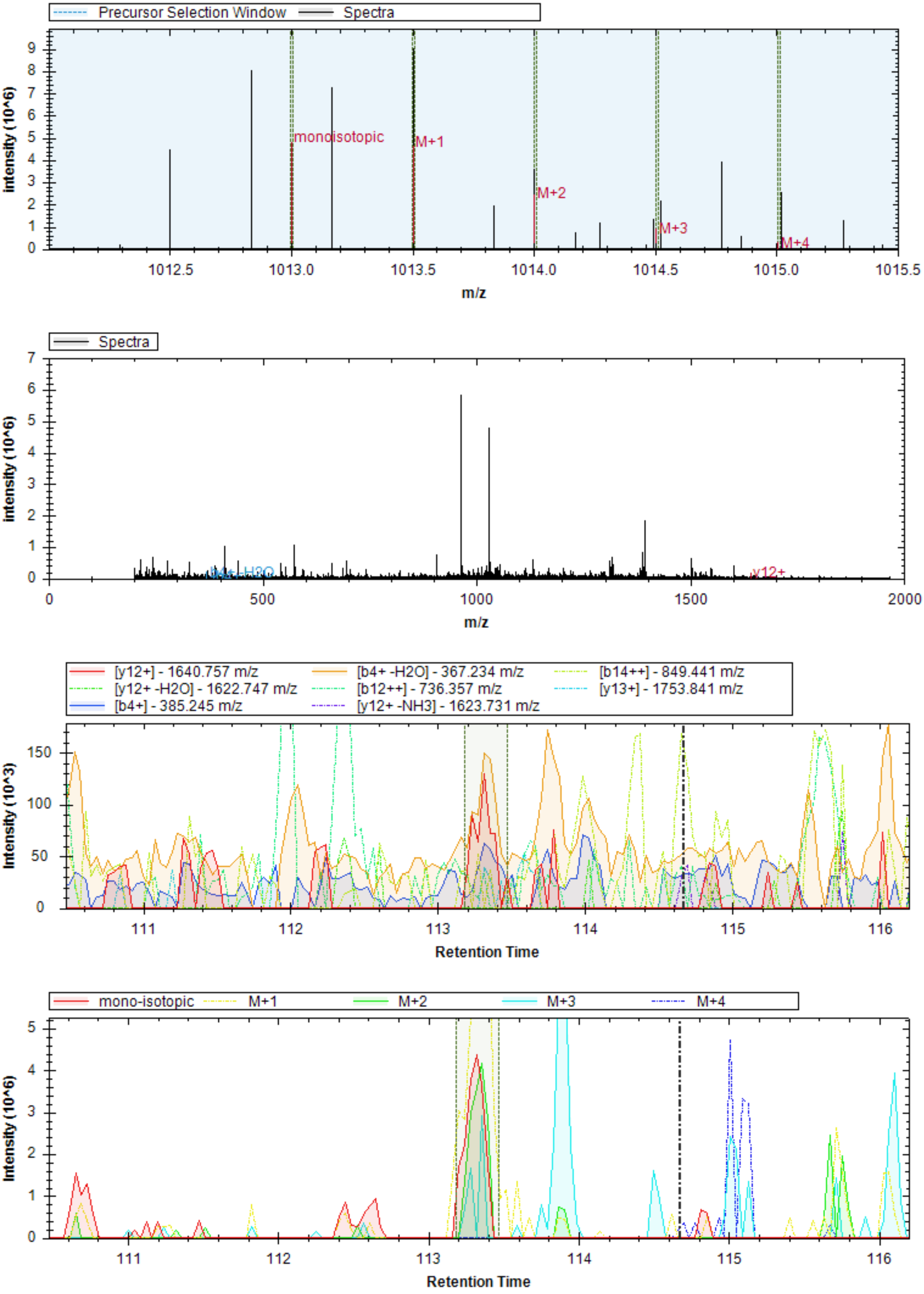

P17742 – _VNPT[+H+O3+P]VFFDITADDEPLGR_.2

Fig4_MP-DIA-barrel-cortex-p30-1_MHRM.htrms

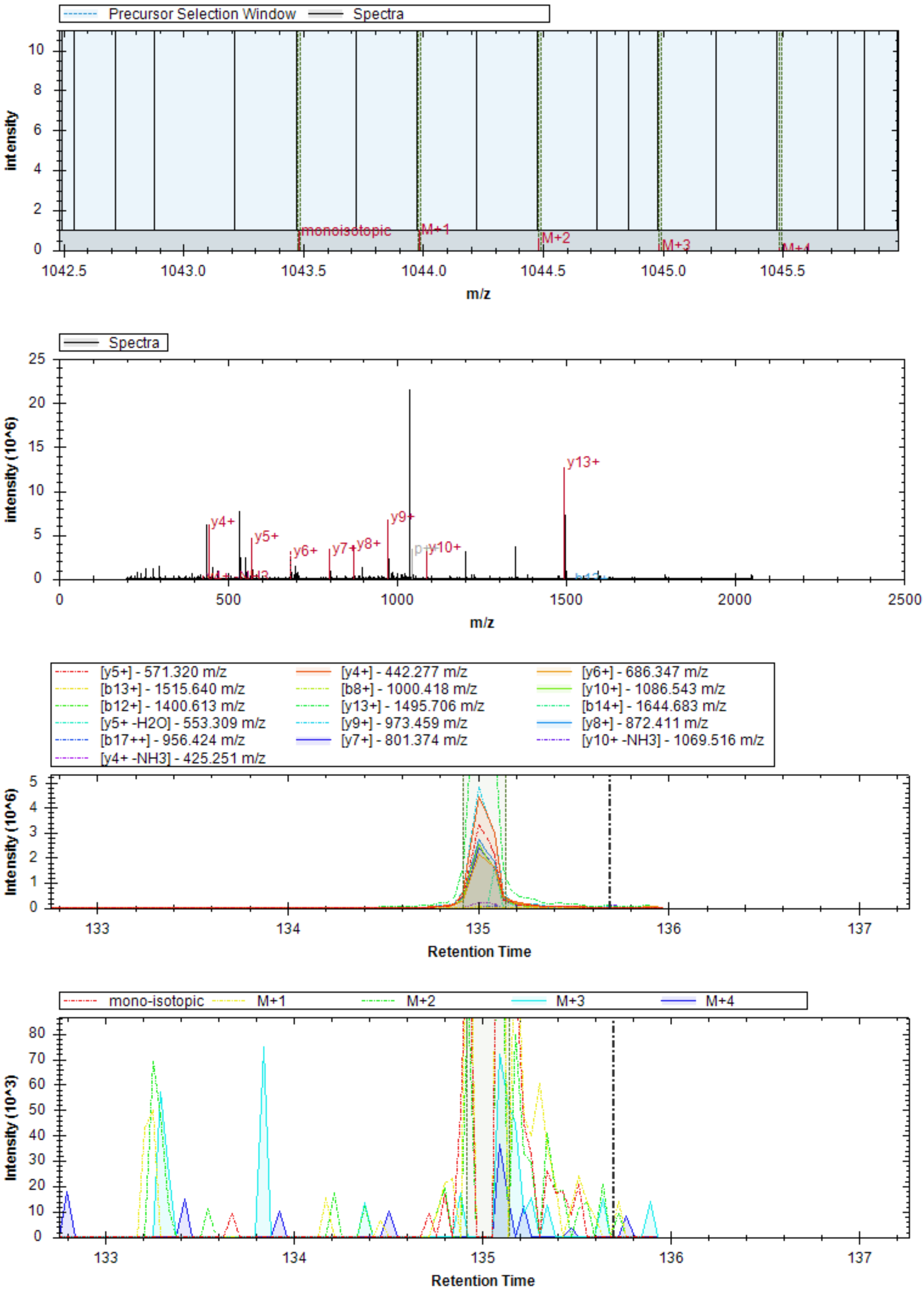

Q8CHK4;Q8CHK4-2;Q8CH[...] – _RGY[+H+O3+P]GKLLIEFS[+H+O3+P]YELSK_.2
Fig4_MP-DIA-barrel-cortex-p30-1_MHRM.htrms

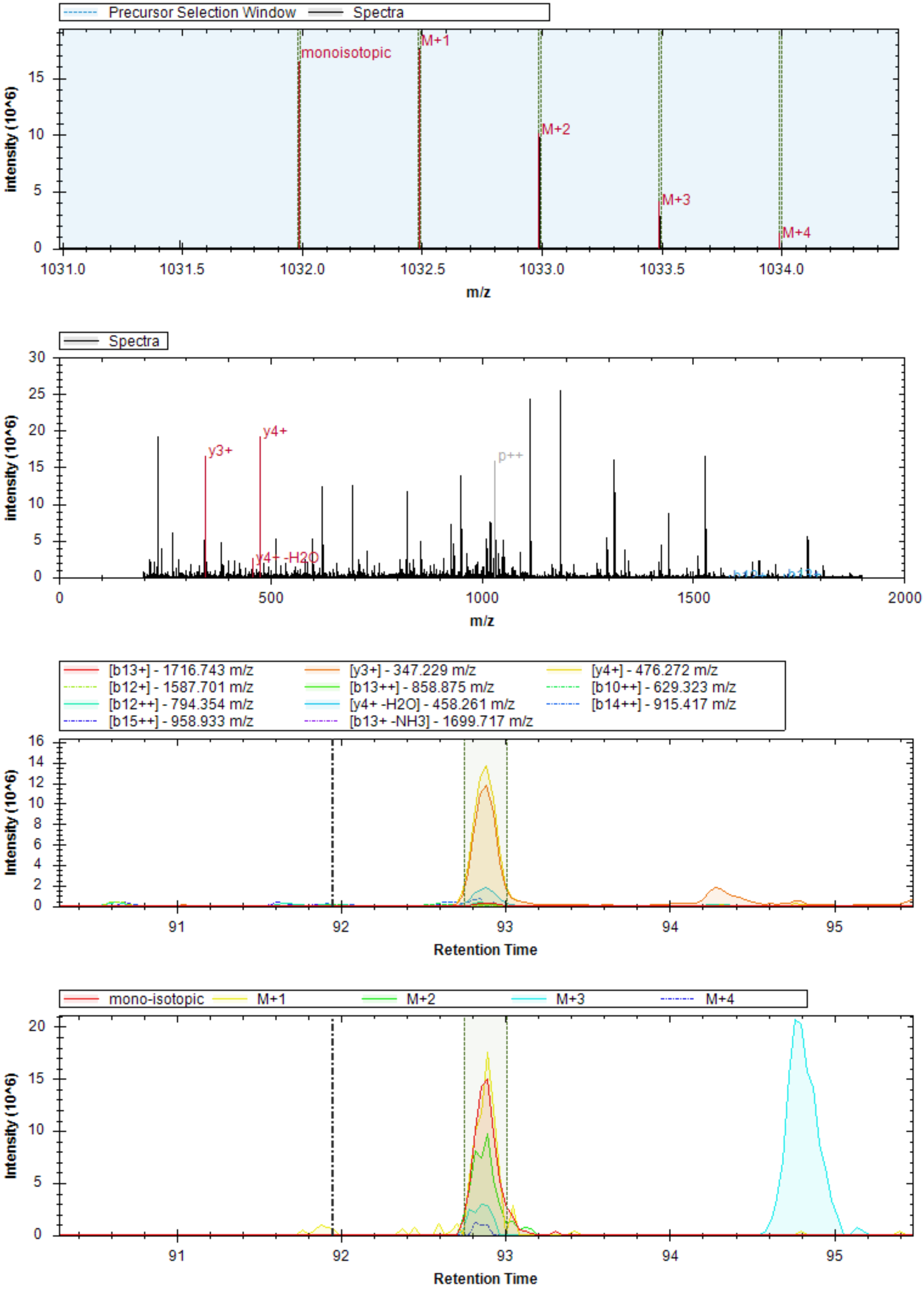

E9Q401 – _MVAEVFIY[+H+O3+P]WSK_.1

Fig4_MP-DIA-barrel-cortex-p30-1_MHRM.htrms

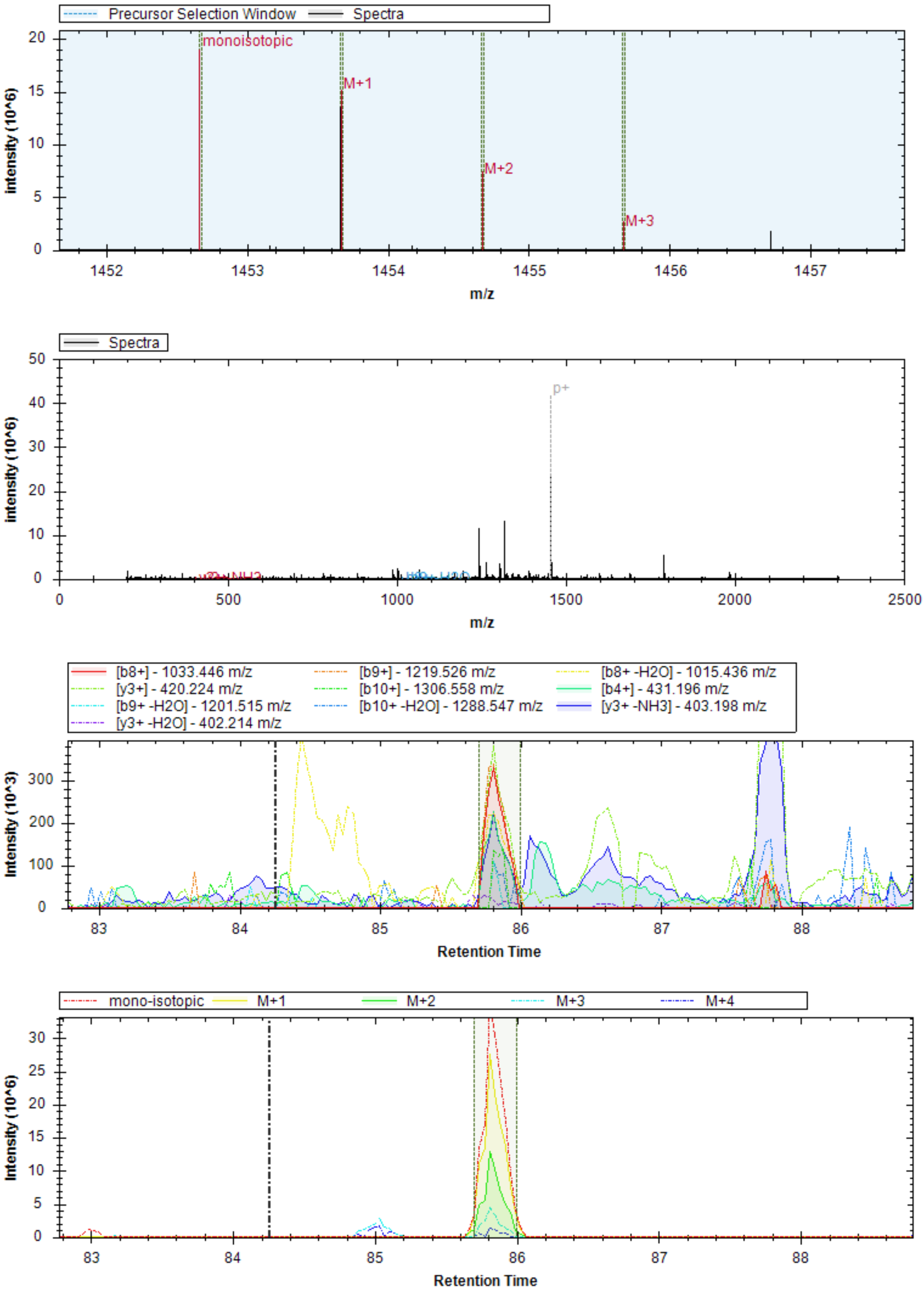

O35526 – _T[+H+O3+P]TTSEELEDMLESGNPAIFASGIIMDSSISK_.3
Fig4_MP-DIA-barrel-cortex-p30-1_MHRM.htrms

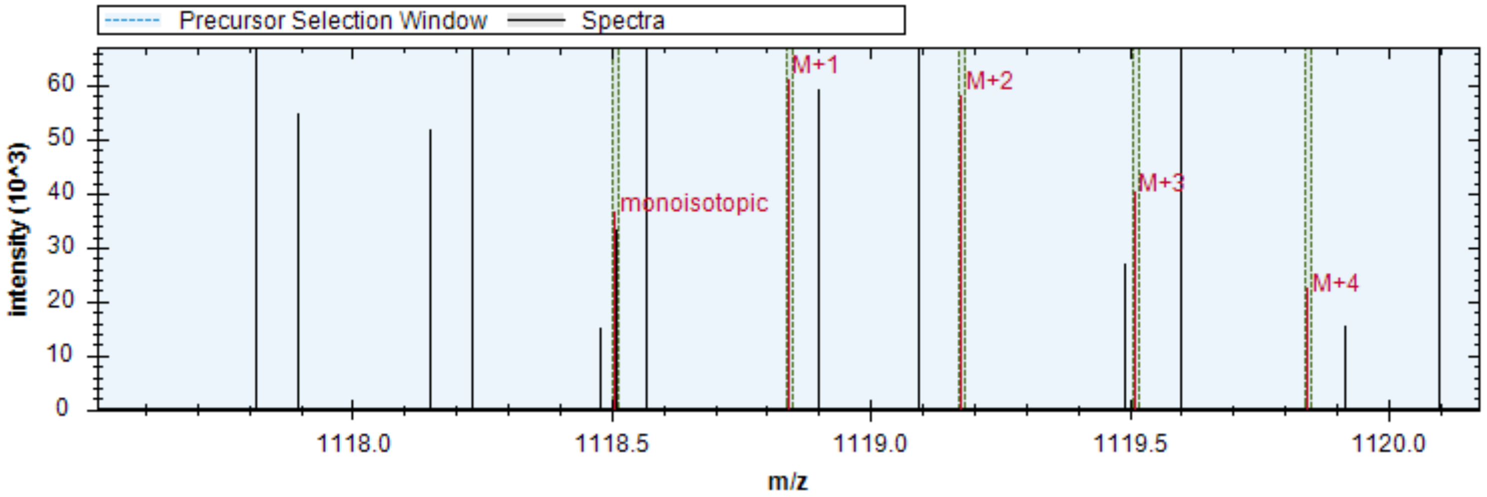

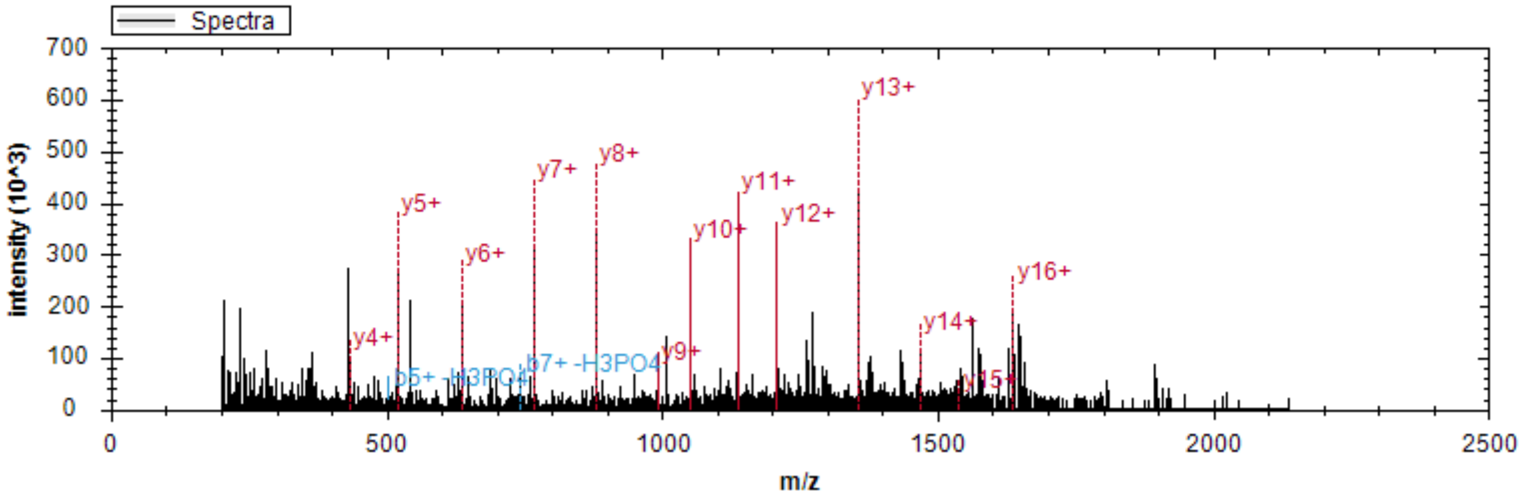

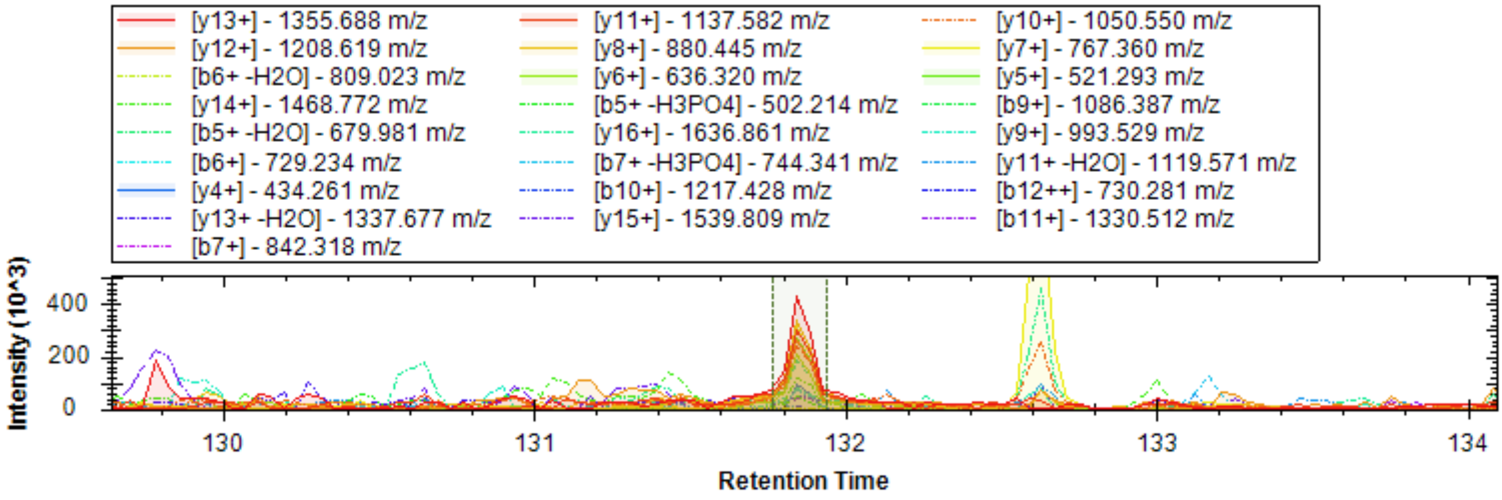

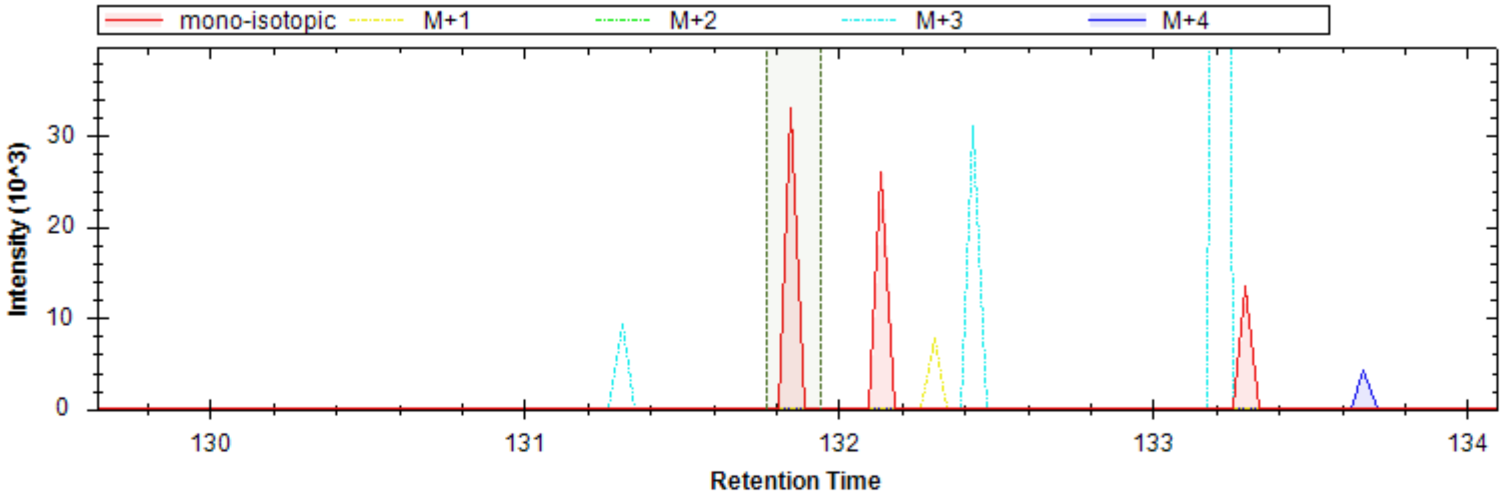

O35526 – _TTTS[+H+O3+P]EELEDMLESGNPAIFASGIIMDSSISK_.3
Fig4_MP-DIA-barrel-cortex-p30-1_MHRM.htrms

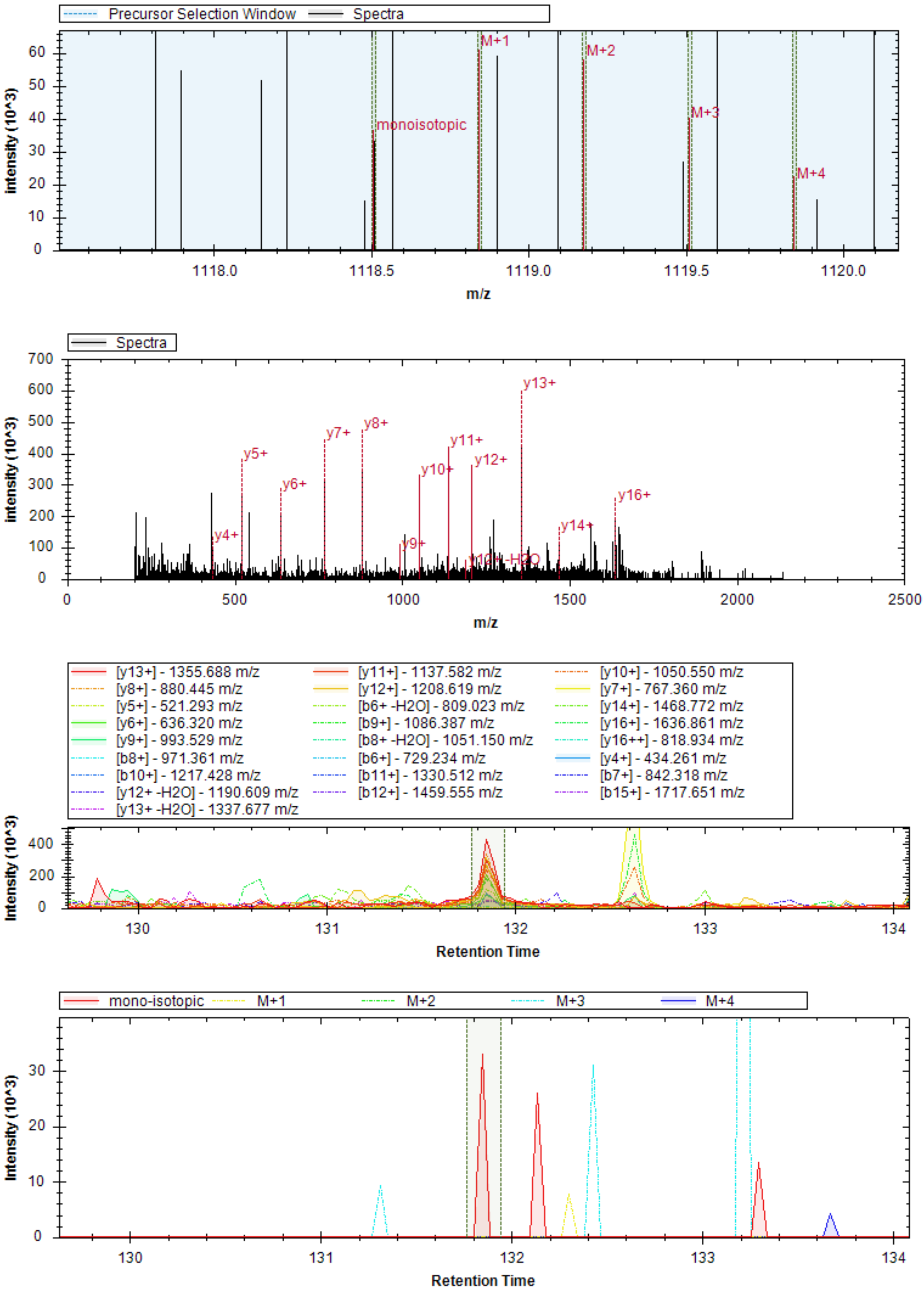

P20357 – _LSNVS[+H+O3+P]SSGSINLLESPQLATLAEDVTAALAK_.2
Fig4_MP-DIA-barrel-cortex-p30-1_MHRM.htrms

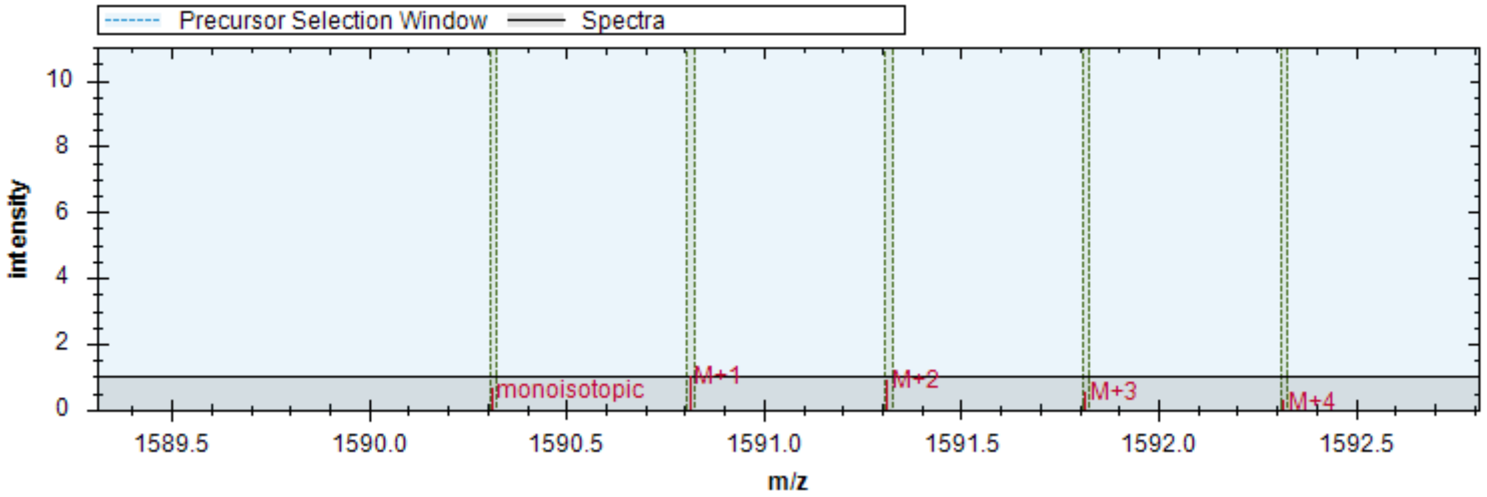

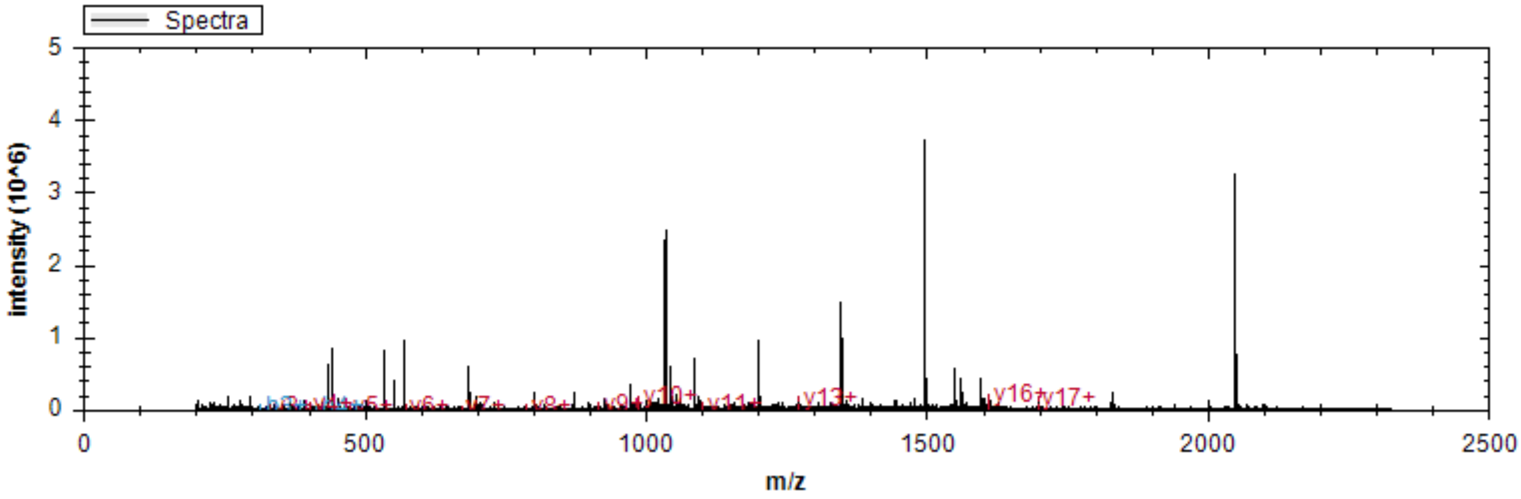

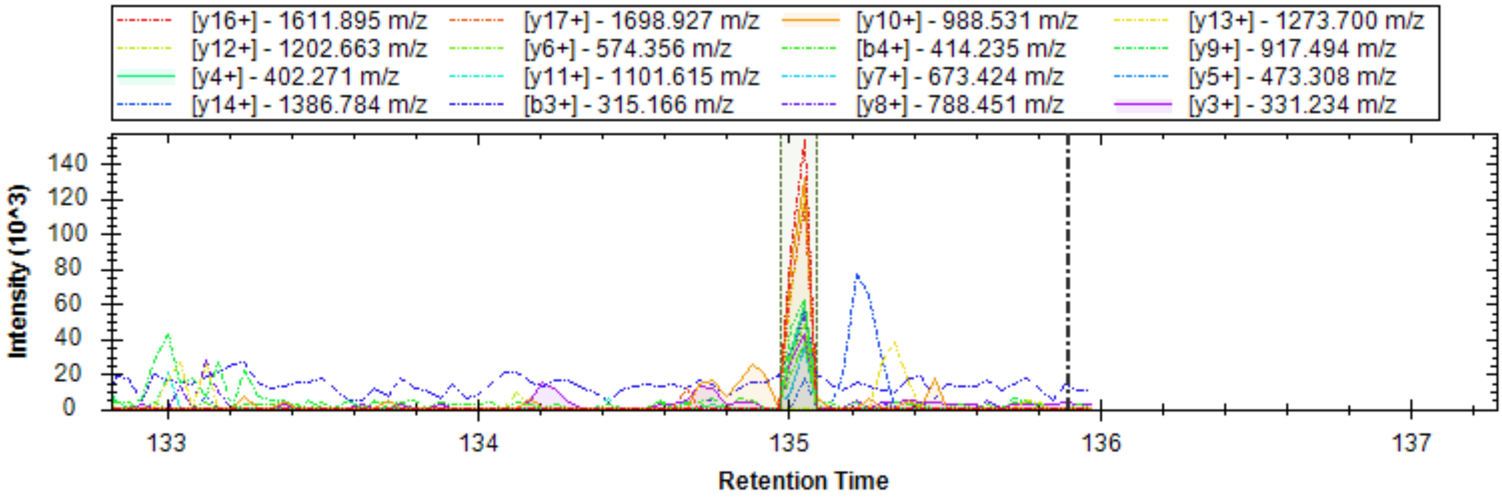

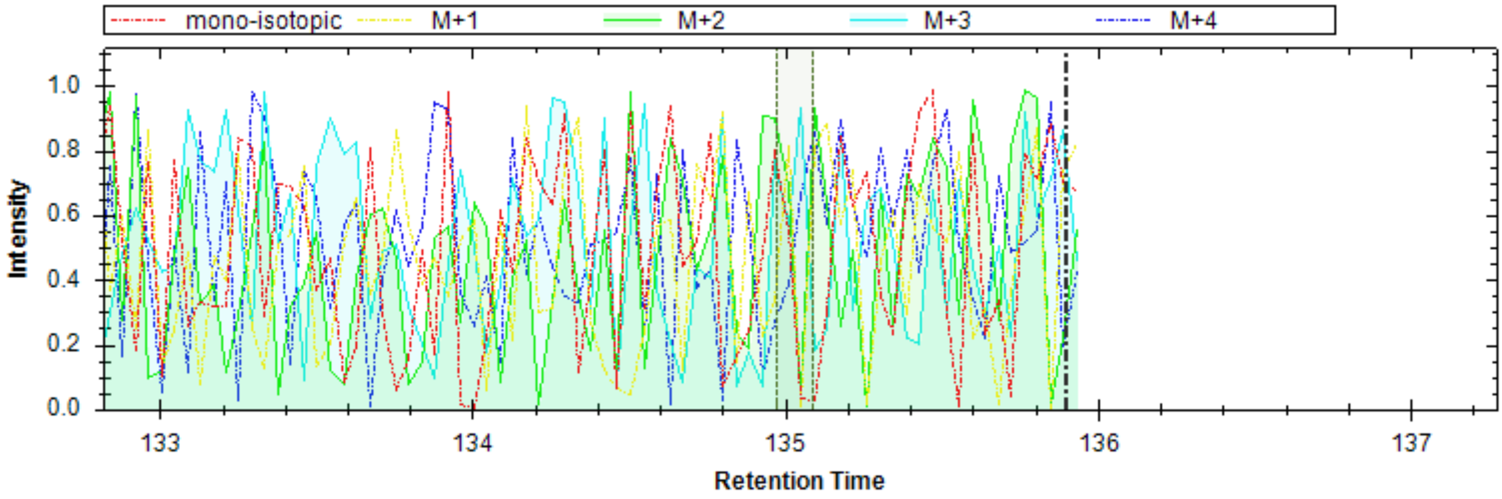

P20357 – _SGTSTPTTPGSTAITPGT[+H+O3+P]PPSYSSR_.2

Fig4_MP-DIA-barrel-cortex-p30-1_MHRM.htrms

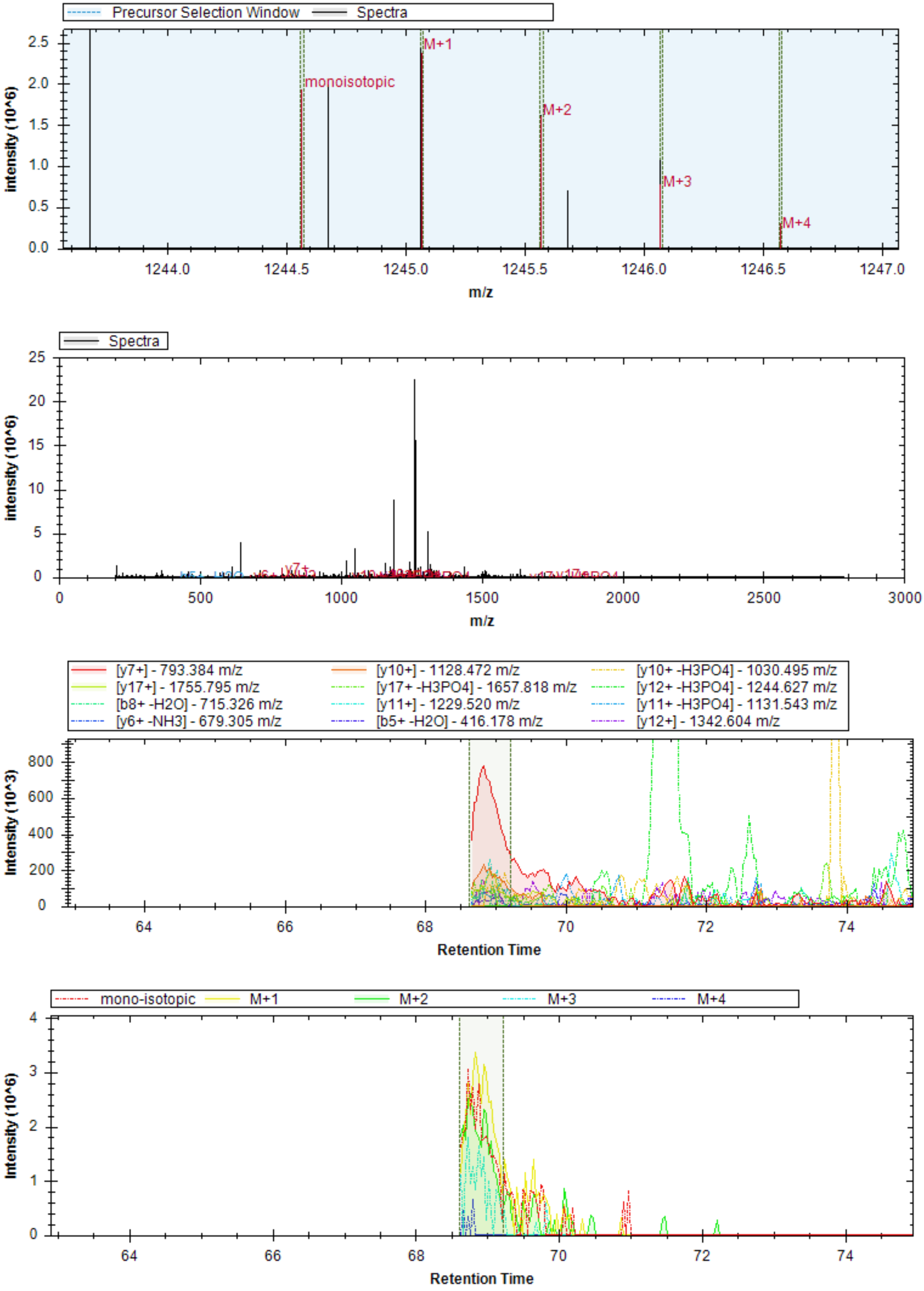

Q8BRT1 – _VLNT[+H+O3+P]GSDVEEAVADALLLGDIR_.2

Fig4_MP-DIA-barrel-cortex-p30-1_MHRM.htrms

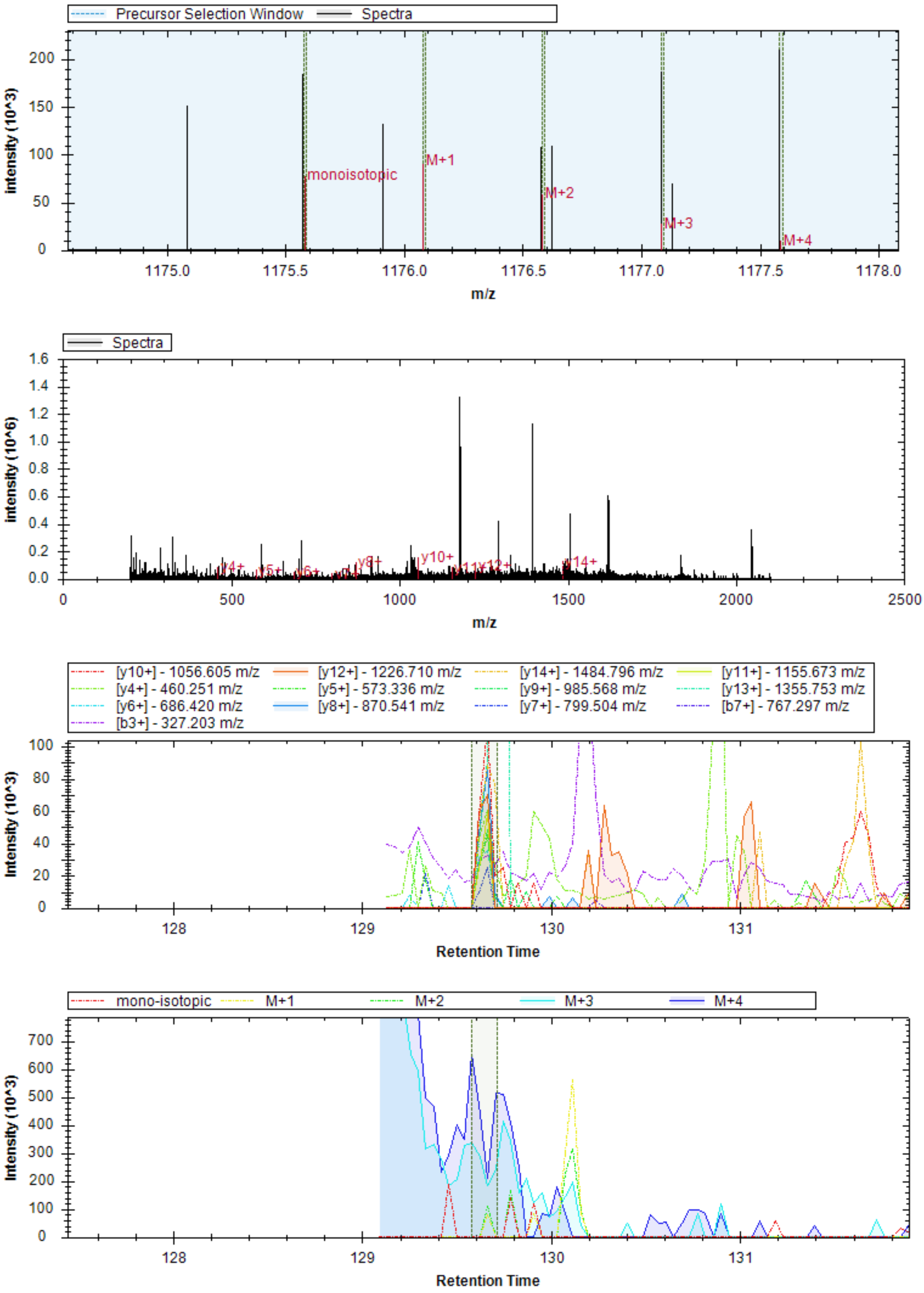

Q8BRT1 – _VLNTGS[+H+O3+P]DVEEAVADALLLGDIR_.2
Fig4_MP-DIA-barrel-cortex-p30-1_MHRM.htrms

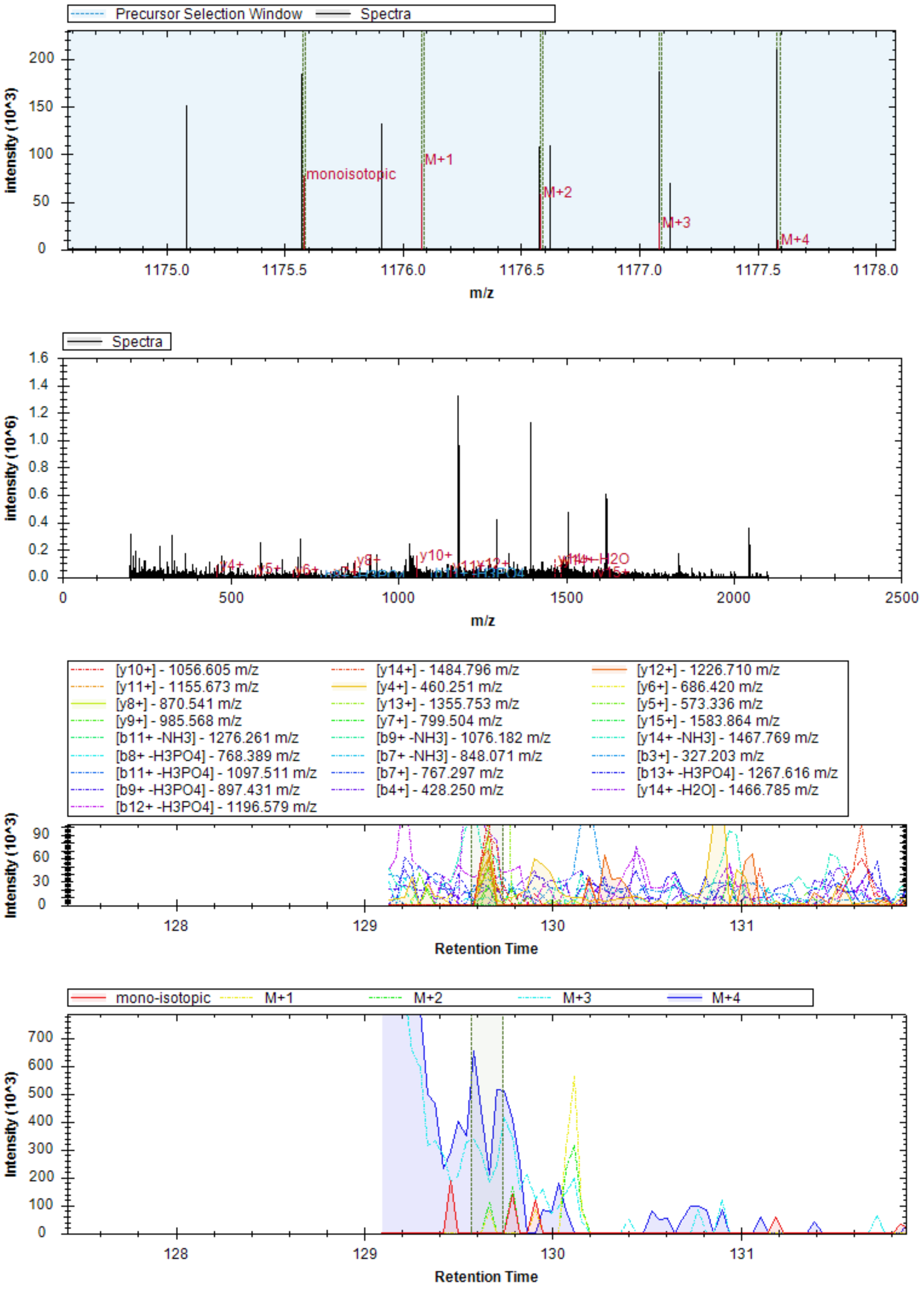

Q8VHY0 – _LLAGPRY[+H+O3+P]GR_.1

Fig4_MP-DIA-barrel-cortex-p30-1_MHRM.htrms

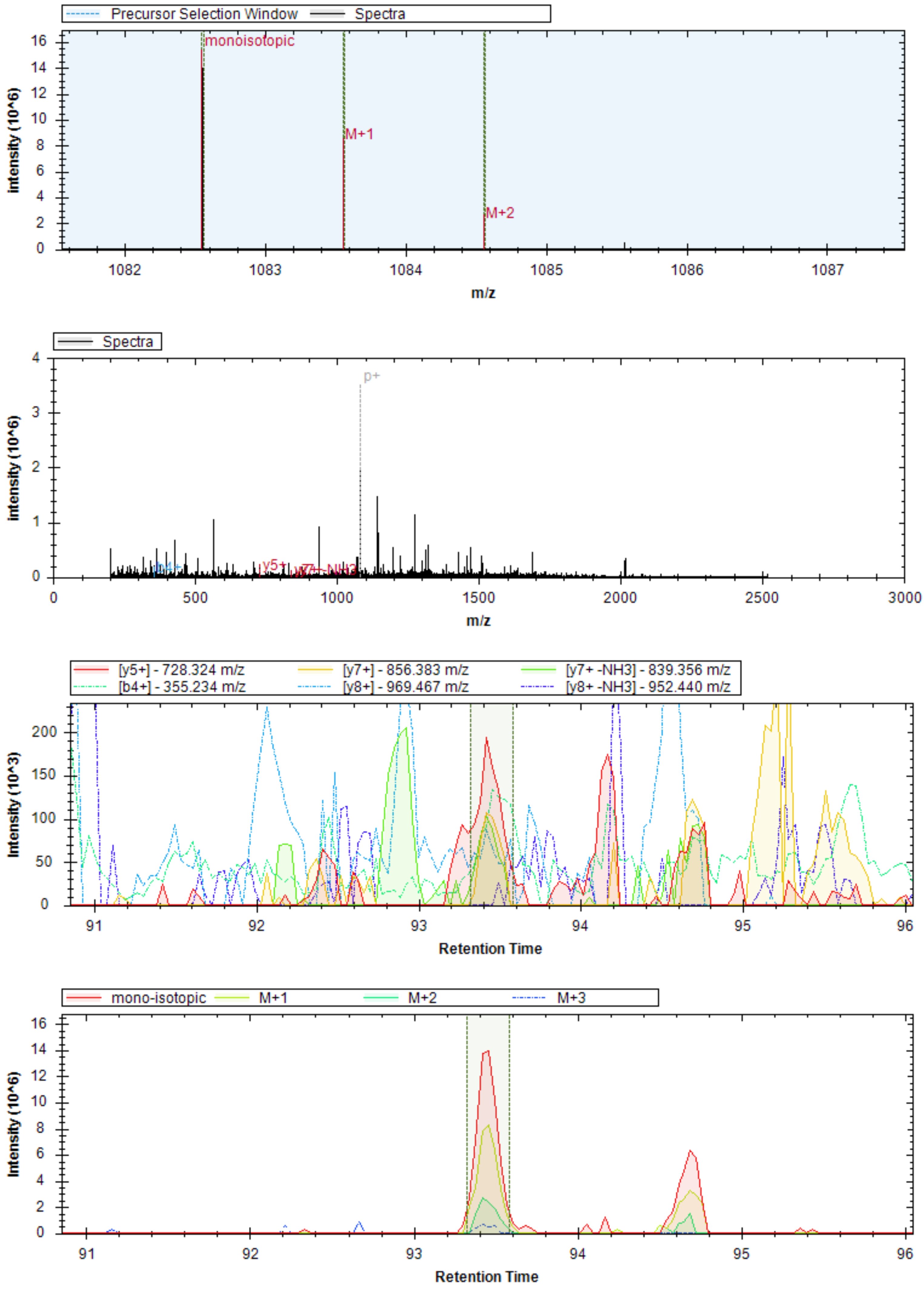

Q921N6;Q921N6-2 – _LIY[+H+O3+P]KPRQAAVTR_.4
Fig4_MP-DIA-barrel-cortex-p30-2_MHRM.htrms

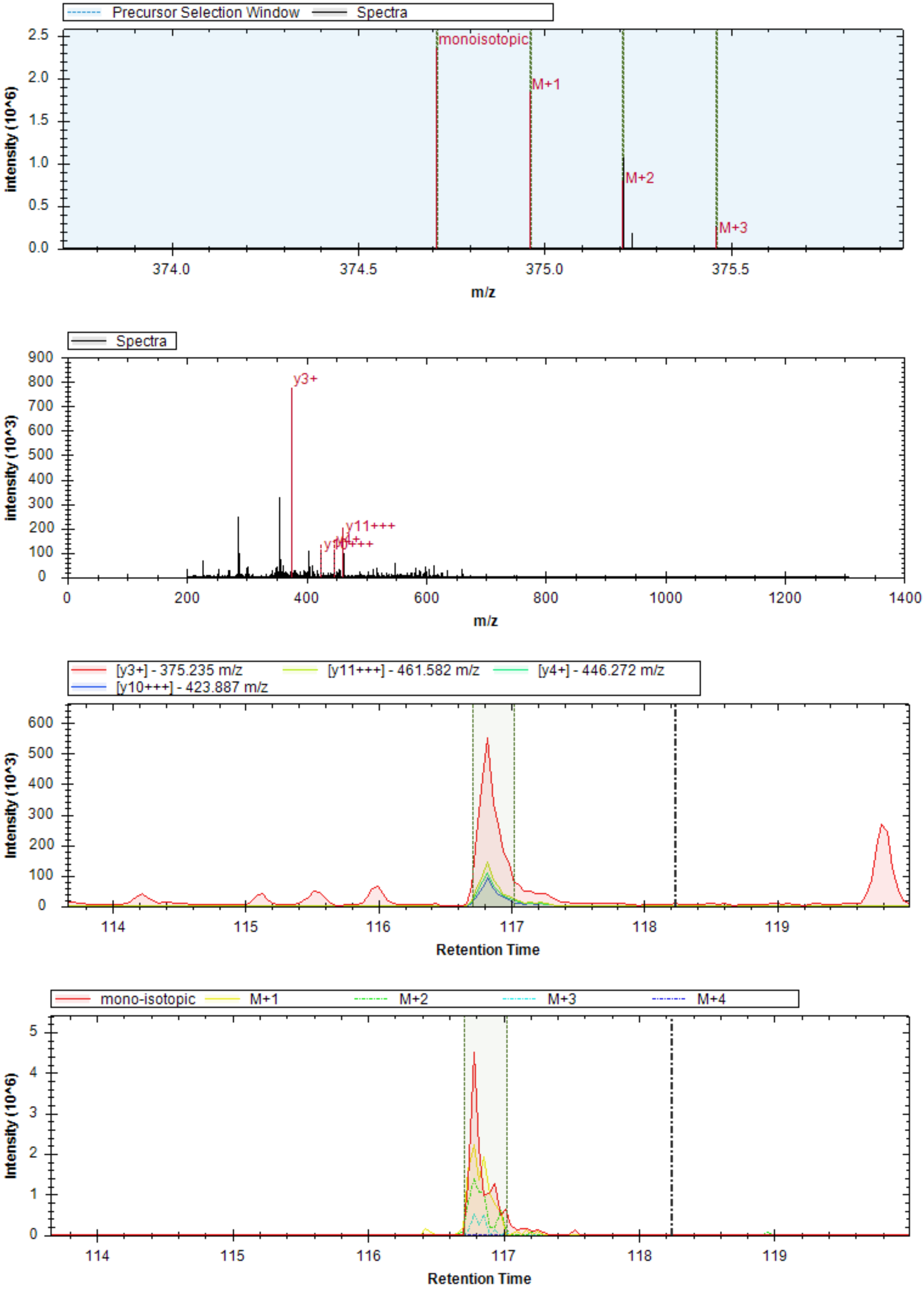

Q9CVB6 – _IIEET[+H+O3+P]LALK_.3
Fig4_MP-DIA-barrel-cortex-p30-2_MHRM.htrms

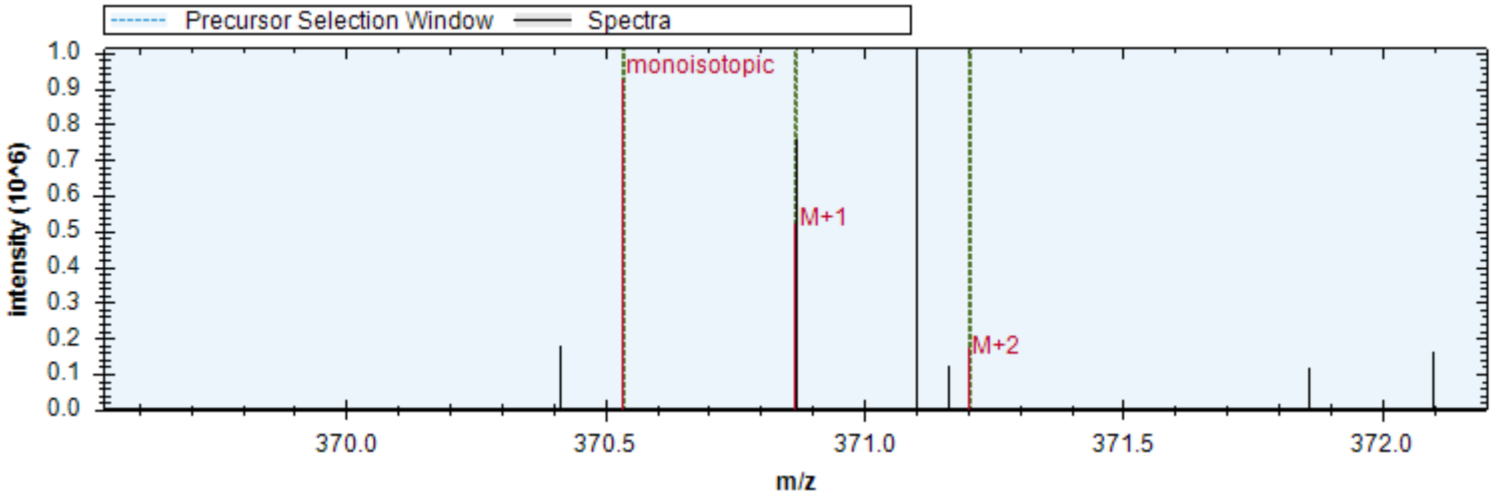

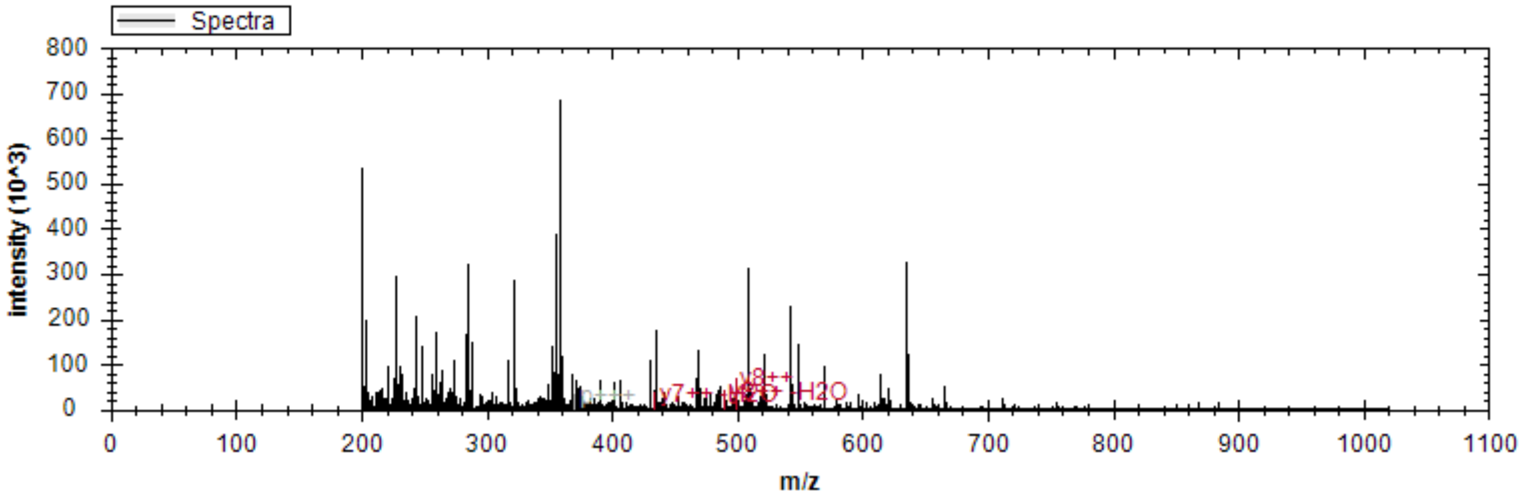

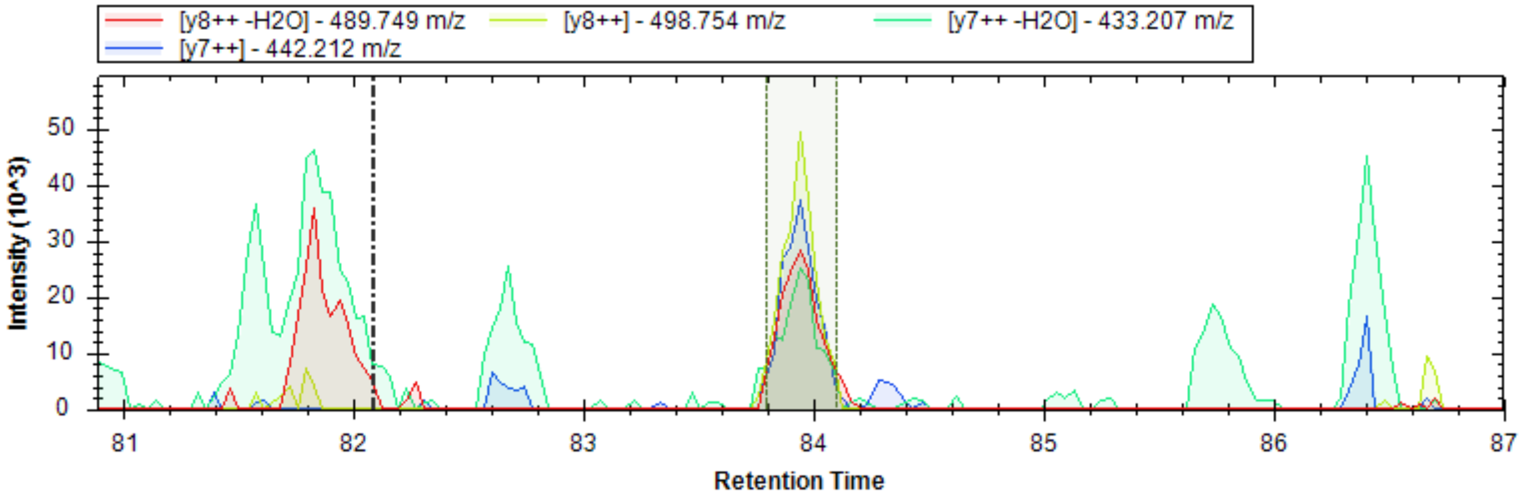

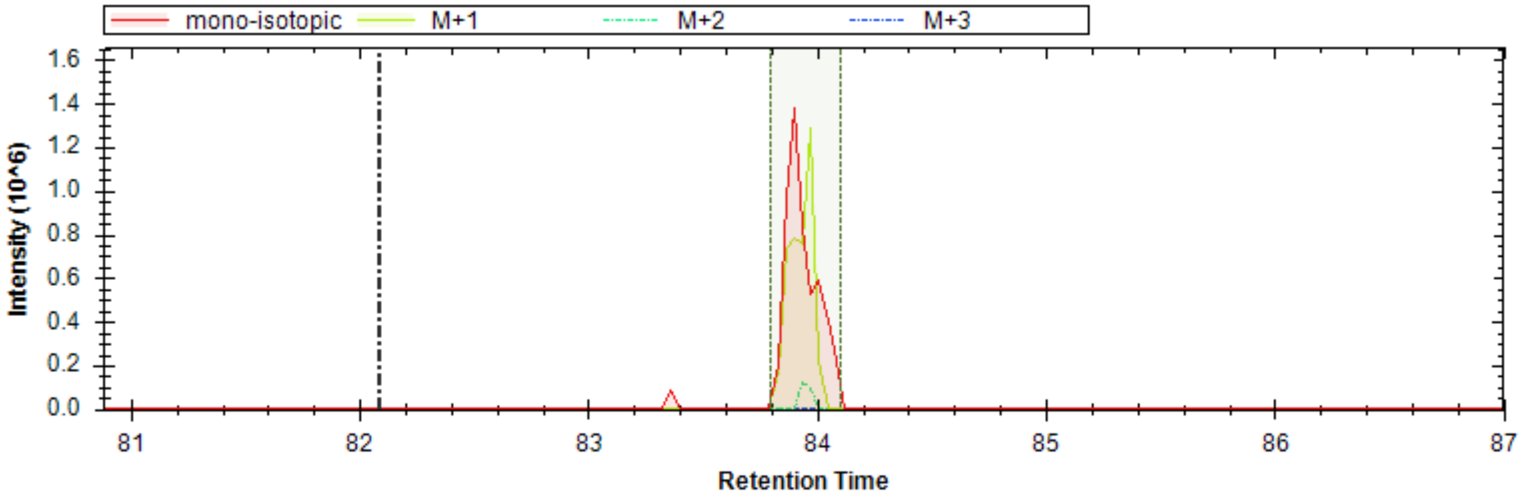

Q9D516;Q9D516-2 – _KLS[+H+O3+P]QGILVIR_.3
Fig4_MP-DIA-barrel-cortex-p30-2_MHRM.htrms

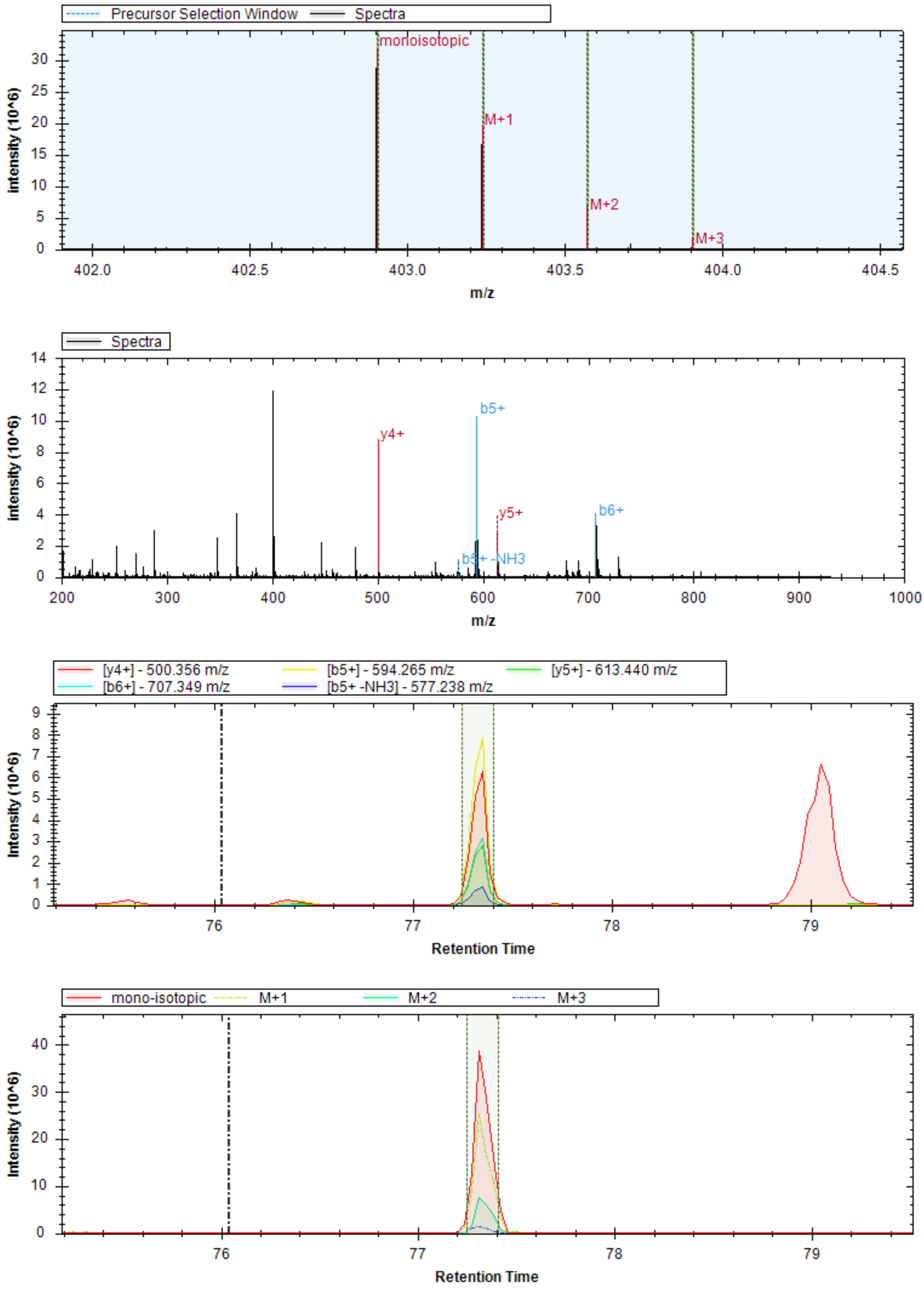

P20357 – _KSEVQAHS[+H+O3+P]PSR_.3
Fig4_MP-DIA-barrel-cortex-p30-2_MHRM.htrms

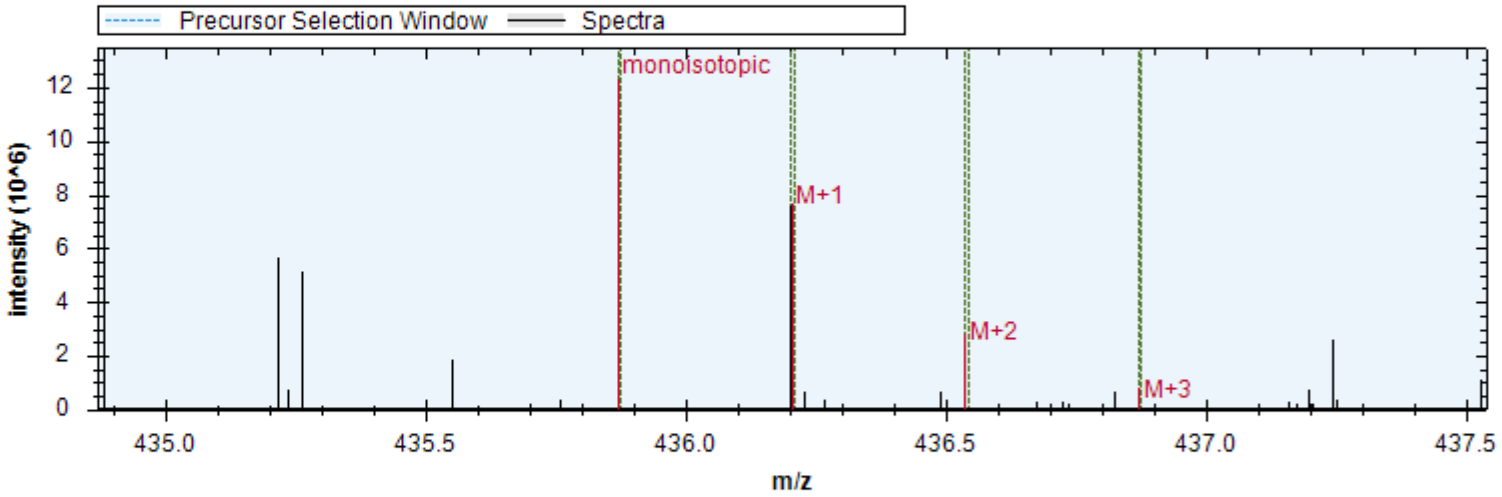

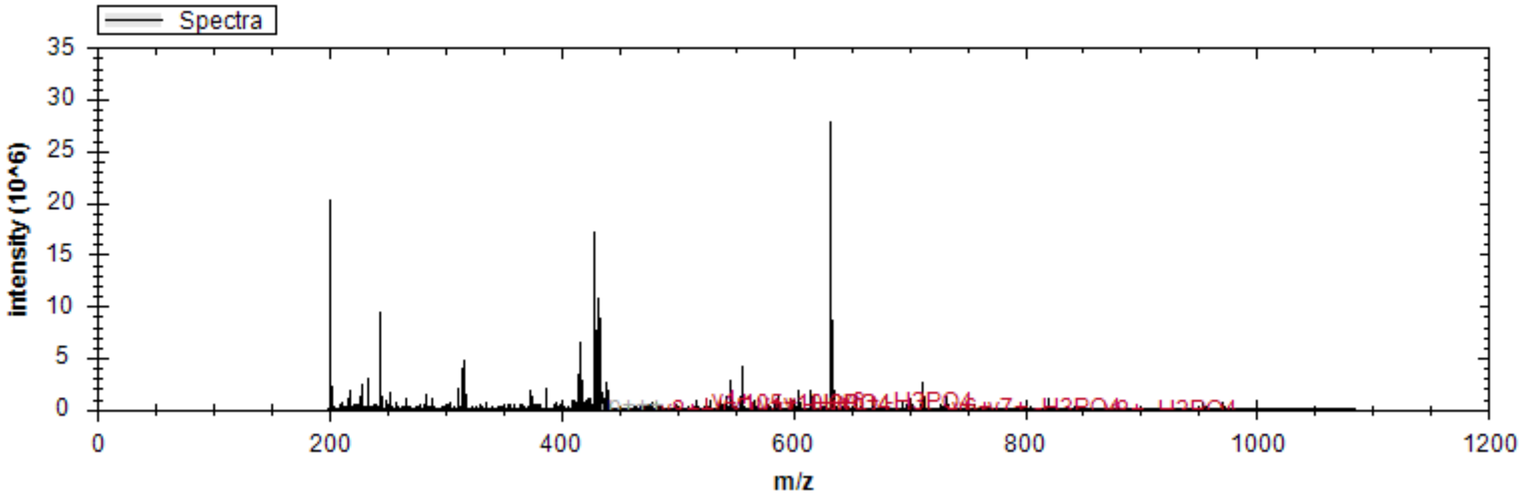

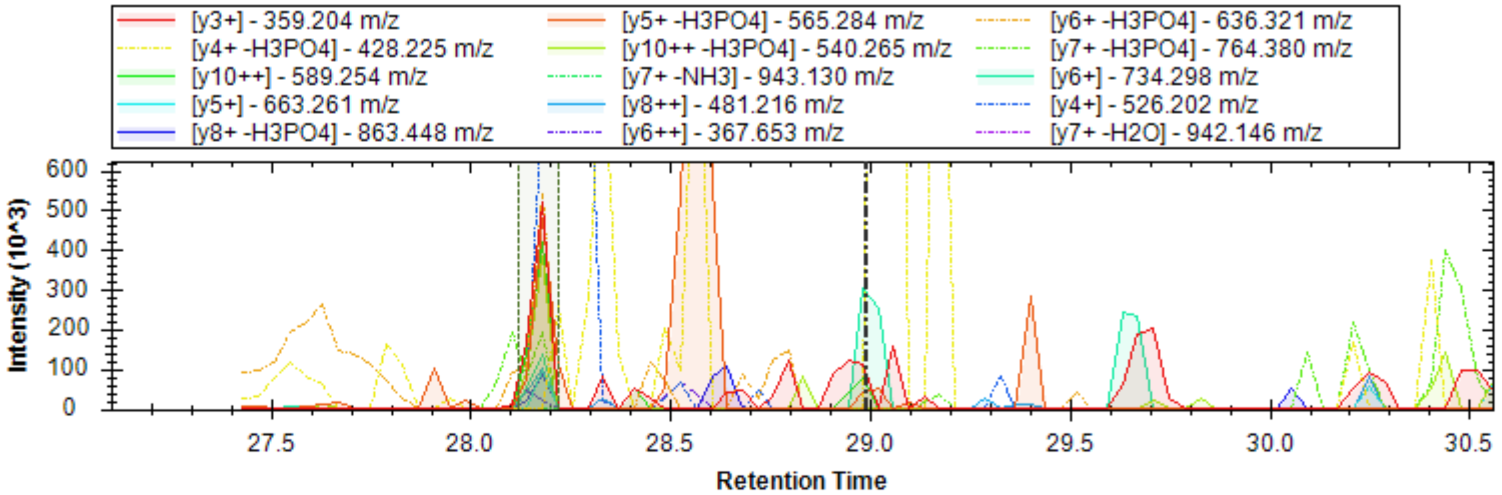

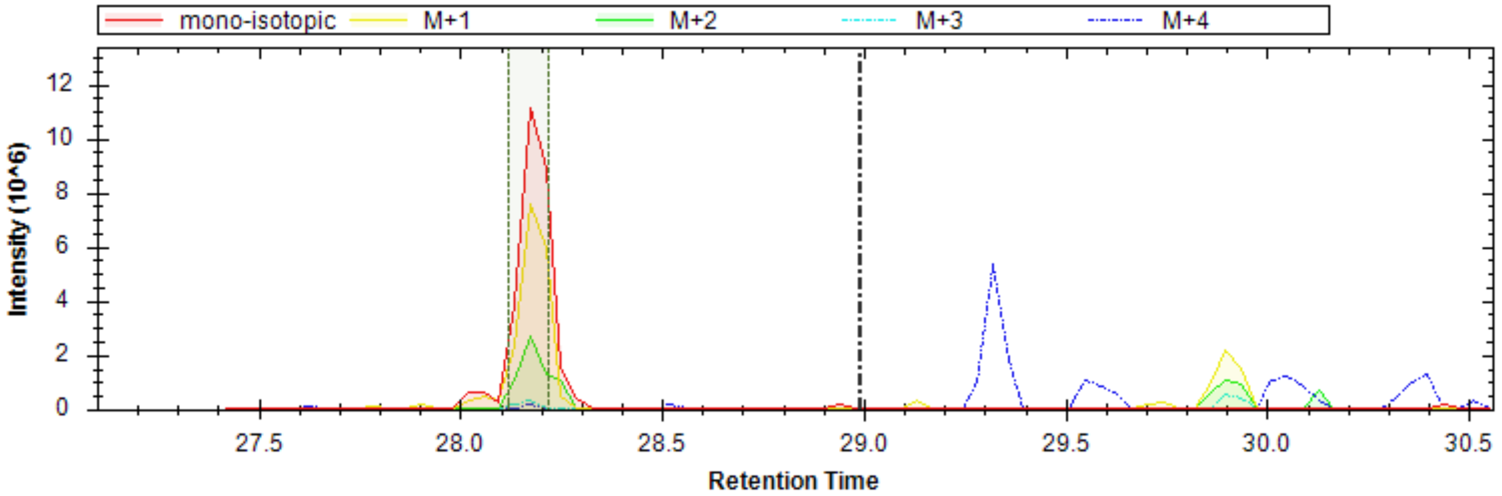

Q03172 – _VALSLLNS[+H+O3+P]KQK_.3
Fig4_MP-DIA-barrel-cortex-p30-2_MHRM.htrms

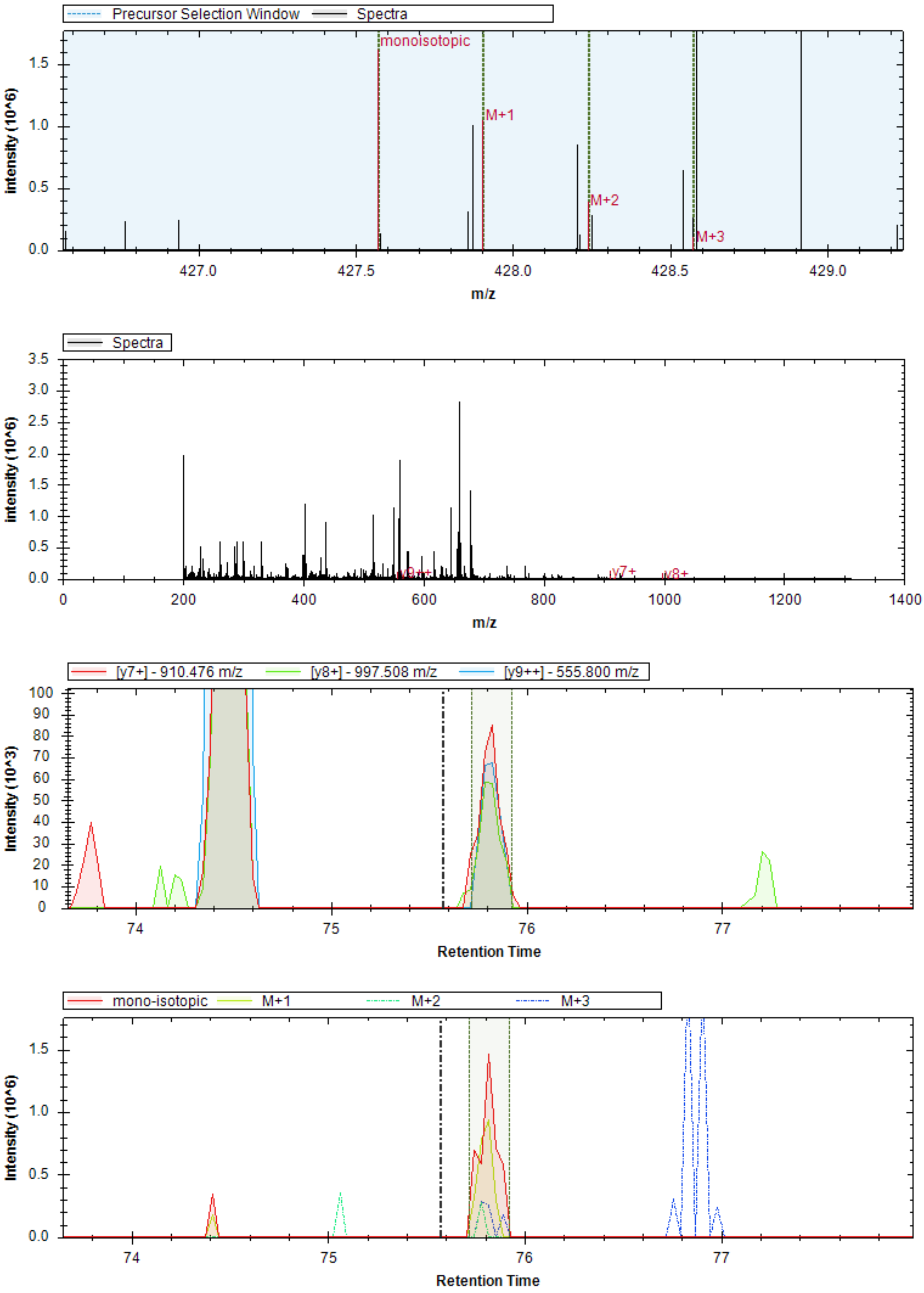

Q62188 – _GS[+H+O3+P]PTRPNPPVR_.3
Fig4_MP-DIA-barrel-cortex-p30-2_MHRM.htrms

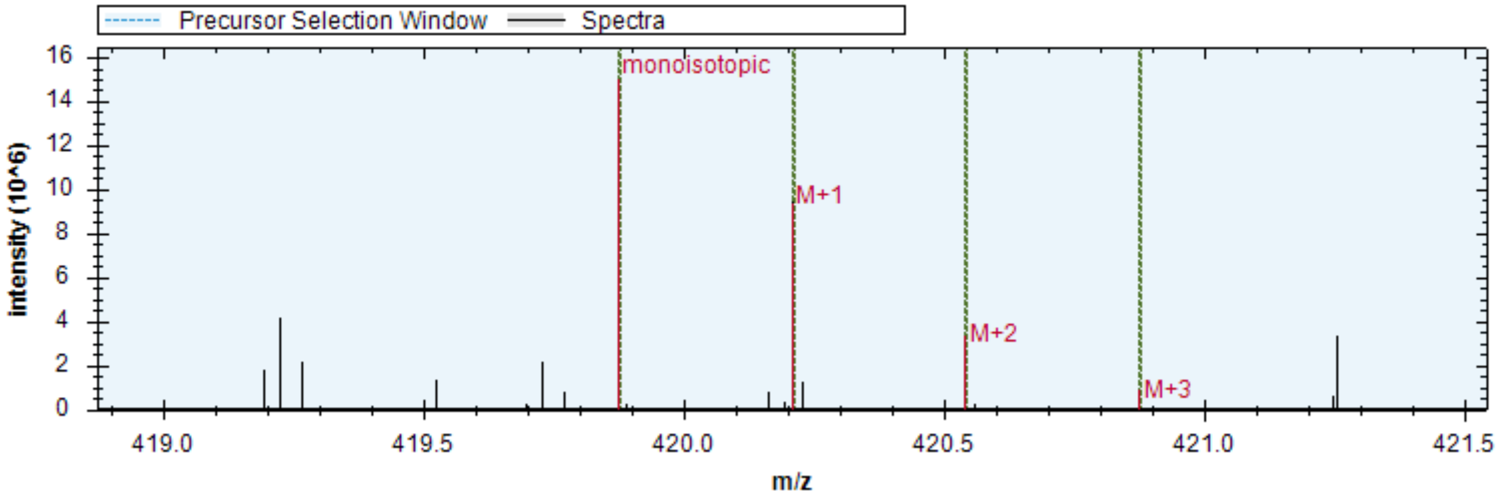

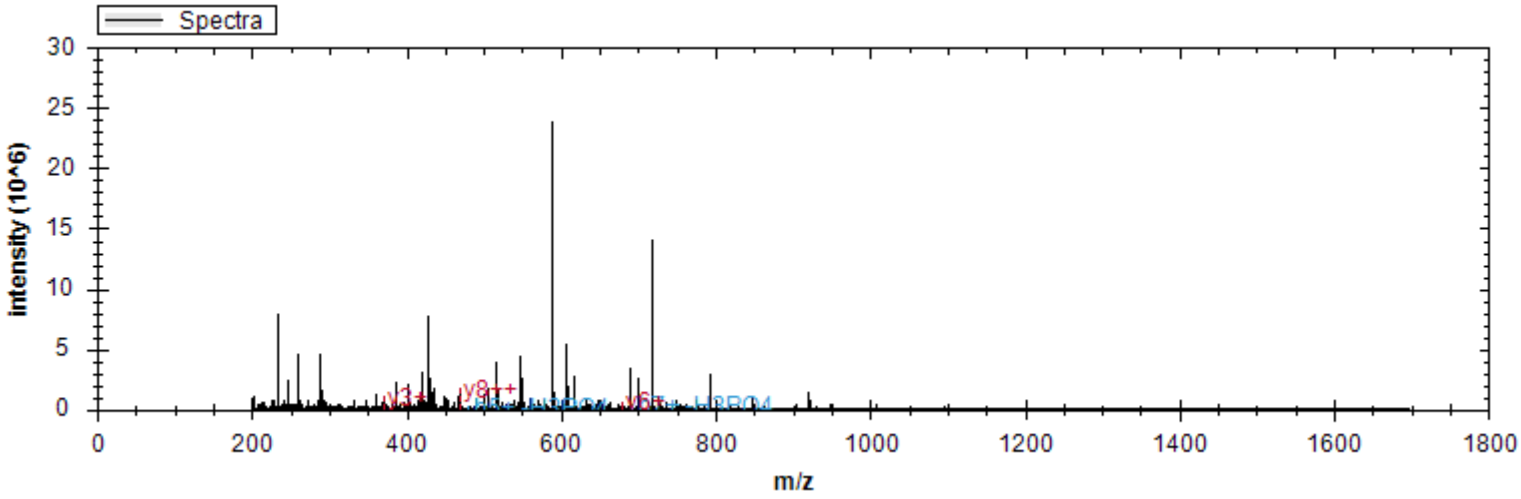

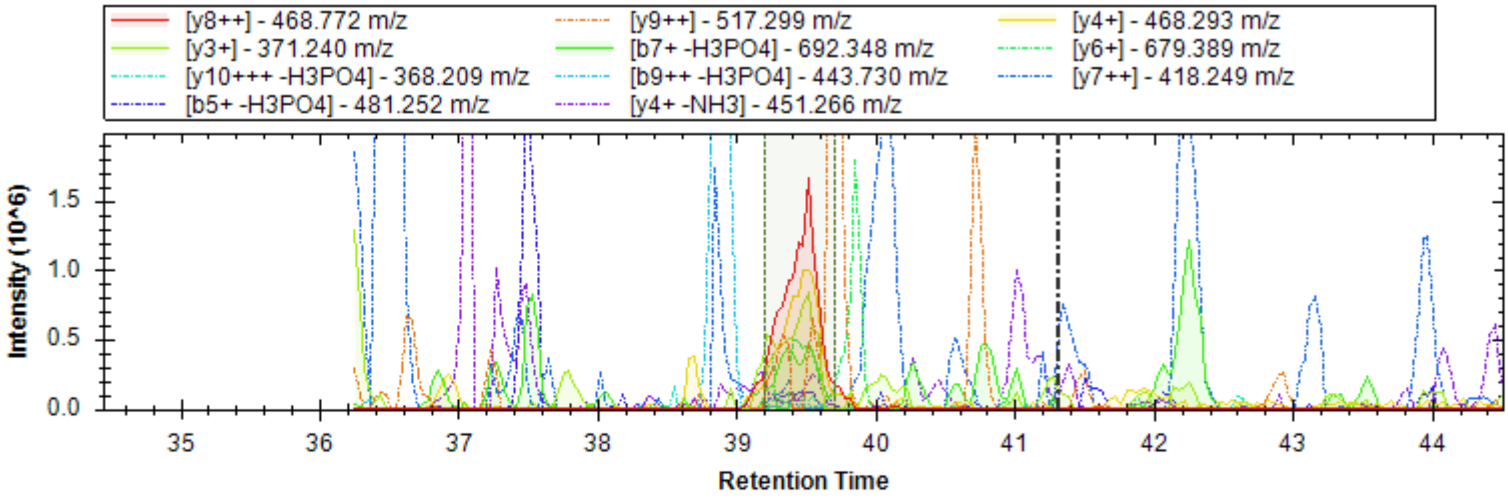

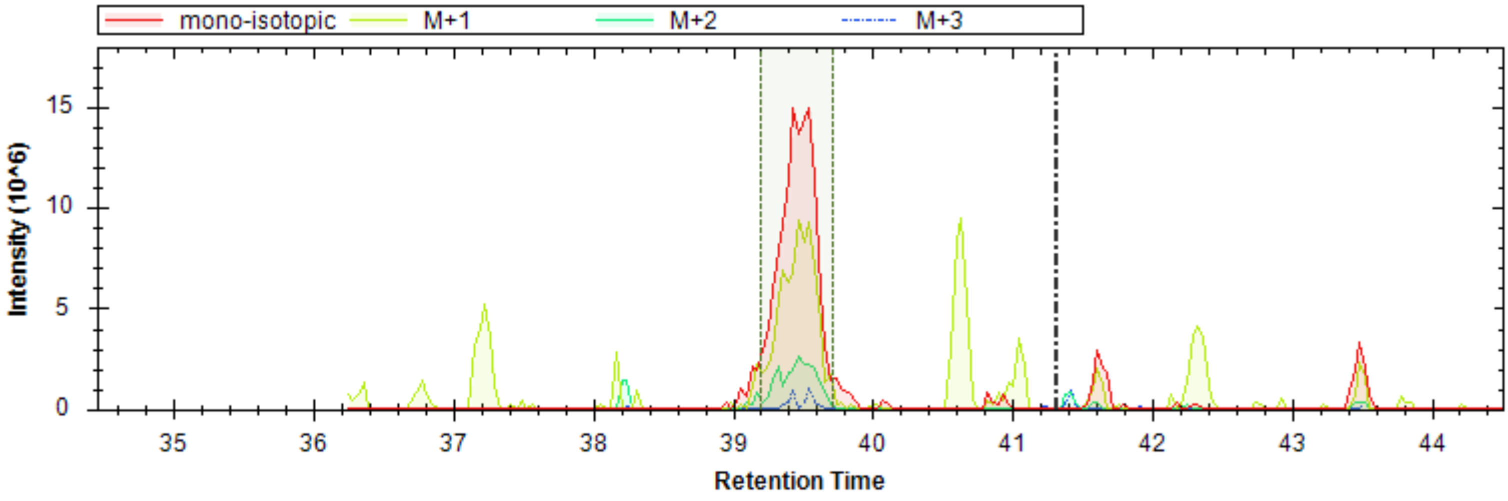

O08553 – _TVT[+H+O3+P]PASSAK_.2

Fig4_MP-DIA-barrel-cortex-p30-2_MHRM.htrms

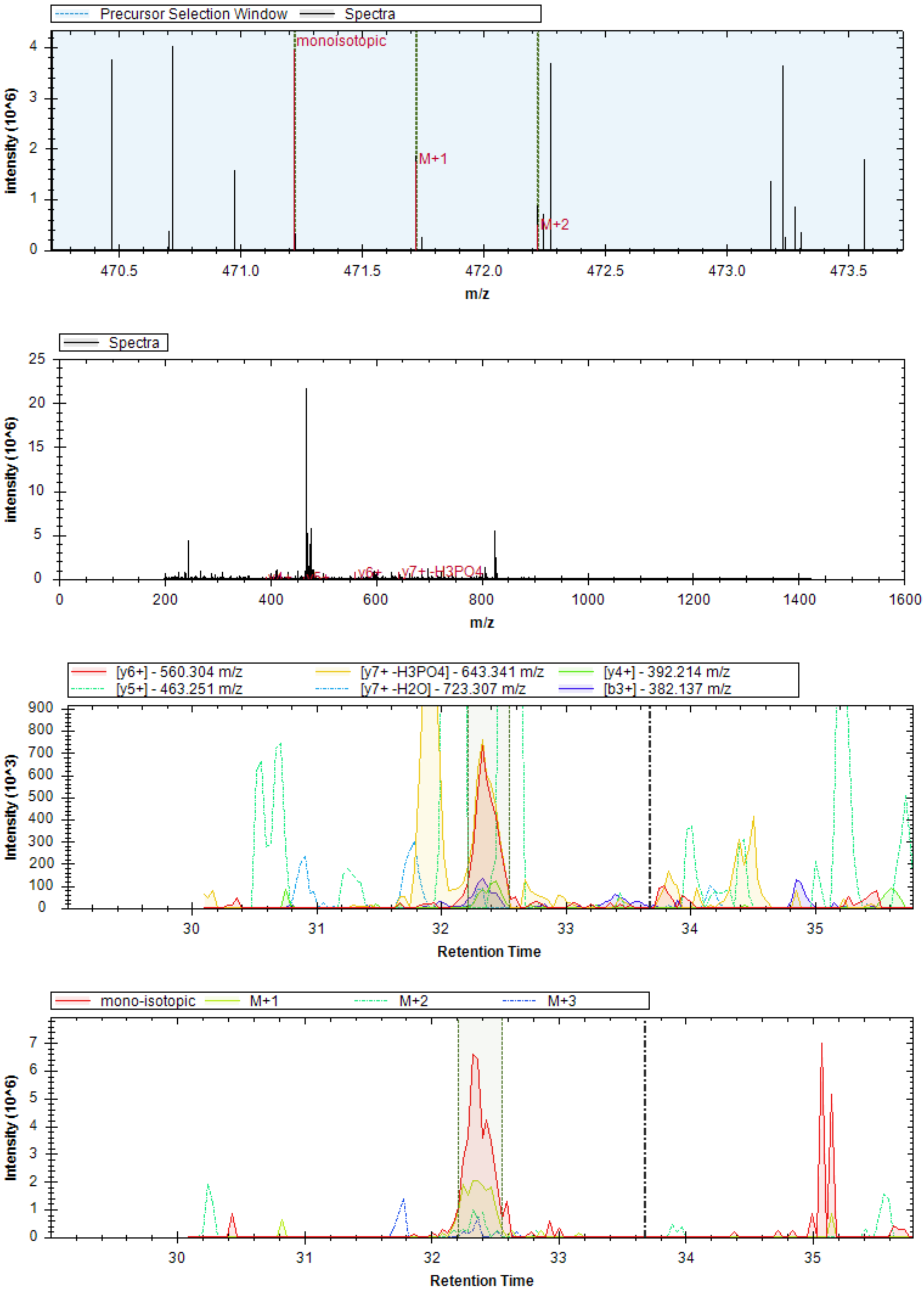

Q9CR81 – _[+C2+H2+O]M[+O]ANHLVKPDS[+H+O3+P]R_.3
Fig4_MP-DIA-barrel-cortex-p30-2_MHRM.htrms

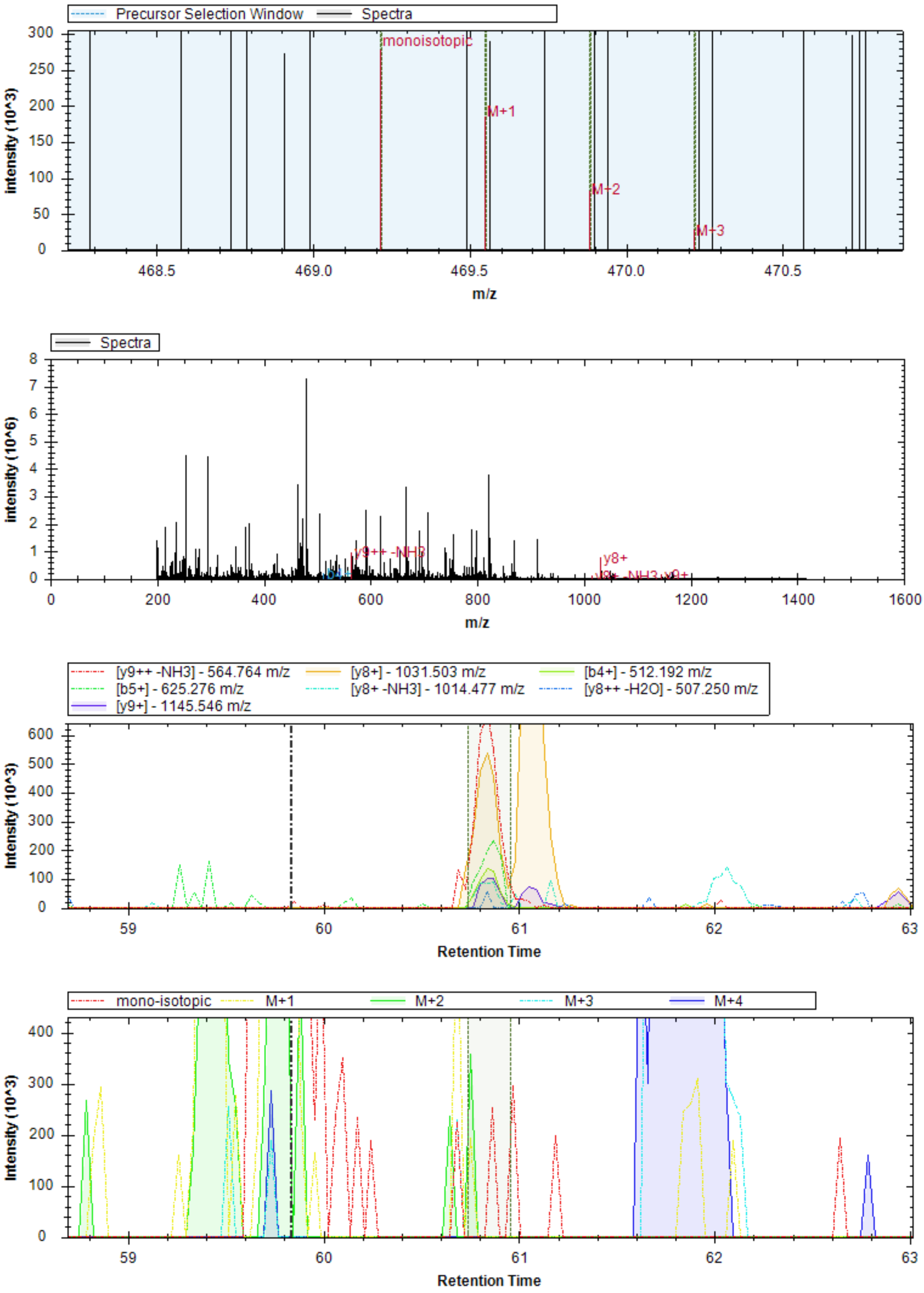

P26645 – _LSGFS[+H+O3+P]FKK_.2
Fig4_MP-DIA-barrel-cortex-p30-2_MHRM.htrms

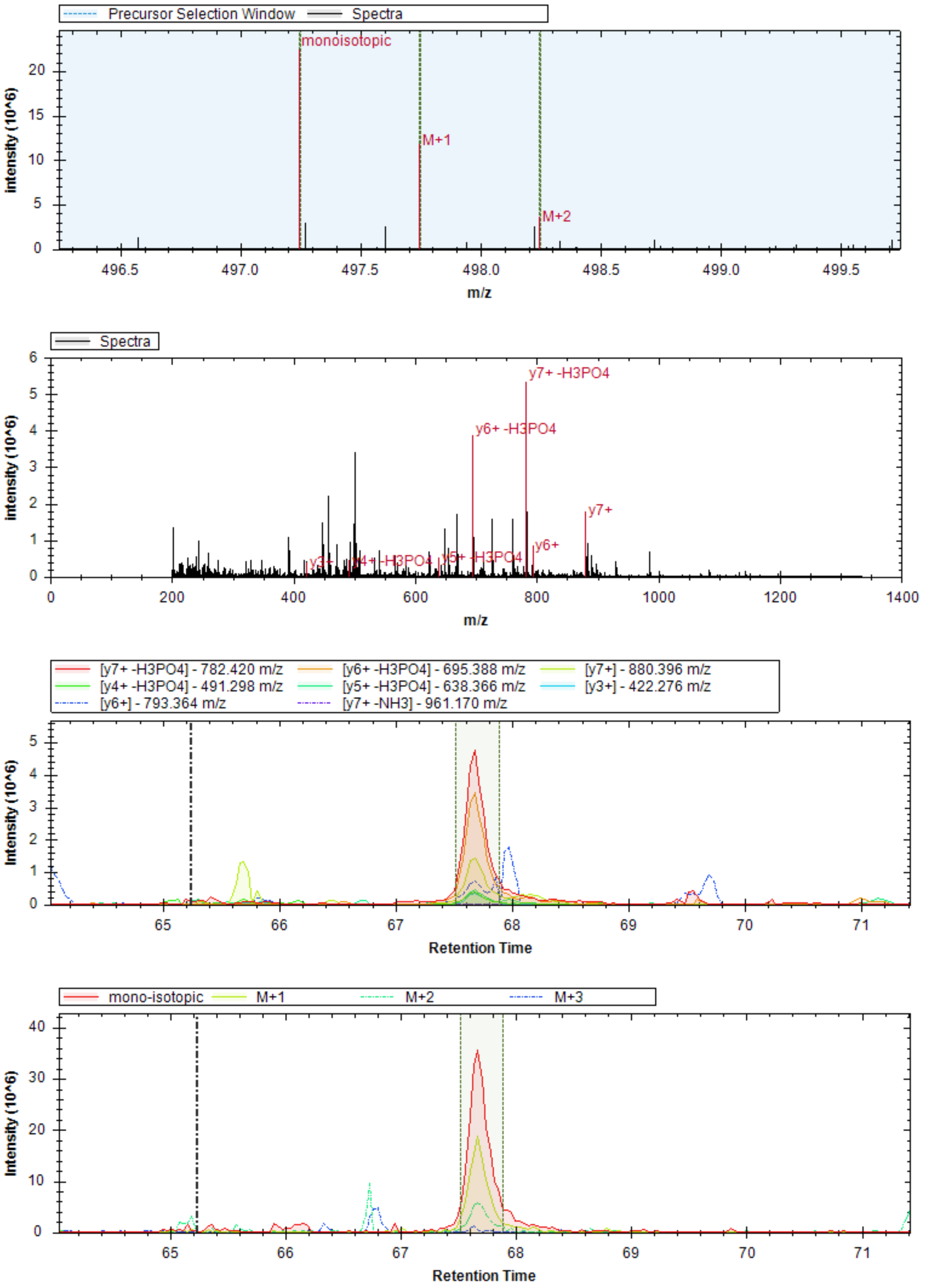

P28667 – _LSGLS[+H+O3+P]FKR_.2
Fig4_MP-DIA-barrel-cortex-p30-2_MHRM.htrms

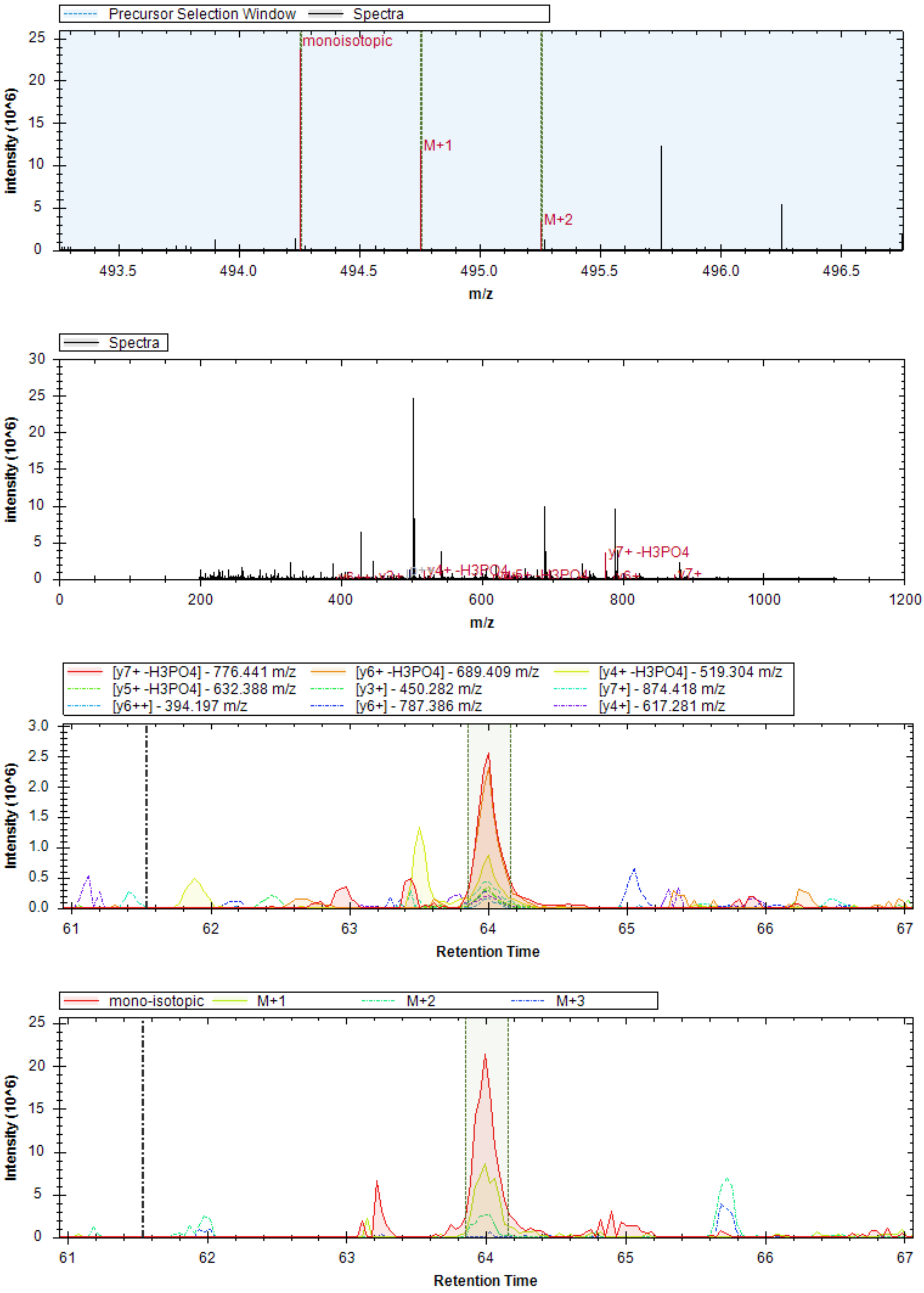

P28738 – _PIRPGHYPAS[+H+O3+P]SPTAVHAVR_.4
Fig4_MP-DIA-barrel-cortex-p30-2_MHRM.htrms

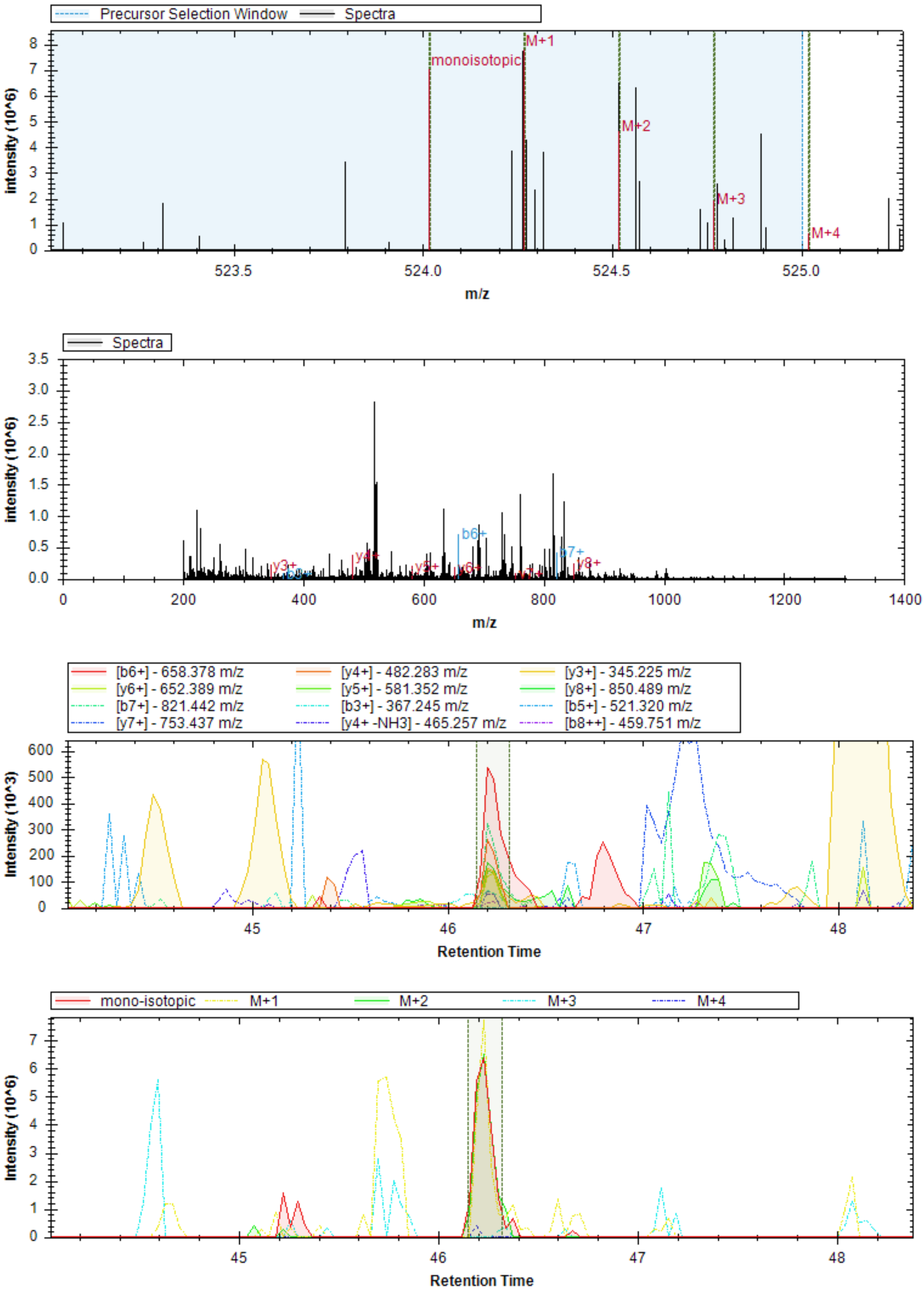

P28738 – _PIRPGHYPASS[+H+O3+P]PTAVHAVR_.4
Fig4_MP-DIA-barrel-cortex-p30-2_MHRM.htrms

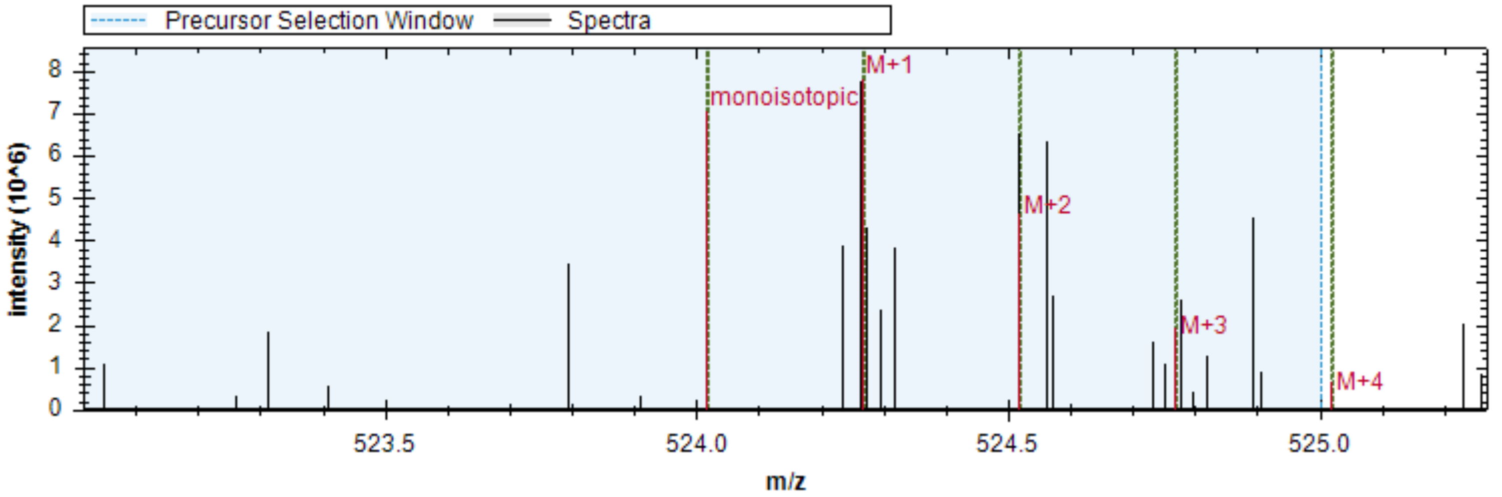

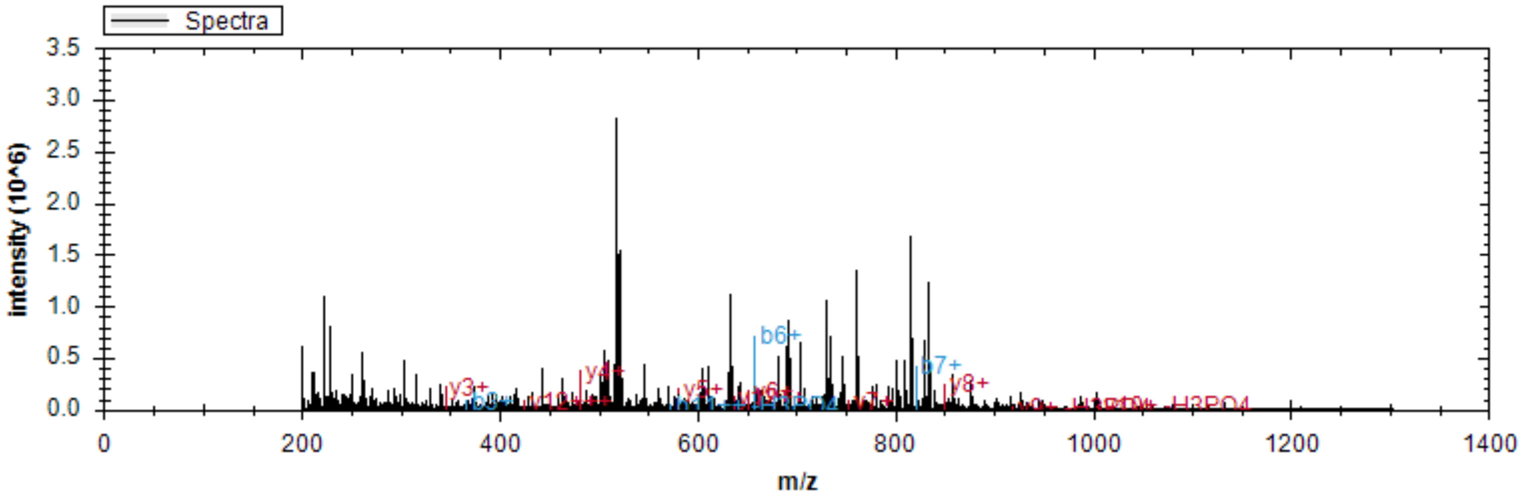

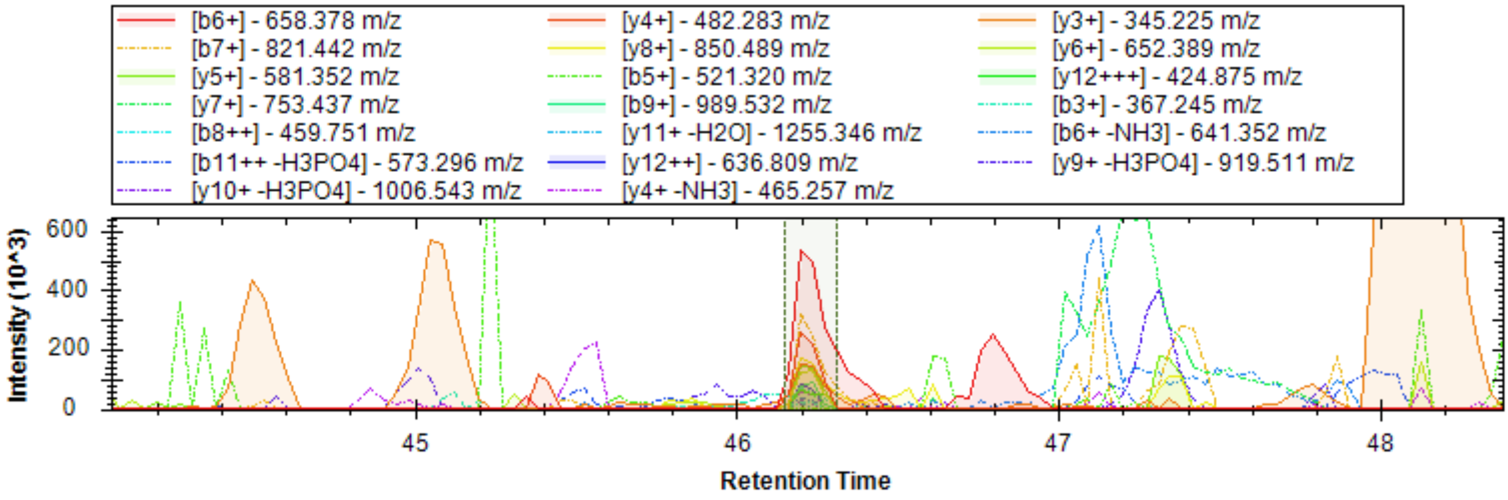

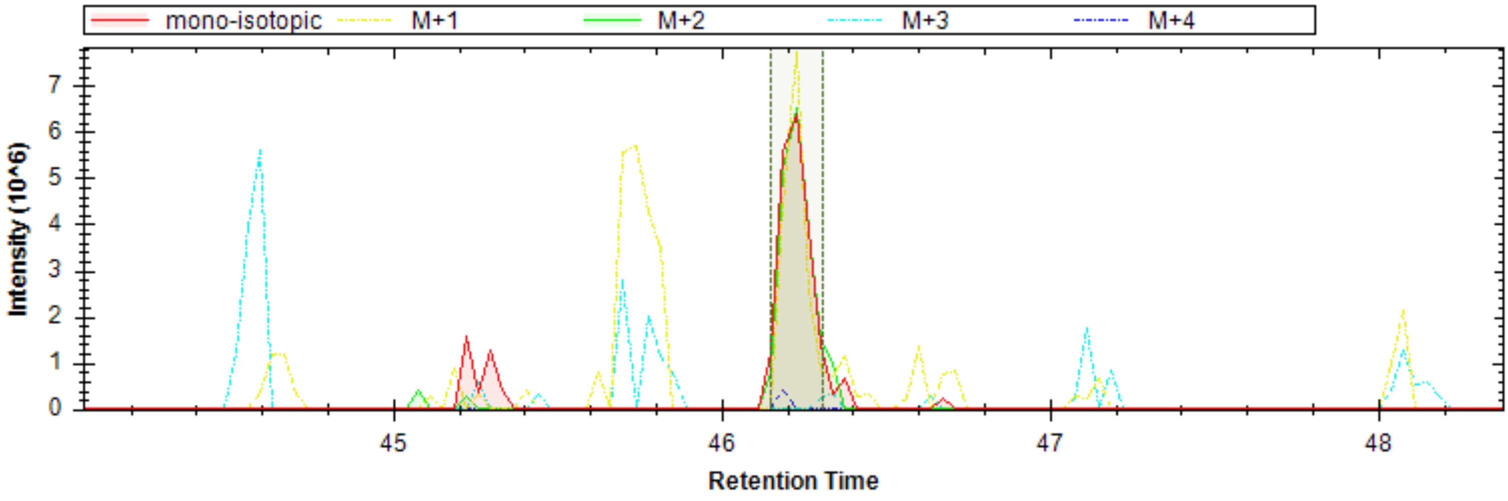

Q8CDY7 – _LVQALC[+C2+H3+N+O]EQIHT[+H+O3+P]R_.3

Fig4_MP-DIA-barrel-cortex-p30-2_MHRM.htrms

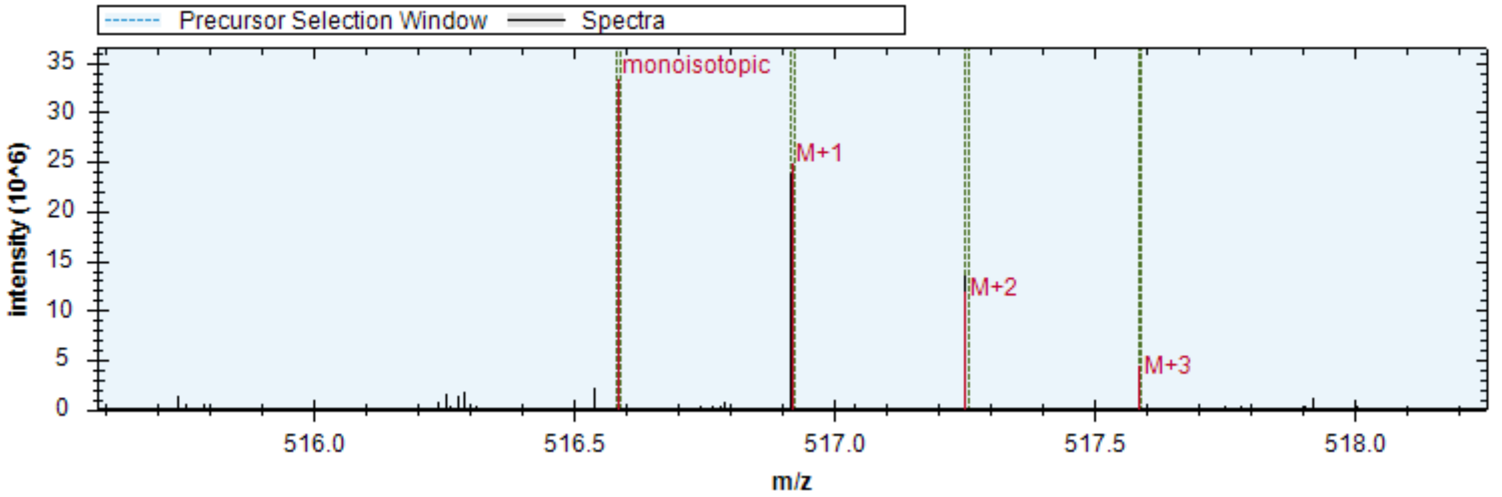

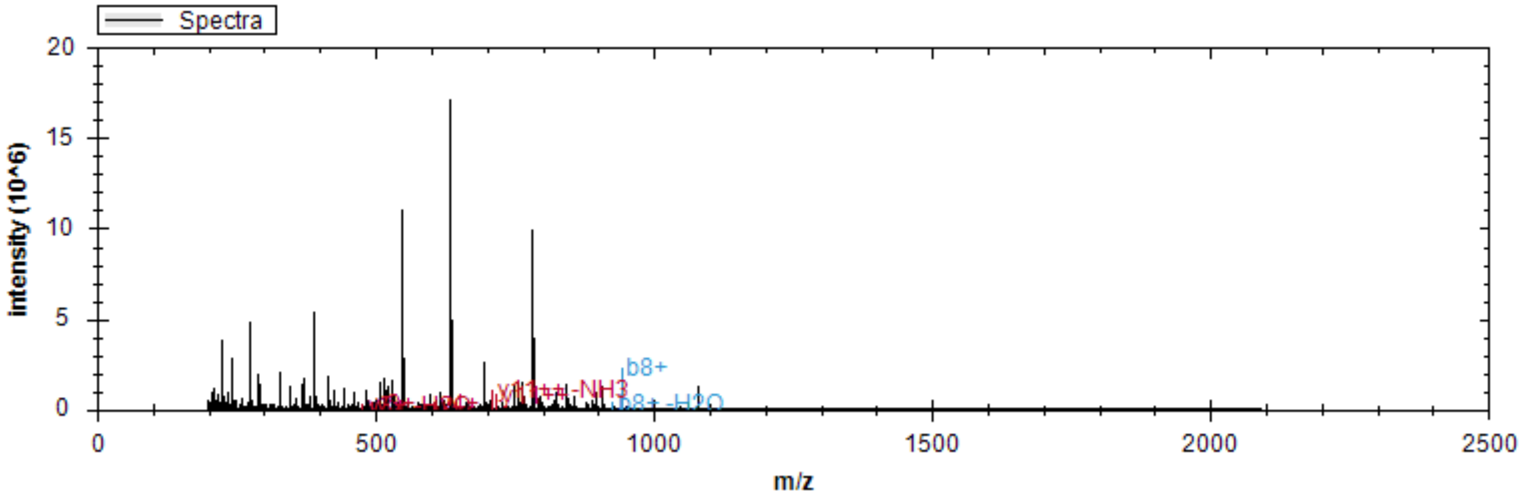

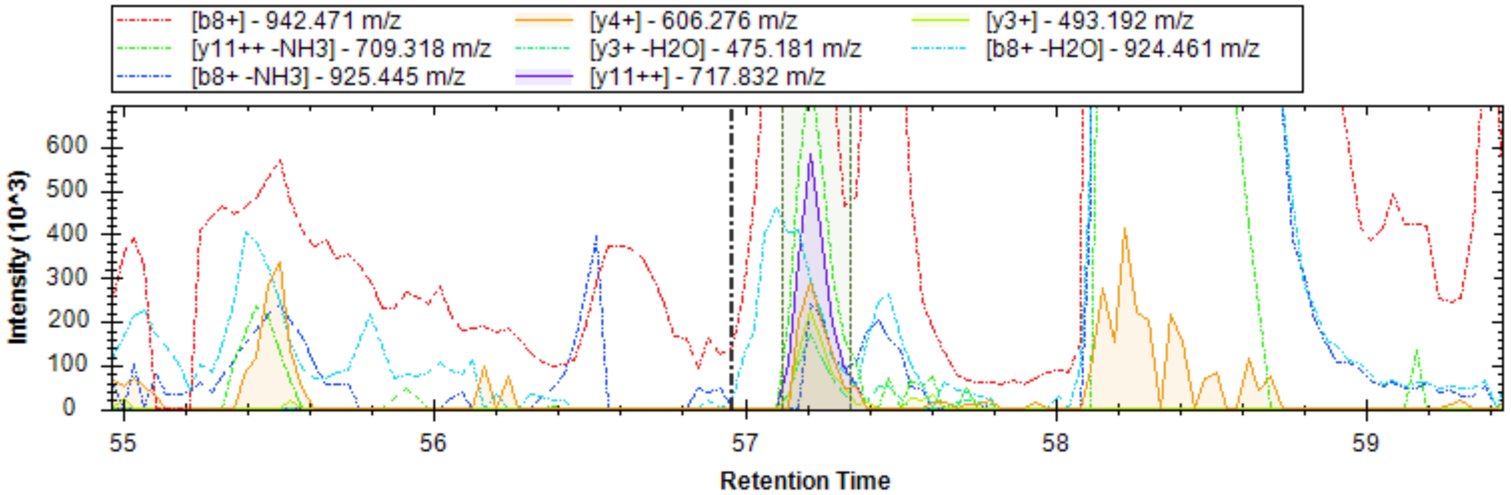

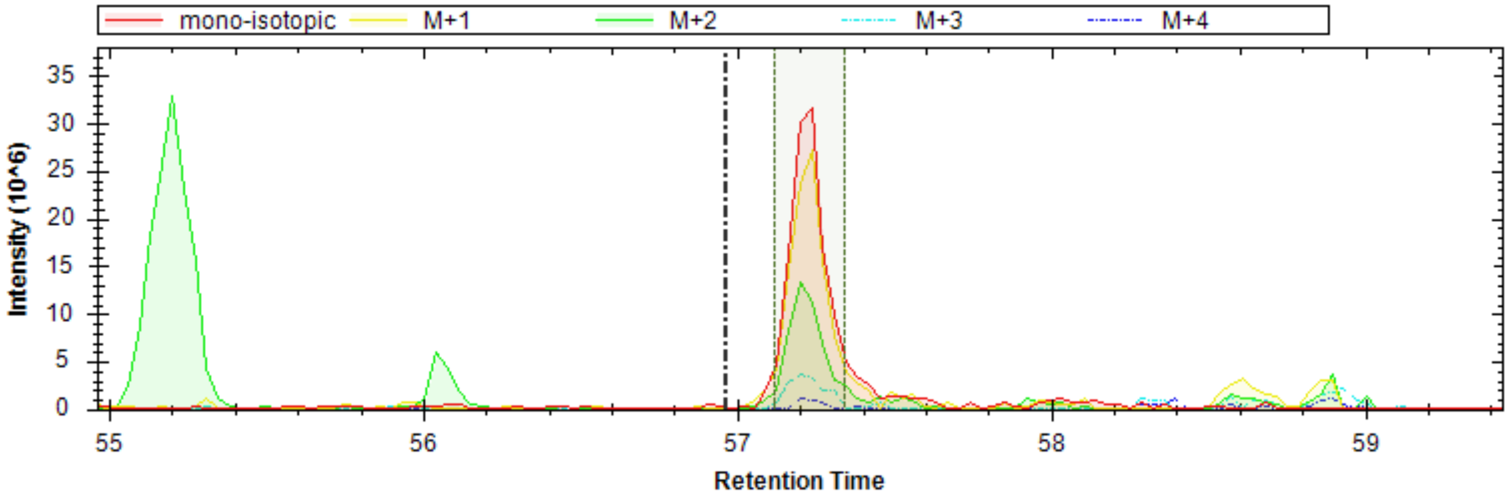

Q9QYC0 – _AAVVT[+H+O3+P]SPPPTTAPHK_.3

Fig4_MP-DIA-barrel-cortex-p30-2_MHRM.htrms

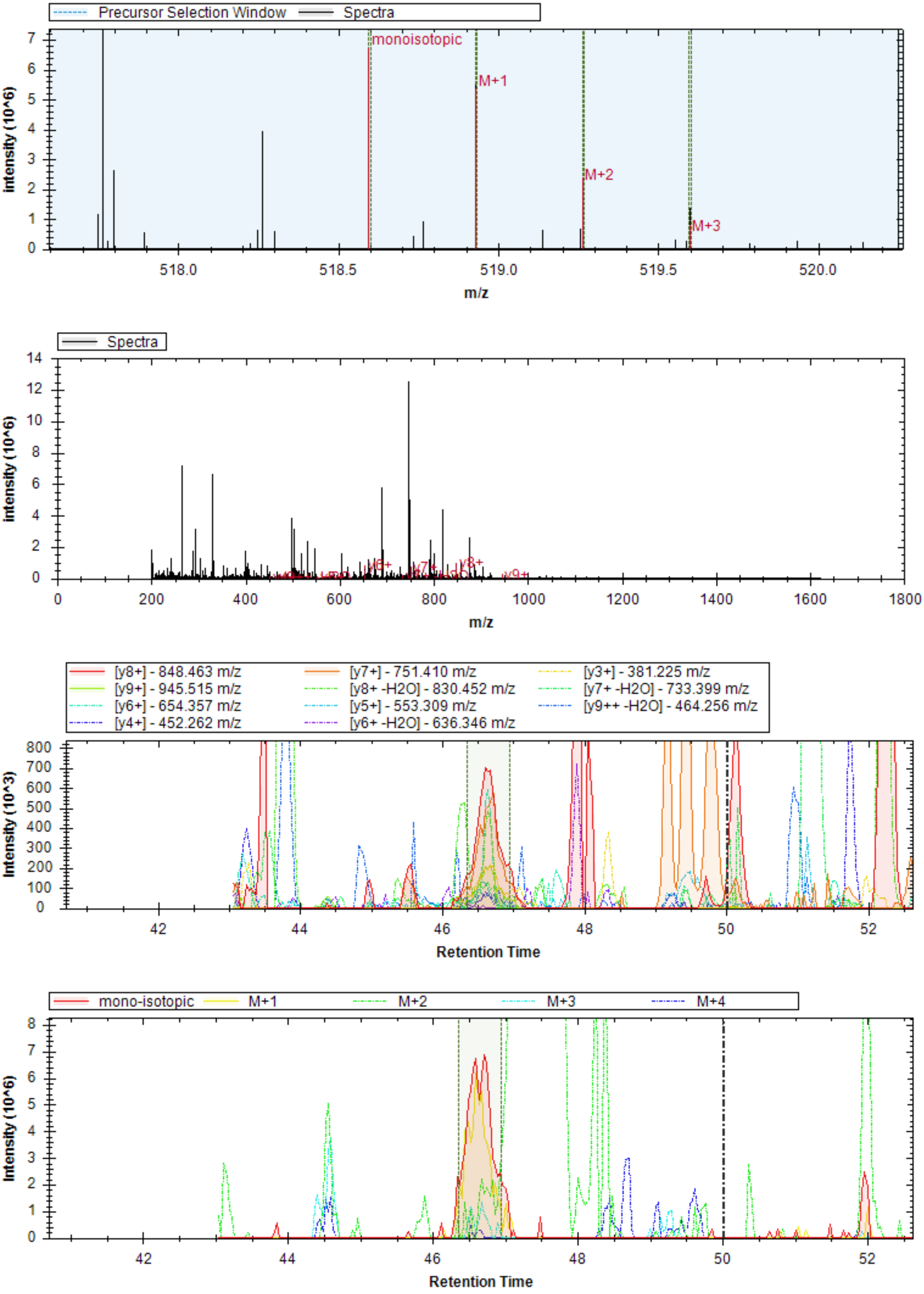

Q9QYC0 – _AAVVTS[+H+O3+P]PPPTTAPHK_.3

Fig4_MP-DIA-barrel-cortex-p30-2_MHRM.htrms

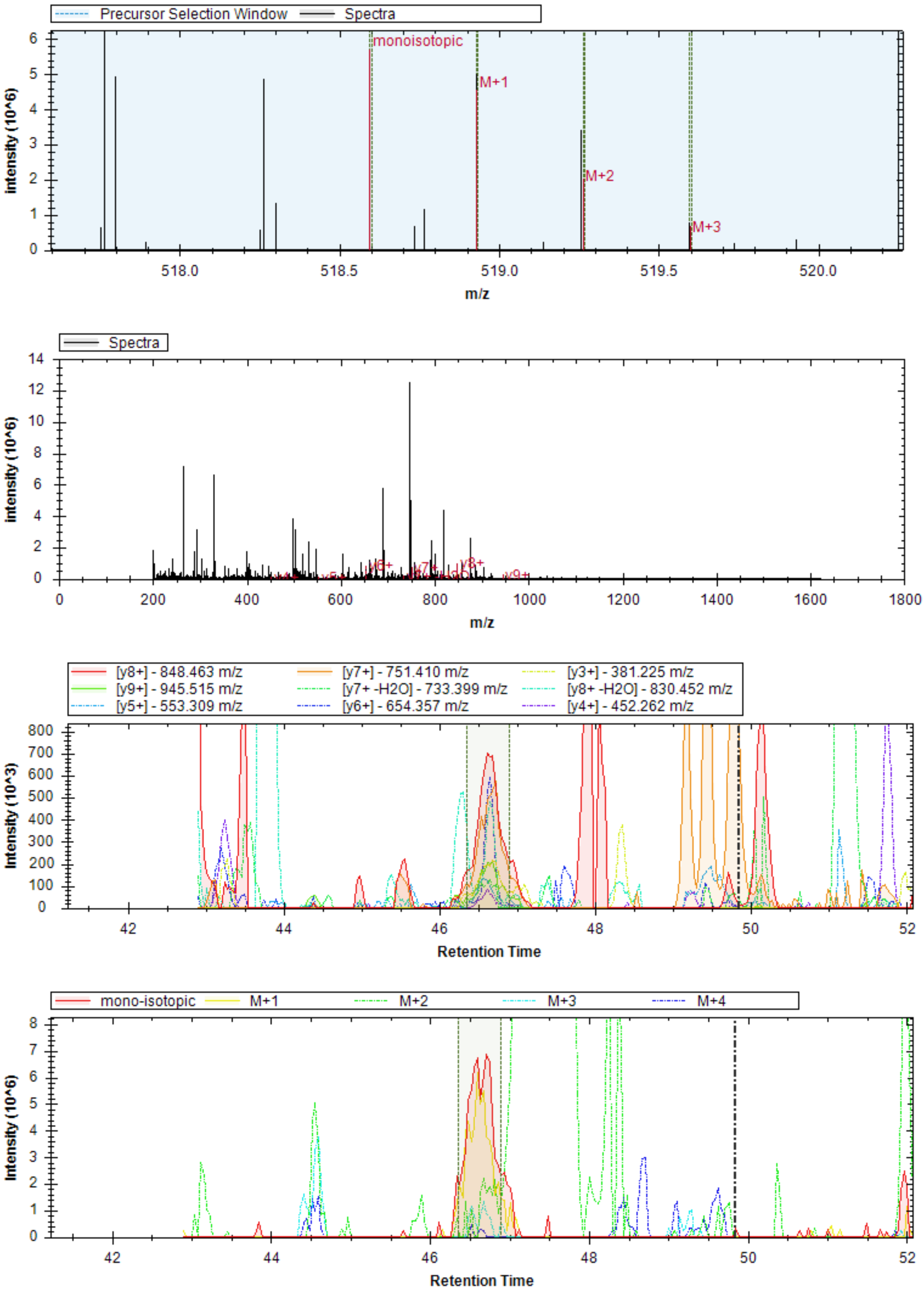

P10637 – _VAVVRT[+H+O3+P]PPKSPSASK_.3
Fig4_MP-DIA-barrel-cortex-p30-2_MHRM.htrms

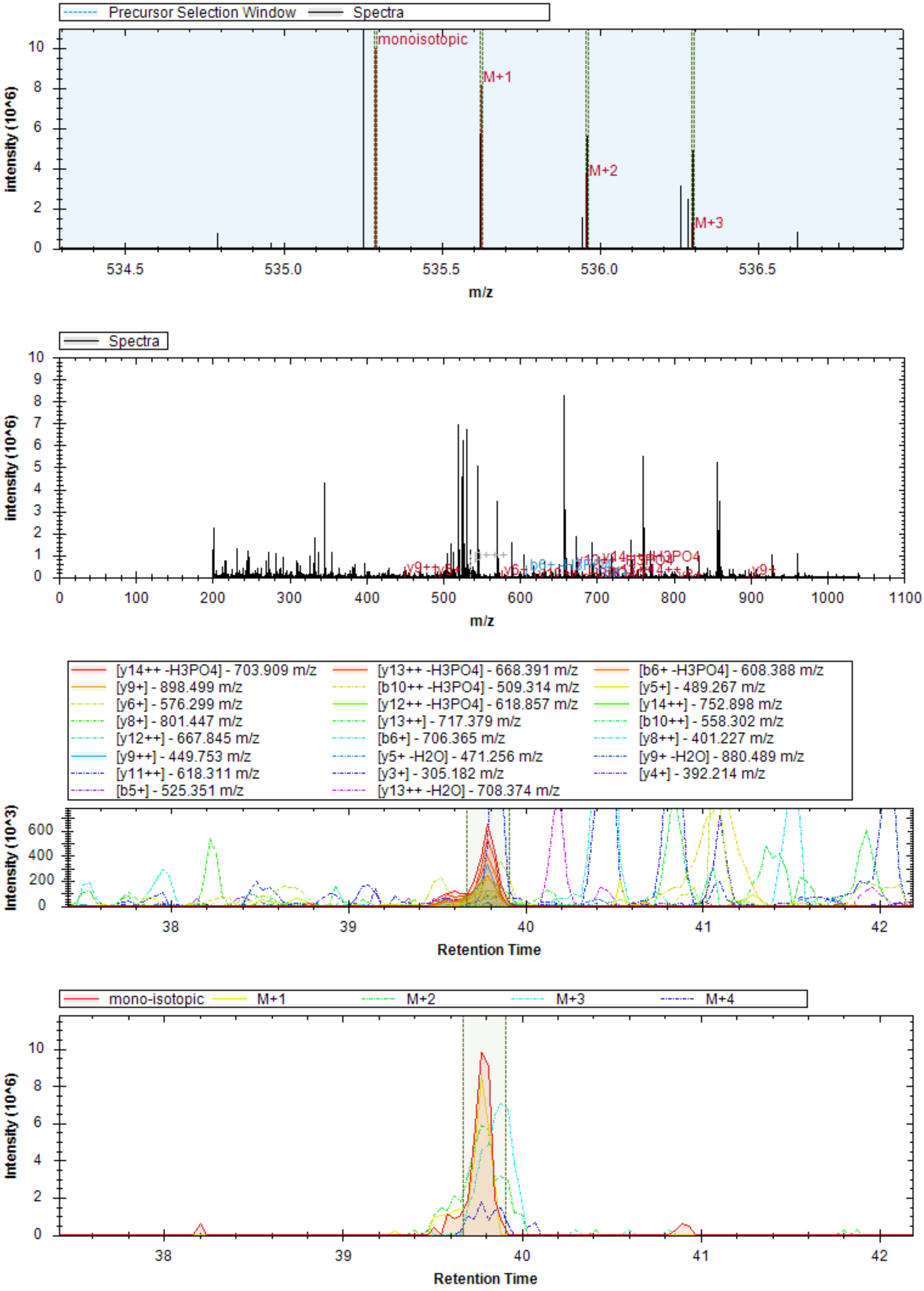

Q7TQD2 – _AANKT[+H+O3+P]PPKSPGDPAR_.3
Fig4_MP-DIA-barrel-cortex-p30-2_MHRM.htrms

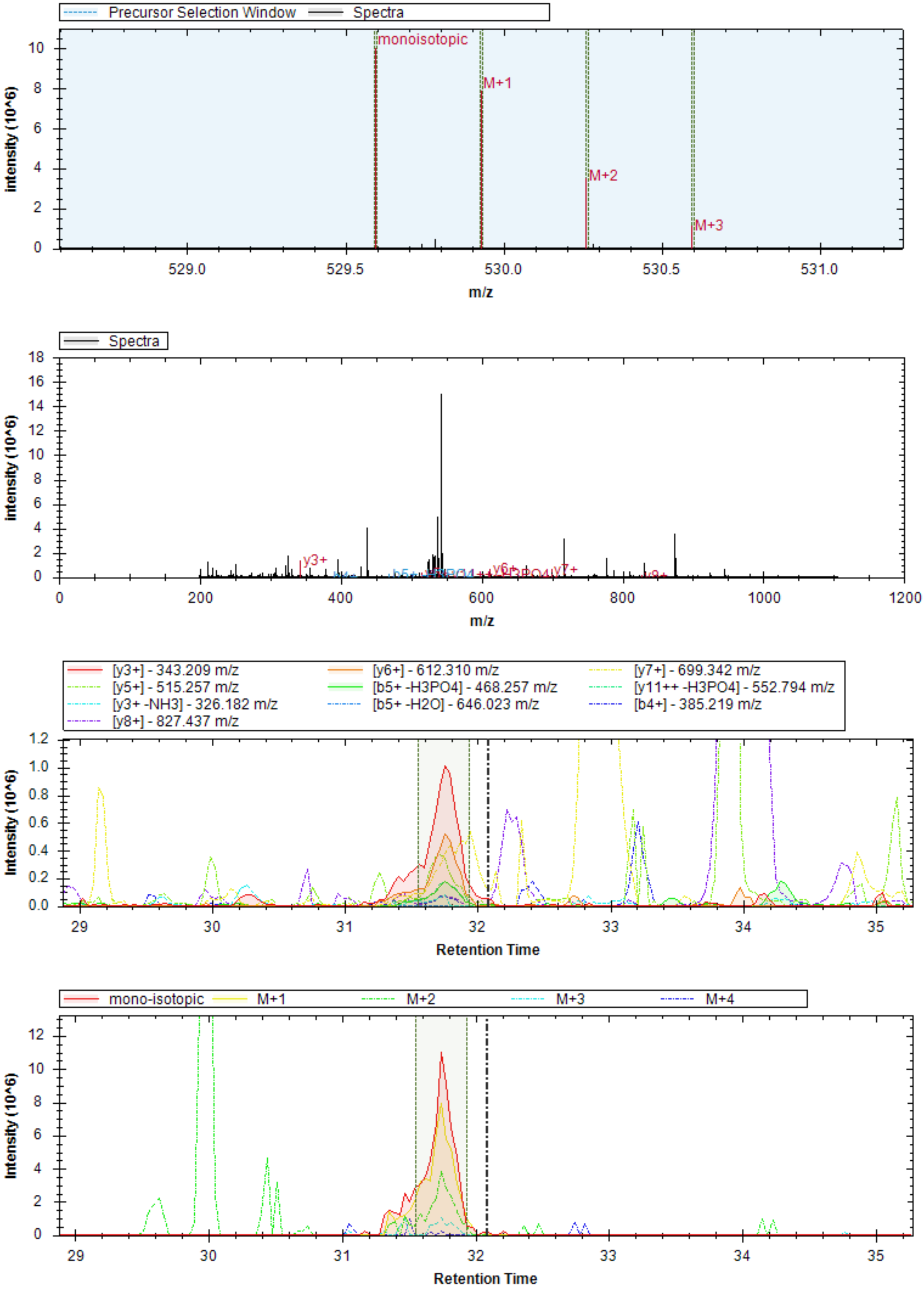

P20357 – _VAIIRT[+H+O3+P]PPKSPATPK_.3

Fig4_MP-DIA-barrel-cortex-p30-2_MHRM.htrms

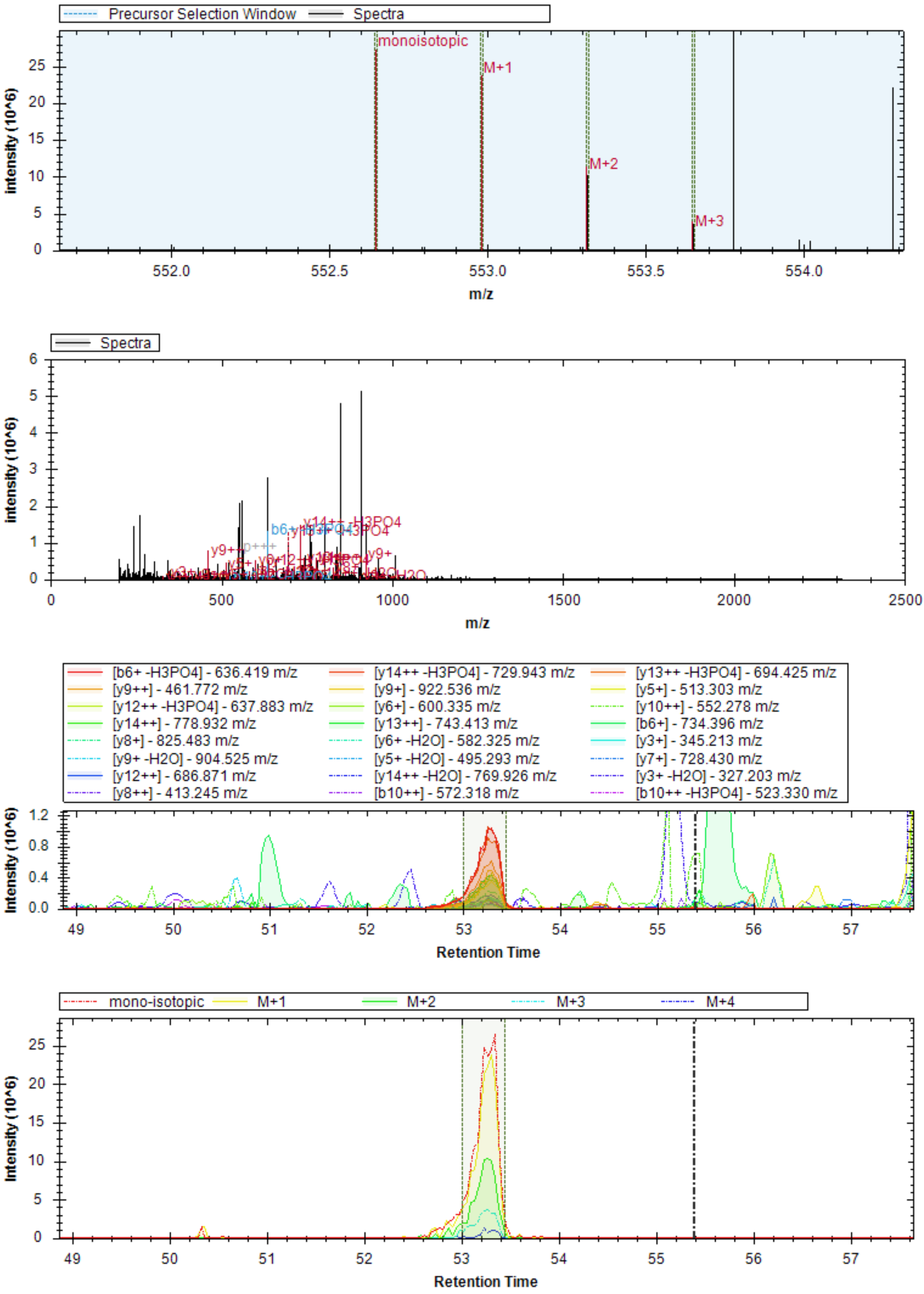

Q9CYT6 – _THTPSPTS[+H+O3+P]PK_.2

Fig4_MP-DIA-barrel-cortex-p30-2_MHRM.htrms

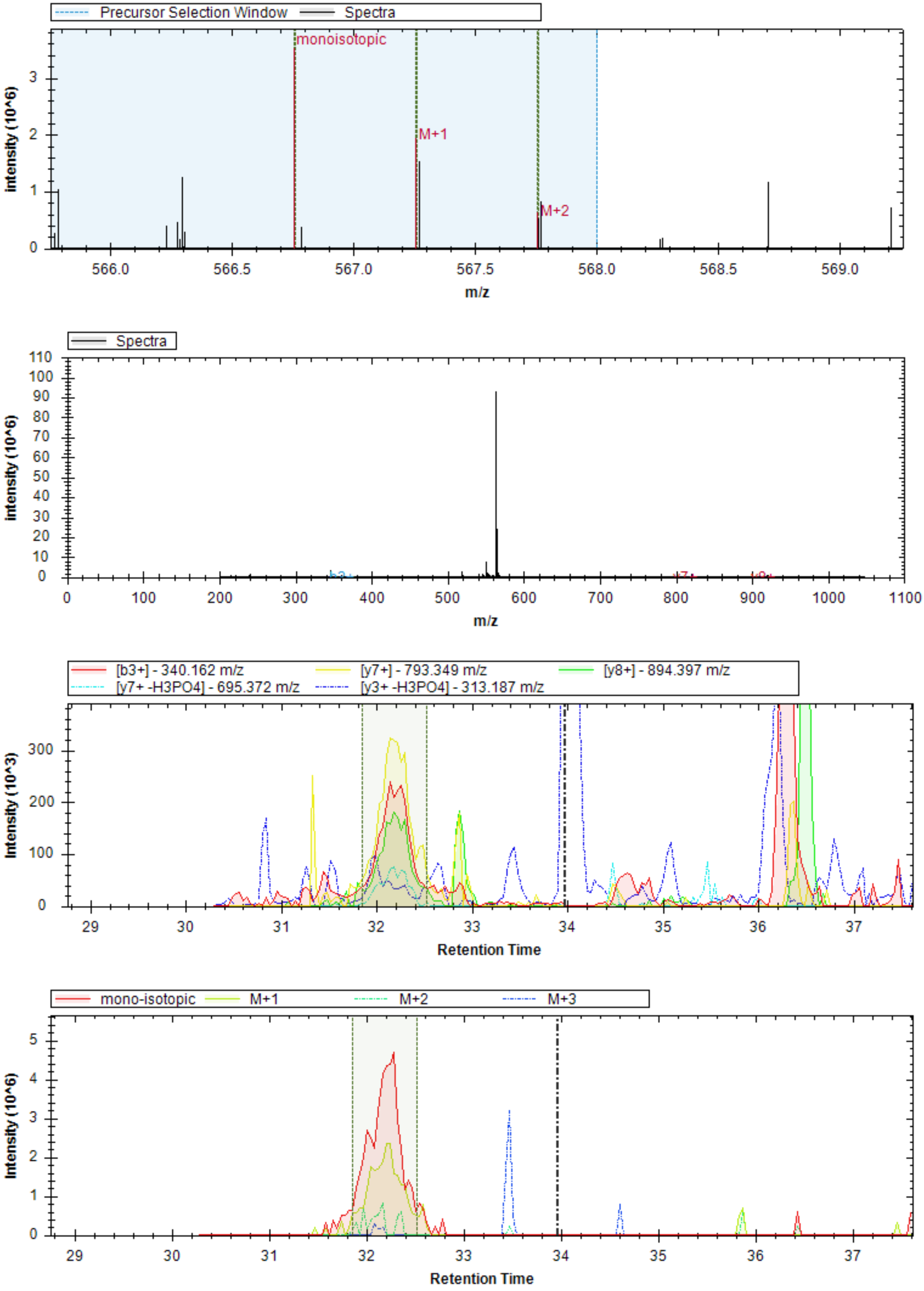

Q9R0K7 – _T[+H+O3+P]SIHNFMAHPEFR_.3
Fig4_MP-DIA-barrel-cortex-p30-2_MHRM.htrms

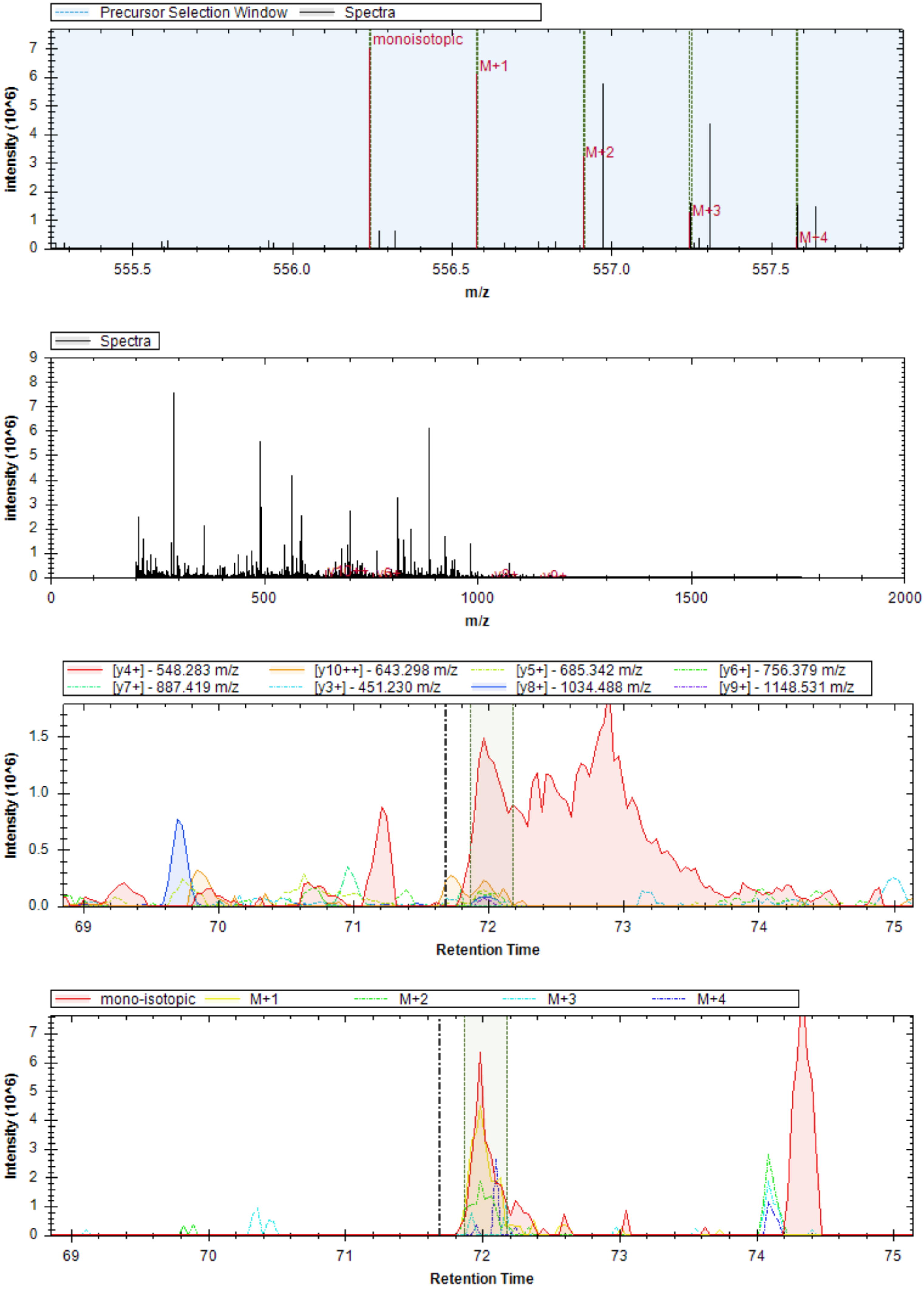

O88935 – _QAS[+H+O3+P]ISGPAPTK_.2
Fig4_MP-DIA-barrel-cortex-p30-2_MHRM.htrms

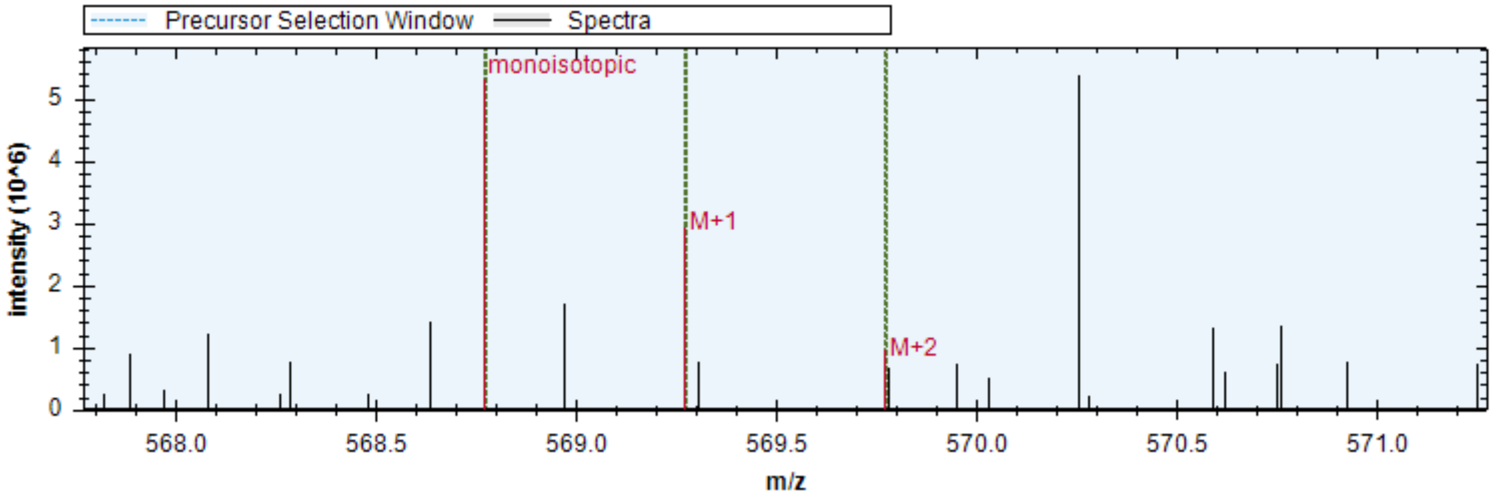

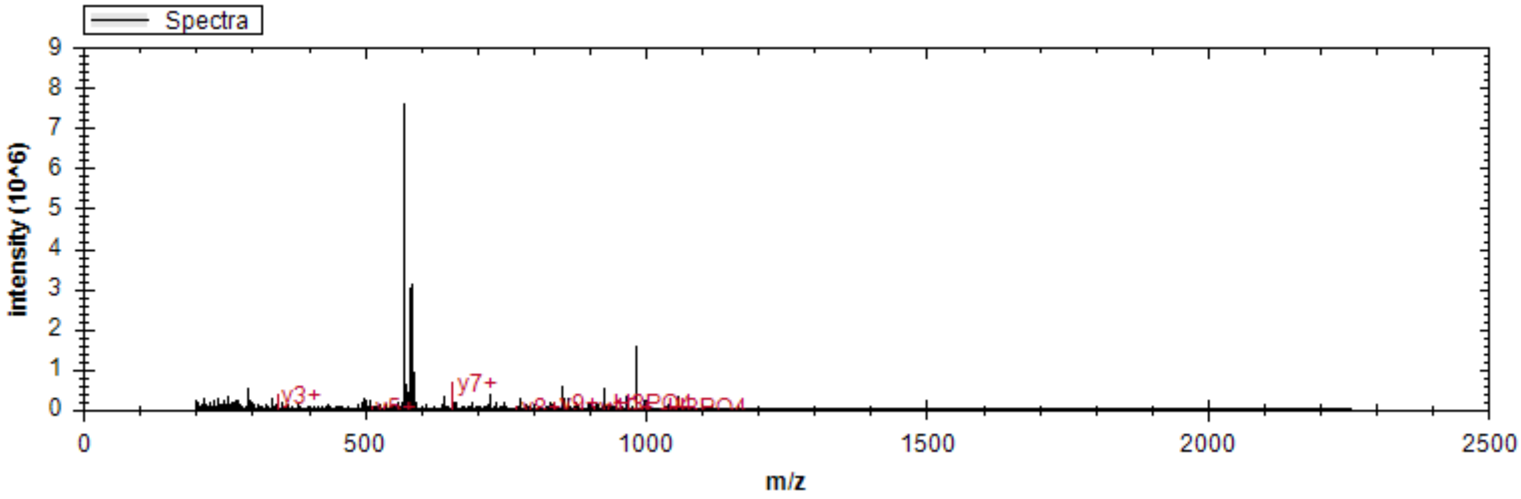

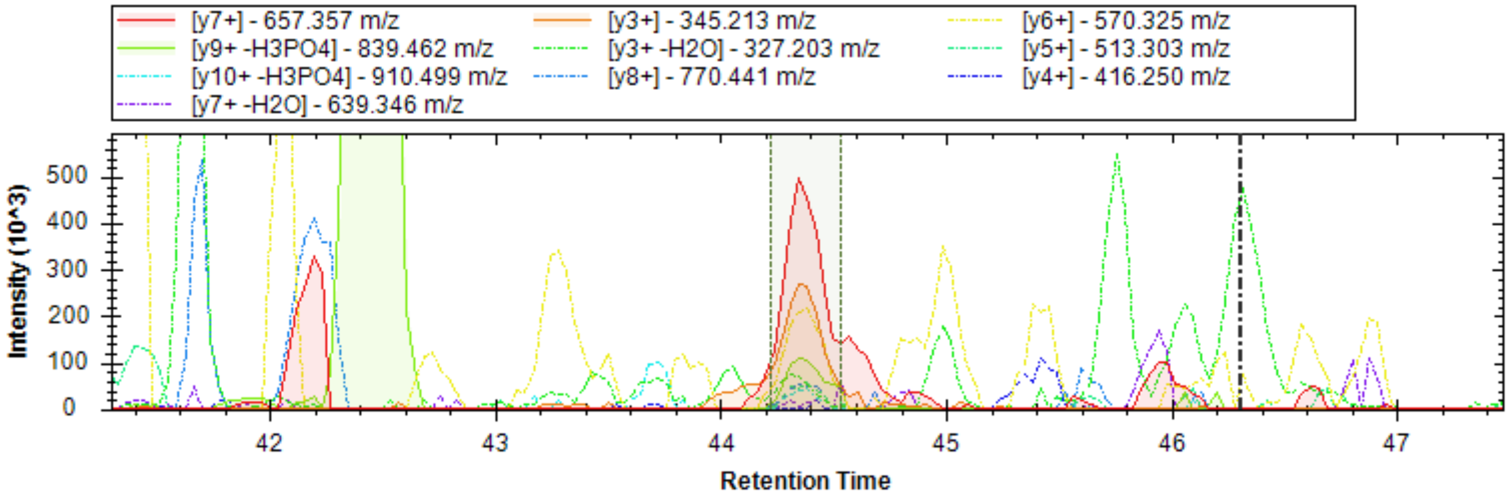

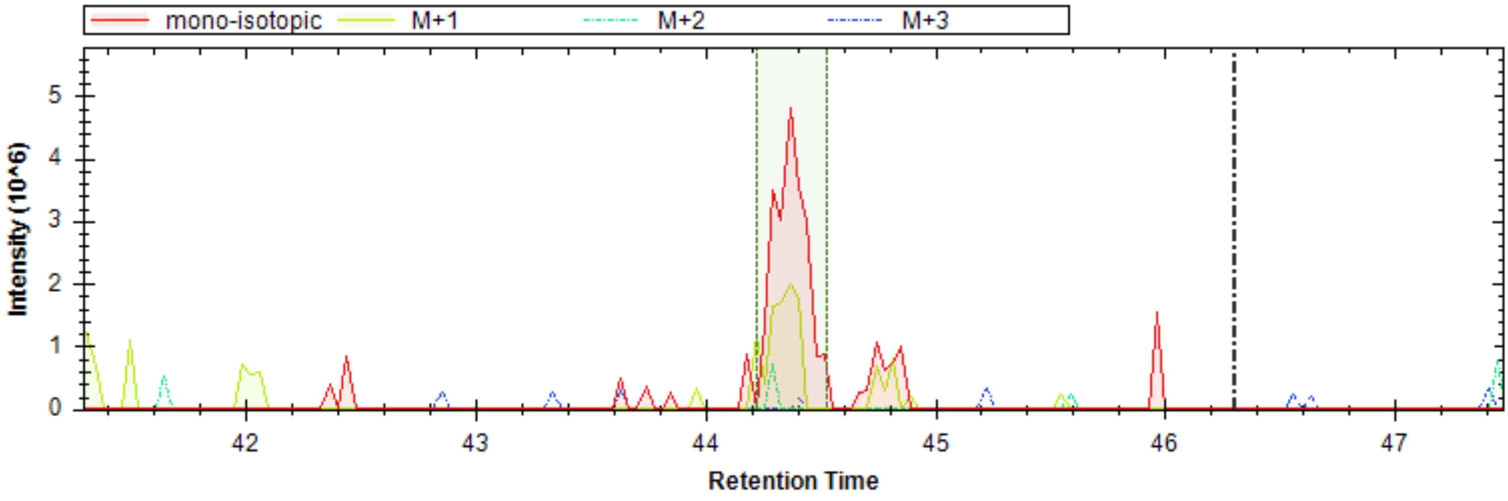

P27546-2 – _T[+H+O3+P]SPSKPSSAPALKPGPK_.3
Fig4_MP-DIA-barrel-cortex-p30-2_MHRM.htrms

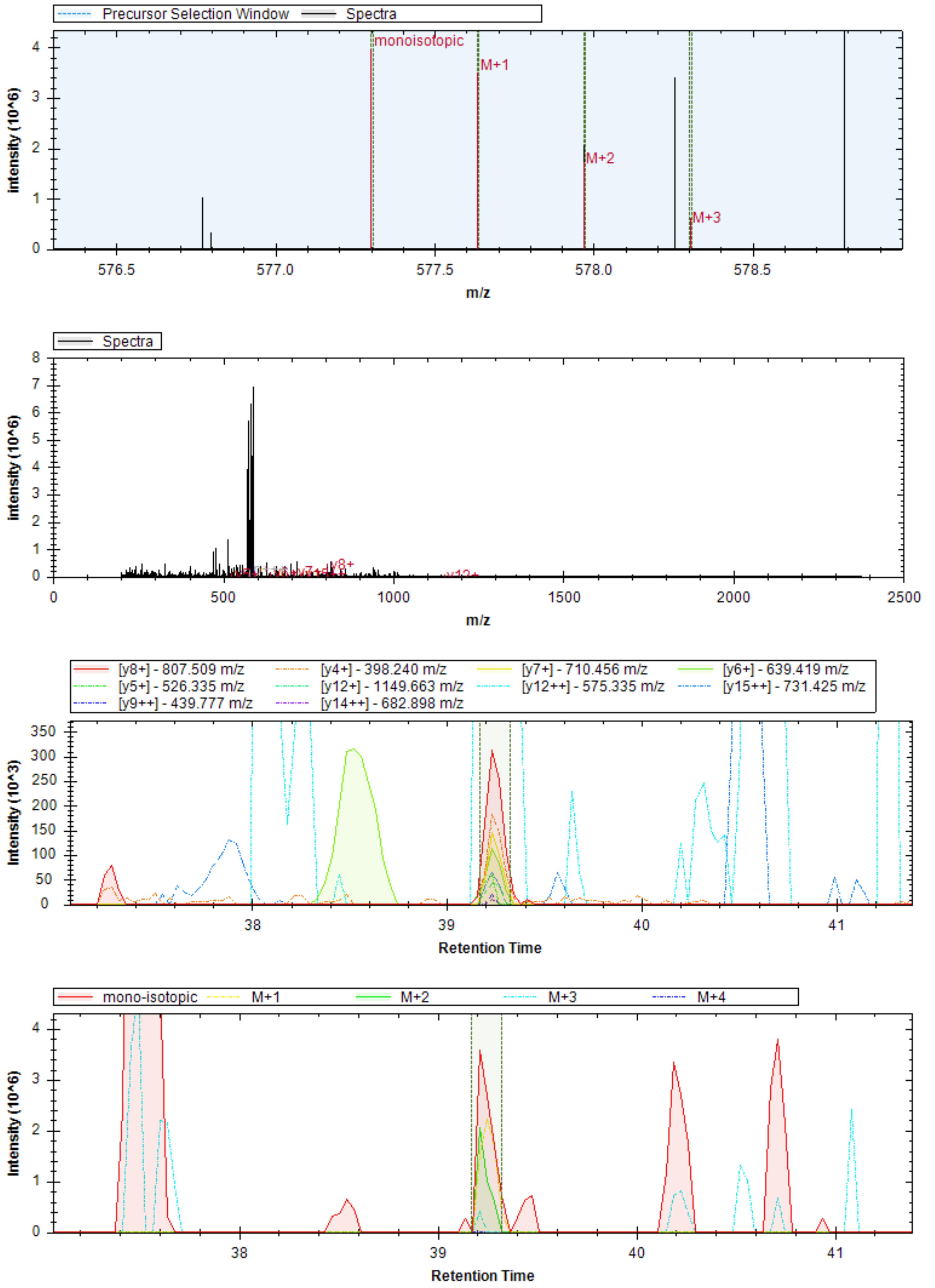

Q8BFU3-4 – _VAS[+H+O3+P]STQVRGASGRELR_.3
Fig4_MP-DIA-barrel-cortex-p30-2_MHRM.htrms

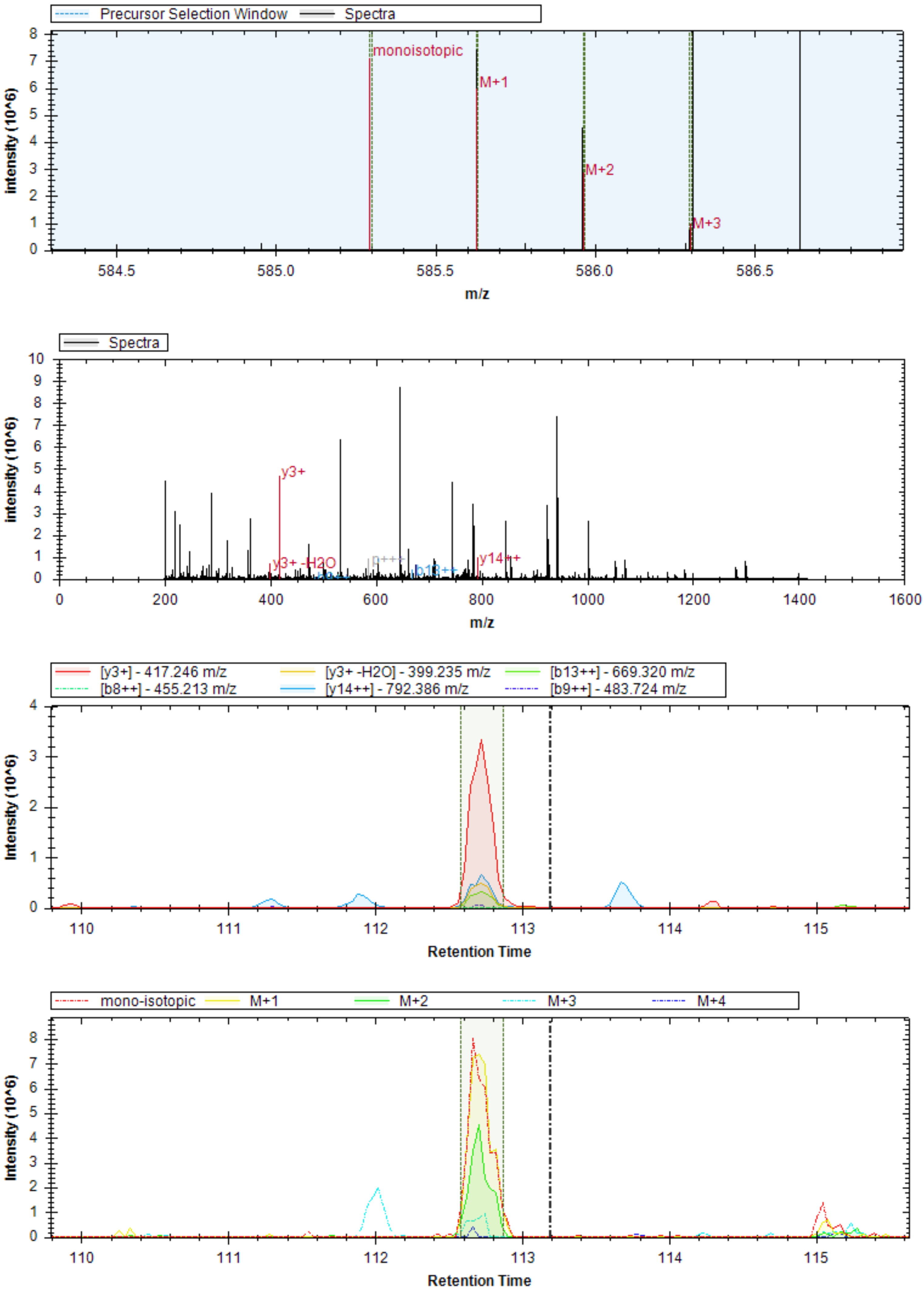

Q9CYT6 – _AQGQIRS[+H+O3+P]PTK_.2

Fig4_MP-DIA-barrel-cortex-p30-2_MHRM.htrms

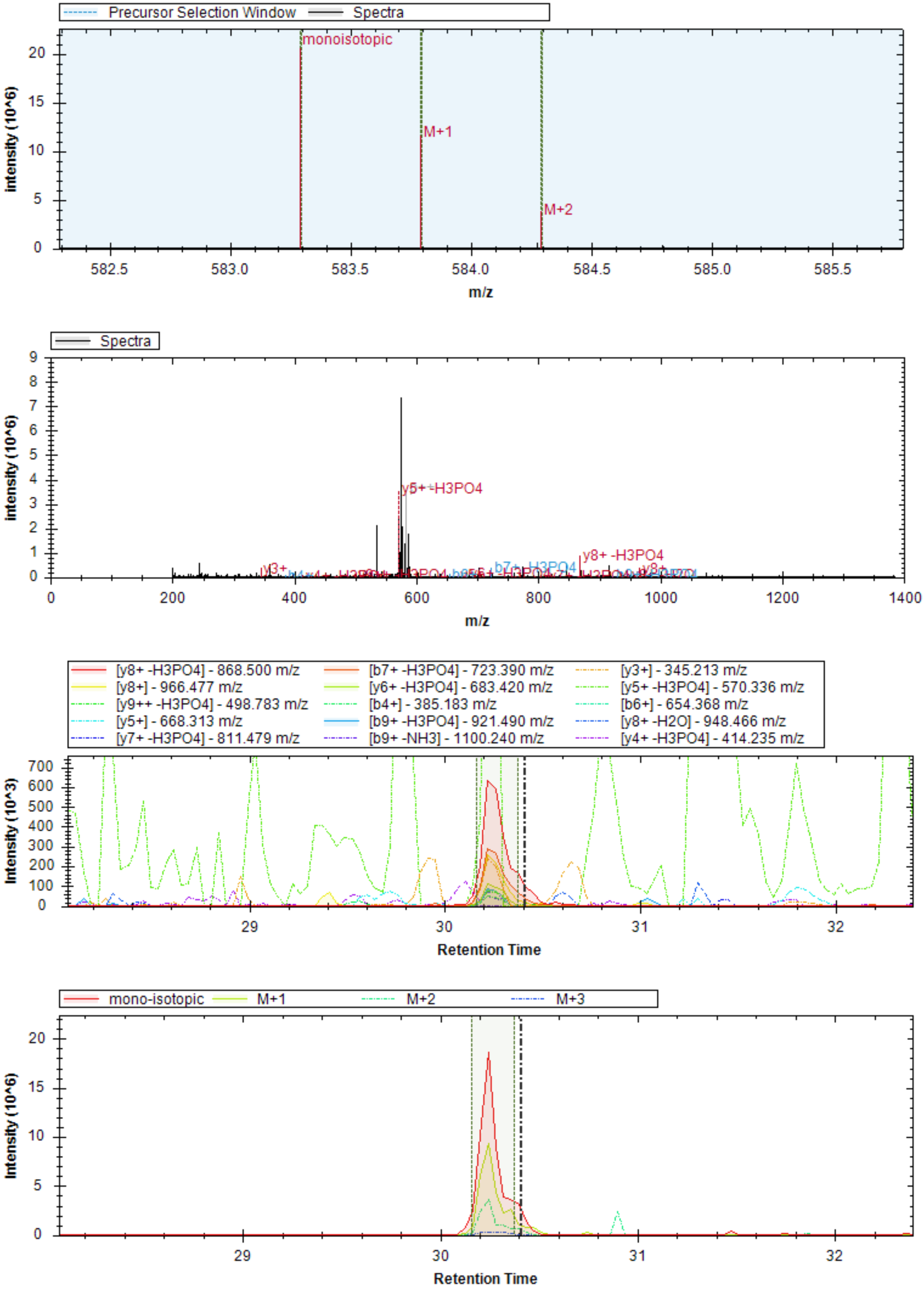

Q9D5Y0 – _ITDT[+H+O3+P]KKTDDLYWR_.3
Fig4_MP-DIA-barrel-cortex-p30-2_MHRM.htrms

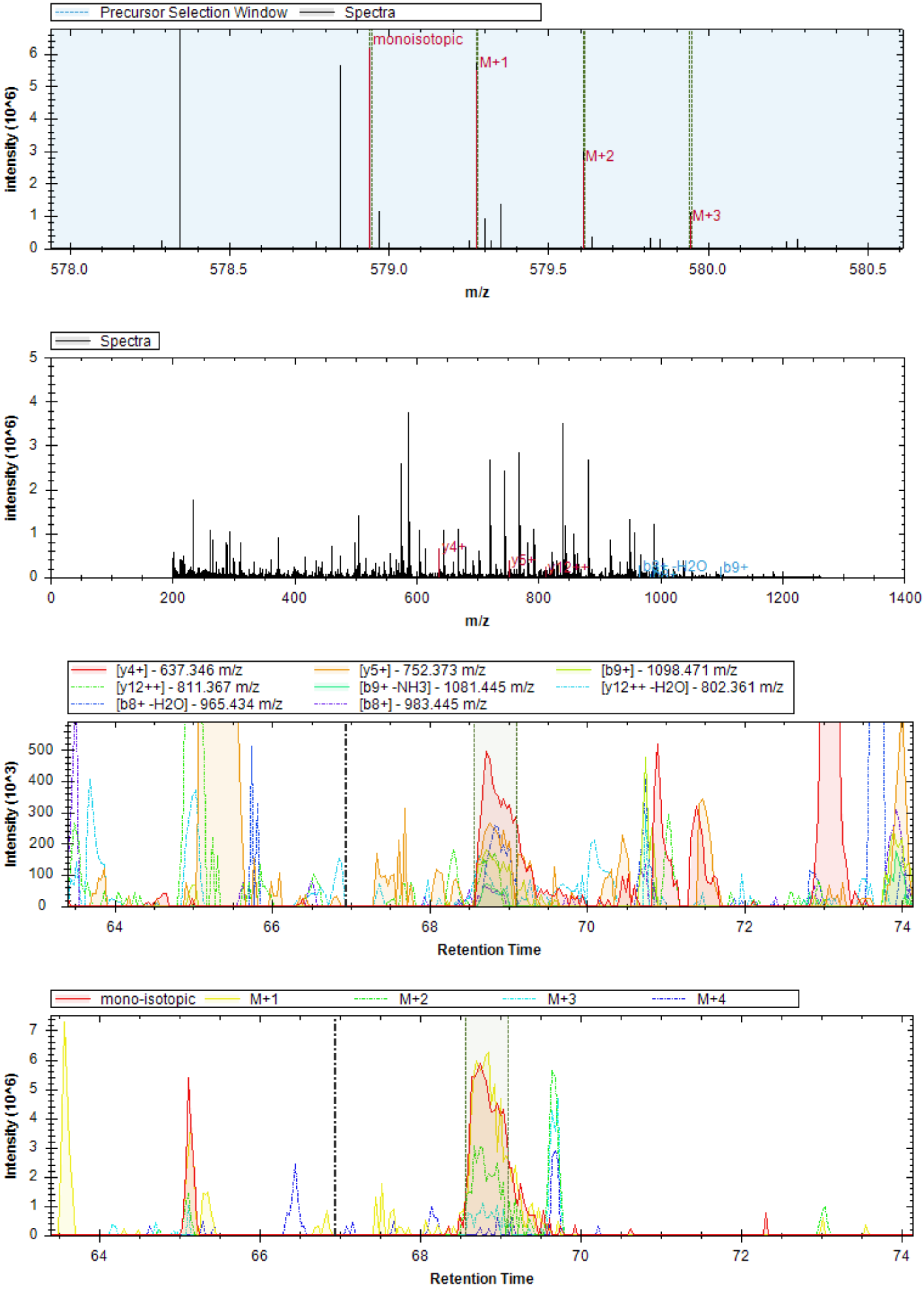

Q62261 – _RPPS[+H+O3+P]PDPNTK_.2

Fig4_MP-DIA-barrel-cortex-p30-2_MHRM.htrms

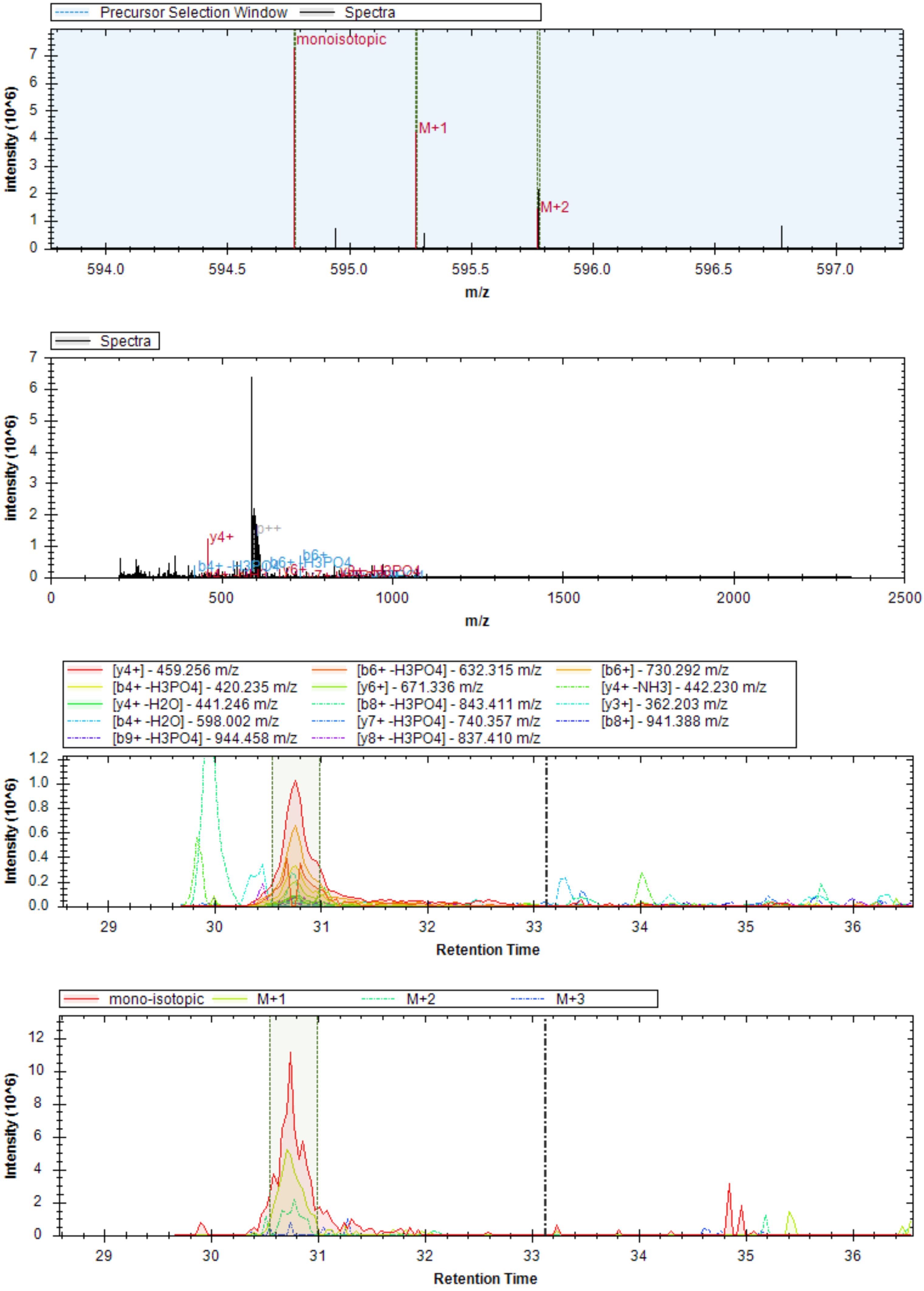

Q80VN0 – _ELTAQIT[+H+O3+P]SIK_.2

Fig4_MP-DIA-barrel-cortex-p30-2_MHRM.htrms

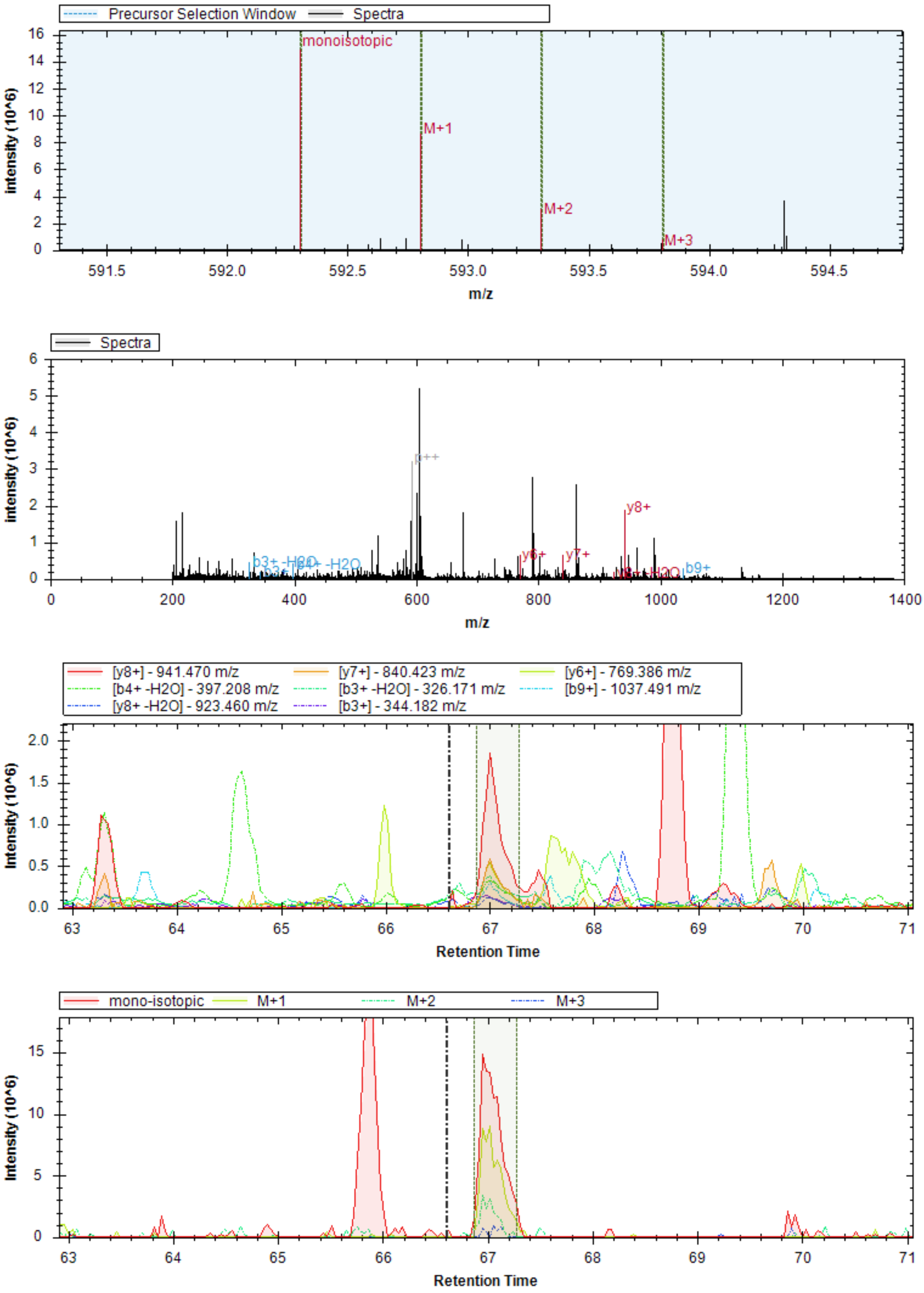

Q99104 – _AIS[+H+O3+P]PTSATSSGR_.2

Fig4_MP-DIA-barrel-cortex-p30-2_MHRM.htrms

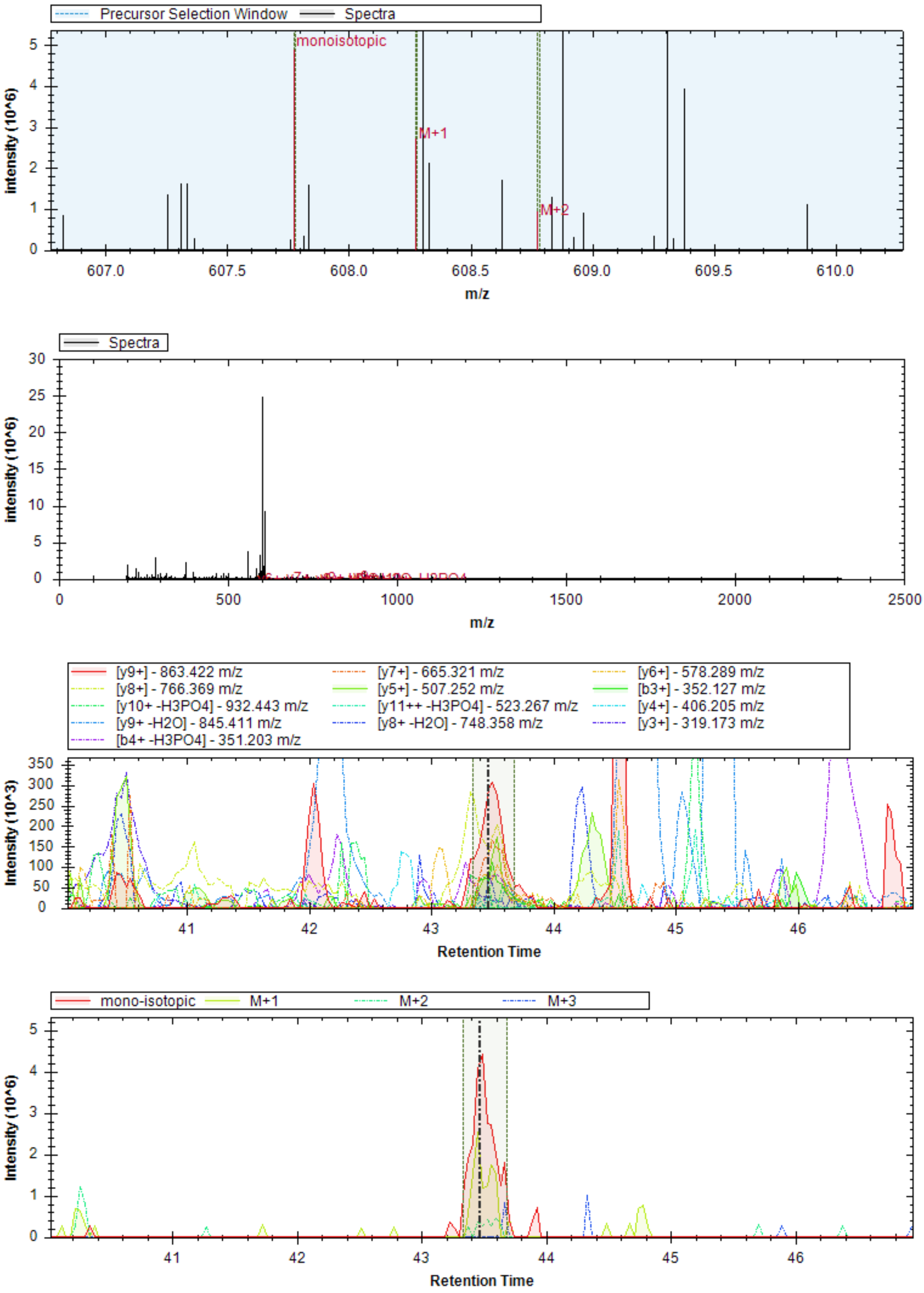

Q9QWI6 – _LSYAGGRPPSYAGS[+H+O3+P]PVHHAAER_.4

Fig4_MP-DIA-barrel-cortex-p30-2_MHRM.htrms

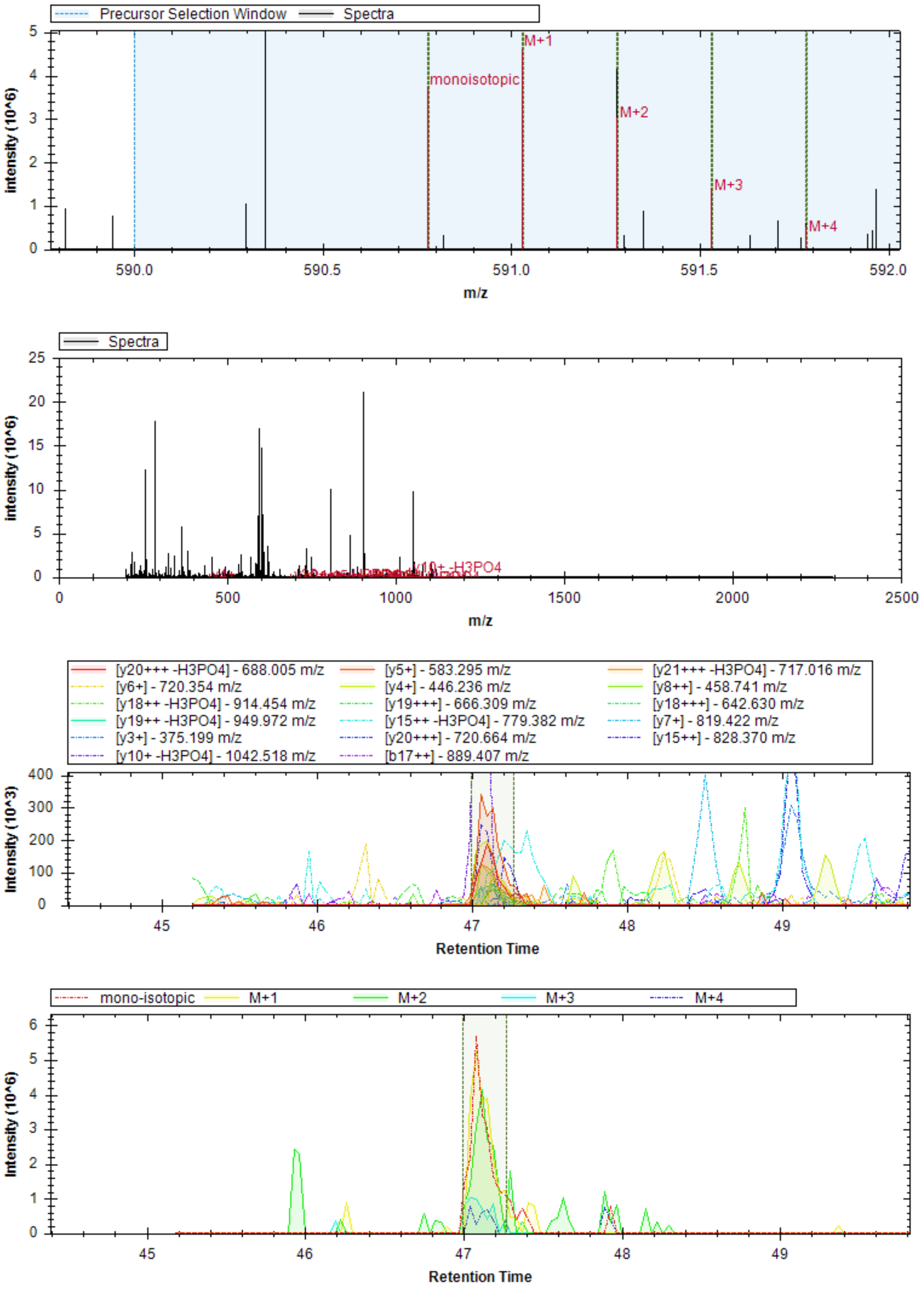

P60904 – _SLS[+H+O3+P]TSGESLYHVLGLDK_.3
Fig4_MP-DIA-barrel-cortex-p30-2_MHRM.htrms

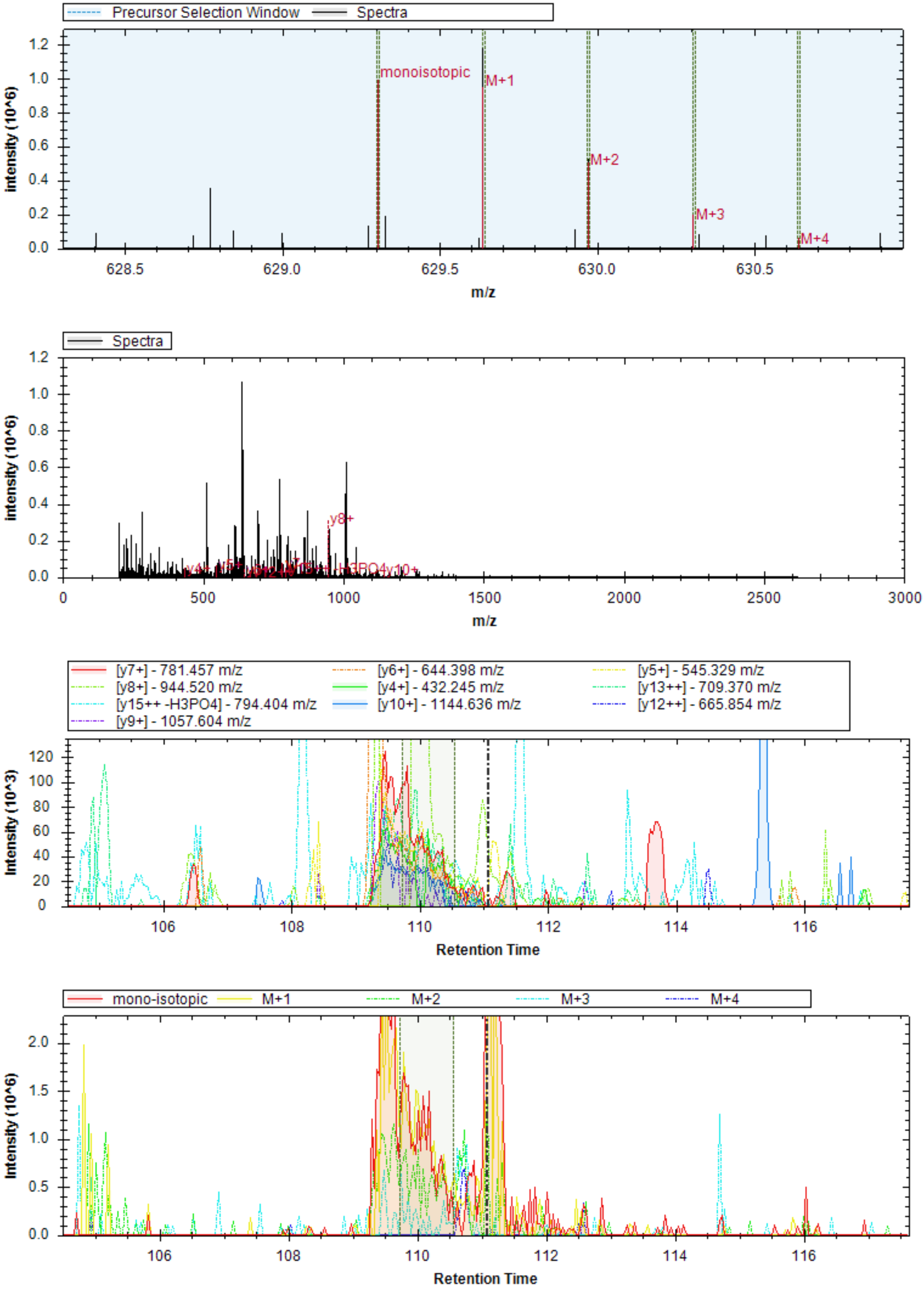

P60904 – _SLST[+H+O3+P]SGESLYHVLGLDK_.3

Fig4_MP-DIA-barrel-cortex-p30-2_MHRM.htrms

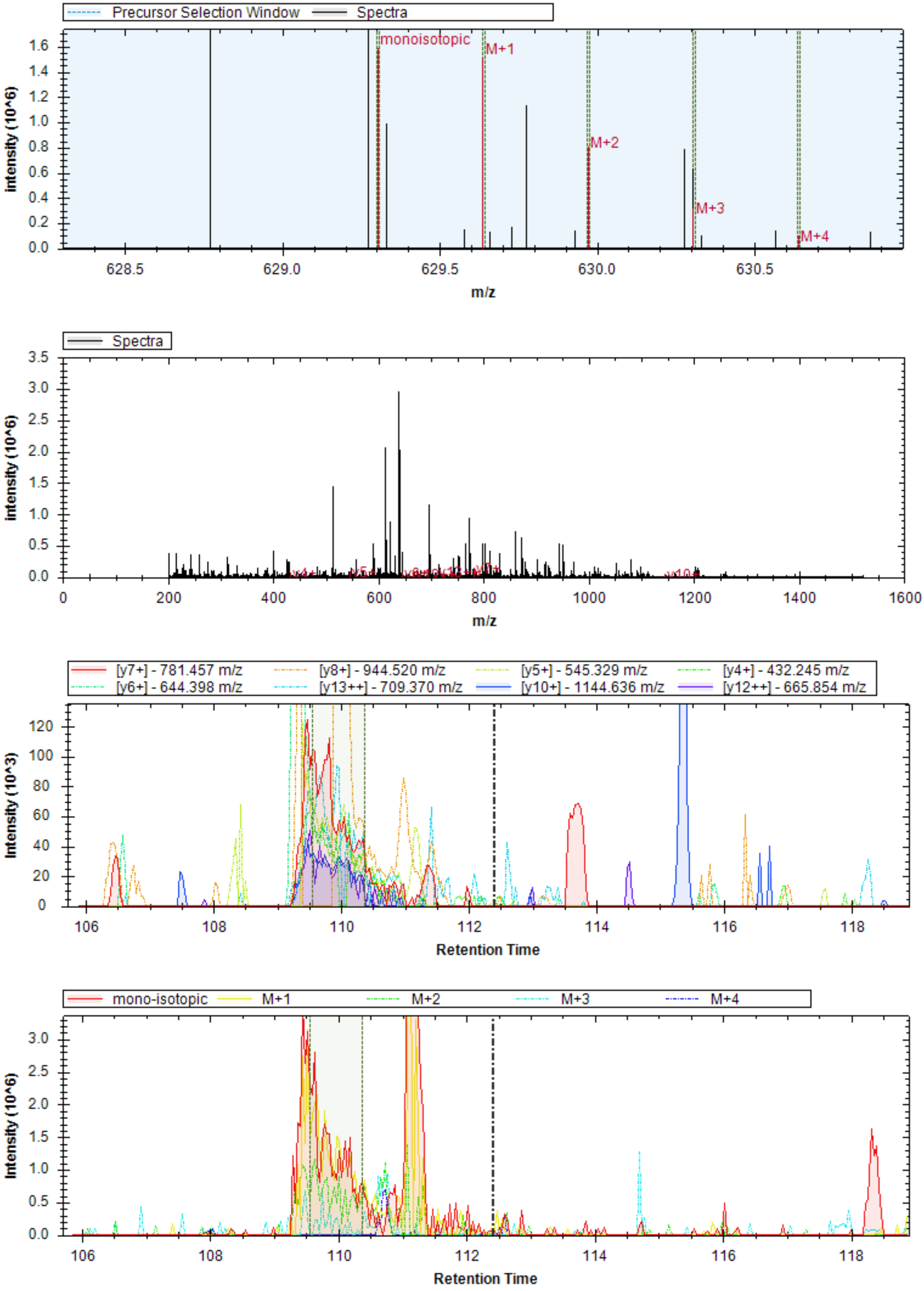

P39447 – _SSGIIRLHT[+H+O3+P]IK_.2
Fig4_MP-DIA-barrel-cortex-p30-2_MHRM.htrms

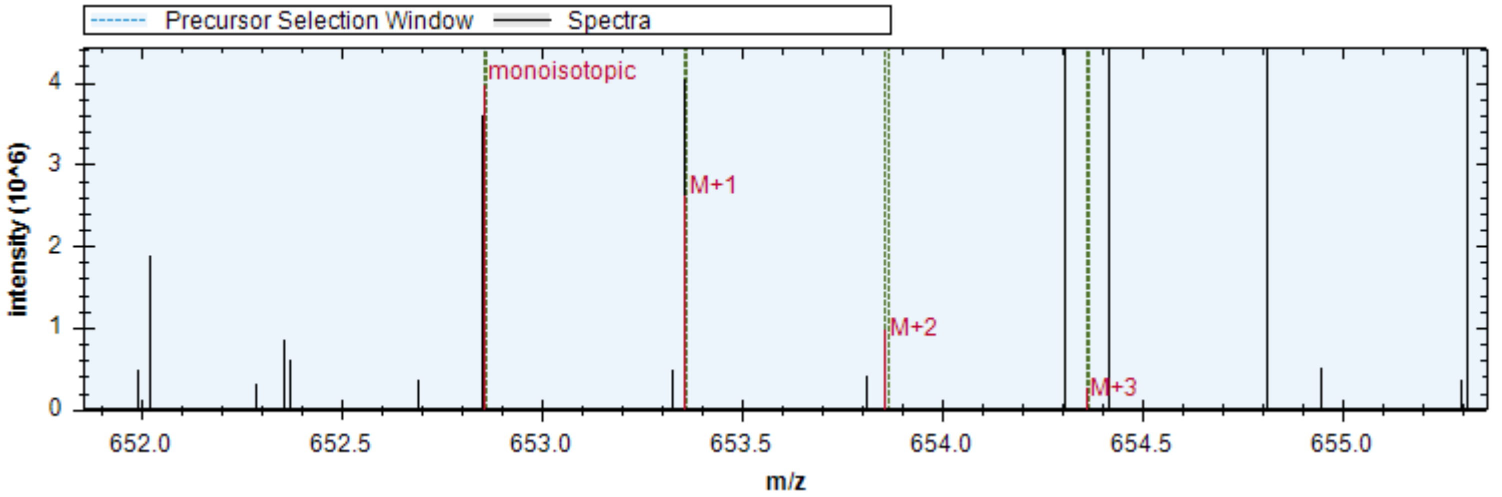

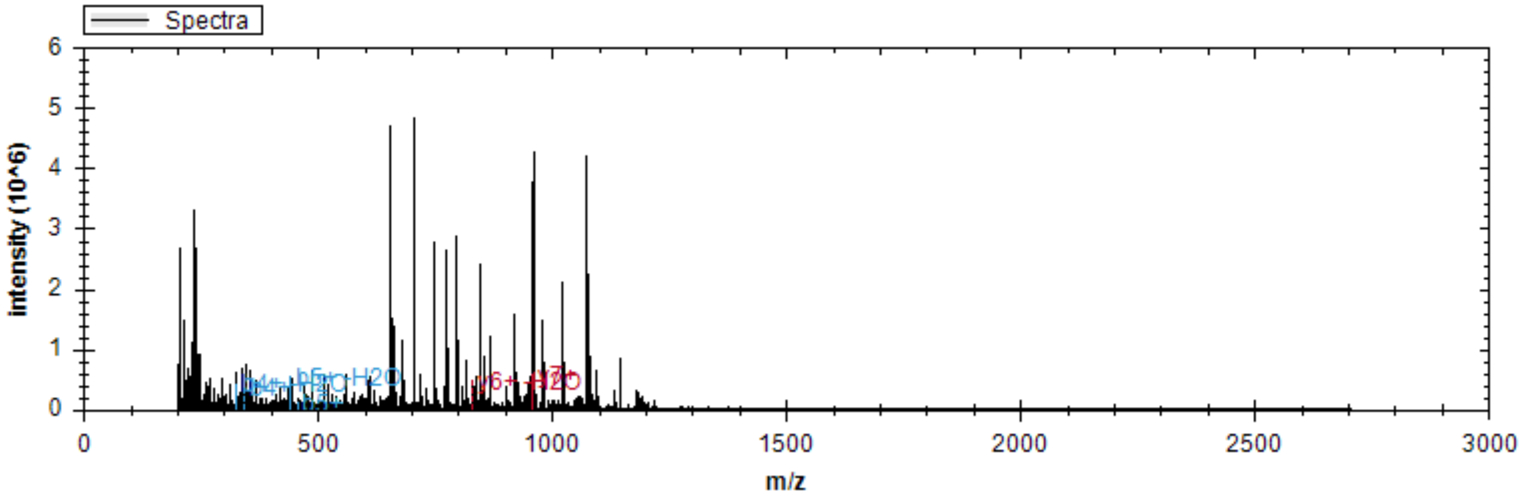

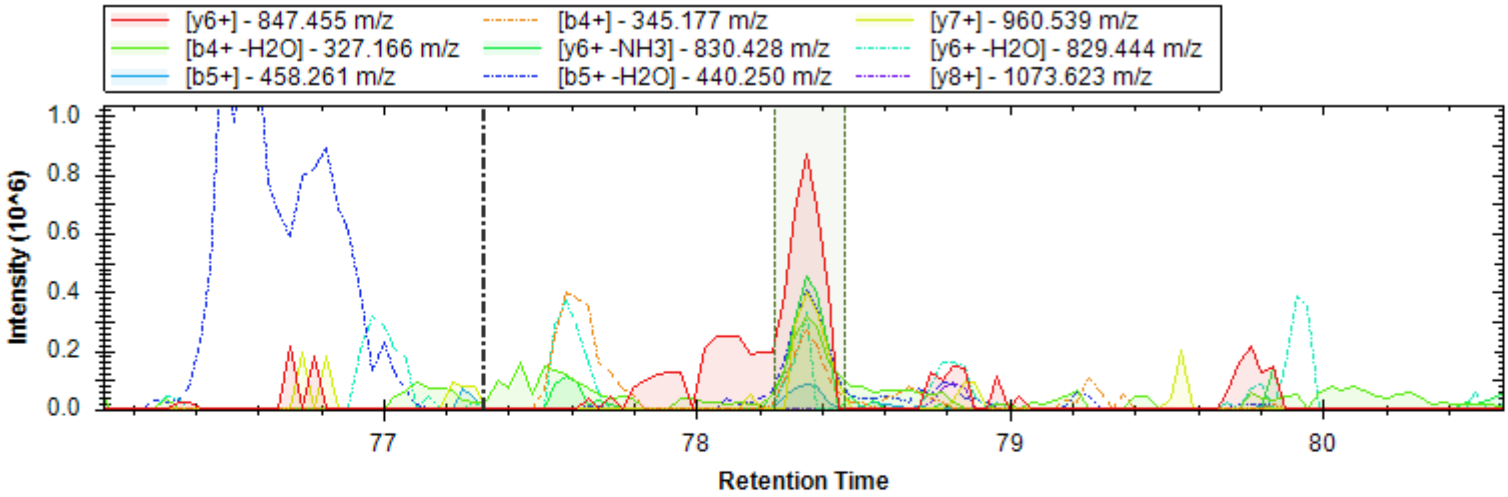

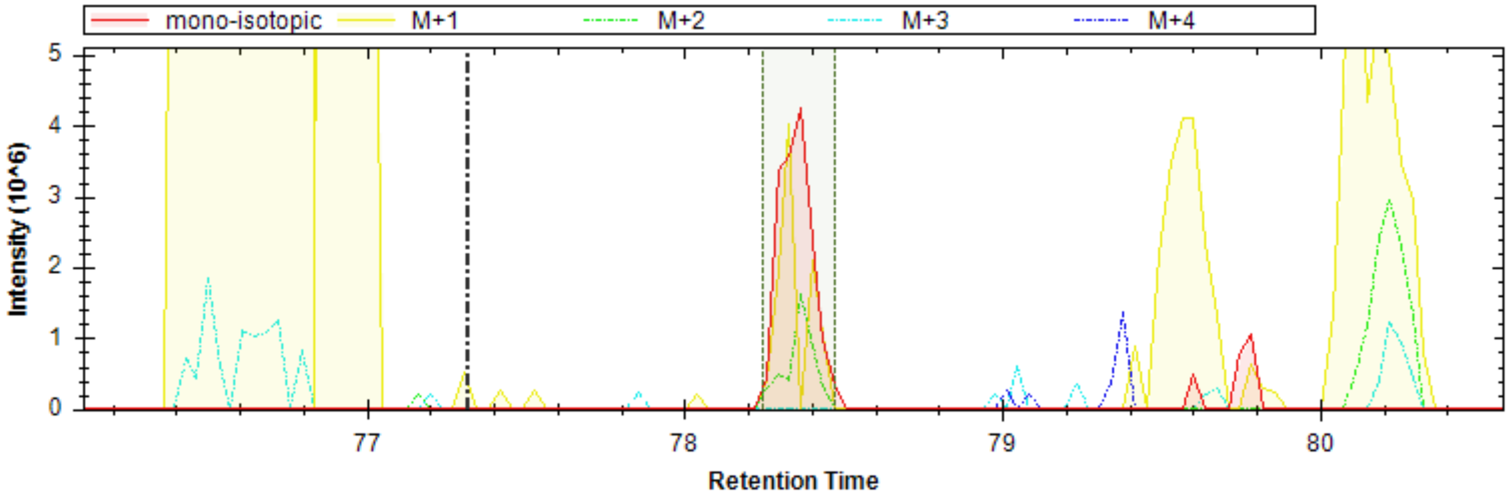

Q8BLJ3 – _M[+O]AS[+H+O3+P]SQGKNELK_.2
Fig4_MP-DIA-barrel-cortex-p30-2_MHRM.htrms

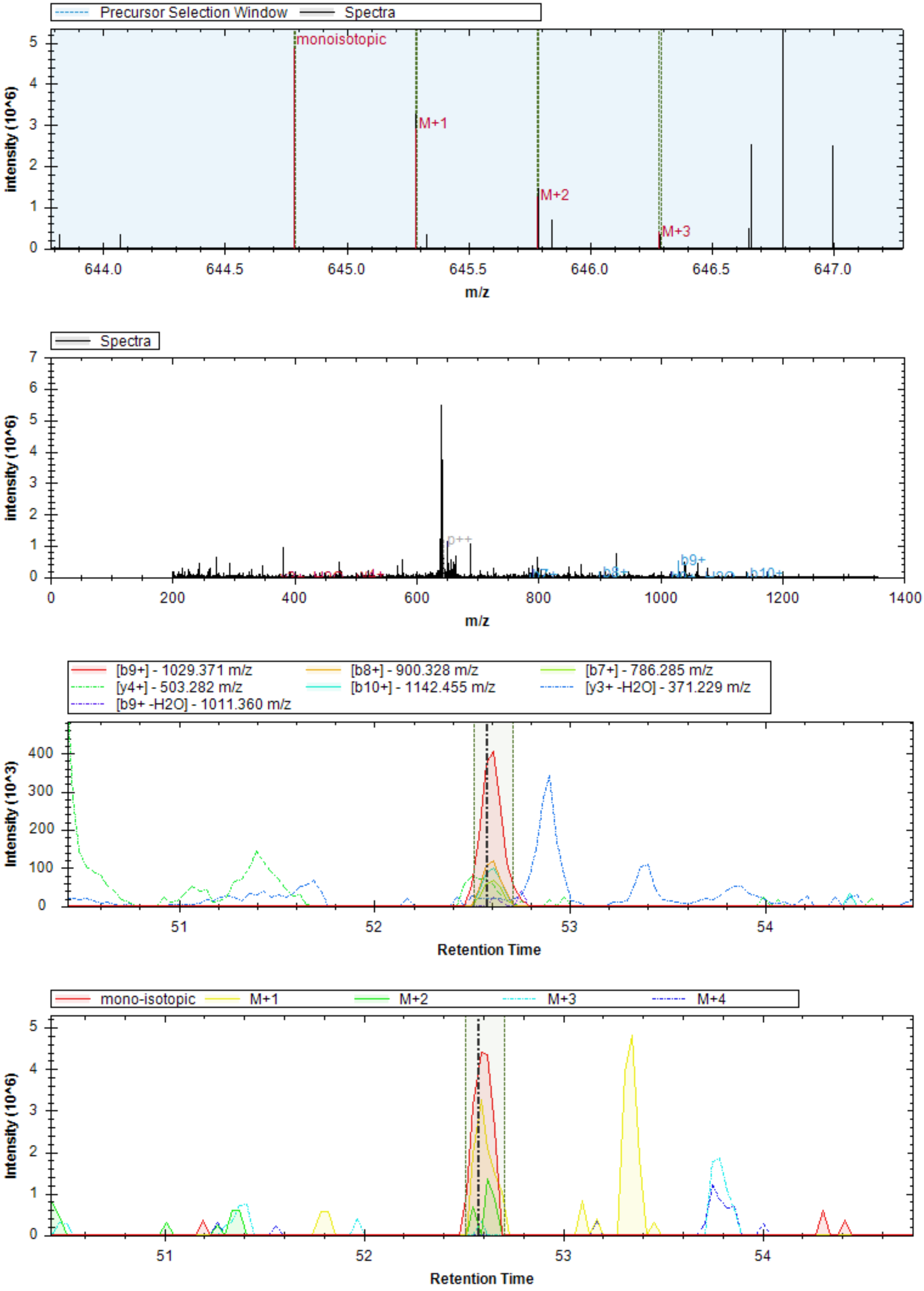

P13595 – _GVTASS[+H+O3+P]SSPASAPK_.2
Fig4_MP-DIA-barrel-cortex-p30-2_MHRM.htrms

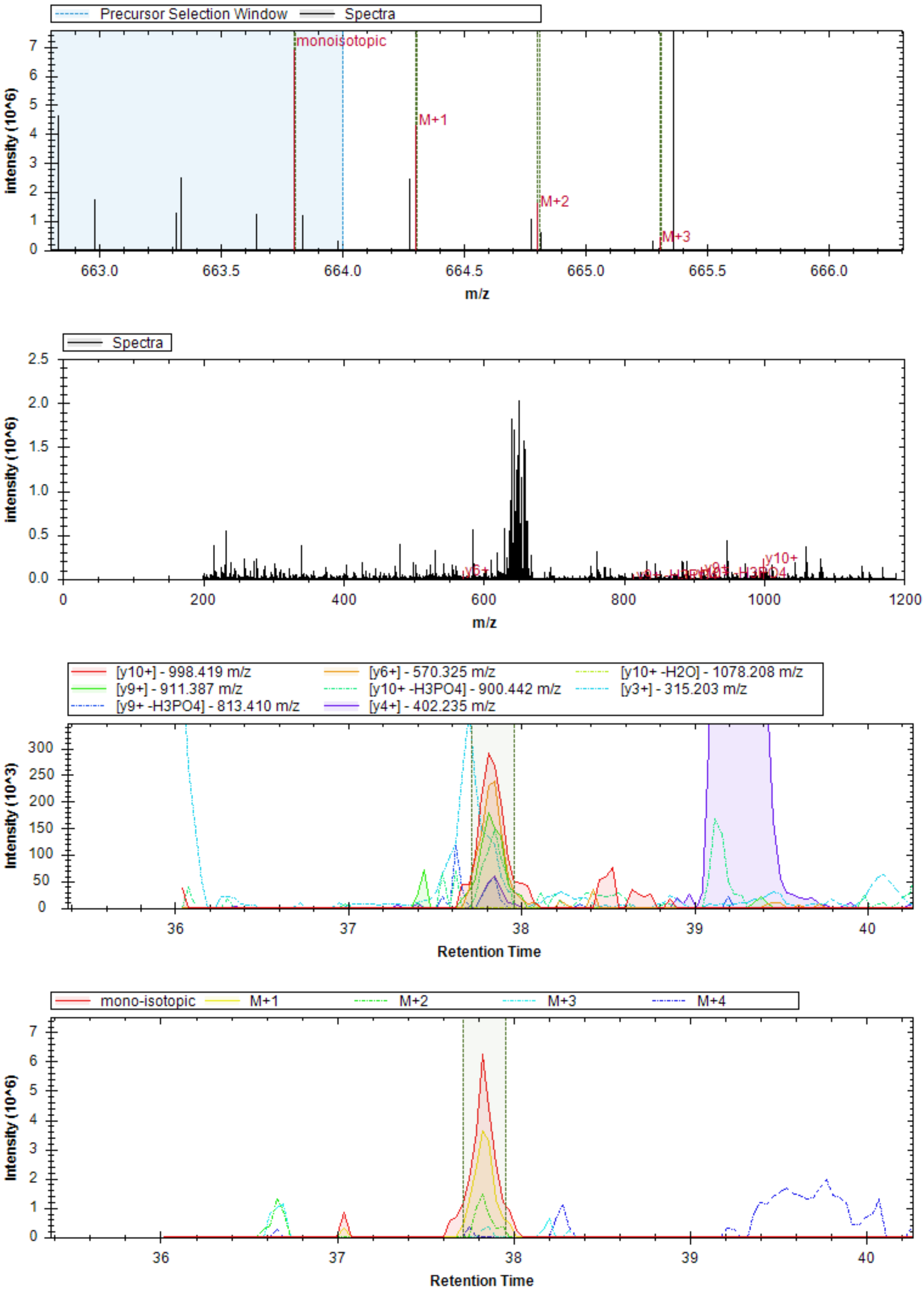

P13595 – _GVTASSS[+H+O3+P]SPASAPK_.2
Fig4_MP-DIA-barrel-cortex-p30-2_MHRM.htrms

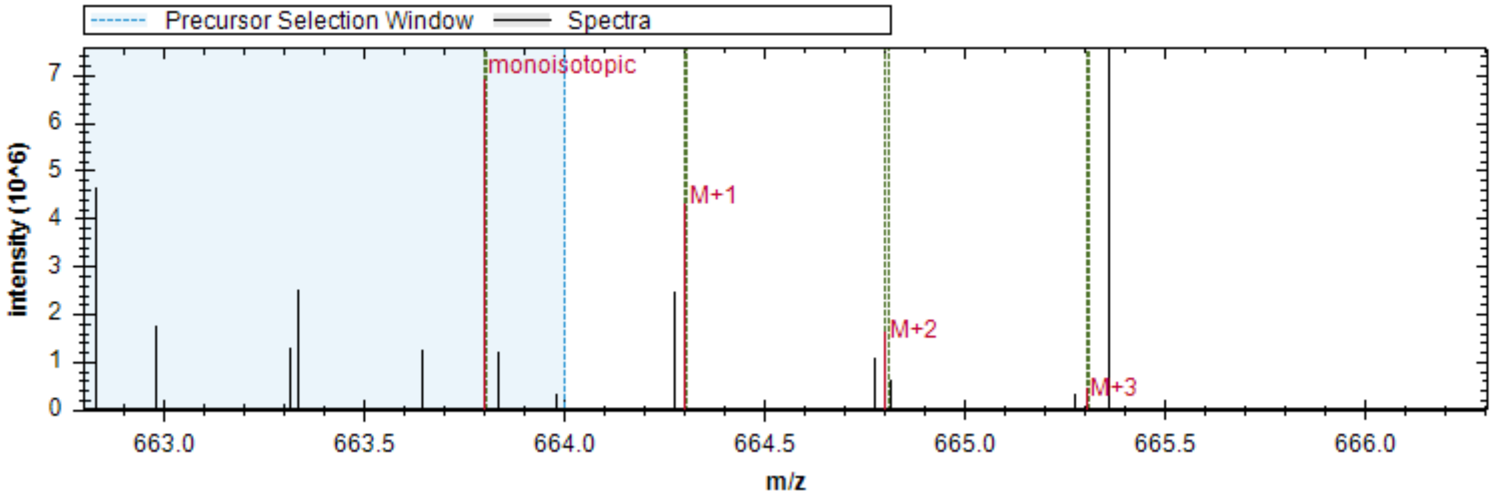

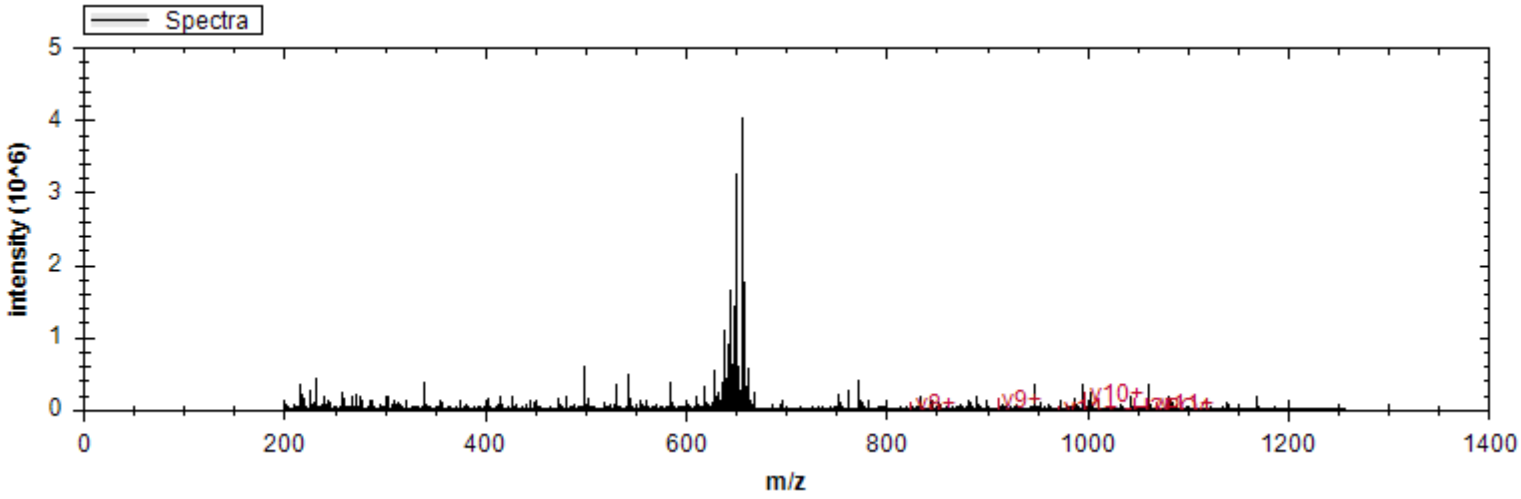

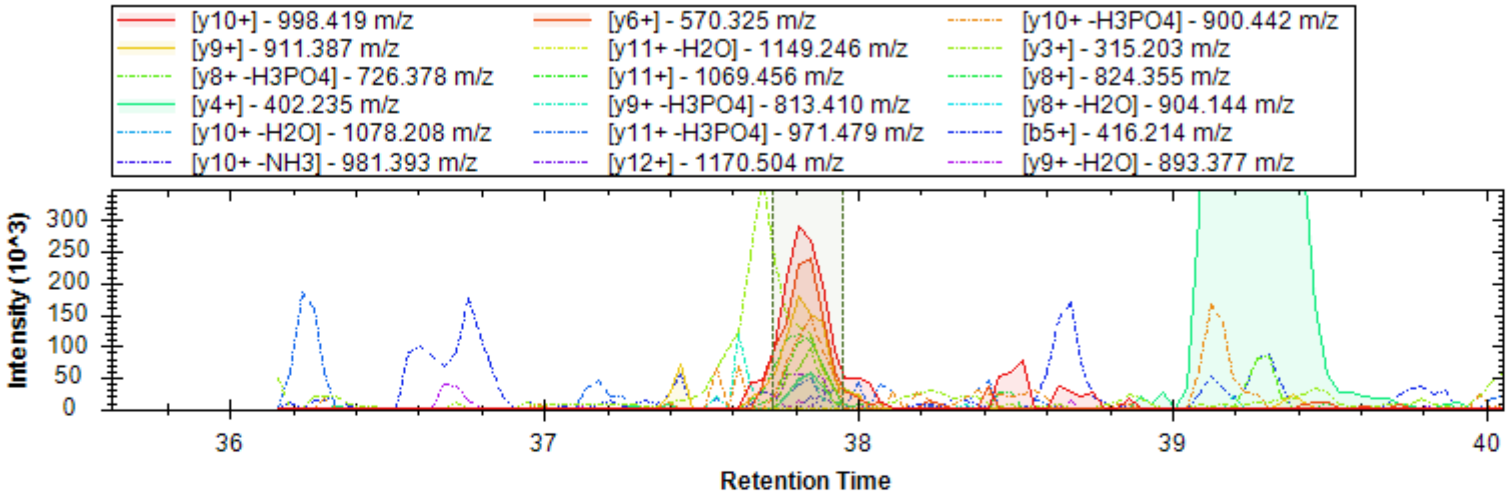

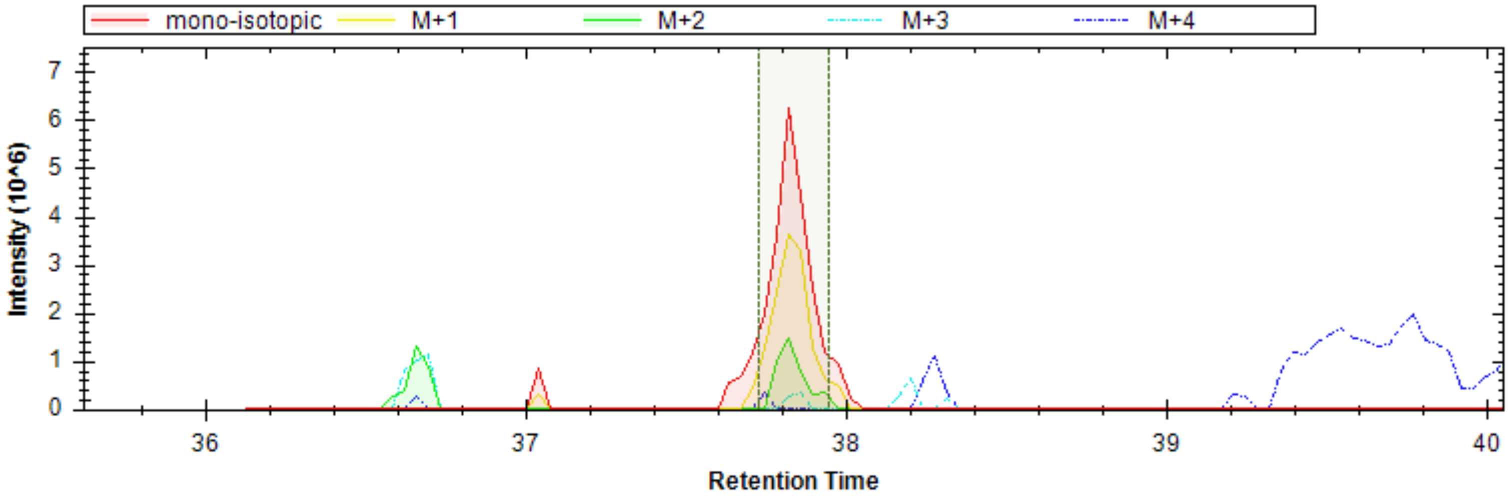

P13595 – _GVTASSSS[+H+O3+P]PASAPK_.2

Fig4_MP-DIA-barrel-cortex-p30-2_MHRM.htrms

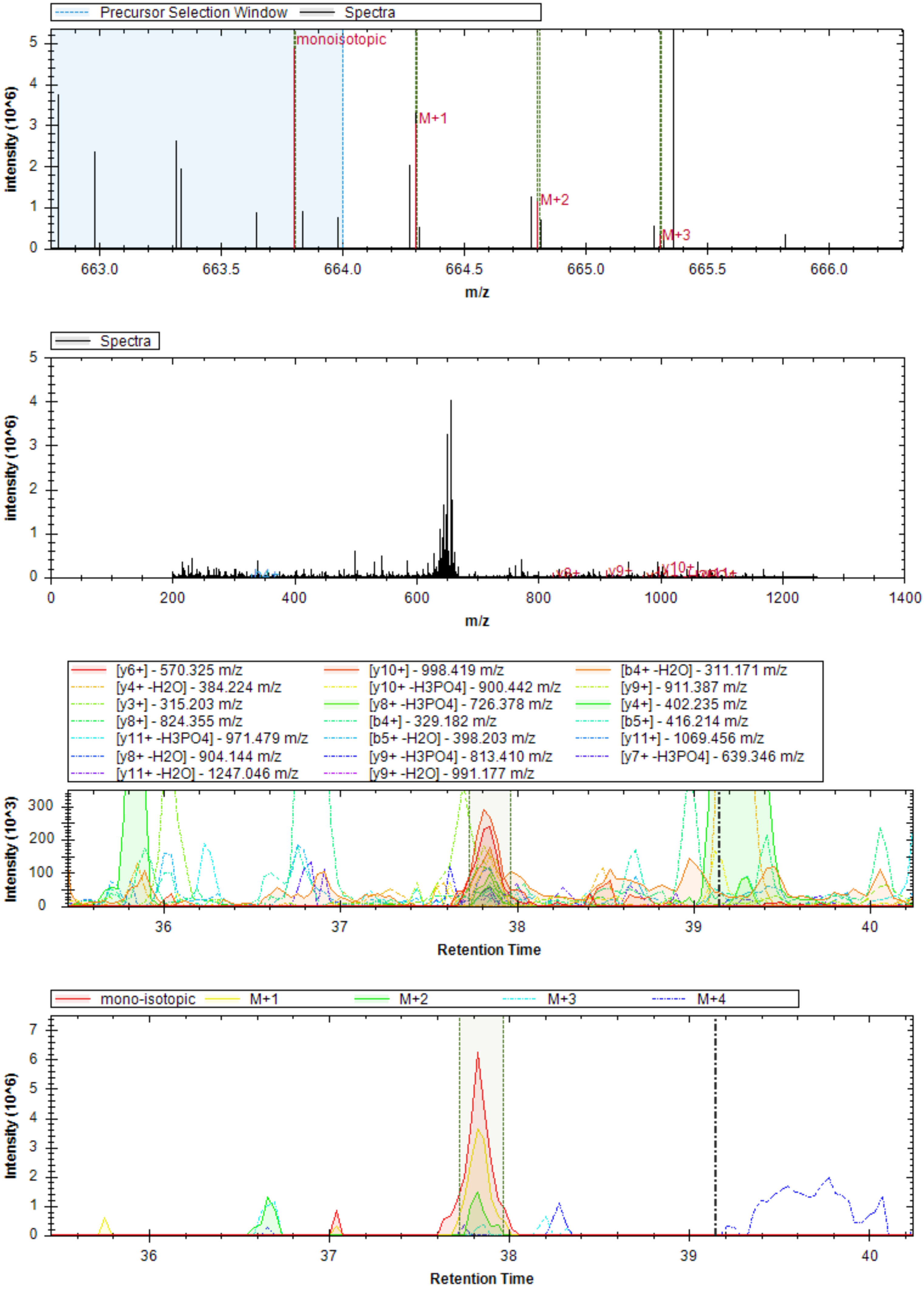

Q6A078 – _T[+H+O3+P]IGLM[+O]KKVVEK_.2
Fig4_MP-DIA-barrel-cortex-p30-2_MHRM.htrms

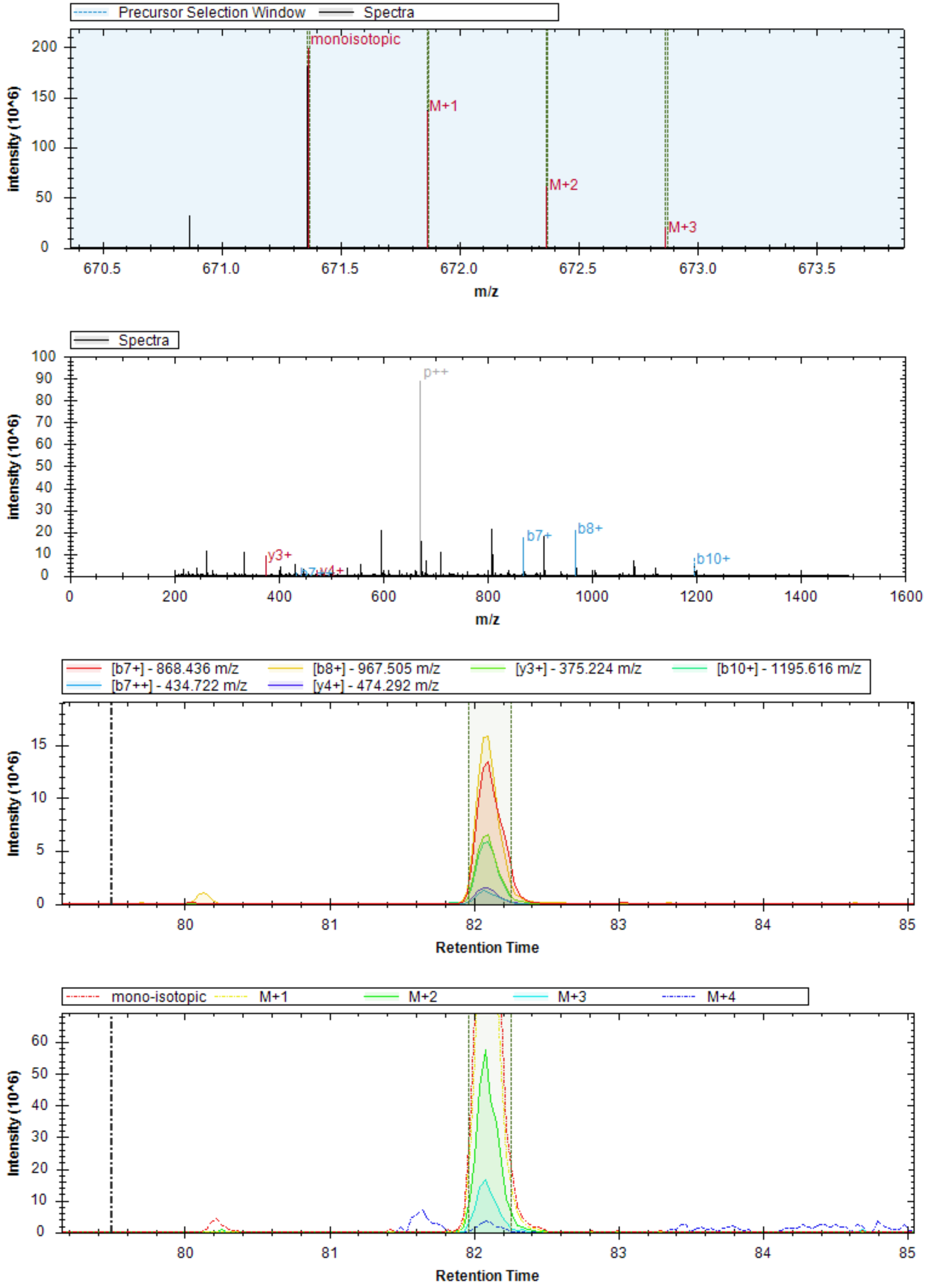

Q8CDU4;Q8CDU4-2 – _[+C2+H2+O]ASLRS[+H+O3+P]ATPRLR_.2
Fig4_MP-DIA-barrel-cortex-p30-2_MHRM.htrms

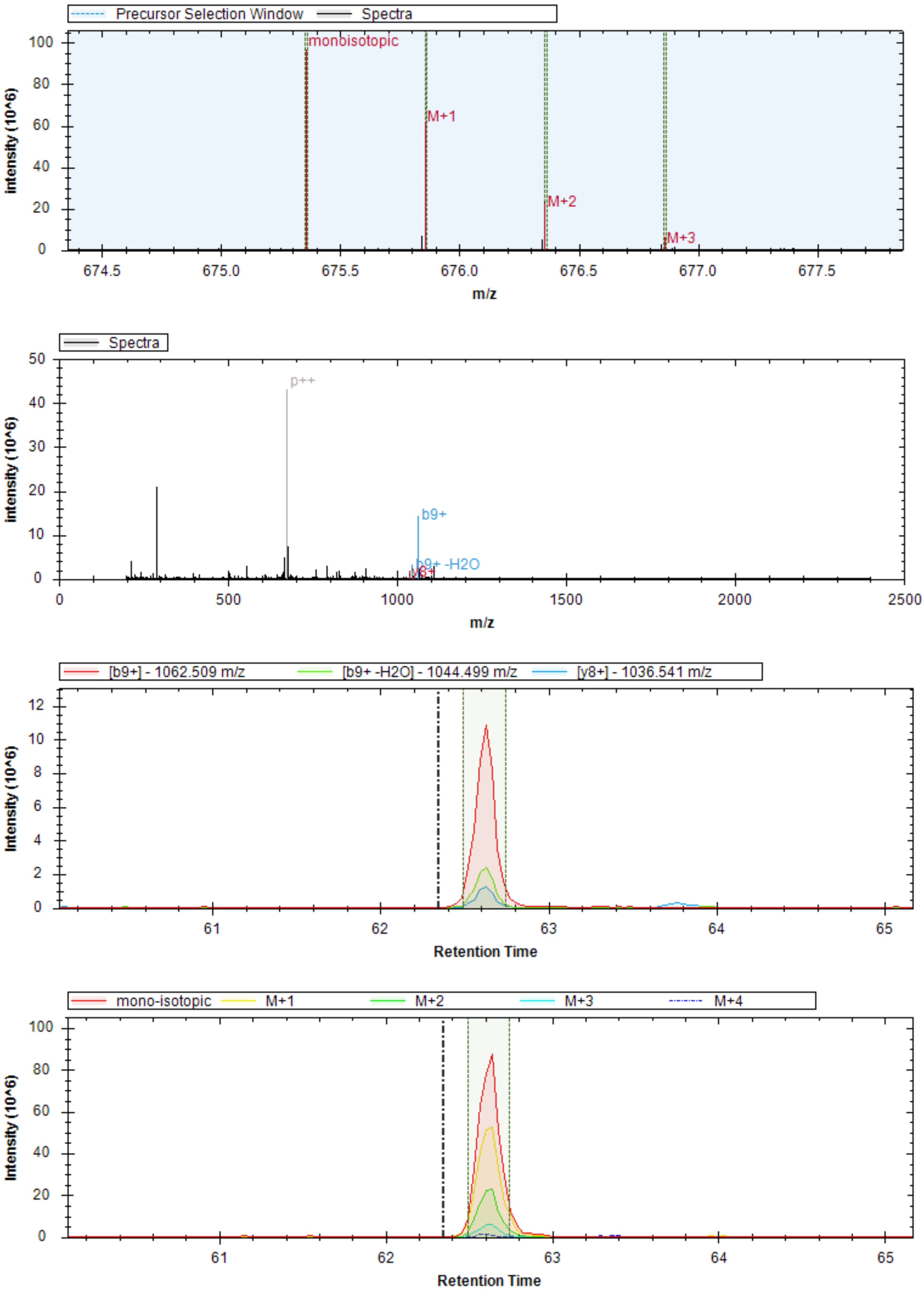

Q8R5L3;Q8R5L3-2 – _LQLY[+H+O3+P]FWKDR_.2

Fig4_MP-DIA-barrel-cortex-p30-2_MHRM.htrms

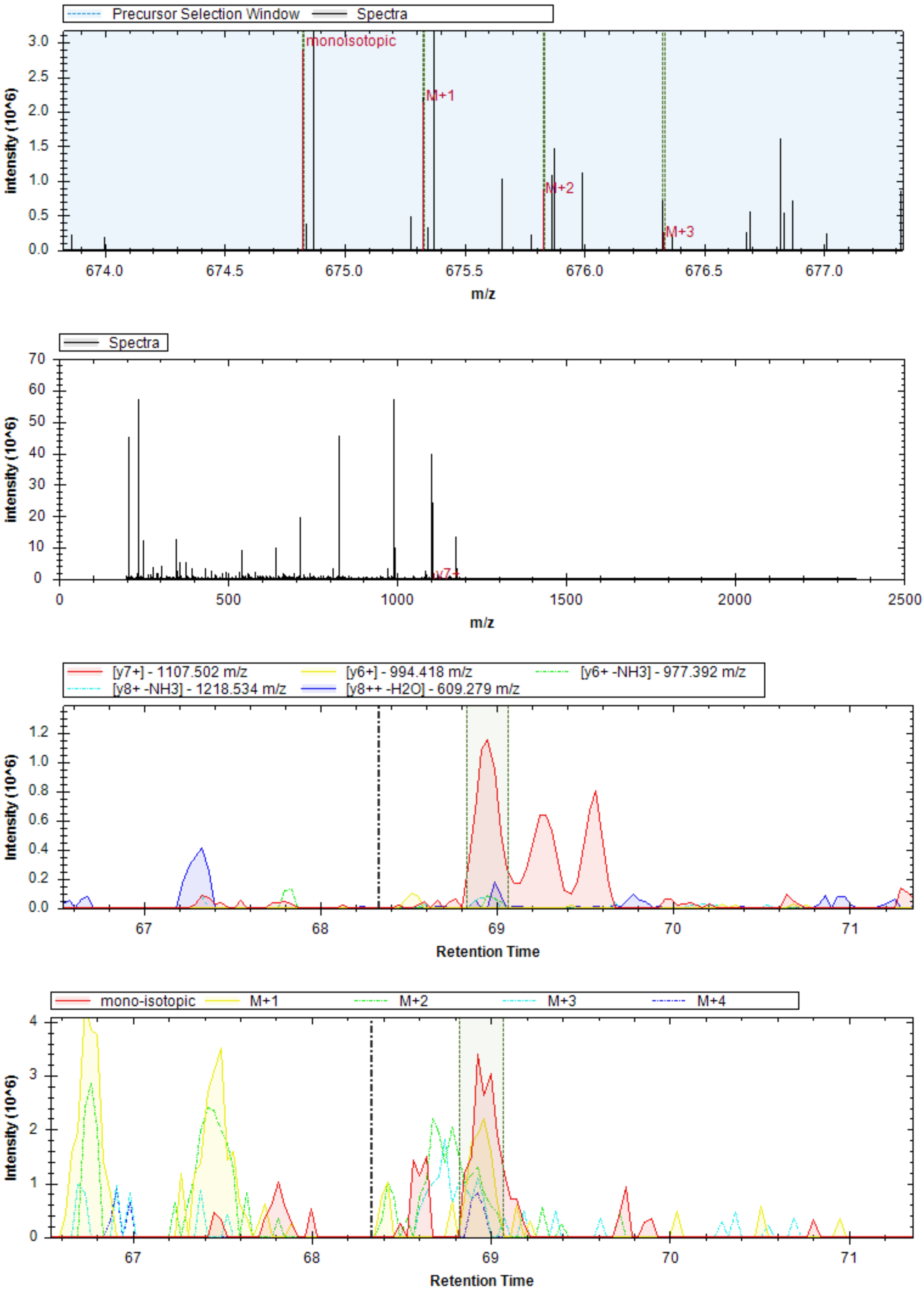

O88935 – _PVAGGPGAPPAARPPAS[+H+O3+P]PSPQR_.3

Fig4_MP-DIA-barrel-cortex-p30-2_MHRM.htrms

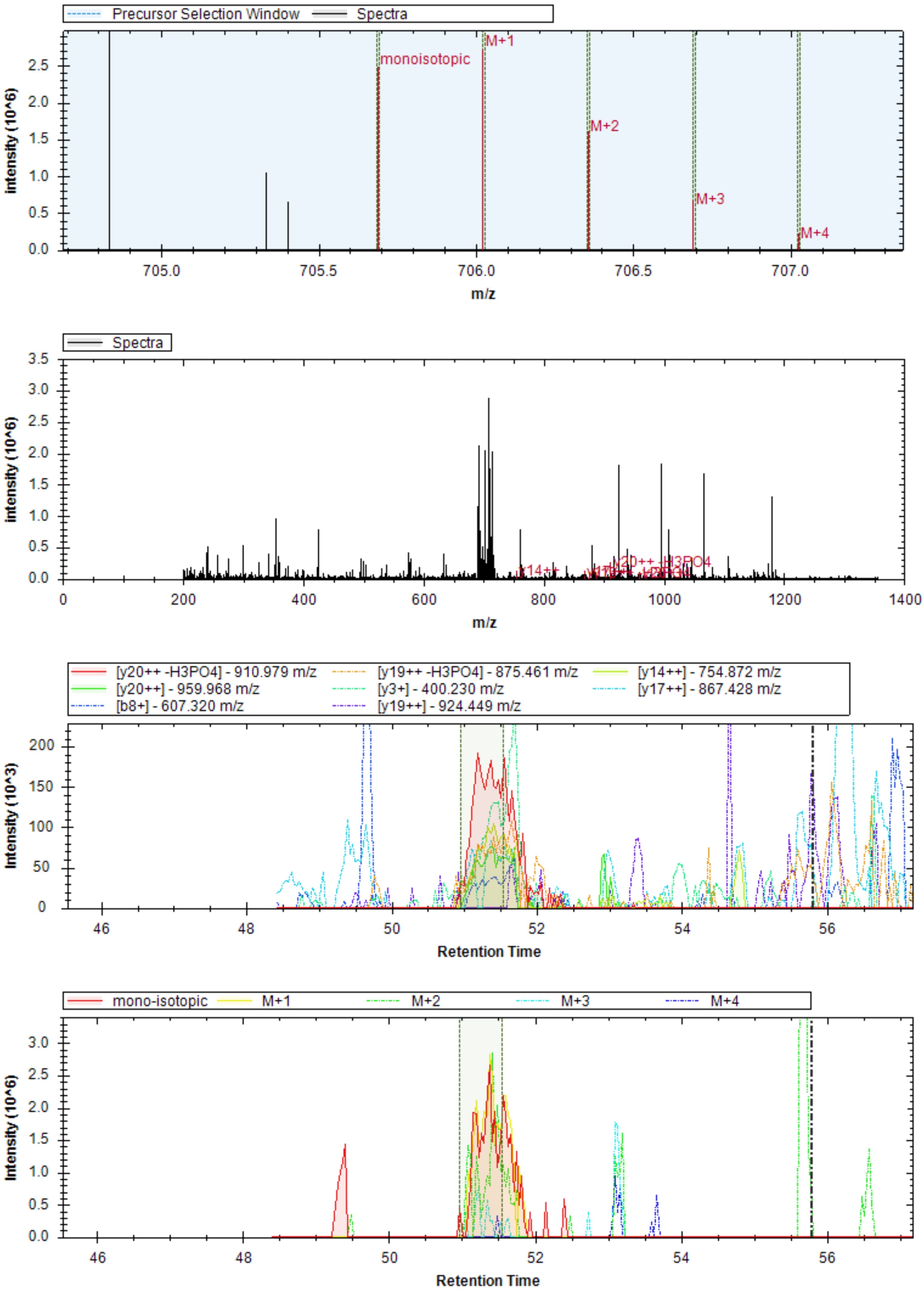

O88935 – _PVAGGPGAPPAARPPASPS[+H+O3+P]PQR_.3
Fig4_MP-DIA-barrel-cortex-p30-2_MHRM.htrms

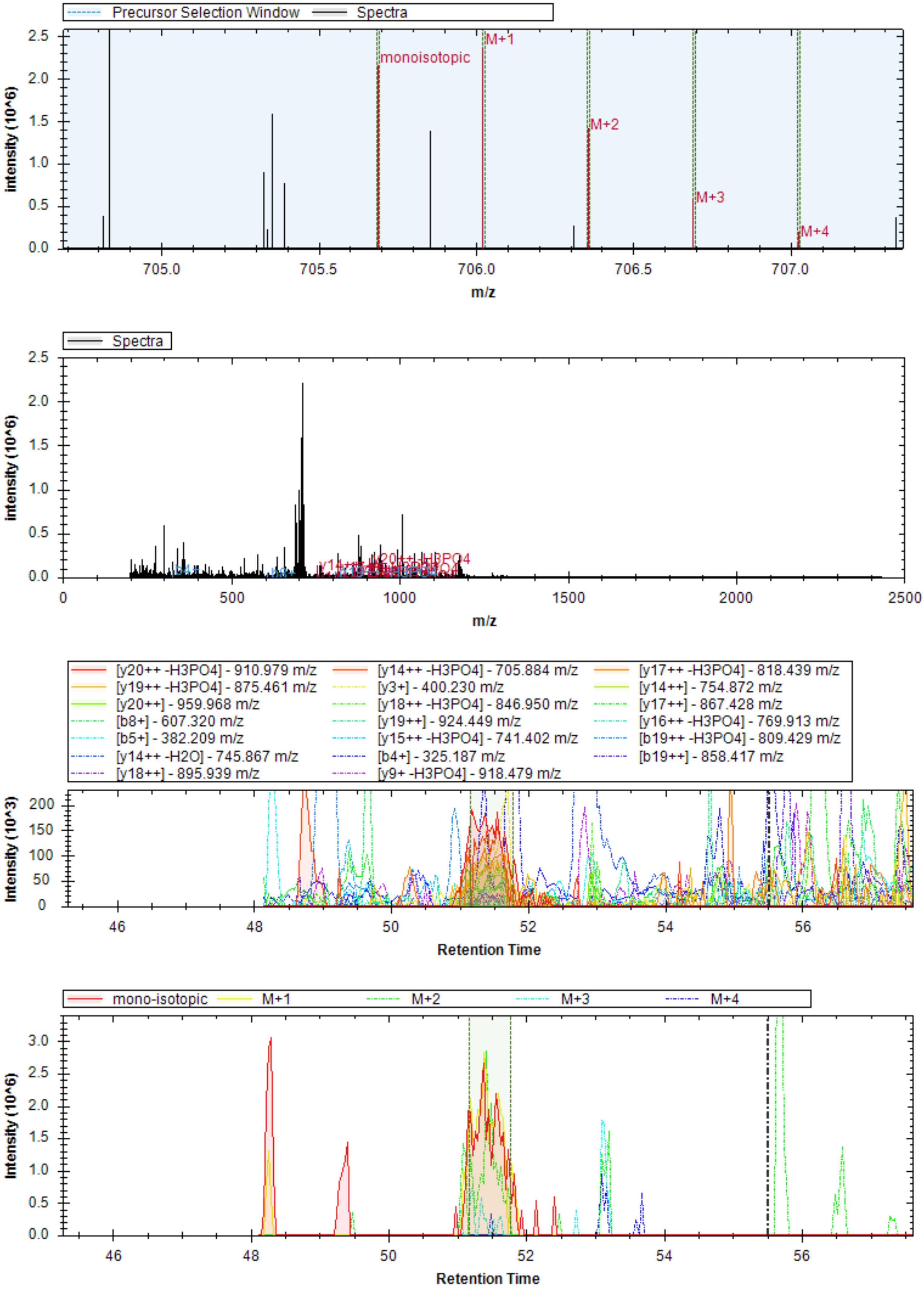

P28738 – _PIRPGHYPAS[+H+O3+P]SPTAVHAVR_.3
Fig4_MP-DIA-barrel-cortex-p30-2_MHRM.htrms

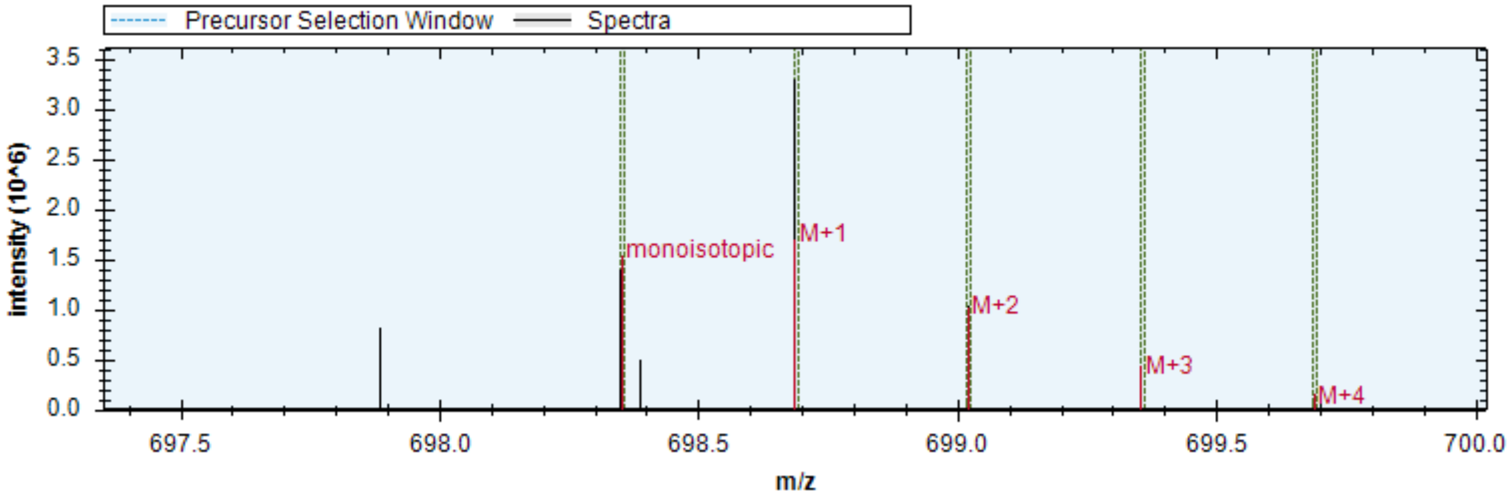

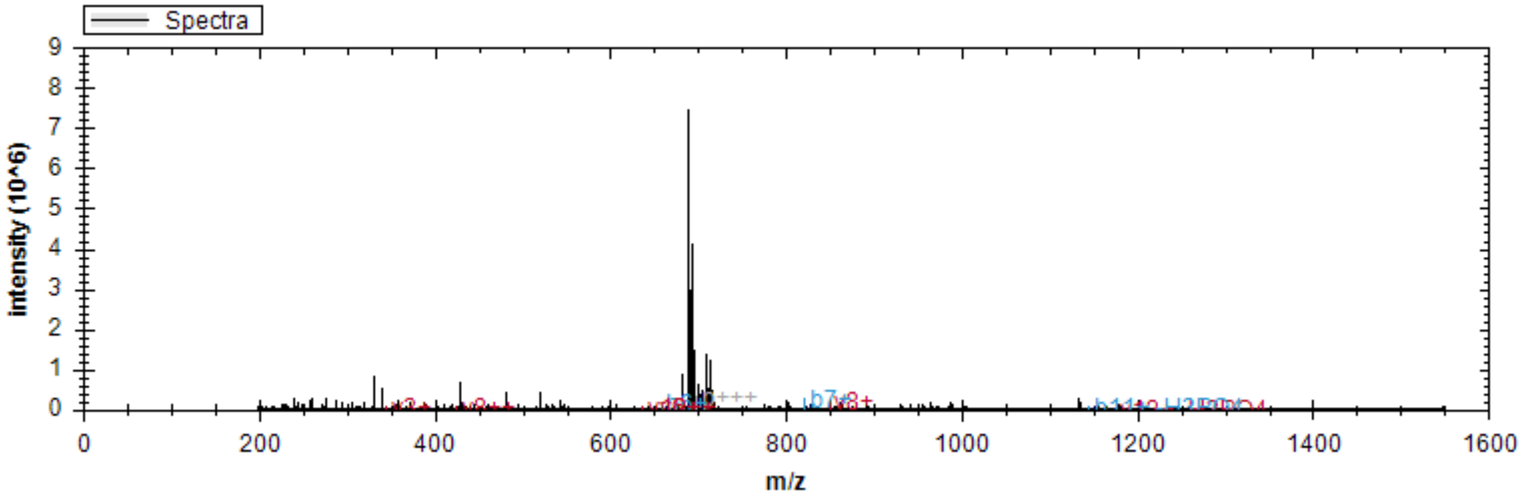

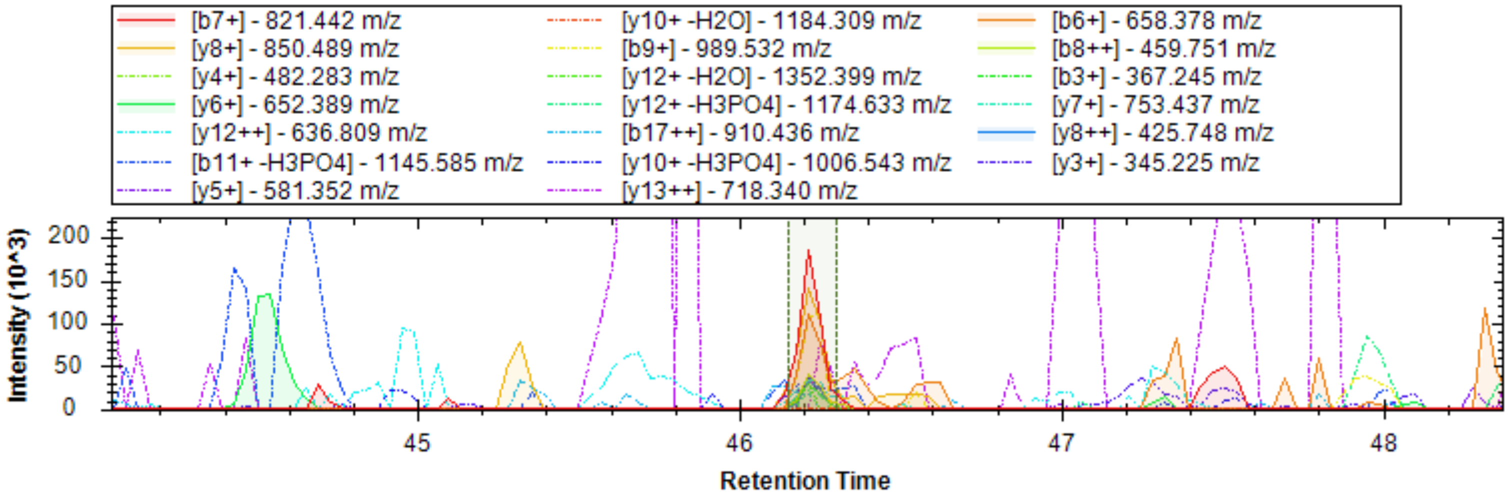

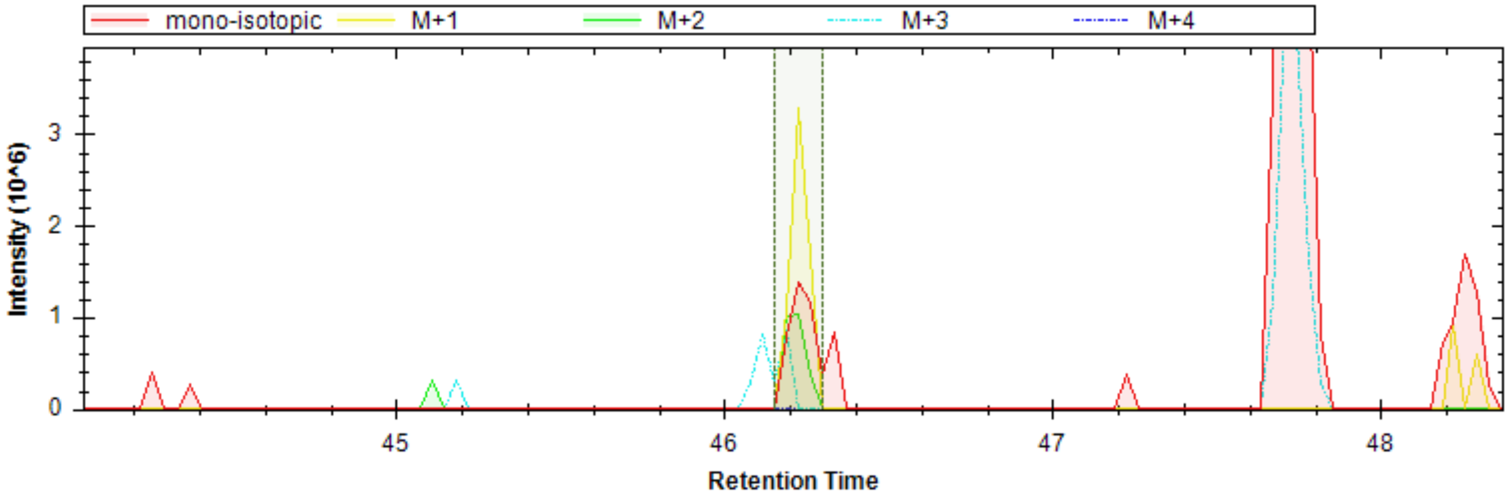

P28738 – _PIRPGHYPASS[+H+O3+P]PTAVHAVR_.3

Fig4_MP-DIA-barrel-cortex-p30-2_MHRM.htrms

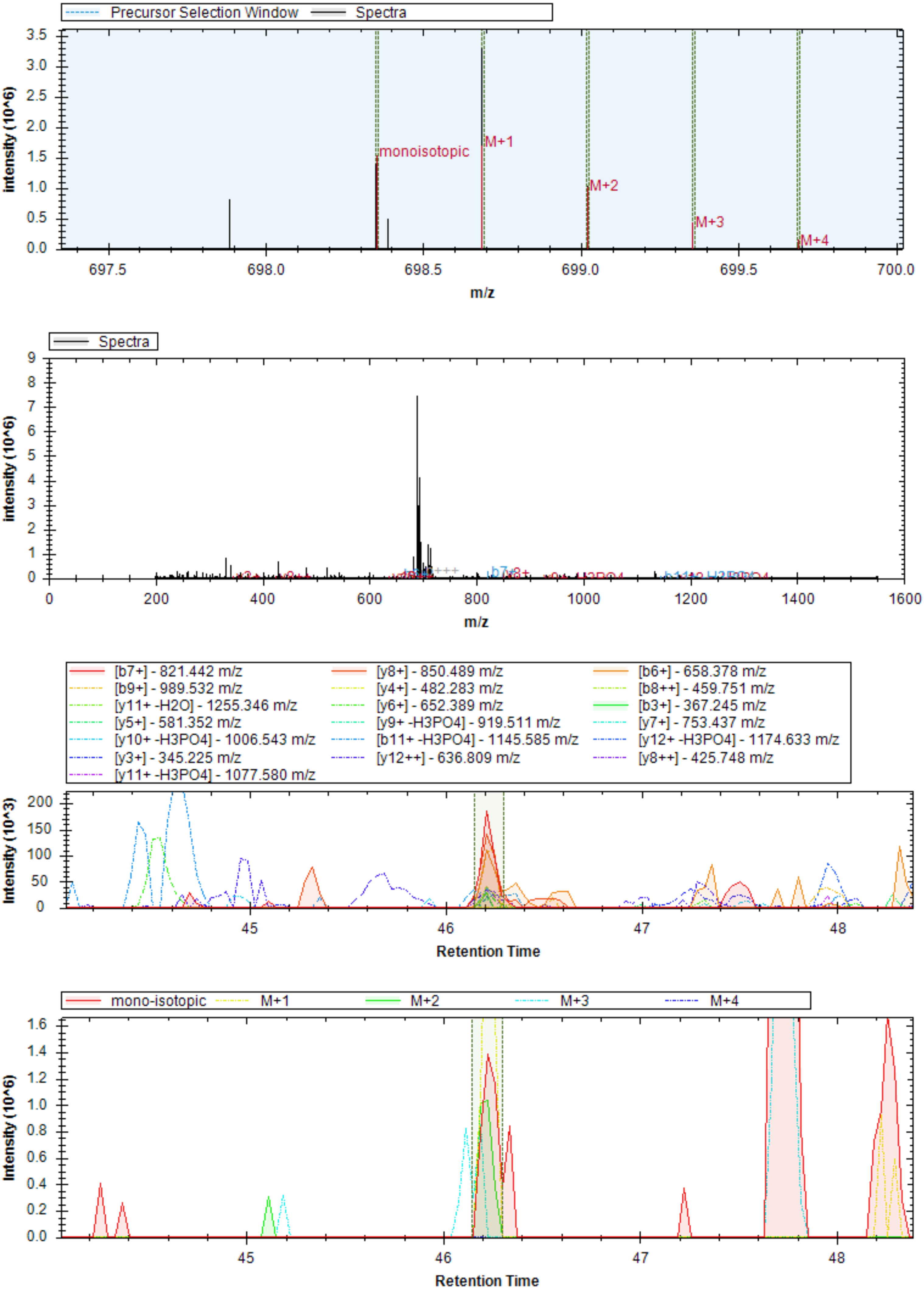

P62751 – _KAY[+H+O3+P]VRLAPDYDALDVANK_.3

Fig4_MP-DIA-barrel-cortex-p30-2_MHRM.htrms

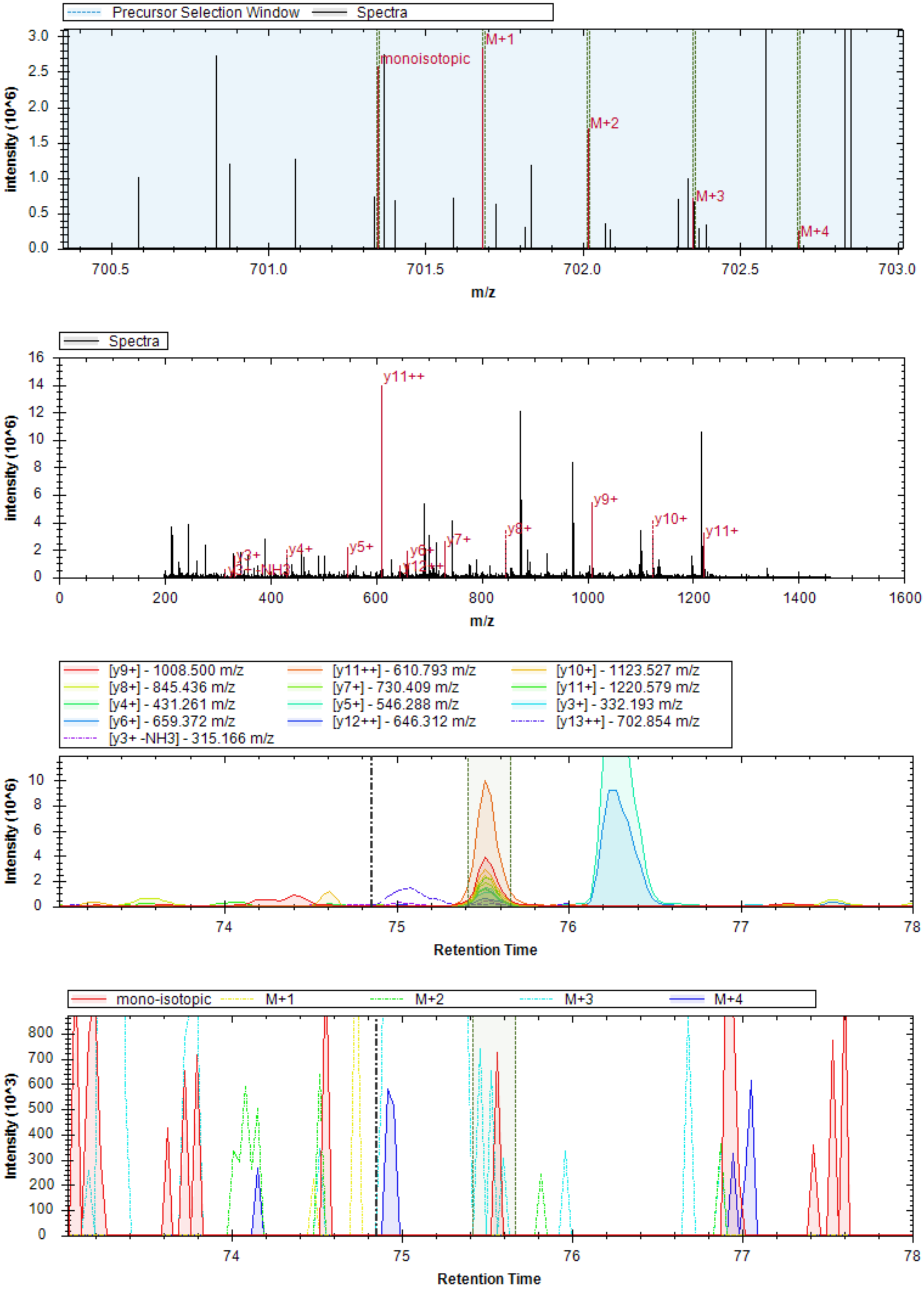

Q3U4B4 – _RHKT[+H+O3+P]LSANEEK_.2
Fig4_MP-DIA-barrel-cortex-p30-2_MHRM.htrms

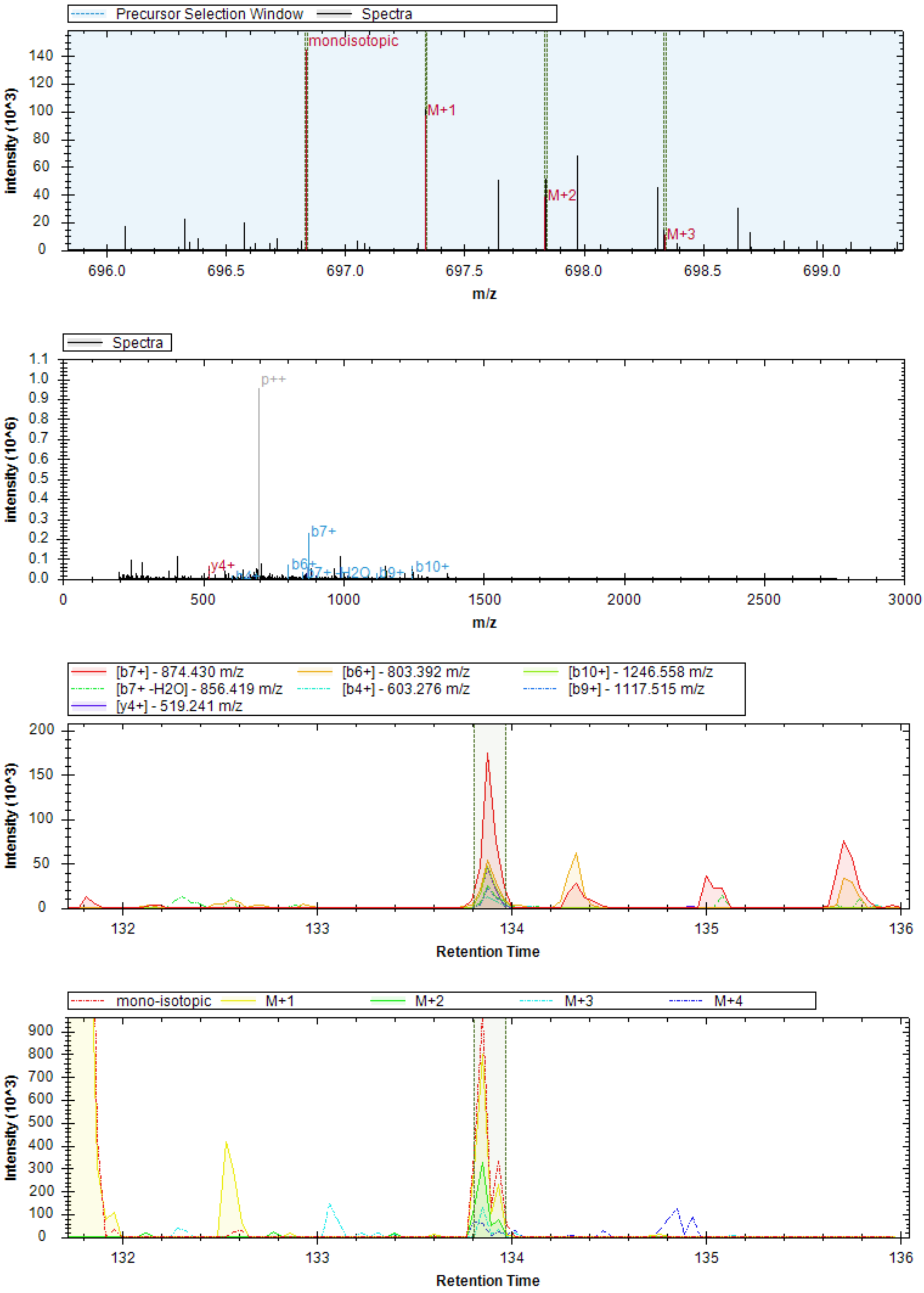

Q8CAA7 – _AVAGVM[+O]ITAS[+H+O3+P]HNR_.2
Fig4_MP-DIA-barrel-cortex-p30-2_MHRM.htrms

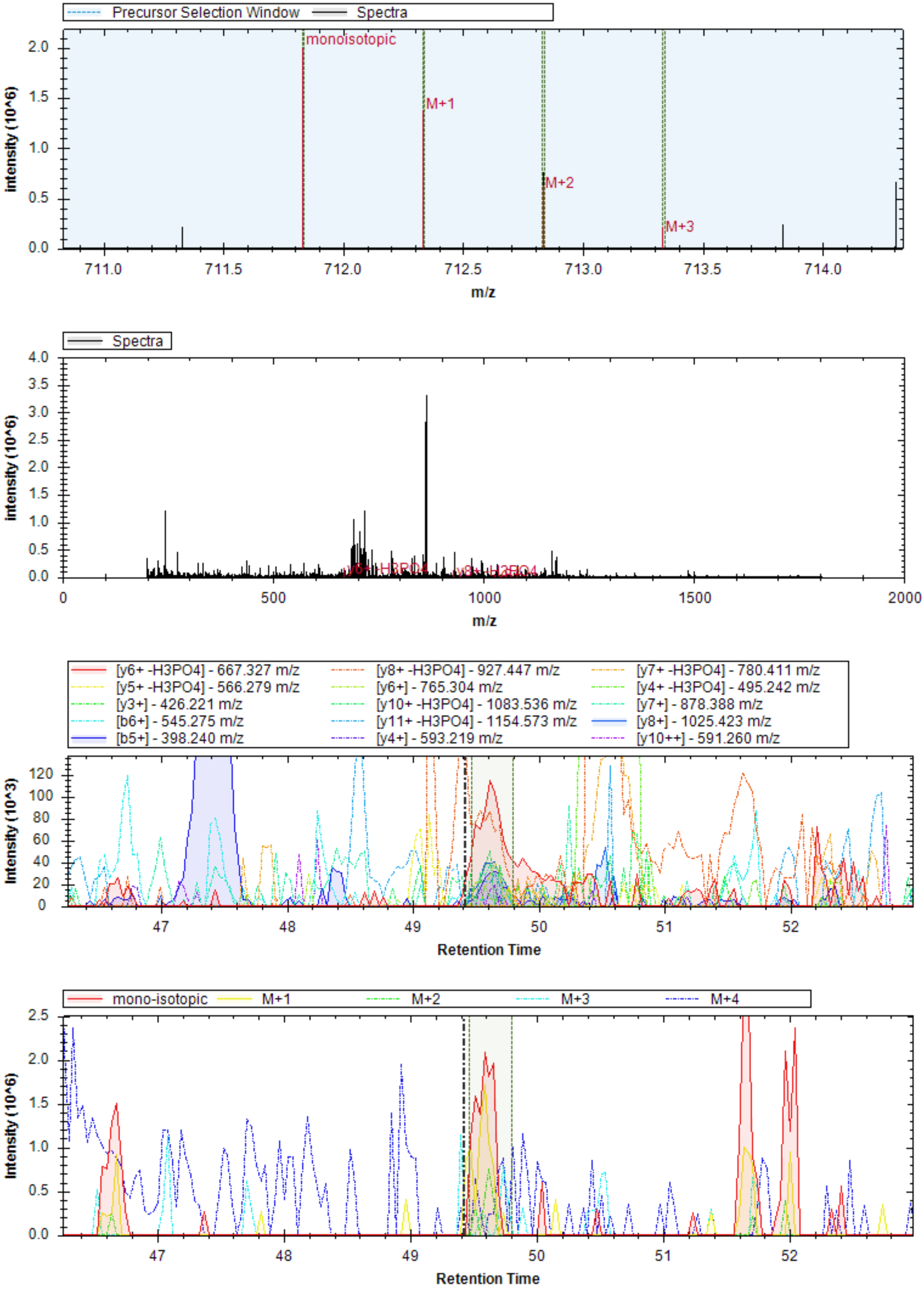

P10637 – _SGYSS[+H+O3+P]PGSPGTPGSR_.2

Fig4_MP-DIA-barrel-cortex-p30-2_MHRM.htrms

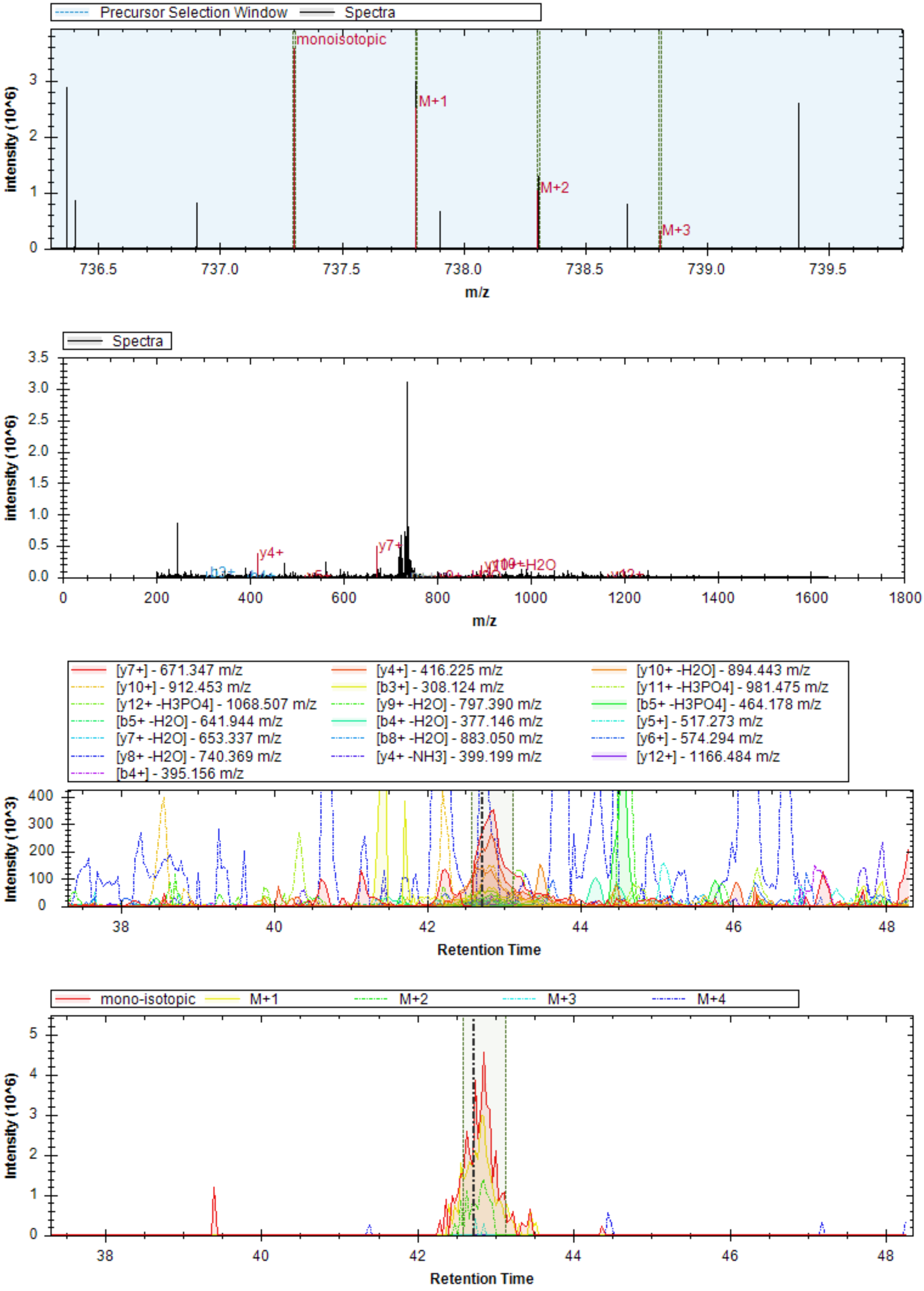

P10637 – _SGYSSPGS[+H+O3+P]PGTPGSR_.2
Fig4_MP-DIA-barrel-cortex-p30-2_MHRM.htrms

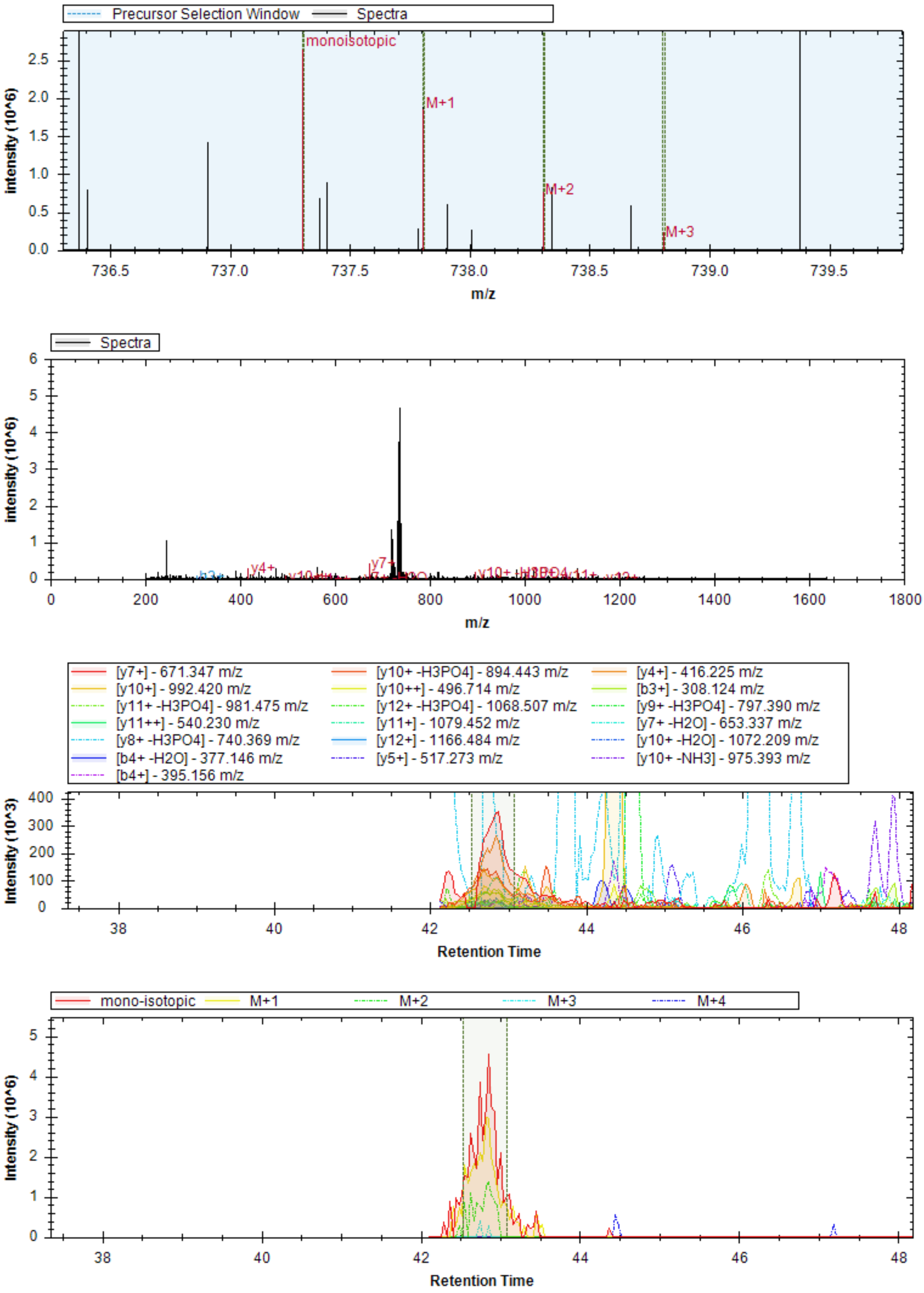

Q8C5R2 – _LAGNEALS[+H+O3+P]PTSPSK_.2
Fig4_MP-DIA-barrel-cortex-p30-2_MHRM.htrms

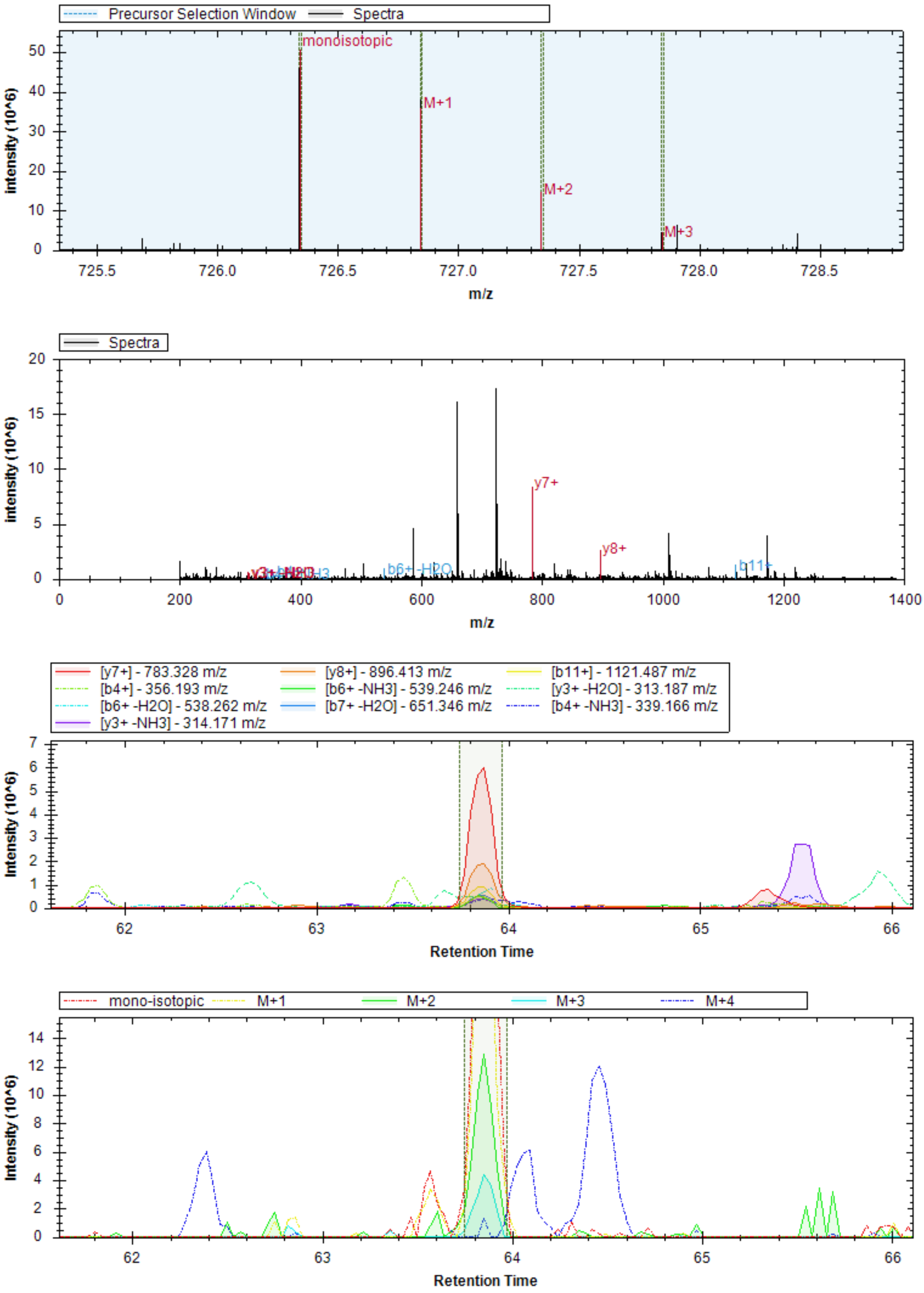

Q9JLM8 – _SPSPSPT[+H+O3+P]SPGSLRK_.2
Fig4_MP-DIA-barrel-cortex-p30-2_MHRM.htrms

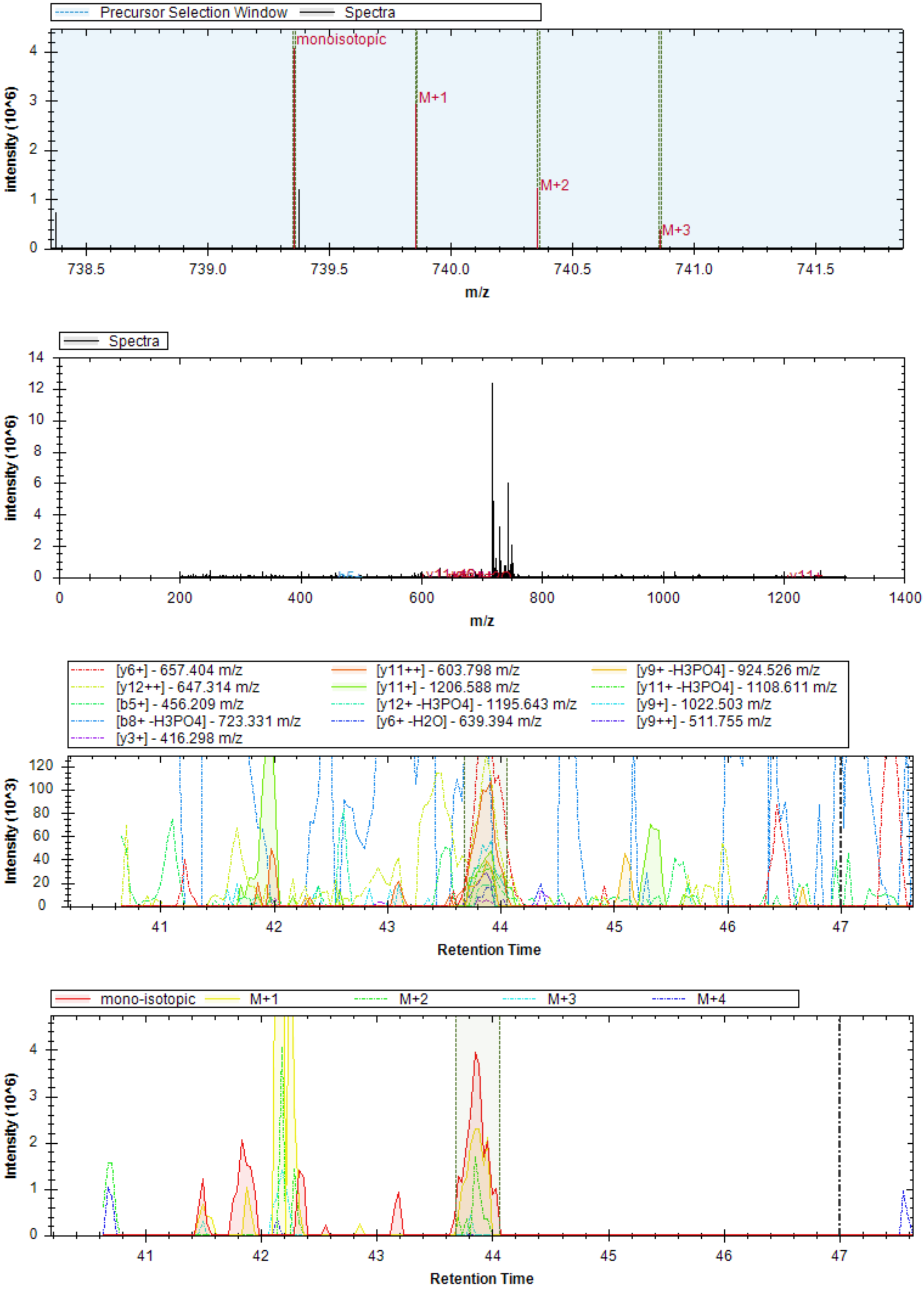

Q9JLM8 – _SPSPSPTS[+H+O3+P]PGSLRK_.2
Fig4_MP-DIA-barrel-cortex-p30-2_MHRM.htrms

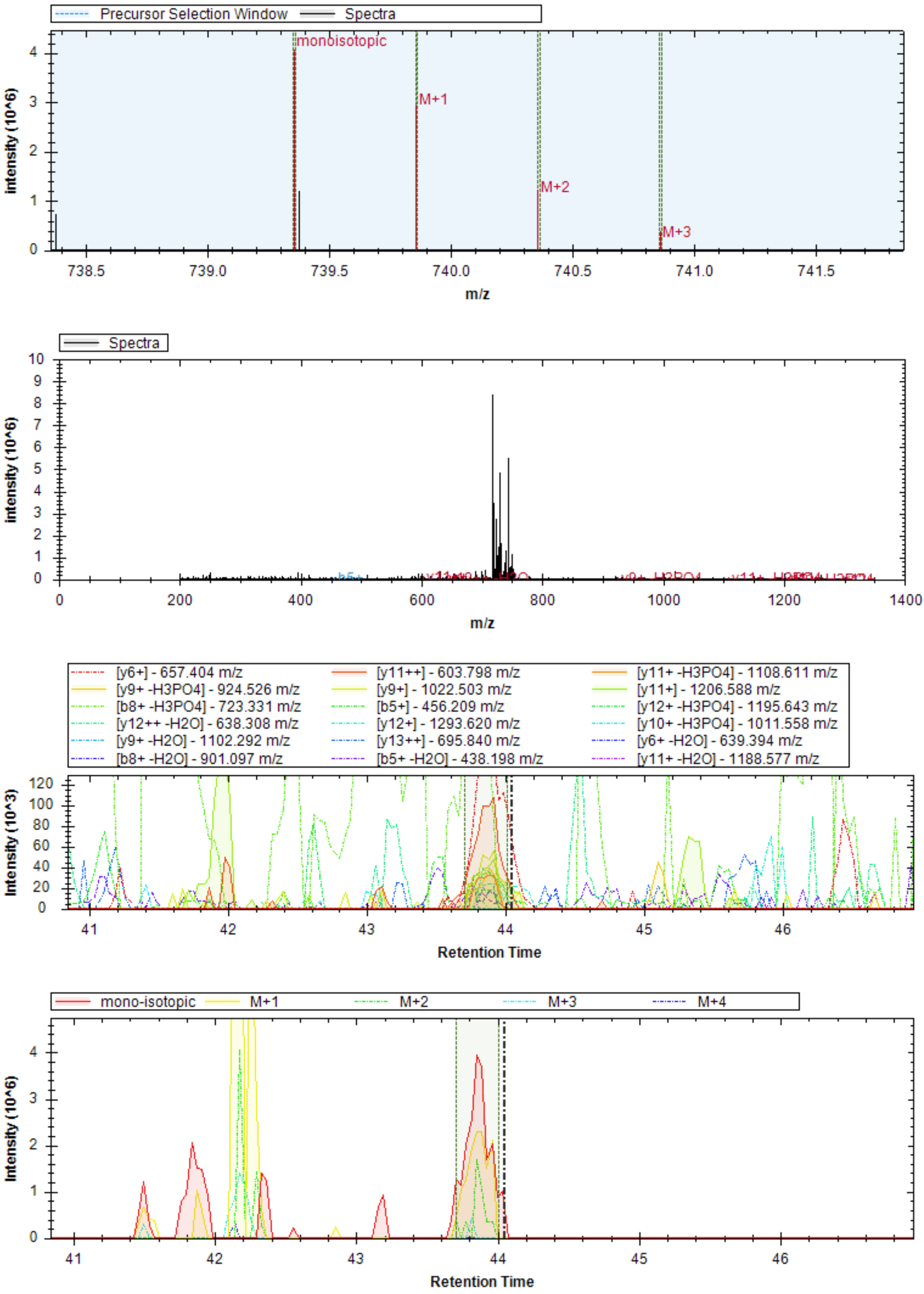

D3Z6S9 – _IPSIEIT[+H+O3+P]ET[+H+O3+P]LPQTQASMLR_.3
Fig4_MP-DIA-barrel-cortex-p30-2_MHRM.htrms

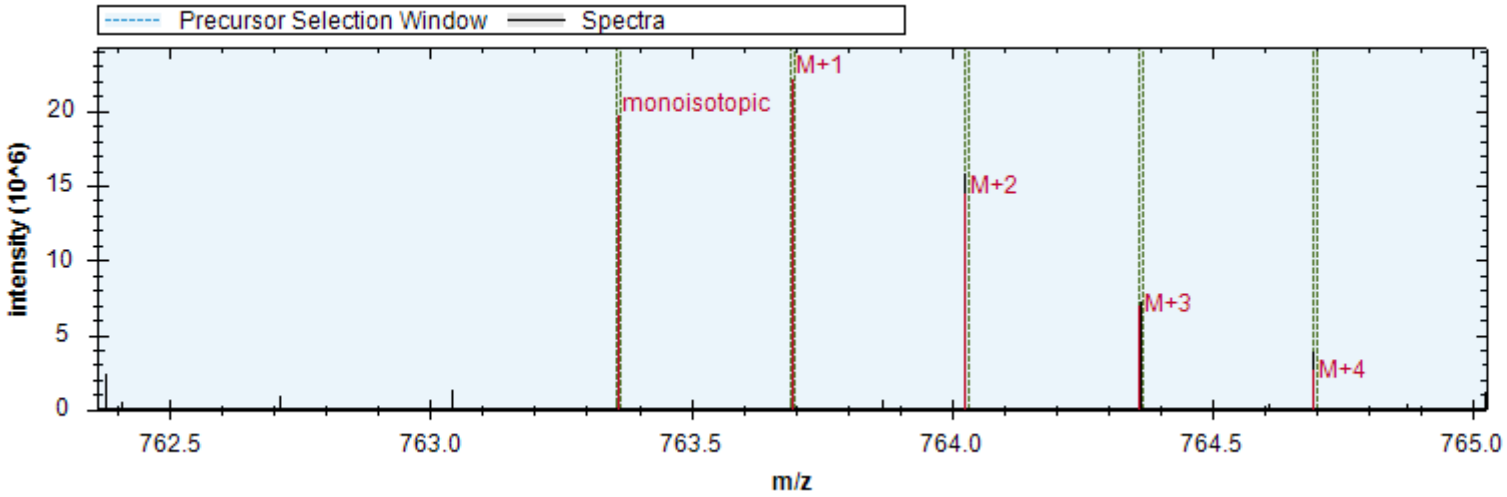

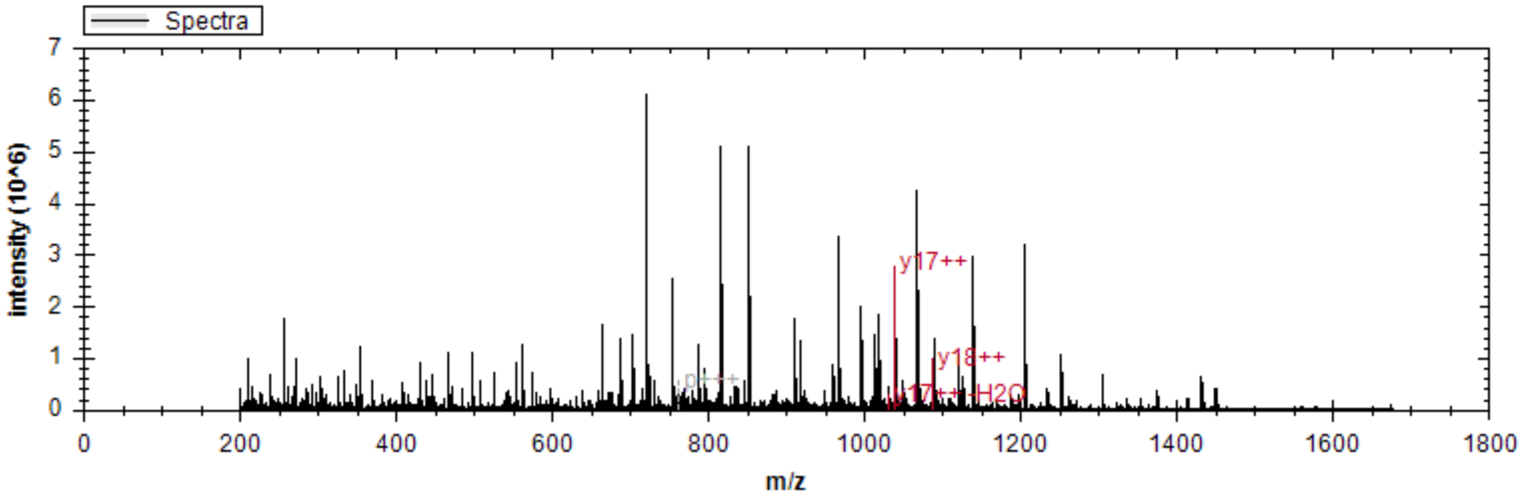

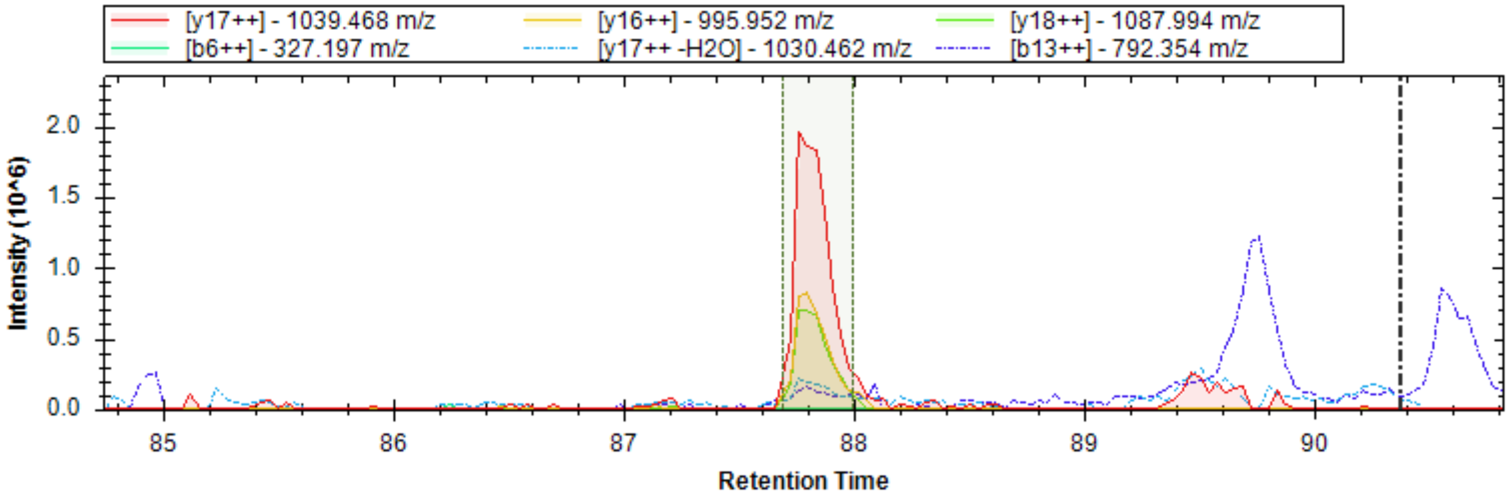

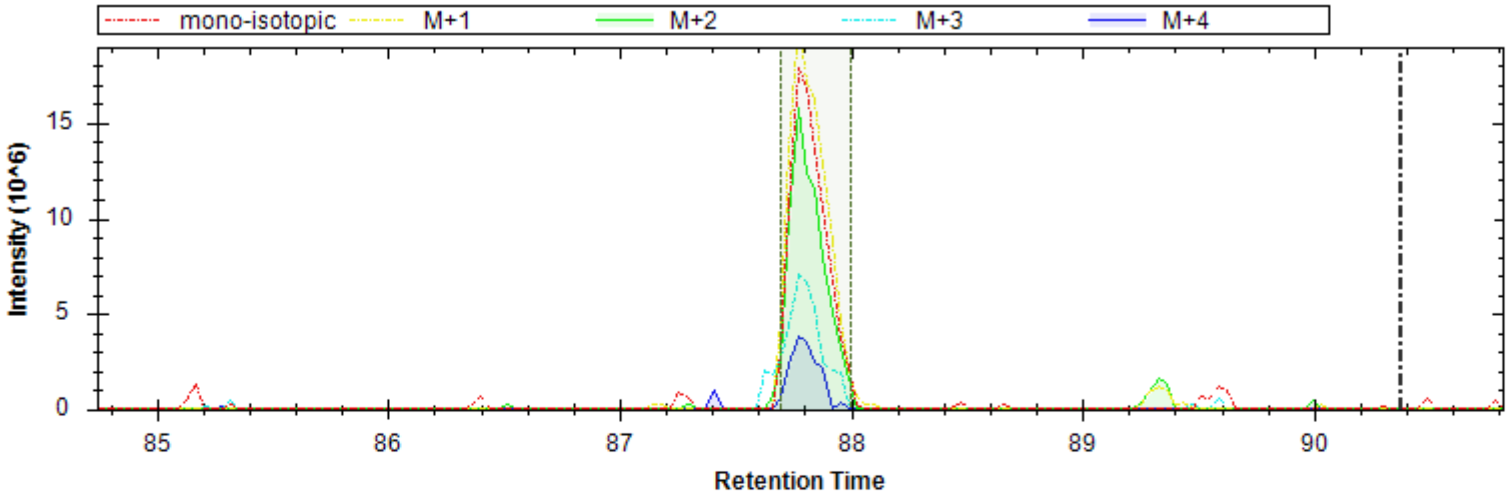

P0C6F1 – _IIDLQM[+O]HDY[+H+O3+P]LR_.2
Fig4_MP-DIA-barrel-cortex-p30-2_MHRM.htrms

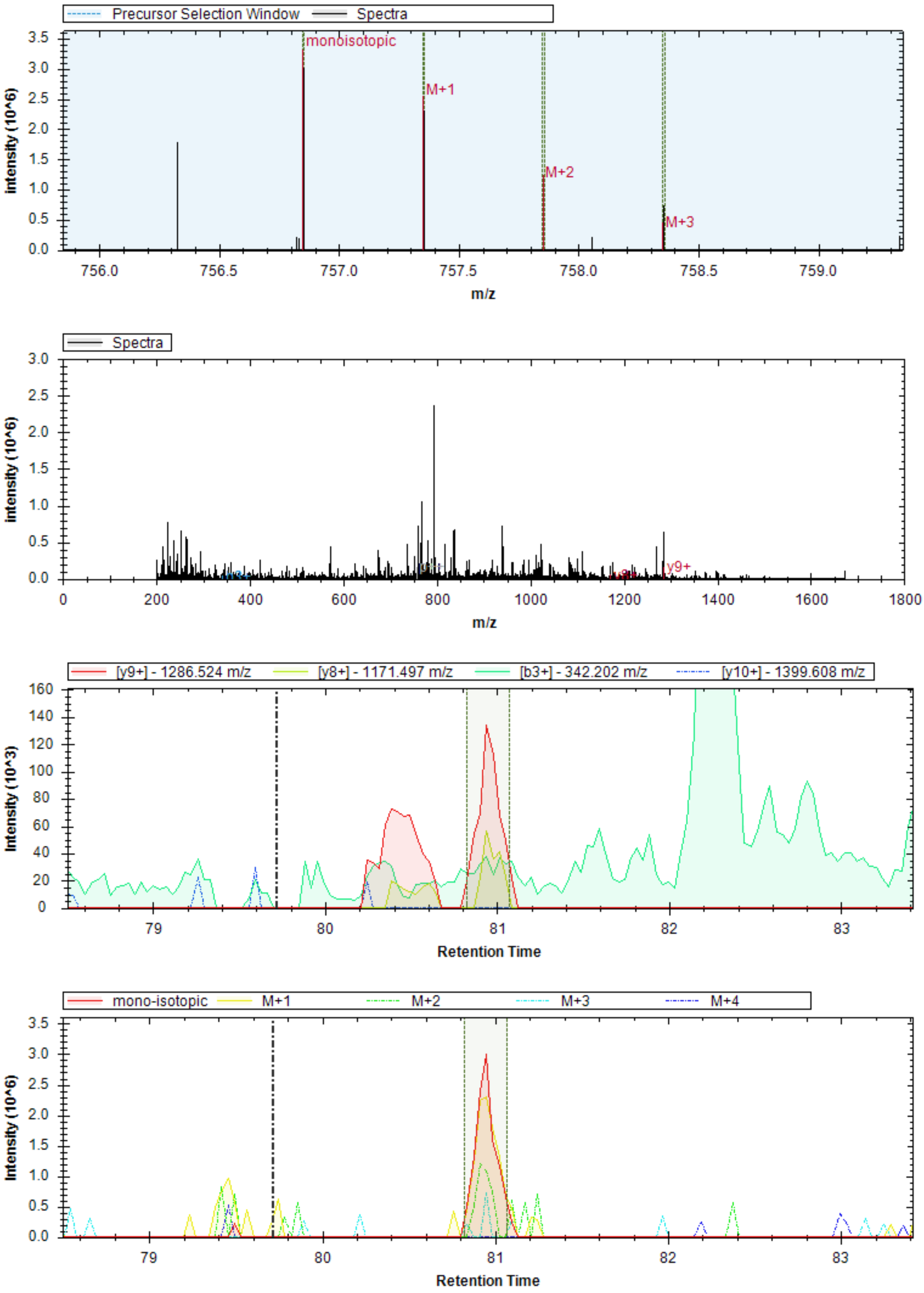

P57776-3 – _ATAPQTQHVS[+H+O3+P]PM[+O]R_.2
Fig4_MP-DIA-barrel-cortex-p30-2_MHRM.htrms

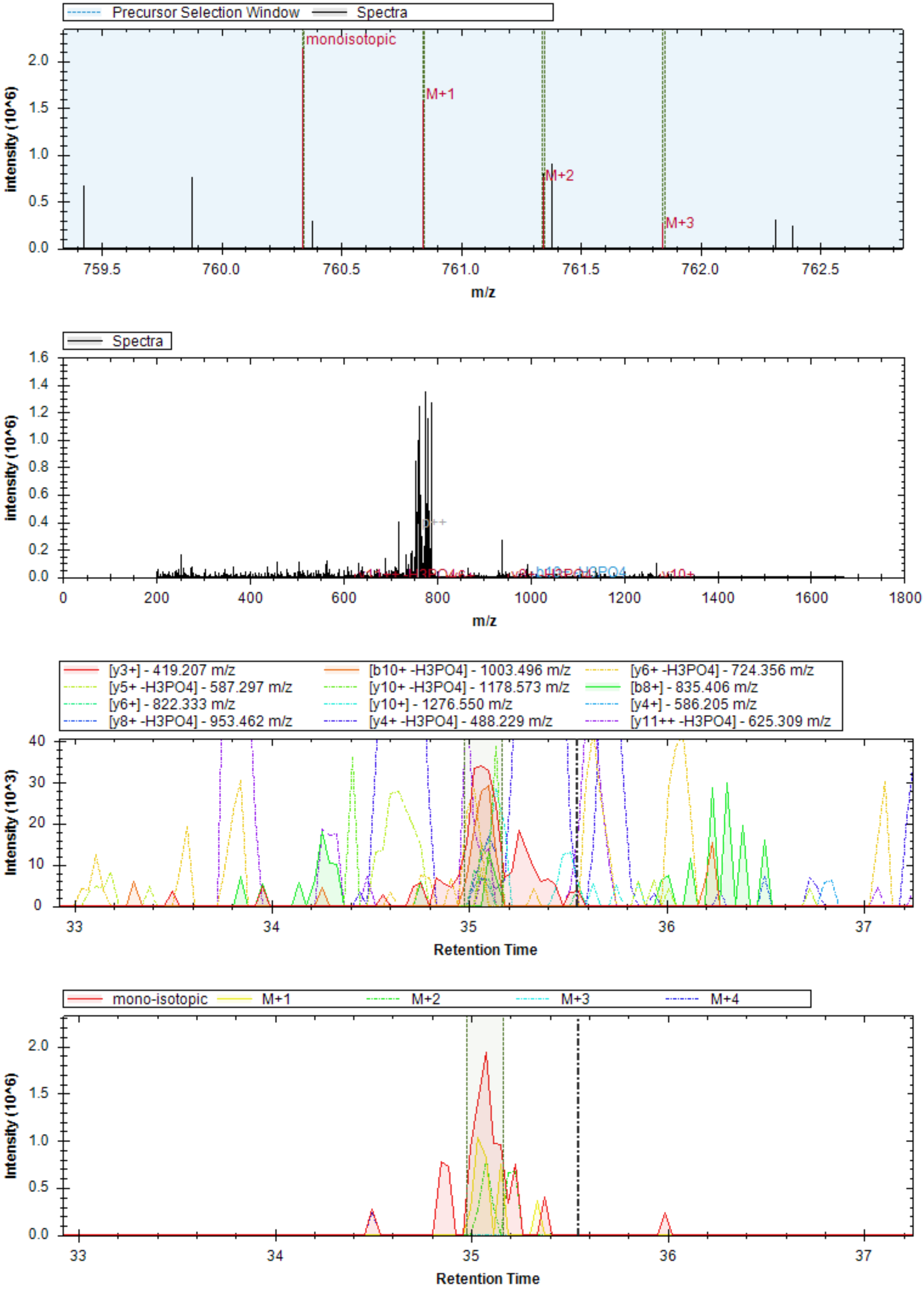

Q68FD9 – _LAQLLS[+H+O3+P]EVPT[+H+O3+P]RR_.2

Fig4_MP-DIA-barrel-cortex-p30-2_MHRM.htrms

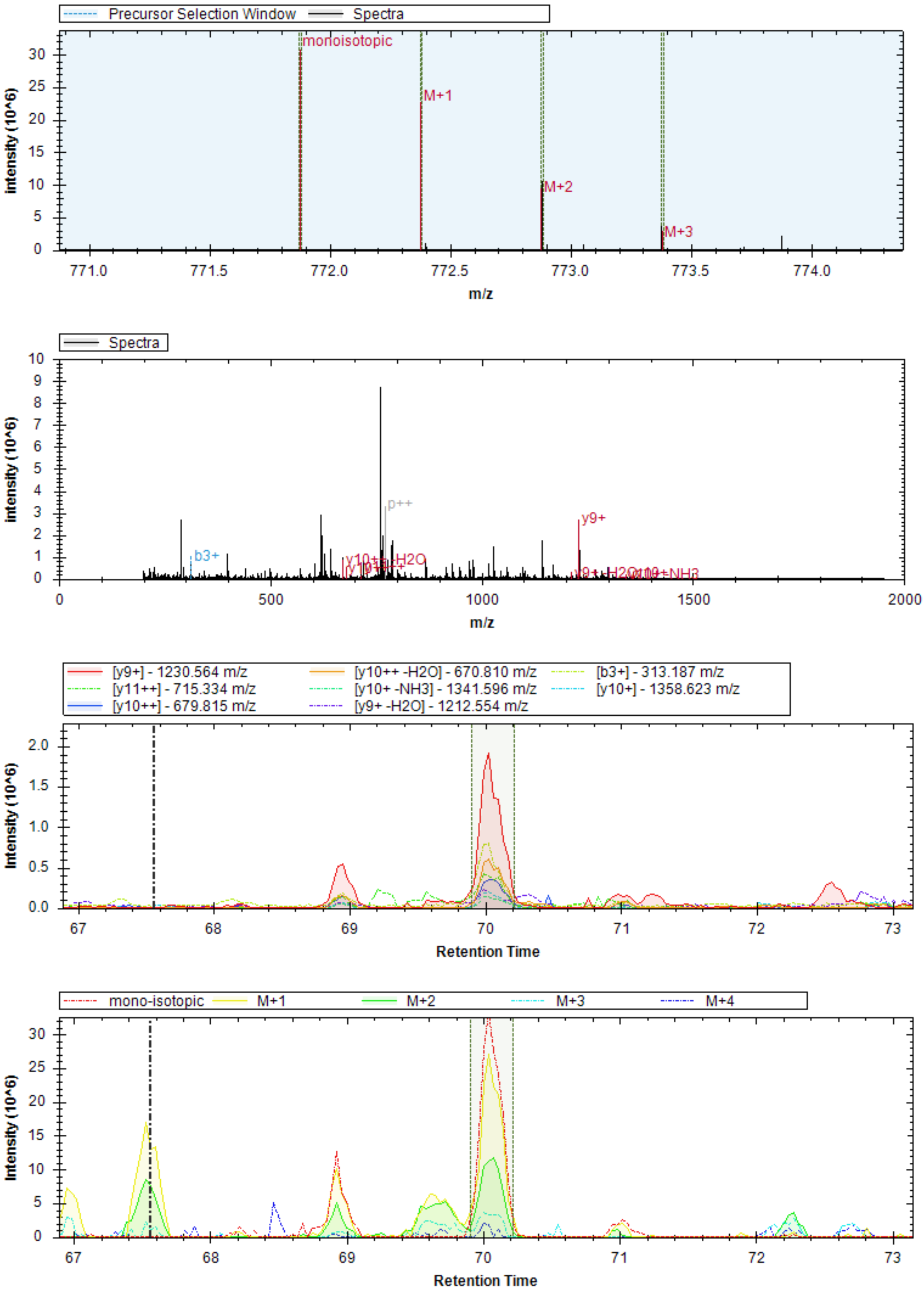

Q8BRT1 – _VLNT[+H+O3+P]GSDVEEAVADALLLGDIR_.3
Fig4_MP-DIA-barrel-cortex-p30-2_MHRM.htrms

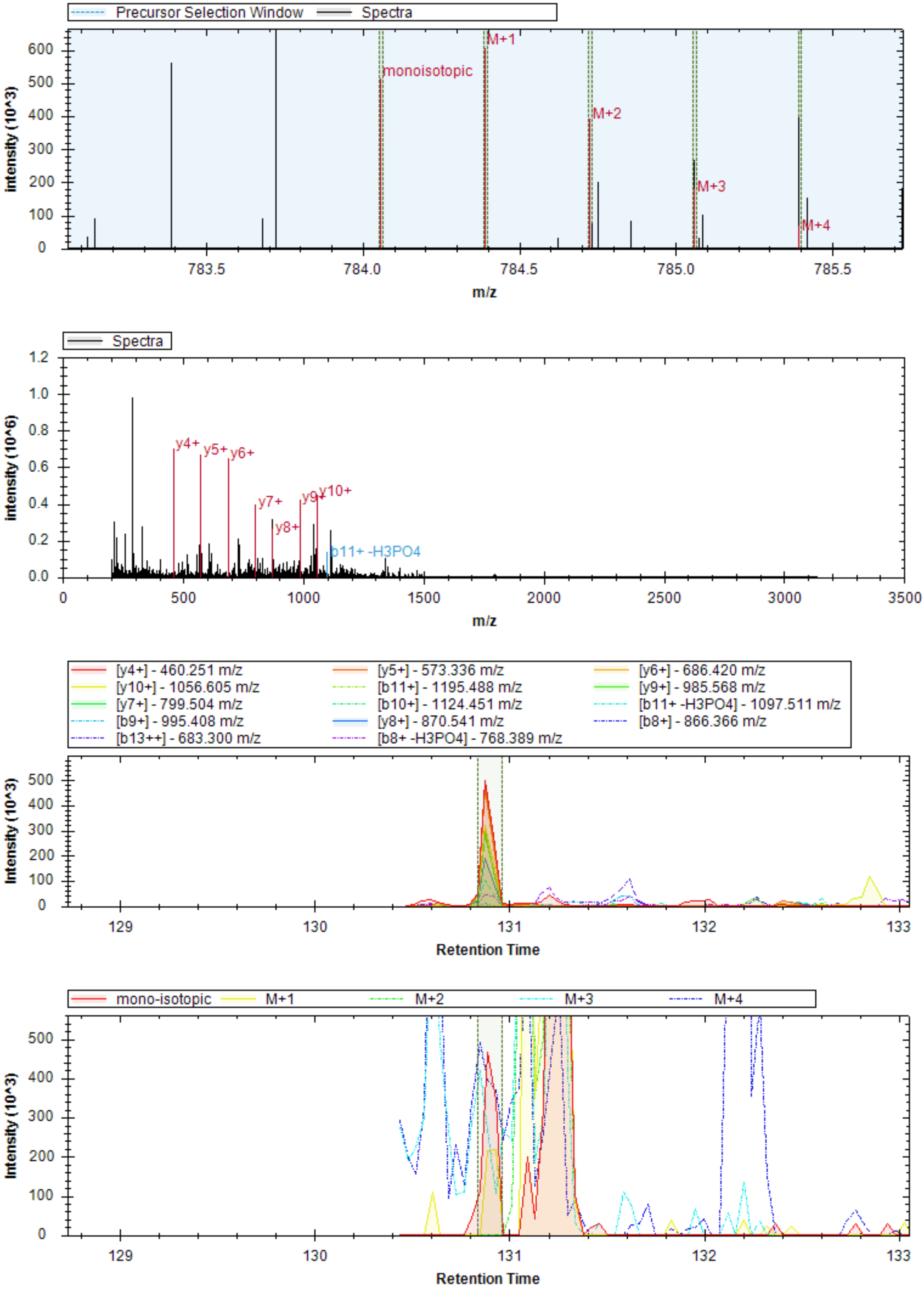

Q9JLM8 – _ISQHGGS[+H+O3+P]STSLSSTK_.2
Fig4_MP-DIA-barrel-cortex-p30-2_MHRM.htrms

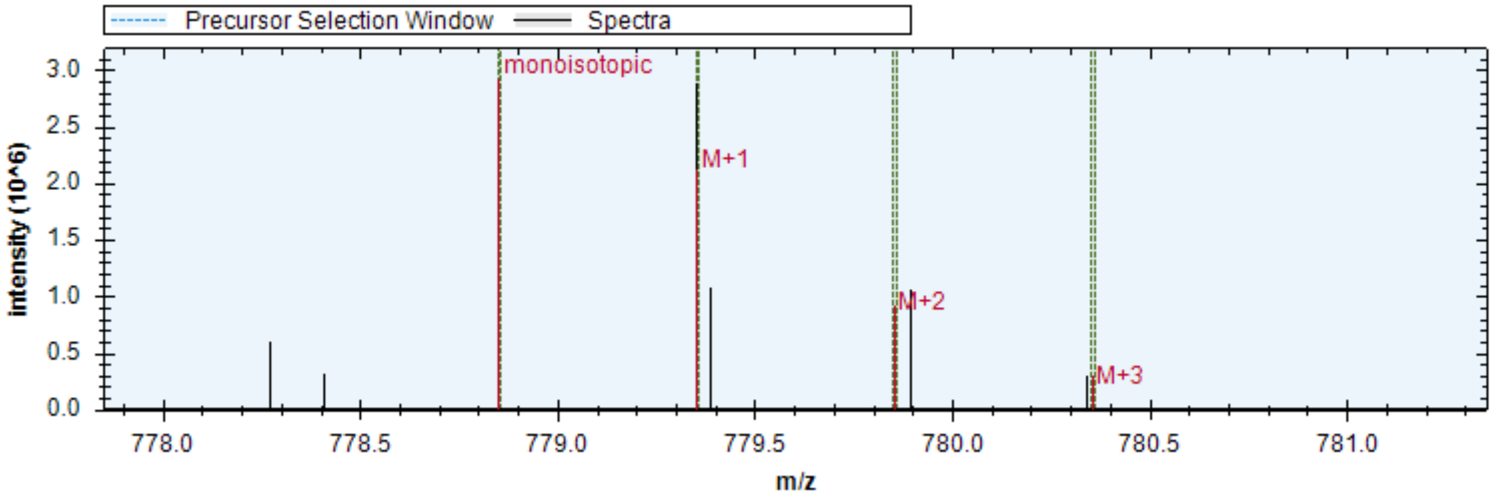

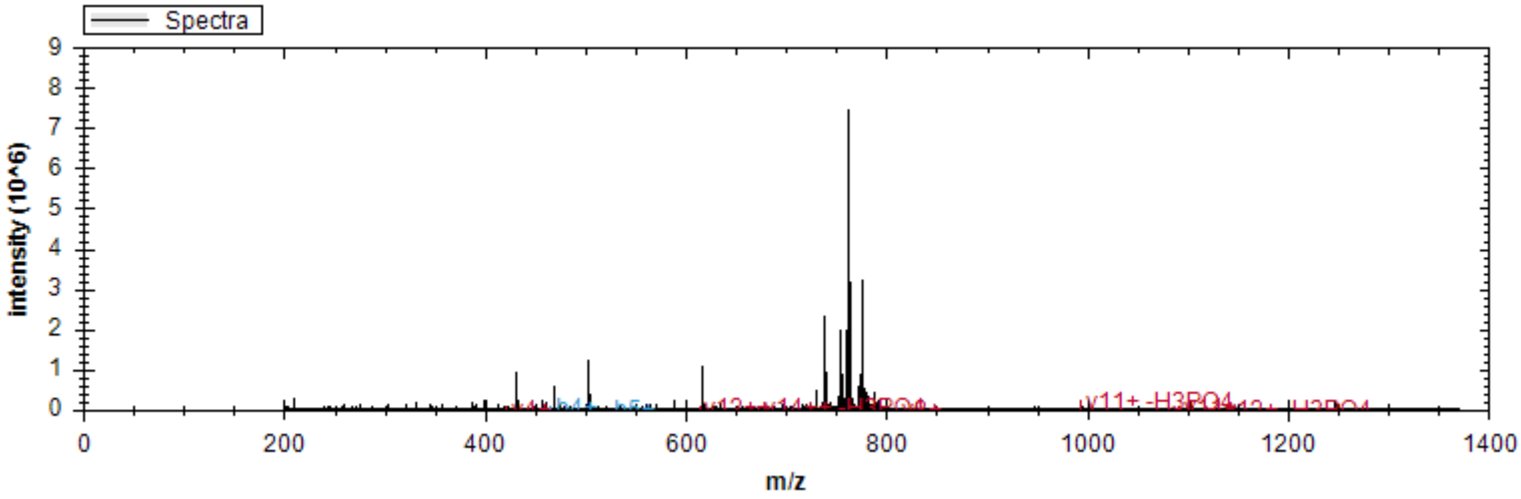

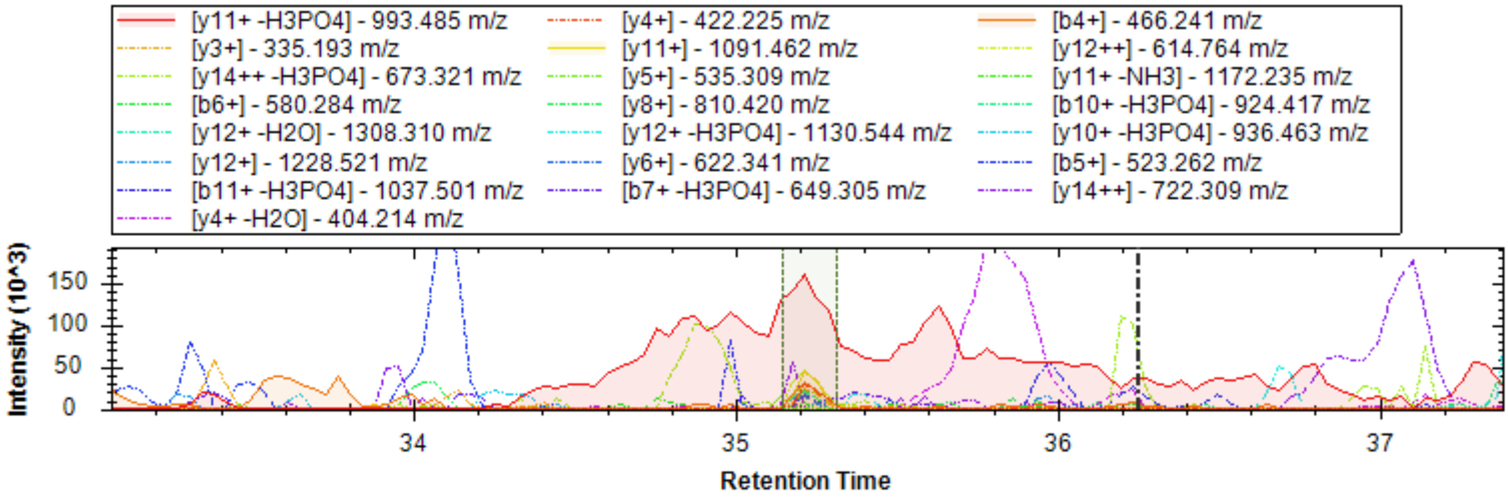

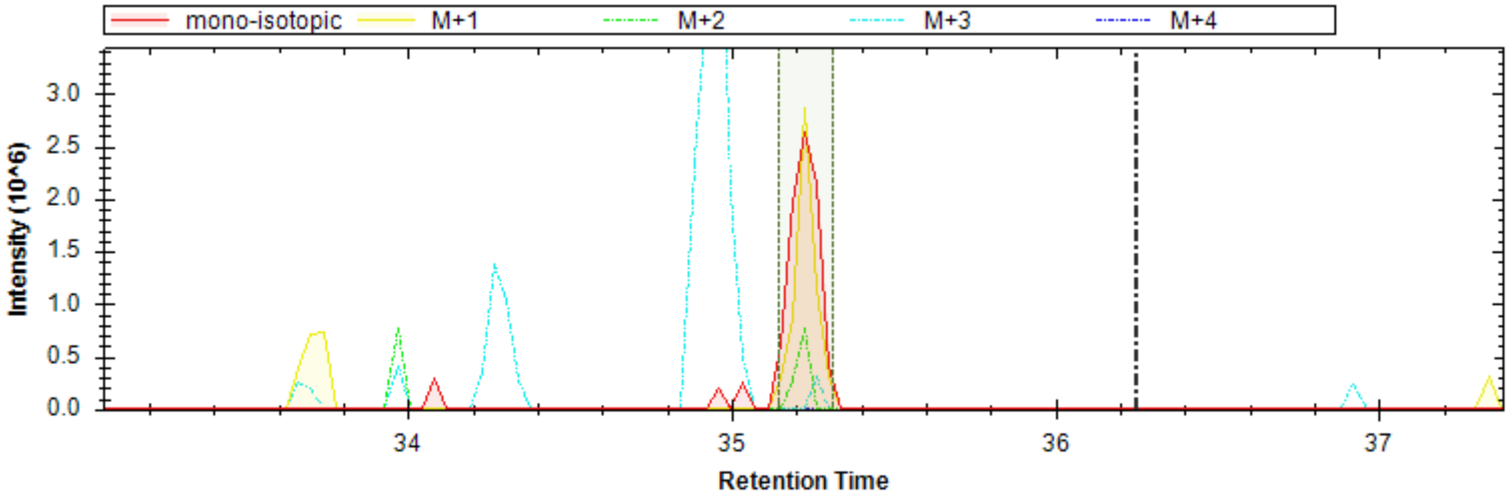

Q9QWI6 – _LSYAGGRPPSYAGS[+H+O3+P]PVHHAAER_.3
Fig4_MP-DIA-barrel-cortex-p30-2_MHRM.htrms

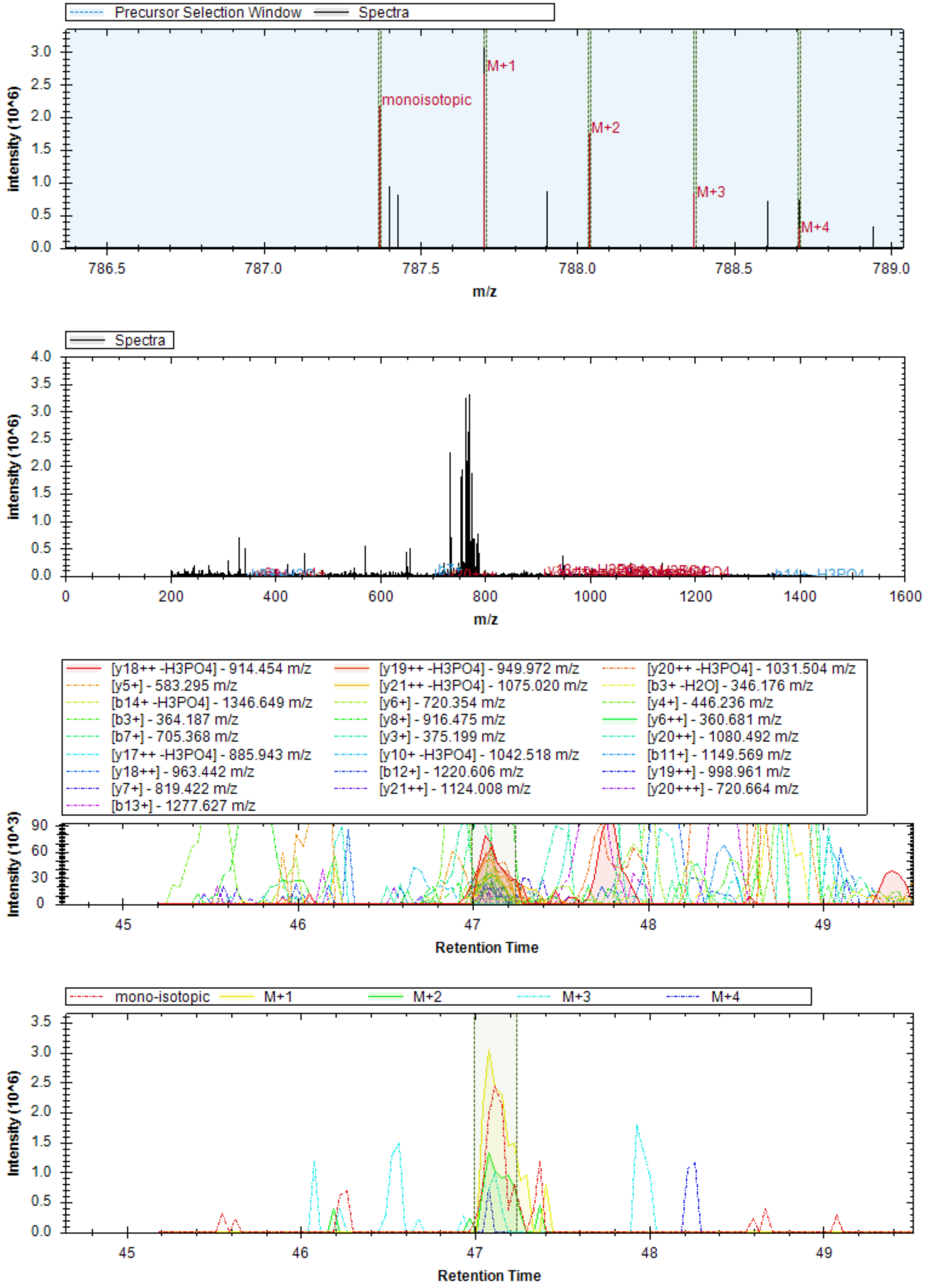

Q9QYC0 – _AAVVTS[+H+O3+P]PPPTTAPHK_.2
Fig4_MP-DIA-barrel-cortex-p30-2_MHRM.htrms

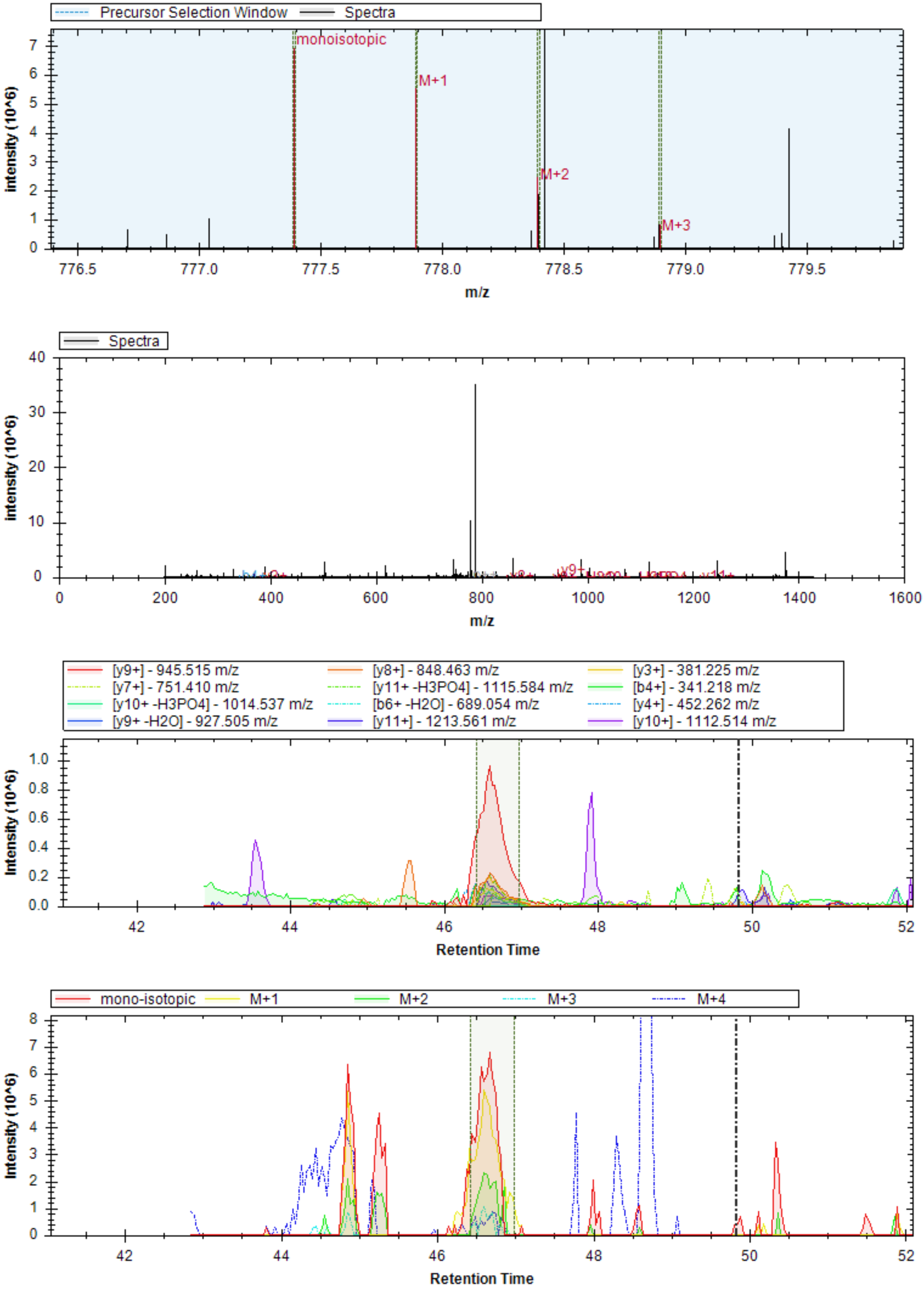

Q9QYR6-2 – _ELSSEPRT[+H+O3+P]PPAQK_.2

Fig4_MP-DIA-barrel-cortex-p30-2_MHRM.htrms

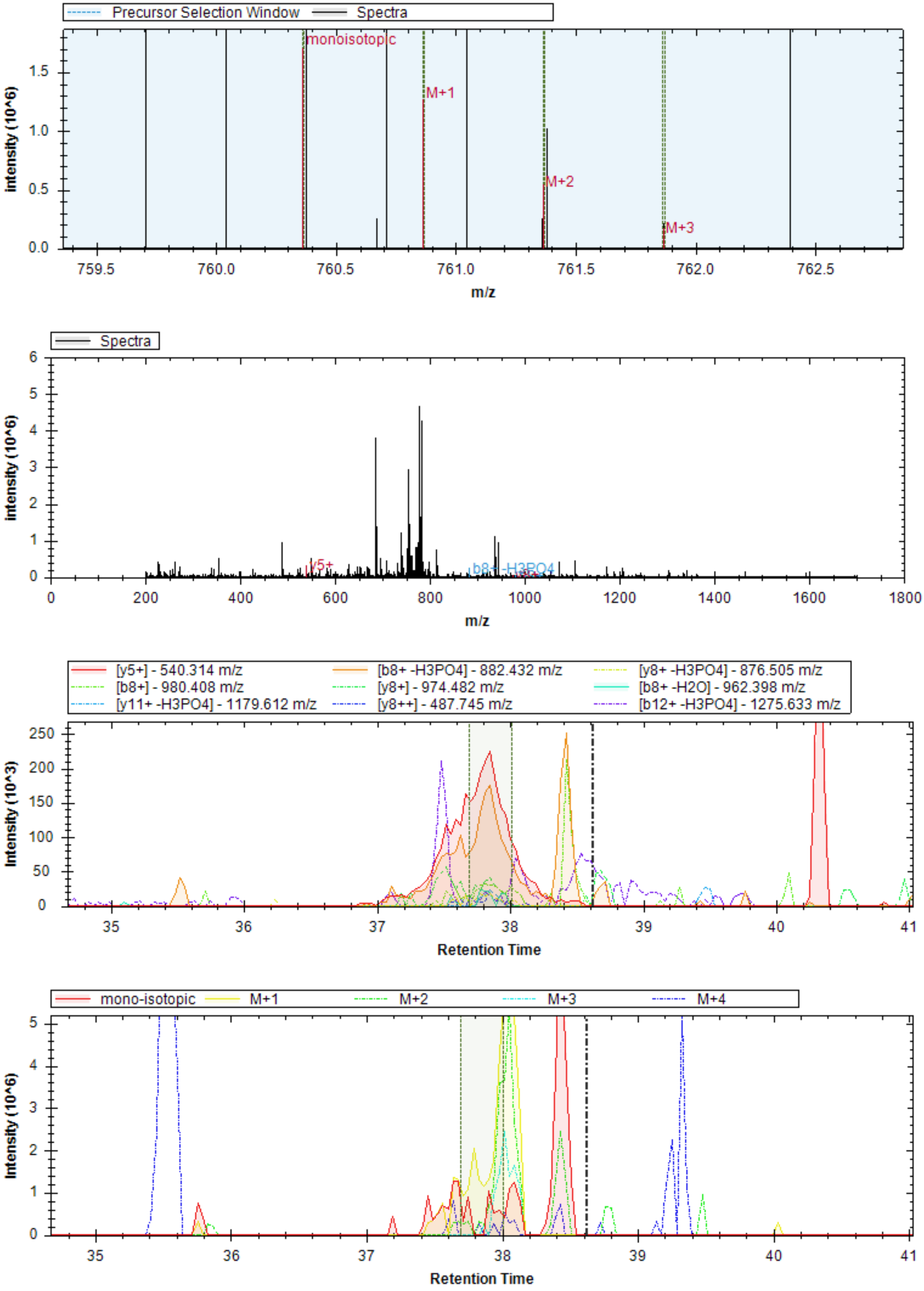

P08249 – _NS[+H+O3+P]PLVSRLTLYDIAHTPGVAADLSHIETR_.4
Fig4_MP-DIA-barrel-cortex-p30-2_MHRM.htrms

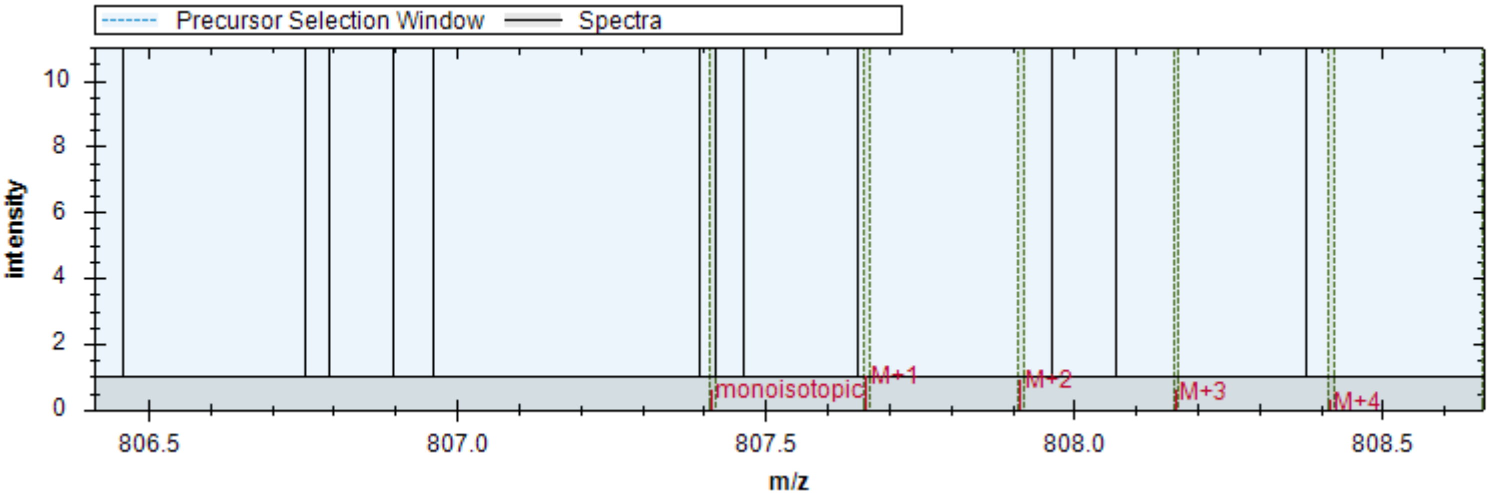

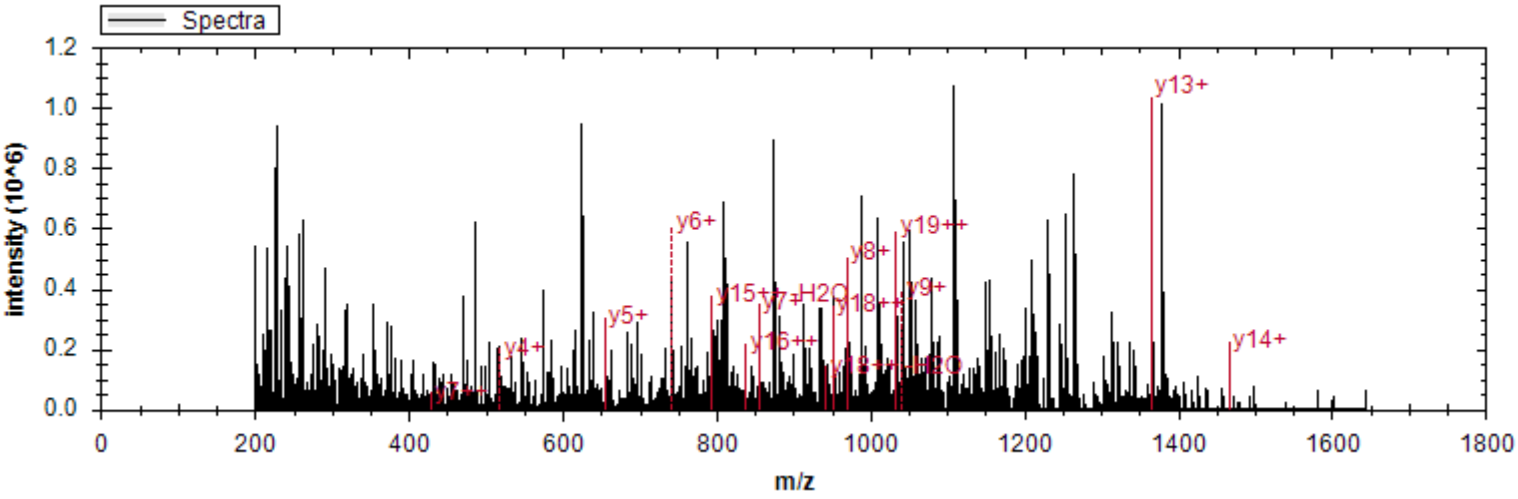

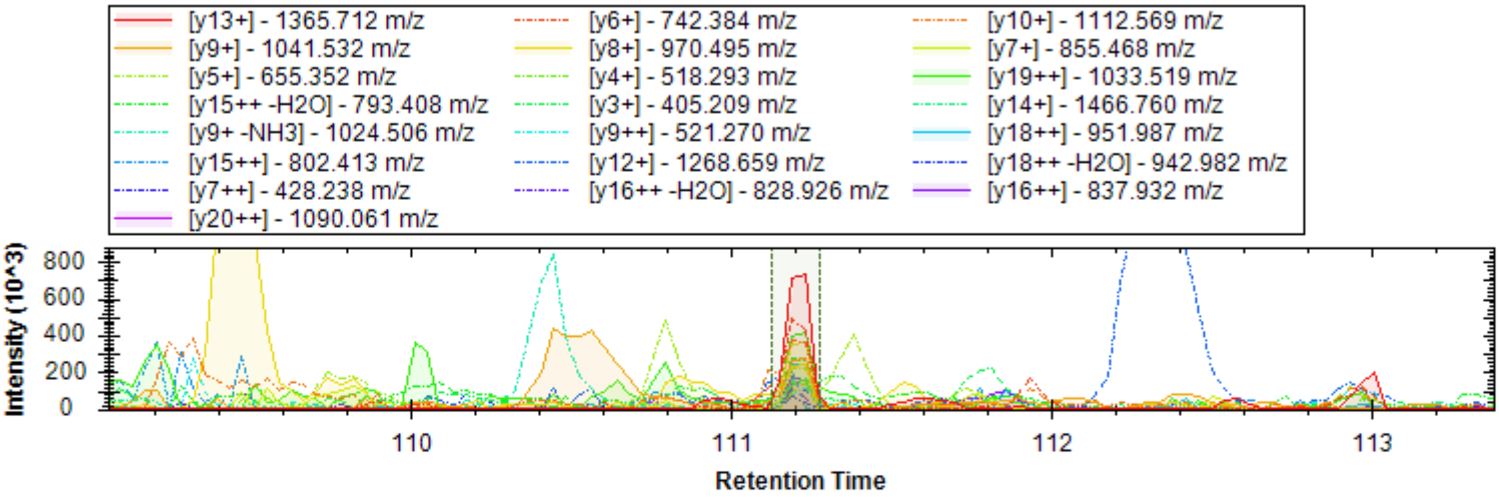

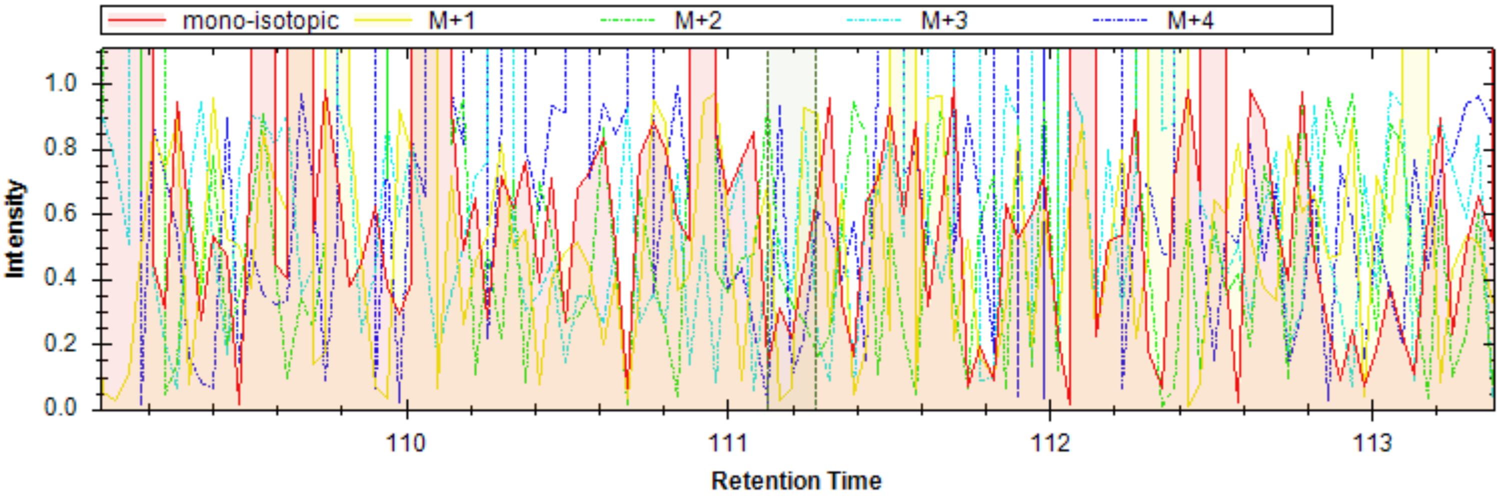

P10637 – _TTPSPKT[+H+O3+P]PPGSGEPPK_.2
Fig4_MP-DIA-barrel-cortex-p30-2_MHRM.htrms

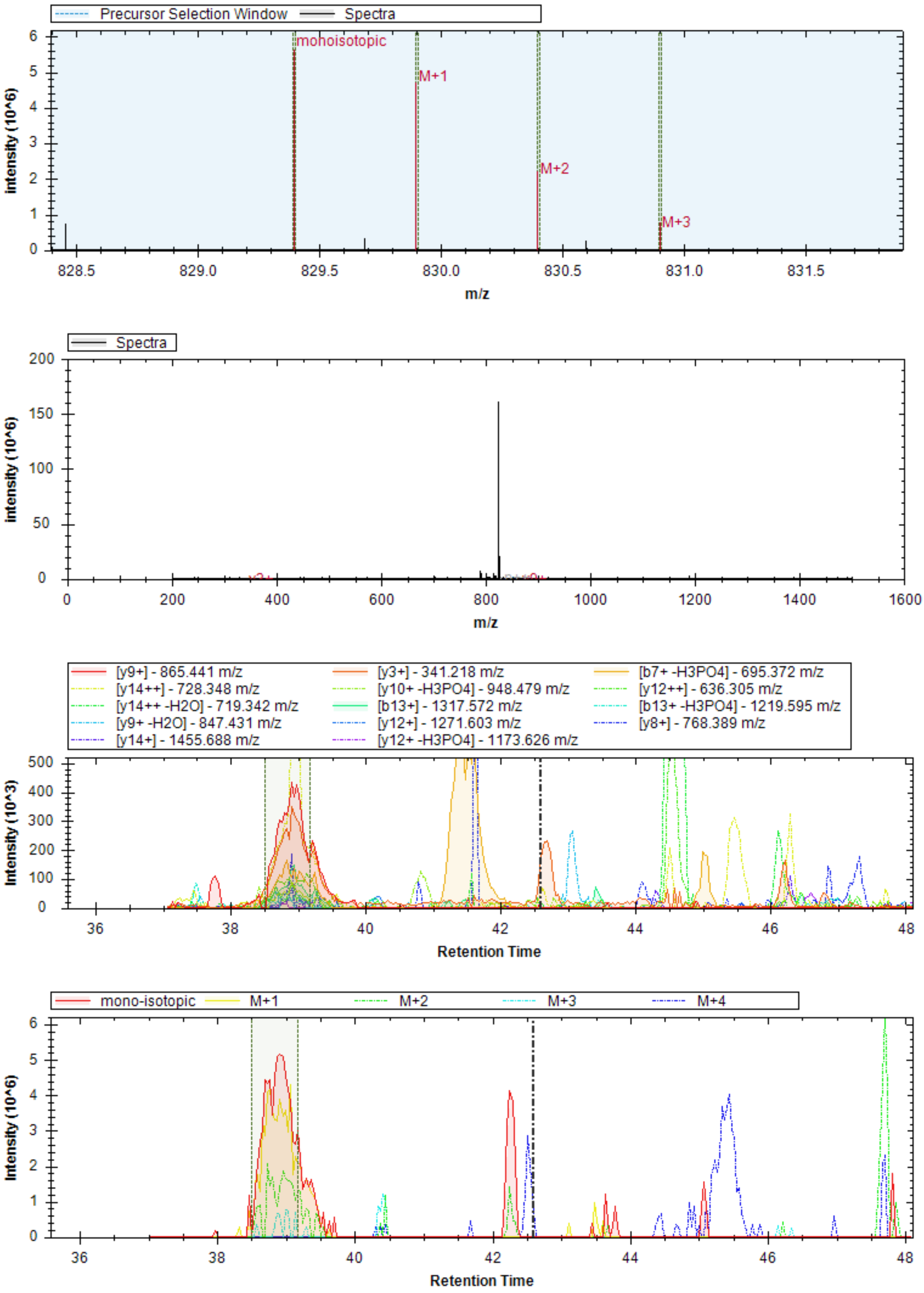

Q60771 – _FYYSS[+H+O3+P]GSSSPTHAK_.2
Fig4_MP-DIA-barrel-cortex-p30-2_MHRM.htrms

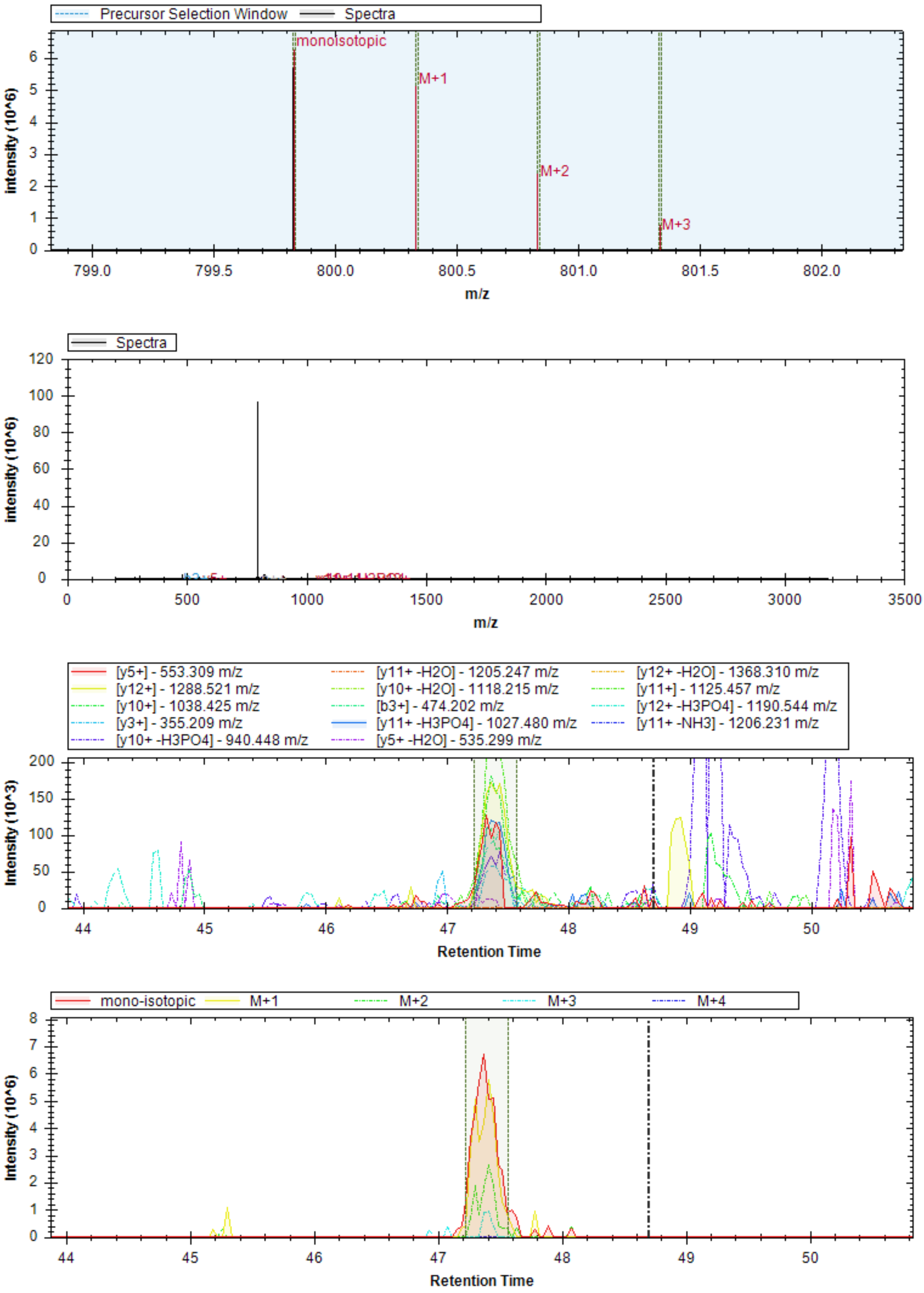

Q60771 – _FYYSSGS[+H+O3+P]SSPTHAK_.2
Fig4_MP-DIA-barrel-cortex-p30-2_MHRM.htrms

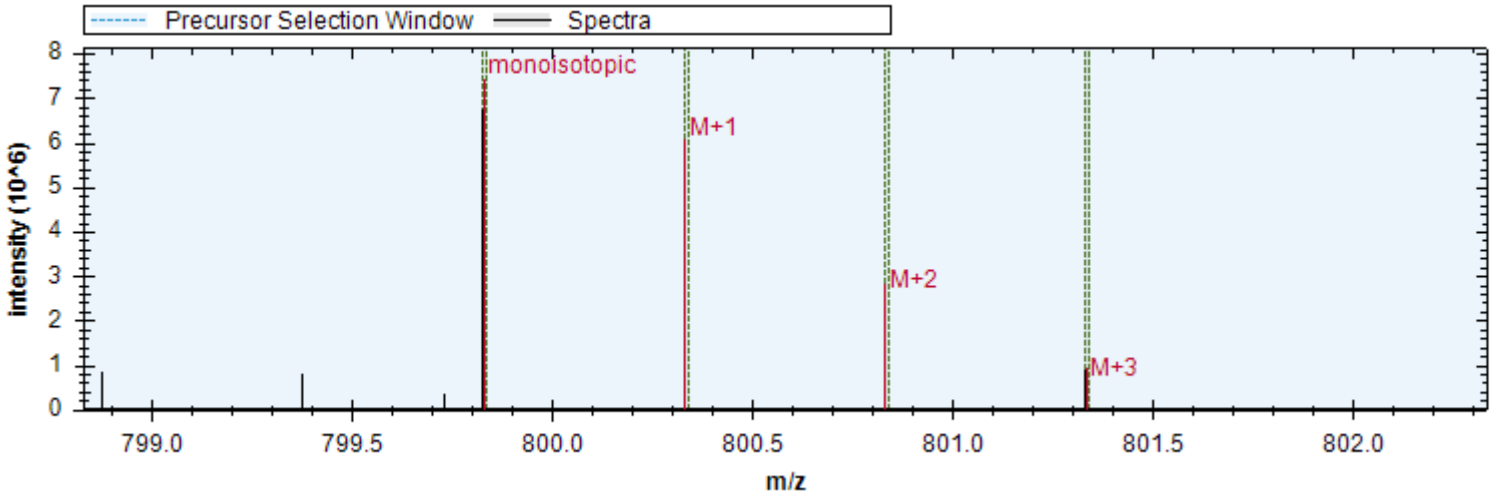

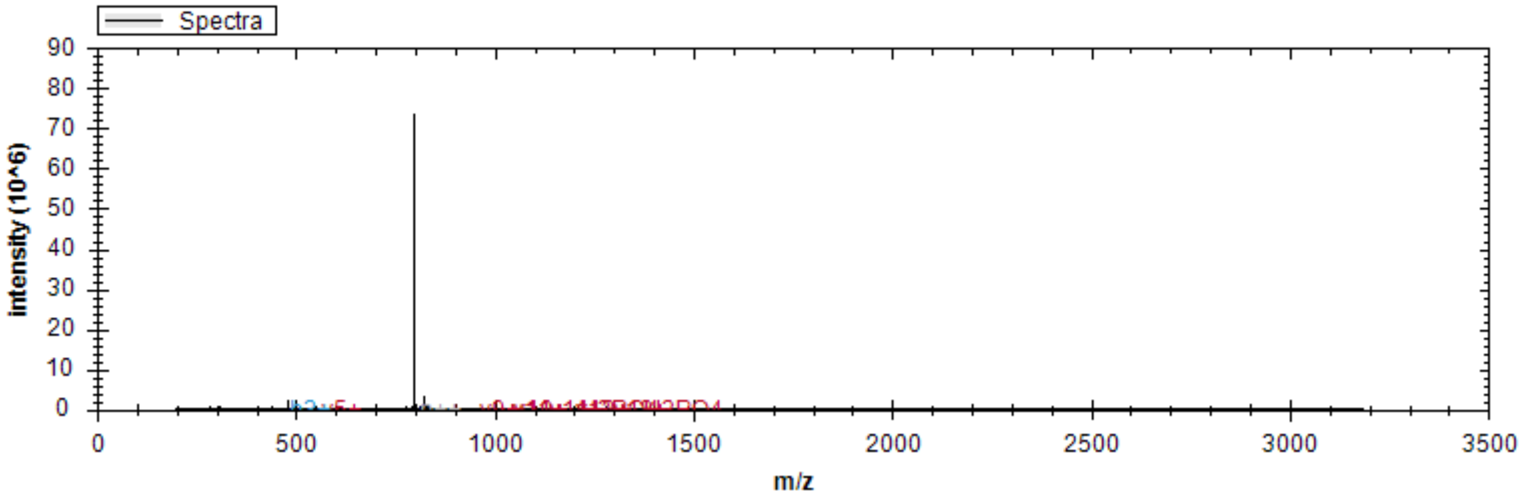

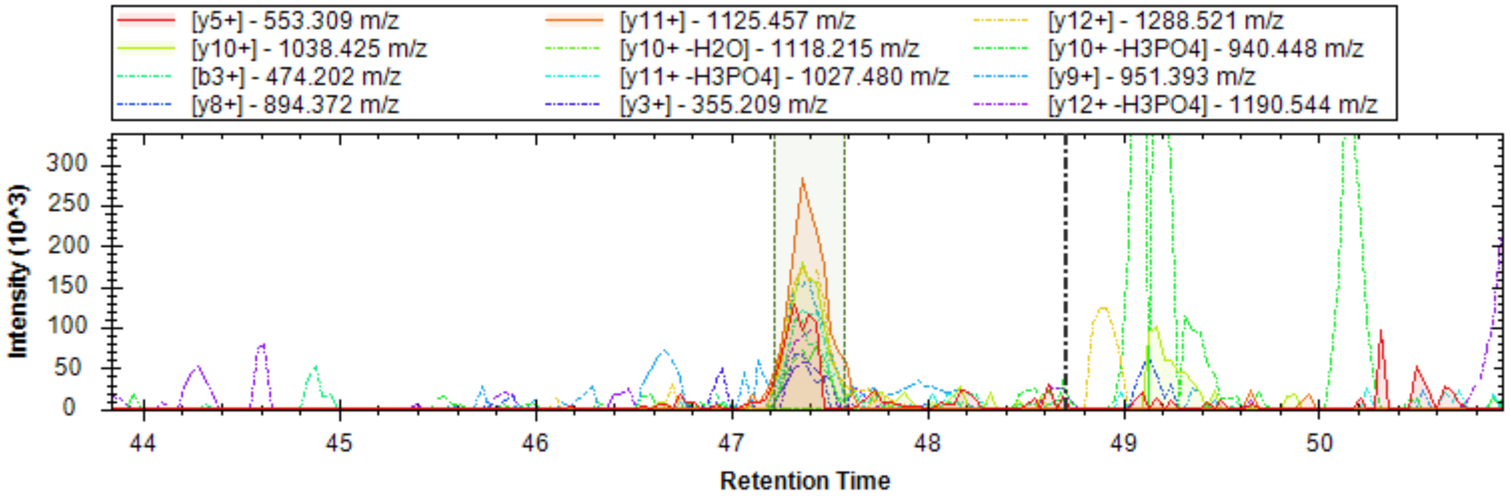

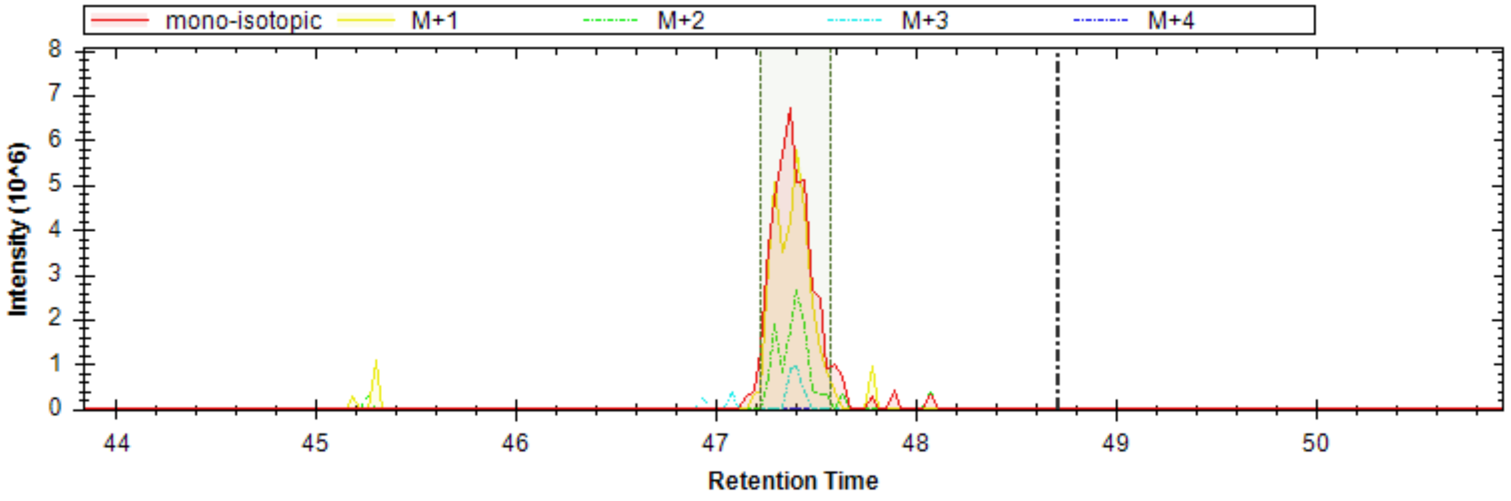

Q60771 – _FYYSSGSSS[+H+O3+P]PTHAK_.2
Fig4_MP-DIA-barrel-cortex-p30-2_MHRM.htrms

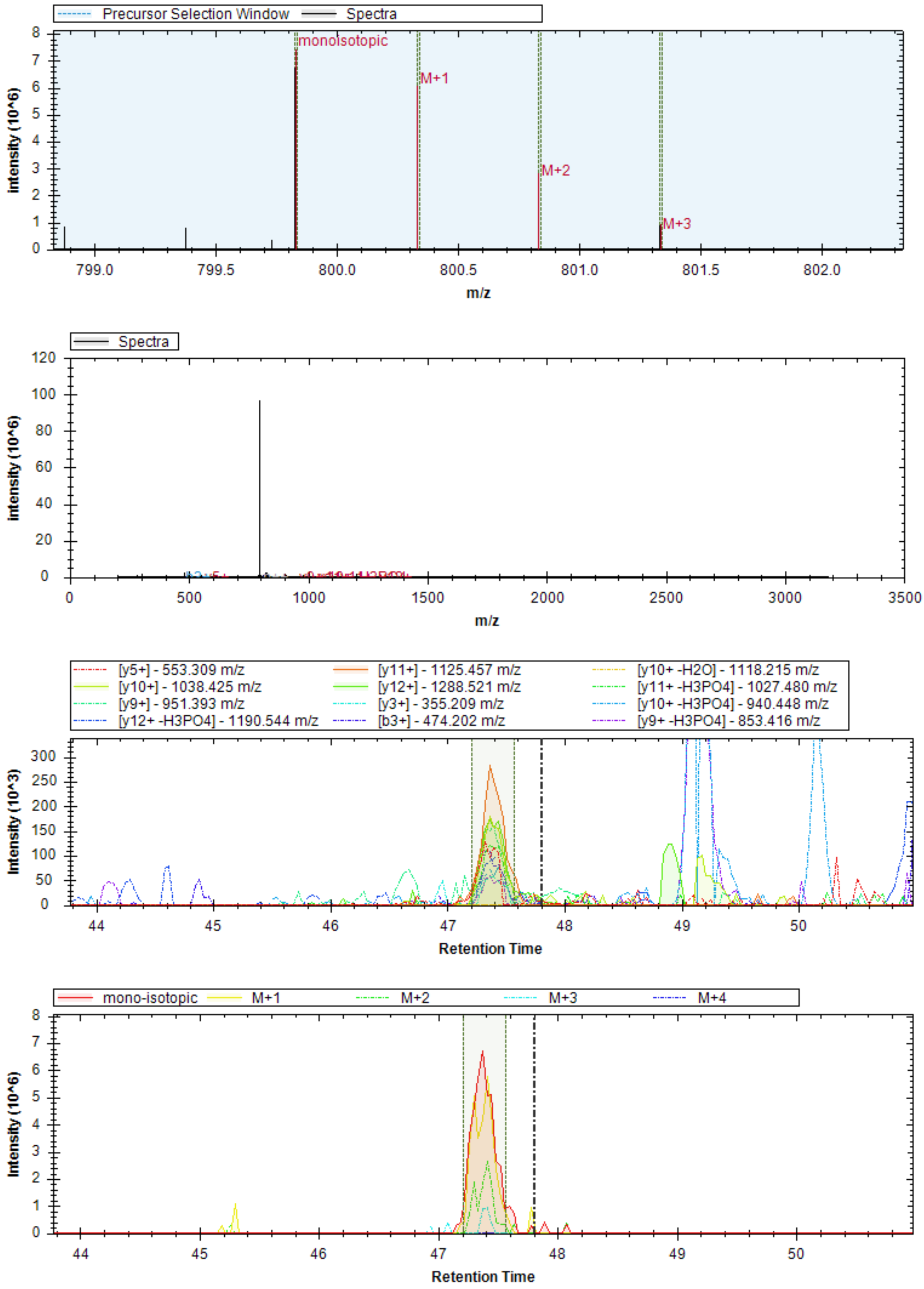

Q64701;Q64701-2 – _LAEILYYKILET[+H+O3+P]IMVQET[+H+O3+P]R_.3
Fig4_MP-DIA-barrel-cortex-p30-2_MHRM.htrms

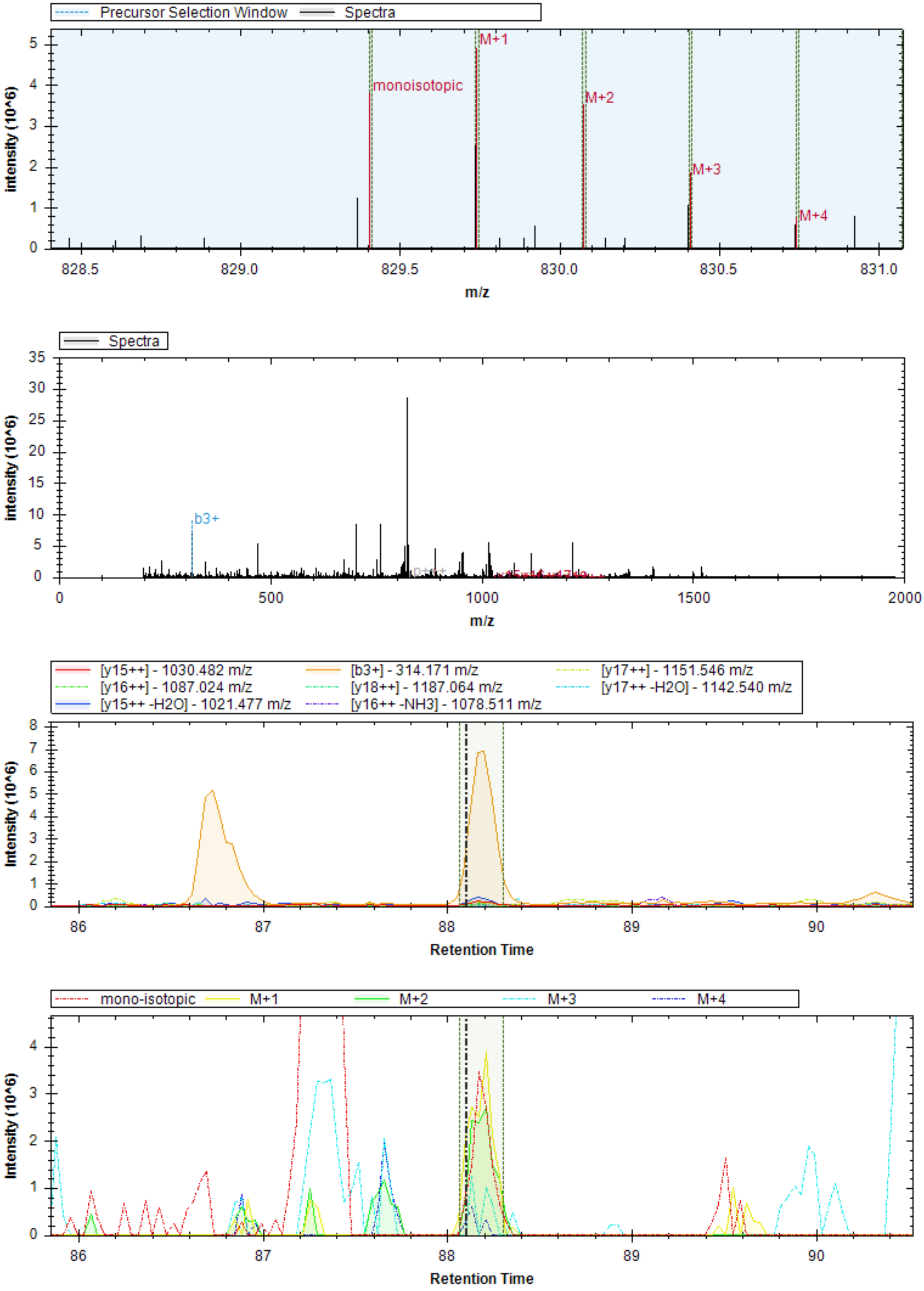

Q7TQD2 – _AANKT[+H+O3+P]PPKSPGDPAR_.2
Fig4_MP-DIA-barrel-cortex-p30-2_MHRM.htrms

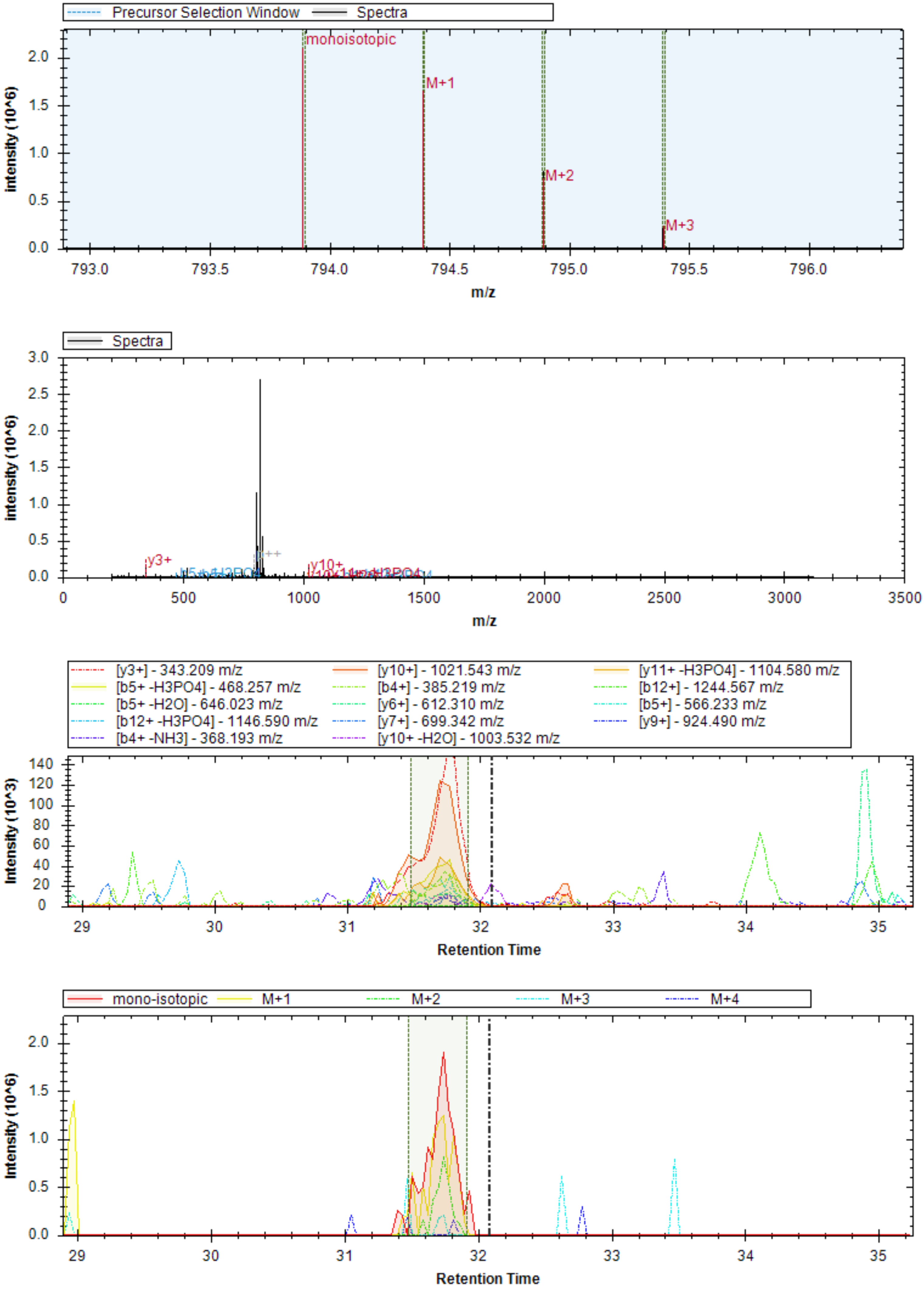

O08784 – _ASAVSPEKAPM[+O]T[+H+O3+P]SKAK_.2

Fig4_MP-DIA-barrel-cortex-p30-2_MHRM.htrms

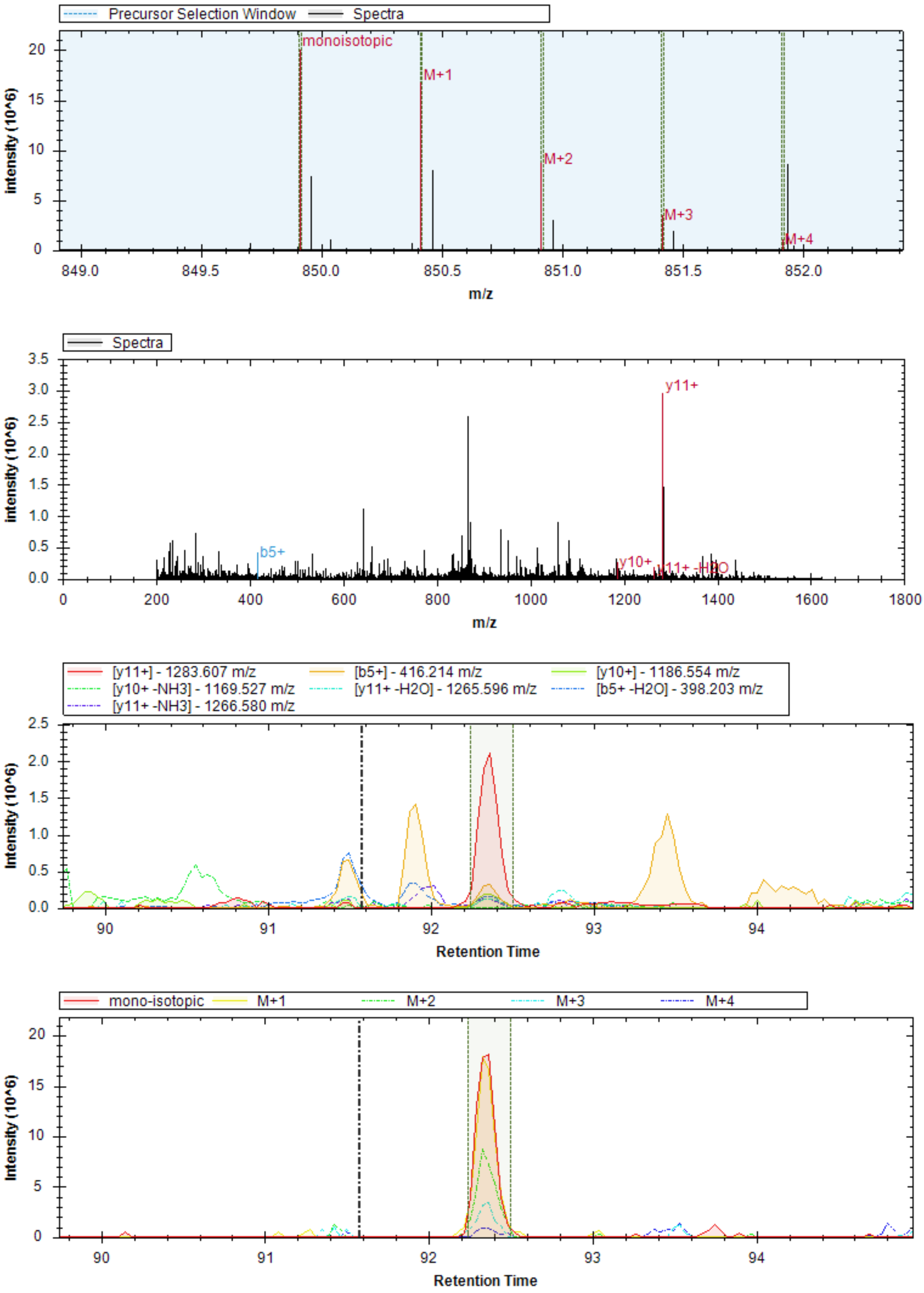

P50538 – _EAEHGYAS[+H+O3+P]M[+O]LPYSK_.2
Fig4_MP-DIA-barrel-cortex-p30-2_MHRM.htrms

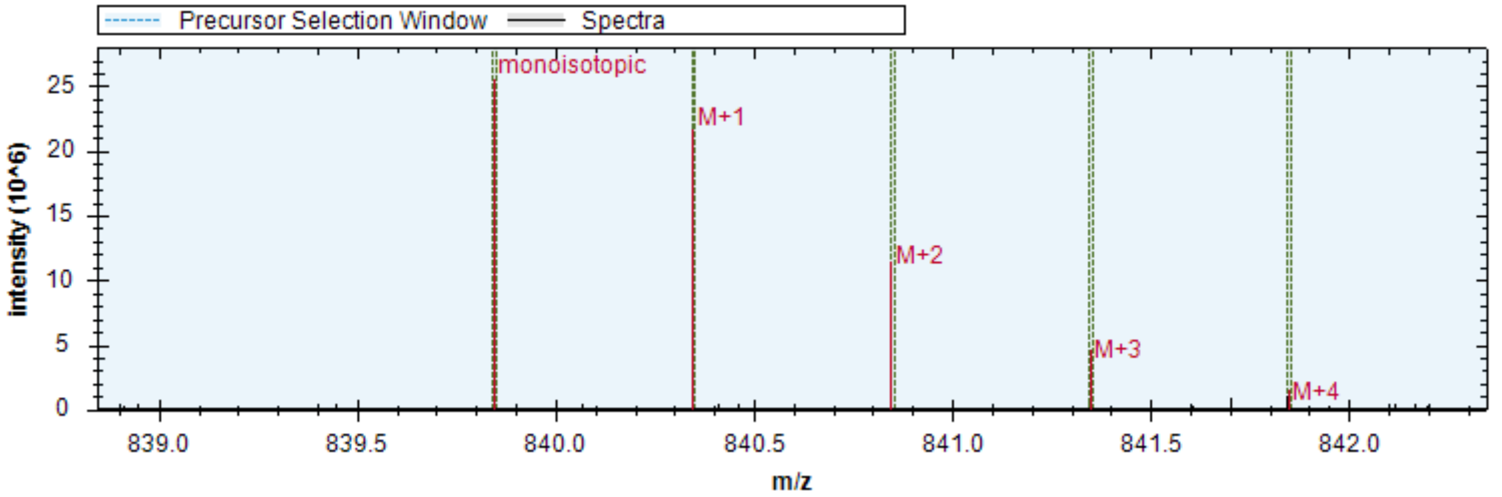

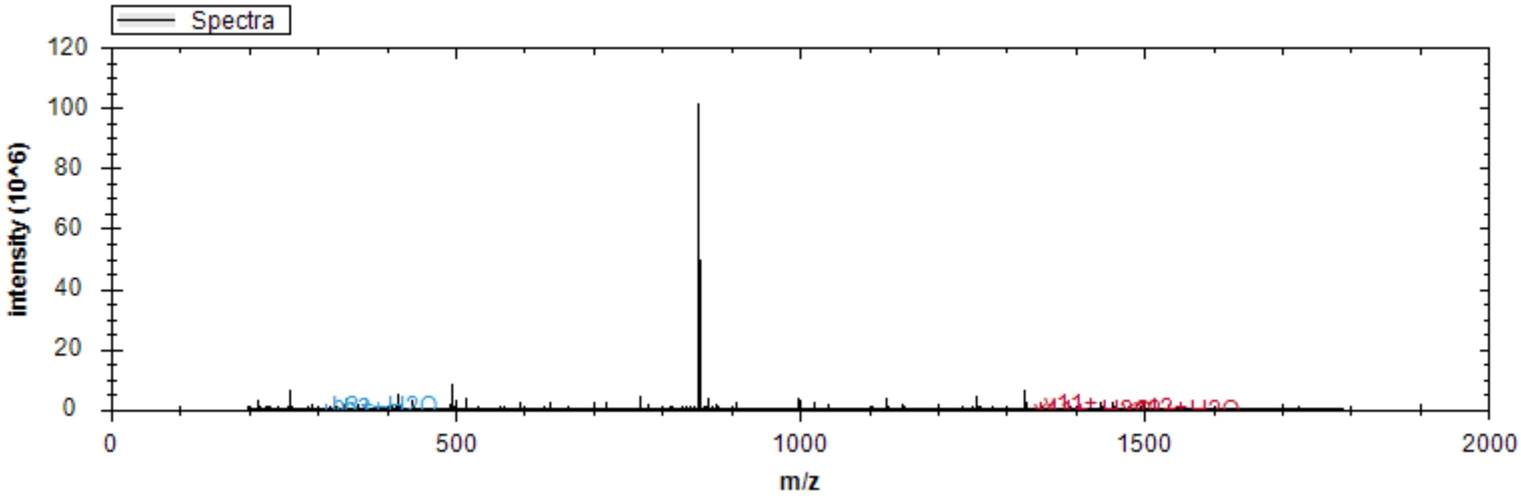

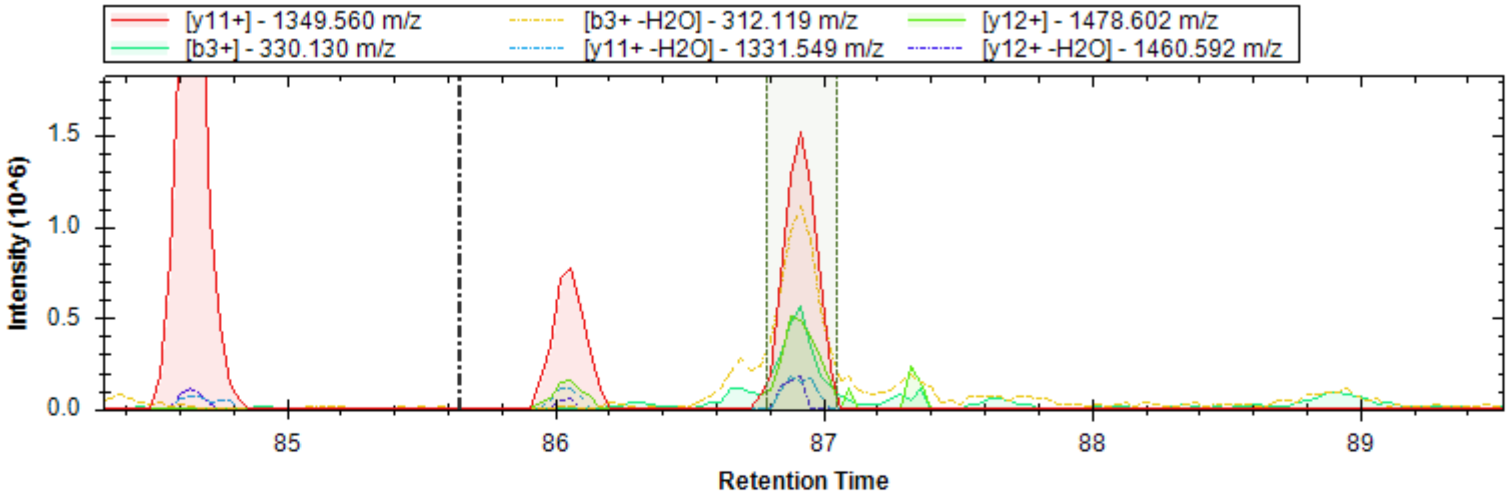

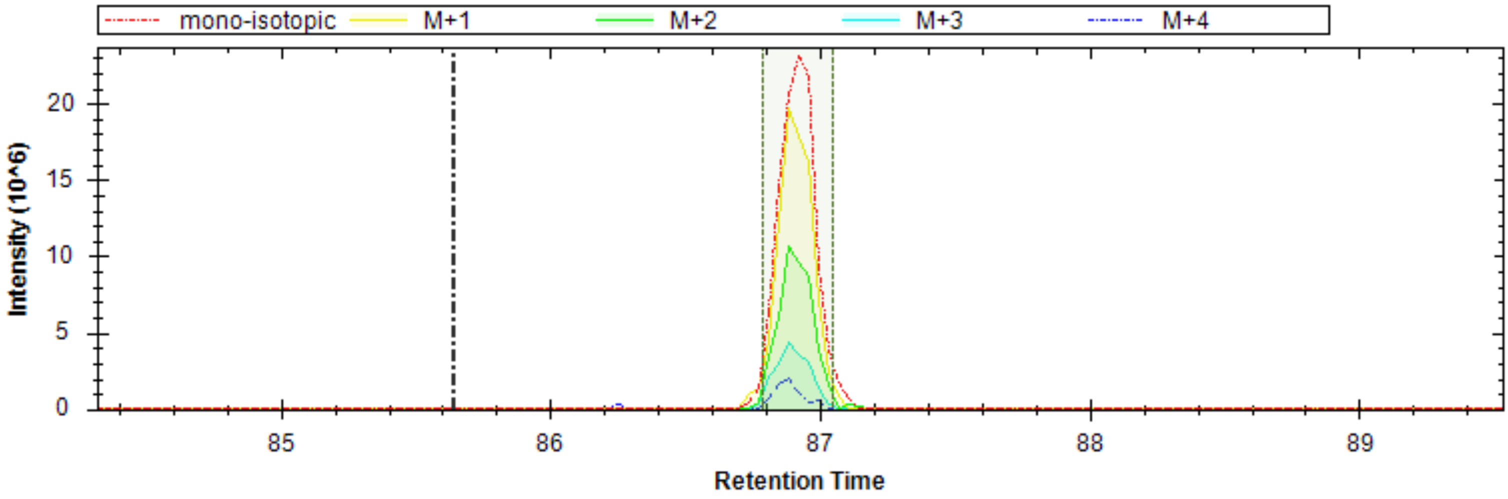

A1A535 – _FLHRGS[+H+O3+P]K_.1

Fig4_MP-DIA-barrel-cortex-p30-2_MHRM.htrms

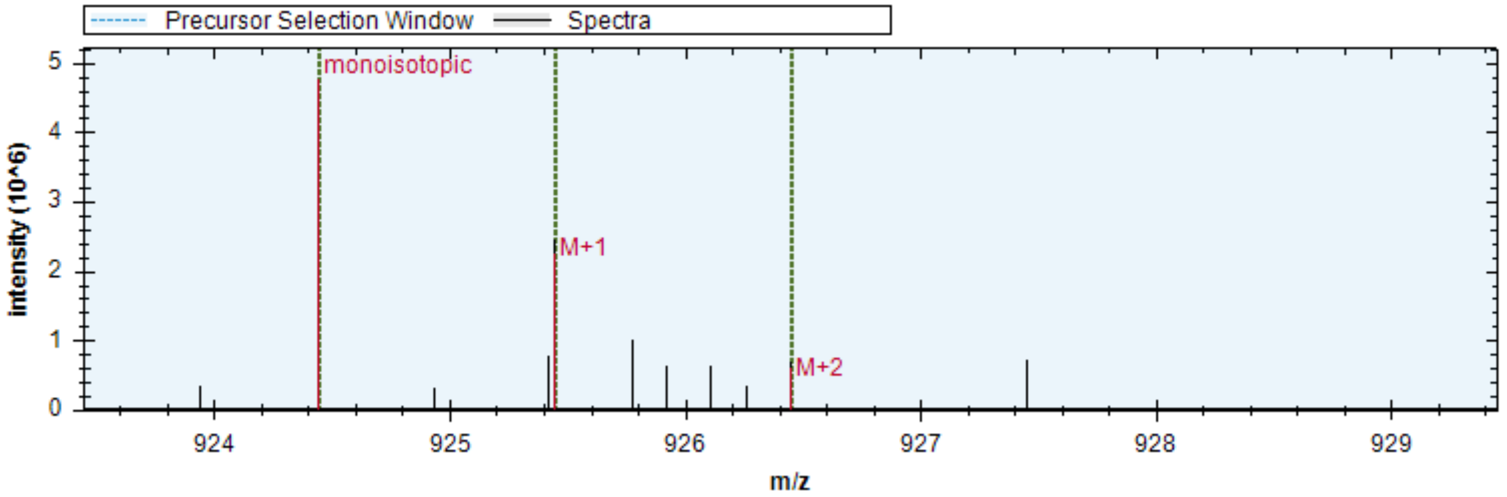

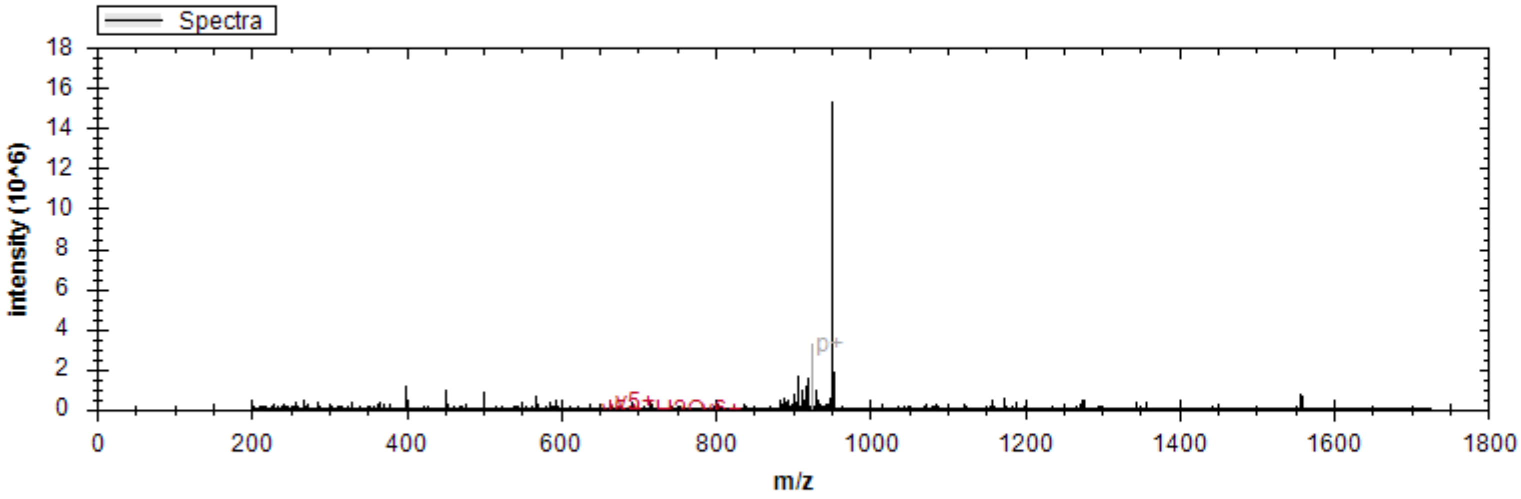

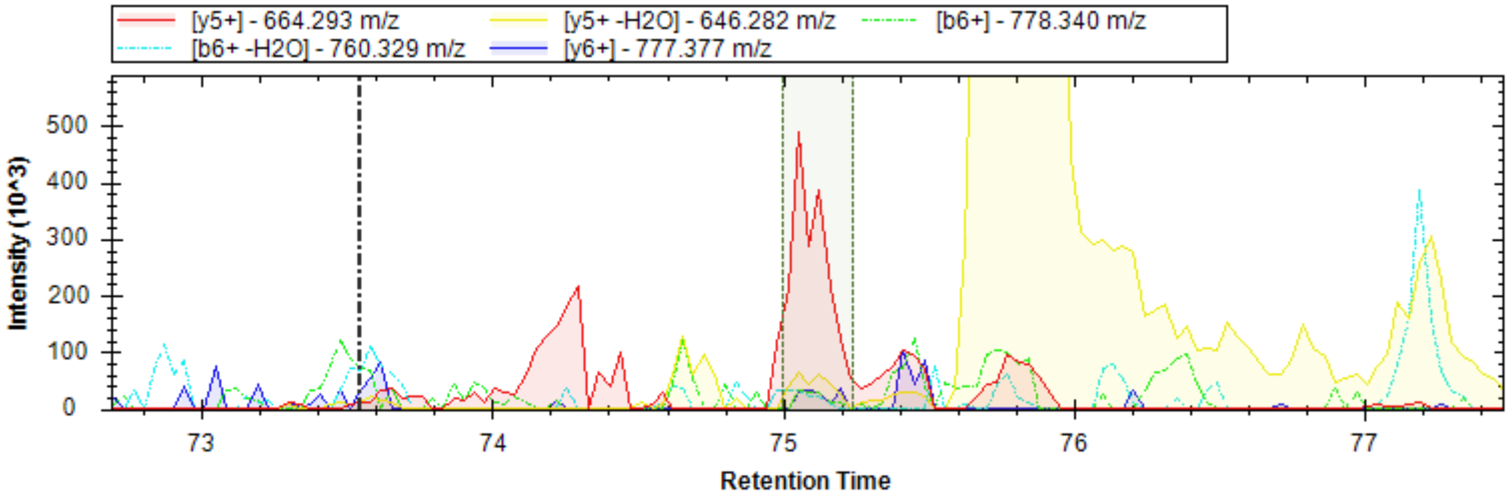

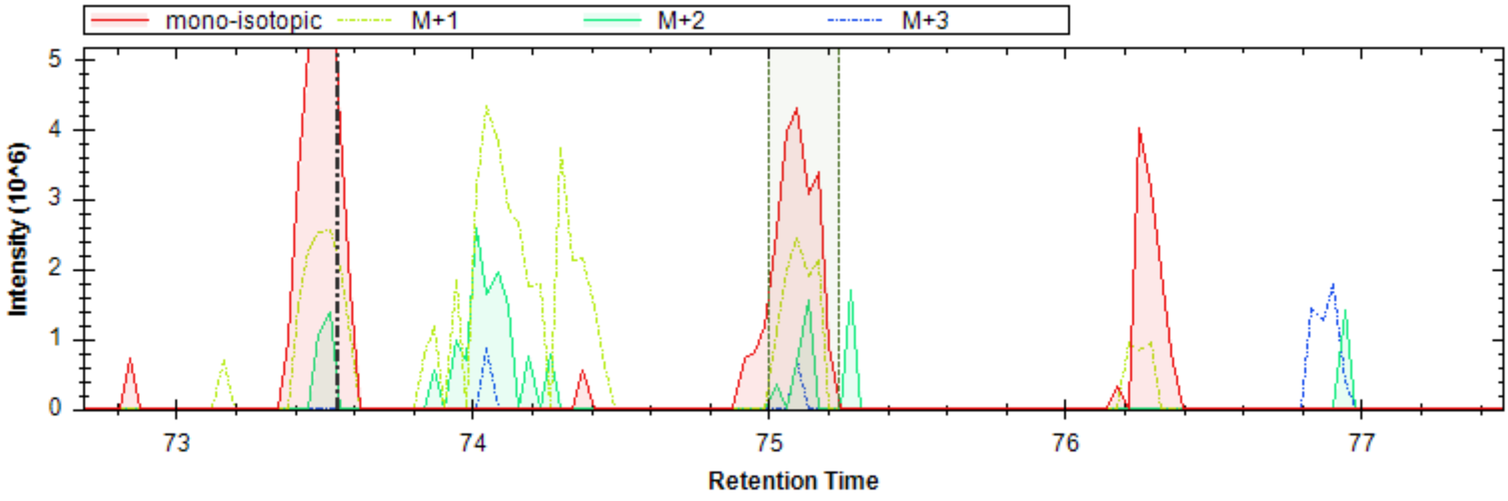

P60904 – _SLS[+H+O3+P]TSGESLYHVLGLDK_.2
Fig4_MP-DIA-barrel-cortex-p30-2_MHRM.htrms

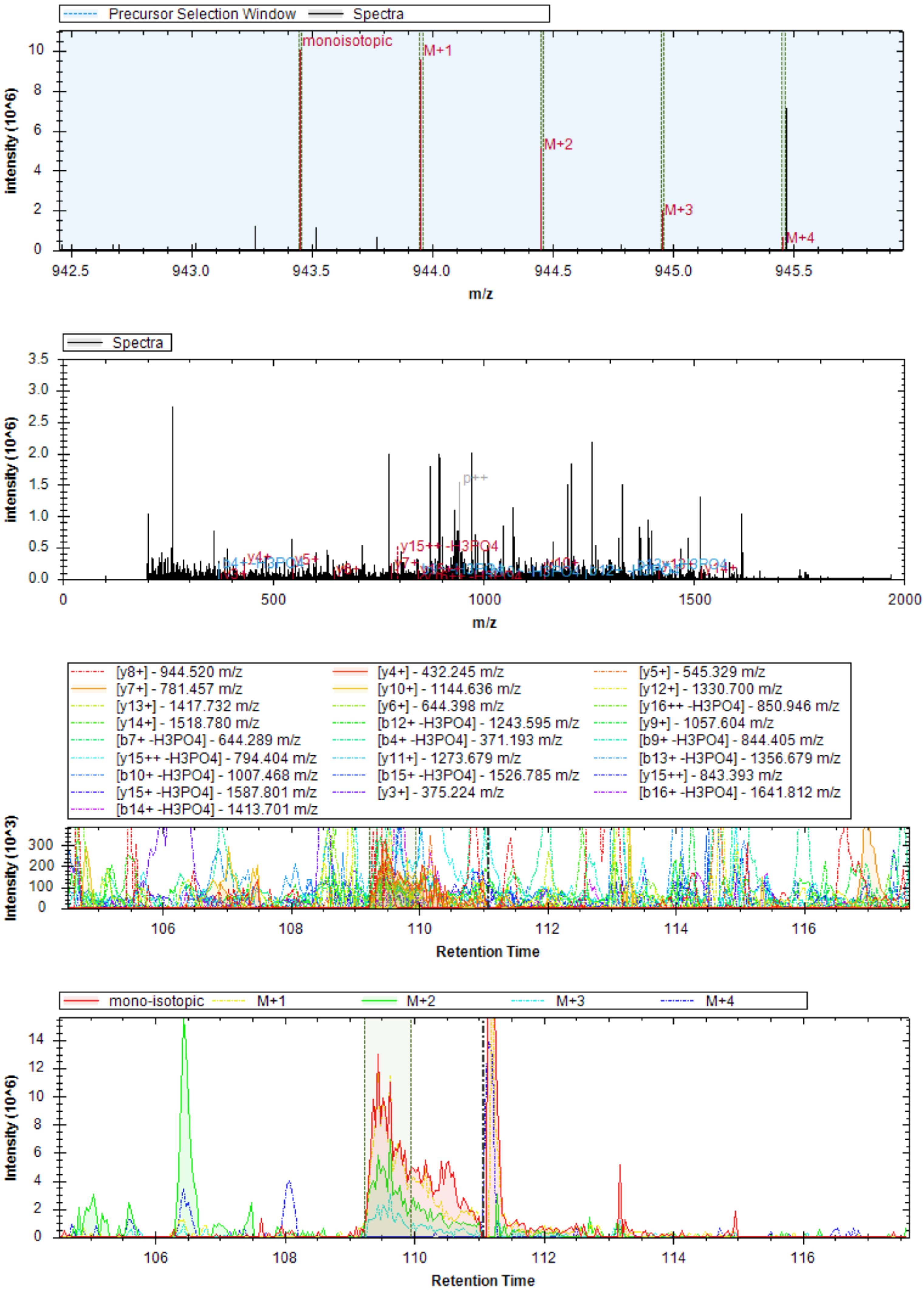

Q8BMA3 – _IIQLC[+C2+H3+N+O]LDLT[+H+O3+P]T[+H+O3+P]AVQK_.2
Fig4_MP-DIA-barrel-cortex-p30-2_MHRM.htrms

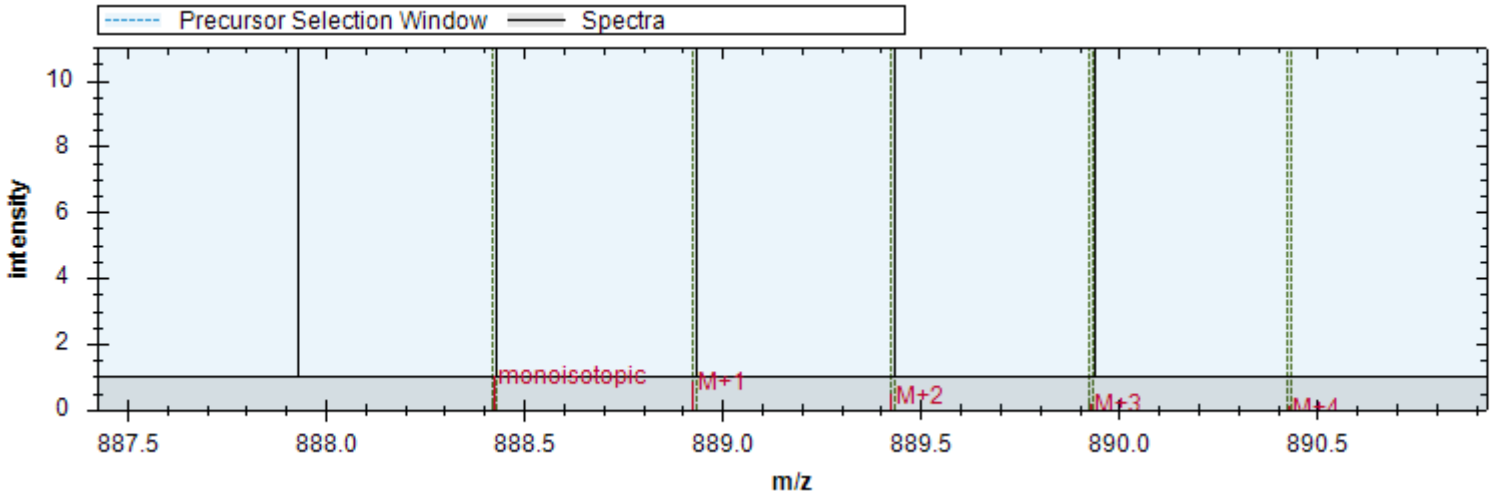

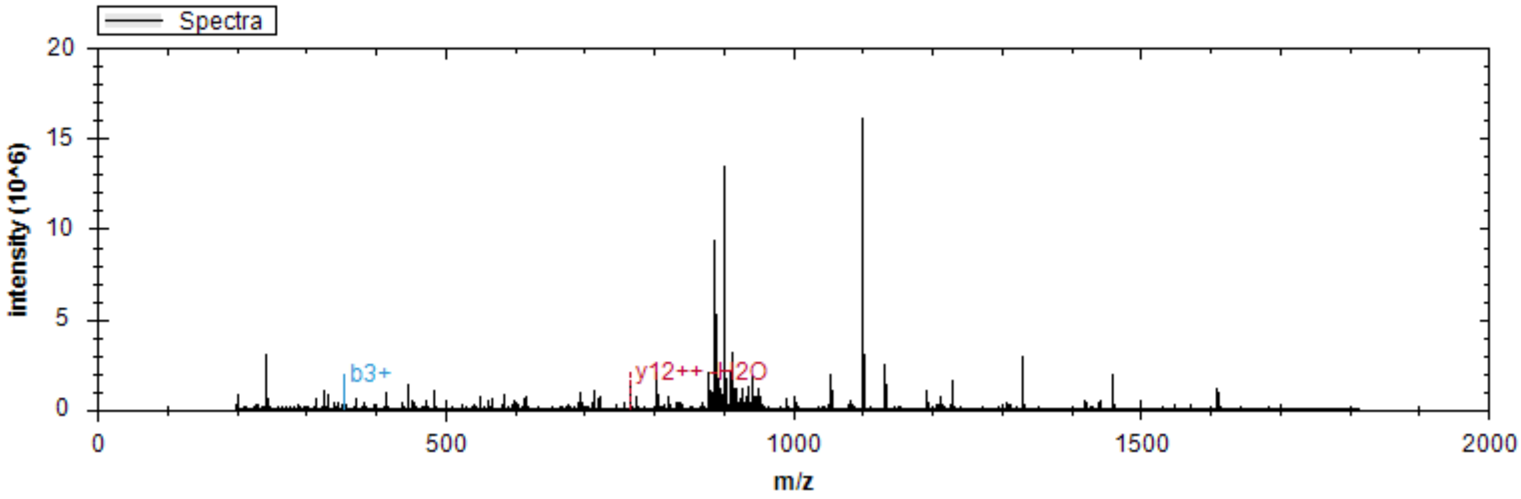

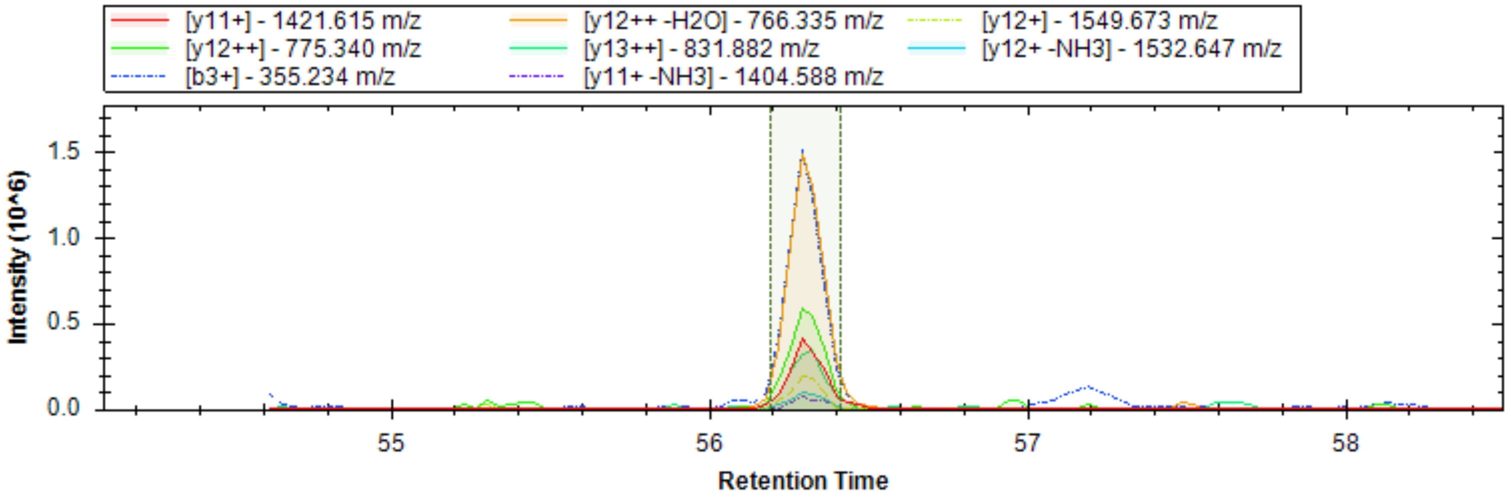

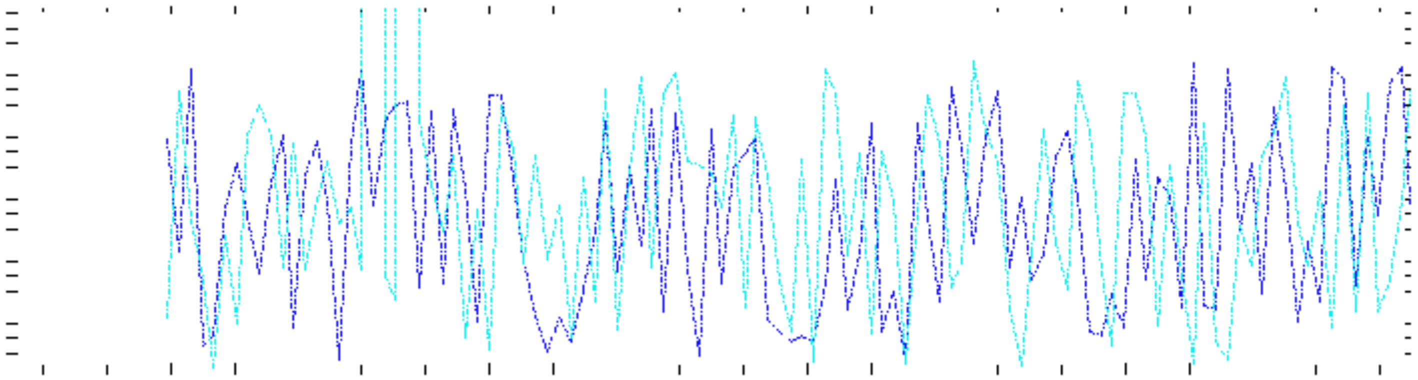

Q8BX90 – _IQALNSLGAGPFS[+H+O3+P]HTIK_.2
Fig4_MP-DIA-barrel-cortex-p30-2_MHRM.htrms

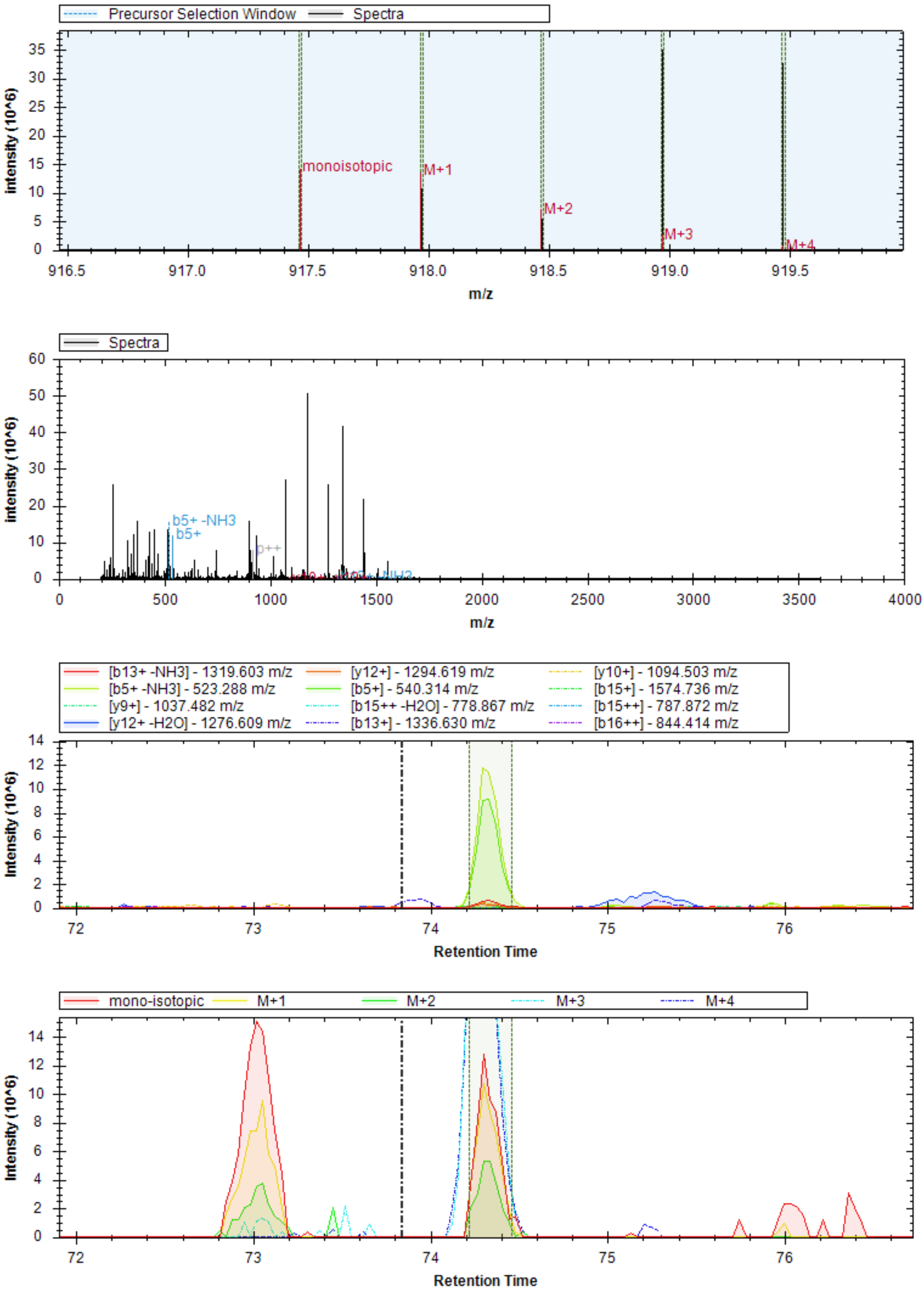

Q91Z31;Q91Z31-2 – _ETSLLAVPGALS[+H+O3+P]PLAIPNAAAAAAAAAAGR_.3
Fig4_MP-DIA-barrel-cortex-p30-2_MHRM.htrms

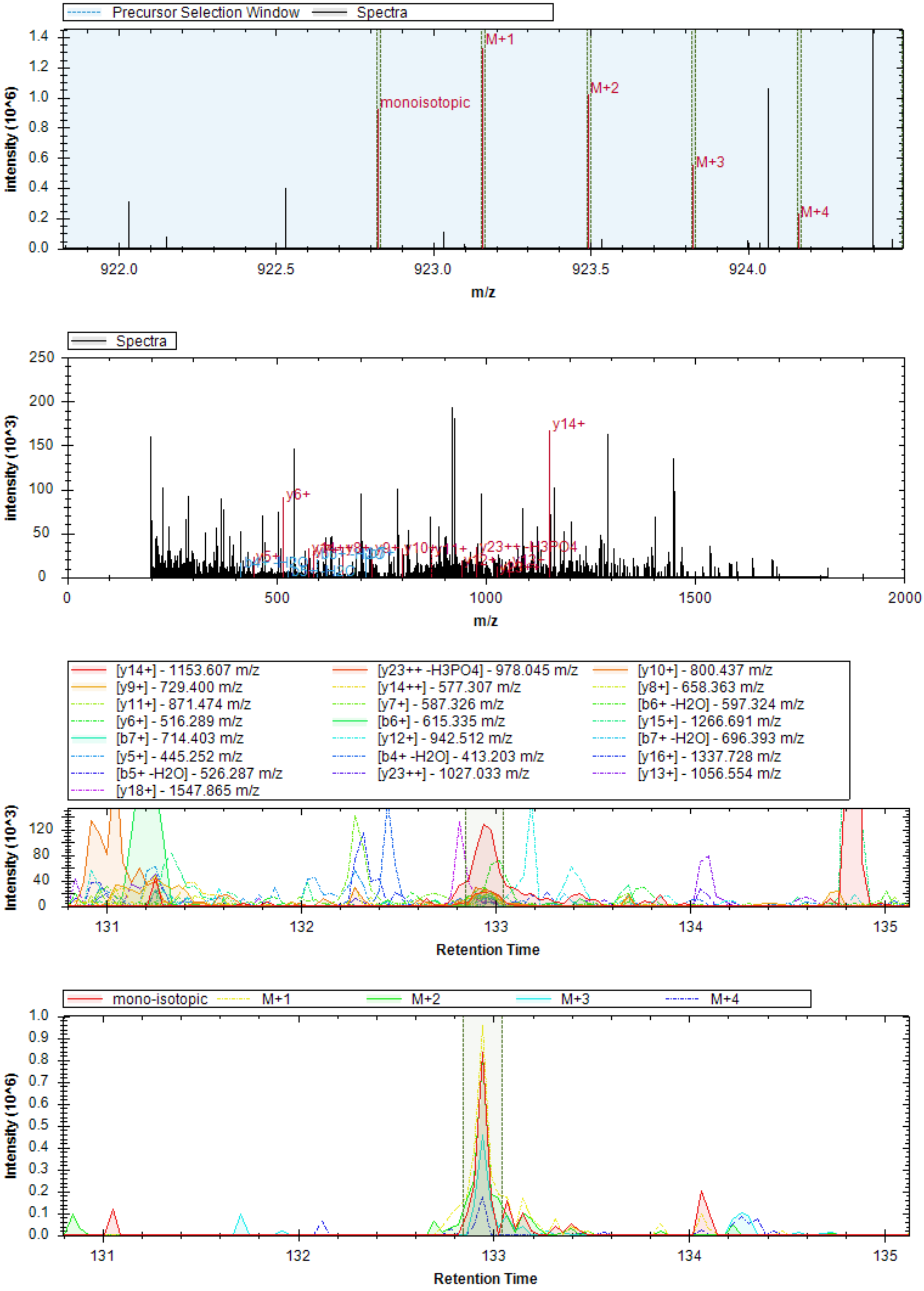

Q9CZT4;Q9CZT4-2 – _KEQM[+O]RAATVLPS[+H+O3+P]VQIK_.2

Fig4_MP-DIA-barrel-cortex-p30-2_MHRM.htrms

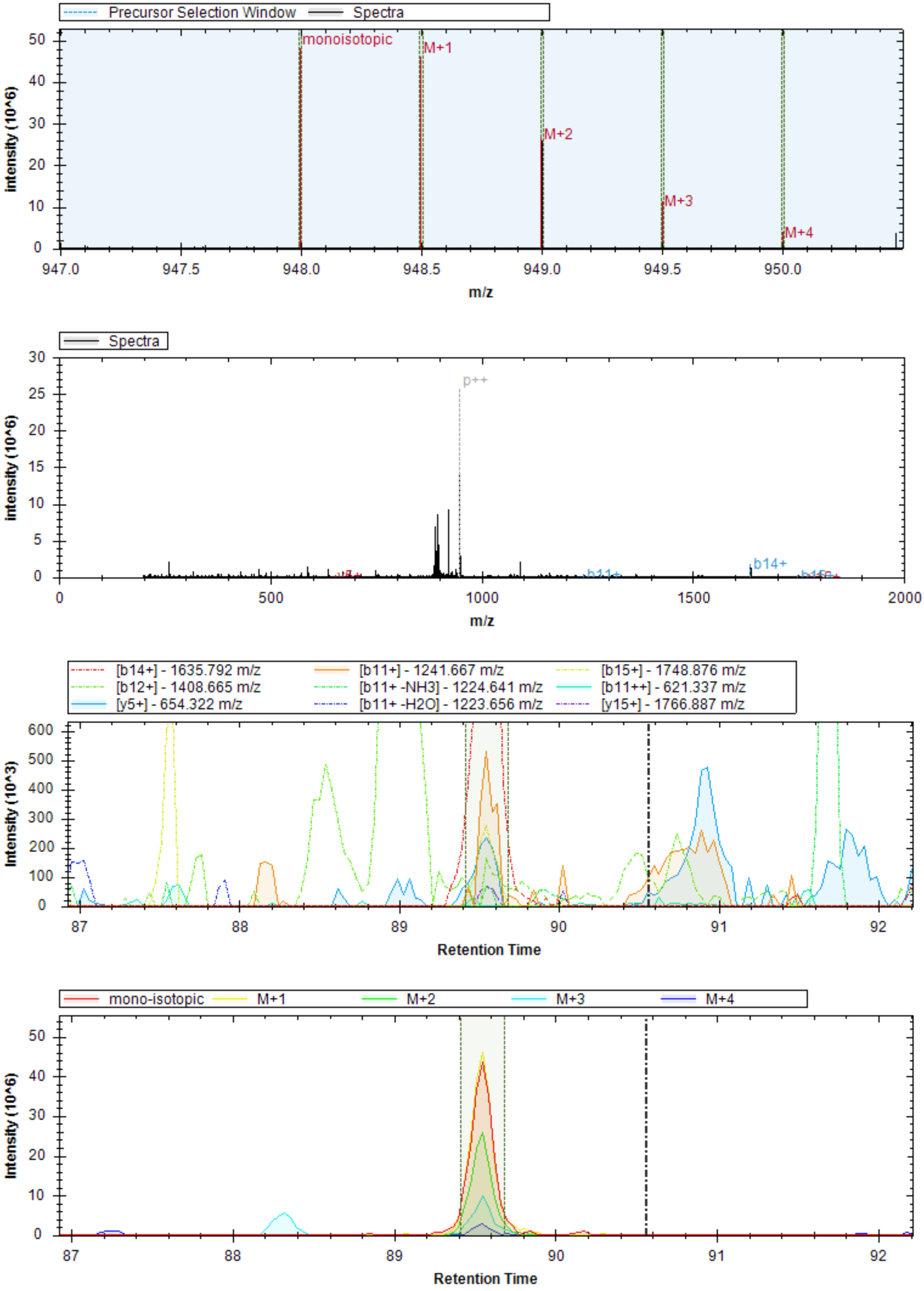

A2A891;A2A891-2;A2A8[...] – _TAVIVQQKLRS[+H+O3+P]S[+H+O3+P]LLT[+H+O3+P]K_.2
Fig4_MP-DIA-barrel-cortex-p30-2_MHRM.htrms

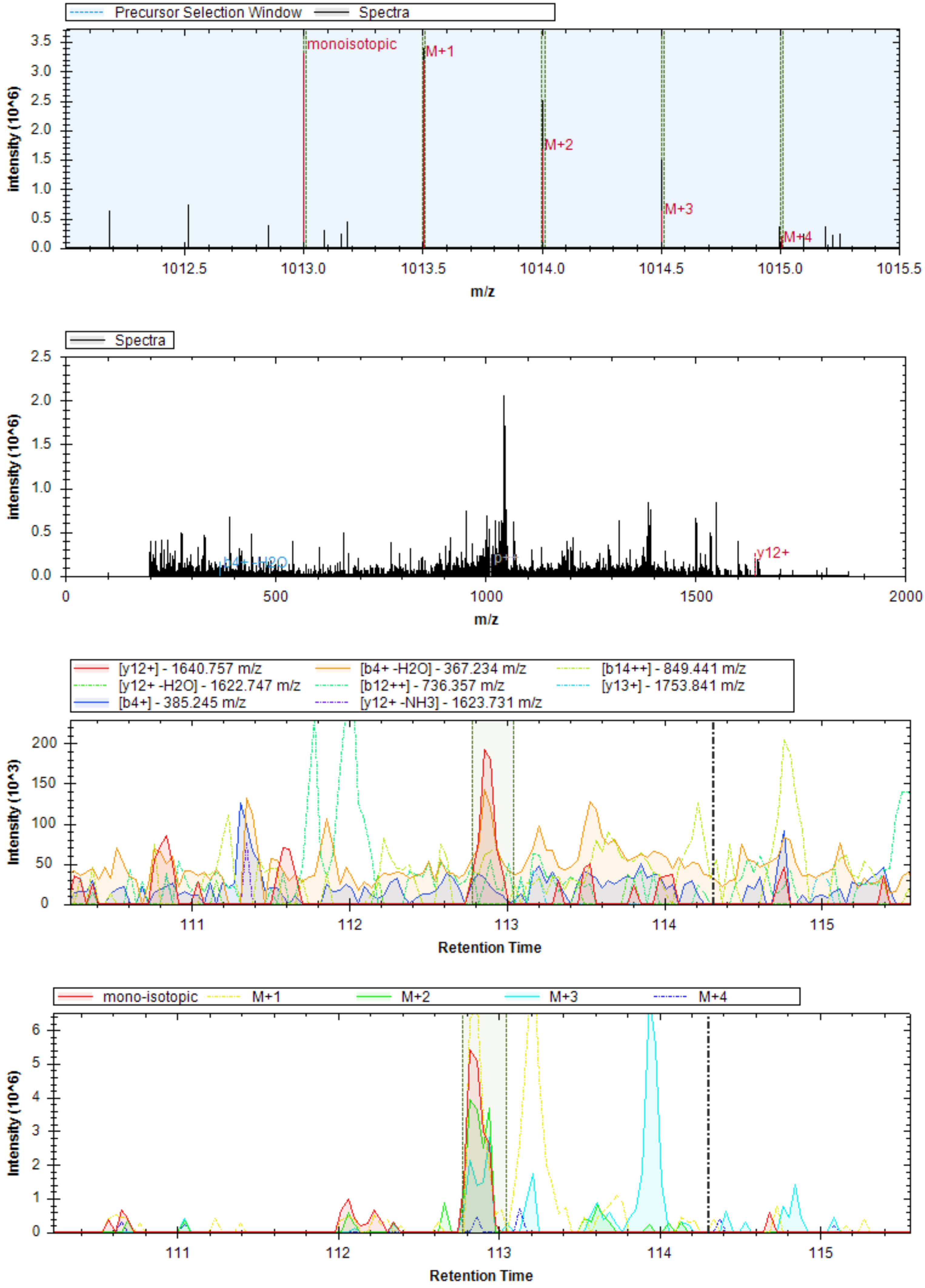

P17742 – _VNPT[+H+O3+P]VFFDITADDEPLGR_.2
Fig4_MP-DIA-barrel-cortex-p30-2_MHRM.htrms

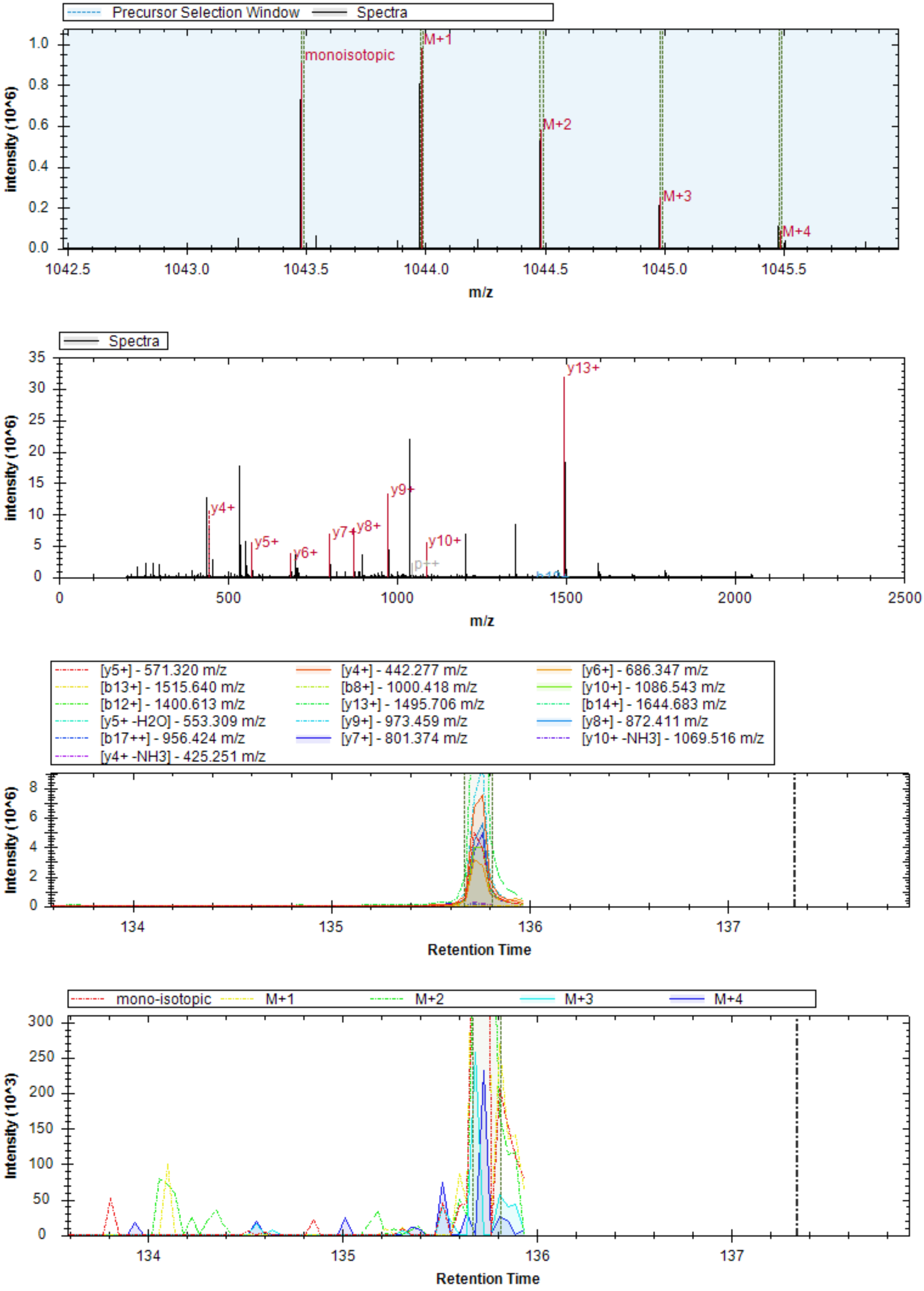

Q8CHK4;Q8CHK4-2;Q8CH[...] – _RGY[+H+O3+P]GKLLIEFS[+H+O3+P]YELSK_.2
Fig4_MP-DIA-barrel-cortex-p30-2_MHRM.htrms

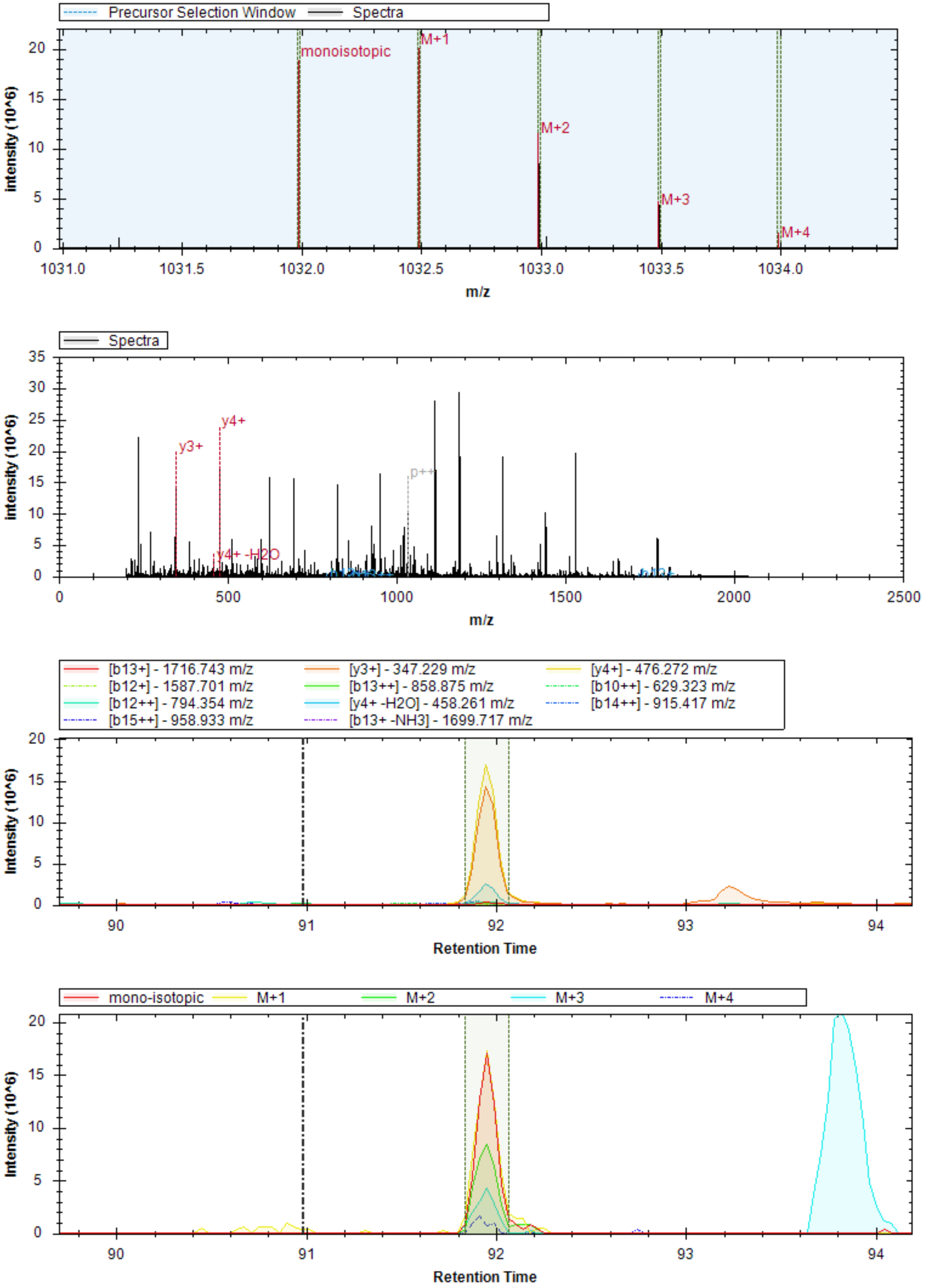

E9Q401 – _MVAEVFIY[+H+O3+P]WSK_.1
Fig4_MP-DIA-barrel-cortex-p30-2_MHRM.htrms

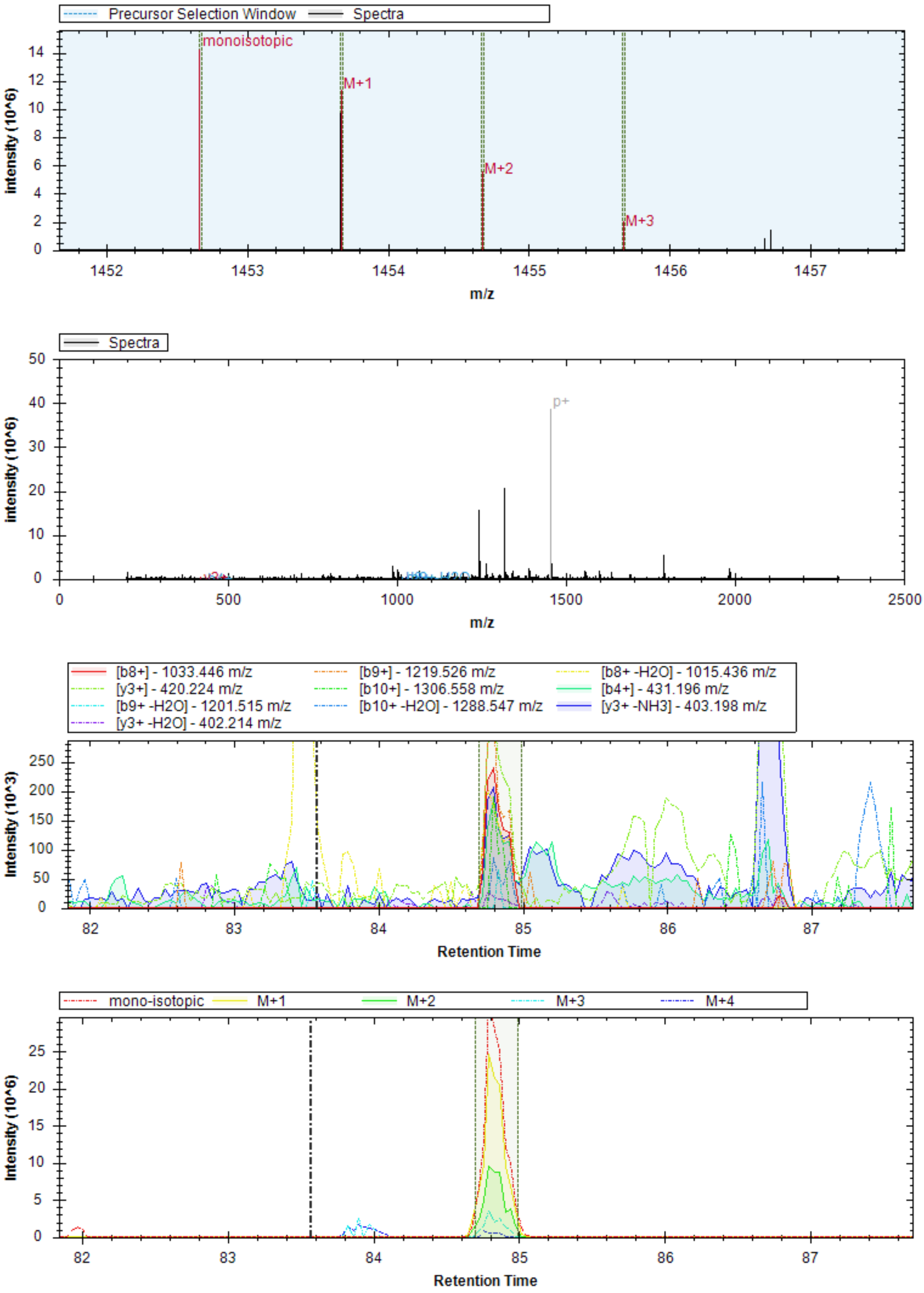

P20357 – _LSNVS[+H+O3+P]SSGSINLLESPQLATLAEDVTAALAK_.2

Fig4_MP-DIA-barrel-cortex-p30-2_MHRM.htrms

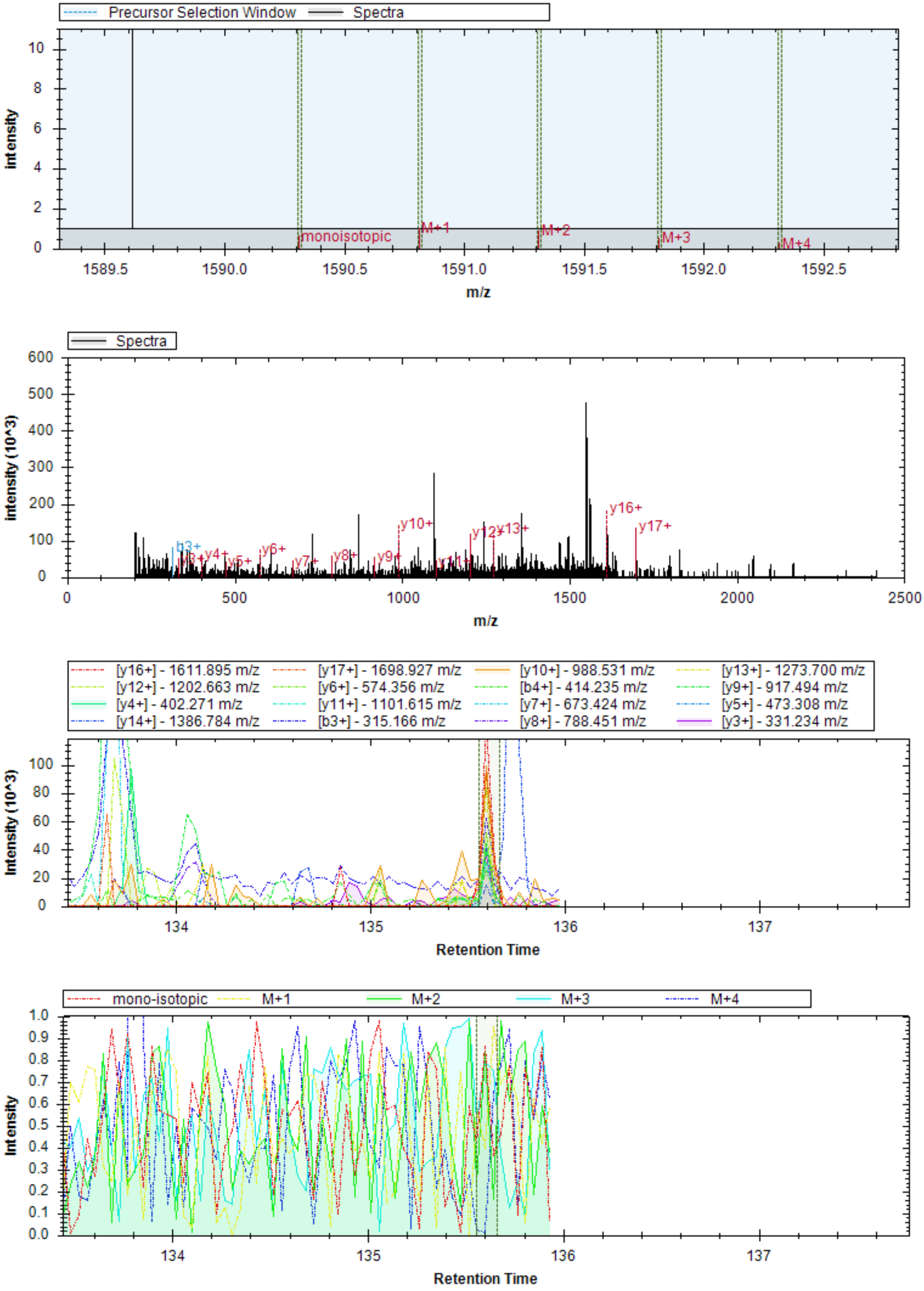

Q8BRT1 – _VLNT[+H+O3+P]GSDVEEAVADALLLGDIR_.2

Fig4_MP-DIA-barrel-cortex-p30-2_MHRM.htrms

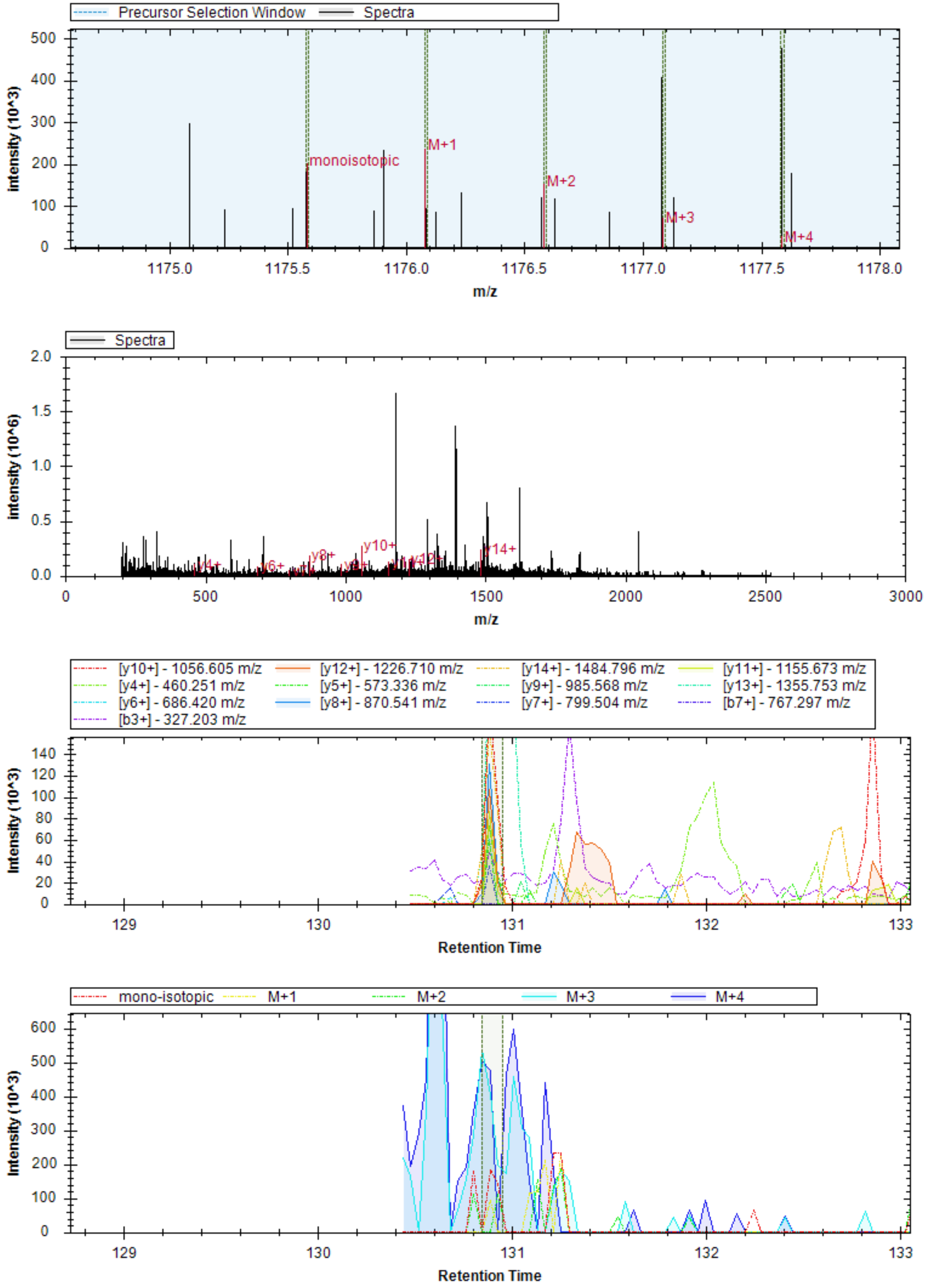

Q8VHY0 – _LLAGPRY[+H+O3+P]GR_.1
Fig4_MP-DIA-barrel-cortex-p30-2_MHRM.htrms

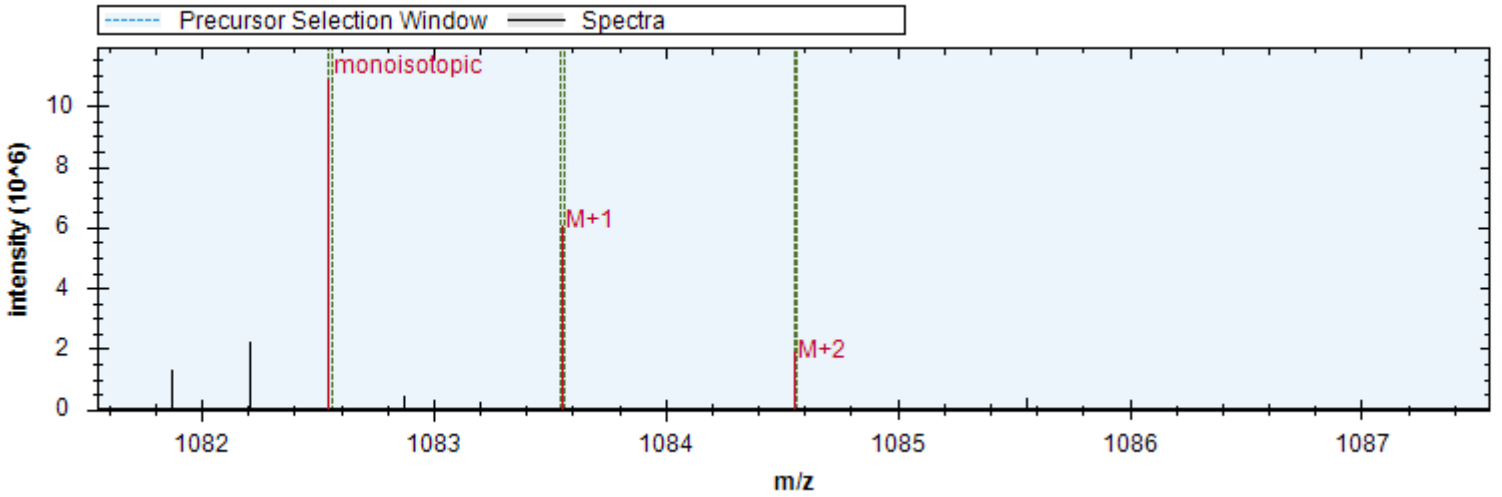

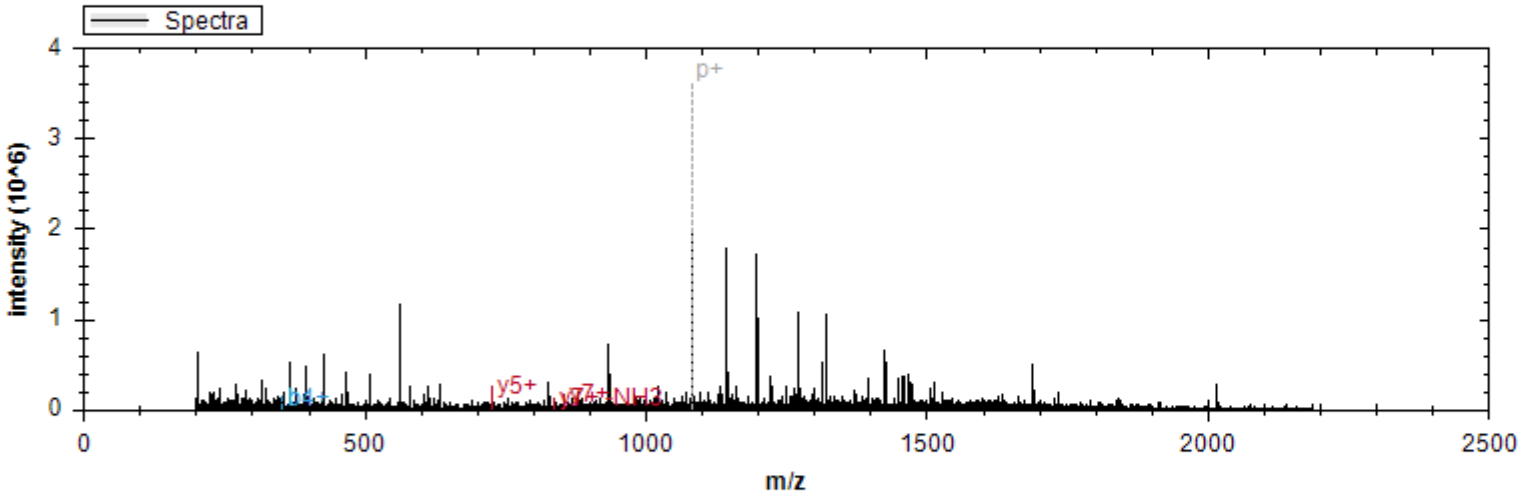

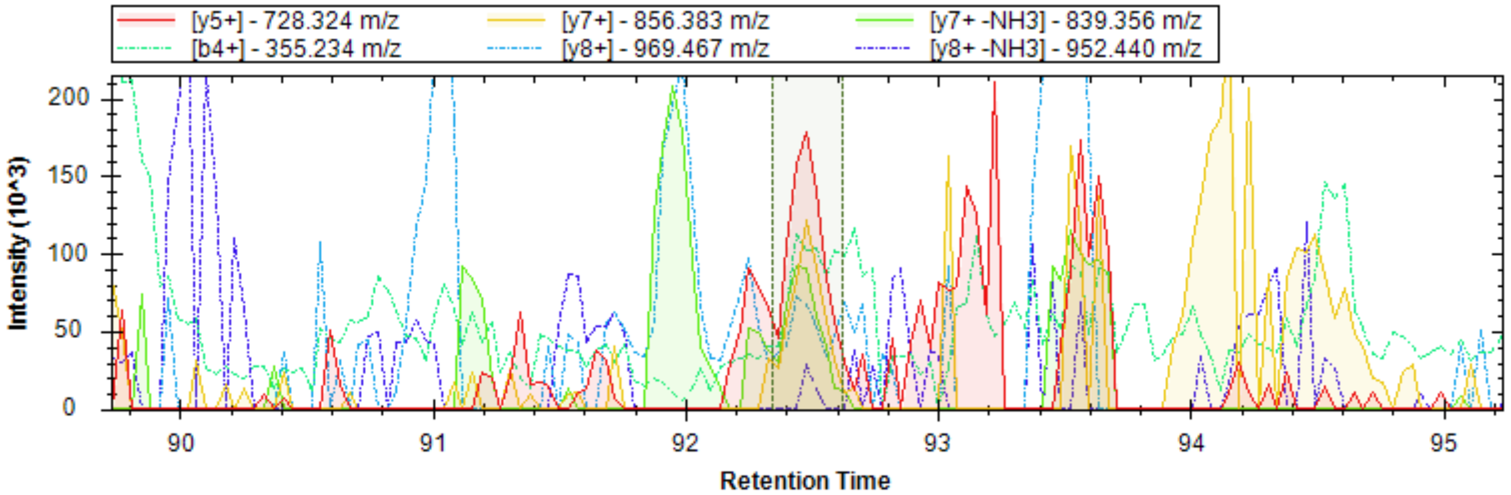

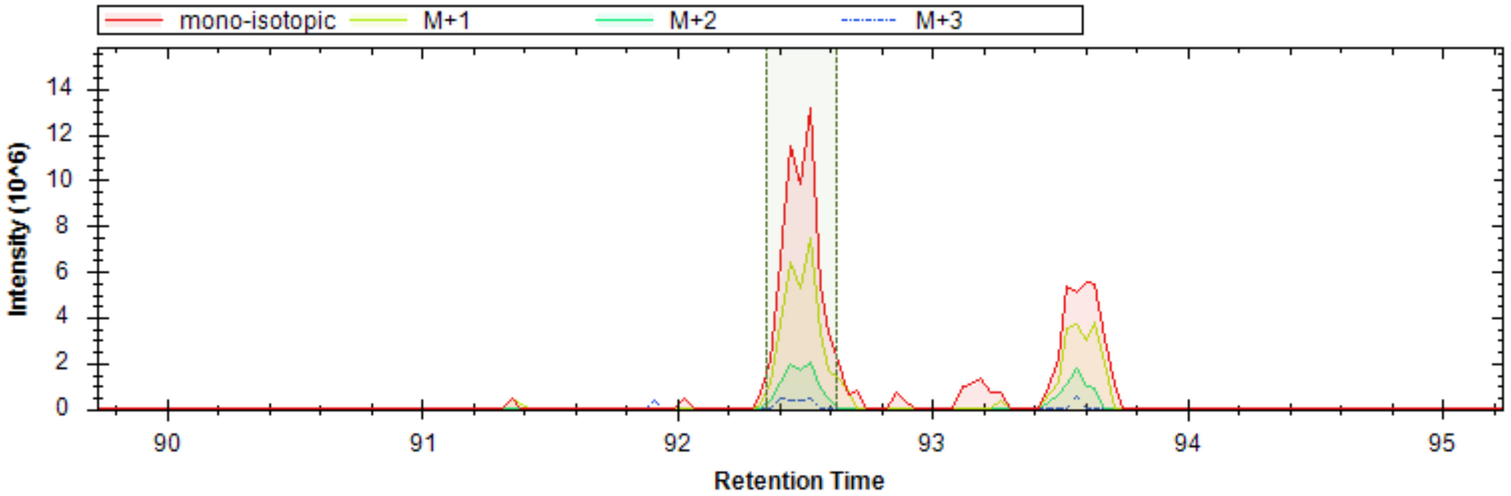

Q921N6;Q921N6-2 – _LIY[+H+O3+P]KPRQAAVTR_.4
Fig4_MP-DIA-barrel-cortex-p30-3_MHRM.htrms

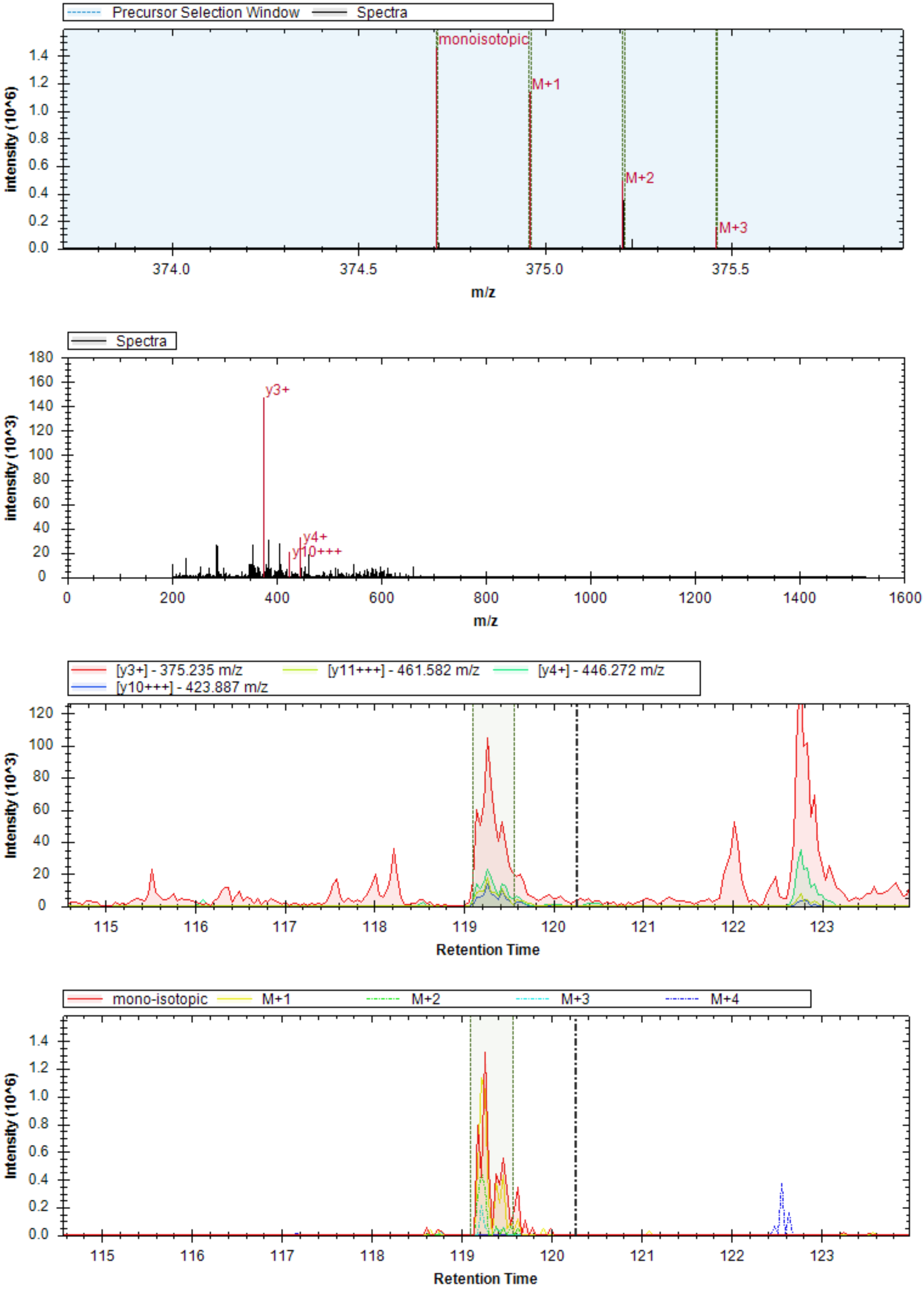

Q9CVB6 – _IIEET[+H+O3+P]LALK_.3
Fig4_MP-DIA-barrel-cortex-p30-3_MHRM.htrms

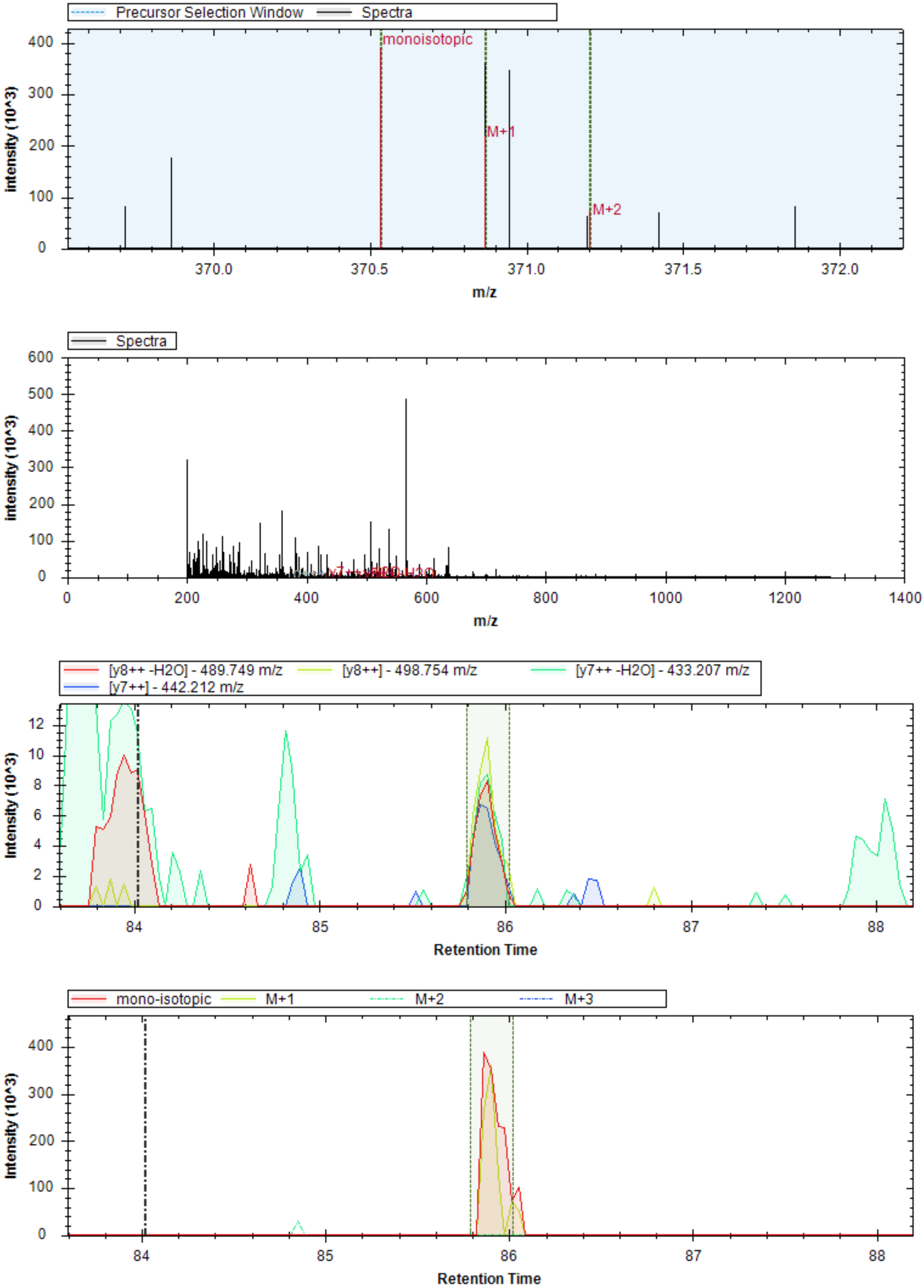

Q9D516;Q9D516-2 – _KLS[+H+O3+P]QGILVIR_.3

Fig4_MP-DIA-barrel-cortex-p30-3_MHRM.htrms

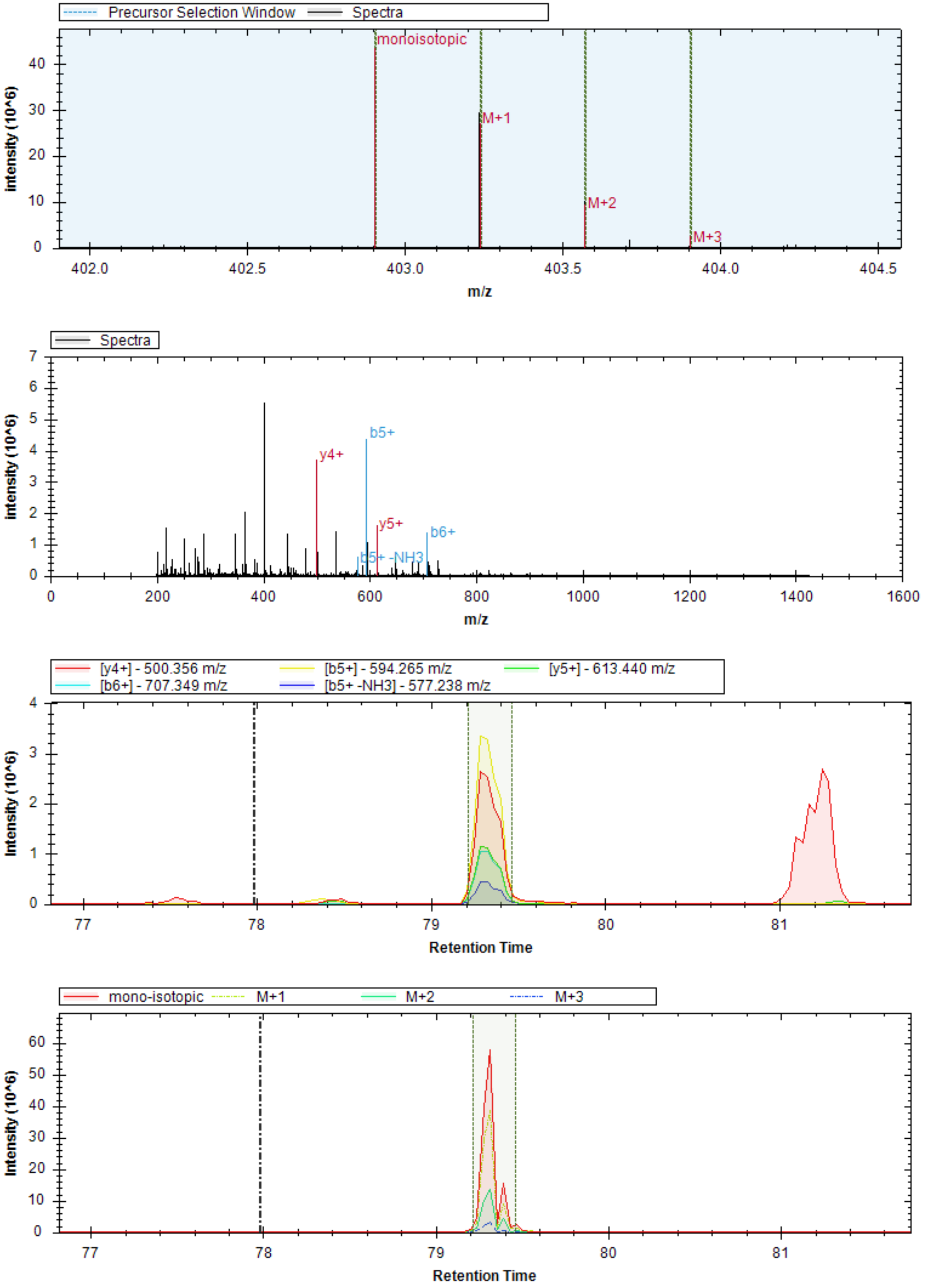

P20357 – _KSEVQAHS[+H+O3+P]PSR_.3

Fig4_MP-DIA-barrel-cortex-p30-3_MHRM.htrms

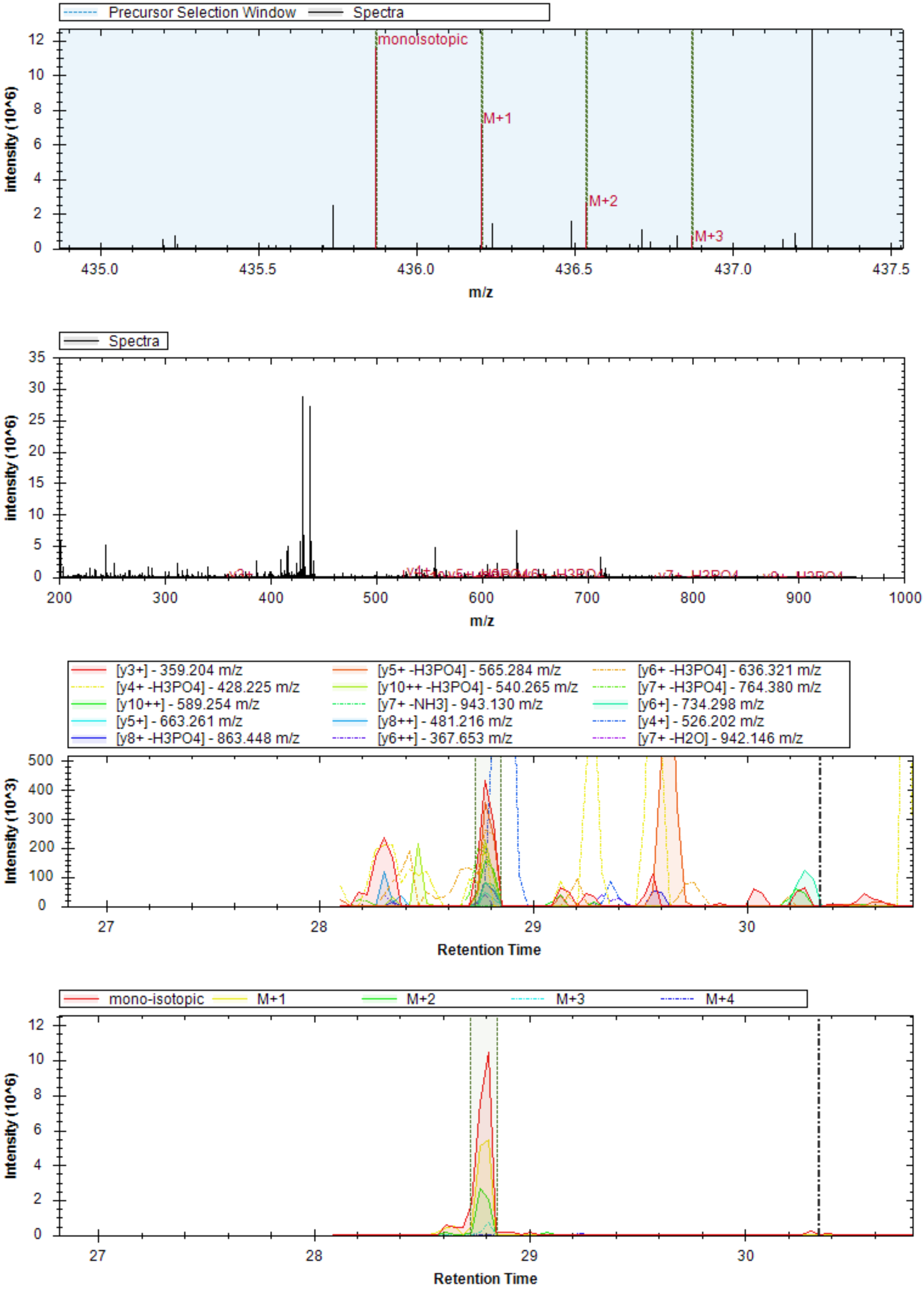

Q03172 – _VALSLLNS[+H+O3+P]KQK_.3
Fig4_MP-DIA-barrel-cortex-p30-3_MHRM.htrms

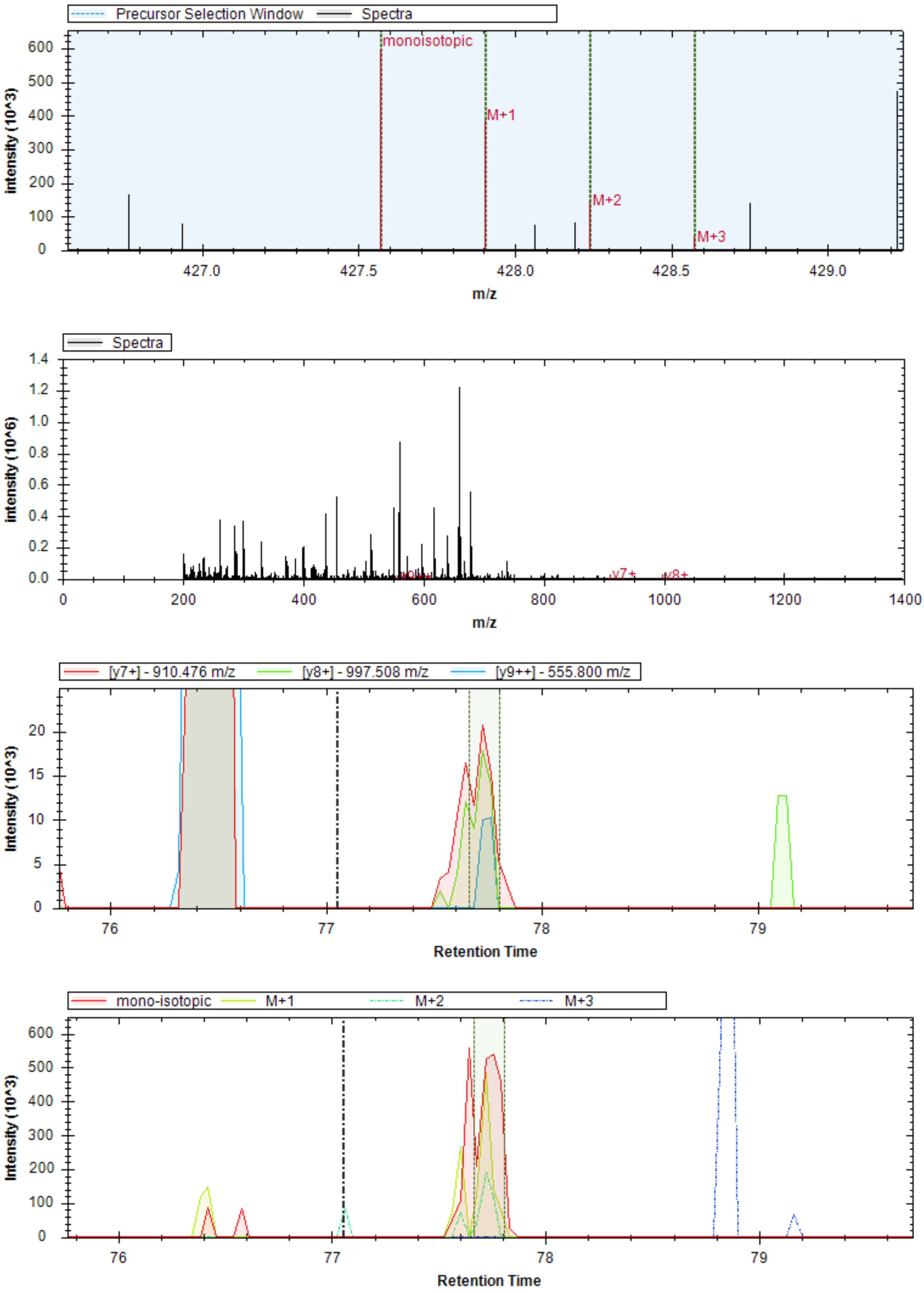

Q62188 – _GS[+H+O3+P]PTRPNPPVR_.3

Fig4_MP-DIA-barrel-cortex-p30-3_MHRM.htrms

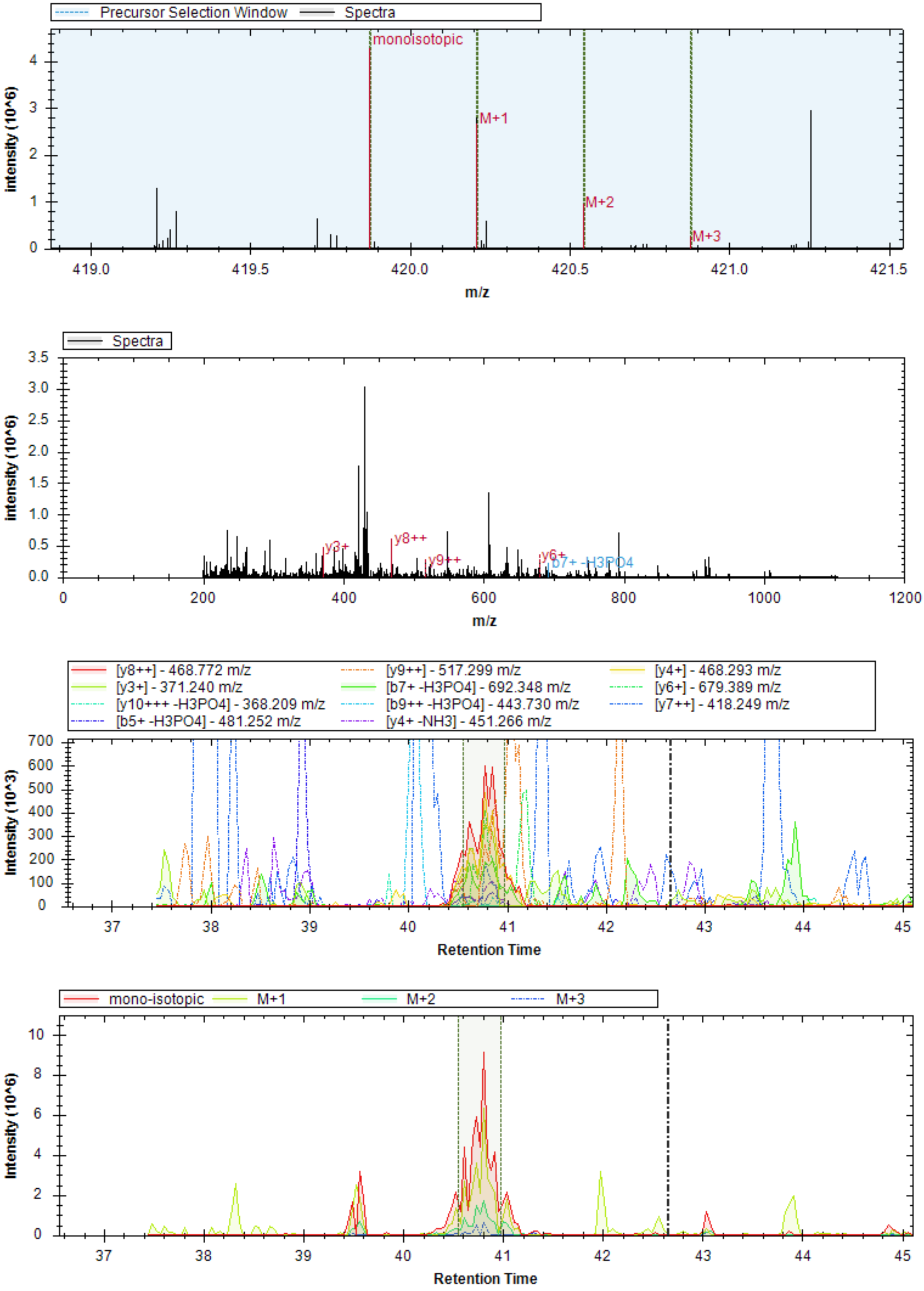

P62484 – _T[+H+O3+P]HKIIAPANLERPVR_.4
Fig4_MP-DIA-barrel-cortex-p30-3_MHRM.htrms

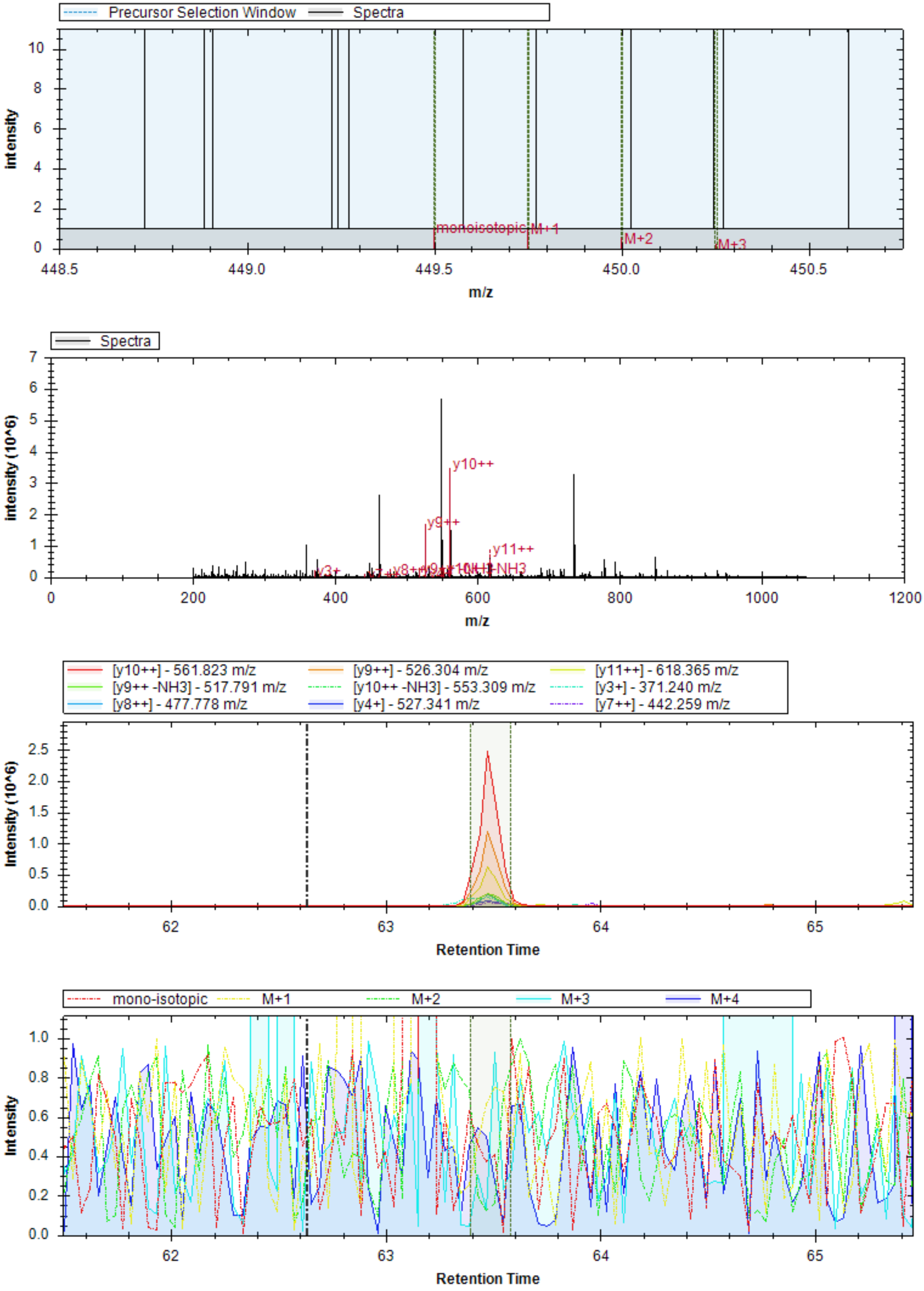

Q8CGI1 – _Y[+H+O3+P]QRS[+H+O3+P]EEELR_.3
Fig4_MP-DIA-barrel-cortex-p30-3_MHRM.htrms

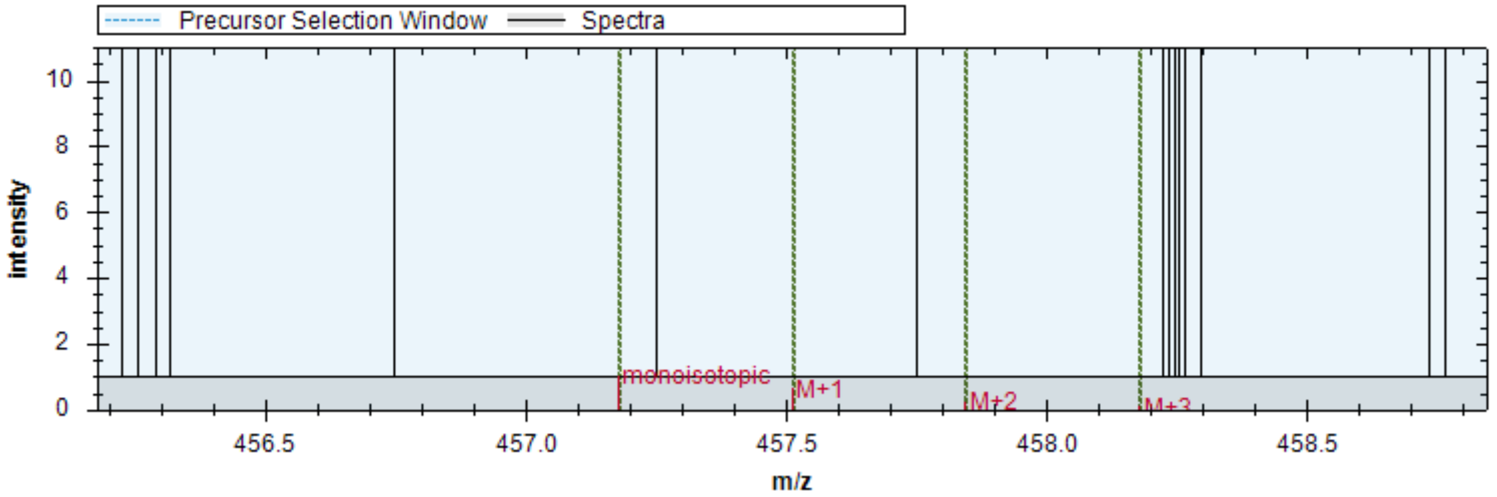

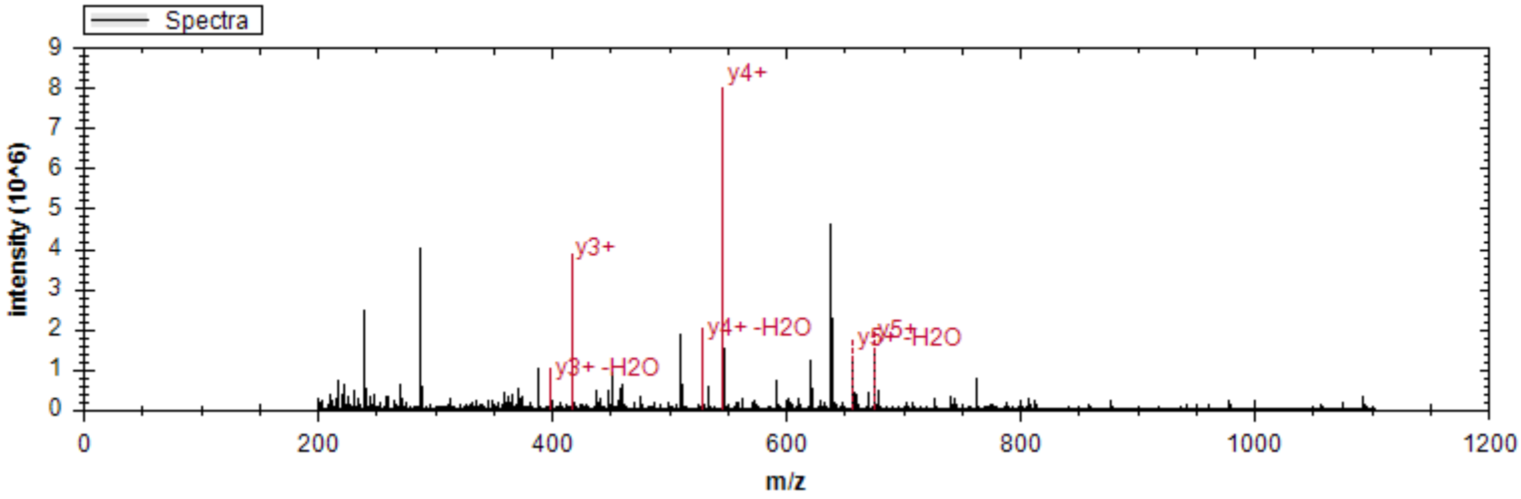

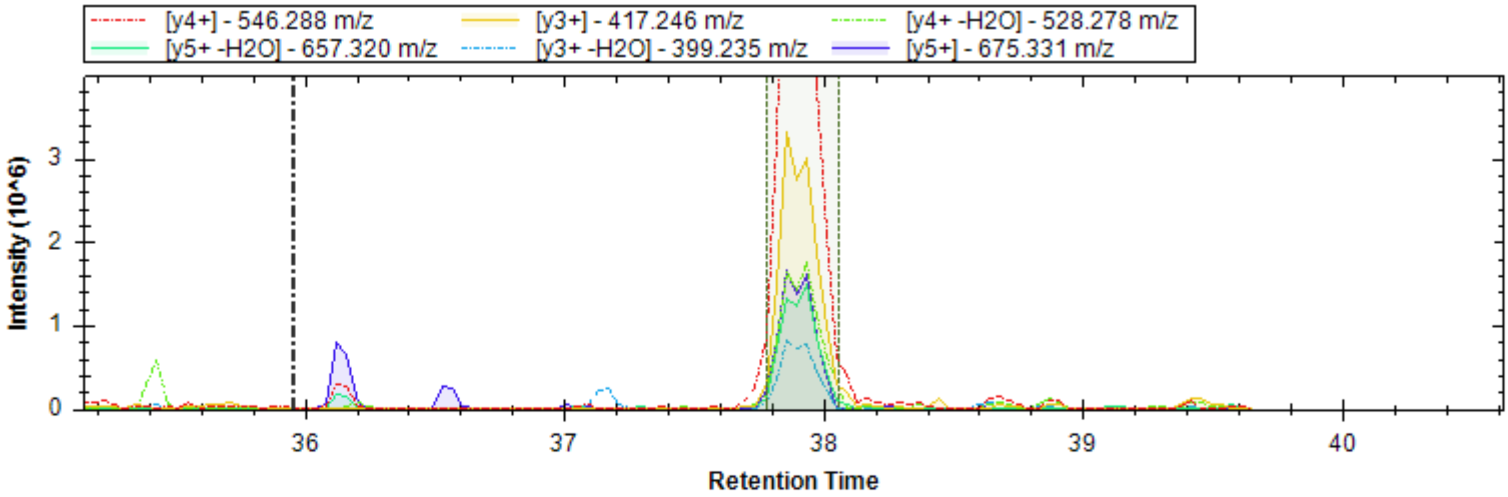

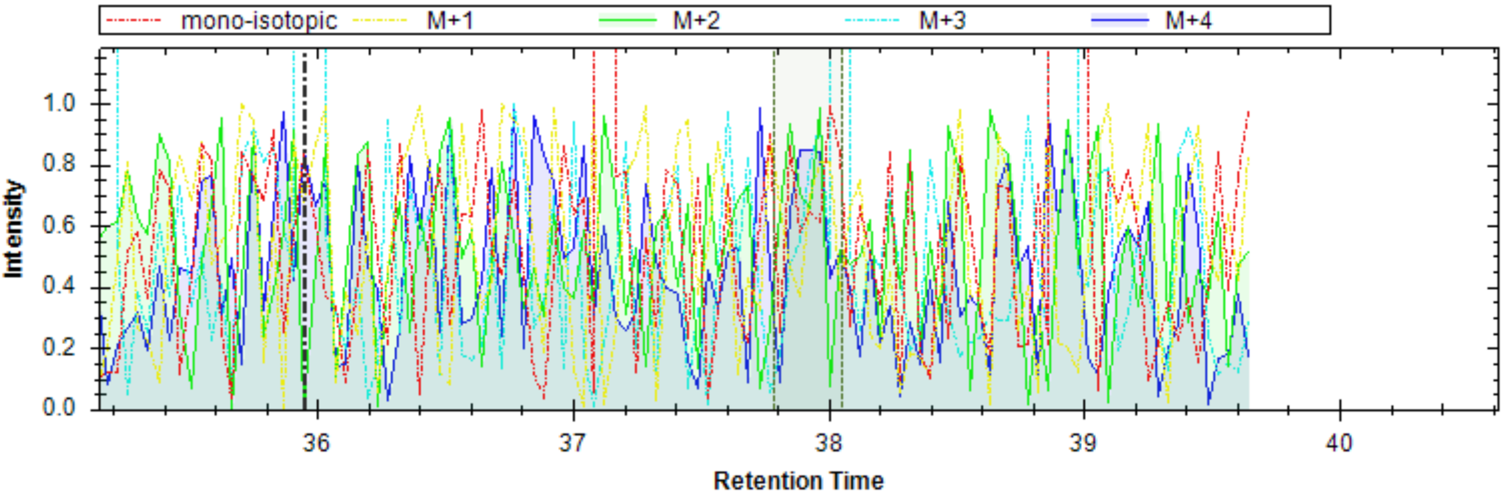

P20357 – _VTDGIS[+H+O3+P]KSPEKR_.3
Fig4_MP-DIA-barrel-cortex-p30-3_MHRM.htrms

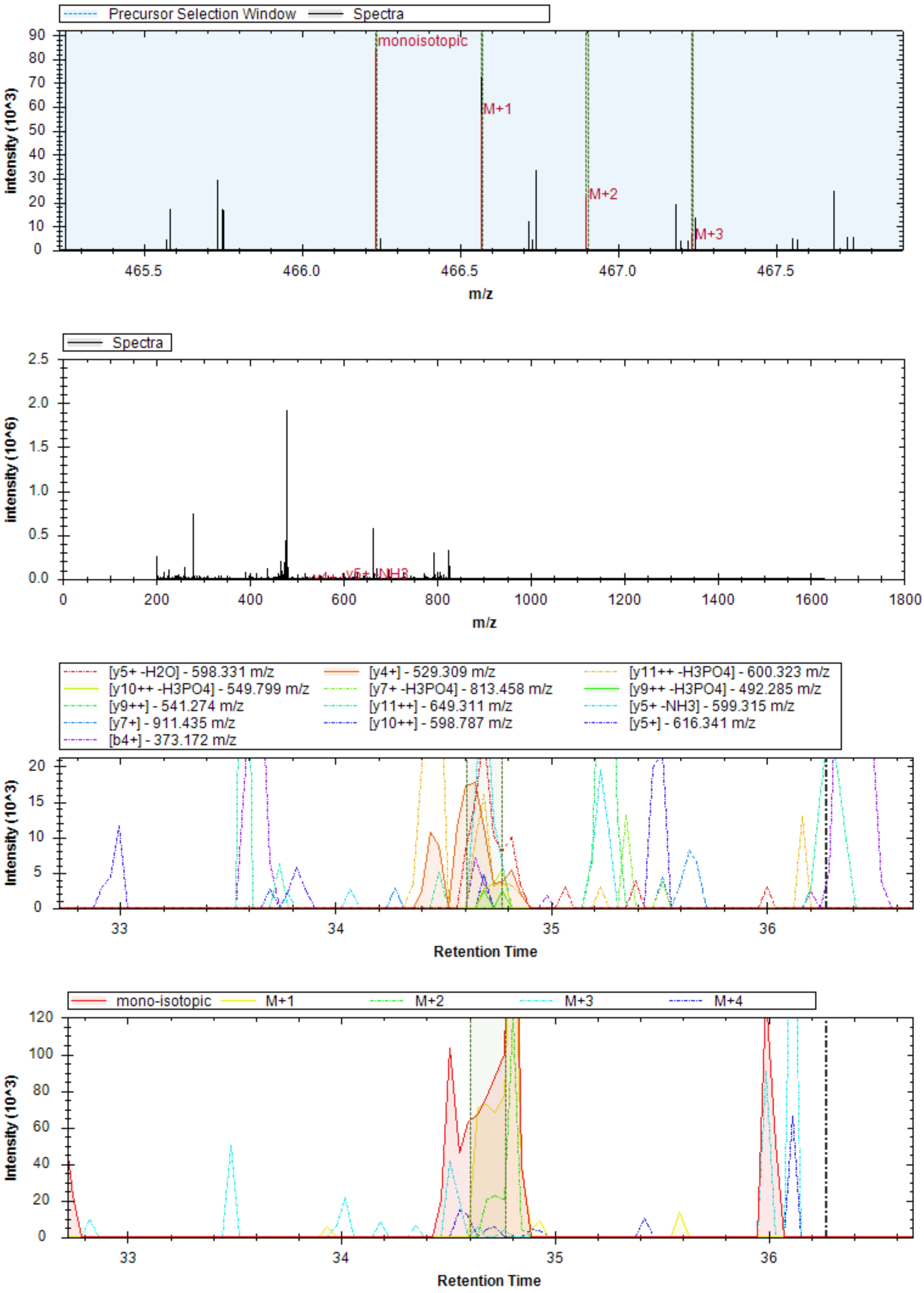

P26645 – _LSGFS[+H+O3+P]FKK_.2
Fig4_MP-DIA-barrel-cortex-p30-3_MHRM.htrms

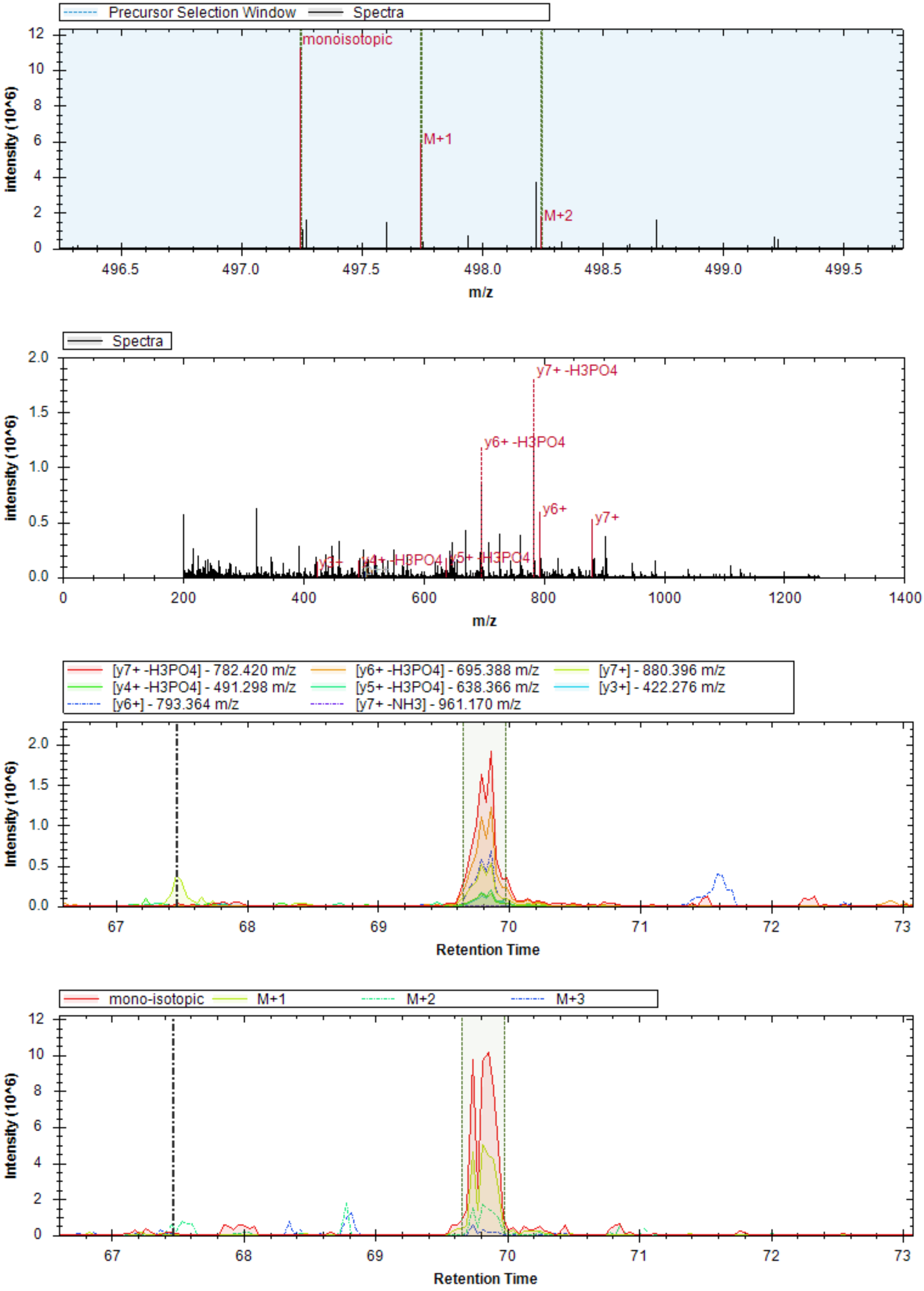

P28667 – _LSGLS[+H+O3+P]FKR_.2
Fig4_MP-DIA-barrel-cortex-p30-3_MHRM.htrms

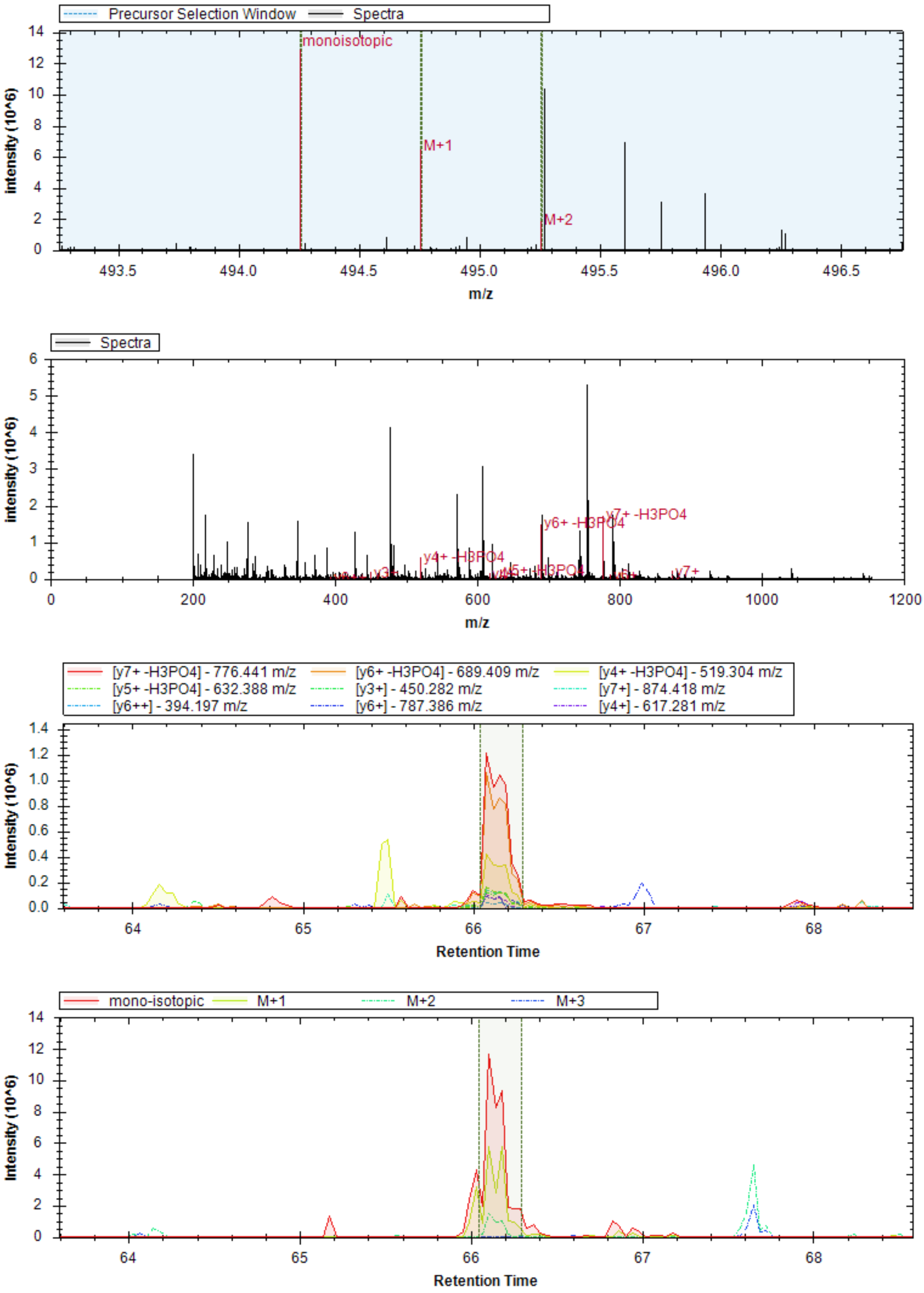

P08553 – _S[+H+O3+P]PVEEVKPKPEAK_.3
Fig4_MP-DIA-barrel-cortex-p30-3_MHRM.htrms

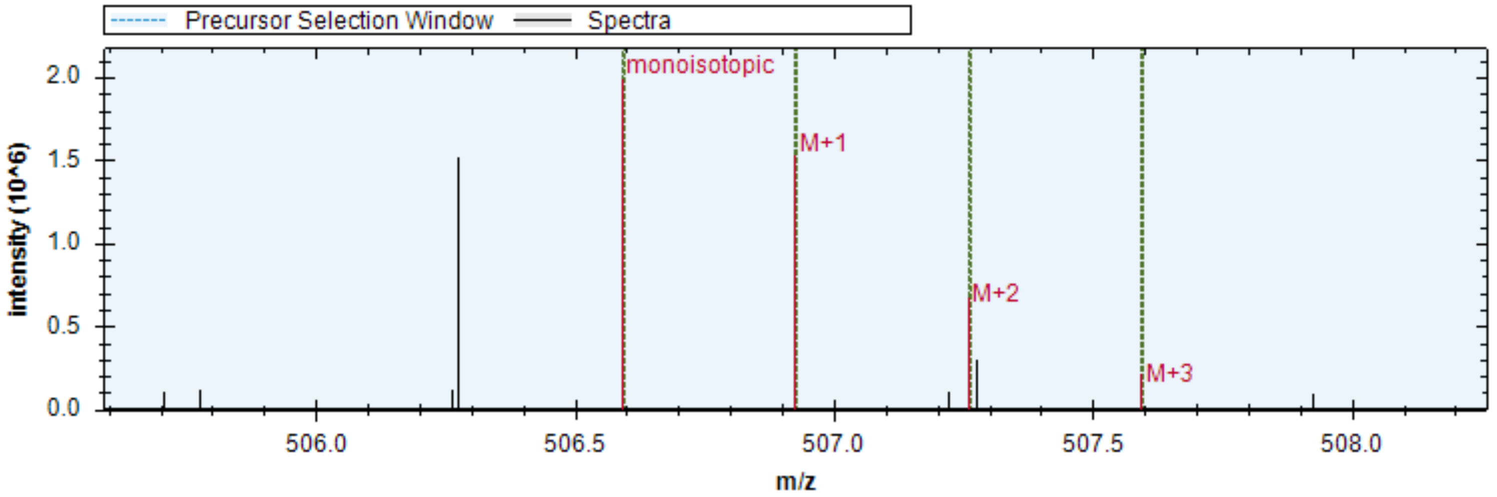

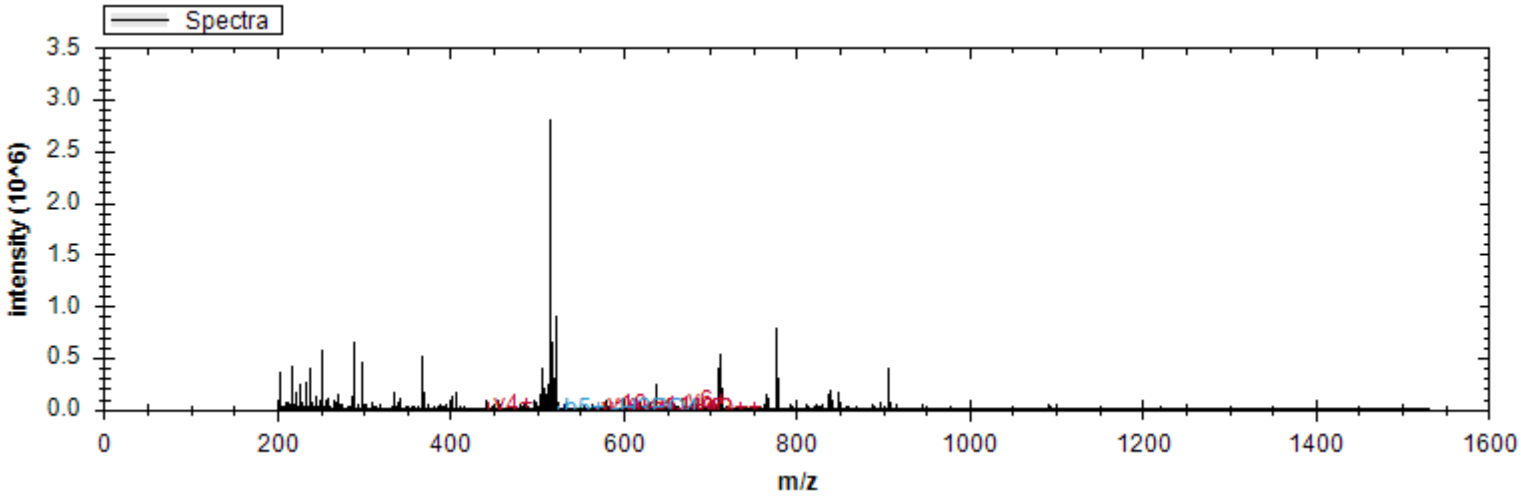

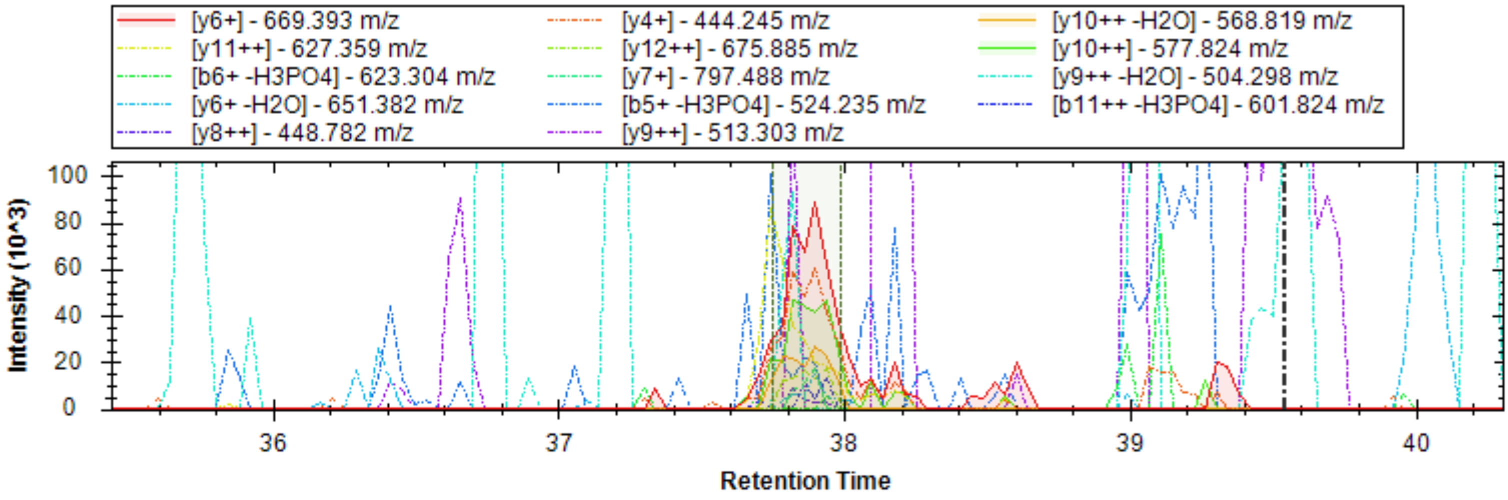

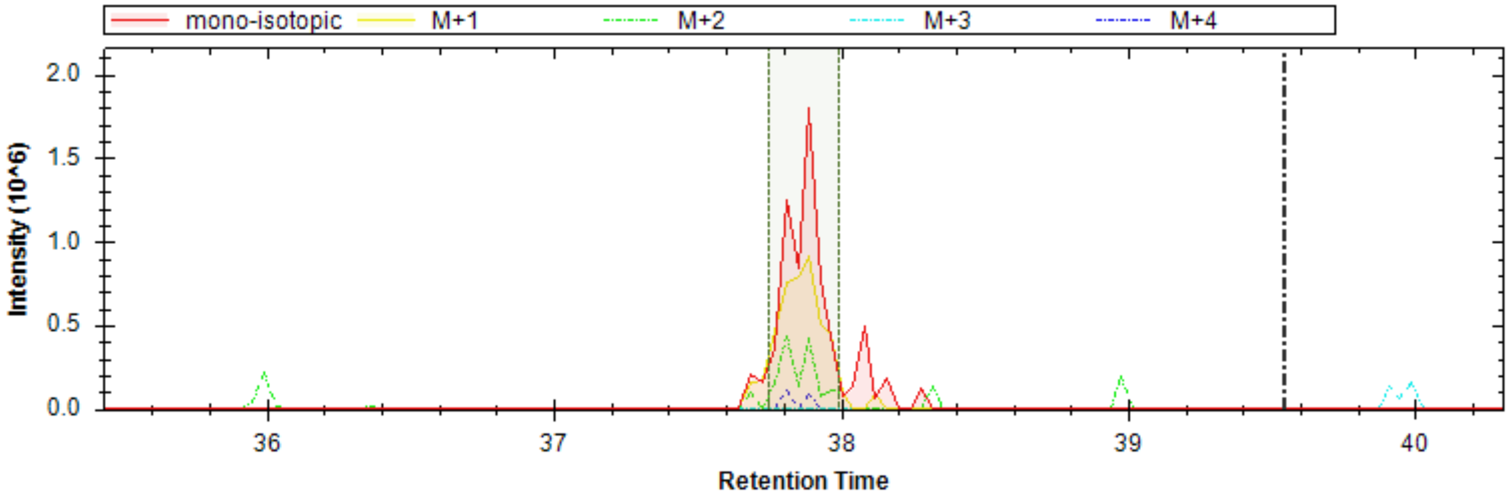

P28738 – _PIRPGHYPAS[+H+O3+P]SPTAVHAVR_.4
Fig4_MP-DIA-barrel-cortex-p30-3_MHRM.htrms

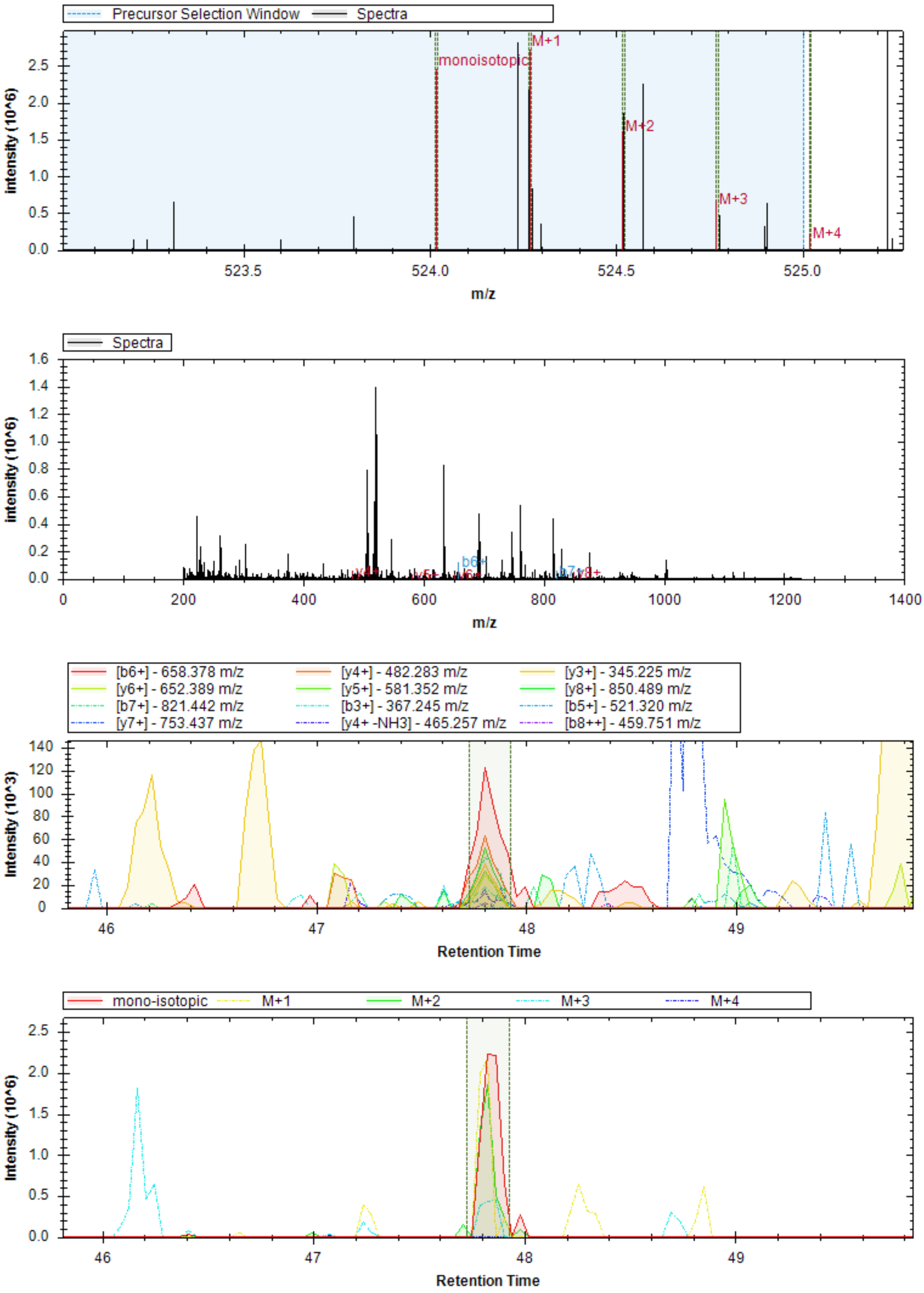

P28738 – _PIRPGHYPASS[+H+O3+P]PTAVHAVR_.4

Fig4_MP-DIA-barrel-cortex-p30-3_MHRM.htrms

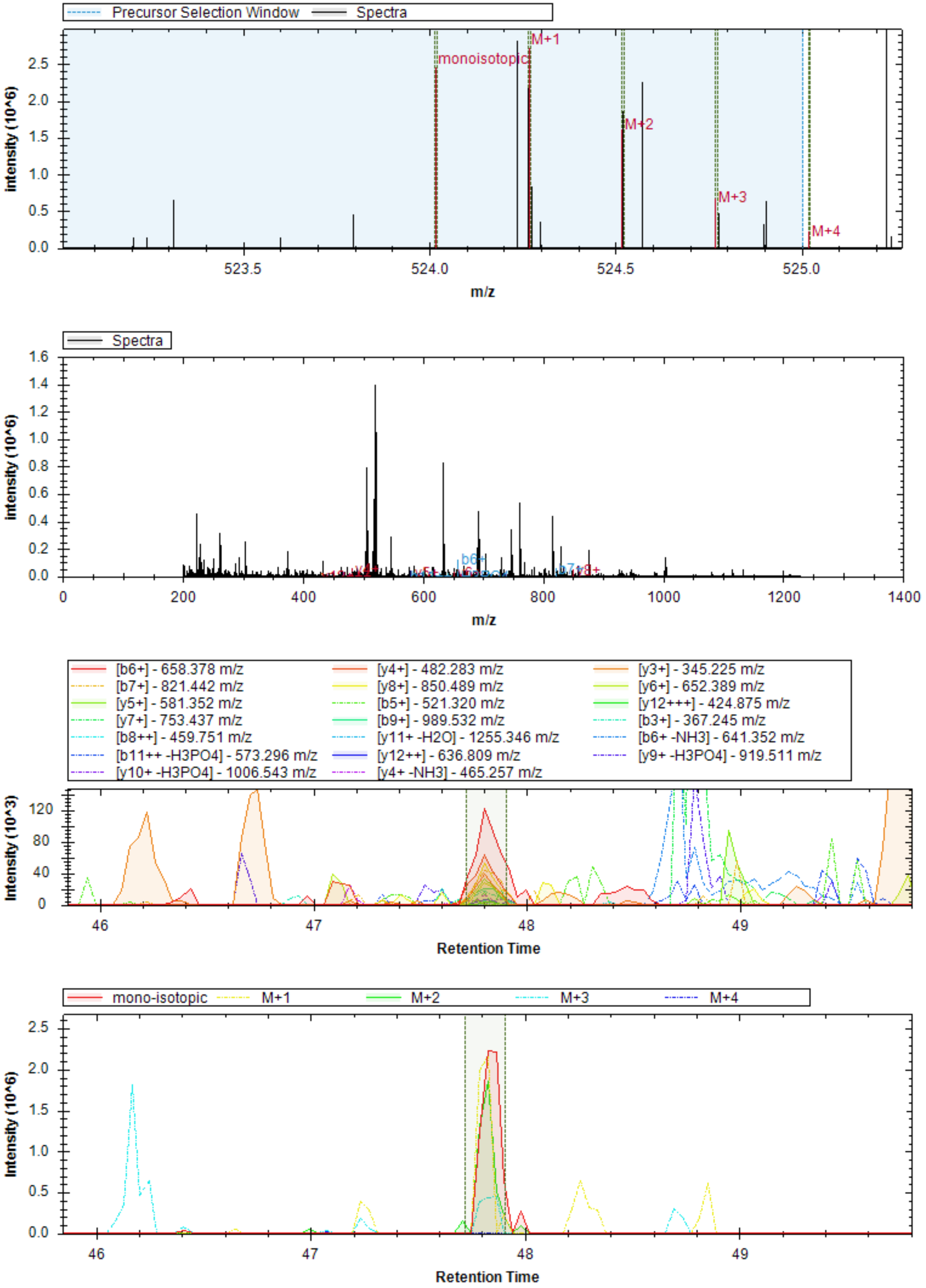

Q8CDY7 – _LVQALC[+C2+H3+N+O]EQIHT[+H+O3+P]R_.3
Fig4_MP-DIA-barrel-cortex-p30-3_MHRM.htrms

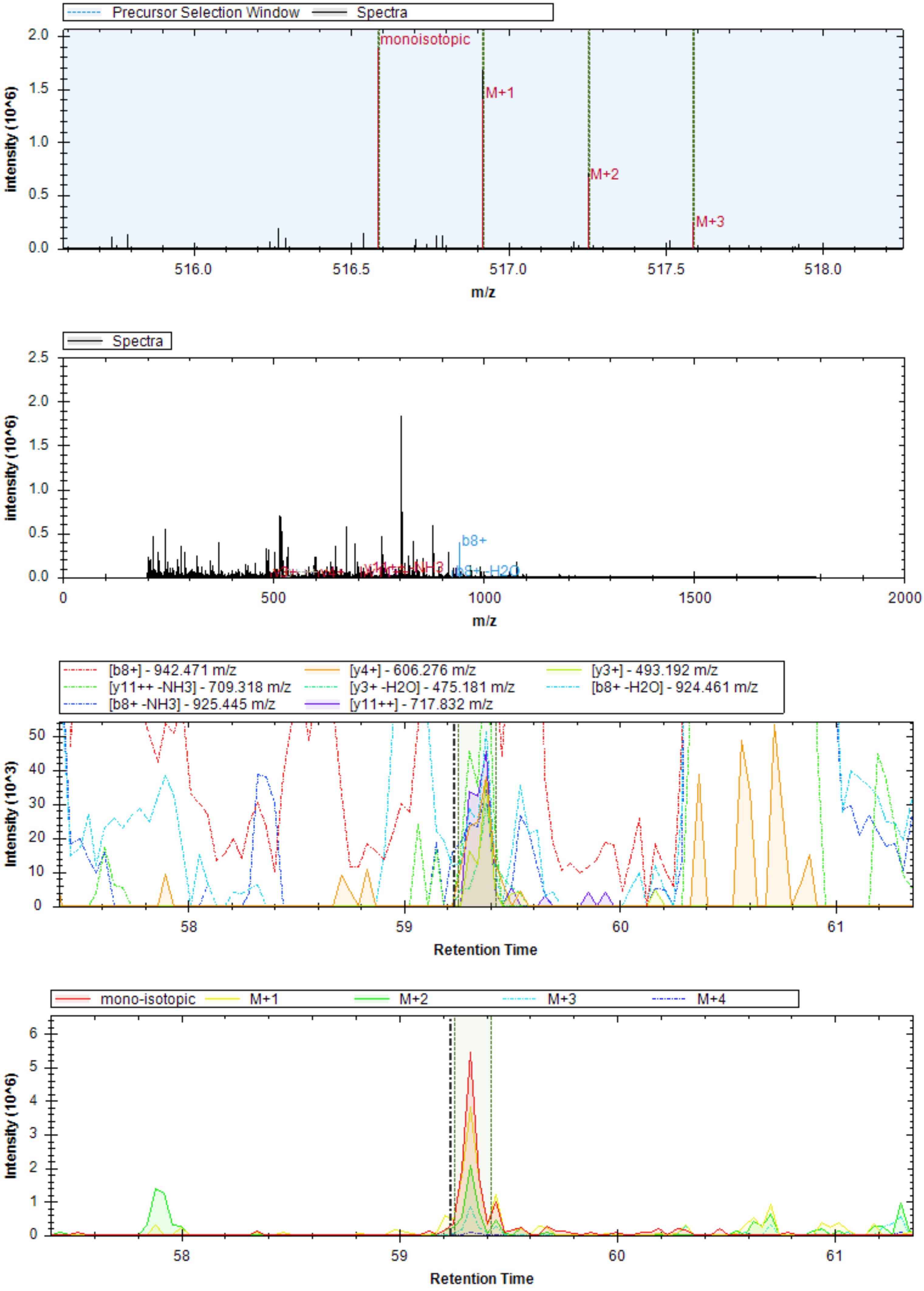

Q9QYC0 – _AAVVT[+H+O3+P]SPPPTTAPHK_.3

Fig4_MP-DIA-barrel-cortex-p30-3_MHRM.htrms

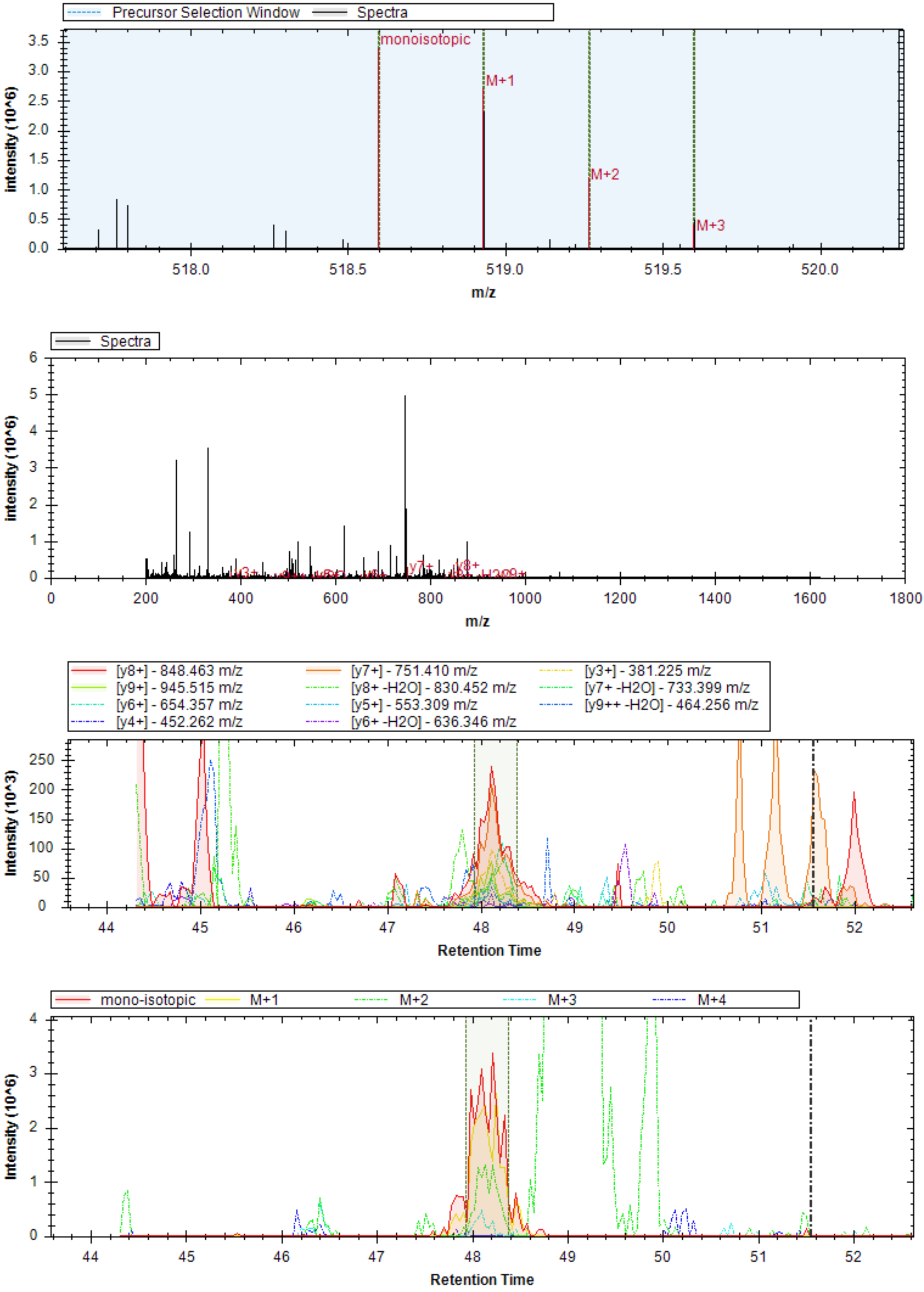

Q9QYC0 – _AAVVTS[+H+O3+P]PPPTTAPHK_.3

Fig4_MP-DIA-barrel-cortex-p30-3_MHRM.htrms

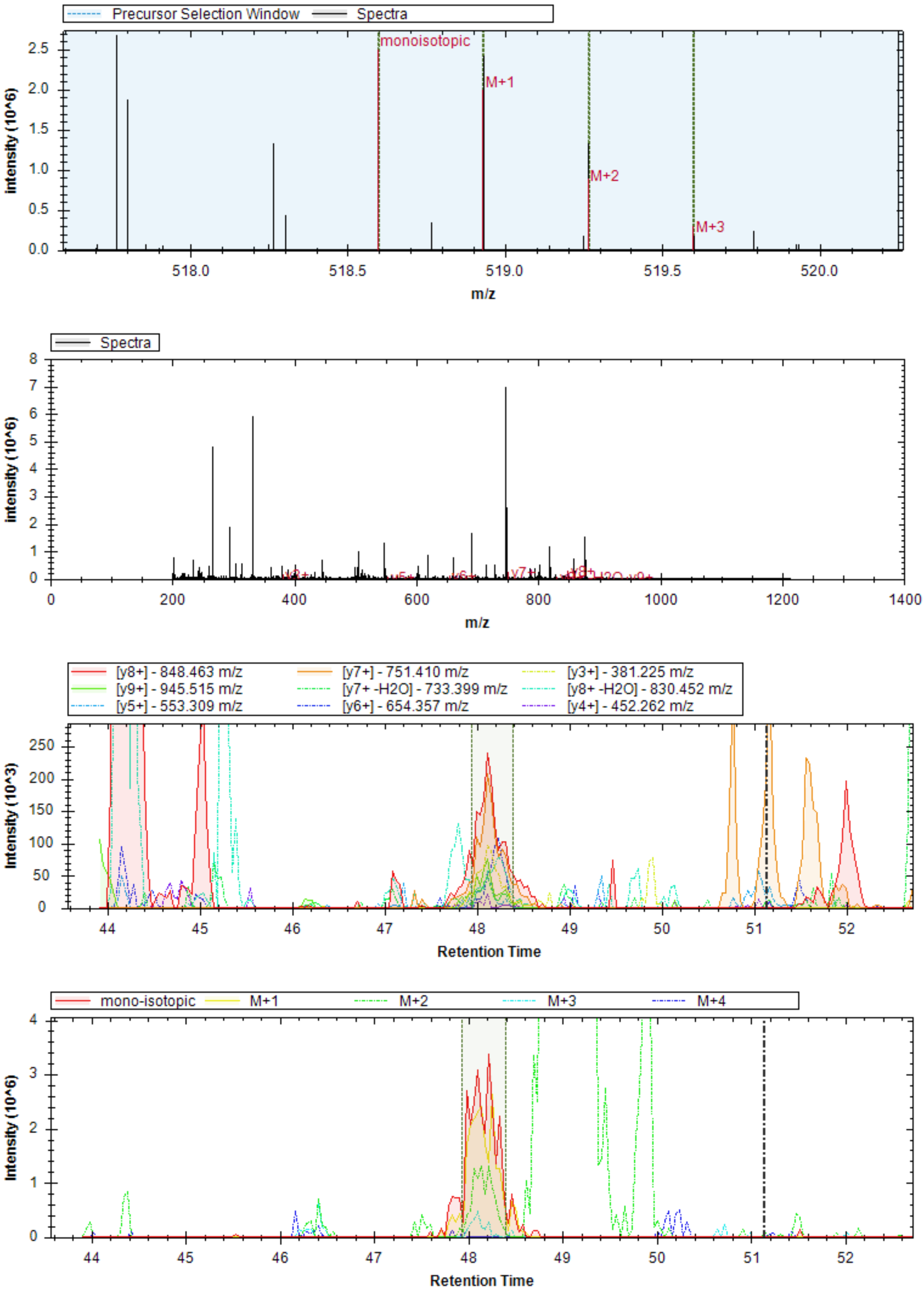

P10637 – _VAVVRT[+H+O3+P]PPKSPSASK_.3

Fig4_MP-DIA-barrel-cortex-p30-3_MHRM.htrms

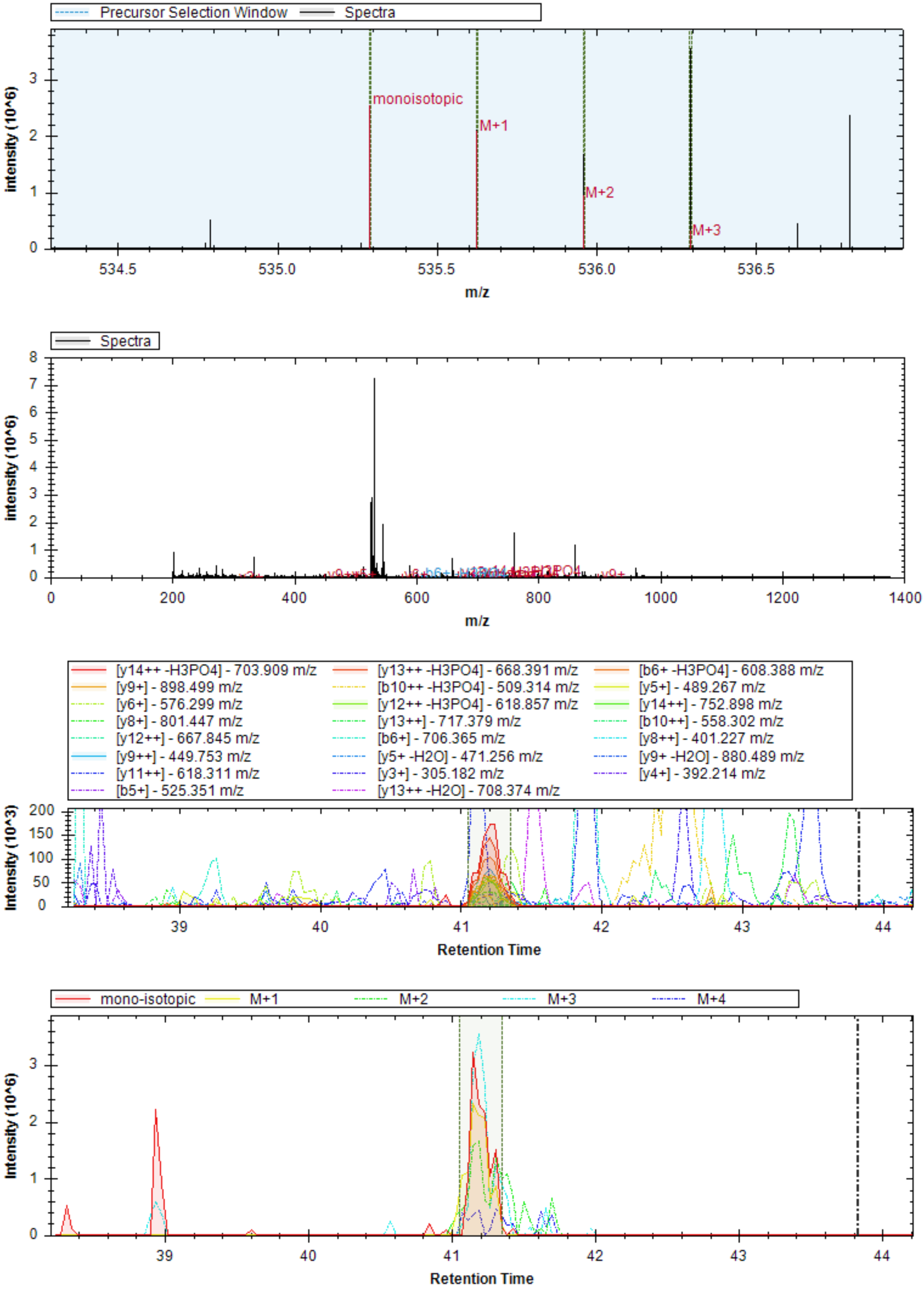

P63318 – _AAPALT[+H+O3+P]PPDR_.2
Fig4_MP-DIA-barrel-cortex-p30-3_MHRM.htrms

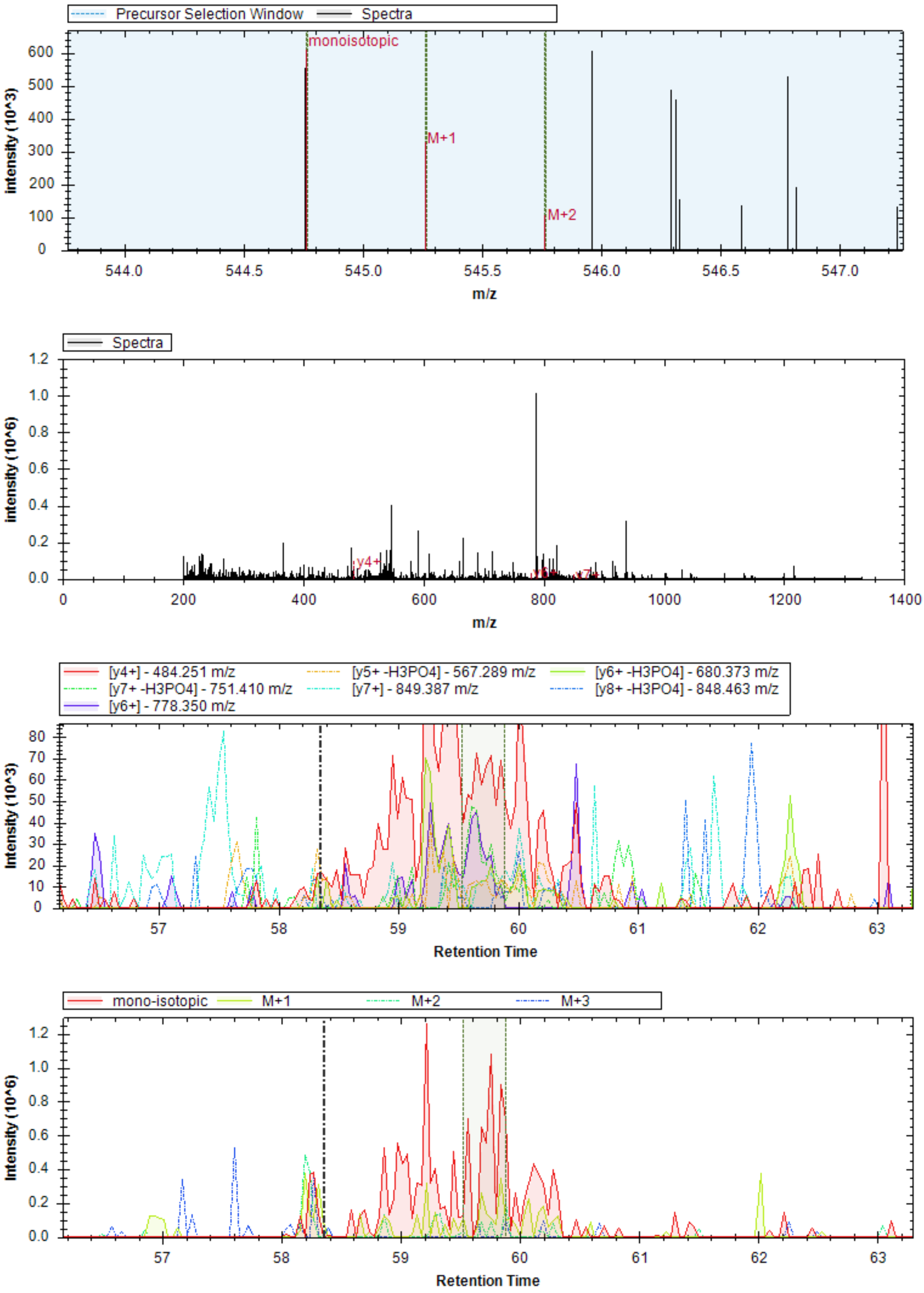

Q7TQD2 – _AANKT[+H+O3+P]PPKSPGDPAR_.3

Fig4_MP-DIA-barrel-cortex-p30-3_MHRM.htrms

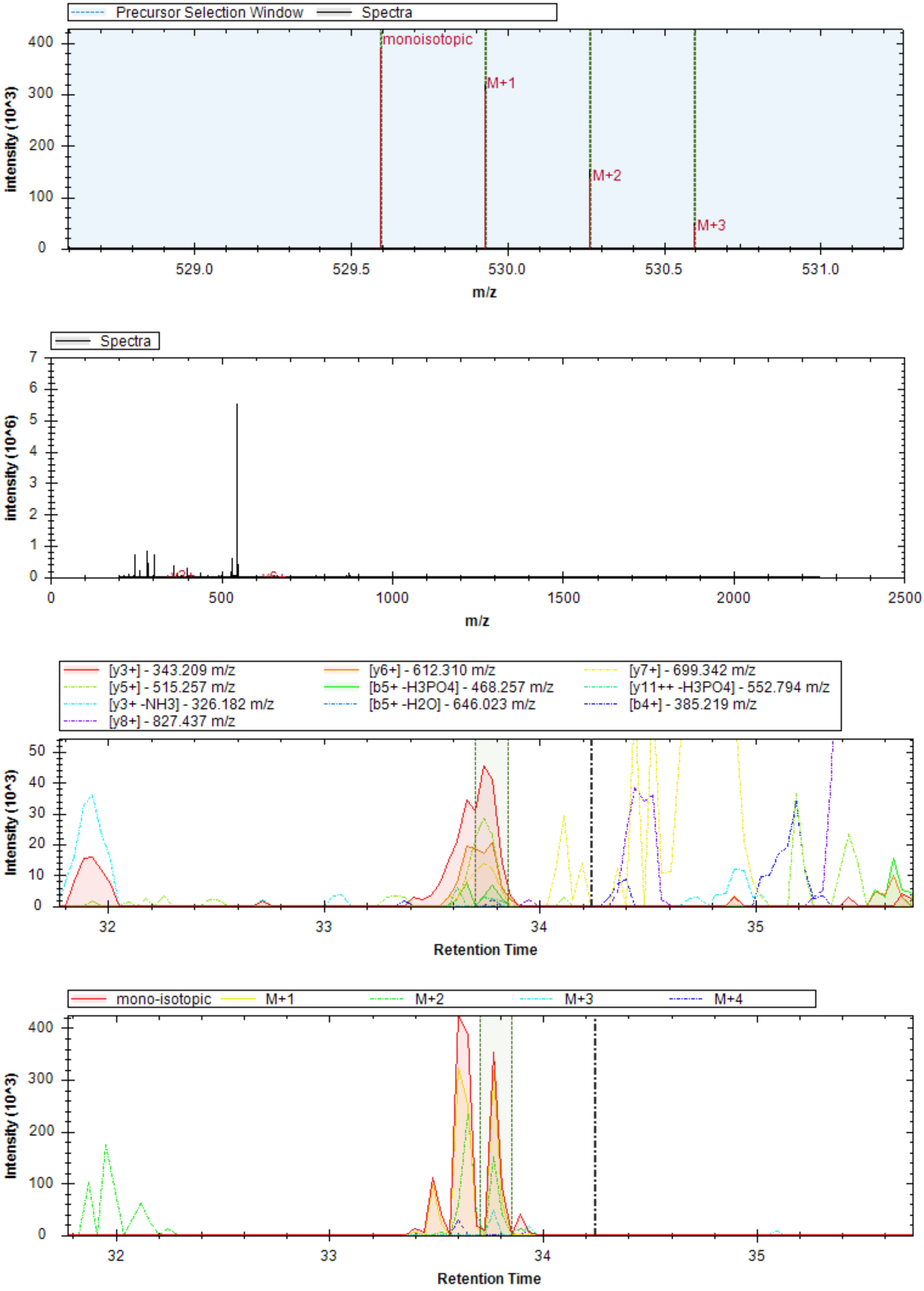

Q9D898 – _AFHAALRNS[+H+O3+P]PINTK_.3
Fig4_MP-DIA-barrel-cortex-p30-3_MHRM.htrms

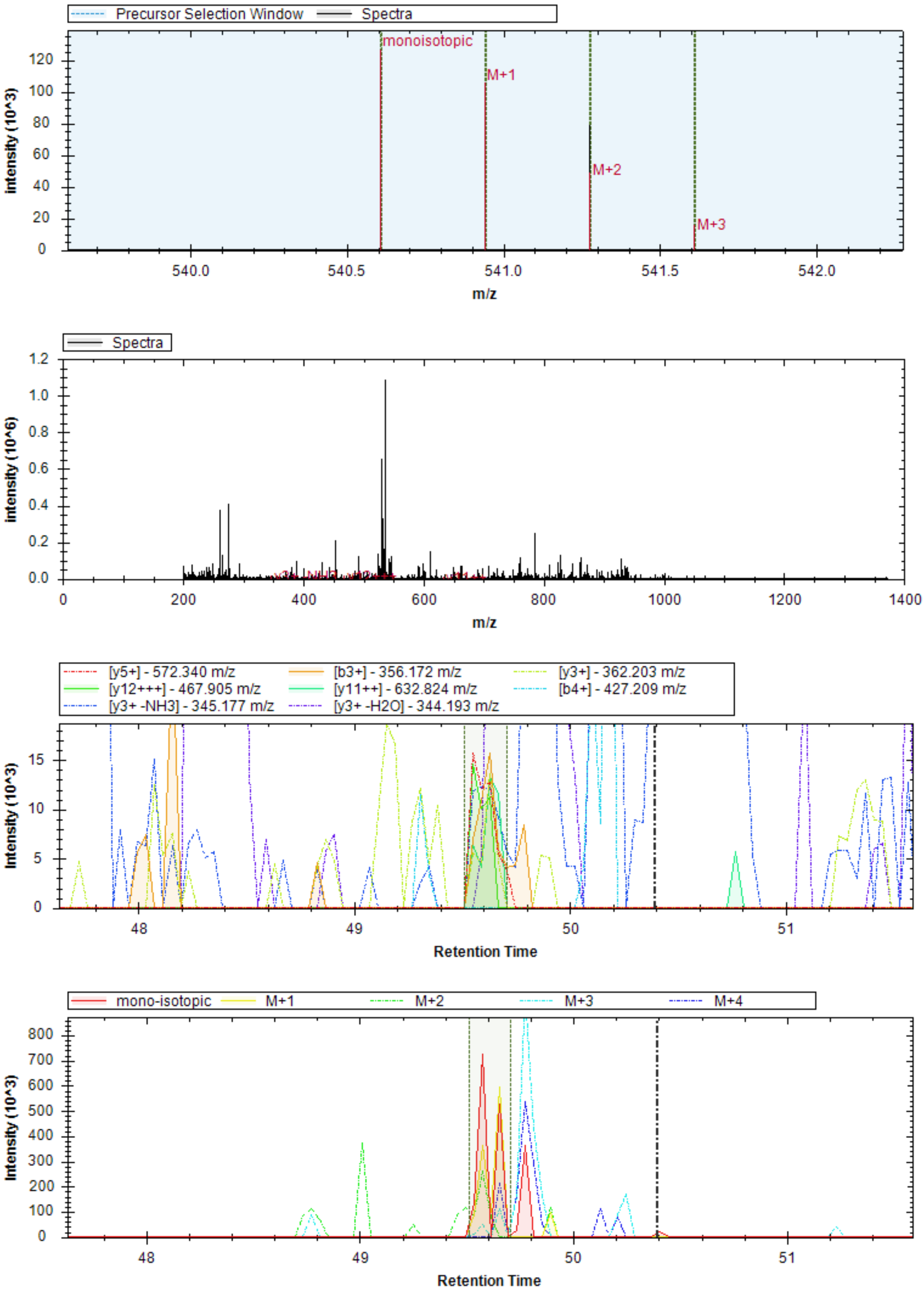

P20357 – _VAIIRT[+H+O3+P]PPKSPATPK_.3

Fig4_MP-DIA-barrel-cortex-p30-3_MHRM.htrms

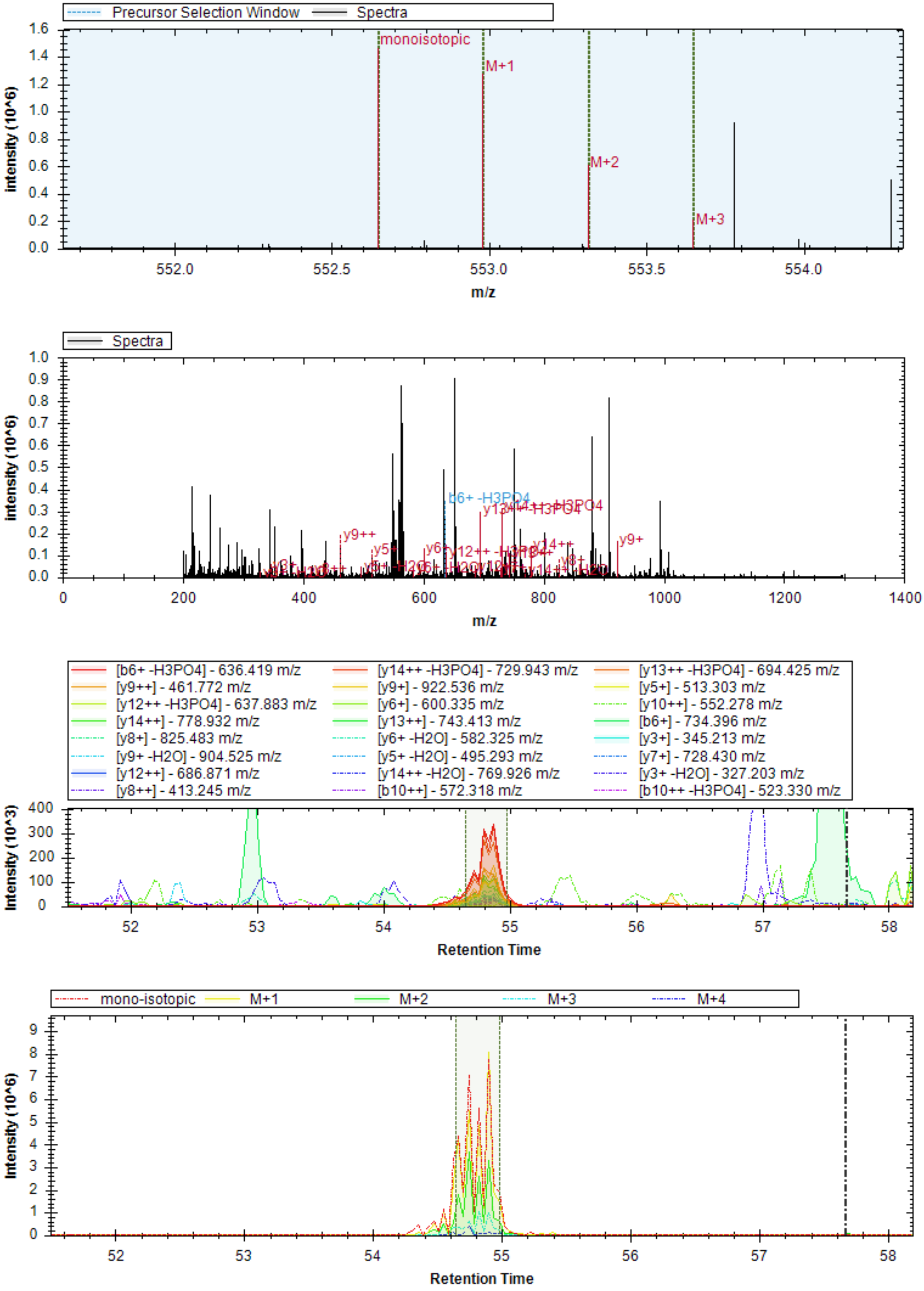

Q9CYT6 – _THTPSPTS[+H+O3+P]PK_.2

Fig4_MP-DIA-barrel-cortex-p30-3_MHRM.htrms

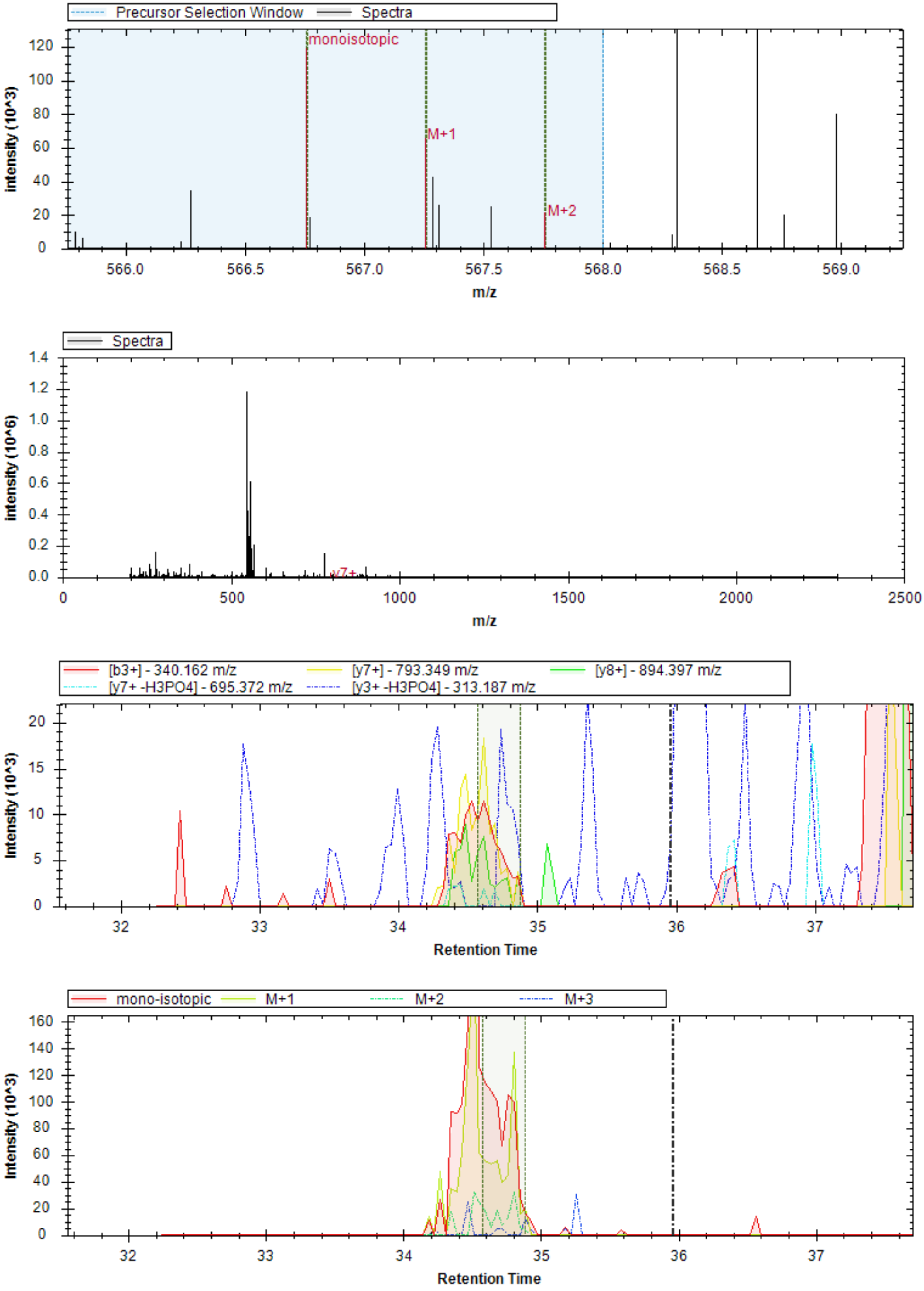

Q9R0K7 – _T[+H+O3+P]SIHNFMAHPEFR_.3
Fig4_MP-DIA-barrel-cortex-p30-3_MHRM.htrms

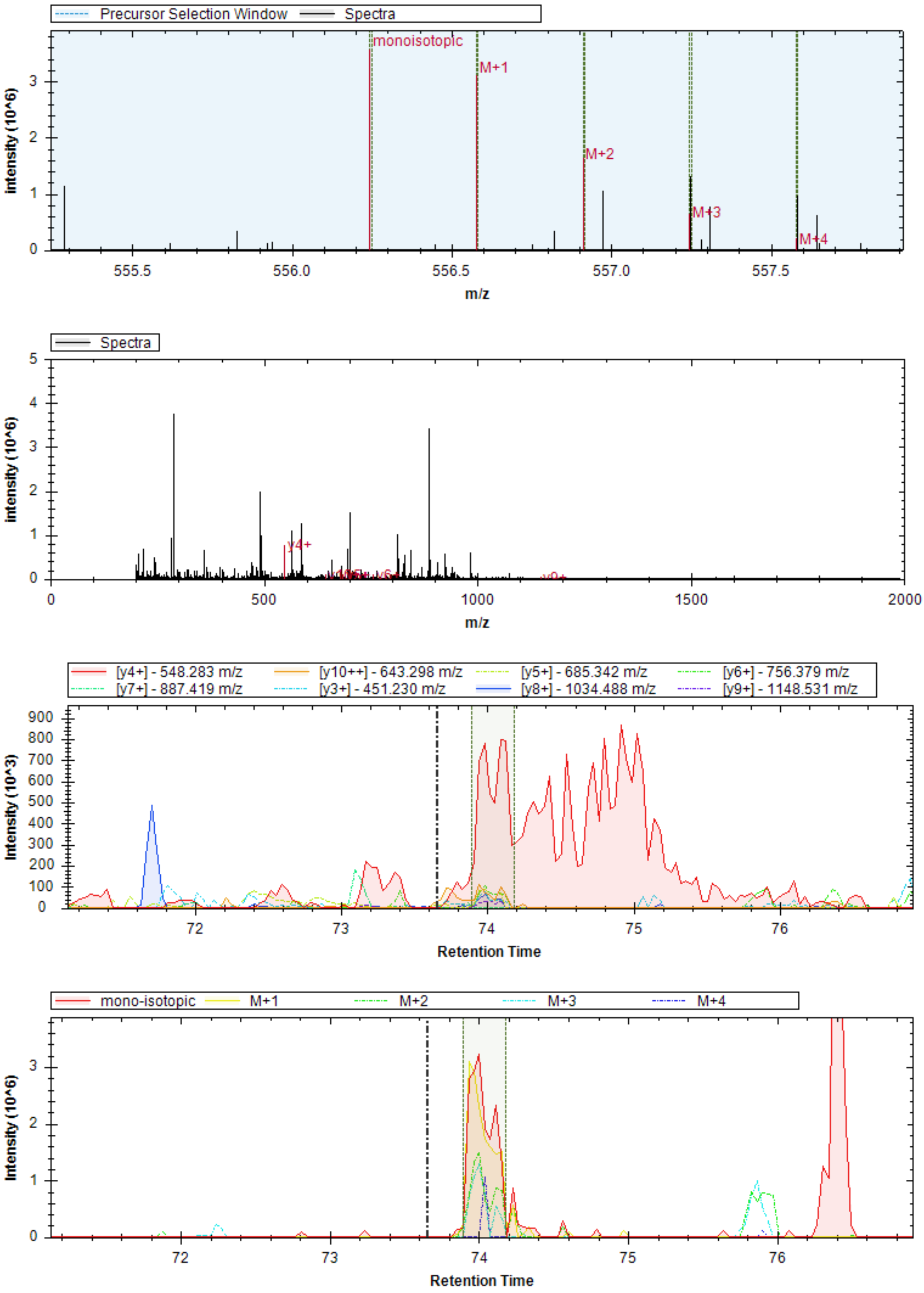

O88935 – _QAS[+H+O3+P]ISGPAPTK_.2
Fig4_MP-DIA-barrel-cortex-p30-3_MHRM.htrms

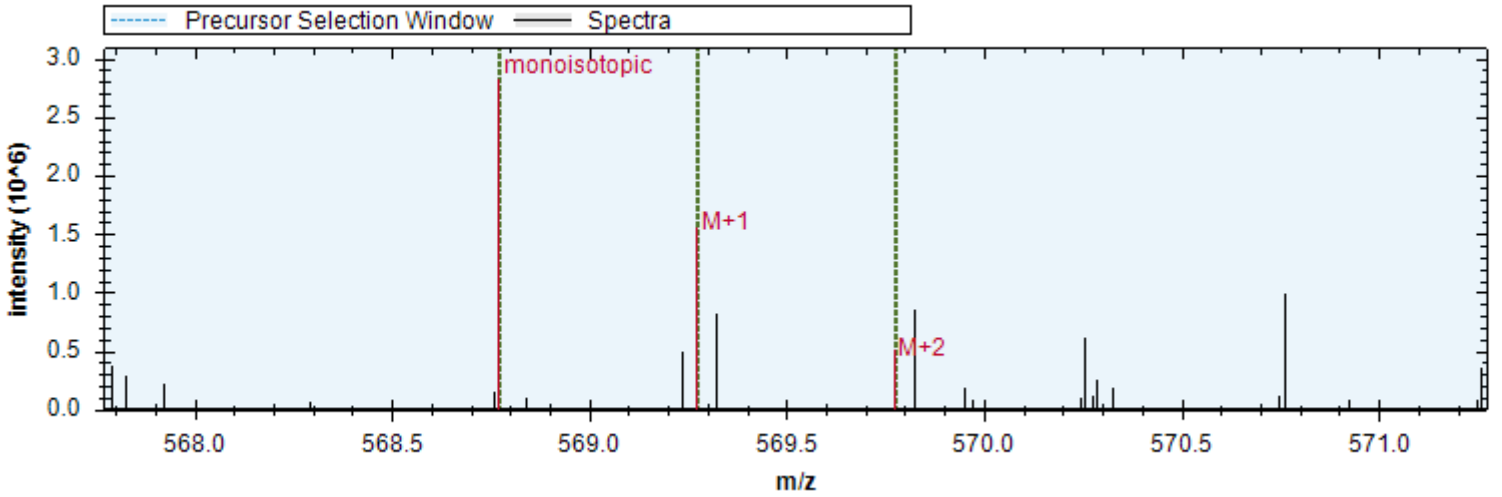

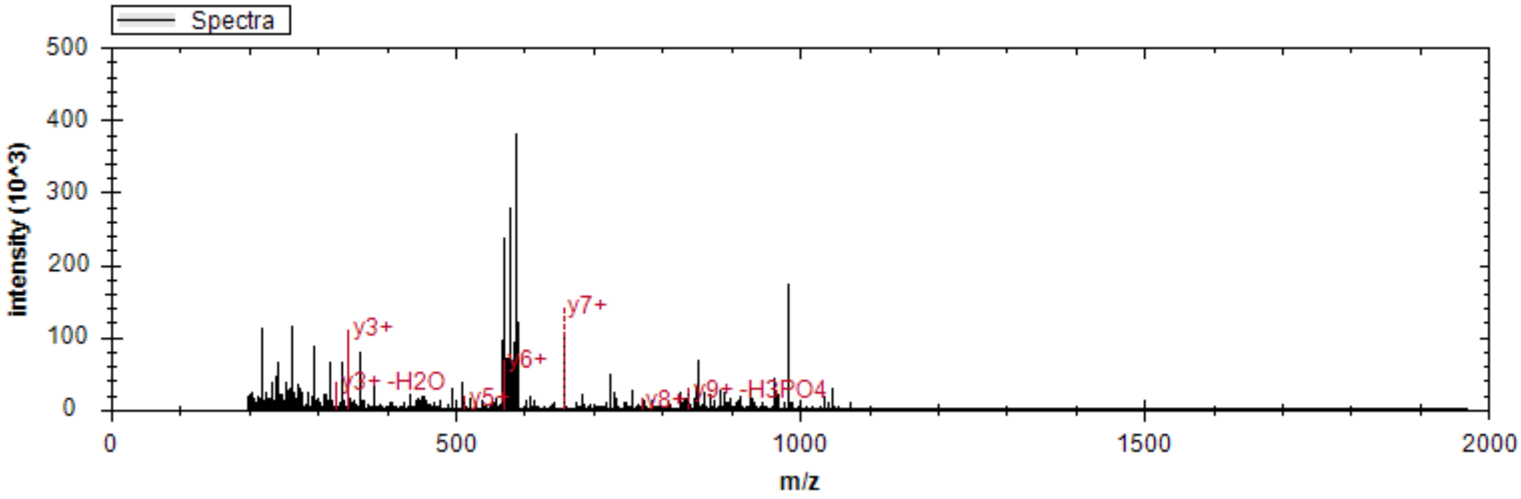

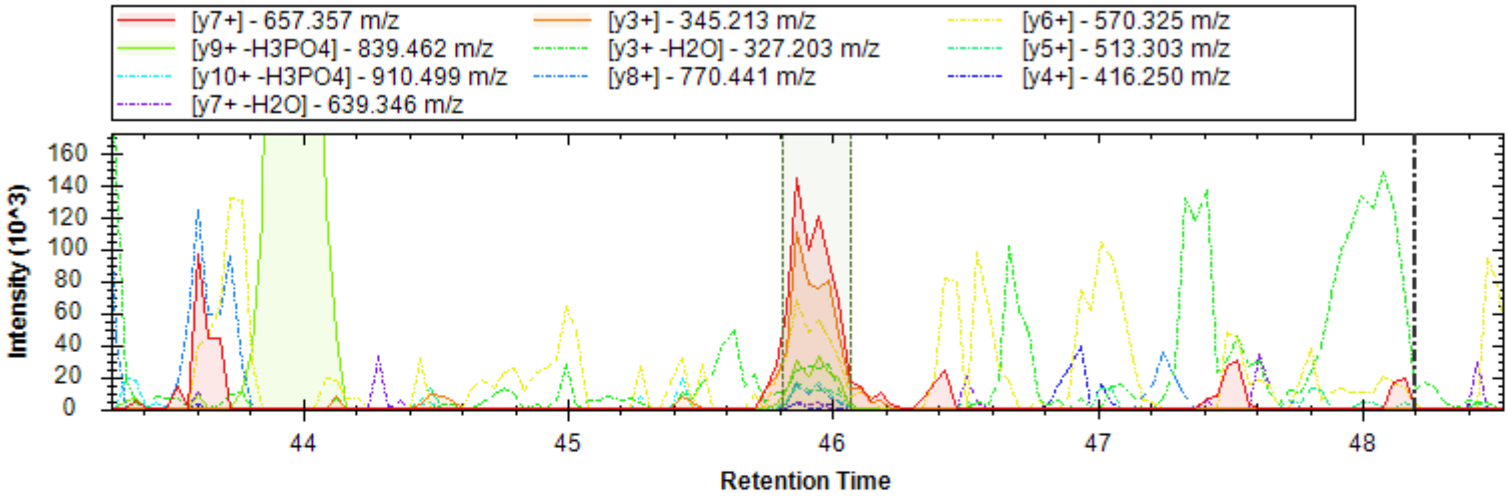

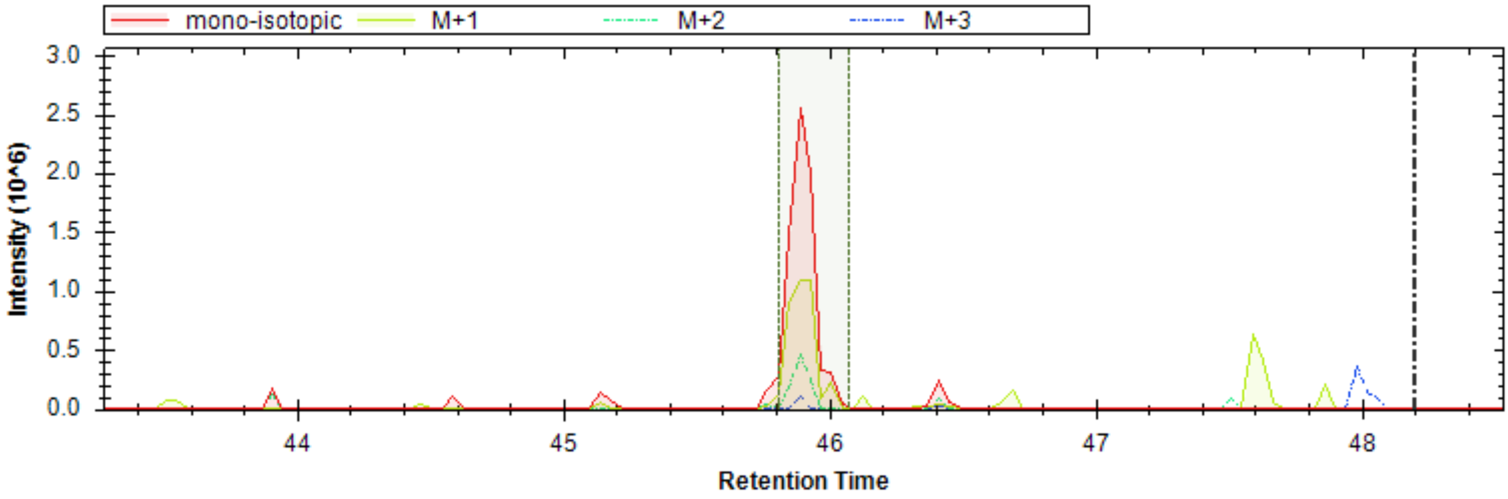

P27546-2 – _T[+H+O3+P]SPSKPSSAPALKPGPK_.3
Fig4_MP-DIA-barrel-cortex-p30-3_MHRM.htrms

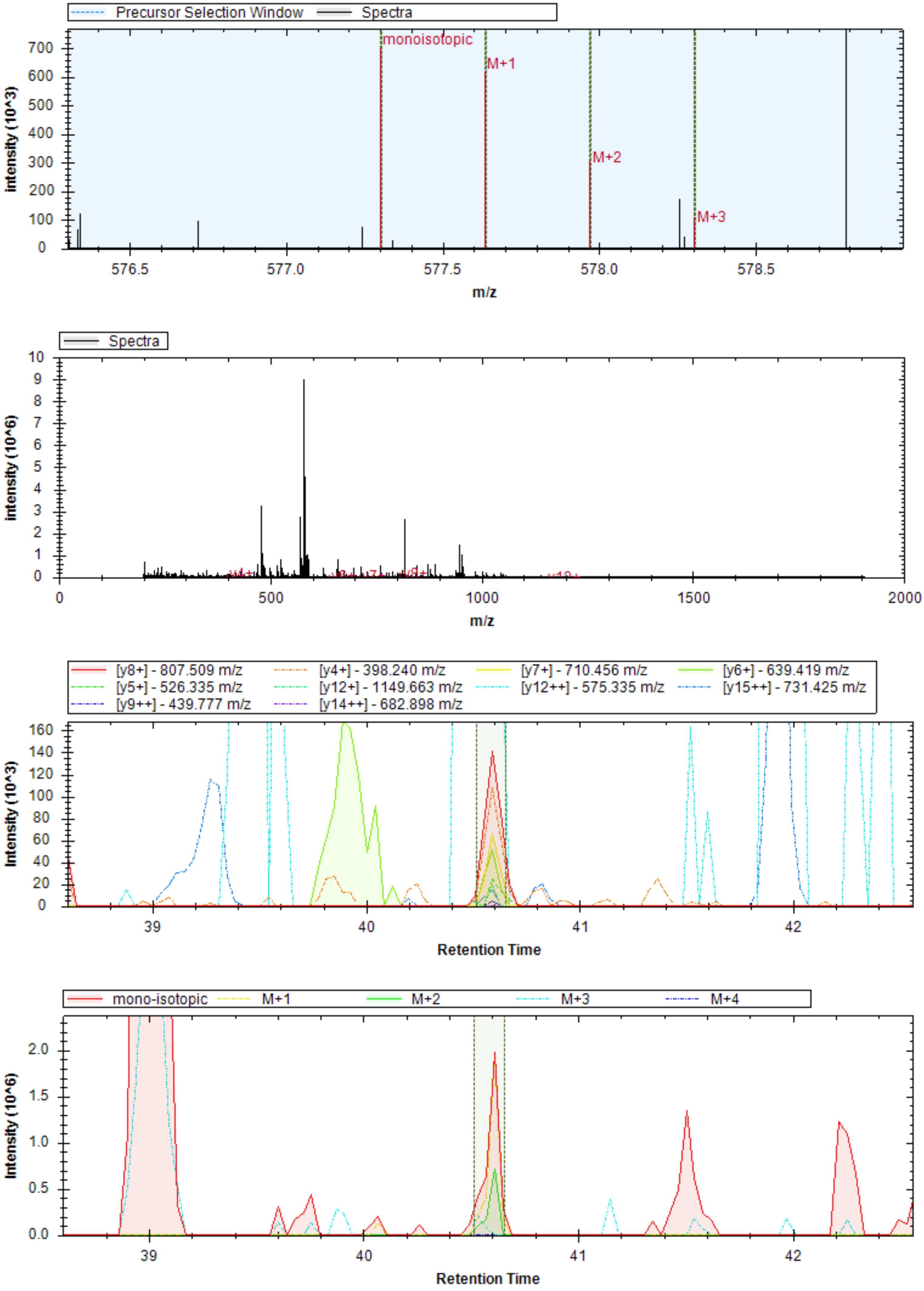

Q64012-2 – _GRLS[+H+O3+P]PVPVPR_.2

Fig4_MP-DIA-barrel-cortex-p30-3_MHRM.htrms

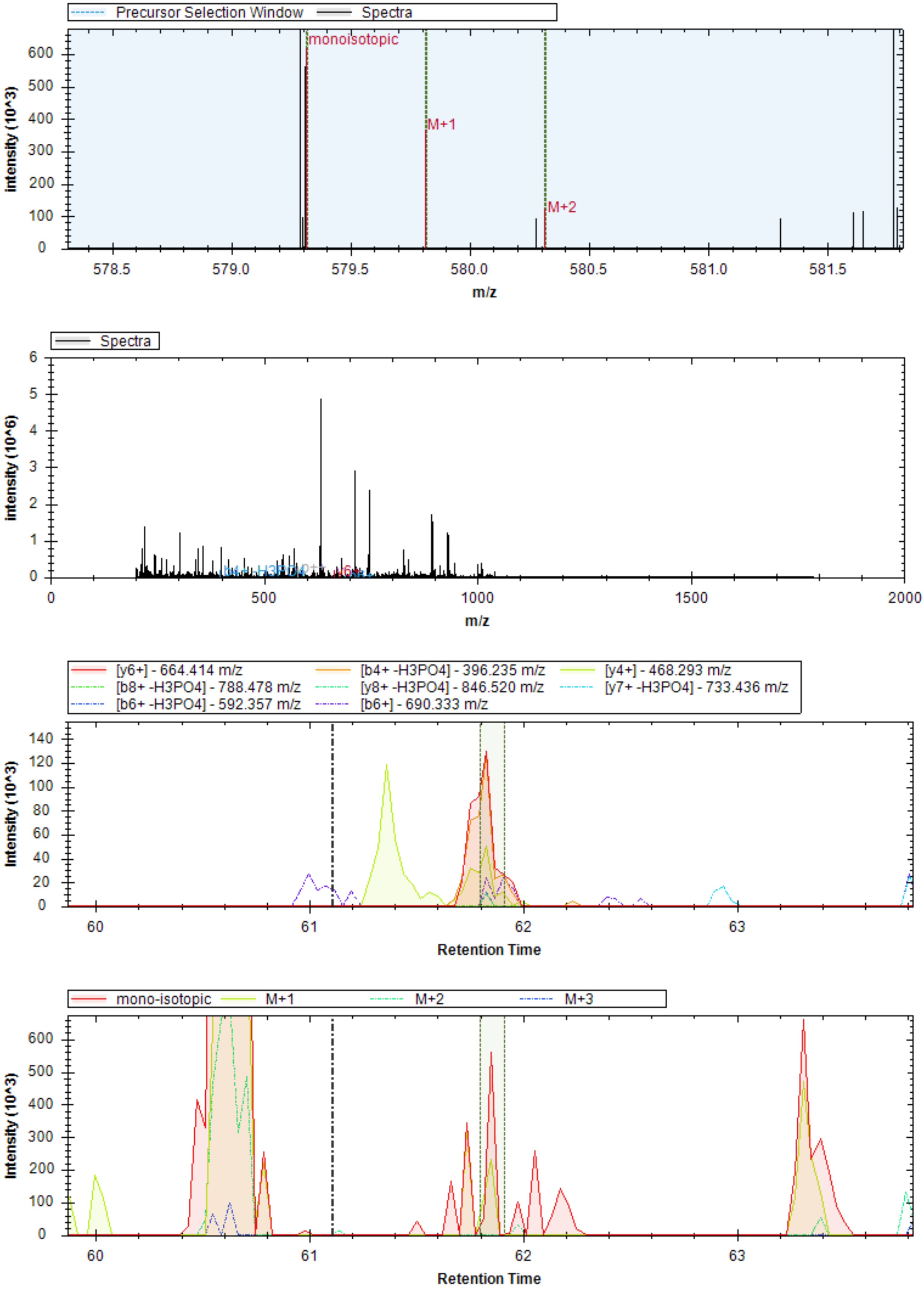

Q8BFU3-4 – _VAS[+H+O3+P]STQVRGASGRELR_.3

Fig4_MP-DIA-barrel-cortex-p30-3_MHRM.htrms

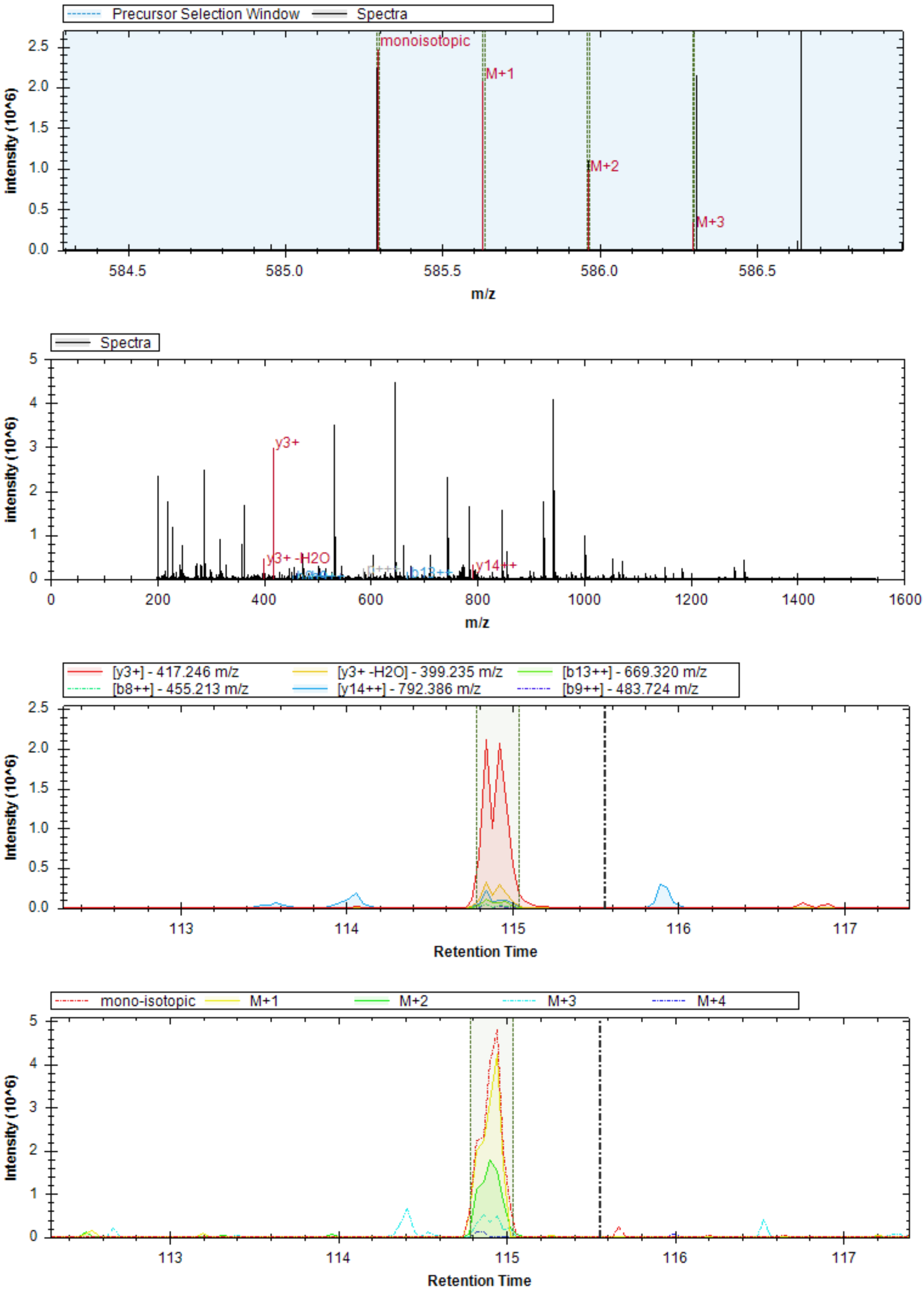

Q9CYT6 – _AQGQIRS[+H+O3+P]PTK_.2
Fig4_MP-DIA-barrel-cortex-p30-3_MHRM.htrms

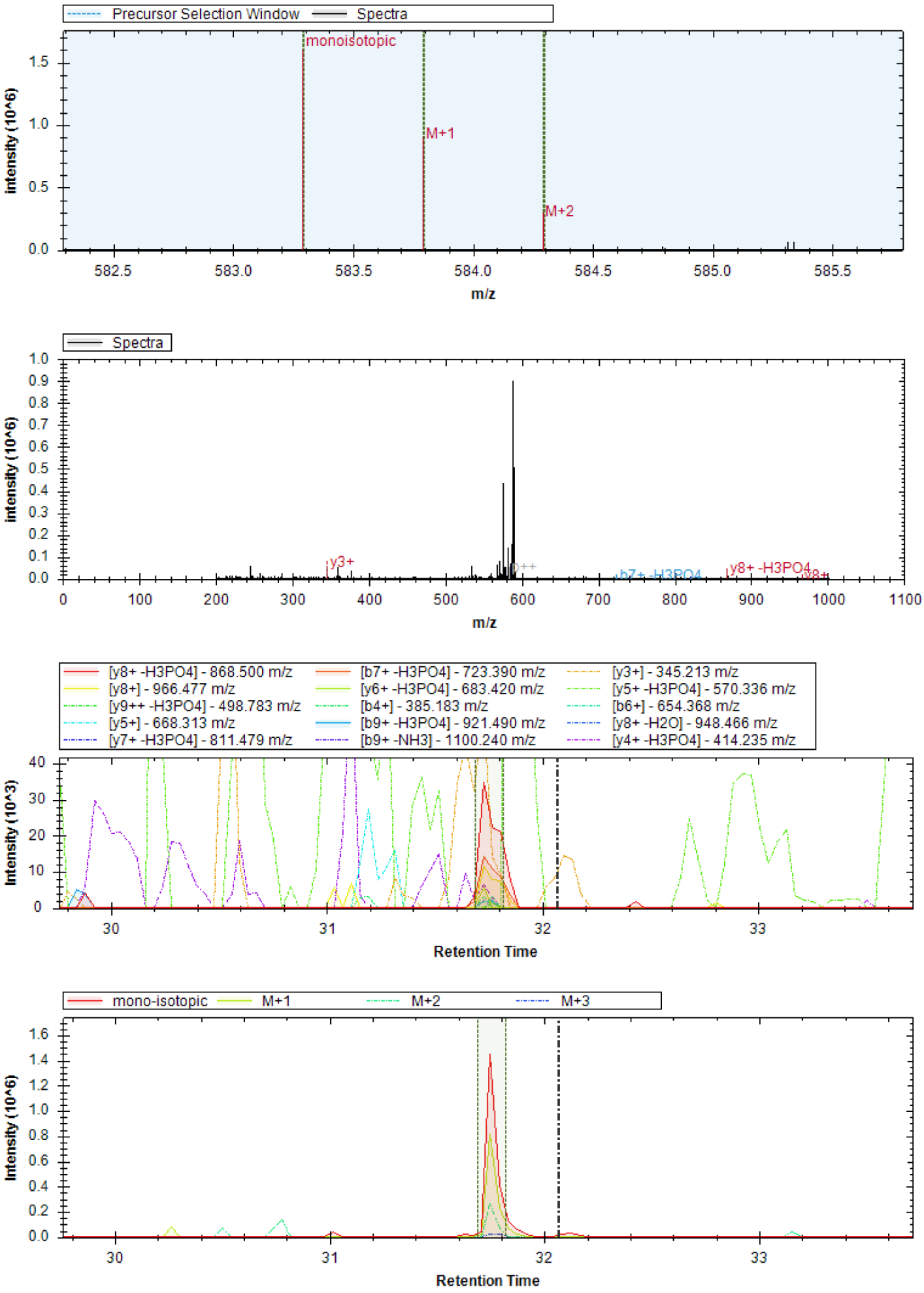

Q9D5Y0 – _ITDT[+H+O3+P]KKTDDLYWR_.3
Fig4_MP-DIA-barrel-cortex-p30-3_MHRM.htrms

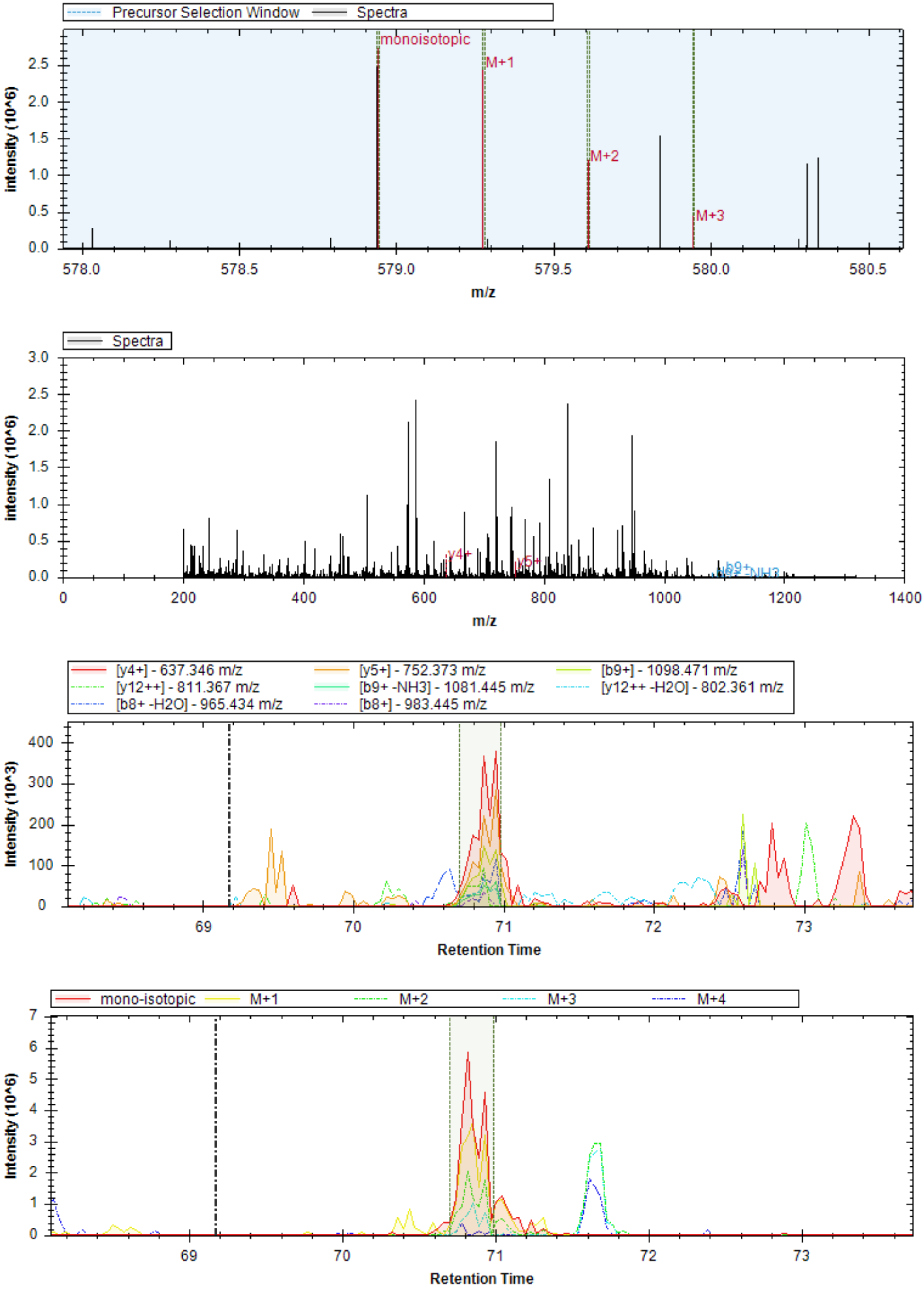

P04370-4 – _TTHYGS[+H+O3+P]LPQK_.2
Fig4_MP-DIA-barrel-cortex-p30-3_MHRM.htrms

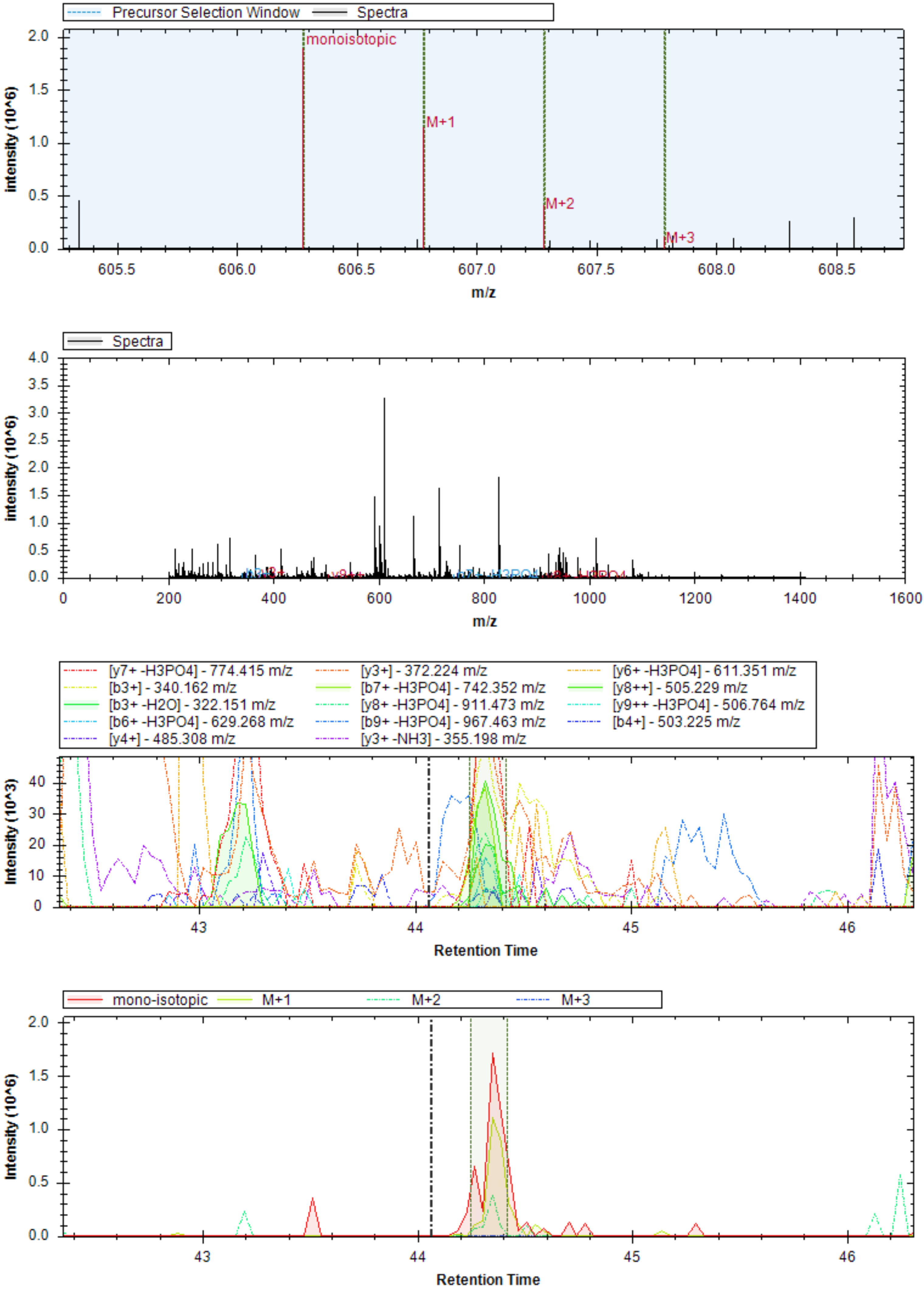

P20357 – _APHWTSASLTEAAAHPHS[+H+O3+P]PEMK_.4
Fig4_MP-DIA-barrel-cortex-p30-3_MHRM.htrms

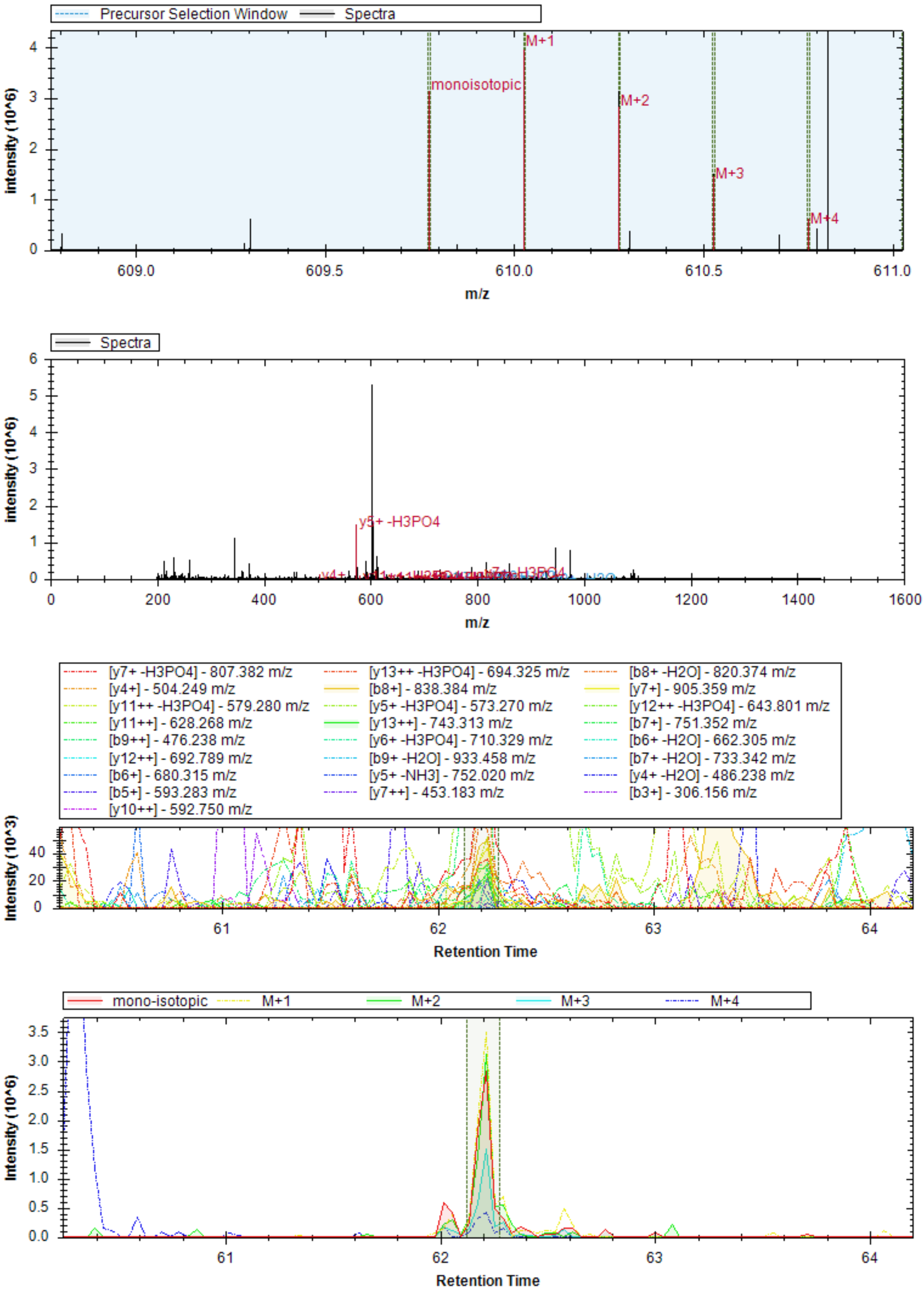

Q62261 – _RPPS[+H+O3+P]PDPNTK_.2

Fig4_MP-DIA-barrel-cortex-p30-3_MHRM.htrms

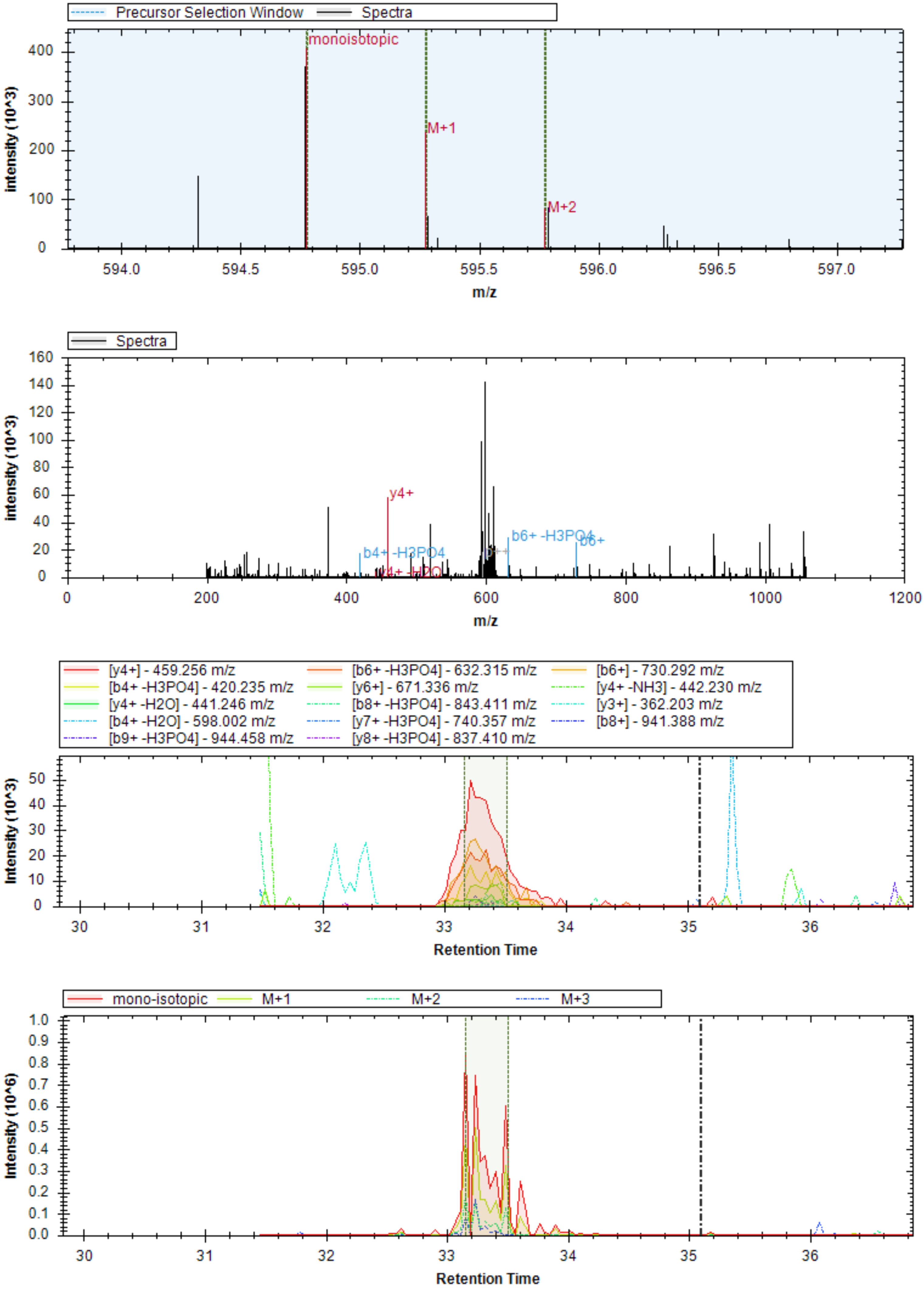

Q80VN0 – _ELTAQIT[+H+O3+P]SIK_.2
Fig4_MP-DIA-barrel-cortex-p30-3_MHRM.htrms

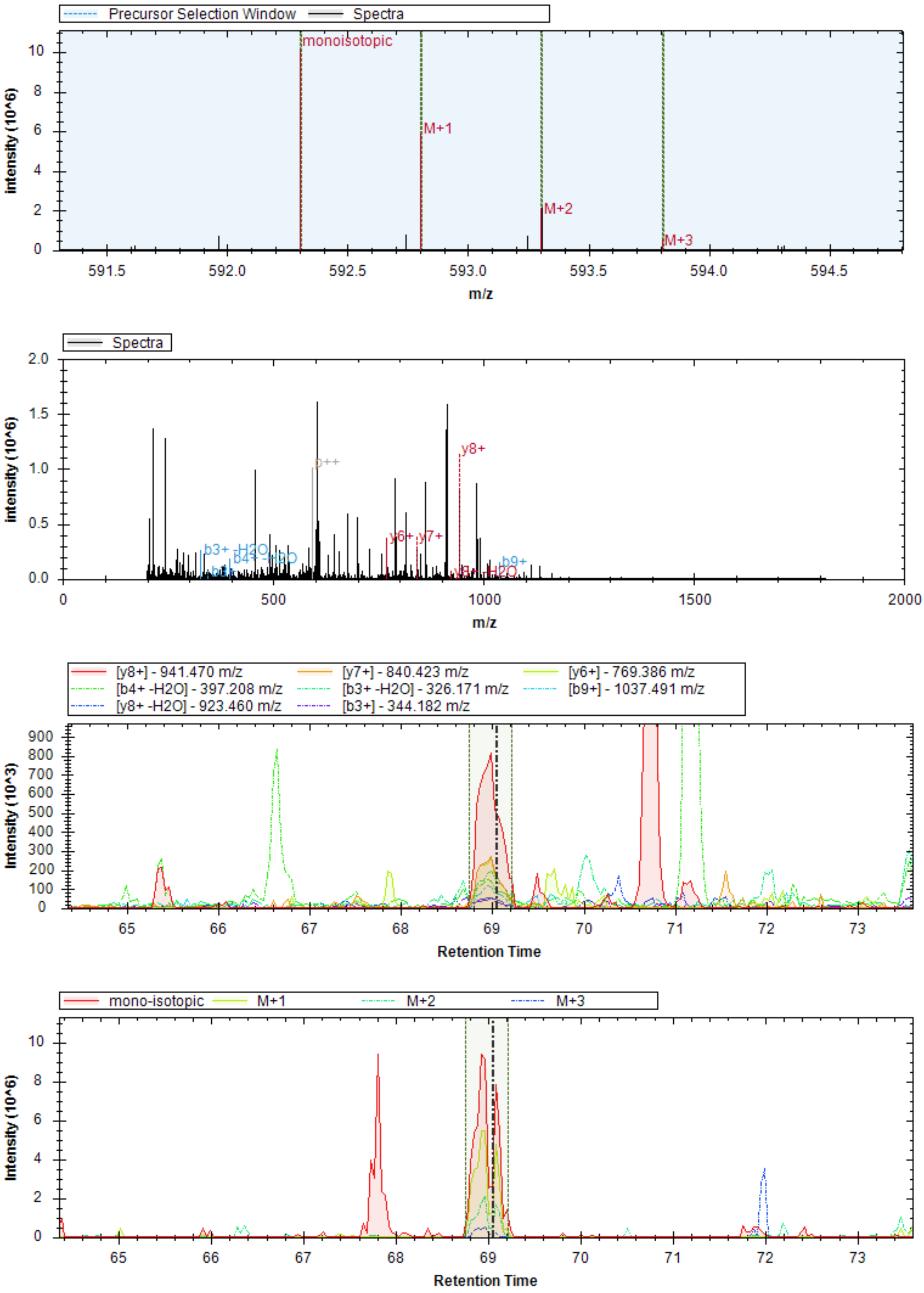

Q99104 – _AIS[+H+O3+P]PTSATSSGR_.2

Fig4_MP-DIA-barrel-cortex-p30-3_MHRM.htrms

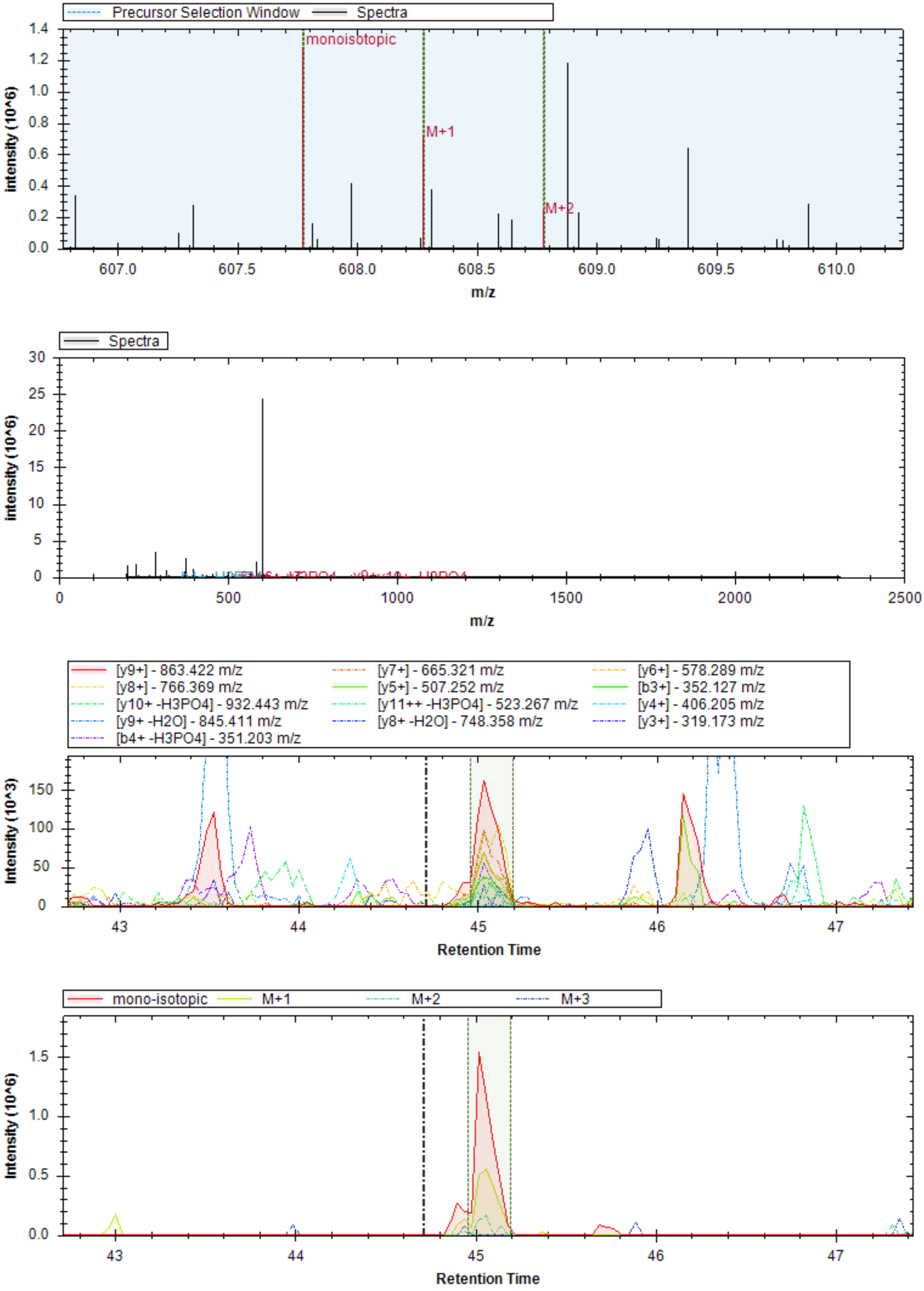

Q9Z2D7 – _KT[+H+O3+P]S[+H+O3+P]LYFSSK_.2
Fig4_MP-DIA-barrel-cortex-p30-3_MHRM.htrms

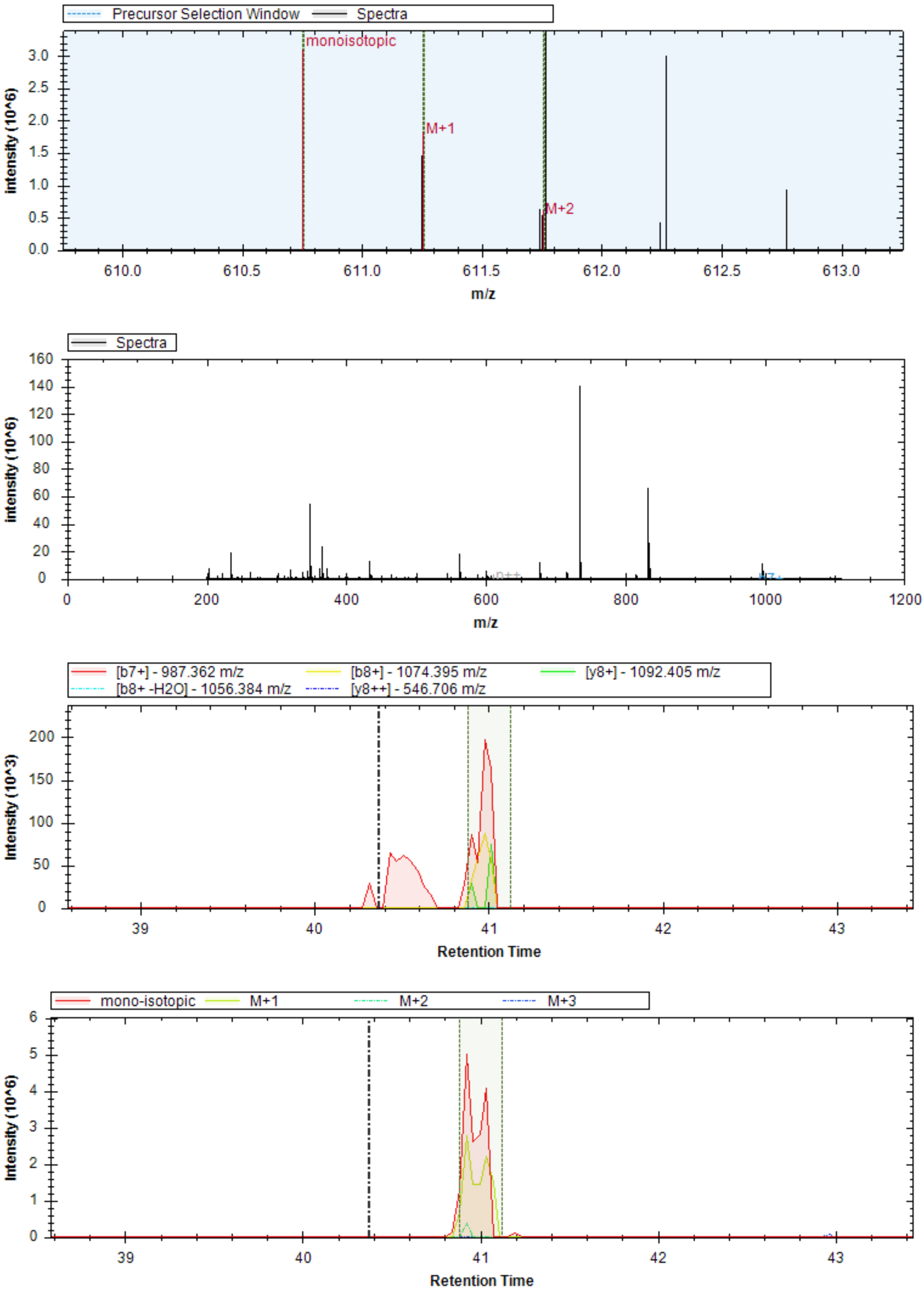

P26645 – _GEATAERPGEAAVASS[+H+O3+P]PSK_.3
Fig4_MP-DIA-barrel-cortex-p30-3_MHRM.htrms

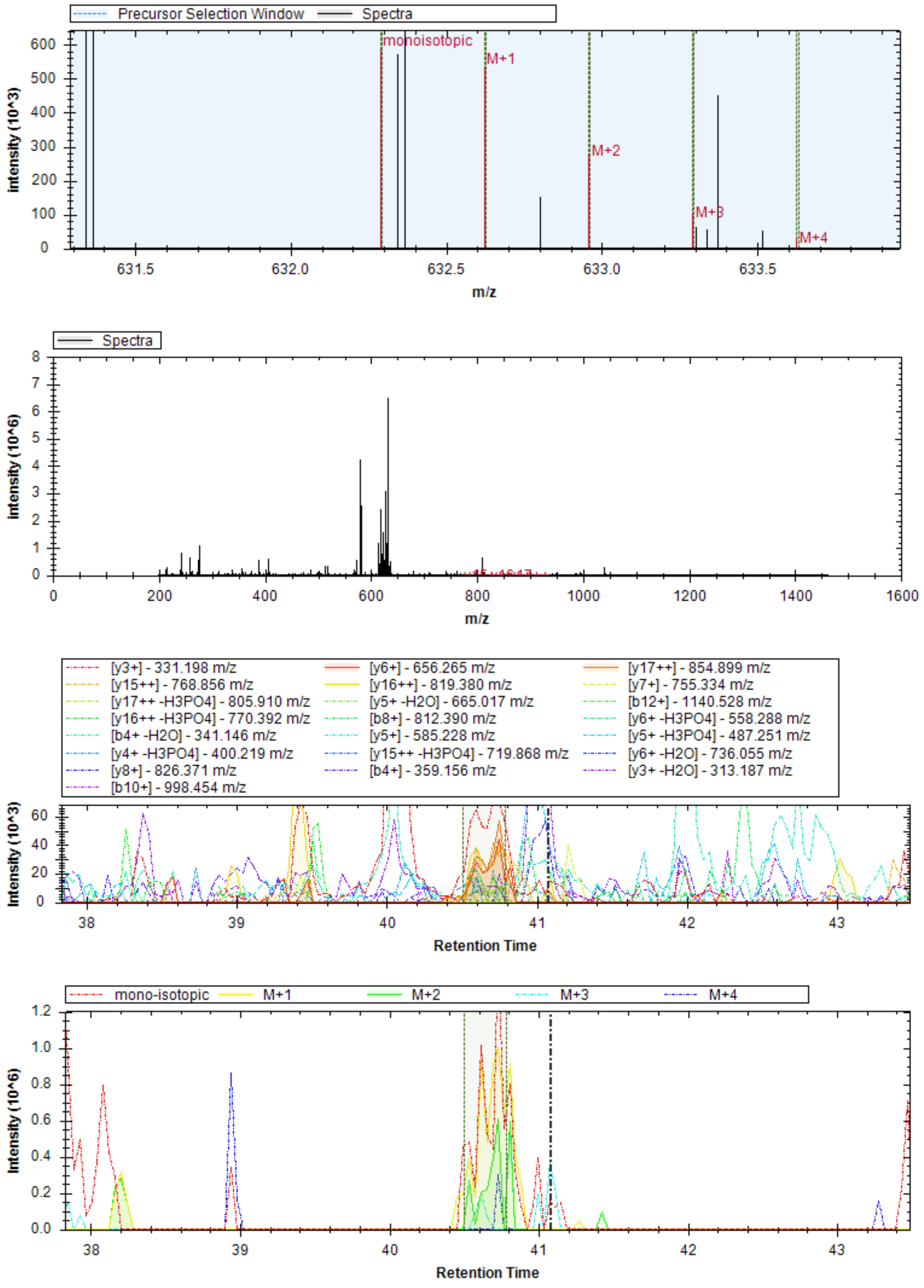

P60904 – _SLS[+H+O3+P]TSGESLYHVLGLDK_.3
Fig4_MP-DIA-barrel-cortex-p30-3_MHRM.htrms

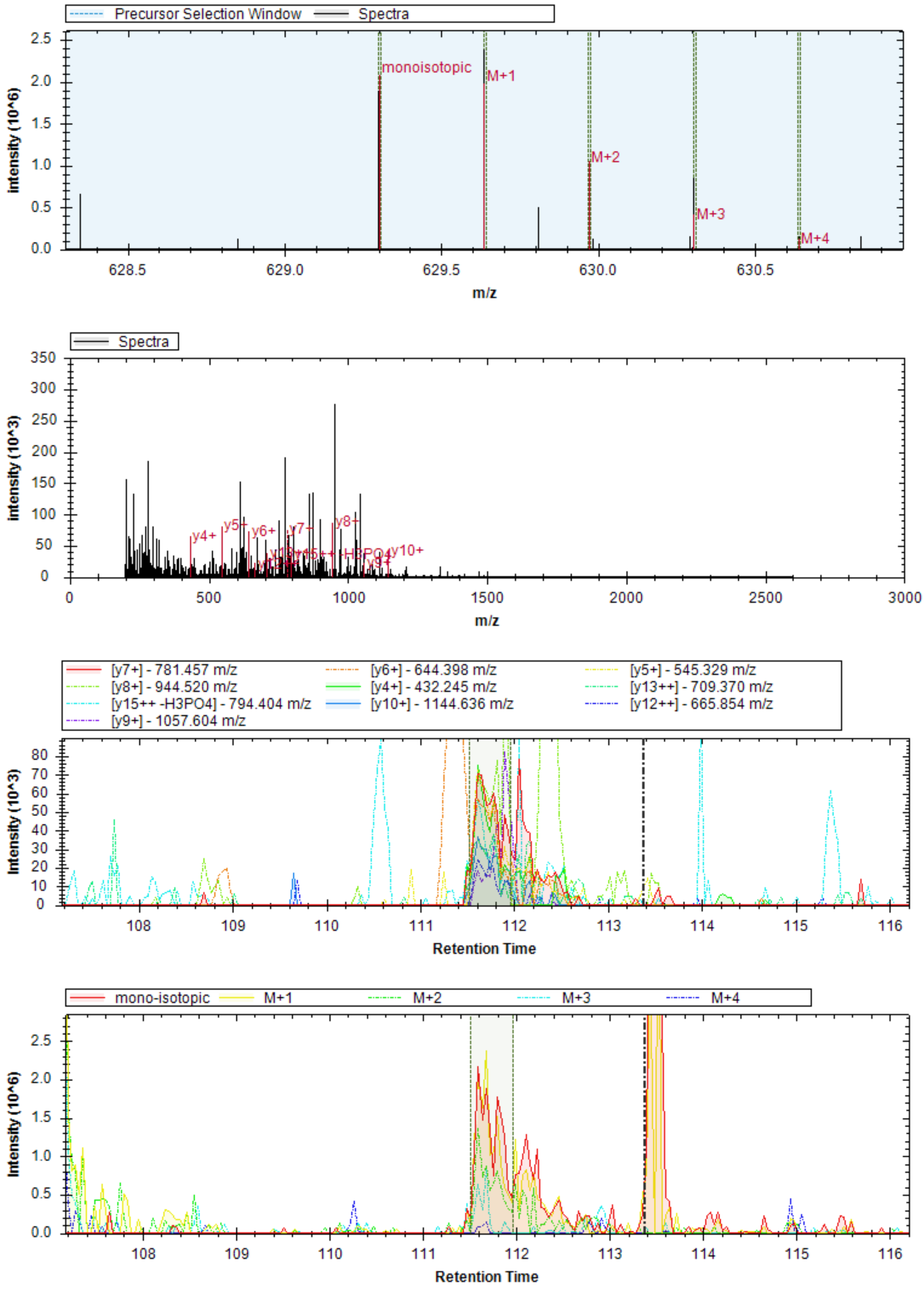

P60904 – _SLST[+H+O3+P]SGESLYHVLGLDK_.3
Fig4_MP-DIA-barrel-cortex-p30-3_MHRM.htrms

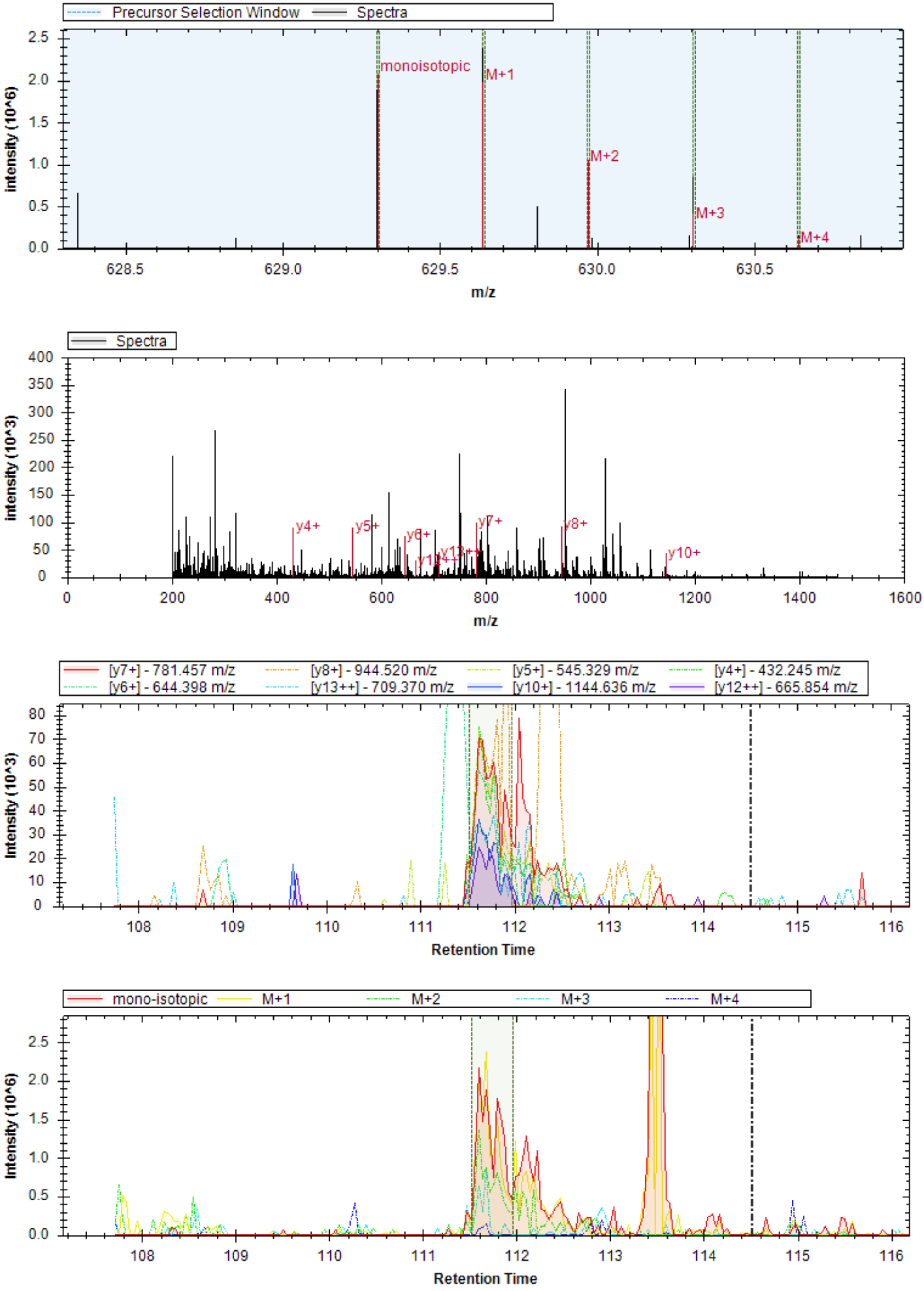

P39447 – _SSGIIRLHT[+H+O3+P]IK_.2
Fig4_MP-DIA-barrel-cortex-p30-3_MHRM.htrms

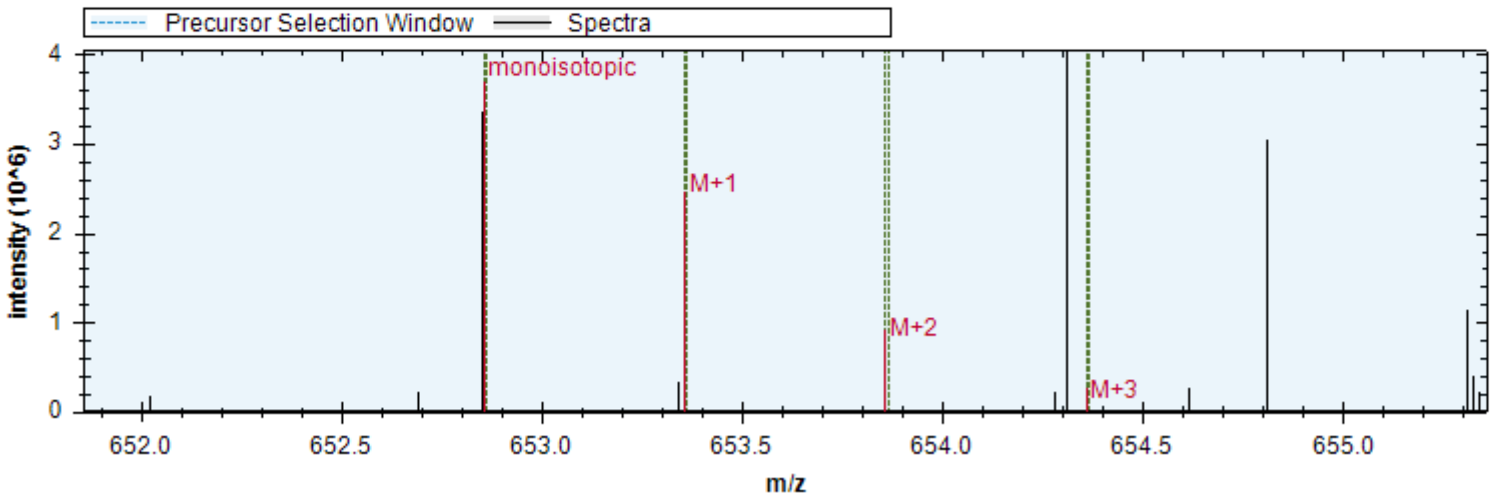

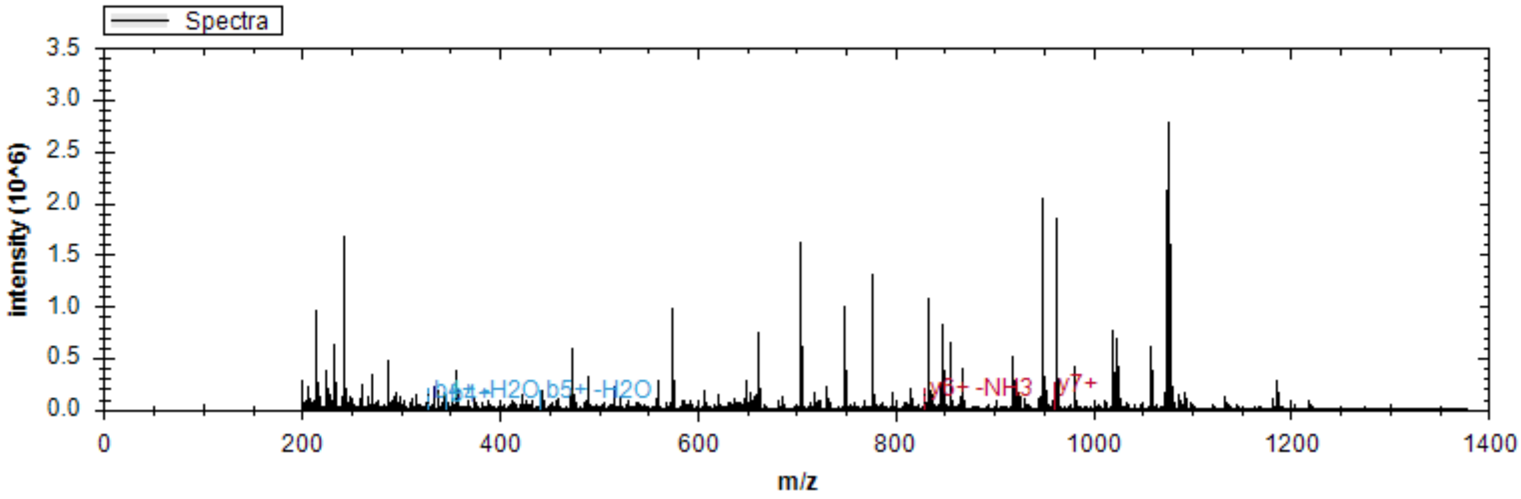

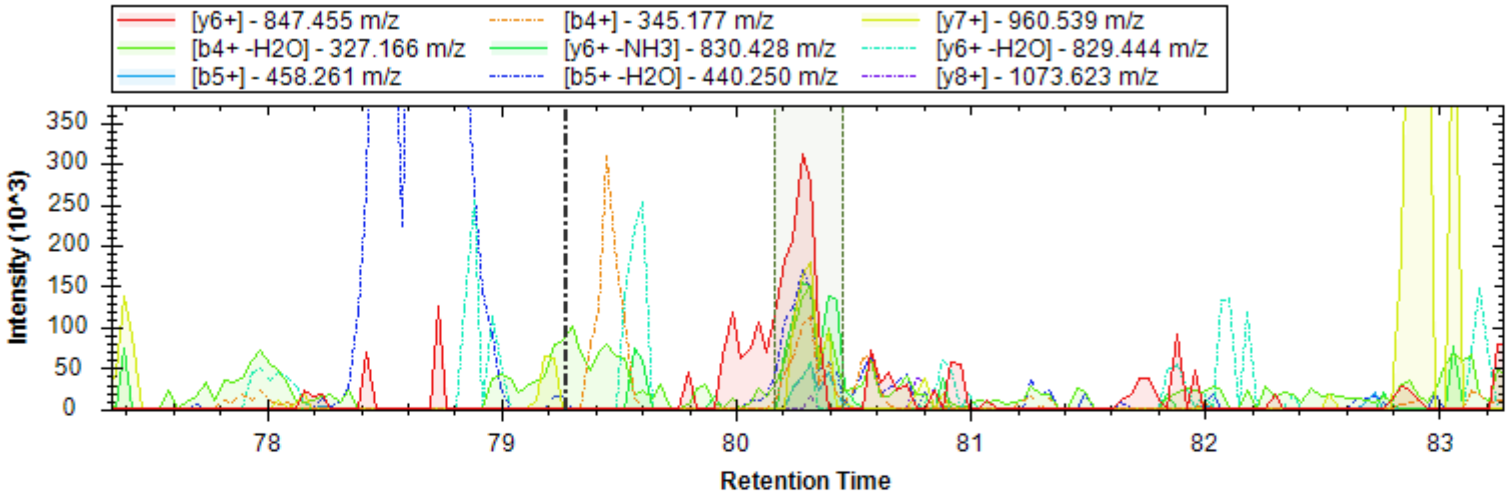

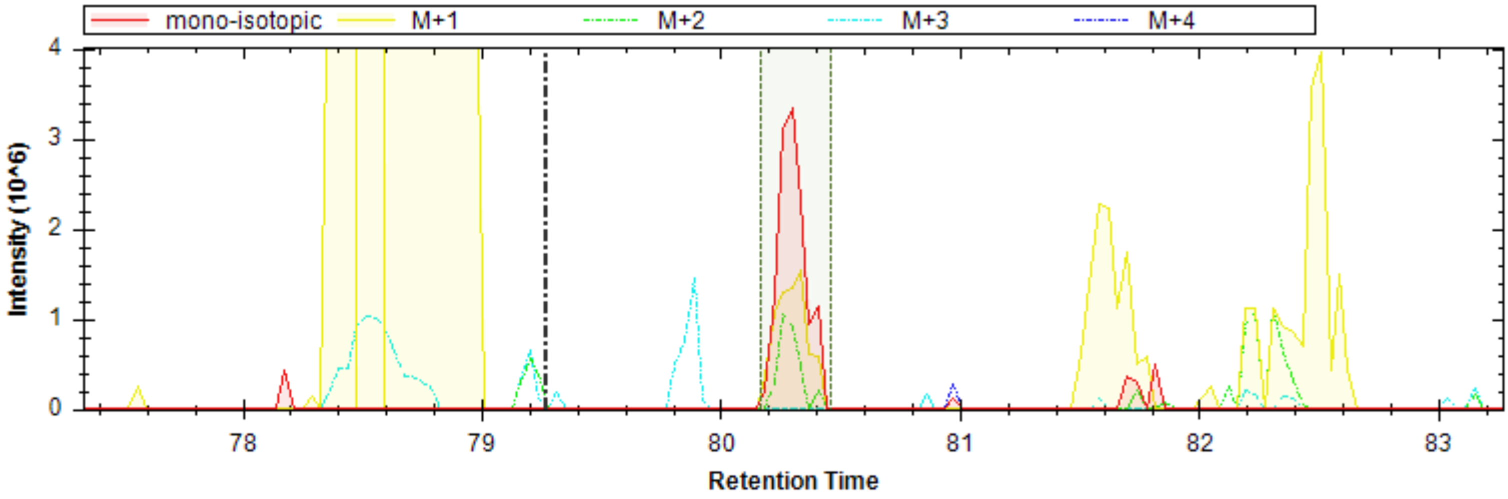

Q8BLJ3 – _M[+O]AS[+H+O3+P]SQGKNELK_.2
Fig4_MP-DIA-barrel-cortex-p30-3_MHRM.htrms

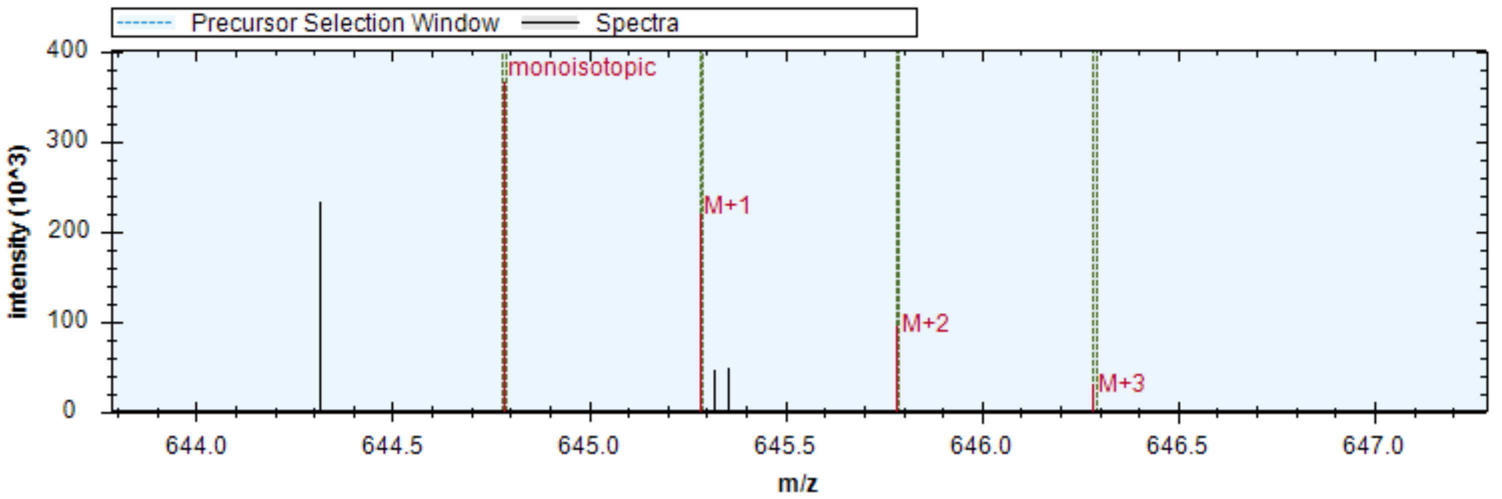

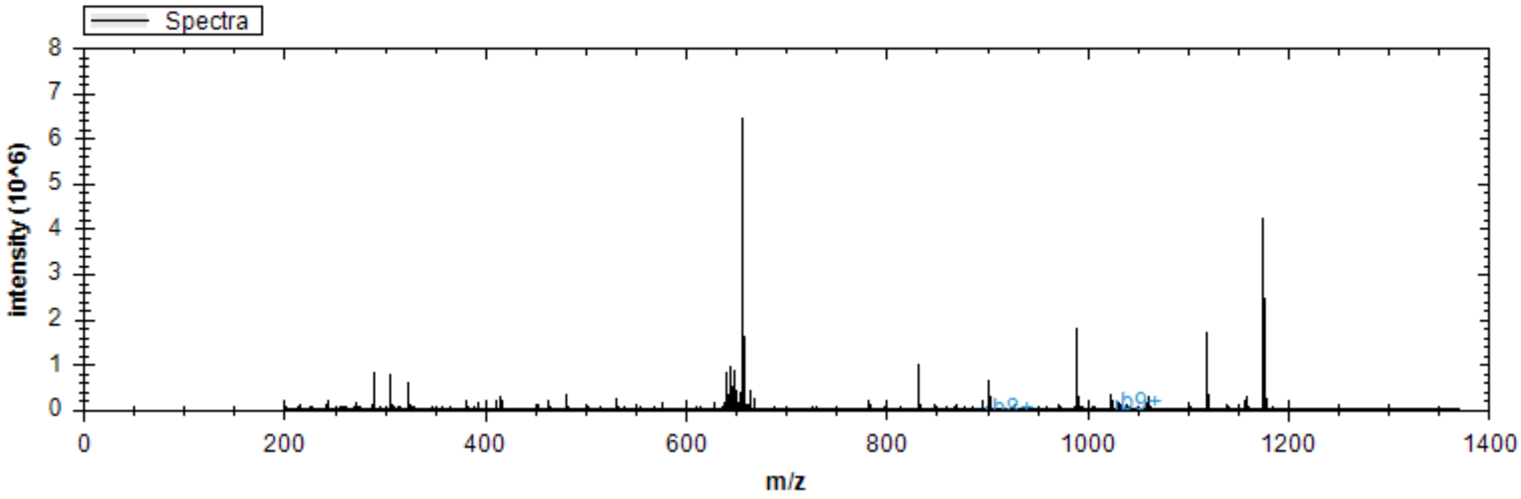

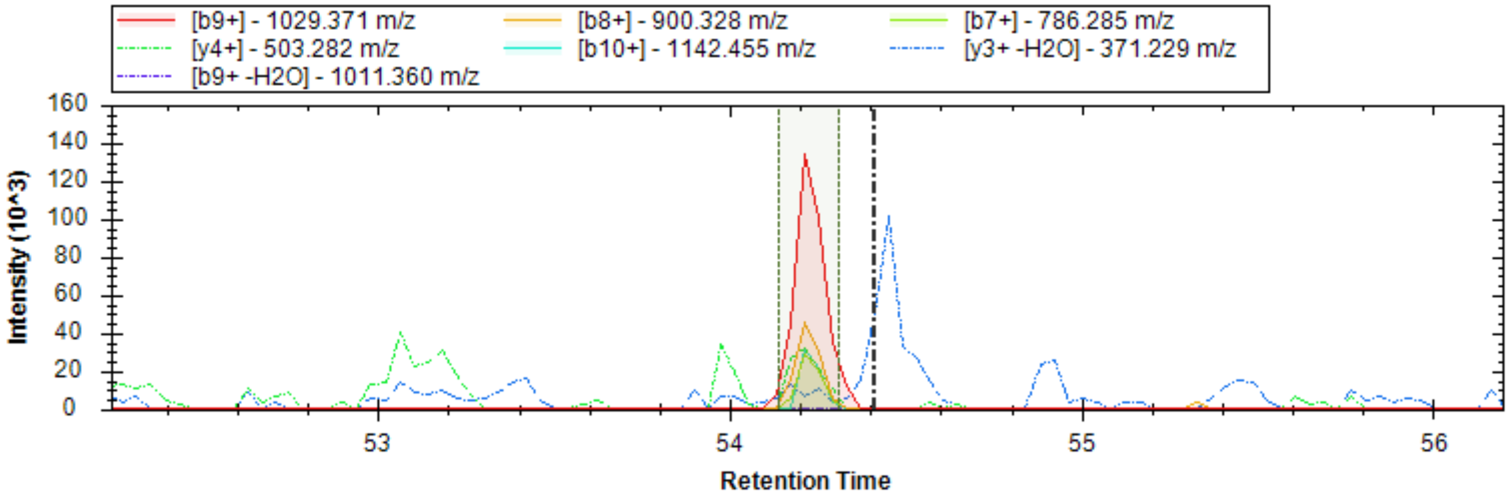

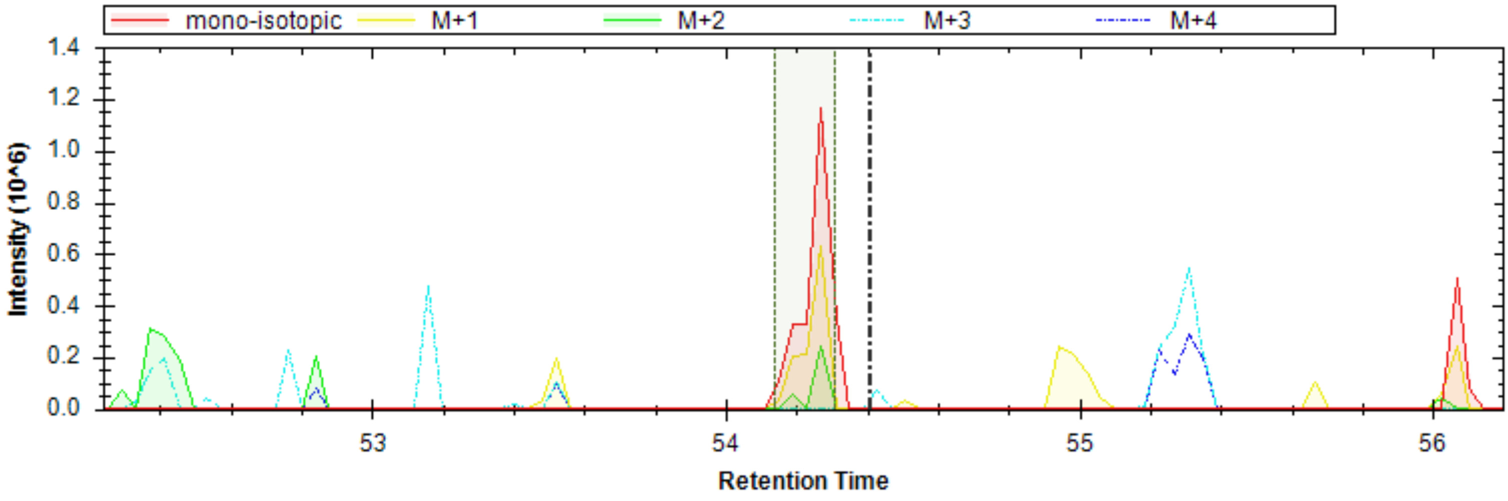

A2AM05;A2AM05-4 – _KDLKMS[+H+O3+P]VLISK_.2
Fig4_MP-DIA-barrel-cortex-p30-3_MHRM.htrms

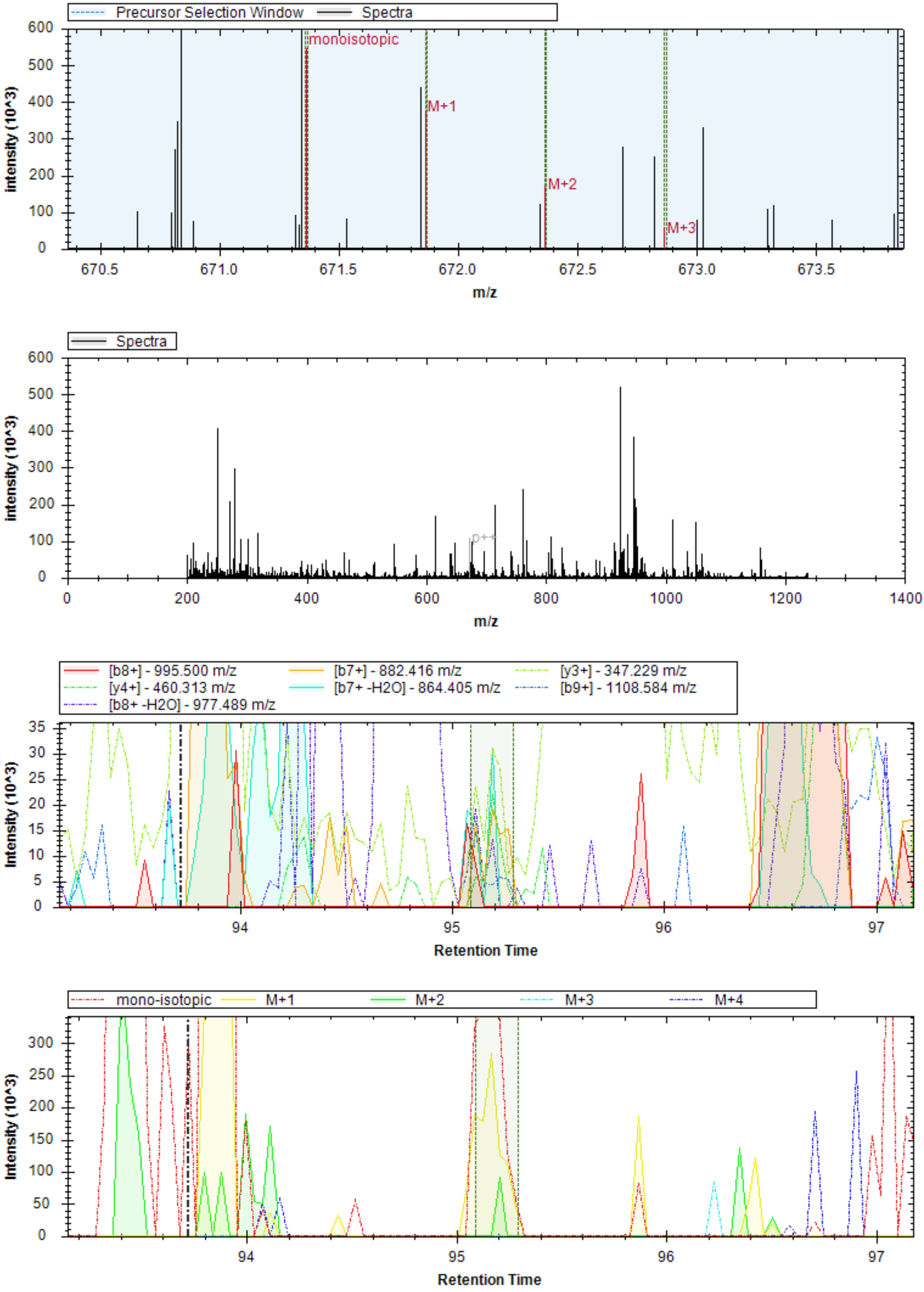

P13595 – _GVTASS[+H+O3+P]SSPASAPK_.2

Fig4_MP-DIA-barrel-cortex-p30-3_MHRM.htrms

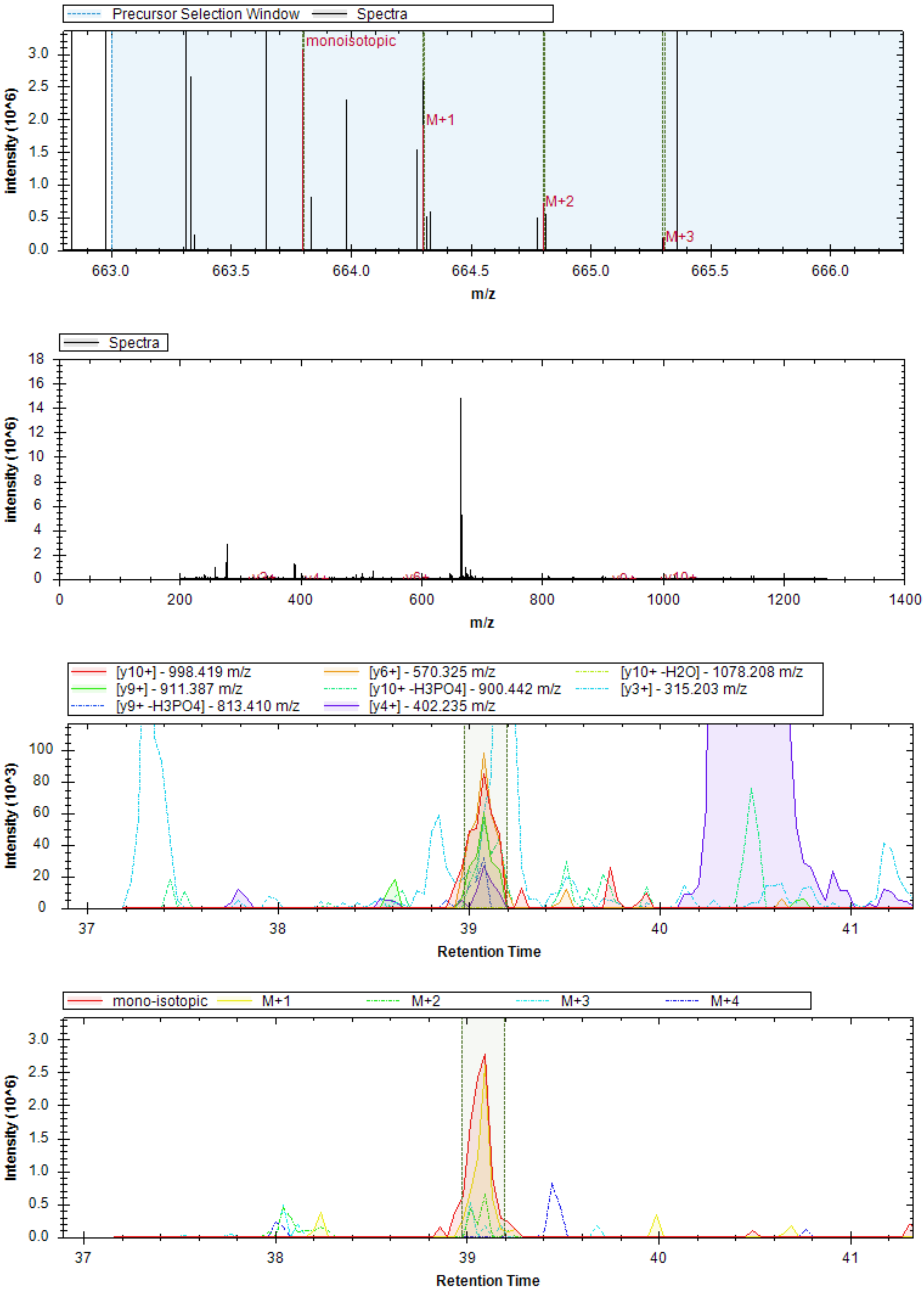

P13595 – _GVTASSS[+H+O3+P]SPASAPK_.2

Fig4_MP-DIA-barrel-cortex-p30-3_MHRM.htrms

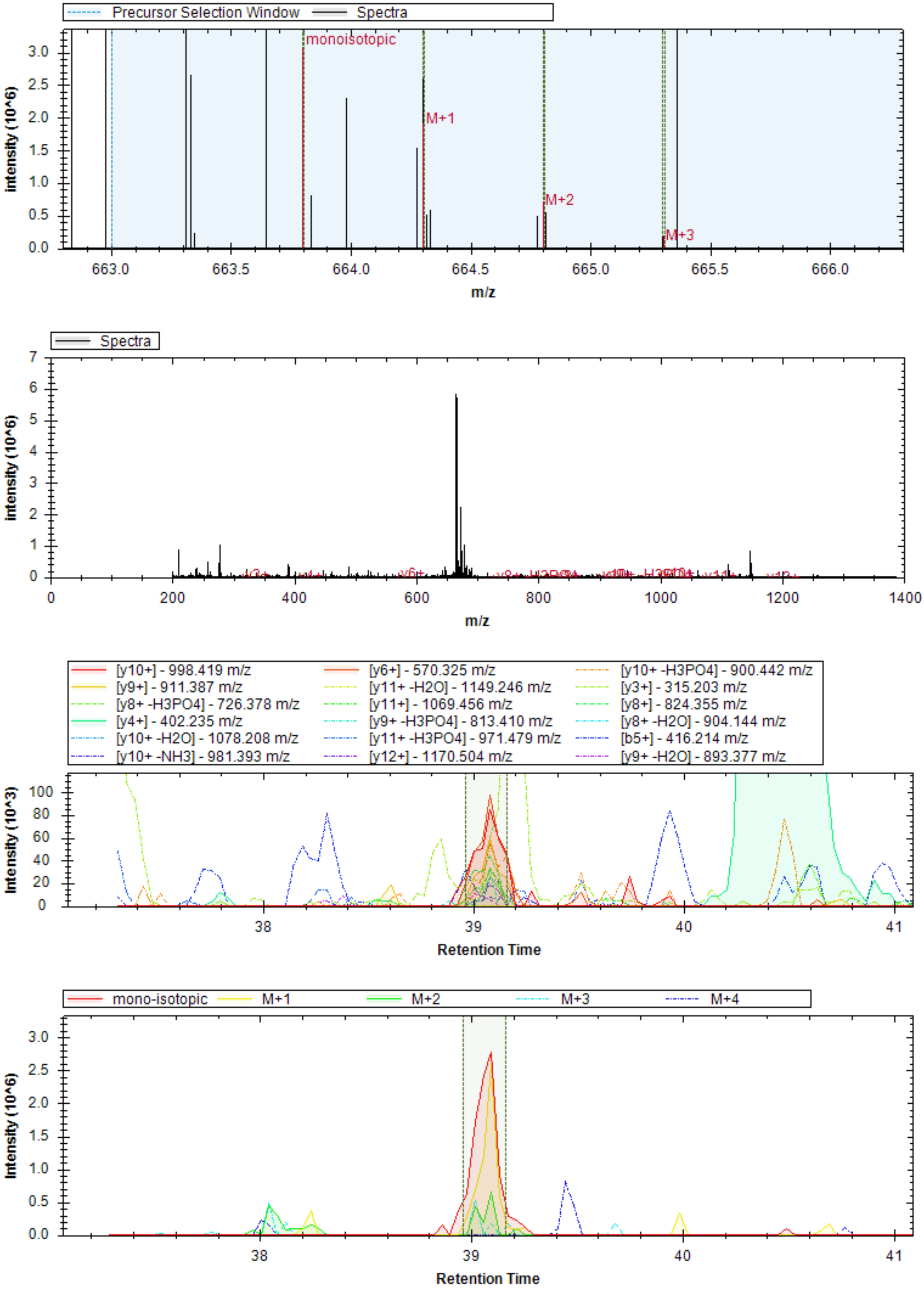

P13595 – _NPPEAATAPAS[+H+O3+P]PK_.2
Fig4_MP-DIA-barrel-cortex-p30-3_MHRM.htrms

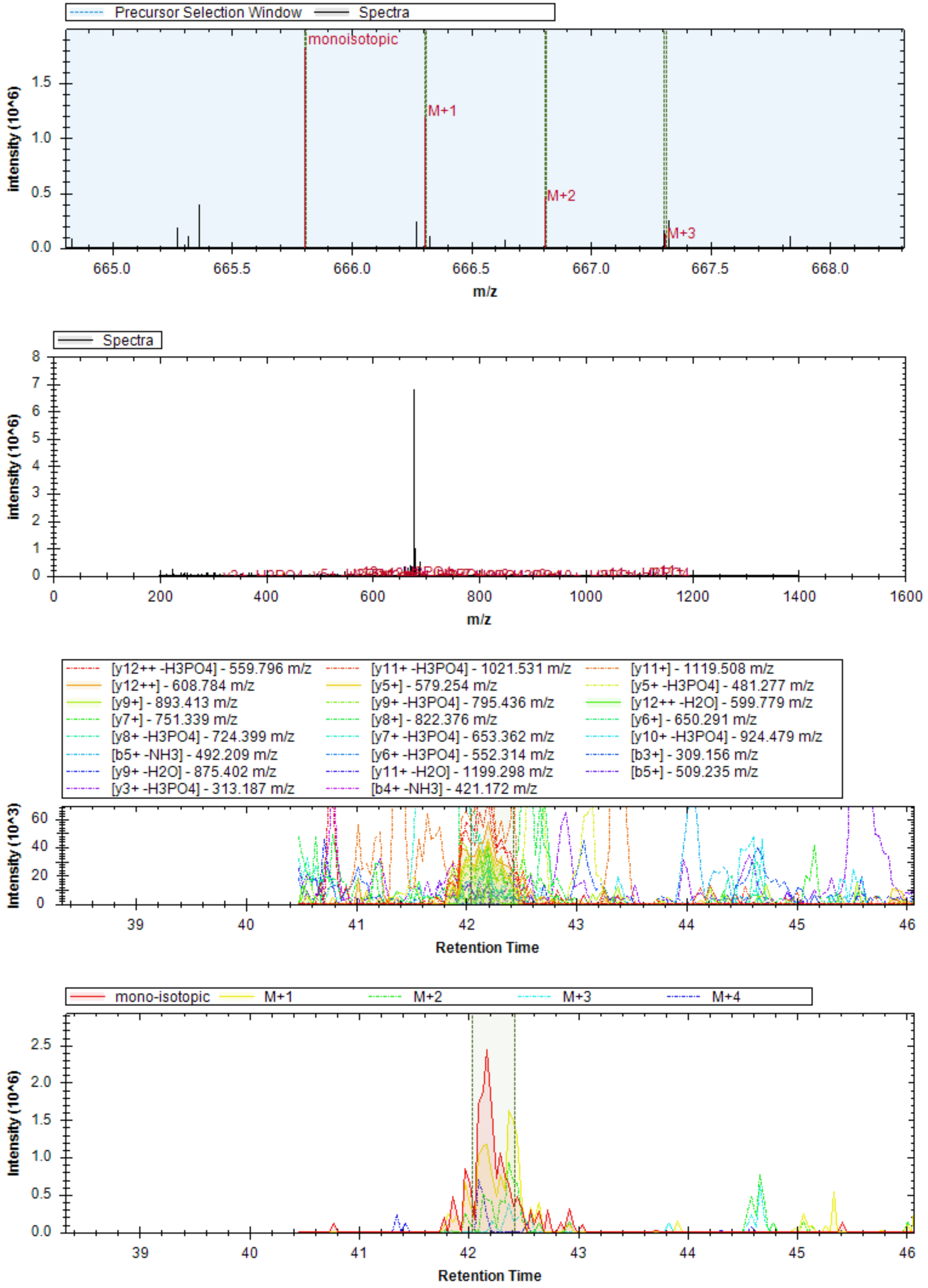

Q6A078 – _T[+H+O3+P]IGLM[+O]KKVVEK_.2
Fig4_MP-DIA-barrel-cortex-p30-3_MHRM.htrms

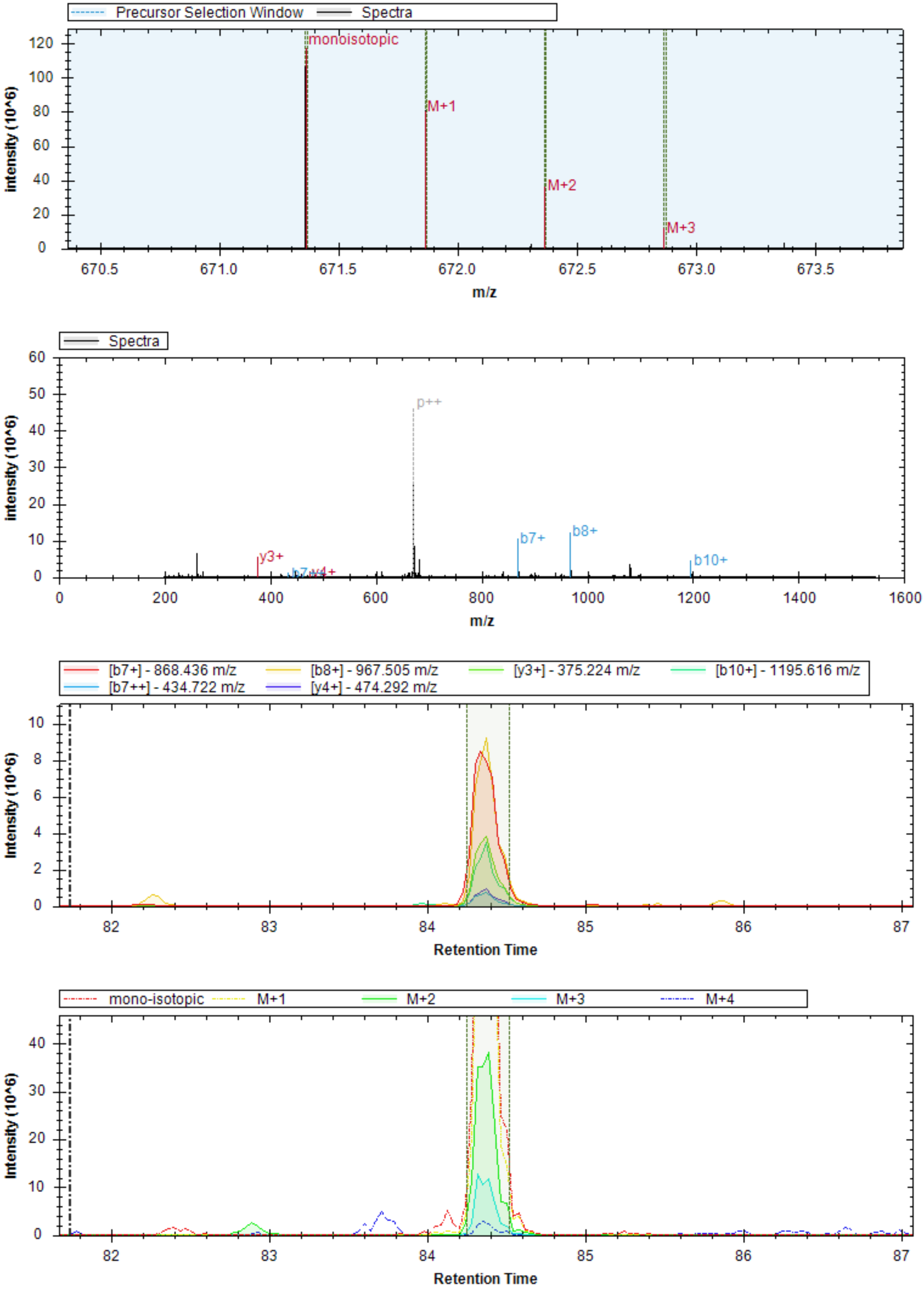

Q8C0E2 – _SMS[+H+O3+P]HQAAIASQR_.2
Fig4_MP-DIA-barrel-cortex-p30-3_MHRM.htrms

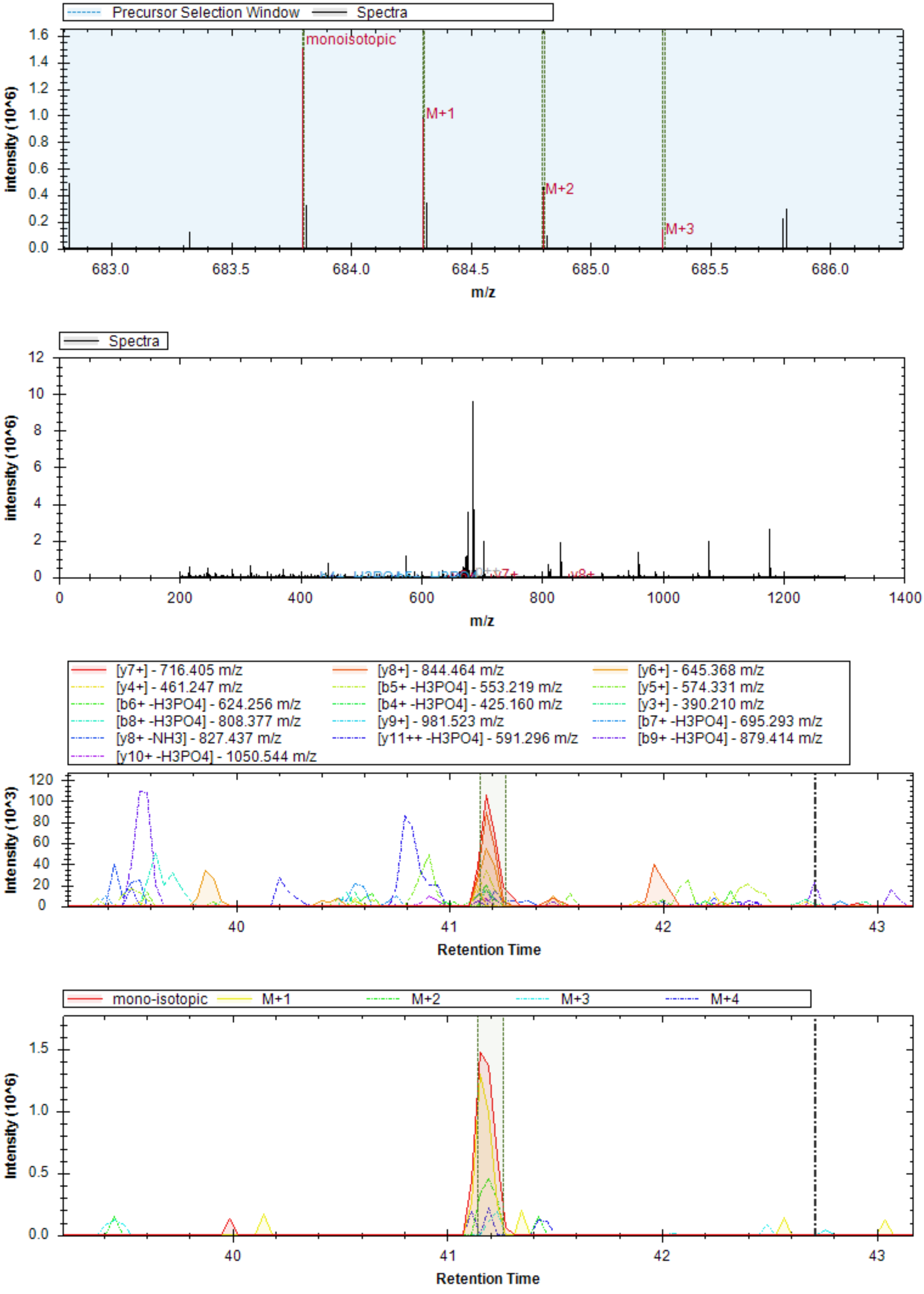

Q8CDU4;Q8CDU4-2 – _[+C2+H2+O]ASLRS[+H+O3+P]ATPRLR_.2

Fig4_MP-DIA-barrel-cortex-p30-3_MHRM.htrms

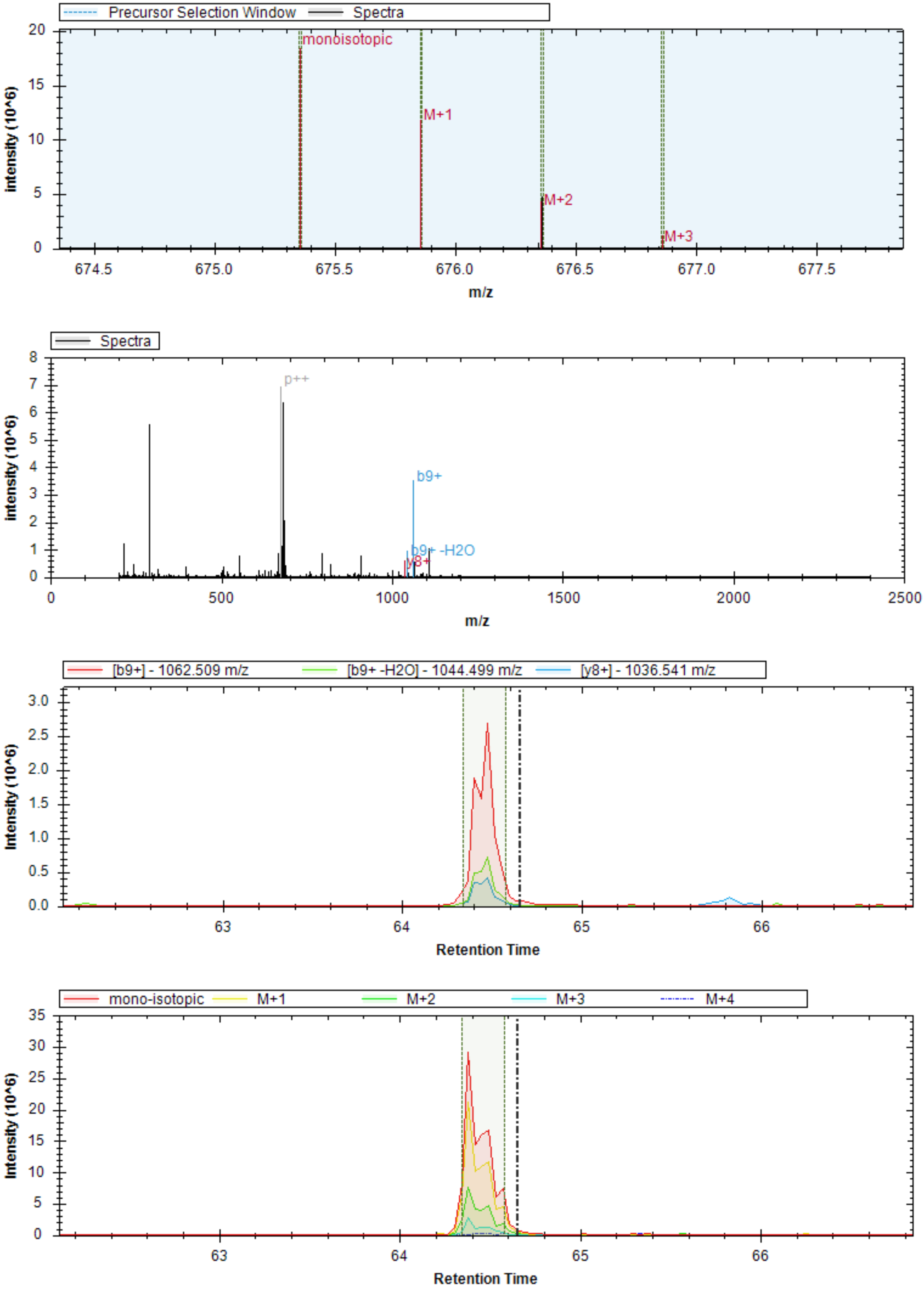

Q8R5L3;Q8R5L3-2 – _LQLY[+H+O3+P]FWKDR_.2
Fig4_MP-DIA-barrel-cortex-p30-3_MHRM.htrms

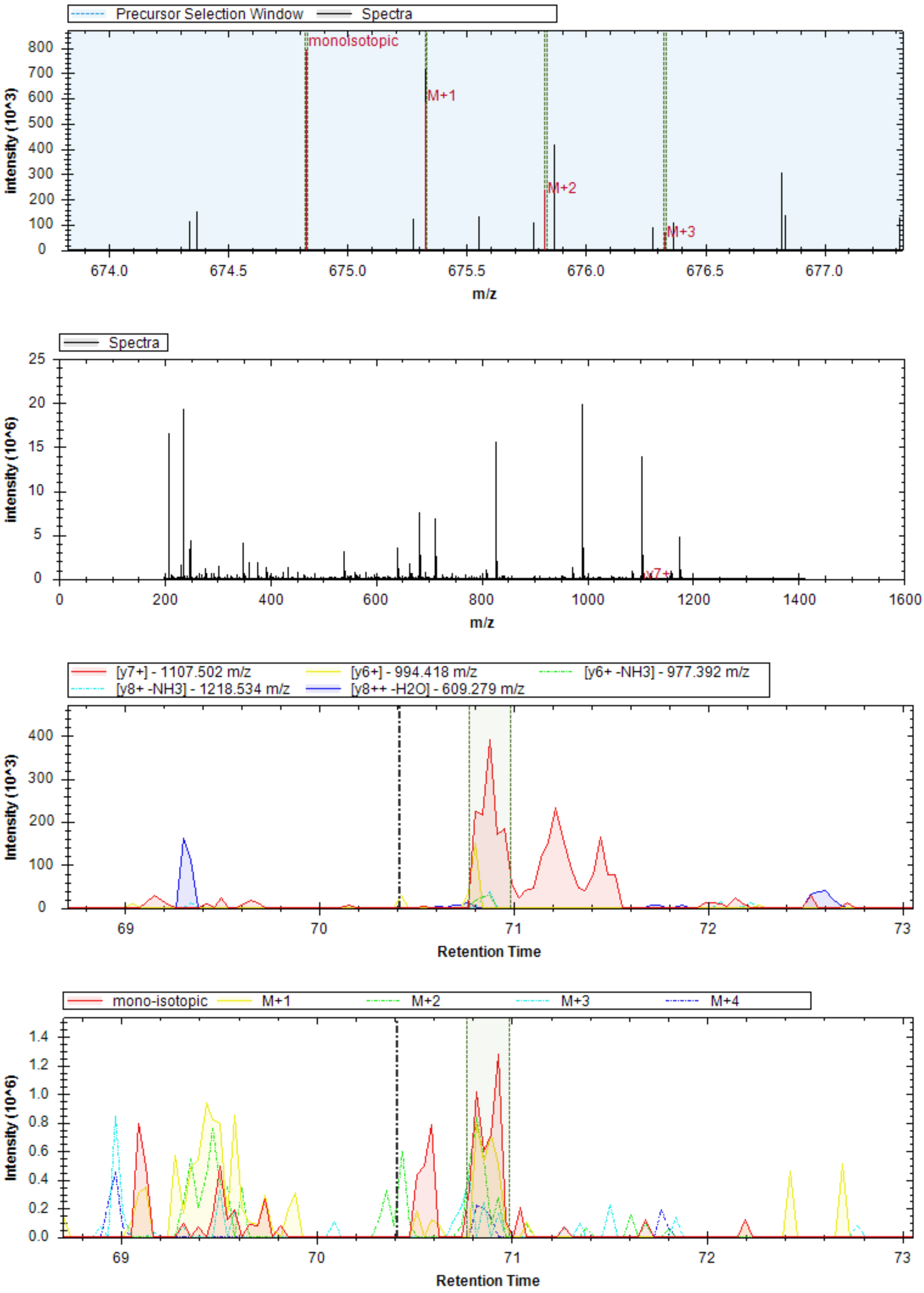

Q9CZU3 – _ESLKNSAT[+H+O3+P]EAAK_.2

Fig4_MP-DIA-barrel-cortex-p30-3_MHRM.htrms

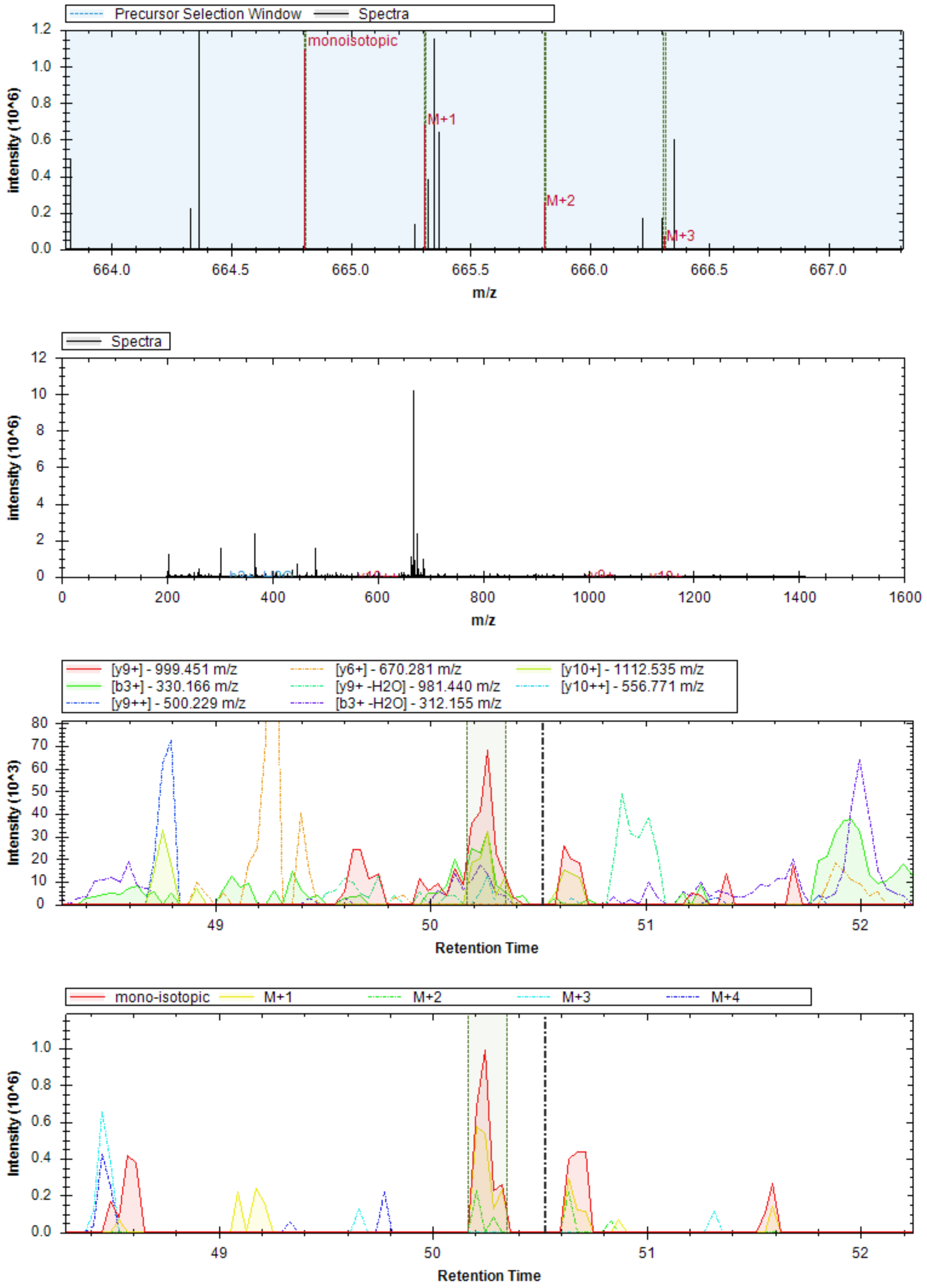

Q9JLM8 – _SPSPSPTS[+H+O3+P]PGSLR_.2
Fig4_MP-DIA-barrel-cortex-p30-3_MHRM.htrms

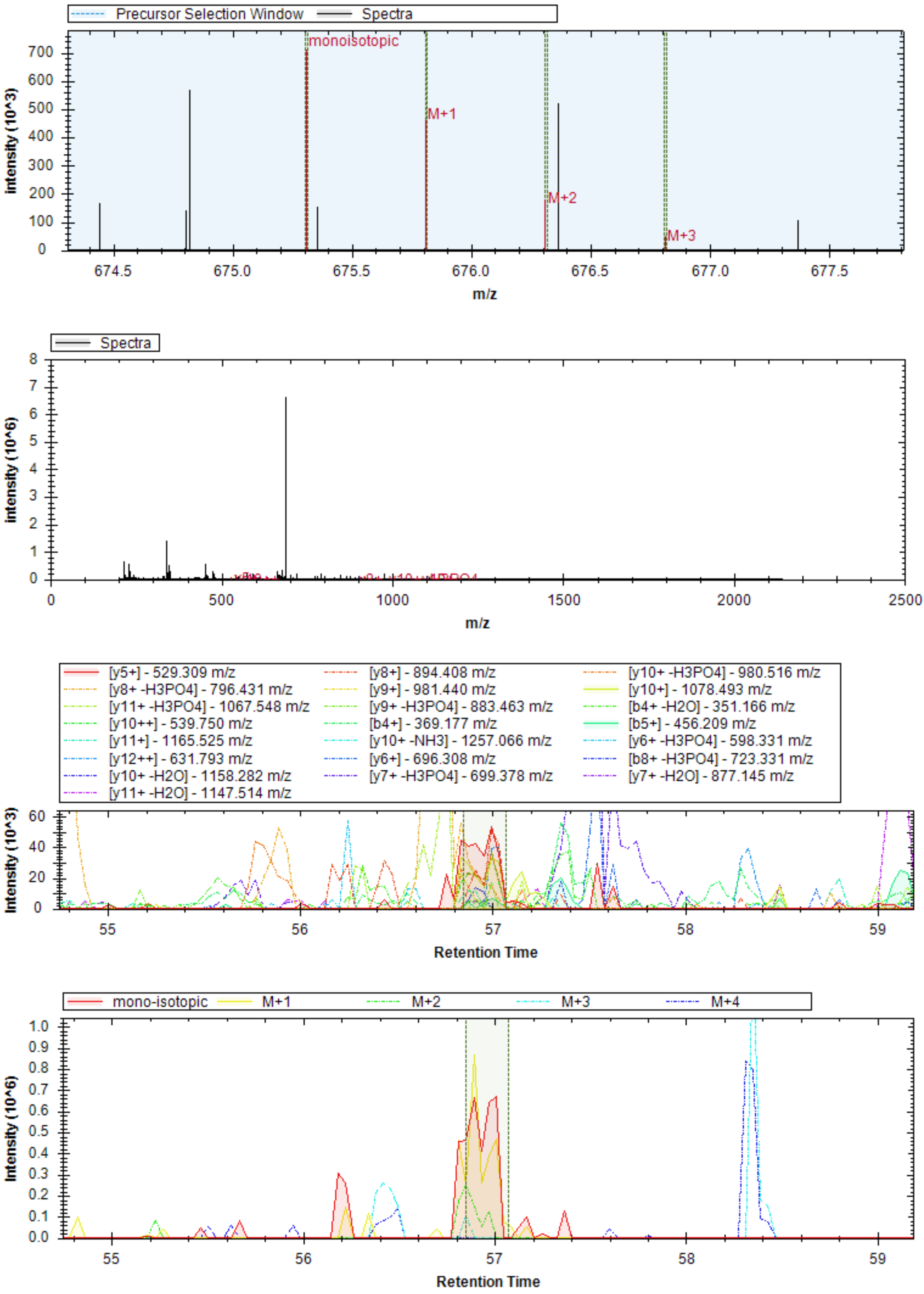

Q9QYR6-2 – _MAS[+H+O3+P]PPPSGPPSAAHTPFHQSPVEEK_.4
Fig4_MP-DIA-barrel-cortex-p30-3_MHRM.htrms

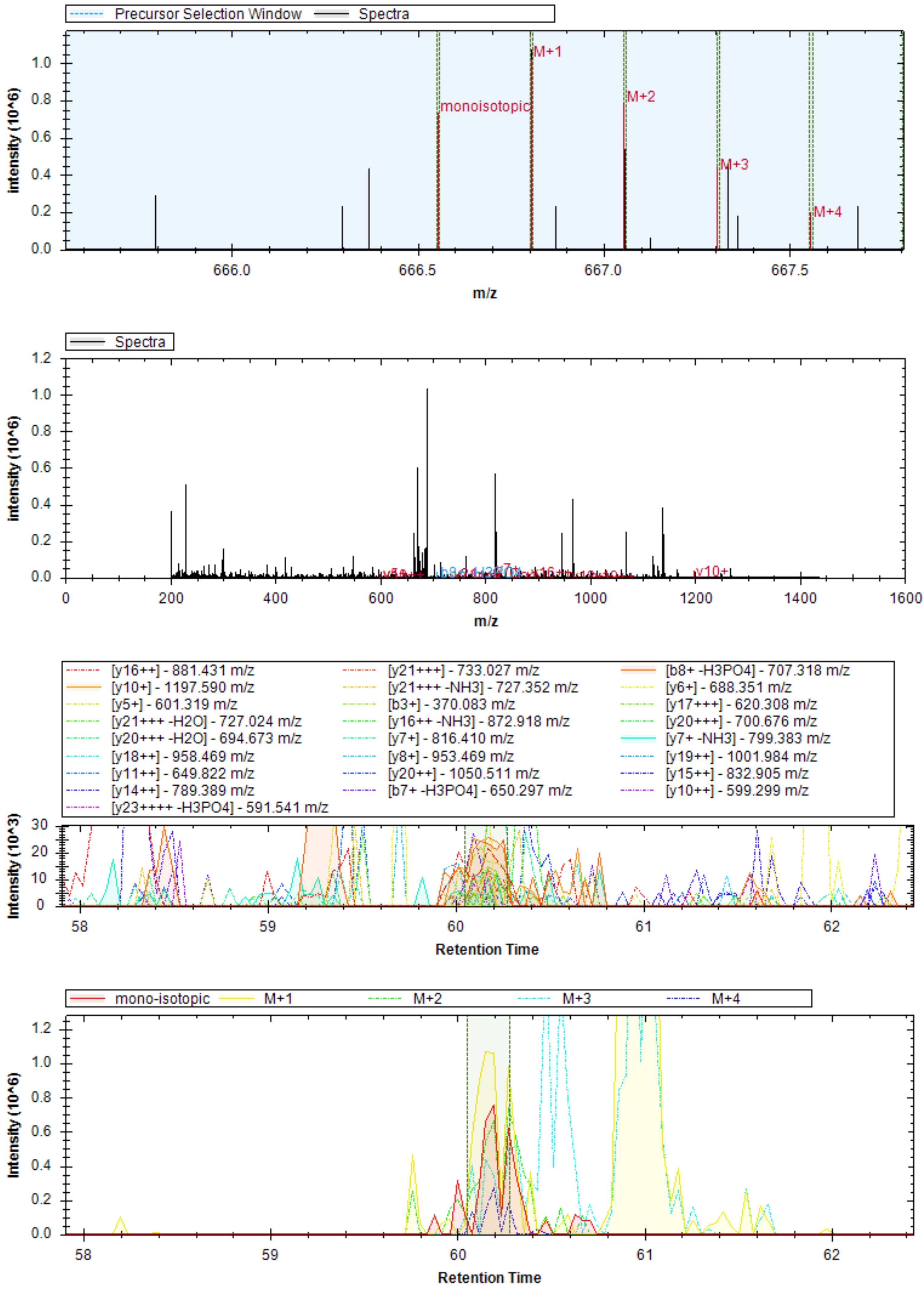

O88935 – _PVAGGPGAPPAARPPAS[+H+O3+P]PSPQR_.3
Fig4_MP-DIA-barrel-cortex-p30-3_MHRM.htrms

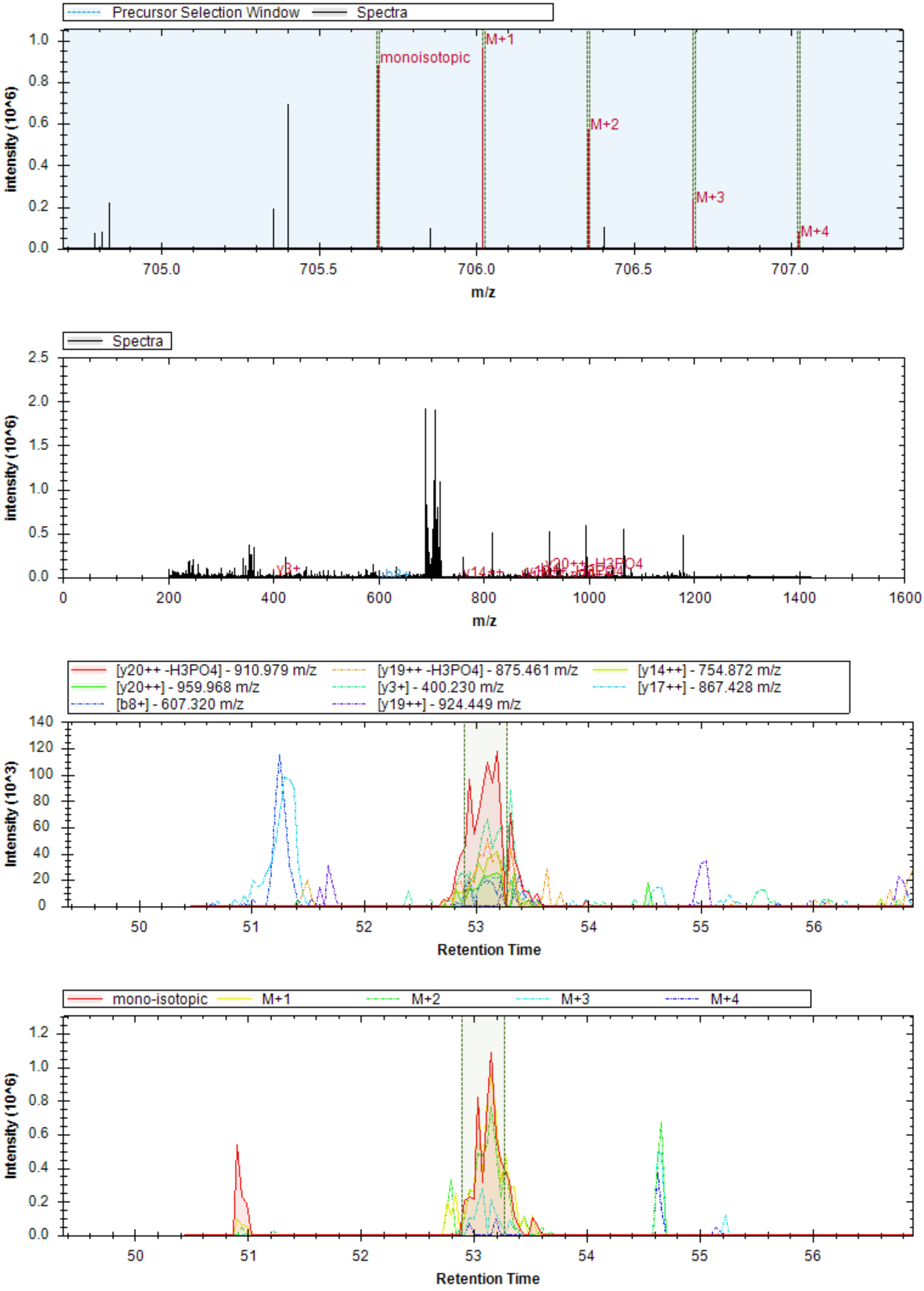

O88935 – _PVAGGPGAPPAARPPASPS[+H+O3+P]PQR_.3
Fig4_MP-DIA-barrel-cortex-p30-3_MHRM.htrms

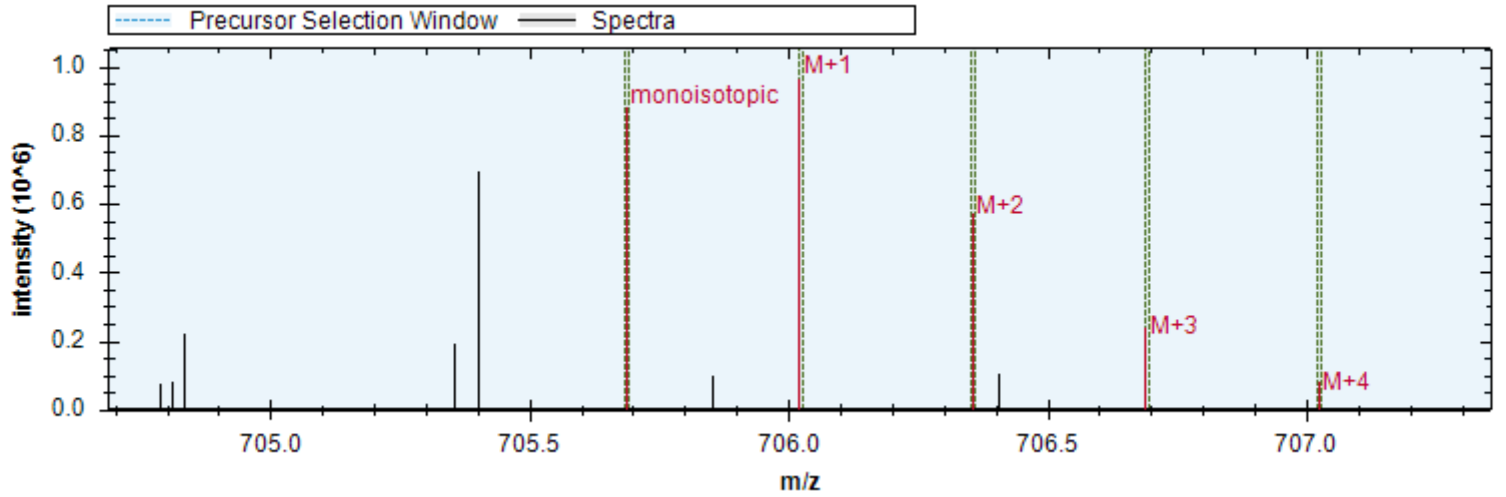

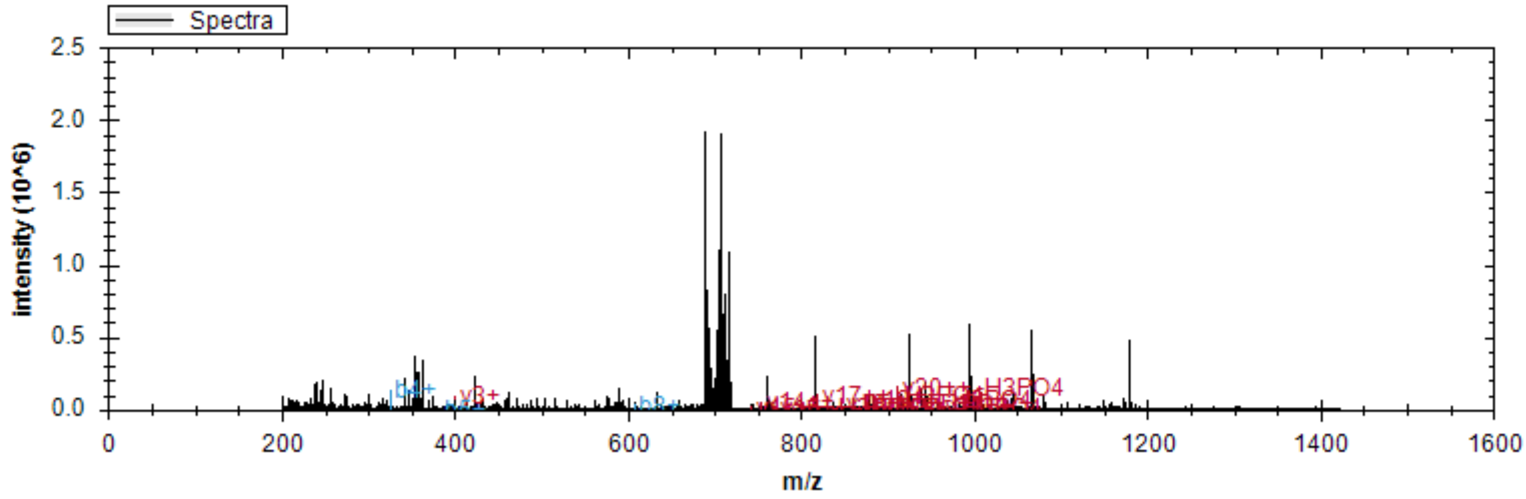

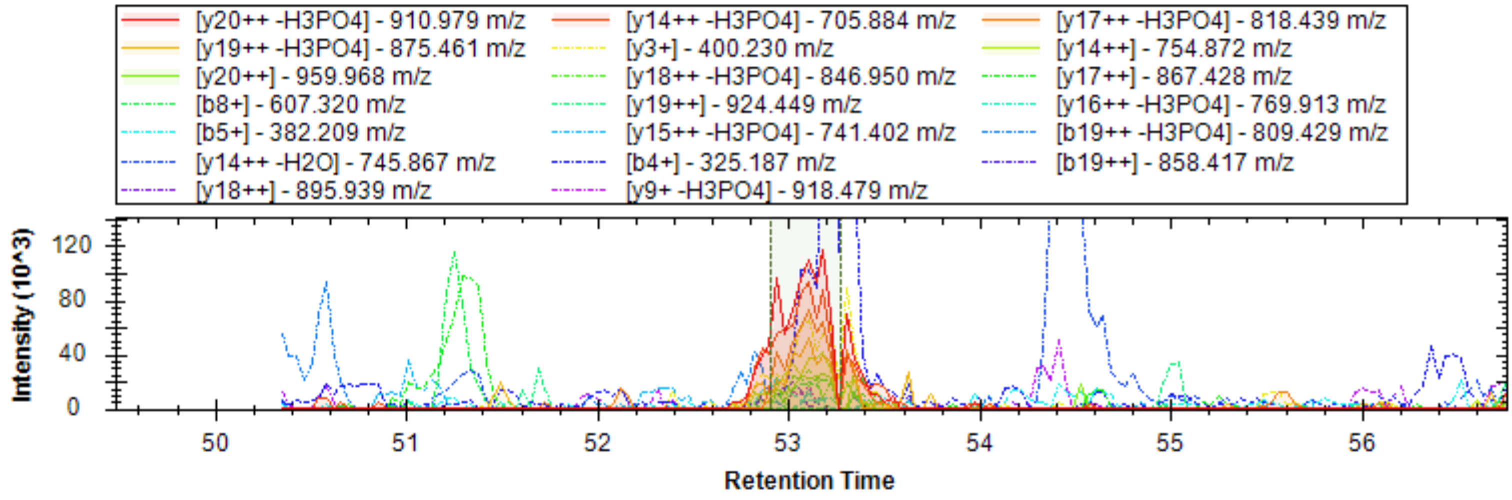

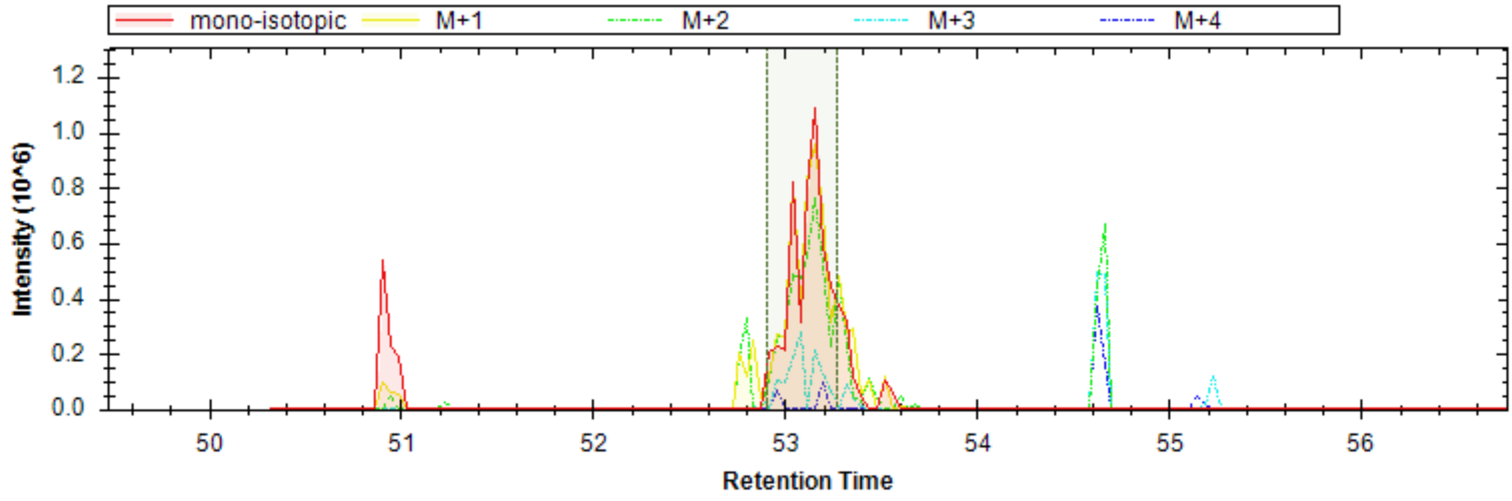

P28738 – _PIRPGHYPAS[+H+O3+P]SPTAVHAVR_.3

Fig4_MP-DIA-barrel-cortex-p30-3_MHRM.htrms

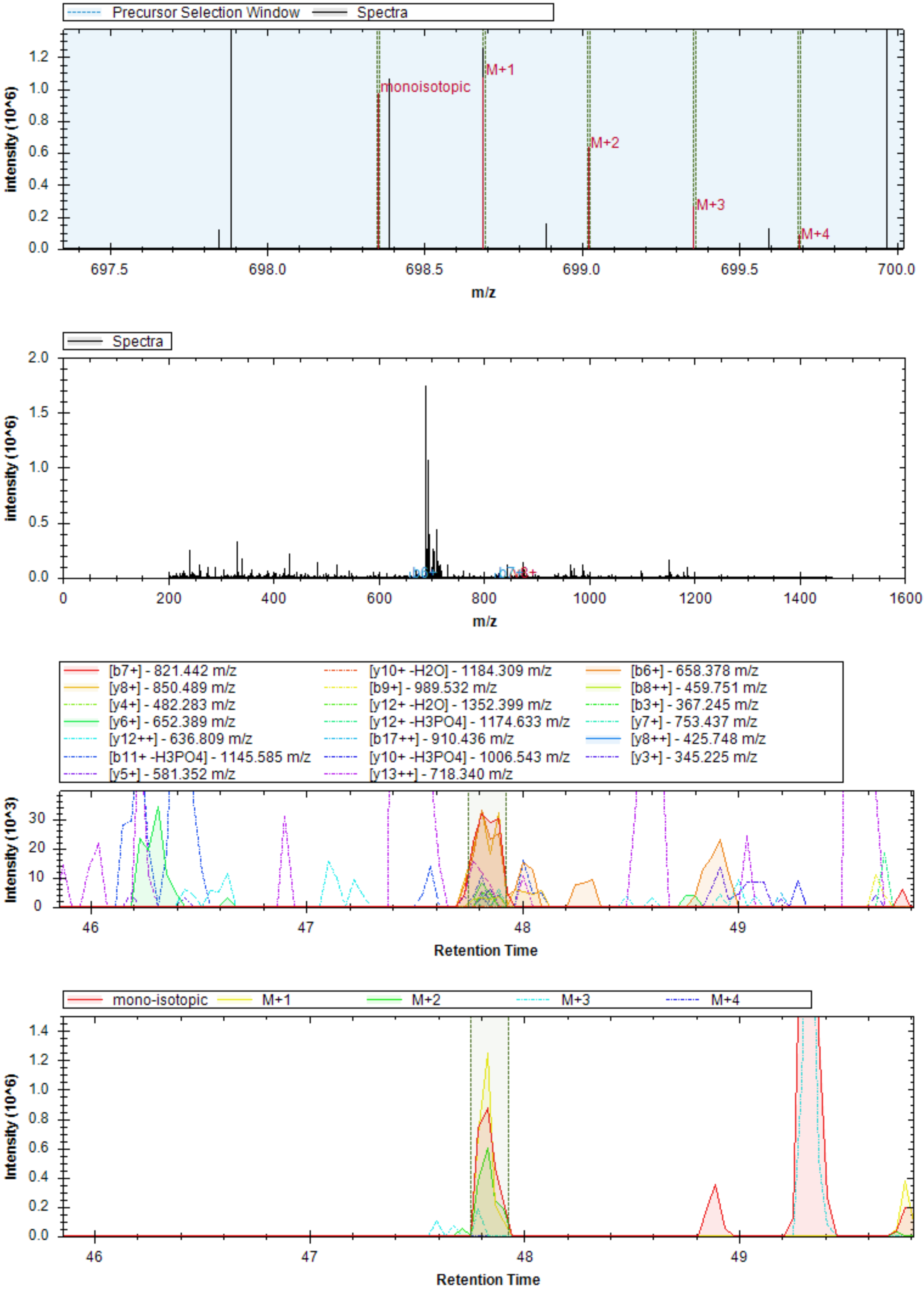

P28738 – _PIRPGHYPASS[+H+O3+P]PTAVHAVR_.3

Fig4_MP-DIA-barrel-cortex-p30-3_MHRM.htrms

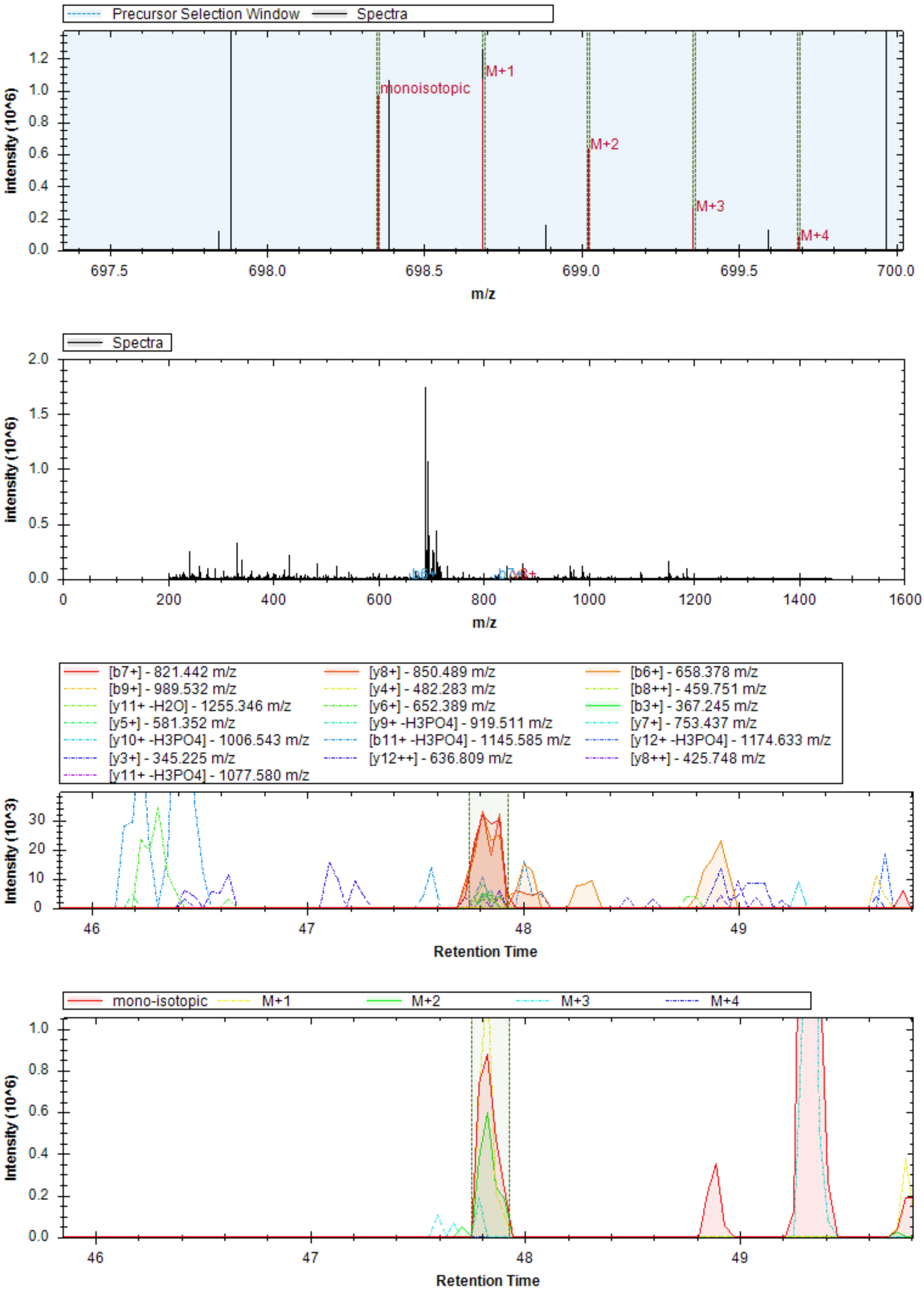

P62751 – _KAY[+H+O3+P]VRLAPDYDALDVANK_.3
Fig4_MP-DIA-barrel-cortex-p30-3_MHRM.htrms

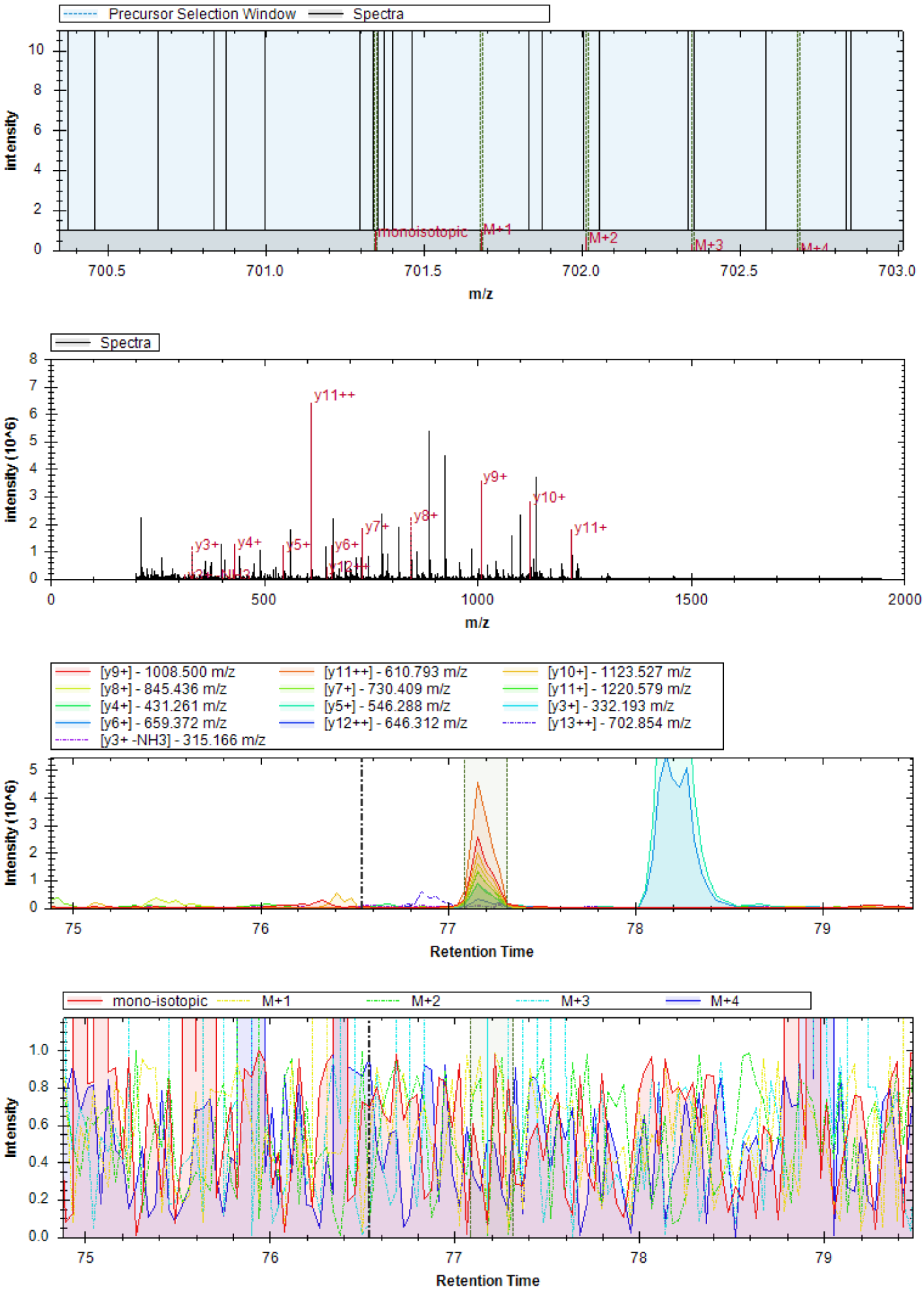

Q3U4B4 – _RHKT[+H+O3+P]LSANEEK_.2
Fig4_MP-DIA-barrel-cortex-p30-3_MHRM.htrms

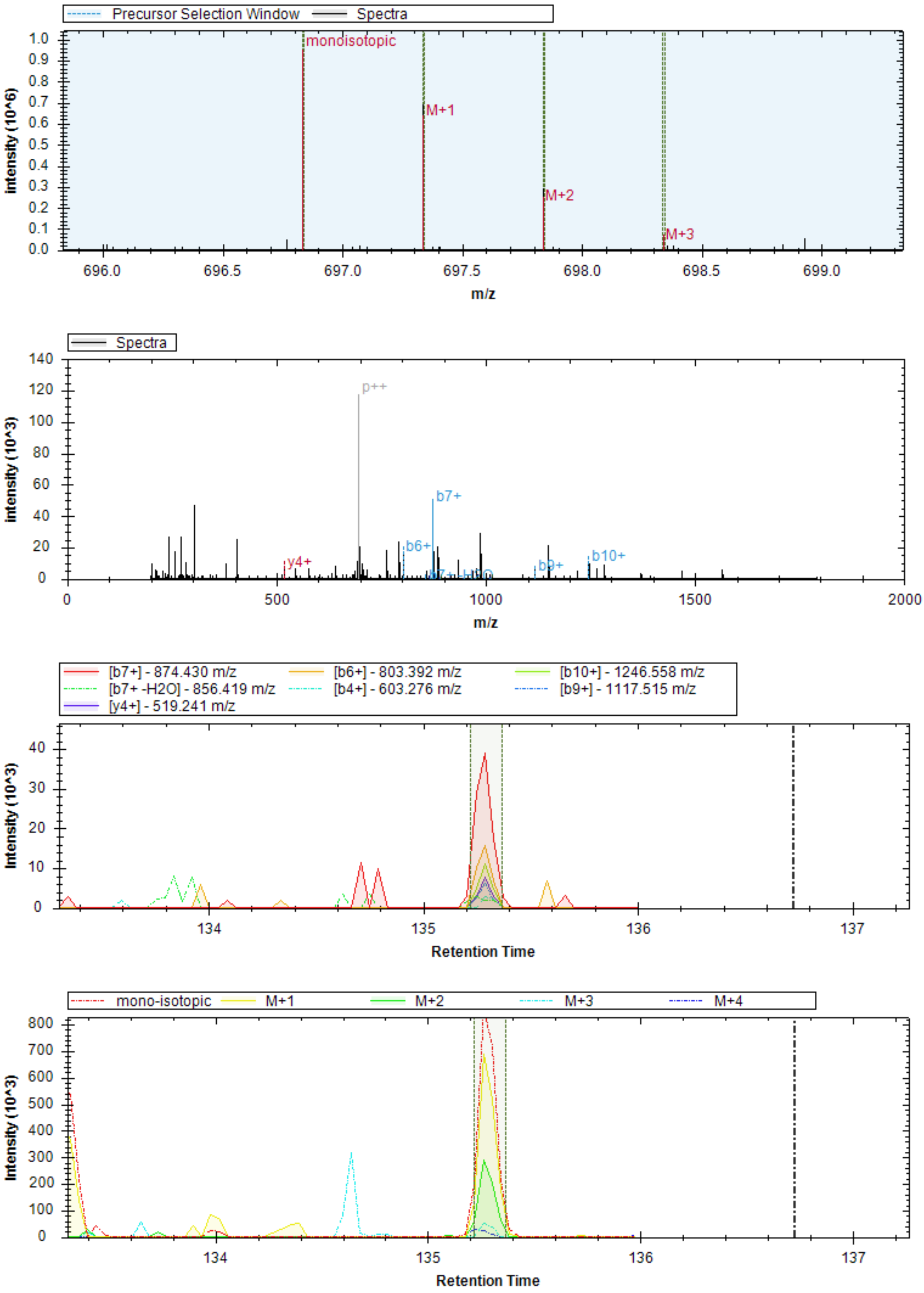

Q8CAA7 – _AVAGVMIT[+H+O3+P]ASHNR_.2
Fig4_MP-DIA-barrel-cortex-p30-3_MHRM.htrms

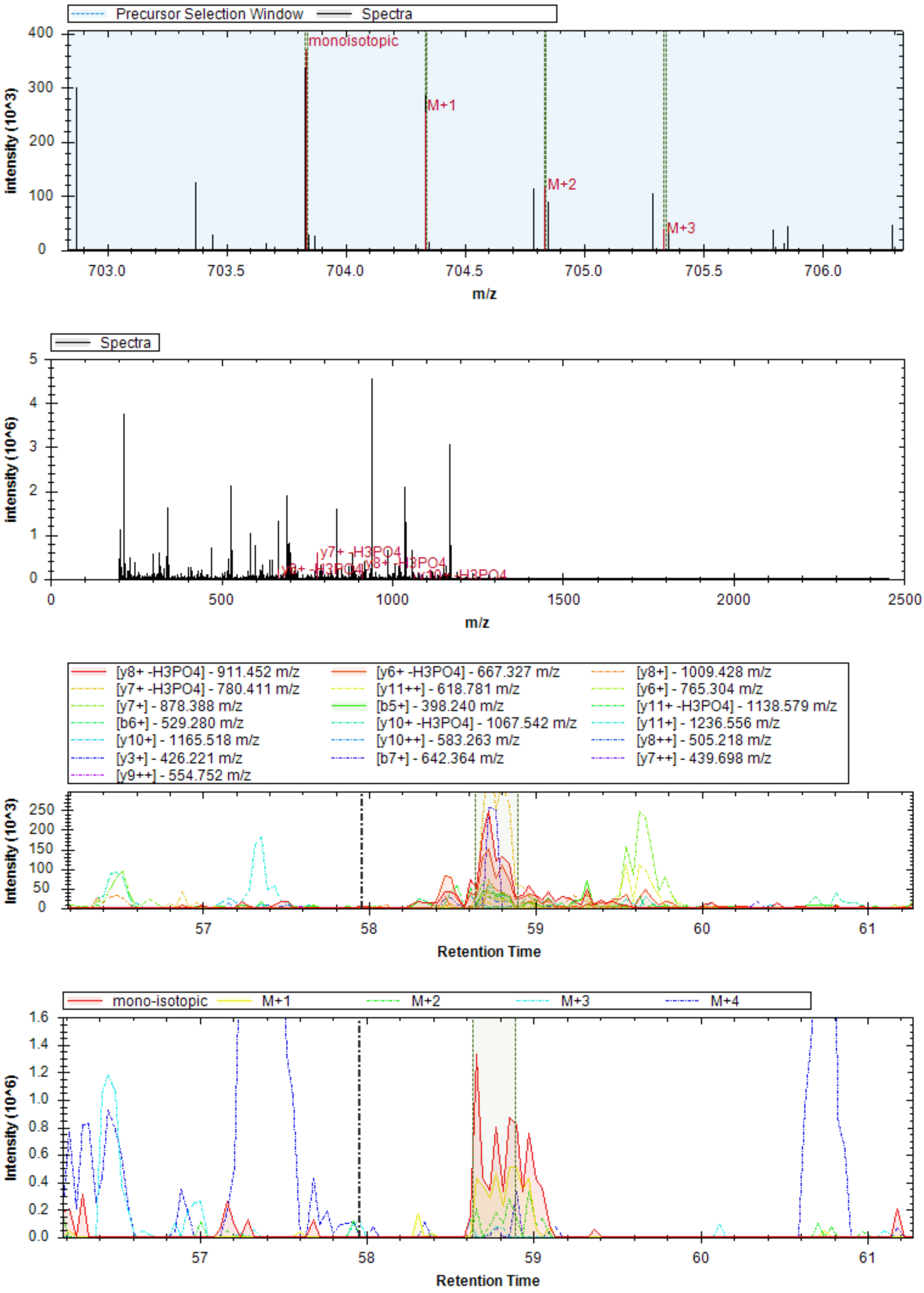

Q8CAA7 – _AVAGVMITAS[+H+O3+P]HNR_.2
Fig4_MP-DIA-barrel-cortex-p30-3_MHRM.htrms

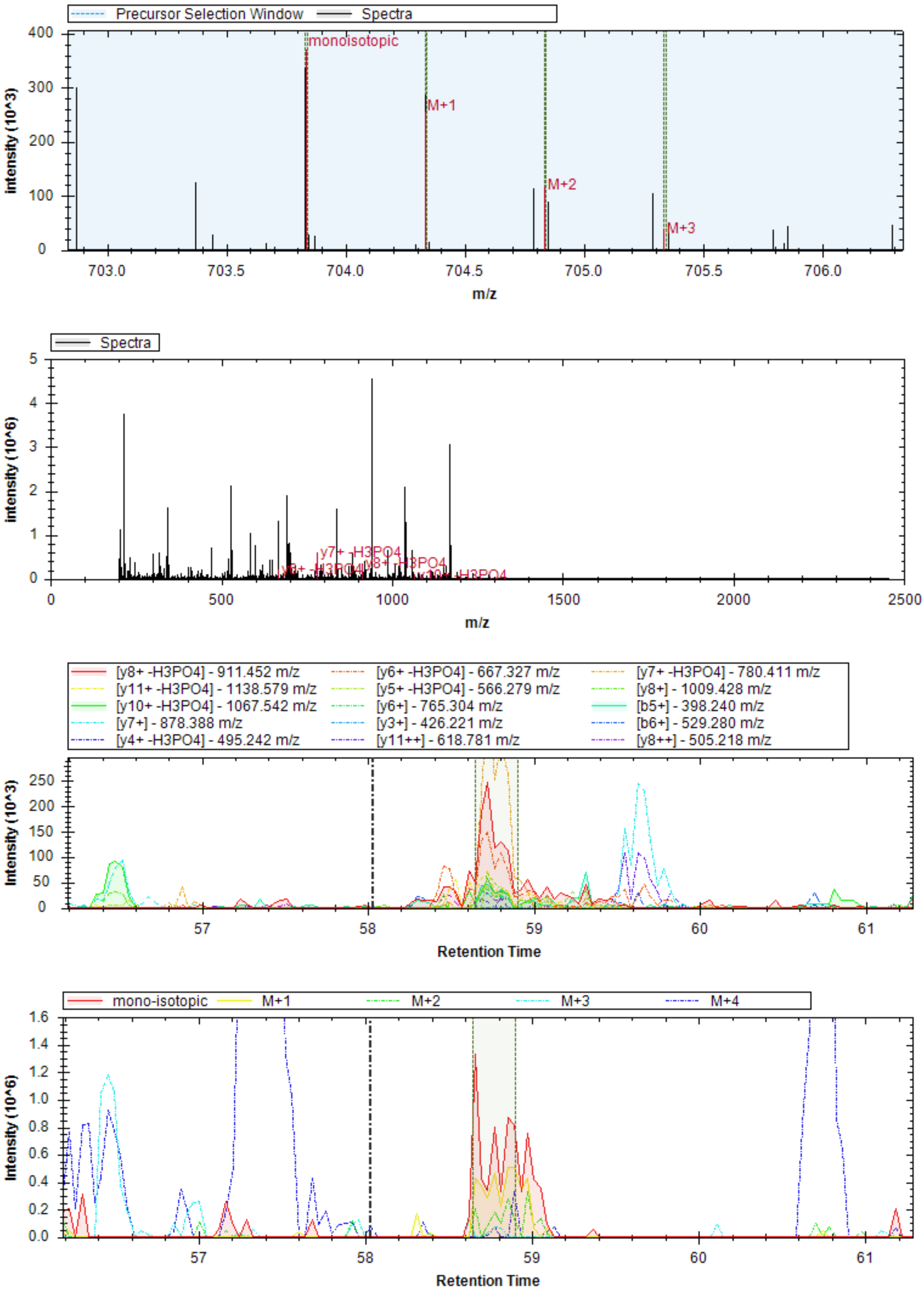

Q9JM52 – _SDSVLPASHGHLPQAGS[+H+O3+P]LER_.3
Fig4_MP-DIA-barrel-cortex-p30-3_MHRM.htrms

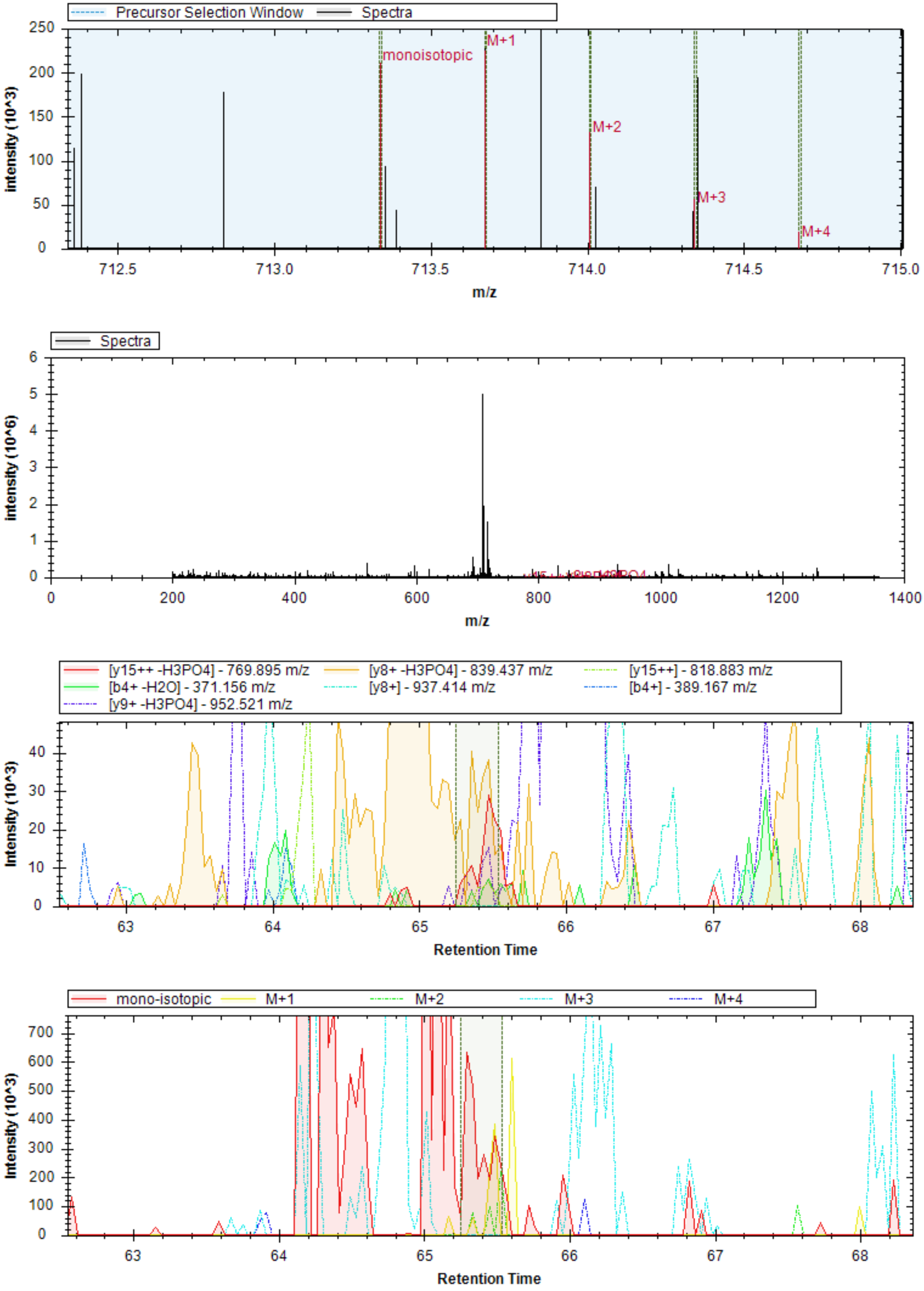

Q9QYC0 – _SPGT[+H+O3+P]PAGEGSGSPPK_.2
Fig4_MP-DIA-barrel-cortex-p30-3_MHRM.htrms

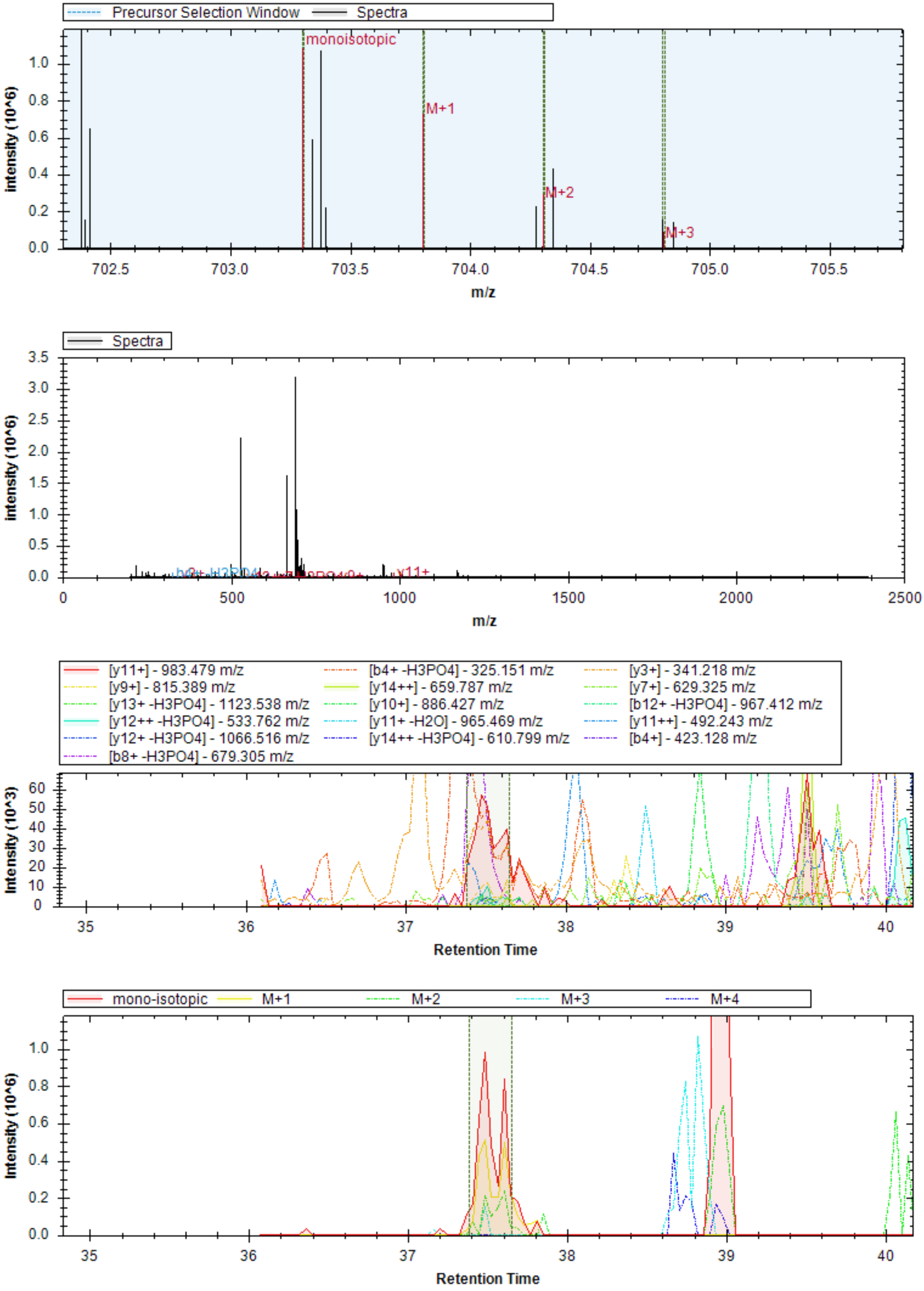

P10637 – _SGYSS[+H+O3+P]PGSPGTPGSR_.2
Fig4_MP-DIA-barrel-cortex-p30-3_MHRM.htrms

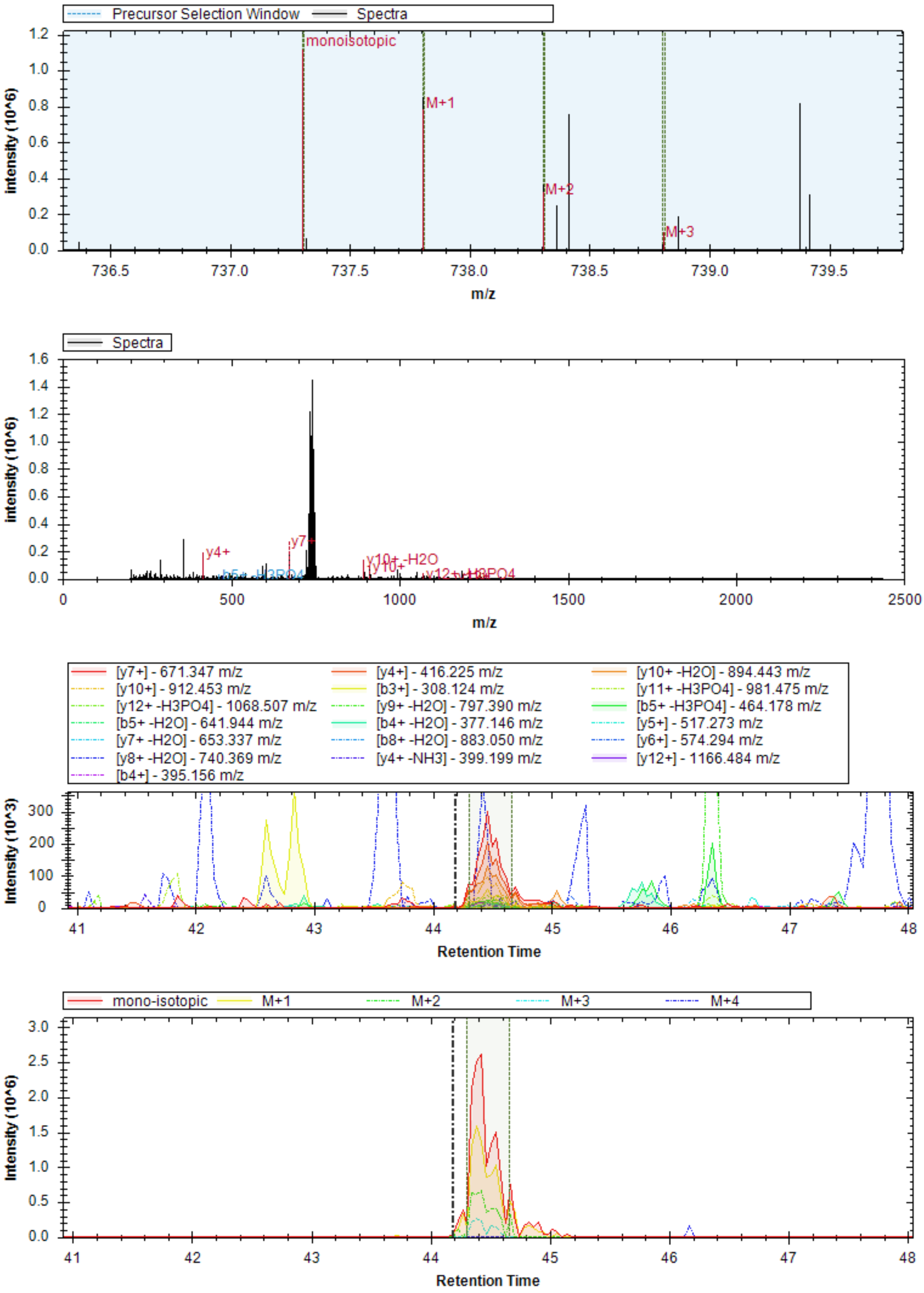

P10637 – _SGYSSPGS[+H+O3+P]PGTPGSR_.2

Fig4_MP-DIA-barrel-cortex-p30-3_MHRM.htrms

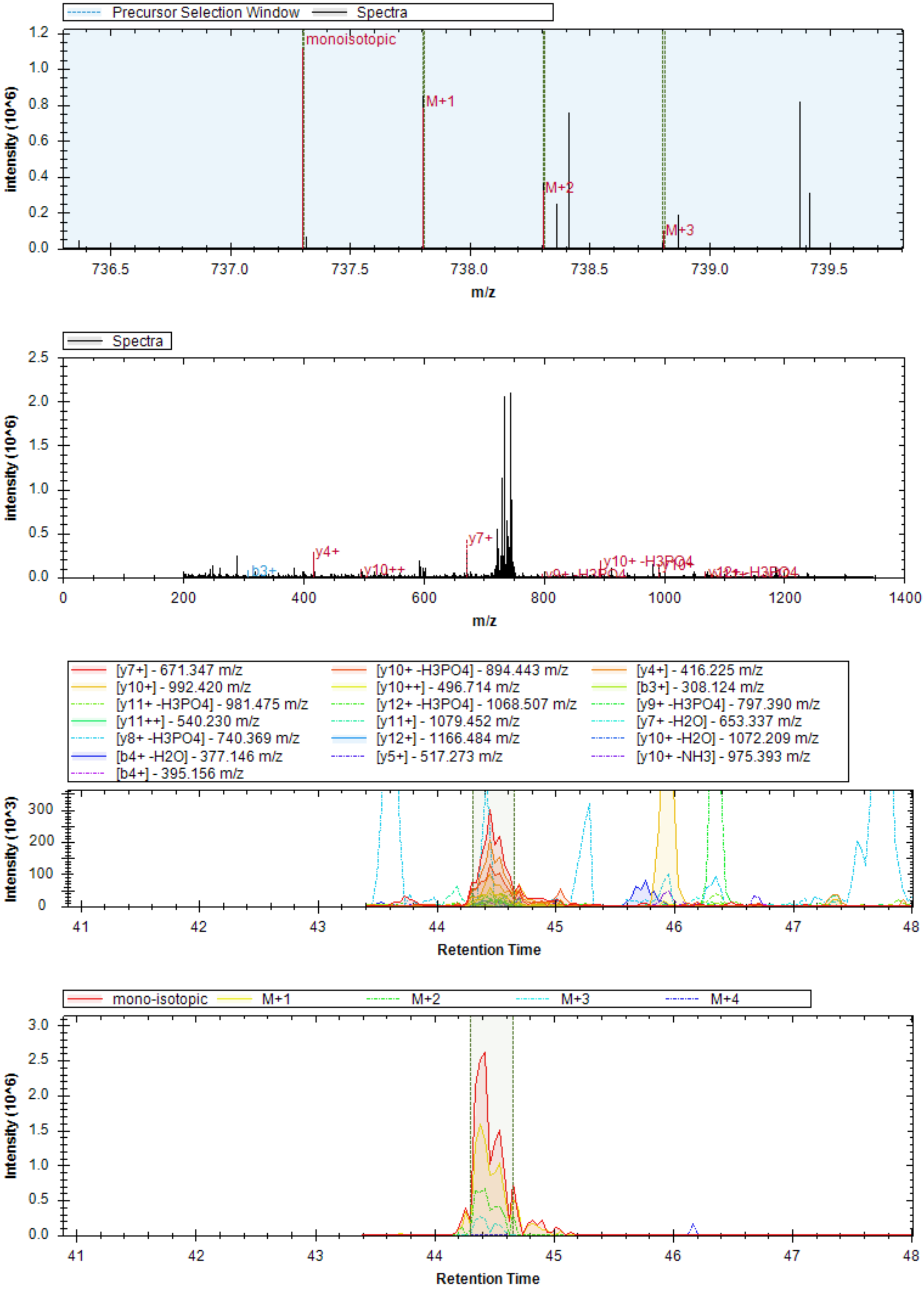

P57776-3 – _ATAPQTQHVS[+H+O3+P]PMR_.2
Fig4_MP-DIA-barrel-cortex-p30-3_MHRM.htrms

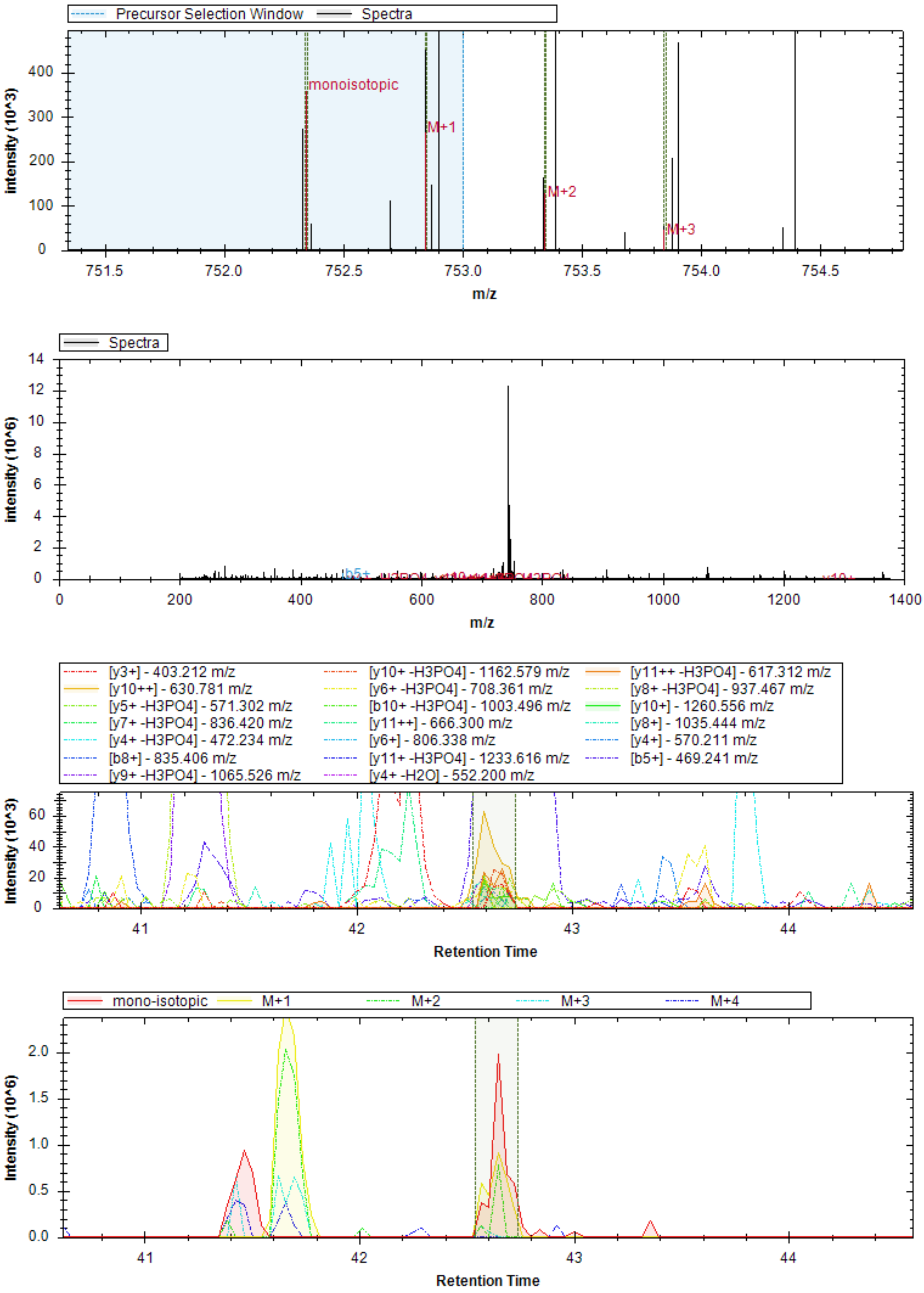

Q8C5R2 – _LAGNEALS[+H+O3+P]PTSPSK_.2

Fig4_MP-DIA-barrel-cortex-p30-3_MHRM.htrms

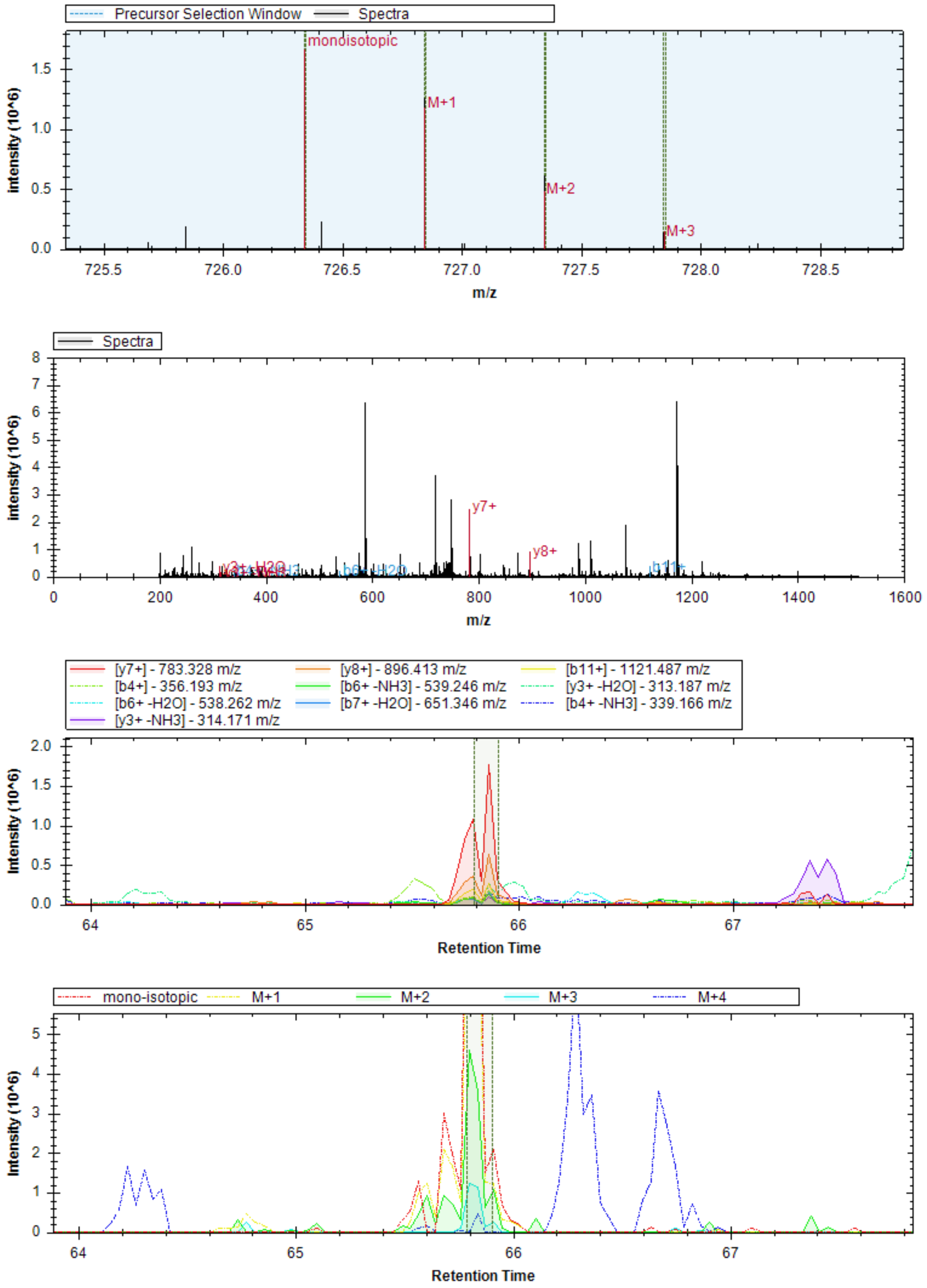

Q8K0U4 – _ETAPTSTYS[+H+O3+P]SPAR_.2
Fig4_MP-DIA-barrel-cortex-p30-3_MHRM.htrms

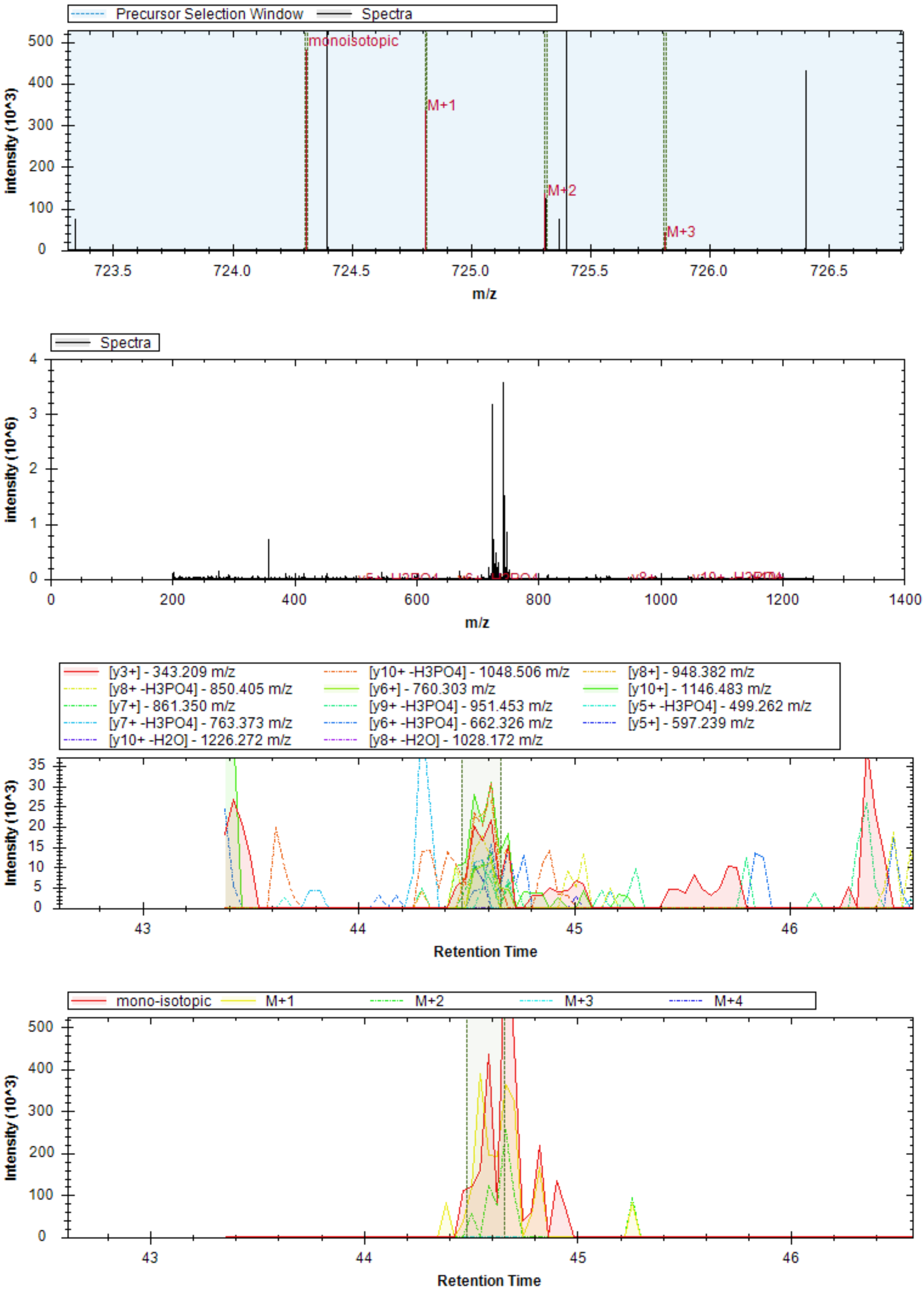

Q9JLM8 – _SPSPSPT[+H+O3+P]SPGSLRK_.2
Fig4_MP-DIA-barrel-cortex-p30-3_MHRM.htrms

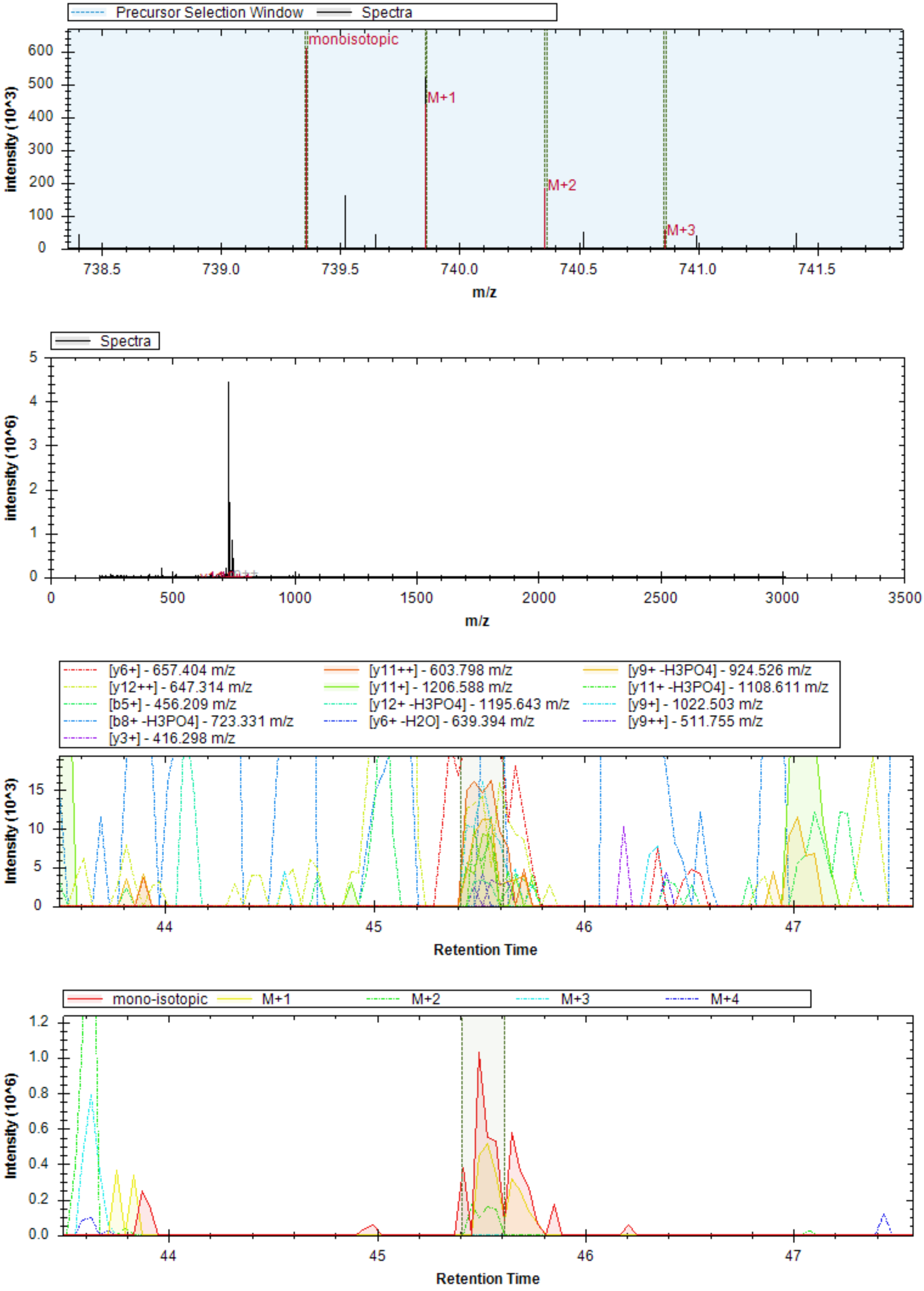

Q9JLM8 – _SPSPSPTS[+H+O3+P]PGSLRK_.2
Fig4_MP-DIA-barrel-cortex-p30-3_MHRM.htrms

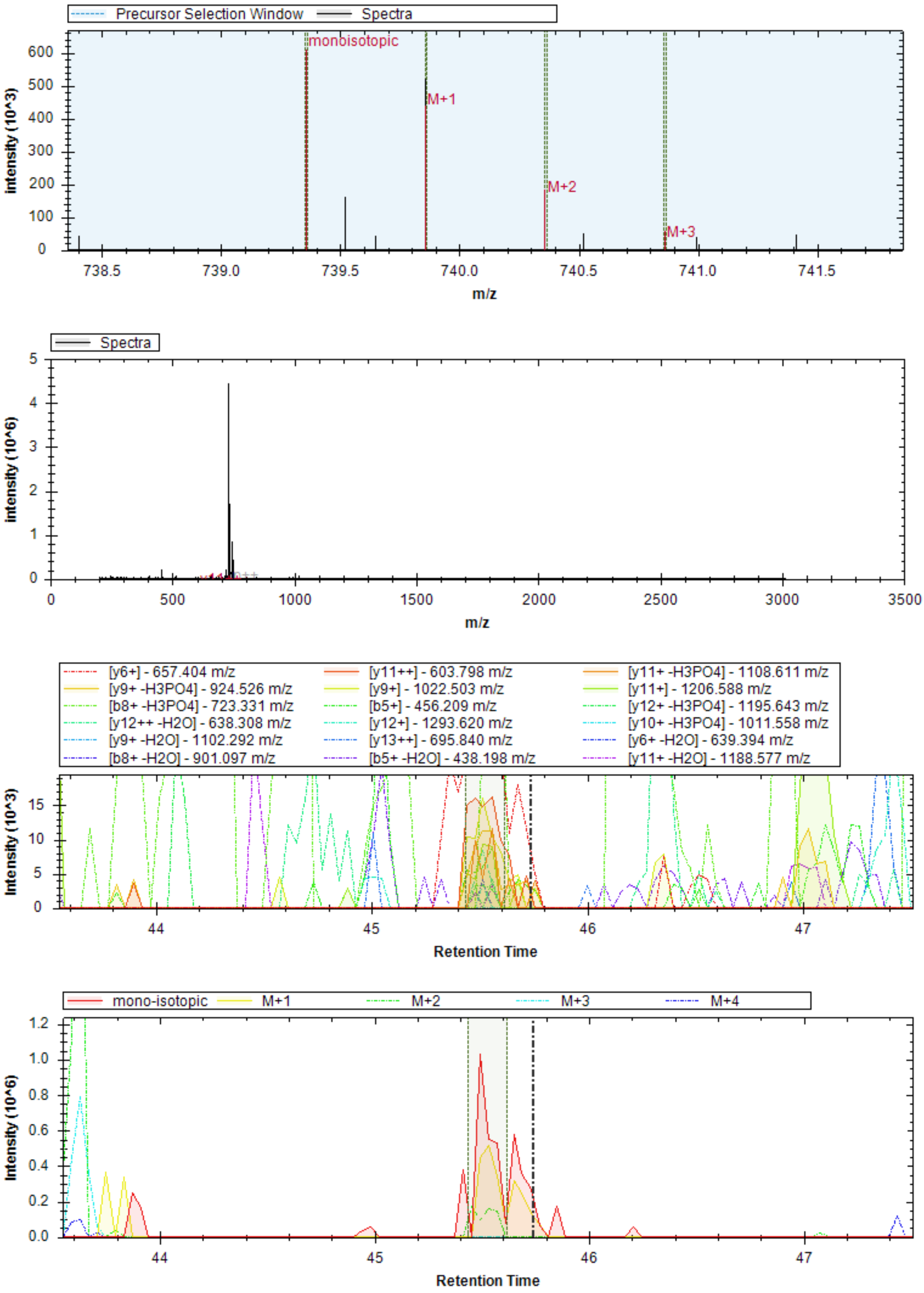

D3Z6S9 – _IPSIEIT[+H+O3+P]ET[+H+O3+P]LPQTQASMLR_.3

Fig4_MP-DIA-barrel-cortex-p30-3_MHRM.htrms

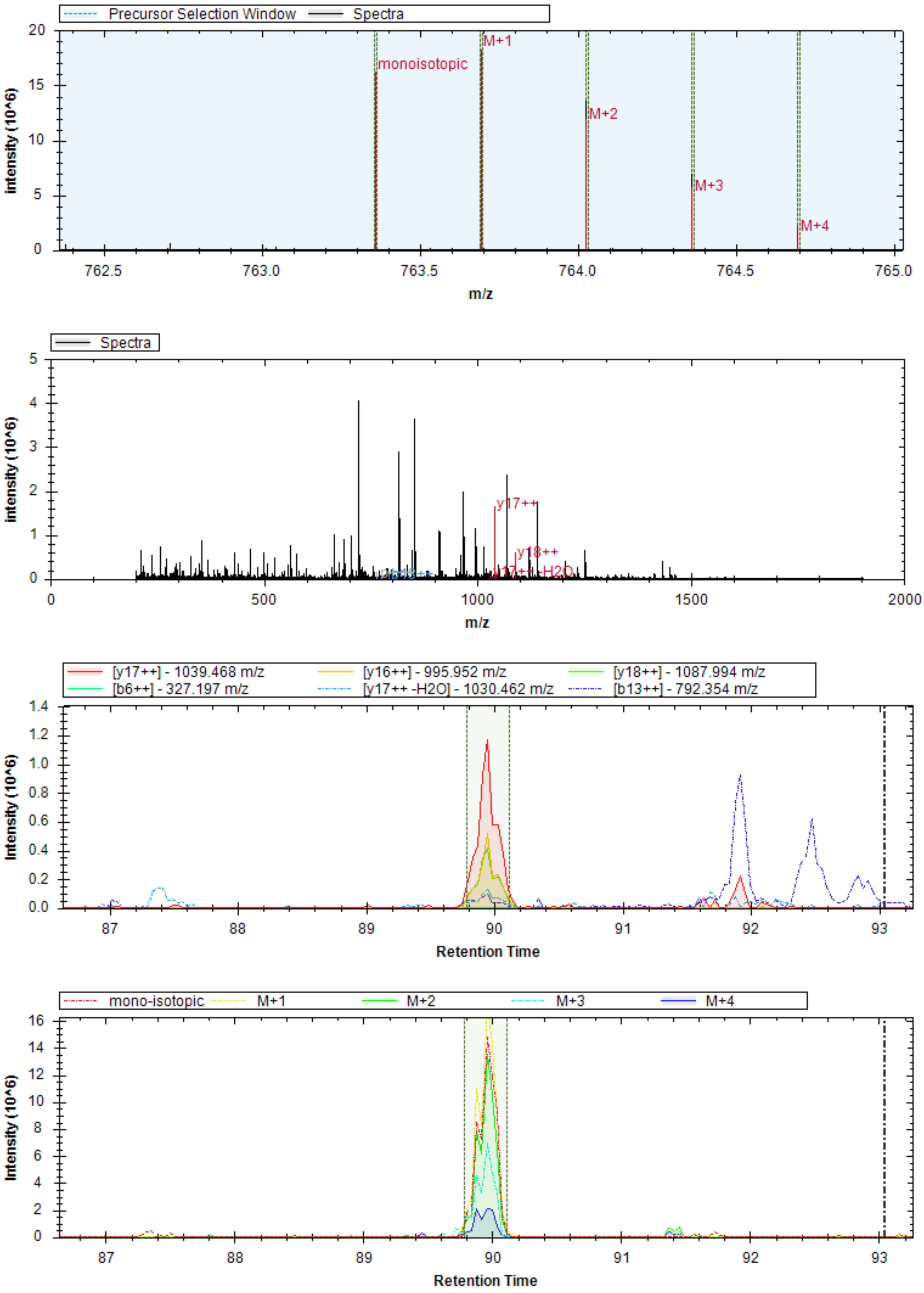

P06837 – _QADVPAAVTDAAAT[+H+O3+P]TPAAEDAATK_.3
Fig4_MP-DIA-barrel-cortex-p30-3_MHRM.htrms

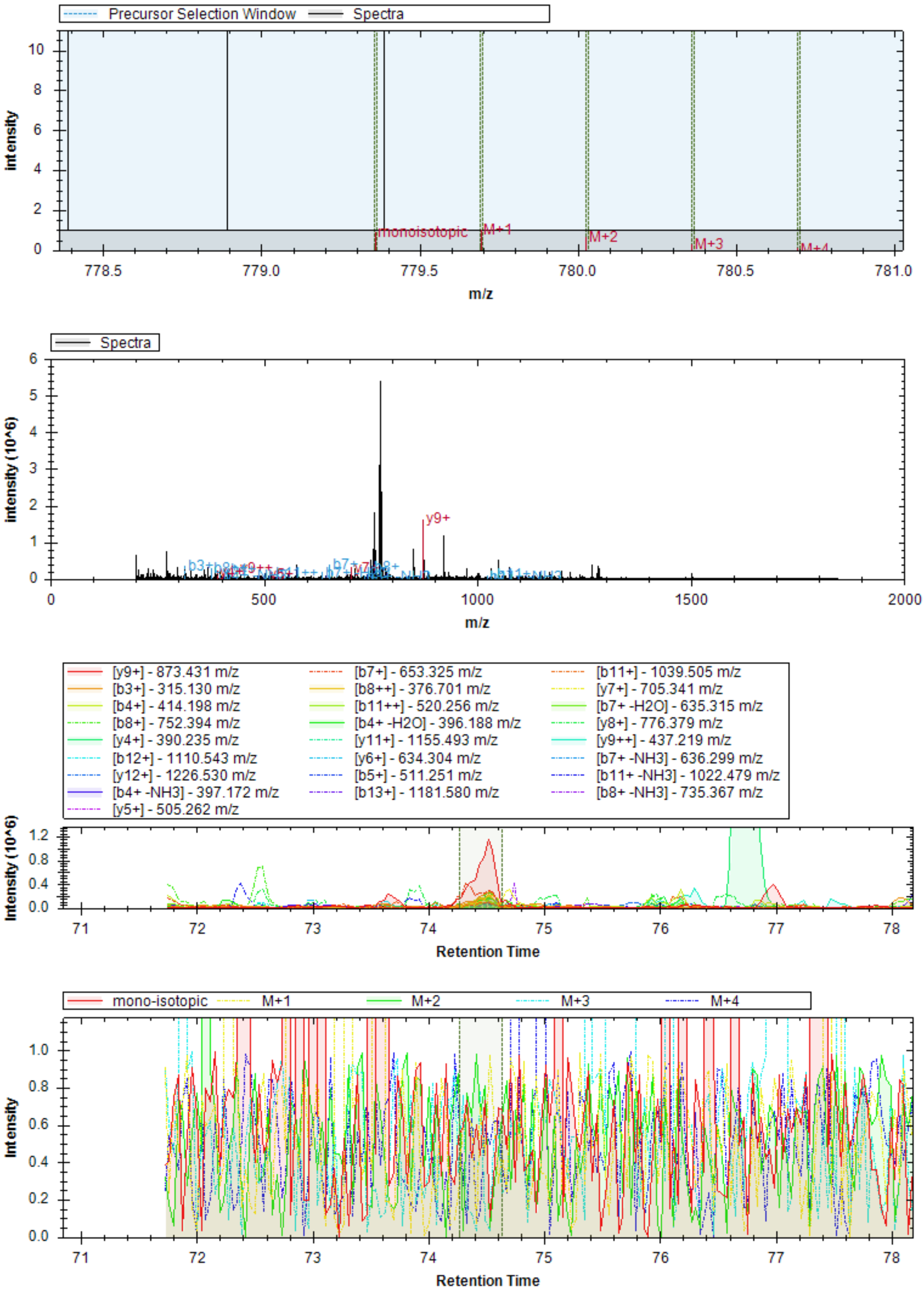

Q68FD9 – _LAQLLS[+H+O3+P]EVPT[+H+O3+P]RR_.2
Fig4_MP-DIA-barrel-cortex-p30-3_MHRM.htrms

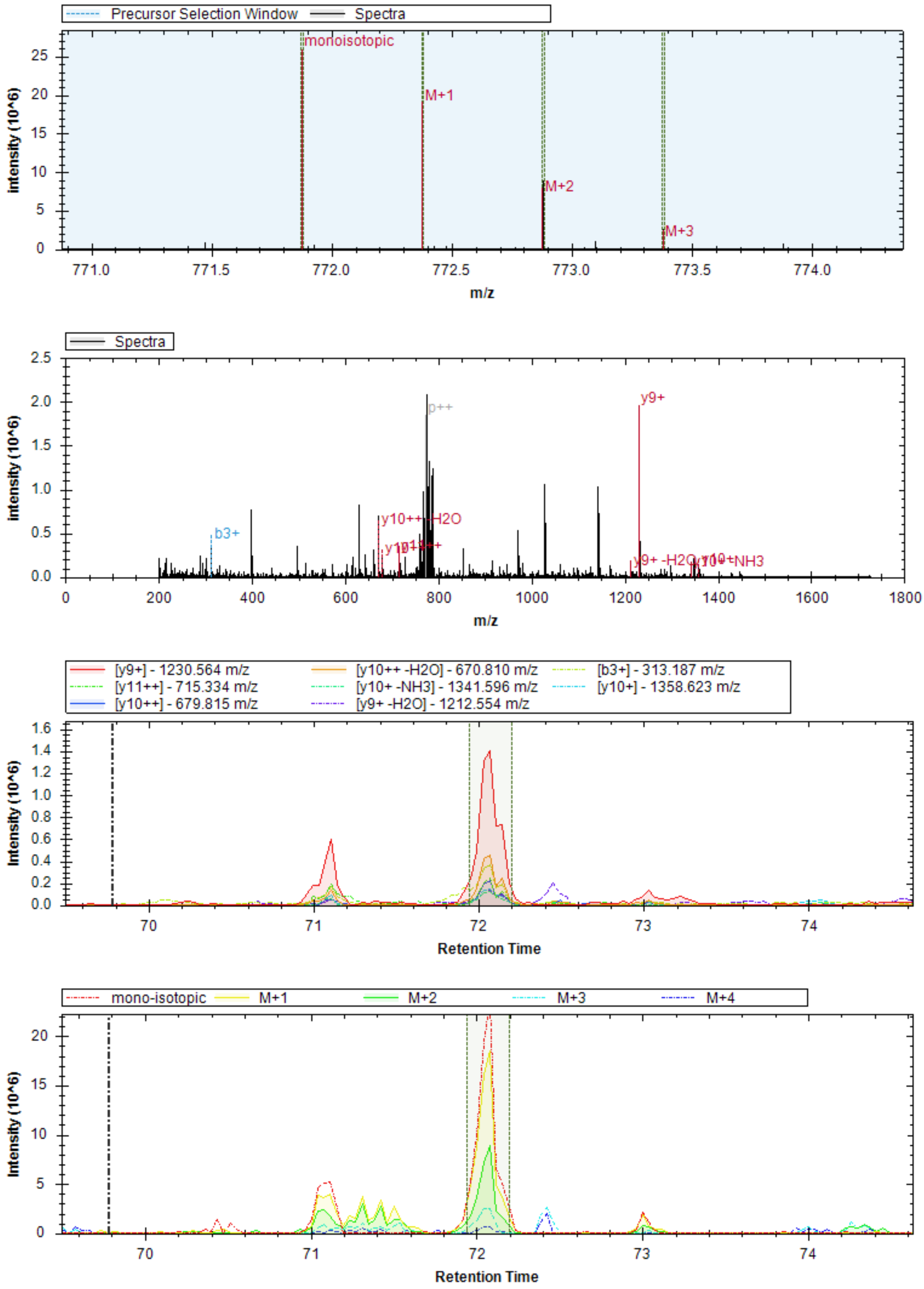

Q8BRT1 – _VLNT[+H+O3+P]GSDVEEAVADALLLGDIR_.3
Fig4_MP-DIA-barrel-cortex-p30-3_MHRM.htrms

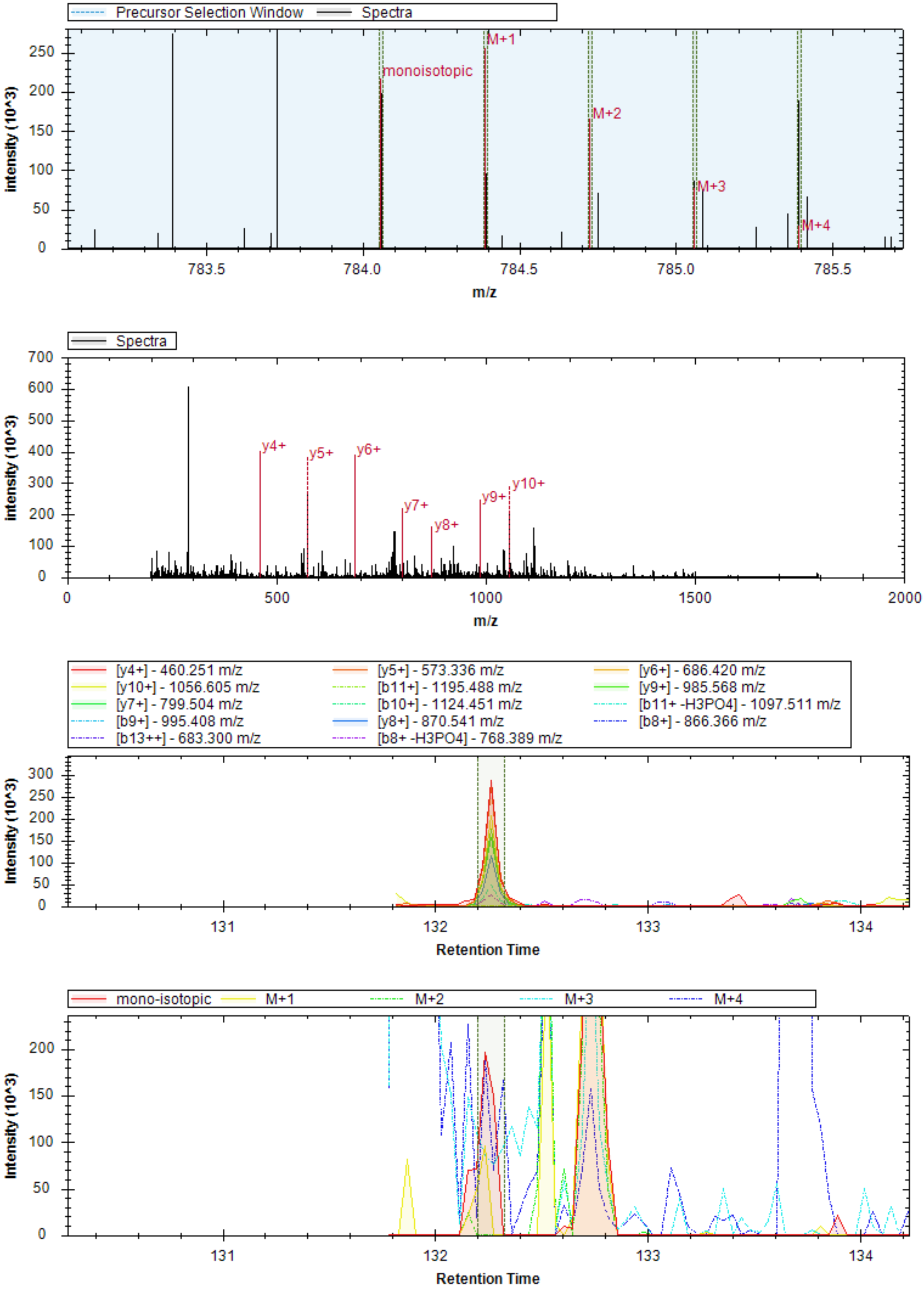

Q9QWI6 – _LSYAGGRPPSYAGS[+H+O3+P]PVHHAAER_.3
Fig4_MP-DIA-barrel-cortex-p30-3_MHRM.htrms

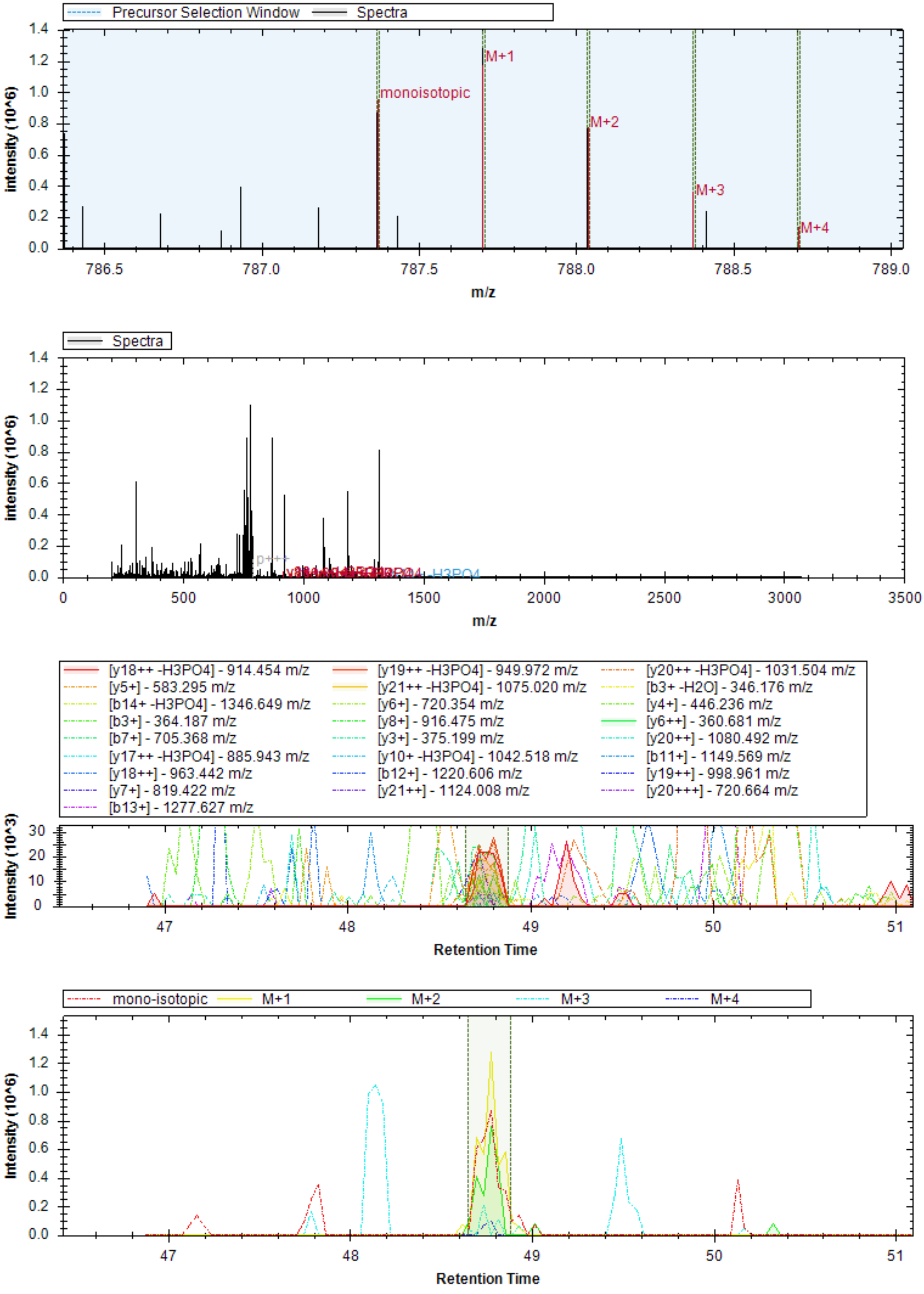

Q9QYC0 – _AAVVTS[+H+O3+P]PPPTTAPHK_.2
Fig4_MP-DIA-barrel-cortex-p30-3_MHRM.htrms

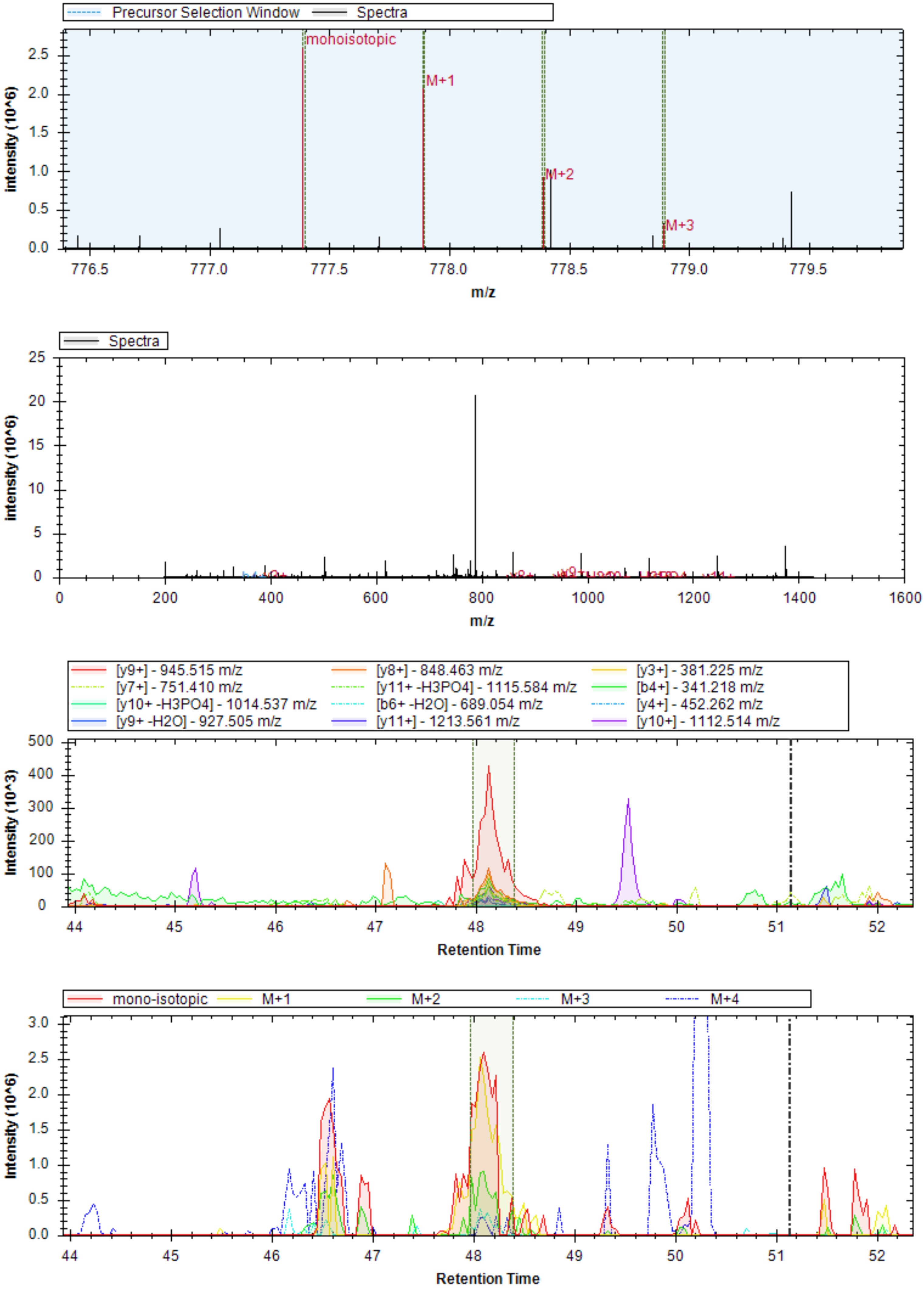

Q9QYR6-2 – _ELSSEPRT[+H+O3+P]PPAQK_.2
Fig4_MP-DIA-barrel-cortex-p30-3_MHRM.htrms

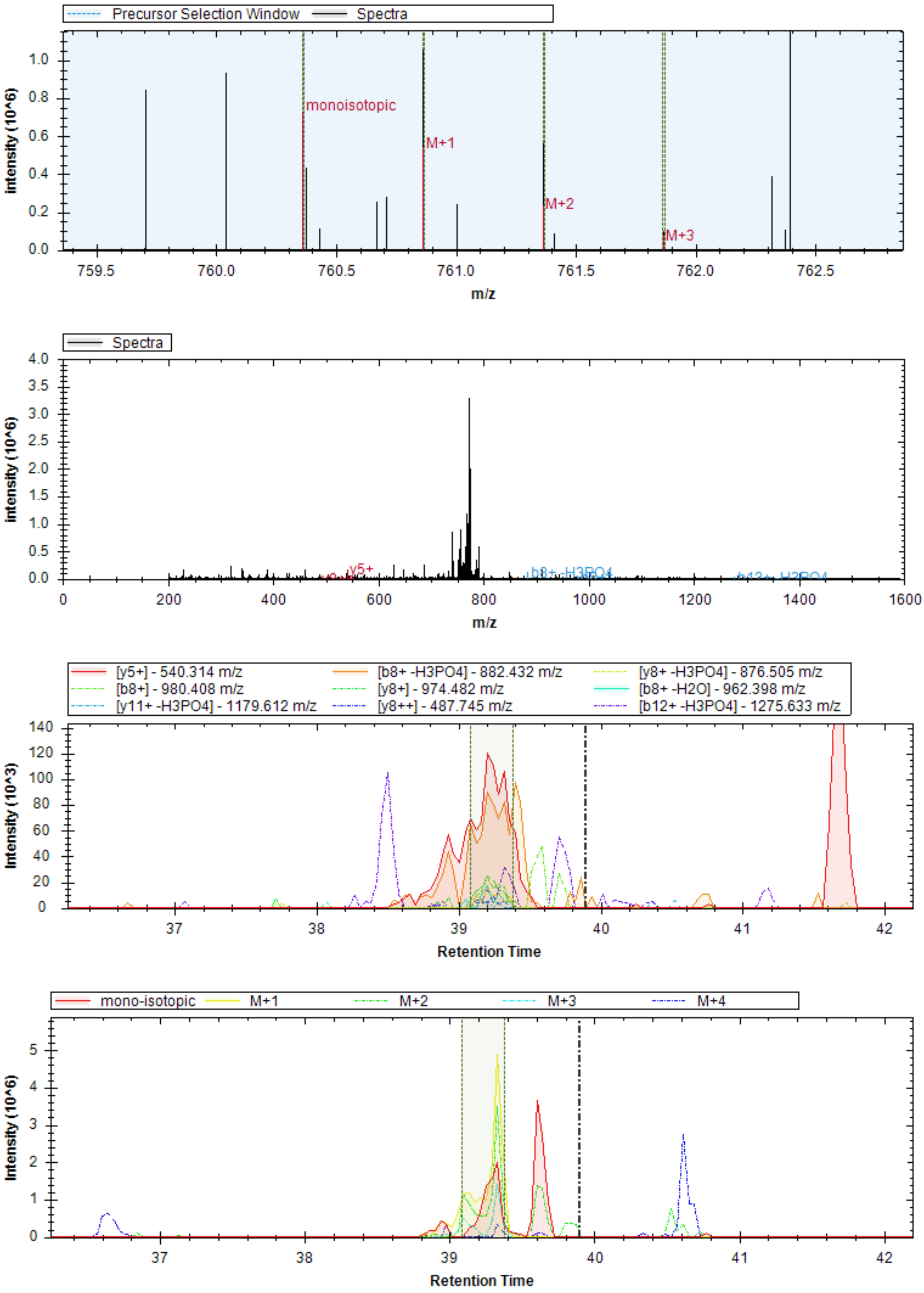

O55023 – _VEKM[+O]LM[+O]S[+H+O3+P]S[+H+O3+P]IKEK_.2
Fig4_MP-DIA-barrel-cortex-p30-3_MHRM.htrms

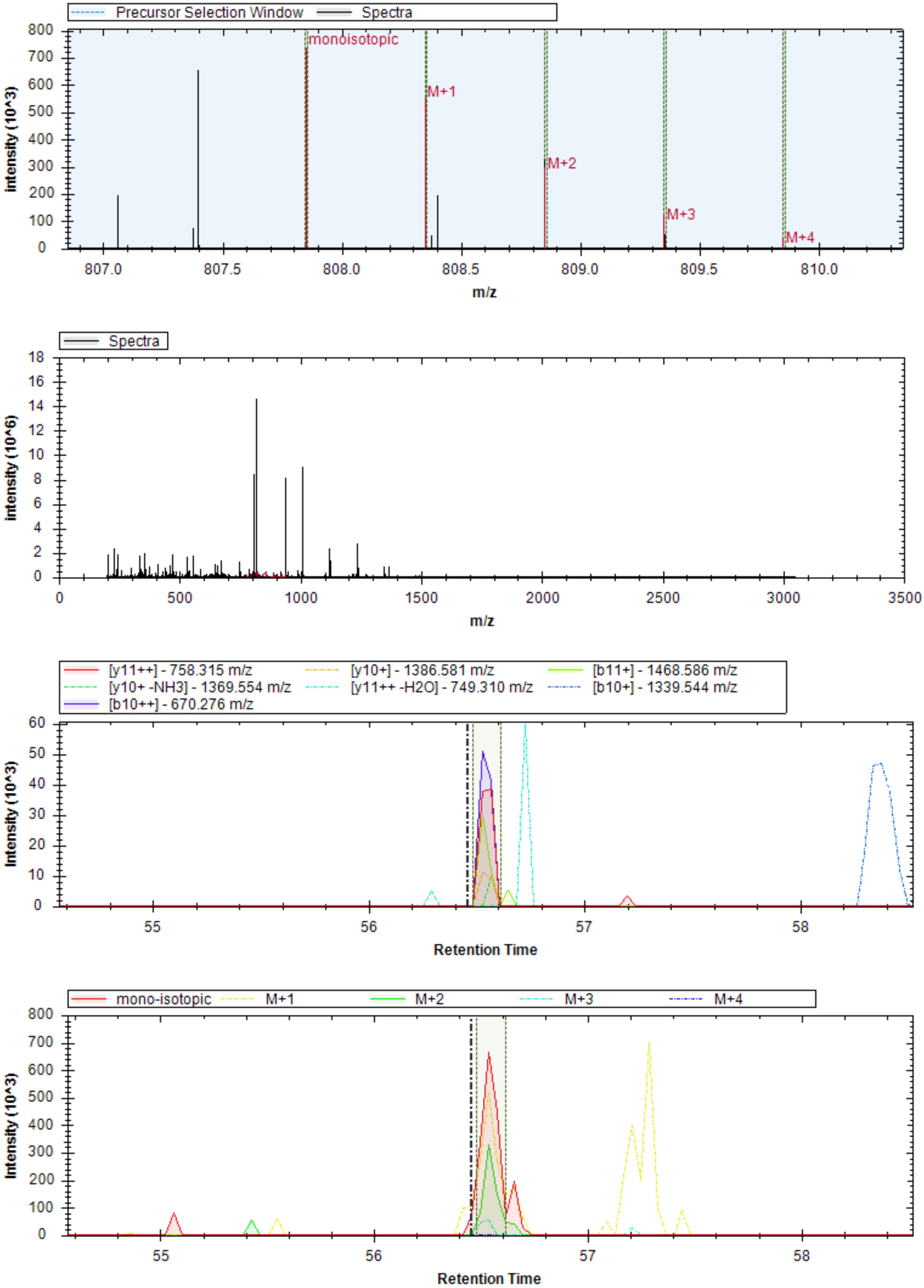

P08249 – _NS[+H+O3+P]PLVSRLTLYDIAHTPGVAADLSHIETR_.4
Fig4_MP-DIA-barrel-cortex-p30-3_MHRM.htrms

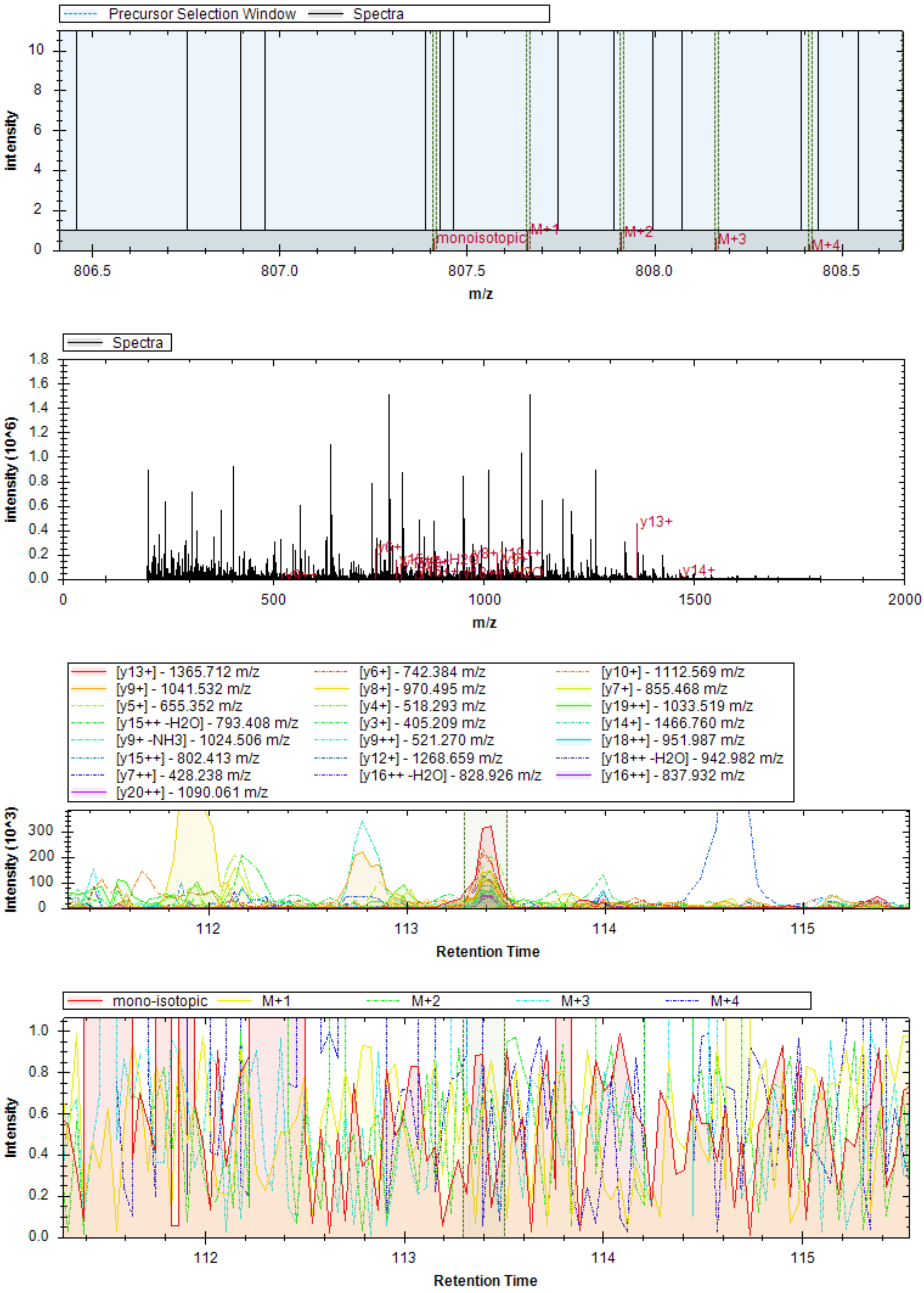

P10637 – _TTPSPKT[+H+O3+P]PPGSGEPPK_.2

Fig4_MP-DIA-barrel-cortex-p30-3_MHRM.htrms

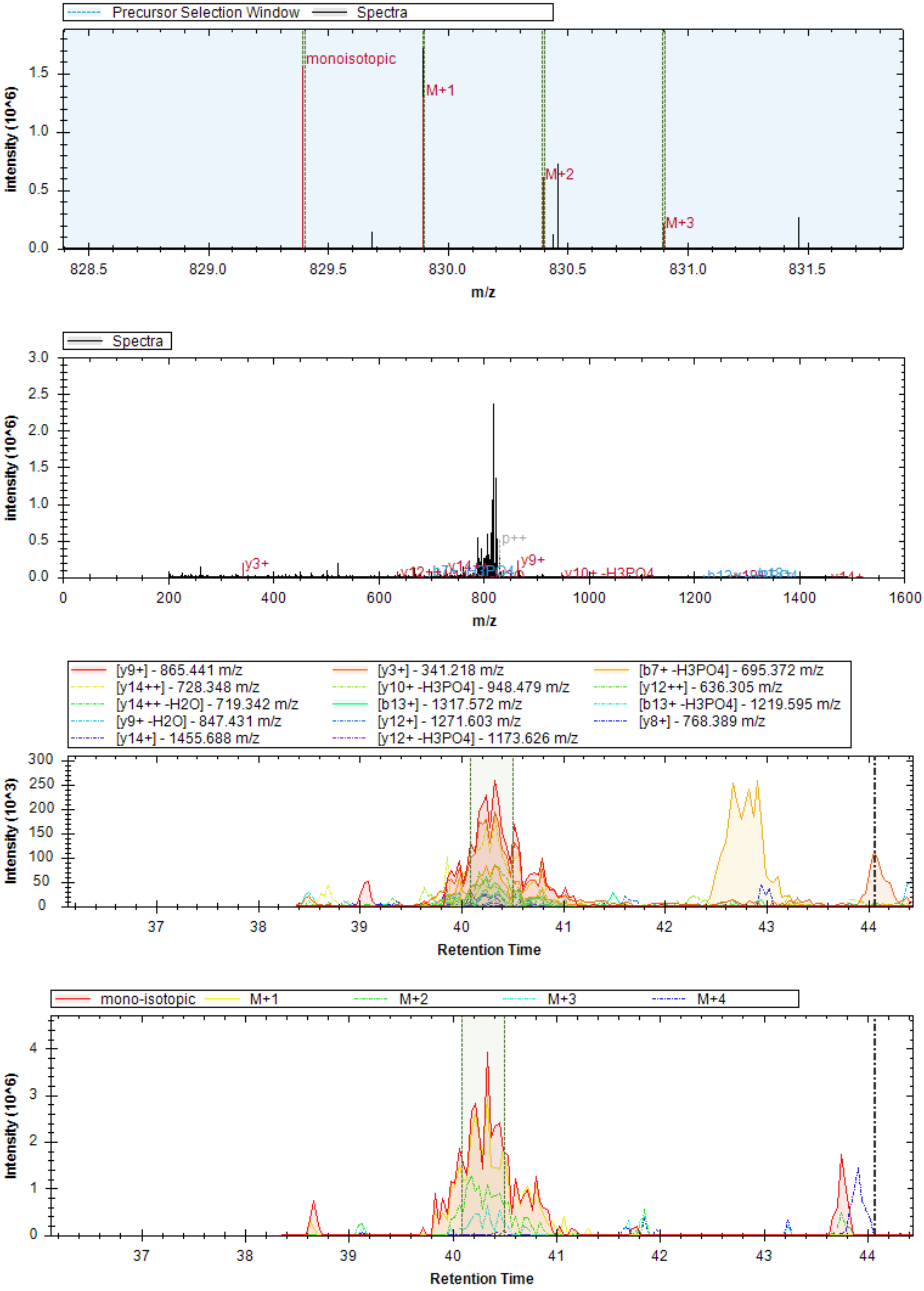

P20357 – _APHWTSASLTEAAAHPHS[+H+O3+P]PEMK_.3
Fig4_MP-DIA-barrel-cortex-p30-3_MHRM.htrms

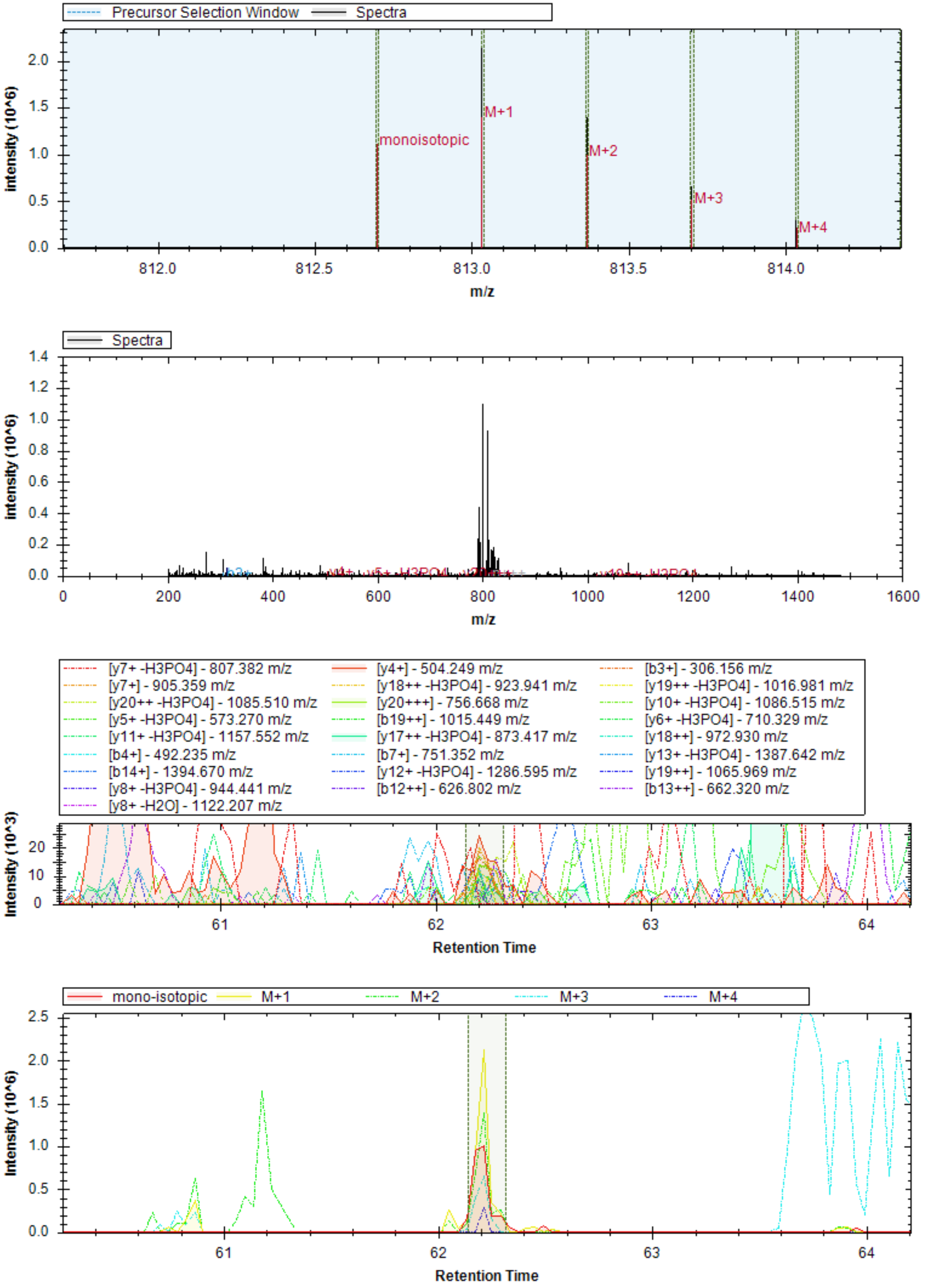

P20357 – _VAIIRT[+H+O3+P]PPKSPATPK_.2
Fig4_MP-DIA-barrel-cortex-p30-3_MHRM.htrms

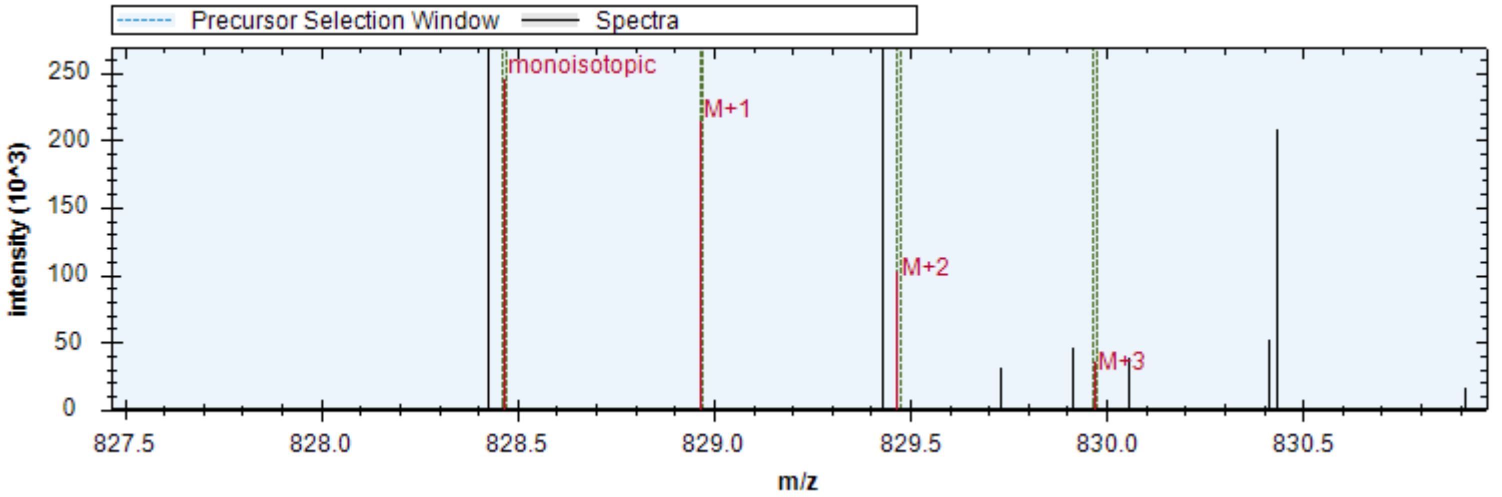

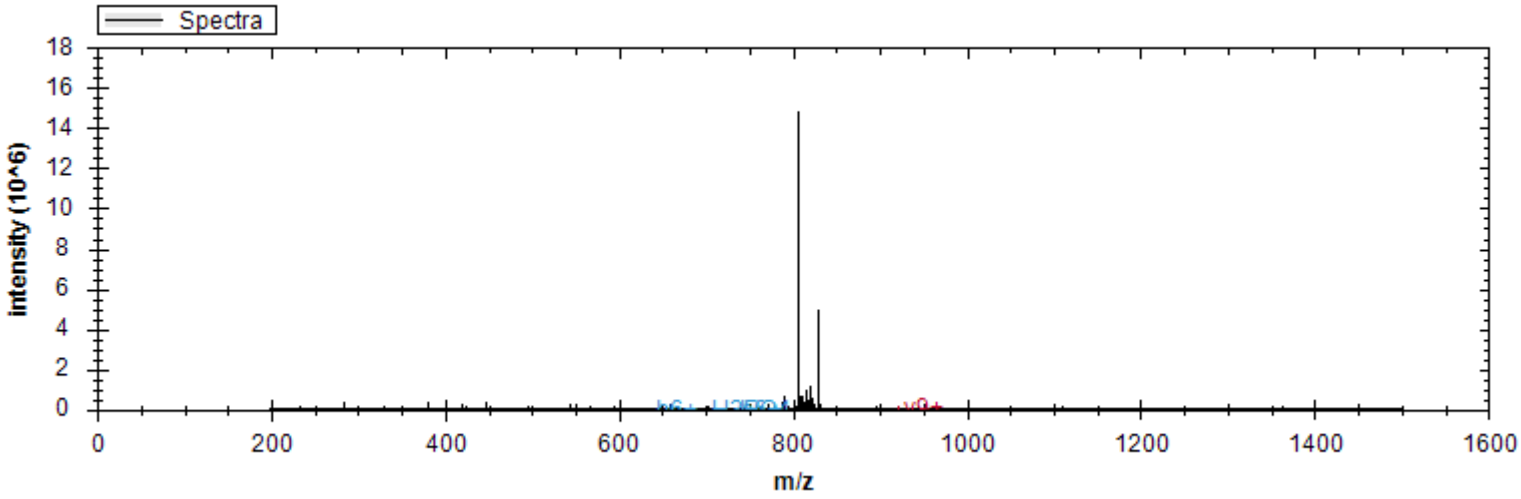

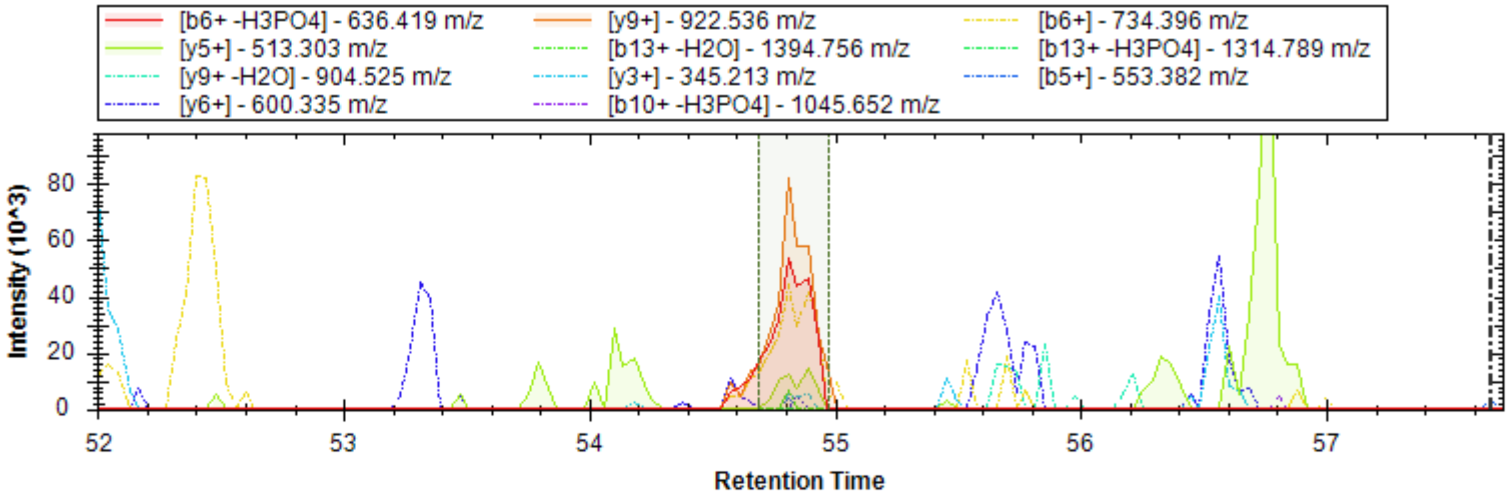

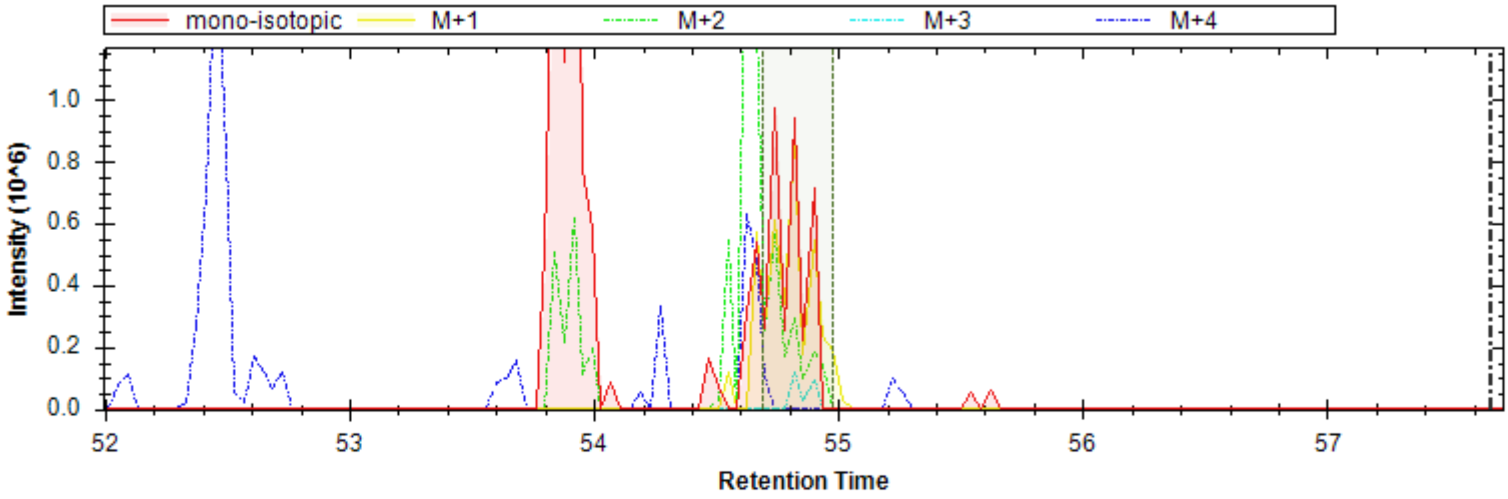

P20357 – _VDHGAEIIT[+H+O3+P]QSPSR_.2
Fig4_MP-DIA-barrel-cortex-p30-3_MHRM.htrms

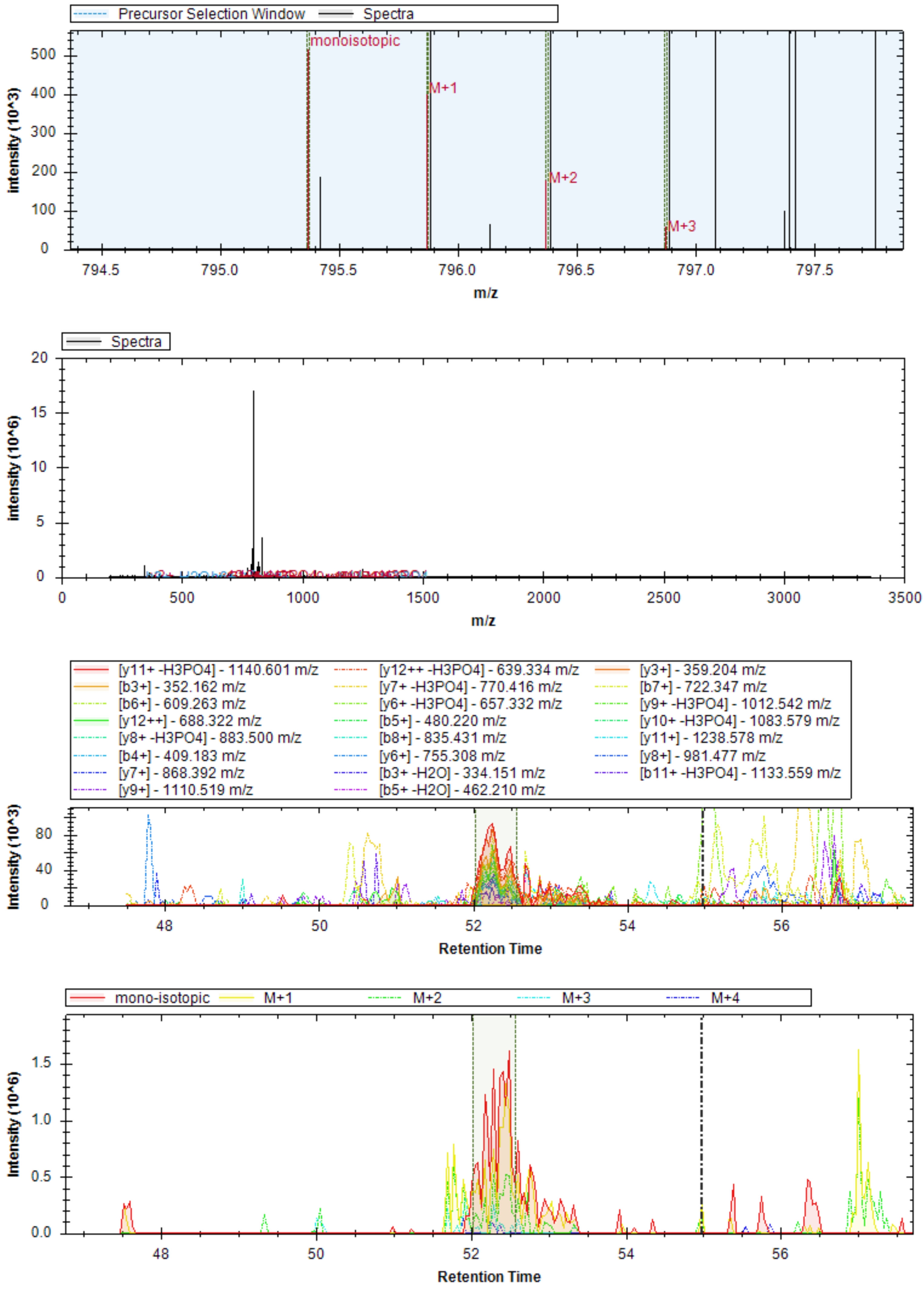

P20357 – _VDHGAEIITQS[+H+O3+P]PSR_.2

Fig4_MP-DIA-barrel-cortex-p30-3_MHRM.htrms

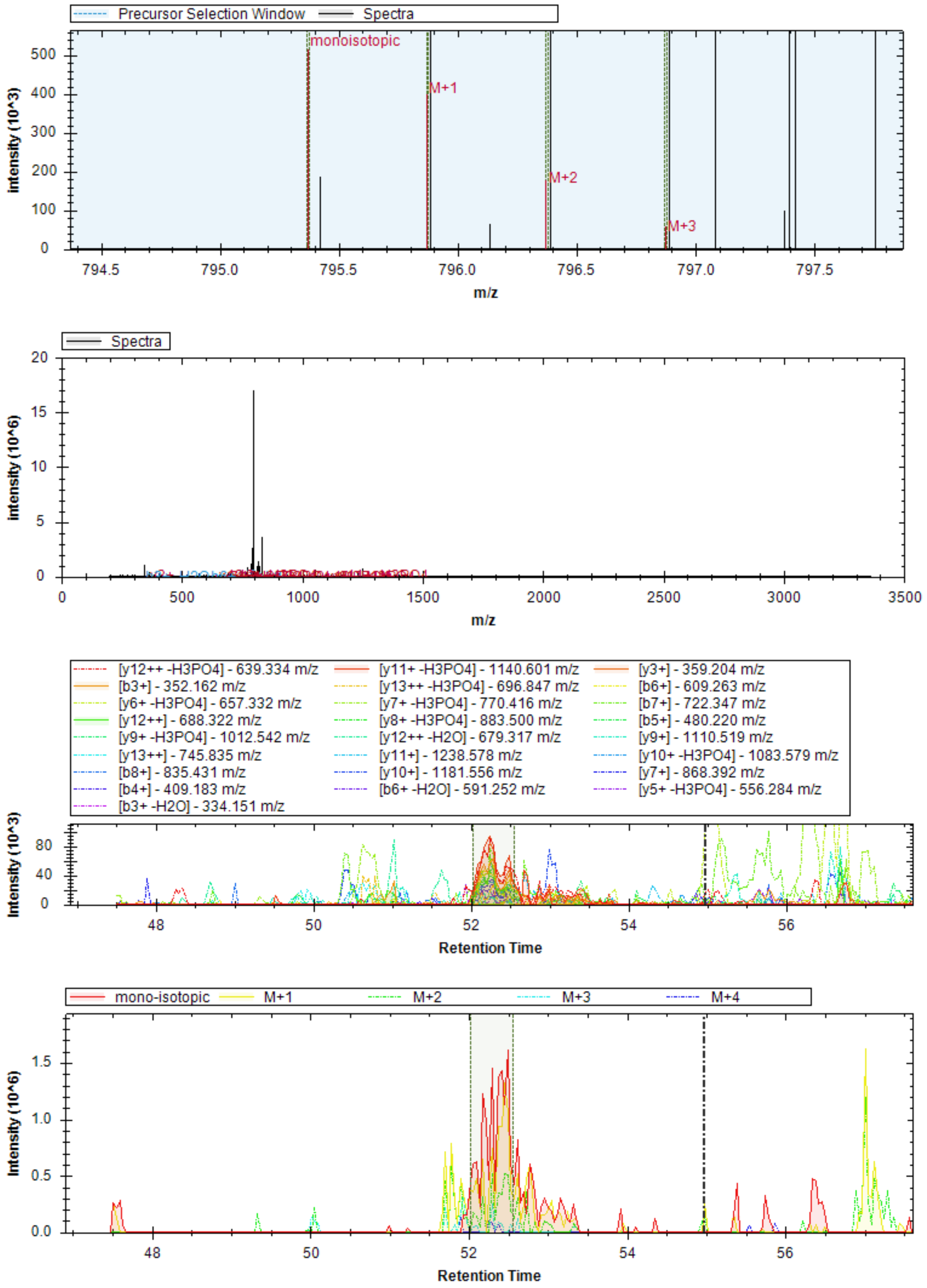

P20357 – _VDHGAEIITQSPS[+H+O3+P]R_.2

Fig4_MP-DIA-barrel-cortex-p30-3_MHRM.htrms

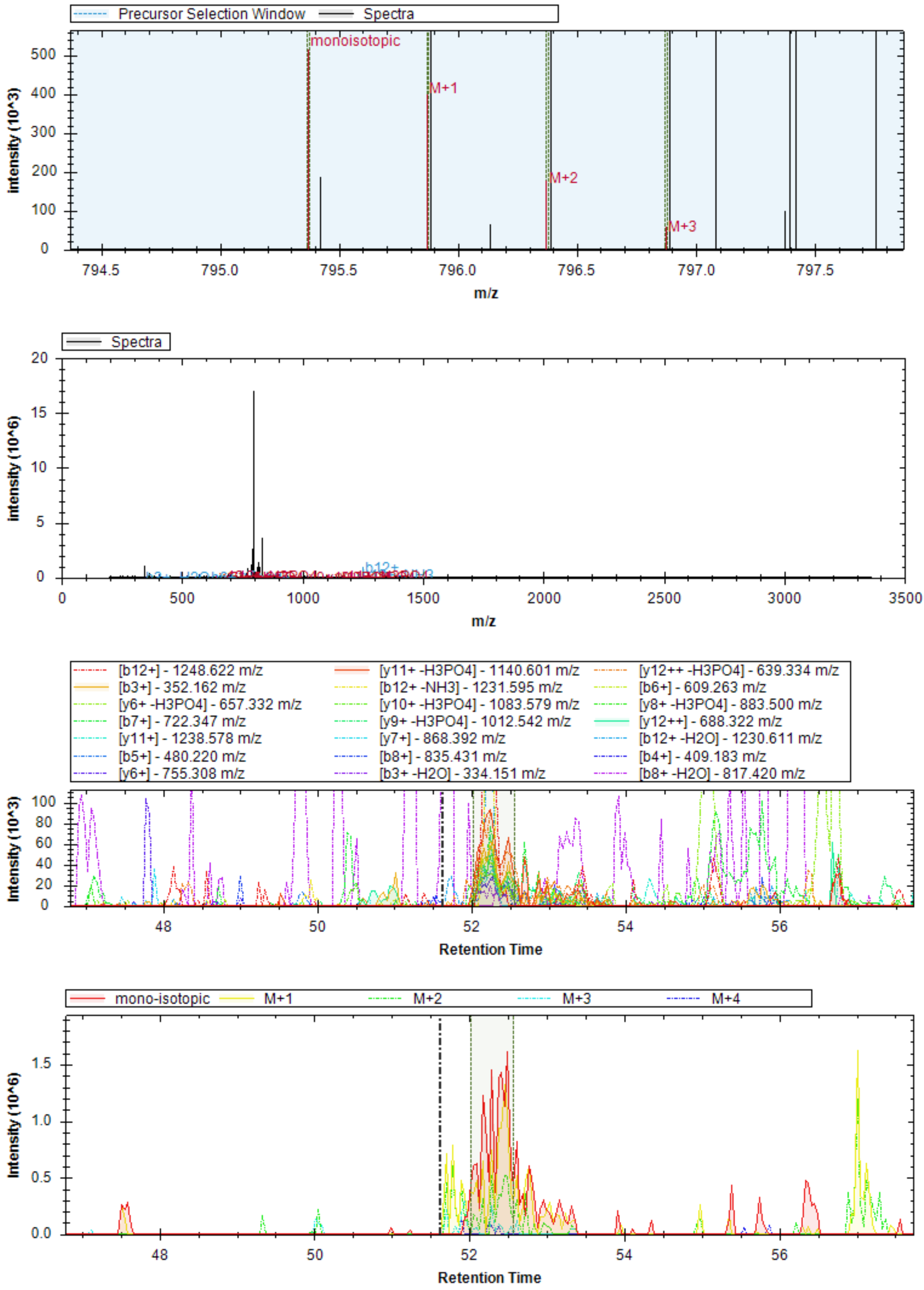

P54227 – _SKESVPDFPLS[+H+O3+P]PPK_.2
Fig4_MP-DIA-barrel-cortex-p30-3_MHRM.htrms

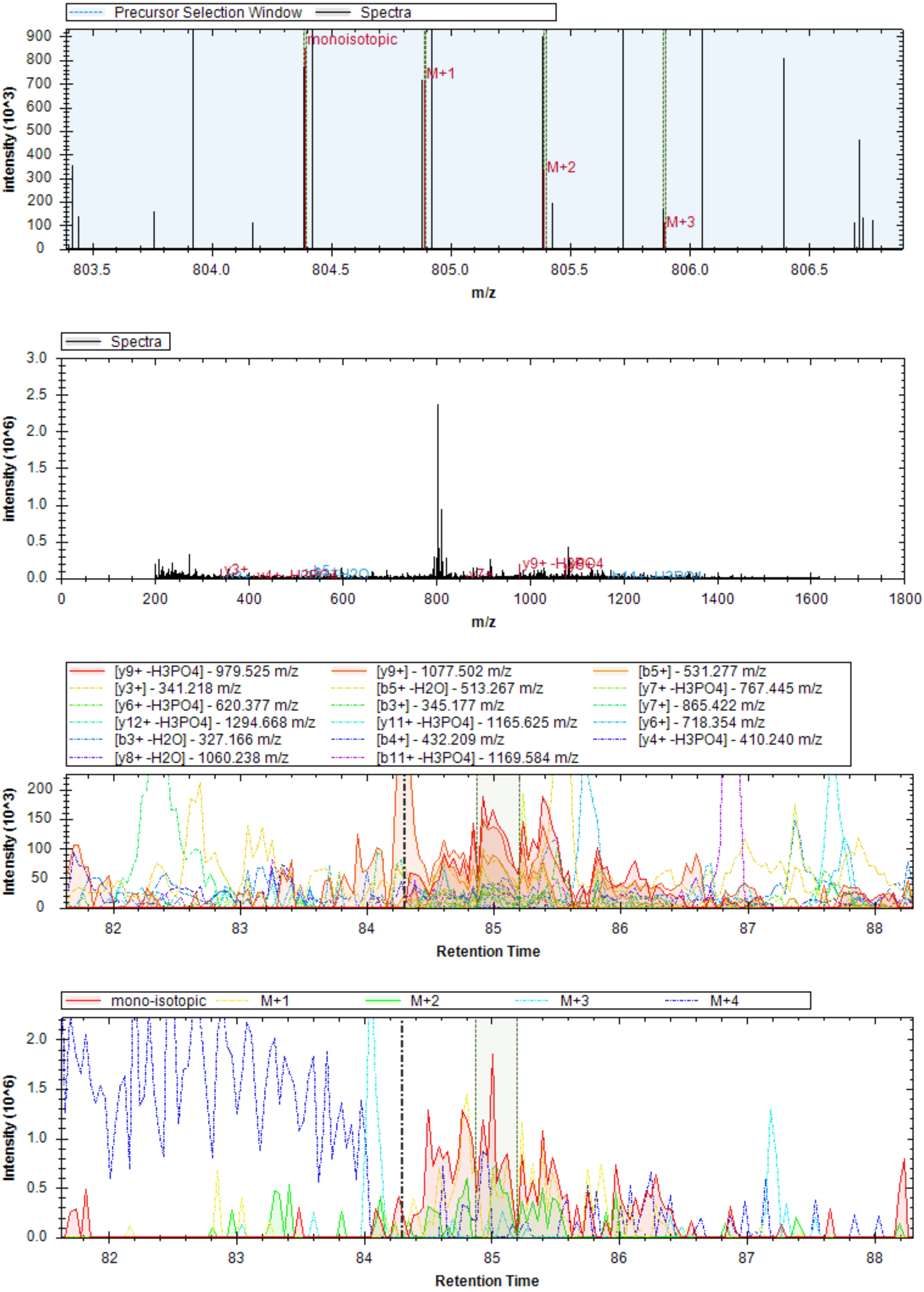

Q60771 – _FYYSS[+H+O3+P]GSSSPTHAK_.2

Fig4_MP-DIA-barrel-cortex-p30-3_MHRM.htrms

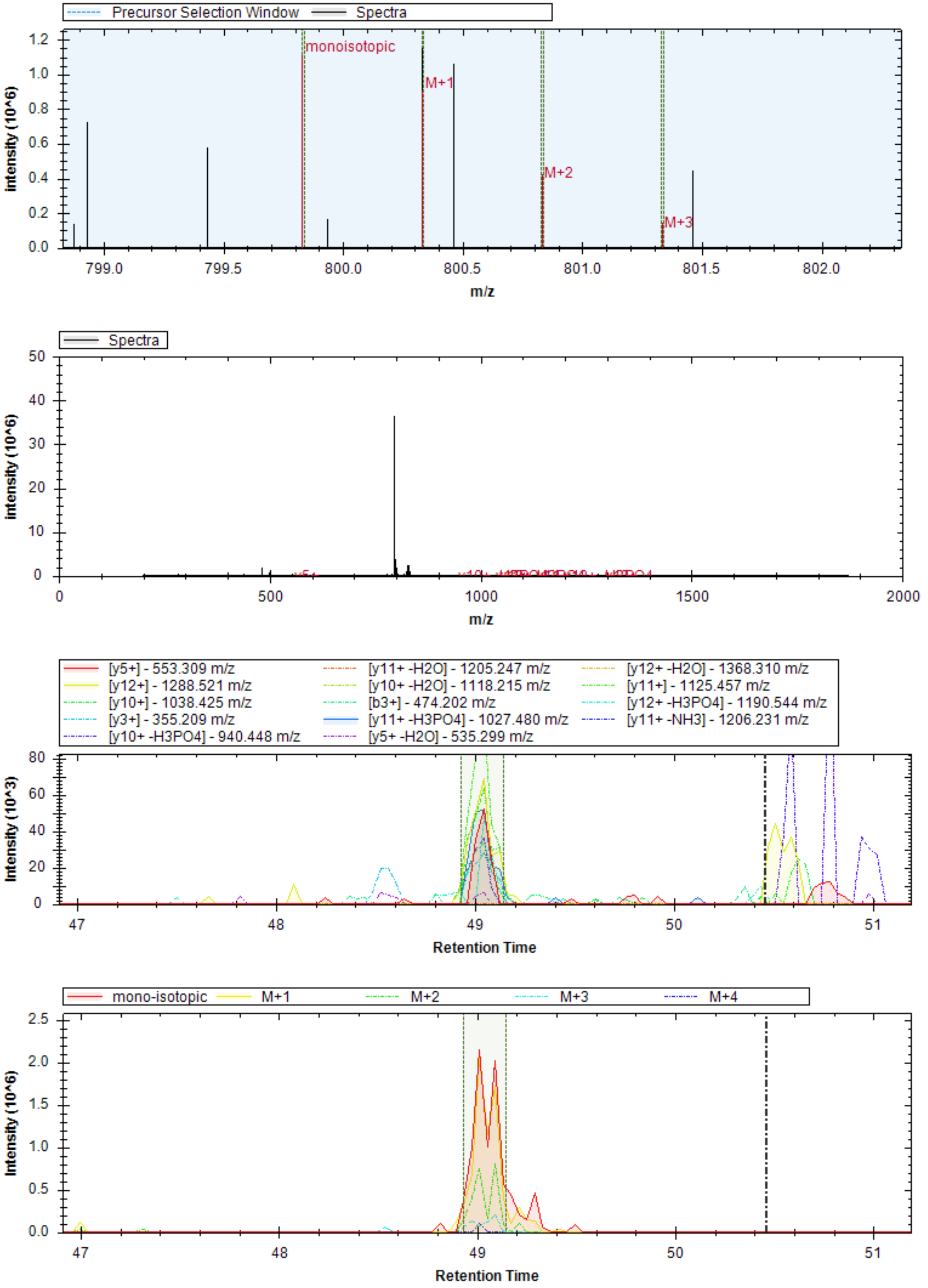

Q60771 – _FYYSSGS[+H+O3+P]SSPTHAK_.2
Fig4_MP-DIA-barrel-cortex-p30-3_MHRM.htrms

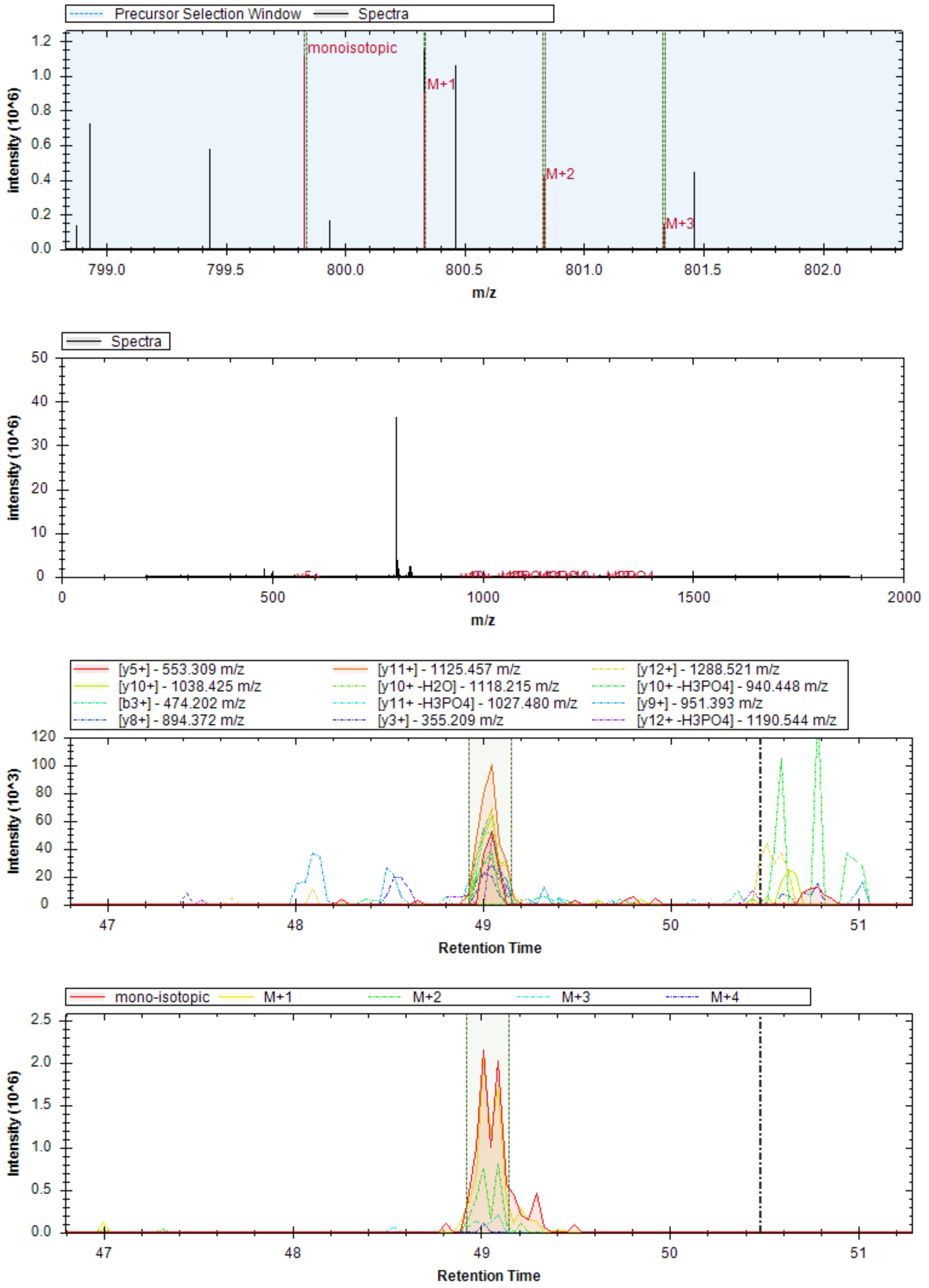

Q60771 – _FYYSSGSSS[+H+O3+P]PTHAK_.2
Fig4_MP-DIA-barrel-cortex-p30-3_MHRM.htrms

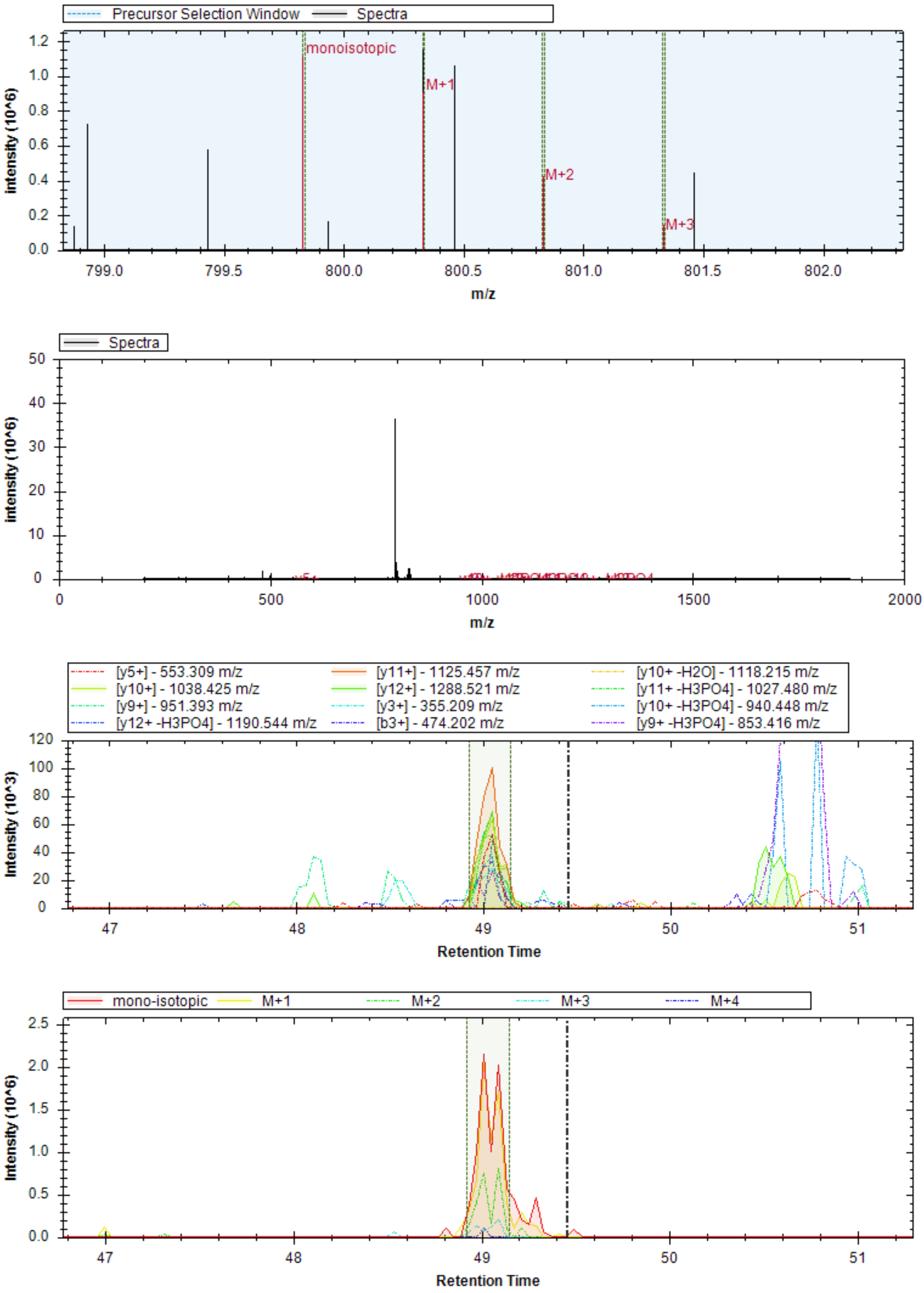

Q61548;Q61548-2;Q61548-3 – _KPGNNEGSGAPS[+H+O3+P]PLSK_.2
Fig4_MP-DIA-barrel-cortex-p30-3_MHRM.htrms

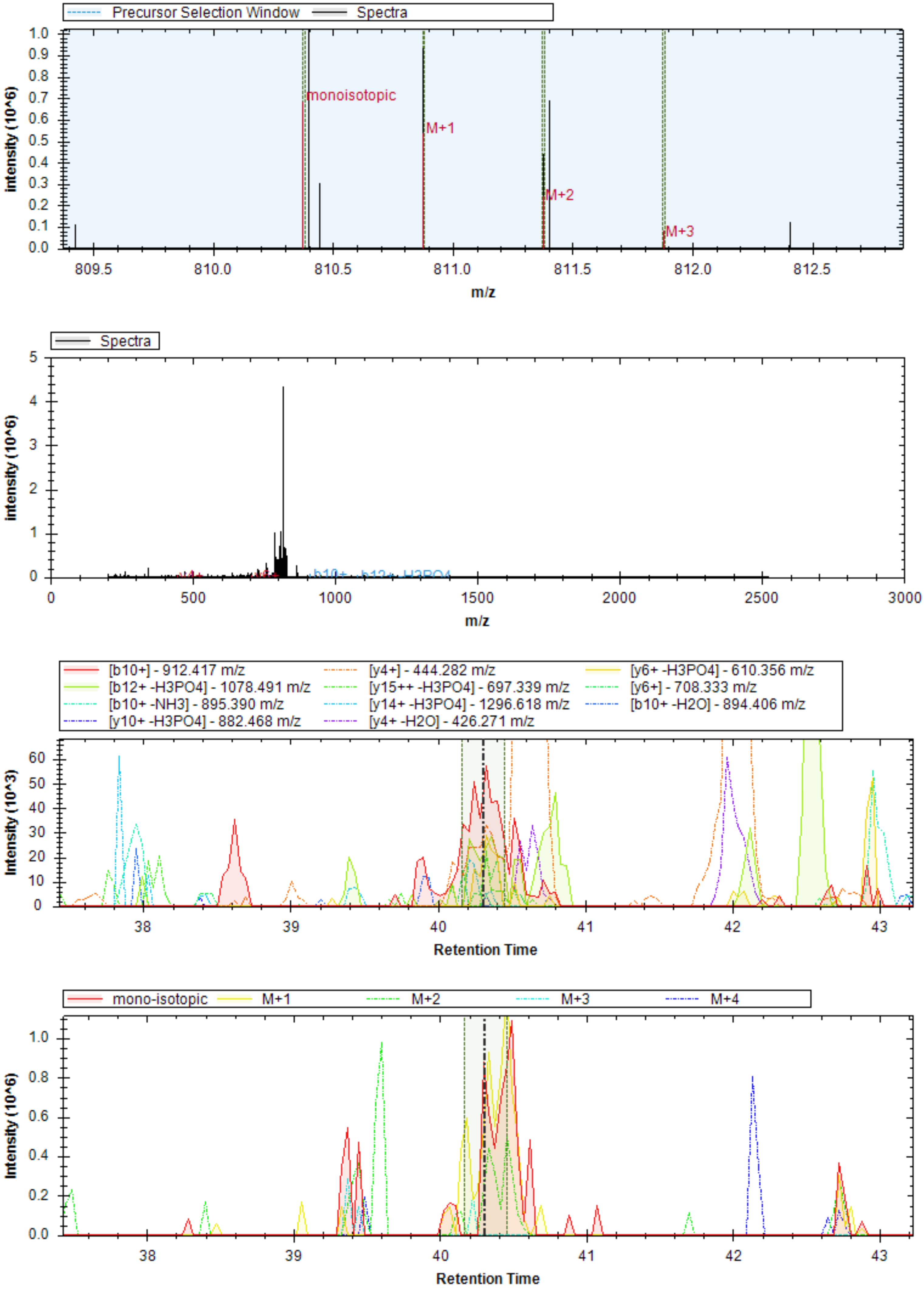

Q64701;Q64701-2 – _LAEILYYKILET[+H+O3+P]IMVQET[+H+O3+P]R_.3
Fig4_MP-DIA-barrel-cortex-p30-3_MHRM.htrms

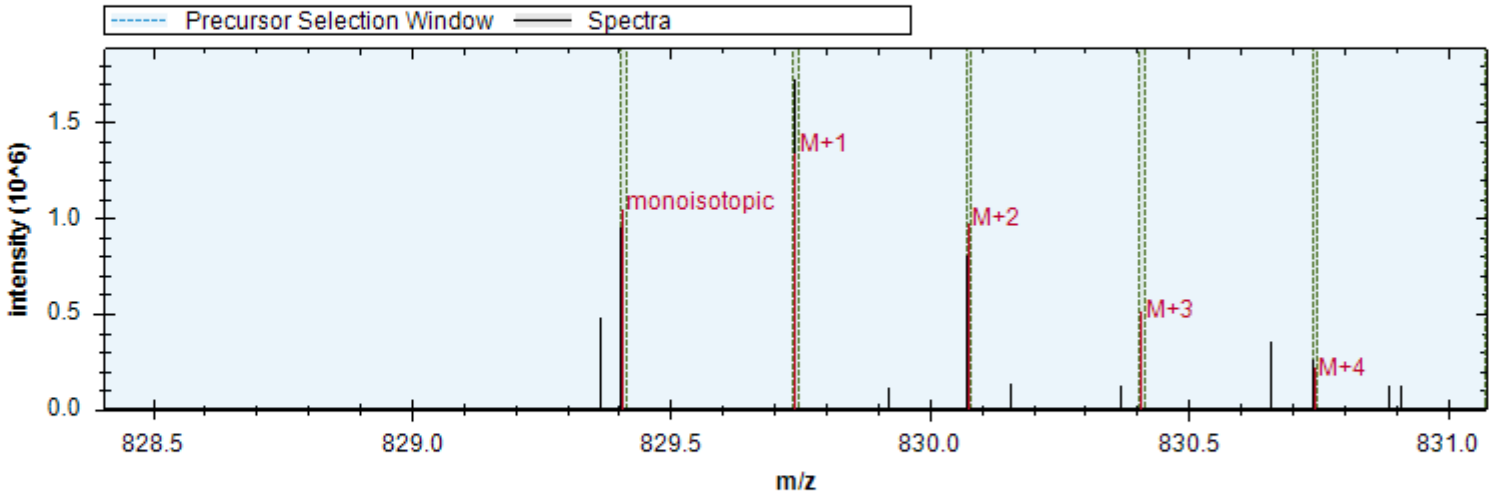

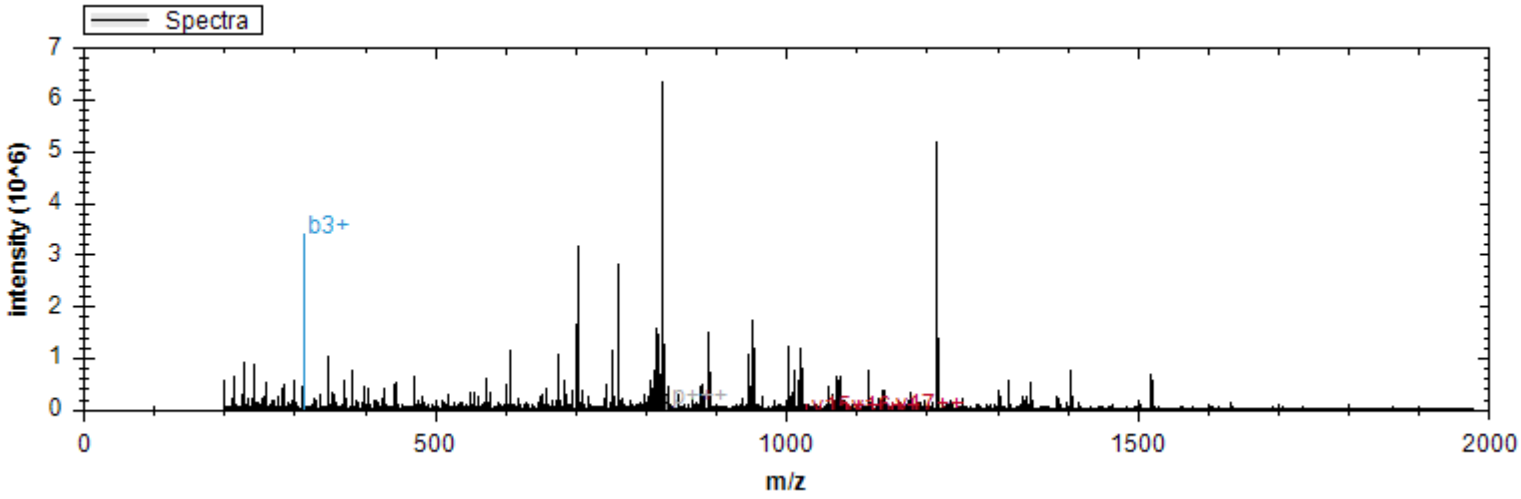

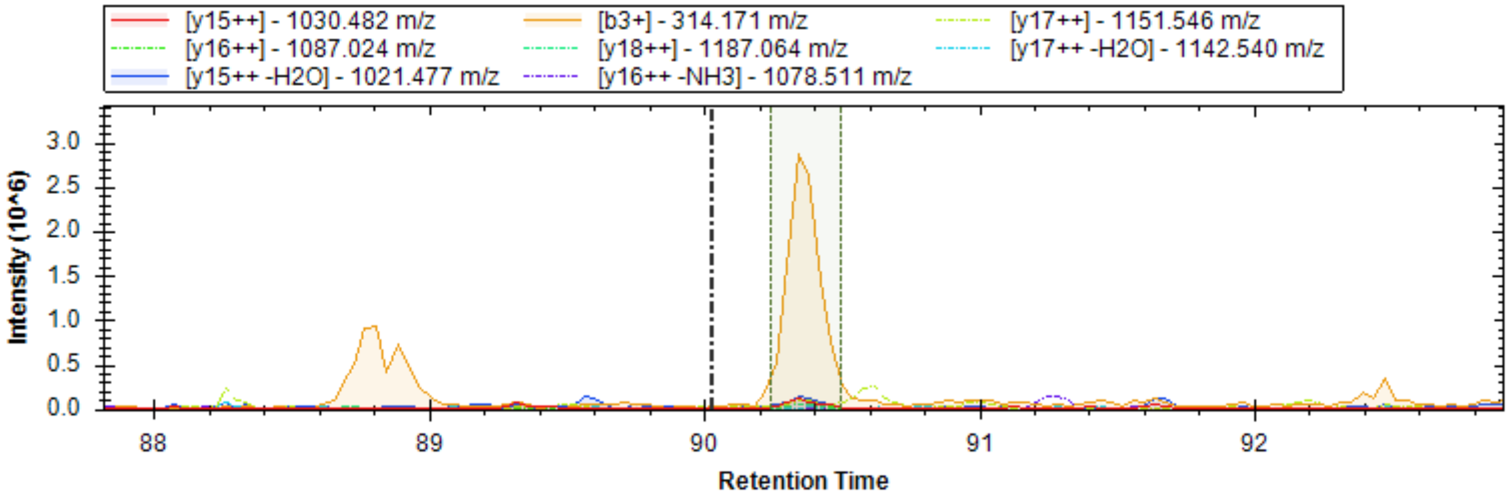

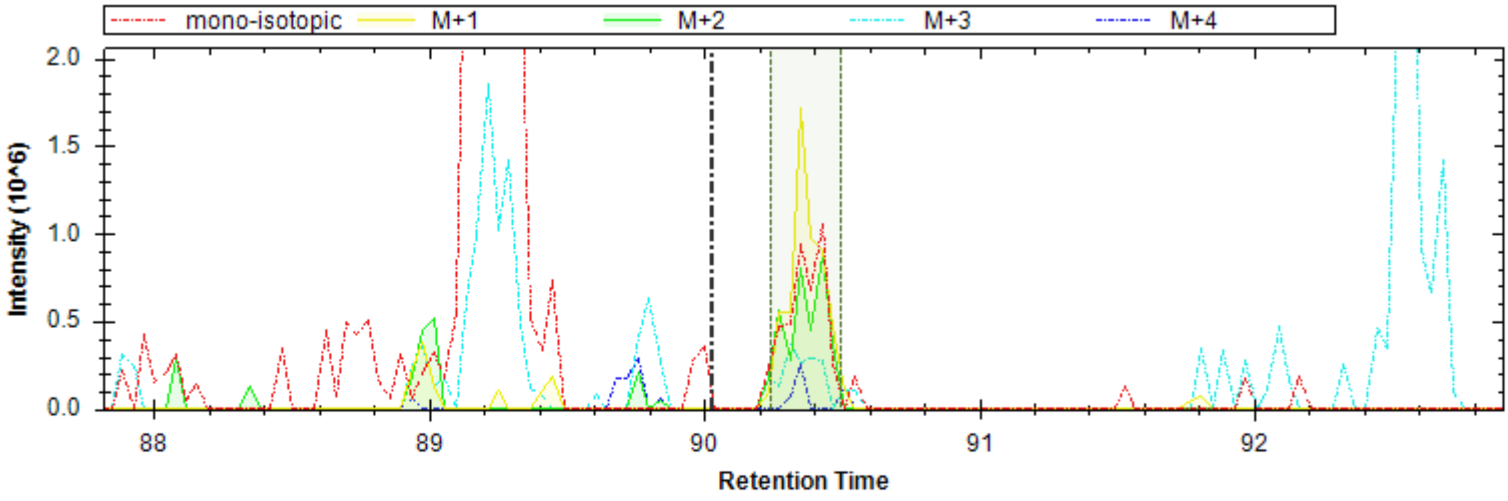

O08784 – _ASAVSPEKAPM[+O]T[+H+O3+P]SKAK_.2
Fig4_MP-DIA-barrel-cortex-p30-3_MHRM.htrms

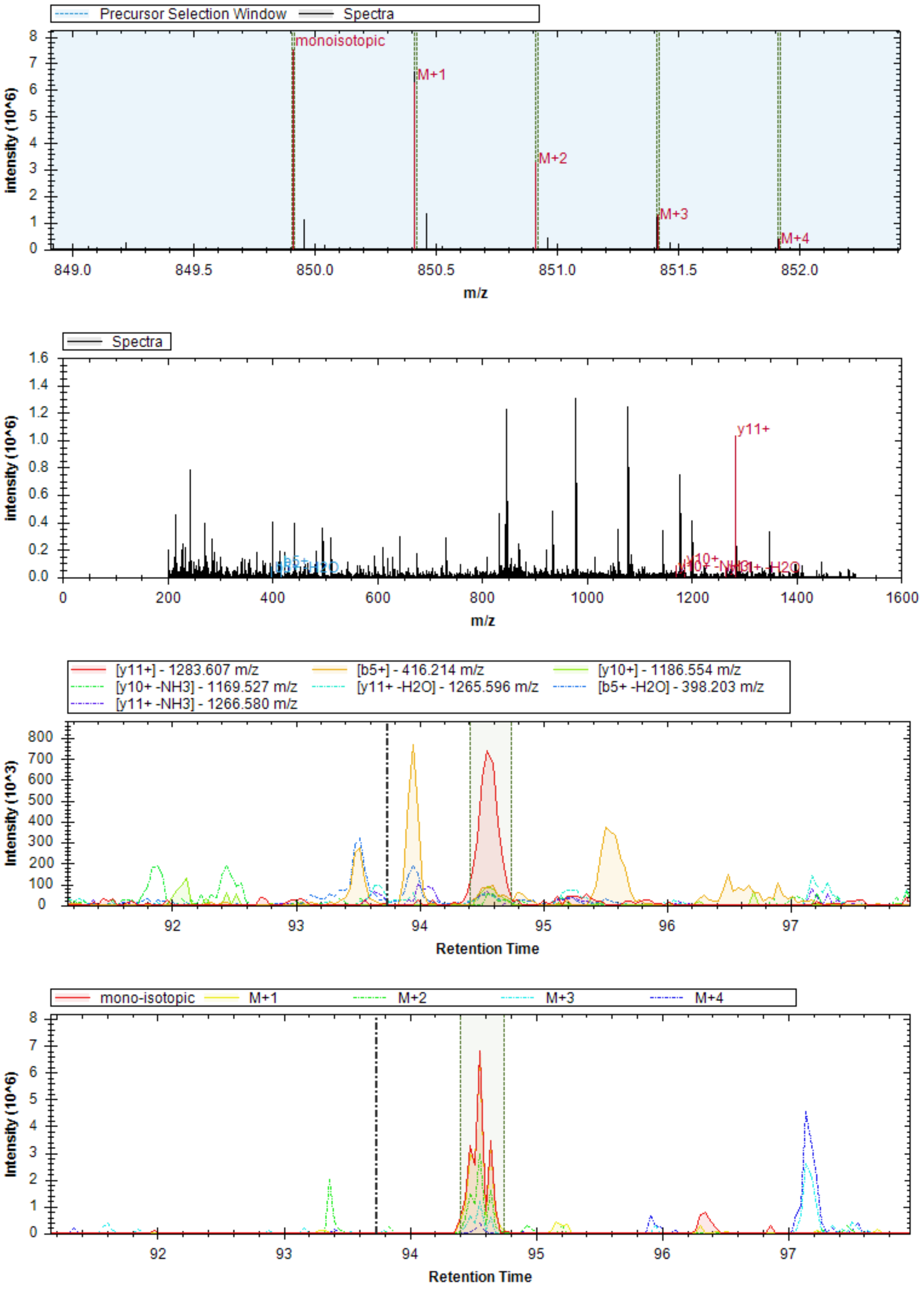

P20917 – _NVTES[+H+O3+P]SSFSGGDNPHVLYSPEFR_.3

Fig4_MP-DIA-barrel-cortex-p30-3_MHRM.htrms

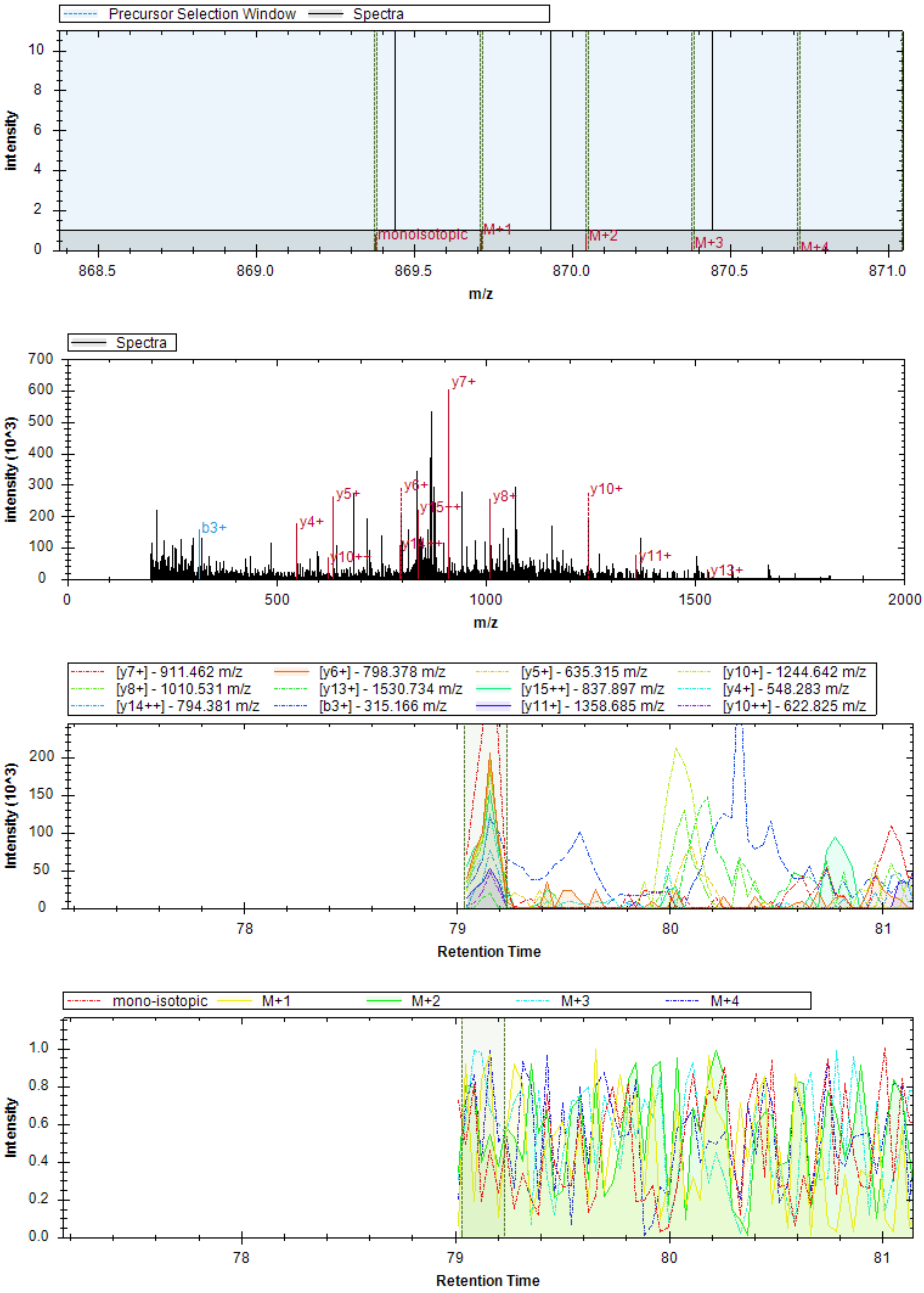

P20917 – _NVTESSS[+H+O3+P]FSGGDNPHVLYSPEFR_.3
Fig4_MP-DIA-barrel-cortex-p30-3_MHRM.htrms

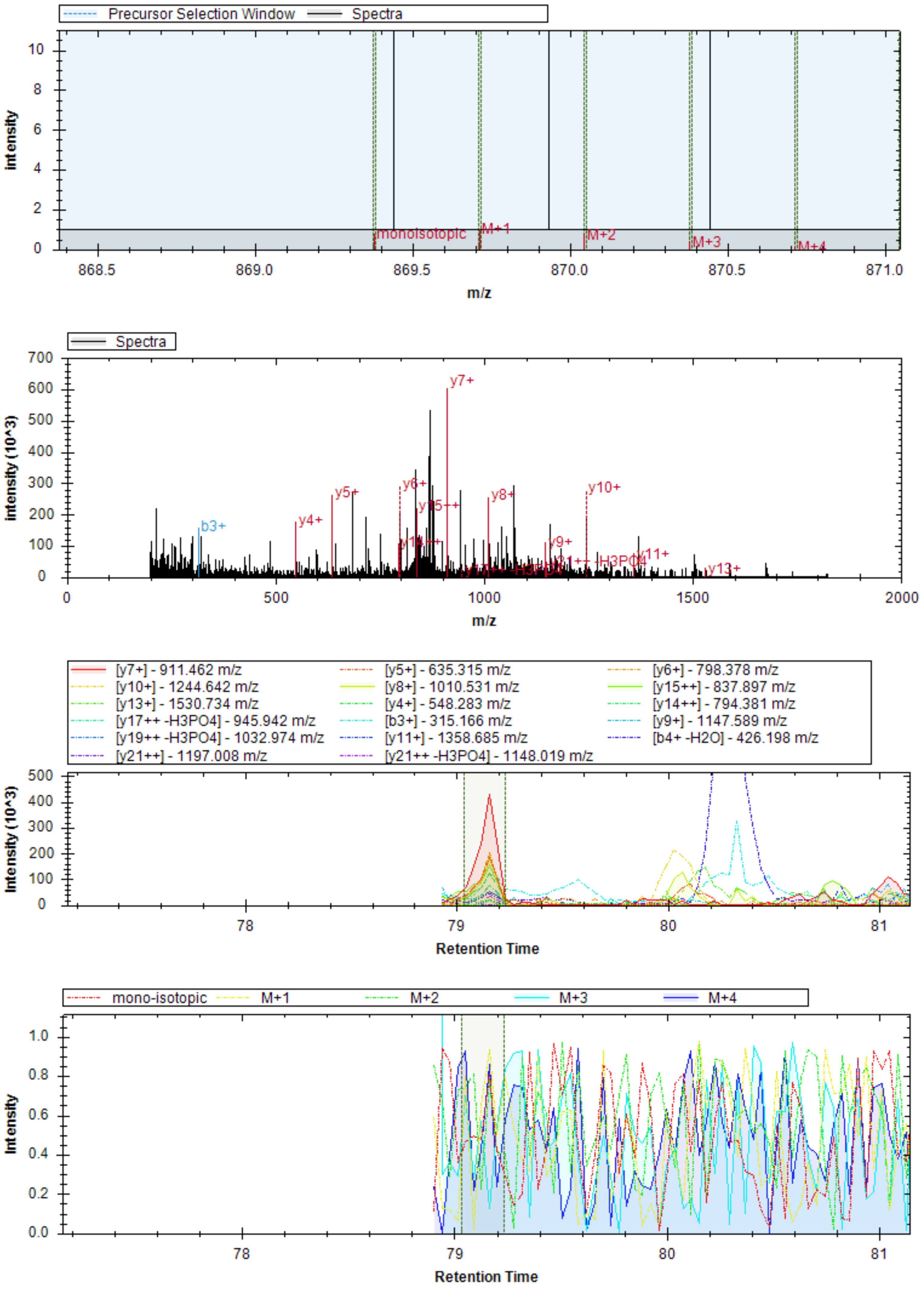

P50538 – _EAEHGYAS[+H+O3+P]M[+O]LPYSK_.2

Fig4_MP-DIA-barrel-cortex-p30-3_MHRM.htrms

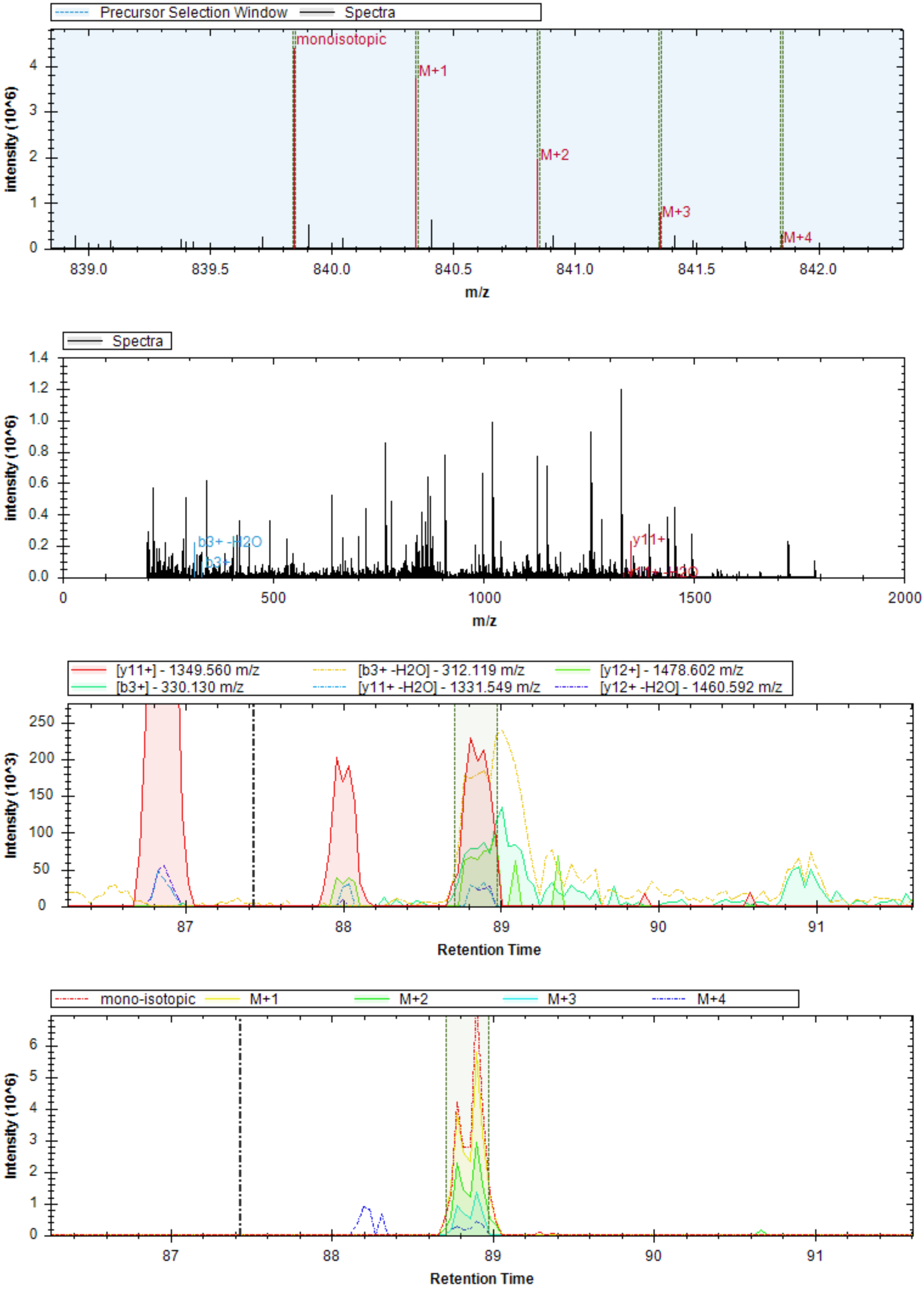

Q61290 – _M[+O]EPS[+H+O3+P]SLPQEIIANAK_.2

Fig4_MP-DIA-barrel-cortex-p30-3_MHRM.htrms

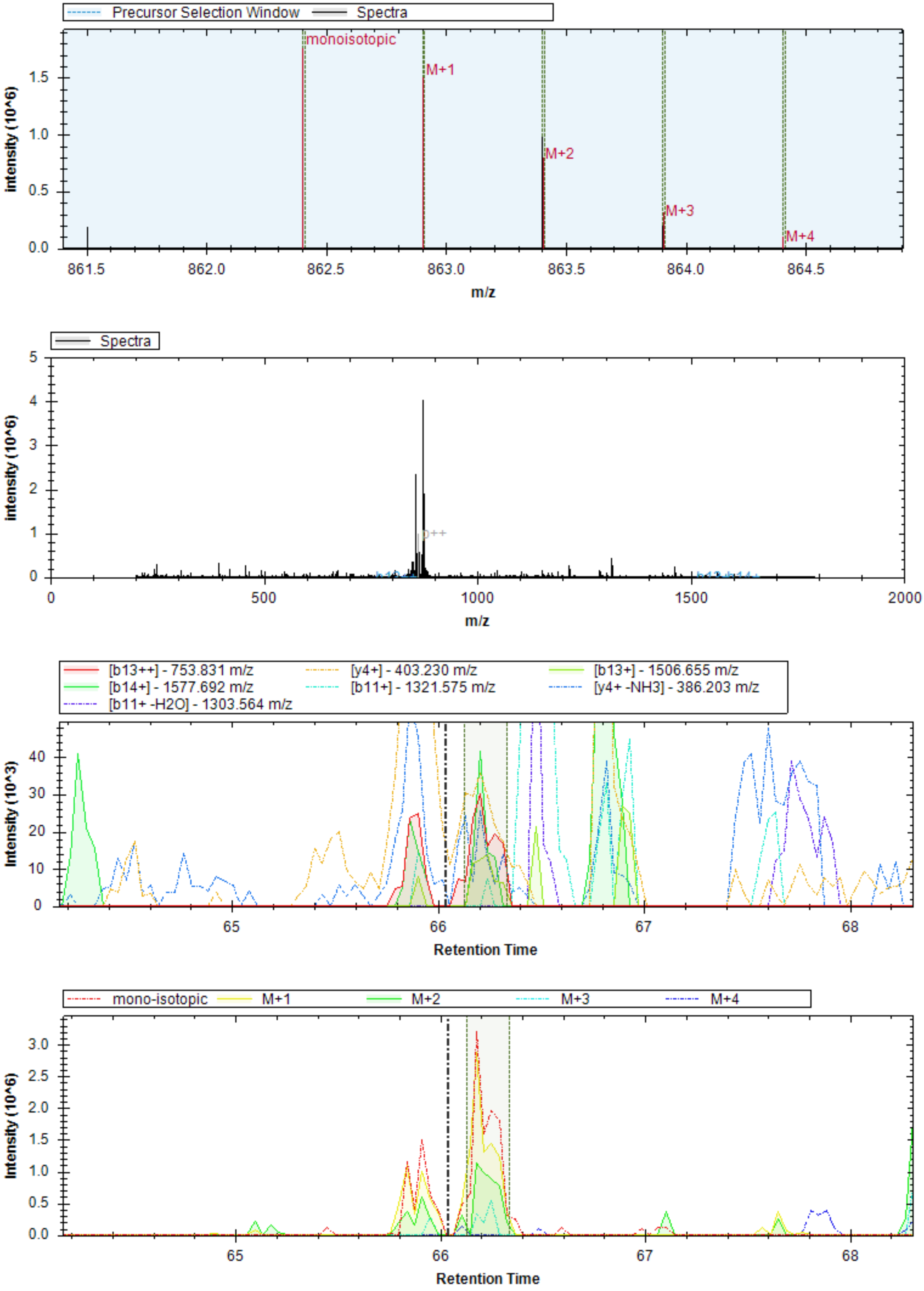

Q7TQF7 – _T[+H+O3+P]PSPPEEPSPLPSPTASPNHTLAPASPAPVRPR_.4
Fig4_MP-DIA-barrel-cortex-p30-3_MHRM.htrms

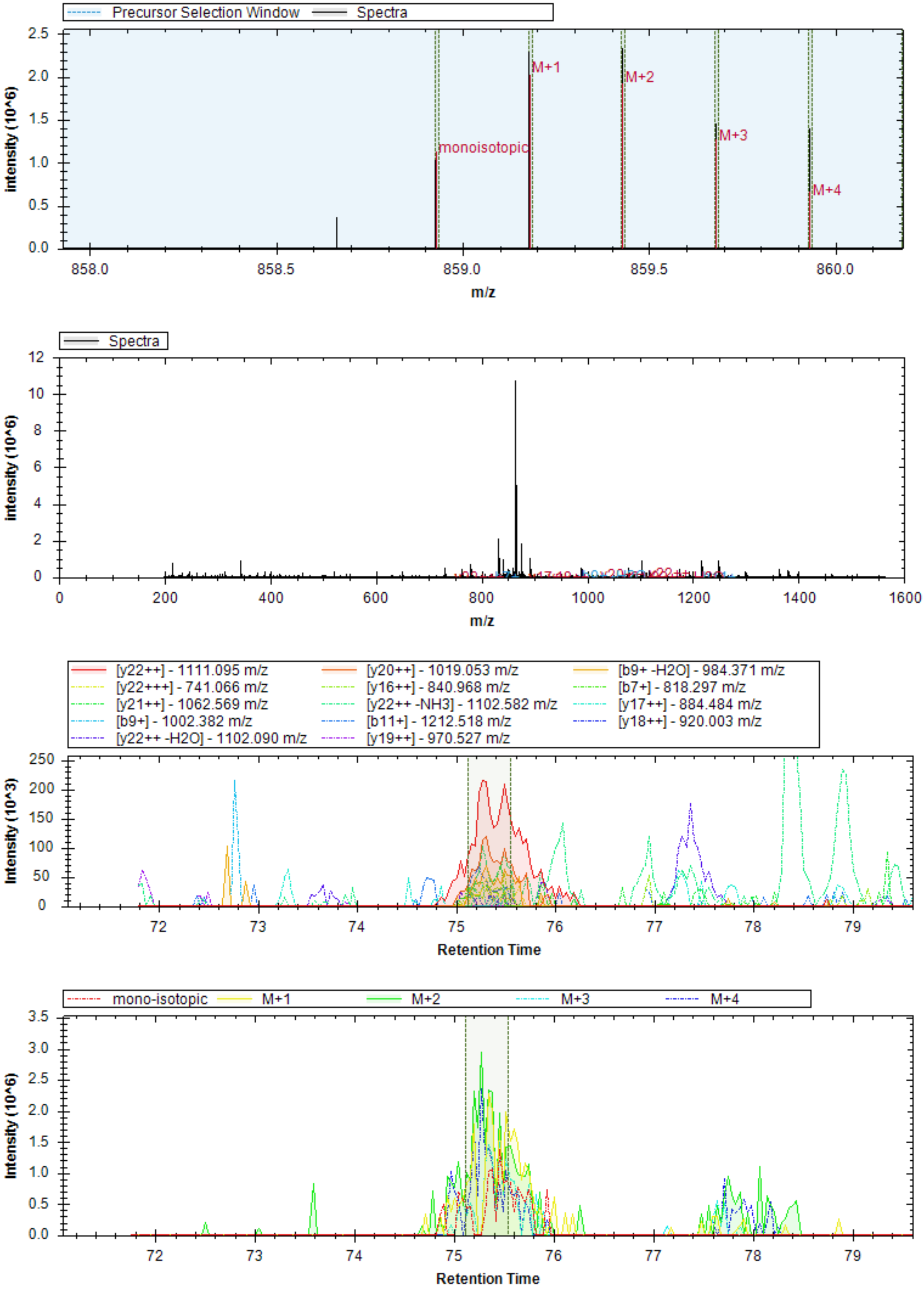

Q9JM96 – _DSSKLPKS[+H+O3+P]LS[+H+O3+P]SSPVK_.2
Fig4_MP-DIA-barrel-cortex-p30-3_MHRM.htrms

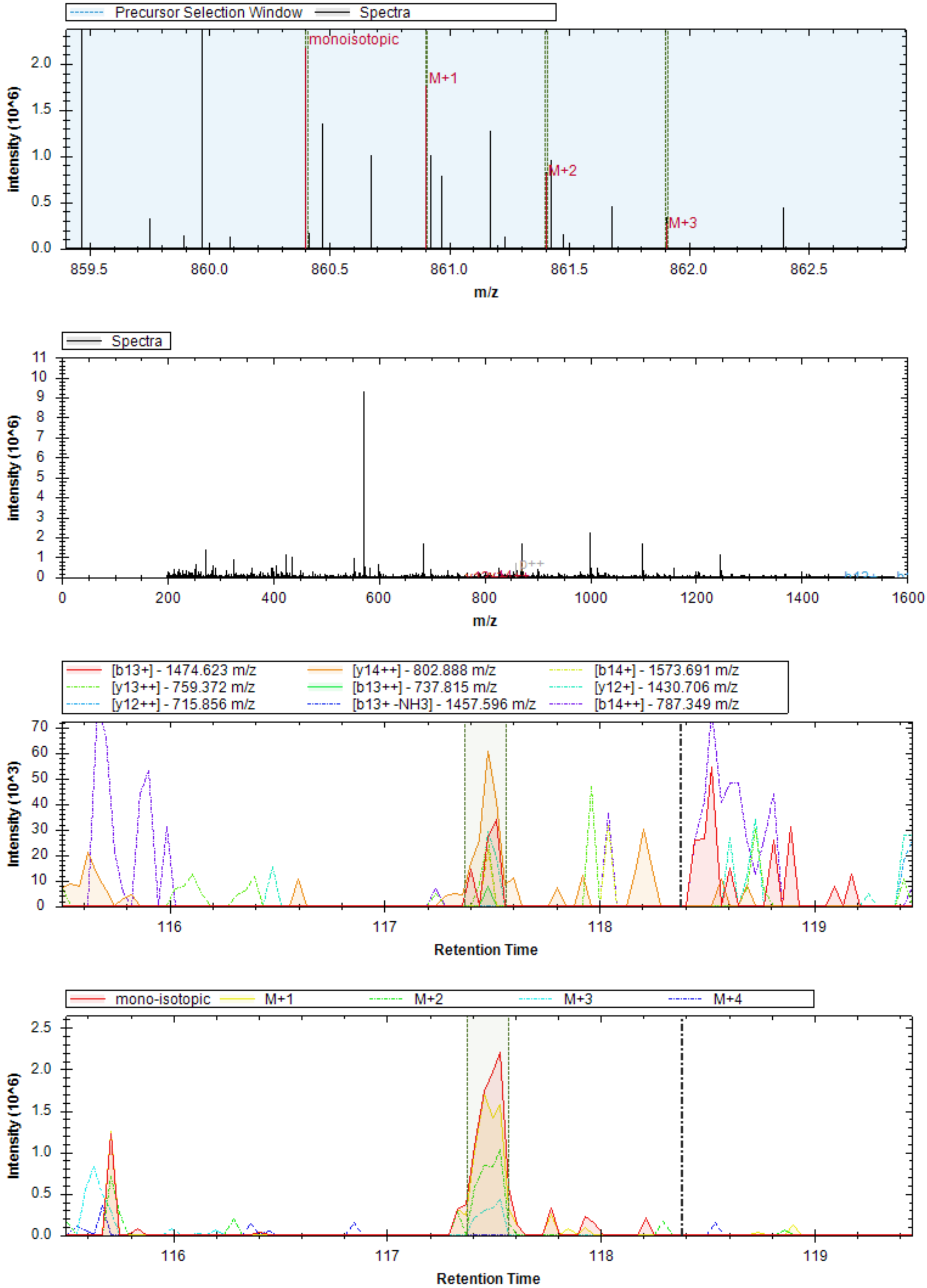

A1A535 – _FLHRGS[+H+O3+P]K_.1
Fig4_MP-DIA-barrel-cortex-p30-3_MHRM.htrms

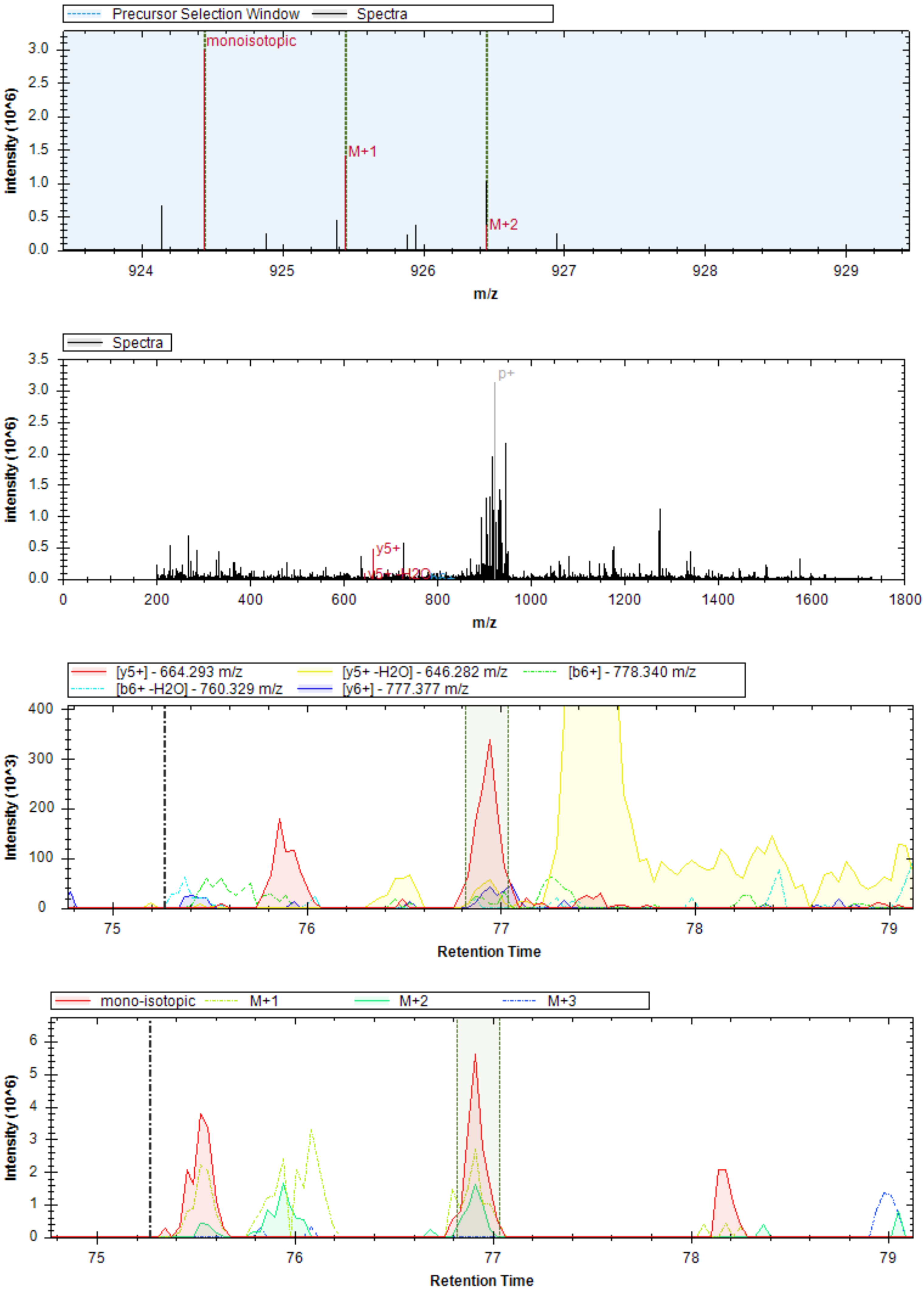

P26645 – _GEATAERPGEAAVASS[+H+O3+P]PSK_.2
Fig4_MP-DIA-barrel-cortex-p30-3_MHRM.htrms

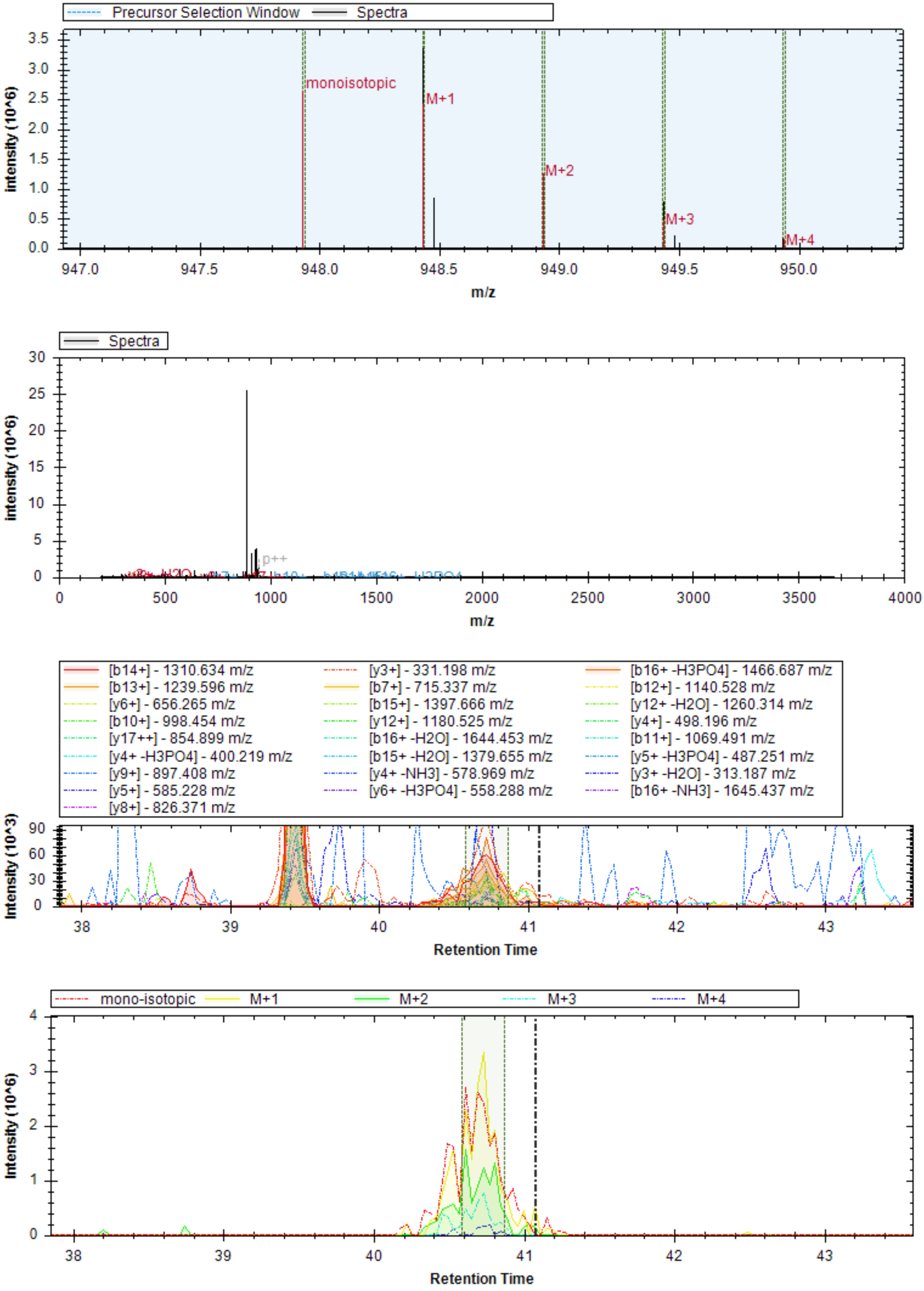

Q8BMA3 – _IIQLC[+C2+H3+N+O]LDLT[+H+O3+P]T[+H+O3+P]AVQK_.2
Fig4_MP-DIA-barrel-cortex-p30-3_MHRM.htrms

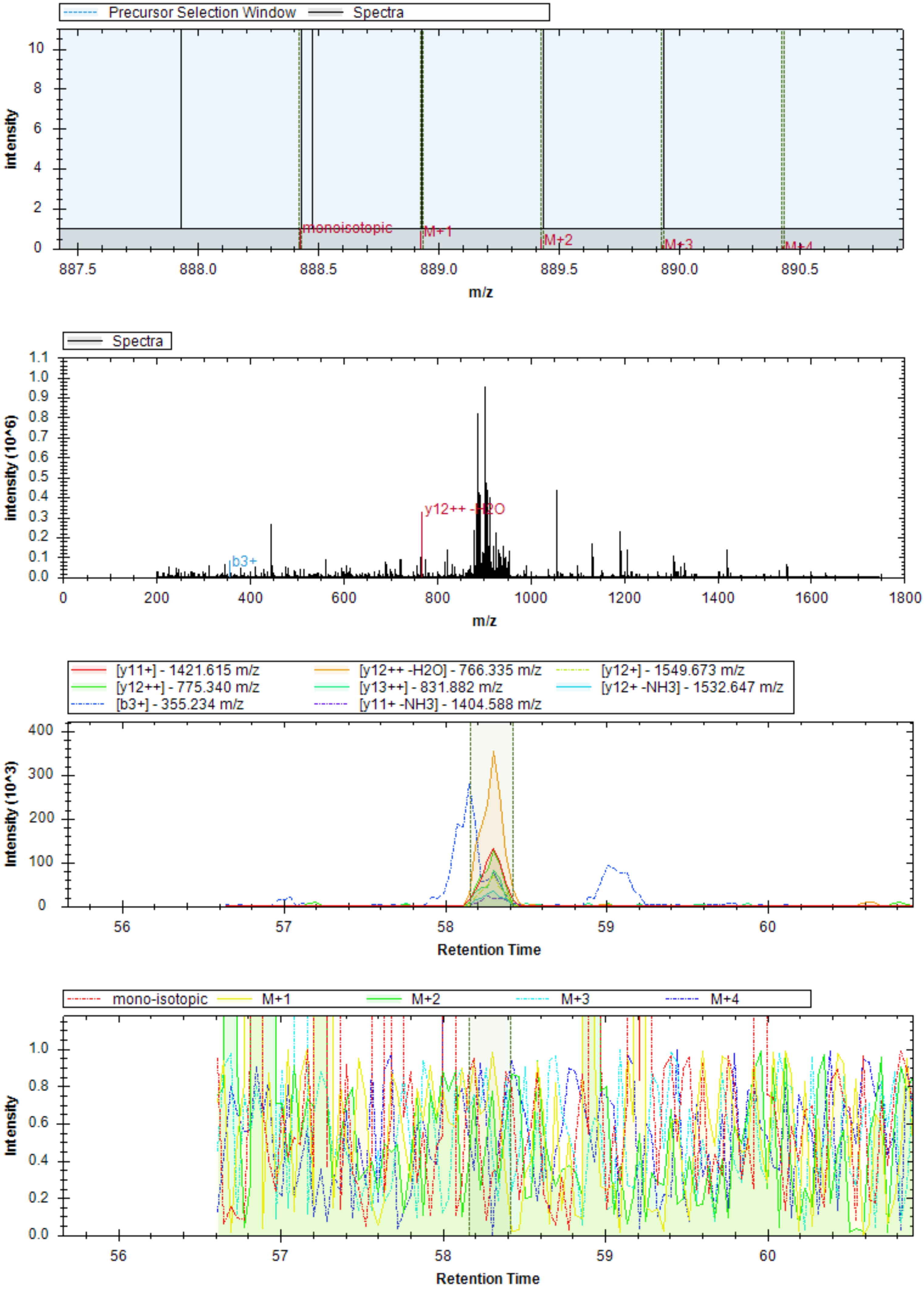

Q8BX90 – _IQALNSLGAGPFS[+H+O3+P]HTIK_.2

Fig4_MP-DIA-barrel-cortex-p30-3_MHRM.htrms

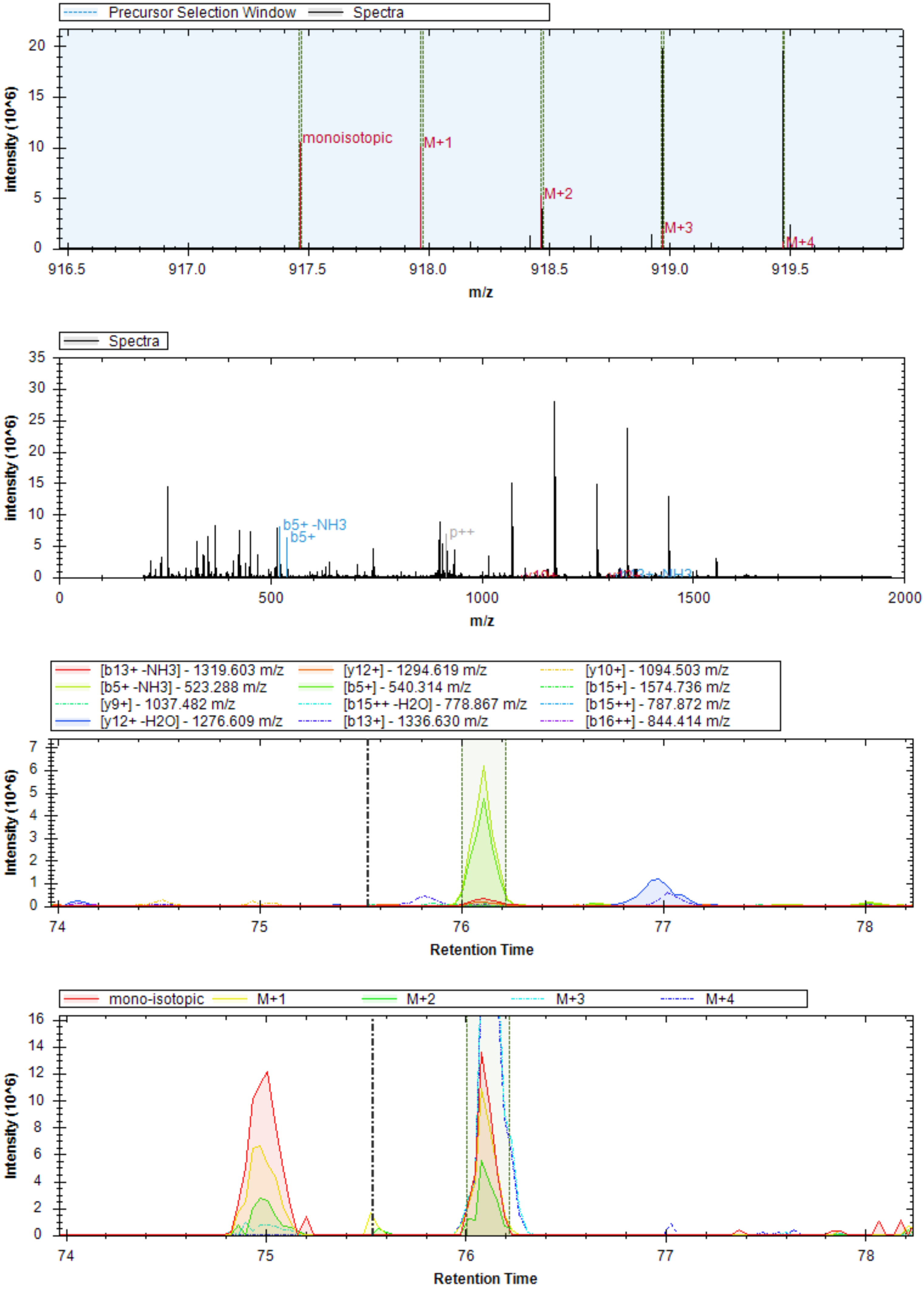

Q91Z31;Q91Z31-2 – _ETSLLAVPGALS[+H+O3+P]PLAIPNAAAAAAAAAAAGR_.3
Fig4_MP-DIA-barrel-cortex-p30-3_MHRM.htrms

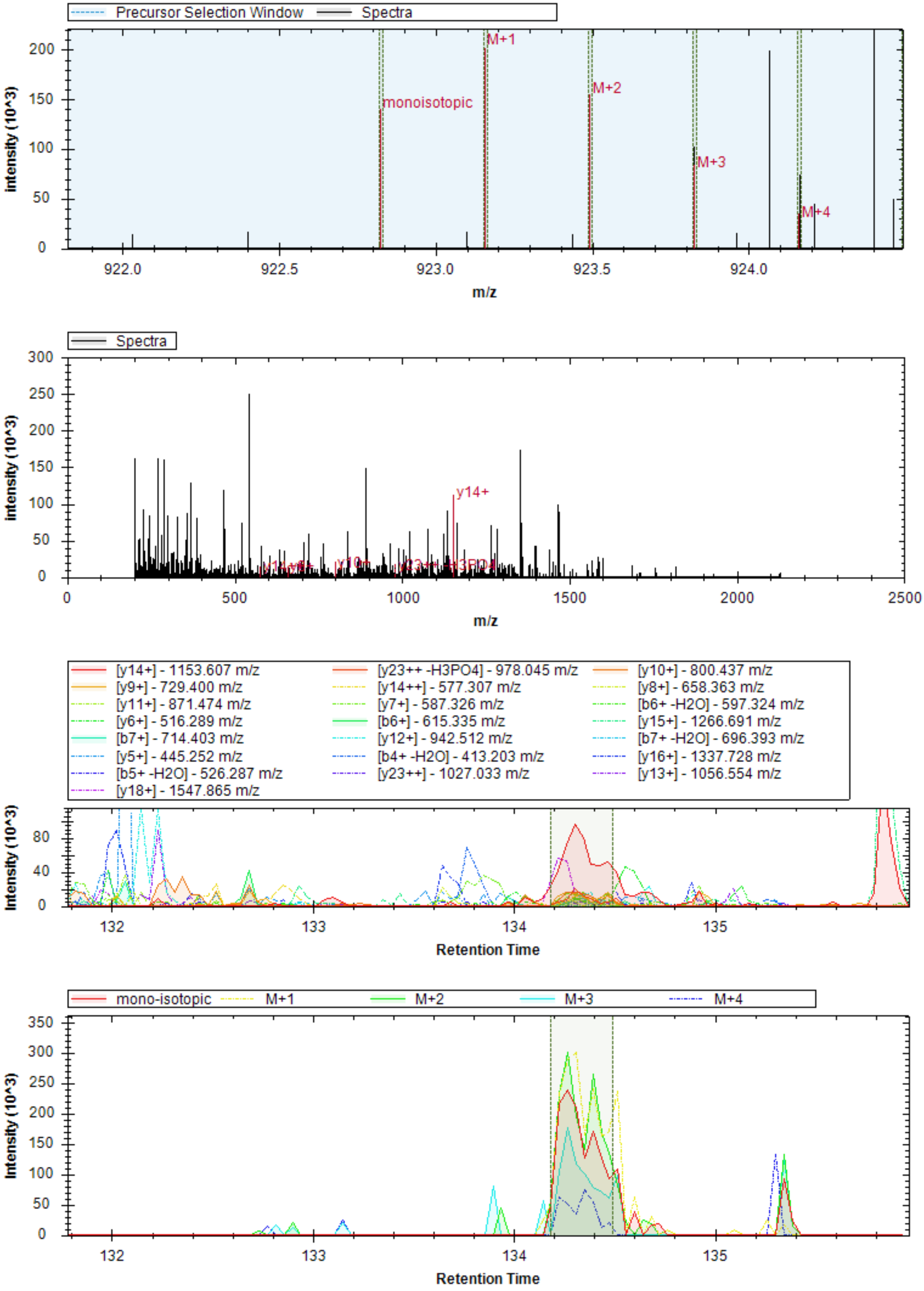

Q9CZT4;Q9CZT4-2 – _KEQM[+O]RAATVLPS[+H+O3+P]VQIK_.2

Fig4_MP-DIA-barrel-cortex-p30-3_MHRM.htrms

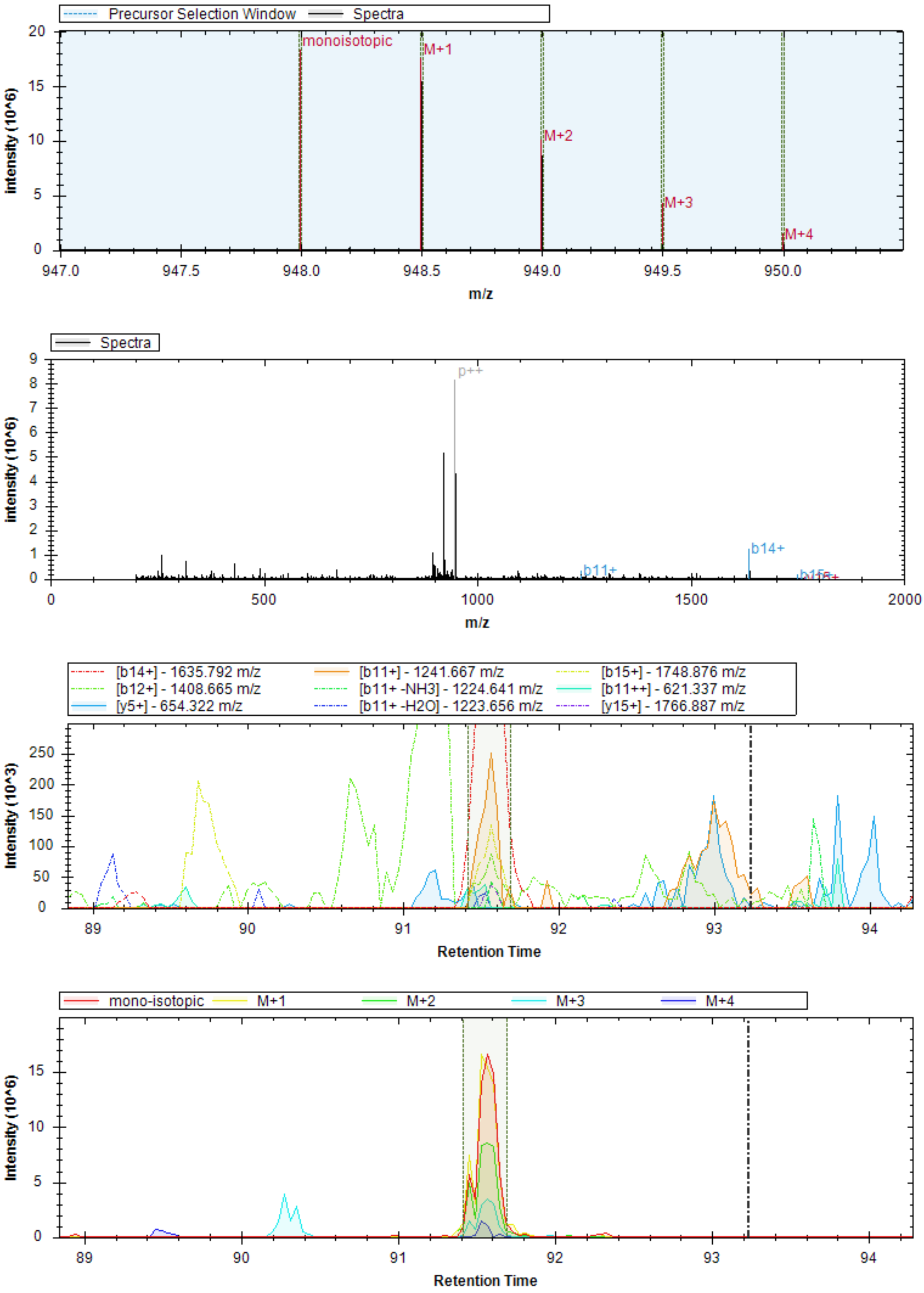

Q9ES97;Q9ES97-2 – _AES[+H+O3+P]SAATQSPSVSSSSSGAEPSALGGGGGSPGAC[+C2+H3+N+O]PALGAK_.4
Fig4_MP-DIA-barrel-cortex-p30-3_MHRM.htrms

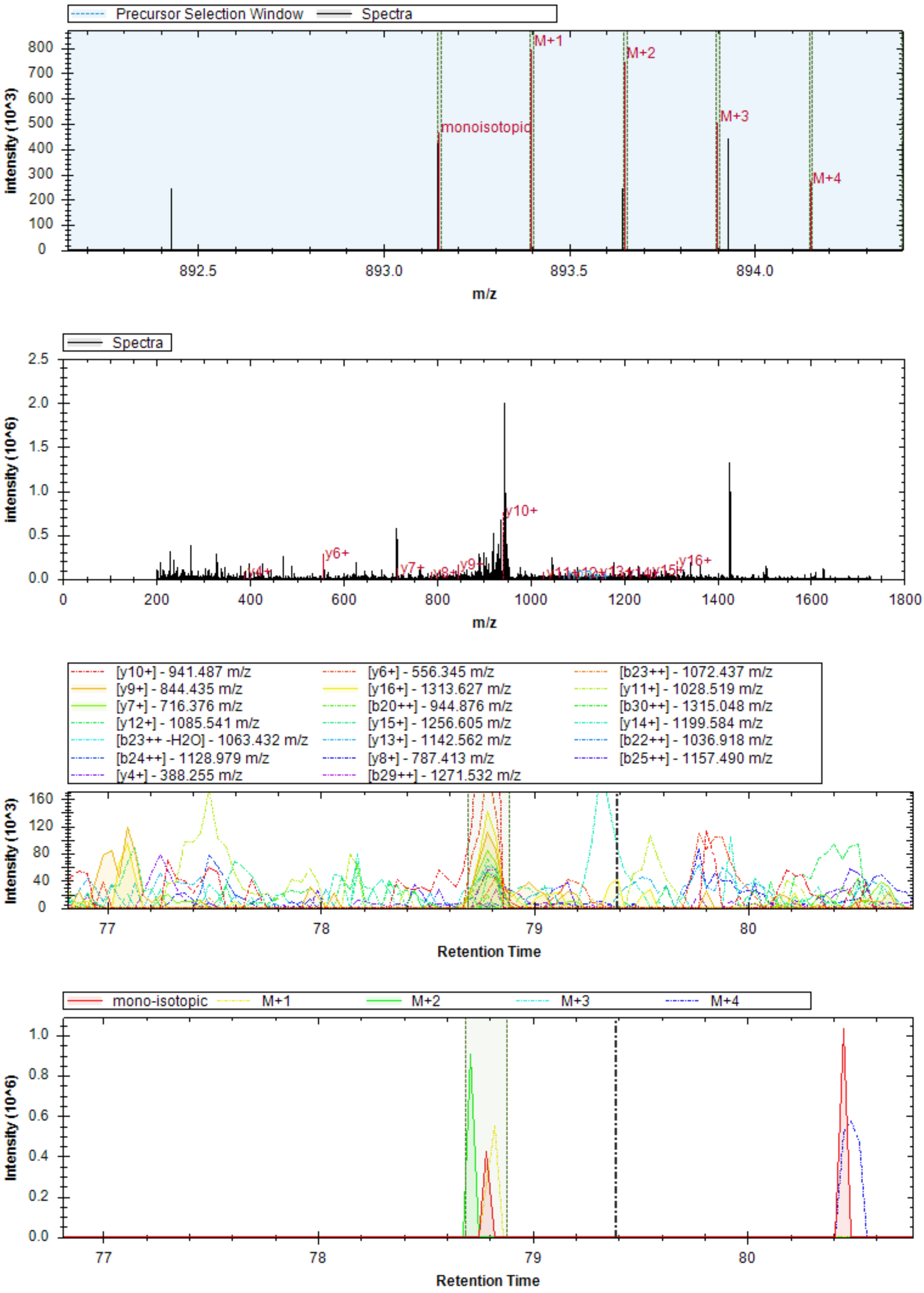

Q9QYR6-2 – _MAS[+H+O3+P]PPPSGPPSAAHTPFHQSPVEEK_.3
Fig4_MP-DIA-barrel-cortex-p30-3_MHRM.htrms

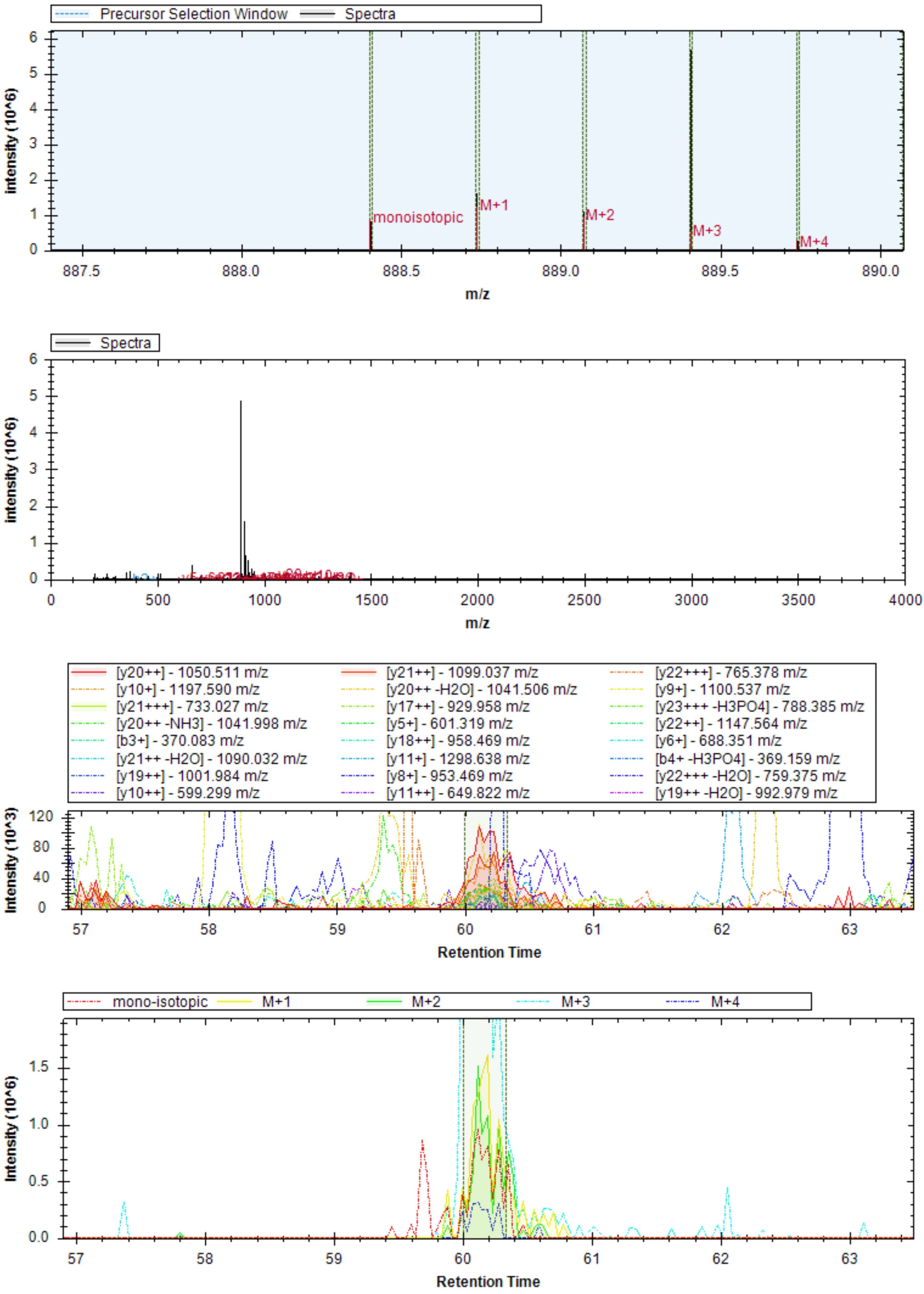

Q3UHL1 – _SATPAT[+H+O3+P]DGRATPATEESTVPATQSSALPAAK_.3
Fig4_MP-DIA-barrel-cortex-p30-3_MHRM.htrms

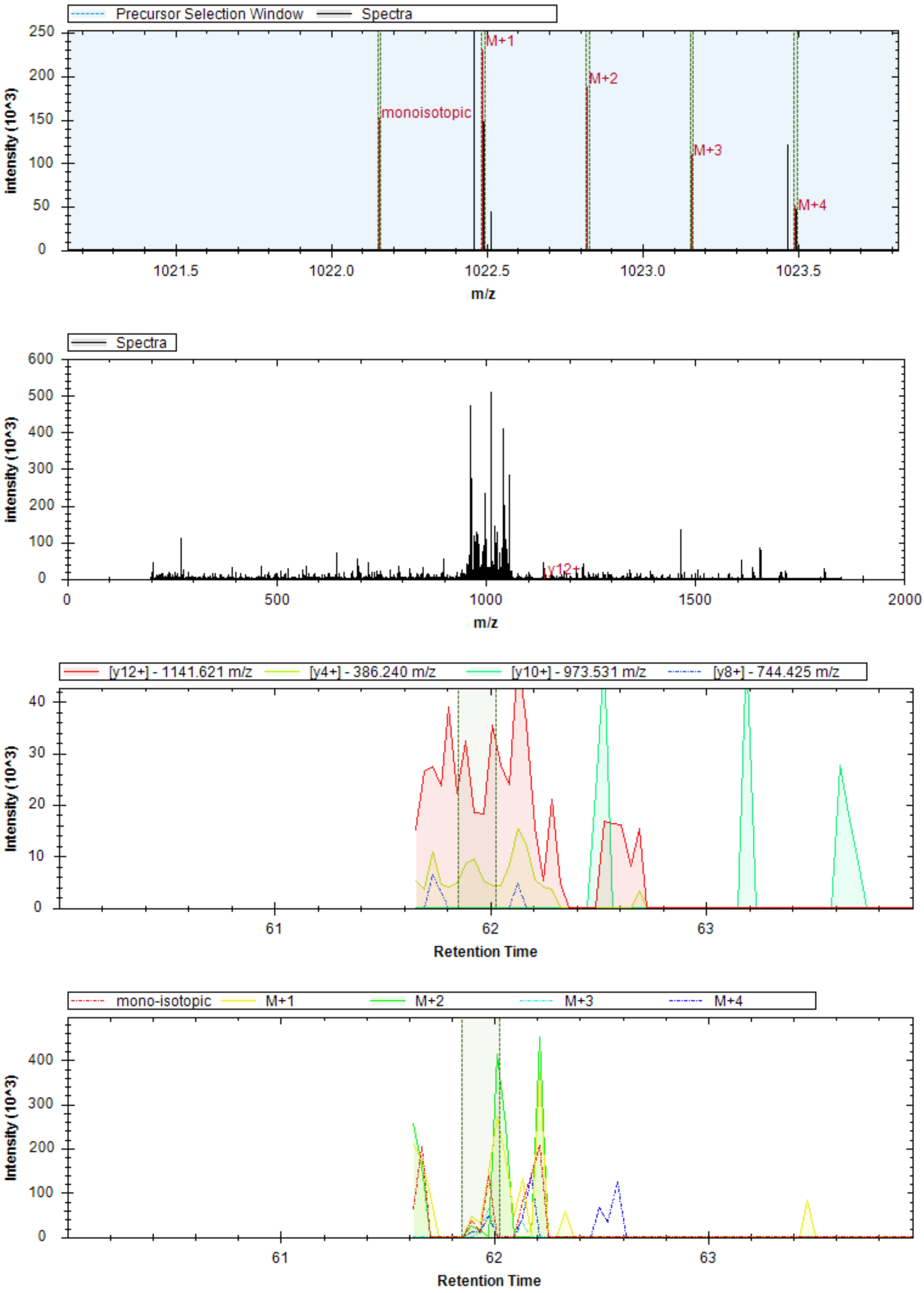

E9Q401 – _MVAEVFIY[+H+O3+P]WSK_.1

Fig4_MP-DIA-barrel-cortex-p30-3_MHRM.htrms

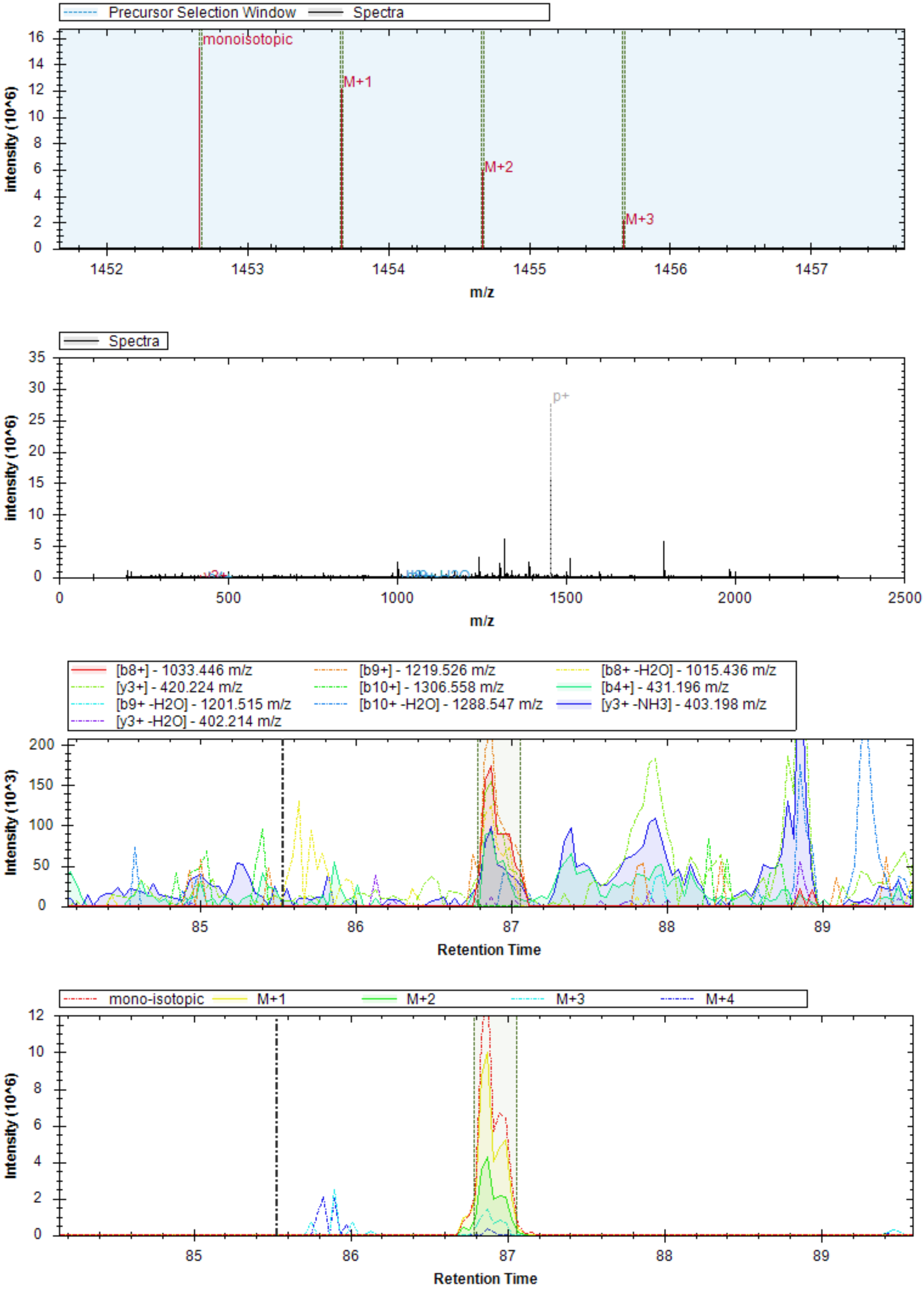

P20357 – _SGTSTPTTPGSTAIT[+H+O3+P]PGTPPSYSSR_.2
Fig4_MP-DIA-barrel-cortex-p30-3_MHRM.htrms

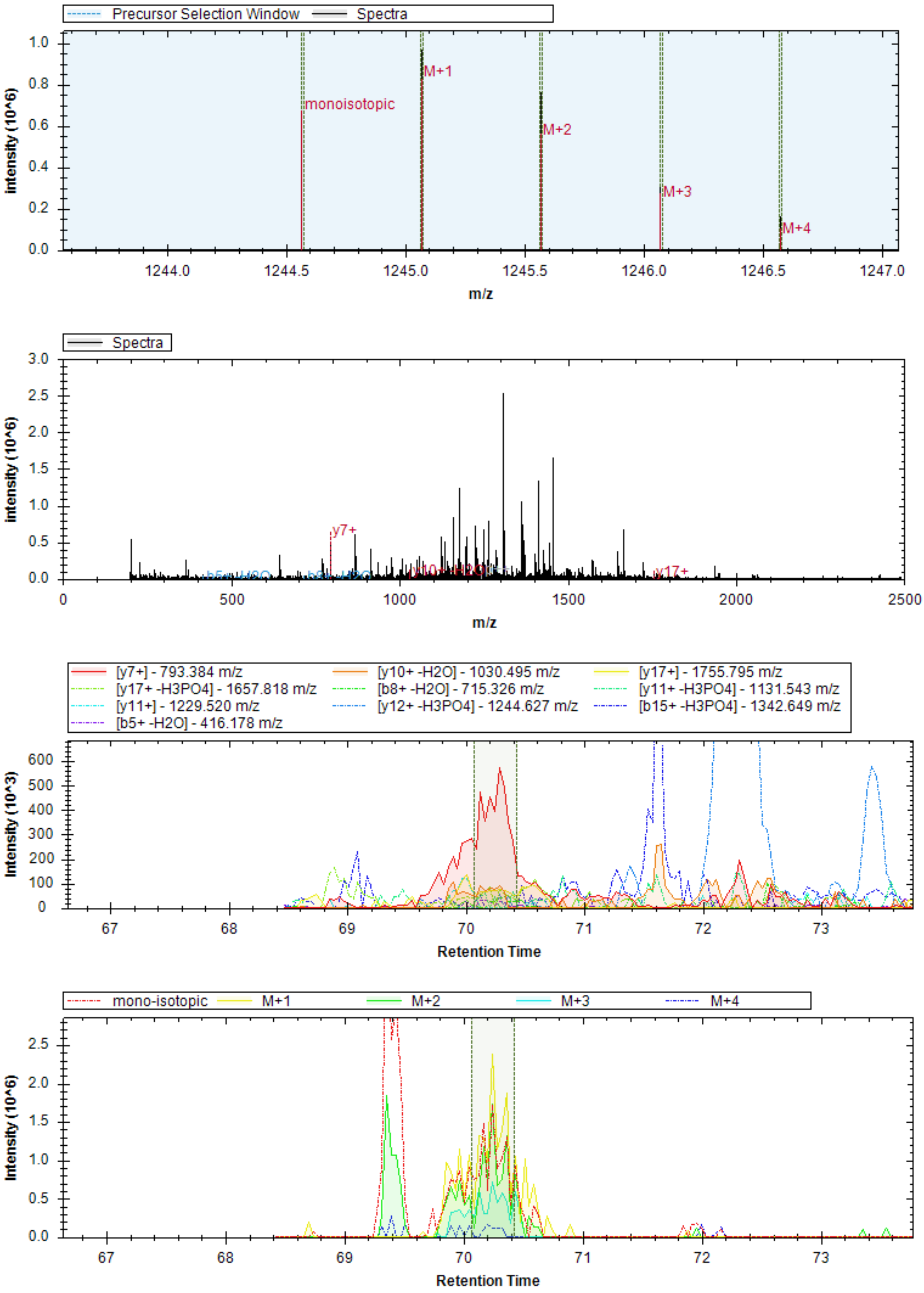

P20357 – _SGTSTPTTPGSTAITPGT[+H+O3+P]PPSYSSR_.2

Fig4_MP-DIA-barrel-cortex-p30-3_MHRM.htrms

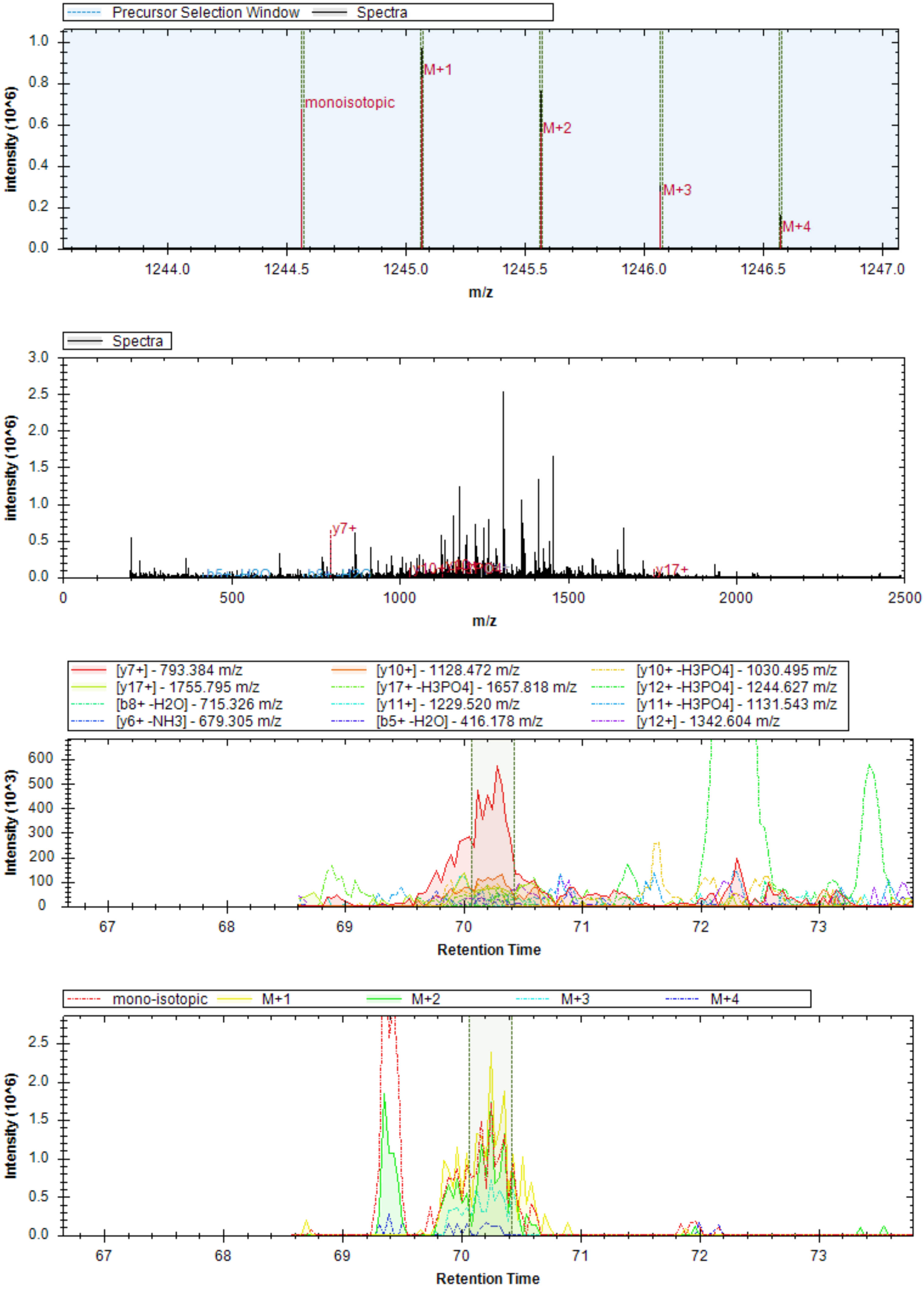

Q8BRT1 – _VLNT[+H+O3+P]GSDVEEAVADALLLGDIR_.2

Fig4_MP-DIA-barrel-cortex-p30-3_MHRM.htrms

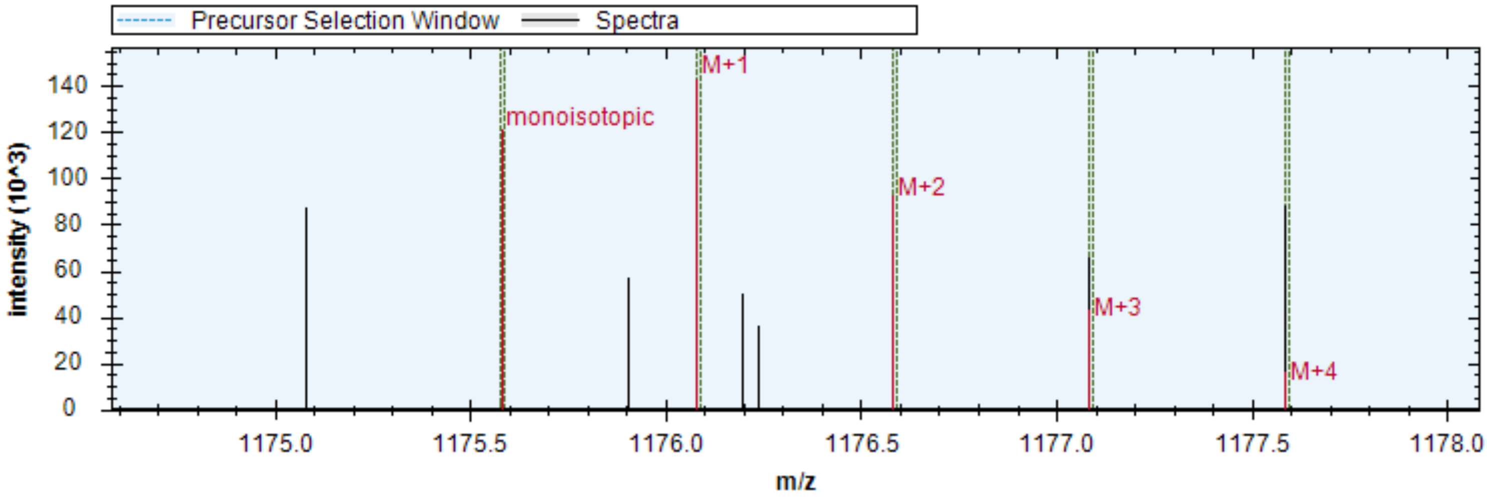

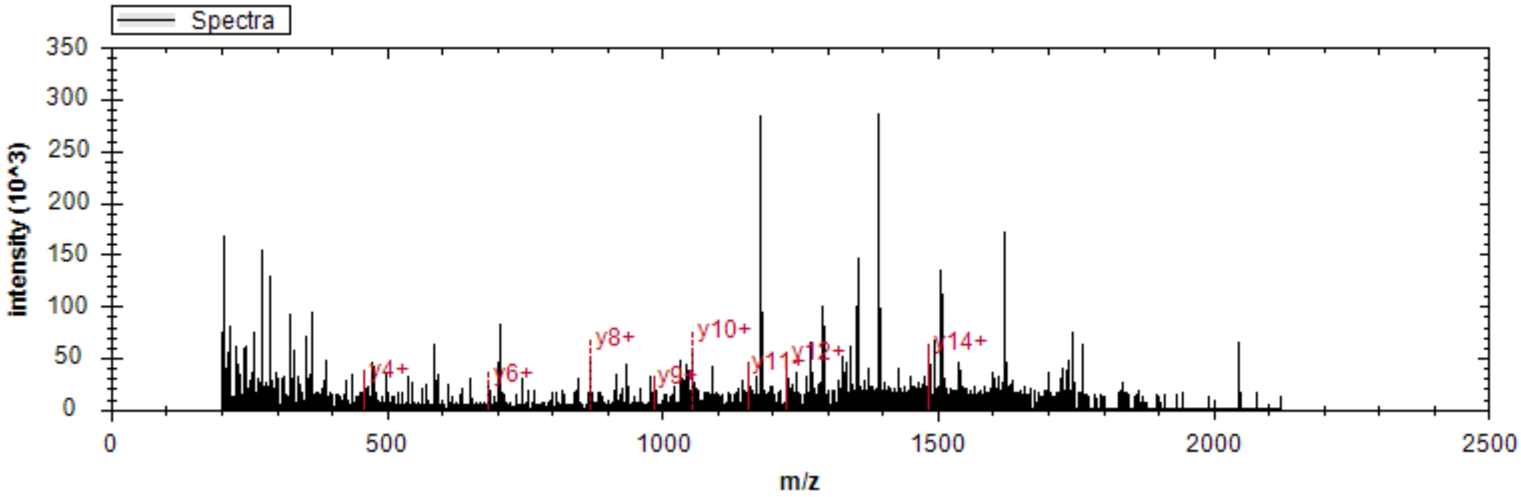

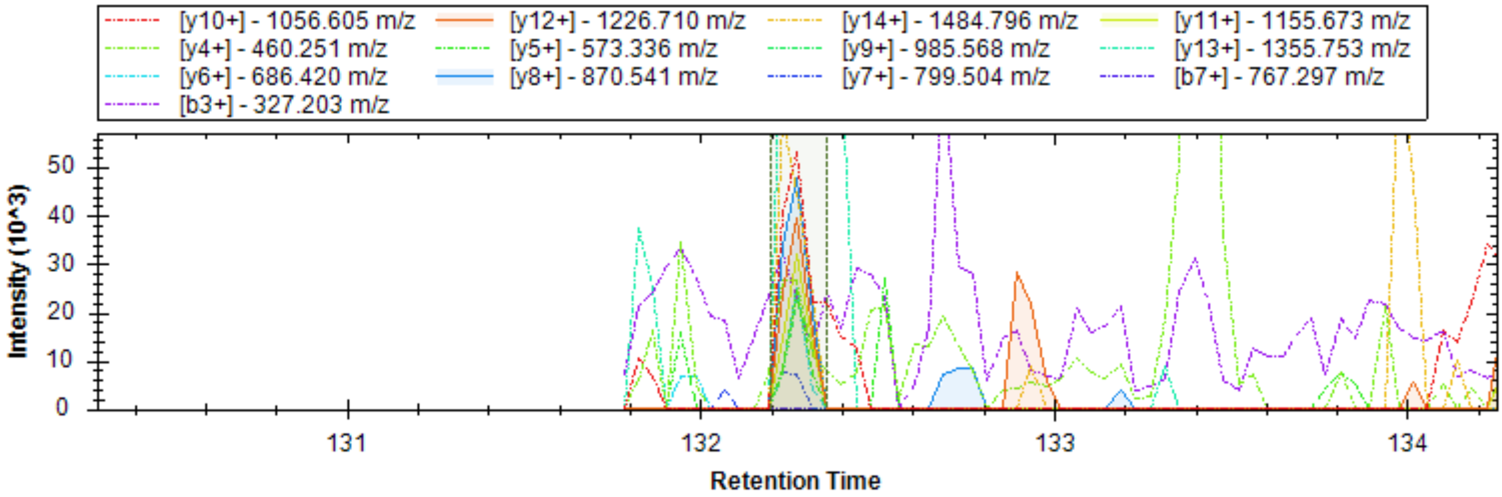

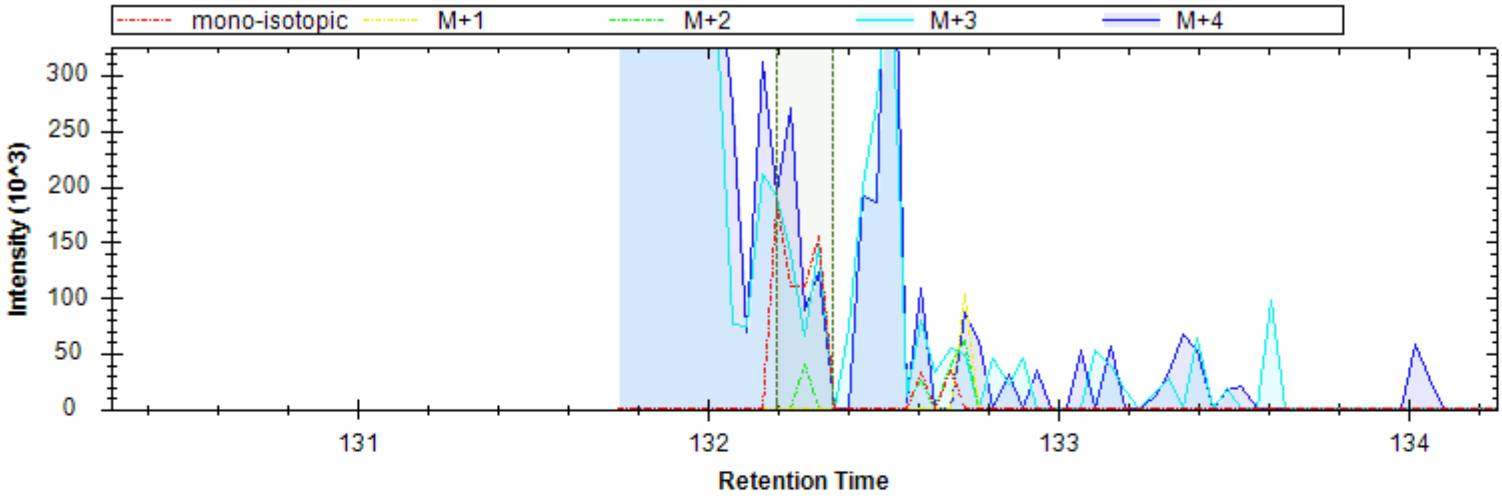

Q8VHY0 – _LLAGPRY[+H+O3+P]GR_.1
Fig4_MP-DIA-barrel-cortex-p30-3_MHRM.htrms

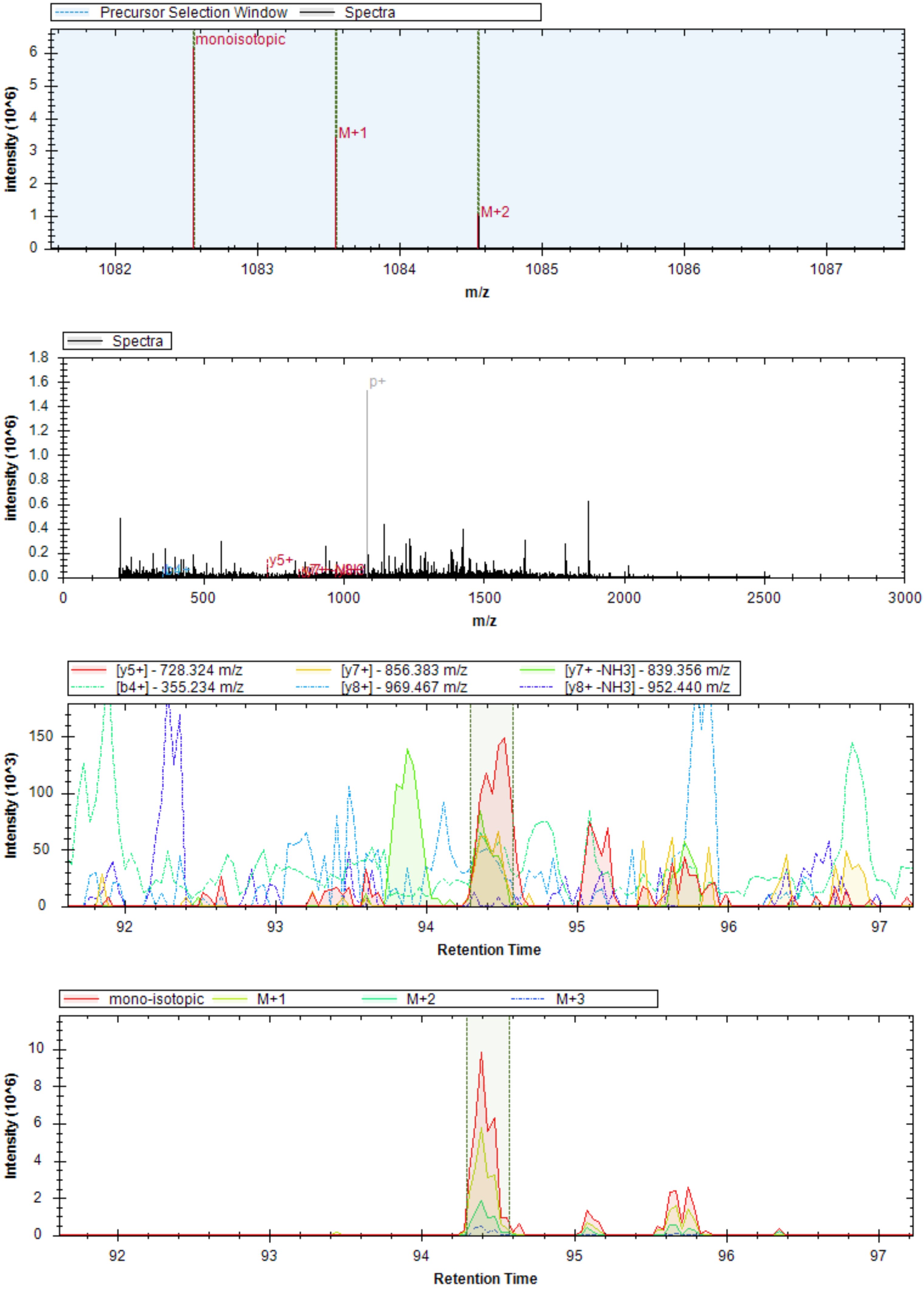

Q921N6;Q921N6-2 – _LIY[+H+O3+P]KPRQAAVTR_.4
Fig4_MP-DIA-barrel-cortex-p54-1_MHRM.htrms

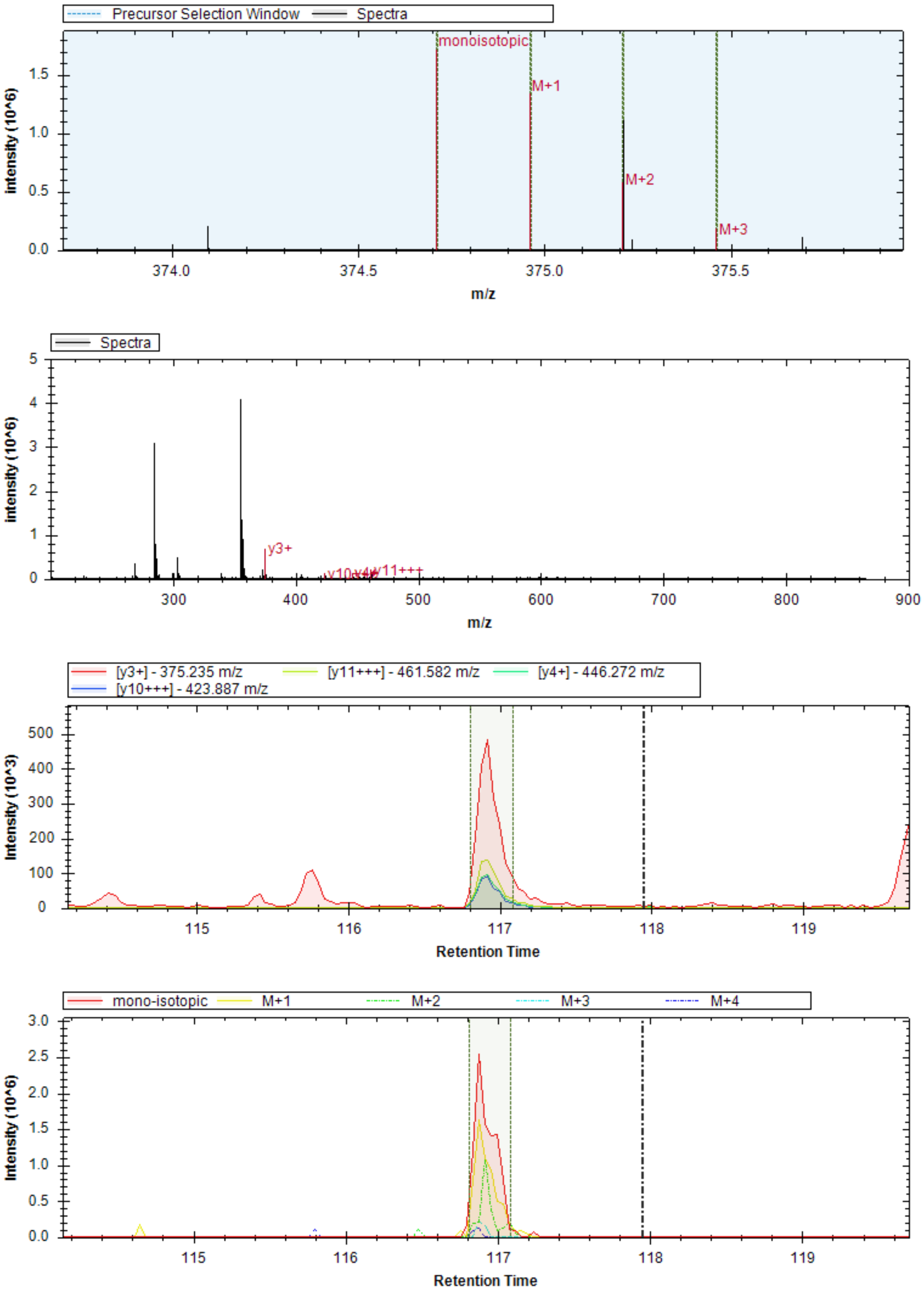

Q9CVB6 – _IIEET[+H+O3+P]LALK_.3
Fig4_MP-DIA-barrel-cortex-p54-1_MHRM.htrms

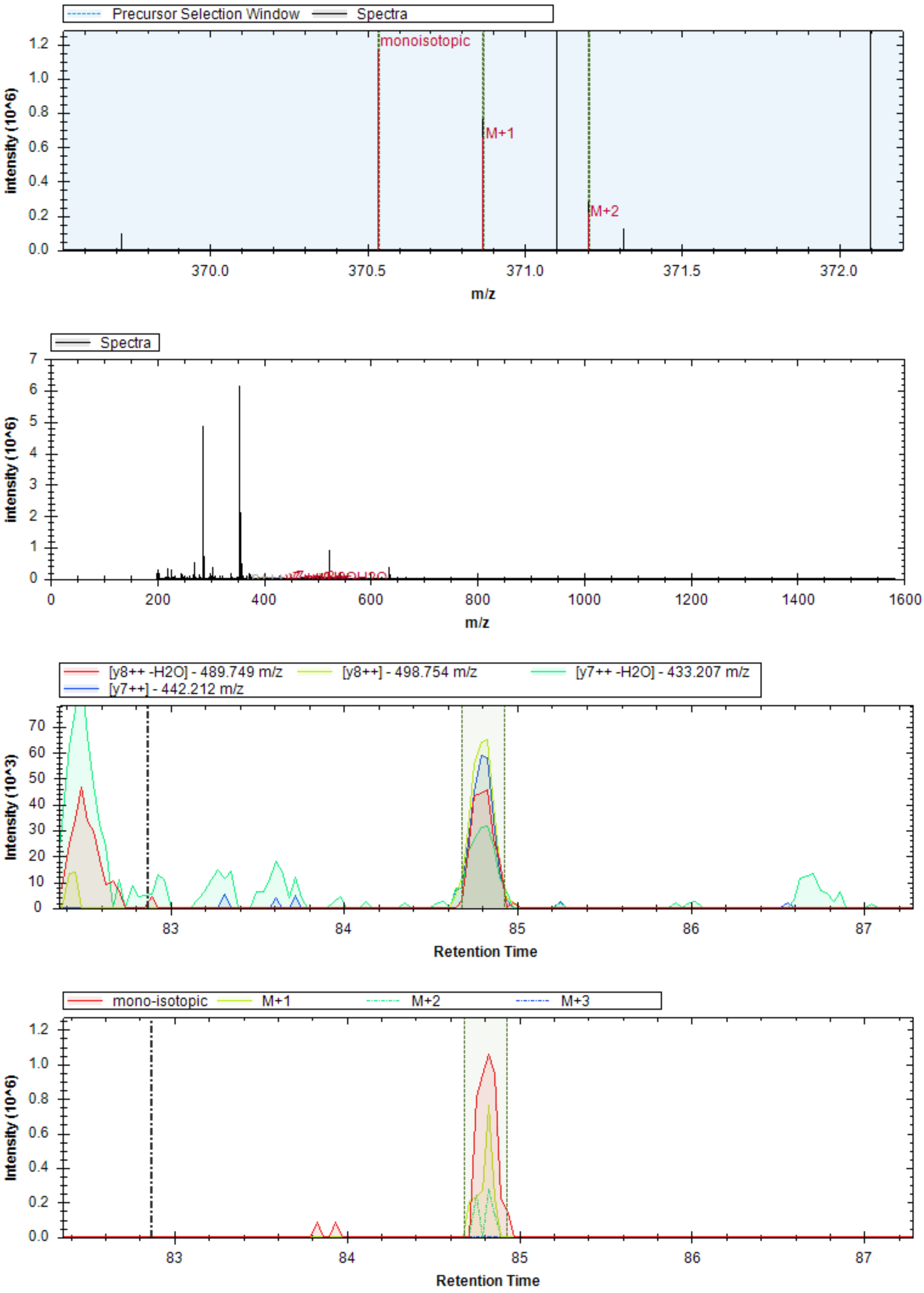

Q9D516;Q9D516-2 – _KLS[+H+O3+P]QGILVIR_.3

Fig4_MP-DIA-barrel-cortex-p54-1_MHRM.htrms

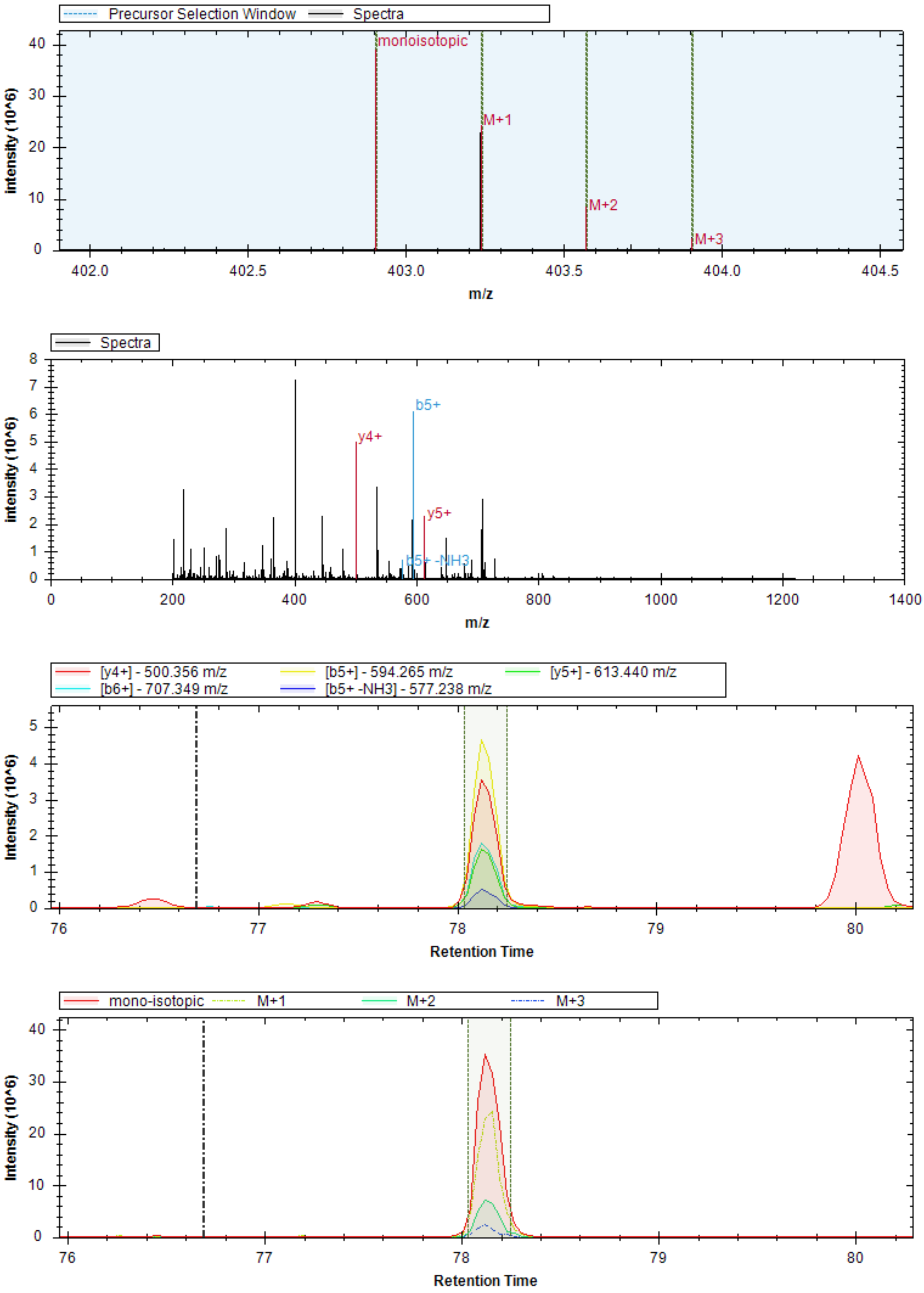

Q03172 – _VALSLLNS[+H+O3+P]KQK_.3
Fig4_MP-DIA-barrel-cortex-p54-1_MHRM.htrms

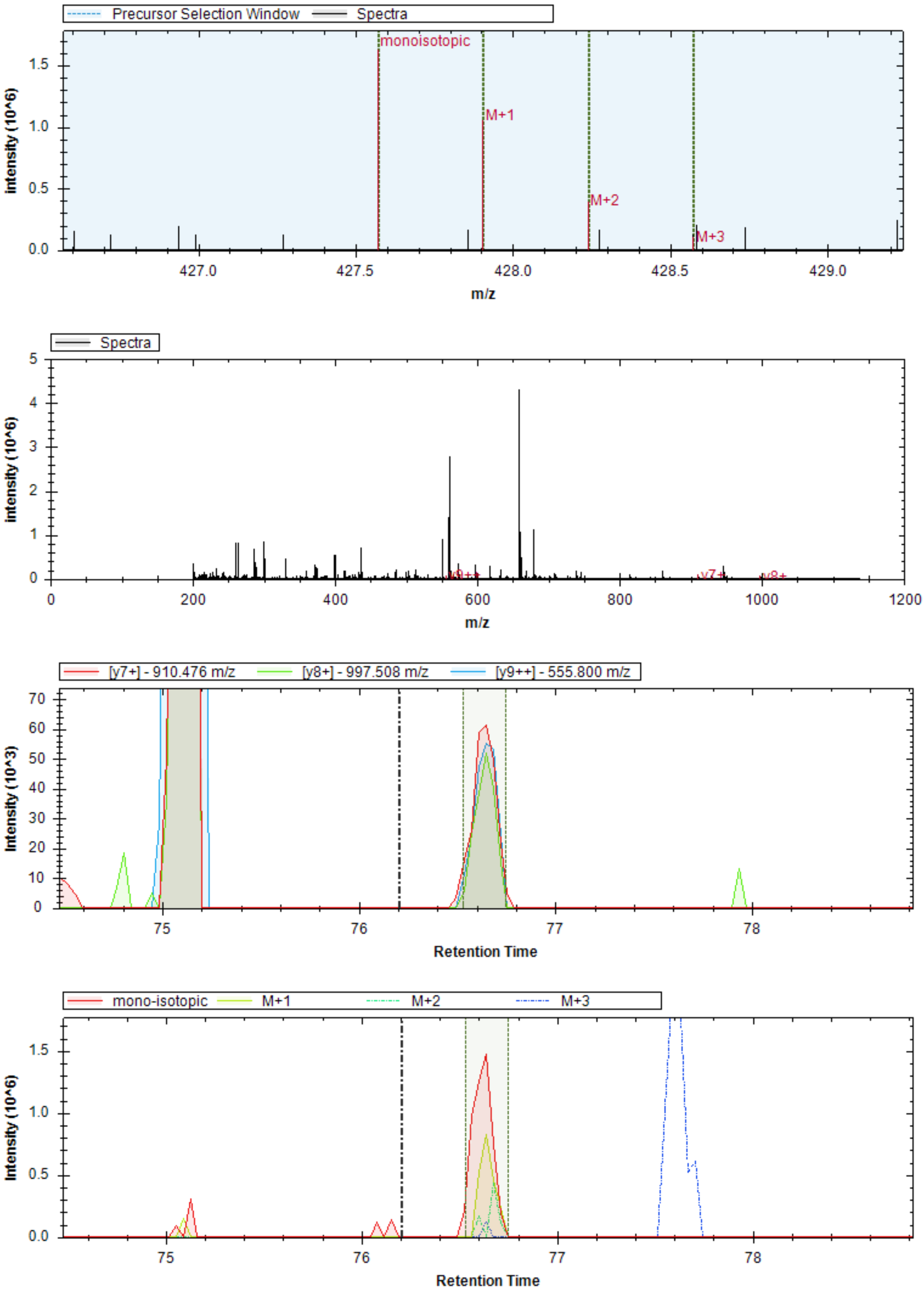

O08553 – _TVT[+H+O3+P]PASSAK_.2

Fig4_MP-DIA-barrel-cortex-p54-1_MHRM.htrms

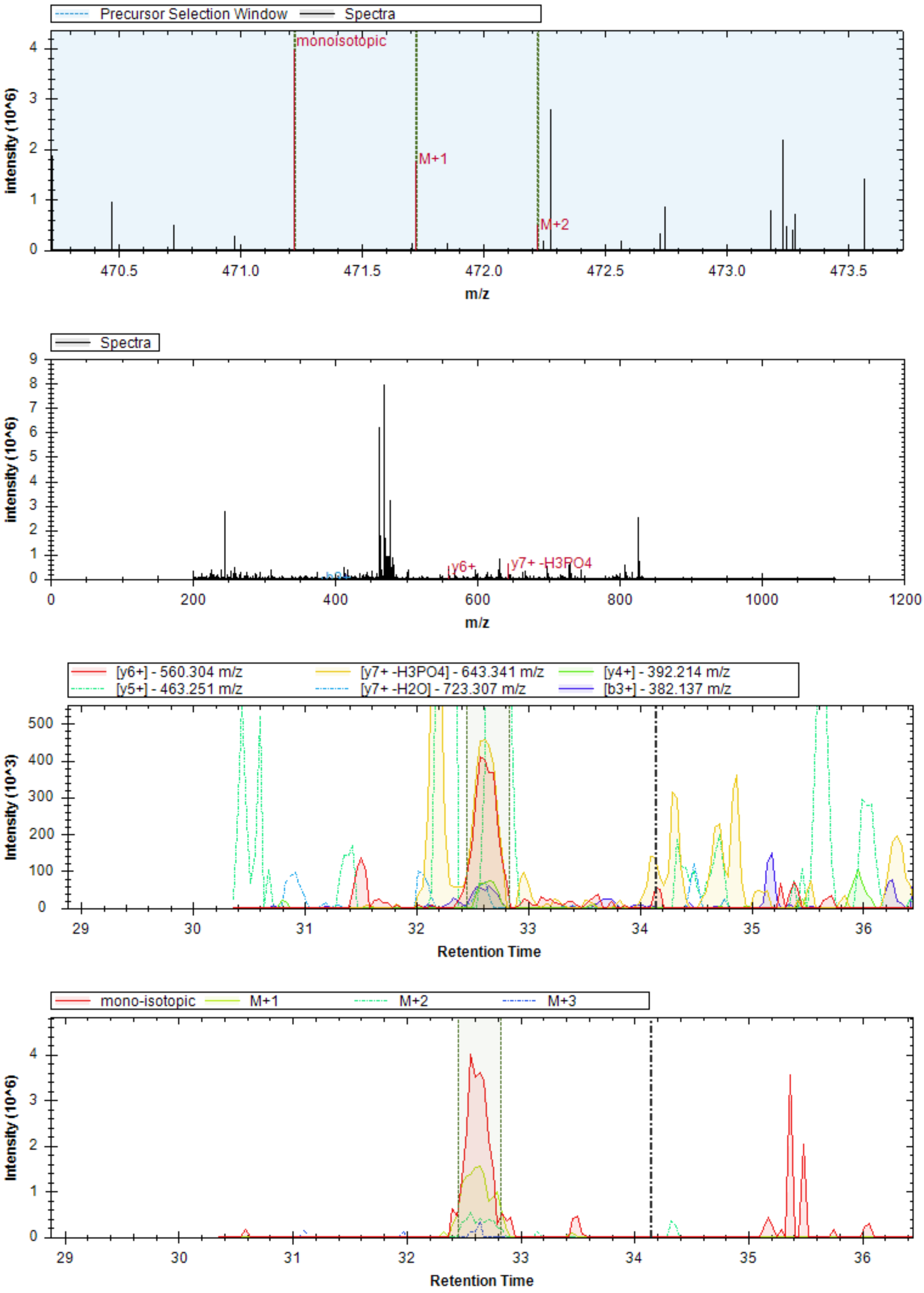

P20357 – _VTDGIS[+H+O3+P]KSPEKR_.3

Fig4_MP-DIA-barrel-cortex-p54-1_MHRM.htrms

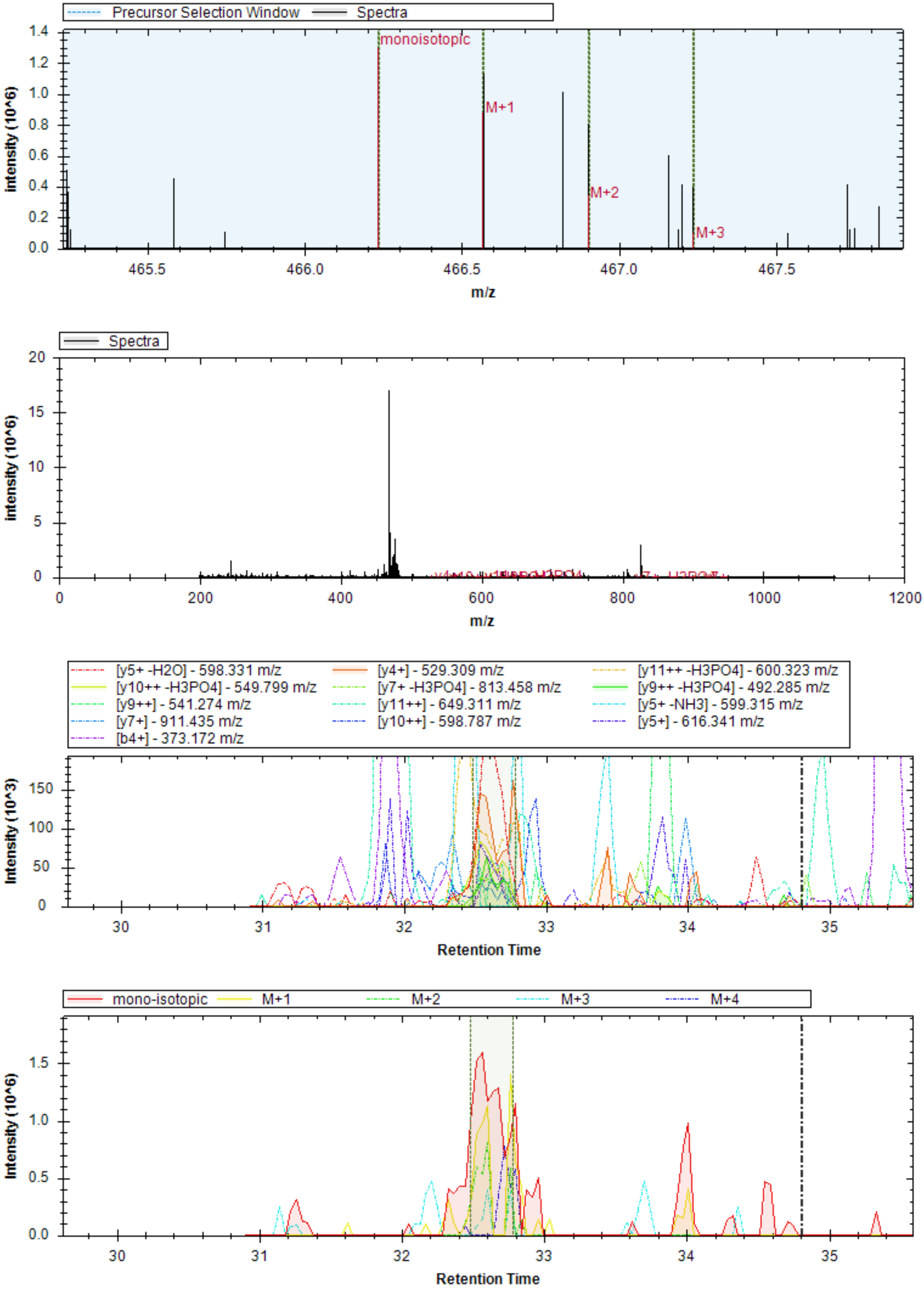

Q8K015;Q8K015-2 – _TVIS[+H+O3+P]EQEVLER_.3
Fig4_MP-DIA-barrel-cortex-p54-1_MHRM.htrms

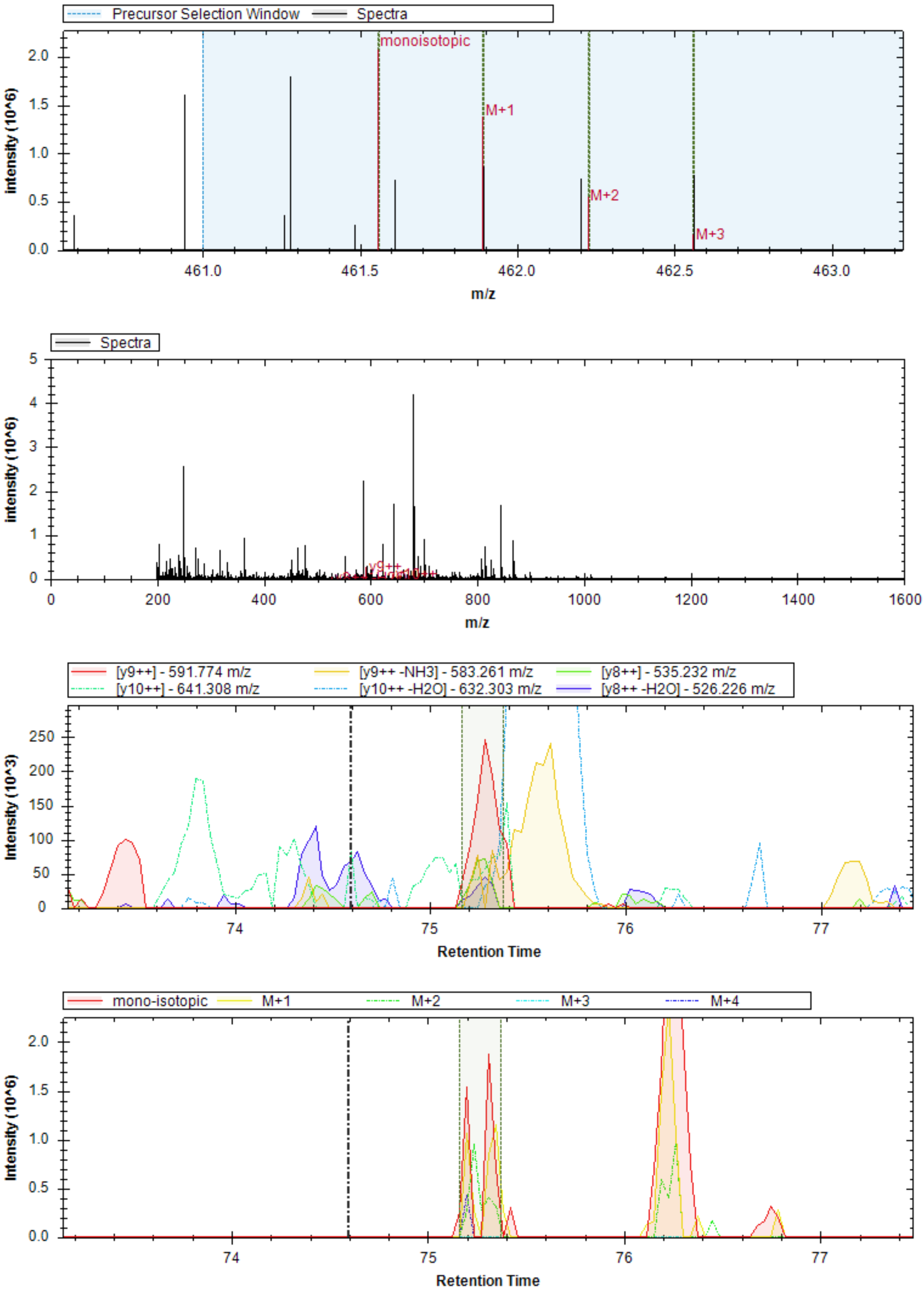

P26645 – _LSGFS[+H+O3+P]FKK_.2
Fig4_MP-DIA-barrel-cortex-p54-1_MHRM.htrms

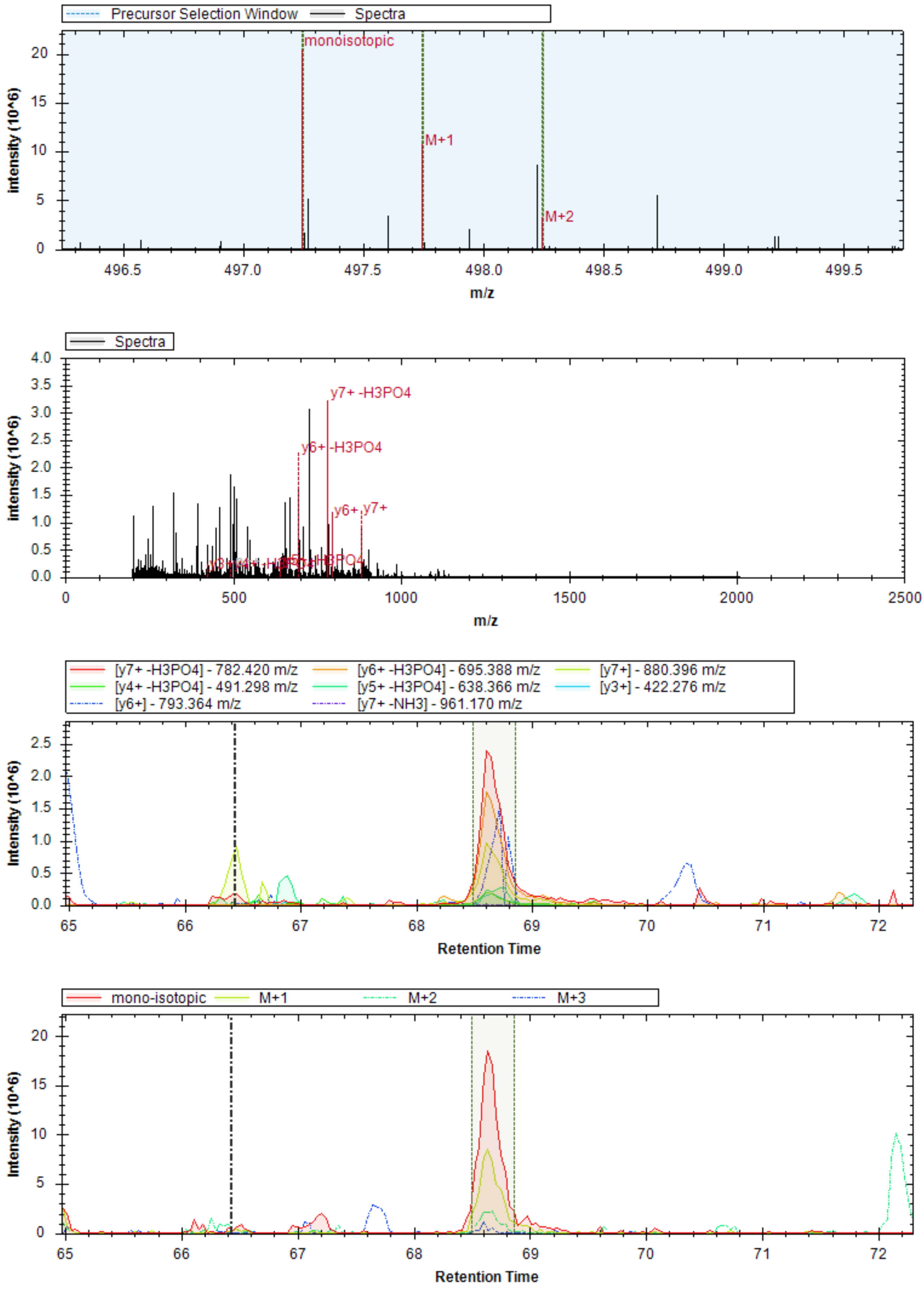

P28667 – _LSGLS[+H+O3+P]FKR_.2

Fig4_MP-DIA-barrel-cortex-p54-1_MHRM.htrms

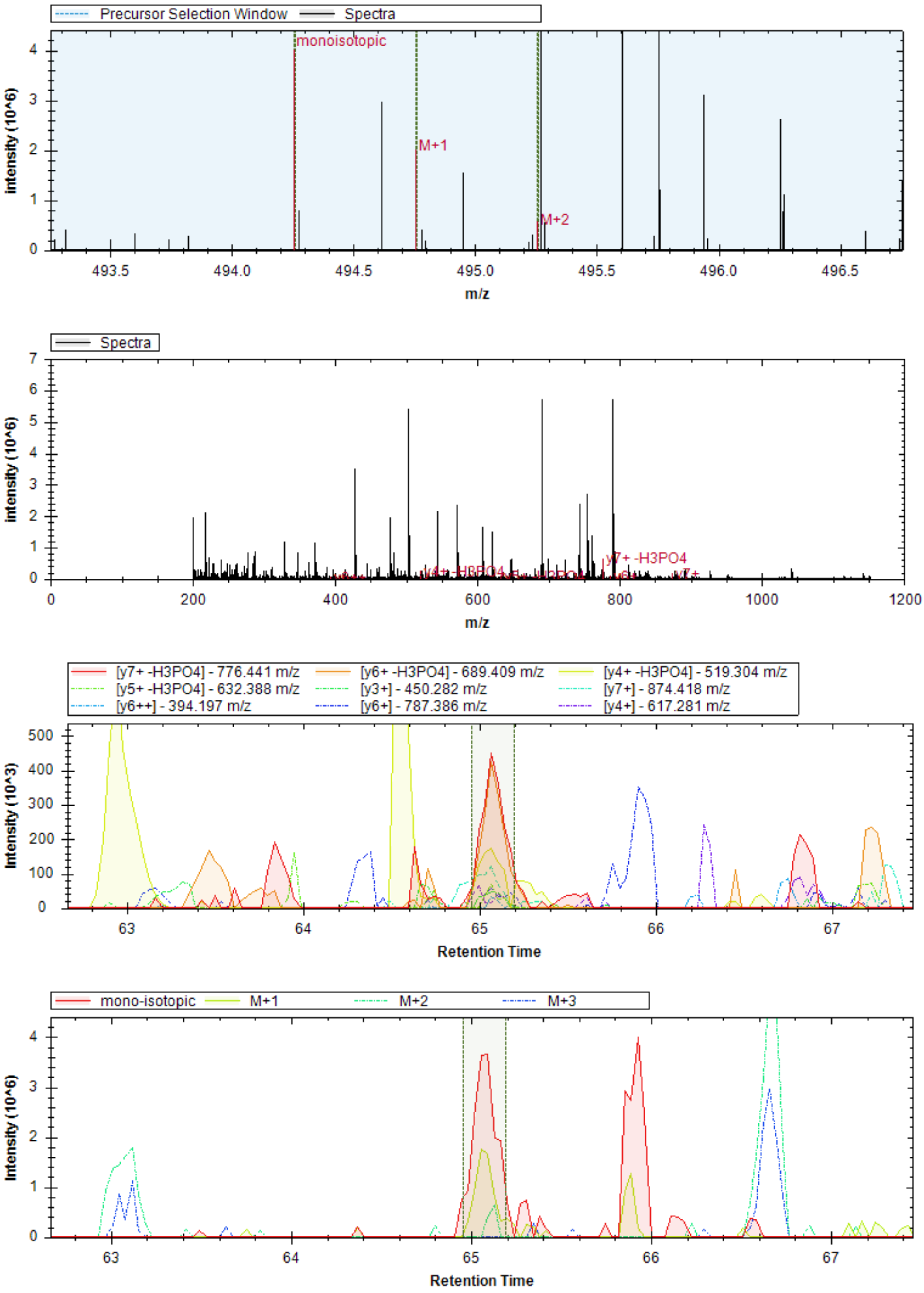

P28738 – _PIRPGHYPAS[+H+O3+P]SPTAVHAVR_.4
Fig4_MP-DIA-barrel-cortex-p54-1_MHRM.htrms

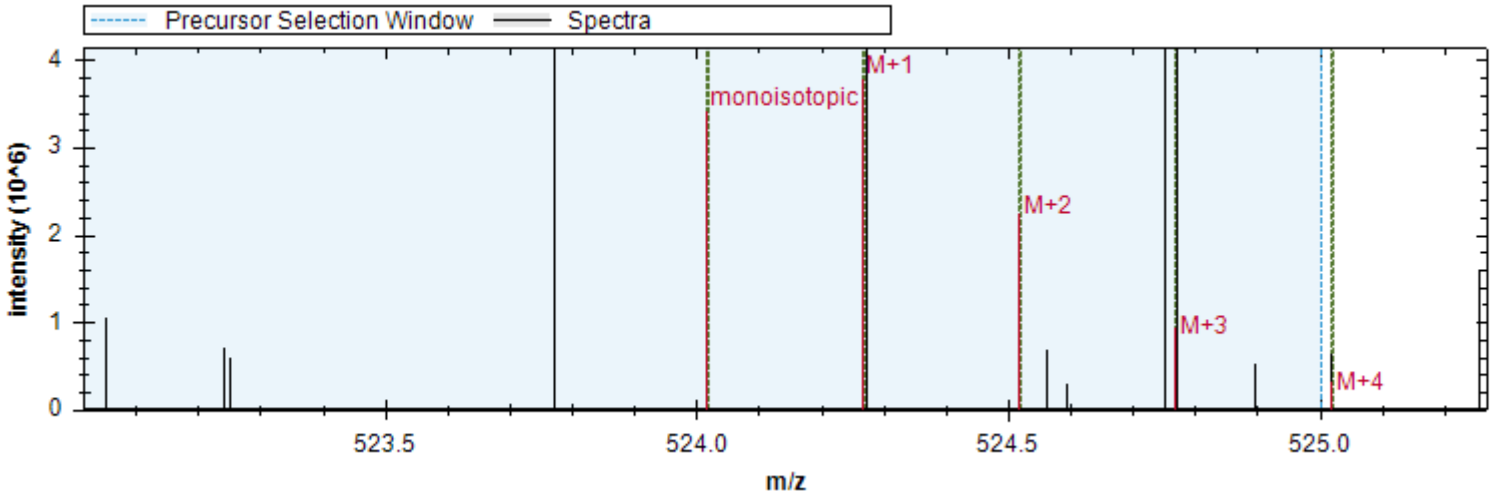

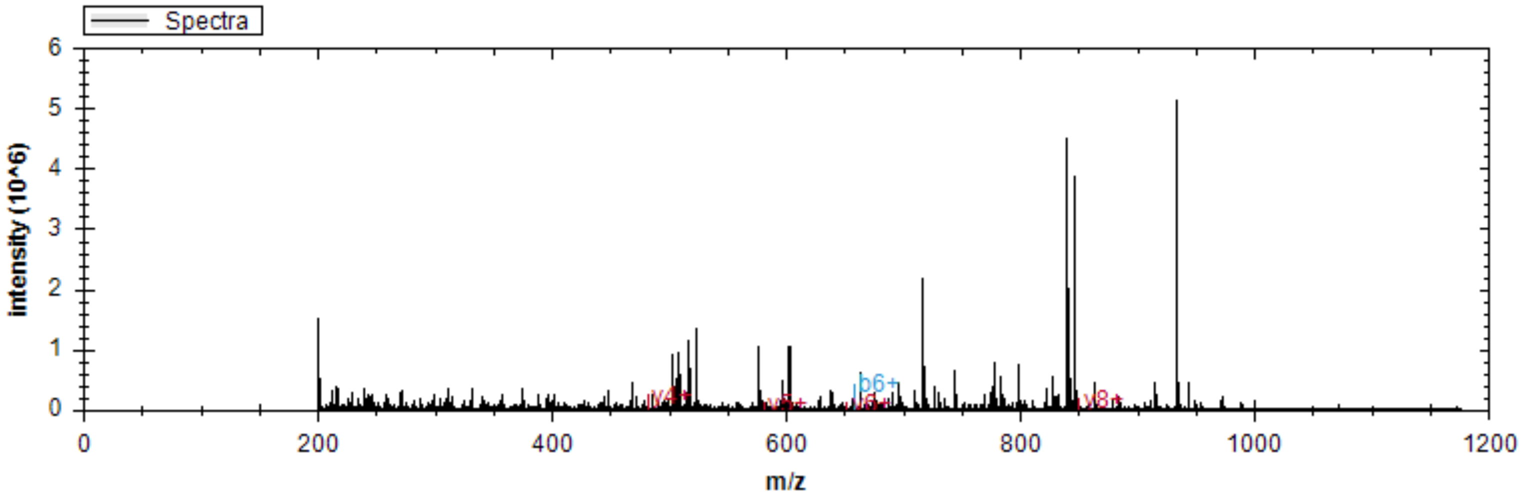

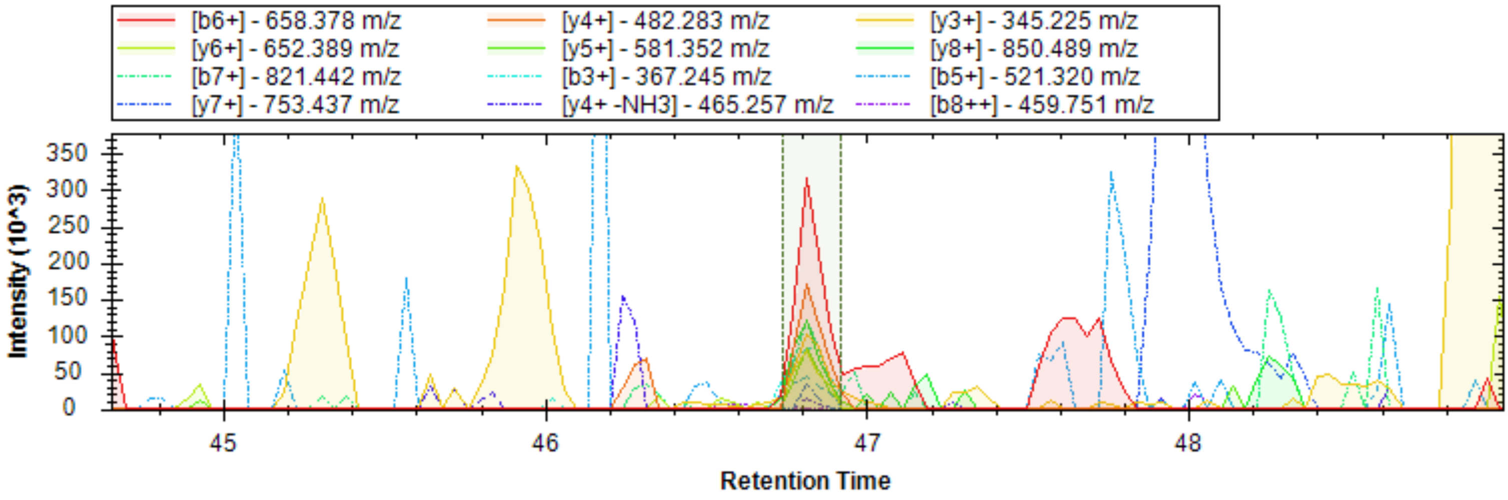

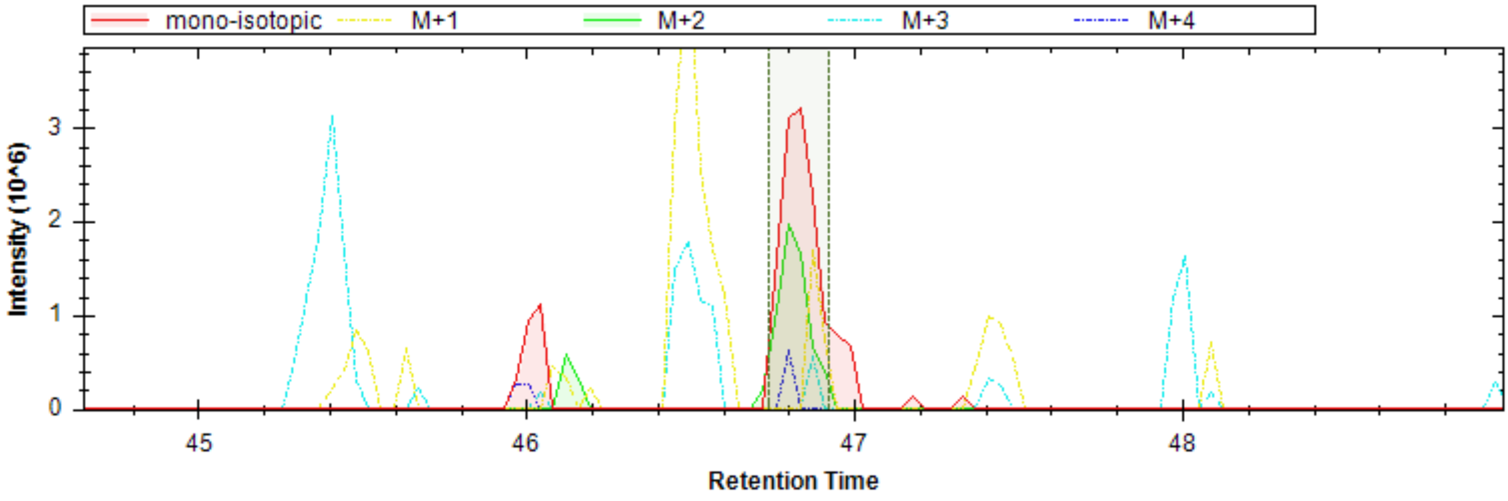

P28738 – _PIRPGHYPASS[+H+O3+P]PTAVHAVR_.4
Fig4_MP-DIA-barrel-cortex-p54-1_MHRM.htrms

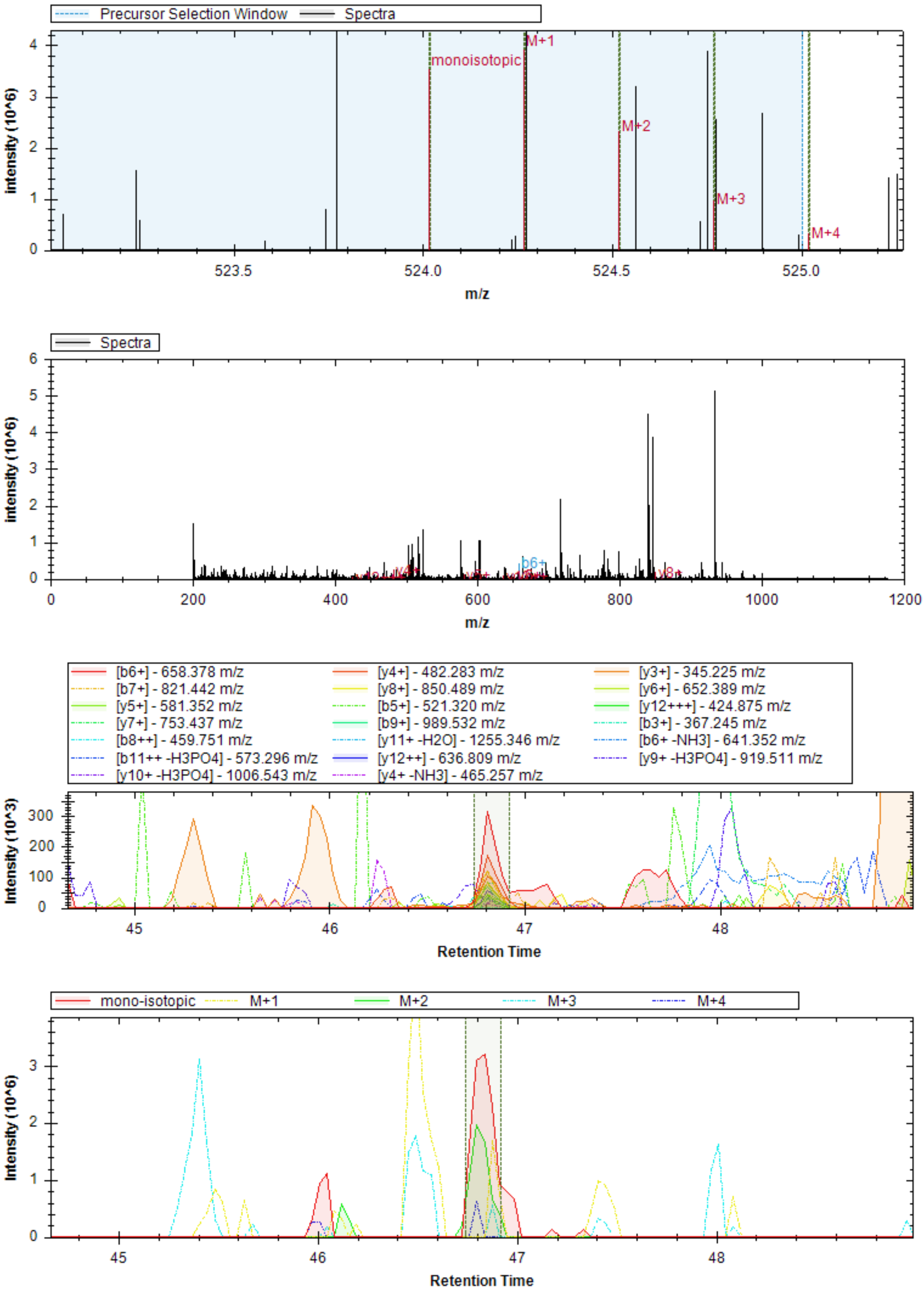

Q8CDY7 – _LVQALC[+C2+H3+N+O]EQIHT[+H+O3+P]R_.3

Fig4_MP-DIA-barrel-cortex-p54-1_MHRM.htrms

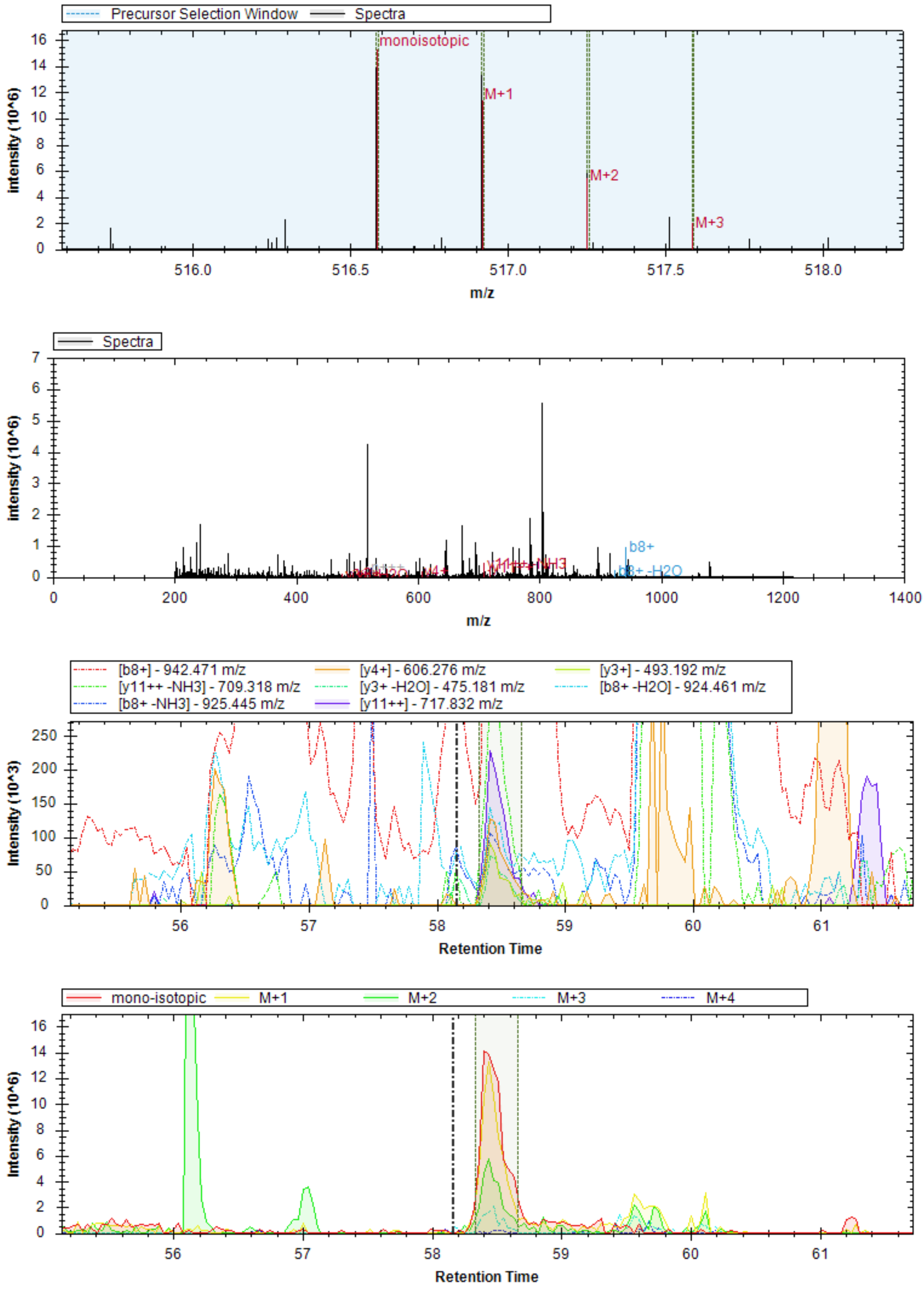

Q9QYC0 – _AAVVT[+H+O3+P]SPPPTTAPHK_.3

Fig4_MP-DIA-barrel-cortex-p54-1_MHRM.htrms

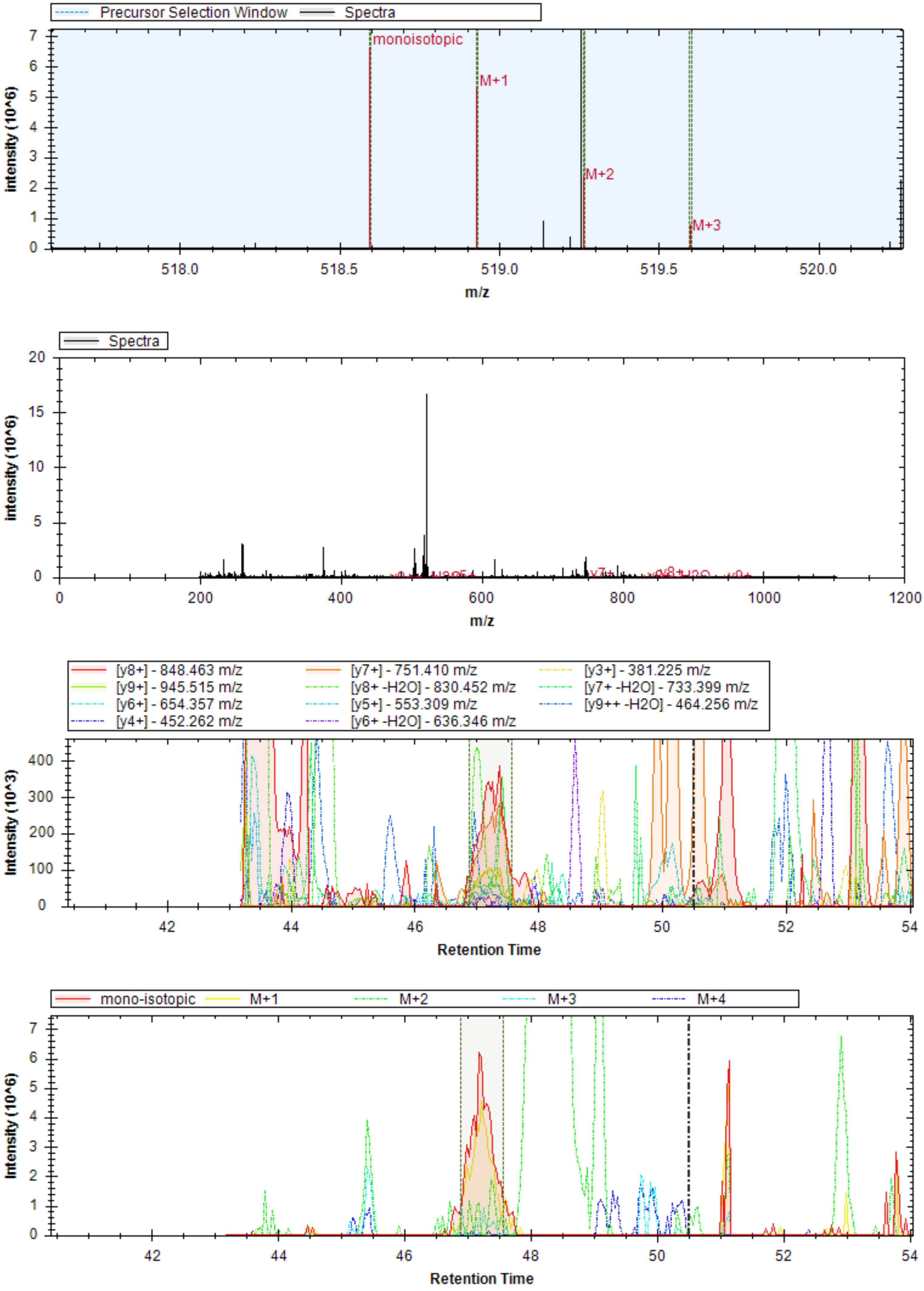

Q9QYC0 – _AAVVTS[+H+O3+P]PPPTTAPHK_.3

Fig4_MP-DIA-barrel-cortex-p54-1_MHRM.htrms

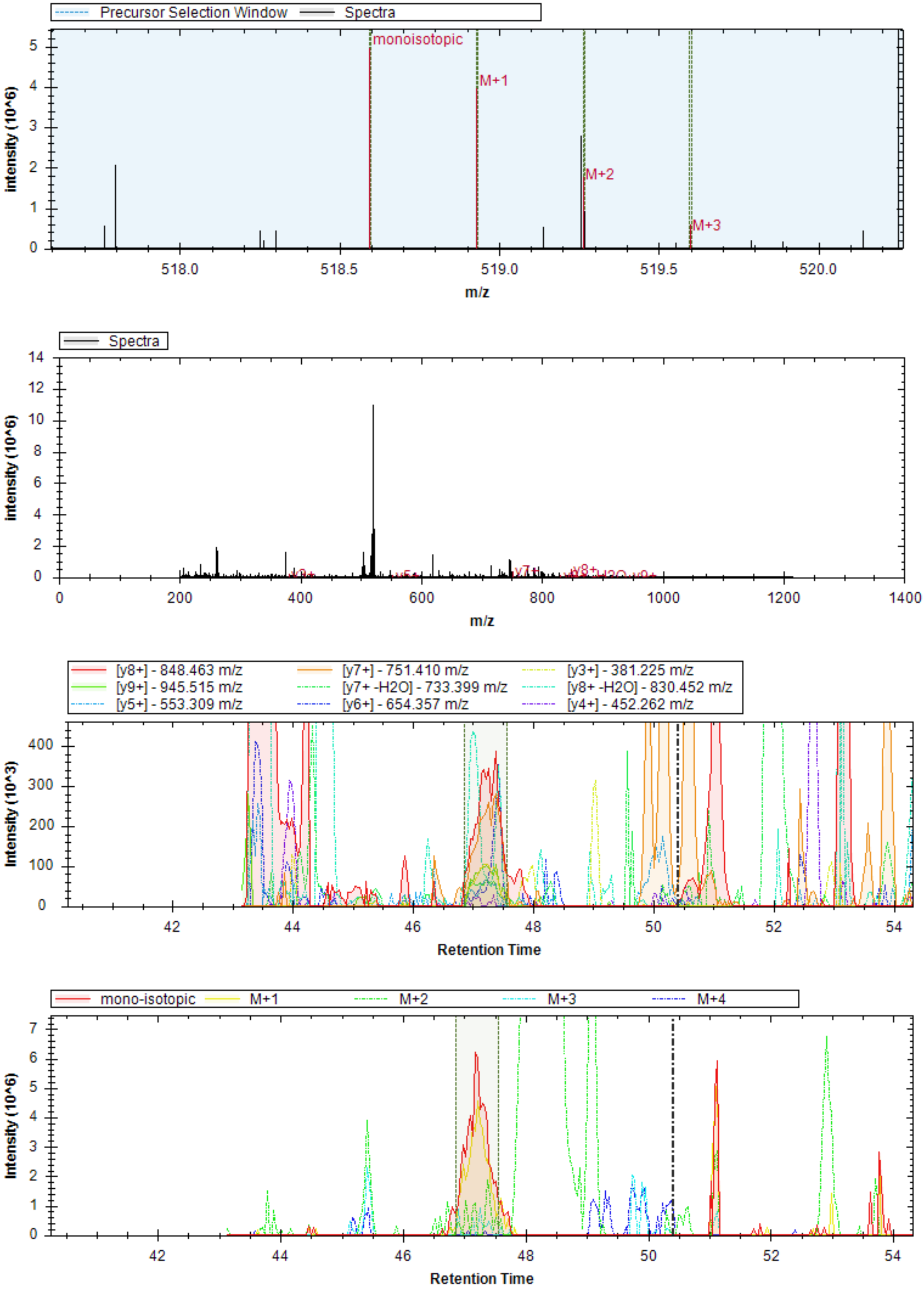

P14152 – _KLS[+H+O3+P]SAMSAAK_.2
Fig4_MP-DIA-barrel-cortex-p54-1_MHRM.htrms

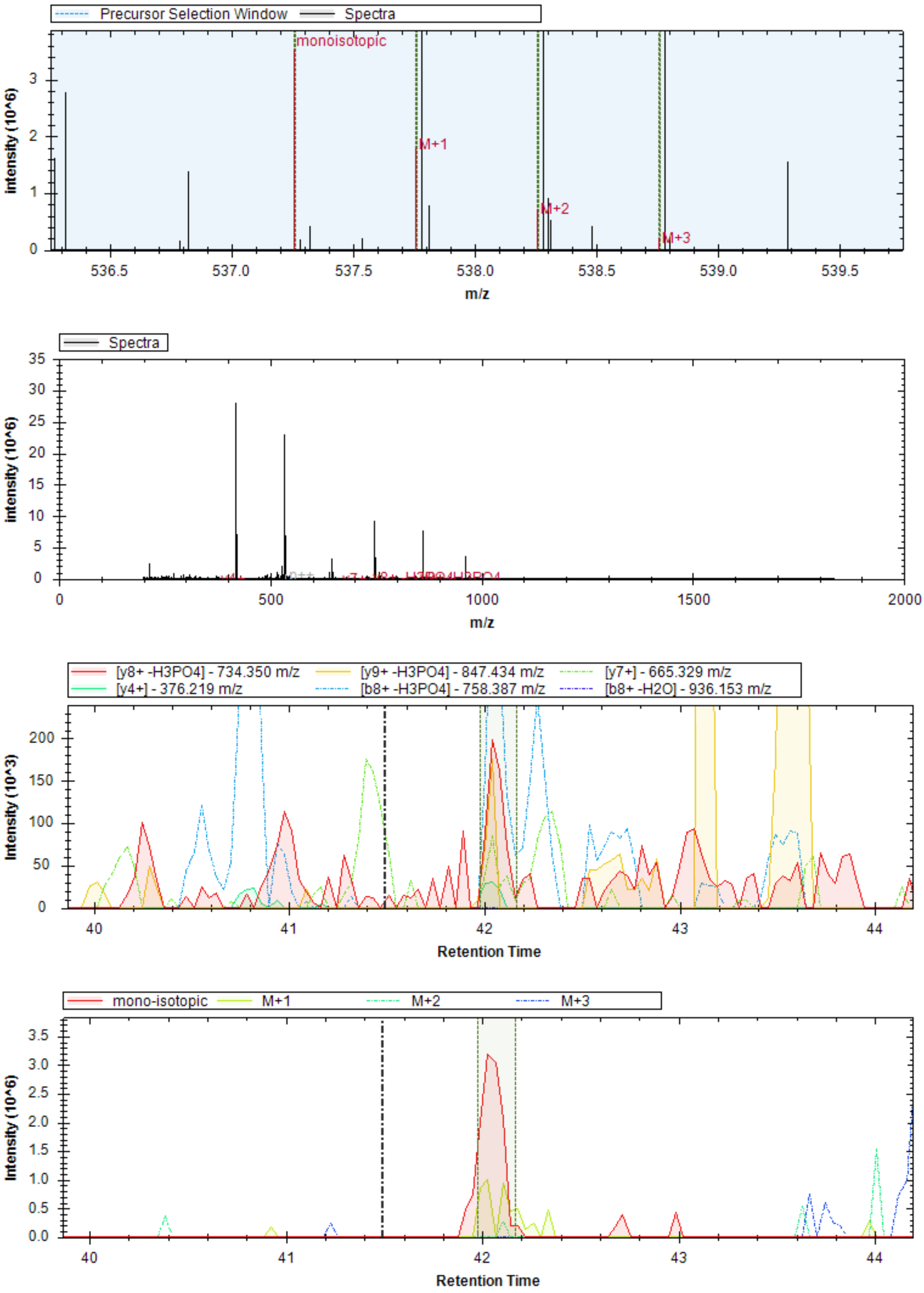

Q7TQD2 – _AANKT[+H+O3+P]PPKSPGDPAR_.3

Fig4_MP-DIA-barrel-cortex-p54-1_MHRM.htrms

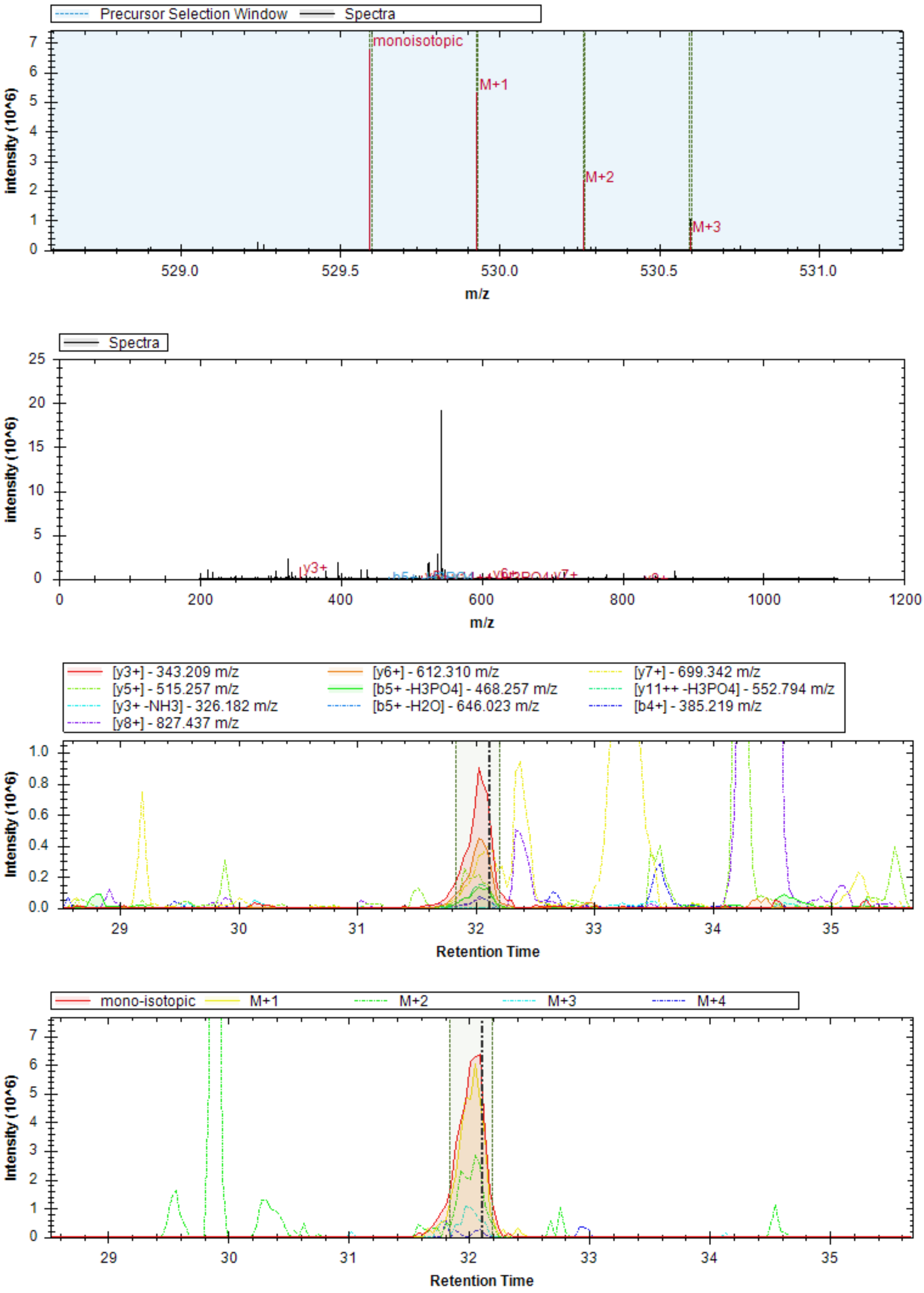

P20357 – _VAIIRT[+H+O3+P]PPKSPATPK_.3
Fig4_MP-DIA-barrel-cortex-p54-1_MHRM.htrms

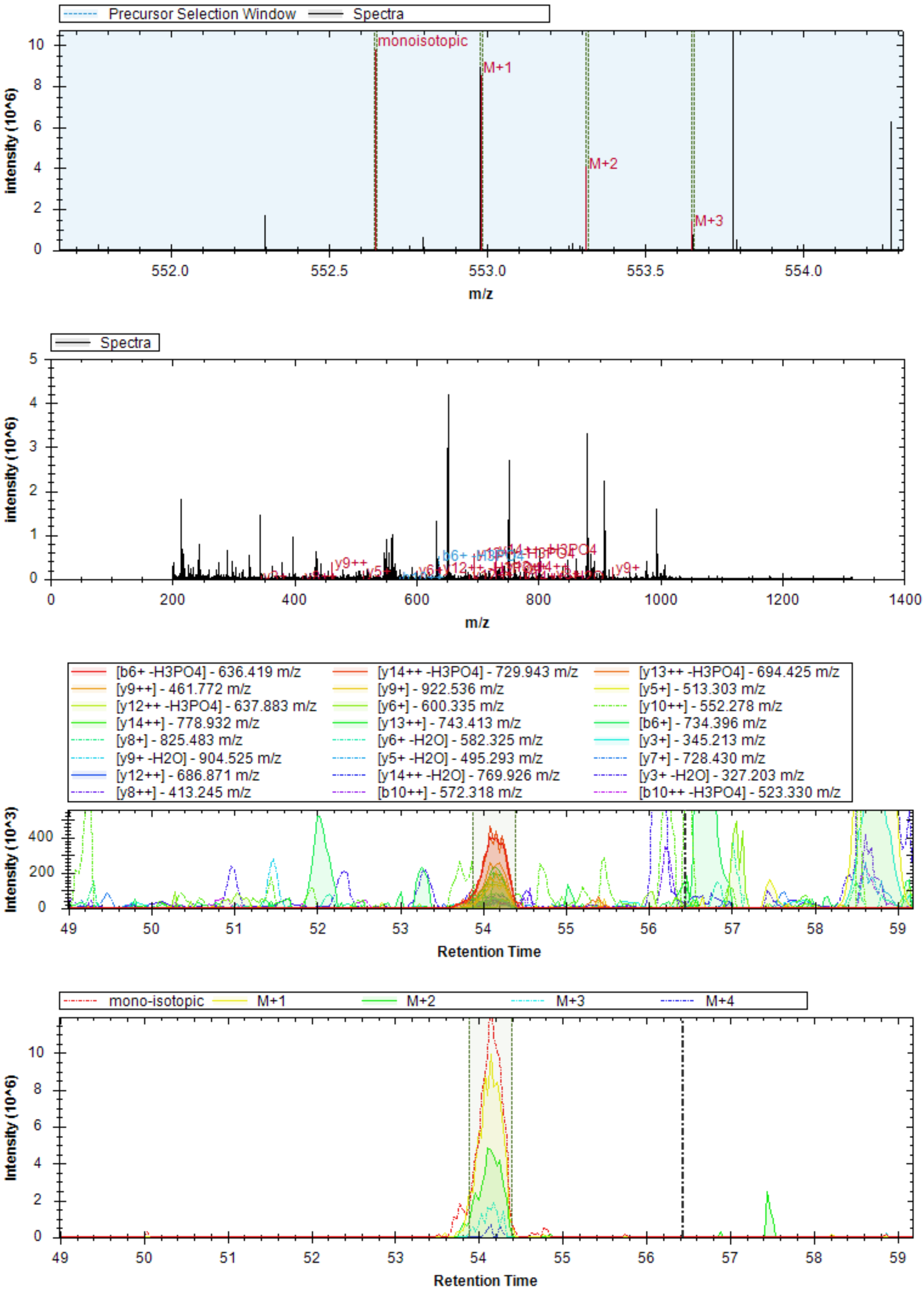

Q9CYT6 – _THTPSPTS[+H+O3+P]PK_.2
Fig4_MP-DIA-barrel-cortex-p54-1_MHRM.htrms

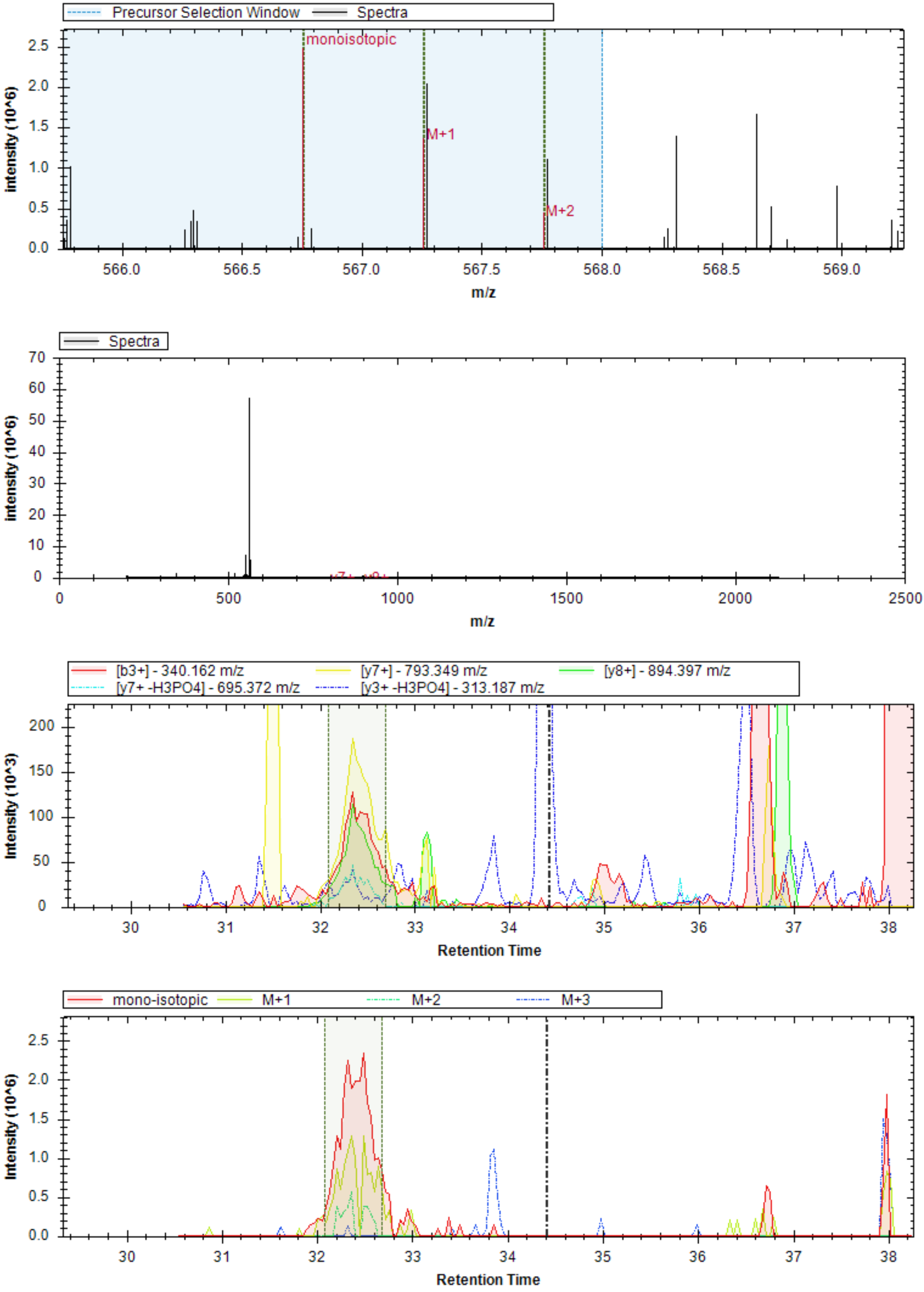

Q8BFU3-4 – _VAS[+H+O3+P]STQVRGASGRELR_.3
Fig4_MP-DIA-barrel-cortex-p54-1_MHRM.htrms

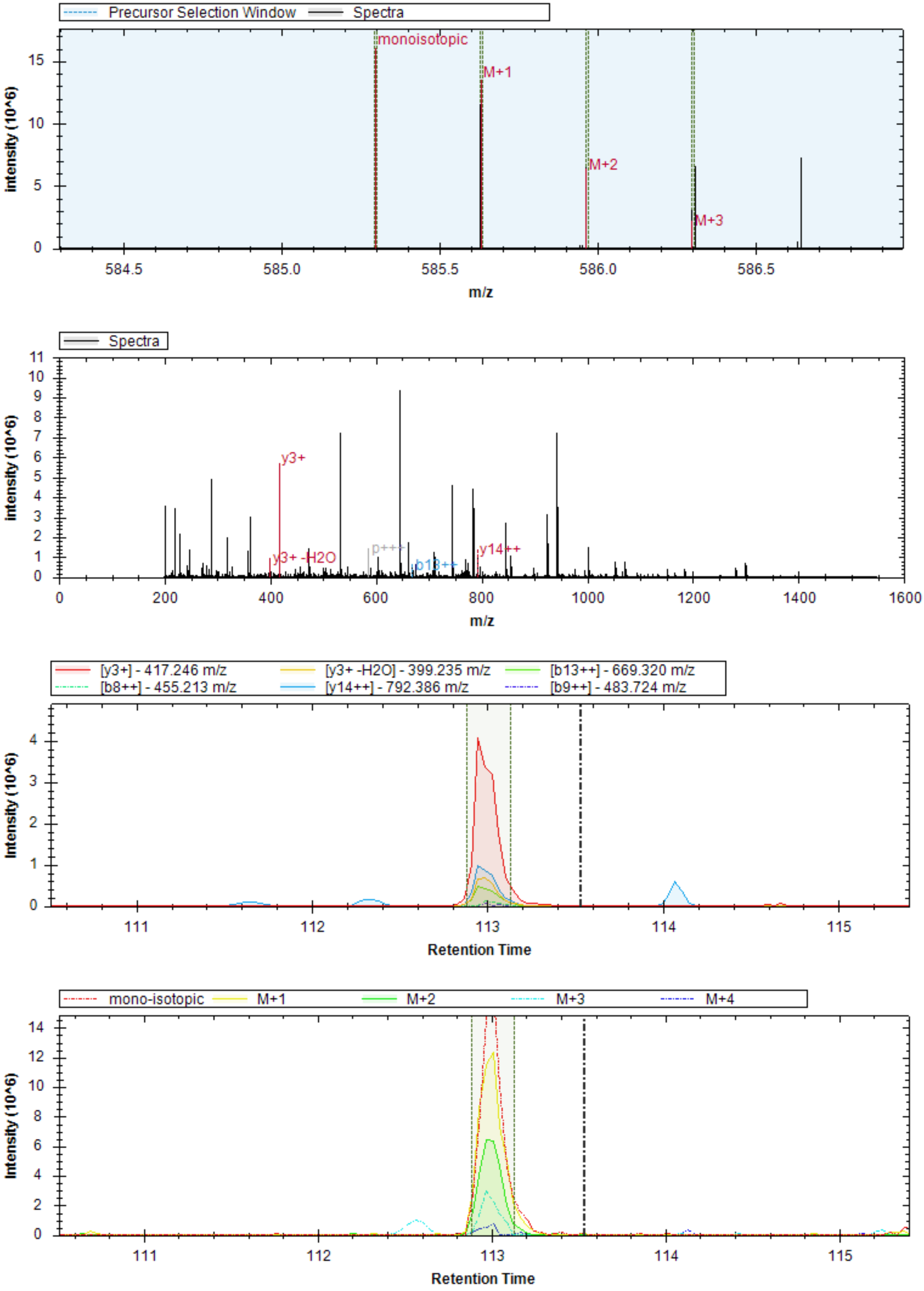

Q9CYT6 – _AQGQIRS[+H+O3+P]PTK_.2
Fig4_MP-DIA-barrel-cortex-p54-1_MHRM.htrms

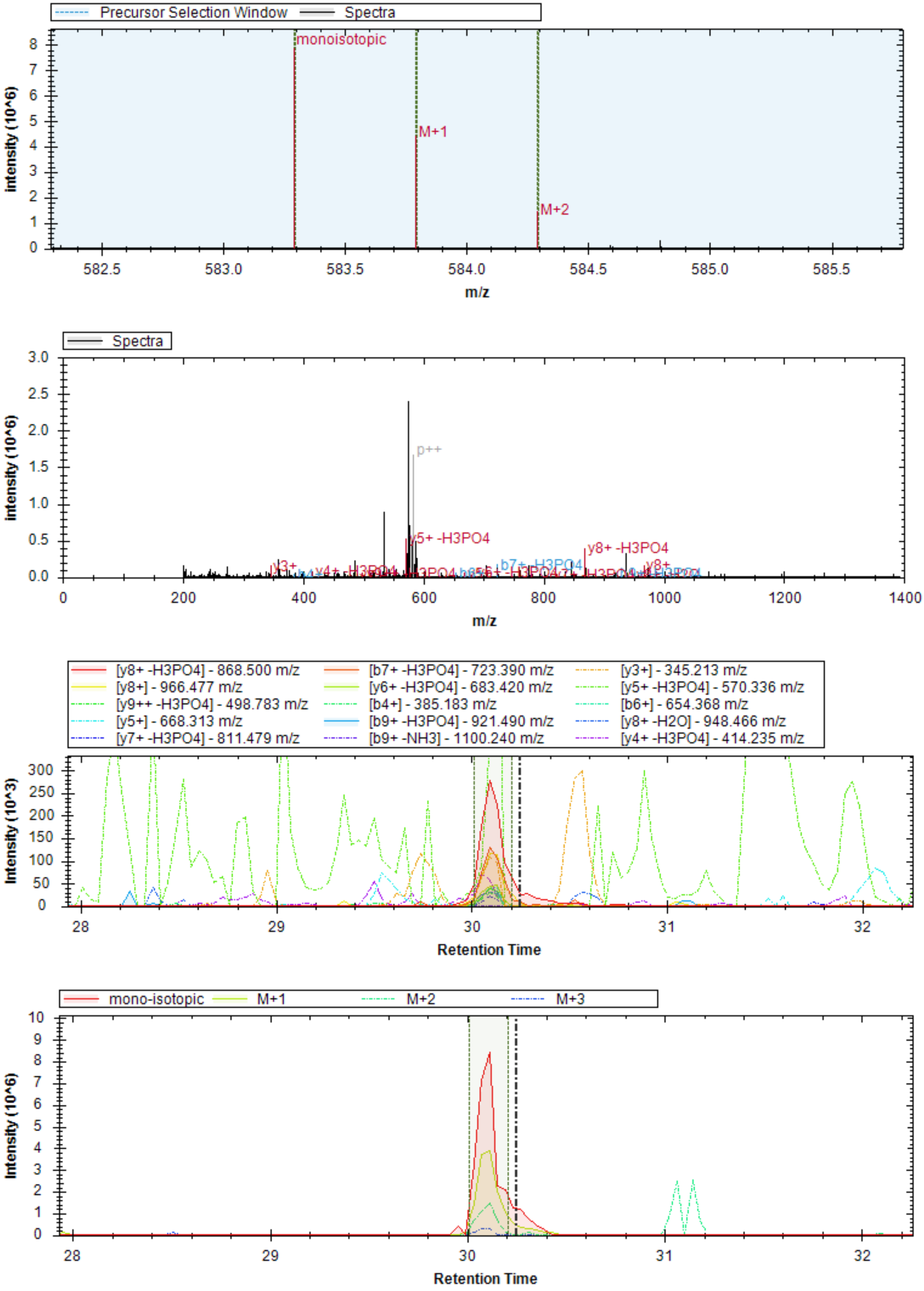

Q9D5Y0 – _ITDT[+H+O3+P]KKTDDLYWR_.3
Fig4_MP-DIA-barrel-cortex-p54-1_MHRM.htrms

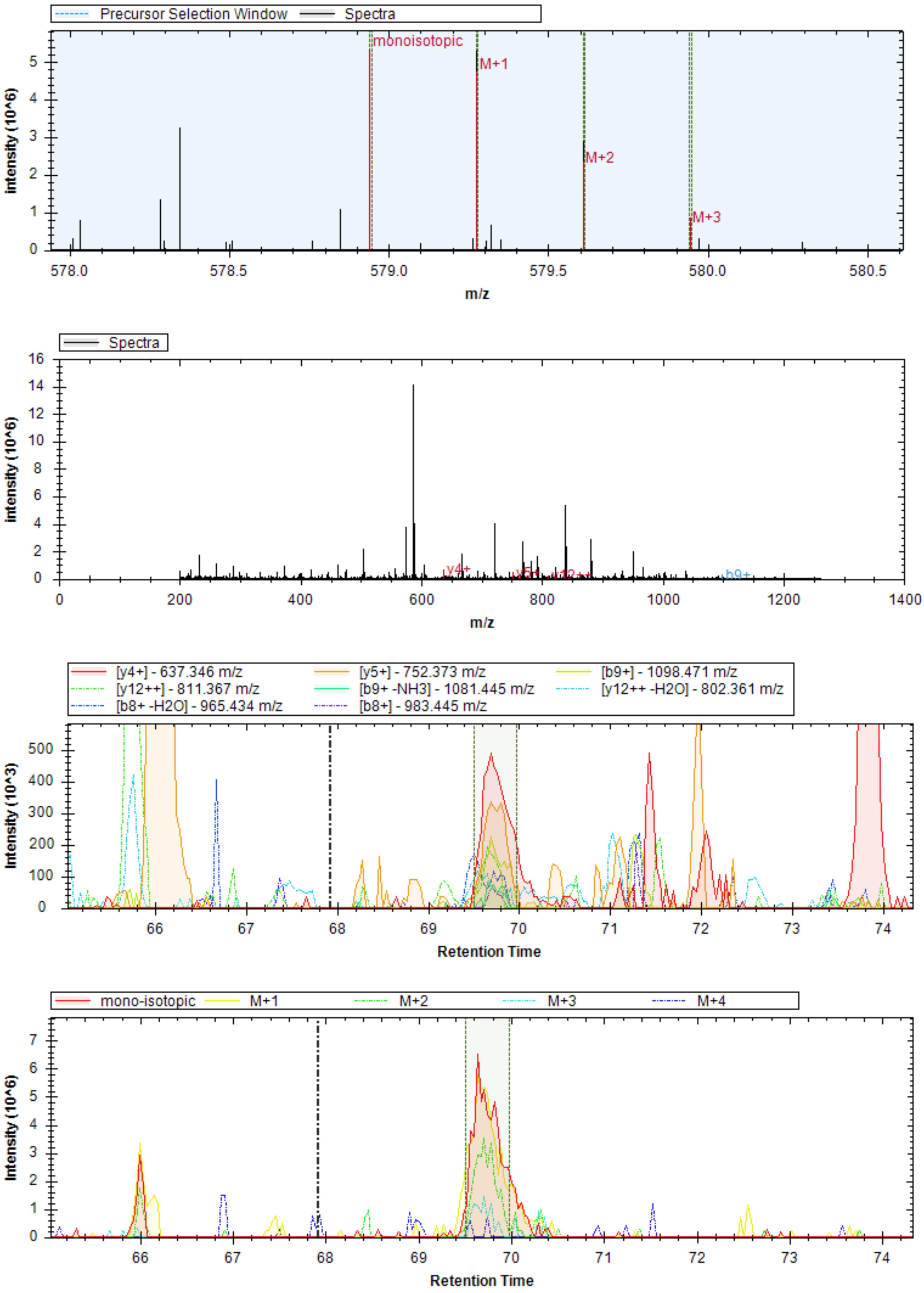

P04370-4 – _TTHYGS[+H+O3+P]LPQK_.2
Fig4_MP-DIA-barrel-cortex-p54-1_MHRM.htrms

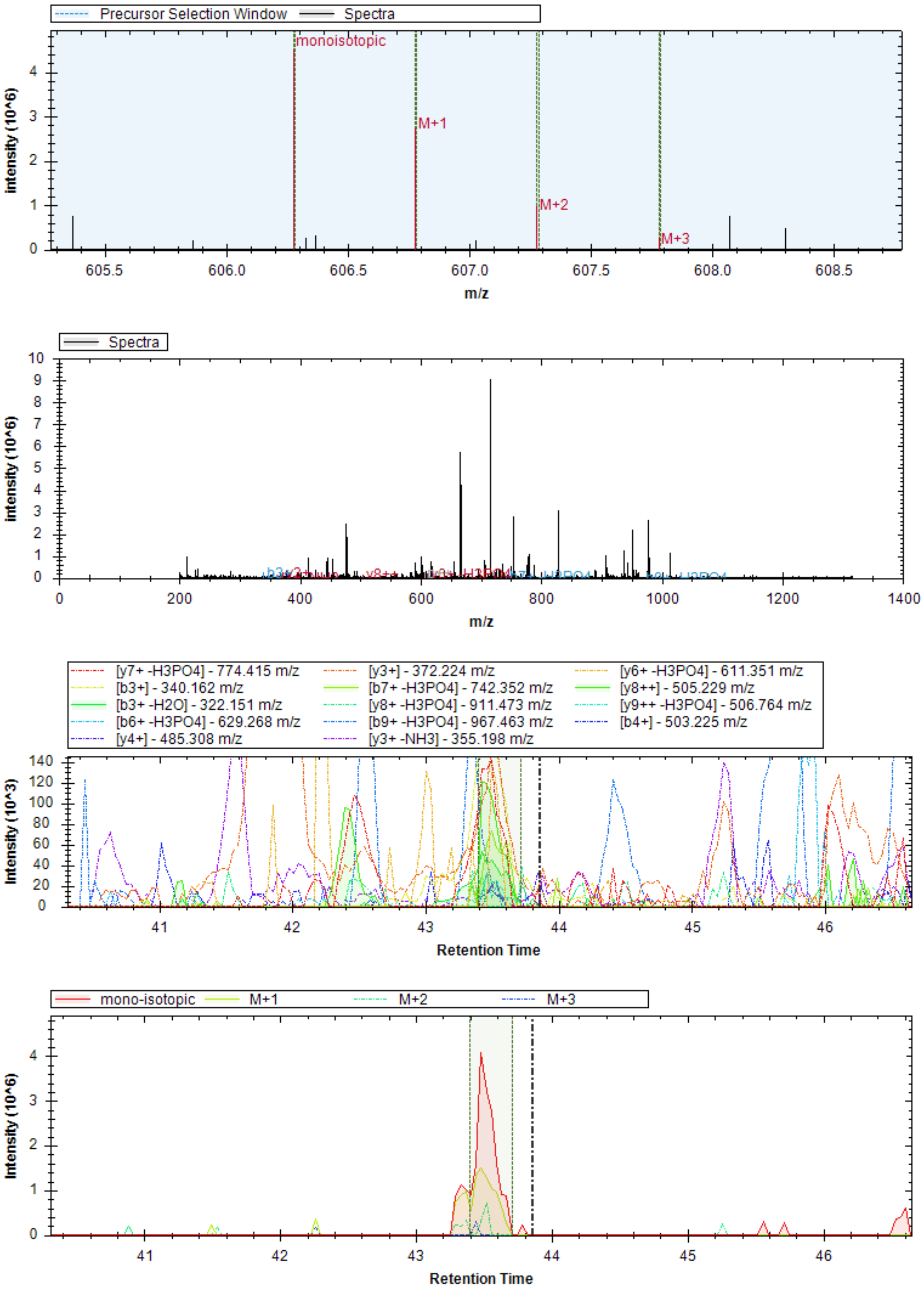

Q62261 – _RPPS[+H+O3+P]PDPNTK_.2
Fig4_MP-DIA-barrel-cortex-p54-1_MHRM.htrms

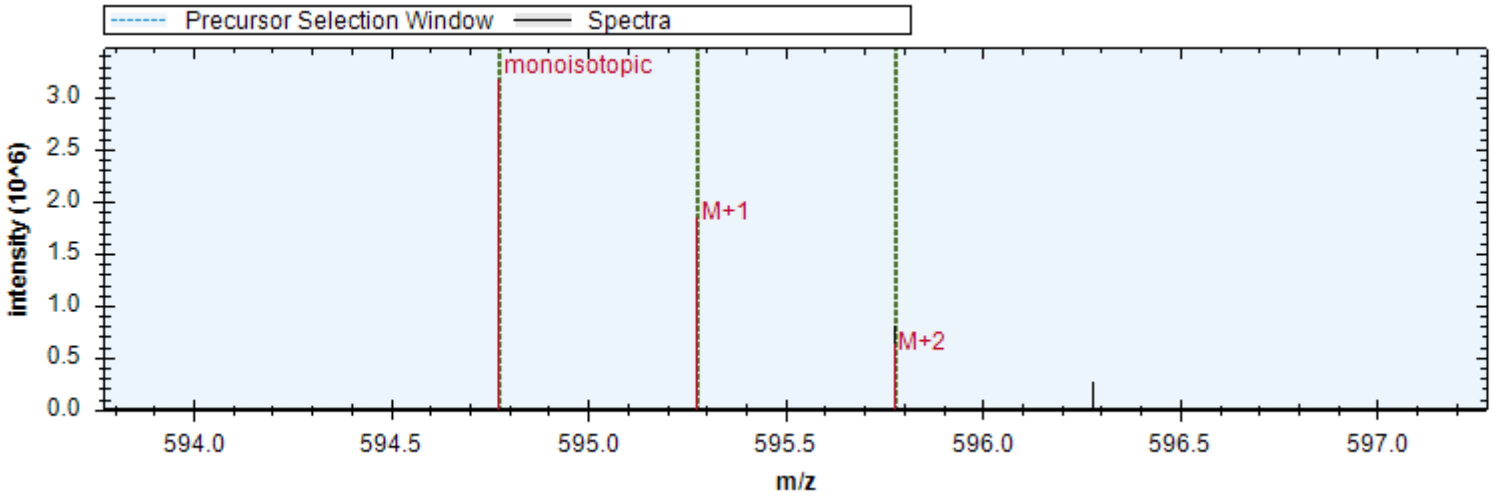

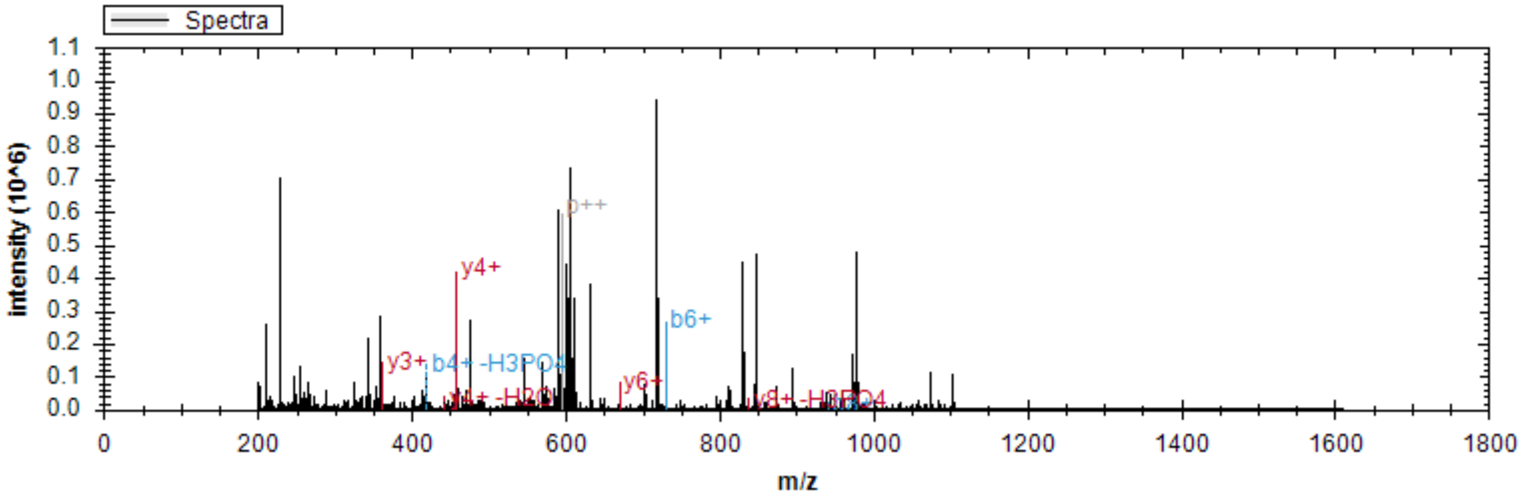

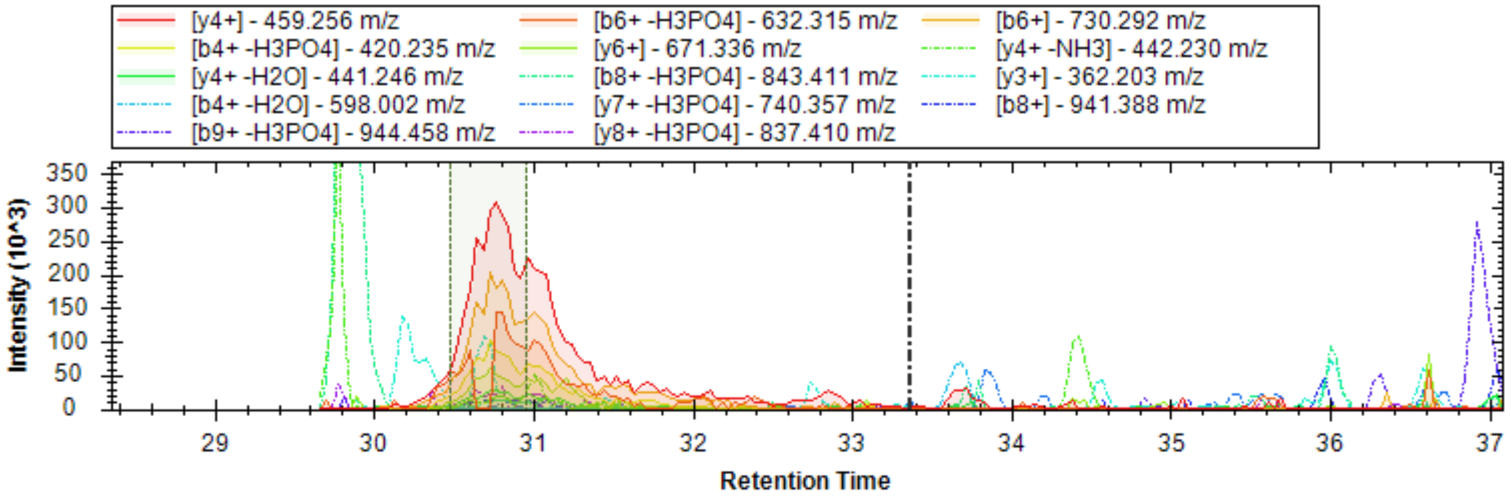

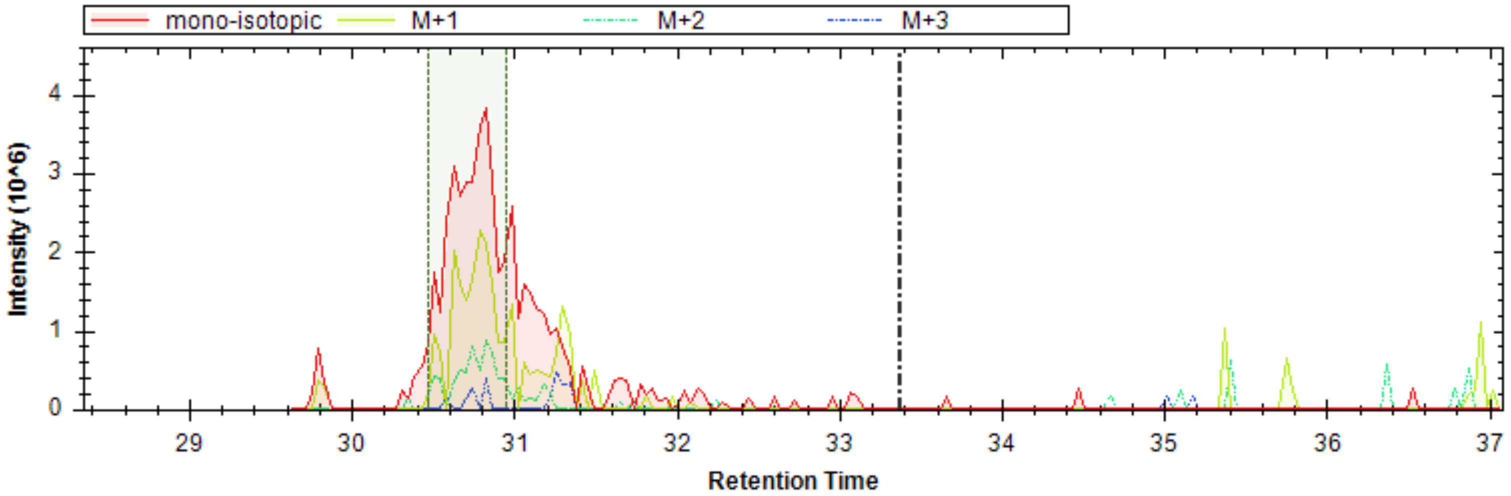

Q80VN0 – _ELTAQIT[+H+O3+P]SIK_.2
Fig4_MP-DIA-barrel-cortex-p54-1_MHRM.htrms

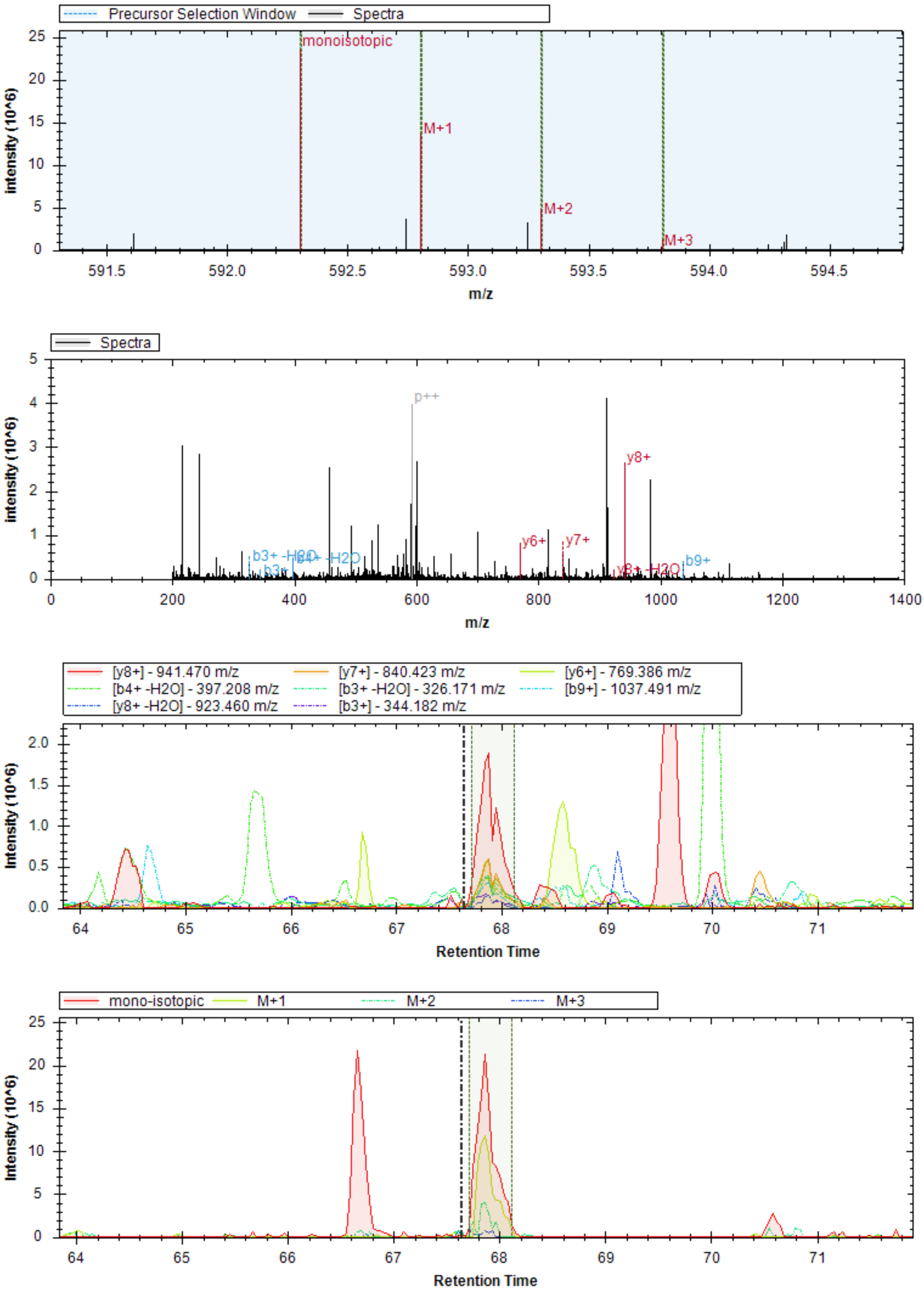

Q99104 – _AIS[+H+O3+P]PTSATSSGR_.2
Fig4_MP-DIA-barrel-cortex-p54-1_MHRM.htrms

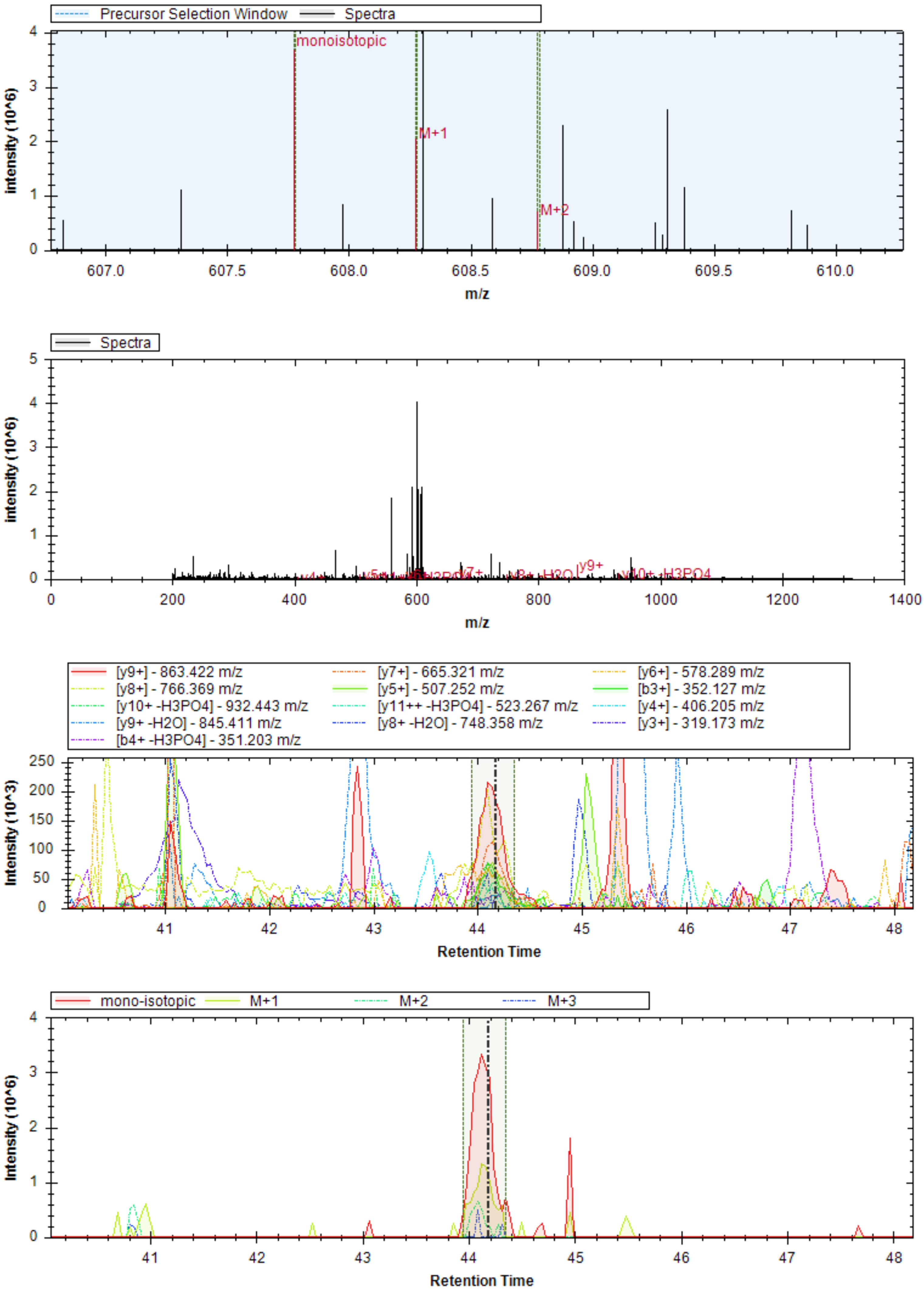

Q9QWI6 – _LSYAGGRPPSYAGS[+H+O3+P]PVHHAAER_.4
Fig4_MP-DIA-barrel-cortex-p54-1_MHRM.htrms

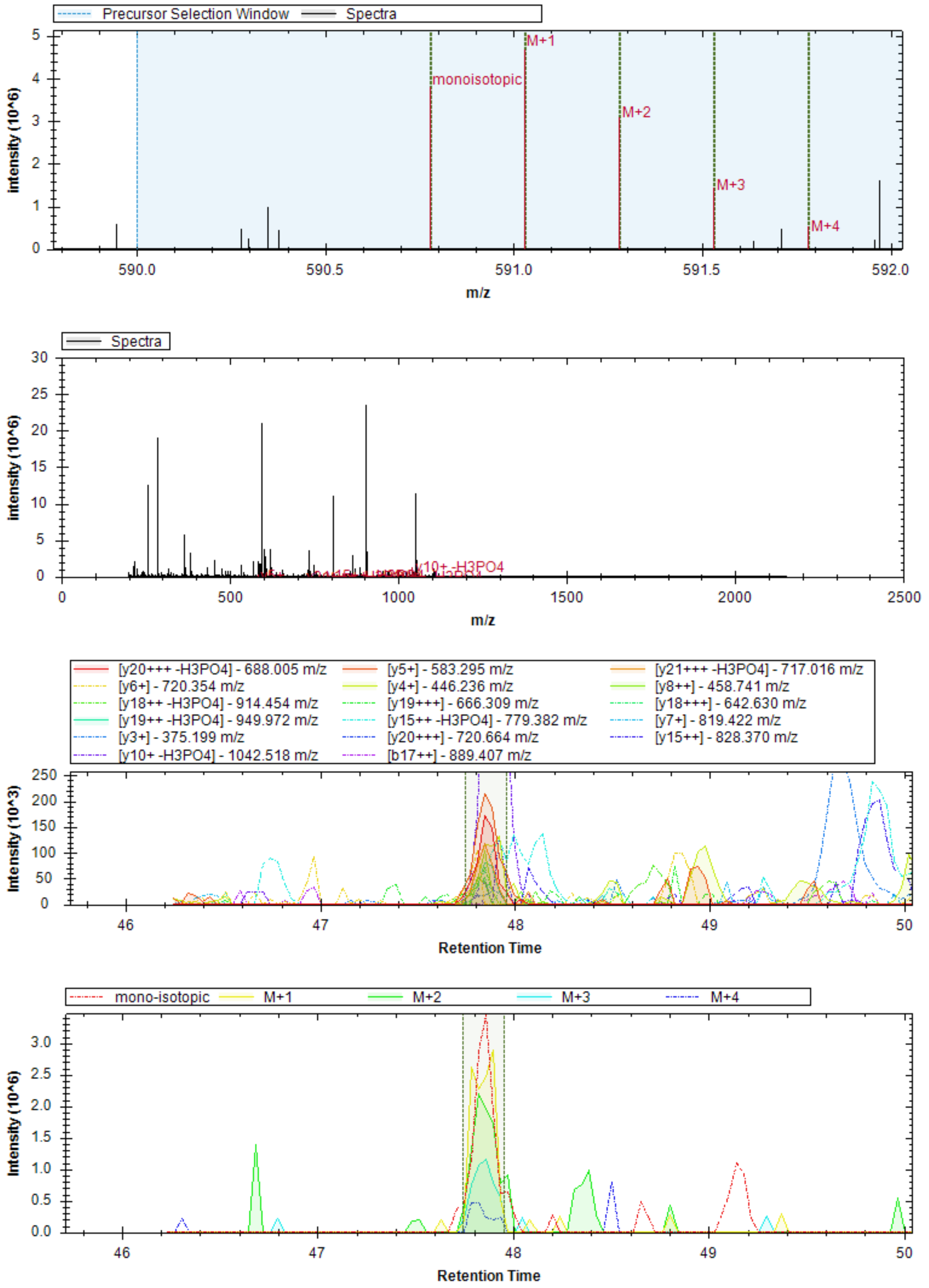

P60904 – _SLS[+H+O3+P]TSGESLYHVLGLDK_.3
Fig4_MP-DIA-barrel-cortex-p54-1_MHRM.htrms

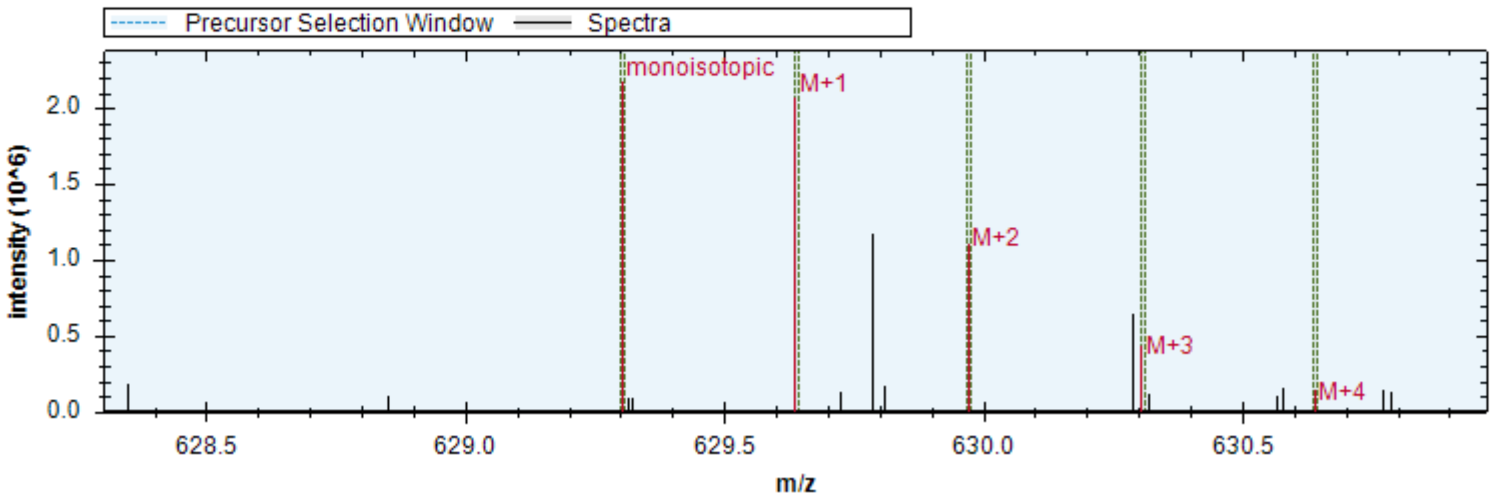

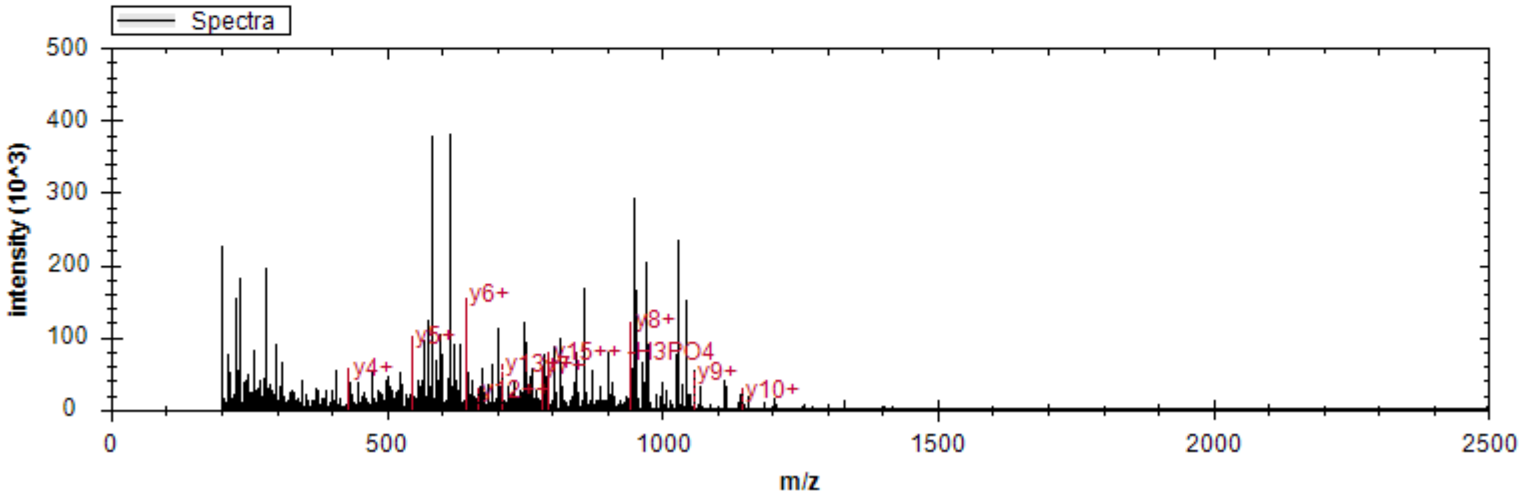

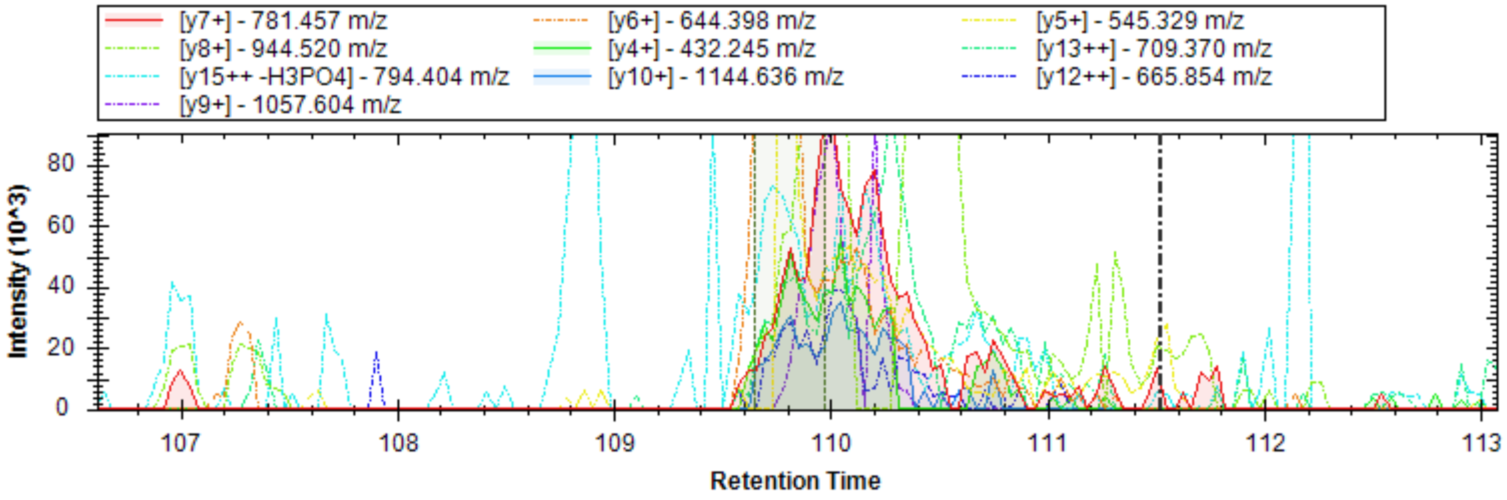

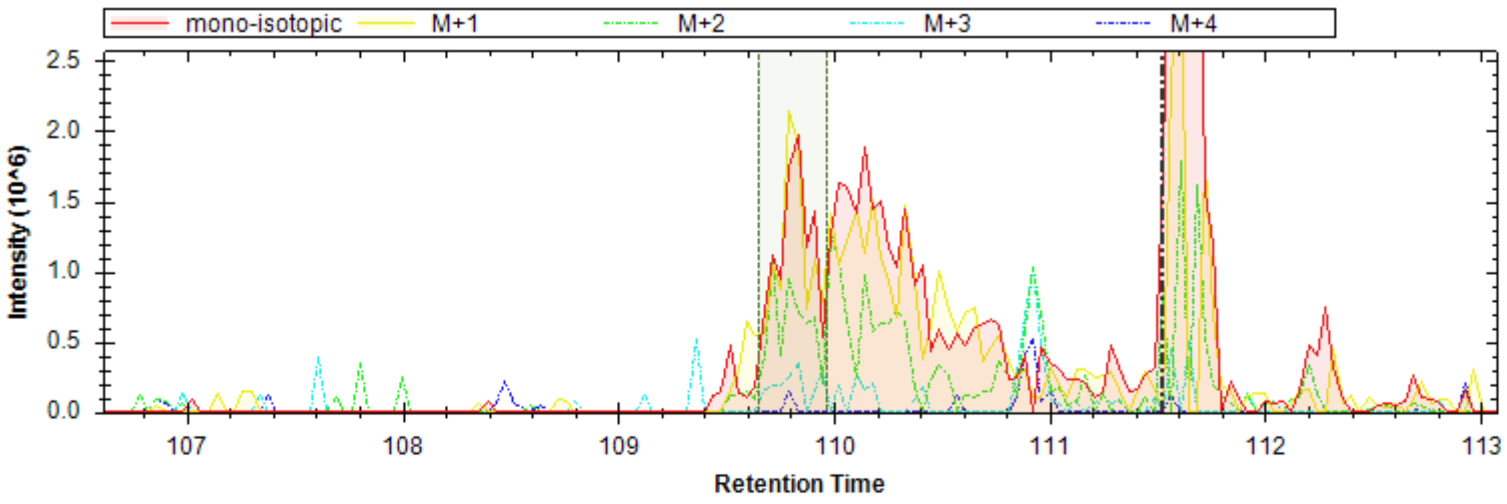

P60904 – _SLST[+H+O3+P]SGESLYHVLGLDK_.3
Fig4_MP-DIA-barrel-cortex-p54-1_MHRM.htrms

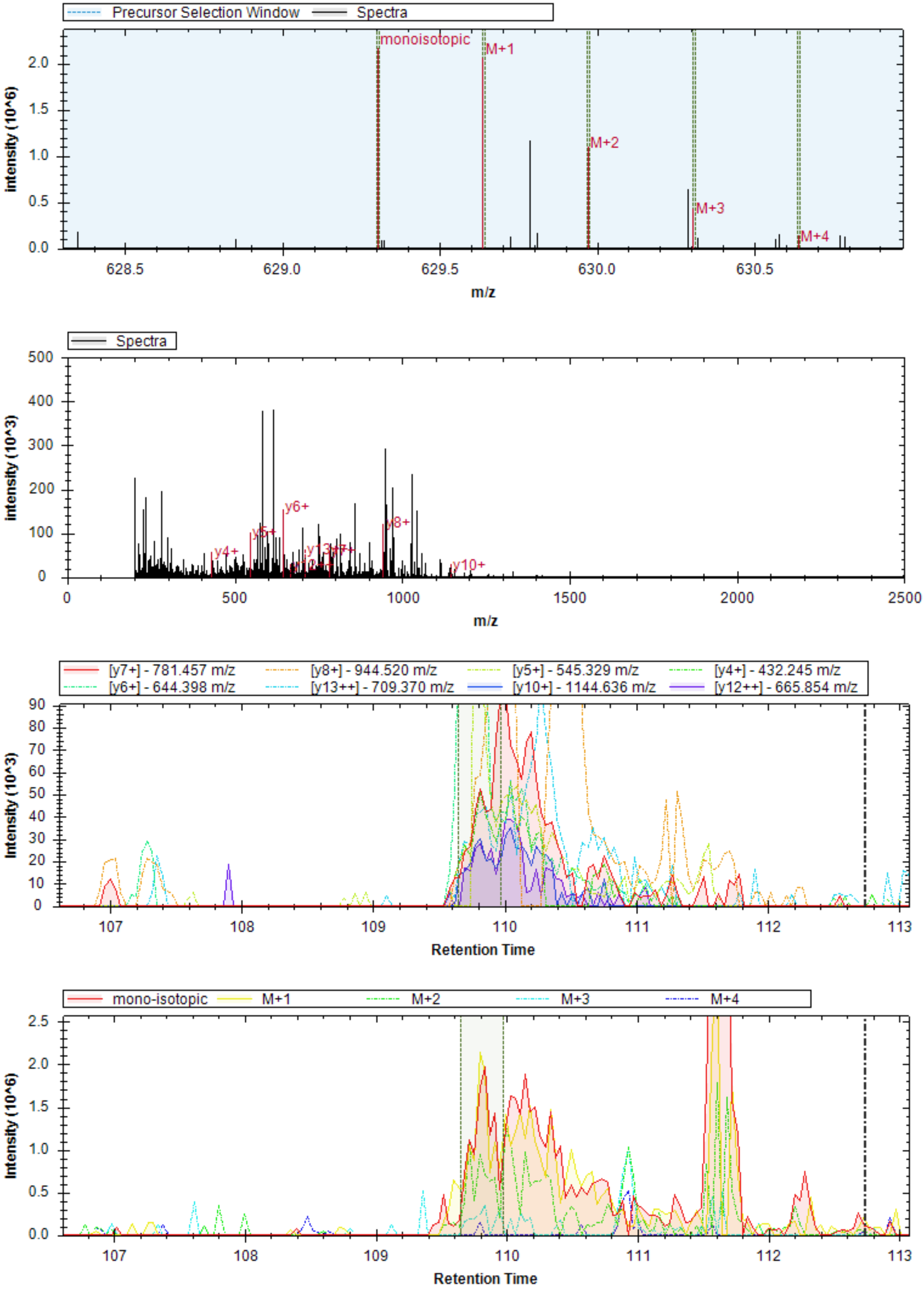

P39447 – _SSGIIRLHT[+H+O3+P]IK_.2

Fig4_MP-DIA-barrel-cortex-p54-1_MHRM.htrms

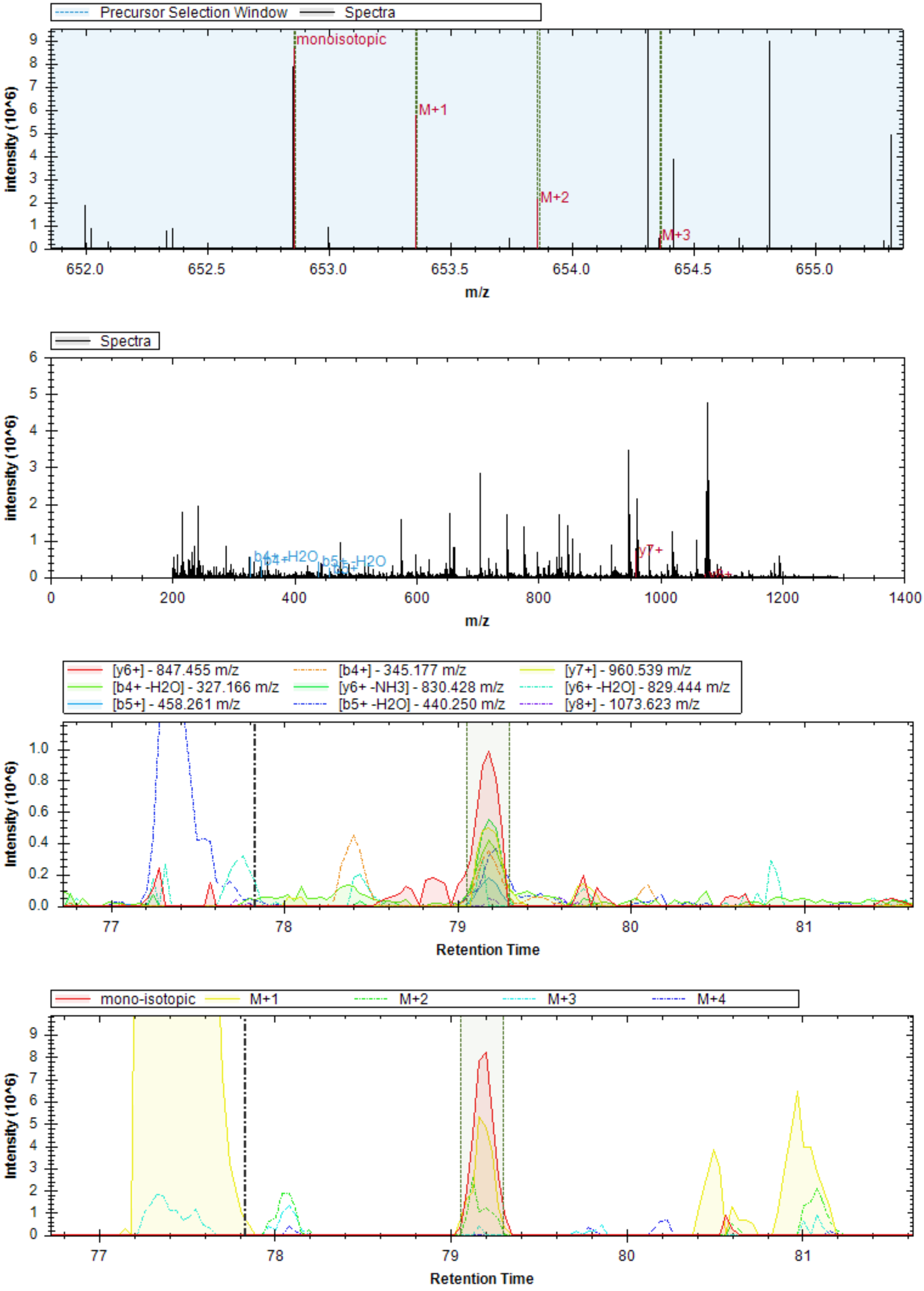

Q8BLJ3 – _M[+O]AS[+H+O3+P]SQGKNELK_.2

Fig4_MP-DIA-barrel-cortex-p54-1_MHRM.htrms

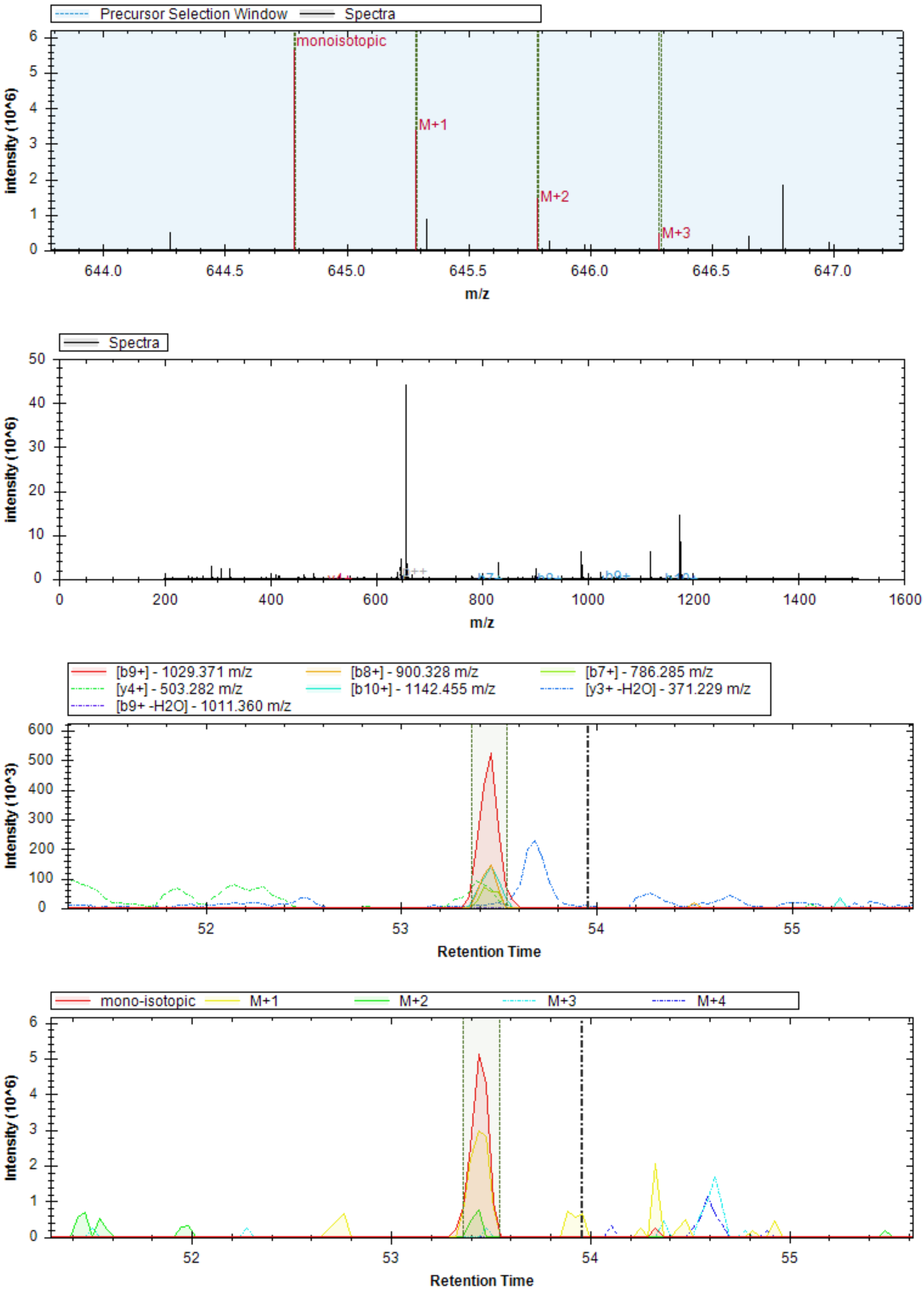

P13595 – _GVTASS[+H+O3+P]SSPASAPK_.2
Fig4_MP-DIA-barrel-cortex-p54-1_MHRM.htrms

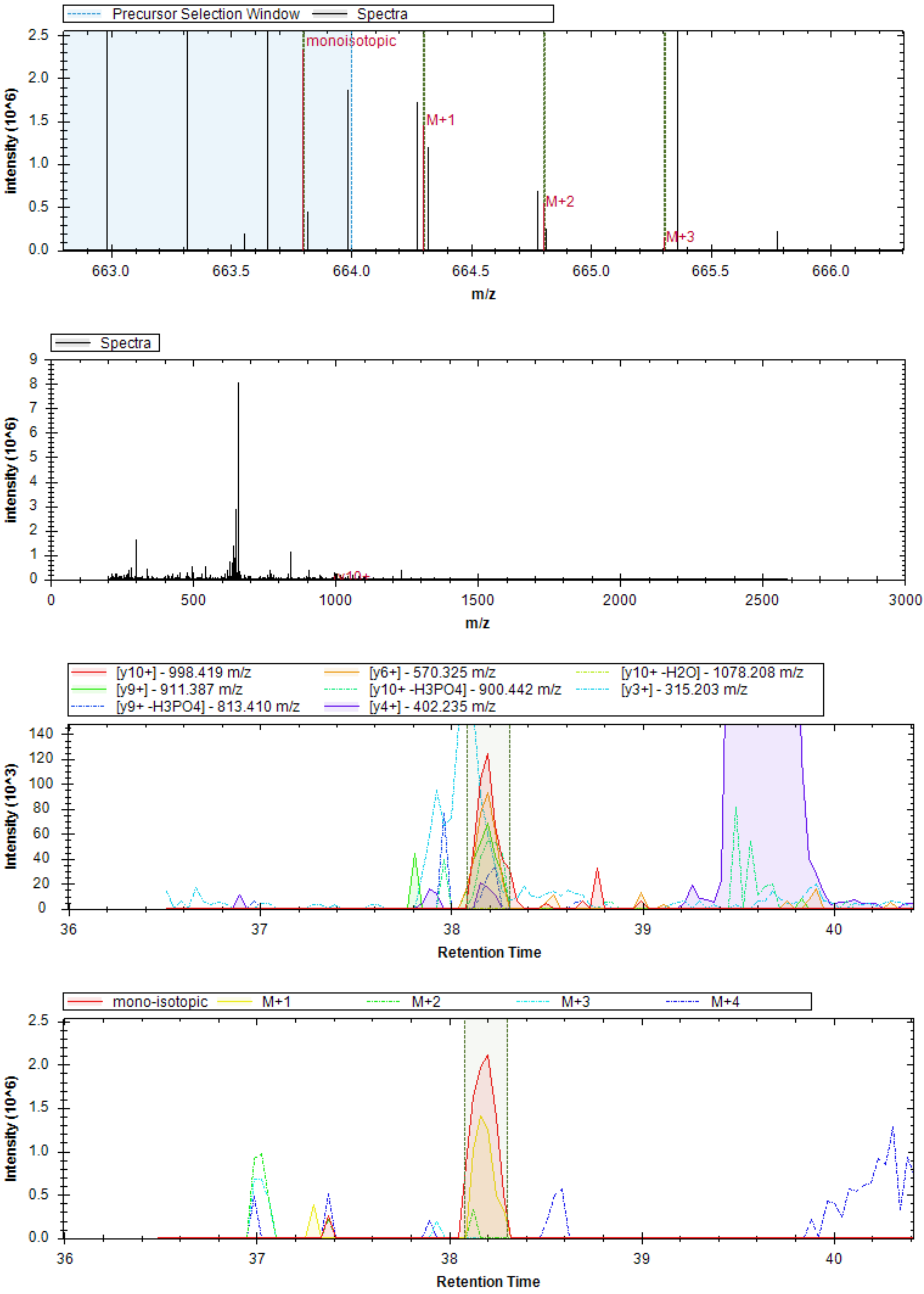

Q6A078 – _T[+H+O3+P]IGLM[+O]KKVVEK_.2

Fig4_MP-DIA-barrel-cortex-p54-1_MHRM.htrms

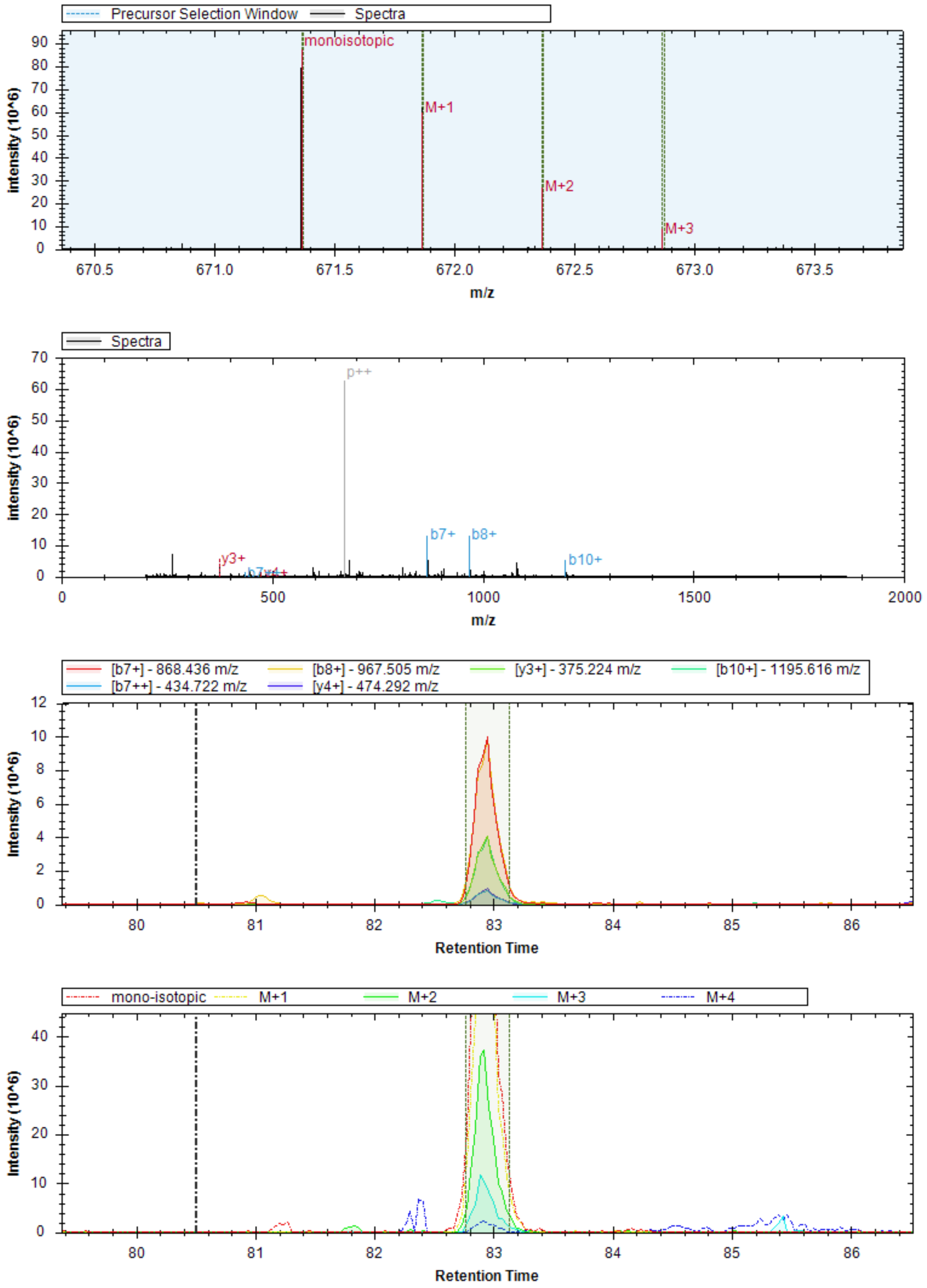

Q8CDU4;Q8CDU4-2 – _[+C2+H2+O]ASLRS[+H+O3+P]ATPRLR_.2

Fig4_MP-DIA-barrel-cortex-p54-1_MHRM.htrms

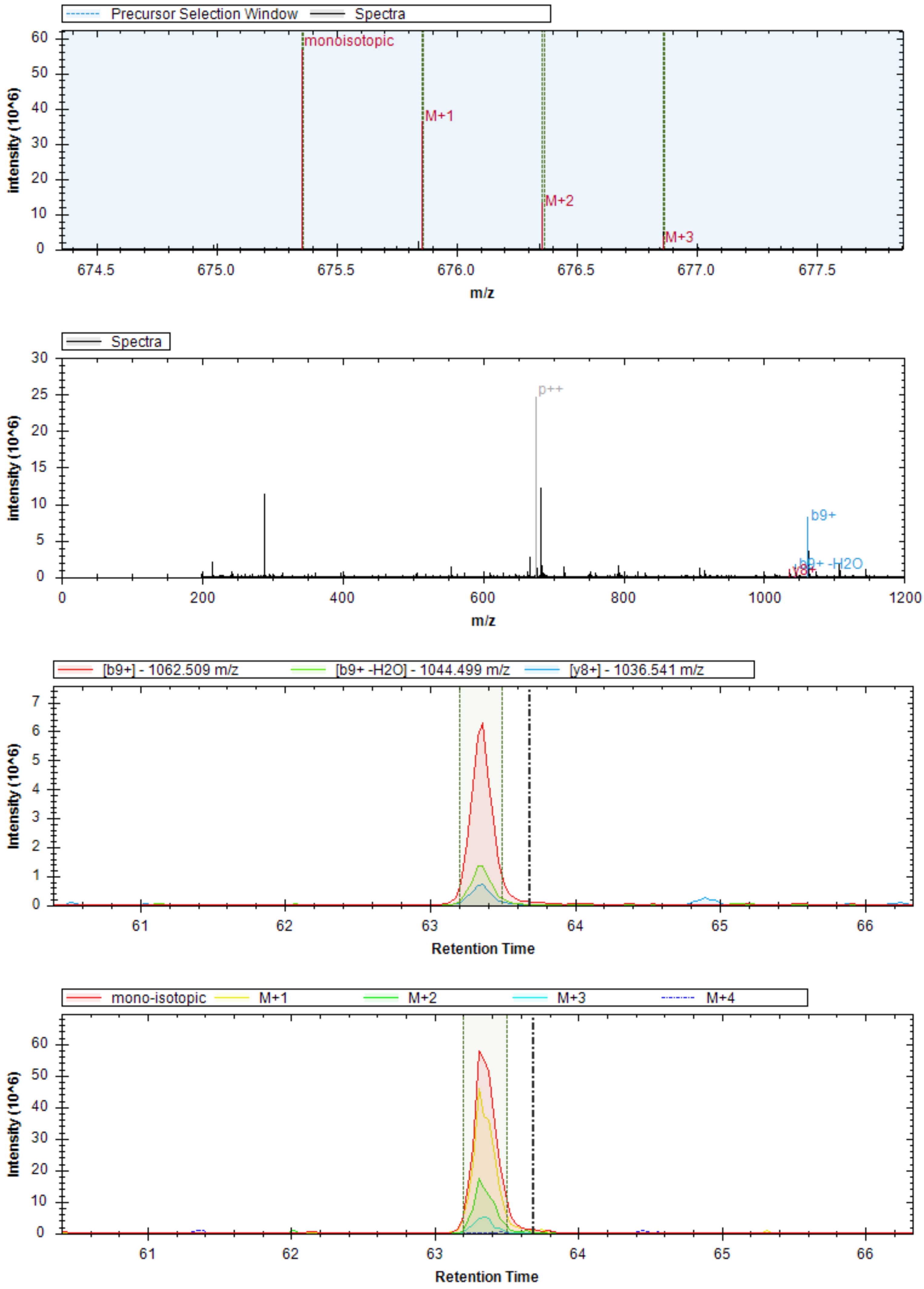

P28738 – _PIRPGHYPAS[+H+O3+P]SPTAVHAVR_.3
Fig4_MP-DIA-barrel-cortex-p54-1_MHRM.htrms

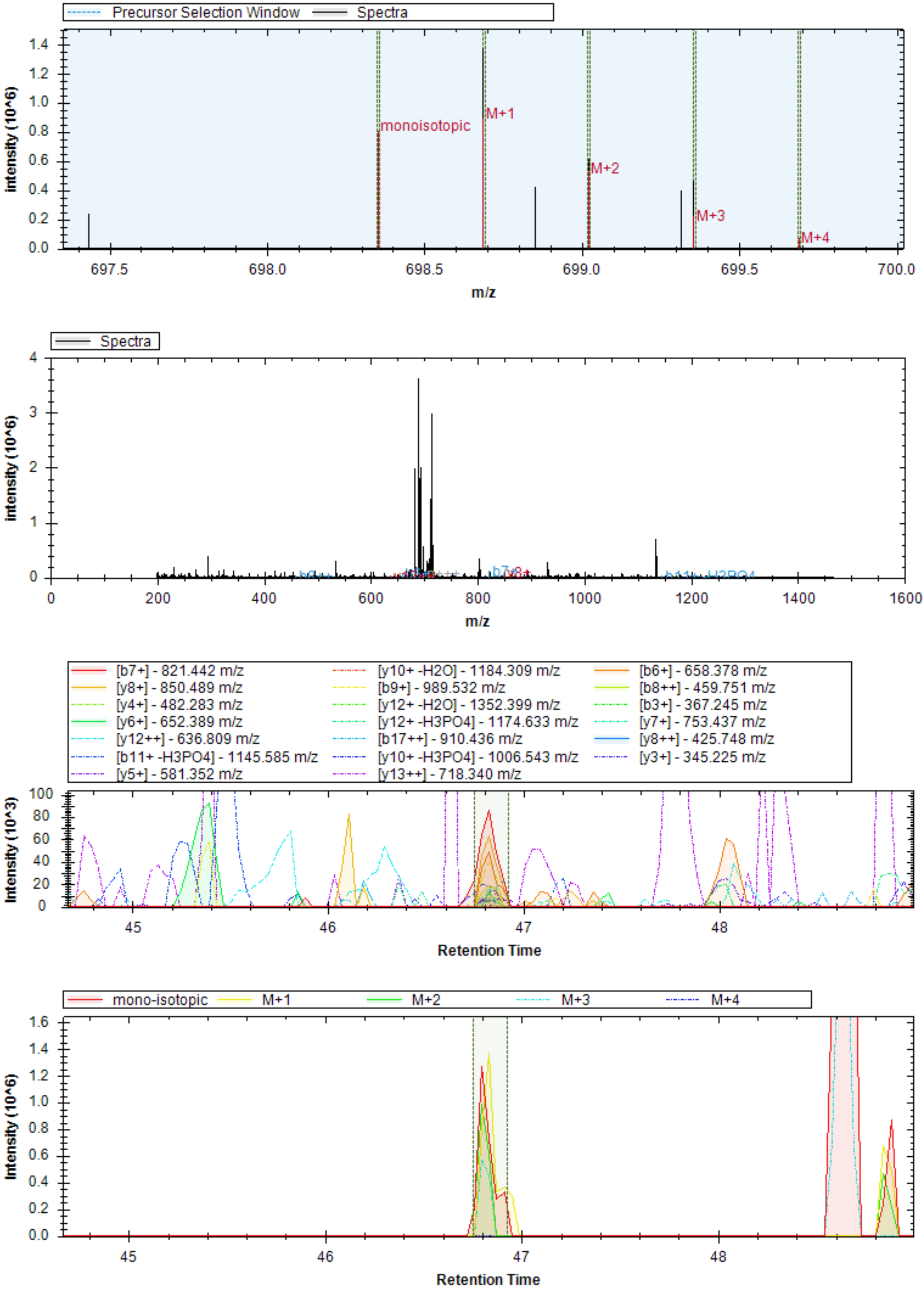

P28738 – _PIRPGHYPASS[+H+O3+P]PTAVHAVR_.3

Fig4_MP-DIA-barrel-cortex-p54-1_MHRM.htrms

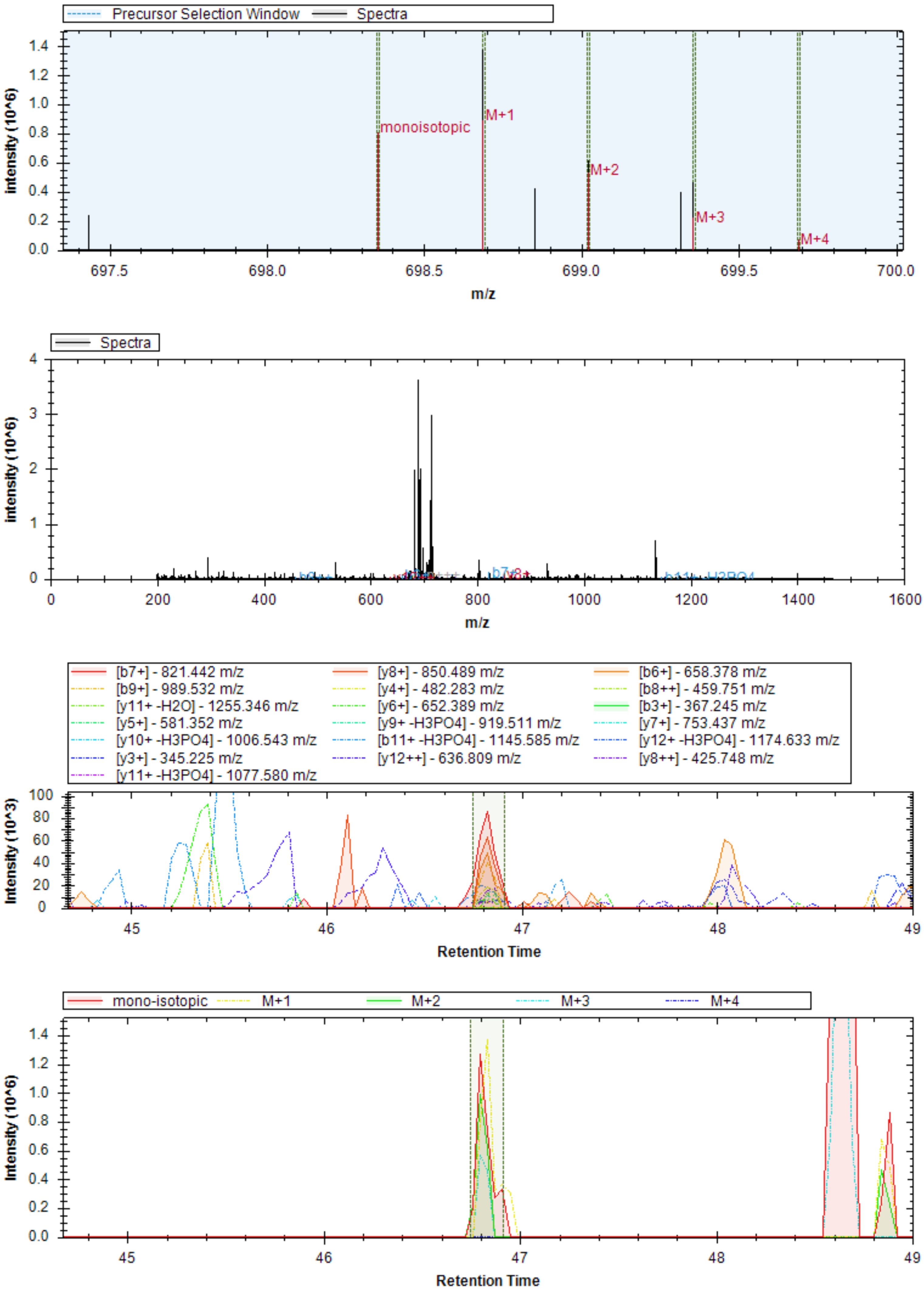

P62751 – _KAY[+H+O3+P]VRLAPDYDALDVANK_.3
Fig4_MP-DIA-barrel-cortex-p54-1_MHRM.htrms

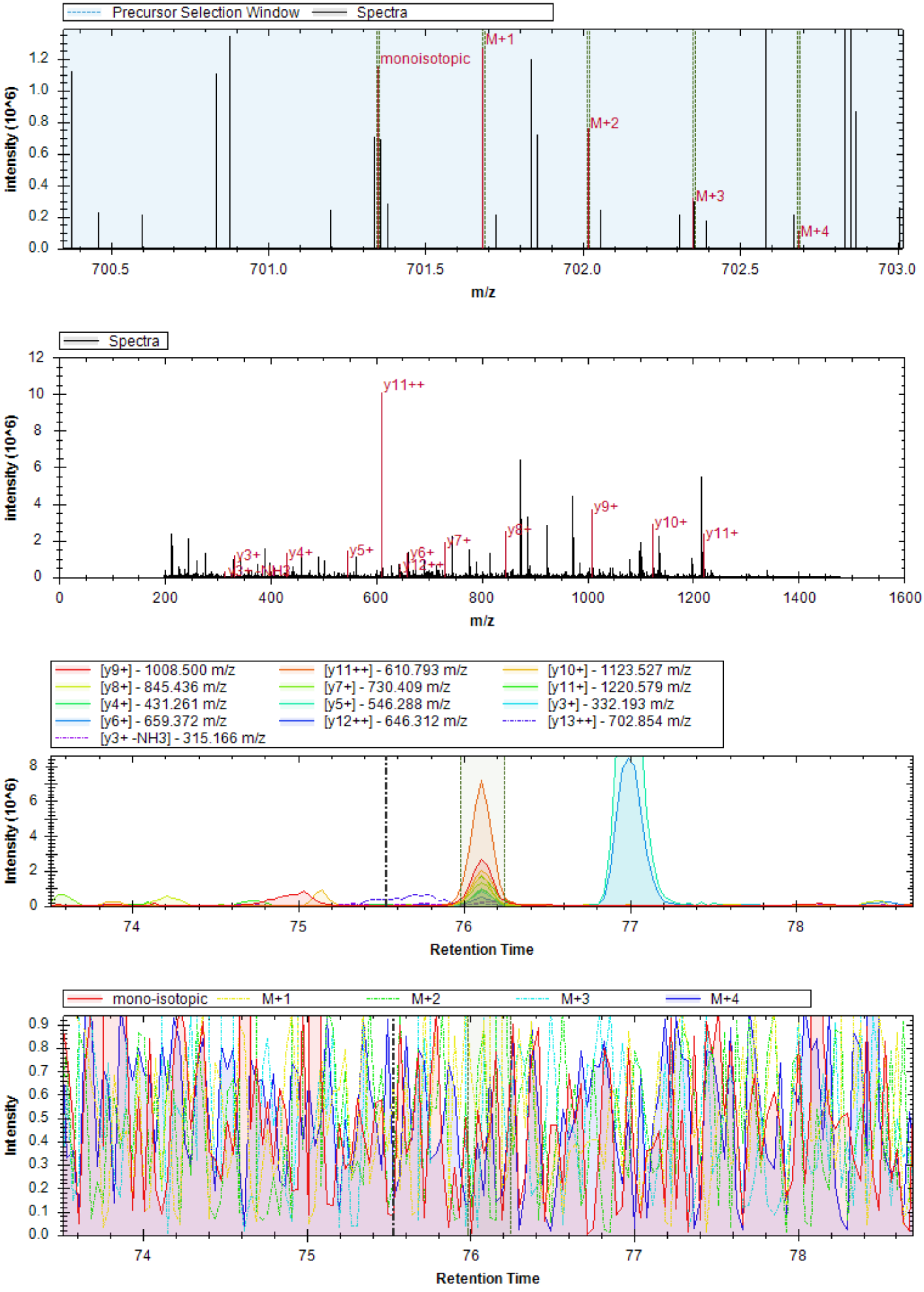

Q3U4B4 – _RHKT[+H+O3+P]LSANEEK_.2
Fig4_MP-DIA-barrel-cortex-p54-1_MHRM.htrms

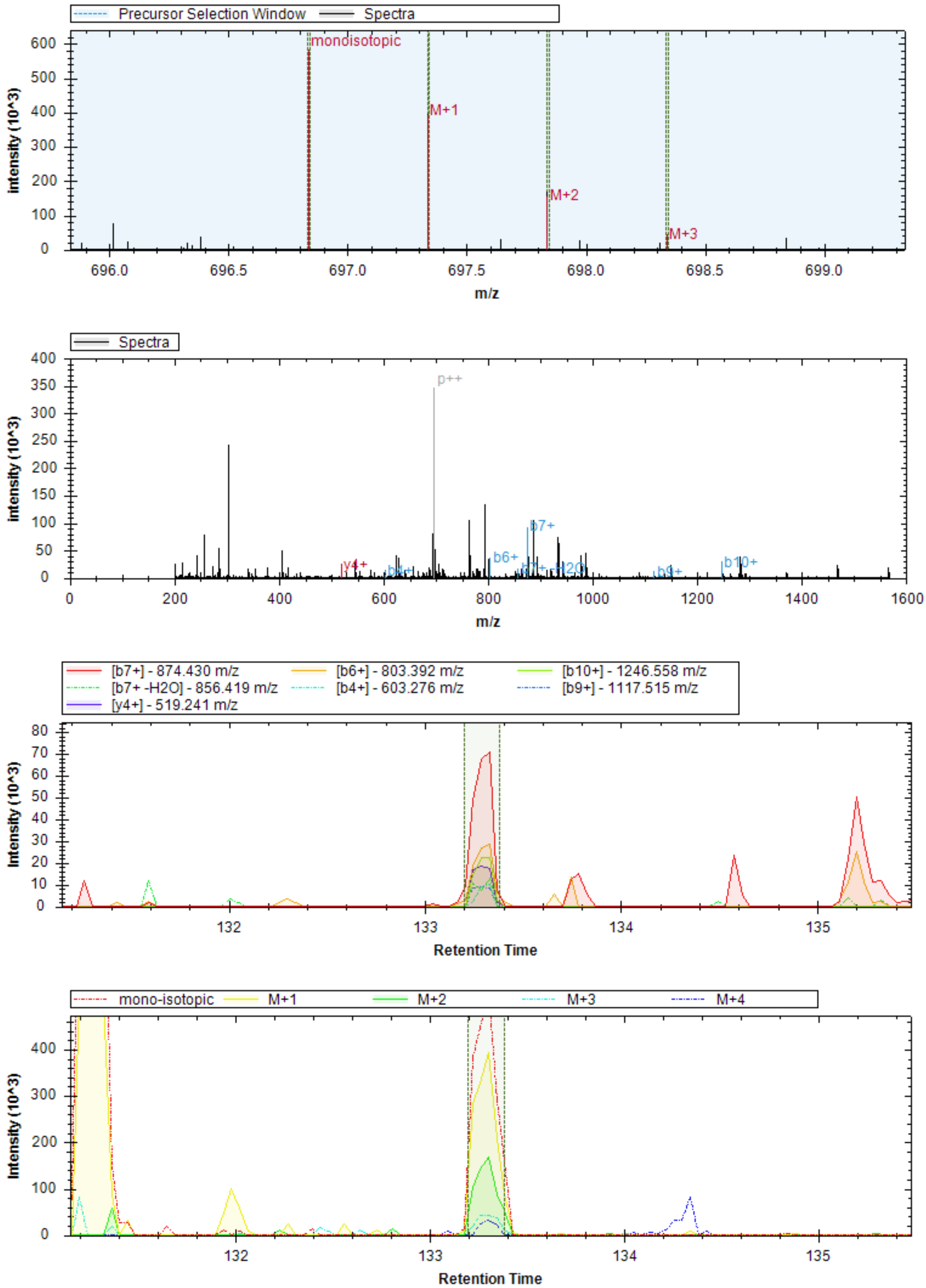

P10637 – _SGYSS[+H+O3+P]PGSPGTPGSR_.2
Fig4_MP-DIA-barrel-cortex-p54-1_MHRM.htrms

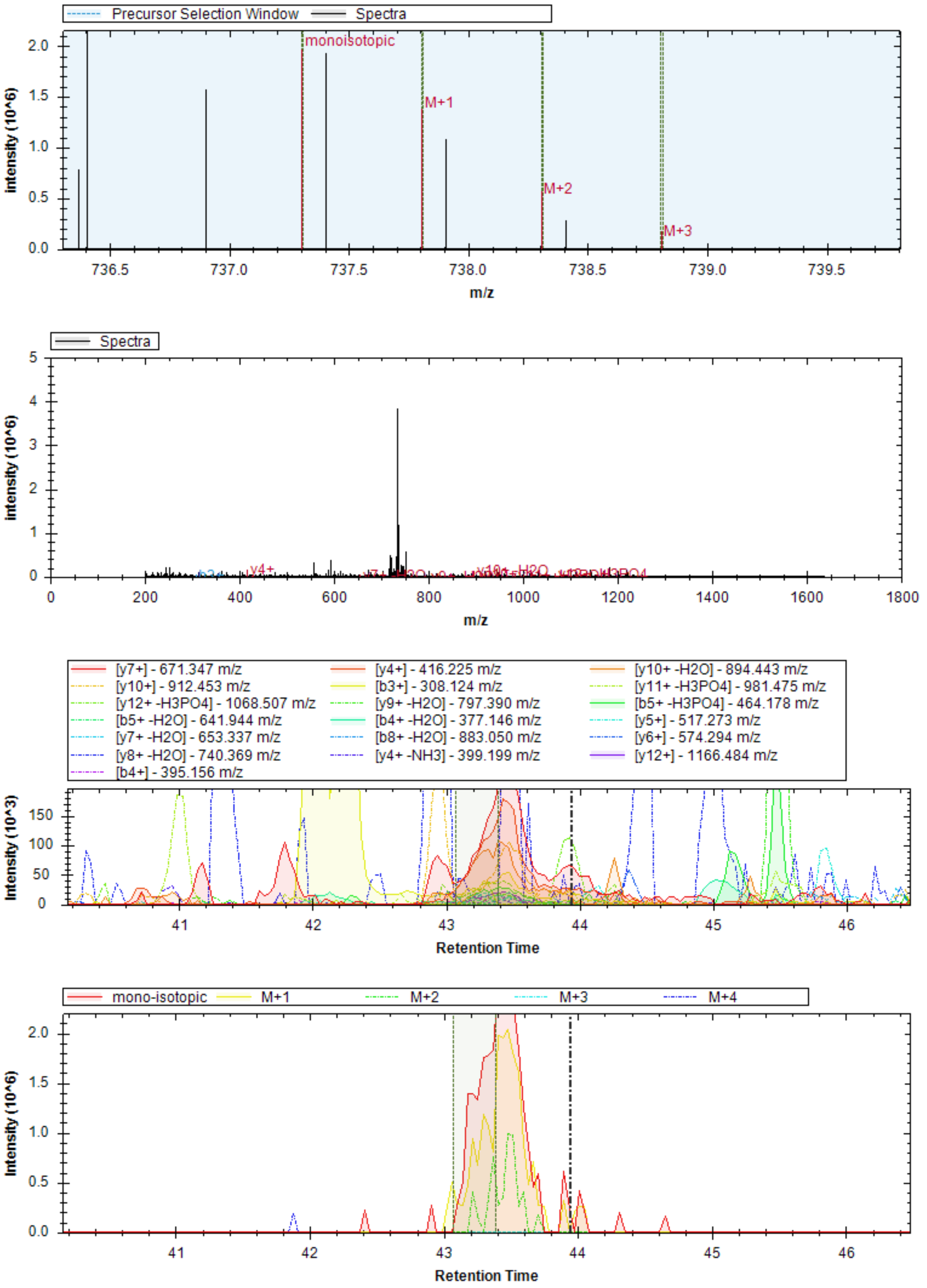

P10637 – _SGYSSPGS[+H+O3+P]PGTPGSR_.2

Fig4_MP-DIA-barrel-cortex-p54-1_MHRM.htrms

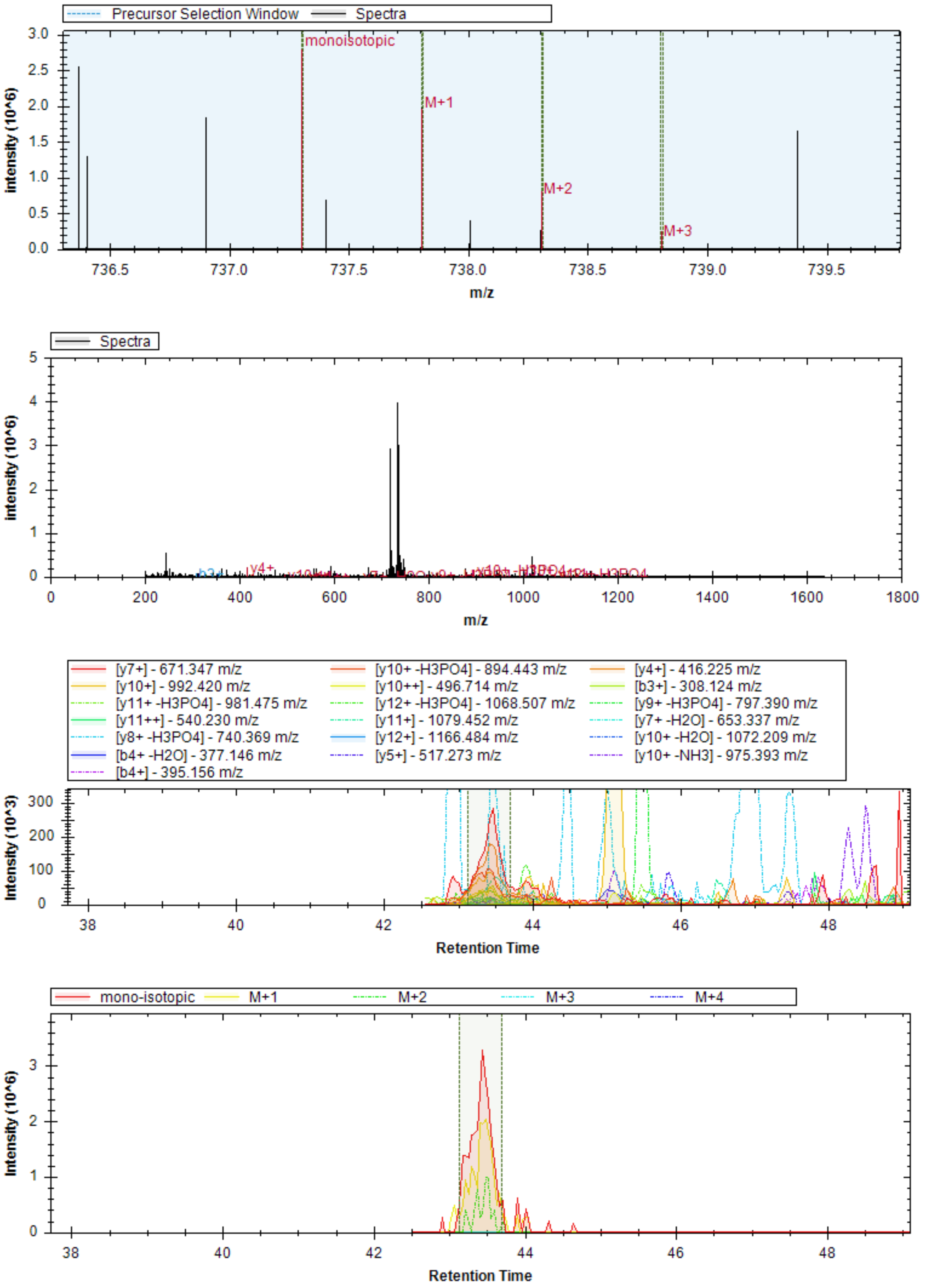

Q8C5R2 – _LAGNEALS[+H+O3+P]PTSPSK_.2

Fig4_MP-DIA-barrel-cortex-p54-1_MHRM.htrms

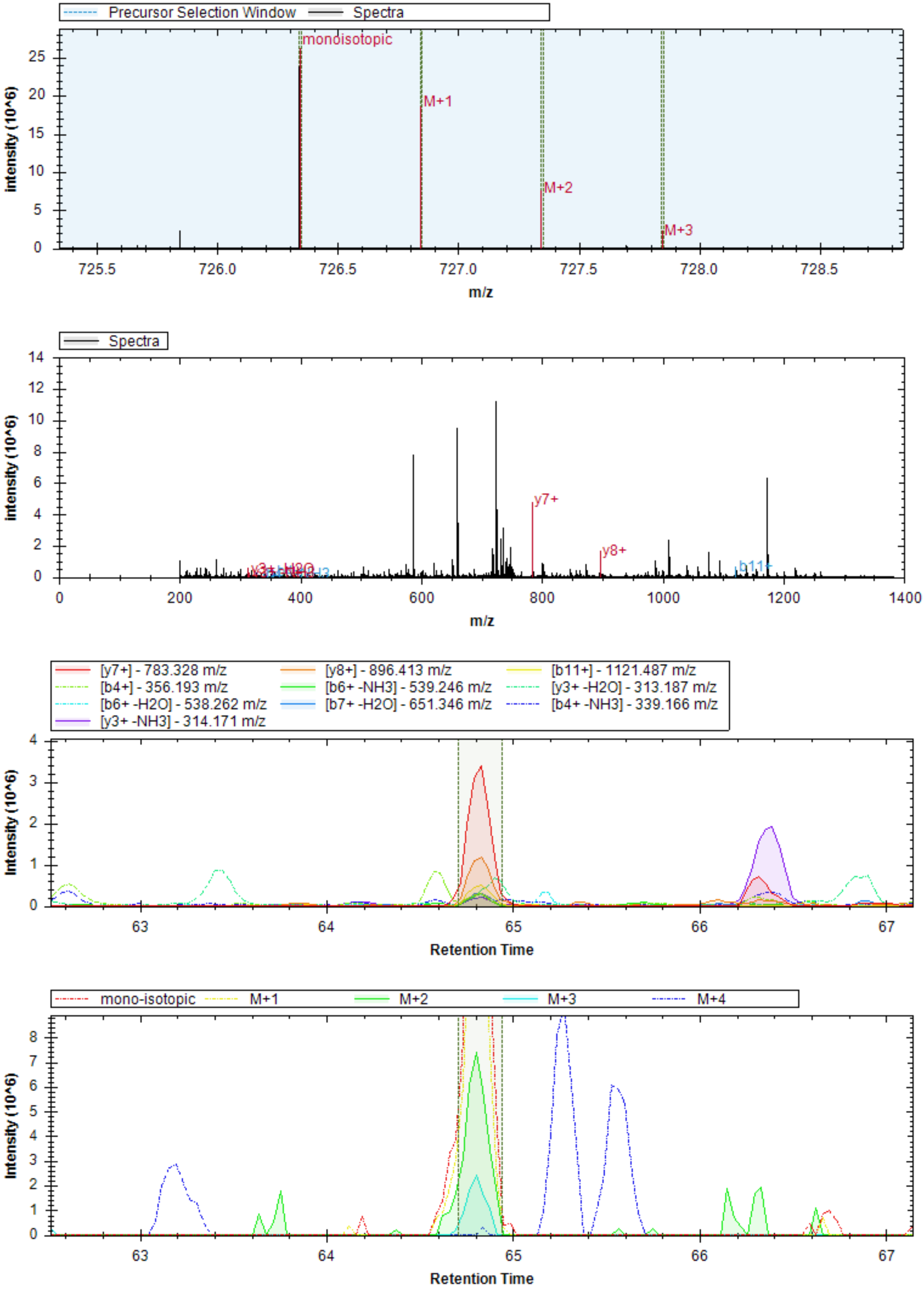

D3Z6S9 – _IPSIEIT[+H+O3+P]ET[+H+O3+P]LPQTQASMLR_.3
Fig4_MP-DIA-barrel-cortex-p54-1_MHRM.htrms

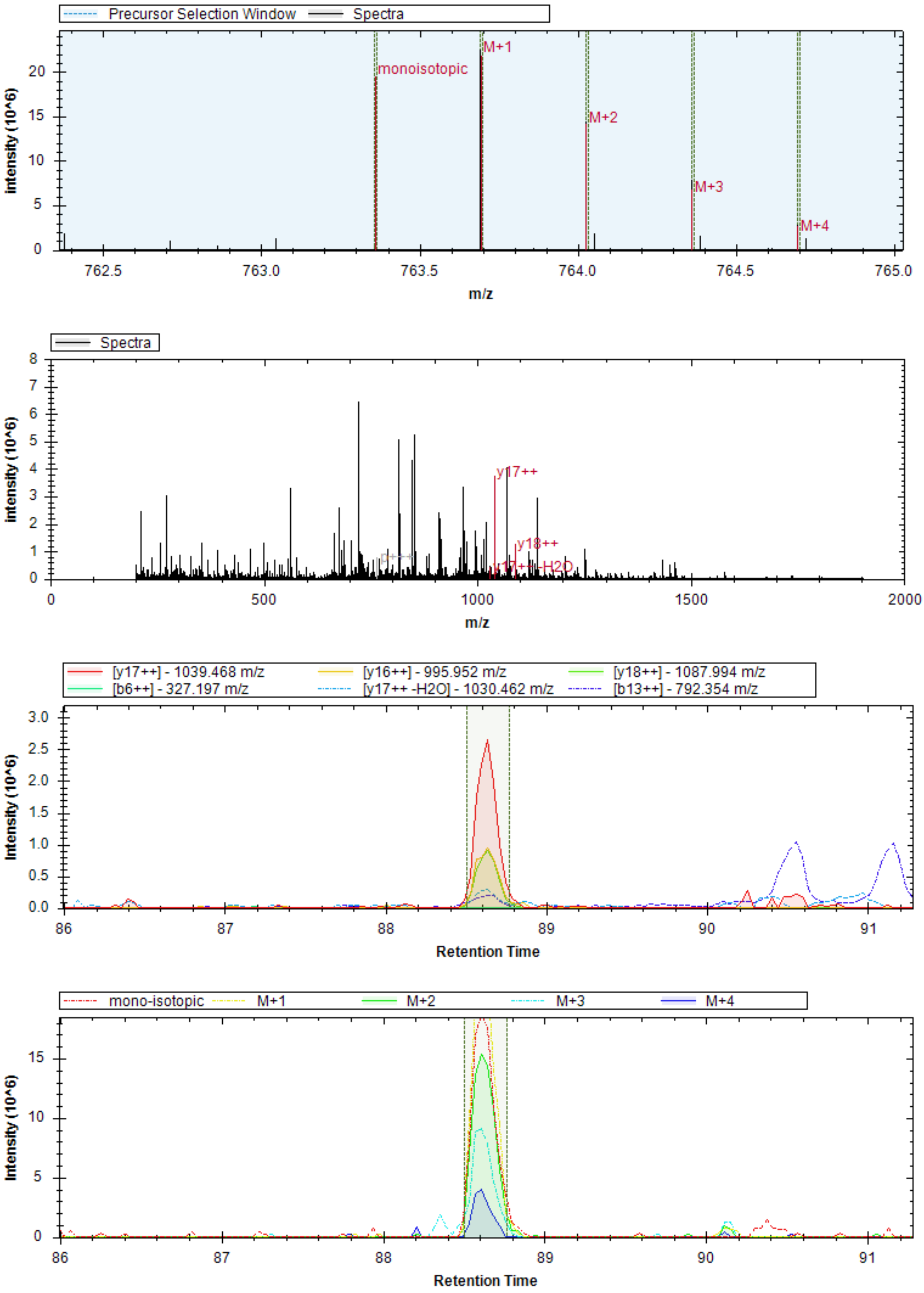

Q68FD9 – _LAQLLS[+H+O3+P]EVPT[+H+O3+P]RR_.2
Fig4_MP-DIA-barrel-cortex-p54-1_MHRM.htrms

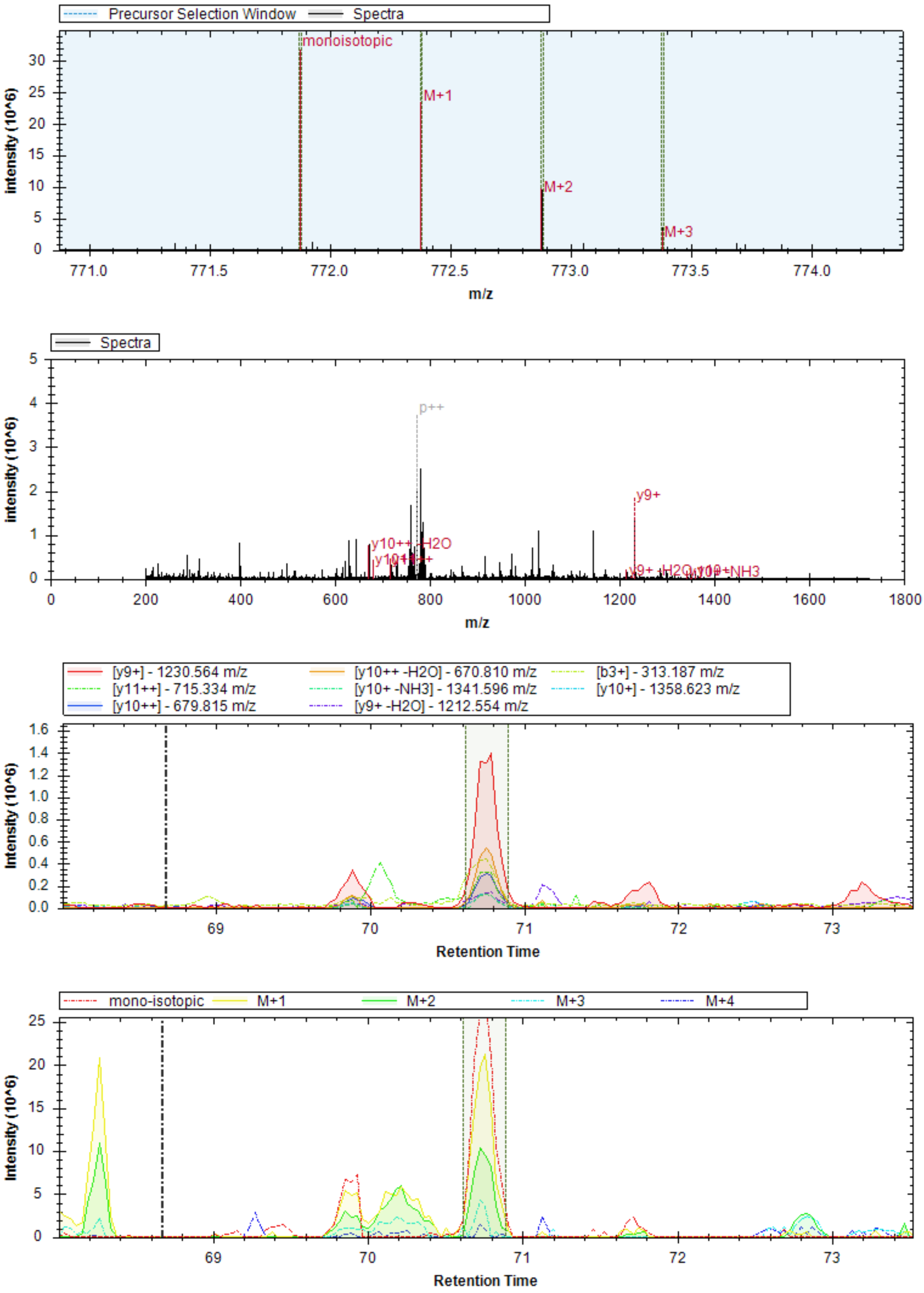

Q6ZQA0;Q6ZQA0-2 – _LHT[+H+O3+P]LLQT[+H+O3+P]AVPTR_.2
Fig4_MP-DIA-barrel-cortex-p54-1_MHRM.htrms

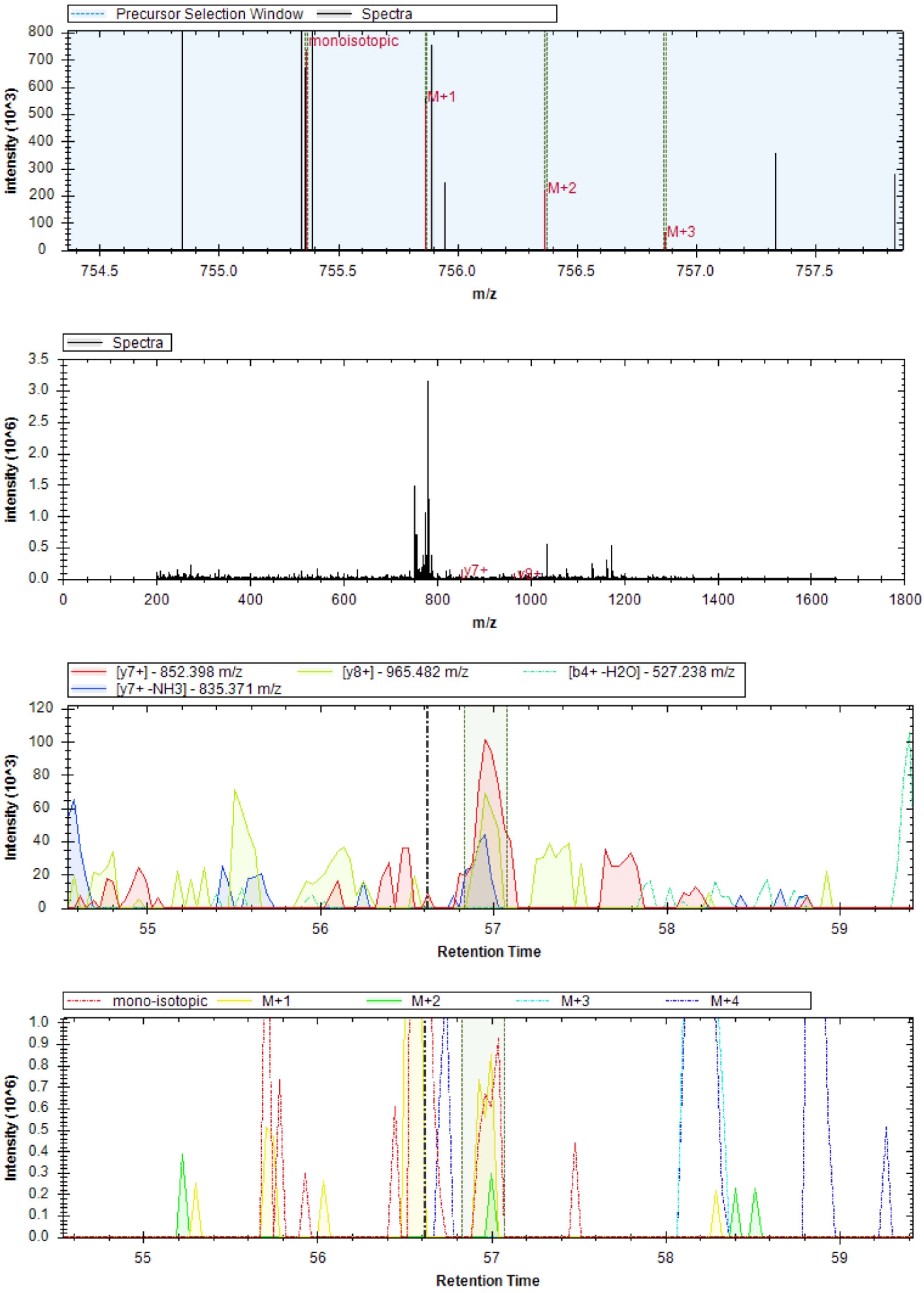

Q9QWI6 – _LSYAGGRPPSYAGS[+H+O3+P]PVHHAAER_.3
Fig4_MP-DIA-barrel-cortex-p54-1_MHRM.htrms

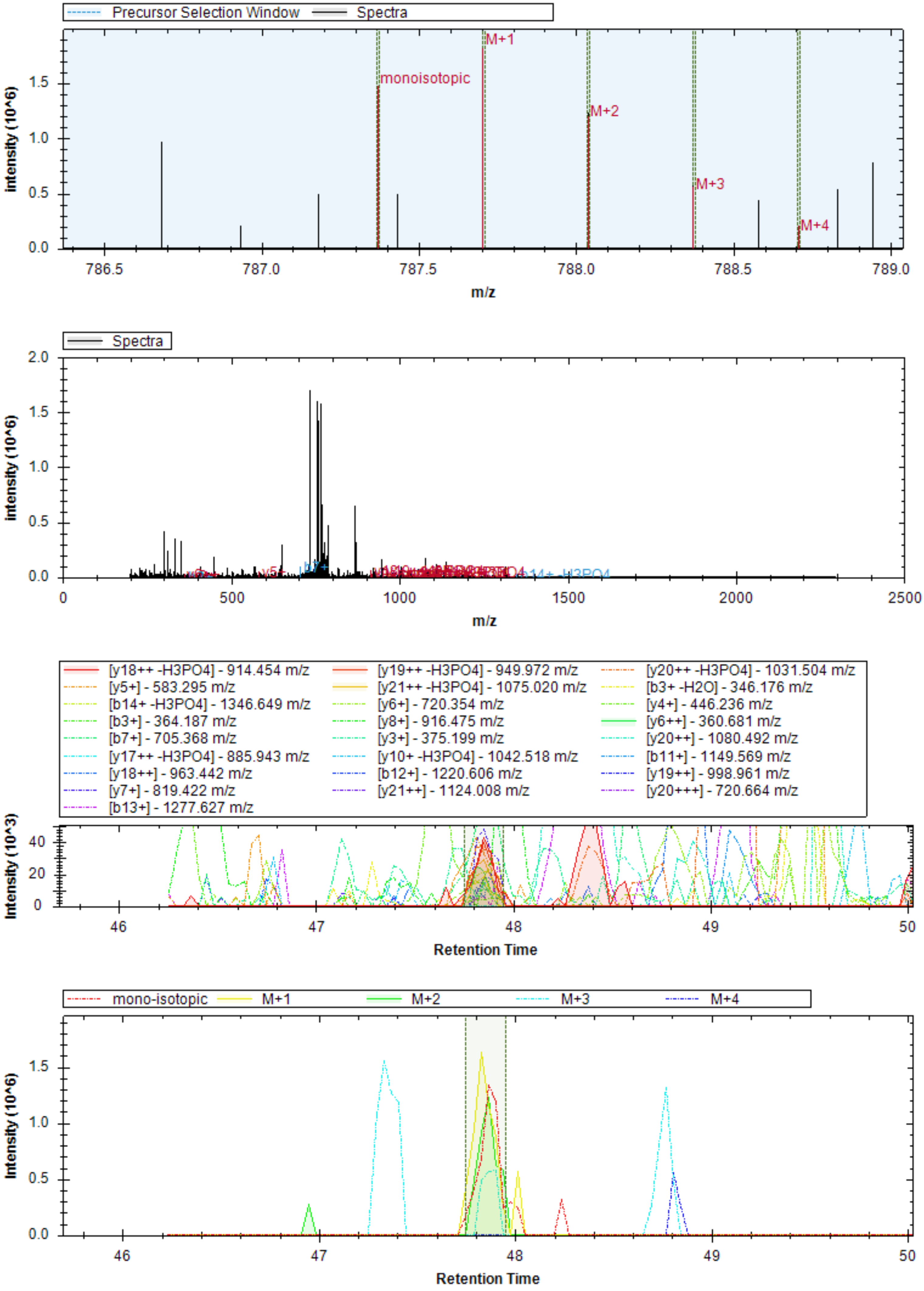

Q9QYC0 – _AAVVTS[+H+O3+P]PPPTTAPHK_.2

Fig4_MP-DIA-barrel-cortex-p54-1_MHRM.htrms

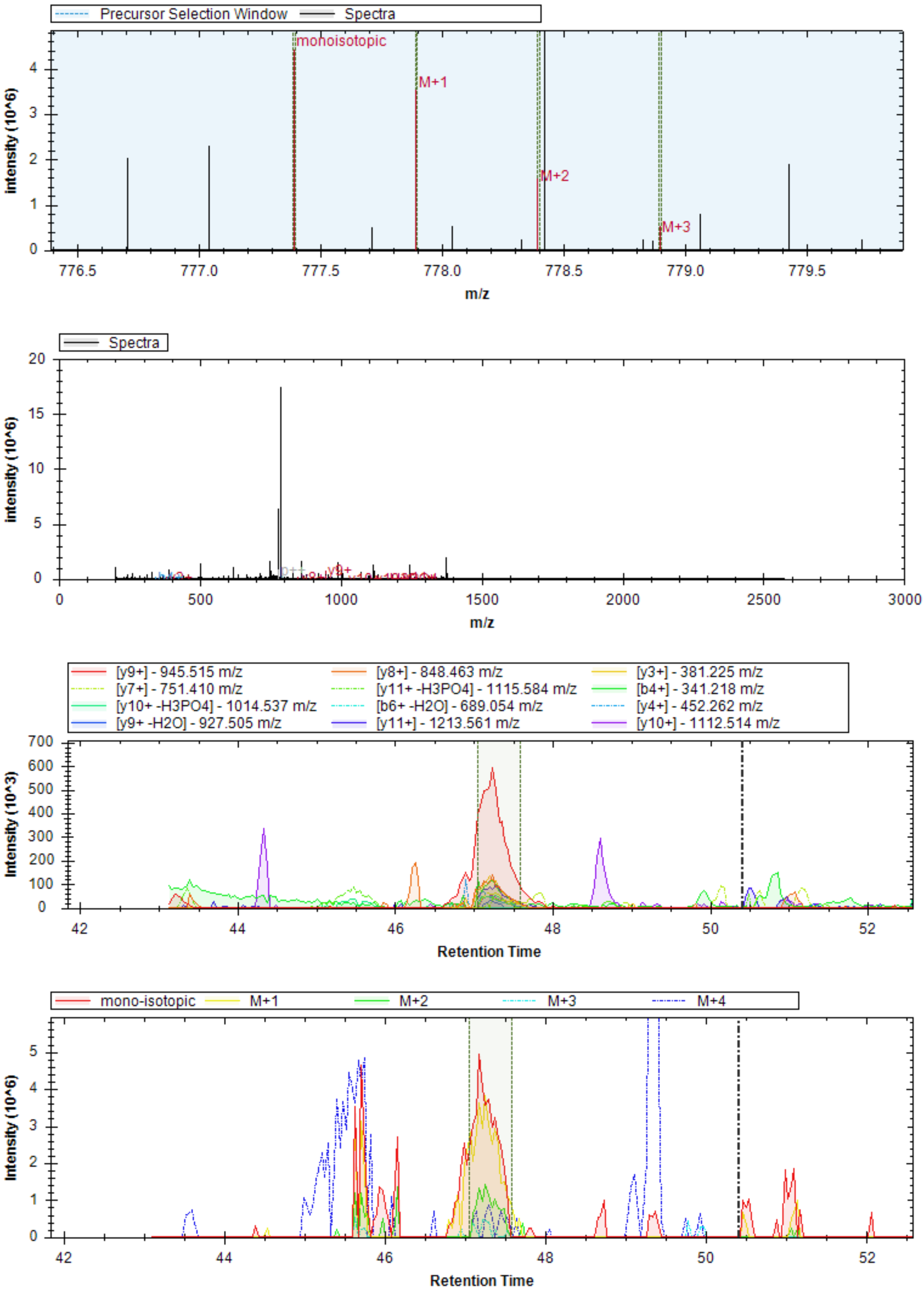

Q9QYR6-2 – _ELSSEPRT[+H+O3+P]PPAQK_.2
Fig4_MP-DIA-barrel-cortex-p54-1_MHRM.htrms

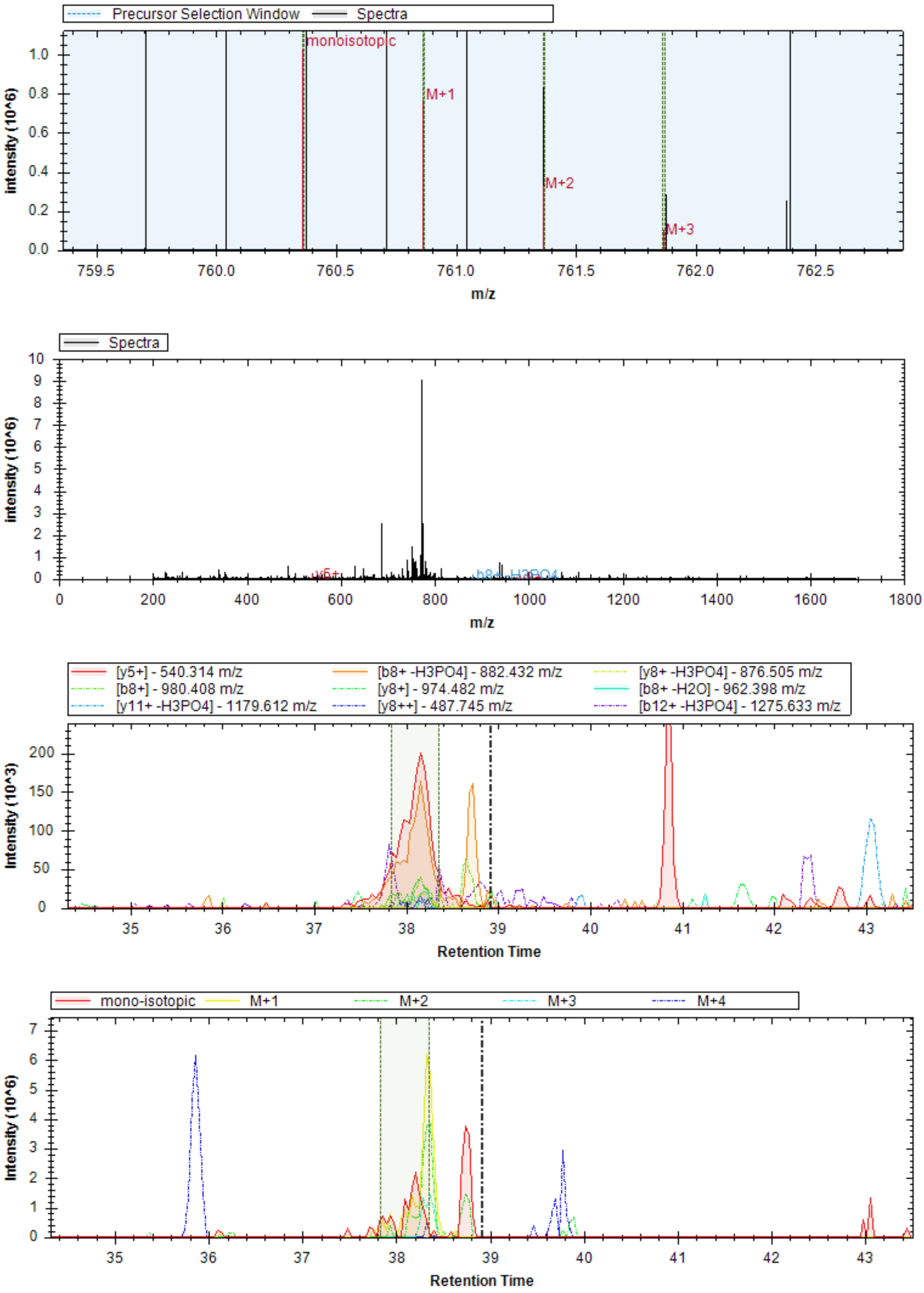

P10637 – _TTPSPKT[+H+O3+P]PPGSGEPPK_.2
Fig4_MP-DIA-barrel-cortex-p54-1_MHRM.htrms

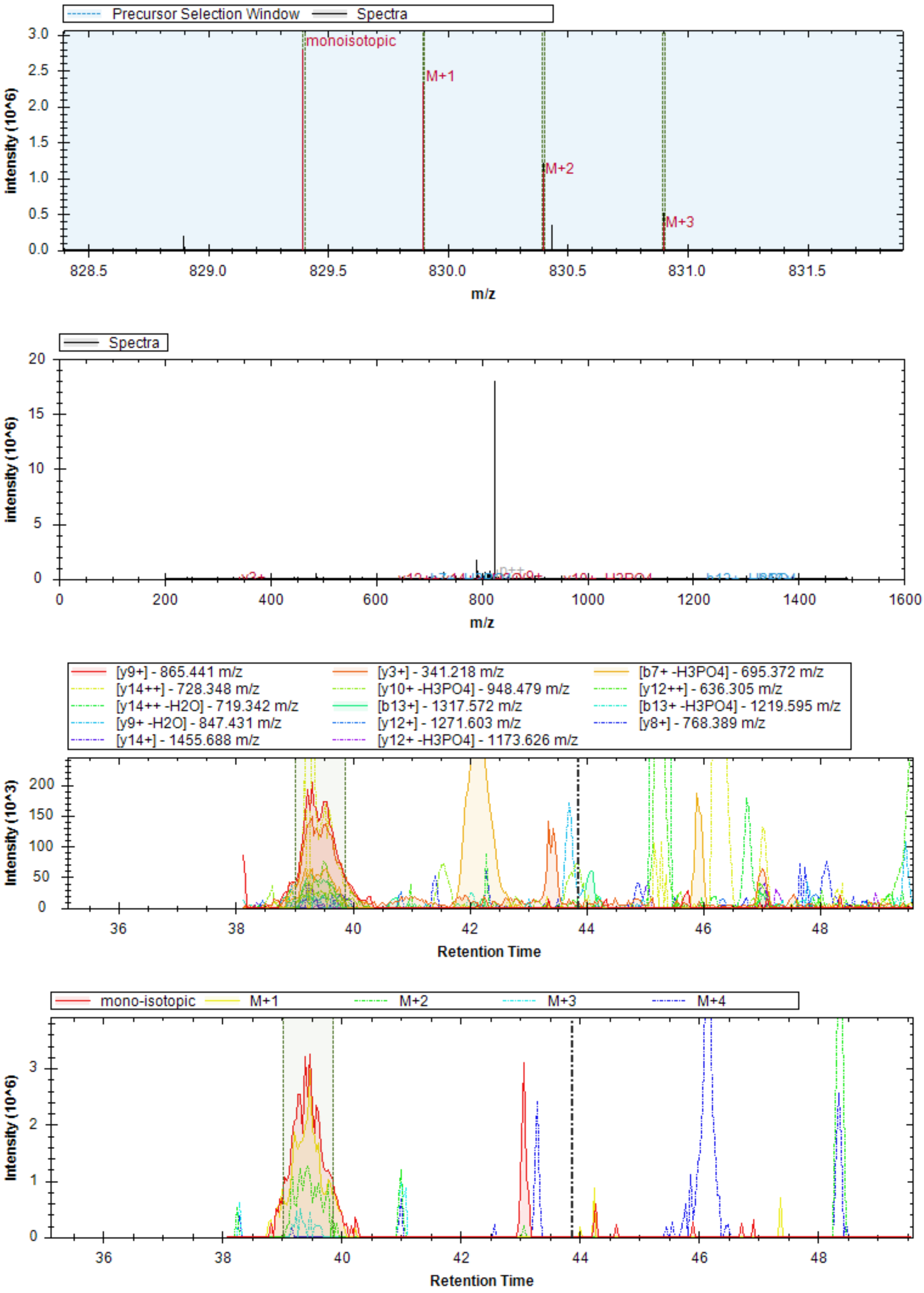

P20357 – _VDHGAEIITQSPS[+H+O3+P]R_.2
Fig4_MP-DIA-barrel-cortex-p54-1_MHRM.htrms

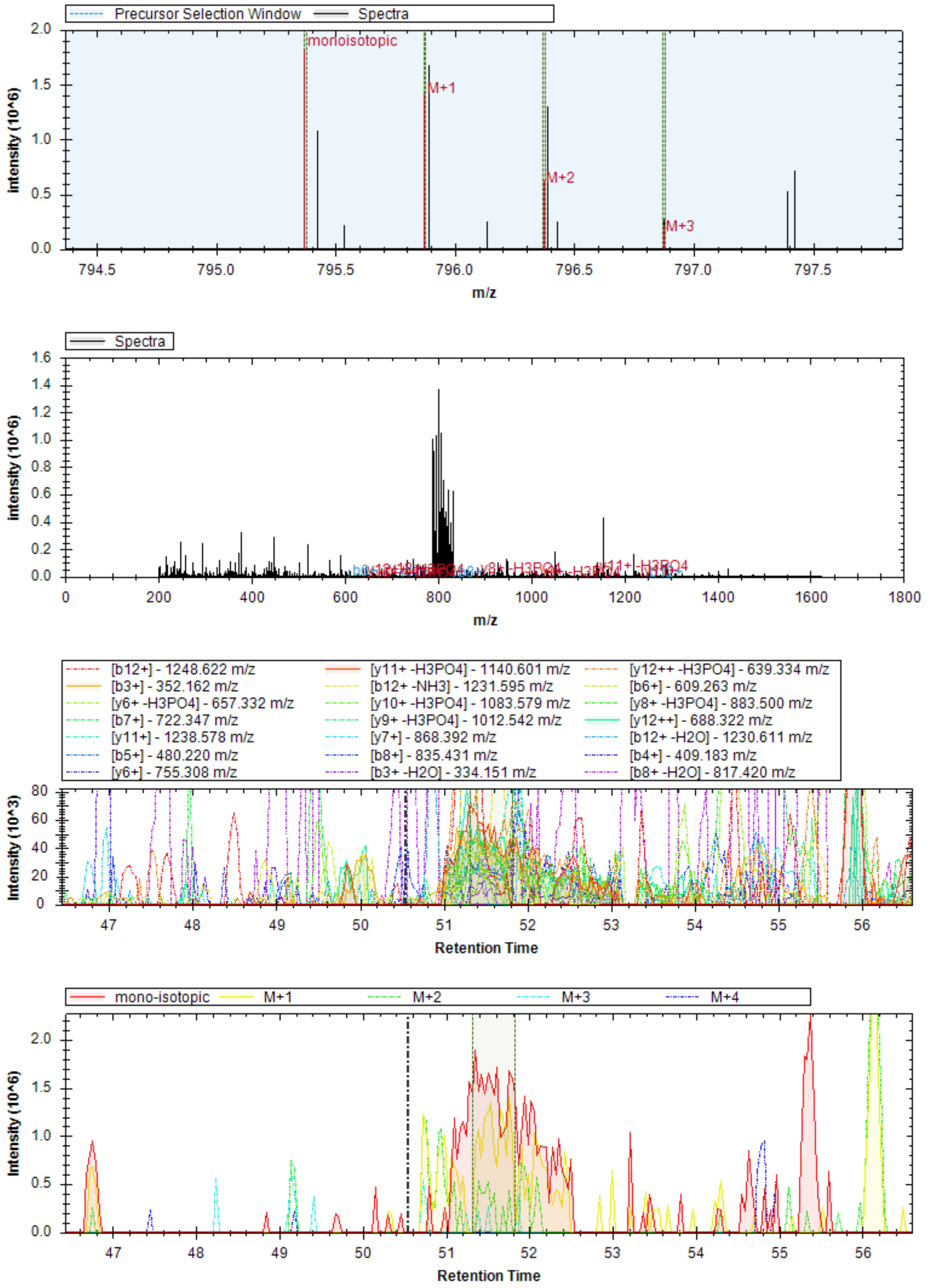

Q60771 – _FYYSS[+H+O3+P]GSSSPTHAK_.2
Fig4_MP-DIA-barrel-cortex-p54-1_MHRM.htrms

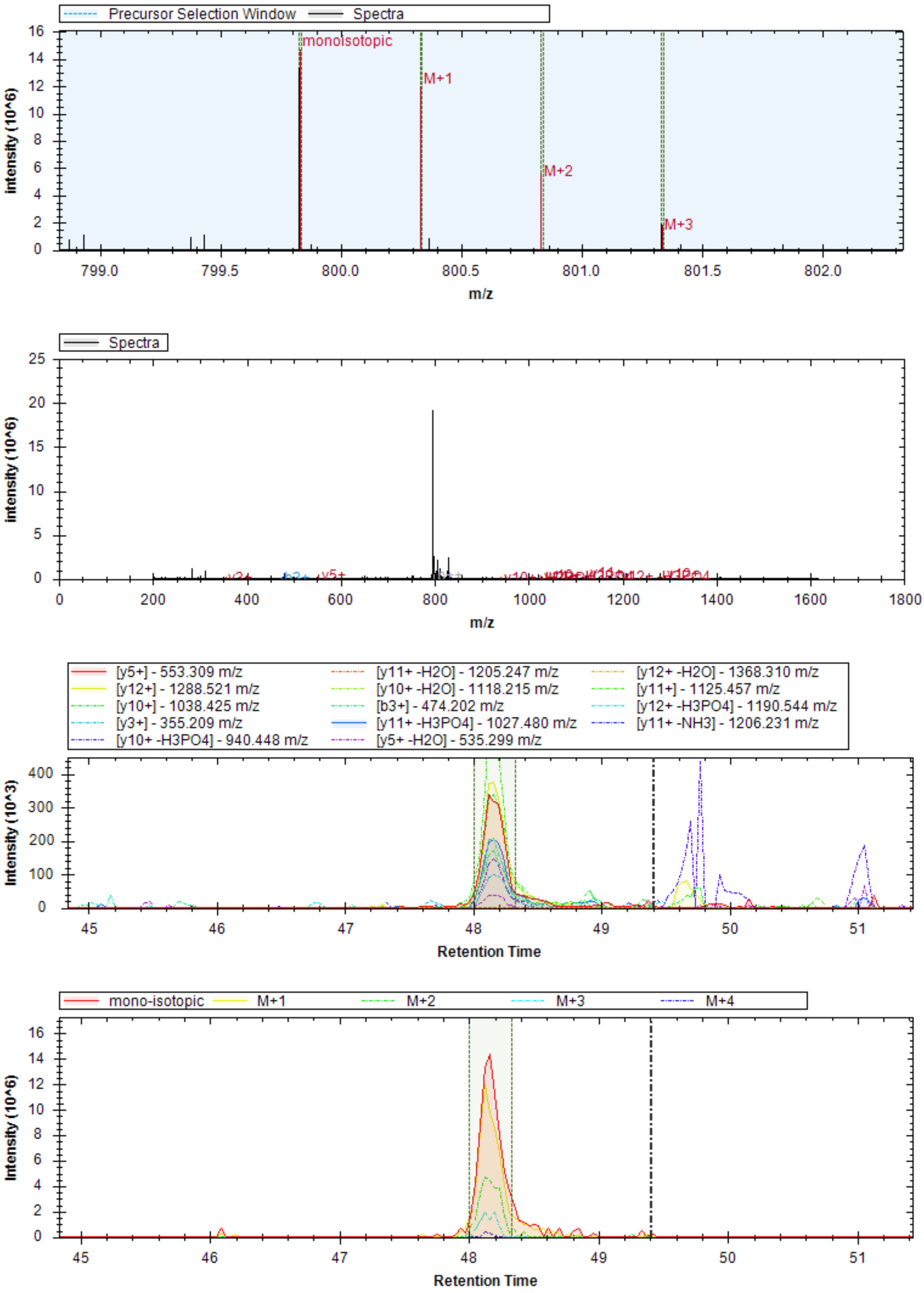

Q60771 – _FYYSSGS[+H+O3+P]SSPTHAK_.2
Fig4_MP-DIA-barrel-cortex-p54-1_MHRM.htrms

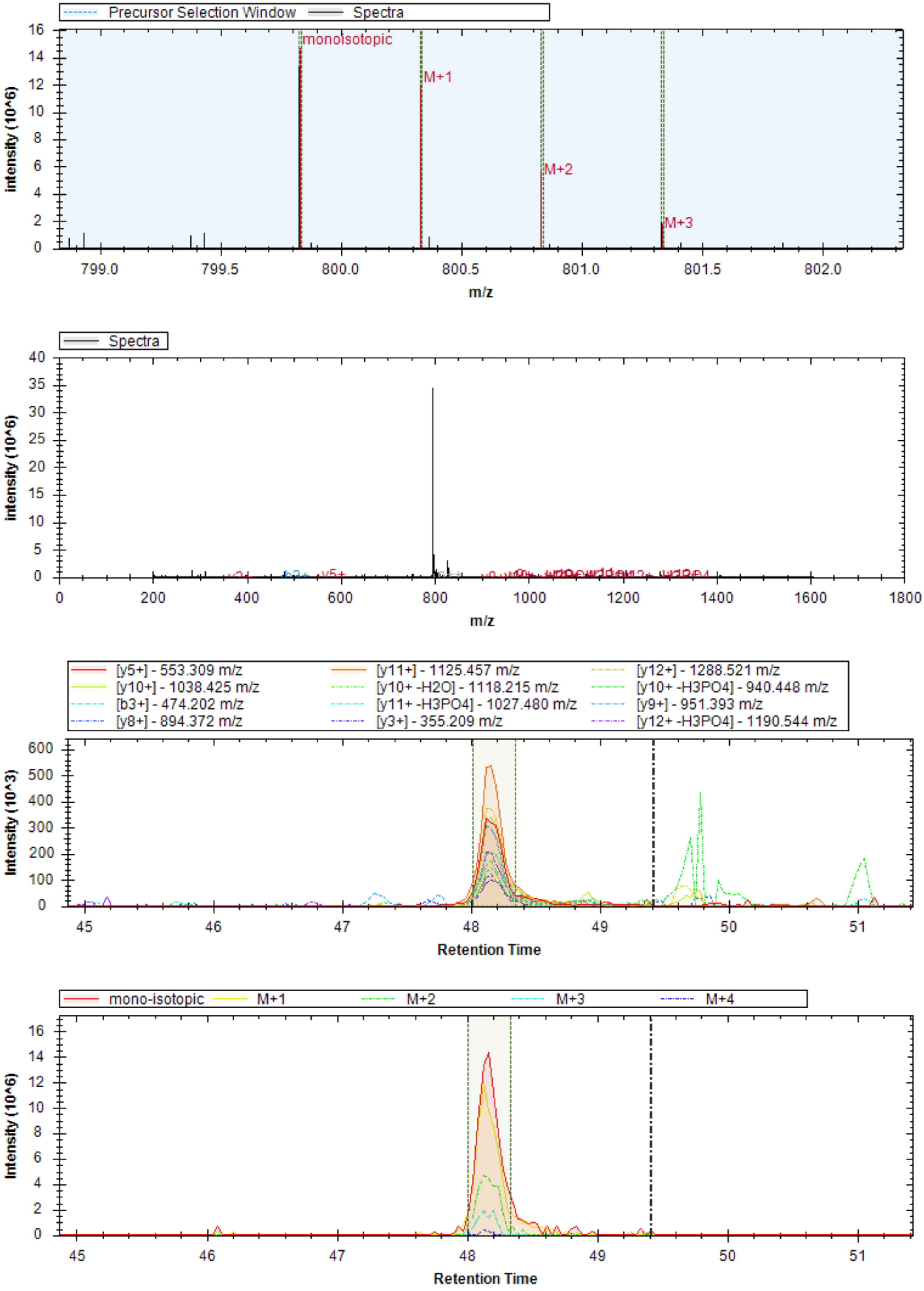

Q60771 – _FYYSSGSSS[+H+O3+P]PTHAK_.2
Fig4_MP-DIA-barrel-cortex-p54-1_MHRM.htrms

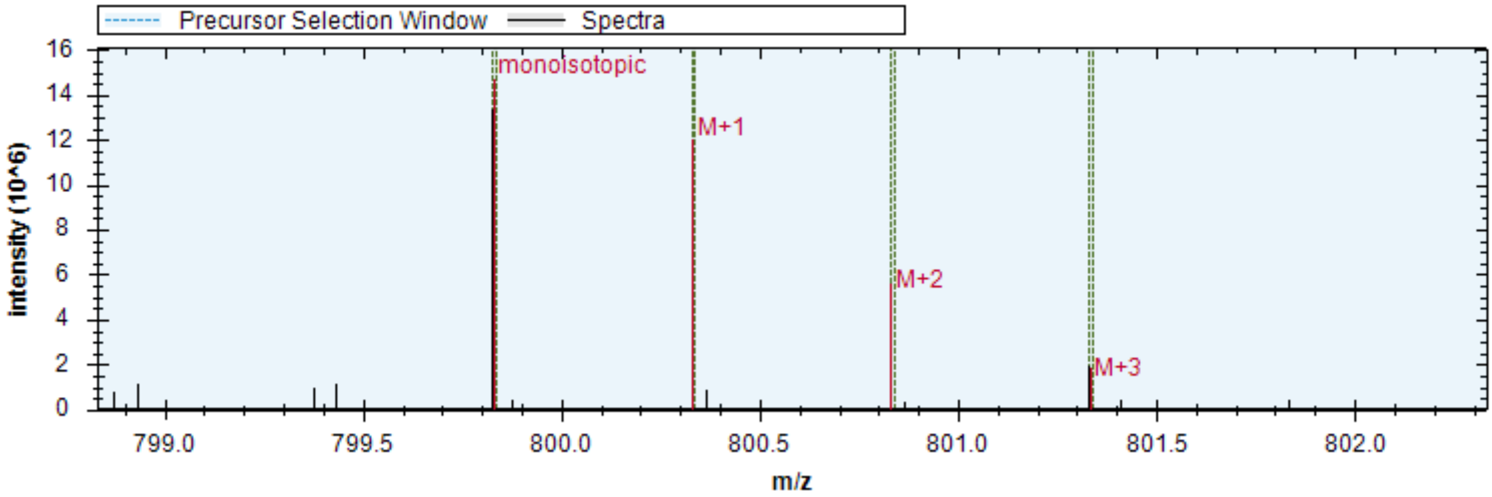

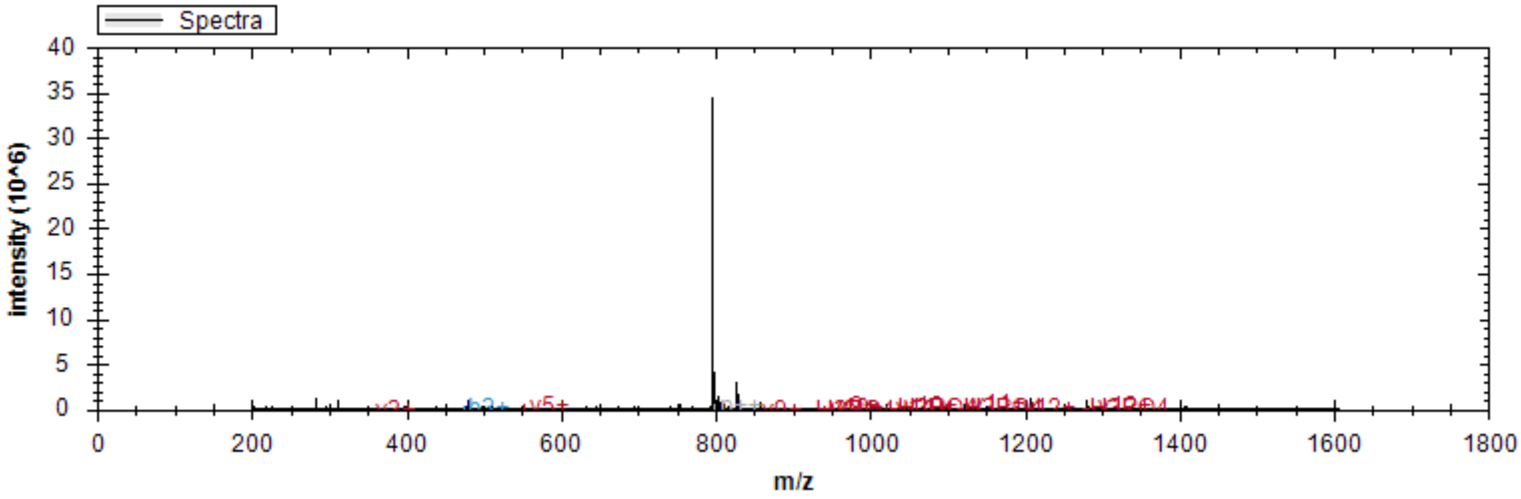

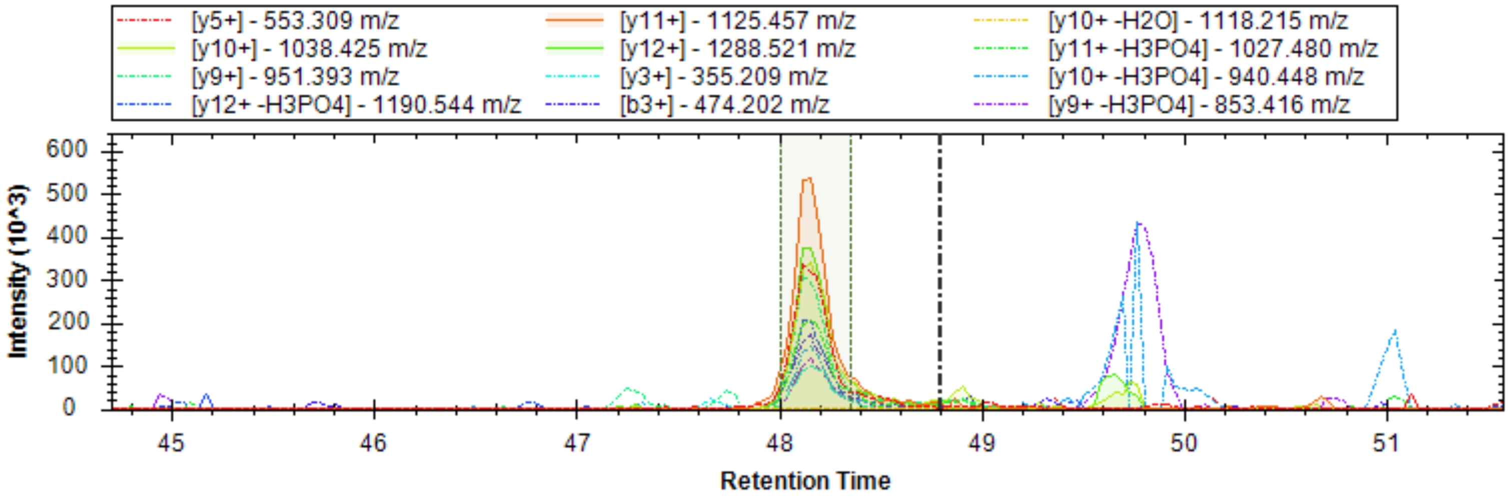

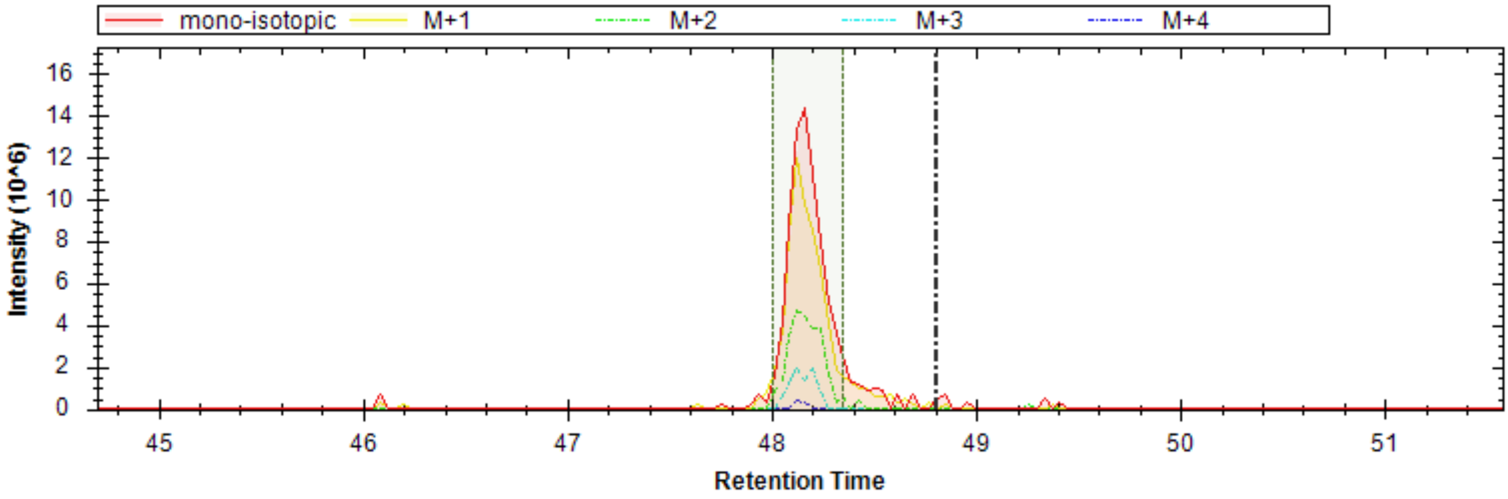

Q7TQD2 – _AANKT[+H+O3+P]PPKSPGDPAR_.2

Fig4_MP-DIA-barrel-cortex-p54-1_MHRM.htrms

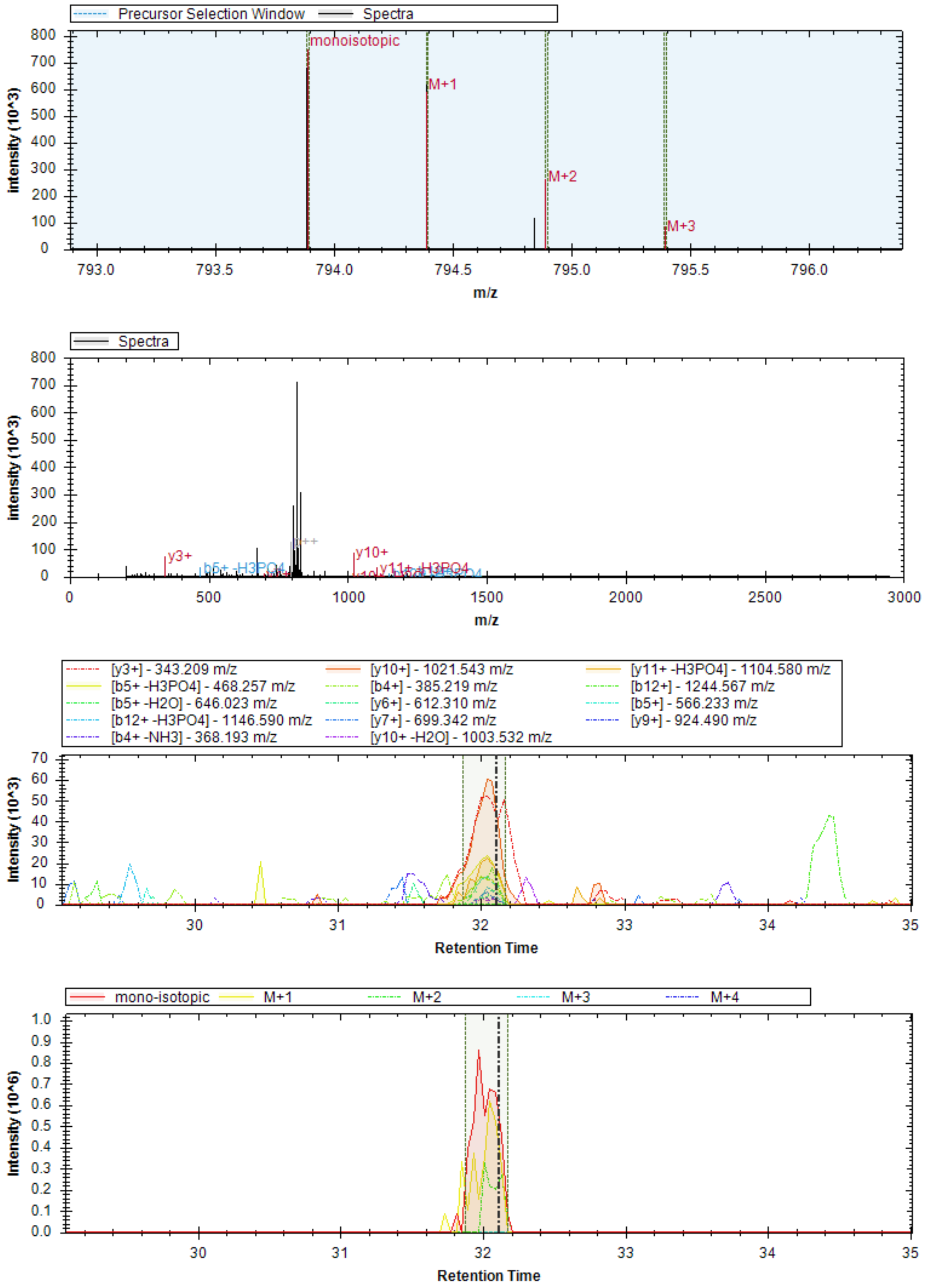

Q91XM9;Q91XM9-2;Q91XM9-7 – _NLSQIENVHGYVLQS[+H+O3+P]HISPLK_.3

Fig4_MP-DIA-barrel-cortex-p54-1_MHRM.htrms

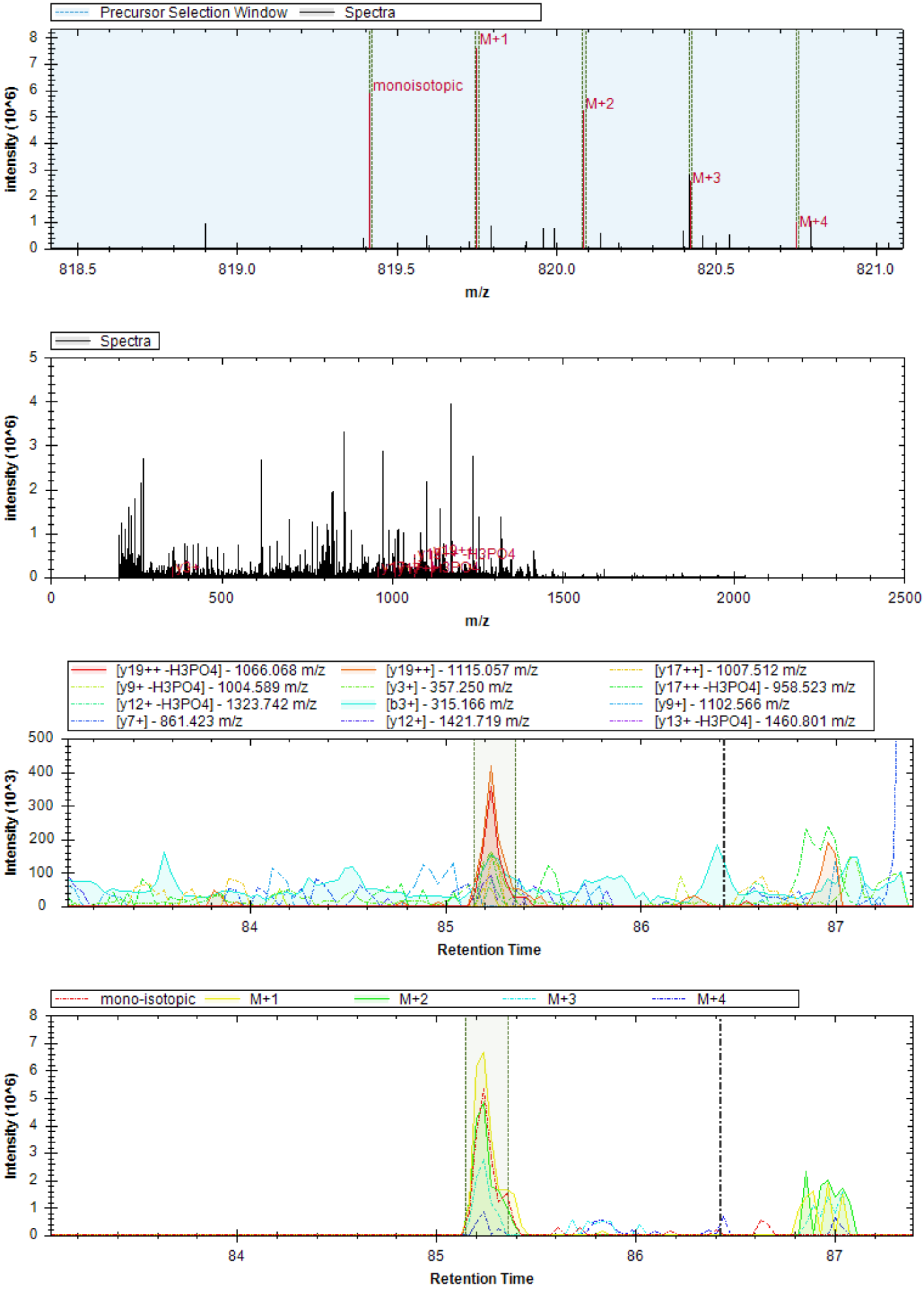

O08784 – _ASAVSPEKAPM[+O]T[+H+O3+P]SKAK_.2

Fig4_MP-DIA-barrel-cortex-p54-1_MHRM.htrms

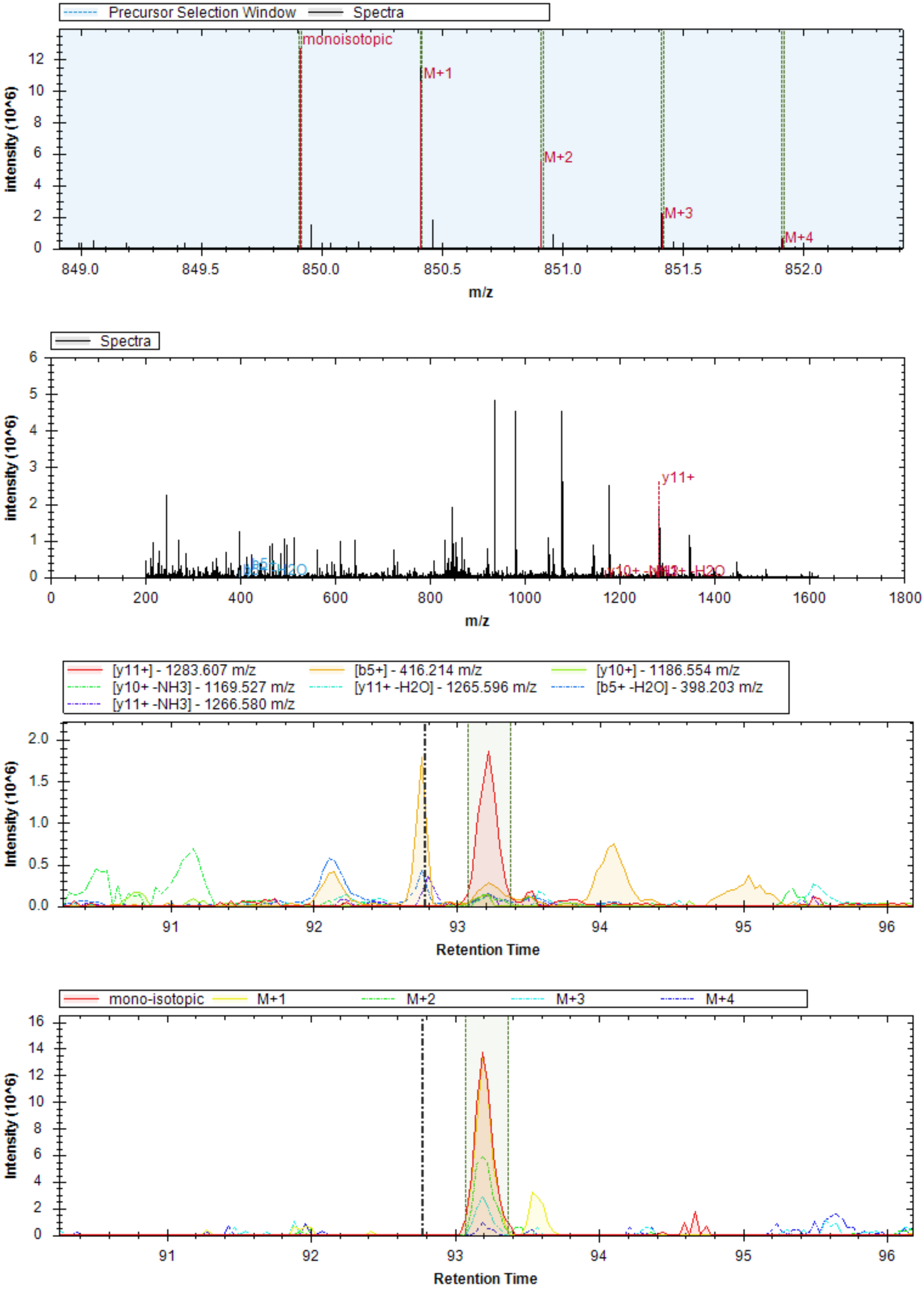

P14873 – _ETAAAHQASS[+H+O3+P]SPPIDAATAEPYGFR_.3

Fig4_MP-DIA-barrel-cortex-p54-1_MHRM.htrms

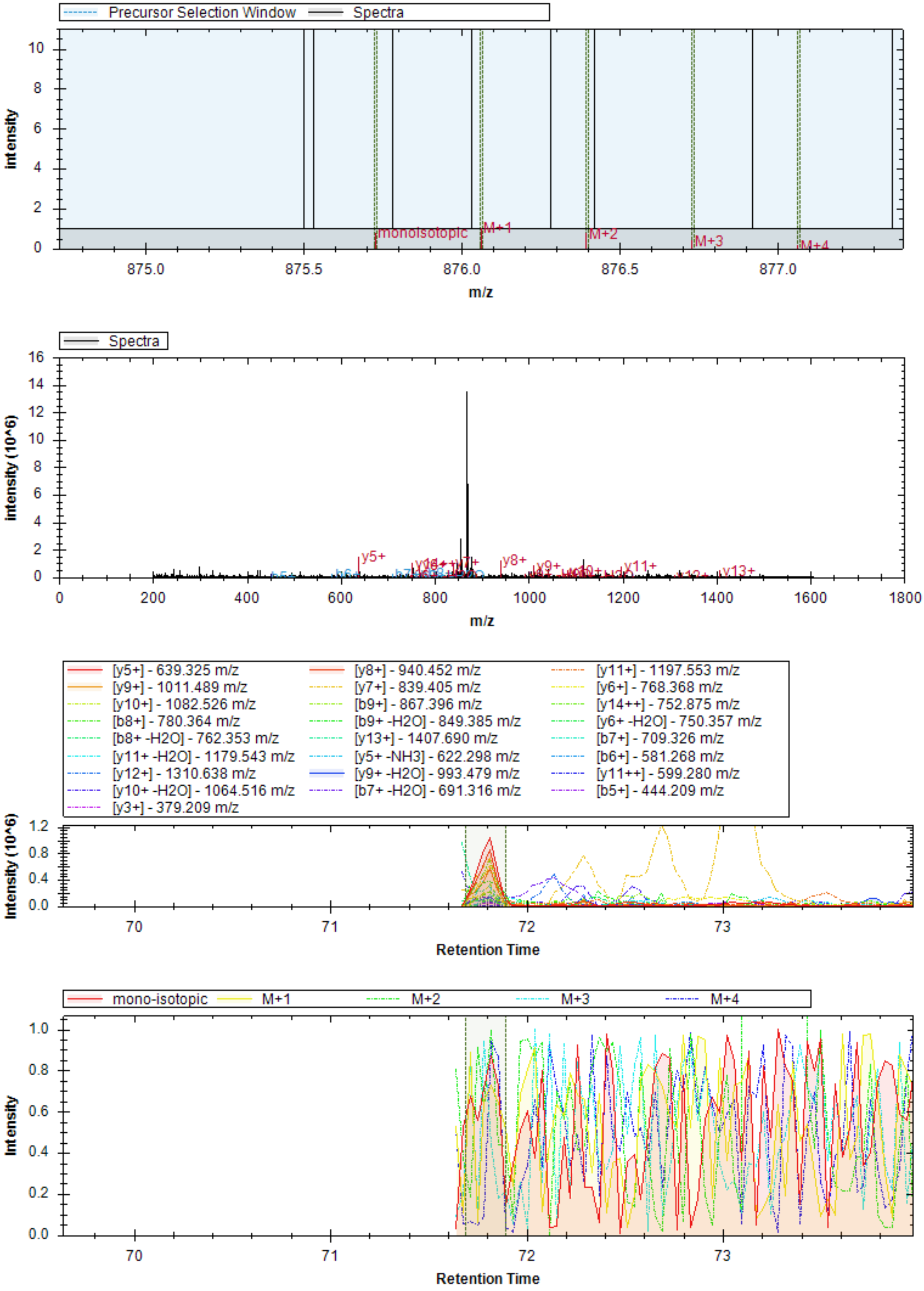

P20917 – _NVTESSSFS[+H+O3+P]GGDNPHVLYSPEFR_.3
Fig4_MP-DIA-barrel-cortex-p54-1_MHRM.htrms

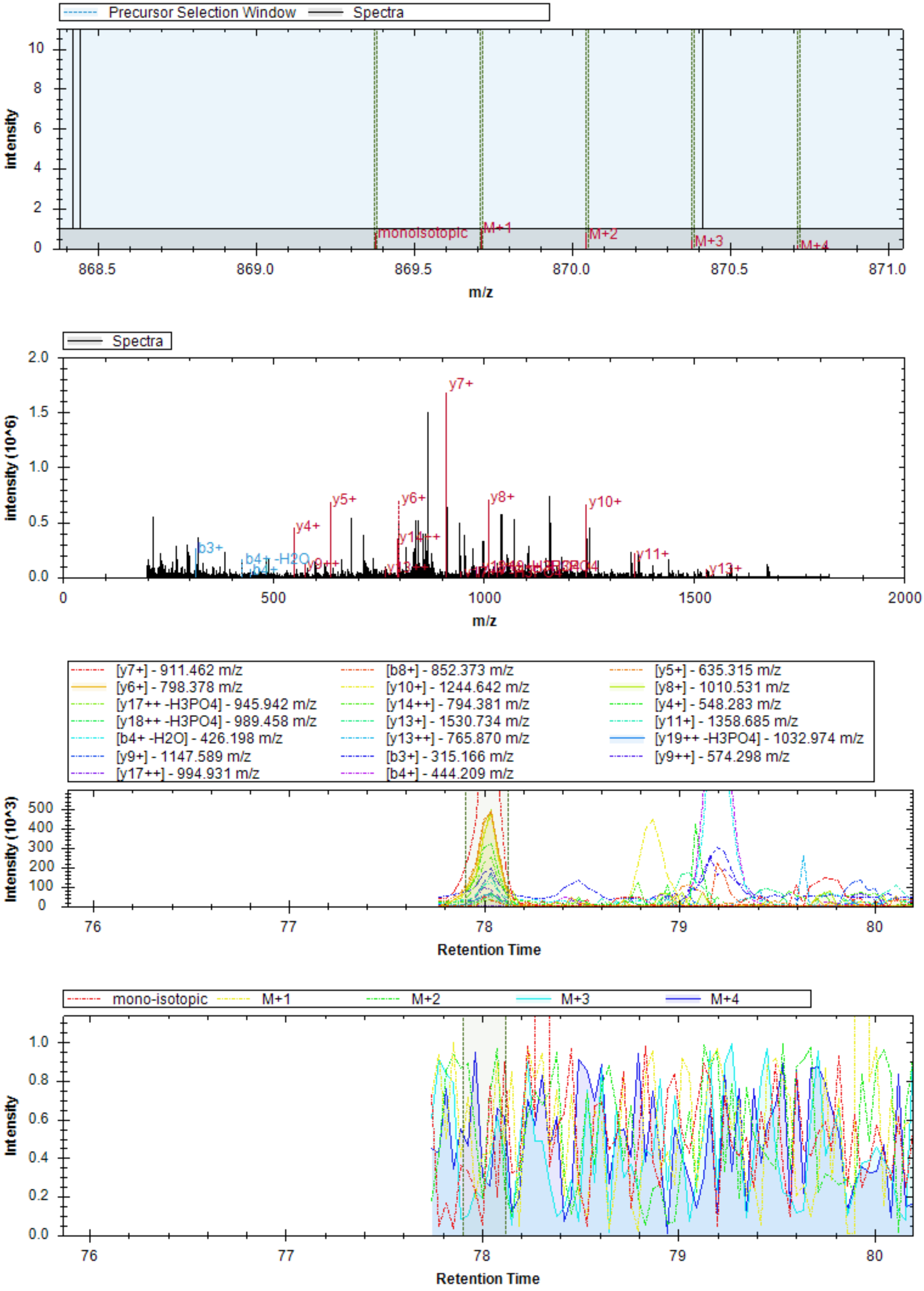

P50538 – _EAEHGYAS[+H+O3+P]M[+O]LPYSK_.2
Fig4_MP-DIA-barrel-cortex-p54-1_MHRM.htrms

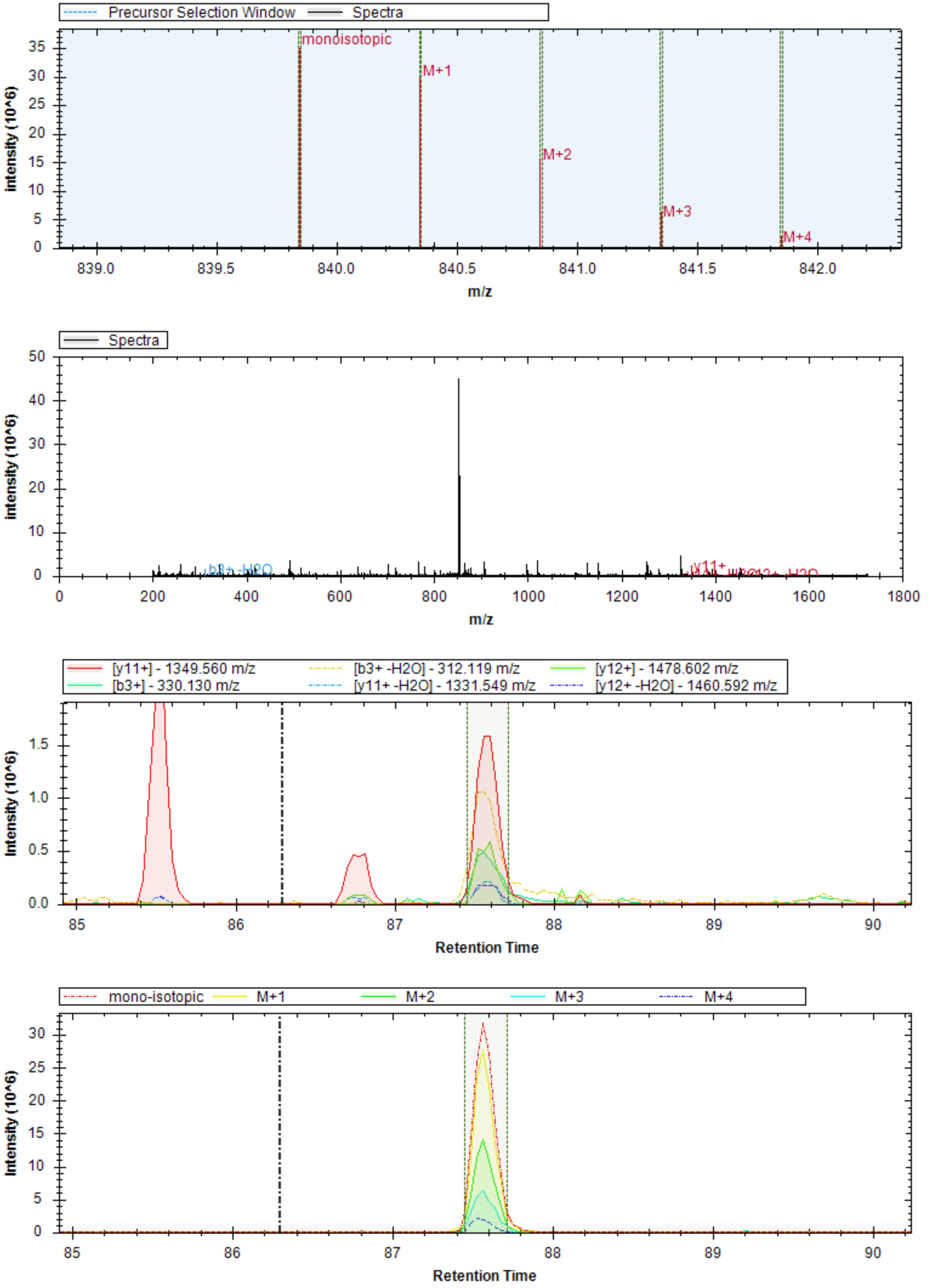

P60904 – _SLS[+H+O3+P]TSGESLYHVLGLDK_.2
Fig4_MP-DIA-barrel-cortex-p54-1_MHRM.htrms

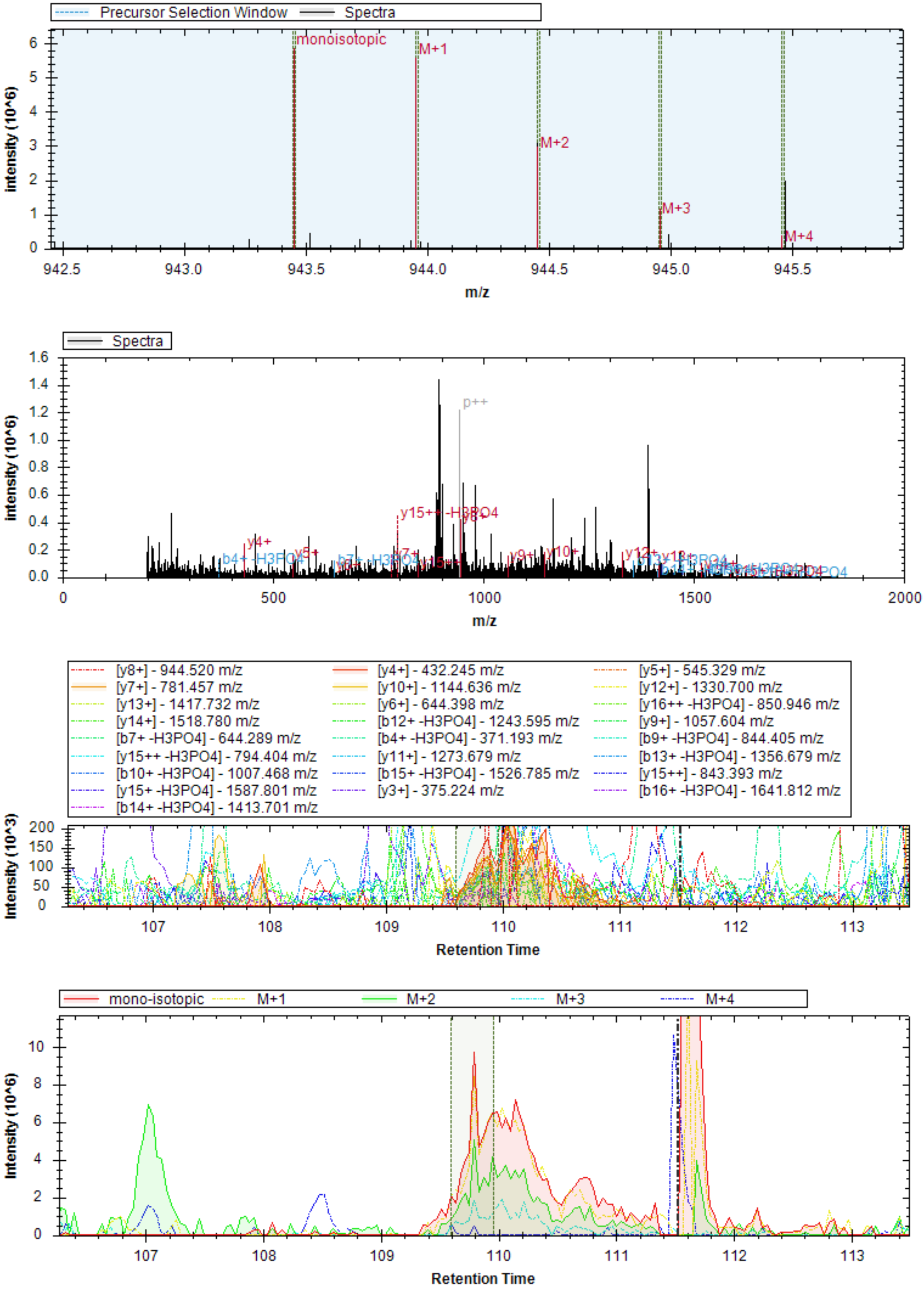

Q91Z31;Q91Z31-2 – _ETSLLAVPGALS[+H+O3+P]PLAIPNAAAAAAAAAAGR_.3

Fig4_MP-DIA-barrel-cortex-p54-1_MHRM.htrms

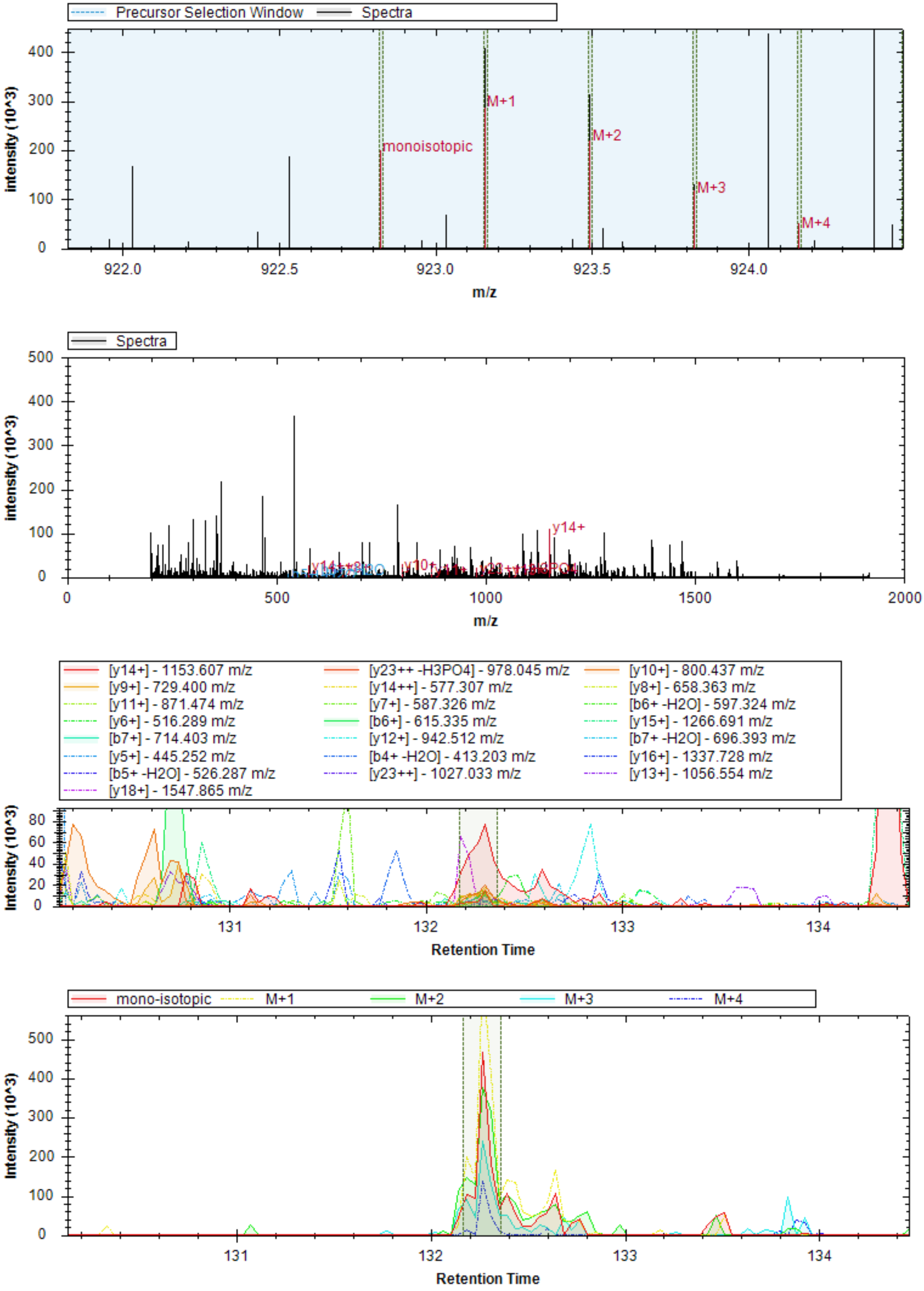

Q9CZT4;Q9CZT4-2 – _KEQM[+O]RAATVLPS[+H+O3+P]VQIK_.2
Fig4_MP-DIA-barrel-cortex-p54-1_MHRM.htrms

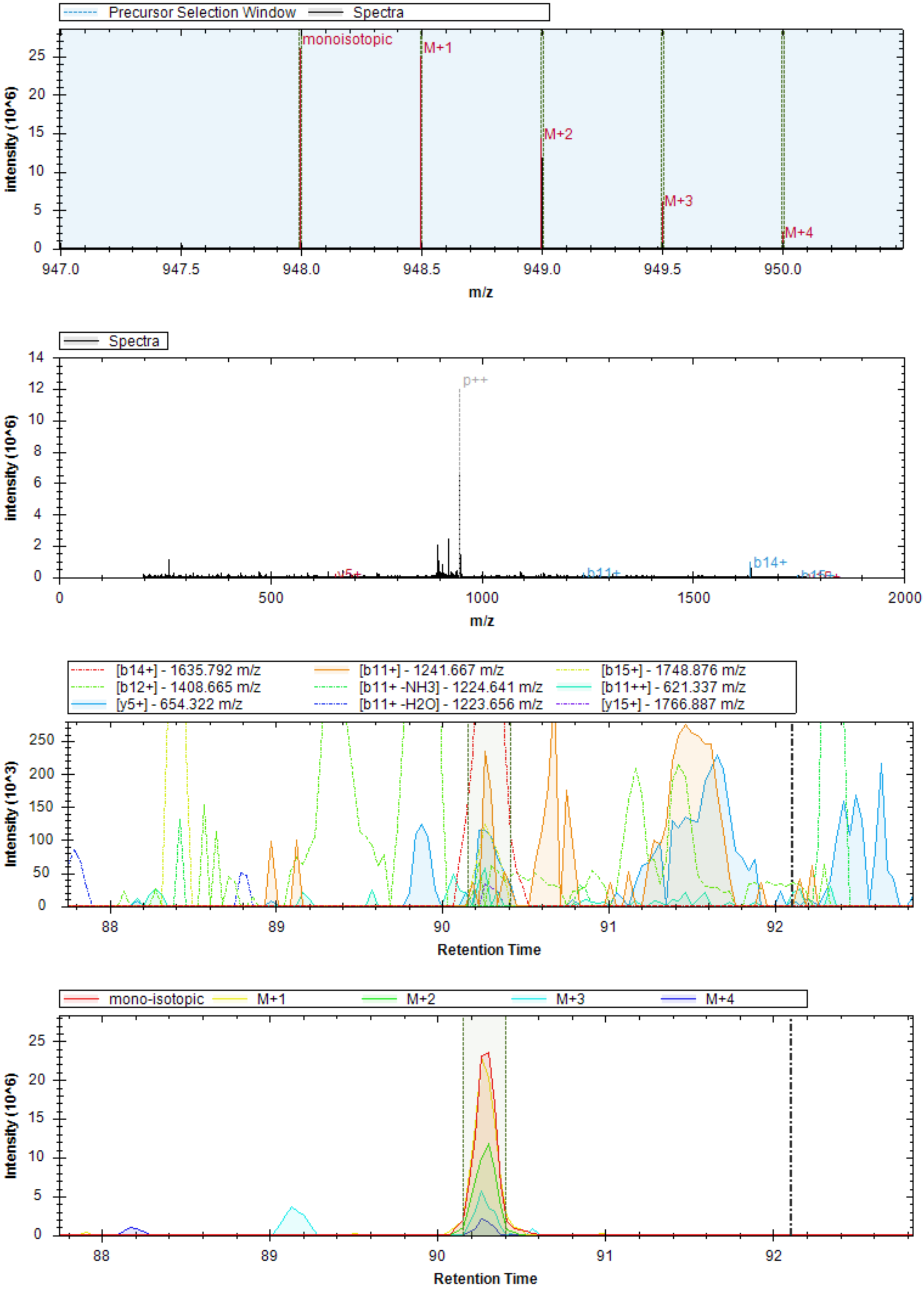

Q9QYR6-2 – _MAS[+H+O3+P]PPPSGPPSAAHTPFHQSPVEEK_.3
Fig4_MP-DIA-barrel-cortex-p54-1_MHRM.htrms

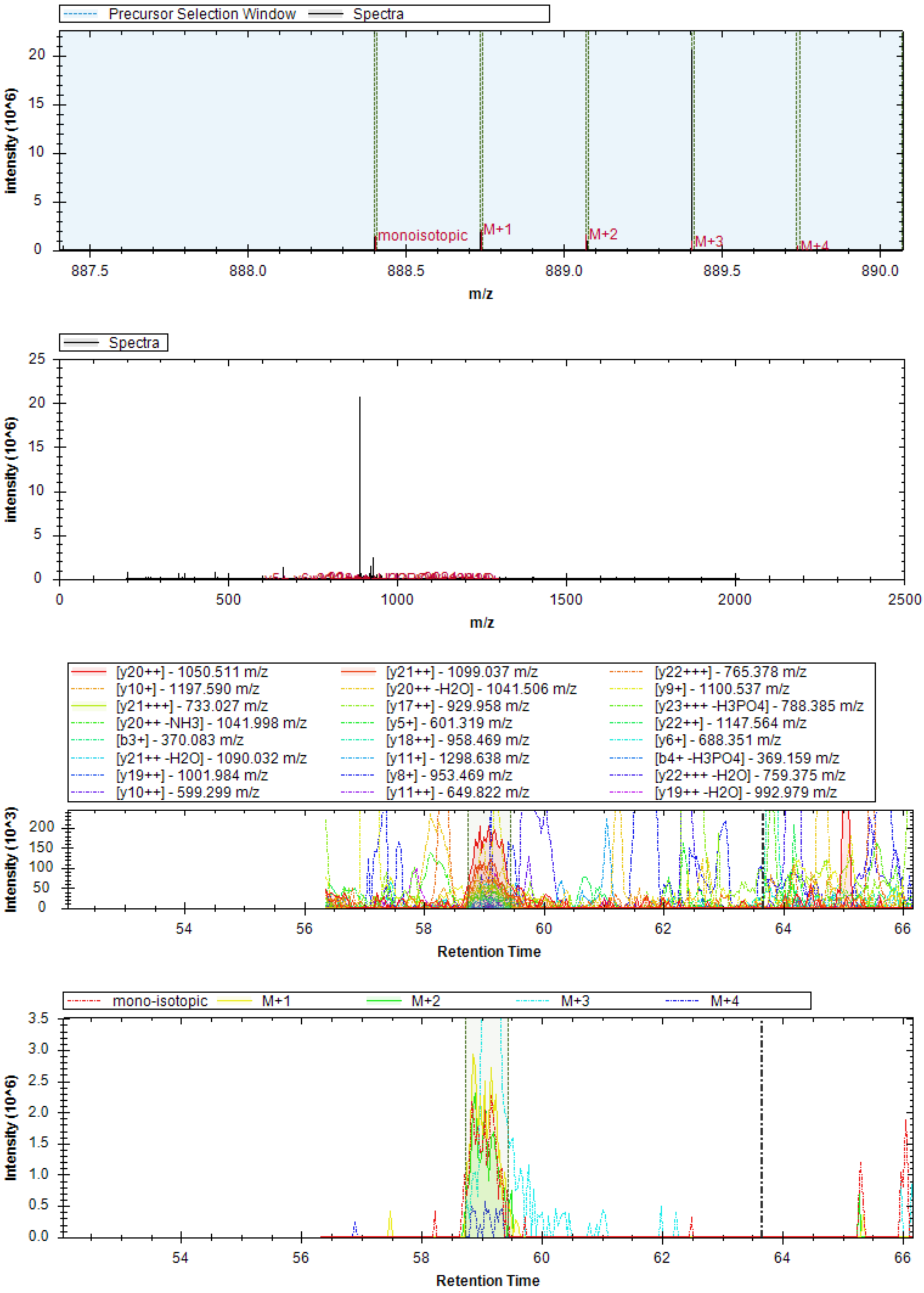

A2A891;A2A891-2;A2A8[...] – _TAVIVQQKLRS[+H+O3+P]S[+H+O3+P]LLT[+H+O3+P]K_.2

Fig4_MP-DIA-barrel-cortex-p54-1_MHRM.htrms

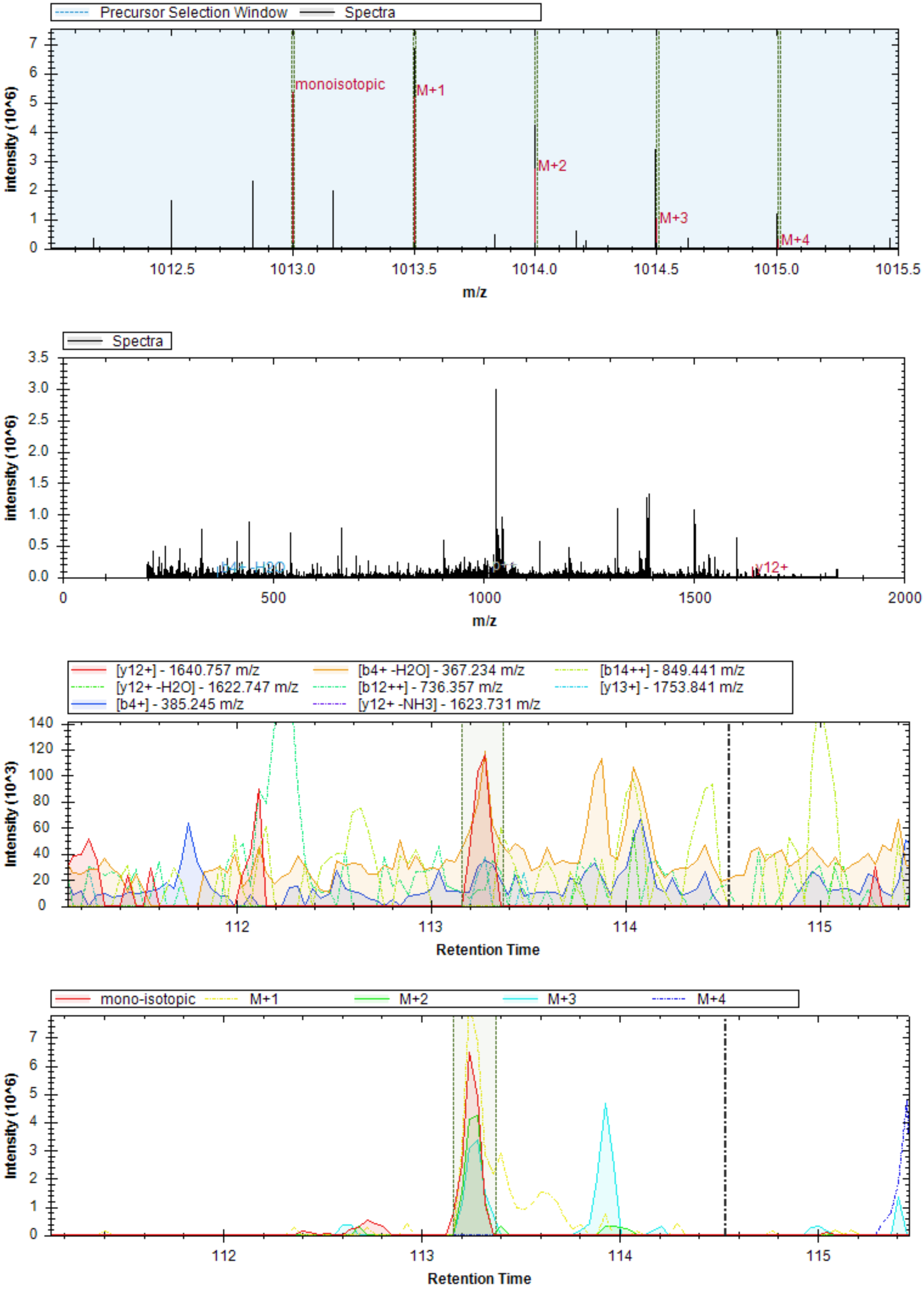

P17742 – _VNPT[+H+O3+P]VFFDITADDEPLGR_.2

Fig4_MP-DIA-barrel-cortex-p54-1_MHRM.htrms

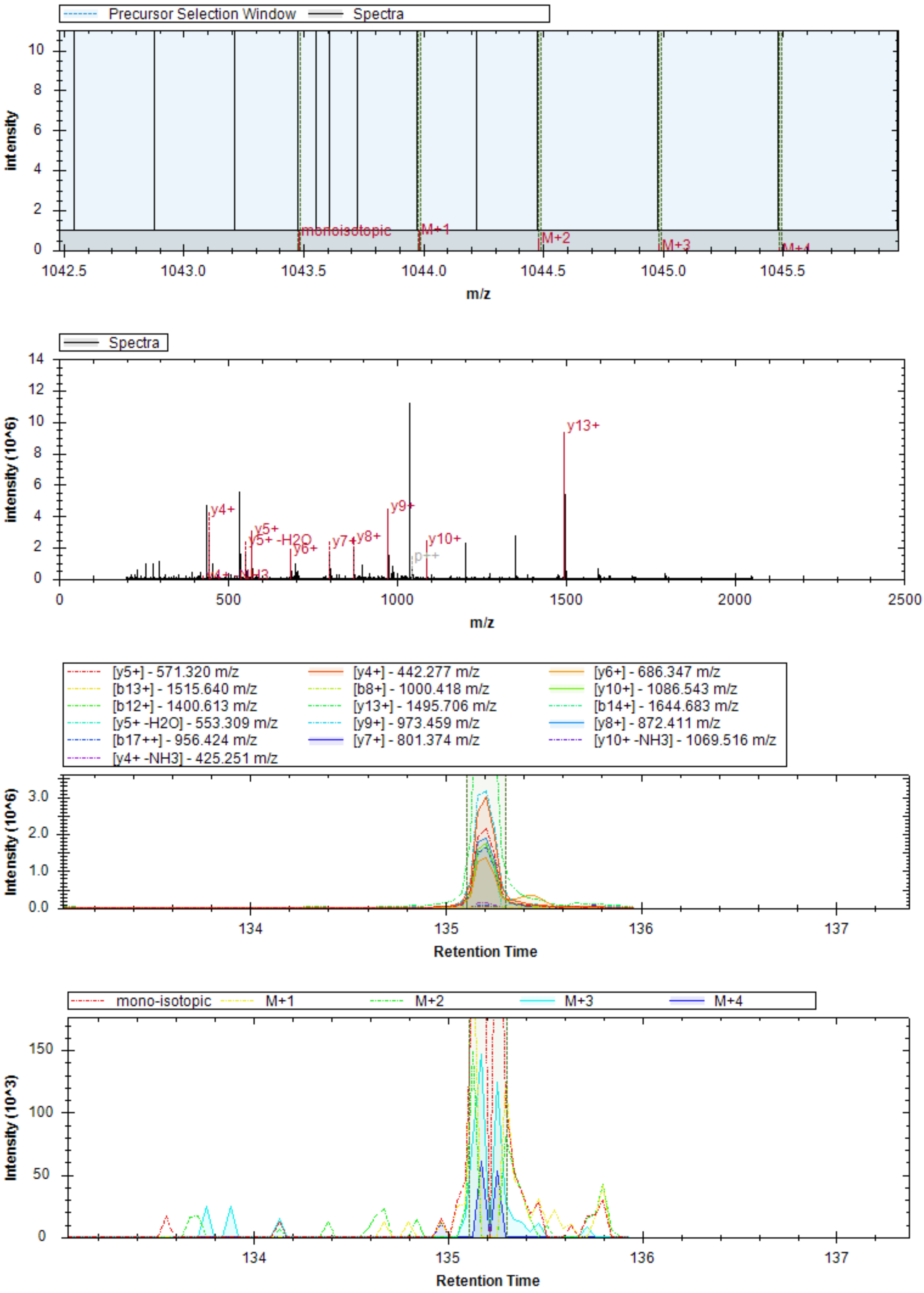

Q8CHK4;Q8CHK4-2;Q8CH[...] – _RGY[+H+O3+P]GKLLIEFS[+H+O3+P]YELSK_.2
Fig4_MP-DIA-barrel-cortex-p54-1_MHRM.htrms

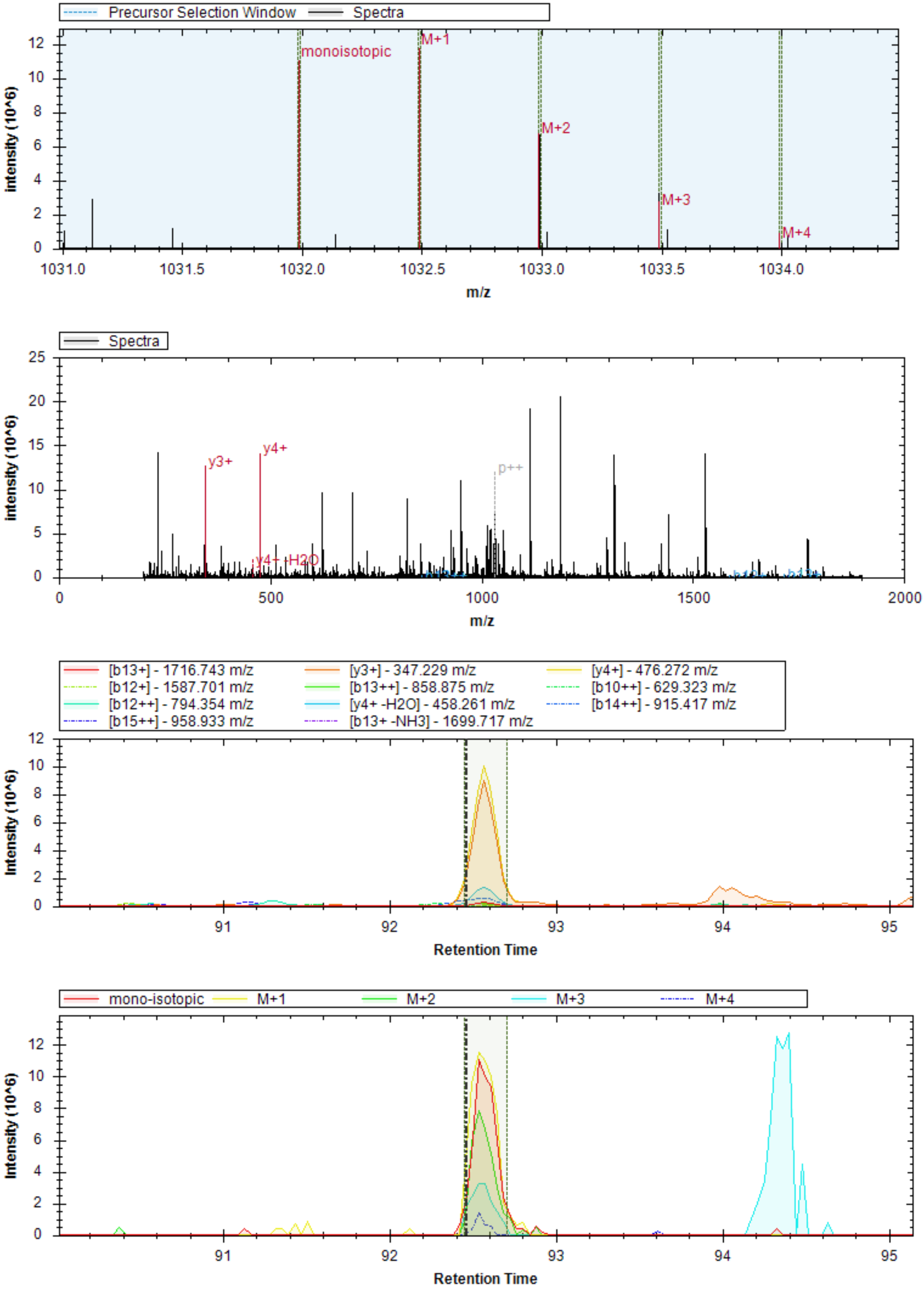

E9Q401 – _MVAEVFIY[+H+O3+P]WSK_.1
Fig4_MP-DIA-barrel-cortex-p54-1_MHRM.htrms

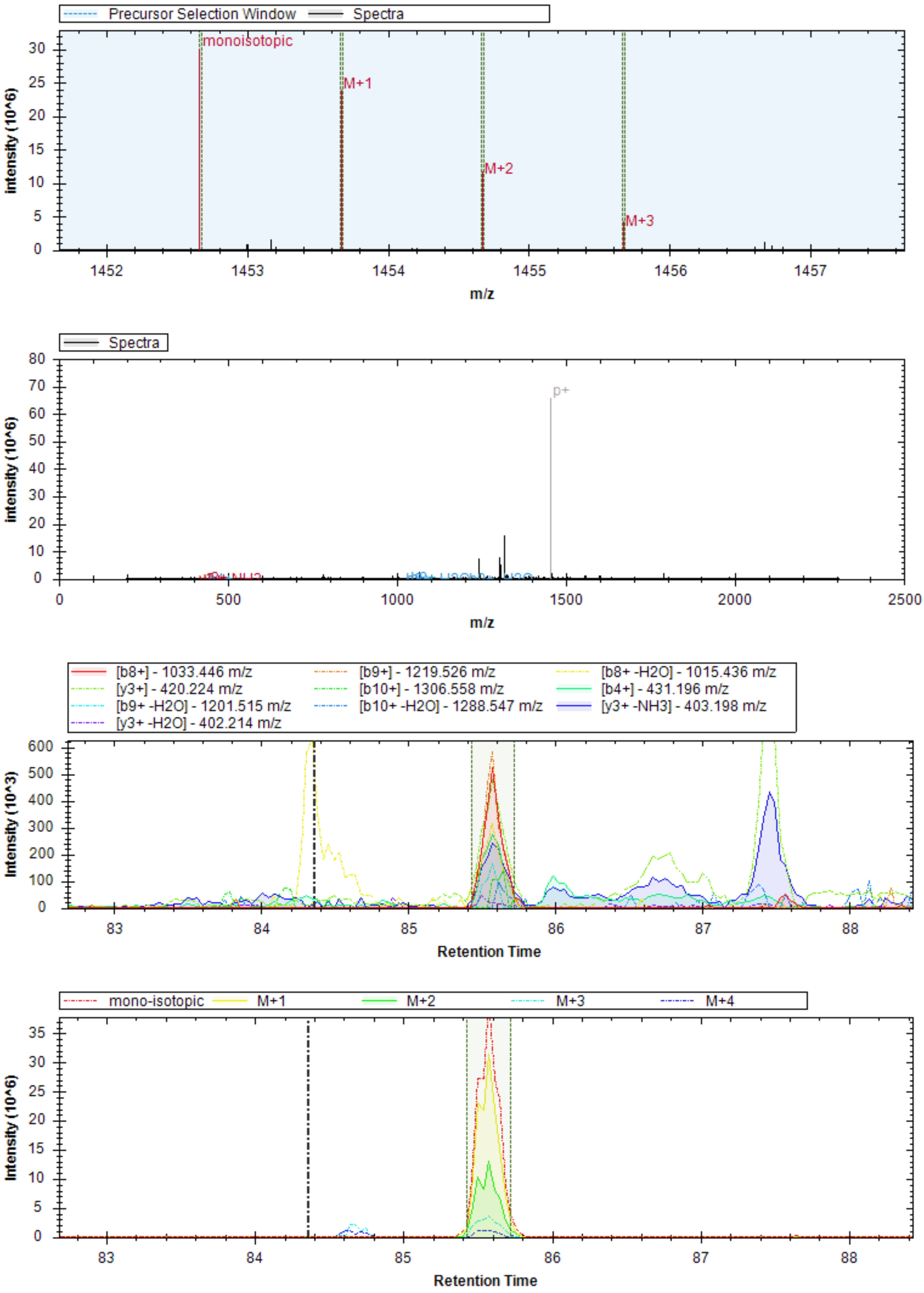

P20357 – _SGTSTPTTPGSTAITPGT[+H+O3+P]PPSYSSR_.2
Fig4_MP-DIA-barrel-cortex-p54-1_MHRM.htrms

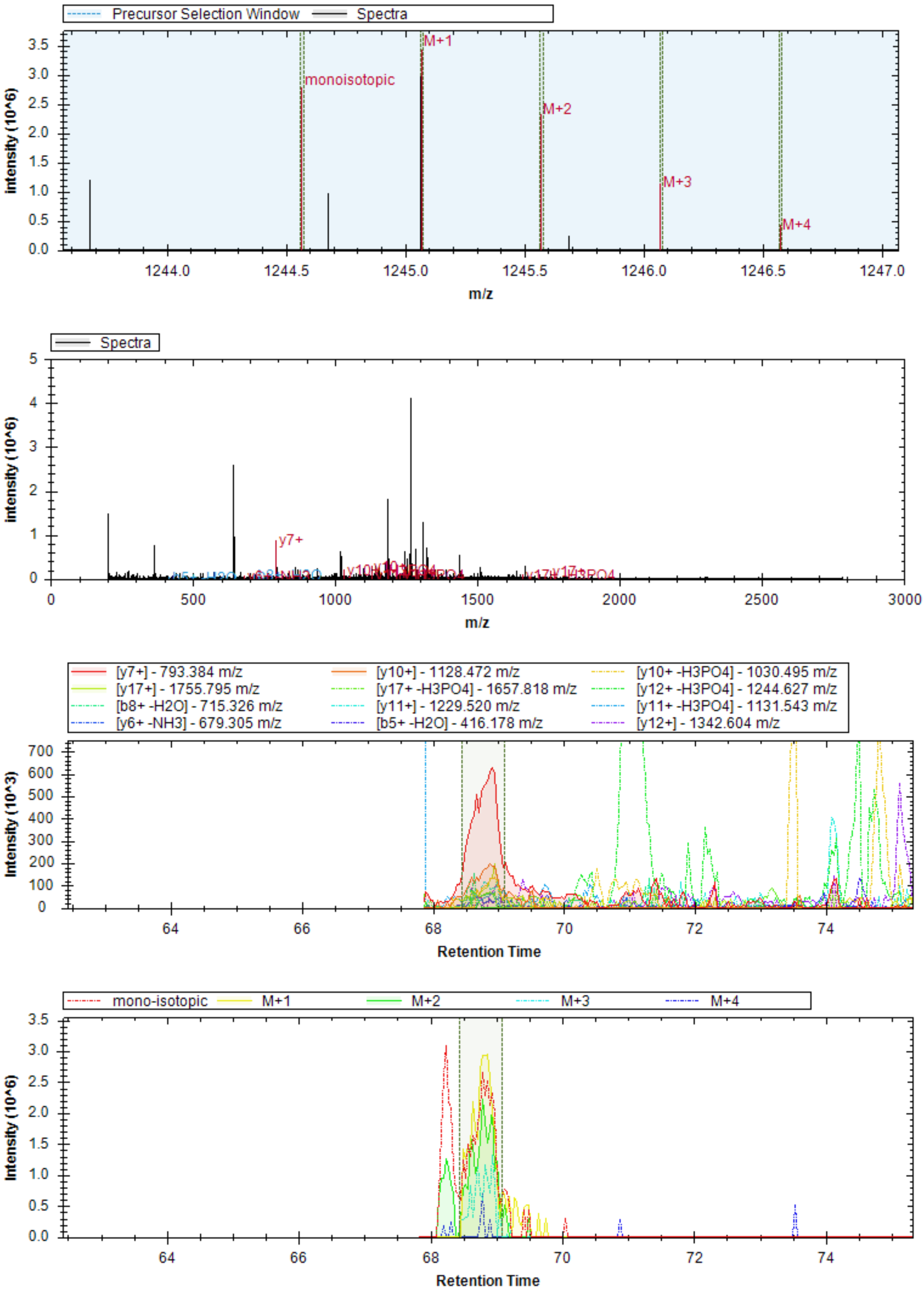

Q8BRT1 – _VLNT[+H+O3+P]GSDVEEAVADALLLGDIR_.2
Fig4_MP-DIA-barrel-cortex-p54-1_MHRM.htrms

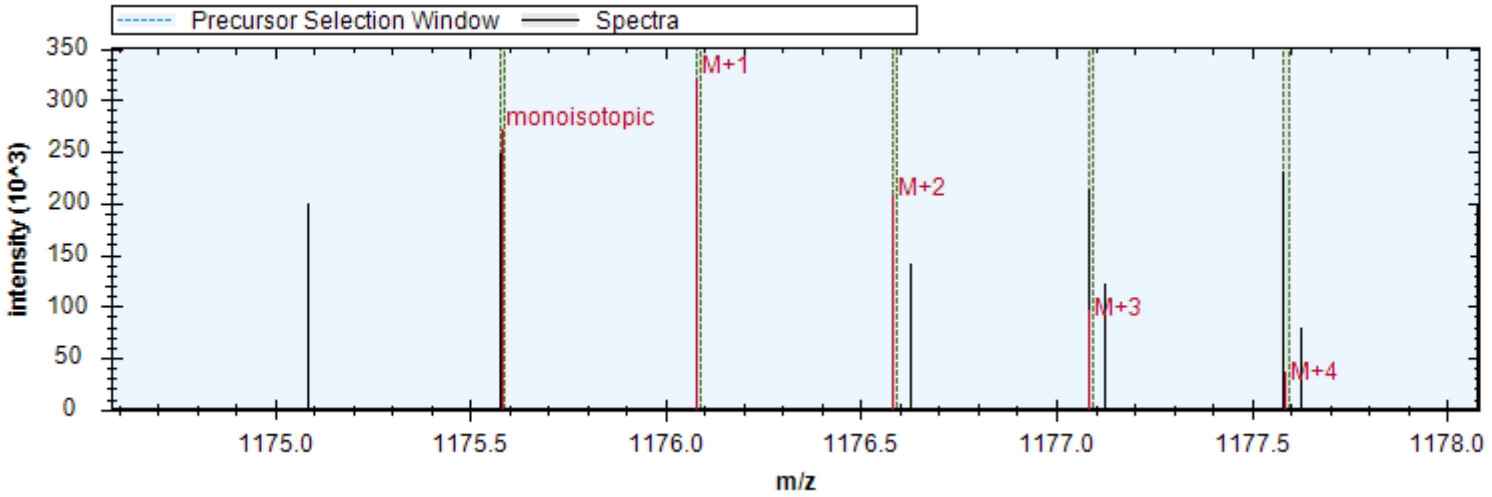

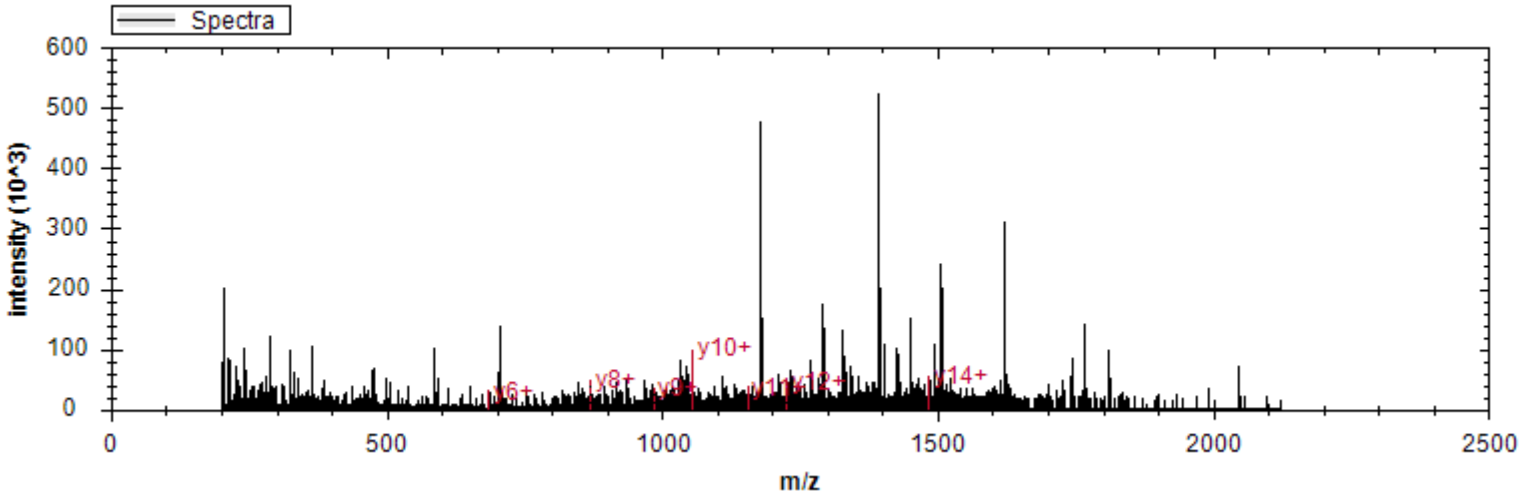

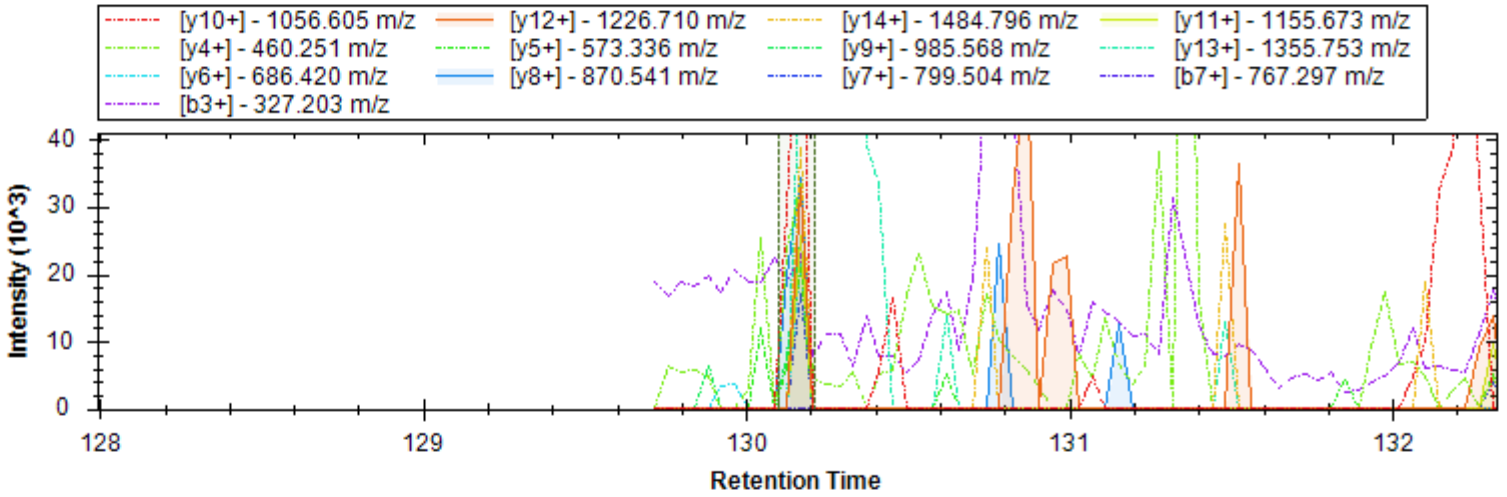

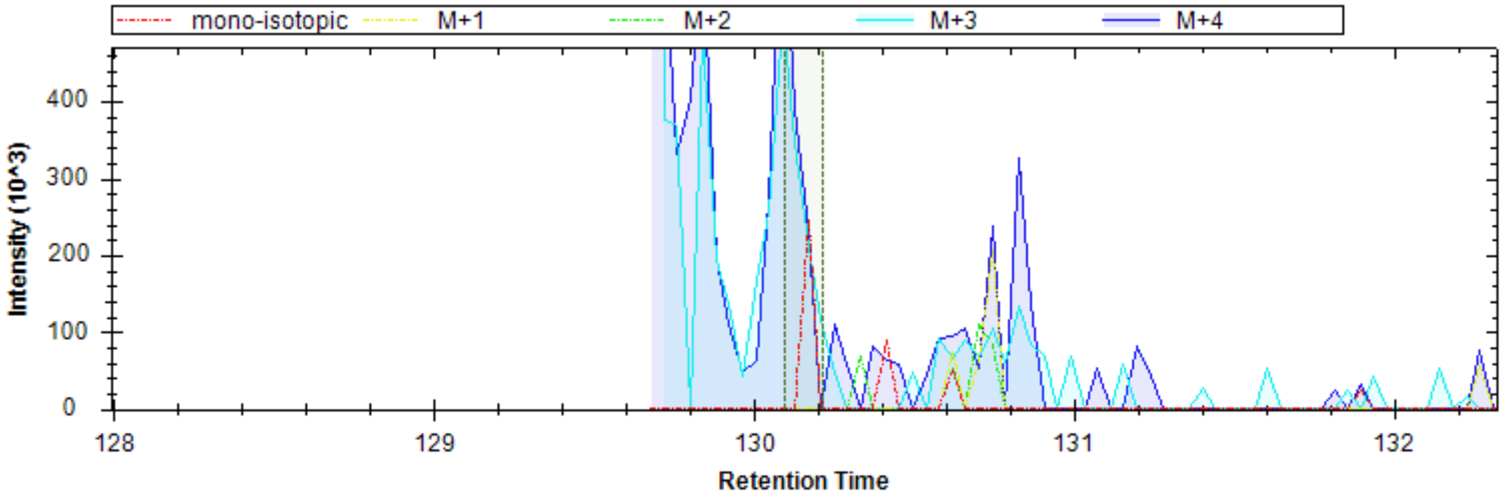

Q8VHY0 – _LLAGPRY[+H+O3+P]GR_.1

Fig4_MP-DIA-barrel-cortex-p54-1_MHRM.htrms

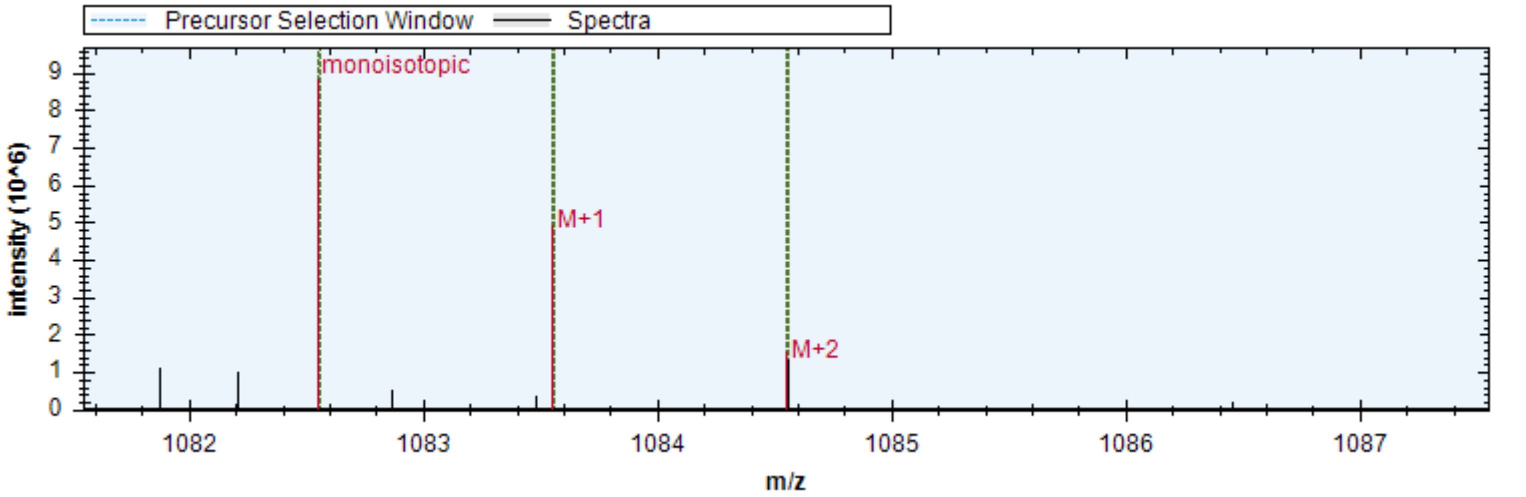

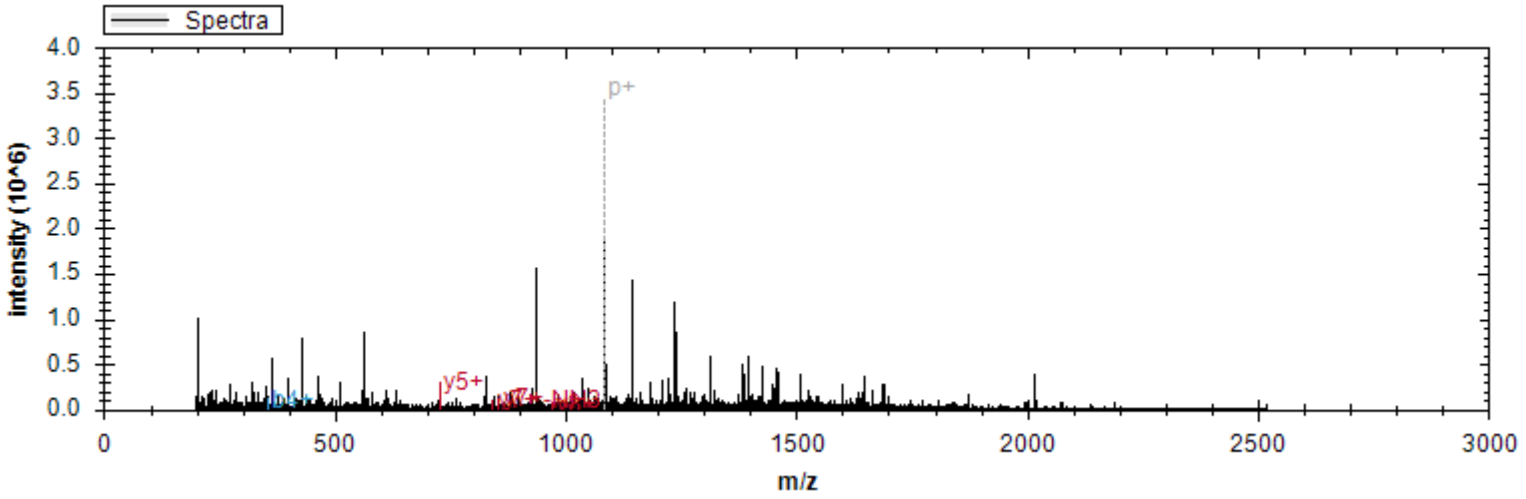

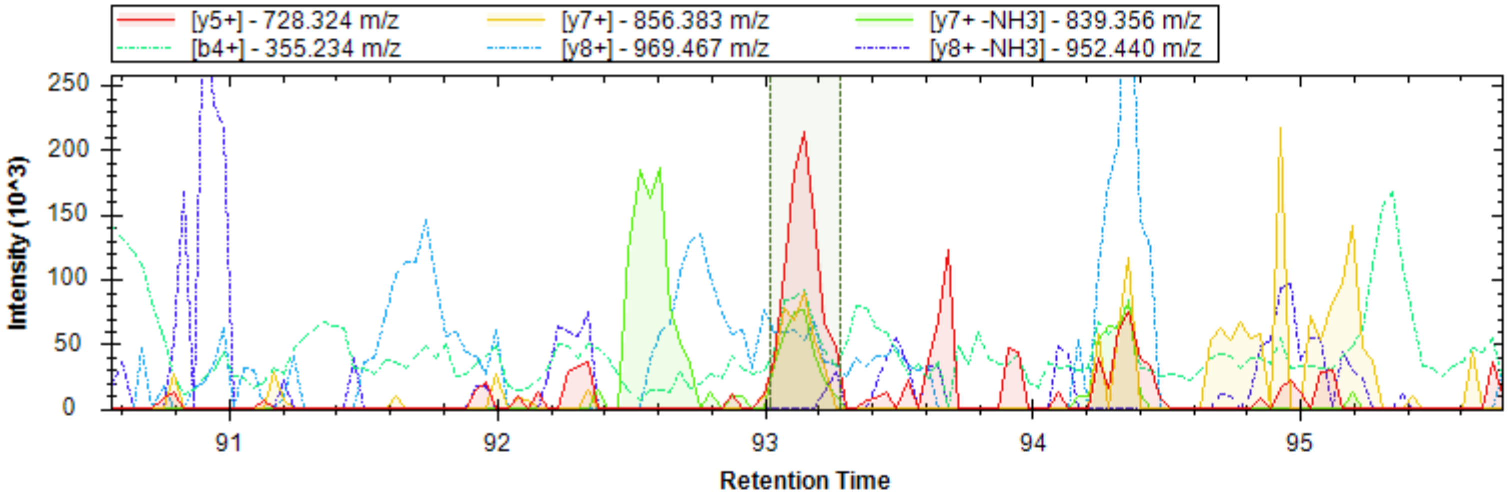

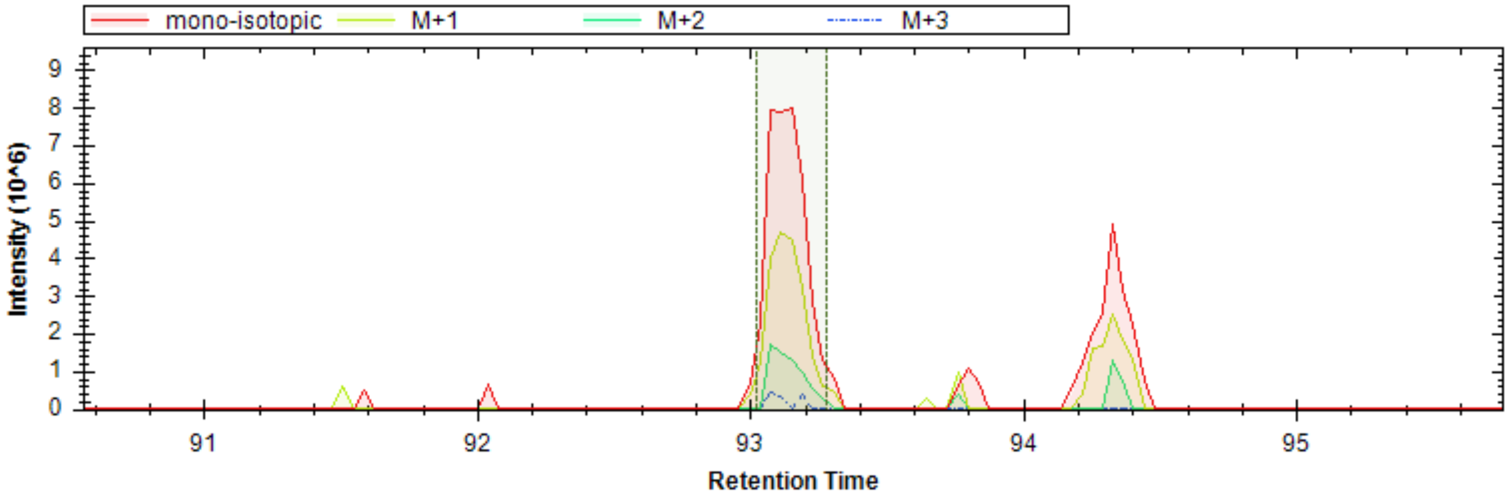

Q921N6;Q921N6-2 – _LIY[+H+O3+P]KPRQAAVTR_.4
Fig4_MP-DIA-barrel-cortex-p54-2_MHRM.htrms

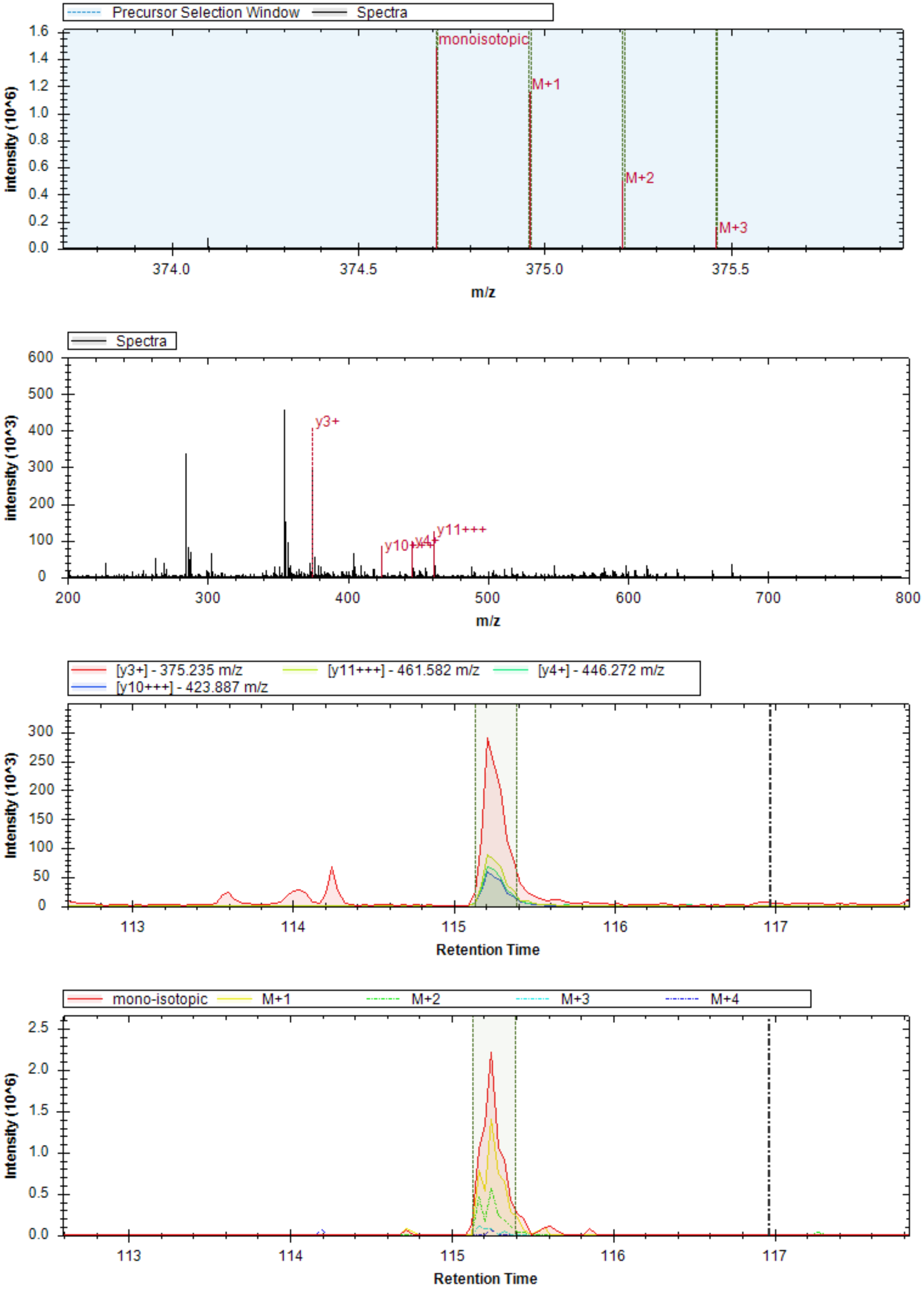

Q9CVB6 – _IIEET[+H+O3+P]LALK_.3

Fig4_MP-DIA-barrel-cortex-p54-2_MHRM.htrms

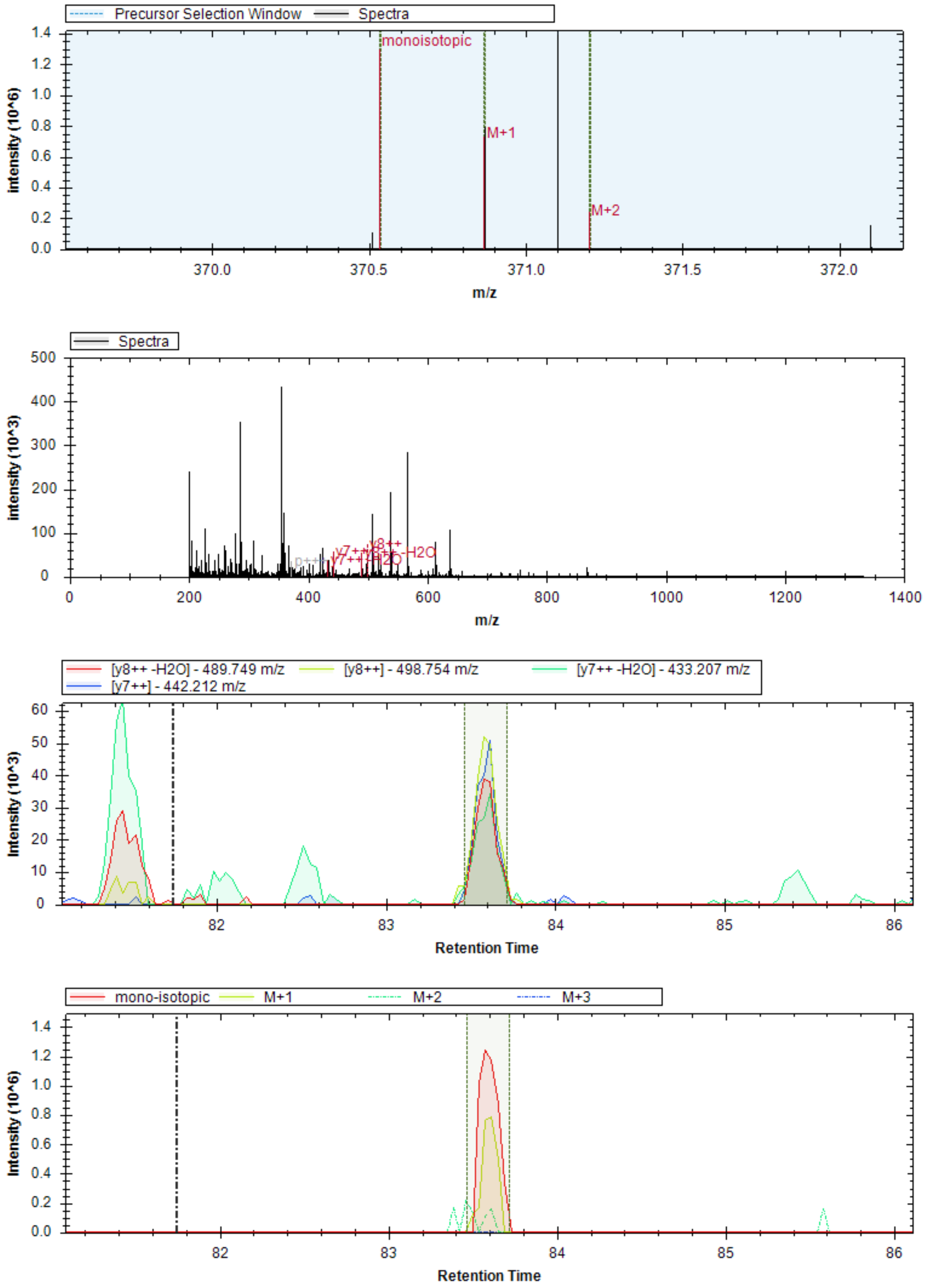

Q9D516;Q9D516-2 – _KLS[+H+O3+P]QGILVIR_.3
Fig4_MP-DIA-barrel-cortex-p54-2_MHRM.htrms

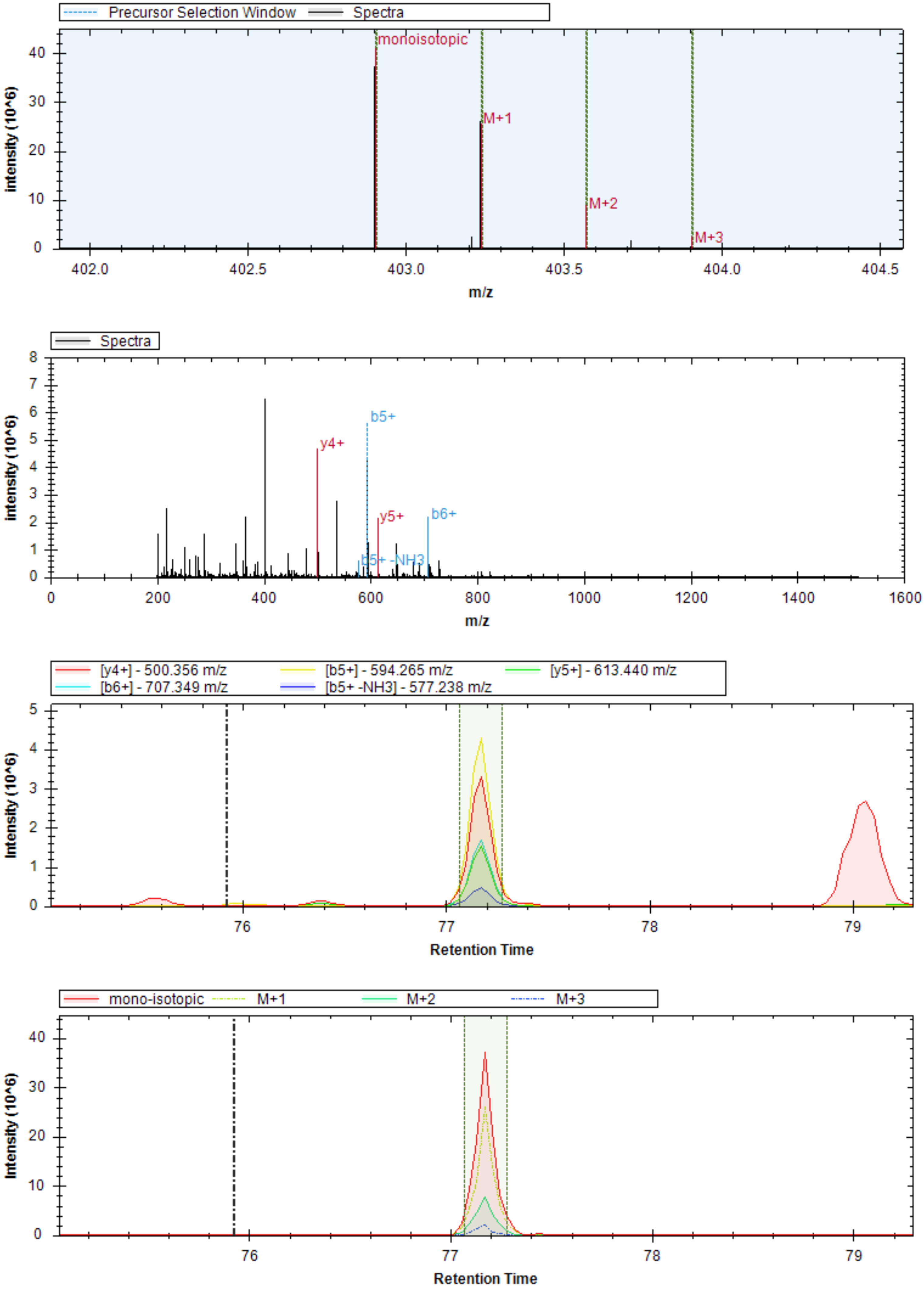

Q03172 – _VALSLLNS[+H+O3+P]KQK_.3

Fig4_MP-DIA-barrel-cortex-p54-2_MHRM.htrms

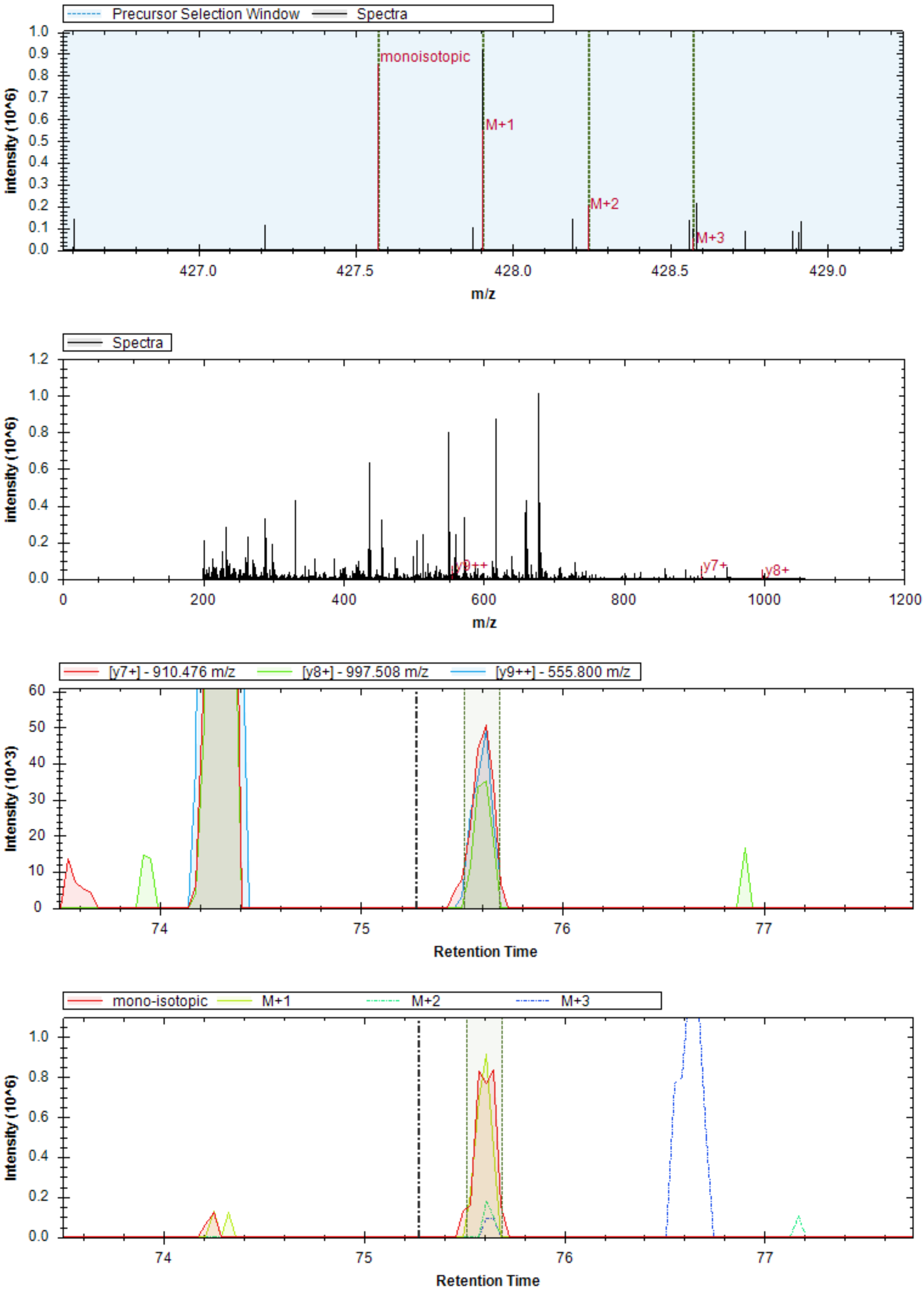

Q8CGI1 – _Y[+H+O3+P]QRS[+H+O3+P]EEELR_.3
Fig4_MP-DIA-barrel-cortex-p54-2_MHRM.htrms

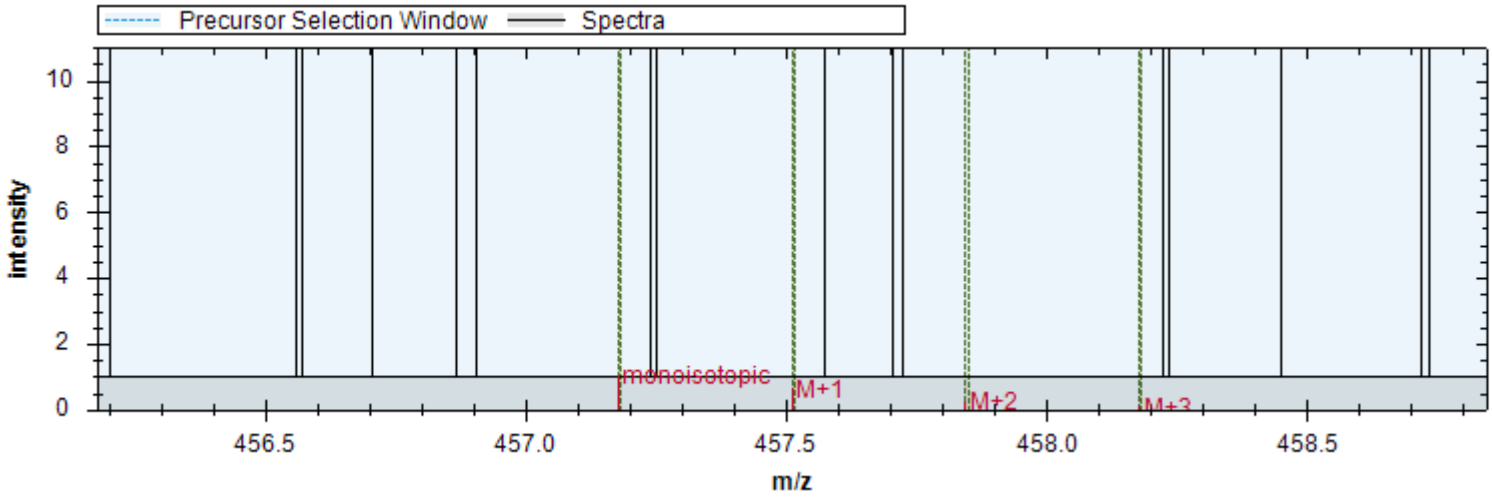

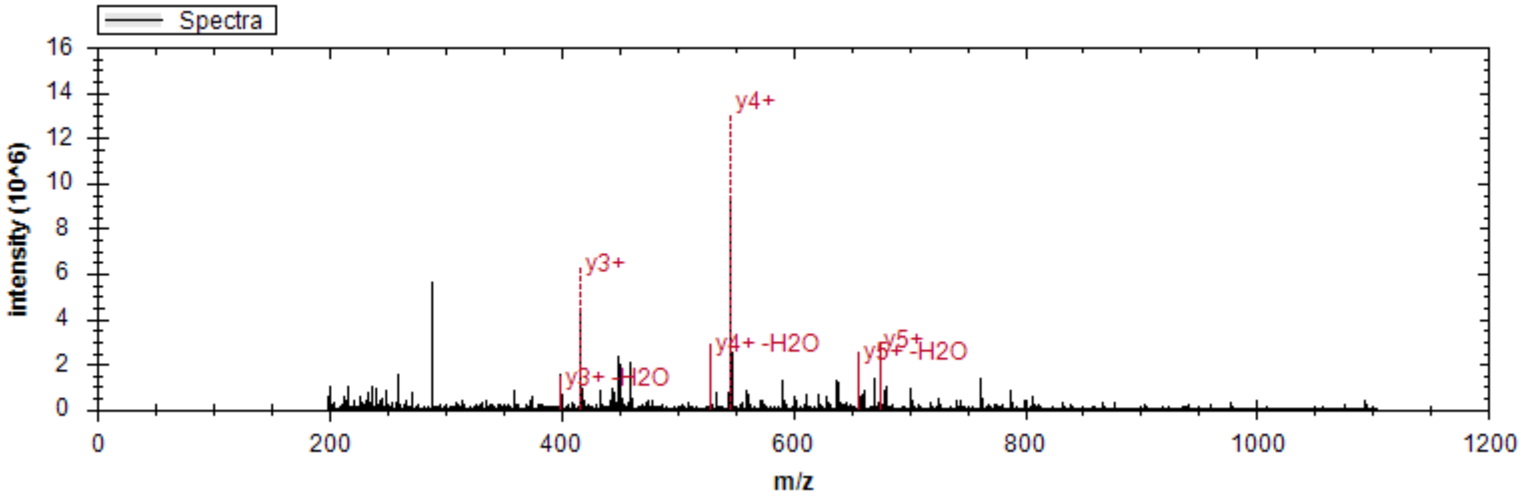

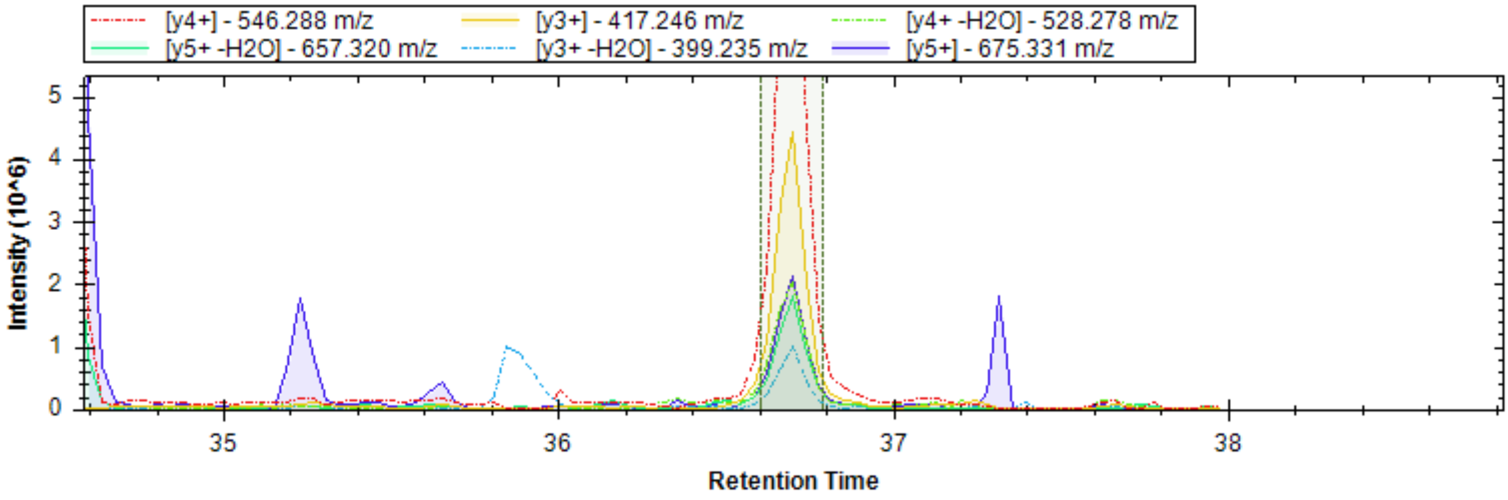

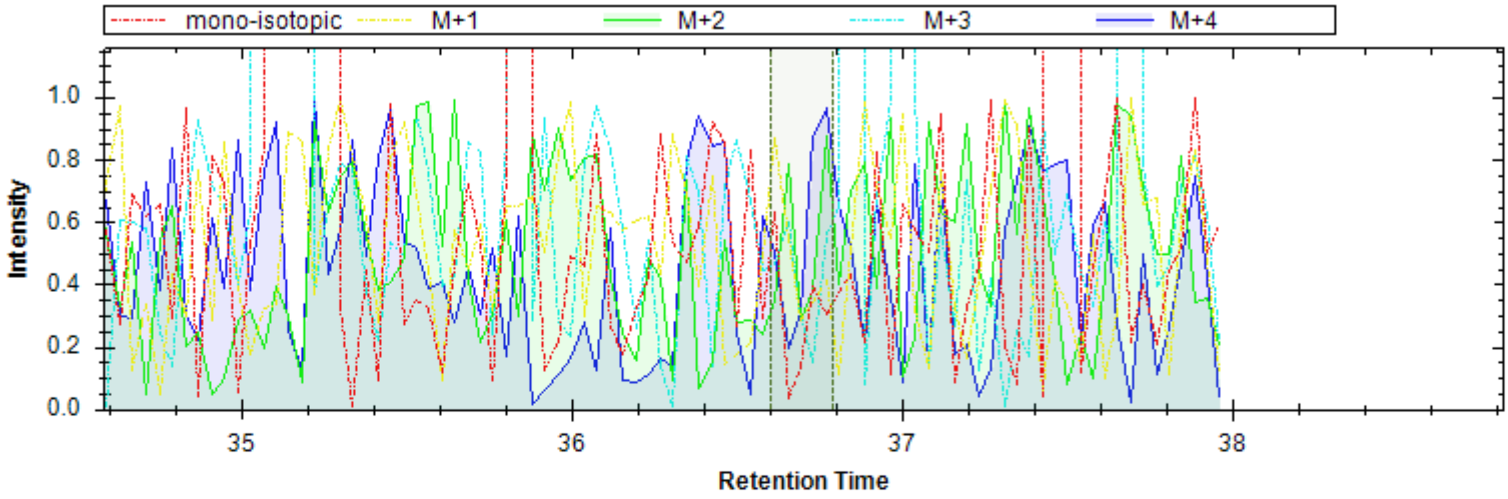

O08553 – _TVT[+H+O3+P]PASSAK_.2
Fig4_MP-DIA-barrel-cortex-p54-2_MHRM.htrms

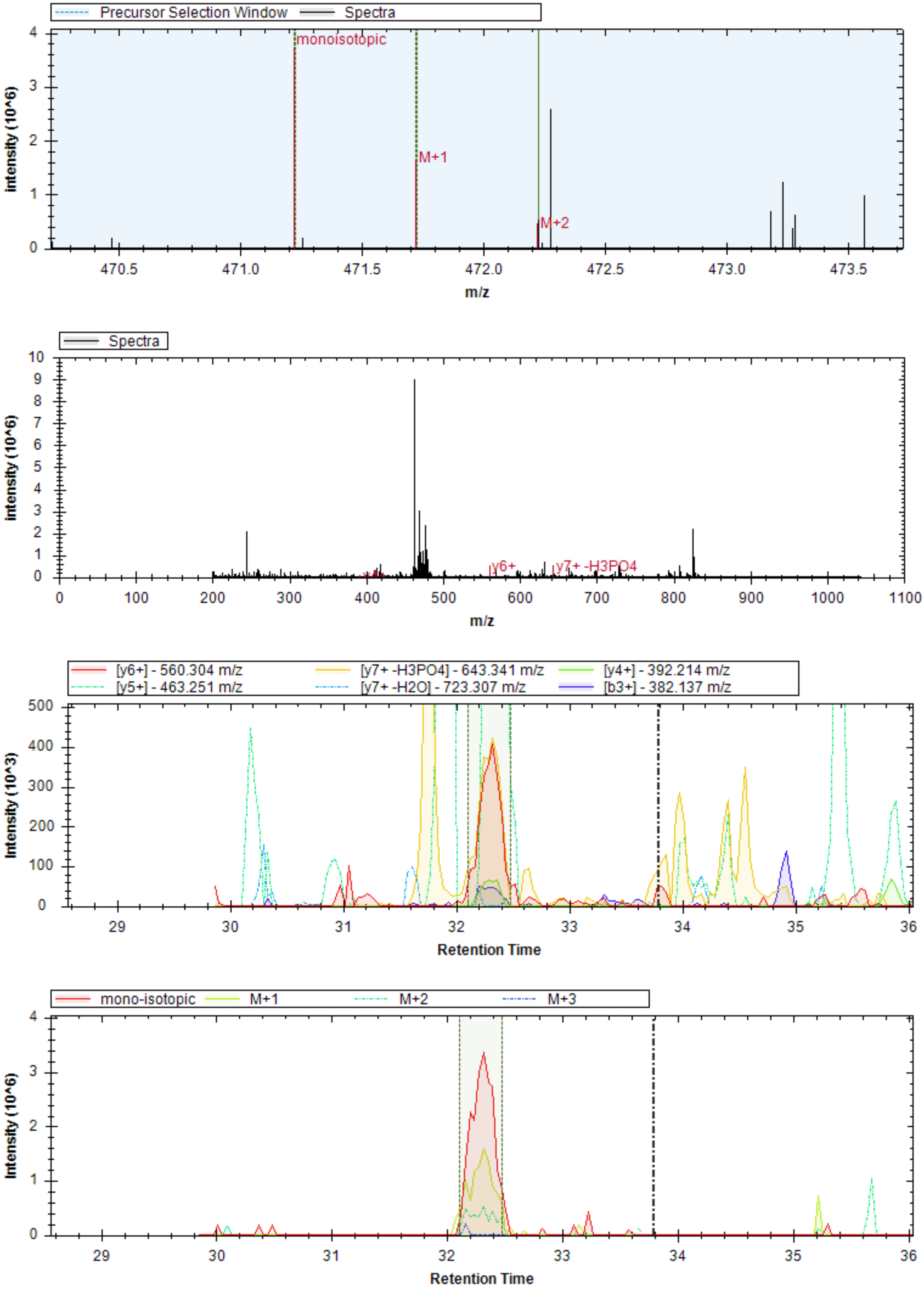

P26645 – _LSGFS[+H+O3+P]FKK_.2
Fig4_MP-DIA-barrel-cortex-p54-2_MHRM.htrms

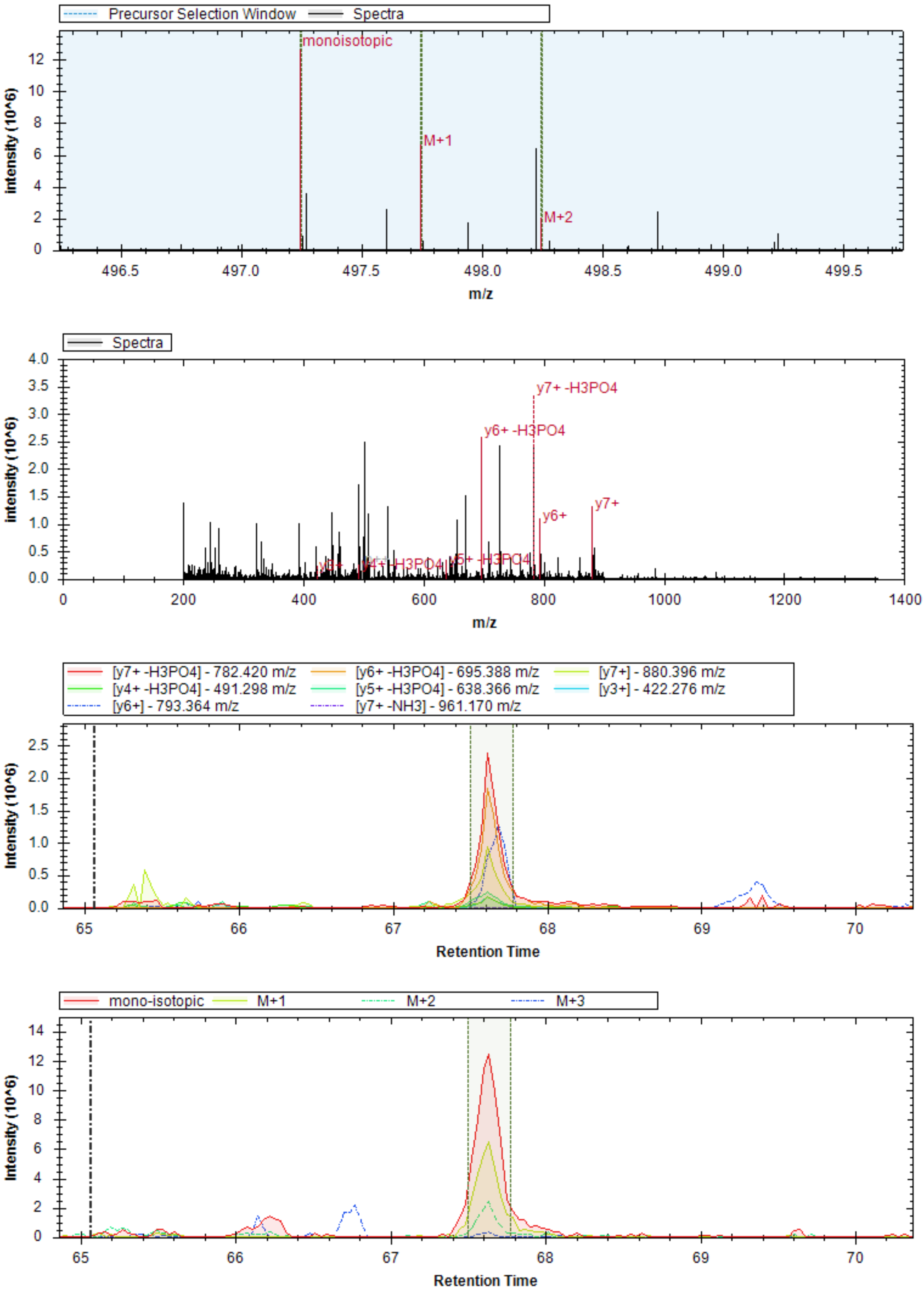

P28667 – _LSGLS[+H+O3+P]FKR_.2

Fig4_MP-DIA-barrel-cortex-p54-2_MHRM.htrms

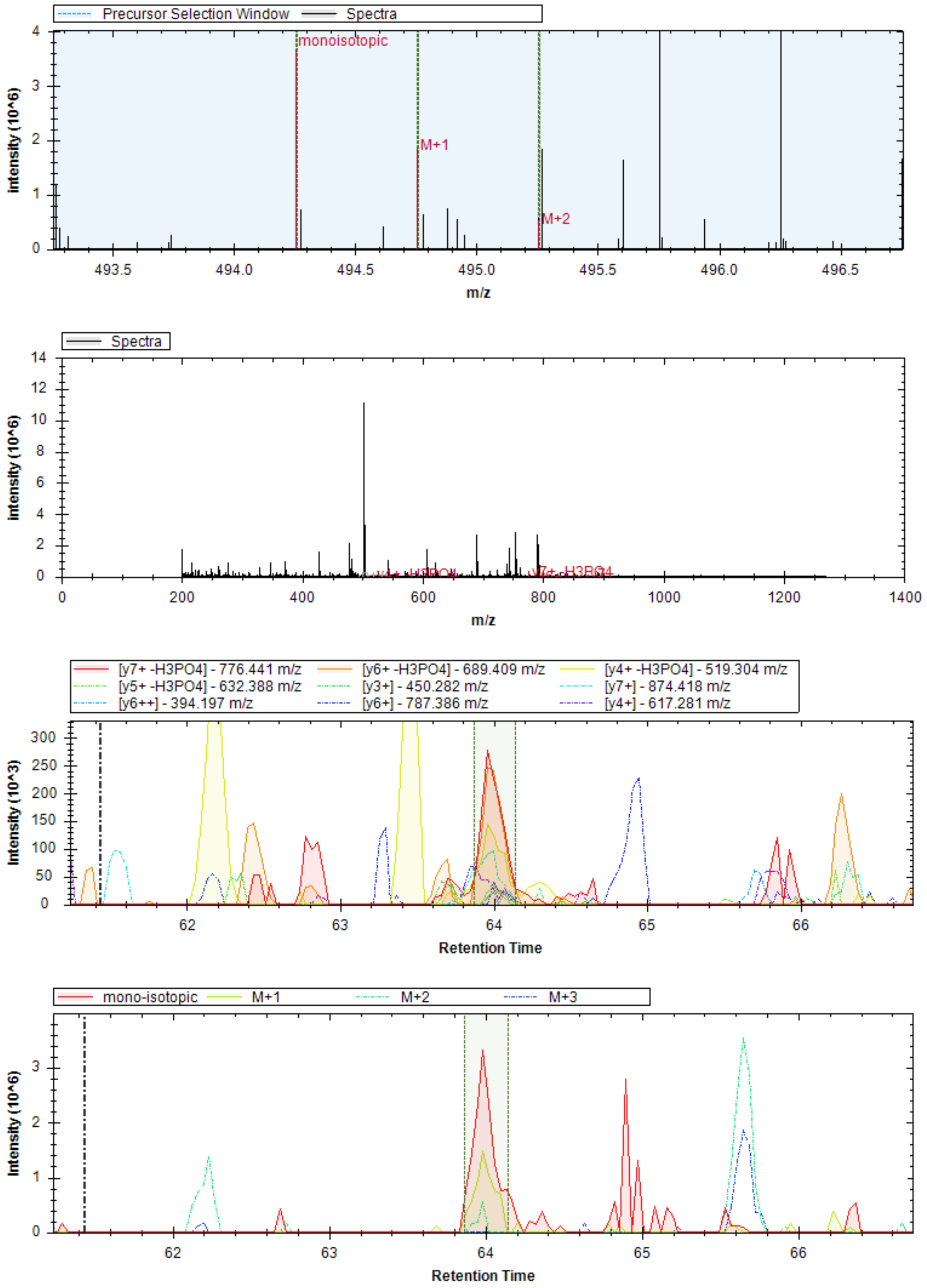

P08553 – _S[+H+O3+P]PVEEVKPKPEAK_.3
Fig4_MP-DIA-barrel-cortex-p54-2_MHRM.htrms

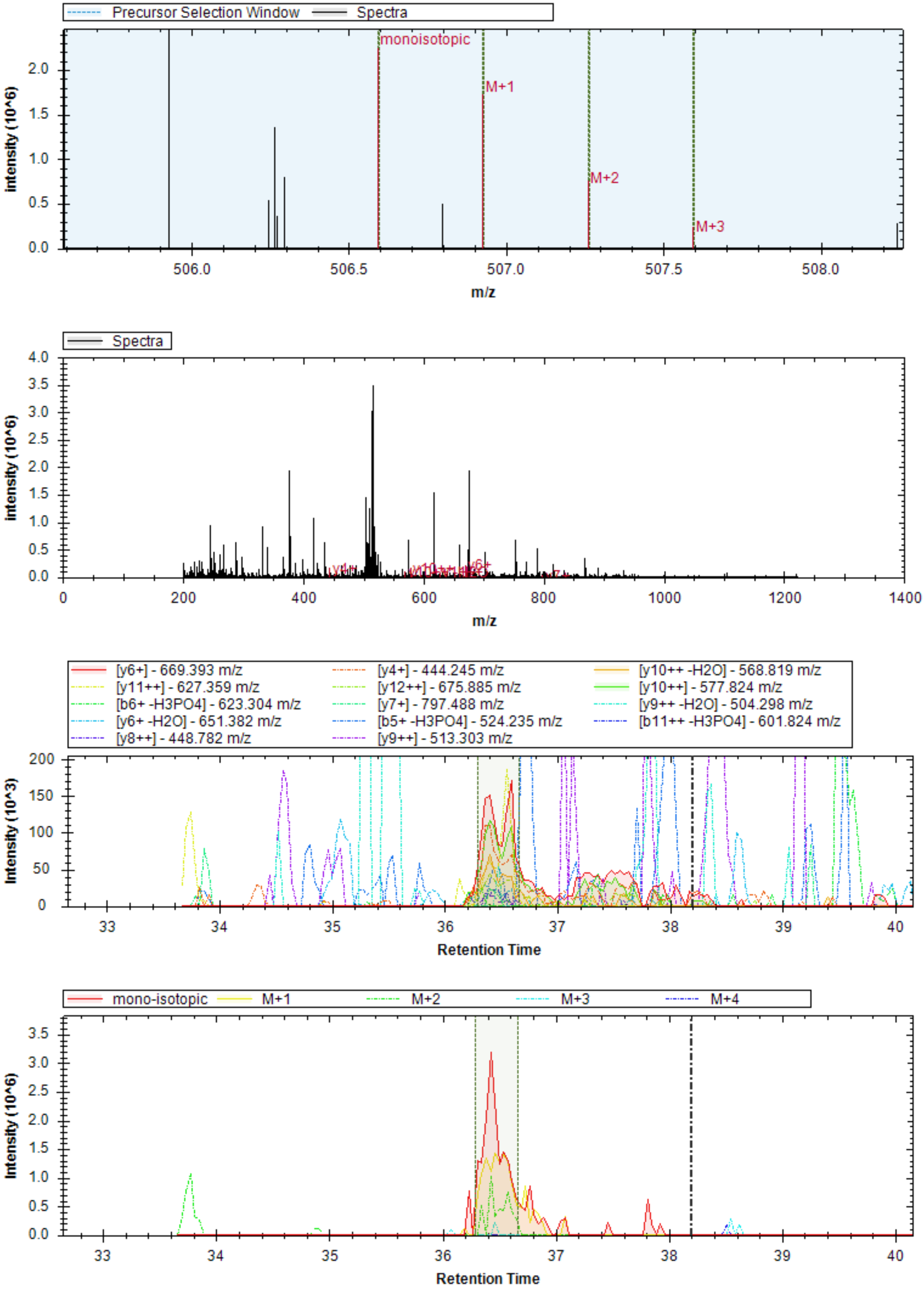

P28738 – _PIRPGHYPAS[+H+O3+P]SPTAVHAVR_.4
Fig4_MP-DIA-barrel-cortex-p54-2_MHRM.htrms

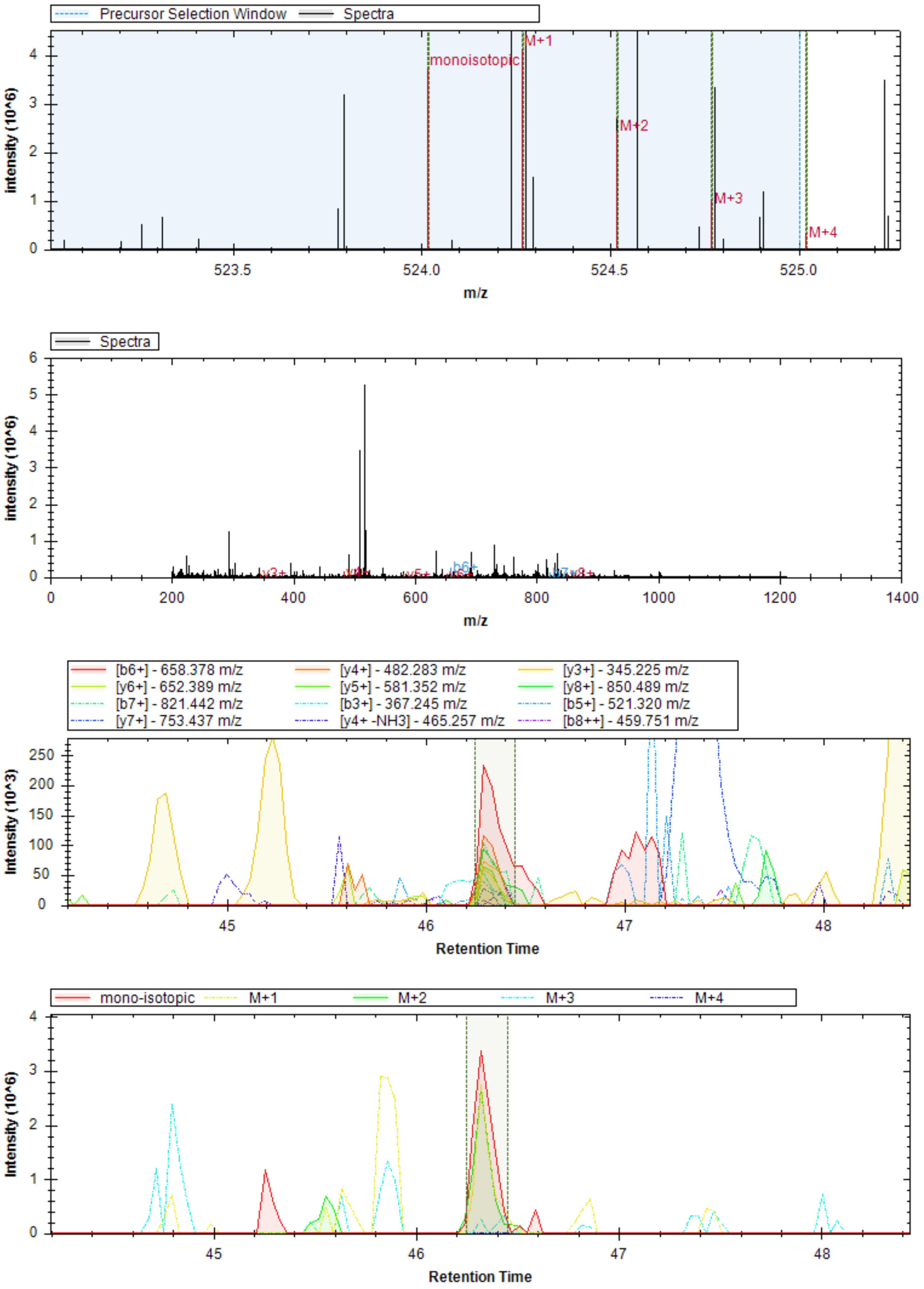

P28738 – _PIRPGHYPASS[+H+O3+P]PTAVHAVR_.4
Fig4_MP-DIA-barrel-cortex-p54-2_MHRM.htrms

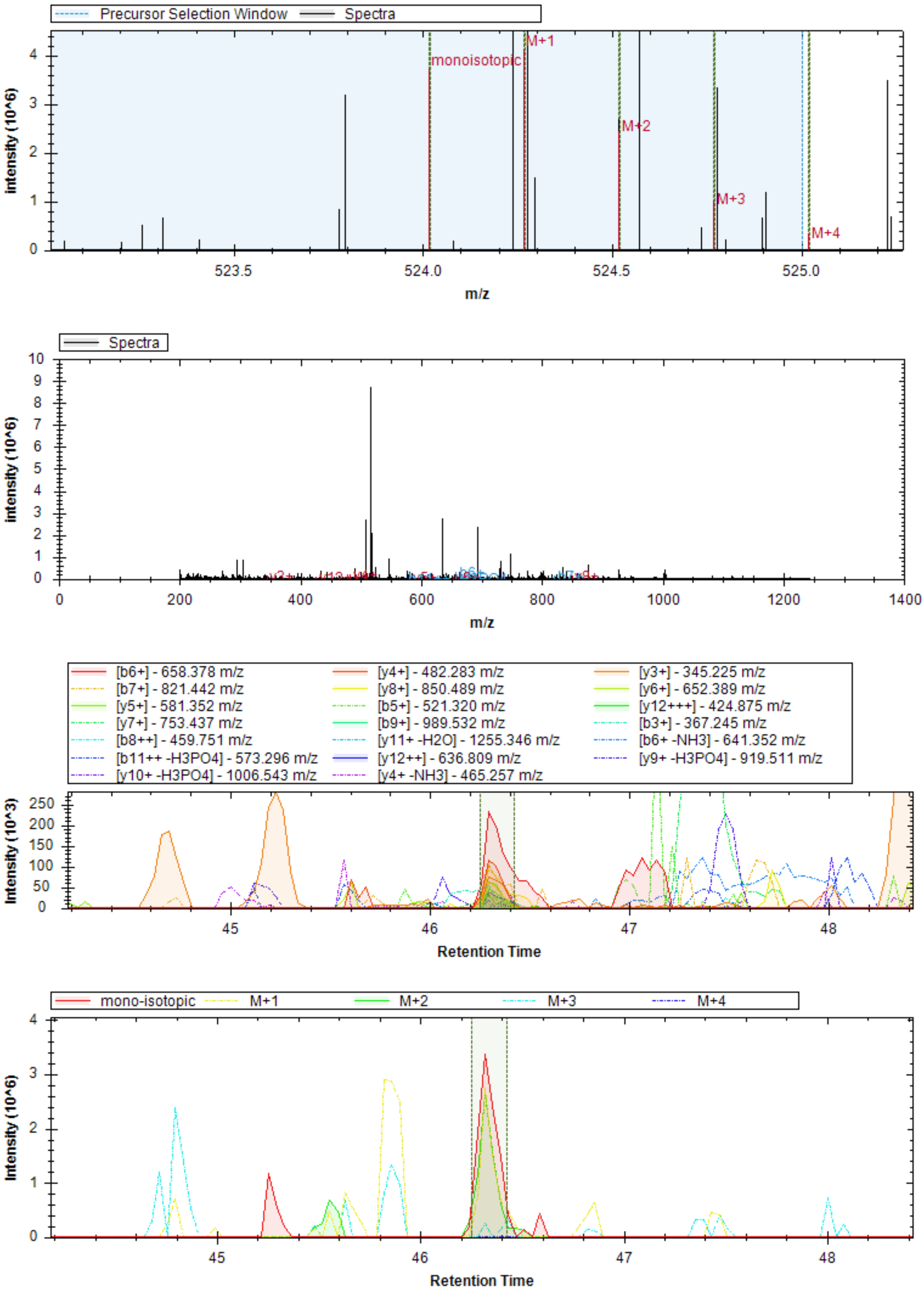

Q8CDY7 – _LVQALC[+C2+H3+N+O]EQIHT[+H+O3+P]R_.3
Fig4_MP-DIA-barrel-cortex-p54-2_MHRM.htrms

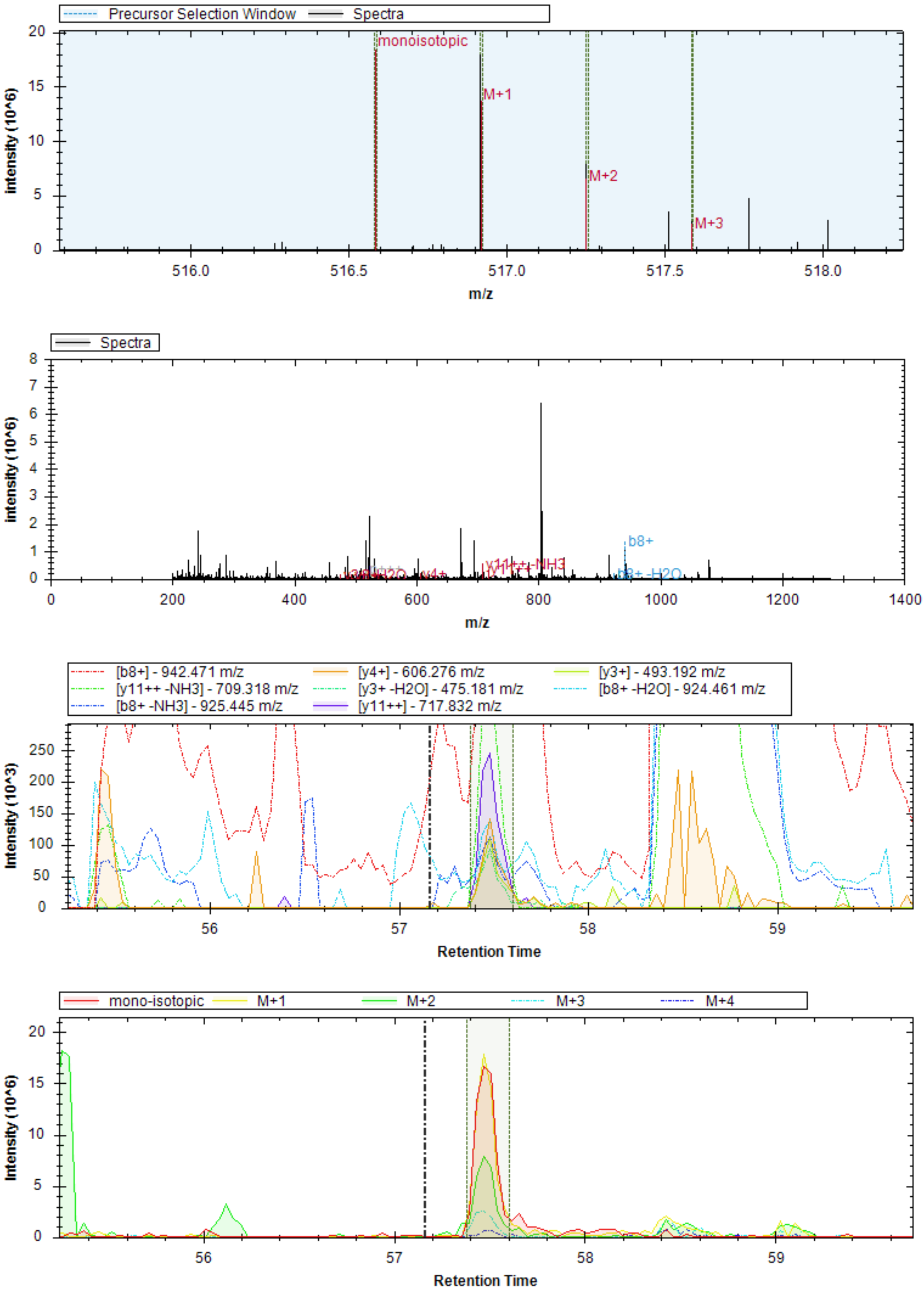

Q9QYC0 – _AAVVT[+H+O3+P]SPPPTTAPHK_.3

Fig4_MP-DIA-barrel-cortex-p54-2_MHRM.htrms

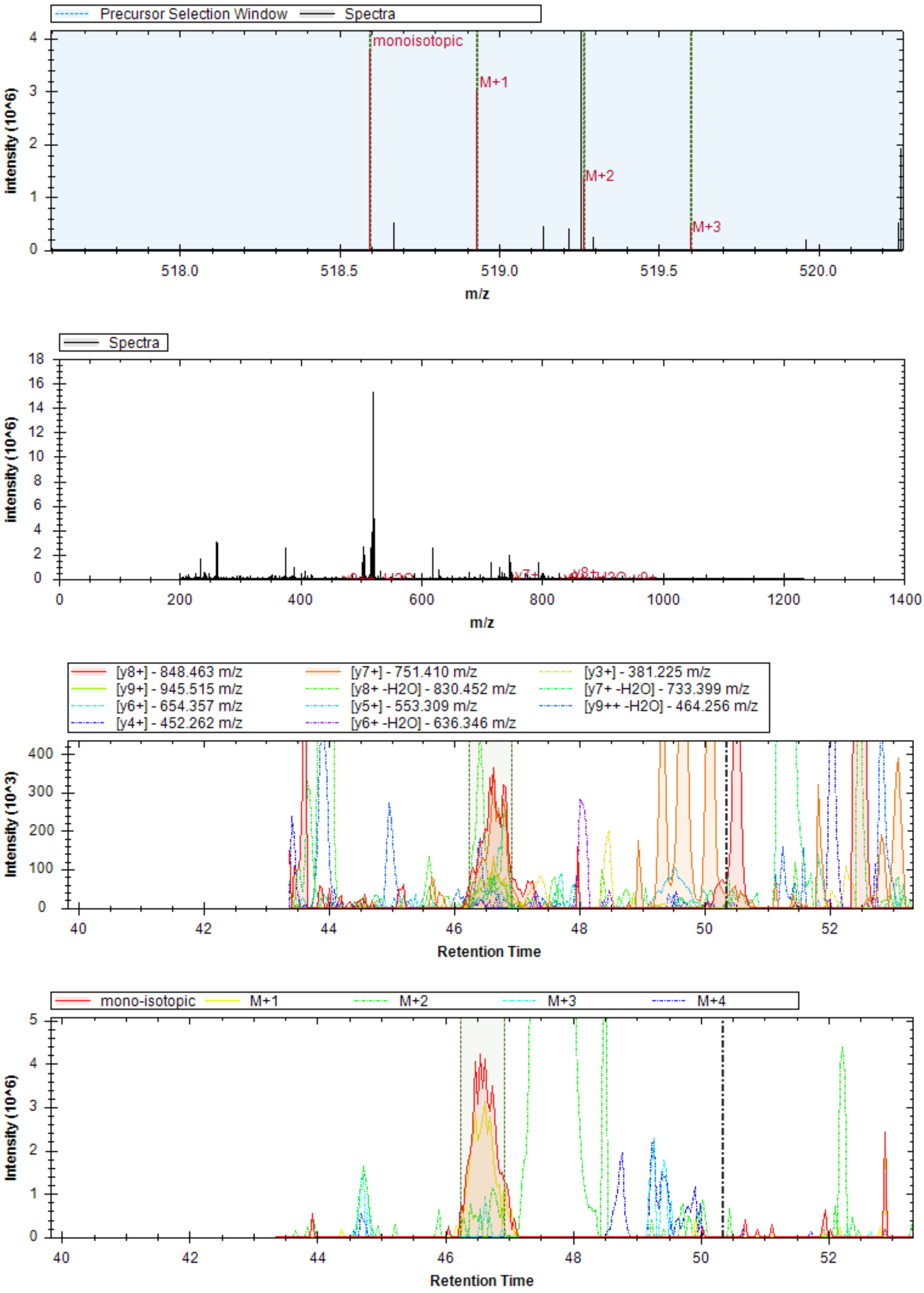

Q9QYC0 – _AAVVTS[+H+O3+P]PPPTTAPHK_.3
Fig4_MP-DIA-barrel-cortex-p54-2_MHRM.htrms

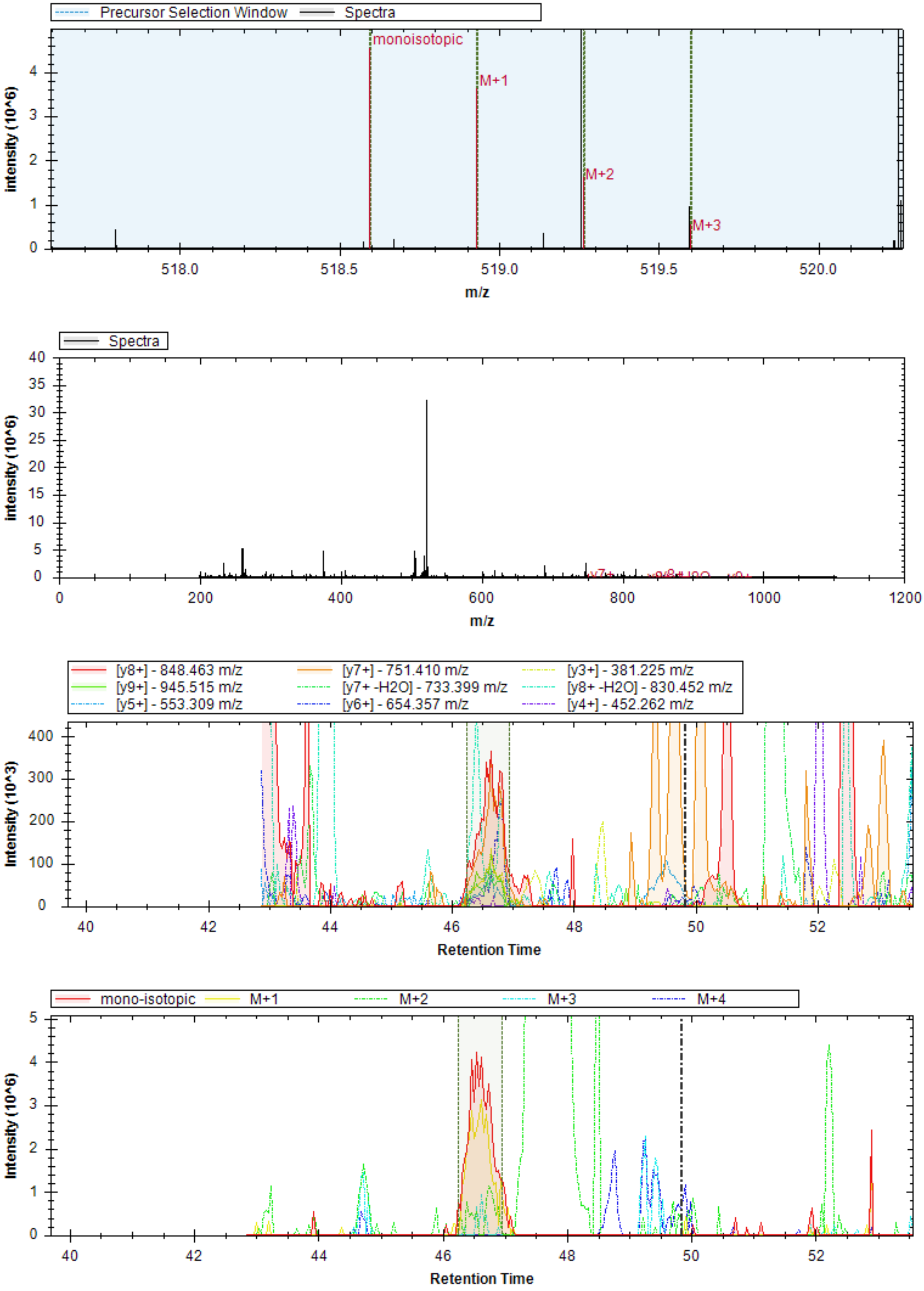

P10637 – _VAVVRT[+H+O3+P]PPKSPSASK_.3

Fig4_MP-DIA-barrel-cortex-p54-2_MHRM.htrms

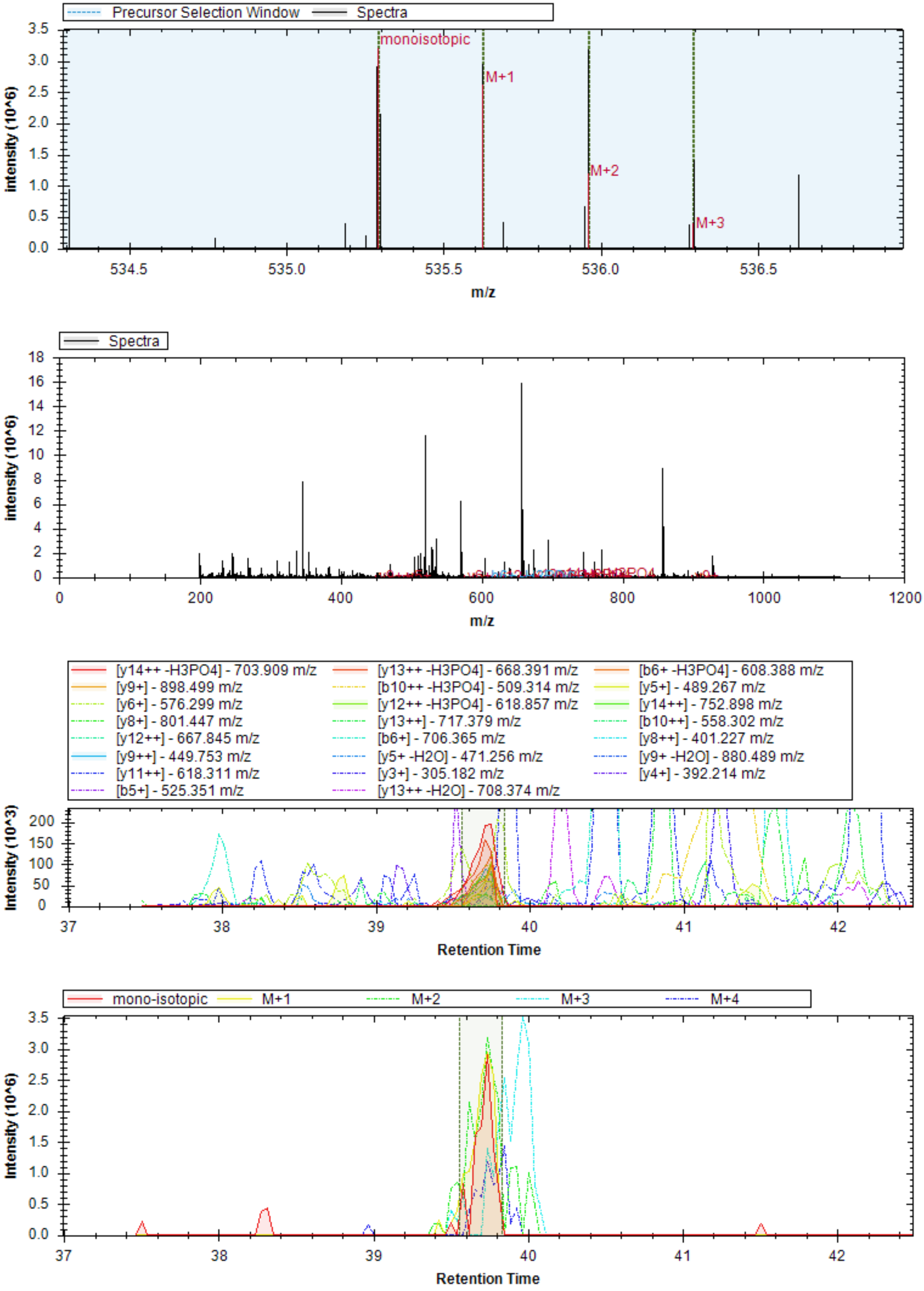

Q60771 – _FYYSSGSSS[+H+O3+P]PTHAK_.3
Fig4_MP-DIA-barrel-cortex-p54-2_MHRM.htrms

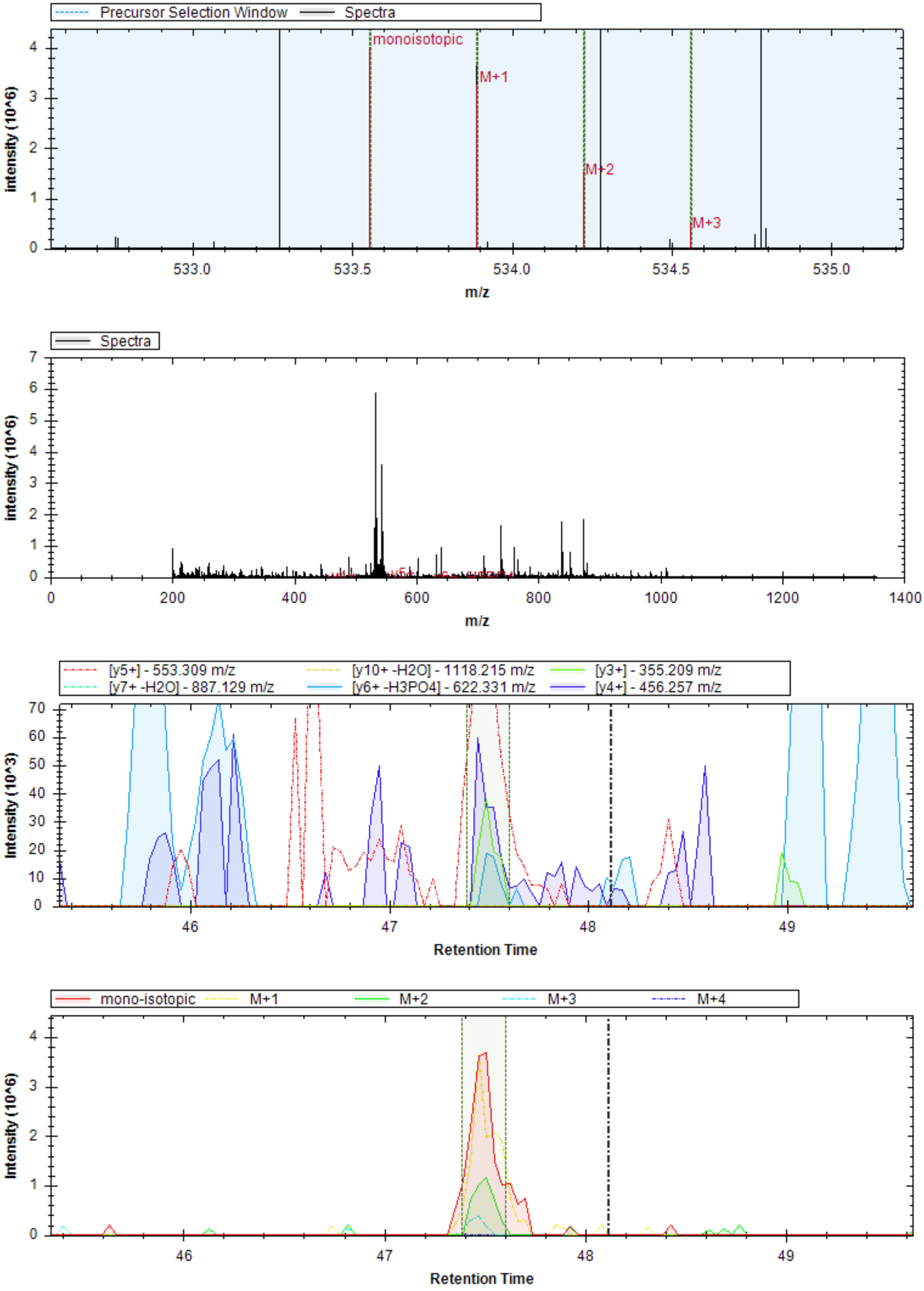

Q7TQD2 – _AANKT[+H+O3+P]PPKSPGDPAR_.3
Fig4_MP-DIA-barrel-cortex-p54-2_MHRM.htrms

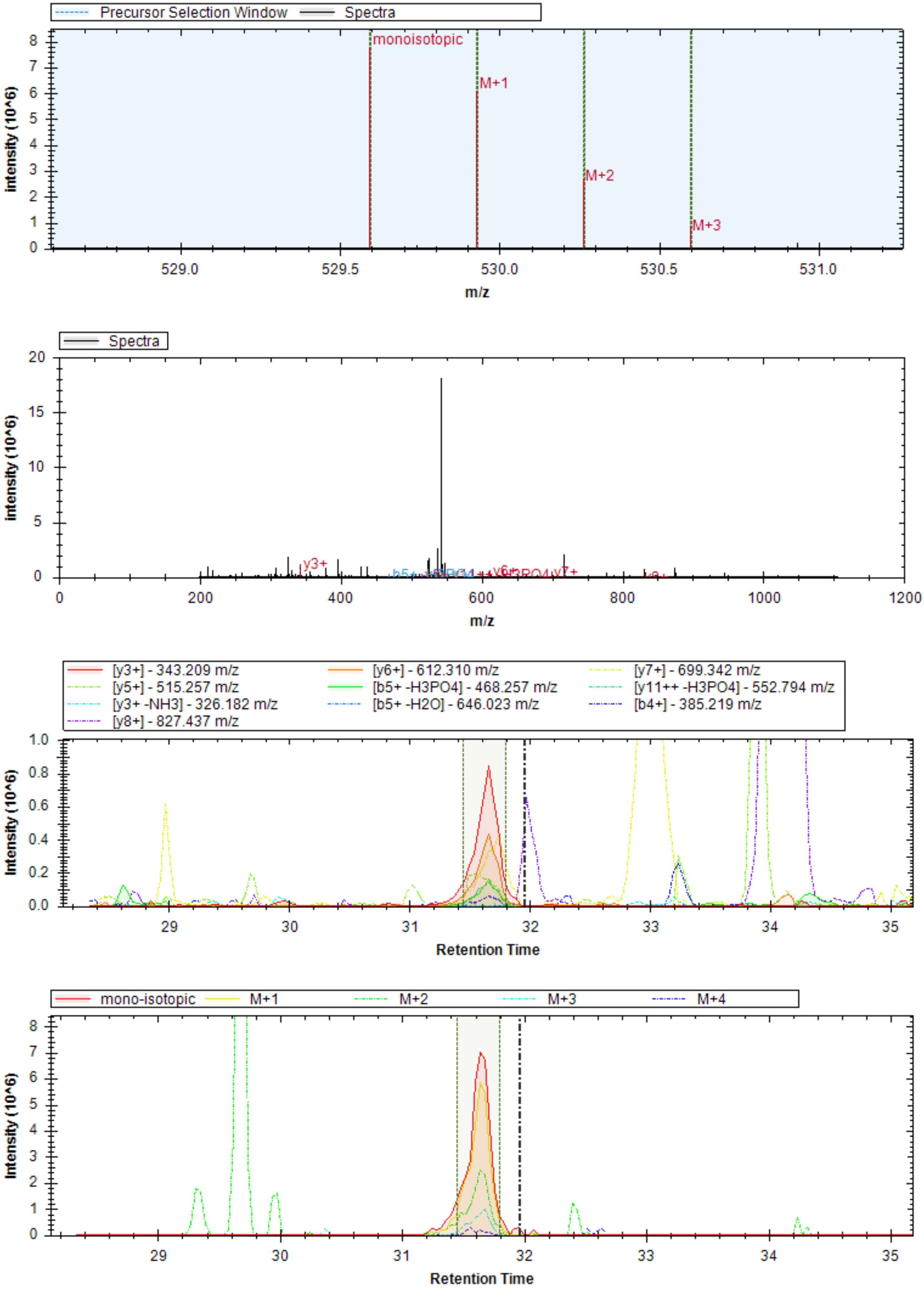

Q9D898 – _AFHAALRNS[+H+O3+P]PINTK_.3

Fig4_MP-DIA-barrel-cortex-p54-2_MHRM.htrms

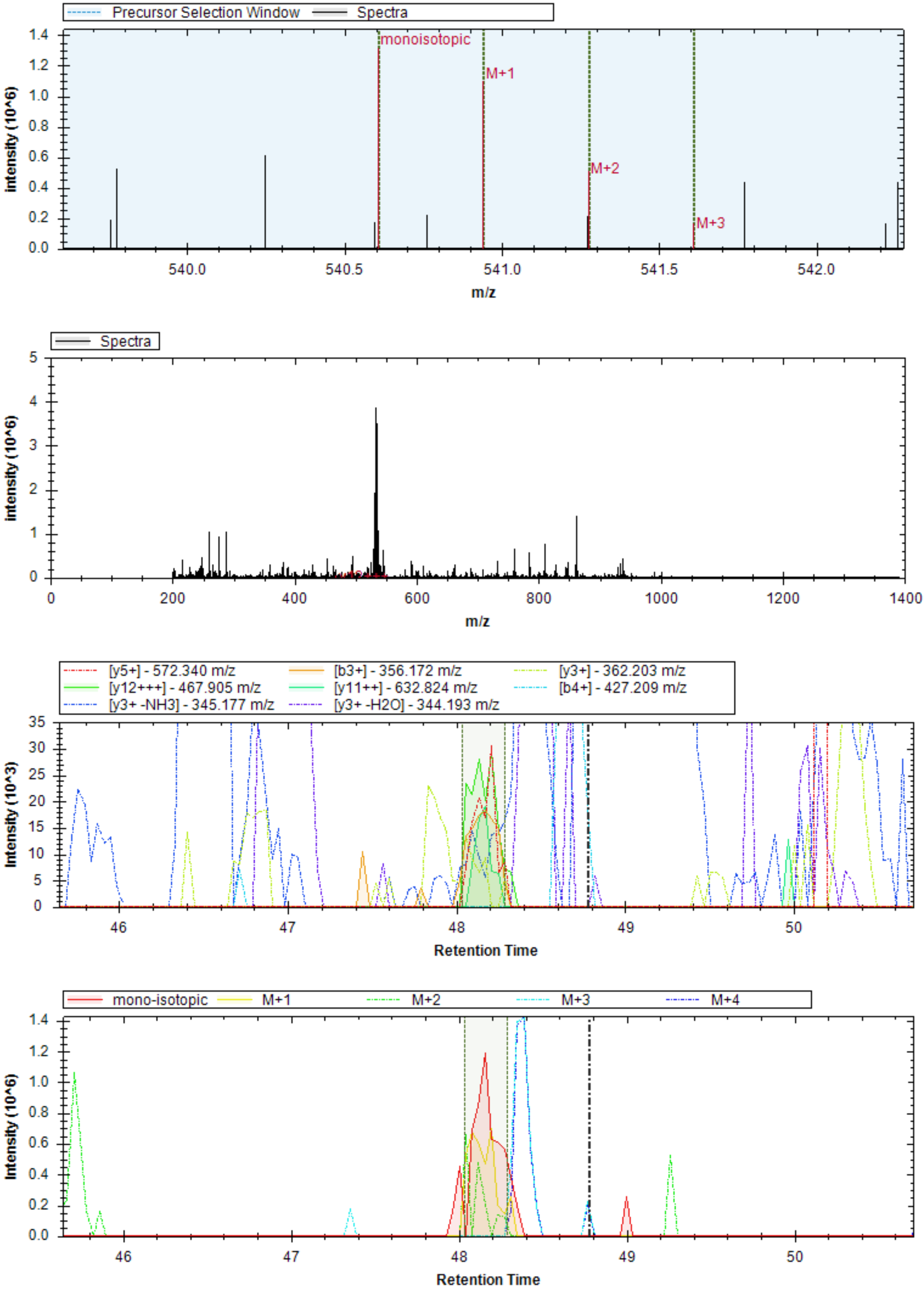

P20357 – _VAIIRT[+H+O3+P]PPKSPATPK_.3
Fig4_MP-DIA-barrel-cortex-p54-2_MHRM.htrms

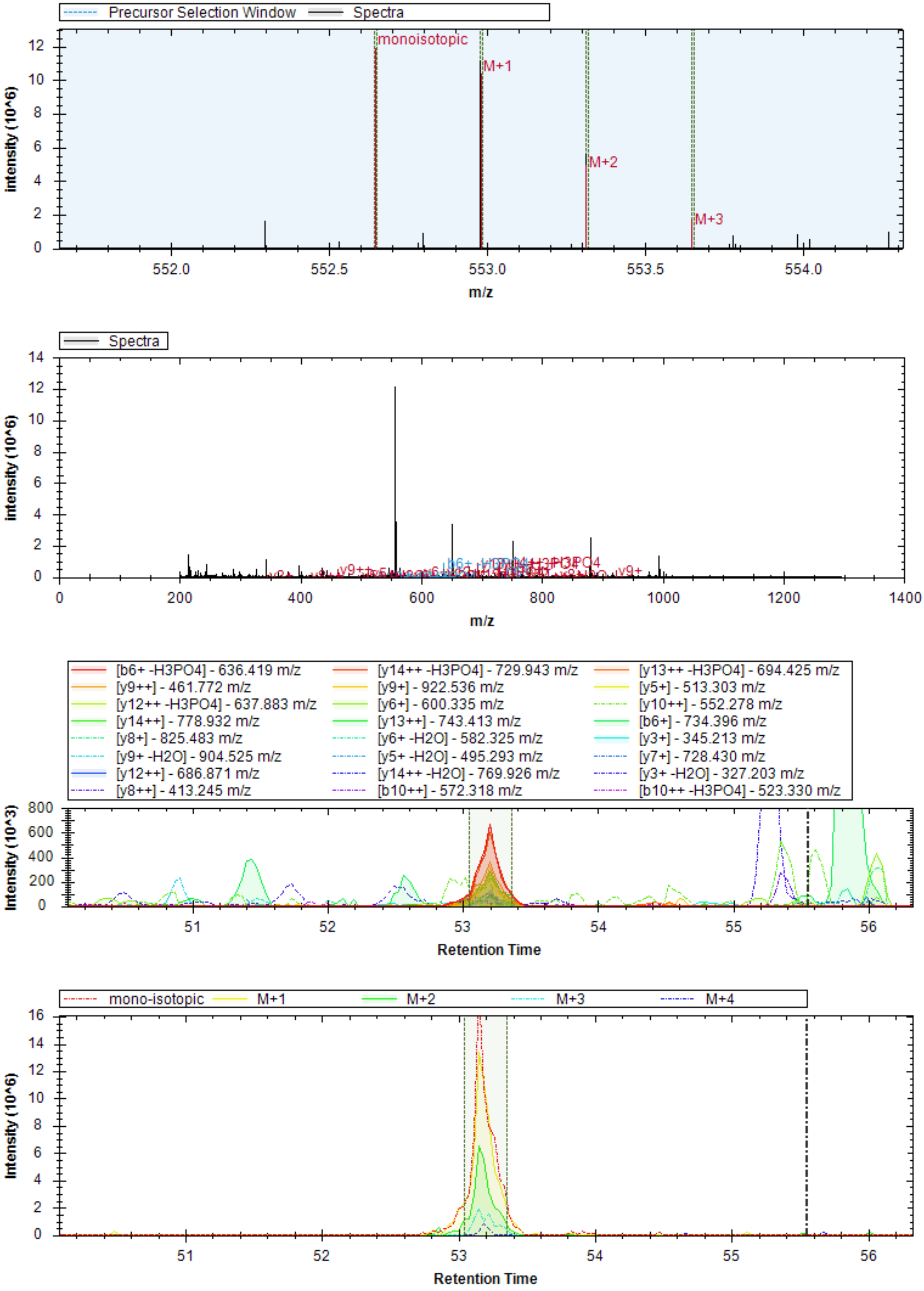

Q9CYT6 – _THTPSPTS[+H+O3+P]PK_.2

Fig4_MP-DIA-barrel-cortex-p54-2_MHRM.htrms

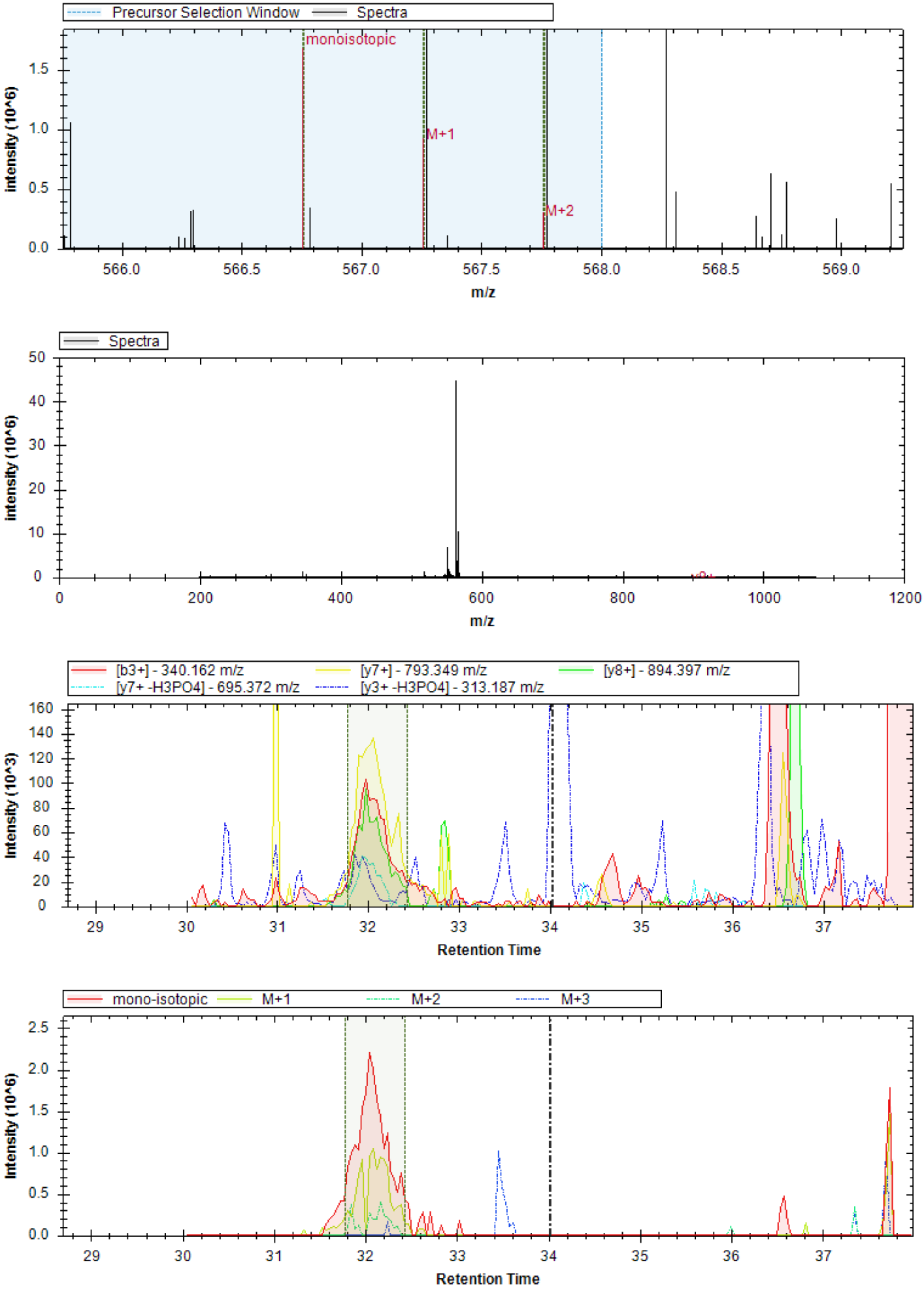

Q9R0K7 – _T[+H+O3+P]SIHNFMAHPEFR_.3
Fig4_MP-DIA-barrel-cortex-p54-2_MHRM.htrms

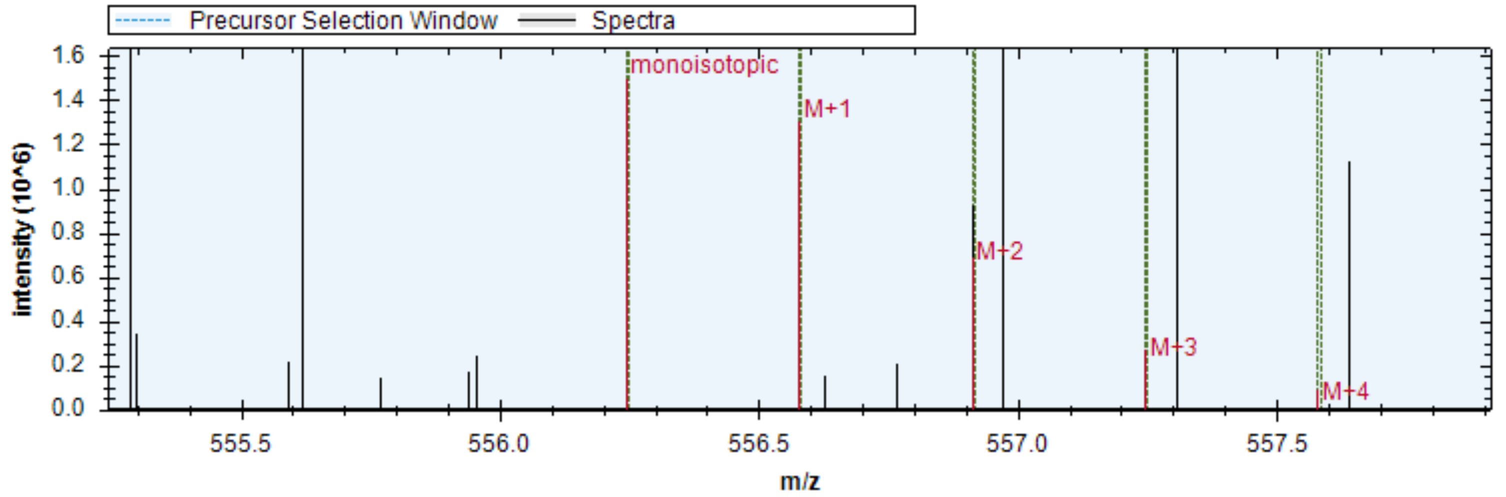

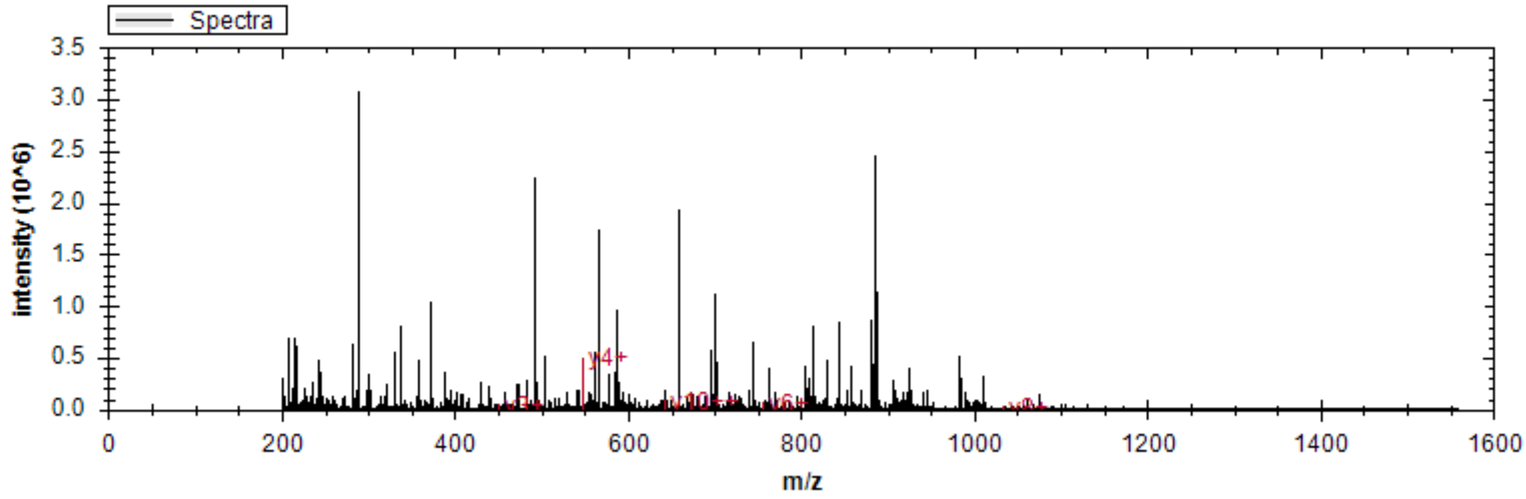

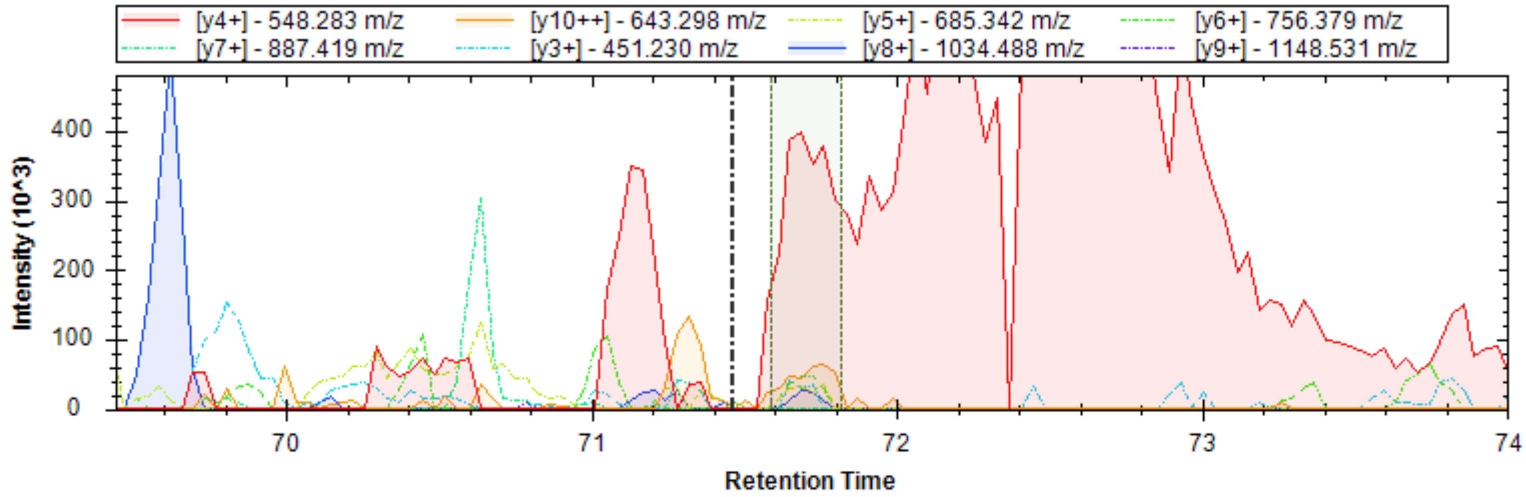

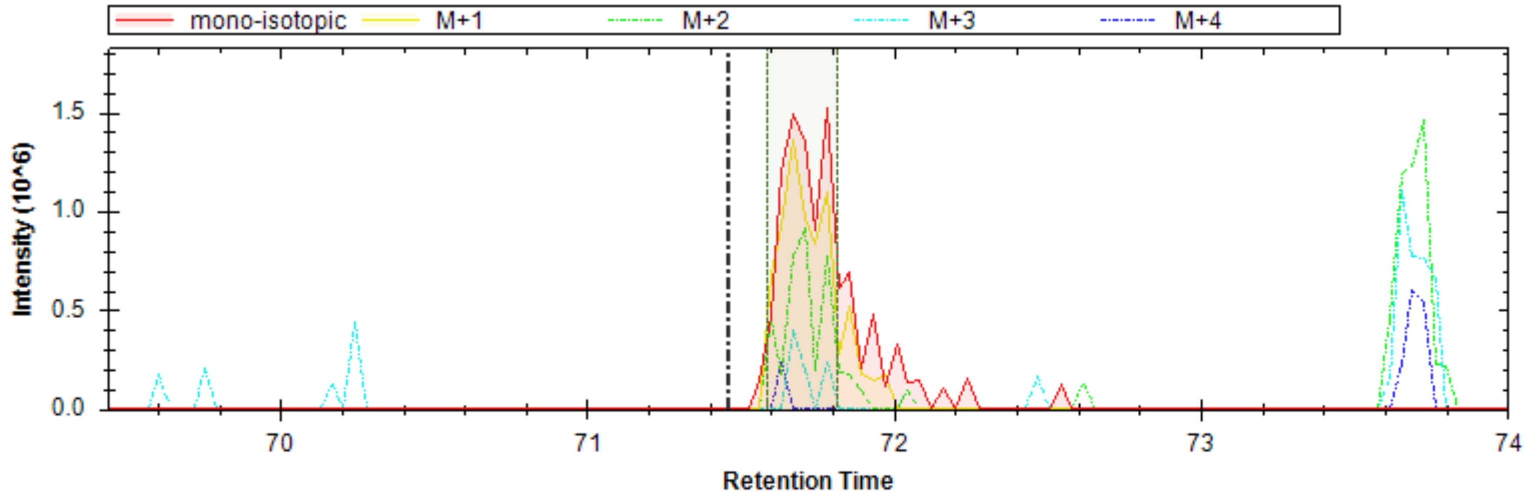

O88935 – _QAS[+H+O3+P]ISGPAPTK_.2

Fig4_MP-DIA-barrel-cortex-p54-2_MHRM.htrms

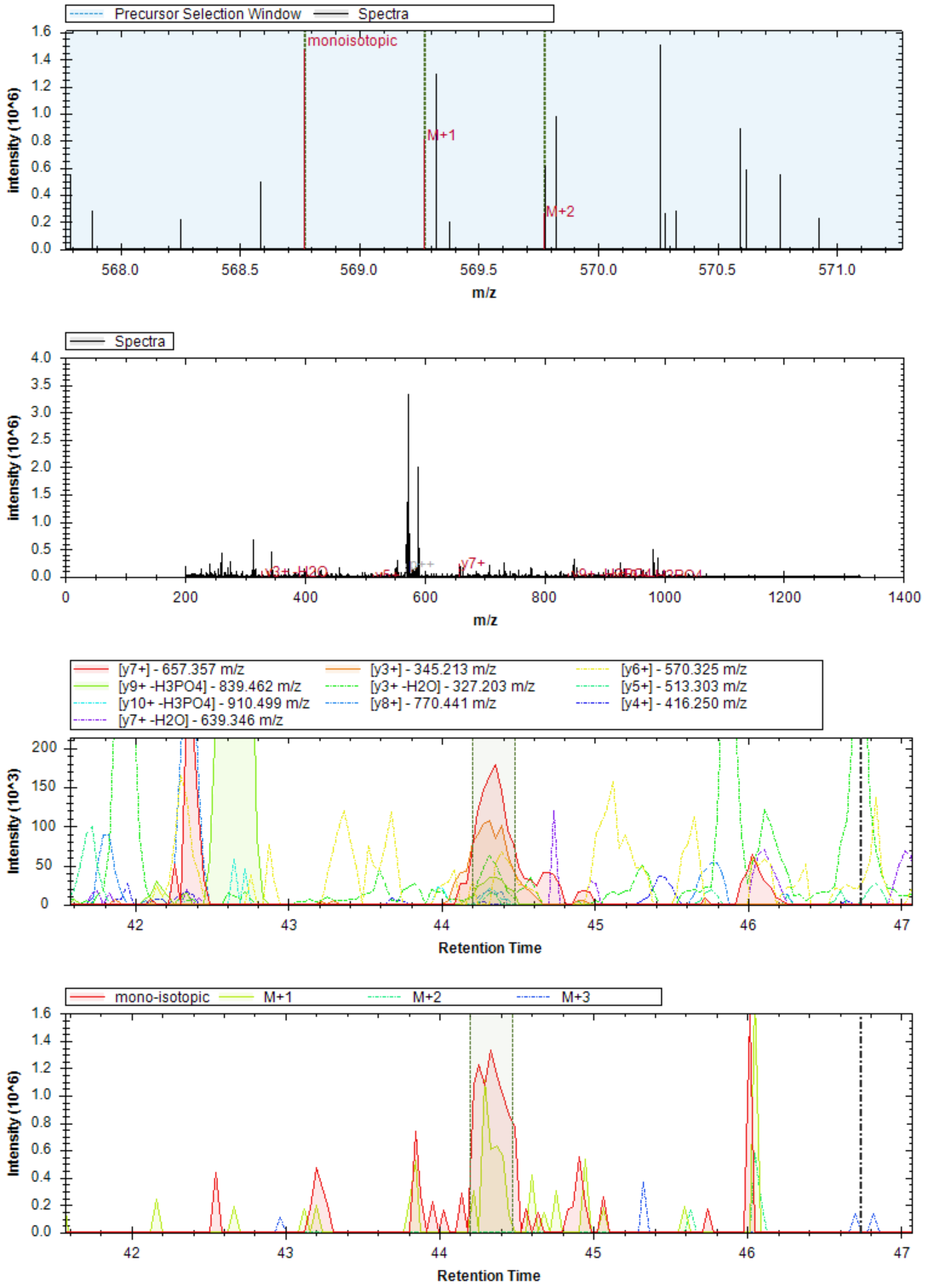

P27546-2 – _T[+H+O3+P]SPSKPSSAPALKPGPK_.3
Fig4_MP-DIA-barrel-cortex-p54-2_MHRM.htrms

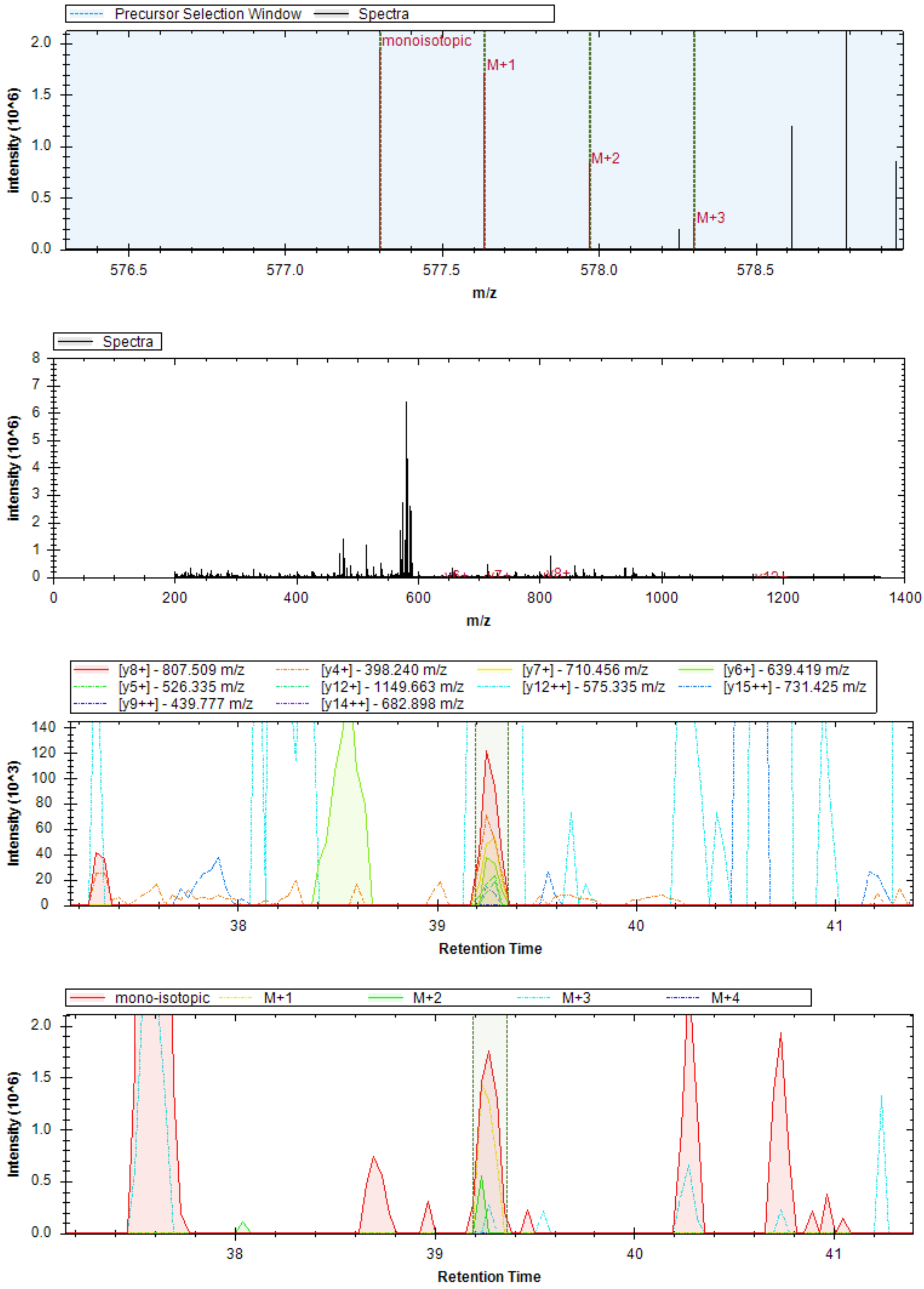

Q8BFU3-4 – _VAS[+H+O3+P]STQVRGASGRELR_.3
Fig4_MP-DIA-barrel-cortex-p54-2_MHRM.htrms

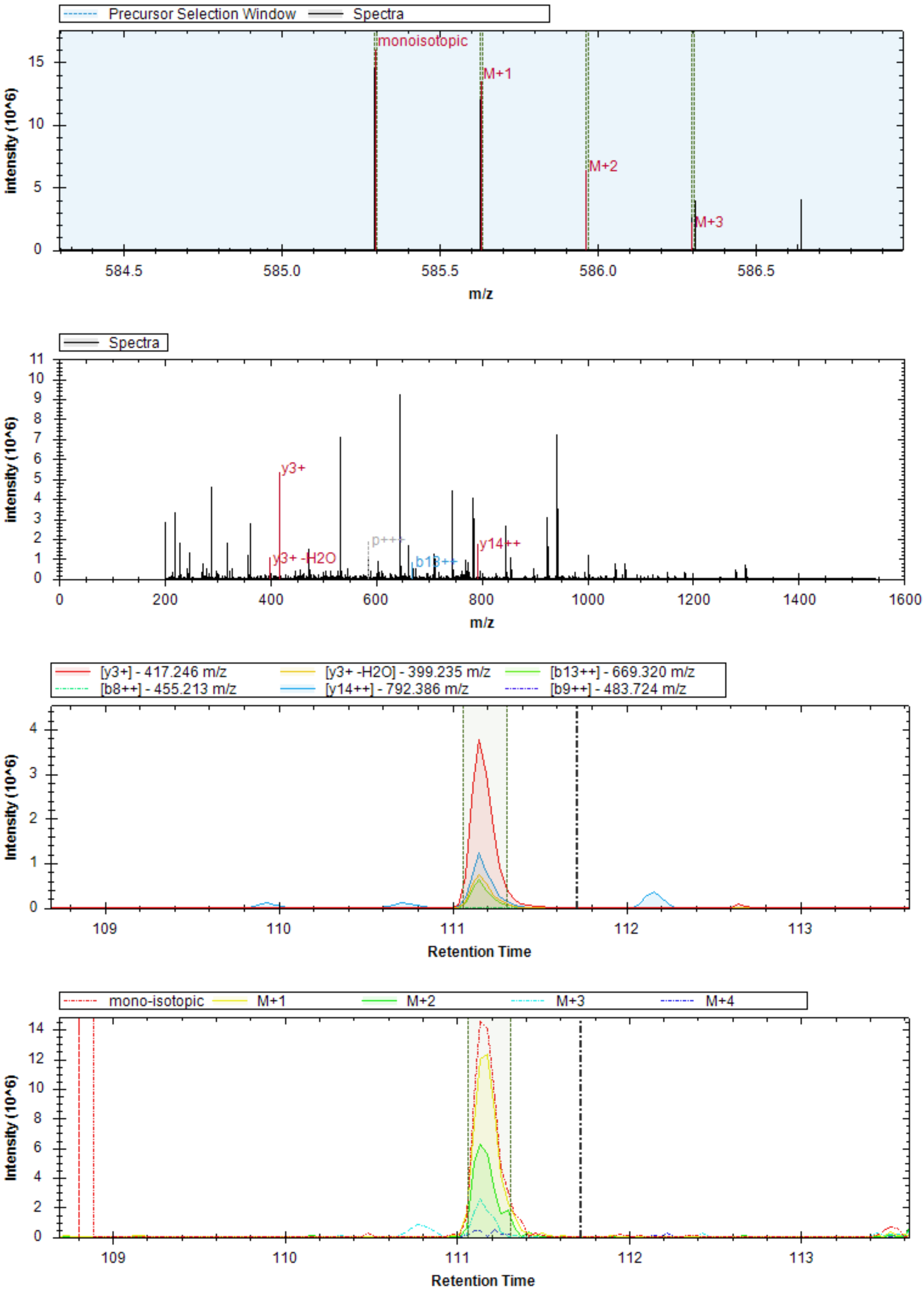

Q9CYT6 – _AQGQIRS[+H+O3+P]PTK_.2
Fig4_MP-DIA-barrel-cortex-p54-2_MHRM.htrms

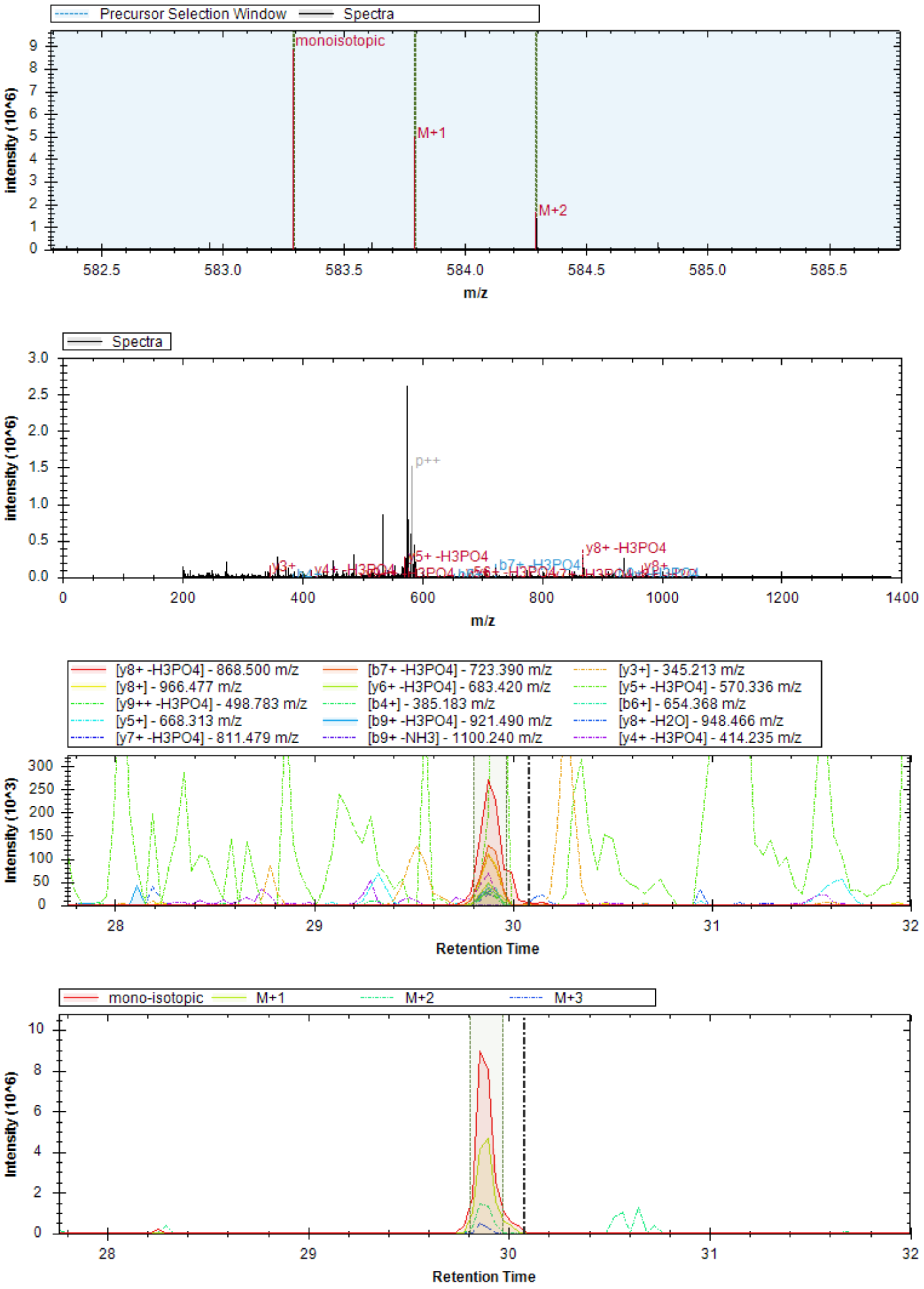

Q9D5Y0 – _ITDT[+H+O3+P]KKTDDLYWR_.3
Fig4_MP-DIA-barrel-cortex-p54-2_MHRM.htrms

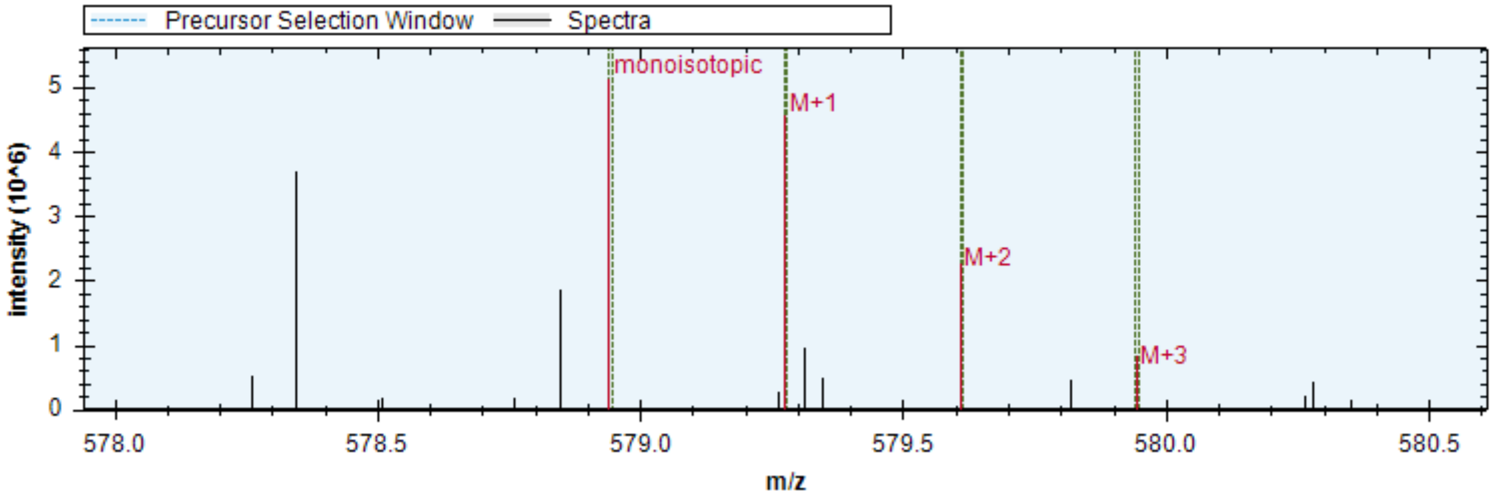

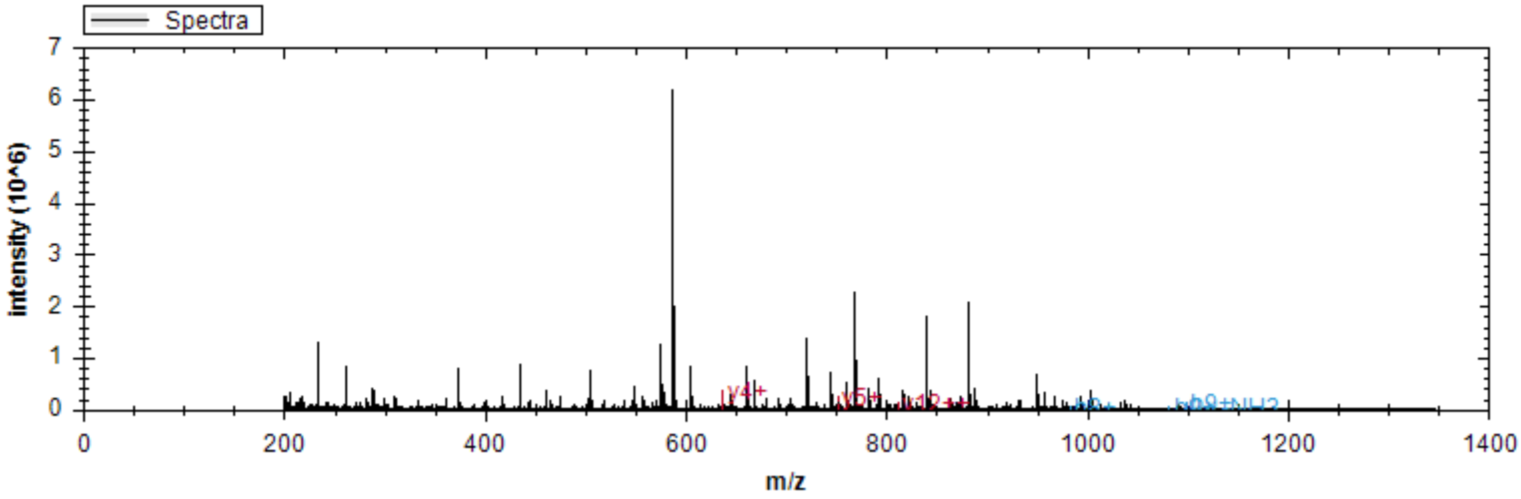

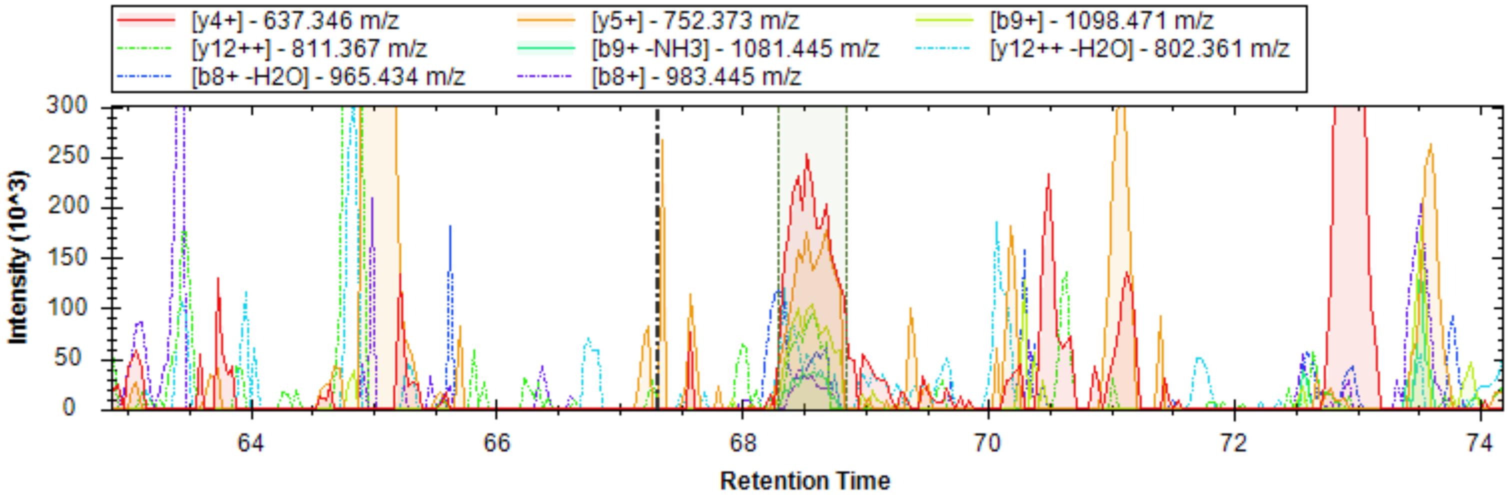

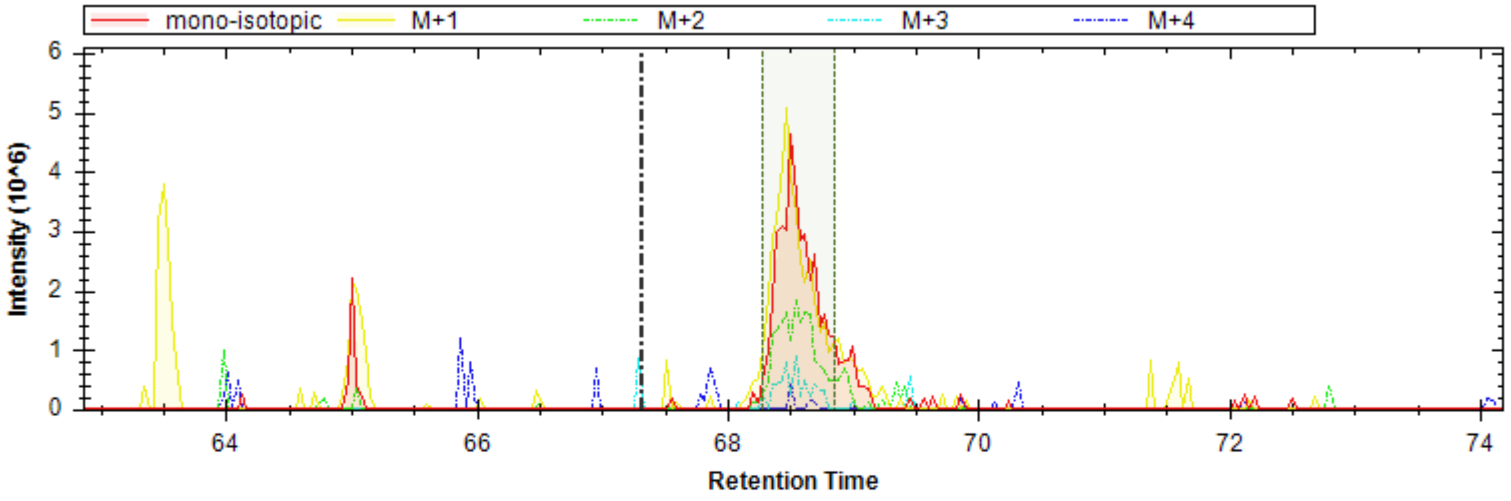

P04370-4 – _TTHYGS[+H+O3+P]LPQK_.2

Fig4_MP-DIA-barrel-cortex-p54-2_MHRM.htrms

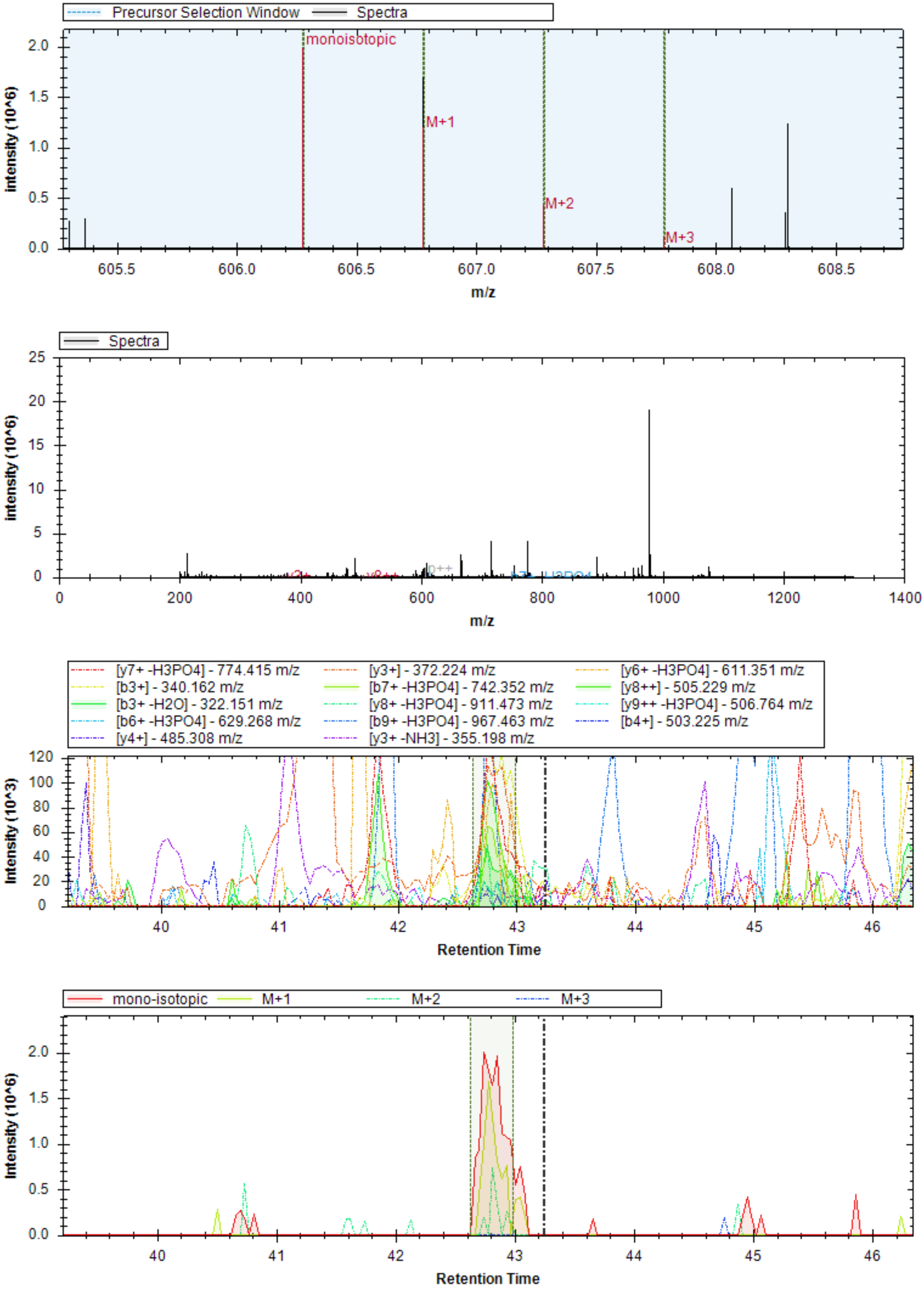

Q62261 – _RPPS[+H+O3+P]PDPNTK_.2
Fig4_MP-DIA-barrel-cortex-p54-2_MHRM.htrms

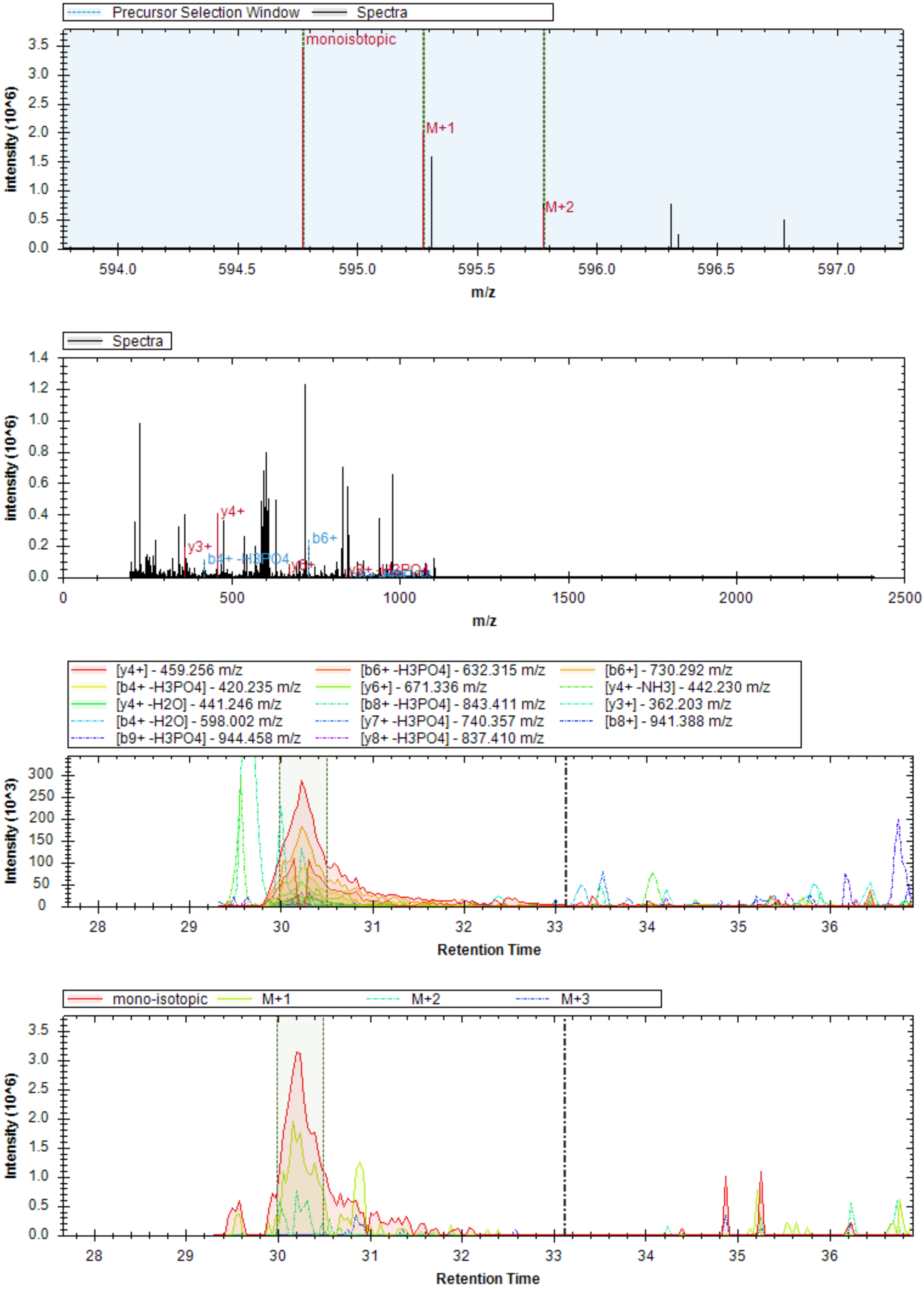

Q80VN0 – _ELTAQIT[+H+O3+P]SIK_.2
Fig4_MP-DIA-barrel-cortex-p54-2_MHRM.htrms

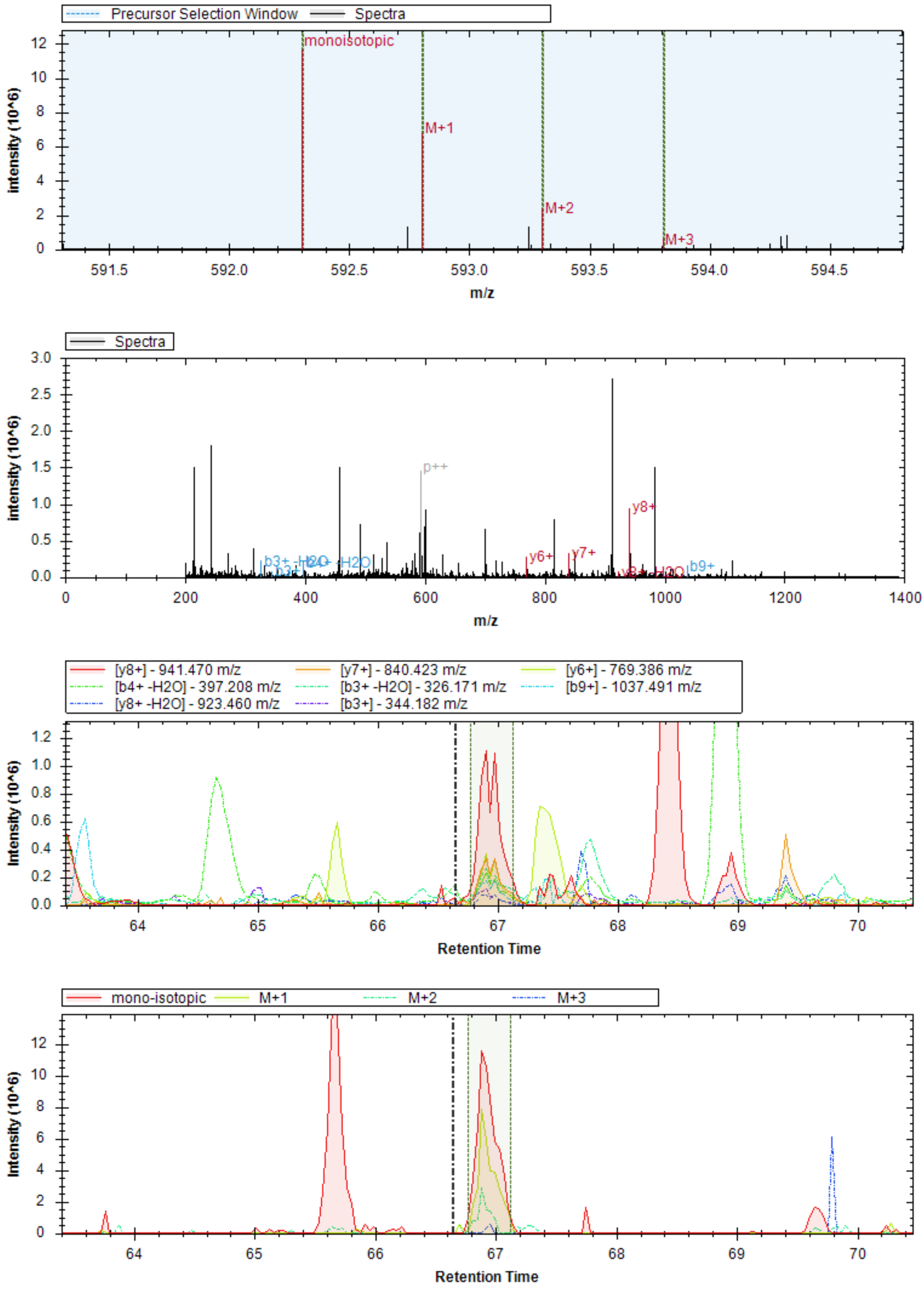

Q99104 – _AIS[+H+O3+P]PTSATSSGR_.2

Fig4_MP-DIA-barrel-cortex-p54-2_MHRM.htrms

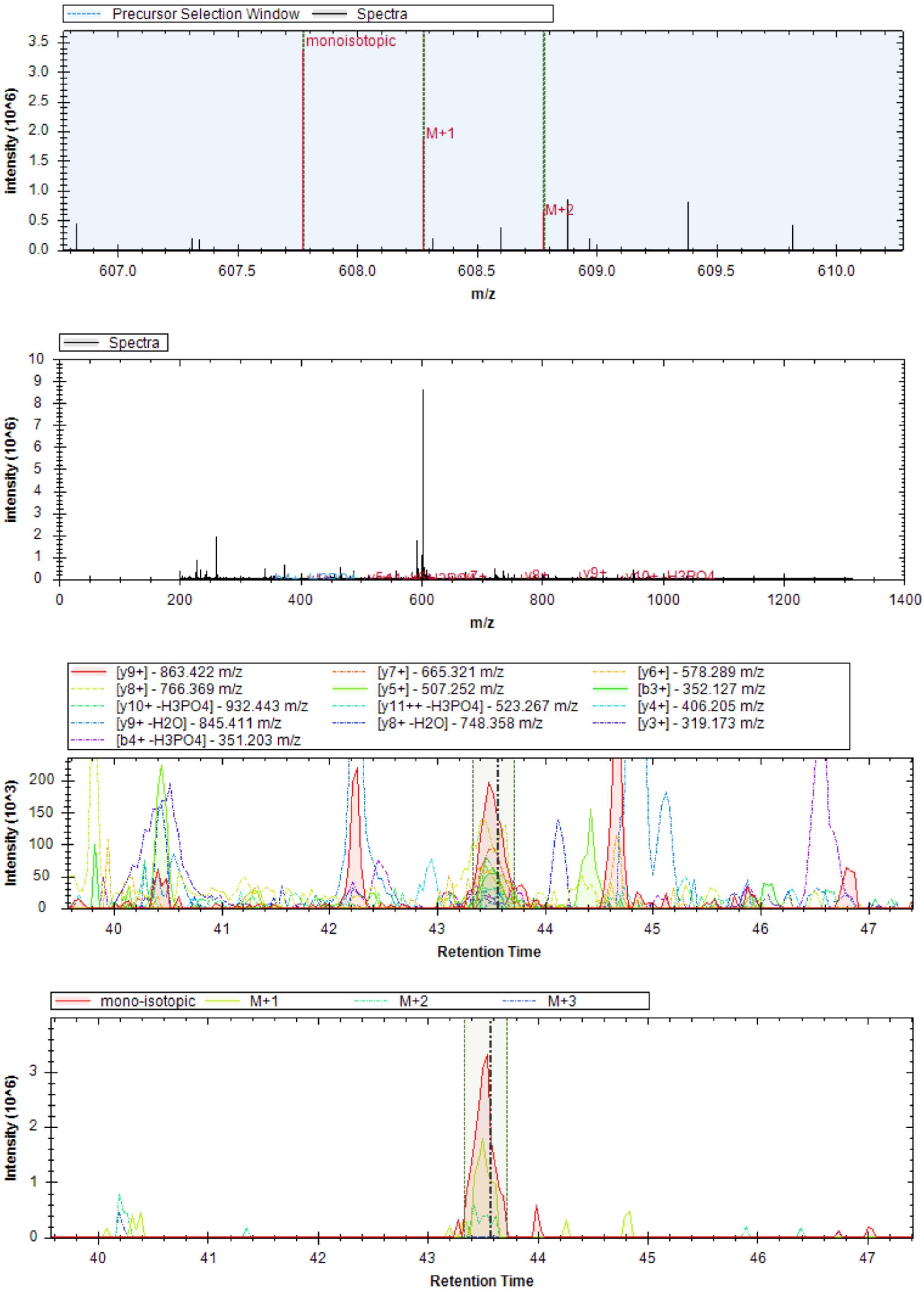

Q9QWI6 – _LSYAGGRPPSYAGS[+H+O3+P]PVHHAAER_.4
Fig4_MP-DIA-barrel-cortex-p54-2_MHRM.htrms

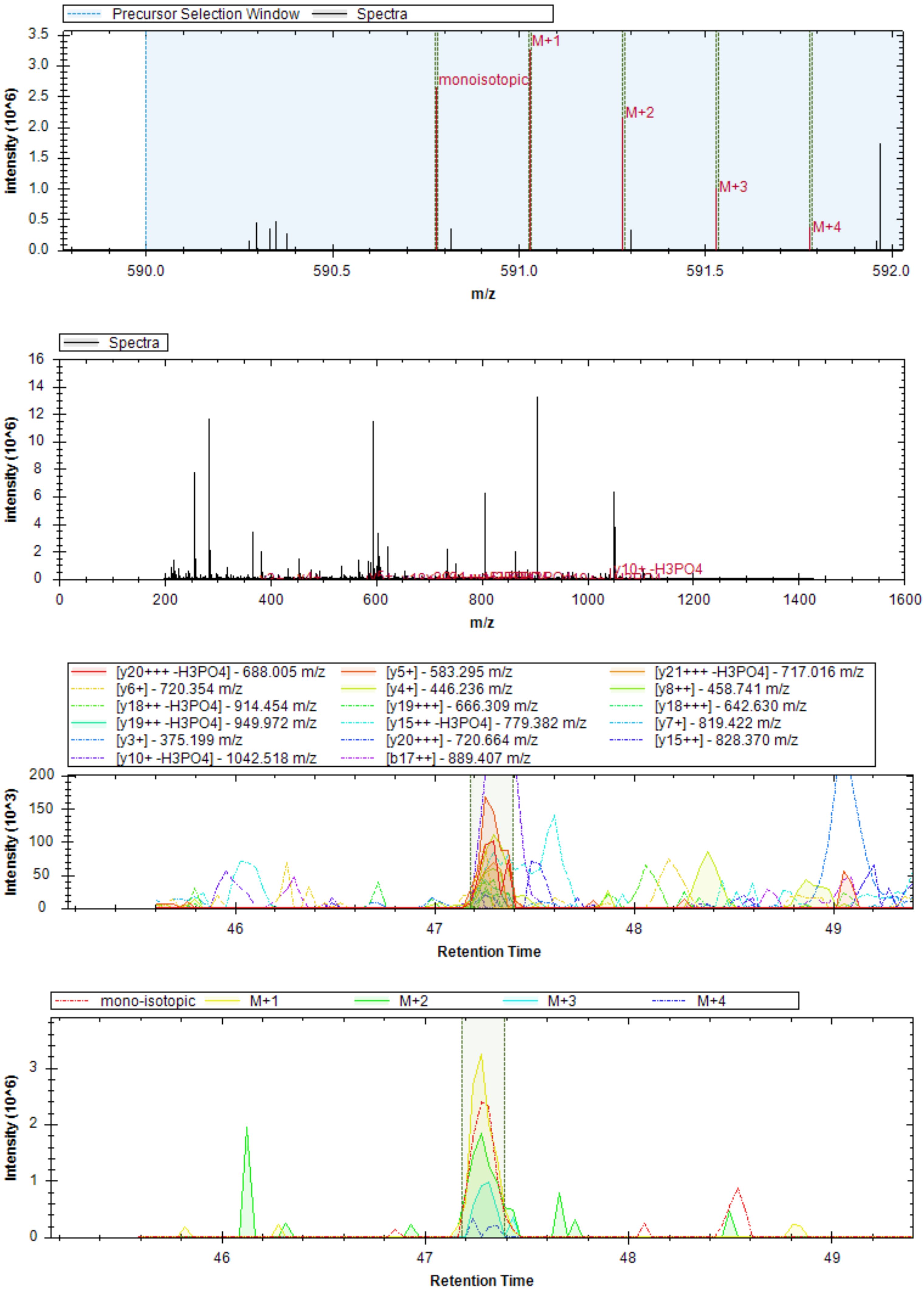

P60904 – _SLS[+H+O3+P]TSGESLYHVLGLDK_.3
Fig4_MP-DIA-barrel-cortex-p54-2_MHRM.htrms

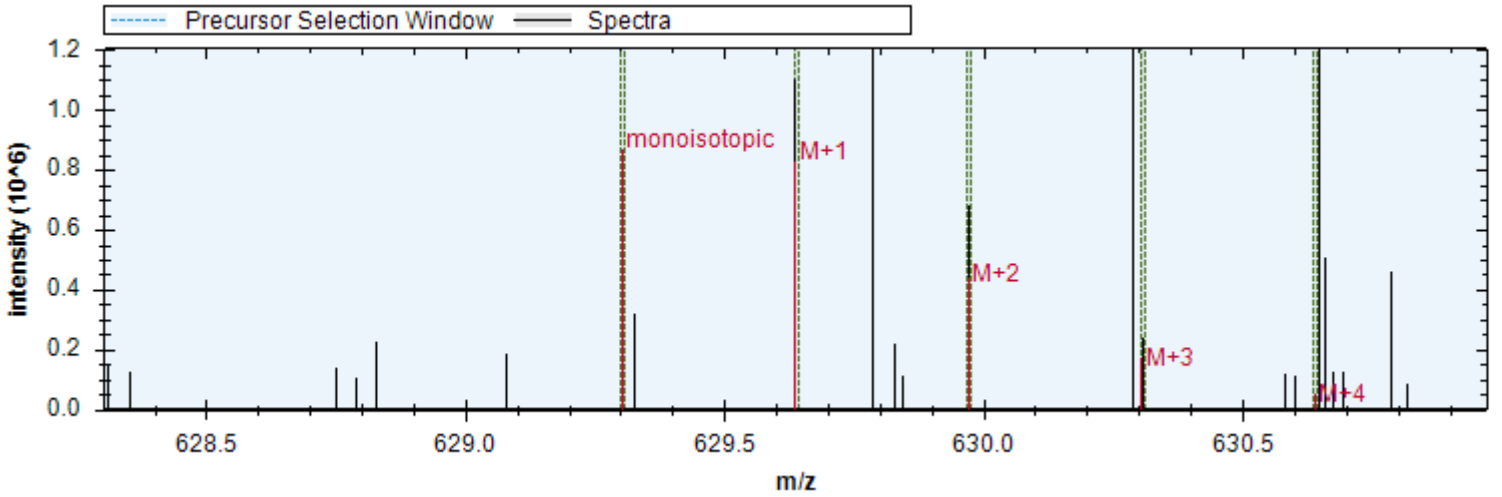

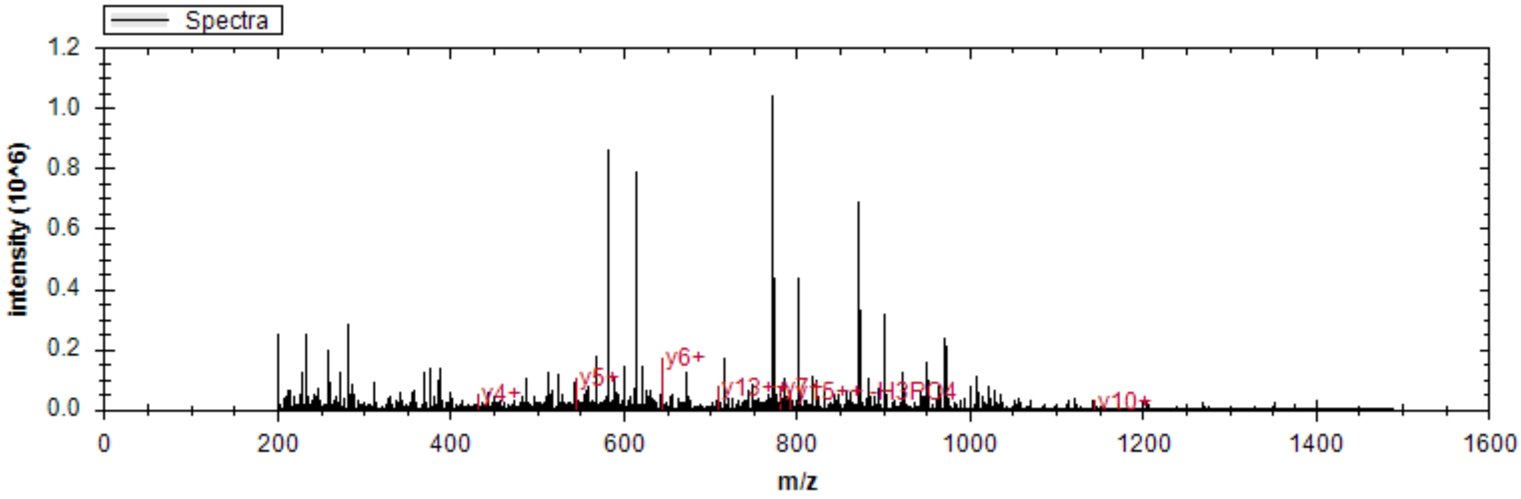

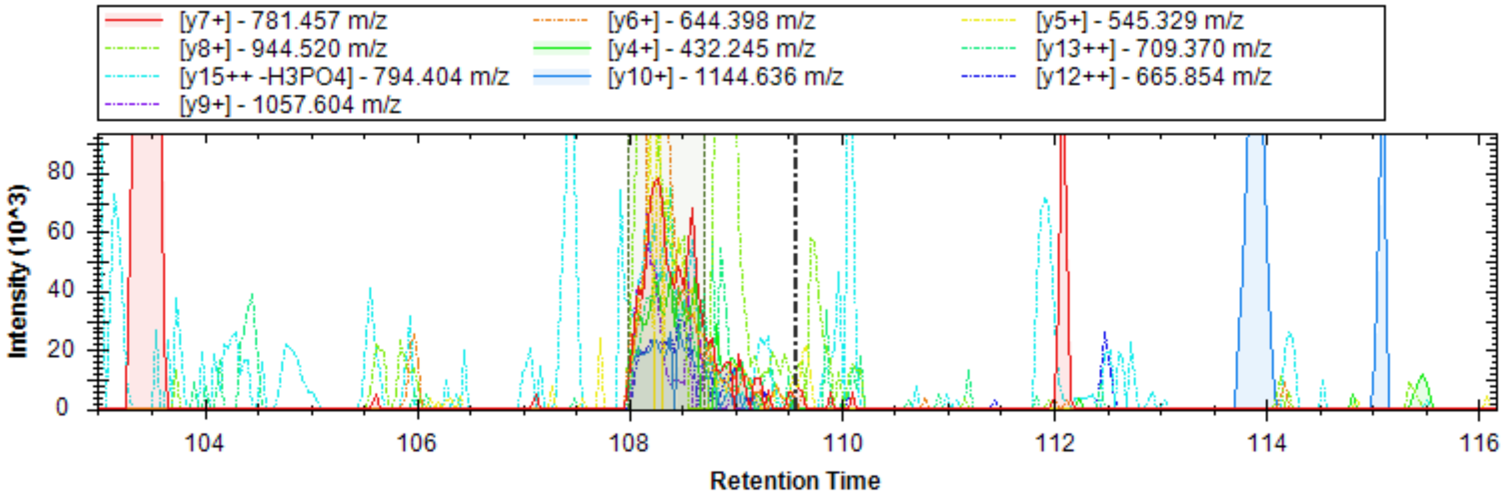

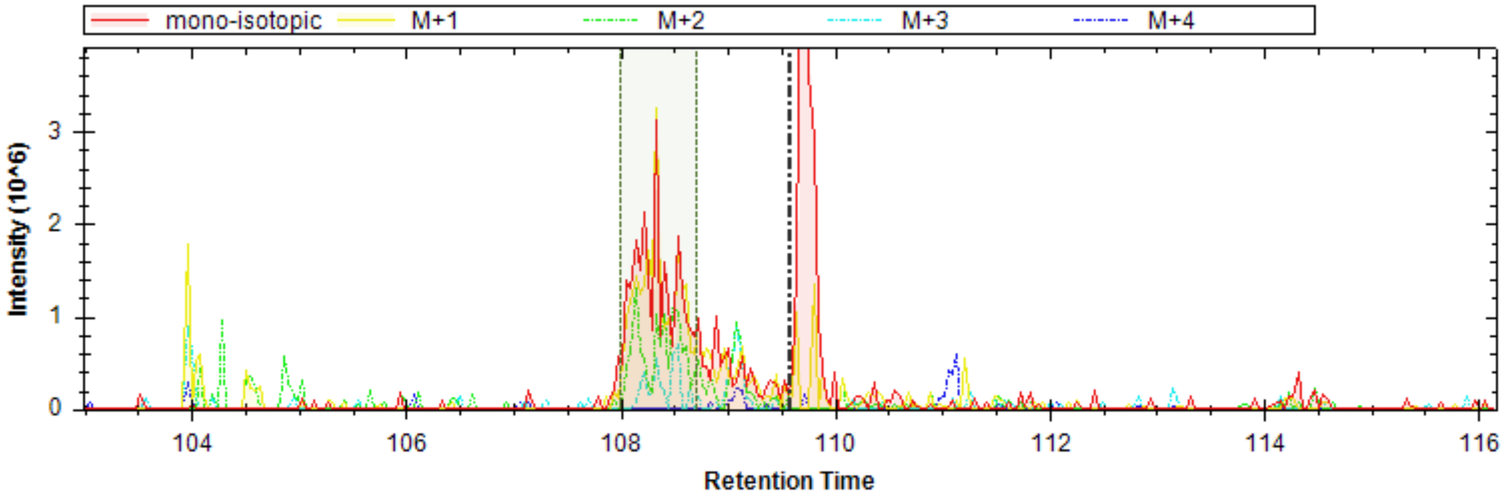

P60904 – _SLST[+H+O3+P]SGESLYHVLGLDK_.3
Fig4_MP-DIA-barrel-cortex-p54-2_MHRM.htrms

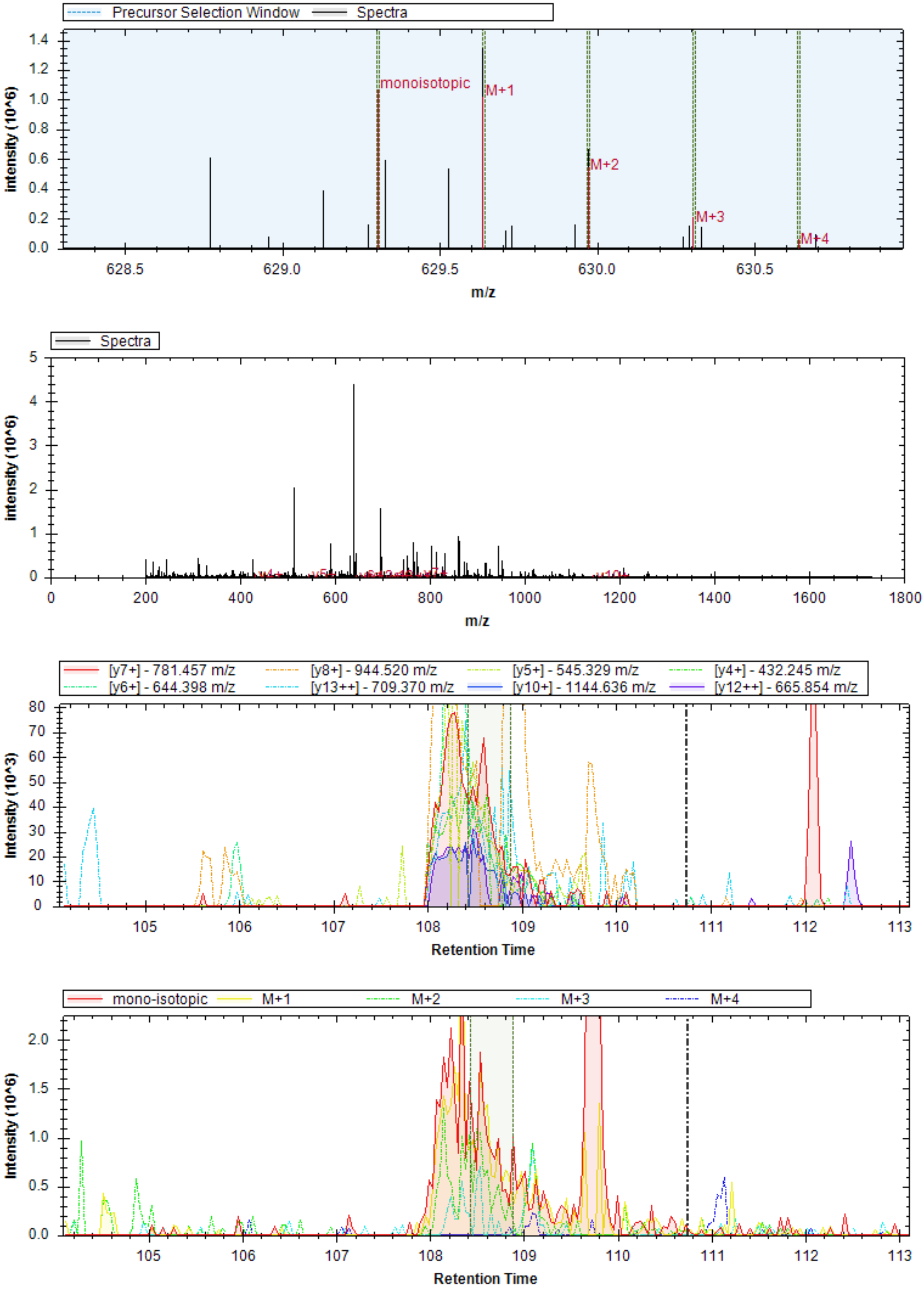

P39447 – _SSGIIRLHT[+H+O3+P]IK_.2

Fig4_MP-DIA-barrel-cortex-p54-2_MHRM.htrms

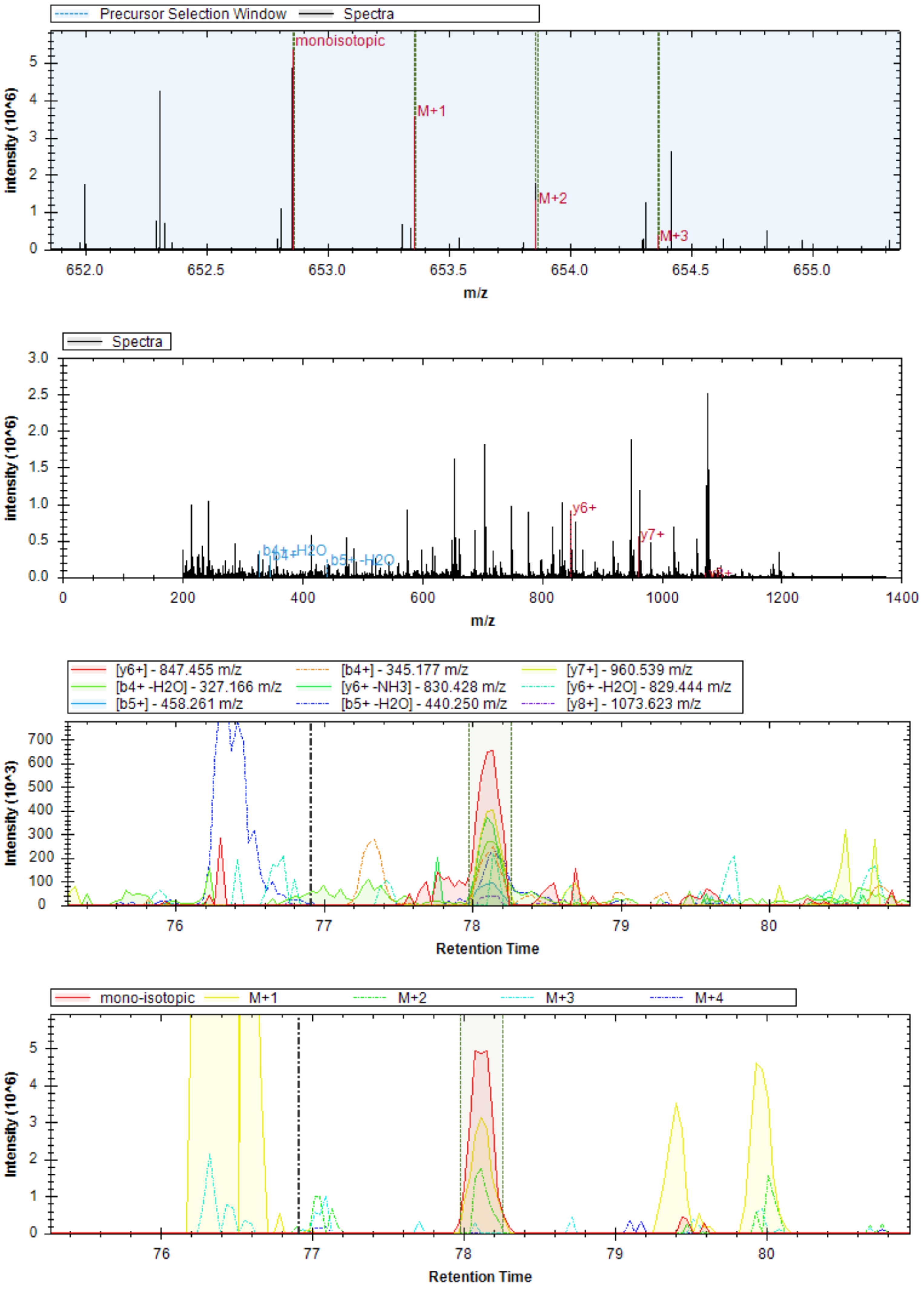

Q8BLJ3 – _M[+O]AS[+H+O3+P]SQGKNELK_.2

Fig4_MP-DIA-barrel-cortex-p54-2_MHRM.htrms

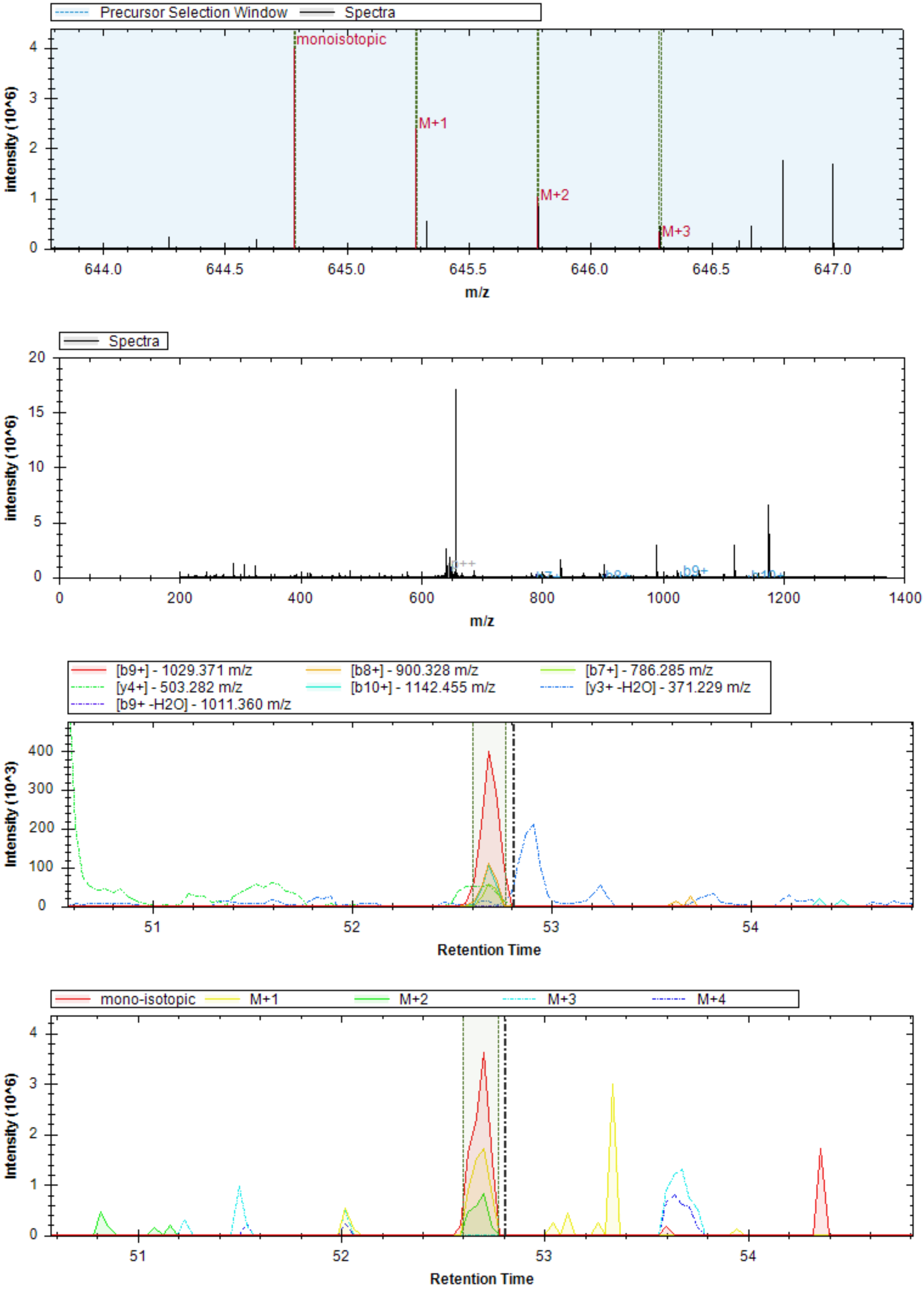

Q8C115;Q8C115-2;Q8C115-3 – _LQELQEKKIS[+H+O3+P]C[+C2+H3+N+O]VSSPK_.3
Fig4_MP-DIA-barrel-cortex-p54-2_MHRM.htrms

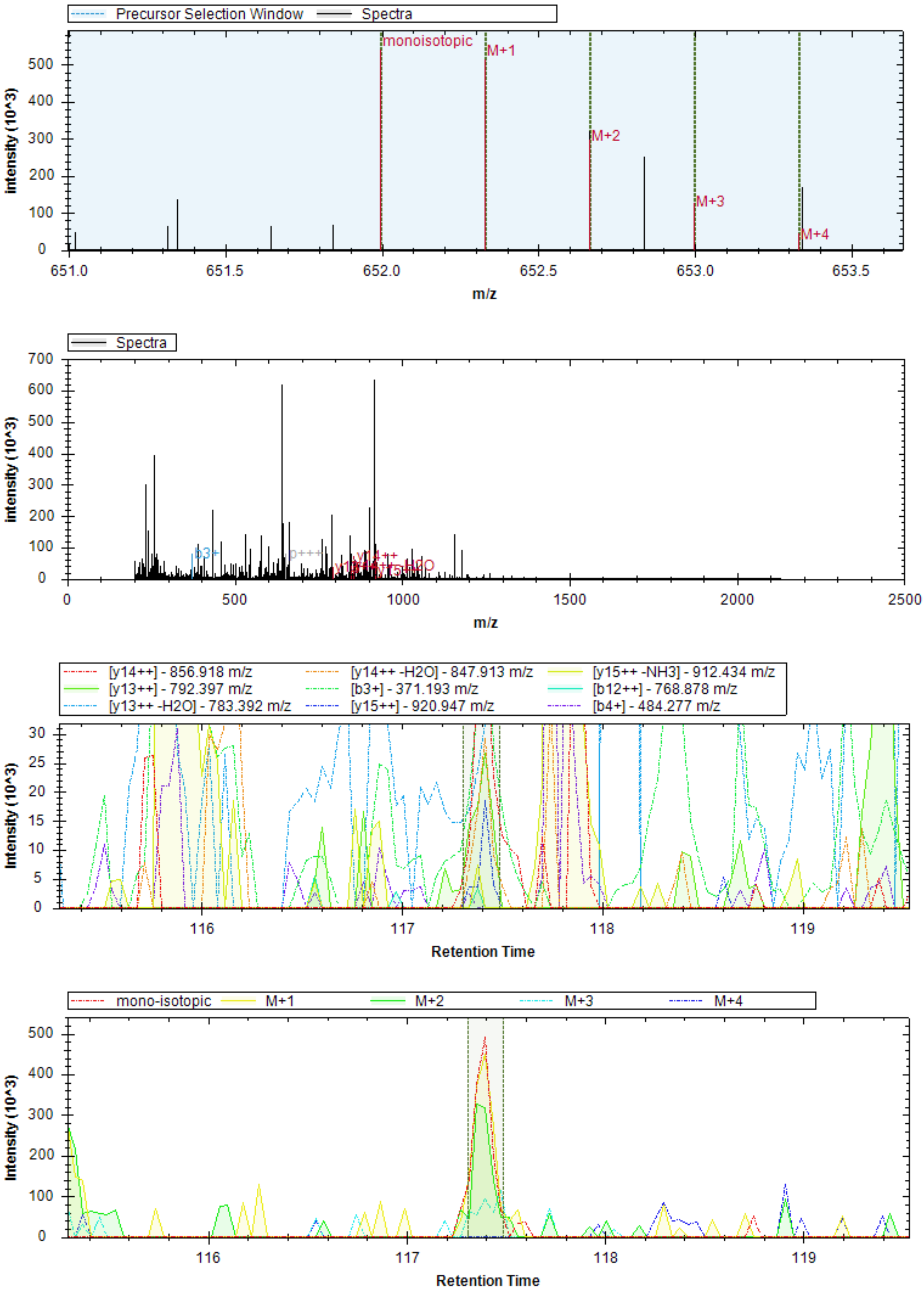

P13595 – _GVTASS[+H+O3+P]SSPASAPK_.2

Fig4_MP-DIA-barrel-cortex-p54-2_MHRM.htrms

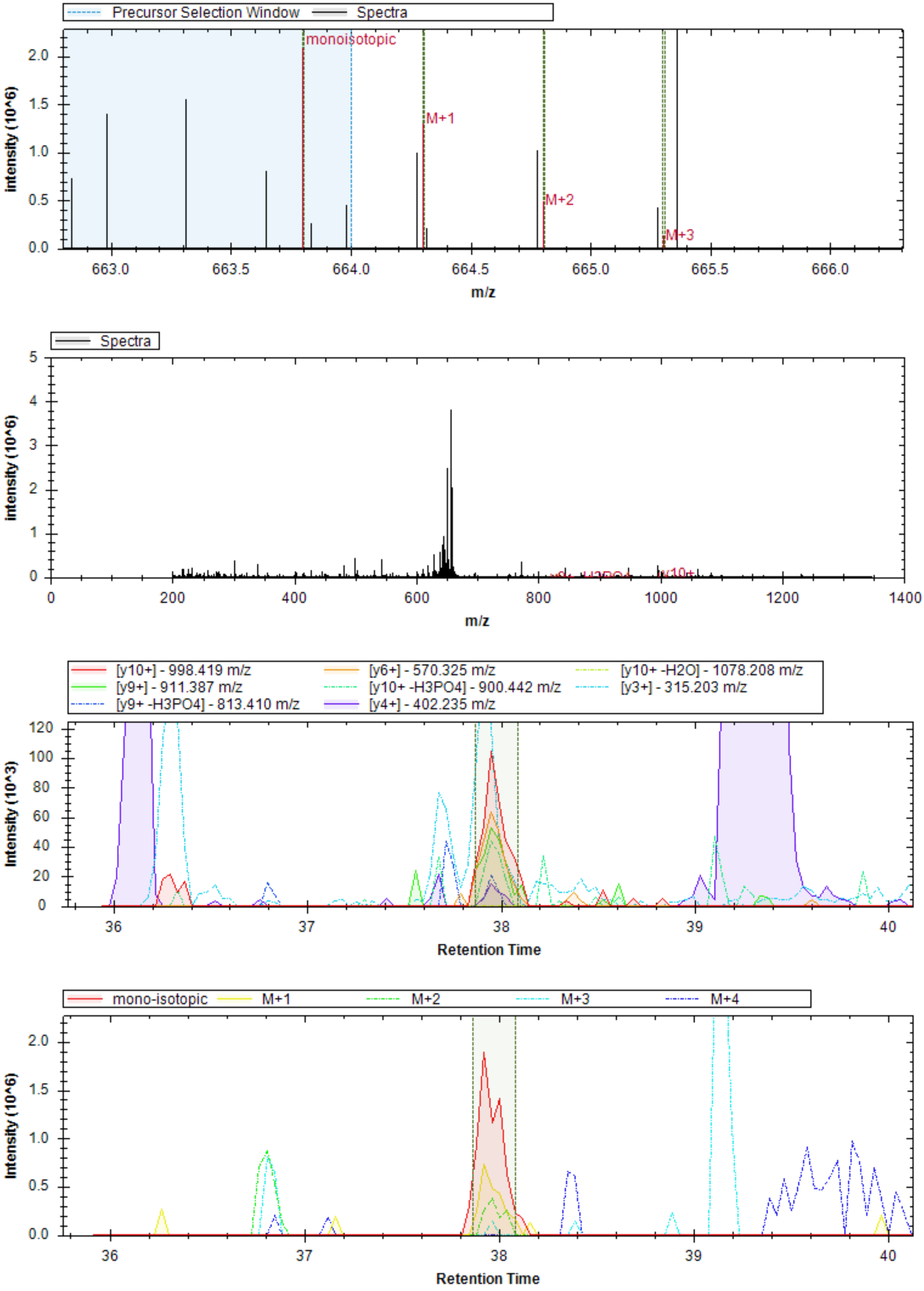

P26361 – _KM[+O]LHS[+H+O3+P]ILHAPMS[+H+O3+P]T[+H+O3+P]ISK_.3
Fig4_MP-DIA-barrel-cortex-p54-2_MHRM.htrms

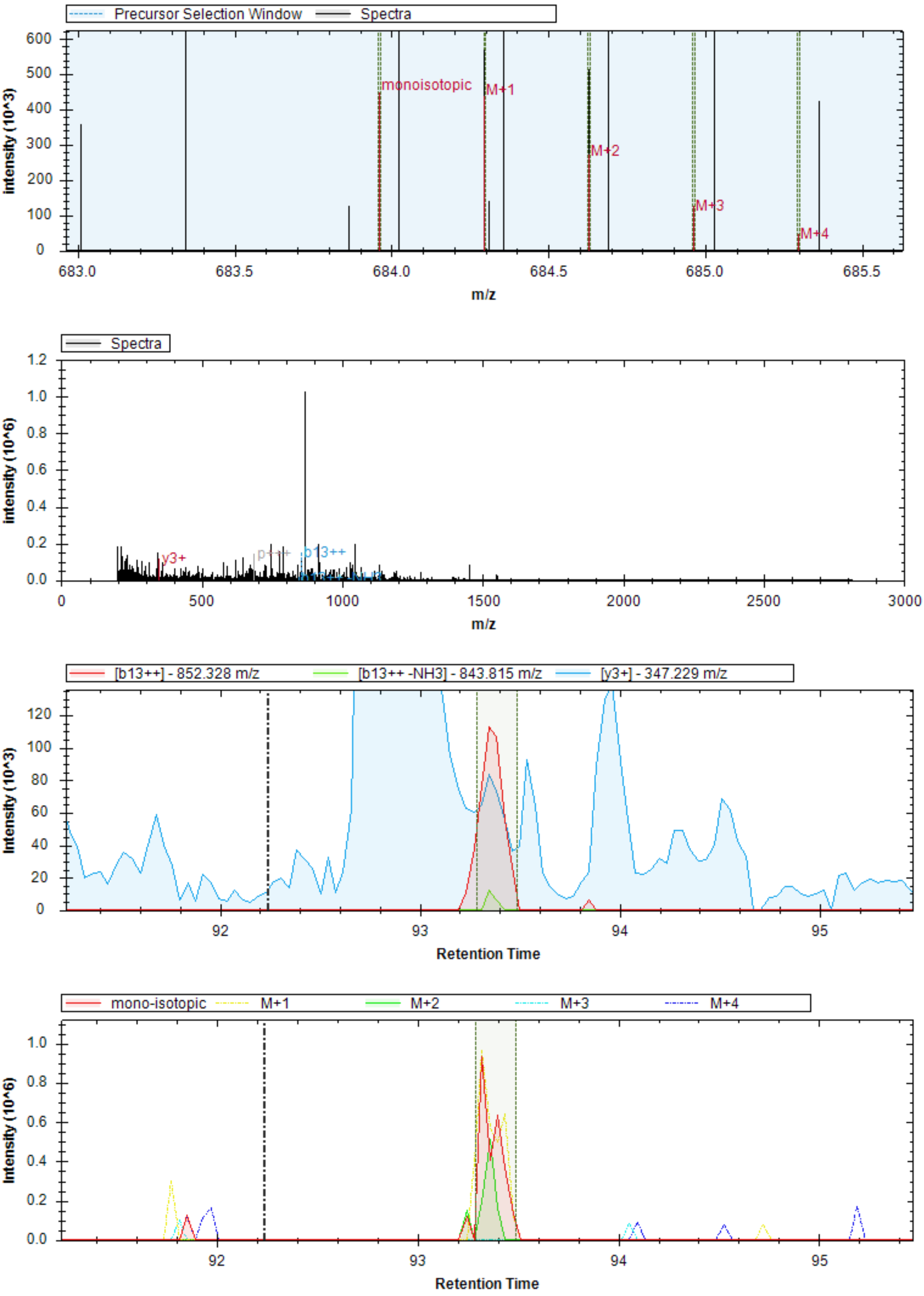

Q6A078 – _T[+H+O3+P]IGLM[+O]KKVVEK_.2
Fig4_MP-DIA-barrel-cortex-p54-2_MHRM.htrms

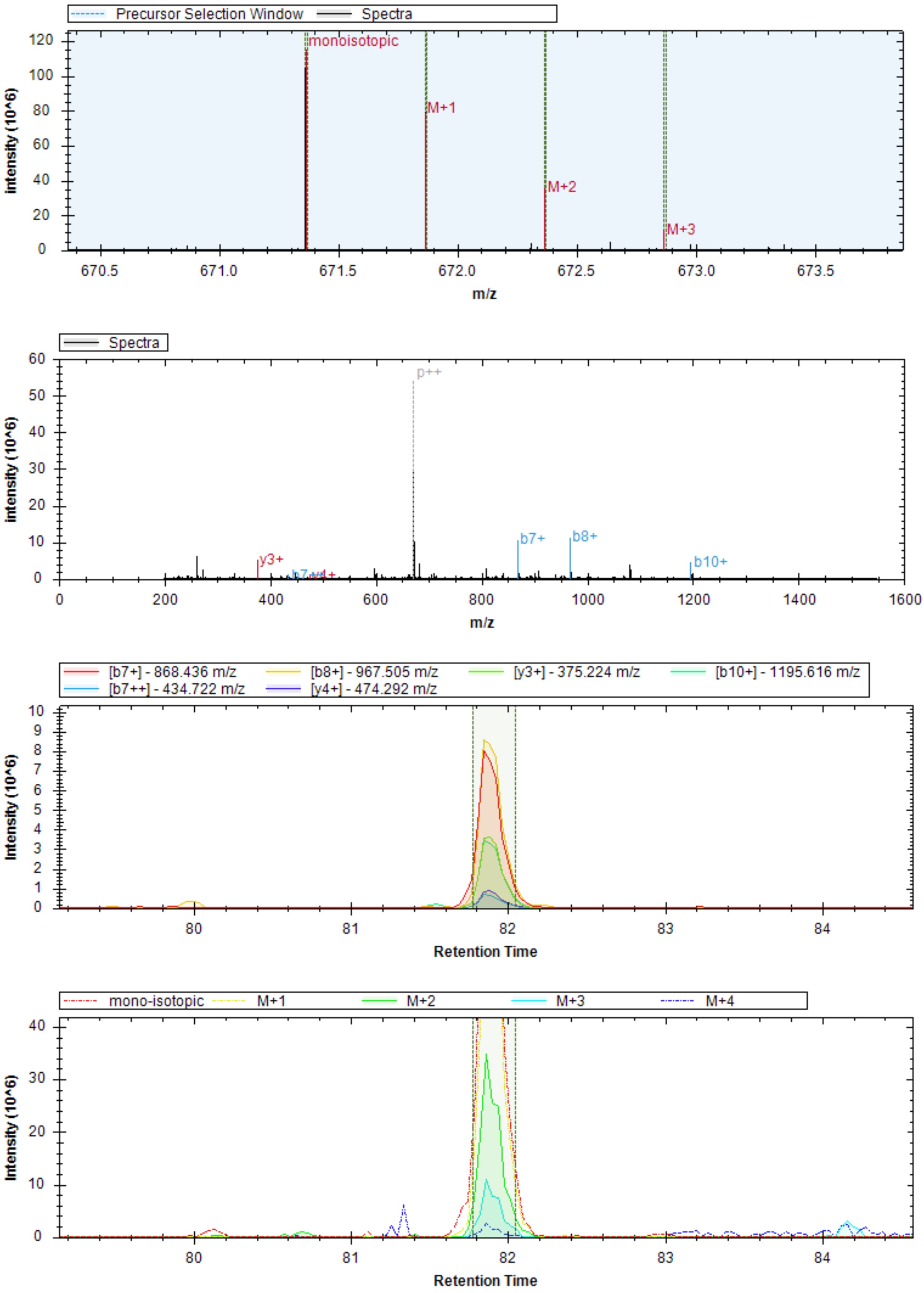

Q8CDU4;Q8CDU4-2 – _[+C2+H2+O]ASLRS[+H+O3+P]ATPRLR_.2
Fig4_MP-DIA-barrel-cortex-p54-2_MHRM.htrms

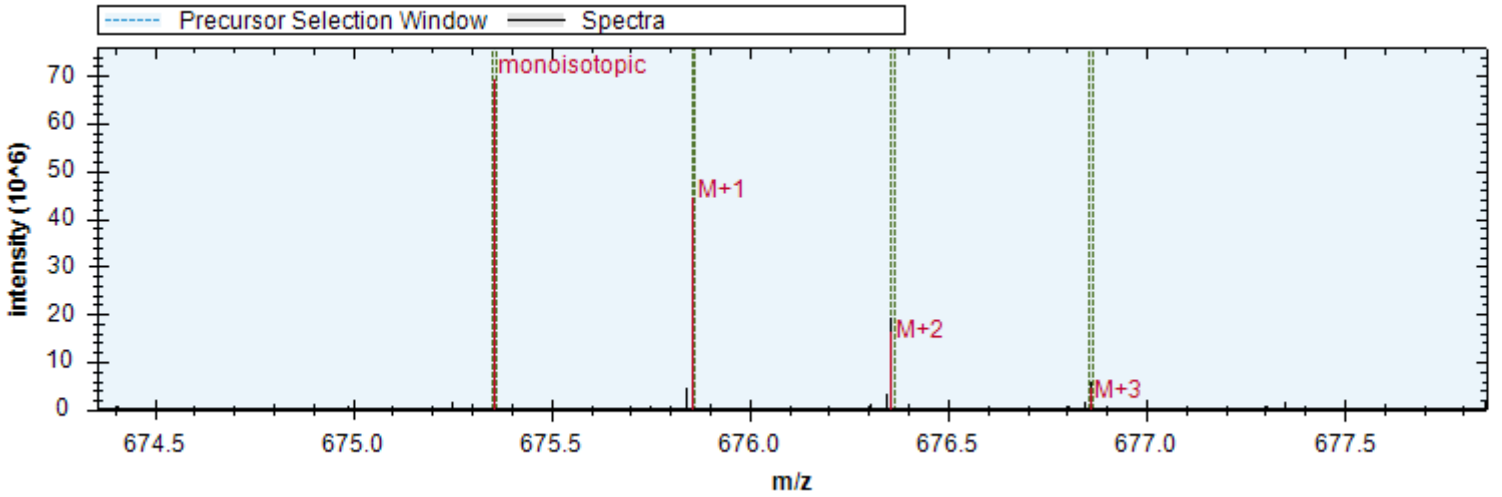

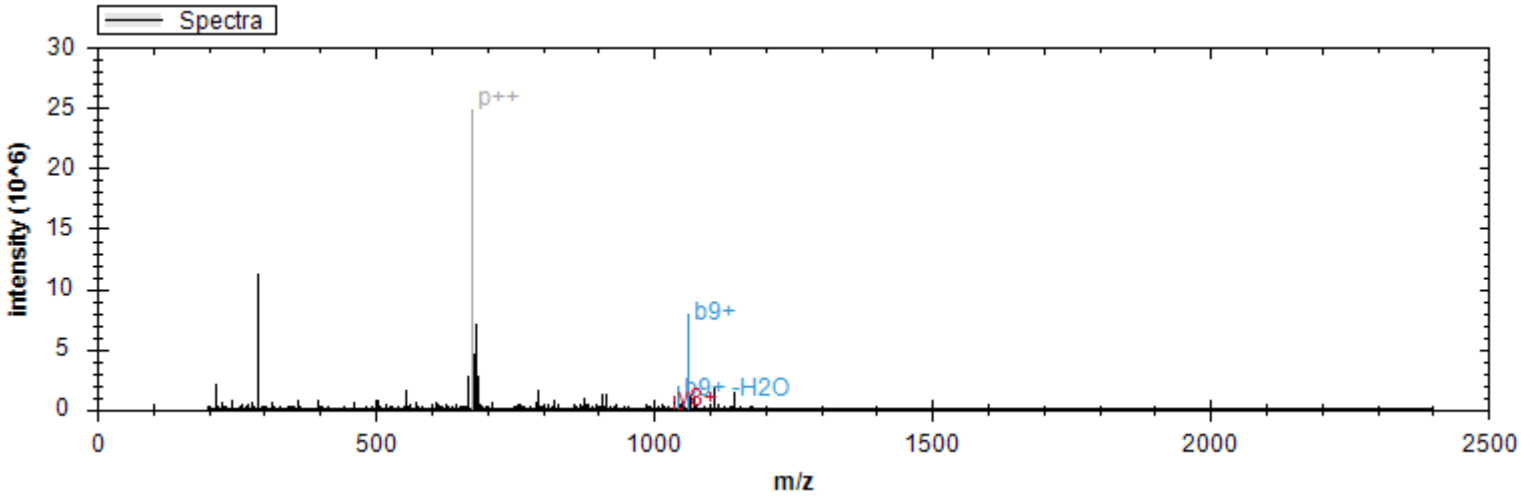

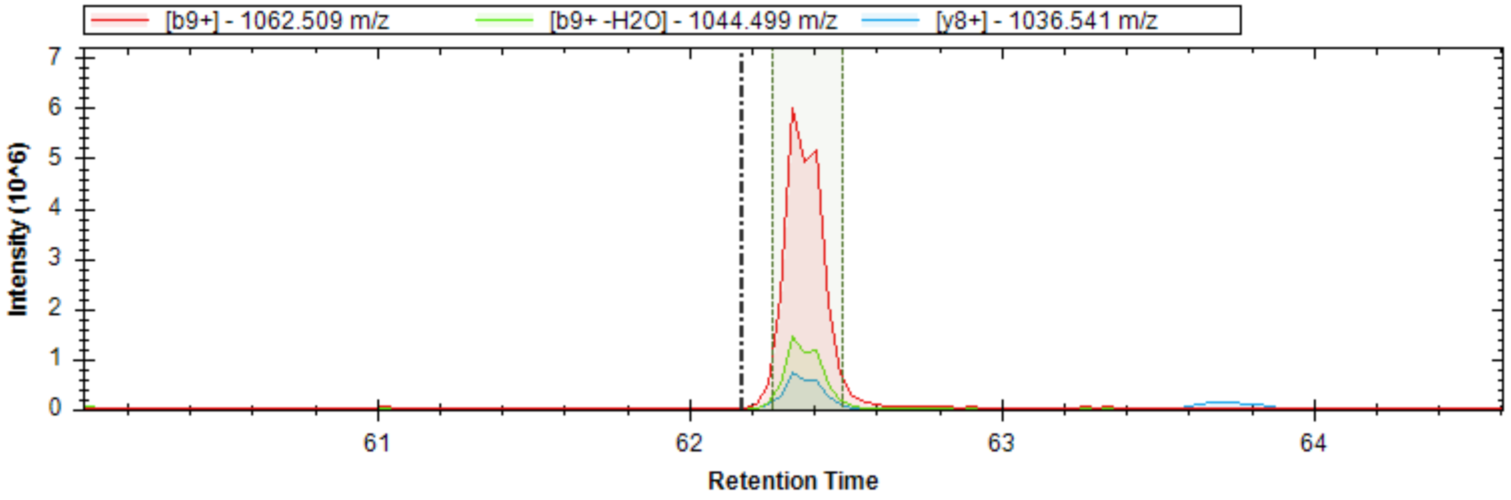

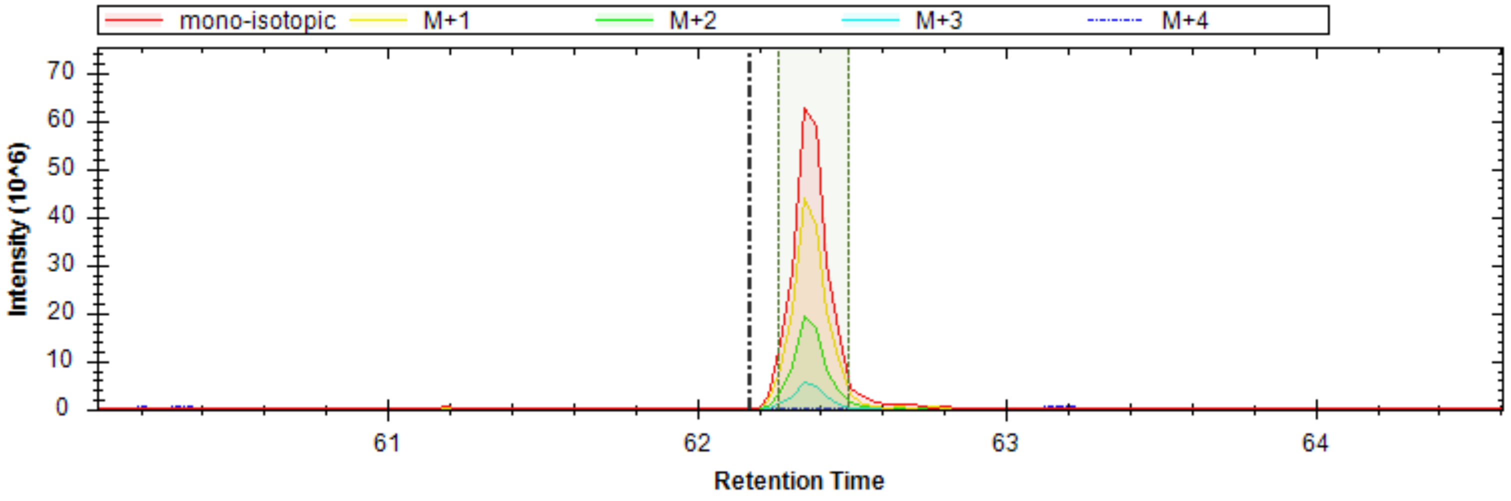

Q8R5L3;Q8R5L3-2 – _LQLY[+H+O3+P]FWKDR_.2
Fig4_MP-DIA-barrel-cortex-p54-2_MHRM.htrms

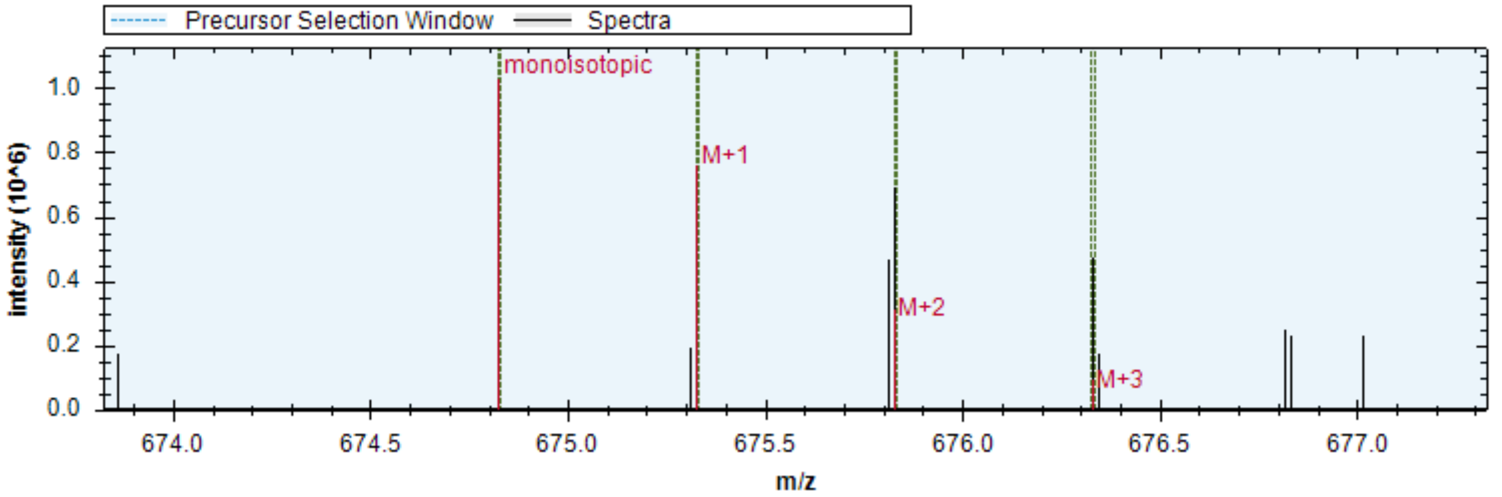

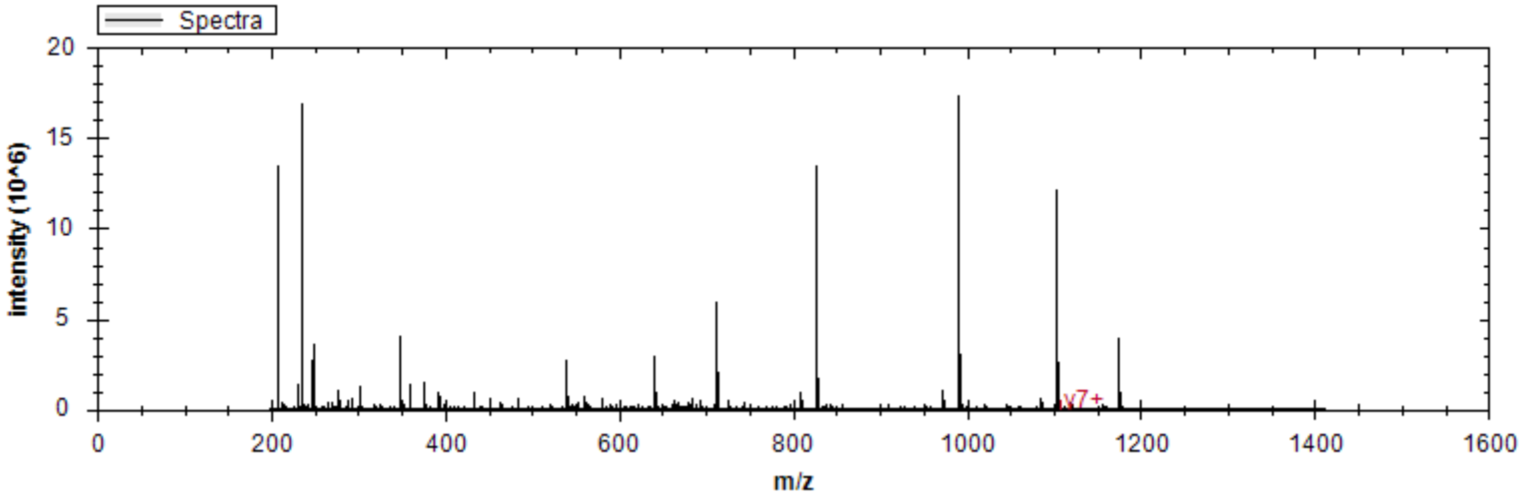

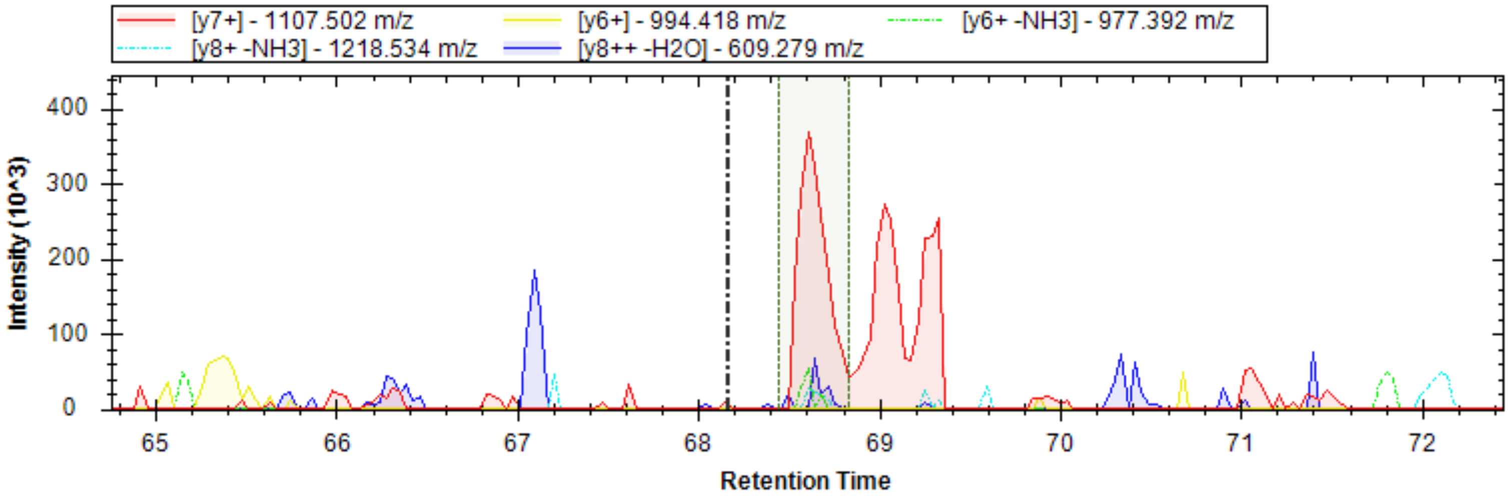

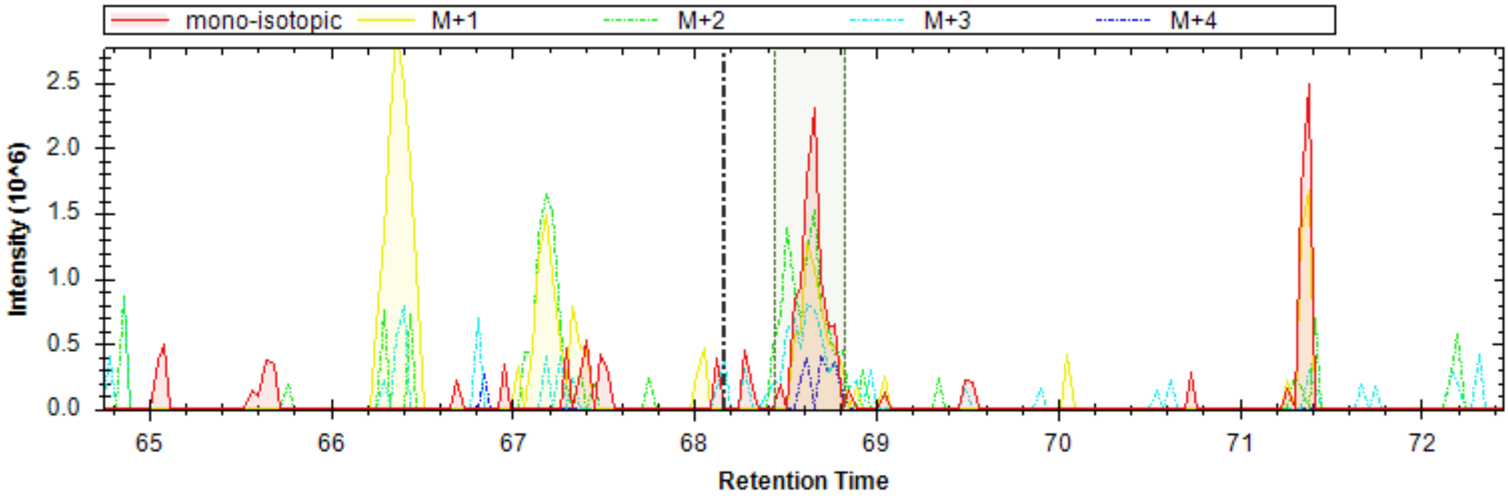

O88935 – _PVAGGPGAPPAARPPAS[+H+O3+P]PSPQR_.3
Fig4_MP-DIA-barrel-cortex-p54-2_MHRM.htrms

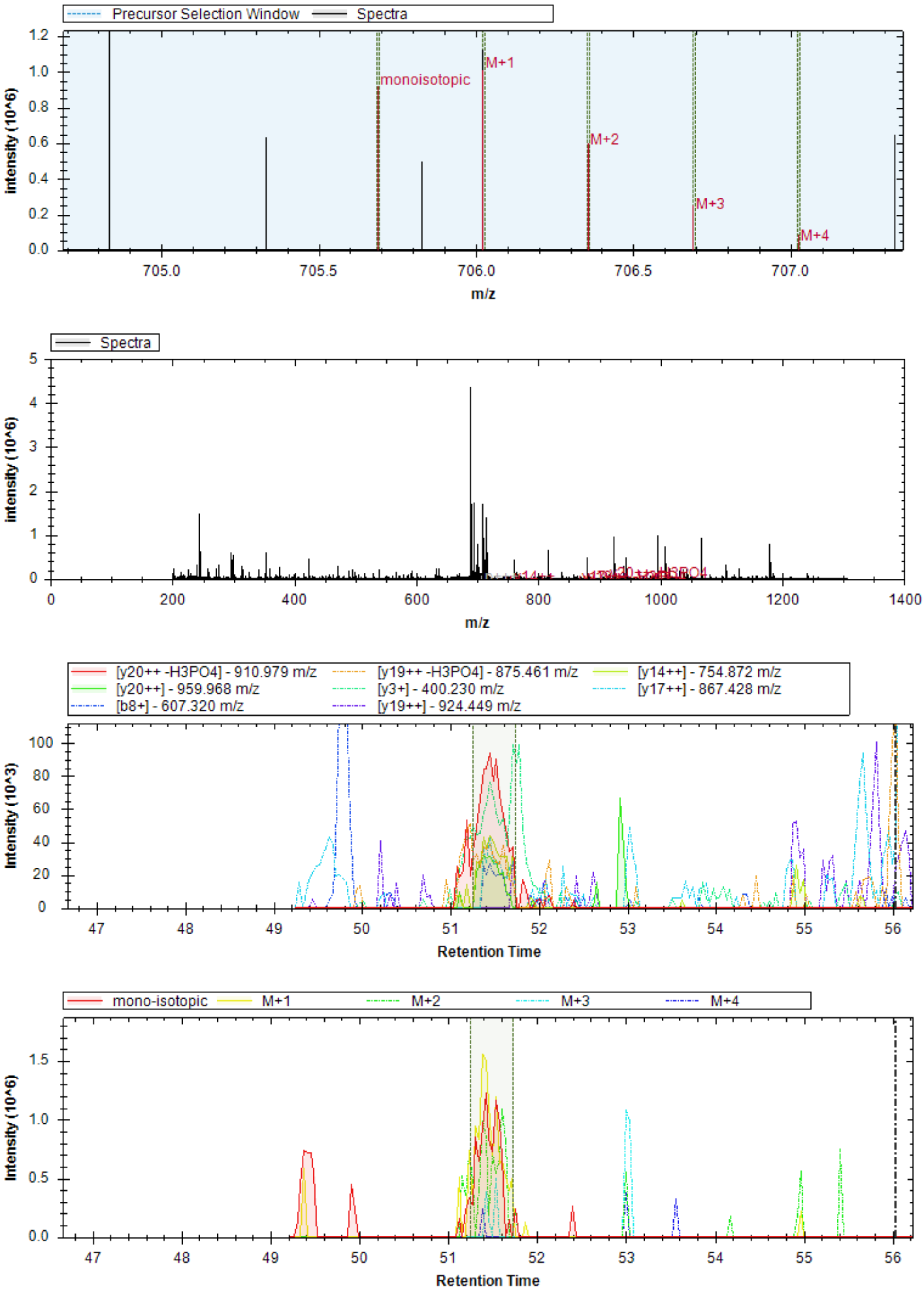

P28738 – _PIRPGHYPAS[+H+O3+P]SPTAVHAVR_.3

Fig4_MP-DIA-barrel-cortex-p54-2_MHRM.htrms

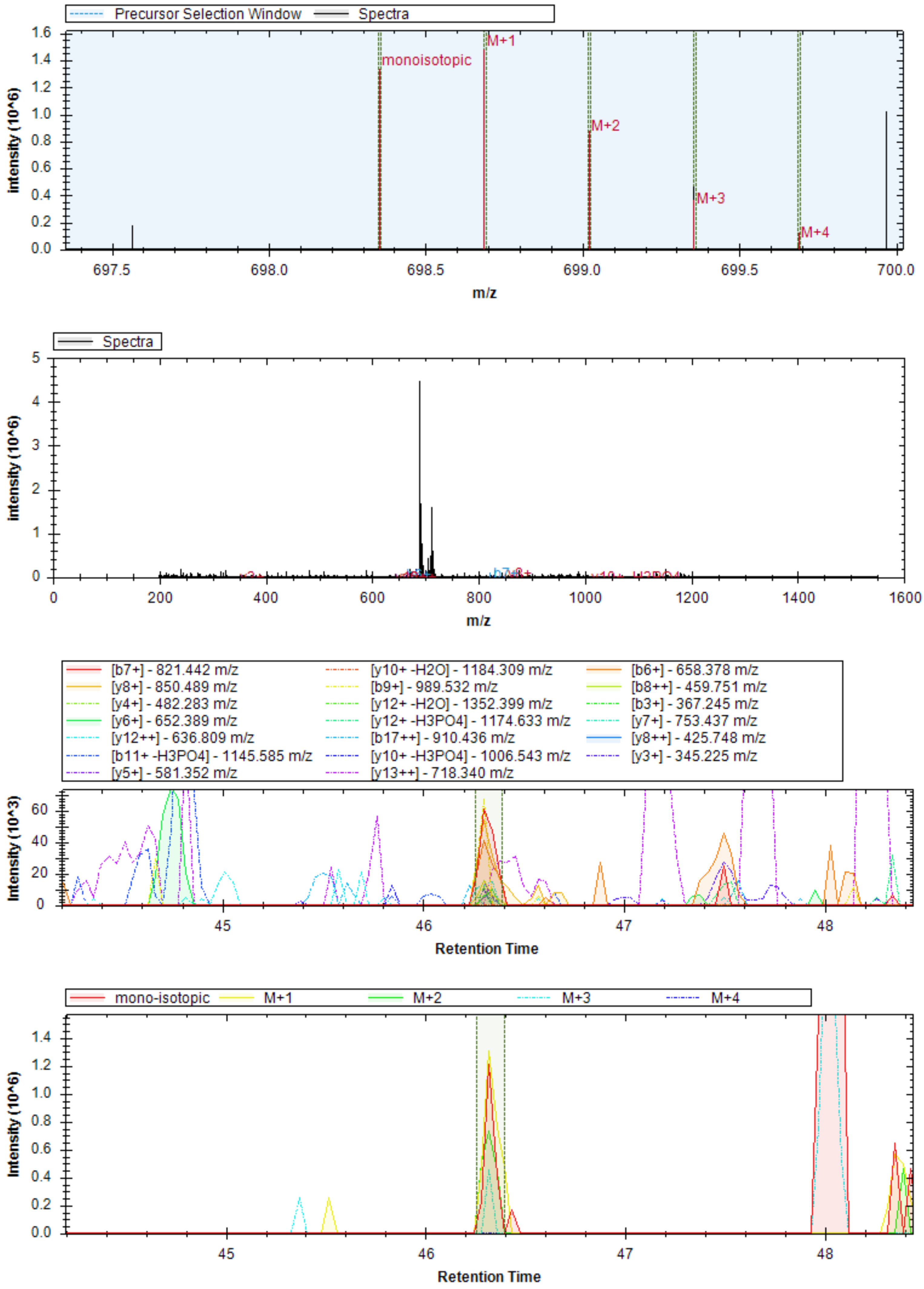

P28738 – _PIRPGHYPASS[+H+O3+P]PTAVHAVR_.3
Fig4_MP-DIA-barrel-cortex-p54-2_MHRM.htrms

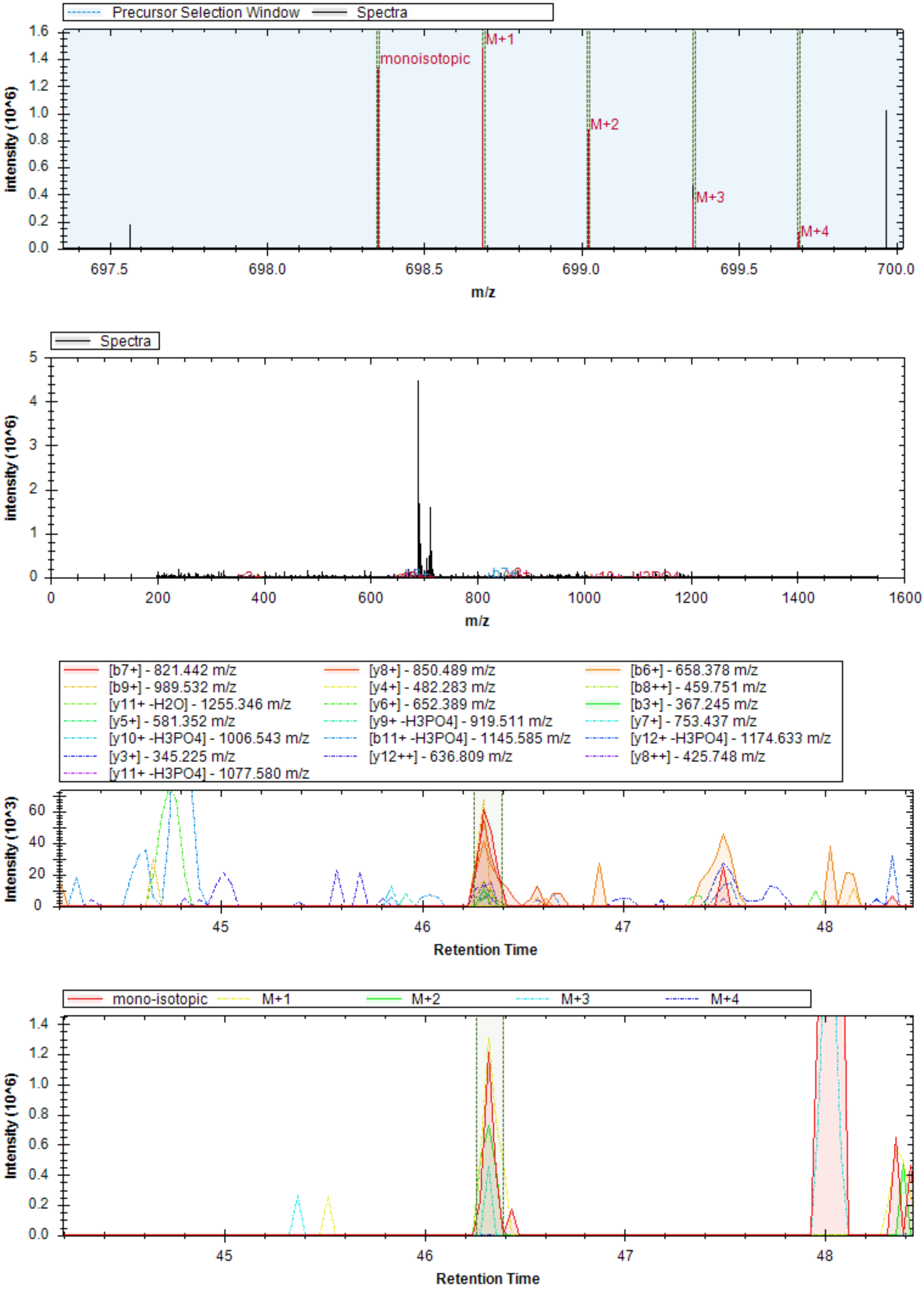

Q3U4B4 – _RHKT[+H+O3+P]LSANEEK_.2
Fig4_MP-DIA-barrel-cortex-p54-2_MHRM.htrms

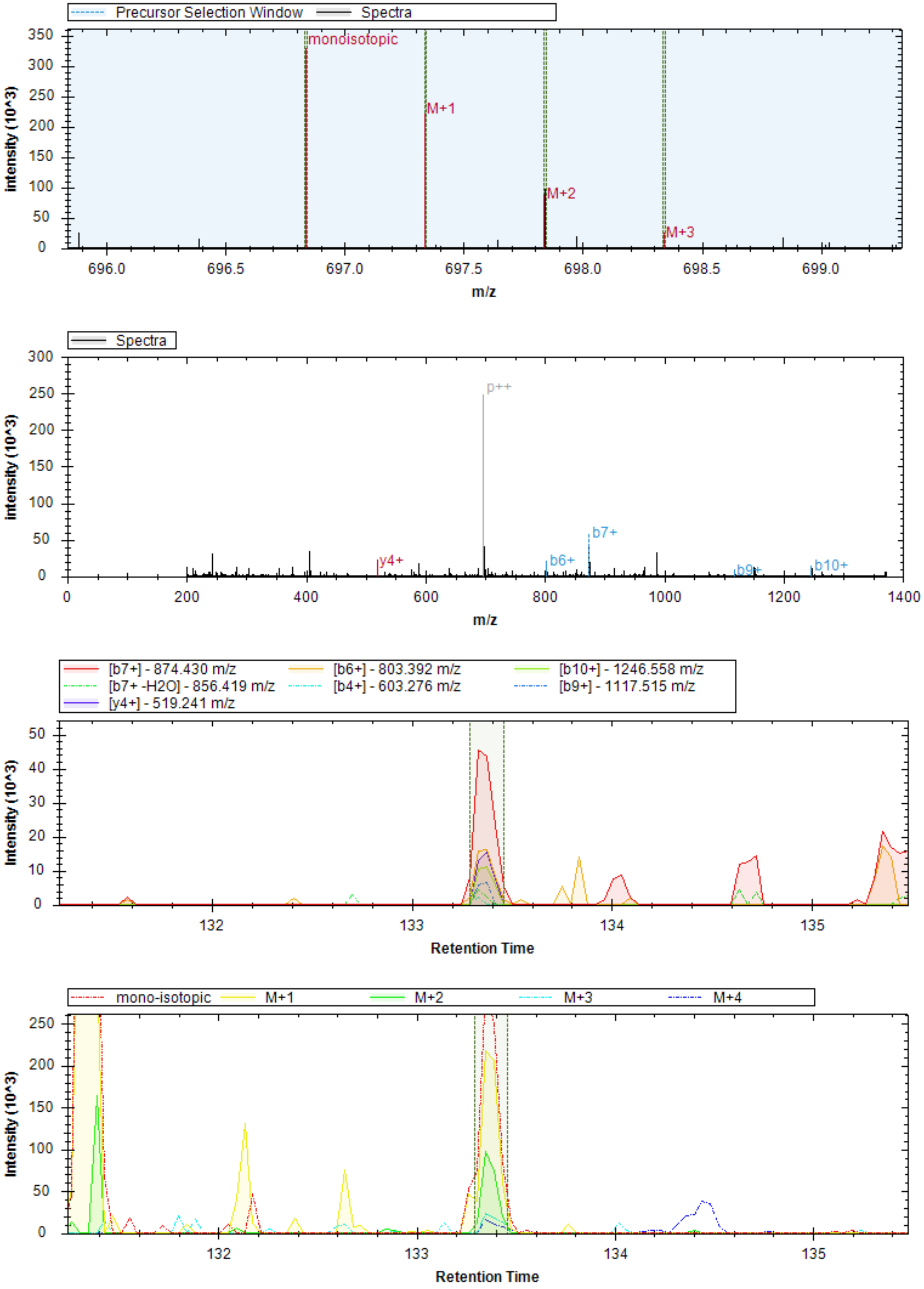

P10637 – _SGYSS[+H+O3+P]PGSPGTPGSR_.2
Fig4_MP-DIA-barrel-cortex-p54-2_MHRM.htrms

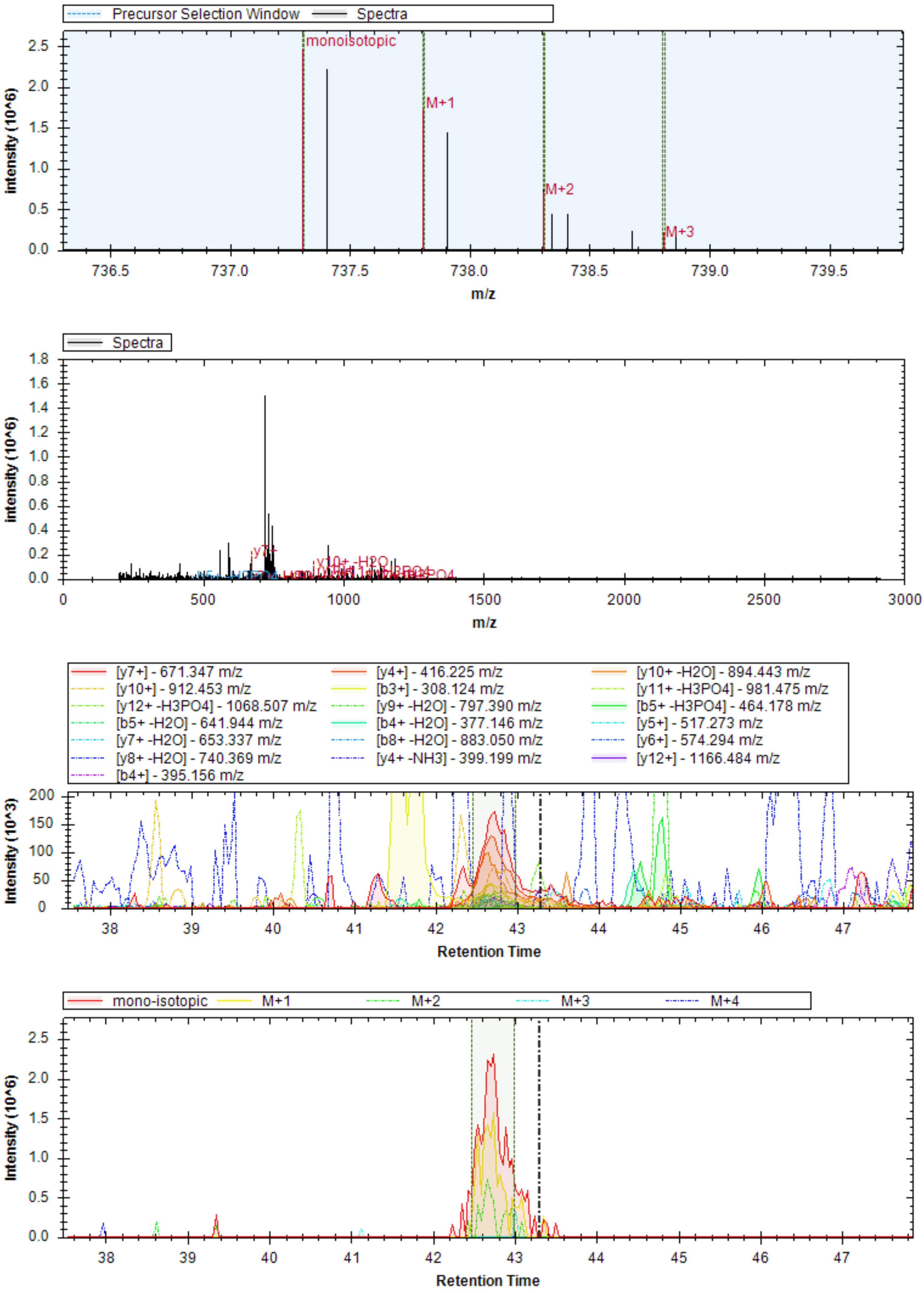

P10637 – _SGYSSPGS[+H+O3+P]PGTPGSR_.2

Fig4_MP-DIA-barrel-cortex-p54-2_MHRM.htrms

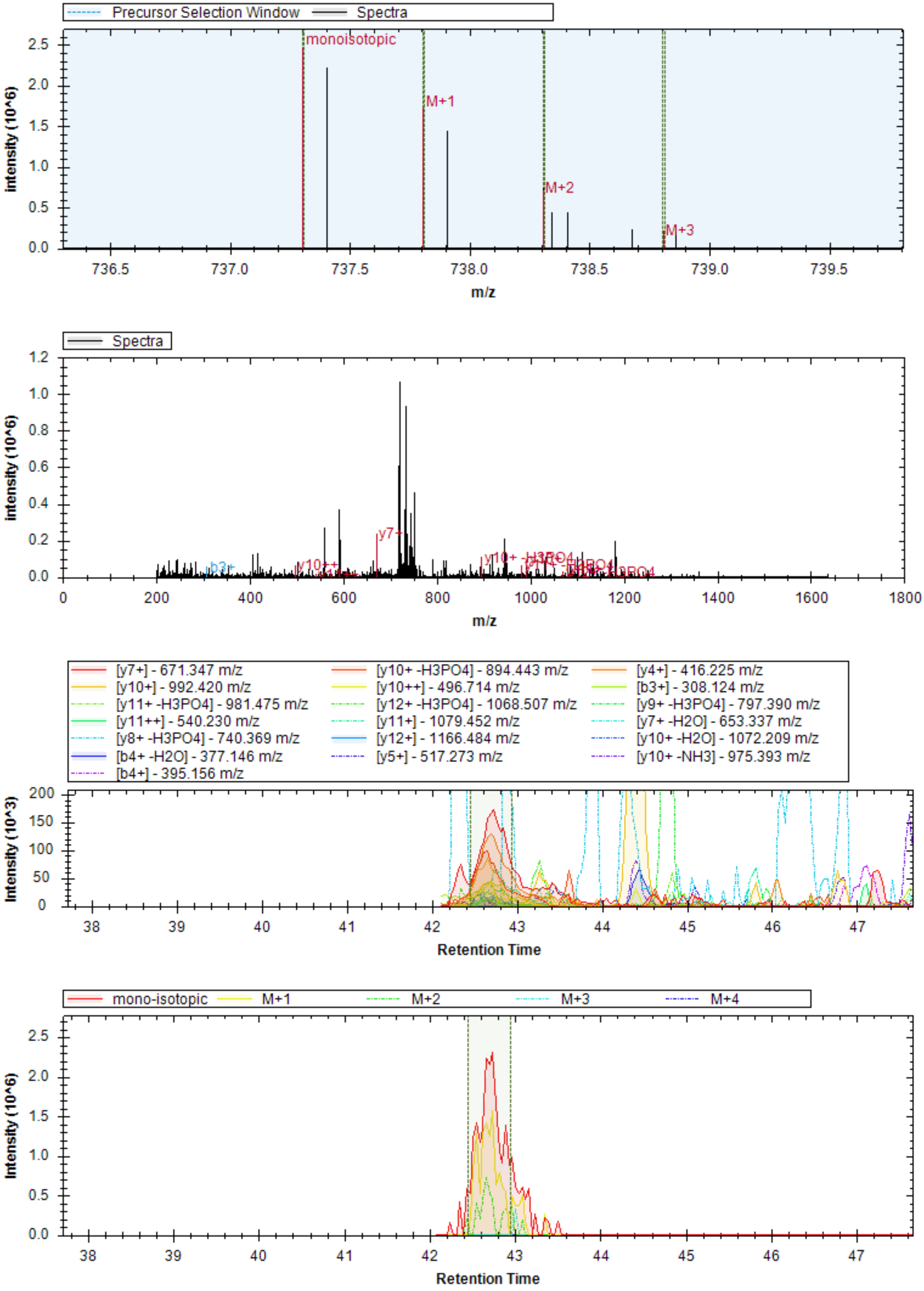

Q8C5R2 – _LAGNEALS[+H+O3+P]PTSPSK_.2
Fig4_MP-DIA-barrel-cortex-p54-2_MHRM.htrms

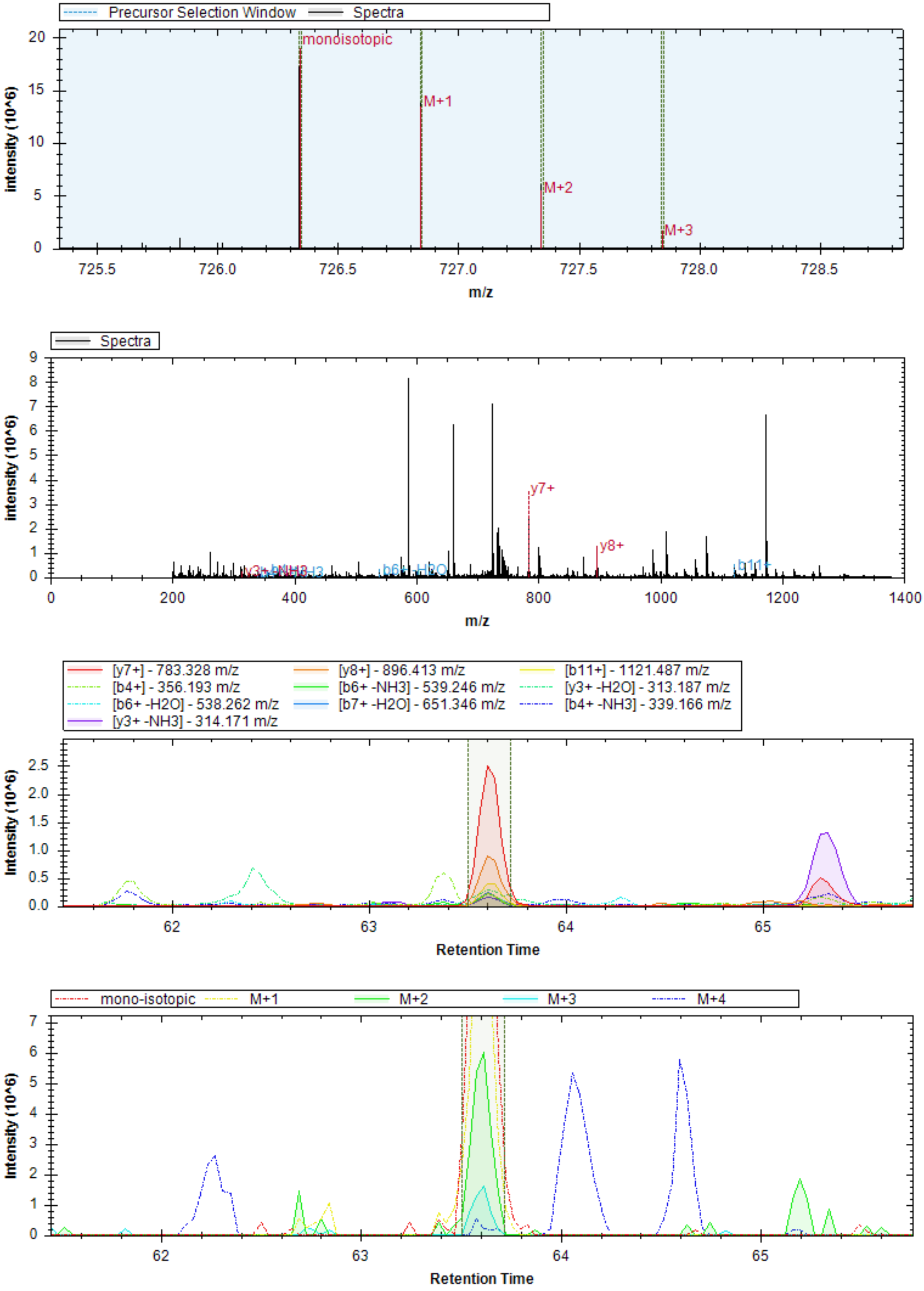

Q9CRB0 – _MEVYIPS[+H+O3+P]FRHEDSDLER_.3

Fig4_MP-DIA-barrel-cortex-p54-2_MHRM.htrms

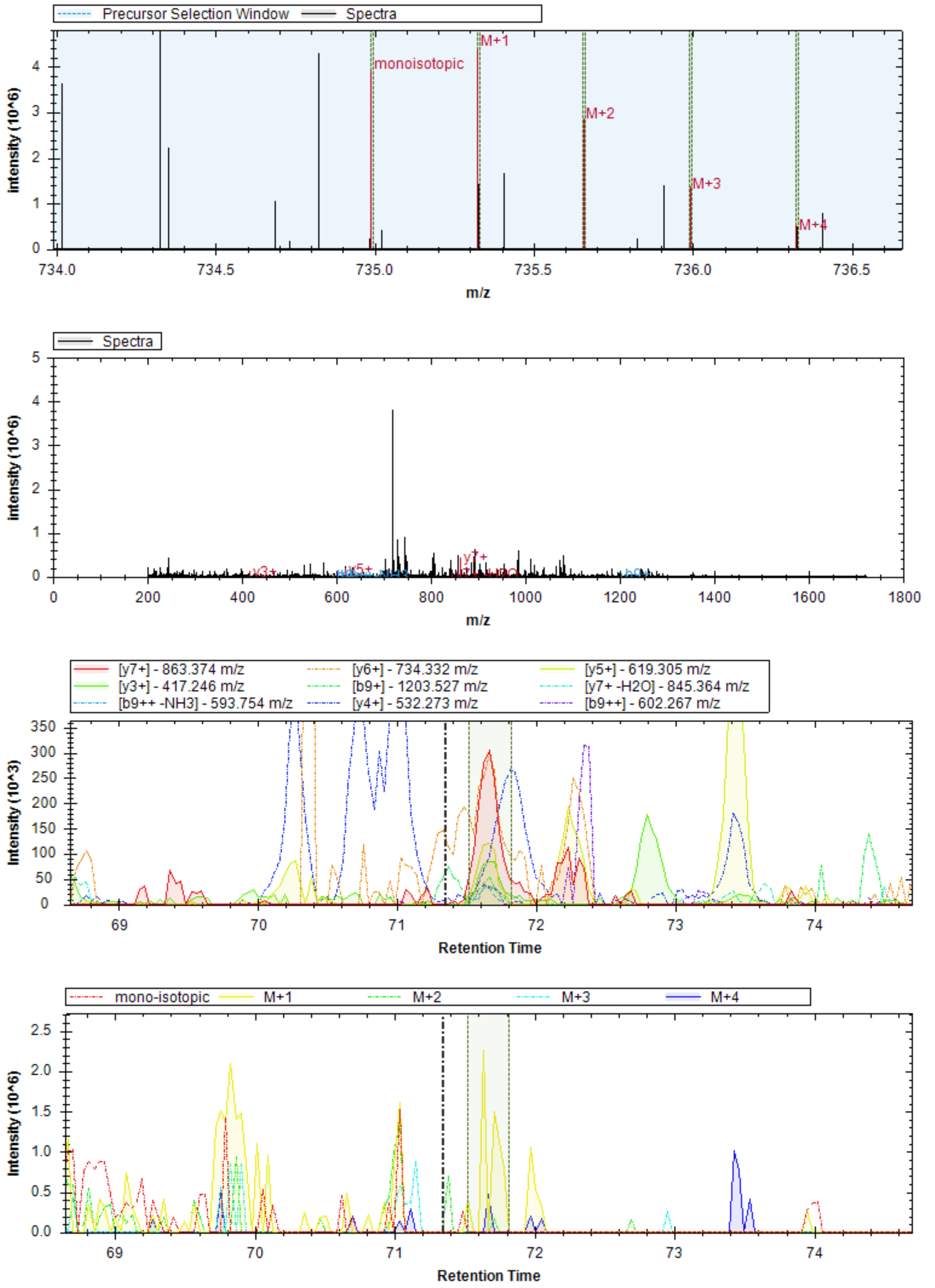

D3Z6S9 – _IPSIEIT[+H+O3+P]ET[+H+O3+P]LPQTQASMLR_.3
Fig4_MP-DIA-barrel-cortex-p54-2_MHRM.htrms

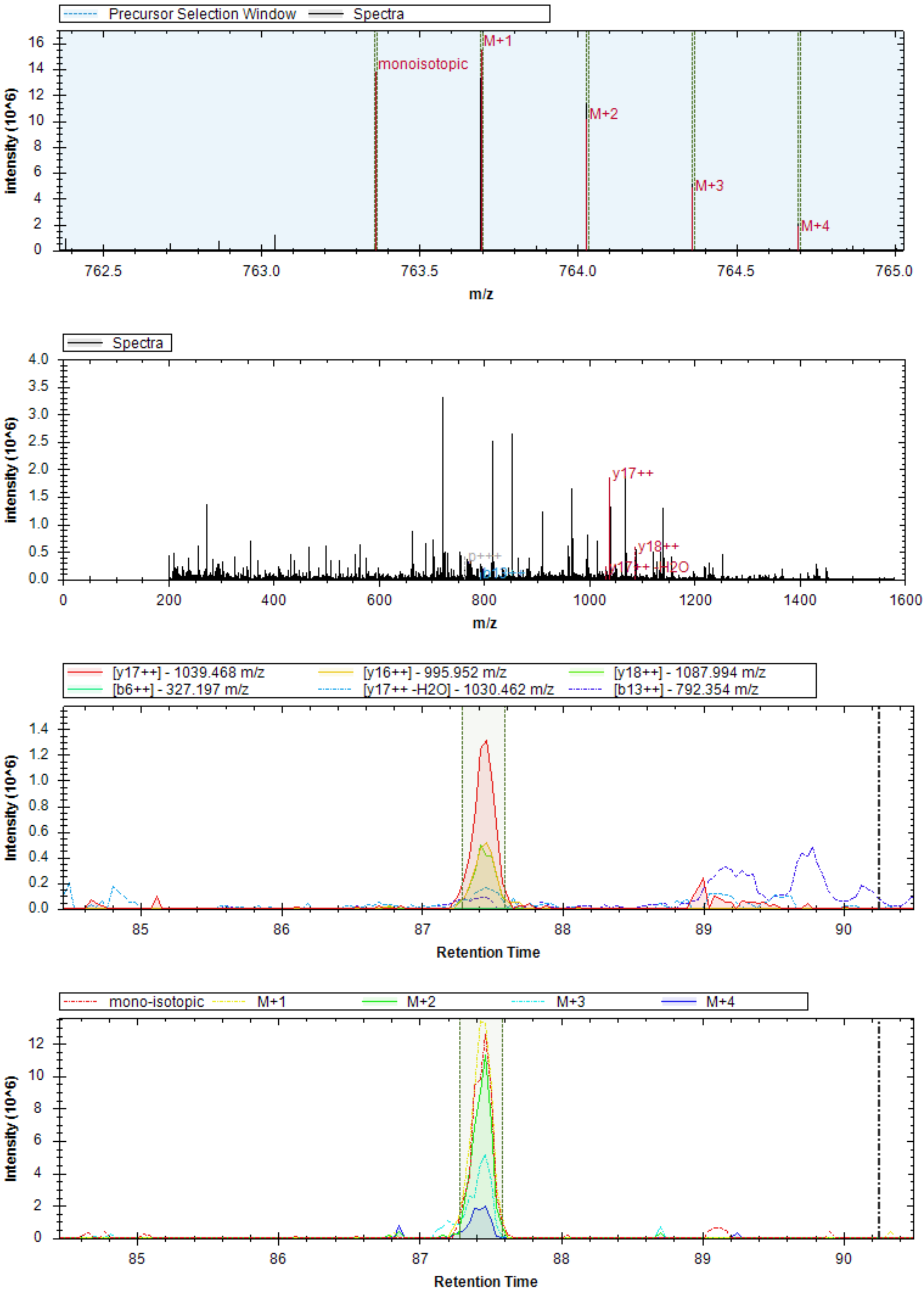

P0C6F1 – _IIDLQM[+O]HDY[+H+O3+P]LR_.2
Fig4_MP-DIA-barrel-cortex-p54-2_MHRM.htrms

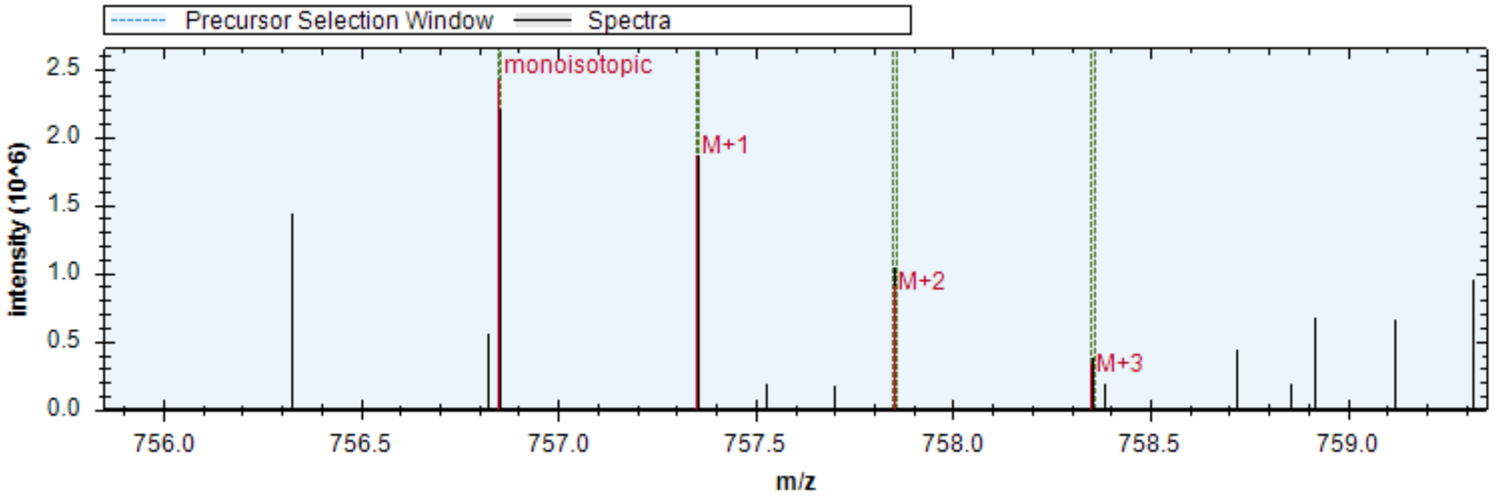

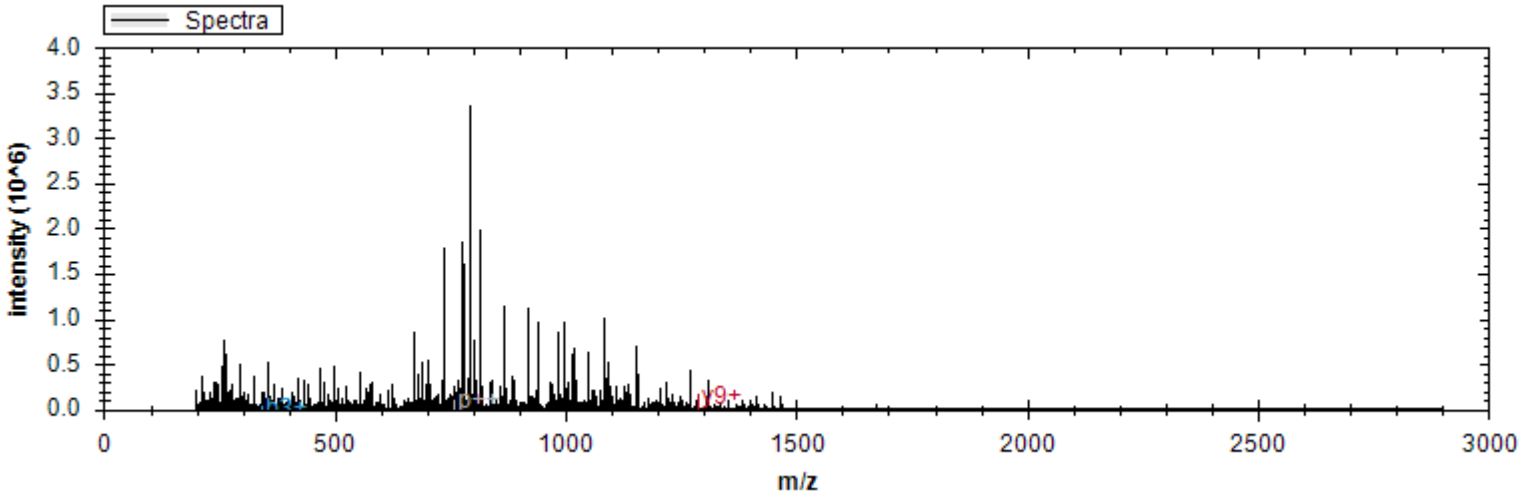

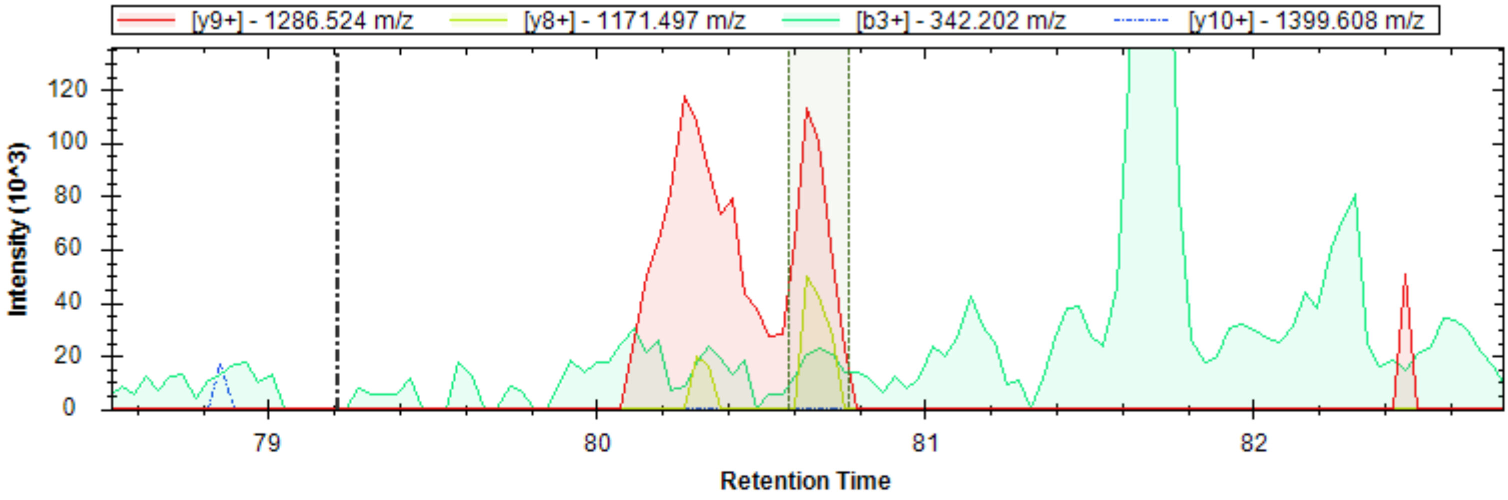

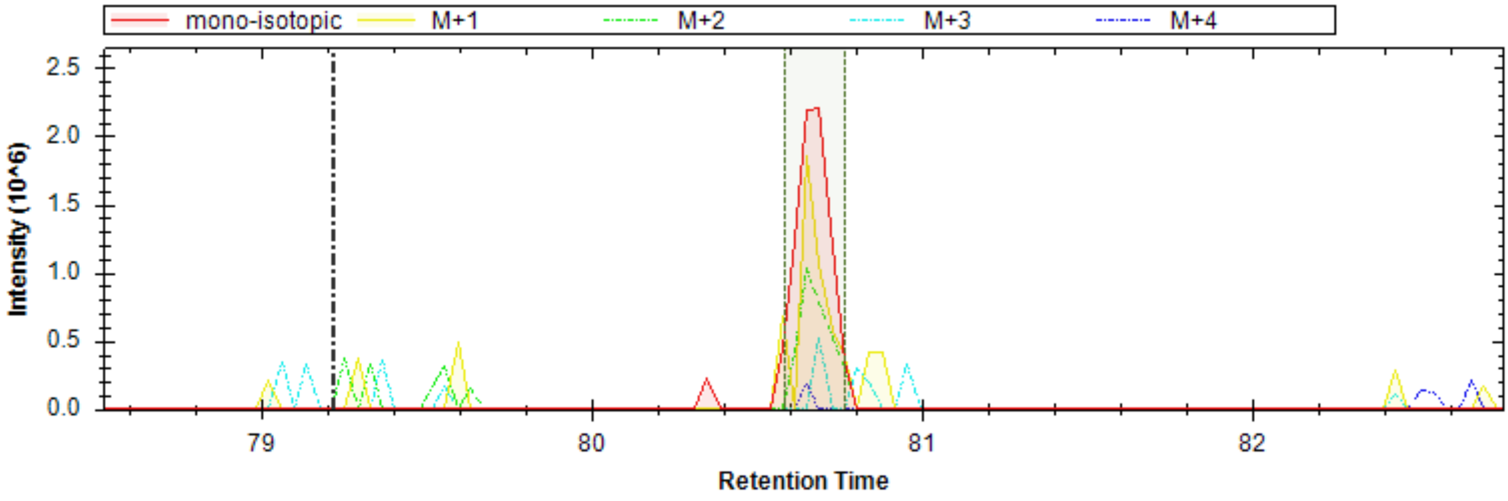

Q68FD9 – _LAQLLS[+H+O3+P]EVPT[+H+O3+P]RR_.2
Fig4_MP-DIA-barrel-cortex-p54-2_MHRM.htrms

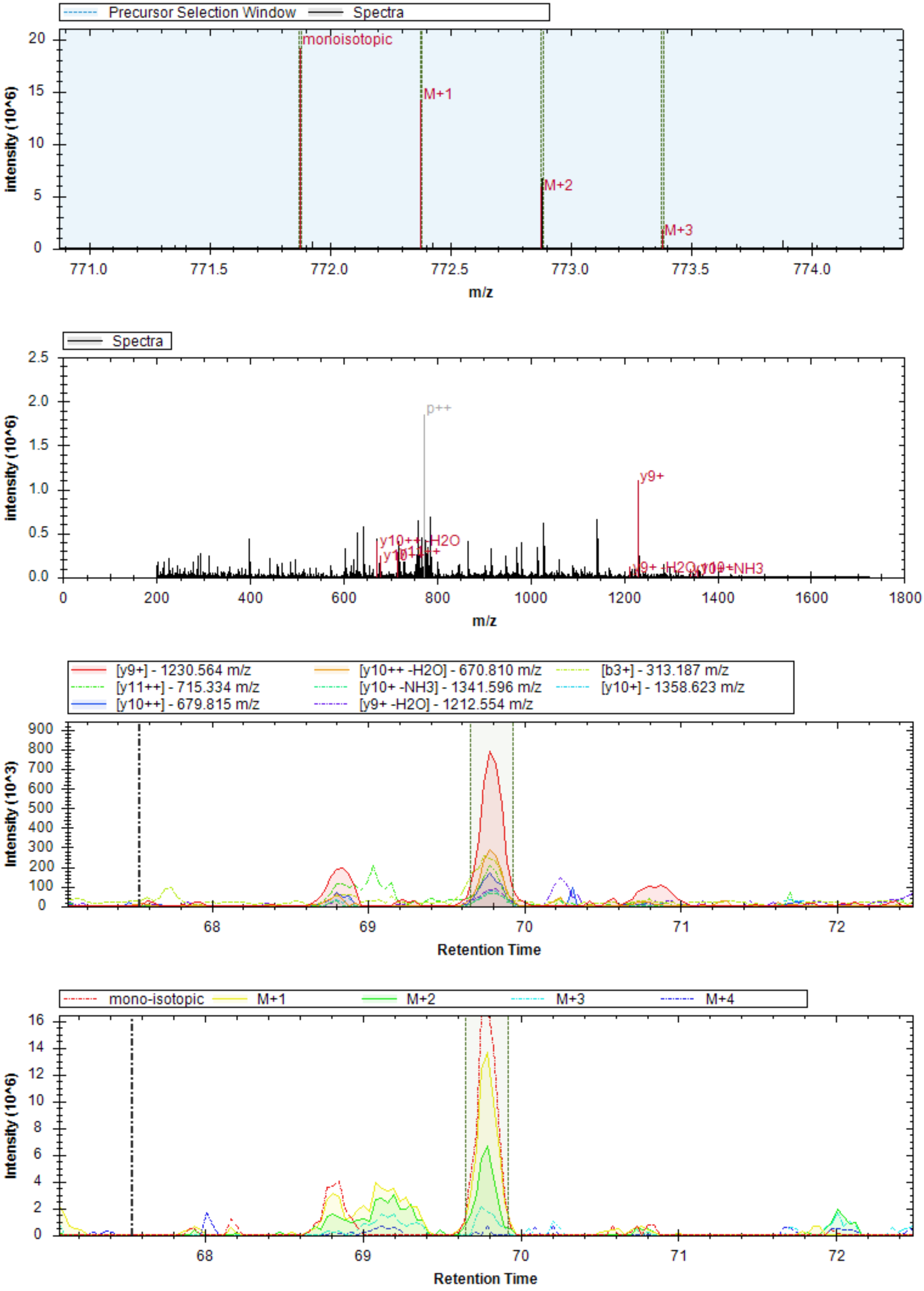

Q8BRT1 – _VLNT[+H+O3+P]GSDVEEAVADALLLGDIR_.3
Fig4_MP-DIA-barrel-cortex-p54-2_MHRM.htrms

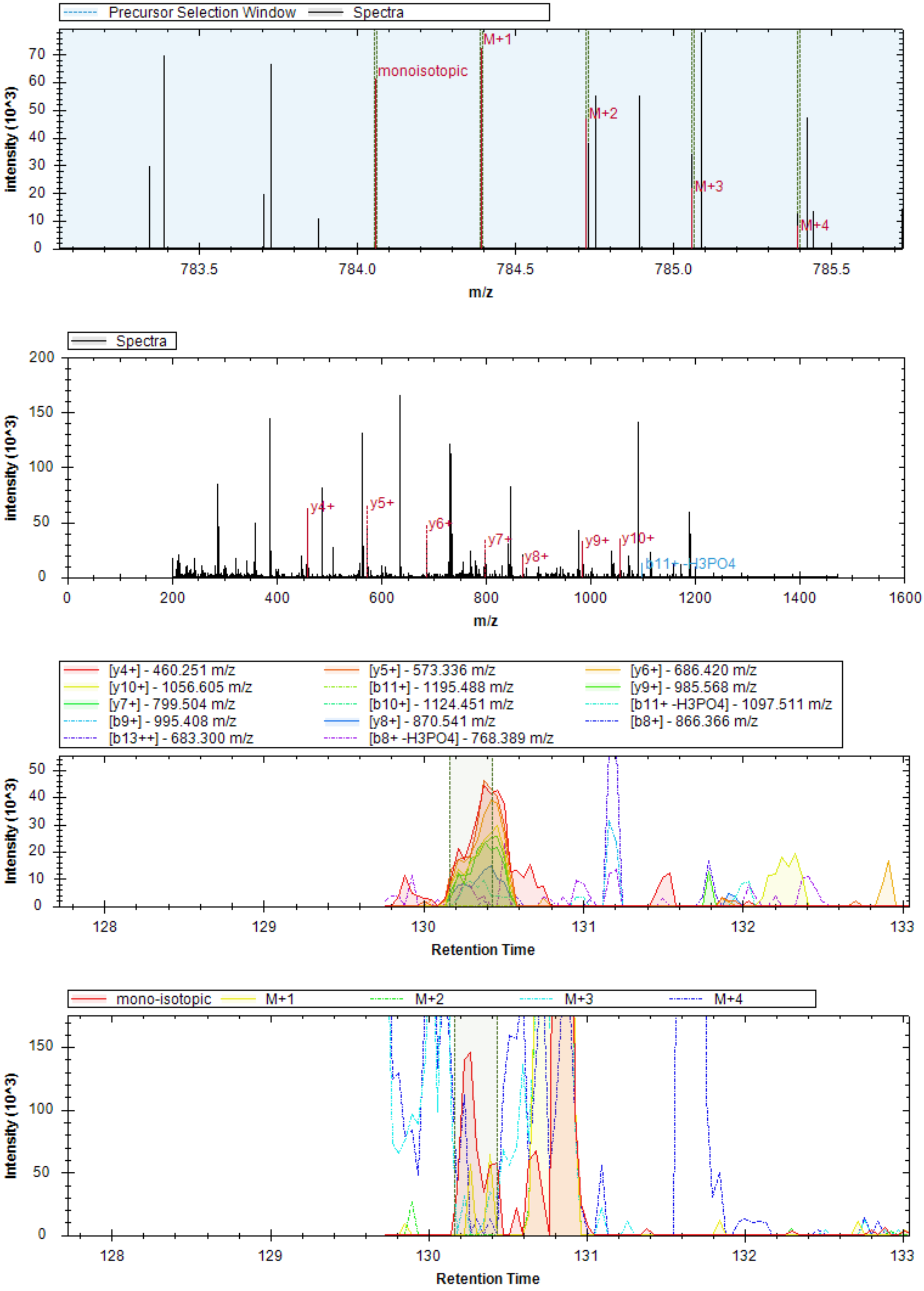

Q9QYC0 – _AAVVTS[+H+O3+P]PPPTTAPHK_.2
Fig4_MP-DIA-barrel-cortex-p54-2_MHRM.htrms

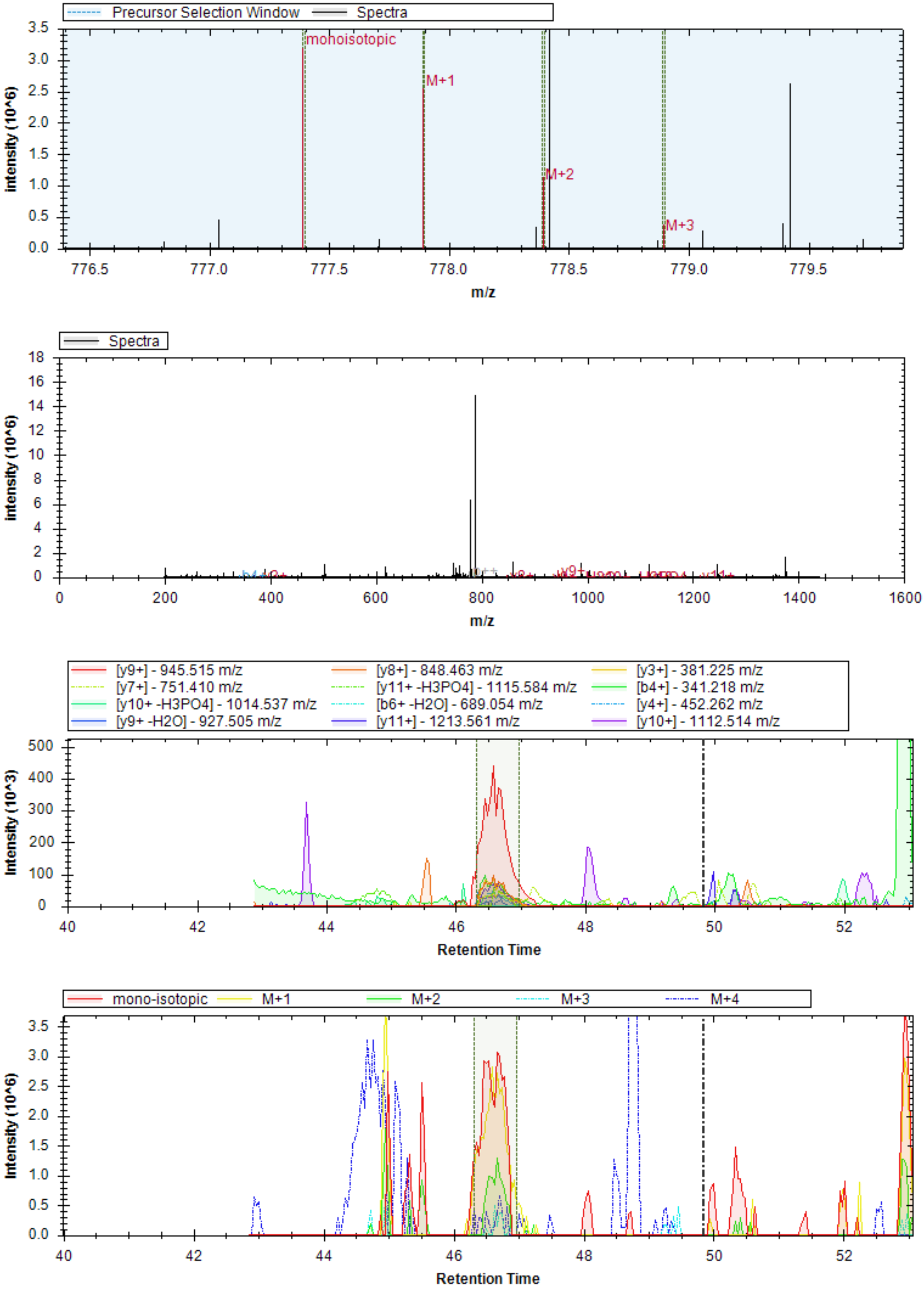

Q9QYR6-2 – _ELSSEPRT[+H+O3+P]PPAQK_.2

Fig4_MP-DIA-barrel-cortex-p54-2_MHRM.htrms

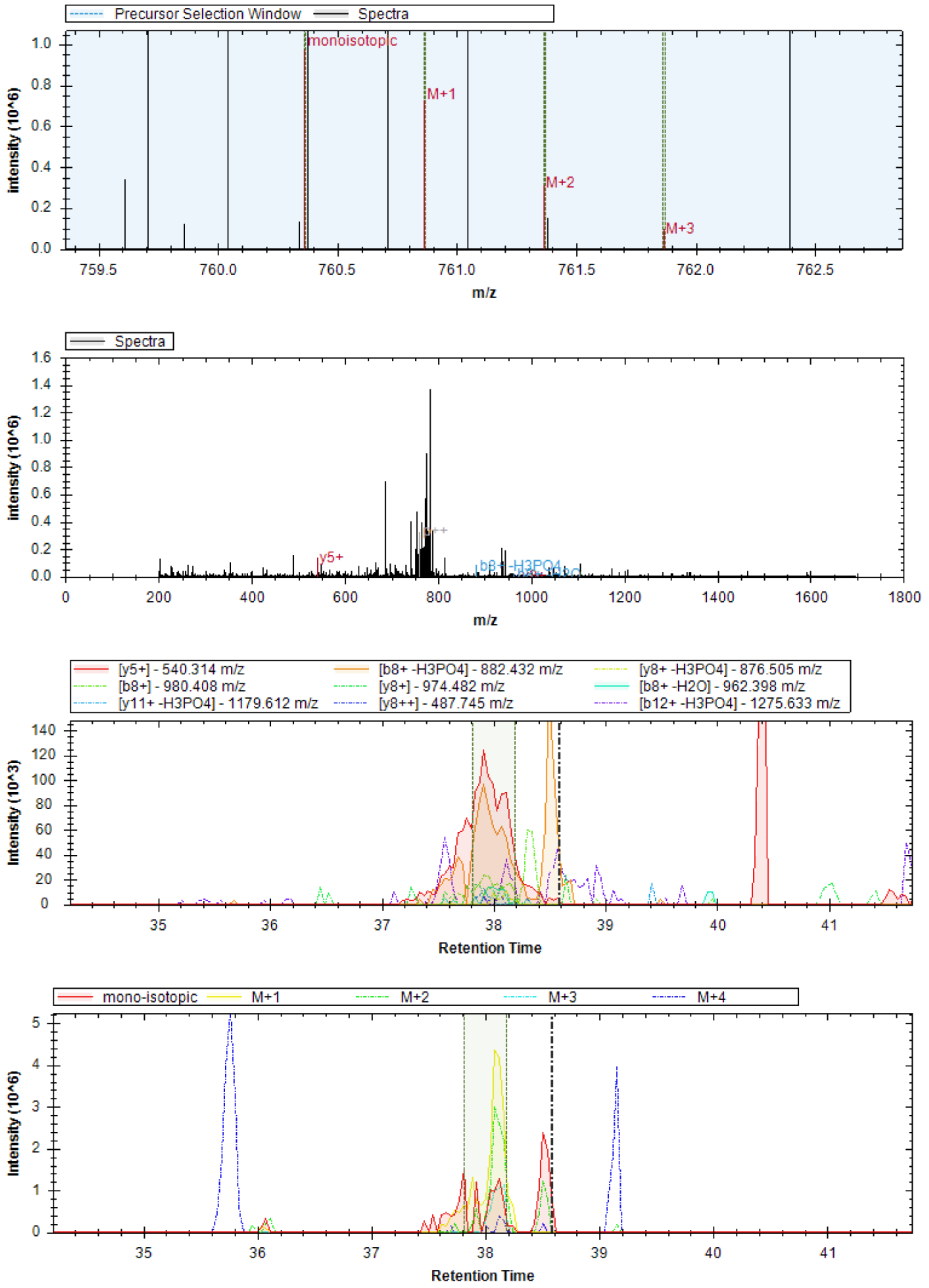

P08249 – _NS[+H+O3+P]PLVSRLTLYDIAHTPGVAADLSHIETR_.4

Fig4_MP-DIA-barrel-cortex-p54-2_MHRM.htrms

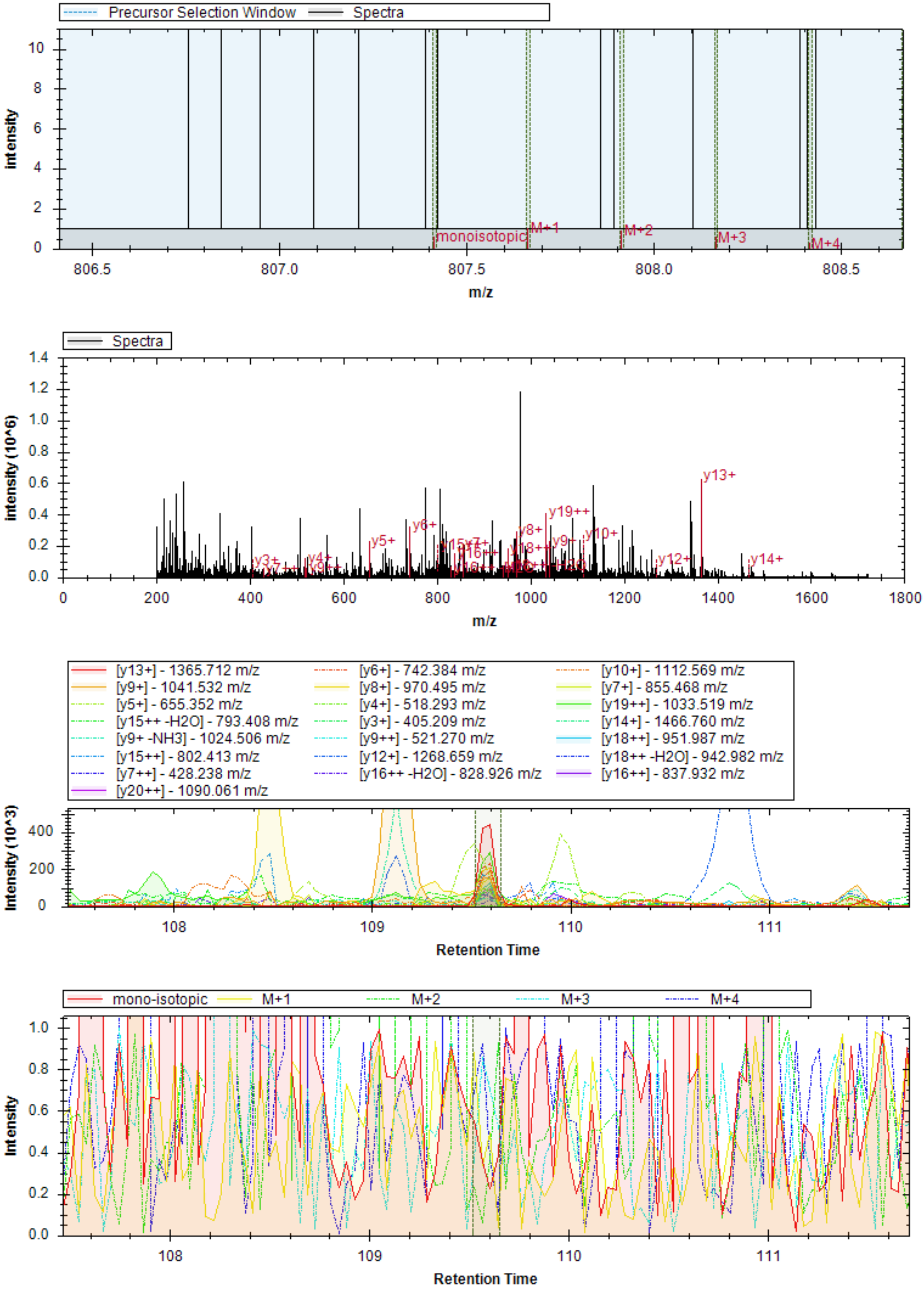

P10637 – _TTPSPKT[+H+O3+P]PPGSGEPPK_.2
Fig4_MP-DIA-barrel-cortex-p54-2_MHRM.htrms

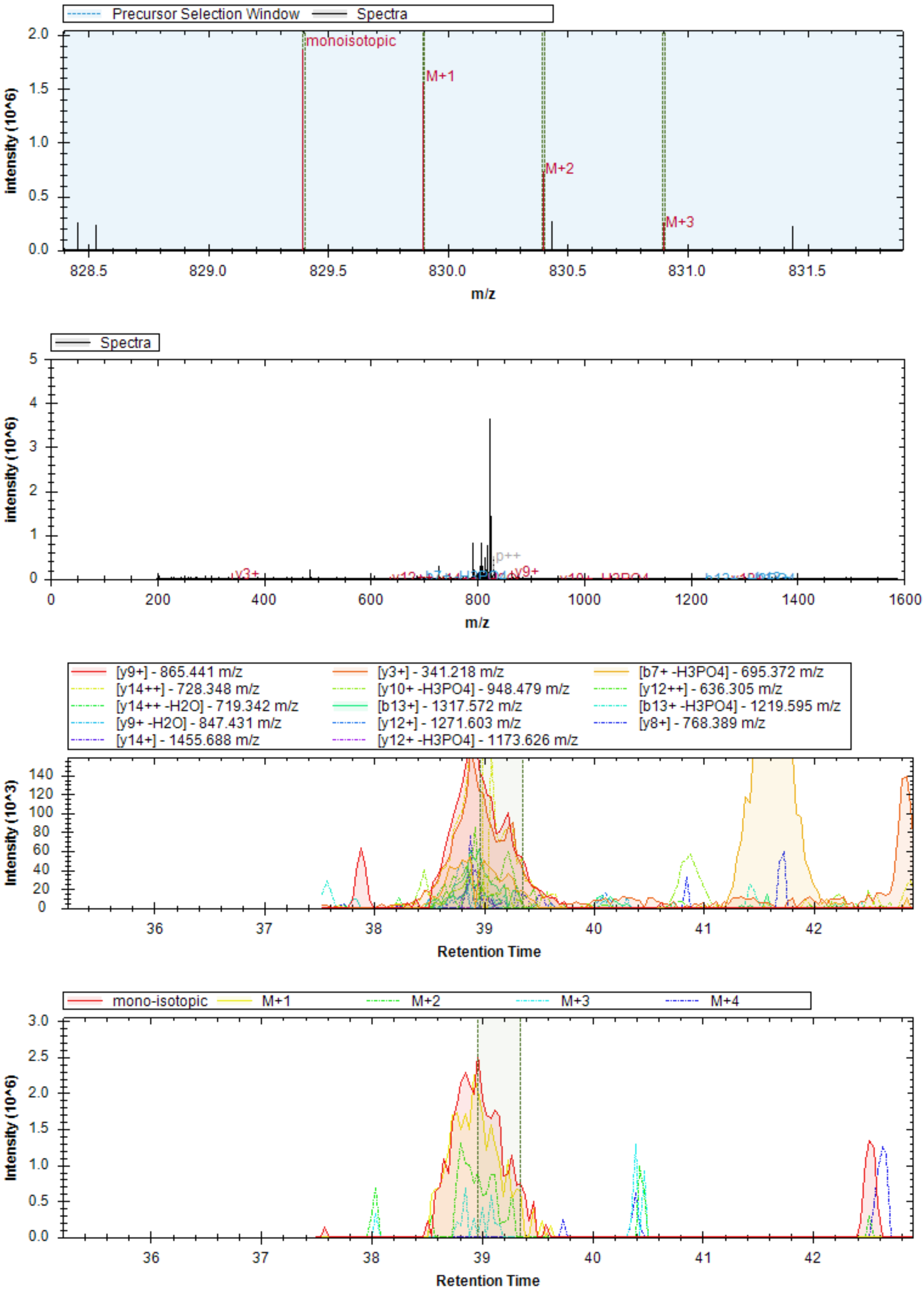

P20357 – _VDHGAEIIT[+H+O3+P]QSPSR_.2

Fig4_MP-DIA-barrel-cortex-p54-2_MHRM.htrms

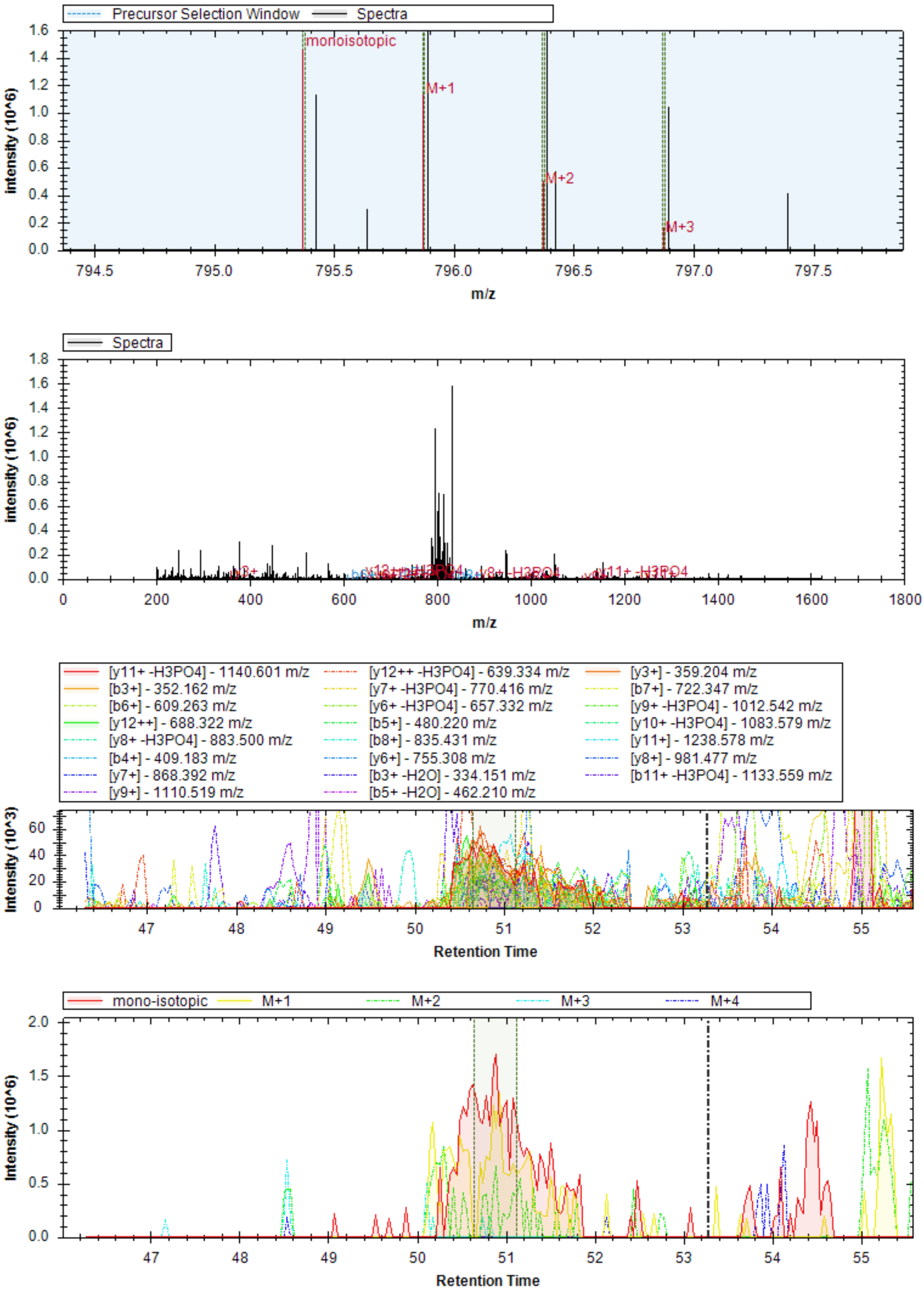

Q60771 – _FYYSS[+H+O3+P]GSSSPTHAK_.2
Fig4_MP-DIA-barrel-cortex-p54-2_MHRM.htrms

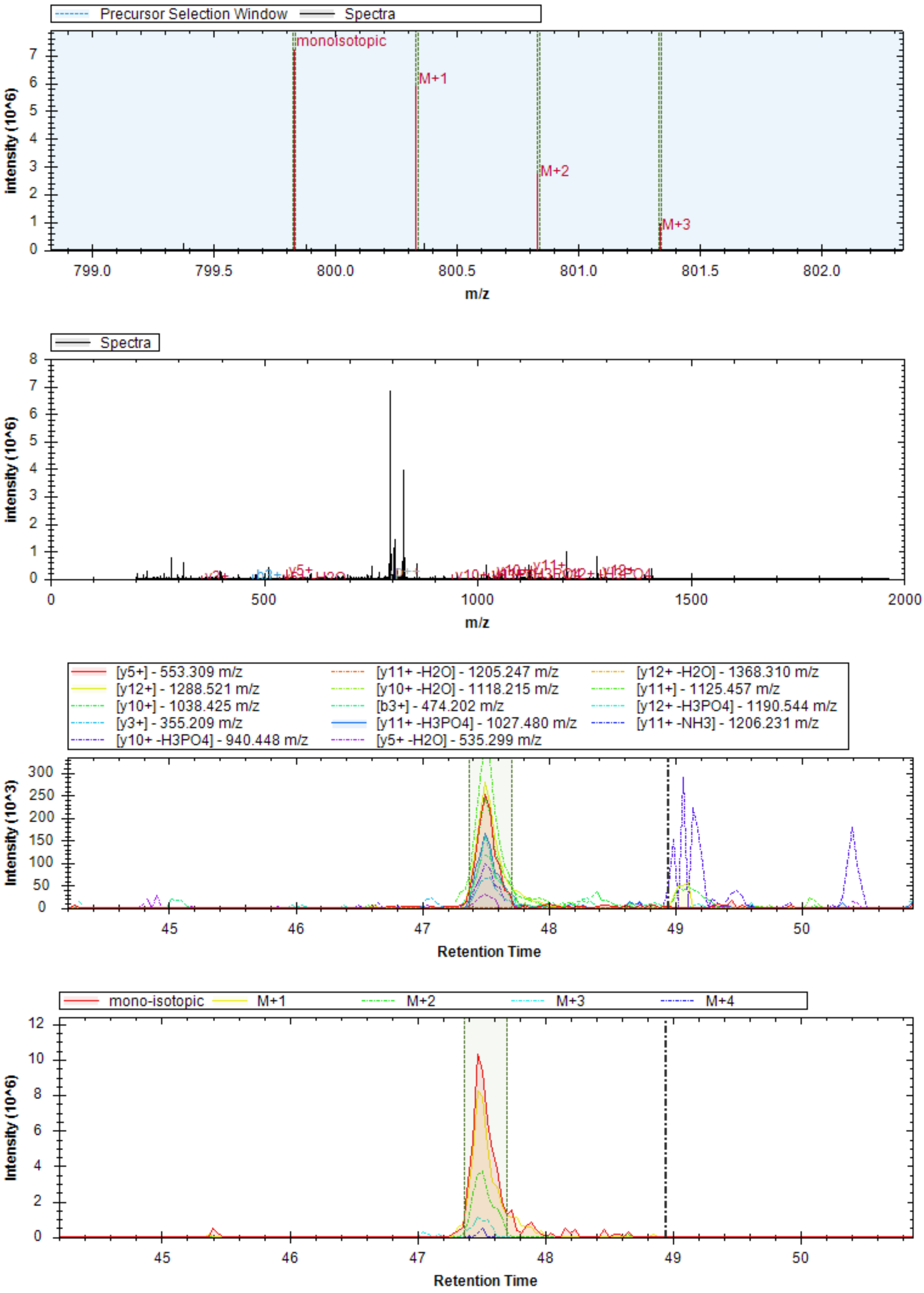

Q60771 – _FYYSSGS[+H+O3+P]SSPTHAK_.2

Fig4_MP-DIA-barrel-cortex-p54-2_MHRM.htrms

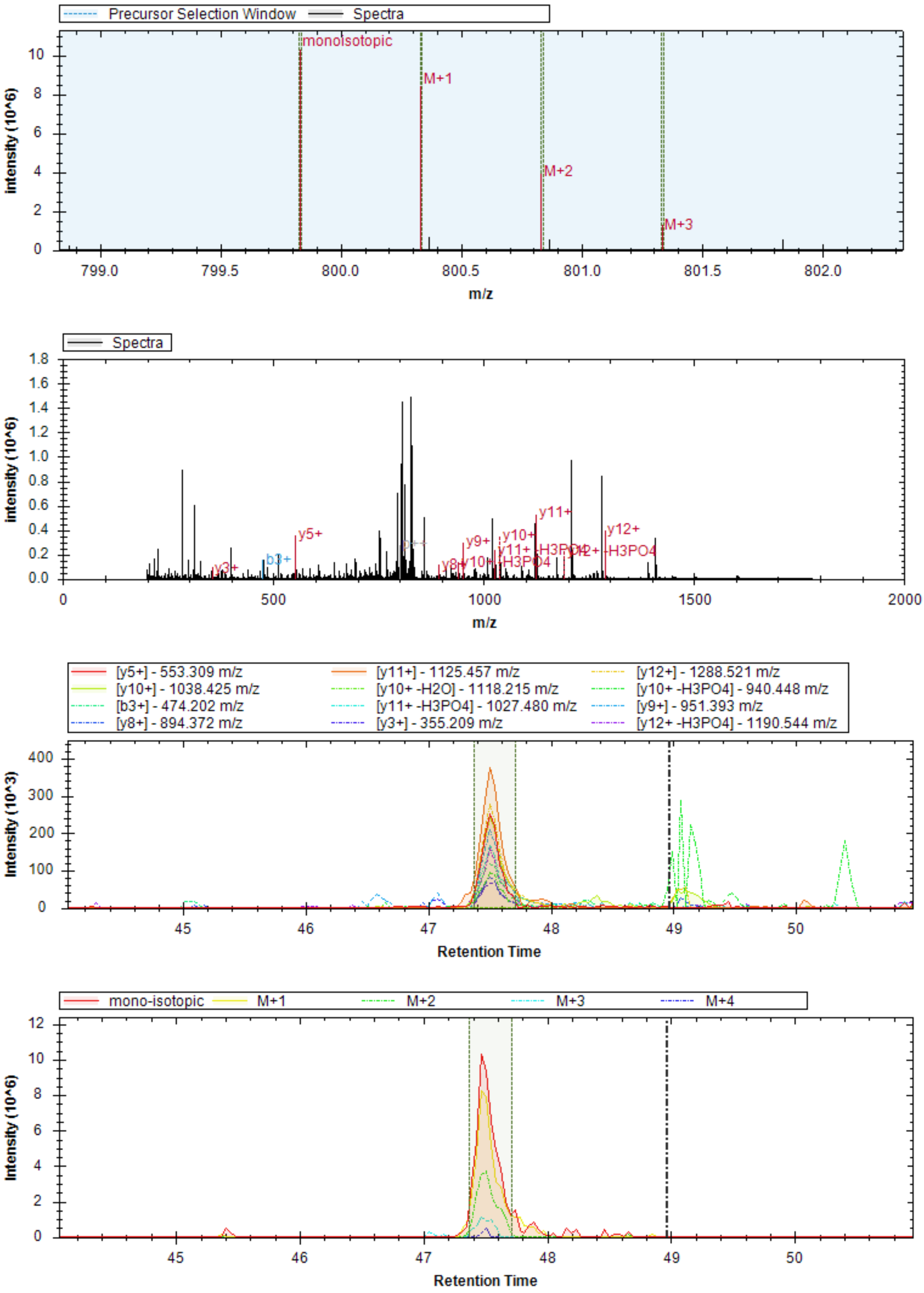

Q60771 – _FYYSSGSSS[+H+O3+P]PTHAK_.2
Fig4_MP-DIA-barrel-cortex-p54-2_MHRM.htrms

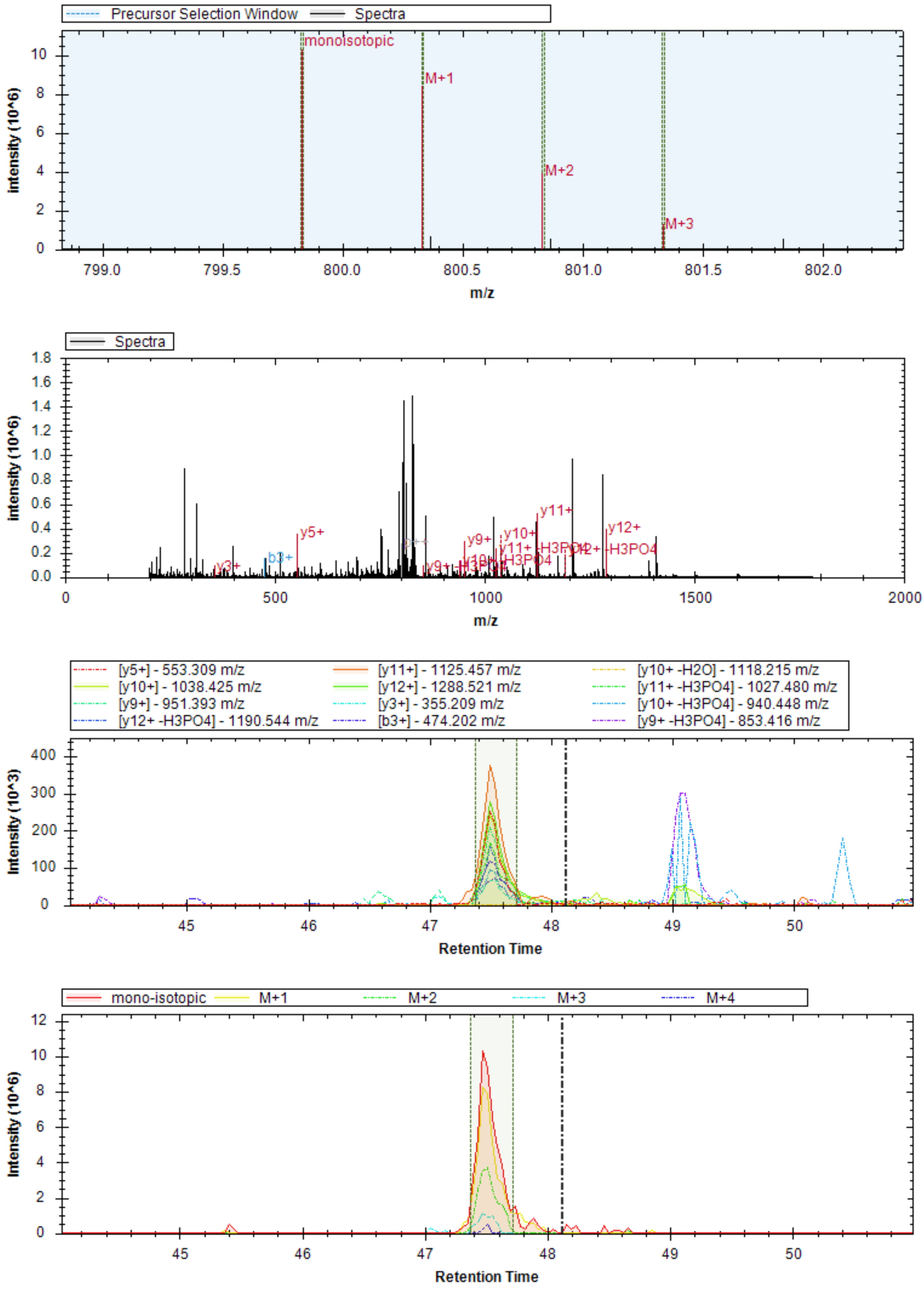

Q64701;Q64701-2 – _LAEILYYKILET[+H+O3+P]IMVQET[+H+O3+P]R_.3
Fig4_MP-DIA-barrel-cortex-p54-2_MHRM.htrms

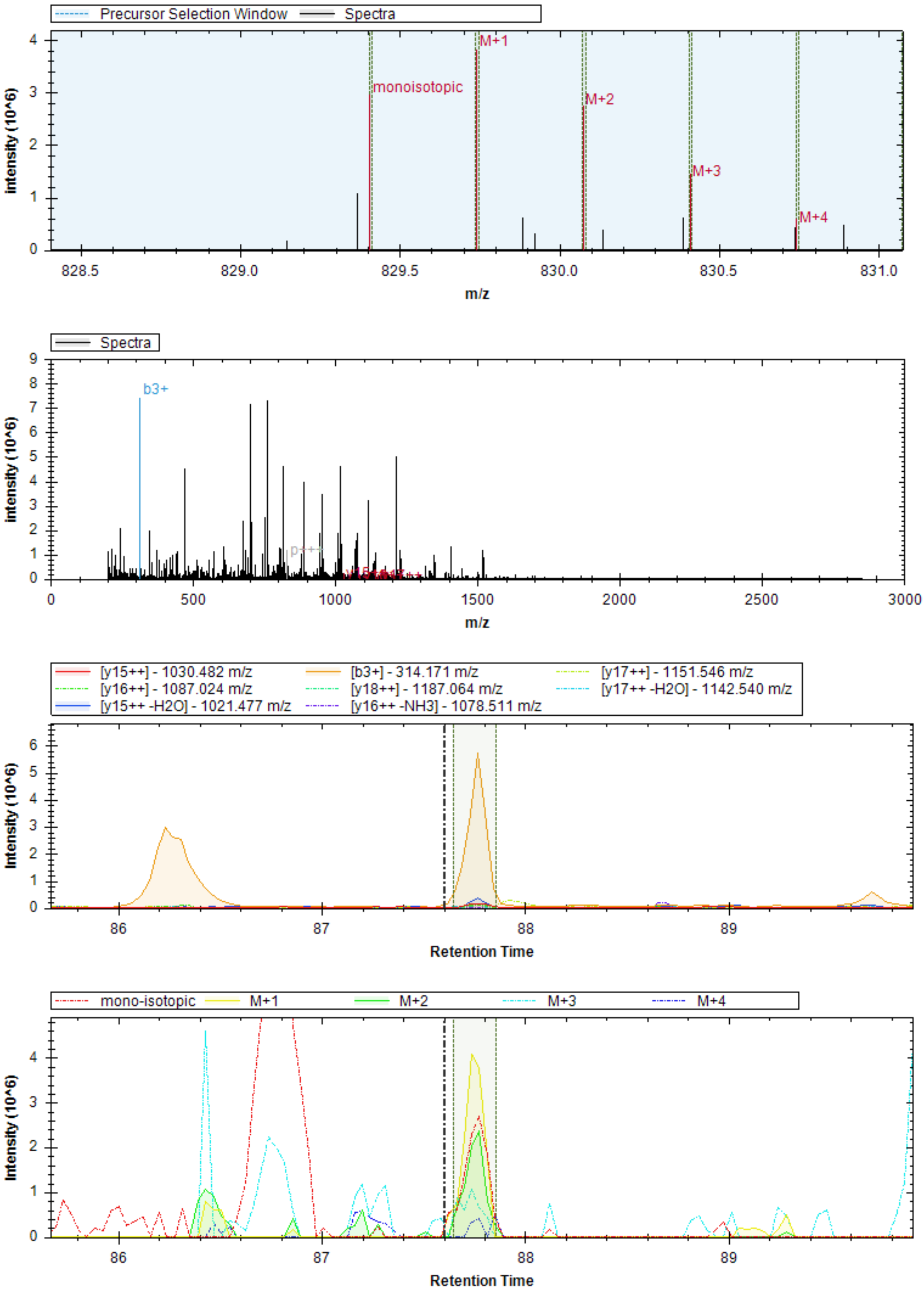

Q7TQD2 – _AANKT[+H+O3+P]PPKSPGDPAR_.2
Fig4_MP-DIA-barrel-cortex-p54-2_MHRM.htrms

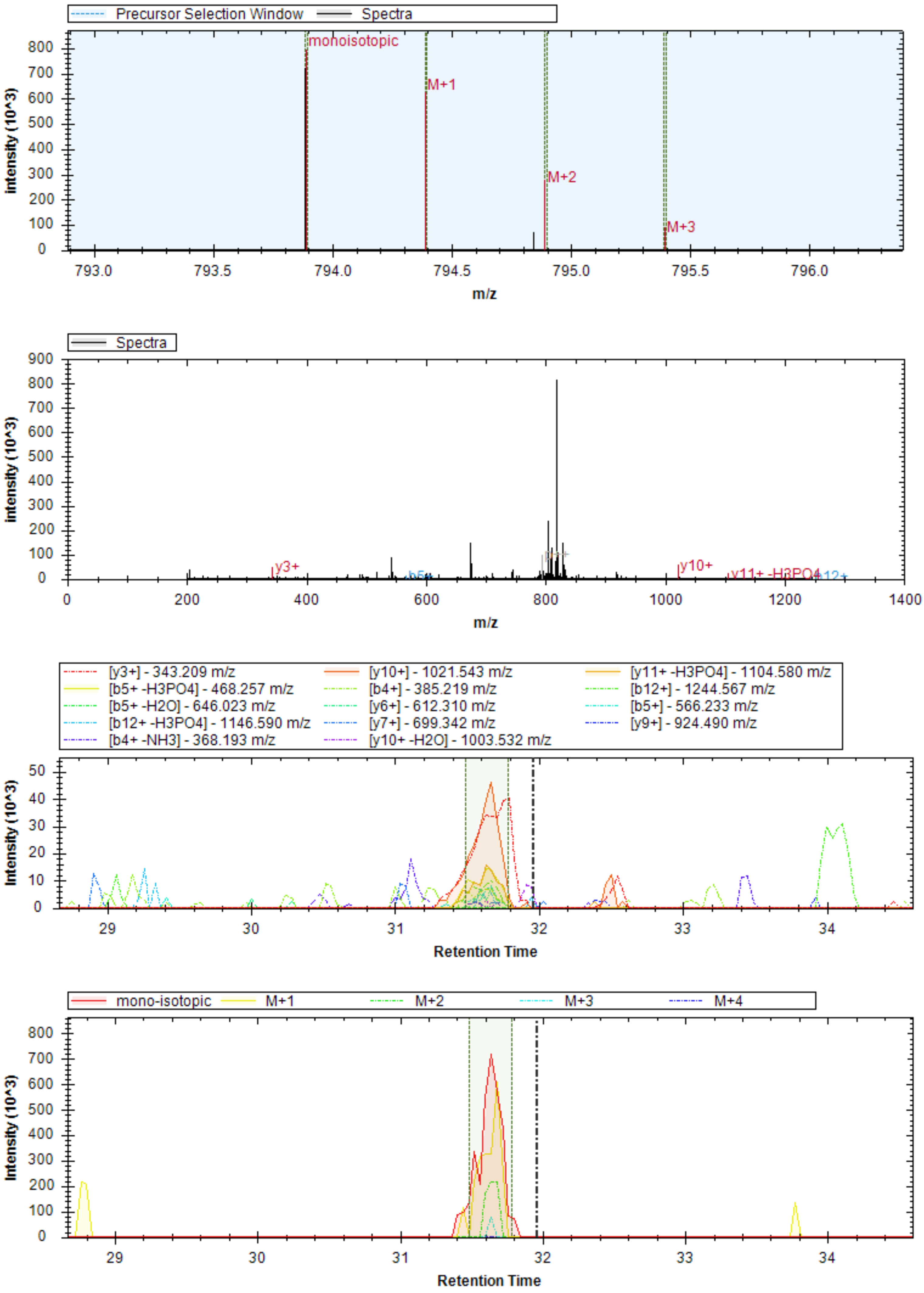

D3YXK2 – _AET[+H+O3+P]LSGLGDASAAGAAAVSSAASETGTR_.3
Fig4_MP-DIA-barrel-cortex-p54-2_MHRM.htrms

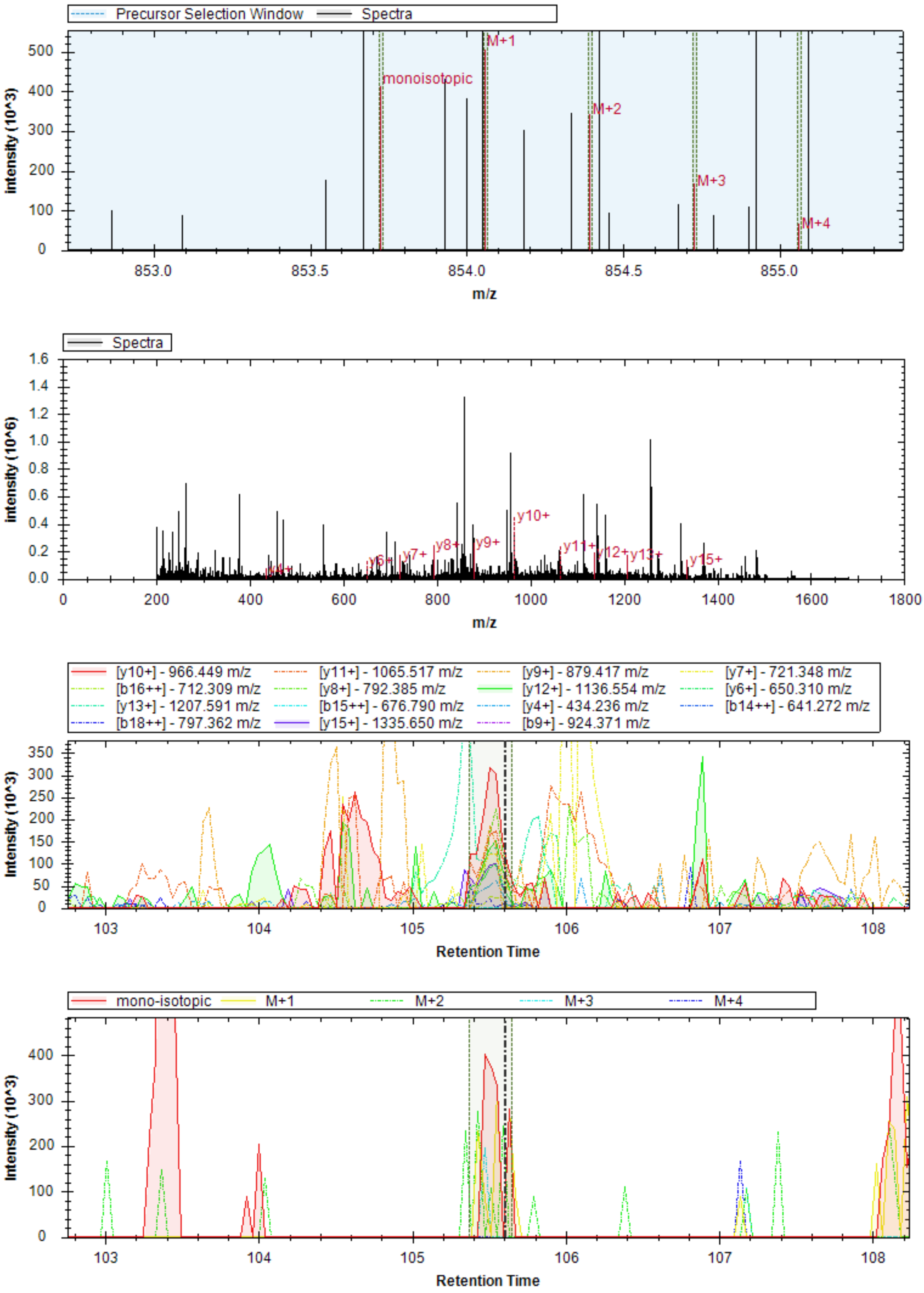

O08784 – _ASAVSPEKAPM[+O]T[+H+O3+P]SKAK_.2
Fig4_MP-DIA-barrel-cortex-p54-2_MHRM.htrms

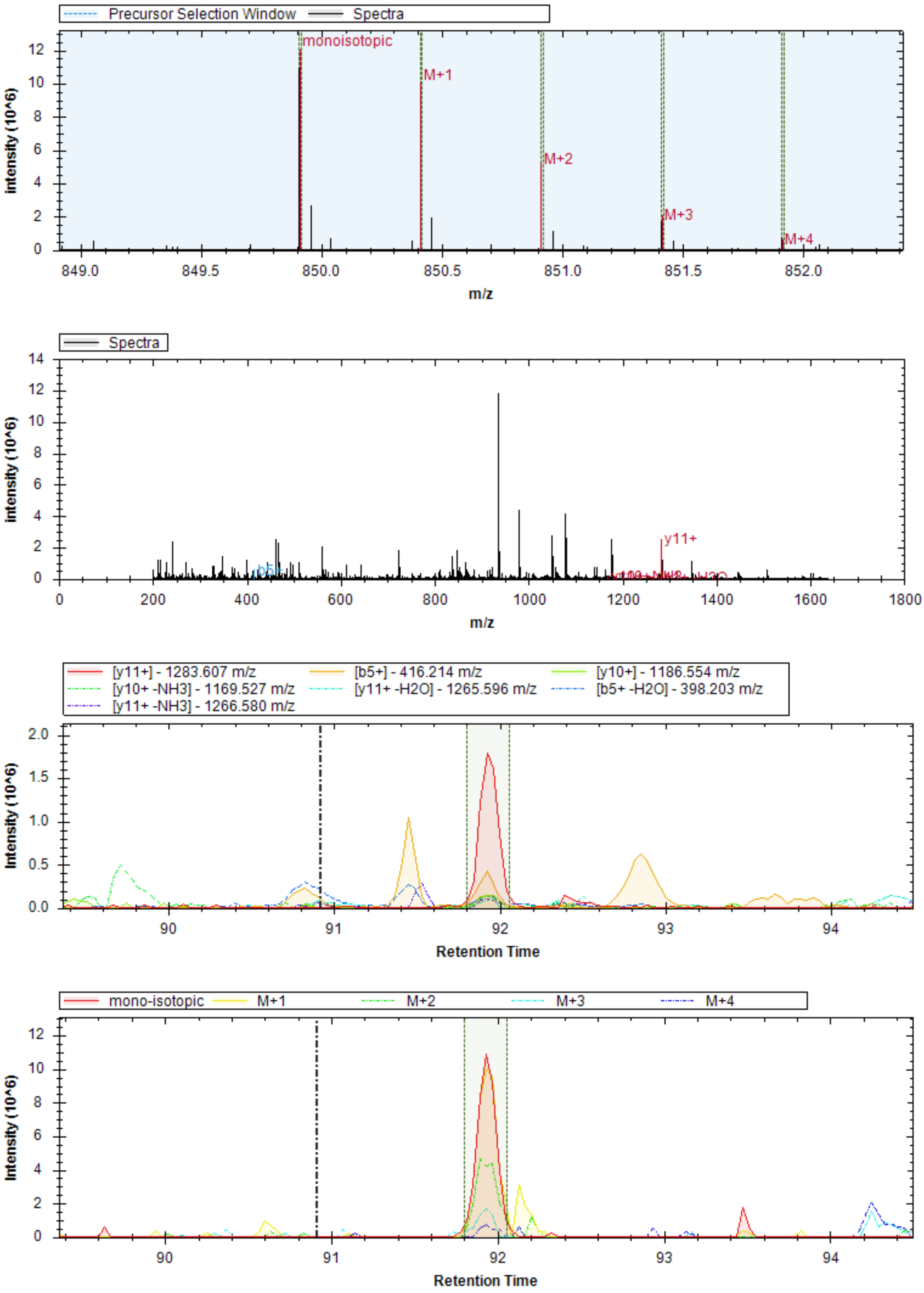

P20917 – _NVTESSS[+H+O3+P]FSGGDNPHVLYSPEFR_.3
Fig4_MP-DIA-barrel-cortex-p54-2_MHRM.htrms

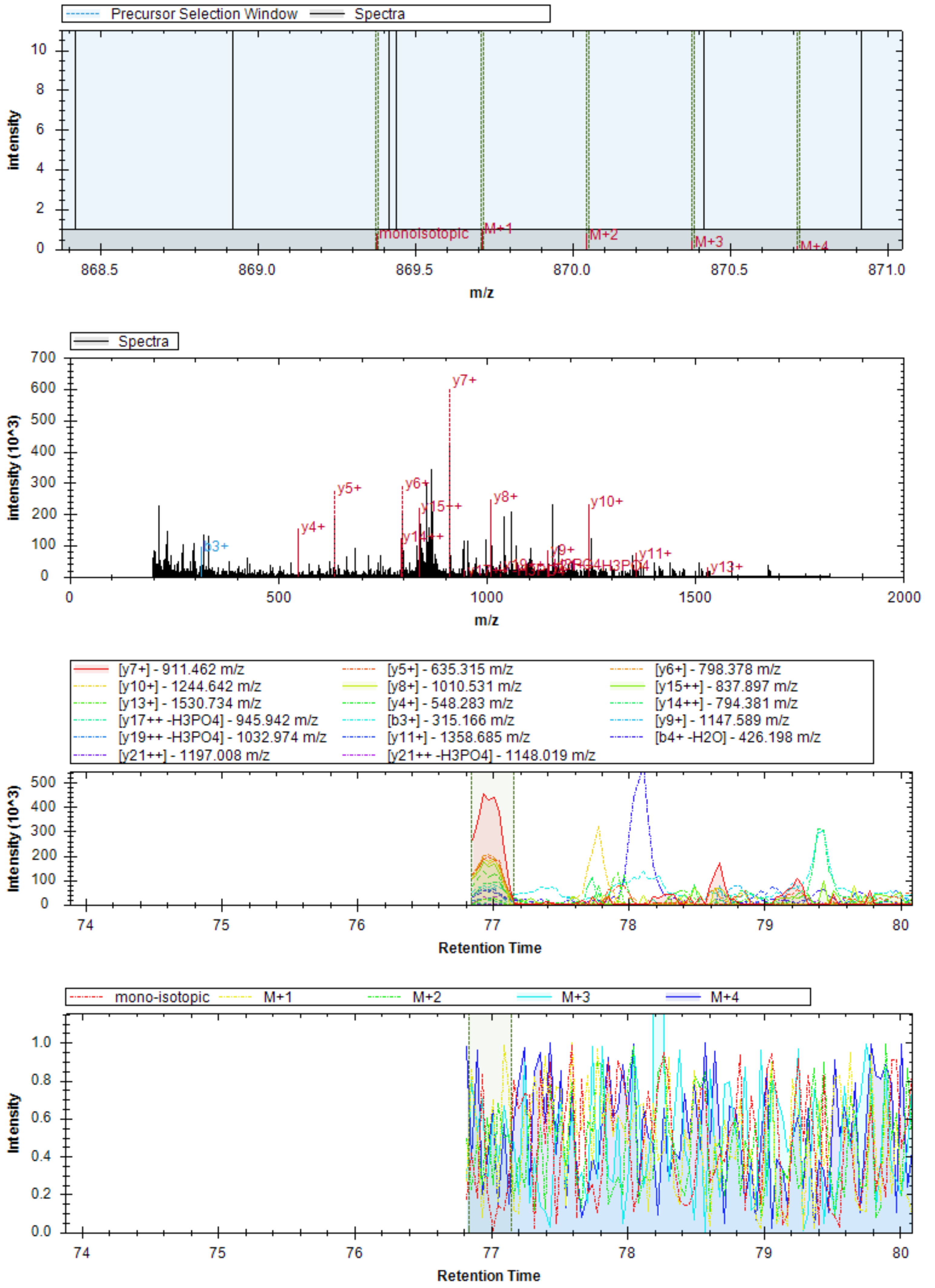

P50538 – _EAEHGYAS[+H+O3+P]M[+O]LPYSK_.2
Fig4_MP-DIA-barrel-cortex-p54-2_MHRM.htrms

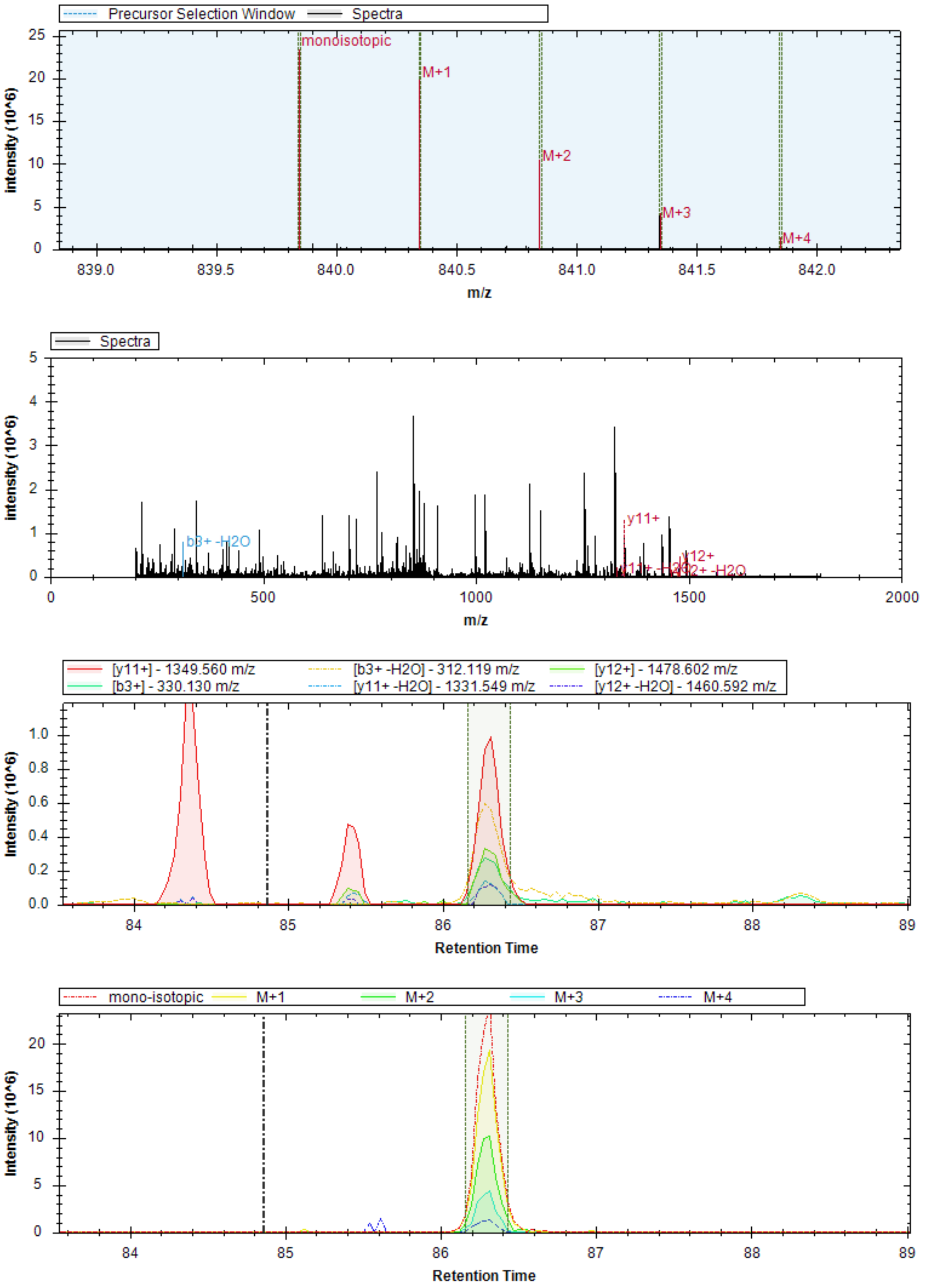

Q9Z0P4 – _SETLVNAQQT[+H+O3+P]PLGTPK_.2
Fig4_MP-DIA-barrel-cortex-p54-2_MHRM.htrms

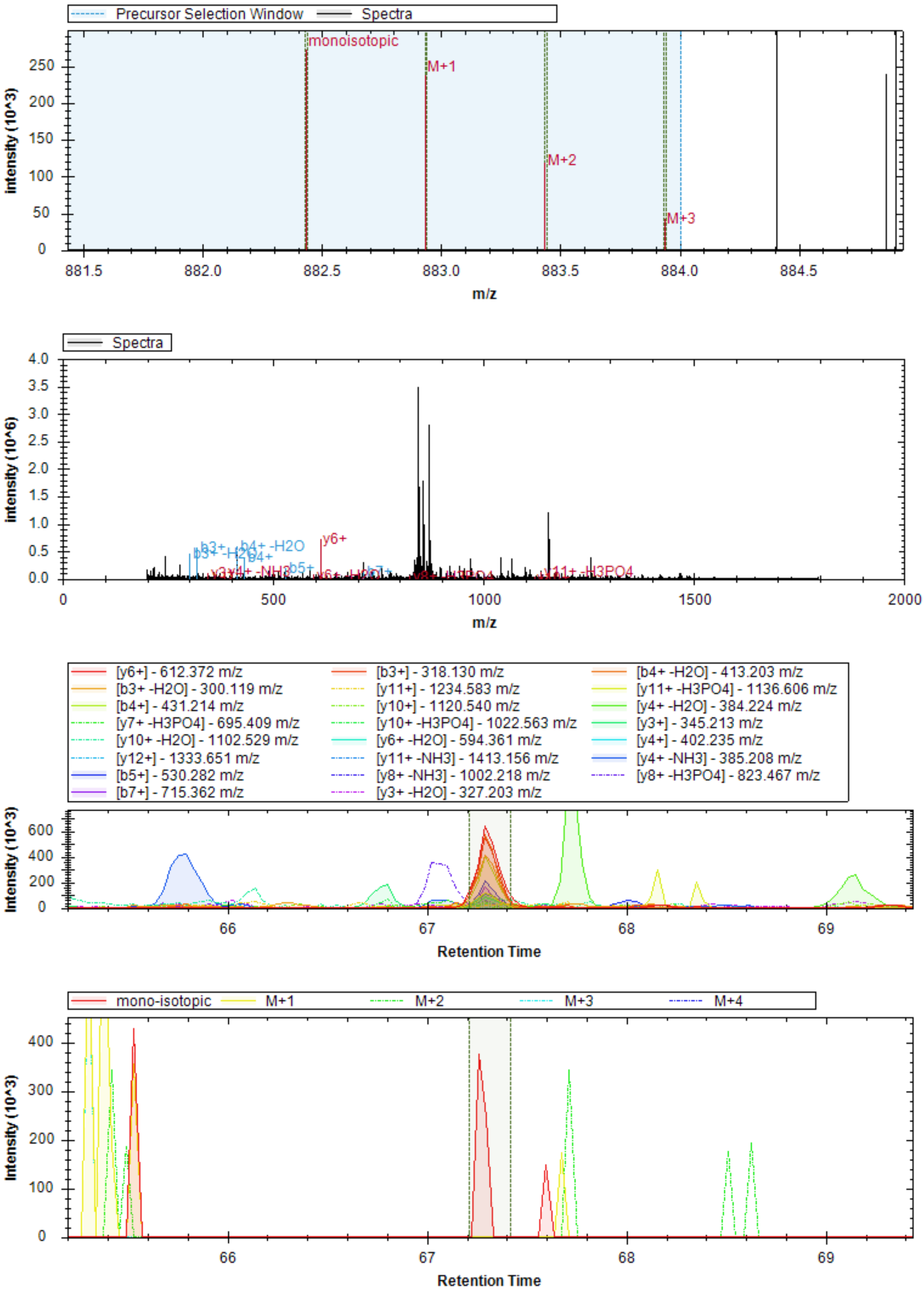

A1A535 – _FLHRGS[+H+O3+P]K_.1
Fig4_MP-DIA-barrel-cortex-p54-2_MHRM.htrms

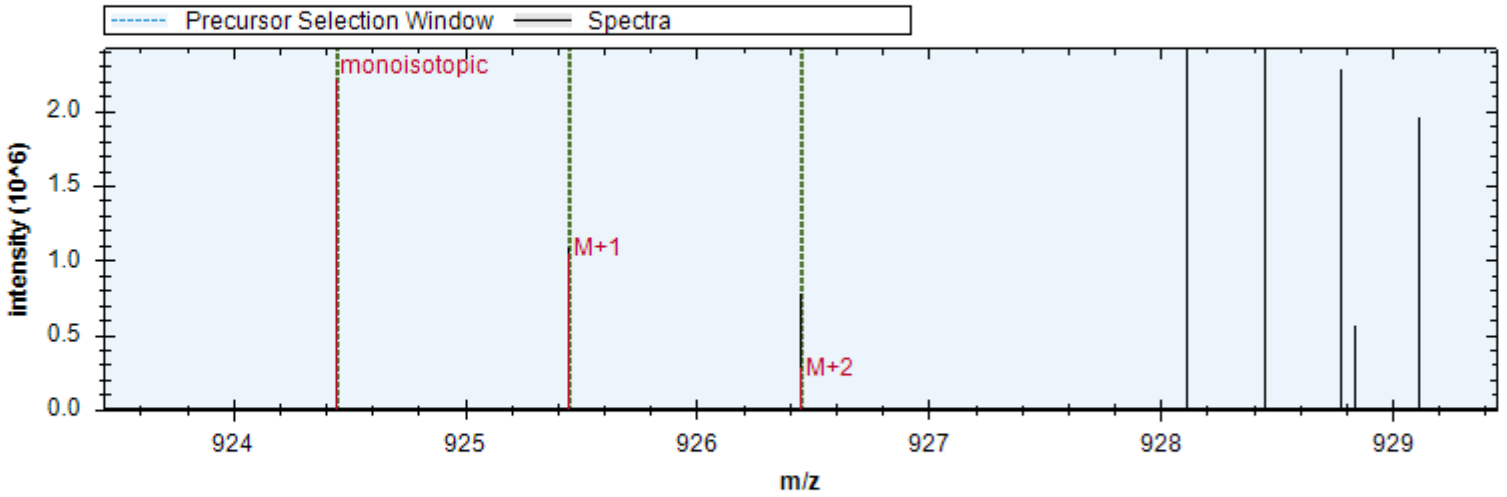

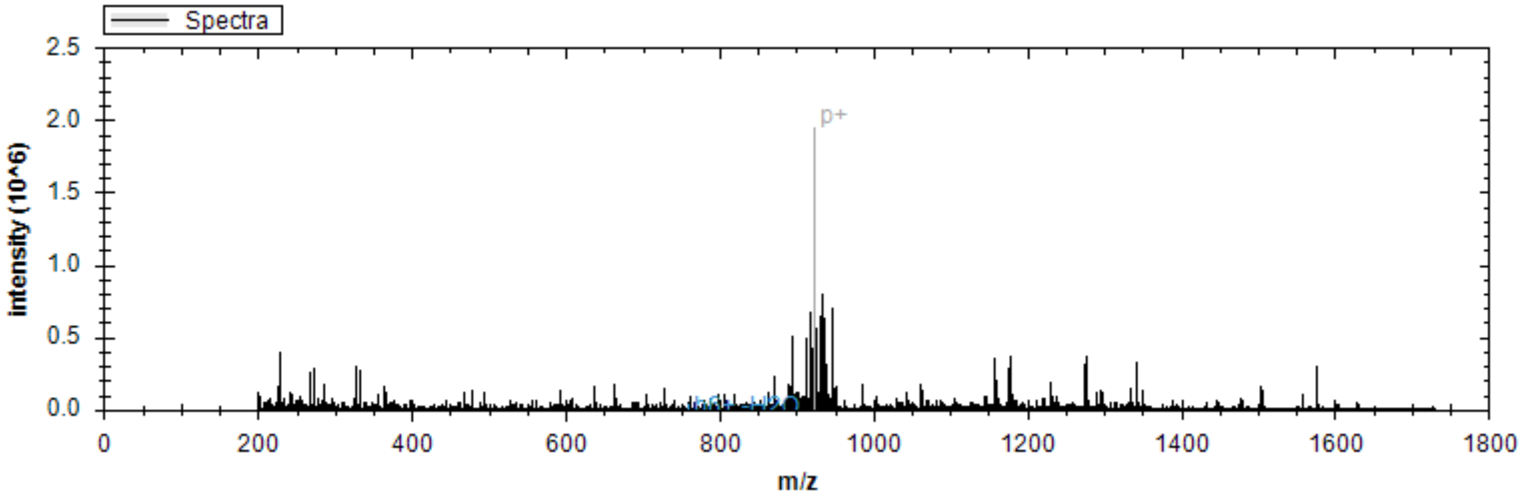

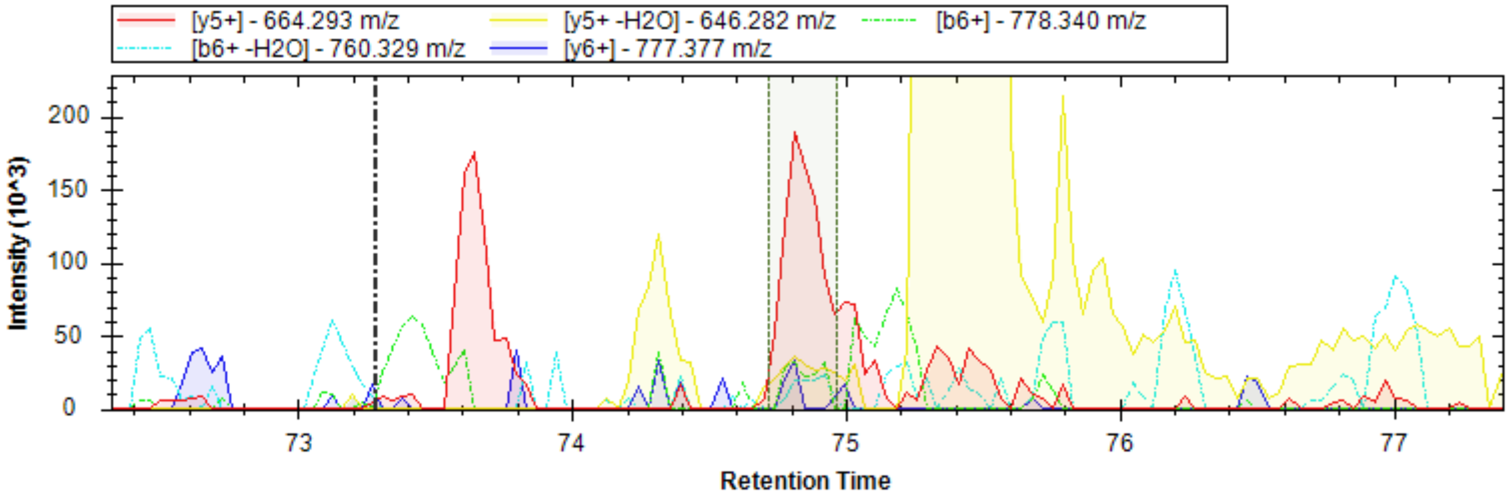

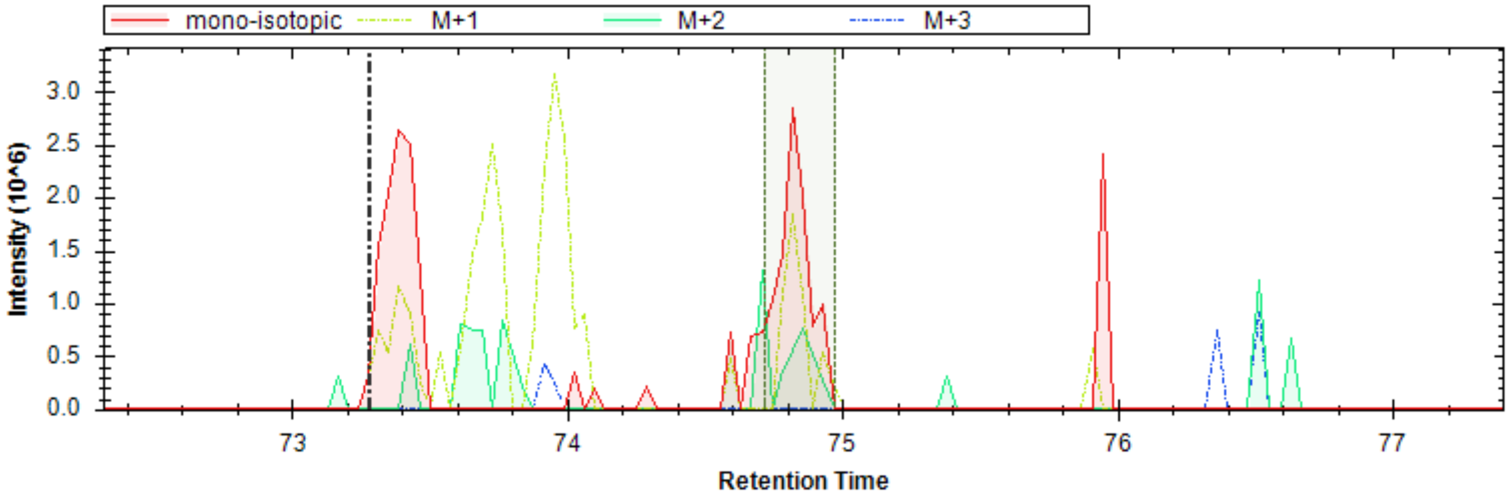

P60904 – _SLS[+H+O3+P]TSGESLYHVLGLDK_.2
Fig4_MP-DIA-barrel-cortex-p54-2_MHRM.htrms

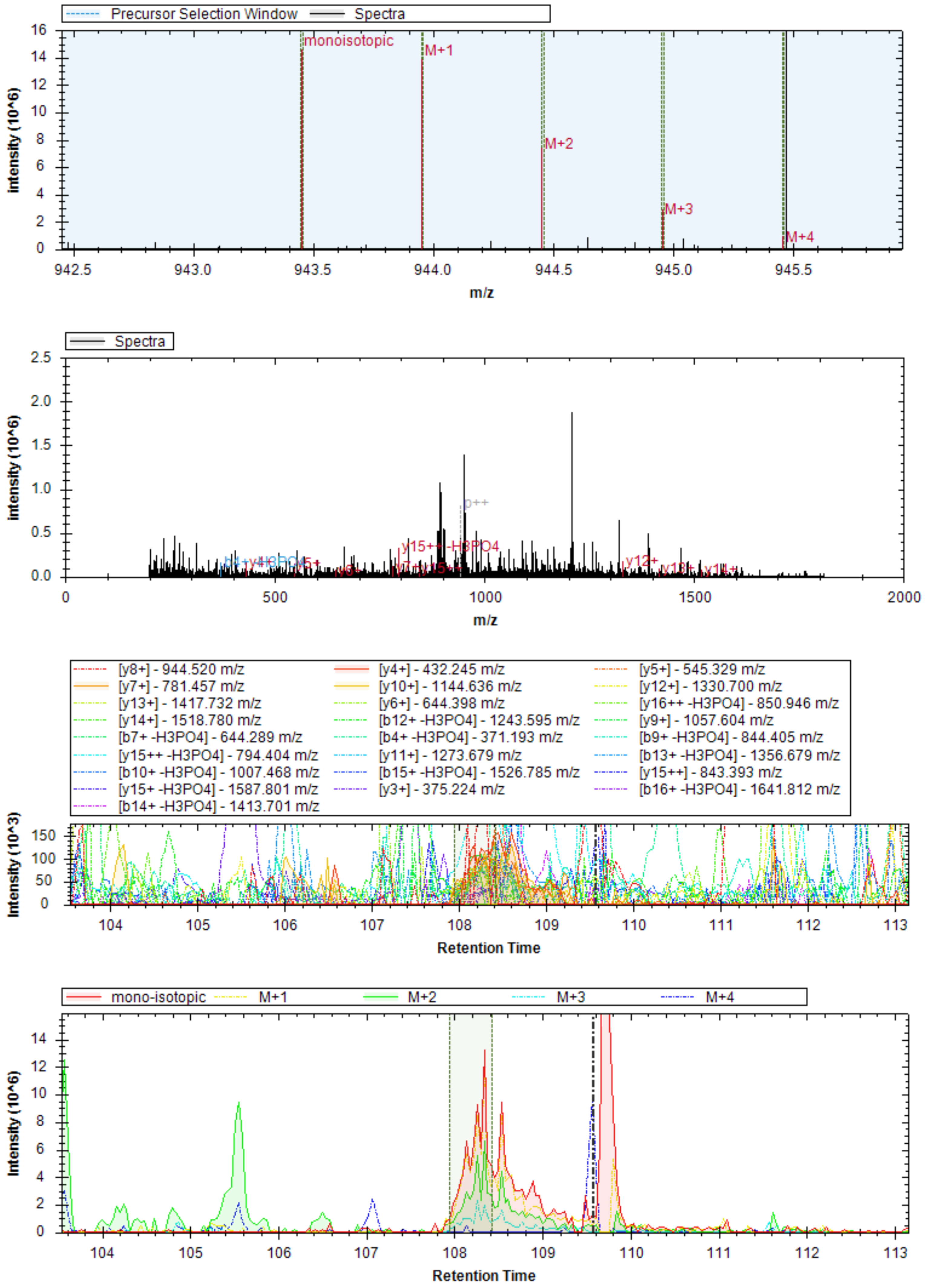

Q91Z31;Q91Z31-2 – _ETSLLAVPGALS[+H+O3+P]PLAIPNAAAAAAAAAAGR_.3

Fig4_MP-DIA-barrel-cortex-p54-2_MHRM.htrms

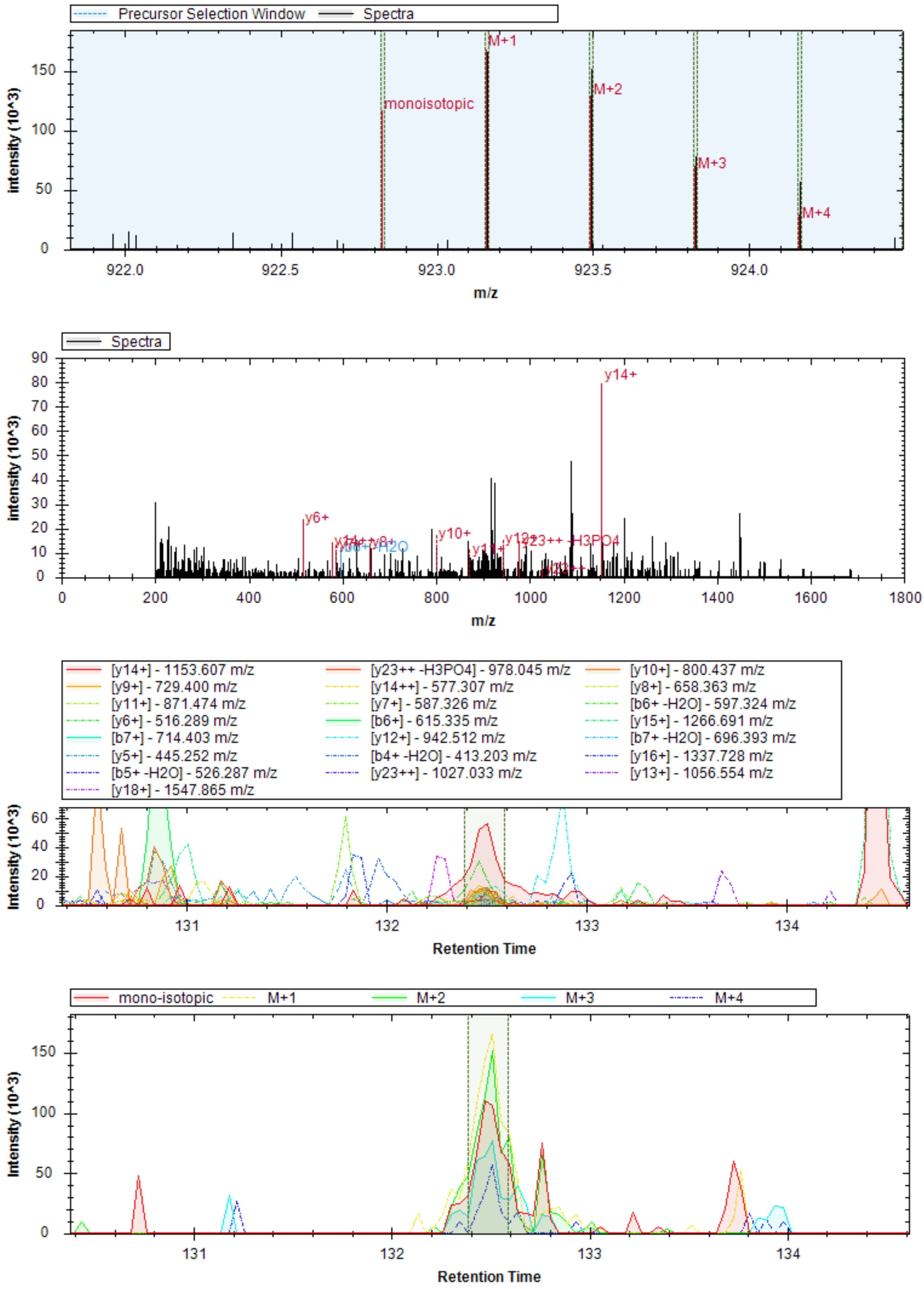

P17742 – _VNPT[+H+O3+P]VFFDITADDEPLGR_.2

Fig4_MP-DIA-barrel-cortex-p54-2_MHRM.htrms

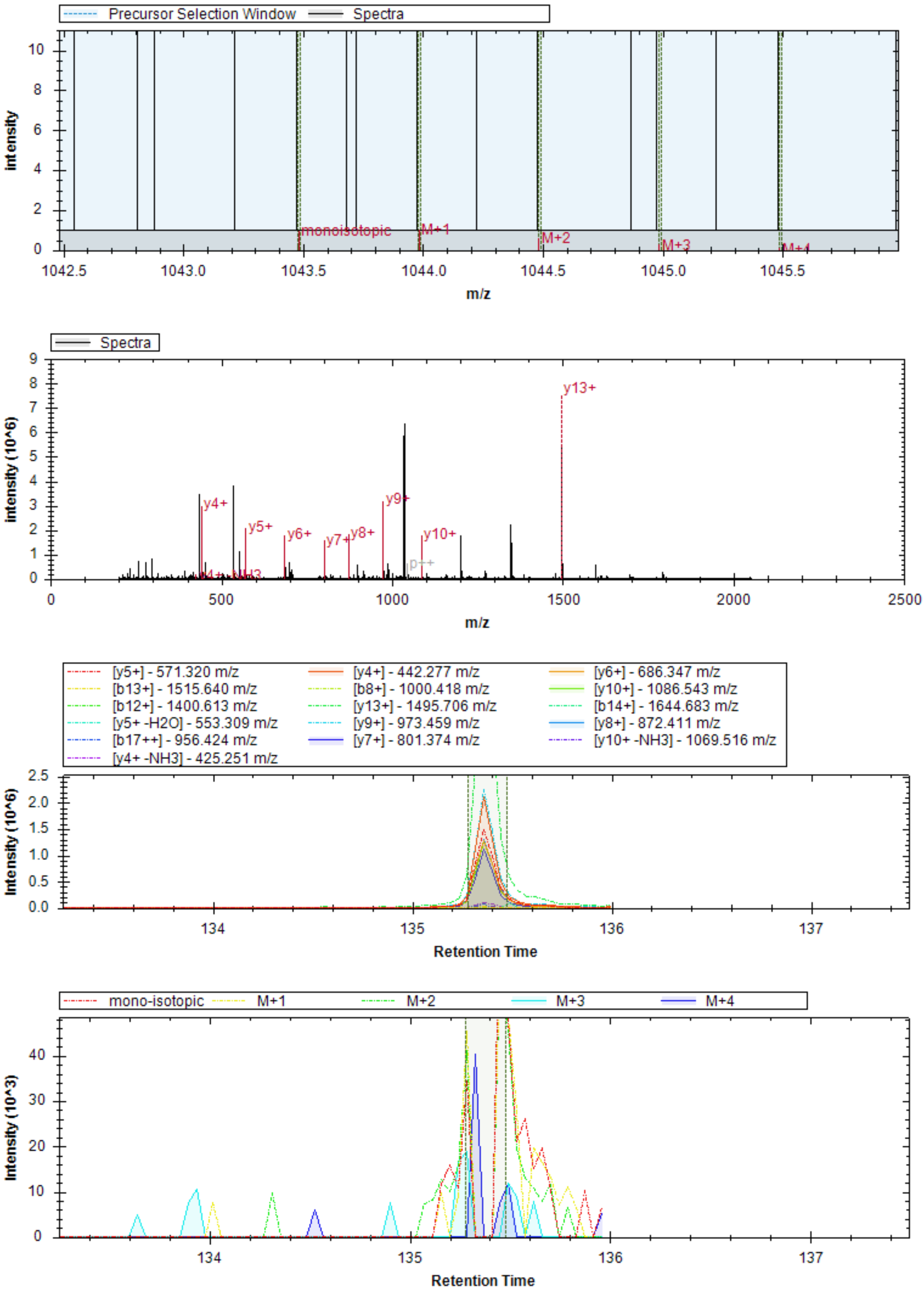

Q8CHK4;Q8CHK4-2;Q8CH[...] – _RGY[+H+O3+P]GKLLIEFS[+H+O3+P]YELSK_.2
Fig4_MP-DIA-barrel-cortex-p54-2_MHRM.htrms

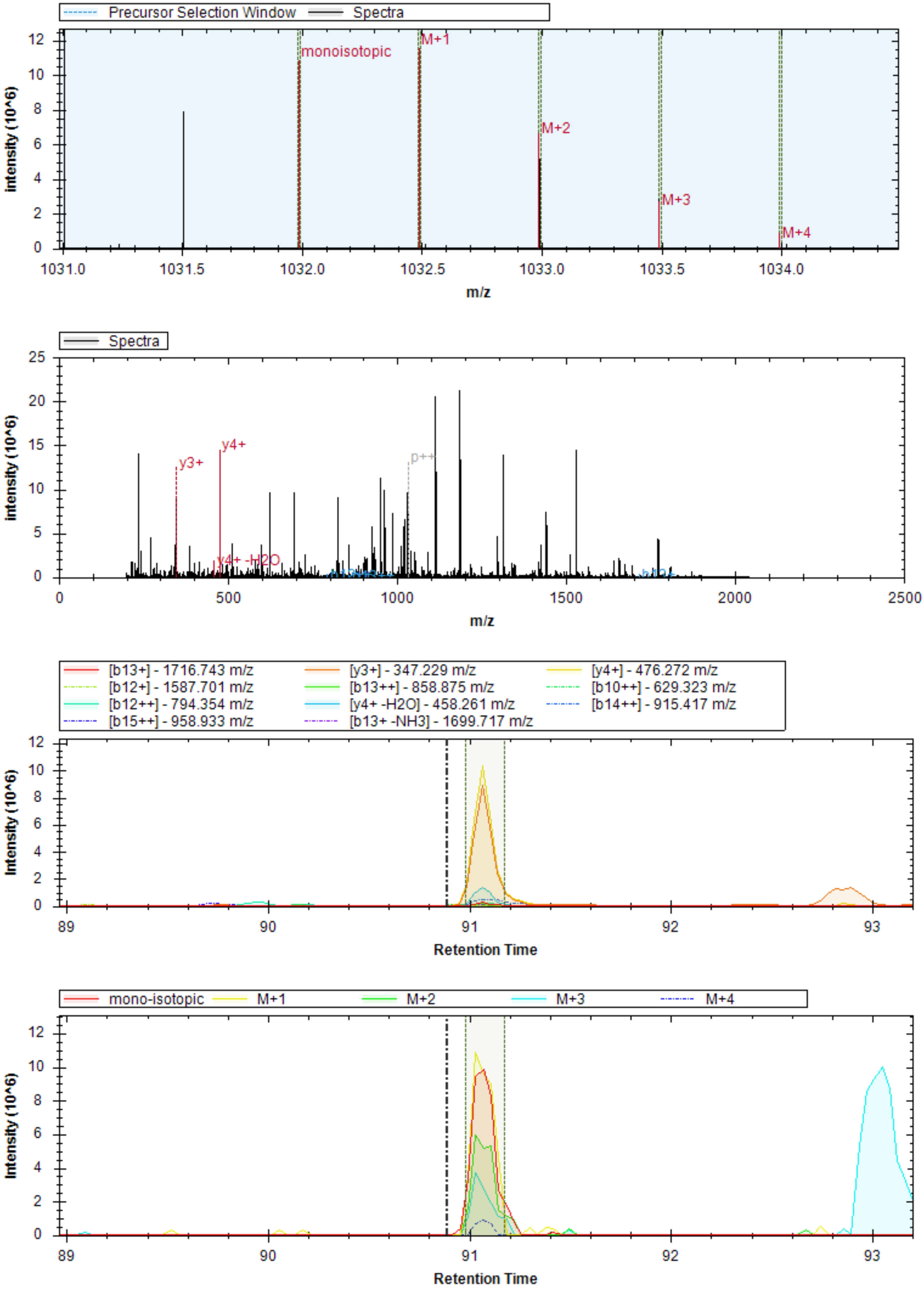

E9Q401 – _MVAEVFIY[+H+O3+P]WSK_.1
Fig4_MP-DIA-barrel-cortex-p54-2_MHRM.htrms

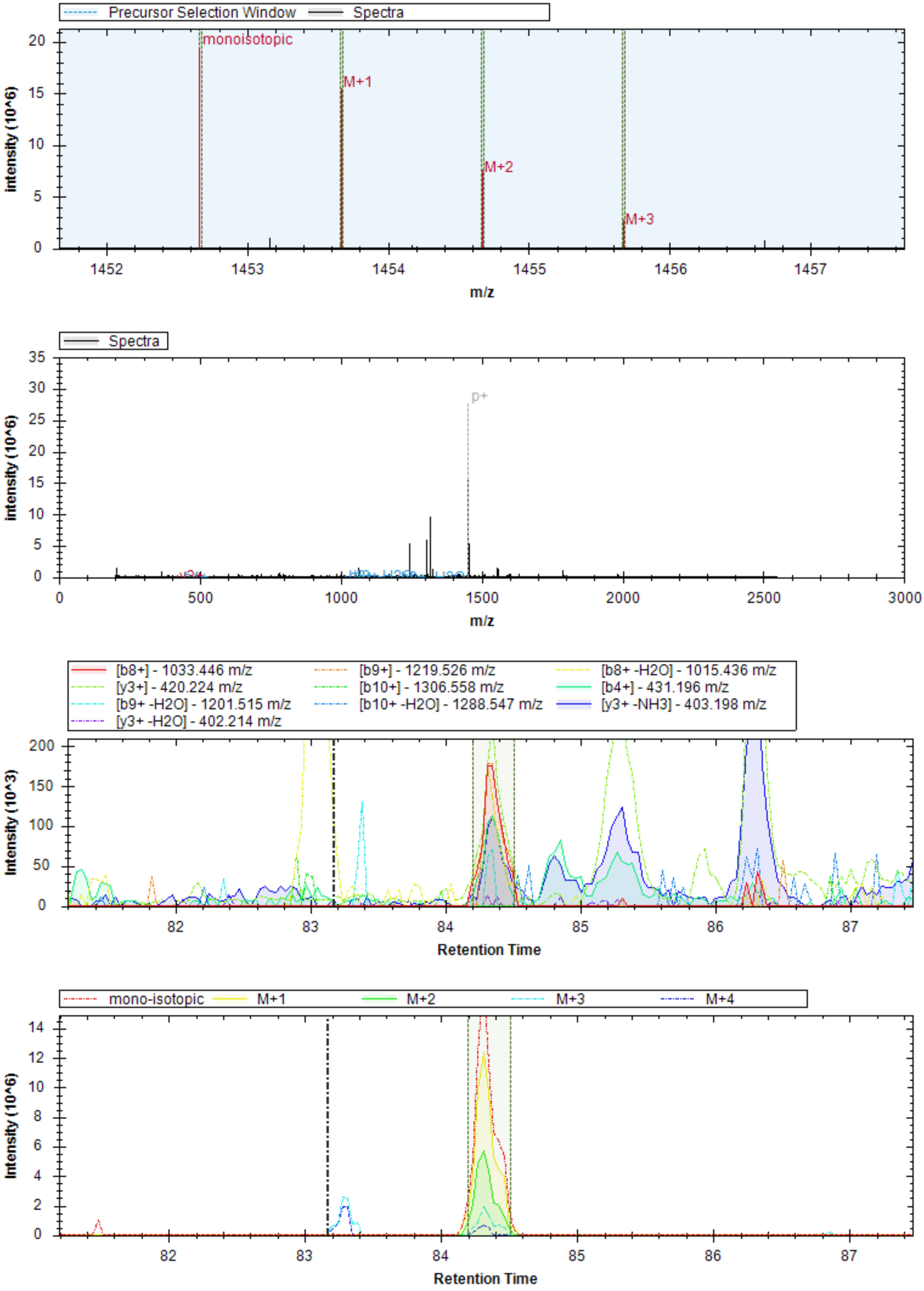

Q8VHY0 – _LLAGPRY[+H+O3+P]GR_.1

Fig4_MP-DIA-barrel-cortex-p54-2_MHRM.htrms

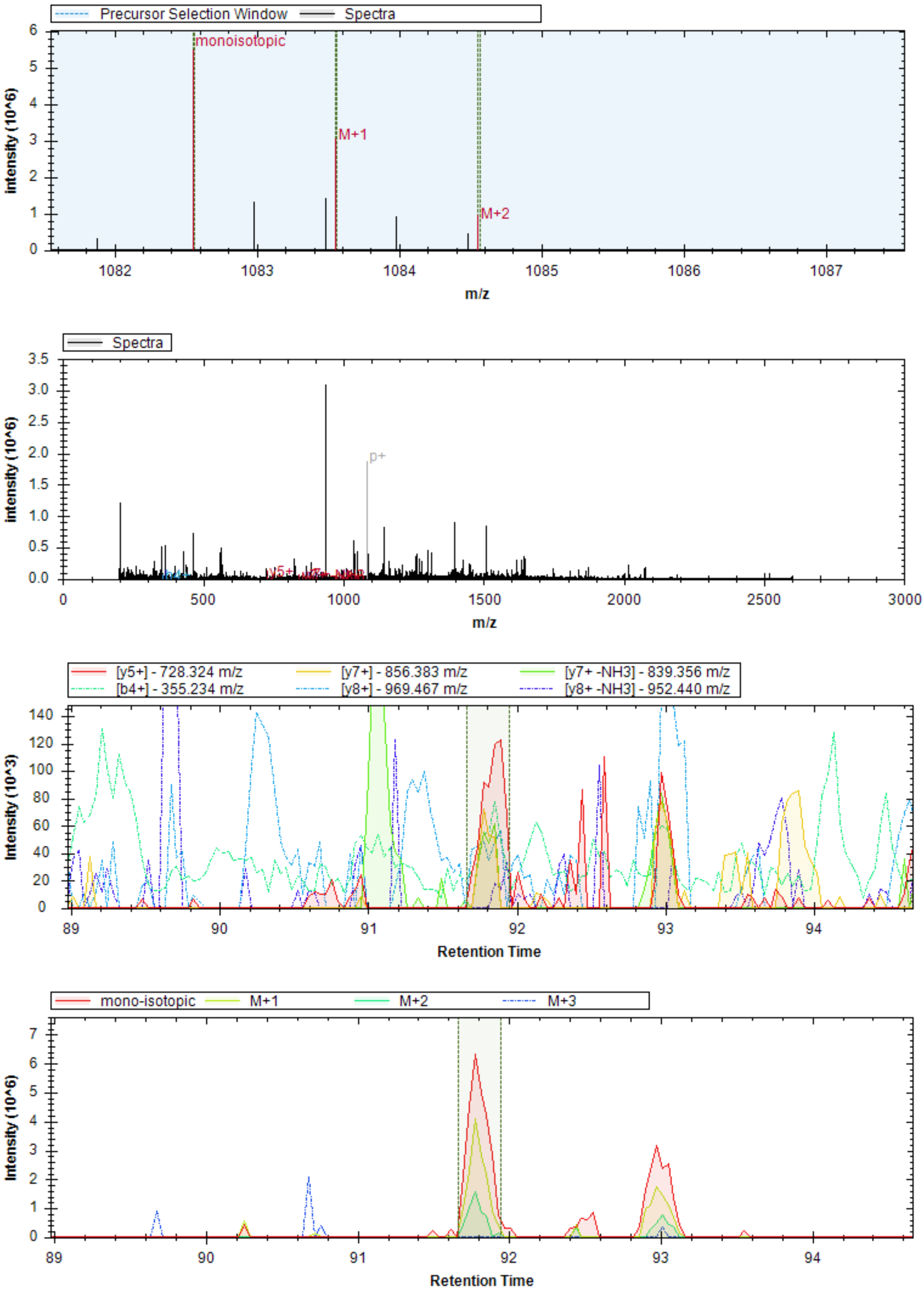

Q921N6;Q921N6-2 – _LIY[+H+O3+P]KPRQAAVTR_.4
Fig4_MP-DIA-barrel-cortex-p54-3_MHRM.htrms

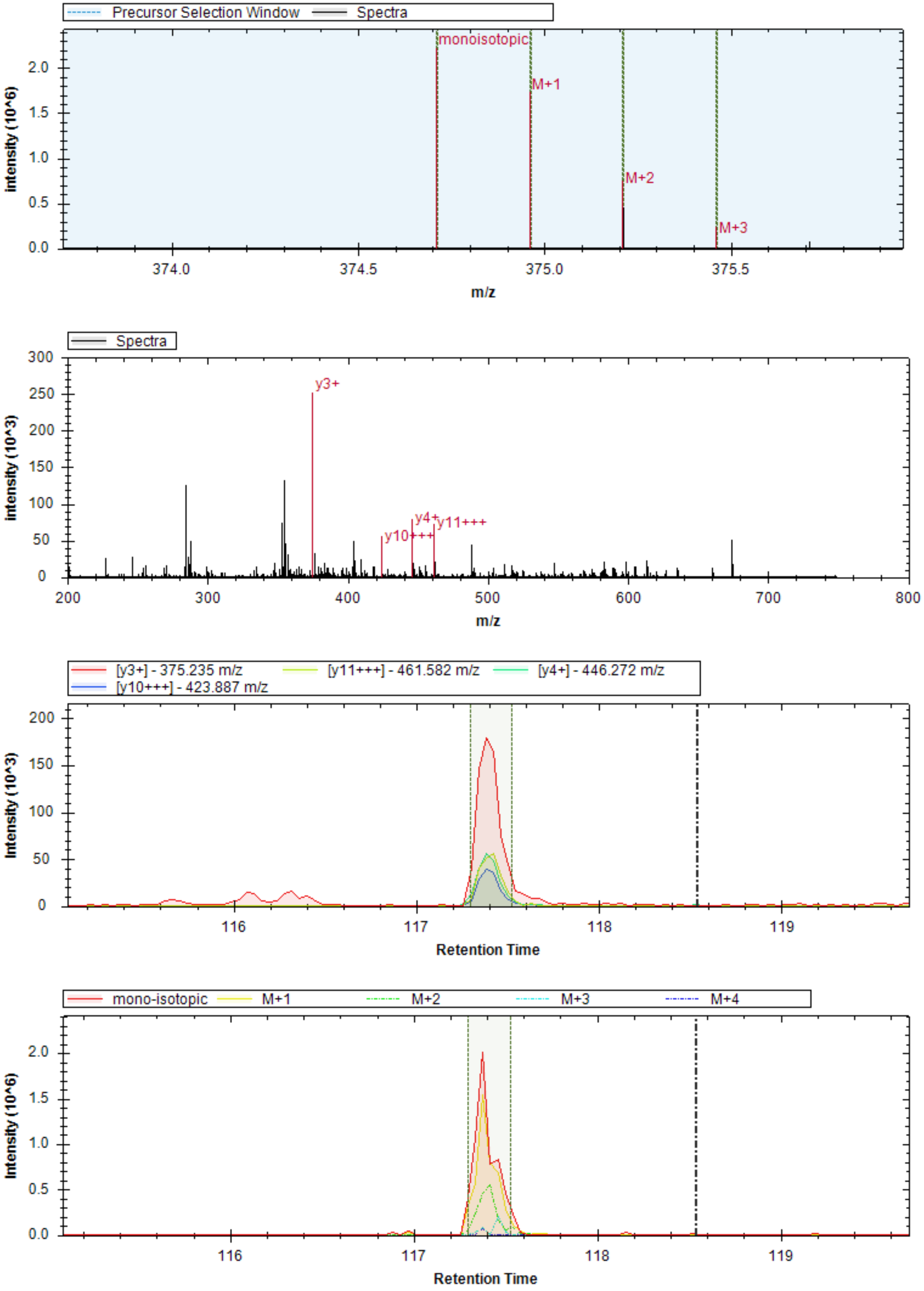

Q9CVB6 – _IIEET[+H+O3+P]LALK_.3

Fig4_MP-DIA-barrel-cortex-p54-3_MHRM.htrms

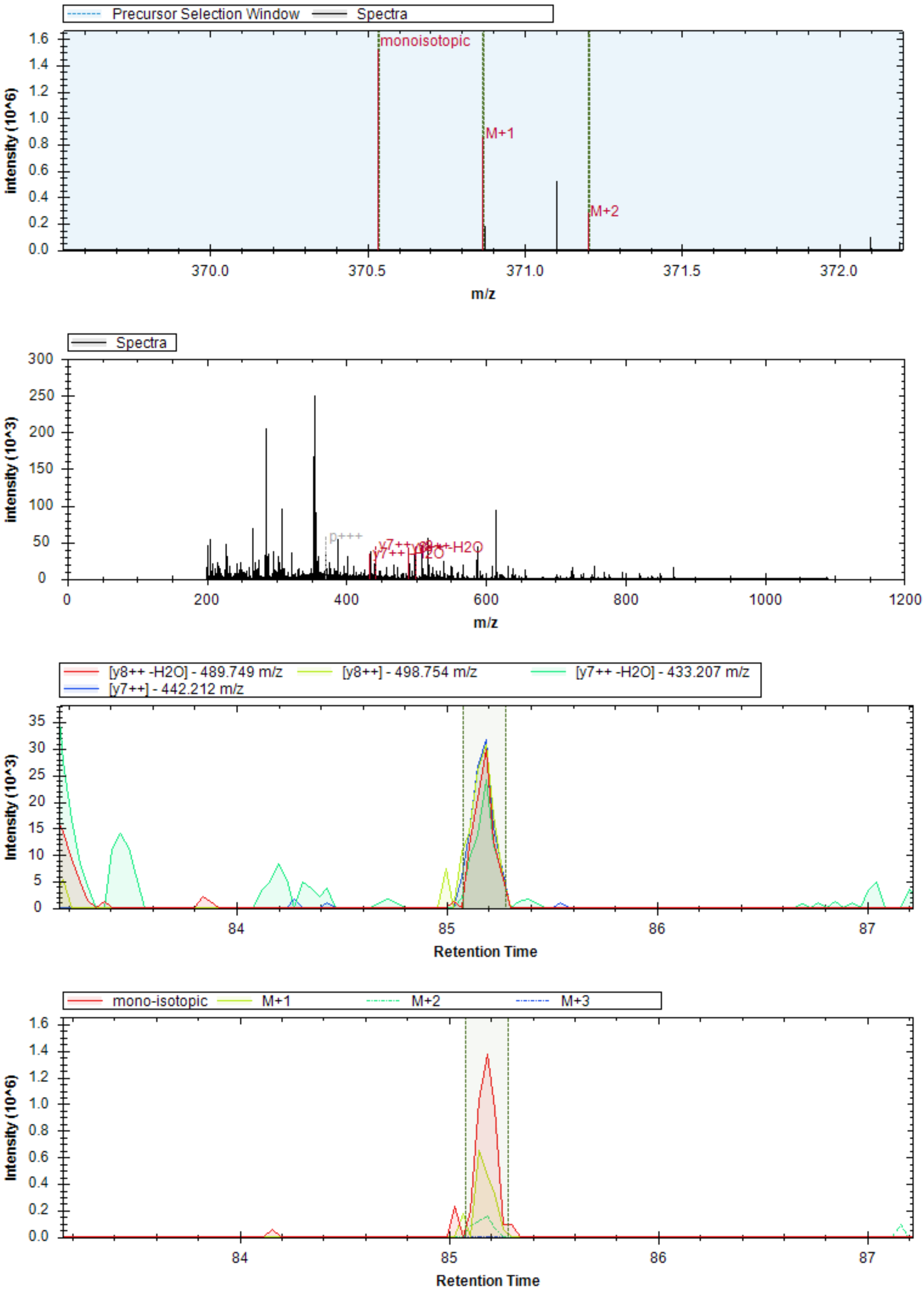

Q9D516;Q9D516-2 – _KLS[+H+O3+P]QGILVIR_.3
Fig4_MP-DIA-barrel-cortex-p54-3_MHRM.htrms

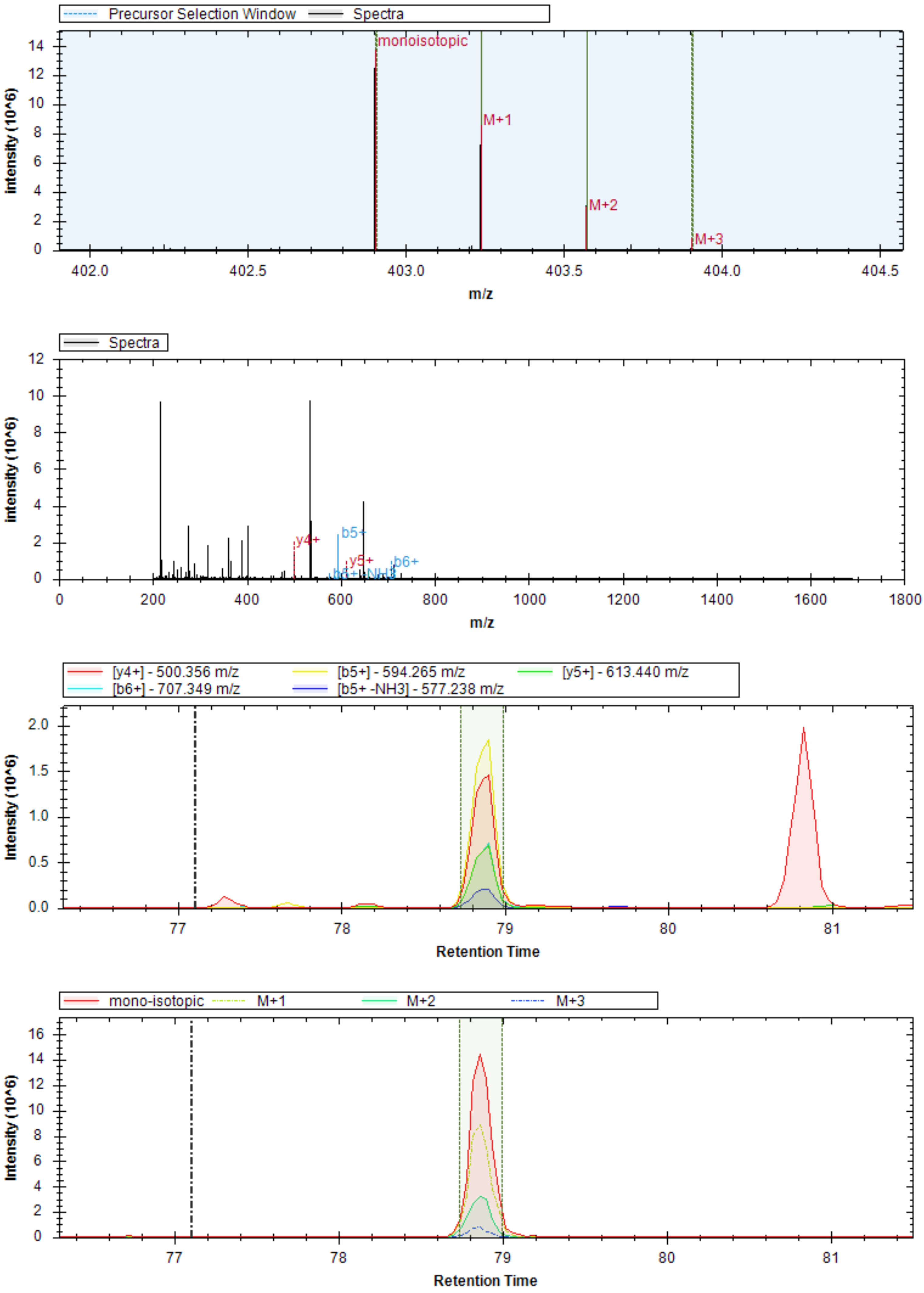

P20357 – _KSEVQAHS[+H+O3+P]PSR_.3
Fig4_MP-DIA-barrel-cortex-p54-3_MHRM.htrms

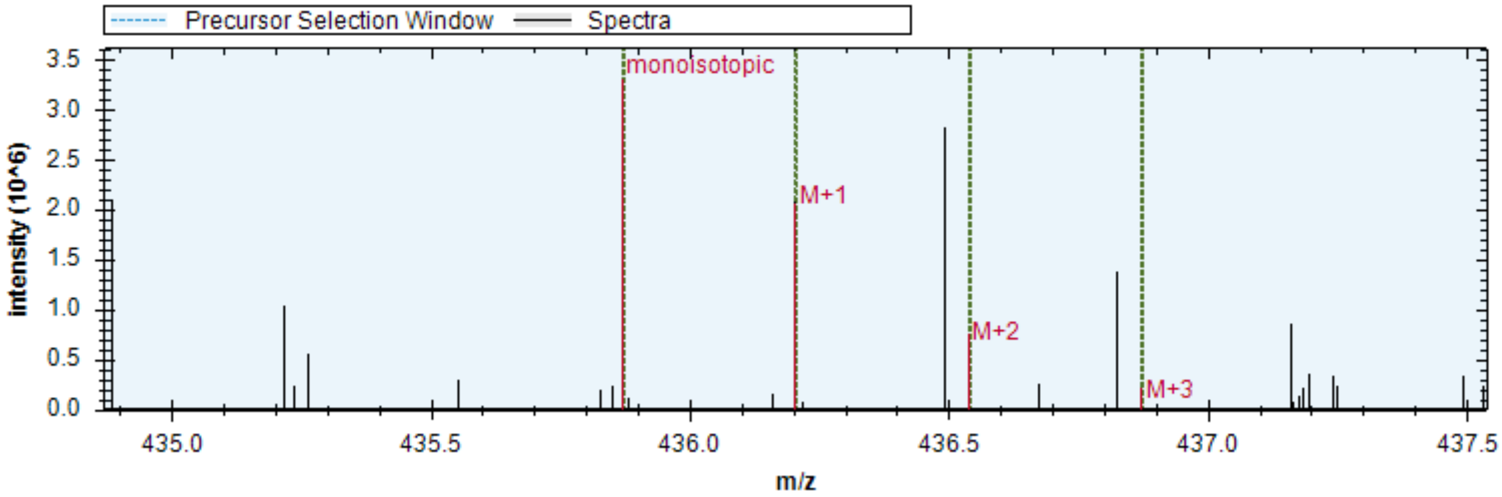

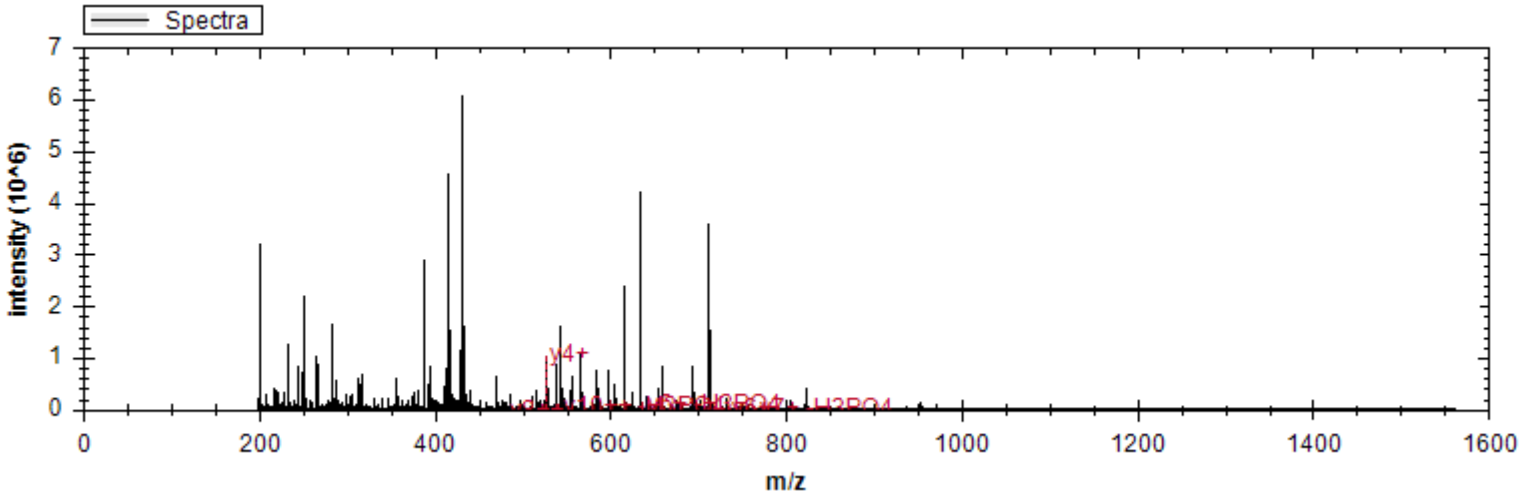

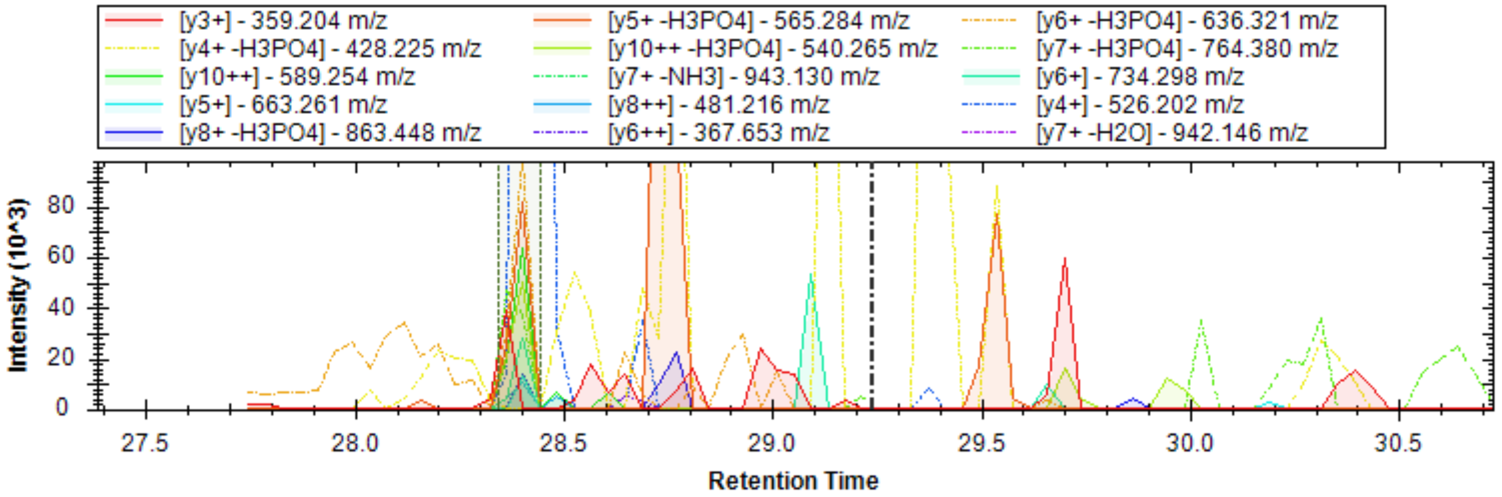

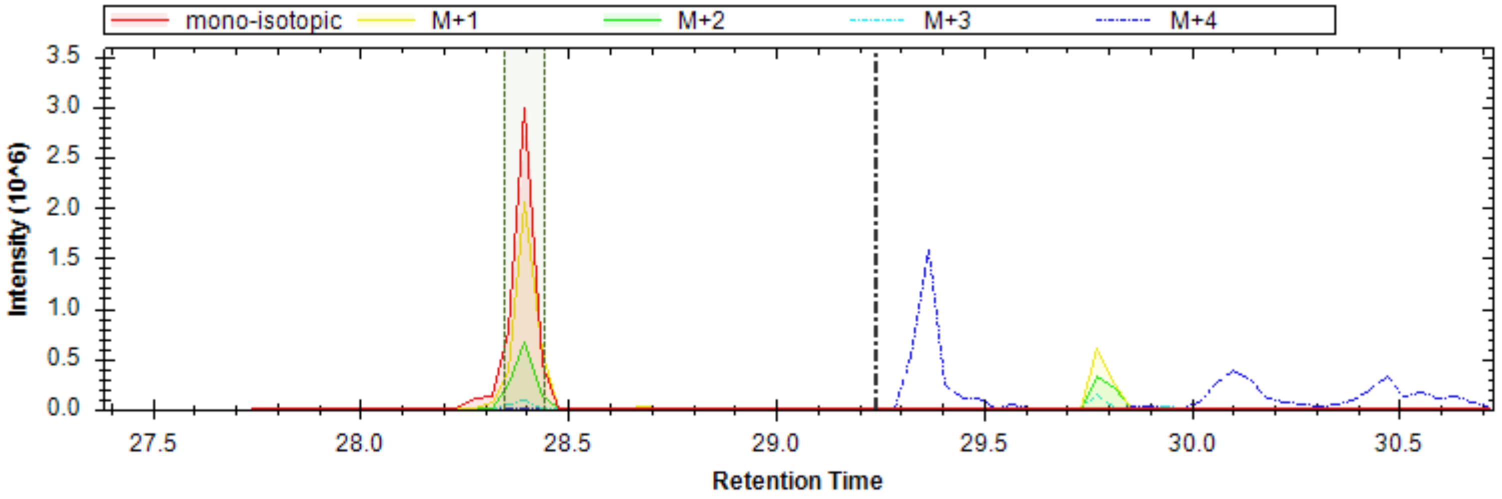

Q03172 – _VALSLLNS[+H+O3+P]KQK_.3
Fig4_MP-DIA-barrel-cortex-p54-3_MHRM.htrms

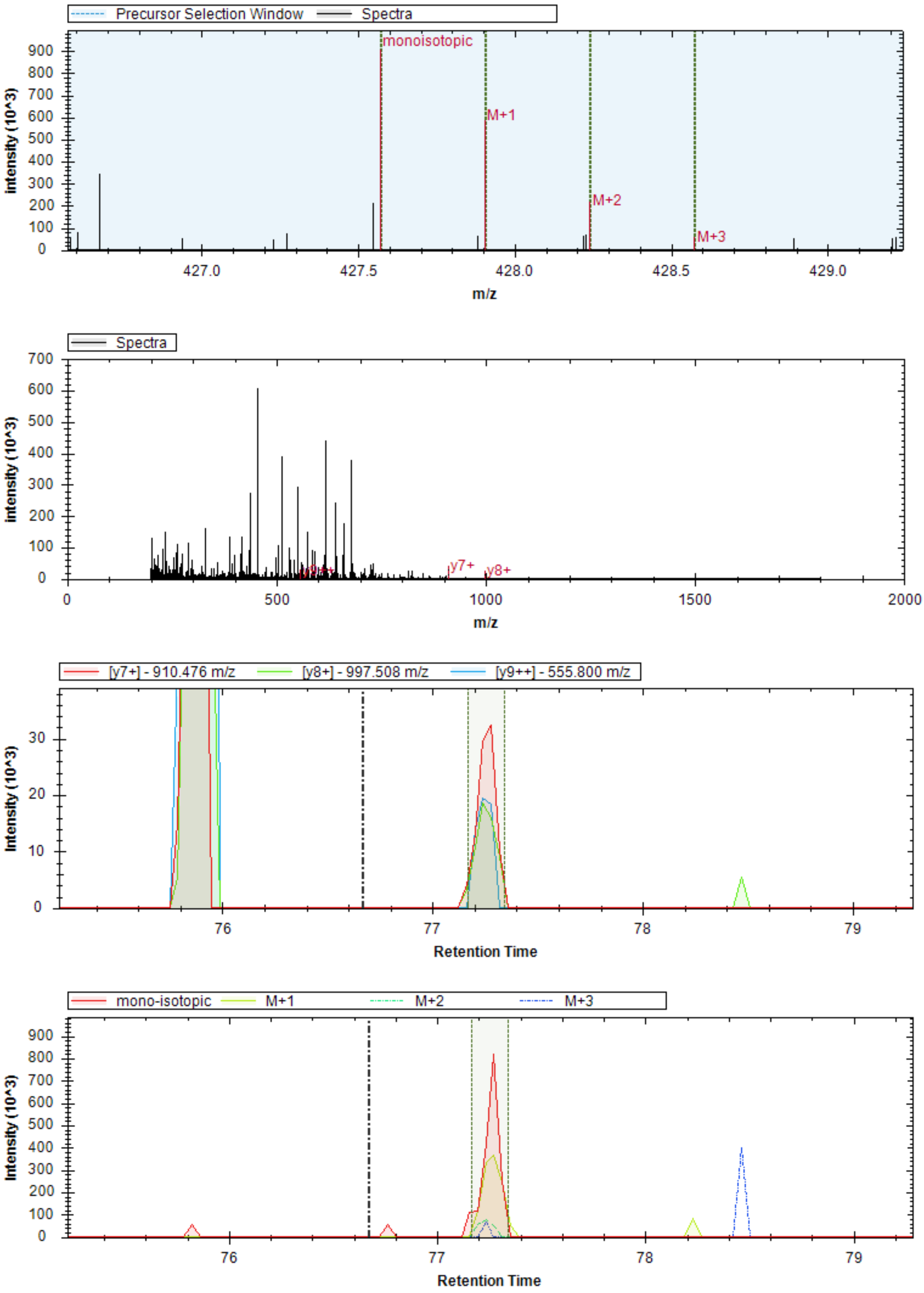

P62484 – _T[+H+O3+P]HKIIAPANLERPVR_.4
Fig4_MP-DIA-barrel-cortex-p54-3_MHRM.htrms

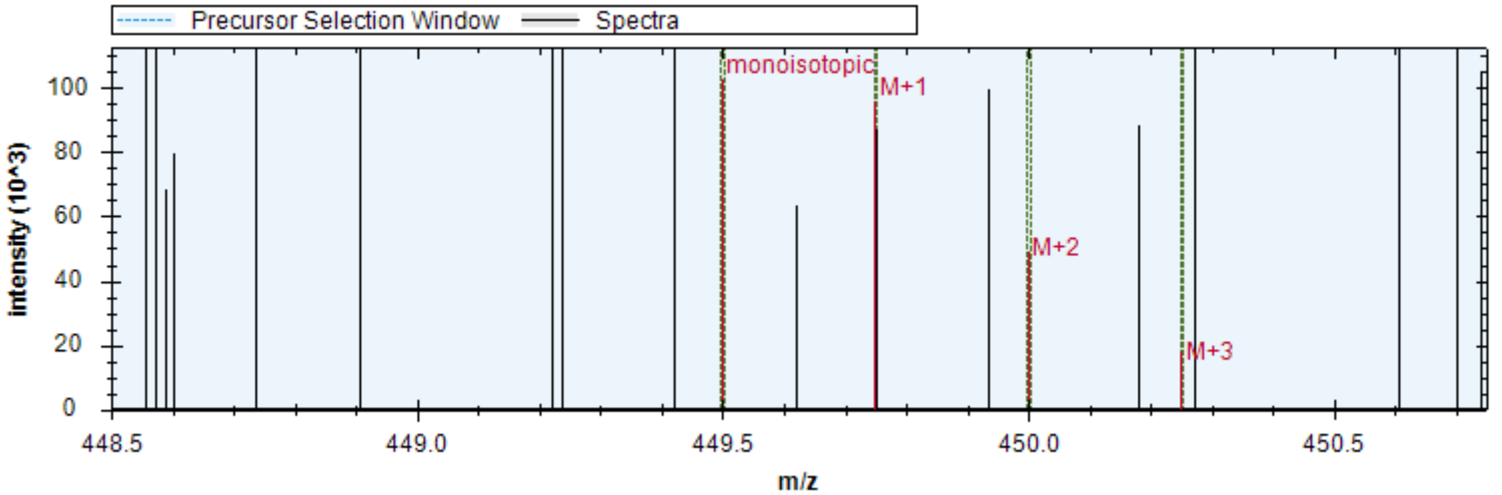

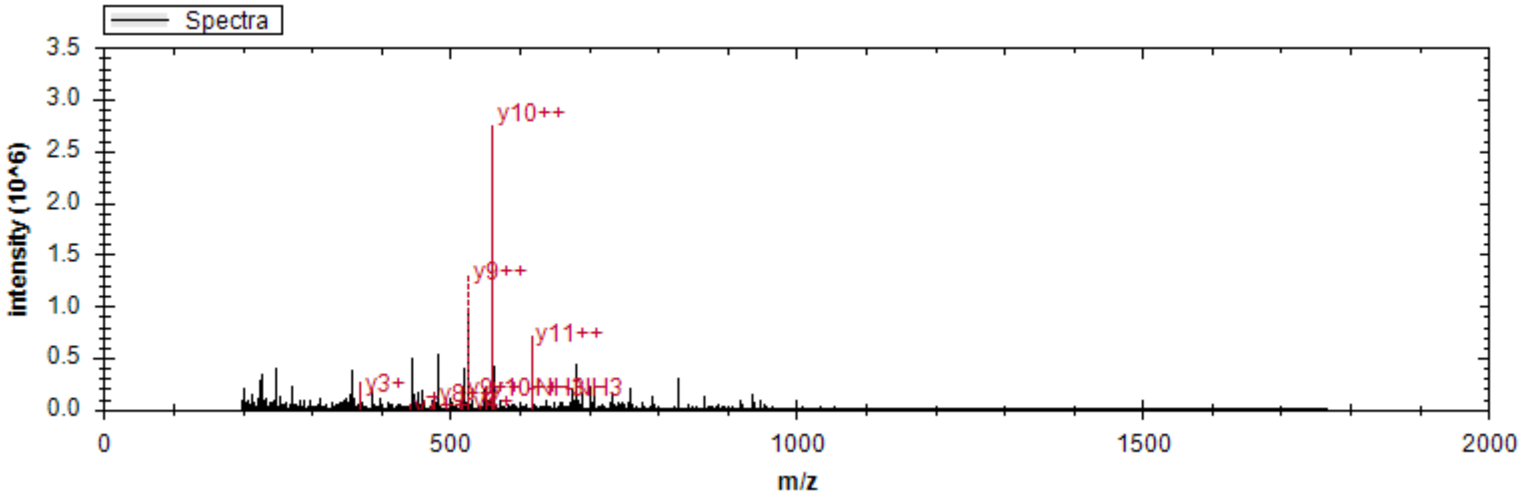

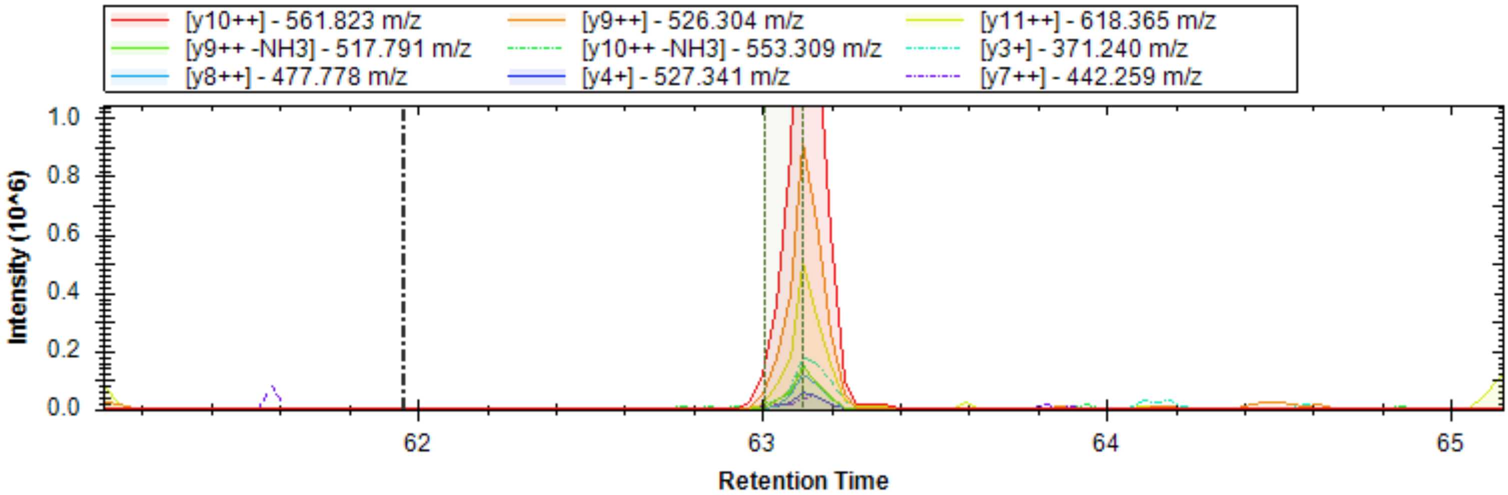

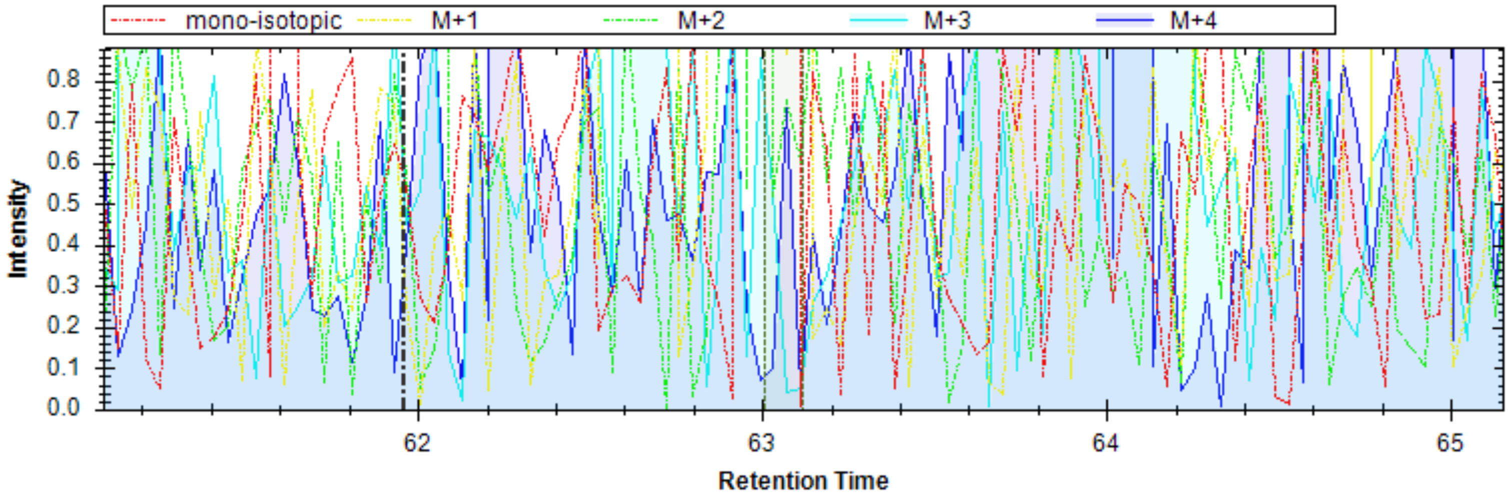

Q8CGI1 – _Y[+H+O3+P]QRS[+H+O3+P]EEELR_.3

Fig4_MP-DIA-barrel-cortex-p54-3_MHRM.htrms

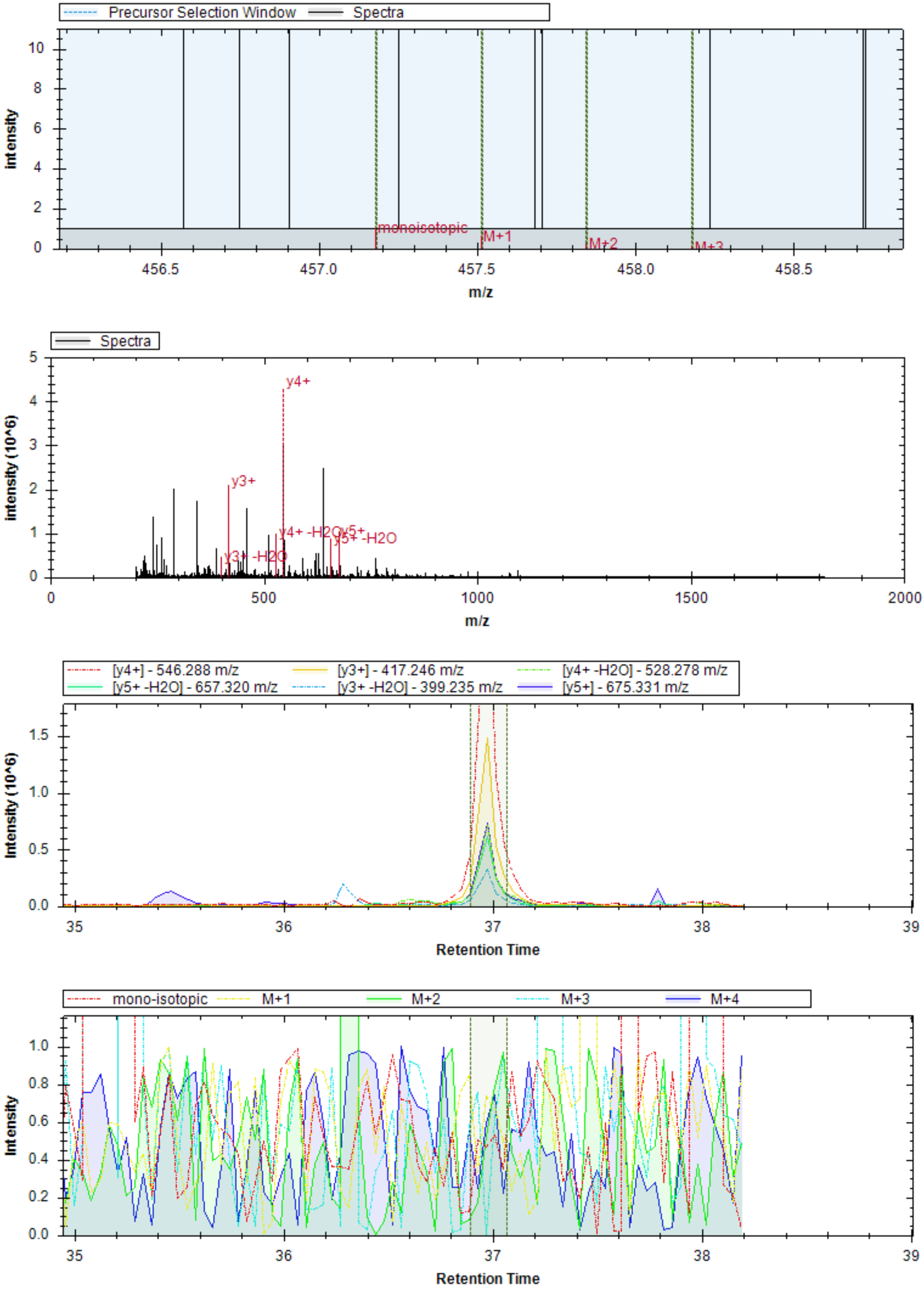

Q9JKK8 – _MLS[+H+O3+P]LWLDFGAK_.3
Fig4_MP-DIA-barrel-cortex-p54-3_MHRM.htrms

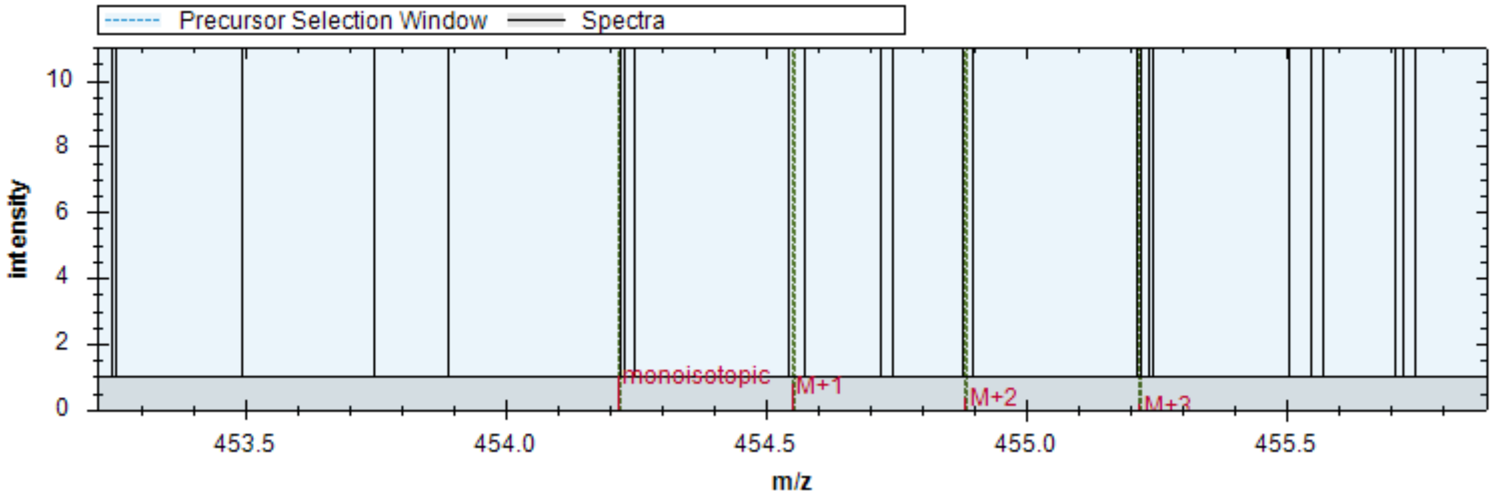

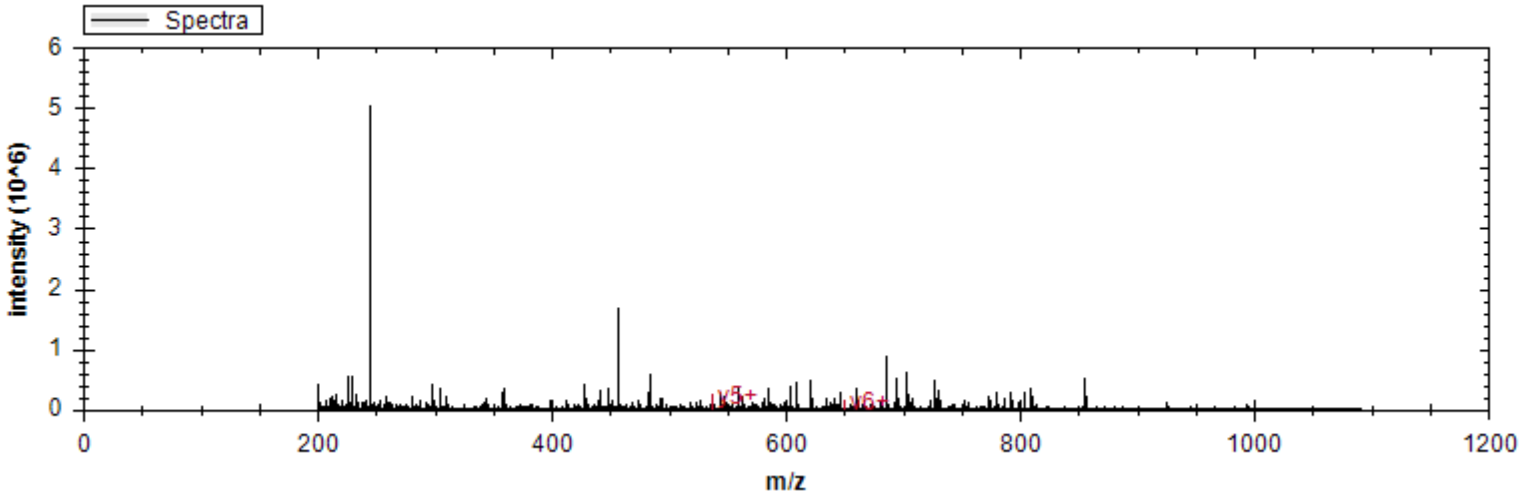

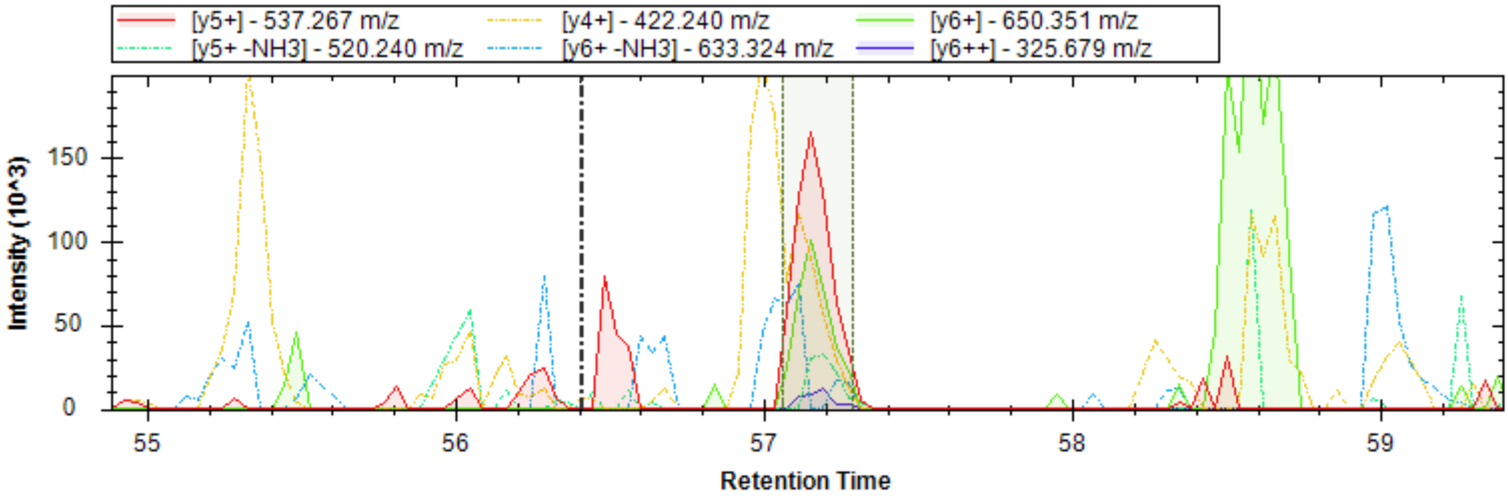

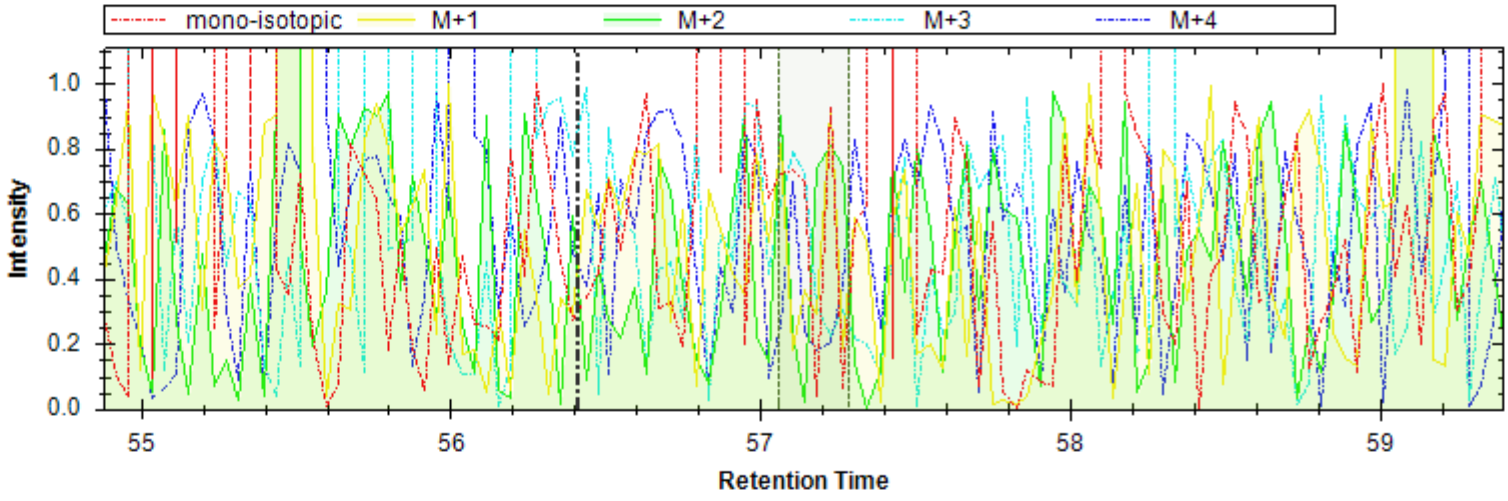

O08553 – _TVT[+H+O3+P]PASSAK_.2

Fig4_MP-DIA-barrel-cortex-p54-3_MHRM.htrms

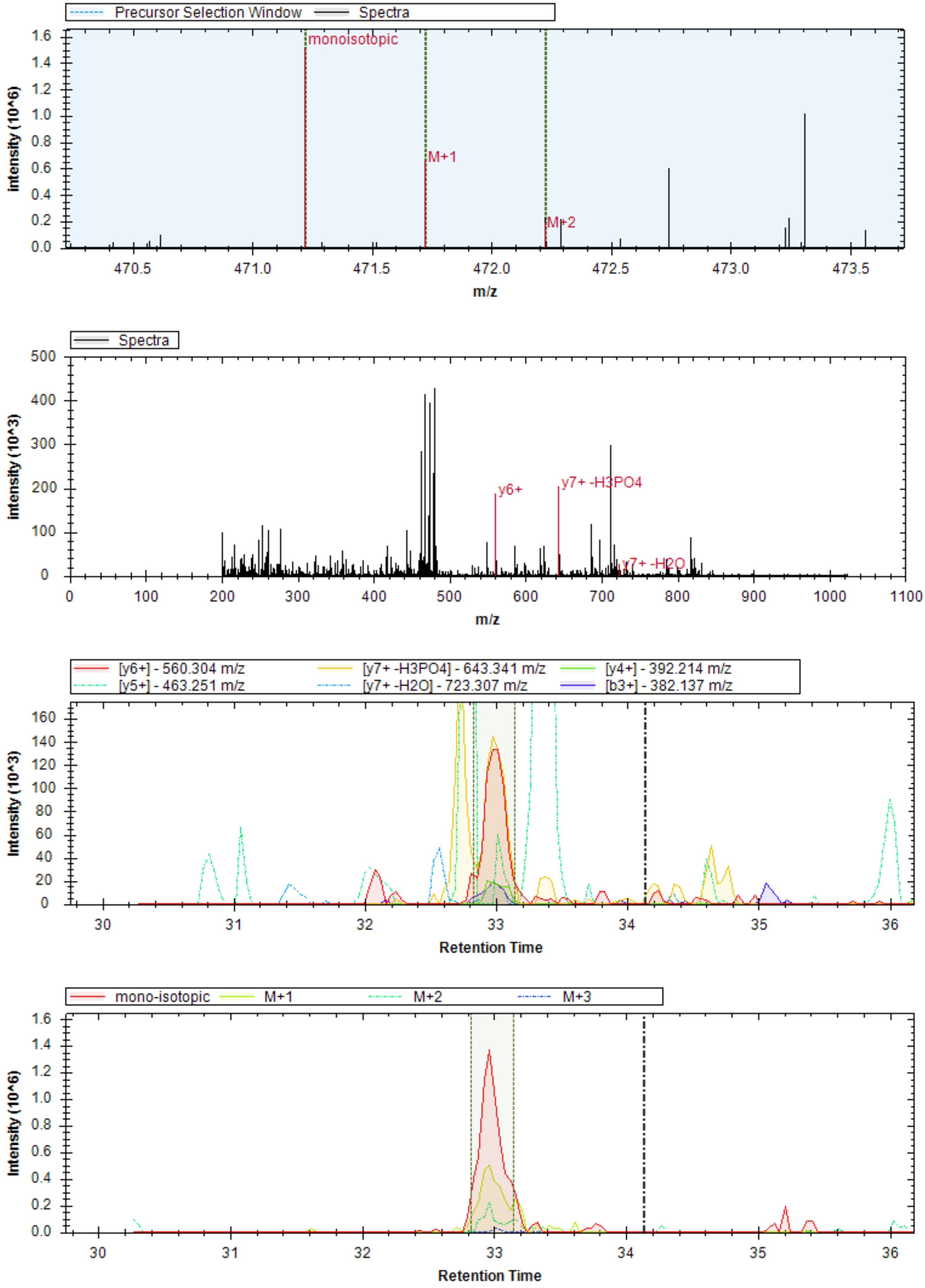

Q8K015;Q8K015-2 – _TVIS[+H+O3+P]EQEVLER_.3
Fig4_MP-DIA-barrel-cortex-p54-3_MHRM.htrms

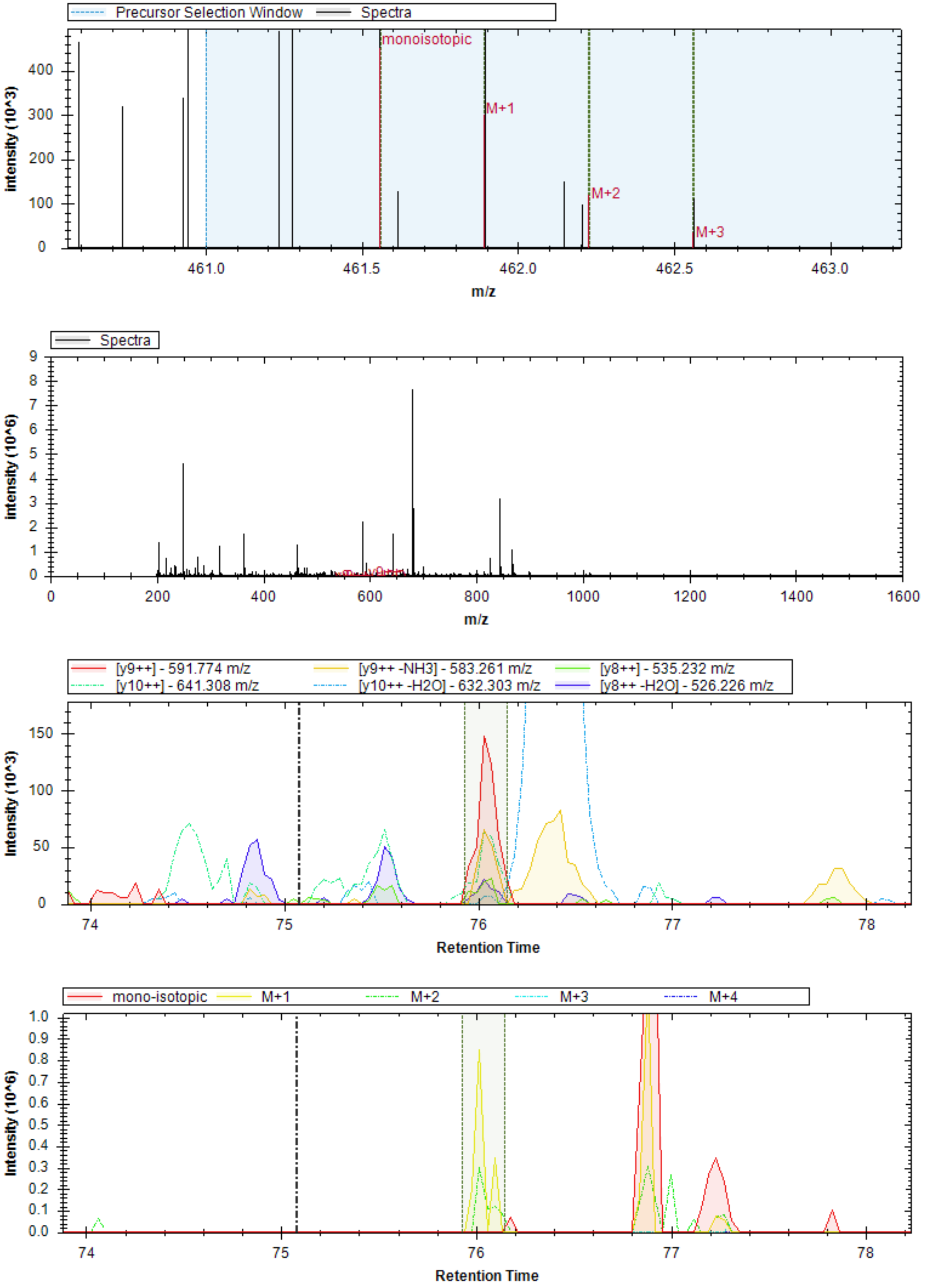

Q9CR81 – _[+C2+H2+O]M[+O]ANHLVKPDS[+H+O3+P]R_.3
Fig4_MP-DIA-barrel-cortex-p54-3_MHRM.htrms

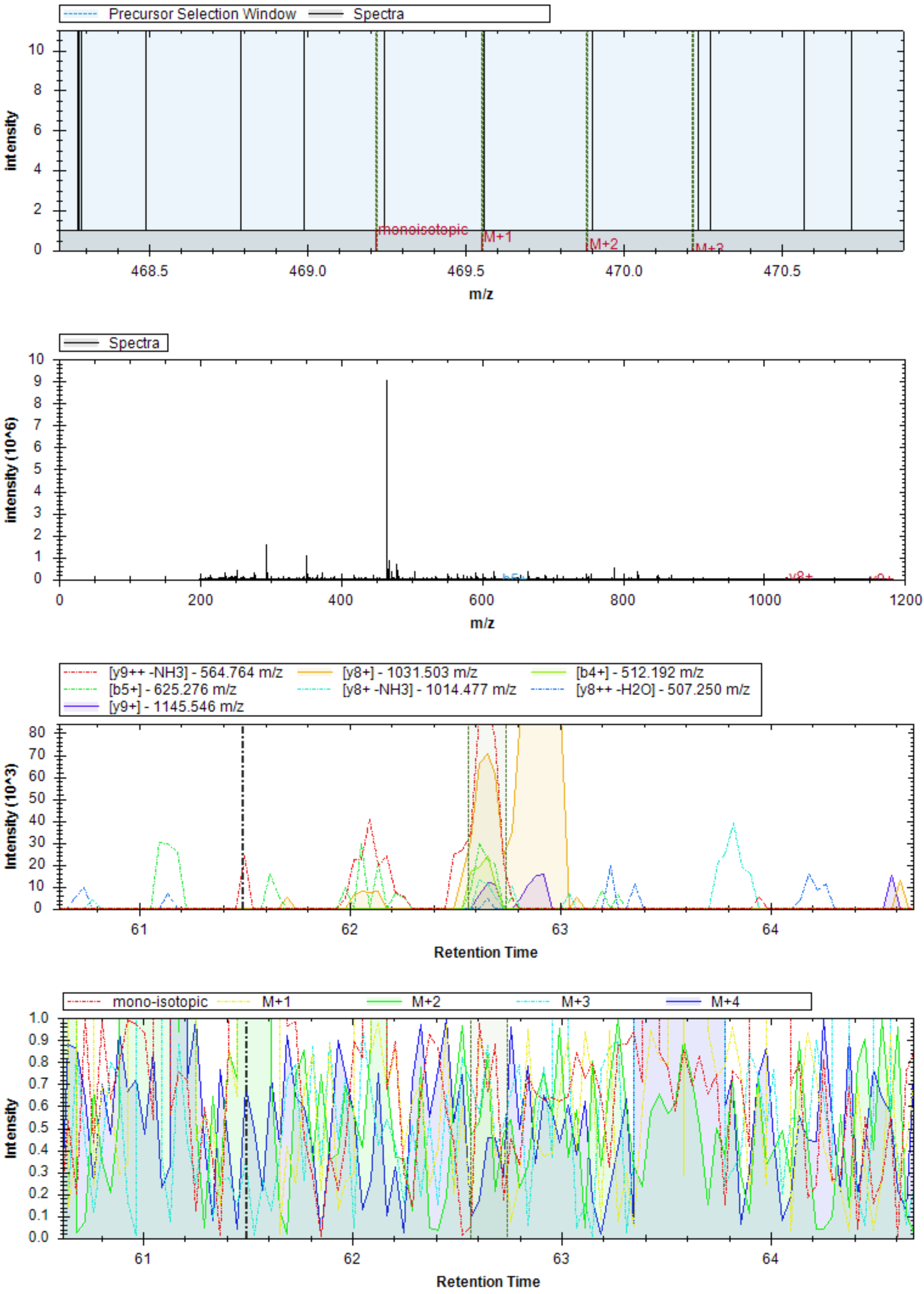

P26645 – _LSGFS[+H+O3+P]FKK_.2

Fig4_MP-DIA-barrel-cortex-p54-3_MHRM.htrms

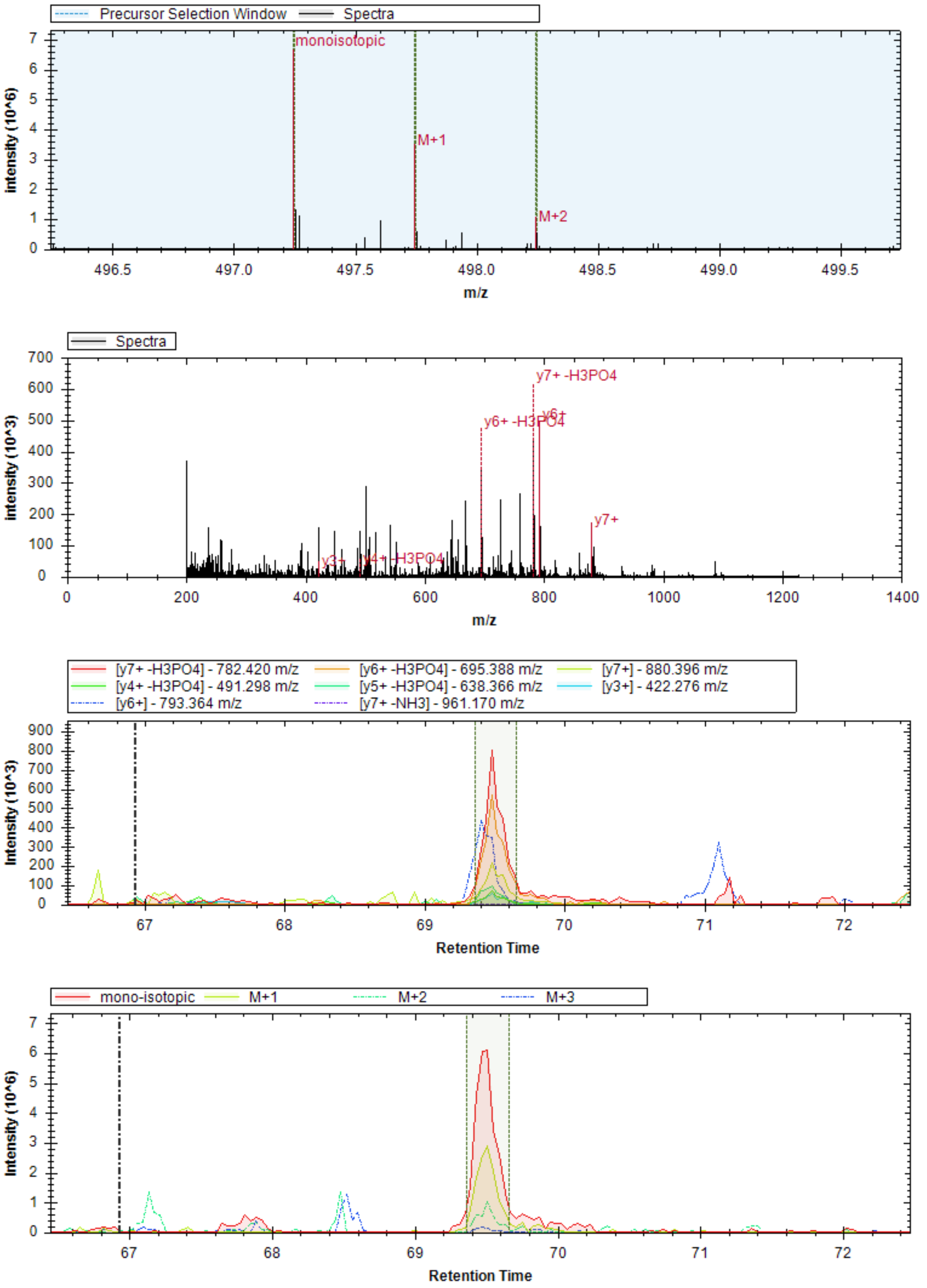

P28667 – _LSGLS[+H+O3+P]FKR_.2
Fig4_MP-DIA-barrel-cortex-p54-3_MHRM.htrms

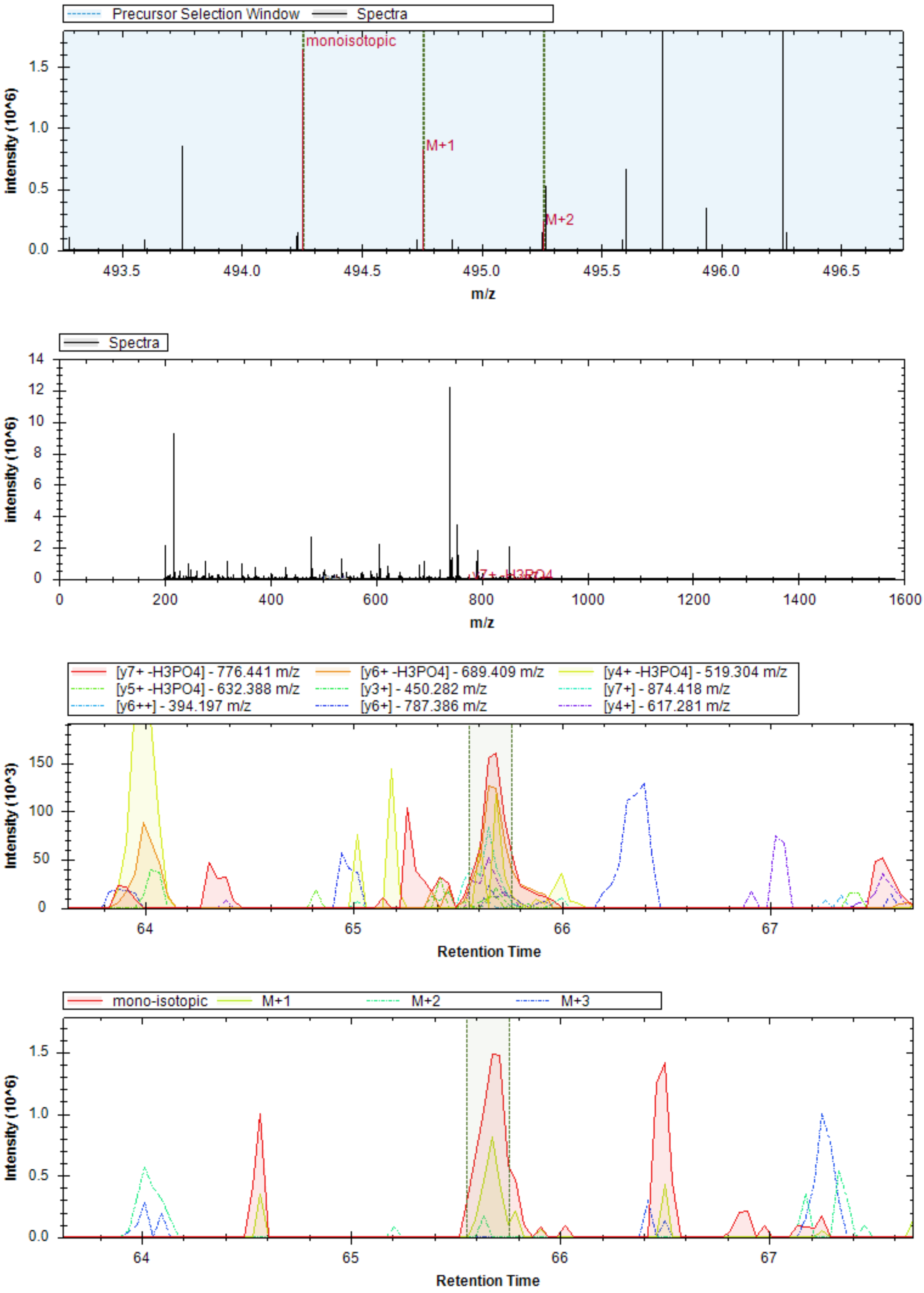

P08553 – _S[+H+O3+P]PVEEVKPKPEAK_.3
Fig4_MP-DIA-barrel-cortex-p54-3_MHRM.htrms

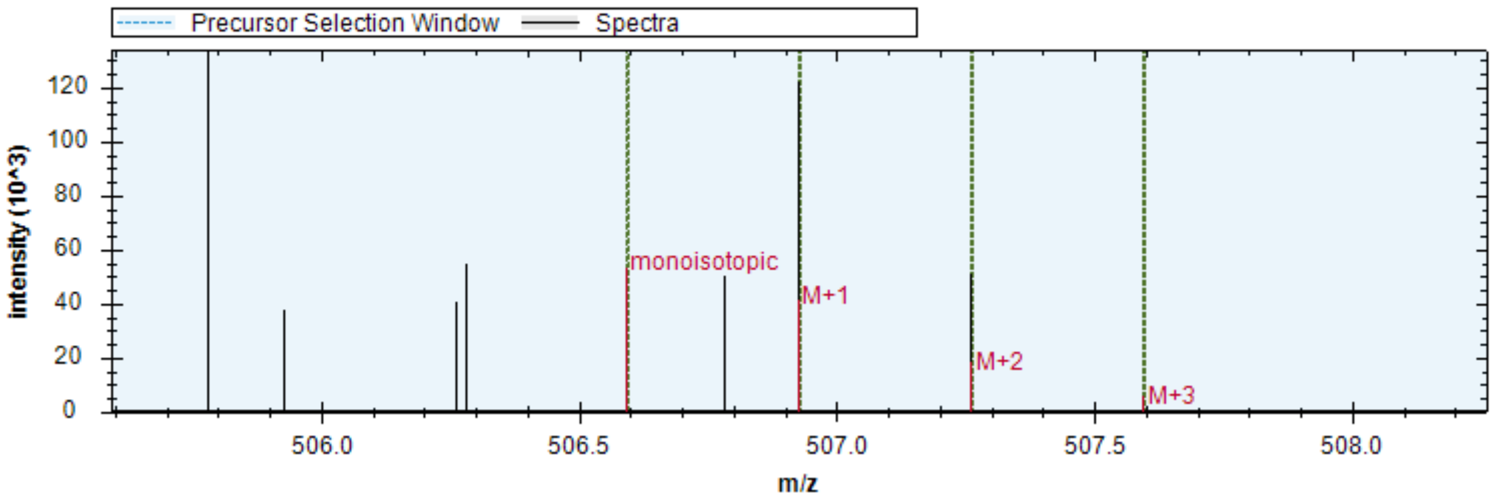

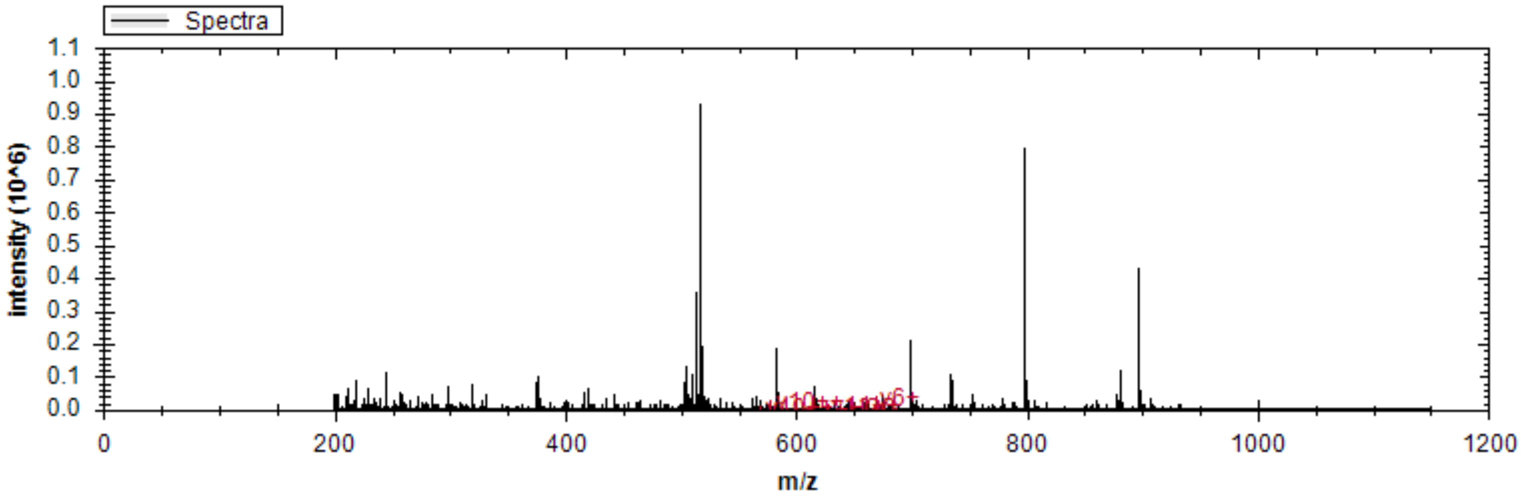

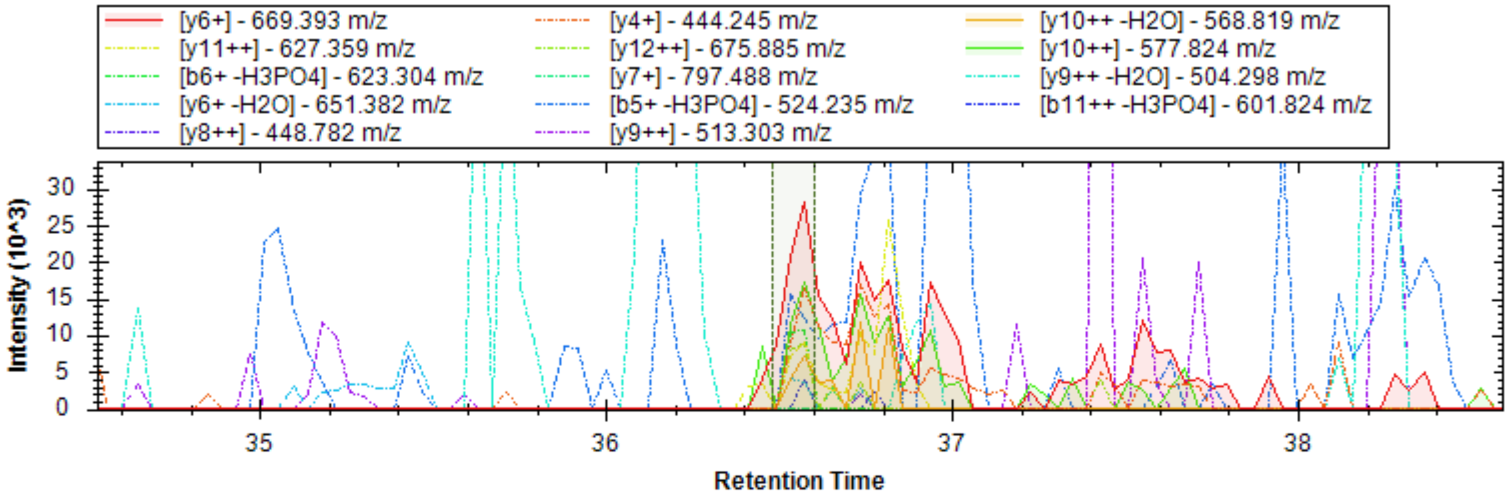

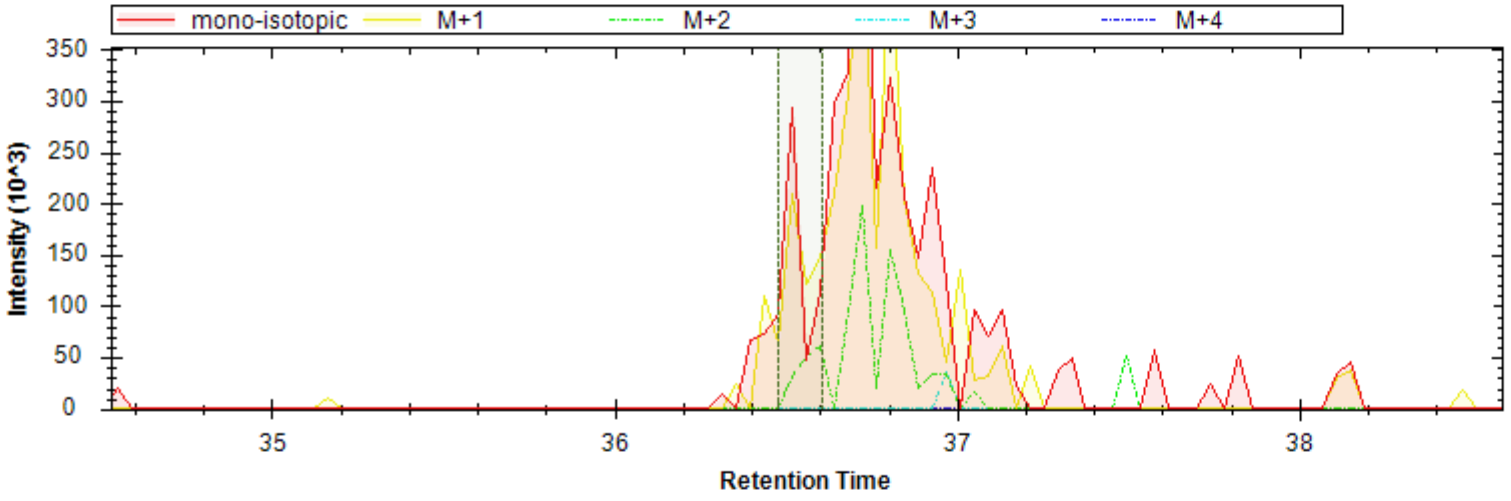

P28738 – _PIRPGHYPAS[+H+O3+P]SPTAVHAVR_.4
Fig4_MP-DIA-barrel-cortex-p54-3_MHRM.htrms

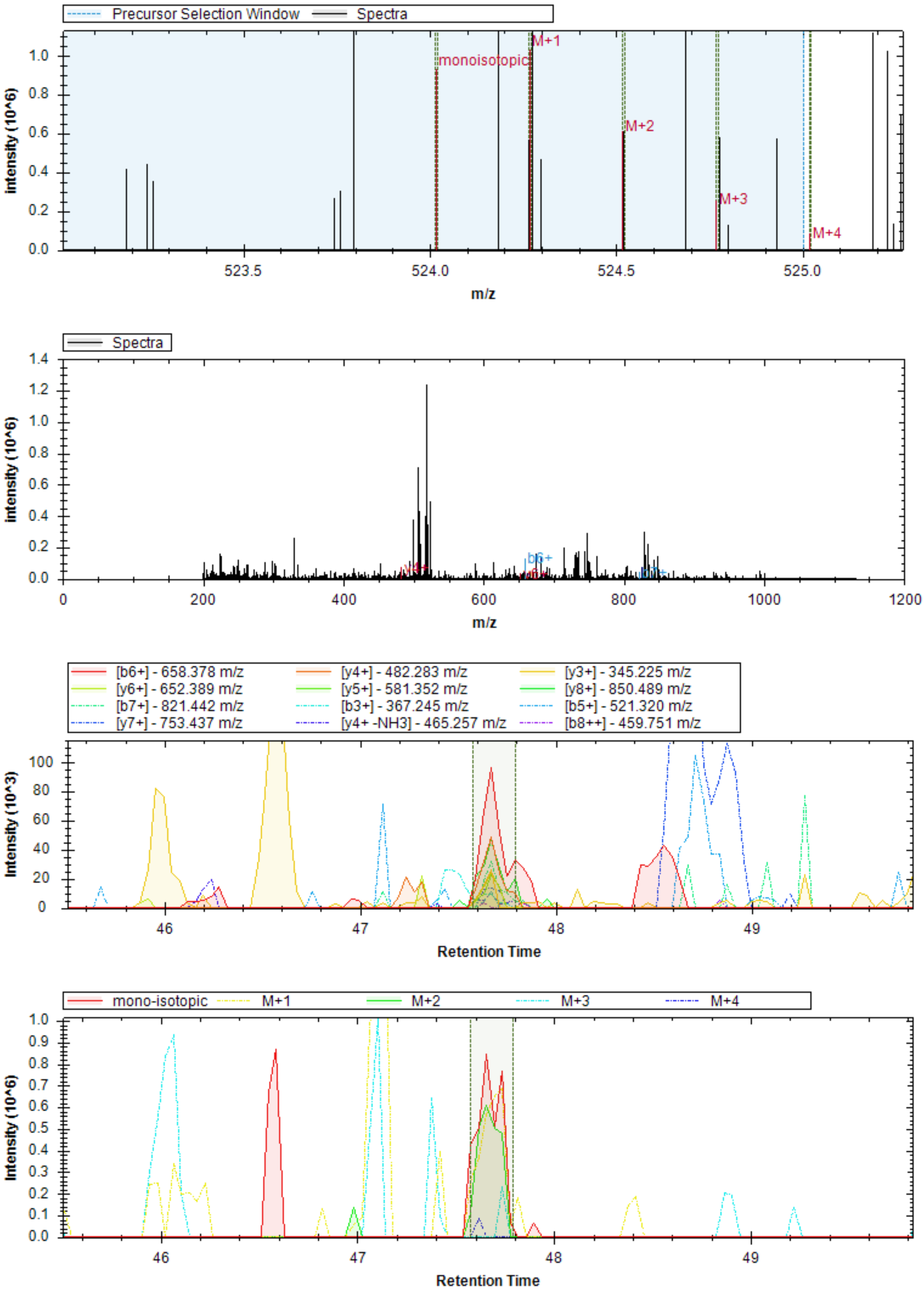

P28738 – _PIRPGHYPASS[+H+O3+P]PTAVHAVR_.4
Fig4_MP-DIA-barrel-cortex-p54-3_MHRM.htrms

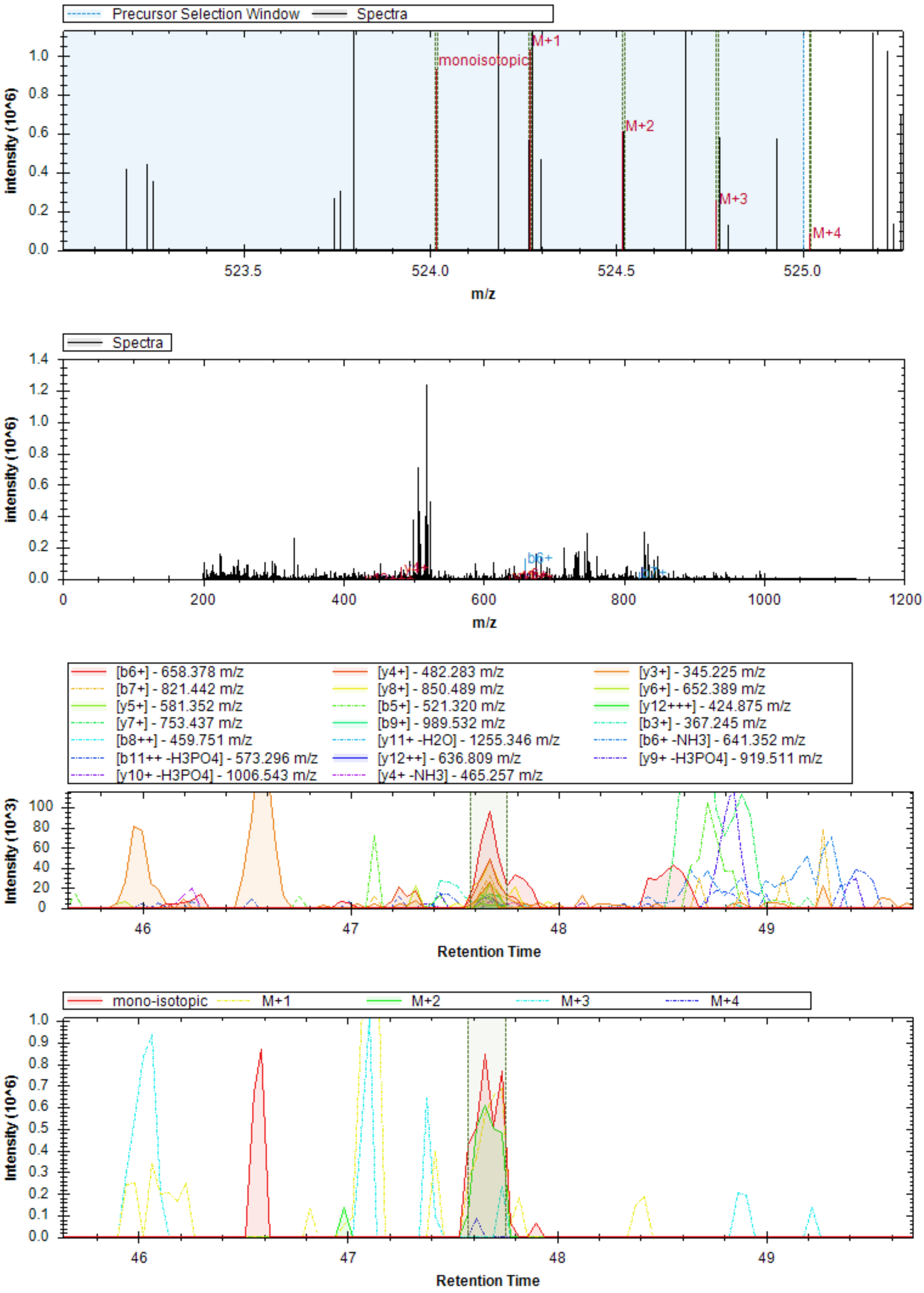

Q8CDY7 – _LVQALC[+C2+H3+N+O]EQIHT[+H+O3+P]R_.3
Fig4_MP-DIA-barrel-cortex-p54-3_MHRM.htrms

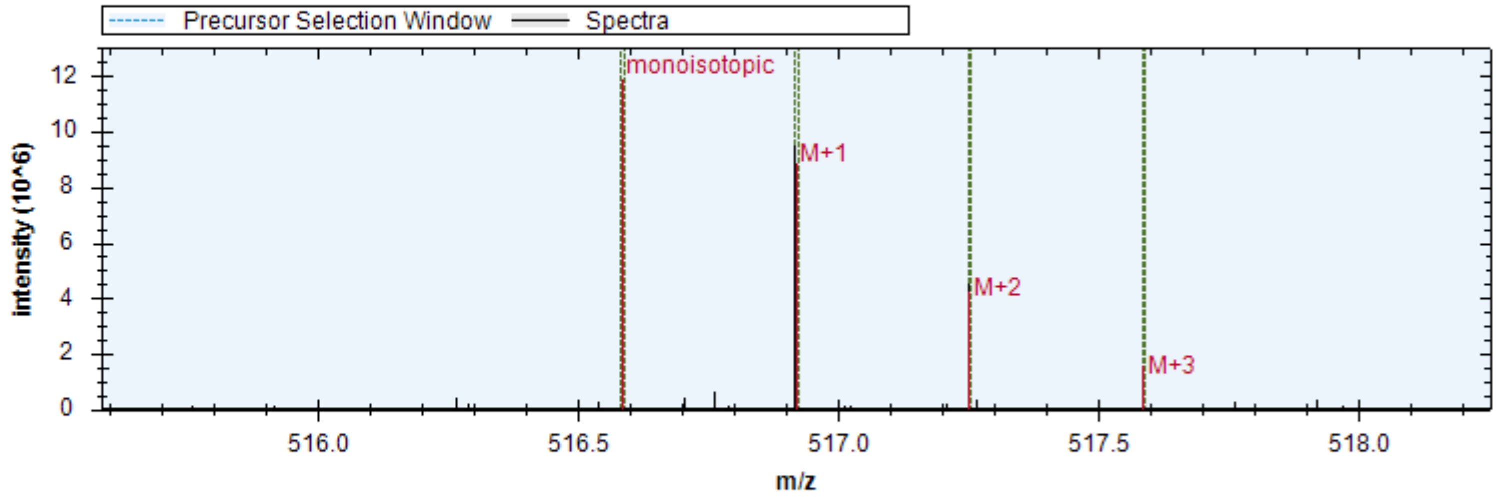

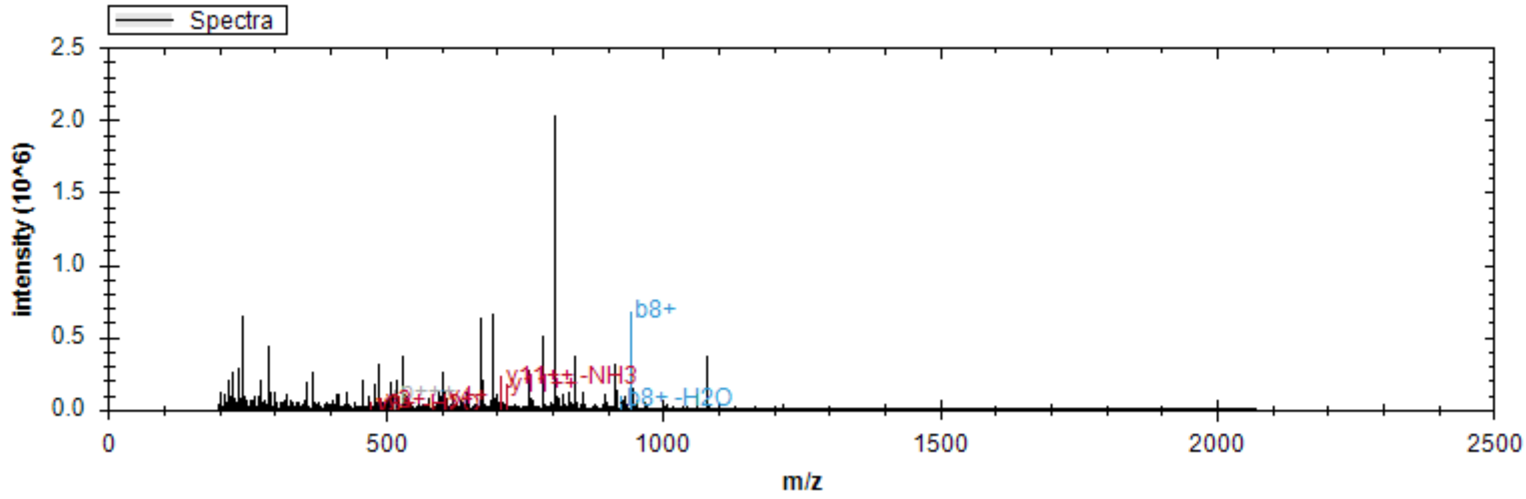

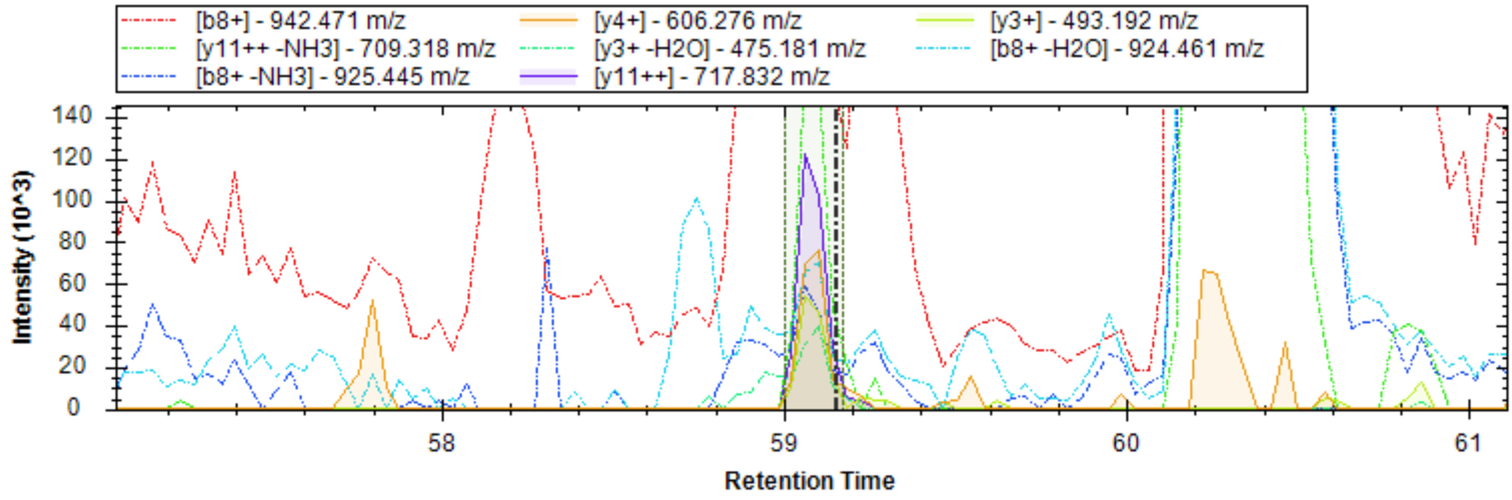

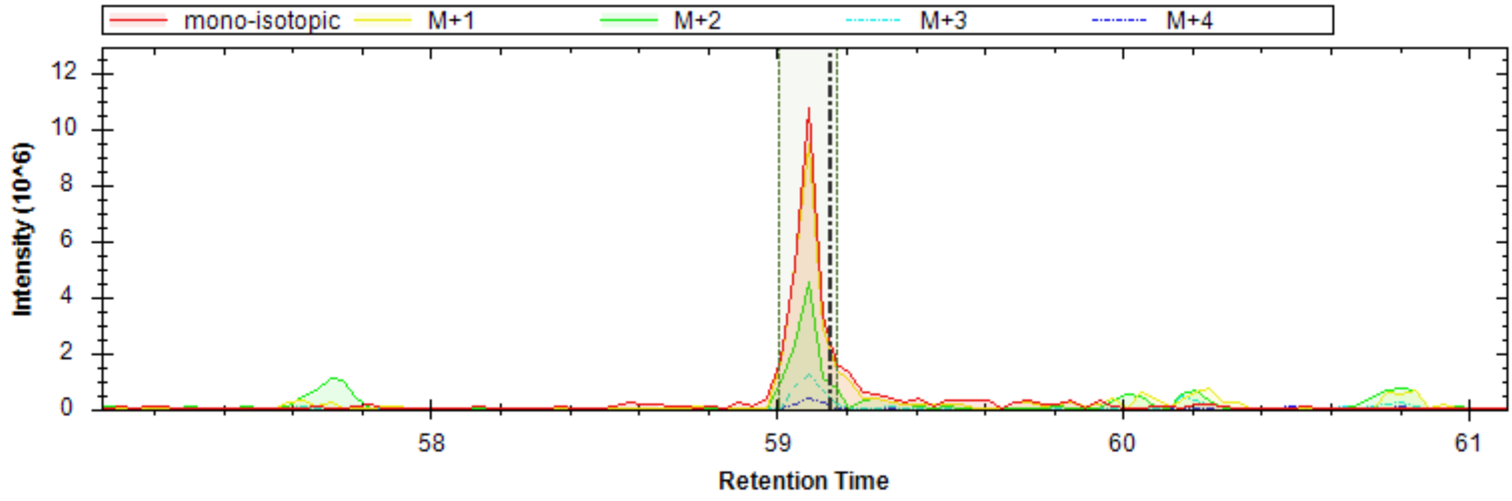

Q9QYC0 – _AAVVT[+H+O3+P]SPPPTTAPHK_.3
Fig4_MP-DIA-barrel-cortex-p54-3_MHRM.htrms

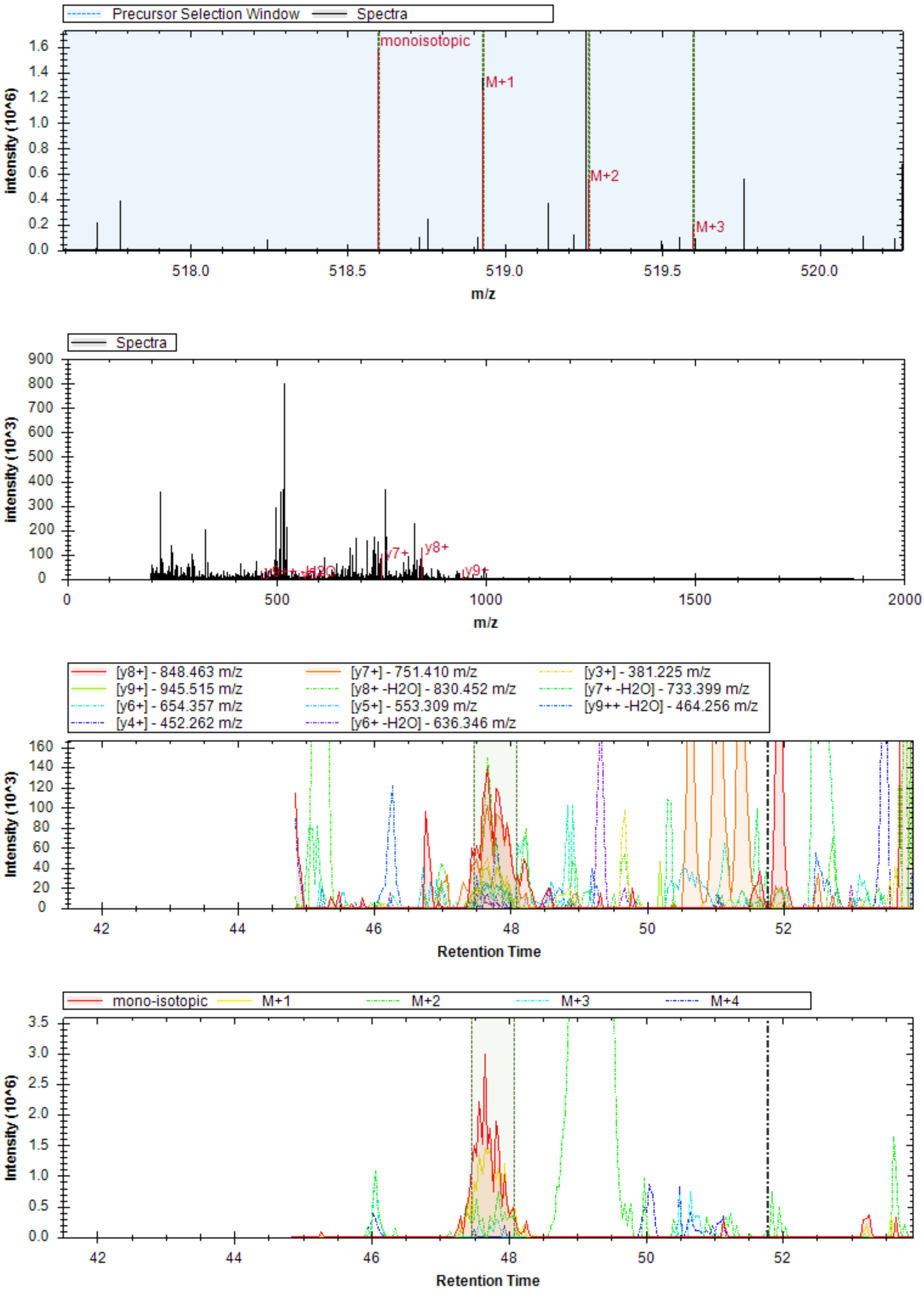

Q9QYC0 – _AAVVTS[+H+O3+P]PPPTTAPHK_.3
Fig4_MP-DIA-barrel-cortex-p54-3_MHRM.htrms

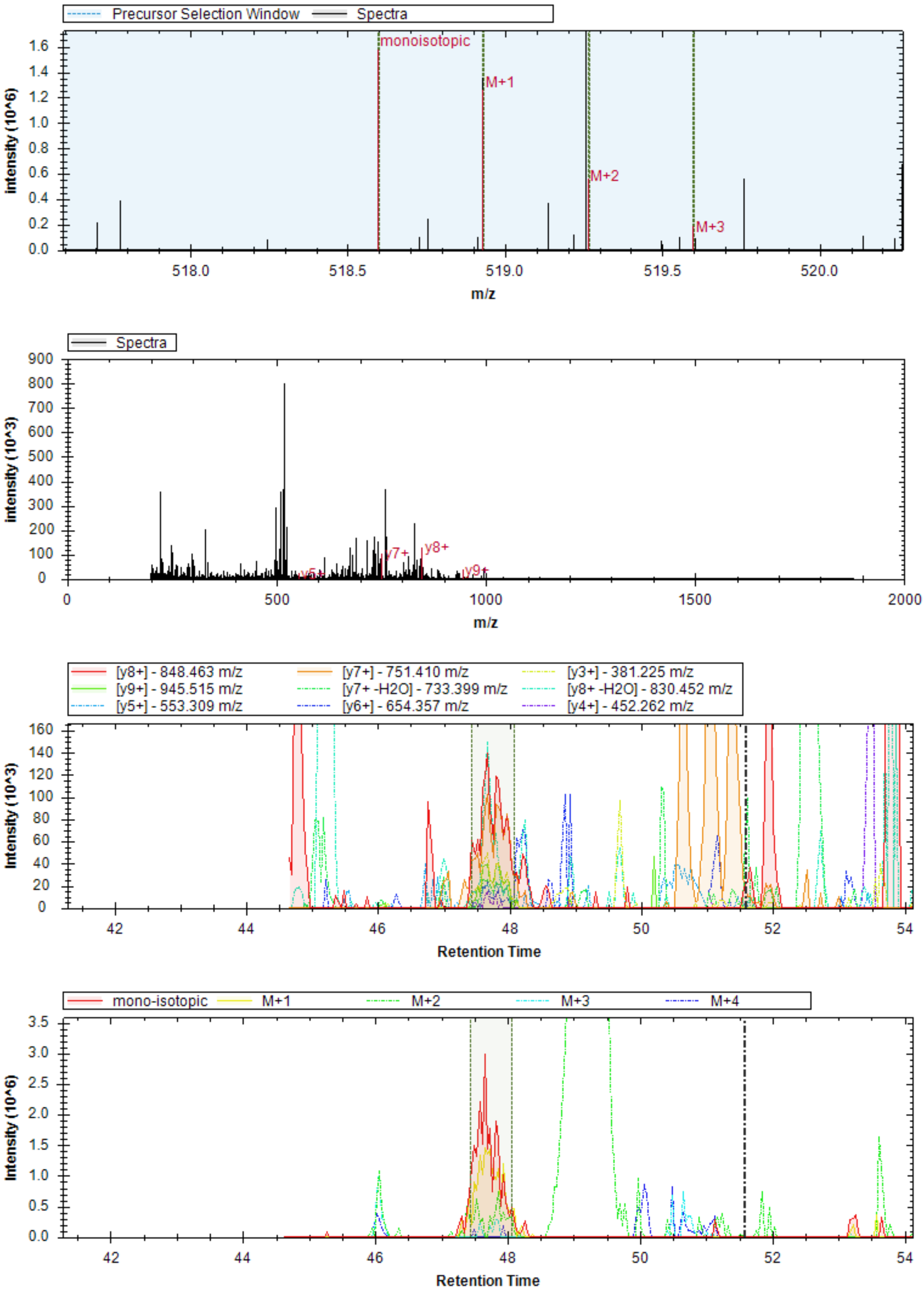

Q7TQD2 – _AANKT[+H+O3+P]PPKSPGDPAR_.3
Fig4_MP-DIA-barrel-cortex-p54-3_MHRM.htrms

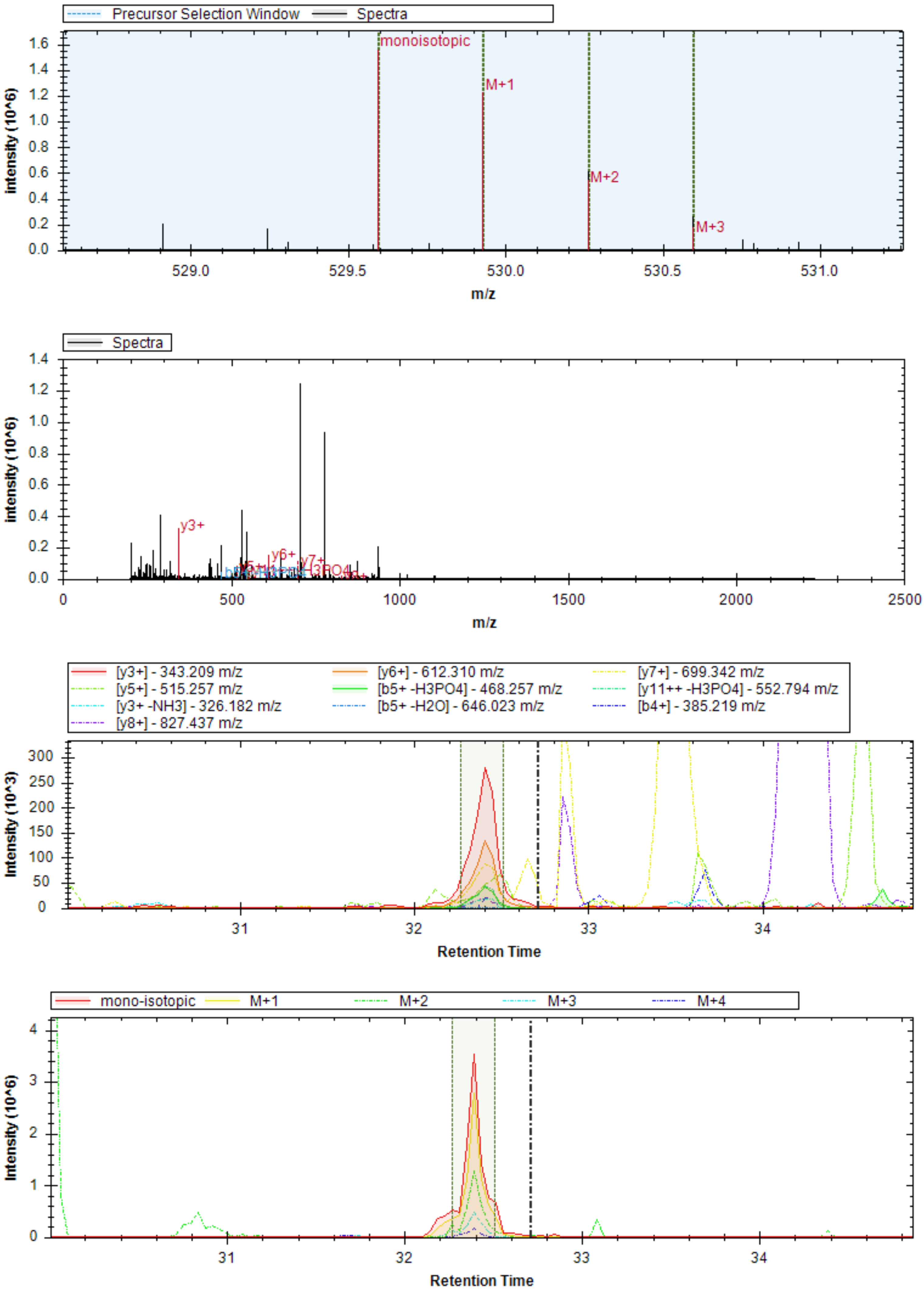

Q9D898 – _AFHAALRNS[+H+O3+P]PINTK_.3

Fig4_MP-DIA-barrel-cortex-p54-3_MHRM.htrms

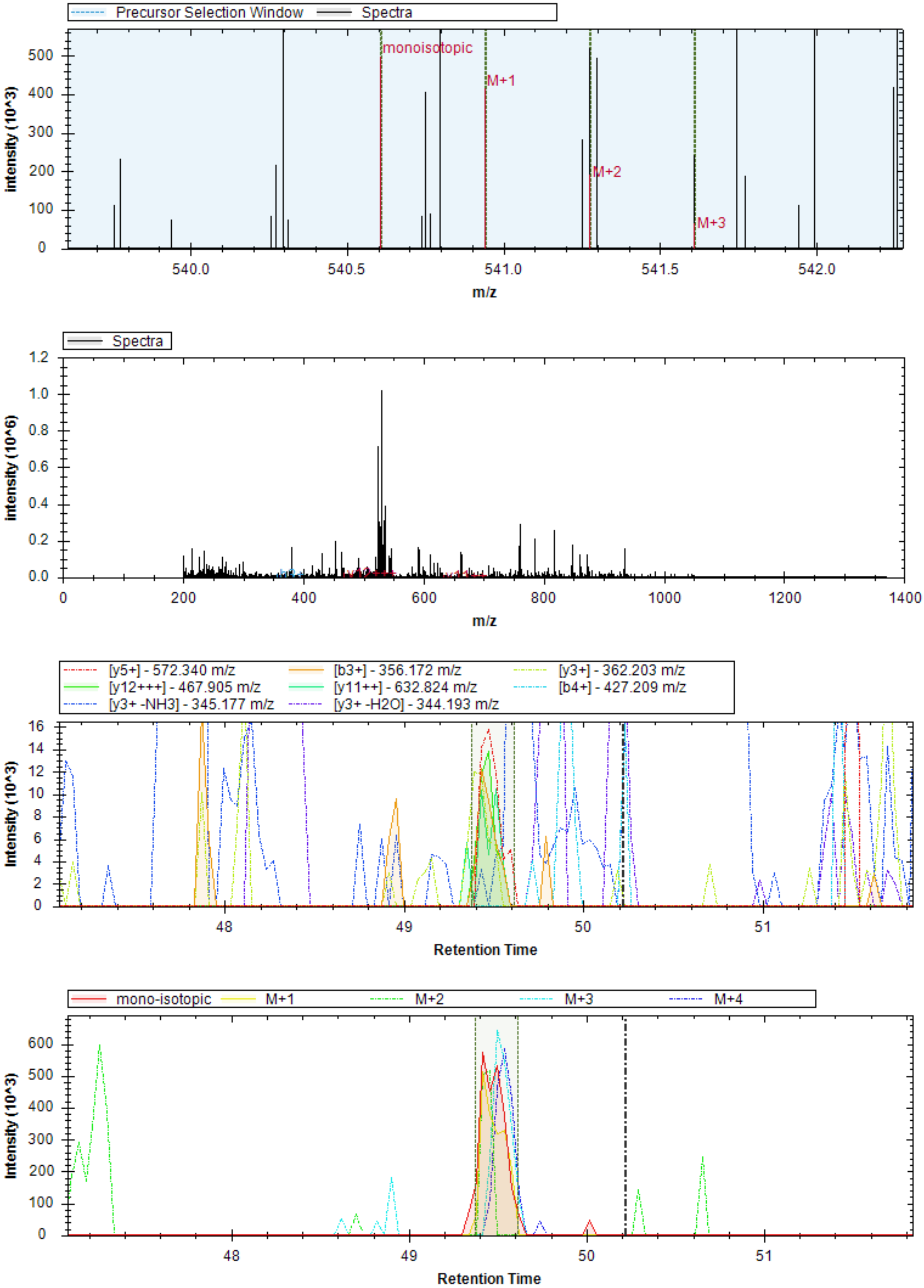

P20357 – _VAIIRT[+H+O3+P]PPKSPATPK_.3

Fig4_MP-DIA-barrel-cortex-p54-3_MHRM.htrms

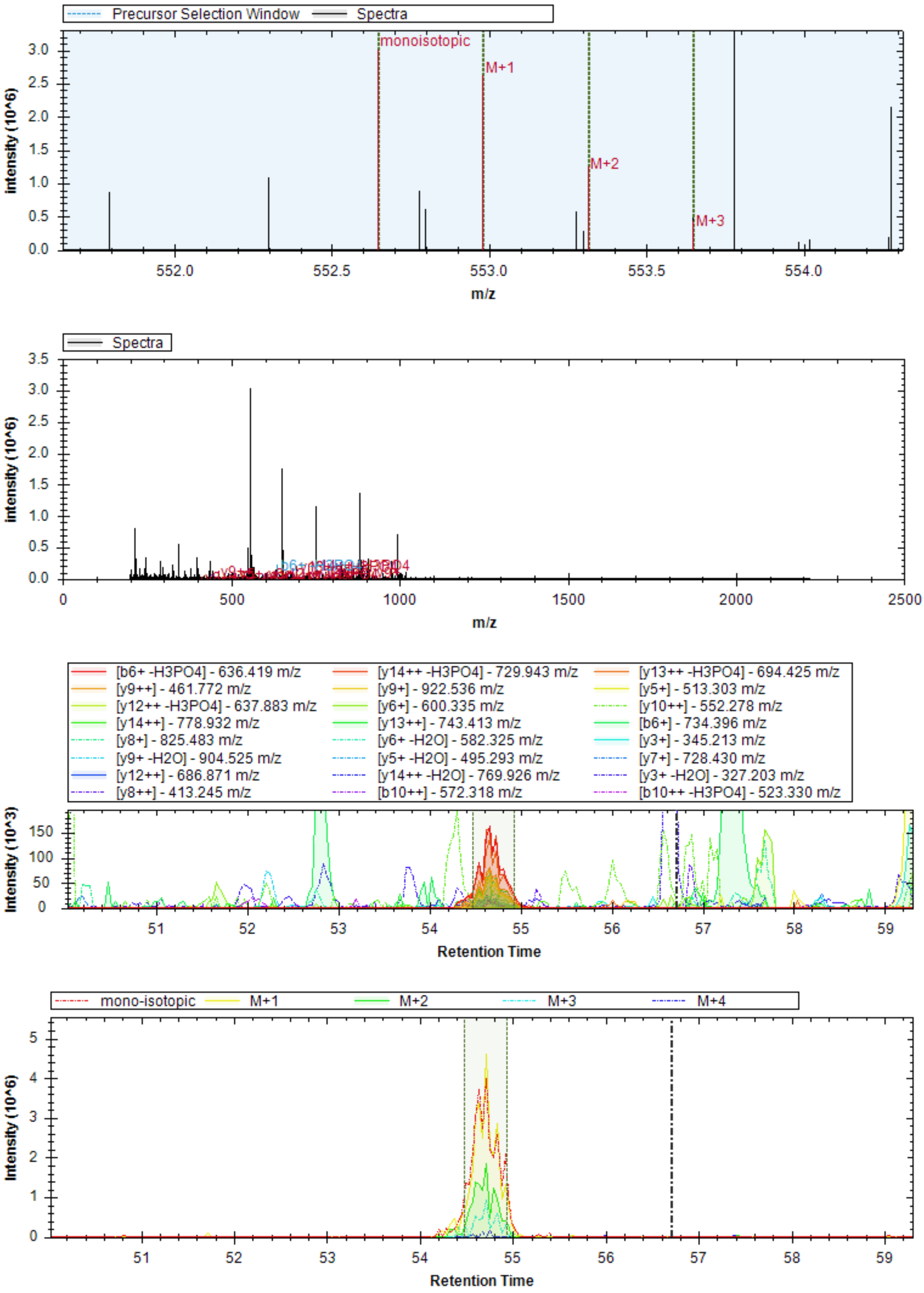

Q9CYT6 – _THTPSPTS[+H+O3+P]PK_.2

Fig4_MP-DIA-barrel-cortex-p54-3_MHRM.htrms

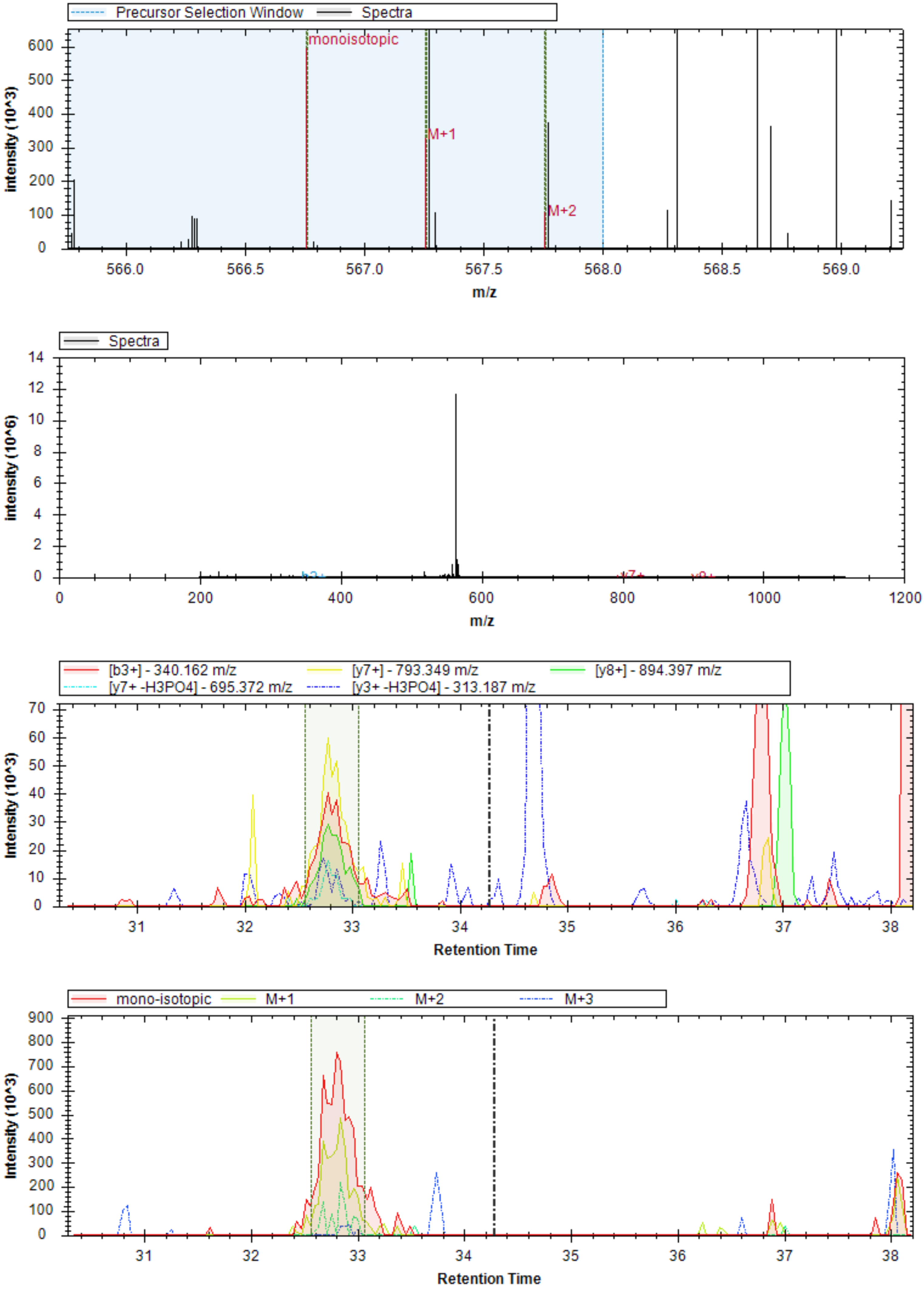

Q8BFU3-4 – _VAS[+H+O3+P]STQVRGASGRELR_.3
Fig4_MP-DIA-barrel-cortex-p54-3_MHRM.htrms

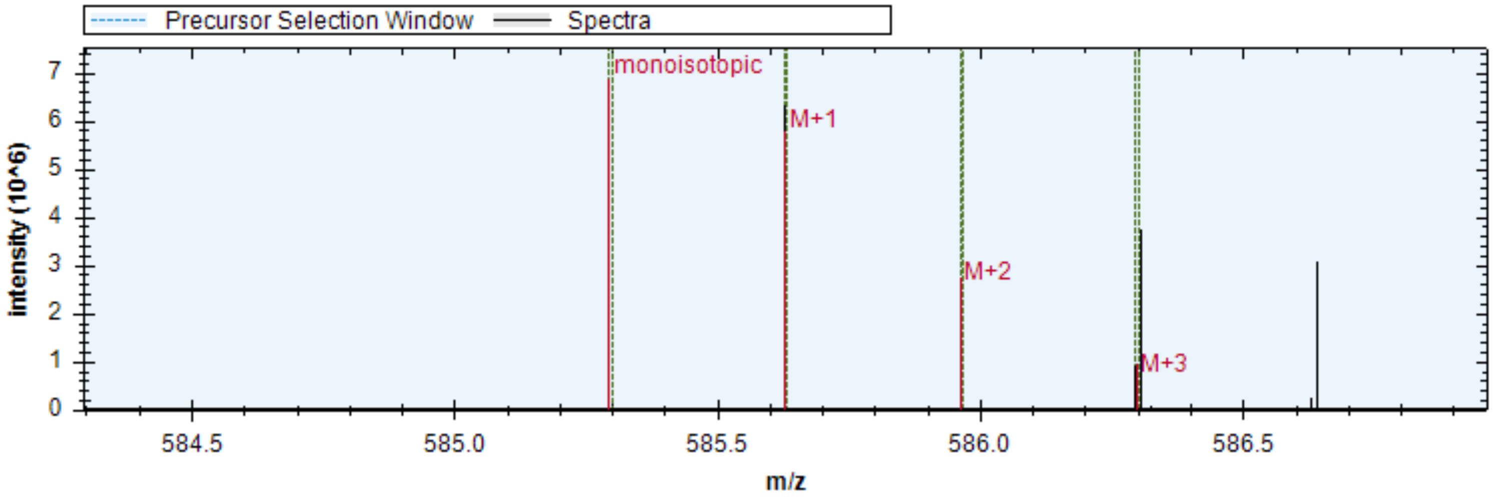

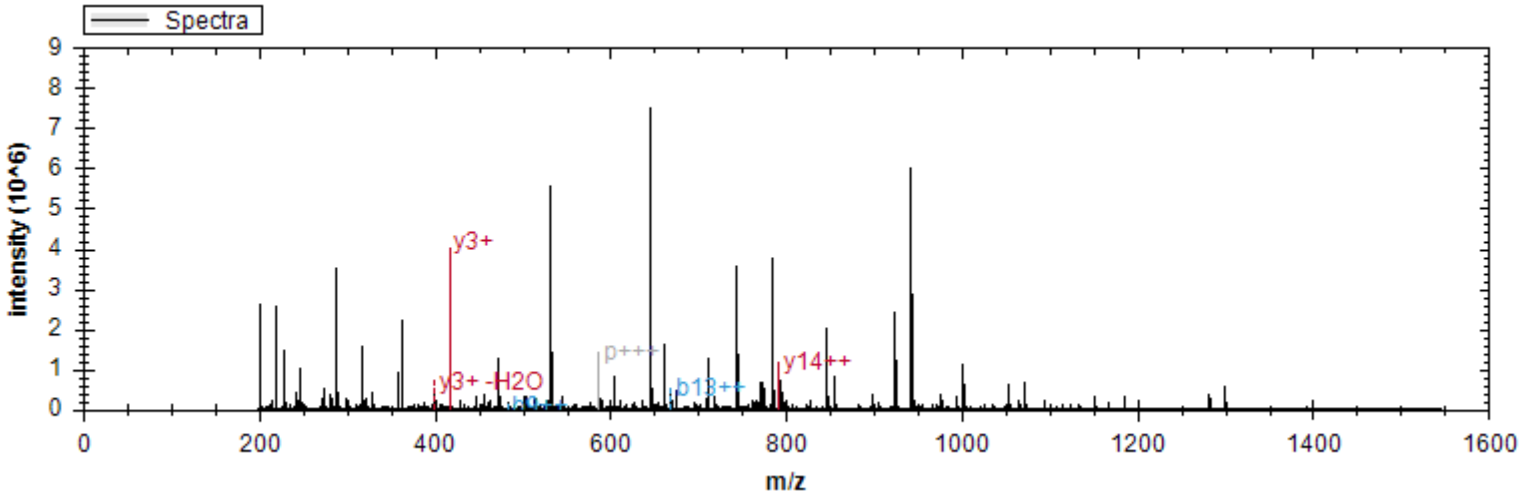

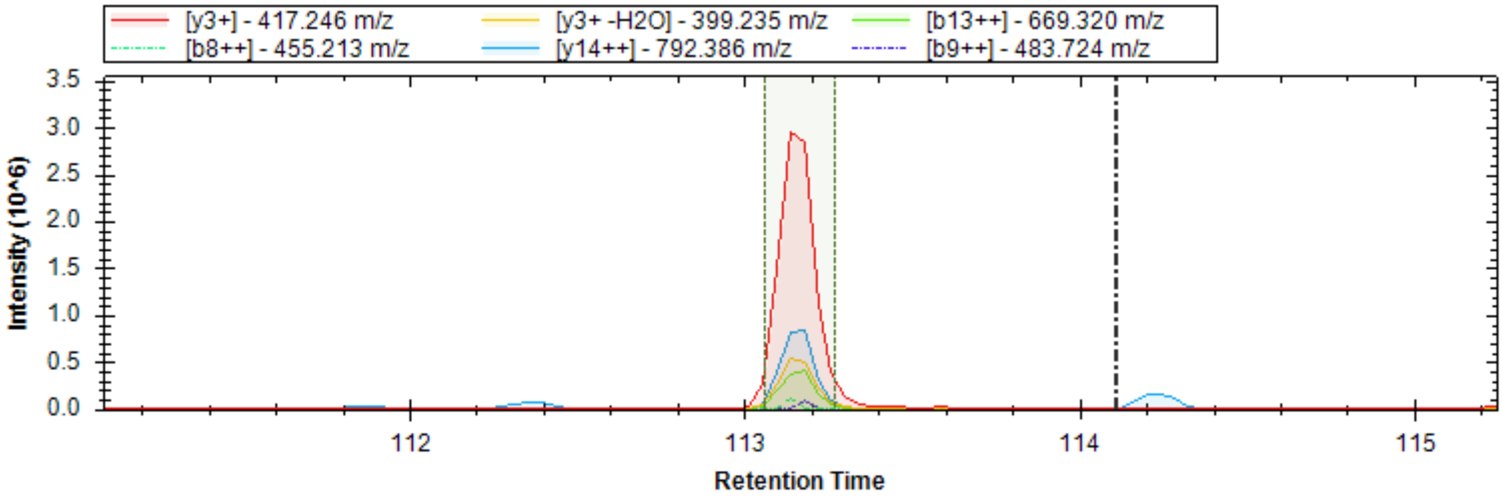

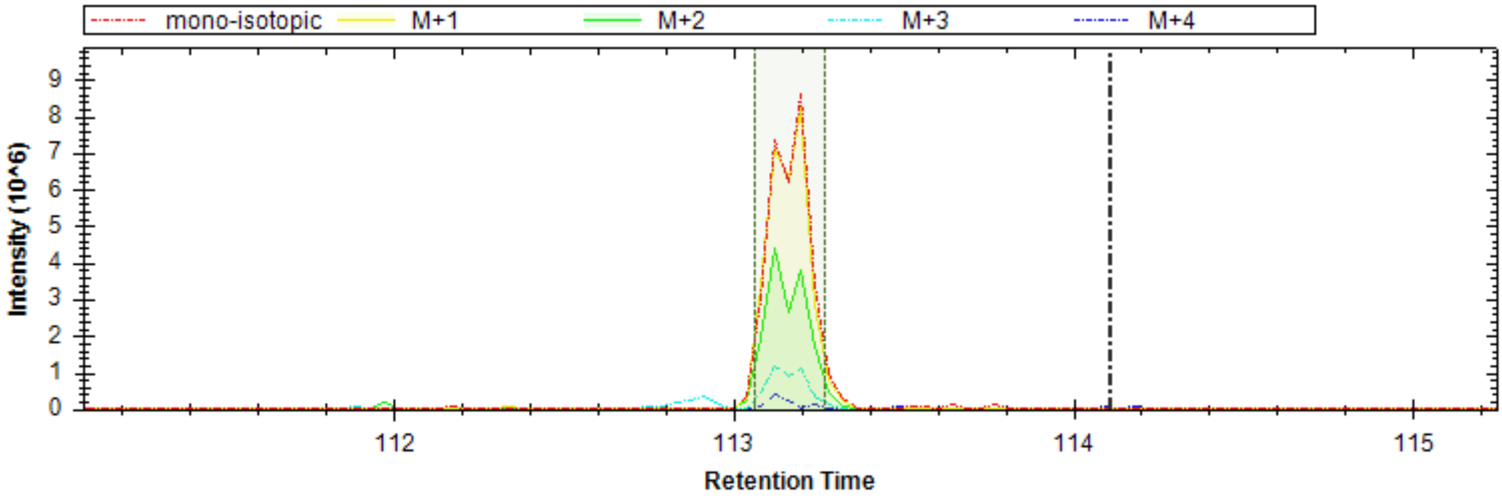

Q9CYT6 – _AQGQIRS[+H+O3+P]PTK_.2

Fig4_MP-DIA-barrel-cortex-p54-3_MHRM.htrms

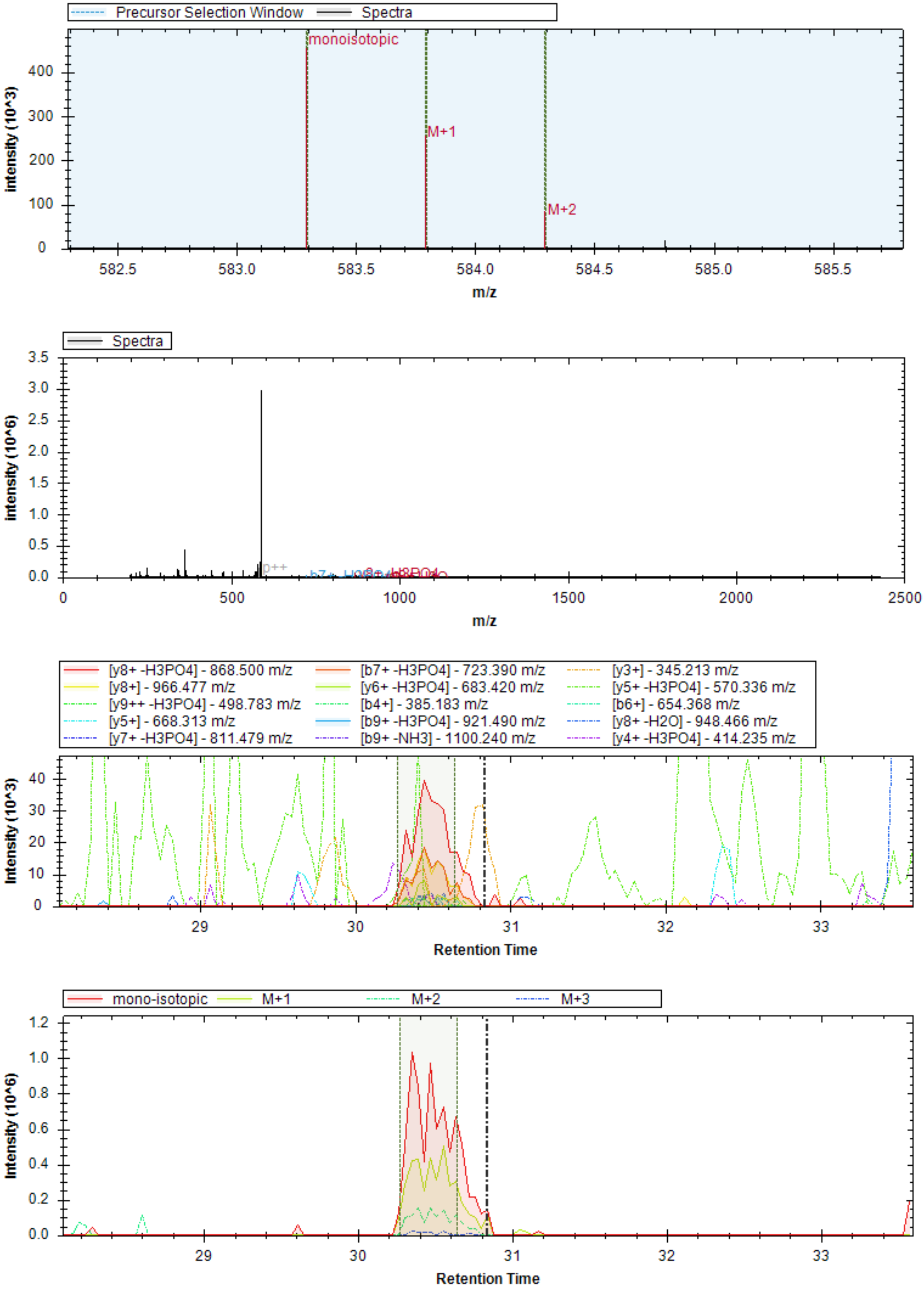

Q9D5Y0 – _ITDT[+H+O3+P]KKTDDLYWR_.3
Fig4_MP-DIA-barrel-cortex-p54-3_MHRM.htrms

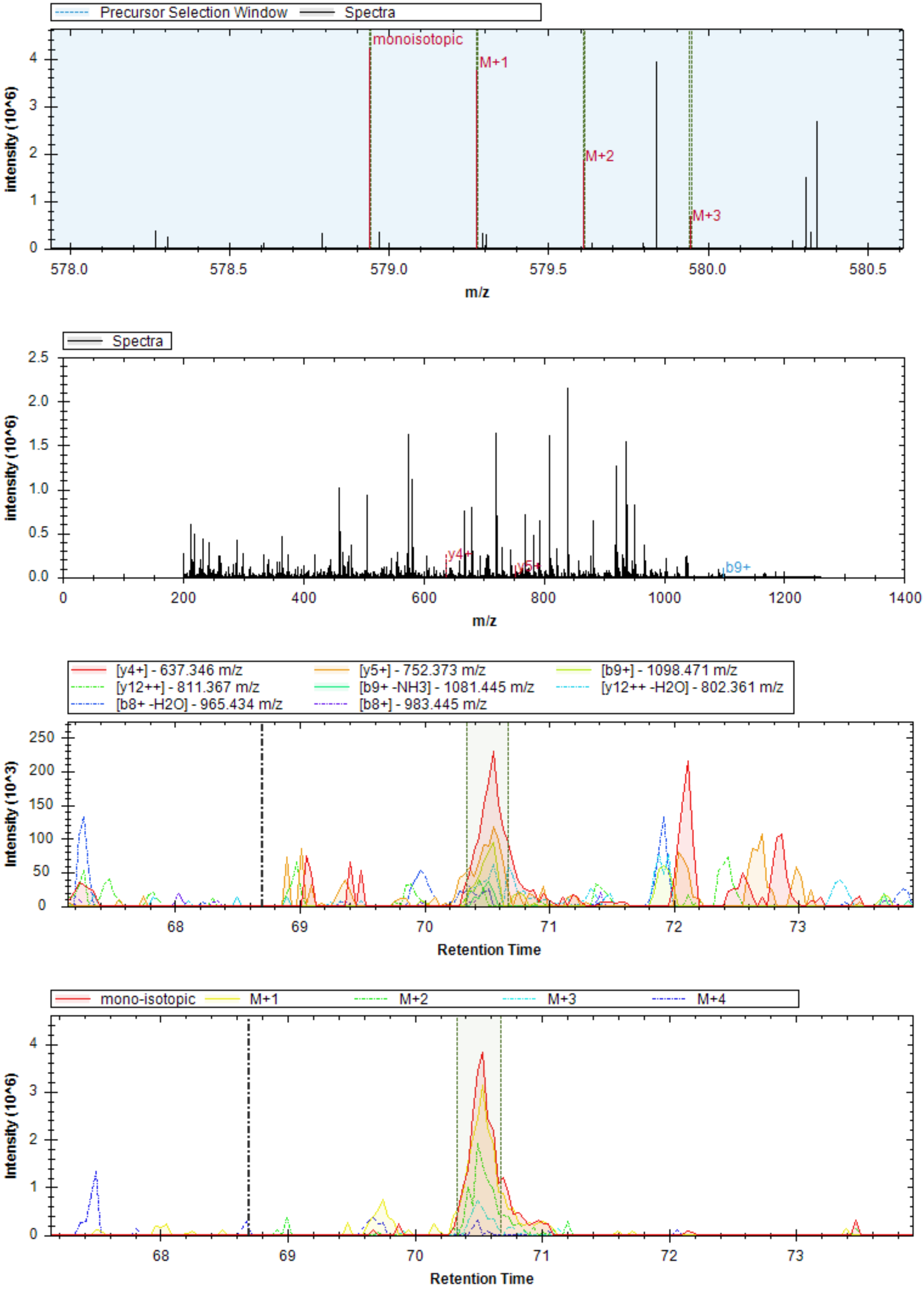

Q9DC63;Q9DC63-2;Q9DC63-3 – _YIDHY[+H+O3+P]AAIK_.2

Fig4_MP-DIA-barrel-cortex-p54-3_MHRM.htrms

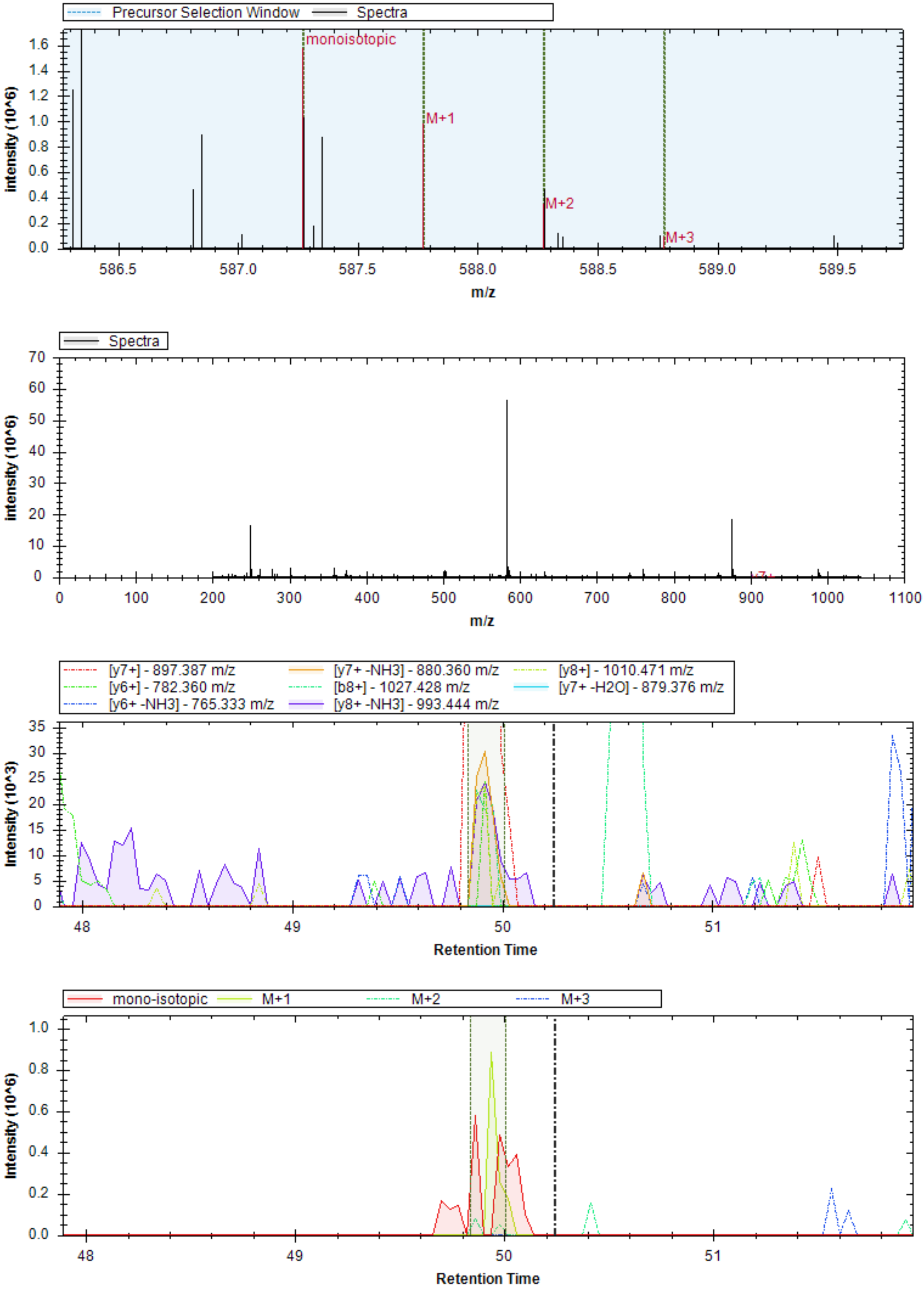

P04370-4 – _TTHYGS[+H+O3+P]LPQK_.2
Fig4_MP-DIA-barrel-cortex-p54-3_MHRM.htrms

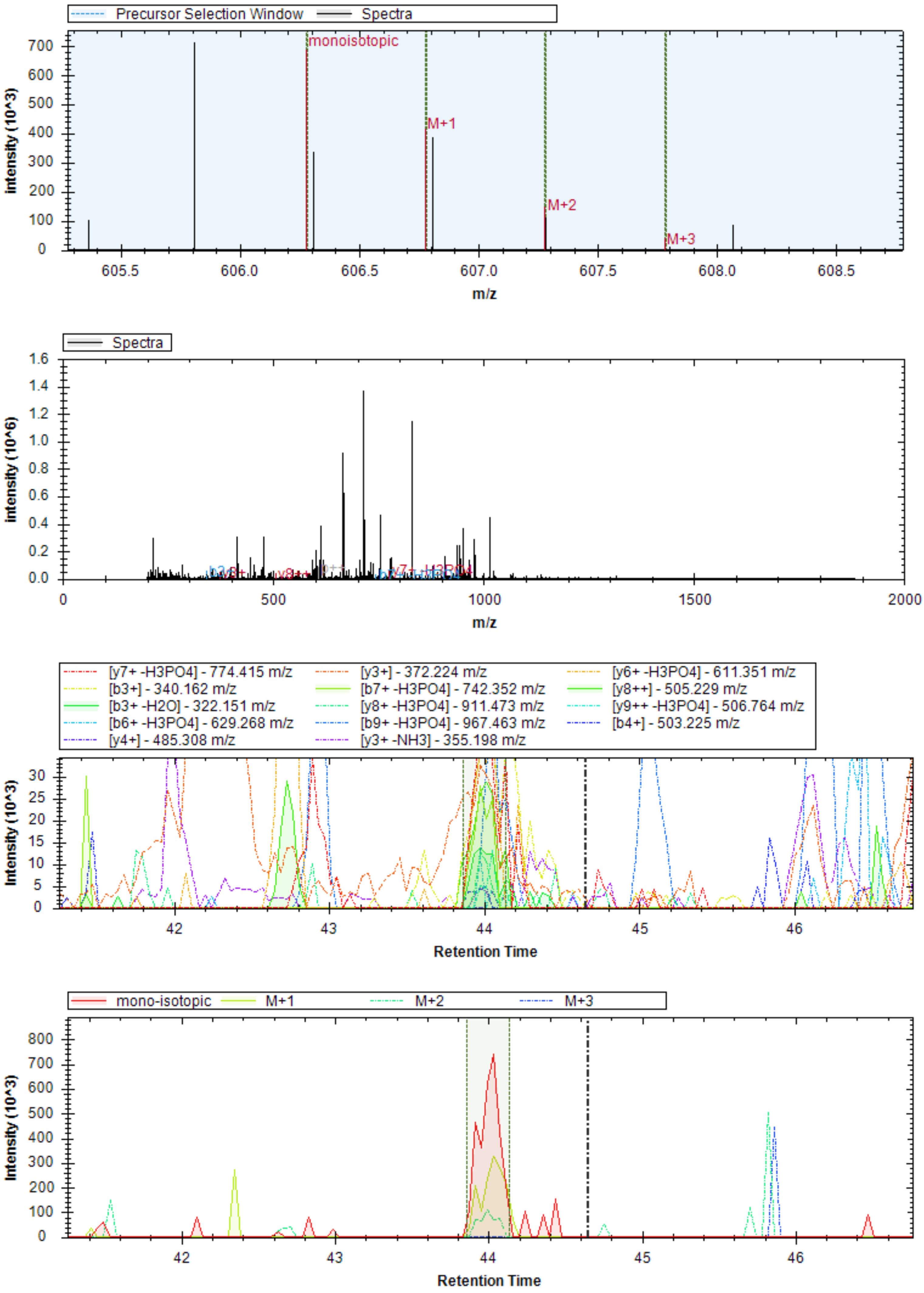

Q62261 – _RPPS[+H+O3+P]PDPNTK_.2

Fig4_MP-DIA-barrel-cortex-p54-3_MHRM.htrms

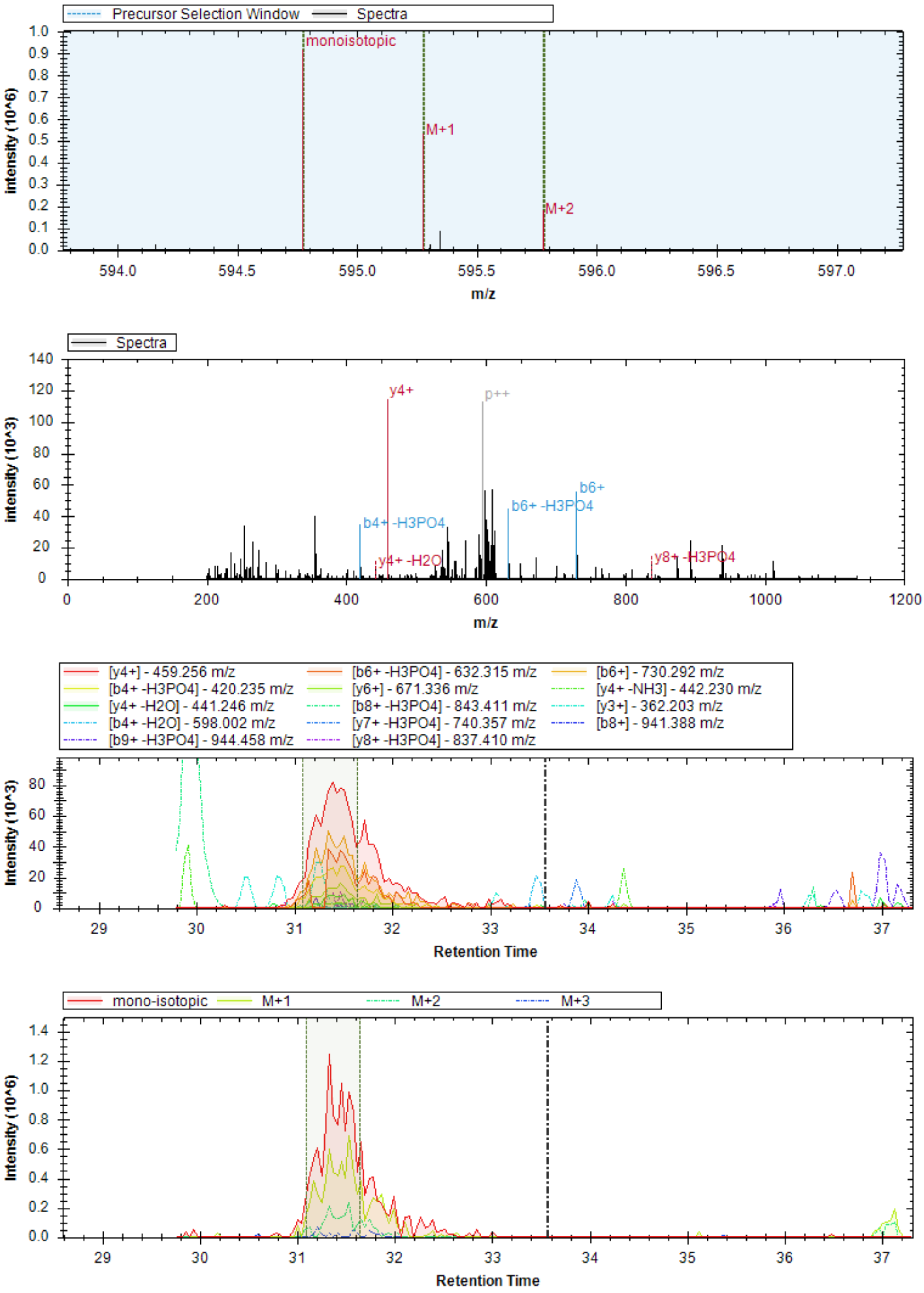

Q80VN0 – _ELTAQIT[+H+O3+P]SIK_.2
Fig4_MP-DIA-barrel-cortex-p54-3_MHRM.htrms

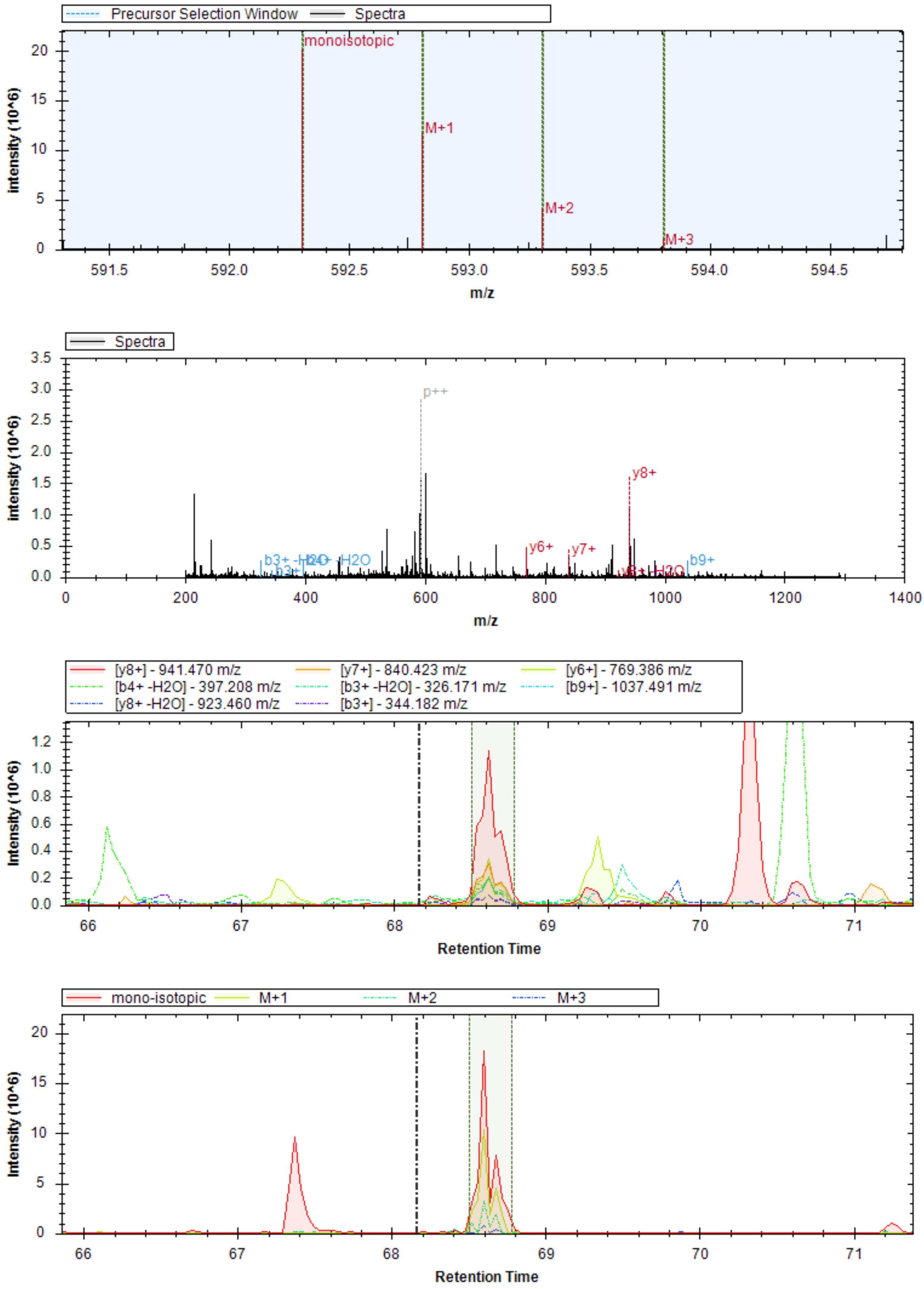

Q9QWI6 – _LSYAGGRPPSYAGS[+H+O3+P]PVHHAAER_.4
Fig4_MP-DIA-barrel-cortex-p54-3_MHRM.htrms

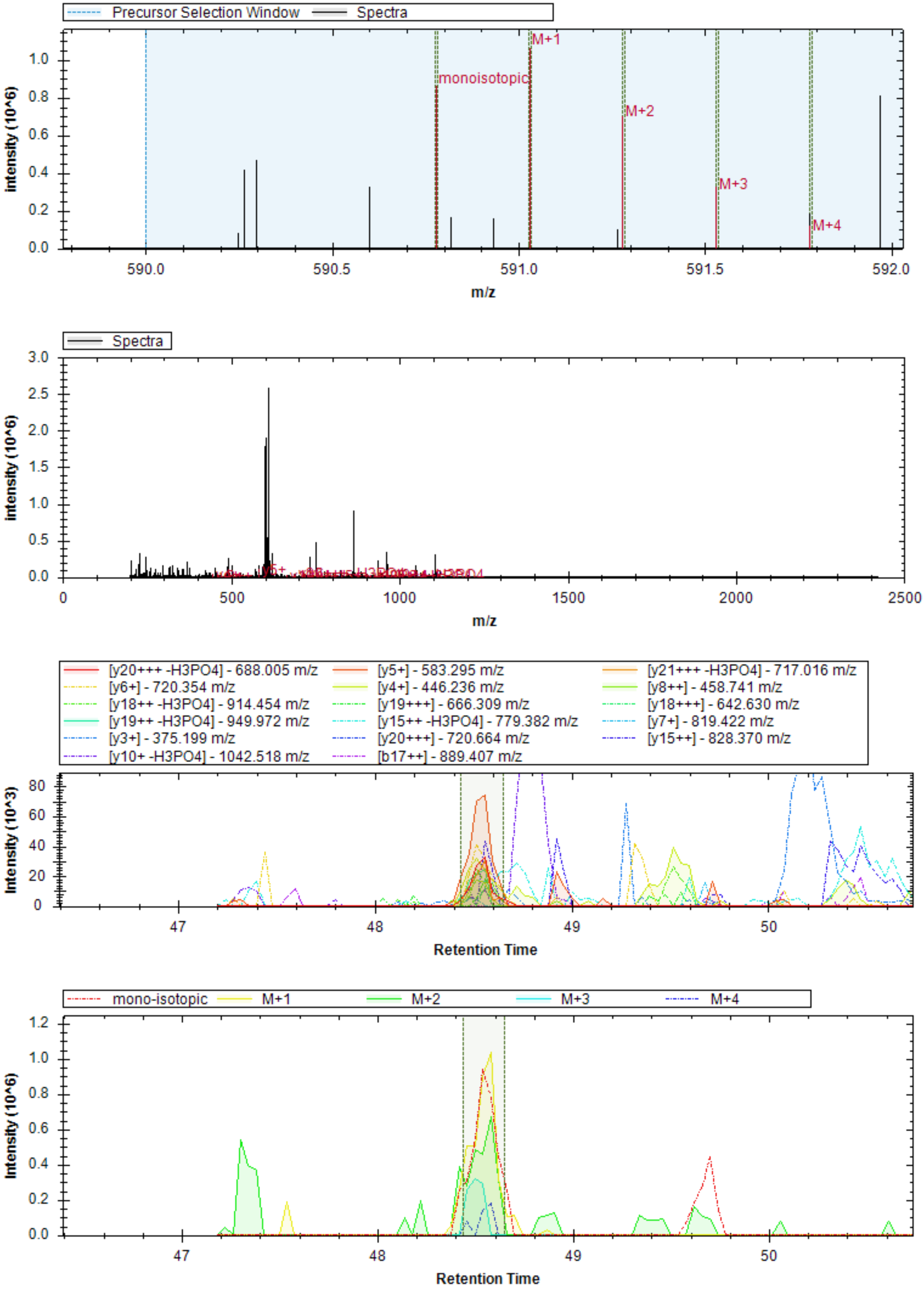

P60904 – _SLS[+H+O3+P]TSGESLYHVLGLDK_.3

Fig4_MP-DIA-barrel-cortex-p54-3_MHRM.htrms

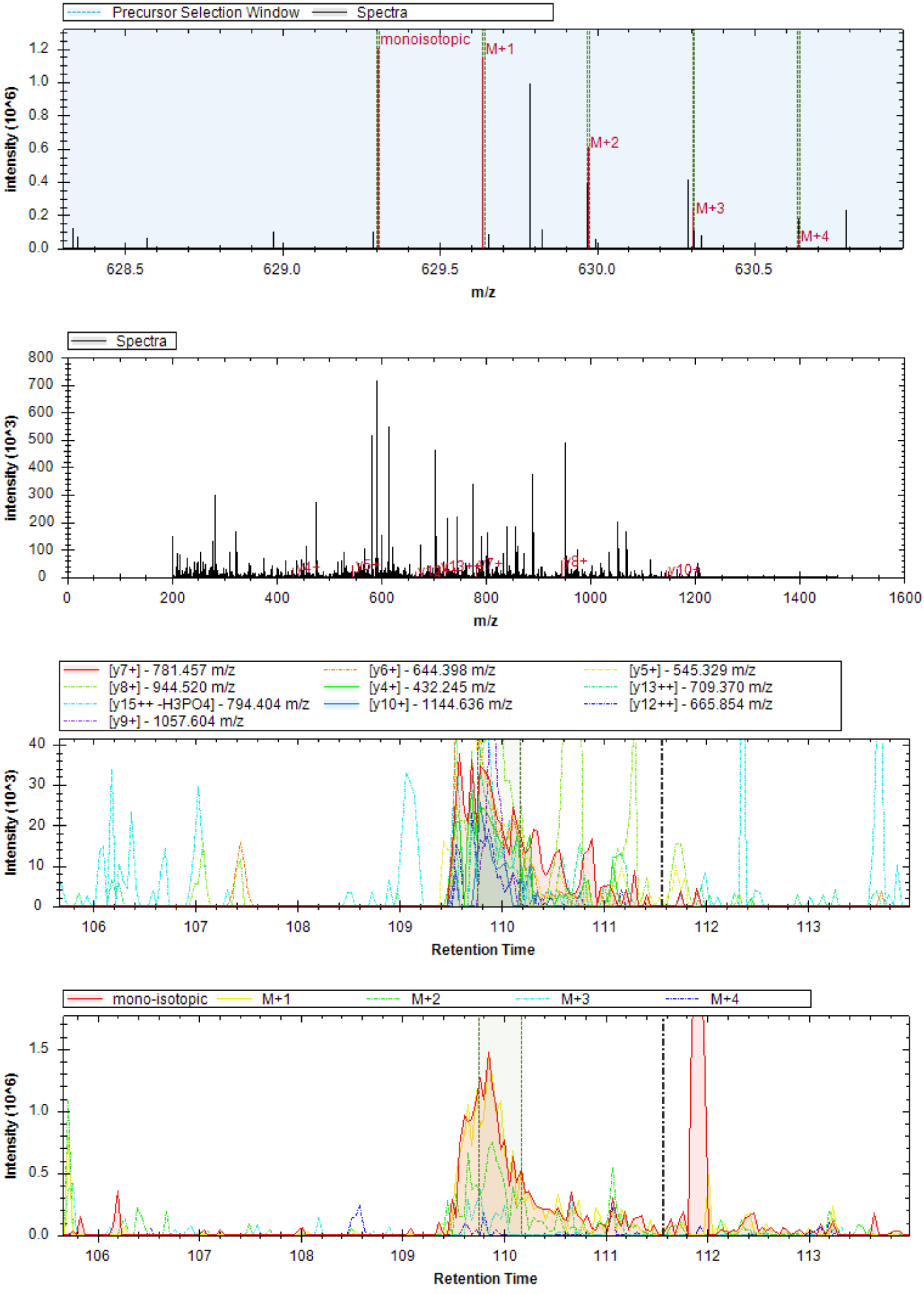

P60904 – _SLST[+H+O3+P]SGESLYHVLGLDK_.3
Fig4_MP-DIA-barrel-cortex-p54-3_MHRM.htrms

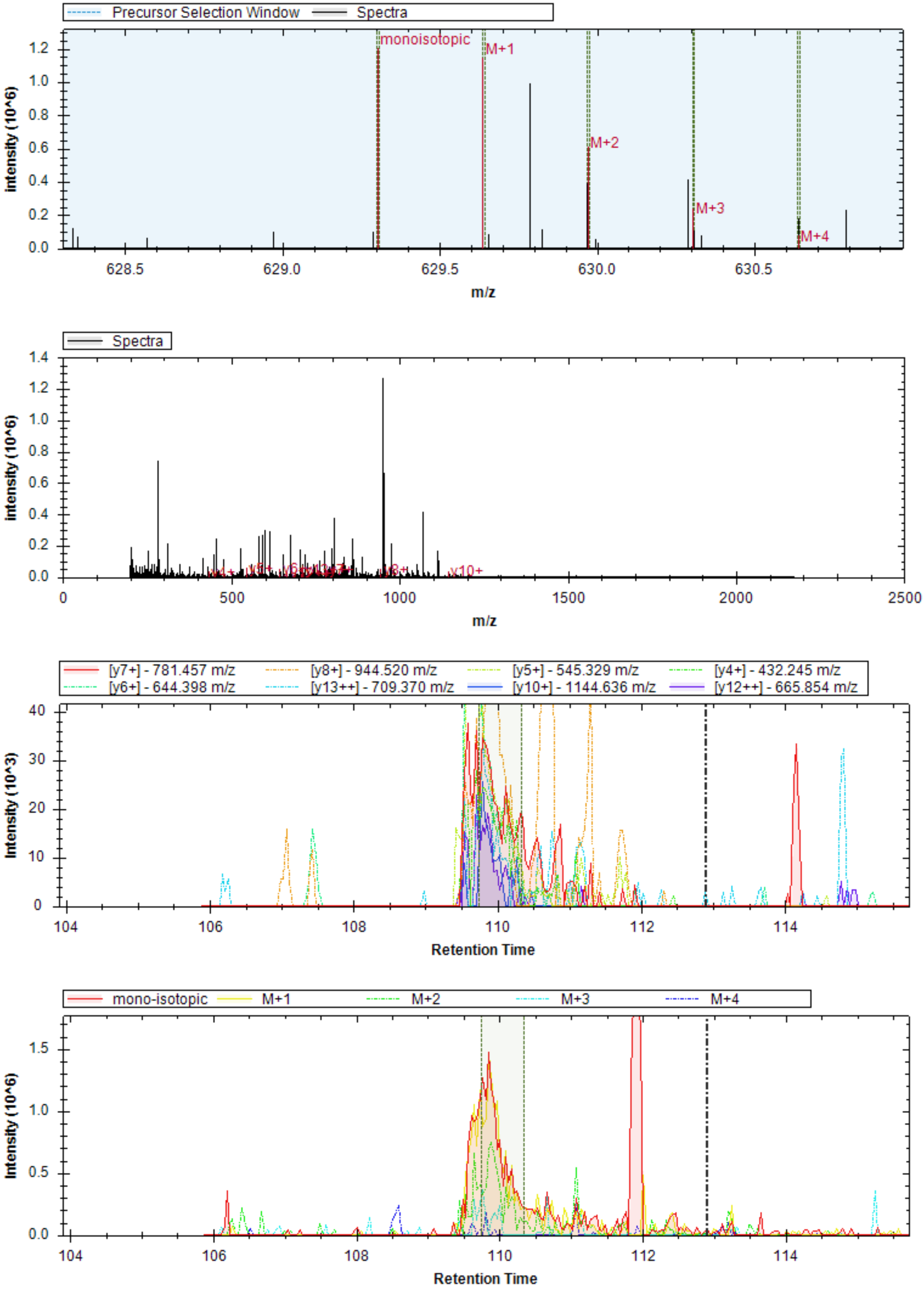

P39447 – _SSGIIRLHT[+H+O3+P]IK_.2

Fig4_MP-DIA-barrel-cortex-p54-3_MHRM.htrms

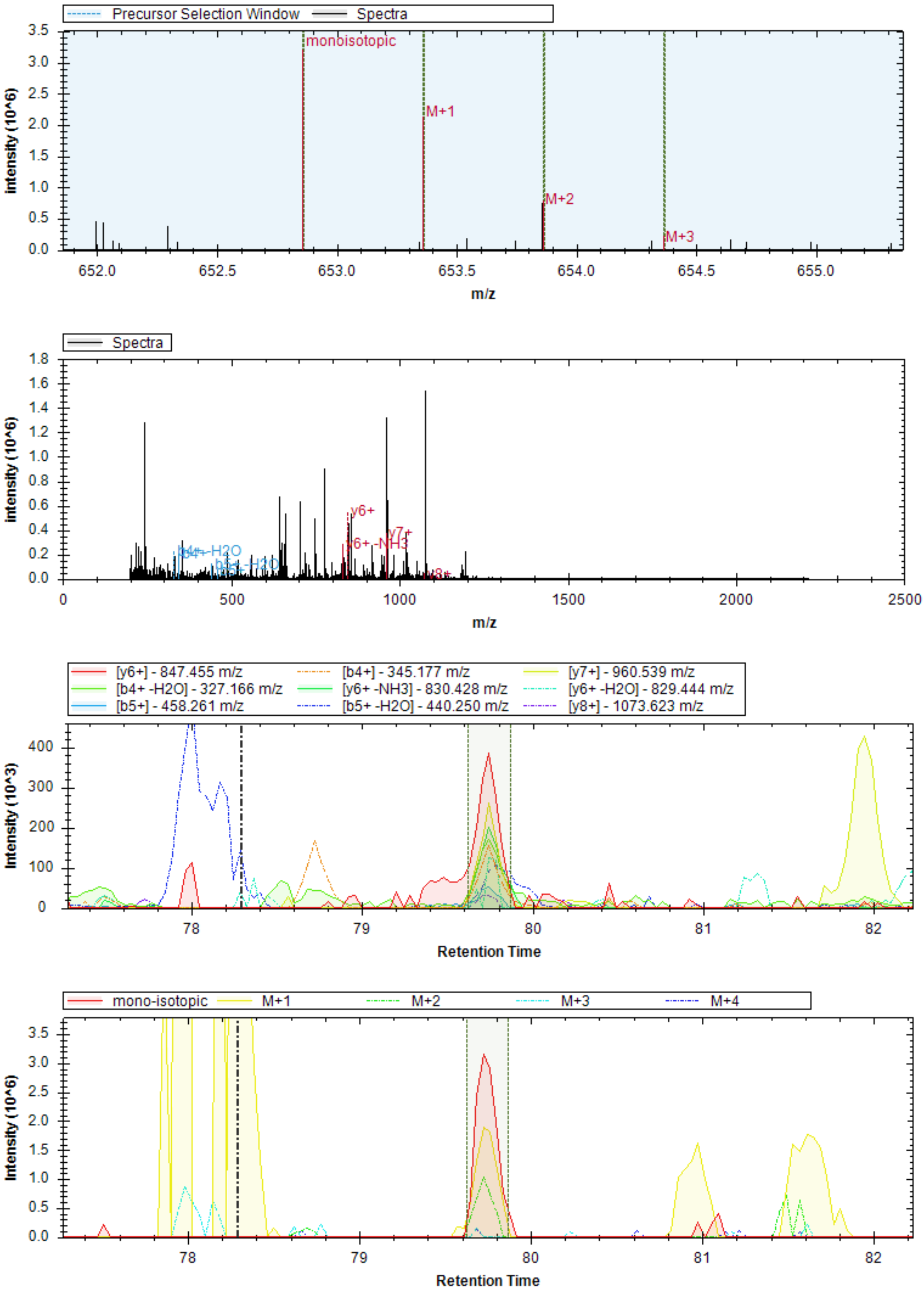

Q8BLJ3 – _M[+O]AS[+H+O3+P]SQGKNELK_.2

Fig4_MP-DIA-barrel-cortex-p54-3_MHRM.htrms

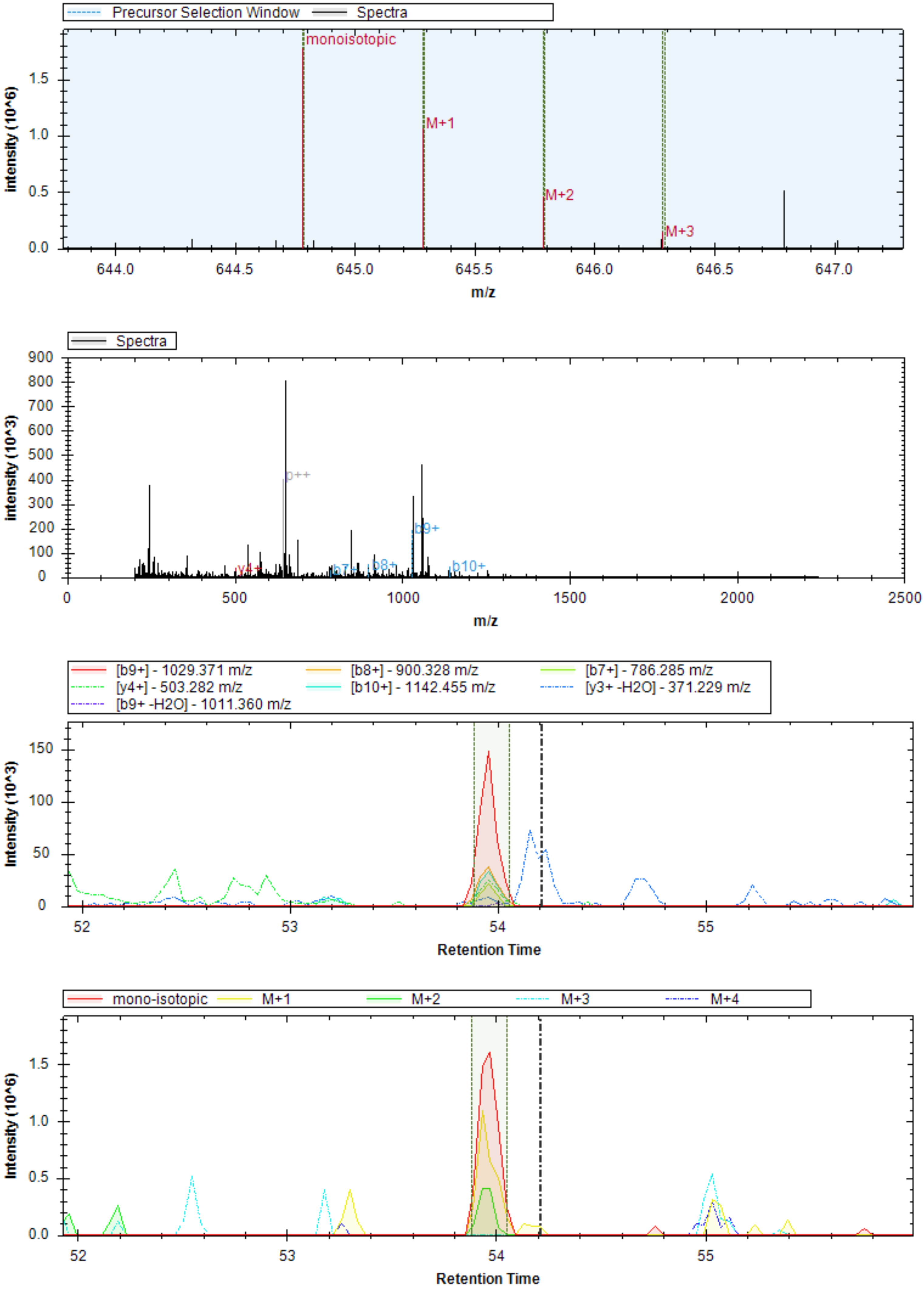

P13595 – _GVTASS[+H+O3+P]SSPASAPK_.2
Fig4_MP-DIA-barrel-cortex-p54-3_MHRM.htrms

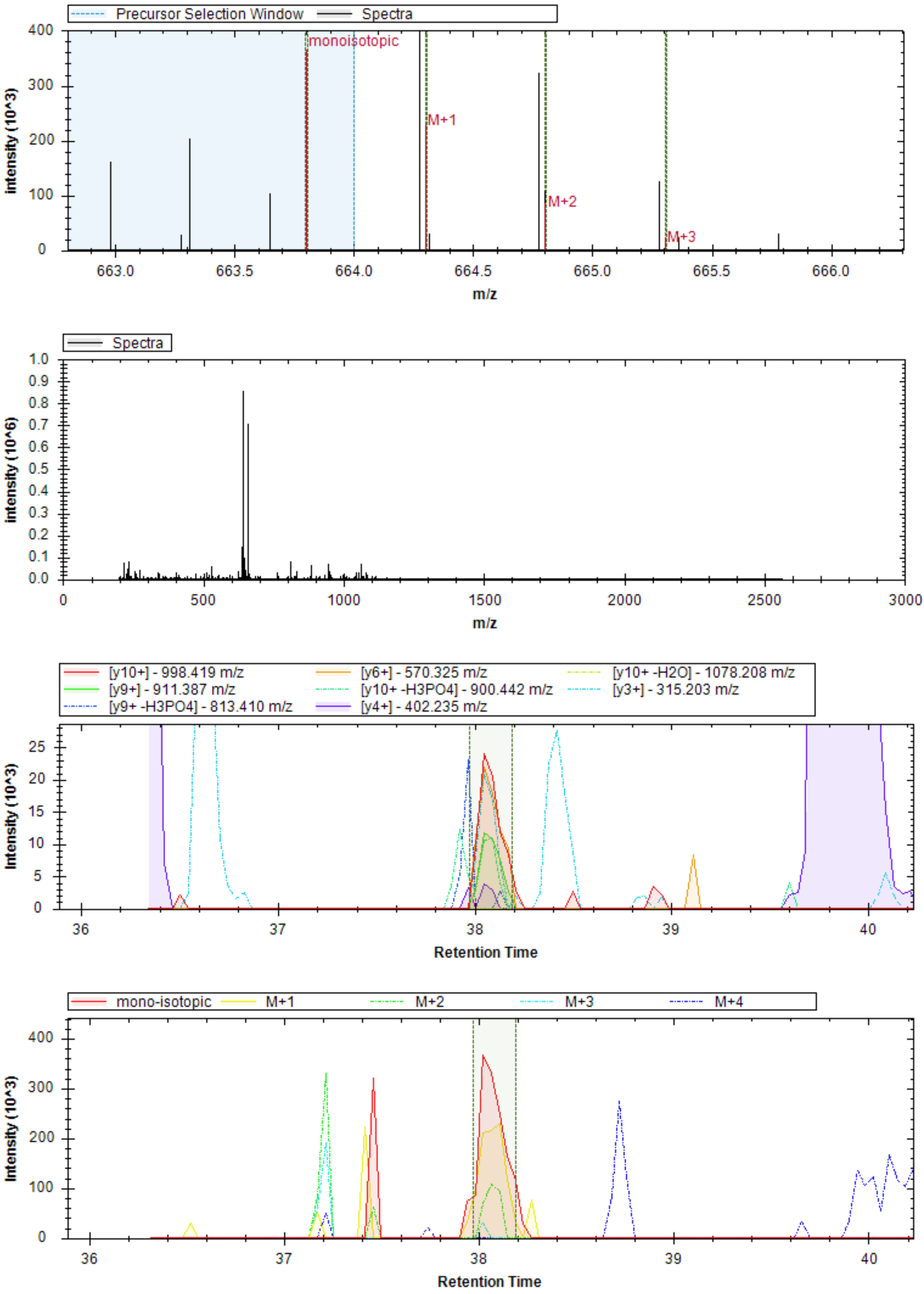

P13595 – _GVTASSS[+H+O3+P]SPASAPK_.2

Fig4_MP-DIA-barrel-cortex-p54-3_MHRM.htrms

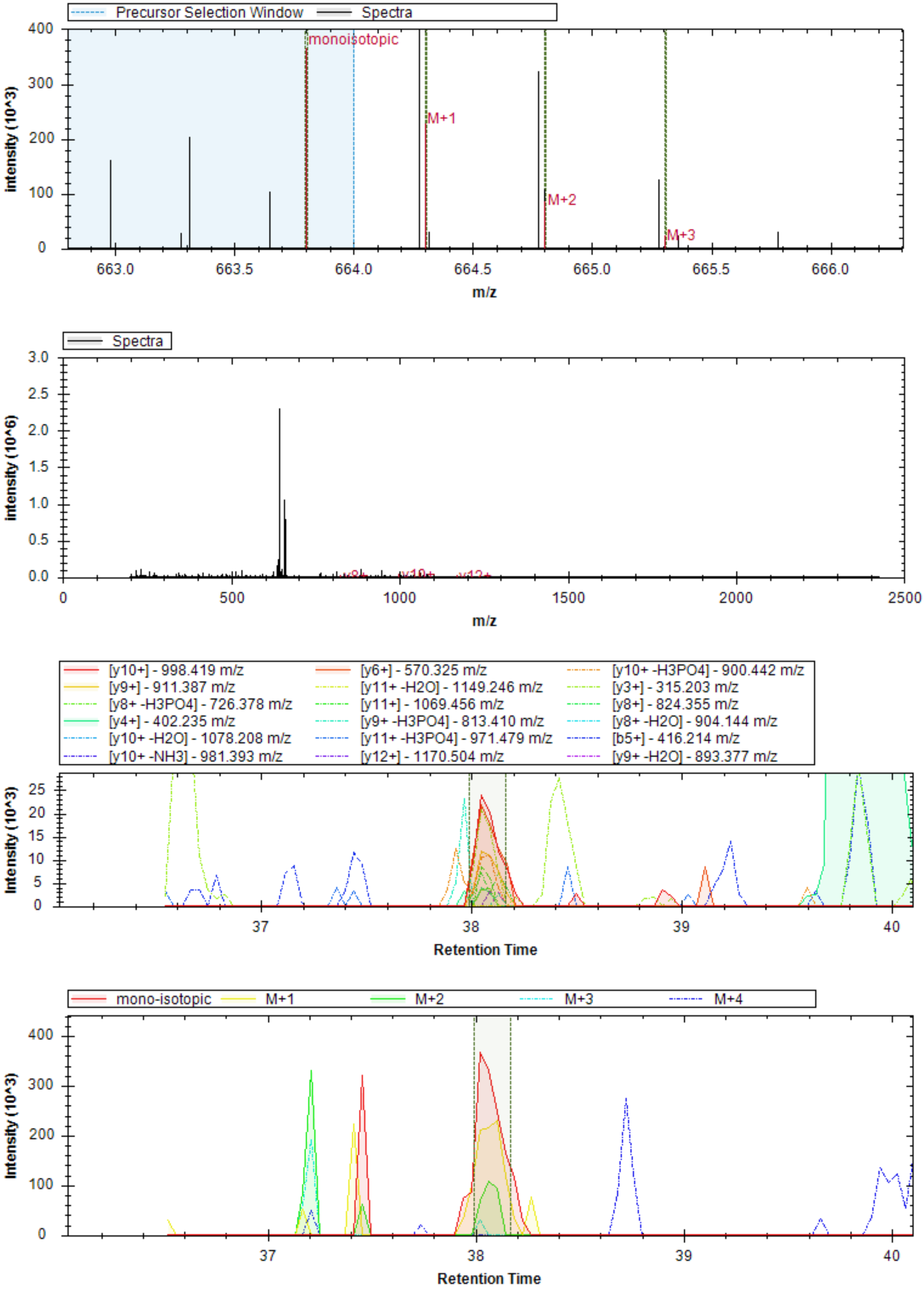

P13595 – _GVTASSSS[+H+O3+P]PASAPK_.2

Fig4_MP-DIA-barrel-cortex-p54-3_MHRM.htrms

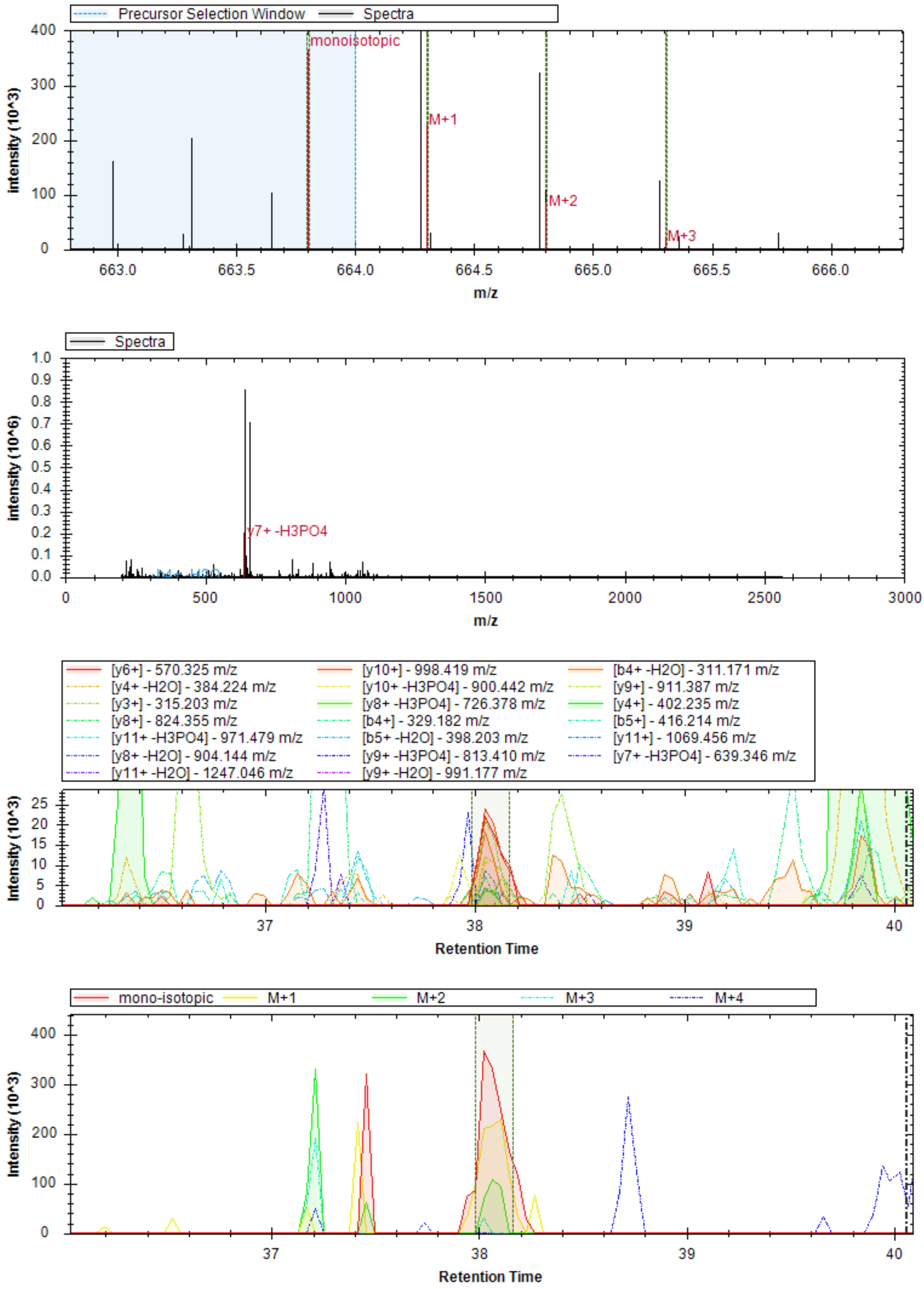

P26361 – _KM[+O]LHS[+H+O3+P]ILHAPMS[+H+O3+P]T[+H+O3+P]ISK_.3
Fig4_MP-DIA-barrel-cortex-p54-3_MHRM.htrms

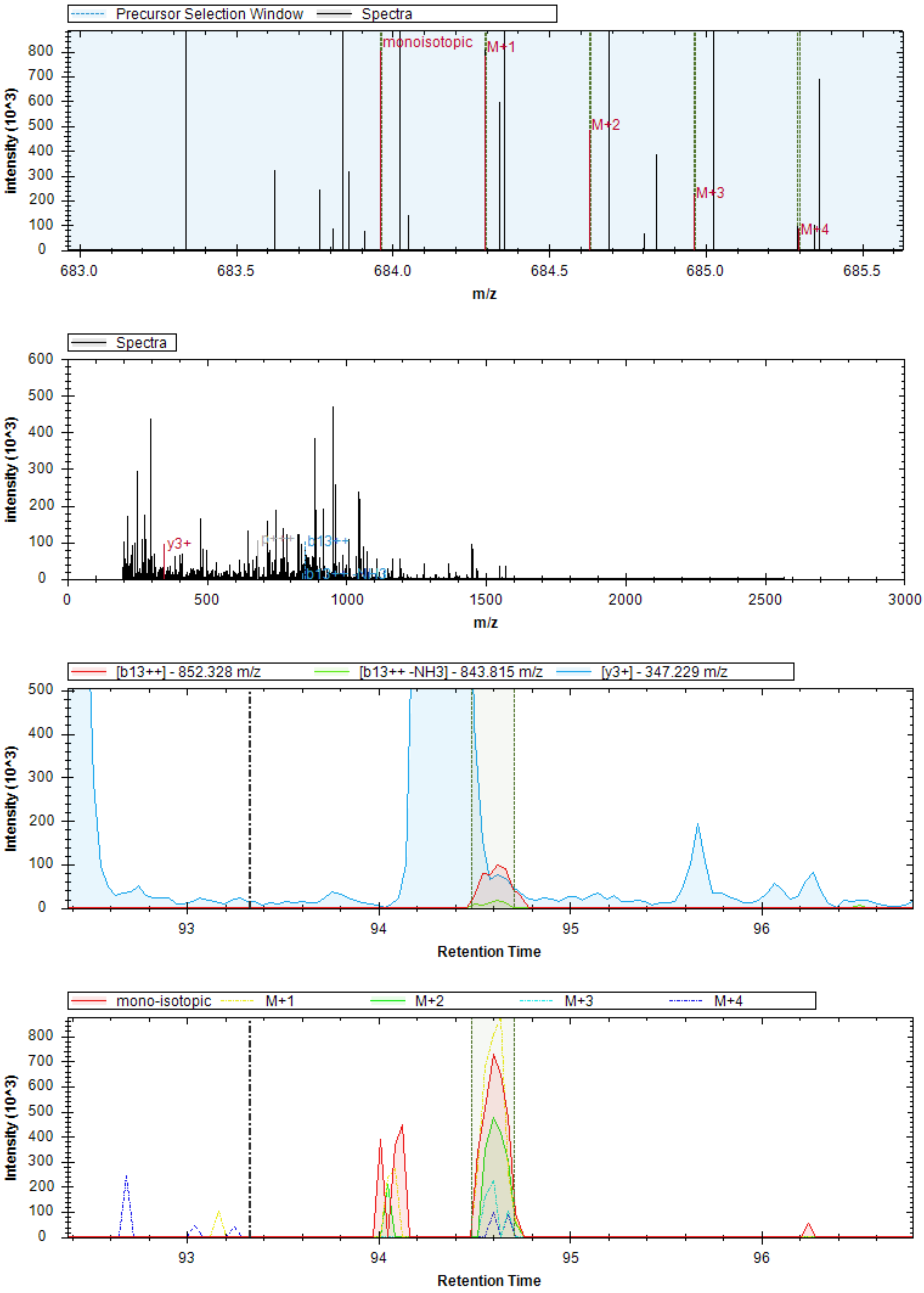

Q6A078 – _T[+H+O3+P]IGLM[+O]KKVVEK_.2

Fig4_MP-DIA-barrel-cortex-p54-3_MHRM.htrms

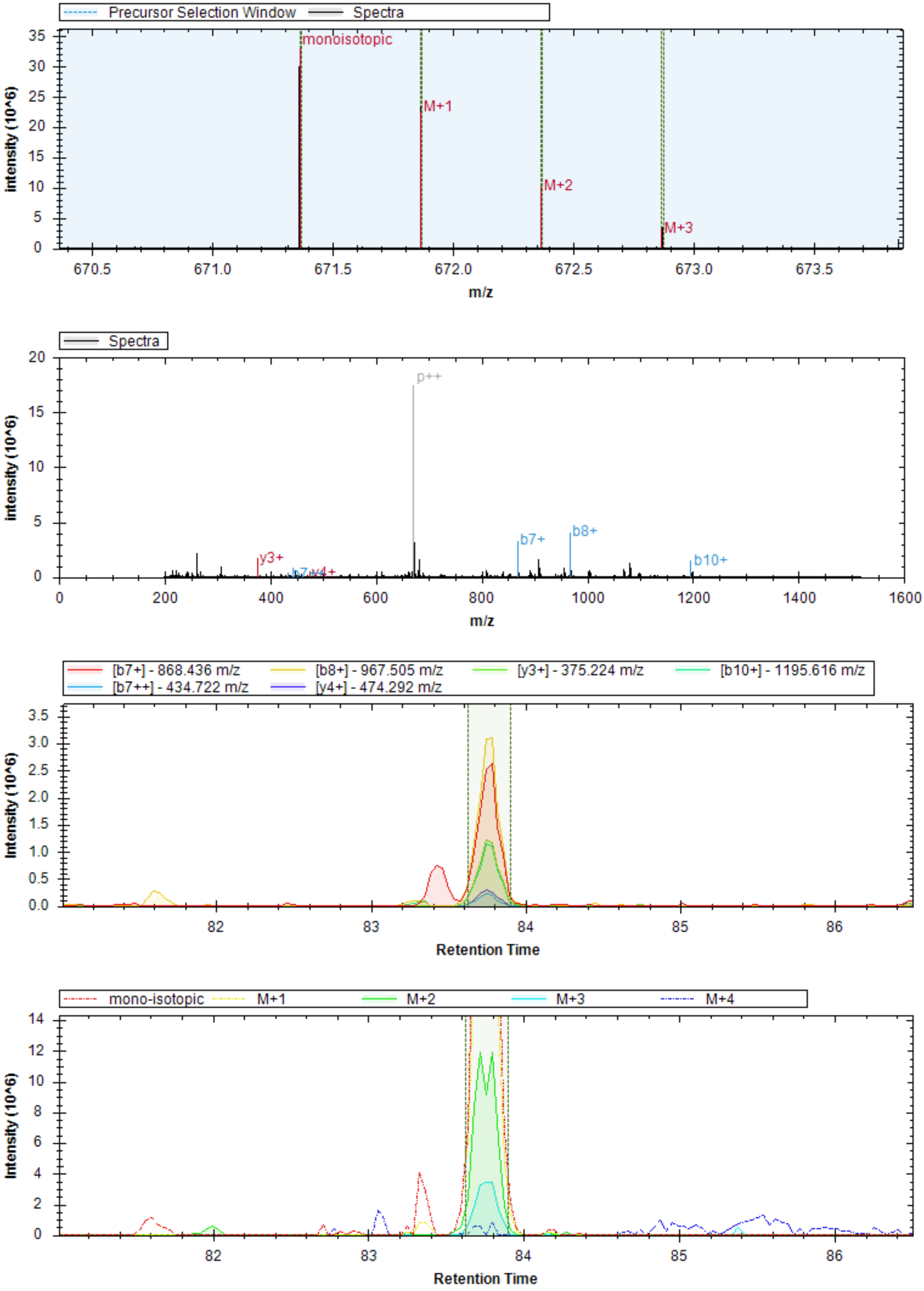

Q8CDU4;Q8CDU4-2 – _[+C2+H2+O]ASLRS[+H+O3+P]ATPRLR_.2
Fig4_MP-DIA-barrel-cortex-p54-3_MHRM.htrms

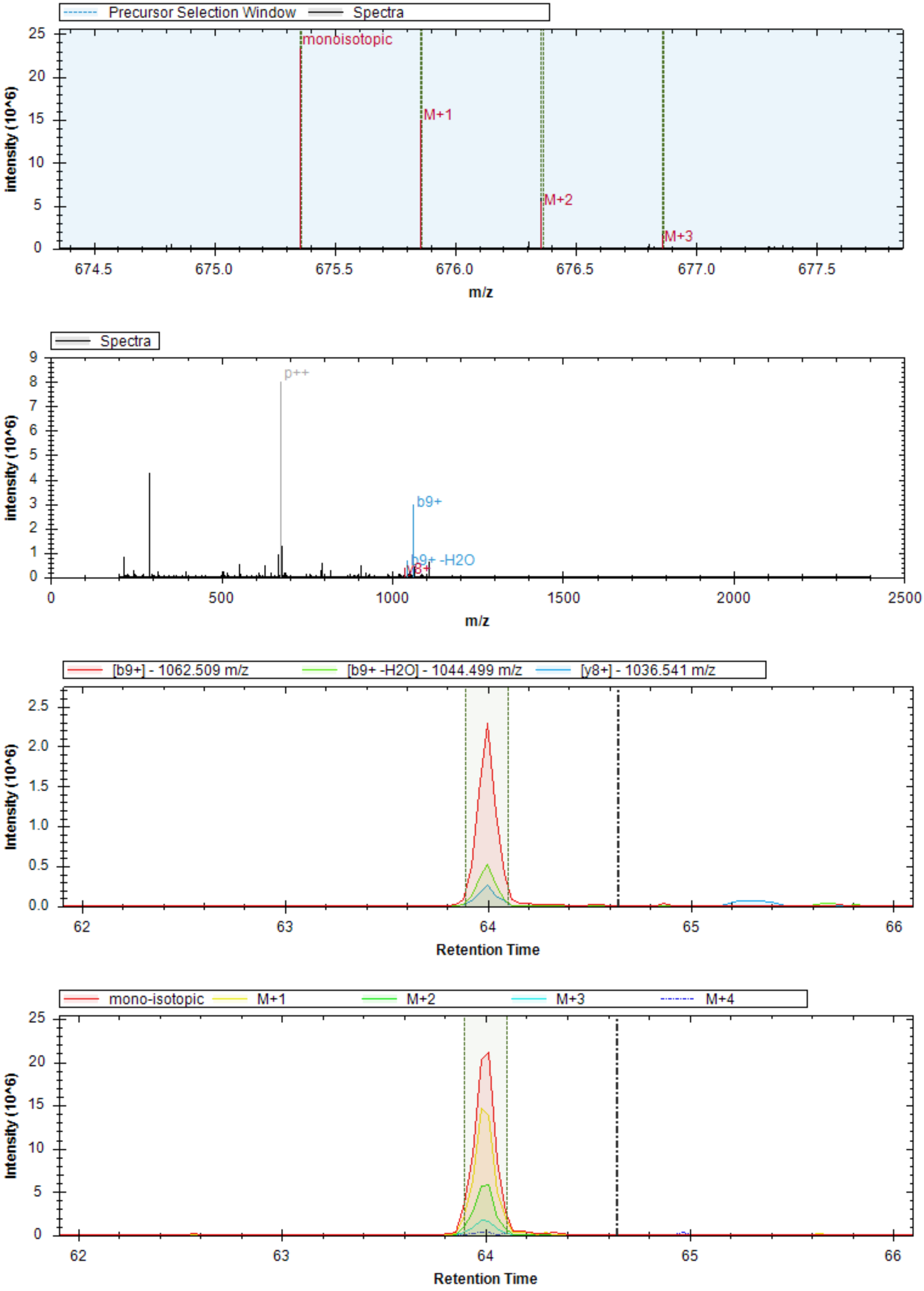

Q8R5L3;Q8R5L3-2 – _LQLY[+H+O3+P]FWKDR_.2
Fig4_MP-DIA-barrel-cortex-p54-3_MHRM.htrms

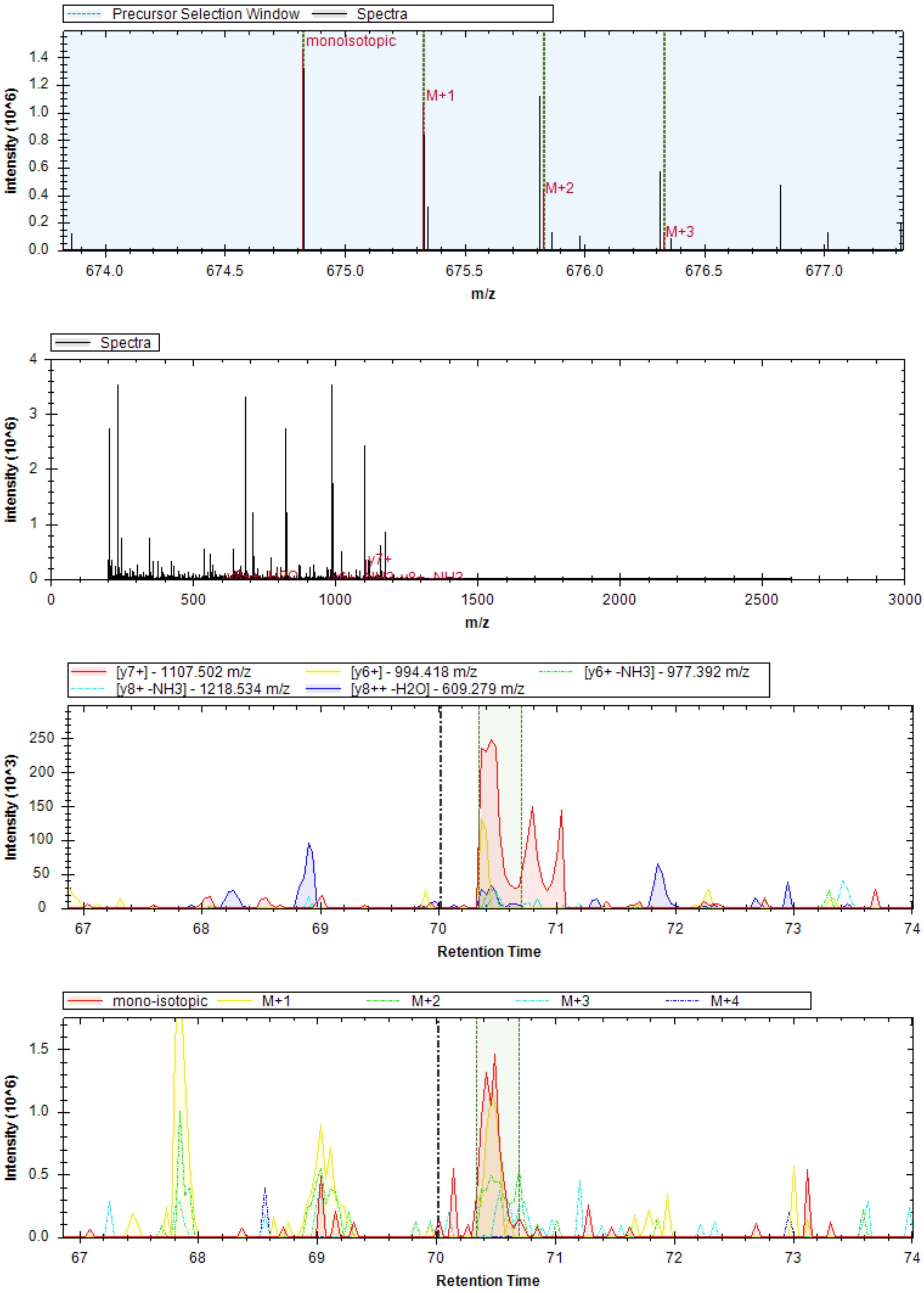

O88935 – _PVAGGPGAPPAARPPAS[+H+O3+P]PSPQR_.3

Fig4_MP-DIA-barrel-cortex-p54-3_MHRM.htrms

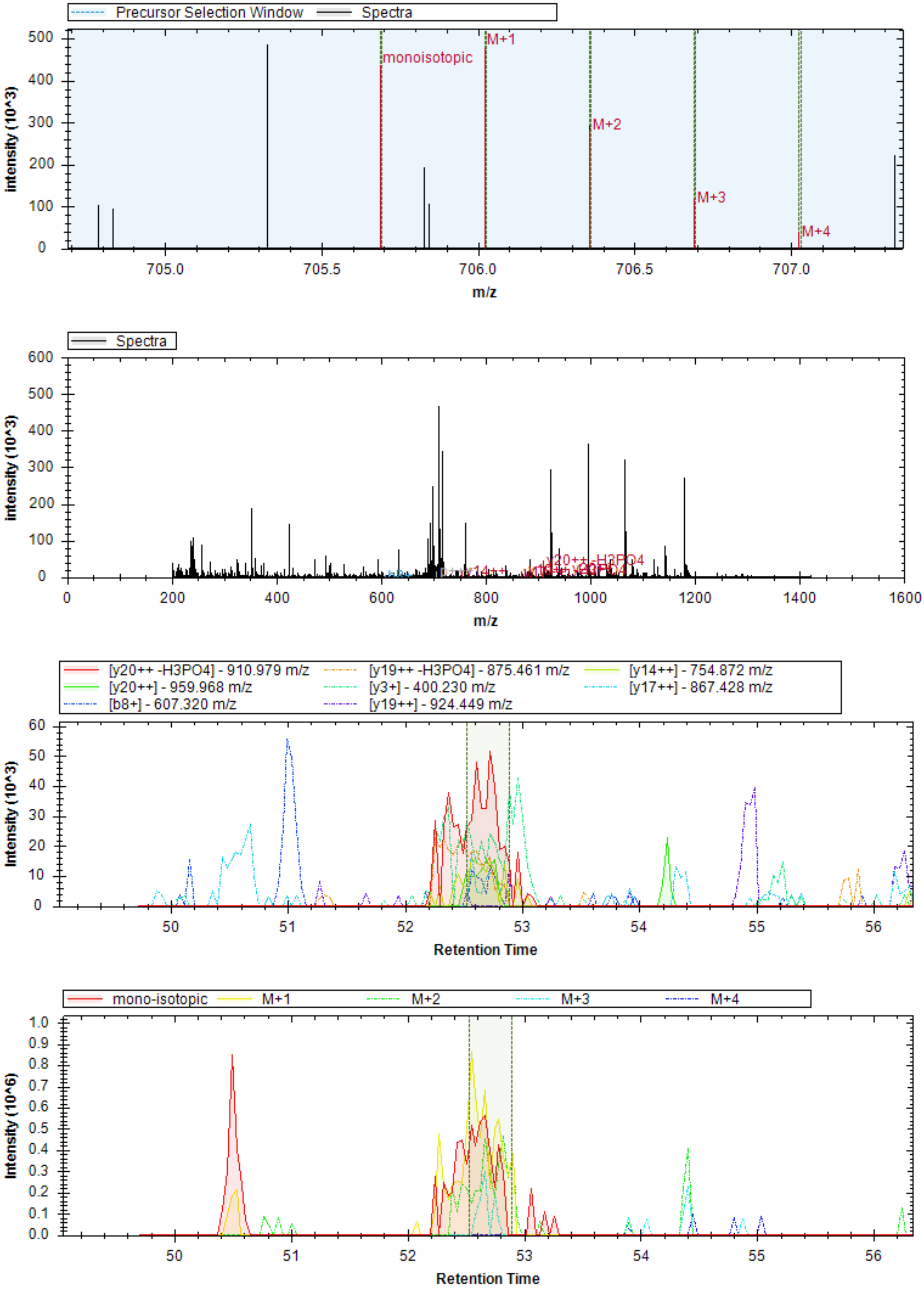

P62751 – _KAY[+H+O3+P]VRLAPDYDALDVANK_.3
Fig4_MP-DIA-barrel-cortex-p54-3_MHRM.htrms

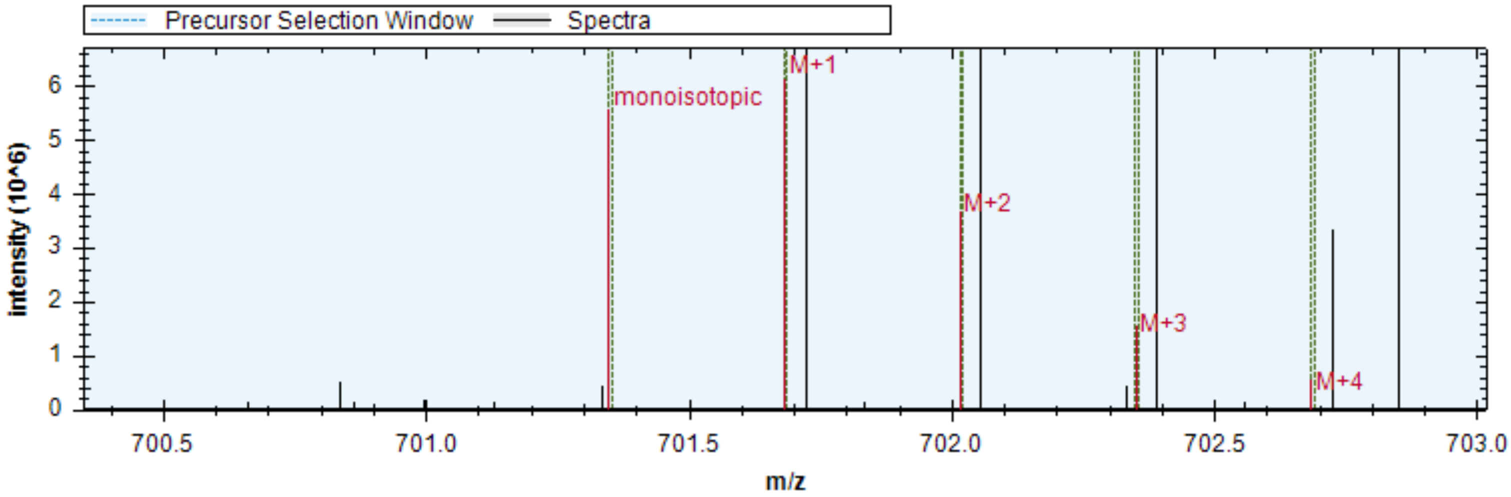

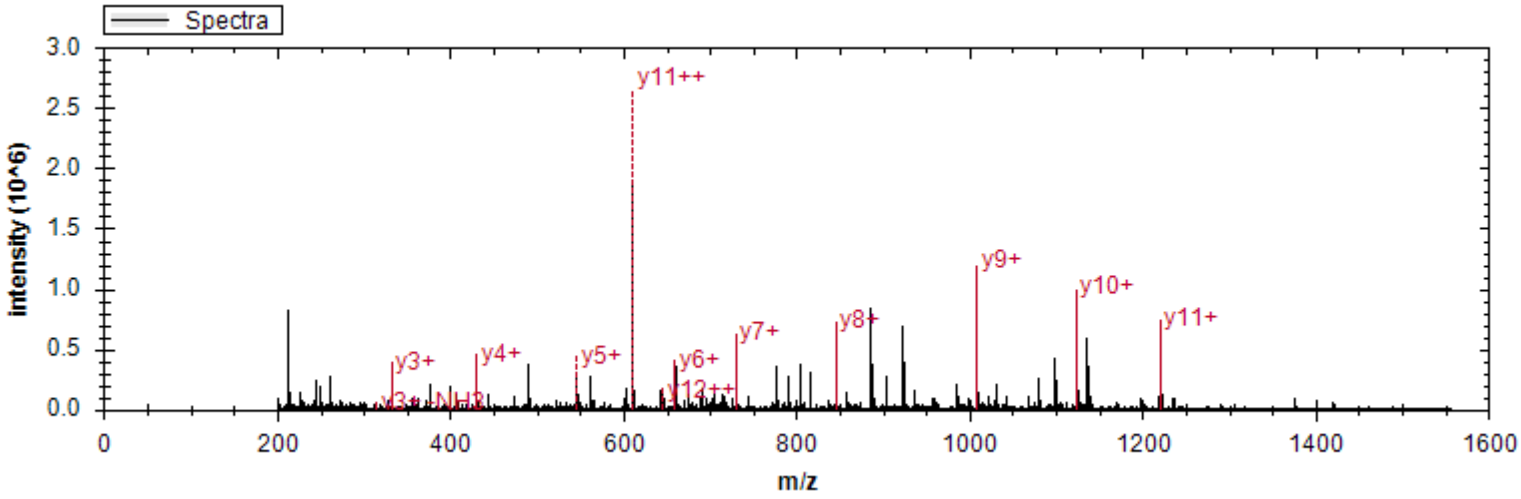

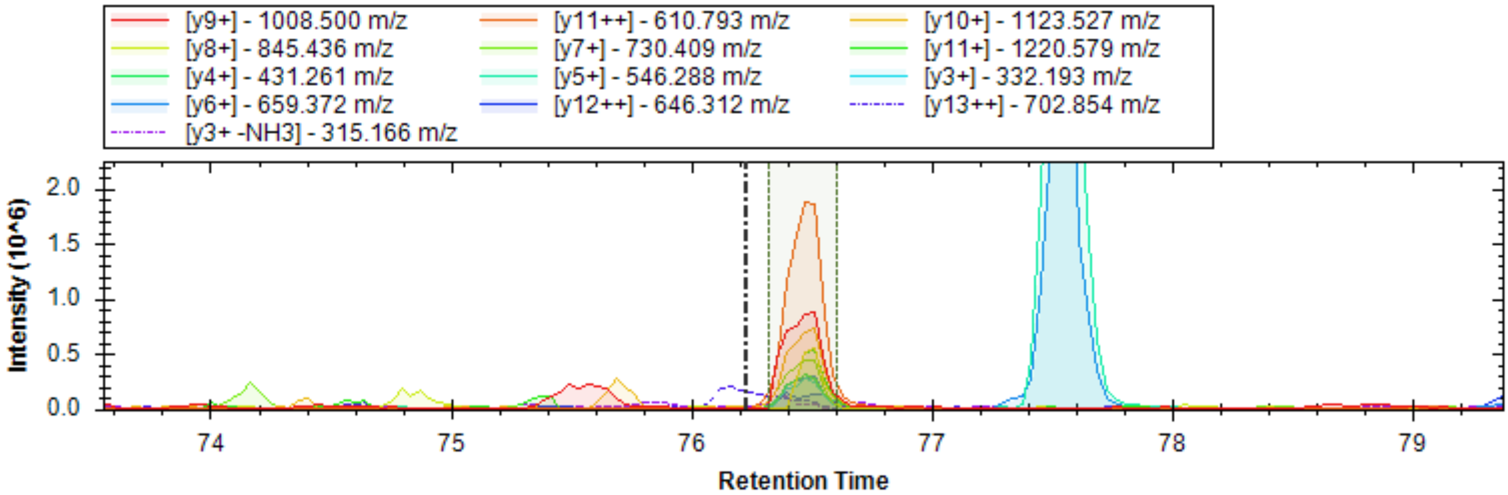

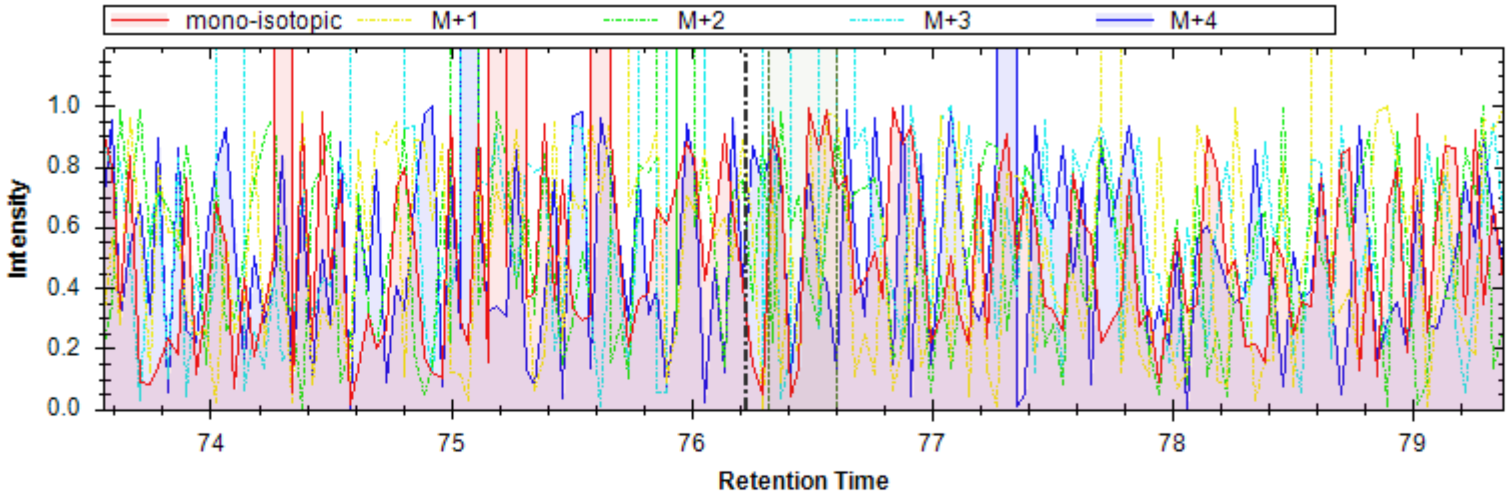

Q3U4B4 – _RHKT[+H+O3+P]LSANEEK_.2
Fig4_MP-DIA-barrel-cortex-p54-3_MHRM.htrms

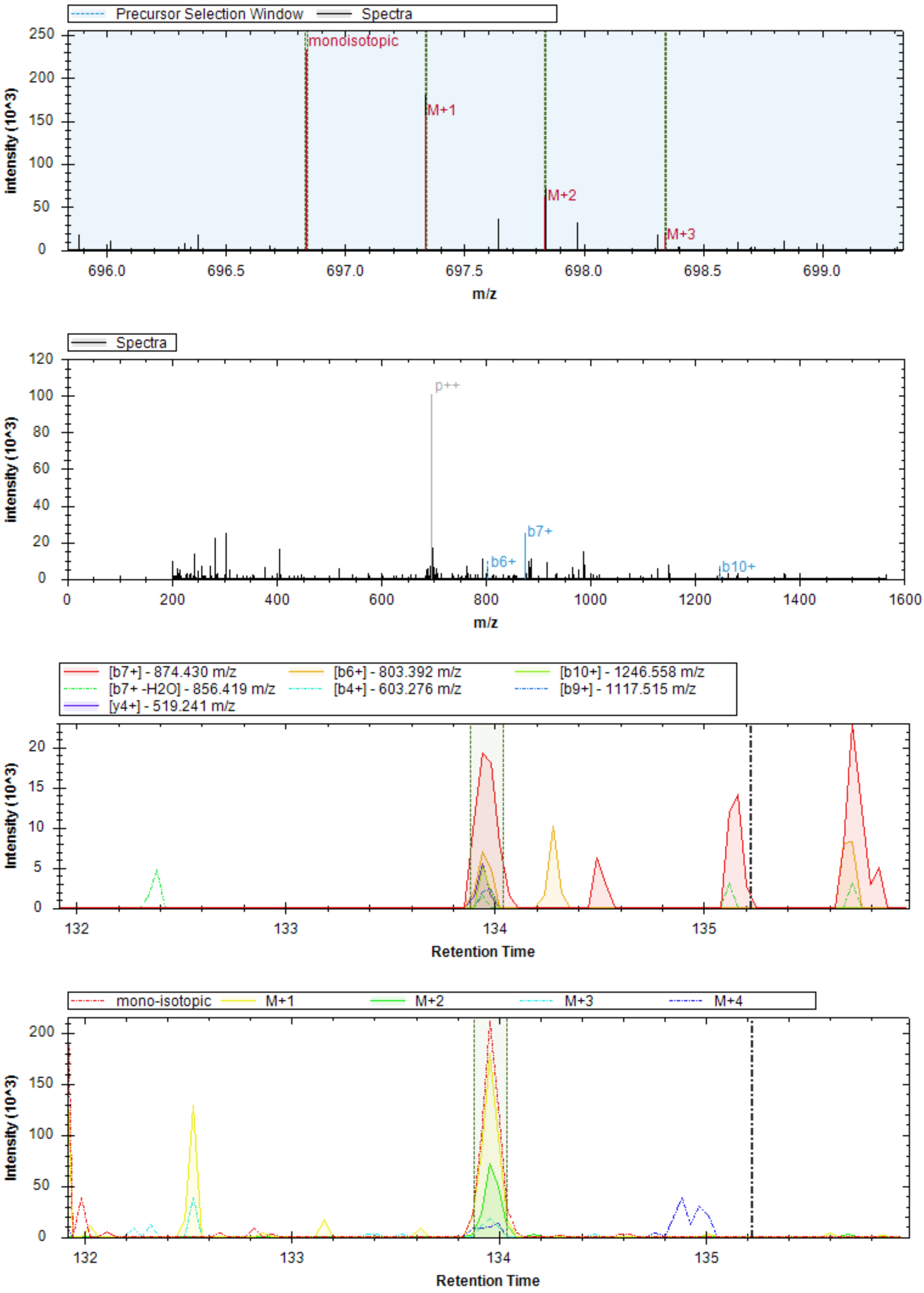

Q99PU8;Q99PU8-2 – _ALGRM[+O]VERS[+H+O3+P]LR_.2
Fig4_MP-DIA-barrel-cortex-p54-3_MHRM.htrms

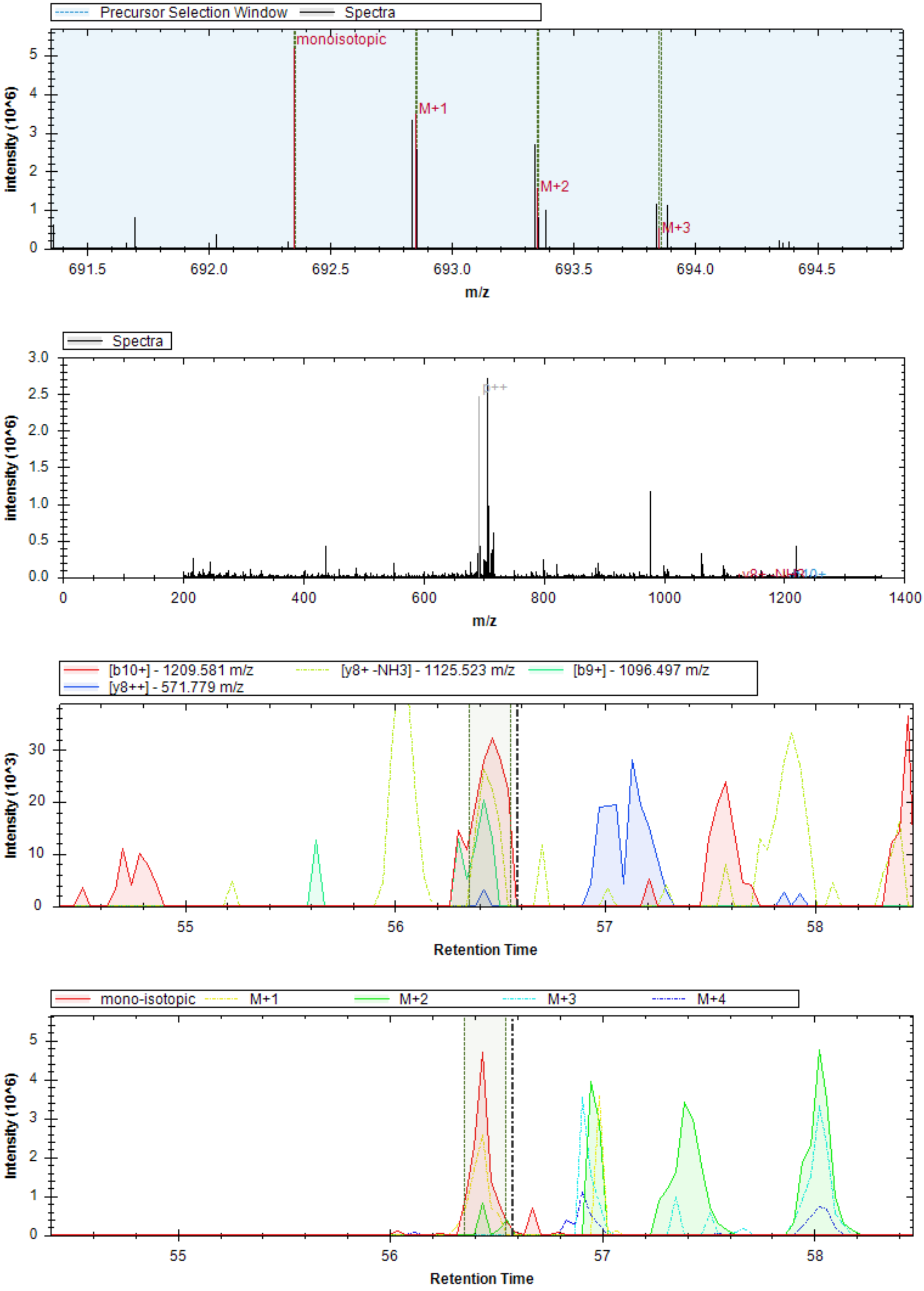

Q8C5R2 – _LAGNEALS[+H+O3+P]PTSPSK_.2
Fig4_MP-DIA-barrel-cortex-p54-3_MHRM.htrms

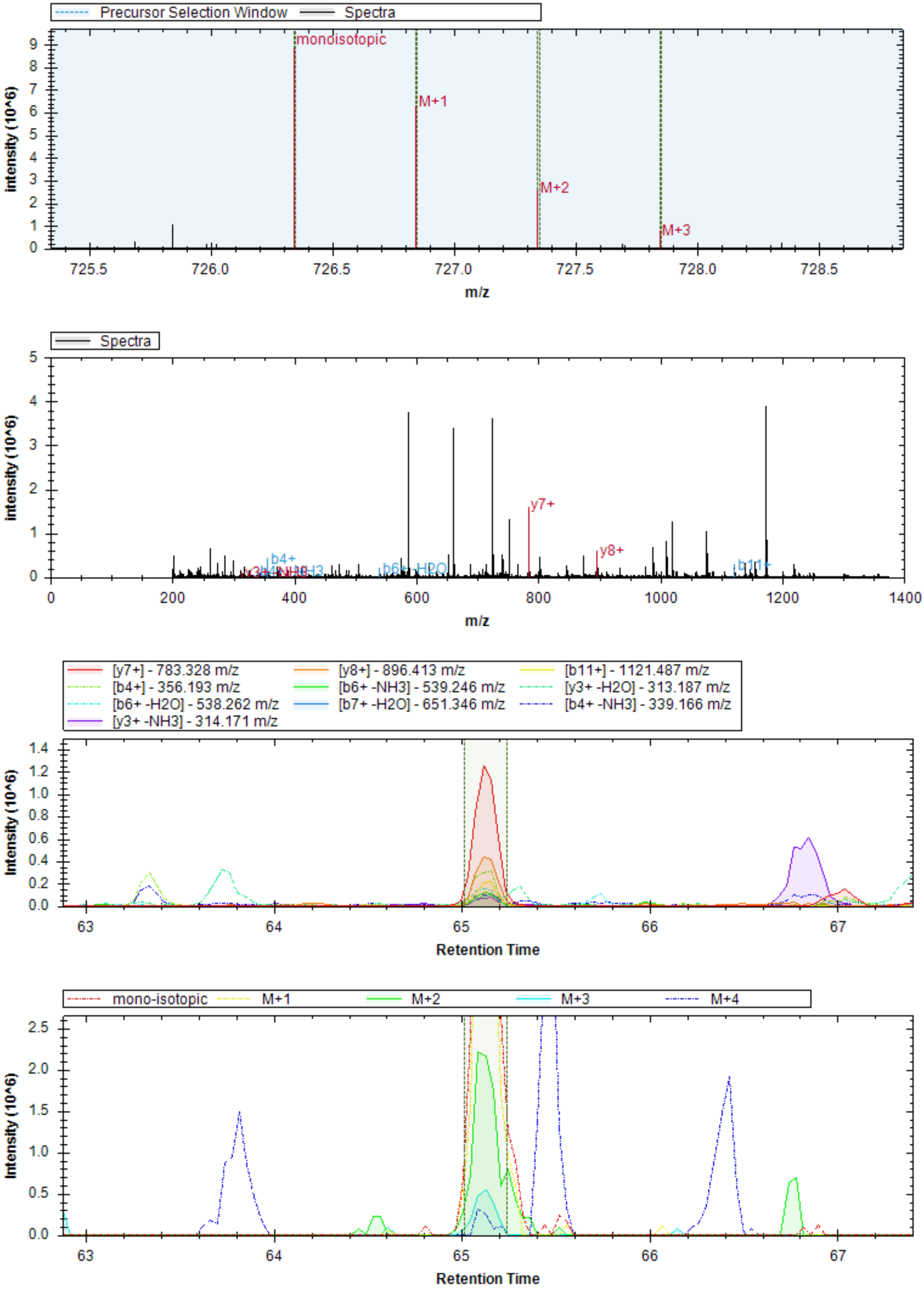

Q9CRB0 – _MEVYIPS[+H+O3+P]FRHEDSDLER_.3
Fig4_MP-DIA-barrel-cortex-p54-3_MHRM.htrms

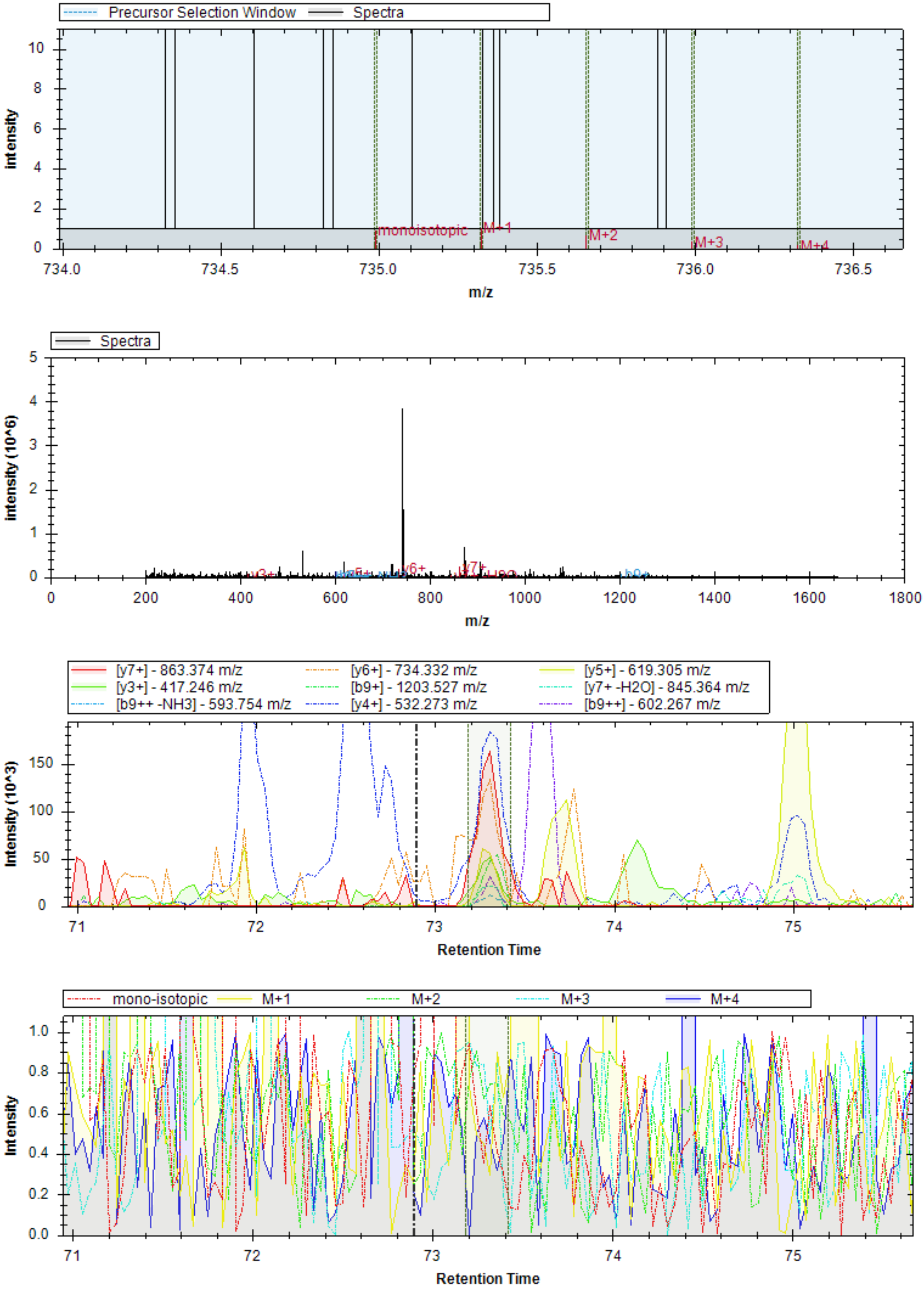

D3Z6S9 – _IPSIEIT[+H+O3+P]ET[+H+O3+P]LPQTQASMLR_.3
Fig4_MP-DIA-barrel-cortex-p54-3_MHRM.htrms

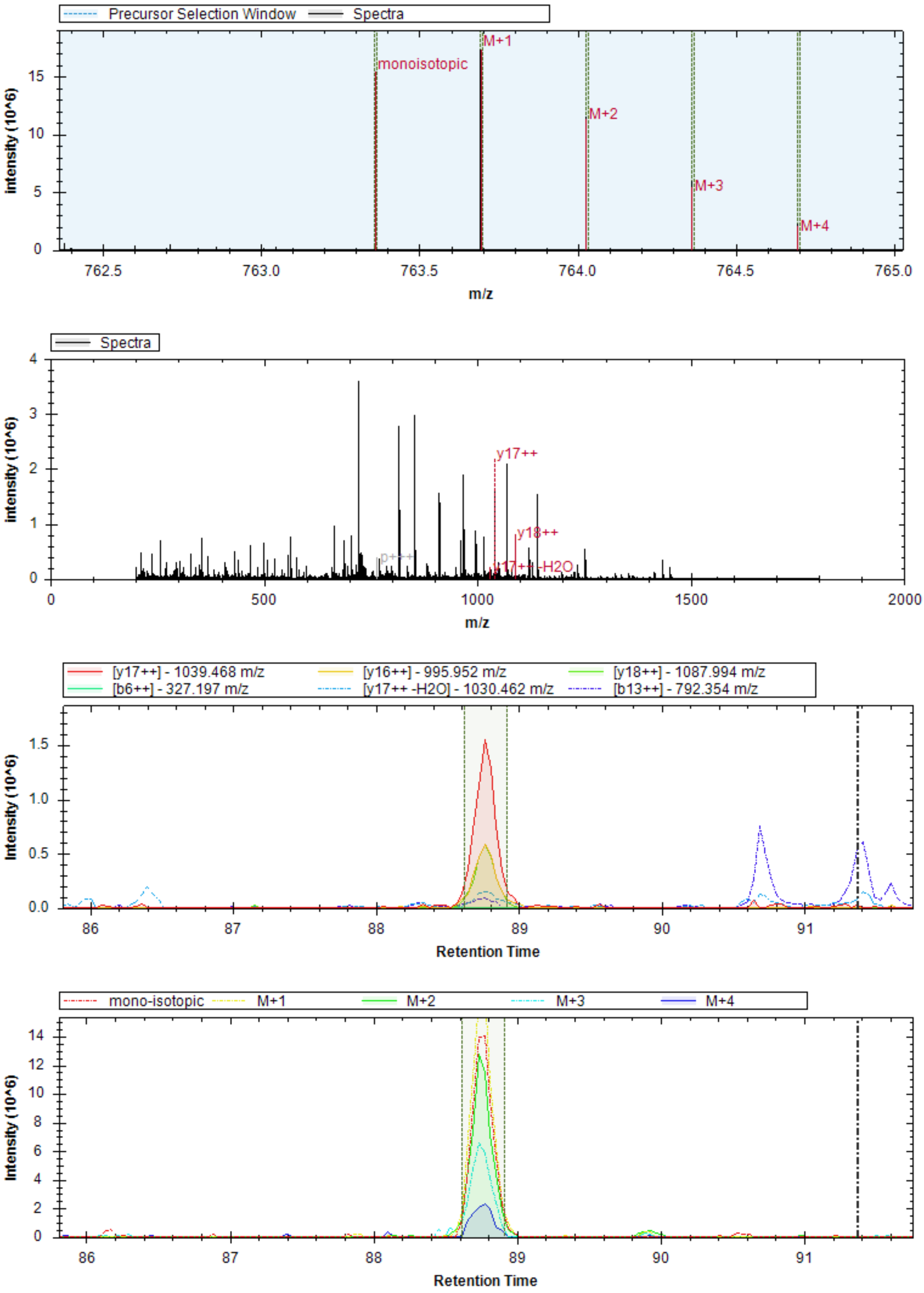

P06837 – _QADVPAAVTDAAAT[+H+O3+P]TPAAEDAATK_.3

Fig4_MP-DIA-barrel-cortex-p54-3_MHRM.htrms

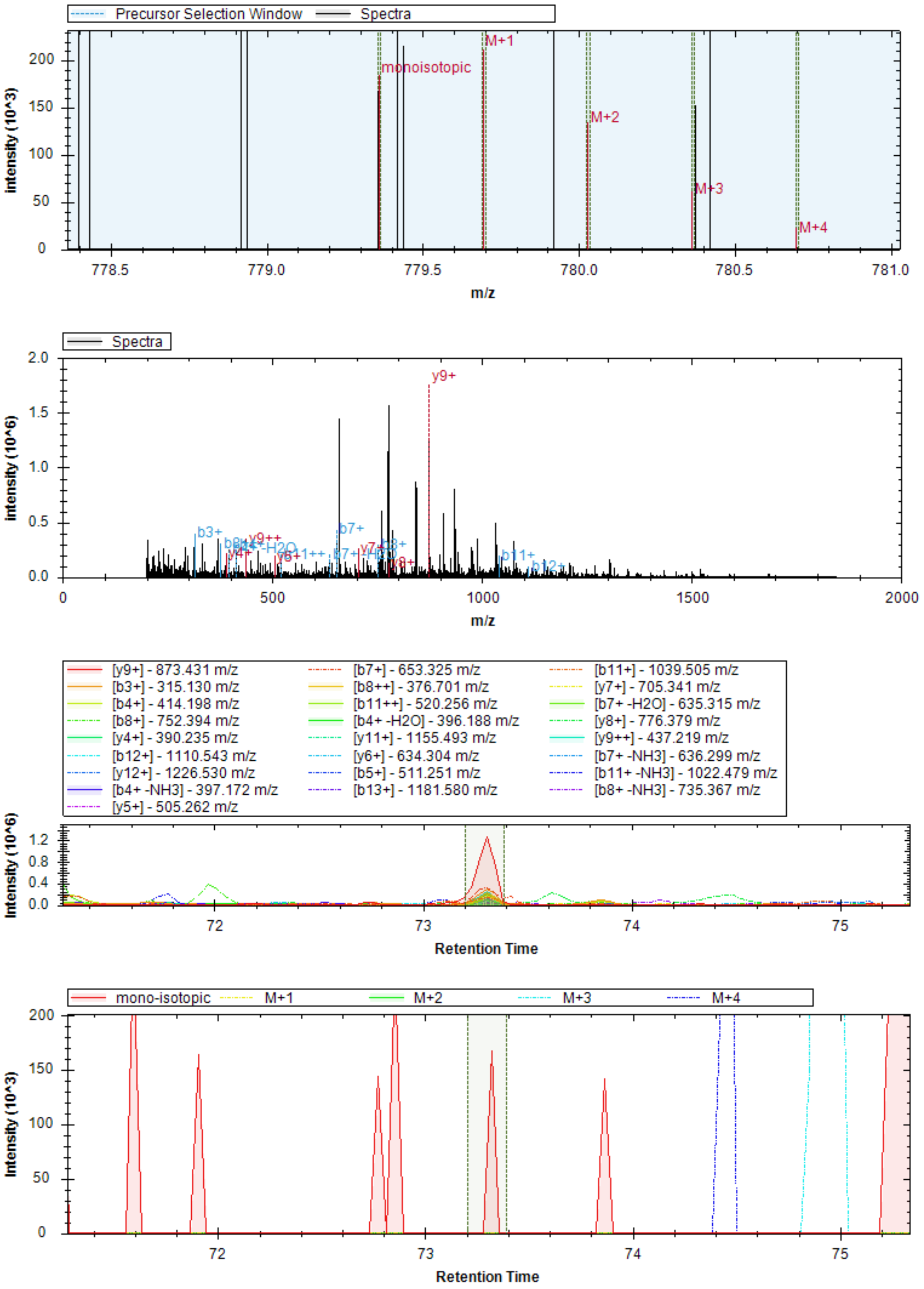

P0C6F1 – _IIDLQM[+O]HDY[+H+O3+P]LR_.2
Fig4_MP-DIA-barrel-cortex-p54-3_MHRM.htrms

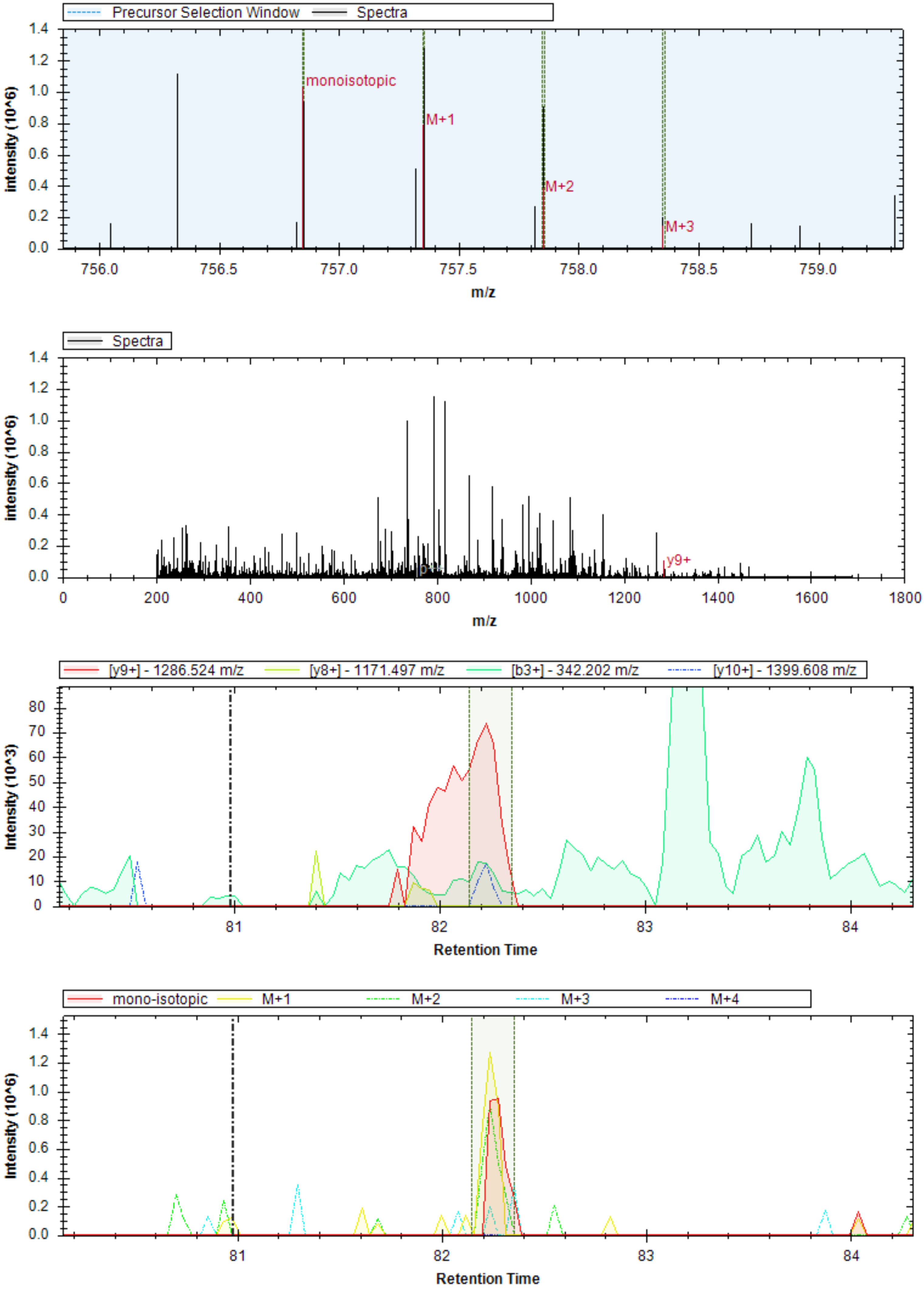

Q68FD9 – _LAQLLS[+H+O3+P]EVPT[+H+O3+P]RR_.2

Fig4_MP-DIA-barrel-cortex-p54-3_MHRM.htrms

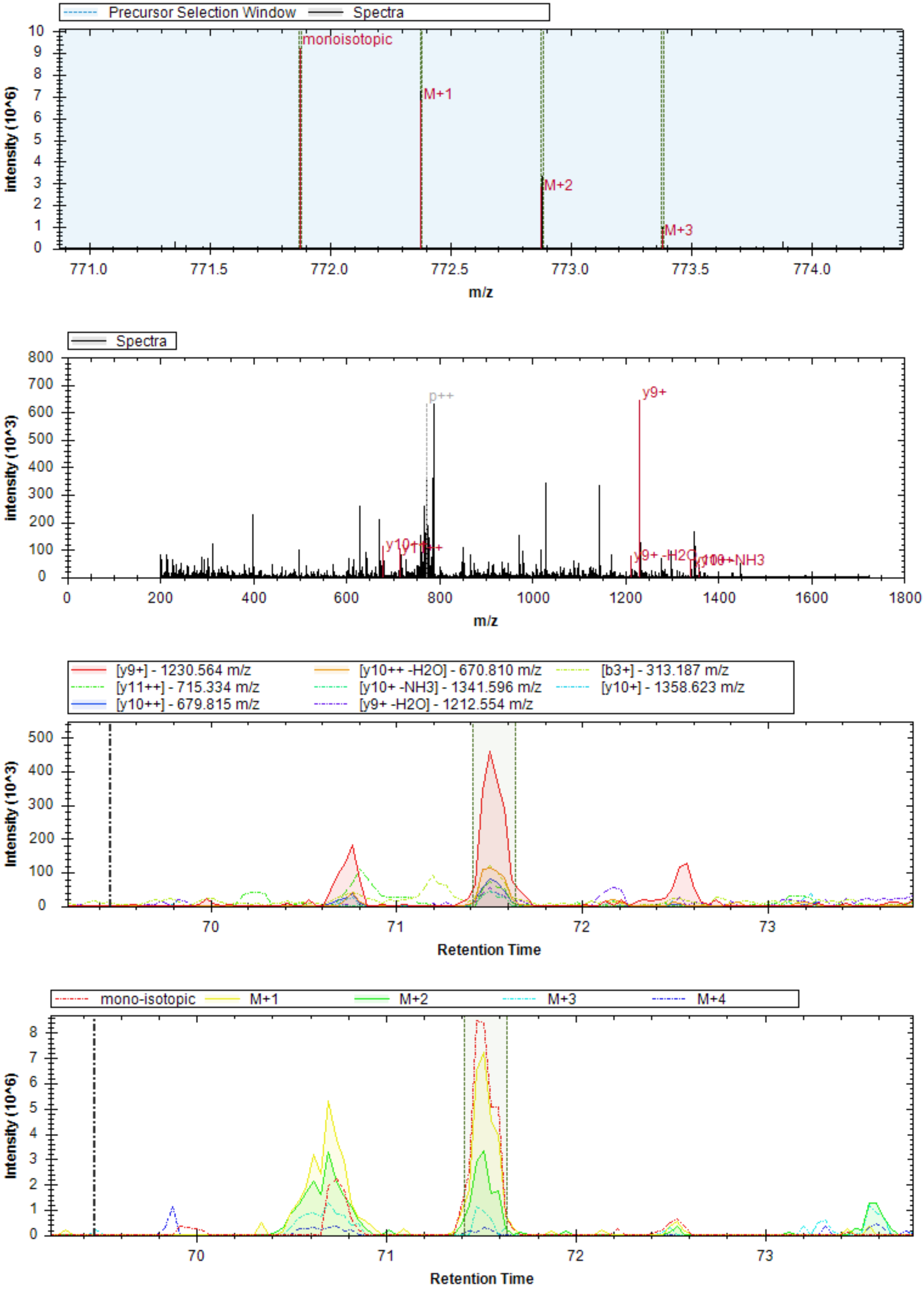

Q9QYC0 – _AAVVTS[+H+O3+P]PPPTTAPHK_.2
Fig4_MP-DIA-barrel-cortex-p54-3_MHRM.htrms

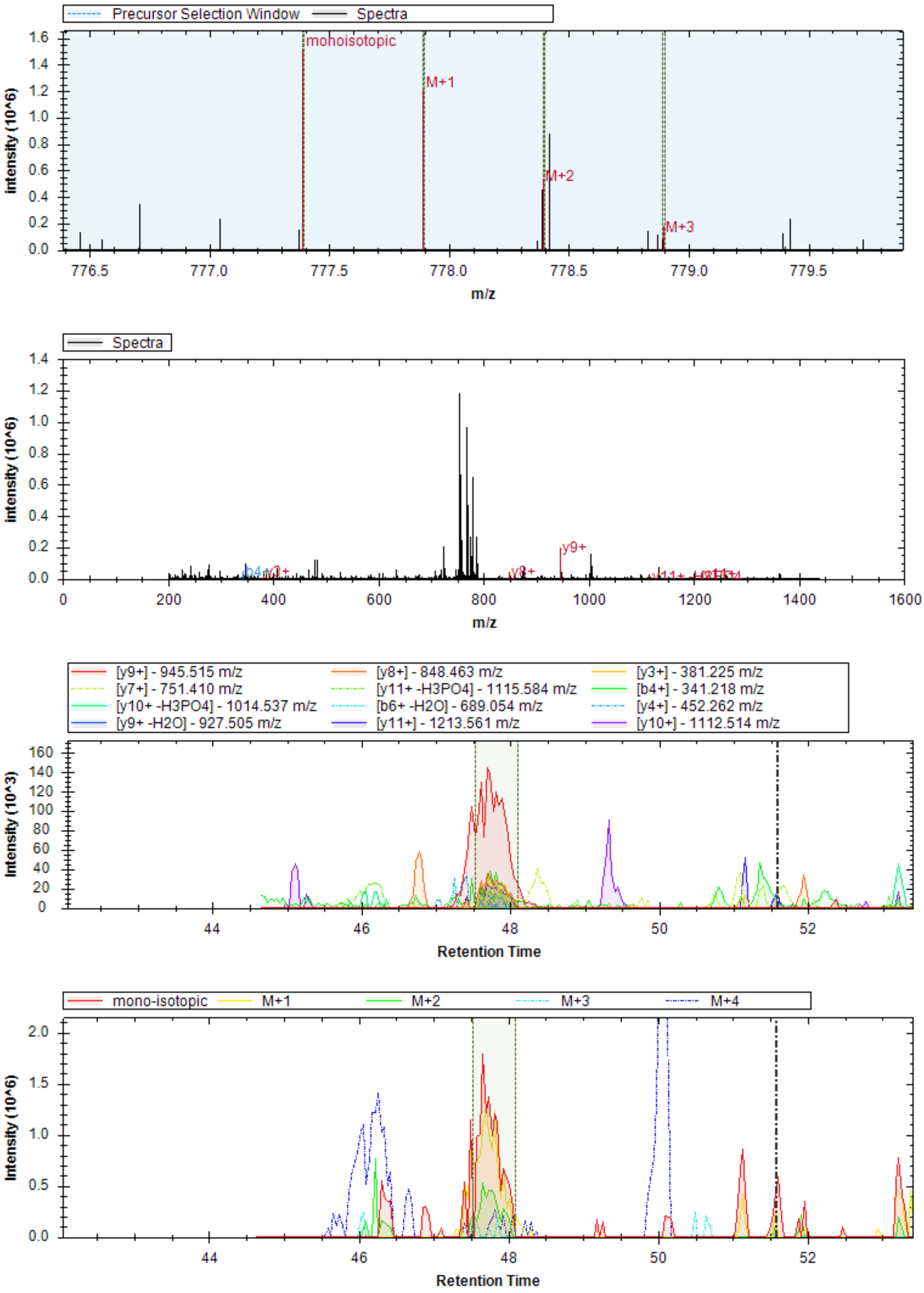

P10637 – _TTPSPKT[+H+O3+P]PPGSGEPPK_.2

Fig4_MP-DIA-barrel-cortex-p54-3_MHRM.htrms

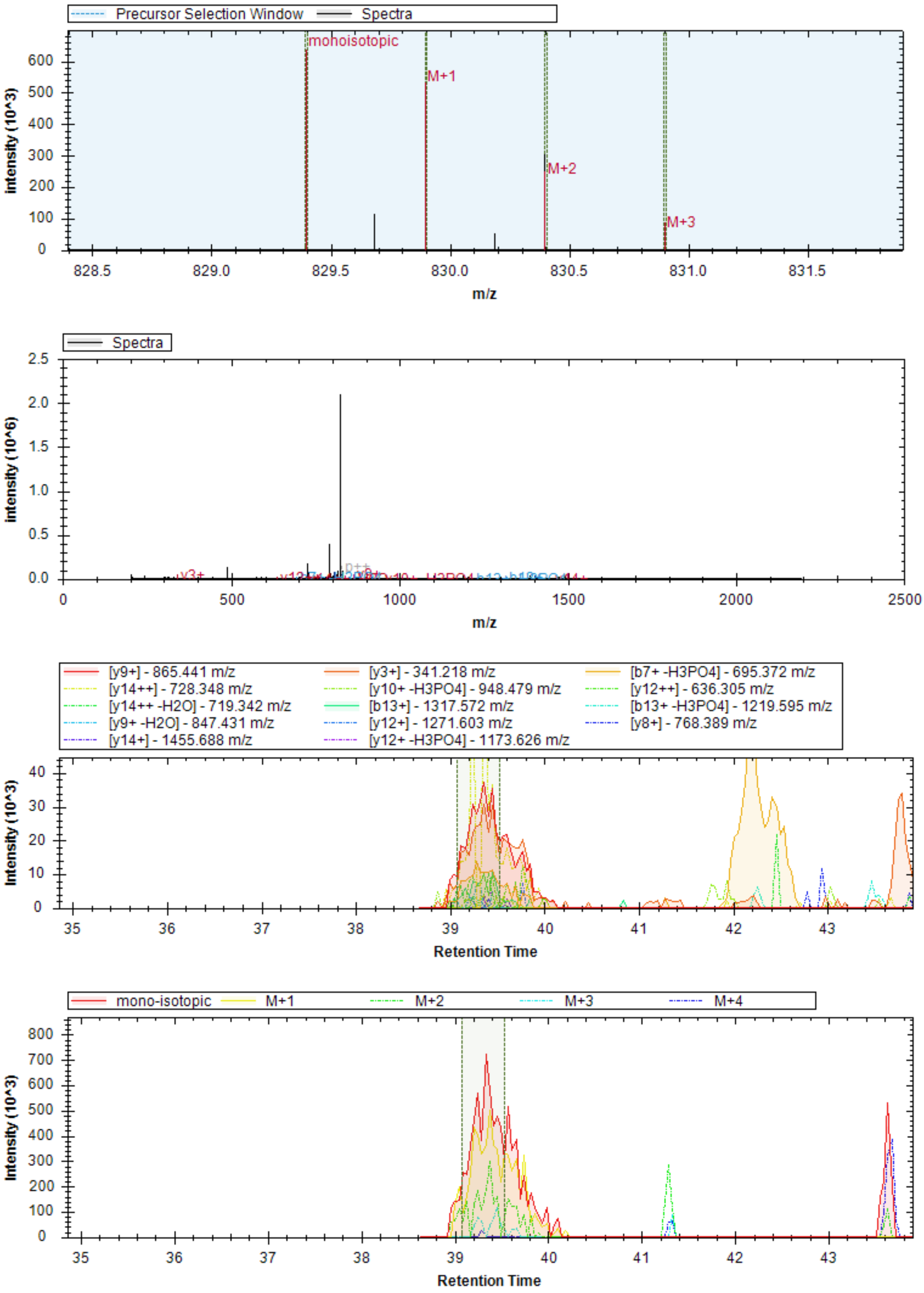

P20357 – _VDHGAEIIT[+H+O3+P]QSPSR_.2

Fig4_MP-DIA-barrel-cortex-p54-3_MHRM.htrms

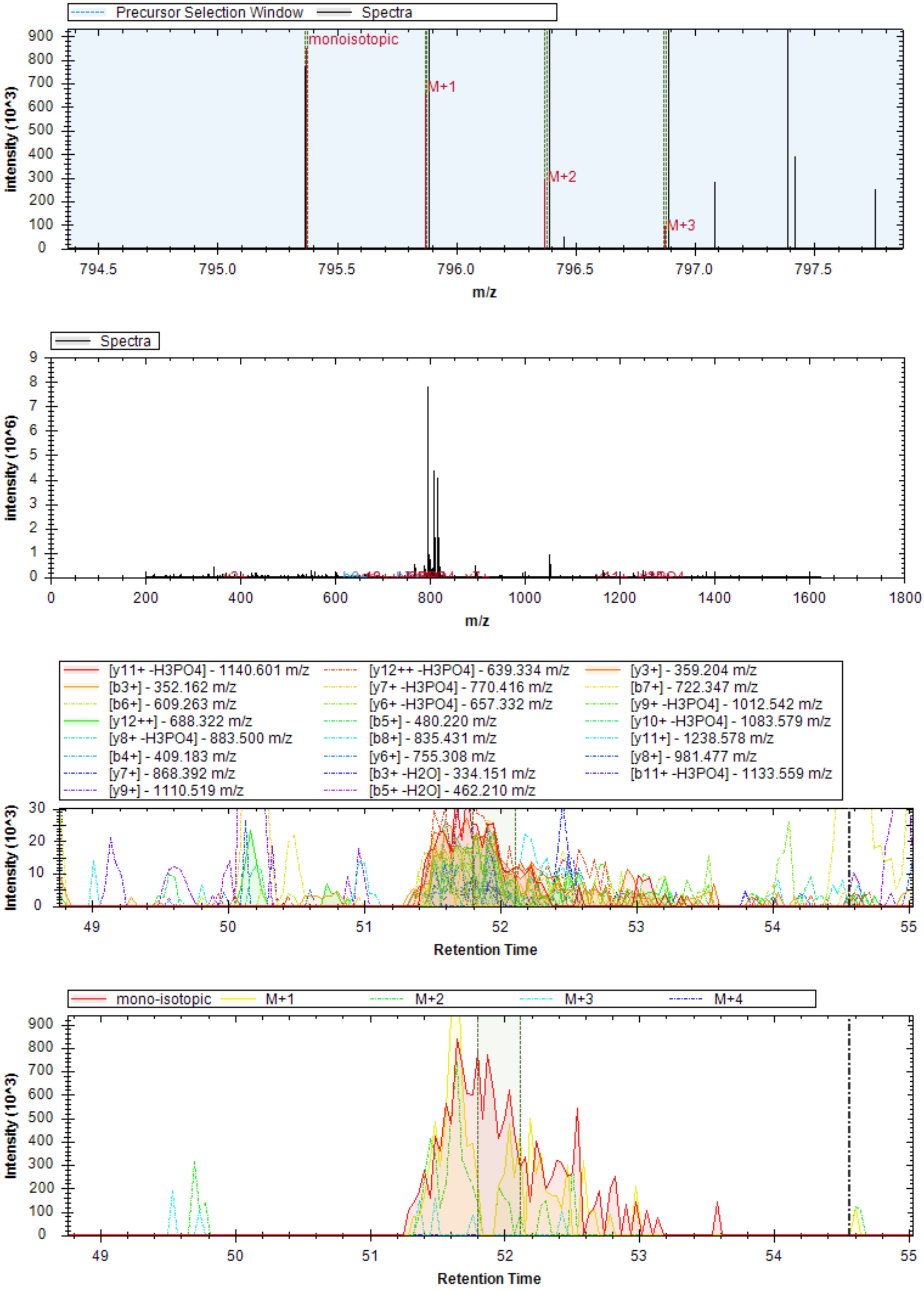

P20357 – _VDHGAEIITQSPS[+H+O3+P]R_.2
Fig4_MP-DIA-barrel-cortex-p54-3_MHRM.htrms

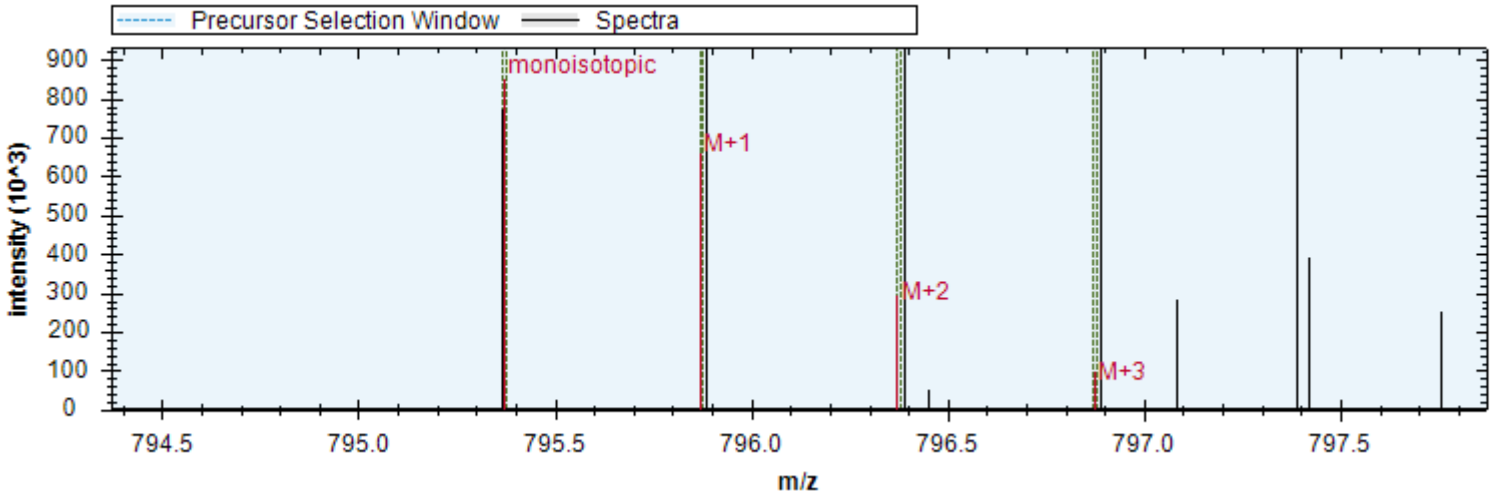

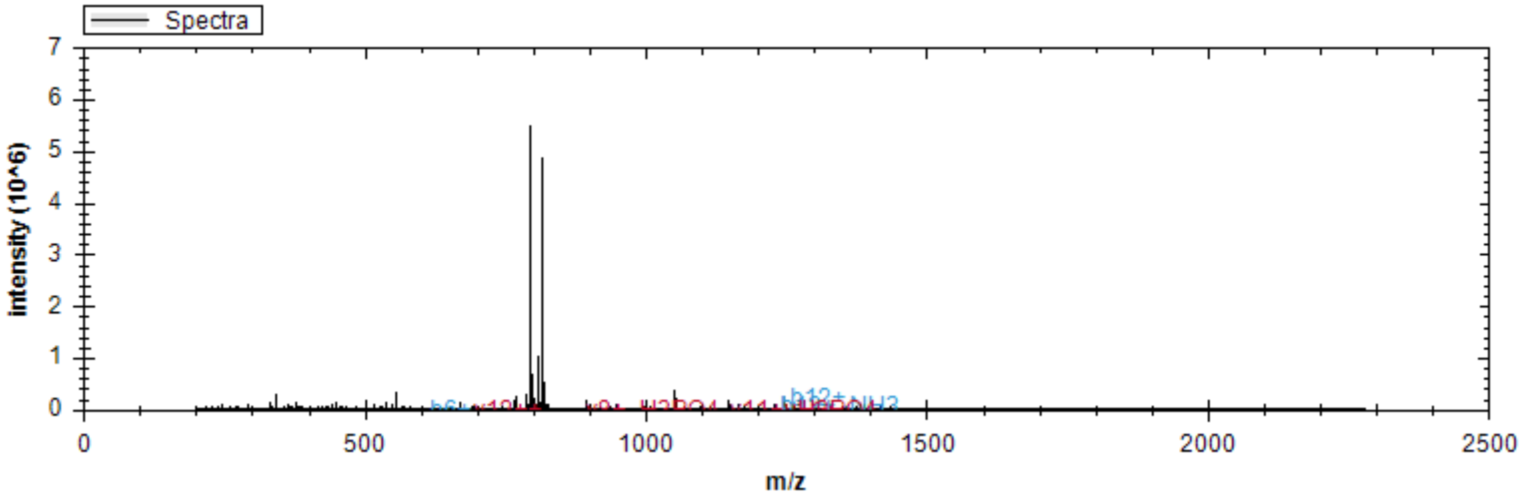

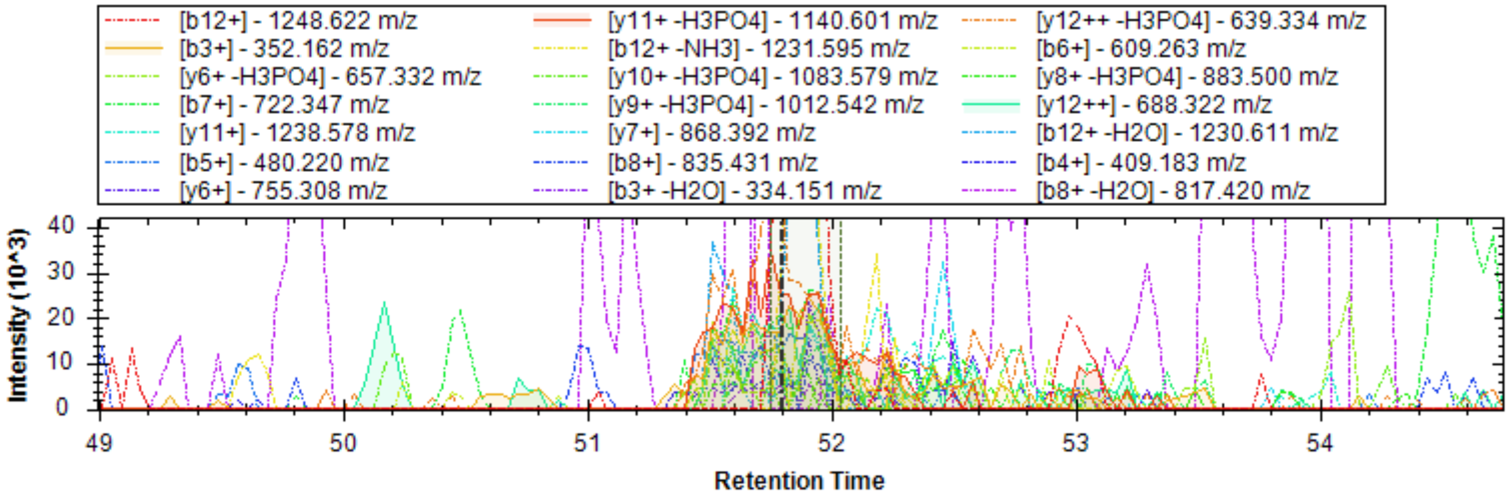

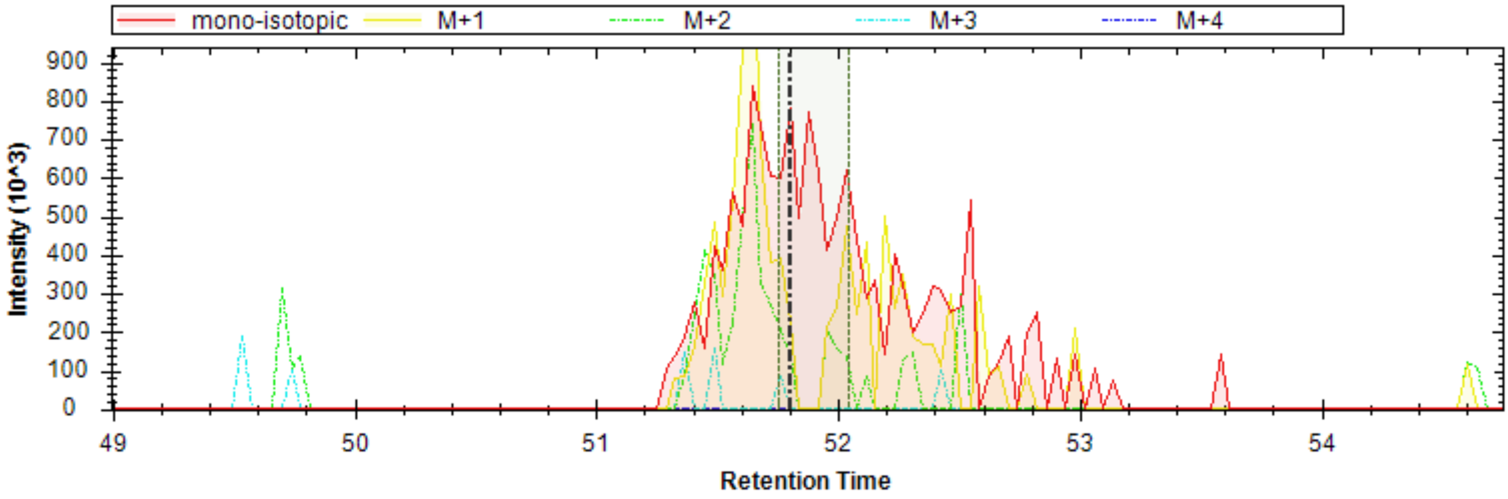

Q60771 – _FYYSS[+H+O3+P]GSSSPTHAK_.2

Fig4_MP-DIA-barrel-cortex-p54-3_MHRM.htrms

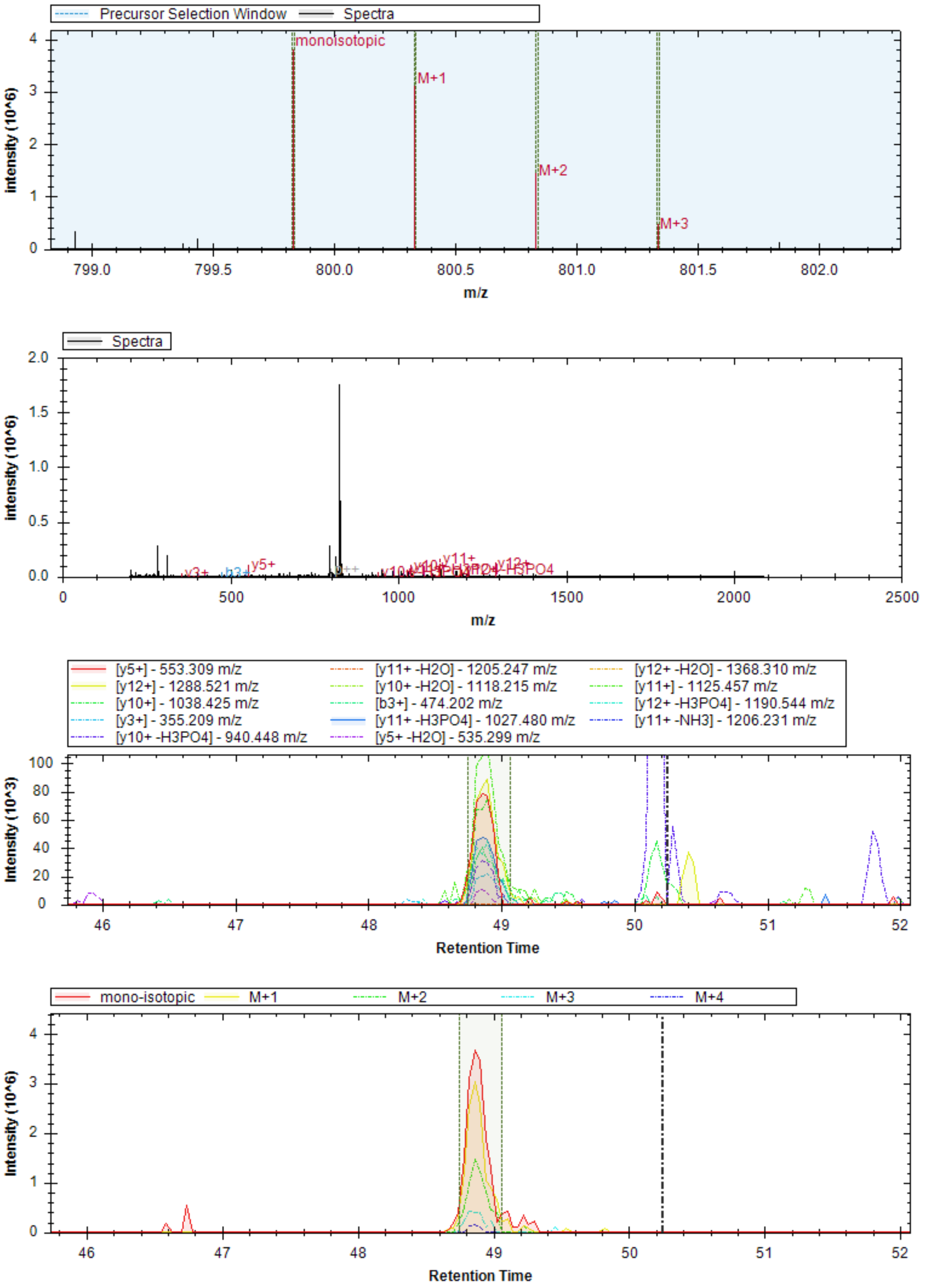

Q60771 – _FYYSSGS[+H+O3+P]SSPTHAK_.2
Fig4_MP-DIA-barrel-cortex-p54-3_MHRM.htrms

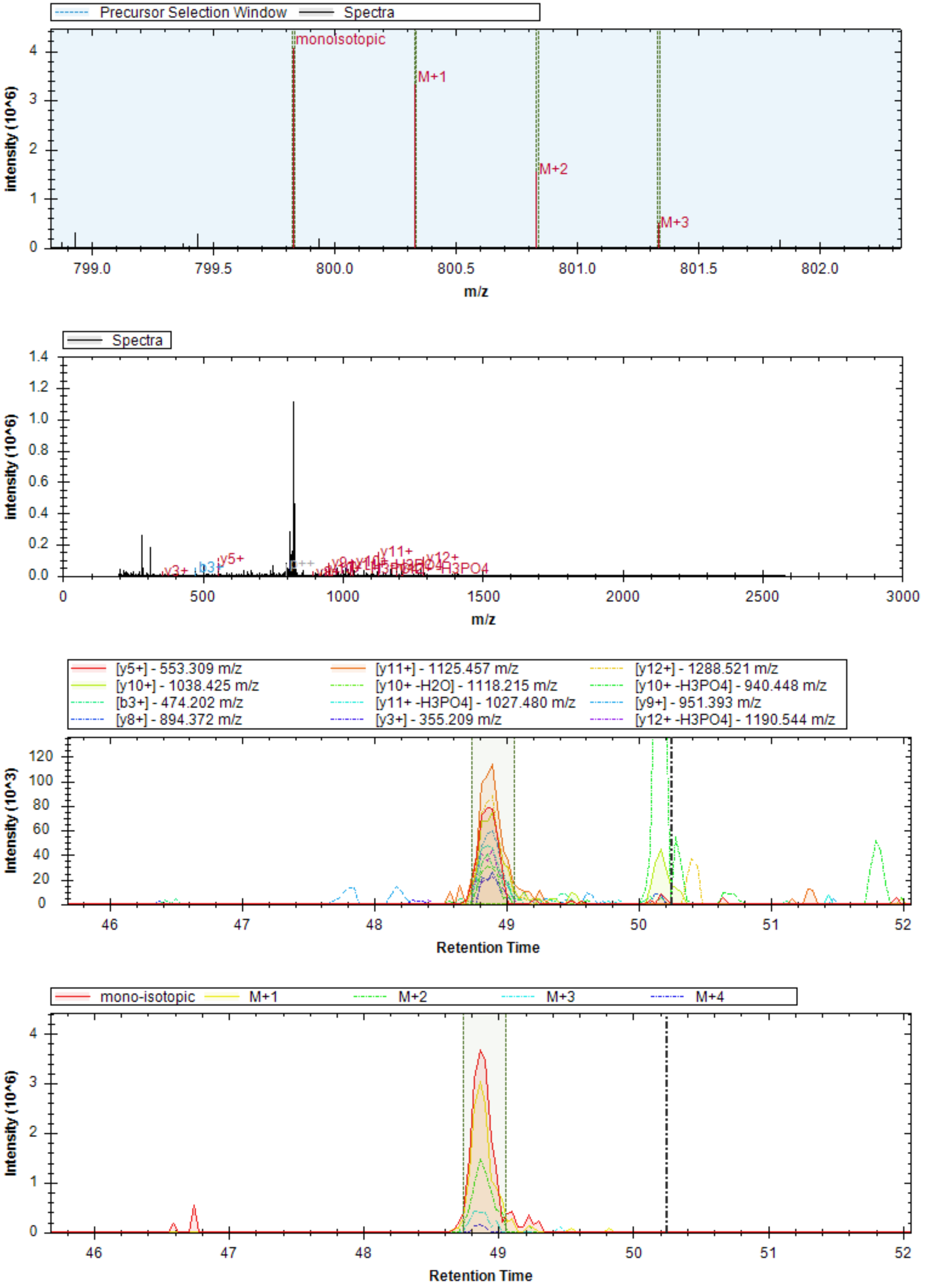

Q60771 – _FYYSSGSSS[+H+O3+P]PTHAK_.2
Fig4_MP-DIA-barrel-cortex-p54-3_MHRM.htrms

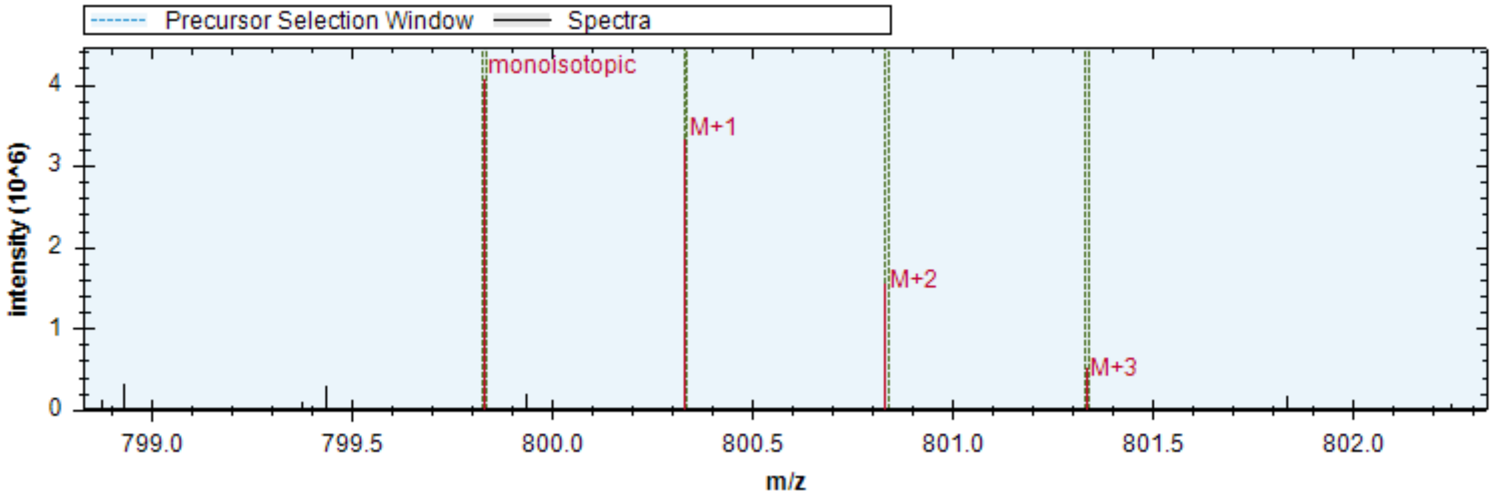

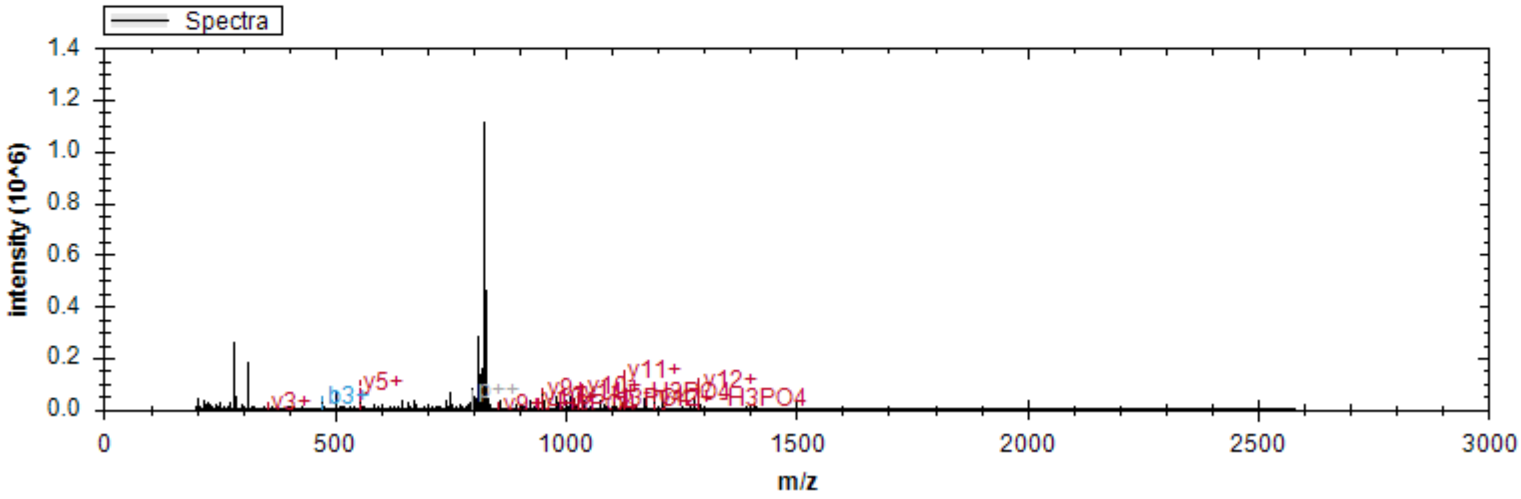

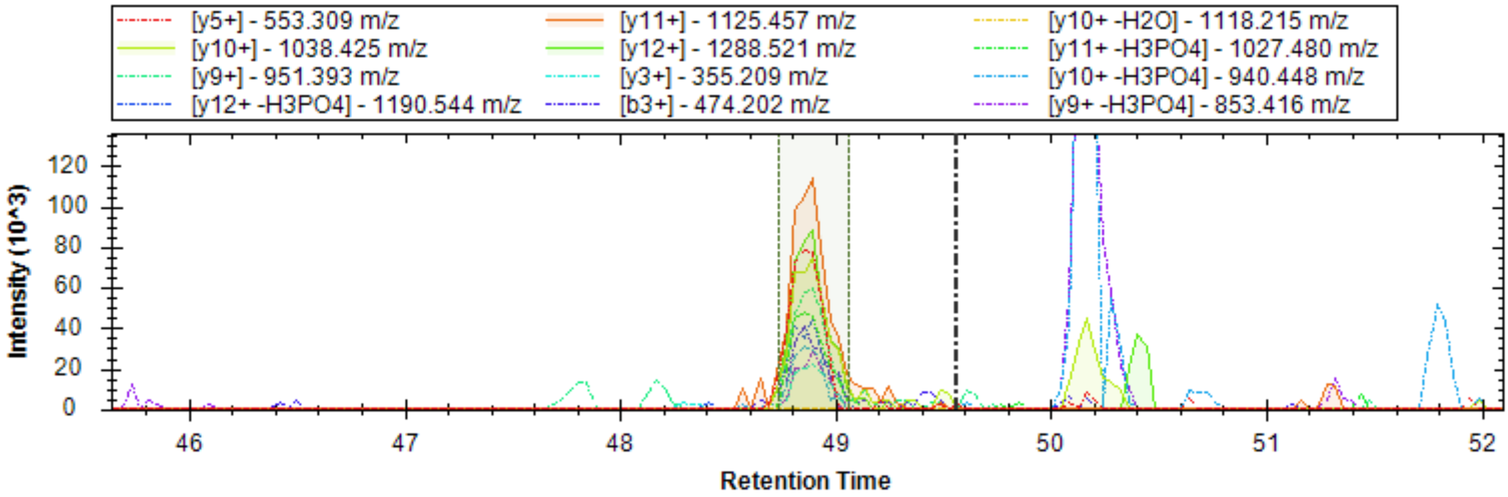

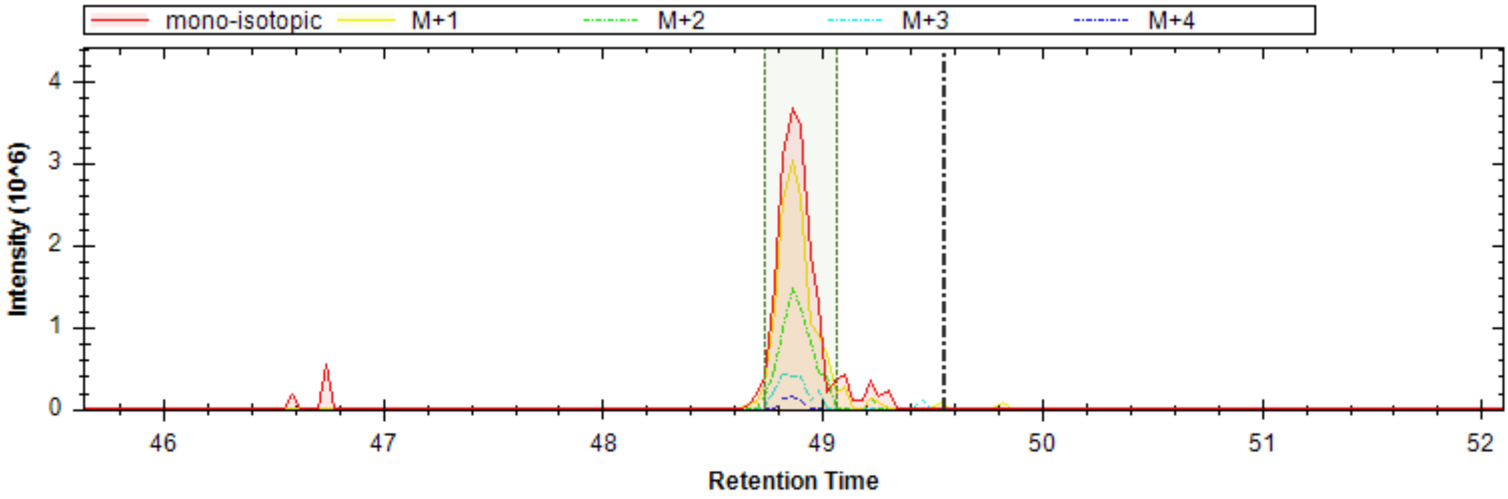

Q64701;Q64701-2 – _LAEILYYKILET[+H+O3+P]IMVQET[+H+O3+P]R_.3
Fig4_MP-DIA-barrel-cortex-p54-3_MHRM.htrms

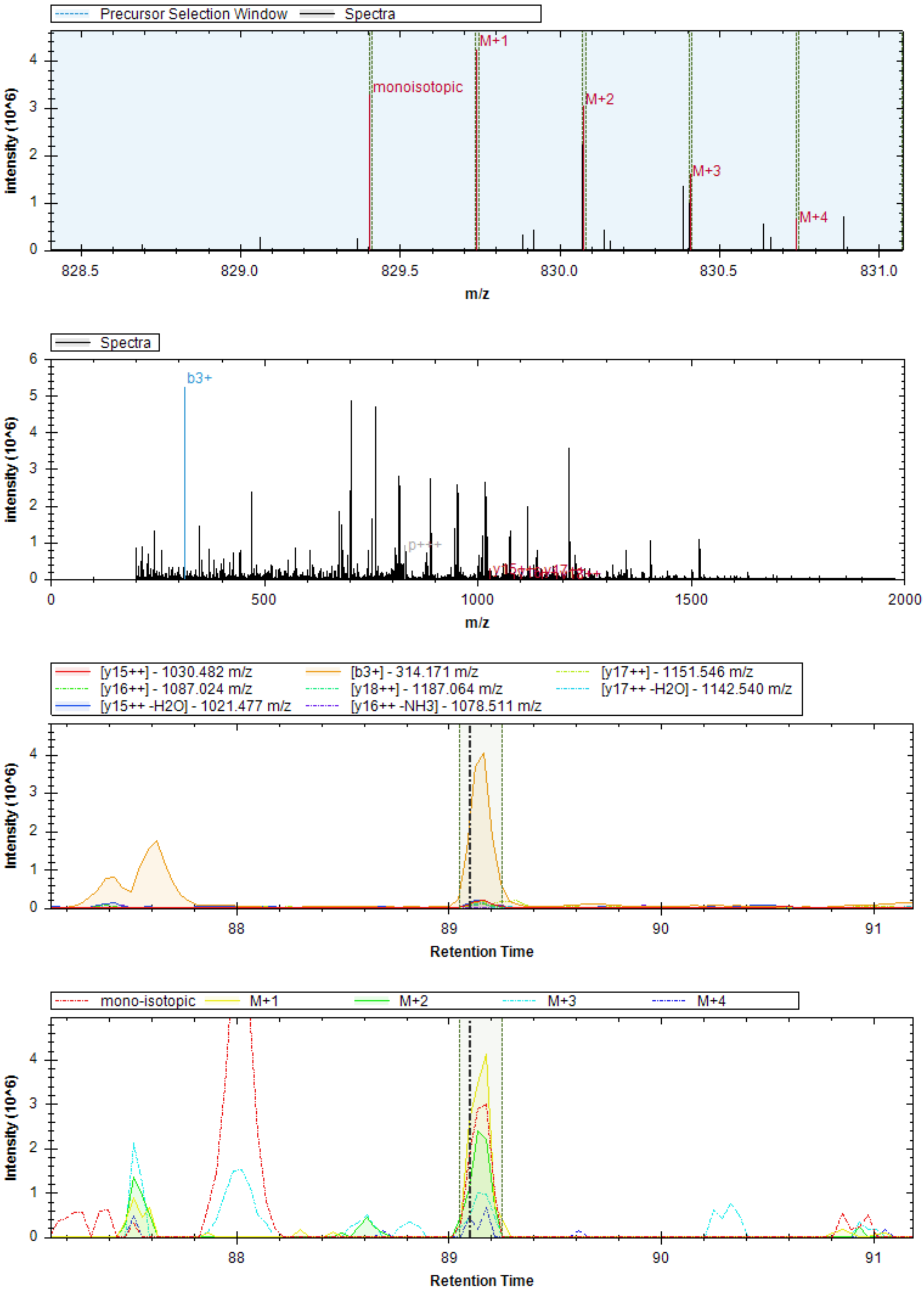

Q7TQD2 – _AANKT[+H+O3+P]PPKSPGDPAR_.2
Fig4_MP-DIA-barrel-cortex-p54-3_MHRM.htrms

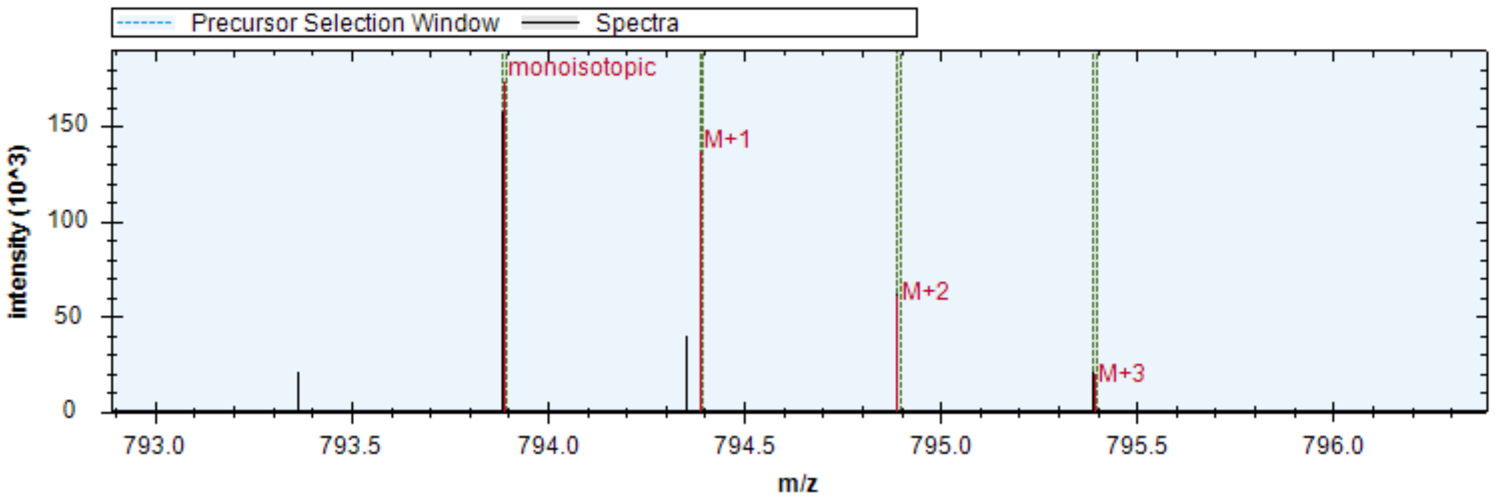

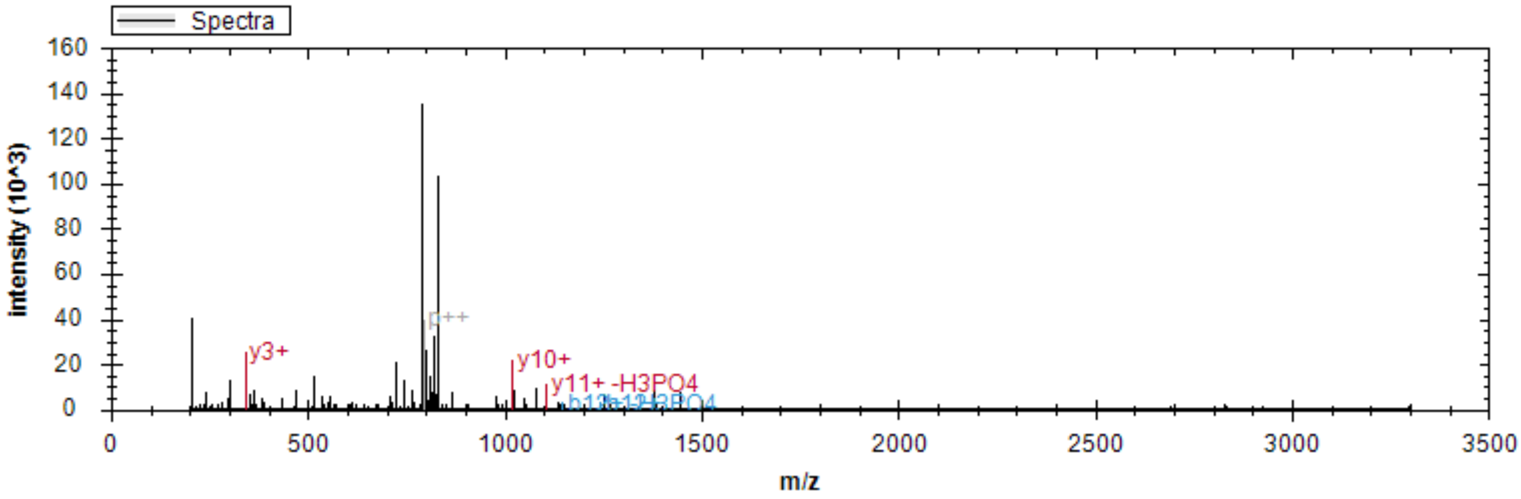

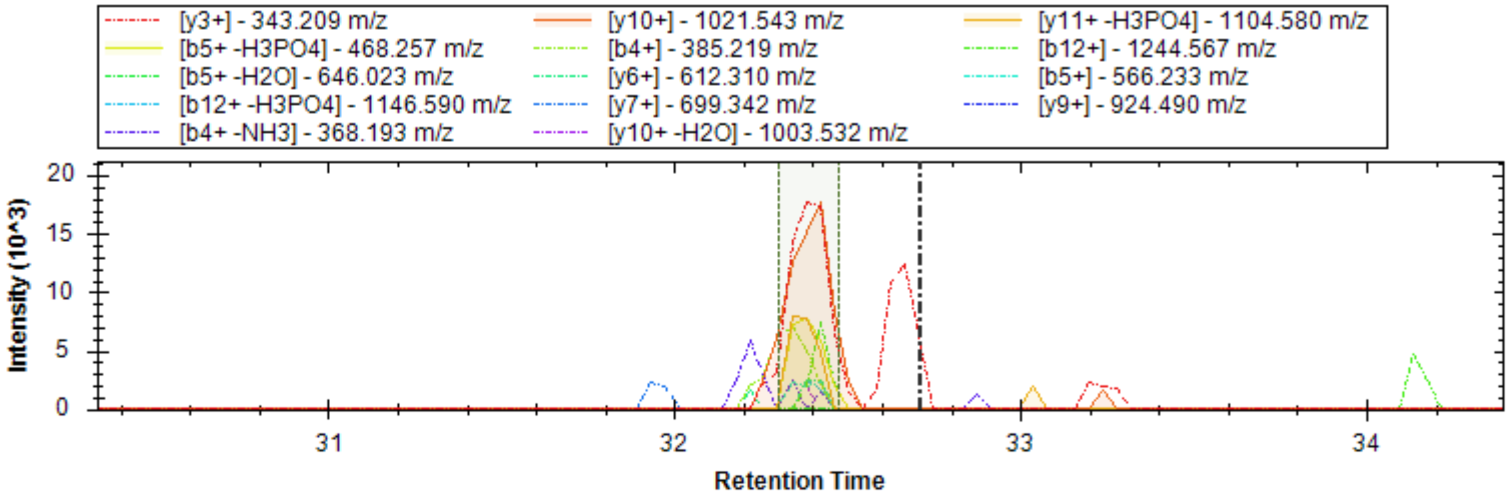

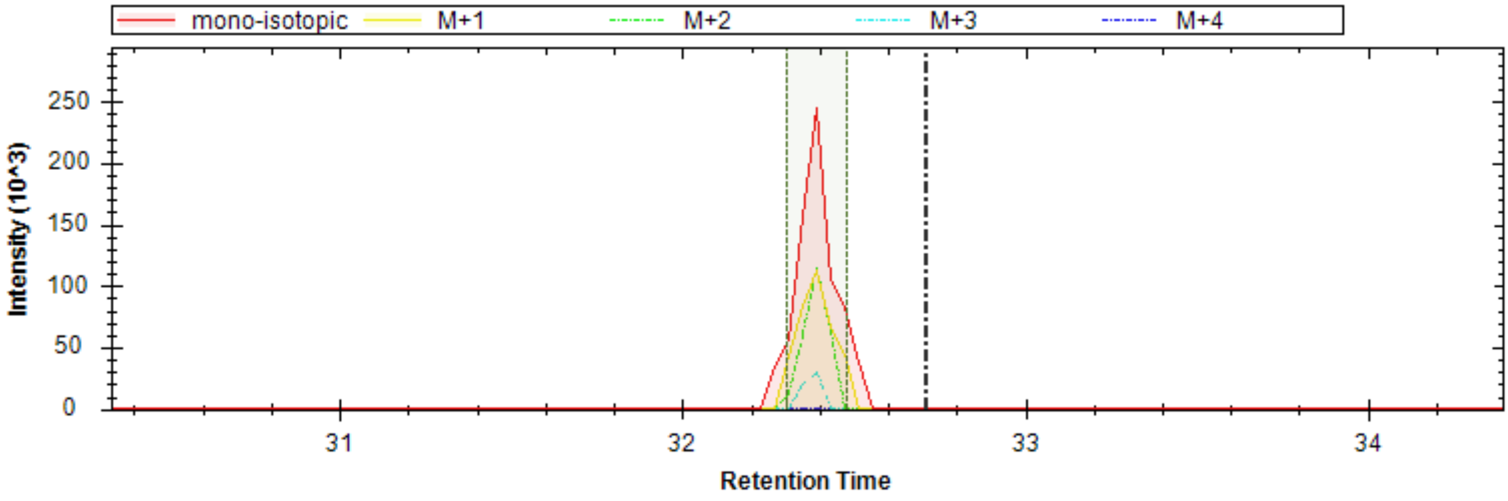

O08784 – _ASAVSPEKAPM[+O]T[+H+O3+P]SKAK_.2

Fig4_MP-DIA-barrel-cortex-p54-3_MHRM.htrms

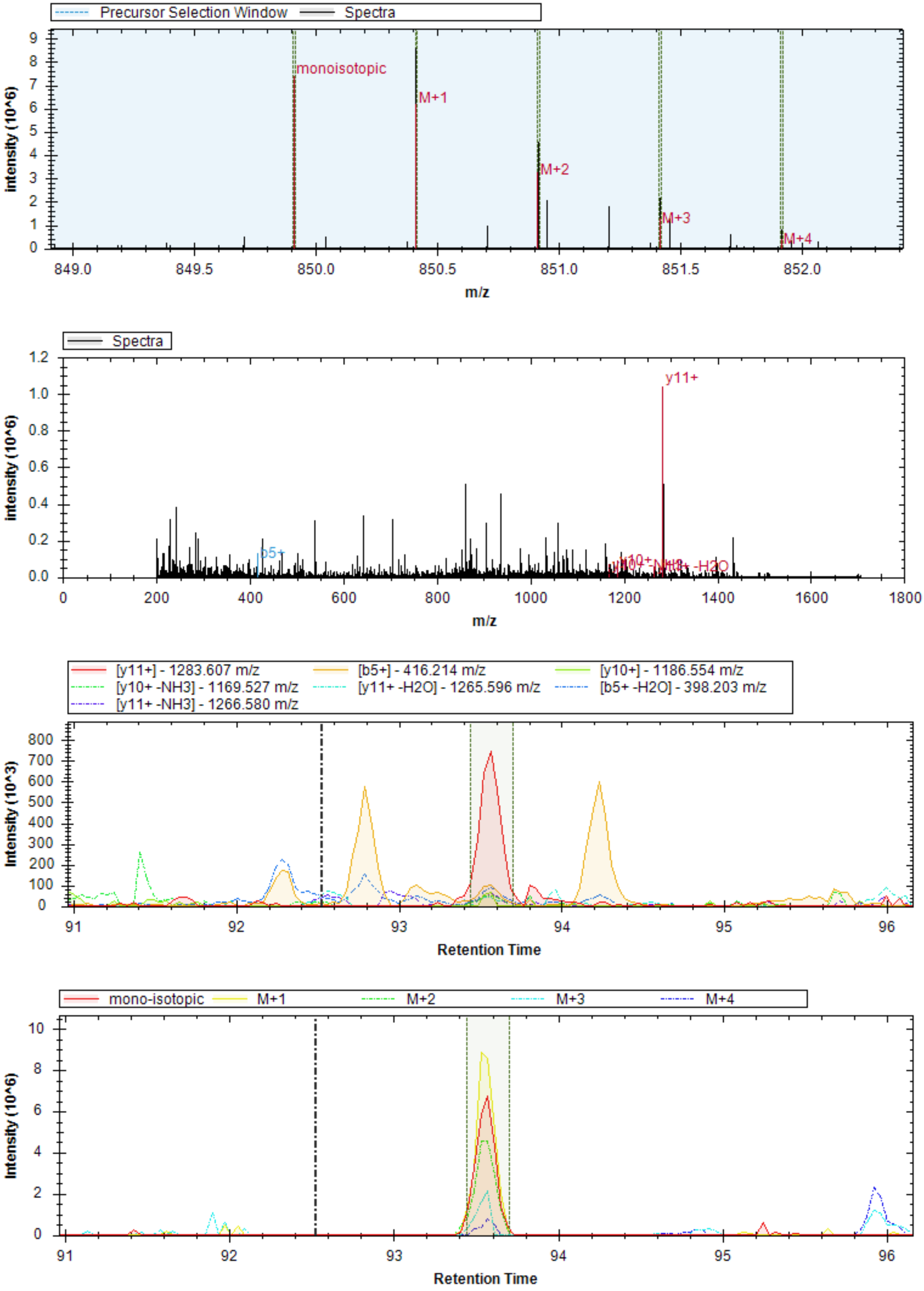

P14873 – _ETAAAHQASS[+H+O3+P]SPPIDAATAEPYGFR_.3
Fig4_MP-DIA-barrel-cortex-p54-3_MHRM.htrms

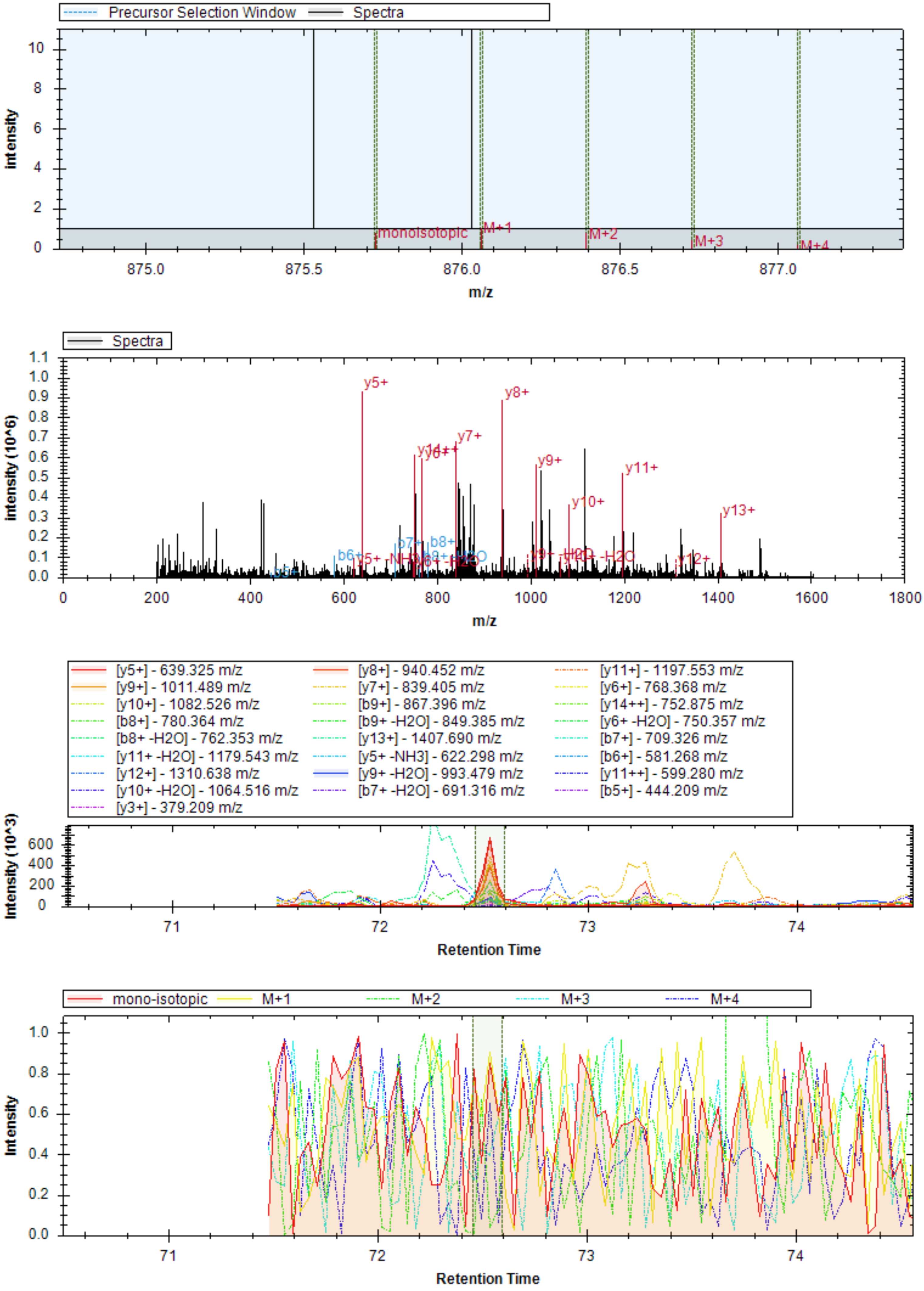

P14873 – _ETAAAHQASSS[+H+O3+P]PPIDAATAEPYGFR_.3

Fig4_MP-DIA-barrel-cortex-p54-3_MHRM.htrms

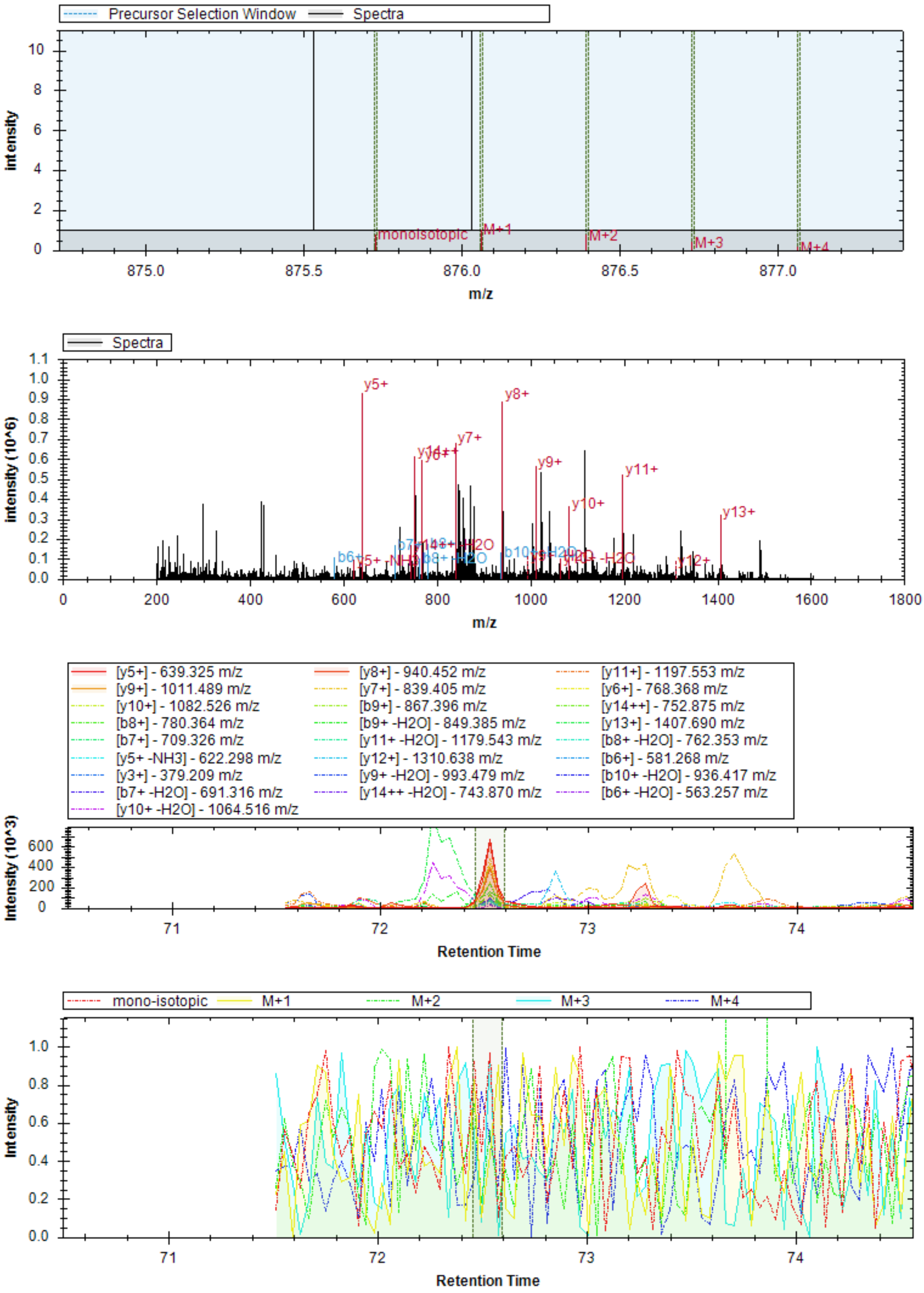

P20917 – _NVTESSS[+H+O3+P]FSGGDNPHVLYSPEFR_.3

Fig4_MP-DIA-barrel-cortex-p54-3_MHRM.htrms

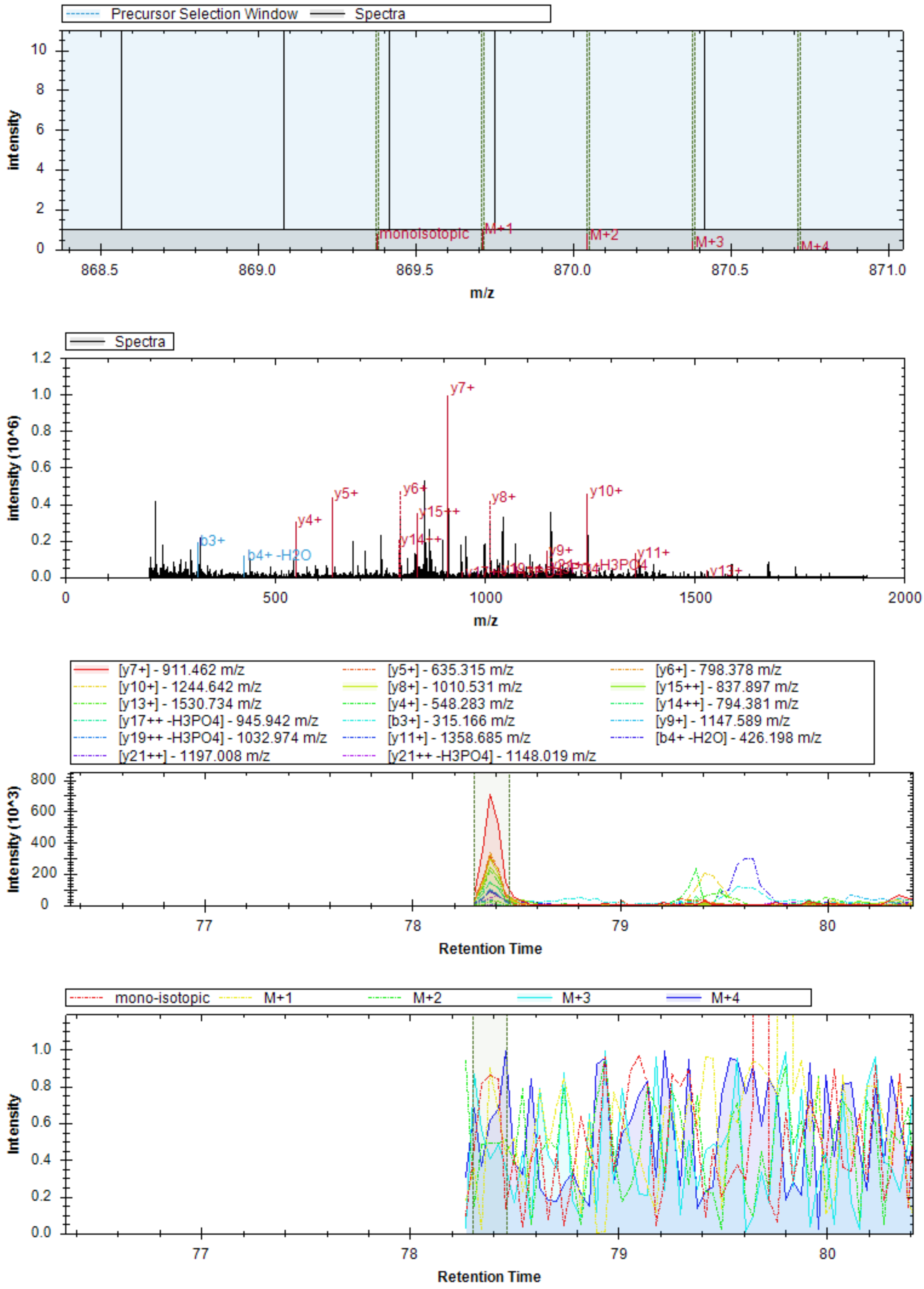

P20917 – _NVTESSSFS[+H+O3+P]GGDNPHVLYSPEFR_.3
Fig4_MP-DIA-barrel-cortex-p54-3_MHRM.htrms

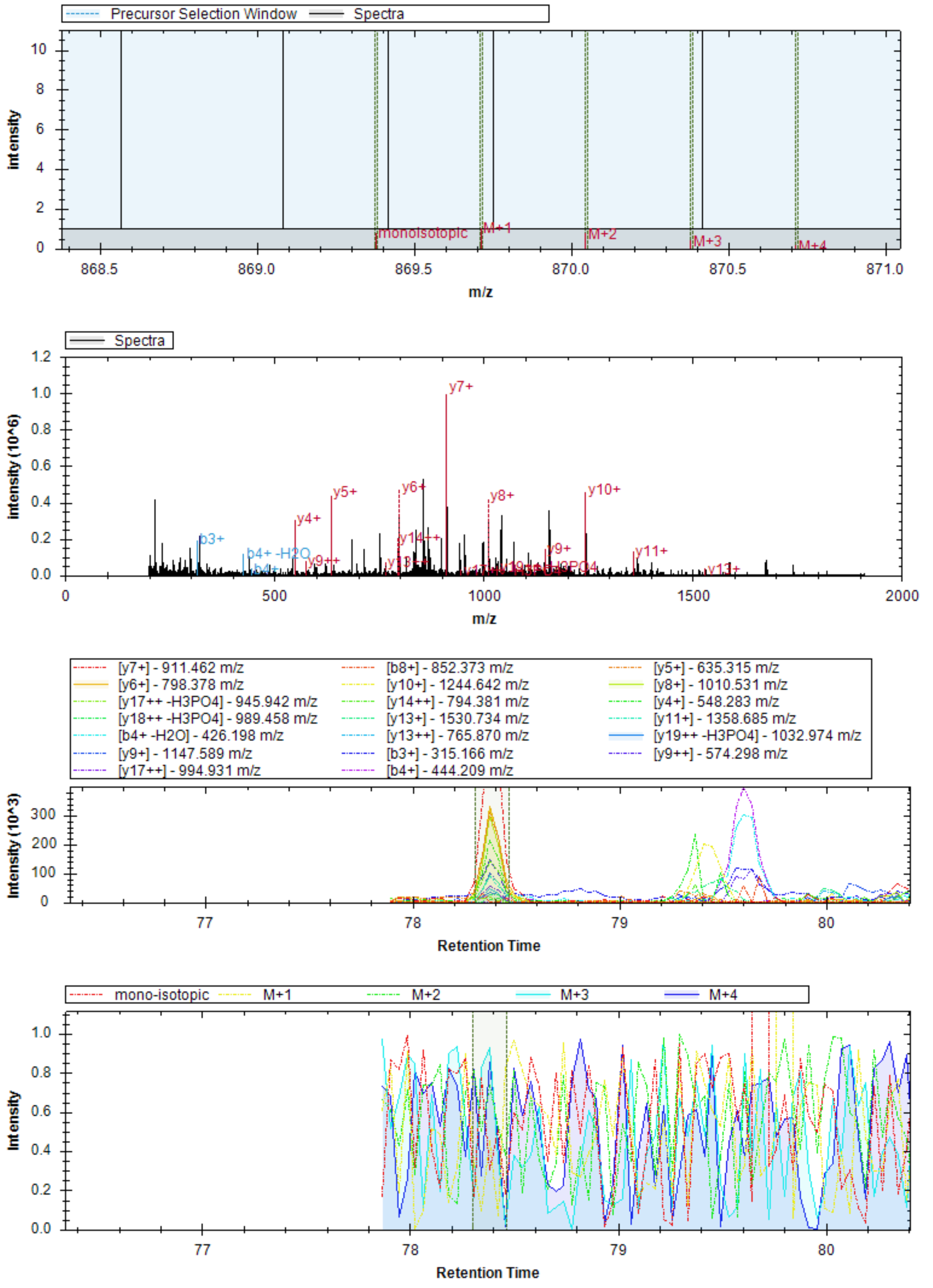

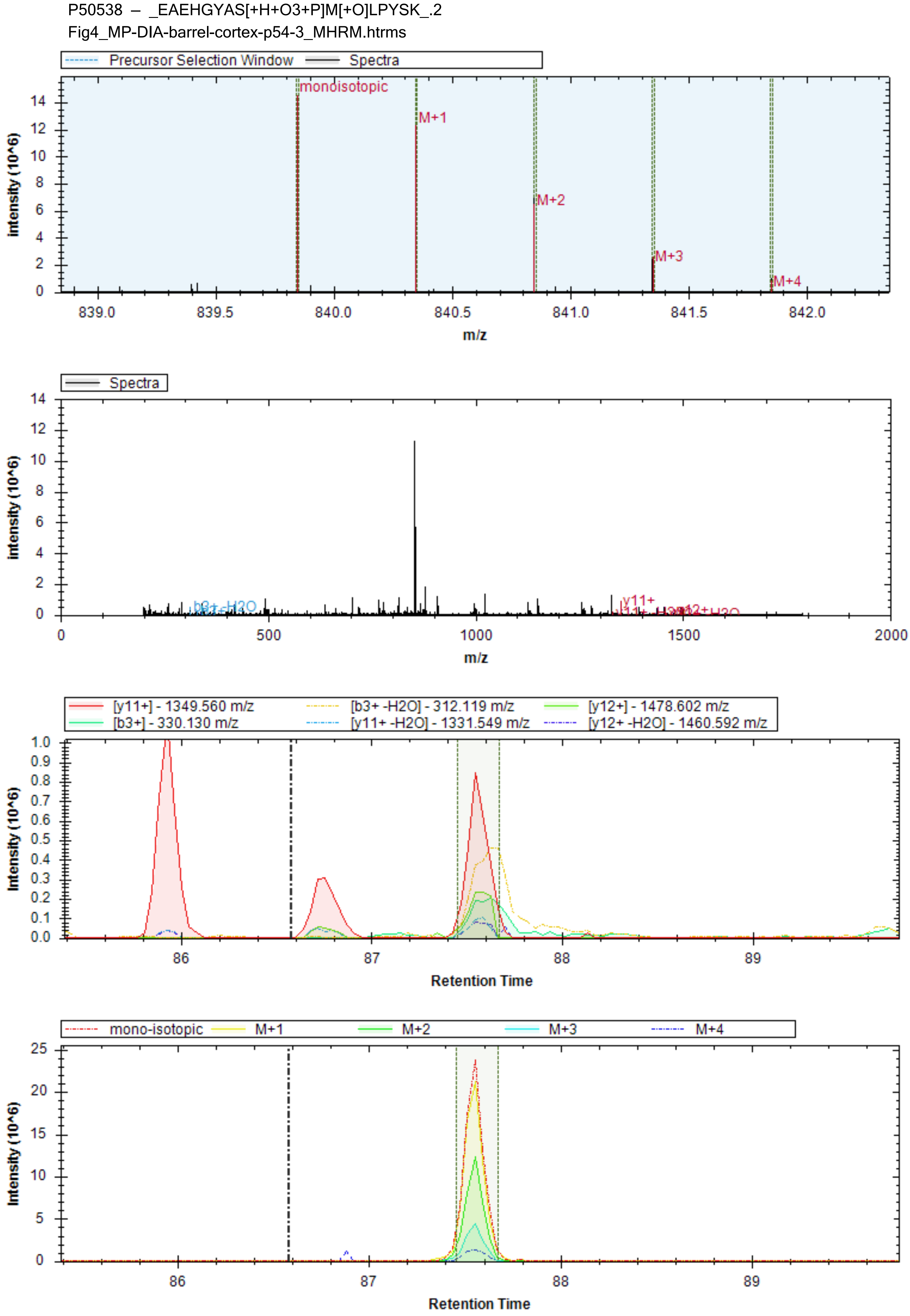
P50538 – _EAEHGYAS[+H+O3+P]M[+O]LPYSK_.2
Fig4_MP-DIA-barrel-cortex-p54-3_MHRM.htrms
Precursor Selection Window
Spectra
monoisotopic
M+1
M+2
M+3
M+4
intensity (10^6)
m/z
Spectra
intensity (10^6)
m/z
[y11+] - 1349.560 m/z
[b3+ -H2O] - 312.119 m/z
[y12+] - 1478.602 m/z
[b3+] - 330.130 m/z
[y11+ -H2O] - 1331.549 m/z
[y12+ -H2O] - 1460.592 m/z
Intensity (10^6)
Retention Time
mono-isotopic
M+1
M+2
M+3
M+4
Intensity (10^6)
Retention Time

Q61029 – _SST[+H+O3+P]PLPTVSSSAENTR_.2

Fig4_MP-DIA-barrel-cortex-p54-3_MHRM.htrms

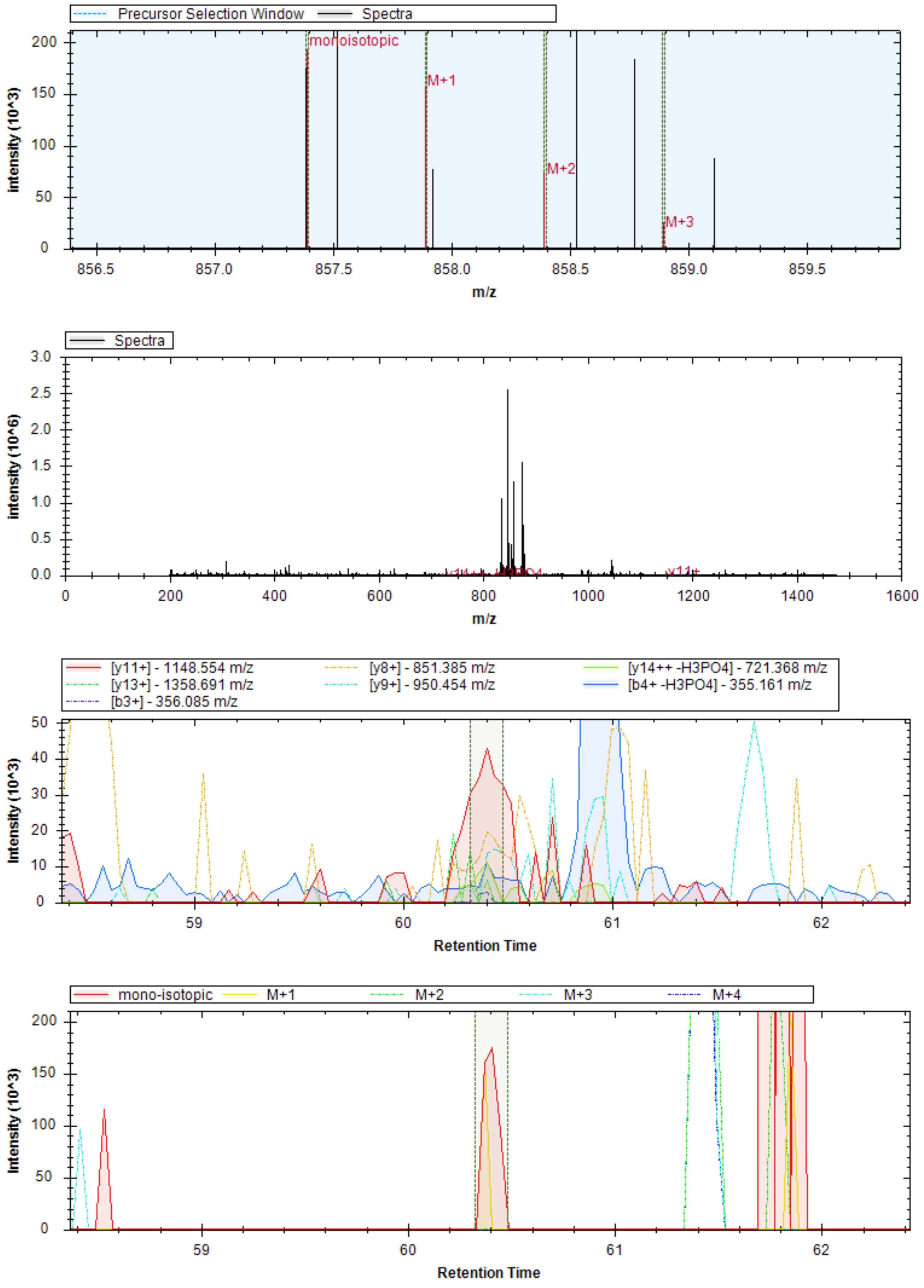

Q61290 – _M[+O]EPS[+H+O3+P]SLPQEIIANAK_.2
Fig4_MP-DIA-barrel-cortex-p54-3_MHRM.htrms

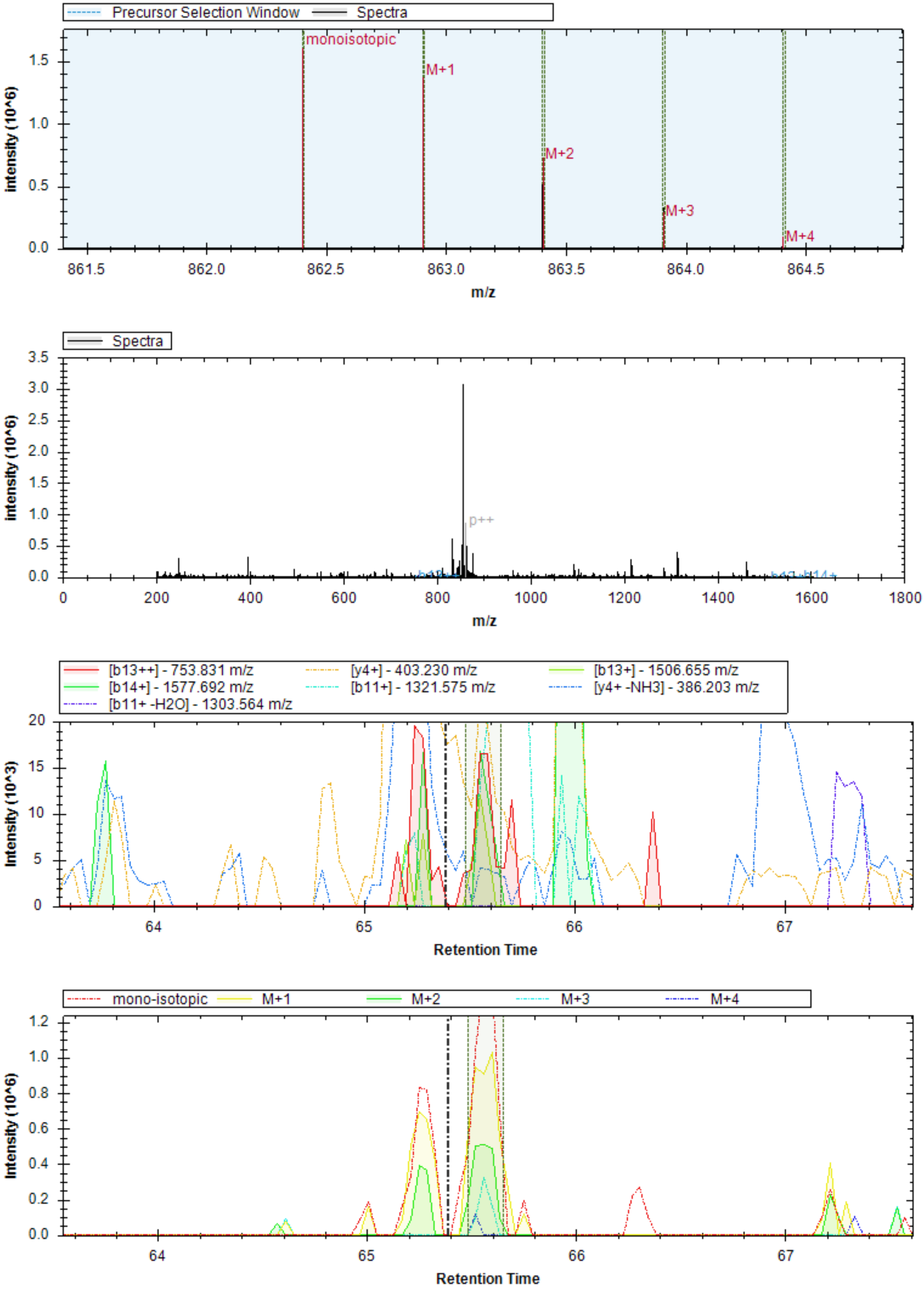

Q7TQF7 – _T[+H+O3+P]PSPPEEPSPLPSPTASPNHTLAPASPAPVRPR_.4

Fig4_MP-DIA-barrel-cortex-p54-3_MHRM.htrms

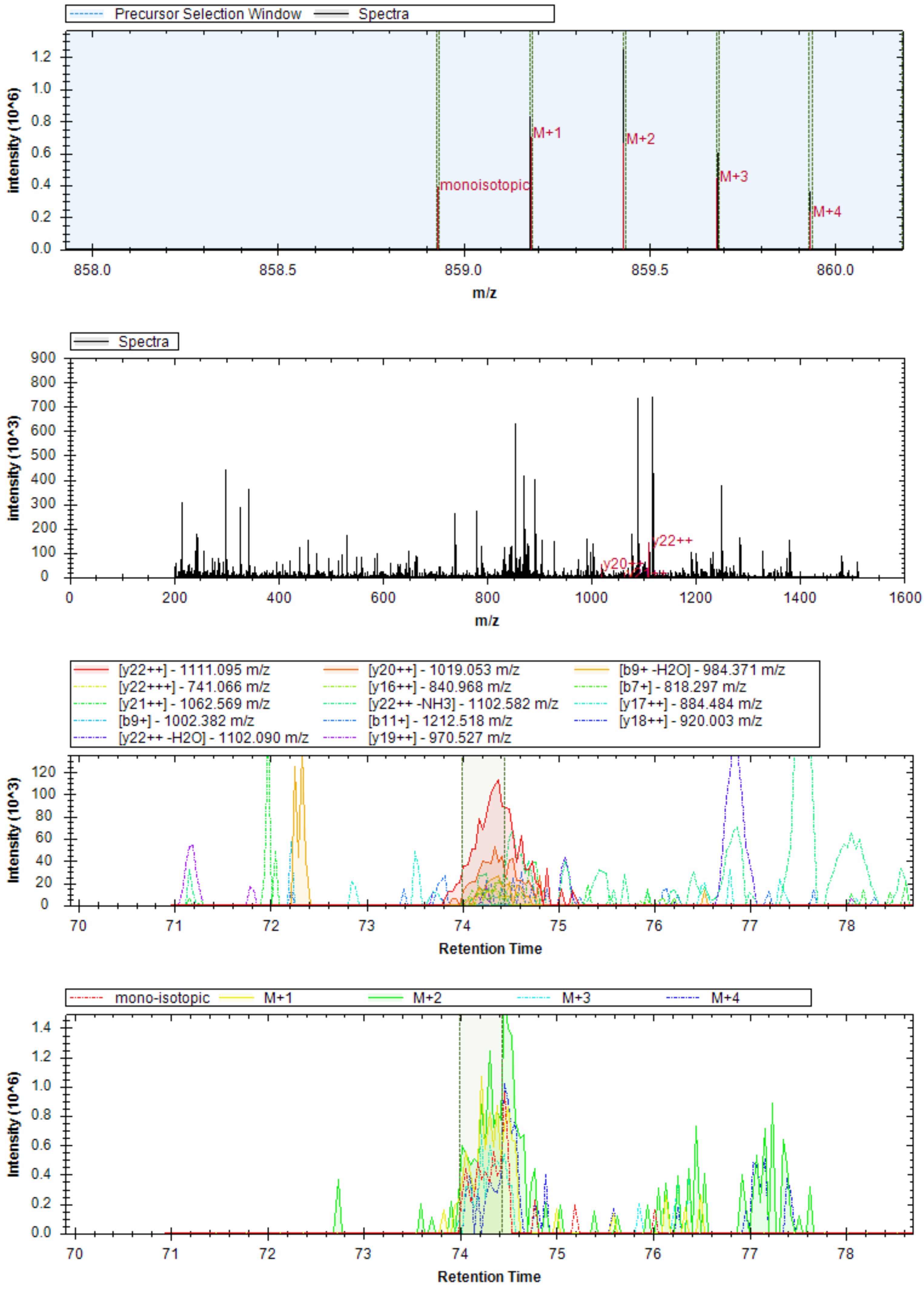

A1A535 – _FLHRGS[+H+O3+P]K_.1
Fig4_MP-DIA-barrel-cortex-p54-3_MHRM.htrms

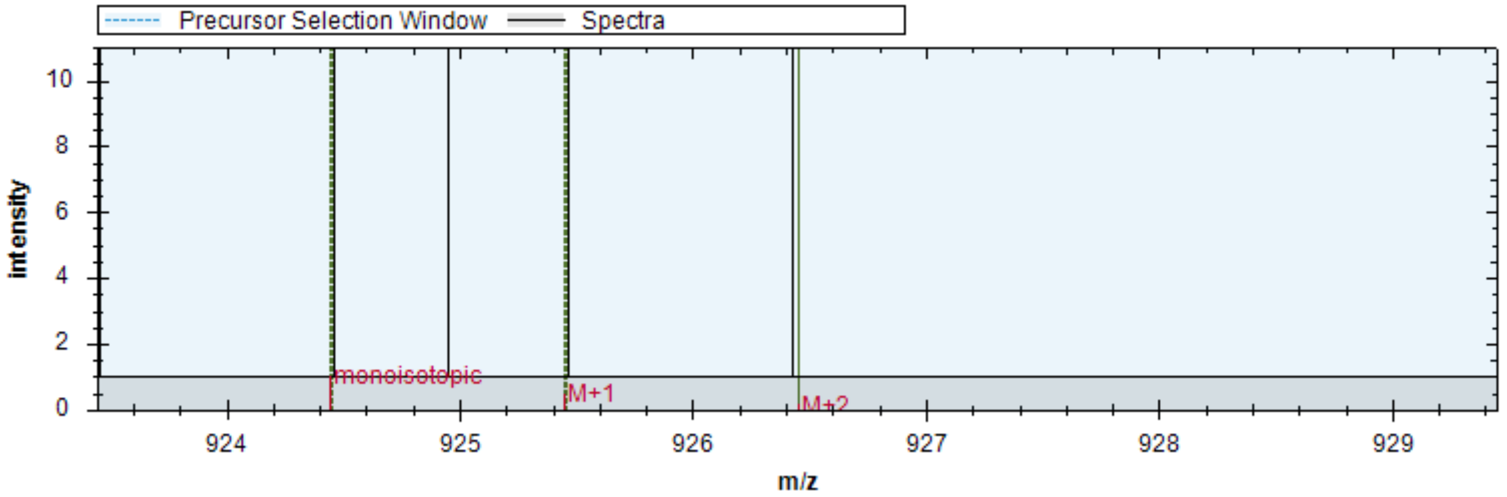

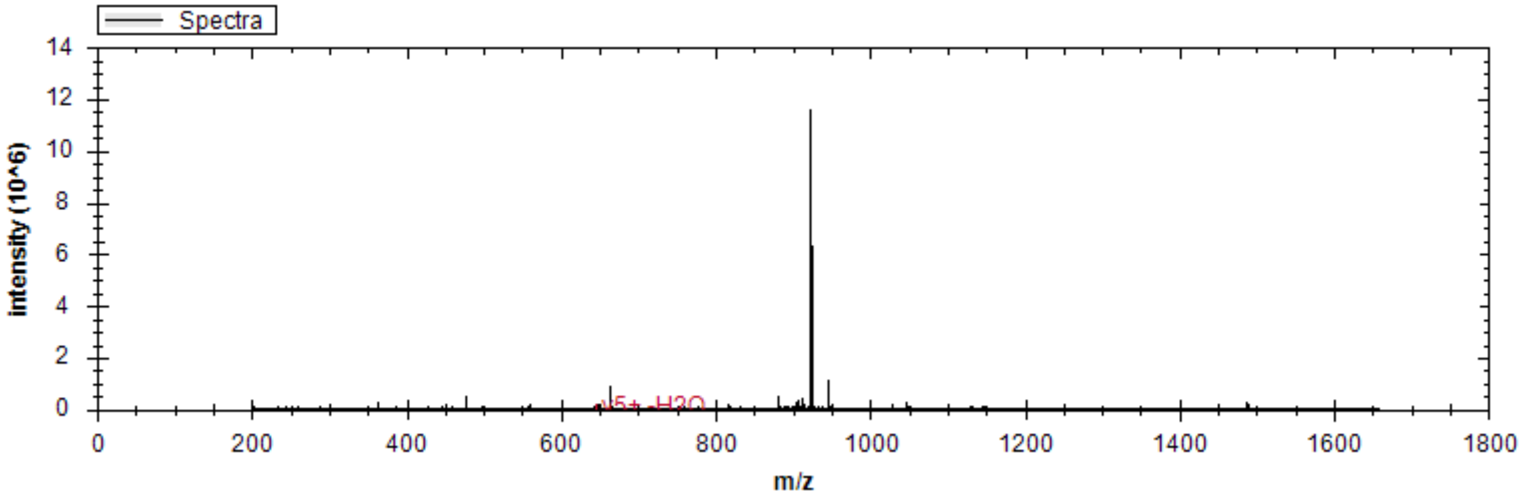

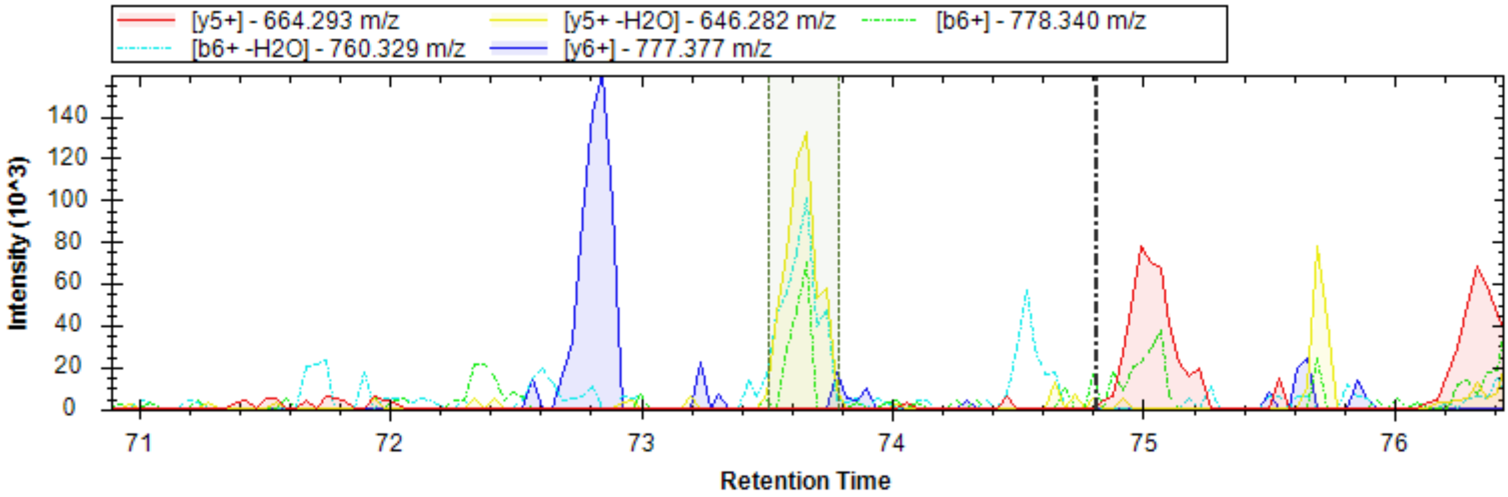

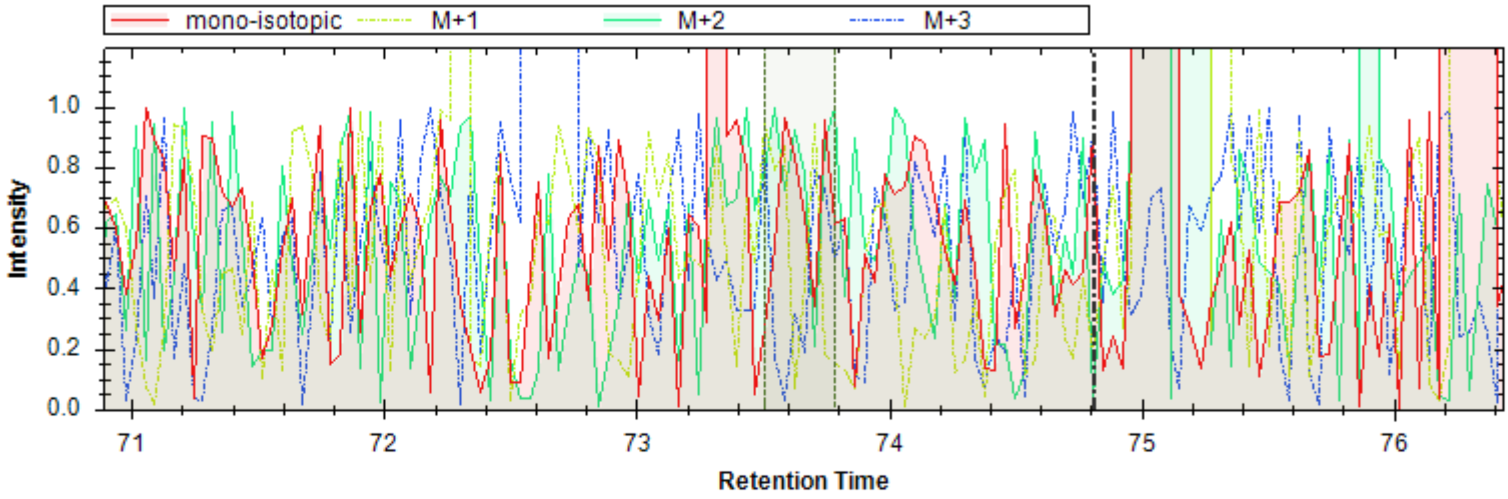

Q8BMA3 – _IIQLC[+C2+H3+N+O]LDLT[+H+O3+P]T[+H+O3+P]AVQK_.2
Fig4_MP-DIA-barrel-cortex-p54-3_MHRM.htrms

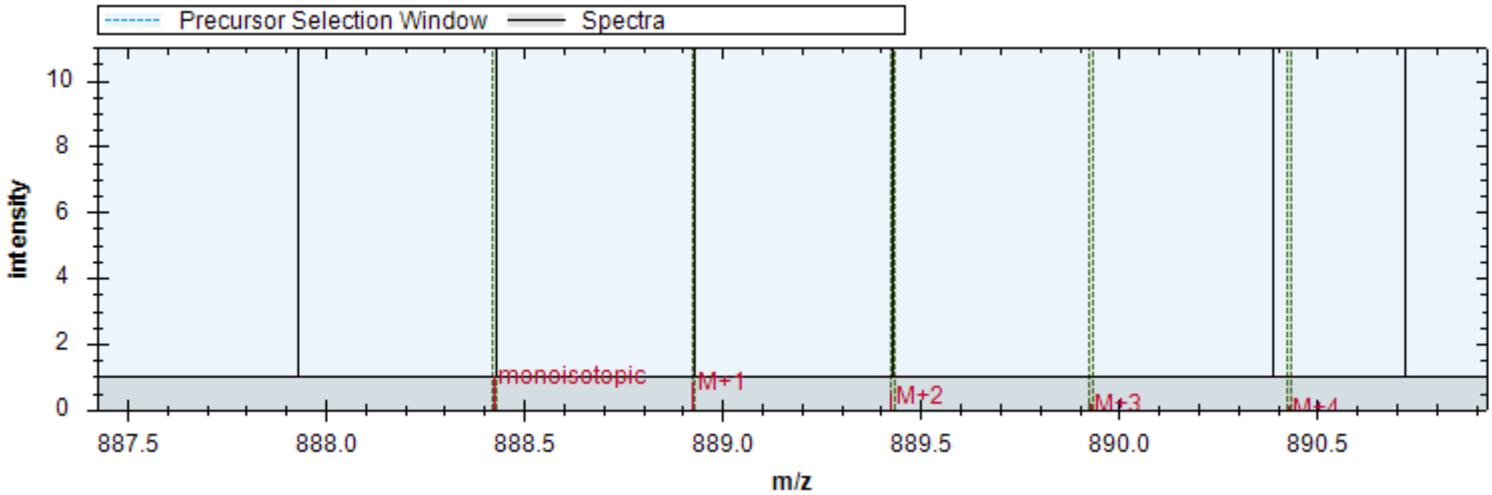

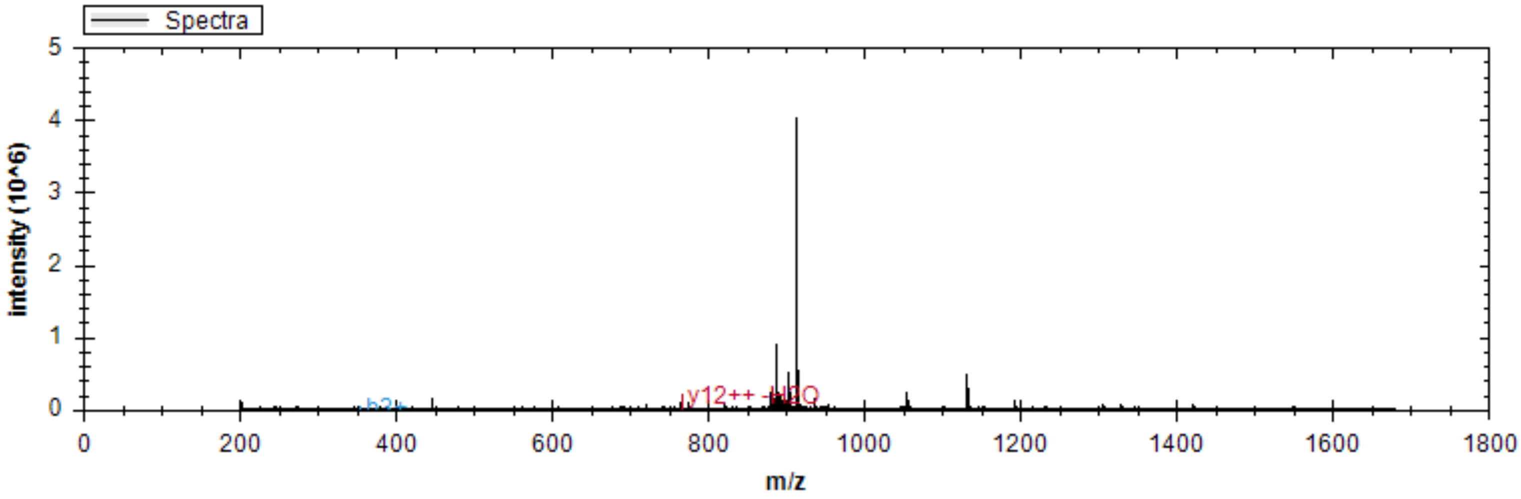

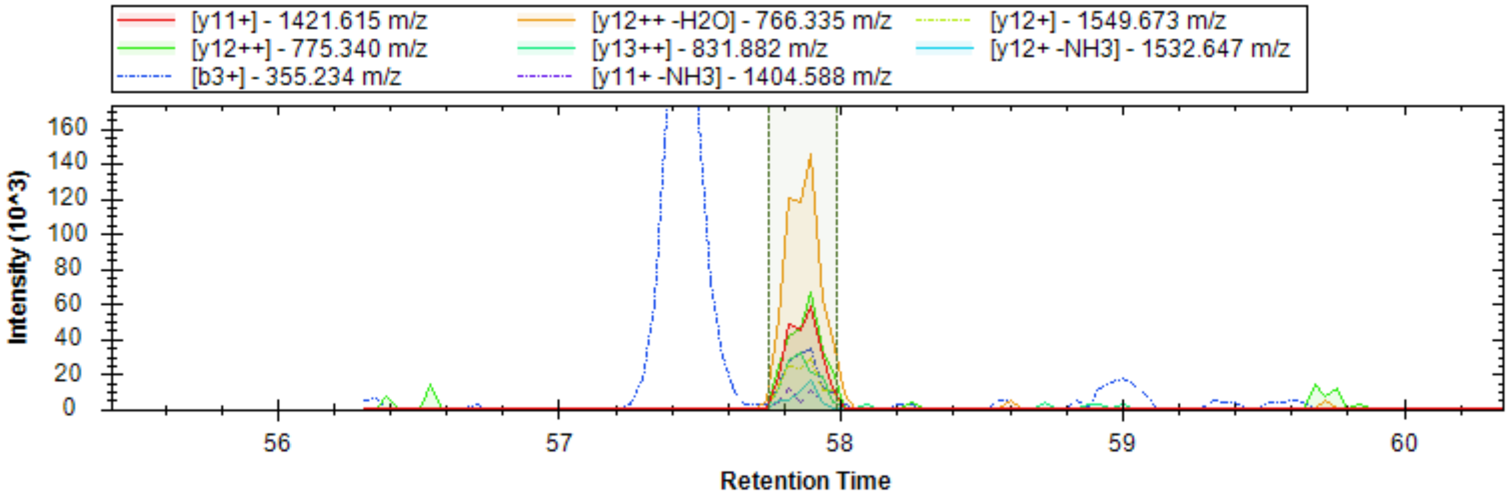

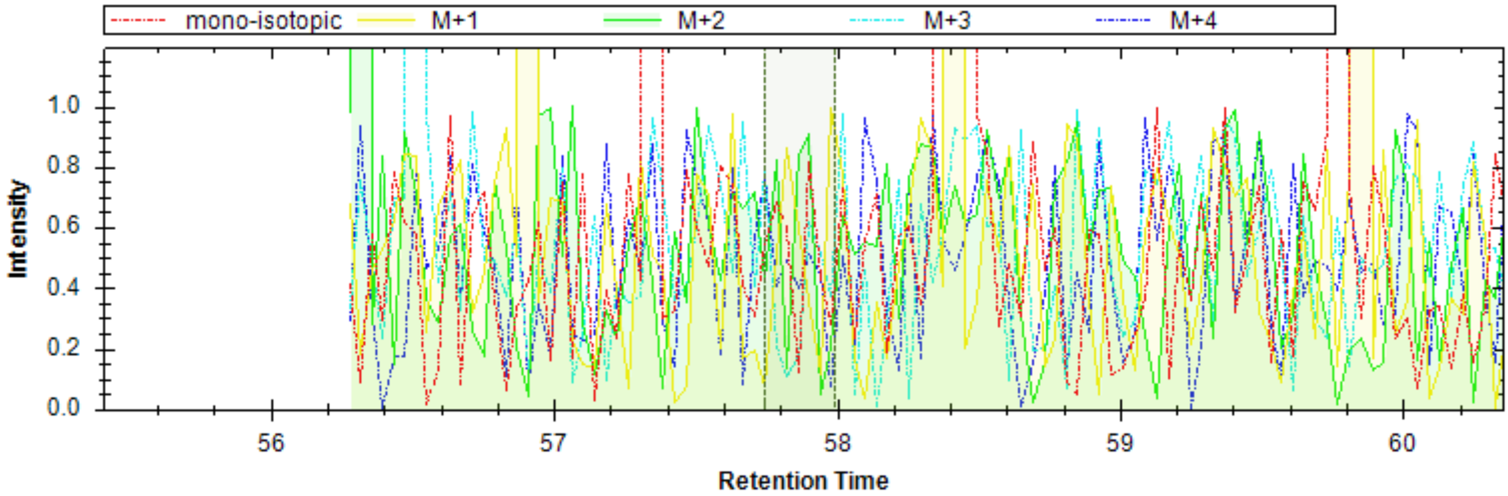

Q8BX90 – _IQALNSLGAGPFS[+H+O3+P]HTIK_.2

Fig4_MP-DIA-barrel-cortex-p54-3_MHRM.htrms

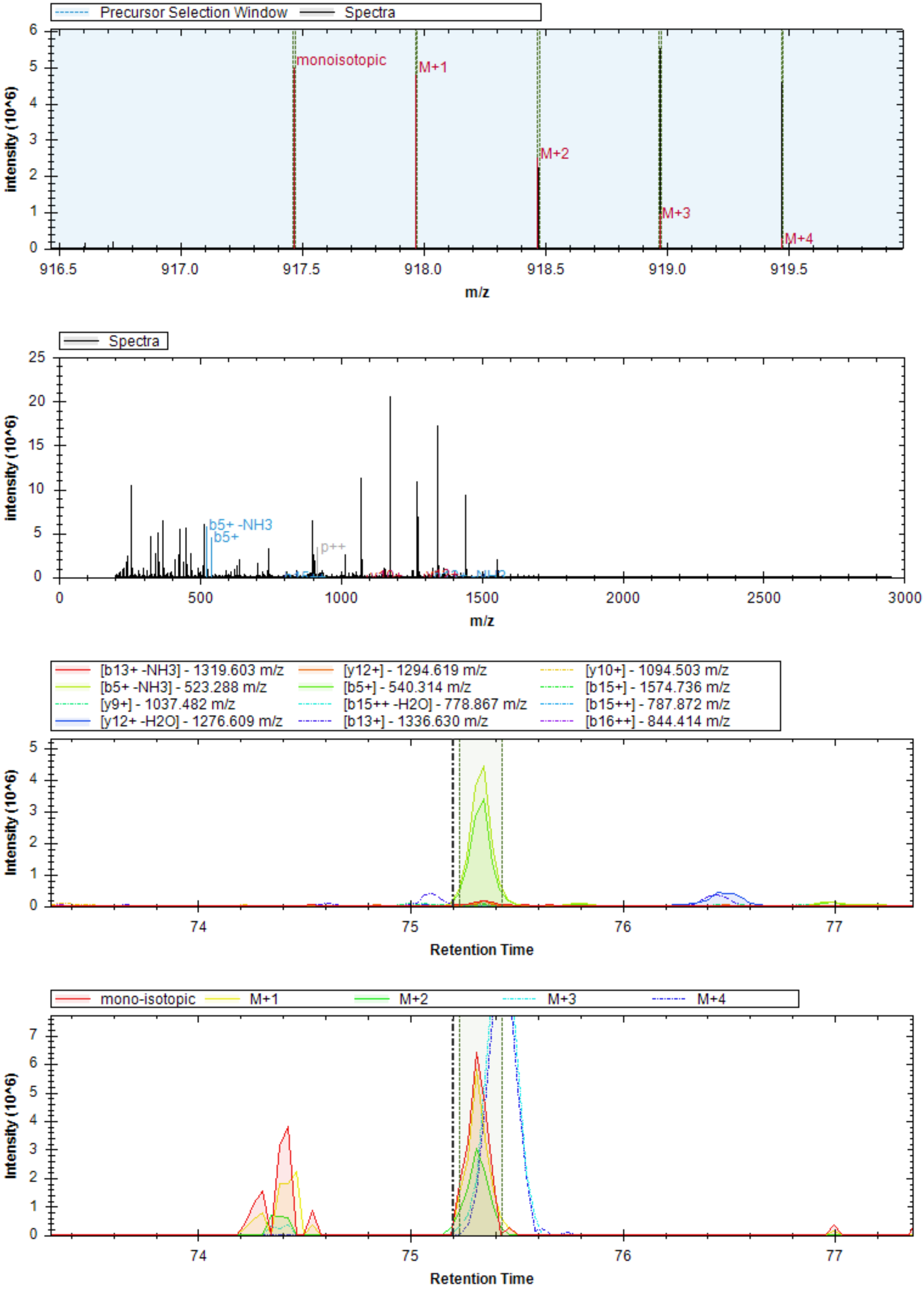

Q91Z31;Q91Z31-2 – _ETSLLAVPGALS[+H+O3+P]PLAIPNAAAAAAAAAAGR_.3
Fig4_MP-DIA-barrel-cortex-p54-3_MHRM.htrms

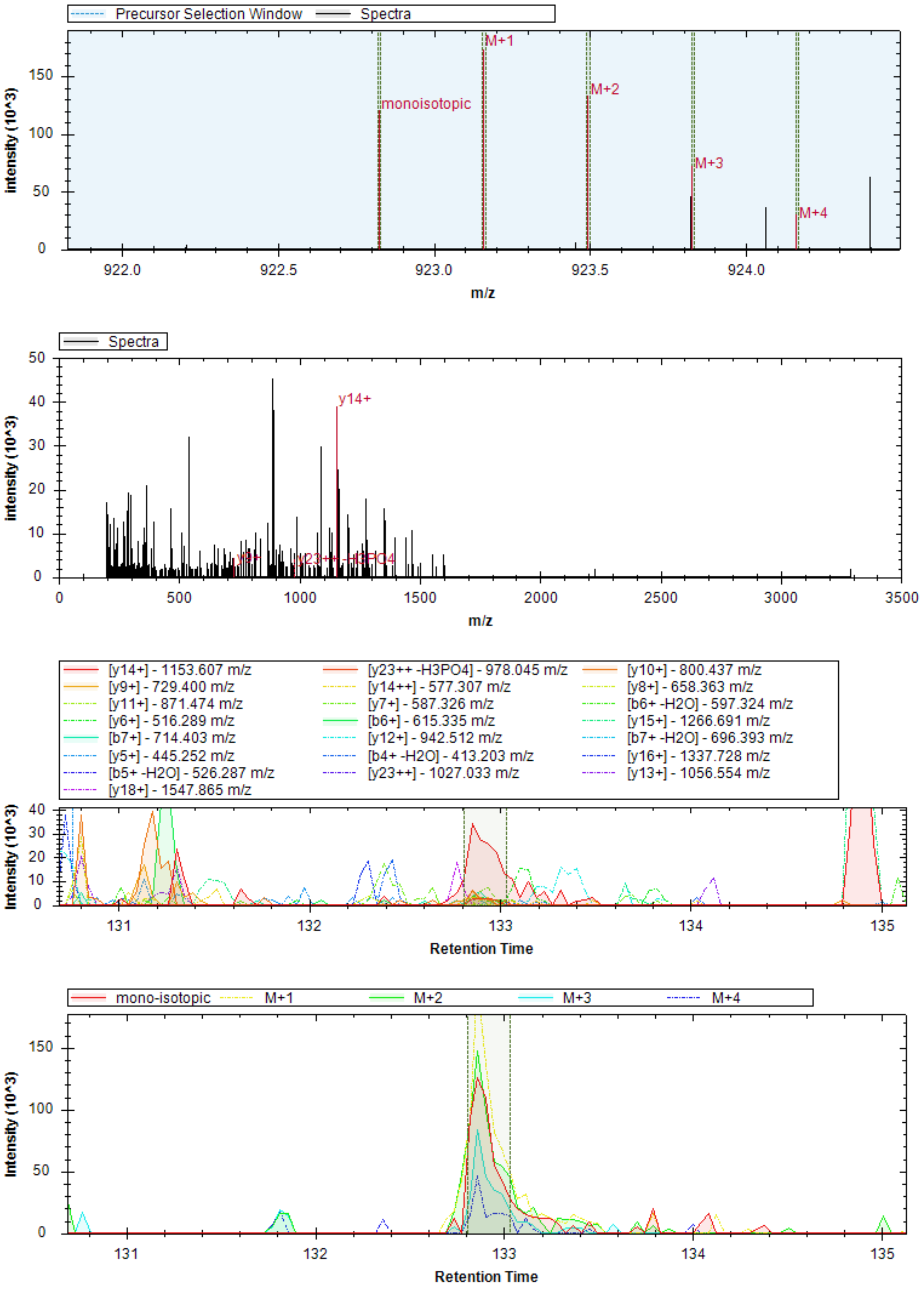

Q9CZT4;Q9CZT4-2 – _KEQM[+O]RAATVLPS[+H+O3+P]VQIK_.2

Fig4_MP-DIA-barrel-cortex-p54-3_MHRM.htrms

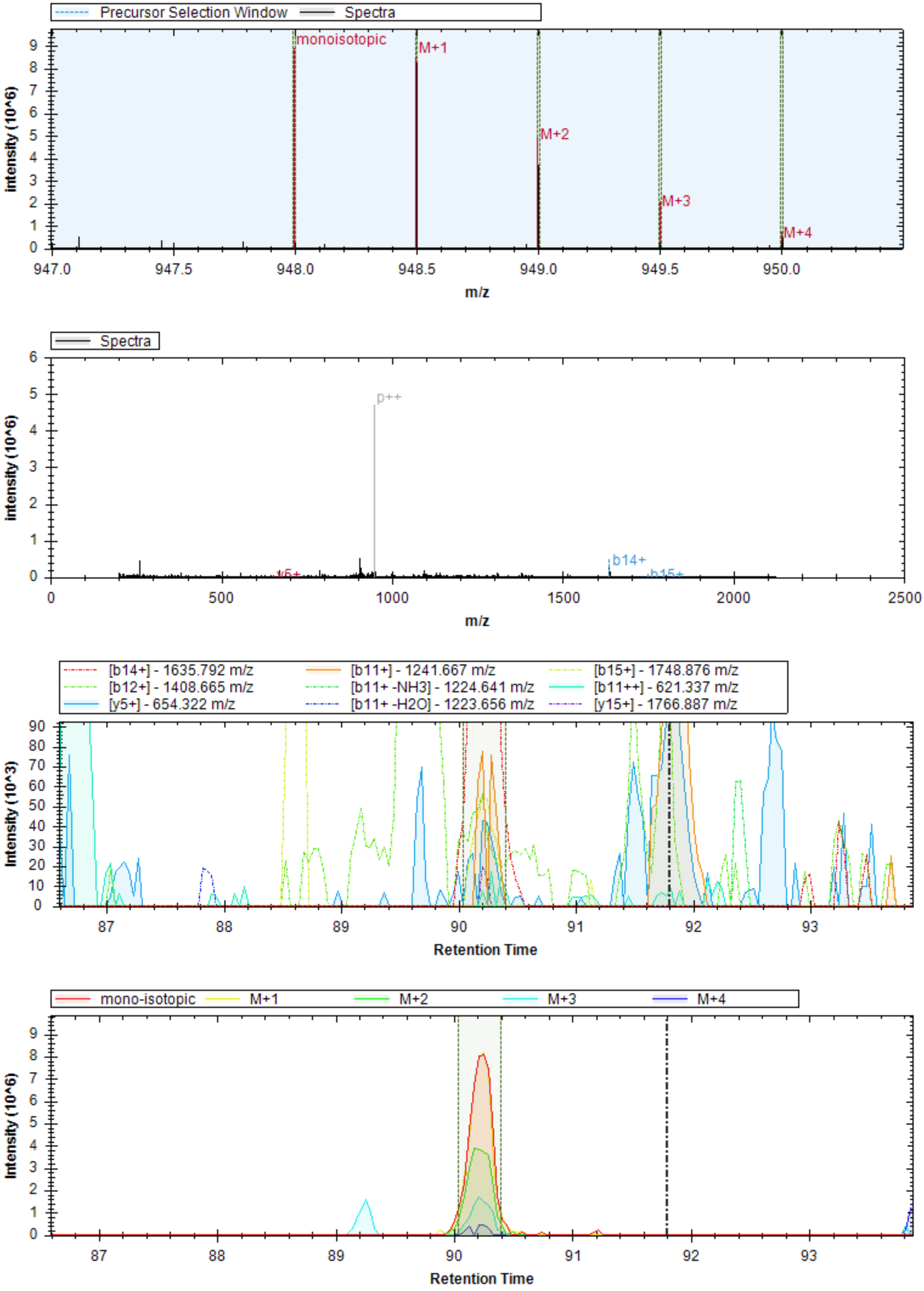

Q9QYR6-2 – _MAS[+H+O3+P]PPPSGPPSAAHTPFHQSPVEEK_.3
Fig4_MP-DIA-barrel-cortex-p54-3_MHRM.htrms

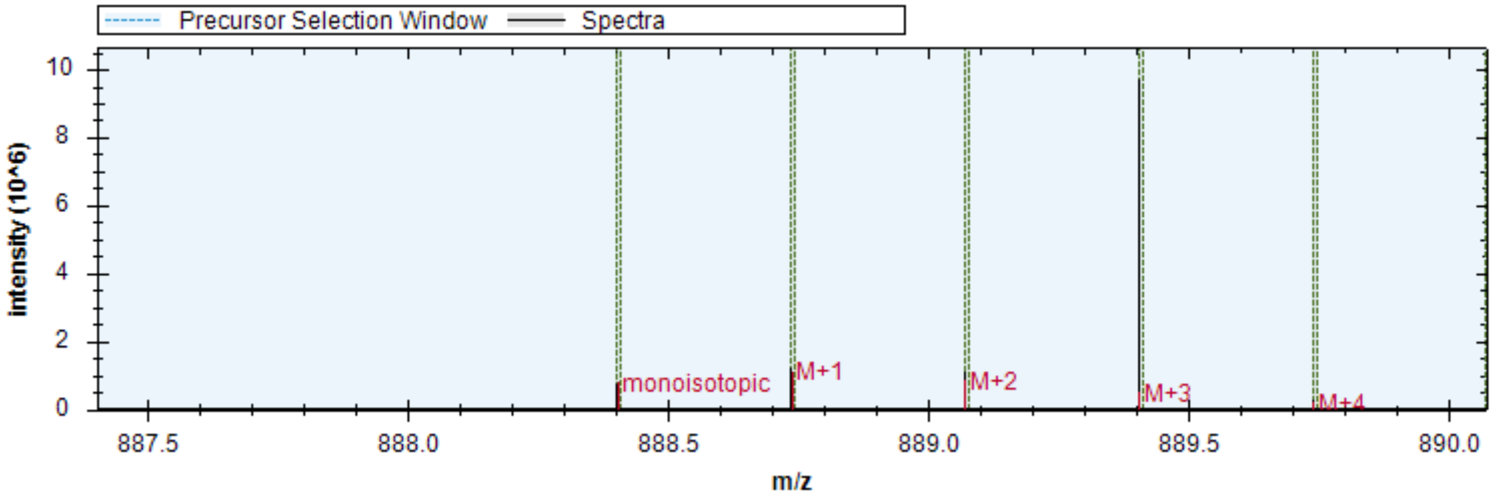

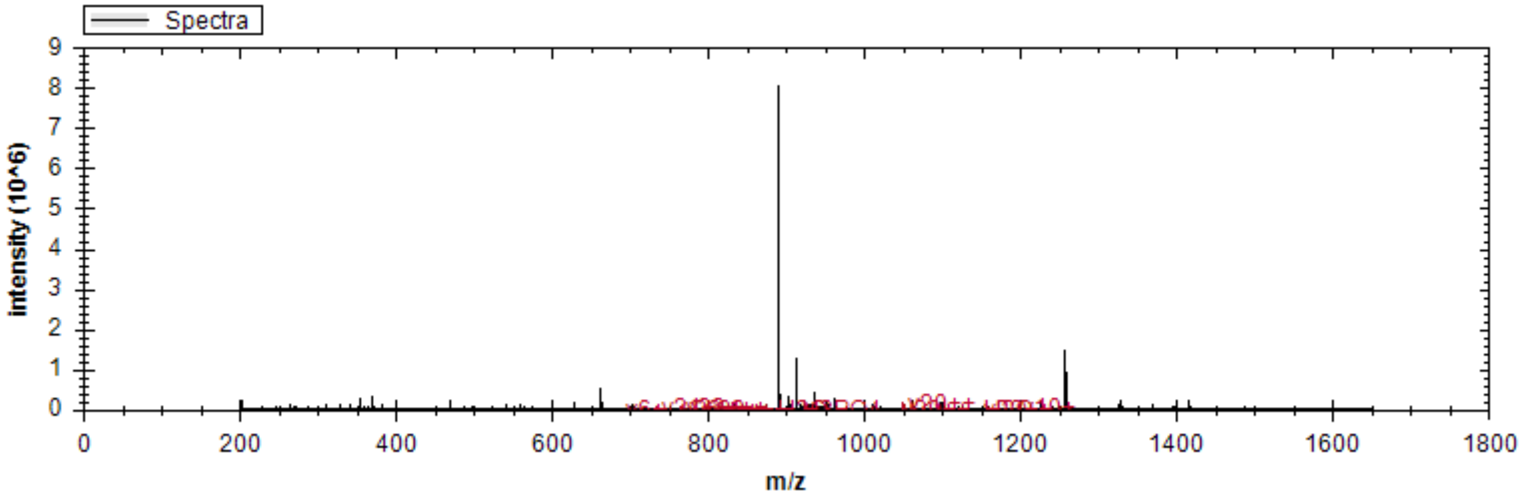

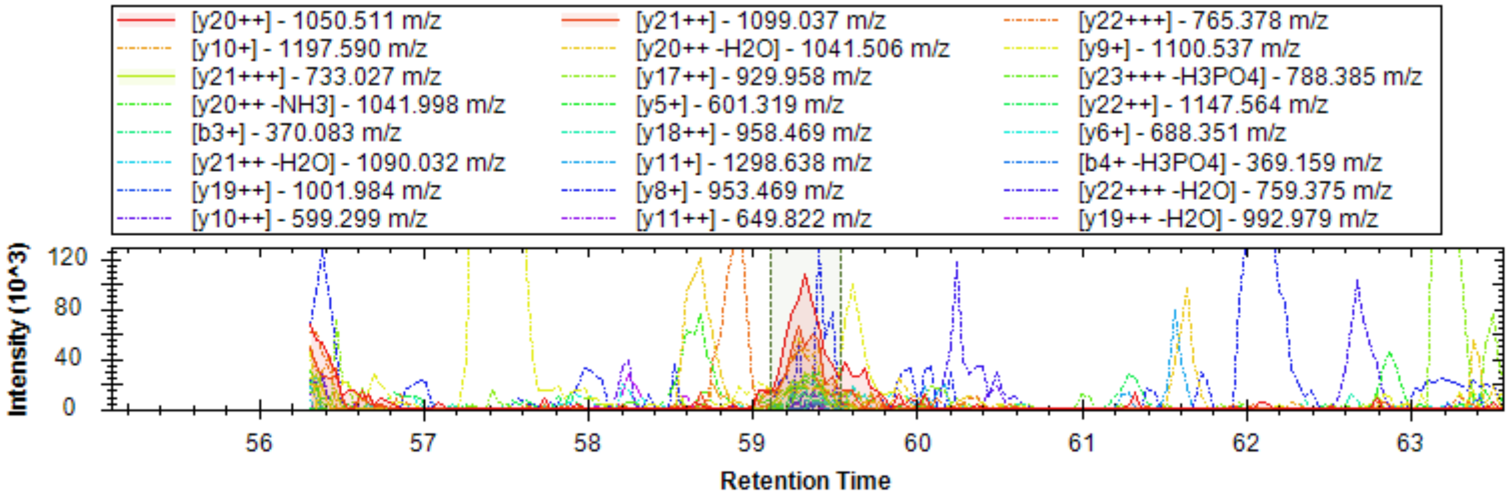

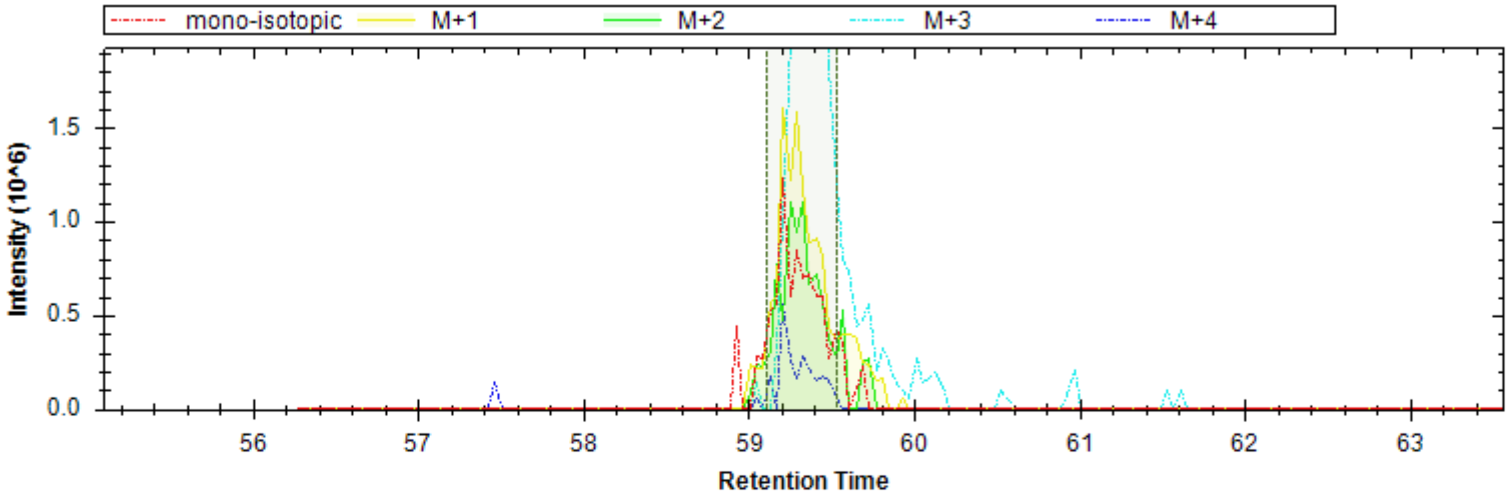

P17742 – _VNPT[+H+O3+P]VFFDITADDEPLGR_.2
Fig4_MP-DIA-barrel-cortex-p54-3_MHRM.htrms

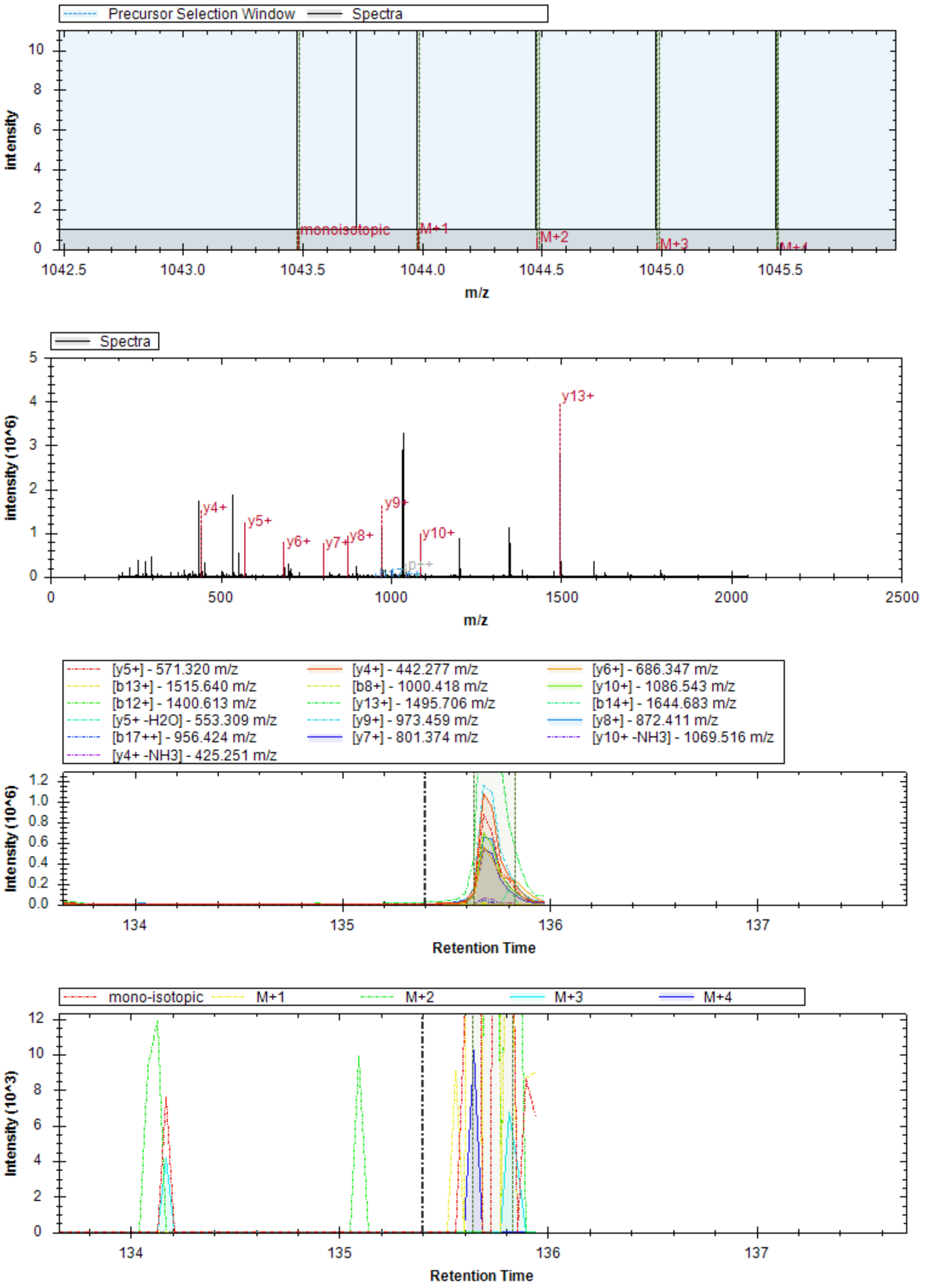

E9Q401 – _MVAEVFIY[+H+O3+P]WSK_.1
Fig4_MP-DIA-barrel-cortex-p54-3_MHRM.htrms

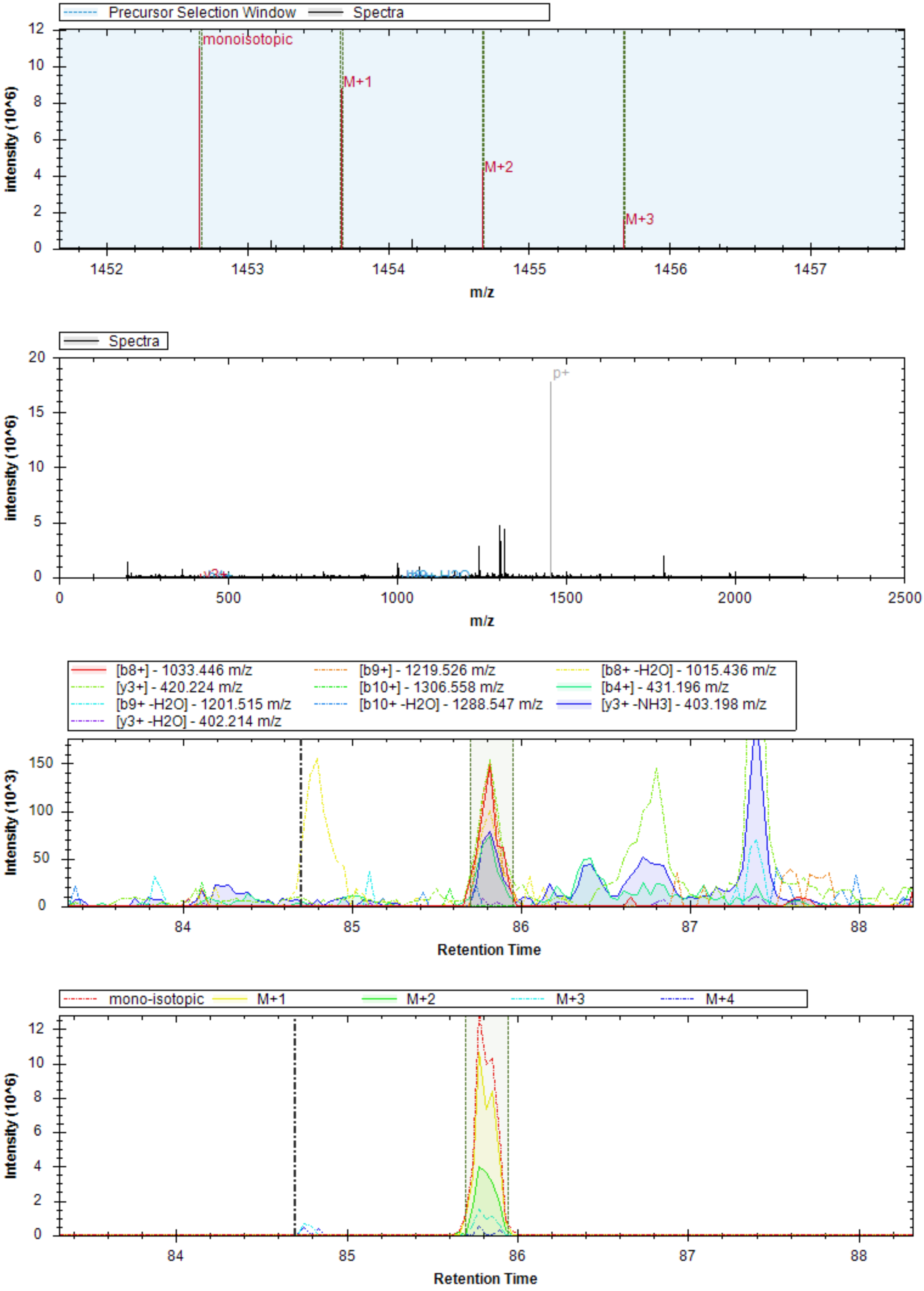

P14873 – _ETAAAHQAS[+H+O3+P]SSPPIDAATAEPYGFR_.2

Fig4_MP-DIA-barrel-cortex-p54-3_MHRM.htrms

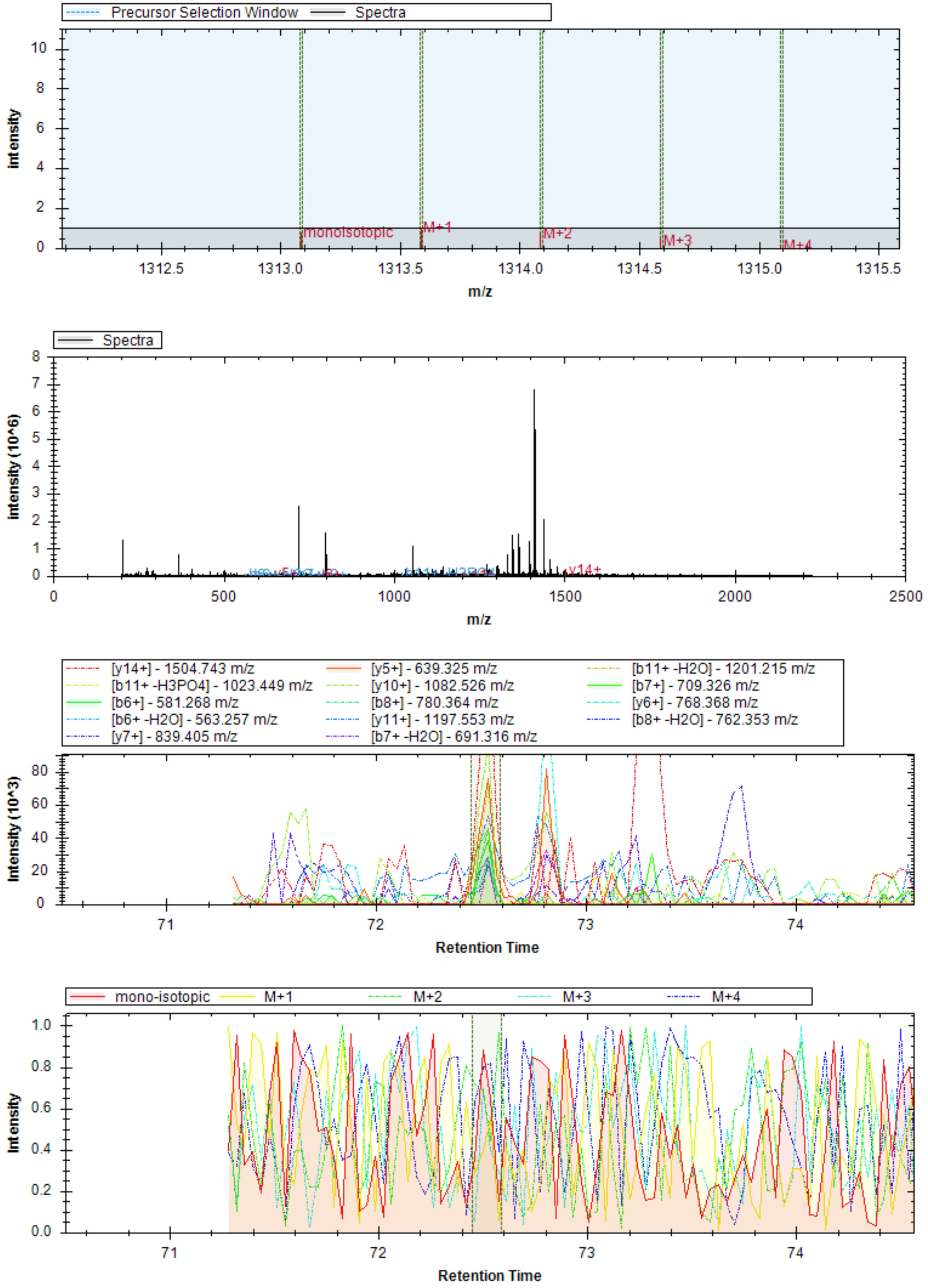

P20357 – _LSNVS[+H+O3+P]SSGSINLLESPQLATLAEDVTAALAK_.2
Fig4_MP-DIA-barrel-cortex-p54-3_MHRM.htrms

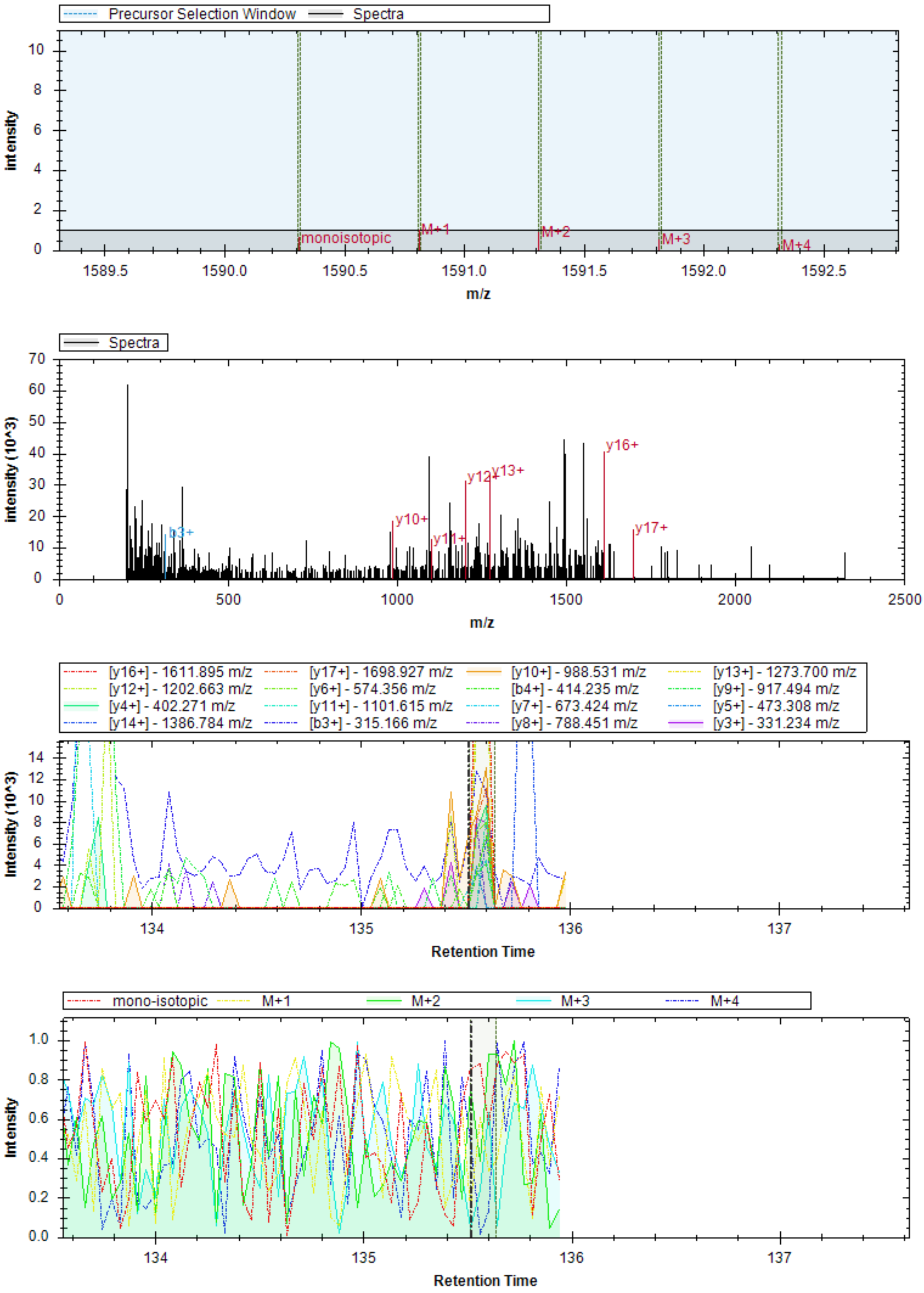

P20357 – _SGTSTPTTPGSTAIT[+H+O3+P]PGTPPSYSSR_.2
Fig4_MP-DIA-barrel-cortex-p54-3_MHRM.htrms

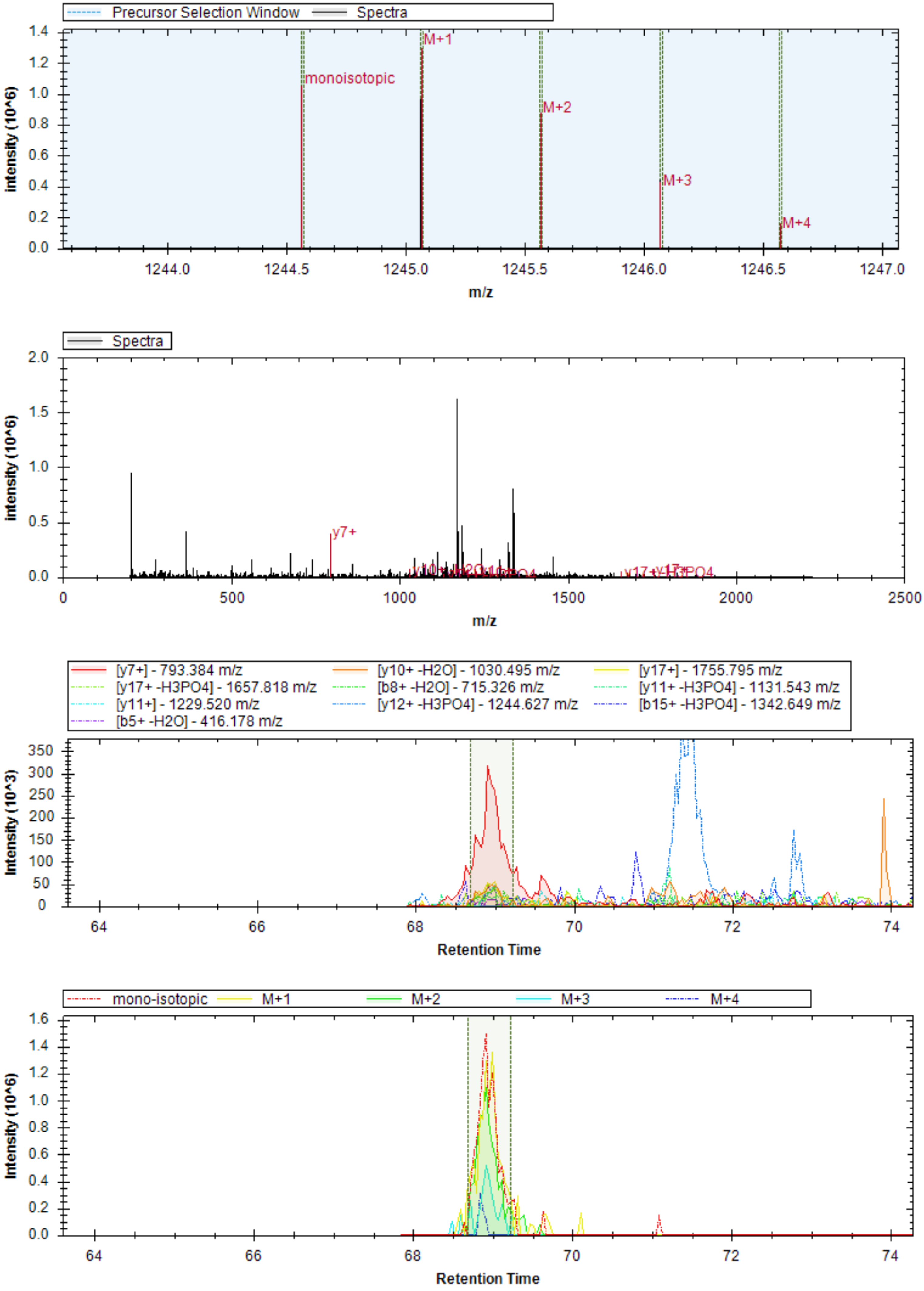

P20357 – _SGTSTPTTPGSTAITPGT[+H+O3+P]PPSYSSR_.2

Fig4_MP-DIA-barrel-cortex-p54-3_MHRM.htrms

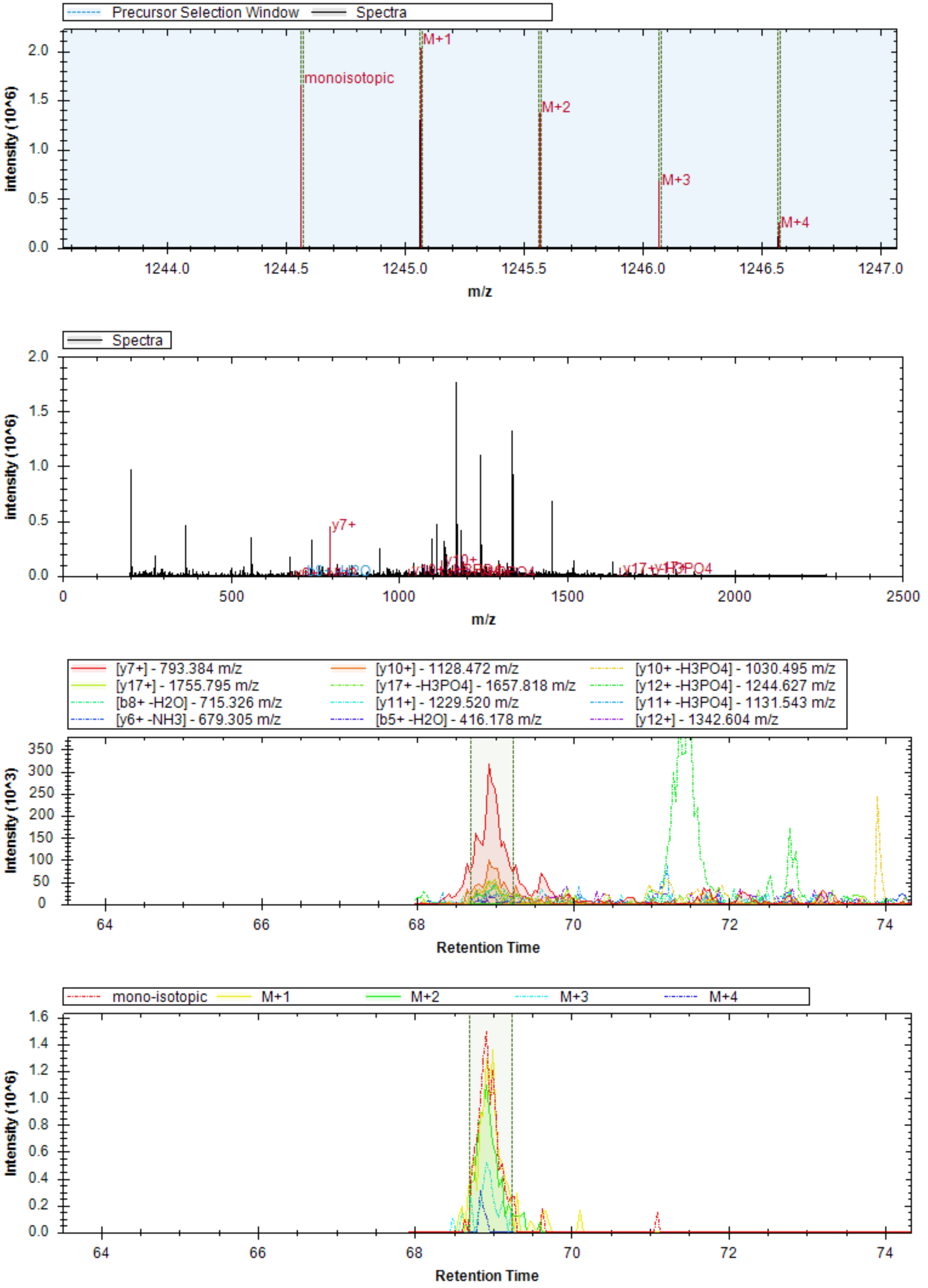

Q8BRT1 – _VLNT[+H+O3+P]GSDVEEAVADALLLGDIR_.2

Fig4_MP-DIA-barrel-cortex-p54-3_MHRM.htrms

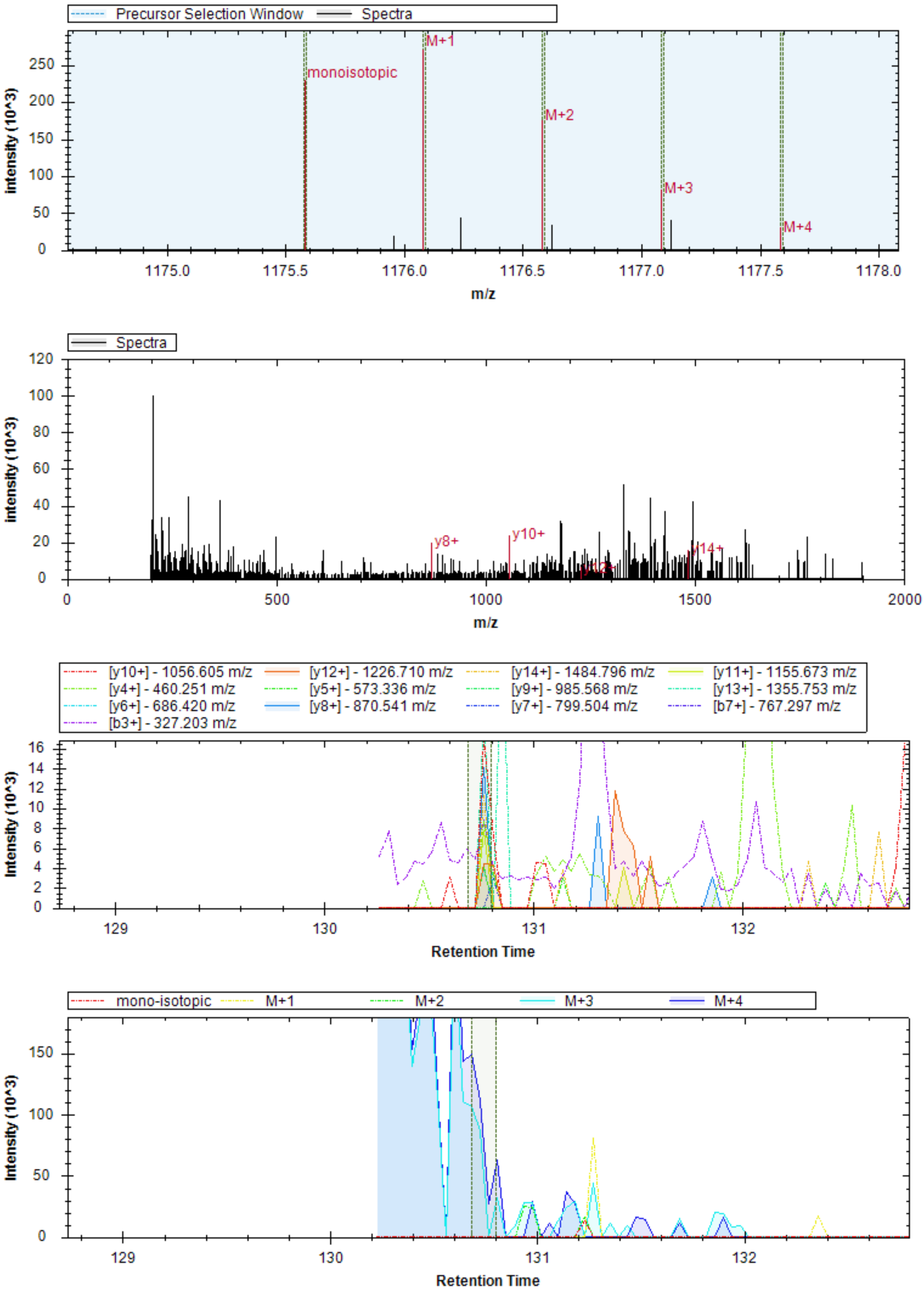

Supplement: Supplemental Data [file supp_RA117.000314_4947_1_supp_14569_ty28nv.pdf]
