# Supplementary material for: Optimization of Experimental Parameters in Data-Independent Mass Spectrometry Significantly Increases Depth and Reproducibility of Results
Source: Mol Cell Proteomics. 2017 Oct 25;16(12):2296–309. doi: 10.1074/mcp.RA117.000314 (PMC5724188; doi:10.1074/mcp.RA117.000314)
Supplement: Supplemental Data [file supp_RA117.000314_4947_1_supp_14434_zy1gl9.pdf]

1. Supplementary information:

1.1 Individual improvements in DIA leading to a leap in performance:

- The MS1 precursor scan resolution was increased from 35,000 (Bruderer et al. MCP 2015) to 120,000 in this study. This increased the identifications by 8% for 2h DIA runs (Figure 1d)
- To increase the dynamic range of the MS1 precursor scans, the m/z space was split into two segments. This increased the identifications by 4% for 4h DIA runs (Figure 3, compared to 4h DIA with only one MS1 segment)
- In this study, high resolution chromatography was used in addition to an improved peak gradient shape resulting in a more uniform peak widths at higher acetonitrile concentrations (Suppl figure 1). The use of Reprosil Pur 1.9um particles instead of ProntoSil 3.5um increased the identification by 10% for 2h DIA runs on the Q Exactive HF of HeLa using the HeLa spectral library of this manuscript.
- Increased sample loading from 1ug (as in Bruderer et al. MCP 2015) to 2ug increased the identifications by 4% in 2h DIA runs of HeLa using the HeLa spectral library of this manuscript.
- The algorithm for spectral library generation in Spectronaut was improved (fragment ion selection, fragment annotation, …). This resulted in an increase of identifications of 23% compared to the version of Spectronaut 5 used in Bruderer et al. MCP 2015 when the spectral library of the 2015 MCP publication data is regenerated with Spectronaut 9 and applied to 2h DIA of HEK-293.
- The targeted analysis of DIA was improved in the new Spectronaut versions. Compared to Spectronaut 5 used in Bruderer et al. MCP 2015, the identifications of 4h DIA runs of HeLa (Figure 3) increased by 12%.

- High field Orbitrap mass analyzer used in the Q Exactive HF improved the identifications by 25% for 2h DIA runs compared to the Q Exactive used Bruderer et al. MCP 2015, using comparable methods (35,000 resolution of QE vs 30,000 resolution of QE HF, identical DIA segments).
- Deep spectral libraries generated by high pH reversed phase fractionation showed improved performance on this high quality DIA data. In HEK-293 DIA of Bruderer et al. MCP 2015 it improved the identifications by 30% when using the HEK-293/HeLa spectral library of this manuscript compared to the spectral library Bruderer et al. MCP.

1.2 Precursor FDR (q-value) estimation in Spectronaut for DIA

Spectronaut uses the mProphet algorithm for FDR estimation which was initially published for MRM data (1). The mProphet algorithm has been shown to be readily applicable to the targeted analysis of DIA/SWATH data because the data structure is identical to MRM (2, 3). In brief Spectronaut will analyze any DIA data not only with the peptide precursors specified in the spectral library ("targets") but also with additionally, on the fly generated decoy peptide precursors ("decoys"). By default, in Spectronaut, the number of decoys will be the maximum of 1000 and 10% of the spectral library size (strategy and number can be adjusted in the Spectronaut settings schema). Due to the large size of spectral libraries used in this study the number of decoys was in the range of 10'000 to 20'000. Extracted ion currents for the precursor isotopic envelope are generated on MS1 for all targets and decoys. Extracted ion currents for the fragment ions are generated on MS2 for all targets and decoys. Extracted ion currents are generated in the retention time window where peptide elution is expected and as determined by the iRT calibration (described in detail in (4). For every target/decoy, candidate peak groups are determined and a discriminant score is computed. For every target/decoy only the candidate peak group with the highest discriminant score is further considered.

The classical assumption for FDR estimation in DDA using the target/decoy approach is: "False peptide-spectrum matches will uniformly distribute with respect to the target and decoy database." This assumption is not valid in the targeted analysis of DIA, i.e. false identifications do **not** uniformly distribute with respect to targets and decoys. Hence, the FDR is estimated with the q-value approach as described in the mProphet paper (5). In brief, the discriminant scores (Cscores) are converted to p-values based on a kernel density fit to the Cscore distribution of the decoys and q-values are estimated based on the p-values of the targets with the Storey method using a lambda of 0.5.

The decoy Cscore distribution should be representative of false identifications to achieve an accurate FDR estimation[1]. We used the "mutated decoy strategy" for decoy generation for all experiments in this manuscript (available as of Spectronaut 11.0.15038.9 in the settings).

The mutated decoy strategy works as follows: Pick N random peptide precursors from the spectral library as templates, where N is the number of decoys to generate. Exchange C-terminal amino acid from K to R, from R to K and from any other amino acid to a random amino acid (I to L and vice versa is avoided). Recalculate the precursor m/z based on this new sequence. Take the original sequence and randomly exchange a variable number of amino acids in the sequence (last amino acid stays the same). Recalculate the fragment ions based on this new sequence. The iRT and relative fragment ion intensities are kept identical to the template. For a more detailed description see the pseudo code below. The mutated decoy model was validated using a two-organism spectral library approach (see below). It was also found to be slightly less optimistic than the scrambled decoy model described in[1] (See Suppl. Figure 1).

[1]Bernhardt, O. M., Bruderer R., Gandhi, T., Miladinovic, S. M., Bober, M., Ehrenberger, T., Rinner, O., and Reiter, L. (2014) General guidelines for validation of decoy models for HRM/DIA/SWATH as exemplified using Spectronaut. Proceedings of the 62nd ASMS Conference on Mass Spectrometry and Allied Topics, 2014, Baltimore, MD, USA.

```
method GenerateDecoys
    N = 5000 //Dependant on settings
    templates = pickNRandomPeptidesForDecoyTemplates (AllPeptides, N)

    foreach template in templates
        sequence = template.getSequenceWithModifications()

        dSequencePrec = exchangeLastAA(sequence)  // Exchange last AA so that decoy has a different precursor m/Z then target
        prec_mz = calculateMz(dSequencePrec)      // This will exchange K <=> R or to a random AA if neither (Never I <=> L)

        dSequenceFrg = Mutate(sequence)

        decoy.Fragments = CutUsingSameFragments(dSequenceFrg, template.Fragments) // Use same Fragment ion definitions as template
        decoy.PrecMz = prec_mz                                                    // but applied to new sequence
    end foreach
end method

methode Mutate (sequence)
    n = NrOfAAs(sequence)-1  //Last AA stays in place and is not swapped
    min = 2
    max = n / 2

    nrOfSwaps = max(min, random(max) +1) //random returns an integer >= 0 and < max

    do
        performRandomSwaps(nrOfSwaps) // pick random positions (a,b) >= 0 and < n
                                      // a and b most be different indices
                                      // a and b can not point to the same AA, if they do -> pick again.
                                      // Retry Max 5 times then throw an exception and skip this decoy
                                      // Swaps are performed including associated modifications
    Repeat nrOfSwaps times
end method
```

1.3 Protein group FDR (q-value) estimation in Spectronaut for DIA

Spectronaut uses a recently published strategy (Rosenberger et al. 2017) for protein group FDR estimation in DIA with slight adaptations. In brief, a unified scoring scale across all runs is established. Since machine learning is performed on a run by run bases, the Cscores which is the primary score used for precursor FDR calculation cannot be directly compared between runs within one experiment. In order to normalize the Cscores across runs a robust normalization is applied. Since the distribution of decoy scores behaves very similar across different runs it can be used as a means of normalization as follows

$$nCscore = \frac{Cscore - Median(DecoyCScores)}{IQR(DecoyCScores)},$$

where DecoyCScores is the set of all Cscores associated with decoy precursors of the respective run and IQR is the interquartile range.

In contrast to using the protein grouping of the spectral library, Spectronaut typically performs its own protein inference on the set of identified peptides (typically performed on all the precursors with FDR of 1%) using the ID picker algorithm (Zhang et al. 2007). That's a small difference to what was published in the Rosenberger et al. approach and has the following subtle effect. Certain protein groups are being "stripped off" all of their peptides. These protein groups are no more representative and removed. If Spectronaut is not asked to perform protein inference on the set of identified peptides this step is not performed. The number of resulting protein groups is typically smaller if Spectronaut protein inference is performed. Hence, in this manuscript the more conservative approach of the two was used throughout.

For protein group FDR estimation, scores for both targets and decoy protein groups are needed. A target protein group score is defined as the best nCscore across all runs associated with this protein group. A decoy protein group is generated for each target protein group. A decoy protein group will be a randomly assembled set of decoy precursors. The number of decoy precursors assigned to a decoy protein is corresponding to its associated target protein groups total precursor count. Similar to a target protein group, the best nCscore of each decoy precursor across all runs will represent the decoy protein group score. Having a set of target and decoy protein groups and protein scores, the Storey method (Storey et al. 2003) can be used to determine a q-value for each target protein group as described Rosenberger et al. 2017 or above for the precursor FDR.

We validated protein group FDR as described below.

1.4 Precursor FDR (q-value) validation

In order to validate the precursor FDR a two-organism spectral library approach was used. Two spectral libraries derived from human Hela cell line and maize were prepared in exactly the same manner and applied to a DIA measurement of Hela. The number of maize precursor identifications can be used to cross

validate the precursor FDR estimate, because it is assumed that all precursors from the Hela library are identified and all maize identifications are false positives.

A Hela and a maize sample was prepared and measured in technical triplicates each using a 2h DDA method on a Q Exactive HF. The Hela and maize DDA data was searched separately with standard settings (variable protein N-terminal acetylation and methionine oxidation, fixed carbamidomethylation of cysteine), UniProt release 2017.06.14 for Hela (20’217 sequences), UniProt and Trembl release 2017.06.14 for maize (131’132 sequences) and filtered with a 0.1% PSM and protein group FDR. A human and a maize spectral library was generated with standard settings using Spectronaut (these libraries are called base libraries in the paragraph “Protein group FDR (q-value) validation”).

Using in house software the spectral libraries were filtered for peptides that occur in both species as follows: Extensive sequence annotation for both species was used combining reviewed, unreviewed and isoforms sequences. The FASTA files comprised 159’692 sequences for human (release 12.7.2017) and 132’460 sequences for maize (release 19.06.2017). The two FASTA files were in silico digested with semi-tryptic specificity and allowing maximally two missed cleavages. After that all peptides occurring in both species were removed. Isoleucine and Leucine were treated as the same amino acid, i.e. peptides only differing in an I to L or L to I exchange were filtered as well. This resulted in a Hela and a maize spectral library with peptides occurring in only the respective species with a very high likelihood.

The remaining precursors in both spectral libraries were sorted by evidence count (the number of PSMs in the DDA acquisitions) and q-value. Precursors with an m/z smaller than 350 and larger than 1650 were removed because this is the range we typically acquire DIA data in. The remaining top 10’000 precursors in the two spectral libraries were retained.

Both spectral libraries were applied to a 2h DIA run of a Hela sample acquired on a Q Exactive HF using Spectronaut with standard settings, except for setting the number of decoys to 10’000. A report with

following columns was generated: PG.FastaFiles and EG.Qvalue without report filters (decoys not exported). The PG.FastaFiles column was used to count the number of maize identifications and hence to determine an “actual” FDR for a given estimated q-value (Suppl. Fig. 1).

In general, a repeated analysis using Spectronaut with unchanged settings and raw data will result in identical results. For this specific analysis here, we performed a 250-fold bootstrap analysis in order to have a more accurate cross validation. This means we repeated the analysis 250 times with various random seeds.

1.5 Protein group FDR (q-value) validation

The base spectral libraries derived from the human Hela cell line and maize as described under “Precursor FDR (q-value) validation” were further filtered for protein group FDR validation as follows: Using in house software the spectral libraries were filtered for proteins that share any peptides between the two species as follows: Extensive sequence annotation for both species was used combining reviewed, unreviewed and isoforms sequences. The FASTA files comprised 159’692 sequences for human (release 12.7.2017) and 132’460 sequences for maize (release 19.06.2017). The two FASTA files were in silico digested with semi-tryptic specificity and allowing maximally two missed cleavages. Isoleucine and Leucine were treated as the same amino acid, i.e. peptides only differing in an I to L or L to I exchange were filtered as well. This resulted in a Hela and a maize spectral library with proteins only yielding peptides that occur in one of the two species with a very high likelihood.

Further, all proteins with less than three precursors were removed. Proteins were sorted by average q-value and the top 1000 protein groups in each library were retained.

Both spectral libraries were applied to a technical triplicate of 2h DIA runs of a Hela sample acquired on a Q Exactive HF using Spectronaut with standard settings. A report with following columns was generated:

PG.FastaFiles, PG.ProteinGroups and PG.Qvalue without report filters (decoys not exported). Duplicated protein groups were removed based on the PG.ProteinGroups column. The PG.FastaFiles column was used to count the number of maize protein identifications for a certain cutoff and hence to determine an "actual" FDR for a given estimated q-value (Suppl. Figure 1).

In general, a repeated analysis using Spectronaut with unchanged settings and raw data will result in identical results. For this specific analysis here, we performed a 250-fold bootstrap analysis in order to have a more accurate cross validation. This means we repeated the analysis 250 times with various random seeds.

1.6 Calculation of the identified "explained" TIC of Suppl figure 10:

First, the scans were centroid the scan. Next, all peak intensities were summed to obtain the Total ion current. Then feature detection was performed (minimally 2 peaks, mono isotopic +1 isotope). Then all precursors that elute for a scan were obtained (Apex RT +/- FWHM *2.55) and fall within the precursor selection window and are identified (Prec and Protein FDR). Use all library fragments for the relevant assays (all detected in DDA of the runs used for the spectral library) and match each fragment to a detected feature (must fall within m/z tolerance from mass-calibration and match the same detected feature charge). Next, all features were collected that matched to any library fragment and all associated peak intensities were summed resulting in the Explained Ion Current

For the control shift analysis, the identified peaks RT by three times the peak width used for explained tic to earlier retention time and get intensities.

Supplementary references:

1. Reiter, L., Rinner, O., Picotti, P., Hüttenhain, R., Beck, M., Brusniak, M.-Y., Hengartner, M. O., and Aebersold, R. (2011) mProphet: automated data processing and statistical validation for large-scale SRM experiments. *Nat. Methods* 8, 430–5

2. Gillet, L. C., Navarro, P., Tate, S., Röst, H., Selevsek, N., Reiter, L., Bonner, R., and Aebersold, R. (2012) Targeted data extraction of the MS/MS spectra generated by data-independent acquisition: a new concept for consistent and accurate proteome analysis. *Mol. Cell. Proteomics* 11, O111.016717

3. Röst, H. L., Rosenberger, G., Navarro, P., Gillet, L., Miladinović, S. M., Schubert, O. T., Wolski, W., Collins, B. C., Malmström, J., Malmström, L., and Aebersold, R. (2014) OpenSWATH enables automated, targeted analysis of data-independent acquisition MS data. *Nat. Biotechnol.* 32, 219–23

4. Bruderer, R., Bernhardt, O. O. M., Gandhi, T., and Reiter, L. (2016) High precision iRT prediction in the targeted analysis of data-independent acquisition and its impact on identification and quantitation. *Proteomics* 16, 1–20

5. Storey, J. D., and Tibshirani, R. (2003) Statistical significance for genomewide studies. *Proc. Natl. Acad. Sci. U. S. A.* 100, 9440–5

1.7 Supplementary figure legends

Suppl Figure 1: **Validation of the new decoy model based on mutated sequences** 2h DIA run of a Hela sample acquired on a Q Exactive HF were analyzed using Spectronaut with a maize spectral library as described in Supplementary Information. The number of maize identifications was counted and used to determine an "actual" FDR for the estimated q-value (< 1%).

Suppl Figure 2: **Characterization of the chromatography** (**a**) The line plot shows the segmented gradient used for the 2h acquisitions. (**b**) The boxplot shows the distribution of full width at half maximum height of the identified chromatographic peaks of the 2h HeLa acquisitions. The time interval for the boxplot calculation was 2.5 min. (**c**) The bar plot shows the average data points per peak of the identified peptides. The time interval for the bar calculation was 2.5 min.

Suppl Figure 3: **Identification reproducibility and quantification analysis** (**a**) The reproducibility of identification of proteins was calculated for the DIA method set with differing data points per peak. (b) The CVs distributions for the 69,968 shared peptides were calculated for the DIA method set with differing data points per peak. (**c**) The reproducibility of identification of proteins was calculated for the DIA method set with varying MS1 resolutions. (**d**) The CV distributions for the 75,740 shared peptides were calculated for the DIA method set with varying MS1 resolutions. (**e**) The reproducibility of identification of proteins was calculated for the DIA method set with varying MS2 resolutions. (**f**) The CV distributions for the 71,570 shared peptides were calculated for the DIA method set with varying MS2 resolutions.

Suppl Figure 4: **MS1 quantification of DIA methods with varying MS1 resolution** (**a**) The CVs of the DIA method set with varying MS1 resolutions were calculated based on peptide intensities. The average identifications of a DIA triplicate analysis of HeLa and the number of identified peptides CVs below defined thresholds were calculated. The error bars display the standard deviations. The asterisk indicates the highest number of peptides with CV below 20%. (**b**) CV comparison of MS1 and MS2 for the method with 120,000 resolution on MS1 (**c**) The boxplot shows the distribution of CVs of identified peptides based on MS1 quantification in 2h HeLa acquisitions plotted against retention time for the method with 120,000 resolution on MS1. (**c**) The boxplot shows the distribution of MS1 based intensities of the identified peptides 2h HeLa acquisitions plotted against retention time for the method with 120,000 resolution on MS1. (**d**) The boxplot shows the distribution of CVs of identified peptides based on MS2 quantification in 2h HeLa acquisitions plotted against retention time for the method with 120,000 resolution on MS1. (**e**) The boxplot shows the distribution of MS2 based intensities of the identified peptides 2h HeLa acquisitions plotted against retention time for the method with 120,000 resolution on MS1. The time interval for the boxplot calculation was 2.5 min.

Suppl Figure 5: **MS2 Fill time analysis for the set of DIA methods with the varying MS2 resolutions** The percentage of MS2 scans, that reached the maximal fill time during mass spectrometric acquisition was calculated for the set of DIA methods with the varying MS2 resolutions.

Suppl Figure 6: **Multiple organism DIA data in depth analysis** (**a**) Reproducibility of protein identification in the mixed proteome sample in a DIA technical triplicate. (**b**) The identifications of the DIA single shot

acquisition with the sample containing peptides from lysates from four organisms were calculated according to their origin (shared peptides were counted only for one organism in this order first to *E.coli*, then *S. cerevisiae* followed by *C. elegans*). (**c**) Significantly differential abundant *C. elegans* proteins (5% FDR by q-value) were calculated using a one sample t-test analysis. (**d**) Significantly differential abundant *S. cerevisiae* proteins (5% FDR by q-value) were calculated using a one sample t-test analysis. (**e**) Significantly differential abundant *E. coli* proteins (5% FDR by q-value) were calculated using a one sample t-test analysis. (**f**) Box plot visualization of percent change (based on MS2 quantification for DIA) of all identified proteins of the large fold change mixed proteome experiment with 60%, 100% and 300% fold change (comparison S2/S1 for *H. sapiens*, *C. elegans* and *S. cerevisiae* and S1/S2 for *E. coli*). Only the overlapping identifications are shown. The theoretical fold changes are indicated for the organisms.

Suppl Figure 7: **Reproducibility of identification and quantification for project specific and resource data based spectral libraries** (**a**) The reproducibility of protein identification was calculated for the analysis of the HEK-293 and the HeLa triplicate DIA analysis. This was performed for the targeted analysis with the project specific spectral library, the published repository derived spectral library (11 common cell lines, see methods) and the panHuman spectral library of Rosenberger et al. (**b**) The CV distributions were calculated for 52,339 shared peptides. (c) Comparison of the retention times of the Cerebellum DIA replicate 1 analyzed with the project specific and the Sharma resource spectral library (105,070 overlapping assays). Pearson correlation is 0.9996. Median abs delta Retention time is 0 and average 0.11 min.

Suppl Figure 8: **Barrel cortex proteome profiling** Four stages normal development of murine barrel cortex were characterized using DIA. (**a**) Reproducibility of identification of proteins within the developmental stages for the targeted analysis with the project specific spectral library. (**b**) Peptide and protein CV distributions were calculated for the developmental stages for the targeted analysis with the project specific spectral library. (**c**) A principal component analysis was performed for the targeted analysis with the project specific spectral library. PC1 and PC2 were plotted. (**d**) Visualization of candidates derived with significant differential abundance from sensory deprivation by postnatal bilateral whisker trimming (Butko et al., 2012) and candidates of the developmental study presented here. (**e**) Fuzzy c-means clustering analysis was performed for the phospho peptides. The black line in the membership bar shows the threshold above which proteins belong to the cluster. (**f**) Proteins profiled by abundance and phosphorylation site. The XICs of MS1 and MS2 are visualized for a phosphorylated peptide and an unmodified peptide.

Suppl Figure 9: **Barrel cortex proteome profiling using the mouse resource spectral library** Four stages of murine barrel cortex development were characterized using DIA and a spectral library generated from published repositories (see methods). (**a**) The protein identifications were calculated per developmental stage and the average with standard deviation are shown. (**b**) Reproducibility of identification of proteins within the developmental stages for the targeted analysis. (**c**) Peptide and protein CV distributions were calculated for the developmental stages for the targeted analysis. (**d**) The average identifications and the

number of peptides CVs below defined thresholds were calculated for analysis with the project specific and repository based spectral library. (**e**) Fuzzy c-means clustering analysis was performed and visualized with enriched GO terms from the targeted analysis of the DIA data with a spectral library generated from published repositories. The black line in the membership bar shows the threshold above which proteins belong to the cluster. (**f**) The DIA-data was analyzed using a spectral library generated from published repositories (see methods). Scatterplot visualization of the overlap of 1,031 which were identified both in the analysis with the project spectral library and the repository spectral library.

Suppl Figure 10: **The TIC identified by the targeted analysis of DIA** (**a**) A random DIA MS1 and corresponding MS2 spectrum was annotated by identified peptides and fragments of the mouse Cerebellum DIA when using the project specific spectral library. The precursor ions were annotated with the respective isotope envelope. The complete spectral library fragment information from the DDA runs (all identified fragments, not only the optimized 6) were taken, additionally, the fragment space was trimmed to >250 m/z, since no fragments below 250 m/z were in the spectral library allowed. Note, that the minimal peptide length for die spectral library DDA runs was set to 7 and a limited set of modifications was used. The analysis was performed as described above. (**b**) A random DIA MS1 and corresponding MS2 spectrum was annotated by identified peptides and fragments of the HEK-293 DIA when using the project specific spectral library. The precursor ions were annotated with the respective isotope envelope. The analysis was performed as described above. (**c**) TIC and identified TIC were calculated for the mouse Cerebellum DIA on MS1 and MS2 level. Additionally, as a control, the identifications were shifted by 3 peak widths to earlier retention time. The analysis was performed as described above. (**d**) TIC and identified TIC were calculated for the HEK-293 DIA on MS1 and MS2 level. The control and analysis was performed as described above.

Suppl. Table 1: Individual improvements in DIA leading to a leap in performance:

The percent improvement on peptide level was calculated for the reported comparisons and the changes

Individual improvements in DIA performance		
**Method**	**Improvement (%)**	**Comparison done**
MS1 scan resolution increase from 35k to 120k or 240k	8	2h DIA, Figure 1d
MS1 dynamic range increase by two-fold slicing MS1 space	4	4h DIA runs, HeLa, Figure 3
High resolution chromatography, improved peak gradient shape	10	ProntoSil 3.5 vs Reprosil Pur 1.9um beads 2h DIA, HeLa
Increased loading	4	2h gradient DIA, HeLa
Spectral library generation algorithm	23	Bruderer et al. MCP 2015, 2h DIA, HEK293
High precision iRT	13	Bruderer et al. Proteomics 2016 2h DIA HeLa
Targeted analysis of DIA with new Spectronaut	12	4h DIA runs, HeLa, compared Spectronaut 9 to Spectronaut version 5 (of Bruderer et al. MCP 2015)
Orbitrap HF mass analyzer	25	Bruderer et al. MCP 2015, 2h DIA QE classic vs QE HF, same DIA method
Fractionation for spectral library generation	30	Bruderer et al. MCP 2015, 2h- DIA, HEK-293

Suppl. Table 2: Mixed proteomes sample generation:

Single sample						
Ratio						
Human	40					
C.Elegans	42					
S.Cereviviae	12					
E.Coli	6					
Small Fold change sample set						
	Ratios			ug used per sample		
	S1	S2			S1	S2
Human	1	1		Human	16.00	16.00
C.Elegans	1	1.1		C.Elegans	16.80	18.48
S.Cereviviae	1	1.2		S.Cereviviae	4.80	5.76
E.Coli	1.3	1		E.Coli	3.64	2.80
Large Fold change sample set						
	Ratios			ug used per sample		
	S1	S2			S1	S2
Human	1	1		Human	16.00	16.00
C.Elegans	1	2		C.Elegans	16.80	33.60
S.Cereviviae	1.6	1		S.Cereviviae	7.68	4.80
E.Coli	1	4		E.Coli	2.40	9.60

Suppl. Table 2: Chromatographic gradient profiles (in min)

Gradient length:	30 min	1h	2h	4 h
percent B				
1	0	0	0	0
4	0.8	1.5	3	6
6	1.5	3	6	12
8	2.5	5	10	20
10	3.8	7.5	15	30
12	5.8	11.5	23	46
17	10.5	21	42	84
26	20.8	41.5	83	166
28	23.0	46	92	184
30	24.5	49	98	196
32	25.8	51.5	103	206
34	26.8	53.5	107	214
36	27.8	55.5	111	222
38	28.5	57	114	228
41	29.3	58.5	117	234
52	30	60	120	240

Suppl. Table 3: Spectral library overview

Spectral library	Sample	Precursors	Sequences	Proteins	In Figure	Based on repository data	Sample Fractionation	Acquisitions	var. Modifications	Comments
HeLa	Hela	91,157	64,356	5,936	1	No	direct digest, SAX, HPRP	17	Oxidation of Methionine, N-terminal acetylation	
HeLa HEK-293	Hela/HEK-293	204,098	107,553	8,119	2,4	No	direct digest, HPRP	20	Oxidation of Methionine, N-terminal acetylation, Deamidation of N,Q	
11 cell lines	see ref.	197,801	127,260	10,403	4	Geiger et al. (2012)	direct digest, SAX	resource	Oxidation of Methionine, N-terminal acetylation, Deamidation of N,Q	
pan human	see ref.	203,313	142,460	10,490	4	Rosenberger et al. (2014)	direct digest, SAX, OGE, 1D-GE, PCT	resource	Oxidation of Methionine, N-terminal acetylation	curated (see methods)
murine tissues	see ref.	451,002	266,828	12,973		Azimifar et at. (2014), Sharma et al. (2015)	direct digest, SAX, SCX	resource	Oxidation of Methionine, N-terminal acetylation	
murine brain tissues	see ref.	445,980	263,909	12,107	4	Sharma et al. (2015)	direct digest, SAX, SCX	resource	Oxidation of Methionine, N-terminal acetylation	
murine barrel cortex	barrel cortex	118,056	81,505	6,459	5	No	direct digest, HPRP	18	Oxidation of Methionine, N-terminal acetylation	
murine barrel cortex phospho peptides	barrel cortex	999	705	402	5	No	direct digest, HPRP	18	Oxidation of Methionine, N-terminal acetylation, Posphorylation of STY	curated (see methods)
C.Elegans	C.Elegans	47,167	33549	4,692	2	Spectronaut 8	direct digest, HPRP	9	Oxidation of Methionine, N-terminal acetylation	
S.Cerevisiae	S.Cerevisiae	32,430	22,286	2,670	2	Spectronaut 8	direct digest	3	Oxidation of Methionine, N-terminal acetylation	
E.Coli	E.Coli	33,890	20,731	1843	2	Spectronaut 8	direct digest	3	Oxidation of Methionine, N-terminal acetylation	

Suppl Table 4: Data set completeness and missing values on protein level for technical replicates

Technical replicates	Hela 2h	Hela 4h	HEK 4h	Cerebellum	4 Mixed proteomes
Missing values proteins	0.4	2.1	1.3	0.3	1.2
Completeness	99.6	97.9	98.7	99.7	98.8

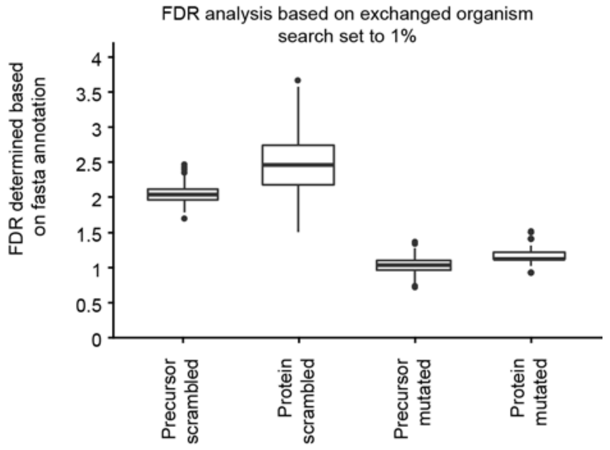

Suppl. Figure 1

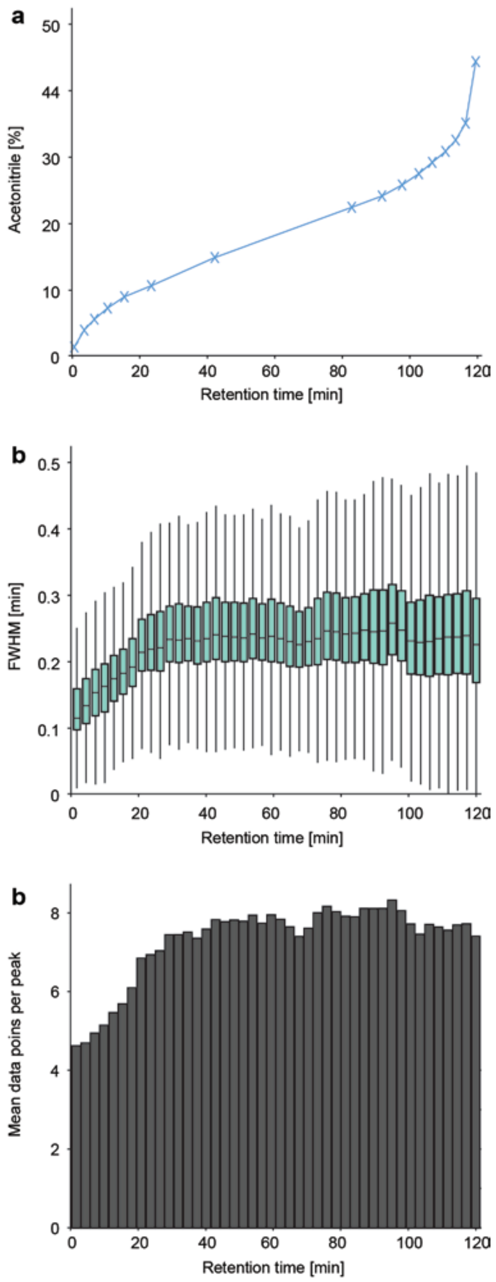

Suppl. Figure 2

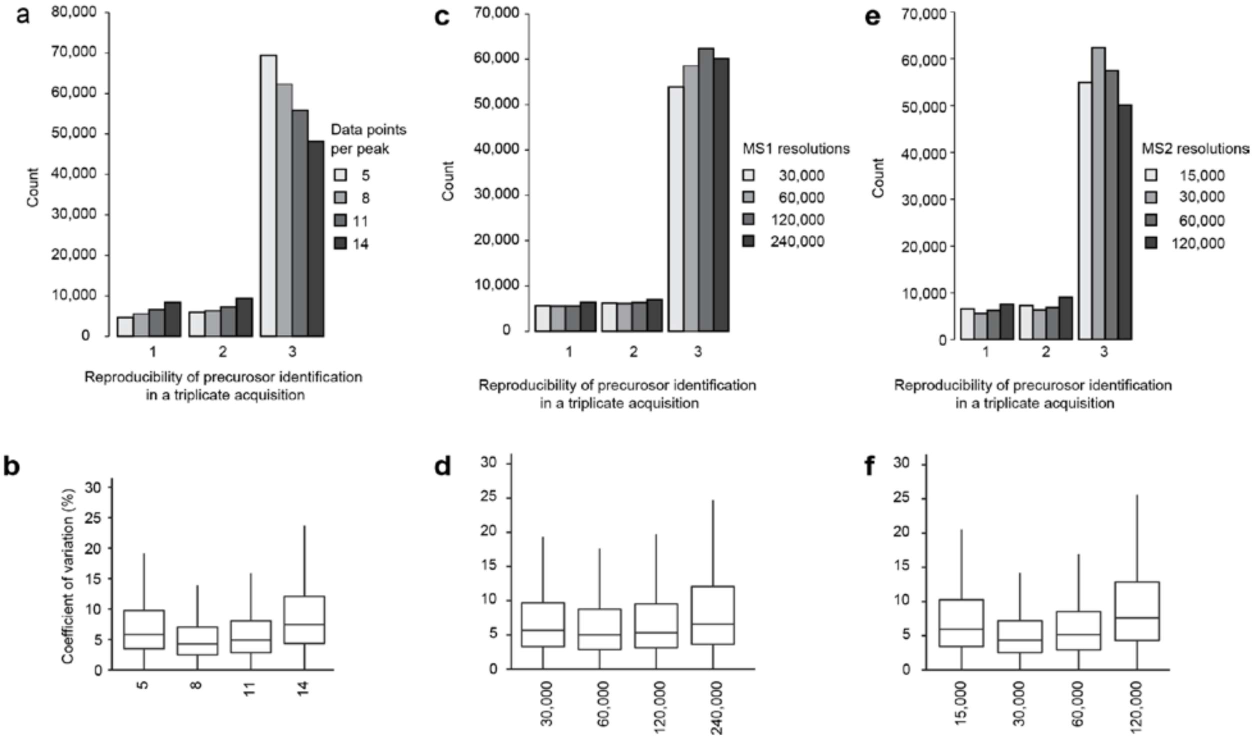

Suppl. Figure 3

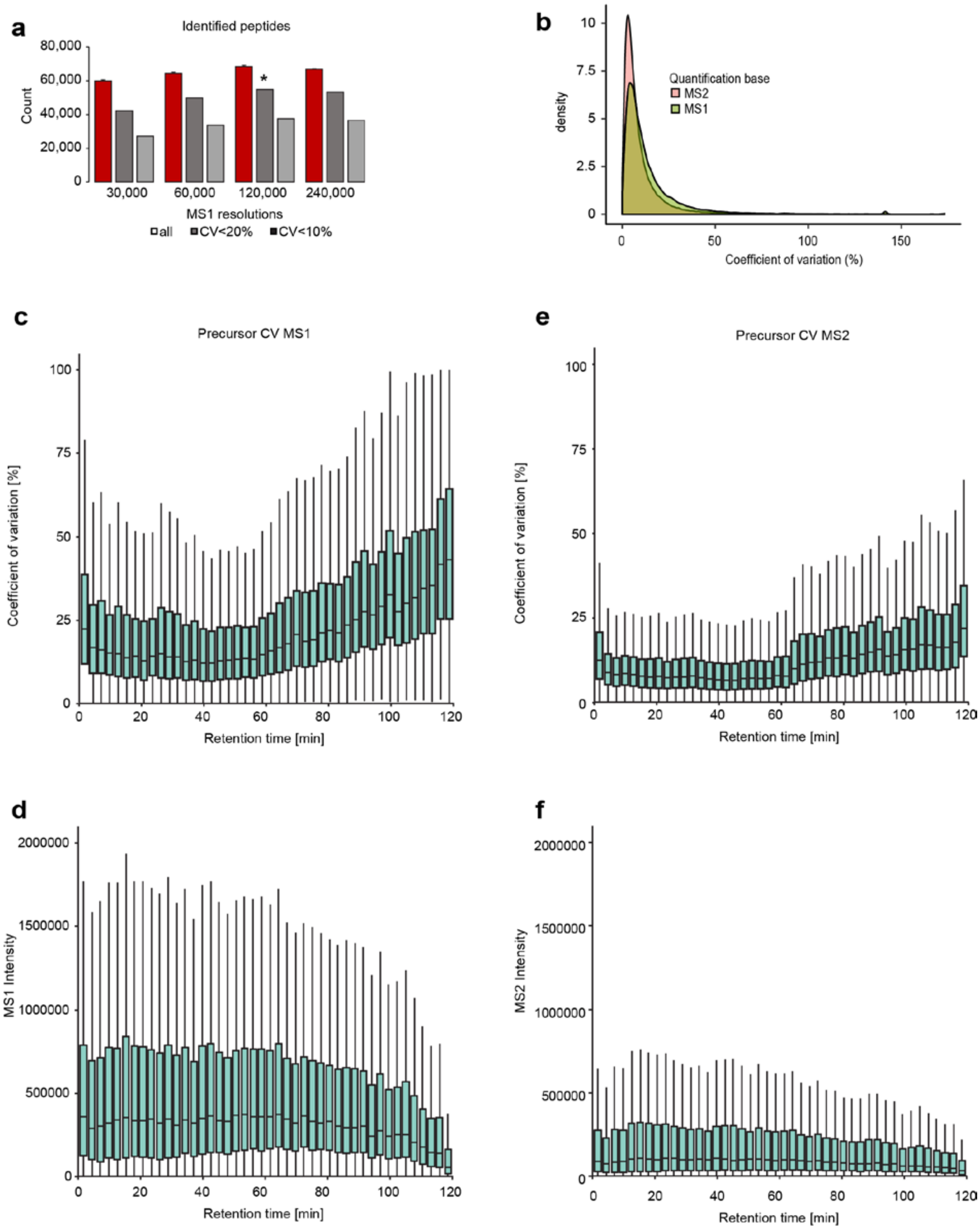

Suppl. Figure 4

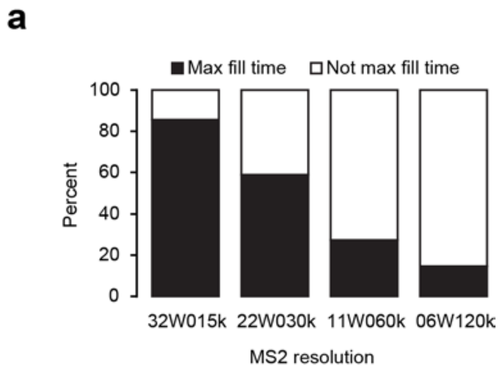

Suppl. Figure 5

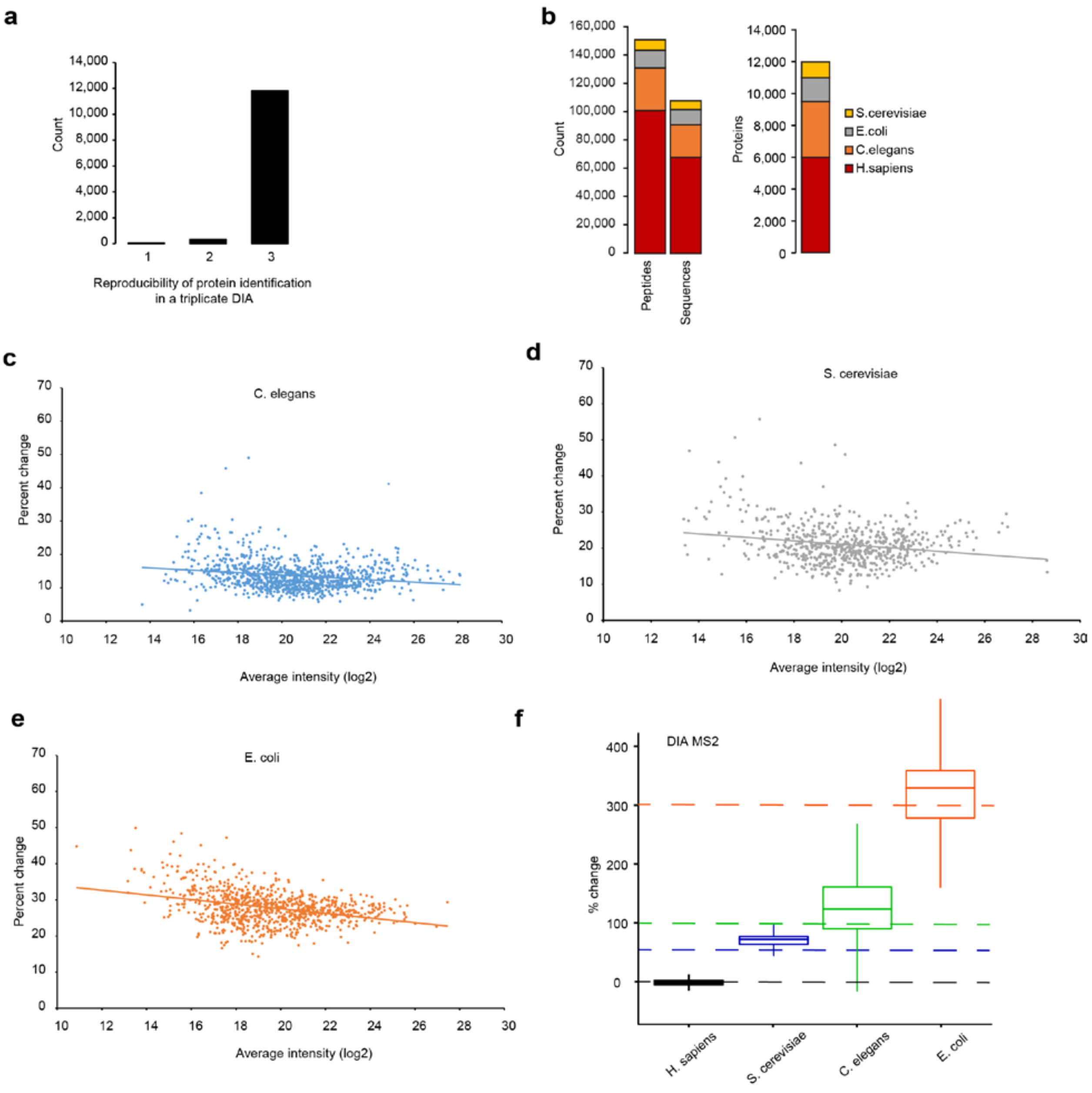

Suppl. Figure 6

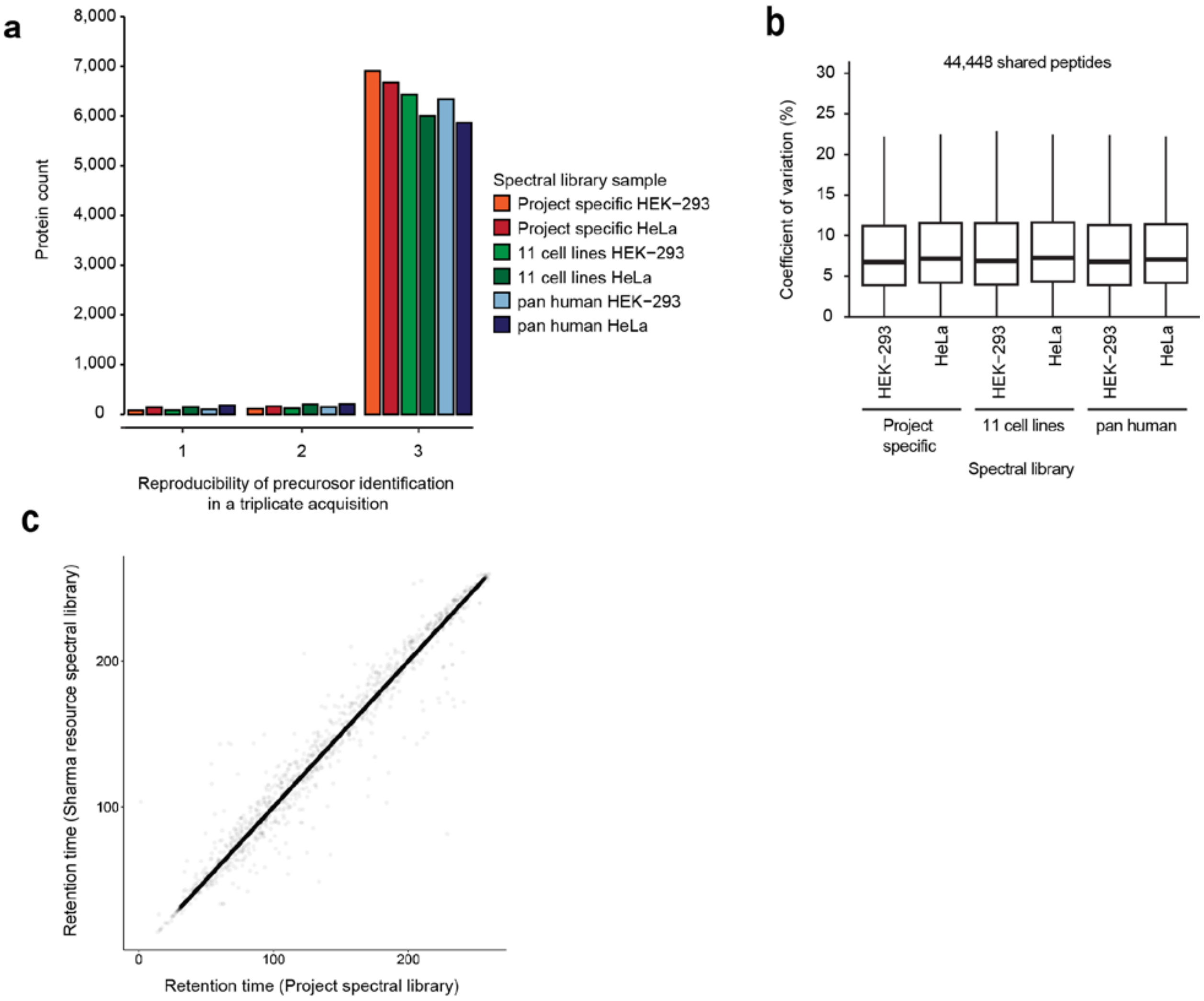

Suppl. Figure 7

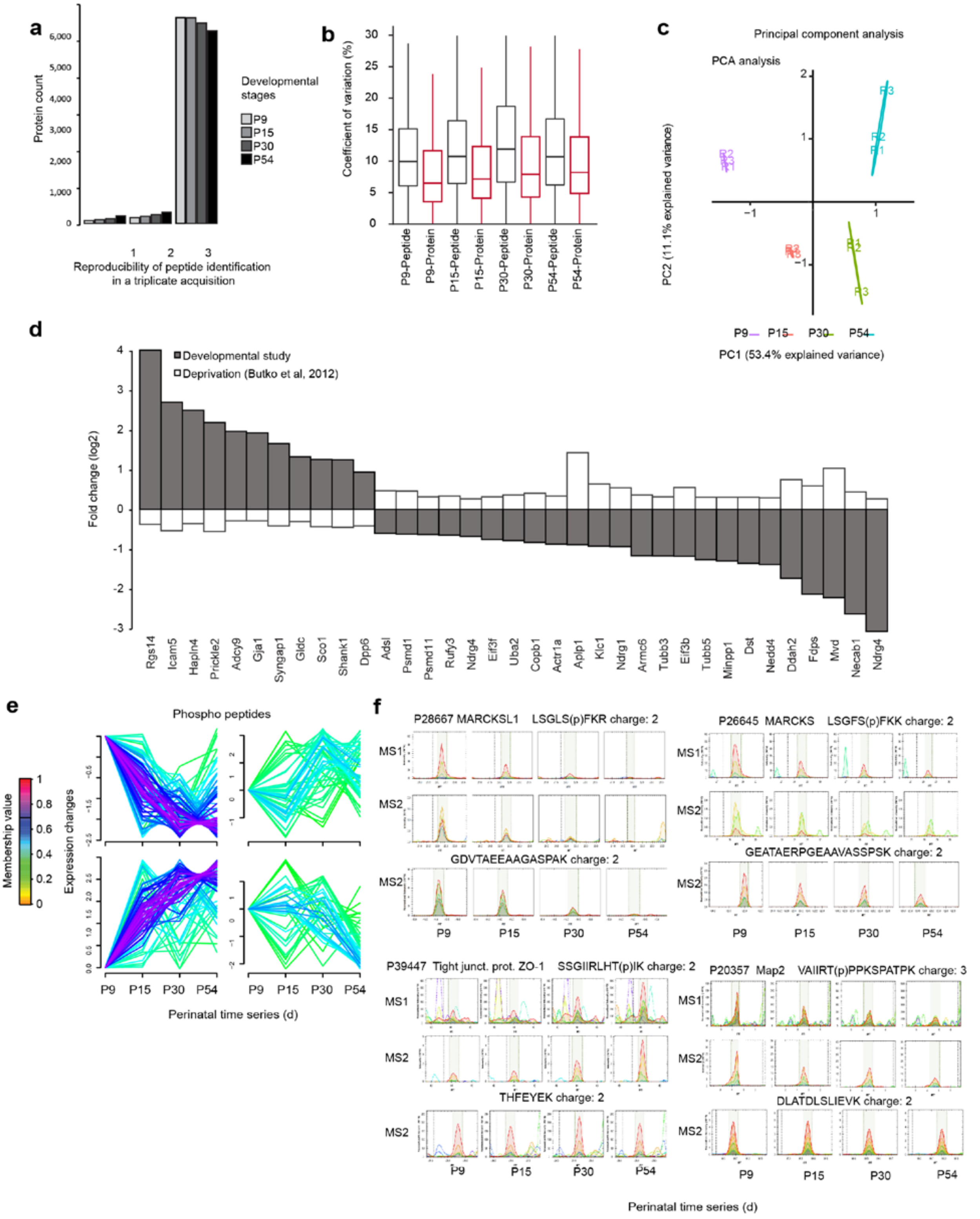

Suppl. Figure 8

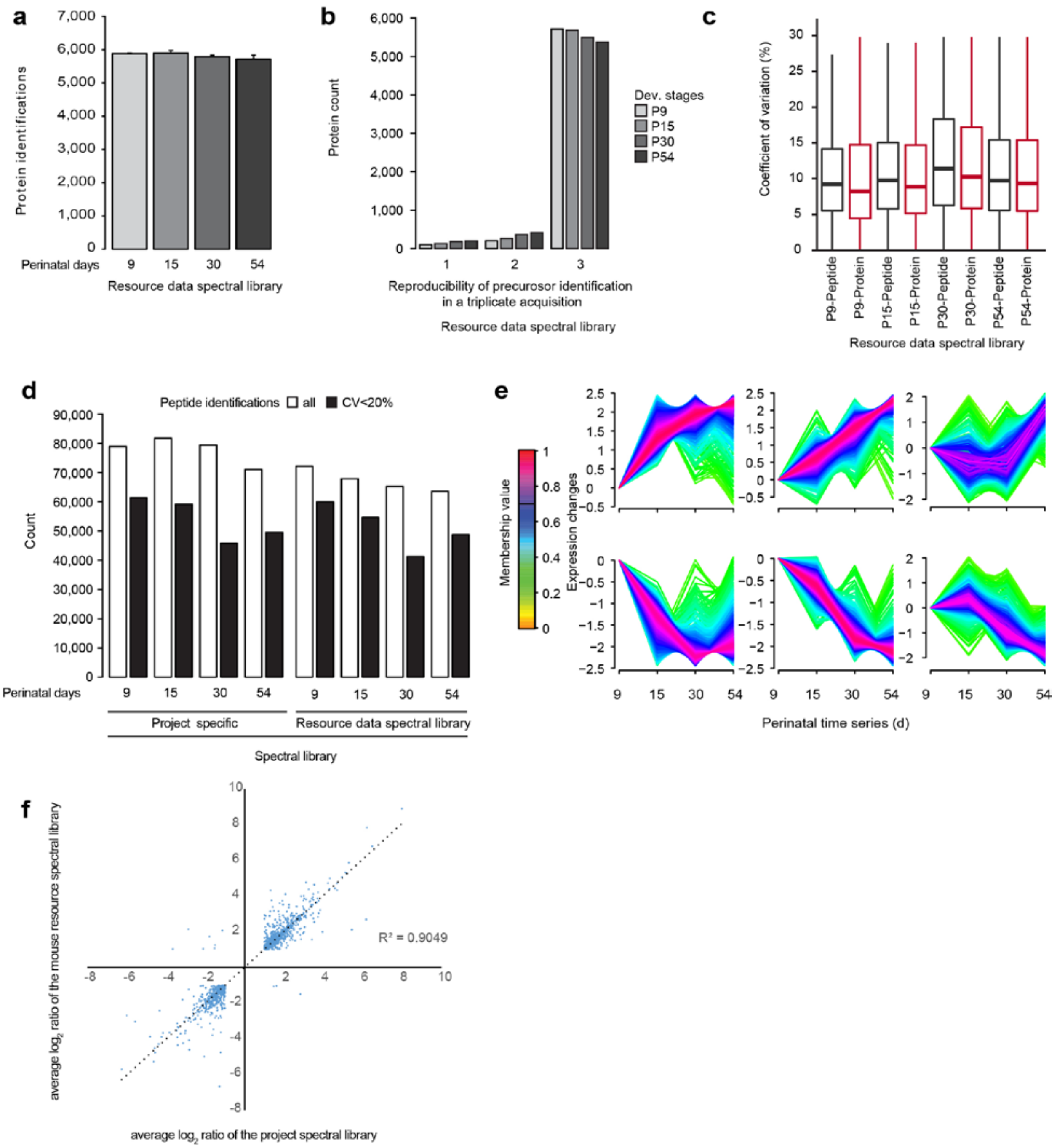

Suppl. Figure 9

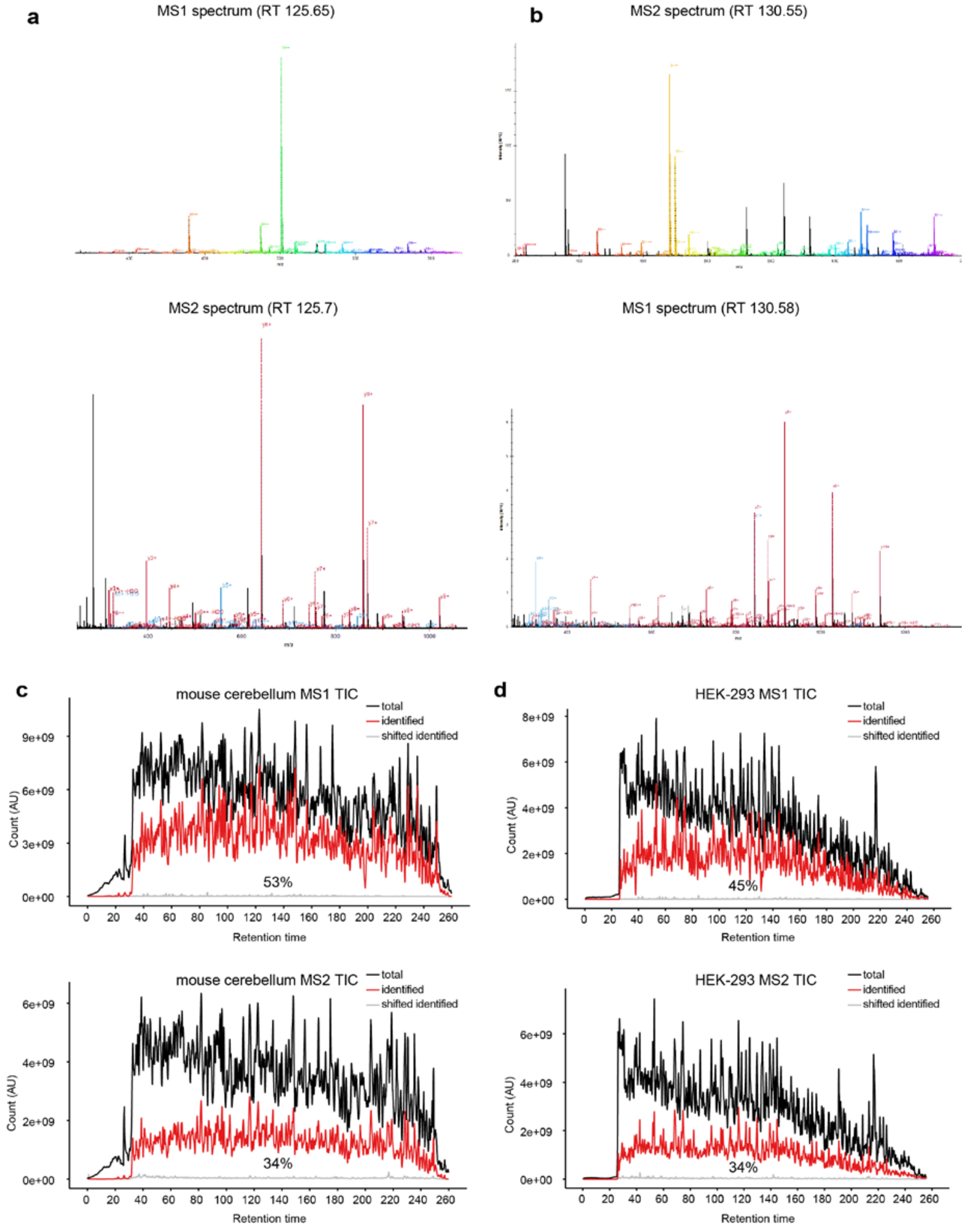

Suppl. Figure 10
